# The transcriptome profile of human trisomy 21 blood cells

**DOI:** 10.1186/s40246-021-00325-4

**Published:** 2021-05-01

**Authors:** Francesca Antonaros, Rossella Zenatelli, Giulia Guerri, Matteo Bertelli, Chiara Locatelli, Beatrice Vione, Francesca Catapano, Alice Gori, Lorenza Vitale, Maria Chiara Pelleri, Giuseppe Ramacieri, Guido Cocchi, Pierluigi Strippoli, Maria Caracausi, Allison Piovesan

**Affiliations:** 1 Department of Experimental, Diagnostic and Specialty Medicine (DIMES), Unit of Histology, Embryology and Applied Biology, University of Bologna, Via Belmeloro 8, 40126 Bologna, BO Italy; 2 Current Address: Department of Molecular and Translational Medicine (DMMT), University of Brescia, Viale Europa 11, 24123 Brescia, BS Italy; 3 MAGI’S Lab, Via delle Maioliche 57/D, 38068 Rovereto, TN Italy; 4 Neonatology Unit, St. Orsola-Malpighi Polyclinic, Via Massarenti 9, 40138 Bologna, BO Italy; 5 Current Address: Department of Medical Biotechnologies, University of Siena, Strada delle Scotte, 4, 53100 Siena, SI Italy; 6 Neonatology Unit, St. Orsola-Malpighi Polyclinic, Department of Medical and Surgical Sciences (DIMEC), University of Bologna, Via Massarenti 9, 40138 Bologna, BO Italy

**Keywords:** Transcriptome, RNA sequencing, Blood cells, Human chromosome 21, Trisomy 21, Down syndrome

## Abstract

**Background:**

Trisomy 21 (T21) is a genetic alteration characterised by the presence of an extra full or partial human chromosome 21 (Hsa21) leading to Down syndrome (DS), the most common form of intellectual disability (ID). It is broadly agreed that the presence of extra genetic material in T21 gives origin to an altered expression of genes located on Hsa21 leading to DS phenotype. The aim of this study was to analyse T21 and normal control blood cell gene expression profiles obtained by total RNA sequencing (RNA-Seq).

**Results:**

The results were elaborated by the TRAM (Transcriptome Mapper) software which generated a differential transcriptome map between human T21 and normal control blood cells providing the gene expression ratios for 17,867 loci. The obtained gene expression profiles were validated through real-time reverse transcription polymerase chain reaction (RT-PCR) assay and compared with previously published data. A post-analysis through transcriptome mapping allowed the identification of the segmental (regional) variation of the expression level across the whole genome (segment-based analysis of expression). Interestingly, the most over-expressed genes encode for interferon-induced proteins, two of them (*MX1* and *MX2* genes) mapping on Hsa21 (21q22.3). The altered expression of genes involved in mitochondrial translation and energy production also emerged, followed by the altered expression of genes encoding for the folate cycle enzyme, *GART*, and the folate transporter, *SLC19A1*.

**Conclusions:**

The alteration of these pathways might be linked and involved in the manifestation of ID in DS.

**Supplementary Information:**

The online version contains supplementary material available at 10.1186/s40246-021-00325-4.

## Background

Down syndrome (DS) is the most common form of intellectual disability (ID) of genetic origin in humans [1, 2]. Also known as trisomy 21, it is caused by the presence of an extra full or partial (partial T21, PT21) human chromosome 21 (Hsa21) [3, 4] in the cells of the affected subjects. Recently, the identification of a highly restricted DS critical region (HR-DSCR) as the minimal region whose duplication is associated to DS because it is shared by all subjects with PT21 diagnosed with DS was confirmed [4].

Hsa21 is the smallest human chromosome, being 46,709,983 bp long [5], and it includes 228 known protein-coding genes and 106 non-coding RNA (ncRNA) genes (retrieved by building the recent GeneBase database up to January 5, 2019, and searching for loci only with a “reviewed” or “validated” gene record including at least one “reviewed” or “validated” RNA) [6–8]. Although it is broadly agreed that the presence of extra genetic material in T21 gives origin to an altered expression of the genes located on Hsa21 leading to DS phenotype, the molecular pathogenesis is still unclear. The third copy of Hsa21 might lead to the over-expression of Hsa21 genes, in theory at 150% of the normal level, i.e. at 3:2 ratio, or to the under-expression of Hsa21 genes in the opposite direction, i.e. at a 2:3 ratio. However, this deregulation affects not only Hsa21 genes but also genes located on other chromosomes [9–11]. As a result of a comprehensive analysis of transcriptome maps of T21 compared to normal control samples, it was demonstrated that most of the gene expression ratios are very close to 1, escaping gene dosage effects, whereas a smaller number of genes has expression ratios close to 3:2 or 2:3, probably due to the stimulatory or inhibitory effects, respectively, of the extra copy of Hsa21 [11].

There are several mechanisms by which the 3:2 DNA template dosage for Hsa21 could affect cellular functions, including the development of the subject with T21 [12]. It has also been suggested that haploinsufficient genes (genes whose loss-of-function results in a recognisable phenotype) are also sensitive to excessive gene dosage and are thus good candidates for contributing to some of the DS phenotypes [12, 13]. This is critical in DS where genes that are present in three copies might contribute to altering cell functions directly, with their downstream effects, or by modification of disomic gene expression.

In recent years, many genes have been suggested to be associated with DS, and several of them mapping on Hsa21 are known to be involved in the alteration of pathways such as one-carbon pathway [14], oxidative metabolism [15] and brain development [16, 17].

Global gene expression profiling techniques have been previously used to analyse the expression levels of Hsa21 genes and of the whole genome aimed to assess their expression in DS and their involvement in the molecular mechanisms that may be related to the pathogenesis of DS [9, 18, 19]. To date, there is no evidence of a clear genotype-phenotype correlation between specific genetic determinants and the main DS symptoms, in particular, ID. Moreover, most of these studies do not analyse the role of genes in the context of T21 but instead provide useful data for each Hsa21 gene product’s roles [20]. The identification of these dose-sensitive genes and genes relevant to DS has become a primary objective of research because it is essential to understand pathogenesis, clarifying the molecular mechanisms underlying the pathology and ultimately developing appropriate therapeutic strategies.

RNA sequencing (RNA-Seq) and microarrays are considered to be the two main kinds of high-throughput technologies to study the gene expression profile [21, 22]. From a technical point of view, it should be considered that a standard protocol for RNA-Seq raw data analysis does not exist while microarray protocols are universally applicable and comparable across platforms [23]. In addition, the analysis of RNA-Seq data also requires extensive experience and bioinformatic competencies for processing data files. However, RNA-Seq does not require probes of known sequence and has the unique ability to discover novel transcripts, novel transcript isoforms and previously unknown changes in the already known transcript sequences [24, 25]. It has the potential to detect rare and low-abundance transcripts [24]. Furthermore, RNA-Seq certainly has a broader dynamic range than that observed with array hybridisation technology (10^5^ vs 10^3^) [26], where the range of the valid gene expression estimation is restricted by background noise at the low end and signal plateau at the high end [27]. By increasing the sequencing coverage depth, very rare transcripts can be detected. Nevertheless, in some cases, the quantification of transcripts by expression microarray has proved more reliable than that observed by RNA-Seq: the ncRNA transcriptome reported by the Illumina Human BodyMap and GTEx projects revealed to be far from comprehensive in comparison with a newer generation microarray platform [28], while it has been shown that an integrated quantitative transcriptome map obtained by processing microarray data correlated better with reverse transcription polymerase chain reaction validation data compared to RNA-Seq data [29]. Therefore, due to the great amount of data from microarray analysis available on public databases, microarray-based transcriptome data still remain a suitable and useful source of gene expression profiling data [29, 30].

The aim of this study was to analyse T21 vs normal control blood cell transcriptome by RNA-Seq. These data were integrated and elaborated by the TRAM (Transcriptome Mapper) software [31] generating a quantitative transcriptome map for each condition and a differential transcriptome map between them for the first time. Gene expression profiles were validated through real-time reverse transcription polymerase chain reaction (RT-PCR) assay and then compared with previously published data whose experimental designs were closer to our kind of analysis [11, 32].

## Results

### RNA sequencing

We generated RNA-Seq paired-end data from rRNA-depleted total RNA isolated from the blood cell samples of 4 T21 and 4 normal control individuals sequenced to a depth range of about 35–53 million reads.

Mapping statistics regarding reads aligned against the *Homo sapiens* (GRCh38) reference genome with STAR aligner [33] are shown in Supplementary Table 1. On average, about 97% of the reads could be mapped on the human genome. Of those, about 69% could be mapped uniquely and about 52% of the reads could be assigned to genes. This percentage of reads might be an indication of residual genomic DNA or a high percentage of pre-mRNAs. In addition, multiple mapping reads are on average 29% and mainly derive from domains highly conserved among gene families and/or expressed transposable elements [34].

FPKM values for each sample are tabulated in Supplementary Table 2. No read map in the HR-DSCR [4].

The Spearman correlation coefficients between each possible pair of T21 samples range from 0.88 to 0.95. The Spearman correlation coefficients between each possible pair of normal control samples range from 0.91 to 0.95. For both groups, the associated *p* values with a sample size gof 58,233 ENSG identifiers are <0.0001. A scatterplot matrix is shown in Supplementary Fig. 1.

### T21 vs normal control blood cell transcriptome maps

The TRAM software was used to convert ENSG identifiers in gene symbols and to perform intra- and inter-sample normalisation of FPKM values to obtain 19,378 genes with an available expression value for T21 blood cell transcriptome map (from 776,003.67 for *RN7SL2*, encoding for RNA component of signal recognition particle 7SL2, to 0.01 for *SCN2A*, encoding for sodium voltage-gated channel alpha subunit 2) and 19,357 genes with an available expression value for normal control blood cell transcriptome map (from 923,210.42 for *RN7SL2* to 0.01 for *FLRT2*, encoding for fibronectin leucine-rich transmembrane protein 2). These values allowed a differential (T21 vs normal control) transcriptome map including the gene expression ratios for 17,867 loci to be obtained. Detailed results for each map are also available in the TRAM software deposited at https://osf.io/ab3np/?view_only=c8cfbaf81a894f379854722a13efb9ec.

Considering the genes expressed in at least two samples (half of the samples for each pool) in both pools, in the T21 vs normal control transcriptome map, we observed the highest expression ratio (ratio=16.79) for *TSPEAR*, a protein that contains an N-terminal thrombospondin-type laminin G domain and several tandem-arranged epilepsy-associated repeats (EARs), an expression ratio >5 for *MX1* gene (ratio=6.76), encoding for MX dynamin-like GTPase 1, *IFI44L* gene (ratio=6.36), encoding for interferon-induced protein 44 like, *IFIT1* gene (ratio=5.76), encoding for interferon-induced protein with tetratricopeptide repeats 1 and *RSAD2* gene (ratio=5.39) encoding for radical *S*-adenosyl methionine domain containing 2. Among the Hsa21 genes, we have found 143 genes with a gene expression ratio of ≥1.30, and in particular, we observed the over-expression of *TSPEAR* and *MX1* genes (cited above), *MX2* gene (ratio=4.32) encoding for MX dynamin-like GTPase 2 and of *SLC19A1* gene (ratio=3.5) encoding for solute carrier family 19 member 1.

Overall, the mean value among all gene expression ratios is 1.30. A T21/normal expression ratio >1.70 (of which 91 on Hsa21) is observed for 2320 genes, 3587 genes (48 on Hsa21) a T21/normal expression ratio between 1.70 and 1.30, 8283 genes (24 on Hsa21) between 1.29 and 0.75, 966 genes (5 on Hsa21, *BACH1-IT3*, *RBM11*, *C21orf62-AS1*, *TMPRSS15*, *LINC01547*) between 0.76 and 0.58, 265 genes have a T21/normal expression ratio <0.58 (4 on Hsa21, *LINC01679*, *ERVH48-1*, *SIK1*, *TEKT4P2*). Detailed results are available in Supplementary Table 3.

In both single transcriptome maps, genes following *RN7SL2* with the highest expression values are *HBB*, *HBA2* and *HBA1*, i.e. genes coding for haemoglobin beta- and alpha-chains.

**Fig. 1:**
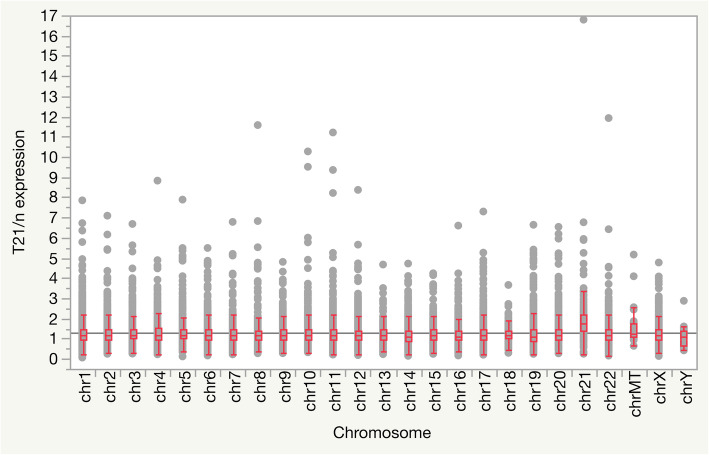
Trisomy 21 (T21) vs normal control (*n*) expression ratio in blood cell transcriptome map divided by chromosome

Considering the chromosomes, Hsa21 has the highest T21 vs normal control mean expression ratio (2.01 with a standard deviation of 1.50) compared to the other chromosomes (Fig. 1). Interestingly, mitochondrial genes have a T21 vs normal control mean expression ratio of 1.56 with a standard deviation of 0.93. Non-parametric Kruskal-Wallis’ test showed a significant difference (*p*<.0001) in T21 vs normal control mean expression ratio among chromosomes. Post hoc analysis with Games-Howell’s test showed a significant difference in the mean expression ratios between Hsa21 and all other chromosomes (*p*=0.0002 with chromosome Y and *p*<.0001 for the other chromosomes), other than mitochondrial genome (*p*=0.8522).

Regarding the analysis of segments in the “Map” mode, in both the single maps, we observed the statistically significant over-expression of the same segments mapping on chromosomes 11, 14, 1, 4, 12 and 2 and on the mitochondrial genome (see Supplementary Table 4). The segment analysis of the differential transcriptome map showed that four segments are statistically significantly over-expressed in T21 compared with the normal control samples (Table 1). The two segments with the highest expression ratios are on Hsa21, while the other two are on chromosomes 22 and 10 (Table 1). No statistically significant under-expressed segments were found.

**Table 1:** List of statistically significant over-expressed segments (*q*<0.05) in trisomy 21 (T21) vs normal control blood cell transcriptome map

Chr	Location	Segment start	Segment end	Expression ratio	Genes
Chr21	21q22.2-q22.3	41,250,001	41,750,000	4.16	*MX2*, *MX1*, *RIPK4*
Chr21	21q22.3	44,000,001	44,500,000	4.11	*PWP2*, *TRPM2*, *TSPEAR*
Chr22	22q11.22	21,750,001	22,250,000	3.65	*IGLV4-69*, *IGLV8-61*, *IGLV10-54*
Chr10	10q23.31	89,000,001	89,500,000	2.78	*IFIT2*, *IFIT3*, *IFIT1*

Segments are sorted by decreasing T21 vs normal control expression ratio. For simplicity, some segments are not shown because they overlap with those highlighted in one of the listed regions

*Chr* chromosome, *Location* segment cytoband derived from that of the first mapped gene within the segment, *Segment start/end* chromosomal coordinates for each segment

The given hypothesis that the one-carbon pathway might be involved in the manifestation of the main symptoms in DS [14], and the experimental confirmation that T21 lymphocytes are more sensitive to the methotrexate effect than normal control cells [35] led us to analyse the expression profiles of the main genes encoding for the key enzymes of the one-carbon pathway. Expression values were selected for genes implicated in the one-carbon metabolic process (Gene Ontology, GO:0006730) and the folic acid-containing compound metabolic process (GO:0006760) [36]. Among 37 genes for which a T21 vs normal control expression ratio was available (derived by expression values detected in at least two samples in both pools), the global mean expression ratio is 1.22 (with a standard deviation of 0.69), and 22 genes are over-expressed and 15 under-expressed (Supplementary Table 5). Among the over-expressed genes, two map on Hsa21: *GART* gene (ratio=1.58), encoding for phosphoribosylglycinamide formyltransferase, phosphoribosylglycinamide synthetase, phosphoribosylaminoimidazole synthetase and *SLC19A1* gene (cited above).

### Real-time RT-PCR validation assay

Real-Time RT-PCR experiments were conducted to validate the T21 vs normal control differential transcriptome map obtained through the elaboration of RNA-Seq data by TRAM analysis. Nineteen genes, selected following the criteria described in the “Materials and methods” section (primer pairs listed in Supplementary Table 6), were analysed by real-time RT-PCR in the same RNA samples used for RNA-Seq analyses (4 subjects with DS and 4 normal controls, see Supplementary Table 7A). For the expression ratio and inter-sample variability values, refer to Supplementary Table 3. Bivariate statistical analysis was performed between the gene expression ratios observed by real-time RT-PCR on the 19 selected genes and the differential expression ratios of the same genes obtained by RNA-Seq (*r*=0.91, *p*=0.0001) (Table 2A).

**Table 2:** Genes selected for the comparison of trisomy 21 (T21) vs normal control (*n*) blood cell transcriptome maps with real-time RT-PCR

Chr	Gene symbol	RNA-Seq T21/*n* ratio	Real-time T21/*n* ratio
A			
chr15	*B2M*	1.31	1.29
chr5	*DHFR*	1.00	0.17
chr21	*DYRK1A*	1.65	1.01
chr21	*ETS2*	2.80	2.81
chr12	***GAPDH***	1.21	1.00
chr19	*HCST*	1.15	0.86
chr11	*LRP5*	0.66	0.60
chr21	*MX1*	6.76	6.96
chr9	*NACC2*	1.15	1.48
chr7	*NDUFA4*	1.41	1.06
chr17	*NPTX1*	0.25	0.13
chr11	*NRGN*	0.87	0.77
chr12	*NUAK1*	4.47	7.84
chr19	*PRDX2*	1.05	0.68
chr1	*RYR2*	1.13	0.37
chr17	*SERPINF1*	2.33	0.67
chr21	*SOD1*	1.41	1.33
chr12	*TUBA1B*	1.13	0.57
chr7	*YKT6*	1.16	1.15
B			
chr10	*IFIT1*	5.76	0.90
chr21	*SLC19A1*	3.50	2.02
chr21	*TSPEAR*	16.79	9.27
chr21	*MX1*	6.76	2.07
chr21	*GART*	1.58	2.80
chr12	***GAPDH***	1.21	1.00

A) Genes randomly selected for the validation by real-time RT-PCR of the RNA-Seq results on the same samples used for RNA-Seq. B) Genes with a statistically significant over-expression in the RNA-Seq results, validated by real-time RT-PCR on a larger cohort of samples. From left to right: chromosome, official gene symbol; T21/*n* expression ratios obtained by TRAM analysis following RNA-Seq data elaboration; T21/*n* expression ratios determined by real-time RT-PCR. The genes in bold are used as a reference

Real-time RT-PCR experiments were also performed on a different and larger cohort of subjects (6 subjects with DS and 6 normal controls), in order to independently confirm the expression level of five genes reported as significantly over-expressed by RNA-Seq (primer pairs were listed in Supplementary Table 6). Selected genes were *IFIT1* mapping on chromosome 10 and *TSPEAR*, *MX1*, *SLC19A1* and *GART* mapping on chromosome 21. *GAPDH*, mapping on chromosome 12, was used as a reference (see Supplementary Table 6). The statistical analysis, reported in the “Materials and methods” section, showed two strong outliers among the *IFIT1* expression values (A10=0.05 and *IFIT1* mean expression value of subjects with DS is 0.016; B8=0.174 and *IFIT1* mean expression value of normal control subjects is 0.018); one strong outlier among the *TSPEAR* expression values (B7=0.001 and *TSPEAR* mean expression value of normal control subjects is 0.0000964); one among the *MX1* expression values (B8=0.1975 and *MX1* mean expression value of normal control subjects is 0.027) and one among the *GART* expression values (B6=0.0121 and *GART* mean expression value of normal control subjects is 0.005). The strong outliers were reported in red in Supplementary Table 8, and we decided to calculate the T21/normal expression ratio after removing these strong outliers from the gene expression values. Bivariate statistical analysis was performed between the gene expression ratios observed by real-time RT-PCR on the six selected genes (including *GAPDH*) in the cohort of 12 subjects (6 subjects with DS and 6 normal controls) and the expression ratios of the same genes in the original group of 8 subjects (4 subjects with DS and 4 normal controls) whose samples had also been subjected to RNA-Seq as described above (*r*=0.88, *p*=0.0186) (Table 2B).

### Pathway analyses

An unbiased functional enrichment analysis of over-/under-expressed genes was performed using ToppFun from the ToppGene Suit Gene Ontology tool. The results obtained submitting a list of human genes with expression values detected in at least two samples in both pools and with a T21/normal expression ratio of ≥1.30 (5845 gene symbols found over 5907) and a list of human genes with a T21/normal expression ratio of ≤0.76 (2022 gene symbols found over 1,533 1,591) are shown in Supplementary Tables 9 and 10, respectively.

Among the genes with a T21/normal expression ratio of ≥1.30, we observed that the most significantly enriched biological process associated to the greatest number of genes (713) is “vesicle organisation”, GO:0016050; the most significantly enriched cellular component associated to the greatest number of genes (645) is “whole membrane”, GO:0098805; the most significantly enriched molecular function associated to the greatest number of genes (599) is “transferase activity, transferring phosphorus-containing groups”, GO:0016772; and finally, the most represented pathway associated to the greatest number of genes (517) is the “innate immune system”, GO:1269203.

Among the genes with a T21/normal expression ratio of ≤0.76, the most significantly enriched biological process associated to the greatest number of genes (149) is “cell cycle”, GO:0007049; the most significantly enriched cellular components associated to the greatest number of genes (143) is “nuclear chromatin”, GO:0000790; the most represented molecular function associated to the greatest number of genes (129) is “DNA-binding transcription factor activity, RNA polymerase II-specific”, GO:0000981; and finally, the most represented pathway associated to the greatest number of genes (79) is the “generic transcription pathway”, 1269650.

### Comparison with publicly available transcriptome maps

The three transcriptome maps (T21, normal control and T21 vs normal control) obtained by the elaboration of the RNA-Seq results were compared with other blood-derived transcriptome maps obtained through publicly available microarray [11] and RNA-Seq meta-analyses for WBC (white blood cells) [32] (Table 3, Supplementary Table 11). The best concordance was found between the normal control WBC transcriptome maps obtained through microarray meta-analyses [11] and RNA-Seq data [32] elaborated here by TRAM for the purpose of this comparison (Supplementary Table 12), as it could be expected due to the similarity of the type of biological samples. Interestingly, no Pearson correlation (*r*<0.07, data not shown) could be seen when our RNA-Seq data were compared with the reference WBC transcriptome previously described following systematic meta-analysis of microarray expression data [11], while a strong correlation emerged when a nonparametric correlation test was applied (Spearman correlation by rank; Table 3). This finding can be explained by the different absolute values produced by the count-based RNA-Seq method and the hybridisation kinetic-based microarray method, so that there is little linear agreement between the values, while the relative levels of the RNAs within each distribution are better conserved. In addition, the expression values obtained with both methods show a strongly skewed distribution, in particular, because *HBB*, *HBA1* and *HBA2* genes have expression values of some orders above the other highly expressed genes also in microarray meta-analysis maps. A strong correlation was also observed between our RNA-Seq results and the RNA-Seq data from Powers and colleagues [32] for both T21 and normal control data and between microarray data from Pelleri and colleagues [11] and RNA-Seq data from Powers and colleagues [32].

**Table 3:** Comparison among trisomy 21 (T21) or normal control (*n*) blood cell transcriptome maps obtained by RNA-Seq (present study) and microarray and RNA-Seq (different studies) experiments and analysed by TRAM. For each comparison, the number of genes in common between the two compared studies, and the number of the unique gene for each study are indicated. For each comparison, Spearman correlation coefficients (*r*) are calculated on common genes (*p*<.0001 by the JMP software for all comparisons). *WBC* white blood cells. Details about the studies used in the following comparisons are listed in Supplementary Table 11

Comparison	Tot first study	Tot second study	First study unique genes	Second study unique genes	Common genes	*r*
T21 blood cells (RNA-Seq) vs T21 WBC (array) [11]	19,378	24,699	4,336	9,657	15,042	0.7353
*n* blood cells (RNA-Seq) vs *n* WBC (array) [11]	19,357	24,699	4,316	9,658	15,041	0.7319
T21/*n* blood cells (RNA-Seq) vs T21/*n* WBC (array) [11]	17,867	24,699	3,414	10,246	14,453	0.1193
T21 blood cells (RNA-Seq) vs T21 WBC (RNA-Seq) [32]	19,378	22,614	3,194	6,430	16,184	0.7137
*n* blood cells (RNA-Seq) vs *n* WBC (RNA-Seq) [32]	19,357	21,099	3,259	5,001	16,098	0.7097
T21/*n* blood cells (RNA-Seq) vs T21/*n* WBC [32]	17,867	20,826	2,517	5,476	15,350	0.1718
T21 WBC (array) [11] vs T21 WBC [32]	24,699	22,614	6,082	3,997	18,617	0.8702
*n* WBC (array) [11] vs *n* WBC [32]	24,699	21,099	6,835	3,235	17,864	0.8721
T21/*n* WBC (array) [11] vs T21/*n* WBC [32]	24,699	20,826	6,939	3,066	17,760	0.3354

## Discussion

In recent years, RNA-Seq analysis has been employed in the study of postnatal gene transcription profile of a few T21 primary tissues, such as endothelial cells [37], fibroblast cells [38, 39] and WBC [32]. In this work, we present a differential quantitative blood cell transcriptome map between T21 and normal control blood cells for 17,867 loci and a post-analysis through transcriptome mapping aimed at identifying the segmental (regional) variation of the expression level across the whole genome (segment-based analysis of expression).

The analysis of RNA-Seq mapping results showed that the percentage of reads assigned to genes is a bit low (52% of the reads) (Supplementary Table 1) compared to other RNA-Seq experiments on human T21 samples [37], even with 97% of the reads mapped on the human genome. A low percentage of reads associated to exonic regions might be an indication of residual genomic DNA or a high percentage of pre-mRNAs. On the other hand, our results are consistent with the analysed biological sample type being that *HBB*, *HBA1* and *HBA2* are among the highest expressed genes.

The analysis of expression observed at the segment level showed that the two most over-expressed segments are on Hsa21 (Table 1), highlighting the presence of the third copy of Hsa21 also seen in the single Hsa21 gene over-expression [11, 32, 38]. The over-expression of the segment on chromosome 10 containing *IFIT2*, *IFIT3* and *IFIT1* genes confirmed previous analyses [11] and the interferon signalling pathway alteration previously found [32, 38]. Interferon not only has an antiviral activity but also performs various kinds of biological activities, including cell differentiation-inducing activity and cell growth inhibition [40].

At a single gene level, the over-expression of *MX1*, *MX2* and *IFIT1* included in the Hsa21 and chr10 over-expressed segments (Table 1) emerged. MX1, MX2 and IFIT1 proteins are regulated by interferons, but only the MX1 and IFIT1 are known to participate in the cellular antiviral response [41–43]. It is interesting to notice that *MX2* (Hsa21 gene) over-expression is involved in the suppression of cell proliferation, migration and invasion in glioblastoma cells [26] but also that the *MX2* gene has an important role in the morphology and function of the mitochondrial membrane [44]. Both aspects, suppression of cell proliferation and mitochondrial membrane structure, are consistent with the two main DS features: reduced incidence of solid tumours and alteration of mitochondrial functions. Another interesting point for the purpose of our study was to have found the over-expression of the *RSAD2* gene whose protein product is another interferon-inducible antiviral protein and belongs to the *S*-adenosyl-l-methionine (SAM) superfamily of enzymes [45]. It plays a role in fatty acid b-oxidation. Focusing also on the first Hsa21 over-/under-expressed genes emerging from the differential transcriptome map analysis, we found the over-expression of the *SLC19A1* gene encoding for a transporter of folate, thus involved in the regulation of intracellular concentrations of folate, and of cGAMP (2′3′-cyclic-GMP-AMP), a second messenger that activates the antiviral stimulator of interferon genes [46]. The over-expression of specific genes located on chromosome 21 supports previous analyses revealing the hyperactivation of the interferon signalling cascade in Down syndrome [47].

The over-expression observed at the chromosomal level of the Hsa21 and mitochondrial genome (2.01 and 1.50, respectively) compared to the other chromosomes (Fig. 1) in T21 supports the hypothesis of the alteration of mitochondrial functions in Down syndrome [48, 49]. In fact, we observed that the majority of the mitochondrial aminacyl-tRNA genes are over-expressed except the mitochondrial transfer RNA gene for the amino acid tryptophan (ratio=0.64) which is under-expressed. We also noticed that the genes encoding for the mitochondrial cytochrome c oxidase I, II and III (*MT-CO1*, *MT-CO2* and *MT-CO3*) and the NADH dehydrogenase 5 and 6 (*MT-ND5*, *MT-NT6*) have gene expression ratios between 1.2 and 1.6 (see Supplementary Table 3).

The unbiased functional enrichment analysis of over-/under-expressed genes highlighted several pathways which may suffer the altered expression of the related genes (Supplementary Tables 9 and 10). There is no GO category directly linked to the one-carbon pathway, probably because the global mean expression ratio of genes involved in it is 1.21 with only a few genes with T21/normal expression ratio ≥1.30 (the chosen cut-off for over-expressed genes in the functional enrichment analysis). However, the over-expression of genes like *GART* (21q22.1, gene expression ratio=1.58), encoding for phosphoribosylglycinamide formyltransferase, phosphoribosylglycinamide synthetase, phosphoribosylaminoimidazole synthetase and *SLC46A1* (17q11.2, gene expression ratio=1.67) and encoding for solute carrier family 46 member 1, and *SLC19A1* (21q22.3, gene expression ratio=3.50), encoding for the solute carrier family 19 member 1, provides interesting hints about the hypothesis regarding the alteration of the one-carbon pathway in the manifestation of ID in DS [35, 36]. Indeed, the first is a key enzyme of the folic acid cycle necessary to convert tetrahydrofolate (THF) to 10-formyl-THF and 5,10-methenyl-THF to THF; the second is the main carrier of folates and antifolates in the nervous system, and the third is the ubiquitously expressed form of folate carriers [36]. A recent study [50] about the transcriptome profile of the whole heart already showed that a coherence at a quantitative level between the transcriptome model and the ratio of gene products found at precise relative amounts in that tissue exists. Nevertheless, the protein levels are often the mirrors of what happens at the transcriptional level and vice versa, thus measuring the activities of the enzymes involved in the one-carbon pathway in T21 vs normal control samples would still be interesting.

The validation by real-time RT-PCR of the RNA-Seq data (*r*=0.91, *p*=0.0001), the further assessment of five significantly over-expressed genes in a larger cohort of samples (*r*=0.88, *p*=0.0186) and the strong correlation of RNA-Seq results with the gene expression data obtained by recently published transcriptome profiles closer to our kind of analysis [11, 32] demonstrated the reliability of the sample choice and treatment methods and of the quantitative gene expression data obtained in this work.

In perspective, a meta-analysis of all the T21 blood cell gene expression profiles conducted by RNA-Seq and available on the public databases might be due, but to date, there is only one available study with this aim [32, 37] conducted on samples from subjects with DS. A limitation of RNA-Seq over microarray in meta-analyses performed by TRAM is the lack of the UniGene clusters, since the UniGene data parsing [51] was not required here while it is performed for microarray analyses giving useful hints to gene characterisation [52].

The lack of reads mapping on the HR-DSCR [4] is likely due to the sample type and to the low depth range; we obtained with this being an intronic region of a new isoform of *KCNJ6* (isoform 2, ENSG00000157542). Actually, the well-known *KCNJ6* isoform 1 seems not to be expressed in blood, and this was confirmed in our dataset where it is present only in two T21 samples with a very low median expression value (0.03). Further research is necessary to better characterise this locus.

## Conclusions

In the present study, we analysed T21 and normal control blood cell gene expression profiles obtained by total RNA sequencing (RNA-Seq). A post-analysis through transcriptome mapping allowed the identification of the segmental (regional) variation of the expression level across the whole genome (segment-based analysis of expression) showing that Hsa21 has the highest T21 vs normal control mean expression ratio compared to the other chromosomes and that mitochondrial genes have a T21 vs normal control mean expression ratio of 1.56, following the 3:2 Hsa21 gene dosage ratio in T21. Interestingly the most over-expressed genes encode for interferon-induced proteins, two of them (*MX1* and *MX2* genes) mapping on Hsa21 (21q22.3). The altered expression of genes involved in mitochondrial translation and energy production also emerged, followed by the altered expression of genes encoding for the folate cycle enzyme, *GART* and the folate transporter, *SLC19A1*.

The alteration of these pathways might be linked and involved in the manifestation of ID in DS.

Finally, the complete quantitative and normalised transcriptome map generated in this work has been released to allow further analysis and comparison with other global gene expression profiles in T21 cells.

## Materials and methods

### Case selection

Subjects were admitted to the Day Hospital of the Neonatology Unit, Sant’Orsola-Malpighi Polyclinic, Bologna, and this study was proposed in the context of the yearly routine follow-up provided for DS. A total of 20 subjects were enrolled in this study: 10 subjects with DS (5 males and 5 females) and 10 normal control subjects (6 males and 4 females). No additional samples were included in this study due to the difficulty in retrieving blood samples of adequate quantity and quality from a larger number of children with DS and a comparable group of normal paediatric controls.

For RNA-Seq analyses, 4 subjects with DS and 4 normal control samples were used. The mean age of subjects with DS was 11.52±0.54, and the mean age of normal control subjects was 7.86±4.33 (Supplementary Table 7A). The unpaired *t* test showed no statistically significant difference (*p* value=0.1448) among the mean ages of the two groups of subjects.

The real-time RT-PCR, performed on RNA samples from 6 children with DS and 6 normal controls, analysed five genes over-expressed in the RNA-Seq experiments. The mean age of subjects with DS (11.07±0.57) and of normal controls (7.78±2.33) is comparable with the mean age of the two groups analysed by RNA-Seq (Supplementary Table 7B). Details about the samples from subjects with DS and normal controls were listed in Supplementary Table 7. Supplementary Table 13 provides the clinical data of subjects with DS.

### Sample processing

Blood samples (3 mL) from DS and normal control recruited donors (Supplementary Table 7) were collected in ethylenediaminetetraacetic acid (EDTA)-coated blood collection tubes, kept at room temperature and treated within 2 h from blood collection. Each sample was transferred into a new tube, and the plasma fraction was isolated by centrifugation at 1200*g* for 10 min, while 5 mL of denaturing solution [53] was added to the remaining blood fraction (buffy coat and red blood cells) and stored at −20°C until RNA extraction.

Total RNA extraction was performed with the method of Chomczynski and Sacchi [53]. The RNA quantity and quality have been verified through electrophoresis on agarose gel (visualisation and quantification with the GelDoc 2000 and Quantity One software, Bio-Rad Laboratories, Hercules, CA, USA) and through Nanodrop spectrophotometer (ND-1000 spectrophotometer, Thermo Scientific, Thermo Fisher Scientific, Waltham, MA, USA).

One or, if necessary, two purification steps were performed using RNA Clean & Concentrator-5 Kit (Zymo Research, 17062 Murphy Ave, Irvine, CA, 92614, USA) following the manufacturer’s instructions in order to remove genomic DNA and salt contamination and to reach the minimum concentration of 50 ng/μL verified through spectrophotometry in a minimum volume of 20 μL. Samples were stored at −80°C until the library preparation process.

### RNA-Seq and data processing

Four T21 and 4 normal control blood samples were used to perform RNA-Seq analyses (see Supplementary table 7A).

Library preparation, sequencing, read mapping and counting were carried out by “Sequentia Biotech SL” (Barcelona, Spain).

TruSeq Stranded Total RNA with Ribo-Zero Gold (Illumina, San Diego, CA) was used for library preparation following the manufacturer’s instructions, starting with 200 ng of RNA as input. This kit allows the depletion of cytoplasmic and mitochondrial ribosomal RNA (rRNA) from total RNA samples using biotinylated probes that selectively bind rRNA species. This process minimises ribosomal contamination and maximises the percentage of uniquely mapped reads covering both mRNA and a broad range of ncRNA species of interest, including long intergenic noncoding RNA (lincRNA), small nuclear (snRNA), small nucleolar (snoRNA) and other RNA species. After the rRNA is depleted, the remaining RNA is purified, fragmented and primed for cDNA synthesis. The RNA was fragmented for 3 minutes at 94°C and every purification step was performed by using 1X Agencourt AMPure XP beads (Beckman Coulter, Brea, CA).

Both RNA samples and final libraries were quantified by using the Qubit 2.0 Fluorometer (Invitrogen, Carlsbad, CA) and quality tested by Agilent 2100 Bioanalyzer (Agilent Technologies, Santa Clara, CA) and Caliper LabChip GX (PerkinElmer, Waltham, MA) assays, RNA integrity number (RIN) >8.

Libraries were then processed with Illumina cBot for cluster generation on the flow cell, following the manufacturer’s instructions and sequenced on paired-end 125 bp mode at the multiplexing level requested on HiSeq2500 (Illumina, San Diego, CA). The CASAVA 1.8.2 version of the Illumina pipeline was used to process raw data for both format conversion and de-multiplexing.

Raw sequencing data were processed with BBDuk (https://jgi.doe.gov/data-and-tools/bbtools/) in order to perform trimming and clipping. Bases with a quality score less than 25 were removed as well as reads shorter than 35 nucleotides. The quality of the reads, before and after trimming, was checked with the software FASTQC (https://www.bioinformatics.babraham.ac.uk/projects/fastqc/). High-quality reads were mapped against the GRCh38 human reference genome and downloaded from Ensembl [54], with the software STAR (version 2.5.2b, https://github.com/alexdobin/STAR). Read summarisation was performed with featureCounts (http://subread.sourceforge.net/). Only reads with a mapping quality higher than 30 were used for this scope. The option “-s 2” was used to indicate that the libraries are strand-specific. The resulting table was imported in the R environment, and the package edgeR (https://bioconductor.org/packages/release/bioc/html/edgeR.html) was used to calculate the fragments per kilobase million (FPKM) values.

Raw and processed files have been deposited in NCBI’s Gene Expression Omnibus [55] and are accessible through GEO Series accession number GSE151282 (https://www.ncbi.nlm.nih.gov/geo/query/acc.cgi?acc=GSE151282).

The Samtools software (http://samtools.sourceforge.net/) was used to identify and extract the reads mapping on the HR-DSCR [4] which has the following coordinates on Hsa21: 37,929,229-37,963,130 (human assembly GRGh38/hg38).

Each possible pair of T21 samples and each possible pair of normal control samples were compared through the nonparametric correlation test (Spearman correlation by rank), transforming FPKM values into a logarithmic scale (log10(FPKM)) and using the JMP 14.2 Pro software (SAS Institute, Campus Drive, Cary, NC, USA).

### Gene expression analysis by TRAM software

The TRAM software [31] is able to import and integrate any gene expression data source in a tabulated text format and map expression values to the relevant genomic region. The software also performs statistical analysis of over- or under-expressed regions compared to the whole genome or to the relative chromosome.

We used the last empty version available of TRAM (TRAM 1.3, http:/apollo11.isto.unibo.it/software/) that was manually configured following the software guide with human chromosome and human gene data downloaded from the National Center for Biotechnology Information (NCBI) Genome and Genes [56], respectively, updated up to January 24, 2019. The Ensembl gene accession number identifier (ENSG) conversion table to official gene symbol was downloaded with BioMart from Ensembl on January 23, 2019, corresponding to the GRCh38 (release 95) and imported in TRAM as a custom identifier data table following the software guide. Since the RNA-Seq technique is able to distinguish and quantify both the genes and their related pseudogenes, manual exclusion of the step for the removal of pseudogenes (necessary if microarray data are treated) from the TRAM set-up pipeline was necessary in order to correctly conduct the analysis. This script modification procedure was carried out using FileMaker Pro 12 software (FileMaker, Santa Clara, CA) and is available upon request.

A single tabulated text file with ENSG identifier and the corresponding FPKM value (excluding those equal to zero) was created for each T21 and normal control sample. These datasets related to T21 (pool A) and normal control (pool B) conditions were imported into TRAM which allowed a differential transcriptome map to be obtained, where the gene expression ratios (A/B) for each locus were shown in addition to the gene expression values of the single pools.

First, TRAM performs intra- and inter-sample normalisations (global normalisation and scaled quantile normalisations) of gene expression values. The value for each locus, in each biological condition, is represented by the mean value of all the values available for that locus. The mean value of the gene expression of the whole genome is used to determine the percentile of expression for each gene [31].

Then, a graphical representation of the gene expression profile is created in two different modes, “Map” or “Cluster”, identifying critical genomic regions (genomic regions including one gene) with significant differential expression comparing two different biological conditions. We mainly focused on the “Map” mode, analysing over-/under-expressed segments of the genome, with a window size of 500,000 bp and a shift of 250,000 bp (default parameters). The expression value for each genomic segment is calculated by the mean of the expression values of the loci included in that segment, considering only loci for which the mean value was derived from at least two biological samples. Over-/under-expression definition and statistical significance have already been explained [57]. A segment was considered to be statistically significant over-/under-expressed for *q* < 0.05, where *q* is the *p* value obtained by the method of hypergeometric distribution [11]. The significance of the over-/under-expression for single genes was determined by running TRAM in “Map” mode with a segment window of 12,500 bp. This window size corresponds to about a quarter of the mean length of a gene, so the significant over-/under-expression of a segment almost always corresponds with that of a gene. When the segment window contains more than one gene, the significance is maintained if the expression value of the over-/under-expressed gene prevails over the others.

Analysis of variance and post hoc test of chromosome mean expression ratios were performed with the JMP 14.2 Pro software (SAS Institute, Campus Drive, Cary, NC, USA) and the add-inn available (https://community.jmp.com/t5/JMP-Add-Ins/Games-Howell-Test-Tukey-HSD-with-Welch-s-correction-for-Unequal/ta-p/213771?trMode=source).

A functional enrichment analysis of over-/under-expressed genes in T21 vs normal control differential transcriptome map was performed using ToppFun from the ToppGene Suite Gene Ontology tool [58].

### Real-time reverse transcription polymerase chain reaction validation of RNA-Seq data

A real-time reverse transcription polymerase chain reaction (RT-PCR) analysis was performed on the same samples used for RNA-Seq analyses (4 subjects with DS and 4 normal control samples, see “Case selection” section). Nineteen genes (Table 2A and Supplementary Table 6) were selected according to their low inter-sample variability (standard deviation or SD as % of expression <100, see Supplementary Table 3) deduced from the RNA-Seq T21 vs normal control blood cell transcriptome map. Four genes were randomly selected among those with a gene expression ratio >1.70 (*ETS2*, *MX1*, *NUAK1*, *SERPINF1*), 4 genes were randomly selected among those with a gene expression ratio between 1.70 and 1.30 (*B2M*, *DYRK1A*, *NDUFA4*, *SOD1*), 9 genes were randomly selected among those with a gene expression ratio between 1.29 and 0.70 (*DHFR*, *GAPDH*, *HCST*, *NACC2*, *NRGN*, *PRDX2*, *RYR2*, *TUBA1B*, *YKT6*), and 2 genes were randomly selected among those with a gene expression ratio <0.70 (*LRP5*, *NPTX1*).

For the validation of the RNA-Seq results, two pools of RNA samples were created, starting from the same RNA samples used for RNA-Seq analysis in this work: one with three T21 blood cell RNA samples (350 ng of each) and the second with four normal control blood cell RNA samples (350 ng of each). Among the T21 samples used for RNA-Seq, one was no more available for the real-time PCR analysis (A3 sample, see Supplementary Table 7A).

Real-time RT-PCR experiments on a different and larger cohort of samples (12 RNA samples, 6 from children with DS and 6 from normal controls, see Supplementary Table 7B) were performed on genes reported by RNA-Seq experiments as statistically significant over-expressed in T21 vs normal control subjects. Four genes mapping on Hsa 21 (*TSPEAR*, *MX1*, *SLC19A1* and *GART*) and one gene mapping on chromosome 10 (*IFIT1*) were selected (Table 2B and Supplementary Table 6) and studied in each individual sample.

The Amplify 3 software [59] was used to design primer pairs so that their length is between 18 and 22 nucleotides; they have a GC content between 40 and 60%, and they have the same melting temperature [60, 61]. Finally, the Primer-BLAST software analysis did not find nonspecific results [61]. Each primer was designed on a different exon, and each primer pair binds to regions common to all splicing isoforms of the same gene [62].

Complementary DNA (cDNA) was obtained by reverse transcription (RT) performed according to [11] from each RNA sample pool.

Real-time RT-PCR assays were performed in triplicate, using the CFX96 instrument (Bio-Rad Laboratories, Hercules, CA, USA).

The reactions were performed in a total volume of 20 μL using Sybr Select Master Mix 2× for CFX (Applied Biosystems, by Life Technologies) according to the manufacturer’s instructions providing the following cycling parameters: 2 min at 50°C (uracil-DNA glycosylase (UDG) activation), 2 min at 95°C (AmpliTaq Fast DNA Polymerase UP activation) and 40 cycles of 15 s at 95°C (denature) and of 1 min at 61°C (anneal and extend). In order to assess the amplification specificity, a melting step consisting of an increase in temperature of 0.5°C/s from 65 to 95°C was performed.

For each gene, we used the primer pair that gave between 90 and 110% efficiency. For the gene expression analysis, we used the Livak method of 2^−∆∆Ct^ (delta delta cycle threshold) [63] by choosing *GAPDH* as a reference gene.

For the validation of five statistically significant over-expressed genes obtained by RNA-Seq analyses, the reverse transcriptions of 1 μg of 6 RNA samples from subjects with DS and 6 RNA samples from normal control subjects were performed, and the cDNA samples were analysed by real-time RT-PCR as cited above. The relative gene expression value of each gene vs a gene chosen as reference (*GAPDH*) was obtained through the 2^−∆Ct^ method in both DS and N groups. The mean among the relative gene expression values was calculated after removing strong outliers (see Supplementary Table 8 for details). Strong outliers, reported in red in Supplementary Table 8, were detected with the SPSS Statistics software as follows: from the leading software Menu, we selected “Analyze” and then “Descriptive statistics”; we then chose “Explore” and included gene expression values in “Dependent List”; finally, in “Statistics section”, we selected the “Outliers” and “Percentiles” options. The SPSS Statistics software indicates strong outliers with an asterisk in the graph. The DS/N ratios obtained after strong outlier removal are listed in Table 2B.

The results obtained by real-time RT-PCR were compared with the gene expression ratios from RNA-Seq analysis through bivariate statistical analyses using the JMP 14.2 Pro software (SAS Institute, Campus Drive, Cary, NC, USA).

### Comparison between RNA-Seq and publicly available gene expression data

We searched for all gene expression studies performed on human blood samples from T21 vs normal controls in the literature in order to compare transcriptome maps obtained here by TRAM elaboration of RNA-Seq analyses with previously published blood transcriptome maps.

In particular, T21 and normal control single transcriptome maps and T21 vs normal control differential transcriptome map were compared with those obtained in the meta-analysis performed by Pelleri and colleagues on WBC samples [11]. These maps were also compared with those obtained in the RNA-Seq experiment performed by Powers and colleagues on WBC samples [32], after reads per kilobase per million (RPKM) value elaboration by TRAM in order to integrate and normalise more samples as a unique pool and to make data fully comparable. Gene expression values were compared performing the nonparametric correlation test (Spearman correlation by rank) using the JMP 14.2 Pro software (SAS Institute, Campus Drive, Cary, NC, USA). It was not possible to use RNA-Seq experiment on peripheral blood cells performed by Costa and colleagues [37] due to the absence of processed RNA-Seq data, such as a list of gene expression values.

## Supplementary Information

**Additional file 1: Supplementary Figure 1.** Scatterplot matrix of each possible pair of trisomy 21 samples (A) and each possible pair of normal control samples (B) obtained with JMP 14.2 Pro software (SAS Institute, Campus Drive, Cary, NC, USA). Fragments per kilobase million (FPKM) values are transformed in logarithmic scale (log10(FPKM)).

**Additional file 2: Supplementary Table 1.** Mapping statitics regarding reads aligned against the Homo sapiens (GRCh38) reference genome with STAR aligner. A: trisomy 21 samples; B: normal control samples.

**Additional file 3: Supplementary Table 2.** Fragments per kilobase million values (FPKM). ID: Ensembl gene accession number identifier. A: trisomy 21 samples, B: normal control samples. FPKM values for each sample are accessible through NCBI's Gene Expression Omnibus (GEO) Series accession number GSE151282 (https://www.ncbi.nlm.nih.gov/geo/query/acc.cgi?acc=GSE151282).

**Additional file 4: Supplementary Table 3.** Differential expression of pool A (trisomy 21 blood) versus pool B (normal control blood cells). Loci were sorted in descending order of expression ratio (Ratio A/B). N/A: not available. SD: standard deviation.

**Additional file 5: Supplementary Table 4.** A) Segment map of the T21 blood cell transcriptome map. B) Segment map of the normal control blood cell transcriptome map. "Genes" column list only over-/under-expressed genes, in red over-expressed genes, in blue under-expressed genes.

**Additional file 6: Supplementary Table 5.** Expression values from RNA sequencing performed here for genes implicated in one-carbon metabolic process (Gene Ontology, GO:0006730) and folic acid-containing compound metabolic process (GO:0006760). Trisomy 21 (T21) vs normal control (N) differential expression ratios between 0.58 and 0.76 are highlighted in blue (under-expression), ratios between 1.30 and 1.70 in red (over-expression). Extreme expression ratios (< 0.58 or > 0.76) are highlighted in green and orange respectively. Sample count represents the number of samples with an available expression value for that gene.

**Additional file 7: Supplementary Table 6.** Primer pairs used for TRAM map experimental validation by Real-Time RT-PCR.

**Additional file 8: Supplementary Table 7.** A) Samples selected for RNA sequencing. B) Samples selected for Real-Time RT-PCR analyses on a larger cohort of samples to validate some Hsa21 genes over-expressed with the RNA-Seq. DS: Down syndrome; n: normal control.

**Additional file 9: Supplementary Table 8.** Real-Time RT-PCR experiments performed on 6 subjects with Down syndrome (A5; A6; A7; A8; A9; A10) and 6 normal controls (B5; B6; B7; B8; B9; B10). The tables on the left report the mean of cycles (Cq mean) for the five genes selected and for the reference gene (GAPDH), the difference of Cq means among the gene and GAPDH gene (∆Cq) and finally the relative expression values of the gene vs GAPDH gene obtained through the 2-∆Ct method (2-∆Ct) for each subject. The presence of strong outliers in the expression values of each gene is reported in red in tables on the left. The following is reported in the tables on the right: mean gene expression values (Mean); standard deviation (S.D.); and ratio between DS and N mean gene expression values (Ratio DS/N) after the exclusion of strong outliers.

**Additional file 10: Supplementary Table 9.** Results of functional enrichment analysis, performed by ToppFun from the ToppGene Suite Gene Ontology tool, of over-expressed genes (with expression ratios ≥1.30) in the trisomy 21 (T21) vs normal control (N) differential transcriptome map. The GO codes are ordered for the number of the column "hit count in query list".

**Additional file 11: Supplementary Table 10.** Results of functional enrichment analysis, performed by ToppFun from the ToppGene Suite Gene Ontology tool, of under-expressed genes (with expression ratios ≤0.76) in the trisomy 21 (T21) vs normal control (N) differential transcriptome map. The GO categories are ordered by the number in the column "hit count in query list".

**Additional file 12: Supplementary Table 11.** Details of transcriptome maps used in the comparisons.

**Additional file 13: Supplementary Table 12.** Differential expression of pool A (trisomy 21 white blood cells) versus pool B (normal control white blood cells) from data available in Powers et al. (2019). Loci were sorted in descending order of expression ratio (Ratio A/B). N/A: not available. SD: standard deviation.

**Additional file 14: Supplementary Table 13.** DS clinical data collected at the moment of enrollment in the study. RNA samples for RNA-seq experiments were collected from subjects A1; A2; A3; A4. RNA samples for Real-time RT-PCR experiments were collected from subjects A5; A6; A7; A8; A9; A10.

## Data Availability

We have submitted raw and processed RNA-Seq data to the NCBI’s Gene Expression Omnibus platform. GEO accession GSE151282. Raw data are available in a fastq format on the NCBI’s Sequence Read Archive (SRA) database under the SRA accession code: SRP264957. Detailed results for each map are also available in the TRAM software deposited at https://osf.io/ab3np/?view_only=c8cfbaf81a894f379854722a13efb9ec.
