# Supplementary material for: The transcriptome profile of human trisomy 21 blood cells
Source: Hum Genomics. 2021 May 1;15:25. doi: 10.1186/s40246-021-00325-4 (PMC8088681; doi:10.1186/s40246-021-00325-4)

"The transcriptome profile of human trisomy 21 blood cells"

Francesca Antonaros, Rossella Zenatelli, Giulia Guerri, Matteo Bertelli, Chiara Locatelli, Beatrice Vione, Francesca Catapano, Alice Gori, Lorenza Vitale, Maria Chiara Pelleri, Giuseppe Ramacieri, Guido Cocchi, Pierluigi Strippoli, Maria Caracausi, Allison Piovesan

Supplementary Figure 1. Scatterplot matrix of each possible pair of trisomy 21 samples (**A**) and each possible pair of normal control samples (**B**) obtained with JMP 14.2 Pro software (SAS Institute, Campus Drive, Cary, NC, USA). Fragments per kilobase million (FPKM) values are transfermed in logarithmic scale (log10(FPKM)).

A

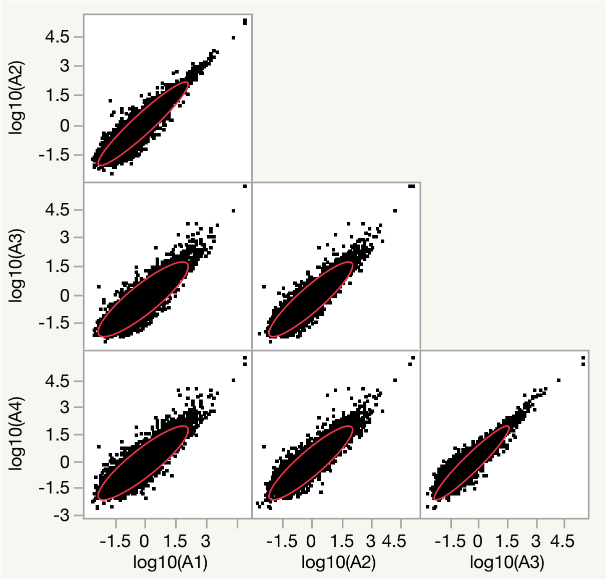

B

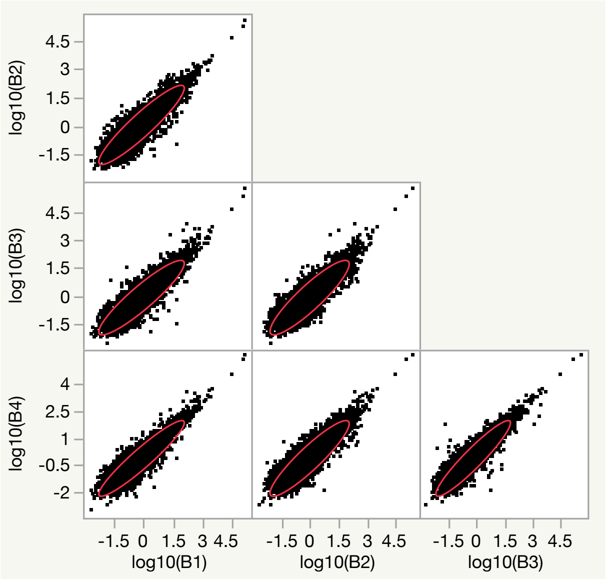

Supplement: Supplementary file 1 — Additional file 1: Supplementary Figure 1. Scatterplot matrix of each possible pair of trisomy 21 samples (A) and each possible pair of normal control samples (B) obtained with JMP 14.2 Pro software (SAS Institute, Campus Drive, Cary, NC, USA). Fragments per kilobase million (FPKM) values are transformed in logarithmic scale (log10(FPKM)). [file 40246_2021_325_MOESM1_ESM.pdf]
