# Supplementary material for: The transcriptome profile of human trisomy 21 blood cells
Source: Hum Genomics. 2021 May 1;15:25. doi: 10.1186/s40246-021-00325-4 (PMC8088681; doi:10.1186/s40246-021-00325-4)
Supplement: Supplementary file 2 — Additional file 2: Supplementary Table 1. Mapping statitics regarding reads aligned against the Homo sapiens (GRCh38) reference genome with STAR aligner. A: trisomy 21 samples; B: normal control samples. [file 40246_2021_325_MOESM2_ESM.pdf]

"The transcriptome profile of human trisomy 21 blood cells"

Francesca Antonaros, Rossella Zenatelli, Giulia Guerri, Matteo Bertelli, Chiara Locatelli, Beatrice Vione, Francesca Catapano, Alice Gori, Lorenza Vitale, Maria Chiara Pelleri, Giuseppe Ramacieri, Guido Cocchi, Pierluigi Strippoli, Maria Caracausi, Allison Piovesan

Supplementary Table 1. Mapping statitics regarding reads aligned against the *Homo sapiens* (GRCh38) reference genome with STAR aligner. A: trisomy 21 samples; B: normal control samples.

**Sample ID**	**Input-reads**	**Unique Mapping**	**% Unique Mapping**	**Multiple Mapping**	**% Multiple Mapping**	**Unmapped**	**% Unmapped**	**Assigned to Genes**	**% Assigned to Genes**	**% Mapped**
A1	24,308,334	18,189,876	74.83	5,746,161	23.64	372,297	1.53	13,642,501	56.12	98.47
A2	15,917,037	11,257,231	70.72	4,246,346	26.68	413,460	2.60	8,196,151	51.49	97.40
A3	16,738,286	11,516,910	68.81	4,871,531	29.10	349,845	2.09	9,283,849	55.46	97.91
A4	19,828,777	12,333,445	62.20	6,777,093	34.18	718,239	3.62	9,192,383	46.36	96.38
B1	20,564,327	14,288,452	69.48	5,693,847	27.69	582,028	2.83	11,221,245	54.57	97.17
B2	16,505,874	10,486,061	63.53	5,623,804	34.07	396,009	2.40	7,733,247	46.85	97.60
B3	16,401,785	11,221,115	68.41	4,673,020	28.49	507,650	3.10	8,184,624	49.90	96.90
B4	20,543,351	14,586,575	71.00	5,459,623	26.58	497,153	2.42	11,111,474	54.09	97.58
**Mean**	18,850,971	12,984,958	68.62	5,386,428	28.80	479,585	2.57	9,820,684	51.86	97.43
