# Supplementary material for: The transcriptome profile of human trisomy 21 blood cells
Source: Hum Genomics. 2021 May 1;15:25. doi: 10.1186/s40246-021-00325-4 (PMC8088681; doi:10.1186/s40246-021-00325-4)
Supplement: Supplementary file 4 — Additional file 4: Supplementary Table 3. Differential expression of pool A (trisomy 21 blood) versus pool B (normal control blood cells). Loci were sorted in descending order of expression ratio (Ratio A/B). N/A: not available. SD: standard deviation. [file 40246_2021_325_MOESM4_ESM.pdf]

"The transcriptome profile of human trisomy 21 blood cells"

Francesca Antonaros, Rossella Zenatelli, Giulia Guerri, Matteo Bertelli, Chiara Locatelli, Beatrice Vione, Francesca Catapano, Alice Gori, Lorenza Vitale, Maria Chiara Pelleri, Giuseppe Ramacieri, Guido Cocchi, Pierluigi Strippoli, Maria Caracausi, Allison Piovesan

Supplementary Table 3. Differential expression of pool A (trisomy 21 blood) versus pool B (normal control blood cells). Loci were sorted in descending order of expression ratio (Ratio A/B). N/A: not available. SD: standard deviation.

Chromosome	txStart	txEnd	Location	Gene name	Expression A	Expression B	Expression AB	Sample Count A	Sample Count B	SD as % of Expression A	SD as %of Expression B
chr11	5244554	5245546	11p15.4	BGLT3	8.180963964	0.320370914	25.53591359	1	1	0	0
chr5	175796539	175884021	5q35.2	CPLX2	0.831101213	0.037046774	22.43383465	1	1	0	0
chr19	55757298	55763421	19q13.42	RFPL4A	6.094814657	0.307294818	19.83376974	3	1	82.25732611	0
chr21	44497892	44711580	21q22.3	TSPEAR	0.987145069	0.058806629	16.78628897	2	3	119.5647294	5.432024533
chr3	180161887	180161977	3q26.33	RNA5SP149	99.5572079	6.300494938	15.80149002	2	1	80.36256937	0
chr22	22098761	22099212	22q11.22	IGLV8-61	13.5594962	1.137986562	11.91533947	3	3	63.39467154	40.25862864
chr8	123179047	123210079	8q24.13	FAM83A	0.800039901	0.069156346	11.5685682	4	3	111.15004	65.87588372
chr11	3559512	3560024	11p15.4	RPS24P14	8.18634259	0.730766818	11.20240053	3	2	120.0148643	6.363834883
chr10	114044047	114046908	10q25.3	ADRB1	0.940630405	0.091640265	10.26437894	2	2	40.53904955	1.914483363
chr2	73644919	73685572	2p13.1	ALMS1P1	1.561118424	0.153470485	10.17210849	1	3	0	6.154201977
chr10	127135426	127196573	10q26.2	INSYN2	0.569939277	0.059974758	9.502985906	3	3	31.08981793	6.015355315
chr11	68044794	68044957	11q13.2	MIR6753	14.16969868	1.516120893	9.346021639	2	3	13.66600427	13.06139472
chr14	28767072	28770277	14q12	FOXG1	0.473364177	0.051973457	9.107806171	1	1	0	0
chr22	21042392	21044249	22q11.21	P2RX6P	4.271741319	0.469294298	9.102478636	1	1	0	0
chr4	185585444	185956716	4q35.1	SORBS2	0.304315608	0.03451251	8.81754494	4	4	96.95633475	64.32810285
chr21	42102134	42108534	21q22.3	UMODL1-AS1	0.959015127	0.11304672	8.483352062	1	1	0	0
chr12	9036324	9037523	12p13.31	VDAC2P2	2.548146144	0.304798391	8.360103658	3	4	72.19757572	12.91914164
chr11	19345200	20121601	11p15.1	NAV2	0.146028377	0.017794882	8.206200854	2	2	64.30472877	0.619239894
chr8	2935353	4995035	8p23.2	CSMD1	0.268270509	0.033169742	8.087808164	1	3	0	92.65540893
chr5	72107268	72209570	5q13.2	MAP1B	1.420526065	0.180304308	7.878492083	3	4	150.7074447	131.1036884
chr1	185734525	186190953	1q25.3-q31.1	HMCN1	0.256143535	0.032664267	7.841704751	4	3	80.81800321	62.62475535
chr1	158285406	158331531	1q23.1	CD1B	2.068500977	0.266652732	7.757284034	1	2	0	52.87365403
chr20	33055331	33073637	20q11.21	BPIFB3	1.422908012	0.183885765	7.737999798	1	1	0	0
chr17	28954905	29006440	17q11.2	SEZ6	0.589636596	0.080903266	7.288167994	2	3	29.05468047	29.75316485
chr17	16137768	16137872	17p11.2	RNU6-862P	14.70475514	2.019952031	7.279754622	1	1	0	0
chr1	92646848	92647730	1p22.1	HMGB3P9	3.037914944	0.428829818	7.084197072	1	3	0	7.220160368
chr2	127535689	127637728	2q14.3	MYO7B	2.56727095	0.362505162	7.082025907	4	4	83.26761105	51.12124816
chr12	8221260	8227618	12p13.31	FAM90A1	0.668979382	0.096671927	6.920099772	1	1	0	0
chr8	7298744	7355363	8p23.1	FAM66B	0.473101833	0.068371732	6.919553149	1	1	0	0
chr10	68499488	68500009	10q21.3	RPL26P27	3.298704935	0.48138445	6.852537376	1	1	0	0
chr8	31639222	32771716	8p12	NRG1	1.968604859	0.288755451	6.817550452	4	4	108.5751584	81.58748397
chr7	157539052	158587802	7q36.3	PTPRN2	5.233267456	0.772427137	6.775095288	4	4	83.98880043	44.25778363
chr21	41420329	41459214	21q22.3	MX1	118.1632247	17.49206052	6.75524902	4	4	83.54084275	65.53248107
chr1	52772153	52772674	1p32.3	RPS13P2	7.040548374	1.048511297	6.7148045	2	3	65.45346402	67.29167955

chr3	151188099	151188223	3q25.1	RNA5SP145	29.67400369	4.447016652	6.672788976	3	3	91.33621779	49.71214561
chr19	11538717	11550323	19p13.2	CNN1	1.551430266	0.233949711	6.631469037	3	3	100.9119	29.36846191
chr16	56865207	56915850	16q13	SLC12A3	0.344546389	0.052270272	6.591631832	4	4	78.3010681	35.9266383
chr20	3686970	3712558	20p13	SIGLEC1	3.087847298	0.472959306	6.528780087	4	4	76.83989088	85.80581936
chr22	22214807	22215271	22q11.22	IGLV10-54	5.762266337	0.898596582	6.412517531	3	2	99.33868406	71.00982828
chr8	132120858	132480757	8q24.22	KCNQ3	0.126868058	0.019898777	6.375671057	1	1	0	0
chr1	78620382	78646145	1p31.1	IFI44L	45.21944714	7.127399549	6.344452395	4	4	100.5487975	60.95211315
chr2	134127125	134127192	2q21.2	MIR3679	23.12993755	3.695176676	6.259494358	2	1	44.86549226	0
chr6	149795207	149795749	6q25.1	BTBD10P2	2.468869658	0.396823385	6.221583088	2	1	27.01149952	0
chr6	32552713	32560002	6p21.32	HLA-DRB6	1.655457182	0.266224125	6.218283863	1	3	0	41.02633825
chr20	50585786	50585879	20q13.13	MIR645	24.39931404	3.948363106	6.179602379	3	4	25.1537673	36.05474417
chr2	26457203	26558698	2p23.3	OTOF	0.468157904	0.075810418	6.175376915	4	3	60.67645914	98.19042674
chr2	89268001	89268475	2p11.2	IGKV1-33	3.642181679	0.592211146	6.150140369	3	2	52.58572127	11.17512189
chr3	53846550	53867388	3p21.1	IL17RB	0.501884853	0.081623787	6.148757283	1	1	0	0
chrX	123184243	123490915	Xq25	GRIA3	0.655953386	0.107509982	6.101325394	1	2	0	93.90104712
chr13	28820348	29505947	13q12.3	MTUS2	0.188842154	0.031267492	6.03956835	1	1	0	0
chr2	231108230	231126172	2q37.1	HTR2B	0.693672661	0.115069608	6.028287362	1	2	0	1.665250866
chr8	136530798	136897601	8q24.23	LINC02055	0.502183132	0.083340314	6.025692789	1	1	0	0
chr10	133160432	133226412	10q26.3	KNDC1	0.529638799	0.088344438	5.995157232	4	4	110.0584082	60.17706411
chr20	23125068	23132636	20p11.21	LINC00656	0.877939621	0.1476693	5.945309036	4	3	52.17401468	10.12223685
chr21	41739369	41767089	21q22.3	RIPK4	0.551487477	0.092986864	5.930810559	3	3	125.3967363	55.55306463
chr21	34446688	34512275	21q22.12	KCNE1	1.236291879	0.210445708	5.874635754	4	4	54.68554635	36.15771158
chr18	9885726	9888159	18p11.22	TXNDC2	0.544092499	0.09335244	5.828369342	1	3	0	32.28340887
chr10	75094432	75109221	10q22.2	DUSP13	0.646326026	0.111813179	5.780410068	2	1	68.21276267	0
chr1	26993663	27000891	1p36.11	TRNP1	1.796756616	0.311010568	5.777156153	2	3	86.87102636	15.35278491
chr10	89392546	89406487	10q23.31	IFIT1	145.2198912	25.20684604	5.761128979	4	4	98.83522645	78.28975313
chr6	41228287	41239386	6p21.1	TREML4	3.263246674	0.571194025	5.713026623	2	1	94.7910785	0
chr12	53046980	53064379	12q13.13	TNS2	0.180768889	0.03200781	5.647649479	2	2	29.81269821	3.270974959
chr5	138439001	138446969	5q31.2	REEP2	0.467421199	0.082873134	5.640201778	1	2	0	6.165781765
chr3	167441789	167474132	3q26.1	SERPINI2	0.512218216	0.09115156	5.619412526	3	3	95.5523098	4.260598349
chr7	75343937	75358997	7q11.23	PMS2P2	1.32956879	0.236828965	5.614046368	1	1	0	0
chr8	28530411	28530519	8p21.1	RNA5SP259	15.96781397	2.892947869	5.519565058	2	2	93.54515732	33.14148159
chr6	37507348	37536280	6p21.2	LINC02520	3.596897663	0.65589633	5.483942354	4	2	109.3278358	37.61967226
chr5	132822141	132831627	5q31.1	SHROOM1	1.093315957	0.199439527	5.481942181	4	3	87.5385496	95.8768648
chr6	1610446	1613897	6p25.3	FOXC1	0.374252488	0.068959186	5.427159334	3	1	55.1476088	0
chr2	114442358	115845752	2q14.1	DPP10	1.068292733	0.19691973	5.425016236	4	3	144.5676607	93.15446512
chr19	57958437	57974534	19q13.43	C19orf18	3.61977011	0.66974277	5.40471696	3	4	110.8981308	18.0337456
chr2	6865733	6898239	2p25.2	RSAD2	116.6597693	21.65992042	5.38597405	4	4	98.71237054	56.76745776
chr22	30542215	30544935	22q12.2	SIRPAP1	0.723499551	0.134431836	5.38190634	1	1	0	0
chr5	31193655	31329146	5p13.3	CDH6	0.172852657	0.032309648	5.34987741	2	2	119.4325843	41.90037705
chr3	112140898	112294258	3q13.2	SLC9C1	3.785237887	0.71342276	5.305743099	4	4	119.2459616	68.42076343
chr8	41261957	41309471	8p11.21	SFRP1	1.050479884	0.198033716	5.304550696	4	1	169.6351242	0
chr17	16683401	16684229	17p11.2	RNASEH1P2	1.92997157	0.366108136	5.27158886	1	1	0	0

chr17	45111640	45132533 17q21.31	PLCD3	0.342128267	0.065133351	5.252735539	2	3	121.0575704	68.99702941
chr19	48047843	48111383 19q13.33	PLA2G4C	0.755804554	0.143948155	5.250533117	4	4	81.91182193	81.90270868
chr11	77734315	77735232 11q14.1	FTH1P16	6.951331683	1.326388791	5.240794954	2	3	112.091397	39.83095509
chr21	32377187	32377322 21q22.11	SNORA80A	18.44635127	3.519894757	5.240597388	3	2	102.6702824	28.93685446
chr20	63528413	63537370 20q13.33	PTK6	0.485897527	0.09301444	5.2238935	3	2	73.78377777	7.630675551
chr3	107841662	107878068 3q13.12	LINC00635	0.291384185	0.0558074	5.221246377	2	1	15.37871847	0
chr11	75422394	75430629 11q13.4	KLHL35	0.530411649	0.10169965	5.215471738	2	1	7.159738959	0
chr7	45912245	45921272 7p12.3	IGFBP3	0.98588272	0.189880126	5.192132227	4	2	76.94535255	10.34111918
chr2	90114887	90115400 2p11.2	IGKV3D-15	2.754766491	0.531232602	5.185612633	3	1	35.77528982	0
chr17	58192724	58205174 17q22	EPX	0.538592412	0.103921402	5.18269001	1	1	0	0
chr10	69801835	69959148 10q22.1	COL13A1	0.597203428	0.115364682	5.176657343	1	2	0	85.69995881
chrMT	9991	10058 N/A	MT-TG	17.29925251	3.354909889	5.156398557	2	2	97.64153185	14.40929045
chr19	54769208	54784326 19q13.42	KIR2DL1	1.188205654	0.231176096	5.139829219	4	2	94.71510481	26.36330273
chr20	38427306	38427443 20q11.23	SNORA71A	80.29357976	15.64736763	5.131443297	3	4	88.61692283	109.9654475
chr5	140332843	140346603 5q31.3	HBEGF	12.10486274	2.369385921	5.108860751	4	4	100.0662469	80.7879347
chr7	151377974	151410705 7q36.1	WDR86	0.334334565	0.06546699	5.106918252	2	2	106.355541	4.26211455
chr9	63372803	63380012 9q13	BMS1P10	2.037553078	0.402005757	5.068467413	1	3	0	76.90199702
chr12	121839527	121888611 12q24.31	HPD	0.599586612	0.118450899	5.061900051	2	1	80.40857119	0
chr5	77180252	77428256 5q13.3	PDE8B	0.308534043	0.060989964	5.058767383	4	3	107.7462949	62.200586
chr1	150982023	150996063 1q21.3	ANXA9	0.698636901	0.139024103	5.025293355	1	1	0	0
chr11	90131693	90193577 11q14.3	NAALAD2	0.175297769	0.03489563	5.023487761	3	3	92.31635779	7.692351969
chr6	33314405	33317942 6p21.32	ZBTB22	0.470645431	0.093914648	5.011416666	1	1	0	0
chr18	22169437	22202528 18q11.2	GATA6	0.784648439	0.157518477	4.981310484	1	1	0	0
chr20	49982976	49988886 20q13.13	SNAI1	0.858255875	0.172387782	4.97863517	4	2	76.33610508	11.59118616
chr19	10109749	10109840 19p13.2	SNORD105B	35.62117427	7.176119872	4.963848836	2	3	11.71307696	97.38899075
chr5	150113839	150155859 5q32	PDGFRB	2.038448375	0.410692782	4.963438519	4	4	31.66052798	110.9857351
chr1	60515716	60622685 1p32.1	LINC01748	0.120945569	0.024508434	4.934854978	2	3	110.0887873	3.654583484
chr4	166733384	167234869 4q32.3	SPOCK3	0.293461274	0.059536517	4.929097108	1	2	0	6.606324697
chr19	55132698	55149354 19q13.42	TNNT1	4.08205499	0.828536243	4.926827312	3	4	109.960471	82.65531217
chr21	41141500	41148064 21q22.2	LINC00323	0.532575212	0.108109862	4.926240803	1	1	0	0
chr10	33177491	33334905 10p11.22	NRP1	1.3723363	0.28000843	4.901053511	4	4	123.5610023	62.19460656
chr17	61590433	61591202 17q23.2	NACA2	1.654501401	0.337992964	4.895076461	2	2	71.62559935	18.03617053
chr4	184893000	184899461 4q35.1	LINC01093	4.421888746	0.905877113	4.881333993	2	3	124.02689	58.6645623
chr6	29726601	29749049 6p22.1	HLA-F-AS1	0.411438958	0.084467232	4.870989001	1	3	0	42.64792048
chr19	51331536	51342124 19q13.41	VSIG10L	0.327612113	0.067518338	4.852194577	3	2	31.19698582	1.18916124
chr6	3224261	3227734 6p25.2	TUBB2B	2.723133418	0.561254356	4.85187044	3	2	95.85717703	113.5927254
chr5	170378161	170389677 5q35.1	KCNMB1	0.698732771	0.144052028	4.850558373	3	4	93.00034064	58.08256941
chr11	187881	194575 11p15.5	SCGB1C1	2.992984471	0.621574777	4.815163972	3	1	53.02701679	0
chr9	136361900	136363811 9q34.3	DNLZ	0.40017032	0.083447328	4.795483941	3	4	52.20044079	6.480331676
chrX	24744441	24744570 Xp22.11	SCARNA23	19.21053834	4.032975204	4.763366341	2	2	52.79567017	38.20009588
chr19	7677057	7679833 19p13.2	MCEMP1	21.70229847	4.557121221	4.762282462	4	4	108.4879245	81.01675024
chr8	142750150	142752534 8q24.3	LYPD2	3.901072515	0.819988586	4.757471726	3	2	58.60388125	49.17739498
chr6	4427963	4428081 6p25.1	RNA5SP202	33.57534025	7.070103904	4.748917513	2	2	50.46008362	93.93364337

chr17	46274184	46361871	17q21.31	ARL17B	0.797731615	0.168059425	4.746723461	4	4	54.61095749	61.08523979
chr14	21397293	21397401	14q11.2	SNORD8	35.80940146	7.566386392	4.732695319	3	4	102.4254483	25.06076573
chr12	12940518	12955908	12p13.1	GPRC5D	1.154363773	0.2444592	4.722112219	4	2	84.17581408	21.23611023
chr14	105765914	105771405	14q32.33	IGHG3	21.08937745	4.475791129	4.711877037	4	4	143.4781796	149.2165178
chr8	130008334	130008453	8q24.21	MIR5194	29.03113338	6.163913675	4.709853984	4	4	56.16477957	71.85716185
chr21	26917912	26967758	21q21.3	ADAMTS5	0.787146217	0.167275276	4.705693731	3	2	58.06767188	93.80022888
chr6	28259297	28260958	6p22.1	NKAPL	0.760607356	0.161749057	4.702391297	2	2	45.18494653	15.19577361
chr20	31605283	31606515	20q11.21	ID1	2.788138428	0.594159523	4.692575515	4	3	157.3751329	33.53823881
chr9	116153752	116402321	9q33.1	PAPPA	0.101829495	0.021732917	4.685496017	1	2	0	1.070923554
chr13	20221962	20232395	13q12.11	GJB6	1.942541772	0.415316548	4.677255896	4	4	71.49535922	75.83371545
chr8	23678693	23682937	8p21.2	NKX3-1	8.682045295	1.861560241	4.663854065	4	4	176.6760355	75.08235199
chr13	20728931	20773958	13q12.11	EEF1AKMT1	90.05040647	19.31178921	4.662975837	4	4	100.3318009	70.60164853
chr1	109113963	109206781	1p13.3	KIAA1324	14.73844329	3.167444355	4.653102515	4	4	100.4841196	49.76152135
chr19	33302821	33305057	19q13.11	CEBPA-DT	0.986063159	0.21208853	4.649299784	2	1	67.6124929	0
chr12	108459425	108473689	12q23.3	LINC01498	0.424005261	0.091236589	4.647316012	1	1	0	0
chr17	78248590	78256185	17q25.3	THA1P	0.945050344	0.203496534	4.644061136	1	1	0	0
chr6	32659464	32666689	6p21.32	HLA-DQB1	17.09994065	3.697391732	4.624865821	3	3	126.5249998	170.7735933
chr4	88456604	88506170	4q22.1	HERC5	57.27600745	12.40625137	4.616705382	4	4	95.21297379	56.35427745
chr1	212352818	212352950	1q32.3	SNORA16B	14.69140685	3.200711919	4.590043473	2	4	51.29304579	69.48378926
chr4	73740506	73743716	4q13.3	CXCL8	1523.159996	332.721928	4.577876804	4	4	91.01968152	65.63996839
chr17	799313	979766	17p13.3	NXN	0.270786502	0.059350964	4.562461723	2	3	14.30080169	42.18477025
chr12	112938433	112973251	12q24.13	OAS3	43.93911885	9.632717782	4.561445673	4	4	74.89911831	74.63421422
chr19	53963036	53990215	19q13.42	CACNG8	0.27850116	0.061074562	4.56001896	2	3	123.256489	90.16805658
chr22	22030994	22031472	22q11.22	IGLV4-69	6.314432275	1.386080123	4.555604088	3	3	60.98250912	44.57135626
chr8	40522766	40897828	8p11.21	ZMAT4	0.391613834	0.086499897	4.527332976	3	3	52.86632594	32.78424462
chr17	72598041	72640472	17q24.3	LINC00511	0.141101234	0.031262194	4.513478342	2	2	14.63127207	3.950262158
chr22	46050459	46054144	22q13.31	PRR34	0.860316729	0.190653101	4.512471741	2	3	91.14148465	54.09431334
chr3	119314293	119322760	3q13.33	ARHGAP31-AS1	1.66182082	0.368661164	4.507718692	1	1	0	0
chr17	36013056	36018179	17q12	CCL23	2.406786896	0.535102444	4.497805839	3	2	96.43446162	51.17862862
chr10	70389366	70390166	10q22.1	CEP57L1P1	3.821085547	0.852058115	4.484536301	2	4	19.11033732	94.07665009
chr3	8750408	8769614	3p25.3	OXTR	3.560974456	0.796105812	4.472991409	4	4	47.95441552	75.9734767
chr12	106063347	106140033	12q23.3	NUAK1	0.437734675	0.097885919	4.471886026	4	3	97.18206331	58.58273144
chr4	7754090	7778928	4p16.1	AFAP1-AS1	0.262207958	0.058640642	4.471437383	4	3	76.57643233	58.47090975
chr17	41486136	41489864	17q21.2	KRT36	0.696137628	0.156020945	4.461821648	1	1	0	0
chr12	95657807	95791206	12q22	NTN4	0.559168673	0.125521738	4.454755664	4	1	77.87495236	0
chr8	97869021	98036720	8q22.1-q22.2	MATN2	0.226979534	0.050979276	4.452388374	2	3	116.2289266	44.28766071
chr5	88538266	88691041	5q14.3	LINC00461	0.186439643	0.04197637	4.441538055	1	1	0	0
chr14	105600066	105601727	14q32.33	IGHE	0.729869677	0.164841113	4.427716278	2	1	24.31607678	0
chr15	93043406	93089214	15q26.1	RGMA	0.089701027	0.020280263	4.423070277	1	2	0	2.660231006
chr12	8602166	8612970	12p13.31	AICDA	0.378581621	0.085621081	4.421593567	1	3	0	2.88378787
chr12	97721716	97721821	12q23.1	RNU6-36P	31.52179992	7.132794645	4.419277645	3	3	62.96841446	43.79329688
chr21	41361944	41408943	21q22.3	MX2	65.22608033	14.84671231	4.39330129	4	4	69.527588	55.69137
chr1	46433827	46445702	1p33	FAAHP1	0.479410408	0.109441385	4.38052211	4	2	73.45172092	13.77835389

chr11	58834065	58905840	11q12.1	GLYATL2	2.145265351	0.490385142	4.374654055	4	4	75.85177073	35.76283855
chr11	27506852	27698171	11p14.1	BDNF-AS	0.571006553	0.130921262	4.361450131	2	4	63.06441043	64.16561149
chr12	113392445	113403887	12q24.13	SDS	0.373640812	0.085806224	4.354472152	2	1	102.6607731	0
chr2	219549408	219571573	2q35	OBSL1	0.118852456	0.027314758	4.351217675	2	3	27.45922662	3.660350589
chr17	68530276	68600954	17q24.2	FAM20A	0.353050078	0.081212937	4.347214741	2	3	11.03803768	51.02140083
chr11	117820075	117828092	11q23.3	FXYD2	0.198123411	0.045661654	4.338945149	1	1	0	0
chr4	73026748	73026872	4q13.3	RNU6ATAC5P	13.17945384	3.044106468	4.329498321	1	1	0	0
chr9	86944362	86947189	9q21.33	GAS1	0.68519723	0.15883377	4.313926622	3	4	40.24483235	24.21525419
chr11	64606174	64723188	11q13.1	NRXN2	0.158199162	0.036732816	4.306752854	2	2	44.87480312	47.54205731
chr6	153420438	153420572	6q25.2	RNA5SP225	10.49443488	2.439126436	4.302538286	2	2	45.9470173	34.58679316
chrMT	577	647	N/A	MT-TF	15.81023018	3.704279096	4.268099074	2	1	104.2519357	0
chr6	143554115	143569339	6q24.2	PHACTR2-AS1	0.789410404	0.185097652	4.264832071	1	1	0	0
chr8	20144855	20183206	8p21.3	SLC18A1	0.280771443	0.065960203	4.256679461	1	2	0	1.283422333
chr3	19940181	19943373	3p24.3	HSPA8P18	0.527678253	0.124257948	4.246635802	1	1	0	0
chr20	44963794	44966458	20q13.12	STK4-AS1	0.524848492	0.123772604	4.240425372	1	1	0	0
chr3	128785147	128785448	3q21.3	RN7SL698P	3.158156375	0.745775481	4.234728087	1	1	0	0
chr15	31216021	31225007	15q13.3	LINC02352	1.726957867	0.408128171	4.231410598	4	4	164.4398037	69.9848335
chr16	67389807	67393535	16q22.1	TPPP3	5.213632451	1.233016018	4.228357439	4	4	87.88370444	80.23024552
chr7	87627656	87832298	7q21.12	RUNDC3B	0.304881921	0.072259044	4.219290821	3	3	124.7392298	36.29451922
chr17	4740015	4746119	17p13.2	ZMYND15	2.020972558	0.479719992	4.212817042	3	4	67.72565995	53.9460051
chr21	13843133	13848364	21q11.2	CYP4F29P	0.674750345	0.160735624	4.197889224	2	2	5.923469869	19.53884783
chr21	17611686	17614114	21q21.1	BTG3-AS1	0.403317261	0.096603016	4.174996587	2	2	98.86656327	4.33814734
chr21	29222254	29223466	21q21.3	GAPDHP14	1.966948365	0.471313556	4.173332893	4	2	61.95015491	67.4729802
chr7	111726108	112206407	7q31.1	DOCK4	13.6459286	3.28648991	4.152128553	4	4	77.35450803	43.22602263
chr15	41838813	41848148	15q15.1	PLA2G4B	0.23887853	0.057746704	4.136660842	3	4	64.38583671	7.664195161
chr11	117285686	117316256	11q23.3	BACE1	1.365211137	0.331665029	4.116234808	4	4	69.90470574	69.83001802
chr1	224398227	224398311	1q42.11	MIR4742	12.30768071	2.991758643	4.113861505	4	1	35.13394421	0
chr19	51795146	51826207	19q13.41	FPR3	4.442389557	1.080677663	4.110744313	4	4	71.14583409	59.05638193
chr22	22922594	22922913	22q11.22	IGLC7	6.083715143	1.481757099	4.105743882	2	3	122.7658004	103.7315561
chr1	59990394	60073770	1p32.1	C1orf87	0.249940978	0.060880524	4.105434057	1	2	0	6.887786039
chr14	100146416	100166886	14q32.2	DEGS2	0.228296835	0.055713563	4.09768862	3	3	7.412237864	4.926696366
chr5	181193924	181218332	5q35.3	TRIM7	0.250999883	0.061309779	4.093961649	3	4	74.5515274	27.26911611
chr6	25652201	25701783	6p22.2	SCGN	1.70811147	0.41730182	4.093227938	2	3	14.39131477	103.2008292
chr5	140726159	140726246	5q31.3	VTRNA1-3	30.80128323	7.536058791	4.087187226	3	4	49.91356547	22.35211755
chrMT	1602	1670	N/A	MT-TV	20.33064515	4.979329691	4.08300844	2	2	12.23596948	22.59623578
chr11	60280541	60308972	11q12.2	MS4A4A	3.553241418	0.871196349	4.078577028	4	4	71.35937623	41.1399519
chrX	136097499	136098347	Xq26.3	E2F6P4	23.03172064	5.653855352	4.073631038	4	3	38.57027839	50.87607525
chr20	46708358	46736347	20q13.12	SLC2A10	0.208719075	0.051383065	4.062020737	4	2	60.37134179	0.257344109
chr1	161606059	161608551	1q23.3	HSPA7	6.112330481	1.505047584	4.061220752	4	4	73.2311731	65.86976129
chr1	78648943	78664078	1p31.1	IFI44	108.1047688	26.69383904	4.049802226	4	4	68.95333491	44.97100366
chr2	219418377	219426739	2q35	DES	0.790204063	0.195308231	4.045933233	2	2	118.4696344	66.1772361
chr6	57029496	57029866	6p12.1	MRPL30P1	2.81384121	0.696834088	4.038036111	1	1	0	0
chr19	52142974	52143386	19q13.41	RPL37P23	3.947921348	0.979480314	4.03062858	4	2	84.1343067	31.18865182

chr1	170461317	170532609	1q24.2	GORAB-AS1	0.328794314	0.081681398	4.025326691	3	1	40.17826443	0
chr12	130953907	131141469	12q24.33	ADGRD1	0.504044434	0.125702947	4.00980603	4	4	57.2053859	46.02138901
chr1	88816779	88816885	1p22.2	RNU6-125P	13.44620851	3.354546194	4.008353957	1	3	0	18.92206409
chr2	166403573	166494264	2q24.3	SCN7A	0.251798863	0.062855996	4.00596407	2	3	114.0267104	50.23842181
chr19	5558167	5568034	19p13.3	TINCR	0.279543577	0.069841146	4.002562889	1	1	0	0
chr3	32818018	32897826	3p22.3	TRIM71	0.175394188	0.043972415	3.988732261	4	2	26.39465663	48.59026572
chr20	45091214	45101112	20q13.12	KCNS1	0.230542408	0.057957921	3.977754965	2	3	94.77169033	5.220729754
chrX	50202713	50351914	Xp11.22	CCNB3	0.237872434	0.05983603	3.975404659	3	4	8.250730407	34.41499277
chr1	209661359	209742562	1q32.2	HSD11B1-AS1	3.619088279	0.911429592	3.970782066	3	4	65.29880893	39.9658163
chr20	35878370	35878930	20q11.23	HIGD1AP16	4.223699041	1.06707433	3.958205086	2	3	71.01676373	19.31253568
chr17	42851182	42858130	17q21.31	AOC3	1.264072462	0.319441053	3.957138417	3	4	66.99989536	95.89544349
chr15	52505029	52505908	15q21.2	EEF1B2P1	1.719431814	0.435013475	3.952594373	1	2	0	11.58963448
chr20	63908955	63909119	20q13.33	RNU1-134P	6.744729017	1.706890253	3.951471984	1	1	0	0
chr6	89090946	89091242	6q15	RN7SL336P	3.753675973	0.950959438	3.947251398	2	4	77.53130685	16.32783913
chr19	55075853	55087923	19q13.42	EPS8L1	0.286637835	0.072730521	3.941094205	3	4	81.08970931	63.9547474
chr1	27666061	27672229	1p35.3	IFI6	120.3967718	30.6004473	3.934477515	4	4	82.64655767	68.11671436
chr6	29672392	29681150	6p22.1	ZFP57	4.272756256	1.087848111	3.927713999	3	3	71.78217101	87.01036007
chr2	199269500	199471266	2q33.1	SATB2	0.247744503	0.063125197	3.92465313	3	4	77.56693976	79.86665204
chr13	77895481	77975723	13q22.3	EDNRB	0.199835682	0.050921541	3.924383998	3	1	57.60508612	0
chr9	121203172	121203435	9q33.2	RN7SL181P	11.06020738	2.819514076	3.922735294	2	4	65.89670045	81.27024721
chr17	7140945	7141592	17p13.1	RPL7AP64	9.413498166	2.402487212	3.91823029	4	4	81.44261405	109.9319683
chr4	96310703	96818415	4q22.3	LINC02267	0.508932516	0.130113815	3.911441042	4	3	95.91768962	30.77761163
chr17	9897710	9905367	17p13.1	RCVRN	0.618277255	0.158182291	3.908637618	2	3	60.5297051	70.54388175
chr3	107240692	107326964	3q13.12	DUBR	0.603100714	0.15445876	3.904606722	4	4	47.61328792	99.21908824
chr2	214846947	214847081	2q35	SNORA70I	8.129971087	2.084325401	3.900528719	1	1	0	0
chr1	90892990	90893913	1p22.2	PHKA1P1	1.72173987	0.445152293	3.867754687	2	3	10.94590146	15.97441441
chr14	74853033	74876663	14q24.3	PROX2	0.215896131	0.055824192	3.867429566	3	3	53.21974661	0.421735657
chr16	31393223	31426513	16p11.2	ITGAD	0.838170327	0.216834259	3.865488473	4	4	97.52966925	66.07092471
chr12	113195441	113221341	12q24.13	IQCD	0.725794466	0.187965744	3.861312436	3	4	46.32058254	81.13864895
chr5	54455601	54456384	5q11.2	HSPB3	2.03844368	0.52795883	3.860989842	3	2	66.31518678	33.51737596
chr10	73422216	73422759	10q22.2	RPL26P6	2.013844221	0.521795561	3.859450655	2	1	89.99528037	0
chr1	1013467	1014540	1p36.33	ISG15	97.7939388	25.36611625	3.855298062	4	4	89.61618578	61.04007023
chr8	143566189	143572758	8q24.3	MROH6	0.589444999	0.15310878	3.849844529	3	4	80.87363999	49.20622218
chr11	2321718	2321827	11p15.5	RNU6-878P	11.71155522	3.046360338	3.844441866	2	4	75.65957267	41.4441674
chr3	157259742	157503777	3q25.31-q25.32	VEPH1	5.835757125	1.518208804	3.843843554	4	4	77.20956161	30.29541278
chr12	51906913	51923361	12q13.13	ACVRL1	0.612335302	0.159561831	3.837605131	3	3	78.80710921	61.48578113
chr1	228628146	228628266	1q42.13	RNA5S9	214.7614068	56.02367052	3.833404788	4	4	106.6721559	128.42712
chrX	66021738	66040125	Xq12	VSIG4	1.557592406	0.406722899	3.829615715	4	4	118.7273236	42.66456569
chr20	45174899	45176544	20q13.12	PI3	65.64481037	17.17973149	3.821061489	4	4	99.383763	69.37197174
chr14	65212893	65222347	14q23.3	LINC02324	2.613911694	0.684713665	3.817525236	3	3	87.46664241	72.9667987
chr19	5680765	5688523	19p13.3	HSD11B1L	0.323116902	0.084689832	3.815297483	4	2	82.44897367	2.887173643
chr9	5091093	5091604	9p24.1	MTND6P5	2.748358222	0.721501235	3.809221783	2	3	99.021368	44.87199195
chr1	161581339	161601220	1q23.3	FCGR2C	17.68416602	4.660204512	3.794718875	4	4	53.41101444	43.87803196

chr2	89862505	89862979	2p11.2	IGKV1D-39	2.293964728	0.604841689	3.792669669	4	1	43.90044169	0
chr6	83512475	83525704	6q14.2	PRSS35	1.063713886	0.281094428	3.784187024	4	1	138.4488772	0
chr9	137299631	137302251	9q34.3	NRARP	0.592716316	0.157232773	3.769674123	1	3	0	11.32806649
chr7	28180457	28241377	7p15.1	JAZF1-AS1	0.665067025	0.176442014	3.769323478	4	4	59.32799174	33.62150276
chr1	27934955	27959157	1p35.3	SMPDL3B	0.968144406	0.256936648	3.768027703	4	3	123.7664233	76.50403762
chr2	240906160	240993311	2q37.3	CROCC2	0.161822203	0.043007399	3.762659651	2	2	14.76174219	1.891742388
chr19	21063924	21064261	19p12	VN1R80P	4.390286312	1.168755294	3.756377689	1	3	0	37.11476598
chr17	39962973	39977766	17q21.1	GSDMA	0.57940312	0.154256736	3.756096075	1	3	0	39.71062598
chr3	9902087	9916402	3p25.3	IL17RE	0.211819594	0.056533987	3.746765552	4	3	54.64031506	5.748224552
chr8	130004528	130004601	8q24.21	RNU7-181P	36.28231419	9.696344452	3.74185492	3	2	79.15728742	91.49813555
chr14	104821201	104823718	14q32.33	LINC00638	0.389756027	0.104215005	3.739922359	2	1	16.97009666	0
chr10	89327819	89340968	10q23.31	IFIT3	437.8926614	117.2373549	3.735095028	4	4	102.1858665	72.56546228
chr9	37915898	38069213	9p13.1	SHB	0.48516289	0.129953936	3.733345094	3	2	51.74850226	40.85902836
chr14	103519658	103522859	14q32.33	CKB	0.73334447	0.196870299	3.725013244	4	4	54.15400481	39.37074637
chr10	93958140	93961915	10q23.33	PIPSL	0.282567914	0.075921421	3.721847005	3	2	93.43572378	3.184972891
chr2	66423342	66433470	2p14	MEIS1-AS3	1.209114065	0.324949504	3.720929104	1	1	0	0
chr10	6149623	6149873	10p15.1	RN7SKP78	3.333668037	0.896722925	3.717612146	3	2	16.44591719	14.71868139
chr3	129397210	129397348	3q21.3	SNORA7B	90.66338862	24.39293818	3.716788357	4	4	102.6396581	90.15260536
chr17	45505883	45520523	17q21.31	LRRC37A4P	0.433308252	0.116725794	3.712189383	4	4	71.91311215	78.09809079
chr19	49969600	49975819	19q13.33	SIGLEC16	0.83849691	0.225876758	3.712187644	1	3	0	53.74360124
chr22	22395052	22395489	22q11.22	IGLV7-43	5.261587891	1.421279255	3.702008506	3	4	36.9479045	63.900555
chrX	155997581	156013017	Xq28 and Yq12	IL9R	0.374768689	0.10133531	3.69830309	3	2	76.16457226	4.725213358
chr15	99014704	99031050	15q26.3	LUNAR1	0.559137736	0.151342286	3.694524179	3	2	79.43678768	31.5426031
chr19	23489866	23505982	19p12	ZNF725P	1.023201121	0.276963299	3.694356347	3	3	114.2962363	40.2026895
chr5	1510762	1510856	5p15.33	MIR6075	9.815080238	2.657515881	3.693328912	2	1	28.44018003	0
chr9	122880213	122880319	9q33.2	SNORD90	9.331242359	2.530219228	3.687918523	1	1	0	0
chr19	41193207	41207539	19q13.2	CYP2S1	1.990150605	0.541063326	3.678221218	4	4	102.6149091	81.06428587
chr5	180899077	180952166	5q35.3	BTNL8	6.157458889	1.683161444	3.658269925	4	4	73.37256105	58.25161588
chr18	7567316	8406861	18p11.23	PTPRM	2.495615845	0.682858976	3.654657742	4	4	44.82065489	59.49007992
chr18	10454628	10488701	18p11.22	APCDD1	0.381576455	0.104461388	3.652799018	2	3	123.2408926	100.9270872
chr16	1944571	1955123	16p13.3	RPL3L	2.508569012	0.689555356	3.637951601	4	3	118.0355743	40.1881567
chr4	118664087	118685341	4q26	METTL14-DT	0.599130457	0.164727127	3.637108653	1	2	0	50.05828107
chr6	133987581	134052651	6q23.2	SLC2A12	0.208378291	0.057309281	3.63603046	2	2	97.95002514	0.515342601
chr7	38349355	38349824	7p14.1	TRGV5	4.764007954	1.310534958	3.635162821	3	4	71.01033929	78.62479888
chr6	27694035	27710222	6p22.1	LINC01012	0.33754137	0.09287655	3.634301339	2	3	50.58556226	40.97432386
chr1	27355179	27368037	1p36.11	MAP3K6	1.235485281	0.340479323	3.62866464	4	4	112.8770423	92.0209497
chr15	73926458	73952141	15q24.1	LOXL1	0.471003847	0.129920824	3.625314508	3	3	83.33196157	40.82004672
chr20	25734229	25735114	20p11.1	VN1R108P	1.197448049	0.330498574	3.623156474	2	4	74.73323011	12.88417768
chr1	228687417	228687879	1q42.13	FTH1P2	5.336283787	1.474491278	3.61906772	3	4	78.08721877	39.91751607
chr1	246945547	247078813	1q44	ZNF670-ZNF695	0.37311612	0.103426428	3.607551061	3	4	101.0664839	41.16215684
chr11	72216773	72221950	11q13.4	FOLR2	3.578412464	0.993604897	3.601444071	3	4	51.86519345	35.75879326
chr5	62775376	62777344	5q12.1	ISCA1P1	2.121730478	0.589317693	3.60031695	3	1	44.93027633	0
chr16	57976435	57988116	16q21	TEPP	0.301844903	0.083924535	3.596622895	1	1	0	0

chr5	140670796	140673586	5q31.3	DND1	0.649669445	0.180979996	3.589730689	1	2	0	11.44330764
chr6	110392180	110415550	6q21	DDO	0.41530783	0.115979519	3.580872161	4	3	33.08590087	3.505294724
chr1	92029976	92063536	1p22.1	EPHX4	0.856243327	0.239203885	3.579554431	4	3	34.32889477	46.19356667
chr15	82293283	82334561	15q25.2	ADAMTS7P1	0.381421442	0.106608121	3.57778975	2	3	117.3731647	45.4689851
chr4	15468660	15601971	4p15.32	CC2D2A	0.215905505	0.060367838	3.576498884	4	2	55.07692392	66.92060852
chrX	102785678	102786604	Xq22.1	MTND1P32	1.084982456	0.304744515	3.560301834	1	2	0	15.27672685
chr10	70458542	70568450	10q22.1	PALD1	0.473320368	0.132955773	3.559983567	2	2	54.93343821	76.38935184
chr19	48170662	48197621	19q13.33	ZSWIM9	0.55048802	0.154774233	3.556716182	4	4	56.23492147	67.38810793
chr19	57230317	57235550	19q13.43	AURKC	0.481989613	0.135564861	3.555417024	2	1	54.80217856	0
chr6	142966421	143037582	6q24.2	LINC01277	0.47645369	0.134543136	3.541270893	2	3	38.1787895	34.40960408
chr3	49121114	49133166	3p21.31	LAMB2	0.941623599	0.265932047	3.540842899	4	4	97.08568805	47.62882581
chr10	89301949	89309276	10q23.31	IFIT2	495.4819365	140.2000537	3.53410661	4	4	104.5181593	73.41607259
chr6	39299001	39314461	6p21.2	KCNK17	0.591647986	0.167974688	3.522244879	2	2	106.3021336	39.33025429
chr12	57009997	57016560	12q13.3	TAC3	2.17520436	0.617973628	3.519898362	1	1	0	0
chr3	134783436	134783555	3q22.2	RNA5SP141	10.39977386	2.968625914	3.503228146	1	4	0	46.5555435
chr13	50711026	50843945	13q14.3	DLEU7	2.032384723	0.580340878	3.502053362	4	4	54.60905521	64.85559073
chr7	20827298	20827820	7p21.1	RPL23P8	2.190251505	0.625545152	3.501348383	2	1	35.47489375	0
chr4	112355537	112359913	4q25	RTEL1P1	1.176882981	0.336205801	3.500483865	4	4	98.00303918	96.85852397
chr20	38429667	38429804	20q11.23	SNORA71C	22.4332721	6.408769488	3.5004024	3	4	73.88731314	73.70826371
chr8	22687661	22693302	8p21.3	EGR3	6.823260778	1.950039794	3.499036685	4	4	98.28998704	63.91373382
chr6	144004916	144008730	6q24.2	HYMAI	0.376809195	0.107708528	3.498415611	3	4	47.33754565	51.94326711
chr21	45502517	45563140	21q22.3	SLC19A1	10.55488957	3.017207767	3.498230943	4	4	88.96007484	40.74003999
chr1	10058673	10059654	1p36.22	PGAM1P11	1.189623357	0.340257496	3.496244372	1	1	0	0
chr2	181694424	181695160	2q31.3	SAP18P2	2.020939991	0.578188764	3.495294474	1	1	0	0
chr3	4490317	4493163	3p26.1	ITPR1-DT	1.141868662	0.326914321	3.492868284	3	3	16.56407848	11.26294867
chrX	103499753	103519489	Xq22.2	RAB40A	0.349502763	0.100221061	3.487318519	3	2	61.62685071	44.98509267
chr15	32441914	32461753	15q13.3	GOLGA8O	0.713923641	0.204790002	3.486125469	3	4	38.05235496	52.36066877
chr1	25293358	25294669	1p36.11	SDHDP6	2.030223477	0.582566727	3.484962982	3	3	71.04231941	17.17829861
chr5	83940595	84384867	5q14.3	EDIL3	1.47423883	0.423152578	3.483941509	4	3	86.83824388	60.11379373
chr6	12716635	13290462	6p24.1	PHACTR1	8.190200283	2.357516719	3.474079405	4	4	101.7593981	63.46512684
chr19	43353659	43366063	19q13.31	CD177	19.15692676	5.518788112	3.471219835	3	3	107.7491156	65.24239795
chr10	92668451	92669847	10q23.33	EIF2S2P3	1.019537509	0.29396408	3.468238393	1	2	0	12.16540434
chr17	55264960	55325176	17q22	HLF	0.346685831	0.100085988	3.463879798	3	3	33.57535807	69.86121609
chr9	14920689	14922433	9p22.3	LDHAP4	0.931626627	0.269003608	3.463249563	1	1	0	0
chr16	57663998	57689378	16q21	ADGRG3	107.0692487	30.91888049	3.462908326	4	4	79.83964667	64.45030555
chr5	659862	700727	5p15.33	TPPP	0.500036758	0.144462011	3.461371995	3	3	78.24211718	120.6938953
chr9	112242550	112242839	9q32	RN7SL57P	3.272046383	0.945890819	3.459222056	2	1	62.40550466	0
chr4	118033608	118258945	4q26	NDST3	1.264863319	0.365769621	3.4580874	4	4	42.71510547	34.08241551
chr15	40835993	40844524	15q15.1	SPINT1-AS1	0.651080874	0.188492307	3.454150903	2	1	81.4802527	0
chr5	73120561	73131817	5q13.2	TMEM171	0.566163176	0.164100204	3.450106477	3	3	56.52449952	6.98919017
chr4	112706018	113383740	4q25-q26	ANK2	0.04779376	0.013881258	3.443042519	1	1	0	0
chr4	184341031	184353977	4q35.1	LINC02363	0.632274291	0.18416651	3.433166494	3	4	60.78471474	45.45186346
chr2	90004827	90005629	2p11.2	IGKV2D-24	2.009606152	0.585892476	3.429991398	1	1	0	0

chr4	86594307	86815176	4q21.3	PTPN13	1.909949763	0.557313791	3.427063524	4	4	51.12718912	42.28799066
chr15	83654086	84039842	15q25.2	ADAMTSL3	0.089399626	0.026155535	3.418000321	1	1	0	0
chr3	13478903	13506424	3p25.1	HDAC11	0.736207064	0.215742233	3.412438327	4	4	59.16006595	51.04267679
chr19	37251885	37265535	19q13.12	LINC01535	0.626777901	0.184045823	3.405553524	1	3	0	61.24712621
chr4	25160672	25198506	4p15.2	SEPSECS-AS1	1.965962379	0.579154281	3.394540011	4	4	62.30144108	68.22978661
chr3	44418427	44439883	3p21.31	C3orf86	30.84065575	9.100642824	3.388843661	4	4	116.8983577	84.63181034
chr21	44350112	44443081	21q22.3	TRPM2	3.418761207	1.009573439	3.386342265	4	4	60.7298252	17.04369763
chr14	87952526	87955403	14q31.3	SHLD2P2	0.334729218	0.098971533	3.382075723	3	1	64.9537466	0
chr17	31345314	31347501	17q11.2	AK4P1	1.28008897	0.379358927	3.374347825	1	1	0	0
chr10	17809343	17911162	10p12.33	MRC1	1.198513689	0.356255049	3.364201271	4	4	123.8270034	46.94259613
chr3	169083499	169663781	3q26.2	MECOM	0.096879835	0.028799775	3.363909506	3	3	85.59369739	0.217539302
chr3	172560901	172595607	3q26.31	LINC02068	0.598035897	0.177838009	3.362812604	1	2	0	79.90074689
chr1	175968397	175968540	1q25.1	SCARNA3	25.33153643	7.535136779	3.361788534	4	4	52.8273222	48.63237023
chr14	22469041	22469614	14q11.2	TRDV3	3.076001133	0.915071106	3.361488647	3	3	58.98706403	56.43541302
chr4	100395341	100518093	4q24	EMCN	0.510905107	0.152107525	3.358841764	3	1	152.8119019	0
chr21	46462471	46469306	21q22.3	DIP2A-IT1	8.747671696	2.606396354	3.356232325	4	4	56.62134496	67.02065516
chr9	34521042	34523039	9p13.3	ENHO	1.433678175	0.427442282	3.354086004	3	2	124.6390208	66.15384161
chr17	16744740	16746762	17p11.2	UPF3AP1	0.795372588	0.237190837	3.353302338	1	1	0	0
chr11	612555	615999	11p15.5	IRF7	31.28344887	9.336790277	3.350557091	4	4	88.64510788	33.16596772
chr1	212686417	212699985	1q32.3	BATF3	0.839804271	0.25069566	3.349895539	4	3	77.07208633	27.75109466
chr15	100551820	100551922	15q26.3	RNU6-322P	8.128875012	2.430428807	3.344625849	1	1	0	0
chr22	42553850	42574382	22q13.2	SERHL2	0.291380759	0.087206201	3.341284849	1	1	0	0
chr12	53103490	53124539	12q13.13	SOAT2	0.431705198	0.129278853	3.339333449	1	1	0	0
chr10	132445189	132448306	10q26.3	C10orf91	0.450320718	0.135025308	3.335083799	2	3	88.69890362	9.995364525
chr17	67740670	67740801	17q24.2	SNORA38B	21.34476635	6.401645149	3.334262655	4	2	100.5470713	5.849260724
chr2	230034974	230068999	2q36.3	SLC16A14	0.974731057	0.292651339	3.33069058	3	4	144.7493745	88.74648291
chr22	18149855	18177397	22q11.21	USP18	5.034716702	1.511977044	3.329889645	4	4	57.63152178	70.19090096
chr8	144051266	144064026	8q24.3	OPLAH	2.019171658	0.606448977	3.329499654	4	4	98.8456357	64.10395017
chr8	21690403	21789296	8p21.3	GFRA2	0.536899257	0.161304893	3.328474705	4	4	84.81255704	74.99765446
chr2	90100263	90100738	2p11.2	IGKV1D-16	2.270269547	0.682320319	3.327278236	3	2	63.62440307	6.027931589
chr21	43805758	43812567	21q22.3	AATBC	4.616605146	1.39082237	3.319334838	4	4	79.89897865	34.99140112
chr18	23598926	23662914	18q11.2	ANKRD29	0.130720031	0.039387535	3.318817239	1	1	0	0
chr3	57197838	57227643	3p14.3	HESX1	0.711815885	0.214689608	3.315558185	3	1	27.01821315	0
chr16	3215562	3216546	16p13.3	OR1F2P	0.899836156	0.271522123	3.314043611	1	3	0	10.10813308
chr21	44107323	44131181	21q22.3	PWP2	0.822465978	0.248182397	3.313957746	4	4	162.9775853	103.099394
chr7	6930780	6939613	7p22.1	FAM86LP	1.467602001	0.443243912	3.311048298	2	2	108.9680737	29.61560955
chr15	28789834	28856574	15q13.1	PDCD6IPP2	0.393488356	0.118895692	3.309525761	4	4	61.70914867	45.72431762
chr2	66574030	66695588	2p14	LINC01798	0.564397154	0.170712472	3.306127246	1	2	0	20.22849451
chr11	118253416	118264536	11q23.3	MPZL2	4.967224403	1.502727718	3.305472006	4	4	98.35945771	43.04454303
chr2	118182922	118186386	2q14.2	THORLNC	0.860999782	0.260497266	3.305216199	1	1	0	0
chr7	20329819	20415759	7p21.1	ITGB8	0.491409415	0.148696479	3.30478179	4	4	41.41178463	38.34708981
chr15	36891021	37101299	15q14	MEIS2	0.214180381	0.064829383	3.303754743	2	3	69.0170347	57.53406154
chr2	236324147	236507535	2q37.2-q37.3	IQCA1	0.136064172	0.041395466	3.286934172	2	2	15.34452991	7.602385736

chr10	133237904	133241930	10q26.3	VENTX	3.012379372	0.916552172	3.286642554	4	4	93.63224416	36.84024039
chr11	134378504	134411986	11q25	B3GAT1	0.841803231	0.256319271	3.284197978	4	4	69.54389552	59.16249618
chr8	37796883	37843986	8p11.23	ADGRA2	0.759263578	0.231318948	3.282323325	4	4	74.15891573	46.7695701
chr1	154467195	154502560	1q21.3	SHE	0.295370228	0.09004627	3.280205053	4	3	93.95696057	115.0821591
chr16	28973999	28984769	16p11.2	SPNS1	0.223321687	0.068082861	3.280145435	3	3	47.35420075	10.10194652
chr1	33306766	33321098	1p35.1	A3GALT2	0.898708751	0.274060983	3.2792291	3	1	43.60259765	0
chr11	2963771	2963893	11p15.4	SNORA54	10.20254831	3.115733964	3.274524858	1	1	0	0
chr1	201896456	201946588	1q32.1	LMOD1	7.293212421	2.22818256	3.273166459	4	4	69.15676376	135.2539639
chr5	72439899	72507422	5q13.2	ZNF366	1.125145108	0.343790774	3.272761205	4	4	70.49832401	82.01586714
chr5	23951348	24178265	5p14.2	C5orf17	6.866786956	2.099278292	3.271022705	1	2	0	1.822954785
chr14	67619862	67651720	14q24.1	ARG2	5.059656408	1.547007321	3.27060922	4	4	61.0886835	63.16656028
chr8	81521520	81522433	8q21.13	FTH1P11	1.409578636	0.431197778	3.268984	2	1	36.85269258	0
chr17	42874674	42898734	17q21.31	LINC00671	1.211533926	0.370708749	3.268155736	4	1	50.19270005	0
chr10	79937740	79982236	10q22.3	SFTPD	1.032409867	0.315969822	3.267431873	2	3	82.99422293	56.14902917
chr9	74497336	74687201	9q21.13	RORB	0.135290886	0.041429336	3.265581772	3	2	66.98163736	41.53078008
chr1	147099482	147114346	1q21.1	NBPF13P	0.59203836	0.181343638	3.264731898	4	4	99.21779294	29.51291604
chr2	30888518	31138726	2p23.1	GALNT14	1.797717558	0.55247635	3.253926722	4	4	84.86389522	94.70719866
chr1	161647243	161678654	1q23.3	FCGR2B	4.075288257	1.252495475	3.253734914	4	4	88.93066695	62.57941916
chr13	93226808	94408020	13q31.3-q32.1	GPC6	0.130328155	0.040064452	3.252962393	2	3	98.68580895	2.940851865
chr18	6256744	6260934	18p11.31	L3MBTL4-AS1	0.628283581	0.193148684	3.252849395	1	4	0	63.2899467
chr4	108808725	109302737	4q25	COL25A1	0.255176469	0.078487204	3.2511856	3	2	95.60846956	70.73683987
chr14	23568035	23578800	14q11.2	JPH4	0.263866651	0.081171311	3.250737843	4	3	59.37086278	34.36769937
chr4	73853296	73854484	4q13.3	PF4V1	208.8331638	64.27691746	3.248960467	4	4	68.93180769	46.73570439
chr11	60396014	60417756	11q12.2	MS4A14	3.464468662	1.067545062	3.245266907	4	4	59.37406685	35.66687585
chr22	24585620	24593103	22q11.23	LRRC75B	0.446548264	0.137676023	3.243471542	3	3	74.8182397	40.74666187
chr2	109614334	109618987	2q13	SOWAHC	1.35536627	0.418155146	3.241299993	4	4	73.61336312	28.05230039
chr10	44993932	45000910	10q11.21	C10orf25	0.512483218	0.158342981	3.236538902	4	4	60.78375388	51.63458444
chr1	202810946	202811913	1q32.1	PCAT6	0.931379175	0.287883037	3.235269379	1	1	0	0
chr4	185362862	185397297	4q35.1	LRP2BP	0.477916137	0.14782771	3.232926601	3	4	25.19125866	22.8161261
chr20	56527124	56555626	20q13.31	FAM209B	2.286741713	0.707966041	3.230016101	3	3	74.05794469	43.82932263
chr7	123790607	123790711	7q31.32	RNU6-11P	11.78014683	3.647422479	3.229718218	1	1	0	0
chr1	183636047	183928551	1q25.3	RGL1	2.405708537	0.745098596	3.228711677	4	4	46.20382796	51.19711182
chr12	120293094	120293237	12q24.23	RNU4-1	804.941917	249.6443606	3.224354498	4	4	115.3450511	71.56763357
chr5	107376889	107670895	5q21.3	EFNA5	0.28719831	0.08913624	3.22201509	4	4	100.4424375	35.7767805
chr2	27996367	27996449	2p23.2	MIR4263	12.18671643	3.788376513	3.216870442	2	3	55.48160527	24.95773058
chr16	25069572	25070301	16p12.1	SCML2P2	1.476709423	0.459506536	3.213685349	3	2	51.02151921	9.797832372
chr10	30692274	30717013	10p11.23	SVIL2P	0.255969562	0.079692636	3.211960044	4	1	122.5345376	0
chr5	140547666	140549578	5q31.3	EIF4EBP3	2.554688187	0.796237557	3.208449746	2	3	26.3014574	55.20793136
chr1	15152418	15220480	1p36.21	TMEM51	1.118352214	0.348613265	3.208002468	3	3	68.82839879	22.75054638
chr16	27268205	27290468	16p12.1	NSMCE1-DT	0.632024982	0.197339076	3.202736101	4	4	65.11489738	58.44693636
chr11	18412298	18451246	11p15.1	LDHC	0.712856694	0.223056198	3.195861409	1	2	0	68.49848071
chr10	45678692	45700553	10q11.22	AGAP10P	2.003248432	0.627087594	3.194527292	3	3	93.96259552	78.57297772
chr15	65285461	65285591	15q22.31	SNORA24B	6.238202295	1.952931513	3.194276017	1	1	0	0

chr7	149125693	149125986	7q36.1	RN7SL521P	3.836767557	1.201846656	3.192393588	2	2	114.9222807	28.60445834
chr1	148263476	148263632	1q21.2	RNU1-120P	14.52254214	4.556047708	3.187530744	4	3	61.69848079	56.11940077
chr3	52777586	52792068	3p21.1	ITIH1	0.195028403	0.061189064	3.187308157	1	1	0	0
chr16	63180375	63181088	16q21	UBE2FP2	2.250786897	0.706999422	3.183576717	3	1	15.09724634	0
chr1	21508982	21578412	1p36.12	ALPL	146.8001335	46.15718372	3.180439569	4	4	113.0918804	95.13089217
chr10	91151995	91153083	10q23.32	NUDT9P1	2.064234374	0.649089493	3.18019995	2	4	11.7026094	57.69124806
chr9	100578079	100588389	9q31.1	CAVIN4	0.31174839	0.098033222	3.180027992	3	1	68.46565972	0
chr12	120916650	120917281	12q24.31	RPL12P33	1.834510055	0.576979812	3.179504754	3	1	66.1989684	0
chr11	4488879	4489908	11p15.4	OR52K1	0.82387842	0.259195446	3.178599135	1	1	0	0
chr21	26836287	26845409	21q21.3	ADAMTS1	1.087092928	0.342110628	3.177606423	4	4	101.402059	71.34016545
chr3	189000779	189325304	3q28	TPRG1	0.809450297	0.254774033	3.177130285	4	4	66.67634839	52.45775985
chr9	5113452	5114909	9p24.1	IGHEP2	0.636013925	0.200209679	3.176739154	4	3	72.80030739	6.860057829
chr11	112961247	113278436	11q23.2	NCAM1	3.072151017	0.967094923	3.176679915	4	4	54.16312892	70.40885015
chr2	73073382	73113018	2p13.2	RAB11FIP5	1.281300707	0.403407932	3.176191159	4	4	19.08117104	50.8073454
chr6	12256463	12297194	6p24.1	EDN1	0.631532993	0.198943578	3.174432675	1	3	0	61.91498434
chr6	159780250	159780381	6q25.3	SNORA20	50.30943802	15.89775679	3.164562063	4	4	108.4941586	58.48901656
chr4	122449479	122456495	4q27	IL2	1.03167011	0.326013633	3.164499906	2	2	40.55032915	37.73676251
chr14	22492851	22492907	14q11.2	TRAJ47	26.90251062	8.51056095	3.161073727	1	4	0	97.71604907
chr1	202168051	202189449	1q32.1	PTPRVP	0.198527831	0.062807396	3.160898944	2	1	50.79311446	0
chr20	17493905	17569220	20p12.1	BFSP1	0.529188908	0.167442887	3.160414388	4	3	93.56084594	12.10136911
chrX	47509795	47513660	Xp11.3	NUS1P1	1.838766355	0.582083912	3.158936912	4	1	120.2704175	0
chr17	34255277	34257203	17q12	CCL2	1.586305994	0.502516731	3.156722743	4	3	120.9884314	43.41255789
chr7	23710140	23832515	7p15.3	STK31	0.515693355	0.163486132	3.154355347	4	4	99.64993727	37.08787717
chr19	54307070	54313139	19q13.42	LILRA5	55.78533686	17.72426602	3.147398984	4	4	94.55064791	48.17222157
chrX	133301036	133415177	Xq26.2	GPC4	0.163209539	0.051879695	3.145923285	3	1	86.50599273	0
chr3	101712434	101713416	3q12.3	PDCL3P4	1.161852235	0.369785486	3.141962784	2	4	4.249483997	13.38866902
chr19	49114339	49118464	19q13.33	LIN7B	0.743324141	0.236872971	3.138070741	3	4	75.64595953	40.26609446
chr2	28488578	28644135	2p23.2	PLB1	6.427252151	2.048409995	3.137678574	4	4	79.16143999	62.87225385
chr2	113099365	113134016	2q14.1	IL1RN	60.14281506	19.18880586	3.134265649	4	4	62.47664424	62.58797166
chr1	12063317	12144213	1p36.22	TNFRSF8	4.978676243	1.589982533	3.131277319	4	4	80.6588558	45.00264407
chr3	141051402	141148611	3q23	SPSB4	0.215042973	0.068695006	3.130401836	1	1	0	0
chr1	156815750	156881850	1q23.1	NTRK1	0.554357304	0.177184618	3.128698809	3	4	53.79593213	31.16726234
chr1	1331324	1335320	1p36.33	TAS1R3	0.382490291	0.122537326	3.121418619	2	3	110.229314	34.32887098
chr10	78943326	79067448	10q22.3	ZMIZ1-AS1	0.283547686	0.090856369	3.120834447	3	2	53.87856681	42.2460853
chr9	99998104	99999209	9q31.1	UPF3AP3	3.317725078	1.065196956	3.114658803	3	4	67.54314783	45.51086364
chr2	74532258	74555719	2p13.1	LOXL3	2.942936469	0.944999351	3.114220624	4	4	58.56875706	34.90005433
chr2	156020535	156254931	2q24.1	LINC01876	0.657422551	0.211281007	3.111602691	2	3	115.0153001	97.71939006
chr21	38238045	38238666	21q22.13	SPATA20P1	1.682649194	0.541178387	3.109232065	2	2	31.11314885	5.012930171
chr1	111483348	111564003	1p13.2	TMIGD3	0.534947688	0.172088734	3.108557293	3	4	24.80191239	37.87786964
chr1	19665287	19680966	1p36.13	HTR6	0.442295621	0.142345516	3.107197417	1	2	0	13.95314494
chrY	25378671	25391610	Yq11.23	LINC00266-4P	0.76192889	0.245251613	3.106723264	1	2	0	11.39795062
chr14	20801356	20802877	14q11.2	RNASE1	0.851655262	0.274147018	3.106564024	4	3	70.85909485	29.79078902
chr2	118942169	118994664	2q14.2	MARCO	10.4497914	3.364528582	3.105870895	4	4	110.3704978	43.50685042

chr9	112252258	112252378	9q32	RNA5SP295	6.799483265	2.190592491	3.103947126	2	2	73.29895842	8.818732138
chr9	123402525	123402603	9q33.3	MIR601	11.37802512	3.665843741	3.103794358	1	1	0	0
chrX	110944241	111227361	Xq23	PAK3	0.076693616	0.024716841	3.102889131	4	1	71.81163587	0
chr17	50271406	50281485	17q21.33	TMEM92	0.327992654	0.105762619	3.101215319	3	3	33.22330819	36.80525266
chr1	148334612	148334767	1q21.2	RNU1-122P	11.22462057	3.620211404	3.100542846	4	4	77.50551081	73.26625013
chr16	67247549	67272204	16q22.1	SLC9A5	0.14634483	0.047214444	3.099577521	3	1	51.67136959	0
chr1	107964443	107994607	1p13.3	VAV3-AS1	1.794745395	0.579465885	3.097240824	3	2	37.08010778	36.73574027
chr13	50807855	50849905	13q14.3	DLEU7-AS1	0.538772028	0.173992835	3.096518475	2	1	82.76010342	0
chr6	56954975	57027652	6p12.1	BEND6	0.230232577	0.074359107	3.096225694	3	3	48.76868303	62.87757138
chr1	56994778	58250547	1p32.2	DAB1	0.164910592	0.053287613	3.094726551	2	4	34.13156361	64.06425688
chrX	41686397	41697277	Xp11.4	GPR34	16.2652473	5.256078768	3.094559276	4	4	78.58889755	74.54783362
chr10	74654951	74656327	10q22.2	POLR3DP1	0.725915796	0.234582496	3.094501119	1	1	0	0
chr19	44846136	44889228	19q13.32	NECTIN2	4.01749893	1.299688857	3.091123624	4	4	57.8581535	61.08963814
chr12	95943293	95968592	12q23.1	AMDHD1	0.471028582	0.152478334	3.089150896	3	4	30.53058717	45.66601138
chr1	26863138	26864457	1p36.11	SFN	1.102371583	0.357089386	3.08710263	2	3	80.01929751	44.34497839
chr17	7101322	7115538	17p13.1	ASGR2	5.094944338	1.650679075	3.086574741	4	4	97.54228299	61.14390154
chr19	11420605	11435782	19p13.2	CCDC151	0.300119361	0.097242819	3.086288163	2	2	91.17360612	8.520952569
chr9	27109141	27230178	9p21.2	TEK	0.758561029	0.245931282	3.084443031	2	4	0.849163658	101.9021548
chr5	53951504	53951599	5q11.2	MIR581	6.800369112	2.206048186	3.082602255	3	1	83.75565597	0
chr5	141943585	141959062	5q31.3	PCDH12	0.492557061	0.160323583	3.072268302	4	3	79.4977887	95.98739975
chr6	75749176	75919537	6q14.1	MYO6	1.324570716	0.43165091	3.068615599	4	4	29.79289839	44.39671247
chr7	1086807	1093815	7p22.3	GPER1	0.240934675	0.078519707	3.068461222	4	1	85.38888129	0
chr1	246945547	247008093	1q44	ZNF695	0.269731809	0.088020469	3.064421414	3	3	87.35790207	38.70172059
chr11	64888458	64893449	11q13.1	MIR194-2HG	0.218156649	0.071217727	3.063235189	1	1	0	0
chr3	167694355	167694642	3q26.1	HMGN1P8	7.551564645	2.46910609	3.058420485	4	4	57.93653718	33.22643801
chr2	144519614	144520899	2q22.3	ZEB2-AS1	1.533447725	0.501423081	3.058191342	4	4	35.74368193	64.91458545
chr5	79991296	80083753	5q14.1	THBS4	0.683995628	0.22380143	3.056261205	3	3	121.7187665	53.67990787
chr1	232397965	232630491	1q42.2	SIPA1L2	22.32259147	7.30698396	3.054966536	4	4	88.73302724	64.80233049
chr8	38166012	38166118	8p11.23	RNU6-323P	11.15783293	3.652738389	3.054648798	1	1	0	0
chr1	22710770	22921500	1p36.12	EPHB2	0.576695388	0.189055874	3.05039656	4	4	115.3892868	45.55503969
chr2	166195185	166375987	2q24.3	SCN9A	1.240567523	0.406835087	3.04931301	4	4	34.52876977	60.14462658
chr19	8630645	8638664	19p13.2	LINC01862	1.551971138	0.509047896	3.048772329	2	1	61.28418758	0
chr5	140631728	140633701	5q31.3	CD14	253.4715953	83.19025445	3.046890492	4	4	87.57176321	47.93505179
chr1	218345284	218444619	1q41	TGFB2	0.488650529	0.16047444	3.045036508	4	4	89.10669851	59.68217695
chr8	90221488	90387959	8q21.3	LINC00534	3.347752292	1.099446627	3.044942983	4	4	68.53321628	97.89851676
chr20	38425195	38425330	20q11.23	SNORA71B	14.02523195	4.612728349	3.040550166	3	4	82.13298416	51.02573333
chr22	22811782	22812281	22q11.22	IGLV3-10	6.401615195	2.107817248	3.037082651	4	3	51.82621655	71.5589953
chr22	39743044	39893884	22q13.1	ENTHD1	0.584451293	0.192678364	3.033300055	3	4	59.04743285	40.55874893
chr12	48656648	48656782	12q13.11	SNORA2A	10.99924697	3.626382178	3.033118528	2	3	17.1094121	69.53965488
chr1	87129765	87169204	1p22.3	LINC01140	0.117105505	0.038610804	3.032972479	1	1	0	0
chr3	131533560	132285696	3q22.1	CPNE4	0.204546118	0.067456739	3.032256259	4	3	81.99956569	70.80452011
chr15	84570649	84580181	15q25.2	LINC00933	0.291658093	0.096316851	3.028110765	1	1	0	0
chr4	7192607	7742837	4p16.1	SORCS2	0.173578234	0.057367396	3.0257297	3	3	71.35185445	50.32378917

chr14	20323179	20323326	14q11.2	SNORA79B	102.779805	34.00813033	3.022212747	4	4	95.71059316	60.57333961
chr16	70529499	70529599	16q22.1	SNORD111B	15.07087642	4.987372687	3.021806744	3	3	101.1743244	26.9079355
chr19	15851993	15864904	19p13.12	LINC01835	0.988884872	0.327444155	3.020010767	2	1	15.30513447	0
chr11	61680184	61747002	11q12.2	DAGLA	0.593075466	0.196448264	3.018990619	4	3	75.91554365	53.81453304
chr12	9127730	9128509	12p13.31	KRT17P8	1.798200002	0.596802874	3.0130552	4	3	18.82044749	41.3173083
chr10	58512903	58831437	10q21.1	BICC1	0.340200908	0.11297067	3.011409155	3	4	95.70207539	65.75354956
chr1	91531954	91535869	1p22.1	WDR82P2	1.025759451	0.34076805	3.010139748	1	1	0	0
chr4	86012296	86594625	4q21.3	MAPK10	0.212626381	0.070659914	3.00915142	4	4	55.83639564	81.29798975
chr7	98212254	98212959	7q21.3	BHLHA15	1.724082674	0.573276112	3.007421099	1	1	0	0
chr17	15782922	15791633	17p12	IL6STP1	0.683337685	0.227646826	3.001744836	1	1	0	0
chr1	16215438	16227316	1p36.13	ANO7L1	3.200498986	1.067098591	2.999253314	4	4	15.37106187	67.41612588
chr7	2527074	2527145	7p22.3	MIR4648	12.00208862	4.002261586	2.99882663	2	1	5.898619186	0
chr1	145959441	145964582	1q21.1	ANKRD34A	0.472916271	0.158024966	2.992668066	4	4	76.36311957	81.24176303
chr11	65170154	65173666	11q13.1	SPDYC	4.586651069	1.534341895	2.989327923	4	3	90.77407196	116.1098357
chr3	39329706	39333680	3p22.1	CCR8	0.787022511	0.263555691	2.986171568	4	3	48.20274319	47.20806408
chrX	96883908	96885467	Xq21.33	RPA4	0.641897857	0.215103197	2.98413909	4	3	61.02912662	44.17425214
chr19	1491005	1497927	19p13.3	REEP6	1.229942886	0.412228665	2.983642312	2	3	74.66143956	31.87214039
chr12	62021488	62022175	12q14.1	RPS3P6	1.132424723	0.379649139	2.982819151	1	1	0	0
chr2	136329300	136330002	2q22.1	UBBP1	3.083798253	1.033915015	2.982641909	1	1	0	0
chr3	158571163	158606460	3q25.32	MLF1	0.328175895	0.110029653	2.982613191	4	4	67.07862893	37.42202587
chr4	110476073	110563337	4q25	ENPEP	0.175284581	0.058824196	2.979804114	4	4	73.36312937	30.53742361
chr12	108288044	108339347	12q23.3	CMKLR1	8.644849157	2.902568456	2.978344624	4	4	51.28335531	57.54857343
chr12	3839459	3846276	12p13.32	OTUD4P1	0.435900362	0.146711168	2.971146417	1	4	0	50.35120401
chr6	33586106	33593338	6p21.31	LINC00336	0.311864899	0.105040176	2.969005863	1	1	0	0
chr1	184821430	184821489	1q25.3	RNU7-13P	38.675187	13.03605427	2.966786283	3	4	59.63361717	83.64261343
chr21	36134901	36146562	21q22.12	CBR3	1.828034798	0.617197119	2.961833006	3	4	41.95659821	78.4186044
chr19	51184746	51185207	19q13.41	SIGLEC20P	0.601202663	0.203496534	2.954363165	1	1	0	0
chr19	41425359	41429024	19q13.2	B3GNT8	22.03473591	7.46236324	2.952782544	4	4	90.39704757	45.03752587
chr4	105552604	105682539	4q24	ARHGEF38	0.217474505	0.073651312	2.952758075	1	2	0	82.68171852
chr1	156149672	156177751	1q22	SEMA4A	27.12394292	9.192322132	2.950717189	4	4	67.50548317	35.98704128
chr1	1449689	1470158	1p36.33	ATAD3C	0.187021622	0.063423649	2.948767921	3	1	12.4547332	0
chr12	781132	781256	12p13.33	RNU4ATAC16P	6.596298021	2.237176672	2.948492224	4	1	57.83603648	0
chrX	47658833	47660111	Xp11.23	UXT-AS1	0.488197852	0.165577379	2.948457427	2	1	17.33789489	0
chr1	156243321	156248522	1q22	PAQR6	0.844385899	0.286503832	2.947206303	4	4	103.4994674	73.55926077
chr1	172418560	172468829	1q24.3	C1orf105	0.62087741	0.210760372	2.945892549	1	3	0	35.78997604
chr1	219080973	219173788	1q41	LYPLAL1-DT	18.64519665	6.332318784	2.944450096	4	4	42.53248563	55.41347184
chr8	39107529	39284917	8p11.22	ADAM32	2.001185335	0.679778442	2.943878789	4	4	114.9636226	75.74801633
chr1	151806071	151832451	1q21.3	RORC	1.367985338	0.465025202	2.941744518	4	4	68.73063032	73.14163136
chr16	74408631	74422248	16q23.1	CLEC18B	0.246720014	0.083869807	2.941702438	2	2	88.35872008	8.734517503
chr20	19757610	20002459	20p11.23	RIN2	7.622806185	2.59204697	2.940844156	4	4	76.69879859	32.12204284
chr10	97713708	97718152	10q24.2	MARVELD1	2.974155922	1.012065847	2.938698043	3	4	61.56945616	30.4558819
chr15	44825747	44884694	15q21.1	SORD2P	0.387017521	0.131753844	2.937428698	2	2	98.93991976	45.33830147
chr14	103120849	103122909	14q32.32	LINC00677	2.651492194	0.902717282	2.937234334	4	3	36.1399594	58.12136882

chr17	41812710	41823217	17q21.2	FKBP10	0.177188042	0.060339479	2.936519253	1	1	0	0
chr17	12081899	12081996	17p12	MIR744	10.17207857	3.465442037	2.93529035	2	2	80.01150588	23.22233525
chrX	37888915	38128820	Xp11.4	SYTL5	0.156784721	0.053449171	2.93334243	2	2	52.80703203	0.16319151
chr2	6840417	6866635	2p25.2	CMPK2	38.45904021	13.11326354	2.932835148	4	4	75.98668917	34.20187299
chr2	10446714	10446849	2p25.1	SNORA80B	9.760028335	3.329310189	2.931546711	3	2	90.03305292	22.67948803
chr11	22688351	22688435	11p14.3	RNA5SP338	9.852934449	3.362288986	2.930424627	1	1	0	0
chr13	36430488	36442882	13q13.3	CCNA1	0.457356571	0.156090596	2.930071272	4	2	27.60642341	49.63398333
chr2	151832771	152099167	2q23.3	CACNB4	0.56167053	0.191729855	2.929489157	4	4	132.7053586	56.47578751
chr12	48814432	48828943	12q13.12	CACNB3	0.220843934	0.075390188	2.929345847	4	4	42.67522013	49.39751484
chr21	33265632	33266260	21q22.11	IL10RB-DT	1.600791122	0.546660769	2.928308033	4	2	79.8244995	48.42658118
chr14	106622357	106622794	14q32.33	IGHV1-58	1.762470581	0.601938262	2.927992276	1	2	0	11.10251177
chr2	96837914	96844021	2q11.2	ANKRD23	0.191735045	0.06551271	2.926684677	4	3	37.73758577	5.923970831
chr2	110732539	111120610	2q13	ACOXL	0.133657492	0.045754037	2.921217489	2	3	87.48113869	3.73416362
chr16	88424805	88440757	16q24.2	ZNF469	0.38968271	0.13345312	2.919997006	4	4	126.2311754	83.86564244
chr11	74845910	74846209	11q13.4	RN7SL239P	7.776217768	2.66411082	2.918879241	3	4	62.85172086	84.72209422
chr19	35641175	35644871	19q13.12	ETV2	0.505261667	0.173180039	2.917551406	2	4	51.91611895	56.66403074
chr3	33150041	33219215	3p22.3	SUSD5	0.149304479	0.051192849	2.916510455	1	1	0	0
chr22	45284987	45295874	22q13.31	UPK3A	1.830691905	0.628246978	2.913968502	3	4	90.96610393	34.72701469
chr17	29614236	29622912	17q11.2	CORO6	0.267425996	0.091865858	2.911048797	4	4	49.47925033	67.7271846
chr21	24428740	24547942	21q21.2	LINC01684	1.435188695	0.493015586	2.911041226	4	4	73.37251111	65.42671256
chr3	33103008	33103305	3p22.3	RN7SL296P	3.322545988	1.142529483	2.908061487	3	4	14.33588336	32.0624057
chr5	77076677	77087246	5q13.3	ZBED3	0.402440061	0.138403366	2.907733189	4	4	70.03795622	107.9118046
chr22	24423597	24442360	22q11.23	ADORA2A	1.276955159	0.439454233	2.90577508	4	4	151.2681147	32.82915807
chrX	37349275	37457295	Xp21.1	PRRG1	0.285169252	0.098200803	2.903940126	4	3	62.76377446	25.49201613
chr19	44671452	44684355	19q13.31	CEACAM19	0.215376911	0.074176856	2.903559448	4	2	61.41058215	46.66937322
chr21	44158773	44159862	21q22.3	LINC01678	0.904140051	0.311662296	2.901024804	2	1	81.19560507	0
chr12	112906777	112932190	12q24.13	OAS1	33.32784204	11.49514125	2.899298175	4	4	78.43681408	49.17619676
chr17	7717354	7833744	17p13.1	DNAH2	0.149008578	0.051419456	2.897902654	3	4	65.24764104	66.09678457
chr7	51016212	51316861	7p12.1	COBL	0.049540246	0.017096107	2.897750056	3	1	101.4489169	0
chr10	73909182	73917501	10q22.2	PLAU	1.030701017	0.356693396	2.889599383	3	4	98.96617178	28.4082311
chr19	5576486	5576951	19p13.3	SNRPEP4	2.956057125	1.023212787	2.888995489	3	3	51.40953522	23.66185138
chr10	6010694	6062370	10p15.1	IL2RA	4.292061338	1.486983115	2.886422378	3	4	81.36288178	72.49831478
chr17	4785285	4786434	17p13.2	VMO1	0.983160054	0.340678318	2.885889716	2	3	63.90203095	13.11163012
chr5	94825890	94826716	5q15	RPL7P18	1.946423288	0.6750476	2.883386726	3	2	63.71617178	11.91778039
chr13	100623673	100624515	13q32.3	COX5BP6	3.837587326	1.330953408	2.883337089	1	1	0	0
chr15	23685407	23687303	15q11.2	NDN	0.67360557	0.233625254	2.883273787	1	4	0	50.27656712
chr11	112052161	112074171	11q23.1	PIH1D2	0.425740111	0.147734015	2.881801528	1	2	0	39.33537082
chr10	104351301	104353575	10q25.1	CFAP58-DT	9.909812673	3.440371174	2.8804487	4	4	54.63637316	73.580889
chrX	153072414	153075019	Xq28	PNMA6A	0.834187404	0.289733824	2.879150911	2	3	116.9190555	41.30582228
chr16	57694801	57731455	16q21	DRC7	0.296674853	0.103096745	2.877635503	4	4	77.29881736	71.15936746
chr4	184843296	184855652	4q35.1	MIR3945HG	10.40601614	3.616490988	2.877379254	4	4	78.41183041	81.3738596
chr16	2737076	2752600	16p13.3	SRRM2-AS1	0.219771845	0.076627111	2.868069057	3	4	40.74014772	54.6281415
chr19	3539157	3544030	19p13.3	C19orf71	3.513369214	1.225650454	2.866534419	2	2	14.29815038	48.30632527

chrY	14522578	14845647	Yq11.221	NLGN4Y	0.258791969	0.090284049	2.866419608	2	2	67.80136207	42.60859342
chr6	31727037	31730265	6p21.33	DDAH2	6.400625031	2.233073373	2.866285143	4	4	46.9405835	29.63471037
chr1	235975830	236065181	1q42.3	NID1	6.91049078	2.416161153	2.860111699	4	4	87.18729162	17.17511667
chr6	113857335	113863475	6q21	MARCKS	112.0443803	39.19317747	2.85877256	4	4	61.86569013	59.16932328
chr3	142724424	142807888	3q23	TRPC1	5.825805398	2.038315934	2.858146425	4	4	82.76008275	70.69272591
chr3	183253207	183273391	3q27.1	B3GNT5	17.20367341	6.026146501	2.85483823	4	4	53.19457445	54.46137172
chr2	86135870	86136006	2p11.2	SNORD94	95.65193649	33.50606301	2.854765016	4	4	118.4910128	83.62122225
chr19	33795540	33815761	19q13.11	KCTD15	1.950831481	0.68339134	2.854633015	4	4	108.0575371	67.91186864
chr20	45206964	45209773	20q13.12	SEMG1	0.915698661	0.32080258	2.854399302	3	3	124.7153879	49.0514602
chr7	43758122	43807342	7p13	BLVRA	30.23815262	10.59428425	2.854194952	4	4	86.23951701	21.3586445
chr20	21087604	21106358	20p11.23	LINC00237	0.421253254	0.14759158	2.854182157	1	1	0	0
chr5	21882517	21884766	5p14.3	HSPD1P1	0.430561061	0.150856194	2.8541159	2	2	93.12160465	2.573766482
chr7	101127089	101139266	7q22.1	SERPINE1	2.480902698	0.869409671	2.853548541	4	4	51.51327135	46.12934404
chr22	23580872	23637732	22q11.23	DRICH1	1.546446312	0.54214316	2.85246855	4	4	98.71774789	49.22748713
chr16	48577487	48578392	16q12.1	RPS2P44	4.64087065	1.628377057	2.849997567	3	4	65.60247031	36.24314347
chr4	54229089	54298247	4q12	PDGFRA	0.303438433	0.106469747	2.84999675	4	4	119.5147724	25.40492363
chr4	112455857	112457986	4q25	TOX4P1	0.787349636	0.276502053	2.847536308	2	3	31.79343536	36.74631522
chr4	55885595	55885701	4q12	RNU6-652P	6.591688611	2.315146201	2.847201877	3	3	17.04665348	14.05416829
chr14	105140981	105168824	14q32.33	JAG2	0.158283101	0.05559253	2.847200891	3	3	61.35208456	40.33654982
chr13	113954565	113975026	13q34	C13orf46	0.359767459	0.126379994	2.84671212	3	4	71.34566786	33.01220451
chr12	8414779	8415774	12p13.31	OR7E140P	0.811788837	0.285175842	2.846625543	2	2	33.1874854	12.23833607
chr3	99833144	100114513	3q12.1	FILIP1L	0.555700654	0.195250279	2.846094026	2	4	7.031893324	107.3720328
chr11	75404421	75404566	11q13.4	SNORD15B	273.8921074	96.28819078	2.844503622	4	4	83.28055602	47.5295669
chr10	12896625	13099773	10p13	CCDC3	0.337882307	0.118828659	2.843441225	4	3	67.23123233	72.74335196
chr1	19643229	19658456	1p36.13	NBL1	0.353341979	0.124413416	2.840063329	2	4	106.1331017	76.15361852
chr3	121593323	121630292	3q13.33	FBXO40	0.310816766	0.109466987	2.839365317	3	3	141.269287	37.70289125
chr3	43346919	43351962	3p22.1	SNRK-AS1	1.007242375	0.354877063	2.838285369	3	1	71.77984363	0
chr1	29236516	29326813	1p35.3	PTPRU	0.108059992	0.038110909	2.835408436	1	2	0	12.332445
chr20	63953384	63956985	20q13.33	UCKL1-AS1	0.554053155	0.195640029	2.832003027	2	4	59.59605183	83.4662955
chr6	68635283	69389511	6q12-q13	ADGRB3	0.215340416	0.076081564	2.830388908	2	1	62.21045674	0
chr22	26169474	26383597	22q12.1	SEZ6L	0.57968254	0.205074137	2.826697453	4	4	80.28134611	60.88805843
chr12	123471073	123533718	12q24.31	RILPL1	1.414352113	0.500601254	2.825306774	4	4	84.09059961	71.63944128
chr12	7470811	7503818	12p13.31	CD163	23.65952438	8.375566743	2.824826679	4	4	75.6799992	55.62180196
chr19	43660500	43660776	19q13.31	RN7SL368P	10.05590097	3.565644952	2.820219373	4	3	127.6824594	59.82331023
chr22	27748277	27801498	22q12.1	MN1	0.141079948	0.050049294	2.818819949	2	4	99.4832164	37.33757386
chr19	21939766	22010943	19p12	ZNF208	0.348652115	0.123841318	2.815313353	3	4	127.3416251	85.6070668
chr2	98087132	98330682	2q11.2	VWA3B	0.084767455	0.030135316	2.812894214	1	1	0	0
chr4	102868978	102892807	4q24	CISD2	165.1134663	58.70198432	2.812740799	4	4	40.72916637	10.35471662
chr22	32190435	32204849	22q12.3	RFPL2	0.360670713	0.12827722	2.81165052	3	3	100.9934946	40.15933801
chr11	825558	831991	11p15.5	CRACR2B	0.402453182	0.143176421	2.810890073	4	3	102.3559024	89.99608662
chrX	153968765	153970091	Xq28	HCFC1-AS1	2.740338822	0.975437814	2.809342412	1	3	0	41.23322038
chr1	158178038	158186427	1q23.1	CD1D	29.0083722	10.32640757	2.809144613	4	4	81.91175457	43.13746057
chr3	128479422	128493187	3q21.3	GATA2	12.73997341	4.537133514	2.80793443	4	4	85.7989139	56.52636232

chr5	42423775	42721878	5p13.1-p12	GHR	0.175433333	0.062542927	2.805006772	1	3	0	65.65206827
chrX	138631573	139222889	Xq26.3-q27.1	FGF13	0.161680987	0.057645556	2.804743321	4	4	76.05010039	141.5038854
chr11	125111997	125433389	11q24.2	PKNOX2	0.216790139	0.077473341	2.798254667	2	2	112.1340933	2.105979203
chr9	131725168	131725467	9q34.13	RN7SL328P	5.711248181	2.042243109	2.796556471	3	4	51.49088845	37.94758087
chr21	38805307	38824955	21q22.2	ETS2	43.1221575	15.42427507	2.795733174	4	4	71.58542856	39.36074227
chr17	81650459	81663418	17q25.3	PDE6G	1.808699151	0.647104603	2.795064574	3	4	50.11888752	67.4978392
chr1	6247346	6260975	1p36.31	GPR153	0.782193928	0.279927617	2.79427209	4	4	55.22436015	77.05672954
chr16	71565016	71572438	16q22.2	TAT-AS1	0.737800657	0.264143215	2.793184208	2	1	46.90421248	0
chr6	4079197	4130788	6p25.2	C6orf201	0.239192979	0.085660693	2.792330666	1	1	0	0
chr8	47667771	47667877	8q11.21	RNU6-665P	5.52192453	1.982509521	2.785320561	1	1	0	0
chr20	2794069	2800646	20p13	CPXM1	0.551096558	0.198066722	2.782378348	1	2	0	62.64436416
chr6	96794122	96837477	6q16.1	GPR63	0.123945587	0.044548328	2.782272494	3	2	47.17957529	2.34069863
chr11	60088261	60098467	11q12.1	MS4A2	4.910136019	1.767126894	2.778598433	4	4	76.27857748	57.15115242
chr6	53334460	53337617	6p12.1	RPS16P5	11.6253242	4.188272067	2.775685058	2	4	49.28805314	56.32992487
chr12	1529856	1647213	12p13.33	WNT5B	0.600768575	0.216548419	2.774292133	4	1	87.19327632	0
chr1	153774354	153780160	1q21.3	SLC27A3	3.188046326	1.149159995	2.774240612	4	4	89.78810095	33.95672627
chrX	49205063	49233404	Xp11.23	CACNA1F	0.208564884	0.07519485	2.773659151	2	3	112.9118947	50.54706194
chr3	87792767	87994856	3p11.2-p11.1	HTR1F	0.211143689	0.076174573	2.771839491	1	1	0	0
chr1	93561741	93847150	1p22.1	BCAR3	0.587843282	0.212078848	2.771814766	3	4	63.67048714	38.82087676
chr5	80500312	80501468	5q14.1	RPL7P24	2.084285892	0.752352023	2.770359922	1	2	0	27.67376195
chrX	103075821	103093094	Xq22.1	NXF3	0.589398577	0.212802617	2.769696094	2	2	118.2018231	51.79690096
chr5	32234767	32234875	5p13.3	RNU6-1079P	5.342903461	1.929218067	2.769465801	2	1	61.52740214	0
chr1	32741839	32774970	1p35.1	KIAA1522	0.217690555	0.078613556	2.769122352	3	4	75.00590205	30.23978616
chrX	120250984	120311536	Xq24	TMEM255A	0.457650383	0.165335446	2.768011312	4	2	55.20336249	84.82087044
chr12	8513492	8522366	12p13.31	CLEC4D	56.79108843	20.52234297	2.767280935	4	4	65.93626626	69.11815375
chr20	45252239	45254564	20q13.12	SLPI	30.25646083	10.9348098	2.766985562	4	4	89.18760687	79.61785206
chr19	46491191	46495912	19q13.32	PNMA8B	0.138811851	0.050185228	2.765990274	2	1	38.80072305	0
chr11	66332063	66337235	11q13.2	RIN1	1.500944398	0.543076389	2.763781356	4	4	82.85189986	47.42639639
chr9	129175552	129178261	9q34.11	IER5L	2.157509177	0.780812269	2.763159934	4	4	110.2215425	89.3069519
chr20	59577497	59849829	20q13.32-q13.33	PHACTR3	0.264062998	0.095568277	2.763082135	4	4	77.75069957	47.26915126
chr1	150270317	150280927	1q21.2	C1orf54	1.956435537	0.708265587	2.762290832	4	4	79.04143324	35.49148454
chr22	47461299	47487111	22q13.31	LINC01644	0.644975796	0.23357286	2.761347344	2	1	76.82418395	0
chr6	3153666	3157549	6p25.2	TUBB2A	87.57300032	31.7332267	2.759662644	4	4	53.63546437	63.60875155
chr6	35943514	36024636	6p21.31	SLC26A8	4.509335975	1.634152822	2.759433459	4	4	63.69589362	80.48145405
chr12	52022832	52059507	12q13.13	NR4A1	3.889460838	1.409658004	2.759152097	4	4	78.6306576	77.20698138
chr4	109555108	109556904	4q25	CDC42P4	1.325450906	0.480815291	2.756673783	2	3	0.750518997	16.30114198
chr5	76849953	76952882	5q13.3	S100Z	3.600401562	1.306429105	2.755910403	4	4	85.87356306	53.45085878
chr1	201519904	201520602	1q32.1	RPS10P7	0.922748323	0.334869126	2.755549109	2	4	84.97212665	61.25279382
chr1	203026491	203078736	1q32.1	PPFIA4	0.146988212	0.053354056	2.754958521	4	3	69.31834807	50.43632582
chr2	74557640	74648338	2p13.1	M1AP	0.487266691	0.176969755	2.75338965	2	4	46.19158028	39.6115501
chr5	138465492	138469315	5q31.2	EGR1	24.45184662	8.880712534	2.7533654	4	4	142.5052577	101.7976587
chr5	176880869	176899373	5q35.2	HK3	42.01288181	15.26428558	2.752364767	4	4	80.11443069	39.0750387
chr8	144048677	144051164	8q24.3	SMPD5	0.336274327	0.122195058	2.751947038	1	1	0	0

chr9	63832050	63859634	9q13	FRG1JP	3.079612095	1.119372021	2.75119624	3	3	102.0578686	100.4038442
chr11	10557866	10568818	11p15.4	LYVE1	0.989930212	0.360071631	2.749259111	4	3	74.17312028	73.52846615
chr3	43314951	43315255	3p22.1	RN7SL517P	8.368313147	3.044043273	2.749078248	4	4	79.12116175	55.71866857
chr19	685521	695461	19p13.3	PRSS57	1.55498188	0.565688535	2.74883047	3	3	38.03060839	70.69579555
chr13	32024046	32031639	13q13.1	FRY-AS1	1.189601178	0.432838701	2.748370639	2	4	107.1266189	58.92624146
chr17	36211063	36212878	17q12	CCL4L2	6.955560175	2.53163033	2.747462808	4	4	88.21117984	140.7136309
chr7	38269491	38269540	7p14.1	TRGJ1	32.78281605	11.94266481	2.745016843	3	3	25.20782245	73.82951447
chr10	71212535	71302879	10q22.1	UNC5B	0.30552682	0.111304825	2.744955742	2	3	54.18978725	87.80986387
chr11	101451470	101583928	11q22.1	TRPC6	1.209381845	0.440709429	2.744170569	4	4	32.26452938	12.38066048
chr11	5248272	5249857	11p15.4	HBG1	2545.06782	928.6149793	2.740713726	4	4	151.5941963	177.6170503
chr2	203040105	203040719	2q33.2	RPL7P14	1.164153605	0.425000793	2.739179839	1	1	0	0
chr15	28698695	28738556	15q13.1	GOLGA8M	0.301503365	0.110084754	2.738829452	3	1	91.63179292	0
chr1	112709994	112715328	1p13.2	PPM1J	0.516842708	0.188800366	2.737509035	3	3	57.62809386	81.97537842
chr10	97828478	97849798	10q24.2	LINC00866	1.6763956	0.612466761	2.737120945	1	1	0	0
chr1	36306348	36329221	1p34.3	SH3D21	1.183731845	0.432553447	2.73661406	4	4	44.69184542	40.72558791
chr13	68984858	68986526	13q21.33	ZDHHC20P4	11.22917609	4.106941757	2.734194142	4	4	115.0717601	52.84607869
chr2	148875223	149026759	2q23.1-q23.2	KIF5C	0.998673971	0.365510104	2.732274595	4	4	60.43569412	63.41479144
chr22	22322508	22322969	22q11.22	IGLV1-51	29.4898094	10.79867384	2.730873237	4	4	47.08777654	123.3096
chr12	8235415	8242564	12p13.31	LINC02449	0.565332423	0.207021877	2.730785903	1	2	0	11.16935871
chr7	38273587	38273648	7p14.1	TRGJP	15.88024237	5.81825169	2.72938388	4	3	65.85677879	53.86799039
chr19	54616930	54637925	19q13.42	LILRB1	4.523841266	1.658865128	2.727069965	4	4	74.51773077	34.26915843
chr2	110083870	110118139	2q13	MALL	0.213319293	0.078259305	2.725801007	2	1	2.296224355	0
chrX	71144389	71175307	Xq13.1	NLGN3	0.857685968	0.314916041	2.723538517	3	4	71.45263778	130.3164184
chr9	21454268	21559798	9p21.3	MIR31HG	1.869225081	0.686409294	2.72319314	1	1	0	0
chr7	48035511	48061297	7p12.3	C7orf57	0.289714987	0.106469236	2.721114547	1	1	0	0
chr19	47713344	47743134	19q13.33	EHD2	0.176569855	0.064936907	2.719098628	2	3	82.77471454	0.514518612
chr3	192796815	192918161	3q29	MB21D2	1.463827725	0.538435229	2.71867004	4	4	46.19099276	46.44378415
chr3	133038288	133397775	3q22.1	TMEM108	0.084362458	0.031066493	2.71554497	1	2	0	1.696545051
chr21	42311667	42315596	21q22.3	TFF3	1.59400809	0.587202598	2.714579423	2	1	64.91198924	0
chr3	39808914	40260321	3p22.1	MYRIP	0.108006415	0.039792472	2.714242388	2	1	76.0189404	0
chr8	41511239	41512590	8p11.21	KRT18P37	0.537527303	0.198071688	2.713801797	2	1	56.42009247	0
chr18	80157232	80247514	18q23	PARD6G	0.338168801	0.124624039	2.713511811	2	3	54.05303335	81.41752932
chr2	198299363	198375097	2q33.1	LINC01923	0.588298789	0.21699295	2.71114241	2	3	91.52464663	26.24632278
chr22	30240447	30257487	22q12.2	LIF	0.355775364	0.131233949	2.711000987	4	2	92.1928532	64.50055835
chr2	9271292	9271628	2p25.1	EIF1P7	1.822291268	0.672353474	2.710317325	2	1	57.43607787	0
chr4	183098640	183320777	4q35.1	WWC2	1.698568338	0.626905931	2.709446912	4	4	64.48060386	44.91748775
chr5	132673986	132682678	5q31.1	IL4	3.680655694	1.358658428	2.70903681	4	3	39.59200828	45.60023511
chr4	122730189	122732458	4q27	CETN4P	2.858266966	1.05533912	2.708387202	1	2	0	67.81856187
chr12	55409686	55414943	12q13.2	PHC1P1	0.4483314	0.165578031	2.707674422	2	4	9.774829372	68.87466025
chr9	86026141	86100201	9q21.33	GOLM1	6.17927871	2.283512025	2.706041677	4	4	65.65913727	63.77166453
chr5	92110	189972	5p15.33	PLEKHG4B	0.054666429	0.020211799	2.704678944	1	2	0	9.491692752
chr3	161083883	161105469	3q26.1	B3GALNT1	2.34670709	0.868538603	2.701903038	4	4	82.35766147	52.16008234
chr12	57128401	57213377	12q13.3	LRP1	15.11768213	5.595816182	2.701604491	4	4	100.5596613	30.53708709

chr1	67092176	67141646	1p31.3	C1orf141	0.125637759	0.046513381	2.701110023	1	1	0	0
chr9	67717498	67719179	9q21.11	FAM27E3	0.531608207	0.196960689	2.699057415	1	1	0	0
chr2	121216587	121285210	2q14.2	TFCP2L1	0.40038058	0.148384967	2.698255685	4	3	66.52903152	61.63125461
chr5	112885122	112886687	5q22.2	XBP1P1	1.008325215	0.373897584	2.696795212	2	2	85.04097719	7.113821755
chr2	64525513	64525565	2p14	MIR4434	23.00320663	8.546627737	2.691495095	4	4	68.97953378	35.1750094
chr9	124887410	124887549	9q33.3	RNU4-82P	4.12338859	1.532806796	2.690090232	1	1	0	0
chr14	20684177	20694186	14q11.2	ANG	0.60673183	0.225550204	2.690007898	4	4	45.55057085	29.05884552
chr4	22726422	22728023	4p15.2	CDC42P6	2.555067028	0.949987863	2.689578601	4	4	43.7296772	67.08497758
chr20	6768098	6780280	20p12.3	BMP2	0.192563877	0.071629651	2.688326356	2	2	82.37462647	0.952195365
chr22	42692112	42721301	22q13.2	A4GALT	0.693562864	0.258005508	2.688170763	3	4	78.23282597	44.35643353
chr19	32978592	33064918	19q13.11	RHPN2	0.146535564	0.054516131	2.687930359	2	4	71.68878971	4.47403592
chr8	16107878	16192791	8p22	MSR1	4.91113089	1.829305965	2.684696264	4	4	29.39732896	64.96722607
chr1	204073096	204127743	1q32.1	SOX13	0.973450734	0.362685707	2.684006329	4	4	36.52781505	94.86452528
chr8	52110832	52409879	8q11.23	ST18	0.098140342	0.03658684	2.682394591	3	2	50.3090193	64.94059789
chr1	10946473	10982037	1p36.22	C1orf127	0.186656147	0.069635918	2.680457905	1	1	0	0
chr11	22192485	22283367	11p14.3	ANO5	0.82678525	0.308532109	2.679738105	4	3	77.43725224	53.62761078
chr4	165252580	165252661	4q32.3	RNU4-87P	9.396979071	3.507104698	2.679412188	4	1	42.73201244	0
chr2	135136628	135136755	2q21.3	SNORA40B	10.53912388	3.935848987	2.677725673	4	4	37.15496874	47.91220779
chr1	194188904	194190542	1q31.3	EEF1A1P14	0.493667267	0.184537583	2.675158413	1	1	0	0
chr21	46598618	46605243	21q22.3	S100B	7.567025351	2.828946544	2.674856252	4	4	98.15429894	104.6594482
chr17	7074537	7080307	17p13.1	CLEC10A	7.673676199	2.870018529	2.673737512	4	4	75.83294489	55.45590384
chr11	18704305	18726939	11p15.1	IGSF22	0.214485044	0.080234735	2.673219328	2	4	67.22039134	64.91543258
chr12	69348354	69354233	12q15	LYZ	3275.681148	1225.591177	2.672735582	4	4	69.44330624	48.22550095
chr2	218259496	218263861	2q35	GPBAR1	7.461627966	2.79540627	2.669246344	4	4	62.88178577	54.35701257
chr1	241652278	241802767	1q43	WDR64	0.138829954	0.05201937	2.668812668	1	1	0	0
chr6	144200240	144201057	6q24.2	TPT1P4	2.198865959	0.824005635	2.668508399	1	4	0	68.01084533
chr4	100811429	100816063	4q24	LINC01218	8.015712464	3.00532084	2.667173619	4	2	161.796962	118.9937198
chr11	45906534	45907282	11p11.2	C11orf94	1.297590314	0.486528135	2.667040653	1	4	0	51.79460731
chr21	6111073	6123778	21p12	SIK1B	1.739759509	0.652839835	2.664910161	4	3	119.7969851	72.29829381
chr8	96493601	96611809	8q22.1	SDC2	1.316540983	0.494045373	2.664817962	3	4	76.9735536	60.35089129
chr4	84687725	84688000	4q21.23	RN7SL552P	1.973137123	0.740476813	2.66468455	2	1	86.12039943	0
chr7	148983755	148983856	7q36.1	RNY3	19.67189634	7.387989527	2.662686009	3	3	94.76776951	66.04272253
chr17	74274210	74314884	17q25.1	DNAI2	0.416809986	0.156574244	2.662059709	2	3	38.97252853	36.6202676
chr3	146515178	146544841	3q24	PLSCR1	65.95516283	24.79393881	2.660132516	4	4	62.8517326	57.57830375
chr17	48548870	48590272	17q21.32	HOXB3	0.340650882	0.128082316	2.65962464	4	4	39.10382055	51.9513603
chrX	102804632	102806120	Xq22.1	MTND5P26	0.379570832	0.142744666	2.659089431	1	2	0	3.059874932
chr21	33266360	33297234	21q22.11	IL10RB	29.47827587	11.08896202	2.658344019	4	4	80.11144393	44.0807796
chr11	2883218	2885804	11p15.4	CDKN1C	2.311394647	0.869553778	2.658138812	4	4	36.41795743	61.53759298
chr22	42074244	42079438	22q13.2	PHETA2	0.459560069	0.172902854	2.657909098	3	3	80.1524293	26.06272424
chr1	146052081	146052244	1q21.1	RNVU1-6	38.80105189	14.60700451	2.65633189	4	4	128.7381614	52.40308052
chr20	31968002	31998453	20q11.21	XKR7	0.200884693	0.075653809	2.655314993	4	2	109.7232346	19.61472431
chr7	142535809	142536292	7q34	TRBV13	1.713251878	0.645386232	2.654614855	3	2	16.26523042	8.139561316
chr11	842824	867116	11p15.5	TSPAN4	1.197126997	0.450983749	2.654479236	4	4	78.17327371	26.60755464

chr1	220747417	220784815	1q41	MARC2	0.509167402	0.191818339	2.654425041	2	2	105.5609565	9.789404928
chr1	17372196	17401699	1p36.13	PADI6	0.3308288	0.124647811	2.654108374	2	1	84.99427855	0
chr1	27612289	27635561	1p35.3	FGR	193.1187621	72.77151724	2.653768526	4	4	83.62028977	31.06113856
chr17	42667914	42677045	17q21.2	PLEKHH3	0.235232898	0.088647108	2.653587946	2	3	73.32680767	29.8293184
chr19	35775515	35788822	19q13.12	ARHGAP33	0.32154204	0.121243158	2.652042768	4	4	95.35616603	30.86074227
chr2	73957967	73981439	2p13.1	DGUOK-AS1	0.834421608	0.314679034	2.651659367	4	3	46.93673238	15.68326977
chr6	132133978	132169374	6q23.2	LINC01013	0.524376457	0.197804097	2.650988856	2	4	15.02801317	136.2456673
chr22	41168217	41168316	22q13.2	RNU6-375P	10.47663245	3.955437723	2.648665756	2	1	27.92928364	0
chr12	116148560	116148654	12q24.21	MIR620	7.038320516	2.657815392	2.64816004	2	1	79.74115687	0
chr6	137943075	137945800	6q23.3	LINC02528	7.526744808	2.843405928	2.647087682	1	3	0	99.99839888
chr4	127781821	127834073	4q28.1	HSPA4L	0.16609406	0.062762721	2.646380818	4	4	60.25107359	46.44997749
chr17	18950676	19020691	17p11.2	SLC5A10	0.108079595	0.040877871	2.643963371	2	2	77.12201837	2.220574014
chr17	42101404	42112733	17q21.2	DHX58	9.804118443	3.710637027	2.642165852	4	4	61.49184206	24.73590563
chr3	52433035	52445113	3p21.1	SEMA3G	0.207412886	0.078515396	2.641684257	3	4	58.90924424	37.39588882
chr19	21123817	21124532	19p12	VN1R81P	4.739494155	1.794979881	2.640416311	3	4	95.88255715	51.2102944
chr4	55595229	55636698	4q12	NMU	2.201082842	0.834943623	2.636205344	2	3	39.23240216	75.77932028
chr11	47331406	47352702	11p11.2	MYBPC3	1.648441809	0.625504912	2.635377879	4	4	75.10665998	62.04692373
chr2	88170295	88186637	2p11.2	THNSL2	0.421910546	0.160147517	2.634511937	3	3	58.28540511	79.47531779
chr19	54216278	54223087	19q13.42	LILRB3	30.97458521	11.76151543	2.633553931	4	4	76.20386838	40.98905069
chr15	38252087	38357249	15q14	SPRED1	1.910509989	0.725489764	2.633407228	4	4	38.89595578	52.66111715
chr19	45212377	45234211	19q13.32	EXOC3L2	0.677769892	0.2573867	2.633274722	2	3	101.0886976	36.41429103
chr1	66752147	66779051	1p31.3	TCTEX1D1	0.34364402	0.130528999	2.632702478	2	3	33.02427262	62.55889143
chr9	12774994	12823060	9p23	LURAP1L	1.048824158	0.398690271	2.630674067	3	4	35.92854664	77.01407489
chr19	1782068	1812276	19p13.3	ATP8B3	0.458148582	0.174165764	2.630531803	4	4	74.9184008	41.37277324
chr6	36390551	36442889	6p21.31	PXT1	1.116757471	0.424739444	2.629276574	4	4	46.62469783	67.03725305
chr2	316284	337054	2p25.3	LINC01865	0.509059204	0.193633642	2.628981194	2	2	17.3293022	11.02344618
chr4	143343460	143343535	4q31.21	MIR3139	9.138098624	3.478642712	2.626914972	3	1	49.28936939	0
chr7	142796560	142796610	7q34	TRBJ2-2	17.7408447	6.756598629	2.625706465	1	2	0	23.0551283
chr21	13943769	13980444	21q11.2	ANKRD20A11P	0.78056854	0.297295149	2.625567693	4	4	78.64605969	66.74112673
chr3	129555213	129606739	3q22.1	PLXND1	8.549148124	3.257717724	2.62427529	4	4	50.97836177	15.82544596
chr3	48403854	48430324	3p21.31	PLXNB1	0.118864633	0.045301766	2.62384106	2	3	0.319004509	36.70222761
chr5	52989326	53094779	5q11.2	ITGA2	1.809442696	0.690051895	2.622183502	4	4	86.4668867	35.75705143
chr2	62491178	62491468	2p15	RN7SL18P	4.44633851	1.696380643	2.621073594	2	1	56.14370955	0
chr20	40685848	40689240	20q12	MAFB	27.05727696	10.32565392	2.620393552	4	4	74.24167575	62.29332688
chrX	48831092	48835638	Xp11.23	PCSK1N	2.47287936	0.944627145	2.617836438	4	3	69.31868982	31.20888375
chr16	67199125	67204029	16q22.1	ELMO3	0.908211437	0.347043423	2.616996541	3	4	111.6661738	56.40625776
chr9	5040908	5042125	9p24.1	CSNK1G2P1	1.86547844	0.713081734	2.616079408	3	3	97.15905026	28.61448047
chr12	51351870	51391955	12q13.13	GALNT6	7.26111042	2.775588425	2.616061645	4	4	27.51684208	41.16475395
chrX	47571901	47619857	Xp11.3-p11.23	SYN1	0.184849443	0.070743873	2.612939251	2	2	77.98338561	6.979913112
chr1	161303594	161309972	1q23.3	MPZ	2.957804295	1.132535562	2.611665713	4	4	84.25223051	15.84493146
chr11	62690262	62727384	11q12.3	HNRNPUL2-BSCL2	0.16465319	0.063055629	2.611236985	2	1	1.89419048	0
chr3	68635685	68637242	3p14.1	PSMC1P1	0.479771952	0.183762155	2.61083112	3	2	45.7579602	5.977080819
chr1	7846345	7913615	1p36.23	UTS2	1.099859388	0.421310941	2.610564503	3	4	76.7709446	57.59891845

chr6	31268749	31272136	6p21.33	HLA-C	424.1172118	162.4743144	2.610364681	4	4	97.27044104	50.71164629
chr2	158968600	159232659	2q24.2	TANC1	0.47489724	0.182204622	2.606395134	4	4	59.77023653	81.50911639
chr14	20780956	20782467	14q11.2	RNASE6	60.6896478	23.29032193	2.605788275	4	4	53.47623653	58.15561271
chr10	26589865	26594324	10p12.1	FAM238A	4.870824718	1.869703085	2.605132739	4	4	37.4780076	38.33027042
chr11	1995176	2001466	11p15.5	H19	0.381779281	0.146585912	2.604474582	3	3	120.2535184	58.41353662
chr3	183122215	183162879	3q27.1	LAMP3	2.214124272	0.850208866	2.604212166	4	4	70.53786331	24.66681434
chr8	130082738	130084768	8q24.21	ASAP1-IT2	0.781073984	0.300339056	2.600640735	3	3	36.06912158	60.85755922
chr19	18173780	18178124	19p13.11	IFI30	7.132650121	2.743460834	2.599873136	4	4	55.44911789	43.3185629
chr7	102636748	102671729	7q22.1	POLR2J2	0.622454042	0.239486225	2.599122522	1	4	0	66.86081309
chr8	70103412	70104275	8q13.3	H2AFZP2	1.551149611	0.596971586	2.598364221	1	1	0	0
chr1	240775193	241357274	1q23.1	RGS7	0.514332954	0.198094747	2.59639875	1	1	0	0
chr11	1839003	1841680	11p15.5	TNNI2	9.819738051	3.782745164	2.595929048	4	4	70.36694139	70.68871993
chr12	72087288	72666662	12q21.1	TRHDE	0.108657863	0.041861175	2.595671568	2	2	37.82990044	2.06912779
chr17	39688084	39728662	17q12	ERBB2	3.186617168	1.227923726	2.59512631	4	4	37.43601052	34.58387508
chr20	34955835	35002437	20q11.22	MYH7B	0.084144303	0.032449348	2.59309685	2	1	8.09822777	0
chr12	111666931	111670379	12q24.12	PCNPP1	5.073845982	1.956779209	2.592957835	3	4	27.0668926	101.206426
chr18	5392276	5630663	18p11.31	EPB41L3	8.096331073	3.123286505	2.59224732	4	4	58.57511761	17.69997497
chr12	126730701	126772262	12q24.32	LINC00944	1.532676074	0.591282298	2.592122373	4	4	45.73912248	24.38411095
chr5	710355	784755	5p15.33	ZDHHC11B	0.483289781	0.186449003	2.592074905	3	4	95.94895116	31.58277852
chr4	173615736	173616842	4q34.1	MORF4	1.299833991	0.501465079	2.592072796	3	4	29.12853304	33.79751644
chr11	6318946	6320510	11p15.4	CAVIN3	0.39327827	0.151764094	2.591378893	1	3	0	6.000193011
chr8	118621001	118726067	8q24.12	SAMD12-AS1	0.805627998	0.311044619	2.590072131	1	2	0	22.20002885
chr1	151036321	151047600	1q21.3	BNIPL	0.292439995	0.112918613	2.589829862	3	4	28.08671936	37.47192773
chr22	38957522	38963183	22q13.1	APOBEC3A	49.2581998	19.02108173	2.589663432	4	4	55.9914056	53.14981933
chr14	21429508	21429806	14q11.2	RN7SL650P	2.700674478	1.043917143	2.58705827	3	2	6.995354309	16.86025929
chr22	20033139	20033220	22q11.21	MIR185	9.08906117	3.513893876	2.586606623	1	1	0	0
chr11	5253191	5254781	11p15.4	HBG2	4989.697649	1929.351077	2.586205128	4	4	137.637249	166.3955575
chr19	8490055	8502654	19p13.2	PRAM1	15.64640384	6.054932447	2.584075707	4	4	86.36586463	49.35734626
chr16	55426797	55462297	16q12.2	MMP2-AS1	0.365442743	0.141552148	2.581682772	2	1	29.62971955	0
chr20	64076955	64077010	20q13.33	MIR6813	13.36262512	5.179255721	2.580028066	2	1	23.21735285	0
chr5	123070783	123071080	5q23.2	RN7SL711P	4.460262731	1.729440943	2.57901997	1	2	0	66.30455508
chr20	64033727	64039008	20q13.33	C20orf204	0.356615683	0.138288825	2.5787744	2	3	17.35521132	46.86407635
chr4	83292461	83335153	4q21.23	HPSE	23.6628993	9.177463963	2.578370168	4	4	48.45424607	10.43594096
chr1	247507058	247577690	1q44	GCSAML	8.190801389	3.183825525	2.572628846	4	4	57.35085027	31.39408105
chr16	22419546	22437715	16p12.2	RRN3P3	0.197330819	0.076728429	2.571808402	1	2	0	1.063654906
chr21	33488055	33491723	21q22.11	DNAJC28	0.374473034	0.1456377	2.571264406	1	2	0	41.15617584
chr16	66552563	66579135	16q21	CKLF-CMTM1	1.788974554	0.69582215	2.571022716	3	3	75.50456903	64.30873743
chr17	44656394	44677086	17q21.31	MEIOC	0.078259903	0.030504464	2.565523005	1	2	0	0.535484412
chr19	51610967	51630474	19q13.41	SIGLEC5	7.901798084	3.081002556	2.56468404	4	4	53.45229043	41.40100382
chr5	69415112	69444022	5q13.2	MARVELD2	0.271257671	0.105788199	2.564158132	4	3	74.16346002	46.23142166
chr10	101825971	101843920	10q24.32	KCNIP2	0.399891845	0.156006581	2.563301132	1	4	0	38.35204679
chr1	228164086	228165512	1q42.13	IBA57-DT	1.666088966	0.650040232	2.563055151	2	3	66.55959835	69.94806988
chr15	80779343	80951771	15q25.1	CEMIP	0.470763506	0.183688084	2.562841835	2	4	101.4171107	101.1719553

chr1	153357854	153361027	1q21.3	S100A9	10925.51435	4266.845508	2.560560098	4	4	105.8890268	67.08668729
chr17	4630919	4641673	17p13.2	ALOX15	7.521831566	2.93790359	2.56027175	4	4	126.7098385	51.56895656
chr7	143290621	143290904	7q34	RN7SL535P	2.63189772	1.028113985	2.559927944	1	3	0	15.52058451
chr18	48026886	48412639	18q21.1	ZBTB7C	0.089171085	0.034847964	2.558860669	3	1	56.27258919	0
chr20	61252426	61940617	20q13.33	CDH4	0.139013604	0.054342503	2.558100884	1	2	0	49.1406087
chr2	166888487	167259753	2q24.3	XIRP2	0.245153623	0.095844013	2.557839729	4	3	61.04005853	17.42685328
chr16	89575768	89597246	16q24.3	CPNE7	0.264555993	0.10343672	2.557660294	2	3	42.52992704	31.14529711
chrMT	10405	10469	N/A	MT-TR	12.85647825	5.031695049	2.555098852	1	2	0	32.64404446
chr6	83210389	83431219	6q14.2	ME1	2.89405911	1.132917114	2.554519722	4	4	67.25335992	58.21103146
chr19	22391097	22422346	19p12	ZNF98	0.475853209	0.186284324	2.554445793	4	3	143.3866896	60.97576087
chr4	110416261	110418701	4q25	ZNF969P	0.638912204	0.250184239	2.553766802	3	4	43.94161731	85.08180415
chr9	33732975	33738416	9p13.3	LINC01251	1.353129634	0.530588439	2.550243341	3	1	36.28017153	0
chrMT	12207	12265	N/A	MT-TS2	19.88035269	7.797082994	2.549716696	3	2	129.118699	17.76858632
chr11	61444149	61444423	11q12.2	RN7SL23P	2.233018924	0.876199263	2.548528648	1	2	0	21.54758931
chrX	21855987	21858727	Xp22.12	YY2	0.246926271	0.097028912	2.544873136	2	3	30.84791701	9.209042242
chr8	92712962	92786060	8q22.1	FLJ46284	0.168916252	0.066382007	2.544608978	4	3	86.0145134	55.1262718
chr3	36380343	36548007	3p22.3-p22.2	STAC	0.579631945	0.227948194	2.542823149	4	4	56.65721597	74.8898864
chr2	11393981	11403077	2p25.1	LINC00570	53.91069473	21.22191387	2.540331426	4	4	99.58461205	76.85183552
chr2	218402646	218402722	2q35	MIR26B	13.11533575	5.165870093	2.538843509	3	2	89.5265509	38.2646873
chr10	132207482	132332559	10q26.3	STK32C	2.25458822	0.8882789	2.538153524	4	4	90.10469079	38.94695722
chr16	72054592	72061056	16q22.2	HP	2.534619936	0.998727947	2.537848214	3	4	95.33454595	52.07245219
chrX	103062653	103064240	Xq22	BEX1	0.794198396	0.312983928	2.537505366	3	1	26.86166791	0
chr2	37099210	37157065	2p22.2	EIF2AK2	53.38286566	21.04270308	2.536882522	4	4	79.37620305	32.55712511
chr5	56457795	56481769	5q11.2	LINC01948	1.980973314	0.781535694	2.534718923	2	4	26.70763093	66.90424884
chr13	33103135	33676835	13q13.1-q13.2	STARD13	0.118849563	0.046934372	2.532250019	2	4	40.55494914	66.29774988
chr17	81035131	81117432	17q25.3	BAIAP2	1.17644593	0.464771589	2.531234607	4	4	99.52661636	63.86757891
chr15	82041778	82046143	15q25.2	MEX3B	0.30298476	0.119710714	2.530974459	3	4	88.69103637	36.53236158
chr12	122714756	122716892	12q24.31	HCAR3	71.44047964	28.2380877	2.529933344	4	4	81.99937258	81.2786594
chr10	75401528	75408982	10q22.2	ZNF503-AS2	0.198664651	0.078552127	2.529080488	1	3	0	7.16641797
chr17	18108706	18180244	17p11.2	MYO15A	0.051237754	0.020262969	2.528640021	1	2	0	34.90449299
chr3	53884976	53892015	3p21.1	SELENOK	166.1340579	65.70302857	2.528560121	4	4	77.29195673	46.2631513
chr17	75721328	75757819	17q25.1	ITGB4	0.648104373	0.256331251	2.528386099	3	4	89.44355483	69.85741581
chr22	20965130	20981360	22q11.21	AIFM3	0.425772837	0.168411441	2.528170496	4	4	85.14660404	21.00196249
chr1	117953595	118185269	1p12	SPAG17	0.067452714	0.026687796	2.527474135	3	1	96.77076031	0
chr7	75324481	75327776	7q11.23	PMS2P10	1.203243125	0.476284838	2.52630995	1	2	0	12.68809868
chr17	30600796	30615980	17q11.2	SMURF2P1	1.533224206	0.607955058	2.521936754	2	3	48.92104996	51.14820592
chr12	8180209	8201000	12p13.31	FAM66C	0.436310579	0.173105288	2.520492498	4	4	57.45832261	31.21418217
chr5	70968483	71025114	5q13.2	NAIP	7.544783569	2.993944563	2.520014453	4	4	35.98007639	70.73946365
chr20	62817062	62841159	20q13.33	COL9A3	0.736469576	0.292276315	2.519771666	4	3	79.92552645	46.49425821
chr17	19190029	19190245	17p11.2	SNORD3C	12.27827973	4.875204864	2.518515646	4	4	103.3757754	93.44073706
chr3	46201709	46208341	3p21.31	CCR1	183.4158879	72.84669279	2.51783411	4	4	71.66474693	52.10051987
chr5	127511262	127512862	5q23.2	HNRNPKP1	0.423785279	0.168329234	2.517597627	2	1	56.02406955	0
chr11	196761	200258	11p15.5	ODF3	0.219755017	0.087350687	2.515778905	2	2	22.35732174	8.187888154

chr3	172237347	172237597	3q26.31	RN7SL141P	6.628698723	2.636151513	2.51453632	4	4	63.83106654	48.5565782
chr1	159283888	159308224	1q23.2	FCER1A	80.17564061	31.88943674	2.514175501	4	4	63.88944345	54.94748841
chr16	28499362	28526730	16p12.1-p11.2	IL27	1.865187228	0.742136798	2.513266061	3	3	61.81781803	68.3712953
chr8	24950955	24956869	8p21.2	NEFL	0.760077871	0.302577058	2.512014216	3	4	50.42571062	46.25038402
chr21	41304229	41357727	21q22.3	FAM3B	1.046494155	0.416704909	2.511355475	2	3	94.41650517	106.016379
chr8	133046481	133046581	8q24.22	MIR7848	24.84126464	9.896422213	2.510125791	4	4	59.62923018	40.52580404
chr1	6206004	6221299	1p36.31	RNF207	0.194097639	0.077362935	2.50892289	2	3	76.83304261	43.82032656
chr6	31530219	31542475	6p21.33	DDX39B	0.088295162	0.03519899	2.508457249	4	2	61.86759435	1.653263249
chr9	62801465	62813486	9q13	LINC01410	6.455780751	2.57368556	2.508379754	4	4	102.2650085	103.5401331
chr14	24311499	24318036	14q12	LTB4R	20.8704197	8.32105104	2.50814706	4	4	49.61278647	34.67504716
chr19	54832676	54848569	19q13.42	KIR2DS4	0.579531293	0.23109923	2.507716248	3	2	83.56198281	32.32206861
chr19	19561707	19618916	19p13.11	PBX4	1.762984803	0.70338098	2.506443666	4	4	88.91306346	8.33958026
chr17	44004546	44009068	17q21.31	NAGS	0.658973274	0.262979865	2.505793645	2	3	34.28415038	33.58549613
chr2	60359216	60391375	2p16.1	MIR4432HG	0.625500313	0.24996525	2.502349078	4	2	60.7571968	54.49690861
chr22	50523568	50526439	22q13.33	SCO2	1.566055491	0.625924856	2.501986421	2	4	57.80705279	105.5571285
chr19	54273821	54281178	19q13.42	LILRB2	23.4536833	9.383661201	2.499417103	4	4	79.97138235	49.35176913
chr1	20184242	20186486	1p36.12	UBXN10-AS1	1.733926509	0.693784428	2.4992295	2	2	111.5617905	35.02526847
chr4	78551588	78610451	4q21.21	ANXA3	33.42141558	13.37386023	2.499010383	4	4	91.88046035	94.28139954
chr19	8848840	9010390	19p13.2	MUC16	0.01733133	0.006943349	2.496105075	1	2	0	22.36257968
chr6	31809619	31815058	6p21.33	HSPA1L	0.252131613	0.101025118	2.495731941	1	1	0	0
chr13	102880099	102896033	13q33.1	METTL21EP	2.552195738	1.023852317	2.492738158	4	4	54.13850963	36.67923913
chr1	155018513	155033781	1q21.3	DCST2	0.216068798	0.086691015	2.492401303	1	1	0	0
chr17	7173431	7179564	17p13.1	ASGR1	1.693379044	0.67955331	2.491900222	4	4	106.4379129	68.64315503
chr14	105939754	105940201	14q32.33	IGHV6-1	3.471159377	1.394307672	2.489521822	4	4	32.59268269	66.63267021
chr16	47150089	47150580	16q12.1	RPL23AP72	2.758678111	1.108279117	2.489154645	1	3	0	49.80646801
chr6	129831244	129861514	6q22.33	TMEM244	0.954004133	0.383267616	2.48913316	3	1	12.8797641	0
chr12	57519163	57519259	12q13.3	MIR616	15.67241082	6.300480643	2.48749448	3	3	46.84567332	82.44628966
chr2	111898479	112039946	2q13	MERTK	1.939190105	0.779908116	2.486434063	4	4	89.72905283	38.74334915
chr1	85318485	85578363	1p22.3	DDAH1	0.526083656	0.211657146	2.485546399	3	3	70.26457326	83.38923723
chr10	89414568	89421002	10q23.31	IFIT5	64.26989552	25.86618957	2.484706738	4	4	72.99384988	51.30825036
chr16	28591943	28597086	16p11.2	SULT1A2	0.959500359	0.386615022	2.481797922	3	4	97.7672477	122.8180242
chr17	74609883	74623958	17q25.1	CD300E	58.4563396	23.57820947	2.479252704	4	4	73.22990189	24.40979957
chr1	1203508	1207901	1p36.33	TNFRSF18	1.369740909	0.552556637	2.478914953	3	4	40.0453144	47.50685531
chr10	23694727	24547848	10p12.2-p12.1	KIAA1217	0.052907279	0.021350032	2.478088916	2	1	71.21771958	0
chr2	162267079	162318708	2q24.2	IFIH1	47.2410095	19.06712399	2.477615896	4	4	78.12488077	48.10058666
chr2	205682500	205798133	2q33.3	NRP2	0.174565219	0.070462978	2.477403355	4	3	114.449619	67.020466
chr19	13836440	13836517	19p13.12	MIR27A	11.86771602	4.793394163	2.47584814	3	3	35.0965903	53.40189031
chr14	104865313	104896770	14q32.33	CEP170B	0.119088843	0.048126592	2.474491485	3	3	56.22087774	41.55255786
chr15	68190705	68206110	15q23	CALML4	0.408580001	0.16513114	2.474275909	4	4	67.29630733	52.83773248
chr15	96325928	96340263	15q26.2	NR2F2	0.09928972	0.040132378	2.474055274	1	1	0	0
chr4	94298556	94342876	4q22.3	HPGDS	0.711909229	0.287946676	2.472364809	2	3	18.19336932	54.47978576
chr5	74369376	74536976	5q13.3	LINC01331	1.145982624	0.463663209	2.471584121	1	1	0	0
chr21	46098071	46132849	21q22.3	COL6A2	6.949496827	2.811917786	2.471443817	4	4	60.90761325	50.87876603

chr15	58434901	58498735	15q21.3	LIPC-AS1	0.758850137	0.307239854	2.469894859	2	1	93.38091561	0
chr14	23346304	23354991	14q11.2	SLC22A17	0.396614004	0.160622953	2.469223701	2	4	56.13189309	92.32768745
chr6	52671086	52686588	6p12.2	TMEM14A	6.523617683	2.642922953	2.468334416	4	4	42.65063185	33.73820099
chr12	104265269	104265892	12q23.3	RPL18AP3	2.80288299	1.135579646	2.468239898	2	4	38.10909294	76.8485241
chr7	27096094	27100258	7p15.2	HOTAIRM1	8.592205647	3.481428376	2.468011609	4	4	103.147171	40.51528465
chr21	46324122	46445769	21q22.3	PCNT	212.8667763	86.26301121	2.467648339	4	4	58.86056589	32.13034302
chr14	22493925	22493990	14q11.2	TRAJ45	19.86043503	8.048611516	2.467560397	2	4	109.6031148	34.59325422
chr12	94548221	94550555	12q22	SUCLG2P2	1.0995207	0.445655275	2.467200013	3	4	75.85948199	82.52455791
chr7	150824875	150861291	7q36.1	AOC1	1.384278033	0.56142577	2.465647476	4	4	58.9717995	136.7886079
chr5	17353713	17354631	5p15.1	FTH1P10	0.725991218	0.29445651	2.465529519	4	4	53.58918018	10.74706566
chr1	38475196	38476484	1p34.3	LINC01685	0.607102384	0.246281003	2.465080035	3	2	55.93203151	33.79400823
chr19	54662524	54670359	19q13.42	LILRB4	2.503593974	1.016555342	2.462821129	4	4	96.84574822	52.62221824
chr3	53812335	53846464	3p21.1	CHDH	0.082795194	0.033634923	2.461584127	2	1	14.318363	0
chr21	31118418	31559977	21q22.11	TIAM1	14.47293076	5.882800339	2.460211111	4	4	63.95894376	21.51044995
chr1	95092517	95197607	1p21.3	TMEM56	52.11416812	21.18830739	2.459572025	4	4	84.76261909	63.30437607
chr2	101272936	101273049	2q11.2	SNORD89	157.8597094	64.19213769	2.459175144	4	4	83.66650893	76.63572873
chr20	21570024	21615722	20p11.22	LINC01727	0.273553113	0.111252629	2.458846277	3	1	22.59955194	0
chr14	24318349	24335071	14q12	ADCY4	0.652552982	0.265511287	2.457722196	4	4	73.7490064	33.23922058
chr3	96349446	96351138	3q11.2	HNRNPKP4	0.650569801	0.264713325	2.457639035	3	3	108.0415092	40.30037047
chr15	58703915	58704154	15q21.3	RN7SKP95	2.227100102	0.906269708	2.457436328	2	1	20.87173373	0
chr4	68053198	68129869	4q13.2	TMPRSS11F	0.359504174	0.146294262	2.457404471	3	1	70.18376934	0
chr11	118133377	118152915	11q23.3	SCN4B	0.101313036	0.041248707	2.456150606	3	1	84.83657147	0
chr14	52314298	52328606	14q22.1	PTGER2	24.58948099	10.01723432	2.454717559	4	4	26.11756601	44.42394265
chr11	123058077	123058156	11q24.1	SNORD14E	17.82332329	7.261790699	2.454397823	4	3	51.15875222	81.76731134
chr16	46702282	46763246	16q11.2	MYLK3	0.067171569	0.027371291	2.454088483	3	1	51.78624141	0
chr9	134164440	134164564	9q34.2	RNU6ATAC	101.2597121	41.27063594	2.453553471	4	4	110.2521188	87.9198245
chr12	132031224	132031360	12q24.33	SNORA49	152.0171731	62.01157795	2.451432105	4	4	110.6825526	85.26127671
chr12	57549992	57586633	12q13.3	KIF5A	0.132081296	0.053892844	2.450813268	4	4	9.614064436	44.36042226
chr10	38383023	38402927	10p11.1	SEPT7P9	0.683411907	0.278935857	2.450068325	2	3	24.26509155	32.44863973
chrX	68647669	68725842	Xq13.1	STARD8	2.808949852	1.146803867	2.449372497	4	4	51.5410205	32.12984336
chr17	20588849	20590815	17p11.2	MEIS3P2	1.210922294	0.494454963	2.449004228	3	4	61.60489415	65.41667445
chr13	98142562	98449773	13q32.2	FARP1	0.019692373	0.008041283	2.448909387	3	1	36.76683394	0
chr15	64739891	64775587	15q22.31	RBPMS2	2.711601192	1.107491042	2.44841817	4	4	70.65286195	47.14982706
chr12	82686952	83134868	12q21.31	TMTC2	2.579492575	1.053797253	2.447807269	4	4	45.86600612	29.89368332
chrX	90112446	90114729	Xq21.31	USP12PX	0.568055375	0.232143391	2.447002142	2	1	73.70407534	0
chr15	81099408	81149175	15q25.1	CFAP161	10.95813836	4.484637094	2.443483861	4	4	65.33082645	28.95370895
chrX	49303646	49319844	Xp11.23	GAGE10	1.205979821	0.493762708	2.442427913	1	1	0	0
chr3	121655475	121655604	3q13.33	RNU4-62P	5.624315909	2.302852711	2.442325505	1	2	0	23.83945038
chr1	155919909	155920042	1q22	SNORA80E	5.211548195	2.134816027	2.44121654	3	3	114.4751003	28.37727146
chr10	101061085	101068141	10q24.31	KAZALD1	0.191775869	0.078627037	2.439057557	2	1	82.24460718	0
chr22	36286847	36286907	22q12.3	MIR6819	13.33305156	5.466750738	2.438935338	2	1	77.44488553	0
chr15	24908864	24909900	15q11.2	RPL5P1	0.628890421	0.257939518	2.438131337	3	1	75.49779056	0
chr11	126067392	126068601	11q24.2	NAP1L1P1	0.631907151	0.259201652	2.437897849	2	2	22.09955469	21.75585749

chr20	6074845	6123609	20p12.3	FERMT1	0.115948467	0.047582298	2.436798383	1	1	0	0
chr9	134379411	134379472	9q34.2	MIR4669	11.37118689	4.668623654	2.435661499	3	1	23.28910415	0
chrMT	4402	4469	N/A	MT-TM	129.106012	53.02251635	2.434928043	4	4	41.12514033	42.37943809
chr3	102385144	102479841	3q12.3	ZPLD1	0.131259847	0.053909616	2.434813248	1	1	0	0
chr12	589533	589588	12p13.33	RNU7-103P	15.84776228	6.510040494	2.434356943	2	3	80.28374163	43.42246189
chr11	11351942	11353357	11p15.4	CSNK2A3	1.09046536	0.447958269	2.434301217	3	3	110.9725699	68.82507478
chr3	126607052	126655124	3q21.3	TXNRD3	1.135863359	0.466871776	2.432923595	2	4	95.139508	51.92835068
chr7	128866348	128872047	7q32.1	ATP6V1FNB	0.251843424	0.103558763	2.431889061	3	3	29.57040962	62.68887438
chr21	29193494	29288205	21q21.3	LINC00189	4.039760535	1.661371581	2.43158158	4	4	25.19107796	87.47401991
chr6	33162692	33193009	6p21.32	COL11A2	0.091425696	0.037628061	2.429721179	3	2	19.45752248	2.758745312
chrX	48960983	48972103	Xp11.23	KCND1	0.277220537	0.114117096	2.429263854	3	3	69.35959504	69.85405933
chr17	4807101	4823432	17p13.2	PLD2	2.514144442	1.034943607	2.429257426	4	4	73.76490087	42.75390081
chr19	33927364	33927625	19q13.11	RN7SL150P	2.059591345	0.847831463	2.429246182	1	1	0	0
chr11	74652562	74653111	11q13.4	RANP3	2.252079257	0.927245407	2.428784484	4	1	63.15574215	0
chr1	161541759	161550737	1q23.3	FCGR3A	181.2329099	74.63250404	2.428337522	4	4	55.93270594	32.50697777
chr16	25066887	25068955	16p12.1	LINC02175	0.921809727	0.379695394	2.427761154	3	4	82.60278213	47.85002755
chr10	102644495	102658319	10q24.32	TRIM8	53.76584121	22.15075238	2.427269299	4	4	79.40432426	43.40528348
chr8	6499651	6563420	8p23.1	ANGPT2	0.135126015	0.055692226	2.426299398	1	2	0	51.09354694
chr19	49948825	49961172	19q13.33	SIGLEC11	0.197549776	0.081436515	2.425813243	4	2	62.94359592	1.205760686
chr5	150053291	150113372	5q32	CSF1R	38.51661326	15.88070785	2.425371313	4	4	65.12280978	36.73227473
chr2	89142574	89143108	2p11.2	IGKV3-20	35.39912755	14.60028513	2.424550427	4	4	48.25529994	104.0202353
chr19	49157741	49211841	19q13.33	TRPM4	0.219510188	0.090554149	2.424076545	4	3	95.76911761	52.60403474
chr5	17216823	17276845	5p15.1	BASP1	191.0022432	78.80458623	2.423745271	4	4	88.46354712	71.97264941
chr20	63627235	63628824	20q13.33	MHENCR	5.680517762	2.343797798	2.423638151	3	4	56.88156799	92.27651354
chr12	10030677	10066030	12p13.2	CLEC9A	1.339379048	0.552935695	2.42230527	4	3	109.4868137	57.72231375
chr22	28800657	29057488	22q12.1	ZNRF3	0.370792662	0.153183057	2.420585347	4	4	64.4342621	35.98508876
chr17	59151136	59151221	17q22	MIR301A	7.588472687	3.136751402	2.41921393	4	2	63.80952689	8.285623042
chr16	85659378	85659675	16q24.1	RN7SL381P	3.722759686	1.540336485	2.416848346	3	4	31.44863511	19.95004054
chr20	646615	653370	20p13	SRXN1	0.736436385	0.304717087	2.416787296	3	4	90.14717627	68.7120966
chr9	95442980	95517057	9q22.32	PTCH1	1.327348391	0.549563599	2.415277127	4	4	47.92747507	50.78878452
chr12	8056844	8066471	12p13.31	C3AR1	37.39938514	15.48582379	2.415072369	4	4	51.73523887	26.66366778
chr19	17323223	17334834	19p13.11	ANO8	1.139962941	0.472101821	2.414654825	4	4	67.75399855	40.08140681
chr22	29312933	29319618	22q12.2	RASL10A	0.27524233	0.114020971	2.413962348	2	1	9.799723273	0
chr1	84811601	84893213	1p22.3	LPAR3	0.479830839	0.19894852	2.411834172	4	4	68.48930751	104.4542527
chr11	66510648	66533613	11q13.2	BBS1	0.171593764	0.071152251	2.411642088	3	4	46.48098129	51.53059002
chr12	116559381	116576597	12q24.22	MAP1LC3B2	0.804179363	0.333457854	2.411637194	4	3	59.63059658	45.21432438
chr12	66794	178460	12p13.33	IQSEC3	0.268676383	0.111410971	2.411579223	1	3	0	70.91355414
chr17	42533120	42534226	17q21.2	PTP4A2P1	1.243783742	0.51584631	2.411151769	2	2	45.64710412	10.07103345
chr8	56917084	56917189	8q12.1	RNU6-13P	6.351989745	2.635910235	2.409789856	3	1	74.06898227	0
chr1	158174353	158178075	1q23.1	ELL2P1	0.797548023	0.331000964	2.409503628	3	3	53.04767796	26.71175217
chr21	32574841	32585545	21q22.11	TCP10L	0.212821577	0.08840429	2.407367073	2	1	73.75497784	0
chr20	38581195	38588463	20q11.23	ADIG	0.622051242	0.258493122	2.406451817	1	1	0	0
chr13	113820549	113864103	13q34	GAS6	0.571535148	0.237588589	2.405566487	3	4	83.55341663	28.61131071

chr9	68543541	68546602	9q21.11	LINC01506	53.42128144	22.22280125	2.403895029	4	4	55.72096266	94.49075617
chr14	92322581	92501481	14q32.12	SLC24A4	3.659861105	1.522621228	2.403658268	4	4	84.23567753	44.81577646
chr6	138422043	138692616	6q24.1	NHSL1	0.418725616	0.174429578	2.400542504	4	4	81.23665653	47.02405409
chr7	128761338	128761598	7q32.1	RN7SL81P	3.955454081	1.649633066	2.397778126	2	1	96.79258951	0
chr9	136363956	136373681	9q34.3	CARD9	1.921066916	0.801338775	2.397321802	4	4	89.215368	52.826998
chr1	21585690	21591187	1p36.12	LINC02596	3.321972309	1.385712342	2.397302967	2	4	38.98539513	66.10684509
chr4	151409143	151670503	4q31.3	FAM160A1	1.142820097	0.476760265	2.397053993	4	4	74.20141577	70.07800123
chr12	48654382	48654518	12q13.11	SNORA2C	5.213959724	2.175389163	2.396794014	1	2	0	39.09438639
chr5	83471674	83582303	5q14.2-q14.3	VCAN	158.3271485	66.16184801	2.393027904	4	4	81.95249605	32.0184724
chr22	22409817	22410282	22q11.22	IGLV1-40	10.75326895	4.494170376	2.392715018	4	4	66.50448268	114.8584225
chr12	6904733	6914242	12p13.31	LRRC23	0.943169961	0.39429196	2.392059838	4	4	103.0052109	49.6566201
chr19	10289351	10296778	19p13.2	ICAM5	0.391363253	0.163663302	2.391270671	3	2	95.08328128	35.5223499
chr11	77128192	77215241	11q13.5	MYO7A	0.499590411	0.208930016	2.391185434	4	4	76.03467073	50.50750068
chr21	14485228	14583402	21q11.2	SAMSN1	100.6704592	42.10356196	2.391020011	4	4	42.22081509	24.26850312
chr11	4449295	4450361	11p15.4	OR52K2	0.902496778	0.37746713	2.390928125	4	4	74.25953068	67.22192222
chr19	49739753	49763328	19q13.33	TSKS	0.242945583	0.10169965	2.388853687	4	1	63.60579173	0
chr4	80321265	80963756	4q21.21	CFAP299	0.135608388	0.056795518	2.387660011	2	1	7.06030542	0
chr18	14748240	14855465	18p11.21	ANKRD30B	0.102857947	0.04310492	2.3862229	1	1	0	0
chr21	39380244	39428528	21q22.2	WRB	14.72209415	6.173151844	2.384858581	4	4	76.73015213	53.92510696
chr5	136975298	137499329	5q31.2	SPOCK1	0.217845282	0.091373449	2.384120156	3	4	87.12631244	43.07663448
chr5	36606355	36688334	5p13.2	SLC1A3	0.291714237	0.122539269	2.380577588	3	3	91.77714815	34.17761798
chr13	108268240	108308484	13q33.3	TNFSF13B	103.0061863	43.27476101	2.380283194	4	4	63.02916273	50.88547585
chr5	112161485	112161617	5q22.1	SNORA13	17.69657998	7.437371269	2.379413282	2	4	13.26806782	71.37806349
chr13	21038529	21038667	13q12.11	RNU4-9P	5.095844587	2.143582649	2.377255941	2	2	4.039807675	39.58901638
chr6	166460663	166465383	6q27	RPS6KA2-IT1	1.058092649	0.445159753	2.376883	1	1	0	0
chr1	156061149	156070514	1q22	RAB25	0.385759834	0.162385524	2.375580182	1	2	0	19.60213269
chr2	38067603	38076181	2p22.2	CYP1B1	15.20736308	6.404722145	2.374398567	4	4	108.3470762	66.25997755
chr4	168216291	168325981	4q32.3	DDX60	56.945381	23.98411193	2.374295999	4	4	57.42861818	39.9106016
chr11	46762389	46762499	11p11.2	SNORD67	12.66486422	5.335471499	2.373710406	4	2	76.58962125	2.742510793
chr2	88966262	88966738	2p11.2	IGKV1-6	3.208688922	1.353837173	2.37007004	3	3	41.10857151	30.91033332
chr1	117776523	117778909	1p12	PNRC2P1	2.339947398	0.987307548	2.370028876	3	3	122.8315733	69.22132475
chr22	36950530	36953724	22q12.3	CSF2RBP1	11.36332477	4.794757111	2.369947946	2	2	51.04178191	10.41137901
chr21	44849585	44873903	21q22.3	PTTG1IP	117.0539585	49.41322426	2.368879187	4	4	37.5350053	20.92345679
chr12	108129343	108250537	12q23.3	WSCD2	0.09235214	0.039000223	2.367990046	1	2	0	2.086296598
chr20	38449369	38449504	20q11.23	SNORA60	17.20290329	7.266596153	2.367394985	3	4	72.21146864	78.29738625
chr5	150094285	150095114	5q32	RPL7P1	1.816019937	0.76720373	2.367063488	3	2	122.149474	63.90001024
chr2	27086743	27100751	2p23.3	KHK	0.613712584	0.259313767	2.366679533	4	4	74.22647362	63.52977251
chr11	27654893	27722058	11p14.1	BDNF	0.075188548	0.031801767	2.364288357	4	1	31.4179532	0
chr10	49154724	49188580	10q11.23	TMEM273	8.211249743	3.473505907	2.363965965	4	4	56.18715068	33.20159539
chr6	41189749	41201233	6p21.1	TREML2	39.16849133	16.57255559	2.363455119	4	4	71.45812254	48.83359117
chrX	1190437	1212762	Xp22.33 and Yp11	CRLF2	0.529172337	0.224046563	2.361885536	2	3	29.23182994	58.43951386
chr16	21349363	21385933	16p12.2	SNX29P1	1.076388365	0.455994307	2.360530271	4	3	55.50053655	41.59494437
chr2	201644874	201700263	2q33.1	MPP4	0.123135446	0.052182297	2.359716834	2	1	61.15318271	0

chr7	142433956	142434394	7q34	TRBV11-2	6.218461337	2.635309778	2.359669967	4	4	116.1608283	71.81601771
chrX	2751104	2816500	Xp22.33	XG	2.83602385	1.201941794	2.3595351	3	4	72.7784539	9.958434828
chr11	126355645	126414641	11q24.2	ST3GAL4	8.02501665	3.404152884	2.357419577	4	4	77.72820522	40.49292745
chr9	133459965	133470787	9q34.2	CACFD1	0.246955748	0.104778453	2.356932551	3	2	107.4627319	46.06383006
chr11	108308519	108469832	11q22.3	C11orf65	3.897516625	1.653892533	2.356571874	4	4	79.13383063	59.38792091
chr3	149369018	149377781	3q25.1	TM4SF1	0.531414639	0.225583314	2.35573558	3	4	97.98074863	102.6882505
chr19	14732697	14778541	19p13.12	ADGRE2	27.46622963	11.66019813	2.355554281	4	4	82.06024312	41.3613482
chr1	186671812	186680427	1q31.1	PTGS2	249.2495112	105.8137805	2.355548682	4	4	42.72701298	94.51376194
chr21	42472486	42496354	21q22.3	RSPH1	0.440024404	0.186812102	2.355438428	2	3	110.1947346	11.20638694
chr12	121018882	121039242	12q24.31	OASL	25.83565138	10.96864221	2.355410168	4	4	84.8256237	55.81306915
chr10	52296848	52314128	10q21.1	PRKG1-AS1	0.227521687	0.09664129	2.35429068	2	3	74.72666848	11.27357487
chr17	27293910	27293996	17q11.1	MIR4522	7.295431588	3.099555041	2.353702868	2	2	45.74079169	8.167589954
chr1	100281241	100281308	1p21.2	MIR553	7.081520001	3.009243467	2.353255919	2	1	33.98045317	0
chr2	189762780	189765556	2q32.2	OSGEPL1-AS1	1.057939915	0.44962507	2.352938003	2	3	33.70884371	61.27277104
chr22	30262828	30266843	22q12.2	OSM	39.56642409	16.82785197	2.351246265	4	4	63.00038541	76.29684527
chr9	107484852	107489720	9q31.2	KLF4	19.86658246	8.457882051	2.348883838	4	4	54.76154302	65.46310924
chr16	89711856	89718165	16q24.3	VPS9D1-AS1	0.349310011	0.148720602	2.348766796	2	2	12.08560753	2.820890487
chr3	151326308	151329549	3q25.1	P2RY13	207.1196148	88.21433476	2.347913356	4	4	66.73750457	53.3082085
chr1	158808402	158809335	1q23.1	OR10AA1P	1.273416788	0.542636466	2.346721735	2	4	60.14424847	66.03575309
chr20	1226040	1255876	20p13	RAD21L1	0.211843899	0.090279762	2.346527	2	3	50.53509405	3.600016744
chr2	132415972	132646596	2q21.2	GPR39	0.196377081	0.083693832	2.346374602	1	1	0	0
chr1	36095236	36126207	1p34.3	COL8A2	0.642551531	0.273854871	2.346321351	4	3	68.01083478	31.55667537
chr6	43770209	43786487	6p21.1	VEGFA	1.698767135	0.72438444	2.3451182	4	4	69.34962047	47.84132373
chr13	44575896	44580432	13q14.11	TSC22D1-AS1	0.520225118	0.222121592	2.342073604	2	4	42.62221952	60.9845677
chr2	74502593	74505065	2p13.1	LBX2-AS1	0.825360418	0.352411162	2.342038239	3	4	91.13985867	83.34520095
chr17	74521174	74531474	17q25.1	CD300LB	27.7133582	11.83337627	2.341965434	4	4	72.21798512	39.96002374
chr17	57618108	57618393	17q22	RN7SL449P	2.066270269	0.882450234	2.341514783	1	1	0	0
chr2	108507527	108534219	2q12.3	GCC2-AS1	1.083646905	0.462847963	2.341258881	1	2	0	54.22265784
chr1	965820	975108	1p36.33	PLEKHN1	0.319220686	0.136346959	2.341238038	3	2	69.80161285	44.50818732
chr21	37065364	37073158	21q22.13	PIGP	2.65085366	1.132450191	2.340812585	4	4	41.89208535	18.79528759
chr17	4733529	4739928	17p13.2	CXCL16	27.3601923	11.69013171	2.340452015	4	4	64.58296617	42.5543035
chr3	9757345	9769987	3p25.3	CAMK1	10.91438944	4.665699127	2.33928274	4	4	79.82157946	62.42590755
chr4	121875980	121952060	4q27	TRPC3	0.138156484	0.059080679	2.338437634	4	1	67.62605473	0
chr9	127612207	127692716	9q34.11	STXBP1	0.87357441	0.373610775	2.338193834	4	4	54.04774116	60.61681918
chr14	20955471	20956435	14q11.2	RNASE2	61.92213249	26.49768941	2.336888003	4	4	33.48088296	73.79339763
chr21	43773665	43776375	21q22.3	CSTB	21.39819549	9.157273654	2.336743041	4	4	59.40012245	63.51786462
chr1	228406877	228416882	1q42.13	TRIM17	0.242179244	0.103652123	2.336461981	1	2	0	41.36732282
chr12	40140926	40159940	12q12	LINC02555	4.621179481	1.978296647	2.33593859	4	4	143.7778121	79.44279285
chr14	61570540	61654713	14q23.1-q23.2	FLJ22447	0.899825818	0.385226498	2.335835732	3	4	22.81182161	53.63591373
chr19	44537058	44556917	19q13.31	CEACAM22P	6.544612752	2.801927303	2.335753945	1	1	0	0
chr3	122055362	122121143	3q13.33	CD86	39.08302263	16.73716833	2.335103637	4	4	62.93468514	20.66211554
chr4	176683534	176792745	4q34.3	VEGFC	1.172365512	0.502193302	2.334490536	4	4	45.97967842	7.694677563
chr4	74036589	74038773	4q13.3	CXCL3	0.477317621	0.204477113	2.334332751	4	4	65.12873821	52.48092897

chr4	84886386	84886486	4q21.23	RNU6-469P	13.31951119	5.706068234	2.334271279	2	1	92.81151454	0
chr15	89784895	89814852	15q26.1	ANPEP	73.16950354	31.35092054	2.333886925	4	4	82.63824762	34.20894312
chrX	103356452	103358469	Xq22.2	TCEAL9	4.510046953	1.932762079	2.333472393	4	4	58.77424557	69.87550305
chr14	24138959	24142602	14q12	EMC9	2.031525462	0.870959777	2.332513528	3	4	71.87351755	100.6276623
chrX	55143102	55161195	Xp11.21	FAM104B	16.8971671	7.245012364	2.332248207	4	4	78.12492741	40.78728926
chr16	21664133	21762625	16p12.2	OTOA	0.080346115	0.034459162	2.331632862	1	1	0	0
chr6	2341357	2342500	6p25.2	HMGN2P28	2.235958689	0.959045888	2.331440776	4	2	21.6407105	2.230271919
chr11	47437757	47449178	11p11.2	RAPSN	0.698109841	0.299434323	2.331428924	2	3	73.4989743	47.04276974
chr2	65307425	65432522	2p14	SPRED2	1.505948061	0.645944386	2.331389657	4	4	42.29142617	36.26237274
chr21	33324443	33360361	21q22.11	IFNAR1	93.92685896	40.28835585	2.331364906	4	4	28.24098309	53.52211923
chr16	47081520	47144025	16q12.1	NETO2	2.037695732	0.874608599	2.329837294	4	4	111.5354875	46.86879727
chr17	1761925	1777574	17p13.3	SERPINF1	2.780296409	1.194192777	2.32818056	4	4	93.21694498	73.65240255
chr7	128241201	128257629	7q32.1	LEP	0.325879706	0.139989796	2.327881857	2	2	104.7000799	65.06019526
chr1	198858873	198858982	1q32.1	MIR181B1	7.595337936	3.263074537	2.327663022	1	3	0	19.08178884
chr11	69294138	69297287	11q13.3	MYEOV	1.968158819	0.845696877	2.327262726	4	4	36.4468226	29.29119329
chr22	18527802	18531920	22q11.21	TMEM191B	0.297415741	0.127878373	2.325770441	3	1	41.77639801	0
chr14	99684262	99727321	14q32.2	CYP46A1	0.063655687	0.027371291	2.325637016	2	1	82.45393528	0
chr2	8670984	8681863	2p25.1	ID2-AS1	0.285464907	0.122750658	2.325567226	4	2	49.23288037	6.526573171
chr1	247619681	247620559	1q44	OR14L1P	0.69851355	0.300403879	2.325248099	3	3	72.64955805	12.14802913
chrX	70962962	70963342	Xq13.1	RPS23P8	4.253153173	1.829391987	2.324899859	3	4	62.4723138	65.51547996
chr22	29608861	29609694	22q12.2	RPEP4	0.887038922	0.381666228	2.324122118	4	1	33.11948984	0
chr1	70411218	70441949	1p31.1	CTH	2.976096285	1.280663938	2.323869827	4	4	44.13266808	85.39819605
chr1	183929662	184051612	1q25.3	COLGALT2	0.569186769	0.245025055	2.322973746	4	4	53.88605837	91.86758724
chr7	27121919	27128760	7p15.2	HOXA-AS2	0.286116959	0.123190379	2.322559286	2	2	103.4047877	13.63242873
chr7	27162438	27165530	7p15.2	HOXA9	0.339011681	0.14601964	2.321685504	3	3	26.38921121	70.47639093
chr1	43172330	43254358	1p34.2	CFAP57	0.101916672	0.043915003	2.32077113	2	1	60.08901689	0
chr3	94075711	94077237	3q11.2	RBBP4P2	0.446049415	0.192209601	2.320640654	1	2	0	6.041929463
chr2	101151660	101155412	2q11.2	TBC1D8-AS1	0.323141917	0.139330855	2.319241612	2	2	11.11219763	33.35467024
chr7	55887277	55942225	7p11.2	ZNF713	0.915140464	0.39520904	2.315585857	4	4	64.56299414	43.66016084
chr7	6754109	6798775	7p22.1	RSPH10B2	0.239833264	0.103629165	2.314341361	3	3	87.170871	83.38278303
chr22	37570246	37580017	22q13.1	LGALS2	29.81818856	12.88877813	2.313500026	4	4	70.15823317	22.55014699
chr4	57031071	57110385	4q12	IGFBP7	6.991901635	3.023930737	2.312189743	4	4	58.59641664	54.76755845
chr8	142771197	142777848	8q24.3	LYNX1	0.618524278	0.267583715	2.311516893	4	4	54.03816004	18.41410982
chr10	17214239	17229985	10p13	VIM-AS1	79.11455498	34.2298184	2.311275919	4	4	49.04683966	47.99155794
chr7	41693919	41779378	7p14.1	INHBA-AS1	0.148911929	0.064448883	2.310543208	2	3	89.95288867	40.37121349
chr16	3012456	3014505	16p13.3	CLDN9	0.539791483	0.233654592	2.310211318	3	2	45.49913651	63.42751429
chr7	65755526	65755659	7q11.21	SNORA22	229.2060154	99.22705788	2.309914456	4	4	39.42499817	27.24178587
chr8	90958471	90985257	8q21.3	C8orf88	19.7429818	8.552229253	2.308518775	4	4	87.27516078	78.23732746
chr6	132680858	132714055	6q23.2	VNN1	19.99106306	8.660549332	2.30829042	4	4	66.55430275	41.66217217
chr2	88947301	88947776	2p11.2	IGKV1-5	21.68005247	9.395165212	2.307575437	4	4	19.37860513	103.2835817
chr11	63506052	63516774	11q12.3	LGALS12	11.47669554	4.974265842	2.30721395	4	4	69.86062297	28.76373988
chr7	100889994	100896133	7q22.1	ACHE	1.337781645	0.580256481	2.305500566	4	4	42.73457912	68.66583997
chr8	143366623	143368635	8q24.3	RHPN1-AS1	0.288575216	0.125201158	2.304892543	2	1	42.68696032	0

chr8	71841542	71844496	8q13.3	MSC	1.152141097	0.500297415	2.302912355	4	4	56.31757098	65.00854855
chr2	65030664	65052505	2p14	LINC02576	0.954044105	0.414295158	2.302812587	1	4	0	34.18409753
chr20	57603846	57620576	20q13.31	ZBP1	13.27436114	5.767801485	2.301459433	4	4	87.0592572	48.6770627
chr21	44885949	44928873	21q22.3	ITGB2	274.3501479	119.2080355	2.301440056	4	4	53.83288334	31.14896395
chr14	70698698	70712153	14q24.2	LINC01269	0.878190672	0.381587547	2.301413344	3	1	85.82267551	0
chr10	88757226	88783662	10q23.31	LIPN	28.54249645	12.40436248	2.301004707	4	4	36.35988911	67.36909245
chr21	39313907	39314915	21q22.2	BRWD1-AS2	0.76065751	0.33072211	2.299989893	4	3	33.76464073	49.27664244
chr6	46700558	46735836	6p12.3	PLA2G7	12.53081115	5.450316275	2.299097982	4	4	107.8522412	52.87024919
chr11	128691672	128696023	11q24.3	SENCR	7.951942143	3.459852319	2.298347273	4	4	30.38879569	23.33569521
chr6	96364771	96521741	6q16.1	UFL1-AS1	2.895946382	1.260545375	2.297375755	3	3	96.44267726	90.20752078
chr15	63594794	63603432	15q22.31	FBXL22	0.163039629	0.070971309	2.297261116	3	1	49.19790189	0
chr22	24219654	24245142	22q11.23	GGT5	0.161370848	0.070286704	2.295894357	4	3	101.6227993	69.1592384
chr9	94347222	94348219	9q22.32	PTMAP12	1.782312909	0.777145493	2.293409568	1	1	0	0
chr7	38362864	38363328	7p14.1	TRGV2	7.572983629	3.302444418	2.293144917	4	4	69.39659483	42.90333471
chr22	22026076	22026553	22q11.22	IGLVI-70	2.569930523	1.121485223	2.291542029	2	1	20.12448024	0
chr4	184798296	184799046	4q35.1	SLED1	9.225152336	4.027046197	2.290798736	4	4	48.4989661	78.6124782
chr21	14582202	14598303	21q11.2	SAMSN1-AS1	0.681195776	0.297386341	2.29060882	3	2	24.46380796	11.59864425
chr3	45159698	45160214	3p21.31	RPS24P8	2.664583296	1.163282456	2.290572924	4	4	66.9967317	49.75408039
chr16	56617461	56618818	16q13	MT1L	13.70097896	5.982361931	2.29022903	4	4	61.88200967	92.78191499
chr6	151239815	151358559	6q25.1	AKAP12	4.440500287	1.93945607	2.289559612	4	4	73.42625827	77.24200708
chr17	81302824	81309248	17q25.3	LINC00482	0.517310546	0.225973477	2.289253374	2	2	98.87144983	68.19709667
chr16	50742305	50742377	16q12.1	MIR3181	10.2792006	4.491482822	2.288598445	2	2	28.43182003	32.27318928
chr13	24121024	24123814	13q12.12	IPO7P2	0.329519818	0.144072611	2.287178777	1	2	0	7.759094304
chr2	20447071	20449445	2p24.1	RHOB	87.45737264	38.25412207	2.28622088	4	4	43.27699239	58.62408387
chrX	17800049	17861346	Xp22.13	RAI2	0.213561189	0.093426687	2.285869237	3	1	47.65564813	0
chr20	34466325	34466418	20q11.22	MIR644A	6.548144827	2.865184968	2.285417835	4	2	74.37667133	8.587391529
chr15	30818036	30827783	15q13.2	HERC2P10	0.528202233	0.231155429	2.285052252	3	2	69.68071666	65.05222745
chr17	74534359	74546171	17q25.1	CD300C	15.22796635	6.664893273	2.284802731	4	4	59.46281253	40.41412422
chr15	74337762	74367740	15q24.1	CYP11A1	0.313856949	0.137386287	2.284485273	1	2	0	65.0055739
chr3	133932696	134030076	3q22.1-q22.2	SLCO2A1	0.16549107	0.072457526	2.283973518	2	2	19.6240935	39.34789385
chr3	50155045	50189075	3p21.31	SEMA3F	0.121426846	0.053169238	2.28378008	1	1	0	0
chr20	36507702	36573275	20q11.23	DLGAP4-AS1	0.571715831	0.250458652	2.282675511	3	4	41.24976458	85.14329351
chr12	112978466	113011723	12q24.13	OAS2	50.6812535	22.20878223	2.282036582	4	4	66.83244477	71.14239544
chr10	59650764	59710079	10q21.2	SLC16A9	0.918568684	0.402845356	2.280201745	4	4	108.7260961	37.19539858
chr4	30719861	31146801	4p15.1	PCDH7	0.047308936	0.0207587	2.278993209	1	1	0	0
chr4	141321124	141332617	4q31.21	LINC02432	3.736622309	1.640156106	2.278211383	4	4	72.75915029	102.1606978
chr11	5527223	5528126	11p15.4	OR52V1P	0.585023839	0.256808264	2.278056904	1	1	0	0
chr2	74135401	74147912	2p13.1	BOLA3	9.848356657	4.324429764	2.277376948	4	4	112.0372098	54.22820309
chr6	33204637	33206831	6p21.32	HSD17B8	1.769263415	0.777123504	2.276682415	3	4	81.04219897	69.21505583
chr9	4676600	4679480	9p24.1	CDC37L1-DT	6.48089003	2.84798365	2.275606473	4	4	88.92356839	14.83690561
chr2	26970371	27027219	2p23.3	MAPRE3	0.780126632	0.342962481	2.27467048	4	4	93.12019217	57.23790587
chrX	49187812	49200202	Xp11.23	SYP	0.285362427	0.125458347	2.274559107	2	3	4.472053831	60.22054316
chr5	122845449	123029318	5q23.2	SNX24	2.077665309	0.913509412	2.274377561	4	4	43.0135603	61.02996706

chr3	190624599	190625166	3q28	GCNT1P3	3.192997759	1.404011476	2.27419634	3	4	86.58237996	63.94904726
chr9	129803143	129811424	9q34.11	TOR1B	30.66279803	13.48374957	2.274055734	4	4	46.82561223	37.92019062
chr7	78017055	79453574	7q21.11	MAGI2	0.354831271	0.15606186	2.273657832	4	4	84.88551559	105.7122662
chr10	98383565	98415221	10q24.2	PYROXD2	1.783739592	0.784672014	2.273229529	4	4	43.87250076	77.20439407
chr1	1020102	1056119	1p36.33	AGRN	0.577165161	0.25394978	2.272753147	4	4	48.50047957	60.41373367
chr10	33073724	33116841	10p11.22	IATPR	2.880905694	1.26778209	2.272398165	4	4	35.23140876	60.42017666
chr18	76528655	76559827	18q23	LINC00908	0.188926272	0.083185297	2.27114981	1	2	0	2.423938598
chr11	66857087	66860475	11q13.2	LRFN4	1.034380004	0.455456811	2.271082522	3	3	73.46939703	84.73479546
chr7	31687017	31710158	7p14.3	PPP1R17	0.495204193	0.218057022	2.27098485	4	4	44.82133177	40.67609228
chr10	114174442	114232669	10q25.3	TDRD1	0.273675435	0.120587989	2.269508239	3	4	22.74313662	75.44895964
chr12	43646926	43661101	12q12	EEF1A1P17	2.724277044	1.200530362	2.269227944	1	1	0	0
chr6	32194843	32224067	6p21.32	NOTCH4	0.116944296	0.051548226	2.268638624	3	2	88.76627741	69.21242703
chr11	119185457	119190223	11q23.3	PDZD3	0.151636552	0.06686552	2.267783916	1	2	0	0.179945309
chr7	75915102	75986855	7q11.23	POR	14.77138997	6.514109433	2.267599297	4	4	88.86855292	58.82235801
chr12	98453840	98457145	12q23.1	SLC9A7P1	3.148948708	1.389221982	2.266699454	4	4	64.82421567	62.73225185
chr11	15073600	15078631	11p15.2	CALCB	0.223294134	0.098518265	2.266525231	3	4	88.0479961	64.15090127
chr11	4474810	4475756	11p15.4	OR52K3P	12.40594597	5.47526005	2.265818583	4	4	53.02261802	45.94720827
chr15	90634666	90635220	15q26.1	HSPE1P3	2.276470891	1.004815563	2.265560941	2	2	72.15232461	16.8350693
chr18	2847030	2914092	18p11.32-p11.31	EMILIN2	27.3269577	12.06441362	2.265087932	4	4	64.27152373	30.23922165
chr9	138150113	138177433	9q34.3	TUBBP5	0.970871262	0.428632922	2.265041278	4	2	94.41454683	46.19125944
chr17	42844583	42850712	17q21.31	AOC2	2.724749954	1.203305067	2.264388333	4	4	79.26874735	52.53618193
chr16	2783953	2787759	16p13.3	PRSS33	10.53162136	4.652828449	2.263487999	4	4	109.8805083	60.03495077
chr9	128149430	128153455	9q34.11	LCN2	38.82548004	17.16395414	2.262035876	4	4	51.0900628	87.08924492
chr14	55129217	55145430	14q22.3	LGALS3	187.2381566	82.77963605	2.261886685	4	4	33.76385583	26.22277277
chr2	121530880	121531009	2q14.2	RNU4ATAC	168.4037449	74.46139237	2.261624978	4	4	80.41510222	44.6282245
chrX	106802689	106876150	Xq22.3	TBC1D8B	0.273232744	0.120816083	2.261559369	4	4	69.47121804	63.41027274
chr21	25385820	25431701	21q21.2	LINC00158	0.502793947	0.222496482	2.259783813	1	2	0	58.28596506
chr5	67001618	67004154	5q12.3	MAST4-AS1	2.241470406	0.992259897	2.258954949	4	4	43.32516333	100.1047042
chr2	98793846	98936221	2q11.2	KIAA1211L	0.726645641	0.321908832	2.257302594	4	4	66.02270582	81.58654864
chr2	74518131	74526281	2p13.1	DQX1	0.278555765	0.123476739	2.255937174	1	2	0	40.16488652
chr19	57651497	57679152	19q13.43	ZSCAN4	1.373521581	0.608932499	2.255622066	3	4	126.8469995	80.14768393
chr4	144109303	144140854	4q31.21	GYPA	271.9975844	120.6432671	2.254560829	4	4	88.08403423	53.22824778
chr7	73242751	73280119	7q11.23	GTF2IRD2P1	3.869778356	1.716470229	2.2544978	4	4	62.8862057	23.52103825
chr6	150599865	150843665	6q25.1	PLEKHG1	3.40620392	1.511003257	2.254266431	4	4	64.66060399	59.46346902
chr15	79411557	79472304	15q25.1	MINAR1	0.540287548	0.239802477	2.253052404	4	4	67.94304463	52.01435967
chr12	8455995	8478330	12p13.31	CLEC6A	3.880704931	1.722551695	2.252881549	4	4	19.19863514	65.48657343
chr7	149776042	149833965	7q36.1	SSPO	0.091441707	0.040589474	2.25284287	3	4	81.47938166	71.48102543
chr10	121928312	121951965	10q26.13	ATE1-AS1	0.898282131	0.398806872	2.252423904	3	4	69.31942663	54.52987519
chr14	106037902	106038345	14q32.33	IGHV2-5	10.43184708	4.631397346	2.252418935	4	3	41.20309593	133.0160569
chrX	70511227	70908717	Xq13.1	TEX11	0.40385431	0.179349993	2.25176652	2	2	38.78831978	80.59691817
chr3	50362613	50504244	3p21.31	CACNA2D2	1.498122102	0.665523256	2.251043955	4	4	46.6403738	73.33566775
chr21	33402896	33437521	21q22.11	IFNGR2	96.42595461	42.83666506	2.25101451	4	4	39.36738549	46.0922908
chr22	23690861	23699176	22q11.23	RGL4	9.983839069	4.436089437	2.250594631	4	4	73.37521454	62.48147593

chr7	150800543	150805120	7q36.1	TMEM176A	5.281522099	2.347325326	2.250017089	4	4	120.8905149	135.861588
chr4	78180226	78182145	4q21.21	SERBP1P5	0.513127142	0.228130599	2.249269252	2	1	10.0888168	0
chrX	18162931	18220904	Xp22.13	BEND2	34.78084745	15.4796564	2.246874643	4	4	36.42695192	37.54357653
chr5	141302629	141304063	5q31.3	SLC25A2	0.633798826	0.282104076	2.246684399	3	2	39.16527313	57.34223337
chr19	38390061	38399884	19q13.2	SPRED3	0.143413263	0.063842306	2.246367206	2	2	99.16428922	45.41642015
chr6	63275951	63567921	6q12	LGSN	0.562002443	0.250230478	2.245939211	4	3	35.70331222	62.63894475
chr4	124246	202306	4p16.3	ZNF718	7.822490583	3.486570959	2.24360573	4	4	76.16033586	41.89004059
chr9	83242990	83585797	9q21.32	FRMD3	14.41213247	6.423834876	2.243540308	4	4	24.09819172	38.72339454
chr17	7445087	7457613	17p13.1	CHRNB1	0.269535319	0.120291451	2.240685567	4	4	69.80384917	40.89480307
chr10	103493788	103592552	10q24.33	NEURL1	1.221875892	0.545340548	2.240574072	4	4	36.21068984	30.8801168
chr22	20206332	20208537	22q11.21	LINC00896	0.844203238	0.376875727	2.240004269	3	4	54.98454326	70.51412398
chr17	64449006	64496469	17q23.3	MILR1	16.91778535	7.553550646	2.239712969	4	4	38.23873887	51.12859069
chr1	53328233	53336509	1p32.3	LINC01771	0.909803485	0.406218299	2.239691039	2	1	26.06510973	0
chr2	218381766	218396894	2q35	SLC11A1	36.32741029	16.22142516	2.239470943	4	4	71.61233064	57.24382343
chr19	41796590	41811553	19q13.2	CEACAM3	31.60949796	14.12399412	2.237999937	4	4	69.84753617	67.89349054
chr19	49118397	49119140	19q13.33	C19orf73	0.762163341	0.340646628	2.237401691	2	1	61.50126383	0
chr4	84669537	84966391	4q21.23	WDFY3	33.57304921	15.00634179	2.2372574	4	4	73.66424177	74.93069991
chr21	33518610	33519108	21q22.11	BTF3P6	1.152335969	0.51528214	2.236320416	1	1	0	0
chr1	161215297	161219248	1q23.3	FCER1G	522.1530835	233.4991934	2.236209367	4	4	70.33005984	42.52407638
chr21	38229915	38303126	21q22.13-q22.2	KCNJ15	74.31614753	33.23345506	2.236184815	4	4	64.07164848	76.21368099
chr14	105707168	105708664	14q32.33	IGHA1	60.76428012	27.17500559	2.236035607	4	4	119.9996485	161.4775259
chr2	201102493	201102990	2q33.1	RPL17P10	1.423143301	0.636730822	2.235078392	2	3	38.74877001	40.54979189
chr15	75649007	75658627	15q24.2	SNX33	0.843334201	0.37734676	2.234905106	4	4	79.15701081	40.90853988
chr16	88677682	88686474	16q24.2	SNAI3	9.181261531	4.108237963	2.234841704	4	4	69.77160126	42.9594276
chr1	78490974	78540701	1p31.1	PTGFR	0.120999862	0.054157627	2.234216452	3	1	43.23976527	0
chr20	50267468	50279795	20q13.13	SMIM25	41.48538629	18.57255032	2.233693573	4	4	72.89704349	61.29536351
chr7	26654772	26864743	7p15.2	SKAP2	96.19601284	43.09320146	2.232278169	4	4	43.19993005	32.72093018
chr15	41503639	41513887	15q15.1	LTK	1.026552803	0.460096648	2.231167751	3	3	75.43599979	115.4945229
chr3	112604386	112641143	3q13.2	CCDC80	0.087190461	0.039094998	2.230220349	2	2	34.65107661	68.99319779
chr18	2652170	2655395	18p11.32	CBX3P2	5.426447078	2.433349828	2.230031628	4	4	47.75515751	47.09588119
chr12	1969552	2697949	12p13.33	CACNA1C	0.032665406	0.014651735	2.229456465	2	2	80.65734398	7.436798902
chr19	21483715	21503238	19p12	LINC00664	0.661901796	0.296951278	2.228991238	3	4	25.59107275	77.41634359
chr2	47369148	47387028	2p21	EPCAM	23.99069476	10.76388462	2.228813816	4	4	97.03655089	49.76294062
chr2	218034934	218090584	2q35	RUFY4	0.329674491	0.147962349	2.228097177	4	2	89.77793752	25.98879604
chr12	122701293	122703357	12q24.31	HCAR2	43.27846484	19.42432156	2.228055415	4	4	86.30528797	77.07096953
chr11	13353208	13353523	11p15.3	RN7SKP151	3.085700633	1.387862812	2.223347009	2	3	28.94569214	63.03876061
chr21	44455486	44462196	21q22.3	LRRC3	0.231361862	0.104065509	2.223232878	3	4	69.73425458	48.86856275
chr7	65065999	65066132	7q11.21	SNORA22C	39.00785323	17.5464077	2.22312475	4	4	61.35696876	19.86996471
chr20	50190583	50192690	20q13.13	CEBPB	95.01585502	42.742576	2.222979144	4	4	37.57602981	61.50260011
chr15	70853239	71050097	15q23	LRRC49	0.084388306	0.037970622	2.222463104	4	2	81.32266273	48.41965161
chr10	21510754	21525682	10p12.31	SKIDA1	0.086743027	0.039033488	2.222271996	1	1	0	0
chr19	7476870	7494977	19p13.2	PEX11G	1.011796164	0.455367289	2.221934224	4	4	80.59544982	48.73529442
chr10	68632337	68632836	10q21.3	COX20P1	3.985587775	1.794686378	2.220771174	2	1	109.6358946	0

chr6	31158524	31164215	6p21.33	TCF19	0.733686241	0.330411401	2.220523376	2	4	49.38896662	18.09854647
chr11	44932348	44951306	11p11.2	TP53I11	6.939809357	3.12536579	2.220479081	4	4	86.93715872	75.32556172
chr5	73626158	73941993	5q13.2	ARHGEF28	0.210474706	0.094788668	2.220462746	4	4	54.78220052	75.78507771
chr7	56055365	56055502	7p11.2	SNORA22B	8.515509646	3.835091635	2.220418821	3	3	104.9203312	100.9729921
chr4	2963504	3040760	4p16.3	GRK4	4.796693546	2.160451541	2.220227325	4	4	129.3591439	44.34715761
chr4	5711197	5829043	4p16.2	EVC	0.457982277	0.206298251	2.220000777	3	2	68.2218812	29.82705274
chr4	88378485	88443097	4q22.1	HERC6	10.19639095	4.595201275	2.218921509	4	4	36.76744572	23.55933279
chr9	136796338	136807741	9q34.3	CCDC183	0.369601473	0.166570542	2.218888575	1	4	0	88.25549594
chr6	132769368	132798656	6q23.2	SLC18B1	23.48960327	10.59693389	2.216641484	4	4	12.3821482	20.51672025
chr14	106116635	106117084	14q32.33	IGHV3-11	3.474602221	1.567665337	2.216418351	4	3	97.58118796	104.5202237
chr7	120988697	121297447	7q31.31	CPED1	3.718170002	1.68064679	2.21234469	4	4	59.20893573	49.23054802
chr2	8559833	8583792	2p25.1	LINC01814	0.171994265	0.077819981	2.210155575	2	4	61.40205315	43.64457088
chr11	125063067	125090516	11q24.2	SLC37A2	4.829299361	2.185130381	2.21007378	4	4	58.58277924	60.59575961
chr20	29746181	29747319	20q11.1	CDC27P3	0.315180727	0.142624465	2.209864397	3	3	68.77650931	13.31544876
chr1	37807801	37809454	1p34.3	C1orf122	3.137083192	1.419739908	2.209618237	4	4	57.36668488	17.99802112
chr1	67138639	67259979	1p31.3	IL23R	0.95682472	0.433044564	2.209529455	4	4	43.99374168	81.28155032
chr1	150549369	150560937	1q21.2	ADAMTSL4	3.2993526	1.493755876	2.208762927	4	4	78.29139163	56.05110842
chr12	32399523	32646050	12p11.21	FGD4	42.51056115	19.24661161	2.20872962	4	4	30.88102454	38.56460762
chr6	159712551	159713985	6q25.3	HNRNPH1P1	0.498977987	0.225986066	2.208003331	2	4	88.22503812	13.15324139
chr7	95583497	95596613	7q21.3	PDK4	29.33419178	13.29542766	2.206336835	4	4	85.72953192	41.11876325
chrX	41570923	41571029	Xp11.4	RNU6-1321P	8.385426466	3.801777974	2.205659174	1	1	0	0
chr2	91636683	91659949	2p11.2	LSP1P4	0.187692951	0.085170789	2.203724451	1	1	0	0
chr19	55769118	55773423	19q13.42	RFPL4AL1	0.766418219	0.347914565	2.202892017	3	1	85.85241217	0
chr21	44133612	44145723	21q22.3	GATD3A	1.557923423	0.707306996	2.202612774	2	2	90.35841984	39.12510741
chr20	23076554	23086830	20p11.21	CD93	99.80732029	45.35127558	2.200761037	4	4	66.29139959	54.96684943
chr6	110858169	110859344	6q21	CNN2P9	1.659057458	0.7540933	2.200069219	3	4	58.93961861	43.09375375
chr18	9334767	9402420	18p11.22	TWSG1	16.17925521	7.354095659	2.200033281	4	4	57.10659098	17.57826388
chr1	33336566	33336864	1p35.1	RN7SKP16	6.664682657	3.029588769	2.199863798	4	4	58.97693594	48.57327552
chr10	32510628	32511819	10p11.22	C1DP1	4.048052261	1.841032689	2.198794343	3	1	135.8014852	0
chr1	8878835	8879885	1p36.23	ENO1-AS1	1.604690292	0.730256626	2.197433388	1	3	0	44.83597535
chr6	52977948	52995380	6p12.2	GSTA4	1.120132457	0.509954795	2.196532846	4	3	104.1697946	50.1125795
chr16	2817163	2821722	16p13.3	PRSS21	0.327367303	0.149064884	2.196139656	2	2	44.30103142	8.299655393
chr19	50311942	50333515	19q13.33	KCNC3	0.305003864	0.13893827	2.19524732	3	4	108.0535461	58.36052788
chr3	119298665	119299012	3q13.32	RPS26P21	3.152598971	1.436174318	2.195136712	2	2	89.19898887	18.14902118
chr12	10116777	10130269	12p13.2	CLEC7A	111.841951	50.96358074	2.194546565	4	4	45.89422104	40.78161077
chr9	72305436	72343210	9q21.13	LINC01504	2.417304145	1.101778647	2.194001628	4	4	47.0517389	94.27015111
chr11	117199294	117207465	11q23.3	TAGLN	1.720697995	0.78429465	2.193943302	4	4	92.77207769	52.08133249
chr5	40841197	40855354	5p13.1	CARD6	18.17126168	8.282729928	2.193873498	4	4	44.07043443	39.76756005
chr17	40309171	40357643	17q21.2	RARA	55.74027097	25.41632299	2.193089496	4	4	76.33339143	53.66771804
chr19	54816438	54830778	19q13.42	KIR3DL1	2.13082222	0.97168398	2.192916899	3	4	29.99536782	61.00216066
chr3	50292828	50299468	3p21.31	HYAL3	2.38687566	1.088540871	2.192729481	4	4	62.45829795	16.97276313
chr11	60171607	60184666	11q12.2	MS4A6A	54.15880442	24.70014566	2.192651216	4	4	64.70079735	43.96526825
chr9	94603133	94640258	9q22.32	FBP1	14.10546543	6.433082599	2.192644851	4	4	102.543091	32.87161861

chrX	63670196	63671502	Xq11.1	ARHGEF9-IT1	1.383183007	0.63095344	2.192210898	2	2	13.21963425	11.25250387
chr7	143809990	143815389	7q35	TCAF2P1	0.404889982	0.184796045	2.19101	1	2	0	58.20718128
chr20	53874498	53875709	20q13.2	SUMO1P1	26.4051095	12.05208099	2.190917031	4	4	27.17855351	68.99921728
chr19	17250021	17264796	19p13.11	USHBP1	0.106863179	0.04878098	2.190673048	1	1	0	0
chr1	231925834	231945205	1q42.2	DISC1-IT1	1.587002883	0.724440327	2.190660601	3	3	90.23457425	9.147350629
chr3	196631504	196632587	3q29	LINC01063	2.424307285	1.10668202	2.190608722	3	3	52.62382626	41.19623788
chr15	92733616	92734276	15q26.1	H2AFVP1	0.632254303	0.288680841	2.190149859	2	1	18.90132221	0
chr16	31355134	31382997	16p11.2	ITGAX	83.48852682	38.12761394	2.189712867	4	4	78.0491886	44.10111433
chr21	33025908	33029196	21q22.11	OLIG2	1.364088875	0.623166033	2.188965385	3	3	21.13074725	60.58123094
chr3	55465715	55505261	3p14.3	WNT5A	0.077507203	0.035417678	2.188376172	1	1	0	0
chr6	43645030	43671011	6p21.1	RSPH9	0.598729937	0.273640893	2.188013389	2	3	63.72309664	106.0611829
chr3	186046317	186109112	3q27.2	ETV5	0.326674317	0.149305937	2.187952629	2	4	39.70817515	35.11050001
chr14	34556226	34556389	14q13.1	RNU1-28P	3.228310416	1.475674988	2.187683902	3	3	58.41400958	17.85464314
chr3	195147871	195152856	3q29	XXYLT1-AS2	1.083284913	0.495232494	2.187426967	3	4	92.49836138	29.61732852
chr5	109699602	109699834	5q21.3	RN7SKP230	5.947162888	2.719542124	2.186825068	3	4	62.72167835	57.39731897
chr5	141364331	141512979	5q31.3	PCDHGA5	0.096771521	0.044252373	2.186809743	1	1	0	0
chr7	22118290	22357133	7p15.3	RAPGEF5	0.237644958	0.108683958	2.186568862	4	3	48.51356651	94.05877117
chr5	172325181	172454523	5q35.1	SH3PXD2B	0.428552477	0.196042631	2.186016758	4	4	74.74824421	59.76109663
chr11	9428766	9428954	11p15.4	SNORA23	449.8909087	205.8247415	2.185796059	4	4	108.6484961	93.9692019
chr2	209424047	209734118	2q34	MAP2	0.226830838	0.103831723	2.184600535	4	2	109.3855156	27.90980155
chr1	201539127	201826974	1q32.1	NAV1	0.309977276	0.141962817	2.183510329	4	4	88.34298059	81.47444456
chr9	91409045	91425063	9q22.31	NFIL3	105.0916911	48.15806591	2.182224081	4	4	58.55564979	40.23016084
chr3	15697298	15697371	3p25.1	MIR3134	7.316144115	3.353232188	2.181818528	3	2	21.32618172	23.59954783
chr17	7854886	7856099	17p13.1	TMEM88	2.604948368	1.193954955	2.181781111	3	3	88.78568118	47.69614276
chr21	36130072	36131916	21q22.12	MEMO1P1	9.991741414	4.582268613	2.18052285	4	4	51.51041479	45.94277627
chr15	64364994	64387687	15q22.31	PCLAF	3.785579434	1.736331111	2.180217478	4	4	136.3303908	36.4779324
chr7	139523928	139544991	7q34	CLEC2L	1.062113469	0.487164504	2.180194698	2	4	66.68654126	40.10573313
chr7	17298652	17346151	7p21.1	AHR	21.06785953	9.664461181	2.179931104	4	4	62.30341451	35.40359905
chr6	130366281	130443424	6q23.1	TMEM200A	0.421661589	0.193546673	2.178604168	4	4	47.88454438	142.2068725
chr10	15075475	15088776	10p13	ACBD7	0.489337886	0.224753282	2.177222425	1	2	0	34.32734654
chr6	79233674	79236800	6q14.1	HMGN3-AS1	0.455204016	0.209092242	2.177048809	4	3	85.1684577	65.52717314
chr3	54122594	55074557	3p21.1-p14.3	CACNA2D3	1.670974248	0.767604881	2.176867669	4	4	46.98891358	79.16301368
chr2	74416811	74418691	2p13.1	HMGA1P8	1.527170788	0.702492624	2.173931421	1	1	0	0
chr19	37548949	37587348	19q13.12	ZNF571-AS1	0.275705402	0.126849087	2.173491416	4	4	69.67333584	73.54566614
chr2	140231423	142132463	2q22.1-q22.2	LRP1B	0.036376079	0.016741417	2.172819551	3	2	53.58642117	4.64809456
chr2	135665397	135665478	2q21.3	MIR128-1	7.618457633	3.506414352	2.172720297	2	1	27.24536801	0
chr17	30959564	30959850	17q11.2	RN7SL138P	2.629591786	1.21046123	2.172388277	3	3	97.48492811	54.11990751
chr17	42025576	42038026	17q21.2	ZNF385C	0.166541859	0.076699111	2.17136622	2	2	74.96586138	43.38546476
chr9	127920886	127931958	9q34.11	PIP5KL1	0.67643969	0.311624513	2.170688315	4	4	85.29251835	19.8566948
chr19	7631614	7637026	19p13.2	PCP2	1.574310722	0.725421405	2.170201639	3	4	50.07811957	79.20529938
chr17	31294650	31297362	17q11.2	OMG	0.566569669	0.261147819	2.169536285	4	4	100.8709145	70.35882005
chr11	10541236	10599932	11p15.4	MRVI1-AS1	1.769754038	0.815859258	2.169190361	4	2	80.46864071	29.75593962
chr7	1022933	1023026	7p22.3	MIR339	4.879058359	2.249991677	2.168478403	1	1	0	0

chr11	77821114	77901281	11q14.1	AAMDC	1.477818112	0.681589529	2.168193683	4	4	58.57682629	21.98032321
chr19	51639478	51646879	19q13.41	SIGLEC14	27.46651653	12.66882509	2.168039761	3	4	58.14900392	31.37058063
chr2	229023973	229271341	2q36.3	PID1	9.137601818	4.214883924	2.167936765	4	4	78.43906022	44.52382318
chr12	111766887	111809985	12q24.12	ALDH2	7.522087437	3.469897832	2.167812368	4	4	69.6270304	35.84917239
chr6	31586179	31588909	6p21.33	LST1	61.5231115	28.38270776	2.167626571	4	4	74.84704645	35.45035232
chr2	27442366	27446481	2p23.3	KRTCAP3	0.705465823	0.325486283	2.167421052	3	3	69.04112025	38.34138135
chr4	43898915	43899409	4p13	NDUFB4P12	1.270571222	0.586240846	2.167319509	1	1	0	0
chr6	36242523	36313955	6p21.31	PNPLA1	1.836901928	0.847657007	2.16703444	4	4	73.85679757	59.70908716
chr1	155208699	155213839	1q22	MTX1	7.871637892	3.632506616	2.166998914	4	4	75.38685542	42.3358265
chr12	12329263	12350243	12p13.2	MANSC1	3.897792684	1.799487111	2.166057573	4	4	78.51126518	85.07084327
chr10	101031239	101041243	10q24.31	SFXN3	9.066937784	4.186965293	2.165515391	4	4	80.8538988	29.50013181
chr10	68674304	68675150	10q21.3	RPS3AP37	0.915000826	0.422628391	2.165024514	1	1	0	0
chr2	89009982	89010457	2p11.2	IGKV1-9	4.307130409	1.98963421	2.164785058	4	3	49.3068697	71.91439402
chr22	36860988	36878017	22q12.3	NCF4	131.2104594	60.61259551	2.164739165	4	4	76.11746489	68.0716529
chr22	17764180	17764259	22q11.21	MIR3198-1	9.368725567	4.329061266	2.164147142	2	2	28.30079869	22.99645451
chr1	248873827	248919146	1q44	PGBD2	18.04224194	8.34088723	2.163108246	4	4	73.00692053	43.54541436
chr11	47354858	47395640	11p11.2	SPI1	199.1109876	92.0530746	2.163002034	4	4	82.42912952	47.76964006
chr19	51752026	51770526	19q13.41	FPR2	187.692441	86.77715543	2.162924563	4	4	86.68563017	66.48596121
chr7	150791287	150801360	7q36.1	TMEM176B	26.49437805	12.251021	2.162626123	4	4	114.8315163	107.0365971
chr4	153152281	153339322	4q31.3	TRIM2	0.486384527	0.224910798	2.162566368	4	4	57.72970693	62.37371638
chr5	77080434	77080571	5q13.3	SNORA47	45.75605559	21.16225076	2.162154494	4	4	85.63961591	77.69416511
chr16	15125139	15154873	16p13.11	PKD1P6	0.230273737	0.106514273	2.161904972	2	4	69.91587697	39.15252682
chr15	79960890	79971301	15q25.1	BCL2A1	618.8773	286.3315801	2.161400778	4	4	67.34600041	65.0719039
chr4	107823645	107915047	4q25	SGMS2	7.765014469	3.59399723	2.160551044	4	4	55.60451318	21.92236859
chr19	4229196	4237539	19p13.3	EBI3	0.390372352	0.180712096	2.160189381	2	1	18.11971732	0
chr19	54803612	54814517	19q13.42	KIR2DL4	0.578853574	0.268091348	2.159165442	3	3	27.87084696	42.88097181
chr3	38548061	38649673	3p22.2	SCN5A	0.056827998	0.026320227	2.159099849	1	1	0	0
chr7	5662432	5680461	7p22.1	RNF216-IT1	1.962574918	0.909259158	2.158432939	2	2	63.40812315	15.93477846
chr20	23627897	23638048	20p11.21	CST3	65.90006127	30.53184971	2.158403828	4	4	68.46371573	35.55172772
chr12	68552925	68553958	12q15	RPSAP12	0.560340534	0.259621609	2.158296976	2	1	59.44806103	0
chr17	81029133	81034719	17q25.3	BAIAP2-DT	5.982396032	2.771946886	2.158192879	4	4	62.15122851	77.3683679
chr9	22012105	22012622	9p21.3	UBA52P6	1.365795952	0.632846535	2.158178765	4	2	76.86947493	7.966016002
chr15	97960703	97973838	15q26.2	ARRDC4	16.30690757	7.557392383	2.157742611	4	4	37.93732669	41.8633042
chr7	102284525	102321711	7q22.1	SH2B2	9.64923578	4.473109872	2.157164938	4	4	76.9485593	40.92022489
chr5	170028488	170028566	5q35.1	MIR378E	9.805850303	4.54611354	2.156974351	3	3	49.66372602	19.39002424
chr1	161623196	161631963	1q23.3	FCGR3B	1036.45605	480.72652	2.156020121	4	4	92.53048474	78.99525398
chr1	150698059	150720910	1q21.3	HORMAD1	4.37325538	2.028962359	2.155414743	4	4	78.33717707	64.66672746
chr12	103843749	103930211	12q23.3	TTC41P	0.455278914	0.211422705	2.153405966	3	4	81.77926253	39.35033311
chr9	134903236	134918003	9q34.3	FCN1	288.8560588	134.1510737	2.153214662	4	4	75.54612016	62.96261176
chr15	32765544	33194765	15q13.3	FMN1	0.869151502	0.403781492	2.152529322	4	4	69.97093481	113.6340265
chrX	40735396	40738701	Xp11.4	MED14OS	1.408181527	0.654246822	2.152370451	4	4	96.22994346	69.54130487
chr21	44300034	44327381	21q22.3	PFKL	14.73096098	6.845320797	2.151975257	4	4	54.52000789	31.74830174
chr20	45640451	45641393	20q13.12	RPS2P7	0.514110197	0.238916134	2.151843779	3	1	48.77258815	0

chr22	30693782	30907824	22q12.2	OSBP2	59.1521308	27.50348161	2.150714285	4	4	27.06896775	33.74854202
chr21	33334613	33335100	21q22.11	USF1P1	2.567633444	1.194435166	2.149663303	4	4	100.980511	58.43130896
chr20	58995187	59007254	20q13.32	CTSZ	177.6010091	82.61808403	2.149662646	4	4	79.83020632	55.06022934
chr8	8228570	8244865	8p23.1	FAM86B3P	4.557813097	2.121507994	2.148383654	4	4	56.39850493	97.99989688
chr1	12191713	12191773	1p36.22	MIR4632	10.2157528	4.755547251	2.148176069	1	1	0	0
chr12	10069504	10099065	12p13.2	CLEC1A	1.090773176	0.507896289	2.147629743	4	4	73.56774914	84.84148179
chrX	66074344	66076363	Xq12	EIF4BP9	0.360991543	0.168138694	2.146986715	2	2	5.754106016	0.384543252
chrX	74159607	74162671	Xq13.2	MKRN5P	0.340906905	0.158801367	2.146750443	2	1	44.86607621	0
chrX	47370600	47413699	Xp11.3	ZNF157	0.577276233	0.268915934	2.146679169	2	4	2.165490633	58.51516961
chr1	116111401	116135240	1p13.1	MAB21L3	0.168737708	0.078625977	2.146080896	3	3	76.78444887	2.243544007
chr5	41870030	41872241	5p13.1	OXCT1-AS1	0.493099576	0.229815229	2.145634894	1	2	0	65.40734296
chr12	102395860	102481839	12q23.2	IGF1	0.070698578	0.032967827	2.144471868	3	3	57.15028191	4.428809649
chr14	94376747	94390692	14q32.13	SERPINA1	158.8949013	74.11989535	2.143755068	4	4	70.27020999	65.92007894
chr7	22419444	22500282	7p15.3	STEAP1B	0.980380774	0.457345654	2.143631988	3	4	87.22515415	48.92636157
chr22	32507820	33058391	22q12.3	SYN3	0.101535731	0.047392523	2.142441987	1	1	0	0
chr11	5596101	5612958	11p15.4	TRIM6	0.589523851	0.275173968	2.142367807	4	4	54.35080047	65.63849599
chr10	103446783	103452405	10q24.33	CALHM2	12.29662544	5.74322535	2.141066159	4	4	55.12122891	29.58357633
chr2	89960015	89960747	2p11.2	IGKV2D-28	1.803461667	0.842749321	2.139974038	3	3	36.92533879	28.51948407
chr21	39175446	39183514	21q22.2	PSMG1	11.14768506	5.21235895	2.13870249	4	4	57.78803875	33.11020559
chr12	6924463	6942321	12p13.31	ATN1	0.876923051	0.410059494	2.138526393	4	4	62.39893207	65.64147872
chr19	46610040	46625097	19q13.32	PTGIR	2.336131957	1.092763811	2.137819659	4	4	53.0440267	35.68387951
chr16	32251603	32255928	16p11.2	TP53TG3D	0.18234277	0.085338264	2.136705872	1	3	0	3.491515992
chr12	57243453	57251193	12q13.3	STAC3	3.642972077	1.705201532	2.136387992	4	4	39.61760259	41.62335784
chr14	49599673	49600913	14q21.3	RHOQP1	2.311788372	1.082540769	2.13552084	2	2	1.946627479	81.92017049
chr11	125445745	125496310	11q24.2	FEZ1	0.492131984	0.230528732	2.134796734	4	4	54.94018539	60.33508535
chr10	30841254	31032407	10p11.23	ZNF438	26.50780861	12.41771463	2.134676903	4	4	48.66008549	54.01881833
chr13	48488963	48535997	13q14.2	RCBTB2	36.50623793	17.10182532	2.134639855	4	4	30.25147406	55.52069962
chr9	98198998	98255721	9q22.33	TBC1D2	7.739144695	3.626751262	2.133905563	4	4	67.88180547	30.01909627
chr1	151838277	151851535	1q21.3	C2CD4D-AS1	6.967705349	3.266293642	2.13321462	4	4	52.75845003	63.64140006
chr1	158831303	158849504	1q23.1	MNDA	2325.036128	1090.367557	2.132341625	4	4	99.56470083	76.280329
chr16	2839573	2842751	16p13.3	PRSS30P	0.193543768	0.090777585	2.132065623	1	2	0	10.77438717
chr1	11934667	11975542	1p36.22	PLOD1	11.7820282	5.530699624	2.130296166	4	4	68.91474443	35.34880816
chr14	69467744	69531551	14q24.1	PLEKHD1	0.134819879	0.06334263	2.128422501	3	4	121.6890336	55.65151684
chrX	103881747	103919548	Xq22.2	TMSB15B-AS1	0.723120747	0.339750741	2.128386078	2	3	16.56485873	25.67772828
chr9	68536580	68540867	9q21.11	TMEM252	3.078607463	1.446977553	2.127612455	3	3	83.82960276	88.95279902
chr12	92257397	92363859	12q22	LINC02391	1.20745811	0.567679174	2.127007938	4	3	71.8789622	26.51565343
chr21	33229895	33264513	21q22.11	IFNAR2	35.43415063	16.66455455	2.1263185	4	4	61.1562083	32.60205379
chr20	41097994	41138000	20q12	PLCG1-AS1	0.677244978	0.318529834	2.126158699	2	3	109.4022278	44.41071491
chr1	166055918	166166788	1q24.1	FAM78B	0.103205261	0.048541355	2.126130618	1	1	0	0
chr8	125430290	125438405	8q24.13	TRIB1	78.29730682	36.83176311	2.125809362	4	4	73.42528806	61.16472722
chr14	61556272	61570653	14q23.1	LINC01303	2.361814559	1.111165027	2.125529962	4	4	73.53177967	63.39960212
chr19	58559186	58574797	19q13.43	MZF1-AS1	0.646196724	0.304261284	2.12382172	3	4	103.4357644	73.23793311
chr21	42199689	42304387	21q22.3	ABCG1	8.805647784	4.146527616	2.123619713	4	4	69.53717303	63.03613696

chr8	78666047	78719765	8q21.13	ZC2HC1A	1.843062354	0.868108859	2.123077462	4	4	61.83669748	93.04308652
chr19	54850320	54867215	19q13.42	KIR3DL2	1.240587101	0.584339821	2.123057603	3	4	46.52930782	68.2605669
chrX	74582662	74583542	Xq13.2	PABPC1P3	9.785643305	4.609833881	2.122775692	4	4	93.88324957	59.11533841
chr10	92046692	92291499	10q23.32	CPEB3	3.639540406	1.715217113	2.121912368	4	4	14.34177482	56.36830832
chr14	23520855	23556315	14q11.2	ZFHX2	0.082573047	0.038930012	2.121064014	3	4	84.01289853	39.75256526
chr13	22210285	22276521	13q12.11	LINC00540	0.510478484	0.240705226	2.120761947	3	3	51.96430279	35.82871753
chr16	14009276	14016018	16p13.12	LINC02185	1.289279983	0.608028273	2.120427684	2	3	1.912303389	74.08960337
chr1	23845077	23868369	1p36.11	FUCA1	18.54373021	8.746156434	2.120214788	4	4	64.11123207	33.09293969
chr14	105741473	105743070	14q32.33	IGHG1	28.55266814	13.47018176	2.119694348	4	4	89.24222299	166.5997765
chr3	138061985	138115862	3q22.3	DZIP1L	0.219377072	0.103508993	2.119401073	1	3	0	52.34754861
chr3	32635165	32635639	3p22.3	RPL30P4	2.876056827	1.357078128	2.119300847	4	2	59.39384347	34.42781641
chr19	51685595	51713157	19q13.41	SPACA6	1.530966293	0.722973356	2.117597116	4	4	110.5842472	54.19912651
chr7	101362369	101559024	7q22.1	COL26A1	0.475969584	0.224768829	2.117596045	2	3	18.43006405	24.87143173
chr10	79382325	79445627	10q22.3	ZCCHC24	2.818525362	1.331084083	2.117466054	4	4	103.5807626	43.98839291
chr20	50292720	50314919	20q13.13	LINC01270	1.270222262	0.60011783	2.116621436	4	4	100.1454538	64.07939582
chr9	35970940	35972457	9p13.3	YBX1P10	4.228710725	1.998545559	2.115894084	4	4	35.6908785	64.31573853
chr3	98782213	98783390	3q12.1	PDLIM1P4	1.646262297	0.778047296	2.115889748	2	3	6.529568506	11.57499293
chr1	27005020	27012842	1p36.11	TENT5B	0.234748623	0.110973496	2.11535754	2	1	46.0032083	0
chr17	44290857	44291151	17q21.31	RN7SL507P	3.271206647	1.546549506	2.115164522	2	2	27.62317742	33.16995911
chr1	37996765	38005691	1p34.3	FHL3	13.26410149	6.271004422	2.115147845	4	4	58.11808271	43.62868725
chr8	33513475	33513578	8p12	SNORD13	255.2939529	120.7420735	2.11437443	4	4	120.9051031	60.42009928
chr3	143517083	143518313	3q24	ST13P15	1.028448028	0.486820176	2.112583003	4	3	61.94461101	16.78140766
chr11	62612741	62615120	11q12.3	ROM1	0.584004414	0.27666264	2.110890052	3	3	56.14382599	73.77555322
chr16	4882507	4937135	16p13.3	PPL	0.127913302	0.060603592	2.110655443	2	3	50.39160259	72.01726853
chr13	41457406	41470877	13q14.11	RGCC	208.3547356	98.77830345	2.109316807	4	4	65.27327308	14.30431817
chr17	29716280	29716725	17q11.2	RPL21P123	7.426476045	3.522491733	2.108301909	4	4	45.67726664	73.24998268
chr1	63548980	63550636	1p31.3	DLEU2L	0.536710804	0.254663649	2.107528133	4	4	26.46955917	44.28517332
chr10	110988926	110989013	10q25.2	MIR548E	6.943221204	3.294630603	2.107435412	4	3	23.93158527	33.48387146
chr17	32040336	32042832	17q11.2	SH3GL1P1	0.582226484	0.276356088	2.106798114	3	4	71.5806308	51.77216816
chr11	119417941	119424985	11q23.3	THY1	0.097881858	0.046460204	2.106789252	1	2	0	1.083483422
chr14	106421709	106422147	14q32.33	IGHV4-39	8.216001688	3.899858349	2.106743618	4	4	99.6476365	103.3251479
chr13	39342892	39603219	13q13.3-q14.11	LHFPL6	0.638664682	0.303231991	2.106191635	3	4	25.36669214	43.19699225
chrX	143622790	143636107	Xq27.3	SLITRK4	0.79003524	0.375193631	2.105673374	4	4	78.54819596	55.46839178
chr12	47841537	47905031	12q13.11	VDR	8.913717513	4.23327405	2.105632049	4	4	61.86926378	38.48175373
chr10	111077032	111080907	10q25.2	ADRA2A	1.125102981	0.534436858	2.105212177	4	4	59.32562427	28.20131129
chr17	73242343	73263778	17q25.1	CPSF4L	0.432961548	0.205671654	2.105110453	1	1	0	0
chr2	28384409	28394672	2p23.2	FLJ31356	0.274874259	0.13073498	2.102530319	2	2	100.6587706	52.0961517
chr19	38801671	38813614	19q13.2	LGALS4	0.208784011	0.099308952	2.102368498	2	2	5.611893634	0.64098155
chr7	99827626	99867138	7q22.1	CYP3A43	0.193874949	0.092247152	2.101690355	4	2	53.94243881	10.44904499
chr7	121083700	121083813	7q31.31	RNA5SP241	5.173716298	2.462493551	2.101007045	1	1	0	0
chr12	6375368	6391571	12p13.31	LTBR	10.48878492	4.993383016	2.100536827	4	4	78.52777084	57.58957046
chr1	51709083	51709939	1p32.3	SLC25A6P3	0.646310487	0.307778876	2.099918279	1	1	0	0
chr21	29298795	29361896	21q21.3	BACH1	213.0637568	101.4885186	2.09938779	4	4	39.18601964	37.41965975

chr2	55014418	55014521	2p16.1	RNU6-433P	5.705294789	2.718146706	2.098964996	1	1	0	0
chr8	144107692	144116392	8q24.3	WDR97	0.214713126	0.102309997	2.098652458	3	3	80.05243263	69.77338443
chr17	57861243	57955323	17q22	CUEDC1	2.030596571	0.967583448	2.098626817	4	4	69.00162712	56.97713062
chr16	84039918	84040033	16q23.3	RNA5SP432	5.077216468	2.419408358	2.098536385	1	1	0	0
chr7	75533298	75738976	7q11.23	HIP1	16.01962661	7.634559725	2.098303922	4	4	70.81366485	47.55882437
chr17	3510502	3557995	17p13.2	TRPV3	0.124634818	0.059429551	2.097185937	4	3	61.85699054	62.85458001
chr17	74213557	74262020	17q25.1	TTYH2	4.609114076	2.197768075	2.097179466	4	4	45.07009111	26.54929608
chr20	38446654	38450921	20q11.23	SNHG11	1.484431206	0.7079247	2.096877261	3	4	42.8446469	83.44584883
chr12	6821797	6827418	12p13.31	GPR162	3.935441798	1.876940386	2.096732442	3	4	117.688306	50.07725624
chr16	67163385	67169945	16q22.1	HSF4	0.275961582	0.131615173	2.096730764	2	3	97.36076488	69.64110301
chr17	40340867	40343136	17q21.2	RARA-AS1	7.273904888	3.469186726	2.096717606	4	4	47.56827489	89.73004307
chrX	78271468	78327691	Xq21.1	CYSLTR1	22.78429724	10.8685398	2.096353113	4	4	38.27181581	35.91754528
chr7	27092993	27096006	7p15.2	HOXA1	0.924842135	0.441374279	2.095369346	4	4	102.0997854	53.40877768
chr10	116662211	116670290	10q25.3	C10orf82	0.308112257	0.147109588	2.094440346	2	2	67.23682917	44.93286877
chr6	47231527	47309947	6p12.3	TNFRSF21	2.368871163	1.131616632	2.093351313	4	4	69.68058951	92.76599304
chr1	1751232	1780509	1p36.33	NADK	85.03562467	40.62184514	2.093347172	4	4	63.30894	47.09278256
chr11	112167372	112172556	11q23.1	TEX12	0.454402057	0.217170115	2.092378396	1	2	0	9.919207877
chr16	1434388	1444489	16p13.3	CCDC154	0.472938168	0.226060891	2.092083088	2	3	45.42170274	60.30213369
chr8	143018485	143022410	8q24.3	LY6E	20.04142287	9.579654898	2.092081926	4	4	65.52511752	94.22713664
chr2	38481775	38482875	2p22.1	RPLP0P6	2.129786492	1.018473042	2.09115647	4	4	45.68540686	84.31863613
chr2	241558731	241574138	2q37.3	BOK	1.007659502	0.482375555	2.088952252	3	3	66.28294027	73.83895807
chr4	140283726	140373392	4q31.1	SCOC-AS1	0.497903792	0.238358808	2.088883547	3	3	14.59564153	59.94296975
chr19	676389	683392	19p13.3	FSTL3	0.880814638	0.42190099	2.087728304	4	4	82.68360518	63.25355307
chr15	70047790	70098171	15q23	TLE3	34.13636466	16.35178399	2.087623264	4	4	78.90778754	48.96186922
chr11	3087116	3165352	11p15.4	OSBPL5	4.692361432	2.248293105	2.087077269	4	4	34.07469454	17.90719389
chr19	48485271	48513926	19q13.33	LMTK3	0.356608083	0.170936767	2.086198829	4	4	85.07977776	60.94205461
chr11	119084864	119093549	11q23.3	HMBS	52.80046977	25.31121162	2.086050663	4	4	96.36653626	64.4858666
chr16	2209253	2211068	16p13.3	BRICD5	1.165531578	0.558806247	2.085752594	2	4	21.39067252	38.07881961
chr17	78890580	78903217	17q25.3	CEP295NL	0.704166314	0.337615184	2.085706887	4	4	98.74568293	67.02030019
chr9	86220265	86259657	9q21.33	C9orf153	3.176256903	1.523602121	2.084702337	4	4	40.11481982	50.09651222
chr3	15254353	15341394	3p25.1	SH3BP5	36.49350398	17.50733891	2.084468929	4	4	50.31116058	55.36110663
chr1	207321472	207360966	1q32.2	CD55	147.263641	70.67646922	2.083630417	4	4	75.24016528	28.57911379
chr22	24583750	24629005	22q11.23	GGT1	3.046060844	1.462027769	2.083449377	4	4	84.11703035	68.70912296
chr14	22544071	22544136	14q11.2	TRAJ2	8.962927055	4.302760705	2.083064262	3	3	92.98418388	38.10438945
chr19	7637101	7647874	19p13.2	STXBP2	18.43868209	8.855535229	2.082164613	4	4	77.98765787	36.97423439
chr17	28897781	28903071	17q11.2	DHRS13	15.51509268	7.451510356	2.082140658	4	4	98.39626792	65.87678825
chr2	9951663	10002284	2p25.1	GRHL1	2.299845396	1.104974376	2.081356315	4	4	45.47768526	65.4962767
chr12	132687606	132704991	12q24.33	PXMP2	0.942873679	0.453309018	2.079979974	2	4	50.50757889	86.82505842
chr5	138753386	138935034	5q31.2	CTNNA1	33.18654574	15.95845116	2.079559313	4	4	48.2728139	24.65325628
chr7	100372659	100400099	7q22.1	PILRA	126.294456	60.74284234	2.079166056	4	4	60.88398858	46.91946495
chr20	32631625	32673941	20q11.21	C20orf203	0.112976109	0.054356139	2.078442506	1	1	0	0
chr4	76958724	76959249	4q21.1	TXNP6	1.673737151	0.805854289	2.076972443	1	2	0	13.60911594
chr4	10115756	10115884	4p16.1	RNA5SP155	6.645037984	3.199501188	2.076898114	4	3	50.72062883	40.09176316

chr22	50600685	50613981	22q13.33	MAPK8IP2	0.117979212	0.056819217	2.076396289	1	3	0	41.44735072
chrX	57229034	57229415	Xp11.21	PPP1R11P2	1.645282698	0.79245271	2.076190386	2	2	29.84691167	15.91577994
chr3	19879472	19947514	3p24.3	EFHB	0.435537573	0.209783781	2.076126055	4	4	106.0795247	68.55106645
chr20	346726	359705	20p13	NRSN2	0.508353474	0.244877098	2.075953516	3	3	18.30196358	63.3643057
chr2	44361714	44772592	2p21	CAMKMT	47.04456277	22.66329993	2.075803741	4	4	74.94102082	42.08037248
chr11	494512	507283	11p15.5	RNH1	7.242395652	3.489068839	2.075738825	4	4	66.20801787	34.12866159
chr1	109910506	109930994	1p13.3	CSF1	6.327722798	3.049727726	2.074848435	4	4	110.4795527	57.02216431
chr4	117574489	117576189	4q26	NT5C3AP1	3.62877518	1.748953279	2.074826826	4	4	73.49255851	25.61543934
chr1	26282282	26318265	1p36.11	UBXN11	7.667034457	3.696141829	2.074334485	4	4	89.16935938	9.10748937
chr7	151877042	151879223	7q36.1	PRKAG2-AS1	1.06654275	0.514515051	2.072908747	3	4	86.3900964	68.40322852
chr8	71843123	72056312	8q13.3-q21.11	MSC-AS1	0.833668045	0.402178482	2.072880781	4	4	19.94447178	44.59781682
chr22	20957092	20964680	22q11.21	LINC01637	0.886009244	0.427466187	2.072700184	3	2	36.98118858	11.47994135
chr8	58411264	58451508	8q12.1	UBXN2B	62.1774337	30.0038497	2.072315197	4	4	80.86772471	43.14551484
chr17	7857727	7866086	17p13.1	CYB5D1	0.550731113	0.265799599	2.071978719	4	4	66.97811771	63.29670958
chr1	158289773	158294774	1q23.1	CD1C	11.30777882	5.458012823	2.071775788	4	4	86.60903972	105.400615
chr3	38137439	38143022	3p22.2	MYD88	48.00493525	23.17419362	2.071482445	4	4	78.60144395	30.05130202
chr7	117710648	117873818	7q31.31	CTTNBP2	0.073605551	0.03553538	2.07133146	3	1	84.21411932	0
chr3	194487140	194488545	3q29	LINC00884	0.436667941	0.21086727	2.070818957	3	4	33.05296734	68.68298873
chr1	11909808	11909927	1p36.22	RNU5E-4P	63.06342754	30.46226923	2.070214371	4	4	91.21552072	46.23096264
chr3	113632704	113632899	3q13.2	RN7SL767P	21.40637697	10.34128161	2.069992654	4	4	106.4424767	56.04869578
chr18	9708187	9862556	18p11.22	RAB31	104.3459529	50.41847571	2.069597533	4	4	59.11470702	21.25346108
chr6	30727709	30742851	6p21.33	FLOT1	42.37841596	20.47774263	2.069486698	4	4	82.34233294	56.08177616
chr1	6466092	6520061	1p36.31	PLEKHG5	0.278420443	0.134559472	2.069125569	3	4	73.79720726	93.10971818
chr10	29457338	29737001	10p11.23	SVIL	27.00961133	13.05724963	2.068552881	4	4	75.70579036	57.16199364
chr2	54051334	54051760	2p16.2	HMGB1P31	2.751920328	1.330360518	2.068552314	2	4	107.2553095	56.58038045
chr4	68537184	68576354	4q13.2	UGT2B17	0.463094177	0.223900986	2.068298962	3	1	73.92155537	0
chr19	54040825	54063966	19q13.42	VSTM1	13.09623564	6.331960771	2.068274917	4	4	68.78193053	55.43441275
chr5	128257909	128538042	5q23.3	FBN2	2.156721536	1.042860434	2.068082618	4	4	63.36161683	40.74169087
chr4	124664048	124712732	4q28.1	ANKRD50	5.234230545	2.531873278	2.067335119	4	4	55.65135134	53.12076768
chr17	6755408	6775647	17p13.1	XAF1	46.17835108	22.33747681	2.067303817	4	4	67.29845383	63.21157406
chr20	38537750	38538459	20q11.23	RPS3P2	1.600078102	0.774176683	2.066812573	4	4	48.42819375	74.14745137
chr19	18929201	18941258	19p13.11	HOMER3	1.261805477	0.610663448	2.066286234	4	4	90.59941453	63.07974123
chr8	33308102	33473146	8p12	FUT10	2.083254033	1.0084488	2.065800499	4	4	58.89094125	56.5864771
chr16	3242028	3256776	16p13.3	MEFV	91.36661163	44.24169269	2.06516989	4	4	57.62952893	45.91274047
chr4	5562408	5709548	4p16.2	EVC2	0.605054039	0.293026387	2.064844897	3	3	77.19459077	92.18800512
chr1	247935814	247937128	1q44	CLK3P2	0.401850376	0.19469245	2.0640265	3	2	67.58214925	14.24546382
chr12	45173034	45216006	12q12	PLEKHA8P1	5.459856017	2.645378405	2.063922502	4	4	55.77181693	15.13171092
chr19	11965054	11980399	19p13.2	ZNF763	1.07517199	0.521299076	2.062485891	4	4	97.97926336	62.24705207
chr6	158947012	159000426	6q25.3	RSPH3	3.71312771	1.801054312	2.061641165	4	4	58.92516713	48.57205778
chrX	24788347	24789092	Xp22.11	EEF1B2P3	2.927474198	1.420276567	2.061200096	4	4	25.99752862	71.74746925
chr22	50274979	50307637	22q13.33	PLXNB2	19.19576578	9.314059473	2.060945159	4	4	81.26761064	26.82936591
chr10	26643108	26653454	10p12.1	FAM238B	0.554290145	0.268983404	2.060685291	2	4	27.17661422	55.31045481
chr19	51366750	51369255	19q13.41	CLDND2	2.414023932	1.171502392	2.060622282	4	4	85.14721913	91.22407759

chr1	8786129	8786951	1p36.23	RPL7P7	1.435024932	0.696418609	2.060578098	1	3	0	43.20336722
chr17	63636601	63637110	17q23.3	EEF1DP7	3.795170494	1.842293351	2.060025072	3	4	53.24428979	42.40485089
chr16	88874855	88977149	16q24.3	CBFA2T3	1.367824768	0.664288917	2.059081121	4	4	45.22932096	70.30032286
chr13	113478915	113479071	13q34	RNU1-16P	4.511901957	2.191347046	2.058962758	1	3	0	9.554395247
chr17	78532813	78533055	17q25.3	RN7SL454P	3.241744616	1.574671171	2.058680362	2	3	78.523994	42.14070285
chr19	49119389	49151030	19q13.33	PPFIA3	0.215638196	0.104751823	2.058562695	3	4	66.24382768	57.38354645
chr5	177437889	177447944	5q35.3	PRR7-AS1	0.615587672	0.299170549	2.05764797	1	3	0	87.51187881
chr21	36156781	36294274	21q22.12	DOP1B	41.02484035	19.93858599	2.057560168	4	4	13.88579664	27.36485782
chr17	81703370	81707526	17q25.3	MRPL12	1.100432484	0.534919999	2.057190768	3	4	94.36528649	108.639719
chr14	23306762	23311759	14q11.2	BCL2L2	2.523271943	1.226795222	2.056799616	4	4	59.59577262	38.72986181
chr13	27177668	27220649	13q12.13-q12.2	LINC02340	3.970063972	1.930221219	2.056792213	3	4	96.18268145	18.31193077
chr5	77426652	77620611	5q13.3-q14.1	WDR41	10.609572	5.159896473	2.056159857	4	4	20.63278404	41.73001877
chr11	67991100	68004125	11q13.2	UNC93B1	22.28790453	10.84109951	2.055871225	4	4	48.49246171	20.93982724
chr22	43039516	43089428	22q13.2	TTLL1	6.24185311	3.036767247	2.055426907	4	4	47.17697275	33.7184195
chr17	4897769	4905019	17p13.2	CHRNE	2.366419345	1.151434436	2.055192437	3	4	104.2755092	49.11432596
chr7	16753726	16784536	7p21.1	TSPAN13	23.28008907	11.328362	2.055026937	4	4	68.86578436	59.01366917
chr19	35904401	35908309	19q13.12	TYROBP	530.5130567	258.1867176	2.0547651	4	4	89.06853668	66.86444123
chr1	203287152	203288798	1q32.1	LINC01353	2.84737829	1.38594157	2.054472102	4	4	46.00970346	95.02402152
chr15	25094036	25094129	15q11.2	SNORD116-24	9.165489733	4.461494102	2.054354332	4	3	84.17798556	39.94460768
chr1	143729407	143729570	1q21.1	RNVU1-18	28.22786735	13.74895753	2.053091465	4	4	58.99050794	107.5387825
chr11	63632194	63632822	11q13.1	IMMP1LP1	0.964582404	0.46989955	2.052741708	1	1	0	0
chr11	63461404	63491208	11q12.3	HRASLS5	1.80919222	0.881789851	2.051727198	4	4	103.2007126	63.57237608
chr3	48094801	48095101	3p21.31	RN7SL664P	4.252084095	2.073204011	2.050972346	3	3	35.87548388	59.32318865
chr19	49635292	49640143	19q13.33	RRAS	9.356541371	4.563905971	2.050117033	4	4	79.86919947	57.44300806
chr21	15729934	15880072	21q21.1	USP25	232.7152476	113.6122665	2.048328536	4	4	42.53290864	16.33542337
chr7	151409161	151413354	7q36.1	WDR86-AS1	2.020296845	0.986418865	2.048112539	3	4	68.2469592	33.49983352
chr11	64746389	64760715	11q13.1	PYGM	1.448964719	0.707499928	2.048006878	4	4	85.76267638	72.42896751
chr7	115935148	116159891	7q31.2	TFEC	15.63594723	7.634885081	2.047961045	4	4	25.69795292	55.38724339
chr21	43719097	43762307	21q22.3	PDXK	5.670855727	2.769046187	2.047945518	4	4	47.37772543	32.69467802
chr1	143972639	144069704	1q21.1	SRGAP2D	2.251419623	1.099774407	2.047164954	3	4	24.87125831	109.238112
chr9	22002903	22009313	9p21.3	CDKN2B	0.712653726	0.348273391	2.04624799	4	4	50.25784149	53.45350738
chr4	9771125	10040248	4p16.1	SLC2A9	1.259416158	0.615482191	2.046226807	4	4	62.78292879	52.08518148
chr5	80066349	80083665	5q14.1	CTD-2201I18.1	0.566766756	0.277042278	2.045777126	3	3	66.04882462	48.51990651
chr1	116987997	117037481	1p13.1	CD101	14.03872499	6.864327727	2.045171144	4	4	48.654525	35.73856874
chr11	73264503	73298625	11q13.4	P2RY6	0.336068974	0.164363099	2.044674119	3	2	53.67237245	31.3922004
chr13	110523066	110561737	13q34	RAB20	17.01042888	8.319811852	2.044568937	4	4	60.11344494	46.53500414
chr6	18155388	18223853	6p22.3	KDM1B	35.30055723	17.26596425	2.044516988	4	4	21.55646921	15.5945348
chr6	27736575	27738514	6p22.1	GPR89P	0.317771983	0.155463635	2.04402775	3	1	37.05686612	0
chr21	44216979	44241004	21q22.3	ICOSLG	0.218173564	0.10677082	2.043381917	1	4	0	98.95521154
chrX	47624213	47630305	Xp11.23	CFP	39.56954214	19.3774468	2.042041067	4	4	77.31069882	33.57735622
chr2	90172899	90173412	2p11.2	IGKV3D-11	1.280355065	0.62714292	2.041568235	2	2	53.55707159	25.47105059
chr14	101120856	101123545	14q32.31	LINC02285	1.272466856	0.623500345	2.040843868	1	3	0	61.11125083
chr6	37632508	37697990	6p21.2	MDGA1	0.450588106	0.220907796	2.039711203	4	3	85.14156773	68.34309323

chr22	50245183	50251405	22q13.33	HDAC10	0.278887231	0.136732913	2.039649594	2	4	6.335362571	74.79503593
chr12	51817892	51848753	12q13.13	FIGNL2	0.190453626	0.093386204	2.039419288	2	2	55.6598919	56.51425725
chr8	56518318	56559823	8q12.1	LINC00968	0.581536805	0.285379198	2.037768729	4	4	74.61358386	54.84243058
chr6	122788456	122809720	6q22.31	SMPDL3A	5.852605262	2.873895325	2.03647127	4	4	28.89656413	43.80599738
chr15	78565520	78595269	15q25.1	CHRNA5	0.716777577	0.351998467	2.036308804	4	4	31.72106668	13.03587369
chr8	8154365	8216916	8p23.1	ENPP7P1	5.392867803	2.648446926	2.036237823	4	4	109.6604814	78.16392493
chr5	120464222	120794587	5q23.1	PRR16	0.488963961	0.240154744	2.03603707	3	2	75.89116175	2.891362629
chr1	152032506	152037035	1q21.3	S100A11	1094.931354	537.7894651	2.03598513	4	4	80.17316863	67.00001696
chr6	132815307	132815379	6q23.2	SNORD101	24.19685002	11.88671786	2.035620791	4	4	58.91576917	43.56700003
chr15	78921750	78945098	15q25.1	CTSH	17.30367401	8.500457548	2.035616778	4	4	83.27019391	20.05341375
chr17	30975335	30976004	17q11.2	DPRXP4	1.538754623	0.756287504	2.034615956	2	4	48.86005121	50.14226379
chr15	57953424	58065923	15q21.3	ALDH1A2	0.477553262	0.234781062	2.03403655	4	4	103.9136085	63.6025052
chr11	319673	320914	11p15.5	IFITM3	245.2687523	120.5922309	2.033868605	4	4	56.2207705	55.96112268
chr21	42893268	42911396	21q22.3	NDUFV3	9.215679687	4.531322214	2.03377276	4	4	35.75372484	24.16532889
chr16	11547722	11636377	16p13.13	LITAF	337.8627166	166.1306571	2.03371685	4	4	68.42602712	50.47575446
chr19	7669086	7670454	19p13.2	RETN	6.461891164	3.178422045	2.033050071	4	4	50.20127045	37.02729217
chr1	45620044	45624787	1p34.1	CCDC17	2.031068535	0.999208821	2.032676746	3	4	57.63644448	20.79935025
chr4	151102057	151102131	4q31.3	SNORD73B	18.02125821	8.866544307	2.032500779	4	4	56.48228052	77.19332632
chr9	84273123	84368634	9q21.32-q21.33	SLC28A3	0.594951217	0.292765676	2.032175442	4	2	38.19810742	60.94271805
chr9	137423372	137434406	9q34.3	NOXA1	1.217578964	0.599176186	2.032088376	3	3	117.3223442	82.74117073
chr2	95274448	95291320	2q11.1	PROM2	0.092019339	0.045285658	2.031975327	1	1	0	0
chr6	52791380	52803967	6p12.2	GSTA1	2.493355641	1.227489607	2.031264157	4	4	140.1163582	98.01849133
chr10	49734641	49762379	10q11.23	OGDHL	0.090865297	0.044797314	2.028364855	1	1	0	0
chr17	30921736	30959398	17q11.2	ADAP2	5.714994748	2.819331095	2.027074705	4	4	79.94390816	48.54136482
chr19	54593576	54602381	19q13.42	LILRA1	10.66736737	5.263417394	2.026699873	4	4	92.1688958	45.34544302
chr10	43194533	43266919	10q11.21	RASGEF1A	1.364241738	0.673215383	2.026456574	4	4	42.84662652	63.84135657
chr7	149102784	149126346	7q36.1	ZNF425	1.050321154	0.518312185	2.026425743	4	4	44.8536069	32.67662094
chr9	117704175	117717491	9q33.1	TLR4	253.4410236	125.1070202	2.025793782	4	4	83.57513194	51.76997133
chr7	5926136	5970850	7p22.1	RSPH10B	0.264685646	0.130684371	2.025381031	1	2	0	80.99213411
chr17	50165517	50175932	17q21.33	SGCA	0.217810083	0.107541543	2.025357602	2	1	9.862846499	0
chr3	57693205	57696651	3p14.3	DENND6A-DT	0.175572748	0.086704344	2.024959059	3	2	62.20843697	3.310100082
chr16	84819981	84909510	16q24.1	CRISPLD2	30.24911091	14.94218104	2.024410683	4	4	66.22878361	31.24409253
chr8	66562175	66613249	8q13.1	MYBL1	26.66089581	13.18517579	2.022035674	4	4	34.99332562	32.40111529
chr17	28727899	28742766	17q11.2	NEK8	0.939323583	0.4645773	2.02188868	4	4	72.72713489	50.52608351
chr13	51813347	51845152	13q14.3	TMEM272	3.185225714	1.575746961	2.021406858	4	4	93.20370302	72.19055363
chr3	139609669	139609939	3q23	RN7SL724P	2.430461876	1.202870669	2.020551284	2	1	66.36726496	0
chr21	34516442	34615142	21q22.12	RCAN1	3.416720136	1.691075334	2.020442299	4	4	45.67021386	4.346845733
chr2	89040224	89040698	2p11.2	IGKV1-12	7.257987765	3.592891696	2.020096451	4	2	53.41774357	57.86562998
chr1	20112650	20119566	1p36.12	PLA2G2D	0.199033206	0.098531757	2.019990426	2	1	39.89424995	0
chr3	53161207	53192717	3p21.1	PRKCD	40.0570007	19.83804581	2.019200938	4	4	71.00459607	41.39839964
chr12	52270507	52309163	12q13.13	KRT86	0.317402275	0.157195441	2.019157004	2	3	87.83253281	58.77421138
chr6	24540128	24646155	6p22.3	KIAA0319	0.785373148	0.388968164	2.019119356	4	4	99.55002668	107.7837005
chr19	43872363	43883964	19q13.31	ZNF404	0.911583503	0.451563222	2.018728407	4	4	69.20461162	99.44510053

chr17	7558292	7561608	17p13.1	TNFSF13	5.302677057	2.626948664	2.018568969	4	4	48.37518227	49.268402
chr9	32566181	32567271	9p21.1	DFFBP1	1.271242426	0.629817008	2.018431401	3	4	21.27198082	54.09478033
chr7	128937032	128950042	7q32.1	IRF5	10.76105399	5.332880932	2.017868789	4	4	52.59721293	43.78476841
chr17	67991099	67993649	17q24.2	C17orf58	1.240424202	0.61488076	2.017341058	4	4	43.17013002	46.74132356
chr5	181039872	181061525	5q35.3	BTNL9	0.085030847	0.042152776	2.01720633	1	1	0	0
chr6	17600287	17611719	6p22.3	FAM8A1	100.8479769	50.00008542	2.016956091	4	4	42.11180893	42.7739072
chrX	153935263	153944780	Xq28	RENBP	9.173259979	4.548188213	2.016904215	4	4	77.20993177	24.80403629
chr1	1352689	1363541	1p36.33	MXRA8	0.193005949	0.095721313	2.01633203	3	3	104.8031736	32.46659828
chr11	101915015	102001065	11q22.1	CEP126	0.800042245	0.396813477	2.01616702	4	4	51.35263209	36.47411196
chr2	240605408	240631259	2q37.3	GPR35	1.074187976	0.532828079	2.016012328	4	4	116.5132694	28.15837428
chr17	47017190	47054412	17q21.32	LRRC37A17P	0.100908554	0.050078411	2.015011099	4	2	55.40393785	0.560086232
chr1	2590639	2633042	1p36.32	MMEL1	0.128266165	0.063657479	2.014942587	3	2	69.14163441	6.794642307
chr17	82290046	82292814	17q25.3	LINC01970	0.608262985	0.301913069	2.014695779	2	3	56.66018067	50.40212163
chr7	102912897	102945111	7q22.1	LRRC17	0.283445294	0.140698554	2.014557268	3	3	93.20699675	57.3350418
chrX	49162569	49168157	Xp11.23	MAGIX	0.371568002	0.184514842	2.013756715	4	4	87.72451783	33.56823044
chr19	38304161	38305006	19q13.2	C19orf33	1.432951615	0.711673894	2.013494703	3	3	29.56178766	26.20580248
chr12	674990	716677	12p13.33	LINC02455	1.146308443	0.569430503	2.013078747	3	3	49.34407156	13.15119499
chr16	67159988	67164174	16q22.1	FBXL8	0.932483521	0.463298324	2.012706443	4	4	38.91692714	40.79395922
chr4	15651521	15690447	4p15.32	FAM200B	17.39590095	8.646203049	2.011969977	4	4	37.8637492	37.06428944
chr4	101330302	101330414	4q24	MIR1255A	4.409094913	2.191702184	2.011721732	2	1	27.85702305	0
chr8	143636013	143653730	8q24.3	ZNF623	0.095223785	0.0473505	2.011040755	4	4	41.49999701	45.1109413
chr1	1598008	1600096	1p36.33	FNDC10	1.555779243	0.773712553	2.01079747	4	4	84.89532182	74.96360151
chr7	134779413	134970729	7q33	CALD1	4.821313402	2.397938808	2.010607354	4	4	79.98700008	30.69587517
chr10	87659719	87747705	10q23.2-q23.31	PAPSS2	2.900446449	1.442802688	2.01028628	4	4	72.30475807	48.41585179
chr15	84816680	84873482	15q25.3	ALPK3	0.131798544	0.065570751	2.010020346	2	3	8.693957312	111.3073408
chr6	13574217	13615158	6p23	SIRT5	56.70993004	28.22462067	2.009236217	4	4	58.47490226	22.36986739
chr1	158353123	158357554	1q23.1	CD1E	0.97442569	0.484991542	2.009160174	4	3	74.08588535	45.41151572
chr5	75847463	76353939	5q13.3	SV2C	0.042190114	0.021002014	2.008860372	1	1	0	0
chr17	67471489	67471585	17q24.2	MIR548AA2	5.214755835	2.595979797	2.008781364	2	1	27.24558174	0
chr15	36617012	36618423	15q14	LARP4P	0.242695167	0.120823093	2.008681952	3	3	47.90310265	13.58303519
chr1	212935543	212992305	1q32.3	VASH2	0.089459352	0.044542855	2.008388389	2	2	91.90921111	49.77911139
chr11	18698735	18704785	11p15.1	TMEM86A	1.146877	0.571341487	2.007340665	3	4	62.04292629	15.0817806
chr1	156699606	156705816	1q23.1	CRABP2	0.444363264	0.22142433	2.006840277	3	2	36.03518985	6.153320566
chr11	60378485	60395954	11q12.2	MS4A7	28.01574387	13.96181185	2.006598009	4	4	64.50161483	41.76745397
chr1	156292687	156295689	1q22	GLMP	5.658564549	2.82050692	2.006222537	4	4	69.38026899	38.5242743
chr21	39315707	39323218	21q22.2	BRWD1-AS1	1.045687655	0.521294931	2.005942494	3	2	85.83349837	36.17560746
chr2	85694291	85698854	2p11.2	GNLY	85.30424071	42.52803724	2.005835356	4	4	28.79998962	35.94790362
chrX	37571569	37684463	Xp21.1	LANCL3	1.557129877	0.776309722	2.005810094	4	4	25.08673867	32.62684067
chr22	20363621	20390758	22q11.21	USP41	0.358772434	0.178878473	2.005676974	3	2	52.66806795	14.43341024
chr5	126751963	126776486	5q23.2	LMNB1-DT	0.862704749	0.430184273	2.005430702	4	2	54.1401453	49.49795505
chr6	151494040	151621193	6q25.1	CCDC170	2.345841761	1.169810793	2.005317248	4	4	133.9493367	62.85057562
chr9	124257606	124352442	9q33.3	NEK6	11.82340628	5.896466533	2.005168046	4	4	65.86981765	34.12589119
chr3	101940859	101997926	3q12.3	LINC02085	0.490650662	0.244860943	2.003793075	4	3	65.70277106	41.7644968

chr19	9621482	9634862	19p13.2	ZNF561-AS1	0.447739809	0.223498921	2.003319777	3	4	60.26335899	69.55164634
chr7	66682091	66811464	7q11.21	RABGEF1	0.907694804	0.453192018	2.002892304	4	4	46.72380314	33.09010042
chr17	82217951	82239499	17q25.3	SLC16A3	24.28143603	12.12521696	2.002556829	4	4	75.08028072	35.12620448
chr3	72035519	72100455	3p13	LINC00877	6.158421899	3.080455926	1.999191693	4	4	88.42350162	34.47146705
chr21	44921035	44929680	21q22.3	ITGB2-AS1	14.29559922	7.15112465	1.999070066	4	4	50.86293651	30.82214973
chr5	42799880	42812076	5p12	SELENOP	0.710529877	0.355437146	1.999031012	4	4	103.2573206	68.63927532
chr1	205657853	205680502	1q32.1	SLC45A3	2.859527116	1.430493128	1.998979974	4	4	70.27313749	66.95355476
chr1	112701127	112707403	1p13.2	RHOC	6.874845688	3.439722182	1.998663067	4	4	67.16991699	26.67785909
chrX	17375420	17735994	Xp22.2-p22.13	NHS	2.669483281	1.335905144	1.998258104	4	4	90.91125782	57.2142806
chr15	30552078	30562501	15q13.2	GOLGA8Q	0.860430961	0.430701021	1.997745348	2	2	12.02704575	100.8614851
chr12	27446482	27506911	12p11.23	SMCO2	0.267354914	0.133840756	1.997559801	1	1	0	0
chr5	145858538	145931673	5q32	GRXCR2	1.390415816	0.69607517	1.997508138	4	4	45.79457857	47.22639745
chr7	91264468	91268817	7q21.13	FZD1	1.687617311	0.84505991	1.997038661	4	4	60.70160585	74.18076481
chr17	74466373	74484798	17q25.1	CD300A	97.00729608	48.58233049	1.996760861	4	4	56.8275032	30.05821363
chrX	56736788	56737584	Xp11.21	UQCRBP1	1.51984349	0.761192244	1.996661818	1	1	0	0
chr9	93121489	93134304	9q22.31	NINJ1	71.89784261	36.00996443	1.996609654	4	4	73.79494895	34.1322117
chr3	172505508	172523507	3q26.31	TNFSF10	189.9037713	95.15876254	1.995651963	4	4	73.506141	50.52563457
chr18	59899960	59904306	18q21.32	PMAIP1	49.49711715	24.81348594	1.994766768	4	4	79.65174127	39.03614699
chr4	168356735	168480514	4q32.3	DDX60L	62.54914841	31.36391764	1.994302788	4	4	44.65270584	60.47273235
chr6	27247701	27256620	6p22.1	PRSS16	0.104443582	0.05240753	1.99291173	1	1	0	0
chr8	116874424	116876868	8q24.11	RAD21-AS1	0.250231893	0.125572598	1.992726895	3	2	37.95739289	7.270987495
chr3	49020618	49020709	3p21.31	MIR191	5.477740732	2.749062693	1.992584871	2	1	26.85409097	0
chr14	22526844	22526906	14q11.2	TRAJ17	14.39933401	7.228055002	1.992145052	1	4	0	60.98420005
chr20	32052188	32101856	20q11.21	HCK	198.3113828	99.58747178	1.991328621	4	4	78.22658473	60.28957413
chr20	1564193	1620447	20p13	SIRPB1	17.82018131	8.949419611	1.991210837	4	4	56.57185988	65.62155994
chr11	73200416	73246743	11q13.4	P2RY2	5.196667249	2.609821305	1.991196577	4	4	95.81651141	58.75349949
chr15	26115813	26133037	15q12	LINC00929	0.309244357	0.155419193	1.98974368	2	1	9.045478381	0
chr19	35447965	35451767	19q13.12	FFAR2	121.9394977	61.29408335	1.989417103	4	4	56.48362664	69.30013652
chr2	24175043	24191698	2p23.3	FAM228A	0.243370983	0.122336586	1.989355682	2	1	27.66887093	0
chr2	28927067	28927144	2p23.2	SNORD53	16.56349371	8.327048372	1.98911943	4	3	41.56364302	93.89650602
chr8	135457461	135656516	8q24.23	KHDRBS3	0.140592888	0.070682115	1.989087182	1	2	0	83.21288775
chr2	96248514	96266013	2q11.2	TMEM127	44.19409729	22.22115628	1.988829777	4	4	76.75294182	13.47961485
chr6	10747759	10759774	6p24.2	TMEM14B	25.44340137	12.79437181	1.988640143	4	4	72.74510126	40.2401488
chr9	127935095	127938484	9q34.11	DPM2	76.90295566	38.67606726	1.988386129	4	4	61.59674361	15.57707706
chr2	131682427	131767404	2q21.2	C2orf27A	0.504443072	0.253833838	1.987296397	3	3	128.5581691	68.53278156
chr16	84292233	84329851	16q24.1	WFDC1	0.0560565	0.028207551	1.987287035	2	2	58.98103582	8.0596106
chr15	99790156	99806927	15q26.3	DNM1P46	0.341053115	0.171638061	1.987048285	3	3	82.79467712	108.660318
chr6	41208554	41217947	6p21.1	TREML3P	10.7310911	5.400656243	1.98699762	4	4	100.954656	49.63774012
chr12	68842193	68963240	12q15	CPM	1.32497709	0.6668618	1.986884074	4	4	70.15664706	70.52579239
chr10	75431646	76557375	10q22.2-q22.3	LRMDA	0.86736231	0.436605859	1.986602544	4	4	76.97661159	49.32302169
chr21	25880550	26171128	21q21.3	APP	35.22061275	17.72911291	1.986597578	4	4	85.80280628	31.0216952
chr1	154697462	154870278	1q21.3	KCNN3	0.115228742	0.058009955	1.986361519	3	3	49.1284801	34.3315894
chr2	101006435	101195321	2q11.2	TBC1D8	7.560201917	3.806504239	1.986127281	4	4	67.72225778	41.61777285

chr17	56978107	57006768 17q22	SCPEP1	28.79318601	14.49777513	1.986041703	4	4	45.68419037	21.60948673
chr9	32455302	32526324 9p21.1	DDX58	98.20313195	49.44753933	1.986006448	4	4	65.49288856	55.60112758
chr19	5720156	5778734 19p13.3	CATSPERD	0.432776622	0.217918115	1.985959826	4	4	50.34454005	45.81594822
chr5	154389769	154445822 5q33.2	SAP30L-AS1	0.788831433	0.397238321	1.98578886	3	2	15.17163181	93.44937675
chr2	37641882	37672535 2p22.2	CDC42EP3	42.09727936	21.20797116	1.984974378	4	4	64.8603807	34.04070166
chr14	102582888	102583177 14q32.31	RN7SL546P	6.781724222	3.416877309	1.98477253	4	4	64.87822255	62.03909722
chr10	70882280	70888786 10q22.1	PCBD1	2.917991784	1.470618188	1.984193999	4	4	80.53081251	65.79647062
chr14	105586937	105588394 14q32.33	IGHA2	2.912862702	1.468377597	1.983728646	4	3	78.24468422	68.312808
chr2	88857361	88857683 2p11.2	IGKC	583.7141941	294.2991221	1.983404469	4	4	63.73174722	125.7379398
chr19	10416773	10469631 19p13.2	PDE4A	1.546586028	0.779846648	1.983192506	4	4	61.11040355	39.76856523
chr6	122471917	122726373 6q22.31	PKIB	0.413653714	0.208586713	1.983125907	3	4	90.39342263	25.37535352
chr1	247990267	247991191 1q44	OR2L1P	0.908519985	0.458386954	1.98199355	2	4	86.6847974	50.73342133
chr8	28698403	28701464 8p21.1	EXTL3-AS1	0.204946608	0.103405634	1.981967529	3	3	74.77722853	31.46512676
chr19	35667948	35673291 19q13.12	UPK1A-AS1	0.57774304	0.291509064	1.981904206	2	2	54.50590062	13.55436067
chr1	39678648	39691710 1p34.2	HPCAL4	1.565673255	0.790011704	1.981835518	3	4	74.90769565	110.4108512
chr2	27078567	27086403 2p23.3	EMILIN1	0.435156077	0.219620985	1.981395713	3	4	22.43653605	19.99060898
chr17	59220467	59275970 17q22	GDPD1	12.51851239	6.31926137	1.9810088	4	4	99.59636654	30.01383698
chr4	74097035	74099280 4q13.3	CXCL2	1.119653152	0.565246836	1.980821617	3	4	46.49573969	70.3927837
chr9	88991409	89005155 9q22.1	S1PR3	1.91290957	0.965841716	1.980562176	4	4	86.11689845	41.9630484
chr6	149818758	149864344 6q25.1	LRP11	3.53099998	1.783204046	1.980143544	4	4	65.00533219	51.22074215
chr3	169764610	169765060 3q26.2	TERC	0.949663569	0.479631982	1.979983833	1	2	0	3.115295969
chr9	127815012	127854773 9q34.11	ENG	2.489028157	1.257192359	1.979830802	4	4	66.55320902	55.84197785
chr2	21123932	21147325 2p24.1	TDRD15	0.08257724	0.041713585	1.97962462	4	2	39.29958823	1.563712987
chr7	38253380	38253429 7p14.1	TRGJ2	30.56326771	15.45086706	1.978094018	4	3	49.60086565	45.64343355
chr19	45668187	45683724 19q13.32	GIPR	0.306808983	0.155112275	1.977980027	4	3	96.19770268	56.73942092
chr1	168055359	168056493 1q24.2	GCSHP5	1.229446205	0.621574777	1.977953822	2	1	26.43168239	0
chr3	112086335	112136379 3q13.2	C3orf52	0.437240903	0.22107753	1.977771795	4	4	70.71076892	66.94943071
chr2	218781733	218815293 2q35	CYP27A1	12.66387445	6.406659346	1.976673609	4	4	96.59456117	38.02481834
chr12	7812512	7891196 12p13.31	SLC2A14	2.099422519	1.062207511	1.976471167	4	4	44.91241464	29.98477789
chrX	134550024	134560398 Xq26.3	LINC00629	0.917326166	0.464313869	1.975659627	1	1	0	0
chr17	36601583	36634698 17q12	MRM1	0.789371854	0.399564659	1.975579763	3	4	72.03779777	72.81237982
chr10	103125017	103125416 10q24.33	RPS15AP29	5.402612563	2.735091634	1.975294903	3	4	44.25526953	46.46404836
chr14	102362912	102502481 14q32.31	TECPR2	17.63743307	8.929361227	1.975217781	4	4	59.41987353	56.24408244
chr15	25135642	25138053 15q11.2	PWAR1	0.233932014	0.118450899	1.974928141	2	1	29.78237478	0
chr2	64347255	64347745 2p14	RPL23AP37	0.930573329	0.471201704	1.97489381	3	1	59.99524639	0
chr6	107490104	107661309 6q21	SOBP	0.110938414	0.056184185	1.974548764	3	4	11.13820697	64.81248737
chr3	32258908	32260289 3p22.3	KRT18P15	0.388403948	0.196768767	1.973910567	2	1	17.97368232	0
chr11	10511678	10541227 11p15	RNF141	40.99384765	20.77016515	1.973689056	4	4	58.94645027	48.77785362
chr1	39955112	39969968 1p34.2	MFSD2A	1.66898445	0.845782052	1.973303222	4	4	71.067658	46.92041041
chr10	26509589	26509704 10p12.1	RNA5SP307	7.413158266	3.757134775	1.973088194	3	2	61.70077093	37.6612611
chr5	175657762	175710756 5q35.2	HRH2	23.19557396	11.76905846	1.970894617	4	4	70.04340141	72.48677454
chr3	15450133	15521751 3p25.1	COLQ	1.762412388	0.894582877	1.970094033	4	4	72.13195568	50.77797665
chr9	129336923	129347477 9q34.11	LINC01503	1.682067157	0.854037086	1.969548142	3	4	43.00325011	146.041912

chr17	7834210	7854796	17p13.1	KDM6B	13.99174761	7.105661036	1.969098657	4	4	85.55980402	34.83865509
chr10	6144878	6254648	10p15.1	PFKFB3	28.40568312	14.42878308	1.968681833	4	4	74.42443799	21.07538264
chr21	29372501	29373980	21q21.3	BACH1-IT2	9.278421203	4.713357844	1.968537402	4	4	12.25236605	43.55179576
chr10	79068994	79316528	10q22.3	ZMIZ1	11.14179523	5.660130676	1.968469612	4	4	74.4809348	29.93951597
chr12	93083293	93083716	12q22	RPL41P5	17.46857363	8.874610633	1.968376344	3	4	83.94173068	18.01351375
chr19	48455707	48466110	19q13.33	KCNJ14	0.115933768	0.058898602	1.968361945	3	3	61.19441795	0.931499177
chr3	21542789	21579959	3p24.3	ZNF385D-AS1	0.694148376	0.352691916	1.968143711	2	2	43.77985899	10.6538849
chr10	132330063	132381506	10q26.3	LRRC27	0.37084892	0.188433447	1.968063136	4	4	73.94941377	42.81163826
chr12	128853323	128984964	12q24.33	GLT1D1	62.75784028	31.9051776	1.967011156	4	4	74.09070121	66.36466608
chr17	7579487	7582111	17p13.1	CD68	0.631693821	0.321165614	1.966878751	3	4	51.70415211	61.97752301
chr17	28714281	28718440	17q11.2	RAB34	2.947394362	1.499051868	1.966172368	4	4	68.17284696	32.49102619
chr14	22520416	22520478	14q11.2	TRAJ23	36.12522799	18.37687588	1.965798116	3	3	52.25219832	59.55820968
chr17	73283624	73312004	17q25.1	CDC42EP4	2.54554568	1.294955684	1.965739609	4	4	65.24988012	16.30946476
chr22	37675606	37679802	22q13.1	LGALS1	61.3895001	31.23486498	1.965415895	4	4	32.50947926	12.44578544
chr1	59296405	59762730	1p32.1	FGGY	7.076274465	3.600800498	1.965194814	4	4	57.65404236	25.52472426
chr15	90206482	90206995	15q26.1	RPS12P26	2.927280209	1.490348031	1.964158805	4	2	66.49856905	61.50442915
chr11	134331874	134376324	11q25	GLB1L2	0.437020811	0.222537823	1.963804648	4	2	76.87342869	58.24886481
chr7	2631969	2664802	7p22.3	TTYH3	7.922000238	4.034430523	1.963598132	4	4	79.617288	30.54681995
chr8	102648777	102655902	8q22.3	KLF10	48.50441379	24.70590458	1.963272125	4	4	49.39550574	52.99223362
chr2	20552934	20651661	2p24.1	HS1BP3	4.325214248	2.203112285	1.963229146	4	4	96.07087087	32.8983136
chr9	137030174	137032840	9q34.3	FUT7	5.672767257	2.890664491	1.962444024	4	4	86.47609853	69.97868763
chr16	67229389	67247522	16q22.1	FHOD1	5.405073672	2.754459285	1.962299353	4	4	66.42629001	26.65137717
chr7	131500262	131556617	7q32.3	PODXL	0.245870339	0.12531957	1.961946887	4	4	75.04660567	64.60719505
chr10	30919008	30921130	10p11.23	DDX10P1	1.714177402	0.873893848	1.961539614	4	4	84.98975691	86.17411988
chr2	185738836	185833289	2q32.1	FSIP2	0.050670865	0.025833392	1.961448399	3	3	67.50395349	69.06057123
chr12	95308420	95308513	12q22	MIR331	6.394353715	3.260053363	1.961426088	1	3	0	27.90616109
chr17	8186945	8187004	17p13.1	MIR4521	8.078183025	4.120029383	1.960710052	2	2	17.89828008	24.29668762
chr15	92617447	92734219	15q26.1	FAM174B	2.023752002	1.032198843	1.960622235	4	4	34.78652974	59.36418291
chr5	56504592	56505114	5q11.2	RPL26P19	3.583582014	1.827961528	1.960425293	4	4	78.76011224	58.85602844
chr6	31353866	31357245	6p21.33	HLA-B	503.4998332	256.8850147	1.96002026	4	4	104.1118688	17.07499705
chr3	150659885	150704563	3q25.1	ERICH6	0.678787894	0.346381133	1.959656083	3	4	123.3469852	88.66090238
chr1	25338298	25362361	1p36.11	TMEM50A	154.3765556	78.78277665	1.959521639	4	4	28.24603718	24.23584321
chr21	37223420	37268108	21q22.13	VPS26C	14.44071338	7.370022585	1.959385228	4	4	42.22148293	19.03288733
chr16	12081237	12081532	16p13.13	RPS23P6	2.678624524	1.367203444	1.959199661	1	2	0	62.33932471
chr1	223108401	223143282	1q41	TLR5	15.72007437	8.025344955	1.958803573	4	4	79.57774531	83.23605669
chrX	37838836	37847636	Xp11.4	DYNLT3	18.6506389	9.522046032	1.958679766	4	4	31.19042279	45.26552154
chr6	138403531	138413445	6q24.1	HEBP2	10.32087209	5.269638579	1.958554071	4	4	60.59758601	29.55985766
chr2	109542797	109614206	2q13	SEPT10	1.405846504	0.717905974	1.958259932	4	4	68.28773316	79.13786771
chr3	129893871	129908912	3q22.1	TMCC1-AS1	0.547711809	0.279737579	1.95794863	4	4	48.31889625	72.52175827
chr20	34990400	34990472	20q11.22	MIR499B	8.212940623	4.19932912	1.955774455	1	3	0	27.24885373
chr19	39369162	39369448	19q13.2	RN7SL566P	1.691542505	0.864938467	1.955679589	3	1	18.07541658	0
chr11	117179223	117197445	11q23.3	SIDT2	7.686314838	3.930492857	1.955560057	4	4	53.3389146	48.08792171
chr12	51589958	51812864	12q13.13	SCN8A	0.14193152	0.072591527	1.955207809	4	4	74.21329597	94.38905978

chr6	143059363	143343594	6q24.2	AIG1	9.255287707	4.734564638	1.954833953	4	4	34.47161066	29.87736292
chr20	43652508	43653340	20q13.12	RPL27AP	1.153757386	0.590266623	1.954637686	1	1	0	0
chr19	41616986	41627087	19q13.2	CEACAM4	13.71213122	7.018311846	1.953764883	4	4	78.1142211	63.02310589
chr4	169790949	169791836	4q33	PTGES3P3	2.59789926	1.329918863	1.953426884	4	4	63.13038127	28.03707831
chr14	106790692	106791153	14q32.33	IGHV3-72	3.576274449	1.831400094	1.952754323	4	2	111.0992356	43.49894555
chr19	39296325	39298672	19q13.2	IFNL1	0.898795102	0.460364953	1.952353446	3	2	49.22360708	50.55407218
chr22	30607083	30627060	22q12.2	TCN2	2.692352272	1.379105331	1.95224557	4	4	52.18132764	26.58950879
chr7	56010487	56051604	7p11.2	PSPH	1.434438271	0.734808815	1.952124474	4	4	54.11575834	62.04630542
chrX	53225784	53321356	Xp11.22	IQSEC2	0.323494272	0.165740405	1.95181297	4	4	67.67199093	73.34495367
chr12	6884682	6885786	12p13.31	DSTNP2	1.678460732	0.860100641	1.951470155	4	4	69.74021007	34.91671988
chr17	64145970	64146309	17q23.3	SNHG25	4.112931184	2.107642377	1.951436936	3	3	101.8753009	45.78559163
chr2	208236227	208255143	2q34	IDH1	12.99372558	6.659025837	1.951295265	4	4	53.00190461	28.33813813
chr9	112486968	112665311	9q32	KIAA1958	1.112172135	0.57001333	1.951133556	4	4	82.70253857	31.8584333
chr10	96750266	96750923	10q24.1	RPL13AP5	4.556720973	2.335529831	1.95104379	4	4	53.69603482	41.26876819
chrX	71911073	72153286	Xq13.1	NHSL2	16.4723911	8.444422242	1.950683023	4	4	61.23407495	55.75295525
chr7	95404862	95435072	7q21.3	PON2	2.192467845	1.124074908	1.950464181	4	4	51.0893649	42.55885987
chr9	87725437	87731469	9q21.33	CTSL	9.631323026	4.939285008	1.949942757	4	4	48.59176575	49.78011749
chr1	42785032	42800818	1p34.2	TMEM269	0.157549289	0.080812416	1.949567857	3	3	78.90773199	42.45825841
chrX	153411461	153426481	Xq28	ZFP92	0.822108858	0.42169515	1.949533586	4	4	80.44338364	53.50573299
chr19	48325372	48330554	19q13.33	EMP3	106.5876774	54.67449179	1.949495531	4	4	47.08049089	26.98894307
chr3	9947404	9954787	3p25.3	PRRT3-AS1	1.52417538	0.781861879	1.949417692	3	2	19.25099848	63.26133149
chr5	173020726	173020929	5q35.1	SNORA74B	240.5380365	123.438762	1.948642652	4	4	67.52878828	29.92985982
chr11	30863603	31369810	11p13	DCDC1	0.076913313	0.039472643	1.948521964	1	1	0	0
chr11	61130256	61161444	11q12.2	VPS37C	5.738198896	2.944955259	1.948484236	4	4	58.51080995	30.19545799
chr4	184851013	184851110	4q35.1	MIR3945	114.2836554	58.65547028	1.948388698	4	4	57.71513735	94.06766427
chr15	69284134	69407637	15q23	PAQR5	0.077132172	0.039591445	1.948202992	2	2	23.58322151	7.607190784
chr19	46838158	46850995	19q13.32	AP2S1	40.43760453	20.7589662	1.947958494	4	4	36.76329533	23.52849357
chr11	102789910	102798235	11q22.2	MMP1	1.44034129	0.739983373	1.946450883	3	4	92.99440748	134.9410201
chr2	30187434	30187566	2p23.1	SNORA10B	3.648540924	1.874502382	1.946405062	1	1	0	0
chr14	68787655	68796243	14q24.1	ZFP36L1	82.36381026	42.33107327	1.945705693	4	4	71.77990391	55.8295038
chr10	90738693	90857914	10q23.31	HTR7	0.526240475	0.270481476	1.945569371	2	3	58.37831288	35.19588906
chr16	28466653	28492302	16p12.1	CLN3	1.288212973	0.662128416	1.945563642	4	4	63.86722849	41.42277447
chr3	190514051	190657197	3q28	IL1RAP	28.34676378	14.57315172	1.945136118	4	4	59.23780463	55.23256624
chr13	79480722	79556077	13q31.1	NDFIP2	3.283856565	1.68908329	1.944164971	4	4	45.59080297	27.13544367
chr16	14200366	14202119	16p13.12	TVP23CP2	0.991609765	0.510090682	1.943987214	1	1	0	0
chr12	51328443	51346679	12q13.13	CELA1	0.559543755	0.287839653	1.943942568	2	2	28.5254448	22.20927102
chr5	140711276	140711373	5q31.3	VTRNA1-1	23.81779354	12.25300846	1.943832293	4	3	75.32702118	72.21343601
chr16	68358327	68448703	16q22.1	SMPD3	5.75636898	2.961380119	1.943812935	4	4	121.0229305	65.61439906
chr5	145936563	146081773	5q32	SH3RF2	0.14047235	0.07228081	1.943425219	3	3	90.6674381	50.70917807
chr14	73711783	73714425	14q24.3	PNMA1	14.94646486	7.691690094	1.943196446	4	4	51.74855784	28.0583162
chr22	22195799	22196276	22q11.22	IGLV6-57	8.7135688	4.484171633	1.943183605	4	4	69.85961152	93.54541087
chr19	12461184	12484818	19p13.2	ZNF709	0.087657077	0.04511224	1.943088539	2	3	64.21087613	1.567420908
chr16	31214021	31225656	16p11.2	TRIM72	0.060342175	0.03105846	1.942857947	2	1	53.84795055	0

chr20	47654569	47786616	20q13.12	SULF2	43.68252713	22.48376797	1.942847266	4	4	86.64447359	20.55200283
chr17	27746040	27755279	17q11.2	LGALS9DP	0.739134217	0.38044587	1.942810464	1	2	0	11.5493853
chrX	78747658	78758714	Xq21.1	LPAR4	0.697709889	0.359186041	1.942474958	4	3	62.7212921	77.50352271
chr19	43460794	43465679	19q13.31	LYPD3	0.488828141	0.25176188	1.941628893	3	2	69.80096833	86.762286
chr1	171864192	171864687	1q24.3	DNM3-IT1	3.850914814	1.983570156	1.941405906	4	2	99.37988041	62.64327034
chr6	29826967	29831130	6p22.1	HLA-G	0.577880157	0.297695616	1.941177919	3	3	122.286074	75.51560132
chr9	87553454	87554459	9q21.33	DAPK1-IT1	0.635417126	0.327444155	1.940535867	2	1	22.87384561	0
chr6	41267385	41286745	6p21.1	TREM1	121.1247175	62.428073	1.940228358	4	4	72.89963446	67.32896304
chr16	31259967	31332892	16p11.2	ITGAM	107.1590436	55.24496932	1.939706817	4	4	73.5166038	45.91118191
chr3	42653684	42667580	3p22.1	ZBTB47	2.941887836	1.516753149	1.939595667	4	4	84.52836796	51.34699178
chr6	29602228	29633135	6p22.1	GABBR1	0.656641434	0.338558597	1.939520781	4	4	56.95322704	70.78480469
chr11	64251523	64269452	11q13.1	PLCB3	3.597953139	1.855457828	1.93911879	4	4	74.64570663	73.36048128
chr20	8248704	8249742	20p12.3	PLCB1-IT1	1.169453032	0.603131599	1.938968268	4	2	90.94190163	30.3053693
chr7	151048292	151052756	7q36.1	ASIC3	0.346663107	0.178853747	1.938249063	3	2	51.17089352	57.2351201
chr13	114173082	114223032	13q34	CFAP97D2	0.421638917	0.217639967	1.937323012	2	2	61.38589154	4.068378421
chr10	87504466	87553461	10q23.2	MINPP1	242.8904066	125.3839347	1.937173269	4	4	88.71932395	61.45944432
chr19	54572997	54587560	19q13.42	LILRA2	16.11179945	8.325048678	1.935339969	4	4	74.17690427	70.62824575
chr4	47485261	47593486	4p12	ATP10D	10.44205264	5.395481832	1.93533274	4	4	50.14156361	44.93511819
chr1	201888669	201899508	1q32.1	SHISA4	32.60752229	16.85028023	1.935132345	4	4	40.06714637	73.11235283
chr12	96000753	96043520	12q23.1	LTA4H	60.39036723	31.21819552	1.934460536	4	4	72.49033053	27.69624435
chr5	173989159	174007524	5q35.2	C5orf47	0.33113892	0.171186133	1.934379349	2	4	18.6185068	85.10063962
chr15	75201880	75212169	15q24.2	C15orf39	42.94416271	22.20247058	1.934206491	4	4	71.9242047	16.60696138
chr11	133908564	133956960	11q25	IGSF9B	0.462843967	0.239302931	1.934134135	3	4	112.4038243	59.72398594
chr22	46576017	46682755	22q13.31	GRAMD4	9.298381304	4.80779091	1.934023646	4	4	53.39333314	29.40603206
chr11	12163683	12163767	11p15.3	MIR6124	6.500138998	3.361324717	1.933802755	2	1	12.42184432	0
chr11	75260122	75351831	11q13.4	ARRB1	23.26390501	12.03207832	1.933490158	4	4	82.67853877	23.51006426
chr21	33577551	33588708	21q22.11	DONSON	4.176503569	2.160306007	1.933292578	4	4	46.27194694	20.83675255
chr3	47562238	47580240	3p21.31	CSPG5	0.120095145	0.062121877	1.93321822	2	1	60.42805546	0
chr7	148987136	148987248	7q36.1	RNY1	10651.14838	5509.589902	1.933201667	4	4	111.2267572	63.87005367
chr15	101175723	101251932	15q26.3	CHSY1	64.72634592	33.48576198	1.932951263	4	4	50.55238531	47.87868901
chr1	21217247	21345541	1p36.12	ECE1	51.5278658	26.6625288	1.932594848	4	4	71.91417392	24.61327016
chr10	44959753	44994724	10q11.21	RASSF4	5.095269805	2.636663618	1.932468659	4	4	63.32734872	28.71693318
chr2	32224449	32265743	2p22.3	NLRC4	26.98096286	13.96631912	1.931859256	4	4	64.64236595	70.6900279
chr11	104994235	105023902	11q22.3	CASP5	23.51290314	12.17218994	1.931690457	4	4	93.93993079	87.47016111
chr16	80540259	80550811	16q23.2	DYNLRB2	0.335290825	0.173645343	1.930894431	1	1	0	0
chr4	83535634	83605875	4q21.23	GPAT3	30.10877397	15.59476894	1.930697024	4	4	61.80521086	63.54033505
chr7	73834590	73842527	7q11.23	METTL27	0.505997014	0.262104461	1.930516599	2	2	78.58695622	26.60275604
chr16	84974460	85011535	16q24.1	ZDHHC7	50.9564912	26.39866106	1.93026802	4	4	75.59230429	33.31842126
chr3	125929272	125995394	3q21.2	ALG1L	1.018616483	0.527779061	1.930005485	1	2	0	68.59271244
chr7	64665010	64711582	7q11.21	ZNF107	23.16829151	12.00554647	1.929798995	4	4	55.15296963	19.72975759
chr8	51817575	51899186	8q11.23	PCMTD1	917.5658165	475.507934	1.929654062	4	4	56.63758864	35.92448866
chr3	135965673	136149615	3q22.2-q22.3	PPP2R3A	0.229677797	0.119028799	1.92959854	4	4	98.20390782	61.03485623
chr13	49531944	49585587	13q14.2	RCBTB1	12.09063836	6.266130018	1.92952242	4	4	27.81130935	21.29364351

chr5	100535305	100586741	5q21.1	FAM174A	24.34548023	12.62390214	1.928522573	4	4	64.12876533	66.7569084
chr4	5014586	5019470	4p16.2	CYTL1	0.972494799	0.504378311	1.928105905	3	1	50.0097273	0
chr21	44654213	44654659	21q22.3	KRTAP12-4	1.415115803	0.734048785	1.927822554	1	1	0	0
chr1	31727105	31764063	1p35.2	ADGRB2	0.158975658	0.082483848	1.927355014	4	3	97.09574309	72.22036308
chr11	72685069	72752389	11q13.4	ARAP1	52.84372392	27.42891284	1.926570121	4	4	66.09731642	25.80084727
chr15	78959947	79090873	15q25.1	RASGRF1	0.05038167	0.026154541	1.926306798	2	2	49.6952214	5.820383496
chr20	62952647	62968597	20q13.33	SLC17A9	0.810637177	0.420893879	1.925989466	4	4	88.79617908	41.43455624
chr5	139387568	139389916	5q31.2	MZB1	3.883512487	2.016424979	1.925939486	4	4	97.20812773	101.9911531
chr19	53793584	53824403	19q13.42	NLRP12	21.72623962	11.2820667	1.925732244	4	4	64.63921434	63.51075015
chr17	82735576	82751197	17q25.3	FN3K	0.570401332	0.296238496	1.925480113	4	4	61.99807429	66.98209167
chr10	128285950	128317726	10q26.2	LINC01163	0.346769337	0.180131307	1.925091991	3	4	75.39836012	82.94439831
chr7	30390885	30404055	7p14.3	LINC01176	2.883229309	1.497778756	1.925003474	4	4	25.38752842	69.3315685
chr1	15792727	15793346	1p36.21	RPL12P14	0.956076759	0.496719673	1.924781341	1	2	0	8.156659852
chr2	218125290	218137253	2q35	CXCR2	649.6313287	337.5234437	1.924699871	4	4	79.91843699	70.75781593
chr12	51124435	51125468	12q13.13	PHBP19	0.881229805	0.458103837	1.923646417	2	3	69.86769116	28.44841096
chr9	6278596	6281993	9p24.1	SELENOTP1	1.044526814	0.543099159	1.923270908	1	1	0	0
chrX	154835788	155022723	Xq28	F8	1.349751188	0.701925829	1.92292566	4	4	75.71653047	45.35908757
chr5	135448868	135452399	5q31.1	TIFAB	0.331056442	0.172256376	1.921882082	4	4	77.58699429	75.42077757
chr1	202010562	202017188	1q32.1	ELF3	0.305093859	0.158759615	1.921734685	1	3	0	87.65987205
chr12	56645027	56645101	12q13.3	SNORD59A	13.98382087	7.279440472	1.921002161	3	3	8.816596089	55.69152064
chr6	49605175	49636874	6p12.3	RHAG	82.30789449	42.84778348	1.920937043	4	4	90.64016924	66.22886527
chr12	54369129	54391610	12q13.13	ZNF385A	4.839760436	2.519811863	1.920683249	4	4	92.54478516	31.65169767
chr10	72273919	72276036	10q22.1	DDIT4	40.91410459	21.30438676	1.920454461	4	4	44.03702715	45.22504451
chr10	48684816	48982956	10q11.23	WDFY4	14.31617012	7.455747913	1.920152114	4	4	72.24137541	59.51059255
chr1	155173381	155184971	1q22	TRIM46	0.108385903	0.056450596	1.920013455	2	2	25.01602532	0.864108055
chr9	89605009	89606555	9q22.2	GADD45G	1.208600346	0.629617169	1.919579714	4	3	108.0265161	118.6000584
chr13	72708357	72727800	13q21.33	MZT1	16.40364951	8.546208428	1.919406676	4	4	55.40386788	28.30470447
chrMT	12138	12206	N/A	MT-TH	23.31731059	12.14826359	1.919394522	2	1	87.27283692	0
chr16	67482571	67483813	16q22.1	AGRP	0.574044233	0.299076494	1.919389332	1	1	0	0
chr4	109827994	109844707	4q25	RRH	0.49604371	0.258506518	1.918882799	4	3	37.23563599	58.33739403
chr3	149981661	149983366	3q25.1	TMEM183B	0.660656619	0.344378144	1.918404611	3	2	67.24641095	26.45634333
chr1	39757182	39788861	1p34.2	BMP8B	0.745246373	0.38848463	1.918341978	4	3	56.73399375	85.45608408
chr3	52245725	52250599	3p21.2	PPM1M	28.2996002	14.75592366	1.917846747	4	4	92.88727881	43.93512518
chr14	95534986	95544718	14q32.13	GLRX5	252.4763174	131.6475168	1.917820583	4	4	35.12131445	34.69882305
chr20	32762385	32809356	20q11.21	DNMT3B	0.396162348	0.206580615	1.917713082	3	4	78.73558406	70.48386584
chr19	35358106	35360491	19q13.12	FFAR3	0.526864359	0.274757385	1.917562143	3	4	73.43123625	50.33077584
chr21	6484623	6499969	21p12	U2AF1L5	0.956328046	0.498918913	1.916800549	4	4	83.53850605	54.80552836
chr11	59208510	59213021	11q12.1	MPEG1	199.6451172	104.21364	1.915729238	4	4	83.36079311	40.10309503
chr2	132261069	132263124	2q21.2	CDC27P1	0.889058797	0.464280449	1.914917588	4	3	65.43125403	31.7242487
chrX	84317874	84502578	Xq21.1	HDX	1.326488568	0.692953486	1.9142534	4	4	21.75000961	28.49874299
chr3	185507045	185552661	3q27.2	LIPH	3.531541295	1.844896844	1.914221549	4	4	39.56698386	44.25326935
chr3	171600404	171810494	3q26.31	PLD1	4.306286825	2.251371138	1.912739643	4	4	31.82930761	37.14046774
chr2	200305881	200482263	2q33.1	SPATS2L	2.308202564	1.207166212	1.912083474	4	4	64.53785785	84.29464991

chr10	65912518	67696217	10q21.3	CTNNA3	0.044785917	0.023427593	1.911673816	1	1	0	0
chr3	39141851	39154602	3p22.2	CSRNP1	67.01442155	35.05699252	1.911585014	4	4	49.4694137	52.42802976
chr3	157436791	157443628	3q25.32	PTX3	1.402289554	0.733647043	1.91139536	4	4	102.0378702	77.29942183
chr12	8102902	8138607	12p13.31	CLEC4A	30.22458626	15.8132342	1.911347539	4	4	46.97012244	41.74984261
chr19	45379634	45406349	19q13.32	PPP1R13L	0.254416327	0.133117539	1.911215666	4	3	73.06746661	51.63753355
chr16	21258518	21303136	16p12.2	CRYM	0.357795716	0.187255153	1.910738959	4	4	90.21875852	74.25488071
chr3	138347648	138405535	3q22.3	MRAS	1.346282178	0.704751956	1.91029222	4	4	73.68751488	39.0216742
chr12	111360651	111369121	12q24.12	PHETA1	1.987409046	1.040516034	1.91002251	4	4	83.39773749	59.08983554
chr2	85544720	85561534	2p11.2	GGCX	2.926997019	1.532668918	1.909738617	4	4	57.81366294	19.48784112
chr11	117836976	117877486	11q23.3	FXYD6	0.701807698	0.367508406	1.909637131	4	4	60.33751865	60.98877117
chr13	20973036	21061583	13q12.11	LATS2	11.84516743	6.203246237	1.909511081	4	4	47.94462331	12.4878899
chr10	45374166	45446121	10q11.21	ALOX5	69.36099497	36.32442781	1.909486237	4	4	79.87916773	57.93213557
chr21	46235125	46286297	21q22.3	MCM3AP	16.66832531	8.730256677	1.909259478	4	4	45.94923957	18.08591727
chr1	109687796	109693745	1p13.3	GSTM1	4.209795145	2.205112834	1.909106455	1	2	0	135.1670554
chr14	22542199	22542261	14q11.2	TRAJ4	14.61289865	7.658280602	1.908117423	4	4	43.1921288	70.61845162
chr18	46334224	46463145	18q21.1	RNF165	0.386140511	0.202369701	1.908094482	4	4	60.07874171	40.93458733
chr21	33642400	33899861	21q22.11	ITSN1	15.80745729	8.284631869	1.90804583	4	4	79.17953821	86.34458394
chr7	141927356	141947009	7q34	CLEC5A	2.558769178	1.341141764	1.907903583	4	4	56.59132312	63.85712987
chrX	46497725	46545466	Xp11.3	ZNF674	3.213452633	1.684637474	1.907503948	4	4	44.58666788	61.50133647
chr6	36027677	36122964	6p21.31	MAPK14	54.98569201	28.83475159	1.906924422	4	4	57.13050809	48.19417041
chr3	53058168	53065117	3p21.1	SERBP1P3	0.538784419	0.282597355	1.906544447	1	1	0	0
chr6	13521213	13522116	6p23	RPS4XP7	0.55872483	0.293059959	1.906520533	1	1	0	0
chr1	158248329	158258269	1q23.1	CD1A	0.914214829	0.479821913	1.905321129	3	3	94.74996078	83.16812788
chr9	133414339	133459403	9q34.2	ADAMTS13	0.08386988	0.0440269	1.904969013	2	1	40.89071223	0
chr16	70579895	70660682	16q22.1	IL34	0.398518801	0.209211904	1.904857199	2	1	95.50550139	0
chr17	28599133	28617377	17q11.2	SPAG5-AS1	0.602695871	0.316492138	1.904299663	3	4	35.17736487	50.46626366
chr1	145891208	145910189	1q21.1	ITGA10	0.192860282	0.101319075	1.903494302	3	2	18.41229969	32.81176569
chr12	123616710	123616840	12q24.31	SNORA9B	4.272663085	2.244823311	1.903340482	3	2	29.69377611	39.30813033
chr11	35943981	36232136	11p13	LDLRAD3	1.834656228	0.963942186	1.903284506	4	4	85.95852723	21.1679582
chr8	143574585	143578351	8q24.3	NAPRT	8.374033158	4.400243157	1.903084184	4	4	78.43945021	43.79268406
chr11	3826978	3840983	11p15.4	RHOG	131.8003467	69.25671414	1.903069591	4	4	63.16861965	19.55182425
chr8	145002811	145006046	8q24.3	ZNF252P-AS1	0.345459806	0.181567722	1.902649894	3	3	66.55452351	49.50913943
chr4	52659406	52661498	4q12	USP46-AS1	1.478228066	0.77696514	1.902566781	3	4	48.31226248	46.72527909
chr12	15620141	15789576	12p12.3	EPS8	1.061978798	0.558267307	1.902276536	4	4	53.47677327	48.0491656
chr21	32628759	32731247	21q22.11	SYNJ1	18.89511071	9.934240536	1.902018644	4	4	24.59386464	17.41348255
chr17	15927782	15975896	17p12	ADORA2B	0.930808834	0.489596017	1.9011773	4	3	56.93543587	85.43256893
chr9	120600918	120714487	9q33.2	MEGF9	160.9927378	84.68078092	1.901172096	4	4	57.6706791	38.02290017
chr12	120522539	120523451	12q24.31	RPL29P24	1.115029147	0.58655579	1.900977139	1	1	0	0
chr7	143381227	143391111	7q34	ZYX	115.1349398	60.59406806	1.900102493	4	4	76.90974274	48.79378884
chr5	180601506	180650298	5q35.3	FLT4	0.261513923	0.137646289	1.899898107	4	3	81.68611172	53.50878291
chr22	36913574	36940449	22q12.3	CSF2RB	204.1173278	107.4437404	1.89976007	4	4	60.8457793	67.91888411
chr11	72135710	72139892	11q13.4	FOLR3	4.928326178	2.59431764	1.899661823	4	4	94.8318362	85.67011708
chr11	9680924	9681238	11p15.4	RN7SKP50	1.794847159	0.944838817	1.899633172	3	2	54.29253218	22.54779045

chr3	69739435	69968337	3p13	MITF	2.346587068	1.235291425	1.899622243	4	4	107.2377632	31.91025435
chr10	114280760	114404778	10q25.3	AFAP1L2	1.147778787	0.604244019	1.899528584	4	4	73.9682351	52.39863331
chr2	185783691	185800151	2q32.1	FSIP2-AS1	1.227620787	0.646361309	1.899279504	2	2	106.8397535	8.537168373
chr11	78215290	78417822	11q14.1	GAB2	52.15052984	27.4587482	1.899231875	4	4	80.44195376	38.46794004
chr15	30604030	30617827	15q13.2	GOLGA8H	0.461715385	0.243319454	1.897568719	1	1	0	0
chr11	8947201	8965011	11p15.4	TMEM9B	43.57905555	22.97688742	1.896647476	4	4	10.52135364	12.58362892
chr12	51987657	51987763	12q13.13	RNU6-574P	3.760238645	1.983139975	1.896103499	3	1	21.22731734	0
chrX	23783158	23786223	Xp22.11	SAT1	584.5327574	308.518782	1.894642374	4	4	53.97298339	74.62860456
chr2	54972189	55137831	2p16.1	RTN4	52.89946135	27.92244567	1.894513897	4	4	23.51318314	18.69232165
chr21	36069924	36073177	21q22.12	CBR1	6.909345642	3.647188238	1.894430776	4	4	56.52219002	59.29740146
chr21	36748625	36990394	21q22.13	HLCS	3.531288976	1.864319719	1.894143445	4	4	29.77360001	21.21856889
chr1	192158462	192187172	1q31.2	RGS18	748.263569	395.0766656	1.893970548	4	4	22.04670743	43.10120719
chr3	195908076	195913264	3q29	TNK2-AS1	4.518042439	2.38555728	1.893914883	4	4	65.28745691	20.4473556
chr1	121167646	121186896	1p11.2	FAM72B	1.988864706	1.050356565	1.893513854	4	4	42.94399606	34.96496647
chr19	38433700	38587564	19q13.2	RYR1	0.237504956	0.125441624	1.89335046	4	4	98.80114155	81.78166049
chr7	143598038	143609179	7q35	TCAF1P1	0.335042981	0.17695981	1.893328107	4	3	86.61591224	51.1685552
chr10	71319253	71363390	10q22.1	SLC29A3	2.359473949	1.246851847	1.892345073	4	4	48.35236298	75.87226103
chr21	39184176	39321595	21q22.2	BRWD1	87.06183825	46.01111827	1.892191312	4	4	65.36460466	30.9638024
chr9	74722495	74888094	9q21.13	TRPM6	8.330816394	4.402974153	1.892088416	4	4	57.43316893	73.44968408
chr15	41899440	41972557	15q15.1	EHD4	4.217792045	2.229382231	1.891910676	4	4	61.62113317	53.40898067
chr2	177623249	178108339	2q31.2	PDE11A	0.081538528	0.04310492	1.891629274	2	1	29.89923208	0
chr4	140641191	140643080	4q31.21	TNRC18P1	0.45751183	0.24190885	1.891257096	3	3	87.13552428	94.25736894
chr3	181231737	181231842	3q26.33	RNU6-4P	60.71088029	32.10258587	1.891152337	4	4	49.36531758	17.01283093
chr3	46353744	46360940	3p21.31	CCR2	44.39350905	23.47734416	1.890908476	4	4	37.63581874	48.64324512
chr11	57460537	57477539	11q12.1	RTN4RL2	0.208968892	0.11053538	1.890515883	2	1	45.14685519	0
chr17	19123189	19159187	17p11.2	GRAPL	0.256816804	0.135854398	1.890382699	2	3	36.70774753	9.950432911
chrX	53422856	53431120	Xp11.22	RIBC1	0.356328743	0.188502548	1.890312601	3	4	62.04376728	63.59673429
chr22	22162198	22162681	22q11.22	IGLV4-60	2.60741801	1.379479031	1.890146898	2	3	27.78035174	12.54406809
chr19	4153601	4173054	19p13.3	CREB3L3	0.637131876	0.337115116	1.889953448	2	2	111.021675	33.11413945
chr17	7380559	7389964	17p13.1	TNK1	0.283646406	0.150152129	1.889060174	1	3	0	56.0479665
chr3	39107661	39139009	3p22.2	TTC21A	0.313358658	0.165888101	1.888976104	4	4	61.98983346	66.81777082
chr1	12166948	12209222	1p36.22	TNFRSF1B	205.0318154	108.5440932	1.888926513	4	4	66.26492206	8.729313442
chr13	76880169	76886405	13q22.3	KCTD12	59.25616865	31.39881331	1.887210452	4	4	53.70231623	18.95605889
chr1	155078872	155087538	1q21.3	EFNA3	0.240378065	0.127386127	1.887003485	2	1	22.086309	0
chr3	97821693	97944986	3q11.2	CRYBG3	8.282921769	4.390811558	1.886421601	4	4	57.21691536	53.08282292
chr3	50317790	50322850	3p21.31	HYAL2	1.165562233	0.617873115	1.886410339	4	4	52.72891823	23.34067779
chr7	35946290	35974692	7p14.2	SEPT7P3	0.98039719	0.519741714	1.886316151	4	4	24.55158631	27.9000756
chr6	39792366	39904877	6p21.2	DAAM2	0.999306924	0.529783911	1.886253819	4	3	164.6872199	124.280368
chr19	893484	893590	19p13.3	RNU6-9	61.13562455	32.4146949	1.886046583	4	4	53.53816647	42.37154846
chr9	137224635	137226311	9q34.3	CYSRT1	0.404531215	0.214489428	1.886019367	1	3	0	11.45953591
chr3	68975212	69013961	3p14.1	EOGT	6.460783002	3.425858769	1.885887141	4	4	45.796113	30.66348418
chr3	129316271	129324569	3q21.3	H1FX-AS1	0.295597472	0.156797986	1.885212175	3	4	93.5181752	12.12235309
chr10	35638249	35641434	10p11.21	FZD8	0.164773703	0.087411675	1.885030834	3	3	49.10482892	40.85588491

chr1	147203124	147204984	1q21.1	CCT8P1	0.736420821	0.390809502	1.884347278	3	3	97.94442068	64.79230048
chr11	5518310	5521785	11p15.4	OLFM5P	0.416050811	0.220885193	1.883561343	2	3	10.22567798	34.7077303
chr4	156761611	156971394	4q32.1	PDGFC	5.383708638	2.859040115	1.883047604	4	4	32.07846014	26.77911476
chr1	151696652	151716821	1q21.3	CELF3	0.108027855	0.057378015	1.882739509	2	2	57.08898897	0.223047261
chr5	139273752	139283184	5q31.2	SNHG4	0.934921644	0.496748131	1.88208387	2	3	0.04972615	46.92965158
chr19	16539688	16539984	19p13.11	RN7SL146P	1.981178546	1.052663803	1.882062003	1	2	0	2.518828076
chrX	103607906	103609927	Xq22.2	TCEAL3	3.36778937	1.790067149	1.881375999	4	4	105.670831	58.83083709
chr20	45833418	45843275	20q13.12	SNX21	1.061657749	0.564311779	1.881331896	4	4	93.5651775	68.11484703
chr16	66579448	66588275	16q21	CMTM2	62.56497651	33.2568148	1.881267851	4	4	55.85507228	94.96183682
chr1	84506301	84556495	1p22.3	SPATA1	2.457327999	1.306425373	1.880955506	4	4	45.82737258	63.96818251
chr2	71068278	71078868	2p13.3	NAGK	10.65338568	5.665212122	1.88049193	4	4	48.00889767	22.66971956
chr19	49688685	49690990	19q13.33	ADM5	0.604712237	0.321620272	1.880205603	2	2	52.2068609	61.02662496
chr20	4686094	4701590	20p13	PRNP	57.61929203	30.64784277	1.880043971	4	4	57.18970646	22.23922749
chr20	49278945	49279023	20q13.13	SNORD12C	45.85826673	24.39312827	1.879966613	4	4	111.9545665	88.74090419
chr16	66566391	66579135	16q21	CMTM1	8.008316663	4.260759378	1.879551496	4	4	100.0757428	66.19719995
chr8	66432483	66468809	8q13.1	ADHFE1	1.283605866	0.683001652	1.879359827	4	4	63.56653337	9.389934403
chr20	36873071	36894235	20q11.23	TLDC2	0.342224992	0.182102336	1.879300396	1	1	0	0
chr14	106810440	106810895	14q32.33	IGHV3-74	3.72521215	1.982326265	1.879212426	4	3	48.18596234	109.2210443
chr2	160099666	160200313	2q24.2	ITGB6	0.32011303	0.170356377	1.879078641	3	3	18.83779541	48.05435988
chr20	50184603	50192069	20q13.13	CEBPB-AS1	0.955885727	0.508751063	1.87888694	4	4	71.58045684	31.50240694
chr1	116500390	116571093	1p13.1	CD58	81.37794321	43.32210093	1.878439445	4	4	18.78895255	25.08782414
chr22	12615367	12626718	22p11.2	FRG1GP	1.129595092	0.601424721	1.878198637	1	3	0	43.40655216
chrX	23836644	23837346	Xp22.11	RPL9P7	0.889762718	0.473744878	1.878147415	1	2	0	8.918435423
chr1	161505415	161524048	1q23.3	FCGR2A	315.6134866	168.0478251	1.878117056	4	4	81.1824882	66.70169783
chr3	58505136	58537202	3p14.3	ACOX2	0.123631195	0.065844043	1.877636756	2	2	55.9790597	0.648626439
chr1	44776492	44776589	1p34.1	SNORD46	20.10254976	10.70820488	1.877303431	4	4	81.54672534	60.27982849
chr10	47535574	47553523	10q11.22	BMS1P2	0.232277799	0.123758685	1.87686059	2	3	44.73064867	3.84463792
chr4	107613666	107720263	4q25	PAPSS1	24.50332345	13.05634861	1.876736305	4	4	21.98470658	27.56954181
chr11	5241954	5243592	11p15.4	HBBP1	5.794082831	3.087318671	1.876736239	3	2	93.68718415	99.84013711
chr12	55720367	55724742	12q13.2	RDH5	0.285278483	0.152035305	1.876396292	3	4	55.63261167	54.28557421
chr2	152335170	152649834	2q23.3	FMNL2	2.397789395	1.278336442	1.875710741	4	4	29.61626033	47.01899046
chr2	161416094	161425867	2q24.2	TBR1	0.050915513	0.027146786	1.875563182	1	2	0	0.180485349
chr8	96645227	97143503	8q22.1	CPQ	43.88714716	23.40597624	1.875040234	4	4	37.77988419	39.16435191
chr10	133379237	133391696	10q26.3	PAOX	1.64150616	0.875828292	1.874232854	4	4	36.65090691	61.12535582
chr1	26321859	26354130	1p36.11	CRYBG2	0.260617034	0.139066765	1.874042539	3	2	77.04820915	54.80850868
chr2	207260445	207271064	2q33.3	LINC01802	1.162566984	0.620493279	1.873617365	1	1	0	0
chr9	91722596	91950206	9q22.31	ROR2	0.087758076	0.046841564	1.873508659	2	2	59.98971676	0.076719246
chr19	40665905	40690658	19q13.2	NUMBL	0.404089201	0.215689358	1.873477695	4	4	70.2340572	78.72177098
chr11	64318088	64321823	11q13.1	PRDX5	265.3657792	141.6763887	1.873041667	4	4	54.40629377	41.11406367
chr7	96219662	96219756	7q21.3	MIR591	5.610454276	2.995514018	1.872952102	2	1	81.79281789	0
chr19	58386770	58386858	19q13.43	MIR4754	5.670503424	3.028341954	1.872477914	1	2	0	8.250566077
chr19	51119830	51120336	19q13.41	SIGLEC18P	1.184933026	0.632875352	1.872300799	4	3	64.44775786	49.28940104
chrX	135020504	135022536	Xq26.3	RTL8B	0.428856739	0.229122643	1.871734425	2	3	94.42114016	46.69423826

chr5	136028895	136063818	5q31.1	TGFBI	27.38896964	14.63314766	1.871707323	4	4	68.15736114	42.81372512
chr19	43646095	43670346	19q13.31	PLAUR	30.52974159	16.31210319	1.871600567	4	4	49.06522293	27.76464528
chr2	131360492	131364158	2q21.1	RAB6D	0.343998878	0.183816731	1.871423106	4	3	70.53462748	92.03333498
chr14	77683190	77684027	14q24.3	RPL21P10	1.771868659	0.947038742	1.87095689	1	2	0	60.77095986
chr13	46169458	46169744	13q14.13	RN7SL288P	11.86268579	6.341126689	1.870753633	4	4	94.92935585	64.46509213
chr6	87472925	87512339	6q15	SLC35A1	12.47970528	6.671624278	1.870564762	4	4	47.03004354	47.30388542
chr10	46337224	46359023	10q11.22	AGAP14P	5.087204412	2.721181287	1.869483829	4	4	97.39768297	60.57199235
chr21	34073523	34143034	21q22.11	MRPS6	8.130625849	4.350100728	1.869066111	4	4	35.79021458	31.79408227
chr15	99251362	99390732	15q26.3	LRRC28	4.535866486	2.427070533	1.868864718	4	4	53.8835278	25.15652865
chr7	28299321	28825894	7p15.1	CREB5	30.91878663	16.54781295	1.868451542	4	4	75.2587388	58.23633165
chr19	36034985	36054762	19q13.12	THAP8	1.228434163	0.657566841	1.868151019	4	4	69.04233235	92.10445604
chr21	33589341	33641854	21q22.11	CRYZL1	5.924503397	3.171371987	1.868119988	4	4	21.9063305	29.78200321
chr17	76673650	76711005	17q25.1	MXRA7	3.432176113	1.837588362	1.867761129	4	4	89.93719404	86.18734204
chr17	74694308	74713000	17q25.1	CD300LF	33.59817187	17.98934136	1.867671039	4	4	69.61760515	32.49906837
chr12	22193391	22334714	12p12.1	ST8SIA1	1.235225134	0.66143372	1.867496467	4	4	75.90875097	49.84337754
chr1	43991247	43996528	1p34.1	CCDC24	0.33569356	0.179784217	1.867202609	3	3	39.32852639	71.79105797
chr5	75611289	75671846	5q13.3	ANKDD1B	0.590119016	0.316127985	1.866709196	4	4	42.81657597	58.99714631
chr15	22867052	22980906	15q11.2	CYFIP1	5.272041777	2.824750151	1.866374545	4	4	51.44863041	23.30459955
chr19	51142260	51153526	19q13.41	SIGLEC7	12.32354125	6.604572668	1.865910464	4	4	61.21796326	51.70953643
chr11	10573091	10693988	11p15.4	MRVI1	14.03566453	7.524860438	1.865239182	4	4	60.64101756	75.72655718
chr19	38319844	38329014	19q13.2	KCNK6	5.892873574	3.159326131	1.86523117	4	4	73.05421645	48.10830424
chr19	571277	583493	19p13.3	BSG	351.0533558	188.2322783	1.865000833	4	4	24.42186962	55.45779688
chr19	35497217	35513678	19q13.12	DMKN	0.065772589	0.035287843	1.863888049	1	1	0	0
chr1	36466043	36483314	1p34.3	CSF3R	180.903866	97.09750604	1.863115474	4	4	72.34889755	59.07138754
chr8	66493256	66518524	8q13.1	VXN	0.192022944	0.103069949	1.863035207	3	4	60.78402337	56.27216259
chrY	10190370	10192415	Yp11.2	CDC27P2	0.232396316	0.124741578	1.863022094	2	1	67.87066568	0
chr11	62433542	62546860	11q12.3	AHNAK	220.3342206	118.292506	1.86262197	4	4	56.85933197	39.06641344
chr2	99161427	99181061	2q11.2	MITD1	92.64220879	49.74292072	1.862419967	4	4	72.06529741	33.58831106
chr8	23102814	23117437	8p21.3	TNFRSF10C	245.8852937	132.0347978	1.862276444	4	4	68.65822386	80.76779429
chr6	163413065	163413950	6q26	CAHM	1.606732235	0.862780697	1.862271885	4	4	108.1618141	51.09424983
chr9	93184156	93327581	9q22.31	WNK2	0.05509144	0.029586861	1.862023845	1	2	0	46.845953
chrX	71301734	71305371	Xq13.1	ITGB1BP2	0.558478277	0.300081304	1.861089876	2	3	65.37522864	65.67926656
chr22	22376014	22376505	22q11.22	IGLV5-45	1.317454208	0.708065469	1.860638973	2	2	64.81651842	26.25075888
chr18	58481247	58629357	18q21.31-q21.32	ALPK2	0.100838803	0.054231198	1.859424215	4	3	81.82052769	85.94083533
chrMT	5826	5891	N/A	MT-TY	18.85957494	10.14316624	1.85933805	4	4	73.92592518	68.79116324
chr22	30080069	30207425	22q12.2	HORMAD2	0.185409992	0.099723852	1.85923416	1	1	0	0
chr6	31859052	31862932	6p21.33	NEU1	2.912689959	1.566652596	1.859180501	4	4	82.96458451	51.34951362
chr8	98064522	98093610	8q22.2	ERICH5	0.280516405	0.150886653	1.859120074	1	1	0	0
chr20	50930984	50945134	20q13.13	ADNP-AS1	1.081440437	0.581837924	1.858662682	3	3	58.95374217	88.8854827
chr4	122892644	122923004	4q28.1	NUDT6	0.394873898	0.212496019	1.858264922	4	4	43.8227208	44.29992178
chr19	859659	863569	19p13.3	CFD	18.786908	10.11306346	1.857687147	4	4	83.02757281	42.1427444
chr6	79631105	79703670	6q14.1	SH3BGRL2	114.0251633	61.38765293	1.857460871	4	4	73.8405561	25.54370541
chr19	17402939	17405648	19p13.11	BST2	51.70436016	27.84122046	1.857115431	4	4	52.47797481	36.76882859

chr19	11355386	11365698	19p13.2	PLPPR2	16.91145975	9.106586075	1.857058134	4	4	64.92066713	31.6292648
chr16	29790713	29805543	16p11.2	KIF22	23.56755843	12.69146666	1.856960985	4	4	74.94481114	31.57176643
chr1	30739156	30739232	1p35.2	MIR4420	22.89372694	12.32873705	1.856940159	4	4	57.90566218	32.57334847
chr15	73917468	73928248	15q24.1	LOXL1-AS1	0.792918545	0.427043047	1.856764908	4	4	52.62956702	30.13147161
chr5	147878711	147882193	5q32	SCGB3A2	0.402299497	0.216677548	1.856673668	2	1	2.623387328	0
chr16	58196862	58283836	16q21	CCDC113	0.119703262	0.064474216	1.856606726	2	2	87.01138123	42.15738257
chr3	121835180	121886526	3q13.33	EAF2	31.54574554	16.99217341	1.856486794	4	4	18.67571124	40.25989955
chr7	47774603	47958913	7p12.3	PKD1L1	0.152295533	0.082035382	1.856461555	2	4	10.34809118	96.56894892
chr1	39491626	39529869	1p34.3	BMP8A	0.260766559	0.140475369	1.856315179	1	1	0	0
chr22	19171706	19172832	22q11.21	LINC01311	0.908435076	0.489421519	1.856140445	4	4	93.62991979	79.65611178
chr1	27959462	27968096	1p35.3	XKR8	26.11889846	14.07298512	1.855960071	4	4	65.22306435	27.90416967
chr16	88663373	88687186	16q24.2	SNAI3-AS1	0.750135502	0.404177898	1.855953791	3	4	30.86520143	71.79827986
chr13	51767993	51804162	13q14.3	DHRS12	6.448251408	3.474771528	1.855733926	4	4	81.62666272	51.31021312
chr1	225810074	225845563	1q42.12	EPHX1	1.348772665	0.726826628	1.855700676	4	4	95.72846649	69.72802929
chr4	70993548	71030914	4q13.3	DCK	112.9352737	60.8661828	1.855468315	4	4	47.48523222	10.07483546
chr17	44345086	44353106	17q21.31	GRN	133.1280929	71.75577958	1.855294356	4	4	62.77979906	29.00526893
chr11	60850925	60855971	11q12.2	PTGDR2	9.983626985	5.382505175	1.854829055	4	4	91.47334268	87.3069419
chr8	25184723	25415716	8p21.2	DOCK5	32.84181959	17.7089942	1.85452766	4	4	61.10128327	50.34611456
chr1	209614870	209652475	1q32.2	LAMB3	0.688534591	0.371321586	1.854281079	4	4	81.03663743	83.54303492
chr19	46759685	46788622	19q13.32	SLC1A5	52.46516608	28.29893372	1.853962647	4	4	50.95214861	40.78264105
chr20	47382766	47412144	20q13.12	LINC01754	0.65837196	0.355145042	1.853811491	1	1	0	0
chr17	72645946	73092714	17q24.3-q25.1	SLC39A11	4.478800411	2.416309639	1.85357056	4	4	41.38794036	16.82724106
chr17	19061912	19062128	17p11.2	SNORD3B-1	18.94766118	10.22330664	1.853378936	4	4	111.9989299	95.24588038
chr15	25054209	25054305	15q11.2	SNORD116-2	7.910003061	4.268355789	1.853173318	2	1	27.00097279	0
chr1	8318085	8344167	1p36.23	SLC45A1	0.298421352	0.1610505	1.852967554	1	1	0	0
chr9	5094100	5095016	9p24.1	MTND1P11	0.983161079	0.530629893	1.852818872	2	3	24.98685403	66.89384888
chr19	34994784	35026471	19q13.11	GRAMD1A	15.01660286	8.105143722	1.852725056	4	4	65.11914634	39.93612819
chr1	198859044	198859153	1q32.1	MIR181A1	5.366063055	2.896503605	1.852600165	1	2	0	31.05690829
chr2	218710835	218757484	2q35	TTLL4	5.345929145	2.886145831	1.852272705	4	4	52.15069657	14.55451016
chr21	32411692	32515400	21q22.11	EVA1C	1.459138034	0.787800382	1.852167208	4	4	66.85574609	55.23214257
chr11	12674422	12944737	11p15.3	TEAD1	0.045581614	0.024612821	1.851945946	2	2	34.54776646	1.313439356
chr22	39315213	39315307	22q13.1	SNORD83A	16.97081277	9.164851471	1.851728075	4	4	63.08563019	66.12513281
chr9	33967877	33968573	9p13.3	OSTCP8	1.064503898	0.574900734	1.851630787	1	1	0	0
chr22	23966222	23972559	22q11.23	DDTL	2.68177944	1.448927391	1.850872209	3	2	57.85243587	97.77634588
chr4	4476121	4481700	4p16.3	STX18-IT1	1.166437603	0.63042507	1.850239875	2	3	75.97997772	27.90142582
chr1	153373708	153375599	1q21.3	S100A12	456.6366324	246.8278108	1.850020996	4	4	88.6017748	72.28113085
chr15	91463339	91494850	15q26.1	CRAT37	0.208762994	0.112914875	1.848852891	2	1	80.21367305	0
chr10	98457077	99235875	10q24.2	HPSE2	0.107040964	0.057915399	1.848229764	1	1	0	0
chr8	133407664	133409525	8q24.22	ST13P6	23.08578118	12.49125157	1.848155971	4	4	62.97593655	21.53337768
chr7	144186083	144195698	7q35	ARHGEF35	0.53445503	0.289218901	1.84792566	1	4	0	81.0991686
chr10	75279726	75401894	10q22.2	ZNF503	0.688009827	0.372333067	1.847834341	3	3	69.171866	31.55683239
chr7	106248285	106285192	7q22.3	NAMPT	1254.105999	678.7166888	1.847760664	4	4	72.34064665	66.28494365
chrX	15825806	15855014	Xp22.2	AP1S2	76.65439749	41.49819489	1.847174261	4	4	44.06482017	14.00504533

chr3	130345516	130484846	3q22.1	COL6A5	0.049636764	0.026875937	1.846884953	2	2	64.82565527	1.607512214
chr1	230321646	230425928	1q42.13	PGBD5	0.042645482	0.023090789	1.846861215	2	3	58.28760513	3.592330966
chr15	45430529	45433449	15q21.1	C15orf48	1.033504832	0.559760647	1.846333495	3	3	33.91448158	58.07495112
chr1	155951273	155951336	1q22	MIR6738	8.353637997	4.524968119	1.846120851	2	1	16.23260769	0
chrX	52947254	52995472	Xp11.22	FAM156A	0.147372581	0.07982969	1.846087343	2	3	15.65104286	49.52867461
chr15	80695311	80755621	15q25.1	ABHD17C	0.820279407	0.444428286	1.845695769	4	4	53.72017783	60.07486539
chr21	39342315	39349121	21q22.2	HMGN1	45.01417538	24.39021121	1.845583665	4	4	50.13448734	24.09679974
chr7	876554	896434	7p22.3	GET4	0.243554577	0.131969596	1.845535514	3	4	86.4988067	45.90681926
chr3	125528858	125595537	3q21.2	OSBPL11	43.78424053	23.72465127	1.845516717	4	4	59.07449748	31.40465448
chr1	148038753	148038916	1q21.2	RNVU1-7	38.60119125	20.92654637	1.844604005	4	4	91.65217913	40.2231146
chr9	74946583	74952886	9q21.13	C9orf40	26.53412403	14.38825624	1.844151479	4	4	59.99959839	48.56774571
chr4	183705931	183706031	4q35.1	RNU6-1053P	8.861453234	4.805706499	1.843943911	1	2	0	26.52757629
chr21	43865160	43987594	21q22.3	AGPAT3	12.71290223	6.894960036	1.843796362	4	4	35.33806377	29.95701208
chr17	60149936	60179021	17q23.1	CA4	5.660597753	3.070168117	1.843741951	4	4	91.07286273	63.25180169
chr19	10286967	10288584	19p13.2	ICAM4	4.172773796	2.263271711	1.84369105	4	4	41.76694413	66.14759349
chr9	35406755	35483029	9p13.3	ATP8B5P	0.076301189	0.04138641	1.843629079	1	1	0	0
chr12	57520710	57530148	12q13.3	MBD6	6.436133672	3.491439346	1.843404119	4	4	54.74249213	25.55762081
chr14	73275210	73458580	14q24.2-q24.3	NUMB	43.72911915	23.72594397	1.843092911	4	4	62.34640266	41.23080175
chr14	52639915	52695931	14q22.1	ERO1A	34.19973048	18.55755246	1.842900919	4	4	54.4342938	32.47515704
chr4	38823680	38857767	4p14	TLR6	36.24616206	19.66971453	1.842739609	4	4	46.26209799	55.91883454
chr11	57761762	57819180	11q12.1	CTNND1	5.648219803	3.065379693	1.842584074	4	4	60.71520166	35.93347477
chr17	55750979	55845001	17q22	PCTP	20.7951686	11.28664608	1.842457756	4	4	11.48579875	17.54613287
chr21	45263928	45287914	21q22.3	POFUT2	3.919148125	2.127238791	1.842363979	4	4	49.931431	23.19658117
chr6	32184733	32190186	6p21.32	PBX2	18.80761873	10.2088056	1.842293748	4	4	76.40971513	27.32797991
chr9	127505338	127579007	9q34.11	FAM129B	6.409632822	3.479497513	1.8421145	4	4	60.11746249	22.80664978
chr21	25734947	25772460	21q21.3	GABPA	37.00587715	20.09320547	1.84171098	4	4	26.69904162	20.40701388
chr18	23689443	23955066	18q11.2	LAMA3	0.033703404	0.018303912	1.841322471	3	2	51.87252529	4.259849118
chr11	9311284	9311689	11p15.4	PRR13P2	2.126006423	1.154619673	1.84130452	2	2	71.27433245	9.181786421
chr1	209785623	209806175	1q32.2	IRF6	0.297628859	0.161644824	1.841252018	4	4	63.37513865	82.76493288
chr3	44861909	44865667	3p21.31	TMEM42	0.295028274	0.160234817	1.841224523	2	4	93.922437	66.69466592
chr12	13196668	13216774	12p13.1	EMP1	0.650984687	0.353587821	1.841083454	4	4	118.615053	46.14885029
chr11	65314818	65322429	11q13.1	CDC42EP2	27.34342873	14.8535192	1.840872076	4	4	84.52738737	51.87734149
chr7	93130054	93148399	7q21.2	SAMD9L	101.9977807	55.42527376	1.840275632	4	4	58.19094639	53.51755981
chr16	4734288	4749396	16p13.3	C16orf71	0.101360557	0.055092166	1.839836117	1	1	0	0
chr16	2616120	2643295	16p13.3	PDPK2P	3.994017716	2.171072726	1.839651739	4	4	117.4495352	78.0867935
chr10	133262416	133276903	10q26.3	ADAM8	88.13890301	47.9118719	1.839604664	4	4	58.72448695	29.54094709
chr2	109760594	109857691	2q13	RGPD5	0.144912333	0.078778974	1.839479811	3	4	137.0196769	12.72804968
chr1	20163083	20177364	1p36.12	PLA2G2C	0.13855056	0.075334411	1.839140412	1	1	0	0
chr7	76959768	77004357	7q11.23	DTX2P1	0.341976511	0.186005796	1.838526099	1	2	0	17.02079846
chr17	3662893	3696404	17p13.2	P2RX5-TAX1BP3	0.274771128	0.149476804	1.838219171	2	4	41.54686207	65.17835673
chr8	18056299	18084998	8p22	ASAH1	46.69632454	25.40347323	1.838186618	4	4	54.17618601	21.56728461
chr11	63998554	64166652	11q13.1	MACROD1	0.480800854	0.261596067	1.837951382	4	4	53.36921512	52.58756493
chr4	183505050	183512428	4q35.1	ING2	8.938005981	4.863511802	1.837767923	4	4	37.69803569	81.49054221

chr17	39737901	39747285	17q12	GRB7	0.127445517	0.069351572	1.837673085	1	1	0	0
chr17	1633858	1645789	17p13.3	SCARF1	6.929395131	3.771330197	1.837387545	4	4	38.86096389	41.66357091
chr7	23105734	23175421	7p15.3	KLHL7	10.12811084	5.513343903	1.837017791	4	4	57.88380087	39.781407
chr3	134485724	134783169	3q22.2	CEP63	18.8687764	10.27172931	1.836961998	4	4	54.31108962	40.98078678
chr15	60419609	60479160	15q22.2	ICE2	22.13204323	12.0495315	1.836755497	4	4	69.36609423	35.59812169
chr3	40173145	40309698	3p22.1	EIF1B-AS1	0.80023478	0.435722769	1.836568657	4	4	32.71610521	47.46089071
chr1	161524540	161526897	1q23.3	HSPA6	55.94184086	30.46394351	1.836329589	4	4	75.08765169	55.37369111
chr20	1894167	1939895	20p13	SIRPA	52.89072259	28.80319687	1.836279592	4	4	72.88735916	52.95726266
chr1	203127705	203167405	1q32.1	ADORA1	0.151614062	0.08257863	1.835996336	3	3	43.91479965	34.01450684
chr15	40844043	40857655	15q15.1	SPINT1	3.82734098	2.086064167	1.834718721	4	4	66.61543829	64.38222522
chr12	76025447	76031776	12q21.2	PHLDA1	0.9163174	0.499566104	1.834226527	4	4	63.10436089	31.04469946
chr11	65892136	65900526	11q13.1	FOSL1	0.796548559	0.434342867	1.83391652	4	4	92.55049825	135.5341219
chr5	88718241	88904105	5q14.3	MEF2C	24.71602847	13.47787989	1.83382169	4	4	78.13326769	43.2337315
chr2	39168045	39248835	2p22.1	CDKL4	1.763648266	0.961972368	1.833366867	4	4	98.28751745	74.60488774
chr8	104339087	104356874	8q22.3	DCSTAMP	0.311336176	0.169842622	1.833086253	4	3	92.02061227	40.64649697
chr19	12646508	12666777	19p13.13	MAN2B1	29.63821459	16.17067875	1.832836769	4	4	73.2776311	37.1032668
chr1	77888515	77948643	1p31.1	NEXN	11.40227764	6.221815389	1.832628731	4	4	70.57299829	37.93503327
chr17	37978155	37989316	17q12	TBC1D3L	0.197330819	0.107703782	1.832162392	1	1	0	0
chr22	31212264	31280080	22q12.2	LIMK2	59.02646489	32.21687698	1.832159738	4	4	96.5476212	49.73235145
chr7	106654362	106661188	7q22.3	CCDC71L	19.48893637	10.6373757	1.832118835	4	4	59.67517262	47.30081283
chr1	205083129	205084460	1q32.1	TMEM81	2.31268313	1.262440019	1.831915255	4	4	87.26278152	87.32738721
chr20	10637684	10674046	20p12.2	JAG1	1.213850058	0.662613135	1.831913667	4	4	57.48256709	45.58608413
chr1	58780791	58784113	1p32.1	JUN	8.554935614	4.670405228	1.83173305	4	4	131.9648107	100.8781886
chr2	219075324	219160865	2q35	NHEJ1	0.234740292	0.12818892	1.83120578	3	2	15.91368975	39.45622366
chr2	218356757	218368393	2q35	CATIP	0.163130882	0.089084507	1.831192505	2	3	9.675464481	35.77857453
chr19	4182618	4224814	19p13.3	ANKRD24	0.103992277	0.056795518	1.830994421	3	1	37.85189776	0
chr1	21812209	21825221	1p36.12	LDLRAD2	0.107043685	0.058466938	1.830841285	2	3	57.56070236	5.398442572
chr2	207753889	207769563	2q33.3	FZD5	2.904829231	1.586955338	1.830441703	4	4	79.1939633	20.23294731
chr15	49125274	49155657	15q21.1	COPS2	58.74076217	32.0950027	1.830215212	4	4	41.38692776	20.11021765
chr13	102394578	102395705	13q33.1	FGF14-AS2	0.79221701	0.433020049	1.829515771	1	3	0	45.27840145
chr1	150887253	150912512	1q21.3	CTXND2	0.399199496	0.218214587	1.829389597	1	1	0	0
chrX	1400531	1415421	Xp22.33 and Yp11	ASMTL-AS1	0.503067514	0.274995916	1.829363584	2	3	82.201674	40.6237641
chr17	9021542	9244000	17p13.1	NTN1	0.1071637	0.058580625	1.82933691	1	2	0	46.12918873
chr9	33677268	33688011	9p13.3	PTENP1-AS	0.762170312	0.416666855	1.829207919	3	2	32.57835936	36.26330739
chr17	16922776	16933176	17p11.2	TBC1D27P	0.225895192	0.123507566	1.828998817	2	4	66.86874378	49.20838683
chr2	218162845	218166993	2q35	CXCR1	272.2066645	148.8332196	1.828937553	4	4	76.3567604	57.85195633
chr4	173370942	173377532	4q34.1	SAP30	11.98324173	6.552477009	1.828810954	4	4	25.27294736	31.39169188
chr7	38353715	38354183	7p14.1	TRGV4	2.788253625	1.52474083	1.828673812	4	4	70.89147023	24.03203367
chr19	45039040	45070956	19q13.32	CLASRP	7.426393663	4.061981586	1.828268668	4	4	71.73176305	28.05811614
chr5	142770370	143229011	5q31.3	ARHGAP26	39.9207714	21.83854007	1.827996344	4	4	34.15563042	29.47428726
chr11	1852970	1892263	11p15.5	LSP1	158.6835037	86.81956929	1.827738896	4	4	72.53989302	38.61741401
chr1	160205319	160215376	1q23.2	PEA15	23.44121533	12.82580565	1.827660264	4	4	63.44790082	41.97875086
chr10	97309530	97310046	10q24.1	RPL12P27	4.125122118	2.257159314	1.827572424	4	4	92.17517992	62.60285125

chr1	203797523	203801074	1q32.1	ZBED6	0.785407858	0.430011025	1.826483073	4	4	94.86954459	72.16252934
chr17	35568114	35574313	17q12	LINC02001	4.138232637	2.26647186	1.82584779	4	4	32.47901577	18.69741421
chr2	23943820	23945078	2p23.3	SDHCP3	0.913036569	0.500089365	1.825746823	2	1	34.49470289	0
chr9	97412020	97496125	9q22.33	TDRD7	16.92550049	9.270815051	1.825675563	4	4	48.51533228	62.47000509
chr1	17513442	17697875	1p36.13	ARHGEF10L	2.358326858	1.291776796	1.825645781	4	4	73.43682971	27.11221195
chr3	43690870	43734371	3p21.33	ABHD5	42.44212172	23.24827667	1.825602918	4	4	55.1698429	39.98309712
chr20	1309909	1329235	20p13	SDCBP2	0.350307063	0.191900774	1.825459349	3	3	19.00642299	47.33270102
chr16	29381318	29404437	16p11.2	NPIPB11	0.311495529	0.170646962	1.825379865	3	3	47.47194916	111.01837
chr5	83052846	83077453	5q14.2	TMEM167A	70.03078011	38.36572618	1.825347441	4	4	20.39566938	26.36489892
chr6	159799422	159820704	6q25.3	PNLDC1	0.529309067	0.289998081	1.825215754	2	4	106.5164017	47.95827092
chr5	473236	480892	5p15.33	SLC9A3-AS1	0.391147422	0.214308821	1.825157833	4	4	17.03159261	89.46626017
chrX	101550531	101554700	Xq22.1	ARMCX1	0.969315189	0.531111712	1.825068374	4	3	63.48360804	64.01977616
chr6	7881514	7910814	6p24.3	TXNDC5	0.685850561	0.37581429	1.824972013	4	4	94.22183345	46.58533919
chr1	74198212	74544432	1p31.1	FPGT-TNNI3K	0.087069281	0.047713009	1.824854104	3	4	102.5825845	54.3094828
chr1	220879401	220885059	1q41	HLX	10.0281385	5.496452659	1.824474643	4	4	82.54695039	46.03154817
chrX	68828997	68842164	Xq13.1	EFNB1	3.476535832	1.905690799	1.824291661	4	4	45.70332591	49.79791804
chr6	41683978	41736259	6p21.1	TFEB	9.407127796	5.156669761	1.824264154	4	4	58.52271975	48.44025006
chr19	14091688	14117747	19p13.12	PRKACA	8.385515121	4.596881387	1.824174786	4	4	78.81339749	49.38426171
chrX	14873405	14922166	Xp22.2	MOSPD2	29.56650824	16.20821286	1.824168309	4	4	44.08608208	61.18276807
chr11	33858576	33892289	11p13	LMO2	23.77701168	13.03468948	1.824133342	4	4	22.41446695	30.38970559
chr6	36697808	36733183	6p21.2	RAB44	2.518507936	1.381188306	1.823435606	4	4	104.4807893	58.22740041
chr19	55379244	55384259	19q13.42	TMEM238	0.44402053	0.243525413	1.823302642	2	2	81.22306462	6.718378654
chr19	16892947	17026818	19p13.11	CPAMD8	0.42473783	0.232969486	1.823147898	4	4	110.8247586	73.09972075
chr11	110577035	110713189	11q22.3-q23.1	ARHGAP20	0.087300556	0.047888014	1.823014746	3	3	93.64164467	39.76385903
chr6	33064569	33080778	6p21.32	HLA-DPA1	62.69831601	34.39936734	1.822658986	4	4	74.63574381	53.01366186
chr16	28494649	28498970	16p12.1	APOBR	43.13272195	23.66535765	1.822610188	4	4	64.94360563	33.59303652
chr21	45070955	45073211	21q22.3	SSR4P1	0.459405739	0.252092921	1.822366683	3	3	85.25179618	18.01964048
chr1	145992426	145996631	1q21.1	TXNIP	1806.276109	991.2171811	1.822280872	4	4	33.98687389	33.63650729
chr19	41354417	41364534	19q13.2	B9D2	6.467929996	3.549925836	1.821990175	4	4	66.6870043	66.1298888
chr6	34685355	34685642	6p21.31	RN7SL200P	2.451853841	1.345940679	1.821665605	1	1	0	0
chr13	43968323	43971710	13q14.11	DGKZP1	0.246393698	0.135285696	1.821284183	2	2	92.41504294	49.43480048
chrX	40934939	40935887	Xp11.4	RPS2P55	0.689398589	0.378602525	1.820903306	3	3	50.12208467	32.14661743
chr15	65304677	65304793	15q22.31	RNU5B-1	337.0014099	185.128919	1.820360707	4	4	88.40996608	74.97384838
chr7	79452957	79471208	7q21.11	MAGI2-AS3	2.219260205	1.219378821	1.819992416	4	4	55.73181929	97.22541612
chr1	2549920	2557011	1p36.32	TNFRSF14-AS1	0.123721338	0.067990427	1.819687629	2	2	26.72257786	53.69694916
chr19	45071500	45076588	19q13.32	ZNF296	0.992470917	0.545420544	1.819643444	4	4	98.03086568	44.93998185
chr17	40818117	40822621	17q21.2	KRT10	4.374048984	2.404292475	1.819266595	3	4	88.86450569	27.37945471
chr7	37796193	37796876	7p14.1	GPR141BP	0.707731235	0.389101495	1.818885928	4	4	65.95201415	36.83134907
chr3	48446710	48465655	3p21.31	ATRIP	1.472777631	0.809757681	1.818788095	4	4	82.34585966	21.13524208
chr11	63552759	63565072	11q12.3	HRASLS2	1.49531332	0.822382748	1.81826932	4	3	52.73228151	95.4927751
chr18	45989653	45989945	18q21.1	RN7SKP26	4.619681381	2.540914645	1.8181175	2	2	14.23192733	20.50705876
chr22	35381067	35394214	22q12.3	HMOX1	21.84493803	12.01593762	1.817996957	4	4	51.06207709	20.81041887
chr6	63528021	63583588	6q12	PTP4A1	42.13034007	23.17579999	1.81785915	4	4	29.41976868	6.24465262

chr11	5689587	5710863	11p15.4	TRIM22	117.0839749	64.40791736	1.817850658	4	4	60.38926477	42.4728999
chr1	22636506	22639682	1p36.12	C1QA	2.97079582	1.634309395	1.817768305	4	4	64.00905175	98.58065557
chr19	4537215	4540024	19p13.3	LRG1	40.10002603	22.07101638	1.816863588	4	4	95.622896	72.03572095
chr3	190642163	190642408	3q28	RN7SKP296	7.45033066	4.101461082	1.816506487	3	4	28.81212856	68.31492355
chr17	50547089	50555852	17q21.33	SPATA20	2.206604256	1.214845851	1.816365635	4	4	76.63657839	30.05418377
chr1	159059226	159132351	1q23.1-q23.2	AIM2	16.60044692	9.140904759	1.816061687	4	4	84.75127381	45.13156002
chr22	50568861	50578667	22q13.33	CPT1B	0.114032704	0.06279609	1.815920447	3	2	72.86720793	0.745148055
chr22	29306650	29312789	22q12.2	GAS2L1	5.247262582	2.889644748	1.81588501	4	4	79.51844721	44.23840082
chr1	154321059	154325325	1q21.3	AQP10	3.145184389	1.732056011	1.815867599	4	4	68.33185657	42.98055537
chr2	161620963	161985276	2q24.2	SLC4A10	4.156027398	2.288941042	1.815698754	4	4	57.88606698	87.30502698
chr19	38409055	38426305	19q13.2	RASGRP4	25.94446718	14.28998986	1.81556932	4	4	62.5696918	48.87592072
chr18	24460629	24479957	18q11.2	HRH4	6.773203533	3.73071781	1.815522877	4	4	32.61559585	49.78800147
chr8	26514193	26658177	8p21.2	DPYSL2	27.81724622	15.32332219	1.815353477	4	4	49.63127702	37.93051883
chr14	21061276	21090248	14q11.2	ARHGEF40	10.31449817	5.683974303	1.814663055	4	4	66.32571718	68.60829832
chr12	124777628	124863973	12q24.31	SCARB1	0.951047633	0.524388363	1.813632226	4	4	112.790469	59.4941978
chr10	50676455	50685882	10q11.23	NUTM2HP	0.264416468	0.145796955	1.813593893	1	2	0	39.49801052
chr15	38068812	38072990	15q14	LINC01852	0.240703341	0.132722515	1.813583331	3	1	46.04591342	0
chr13	31846783	31959584	13q13.1	EEF1DP3	2.441933994	1.346605619	1.813399527	4	4	21.95747857	56.87937589
chr4	73869392	73871302	4q13.3	CXCL1	62.67051167	34.56674335	1.813029103	4	4	58.93859755	79.7699064
chrX	154424380	154428479	Xq28	CH17-340M24.3	1.630660682	0.899510375	1.812831432	2	3	25.53050868	94.94962858
chr17	58205436	58219605	17q22	MKS1	9.112354721	5.026857183	1.812733959	4	4	45.47926702	29.69295723
chr21	17819329	18267371	21q21.1	CHODL	0.290531487	0.160285784	1.812584239	2	3	31.35242047	44.88798035
chr6	20421657	20421887	6p22.3	RN7SL128P	176.7634448	97.52229449	1.812543949	4	4	54.58233912	20.01499982
chr7	127997509	128000077	7q32.1	SND1-IT1	0.518692355	0.286202547	1.812326131	3	4	56.07573165	47.99765541
chr3	129401321	129428651	3q21.3	EFCAB12	0.154109968	0.085038716	1.812233012	3	4	59.13740946	47.58797205
chr7	142384379	142384841	7q34	TRBV7-3	3.075733976	1.697438975	1.811985009	2	4	11.77843335	80.42267875
chr16	14079	18091	16p13.3	WASH4P	1.671595856	0.922680375	1.811673795	4	4	113.9565593	13.98139408
chr2	159768628	159904756	2q24.2	LY75-CD302	0.186993047	0.103254492	1.810991887	2	1	64.29305956	0
chr19	2274632	2282182	19p13.3	PEAK3	6.744688961	3.724605717	1.810846429	4	4	72.99709408	67.09167305
chr1	35350645	35351666	1p34.3	RPL5P4	1.697095265	0.937803918	1.809648299	2	2	33.19274579	97.84565555
chr6	17281508	17293875	6p22.3	RBM24	0.132661717	0.073332771	1.809037275	1	2	0	2.496271535
chr2	190343470	190371665	2q32.2	INPP1	3.640436355	2.012406992	1.808996078	4	4	64.33332996	32.48781413
chr1	143874743	143883733	1q21.1	FCGR1CP	9.929004046	5.488949872	1.808907765	4	4	94.37900513	55.98749842
chr1	181088502	181090843	1q25.3	IER5	17.86273625	9.874934998	1.80889659	4	4	52.21641578	49.33683501
chr13	48400897	48444704	13q14.2	LPAR6	41.17899478	22.76483122	1.808886452	4	4	23.13161531	27.26406446
chrX	5890026	6228882	Xp22.32-p22.31	NLGN4X	0.095076978	0.052564639	1.808763068	1	2	0	45.61394272
chr2	229236340	229236609	2q36.3	RN7SKP283	2.497089726	1.380688471	1.808583021	4	2	50.63207489	47.29754829
chr6	2832332	2842049	6p25.2	SERPINB1	151.9027548	83.99449621	1.808484623	4	4	61.70024903	32.21729396
chr1	185157060	185291781	1q25.3	SWT1	785.2347319	434.2309444	1.808334348	4	4	57.20516167	24.97835641
chr2	8852690	9003750	2p25.1	MBOAT2	473.2826951	261.7311497	1.808278058	4	4	103.5152458	63.1115926
chr4	168497039	168928457	4q32.3	PALLD	1.355066253	0.749529482	1.807889196	4	4	53.23367955	38.56475243
chr19	11296139	11326996	19p13.2	TSPAN16	0.336590426	0.186200192	1.807680342	1	2	0	11.50519614
chr1	48221113	48248644	1p33	SLC5A9	0.267877012	0.148230991	1.807159283	4	3	72.12008902	22.25067233

chr19	1554669	1568058	19p13.3	MEX3D	0.570771695	0.315841919	1.807143575	3	4	102.3783117	27.15225973
chr20	17962710	17962946	20p11.23	SNORD17	10183.42579	5637.13299	1.806490252	4	4	35.89742538	51.26672546
chr11	59852808	59866568	11q12.1	TCN1	9.124360985	5.051234345	1.806362636	4	4	52.32449679	56.335685
chr12	120762510	120904352	12q24.31	SPPL3	78.81089204	43.64511609	1.805720756	4	4	76.72411638	39.24360529
chr21	17788967	17819386	21q21.1	C21orf91	44.46030884	24.62383483	1.805580209	4	4	20.39345836	33.42488166
chr1	183555562	183590921	1q25.3	NCF2	542.337425	300.3818759	1.805493169	4	4	68.72013793	53.84650519
chr3	167478684	167658003	3q26.1	WDR49	3.895084992	2.157849634	1.805077115	4	4	33.48030773	21.49486186
chr19	40808443	40811100	19q13.2	CYP2T1P	1.129526616	0.625760716	1.805045583	3	3	94.00746305	69.23441375
chr17	31303770	31314149	17q11.2	EVI2B	467.5258351	259.0240288	1.8049516	4	4	70.38721115	44.31237436
chr14	59595976	59871288	14q23.1	RTN1	5.724813912	3.172210417	1.804676601	4	4	66.53681633	30.50314854
chr17	41801952	41812710	17q21.2	P3H4	0.410497765	0.227475577	1.804579507	3	3	65.75417074	80.09389407
chr2	85598547	85602699	2p11.2	TMEM150A	0.977300047	0.54165747	1.804276872	4	4	68.89687935	88.81664817
chrX	153724851	153744762	Xq28	ABCD1	6.027109845	3.340461278	1.804274723	4	4	77.39917749	29.10153394
chr19	46633953	46639326	19q13.32	GNG8	7.723384751	4.280836901	1.804176363	3	3	67.47611521	46.89229422
chr17	7435443	7437679	17p13.1	TMEM102	0.350246998	0.19423135	1.803246478	4	3	69.79690053	31.79630825
chr1	115098324	115105345	1p13.2	LINC01765	5.707023405	3.165456504	1.80290691	4	4	72.35816783	84.21506616
chr7	36153227	36301543	7p14.2	EEPD1	6.975171825	3.868949095	1.80285955	4	4	76.59660748	47.11346594
chr15	79898840	79923754	15q25.1	ST20	6.913621738	3.834852715	1.802838923	4	4	69.18019787	80.57174397
chr15	89830599	89894385	15q26.1	AP3S2	3.695598738	2.050125635	1.802620618	4	4	33.21795226	26.90145671
chr1	15438442	15449247	1p36.21	CTRC	0.131327474	0.072864985	1.802339957	1	2	0	2.882659541
chr19	20398658	20424965	19p12	ZNF826P	0.715671205	0.397141916	1.802054067	4	4	69.70712187	22.81216032
chr19	1267471	1270260	19p13.3	CIRBP-AS1	0.441660456	0.245190562	1.801294685	1	2	0	6.700874605
chr11	8693352	8910951	11p15.4	ST5	0.105717688	0.058702184	1.80091575	3	4	10.72984454	43.86385259
chr15	101648206	101648312	15q26.3	RNU6-807P	7.883326655	4.377433142	1.80090167	4	2	56.31340496	18.52484206
chr3	183447608	183456013	3q27.1	LINC00888	1.072416256	0.59552199	1.800800431	4	4	60.3223447	68.27974558
chr14	105486920	105488789	14q32.33	CRIP1	1.973737484	1.096449862	1.800116496	4	4	93.32047971	83.63459866
chr9	5855786	5856426	9p24.1	AK4P4	0.742990328	0.412769813	1.800011301	2	1	41.4867773	0
chr8	141128562	141195804	8q24.3	DENND3	23.0100557	12.78386994	1.799928801	4	4	58.59440936	46.12652572
chr7	38276259	38276318	7p14.1	TRGJP1	24.16860334	13.42814028	1.799847397	2	3	36.25744787	1.651644802
chr3	40387156	40428626	3p22.1	ENTPD3	0.136170296	0.075660826	1.799746343	1	2	0	3.180588826
chr4	55366601	55385580	4q12	SRD5A3-AS1	0.13507942	0.07507391	1.799285796	2	4	13.78770294	36.77612001
chr4	70655541	70666631	4q13.3	JCHAIN	115.2440914	64.06201726	1.798945715	4	4	55.73459967	101.897727
chrX	85003852	85093317	Xq21.1	APOOL	15.46892986	8.600008002	1.7987111	4	4	67.88621925	45.60095498
chr3	136721394	136721495	3q22.3	RNU6-789P	4.248815588	2.362494028	1.798445007	1	1	0	0
chr17	3860315	3895633	17p13.2	CAMKK1	1.939691797	1.078674344	1.798218163	4	4	105.6619564	25.50309702
chr11	105080453	105101492	11q22.3	CARD17	2.565184704	1.426875053	1.797764071	4	4	61.9167925	103.494875
chr20	44355801	44432845	20q13.12	HNF4A	0.082972915	0.046153614	1.797755521	1	3	0	42.04096331
chr3	54626153	54626699	3p14.3	RPS15P5	1.112956178	0.619527981	1.796458291	1	1	0	0
chr4	74365143	74388760	4q13.3	EREG	2.063604163	1.148979432	1.796032292	4	4	44.21269624	80.10578904
chr9	121815674	122093606	9q33.2	TTLL11	4.429548307	2.466534312	1.795859188	4	4	75.54660336	37.25519106
chr10	103088017	103193306	10q24.32-q24.33	NT5C2	68.56665641	38.18358194	1.795710432	4	4	60.09816886	35.51905485
chr2	142877626	143042316	2q22.2	KYNU	3.807912386	2.12057265	1.795700037	4	4	64.45641489	21.58654397
chr12	9065177	9068055	12p13.31	A2M-AS1	3.587417859	1.998062843	1.795447962	4	4	30.99501367	17.58091656

chr5	160251652	160339592	5q33.3	CCNJL	16.58429235	9.242662048	1.79431989	4	4	82.04906416	75.41588904
chr14	100537147	100587404	14q32.2	BEGAIN	0.212558094	0.118477619	1.794078033	4	3	106.1408686	44.2584574
chr6	42960754	42963880	6p21.1	GNMT	0.631513048	0.35206294	1.793750422	3	3	43.70025002	44.98250451
chr2	207711540	207756174	2q33.3	CCNYL1	56.22526735	31.35142345	1.793388024	4	4	71.715883	45.89890044
chr11	308107	309410	11p15.5	IFITM2	856.3819612	477.5616955	1.79323838	4	4	66.44883658	58.90948282
chr11	59263400	59263706	11q12.1	SLC25A47P1	0.994574835	0.554625955	1.793235289	1	1	0	0
chrMT	12266	12336	N/A	MT-TL2	13.36209007	7.452066675	1.793071728	2	3	18.11016101	42.18283736
chr14	64704128	64750247	14q23.3	PLEKHG3	18.59820177	10.37336194	1.79288083	4	4	52.17147437	52.96288996
chr6	32190766	32195523	6p21.32	GPSM3	67.79971745	37.81850515	1.792765663	4	4	78.31265007	62.90724135
chr10	50264888	50267112	10q11.23	DYNC1I2P1	0.67210919	0.374988015	1.792348456	2	1	0.703587408	0
chr19	14381144	14408725	19p13.12	ADGRE5	117.7078374	65.68334809	1.7920499	4	4	53.37015444	25.88246888
chr7	144646989	144648638	7q35	EEF1A1P10	1.03106043	0.57545367	1.79173491	3	3	75.64681856	58.06590948
chr12	6976185	7018538	12p13.31	LPCAT3	12.88074443	7.189321651	1.791649484	4	4	39.88124204	25.47224685
chr9	136977504	136981742	9q34.3	PTGDS	1.094102484	0.61077191	1.791343817	3	3	8.63519333	44.63938125
chr11	112143251	112164117	11q23.1	IL18	3.193009819	1.782525088	1.791284645	4	4	75.65050904	46.36788998
chr2	137964068	138016364	2q22.1	HNMT	7.644780405	4.268704651	1.790889984	4	4	55.91191294	38.05618046
chr7	81699006	81770438	7q21.11	HGF	3.154878079	1.761725092	1.790789092	4	4	65.03806878	54.19949793
chr4	82483170	82562560	4q21.22	TMEM150C	0.258495362	0.144369065	1.790517678	3	1	60.2630716	0
chr4	120684906	120922866	4q27	PRDM5	3.689409159	2.060975001	1.790128049	4	4	34.11788147	58.44604502
chr4	101347777	101348883	4q24	FLJ20021	2.223142352	1.241994737	1.789977272	4	3	70.18040526	80.63310107
chr16	75499588	75505292	16q23.1	TMEM231P1	0.522610934	0.291996254	1.789786436	1	2	0	7.382870999
chr11	65597755	65614249	11q13.1	MAP3K11	10.61702561	5.932210868	1.789724918	4	4	52.18237081	33.17469704
chr3	9917074	9933627	3p25.3-p24.1	IL17RC	0.360785702	0.201588546	1.789713303	4	4	65.53564122	19.20669973
chr20	3039061	3045896	20p13	GNRH2	0.526253239	0.294047466	1.789688061	1	2	0	7.567177364
chr12	57693955	57721557	12q13.3-q14.1	OS9	75.44376691	42.15912132	1.789500458	4	4	59.86230338	29.43648878
chr10	1022637	1044201	10p15.3	IDI2-AS1	2.017496629	1.128091483	1.788415798	4	4	31.69584045	133.8998268
chr19	15508487	15552317	19p13.12	CYP4F22	1.178611469	0.659101504	1.788209345	4	4	40.41168737	115.3627097
chr14	77274956	77320885	14q24.3	POMT2	0.461381464	0.258036668	1.788046125	3	4	19.69702913	22.53510909
chr21	45405137	45513720	21q22.3	COL18A1	6.075317444	3.398006761	1.787906226	4	4	59.60115511	49.50834724
chr14	64345473	64347611	14q23.3	TEX21P	1.049594806	0.587301766	1.787147371	4	4	66.84622997	103.2693406
chr7	73006080	73016375	7q11.23	PMS2P7	0.465519448	0.260504044	1.786995091	3	3	14.83694986	46.79679878
chr19	53772495	53773288	19q13.42	SEPT7P8	0.66645513	0.373055916	1.786475166	3	1	36.77526167	0
chr12	89011126	89019692	12q21.33	LINC02458	1.353742984	0.757837737	1.786323006	4	3	63.07234375	30.86215071
chr15	49858238	50181851	15q21.2	ATP8B4	16.11081226	9.019711217	1.786178279	4	4	24.48125817	51.24959666
chr15	42548813	42569994	15q15.2	HAUS2	39.43606096	22.07938012	1.786103629	4	4	87.92957591	48.43195993
chr19	42268537	42295796	19q13.2	CIC	11.62945675	6.511422069	1.786008744	4	4	99.79095879	33.2382349
chr11	440399	440693	11p15.5	RN7SL838P	1.527118092	0.855048211	1.786002323	2	1	24.147276	0
chr8	37695751	37700021	8p11.23	ZNF703	0.855910582	0.479256365	1.785913855	4	4	41.77162776	96.43350072
chr3	3066324	3110414	3p26.2	IL5RA	9.01704953	5.04915957	1.785851567	4	4	48.06967123	91.89637305
chr6	108211217	108261260	6q21	SNX3	517.8189241	289.9618992	1.785817122	4	4	22.18995021	17.47280411
chr6	130827955	131063322	6q23.1-q23.2	EPB41L2	2.449997981	1.372063619	1.785630015	4	4	62.17909194	54.56273231
chr9	96385941	96418639	9q22.32	ZNF367	13.02388626	7.295389592	1.785221488	4	4	35.26370045	27.04388418
chr11	46277638	46321422	11p11.2	CREB3L1	0.115797257	0.064868293	1.785113362	2	2	54.85394497	3.893206877

chr1	113979391	113982254	1p13.2	OLFML3	0.464601019	0.260323052	1.784709484	1	3	0	56.16843438	
chr10	71816283	71851375	10q22.1	PSAP	841.39464	471.5124061	1.784459177	4	4	68.60128417	33.95907405	
chr4	8269712	8307111	4p16.1	HTRA3	0.212259881	0.11896386	1.784238344	2	2	69.81523562	49.52242929	
chr6	88275295	88277074	6q15	ACTBP8	0.509810727	0.285802861	1.783784546	1	1	0	0	
chr3	71753724	71756380	3p13	GPR27	21.92829772	12.29429763	1.783615329	4	4	62.55191387	78.49875877	
chr4	3241598	3256615	4p16.3	MSANTD1	0.123053596	0.069002114	1.783330821	2	3	49.19591238	50.40847622	
chr17	44409059	44409358	17q21.31	RN7SL258P	1.493757198	0.837629155	1.783315671	3	3	24.80429301	9.886923095	
chr19	51124880	51140480	19q13.41	SIGLEC9	35.18360265	19.72941807	1.783306661	4	4	66.31287094	69.13726357	
chr17	36591796	36600804	17q12	DHRS11	0.143685947	0.080585472	1.783025439	4	4	72.29193698	54.64411355	
chr1	186828900	186988981	1q31.1	PLA2G4A	12.1913547	6.837957912	1.782894082	4	4	13.62261156	24.82420655	
chr10	71396934	71815947	10q22.1	CDH23	1.714305616	0.961746586	1.782492022	4	4	83.52845817	44.50171573	
chrX	48058178	48071658	Xp11.23	ZNF630	1.243012975	0.697393828	1.782368765	4	3	52.58292199	32.85240382	
chr16	31073665	31083512	16p11.2	ZNF646	11.92942592	6.694765531	1.781903469	4	4	80.48823662	41.38920724	
chr1	36417906	36450485	1p34.3	OSCP1	0.314155668	0.176307059	1.781866646	1	3	0	76.57645267	
chr12	91875303	91880655	12q21.33	LINC02404	0.411600645	0.231091007	1.78111927	3	2	58.72068433	10.8454229	
chr9	108942569	109013594	9q31.3	CTNNAL1	34.16689769	19.18466769	1.780948112	4	4	55.16027483	29.06334548	
chr10	28677495	28682939	10p12.1	BAMBI	1.008645747	0.56636086	1.780924174	4	4	90.89712567	70.29568785	
chr2	199663332	199851180	2q33.1	FTCDNL1	0.431435969	0.242273787	1.780778577	4	4	120.8226027	117.7334468	
chr10	133394117	133422529	10q26.3	MTG1	0.874295212	0.490967442	1.780760063	3	4	20.58755212	67.0278527	
chr5	69351726	69370013	5q13.2	AK6	9.775477883	5.490925333	1.780296997	4	4	92.42859533	59.39368099	
chr11	61816203	61867354	11q12.2	FADS2	2.475885487	1.391050419	1.77986754	4	4	101.3508284	79.94640753	
chr14	59484443	59505407	14q23.1	JKAMP	20.63959799	11.59745913	1.779665507	4	4	14.1600482	17.85897369	
chrMT	5761	5826	N/A	MT-TC	9.506476926	5.342367646	1.779450153	2	3	2.051525438	16.48749434	
chr20	17613678	17682283	20p12.1	RRBP1	15.23875519	8.56392125	1.779413279	4	4	60.09067349	44.43746906	
chr1	33332393	33332492	1p35.1	MIR3605	13.43156497	7.549069753	1.77923445	4	3	90.01399473	66.03185049	
chr11	2295640	2303049	11p15.5	C11orf21	13.61744137	7.653667619	1.779204696	4	4	48.35077519	45.50273245	
chr19	40348395	40378490	19q13.2	PLD3	14.83371405	8.337886601	1.779073614	4	4	90.53434274	27.91733896	
chr8	85438827	85449040	8q21.2	CA3	3.54495768	1.993690917	1.778087892	4	4	149.8037916	43.9683394	
chr14	61695401	61748259	14q23.2	HIF1A	185.8555777	104.5337613	1.777947865	4	4	63.00899385	20.47141429	
chr17	69414697	69553861	17q24.3	MAP2K6	6.383257221	3.590675873	1.777731393	4	4	40.71345384	23.83707865	
chr7	29212113	29213887	7p14.3	NANOGP4	0.987369665	0.55545678	1.777581445	3	3	56.57155354	91.36995516	
chr21	43092956	43108291	21q22.3	U2AF1	1.615787743	0.908993608	1.777556772	4	4	54.6992258	64.21014684	
chr1	40784012	40840457	1p34.2	KCNQ4	0.108698943	0.06116847	1.777042049	2	3	58.45763497	1.533492061	
chr22	43528549	43812977	22q13.2-q13.31	EFCAB6	0.335331186	0.188725868	1.776816233	4	4	90.61989874	63.85101418	
chr21	26466209	26573404	21q21.3	CYYR1	0.954490294	0.537252947	1.776612487	4	3	45.31304538	61.47352455	
chr20	3753499	3768388	20p13	C20orf27	7.09915672	3.996378719	1.776397389	4	4	48.2712553	21.79077896	
chr5	143192500	143194166	5q31.3	ARHGAP26-IT1	8.366331915	4.710437398	1.776126336	3	4	33.93487064	44.70455705	
chr13	54440678	54441337	13q14.3	RPL13AP25	1.561684348	0.879373806	1.775905011	3	4	86.70081604	35.29984444	
chr21	42499632	42581440	21q22.3	SLC37A1	6.124371534	3.448657611	1.775871143	4	4	63.34138454	26.12666599	
chr22	42058334	42070896	22q13.2	NAGA	21.94313675	12.35770671	1.775664147	4	4	65.2895145	28.239804	
chr22	50525752	50530085	22q13.33	TYMP	71.33172773	40.17610046	1.775476637	4	4	63.12541762	33.66820757	
chr3	48467798	48504826	3p21.31	SHISA5	86.72654888	48.85238775	1.775277584	4	4	46.8085549	27.05265886	
chr20	2692878	2760108	20p13	EBF4	0.354792666	0.199875439	1.775068855	2	3	28.0133734	32.50465665	

chr12	6539528	6556097	12p13.31	IFFO1	3.787414749	2.134046186	1.774757628	4	4	55.83890065	27.10065535
chr6	30676389	30687895	6p21.33	PPP1R18	12.04965527	6.790033406	1.774609129	4	4	91.30946952	44.74053525
chr17	28546707	28552663	17q11.2	UNC119	13.00507091	7.329696838	1.774298609	4	4	72.08634541	29.16141185
chr10	58191517	58269961	10q21.1	IPMK	54.82004579	30.89688937	1.774290127	4	4	57.81589643	50.89140757
chr14	77024543	77028699	14q24.3	IRF2BPL	15.53977623	8.758606536	1.774229287	4	4	79.59883079	31.57646115
chr2	120252838	120294711	2q14.2	RALB	114.5323721	64.55712776	1.774124346	4	4	61.88152067	54.18567191
chr10	79827273	79851338	10q22.3	NUTM2E	0.25513224	0.143814413	1.774038049	2	2	94.7816637	64.46427738
chr6	6346465	6622826	6p25.1	LY86-AS1	0.783238465	0.441507775	1.774008317	4	4	75.58488816	27.84347129
chr17	8002670	8020340	17p13.1	GUCY2D	0.16492157	0.092970213	1.773918388	4	3	45.81070894	55.08897806
chr1	28578538	28582983	1p35.3	SNHG12	20.6149647	11.62208222	1.7737755	4	4	54.74355031	19.44961756
chrX	30653348	30731462	Xp21.2	GK	61.59464902	34.7284164	1.773609494	4	4	85.03903541	62.8496075
chr7	26152227	26187137	7p15.2	NFE2L3	5.986342307	3.375593733	1.773419072	4	4	34.5687402	45.73702639
chr1	39567374	39567510	1p34.3	SNORA55	5.516278259	3.111148072	1.773068376	3	3	31.20313383	77.56664663
chr11	74330312	74398751	11q13.4	PGM2L1	19.85918054	11.20571137	1.772237377	4	4	48.39997451	38.73607495
chr6	119177201	119350619	6q22.31	MAN1A1	93.97744036	53.0333503	1.772044192	4	4	21.85266991	16.93978036
chr17	50426158	50474845	17q21.33	ACSF2	1.818159474	1.026162838	1.771804052	4	4	78.26521576	28.99696747
chr1	233614004	233672512	1q42.2	KCNK1	0.701665813	0.396023254	1.771779322	4	4	143.7513836	104.6208634
chr14	22773222	22819811	14q11.2	SLC7A7	24.72159175	13.95395288	1.771655097	4	4	48.30240066	22.56992387
chr15	49326997	49620931	15q21.2	FAM227B	6.599471701	3.725120183	1.771613096	4	4	76.34634661	60.8394863
chrX	53374149	53422728	Xp11.22	SMC1A	79.7527512	45.01906199	1.771532939	4	4	51.39277815	19.26973472
chr19	35143250	35154302	19q13.12	FXYD7	0.339969995	0.191918049	1.771433151	2	2	11.1110281	32.37276972
chr7	144250674	144251603	7q35	OR2A20P	0.15378053	0.086814015	1.771379085	1	2	0	3.255780005
chr2	174348150	174395715	2q31.1	CIR1	77.9312688	43.99792095	1.771248893	4	4	50.60444758	22.09544779
chr2	218274192	218292577	2q35	TMBIM1	54.79718227	30.93808047	1.771188821	4	4	44.7380015	18.65967013
chr6	32844086	32846500	6p21.32	PSMB8-AS1	3.650838644	2.061330697	1.771107688	4	4	52.30245343	53.49810107
chr14	77505997	77616767	14q24.3	SPTLC2	39.50473032	22.30514095	1.771104267	4	4	21.30098045	13.94614656
chr2	187341963	187448304	2q32.1	CALCRL	1.57971303	0.891953177	1.771071701	4	4	37.44245024	73.04233663
chr15	70654554	70778823	15q23	UACA	1.010200402	0.570506562	1.77070777	4	4	40.45022175	55.36237429
chr12	45874035	45874330	12q12	RN7SL246P	1.520166314	0.85855082	1.770618906	2	2	24.24334822	1.001472418
chr7	29920103	29990289	7p14.3	SCRN1	10.07521688	5.690814944	1.770434811	4	4	75.30703934	37.46290528
chr3	119294289	119419476	3q13.32-q13.33	ARHGAP31	4.896200465	2.766058425	1.770100161	4	4	73.03117626	44.59941893
chr15	43793659	43793759	15q15.3	MIR1282	10.91994032	6.169358059	1.770028618	3	4	108.5702518	108.9226363
chr7	148963315	148963410	7q36.1	RNY4	499.3533042	282.1239459	1.769978449	4	4	113.5138759	96.96057013
chr11	61964285	61967660	11q12.3	FTH1	2154.765924	1217.560431	1.769740432	4	4	74.0568424	37.58515591
chr5	10441862	10482807	5p15.2	ROPN1L	5.87245707	3.318481973	1.769621507	4	4	66.7028263	76.1592958
chr19	45641494	45642840	19q13.32	EML2-AS1	0.46477427	0.262658225	1.769502054	3	4	71.92630322	29.09349146
chr17	56834099	56869567	17q22	DGKE	12.20064178	6.8955306	1.769355034	4	4	66.37602665	30.02085302
chr6	44158811	44184402	6p21.1	CAPN11	0.396146936	0.223897986	1.769318887	3	3	64.8530099	30.17925065
chr1	233327732	233385148	1q42.2	MAP3K21	0.740657469	0.418612736	1.769314226	4	4	47.19009284	54.32329092
chr9	27937617	29215243	9p21.2-p21.1	LINGO2	0.526781758	0.297785301	1.768998522	3	4	21.14373248	27.37654045
chr2	178413659	178440243	2q31.2	PRKRA-AS1	1.826442054	1.032640664	1.76871018	4	4	31.9930432	58.56604599
chr16	18791667	18801678	16p12.3	ARL6IP1	174.7354423	98.79325188	1.768698155	4	4	54.02821575	16.2271725
chr4	76158737	76213899	4q21.1	SCARB2	6.36876724	3.601065794	1.768578417	4	4	24.36151676	34.6327312

chr6	26087281	26096216	6p22.2	HFE	1.288417864	0.728997903	1.767382127	4	4	66.45539101	60.4838718
chr20	45791937	45811427	20q13.12	DNTTIP1	24.08027136	13.62646214	1.767169726	4	4	74.37426485	37.24222038
chr4	159128805	159128891	4q32.1	MIR3688-2	5.784791353	3.274116815	1.766824973	1	1	0	0
chr18	23994213	24015460	18q11.2	TTC39C-AS1	1.075458255	0.608723527	1.76674337	3	2	28.42951494	11.15116681
chr21	44768580	44801836	21q22.3	UBE2G2	11.9752619	6.778687957	1.766604685	4	4	31.94042192	15.27125118
chr12	32790746	32896846	12p11.21	PKP2	0.458811542	0.259727225	1.766513084	4	4	73.20528429	99.67179047
chr10	133453928	133469183	10q26.3	SCART1	0.582079648	0.32951445	1.766476851	4	4	79.89059701	41.42607338
chr18	48539056	48863217	18q21.1	CTIF	0.684644329	0.387579443	1.766461926	4	4	69.93827056	47.3934876
chr7	124929873	125145234	7q31.33	POT1-AS1	0.359938554	0.203764228	1.766446237	4	4	75.11532728	33.30332109
chr2	27625638	27629031	2p23.3	CCDC121	1.390458919	0.787224589	1.766279839	4	4	58.21130401	58.89978437
chr6	33075926	33089696	6p21.32	HLA-DPB1	59.12574146	33.48218801	1.76588643	4	4	84.68081149	67.13605098
chr8	38996767	39105261	8p11.22	ADAM9	9.5670664	5.417908387	1.765822844	4	4	41.4578212	22.37377896
chr10	73126080	73126207	10q22.1	SNORA11F	7.401566394	4.191852598	1.765702925	4	4	90.23423184	65.79483553
chr6	31575567	31578336	6p21.33	TNF	2.133932045	1.208604542	1.765616436	4	4	110.2035551	78.72915409
chr3	155762612	155806287	3q25.31	C3orf33	2.71271253	1.53641599	1.765610712	4	4	50.13333351	56.93660639
chr9	92711363	92764841	9q22.31	BICD2	34.22345235	19.3836889	1.765579943	4	4	61.65601949	34.31216747
chr14	106153622	106154083	14q32.33	IGHV3-15	7.93826397	4.496618636	1.765385195	4	3	25.01251673	75.62513162
chr2	60935930	60938049	2p16.1	NONOP2	0.783181308	0.443643006	1.76534127	2	4	93.10194061	73.74288972
chr22	36253071	36267531	22q12.3	APOL1	14.94466269	8.466331994	1.765187415	4	4	50.24104834	46.37712551
chr2	26042675	26042781	2p23.3	RNU6-942P	4.377053194	2.4801033	1.764867291	2	2	82.34166523	8.524858901
chr1	44674696	44725591	1p34.1	ARMH1	2.198632209	1.24589609	1.764699502	4	4	110.3942298	65.47978373
chr12	11548030	11564403	12p13.2	LINC01252	0.132242418	0.07494213	1.764593787	2	1	42.42426744	0
chr2	11155372	11182028	2p25.1	PQLC3	21.53001818	12.20144866	1.764546061	4	4	52.39589331	25.77421481
chr5	140558268	140564604	5q31.3	APBB3	2.592622207	1.469672638	1.764081429	4	4	60.43407814	29.60306215
chr3	58192626	58211003	3p14.3	DNASE1L3	0.73463051	0.41644616	1.764046786	4	3	78.94498436	19.69651977
chr6	37005647	37029072	6p21.2	FGD2	7.865306268	4.459362124	1.763773842	4	4	47.82045846	32.97129511
chr10	96593312	96720522	10q24.1	PIK3AP1	128.2883069	72.74922466	1.763431947	4	4	47.38212961	35.34858048
chr5	141398128	141512979	5q31.3	PCDHGB5	0.097077505	0.055059202	1.763147696	1	2	0	6.37130383
chr15	73051715	73305206	15q24.1	NEO1	1.472530685	0.835232212	1.763019511	4	4	24.74798775	58.21428929
chr5	140175068	140243789	5q31.3	CYSTM1	39.52954617	22.43421815	1.762020228	4	4	32.9671893	25.95588163
chr17	4710494	4721500	17p13.2	ARRB2	156.0369631	88.55672317	1.76200019	4	4	57.17269068	44.08414955
chr7	140453040	140479569	7q34	MKRN1	1069.044126	606.7484403	1.761923154	4	4	16.3442483	16.51524609
chr12	131929200	131944040	12q24.33	PUS1	1.294649897	0.734830581	1.761834538	4	4	98.71509904	56.18627709
chr5	73508609	73509715	5q13.2	FUNDC2P1	1.070657117	0.607718372	1.761765263	3	3	66.62782192	51.13484596
chr16	21641284	21652666	16p12.2	IGSF6	57.35391135	32.55627337	1.761685396	4	4	76.83843675	61.77564701
chr11	64340203	64357534	11q13.1	CCDC88B	23.26087272	13.20476929	1.761550862	4	4	66.03031566	26.40116596
chr11	45804072	45813016	11p11.2	SLC35C1	2.284844983	1.297410565	1.761080913	4	4	92.07383846	27.77057405
chr1	17066761	17119453	1p36.13	PADI2	53.95072504	30.63528487	1.761064905	4	4	75.97436151	76.82386702
chr6	169751622	169781584	6q27	ERMARD	14.44019029	8.200752202	1.760837291	4	4	69.14428046	17.33023123
chr16	30949084	30954938	16p11.2	ORAI3	4.089158949	2.322487021	1.760681077	4	4	61.69535323	61.915497
chr12	94460003	94462570	12q22	CEP83-DT	0.181957244	0.103358793	1.760442815	2	1	24.50032807	0
chr11	74490511	74493713	11q13.4	LIPT2	1.127528622	0.640623065	1.760049994	4	4	97.10832599	48.88675769
chr3	58237502	58294736	3p14.3	ABHD6	2.56064666	1.455066607	1.759814051	4	4	46.69275207	51.6691082

chr17	78852977	78925390	17q25.3	TIMP2	25.97673972	14.76188663	1.759716788	4	4	51.60163167	41.31796648
chr3	187216085	187293529	3q27.3	MASP1	0.038529051	0.021897491	1.759518976	1	1	0	0
chr5	177305500	177312291	5q35.3	MXD3	4.36434729	2.480775934	1.759267022	4	4	60.14955473	31.64486801
chr9	35658290	35661503	9p13.3	CCDC107	5.390277029	3.064022724	1.759215748	4	4	56.12782031	24.86066417
chr1	224257004	224257141	1q42.11	MIR320B2	4.194422622	2.384434203	1.759085076	1	2	0	34.98150118
chr14	58408494	58427614	14q23.1	TIMM9	21.85454601	12.4246782	1.75896274	4	4	84.19340256	33.74812732
chr8	142702252	142726973	8q24.3	LNCOC1	0.082972915	0.047176613	1.758772171	1	2	0	3.577464064
chr3	139560180	139678050	3q23	NMNAT3	2.211663868	1.258422561	1.757489048	4	4	29.76041669	75.24876269
chr19	50333800	50344748	19q13.33	NAPSB	20.36890342	11.59067286	1.757352974	4	4	54.11753443	44.88474049
chr3	195066970	195271166	3q29	XXYLT1	2.277641839	1.296380716	1.756923572	4	4	59.43956721	17.4774073
chr11	105040218	105045416	11q22.3	CARD16	84.28912423	47.97780562	1.756835752	4	4	70.9590061	52.25535686
chr19	18391137	18397645	19p13.11	LRRC25	91.81678702	52.2706447	1.756565038	4	4	64.82108122	43.36332339
chr16	30114105	30123309	16p11.2	MAPK3	20.89334593	11.89609071	1.756320327	4	4	82.2856009	32.62343318
chr19	531720	542088	19p13.3	CDC34	169.851482	96.71601118	1.756187832	4	4	42.86919598	23.85445552
chr2	26149092	26151272	2p23.3	SMARCE1P6	0.525534436	0.299311091	1.755813439	3	2	34.24728305	37.25702489
chr1	223701593	223776018	1q41	CAPN2	35.80293824	20.39224761	1.755713197	4	4	54.45536266	44.18704412
chr17	29042900	29057227	17q11.2	PIPOX	0.774740344	0.441417345	1.755119849	4	4	53.81931516	52.48408625
chr2	207166400	207166498	2q33.3	MIR7845	9.377199327	5.344004823	1.754713859	4	4	67.01597151	83.62365436
chr16	67175602	67183980	16q22.1	KIAA0895L	0.527507731	0.300720552	1.754145925	4	4	55.44659257	44.38338967
chr7	53187132	53188942	7p12.1	HAUS6P1	4.062812019	2.316807717	1.753625037	4	4	121.1516376	98.2528501
chr1	230837119	230869589	1q42.2	C1orf198	6.309927962	3.598329547	1.75357145	4	4	38.64679401	10.1729282
chr20	32192504	32207743	20q11.21	PLAGL2	7.159342273	4.084327426	1.75288157	4	4	95.99305253	20.60554786
chr16	57603098	57605538	16q21	HMGB3P32	1.47922438	0.844037821	1.752556986	3	4	44.0102335	54.48645509
chr11	65576034	65592650	11q13.1	EHBP1L1	53.51161925	30.53570786	1.752427666	4	4	66.65640757	32.21002974
chr6	153010891	153131284	6q25.2	RGS17	0.098679696	0.056327203	1.751901213	4	3	54.50794649	36.84549776
chr8	86481561	86483028	8q21.3	NTAN1P2	0.978220517	0.558464305	1.751625858	4	4	34.46087674	10.88760369
chr22	37282383	37315348	22q13.1	CYTH4	70.98219266	40.52729967	1.751466129	4	4	66.77474563	41.55319197
chr12	57472255	57488814	12q13.3	ARHGAP9	67.64193334	38.62066691	1.751443948	4	4	24.50057969	27.6640724
chr3	52691378	52691444	3p21.1	SNORD19C	6.866788426	3.921041447	1.751266473	3	3	20.14736604	32.22736246
chr11	70467856	71252724	11q13.3-q13.4	SHANK2	0.028354225	0.016191177	1.751214528	2	1	63.56779273	0
chr11	121452203	121633762	11q24.1	SORL1	207.4833702	118.4828694	1.751167669	4	4	77.69037115	56.72502094
chr3	134599923	134656053	3q22.2	KY	0.158197029	0.090381283	1.750329537	3	1	65.76550883	0
chrX	47582291	47586791	Xp11.3	TIMP1	98.59398981	56.33531045	1.750127744	4	4	69.43458375	31.3075608
chr20	4023718	4024606	20p13	FTLP3	3.176613201	1.815617276	1.749605075	4	4	16.12002776	52.58863005
chr12	63142759	63152810	12q14.2	AVPR1A	4.032510442	2.304826154	1.749594187	4	4	10.80692096	40.05470346
chr14	50533082	50633068	14q22.1	ATL1	1.751689377	1.001510532	1.749047385	4	4	47.02204169	16.11825855
chr2	196804415	196810276	2q33.1	C2orf66	0.279486316	0.159844364	1.748490275	3	1	35.94073885	0
chr17	15229777	15265373	17p12	PMP22	2.135204205	1.221249727	1.748376403	4	4	95.53931394	59.64223078
chr14	89417354	89419793	14q32.11	FOXN3-AS1	0.907856403	0.519278304	1.748304129	2	3	48.02285557	56.09086204
chr20	34049119	34049190	20q11.22	MIR4755	9.631838698	5.509387794	1.748259345	2	1	19.25842621	0
chr11	1732410	1750594	11p15.5	IFITM10	0.100273563	0.057362343	1.748073012	2	1	56.86457522	0
chr2	143094233	143094715	2q22.2	MTND6P11	1.195402873	0.684035373	1.747574643	3	3	30.68398531	20.99942271
chr19	45485288	45497055	19q13.32	RTN2	2.429358973	1.390311649	1.747348498	4	4	45.53695622	44.95953267

chr2	109127327	109130119	2q13	SH3RF3-AS1	0.273512998	0.156607278	1.746489694	1	2	0	9.073031238
chr1	39895424	39902015	1p34.2	MYCL	6.049604527	3.463926833	1.746458519	4	4	67.07203781	43.74241503
chr19	11322046	11339668	19p13.2	RAB3D	65.7434777	37.64513283	1.746400471	4	4	64.02678761	55.42132008
chr7	36597834	36600120	7p14.2	AOAH-IT1	7.761700007	4.444961604	1.746179315	4	4	39.04497148	25.61889331
chr12	56520406	56596196	12q13.3	RBMS2	0.9135914	0.52329998	1.745827315	4	4	81.25108078	46.60316355
chr6	150934814	150935885	6q25.1	ARL4AP5	0.780284997	0.447050198	1.745408011	3	3	55.77363503	16.78031963
chr5	122063195	122078501	5q23.1	LOX	0.638363514	0.365744813	1.745379543	4	3	86.84331018	63.92443831
chr22	21919419	21952878	22q11.22	PPM1F	20.06352574	11.49522857	1.745378582	4	4	84.68325027	61.44466773
chr7	128392277	128409989	7q32.1	IMPDH1	49.88348787	28.58866475	1.744869455	4	4	84.88672793	51.69349002
chr3	71771655	71785206	3p13	PROK2	139.0578816	79.70482445	1.744660785	4	4	53.91375317	90.7991987
chr11	22812429	22829865	11p14.3	SVIP	22.08682121	12.65967151	1.744659899	4	4	20.53123425	15.98680124
chr2	108448561	108509398	2q12.3	GCC2	27.02611018	15.49085424	1.744649441	4	4	67.71150005	12.51034859
chr3	169793011	169812904	3q26.2	LRRC34	0.652621304	0.374113267	1.744448435	4	4	62.0598685	39.68333669
chr16	14974591	15153343	16p13.11	PDXDC1	13.15681044	7.542120619	1.744444448	4	4	55.72690363	7.261389841
chr4	82629537	82798857	4q21.22	SCD5	0.83604839	0.479271735	1.744414136	4	4	36.54629381	52.69711051
chr13	45389294	45391426	13q14.13	RCN1P2	0.602546991	0.345496809	1.744001608	3	1	35.1448152	0
chr22	36487186	36507101	22q12.3	FOXRED2	0.533555919	0.305947429	1.743946405	4	4	31.21768467	75.46326781
chr12	62603686	62603769	12q14.1	MIRLET7I	4.351231321	2.495126943	1.743891762	2	1	66.251173	0
chr9	13105296	13279713	9p23	MPDZ	0.060222057	0.034538309	1.743630725	1	2	0	47.9412269
chr17	30194319	30235968	17q11.2	SLC6A4	1.295391838	0.742996427	1.743469809	4	4	69.59175565	21.77153483
chr10	21321961	21322066	10p12.31	RNU6-15P	3.764187673	2.159079698	1.74342229	1	2	0	11.55389842
chr2	169527880	169573921	2q31.1	FASTKD1	37.83549859	21.70337061	1.743300581	4	4	76.08394153	36.22520546
chr17	50862223	50867978	17q21.33	TOB1	33.86757945	19.42887637	1.743156877	4	4	51.78100532	32.28198396
chr10	5638857	5666595	10p15.1	ASB13	2.440750354	1.400192921	1.743152902	4	4	64.63516425	35.45128488
chr16	535316	535368	16p13.3	MIR5587	6.687812518	3.836720677	1.74310644	2	1	66.39201863	0
chr10	112950220	113167678	10q25.2-q25.3	TCF7L2	3.324088354	1.907089919	1.743016059	4	4	57.47799334	21.14977951
chr15	60347151	60398025	15q22.2	ANXA2	33.68935121	19.3283083	1.743005683	4	4	49.36420113	32.56712375
chr16	84145260	84178761	16q24.1	DNAAF1	0.058434522	0.033532066	1.742646019	2	3	55.1625401	1.309917387
chr21	44805617	44818129	21q22.3	SUMO3	38.21967637	21.93250229	1.742604463	4	4	66.67978975	38.43404627
chr14	45110875	45110975	14q21.2	SNORD127	5.870863871	3.369323771	1.742445746	1	1	0	0
chr7	45104906	45105042	7p13	SNORA5C	36.92644581	21.19249301	1.742430482	4	4	37.18088032	45.88662918
chr17	30709608	30792830	17q11.2	SUZ12P1	135.5279443	77.78362191	1.742371222	4	4	86.68531013	54.58418291
chr6	42928002	42939287	6p21.1	CNPY3	100.5332374	57.70182201	1.742288785	4	4	66.49112441	36.95408382
chr16	50693587	50733077	16q12.1	NOD2	11.05003482	6.343290653	1.742003547	4	4	73.0828874	16.95179037
chr12	66189198	66254622	12q14.3	IRAK3	50.43276245	28.95113111	1.741996272	4	4	43.03357539	32.64100467
chr17	81395431	81466332	17q25.3	BAHCC1	0.241271677	0.138508891	1.741921951	4	4	74.39822535	65.03812708
chr9	132161234	132244534	9q34.13	NTNG2	3.195686345	1.834657602	1.74184346	4	4	70.90256274	47.37980806
chr21	14371115	14383188	21q11.2	HSPA13	37.42983047	21.49134612	1.741623362	4	4	23.95537036	21.36481465
chrX	12906620	12923169	Xp22.2	TLR8	155.0176585	89.01232709	1.74153023	4	4	78.53359725	57.0164519
chr10	74371453	74372486	10q22.2	RPSAP6	0.494574556	0.283992971	1.741502806	1	2	0	11.30185743
chr2	112368369	112433645	2q14.1	RGPD8	11.48216673	6.594143311	1.741267393	4	4	106.5691457	60.13623887
chr13	43879178	43893932	13q14.11	LACC1	2.591323157	1.48842867	1.740979067	4	4	62.87951939	65.1359719
chr1	246724076	246768137	1q44	SCCPDH	28.56694609	16.41103509	1.74071568	4	4	37.54948302	37.29657569

chr11	68008547	68029282	11q13.2	ALDH3B1	5.616284225	3.226780276	1.740522671	4	4	59.98153126	21.60971971
chr3	197001740	197029816	3q29	MELTF	0.147052428	0.084493887	1.740391322	4	4	64.32808974	84.03746631
chr12	190077	214205	12p13.33	SLC6A12	1.322779669	0.760079716	1.740317023	4	4	87.01372162	81.818002
chr3	122325164	122341969	3q21.1	CSTA	75.35752005	43.3026886	1.740250374	4	4	62.30163263	60.14805725
chr21	32154682	32283954	21q22.11	MIS18A	6.725814885	3.865169683	1.740108569	4	4	28.98108726	13.40662365
chr20	23361585	23375399	20p11.21	GZF1	8.082248267	4.645051429	1.739969598	4	4	47.68366076	18.60862852
chr16	27550128	27780371	16p12.1	KIAA0556	4.170559856	2.397290237	1.73969751	4	4	58.60444089	24.86351329
chr4	15002426	15070153	4p15.32	CPEB2	13.36903392	7.684760181	1.739681344	4	4	30.34094903	30.26444228
chr13	25096138	25098567	13q12.13	PABPC3	0.369898304	0.212625408	1.739671227	4	4	32.48532487	30.30143676
chr11	44047981	44060667	11p11.2	ACCSL	1.352685412	0.777638648	1.739478117	4	3	121.099431	93.91941814
chr11	124635789	124676303	11q24.2	SIAE	5.577375703	3.206671256	1.739303863	4	4	31.90109824	24.60151902
chr19	15640897	15661606	19p13.12	CYP4F3	49.11715668	28.24537948	1.738944832	4	4	60.26489806	56.70632402
chr2	26401912	26456713	2p23.3	DRC1	0.406424492	0.233723052	1.738914871	3	2	18.16884333	92.1729408
chr3	197889075	197960111	3q29	IQCG	2.428654795	1.396857061	1.738656633	4	4	64.31298558	19.29557254
chr1	230979049	231000733	1q42.2	ARV1	23.94291001	13.7729899	1.73839596	4	4	73.11621845	23.81675922
chr20	23045633	23049664	20p11.21	THBD	10.96773971	6.31038433	1.738046233	4	4	51.00346294	56.03038741
chr17	75757937	75765199	17q25.1	GALK1	2.853915262	1.642241922	1.737816593	4	4	90.25261381	54.48449688
chr19	6677835	6720682	19p13.3	C3	0.693699729	0.399241431	1.73754444	3	4	87.38550714	39.46676933
chr5	134371176	134392108	5q31.1	UBE2B	123.2163894	70.92020764	1.737394651	4	4	58.76660716	56.87766171
chrMT	15956	16023	N/A	MT-TP	532.9309269	306.7676508	1.737246172	4	4	24.78883186	14.73740394
chr6	125780943	125932030	6q22.31-q22.32	NCOA7	25.78776594	14.84590331	1.737029092	4	4	20.46565084	12.24902019
chr1	244452013	244640362	1q44	CATSPERE	0.800516809	0.460958762	1.736634324	4	4	43.23660487	83.37851694
chr9	111602831	111613553	9q31.3	LRRC37A5P	0.195266765	0.112449499	1.736484092	1	1	0	0
chr2	102355801	102398777	2q12.1	IL18R1	15.62434005	8.998511917	1.736324871	4	4	50.81165084	54.26627256
chr5	31400494	31532175	5p13.3	DROSHA	26.80027602	15.43519973	1.736308987	4	4	52.03509492	17.04920559
chr5	132369704	132395614	5q31.1	SLC22A5	0.386986027	0.222879288	1.736303229	4	4	42.97865966	7.344572064
chr12	50924751	50932517	12q13.12	METTL7A	34.1852909	19.69255773	1.735949762	4	4	32.22837888	30.97145928
chr20	63558086	63574239	20q13.33	HELZ2	14.15768527	8.155881517	1.735886579	4	4	68.1838718	35.91443512
chr10	69057533	69104811	10q22.1	SRGN	3259.146318	1878.354339	1.735107296	4	4	63.29480167	42.42278425
chr7	155003433	155005703	7q36.2	PAXIP1-AS1	1.930834757	1.112959538	1.734865187	3	4	26.00210127	26.21370887
chr1	156082546	156140089	1q22	LMNA	5.019371611	2.893327137	1.734809571	4	4	56.45553496	11.53709066
chr9	36136536	36163913	9p13.3	GLIPR2	80.5439809	46.42831705	1.73480294	4	4	64.431344	37.51568016
chr17	7921859	7930346	17p13.1	KCNAB3	0.501403646	0.289055982	1.734624696	4	4	69.72897039	47.92554472
chr1	26361632	26374536	1p36.11	ZNF683	5.358454687	3.089136736	1.734612335	4	4	99.12053178	94.61583675
chr3	132317367	132368765	3q22.1	ACPP	13.94215306	8.038070482	1.734514905	4	4	74.80932324	58.60714814
chr8	55876041	56012447	8q12.1	LYN	204.0731701	117.6561683	1.734487643	4	4	58.46452365	45.39338663
chr11	66362521	66372476	11q13.2	SLC29A2	0.544697267	0.314053735	1.73440786	3	4	14.09334688	31.74541813
chr9	97983207	98015943	9q22.33	ANP32B	436.6747252	251.7733096	1.734396414	4	4	83.4791745	76.65489048
chr16	30757226	30762272	16p11.2	CCDC189	0.947690085	0.546483902	1.734159198	3	4	62.90311721	99.22422458
chr12	8950044	9215657	12p13.31	KLRG1	21.95174084	12.65898807	1.734083382	4	4	32.57258062	39.38652358
chr14	95686417	95692643	14q32.13	TCL1B	0.23529046	0.135690941	1.734017448	1	2	0	19.95237896
chr2	37826247	37875863	2p22.2	LINC00211	8.411669051	4.851289975	1.733903579	4	4	36.42466239	36.92106229
chr10	68991128	68991868	10q22.1	MED28P1	0.783641036	0.451968683	1.733839237	1	1	0	0

chr2	8007771	8328419	2p25.1	LINC00299	8.012351847	4.621321966	1.733779188	4	4	120.4787083	26.38797795
chr7	151556114	151877231	7q36.1	PRKAG2	8.716321488	5.030272751	1.732773136	4	4	57.05568582	22.48157596
chr1	236142529	236213535	1q42.3	GPR137B	10.94540306	6.317013379	1.732686383	4	4	35.8328821	20.20974923
chr22	23741584	23751092	22q11.23	ZNF70	0.84370247	0.486953247	1.732614938	4	4	68.00462083	59.24055393
chr10	29409534	29487856	10p11.23	SVIL-AS1	1.932966384	1.115775133	1.732397798	4	4	33.11725768	46.03106031
chr10	26790540	26790646	10p12.1	RNU6-946P	11.16025016	6.442166197	1.732375387	1	2	0	22.50988507
chr5	90473929	90514555	5q14.3	POLR3G	9.017789756	5.206543327	1.732010893	4	4	20.80896298	15.27484399
chr1	235853000	235853060	1q42.3	MIR1537	26.32807081	15.20289916	1.731779612	4	4	71.19888839	74.79489481
chr10	96620877	96621707	10q24.1	RPS2P36	3.85643059	2.228289332	1.730668695	4	4	64.28721449	83.38959915
chr22	21632121	21637327	22q11.21	CCDC116	0.152098806	0.087897934	1.730402517	1	1	0	0
chr5	103548855	103562805	5q21.2	NUDT12	1.266215869	0.732047441	1.729690998	3	4	21.37877762	61.05842056
chr17	15436015	15467611	17p12	CDRT4	0.142720504	0.082528395	1.729350299	3	3	21.82165649	5.773642219
chr8	144098633	144104248	8q24.3	SHARPIN	43.56931046	25.19442073	1.729323763	4	4	18.95183557	34.33685728
chr21	43453987	43659499	21q22.3	HSF2BP	0.502594987	0.290664921	1.729121581	4	3	111.1995782	48.53650071
chr1	99472420	99487804	1p21.2	LINC01708	0.713285007	0.412527266	1.729061486	3	2	90.80794247	71.58057473
chr18	62325287	62388096	18q21.33	TNFRSF11A	0.405854994	0.234744635	1.728921282	4	4	50.85088663	60.52472602
chr4	158170752	158202877	4q32.1	FAM198B-AS1	0.46509255	0.269105378	1.728291548	4	4	113.6507056	10.95092302
chr2	74497517	74503316	2p13.1	LBX2	0.246151023	0.142429299	1.728233053	2	3	47.78741979	32.78884271
chr12	47341614	47342089	12q13.11	PPIAP45	1.395523153	0.807754966	1.727656543	2	2	67.00246563	44.31524963
chr8	101368893	101369595	8q22.3	NACA4P	0.507179743	0.293587463	1.727525211	4	4	9.766204286	53.4666294
chr9	5109523	5110754	9p24.1	MTND5P14	1.047610374	0.606492397	1.727326474	4	1	53.65890631	0
chr11	93730194	93741537	11q21	TAF1D	49.217627	28.49364436	1.727319481	4	4	51.65301364	29.68656538
chr1	168540765	168543997	1q24.2	XCL2	10.76854119	6.234729498	1.727186592	4	4	60.52571581	75.49224012
chr4	184755595	184826593	4q35.1	ACSL1	256.9418826	148.7972584	1.726791779	4	4	55.14941639	81.89635863
chr14	22522040	22522102	14q11.2	TRAJ22	38.73016483	22.43081997	1.726649533	2	4	54.29506661	73.35396507
chr16	67394419	67416494	16q22.1	ZDHHC1	0.339412767	0.196576216	1.72662174	4	4	108.2286347	44.93471672
chr4	55540502	55540835	4q12	RN7SKP30	1.467641235	0.850034288	1.7265671	2	1	25.17634307	0
chr16	30104810	30113557	16p11.2	GDPD3	1.806776789	1.046547784	1.726415953	4	4	44.09243141	47.97680703
chr7	101205977	101217730	7q22.1	PLOD3	3.829949818	2.21851382	1.726358332	4	4	75.14213861	44.52119324
chrX	148052012	148052923	Xq28	FTH1P8	3.217633675	1.863879653	1.72630978	4	4	44.38943035	31.47125971
chrX	63347228	63351339	Xq11.1	SPIN4	5.36494511	3.107935787	1.72620848	4	4	81.47026022	31.50374942
chr7	73220639	73235945	7q11.23	NCF1B	49.82399033	28.86436668	1.726141816	4	4	81.23004573	16.59460656
chr2	201487421	201619182	2q33.1	C2CD6	0.26998877	0.15642369	1.726009464	4	4	71.28660456	35.58387212
chr1	151790749	151794402	1q21.3	TDRKH-AS1	0.367869705	0.213152196	1.725854632	1	2	0	5.906770162
chr19	18196784	18204064	19p13.11	RAB3A	0.933932297	0.541306461	1.725330038	4	3	41.4974812	50.48662768
chr7	74209757	74229834	7q11.23	LAT2	43.16247759	25.01729612	1.72530546	4	4	62.59598193	48.26003708
chrX	69672479	69672597	Xq13.1	RNA5SP506	34.03370275	19.72645161	1.725282551	2	2	21.91344596	69.75583033
chr11	103109431	103479863	11q22.3	DYNC2H1	1.07399131	0.622509857	1.725259927	4	4	44.78511301	26.60666273
chr16	85027709	85094230	16q24.1	KIAA0513	25.037165	14.51284698	1.725172534	4	4	53.81680129	28.89091981
chr2	73729837	73737406	2p13.1	TPRKB	17.38742035	10.07911919	1.725093237	4	4	9.396187261	15.60000781
chr6	41770176	41779905	6p21.1	FRS3	1.176094355	0.68199188	1.72449906	4	4	58.22144186	40.6024565
chr14	34432938	34462262	14q13.1	SPTSSA	15.51212766	8.996528845	1.72423475	4	4	37.674982	28.66497826
chr5	100806935	100903285	5q21.1	ST8SIA4	72.6322376	42.12851379	1.724063611	4	4	47.92409623	50.9104747

chr7	132123332	132648688	7q32.3	PLXNA4	0.563563643	0.326963162	1.723630391	4	4	110.8995919	35.56302452
chr7	149764182	149776323	7q36.1	ZNF467	13.23511764	7.681589523	1.722966009	4	4	92.89546447	61.13144937
chr9	33500756	33511166	9p13.3	SUGT1P1	26.00790592	15.09781589	1.722627042	4	4	76.59018691	103.1335393
chr14	74479940	74493381	14q24.3	NPC2	37.12829262	21.5540699	1.722565288	4	4	52.35914853	26.36777403
chr17	4949182	4957131	17p13.2	ENO3	0.56257669	0.326681946	1.72209299	4	4	58.4595781	13.78833429
chr4	87975650	87983411	4q22.1	SPP1	4.649873239	2.700208335	1.722042399	4	3	135.5778149	64.48134457
chr13	21372571	21459370	13q12.11	ZDHHC20	206.7212258	120.1065611	1.721148485	4	4	62.68850896	23.73397747
chrX	136146702	136211359	Xq26.3	FHL1	9.674873236	5.622837045	1.720639094	4	4	73.02564043	28.17092356
chr20	833697	846279	20p13	FAM110A	12.36119007	7.184362158	1.720568896	4	4	71.0522936	28.47723447
chr12	93569822	93626236	12q22	SOCS2	2.627887044	1.527341531	1.720562815	4	4	51.76084	29.91087029
chr14	92936914	93115919	14q32.12	ITPK1	12.63969613	7.346639022	1.720473279	4	4	69.28491155	25.93836268
chr1	228644647	228746669	1q42.13	RHOU	11.39449742	6.623665595	1.720270635	4	4	31.97325908	15.65601033
chr2	72887383	72892160	2p13.2	SPR	0.684772571	0.398136099	1.719945949	2	2	7.499279391	23.93227314
chr5	94150851	94618604	5q15	KIAA0825	3.670365778	2.134070062	1.719890009	4	4	59.70948206	41.72255427
chr6	31197760	31203968	6p21.33	HCG27	0.838757852	0.487731112	1.719713652	1	1	0	0
chr14	20365667	20413540	14q11.2	TEP1	14.72344425	8.562632173	1.719499793	4	4	49.97986713	26.99344852
chr2	26033860	26137454	2p23.3	RAB10	129.8247122	75.51029764	1.719298112	4	4	15.28723838	17.36184856
chr19	47309847	47322070	19q13.32	C5AR1	186.7632366	108.6321256	1.71922657	4	4	64.50256944	55.81148615
chr12	54395261	54419266	12q13.13	ITGA5	32.65449389	18.99755122	1.718879107	4	4	58.51775599	53.37599369
chr4	152626114	152680165	4q31.3	TMEM154	105.0766469	61.13979961	1.718629232	4	4	57.68915377	44.1644758
chr11	89852840	89863065	11q14.3	TRIM51BP	1.097987541	0.63888809	1.718591344	4	3	42.97129872	72.29104729
chr1	28578743	28578822	1p35.3	SNORD99	13.40121917	7.797996079	1.718546538	4	4	102.7330281	35.26307636
chrX	49171837	49175120	Xp11.23	PLP2	85.75227894	49.8988296	1.718522852	4	4	50.19803977	39.34087773
chr1	69567130	70152426	1p31.1	LRRC7	0.093012345	0.054132123	1.718246767	3	4	70.27819695	61.77242498
chr4	153684080	153710643	4q31.3	TLR2	87.34900655	50.84209904	1.718044852	4	4	39.53709227	49.94311158
chr19	44643798	44666161	19q13.31	PVR	3.026219264	1.761788823	1.717696936	4	4	73.0723354	70.48332124
chr19	14489267	14489890	19p13.12	SNRPGP15	4.363236341	2.540212609	1.717665807	2	3	37.55543873	34.62361619
chr13	110712623	110721074	13q34	ING1	7.133032177	4.153542877	1.717336835	4	4	65.40385675	53.54898497
chr3	195274736	195443176	3q29	ACAP2	120.6706965	70.26816887	1.71728819	4	4	34.79072357	25.96800645
chr5	14796181	14798497	5p15.2	RBBP4P1	0.697823039	0.406391196	1.717121446	3	3	43.46015811	49.3396093
chr17	8802723	8870003	17p13.1	PIK3R6	3.234605579	1.883942017	1.716934783	4	4	70.98674934	57.01462085
chr11	82822940	82901740	11q14.1	PRCP	12.95717601	7.547801013	1.716682248	4	4	54.50411592	32.99906969
chr13	41121885	41122171	13q14.11	RN7SL597P	3.486526097	2.031119195	1.716554157	2	2	3.741089013	59.0187084
chr12	70222190	70243360	12q15	LINC01481	8.692395642	5.064438387	1.716359244	4	4	94.9396861	66.32854677
chr8	143612618	143618043	8q24.3	TSTA3	20.76829262	12.10026034	1.716350891	4	4	56.71320658	67.58941447
chr2	11584773	11584972	2p25.1	RN7SL674P	116.6526703	67.96824334	1.716281966	4	4	59.84921691	25.28819427
chr9	137250219	137253497	9q34.3	STPG3-AS1	0.258988462	0.150907301	1.716208966	1	1	0	0
chr6	25081067	25138392	6p22.3	CMAHP	12.13891851	7.07337068	1.716143415	4	4	41.63599491	17.30390476
chr9	136494433	136545786	9q34.3	NOTCH1	34.2140777	19.94382954	1.715521968	4	4	81.99021318	28.7054795
chr9	130444707	130501274	9q34.11	ASS1	0.217765611	0.126938959	1.715514393	1	2	0	43.85895674
chr22	22771833	22772582	22q11.22	IGLV3-12	4.187688008	2.441298164	1.71535295	3	3	30.81864715	77.61529733
chr9	112997120	113012192	9q32	ZNF883	0.211312139	0.123193757	1.715282852	4	4	124.633564	60.65722342
chr3	58633943	58666843	3p14.2	FAM3D	0.274948322	0.160315148	1.715048925	3	2	43.01688605	2.1814781

chr2	42712740	42712847	2p21	RNU6-137P	5.466607225	3.187957932	1.71476768	1	2	0	25.73704495
chr5	176308055	176309210	5q35.2	BRCC3P1	0.723741443	0.422073578	1.714728145	2	2	67.79698753	36.49082768
chr22	44752558	44862784	22q13.31	ARHGAP8	0.088540333	0.051640173	1.714563054	3	1	48.42780043	0
chr6	87674860	87702267	6q15	AKIRIN2	47.71140346	27.83086433	1.714334233	4	4	28.8201772	11.74783564
chr3	9703807	9729908	3p25.3	CPNE9	0.178624481	0.104195744	1.714316483	2	1	53.1718151	0
chr12	52644558	52652175	12q13.13	KRT2	0.311946777	0.182052597	1.713498091	3	4	43.45696043	50.56733081
chr1	27872531	27886685	1p35.3	THEMIS2	119.2142693	69.57856603	1.713376347	4	4	66.87982788	22.74032216
chr1	181317712	181808084	1q25.3	CACNA1E	0.438890467	0.256182224	1.713196413	4	4	110.7377012	72.26609142
chr15	41387349	41402501	15q15.1	NDUFAF1	10.42098953	6.082832737	1.713180352	4	4	50.67961535	27.25699279
chr17	40121409	40136790	17q21.1	MSL1	64.55183407	37.67964348	1.713175288	4	4	50.5411289	44.71249914
chr9	86264546	86282575	9q21.33	ISCA1	142.8337576	83.39645883	1.712707705	4	4	28.38083916	7.606065635
chr5	76403255	76708132	5q13.3	IQGAP2	97.16877107	56.74722346	1.712308817	4	4	43.64493938	12.4497943
chr2	46297402	46386703	2p21	EPAS1	1.53253442	0.895268234	1.711815926	4	4	99.79367214	44.96328656
chr4	40423255	40630911	4p14	RBM47	29.48737773	17.22663961	1.711731272	4	4	64.8757587	54.74253439
chr19	48813014	48836688	19q13.33	HSD17B14	0.341248116	0.199367707	1.711651904	1	1	0	0
chr11	73376360	73397474	11q13.4	RELT	14.05057596	8.209120628	1.71158113	4	4	59.49048865	39.05543518
chr8	117799363	118111819	8q24.11	EXT1	3.923820116	2.292542937	1.711557961	4	4	77.08444495	18.6766904
chr19	54923509	54965184	19q13.42	NLRP7	0.19809762	0.115745228	1.711497084	3	3	54.7280154	41.00327872
chr19	49838645	49851676	19q13.33	PTOV1-AS1	0.45404332	0.265309734	1.711370758	3	4	47.2441261	76.74302903
chr14	73932031	73950414	14q24.3	FAM161B	1.163473732	0.680088715	1.71076759	4	4	62.64966099	66.00015592
chr2	178450592	178467549	2q31.2	PJVK	0.188848559	0.110399921	1.710586001	3	2	40.29564145	14.05102305
chr6	71288296	71309059	6q13	OGFRL1	138.4778028	80.95515892	1.710549453	4	4	44.96813943	42.63844207
chr6	44258166	44265788	6p21.1	NFKBIE	9.110527843	5.326463955	1.710427015	4	4	75.26273698	21.60948436
chr11	119638098	119728725	11q23.3	NECTIN1	1.6798432	0.982167329	1.710343187	4	4	66.37063047	60.00528304
chr14	30559123	30620064	14q12	G2E3	24.39593774	14.26452903	1.710251891	4	4	81.31083689	36.82751149
chr4	78971748	79308799	4q21.21	LINC01088	2.060046731	1.204537422	1.71023888	4	4	73.45957492	61.21255621
chrX	40834254	40837807	Xp11.4	MKRN4P	0.758977659	0.443813974	1.710125647	4	3	131.7193431	55.62497858
chr6	133952170	133987500	6q23.2	TBPL1	53.8764606	31.50549595	1.710065466	4	4	58.33369701	37.79123969
chr1	114584575	114670111	1p13.2	DENND2C	2.821075842	1.650147882	1.709589712	4	4	47.82650083	61.66641505
chr17	48655662	48707346	17q21.32	LINC02086	0.113237003	0.066245059	1.709365275	3	1	46.07104002	0
chr1	143955364	143973519	1q21.1	FAM72C	0.840220819	0.491560856	1.709291554	4	4	88.85745169	94.46721064
chr16	77190919	77199646	16q23.1	MON1B	17.66072503	10.33411904	1.70897248	4	4	69.90829473	38.05295773
chr16	722578	726880	16p13.3	CCDC78	0.248699399	0.145528085	1.708944349	2	3	100.5234599	95.42105742
chr7	23129100	23129916	7p15.3	AK3P3	1.418052527	0.829815998	1.70887586	3	4	61.13992624	64.91862878
chr20	38433862	38433999	20q11.23	SNORA71D	20.95957799	12.26553498	1.708818899	4	4	96.65760222	93.98519006
chr16	72782885	73058635	16q22.2-q22.3	ZFHX3	1.039601828	0.608458534	1.708582869	4	4	95.39579136	44.66079609
chr4	48483343	48489524	4p11	SLC10A4	0.533824819	0.312439994	1.708567502	2	4	55.93951711	65.75947352
chr12	57941504	57957269	12q14.1	ATP23	203.0306549	118.8475368	1.708328674	4	4	92.09685359	33.24946092
chr15	40087890	40108884	15q15.1	BMF	7.567875269	4.430547258	1.708112977	4	4	84.34776813	39.57688907
chr7	87934143	88202889	7q21.12	ADAM22	1.420971103	0.831949908	1.708000792	4	4	82.51567207	43.97612511
chr2	10043555	10054836	2p25.1	KLF11	13.09369699	7.666363614	1.707941033	4	4	85.75838558	22.27602758
chr1	111462027	111478512	1p13.2	C1orf162	66.57970038	38.9831519	1.707909626	4	4	65.6690536	44.62491261
chr19	9827880	9830121	19p13.2	UBL5	38.10534896	22.31122266	1.707900528	4	4	41.06568512	18.64777635

chr2	69960089	69962265	2p13.3	ASPRV1	3.454739686	2.022935073	1.707785748	4	4	31.87134681	100.2332056
chr10	73812501	73874591	10q22.2	CAMK2G	14.42326651	8.445794385	1.7077454	4	4	65.98551132	43.96082732
chr19	13144058	13150374	19p13.13	STX10	29.31478689	17.16704181	1.707620172	4	4	64.09881702	29.69554463
chr20	36306323	36528637	20q11.23	DLGAP4	3.209472244	1.879528673	1.707594191	4	4	79.7271711	55.27413712
chr1	207496128	207641765	1q32.2	CR1	30.68715309	17.97114315	1.707579359	4	4	42.65202883	48.77633552
chr14	58564058	58564738	14q23.1	RPL9P5	1.006783748	0.589615826	1.707524974	1	2	0	24.62805939
chr9	121338988	121370304	9q33.2	STOM	287.2175192	168.2181151	1.707411351	4	4	34.46704294	30.55034596
chr11	86945675	86955398	11q14.2	FZD4	0.060670027	0.03553538	1.707313304	2	1	73.07499866	0
chr17	76561108	76561193	17q25.1	SNORD1B	4.850491711	2.841029682	1.707300611	1	2	0	28.43152469
chr17	17200990	17206905	17p11.2	PLD6	1.958310961	1.147068541	1.70723099	4	4	57.78143566	36.79584752
chr5	94703690	95284575	5q15	MCTP1	18.13499564	10.6244486	1.706911701	4	4	37.74389444	31.48003451
chr6	75644084	75644177	6q14.1	RNU6-1016P	30.56627697	17.90737693	1.706909788	4	3	70.35859697	56.04400612
chr21	33543038	33577514	21q22.11	SON	59.93507423	35.11524704	1.706810554	4	4	28.95346932	7.153165759
chr11	4418060	4419008	11p15.4	OR51R1P	2.043170828	1.197478743	1.706227222	4	4	69.37060394	89.52250955
chr6	116254152	116441261	6q22.1	DSE	9.707606841	5.690171406	1.706030653	4	4	56.67994621	21.91145489
chr11	10450321	10507579	11p15.4	AMPD3	11.99713081	7.032740798	1.705896912	4	4	70.01975565	49.58297139
chr19	13150468	13154908	19p13.13	IER2	52.48720564	30.7689419	1.705850199	4	4	56.70733458	42.70704179
chr10	27504174	27542237	10p12.1	RAB18	62.57771704	36.68545706	1.705790852	4	4	33.45857044	25.96037805
chr11	102396325	102453044	11q22.2	TMEM123	253.8054082	148.8170487	1.705486102	4	4	46.48392318	18.5988047
chr1	151280024	151281929	1q21.3	ZNF687-AS1	1.510899464	0.886024949	1.705256116	4	3	80.29921383	81.70650644
chr19	3607247	3613930	19p13.3	CACTIN-AS1	0.231781332	0.135936627	1.705069031	1	2	0	2.980156366
chr3	50337511	50338300	3p21.31	RASSF1-AS1	2.471427089	1.449532347	1.704982365	4	4	66.24013749	71.29220814
chr16	88696495	88706421	16q24.2-q24.3	RNF166	25.4729776	14.94434789	1.704522525	4	4	73.20236366	18.076987
chr14	24305187	24311377	14q12	CIDEB	12.89143841	7.56441953	1.704220444	4	4	77.74566282	46.10977798
chr6	31897785	31945674	6p21.33	C2	0.369273227	0.216705894	1.704029458	4	4	52.8516223	111.3198908
chr7	105456501	105522258	7q22.3	PUS7	28.67716718	16.83022563	1.703908659	4	4	71.77232039	41.45384678
chr14	64540468	64543237	14q23.3	HSPA2	0.496679477	0.291521593	1.703748502	3	4	85.51068941	47.87637029
chr21	36320189	36376646	21q22.12	MORC3	1901.779117	1116.317569	1.70361837	4	4	41.0907844	21.91963814
chr12	109783087	109833407	12q24.11	TRPV4	0.230896532	0.135569948	1.703154241	3	2	25.52500766	70.01673504
chr11	61799625	61817057	11q12.2	FADS1	2.240673733	1.315915843	1.702748505	4	4	35.85491787	38.83915562
chr12	2668500	2672220	12p13.33	CACNA1C-AS2	1.339449973	0.786681632	1.702658253	2	2	92.45396213	8.557641372
chr11	30384493	30586995	11p14.1	MPPED2	0.113883738	0.066898915	1.702325634	2	3	54.9751901	57.69382574
chr15	42723544	42737133	15q15.2	CDAN1	8.734108548	5.131422974	1.702083144	4	4	71.98496426	65.12267152
chr15	64841883	64868002	15q22.31	PLEKHO2	43.76266652	25.71249752	1.701999834	4	4	53.40372914	42.76006123
chr1	147183963	147225798	1q21.1	FMO5	1.874876567	1.101586098	1.701979147	4	4	65.8309046	41.33391932
chr19	54173412	54189882	19q13.42	MBOAT7	25.84459094	15.18745032	1.701707028	4	4	78.12114864	51.74279841
chr10	71747556	71773580	10q22.1	VSIR	189.3343799	111.2844438	1.701355314	4	4	70.34193738	25.75163119
chr7	135662473	135676412	7q33	STMP1	110.5827588	65.00451227	1.701155118	4	4	51.90780629	50.50673292
chr1	153618799	153634092	1q21.3	S100A13	0.463813963	0.272687218	1.700901003	3	3	80.75104404	104.5233362
chr16	3611737	3665472	16p13.3	DNASE1	1.895347368	1.114589382	1.700489344	4	4	72.62793823	60.54791803
chr11	64894532	64917250	11q13.1	ATG2A	19.13908729	11.25708351	1.700181692	4	4	89.9018424	5.919218885
chr16	684622	690661	16p13.3	WDR24	2.459545228	1.446641805	1.70017569	4	4	62.25749111	40.7499682
chr3	39263494	39281735	3p22.2	CX3CR1	112.9701008	66.45308141	1.69999793	4	4	56.14538283	73.20786179

chr7	137874979	138002101	7q33	CREB3L2	5.406570637	3.180655764	1.699828915	4	4	20.87963199	33.91159634
chrX	39786524	39786813	Xp11.4	RN7SL732P	2.881681059	1.69543645	1.699669167	4	3	83.43853966	47.68457548
chr16	69950232	69968478	16q22.1	CLEC18A	0.138273511	0.081361575	1.699494036	3	3	66.99469188	46.98692054
chr11	120210975	120230332	11q23.3	OAF	3.728056176	2.193628398	1.699493032	4	4	66.48722134	30.02128191
chr13	113759217	113813797	13q34	TMEM255B	0.122793931	0.072255967	1.699429613	3	2	60.61742706	70.73260292
chr3	101662065	101662164	3q12.3	RNY1P12	7.102315223	4.179555982	1.699298981	2	2	28.82945854	73.1396675
chr19	3538270	3557592	19p13.3	MFSD12	7.28685527	4.288546494	1.699143353	4	4	26.01514702	36.90050699
chr1	94168907	94275068	1p22.1	ARHGAP29	0.122824624	0.072286717	1.699131305	4	4	107.1613811	91.33345868
chr13	21358376	21359311	13q12.11	GAPDHP52	0.420683901	0.247624177	1.698880563	1	2	0	13.05709377
chr17	6657719	6661382	17p13.1	ALOX15P1	0.386100856	0.227370549	1.698112874	3	4	47.24365722	84.92425135
chr9	135477650	135488894	9q34.3	PPP1R26	1.049209717	0.617888103	1.698057807	4	4	59.60854451	56.48670212
chr12	9852369	9869859	12p13.31	CLEC2B	188.8585555	111.2331888	1.69786156	4	4	60.45567737	36.55906105
chr2	89176328	89177130	2p11.2	IGKV2-24	4.319403377	2.54468819	1.697419509	3	2	27.33793358	20.5351357
chr13	100089015	100530437	13q32.3	PCCA	5.812670078	3.424850039	1.697204261	4	4	73.11189853	23.12076379
chr19	41998316	42071804	19q13.2	GRIK5	0.143993774	0.08484498	1.697139581	2	2	75.18408734	55.93085865
chr17	30631755	30637477	17q11.2	LRRC37BP1	1.255559377	0.739913946	1.696899191	4	4	57.33568382	51.53574104
chr3	98732236	98795845	3q12.1	ST3GAL6	6.501101312	3.831217279	1.696876172	4	4	11.57260512	62.87558112
chr1	6424776	6461367	1p36.31	ESPN	3.579249802	2.109387323	1.696819623	4	4	77.88572113	44.24316326
chr19	4838334	4867768	19p13.3	PLIN3	24.16465049	14.24169615	1.69675369	4	4	75.97792417	47.70285502
chr1	160317265	160318474	1q23.2	SUMO1P3	1.573609959	0.927615018	1.696404142	2	3	32.41182456	21.79982676
chr12	120291763	120291903	12q24.23	RNU4-2	2545.159527	1500.456072	1.696257274	4	4	96.42164318	78.11413062
chr1	35807624	35857890	1p34.3	AGO4	58.8575302	34.70344382	1.696014105	4	4	70.84106888	44.63573223
chr15	65296051	65296167	15q22.31	RNU5A-1	725.7709523	427.9580221	1.695892856	4	4	95.41314441	107.1940119
chr16	75111860	75116795	16q23.1	LDHD	0.196407168	0.115816572	1.695846847	3	3	40.09231511	10.52381844
chr8	10474565	10481974	8p23.1	LINCR-0001	0.585277886	0.34517012	1.695621528	4	2	77.85084618	62.98221398
chr15	68930500	68946811	15q23	SPESP1	0.909186166	0.536234214	1.695501968	3	2	70.67769033	36.74888753
chr6	7326654	7389709	6p24.3	CAGE1	0.256868228	0.151510839	1.695378561	3	2	34.81429965	25.69289735
chr11	2608328	2699998	11p15.5	KCNQ1OT1	0.111886472	0.066002126	1.695194977	4	4	75.53777795	27.9931044
chr3	14124940	14143680	3p25.1	TMEM43	53.86081192	31.79038654	1.694248412	4	4	59.70839799	37.28390881
chr12	9239326	9240496	12p13.31	PTMAP4	2.023867115	1.194736586	1.693986054	2	3	52.41127013	69.44009041
chr2	55172547	55232563	2p16.1	CLHC1	10.59608339	6.25526758	1.693945663	4	4	57.67898807	52.46414526
chr9	92836766	92837741	9q22.31	EEF1DP2	1.603206062	0.946488484	1.693846348	3	4	86.68451396	76.50425121
chr12	4097373	4099720	12p13.32	HSPA8P5	0.317158942	0.187245541	1.693813058	1	2	0	53.27247239
chr19	9683239	9684096	19p13.2	RPS4XP22	0.549167396	0.324246692	1.693671544	2	1	60.29861898	0
chr12	127881617	127898639	12q24.32	LINC02393	0.192415303	0.113609238	1.693658955	1	1	0	0
chr19	53223908	53224879	19q13.42	NDUFV2P1	0.808987899	0.47775624	1.69330682	3	3	15.08919702	64.48753186
chr13	49444293	49495003	13q14.2	SETDB2	60.67512799	35.83302062	1.693274163	4	4	71.04638483	32.45761818
chrX	24146225	24149654	Xp22.11	ZFX-AS1	1.777488162	1.049759926	1.693233013	2	1	35.5223687	0
chr1	151612027	151699083	1q21.3	SNX27	27.86556416	16.45775394	1.693157174	4	4	36.77809152	25.1447074
chr4	44619578	44656868	4p12	YIPF7	0.222321499	0.131307161	1.693140699	1	1	0	0
chr5	140596529	140647411	5q31.3	TMCO6	3.464985262	2.046781554	1.692894513	4	4	86.16548068	27.74435938
chr13	102729367	102759072	13q33.1	CCDC168	0.085423355	0.050462015	1.692824887	3	3	43.68114842	47.69761727
chr7	31616777	32428224	7p14.3	PDE1C	0.029271515	0.017291657	1.692811444	2	1	62.23457575	0

chr19	42507306	42528509	19q13.2	CEACAM1	8.016960921	4.736628019	1.692546024	4	4	64.65216874	46.16357832
chr17	45159948	45170040	17q21.31	HEXIM2	2.260354931	1.335661933	1.692310663	4	4	48.62915995	11.01795051
chr13	22695862	22696791	13q12.12	FTH1P7	0.861176327	0.508882939	1.69228768	4	3	47.22875091	11.98632026
chr1	192809039	192812277	1q31.2	RGS2	1770.568623	1046.395759	1.692064028	4	4	72.86914512	68.58418236
chr4	108652150	108652255	4q25	RNU6-431P	4.453258562	2.632092076	1.69190835	2	1	22.11446838	0
chr14	24336021	24340036	14q12	RIPK3	5.511052259	3.257458064	1.691826004	4	4	32.46343714	25.19625652
chrX	154401236	154412101	Xq28	DNASE1L1	6.265736008	3.70377976	1.691713982	4	4	55.63902952	32.16789551
chr4	46723670	46724607	4p12	RAC1P2	3.433918545	2.029939493	1.691635912	4	4	90.92641964	45.96116265
chr14	50117119	50231693	14q21.3	SOS2	125.7917205	74.38228022	1.691151712	4	4	16.25125499	28.64840975
chr22	22117481	22118005	22q11.22	ABHD17AP5	1.625078608	0.961157299	1.690751981	2	2	24.65004444	56.81386523
chr17	50111309	50129882	17q21.33	SAMD14	0.47702594	0.28217125	1.690554723	4	4	79.74480823	28.90334784
chr21	29077471	29175889	21q21.3	MAP3K7CL	119.0155864	70.40155502	1.690524965	4	4	17.59326644	36.76699273
chr22	18533864	18548828	22q11.21	PI4KAP1	2.439746122	1.443212993	1.690496229	4	4	47.31962006	111.7142268
chr5	181223263	181235808	5q35.3	TRIM41	2.032156799	1.202250152	1.690294483	4	4	77.14045372	30.64890952
chr8	21908873	21913717	8p21.3	DOK2	48.15260788	28.4912486	1.690084157	4	4	58.59893149	36.58422788
chr3	156826287	157047659	3q25.31	LEKR1	0.54852336	0.324556382	1.690071097	3	4	94.28271491	36.39457495
chr1	185292367	185317329	1q25.3	IVNS1ABP	95.67550527	56.61683801	1.689877228	4	4	31.19273367	39.21515531
chr4	87386883	87387174	4q22.1	RN7SL681P	3.161623425	1.870934643	1.689863105	3	3	84.92830074	47.0535002
chr12	57460135	57472451	12q13.3	GLI1	0.191959262	0.113612145	1.689601592	2	2	9.26177589	65.59251444
chr10	58334979	58370753	10q21.1	UBE2D1	68.74535782	40.69244206	1.689388848	4	4	45.19224436	51.68753982
chr4	53985486	53987282	4q12	RPL21P44	2.425201643	1.435728244	1.689178752	4	4	35.66205456	89.2065157
chr5	61332273	61546172	5q12.1	ZSWIM6	16.243026	9.616284611	1.689116604	4	4	50.35522942	27.81035044
chr1	116909923	116990358	1p13.1	PTGFRN	0.318928685	0.18884506	1.688837844	4	4	90.42083059	38.53308675
chr4	79901617	80073472	4q21.21	ANTXR2	26.5852744	15.74184948	1.688827888	4	4	53.99164737	38.5366816
chr4	6687448	6690519	4p16.1	LINC002481	1.860679145	1.101850574	1.688685552	4	4	50.17191518	63.86160347
chr6	33291654	33299388	6p21.32	RGL2	11.11134711	6.580049474	1.68864188	4	4	77.58011275	46.15798032
chr22	50530409	50532579	22q13.33	ODF3B	9.70599821	5.748957495	1.688305787	4	4	58.28833765	41.36459648
chr8	95268836	95810143	8q22.1	C8orf37-AS1	0.304118868	0.180147452	1.688166363	3	2	73.09910092	3.426759346
chr6	40391589	40587464	6p21.2-p21.1	LRFN2	0.290043528	0.171843291	1.687837366	4	3	75.50862367	58.83077375
chr10	68282660	68332958	10q21.3	PBLD	2.152790795	1.275774398	1.687438467	4	4	49.22793911	11.06832558
chr11	6926361	6991144	11p15.4	ZNF215	0.546383606	0.323807806	1.687370088	3	4	109.6875009	97.91779481
chr22	35299275	35347994	22q12.3	TOM1	11.77679339	6.980417888	1.687118677	4	4	51.77394296	20.01541126
chr12	98613404	98645051	12q23.1	IKBIP	21.38114884	12.67471161	1.68691403	4	4	43.5952584	62.76436495
chr15	30103732	30131748	15q13.2	ULK4P3	0.304067966	0.180252673	1.686898512	4	1	35.79365485	0
chr6	159679064	159762529	6q25.3	SOD2	292.116134	173.1700609	1.68687435	4	4	78.85220007	56.67952396
chr15	83107486	83145403	15q25.2	TM6SF1	23.17435586	13.73887656	1.68677226	4	4	23.13069535	45.66465271
chr1	63367590	63438562	1p31.3	ALG6	16.64238909	9.868327899	1.68644468	4	4	67.19213064	37.00008987
chr20	37289648	37317260	20q11.23	MANBAL	2.991051123	1.773938951	1.686107135	4	4	63.76846588	66.57263062
chr2	217760390	217761311	2q35	RPL7L1P9	0.517026382	0.306677787	1.685894457	3	1	15.00899707	0
chr6	109444062	109465968	6q21	MICAL1	11.38331265	6.755122271	1.685137914	4	4	46.96105528	29.20244045
chr11	130069821	130144811	11q24.3	APLP2	102.5483866	60.85818101	1.685038641	4	4	75.52645875	44.40621523
chr5	172253780	172255740	5q35.1	KLF3P1	0.401823061	0.23847279	1.684984948	3	2	58.79840411	6.567238421
chr1	162631265	162786573	1q23.3	DDR2	0.109979738	0.065273554	1.684905013	4	4	70.3679342	79.89158732

chr6	10722915	10731129	6p24.2	TMEM14C	38.10155368	22.61596371	1.684719438	4	4	50.41213572	27.25487003
chr2	20590775	20592548	2p24.1	HS1BP3-IT1	0.742016587	0.440463775	1.684625681	2	1	21.86339502	0
chr11	27365961	27472775	11p14.1	LGR4	3.142433536	1.865533537	1.684469067	4	4	71.26886414	52.80394303
chr17	43006725	43014459	17q21.31	IFI35	22.39294502	13.29435993	1.68439437	4	4	85.40550003	45.17238531
chr13	29764408	29850683	13q12.3	UBL3	46.29848342	27.48771542	1.684333627	4	4	79.05822502	20.20352476
chr13	113584688	113641473	13q34	TFDP1	112.3086232	66.69037907	1.684030362	4	4	50.2889879	13.05336927
chr17	8390704	8397835	17p13.1	RNF222	0.139290093	0.082719529	1.683884024	2	2	35.5992632	3.583197967
chr16	83807903	83813002	16q23.3	HSBP1	15.12473461	8.983936417	1.683530906	4	4	46.00378798	28.89504735
chr10	124006059	124093684	10q26.13	CHST15	57.8311042	34.35177871	1.683496645	4	4	65.60182007	49.40059906
chr1	109619813	109632055	1p13.3	AMPD2	37.99520901	22.56999598	1.683438891	4	4	64.91817546	39.18673386
chr9	124878294	124941407	9q33.3	GOLGA1	41.49952397	24.65610533	1.683133789	4	4	76.84011628	49.65800119
chr7	102396357	102426682	7q22.1	PRKRIP1	17.21304811	10.22953396	1.682681555	4	4	49.7904964	13.75884918
chr17	45220268	45247322	17q21.31	FMNL1	59.48053605	35.35070674	1.682584071	4	4	57.55791533	21.95123602
chr10	95753206	96090235	10q24.1	ENTPD1-AS1	1.295541306	0.770091884	1.682320426	4	4	28.04016365	34.73347323
chr17	19770828	19867926	17p11.2	ULK2	4.642086172	2.759410985	1.682274296	4	4	21.98465095	33.85393762
chr18	63907969	63936111	18q22.1	SERPINB10	1.584852155	0.94226761	1.681955464	4	4	86.13078419	82.55641932
chr1	144887191	145095321	1q21.1	SRGAP2B	2.176669741	1.294426076	1.681571301	4	4	48.65909308	38.63368456
chr2	96325324	96330507	2q11.2	ITPRIPL1	3.045173351	1.811296274	1.681212176	4	4	29.27291679	62.23823693
chr19	11377205	11384342	19p13.2	EPOR	6.920869023	4.116782993	1.681135254	4	4	23.26756731	36.12060726
chr5	39168934	39170362	5p13.1	GOLGA5P1	2.880471827	1.713668129	1.680880783	4	4	68.70932161	51.75780504
chr2	6729168	6770311	2p25.2	LINC00487	0.199844627	0.118893095	1.680876655	1	1	0	0
chr19	197016	202209	19p13.3	LINC01002	1.308826984	0.778696783	1.680791563	4	4	84.57492316	62.07821946
chr13	27545913	27621106	13q12.2	LNX2	41.92970695	24.94792098	1.680689424	4	4	68.8741554	63.95694826
chr9	32976389	32977202	9p21.1	TCEA1P4	1.11408671	0.662888234	1.680655429	2	2	12.21678568	25.55026771
chr20	1474591	1491762	20p13	SIRPB2	16.95673842	10.09225651	1.68017315	4	4	73.50485563	58.00475019
chr5	177483394	177497605	5q35.3	PDLIM7	14.77614347	8.796284117	1.679816531	4	4	56.22430735	35.28720764
chr10	23095498	23122013	10p12.2	MSRB2	5.668605698	3.37474466	1.679713954	4	4	71.69242334	52.26819532
chr1	211575039	211578757	1q32.3	SLC30A1	31.94215093	19.01891912	1.679493495	4	4	28.5435633	31.27812486
chr16	88715338	88785220	16q24.3	PIEZO1	7.377161791	4.392888784	1.679341807	4	4	55.62815115	31.06695638
chr10	122980040	122990407	10q26.13	PSTK	0.704299332	0.41953624	1.67875684	3	4	62.7280329	20.89541221
chr3	10164879	10243743	3p25.3	IRAK2	5.58299465	3.326207709	1.678486475	4	4	24.75374607	36.59991763
chr6	30743199	30744550	6p21.33	IER3	3.002853478	1.789212164	1.678310453	3	4	119.5487101	50.05938176
chr16	84565596	84618096	16q24.1	COTL1	142.6269454	84.98998677	1.678161756	4	4	72.35289994	45.72241063
chr8	30729131	30767006	8p12	UBXN8	5.013923145	2.988210657	1.677901501	4	4	72.85619879	43.33031223
chr3	33113958	33147773	3p22.3	CRTAP	13.05405729	7.781735886	1.677525102	4	4	64.1683525	26.96990052
chr1	84498323	84506579	1p22.3	GNG5	69.93116671	41.68731362	1.677516746	4	4	46.01727742	28.37386955
chr12	53097652	53102344	12q13.13	IGFBP6	0.341729098	0.203733496	1.677333893	1	2	0	15.27792481
chr13	113421940	113453524	13q34	ADPRHL1	0.054091571	0.03224866	1.677327727	2	3	21.5683219	49.1352837
chr22	19036282	19122454	22q11.21	DGCR2	25.3979083	15.14204215	1.677310633	4	4	51.82559864	28.44639029
chr2	207580631	207626053	2q33.3	METTL21A	4.316289073	2.573416993	1.677259879	4	4	38.30525258	23.85635487
chr9	92297358	92325994	9q22.31	NOL8	26.16689329	15.60174481	1.677177368	4	4	75.36464439	49.64878642
chr3	146192335	146251179	3q24	PLSCR4	9.161206138	5.462377077	1.677146416	4	4	106.3131758	35.67854378
chr3	12897043	13073410	3p25.2-p25.1	IQSEC1	35.62349096	21.24142121	1.677076623	4	4	64.03469677	7.77341598

chr13	48296513	48303661	13q14.2	RB1-DT	0.986311689	0.588149294	1.676975046	4	4	27.67369663	101.3630337
chr3	8980591	9363446	3p25.3	SRGAP3	0.319339162	0.190453253	1.676732521	4	4	82.44643268	78.21830871
chr8	85441806	85464915	8q21.2	CA3-AS1	8.799754242	5.248599223	1.676591004	4	4	75.53750284	52.70030788
chrX	23701055	23743290	Xp22.11	ACOT9	11.94222999	7.123443563	1.676468675	4	4	49.27745939	25.55113196
chr3	124965710	125055958	3q21.2	HEG1	4.926706904	2.938950341	1.67634915	4	4	39.87377119	16.28409332
chr19	11374707	11376951	19p13.2	SWSAP1	2.657020178	1.585068962	1.676280491	4	4	49.26127833	17.93781671
chr1	151156629	151159749	1q21.3	TNFAIP8L2	38.24198515	22.81819271	1.675942773	4	4	45.75049467	49.3608791
chr19	12096380	12097109	19p13.2	RSL24D1P8	0.782283309	0.466832074	1.675727423	2	1	13.14431759	0
chr7	103152007	103152325	7q22.1	RPL23AP95	1.75039727	1.044650425	1.675581829	1	1	0	0
chr20	49363877	49484033	20q13.13	KCNB1	0.025733121	0.015357751	1.675578757	2	2	64.28803426	13.59624227
chr9	63819574	63819654	9q13	MIR4477B	26.26363436	15.67548392	1.675459239	3	3	120.8847955	79.56102316
chr21	14961235	15065903	21q11.2-q21.1	NRIP1	34.93378139	20.85083529	1.675414002	4	4	24.29204099	13.87506738
chr7	35081287	35186162	7p14.2	DPY19L2P1	0.290103636	0.173178531	1.675170905	4	3	116.3094261	78.75303736
chr8	143990058	143993415	8q24.3	GRINA	116.7731399	69.71358356	1.675041418	4	4	26.07416814	35.773357
chr1	95117923	95244955	1p21.3	TMEM56-RWDD3	1.1268339	0.672735728	1.675002312	4	4	95.65808661	81.87149674
chr20	48624252	48827883	20q13.13	PREX1	153.2056427	91.47843706	1.674773286	4	4	65.17962491	30.51904901
chr3	138777760	138779011	3q22.3	GAPDHP39	0.448665983	0.267906778	1.674709336	1	2	0	3.864716854
chr19	11665736	11667089	19p13.2	HNRNPA1P10	0.438117031	0.261611741	1.674684132	3	2	58.77071906	1.841256299
chr12	62601751	62603434	12q14.1	LINC01465	0.825292064	0.492876099	1.674441233	4	3	51.66731924	56.59095789
chr9	127738317	127778769	9q34.11	SH2D3C	28.60410688	17.08280151	1.674438871	4	4	51.06820481	42.84610802
chr2	184598366	184939487	2q32.1	ZNF804A	0.357514511	0.213513532	1.674434908	3	4	87.47164226	78.97438042
chr1	178006136	178038007	1q25.2	CRYZL2P	0.737289352	0.440344901	1.674345157	4	4	77.77865284	27.38022683
chr1	193096465	193106535	1q31.2	GLRX2	12.35748352	7.380562929	1.674328048	4	4	31.3371461	26.48049708
chr20	3927309	4015591	20p13	RNF24	55.43619279	33.10989801	1.674308776	4	4	25.79878219	48.07757199
chr9	65666374	65734041	9q21.11	CBWD5	2.41711441	1.443676812	1.674276673	4	4	18.304504	23.50854927
chr4	152489327	152489416	4q31.3	MIR3140	21.0402324	12.56784231	1.674132431	4	4	50.9732356	22.29991404
chr3	49277144	49340103	3p21.31	USP4	68.93637131	41.1790101	1.674065771	4	4	36.27474277	18.0822679
chr13	100603633	100675631	13q32.3	TMTC4	1.557397745	0.930579243	1.673578857	4	4	39.26275728	11.3656513
chr12	131828393	131851771	12q24.33	MMP17	0.904429111	0.540569002	1.673105761	4	4	67.46315647	45.91006949
chr21	28928144	28992988	21q21.3	LTN1	31.10747843	18.59541717	1.672857249	4	4	13.99902123	24.0046146
chr19	54189970	54194536	19q13.42	TSEN34	8.507077504	5.085664519	1.672756328	4	4	72.8542542	52.76207143
chr19	39863323	39934626	19q13.2	FCGBP	0.343438224	0.20535595	1.672404546	4	4	149.7586838	58.59002251
chr2	8681983	8684453	2p25.1	ID2	102.6076272	61.35968453	1.672231987	4	4	44.95761607	11.91333625
chr9	35489833	35561898	9p13.3	RUSC2	1.223301676	0.731575772	1.672146239	4	4	101.9148218	53.63635348
chr19	42086110	42197931	19q13.2	POU2F2	5.935587673	3.549713191	1.672131621	4	4	86.54311838	40.45122756
chr17	75776434	75779935	17q25.1	H3F3B	268.0002182	160.2917202	1.671952973	4	4	57.63857665	36.98091834
chr14	68979682	68987463	14q24.1	ACTN1-AS1	1.223665788	0.731947823	1.671793738	2	4	100.7220038	78.58517856
chr2	119222476	119265652	2q14.2	STEAP3	1.211488844	0.724667322	1.671786221	4	4	62.12450675	97.24211721
chr15	90884421	90895776	15q26.1	FES	20.91057432	12.50897203	1.671646102	4	4	72.43458664	40.002891
chr11	62337302	62401401	11q12.3	ASRGL1	1.679361429	1.004639068	1.671606733	4	4	75.64037806	53.04901265
chr10	58211915	58213456	10q21.1	TPT1P10	4.30440827	2.575190586	1.671491149	4	4	78.01567562	40.33685001
chr21	32599354	32612871	21q22.11	CFAP298	1.808757425	1.082574602	1.670792407	4	4	24.93241319	63.41740822
chr1	11806096	11843144	1p36.22	CLCN6	2.115201869	1.266137784	1.670593751	4	4	63.53556049	37.53504346

chr4	88695913	88698235	4q21-q22	NAP1L5	5.488178571	3.285622809	1.670361721	4	4	96.52891432	26.55196064
chr14	75426692	75472701	14q24.3	JDP2	6.189313338	3.70541182	1.670344253	4	4	76.36367551	32.96251176
chr22	50170731	50180295	22q13.33	PANX2	3.801773612	2.276140623	1.670271851	4	4	119.2684254	56.09786498
chr3	136957865	137011085	3q22.3	IL20RB	0.437804685	0.262149059	1.670060107	4	4	27.39238421	51.91648537
chr4	41935120	41960807	4p13	TMEM33	34.36809457	20.58164886	1.669841653	4	4	37.4596162	23.74728726
chr15	24954893	24978723	15q11.2	SNURF	1.247275365	0.747098008	1.669493629	4	4	53.36314968	42.74503298
chr2	151357592	151380048	2q23.3	TNFAIP6	42.05359809	25.1902437	1.669439907	4	4	84.56021189	103.1039705
chr16	4846665	4882401	16p13.3	UBN1	31.80422748	19.05671998	1.668924532	4	4	58.62261525	37.5499118
chr1	196651878	196747504	1q31.3	CFH	0.417605082	0.2502323	1.668869615	4	4	33.18705388	68.48901771
chr20	62708770	62762771	20q13.33	NTSR1	0.20542667	0.123097187	1.668816933	2	2	1.829158453	65.39958231
chr8	133036728	133103066	8q24.22	SLA	64.73621287	38.79378484	1.668726399	4	4	56.3769686	10.97823079
chr17	68259182	68452005	17q24.2	ARSG	5.43802442	3.258915904	1.668660555	4	4	75.90171376	42.59953884
chr19	11342512	11346459	19p13.2	TMEM205	6.515233494	3.904557043	1.668622951	4	4	58.68183257	38.97110439
chr19	1597155	1605484	19p13.3	UQCR11	8.745494719	5.241394669	1.668543445	4	4	9.537125204	24.72939482
chr19	54874093	54891420	19q13.42	FCAR	11.03986932	6.618001123	1.668157669	4	4	57.01114133	27.06258301
chr5	96662037	96774683	5q15	CAST	45.52174449	27.29180614	1.667963793	4	4	20.75153577	15.10221875
chr11	66466327	66477337	11q13.2	PELI3	1.764621596	1.058047346	1.667809672	4	4	102.2447844	48.16157188
chr1	155002628	155018525	1q21.3	ZBTB7B	37.30261494	22.36987339	1.667538045	4	4	52.34287009	23.87360175
chr17	2557753	2558094	17p13.3	RN7SL33P	1.48653507	0.891524919	1.667407201	1	2	0	16.0870436
chr15	75023544	75060180	15q24.2	PPCDC	11.24920843	6.746571156	1.667396395	4	4	64.80248543	47.44432213
chr17	28394642	28406630	17q11.2	SLC46A1	0.676931958	0.40599529	1.667339437	4	4	71.60067562	40.6732074
chr19	41330323	41353883	19q13.2	TGFB1	91.17038203	54.69079582	1.667015092	4	4	75.15166442	52.82234924
chr12	6328757	6342117	12p13.31	TNFRSF1A	77.43813197	46.45563894	1.666926421	4	4	68.92820139	53.96572236
chr7	112423144	112477940	7q31.1	IFRD1	40.58821501	24.35894249	1.666255217	4	4	51.2495096	62.46025767
chr2	135164218	135531236	2q21.3	ZRANB3	8.930264514	5.359747503	1.666172615	4	4	109.3711649	65.05036064
chr1	37793816	37801606	1p34.3	MANEAL	0.39616372	0.237796029	1.665981223	4	4	49.78504428	19.58020543
chr2	950868	1367991	2p25.3	SNTG2	0.225922065	0.135617218	1.665880403	2	3	31.7479491	26.41292482
chr1	84553121	84574480	1p22.3	CTBS	30.29845969	18.1962927	1.665089708	4	4	55.8949597	41.98230972
chr1	155234448	155244862	1q22	GBA	10.80750803	6.490731785	1.665067729	4	4	55.76256057	35.10028398
chr22	23648788	23653359	22q11.23	ASLP1	1.179885243	0.708643275	1.664991803	1	1	0	0
chr12	128793194	128823996	12q24.33	SLC15A4	32.8203881	19.71419863	1.664809649	4	4	17.29865716	38.95954125
chr12	68431842	68451484	12q15	LINC02384	7.137594762	4.28763767	1.664691681	4	4	61.5539197	55.43480851
chr1	151540294	151583583	1q21.3	TUFT1	1.185722823	0.712462271	1.664260511	4	4	57.21981801	36.66962653
chr15	34341716	34343161	15q14	NOP10	174.7061125	104.9956109	1.663937293	4	4	31.78112998	30.34827173
chr20	37344685	37405432	20q11.23	SRC	4.999146172	3.004555047	1.663855743	4	4	70.0019142	23.47668965
chr17	27631148	27649560	17q11.2	LGALS9	12.84383514	7.720783068	1.663540476	4	4	52.58434514	47.78384753
chr11	86791059	86952910	11q14.2	PRSS23	3.250893564	1.954342753	1.66342038	4	4	17.75124519	21.93249426
chr5	177501904	177510508	5q35.3	DOK3	17.33864241	10.4237782	1.663374074	4	4	74.12911877	43.35760449
chr1	153929501	153946681	1q21.3	DENND4B	12.63986729	7.599317268	1.663289852	4	4	57.95151897	15.31746713
chr1	54548134	54634744	1p32.3	ACOT11	0.155860337	0.093743789	1.662620419	4	4	117.8126029	76.23539402
chr7	100574011	100586188	7q22.1	LRCH4	8.226238647	4.948036986	1.662525698	4	4	58.59586582	27.39219405
chr2	177229097	177229520	2q31.2	DNAJC19P5	14.17347796	8.526472832	1.662290872	4	4	66.01210894	75.37753844
chr12	4615508	4649047	12p13.32	AKAP3	0.597508222	0.359498352	1.662061087	2	2	10.55142921	31.97229947

chr12	14825569	14843533	12p12.3	ART4	3.972814033	2.390315876	1.662045621	4	4	104.8688024	64.58942561
chr17	41754607	41786768	17q21.2	JUP	9.775066511	5.881830696	1.661908854	4	4	100.9884058	152.446678
chr12	7919228	7936296	12p13.31	SLC2A3	213.8957195	128.7057213	1.661897524	4	4	63.00189517	28.54287121
chr18	58690356	58691602	18q21.32	MRPL37P1	0.544946755	0.327941348	1.66172018	4	3	63.79179102	66.29638927
chr2	219236598	219245441	2q35	GLB1L	0.926915507	0.557874217	1.661513434	4	4	78.76444613	52.17445425
chr4	139015759	139045939	4q31.1	NOCT	2.247099257	1.35264648	1.661261304	4	4	96.21291776	55.02710177
chr17	40922696	40937643	17q21.2	KRT23	17.22195762	10.36787044	1.661089201	4	4	102.5567456	61.0507121
chr6	31399784	31415315	6p21.33	MICA	1.539280062	0.92690157	1.660672623	4	4	65.05018642	57.7912192
chr1	11736085	11750771	1p36.22	AGTRAP	28.99558339	17.46033698	1.660654283	4	4	64.82881992	29.3642111
chr1	201982638	202006147	1q32.1	RNPEP	13.96924132	8.411959405	1.660640601	4	4	50.64414321	14.53206561
chr16	68822449	68823232	16q22.1	FTLP14	1.562947823	0.941219442	1.660556246	1	2	0	59.4929103
chr10	36521736	36524236	10p11.21	NAMPTP1	10.21677546	6.153835341	1.66022893	4	4	36.76983766	106.7154369
chr17	7240419	7242434	17p13.1	GABARAP	47.35605836	28.52749384	1.660014673	4	4	12.4293133	30.44759332
chr19	3982507	3982572	19p13.3	SNORD37	10.28420437	6.196591019	1.659655179	2	3	12.65070261	39.55021539
chr1	4654775	4792518	1p36.32	AJAP1	0.18993039	0.114447015	1.65954865	2	3	126.7100687	26.51438864
chrX	48683753	48691427	Xp11.23	WAS	84.91004898	51.17495663	1.659210961	4	4	59.99501738	26.95787278
chr21	44012325	44106552	21q22.3	TRAPPC10	19.63725816	11.83540063	1.659196741	4	4	25.06042821	11.34663122
chr11	65773898	65780591	11q13.1	AP5B1	36.67860555	22.10836977	1.659037095	4	4	65.37114459	28.22670348
chr11	61946722	61964465	11q12.3	BEST1	9.26138889	5.58308251	1.65883074	4	4	67.23216016	71.47020233
chr19	46719509	46746475	19q13.32	STRN4	11.65560727	7.027171906	1.658648376	4	4	74.68174522	20.22794534
chr7	44028887	44040623	7p13	RASA4CP	1.078140467	0.650393369	1.657674445	4	4	114.8018647	30.10963944
chr19	7920309	7923251	19p13.2	SNAPC2	3.710355663	2.238298118	1.657668223	4	4	79.95278068	59.18333506
chr1	212285537	212361863	1q32.3	PPP2R5A	78.02998676	47.07505375	1.657565537	4	4	25.20992786	30.26340045
chr4	118280038	118353042	4q26	PRSS12	0.107946957	0.06513406	1.657304281	1	2	0	45.07104944
chr7	33129244	33637238	7p14.3	BBS9	17.18659949	10.37045414	1.65726585	4	4	60.54396297	11.34051999
chr4	75910655	75941013	4q21.1	NAAA	14.77453047	8.915006453	1.657265258	4	4	54.9030221	35.88911432
chr1	42456070	42654190	1p34.2	CCDC30	0.929156649	0.560672374	1.657218532	4	4	22.92350894	35.71819445
chr22	22899618	22899655	22q11.22	IGLJ2	2.063376119	1.245234041	1.657018722	2	1	11.42908807	0
chr17	6451263	6556557	17p13.2-p13.1	PITPNM3	0.040799385	0.024622494	1.656996464	2	2	47.55160812	1.997608861
chr12	13000442	13004830	12p13.1	HTR7P1	1.004964858	0.60652759	1.656915324	4	4	70.95323609	63.5784082
chr4	169094256	169271098	4q32.3-q33	SH3RF1	1.858400735	1.121862297	1.656531948	4	4	30.66493585	36.25322645
chr12	106237881	106247935	12q23.3	CKAP4	26.52311629	16.01128365	1.656526539	4	4	64.09994554	49.11976695
chr15	76994680	77037475	15q24.3	PSTPIP1	18.55451958	11.20385143	1.656084044	4	4	60.23020818	32.01667931
chr15	90001491	90082200	15q26.1	ZNF710	12.22191525	7.380589699	1.655953758	4	4	65.33875489	28.40836296
chr17	31937010	32001045	17q11.2	SUZ12	150.4583733	90.8607234	1.655923128	4	4	86.97188671	27.47490514
chr7	6161779	6272611	7p22.1	CYTH3	4.983387541	3.010217583	1.655490809	4	4	40.54391845	32.13150991
chr12	4720586	4772726	12p13.32	GALNT8	0.062295398	0.037635906	1.65521187	3	3	6.077809596	41.81463405
chr18	57646426	57803822	18q21.31	ATP8B1	0.064447373	0.038936837	1.655177402	2	1	58.47043099	0
chr15	74409284	74433959	15q24.1	SEMA7A	4.757215153	2.874263844	1.655107329	4	4	56.21702181	54.79207138
chr19	1040103	1065572	19p13.3	ABCA7	11.84074307	7.154790649	1.654939138	4	4	62.56239161	31.05061948
chr20	59028678	59032367	20q13.32	ATP5F1E	118.5053993	71.62017359	1.654637142	4	4	44.56833991	27.63300746
chr15	82543201	82648016	15q25.2	CPEB1	0.788944727	0.476818845	1.654600559	3	2	112.7590344	98.66351058
chr22	23145298	23169285	22q11.23	RAB36	0.601190753	0.363418633	1.654265079	4	4	84.89962831	75.80903871

chr6	127317883	127317973	6q22.33	RNA5SP217	5.049641977	3.052596314	1.654212171	1	1	0	0
chr7	28995231	29195451	7p14.3	CPVL	55.10531181	33.31757083	1.653941462	4	4	52.21839054	19.07139097
chrX	48893447	48898143	Xp11.23	TIMM17B	11.59975716	7.014101596	1.65377661	4	4	18.2766253	55.79181055
chr16	4510675	4538815	16p13.3	CDIP1	1.915590773	1.158574958	1.653402535	4	4	90.51562227	49.43381226
chrX	103918896	103974426	Xq22.2	TMSB15B	0.12506327	0.075648995	1.653204651	1	3	0	52.19041203
chr17	56887909	56914048	17q22	TRIM25	39.51678746	23.90372945	1.653164103	4	4	52.3112982	42.51652565
chr1	32932498	32965266	1p35.1	RNF19B	90.2688118	54.60503294	1.653122559	4	4	49.98019708	16.21416087
chr1	108560349	108639327	1p13.3	FAM102B	8.796974201	5.321576795	1.653076624	4	4	22.73039868	10.26512176
chr16	784974	788383	16p13.3	RPUSD1	5.269654065	3.187831329	1.653052976	4	4	87.18878765	43.17633564
chr11	66848522	66958418	11q13.2	PC	0.473532621	0.286493167	1.652858345	4	4	64.5490057	61.97118724
chr2	219289623	219309573	2q35	PTPRN	0.285893673	0.172987628	1.652682772	3	4	36.69475735	29.93817311
chr4	88090264	88231626	4q22.1	ABCG2	39.64205627	23.987853	1.652588761	4	4	51.08988401	50.4964462
chr17	75845699	75856436	17q25.1	WBP2	81.40139917	49.26083004	1.652456914	4	4	14.22210116	28.12136498
chr10	92689924	92695651	10q23.33	HHEX	25.86605316	15.65396002	1.652364841	4	4	44.28378359	56.85965055
chr8	124550770	124728507	8q24.13	MTSS1	12.85630937	7.780687639	1.652335882	4	4	32.43921297	30.15259661
chr2	202376310	202567751	2q33.1-q33.2	BMPR2	18.74723568	11.34755817	1.652094256	4	4	45.08018562	27.94390808
chr8	109085745	109119785	8q23.1	TRHR	0.544897435	0.329899424	1.651707748	4	2	80.76935774	61.14746167
chr10	79347464	79355334	10q22.3	PPIF	27.98554947	16.94420518	1.65162952	4	4	83.208085	12.4858403
chr19	45507430	45526989	19q13.32	VASP	141.0316148	85.39577128	1.651505838	4	4	60.00267255	32.901843
chr19	16134028	16158575	19p13.11	HSH2D	36.64303599	22.18834059	1.651454549	4	4	50.48372683	24.17665644
chr10	97332497	97334701	10q24.1	FRAT2	157.9749172	95.66044997	1.651413068	4	4	77.90461029	60.15022748
chr17	29390925	29551904	17q11.2	TAOK1	50.84081672	30.79084781	1.65116651	4	4	14.39295331	10.7618204
chr1	35713876	35719189	1p34.3	C1orf216	1.971054866	1.19376117	1.651129988	4	4	34.56984872	45.24478261
chr19	40440841	40444375	19q13.2	SERTAD3	16.66717374	10.09705018	1.650697327	4	4	42.99284838	31.02812921
chr19	54738509	54753052	19q13.42	KIR2DL3	0.609855652	0.369464765	1.650646314	4	3	107.3648332	31.40108378
chr3	119579433	119589954	3q13.33	ADPRH	3.83948336	2.326082301	1.650622318	4	4	40.42226147	42.58801683
chr16	88862623	88870152	16q24.3	PABPN1L	0.515445586	0.312296386	1.650501283	1	4	0	42.21405665
chrX	72127110	72131901	Xq13.1	RTL5	0.388922276	0.235646571	1.650447426	4	4	71.83963964	48.93181979
chr22	22357795	22358262	22q11.22	IGLV1-47	14.71093911	8.913550721	1.650401683	4	4	85.04158698	109.914004
chr2	37344610	37373322	2p22.2	QPCT	73.87944186	44.76684545	1.650316012	4	4	96.39451248	65.90901923
chr12	27332854	27425813	12p11.23	ARNTL2	2.184756182	1.323932055	1.650202647	4	4	27.41002549	23.41437048
chr2	68042200	68063027	2p14	C1D	16.18785423	9.809818975	1.650168497	4	4	38.77016597	37.40390601
chr9	96313437	96383710	9q22.32	SLC35D2	50.70529355	30.72819194	1.650122911	4	4	55.48485011	44.83541498
chr3	196639696	196662004	3q29	NRROS	8.774548185	5.317514832	1.650121995	4	4	52.5082883	21.93764402
chr12	564296	663741	12p13.33	NINJ2	32.11004928	19.45952882	1.650093873	4	4	60.72732709	36.94357665
chr7	102678708	102748694	7q22.1	RASA4DP	0.348532826	0.21122055	1.650089568	1	1	0	0
chr3	49803130	49805030	3p21.31	INKA1	1.852843157	1.123076122	1.649793029	3	4	27.49025015	79.32657873
chr20	36777442	36863684	20q11.23	SOGA1	0.87328571	0.529344908	1.649748013	4	4	89.89933416	77.12035467
chr5	160393148	160400097	5q33.3	ZBED8	0.361645852	0.219239385	1.649547829	4	4	64.27318984	43.65892909
chr7	151085831	151144436	7q36.1	AGAP3	1.484404789	0.900042833	1.649260162	4	4	55.717161	26.74378178
chr3	48663814	48685941	3p21.31	NCKIPSD	2.699928936	1.637312882	1.648999996	4	4	59.05829321	52.56406144
chrX	153929827	153935154	Xq28	NAA10	1.855590426	1.125347413	1.648904511	4	4	41.50951808	48.94345289
chr1	162069791	162370023	1q23.3	NOS1AP	0.128108099	0.077694513	1.64886933	4	4	44.34899876	21.68009623

chr20	17569173	17608242	20p12.1	DSTN	22.52912623	13.66477906	1.648700366	4	4	19.26822128	13.84923848
chr15	82750564	82757206	15q25.2	SNHG21	0.711581821	0.431684896	1.648382484	3	4	89.524181	83.52241609
chr3	172630645	172711218	3q26.31	NCEH1	12.47137894	7.56718517	1.648086925	4	4	32.85455916	40.48114118
chr11	118593988	118594093	11q23.3	RNU6-1157P	4.783332425	2.902703435	1.647888781	2	3	60.58330385	24.00859104
chr17	68974632	69078978	17q24.2	ABCA9	0.065767232	0.039911087	1.647843675	3	2	54.44283946	39.38471012
chrX	135050932	135052191	Xq26.3	RTL8A	2.636053907	1.600078001	1.647453378	4	4	89.55605111	63.74147056
chr9	75060577	75088324	9q21.13	NMRK1	8.283404667	5.02850146	1.647290894	4	4	31.03791016	27.53573296
chr1	209675325	209676390	1q32.2	G0S2	268.7720588	163.1729811	1.647160314	4	4	66.10708181	110.4896039
chr4	1974636	1974760	4p16.3	SCARNA22	47.72199506	28.97333775	1.647100361	4	4	110.5541283	100.9115801
chr14	102932379	103057405	14q32.32	CDC42BPB	4.114044921	2.497998413	1.646936563	4	4	52.8433093	25.32031996
chr19	7534152	7561767	19p13.2	PNPLA6	11.99952571	7.28637224	1.646845003	4	4	65.34413116	19.38567155
chr1	221913645	221978520	1q41	LINC02257	0.449964323	0.273232794	1.646816681	2	2	7.20767191	2.275156033
chr4	1341998	1388049	4p16.3	UVSSA	2.573275861	1.562753795	1.646629091	4	4	68.33014607	24.96703917
chr17	15704079	15705093	17p12	UBE2SP1	0.639970775	0.388662068	1.646599524	1	3	0	2.77369358
chr6	109094622	109167696	6q21	CEP57L1	82.50213994	50.10580373	1.646558558	4	4	87.44383372	67.87497159
chr15	75933219	76069830	15q24.2	NRG4	0.367661057	0.223291987	1.646548367	4	3	94.45427116	42.39807371
chr22	19179473	19291716	22q11.21	CLTCL1	0.748446982	0.454566297	1.646507862	4	4	57.77378544	55.3473095
chrX	75384306	75384863	Xq13.3	RPL21P134	1.108485182	0.673251915	1.646464209	1	1	0	0
chr21	37365573	37515376	21q22.13	DYRK1A	79.29721424	48.16458786	1.646380002	4	4	19.24220348	14.16999017
chr17	59952362	59964756	17q23.1	RNFT1	8.927854713	5.422811122	1.646351774	4	4	8.020502052	25.90532621
chr2	20032650	20052028	2p24.1	LAPTM4A	51.81769203	31.47549404	1.646286853	4	4	36.40244484	26.97849155
chr9	128068200	128109258	9q34.11	SLC25A25	4.176558752	2.537122876	1.646179139	4	4	55.46890048	50.74989633
chr6	31797392	31806984	6p21.33	LSM2	6.465985014	3.928139283	1.646068163	4	4	67.53707419	54.76976604
chr15	85255366	85270854	15q25.3	ADAMTS7P4	0.099709191	0.060592298	1.645575343	3	3	34.22251206	40.33537374
chr1	25242237	25338254	1p36.11	RSRP1	53.57185018	32.5561197	1.645523197	4	4	11.48436206	34.53708111
chr11	10507887	10509176	11p15.4	MTRNR2L8	0.493639596	0.300059324	1.645140001	2	4	92.50877936	33.16775453
chr3	40309682	40312424	3p22.1	EIF1B	104.1047709	63.28485156	1.645018805	4	4	38.7657543	55.35353591
chr19	38264458	38292614	19q13.2	SPINT2	7.863298904	4.780130441	1.644996721	4	4	62.54403254	34.79607241
chr3	196706273	196712294	3q29	CEP19	27.31115172	16.60670569	1.644585761	4	4	66.58200888	74.94131317
chr3	138643309	138644721	3q22.3	PPIAP72	1.748395909	1.063126263	1.644579737	4	2	49.27201882	80.81542495
chr10	132397196	132417863	10q26.3	PWWP2B	3.498965621	2.127789047	1.644413776	4	4	74.73220286	14.81651732
chr21	46229231	46251701	21q22.3	MCM3AP-AS1	0.491052094	0.298619259	1.644408658	4	3	77.36443822	54.90553418
chr19	45890020	45902619	19q13.32	MYPOP	2.041432973	1.241447298	1.644397613	4	4	46.22997929	54.68482167
chr1	160830160	160862902	1q23.3	CD244	24.1364289	14.67837869	1.644352513	4	4	48.85255813	19.12348765
chr9	99979179	100099052	9q31.1	ERP44	45.52716358	27.68998055	1.644174632	4	4	31.49904239	13.63393492
chr5	177303762	177306959	5q35.3	PRELID1	82.12887987	49.95876815	1.643933245	4	4	82.68577763	31.38599434
chr7	142111703	142223733	7q34	MGAM2	0.83232516	0.506450385	1.643448569	4	4	87.24987363	90.62153671
chr3	172039628	172400703	3q26.31	FNDC3B	33.81431875	20.57645669	1.643349935	4	4	50.10767858	36.88689688
chr19	49512279	49526428	19q13.33	FCGRT	38.86538966	23.65293764	1.643152755	4	4	76.94766204	40.39330123
chr11	124865409	124881474	11q24.2	ROBO3	0.346369142	0.21080924	1.643045351	4	4	68.37579566	79.06585519
chr12	101745497	101830867	12q23.2	GNPTAB	20.86605413	12.69979846	1.643022461	4	4	15.42673263	21.00679001
chr16	57092543	57147966	16q13	CPNE2	3.61743112	2.202002643	1.642791452	4	4	83.42039731	50.4896183
chr5	134156391	134177033	5q31.1	SKP1	63.516602	38.66684006	1.642663375	4	4	66.96196904	41.21782555

chr17	44557444	44561262	17q21.31	FZD2	1.045372705	0.636543391	1.642264643	4	4	58.06412617	93.9529231
chr6	131637235	131747413	6q23.2	ENPP3	1.160021466	0.706639099	1.641603851	4	4	94.17534079	62.02741838
chr2	55287817	55419921	2p16.1	CCDC88A	17.4241285	10.61438356	1.641558213	4	4	20.76822637	30.03686218
chr14	70907405	71115382	14q24.2	PCNX1	36.94408709	22.50621814	1.641505777	4	4	35.86218134	27.88065665
chr1	46557407	46604311	1p33	MKNK1	10.77282904	6.563493056	1.641325579	4	4	49.66156257	29.38294385
chrX	75201470	75201975	Xq13.3	BUD31P2	1.230973179	0.750088978	1.641102876	1	1	0	0
chr5	34915715	34925682	5p13.2	BRIX1	22.56082231	13.74756979	1.641077125	4	4	61.54433613	30.03640584
chr1	10398992	10420511	1p36.22	PGD	146.5451532	89.30418776	1.640966196	4	4	75.94548688	54.4783991
chr1	53062052	53085502	1p32.3	PODN	0.148805152	0.090716	1.640340753	4	3	61.71308757	69.49915718
chr4	57002692	57002792	4q12	RNU6-998P	5.41948961	3.304101351	1.640231044	2	4	23.12024601	23.3502415
chrX	37685686	37732130	Xp21.1	XK	69.16965717	42.17618041	1.640017102	4	4	54.46576115	34.10376726
chr2	120217471	120223408	2q14.2	TMEM185B	13.56142182	8.269698675	1.639893103	4	4	79.47060308	50.01683821
chr2	46542409	46584688	2p21	RHOQ	39.11317467	23.85322544	1.639743638	4	4	55.75383565	26.62587781
chr12	38652200	38906269	12q12	CPNE8	5.544453065	3.38130153	1.639739318	4	4	27.3503471	10.80151465
chr6	116496483	116545610	6q22.1	TRAPPC3L	9.628042938	5.872017757	1.639648131	4	4	14.52861976	34.22836704
chr14	80954989	81146302	14q31.1	TSHR	0.177924845	0.108523831	1.639500218	2	2	100.9517925	88.74803061
chr8	1970718	2006943	8p23.3	KBTBD11	0.084806838	0.051728201	1.639470102	1	3	0	42.19463005
chr1	100872372	100895411	1p21.2	EXTL2	1.302119082	0.794338527	1.639249562	4	4	29.97385016	25.81615602
chr1	12507246	12507397	1p36.21	SNORA59A	13.6233406	8.311601532	1.639075279	4	4	54.24639285	45.48228571
chr16	55802851	55833163	16q12.2	CES1	3.488324259	2.128771715	1.638655866	4	4	65.20284164	41.10404075
chr1	153534599	153536241	1q21.3	S100A6	368.7749539	225.0734477	1.638464944	4	4	48.98116749	40.96921059
chr1	32333829	32336239	1p35.1	MARCKSL1	24.67414009	15.0597295	1.638418545	4	4	65.89552712	31.92732377
chr4	150078274	150257457	4q31.23-q31.3	DCLK2	0.296735436	0.181173102	1.637855914	4	4	98.3520778	51.40436431
chr8	8100762	8227503	8p23.1	FAM85B	0.651802577	0.397988809	1.637740966	4	1	36.20935433	0
chr3	53495049	53813151	3p21.1	CACNA1D	0.525202314	0.320687936	1.637736428	4	4	63.23023386	92.13817855
chr21	34073523	34106262	21q22.11	SLC5A3	14.62432355	8.93274952	1.637158135	4	4	23.17095446	15.44511946
chr7	77193371	77199833	7q11.23	FGL2	541.1999696	330.5788402	1.637128285	4	4	58.19487581	37.67661917
chr5	178237616	178590555	5q35.3	COL23A1	0.129360802	0.079031267	1.636830673	3	1	24.48039366	0
chr6	161129980	161274108	6q26	AGPAT4	6.452308418	3.942383859	1.63665149	4	4	43.78107231	35.15265148
chr3	149812688	149962139	3q25.1	RNF13	260.9993813	159.4845205	1.636518582	4	4	10.47517052	21.35738126
chr1	153390032	153422583	1q21.3	S100A8	1952.274751	1193.08395	1.636326388	4	4	79.27521256	61.6187084
chr8	23296897	23404209	8p21.3	LOXL2	0.458088193	0.279966147	1.636227084	2	4	110.2167853	98.30010725
chr14	34916480	34982566	14q13.2	SRP54-AS1	18.77536609	11.4761791	1.636029373	4	4	56.08747361	42.40658008
chr18	57544841	57586737	18q21.31	FECH	291.2656939	178.0632085	1.635743264	4	4	60.51812051	37.39319887
chr8	47736914	47738164	8q11.21	CEBPD	59.79028809	36.56005823	1.635399148	4	4	57.9266781	60.19958826
chr1	235127803	235127937	1q42.3	SNORA14B	29.71126798	18.16969783	1.635209801	4	3	39.8925177	1.626962403
chr16	1706183	1770317	16p13.3	MAPK8IP3	5.592417987	3.420266342	1.635082601	4	4	65.4010222	15.67813361
chr1	153959099	153968184	1q21.3	SLC39A1	16.41985759	10.0426391	1.635014206	4	4	69.05685797	48.61993777
chr10	28519917	28532354	10p12.1	WAC-AS1	18.3934015	11.24996902	1.634973524	4	4	85.69807085	26.26889847
chr4	51993640	52020311	4q12	LRRC66	0.126141706	0.077164475	1.634712167	3	1	50.54875154	0
chr17	20775448	20776959	17p11.2	OLA1P2	0.359458103	0.219895907	1.634673914	3	2	50.30777915	5.67680781
chr5	138869390	138875368	5q31.2	LRRTM2	0.091139131	0.055754322	1.634655882	2	3	55.82909706	46.54523161
chr14	24366911	24379604	14q12	NFATC4	0.09882798	0.0604615	1.634560512	1	1	0	0

chr5	98769618	98773084	5q15	RGMB-AS1	0.190271638	0.116412467	1.634461005	2	3	66.81040757	9.042829096
chr22	31681347	31750172	22q12.2	PRR14L	20.48663336	12.53446982	1.634423605	4	4	67.44830919	26.45243156
chr1	222712553	222735196	1q41	BROX	90.4961716	55.38076785	1.634072172	4	4	39.87755983	23.58358486
chr1	156193932	156212796	1q22	SLC25A44	13.41355158	8.208890741	1.634027301	4	4	68.30673536	48.76755929
chrX	101349447	101390796	Xq22.1	BTK	31.66710691	19.38136554	1.633894518	4	4	42.27650183	45.21866572
chr7	103175013	103280466	7q22.1	DPY19L2P2	7.406711172	4.533257957	1.633860513	4	4	102.5743735	47.61740244
chr1	247895587	247896531	1q44	OR2W3	114.8748553	70.32102893	1.633577567	4	4	40.99126032	91.41791427
chr19	49766923	49807114	19q13.33	AP2A1	56.72001096	34.72161689	1.633564794	4	4	32.56497319	46.14342739
chr20	33407957	33443892	20q11.21	SNTA1	0.927258521	0.567840329	1.632956438	4	4	53.04826875	52.78015794
chr1	158742568	158743415	1q23.1	OR6K5P	0.776157444	0.475384879	1.632692747	3	2	44.70051277	41.8366399
chr11	6603708	6610874	11p15.4	ILK	44.24995897	27.10874291	1.632313203	4	4	57.04323981	25.53139486
chr12	121209861	121234106	12q24.31	P2RX4	2.77282862	1.698977757	1.632057046	4	4	75.74194409	30.05141813
chr15	32593456	32607310	15q13.3	GOLGA8N	0.263839159	0.161673233	1.631928517	4	3	58.76031999	50.51391908
chr2	196260024	196264204	2q32.3	HECW2-AS1	6.470709305	3.965177719	1.631883806	4	4	43.74470302	51.72755219
chr7	100673740	100679169	7q22.1	GNB2	61.92117465	37.94481287	1.631874556	4	4	53.43228968	27.0315265
chr3	129167815	129183967	3q21.3	CNBP	384.4172022	235.6008097	1.631646354	4	4	64.29534085	33.72277054
chr10	19816215	20289856	10p12.31	PLXDC2	37.8676111	23.20831935	1.631639522	4	4	42.53434342	28.22090625
chr7	114922154	115019916	7q31.1-q31.2	MDFIC	25.6338976	15.71109447	1.631579369	4	4	26.57112845	21.1480083
chr1	115048011	115089501	1p13.2	TSPAN2	18.09019782	11.08965874	1.631267313	4	4	14.39569869	36.92464942
chr1	37860697	37947095	1p34.3	INPP5B	7.866679468	4.823561458	1.630886128	4	4	52.31300937	19.6976983
chr20	9068710	9480816	20p12.3-p12.2	PLCB4	0.807527072	0.495146577	1.630884892	4	4	45.24699818	17.05615468
chrX	134796413	134855137	Xq26.3	FAM122C	5.159385716	3.16357151	1.630873745	4	4	56.78536935	31.05840786
chr5	138337535	138349729	5q31.2	FAM53C	32.4525171	19.89938934	1.630829798	4	4	49.9025704	28.89716463
chrX	1268800	1325097	Xp22.33 and Yp11.	CSF2RA	33.32133911	20.43837064	1.630332461	4	4	54.14325357	56.12136303
chr2	180872705	180873565	2q31.3	FTH1P20	1.421762266	0.872187113	1.630111525	4	2	89.99464149	9.692890083
chr20	56524729	56533590	20q13.31	FAM209A	1.481315435	0.908949416	1.62970063	3	3	58.48830769	66.58048953
chr13	44110549	44154079	13q14.11	SMIM2-AS1	0.324917168	0.199381136	1.629628427	2	3	92.2105244	54.82982538
chr11	65879813	65883741	11q13.1	CTSW	93.11256543	57.15077354	1.629244184	4	4	48.04634758	37.89284047
chr14	103715744	103733668	14q32.33	ZFYVE21	2.884518075	1.77048022	1.629229202	4	4	20.48150182	25.11101109
chr8	43140449	43202827	8p11.21-p11.1	HGSNAT	5.549679456	3.406475469	1.629155855	4	4	59.5690917	40.47441356
chr8	37858947	37899497	8p11.23	RAB11FIP1	68.85838207	42.27291858	1.628900591	4	4	45.55474867	41.45528796
chr12	8940361	8949761	12p13.31	M6PR	64.91085974	39.85586961	1.628639906	4	4	77.33103415	36.55584525
chr19	36685439	36687449	19q13.12	LINC01534	1.707624819	1.048552374	1.628554626	4	4	82.03791942	52.5060449
chr5	177301190	177303744	5q35.3	RAB24	6.298866346	3.867832018	1.628526347	4	4	95.50786289	21.08639255
chr10	116883377	117126586	10q25.3	SHTN1	4.487418718	2.756017061	1.62822603	4	4	59.69081868	48.71761782
chr1	144412576	144412740	1q21.1	RNVU1-15	89.87025354	55.19690595	1.628175565	4	4	44.69863467	78.0140975
chr7	137384401	137846864	7q33	DGKI	0.13208227	0.081129521	1.628042027	1	3	0	84.62546184
chr6	42727576	42728194	6p21.1	ATP6V0CP3	0.898691152	0.552068091	1.62786288	1	1	0	0
chr11	33076150	33079454	11p13	LINC00294	3.398807686	2.087897431	1.627861424	4	4	36.60641181	21.51266602
chrX	155612565	155782459	Xq28 and Yq12	SPRY3	0.528490629	0.32467382	1.627758682	4	4	90.93716163	76.7144082
chr2	188291669	188595926	2q32.1-q32.2	GULP1	0.075252234	0.046232342	1.627696789	2	2	89.70082205	75.4453714
chr5	112141829	112419316	5q22.1-q22.2	EPB41L4A	18.46083763	11.34228652	1.627611647	4	4	67.99394164	45.01964882
chr17	3565446	3609411	17p13.2	TRPV1	0.311296931	0.191266888	1.627552655	3	4	26.84884531	57.43515236

chr9	114893343	114930674	9q32-q33.1	TNFSF8	19.95760288	12.26325112	1.627431641	4	4	61.18189855	10.74748973
chrX	55089052	55092836	Xp11.21	PAGE2	1.28631595	0.790562865	1.627088756	3	3	92.55221723	54.59496324
chr5	69639463	69710527	5q13.2	GUSBP3	0.741917464	0.456022346	1.626932255	2	4	15.62307163	105.9408348
chr16	67662945	67667382	16q22.1	ENKD1	1.783427508	1.096265329	1.626821045	4	4	98.40580951	37.03712494
chr6	41041344	41044337	6p21.1	TSPO2	5.158559893	3.171316671	1.626630333	4	4	113.2499047	40.67881413
chr16	67154185	67159909	16q22.1	TRADD	8.213161617	5.049370646	1.626571348	4	4	63.00680466	50.85963866
chr21	29056322	29073797	21q21.3	CCT8	70.43146621	43.30367494	1.626454713	4	4	37.29522966	31.33058466
chr21	33903453	33915854	21q22.11	ATP5PO	13.48834944	8.293756538	1.626325704	4	4	55.99429995	14.70666184
chr7	113116718	113118582	7q31.1	SMIM30	12.08855354	7.433332904	1.626262902	4	4	37.01549308	19.08679063
chr14	91060283	91225396	14q32.11	DGLUCY	14.14409734	8.699090762	1.625928241	4	4	53.53842918	39.32833599
chr20	34874942	34927966	20q11.22	ACSS2	6.757490611	4.156500176	1.625764544	4	4	62.01241128	33.33195359
chr3	151336843	151384812	3q25.1	P2RY12	34.10254865	20.97822166	1.625616757	4	4	28.12553287	38.62677092
chr14	24180219	24188918	14q12	IPO4	0.230603212	0.141859027	1.625580106	4	4	57.81261736	64.18749144
chr14	73136435	73223691	14q24.2	PSEN1	39.4891044	24.29292364	1.625539395	4	4	38.80233712	23.72442091
chrX	37780017	37813461	Xp21.1-p11.4	CYBB	355.090454	218.5111309	1.625045152	4	4	63.69265556	28.541352
chr14	103121175	103137439	14q32.32	TNFAIP2	76.04358425	46.79935945	1.624885151	4	4	55.60480371	35.15753943
chr3	42770612	42773635	3p22.1	LINC02158	5.619915884	3.458774648	1.624828575	4	4	69.33373372	71.3178756
chr4	76306026	76311130	4q21.1	STBD1	0.216630276	0.133328894	1.624781159	3	4	74.90534582	52.34743351
chr14	21887959	21888502	14q11.2	TRAV12-2	18.62650476	11.46545178	1.624576607	4	4	114.2050932	72.38753824
chr12	53452102	53481162	12q13.13	PCBP2	29.71232461	18.29174041	1.624357439	4	4	20.84987466	6.881651691
chr6	52497402	52577064	6p12.2	TRAM2	3.780702722	2.327578762	1.624307105	4	4	48.30896829	29.84359135
chr13	36301638	36370180	13q13.3	SPART	14.92836188	9.193386691	1.62381529	4	4	31.07676634	34.69110318
chr1	160995206	161021343	1q23.3	F11R	21.48632097	13.23507434	1.623437875	4	4	37.2633554	58.11289863
chr11	66016752	66026518	11q13.1	CATSPER1	0.906296913	0.558386212	1.623064635	4	4	62.28540735	35.02398316
chr12	8356963	8390752	12p13.31	LINC00937	0.998202664	0.615156002	1.622682151	4	4	105.3454205	33.58265032
chr11	68891276	68903835	11q13.3	MRPL21	3.302266746	2.035103184	1.622653226	4	4	19.71074127	34.24222496
chr12	120844913	120845750	12q24.31	ARF1P2	0.790422508	0.487176376	1.622456562	1	2	0	1.916177957
chr2	230690921	230700529	2q37.1	LINC01907	0.222174114	0.136947923	1.622325544	2	3	71.88085646	43.01015857
chr3	142596387	142713664	3q23	PLS1	3.870427937	2.386061888	1.622098721	4	4	62.58061599	29.12750755
chr22	43151514	43163242	22q13.2	TSPO	35.93466835	22.15347281	1.622078338	4	4	48.90586534	24.42692193
chr10	43077027	43130351	10q11.21	RET	0.14705799	0.090663594	1.622018095	4	3	122.594489	50.67042644
chr13	96985719	96994730	13q32.1	OXGR1	0.137490288	0.084765121	1.622014879	2	1	30.85388812	0
chr6	33193585	33200991	6p21.32	RXRB	3.699829956	2.28112178	1.621934431	4	4	92.12785393	54.5035661
chr11	22627758	22885930	11p14.3	GAS2	0.474464432	0.292545105	1.621850526	4	4	114.4650373	86.5629217
chr17	43211827	43221335	17q21.31	TMEM106A	3.226960872	1.989770966	1.621775032	4	4	64.54281642	19.95485834
chr2	109129439	109504636	2q13	SH3RF3	1.211714612	0.747233159	1.621601767	4	4	98.37150773	73.32079801
chr11	68706674	68751564	11q13.3	TESMIN	0.83571332	0.515385591	1.621530239	4	4	52.80872135	61.90327147
chr6	31615209	31617025	6p21.33	AIF1	168.6669901	104.0321648	1.621296552	4	4	73.98820395	60.18081949
chr4	51843000	51916837	4q12	DCUN1D4	68.83724225	42.47276019	1.620738609	4	4	72.42656612	31.07117422
chr9	42852563	42895739	9p11.1	ANKRD20A7P	0.621148691	0.383329565	1.620403817	1	3	0	11.91677076
chr1	183460874	183471982	1q25.3	SMG7-AS1	0.332533491	0.205237049	1.620241041	4	4	68.8804776	60.56033618
chr18	54222284	54222412	18q21.2	SNORA37	24.54293482	15.14776324	1.620234911	4	3	96.45912219	42.22219934
chr2	89330116	89330430	2p11.2	IGKV2-40	1.142434213	0.705330539	1.619714657	1	1	0	0

chr6	5997999	6007605	6p25.1	NRN1	0.37742596	0.233029701	1.619647451	3	4	56.73306178	61.3472538
chr13	72727739	72756198	13q21.33	BORA	26.00100708	16.06195473	1.618794693	4	4	67.01217274	36.42027631
chr7	6461081	6484218	7p22.1	KDELR2	23.93881362	14.78946233	1.618639886	4	4	17.02684558	16.61457924
chr14	102592593	102730576	14q32.31-q32.32	RCOR1	36.13186994	22.32479503	1.618463681	4	4	45.19934936	27.6649558
chr14	58292293	58292388	14q23.1	RNU6-341P	9.1595579	5.659451388	1.618453322	3	4	37.8739615	91.95046503
chr2	69915041	69942945	2p13.3	MXD1	214.6091687	132.6026779	1.618437667	4	4	67.55529588	46.272178
chr15	65869459	65891991	15q22.31	RAB11A	90.45043172	55.88941806	1.618382063	4	4	21.09632287	24.35779399
chr1	166921121	167022214	1q24.1	MAEL	0.358792331	0.221748974	1.618011236	2	2	19.15588645	13.92217211
chr1	23795599	23800804	1p36.11	GALE	1.264861261	0.781738603	1.618010491	3	4	78.87822716	49.70787433
chr5	88268882	88436674	5q14.3	TMEM161B-AS1	7.376916442	4.559619042	1.617879997	4	4	84.147501	25.63784314
chr1	51236273	51273447	1p32.3	RNF11	643.1809743	397.5636625	1.617806241	4	4	53.44812131	66.33859746
chr7	64974452	64991036	7q11.21	ZNF117	14.2529611	8.813653025	1.617145701	4	4	35.1007326	63.10911749
chr5	55625845	55712343	5q11.2	SLC38A9	21.37375029	13.21702151	1.617138194	4	4	54.15250365	33.14439327
chr2	218059155	218061290	2q35	CXCR2P1	102.2138502	63.21762194	1.616856931	4	4	47.10197023	15.37385616
chr2	73982036	74135394	2p13.1	TET3	20.33930026	12.58197548	1.616542672	4	4	37.22517259	8.066426265
chr4	139794125	139794433	4q31.1	RN7SKP253	1.255253701	0.776506455	1.616539943	2	2	47.93095783	8.338661214
chr19	35887630	35902301	19q13.12	NFKBID	8.502734415	5.26020014	1.616427928	4	4	92.58523518	19.17030904
chr9	69121006	69255208	9q21.11	TJP2	8.257752262	5.108900972	1.61634612	4	4	65.17842709	22.42880191
chr19	41549236	41586844	19q13.2	CEACAM21	2.332420351	1.44315861	1.616191273	4	4	74.04728112	61.92234373
chr9	137027464	137037770	9q34.3	C9orf139	1.274797427	0.788827884	1.616065371	4	4	102.6770131	60.64799846
chr19	7522610	7534009	19p13.2	MCOLN1	17.46639892	10.80871378	1.615955356	4	4	24.61120854	28.45695487
chr2	97663922	97703066	2q11.2	C2orf92	0.373228646	0.230975925	1.615876833	4	4	22.57457873	35.80502068
chr12	106015813	106018409	12q23.3	ST13P3	0.339949538	0.210399648	1.615732448	2	1	17.27601225	0
chr16	31108294	31117651	16p11.2	BCKDK	10.4388932	6.461326121	1.615596087	4	4	77.69246218	24.75838689
chr19	2100988	2151626	19p13.3	AP3D1	16.38536591	10.14204203	1.615588444	4	4	83.12460715	49.92457333
chr19	23074989	23147886	19p12	ZNF730	10.63560138	6.583458828	1.615503591	4	4	91.52717174	41.20876601
chr11	117986391	118001483	11q23.3	IL10RA	58.24687416	36.05595802	1.615457676	4	4	65.81306848	28.71868027
chr19	6375148	6375916	19p13.3	PSPN	0.566671518	0.35082814	1.615239637	4	4	68.91533134	46.16553676
chr19	1505018	1513189	19p13.3	ADAMTSL5	0.225747293	0.139772994	1.615099494	2	1	41.41930481	0
chr3	51707033	51718616	3p21.2	GRM2	0.059952339	0.0371228	1.614973504	2	1	51.27181573	0
chr15	23565307	23568020	15q11.2	MKRN3	0.372193372	0.23047288	1.614911793	4	4	132.4086453	49.76658855
chr15	90388241	90502243	15q26.1	IQGAP1	219.265837	135.7763358	1.614904658	4	4	67.36935521	36.06520652
chr11	5590906	5685109	11p15.4	TRIM5	17.60420131	10.90186889	1.614787472	4	4	45.2746051	37.98477766
chr7	48088308	48108746	7p12.3	UPP1	9.131294234	5.655619211	1.614552517	4	4	67.50422855	32.35052743
chr6	26521706	26527393	6p22.2	HCG11	5.869365548	3.635415615	1.614496435	4	4	48.67159791	17.03350478
chr19	56810077	56840726	19q13.43	PEG3	0.048016166	0.02974078	1.614489109	3	1	53.22348388	0
chr9	124777158	124814891	9q33.3	OLFML2A	0.170352277	0.105517146	1.614451137	3	2	62.8067668	88.70502401
chr5	56815073	56896152	5q11.2	MAP3K1	119.4983773	74.02774848	1.614237631	4	4	46.29805431	28.01907382
chr6	43305472	43312308	6p21.1	CRIP3	0.266146386	0.164885057	1.614132843	2	4	69.04447234	44.58348026
chr14	49690462	49753152	14q21.3	KLHDC1	20.01848382	12.40228746	1.6140961	4	4	71.57923345	51.88311077
chr19	60951	70966	19p13.3	WASH5P	0.393562903	0.243829396	1.614091286	3	4	107.1988425	25.44931046
chr1	151982910	151994238	1q21.3	S100A10	64.88415489	40.20586007	1.613798456	4	4	56.16199081	19.65975321
chr14	22501601	22501663	14q11.2	TRAJ39	37.06519428	22.96795483	1.613778613	4	4	75.88849707	45.48594922

chr22	42869766	43016174	22q13.2	PACSIN2	49.63444097	30.75714089	1.613753409	4	4	53.56228079	35.43433409
chr12	50763682	50821162	12q13.12	ATF1	37.19052574	23.04648725	1.613717759	4	4	35.63286493	9.420958068
chr2	71453154	71686763	2p13.2	DYSF	52.68248347	32.65165317	1.613470632	4	4	95.46558298	59.34657977
chr5	148451032	148654527	5q32	HTR4	0.060353922	0.037413951	1.613139509	2	1	48.71530129	0
chr6	32014795	32035418	6p21.33	C4B	0.062891512	0.03898735	1.613126124	2	1	39.36382274	0
chr12	87782885	87784711	12q21.32	MKRN9P	0.230475269	0.14289127	1.612941568	3	4	70.03686245	56.05237922
chr12	16990670	16992082	12p12.3	EEF1A1P16	2.698452734	1.673018206	1.612924906	2	1	129.2485494	0
chr22	29730956	29767011	22q12.2	ZMAT5	4.181829434	2.592847348	1.612832871	4	4	102.0448117	58.77265294
chr6	159969099	160111504	6q25.3	IGF2R	92.30909063	57.23618852	1.612774942	4	4	68.40844967	45.00937987
chr1	202133404	202144743	1q32.1	ARL8A	29.97444339	18.58785275	1.612582356	4	4	74.08842643	30.75794311
chr15	42528332	42548804	15q15.2	LRRC57	4.196277824	2.602303345	1.612524471	4	4	22.18573698	28.08635567
chr11	28020616	28108199	11p14.1	KIF18A	45.8865125	28.45929163	1.612356102	4	4	82.56556462	44.50665251
chr17	76565379	76586128	17q25.1	ST6GALNAC2	4.707712607	2.920462683	1.611974923	4	4	103.3761152	49.37237482
chr10	73007217	73096974	10q22.1	P4HA1	23.9703869	14.8702228	1.611972277	4	4	45.94769259	15.02691979
chr12	57253764	57431005	12q13.3	R3HDM2	11.59593462	7.195918636	1.611459942	4	4	31.17293413	15.6625959
chr15	55317184	55348725	15q21.3	PIGBOS1	10.24433464	6.357969454	1.611258864	4	4	26.61451998	30.24836556
chr10	43859411	43895433	10q11.21	LINC00840	0.253540172	0.157359016	1.611221135	2	1	59.46898872	0
chr12	39293228	39443390	12q12	KIF21A	2.872589218	1.782890831	1.611197483	4	4	33.24153099	28.64968813
chr8	92883530	92966150	8q22.1	TRIQK	17.54375971	10.88927034	1.611105167	4	4	20.27298103	35.80166835
chr3	46210699	46266706	3p21.31	CCR3	18.52726688	11.50139327	1.610871521	4	4	66.36472566	78.4997747
chr1	87328468	87348923	1p22.3	LMO4	7.69572401	4.777653737	1.610774751	4	4	41.60173443	40.54572085
chr7	80742536	80922379	7q21.11	SEMA3C	3.468882642	2.153736061	1.610634982	4	4	47.8588629	57.75978188
chr14	24158603	24158722	14q12	RNA5SP383	3.931705827	2.441204038	1.610560103	2	3	35.24887076	29.32603703
chr4	89724099	89838324	4q22.1	SNCA	1310.887933	813.9388763	1.610548373	4	4	28.08023745	27.28754258
chr18	14222008	14222340	18p11.21	RHOT1P1	11.03782063	6.854101854	1.610396353	4	4	84.43564142	70.76081488
chr3	13818108	13880121	3p25.1	WNT7A	0.470215419	0.292003353	1.610308289	3	4	74.07416059	57.9525009
chr19	42198593	42220152	19q13.2	DEDD2	44.33101961	27.53055687	1.610247835	4	4	59.23721306	28.54945825
chr11	65961689	65980136	11q13.1	SART1	24.12989517	14.98706301	1.61004829	4	4	92.91339163	58.95155053
chr1	205504556	205532793	1q32.1	CDK18	0.263025107	0.163446584	1.609241994	4	4	113.9594583	139.04949
chr12	122364742	122365674	12q24.31	RPL21P1	0.774469836	0.481306795	1.609098072	3	1	25.85041648	0
chr19	8520790	8577447	19p13.2	MYO1F	128.1859421	79.66730566	1.609015656	4	4	66.90895319	28.45159175
chr10	86755786	86927969	10q23.2	BMPR1A	1.123835177	0.698500411	1.608925578	4	4	36.0837664	41.2949489
chr3	151212115	151279167	3q25.1	P2RY14	34.62709263	21.52669395	1.608565287	4	4	30.99814421	55.83790739
chr15	43517608	43531620	15q15.3	MAP1A	3.48511647	2.166942655	1.608310428	4	4	74.45810078	29.01501151
chr19	1103926	1106789	19p13.3	GPX4	53.72093847	33.40337467	1.608248837	4	4	31.74911699	49.66881264
chr1	42807052	42817388	1p34.2	SVBP	43.87584894	27.28190766	1.608239772	4	4	34.36609697	32.80754872
chr3	169483618	169484020	3q26.2	RPL22P1	7.299448689	4.539644363	1.607934037	4	4	117.3974779	20.23877205
chr2	46916129	47076136	2p21	TTC7A	9.643474863	5.997437919	1.607932419	4	4	67.03654514	31.30010816
chr3	134595264	135260467	3q22.2	EPHB1	6.895674905	4.288596614	1.607909422	4	4	56.9131136	42.39865137
chr17	7588180	7590170	17p13.1	SOX15	0.243802262	0.151630115	1.607874933	1	1	0	0
chr16	853632	981613	16p13.3	LMF1	0.970587808	0.603700749	1.607730003	4	4	33.88934729	31.7363338
chr12	27022522	27030673	12p11.23	MED21	38.90844146	24.20320485	1.607573944	4	4	61.62683895	23.1325831
chr10	64168953	64169690	10q21.3	DBF4P1	5.623916717	3.498523938	1.607511287	4	4	83.94646337	53.87614354

chr2	101697703	101894690	2q11.2	MAP4K4	54.62508086	33.98159622	1.60749014	4	4	65.25332883	38.44038605
chr2	106801600	106887410	2q12.3	ST6GAL2	2.130583219	1.325517467	1.607359595	4	4	30.86173718	32.09107898
chr17	45393902	45432916	17q21.31	ARHGAP27	7.139344072	4.441771499	1.607319078	4	4	75.94085849	45.51197279
chr12	64622509	64622605	12q14.2	MIR548C	9.887570554	6.15216624	1.60716895	2	2	35.1594188	47.86358383
chr1	207762589	207764587	1q32.2	CDCA4P4	2.849290062	1.772922734	1.607114629	4	4	69.4866694	44.4798408
chr7	27830573	28180818	7p15.2-p15.1	JAZF1	96.68366863	60.16859065	1.606879396	4	4	55.2130512	18.22534895
chr13	30340266	30373914	13q12.3	LINC00426	2.070485819	1.288658807	1.606698226	4	4	35.76908434	52.19449401
chr11	82689530	82689904	11q14.1	RPS28P7	11.34181283	7.0598897	1.606514168	1	3	0	30.13186435
chr3	15667236	15859813	3p25.1	ANKRD28	20.79005207	12.94228528	1.606366389	4	4	36.32808245	31.97710431
chr1	146151908	146229032	1q21.1	NOTCH2NLA	1.092089093	0.679863383	1.606336096	4	4	24.71213743	38.08665732
chr19	58401472	58431396	19q13.43	ZNF584	0.723421764	0.450379921	1.606247815	4	4	49.16684499	47.88972924
chr3	4979412	4985181	3p26.1	BHLHE40	45.18418466	28.13370776	1.606051539	4	4	90.22455857	44.03913954
chr14	92513773	92688994	14q32.12	RIN3	12.71083266	7.914369634	1.606044859	4	4	55.76703762	31.19152201
chr9	72351425	72365247	9q21.13	ZFAND5	57.83018927	36.01200384	1.605858689	4	4	7.891121083	18.49610058
chr22	38219291	38273034	22q13.1	TMEM184B	6.297516299	3.921619382	1.605845873	4	4	59.76986821	25.45748239
chr2	201116104	201172688	2q33.1	CFLAR	48.52556388	30.2192796	1.605781624	4	4	51.89089293	39.0035398
chr1	112466541	112521288	1p13.2	WNT2B	0.065826108	0.040998853	1.605559751	4	2	53.71145132	71.99241607
chr1	40757284	40757372	1p34.2	MIR30C1	4.57423192	2.849776205	1.605119697	1	1	0	0
chr19	58069970	58070288	19q13.43	RN7SL526P	1.259288094	0.784628639	1.60494791	1	1	0	0
chr9	98056677	98083083	9q22.33	NANS	7.897570759	4.920779904	1.604942898	4	4	64.94266596	16.57425158
chr6	141486141	141486412	6q24.1	RN7SKP106	2.2539265	1.404458621	1.60483653	2	3	75.15994327	45.0062292
chr17	28879335	28897736	17q11.2	FLOT2	102.3973706	63.8089584	1.604749132	4	4	67.39746846	47.22150255
chr2	159003650	159004718	2q24.2	BTF3L4P2	1.047485587	0.652745786	1.604737419	1	1	0	0
chr5	132294384	132344206	5q31.1	SLC22A4	16.09034177	10.02712579	1.604681352	4	4	35.30319044	35.46688025
chr6	138161690	138344663	6q23.3-q24.1	ARFGEF3	0.028118831	0.017523217	1.604661468	4	2	60.81640244	10.75024276
chr20	44496221	44522116	20q13.12	SERINC3	138.5037445	86.31900396	1.604556797	4	4	53.72571742	16.95221783
chr17	29073510	29180389	17q11.2	MYO18A	6.815088831	4.247785383	1.604386337	4	4	63.24242604	55.15417403
chr16	66508003	66550412	16q21	TK2	4.230763671	2.637150449	1.604293631	4	4	45.29669172	27.5233843
chr16	50246138	50318135	16q12.1	ADCY7	31.65104191	19.7334087	1.603931809	4	4	28.7421049	31.68280692
chr7	38291616	38292078	7p14.1	TRGV11	0.916507079	0.571468731	1.603774676	1	2	0	18.77475526
chr3	84958982	86074429	3p12.1	CADM2	0.375241055	0.234009252	1.603530851	3	3	63.99407743	50.99035112
chr11	119206276	119308149	11q23.3	CBL	26.11871975	16.28842664	1.603513976	4	4	42.93451387	34.03658364
chr5	56909260	56928900	5q11.2	SETD9	2.645937436	1.650178833	1.603424661	4	4	35.03259991	32.36767229
chr19	17468745	17506169	19p13.11	SLC27A1	1.02702952	0.640601682	1.603226387	4	4	91.64510456	57.88298164
chr14	106268606	106269061	14q32.33	IGHV3-23	24.70718843	15.41118126	1.603198872	4	4	55.88631732	127.8262494
chr14	50242434	50312229	14q21.3	L2HGDH	1.741663351	1.08642888	1.60310848	4	4	59.83546631	12.38480676
chr5	139561166	139628434	5q31.2	UBE2D2	216.6702157	135.1731686	1.602908462	4	4	60.50289333	39.72834373
chr2	101271213	101308716	2q11.2	RNF149	193.7202933	120.8646234	1.602787382	4	4	46.22399759	59.41350772
chr17	76271296	76308811	17q25.1	QRICH2	0.833818355	0.520290871	1.602600393	3	4	66.95050446	56.30643343
chr6	42956334	42956766	6p21.1	RPL24P4	1.89221143	1.180757505	1.602540252	2	3	11.7787304	47.89589458
chr2	147844517	147930827	2q22.3-q23.1	ACVR2A	14.89532565	9.29588594	1.602356758	4	4	29.14623871	15.93244157
chr6	21666413	22195820	6p22.3	CASC15	0.300066228	0.187295881	1.602097316	4	4	90.21878756	58.10792176
chr19	47274402	47275723	19q13.32	INAFM1	4.114127464	2.568187717	1.601957457	4	4	102.2636076	42.30798139

chrX	153447665	153495525	Xq28	HAUS7	0.219087836	0.136786255	1.601680202	3	4	25.66695748	40.9767294
chr4	121824329	121870497	4q27	BBS7	9.380015252	5.856546979	1.601628961	4	4	32.26600751	27.87891313
chr21	46635156	46665685	21q22.3	PRMT2	25.80248311	16.11060218	1.601584027	4	4	45.89925384	39.66593732
chr8	144477982	144502121	8q24.3	PPP1R16A	0.755599296	0.471805728	1.601505134	3	4	15.65660149	15.15479555
chr3	125827237	125886077	3q21.2	LINC02614	0.089478475	0.055875934	1.60137771	2	4	73.69306901	62.274739
chr7	23619308	23622893	7p15.3	FCF1P1	1.179646735	0.736674499	1.601313384	2	3	69.81935392	66.73128494
chr12	111405107	111451623	12q24.12	SH2B3	30.79794671	19.23737664	1.600943169	4	4	28.25479644	10.29481553
chr17	83079691	83095126	17q25.3	METRNL	10.84310225	6.773080559	1.600911455	4	4	107.7706333	48.46236284
chr11	18529606	18588838	11p15.1	UEVLD	10.00830071	6.25189076	1.600843824	4	4	17.81469799	19.61457438
chr21	36380145	36416827	21q22.12-q22.13	CHAF1B	3.053428225	1.907417711	1.600817801	4	4	36.175568	71.00031823
chr4	174490177	174522898	4q34.1	HPGD	5.308794112	3.316476516	1.600733214	4	4	54.88391889	53.8658002
chr3	155458603	155745724	3q25.31	PLCH1	0.785361678	0.490656735	1.600633644	4	4	21.23471417	57.99659059
chr1	154944080	154956163	1q21.3	PBXIP1	89.60782063	55.98313184	1.600621789	4	4	63.28826403	30.96086914
chr1	33323623	33375593	1p35.1	PHC2	43.58959782	27.23773727	1.600338435	4	4	57.15666208	37.75744038
chrX	68042344	68433841	Xq12	OPHN1	2.897806294	1.810899538	1.60020268	4	4	28.88942428	41.42319638
chr22	17116299	17121367	22q11.1	TMEM121B	2.018686029	1.26168788	1.599988445	4	4	95.05450849	68.36077102
chr12	108621895	108633894	12q24.11	SELPLG	239.6734355	149.8094208	1.599855564	4	4	68.07007061	48.89287988
chr12	68686951	68745814	12q15	NUP107	34.50385695	21.56695873	1.599848054	4	4	88.76723799	37.54606
chr19	17226204	17229219	19p13.11	OCEL1	4.734008512	2.959411734	1.599645111	4	4	86.33237887	55.203154
chr1	231363751	231425044	1q42.2	EGLN1	47.89815989	29.95236929	1.599144275	4	4	58.00613529	56.56640164
chr19	39385629	39391195	19q13.2	PAF1	30.19580194	18.8828266	1.599114507	4	4	76.89952972	52.25088574
chr2	196194370	196593692	2q32.3	HECW2	2.038309127	1.274662808	1.599096729	4	4	46.8823439	83.19883075
chr1	241531883	241595647	1q43	KMO	3.320296557	2.076457353	1.599019865	4	4	36.58546822	60.37229853
chr10	46634502	46635846	10q11.22	RHEBP1	12.49681233	7.815405719	1.598997259	4	4	95.33833707	46.95864377
chr3	48241088	48275293	3p21.31	ZNF589	4.051353794	2.533710766	1.598980377	4	4	64.35316775	22.28798619
chr19	51211054	51240019	19q13.41	CD33	5.194628802	3.248944354	1.598866658	4	4	71.22485436	33.49658865
chr12	220621	262930	12p13.33	SLC6A13	0.094850403	0.059325336	1.598817792	1	1	0	0
chr16	67929570	67931875	16q22.1	CTRL	0.163105505	0.102019541	1.59876728	3	2	29.62277423	8.593440466
chr11	74000281	74009237	11q13.4	UCP3	0.705082325	0.441042998	1.598670262	3	4	34.84573556	62.79892994
chr17	4899653	4902932	17p13.2	C17orf107	2.904341365	1.816754905	1.598642369	4	4	85.60222362	63.7412899
chr14	101632321	101731343	14q32.31	LINC02320	1.951364068	1.220721847	1.598532928	3	3	9.838851294	53.26278024
chr12	52514575	52520459	12q13.13	KRT5	0.533196679	0.33355633	1.598520641	4	3	72.03573511	44.8301718
chr10	97498882	97571210	10q24.1-q24.2	UBTD1	4.111357286	2.572346682	1.598290508	4	4	90.07860447	56.92954345
chr12	121237692	121298308	12q24.31	CAMKK2	15.62606497	9.777539516	1.598159225	4	4	56.59945913	35.79035492
chrY	13751655	13920022	Yq11.221	ANOS2P	1.626126429	1.017509427	1.598143846	2	2	91.75441514	12.54559402
chrX	2903970	2929375	Xp22.33	ARSD	4.351175492	2.722969862	1.59795213	4	4	52.16365848	24.63685609
chr6	31971175	31981446	6p21.33	STK19	0.701640141	0.439107555	1.597877634	4	4	71.06976903	57.34531969
chr1	229516582	229558988	1q42.13	ABCB10	100.7408055	63.04963976	1.597801444	4	4	78.43052319	45.91823542
chr8	19404161	19758029	8p21.3	CSGALNACT1	7.335701442	4.591209431	1.597771034	4	4	46.89488817	31.22895213
chr8	143977152	143986471	8q24.3	PARP10	17.35381879	10.86319458	1.597487614	4	4	40.59827416	49.10385879
chr5	74777574	74867994	5q13.3	FAM169A	6.681487444	4.182636491	1.597434408	4	4	53.56999367	28.56179199
chr4	17632073	17781512	4p15.32-p15.31	FAM184B	0.060583192	0.03793083	1.597201861	2	1	63.98569057	0
chr12	53380195	53416446	12q13.13	SP1	73.77170972	46.1928501	1.597037411	4	4	62.69418141	31.82499249

chr10	124987849	125161061	10q26.13	CTBP2	10.38961506	6.506471315	1.596812552	4	4	28.28315286	46.99718958
chr13	113208193	113267108	13q34	CUL4A	32.25115578	20.19928487	1.596648395	4	4	18.6753968	9.489728056
chr17	49968970	49974959	17q21.33	DLX4	0.109608148	0.068651862	1.596579397	2	1	21.28094928	0
chr6	57314805	57646852	6p11.2	PRIM2	14.53127701	9.101993313	1.596493922	4	4	82.40990289	30.96073881
chr1	27098809	27155130	1p36.11	SLC9A1	3.735232336	2.339805554	1.596385789	4	4	69.74222356	28.19324787
chr15	91853708	92172435	15q26.1	SLCO3A1	9.498042547	5.951206798	1.595985969	4	4	62.62869391	30.71871283
chr1	146066318	146144804	1q21.1	NBPF10	1.187425224	0.744019753	1.595959274	4	4	87.89521275	82.96907733
chr4	155924118	155953896	4q32.1	CTSO	7.624186511	4.77762916	1.595809607	4	4	76.40265129	58.23755553
chr17	63622209	63696310	17q23.3	MAP3K3	40.8810556	25.62710521	1.595227212	4	4	62.90436061	34.53186142
chr1	15236560	15400283	1p36.21	FHAD1	0.064803662	0.040628151	1.595043355	1	1	0	0
chr17	35350305	35373914	17q12	SLFN11	13.80749865	8.656939237	1.5949631	4	4	22.88828084	20.5716172
chr3	48517684	48562383	3p21.31	PFKFB4	13.42838564	8.419581976	1.594899328	4	4	44.89437952	59.34506154
chr17	78185102	78207702	17q25.3	AFMID	1.381413574	0.866285532	1.594640015	4	4	37.60039116	46.15144519
chr13	73054976	73077542	13q22.1	KLF5	4.077964825	2.557482676	1.594522952	4	4	59.46821499	47.97577527
chr20	64073181	64080004	20q13.33	RGS19	60.85744444	38.1709756	1.594338198	4	4	45.38602476	33.93886356
chr6	73696085	73828317	6q13	CD109	2.919831879	1.831551444	1.594185022	4	4	48.97639636	43.34080646
chr21	25724480	25735654	21q21.3	ATP5PF	27.81450617	17.44807965	1.594129941	4	4	44.42462524	39.95754505
chr9	83938311	83956986	9q21.32	C9orf64	4.932739588	3.094328232	1.59412293	4	4	32.78136781	64.20729398
chr10	127877841	127892947	10q26.2	CLRN3	0.370979383	0.232723267	1.594079478	2	1	32.41950595	0
chr2	55634063	55693910	2p16.1	PNPT1	31.79784466	19.94747519	1.594078667	4	4	48.11452857	26.91247278
chr5	150475516	150485992	5q33.1	NDST1-AS1	1.10613068	0.6942837	1.59319696	1	1	0	0
chr1	15409895	15430343	1p36.21	EFHD2	138.7971169	87.12994216	1.592989889	4	4	52.71646488	30.42482747
chr3	196323546	196338420	3q29	TM4SF19	0.223314631	0.140212166	1.59269083	2	1	50.15522786	0
chr12	123936409	123972985	12q24.31	CCDC92	0.657118093	0.412622201	1.592541777	4	4	65.13498074	31.71384945
chr21	46458942	46571226	21q22.3	DIP2A	8.988315121	5.644145635	1.592502338	4	4	34.69511934	30.31107071
chr18	49488481	49492565	18q21.1	RPL17	21.76174804	13.6656194	1.592445055	4	4	42.83308865	35.35984002
chrX	46573687	46598496	Xp11.3	CHST7	3.866136202	2.427899447	1.592379045	4	4	68.59929426	11.41736489
chr5	74640023	74721288	5q13.3	HEXB	30.21749568	18.98193223	1.591908312	4	4	20.50992474	25.63361121
chr6	161160114	161161982	6q26	AGPAT4-IT1	0.902333198	0.566886319	1.59173571	4	4	65.26024253	28.43396312
chr5	55840334	55922854	5q11.2	IL31RA	0.638568632	0.401185802	1.591702967	4	3	59.37441708	12.53097222
chr11	67289291	67302484	11q13.2	ANKRD13D	13.7960454	8.669415382	1.591346682	4	4	46.39204546	36.66582921
chr3	129382834	129399439	3q21.3	RPL32P3	2.362030243	1.484430845	1.591202616	4	4	46.9848522	21.79934715
chr6	26198784	26199293	6p22.2	HIST1H2AD	1.085768905	0.682531688	1.590796331	2	1	22.47152061	0
chr17	28703197	28712065	17q11.2	PROCA1	0.346828847	0.218054731	1.590558692	4	4	95.01016843	69.33938263
chr10	87342411	87357302	10q23.2	LINC00863	1.826169901	1.148147735	1.590535647	4	4	59.38727869	33.56736489
chr15	88635614	88656344	15q26.1	ISG20	16.7435753	10.52829991	1.590339888	4	4	79.1518427	30.32290237
chr1	108134722	108200358	1p13.3	SLC25A24	7.908365686	4.973135524	1.590217207	4	4	61.84184262	33.0476647
chr15	76736641	76736879	15q24.3	RN7SKP217	1.325186457	0.833483035	1.589938129	2	2	43.45379082	28.288358
chr1	180154833	180198034	1q25.2	QSOX1	5.381992756	3.385183917	1.589867165	4	4	62.38566115	20.19871807
chr20	46008908	46016561	20q13.12	MMP9	63.87767622	40.19025439	1.589382232	4	4	76.72478161	39.88276979
chr2	233307663	233347066	2q37.1	SAG	0.036487726	0.02295733	1.589371528	1	1	0	0
chr19	11394056	11419342	19p13.2	RGL3	0.184443052	0.116065556	1.589128231	2	2	90.99034984	5.7879526
chr4	83138564	83285134	4q21.22-q21.23	COQ2	13.2447295	8.334794708	1.589088869	4	4	54.57013507	16.55936686

chr3	32996608	33097230	3p22.3	GLB1	17.87374625	11.24940868	1.588860957	4	4	33.74857196	6.200847631
chr21	44675528	44678165	21q22.3	IMMTP1	0.186942481	0.117661975	1.588809649	1	1	0	0
chr19	1383884	1395589	19p13.3	NDUFS7	2.001181479	1.259730326	1.588579268	4	4	73.05320232	37.77528328
chr1	71402942	72282594	1p31.1	NEGR1	0.314894655	0.198228847	1.588541022	4	3	104.1978625	71.84643741
chrX	44844003	44844888	Xp11.3	DUSP21	0.485404231	0.305618859	1.588266615	1	1	0	0
chr7	98881650	98881729	7q22.1	MIR3609	1454.862153	916.0237033	1.588236361	4	4	84.96061253	61.12342121
chr19	13931187	13953392	19p13.12	PODNL1	0.182380679	0.1148328	1.588228091	3	2	54.54195596	65.08536965
chr19	1481428	1490874	19p13.3	PCSK4	0.175765659	0.110685233	1.587977493	1	2	0	38.30507084
chr17	49845910	49848837	17q21.33	FLJ45513	0.699448402	0.440513978	1.587800698	2	4	32.17885711	41.11848896
chr20	1534251	1557697	20p13	SIRPD	2.151444494	1.355238931	1.587501986	4	4	69.62383408	68.64519361
chr11	128899565	128909520	11q24.3	C11orf45	0.108707229	0.068479355	1.5874453	2	1	18.91372478	0
chr4	62816826	62818791	4q13.1	EXOC5P1	0.836319394	0.52683671	1.587435684	3	3	41.52195547	85.50852339
chr2	10440371	10448554	2p25.1	ODC1	188.9893791	119.0542616	1.587422211	4	4	36.94215577	18.07450714
chr8	119833941	119855930	8q24.12	DSCC1	8.072851203	5.085863842	1.587311704	4	4	98.26415084	58.26794182
chr2	69643805	69826477	2p13.3	ANXA4	3.248890622	2.046888263	1.587233989	4	4	30.91295758	29.43136599
chr10	45699382	45723248	10q11.22	FAM21FP	0.838858351	0.528583473	1.58699315	4	4	22.31503232	34.57881606
chr16	29816207	29822495	16p11.2	PAGR1	1.903295593	1.199449243	1.586807948	4	4	42.92190972	79.59782446
chr3	10115649	10127190	3p25.3	BRK1	145.420254	91.64338032	1.586805872	4	4	46.51660844	22.12390898
chrX	101408133	101414140	Xq22.1	HNRNPH2	136.1524592	85.8080032	1.586710494	4	4	58.77936209	38.78608675
chr19	12833931	12874953	19p13.13	MAST1	0.124962556	0.078764135	1.586541336	3	1	70.32230235	0
chr16	35802090	35803283	16p11.1	HMGN2P41	0.984389527	0.620493279	1.586462835	2	1	28.52671786	0
chr6	151452258	151470101	6q25.1	ARMT1	22.51175828	14.19059046	1.586386299	4	4	27.21843309	31.76297104
chr20	36890882	36951843	20q11.23	SAMHD1	200.6164106	126.4700852	1.586275603	4	4	35.24116495	33.80895162
chr2	201140289	201157792	2q33.1	CFLAR-AS1	0.257637711	0.162419563	1.586248028	2	2	75.30367693	19.2585199
chr1	207645079	207657411	1q32.2	CD46P1	216.9866275	136.8046594	1.586105535	4	4	106.3905869	55.81759526
chr2	108377911	108388989	2q12.3	SULT1C4	0.323346101	0.203873488	1.586013483	3	3	71.75131538	48.53835328
chr19	16111680	16133635	19p13.11	RAB8A	54.79649516	34.55681688	1.585692784	4	4	64.65051215	45.22835824
chr6	109366514	109382812	6q21	CD164	93.23276191	58.80303927	1.585509237	4	4	20.69285894	11.48348708
chrX	68498362	68572459	Xq12-q13.1	YIPF6	39.50388727	24.918057	1.58535183	4	4	62.24565001	57.18448238
chr11	100687240	100993945	11q22.1	ARHGAP42	18.7153175	11.80526813	1.58533608	4	4	98.69151775	60.13155316
chr4	139665725	139754608	4q31.1	MGST2	9.657999282	6.092297882	1.585280213	4	4	19.44801162	29.33028611
chr4	3503597	3532497	4p16.3	LRPAP1	10.14347792	6.398625214	1.585258954	4	4	72.10294707	34.74483574
chr9	104781002	104928246	9q31.1	ABCA1	14.26551098	9.000049092	1.585048129	4	4	53.95166965	40.13924128
chr5	83064204	83064337	5q14.2	SCARNA18	11.30519035	7.132580979	1.585006939	4	3	53.64301853	65.03935517
chr9	19053137	19102942	9p22.1	HAUS6	61.6693487	38.91002817	1.584921718	4	4	75.97361807	39.74889089
chr16	81445170	81711762	16q23.2-q23.3	CMIP	13.75451318	8.679728308	1.584670935	4	4	59.04239258	19.0344672
chr1	7771269	7781432	1p36.23	VAMP3	130.8482574	82.57542566	1.584590771	4	4	17.78016677	9.614266155
chr19	6658126	6670595	19p13.3	TNFSF14	10.80285437	6.817461553	1.584586035	4	4	73.36024451	49.64944382
chr19	43951196	43970207	19q13.31	ZNF221	0.877582826	0.553905927	1.584353559	4	4	31.49993878	67.83223024
chr2	159315312	159712439	2q24.2	BAZ2B	24.69029215	15.5846011	1.584274888	4	4	55.32170099	37.48307802
chr6	70513295	70513353	6q13	RNU7-48P	7.302081924	4.609657839	1.584083283	2	1	50.37579391	0
chr7	50590063	50793462	7p12.1	GRB10	3.328448815	2.101190643	1.584077497	4	4	27.21516116	44.65480899
chr13	33016063	33066145	13q13.1	KL	0.342942902	0.216505072	1.58399477	3	4	49.48966806	61.20740815

chr15	85380616	85749358	15q25.3	AKAP13	73.00807231	46.09576025	1.583834867	4	4	64.03710184	24.76515786
chr11	95165513	95232541	11q21	SESN3	120.6979344	76.2193074	1.583561154	4	4	34.71844423	31.24484714
chr1	111722064	111755797	1p13.2	INKA2	9.180024696	5.797177419	1.583533508	4	4	68.01523248	61.50642645
chr19	19629473	19643667	19p13.11	GMIP	48.74754037	30.78436188	1.583516351	4	4	56.12825771	24.58381132
chr11	8612037	8683217	11p15.4	TRIM66	0.992430914	0.626763828	1.58342085	4	4	68.29332411	25.81211221
chrX	45848074	45851490	Xp11.3	LINC02595	0.960229821	0.606492397	1.583251208	1	1	0	0
chr16	30524001	30532367	16p11.2	ZNF768	3.218977721	2.033507853	1.582967932	4	4	95.60723617	48.7140472
chr19	12995512	13098796	19p13.13	NFIX	36.78916024	23.24415293	1.582727508	4	4	39.74572236	39.68814564
chr21	17777404	17792509	21q21.1	C21orf91-OT1	1.055140018	0.666705668	1.582617441	3	4	59.65822871	62.90802993
chr1	6579991	6589280	1p36.31	ZBTB48	4.596540884	2.904548974	1.58253172	4	4	59.73234713	27.21922925
chr1	145093805	145112692	1q21.1	FAM72D	1.204205766	0.760955146	1.582492441	4	4	59.09865248	46.23462187
chr11	130159787	130210362	11q24.3	ST14	4.271448112	2.699839048	1.582112132	4	4	59.45106904	37.16525329
chr15	67254772	67501804	15q23	IQCH	0.69536016	0.4395251	1.582071558	4	4	96.36435061	75.54872045
chr15	69160555	69272209	15q23	GLCE	6.935369421	4.383734876	1.582068628	4	4	36.24794068	16.58502072
chr19	15159633	15200981	19p13.12	NOTCH3	0.043857242	0.02772261	1.582002641	2	2	42.5589681	?
chr3	187721377	187745725	3q27.3	BCL6	73.01345727	46.15374742	1.581961625	4	4	74.90864233	50.11223617
chr21	33503931	33542892	21q22.11	GART	10.93055598	6.909581994	1.581941713	4	4	40.05188178	53.57721238
chr1	1471732	1509466	1p36.33	ATAD3B	2.023937286	1.279436162	1.581897829	4	4	23.73655057	60.86374961
chr1	45016399	45206586	1p34.1	ZSWIM5	0.329150052	0.208083133	1.581819951	4	4	77.74473965	50.75960796
chr19	50505997	50511355	19q13.33	JOSD2	2.563152939	1.620405648	1.58179709	4	4	51.42216638	51.59460201
chr3	112561320	112585577	3q13.2	SLC35A5	22.68823143	14.34399135	1.581723726	4	4	7.998037812	27.93774557
chr9	68241866	68300035	9q21.11	CBWD3	0.891631467	0.563837649	1.581362062	4	4	40.60381853	45.36037509
chr18	31621036	31724670	18q12.1	B4GALT6	3.415542496	2.160024891	1.581251453	4	4	56.39077906	40.12927056
chr2	218672026	218702717	2q35	STK36	0.886951471	0.560967545	1.581110135	4	4	91.15695522	42.00108015
chr4	52020695	52038319	4q12	SGCB	0.979217988	0.619325914	1.581102883	4	4	69.26334448	89.04210513
chrX	101397791	101408013	Xq22.1	GLA	16.71209592	10.5702234	1.581054183	4	4	11.49098452	25.08361737
chr21	42062959	42142996	21q22.3	UMODL1	0.171665636	0.108602184	1.580683098	4	4	56.45899453	69.84181001
chr22	19031528	19034922	N/A	CA15P1	2.834845613	1.793584219	1.580547812	4	4	63.24285884	45.4384737
chr1	19210386	19240704	1p36.13	EMC1-AS1	2.885017717	1.825394688	1.580489817	2	4	28.70687973	43.64539965
chr15	58138169	58185911	15q21.3	AQP9	505.5106674	319.8504311	1.580459547	4	4	73.74937523	70.78672352
chr20	1442162	1467793	20p13	NSFL1C	17.03295516	10.77831628	1.580298325	4	4	79.7824199	37.75769183
chr2	53767783	53775196	2p16.2	CHAC2	8.276280908	5.237554704	1.580180327	4	4	109.5416831	46.7767551
chr13	99215172	99215862	13q32.3	H2AFZP3	0.931379175	0.589453279	1.58007294	1	1	0	0
chr1	150730196	150765957	1q21.3	CTSS	878.0991377	555.7349212	1.58006831	4	4	61.77985992	45.63598788
chr4	79492593	79576460	4q21.21	LINC00989	65.22155735	41.2850679	1.579785638	4	4	38.58934422	56.04309613
chr13	52132643	52159861	13q14.3	NEK3	2.258651255	1.429746309	1.579756661	4	4	38.71083105	35.00807909
chr14	20343041	20357904	14q11.2	PARP2	10.86260381	6.876316433	1.579712615	4	4	70.25711365	40.96489918
chr5	10564323	10657816	5p15.2	ANKRD33B	3.367230476	2.131690582	1.579605645	4	4	38.09854664	29.86157804
chr9	35697337	35732395	9p13.3	TLN1	208.0957399	131.7575175	1.579384189	4	4	67.66237953	23.19854303
chr9	122546009	122576960	9q33.2	OR1L8	0.458050249	0.290024966	1.579347647	1	1	0	0
chr9	133471094	133479154	9q34.2	SLC2A6	2.02555243	1.282526141	1.579345922	4	4	75.17392785	12.13301259
chr18	5954703	6414919	18p11.31	L3MBTL4	1.726799572	1.093386723	1.579312731	4	4	57.20394814	31.86235021
chr17	31316410	31321749	17q11.2	EVI2A	216.9841661	137.4108416	1.579090585	4	4	26.1492333	44.523676

chr19	12791496	12793311	19p13.13	JUNB	374.6226385	237.2479874	1.579034	4	4	73.7830323	64.92654901
chr8	38728186	38853030	8p11.22	TACC1	34.12235871	21.61034176	1.578982835	4	4	38.29784208	11.99514625
chr22	17084959	17115694	22q11.1	IL17RA	72.6418286	46.01029216	1.578816938	4	4	56.87503418	52.23996899
chr7	38240024	38249572	7p14.1	TRGC2	76.35434137	48.37622315	1.57834441	4	4	26.01139237	40.14316996
chr3	151085665	151436677	3q25.1	MED12L	6.445469959	4.084229418	1.578136118	4	4	48.49658717	28.69800668
chr13	99196374	99200757	13q32.3	UBAC2-AS1	0.899075913	0.569751196	1.578014963	4	4	60.92695314	54.71143764
chr14	22871613	22881713	14q11.2	LRP10	97.30983197	61.67166648	1.577869345	4	4	42.84930913	14.01449841
chr1	41014590	41043890	1p34.2	SLFNL1-AS1	0.219391296	0.139051244	1.577772981	3	4	51.17579539	78.38764261
chr7	102464883	102473168	7q22.1	LRWD1	1.928984196	1.222772275	1.57754983	4	4	43.91236269	33.81596277
chr11	85020781	85021843	11q14.1	HNRNPCP6	0.494680148	0.313585578	1.577496488	1	2	0	21.98549762
chr10	96998038	97185926	10q24.1	SLIT1	0.17143493	0.10867555	1.577493098	4	4	113.3191369	33.02415939
chr19	10270841	10286615	19p13.2	ICAM1	35.92458086	22.77484988	1.57737948	4	4	72.17776421	11.14673313
chr14	43300260	43300910	14q21.2	HNRNPUP1	1.073810218	0.680845531	1.577171575	2	1	18.06393724	0
chr19	36445119	36489902	19q13.12	ZNF566	18.20154326	11.54346089	1.576783899	4	4	72.06959742	45.58134653
chr22	42380407	42438155	22q13.2	NFAM1	53.54254138	33.95835703	1.576711775	4	4	70.9916054	41.5980563
chr17	59976015	60002367	17q23.1	DHX40P1	0.591517834	0.375211369	1.576492297	3	3	108.0327185	89.52797217
chr14	22495913	22495966	14q11.2	TRAJ43	6.649439632	4.218352989	1.576311809	2	2	23.51770459	15.02213559
chr17	7217125	7225267	17p13.1	ACADVL	22.3679454	14.19385699	1.575889162	4	4	74.13978273	20.8750176
chr12	112013340	112023220	12q24.13	ERP29	51.14334235	32.456459	1.575752375	4	4	57.82272655	42.3105876
chr12	98645141	98735433	12q23.1	APAF1	40.67858532	25.81785705	1.575598828	4	4	42.90213232	46.07844562
chr20	319687	325252	20p13	NRSN2-AS1	3.334729388	2.116632518	1.575488121	3	4	76.27367981	35.89107403
chr4	139716391	140154079	4q31.1	MAML3	10.72043936	6.804544831	1.575482215	4	4	74.23603121	19.68490752
chr2	218419115	218449525	2q35	VIL1	2.093068047	1.328761721	1.575201945	4	4	91.40113687	35.76978062
chr5	1461423	1523977	5p15.33	LPCAT1	9.31393274	5.91300322	1.575161114	4	4	56.01523529	21.89807268
chr5	77545371	77546313	5q13.3	RPS2P24	0.383260456	0.243329915	1.575065093	1	1	0	0
chr11	59755059	59805882	11q12.1	STX3	40.76911649	25.88646633	1.574920113	4	4	46.95077838	68.18780348
chr9	129094794	129110798	9q34.11	CRAT	13.54520785	8.600869988	1.574864853	4	4	36.78560115	65.78945121
chrX	96684663	97600598	Xq21.33	DIAPH2	9.320332334	5.919365948	1.574549101	4	4	49.25141523	22.28986548
chr13	112690034	112887168	13q34	ATP11A	9.473210407	6.016629878	1.574504432	4	4	64.11466085	26.53061952
chr11	278365	285942	11p15.5	NLRP6	5.846616019	3.713839313	1.574278133	4	4	89.12383813	41.54243423
chr1	196225779	196608576	1q31.3	KCNT2	0.210090663	0.133456823	1.574221967	4	3	71.10553798	76.65053007
chr1	231018958	231040249	1q42.2	FAM89A	1.692910203	1.075436279	1.57416133	4	4	36.28341875	34.77592462
chr18	3878180	3897069	18p11.31	DLGAP1-AS3	0.328766596	0.208868343	1.57403746	1	1	0	0
chr19	48382570	48391553	19q13.33	KDELR1	16.77354294	10.65714854	1.573924102	4	4	53.16839808	39.91557408
chr14	101730437	101732525	14q32.31	LINC00239	2.711730864	1.722971513	1.573868658	4	4	50.96570982	58.16805657
chr1	184791025	184974586	1q25.3	FAM129A	185.6760006	117.9782872	1.573815021	4	4	77.20276783	66.96763025
chr16	370776	381950	16p13.3	TMEM8A	14.76332174	9.381154618	1.573721183	4	4	53.6356576	37.37382229
chr11	64240941	64244135	11q13.1	FKBP2	14.30478986	9.090687672	1.57356521	4	4	62.31112081	59.75131602
chr11	14673382	14674489	11p15.2	MORF4L1P3	0.759173959	0.482542204	1.573279918	1	1	0	0
chr12	64610513	64697567	12q14.2	RASSF3	122.4144144	77.80846289	1.573278919	4	4	66.41863223	44.56977216
chr4	2793023	2841096	4p16.3	SH3BP2	13.72621479	8.72473628	1.573252686	4	4	58.46829366	19.61801598
chr2	239401436	239402364	2q37.3	HDAC4-AS1	3.363059716	2.137715859	1.573202398	3	4	62.03254617	14.94788298
chr11	46380784	46383837	11p11.2	MDK	0.684010459	0.434789048	1.573200757	4	3	100.0555254	80.1130963

chr6	108559823	108684769	6q21	FOXO3	111.8952387	71.13860747	1.572918598	4	4	17.82088849	22.54009789
chr7	107579977	107623317	7q22.3	BCAP29	7.474260322	4.752125022	1.57282485	4	4	60.74693496	41.07047596
chr6	45898451	46129809	6p21.1	CLIC5	0.5996363	0.381271541	1.572727664	4	3	33.88701345	35.12730547
chr7	23597379	23644708	7p15.3	CCDC126	66.74220977	42.43966401	1.572637563	4	4	38.75420088	44.16122994
chr19	45047460	45047560	19q13.32	RNU6-611P	4.034655942	2.565697901	1.572537414	2	2	43.81179237	8.663457077
chr3	184346185	184361651	3q27.1	CLCN2	0.129702578	0.082506064	1.572036912	3	4	62.79794211	55.39253446
chr16	67148102	67150999	16q22.1	B3GNT9	1.272613418	0.809534263	1.572031569	3	4	49.83380998	51.75568647
chr12	8533305	8541327	12p13.31	CLEC4E	69.50811987	44.21550668	1.572030382	4	4	33.37481649	49.99433004
chr16	70011002	70065962	N/A	PDXDC2P	0.344380276	0.219160712	1.571359546	4	4	88.98742613	64.91524793
chr19	40592888	40629820	19q13.2	LTBP4	0.95778734	0.609586881	1.571207272	4	4	42.88487946	33.74907423
chr10	91806043	91807533	10q23.32	SRP9P1	5.40458512	3.439871374	1.571159073	3	3	93.61290655	105.3779466
chr17	74776483	74839783	17q25.1	TMEM104	4.363478314	2.777348767	1.571094839	4	4	82.7310479	63.881849
chr15	59135969	59372872	15q22.2	MYO1E	2.183278367	1.389797569	1.570932643	4	4	63.8013999	54.0147007
chr16	19113932	19121630	16p12.3	ITPRIPL2	3.814601488	2.428978206	1.570455214	4	4	49.94801716	41.2445989
chr8	61500556	61714640	8q12.3	ASPH	10.88190846	6.929258529	1.570428988	4	4	13.07260677	35.45418072
chr7	1433639	1459497	7p22.3	MICALL2	0.458510161	0.291968462	1.570409892	4	4	37.1557079	77.9036165
chr18	9785127	9787866	18p11.22	ZNF415P1	2.362332995	1.504300423	1.570386447	4	4	23.58827425	42.04429014
chr13	113323252	113364158	13q34	GRTP1	0.512923239	0.326637776	1.570312059	3	3	95.62305167	36.59272128
chr2	159768628	159798255	2q24.2	CD302	77.13620104	49.12314779	1.570261771	4	4	78.05325907	38.54981207
chr10	48429831	48656265	10q11.22-q11.23	ARHGAP22	0.141127722	0.089876183	1.570246052	4	3	98.18040374	67.46586264
chr15	63907036	64046470	15q22.31	DAPK2	2.740356521	1.745262982	1.570168249	4	4	92.39704024	50.13954421
chr22	21001922	21010374	22q11.21	THAP7-AS1	0.539287517	0.343465813	1.570134481	3	4	90.95637261	26.23233733
chr17	47323961	47441312	17q21.32	EFCAB13	55.23740687	35.182697	1.570016274	4	4	57.49327943	40.35908342
chr2	174719800	174720858	2q31.1	H3F3AP4	18.9495938	12.06994793	1.569981405	4	4	51.19653927	39.75354456
chr3	52551846	52551943	3p21.1	RNU6-856P	4.282445373	2.727786976	1.569933947	2	2	43.30659717	8.508221206
chr3	47164370	47244116	3p21.31	KIF9-AS1	4.241438161	2.701801789	1.569855412	4	4	68.62970351	66.93071756
chr1	201688256	201829559	1q32.1	IPO9-AS1	0.439109674	0.279728485	1.569771035	2	4	74.63504417	44.3321573
chr21	42371887	42396846	21q22.3	TMPRSS3	0.389148655	0.247916696	1.569675061	4	3	64.51356548	63.40927966
chr3	98569837	98593684	3q11.2	CPOX	17.13249468	10.91673145	1.569379512	4	4	73.18006578	43.49716689
chr14	67676800	67695793	14q24.1	RDH11	22.02493066	14.03455178	1.569336236	4	4	21.55769522	17.63073456
chr1	37454879	37474443	1p34.3	LINC01137	2.07046784	1.319358415	1.569298999	4	4	55.76012802	33.94463417
chr11	117232625	117285688	11q23.3	RNF214	19.24240014	12.26430142	1.568976453	4	4	49.51008614	14.39044718
chrY	7699357	7701247	Yp11.2	TTTY16	2.039611823	1.300042123	1.568881336	1	2	0	17.7284516
chr2	30146568	30160533	2p23.1	YPEL5	185.1407433	118.0308196	1.568579664	4	4	22.023897	23.2495827
chr1	10210706	10381603	1p36.22	KIF1B	7.827460479	4.991269964	1.568230237	4	4	53.77987824	39.6803339
chr2	101991805	102028544	2q11.2	IL1R2	24.0008512	15.30499121	1.568171512	4	4	54.18793875	64.90374647
chr16	48538726	48610214	16q12.1	N4BP1	37.30984337	23.7919702	1.568169557	4	4	64.18830943	38.18677939
chr12	101594849	101695841	12q23.2	MYBPC1	0.056626619	0.036116782	1.567875543	1	1	0	0
chr2	147930397	148021604	2q23.1	ORC4	56.59226725	36.0982251	1.567729912	4	4	85.04434962	59.34306119
chrX	154444126	154450654	Xq28	FAM50A	10.78197831	6.8777755	1.567654878	4	4	67.23495647	30.3363851
chr8	80776666	80776962	8q21.13	RN7SL308P	2.584606021	1.648821481	1.567547519	2	4	35.45310395	41.46442497
chr3	52495338	52524496	3p21.1	STAB1	5.088285446	3.246080125	1.567516897	4	4	89.11782825	27.54323866
chr16	11668433	11679159	16p13.13	SNN	99.20156289	63.28860614	1.567447427	4	4	72.76458512	25.64564956

chr7	116524785	116561185	7q31.2	CAV1	0.400605666	0.255590671	1.567372018	3	4	102.6084692	72.13631194
chr18	9121265	9136720	18p11.22	NDUFV2-AS1	2.505344001	1.598729813	1.567084056	4	4	21.49312343	44.96292512
chr11	67452406	67461774	11q13.2	CABP4	0.344206645	0.219678734	1.566863752	3	4	87.26140755	65.77324203
chr7	135148014	135166215	7q33	TMEM140	68.14496224	43.49367813	1.566778556	4	4	52.76394308	38.78548033
chr4	83407343	83456046	4q21.23	HELQ	31.96992041	20.40561004	1.566722109	4	4	74.90461305	44.42490558
chr1	27834401	27834566	1p35.3	SCARNA1	29.38925615	18.75885839	1.566686818	4	4	67.0665498	61.86983774
chr5	100816482	100816565	5q21.1	MIR548P	6.438584444	4.109770386	1.566653083	4	2	17.38857926	24.04792888
chrMT	14149	14673	N/A	MT-ND6	17.28762713	11.03528116	1.566577859	4	4	69.25943116	42.0719888
chr5	88883330	89467581	5q14.3	MEF2C-AS1	2.143950143	1.368603887	1.566523494	4	4	55.80509775	42.16960844
chr9	33673504	33677420	9p13.3	PTENP1	2.229640509	1.423341245	1.566483454	4	4	71.51608734	49.6727937
chr5	148826593	148828634	5q32	ADRB2	13.38205702	8.542767484	1.566477964	4	4	63.24639542	26.38935215
chr18	80067256	80081304	18q23	RBFADN	0.062130075	0.039663255	1.566439148	2	2	25.38368371	1.548061643
chr7	6016877	6017011	7p22.1	SNORA80D	21.41823949	13.6737625	1.566374982	4	4	66.58986145	71.38566813
chr6	7726099	7881728	6p24.3	BMP6	14.87098025	9.495123829	1.566170228	4	4	44.18616362	10.72433679
chr11	74454841	74467729	11q13.4	KCNE3	31.38132397	20.03930491	1.565988647	4	4	63.98140723	56.77385591
chr1	113904619	113914086	1p13.2	DCLRE1B	22.48532937	14.35962653	1.565871461	4	4	71.8629809	5.910207796
chr21	32733899	32771858	21q22.11	PAXBP1	14.51406846	9.271239985	1.565493772	4	4	25.51231746	15.8413923
chr5	90515626	90529616	5q14.3	LYSMD3	44.41062973	28.37128556	1.565337236	4	4	16.94339459	24.08297664
chr6	2999816	3019876	6p25.2	NQO2	3.548767339	2.267258459	1.565223993	4	4	74.48247993	39.96052352
chr19	925733	975934	19p13.3	ARID3A	9.349926049	5.973569106	1.565216018	4	4	65.4340703	51.62285023
chr18	11981428	12030886	18p11.21	IMPA2	12.99280319	8.302784308	1.564873024	4	4	100.2793769	61.13300293
chr16	88813734	88856970	16q24.3	GALNS	3.257698698	2.081798468	1.564848254	4	4	66.33489712	34.84032974
chr4	169986597	170033028	4q33	MFAP3L	27.95474594	17.86694537	1.564606896	4	4	26.47372272	52.91629945
chrX	80669488	80809736	Xq21.1	BRWD3	30.65373157	19.5922452	1.564584929	4	4	45.99075789	30.01781769
chr11	74748856	74842413	11q13.4	RNF169	46.05610593	29.43751026	1.564538085	4	4	14.29568974	18.02704152
chr3	197960200	198043720	3q29	LMLN	1.031470992	0.659305229	1.56448174	4	4	10.63808168	5.490138853
chr16	3045963	3060729	16p13.3	MMP25	60.73820185	38.82471381	1.564421109	4	4	69.55190977	73.40293394
chr2	102473282	102533974	2q12.1	SLC9A4	0.088789253	0.056755342	1.564421075	1	1	0	0
chr3	98515473	98523066	3q11.2	CLDND1	26.66812558	17.04828591	1.564270198	4	4	44.23708742	18.79674843
chr13	37934499	38065571	13q13.3	LINC02334	0.29649395	0.189546831	1.564225305	3	3	119.4453986	72.42236375
chr8	38030502	38060365	8p11.23	EIF4EBP1	9.875943722	6.314062109	1.564118875	4	4	73.2833333	27.01334969
chr19	51410021	51417803	19q13.41	SIGLEC10	32.75835174	20.94565171	1.563969085	4	4	81.16082592	30.77361817
chr11	63574462	63616926	11q12.3-q13.1	PLA2G16	1.524172956	0.97464291	1.56382706	4	4	96.15138062	78.12920388
chr1	53851719	53889831	1p32.3	YIPF1	26.54834942	16.97693954	1.563788889	4	4	46.74999977	36.35322739
chr5	115124762	115180546	5q22.3	TRIM36	0.658432975	0.421073349	1.563701376	4	4	47.3250289	83.67841
chr11	122881640	122963862	11q24.1	JHY	1.22621294	0.784198053	1.56365211	4	4	44.79720741	50.91102262
chr17	28905250	28951490	17q11.2	PHF12	13.02284333	8.329345523	1.563489387	4	4	69.41843961	25.249821
chr2	24033205	24067743	2p23.3	FKBP1B	7.204822757	4.608398984	1.563411237	4	4	28.60097384	34.51588293
chr7	37683560	37743835	7p14.1	GPR141	8.209969677	5.251496289	1.563358179	4	4	81.82540004	74.47448465
chr1	23778386	23788232	1p36.11	PITHD1	185.850127	118.8892025	1.563221244	4	4	58.11644976	38.69798841
chr1	161171310	161185007	1q23.3	B4GALT3	22.34462581	14.29454927	1.563157074	4	4	28.8437368	42.57931841
chrX	135032303	135033546	Xq26.3	RTL8C	8.969454624	5.738323122	1.563079393	4	4	62.67400915	59.26610848
chr11	3665587	3673629	11p15.4	CHRNA10	0.432238456	0.276530995	1.563074174	3	4	19.06134614	53.31919837

chr1	65147342	65232145	1p31.3	AK4	0.302006797	0.193218737	1.56303059	2	4	69.75165292	12.51422953
chr9	129738336	129777579	9q34.11	PTGES	0.905985233	0.579675213	1.562918705	3	3	98.77158612	68.24274925
chr11	65638107	65650930	11q13.1	SIPA1	47.10483043	30.13903166	1.562917846	4	4	57.77854983	33.54223984
chr5	135033280	135344680	5q31.1	C5orf66	0.371884876	0.237949892	1.562870541	3	3	62.525113	65.18296899
chr19	1000419	1009748	19p13.3	GRIN3B	0.118633174	0.075911407	1.562784552	2	1	24.75743056	0
chr19	45497964	45502694	19q13.32	PPM1N	0.804689739	0.514925483	1.562730462	3	4	118.0666188	69.68017236
chr3	46979683	47009704	3p21.31	NBEAL2	36.18656521	23.15925113	1.562510161	4	4	68.92056494	26.42834958
chr7	35754964	35800625	7p14.2	SEPT7-AS1	6.087330273	3.89647194	1.562267191	4	4	93.24448659	43.95081536
chr7	55951918	55955341	7p11.2	MRPS17	1.764221243	1.129288932	1.562240799	4	4	80.58706282	83.16843814
chr1	229593111	229626047	1q42.13	TAF5L	4.902953832	3.139037344	1.561929118	4	4	68.88640757	29.71447622
chr19	35717818	35738880	19q13.12	KMT2B	5.402586238	3.458962754	1.561909342	4	4	70.4401945	36.90433968
chr19	53865584	53876435	19q13.42	MYADM	51.67378061	33.08518589	1.561840419	4	4	63.4235934	60.78086108
chr7	75995820	76049344	7q11.23	STYXL1	11.83932872	7.581295969	1.561649718	4	4	45.04889788	11.60831567
chr11	10304980	10307402	11p15.4	ADM	24.68508714	15.80752591	1.561603459	4	4	76.64643394	64.96657132
chr2	127298669	127387533	2q14.3	MAP3K2	63.01497098	40.35505727	1.561513606	4	4	30.99269277	32.54112888
chr3	37275537	37278152	3p22.2	TCEA1P2	56.50183614	36.18612207	1.561422803	4	4	73.40192217	47.12407926
chr14	21459020	21476973	14q11.2	RAB2B	187.3248604	119.9825567	1.561267451	4	4	61.52873931	22.89195359
chr1	116531756	116534111	1p13.1	NAP1L4P1	8.445913539	5.40988206	1.561201048	4	4	52.05944075	55.2462645
chr16	8621640	8673565	16p13.2	METTL22	3.123885857	2.001195574	1.561009777	4	4	37.43868195	20.56862688
chr2	171522247	171558133	2q31.1	CYBRD1	23.29177801	14.92163911	1.560939641	4	4	21.16675878	27.6861339
chr13	30419439	30422244	13q12.3	LINC01058	0.926958227	0.593922891	1.560738339	1	1	0	0
chr2	89914071	89914545	2p11.2	IGKV1D-33	1.311751705	0.840485024	1.560708004	2	3	82.12853386	30.94598167
chr2	210477682	210679107	2q34	CPS1	0.03909237	0.025057336	1.560116762	2	2	30.50887635	5.75004132
chr13	20187463	20192975	13q12.11	GJB2	0.183393221	0.117553509	1.560082913	1	1	0	0
chr1	17308195	17364004	1p36.13	PADI4	24.93750999	15.9867769	1.559883531	4	4	63.84633221	45.09605129
chr7	47275154	47582553	7p12.3	TNS3	3.280027027	2.102861583	1.559792168	4	4	53.62864577	16.21916851
chr9	114784635	114806128	9q32	TNFSF15	0.536062364	0.343678769	1.559777363	4	3	46.97653911	83.71566565
chr9	92031134	92115474	9q22.31	SPTLC1	37.068127	23.76551204	1.559744513	4	4	25.5943239	8.762388225
chr6	31730581	31737318	6p21.33	CLIC1	161.1209824	103.314812	1.559514838	4	4	56.03769446	24.12286182
chr12	6963245	6964447	12p13.31	MIR200CHG	2.025581624	1.298892867	1.559467818	1	2	0	57.36217662
chr12	69738007	69823204	12q15	RAB3IP	26.25295682	16.83504211	1.559423294	4	4	78.97800838	49.15722384
chr9	35104121	35116341	9p13.3	FAM214B	25.97856472	16.66207595	1.559143339	4	4	8.89588073	6.362141153
chr19	52336245	52367123	19q13.41	ZNF610	0.730831966	0.468743622	1.559129406	4	4	71.24704833	66.86993041
chr1	109309565	109397945	1p21.3-p13.1	SORT1	13.52602266	8.675407001	1.559122547	4	4	48.39709389	19.65512449
chr5	60459669	60460805	5q12.1	SETP21	0.369601473	0.237123631	1.558686795	1	1	0	0
chr15	58595204	58749978	15q21.3	ADAM10	41.79603381	26.81520256	1.55866933	4	4	42.67839594	27.11028712
chr8	58552924	58582860	8q12.1	SDCBP	244.9716162	157.1705915	1.558635199	4	4	47.85122039	49.26196002
chr8	81656916	81686354	8q21.13	IMPA1	16.50100349	10.58753085	1.558531798	4	4	21.92676053	38.47669808
chr19	3136032	3163769	19p13.3	GNA15	9.607535566	6.164524261	1.558520197	4	4	74.96109299	14.02006763
chr15	55826917	55993746	15q21.3	NEDD4	5.440642912	3.491131153	1.558418368	4	4	22.62292162	15.5107432
chr19	47520685	47573248	19q13.33	ZNF541	0.118718451	0.076186572	1.558259517	3	3	32.73692633	37.79350831
chr9	36169392	36171334	9p13.3	CCIN	0.637890989	0.40944038	1.55795818	3	3	94.02974363	85.25607421
chr1	31409565	31434680	1p35.2	SERINC2	0.895509447	0.57481228	1.557916345	4	4	76.42791756	38.85126242

chr12	116856144	116881427	12q24.22	HRK	0.257404046	0.165241509	1.557744465	1	4	0	96.47694086
chr15	42427591	42427697	15q15.1	RNU6-188P	3.51322323	2.255445298	1.557662796	1	1	0	0
chr6	69675749	69797157	6q13	LMBRD1	87.40757029	56.11619756	1.557617481	4	4	27.67793706	35.52434878
chr12	12974864	13000309	12p13.1	HEBP1	6.110288853	3.922887599	1.557599778	4	4	89.73702454	58.12460714
chr12	123409890	123436733	12q24.31	RILPL2	17.49543994	11.23329278	1.557463184	4	4	54.86413889	20.43088382
chr11	118193727	118225094	11q23.3	JAML	97.20295643	62.41829966	1.557282992	4	4	64.36947363	63.20275318
chr1	247937029	248100922	1q44	OR2L13	0.535645324	0.343994783	1.557132112	3	4	106.826736	16.30199585
chr1	74705470	74733408	1p31.1	CRYZ	3.928000763	2.522840378	1.556975542	4	4	60.19053457	37.91737396
chr16	29662950	29670876	16p11.2	SPN	46.15227292	29.64518524	1.556821877	4	4	43.86565398	46.65364339
chr19	33080880	33130412	19q13.11	GPATCH1	41.4447722	26.62155262	1.556812737	4	4	53.53399168	11.2477837
chr9	33817164	33920403	9p13.3	UBE2R2	54.96413543	35.31408442	1.556436655	4	4	50.01400093	25.2337183
chr14	77377378	77391300	14q24.3	SAMD15	0.533148571	0.342551614	1.556403616	2	3	53.61156636	32.01640119
chr9	37800554	37867669	9p13.2	DCAF10	15.95850831	10.25425885	1.556281009	4	4	24.25747991	30.27586778
chr8	22544990	22575495	8p21.3	SORBS3	1.331945905	0.855877879	1.55623359	4	4	78.35313814	34.56063556
chr22	30488150	30505711	22q12.2	SEC14L4	5.612738617	3.606826353	1.556143287	4	4	68.01615862	49.66298188
chr19	1248552	1259143	19p13.3	MIDN	30.75535701	19.76485186	1.556063117	4	4	73.6605719	31.43752049
chr10	22534837	22714574	10p12.2	PIP4K2A	546.3060588	351.1413247	1.555801099	4	4	17.6293102	19.51020753
chr18	79069275	79378391	18q23	ATP9B	21.28758803	13.68446315	1.555602716	4	4	72.58176071	33.29131907
chr6	158644867	158766298	6q25.3	SYTL3	26.15158647	16.81160925	1.555567113	4	4	23.06635101	22.67449662
chr11	560971	564025	11p15.5	RASSF7	1.039840544	0.66853096	1.555411202	3	4	11.72803062	33.67174502
chr19	48394875	48444937	19q13.33	GRIN2D	0.081491652	0.052401712	1.555133376	4	2	37.40800663	5.054674513
chr14	24189143	24195736	14q12	TM9SF1	0.8794745	0.565708511	1.554642512	3	4	68.83494439	44.77142316
chr4	80183418	80204329	4q21.21	PRDM8	2.110471271	1.35771092	1.554433451	4	4	90.16912869	68.20160673
chr12	89588049	89709353	12q21.33	ATP2B1	56.7152493	36.48677828	1.554405513	4	4	9.92489456	25.84887185
chr21	46286056	46314188	21q22.3	YBEY	6.492872315	4.177087494	1.554401799	4	4	22.14319744	29.14266524
chr3	71679289	71754773	3p13	EIF4E3	35.31883928	22.72197846	1.55439102	4	4	23.84444043	34.21496798
chr12	125065367	125143333	12q24.31	AACS	0.472380564	0.303905122	1.55436855	4	4	62.00326013	26.45609982
chr6	108041409	108074737	6q21	OSTM1	35.71951717	22.98017121	1.554362534	4	4	25.04361028	36.45515596
chr9	137062127	137070576	9q34.3	SAPCD2	0.672918088	0.43301824	1.55401788	3	4	106.6999887	108.6627035
chr16	15703135	15857032	16p13.11	MYH11	0.037030101	0.023829352	1.553970149	3	2	89.13543602	40.13001192
chr3	42782908	42804535	3p22.1	HIGD1A	53.42905089	34.38260351	1.553955938	4	4	62.49519763	39.91542225
chr14	22487183	22487245	14q11.2	TRAJ51	8.585479809	5.525314891	1.553844437	1	2	0	39.08461492
chr2	186033531	186086317	2q32.1	LINC01473	32.12352673	20.67424119	1.553794717	4	4	50.18024468	38.16950617
chr3	122527910	122564676	3q21.1	PARP9	59.72426187	38.44866327	1.5533508	4	4	46.26985282	47.72820322
chr1	247416156	247449108	1q44	NLRP3	20.46906261	13.17756204	1.553326977	4	4	51.78633214	33.0462764
chr7	88276429	88306913	7q21.12	STEAP4	49.68396735	31.9874142	1.553234877	4	4	45.67997365	74.45742775
chr16	89917879	89920977	16q24.3	MC1R	0.290654841	0.187151034	1.553049614	4	4	122.9739646	72.43529709
chr2	43097746	43102445	2p21	LINC02580	1.140362203	0.734382696	1.552817365	4	3	42.86404898	21.37431431
chr8	20229779	20229877	8p21.3	RNU6-892P	7.209932108	4.643254219	1.552775655	3	3	45.73196116	22.21968604
chr3	14402576	14489349	3p25.1	SLC6A6	48.87629506	31.47749366	1.552737826	4	4	59.57240939	38.23353589
chr5	10440735	10441851	5p15.2	ROPN1L-AS1	2.550907648	1.642892775	1.552692718	1	2	0	28.28349299
chr2	169067035	169096167	2q31.1	DHRS9	7.015748734	4.519050466	1.552482936	4	4	31.85370525	61.12480008
chr9	41131309	41198865	9p11.2	CBWD6	2.084797592	1.342987595	1.552358041	4	4	28.09730437	43.08601623

chr6	655939	656964	6p25.3	HUS1B	0.617531891	0.397921384	1.551894208	3	3	58.12015472	57.75677378
chr3	50611862	50649297	3p21.2	MAPKAPK3	37.49883935	24.16328122	1.551893512	4	4	55.67669574	24.33510513
chr12	110452486	110468721	12q24.11	GPN3	13.6011997	8.765100419	1.55174488	4	4	81.42543868	58.89587554
chr3	44555175	44583464	3p21.31	ZKSCAN7	0.416035512	0.268159355	1.55144881	4	4	71.89947916	51.64041695
chr8	132709945	132760668	8q24.22	TMEM71	96.70861865	62.33794988	1.551360268	4	4	30.39652164	34.35516523
chr8	73991275	74099807	8q21.11	LY96	164.4042083	105.9954101	1.55105026	4	4	51.73115725	72.0556632
chr6	158764661	158764753	6q25.3	MIR3918	4.987676867	3.216004312	1.550892469	4	3	30.64663042	31.1858811
chr4	87420002	87459454	4q22.1	NUDT9	3.797022054	2.448704628	1.550624772	4	4	26.45985644	26.39817942
chr12	68610839	68660605	12q15	RAP1B	78.83657533	50.84192935	1.550621236	4	4	26.24961321	8.132371492
chr1	229042092	229042589	1q42.13	ISCA1P2	0.845501629	0.54526875	1.550614496	1	1	0	0
chr19	6887549	6940453	19p13.3-p13.2	ADGRE1	31.03336138	20.01401858	1.550581222	4	4	72.75859698	58.83748893
chr9	83980359	84004074	9q21.32	RMI1	7.247573031	4.674991537	1.550285807	4	4	29.06671567	35.9012427
chr7	140023744	140063721	7q34	PARP12	13.75520036	8.872768779	1.550271477	4	4	41.97974159	35.12561737
chr12	95972662	95996365	12q23.1	HAL	14.70868091	9.488988001	1.550078987	4	4	65.46042518	55.34463739
chr1	227563519	227677443	1q42.13	ZNF678	6.442000514	4.15624716	1.54995607	4	4	18.07744473	12.43109165
chr3	111592900	111595335	3q13.13	ZBED2	0.162641731	0.104946657	1.549756182	1	1	0	0
chr3	53224707	53256114	3p21.1	TKT	60.70594619	39.17266074	1.549701885	4	4	83.82399351	60.23858139
chr9	137084946	137086817	9q34.3	MAN1B1-DT	0.872006415	0.562712002	1.549649575	2	4	19.17232799	46.82784694
chr6	35664717	35664789	6p21.31	MIR5690	14.48023128	9.345539686	1.549426974	3	3	49.59572867	67.84174186
chr3	42704118	42773253	3p22.1	CCDC13	0.287847689	0.185777475	1.549421898	4	4	56.14510056	100.5143662
chr17	9910609	10198551	17p13.1	GAS7	19.00035141	12.26316166	1.549384403	4	4	66.90003989	36.42507796
chr1	32352720	32365265	1p35.1	TSSK3	0.359497414	0.232065047	1.549123486	1	1	0	0
chr1	149832657	149833052	1q21.2	HIST2H4A	0.864967025	0.558437529	1.548905615	4	4	33.15528181	85.50224005
chr1	43363397	43368074	1p34.2	ELOVL1	21.78093025	14.06567563	1.548516461	4	4	38.32571581	26.94214665
chr1	224183208	224193441	1q42.11	DEGS1	39.98443608	25.82141685	1.548498919	4	4	29.69268896	34.60019116
chr1	212830141	212847649	1q32.3	SPATA45	0.992915628	0.641331608	1.548209407	4	1	66.82771994	0
chr9	75088480	75147198	9q21.13	OSTF1	166.1621838	107.3288408	1.548159679	4	4	55.42948697	37.27013174
chr6	143494812	143511883	6q24.2	FUCA2	9.393879559	6.068103834	1.548074953	4	4	48.71953883	19.00854584
chr1	150996737	151008378	1q21.3	MINDY1	28.59366433	18.47126661	1.548007775	4	4	49.83618783	38.63669096
chr20	5496067	5504596	20p12.3	LINC00654	0.780011154	0.503882723	1.548001388	4	4	78.97272806	88.73818061
chr10	75233656	75236066	10q22.2	COMTD1	1.217032309	0.786326311	1.54774461	4	4	96.49092348	19.12593026
chr2	43027853	43039543	2p21	LINC01819	0.164116597	0.106038522	1.547707321	2	1	35.68361672	0
chr10	119033670	119080884	10q26.11	EIF3A	83.72636023	54.09942781	1.547638554	4	4	49.08610118	35.81854894
chrX	54933134	55000052	Xp11.21	PFKFB1	1.059834515	0.684807942	1.547637591	4	4	62.87647483	42.36295212
chr19	54094668	54102686	19q13.42	OSCAR	2.316553931	1.496905736	1.547561664	4	4	7.808614309	64.59741205
chr22	31618491	31662564	22q12.2	PISD	19.57281954	12.65006618	1.547250367	4	4	68.15653714	42.52189907
chr7	112735000	112738438	7q31.1	MIPEPP1	0.218209325	0.141037616	1.547171107	1	2	0	10.38607322
chr16	66934444	66945096	16q22.1	CES2	6.25563444	4.043665158	1.547020882	4	4	96.05857625	30.13247048
chr12	10407384	10409757	12p13.2	KLRC4	7.489673264	4.841496659	1.546974787	4	4	53.2843497	53.78480873
chr19	12751691	12758458	19p13.13	BEST2	0.147847916	0.095593403	1.546633048	2	2	19.72719421	4.712441687
chr1	40620680	40692104	1p34.2	RIMS3	0.680902983	0.440264593	1.546576749	4	4	87.78525044	66.09106399
chr5	44744328	44808793	5p12	MRPS30-DT	0.241269938	0.15600589	1.546543778	4	3	51.2531728	47.92840088
chr7	149838367	149867479	7q36.1	ZNF862	2.459079257	1.590372389	1.54622859	4	4	67.86985437	39.93383915

chr7	77537190	77640071	7q11.23	PTPN12	87.92614861	56.86540809	1.546215029	4	4	30.22339412	16.30406443
chr12	124935480	124935865	12q24.31	RPL22P19	0.801454313	0.518401718	1.546010139	1	1	0	0
chr11	68038995	68053846	11q13.2	TCIRG1	32.29136608	20.88874137	1.545874186	4	4	71.31580698	29.13187082
chr6	53267398	53349179	6p12.1	ELOVL5	169.6093189	109.7209741	1.545824035	4	4	44.65559843	38.85044224
chr6	18128311	18155169	6p22.3	TPMT	17.24757106	11.15774754	1.54579327	4	4	55.6666393	21.94304648
chr16	28097976	28212031	16p12.1	XPO6	118.9669405	76.97360903	1.545554925	4	4	50.14431954	40.84633177
chr18	14179097	14227050	18p11.21	ANKRD20A5P	0.43920649	0.284177805	1.545534109	4	4	64.83237135	39.74530169
chr17	76382977	76387860	17q25.1	SPHK1	0.996834173	0.645081758	1.545283464	4	4	74.77364193	23.41036665
chrX	46836912	46882358	Xp11.3	RP2	65.01111227	42.07331289	1.545186433	4	4	30.8090767	36.61263603
chr6	10521283	10629368	6p24.3-p24.2	GCNT2	5.595649578	3.621346453	1.545184823	4	4	48.24644206	74.82555238
chr16	88643289	88651084	16q24.2	CYBA	81.27288002	52.60810782	1.544873659	4	4	72.06135659	57.35007502
chr9	136673143	136687459	9q34.3	AGPAT2	6.491116422	4.202364039	1.544634487	4	4	77.25351899	51.47518902
chr5	1201595	1225117	5p15.33	SLC6A19	1.012148633	0.655272518	1.544622437	4	4	117.3221866	54.0494702
chr3	52048919	52056445	3p21.2	DUSP7	4.776322827	3.092616257	1.544427898	4	4	70.58873146	54.01037144
chr12	88035491	88050160	12q21.32	C12orf29	10.9006811	7.05827815	1.544382479	4	4	41.57347572	29.39811048
chr17	42313324	42388503	17q21.2	STAT3	68.22539995	44.17819383	1.54432298	4	4	47.49810089	27.37787664
chr7	150944956	150978314	7q36.1	KCNH2	1.67945578	1.087503672	1.54432194	4	4	30.87005944	26.31900055
chr3	50287732	50292595	3p21.31	IFRD2	4.6311581	2.998871487	1.544300288	4	4	12.23087478	22.55927549
chr6	3020156	3024771	6p25.2	HTATSF1P2	18.19817527	11.78494037	1.544188999	4	4	83.99248433	63.21199288
chrX	72578814	72714181	Xq13.1	PHKA1	0.172308465	0.111599939	1.543983507	3	2	101.3373683	90.91343075
chr3	197668867	197749727	3q29	RUBCN	7.506028543	4.861602542	1.543941217	4	4	32.86086606	29.17986709
chr2	144384375	144520391	2q22.3	ZEB2	27.10770773	17.55750952	1.543938092	4	4	61.45197586	25.01050025
chr8	94925972	94949387	8q22.1	TP53INP1	41.63478822	26.96760314	1.543881672	4	4	46.66157427	21.84110902
chr15	89883931	89884030	15q26.1	MIR5009	4.533545196	2.936901513	1.543649038	2	2	29.5101248	48.78320109
chr8	85328229	85378154	8q21.2	CA1	297.6451243	192.8382515	1.543496283	4	4	84.19958387	46.86787307
chr1	1216908	1232067	1p36.33	SDF4	36.85645387	23.8854276	1.543051876	4	4	18.20363032	14.91230347
chr10	132537779	132783480	10q26.3	INPP5A	11.21549238	7.268809715	1.542961341	4	4	35.03580241	34.50479977
chr1	100038078	100083377	1p21.2	MFSD14A	3.388456714	2.196185085	1.542883037	4	4	74.98691829	36.5160076
chr12	57516588	57520517	12q13.3	DDIT3	23.9958013	15.55258525	1.542881837	4	4	47.63688367	30.37478275
chr19	10115014	10119924	19p13.2	EIF3G	46.1472438	29.91012537	1.542863603	4	4	82.46240909	54.62282869
chr6	13786549	13814994	6p23	MCUR1	16.26323368	10.54318297	1.542535468	4	4	57.23279343	21.61461432
chr1	200404947	200410058	1q32.1	ZNF281	32.85110933	21.29734211	1.542498081	4	4	78.01715854	34.79349827
chr4	144642916	144646019	4q31.21	HHIP-AS1	0.213006654	0.138104347	1.542360236	1	1	0	0
chr15	35298047	35299610	15q14	RBM17P4	0.27633864	0.179172221	1.542307389	1	1	0	0
chr1	26826710	26855720	1p36.11	ZDHHC18	57.59468334	37.35350248	1.541881738	4	4	46.15442883	48.33806058
chr6	81745730	81752711	6q14.1	TENT5A	12.77503946	8.285367276	1.541879682	4	4	39.53656889	24.82919091
chr16	1351899	1363351	16p13.3	GNPTG	7.158790146	4.643042215	1.541831802	4	4	61.64642282	51.31082948
chr1	155051272	155062776	1q21.3	ADAM15	3.759746482	2.43854443	1.541799458	4	4	67.29618332	33.08355038
chr4	118722823	118838683	4q26	SEC24D	8.581234108	5.566289284	1.541643574	4	4	54.84850422	36.69939372
chr1	68474152	68497221	1p31.3	DEPDC1	13.85919605	8.990055449	1.541614079	4	4	79.77088699	32.24847114
chrX	119574467	119584429	Xq24	UBE2A	13.03133267	8.453702374	1.54149414	4	4	36.94599247	33.60885399
chr9	62844712	62845611	9q13	CDK2AP2P2	0.875298833	0.567845267	1.541438987	2	1	17.32069054	0
chr1	45343883	45491342	1p34.1	TESK2	25.40667411	16.48353606	1.541336399	4	4	54.1145318	28.86595043

chr7	77793728	77798430	7q11.23	TMEM60	19.5693558	12.69663532	1.541302503	4	4	27.4380683	45.16490946
chr17	60231516	60231645	17q23.2	SCARNA20	8.917814269	5.786761624	1.541071648	4	3	45.39057284	85.84822395
chr2	88691658	88750935	2p11.2	RPIA	112.8144546	73.21201674	1.54092811	4	4	35.62962849	23.14292052
chr15	101270909	101277520	15q26.3	SELENOS	6.448762896	4.185164717	1.540862387	4	4	49.88604204	19.45763955
chr15	51723064	51751453	15q21.2	LYSMD2	19.71789273	12.7967476	1.540851891	4	4	28.1012595	40.56973648
chr13	44373523	44397714	13q14.11	SERP2	0.583045931	0.378409077	1.540782097	2	2	30.98171624	7.163388693
chr16	31472155	31477960	16p11.2	TGFB1I1	0.90910041	0.590078956	1.540641978	4	4	76.72124867	56.7367761
chr20	36541484	36549823	20q11.23	MYL9	33.18228869	21.54123324	1.540408032	4	4	71.94874579	57.72352898
chr12	122980060	122982913	12q24.31	ARL6IP4	0.293464369	0.19053801	1.54018807	2	4	86.38435273	40.89640724
chr1	212565334	212620777	1q32.3	ATF3	1.534740081	0.996606801	1.539965491	4	4	66.23558485	77.8074278
chr5	58491435	58496358	5q11.2	GAPT	59.2316407	38.46607235	1.53984114	4	4	56.76646845	60.76080066
chr5	172834220	172952685	5q35.1	ERGIC1	19.1611095	12.44362727	1.539833127	4	4	70.19890818	36.03264544
chrY	3682558	3684842	Yp11.2	USP12PY	0.622833541	0.404490507	1.539797671	2	2	39.81874346	53.32615511
chr5	141653401	141682233	5q31.3	ARAP3	9.574529773	6.218230376	1.539751536	4	4	72.49223827	51.24722564
chrX	118726954	118794533	Xq24	IL13RA1	77.71581832	50.47646435	1.539644651	4	4	56.18578231	54.66723617
chr15	67542683	67807117	15q23	MAP2K5	15.97504232	10.37583697	1.539638909	4	4	62.82837325	23.9295917
chr1	44799952	44805995	1p34.1	PLK3	16.51327155	10.72585492	1.539576255	4	4	94.09870164	42.54367723
chr22	49960495	49964072	22q13.33	PIM3	32.44297005	21.07411757	1.539469918	4	4	80.55154902	34.5803176
chr2	152717693	152761253	2q23.3	ARL6IP6	23.34552199	15.17189422	1.538734824	4	4	51.28905862	21.49515546
chr8	104379424	104467074	8q22.3	DPYS	0.103590398	0.067323772	1.538689751	2	2	66.22355825	0.150181043
chr3	39096599	39108363	3p22.2	GORASP1	3.948240525	2.566140255	1.538591087	4	4	47.14733936	36.58104849
chr20	35115357	35147358	20q11.22	EDEM2	22.17573553	14.4157761	1.538296334	4	4	62.96541429	34.5106873
chr11	313991	315272	11p15.5	IFITM1	217.1005223	141.1562941	1.538015175	4	4	45.24597008	33.43620412
chr1	235661031	235883708	1q42.3	LYST	79.47454702	51.67383753	1.538003578	4	4	41.04141793	24.67646237
chr8	22165140	22212326	8p21.3	BMP1	0.365355918	0.237554111	1.537990298	4	4	98.37940334	65.50407217
chr7	97116593	97181763	7q21.3	SDHAF3	8.133380936	5.288420095	1.537960447	4	4	35.24035183	18.06468963
chr15	98648539	98964530	15q26.3	IGF1R	9.437609829	6.136516366	1.537942583	4	4	71.89885119	33.21854356
chr19	4502180	4520285	19p13.3	PLIN4	0.884388287	0.575224265	1.537466933	4	4	90.77744132	76.60724466
chr7	77798792	77957504	7q11.23-q21.11	PHTF2	9.851093702	6.407382934	1.537459803	4	4	2.229836807	19.64790939
chr1	42430329	42456267	1p34.2	ZMYND12	1.400340676	0.910820082	1.537450375	4	4	62.81427097	44.14578157
chr12	51245349	51270418	12q13.13	SMAGP	1.305016005	0.848821422	1.53744471	4	4	81.75300461	8.802168877
chr6	43620481	43631309	6p21.1	GTPBP2	19.14626443	12.45438166	1.537311522	4	4	50.7955545	22.52780849
chr15	74995535	75021495	15q24.2	SCAMP5	0.543846466	0.353764818	1.537310772	4	4	39.92621553	67.11426455
chr14	60981114	61083733	14q23.1	SLC38A6	7.442901916	4.841794803	1.53721961	4	4	61.8980065	68.26972052
chr9	110873252	111039259	9q31.3	LPAR1	6.252699057	4.067580887	1.537203372	4	4	31.34463777	42.32794544
chr6	166319728	166342606	6q27	SFT2D1	17.50270797	11.38614353	1.537193688	4	4	7.550981563	44.88340571
chr16	3953387	4116185	16p13.3	ADCY9	1.835921031	1.194392144	1.537117471	4	4	67.59804293	46.56747576
chr3	195863364	195909009	3q29	TNK2	6.580825382	4.281426401	1.537063765	4	4	39.29337813	25.90303302
chr4	99816824	99872290	4q23	DAPP1	80.34809499	52.28041455	1.536867978	4	4	23.71081395	48.81528591
chr5	75368486	75511981	5q13.3	COL4A3BP	22.38428582	14.56490756	1.536864256	4	4	34.38167469	27.26610507
chr1	200738905	200860707	1q32.1	CAMSAP2	1.815002253	1.181045046	1.536776484	4	4	75.75416943	63.8931595
chr10	28532588	28623112	10p12.1-p11.2	WAC	70.3887583	45.82912725	1.535895674	4	4	44.06861611	14.92194011
chr19	36139926	36150353	19q13.12	CAPNS1	72.43238983	47.16119649	1.535847163	4	4	51.69877457	40.336639

chr15	73994673	74047819	15q24.1	PML	4.743314101	3.089050379	1.535525006	4	4	60.96835005	42.44072864
chr2	72175984	72826042	2p13.2	EXOC6B	9.892757433	6.442818056	1.535470558	4	4	51.29624919	34.17054327
chr16	69565952	69704666	16q22.1	NFAT5	36.75024878	23.93719592	1.535277937	4	4	44.55944196	6.422083697
chr7	134646779	134679816	7q33	BPGM	392.7540201	255.8393281	1.535158894	4	4	57.4945921	58.98697185
chr1	2050470	2185399	1p36.33	PRKCZ	1.826267118	1.189715615	1.535045094	4	4	79.79432426	59.06278466
chr7	83956846	84515189	7q21.11	SEMA3A	0.099149316	0.064607136	1.53464962	4	3	89.84352388	20.15626116
chr3	81489699	81761799	3p12.2	GBE1	9.826714773	6.403877649	1.53449446	4	4	55.53183938	38.55977111
chr7	66376044	66400408	7q11.21	LINC00174	3.007281223	1.95981967	1.534468333	4	4	55.19088375	36.78809723
chr2	69893943	69905236	2p13.3	SNRNP27	20.64021756	13.45287434	1.534260787	4	4	51.0738213	16.81668257
chr7	23310209	23470674	7p15.3	IGF2BP3	7.691074612	5.01435013	1.53381284	4	4	21.64826144	35.49881036
chr6	70566922	70588903	6q13	SDHAF4	4.618033128	3.010995422	1.533723065	4	4	55.85996674	29.9570717
chr19	38647616	38730532	19q13.2	ACTN4	35.04459557	22.849432	1.533718456	4	4	70.48755612	43.9327186
chr17	8729928	8744836	17p13.1	CCDC42	0.222595294	0.145137621	1.533684329	3	1	26.1656225	0
chr7	90403334	90415954	7q21.13	CLDN12	0.342737256	0.223483715	1.533611773	4	4	52.68487785	46.0229403
chr9	73151731	73170393	9q21.13	ANXA1	103.5376605	67.5123161	1.533611443	4	4	34.91632888	18.36926123
chr13	27620743	27667422	13q12.2	POLR1D	25.1631459	16.4088496	1.533510668	4	4	22.53344592	35.84935431
chr16	2154782	2155358	16p13.3	SNHG19	5.614741472	3.661703975	1.533368484	4	4	65.19227533	58.66460465
chr14	67386979	67412200	14q23.3-q24.1	PLEK2	42.56414781	27.76065405	1.533254502	4	4	62.76557765	15.86039938
chr16	58548499	58548633	16q21	SNORA46	11.98462183	7.817288363	1.533091946	4	4	54.28428197	66.62006426
chr19	13906143	13930880	19p13.12	CC2D1A	4.43694452	2.894569084	1.532851485	4	4	48.23104997	46.05341622
chr13	19201437	19227435	13q12.11	PSPC1P1	17.47373328	11.40243316	1.532456541	4	4	75.81899321	46.64609068
chr1	11919588	11926428	1p36.22	KIAA2013	22.94727136	14.97433486	1.53244011	4	4	62.37488584	35.96557583
chr1	150796208	150808441	1q21.3	CTSK	2.249354638	1.467830276	1.532435101	4	4	59.52749824	54.44561678
chr8	133237171	133297587	8q24.22	NDRG1	13.80077323	9.007427813	1.532154741	4	4	64.43491167	35.11210145
chr5	95813849	95822873	5q15	GLRX	18.68271531	12.19485793	1.532015822	4	4	35.71998465	32.17001251
chr19	18442663	18522127	19p13.11	ELL	14.07524749	9.187636775	1.53197692	4	4	69.84257112	29.11200957
chrX	55009055	55031064	Xp11.21	ALAS2	3135.759584	2047.430935	1.531558174	4	4	60.76171994	77.04023197
chr14	99604476	99680569	14q32.2	HHIPL1	0.050554359	0.033012292	1.531379878	1	3	0	2.446932834
chr10	63133247	63155031	10q21.3	NRBF2	103.2266029	67.40931389	1.531340358	4	4	69.80087924	66.557495
chr19	38251237	38256591	19q13.2	PPP1R14A	0.793502942	0.518184809	1.531312627	4	3	80.99377767	33.85239924
chr16	90102264	90177606	16q24.3	FAM157C	1.823364746	1.190729628	1.531300392	4	4	77.31517347	69.35012356
chr17	75525080	75575209	17q25.1	LLGL2	2.055073316	1.342063245	1.531279039	4	4	60.72166165	34.28675673
chr8	9136254	9151642	8p23.1	PPP1R3B	41.32013571	26.99072122	1.530901504	4	4	68.94393163	61.8318096
chr11	46384792	46386608	11p11.2	CHRM4	0.835767556	0.545981174	1.530762591	1	2	0	33.68838499
chr1	149928651	149938329	1q21.2	MTMR11	4.184076616	2.733454301	1.530691994	4	4	37.60486858	40.67100367
chr1	173791549	173791887	1q25.1	RN7SKP160	1.164041753	0.760468262	1.530690775	2	2	40.39650961	6.905781833
chr2	74832655	74893354	2p12	HK2	16.94915786	11.073467	1.530609868	4	4	77.68285413	51.05861088
chr2	95807052	95826981	2q11.1	LINC00342	1.401840447	0.915944144	1.530486827	4	4	48.63961432	13.81866484
chr22	25564894	25729294	22q12.1	GRK3	22.34925672	14.60424181	1.530326395	4	4	53.82390359	29.72239214
chr6	163414486	163578596	6q26	QKI	34.27319898	22.39657043	1.530287821	4	4	29.68238866	26.7126066
chr1	225401502	225428855	1q42.12	LBR	153.2833834	100.1716439	1.530207326	4	4	38.20837564	24.19051318
chr10	99875571	100009953	10q24.2	DNMBP	3.250002467	2.123916764	1.530192954	4	4	50.65568598	36.68823944
chr7	75986837	75994674	7q11.23	TMEM120A	10.67015219	6.973250424	1.53015474	4	4	66.11157931	30.63391239

chr4	15701866	15774178	4p15.32	BST1	36.71303377	23.99435484	1.530069636	4	4	56.31368232	47.36629673
chr16	47154387	47461274	16q12.1	ITFG1	21.36996885	13.96730531	1.529999408	4	4	19.10083971	11.65032961
chr7	100140177	100154212	7q22.1	LAMTOR4	25.58101352	16.72213815	1.529769296	4	4	64.88349864	34.77765637
chr21	37073184	37203118	21q22.13	TTC3	20.96346991	13.70649799	1.529454856	4	4	33.67819288	25.84973397
chr7	5306790	5423843	7p22.1	TNRC18	11.83871901	7.740813169	1.529389581	4	4	85.59326282	40.18942736
chr8	9741672	9741768	8p23.1	MIR597	6.215495856	4.065315481	1.528908614	1	1	0	0
chr19	36182060	36215084	19q13.12	ZNF565	0.977702694	0.639530349	1.528782326	4	4	81.56175787	32.82031702
chr14	22503750	22503811	14q11.2	TRAJ37	21.92395835	14.34231994	1.528620087	3	4	10.1517633	58.39131692
chr5	78360503	78480739	5q14.1	SCAMP1	19.77880781	12.93913502	1.528603557	4	4	30.85748681	17.4364124
chr16	56191339	56357444	16q13	GNAO1	0.312263783	0.204291232	1.528522688	4	4	71.90452975	49.5259079
chr11	63624082	63671974	11q13.1	ATL3	27.92989175	18.27284056	1.528492062	4	4	19.89793154	23.33863155
chr2	2895048	3126026	2p25.3	LINC01250	0.196103587	0.12830119	1.528462727	2	1	69.05813957	0
chr11	9280654	9314768	11p15.4	TMEM41B	25.54752501	16.71524748	1.528396457	4	4	69.29298794	46.03556134
chr3	129087569	129122150	3q21.3	RAB43	1.492041757	0.976335061	1.528206675	4	4	85.46885789	47.8968469
chr1	65420448	65436007	1p31.3	LEPROT	40.35822448	26.41716561	1.527727277	4	4	16.40548474	19.33824003
chr4	144967117	145098541	4q31.21	ANAPC10	15.92142384	10.42206508	1.527664979	4	4	68.63360054	17.73494932
chr11	44565591	44620363	11p11.2	CD82	17.16486362	11.23621765	1.52763716	4	4	47.01732469	26.53993372
chr10	95036772	95069497	10q23.33	CYP2C8	0.15916096	0.104209063	1.527323582	3	3	34.16597434	9.247059807
chr19	45995457	46018616	19q13.32	CCDC61	1.629642775	1.067177501	1.527058782	4	4	91.81888661	57.61280102
chr9	34989641	34998433	9p13.3	DNAJB5	0.538369362	0.352558192	1.52703688	4	4	99.37055661	82.77662767
chr3	160100691	160102912	3q25.33	BRD7P2	0.338055218	0.221426389	1.526716033	1	3	0	67.72057889
chr15	82986203	82991057	15q25.2	RAMAC	19.15898985	12.5500488	1.526606801	4	4	56.71123258	18.67268937
chr15	100919017	101072890	15q26.3	LRRK1	1.941953194	1.272136588	1.526528843	4	4	43.95975997	35.92777364
chr5	71035124	71109113	5q13.2	GTF2H2	10.64714943	6.974802165	1.526516334	4	4	78.39872704	53.62981067
chr10	63521353	63625123	10q21.3	REEP3	18.1020922	11.86125897	1.526152684	4	4	12.84872232	23.1459946
chr5	139278780	139278979	5q31.2	SNORA74A	276.442564	181.1411125	1.526117181	4	4	69.23027922	18.60204612
chr6	63719980	65707225	6q12	EYS	0.090651173	0.059401464	1.526076394	4	3	96.56795395	63.94880498
chr10	87863438	87971930	10q23.31	PTEN	58.02940283	38.03205657	1.525802391	4	4	13.55999131	30.78393056
chr12	51391317	51515763	12q13.13	SLC4A8	0.53135417	0.34825338	1.525768885	4	4	55.78538134	44.27123201
chr4	184365561	184382306	4q35.1	LINC02362	1.79877031	1.178936809	1.525756339	4	4	92.04155628	66.1753093
chr10	104339124	104455090	10q25.1	CFAP58	1.598666101	1.047939102	1.5255334	4	4	67.62455311	23.39287922
chr3	32685145	32773875	3p22.3	CNOT10	44.85901152	29.40896587	1.525351544	4	4	64.08645967	23.31212092
chr1	42176539	42336805	1p34.2	FOXJ3	45.88794917	30.08423436	1.525315507	4	4	45.80687936	29.75135025
chr9	81583683	81689535	9q21.32	TLE1	4.021048252	2.636293089	1.525266014	4	4	57.83780945	29.54434198
chr14	61681041	61695823	14q23.2	HIF1A-AS1	0.640648045	0.420063509	1.525121873	3	2	38.83908678	21.85587995
chr7	100826874	100867012	7q22.1	SLC12A9	10.76572324	7.059397416	1.525020141	4	4	63.30254206	42.0955946
chr17	64039142	64132469	17q23.3	ERN1	14.17806959	9.297080276	1.525002384	4	4	38.00188982	9.287032675
chr11	532242	535567	11p15.5	HRAS	1.300861886	0.853219483	1.524650939	4	4	91.05640722	41.05680055
chr9	32384603	32450836	9p21.1	ACO1	1.939861471	1.272374879	1.524599002	4	4	39.96322809	54.84980921
chr4	102631486	102760994	4q24	MANBA	36.46953521	23.92562586	1.524287616	4	4	22.59266246	35.25053912
chr16	11965107	11968068	16p13.13	TNFRSF17	2.739257673	1.797135199	1.524235725	4	4	48.53789036	93.56667334
chr21	45074578	45226563	21q22.3	ADARB1	2.835984149	1.860736254	1.52411936	4	4	28.90498697	32.47343247
chr2	113235527	113267023	2q14.1	PAX8-AS1	0.958916521	0.629302324	1.523777182	3	4	61.23724382	64.4240304

chr2	68834374	68837726	2p13.3	LINC01888	3.916366638	2.570244324	1.523733211	4	3	57.01141066	34.1408327
chr8	94371960	94475115	8q22.1	RAD54B	1.013111352	0.664902171	1.52369987	3	4	85.77828872	42.95822364
chr4	6670728	6673881	4p16.1	LINC02482	2.309961337	1.51610079	1.523619902	3	4	52.28419013	58.10346816
chr1	52368480	52369345	1p32.3	PLA2G12AP1	0.851023509	0.558635118	1.523397798	1	1	0	0
chr17	7012417	7014533	17p13.1	RNASEK	5.086321534	3.338825872	1.523386283	4	4	60.63544691	38.00766672
chr7	897900	955407	7p22.3	ADAP1	1.653364982	1.085392093	1.523288214	4	4	67.7067307	32.35973542
chrX	19343893	19361707	Xp22.12	PDHA1	10.73142009	7.046802053	1.522878039	4	4	55.64445844	42.74301609
chr4	3941760	3955428	4p16.3	FAM86EP	0.562966321	0.369721193	1.522677985	2	3	82.49775358	121.6531376
chr20	4780023	4823668	20p13	RASSF2	202.2817753	132.8538189	1.52258909	4	4	74.41665495	48.9316311
chr8	22578741	22598025	8p21.3	PDLIM2	3.054255279	2.006331639	1.522308286	4	4	60.99713428	36.02154595
chr2	200853009	200864744	2q33.1	CLK1	177.9780452	116.9136977	1.522302765	4	4	58.08688465	42.52805312
chr2	49918505	51032536	2p16.3	NRXN1	0.293446589	0.192789657	1.522107533	4	3	23.2387541	22.23882476
chr8	42836674	42843331	8p11.21	THAP1	15.72076157	10.32850441	1.522075312	4	4	36.59219076	33.98896996
chr12	56510941	56513190	12q13.3	HSPD1P4	0.212503804	0.139620967	1.522004955	2	1	30.85568824	0
chr6	73394562	73417566	6q13	DDX43	1.006171882	0.66110154	1.521962696	4	4	63.55477427	77.36123982
chr20	50081124	50115959	20q13.13	UBE2V1	4.898463669	3.218537361	1.521953335	4	4	64.81438431	51.07780987
chr7	143407813	143523449	7q35	EPHA1-AS1	0.603578613	0.3965834	1.521946237	4	4	113.7209868	128.2765281
chr3	136430084	136430183	3q22.3	RNU6-1284P	4.320941766	2.839149986	1.521913878	3	1	36.05102318	0
chr18	8706054	8832778	18p11.22	MTCL1	0.190826204	0.12539176	1.521840066	3	4	42.8138946	88.10456557
chr19	16355244	16472012	19p13.11	EPS15L1	10.73993552	7.057585704	1.521757718	4	4	42.96283905	17.31764059
chr12	101877327	101923623	12q23.2	DRAM1	9.651553765	6.345242307	1.521069377	4	4	24.50848203	30.7105659
chr4	82430480	82461175	4q21.22	ENOPH1	31.85013089	20.94016236	1.521006874	4	4	52.03094933	23.76277494
chrX	141118031	141118136	Xq27.1	RNU6-3P	43.83335443	28.81906394	1.520984669	4	4	32.08278054	50.35834562
chr14	103733751	103849327	14q32.33	PPP1R13B	1.114986443	0.733082803	1.520955667	4	4	45.68248614	49.56740256
chr4	140620782	140756317	4q31.21	TBC1D9	12.18579592	8.012152802	1.520914068	4	4	74.03722979	38.90338322
chr1	156033129	156053825	1q22	UBQLN4	4.781727991	3.14399302	1.520909226	4	4	31.33742951	45.93145453
chr17	4731408	4733610	17p13.2	MED11	9.370583543	6.16120265	1.520901693	4	4	63.12612304	46.21619963
chr10	1177313	1737476	10p15.3	ADARB2	0.523471207	0.344189168	1.520882283	2	2	134.0062523	55.45981556
chr3	44337465	44409448	3p21.31	TCAIM	4.401435665	2.894178774	1.520789146	4	4	29.41551177	14.15238753
chr14	50905217	50944530	14q22.1	PYGL	102.3136315	67.28173073	1.520674787	4	4	69.34166781	63.35239575
chr16	75566351	75577881	16q23.1	GABARAPL2	234.7049934	154.3498721	1.520603744	4	4	35.04840976	42.49607089
chr14	75013764	75051532	14q24.3	MLH3	11.74977085	7.728341102	1.520348377	4	4	51.32719791	37.71177277
chr17	7626234	7627878	17p13.1	SAT2	9.652021671	6.348720126	1.520309839	4	4	64.47044286	8.774755827
chr12	52821447	52834293	12q13.13	KRT79	1.20142617	0.790314755	1.520186942	4	3	86.15747156	55.2649558
chr2	105037469	105099960	2q12.1	MRPS9	34.08903527	22.42789482	1.519939145	4	4	58.55896035	19.52303093
chr15	66334974	66387273	15q22.31	TIPIN	33.66742229	22.15113791	1.519895837	4	4	84.74024255	46.46686466
chr10	117280489	117375492	10q25.3-q26.11	PDZD8	36.7763084	24.19846922	1.5197783	4	4	30.4794232	29.77573842
chr22	40410281	40636862	22q13.1-q13.2	MRTFA	20.26156316	13.33209248	1.519758672	4	4	46.30949452	30.61406569
chr6	34889261	35097992	6p21.31	ANKS1A	4.584000833	3.016645539	1.51956893	4	4	50.25408238	40.51029247
chr1	31518435	31524245	1p35.2	LINC01226	0.066146524	0.043529866	1.519566472	2	3	16.10681561	32.42796474
chr1	186680654	186681446	1q31.1	PACERR	3.601218328	2.369986515	1.51951005	4	4	46.0489172	112.2123924
chr16	66552563	66566287	16q21	CKLF	14.19869405	9.34572223	1.519271994	4	4	19.33319309	78.90106316
chr6	132743503	132763489	6q23.2	VNN2	502.9971875	331.0903263	1.519214388	4	4	92.00874564	71.55721144

chr3	50267558	50277171	3p21.31	SEMA3B	0.227047085	0.149493094	1.518779757	2	2	44.74976902	11.16941069
chr19	15380048	15419121	19p13.12	AKAP8L	19.88797056	13.09513344	1.518729889	4	4	71.2448709	13.08996379
chr2	189923169	189924890	2q32.2	HNRNPCP2	3.292723996	2.168099442	1.51871447	4	4	27.6823076	53.45259629
chr7	93099513	93118023	7q21.2	SAMD9	191.8556958	126.3331537	1.518648828	4	4	22.67307965	17.35720873
chr3	196347659	196454323	3q29	UBXN7	142.4304176	93.79630509	1.518507765	4	4	40.7188025	15.18827106
chr11	65497738	65506516	11q13.1	MALAT1	1539.447356	1013.844768	1.518425112	4	4	54.30734218	31.93368207
chr18	12912803	12914106	18p11.21	EIF4A2P1	0.798786811	0.526073981	1.518392545	1	4	0	67.47567239
chr12	55725443	55729673	12q13.2	CD63	46.9083187	30.89388906	1.518368847	4	4	41.80578346	26.70830224
chr9	5450503	5470567	9p24.1	CD274	11.56907143	7.619515563	1.518347372	4	4	100.9921449	85.22737793
chr9	112217715	112333669	9q32	PTBP3	155.9533154	102.7238361	1.518180407	4	4	31.35616104	18.43130737
chr15	43800421	43802589	15q15.3	HYPK	1.394910439	0.918909929	1.518005622	4	4	86.42079529	46.52276681
chr7	100466519	100479279	7q22.1	TSC22D4	40.63472001	26.77142233	1.517839415	4	4	53.54053509	23.46124397
chr11	70283955	70284070	11q13.3	MIR548K	3.250385615	2.141470762	1.517828622	1	1	0	0
chr18	46104332	46128333	18q21.1	HAUS1	97.12402163	63.99366576	1.51771305	4	4	92.32687022	25.72761983
chr3	44648741	44660791	3p21.31	ZNF35	0.487206068	0.321043892	1.517568408	2	3	110.2754225	84.40645997
chr15	41828085	41848148	15q15.1	JMJD7-PLA2G4B	0.388289714	0.255895599	1.517375508	3	4	24.84346694	16.97553293
chr2	27123780	27131114	2p23.3	ABHD1	0.088022536	0.058015561	1.517222867	2	2	38.88709406	6.754229747
chr10	119104065	119137984	10q26.11	FAM45A	11.52627937	7.59775508	1.517063823	4	4	45.88950693	37.21897063
chr4	121667997	121696994	4q27	ANXA5	105.9814017	69.86089808	1.51703463	4	4	68.2042963	46.26774739
chr1	109603091	109619733	1p13.3	GNAT2	0.387766617	0.255644092	1.516822133	2	2	25.75114194	43.88087922
chr2	187464230	187554505	2q32.1	TFPI	7.289538866	4.805952865	1.516772859	4	4	47.35997345	54.77045236
chr3	38038595	38122741	3p22.2	DLEC1	0.397140707	0.261871775	1.516546437	4	4	37.08543454	43.27432117
chr4	38791055	38806262	4p14	TLR1	147.3304377	97.1582471	1.516396622	4	4	56.04323878	47.48973403
chr8	78533892	78558503	8q21.13	PKIA-AS1	0.216068798	0.142527628	1.515978353	1	2	0	31.7167931
chr8	79764010	79767863	8q21.13	HEY1	0.736584018	0.485907043	1.515894918	4	4	68.22597691	65.35762714
chr9	34634722	34637826	9p13.3	SIGMAR1	7.87334119	5.193895719	1.515883571	4	4	41.37484478	48.79746769
chr6	11183298	11382348	6p24.2	NEDD9	31.87924278	21.03177382	1.515765767	4	4	47.25202706	31.07530931
chr1	120942600	120942763	1p11.2	RNVU1-4	17.12415142	11.29899623	1.515546255	4	4	99.88135766	44.32580784
chr1	201483530	201507259	1q32.1	CSRP1	4.504102861	2.971949101	1.515538358	4	4	61.58394273	35.72313632
chr14	63684216	63728108	14q23.2	SGPP1	28.47483278	18.7904498	1.515388567	4	4	12.71687196	21.9592351
chr19	6210381	6279948	19p13.3	MLLT1	9.82720059	6.485213263	1.515324198	4	4	77.69276443	25.65151983
chr2	110513802	110610903	2q13	RGPD6	2.870259905	1.8943114	1.515199615	4	4	97.30972774	50.94849121
chr5	39105252	39274500	5p13.1	FYB1	320.1606285	211.3210614	1.515043633	4	4	63.32341121	34.69287826
chr6	31686949	31703360	6p21.33	ABHD16A	0.84605832	0.558439556	1.515040098	4	4	38.30799343	26.95979643
chr4	52590960	52659335	4q12	USP46	2.027334859	1.338292596	1.514866678	4	4	53.1798646	33.93199716
chr10	97766751	97771999	10q24.2	SFRP5	0.239106571	0.157842226	1.514845408	2	1	11.965316	0
chrX	15790451	15826807	Xp22.2	ZRSR2	20.45029656	13.50003655	1.51483268	4	4	41.50203183	14.82642335
chr19	36743478	36772814	19q13.12	ZNF850	7.784934258	5.139162919	1.514825348	4	4	82.90748686	50.0103504
chr1	166913204	166975324	1q24.1	ILDR2	0.185427042	0.122411171	1.514788569	3	4	70.34522656	63.75169805
chr12	104802553	104958744	12q23.3	SLC41A2	1.466496038	0.96830848	1.514492611	4	4	50.21326192	58.69086176
chr6	146543692	146554956	6q24.3	RAB32	28.73232455	18.97168173	1.51448485	4	4	39.0626308	17.5797309
chr1	46607715	46616891	1p33	MOB3C	9.18928257	6.069143823	1.514098667	4	4	44.68792618	25.49185601
chr6	108294874	108525784	6q21	AFG1L	2.408757126	1.590943742	1.514042931	4	4	49.3688867	44.06473128

chr10	119140913	119165728	10q26.11	SFXN4	6.880614719	4.545466436	1.513731278	4	4	38.10414719	26.88611415
chr4	37891084	38139175	4p14	TBC1D1	26.93650237	17.80258236	1.51306714	4	4	26.30825177	25.19994521
chr3	19148525	19535643	3p24.3	KCNH8	0.226655163	0.149804973	1.513001595	3	4	78.02052088	75.17510814
chr1	16467436	16492701	1p36.13	CROCCP3	0.493902199	0.326460131	1.512902045	4	4	24.90369281	28.63216047
chrX	101623130	101631910	Xq22.1	ARMCX3	14.35888886	9.492185973	1.51270623	4	4	26.78778629	22.04232558
chr3	28322353	28349278	3p24.1	AZI2	14.62764513	9.671419027	1.512461107	4	4	34.06219027	25.02664951
chr5	91302986	91314402	5q14.3	LUCAT1	18.66053407	12.33802851	1.512440505	4	4	68.24488596	61.65798123
chr1	161100556	161118076	1q23.3	PFDN2	24.24433433	16.03175693	1.512269331	4	4	34.48959282	18.31243094
chr8	73780097	73878910	8q21.11	UBE2W	21.31651858	14.09752322	1.512075437	4	4	26.74510093	46.69359979
chr17	82942149	83051845	17q25.3	B3GNTL1	1.595728566	1.055536703	1.511769853	4	4	78.51040782	56.78865874
chr4	24803514	24980204	4p15.2	CCDC149	1.204102781	0.796500783	1.511740862	4	4	89.35811975	74.74435693
chr5	111729303	111976931	5q22.1	NREP	2.338253189	1.547061678	1.511415623	4	4	42.87979442	54.05006086
chr13	19557832	19565196	13q12.11	CASC4P1	0.307225973	0.20327877	1.511352973	2	1	3.862850994	0
chr4	94451857	94668227	4q22.3	PDLIM5	12.82062201	8.483180473	1.511298982	4	4	27.22310139	19.64829113
chr21	46161148	46185163	21q22.3	SPATC1L	0.939914177	0.621966822	1.511196648	4	4	66.03519376	72.89001599
chr9	113165036	113221361	9q32	FKBP15	15.12665337	10.01042827	1.511089532	4	4	45.53738635	31.66839652
chr7	72574507	72828234	7q11.22-q11.23	TYW1B	10.27465522	6.799675917	1.511050724	4	4	57.100111	29.52351518
chr10	77790787	77926590	10q22.3	DLG5	0.487683474	0.322765207	1.510954288	4	4	75.61242014	34.31544466
chr22	22900976	22901295	22q11.22	IGLC2	144.6105934	95.72492252	1.510689062	4	4	88.63787483	139.8574211
chr4	74445098	74455009	4q13.3	AREG	6.732155586	4.456967545	1.510478934	4	4	106.5804418	79.07418543
chr7	6496778	6551436	7p22.1	GRID2IP	0.092635893	0.061340249	1.510197543	2	2	31.11529015	1.465306261
chr10	73998114	74120160	10q22.2	VCL	86.31543022	57.15508911	1.510196757	4	4	65.96088342	12.57074698
chr1	40804846	40805141	1p34.2	RN7SL326P	1.400917869	0.927746414	1.510022402	1	1	0	0
chrX	72301638	72307187	Xq13.1	CITED1	0.218663053	0.144817543	1.509921024	2	1	21.04757545	0
chr17	75898643	75905100	17q25.1	MRPL38	0.454256074	0.3008595	1.509861161	4	4	62.24047001	37.92442285
chr16	15643267	15726353	16p13.11	NDE1	29.37052119	19.45264445	1.509847222	4	4	14.1581195	10.42278654
chr3	44498970	44510640	3p21.31	ZNF852	0.487614156	0.322958325	1.509836156	4	4	58.21380101	81.17407019
chr4	77048012	77075972	4q21.1	CCNI	288.6595004	191.1871684	1.509826746	4	4	10.3089897	12.95382769
chr12	108645109	108759955	12q24.11	CORO1C	153.0249903	101.3729729	1.509524539	4	4	45.43364196	35.81073386
chr6	28281537	28303681	6p22.1	PGBD1	0.465380237	0.308325396	1.509380164	3	4	41.02427886	45.74408466
chr21	33432486	33480009	21q22.11	TMEM50B	5.967090872	3.954935979	1.508770535	4	4	45.39044722	35.20148892
chr14	106762092	106762530	14q32.33	IGHV1-69D	9.366494204	6.208368208	1.508688578	2	2	9.53370972	54.33890099
chr4	89879539	89954629	4q22.1	MMRN1	21.09523943	13.98362262	1.508567558	4	4	28.10594726	24.38840913
chr12	55992547	56005525	12q13.2	SUOX	1.493429844	0.989987758	1.508533647	4	4	71.06553621	69.35171315
chr15	41231156	41281887	15q15.1	CHP1	54.38423235	36.05209643	1.50849015	4	4	32.39742537	11.48064436
chr7	98281667	98322398	7q21.3	BRI3	10.89086986	7.220057897	1.508418633	4	4	46.41341597	50.87987877
chr1	160027672	160031993	1q23.2	PIGM	3.543295263	2.349225931	1.508282033	4	4	47.12507292	17.15860884
chr10	131092392	131311721	10q26.3	TCERG1L	0.241072381	0.159872827	1.507900903	1	2	0	94.91096297
chr4	89836401	89841991	4q22.1	SNCA-AS1	0.413861579	0.274469719	1.507858789	3	4	110.0707743	63.81256034
chr1	162395266	162412138	1q23.3	SH2D1B	16.227578	10.76218766	1.507832656	4	4	63.65136142	35.57086574
chr19	11346483	11354951	19p13.2	CCDC159	2.891351213	1.917767802	1.507664906	4	4	66.32546462	31.8604983
chr7	100867328	100873454	7q22.1	TRIP6	0.754650029	0.500689935	1.507220291	4	4	74.31994568	61.35329831
chr17	78168545	78172928	17q25.3	SYNGR2	13.88368717	9.211677791	1.507183326	4	4	68.95563617	39.03109036

chr12	9239467	9239551	12p13.31	MIR1244-3	4.79158882	3.179338771	1.507102315	1	2	0	8.151343044
chr16	30357101	30370466	16p11.2	TBC1D10B	7.441400113	4.938035685	1.506955516	4	4	62.57226443	40.25511931
chr10	75210154	75231448	10q22.2	VDAC2	38.46464686	25.53108739	1.506580831	4	4	51.57112984	11.87110333
chr7	128790762	128822133	7q32.1	CCDC136	0.399877966	0.265432065	1.506517179	4	4	64.08923432	41.77050267
chr3	196708859	196708964	3q29	RNU6-646P	3.547710811	2.355371643	1.506221246	1	1	0	0
chrX	38269163	38327542	Xp11.4	RPGR	10.28943818	6.831416222	1.506194008	4	4	30.01743922	41.24301341
chr1	151847101	151853697	1q21.3	THEM5	3.294898506	2.187942811	1.505934474	4	4	73.32439751	47.73192917
chr10	100237156	100237302	10q24.31	SNORA12	1074.105413	713.2702165	1.505888496	4	4	34.50390789	21.84513245
chr2	86720291	86778041	2p11.2	RMND5A	94.8305696	62.97432854	1.50586075	4	4	13.40808807	13.73252776
chr19	4360367	4400568	19p13.3	SH3GL1	16.34217406	10.85278945	1.505804027	4	4	18.66575249	33.04215045
chr17	76617769	76643838	17q25.1	ST6GALNAC1	0.863778768	0.573698843	1.505631009	4	4	60.85771355	42.32612195
chr19	3359563	3469217	19p13.3	NFIC	1.635544183	1.086320076	1.505582213	4	4	88.93307108	45.45829717
chrX	111617654	111621516	Xq23	EIF4BP7	0.604035829	0.401291086	1.505231118	4	4	87.30192685	50.87676794
chr12	116533422	116536513	12q24.22	LINC00173	1.716342346	1.140379381	1.505062591	4	4	65.0741328	28.58676441
chr2	25160853	25168851	2p23.3	POMC	0.948461726	0.630366225	1.504620153	4	4	111.0743852	81.40249049
chr6	43182170	43224587	6p21.1	CUL9	2.857131251	1.898906022	1.504619617	4	4	56.66134847	14.7494244
chr1	99968401	100026979	1p21.2	SLC35A3	2.221058634	1.476254945	1.5045224	4	4	44.75817887	28.07138029
chr3	130516709	130678137	3q22.1	COL6A6	0.081848252	0.05440172	1.504515892	1	3	0	48.90303959
chr11	7513765	7666642	11p15.4	PPFIBP2	2.07245322	1.377632929	1.504358074	4	4	69.33406713	32.2127007
chr12	110360452	110360746	12q24.11	RN7SL769P	1.789150434	1.189324379	1.504341847	1	1	0	0
chrX	129980285	130058083	Xq26.1	BCORL1	1.229890177	0.817573933	1.504316769	4	4	84.17933746	59.18838607
chr2	231198546	231394991	2q37.1	ARMC9	0.106148175	0.07056599	1.504239868	4	4	30.04289481	54.17976436
chr3	45689241	45745425	3p21.31	SACM1L	25.81070628	17.15924027	1.504187008	4	4	28.61502994	18.71905111
chr4	139453232	139475916	4q31.1	RAB33B	22.8971546	15.22265385	1.504149987	4	4	46.74626775	60.34113172
chr1	9629889	9728925	1p36.22	PIK3CD	81.30445509	54.05823982	1.504015953	4	4	61.3740225	32.34709368
chr20	45363169	45410610	20q13.12	SYS1-DBNDD2	0.280268582	0.186349121	1.50399734	1	2	0	2.654126003
chr2	60756230	60802086	2p16.1	PAPOLG	29.75712246	19.78554006	1.503983331	4	4	26.93249599	14.03598435
chr2	89085177	89085690	2p11.2	IGKV3-15	8.412930756	5.593830031	1.503966104	4	3	58.30727878	116.679407
chr7	151232489	151238827	7q36.1	CHPF2	16.32575105	10.85530372	1.503942356	4	4	45.7710859	10.75286151
chr11	71997944	72002919	11q13.4	IL18BP	1.951535262	1.297636619	1.503915067	4	4	79.03698605	40.19822344
chr11	2268498	2270952	11p15.5	ASCL2	4.188622564	2.785496201	1.503725822	4	4	40.81407081	40.75510888
chr14	106210936	106211391	14q32.33	IGHV3-20	1.767742212	1.175974077	1.503215288	3	2	90.4958365	45.30033369
chr17	64319415	64413844	17q23.3	PECAM1	115.1149716	76.58397276	1.50312092	4	4	70.94976151	43.87376058
chr7	141986517	142106747	7q34	MGAM	54.43052365	36.21315027	1.503059614	4	4	64.3723579	75.05759448
chr12	102073962	102120131	12q23.2	NUP37	13.25699325	8.821473926	1.502809323	4	4	53.21366375	19.71783877
chr10	26438324	26567879	10p12.1	APBB1IP	98.92364733	65.83981523	1.502489747	4	4	36.7897708	27.34319335
chr2	151372012	151372284	2q23.3	RN7SL124P	1.46285739	0.973632496	1.502473876	2	3	23.72236228	24.83764712
chr17	7459366	7484249	17p13.1	ZBTB4	0.671750709	0.44710107	1.502458291	4	4	77.14597143	57.13114737
chr20	63553020	63556712	20q13.33	FNDC11	0.152625232	0.101585647	1.502429105	3	2	39.49275243	41.64392752
chr10	73436428	73496024	10q22.2	PPP3CB	35.740574	23.78892361	1.502404001	4	4	45.44520144	15.48640994
chr2	69962263	70087015	2p13.3	PCBP1-AS1	5.917401152	3.939307944	1.502142315	4	4	18.85909269	45.56747363
chr10	127907053	128085855	10q26.2	PTPRE	38.06159258	25.33880515	1.502106842	4	4	59.51438281	21.23562214
chr2	143945045	144333069	2q22.3	GTDC1	28.514567	18.98341143	1.502078123	4	4	65.32300197	33.41400378

chr20	43590613	43647296	20q13.12	IFT52	20.37049412	13.56187616	1.502041007	4	4	59.70685284	23.26783144
chr15	40285496	40307973	15q15.1	PLCB2	26.60762953	17.71460794	1.502016281	4	4	68.15420185	25.22729187
chr19	40691529	40716885	19q13.2	COQ8B	3.435733645	2.287472194	1.501978321	4	4	58.29092263	49.43723695
chr1	104153329	104154037	1p21.1	FTLP17	0.660633983	0.43984798	1.501959796	1	1	0	0
chr15	74541177	74598131	15q24.1	ARID3B	7.244756835	4.823659713	1.50192121	4	4	48.69029947	40.45073623
chr6	109165831	109354948	6q21	CCDC162P	0.162387622	0.10812223	1.501889312	4	4	43.71062192	92.1077026
chr19	6729914	6737622	19p13.3	GPR108	16.52619904	11.00378482	1.501864976	4	4	66.73300296	24.79740553
chr17	29560661	29567024	17q11.2	ABHD15	7.313864905	4.86993653	1.501839882	4	4	47.6527704	13.49938622
chr1	46635039	46668427	1p33	ATPAF1	7.891823538	5.254889825	1.501805709	4	4	18.89153856	5.638063072
chr6	26195566	26195771	6p22.2	HIST1H1PS1	1.798658432	1.197675251	1.501791433	3	4	76.77088818	51.71126999
chr17	31518441	31518729	17q11.2	RN7SL45P	1.989011555	1.324474459	1.501736437	3	2	60.50913665	60.76573999
chr3	27372721	27484420	3p24.1	SLC4A7	15.64839506	10.42058149	1.50168156	4	4	22.87373362	20.01359044
chr6	30600400	30618607	6p21.33	PPP1R10	12.18741434	8.115885167	1.501674074	4	4	46.6723831	33.31370514
chr3	24117153	24495281	3p24.2	THRB	0.791767715	0.527257006	1.501673197	4	4	44.8548237	79.6534647
chr15	41828085	41837587	15q15.1	JMJD7	0.532978575	0.354929507	1.501646282	3	4	18.20812914	47.64846942
chr1	161039251	161045979	1q23.3	USF1	41.79219851	27.83093571	1.501645469	4	4	71.74002392	30.08017667
chr5	134905120	134962646	5q31.1	PCBD2	4.257436288	2.835624728	1.501410341	4	4	78.86100432	51.62726652
chr22	29437015	29442129	22q12.2	RFPL1S	0.263782002	0.175694977	1.501363366	4	4	69.37330578	58.82724134
chr13	109753837	109786567	13q34	IRS2	16.0516323	10.69195213	1.501281722	4	4	57.98151761	63.86274855
chr1	77847080	77848999	1p31.1	NSRP1P1	0.94810187	0.631668589	1.500948261	3	4	69.35067755	58.52064243
chr8	143039209	143054303	8q24.3	C8orf31	0.260598654	0.173626769	1.500912882	2	1	18.39582877	0
chr6	116452817	116453655	6q22.1	CBX3P9	0.718358322	0.478653009	1.500791406	2	1	19.39325596	0
chr14	71448689	71448958	14q24.2	RN7SL683P	3.670524019	2.445895622	1.500687104	4	3	78.55911771	74.62858571
chr19	35738801	35742450	19q13.12	IGFLR1	4.451980381	2.966885854	1.500556678	4	4	89.38315871	60.41547865
chr14	60144119	60169864	14q23.1	DHRS7	37.86714776	25.23715984	1.500452032	4	4	52.37773979	41.06271483
chr12	8254427	8266638	12p13.31	ENPP7P5	0.542573078	0.361658662	1.500235262	3	2	73.01638416	60.86462603
chr12	112418898	112509918	12q24.13	PTPN11	60.5598388	40.37894346	1.499787602	4	4	27.73935329	13.08966184
chr7	33036212	33036382	7p14.3	RPS29P14	5.117274344	3.412333401	1.499640786	3	4	60.98261963	50.91492289
chr10	27110111	27155051	10p12.1	YME1L1	87.04730004	58.04604727	1.499624938	4	4	48.77965145	21.10873988
chr10	29545004	29545097	10p11.23	MIR604	6.461065018	4.308568022	1.499585242	2	1	55.05173879	0
chr19	56087363	56121373	19q13.43	ZNF787	5.986956803	3.993352736	1.499230646	4	4	74.45760572	22.39241357
chr1	39084167	39487138	1p34.3	MACF1	27.6848681	18.466631	1.499183479	4	4	59.77813464	20.25555055
chr16	80975795	81007036	16q23.2	CMC2	2.45836904	1.63989334	1.499103009	4	4	35.11652949	22.9761778
chr15	28737583	28758362	15q13.1	WHAMMP2	8.344661087	5.566754113	1.499017366	4	4	31.14134006	42.92935631
chr9	87497228	87708634	9q21.33	DAPK1	11.04037146	7.366890764	1.498647369	4	4	76.81673997	59.0815417
chr1	154405193	154469450	1q21.3	IL6R	91.97134102	61.37119024	1.498607745	4	4	57.74345785	48.89692918
chr19	38899503	38908894	19q13.2	NFKBIB	3.187697146	2.127647596	1.498226093	4	4	59.02098665	82.44660314
chr22	40586625	40588023	22q13.1	RPL4P6	2.944688495	1.965512186	1.498178701	4	4	21.50823861	46.89312392
chr4	40502040	40502119	4p14	MIR4802	4.451661756	2.971407392	1.498166077	1	1	0	0
chrY	12903999	12920478	Yq11.221	DDX3Y	102.9772207	68.73697781	1.49813425	2	2	50.52758723	19.01108454
chr11	4384897	4393696	11p15.4	TRIM21	68.8110811	45.93397286	1.498043318	4	4	70.6406069	48.66975663
chr6	32178385	32180793	6p21.32	RNF5	13.76199301	9.186811741	1.498016222	4	4	14.69094204	44.6652708
chr2	233059967	233207903	2q37.1	INPP5D	31.43456961	20.98546305	1.497921182	4	4	45.5344656	36.76403925

chr12	94567124	94650562	12q22	TMCC3	29.43224479	19.65023815	1.497806009	4	4	50.67145646	53.8465453
chr1	151148496	151165948	1q21.3	LYSMD1	0.894946234	0.597517752	1.497773467	3	4	54.32438272	83.707061
chr14	21392151	21392253	14q11.2	SNORD9	24.29465322	16.22106056	1.497722861	3	3	93.97721529	41.26360994
chr2	219627570	219641980	2q35	SLC4A3	0.159070835	0.106210492	1.497694179	2	2	89.72325221	12.88142967
chr5	140401814	140549578	5q31.3	ANKHD1-EIF4EBP3	0.458611813	0.306212367	1.497692	4	4	51.64032526	57.26160492
chr2	195737604	196068812	2q32.3	DNAH7	0.084358046	0.056329774	1.497574719	4	4	70.67220988	60.77844193
chr14	91126426	91126552	14q32.11	SNORA11B	4.138731592	2.76422498	1.497248459	2	4	12.75131235	46.38014795
chr8	2045043	2145456	8p23.3	MYOM2	1.988826466	1.328434632	1.497120308	4	4	74.91913102	134.1143562
chr9	68780006	68783879	9q21.11	FAM122A	15.94162871	10.64853991	1.49707179	4	4	10.38027789	16.72692144
chr9	33110641	33167358	9p21.1	B4GALT1	30.11000513	20.1147788	1.496909583	4	4	62.4091448	28.19676879
chr5	95652181	95731848	5q15	SPATA9	0.35009882	0.233881535	1.496906628	2	2	47.02710825	88.57640135
chr3	170222432	170305982	3q26.2	PRKCI	7.497649224	5.00877898	1.496901591	4	4	48.14879706	40.27637019
chr8	106697427	106698040	8q23.1	TAGLN2P1	0.672643232	0.449379246	1.496827542	2	1	63.03112216	0
chr10	79703227	79712757	10q22.3	NUTM2B	0.075024517	0.050128284	1.496650415	2	1	33.4967931	0
chr17	49289206	49362473	17q21.32-q21.33	ZNF652	39.04126812	26.08792129	1.496526599	4	4	14.9431083	20.76809112
chr6	149566208	149591751	6q25.1	GINM1	53.99185514	36.07880918	1.496497705	4	4	44.0605849	39.65036321
chr3	37987974	38007188	3p22.2	VILL	1.776307618	1.187090084	1.496354524	4	4	65.72478135	15.01190688
chr12	25107057	25195162	12p12.1	CASC1	1.184622514	0.79168705	1.496326756	4	4	58.89796772	79.03175213
chr12	56202504	56221982	12q13.3	RNF41	10.78060987	7.206241278	1.496010119	4	4	46.73117	17.27438216
chr19	4815924	4831742	19p13.3	TICAM1	3.738269015	2.498855122	1.495992698	4	4	49.28789901	59.18937266
chr12	113221429	113298589	12q24.13	TPCN1	3.769069944	2.519792155	1.495786046	4	4	103.6394912	29.50943135
chr1	61077257	61462788	1p31.3	NFIA	12.83889976	8.584983696	1.495506597	4	4	65.4124612	30.20451895
chr1	248850004	248860761	1q44	ZNF692	2.311672693	1.5460999	1.495163858	4	4	51.36427783	22.52047835
chr15	42412437	42491197	15q15.1	ZNF106	46.69964196	31.23506935	1.495102874	4	4	56.91351487	33.11622415
chr10	37793938	37795175	10p11.21	ZNF33BP1	0.198005365	0.132445677	1.494993031	2	1	14.99662713	0
chr6	11538227	11583524	6p24.2	TMEM170B	27.33132768	18.28303517	1.494901007	4	4	60.50630163	40.2559146
chr3	193398967	193593114	3q29	ATP13A4	0.666101405	0.445597286	1.494850676	4	4	31.78077143	58.33316376
chr18	76619777	76623559	18q23	LINC00683	0.198171007	0.132597659	1.494528701	2	2	49.82995248	81.34610849
chr17	42039705	42050599	17q21.2	C17orf113	0.191110053	0.127878373	1.494467343	2	1	28.12457952	0
chr21	39235328	39236049	21q22.2	METTL21AP1	1.247113167	0.834603939	1.494257465	4	2	44.19604602	21.03727239
chr13	36168208	36297855	13q13.3	CCDC169-SOHLH2	0.280875216	0.187973552	1.494227317	1	2	0	28.8365223
chrX	41724155	41730135	Xp11.4	GPR82	1.939138814	1.297889839	1.494070418	4	4	45.63103908	66.73323995
chr7	100320640	100336307	7q22.1	PMS2P1	23.79508948	15.92865127	1.493854632	4	4	56.16308131	15.33442822
chr16	30919319	30923269	16p11.2	FBXL19-AS1	0.246093858	0.16474295	1.493805095	4	4	59.8173819	74.19368977
chr19	46688797	46689068	19q13.32	RN7SL364P	2.158966563	1.445441969	1.493637662	1	2	0	61.32347897
chr2	203238441	203305840	2q33.2	CYP20A1	26.70867223	17.88240657	1.493572586	4	4	67.40667426	25.7521921
chr17	64749662	64781999	N/A	ARHGAP27P1-BPT	1.842746317	1.233988792	1.493325003	4	4	50.35455873	69.19249875
chr8	144522377	144527032	8q24.3	LRRC24	0.674725141	0.451860962	1.493214057	4	2	29.87127715	96.57434552
chr5	75336334	75362101	5q13.3	HMGCR	22.91473642	15.34674512	1.493133315	4	4	33.12983291	37.90247729
chrX	109641330	109733403	Xq23	ACSL4	81.6466201	54.68420917	1.493056613	4	4	50.6275999	44.32934936
chr4	98470367	98658661	4q23	TSPAN5	96.61211424	64.71502063	1.492885474	4	4	35.66173034	24.36296884
chr15	72241181	72272575	15q23	PARP6	3.976839793	2.664204299	1.492693257	4	4	47.5346809	24.9138274
chr7	123536997	123601651	7q31.32	NDUFA5	16.72064519	11.20383369	1.492403909	4	4	39.50977085	27.70325923

chr12	94148723	94307675	12q22	PLXNC1	108.4326261	72.65867199	1.49235629	4	4	44.8715905	56.05582469
chr6	41934933	42048894	6p21.1	CCND3	44.29835383	29.68606525	1.492227193	4	4	31.40533492	14.85115044
chr11	93721514	93721866	11q21	SCARNA9	380.4104492	254.9372424	1.49217292	4	4	82.10810496	35.865864
chr5	140550067	140558093	5q31.3	SRA1	10.43183651	6.991299416	1.492116971	4	4	56.75595995	23.59211072
chr14	23117306	23119611	14q11.2	CEBPE	3.206935533	2.149680869	1.491819357	4	4	126.5724562	63.25338227
chr12	6946577	6961316	12p13.31	PTPN6	112.666226	75.52589768	1.491756198	4	4	69.53493156	45.2528338
chr16	89720368	89740925	16q24.3	ZNF276	13.64642475	9.149663799	1.491467343	4	4	54.17694683	23.00689325
chr1	151364302	151372734	1q21.3	SELENBP1	160.9535539	107.9239635	1.491360664	4	4	55.8480591	42.59483328
chr4	129093674	129112688	4q28.2	C4orf33	4.010380421	2.689329646	1.491219355	4	4	38.34913211	26.83929148
chr22	16601911	16648830	22q11.1	TPTEP1	10.33283866	6.931287454	1.490753158	4	4	67.08427113	61.20474688
chr19	8374376	8390691	19p13.2	RAB11B-AS1	1.607263928	1.078169808	1.490733571	2	4	119.6000825	18.33033383
chr8	53966554	54022456	8q11.23	TCEA1	176.1233301	118.1484576	1.490695128	4	4	47.22473463	17.26044135
chr18	23503470	23531807	18q11.2	RMC1	43.78601654	29.37473362	1.490601314	4	4	36.62877145	16.00604984
chr5	177445936	177456701	5q35.3	PRR7	2.589379653	1.737198941	1.490548717	4	4	81.88968434	84.96281039
chr11	6612766	6619461	11p15.4	TPP1	25.59494047	17.17462231	1.490276759	4	4	50.64670916	20.10663506
chr2	135985176	136007542	2q21.3	DARS-AS1	0.600425021	0.402900843	1.490255063	4	4	53.80190309	64.96445961
chr12	7094550	7109278	12p13.31	C1RL	17.19844773	11.54085346	1.490223213	4	4	69.92231956	51.82585437
chr19	42387019	42390318	19q13.2	CNFN	1.209405853	0.811591832	1.490165137	2	1	86.66719524	0
chr9	77716274	78031449	9q21.2	GNAQ	84.68497245	56.83060146	1.490129794	4	4	34.10169241	33.80806615
chr17	81843158	81860668	17q25.3	P4HB	45.53550904	30.55827873	1.490120221	4	4	63.26483756	34.94387298
chr1	148388490	148388651	1q21.2	RNU1-13P	12.76134714	8.564020219	1.490111748	3	4	106.4322368	50.02571144
chr3	122680726	122730840	3q21.1	PARP14	77.22560021	51.8280624	1.490034484	4	4	43.37182161	55.15346358
chr3	43079229	43106085	3p22.1	POMGNT2	2.977104535	1.998178931	1.489908881	4	4	62.47594236	23.48425644
chr22	30356635	30378655	22q12.2	CCDC157	0.326344339	0.219037659	1.489900601	4	4	80.30045555	52.25149171
chr17	21197950	21214595	17p11.2	TMEM11	6.096583228	4.092281553	1.489776094	4	4	57.49302628	30.4103638
chr19	38403124	38409088	19q13.2	FAM98C	1.172503582	0.787056853	1.489731749	4	4	59.52306897	54.62113839
chr5	150930436	150946584	5q33.1	ZNF300P1	0.121938258	0.081857844	1.48963438	2	1	27.03326704	0
chr11	60201253	60243124	11q12.2	MS4A4E	2.137228735	1.434883848	1.489478565	4	4	52.80183348	44.72754656
chr2	128265480	128318597	2q14.3	HS6ST1	3.188273898	2.140635658	1.489405209	4	4	62.07151629	18.44750289
chr22	17178790	17221854	22q11.1	ADA2	25.37006337	17.03369443	1.489404631	4	4	47.93361422	35.34983639
chr4	26163489	26435131	4p15.2	RBPJ	34.61776603	23.24377923	1.48933466	4	4	53.80695208	50.44092932
chr15	29268149	29269816	15q13.1	NSMCE3	0.120329662	0.080801326	1.489204051	2	2	68.90259275	49.04761504
chr10	11460510	11611789	10p14	USP6NL	37.7537607	25.35251836	1.489152287	4	4	93.94503993	50.15019634
chr17	74950742	74972805	17q25.1	HID1	0.175468834	0.117862779	1.488755277	4	4	83.62426525	102.3587767
chr7	110662645	111562531	7q31.1	IMMP2L	15.29286871	10.27227187	1.488752332	4	4	25.00213817	58.88199347
chr11	72754729	72793705	11q13.4	STARD10	2.650759273	1.780741145	1.488570802	4	4	84.34135486	56.24135706
chr2	62205826	62205917	2p15	MIR5192	9.16733545	6.158736219	1.488509188	2	2	108.604876	50.93849796
chr20	57391398	57409333	20q13.31	RBM38	421.6229765	283.2693779	1.488417067	4	4	21.71204434	50.48293534
chr13	106489731	106535040	13q33.3	EFNB2	0.399718935	0.268561044	1.488372736	3	4	39.08621476	99.06857897
chr5	180790541	180815635	5q35.3	MGAT1	16.9377186	11.38056673	1.488301858	4	4	59.22357279	19.23361259
chr19	16829387	16880355	19p13.11	SIN3B	4.603199446	3.093023326	1.488252419	4	4	95.05898561	27.59175344
chr16	12659799	12803887	16p13.12	CPPED1	78.30049853	52.62034197	1.48802717	4	4	73.25698911	52.18125482
chr6	132457524	132513472	6q23.2	STX7	24.27727691	16.31533648	1.488003446	4	4	27.04009217	42.66469129

chr1	179081977	179095996	1q25.2	TOR3A	5.632054503	3.785112271	1.487949128	4	4	55.52227363	33.81850095
chr17	64002595	64020634	17q23.3	ICAM2	16.41229131	11.03046659	1.487905446	4	4	52.04937953	29.8939735
chr2	208266178	208358751	2q34	PIKFYVE	28.40487023	19.09123412	1.487848823	4	4	26.63599367	17.89737673
chr8	143915147	143976800	8q24.3	PLEC	21.24345968	14.27856962	1.487786259	4	4	76.73951115	46.20859263
chr13	110863987	110870308	13q34	LINC00346	0.146461142	0.098453092	1.487623586	3	4	28.84483452	62.19294129
chr19	17405686	17415736	19p13.11	BISPR	7.796091242	5.241276028	1.487441455	4	4	61.60727447	48.80630243
chr15	45631148	45691294	15q21.1	SQOR	19.75857423	13.2838805	1.487409814	4	4	40.59220538	25.38518888
chr19	33386949	33521893	19q13.11	PEPD	6.470611701	4.350432059	1.487349213	4	4	76.64833316	17.37475258
chr6	158609707	158609790	6q25.3	MIR7161	15.7641837	10.59950911	1.487256018	4	4	51.00478293	39.8572346
chr12	57095404	57111413	12q13.3	STAT6	60.37911682	40.597903	1.487247182	4	4	65.12719902	24.584049
chr19	1107634	1174283	19p13.3	SBNO2	26.46024177	17.79218093	1.487183717	4	4	84.17558815	39.63487114
chr17	75109952	75130265	17q25.1	ARMC7	2.930541021	1.97085483	1.486939056	4	4	77.18971413	35.84270707
chr4	147732063	148072781	4q31.23	ARHGAP10	7.460943213	5.018492768	1.48669004	4	4	14.50708562	18.46370105
chr5	39371674	39425233	5p13.1	DAB2	12.07028868	8.120166471	1.48645828	4	4	49.56360194	11.76863924
chr17	28648356	28662189	17q11.2	SDF2	12.13855387	8.166383316	1.486405107	4	4	43.22379256	32.72240234
chr7	112819147	112939877	7q31.1	BMT2	21.70302035	14.60184765	1.486320147	4	4	14.65999665	30.66751484
chr2	237627576	237781647	2q37.3	LRRFIP1	46.10865173	31.02284042	1.486280789	4	4	57.89700109	39.70613969
chr14	106335080	106335533	14q32.33	IGHV3-30	6.558176072	4.412628716	1.486228843	4	4	58.14746371	94.62245008
chr20	20389558	20712638	20p11.23	RALGAPA2	16.45342328	11.07064526	1.486220802	4	4	50.84247018	35.90990167
chr16	87830016	87869499	16q24.2	SLC7A5	19.91244287	13.39814282	1.486209182	4	4	48.84401554	46.84768278
chr17	75941507	75979434	17q25.1	ACOX1	34.63407184	23.30573097	1.48607533	4	4	55.12847517	57.469903
chr3	66133610	66380021	3p14.1	SLC25A26	6.560117682	4.414562573	1.4860176	4	4	81.40019082	39.06013897
chr17	15786850	15789705	17p12	MEIS3P1	1.217640806	0.819476048	1.485877236	3	2	94.75949448	58.8433093
chr19	51745770	51751897	19q13.41	FPR1	418.7355962	281.8742206	1.485540591	4	4	62.57578956	64.12663984
chr2	46581274	46617112	2p21	PIGF	4.494262593	3.025429716	1.485495621	4	4	68.76145354	43.70667056
chr13	49628299	49633872	13q14.2	ARL11	7.978700155	5.37123453	1.485449967	4	4	74.87071295	65.20706184
chr1	1292384	1307925	1p36.33	ACAP3	1.814170416	1.221320216	1.485417495	4	4	65.4145579	34.6673639
chr11	13962637	14268133	11p15.2	SPON1	0.557963062	0.375688028	1.485176585	4	4	86.10437616	51.15315701
chr14	22499689	22499749	14q11.2	TRAJ40	21.7064513	14.61576777	1.485139313	4	2	71.09534994	77.75969168
chr13	44432143	44576825	13q14.11	TSC22D1	32.70243889	22.01978401	1.485138949	4	4	9.76971251	25.59446123
chr4	42029085	42031265	4p13	ATP1B1P1	1.309967981	0.882153797	1.48496553	1	4	0	61.78990685
chr2	202293051	202293134	2q33.1	SNORD11	14.8213275	9.981338234	1.484903843	4	4	65.36592865	30.39628596
chr4	47450787	47463730	4p12	COMMD8	20.06533175	13.5141044	1.484769626	4	4	8.286427053	47.75931129
chr14	68874123	68979366	14q22-q24	ACTN1	44.11436232	29.71186936	1.484738701	4	4	59.13160065	37.80794964
chr14	35085467	35122662	14q13.2	PPP2R3C	51.83173678	34.91485418	1.484518208	4	4	29.36535596	38.72345763
chr6	44219505	44234151	6p21.1	SLC29A1	7.811249216	5.261987787	1.484467379	4	4	108.2382932	29.13572015
chr16	68209381	68209824	16q22.1	RPS12P27	3.41610272	2.301358765	1.484385126	2	4	2.778585712	61.54401381
chr3	196554177	196568674	3q29	WDR53	6.207782463	4.182778136	1.484129031	4	4	46.33077248	23.84348232
chr1	158999970	159055155	1q23.1	IFI16	104.0330825	70.09780914	1.484113181	4	4	74.42440279	42.53775948
chr1	1081811	1116361	1p36.33	C1orf159	0.618917541	0.417066178	1.483979216	4	4	89.87417024	20.4165766
chr14	69767087	69772005	14q24.1	SRSF5	118.1181993	79.59808053	1.483932759	4	4	53.41346333	46.14973331
chr14	91580774	91732086	14q32.12	CATSPERB	2.136634877	1.439871186	1.483906962	4	4	116.2551614	37.97656086
chr17	74209980	74213321	17q25.1	MGC16275	0.925143205	0.62349347	1.483805765	4	4	106.1896414	41.72663372

chr3	121987323	122060554	3q13.33	ILDR1	0.1772008	0.119459028	1.483360476	4	2	73.02309075	45.05661065
chr12	51951667	51997079	12q13.13	ACVR1B	4.149893485	2.797875057	1.483230452	4	4	57.57991053	38.3215259
chr2	178194465	178399433	2q31.2	OSBPL6	0.121414177	0.081858701	1.483216521	4	4	70.37921313	97.84689937
chr5	69189548	69210357	5q13.2	CENPH	101.0199595	68.11292232	1.48312473	4	4	76.56231668	63.68851198
chr19	47487634	47515258	19q13.32-q13.33	NAPA	25.62984269	17.2817508	1.483058226	4	4	11.25360391	13.36412994
chr6	29942470	29945884	6p22.1	HLA-A	385.019116	259.6140779	1.48304406	4	4	91.84170679	50.43066077
chr10	87050202	87095047	10q23.2	GLUD1	50.39476492	33.98480498	1.482861677	4	4	36.51302882	19.59042972
chr11	94973681	94999514	11q21	KDM4D	0.385795431	0.260194207	1.482721058	2	4	95.75935921	73.45148305
chr9	128405994	128437351	9q34.11	CERCAM	0.271147467	0.182876174	1.482683397	4	4	72.51598917	88.29777652
chr22	17359949	17558155	22q11.1-q11.21	CECR2	0.17831493	0.120285793	1.482427193	3	4	41.56320914	52.34446107
chr14	22221896	22222475	14q11.2	TRAV35	1.876038583	1.26555217	1.482387393	4	3	58.71872177	36.80490028
chr9	68356584	68531061	9q21.11	PGM5	1.022499411	0.689997197	1.481889223	4	3	111.1890068	48.94551299
chr3	167683893	167735810	3q26.1	PDCD10	165.8444031	111.9299329	1.481680537	4	4	50.53972251	43.23849912
chr1	112517444	112619881	1p13.2	ST7L	19.96288304	13.47342303	1.481648947	4	4	64.17489762	34.8111607
chr3	69168782	69541340	3p14.1	FRMD4B	8.04015757	5.427373408	1.481408587	4	4	29.11594595	18.32175453
chr18	2655887	2805017	18p11.32	SMCHD1	280.0032827	189.0260965	1.481294318	4	4	39.2822922	34.79737773
chr19	15611641	15612609	19p13.12	RPL23AP2	0.8282539	0.559227202	1.48106869	2	2	12.36062499	17.07208919
chr17	50464492	50469881	17q21.33	CHAD	0.218103353	0.147271725	1.480958772	2	1	17.57708039	0
chr18	63970028	64019779	18q22.1	SERPINB8	3.822901166	2.581400859	1.480940533	4	4	61.29849928	30.15692868
chr1	247857199	247880138	1q44	TRIM58	332.0774227	224.2466461	1.480857924	4	4	36.58018813	28.91645801
chr14	52430590	52552583	14q22.1	TXNDC16	174.2076425	117.6491715	1.480738371	4	4	65.79578799	38.33417166
chr19	47349281	47382704	19q13.32	DHX34	10.55312161	7.127903704	1.480536501	4	4	57.34135481	41.78742142
chr13	50882378	50910712	13q14.3	RNASEH2B-AS1	0.354844533	0.239674411	1.480527406	4	4	46.35529016	89.724039
chr3	94014365	94029281	3q11.2	STX19	0.588029317	0.397176732	1.480523077	1	1	0	0
chr14	77181759	77259495	14q24.3	TMEM63C	0.550026588	0.371529046	1.480440342	4	4	86.11817491	70.06339043
chr1	16740516	16740679	1p36.13	RNU1-4	12.54370325	8.472975628	1.480436602	4	3	45.76663789	44.0652729
chr15	63121800	63142065	15q22.2	LACTB	19.26695324	13.0149688	1.480368761	4	4	66.91234527	39.00786515
chr1	23873075	23913327	1p36.11	CNR2	4.95922762	3.350321673	1.48022432	4	4	69.60509507	65.04255299
chr5	177357671	177372601	5q35.3	RGS14	24.84085326	16.7829207	1.480126952	4	4	60.93542161	59.62476687
chr2	85394748	85418467	2p11.2	CAPG	19.00621836	12.84234	1.479965361	4	4	68.45041694	53.92799777
chr1	28095035	28096458	1p35.3	SPCS2P4	1.654659149	1.11810718	1.479875256	4	3	56.004146	87.64134692
chr7	142308589	142309048	7q34	TRBV3-1	7.446640476	5.032433773	1.479729453	3	4	48.09567391	122.1120646
chr22	21759657	21867680	22q11.22	MAPK1	99.76157078	67.42094189	1.479682247	4	4	25.15716435	8.075225715
chr12	56341597	56360253	12q13.3	STAT2	44.65018614	30.17759155	1.479580837	4	4	38.52103679	44.04525474
chr9	89360791	89498065	9q22.2	SEMA4D	41.11522733	27.78999507	1.479497467	4	4	59.81656491	12.95342985
chr22	22484421	22509187	22q11.22	ZNF280B	0.127224266	0.085994317	1.479449697	1	1	0	0
chr5	180300690	180353387	5q35.3	GFPT2	0.290680899	0.196480483	1.479439051	4	3	95.99280379	103.9609409
chr19	6389638	6389703	19p13.3	MIR6885	5.537233121	3.743551348	1.479139087	1	2	0	23.84129935
chr2	151485334	151734487	2q23.3	NEB	0.138454998	0.093607884	1.479095476	4	4	84.67201842	68.53767152
chr10	50991151	52298350	10q11.23-q21.1	PRKG1	2.425195883	1.639987328	1.478789403	4	4	26.78664384	32.40049137
chr15	77100829	77420699	15q24.3	PEAK1	20.12052943	13.60718553	1.478669442	4	4	66.29417905	47.52066611
chr1	154582057	154631560	1q21.3	ADAR	143.8072167	97.26813878	1.478461689	4	4	52.56178275	32.0174113
chr7	128027071	128032107	7q32.1	LRRC4	16.36627274	11.07079851	1.478328119	4	4	56.06914348	54.09074887

chr8	18527303	19086909	8p22	PSD3	1.628219325	1.101414664	1.478298209	4	4	63.02396592	45.62541835
chr10	12456483	12456631	10p13	RNU6ATAC39P	3.285293816	2.222370124	1.478283829	2	2	29.03365878	20.99802165
chr11	66337333	66345125	11q13.2	BRMS1	13.69232206	9.263599931	1.478077871	4	4	57.88766645	23.12640993
chr19	49334961	49340608	19q13.33	CD37	47.2111865	31.94311558	1.477976886	4	4	46.29612998	30.51257035
chr5	54320944	54415125	5q11.2	LINC01033	0.414394128	0.280423429	1.477744318	1	3	0	56.35778471
chr6	29723340	29740355	6p22.1	HLA-F	29.79086661	20.16068375	1.477671441	4	4	61.38037688	31.33374282
chr6	26365159	26378320	6p22.2	BTN3A2	33.78797741	22.86672748	1.477604412	4	4	63.62512793	32.77871997
chr6	24649977	24666887	6p22.3	TDP2	63.23237108	42.79556791	1.477544853	4	4	41.38981201	27.58433017
chr17	19648146	19677596	17p11.2	ALDH3A2	5.685041065	3.847716084	1.477510539	4	4	73.64170372	38.92361107
chr10	37949572	37976655	10p11.21	ZNF25	10.73133709	7.26391962	1.477347996	4	4	23.38941215	6.549589906
chr2	237966945	238042782	2q37.3	UBE2F	16.36868291	11.08025816	1.477283533	4	4	32.34066317	41.05219527
chr5	132750817	132778216	5q31.1	SEPT8	1.015975198	0.687737129	1.477272574	4	4	40.7454087	27.16751882
chr1	2586460	2591469	1p36.32	PRXL2B	0.508541357	0.344244818	1.477266555	3	3	64.56665829	36.95425595
chr18	27932878	28177446	18q12.1	CDH2	0.565109636	0.382614804	1.476967512	4	4	69.27273362	130.2901949
chr17	63745250	63773728	17q23.3	CCDC47	53.66501293	36.33788389	1.476833739	4	4	15.62750233	15.08385879
chr22	40951347	40973015	22q13.2	RBX1	58.09236407	39.33809257	1.476745828	4	4	77.09984337	42.51503772
chr19	46019153	46023298	19q13.32	PGLYRP1	27.1462464	18.38293084	1.47670938	4	4	78.1776225	46.78152273
chr22	24806221	24926846	22q11.23	SGSM1	0.278328686	0.188503088	1.476520559	4	4	65.73921301	33.75187544
chr13	109163905	109167303	13q33.3	MYO16-AS1	3.534362782	2.393799471	1.476465688	2	2	19.21079266	26.46405354
chr1	111139383	111185104	1p13.3	CEPT1	17.80488955	12.05970402	1.476395235	4	4	25.57540279	9.453599406
chr9	112749723	112875847	9q32	SNX30	8.084474635	5.476139498	1.476309111	4	4	72.29677547	30.99822425
chr1	180632010	180890279	1q25.3	XPR1	14.71621636	9.968715569	1.476239969	4	4	8.702506711	7.942544157
chr9	118082693	118083475	9q33.1	TPT1P9	3.487469228	2.362629859	1.476096315	2	4	55.8324587	72.89879398
chr19	35030688	35040449	19q13.11	SCN1B	0.821101985	0.556271392	1.476081633	4	4	56.95387278	41.44735712
chr1	48258643	48472208	1p33	SPATA6	1.75148785	1.186622248	1.476028158	4	4	33.20365504	60.28968142
chr19	43531574	43536138	19q13.31	ZNF575	0.235606637	0.159622714	1.476021994	3	1	68.78293966	0
chr3	179396088	179451583	3q26.33	GNB4	42.56247957	28.84287761	1.475666892	4	4	26.36162348	20.78809474
chr1	113813811	113871761	1p13.2	PTPN22	105.4208731	71.44082359	1.475639107	4	4	11.55290111	21.08206458
chr1	229431245	229434096	1q42.13	ACTA1	0.450954243	0.305618859	1.475544553	1	1	0	0
chr17	3714625	3803766	17p13.2	ITGAE	1.013894776	0.687175158	1.475453186	4	4	64.01686901	31.04387947
chr19	3490821	3500940	19p13.3	DOHH	2.07716939	1.407877202	1.475391026	4	4	52.69751614	68.0469169
chr2	230165473	230225729	2q37.1	SP110	45.71824333	30.98750212	1.475376852	4	4	45.71622934	36.30979912
chr17	4940008	4945223	17p13.2	RNF167	42.96451174	29.12141739	1.47535785	4	4	69.16516042	48.7017252
chr17	4433924	4488204	17p13.2	SPNS3	2.206428676	1.495531288	1.47534772	4	4	103.981662	53.30127199
chr5	50665831	50846522	5q11.1	PARP8	62.65028283	42.47055004	1.47514649	4	4	31.92502206	19.80361958
chr4	102501266	102617302	4q24	NFKB1	32.43461155	21.98816659	1.475093952	4	4	46.06172378	28.77885461
chr12	52069923	52077495	12q13.13	ATG101	4.609312292	3.124770524	1.475088253	4	4	61.51741415	36.02067188
chr12	19089131	19089951	12p12.3	RPL7P6	0.456533108	0.309521225	1.474965433	1	1	0	0
chr22	46295143	46296660	22q13.31	GTSE1-DT	0.489365433	0.33178296	1.474956498	3	3	61.98017836	37.21558033
chr7	42916861	42932206	7p14.1	PSMA2	2.492286019	1.68981204	1.47488949	4	4	36.28941998	37.56915992
chr12	6570082	6607439	12p13.31	CHD4	51.77325586	35.105856	1.474775487	4	4	62.71945984	34.2656804
chr9	33264879	33282069	9p13.3	CHMP5	121.1852402	82.17257119	1.474765101	4	4	38.16327906	34.68628629
chr3	183487528	183555712	3q27.1	KLHL6	12.03592356	8.161534213	1.474713362	4	4	50.9434075	37.44388984

chr17	8172460	8176411	17p13.1	TMEM107	5.366855725	3.640261871	1.474304848	4	4	17.80216028	39.37770444
chr1	86571181	86704493	1p22.3	CLCA4-AS1	1.00154803	0.679341215	1.474293046	2	2	47.60875681	35.58266723
chr2	151270465	151289916	2q23.3	NMI	70.65745991	47.93132065	1.474139643	4	4	37.73389803	28.64657376
chr2	102069638	102179874	2q11.2-q12.1	IL1R1	5.438242548	3.689239505	1.474082271	4	4	26.15559075	62.48924608
chr14	22202583	22203368	14q11.2	TRAV26-2	6.768431889	4.591817022	1.474020384	4	4	95.03214462	79.19709002
chr1	160343273	160358952	1q23.2	NCSTN	27.16616063	18.4305892	1.473971361	4	4	64.00287023	26.44954051
chr7	29146547	29514335	7p14.3	CHN2	2.322811252	1.576099287	1.473772161	4	4	41.60122775	14.59162178
chr7	65865772	65959563	7q11.21	VKORC1L1	7.444411009	5.051339845	1.473749785	4	4	23.20592466	18.52918773
chr2	28392855	28417312	2p23.2	FOSL2	54.42649726	36.93635858	1.473520925	4	4	58.25089435	6.94016657
chr1	25222276	25232522	1p36.11	SYF2	65.7995264	44.65524119	1.473500638	4	4	23.12714188	21.15990497
chr4	70688479	70690551	4q13.3	UTP3	22.44964752	15.23666544	1.473396368	4	4	52.8973082	20.22793756
chr17	5208920	5234860	17p13.2	SCIMP	12.47341969	8.467303892	1.473127674	4	4	59.46040439	47.12178719
chr1	193178730	193186613	1q31.2	B3GALT2	1.541554184	1.046629114	1.472875313	4	4	32.03618673	73.85089181
chr19	41309855	41324883	19q13.2	CCDC97	8.005495791	5.435975266	1.472688046	4	4	61.89479425	32.23487633
chr6	125956715	125980244	6q22.32	HINT3	17.88708507	12.14720372	1.472526968	4	4	27.49720432	22.72998004
chr11	77322015	77514957	11q13.5-q14.1	PAK1	50.04144887	33.98350314	1.47252179	4	4	37.0616764	32.01319482
chr10	110207605	110287365	10q25.2	MXI1	300.0410889	203.7783891	1.472389149	4	4	31.92061129	53.41242149
chr3	12004360	12192032	3p25.2	SYN2	0.125930314	0.085532875	1.472303068	3	2	9.597886539	67.59250197
chr10	100273278	100286712	10q24.31	BLOC1S2	28.28497925	19.21300895	1.472178529	4	4	9.874027787	33.4788893
chr19	32405749	32485893	19q13.11	DPY19L3	4.275261665	2.904220805	1.472085613	4	4	47.10459061	35.23100388
chr12	51238724	51246717	12q13.13	DAZAP2	170.7646668	116.0063864	1.472028155	4	4	58.54868173	26.58778782
chr1	169690665	169711702	1q24.2	SELL	937.9537099	637.2336026	1.471915018	4	4	66.44786857	44.29383302
chr10	104268336	104268405	10q25.1	MIR4482	18.09260311	12.29200723	1.47189981	4	3	99.97147393	92.09248256
chr20	44302838	44311252	20q13.12	FITM2	1.157753584	0.786763467	1.471539583	3	4	79.72642063	37.50183868
chr2	138501780	138573235	2q22.1	SPOPL	51.04365983	34.68742859	1.471531962	4	4	43.07236031	32.84680177
chr10	45825592	45853877	10q11.22	AGAP4	4.500741285	3.05862711	1.47149068	4	4	58.07962527	57.4397145
chr8	143309324	143359977	8q24.3	TOP1MT	2.734946352	1.858923146	1.471253052	4	4	109.3170256	79.77600969
chr2	85753786	85791383	2p11.2	ATOH8	0.329486413	0.223998574	1.470930848	3	3	13.40273431	122.6306523
chr4	163524298	164384050	4q32.2-q32.3	MARCH1	23.98321939	16.31123719	1.470349496	4	4	46.65422283	29.79419354
chr19	52281797	52281893	19q13.41	MIR643	5.458664406	3.712596771	1.47030899	2	1	18.17648943	0
chr15	74788672	74788757	15q24.1	MIR4513	13.33267362	9.068998924	1.470137303	3	4	55.55798367	77.90430395
chr3	196397508	196397778	3q29	RN7SL434P	2.63300327	1.791179278	1.469983101	2	3	10.64884228	60.2162841
chr13	108207442	108218368	13q33.3	LIG4	15.04851481	10.2395046	1.469652624	4	4	30.90487399	40.45623676
chr2	74483052	74494564	2p13.1	TTC31	3.46666727	2.359016061	1.469539495	4	4	34.59701102	23.53418277
chr14	23185284	23273478	14q11.2	RNF212B	0.478487253	0.325624099	1.469446687	4	4	110.6085354	26.55087944
chr9	131860110	132079887	9q34.13	MED27	26.52608743	18.05277803	1.469363185	4	4	75.02411662	8.884782083
chr2	70447280	70554015	2p13.3	TGFA	10.03639051	6.83151898	1.469130151	4	4	71.64857056	88.86961564
chr17	41688875	41691646	17q21.2	EIF1	161.7033289	110.068103	1.469120704	4	4	26.96929488	13.54876504
chrX	107713221	107777329	Xq22.3	TSC22D3	139.0231515	94.64182724	1.46893985	4	4	33.03719159	31.52267864
chr9	112683948	112718149	9q32	INIP	14.47811743	9.856534918	1.468885116	4	4	62.17320629	26.72599168
chrX	30630097	30630975	Xp21.2	FTLP2	1.419337568	0.966294379	1.468845931	2	3	62.53296761	51.10134188
chr11	13668659	13732346	11p15.3	FAR1	33.60901675	22.88256418	1.468760952	4	4	12.7626219	33.54239842
chr1	155213825	155227534	1q22	GBAP1	0.310022025	0.211078991	1.46874885	4	3	71.0516758	55.27308528

chr14	69611593	69715144	14q24.1	SUSD6	39.90817057	27.17426266	1.468601782	4	4	53.59186915	30.22955344
chr22	37010859	37020183	22q12.3	TST	4.524630196	3.081093085	1.468514605	4	4	57.09821649	74.15232709
chr2	200870907	200889333	2q33.1	PPIL3	24.91772373	16.96983208	1.468354172	4	4	68.29276719	17.31860765
chrX	47438736	47439857	Xp11.3	NPM1P49	0.471377549	0.321035283	1.468304495	1	1	0	0
chr10	32900318	32958365	10p11.22	ITGB1	134.6491708	91.72859933	1.467908284	4	4	48.5827553	20.22419272
chr20	35433347	35454746	20q11.22	GDF5	0.142003958	0.096741173	1.467875086	2	1	2.604913164	0
chr12	109880667	109918069	12q24.11	TCHP	4.038753701	2.751448968	1.467864295	4	4	50.02047151	42.05594707
chr1	18871430	18902799	1p36.13	ALDH4A1	0.332475683	0.226517145	1.467772709	3	3	42.3941748	35.22353128
chr18	69860228	69962086	18q22.2	CD226	35.81700289	24.40268606	1.467748378	4	4	18.40886653	20.31047713
chr20	38872770	38873074	20q11.23	RN7SL116P	2.559388581	1.743894727	1.467627914	4	4	23.88419522	91.90831215
chr8	89901868	89935614	8q21.3	OSGIN2	31.60073236	21.53369439	1.467501664	4	4	46.69368472	43.53238658
chr17	35918080	35931821	17q12	RDM1	0.321480175	0.219074698	1.467445476	3	3	34.78371173	67.600258
chr1	121519112	121571888	1p11.2	EMBP1	3.283395781	2.237796425	1.467245074	4	4	81.11364826	46.92020791
chr12	49064676	49070025	12q13.12	RHEBL1	1.296267207	0.883536763	1.467134432	4	3	45.18047424	68.50304248
chrX	41683168	41683466	Xp11.4	RN7SL144P	1.526527707	1.040536253	1.467058647	2	1	24.16366443	0
chr17	64966550	64975585	17q24.1	AMZ2P1	2.327747596	1.586684057	1.467051734	4	4	88.40887119	35.03648884
chr1	6221193	6235984	1p36.31	ICMT	8.841162831	6.026558968	1.467033323	4	4	34.36518344	37.67707382
chr5	77582354	77583200	5q13.3	RPL7P23	0.893468552	0.609043544	1.467002747	2	1	69.97601747	0
chr9	132261356	132356726	9q34.13	SETX	112.0601197	76.3974998	1.466803494	4	4	35.25562423	19.74157139
chr3	87227263	87255548	3p11.2	CHMP2B	41.80249468	28.50157051	1.466673378	4	4	34.65553384	22.09806537
chr1	19312246	19332611	1p36.13	PQLC2	1.949091652	1.329013499	1.466570245	4	4	62.50423269	27.81097566
chr11	9778667	10294207	11p15.4	SBF2	8.12316032	5.539665366	1.466362999	4	4	55.34857664	43.76488601
chr4	158201597	158255287	4q32.1	TMEM144	0.672903397	0.458945016	1.46619611	4	4	47.58219446	61.23413893
chr12	89525570	89541542	12q21.33	POC1B-AS1	1.317182639	0.898373222	1.466186444	4	4	54.32026337	33.70315783
chr19	10566462	10568979	19p13.2	CDKN2D	89.42431234	60.99386825	1.466119708	4	4	27.2525196	25.38100585
chr2	189784085	189877629	2q32.2	PMS1	16.60129999	11.32354292	1.466087081	4	4	48.67680198	20.38601092
chr3	112532507	112561963	3q13.2	ATG3	55.56487567	37.9040208	1.465936185	4	4	12.5044625	23.8684515
chr3	143119776	143124014	3q24	CHST2	14.69284986	10.02397225	1.465771203	4	4	36.33925716	15.67799973
chr2	66435400	66572759	2p14	MEIS1	4.168536032	2.84412553	1.465665276	4	4	39.93552544	17.21699717
chr5	103258702	103278660	5q21.1	C5orf30	5.95341547	4.062607289	1.465417415	4	4	39.22262019	12.35154909
chr7	100602259	100608175	7q22.1	PCOLCE	0.303461783	0.207088224	1.46537441	3	2	49.89241113	5.969799418
chr1	167721950	167791919	1q24.2	MPZL1	13.50987178	9.219994931	1.46527974	4	4	99.22200135	46.80186333
chr22	50200979	50217616	22q13.33	SELENOO	4.598943047	3.138757207	1.465211465	4	4	63.81212449	21.14862418
chr9	101569352	101738580	9q31.1	GRIN3A	0.218099045	0.148857794	1.46515032	4	3	40.00203129	84.93395475
chr10	110898730	110919366	10q25.2	BBIP1	6.113828027	4.172857058	1.465141974	4	4	30.79450543	27.25741809
chr1	68496676	68538627	1p31.3-p31.2	DEPDC1-AS1	0.315083893	0.215061611	1.465086639	1	1	0	0
chr6	158536432	158635435	6q25.3	TMEM181	41.29792257	28.18850565	1.465062501	4	4	58.61227422	34.68666914
chrX	23907932	23939520	Xp22.11	CXorf58	0.188937056	0.128967908	1.464992797	2	2	14.95474887	3.679446162
chr1	38991244	39006065	1p34.3	AKIRIN1	59.97732069	40.94192175	1.464936626	4	4	33.52163639	17.85227324
chr1	30732468	30757840	1p35.2	LAPTM5	620.6929434	423.7066699	1.464911901	4	4	62.97721866	32.04190391
chr11	118544528	118576456	11q23.3	IFT46	4.044358382	2.760854955	1.464893465	4	4	29.16981998	17.26926191
chr2	182715979	182780025	2q32.1	DNAJC10	8.676567605	5.923132438	1.46486132	4	4	45.77209499	28.72263602
chr17	48765554	48765847	17q21.32	RN7SL125P	1.273340694	0.869289766	1.464805804	1	1	0	0

chr5	58453982	58460139	5q11.2	PLK2	1.055480721	0.720599935	1.464724974	4	4	33.69876366	66.3612167
chr19	32719773	32829580	19q13.11	TDRD12	0.200432207	0.136842583	1.464691785	3	3	20.80709693	99.49744963
chr1	206009264	206023909	1q32.1	CTSE	14.17632252	9.68084551	1.464368222	4	4	90.17306539	34.40534003
chr9	127451486	127503501	9q33.3-q34.11	LRSAM1	3.570346969	2.438194984	1.464340216	4	4	48.18085505	24.26912217
chr12	49538541	49558312	12q13.12	KCNH3	0.464441503	0.317198986	1.464196052	4	4	68.36052053	38.80852502
chr7	36509313	36724549	7p14.2	AOAH	75.84131111	51.80003419	1.464117009	4	4	35.97255789	28.4636535
chr14	32202045	32202151	14q12	RNU6-7	3.539072849	2.417439287	1.463975897	3	2	52.61713497	25.401792
chr19	33299934	33302564	19q13.11	CEBPA	8.619567	5.887829174	1.463963499	4	4	32.91369152	46.43353965
chr17	78991716	79009817	17q25.3	CANT1	19.54991338	13.35554205	1.463805311	4	4	53.74135601	35.10253282
chr5	131641713	131797063	5q31.1	FNIP1	51.6202911	35.26448042	1.463804102	4	4	15.6155941	26.05883693
chr9	127448502	127448637	9q33.3	SNORA65	8.350105318	5.704529754	1.463767511	4	4	61.77870945	59.46864929
chr11	60056628	60071116	11q12.1	MS4A3	11.08256093	7.571314109	1.463756591	4	4	29.04010666	20.50044302
chr19	23914874	23944713	19p12	ZNF726	1.189313147	0.812614304	1.46356413	4	4	54.09198234	28.07451597
chr16	66754410	66801620	16q22.1	TERB1	0.791892735	0.541121352	1.463429103	3	3	126.3778271	67.0890859
chr3	127571232	127598265	3q21.3	TPRA1	4.776888177	3.264264939	1.463388624	4	4	34.25049235	26.14298491
chr7	131054886	131054992	7q32.3	RNU6-1010P	7.180691972	4.907537516	1.463196552	2	3	23.70633628	22.3613814
chr5	32124711	32174319	5p13.3	GOLPH3	73.45946433	50.20692818	1.463134013	4	4	22.86498018	13.12480046
chr3	113747019	113812058	3q13.31	ATP6V1A	77.27156059	52.8183046	1.462969347	4	4	47.60011533	32.65593409
chr17	50346032	50361185	17q21.33	XYLT2	3.326208843	2.27370398	1.462903206	4	4	44.54756731	39.53028006
chr5	54517759	54619249	5q11.2	SNX18	52.92566356	36.17910399	1.462879334	4	4	72.28231556	39.21152264
chr21	5097172	5128463	21p12	GATD3B	0.737834362	0.504399225	1.462798366	4	4	60.78511712	78.74321842
chr17	80260820	80398786	17q25.3	RNF213	71.58919316	48.94184099	1.462740096	4	4	41.90575247	50.10725497
chr12	53210566	53232256	12q13.13	RARG	1.554500354	1.062733242	1.462738053	4	4	73.86652611	42.14279799
chr1	35568739	35595591	1p34.3	TFAP2E	0.384619161	0.262951306	1.462701085	3	4	74.40091242	37.93076619
chr20	64164474	64242253	20q13.33	MYT1	0.114804097	0.07848863	1.462684426	2	1	83.80434616	0
chr1	28643256	28648801	1p35.3	LINC01715	1.239391947	0.847495175	1.462417703	3	3	28.39227363	92.40538998
chr11	32829756	32858123	11p13	PRRG4	10.26012094	7.015880273	1.462413916	4	4	64.37203423	58.85788307
chr9	97926791	97944915	9q22.33	HEMGN	1466.592735	1002.89879	1.462353679	4	4	50.63630155	47.15604655
chr7	104126341	104208047	7q22.1-q22.2	ORC5	50.73157573	34.69493055	1.462218685	4	4	95.17147213	45.05439398
chr1	42499781	42500417	1p34.2	TMSB4XP1	3.587538963	2.453665709	1.462113991	1	2	0	21.37415732
chr2	96705929	96740092	2q11.2	LMAN2L	3.188412982	2.180776536	1.462053965	4	4	55.31071432	61.18856667
chr7	94907202	95296415	7q21.3	PPP1R9A	0.734306025	0.502248154	1.462038274	3	4	85.79287696	52.23728719
chr17	1569254	1630014	17p13.3	SLC43A2	7.117526711	4.868339831	1.462002851	4	4	58.90856684	37.94713855
chr4	4267701	4290195	4p16.3	LYAR	36.5076313	24.97288358	1.461890902	4	4	49.75513765	45.47649843
chr2	45651315	46187990	2p21	PRKCE	2.742789303	1.876341945	1.461774764	4	4	38.08695034	28.06655852
chr10	133527363	133539116	10q26.3	CYP2E1	0.089498697	0.061227321	1.46174447	3	3	113.3940126	22.76756542
chr2	240468632	240561216	2q37.3	ANKMY1	1.885556979	1.28995917	1.461718342	4	4	63.9485327	38.17815618
chr17	81117295	81166072	17q25.3	AATK	7.069017172	4.836649089	1.461552625	4	4	57.20686594	65.06359794
chr19	54137689	54155708	19q13.42	CNOT3	6.281862527	4.298109071	1.461540976	4	4	70.87978402	38.32432934
chr18	54269479	54321266	18q21.2	POLI	9.006826293	6.162790135	1.461485155	4	4	73.63763924	47.06077185
chr2	9843442	9934416	2p25.1	TAF1B	6.439318434	4.406395083	1.461357484	4	4	47.71393281	31.61477236
chr17	65137338	65227703	17q24.1	RGS9	1.154659097	0.790191576	1.461239442	4	4	27.67617204	12.02048351
chr4	55556523	55592274	4q12	PDCL2	2.260320056	1.546883691	1.461208796	3	3	50.56122779	39.9275927

chrX	119758613	119760202	Xq24	SOWAHD	1.690092571	1.156717436	1.461111	4	4	104.2533631	44.06900172
chr14	22456689	22456742	14q11.2	TRDJ2	13.65565754	9.346725836	1.461009746	4	3	19.87755473	19.77566875
chr10	96518110	96587052	10q24.1	TM9SF3	89.25715247	61.10698126	1.460670297	4	4	26.09068691	11.89895917
chr17	8287763	8295689	17p13.1	SLC25A35	0.877344412	0.600738499	1.460443129	4	4	82.01518917	54.12838672
chr4	141220294	141234697	4q31.21	ZNF330	23.13885291	15.84519692	1.460307059	4	4	41.14458488	17.10744286
chr14	22525263	22525322	14q11.2	TRAJ19	36.17608397	24.77474439	1.460200089	3	4	55.25050916	16.59023776
chr4	15604384	15681544	4p15.32	FBXL5	237.9050132	162.9291039	1.460175055	4	4	37.9039614	48.60377441
chr22	36758202	36776133	22q12.3	IFT27	0.847311227	0.580289541	1.460152505	4	4	94.85194598	47.22724824
chr6	85427251	85428054	6q14.3	TPT1P6	3.487245573	2.388287388	1.460144868	3	3	88.75818617	34.90378466
chr21	41798225	41879482	21q22.3	PRDM15	1.063522532	0.728429098	1.460022033	4	4	67.58817956	34.21030557
chr1	15617458	15661057	1p36.21	DDI2	85.5137232	58.58254113	1.45971345	4	4	74.03140777	36.18762508
chr14	102348282	102362916	14q32.31	CINP	5.740030413	3.932323114	1.45970467	4	4	61.8912482	24.82751634
chr6	116511645	116524792	6q22.1	CALHM5	0.60194427	0.412377726	1.45969152	4	4	33.74449214	44.78198237
chr13	43213133	43786980	13q14.11	ENOX1	0.116825741	0.080042889	1.459539294	1	1	0	0
chr20	25195670	25228075	20p11.21	ENTPD6	4.065578072	2.785597038	1.459499711	4	4	73.64579661	33.16258096
chr8	108443583	108489188	8q23.1	EMC2	61.83870611	42.37222693	1.459416004	4	4	60.48766282	13.69848343
chr15	66386873	66491544	15q22.31	MAP2K1	15.69516931	10.75601076	1.459199852	4	4	41.41975507	8.029105655
chr6	137197484	137220351	6q23.3	IFNGR1	189.106375	129.6070979	1.459074217	4	4	51.35871779	40.19090626
chr4	40810027	41216714	4p14-p13	APBB2	0.1936587	0.132731836	1.459022239	3	4	50.1238707	102.6995342
chrX	154542240	154565046	Xq28	IKBKG	3.014980231	2.066524443	1.458961805	4	4	57.6928056	29.75477841
chr11	118000027	118089911	11q23.3	SMIM35	0.335266754	0.229815976	1.45884877	3	4	66.30176626	21.76075836
chr1	52726454	52827342	1p32.3	ZYG11B	14.47283833	9.921622927	1.458716829	4	4	37.12149998	38.8340729
chr12	9134760	9135797	12p13.31	BTG1P1	3.646123843	2.499621552	1.45867035	4	4	46.74307808	38.14267457
chr13	110148958	110307157	13q34	COL4A1	0.290601852	0.199247875	1.458494104	3	4	52.81071013	105.3994031
chr2	47160082	47176936	2p21	CALM2	117.9925203	80.90043087	1.458490629	4	4	11.77900591	22.57811771
chr1	165630873	165656136	1q24.1	MGST3	2.85286659	1.956041395	1.458489885	4	4	34.7947744	31.44503351
chr6	160466555	160511124	6q25.3	LPAL2	0.638410607	0.437723831	1.458478068	4	3	90.4541691	63.93414098
chr11	33256672	33357023	11p13	HIPK3	158.3731294	108.5939301	1.458397622	4	4	42.87224952	13.36340206
chr16	58665109	58684777	16q21	SLC38A7	0.928818302	0.636941946	1.458246403	4	4	53.34896914	38.86992729
chr17	18039297	18068405	17p11.2	GID4	13.28016402	9.108592966	1.457981938	4	4	19.9494022	24.01896567
chr1	44731069	44731168	1p34.1	RNU5D-1	231.7165856	158.9349776	1.457933232	4	4	38.29751805	39.04808596
chr5	10277595	10308056	5p15.2	CMBL	4.562254573	3.129267996	1.457930282	4	4	74.36761218	147.1581657
chr5	170861882	171300015	5q35.1	RANBP17	21.94580596	15.05416512	1.45778964	4	4	96.14952333	67.11979759
chr18	45983536	46072327	tdb7990	PSTPIP2	39.24422861	26.9211853	1.457745199	4	4	31.64291173	32.3704623
chr9	127731524	127735349	9q34.11	TOR2A	4.154095194	2.849925967	1.457615125	4	4	63.48451685	59.31159219
chr7	122318424	122886759	7q31.32	CADPS2	0.057929571	0.039745207	1.45752347	1	1	0	0
chr7	30284307	30367692	7p14.3	ZNRF2	18.22068438	12.50188196	1.457435324	4	4	9.841248203	24.27939295
chr8	144526219	144529074	8q24.3	C8orf82	1.871733374	1.284282583	1.457415524	4	4	54.3708102	20.6762147
chr2	42762499	42764261	2p21	OXER1	5.80187357	3.980935078	1.457414767	4	4	59.51930553	51.85870667
chr2	170178145	170655164	2q31.1	MYO3B	0.577449556	0.396264083	1.457234154	4	4	69.83229398	107.3432269
chr10	80046233	80079193	10q22.3	TMEM254-AS1	0.176016463	0.120798745	1.457105065	2	3	48.76122012	57.19105317
chr19	54754756	54767881	19q13.42	KIR2DP1	0.272345751	0.186962821	1.456684004	2	2	29.5124985	9.692338104
chr3	177019355	177197357	3q26.32	TBL1XR1	63.76625307	43.78135707	1.456470456	4	4	16.83872011	10.11420168

chr15	89135081	89136674	15q26.1	HMGB1P8	1.339991192	0.920140765	1.456289346	3	3	4.782502366	84.31729101
chr1	246771878	246784760	N/A	KIF28P	3.043899972	2.090294495	1.456206281	4	4	32.51027008	32.0862923
chr5	149730252	149857861	5q32	PPARGC1B	1.021221539	0.701406628	1.455962202	4	4	75.15639803	53.13006777
chr12	101719808	101719918	12q23.2	RNY1P16	9.007326281	6.186518536	1.455960445	3	3	23.21504627	52.9052447
chr16	27460613	27549930	16p12.1	GTF3C1	13.48177205	9.260195428	1.455884182	4	4	60.07725255	45.49829922
chr21	33070144	33072422	21q22.11	OLIG1	2.353500845	1.61661895	1.455816688	4	3	110.0370616	70.15772398
chr4	48830924	48861817	4p11	OCIAD1	41.99506246	28.84679764	1.455796341	4	4	37.70248426	34.19556063
chr22	24495060	24528681	22q11.23	UPB1	0.279014223	0.191678925	1.455633284	4	4	52.86125711	62.86979112
chr10	84390899	84392246	10q23.1	TNPO1P1	1.624964336	1.116576302	1.455309712	4	4	55.04335882	64.8186412
chr4	128269237	128288829	4q28.2	PGRMC2	12.09177852	8.308838663	1.455291047	4	4	62.00476766	36.27431292
chr10	89378056	89385205	10q23.31	IFIT1B	244.9825425	168.342027	1.455266679	4	4	43.72460038	95.15526232
chr17	78378618	78424659	17q25.3	PGS1	15.9677788	10.97359238	1.455109526	4	4	64.97317184	50.20317424
chr17	4967992	4988281	17p13.2	CAMTA2	5.469785037	3.75906979	1.455090047	4	4	60.88386723	36.09925246
chr17	16620734	16655210	17p11.2	ZNF624	4.669956724	3.209632965	1.454981543	4	4	35.30098461	41.62934176
chr10	32446082	32882864	10p11.22	CCDC7	102.0301317	70.12620002	1.454950242	4	4	28.62497544	22.79401351
chr5	163460511	163491945	5q34	HMMR	35.68019608	24.52454342	1.454877078	4	4	58.50525827	37.17062215
chr14	75578597	75648169	14q24.3	FLVCR2	2.582752441	1.775280359	1.454842007	4	4	62.48303871	36.47412205
chr10	125823535	125853695	10q26.2	BCCIP	12.14813715	8.3502975	1.454814891	4	4	24.78577353	12.08570378
chr16	83968632	84002776	16q23.3	NECAB2	1.155246036	0.794165284	1.454667007	4	3	96.14887946	78.96886155
chr11	66070276	66244747	11q13.1-q13.2	PACS1	14.53744392	9.994920036	1.454483264	4	4	78.57407421	29.97336341
chr22	43132206	43143397	22q13.2	MCAT	1.511927678	1.039530137	1.454433714	4	4	68.16218046	29.34782601
chr1	167630193	167708696	1q24.2	RCSD1	65.44078958	44.99474651	1.454409562	4	4	62.19295718	39.25901761
chr1	180269663	180502887	1q25.2-q25.3	ACBD6	9.113925121	6.266675557	1.45434769	4	4	33.96420143	33.3210368
chr2	40112117	40512452	2p22.1	SLC8A1	8.948101684	6.152995378	1.454267578	4	4	49.74542825	38.39756113
chr17	30116807	30186475	17q11.2	NSRP1	10.14537996	6.976768724	1.454166014	4	4	90.88574115	32.36394068
chr12	100267140	100341724	12q23.1	SCYL2	65.9862891	45.37844178	1.454132987	4	4	28.36686247	18.69185844
chr19	1941149	1981338	19p13.3	CSNK1G2	31.68106651	21.78699323	1.454127524	4	4	66.48333081	24.13091747
chr6	134169246	134318112	6q23.2	SGK1	33.06542641	22.73921284	1.454114821	4	4	64.63510069	48.13919447
chr7	74083777	74122525	7q11.23	LIMK1	6.790391354	4.670034125	1.454034633	4	4	60.91723527	44.12957838
chr8	124306189	124372699	8q24.13	TMEM65	7.696618007	5.293487832	1.453978596	4	4	30.13126519	51.1151758
chr7	43866558	43869546	7p13	MRPS24	0.521602908	0.358749312	1.453948176	2	4	5.698435873	64.89837753
chrX	118974614	119022925	Xq24	LONRF3	1.262492419	0.868352934	1.453893192	4	4	71.25652454	29.07167889
chr1	16895980	16896143	1p36.13	RNU1-2	74.10573943	50.97264806	1.453833424	4	4	36.61979926	37.76688221
chr11	18020537	18040788	11p15.1	TPH1	0.143202993	0.098501895	1.453809532	4	2	31.59570294	7.771672497
chrMT	5904	7445	N/A	MT-CO1	3567.537163	2454.281365	1.453597462	4	4	93.51506018	63.01473884
chr14	49643552	49688417	14q21.3	POLE2	319.742181	220.0181854	1.453253423	4	4	89.96912825	46.52259552
chr17	996893	1002183	17p13.3	TIMM22	2.563713361	1.764164552	1.453216684	4	4	48.09525489	48.78357954
chr1	111499429	111504121	1p13.2	ADORA3	2.003783187	1.378932682	1.453140689	4	4	74.13260564	84.12257106
chr14	81175452	81221231	14q31.1	GTF2A1	29.03816836	19.98394213	1.453075082	4	4	17.47367632	22.20466339
chr6	28224702	28233487	6p22.1	ZSCAN9	2.138083205	1.471562937	1.452933579	4	4	63.02638792	42.64071469
chr19	11648348	11652160	19p13.2	ZNF887P	0.5946375	0.409268954	1.45292599	2	3	3.98851353	52.24947392
chr16	74671855	74701148	16q23.1	MLKL	15.97640344	10.99844601	1.452605525	4	4	77.02926197	48.77510005
chr16	57999599	58021623	16q21	USB1	2.486190511	1.711677581	1.452487629	4	4	72.99342217	30.9482915

chr3	72374593	72446623	3p13	RYBP	6.082393614	4.187639937	1.45246337	4	4	71.15407595	33.66033958
chr13	51584194	51766799	13q14.3	WDFY2	4.025317228	2.771551966	1.452369386	4	4	56.4761678	38.41281187
chr3	25718944	25790039	3p24.2	NGLY1	10.57618625	7.282916039	1.452191155	4	4	33.00382659	20.16318476
chr16	31083424	31089652	16p11.2	PRSS53	0.239286375	0.164785319	1.452109792	1	4	0	86.88254665
chr3	50226688	50259354	3p21.31	GNAI2	102.5250058	70.60471348	1.452098603	4	4	64.43234359	30.04370035
chr2	264869	278283	2p25.3	ACP1	54.1684419	37.30417105	1.452074671	4	4	59.70402868	46.58576859
chr2	25994678	25995271	2p23.3	UQCRHP2	1.383841547	0.953065726	1.451989625	1	1	0	0
chr2	240997410	241095568	2q37.3	SNED1	0.455001658	0.313370047	1.451962823	4	4	127.6686428	60.37268225
chr1	171841478	172418466	1q24.3	DNM3	18.38063911	12.66082722	1.451772367	4	4	24.89948803	39.59946602
chr12	49791139	49828750	12q13.12	NCKAP5L	3.221000169	2.218848194	1.451654141	4	4	79.5183728	43.49810668
chr13	80655397	80657198	13q31.1	PWWP2AP1	0.173689565	0.119652043	1.451622228	3	2	58.39145892	15.37609893
chr9	21003621	21031712	9p21.3	HACD4	24.10068553	16.60275803	1.451607347	4	4	34.61861557	18.35373307
chr2	201071739	201085750	2q33.1	NDUFB3	47.47639667	32.70990384	1.451437977	4	4	47.74764604	27.267702
chr21	17593650	17612950	21q21.1	BTG3	4.215390264	2.904482503	1.451339528	4	4	15.51096543	34.49619126
chr13	48233138	48262096	13q14.2	ITM2B	225.2555531	155.2074471	1.451319234	4	4	33.95963668	28.98238119
chrX	112774877	112840946	Xq23	AMOT	0.720543894	0.496555384	1.451084649	4	4	36.79863825	86.34726231
chr1	97077743	97921059	1p21.3	DPYD	57.45929582	39.59954597	1.451008955	4	4	41.92043654	20.70849562
chr12	1691027	1788679	12p13.33	ADIPOR2	65.69702204	45.28018864	1.450899919	4	4	50.68973765	40.96191911
chr17	40092784	40100725	17q21.1	NR1D1	1.602176821	1.104292	1.450863377	4	4	99.83125439	51.2165679
chr2	189879479	190203484	2q32.2	C2orf88	68.85580912	47.45870905	1.450857187	4	4	22.84117033	22.45037278
chr16	22091929	22156966	16p12.2	VWA3A	0.147671799	0.101795082	1.450677138	1	1	0	0
chr10	46911372	47003829	10q11.22	PTPN20	0.203770296	0.140477529	1.450554386	3	4	78.36203371	44.99686478
chr12	49184795	49189324	12q13.12	TUBA1A	172.8638016	119.1777078	1.450470938	4	4	63.31477172	95.17916547
chr1	7915871	7943165	1p36.23	TNFRSF9	1.790776677	1.234657352	1.450424017	4	4	72.17072288	74.04112569
chr11	63681315	63759891	11q13.1	RTN3	59.15868073	40.78987294	1.450327654	4	4	44.58135837	35.90109185
chr5	56192368	56193835	5q11.2	C1GALT1P2	0.554776846	0.382543468	1.450232175	4	2	25.10098223	35.47548615
chr3	160755532	161078907	3q25.33-q26.1	PPM1L	7.267083425	5.012634724	1.449753238	4	4	24.65896756	27.86532873
chr19	7680821	7682862	19p13.2	TRAPPC5	0.527170527	0.363641477	1.449698564	4	4	90.33658534	53.07906265
chr9	133019423	133021169	9q34.13	EEF1A1P5	27.96216838	19.29200362	1.449417537	4	4	45.30394483	24.0379504
chr4	39044674	39143101	4p14	KLHL5	17.91560979	12.36118443	1.449344106	4	4	54.09624533	10.6273604
chr16	70379435	70439088	16q22.1	ST3GAL2	11.71433164	8.082885297	1.449275996	4	4	50.96054687	26.78006599
chr17	48026167	48048086	17q21.32	COPZ2	0.932547585	0.643482835	1.449219055	3	3	31.05492706	69.2941905
chr17	75087727	75106162	17q25.1	SLC16A5	4.423405202	3.052626976	1.449048717	4	4	58.16040065	32.66329088
chr10	69124153	69174416	10q22.1	VPS26A	35.63935925	24.59567902	1.449008959	4	4	27.79208522	35.68737597
chr1	23281309	23285518	1p36.12	LINC01355	0.179391694	0.123809665	1.44893126	1	2	0	18.0043259
chr10	89579497	89645572	10q23.31	PANK1	0.508291135	0.350832502	1.44881427	4	4	47.39337718	94.71210166
chr9	136547842	136549744	9q34.3	NALT1	0.820679393	0.566514867	1.448645818	2	3	51.72005201	69.73291665
chrX	18890296	18894975	Xp22.13	PHKA2-AS1	0.56678299	0.391291721	1.44849216	1	2	0	36.60316122
chr6	46652915	46678190	6p12.3	SLC25A27	0.241955054	0.167040047	1.44848531	1	2	0	3.72458919
chr4	56647988	56681866	4q12	HOPX	3.805243036	2.627508493	1.44823244	4	4	27.1535829	22.84239053
chr2	47806920	47905793	2p16.3	FBXO11	40.85140202	28.21094982	1.448069004	4	4	18.10967571	15.43939485
chrX	74218377	74218461	Xq13.2	MIR421	9.741741968	6.72769047	1.448006862	3	4	100.2003944	48.51299466
chr7	116862509	116919261	7q31.2	CAPZA2	76.96144222	53.15171203	1.447957917	4	4	4.461838727	33.41595519

chr1	38862490	38873378	1p34.3	MYCBP	11.05451469	7.63473456	1.447923906	4	4	17.41201234	31.65429387
chr4	76313039	76421920	4q21.1	CCDC158	0.153051407	0.105708841	1.447858143	1	2	0	22.26442898
chr15	43185118	43197177	15q15.2	CCNDBP1	58.01324775	40.07089332	1.447765271	4	4	27.04764196	27.12612681
chr14	35401510	35404754	14q13.2	NFKBIA	135.69316	93.73380675	1.447643755	4	4	69.74500348	38.85530728
chr22	50578958	50582999	22q13.33	CHKB	0.723134912	0.499534785	1.447616732	4	4	26.96853171	47.0179523
chr6	30489406	30494205	6p22.1	HLA-E	399.1375045	275.7341775	1.447544545	4	4	61.14142324	25.17678341
chr2	69319769	69387254	2p13.3	GFPT1	10.95537475	7.568305643	1.44753334	4	4	30.5220912	19.17381803
chr6	75601509	75718285	6q14.1	SENP6	83.48676954	57.67637824	1.447503676	4	4	25.24837357	17.23447371
chr12	112125545	112153604	12q24.13	TRAFD1	39.52546791	27.30691272	1.447452823	4	4	51.35569409	30.68373674
chr10	96364606	96513926	10q24.1	TLL2	0.047205118	0.032613597	1.447406089	2	1	38.49189837	0
chr20	36996349	37096215	20q11.23	RBL1	136.2344637	94.12398639	1.447393687	4	4	74.35584671	33.30751263
chr6	89254464	89315299	6q15	GABRR2	0.285832724	0.197490752	1.447322073	3	3	44.10740237	62.25550531
chr9	130400269	130400565	9q34.11	RN7SL665P	1.226684034	0.847580906	1.447276627	2	1	31.81967346	0
chr13	48987911	48988016	13q14.2	RNU6-60P	9.73108161	6.723811748	1.447256701	4	4	74.57513667	50.31061778
chr17	27294021	27313631	17q11.1	WSB1	32.23495834	22.27316272	1.447255549	4	4	15.58129915	37.49886981
chr18	26687621	26703638	18q11.2	PCAT18	0.145538743	0.100582457	1.446959514	1	1	0	0
chr2	173075435	173268009	2q31.1	MAP3K20	4.619422509	3.192660487	1.446888113	4	4	65.94544393	26.27673647
chr10	103245887	103248016	10q24.33	RPEL1	0.164392488	0.113640278	1.446604062	2	1	12.31981162	0
chr1	207018521	207032761	1q32.1	C1orf116	2.069552893	1.430637847	1.446594536	4	4	28.1200791	21.86424019
chr10	93566178	93590072	10q23.33	FFAR4	0.090659886	0.062672825	1.446558148	3	2	24.97724445	1.572811057
chr2	85341955	85354698	2p11.2	RETSAT	3.527323919	2.438564237	1.446475703	4	4	49.68203512	39.90566752
chr19	14600175	14674918	19p13.12	ADGRE3	79.40260517	54.90212611	1.446257382	4	4	65.83186734	59.91022056
chr17	47694065	47712063	17q21.32	TBKBP1	4.336962922	2.998807125	1.446229364	4	4	55.99520426	56.18386475
chr11	31509729	31784525	11p13	ELP4	2.322780771	1.60614286	1.44618566	4	4	37.31117621	19.43806663
chrX	20149911	20267514	Xp22.12	RPS6KA3	56.7404053	39.23648465	1.446113377	4	4	37.17832007	13.29424638
chr11	66284580	66289167	11q13.2	YIF1A	3.667130925	2.536317385	1.445848594	4	4	60.09975342	41.81943469
chr3	131361845	131365122	3q22.1	NUDT16P1	3.769593859	2.60726017	1.445806561	4	4	37.60700835	65.63550177
chrX	65407877	65408674	Xq12	TLE1P1	0.916000095	0.633665505	1.445557773	2	4	84.03675869	50.92937417
chr17	35009112	35089329	17q12	RFFL	16.79454272	11.61920956	1.445411811	4	4	41.95590652	26.93682154
chr16	28878488	28904509	16p11.2	ATP2A1	0.23707314	0.164028838	1.445313783	3	4	104.3346265	55.0193579
chr3	170037947	170085417	3q26.2	GPR160	15.56498224	10.76985498	1.445236009	4	4	61.51146746	52.03034935
chr5	134236120	134372380	5q31.1	CDKL3	1.964174047	1.359386141	1.444897802	4	4	131.0724047	38.65908951
chr1	155946839	155991262	1q22	ARHGEF2	31.73887221	21.96851786	1.444743447	4	4	47.40673138	39.5357359
chr1	14362	29370	1p36.33	WASH7P	2.647194123	1.83261483	1.444490178	4	4	128.4247502	61.2992195
chr12	98512973	98516226	12q23.1	TMPO-AS1	1.255963384	0.869771799	1.444014839	4	4	60.04125298	81.0280606
chr8	73420369	73747708	8q21.11	STAU2	20.47000289	14.17590824	1.443999393	4	4	13.01546809	20.72263289
chr11	103907186	104164299	11q22.3	PDGFD	6.346115516	4.395107231	1.443904593	4	4	60.1229953	22.61471491
chr9	111631352	111654351	9q31.3	DNAJC25	3.340396932	2.313510535	1.443865019	4	4	35.18616987	36.34533508
chr20	49632945	49713884	20q13.13	B4GALT5	54.99840295	38.09120254	1.443861031	4	4	52.72323827	16.84659761
chr22	22668326	22668808	22q11.22	IGLV3-27	1.698504782	1.176508948	1.443681992	2	3	31.7345513	43.14238577
chr19	19176904	19192591	19p13.11	BORCS8	1.608352789	1.114110441	1.443620605	4	4	75.12356426	48.11372169
chr8	66712418	66862022	8q13.1	SGK3	3.28701761	2.276940139	1.443611781	4	4	63.71383687	59.23953623
chr6	56457987	56954671	6p12.1	DST	0.996317553	0.690168812	1.443585303	4	4	61.05792644	52.33754198

chr2	85318018	85328296	2p11.2	TGOLN2	53.81432719	37.28293389	1.443403766	4	4	41.85300479	26.90383631
chr19	1021522	1021628	19p13.3	RNU6-2	92.84493459	64.33121216	1.443233098	4	4	31.60371414	57.78582892
chrX	47635520	47650604	Xp11.23	ELK1	5.256618236	3.642279421	1.443222122	4	4	70.99840499	38.28569412
chr15	82836946	82986176	15q25.2	HOMER2	0.793919593	0.550110268	1.443200825	4	4	103.3092856	31.13860438
chr10	50184861	50251564	10q11.23	ASAH2	0.165133172	0.114426839	1.443133208	3	3	38.0910223	90.70149536
chr6	30653119	30673053	6p21.33	DHX16	10.82250421	7.499843555	1.443030662	4	4	56.37506737	37.16279478
chr20	56411550	56460240	20q13.31	CASS4	21.41053545	14.83748878	1.443002637	4	4	58.15768246	40.07178781
chr12	48334380	48351414	12q13.11	ZNF641	17.49699582	12.12548128	1.442993925	4	4	60.53120073	52.10024379
chr11	62856012	62888883	11q12.3	SLC3A2	8.057008225	5.584610239	1.442716301	4	4	36.71645396	15.58177889
chr11	31432399	31509644	11p13	IMMP1L	23.62125092	16.37294132	1.442700518	4	4	83.98483991	66.49293031
chr8	51319575	51809445	8q11.22-q11.23	PXDNL	0.143006162	0.099126862	1.442658013	3	3	69.57940546	45.67099497
chr12	10372353	10410146	12p13.2	KLRC4-KLRK1	4.325295152	2.998371212	1.442548252	4	4	43.95571996	25.75491688
chr17	1003518	1229712	17p13.3	ABR	6.17003149	4.277199665	1.442539973	4	4	60.71512664	30.51026162
chr17	36103827	36105621	17q12	CCL4	14.49077453	10.04841255	1.442095899	4	4	64.69655867	49.04085853
chr17	7549058	7557890	17p13.1	TNFSF12	5.458203173	3.78519263	1.441988217	4	4	65.83059125	46.56431043
chrX	154010506	154019984	Xq28	IRAK1	16.80134367	11.65151779	1.441987556	4	4	69.54201338	24.63769389
chr6	30647039	30653210	6p21.33	C6orf136	4.197103097	2.91077975	1.4419171	4	4	27.80650711	24.25580099
chr14	93182196	93184904	14q32.12	MOAP1	1.387836383	0.962558785	1.441819871	4	4	79.10629947	87.87048193
chr5	102233986	102296549	5q21.1	SLCO4C1	14.45674946	10.02821514	1.441607431	4	4	22.5610211	50.38713321
chr19	18990887	19034052	19p13.11	SUGP2	8.76589575	6.08081455	1.44156604	4	4	32.87827284	37.6601886
chr3	71541970	71542089	3p13	MIR1284	4.909551975	3.406147305	1.441379816	1	1	0	0
chr1	99978939	99979045	1p21.2	RNU6-750P	4.632364685	3.213915	1.441346359	2	2	26.84859485	25.7524793
chr4	112145397	112189081	4q25	FAM241A	3.161219188	2.193397849	1.44124295	4	4	39.39861415	41.97488467
chr9	14081843	14398983	9p23-p22.3	NFIB	0.691744372	0.480012429	1.441096793	4	4	30.5380121	26.03549187
chr17	42458844	42522579	17q21.2	ATP6V0A1	53.19496388	36.91481581	1.44101935	4	4	65.34170829	65.09824703
chr2	159803355	159904756	2q24.2	LY75	6.980696632	4.844446021	1.440969019	4	4	36.71554626	30.26158774
chr22	19966727	20016802	22q11.21	ARVCF	0.544052402	0.377606868	1.440790537	3	4	78.56732574	38.06110069
chr1	206203556	206464443	1q32.1	SRGAP2	3.615571821	2.509523348	1.440740459	4	4	48.29005758	28.58805734
chr21	32728114	32743122	21q22.11	PAXBP1-AS1	1.382110665	0.959328521	1.440706322	4	4	45.59033618	46.40857917
chr21	37208503	37221736	21q22.13	DSCR9	0.236548063	0.164212487	1.440499851	2	1	60.62183636	0
chr1	24745269	24844324	1p36.11	CLIC4	37.00389152	25.69061749	1.440365983	4	4	4.00675948	20.74331018
chr13	26254104	26405238	13q12.13	CDK8	106.6689716	74.06912558	1.440127324	4	4	45.55293135	14.04562651
chr15	28111037	28322173	15q13.1	HERC2	4.135107705	2.871893985	1.439853883	4	4	36.63824358	23.16170622
chr5	14581782	14616180	5p15.2	OTULINL	19.13350598	13.28986441	1.439706636	4	4	47.27458976	22.78789865
chr22	23262767	23265005	22q11.23	FBXW4P1	0.709320672	0.492733307	1.439563068	3	4	66.89778314	47.45229701
chr12	7080209	7092607	12p13.31	C1R	0.308816501	0.214528643	1.439511748	4	4	102.0441488	62.38167255
chr2	27663471	27694980	2p23.3	SLC4A1AP	25.50211828	17.71727925	1.439392466	4	4	23.19088632	13.72850451
chr2	37920789	38069246	2p22.2	RMDN2	7.598008163	5.278863202	1.439326588	4	4	59.17689608	57.04214822
chr3	196237745	196287752	3q29	PCYT1A	14.78302638	10.27146506	1.439232504	4	4	37.44997088	34.39766337
chr3	120805887	120806662	3q13.33	NAP1L1P3	0.377197847	0.262093529	1.439172679	1	1	0	0
chr22	50059391	50085929	22q13.33	MLC1	6.284490124	4.366910707	1.439115783	4	4	48.27585695	27.97612488
chrX	129532736	129592561	Xq26.1	OCRL	2.36473249	1.643329494	1.43898865	4	4	16.99703834	32.14447127
chr3	196946343	196969411	3q29	PIGZ	0.246006989	0.170969872	1.438890882	3	2	61.83169612	20.31970626

chr5	77030385	77065234	5q13.3	AGGF1	106.3897633	73.94095804	1.43884751	4	4	38.86053274	23.76411526
chr9	96641251	96655331	9q22.33	PRXL2C	5.902160936	4.102114591	1.438809376	4	4	35.72958359	24.70257195
chr10	67825310	67825594	10q21.3	RN7SL394P	1.551279636	1.078272768	1.438670884	2	2	24.31269555	17.81453191
chr8	38382505	38409527	8p11.23	LETM2	1.390711016	0.966716809	1.438591946	4	4	65.56114659	55.04107056
chr3	23889949	23917046	3p24.2	NKIRAS1	2.619678671	1.821095476	1.438518027	4	4	37.40708602	19.14733069
chr1	39560813	39576849	1p34.3	PABPC4	14.28386506	9.929979749	1.438458629	4	4	42.34155099	21.50975736
chr12	14973022	14981865	12p12.3	PDE6H	1.941052681	1.349584394	1.438259578	1	2	0	103.9285588
chr19	45609396	45645629	19q13.32	EML2	2.563909302	1.78277143	1.438159294	4	4	61.87743602	35.11686323
chr9	35817017	35829148	9p13.3	FAM221B	0.103335802	0.071855416	1.43810734	2	1	19.86106548	0
chr19	35540738	35546029	19q13.12	TMEM147-AS1	0.695576308	0.483721418	1.437968803	4	4	74.34999488	74.59267271
chr19	55230885	55230952	19q13.42	MIR6804	6.691211086	4.653486627	1.437891977	3	2	73.52445862	36.83175797
chr3	47250525	47250626	3p21.31	SNORD13P3	3.909124902	2.718751804	1.43783809	1	1	0	0
chr1	65792510	66374579	1p31.3	PDE4B	47.81481319	33.25669822	1.437749859	4	4	41.02029362	11.04174711
chr1	99850077	99924023	1p21.2	AGL	9.109296146	6.336279016	1.43764126	4	4	45.46188442	25.5232503
chr19	18097764	18151692	19p13.11	MAST3	22.54249768	15.68122224	1.437547235	4	4	58.46353228	41.66879509
chr9	114334149	114398498	9q32	AKNA	73.20861024	50.92609156	1.437546216	4	4	82.18531906	42.83340287
chr8	97643972	97730260	8q22.1	MTDH	76.2867927	53.06901503	1.437501575	4	4	18.25740427	9.024890485
chr12	71686098	71704060	12q21.1	TMEM19	4.798520171	3.338101003	1.437499993	4	4	35.17806895	36.52169504
chrX	152827327	152830757	Xq28	CETN2	22.66366392	15.76702915	1.437408639	4	4	25.82632643	8.99207096
chr2	231433572	231434430	2q37.1	ZBTB8OSP2	0.69392673	0.48279	1.437326228	1	2	0	7.978769886
chr1	162497174	162529629	1q23.3	UHMK1	74.06950486	51.53291751	1.437324111	4	4	13.88426302	10.42542651
chr4	118850688	119061247	4q26	SYNPO2	0.192697992	0.134068894	1.437305747	4	4	34.03494762	17.52763224
chr6	113969701	114340729	6q21-q22.1	HDAC2-AS2	0.107648488	0.074899189	1.437245041	2	4	44.05133712	89.02843697
chr17	39665346	39666554	17q12	TCAP	0.855423313	0.595235682	1.437116993	4	4	94.12706974	33.44966374
chr6	6169304	6169435	6p25.1	MIR7853	6.109751897	4.251529826	1.437071395	2	2	79.44355097	78.66005807
chr13	30200026	30307501	13q12.3	KATNAL1	24.20890189	16.84805508	1.436895937	4	4	112.7672974	41.45839771
chr4	165207618	165323156	4q32.3	KLHL2	30.6172207	21.3081962	1.436875295	4	4	43.87090849	54.44453913
chr12	53507856	53626415	12q13.13	ATF7	2.679648731	1.865074582	1.436751515	4	4	73.93795977	25.02864382
chrX	63634967	63785546	Xq11.1	ARHGEF9	1.899037844	1.321851618	1.436649786	4	4	57.79859913	26.10050429
chr3	121631398	121660948	3q13.33	HCLS1	157.0629378	109.3369509	1.43650373	4	4	70.49083621	46.01197428
chr11	66052345	66068911	11q13.1	SF3B2	25.76831357	17.9392618	1.436419952	4	4	73.99255484	36.73386393
chr4	121131408	121227609	4q27	TNIP3	0.887250052	0.617887181	1.435941835	4	4	55.63497697	125.2806827
chrX	12902817	12943300	Xp22.2	TLR8-AS1	0.886085835	0.617142147	1.435788885	3	2	96.81478477	94.07099237
chr2	164492406	164622959	2q24.3	GRB14	3.288461146	2.290485801	1.435704663	4	4	29.33312584	22.21094761
chr5	172953677	172969771	5q35.1	RPL26L1	7.318284107	5.098299766	1.435436213	4	4	22.80999984	37.21646475
chr11	126341716	126355587	11q24.2	GSEC	2.255865526	1.571596893	1.435397038	4	4	79.55048125	73.74637286
chr5	69217746	69230158	5q13.2	MRPS36	15.15388282	10.55745997	1.435372037	4	4	53.34537558	43.40189613
chr4	99878338	99894546	4q23	LAMTOR3	25.82952083	17.99505221	1.435367929	4	4	11.4458236	28.14149679
chr12	75480733	75501936	12q21.2	GLIPR1	69.72328708	48.57590735	1.435347086	4	4	70.02733201	28.17073431
chr11	62602218	62612765	11q12.3	EML3	7.190717968	5.009805448	1.435328785	4	4	72.27597044	26.74018838
chr15	42210452	42273584	15q15.1	TMEM87A	34.48129025	24.02412322	1.435277781	4	4	38.89323848	29.46752775
chr10	68341107	68407302	10q21.3	RUFY2	36.23803083	25.25021769	1.435157165	4	4	65.26400733	42.42327177
chr2	24492050	24770702	2p23.3	NCOA1	103.2855979	71.97237291	1.435072844	4	4	17.5587541	13.40227296

chr20	59863277	59933700	20q13.33	SYCP2	2.548233731	1.775933163	1.43487029	4	4	18.62313644	44.18042701
chr9	94038030	94038345	9q22.32	CYCSP24	1.813107141	1.26392039	1.434510555	3	3	86.95704057	44.51789024
chr12	19129680	19376400	12p12.3	PLEKHA5	1.603936938	1.118321957	1.434235398	4	4	26.15119506	32.01494724
chr5	40679930	40740936	5p13.1	PTGER4	41.40689835	28.87088495	1.43420953	4	4	48.57926626	26.83425936
chr6	3722602	3752297	6p25.2	PXDC1	0.715744755	0.499059859	1.434186183	3	4	11.67496772	50.2476281
chr1	70144802	70205678	1p31.1	LRRC40	32.59213962	22.72749908	1.434039861	4	4	52.4520141	29.87952872
chr2	112055223	112119318	2q13	TMEM87B	21.17113947	14.76517816	1.433856012	4	4	15.07534935	20.02427291
chrX	30558824	30577916	Xp21.2	CXorf21	27.24495288	19.00244235	1.433760586	4	4	57.89434518	51.04650737
chr7	6798935	6826295	7p22.1	CCZ1B	27.88840247	19.45344654	1.433596993	4	4	71.81333164	55.78083024
chr3	149984254	149984989	3q25.1	PPIAP73	0.95192618	0.664032943	1.433552643	2	1	9.61049569	0
chr9	76441666	76507416	9q21.13	GCNT1	5.960380882	4.15781789	1.433535821	4	4	31.54194245	43.23646996
chr4	109673779	109674321	4q25	HIGD1AP14	3.365265999	2.347603742	1.433489792	3	4	78.12490979	48.17938
chr6	112087471	112102790	6q21	FAM229B	0.437731853	0.305400964	1.433302132	2	2	58.17886982	26.59844705
chr6	109492855	109691212	6q21	AK9	5.896167639	4.114181675	1.433132541	4	4	49.01054526	27.18561412
chr1	161983192	162024465	1q23.3	OLFML2B	0.481333321	0.335883892	1.433034842	4	3	98.15152296	65.215376
chr3	49268597	49277075	3p21.31	C3orf62	6.155826292	4.295791569	1.432989984	4	4	76.91192769	48.7732852
chr5	127517609	127555089	5q23.2	PRRC1	9.9496376	6.943573275	1.432927573	4	4	37.3741615	26.74765236
chr3	150741123	150763148	3q25.1	SIAH2	183.7821902	128.2572239	1.432918822	4	4	45.15967814	72.79834347
chr17	82047902	82051774	17q25.3	RFNG	4.616150615	3.221501852	1.432918815	4	4	78.99063072	50.8920079
chr9	38621088	38623284	9p13.1	FAM201A	0.142048108	0.099136004	1.432860941	2	2	66.95438103	36.32243149
chr16	69033630	69034561	16q22.1	RPS2P45	0.199717926	0.139414301	1.43254978	2	4	55.5503236	31.19233587
chr11	65569220	65570413	11q13.1	SSSCA1-AS1	0.583097948	0.40704877	1.432501437	4	4	55.57734705	44.80707053
chr9	25676389	25678858	9p21.2	TUSC1	0.356198154	0.248671587	1.432403914	2	2	55.85689851	70.56159641
chr7	44044640	44061913	7p13	DBNL	20.33191588	14.19522386	1.432306815	4	4	64.72352656	26.06477962
chr2	199911256	199928273	2q33.1	C2orf69	12.13438264	8.472028589	1.432287735	4	4	22.73415756	16.30767432
chr15	56244043	56445997	15q21.3	TEX9	57.07771092	39.8537766	1.432178222	4	4	48.20725987	37.36874932
chrX	81201804	81298547	Xq21.1	SH3BGRL	343.1517128	239.6203321	1.432064256	4	4	31.61302684	25.62555037
chr10	103396615	103446295	10q24.33	PDCD11	17.93809392	12.52857078	1.431774959	4	4	38.63159594	17.02874289
chr11	9138825	9265326	11p15.4	DENND5A	43.34661261	30.27550574	1.43173868	4	4	56.31553184	45.18232395
chr6	16129086	16151015	6p22.3	MYLIP	35.97421704	25.12873402	1.431596873	4	4	34.47108161	25.2888195
chr7	6615617	6624290	7p22.1	ZNF853	1.356127522	0.947291811	1.431583707	3	4	109.6968461	96.43832289
chr1	167541013	167553819	1q24.2	CREG1	133.25484	93.09174344	1.431435647	4	4	19.52683705	37.49627852
chr14	24232422	24239242	14q12	GMPR2	21.48457776	15.0114135	1.431216172	4	4	62.74823152	36.41685807
chr1	206186179	206205346	1q32.1	FAM72A	2.085398523	1.45718593	1.431113546	4	4	58.56474065	40.75375045
chr14	50719763	50831140	14q22.1	NIN	73.65210358	51.46609477	1.431080091	4	4	52.53021293	28.1691226
chr14	87933014	87993665	14q31.3	GALC	17.25629406	12.05829525	1.431072445	4	4	18.0719119	22.39666188
chr16	1328626	1329809	16p13.3	RPS20P2	0.924276997	0.645884527	1.431025142	1	3	0	8.251948374
chr1	161118072	161125446	1q23.3	NIT1	5.868588573	4.101190557	1.430947548	4	4	41.06396922	29.64437813
chr15	90184920	90229660	15q26.1	SEMA4B	9.615312025	6.720669581	1.430707448	4	4	53.64964968	29.83475424
chr3	17157162	17742739	3p24.3	TBC1D5	30.76013427	21.49999773	1.430704071	4	4	35.45717005	29.71369775
chr17	8220630	8224043	17p13.1	LINC00324	6.221746062	4.349066433	1.430593475	4	4	47.79110862	46.13769742
chr12	124915547	124915659	12q24.31	MIR5188	3.60411627	2.519560424	1.43045439	2	3	63.24343912	31.72308892
chr11	67364168	67374177	11q13.2	CLCF1	1.687414336	1.179647767	1.430439139	4	4	79.16061887	67.74057666

chr11	6211334	6234711	11p15.4	FAM160A2	7.884021268	5.511734993	1.430406447	4	4	39.55021332	42.87918346
chr1	1211326	1216812	1p36.33	TNFRSF4	1.180392429	0.825228143	1.430383148	4	4	75.39788459	79.25015099
chr16	20899626	20925006	16p12.3	LYRM1	20.42414064	14.27887296	1.43037484	4	4	10.56803125	17.29095356
chrX	49922615	50099235	Xp11.23	CLCN5	1.735215066	1.213259598	1.430209222	4	4	47.57979499	40.65618315
chr1	153543619	153545806	1q21.3	S100A4	279.9394915	195.74338	1.430135167	4	4	13.46879789	43.19695918
chr14	81471547	81533861	14q31.1	SEL1L	38.54651659	26.95474634	1.430045607	4	4	56.17590697	20.87787778
chr4	1692731	1715876	4p16.3	SLBP	72.69471815	50.8436002	1.429771257	4	4	20.80887864	13.54063109
chr2	63840964	63891562	2p15	UGP2	54.0956019	37.83724216	1.429691986	4	4	20.24068508	10.21440301
chr1	27341992	27353932	1p36.11	SYTL1	9.199944861	6.435115222	1.429647263	4	4	42.20456069	32.20434353
chr5	95646744	95658082	5q15	RFESD	14.3590343	10.04376514	1.429646562	4	4	76.95197868	76.31009622
chr19	41844743	41852330	19q13.2	DMRTC2	0.11392795	0.079693613	1.429574415	3	2	16.2132216	7.010038769
chrX	10156945	10237660	Xp22.2	CLCN4	4.226327764	2.95648409	1.429511418	4	4	47.51808559	31.52332696
chr15	44562696	44663678	15q21.1	SPG11	46.61169248	32.61018884	1.429359784	4	4	27.79714095	7.055551545
chr5	91368632	91383373	5q14.3	ARRDC3	146.4352181	102.4491068	1.429345972	4	4	53.80924575	33.33280531
chr7	92198969	92246100	7q21.2	KRIT1	63.90012134	44.71059757	1.42919408	4	4	56.37601722	45.34725007
chr8	13083361	13604616	8p22	DLC1	0.338287071	0.236706493	1.429141494	4	4	7.943049822	41.9289026
chr7	22895848	23014151	7p15.3	FAM126A	14.21358021	9.946025368	1.429071381	4	4	10.85560299	23.69841603
chr10	101588288	101609664	10q24.32	DPCD	6.27302289	4.389901561	1.428966642	4	4	51.99817486	66.39096637
chr22	20982269	20999037	22q11.1-q11.2	LZTR1	3.013626356	2.109367182	1.42868742	4	4	65.10270563	27.17673516
chr1	155859469	155885199	1q22	SYT11	10.55554821	7.38879864	1.428587883	4	4	29.9819776	50.16970225
chr3	134477704	134486023	3q22.2	ANAPC13	27.16701927	19.0189221	1.428420556	4	4	30.79452344	16.88819655
chr8	127079874	127092595	8q24.21	PRNCR1	0.08871492	0.062109573	1.428361457	4	3	74.47813819	5.653459805
chr9	105244605	105439775	9q31.1-q31.2	SLC44A1	7.358660265	5.151899929	1.42833913	4	4	41.5437663	23.33678436
chr2	73457103	73459482	2p13.1	ALMS1-IT1	1.191344851	0.834086722	1.428322524	4	4	65.71985261	31.20561153
chrX	85277389	85379743	Xq21.1	POF1B	0.140775508	0.09856787	1.428208881	4	4	59.42912364	67.24434391
chrX	78129748	78139682	Xq21.1	TAF9B	12.42200799	8.698091206	1.428130344	4	4	50.08266952	14.25834214
chr22	31757202	31757498	22q12.2	RN7SL20P	1.793033848	1.255558881	1.428076273	1	2	0	45.71286764
chr1	109548564	109595843	1p13.3	GNAI3	15.75212462	11.03057599	1.428041893	4	4	31.66813568	21.50821157
chr16	70289767	70335213	16q22.1	DDX19B	3.438937913	2.408172901	1.428027826	4	4	48.55730135	35.54478521
chr1	220690286	220699157	1q41	C1orf115	0.56898854	0.398444509	1.428024549	4	4	93.85103304	44.38982204
chr5	134647757	134727911	5q31.1	SEC24A	20.33882223	14.24334007	1.427953143	4	4	34.9260625	7.168803482
chr4	676205	690748	4p16.3	SLC49A3	1.531743168	1.072692122	1.427942964	4	4	53.31216107	48.55781834
chr6	129576145	129710225	6q22.33	ARHGAP18	35.78334264	25.06062058	1.42787137	4	4	28.74476003	10.79118689
chr14	104769349	104795743	14q32.33	AKT1	13.42700311	9.40385337	1.427819276	4	4	66.07573363	24.84591027
chr2	218606871	218637186	2q35	PLCD4	0.217224988	0.152141403	1.427783516	4	3	49.88351975	39.60360834
chr14	88180108	88326912	14q31.3	KCNK10	0.169428568	0.118670505	1.427722646	2	2	83.61257483	59.84825811
chr19	2071036	2096664	19p13.3	MOB3A	63.7509625	44.65249355	1.42771338	4	4	61.05324293	37.78278787
chr6	146841388	147204614	6q24.3	STXBP5-AS1	1.995537632	1.397845852	1.427580608	4	4	29.03993425	37.98774886
chr7	138591689	138701547	7q34	SVOPL	0.060993992	0.042725594	1.427575066	2	1	35.13156091	0
chr22	41561010	41576666	22q13.2	CSDC2	0.119193815	0.083494237	1.427569367	2	2	8.671869728	7.458919478
chr4	47847233	47914667	4p12	NFXL1	8.343041968	5.845296874	1.427308509	4	4	40.07427773	44.61251562
chr5	172209645	172283797	5q35.1	UBTD2	4.03338165	2.826078231	1.427200991	4	4	36.78991496	45.06090092
chr1	25861484	25876707	1p36.11	PAQR7	2.603554774	1.824248837	1.427192783	4	4	73.65144	27.56364055

chr1	198808003	198937429	1q32.1	MIR181A1HG	2.968811459	2.08018987	1.427182923	4	4	65.27845108	45.01224908
chr17	58692140	58734342	17q22	RAD51C	17.20482672	12.05674643	1.426987523	4	4	89.23910362	44.93323805
chr15	66702110	66782000	15q22.31	SMAD6	0.209381494	0.146733748	1.42694844	3	3	68.18548957	69.16389534
chrX	130064920	130110713	Xq26.1	ELF4	31.69183794	22.21006425	1.426913384	4	4	60.99326174	38.09796332
chr17	77088643	77217101	17q25.2-q25.3	SEC14L1	77.7715281	54.50947643	1.426752433	4	4	36.99515385	29.5406748
chr11	75768733	75801536	11q13.5	DGAT2	17.83240937	12.49890217	1.426718053	4	4	61.34925343	61.37063635
chr15	64671263	64671368	15q22.31	RNU6-549P	3.766158094	2.639832042	1.426665801	1	1	0	0
chr20	49936377	49953885	20q13.13	RNF114	21.83801559	15.30895965	1.426485933	4	4	18.31099544	20.96492336
chr6	292057	351355	6p25.3	DUSP22	10.67654313	7.485387019	1.426318119	4	4	44.33774938	36.07146501
chr3	187368380	187371584	3q27.3	RTP4	10.64419765	7.462803583	1.42630012	4	4	80.86171749	62.27869049
chr20	6005938	6040053	20p12.3	CRLS1	15.67229409	10.9888959	1.426193698	4	4	61.77269121	32.08924434
chr15	90954864	90964858	15q26.1	RCCD1	8.534694354	5.98426527	1.426189176	4	4	27.75786922	26.11576615
chr12	14503663	14567857	12p13.1	PLBD1	95.26539278	66.80644855	1.425990976	4	4	64.23566261	52.08289082
chr22	31160152	31207019	22q12.2	RNF185	12.61119367	8.844049974	1.425952331	4	4	54.11110314	39.00067284
chr2	206651621	206718396	2q33.3	DYTN	1.788586877	1.254318128	1.425943576	4	4	66.22081078	70.98632184
chr11	61897296	61946312	11q12.2-q12.3	RAB3IL1	3.573254401	2.506115638	1.425813856	4	4	49.11217668	60.89914156
chr1	44807442	44814199	1p34.1	BTBD19	1.556112046	1.091426312	1.425760062	4	4	91.9902077	95.77737886
chr4	110045851	110199199	4q25	ELOVL6	2.071264439	1.45281113	1.425694225	4	4	56.00949224	52.26477713
chr6	158636474	158644786	6q25.3	DYNLT1	34.10154821	23.92037457	1.425627684	4	4	14.37498442	38.66043314
chr10	126012385	126388558	10q26.2	ADAM12	0.341614693	0.239626299	1.425614363	4	4	90.98410417	90.04246876
chr17	1517989	1562816	17p13.3	PITPNA	24.49597489	17.18371384	1.425534382	4	4	44.96415959	21.41277547
chr12	108515274	108519603	12q23.3	FICD	1.710175222	1.199683523	1.425521973	4	4	49.46092422	35.88240436
chr12	6789472	6820810	12p13.31	CD4	39.62347847	27.79677254	1.425470472	4	4	55.89759914	32.74846865
chr8	94880224	94896671	8q22.1	CCNE2	26.50399428	18.59318462	1.425468247	4	4	99.49212287	73.37708895
chr9	104763741	104777764	9q31.1	NIPSNAP3B	1.397177147	0.980156948	1.425462676	3	4	38.4950484	38.76970384
chr16	50025206	50037088	16q12.1	CNEP1R1	15.10850815	10.59931772	1.425422707	4	4	41.15255294	44.71920577
chr14	63371356	63543395	14q23.2	PPP2R5E	13.63529969	9.565828728	1.425417502	4	4	30.5921895	6.347822738
chr13	95019829	95301446	13q32.1	ABCC4	113.9703235	79.95869164	1.425365037	4	4	43.05548888	35.81566115
chr2	158171356	158456753	2q24.1	CCDC148	0.730000986	0.512151314	1.425361933	4	3	19.48656969	22.38879066
chr1	161046942	161069971	1q23.3	ARHGAP30	132.6084974	93.0360713	1.425344983	4	4	52.36170301	33.48406208
chr1	28147166	28193936	1p35.3	PTAFR	94.50703866	66.30917488	1.425248298	4	4	81.96995707	67.37906464
chr13	97434221	97467998	13q32.1	RAP2A	24.00104409	16.84253168	1.425025914	4	4	14.1837147	23.52461867
chr12	64713442	64759446	12q14.3	GNS	66.32532423	46.5488982	1.424852721	4	4	33.15366455	31.08726334
chr5	179911651	180072118	5q35.3	RNF130	18.87506562	13.24749159	1.424802989	4	4	56.4233611	46.74099965
chr4	88725954	89111398	4q22.1	FAM13A	31.01769645	21.77056104	1.424754116	4	4	76.66183465	43.68757357
chr1	150508011	150513789	1q21.2	ECM1	0.371942734	0.261062983	1.424724142	2	3	100.571518	75.08743807
chr19	51211069	51211601	19q13.41	SIGLEC22P	3.864398642	2.71251297	1.424656282	4	4	63.74092017	68.80913842
chr1	212051486	212052051	1q32.3	RPL21P28	5.378282269	3.775479793	1.424529481	3	4	19.32062766	83.05806241
chr13	26132115	26223085	13q12.13	RNF6	39.54741517	27.7643219	1.42439694	4	4	43.23670156	19.7773493
chr19	10333776	10339834	19p13.2	ICAM3	154.263764	108.3036797	1.424363092	4	4	71.98540801	58.03870975
chr6	28324737	28359157	6p22.3-p22.1	ZSCAN31	0.29087871	0.204220948	1.424333361	4	4	33.04114534	75.99329423
chr17	38869859	38921770	17q12	LASP1	61.63007081	43.27108946	1.424278232	4	4	62.01967063	49.25896635
chr6	106184476	106325820	6q21	ATG5	19.91699627	13.98399183	1.424271161	4	4	32.19320513	30.40848149

chr17	18244815	18258916	17p11.2	FLII	30.01051806	21.07265005	1.42414542	4	4	66.67448537	41.99482093
chr12	120725738	120740008	12q24.31	ACADS	2.979162937	2.091927094	1.424123692	4	4	25.3727193	54.51050539
chrY	20575711	20593154	Yq11.223	EIF1AY	153.1524859	107.5521506	1.423983482	2	3	15.00194028	86.81108687
chr15	25081286	25081379	15q11.2	SNORD116-15	8.979031888	6.306236054	1.423833775	2	1	36.98434607	0
chr16	367384	370569	16p13.3	MRPL28	11.34569589	7.970266924	1.423502625	4	4	83.47082401	35.03710972
chrX	120625674	120630150	Xq24	C1GALT1C1	21.12063679	14.83781113	1.423433458	4	4	41.09406812	46.45138677
chr6	157981856	158099176	6q25.3	SYNJ2	2.714384714	1.906956887	1.423411684	4	4	50.0076432	54.20882239
chr1	26177490	26189884	1p36.11	CNKSR1	0.211858274	0.148843219	1.423365307	3	2	75.72423657	76.6381376
chr8	70608577	70663279	8q13.3	LACTB2-AS1	0.286284645	0.201132386	1.423364236	2	4	35.96459161	45.1602315
chr9	16409503	16870788	9p22.3-p22.2	BNC2	1.119049953	0.786210749	1.423346036	4	4	68.97765381	56.34034765
chr19	38867825	38878279	19q13.2	RINL	2.556391424	1.796045661	1.423344338	4	4	64.12823183	47.20142571
chr6	36493882	36547516	6p21.31	STK38	125.7036658	88.31798364	1.423307696	4	4	39.97774642	36.49480563
chr18	54717842	54895516	18q21.2	RAB27B	59.09997047	41.52732341	1.423158673	4	4	38.46702487	43.50398858
chr2	89099859	89100334	2p11.2	IGKV1-16	5.502723546	3.866762427	1.423082915	4	3	65.56096189	114.3124969
chr3	150408335	150460119	3q25.1	TSC22D2	5.705519942	4.009461791	1.42301392	4	4	34.38966442	24.91744051
chr2	70295976	70302088	2p13.3	FAM136A	6.261011386	4.399934261	1.422978394	4	4	41.12390904	25.84449177
chr1	23901049	23903008	1p36.11	BTBD6P1	0.520879744	0.366049056	1.422977974	4	2	95.61677431	51.87783447
chr14	24146875	24160661	14q12	RNF31	8.365323384	5.878947379	1.422928774	4	4	68.70489452	37.59505935
chr22	39155536	39156096	22q13.1	FUNDC2P4	0.821054628	0.577049054	1.422850661	2	2	24.94154722	7.726140994
chr9	137077475	137084538	9q34.3	UAP1L1	0.964832014	0.678142895	1.422756208	4	4	88.73273298	41.33906381
chr8	95133721	95156685	8q22.1	PLEKHF2	52.7355005	37.06616066	1.422739759	4	4	23.47895698	39.43777221
chr9	129812942	129824162	9q34.11	TOR1A	16.86781651	11.8566483	1.422646273	4	4	39.54874637	13.90538718
chr11	64316492	64318084	11q13.1	TRMT112	26.67016917	18.74713828	1.422626151	4	4	32.88920741	27.08398642
chr16	24539587	24572863	16p12.1	RBBP6	16.98403607	11.93859082	1.422616482	4	4	55.5343024	9.650646309
chr17	43170310	43211689	17q21.31	NBR1	51.4660387	36.17874615	1.422548987	4	4	46.78786084	18.56109361
chr21	29024617	29054488	21q21.3	USP16	26.03736774	18.3033827	1.422544027	4	4	29.82863648	15.28102753
chr15	74598476	74645418	15q24.1	CLK3	7.704410236	5.416309773	1.422446381	4	4	43.042287	36.73551578
chr11	72223861	72239147	11q13.4	INPPL1	13.60788993	9.566941393	1.422386672	4	4	56.47993305	36.32491306
chr11	47716494	47767478	11p11.2	FNBP4	29.54148011	20.77079184	1.42226066	4	4	46.95755456	16.26786391
chr5	148383935	148442836	5q32	FBXO38	18.80216464	13.22303738	1.421924789	4	4	50.29615214	14.02591016
chr11	6234713	6245924	11p15.4	CNGA4	0.166049037	0.116785995	1.4218232	2	2	31.88893283	5.99201937
chr6	131808016	131895155	6q23.2	ENPP1	0.107415029	0.075554076	1.421697342	3	2	62.10146614	24.44570251
chr18	3247482	3256237	18p11.31	MYL12A	304.8554067	214.4362326	1.421659964	4	4	21.5395649	19.86105352
chr9	111896766	111935374	9q31.3	UGCG	43.37729289	30.51205394	1.421644475	4	4	38.19946174	19.2030967
chr18	23237252	23437969	18q11.2	TMEM241	1.212560591	0.853167565	1.421245533	4	4	58.73432128	25.40174267
chr22	42160013	42283927	22q13.3	TCF20	1.051188567	0.739813969	1.42088229	4	4	86.15212191	59.70733786
chr16	67828157	67847458	16q22.1	CENPT	3.175494942	2.234988313	1.420810536	4	4	66.93616237	23.47167256
chr1	9997197	10016020	1p36.22	RBP7	25.48798224	17.93946428	1.420777223	4	4	71.67754535	34.45217389
chr6	73593379	73654014	6q13	SLC17A5	12.96682855	9.126872871	1.420730708	4	4	31.35652858	24.7088481
chr10	69220303	69267559	10q22.1	HKDC1	0.939820218	0.661554336	1.42062438	4	4	85.97735702	72.65699829
chr1	7784285	7845181	1p36.23	PER3	2.028349377	1.427838704	1.420573186	4	4	43.61048024	24.60358235
chr11	67438047	67443821	11q13.2	CORO1B	7.446749052	5.242110416	1.420563182	4	4	86.77384695	37.42639701
chr21	36034541	36061037	21q22.12	SETD4	1.761835868	1.24023821	1.42056248	4	4	37.76162376	29.48332428

chr6	122745280	122745529	6q22.31	RN7SL564P	5.189660322	3.653543516	1.42044574	3	4	89.06391174	138.8649488
chr11	128685263	128813267	11q24.3	FLI1	60.46509948	42.56771607	1.420445001	4	4	70.63502415	42.71954233
chr7	139043519	139109719	7q34	ZC3HAV1	105.6936304	74.40931958	1.420435384	4	4	17.51037437	5.1958804
chr9	4792834	4861077	9p24.1	RCL1	166.6881828	117.3513108	1.420420289	4	4	91.46788072	51.44130878
chr5	134601149	134632843	5q31.1	SAR1B	8.917647888	6.278310764	1.420389691	4	4	42.96318018	32.26562114
chr4	143870866	143912131	4q31.21	GYPE	47.55214281	33.47968551	1.42032824	4	4	89.40759059	90.86472642
chr5	112876384	112922334	5q22.2	REEP5	36.94543443	26.01366134	1.420232006	4	4	39.314023	26.31616269
chr3	15738515	15738786	3p25.1	RN7SL4P	6063.698273	4269.562178	1.420215474	4	4	52.69578848	35.19926492
chr6	36676460	36687339	6p21.2	CDKN1A	19.60421672	13.80495875	1.420085136	4	4	32.68337717	13.61102992
chr7	69598475	70793068	7q11.22	AUTS2	2.293086986	1.614915812	1.419942123	4	4	47.07188088	51.77540556
chr8	144358547	144361286	8q24.3	SLC52A2	2.570681403	1.810556664	1.419829301	4	4	58.20313684	39.06401432
chr20	2840697	2866732	20p13	VPS16	8.165600518	5.751718476	1.419680145	4	4	29.48796668	36.81390257
chr20	29080390	29096832	20q11.1	FRG1DP	4.587682499	3.231555974	1.419651257	3	4	80.25371126	52.08806816
chr1	247919196	247923152	1q44	OR2T8	1.535878562	1.081871918	1.419649162	4	4	46.3416784	26.46716
chr1	226062707	226072002	1q42.12	H3F3A	121.3856414	85.50864224	1.419571615	4	4	59.54134678	29.01377024
chr14	34752731	34875647	14q13.1-q13.2	BAZ1A	120.9118694	85.1792732	1.419498722	4	4	30.84533403	33.46241136
chr8	144412414	144417146	8q24.3	SLC39A4	0.588758324	0.414766572	1.419493187	3	4	96.28015438	82.94130414
chr1	198156978	198322420	1q31.3	NEK7	100.1427318	70.55944049	1.419267657	4	4	11.54961179	24.14006345
chr11	8685439	8685569	11p15.4	SNORA3B	16.52743032	11.64565953	1.419192299	4	4	77.62941501	69.41731669
chr12	8032680	8055522	12p13.31	FOXJ2	10.50857386	7.404943194	1.419129571	4	4	26.63934346	21.96433589
chr17	39404285	39451281	17q12	MED1	43.08316301	30.36211672	1.418977584	4	4	56.29735811	11.20446362
chr7	150541564	150542126	7q36.1	ALDH7A1P3	1.050153713	0.74009608	1.418942407	1	1	0	0
chr1	55376622	55376851	1p32.3	RN7SKP291	1.114544618	0.785486614	1.418922485	1	1	0	0
chr2	85839133	85889034	2p11.2	ST3GAL5	2.709345284	1.909742984	1.418696289	4	4	18.91778128	16.08735668
chr22	46244013	46250780	22q13.31	CDPF1	2.191741693	1.544992674	1.418609764	3	4	48.63956106	70.1858478
chr19	4324043	4338877	19p13.3	STAP2	0.269494263	0.189981594	1.418528278	3	4	20.85540236	69.34837927
chr17	6776233	6787646	17p13.1	FBXO39	0.493554295	0.347947895	1.418471854	2	1	108.572842	0
chr19	21397119	21427495	19p12	ZNF493	8.828005921	6.223718023	1.418445676	4	4	57.08005299	33.21114496
chr19	13116848	13141147	19p13.13	NACC1	8.849747594	6.239279673	1.418392516	4	4	58.26616006	48.84587778
chr12	110501651	110534632	12q24.11	RAD9B	6.24703637	4.404923177	1.41819417	4	4	106.6448179	93.19552136
chr18	49783504	49813881	18q21.1	ACAA2	9.83492836	6.93524112	1.418109074	4	4	70.44892747	39.83600095
chr17	34319047	34321402	17q12	CCL8	0.477141526	0.336487891	1.41800504	2	2	66.1613863	36.17171118
chr1	58480719	58546799	1p32.2-p32.1	OMA1	50.45444209	35.58484077	1.417863365	4	4	31.26394702	21.45009952
chr16	69328621	69339623	16q22.1	COG8	1.47981089	1.043921358	1.417550162	4	4	63.88453833	50.34988488
chr3	196739857	196832647	3q29	PAK2	86.99713745	61.37347261	1.417503911	4	4	34.46447777	13.88962965
chr1	54026681	54053573	1p32.3	TMEM59	35.41897116	24.99089009	1.417275297	4	4	30.8575816	31.61743847
chr11	59171430	59208587	11q12.1	DTX4	2.605631449	1.83894615	1.416915579	4	4	98.42982599	47.24756114
chr17	75135243	75154683	17q25.1	JPT1	33.48809384	23.6356187	1.416848624	4	4	39.08655955	33.90132162
chr11	110429937	110464884	11q22.3	FDX1	5.65586642	3.992083196	1.416770679	4	4	17.88411074	18.06836527
chr6	45328317	45551082	6p21.1	RUNX2	5.360513728	3.784560749	1.416416351	4	4	53.32314355	37.49613741
chr3	108380369	108549462	3q13.13	MYH15	0.184905077	0.130552207	1.416330542	3	4	67.07256074	44.24008204
chr15	84748556	84806432	15q25.3	ZNF592	15.98603145	11.28756085	1.416252072	4	4	49.87152771	32.6292785
chr12	76351797	76559809	12q21.2	OSBPL8	149.3241292	105.4371318	1.416238536	4	4	41.39689304	37.19774353

chr17	74367506	74372600	17q25.1	GPR142	0.206955264	0.146133437	1.416207465	1	1	0	0
chr9	36572862	36677683	9p13.2	MELK	60.55110792	42.76025115	1.416060624	4	4	82.18919896	42.34958642
chr3	112736449	112749319	3q13.2	LINC02042	0.324795075	0.229380847	1.415964234	1	1	0	0
chr3	36826817	36945662	3p22.2	TRANK1	45.43479642	32.09906056	1.415455643	4	4	55.25899397	46.33774938
chr17	79074812	79088603	17q25.3	ENGASE	3.720875679	2.628796662	1.415429247	4	4	45.1581148	31.2224997
chr2	97756336	97996182	2q11.2	TMEM131	20.37004262	14.39274313	1.415299532	4	4	21.01792983	23.42173248
chr16	11903753	11904193	16p13.13	COX6CP1	3.130574848	2.21196826	1.415289227	3	4	50.44306669	55.90302422
chr21	20256862	20258685	21q21.1	LINC02573	6.695195363	4.730715088	1.415260746	3	4	85.10539526	113.3734503
chrX	156021658	156025672	Xq28 and Yq12	WASH6P	2.049150061	1.448069986	1.415090487	3	4	69.33788189	61.34446738
chr3	40502604	40518221	3p22.1	ZNF620	1.348014786	0.952673157	1.414981387	4	4	38.69500552	33.23173008
chr7	44962661	44979105	7p13	MYO1G	27.93503612	19.74268662	1.414956164	4	4	81.53385111	55.5986985
chr10	27088321	27088427	10p12.1	RNU6-490P	3.765093614	2.660945312	1.414945883	1	1	0	0
chr5	153887428	153898987	5q33.2	LINC01861	0.774192815	0.547184422	1.414866331	1	2	0	13.59360487
chr1	156212988	156240077	1q22	PMF1	4.529646455	3.201502924	1.414850013	4	4	65.60328728	35.81020164
chr17	75827225	75844717	17q25.1	UNC13D	21.14317815	14.94423987	1.414804522	4	4	54.25851876	25.35632085
chr1	45694324	45750813	1p34.1	IPP	5.020856834	3.549212046	1.414639861	4	4	54.22489572	11.57429426
chr14	95533355	95533629	14q32.13	SCARNA13	2164.145321	1529.930998	1.414537861	4	4	60.43211759	59.47311095
chr5	132410773	132476044	5q31.1	C5orf56	3.143838288	2.222615149	1.414477126	4	4	55.93307932	30.68593469
chr22	38344665	38398926	22q13.1	TPTEP2	1.581473762	1.118237895	1.414255203	4	4	49.70072109	47.05742946
chr22	42796526	42857402	22q13.2	ARFGAP3	32.14636523	22.736336	1.413876239	4	4	23.27416985	30.79031688
chr2	43091388	43091494	2p21	RNU6-242P	4.450822417	3.148034587	1.413841651	2	2	22.0949617	22.11332904
chrX	83748625	83750102	Xq21.1	TERF1P4	0.296351837	0.209613529	1.413801095	1	1	0	0
chr17	82321024	82334045	17q25.3	SECTM1	71.43536874	50.52914376	1.413745879	4	4	69.97400233	42.9629608
chr9	113221528	113264492	9q32	SLC31A1	12.05693499	8.528882234	1.413659452	4	4	37.21223486	16.84877562
chr6	7281143	7313314	6p24.3	SSR1	32.25444604	22.8167964	1.413627289	4	4	22.81462086	6.86899668
chr17	27456441	27626438	17q11.2	KSR1	3.550054719	2.511326536	1.413617333	4	4	59.43145508	19.28909785
chr4	8182072	8241103	4p16.1	SH3TC1	4.859222774	3.437455532	1.413610366	4	4	70.93379027	20.11636412
chr1	36224412	36305357	1p34.3	THRAP3	115.1533951	81.46391308	1.413550991	4	4	57.81647479	37.23554845
chr9	113267008	113275589	9q32	CDC26	19.59339077	13.86115987	1.413546266	4	4	63.41709103	38.71959553
chr14	22490491	22490553	14q11.2	TRAJ48	9.910688795	7.012467773	1.41329545	2	4	74.31170614	58.03145102
chr1	37566812	37595985	1p34.3	GNL2	13.63737363	9.649419662	1.413284333	4	4	24.37225001	6.445686245
chrX	118495664	118686160	Xq24	DOCK11	127.9588093	90.5441507	1.413220051	4	4	20.44067964	25.03270787
chr16	82148162	82170224	16q23.3	MPHOSPH6	8.376483566	5.927345577	1.413193049	4	4	21.48366729	17.66039987
chr2	63643198	63644068	2p15	RPS4XP5	0.209674235	0.148382996	1.413061064	1	1	0	0
chr1	211382755	211432535	1q32.3	LINC00467	0.941097667	0.666050221	1.412953014	4	4	56.33448298	55.53125159
chr13	100530164	100588792	13q32.3	GGACT	0.817375657	0.578496401	1.412931275	4	4	77.27601997	60.591994
chr17	18476785	18494945	17p11.2	LGALS9C	0.412837599	0.29218689	1.412923072	4	4	94.66487516	54.37815318
chr2	135531429	135725270	2q21.3	R3HDM1	43.66499525	30.90412544	1.412918004	4	4	44.02859221	9.388816803
chr19	3185877	3209575	19p13.3	NCLN	6.891642795	4.877741423	1.412875796	4	4	74.23477609	31.96805055
chr17	3896591	3916666	17p13.2	P2RX1	12.72810518	9.008837517	1.412846569	4	4	62.56804686	40.14948537
chr18	3262113	3278284	18p11.31	MYL12B	230.0124454	162.801415	1.412840579	4	4	42.41942661	31.27022843
chr19	13862063	13880757	19p13.12	NANOS3	0.499741964	0.353723556	1.412803742	1	3	0	28.15135939
chr4	103018033	103077371	4q24	SLC9B2	6.675940696	4.725556031	1.412731254	4	4	64.04535236	56.87980041

chr12	69359705	69427077	12q15	YEATS4	38.3634258	27.15806361	1.41259798	4	4	71.27523552	21.39018011
chr1	15807162	15807699	1p36.21	UQCRHL	1.48218582	1.04940441	1.412406701	4	4	37.15686063	24.0555792
chr2	28751748	28802940	2p23.2	PPP1CB	156.2353827	110.618163	1.412384535	4	4	19.0308256	22.70182352
chr22	25647141	25652035	22q12.1	YES1P1	0.923382718	0.653785592	1.412363211	4	4	46.21950861	21.59201948
chr4	499200	540196	4p16.3	PIGG	3.100399057	2.19527354	1.412306485	4	4	36.55207474	19.92785378
chr20	62774121	62776856	20q13.33	LINC00659	0.713678731	0.505348561	1.412250447	2	3	14.83863155	16.8911376
chrX	120010717	120015551	Xq24	RHOXF1P1	2.733925124	1.935924422	1.412206537	4	3	112.6498853	72.41863256
chr7	10931953	10940186	7p21.3	NDUFA4	24.97750029	17.68736078	1.412166609	4	4	26.93188809	9.386286654
chr4	16226663	16258187	4p15.32	TAPT1-AS1	1.300683532	0.921133056	1.412047394	4	4	77.76456172	25.79372158
chr16	57447425	57461275	16q21	COQ9	3.14258351	2.225620711	1.412003175	4	4	66.21571295	47.21904724
chr4	2269585	2418698	4p16.3	ZFYVE28	0.952557464	0.67463587	1.411957926	4	4	45.18135892	45.94032996
chr11	67303448	67312607	11q13.2	SSH3	2.78098705	1.96974355	1.411852345	4	4	84.99930583	23.70971621
chr10	45727200	45792964	10q11.22	WASHC2C	39.60887522	28.05553182	1.411802687	4	4	32.78219869	16.04836462
chr11	65919257	65921577	11q13.1	DRAP1	33.07451938	23.42970585	1.411648938	4	4	61.46142929	58.23992837
chr20	49113339	49219295	20q13.13	STAU1	82.60703969	58.5196911	1.411611	4	4	11.14137021	11.31738397
chr1	206507530	206589284	1q32.1	RASSF5	86.27464422	61.11879892	1.411589327	4	4	39.75166863	8.839319892
chr2	201182881	201229406	2q33.1	CASP10	9.227233163	6.537586114	1.411412867	4	4	22.79886929	33.99757729
chr3	177441921	177752704	3q26.32	LINC00578	0.267325826	0.189425388	1.411246028	1	1	0	0
chr11	47239302	47248847	11p12-p11	ACP2	5.901303331	4.181648523	1.411238486	4	4	37.85716935	29.63401144
chr20	24949230	24959928	20p11.21	CST7	198.3610304	140.5592848	1.411226805	4	4	75.50462056	68.90080097
chr7	107660635	107717809	7q22.3	SLC26A4	0.098088991	0.06950686	1.411213092	3	4	42.60003685	42.06916048
chr11	20363685	20383783	11p15.1	HTATIP2	20.72577714	14.68952409	1.410922302	4	4	46.50706673	16.04818975
chr22	22380880	22381347	22q11.22	IGLV1-44	6.877130517	4.874236585	1.410914386	4	4	51.61226557	119.8623385
chr6	139159162	139324029	6q24.1	TXLNB	0.465819716	0.330218259	1.410641911	4	4	40.67148638	48.61734977
chr7	106865278	106908978	7q22.3	PIK3CG	49.83668366	35.32941274	1.410628702	4	4	42.84780626	25.33075961
chr15	52307283	52529050	15q21.2	MYO5A	15.33936471	10.87596236	1.410391485	4	4	44.81258241	26.13721177
chr14	22459098	22459156	14q11.2	TRDJ3	9.243321771	6.554127105	1.410305541	3	2	21.11020991	66.63819965
chr15	55408525	55418712	15q21.3	C15orf65	1.647333216	1.168173462	1.410178599	3	3	62.43531844	62.80613848
chr7	144258996	144286003	7q35	ARHGEF34P	0.313361402	0.222226893	1.41009667	2	4	29.1988975	65.70511003
chr1	1635226	1659619	1p36.33	CDK11B	21.58096575	15.30537075	1.410025677	4	4	72.08335967	10.3719996
chr14	23559563	23567939	14q11.2	AP1G2	7.584218333	5.378965894	1.409977026	4	4	62.99631005	30.59525172
chr11	117316346	117413266	11q23.3	CEP164	2.841873281	2.015680283	1.409882959	4	4	70.63835096	41.82391853
chr1	107571160	107964923	1p13.3	VAV3	21.16645858	15.01441083	1.409742867	4	4	21.40139118	35.69682928
chr1	109711751	109718268	1p13.3	GSTM5	0.19069798	0.135277912	1.409675662	4	3	67.91097867	60.31616468
chr19	45692400	45703990	19q13.32	QPCTL	0.747975321	0.530637907	1.409577625	4	4	58.86091734	38.59712268
chr3	197791226	197889494	3q29	LRCH3	59.03256921	41.88112133	1.40952695	4	4	65.5942268	22.91776965
chr2	97655939	97664098	2q11.2	ACTR1B	10.21011166	7.243780572	1.409500407	4	4	31.02839572	14.13894147
chr19	5913644	5916211	19p13.3	CAPS	1.161654132	0.824191828	1.409446313	4	4	66.75008684	57.00585396
chr4	84965616	85008743	4q21.23	WDFY3-AS2	0.326740898	0.23183788	1.409350781	2	3	74.83829672	141.350825
chr17	68413623	68551316	17q24.2	PRKAR1A	159.9560686	113.4997823	1.409307273	4	4	52.48100172	37.3916151
chr14	103629188	103701551	14q32.33	KLC1	1.674732339	1.188370588	1.409267744	4	4	44.59258974	23.00790363
chr3	32391671	32455528	3p22.3	CMTM7	13.47208707	9.560879523	1.409084493	4	4	57.53786542	37.08162933
chr10	102742708	102816267	10q24.32	WBP1L	31.63776526	22.45336286	1.409043512	4	4	69.77380912	38.39903248

chr2	231519903	231530471	2q37.1	NMUR1	2.278393124	1.617015362	1.409011428	4	4	58.96202199	33.68238942
chr2	152116801	152175992	2q23.3	STAM2	19.83636475	14.07854881	1.408977944	4	4	16.59310302	21.58760512
chr21	41885110	41953968	21q22.3	C2CD2	1.515452267	1.075581125	1.408961381	4	4	79.70086263	36.56158748
chr12	11649601	11895402	12p13.2	ETV6	17.84167853	12.6641437	1.408834182	4	4	55.29002548	19.37064331
chr5	172768090	172771200	5q35.1	DUSP1	542.5329518	385.1071112	1.408784559	4	4	51.91425291	85.93676822
chr1	11908154	11908272	1p36.22	RNU5E-1	331.8581474	235.5744081	1.408719012	4	4	20.53940047	45.30568699
chr1	110210714	110282649	1p13.3	KCNC4	0.100725474	0.071502573	1.408697187	4	4	51.30405587	31.0046115
chr1	2319202	2391751	1p36.32	MORN1	0.272394315	0.193376535	1.408621347	4	4	96.04750604	26.79114702
chr3	3126916	3153435	3p26.2	TRNT1	46.22442449	32.81559832	1.408611357	4	4	63.04713026	38.33951422
chrX	78104169	78126827	Xq21.1	PGK1	95.73766912	67.9665624	1.408599549	4	4	63.84301982	21.97673858
chr2	88027203	88055801	2p11.2	KRCC1	55.51993743	39.4150868	1.408596097	4	4	53.95380632	52.6819121
chr2	37196461	37216193	2p22.2	CEBPZOS	13.17757605	9.355369785	1.408557476	4	4	40.54976699	35.03983072
chr12	50504985	50748667	12q13.12	DIP2B	29.37826107	20.8589701	1.408423375	4	4	45.37236214	36.4926229
chr14	19402764	19434341	14q11.2	POTEG	0.126342402	0.089707442	1.408382624	1	1	0	0
chr19	39731253	39738029	19q13.2	CLC	221.3939106	157.2185927	1.408191658	4	4	75.56478202	46.47455934
chr4	38867733	38945744	4p14	FAM114A1	0.97198581	0.690265132	1.408134012	4	4	60.02264322	53.20951896
chr2	43774039	43828320	2p21	DYNC2LI1	4.460206197	3.167666407	1.408041638	4	4	80.78428266	44.430251
chr2	46511835	46542557	2p16-p12	ATP6V1E2	1.042490452	0.740387373	1.408033807	4	4	77.09917008	50.96927812
chr15	32536759	32580609	15q13.3	LINC02256	0.525380771	0.373150245	1.407960408	3	3	44.34891784	49.30708463
chr12	68746086	68768690	12q15	SLC35E3	1.148737436	0.815925895	1.407894323	4	4	49.72235319	22.46034965
chr7	73699205	73719687	7q11.23	STX1A	0.925324394	0.657241265	1.407891506	4	4	50.28612638	41.17718028
chr5	141382739	141512979	5q31.3	PCDHGA7	0.134630932	0.095627649	1.407866178	2	1	30.77763121	0
chr1	174159414	174995308	1q25.1	RABGAP1L	75.05754838	53.31949751	1.407694219	4	4	33.66193855	38.62364806
chr19	17555594	17583162	19p13.11	COLGALT1	11.93620393	8.479803484	1.407603839	4	4	65.19538347	30.02465967
chr14	39147811	39170430	14q21.1	TRAPPC6B	56.46820338	40.1178341	1.407558624	4	4	47.69297034	25.49388685
chr6	2245753	2413591	6p25.3-p25.2	GMDS-DT	1.250163253	0.888226768	1.407482074	4	4	43.40228858	27.1276886
chr1	185045419	185102608	1q25.3	RNF2	29.21673892	20.76003056	1.407355294	4	4	22.08044014	15.66629181
chr7	93962770	94004382	7q21.3	BET1	9.630559647	6.843345585	1.407288223	4	4	25.00307595	45.36061722
chr1	3812081	3857233	1p36.32	CEP104	5.830914662	4.143617091	1.407204028	4	4	21.51180585	23.2361536
chr17	50133735	50151027	17q21.33	PPP1R9B	62.46919878	44.39255452	1.407199911	4	4	68.63905271	51.83877573
chr2	239892450	240025402	2q37.3	NDUFA10	22.72811071	16.15278944	1.407070327	4	4	55.0394657	21.74112388
chr9	37878061	37912435	9p13.2-p13.1	PAICSP1	0.450725906	0.320338174	1.407031513	2	4	34.35013901	32.7510393
chr5	141515016	141619055	5q31.3	DIAPH1	58.5393579	41.60516341	1.407021463	4	4	41.90589824	29.14873653
chr9	136206333	136245841	9q34.3	QSOX2	57.85247325	41.12186209	1.406854415	4	4	62.39698542	21.54151246
chr5	172042070	172188342	5q35.1	STK10	69.09208002	49.11537974	1.406730038	4	4	42.20214058	27.52515538
chr2	113889934	113961554	2q14.1	ACTR3	112.5187064	80.00318255	1.40642788	4	4	27.70188867	23.02089911
chr9	76385517	76394528	9q21.13	RFK	9.45086934	6.720402509	1.406295133	4	4	19.23778853	26.40841263
chrX	65588382	65741931	Xq12	MSN	396.0074881	281.6130858	1.406211246	4	4	65.81783311	36.48905272
chr12	120210439	120265771	12q24.23	PXN	37.39812203	26.60226122	1.40582493	4	4	54.14376424	27.60729504
chr1	197746555	197747503	1q31.3	FAM204BP	0.897745492	0.638646522	1.405700119	1	4	0	49.24702864
chr12	118373189	118418035	12q24.23	SUDS3	31.54935077	22.44458844	1.405655125	4	4	12.73376764	10.90944428
chr19	45251258	45305283	19q13.32	MARK4	5.84418142	4.157621215	1.405655089	4	4	13.9571955	8.701001492
chr3	129647792	129893603	3q22.1	TMCC1	19.83296642	14.11011142	1.405585387	4	4	21.70982319	42.26424866

chr5	53883942	54310590	5q11.2	ARL15	13.45452634	9.572633753	1.405519807	4	4	36.9182037	32.32611658
chr5	141408203	141512979	5q31.3	PCDHGB6	0.080825873	0.057506261	1.4055143	4	2	95.17692615	46.34287167
chr2	201233443	201287711	2q33.1	CASP8	44.75170673	31.84011928	1.405513162	4	4	47.10994435	31.22455559
chr21	31659622	31668931	21q22.11	SOD1	48.71121673	34.66030036	1.405389342	4	4	19.40307006	22.47783722
chr19	5993164	6110653	19p13.3	RFX2	2.827716343	2.01223033	1.405264746	4	4	84.89160143	73.43836413
chr1	168226017	168242850	1q24.2	SFT2D2	23.73637106	16.89160939	1.405216668	4	4	30.31983708	10.02177312
chr10	47689700	47731218	10q11.22	SHLD2P3	0.42487196	0.302362257	1.405175247	4	2	52.91956254	22.13849054
chr5	90393335	90409786	5q14.3	CETN3	6.624791321	4.714609068	1.405162385	4	4	70.7287884	25.36583669
chr14	75294404	75296410	14q24.3	LINC01220	4.574253878	3.255340271	1.405153839	4	4	33.11357478	58.50333943
chr11	61789130	61792613	11q12.2	TMEM258	9.507082994	6.767251329	1.404866249	4	4	17.45903987	29.4200463
chr17	75318076	75405709	17q25.1	GRB2	76.71568017	54.61375259	1.404695274	4	4	48.45598998	34.43412816
chr4	38664079	38701508	4p14	KLF3	119.5828654	85.13110348	1.404690653	4	4	36.65498569	13.83808003
chr4	69726848	69760712	4q13.3	SULT1B1	32.43331333	23.09239067	1.404502193	4	4	50.45455704	70.91114077
chr4	6575174	6622403	4p16.1	MAN2B2	10.32687194	7.352726732	1.404495546	4	4	76.69613782	41.49009928
chr6	143608180	143831185	6q24.2	PHACTR2	16.53744255	11.77607078	1.404326016	4	4	25.01648747	45.67738227
chr7	6374495	6403967	7p22.1	RAC1	69.11792305	49.22194647	1.404209464	4	4	40.17796491	27.37115816
chr5	80487981	80542563	5q14.1	FAM151B	2.036514852	1.450301743	1.404200789	4	4	46.95620874	42.15773199
chr3	184314495	184335358	3q27.1	EIF4G1	35.69001418	25.42495934	1.403739283	4	4	53.21772187	45.87379863
chr20	3888823	3933087	20p13	PANK2	16.65946329	11.86811429	1.403716116	4	4	30.62474964	18.99119845
chr17	29625938	29930239	17q11.2	SSH2	68.80873253	49.02137712	1.403647481	4	4	41.13473553	34.17968725
chr2	186590063	186680902	2q32.1	ITGAV	5.16856655	3.682344397	1.403607591	4	4	22.22887607	19.55070318
chr20	44966470	45079977	20q13.12	STK4	121.1216575	86.29791619	1.403529342	4	4	38.738679	21.79114115
chr14	95181939	95319908	14q32.13	CLMN	1.664978984	1.186289568	1.403518188	4	4	70.81963094	64.33638676
chr11	104942866	104968598	11q22.3	CASP4	65.6466767	46.77348134	1.403502045	4	4	63.00701187	39.34717363
chr15	32406611	32435049	15q13.3	ULK4P1	0.261667041	0.186447708	1.403433938	1	1	0	0
chr11	35138870	35232402	11p13	CD44	96.56955832	68.80973791	1.403428661	4	4	40.52808686	24.56768774
chr14	33924215	33951078	14q13.1	EGLN3	1.146396271	0.816860528	1.403417392	4	4	57.01438518	29.55720249
chr4	185151723	185205443	4q35.1	CFAP97	21.00311666	14.96611711	1.40337781	4	4	39.09275752	25.80984075
chr7	32580939	32719168	7p14.3	DPY19L1P1	111.5187927	79.46575517	1.403356609	4	4	76.38861835	56.73315183
chr3	188941715	188947639	3q28	TPRG1-AS1	1.665445961	1.186835784	1.403265711	2	2	78.38769169	28.95946292
chr3	171106664	171106759	3q26.2	MIR569	5.763143207	4.10697338	1.403257989	2	1	46.33373045	0
chr16	50548330	50649249	16q12.1	NKD1	0.178811261	0.127428747	1.403225454	3	4	125.7370786	128.5813158
chr8	47773108	47960183	8q11.21	PRKDC	37.38169613	26.64062044	1.403184142	4	4	29.6003022	3.846692662
chr7	112479542	112491062	7q31.1	LSMEM1	2.87931917	2.05203187	1.403155191	4	4	48.32727756	38.21509414
chr7	35800986	35915763	7p14.2	SEPT7	59.57263234	42.45650469	1.403145001	4	4	28.261838	11.80300998
chr16	631012	634116	16p13.3	WFIKKN1	0.110678248	0.078880066	1.403120622	4	2	47.58415881	46.96734802
chr11	78171243	78188854	11q14.1	KCTD21	3.37976089	2.408794647	1.403092163	4	4	50.24106134	67.50536954
chr7	72997295	73005943	7q11.23	STAG3L3	3.578975422	2.551045674	1.40294447	4	4	11.98925164	27.3516177
chr11	88293592	88337787	11q14.2	CTSC	25.29833824	18.03397187	1.402815665	4	4	39.01328582	26.09391124
chr12	6747992	6753470	12p13.31	MLF2	21.16088891	15.08523618	1.402754896	4	4	53.22932705	40.31043341
chr1	119911553	120069703	1p12	NOTCH2	50.66855971	36.12313556	1.402662281	4	4	57.49995297	31.71252236
chr16	84368523	84465777	16q24.1	ATP2C2	0.150737119	0.107467972	1.402623646	3	3	68.99774287	91.07431259
chr7	106090368	106112647	7q22.3	SYPL1	28.46946154	20.29853306	1.402537881	4	4	25.29963494	21.4469065

chrMT	15888	15953	N/A	MT-TT	519.3415167	370.3075116	1.402460119	4	4	69.53166588	41.16059375
chr16	1528741	1555580	16p13.3	TMEM204	4.851828383	3.45954527	1.402446855	4	4	56.5381658	35.76399453
chr19	13836287	13836359	19p13.12	MIR24-2	5.718126404	4.077396069	1.402396605	2	3	41.60914476	32.0982305
chr13	113926514	113928991	13q34	LINC00565	0.723686508	0.516057132	1.402337964	3	4	36.98003344	62.73597769
chr2	68178857	68252519	2p14	PPP3R1	129.9983231	92.70346483	1.402302744	4	4	25.06874883	40.47879914
chr4	88684848	88684954	N/A	RNU6-33P	22.37472187	15.95617678	1.402260842	4	4	49.6954026	68.11810105
chr4	17486393	17512234	4p15.32	QDPR	1.262015044	0.89999983	1.402239202	4	4	81.74228766	89.23682763
chr1	94529176	94541857	1p21.3	F3	0.226471308	0.16150856	1.402224799	2	4	72.13961024	57.51626935
chr18	74253292	74292016	18q22.3	CYB5A	1.177921312	0.840063101	1.402181943	4	4	15.88007276	35.69559978
chr6	99369401	99394223	6q16.2	COQ3	1.811443681	1.291900045	1.402154671	4	4	32.25237589	12.43401424
chr12	109477410	109543628	12q24.11	UBE3B	6.99534561	4.989579521	1.401991006	4	4	26.09348005	22.2595112
chr22	29767369	29770413	22q12.2	UQCR10	35.74450558	25.49993778	1.401748737	4	4	33.13352817	32.93398001
chr11	63838928	63911020	11q13.1	MARK2	17.70983801	12.63447934	1.401706991	4	4	43.40262563	41.64176439
chr10	62374157	62672011	10q21.2	ZNF365	0.213954263	0.152652935	1.401573201	4	4	66.26711983	101.1539643
chr4	87336522	87391303	4q22.1	HSD17B11	160.2172331	114.3202966	1.40147671	4	4	46.87450152	42.92233397
chr16	1984149	1987749	16p13.3	GFER	2.586941799	1.845872887	1.401473426	4	4	61.29243334	29.3420556
chr14	88005124	88014811	14q31.3	GPR65	67.5513047	48.2145593	1.401056147	4	4	26.43079653	39.12717569
chr2	27781364	27890396	2p23.2	RBKS	3.799760559	2.712100939	1.401039506	4	4	30.83788945	46.0119012
chr8	85245487	85284073	8q21.2	CA13	2.176265496	1.553559936	1.400824935	4	4	21.96182419	14.48382711
chr3	158801264	158829719	3q25.32	MFSD1	54.18140608	38.6790139	1.400795951	4	4	31.04682436	23.10471639
chr11	60937083	60952081	11q12.2	SLC15A3	10.0790835	7.195437593	1.400760325	4	4	60.318917	25.03271657
chrX	110002413	110184232	Xq23	TMEM164	28.06696277	20.03770072	1.400707754	4	4	53.48218704	20.60240465
chr17	76736565	76803805	17q25.1-q25.2	MFSD11	10.30783481	7.359277738	1.400658486	4	4	54.76563891	43.63525987
chr2	68643588	68655576	2p13.3	PROKR1	0.082507793	0.058908967	1.40059819	1	1	0	0
chr2	98608548	98618515	2q11.2	UNC50	11.96959011	8.547044993	1.400436071	4	4	37.5251541	36.20125322
chr9	121201483	121332844	9q33.2	GSN	14.29625197	10.20894872	1.400364755	4	4	48.58352679	19.15203366
chr19	18279694	18281656	19p13.11	JUND	80.60804222	57.56528469	1.400289127	4	4	31.01613883	25.52614117
chr22	29480192	29491290	22q12.2	NEFH	1.206084865	0.861326569	1.400264323	3	4	72.04527462	91.35586573
chr3	43366326	43691594	3p22.1-p21.33	ANO10	14.24999719	10.1769526	1.400222419	4	4	35.596505	40.79646338
chr3	123612296	123884302	3q21.1	MYLK	4.969144837	3.548840474	1.400216458	4	4	55.28050857	18.16237276
chr7	7182547	7248651	7p22.1-p21.3	C1GALT1	43.4359196	31.02578296	1.39999431	4	4	26.75346691	30.99211145
chr12	49018975	49060884	12q13.12	KMT2D	8.315172023	5.939461361	1.399987561	4	4	75.18730955	25.94520724
chr5	173888328	173960991	5q35.2	CPEB4	48.64858074	34.7496475	1.399973359	4	4	10.10832533	33.3519428
chr5	65488919	65563194	5q12.3	CENPK	135.1993014	96.57405505	1.399954691	4	4	89.9345857	60.01251684
chr1	52686342	52698366	1p32.3	COA7	1.757698458	1.255569558	1.399921212	4	4	43.07688445	38.41960518
chr21	41986831	42010418	21q22.3	ZBTB21	7.982386092	5.702273124	1.399860357	4	4	68.65776586	16.30702689
chr4	55427901	55547138	4q12	CLOCK	4.939229239	3.528421037	1.399841228	4	4	21.31690427	2.646329462
chr13	46125923	46182324	13q14.13	LCP1	942.4375284	673.2997405	1.399729529	4	4	65.16774237	26.68392014
chr15	22786225	22829789	15q11.2	NIPA1	3.770500857	2.693779159	1.399706744	4	4	41.31150512	38.96536732
chr2	97080738	97094882	2q11.2	FAHD2B	0.592341831	0.423191222	1.399702547	3	4	102.3330997	45.5614031
chr5	172983760	173034897	5q35.1	ATP6V0E1	143.4957681	102.520522	1.399678477	4	4	57.70161899	27.9950113
chr21	43659551	43696079	21q22.3	RRP1B	15.30943178	10.93969731	1.399438334	4	4	48.41898177	39.96741919
chr4	122152333	122362759	4q27	KIAA1109	33.27862979	23.78059103	1.399402973	4	4	18.19790781	10.52107583

chr16	81779258	81962693	16q23.3	PLCG2	17.87406833	12.77269691	1.399396576	4	4	58.91242136	27.02622474
chr9	132878320	132890314	9q34.13	SPACA9	0.896716983	0.640822891	1.399321084	4	4	69.99615645	81.59953072
chr4	109912883	110013079	4q25	EGF	11.89483341	8.50052903	1.399305074	4	4	25.69970672	35.76044941
chr12	62260340	62409721	12q14.1	USP15	110.5717448	79.02882657	1.399131806	4	4	27.58011315	35.13492057
chr19	55090913	55117600	19q13.42	PPP1R12C	6.517531698	4.658649938	1.399017266	4	4	71.33173782	13.80113351
chr18	76978833	77133708	18q23	MBP	37.93550745	27.1167016	1.398972043	4	4	37.60712895	14.35932606
chrX	135520626	135582535	Xq26.3	INTS6L	28.95074308	20.69612656	1.398848379	4	4	52.3396158	24.51408601
chr7	96120220	96322147	7q21.3	SLC25A13	9.652349345	6.900321741	1.398825983	4	4	27.25609328	37.39399144
chr2	197568224	197675860	2q33.1	RFTN2	0.061565805	0.0440207	1.398564857	3	2	25.43932566	2.187394577
chr17	50507778	50531502	17q21.33	MYCBPAP	0.111244764	0.079544116	1.398529142	1	2	0	0.963152221
chr6	33299694	33314387	6p21.32	TAPBP	32.71586822	23.3953171	1.398393879	4	4	75.98076229	63.66304229
chr3	197002906	197004744	3q29	MELTF-AS1	0.357691705	0.255794549	1.398355459	2	2	4.502525599	11.5871251
chr18	45724123	45752520	18q12.3	SLC14A1	49.36699203	35.3036885	1.398352244	4	4	68.16221907	44.57552685
chr19	48255675	48258194	19q13.33	CARD8-AS1	27.53464962	19.69122678	1.39832068	4	4	45.15923854	27.01793651
chr16	90019629	90044971	16q24.3	GAS8	0.513241752	0.367063755	1.398235988	4	4	89.63047301	33.7432029
chr11	62806860	62832091	11q12.3	STX5	23.09059283	16.51483242	1.398173003	4	4	38.90069264	25.44026681
chr7	92447448	92494631	7q21.2	GATAD1	5.88865912	4.211790463	1.398136772	4	4	46.7709367	42.83323784
chr8	90621995	90645905	8q21.3	TMEM64	13.75606777	9.839523825	1.398042021	4	4	51.90191306	29.5201435
chr7	77310751	77416419	7q11.23	GSAP	15.94107087	11.40258774	1.398022207	4	4	51.03772481	33.43405987
chr16	8674587	8784575	16p13.2	ABAT	4.487588028	3.210144825	1.39793943	4	4	63.05771502	41.2906527
chr7	93232340	93361123	7q21.2-q21.3	VPS50	45.0276837	32.21013057	1.39793546	4	4	69.89306519	29.27050988
chr17	35407171	35433310	17q12	SLFN12	6.232311154	4.458417701	1.397875115	4	4	7.278891161	21.87863713
chr9	135075243	135121184	9q34.3	OLFM1	1.221785096	0.874060213	1.397827149	3	2	84.89563194	107.9873627
chr2	25926586	25982740	2p23.3	KIF3C	2.322610244	1.661590513	1.397823486	4	4	62.85670245	50.6233965
chr12	55835430	55842983	12q13.2	MMP19	0.145466613	0.104071414	1.397757624	3	1	56.26575895	0
chr5	168616329	168617055	5q34	RPL10P9	0.593005826	0.424308097	1.397583101	2	2	36.93226403	22.10467176
chr8	47260878	47736001	8q11.21	SPIDR	45.00024044	32.20093625	1.397482362	4	4	61.68163387	31.39078749
chr12	106991364	107093872	12q23.3	CRY1	9.121416615	6.527240077	1.397438505	4	4	68.89136823	17.02917122
chr15	25067787	25067883	15q11.2	SNORD116-7	3.543711657	2.535865311	1.397436859	1	1	0	0
chr14	49598697	49614672	14q21.3	LRR1	50.18501752	35.91868599	1.397184116	4	4	89.72899871	62.55733012
chr19	16324826	16328662	19p13.11	KLF2	151.8580081	108.701276	1.397021394	4	4	56.29455311	55.13354573
chr4	143995172	144021861	4q31.21	GYPB	231.3955489	165.6477385	1.39691342	4	4	109.9246361	68.46703385
chr12	55901413	55927913	12q13.2	PYM1	9.265600215	6.633129278	1.396867124	4	4	46.03538995	16.59917377
chr22	42776345	42777370	22q13.2	RPL5P34	0.91714407	0.656620914	1.396763415	4	2	44.76823685	17.21283025
chr2	70087453	70089203	2p13.3	PCBP1	152.6350517	109.2992983	1.396487023	4	4	40.10496274	18.54758151
chr13	48303747	48481890	13q14.2	RB1	50.13021123	35.89749323	1.396482225	4	4	49.44403774	17.7100936
chr2	31867188	32011052	2p22.3	MEMO1	0.969101736	0.693960134	1.396480415	4	4	75.07299712	45.72340104
chr6	98868535	98948006	6q16.1-q16.2	FBXL4	25.53680364	18.29070409	1.396162964	4	4	23.75901519	28.01682038
chr7	131110096	131496639	7q32.3	MKLN1	101.8182674	72.9291498	1.396125797	4	4	55.14255751	40.79864201
chr12	110041177	110073639	12q24.11	C12orf76	3.777506726	2.70582034	1.396067089	4	4	29.8143836	7.360683458
chr19	48932790	48944969	19q13.33	DHDH	0.493020805	0.353180399	1.395946112	2	2	65.04674572	55.03750137
chr21	32393133	32393960	21q22.11	URB1-AS1	2.42958182	1.74047899	1.395927118	4	4	71.64457624	54.14604832
chr17	4997948	5028399	17p13.2	KIF1C	5.85714254	4.19622187	1.395813358	4	4	81.32781892	38.12919794

chr5	6713938	6757048	5p15.31	TENT4A	8.337612747	5.97385776	1.395683172	4	4	31.52220888	16.99135536
chr17	39173456	39197703	17q12	CACNB1	0.554066899	0.396987016	1.39568015	4	4	79.6576392	51.56272032
chr18	48919853	48950711	18q21.1	SMAD7	7.464157141	5.348131482	1.395657	4	4	56.60831666	9.032885639
chr13	43821959	43880023	13q14.11	CCDC122	1.083590033	0.776506804	1.395467532	4	4	97.38212639	83.96658692
chr2	200973718	201071671	2q33.1	FAM126B	34.39031796	24.64625218	1.395356896	4	4	48.32558593	47.812705
chr20	408050	432139	20p13	RBCK1	21.36593762	15.31530973	1.395070553	4	4	55.34333923	33.0132729
chr17	44076753	44123651	17q21.31	HDAC5	8.728792829	6.256993894	1.395045764	4	4	63.29222077	25.09524895
chr6	150061046	150069147	6q25.1	ULBP3	1.596230238	1.144228472	1.395027547	1	2	0	107.6802789
chr12	53441649	53446643	12q13.13	PRR13	48.49649418	34.76555448	1.394958168	4	4	47.57338128	34.50808395
chr2	127480807	127526886	2q14.3	IWS1	20.81064557	14.91856248	1.394949789	4	4	49.20830863	29.19876002
chr16	71929538	72000401	16q22.2	PKD1L3	4.65569824	3.337603629	1.394922453	4	4	55.56579607	54.03280903
chr14	34561093	34630183	14q13.1	SNX6	87.76377236	62.91676666	1.394918668	4	4	43.4225795	23.35457076
chr9	90801680	90898560	9q22.2	SYK	69.34691934	49.71531693	1.394880363	4	4	68.48608376	40.39817855
chr16	30407414	30418595	16p11.2	ZNF771	0.184719989	0.132432766	1.394820889	3	3	68.8596587	28.68717243
chr5	181191046	181191908	5q35.3	LINC01962	0.867496739	0.62197978	1.394734632	1	1	0	0
chr9	35812960	35815479	9p13.3	HINT2	2.329717031	1.670371096	1.39473021	4	4	43.52457303	88.84414156
chr14	92121954	92164199	14q32.12	CPSF2	24.48257929	17.55370211	1.394724551	4	4	25.01531953	15.65018171
chr7	139778248	140020325	7q34	TBXAS1	26.96343898	19.33251677	1.394719544	4	4	55.75979632	50.8934847
chr1	158724113	158725067	1q23.1	OR6K4P	0.889430916	0.637734171	1.394673449	3	3	107.9692114	89.93644485
chr15	75353611	75353685	15q24.2	MIR631	4.91555581	3.524719349	1.394594951	1	1	0	0
chr11	67350765	67353596	11q13.2	POLD4	3.640992827	2.610912586	1.394528812	4	4	50.3588347	33.71493309
chr10	104122058	104126385	10q25.1	SFR1	8.863889973	6.357155144	1.394317076	4	4	46.23972513	38.8304545
chr11	59142746	59155039	11q12.1	FAM111A	21.51472921	15.43093697	1.394259419	4	4	26.4598326	34.64742326
chr6	28903002	28923991	6p22.1	TRIM27	4.49549069	3.224461789	1.394183273	4	4	77.3254995	59.06324816
chr4	26860691	27025381	4p15.2	STIM2	20.71374625	14.85746768	1.394163978	4	4	29.1134663	9.117363016
chr8	90791693	90959402	8q21.3	NECAB1	0.149191331	0.107012368	1.394150359	2	4	55.23230014	27.85563922
chr1	16985958	17011972	1p36.13	ATP13A2	4.850278155	3.479037327	1.394143753	4	4	50.20742038	43.82060669
chr8	27771949	27812430	8p21.1	ESCO2	4.618957427	3.313115573	1.394143164	4	4	108.289982	32.64142877
chr15	63504511	63594640	15q22.31	USP3	57.75484596	41.42703188	1.394134297	4	4	24.99285741	29.36207394
chr7	101232092	101238820	7q22.1	CLDN15	2.430092255	1.743255094	1.393996933	4	4	70.34899344	36.54764693
chr11	65649192	65649253	11q13.1	MIR4489	10.29023149	7.382115325	1.393940766	3	2	64.46668157	17.0494003
chr15	92882843	92898747	15q26.1	LINC01578	41.03637815	29.4419191	1.393807857	4	4	21.67705921	37.14420299
chr19	49848554	49860744	19q13.33	PTOV1	3.961941519	2.842533411	1.393806491	4	4	48.71731175	23.18563324
chr1	110401253	110407924	1p13.3	LAMTOR5	24.01420934	17.23035552	1.393715256	4	4	34.58118231	16.90167868
chr8	120445398	120523635	8q24.12	MTBP	13.64458301	9.790634735	1.393636203	4	4	105.4284888	68.31127755
chr12	22046174	22065674	12p12.1	CMAS	38.8000763	27.84130676	1.393615488	4	4	49.6775706	40.75076497
chr2	232782665	232782766	2q37.1	RNU6-107P	4.929725926	3.53746342	1.393576509	4	3	38.26360118	18.73230253
chr12	49366905	49527441	12q13.12	SPATS2	5.358729237	3.845346922	1.393561971	4	4	72.2508107	33.57035772
chr11	63813374	63827718	11q13.1	SPINDOC	2.340965679	1.679863148	1.393545469	4	4	51.5003208	62.71771124
chr15	81324333	81443076	15q25.1-q25.2	TMC3-AS1	0.739897914	0.530975976	1.39346778	1	4	0	35.38357028
chr3	12287485	12471054	3p25.2	PPARG	0.19922416	0.142979682	1.393373922	3	1	63.29515734	0
chr1	161401289	161401395	1q23.3	RNU6-481P	4.157869865	2.984420884	1.393191519	3	2	41.45863411	30.99140458
chr10	3137727	3172841	10p15.2	PITRM1	15.59462447	11.19554366	1.392931414	4	4	26.8665743	9.406806405

chr5	132747445	132754403	5q31.1	CCNI2	0.300242635	0.21556467	1.39281931	2	4	95.69556403	63.28444141
chr16	10743164	10769351	16p13.13	NUBP1	18.8456315	13.53074333	1.392800901	4	4	18.95689384	29.49489605
chr10	71844737	71844792	10q22.1	RNU7-38P	6.728166442	4.830719055	1.392787775	2	1	37.37050429	0
chr5	141318490	141320784	5q31.3	TAF7	84.16524924	60.43096181	1.39275045	4	4	51.08250278	27.52433303
chr8	124488494	124540486	8q24.13	TATDN1	13.80188013	9.911176085	1.392557251	4	4	54.01475118	32.09212461
chr11	8980576	9004651	11p15.4	NRIP3	0.953828046	0.684962141	1.392526665	4	4	47.13788282	41.48501235
chr1	169921326	170085203	1q24.2	KIFAP3	21.00803618	15.08676267	1.392481385	4	4	27.90449195	22.45767097
chr18	9102630	9134345	18p11.22	NDUFV2	8.193099648	5.884320666	1.392361177	4	4	81.03631376	23.76619777
chr11	126303752	126350005	11q24.2	DCPS	4.720889276	3.390994581	1.392184259	4	4	52.2661038	43.97205098
chr12	56951431	56959374	12q13.3	RDH16	0.220383619	0.158318432	1.392027546	3	4	35.93093906	47.57849219
chr19	43827292	43853286	19q13.31	ZNF283	2.039076824	1.464894019	1.39196201	4	4	31.29840182	32.16399106
chr19	54333185	54339169	19q13.42	LILRA4	1.051425592	0.755360581	1.391951895	4	4	52.02733465	48.6496795
chr13	79406309	79424336	13q31.1	RBM26-AS1	0.928367538	0.667019147	1.391815426	4	4	84.99003017	35.7612885
chr2	68365190	68397453	2p14	PLEK	243.5144619	174.9798448	1.391671493	4	4	57.31460449	31.50079477
chr18	46476972	46657115	18q21.1	LOXHD1	1.778492673	1.27812545	1.39148522	4	4	83.16567	63.48926995
chr3	193593144	193697811	3q29	OPA1	109.8289749	78.93018703	1.391469842	4	4	69.8787065	52.94021019
chr18	24426645	24453531	18q11.2	IMPACT	10.3502799	7.438382076	1.391469246	4	4	35.20202435	18.06736171
chr5	140647799	140662480	5q31.3	IK	71.04409256	51.05825506	1.391432051	4	4	66.0382914	37.41830946
chr15	40470961	40473158	15q15.1	CHST14	1.664727184	1.196428093	1.391414322	4	4	51.2572355	34.90380795
chr10	5861726	5889897	10p15.1	ANKRD16	0.68788968	0.494397059	1.391370899	4	4	68.79034759	64.78218959
chr10	73772291	73779104	10q22.2	FUT11	10.41199701	7.483518568	1.391323736	4	4	37.17037184	27.7471404
chr2	157412782	157444092	2q24.1	CYTIP	219.0733668	157.4588312	1.391305684	4	4	33.97693917	9.996318812
chr1	155063737	155069553	1q21.3	EFNA4	2.634536006	1.893590691	1.391291169	3	3	64.90375819	85.79591289
chr14	81260650	81436464	14q31.1	STON2	12.6028364	9.058453598	1.391279015	4	4	23.92307274	14.71712239
chr18	9923920	9924021	18p11.22	RNA5SP450	5.384862974	3.870557585	1.391237013	3	2	31.92417887	65.45446864
chr5	17498231	17498827	5p15.1	TAF11L2	0.886420395	0.637289429	1.390922798	2	2	22.64046896	62.973807
chr11	77066945	77126155	11q13.5	CAPN5	3.535158807	2.541705962	1.39086065	4	4	15.44180342	74.33257391
chr10	5765222	5813549	10p15.1	GDI2	224.969055	161.7544303	1.390806141	4	4	12.7405782	7.506944804
chr17	7484379	7514618	17p13.1	POLR2A	9.348136708	6.721608535	1.390758873	4	4	68.81486629	33.68387526
chr9	79571773	79726882	9q21.31	TLE4	23.43934848	16.85455864	1.390683018	4	4	34.19015019	28.30459293
chr7	107044728	107161811	7q22.3	PRKAR2B	66.91516854	48.11819174	1.390641795	4	4	36.67238885	20.88240258
chr3	160494995	160565588	3q25.33	KPNA4	29.88726995	21.49254071	1.390588035	4	4	26.49178334	15.00387556
chr15	84669537	84716460	15q25.2-q25.3	SEC11A	44.62182231	32.08954806	1.39054069	4	4	43.56950827	33.12179976
chr7	75922713	75923644	7q11.23	RPL7L1P3	0.958325193	0.689269419	1.390349211	4	3	49.92284881	84.43422293
chr6	52420196	52495785	6p12.2	EFHC1	0.496916212	0.357457021	1.390142543	4	4	29.09010068	27.49416107
chr1	25272393	25330445	1p36.11	RHD	10.45421198	7.520786549	1.390042373	4	4	54.12709817	125.727878
chr21	42974510	43033931	21q22.3	PKNOX1	3.99975912	2.877441975	1.390039887	4	4	50.46140451	27.2505062
chr1	6783892	6784980	1p36.31	CAMTA1-DT	2.083444091	1.498953571	1.389932372	2	3	17.22649047	64.05936474
chr13	113297154	113323426	13q34	LAMP1	31.1740225	22.42919229	1.389886096	4	4	43.698423	16.63868835
chr16	2275878	2340746	16p13.3	ABCA3	0.660565883	0.475343769	1.389659287	4	4	58.60758103	53.33522905
chr4	14111968	14140052	4p15.33	LINC01085	0.156446029	0.112583658	1.389598033	2	2	30.18157489	11.34804567
chr1	18904280	18956672	1p36.13	IFFO2	6.144453529	4.421822109	1.389575016	4	4	36.89403595	47.532233
chr1	12567910	12617731	1p36.21	DHRS3	5.225113715	3.760412035	1.389505635	4	4	48.21201731	34.41032593

chr8	144064061	144080648	8q24.3	EXOSC4	3.864245192	2.781084188	1.389474367	4	4	45.88952558	68.6881798
chr22	30634148	30669016	22q12.2	SLC35E4	0.233013908	0.167699387	1.389473819	4	4	90.21812764	27.63665099
chr16	57994668	58000690	16q21	ZNF319	7.439515005	5.354356984	1.389432014	4	4	60.13731848	28.74114025
chr12	1791957	1922606	12p13.33	CACNA2D4	0.961701149	0.692178279	1.389383599	4	4	67.01967515	44.73035068
chr1	1785285	1891117	1p36.33	GNB1	89.68393589	64.55438156	1.389277284	4	4	23.4276569	20.93105731
chr18	70630519	70650857	18q22.2	GTSCR1	0.820792839	0.590806038	1.389276321	2	2	12.42768196	24.57112239
chr9	100099185	100302175	9q31.1	INVS	4.174081001	3.004892513	1.389094945	4	4	25.32097924	25.89972191
chr12	69585428	69601577	12q15	CCT2	213.4047567	153.6419059	1.388974938	4	4	69.44459771	27.67083836
chr7	156669012	156893208	7q36.3	LMBR1	15.61294086	11.24096262	1.388932727	4	4	45.84362578	35.87585874
chr22	42617840	42649399	22q13.2	CYB5R3	21.05279989	15.15775349	1.388912935	4	4	17.3791362	50.99264089
chr16	28928	36491	16p13.3	IL9RP3	0.547153947	0.393949339	1.388894187	3	4	67.75225258	68.34788841
chr1	211743457	211830772	1q32.3	LPGAT1	63.24480941	45.53658018	1.388879208	4	4	34.01767826	26.79340813
chr1	156742107	156752448	1q23.1	HDGF	164.9510053	118.771159	1.388813636	4	4	11.74647833	47.286827
chr1	154325560	154351304	1q21.3	ATP8B2	14.63899455	10.54178976	1.388663109	4	4	67.04475586	36.65591958
chr1	120805556	120842215	1p11.2	NBPF26	7.20577493	5.190053599	1.388381602	4	4	73.31338514	45.76240727
chr7	39852697	39855260	7p14.1	RWDD4P2	9.064304993	6.528687529	1.388380889	4	4	64.29151896	11.25637186
chr3	122359587	122383231	3q21.1	CCDC58	6.518044827	4.694891333	1.388327091	4	4	19.58009781	11.68771229
chr5	41918061	42040230	5p13.1	FBXO4	6.309626916	4.544937806	1.388275744	4	4	35.05956123	13.32707497
chr15	55355223	55408510	15q21.3	CCPG1	15.02581873	10.8235484	1.388252557	4	4	34.74802503	45.48159004
chr14	103385364	103503831	14q32.32-q32.33	MARK3	141.4566933	101.8962452	1.388242452	4	4	52.66435418	37.53002299
chr4	10074339	10117034	4p16.1	WDR1	83.11458285	59.87579022	1.388116675	4	4	61.37974515	33.04956339
chr5	6371926	6378526	5p15.31	MED10	8.399198053	6.050992256	1.388069543	4	4	37.78861267	24.08029554
chr1	150809705	150876768	1q21.3	ARNT	22.26082894	16.03905654	1.387913864	4	4	44.59636801	12.30402247
chr20	62122454	62135378	20q13.33	LSM14B	12.15194921	8.755651215	1.387897817	4	4	39.40642866	30.70143507
chr19	44002925	44013920	19q13.31	ZNF230	4.820785149	3.47351673	1.3878687	4	4	41.7342646	26.91309801
chr5	87267845	87391926	5q14.3	RASA1	38.18342177	27.51464842	1.387748853	4	4	32.27087254	9.479963769
chr18	77432057	77437249	18q23	BDP1P	0.172003318	0.123945395	1.387734635	1	2	0	4.383015632
chr1	203178931	203186794	1q32.1	CHI3L1	31.35378883	22.59617329	1.387570737	4	4	64.51177189	70.97955497
chr21	36131767	36156308	21q22.12	CBR3-AS1	0.207189739	0.149328937	1.387472134	3	3	69.32393575	70.69017907
chr19	16185184	16192046	19p13.11	FAM32A	42.69952305	30.77788081	1.38734448	4	4	60.31183262	29.97985974
chr13	37009312	37059714	13q13.3	SUPT20H	20.66295361	14.89398348	1.387335607	4	4	10.61336298	13.00653929
chr6	30688047	30691420	6p21.33	NRM	0.718688577	0.518043666	1.387312738	4	4	112.4349009	47.04821045
chr16	15363613	15381467	16p13.11	NPIPA5	0.888586667	0.64050953	1.38731217	3	3	87.28864523	76.8155973
chr19	49861203	49867565	19q13.33	PNKP	4.76068642	3.432424916	1.386974671	4	4	84.17234627	26.86331986
chr15	51447724	51622833	15q21.2	DMXL2	26.1054896	18.82213649	1.386956769	4	4	46.61077038	15.44555932
chr5	107859033	108382098	5q21.3	FBXL17	9.854250143	7.105569779	1.386834617	4	4	31.85584955	19.48826718
chr1	31296982	31365722	1p35.2	ZCCHC17	53.20202023	38.3623898	1.38682758	4	4	24.42126225	7.925624007
chr18	58044234	58401540	18q21.31	NEDD4L	8.82032545	6.360692457	1.386692645	4	4	77.2381966	42.44562068
chr12	48965340	48979587	12q13.12	WNT10B	0.379799167	0.27390504	1.386608904	2	3	100.9675236	43.45945236
chr14	22598238	22612864	14q11.2	ABHD4	9.44599611	6.812462651	1.386575838	4	4	53.51715491	9.231027738
chr3	11272324	11564652	3p25.3	ATG7	8.578495626	6.187221781	1.386485879	4	4	22.26810823	22.59939696
chr19	56272769	56310426	19q13.43	EDDM13	0.151962925	0.109606434	1.38644165	2	1	14.69710522	0
chr17	47649838	47683638	17q21.32	KPNB1	61.34605323	44.24903821	1.386381619	4	4	50.69950977	27.6514161

chr17	76712830	76726799	17q25.1	JMJD6	8.855696367	6.387719594	1.386362729	4	4	49.17991433	32.23444431
chr1	207043849	207052980	1q32.1	YOD1	395.0313759	284.9656303	1.386242178	4	4	46.3773657	85.1019321
chr3	37753689	37861780	3p22.2	ITGA9-AS1	0.635284113	0.458313639	1.38613399	4	4	72.4860023	57.10810994
chr7	129225014	129430211	7q32.1	AHCYL2	3.173272447	2.289383627	1.386081568	4	4	58.05019105	25.10225286
chr7	2512529	2529177	7p22.3	LFNG	22.08174956	15.93125772	1.386064424	4	4	55.04036691	33.9288252
chr9	121178137	121202087	9q33.2	RAB14	35.39165752	25.53642672	1.385928341	4	4	14.24247245	15.95332026
chrX	154436913	154443467	Xq28	GDI1	17.33470485	12.50842353	1.385842493	4	4	47.52978209	31.44684009
chr22	37051725	37063390	22q12.3	KCTD17	1.232074341	0.889126746	1.385712831	4	4	68.8364389	39.45462764
chr16	82192505	82192790	16q23.3	RN7SKP190	2.735420246	1.9740922	1.385659822	1	1	0	0
chr3	132417660	132539032	3q22.1	DNAJC13	27.37996947	19.75956698	1.385656351	4	4	27.90399807	19.29648654
chr19	58476026	58489533	19q13.43	ZNF446	1.285470169	0.927719385	1.385623917	4	4	85.62482348	33.84065579
chr7	142425016	142425465	7q34	TRBV10-2	1.215780726	0.877504708	1.385497667	3	2	23.01922238	36.98655694
chr22	31104772	31107587	22q12.2	SELENOM	0.60409377	0.436021742	1.385467079	4	4	80.75917038	52.30014355
chrX	119399295	119454478	Xq24	SLC25A43	3.771163501	2.722101623	1.385386743	4	4	34.81233654	39.86681669
chr8	78759445	78762560	8q21.13	THAP12P7	0.363402467	0.262324791	1.385314999	3	4	53.73196293	25.032063
chr9	126914774	127223166	9q33.3	RALGPS1	0.773725823	0.558611238	1.385088181	4	4	43.12679557	29.39237827
chr5	17444010	17483946	5p15.1	LINC02218	11.13295015	8.038458951	1.384960752	4	3	40.91147925	68.68952899
chr3	38122710	38137242	3p22.2	ACAA1	5.808249229	4.193900585	1.384927733	4	4	65.78302024	15.32151632
chr13	39038311	39050109	13q13.3	NHLRC3	6.342715213	4.579885113	1.384907057	4	4	45.27350621	23.59870161
chr8	64586593	64798791	8q12.3	CYP7B1	0.698352419	0.504302662	1.384788286	4	4	79.07664642	86.82987349
chr3	185190624	185254098	3q27.2	EHHADH	0.858206764	0.619834057	1.384575039	4	4	52.26318172	58.93268975
chr6	31863192	31879046	6p21.33	SLC44A4	1.15485673	0.83410266	1.384549871	3	3	90.88902527	86.97704044
chr1	226144679	226186722	1q42.12	ACBD3	26.58548741	19.20303112	1.38444224	4	4	51.40583029	31.76898014
chr15	40239091	40277487	15q15.1	PAK6	0.074991093	0.054171095	1.384337787	1	3	0	42.60738598
chr8	73976142	73982783	8q21.11	TMEM70	7.050149487	5.093111034	1.384252069	4	4	56.29588102	36.44230062
chr6	27937404	27938401	6p22.1	OR2W6P	0.466823985	0.33724162	1.384241914	1	1	0	0
chrX	149476990	149505354	Xq28	IDS	28.83038084	20.82760526	1.384238874	4	4	60.68057535	29.70427401
chr2	68710510	68826845	2p13.3	ARHGAP25	92.61814664	66.91092145	1.384200735	4	4	60.40393917	41.88029926
chr5	115578496	115602479	5q22.3	TICAM2	0.373461529	0.269804356	1.384193845	3	2	97.99142522	65.24030836
chr3	27712181	27714006	3p24.1	LINC02084	3.482666041	2.516106084	1.384149128	4	4	69.62282996	62.81389212
chr16	89707133	89721231	16q24.3	VPS9D1	5.319348019	3.843594184	1.383951521	4	4	68.52370731	49.89104208
chr12	132825681	132826237	12q24.33	RPS11P5	0.679435781	0.490940191	1.383948173	4	2	29.12263636	15.60911426
chr2	32201498	32204218	2p22.3	DDX50P1	0.576010659	0.416215459	1.383924229	4	4	82.00001848	47.46945684
chr19	10836558	10869796	19p13.2	C19orf38	35.73716933	25.82450517	1.383847206	4	4	59.97530284	43.08169177
chr4	98261376	98443861	4q23	RAP1GDS1	11.17476044	8.075282505	1.383822849	4	4	26.20307401	14.76625347
chr5	177426496	177442849	5q35.3	GRK6	54.33276667	39.26632999	1.383698621	4	4	57.35941223	36.86989638
chr9	92493547	92536841	9q22.31	ECM2	0.135907058	0.098231763	1.383534755	1	1	0	0
chr15	74843730	74873379	15q24.1	SCAMP2	21.3101623	15.40331194	1.38347924	4	4	60.75300321	52.10079987
chr15	99565547	99716488	15q26.3	MEF2A	28.29789828	20.45513321	1.383413053	4	4	22.13254291	21.62585546
chr5	132311276	132369916	5q31.1	MIR3936HG	0.830288173	0.600191178	1.383372837	3	3	81.08564052	41.57275554
chr2	71276593	71435061	2p13.3-p13.2	ZNF638	70.87777117	51.24001191	1.383250482	4	4	29.29752242	31.44029128
chr2	29097676	29183813	2p23.2	CLIP4	9.60811222	6.946124657	1.383233485	4	4	29.82921165	12.97432838
chr4	3292978	3439913	4p16.3	RGS12	0.485560443	0.351036285	1.383220093	4	4	95.65806659	47.66030861

chr9	36190855	36212062	9p13.3	CLTA	41.04992695	29.67773043	1.383189562	4	4	34.62940048	12.84092091
chr14	106578742	106579177	14q32.33	IGHV5-51	10.97857781	7.937147749	1.383189297	3	4	12.86049212	133.4095068
chr6	41789896	41895361	6p21.1	USP49	9.967722679	7.206846823	1.383090681	4	4	70.3748215	63.6892622
chr17	68267026	68291439	17q24.2	SLC16A6	11.60169905	8.388251926	1.383089009	4	4	35.45742655	33.58915652
chr10	102673727	102714433	10q24.32	ARL3	2.038080169	1.473601252	1.383060829	4	4	48.21757676	64.09597034
chr7	139133744	139191986	7q34	TTC26	2.452904856	1.773614619	1.382997654	4	4	48.96352956	63.31005275
chr2	58251429	58252687	2p16.1	EIF3FP3	0.868267017	0.627839084	1.382945151	4	4	99.59229055	43.10607848
chr2	230712842	230821075	2q37.1	CAB39	93.78377716	67.81548785	1.382925643	4	4	56.79733723	36.3372574
chr16	30183410	30189076	16p11.2	CORO1A	129.5122614	93.65140179	1.382918557	4	4	51.1184535	35.93898502
chr9	127785538	127790782	9q34.11	CDK9	14.94059721	10.80417342	1.382854257	4	4	54.13084711	13.27604222
chr20	60308474	60308567	20q13.33	MIR646	5.830863746	4.216640152	1.382822232	1	1	0	0
chr2	229922491	230013913	2q36.3	FBXO36	0.690405232	0.499294738	1.382760882	4	4	53.07809296	17.53276987
chr17	2384060	2400964	17p13.3	MNT	3.638195137	2.631176523	1.3827256	4	4	82.0113397	24.7171581
chr10	75099575	75182123	10q22.2	SAMD8	15.15127372	10.9575536	1.382724126	4	4	36.79007375	23.25329652
chr14	103337849	103337974	14q32.32	SNORA28	305.7297972	221.1139593	1.382679764	4	4	53.42333889	19.02628578
chr13	94574054	94596275	13q32.1	TGDS	7.598712249	5.49618953	1.382541888	4	4	38.92330225	18.30489624
chr3	146069437	146161495	3q24	PLOD2	4.163484667	3.011605259	1.382480209	4	4	48.8578003	49.43075744
chr9	213108	215893	9p24.3	C9orf66	0.693931446	0.501961383	1.382439906	4	4	110.6219371	58.43743932
chr5	115613208	115626179	5q22.3	TMED7	47.71669479	34.51925053	1.382321286	4	4	13.64045151	10.53091172
chr17	64784841	64837184	17q24.1	PLEKHM1P1	6.460035746	4.673606979	1.382237697	4	4	31.87861477	32.00496691
chr1	193121958	193254815	1q31.2	CDC73	53.37935408	38.61981648	1.382175239	4	4	35.86803489	18.80550013
chr2	241315187	241354027	2q37.3	SEPT2	47.89198951	34.65163359	1.38209904	4	4	20.50293086	11.08392096
chr19	11553043	11559236	19p13.2	ELOF1	25.36525419	18.35357488	1.382033438	4	4	51.31139598	22.07914321
chr16	30985189	30989152	16p11.2	HSD3B7	0.920290209	0.665903226	1.382017946	4	4	90.0157688	30.29418778
chr1	20482391	20486235	1p36.12	CAMK2N1	0.431495643	0.312223454	1.382009061	1	2	0	96.2056881
chr7	128739292	128773423	7q32.1	CALU	16.48498293	11.92870647	1.381958972	4	4	30.12256206	10.86671858
chr9	12972560	12973506	9p23	PRDX1P1	1.043010805	0.754740406	1.381946425	2	1	40.31864675	0
chr16	18499924	18562143	16p12.3	NOMO2	2.61669001	1.893504687	1.381929513	4	4	83.50809612	35.18616788
chr1	16683091	16686249	1p36.13	EIF1AXP1	0.93018847	0.673198044	1.381745651	3	2	19.82264339	17.59259164
chr7	75359200	75367809	7q11.23	STAG3L1	1.053736245	0.76263491	1.381704708	4	4	23.68178813	76.64303643
chr1	159918107	159925542	1q23.2	TAGLN2	269.7487809	195.2445014	1.381594764	4	4	59.78715001	22.51882715
chr16	30748299	30761176	16p11.2	PHKG2	2.622582677	1.898231119	1.381592922	4	4	76.79090738	31.41402835
chr17	42199177	42288437	17q21.2	STAT5B	59.65079299	43.17809795	1.381505806	4	4	60.59931625	40.7692064
chr10	96128906	96205291	10q24.1	ZNF518A	15.13048027	10.95231427	1.381487044	4	4	25.89856775	25.34430006
chrX	39786931	39787828	Xp11.4	GAPDHP1	0.36206233	0.262093546	1.381424056	2	2	29.40516165	9.467500306
chr3	130850500	131016712	3q22.1	ATP2C1	21.3187387	15.4342896	1.381258176	4	4	33.25760621	25.20252193
chr3	196784981	196785086	3q29	RNU6-42P	10.94607576	7.925107719	1.381189524	3	3	45.61994744	119.0022363
chr16	29820394	29848039	16p11.2	MVP	40.15994129	29.0768282	1.381166509	4	4	52.98718989	26.80968649
chr16	30092314	30096216	16p11.2	YPEL3	177.7563605	128.7052586	1.381111871	4	4	13.58568304	8.189551047
chr5	122774996	122834539	5q23.2	SNX2	56.78932333	41.12179908	1.381002889	4	4	13.43743341	22.76715541
chr6	26457904	26476621	6p22.2	BTN2A1	19.3502715	14.01241493	1.380937661	4	4	52.39266602	30.32162964
chr2	70258099	70281185	2p13.3	PCYOX1	5.579253609	4.040196106	1.380936336	4	4	44.24614183	41.66674266
chr1	43522238	43623672	1p34.2	PTPRF	0.869203982	0.629486645	1.380814016	4	4	61.97655315	13.66883755

chr14	61277370	61281812	14q23.1	TMEM30B	0.345898895	0.250524445	1.380699177	4	4	92.84289745	55.31275201
chr19	44326553	44367215	19q13.31	ZNF112	0.143687696	0.10407889	1.380565224	3	2	62.57257395	45.39729971
chr1	212858255	212899363	1q32.3	FLVCR1	26.16081652	18.9510557	1.380441119	4	4	62.17471205	45.2229301
chr2	8721134	8837639	2p25.1	KIDINS220	34.59648967	25.06222873	1.380423507	4	4	15.54746583	12.72837659
chr6	41101034	41140835	6p21.1	ADCY10P1	1.252188986	0.907356175	1.38004129	4	4	61.62365062	13.13569031
chr10	13277796	13300130	10p13	PHYH	2.617299054	1.89676285	1.379876801	4	4	48.43145927	23.06392511
chr1	45511035	45522890	1p34.1	PRDX1	46.39617295	33.62430759	1.379840249	4	4	26.94730338	15.49545229
chr4	6909444	7033118	4p16.1	TBC1D14	61.2865762	44.41645978	1.379816773	4	4	32.2077142	30.6996868
chr8	109539700	109565996	8q23.2	EBAG9	7.62104209	5.523842671	1.379663134	4	4	42.9378598	18.60067653
chr1	230906241	230978872	1q42.2	TTC13	51.37506166	37.24499806	1.37938151	4	4	58.00411609	44.94862968
chr22	20604895	20626185	22q11.21	SMPD4P1	1.643519037	1.191563518	1.379296203	4	4	20.99259071	140.4817902
chr9	32540544	32552628	9p21.1	TOPORS	59.35630452	43.03545425	1.379241966	4	4	62.20850043	34.66383991
chr1	31906421	31938387	1p35.2	PTP4A2	145.5911117	105.5640195	1.379173627	4	4	24.08564489	24.98817765
chr22	17777290	17779481	22q11.21	LINC00528	6.94555585	5.036181615	1.37913133	4	4	79.49520181	28.65181571
chr19	12945814	12953643	19p13.13	RAD23A	77.50897623	56.20181164	1.379118821	4	4	19.7084897	51.14481591
chr6	79201245	79234738	6q14.1	HMGN3	36.06620216	26.15406961	1.378990065	4	4	68.15843842	32.06162291
chr18	31829173	31943128	18q12.1	TRAPPC8	44.89991492	32.566838	1.378700472	4	4	28.66015628	22.64592401
chr19	48203148	48256264	19q13.33	CARD8	28.13062355	20.40382937	1.378693334	4	4	32.67132854	47.74274936
chr8	142982031	143018390	8q24.3	LY6E-DT	0.136413881	0.098953073	1.378571447	2	2	37.83351167	47.60619599
chr3	57625454	57693118	3p14.3	DENND6A	50.89640098	36.91990188	1.378562737	4	4	42.56542523	29.10006552
chr1	26529758	26575029	1p36.11	RPS6KA1	32.24492426	23.39258598	1.378424954	4	4	49.81870616	36.99445312
chr16	66802878	66830977	16q22.1	NAE1	17.22593042	12.49702248	1.378402771	4	4	56.33622929	29.33412199
chr4	101023430	101348295	4q24	PPP3CA	35.9823165	26.10977084	1.378116902	4	4	49.68957397	42.47746636
chr18	24162045	24397882	18q11.2	OSBPL1A	3.329559662	2.416082252	1.378082083	4	4	21.68681165	33.86109314
chr15	73560003	73633412	15q24.1	NPTN	37.35236331	27.10739286	1.377940088	4	4	34.75409763	18.10268251
chr15	90230245	90265759	15q26.1	CIB1	19.95361143	14.48167439	1.377852511	4	4	50.08644058	21.33539084
chr5	72816591	72914388	5q13.2	TNPO1	44.04592425	31.96742948	1.377837536	4	4	16.16203181	22.73356049
chr2	197515571	197553699	2q33.1	MOB4	19.15620684	13.90383971	1.377763786	4	4	12.53934552	13.59402156
chr1	175156986	175193295	1q25.1	KIAA0040	63.92451003	46.40218761	1.377618456	4	4	71.93309739	52.69253518
chrX	71103249	71106973	Xq13.1	CXorf65	3.448004256	2.502883181	1.377612939	4	4	55.53147645	19.59508654
chr5	77691169	77776361	5q14.1	TBCA	24.54534663	17.81767368	1.37758425	4	4	36.710954	16.81358976
chr17	40062193	40093867	17q21.1	THRA	3.258673374	2.365600413	1.377524858	4	4	69.14987798	37.53527041
chrMT	8295	8364	N/A	MT-TK	4.500065006	3.267014237	1.377424364	2	2	42.11674926	14.28782837
chr22	30447959	30472047	22q12.2	SEC14L3	0.126961673	0.092184804	1.377251652	2	3	34.42915669	35.81648559
chr9	64439032	64440151	9q13	SNX18P9	0.682220874	0.495375485	1.377179323	1	2	0	33.62894936
chr14	23047061	23057619	14q11.2	CDH24	0.254923854	0.185105805	1.377179146	2	3	10.40292065	62.55277782
chr14	92116123	92121809	14q32.12	NDUFB1	25.04857215	18.18874603	1.377146731	4	4	65.92091211	39.38868431
chr7	150450630	150479395	7q36.1	GIMAP8	36.24943021	26.32408029	1.377044509	4	4	47.89756207	43.88394659
chr4	184627696	184649475	4q35.1	CASP3	38.54102528	27.99463186	1.376729134	4	4	13.14920127	16.39281886
chr13	36819202	36829603	13q13.3	RFXAP	5.202585178	3.779359854	1.37657841	4	4	67.08825241	36.19203848
chr4	143513463	143557489	4q31.21	SMARCA5	395.6091969	287.4191398	1.376419111	4	4	43.74900645	16.67803821
chr3	197749553	197784446	3q29	FYTTD1	19.31491993	14.03304194	1.37638867	4	4	45.36539867	18.40520636
chr3	142157527	142225607	3q23	GK5	5.738626457	4.17004467	1.376154673	4	4	42.35326583	34.66679882

chr10	97446131	97457370	10q24.1	ZDHHC16	4.463727194	3.243652098	1.376142403	4	4	58.33738978	44.46435583
chr1	109984765	110023742	1p13.3	AHCYL1	17.19612313	12.49755447	1.375959047	4	4	51.85653516	23.72683188
chr11	34438925	34472060	11p13	CAT	137.6981675	100.0748668	1.375951544	4	4	34.3839251	26.16958872
chr11	706120	727727	11p15.5	EPS8L2	0.343682821	0.249785173	1.375913619	4	4	77.46273365	77.2293302
chr7	20134655	20217390	7p21.1	MACC1	0.47066959	0.342085197	1.375884118	4	4	98.78825572	27.3982133
chr5	25909307	25913278	5p14.1	MSNP1	0.393493088	0.285999098	1.375854295	2	1	93.94730766	0
chr12	110434890	110450411	12q24.11	ARPC3	155.4799481	113.0080871	1.37583028	4	4	49.09480394	31.28748764
chr6	109691221	109825431	6q21	FIG4	16.37492194	11.90243318	1.375762559	4	4	17.29882986	22.82847033
chr2	68284171	68320051	2p14	CNRIP1	0.574093779	0.417315123	1.375684101	4	3	68.14270882	42.59967752
chr1	36136569	36156053	1p34.3	TRAPPC3	12.34933796	8.977611705	1.375570515	4	4	40.21210961	7.50395209
chr5	10353639	10440388	5p15.2	MARCH6	28.79837414	20.9358393	1.37555384	4	4	21.63717524	20.67420055
chr13	28659004	28678957	13q12.3	POMP	55.59575875	40.42198204	1.375384282	4	4	19.1209655	16.81179218
chr11	107928475	107963482	11q22.3	RAB39A	3.040112675	2.210500367	1.375305211	4	4	55.92341777	62.40207206
chr15	51751561	51816363	15q21.2	TMOD2	5.419141062	3.940771064	1.375147395	4	4	16.52802063	49.60147754
chr9	136982993	136985758	9q34.3	LCNL1	0.090673593	0.065938787	1.375117702	3	2	25.10266511	8.209050471
chr2	99284238	99304775	2q11.2	LYG1	1.152216646	0.838015762	1.374934337	3	4	101.815657	22.26761913
chrX	48973720	49002264	Xp11.23	GRIPAP1	10.61426551	7.719990465	1.374906556	4	4	70.43752499	34.87092919
chr13	48653711	48711226	13q14.2	CYSLTR2	8.631728831	6.278283572	1.374854884	4	4	54.75057906	41.07751501
chr19	38878555	38899862	19q13.2	SIRT2	4.61840631	3.359670888	1.374660335	4	4	52.6382563	30.21775331
chr4	112285995	112442618	4q25	ALPK1	12.48492895	9.083282546	1.374495276	4	4	68.32600723	61.24143608
chr14	67728892	67817293	14q24.1	ZFYVE26	5.003279258	3.6401114	1.374485203	4	4	35.43734759	24.62891844
chr11	112226365	112233973	11q23.1	PTS	5.207923098	3.789488277	1.374307748	4	4	24.99860862	26.26153326
chr7	33014113	33062821	7p14.3	NT5C3A	183.8303699	133.764141	1.374287373	4	4	35.13585058	63.65832632
chr4	39547297	39638998	4p14	SMIM14	14.33576398	10.43162636	1.374259726	4	4	28.7778634	46.30017197
chr8	123768439	123815452	8q24.13	FAM91A1	23.09909627	16.80873667	1.374231552	4	4	32.58060012	22.99991426
chr9	99104038	99154192	9q22.33	TGFBR1	43.50981185	31.66473358	1.374077939	4	4	13.15579402	24.20684886
chr22	31944493	31957603	22q12.3	YWHAH	63.42060966	46.15753844	1.37400329	4	4	49.5666172	39.34913465
chrX	119870532	119871828	Xq24	RNF113A	16.93960734	12.32951621	1.373906895	4	4	65.19389192	48.24347912
chr2	234951984	235055714	2q37.2	SH3BP4	0.435022838	0.316633903	1.373898478	4	4	111.2290142	67.51351006
chr5	75511308	75605820	5q13.3	POLK	18.10590256	13.17964016	1.373778217	4	4	37.52933178	15.93566609
chr19	7112255	7294405	19p13.2	INSR	4.377372658	3.186624574	1.373670652	4	4	33.16473346	18.1430242
chr6	89326625	89353155	6q15	UBE2J1	75.22360713	54.7651906	1.373566061	4	4	51.72165959	35.71581046
chr6	6588639	6654983	6p25.1	LY86	20.81758863	15.15635107	1.373522461	4	4	65.17837691	55.0292515
chr16	22206271	22288745	16p12.2	EEF2K	1.430383869	1.041398292	1.373522388	4	4	98.1179903	54.47030739
chr4	102794383	102868893	4q24	UBE2D3	169.7020518	123.5589585	1.373450002	4	4	11.0200632	7.677236844
chr1	50968695	50974637	1p32.3	CDKN2C	4.923326517	3.584663144	1.373441888	4	4	19.96941017	48.11460287
chr6	26402237	26415216	6p22.2	BTN3A1	40.37950927	29.40645152	1.373151372	4	4	21.08588862	39.57647865
chr1	154924732	154942689	1q21.3	PMVK	4.890179838	3.561331767	1.373132344	4	4	38.52995848	76.13080652
chr17	63432303	63446361	17q23.3	CYB561	2.49864998	1.820065572	1.372835143	4	4	35.19523205	48.80159924
chr19	35115818	35124324	19q13.12	FXYD3	0.102279537	0.074513996	1.372621816	1	1	0	0
chr2	39917634	40255209	2p22.1	SLC8A1-AS1	0.04054246	0.029536776	1.372609497	1	1	0	0
chr10	102394110	102402529	10q24.32	NFKB2	16.62202195	12.10996762	1.372590123	4	4	54.53168279	29.73294489
chr15	98437162	98514382	15q26.3	FAM169B	0.291576118	0.212447206	1.372463881	3	3	73.82866758	68.21233924

chr14	44897304	44907257	14q21.2	C14orf28	2.279845084	1.661226821	1.372386392	4	4	25.31307973	54.19843221
chr1	147626182	147670543	1q21.2	ACP6	0.618944646	0.451002702	1.372374585	4	4	56.4811642	54.08224443
chr1	36155965	36180849	1p34.3	MAP7D1	24.90384173	18.14708092	1.372333205	4	4	59.85833359	18.83958026
chr11	16738601	16758354	11p15.2	C11orf58	35.83135906	26.11226009	1.372204433	4	4	14.82921197	7.534391618
chr1	186375758	186421375	1q31.1	ODR4	37.06967962	27.01525062	1.37217604	4	4	58.05985208	39.04618718
chr22	45192242	45240769	22q13.31	KIAA0930	10.95277997	7.982569148	1.372087077	4	4	61.08320544	32.15000457
chr5	149495894	149551552	5q32	CSNK1A1	35.5718303	25.92796976	1.371948156	4	4	26.35759027	6.093234764
chr13	28232501	28232607	13q12.2	RNU6-82P	7.583155886	5.527312442	1.371942687	3	3	23.71248397	50.2160322
chr13	50015254	50020922	13q14.2	KCNRG	0.504490971	0.367729133	1.371909172	2	2	27.82359049	42.12056767
chr2	26764274	26781231	2p23.3	SLC35F6	8.839378572	6.444316184	1.371655009	4	4	55.18758263	24.93954405
chr1	111739841	111747798	1p13.2	INKA2-AS1	4.248214972	3.097214472	1.37162441	4	4	70.0460607	32.00152728
chr8	30156297	30183639	8p12	DCTN6	27.72412603	20.21387785	1.371539209	4	4	70.26470469	29.70354323
chr17	69244435	69327182	17q24.3	ABCA5	3.463582863	2.525581924	1.371399926	4	4	51.92739405	45.89315092
chr1	75724347	75763679	1p31.1	ACADM	37.08889677	27.04522828	1.371365639	4	4	44.75969738	15.52347642
chr11	105025508	105035591	11q22.3	CASP1	69.1721384	50.44066661	1.371356547	4	4	54.36955151	44.71010592
chr9	128340516	128361470	9q34.11	SLC27A4	3.531980864	2.575703193	1.371268582	4	4	69.47728957	44.62896427
chr1	93345921	93362592	1p22.1	DR1	26.4421463	19.28383928	1.371207564	4	4	23.39441871	26.41341075
chr1	226231149	226310082	1q42.12	LIN9	39.53577997	28.83283784	1.371206684	4	4	86.42958595	31.600636
chr2	95085391	95122027	2q11.1	MRPS5	6.120215161	4.464163523	1.370965721	4	4	35.61622416	43.72331392
chr1	206684912	206734285	1q32.1	MAPKAPK2	42.49493344	31.00084678	1.370766861	4	4	49.03831049	25.8839048
chr6	89363509	89412276	6q15	RRAGD	10.98186484	8.011740421	1.370721499	4	4	27.68922329	50.35231431
chr20	63939829	63956447	20q13.33	UCKL1	4.149201016	3.027199886	1.370639922	4	4	63.46539303	29.29145154
chr19	19385803	19508932	19p13.11	GATAD2A	33.54069924	24.47110289	1.370624748	4	4	36.06058858	33.94899217
chr1	227410173	227423782	1q42.13	NUCKS1P1	0.484514998	0.353534462	1.370488738	1	2	0	10.62403708
chrX	101098159	101181847	Xq22.1	CENPI	20.75159602	15.14210271	1.370456694	4	4	86.10494184	66.90826755
chrX	48476021	48486364	Xp11.23	FTSJ1	10.06725606	7.346573231	1.370333589	4	4	44.51240906	60.62376388
chr1	2228695	2310213	1p36.33-p36.32	SKI	10.20724095	7.448846349	1.370311653	4	4	76.65854972	23.28971587
chr12	104456914	104762014	12q23.3	CHST11	36.17411088	26.39882922	1.370292242	4	4	51.20596924	20.94674011
chr12	121888731	121917865	12q24.31	PSMD9	0.794204417	0.579660562	1.370119805	4	4	55.94000337	35.81538307
chr2	169479496	169506655	2q31.1	BBS5	0.181275773	0.132310065	1.370083022	2	2	48.02891314	77.2497072
chr1	6624616	6635586	1p36.31	THAP3	1.323836541	0.966308226	1.369994072	4	4	48.91763358	69.09169919
chr8	144104402	144107612	8q24.3	MAF1	95.12944101	69.44178026	1.369916506	4	4	18.14667272	55.95774005
chr11	839721	842529	11p15.5	POLR2L	11.6997021	8.540687244	1.369878298	4	4	43.39480928	57.14145704
chr7	142563799	142564245	7q34	TRBV12-4	5.759809354	4.204710347	1.369846881	4	4	59.01490275	66.08691247
chr2	160272151	160493983	2q24.2	RBMS1	25.73402954	18.78709571	1.369771567	4	4	28.25193976	36.43603023
chr14	51240168	51257655	14q22.1	TMX1	40.9055109	29.86406229	1.369723599	4	4	20.50245851	18.32376542
chr5	37208999	37212497	5p13.2	OFD1P17	0.121865141	0.088975594	1.369646842	1	2	0	10.87931879
chr6	149724315	149746572	6q25.1	NUP43	21.22513001	15.49808391	1.369532526	4	4	86.65872661	34.13748201
chr1	633696	634376	1p36.33	MTATP6P1	55.00549964	40.16689694	1.369423676	4	4	86.0877559	40.64737711
chr11	116843402	117098453	11q23.3	SIK3	16.46043129	12.02037383	1.369377651	4	4	30.43763775	10.91587063
chr11	119168334	119184016	11q23.3	NLRX1	3.127141524	2.283707162	1.369326846	4	4	54.1943512	44.81664988
chr22	18606124	18611919	22q11.21	RIMBP3	0.181598482	0.132633988	1.36917003	4	3	25.74203012	69.52794664
chr8	17156000	17230706	8p22	ZDHHC2	54.53218129	39.83105349	1.369087094	4	4	58.84653146	30.70657519

chr3	49683947	49689523	3p21.31	MST1	0.17566932	0.128329341	1.368894434	4	4	45.76447704	19.08138153
chr17	17246624	17281303	17p11.2	COPS3	80.51793672	58.82297671	1.368817786	4	4	46.43521941	21.41743149
chr6	44113454	44127491	6p21.1	MRPL14	12.24465202	8.945453714	1.368812853	4	4	19.55086145	1.850288477
chr8	127289676	127482139	8q24.21	CASC8	0.427764811	0.312544535	1.368652347	3	3	89.27777374	74.53069881
chr11	117291346	117292170	11q23.3	BACE1-AS	1.53939067	1.124778056	1.368617268	3	4	120.5118324	59.50089663
chr13	27270327	27273767	13q12.2	RASL11A	1.476932024	1.079333681	1.368373887	4	4	55.29211757	36.40081839
chr1	89106132	89126159	1p22.2	GBP2	165.243621	120.7648048	1.368309428	4	4	97.8972307	36.21092393
chr8	144355428	144358523	8q24.3	FBXL6	0.808771803	0.591118115	1.368206763	4	4	66.25697929	45.59785474
chr15	90930918	90932569	15q26.1	HDDC3	3.978232435	2.907741126	1.368152206	4	4	41.83299958	43.79992122
chr11	45885496	45906465	11p11.2	MAPK8IP1	0.190863086	0.139508488	1.36811092	1	3	0	77.2322966
chrX	49074433	49101121	Xp11.23	WDR45	29.55148885	21.60104694	1.368058175	4	4	46.34906804	29.88239385
chr1	42746335	42767084	1p34.2	P3H1	1.802330383	1.317445786	1.368048995	4	4	77.10105281	31.74547762
chr20	63865228	63891545	20q13.33	TPD52L2	26.85299153	19.62968934	1.367978426	4	4	57.61247635	41.62830111
chr17	46295131	46337794	17q21.31	LRRC37A	2.084695861	1.523966045	1.367941148	4	4	107.7023486	57.21893021
chr2	151409883	151534200	2q23.3	RIF1	28.73599853	21.00884252	1.367804938	4	4	37.57310303	19.12856116
chr9	126326844	126507041	9q33.3	MVB12B	3.12952596	2.288206017	1.367676658	4	4	19.84963359	56.1667715
chr19	31149870	31349525	19q12	TSHZ3	4.358350094	3.186771633	1.367638035	4	4	58.19965013	45.19147441
chrX	131646640	131648237	Xq26.2	HDGFP1	1.502791118	1.099114916	1.367273882	1	1	0	0
chr13	94712716	94715945	13q32.1	SOX21-AS1	0.319778898	0.233881794	1.367267165	1	3	0	18.72230392
chr11	77660009	77872232	11q14.1	RSF1	37.08941172	27.12799807	1.36720047	4	4	44.43936485	20.25015761
chr1	182573634	182589285	1q25.3	RNASEL	17.32048358	12.6688192	1.367174266	4	4	40.75842949	62.02624812
chr1	36339619	36385927	1p34.3	STK40	39.44295328	28.85109327	1.367121617	4	4	37.99681467	21.107758
chr20	13008954	13169103	20p12.1	SPTLC3	0.167128673	0.122253431	1.36706735	3	3	49.86484042	105.4856856
chr12	56041855	56044697	12q13.2	RPS26	83.65475436	61.19301349	1.367063813	4	4	146.9431518	105.9722535
chr4	102727274	102730721	4q24	LRRC37A15P	7.04133924	5.150799033	1.367038239	4	4	53.13733351	38.58900405
chr2	64092652	64144471	2p14	PELI1	92.95812246	67.99988963	1.367033431	4	4	57.59040508	45.49267848
chr6	147204184	147390476	6q24.3	STXBP5	27.99409019	20.47958499	1.366926635	4	4	35.13854493	29.80919917
chrX	38801432	38806532	Xp11.4	MID1IP1	14.02445957	10.25992655	1.366916176	4	4	51.64587855	49.13109227
chr1	184690491	184754907	1q25.3	EDEM3	34.25073917	25.05884262	1.366812494	4	4	19.87947792	22.67407527
chr11	64359153	64372215	11q13.1	RPS6KA4	8.580363359	6.277965144	1.366742752	4	4	60.55129526	24.37764947
chr10	5048766	5107686	10p15.1	AKR1C3	2.762027065	2.021075125	1.366612765	4	4	63.87851389	68.35483152
chr6	32168206	32178111	6p21.32	AGPAT1	4.58026778	3.352147058	1.366368391	4	4	28.26685636	11.79179871
chr3	121663199	121751169	3q13.33	GOLGB1	27.17534512	19.88904483	1.366347422	4	4	59.36689183	16.0883448
chr10	104031288	104085880	10q25.1	COL17A1	0.150751717	0.110334958	1.366309641	3	4	92.70676984	102.660019
chr12	21526257	21532947	12p12.1	SPX	14.28467462	10.45563982	1.366217167	4	4	59.02399078	19.71465924
chr1	226870572	226903829	1q42.13	PSEN2	1.137980871	0.832952286	1.36620175	4	4	107.683927	28.53397672
chr5	79612417	79688243	5q14.1	TENT2	151.5718507	110.9444277	1.366196156	4	4	52.9271012	35.46854081
chr8	73063077	73093272	8q21.11	SBSPON	0.079739016	0.058369955	1.366096936	1	1	0	0
chr17	77879001	77884087	17q25.3	LINC01973	0.277064459	0.202815153	1.366093482	3	2	95.64215664	63.13517727
chr17	30782684	30824760	17q11.2	CRLF3	91.75575851	67.16972009	1.366028597	4	4	23.22679409	16.18871849
chr22	30652051	30667890	22q12.2	DUSP18	3.205448398	2.346587456	1.366004233	4	4	40.47843605	40.53535043
chr11	78100942	78139653	11q14.1	ALG8	10.99261706	8.047291066	1.36600217	4	4	45.79166247	15.66878079
chr14	103912288	103921566	14q32.33	ATP5MPL	22.45859924	16.44151273	1.365969155	4	4	13.49448484	10.66336559

chr15	67200675	67255198	15q23	AAGAB	18.84198373	13.79436338	1.365919051	4	4	7.544116146	19.89459995
chrX	1591593	1602520	Xp22.33 and Yp11	AKAP17A	16.45379761	12.0463894	1.365869646	4	4	74.84817879	34.62397302
chr15	59659146	59689534	15q22.2	BNIP2	74.36815559	54.4481543	1.365852645	4	4	29.59198211	15.40096265
chr7	74289437	74405943	7q11.23	CLIP2	4.041907011	2.959531282	1.365725389	4	4	63.56341374	41.14338307
chr9	137551857	137552555	9q34.3	MRPL41	4.664907574	3.415996995	1.365606463	4	4	76.41576988	20.15111422
chr14	24161213	24166565	14q12	IRF9	6.246260813	4.575026474	1.36529501	4	4	75.43057044	43.28180347
chr8	8702156	8704107	8p23.1	CLDN23	0.326804707	0.239450081	1.364813516	3	4	49.68735055	61.88316937
chr22	37639475	37656119	22q13.1	SH3BP1	12.99048724	9.519366976	1.36463772	4	4	72.40049816	41.92265874
chr10	103302796	103351134	10q24.33	PCGF6	39.32759397	28.82034013	1.364577718	4	4	89.20001981	51.64544712
chr12	93467490	93503772	12q22	MRPL42	6.979121055	5.114964012	1.364451644	4	4	54.95396962	20.03273598
chr12	56266858	56270966	12q13.3	COQ10A	1.4336275	1.050838278	1.364270344	4	4	64.7360461	46.73609219
chr16	67438014	67481186	16q22.1	ATP6V0D1	20.42172863	14.96974744	1.364199945	4	4	53.94713489	29.06999255
chr16	1333605	1349441	16p13.3	BAIAP3	1.038502971	0.761255691	1.364197317	3	4	61.88571158	56.61924141
chr1	111542223	111716695	1p13.2	RAP1A	45.46069537	33.32524304	1.364151953	4	4	15.93381922	28.33477139
chr16	21597221	21657473	16p12.2	METTL9	15.31447144	11.22747865	1.364016973	4	4	31.83548825	29.00714553
chr1	146052596	146058799	1q21.1	LINC01719	0.158301713	0.116058015	1.363987763	1	1	0	0
chr8	80967810	81112068	8q21.13	PAG1	35.77729914	26.23037062	1.363964683	4	4	37.9182415	11.86233608
chr13	94601850	94634645	13q32.1	GPR180	3.765319941	2.760592427	1.363953586	4	4	26.60672703	21.16287513
chr12	102012840	102062124	12q23.2	WASHC3	43.57974777	31.95258994	1.363887806	4	4	47.69439659	11.10314646
chr20	45880920	45885266	20q13.12	ZSWIM1	4.397344077	3.224346134	1.363794051	4	4	36.51459758	20.70535972
chr12	120290639	120313242	12q24.23-q24.31	SIRT4	0.658191319	0.482631612	1.363755093	2	3	39.54490209	99.47843773
chr19	13961530	14007514	19p13.12	RFX1	2.539931204	1.862790908	1.363508483	4	4	62.66683271	21.72179743
chr4	24520192	24520290	4p15.2	MIR573	12.46279233	9.140644619	1.363447858	4	4	50.84328707	21.62225379
chr8	90994271	91040975	8q21.3	PIP4P2	33.28141492	24.41098045	1.363378869	4	4	18.41080913	36.59769956
chr3	186570676	186585800	3q27.3	DNAJB11	6.919359994	5.075638766	1.363249103	4	4	59.55404861	22.57068589
chr2	117915478	118014163	2q14.1	CCDC93	11.49762954	8.435309627	1.363035864	4	4	45.64541473	13.41035703
chr19	2037471	2051278	19p13.3	MKNK2	74.5892847	54.72344972	1.363022344	4	4	41.45290832	35.07547383
chr17	58679527	58679690	17q22	RNU1-85P	2.341145244	1.717771291	1.362896944	1	1	0	0
chr3	138652698	138834938	3q22.3	PIK3CB	15.82310554	11.61068611	1.362805384	4	4	4.759242542	22.88397522
chr17	47450227	47492620	17q21.32	MRPL45P2	5.156194225	3.783700789	1.362738364	4	4	57.76844665	17.07769053
chr6	116677643	116741867	6q22.1	KPNA5	26.54905802	19.48225889	1.362729968	4	4	77.28349639	45.75641626
chr12	6638076	6647414	12p13.31	ACRBP	24.43520999	17.93116801	1.362722717	4	4	31.16539959	28.2999697
chr16	89267619	89490561	16q24.3	ANKRD11	9.829544181	7.214255986	1.362516689	4	4	74.04363858	31.65236671
chr16	67227113	67229279	16q22.1	TMEM208	5.24398201	3.84955713	1.362229948	4	4	68.30448841	17.74765051
chr2	127645864	127652639	2q14.3	GPR17	0.271132983	0.199044258	1.36217435	2	1	29.48724115	0
chr18	26226445	26391685	18q11.2	TAF4B	29.27796239	21.49482099	1.362093799	4	4	103.9549325	45.72152396
chr14	106088083	106088573	14q32.33	IGHV3-64D	3.696708852	2.71444927	1.361863304	2	1	18.6019755	0
chr22	25351418	25405410	22q11.23	LRP5L	0.793447637	0.582649746	1.361791785	4	4	74.56925693	46.21441239
chr4	86876205	86897256	4q21.3	C4orf36	0.52039612	0.382144525	1.361778295	4	4	36.54631507	40.41077686
chr17	6993791	7010736	17p13.1	ALOX12	9.210386946	6.763640262	1.361749973	4	4	76.7369814	23.16106574
chr6	110609978	110816531	6q21	CDK19	25.64213523	18.83080128	1.36171238	4	4	26.51927154	30.31210171
chr22	20481935	20482213	22q11.21	RN7SL812P	1.219348787	0.895482876	1.361666223	1	2	0	15.45794161
chr13	98450199	98577151	13q32.2	STK24	24.60107552	18.06758157	1.361614194	4	4	17.89682932	12.74899524

chr20	18507482	18561415	20p11.23	SEC23B	25.59314963	18.79641411	1.361597456	4	4	41.61977995	21.96072012
chr3	72996743	73069201	3p13	PPP4R2	52.28798543	38.40955053	1.361327709	4	4	9.759247859	23.655002
chr15	82429363	82439387	15q25.2	GOLGA6L9	0.088077158	0.064702907	1.361255043	3	3	80.11925895	32.38469775
chr15	66505083	66549485	15q22.31	ZWILCH	50.6327307	37.19830674	1.361156868	4	4	81.28815949	34.54270464
chr9	136654753	136672678	9q34.3	EGFL7	0.970237841	0.712840442	1.361086974	4	3	47.84023851	35.02881131
chr1	45786992	46036124	1p34.1	MAST2	2.190976434	1.609794571	1.361028589	4	4	39.72438314	18.49278534
chr5	180494373	180578405	5q35.3	CNOT6	41.75085465	30.67786149	1.360944102	4	4	41.21379565	21.07162478
chr19	13795424	13832254	19p13.13-p13.12	ZSWIM4	0.863588762	0.634569126	1.360905735	4	4	91.80213708	74.61578003
chr11	93144171	93386038	11q21	SLC36A4	3.874719843	2.847187864	1.360893635	4	4	48.70098881	18.26067547
chr17	45263119	45317064	17q21.31	MAP3K14	4.636975124	3.407345484	1.360876126	4	4	106.9952734	20.2434949
chr17	41966741	41977740	17q21.2	CNP	5.071602441	3.726747604	1.360865554	4	4	45.03815501	49.85421103
chr7	48901756	48904003	7p12.3	GDI2P1	0.353544445	0.259817226	1.3607429	3	3	38.41978841	29.79310335
chr14	94110733	94116699	14q32.12	IFI27	5.727055934	4.2087931	1.360735916	4	4	65.49392377	153.9811677
chr8	10054224	10428891	8p23.1	MSRA	3.382389849	2.485803981	1.360682449	4	4	62.34204841	42.77577726
chr21	38367261	38661780	21q22.2	ERG	0.300580148	0.220914134	1.360619811	4	4	20.52217081	22.32138864
chr8	98102339	98117190	8q22.2	RIDA	9.402473486	6.910599991	1.360587141	4	4	66.09286469	24.82289699
chr10	7158624	7411486	10p14	SFMBT2	11.35806872	8.347961982	1.360579833	4	4	41.45616493	37.33296742
chr17	48048305	48061545	17q21.32	NFE2L1	15.61868505	11.48015165	1.360494663	4	4	50.86700133	35.38594052
chr22	36226209	36239954	22q12.3	APOL2	31.53647242	23.18237535	1.360364153	4	4	61.1130415	45.13444965
chr17	40219304	40284136	17q21.2	WIPF2	11.30734147	8.313844307	1.36006173	4	4	43.59355567	36.79610128
chr2	24067584	24076444	2p23.3	SF3B6	66.99562771	49.26142825	1.360001732	4	4	14.39925325	15.08856808
chr11	36296175	36465204	11p13-p12	PRR5L	11.76756272	8.653074242	1.359928552	4	4	21.68478552	59.91247103
chr14	71398337	71398407	14q24.2	SNORD56B	5.684495329	4.180114931	1.359889721	3	3	69.80557396	32.24577827
chr8	70637266	70669212	8q13.3	LACTB2	18.03065776	13.26067791	1.359708597	4	4	66.43441882	46.37098912
chr7	77696426	77779803	7q11.23	RSBN1L	49.00799831	36.05077736	1.359415855	4	4	23.96528542	29.04888083
chr3	128909866	129032431	3q21.3	KIAA1257	0.732809926	0.539121026	1.359267938	4	4	86.41924559	45.51175347
chr3	49468703	49535618	3p21.31	DAG1	3.130524283	2.303384075	1.359097824	4	4	68.2074325	52.7652629
chr7	66987677	66995696	7q11.21	SBDS	197.1718133	145.0819185	1.359037813	4	4	51.97872557	40.45607273
chr11	62662817	62671769	11q12.3	LBHD1	1.18374271	0.871082406	1.358933095	4	4	60.9714589	53.70122688
chr12	57816702	57846964	12q14.1	CTDSP2	63.0370224	46.38806551	1.35890604	4	4	40.49438047	28.79538573
chr12	26373598	26373733	12p11.23	RNA5SP354	3.310175351	2.43608844	1.358807545	3	2	44.00549422	20.9750342
chrX	134887626	134915344	Xq26.3	MOSPD1	31.11292667	22.89917324	1.358692139	4	4	50.89694215	58.99191354
chr1	161037631	161038984	1q23.3	TSTD1	10.11811166	7.447572162	1.358578532	4	4	28.66466072	38.80460672
chr16	72283301	72665009	16q22.2	LINC01572	11.09841939	8.169953442	1.358443408	4	4	88.63092509	63.52013923
chr4	47593998	47838106	4p12	CORIN	0.164193997	0.120874642	1.358382488	4	3	82.98427912	70.17646645
chr22	39349949	39385588	22q13.1	SYNGR1	0.448488413	0.330170382	1.358354465	3	3	43.37109897	34.79772636
chr21	43789537	43804102	21q22.3	RRP1	2.556955196	1.882433059	1.358324634	4	4	23.20874209	45.29769108
chr12	93377923	93403248	12q22	NUDT4	24.16876138	17.79342892	1.358297014	4	4	28.09957134	21.15098299
chr19	47256474	47271958	19q13.32	CCDC9	12.43049316	9.152388211	1.358169352	4	4	41.44643388	12.21816242
chrX	101219786	101264497	Xq22.1	DRP2	0.162804234	0.119872166	1.358148763	2	3	3.661353661	27.04868704
chr10	113834724	113854404	10q25.3	DCLRE1A	6.785136363	4.996494618	1.35797932	4	4	32.06032553	32.0000117
chr11	94128928	94185596	11q21	PANX1	15.16246413	11.16595957	1.357918594	4	4	14.73959959	16.49128332
chr1	244048939	244057476	1q44	ZBTB18	42.96218196	31.64135328	1.357785856	4	4	56.97498401	38.75577392

chr17	75266228	75271292	17q25.1	MIF4GD	7.082674623	5.2164258	1.357763897	4	4	33.34367353	34.9200664
chr4	119296419	119304445	4q26	C4orf3	82.22023224	60.56181601	1.357624947	4	4	37.83330112	42.45121431
chrX	54814370	54814500	Xp11.21	SNORA11	7.856783267	5.78760375	1.357519209	3	2	90.78804376	25.83504362
chr11	34882295	34916411	11p13	APIP	4.734221513	3.487426935	1.357511312	4	4	33.84831544	31.32368642
chr5	157477304	157575823	5q33.3	ADAM19	21.37930007	15.74918204	1.357486377	4	4	60.39220587	60.6538929
chr19	11199293	11262501	19p13.2	DOCK6	0.097742176	0.072002375	1.357485445	3	3	85.72864102	74.3073995
chr3	179239749	179267050	3q26.32	KCNMB3	0.141918513	0.104562754	1.357256843	3	4	54.77743337	67.01083454
chr2	241149573	241183650	2q37.3	PPP1R7	29.5350739	21.76176497	1.357200298	4	4	47.83720135	22.88245489
chr17	70166961	70169402	17q24.3	KCNJ2-AS1	1.37728158	1.014801447	1.357193156	4	4	79.53384434	34.83387588
chr11	747432	765024	11p15.5	TALDO1	189.5222351	139.6453277	1.357168466	4	4	43.32895717	41.87980269
chr2	74958643	74969732	2p12	POLE4	3.47286259	2.559008372	1.357112633	4	4	82.22254925	38.68353996
chr15	67232407	67232914	15q23	RPS24P16	1.942107074	1.431120843	1.357053168	1	1	0	0
chr4	10486405	10734853	4p16.1	CLNK	0.177095024	0.130508174	1.356965	3	4	31.83798456	63.22377477
chr10	43436482	43483184	10q11.21	ZNF487	4.137469803	3.049198185	1.356904193	4	4	44.74472559	19.45338108
chr12	10604190	10613609	12p13.2	MAGOHB	4.400791095	3.243737279	1.356703924	4	4	56.36917091	15.88504165
chr1	160288587	160343564	1q23.2	COPA	55.04721397	40.57496	1.356679439	4	4	32.95364528	26.81461913
chr17	19904302	19977856	17p11.2	AKAP10	34.34931275	25.31991346	1.356612565	4	4	16.78100186	13.25105339
chr2	174559574	174682899	2q31.1	WIPF1	112.0307193	82.58260266	1.356589835	4	4	41.5529229	29.00674722
chr7	72829373	72837399	7q11.23	SBDSP1	142.9349459	105.3659336	1.356557486	4	4	73.41472156	55.12986878
chr11	118998117	119015793	11q23.3	CCDC84	0.794441313	0.58563623	1.356543998	4	4	81.78438125	42.15037264
chr19	35754566	35757029	19q13.12	HSPB6	0.187611358	0.138321242	1.356345242	1	1	0	0
chr11	86244384	86285420	11q14.2	EED	12.19801907	8.99365682	1.356291364	4	4	70.44039476	60.28036543
chr14	93202892	93207114	14q32.12	GON7	1.093714234	0.806417364	1.35626325	4	4	45.92965556	30.01870636
chr5	146589504	147081520	5q32	PPP2R2B	1.229222667	0.906540374	1.355949168	4	4	42.82506304	70.14556264
chr17	67070438	67245989	17q24.2	HELZ	29.57380461	21.81042026	1.355948407	4	4	24.83949335	12.73145999
chr6	46129964	46146699	6p21.1	ENPP4	15.96229827	11.7733125	1.355803498	4	4	22.07071793	19.33986166
chr6	18387329	18468874	6p22.3	RNF144B	30.17340639	22.25506141	1.355799737	4	4	48.74672313	37.56412812
chr1	149851061	149851594	1q21.2	HIST2H2AA4	1.737530882	1.281702544	1.355642844	4	4	80.26700531	56.42325118
chr7	129144716	129169694	7q32.1	TSPAN33	20.29217469	14.96907223	1.355606705	4	4	41.6563705	17.2373116
chr2	102418550	102452568	2q12.1	IL18RAP	60.56516164	44.67947398	1.355547777	4	4	52.49547505	81.80518995
chr1	47333797	47378839	1p33	CMPK1	164.038022	121.0169735	1.355495988	4	4	21.19600032	8.256267477
chr19	18257096	18274509	19p13.11	IQCN	0.970321905	0.715883729	1.355418297	4	4	106.1511134	63.86957213
chr3	149030064	149086554	3q24	HLTF	30.25548698	22.32232491	1.35539139	4	4	75.17839377	28.61630399
chr1	234604269	234609525	1q42.3	IRF2BP2	43.36327301	31.99423724	1.355346361	4	4	35.98745181	15.71369108
chr9	112379909	112472410	9q32	HSDL2	55.4962711	40.94657424	1.355333679	4	4	10.87608659	31.55501835
chr11	126211414	126264540	11q24.2	FAM118B	3.766968786	2.779386719	1.355323734	4	4	32.45006514	34.82230877
chr17	75587545	75626851	17q25.1	MYO15B	3.119447257	2.301796309	1.355222982	4	4	82.31832902	52.97429354
chr14	58200115	58235636	14q23.1	ACTR10	29.2319382	21.57001889	1.355211525	4	4	47.96220936	28.2734698
chr1	44653247	44674608	1p34.1	TMEM53	0.67130228	0.495383477	1.355116411	3	3	73.3236485	77.27612977
chr21	34787801	35049334	21q22.12	RUNX1	8.143160336	6.0100398	1.354926191	4	4	33.10590289	29.72334042
chr11	64898363	64898437	11q13.1	MIR6750	6.210315963	4.583738569	1.354858238	2	2	11.65621964	22.0879531
chr7	65751105	65763675	7q11.21	CCT6P1	12.87969587	9.506366347	1.354849518	4	4	35.69483919	79.2710924
chr11	67266400	67286558	11q13.2	GRK2	52.48251528	38.73840775	1.354792784	4	4	41.27788436	24.72119068

chr2	45388680	45611294	2p21	SRBD1	34.65838689	25.58306255	1.35473956	4	4	25.71908787	14.00398269
chr1	159834478	159855037	1q23.2	SNHG28	0.312890433	0.230972064	1.354667868	2	4	39.21787505	44.46595834
chr21	17512906	17636213	21q21.1	CXADR	0.333532572	0.246213652	1.354646945	4	4	108.6875482	79.34904325
chr10	92451684	92574095	10q23.33	IDE	23.20328124	17.13064252	1.35448984	4	4	38.27834042	29.26298721
chr20	58232777	58309439	20q13.32	PPP4R1L	3.235227885	2.38853828	1.354480232	4	4	42.49997615	47.23588461
chr19	35232290	35233003	19q13.12	FAM187B2P	1.056530313	0.780047458	1.354443634	4	3	47.945043	88.71226209
chr20	31514410	31569567	20q11.21	HM13	8.584601151	6.338234996	1.354415094	4	4	41.43010612	35.41427304
chr15	89088150	89202360	15q26.1	ABHD2	101.8559774	75.20420536	1.354392044	4	4	21.42374962	25.08464977
chrX	101615118	101618001	Xq22.1	ARMCX6	6.510894712	4.807343402	1.354364389	4	4	36.15985782	6.327052605
chr16	27203486	27222305	16p12.1	KDM8	4.971158162	3.6705065	1.354352093	4	4	52.70102506	23.49963398
chr7	72879335	72951440	7q11.23	POM121	13.63461669	10.06758049	1.354309181	4	4	34.78531126	9.934784627
chr10	70202830	70233434	10q22.1	PPA1	17.86811322	13.19420087	1.354239896	4	4	39.78427941	29.97462855
chr19	2096669	2099588	19p13.3	IZUMO4	0.389587121	0.287700324	1.354142101	3	3	51.94685542	89.02984671
chr20	25612935	25624012	20p11.21	NANP	1.503575595	1.110414593	1.354066854	4	4	19.82915235	26.72966549
chr8	23135588	23164030	8p21.3	TNFRSF10D	3.900240759	2.880477536	1.354025751	4	4	79.09548256	41.40737307
chr12	96278261	96400588	12q23.1	CDK17	80.52797839	59.47376697	1.354008372	4	4	43.96280442	16.91663533
chr19	46674316	46717127	19q13.32	PRKD2	15.57681273	11.50542271	1.353867052	4	4	50.54129417	33.8963444
chr18	54151601	54224788	18q21.2	MBD2	38.71391421	28.59577404	1.353833408	4	4	39.57345132	26.25930651
chr12	29149003	29335617	12p11.22	FAR2	12.45117962	9.197480119	1.353759885	4	4	22.85831597	27.79900106
chr14	91232532	91264581	14q32.11	GPR68	2.051801613	1.515659042	1.353735607	4	4	40.96615632	51.98113199
chr16	46955362	46973714	16q11.2	DNAJA2	34.61098981	25.57075008	1.353538308	4	4	39.28536829	17.65330002
chrX	49235467	49250526	Xp11.23	CCDC22	5.5348165	4.090480407	1.353096934	4	4	18.87337361	32.34358054
chr10	112987482	112987864	10q25.2	RPS15AP30	1.047859704	0.774416068	1.353096543	1	2	0	15.39069734
chr20	44210960	44226027	20q13.12	OSER1-DT	8.434106349	6.233515132	1.353025728	4	4	28.91310849	36.44024462
chrX	154531390	154547586	Xq28	G6PD	28.63941424	21.16710479	1.353015187	4	4	48.61588818	32.75188429
chr2	161136955	161236176	2q24.2	TANK	138.0777675	102.0565258	1.35295383	4	4	37.9957321	29.92952744
chr7	23680114	23703249	7p15.3	FAM221A	1.298130626	0.959526696	1.352886408	4	4	93.86036889	31.00920896
chr1	173477347	173488807	1q25.1	PRDX6	278.4156475	205.8060036	1.352806248	4	4	54.67479767	81.92520351
chr19	45001449	45038198	19q13.32	RELB	11.34696259	8.387732041	1.352804613	4	4	45.53824409	28.32631708
chr2	89159751	89160302	2p11.2	IGKV6-21	2.158308964	1.59545155	1.352788785	4	1	40.86801361	0
chr19	17215346	17219831	19p13.11	USE1	1.718362299	1.27031965	1.352700715	4	4	82.79963685	33.02425541
chr20	62841014	62861763	20q13.33	TCFL5	5.228652247	3.865666672	1.352587455	4	4	34.57480173	41.32911735
chr1	26787963	26798403	1p36.11	PIGV	4.677375879	3.458363303	1.35248251	4	4	38.28299409	50.93031641
chr1	145959037	145978848	1q21.1	POLR3GL	15.20258564	11.24052913	1.352479538	4	4	67.88736666	41.95891218
chr22	43166622	43187131	22q13.2	TTLL12	5.037822434	3.725116249	1.352393348	4	4	73.58111183	42.23329662
chr12	98485544	98503855	12q23.1	LINC02453	0.576136064	0.42602355	1.352357314	3	3	22.99211498	49.08633821
chr1	229321005	229343795	1q42.13	CCSAP	13.4218633	9.925408064	1.352273198	4	4	19.21569882	31.20154277
chr2	105744649	105894274	2q12.2	NCK2	40.07970408	29.64014846	1.352209964	4	4	56.38752903	29.58960741
chr6	118894077	118909171	6q22.31	ASF1A	30.16472793	22.30777141	1.352207147	4	4	35.67869419	17.35630662
chr21	45643725	45942454	21q22.3	PCBP3	0.708901505	0.524355775	1.35194755	4	4	21.03607211	47.76532827
chr12	130789600	130839274	12q24.33	STX2	18.86758265	13.95624957	1.351909233	4	4	52.04846397	10.30769076
chr7	112206588	112343934	7q31.1	ZNF277	21.46047434	15.87482	1.351856232	4	4	15.99685451	18.72936307
chr5	126776623	126837020	5q23.2	LMNB1	70.21030513	51.93981509	1.351762709	4	4	74.79070962	39.56211225

chr6	10690865	10709782	6p24.2	PAK1IP1	65.20016989	48.2360272	1.351690296	4	4	82.74444704	62.73612952
chr10	73785577	73801798	10q22.2	ZSWIM8	10.65275256	7.881333343	1.351643446	4	4	72.61713315	22.99762222
chr1	37809567	37859620	1p34.3	MTF1	9.370516931	6.933654292	1.351454303	4	4	53.47653536	20.83326886
chr1	108746635	108809526	1p13.3	STXBP3	45.63769164	33.77605615	1.35118474	4	4	6.184351402	22.88742433
chr12	104215779	104350307	12q23.3	TXNRD1	31.74251945	23.49292078	1.351152534	4	4	46.62411317	24.99394474
chr17	43014605	43022442	17q21.31	VAT1	5.406907607	4.00187184	1.351094643	4	4	71.84607613	43.24421263
chr20	1368977	1393172	20p13	FKBP1A	61.83945289	45.77574621	1.350921788	4	4	28.88886327	29.26765616
chr17	6643313	6651634	17p13.1	MED31	6.226532389	4.609254124	1.350876351	4	4	15.99324804	20.00321048
chr20	56468585	56518886	20q13.31	RTF2	38.48083243	28.48819223	1.350764278	4	4	30.67438687	29.04242163
chr22	23786931	23834518	22q11	SMARCB1	8.646578502	6.402989165	1.350397178	4	4	53.10194184	49.79406421
chr2	11179759	11345407	2p25.1	ROCK2	26.38087437	19.53602195	1.35037084	4	4	22.00935688	18.083813
chr8	33547754	33567128	8p12	RNF122	6.472733711	4.793363748	1.350353124	4	4	51.07902182	28.22508421
chr4	75513944	75547334	4q21.1	THAP6	4.890975052	3.62226593	1.350252893	4	4	25.26410579	40.57525499
chr5	115267188	115296761	5q22.3	CCDC112	7.33969873	5.435870138	1.350234377	4	4	45.44944025	40.02932418
chr4	55395653	55453397	4q12	TMEM165	30.02345928	22.23636512	1.350196362	4	4	32.45197	20.95964897
chr7	142554881	142555318	7q34	TRBV11-3	0.881971933	0.653278465	1.350070422	2	1	25.7124643	0
chr2	23927172	24001000	2p23.3	UBXN2A	46.15737722	34.19643458	1.349771629	4	4	83.8176131	52.4974112
chr11	44095549	44245430	11p11.2	EXT2	6.699071597	4.964096869	1.349504607	4	4	39.53993404	13.59564871
chr10	16590616	16817454	10p13	RSU1	121.3829672	89.95308581	1.349403037	4	4	10.27251388	8.37350279
chr6	144140044	144191939	6q24.2	STX11	106.1970459	78.70073238	1.349378115	4	4	68.79383268	37.15365582
chr1	9942923	9996884	1p36.22	NMNAT1	4.076948569	3.021382194	1.349365392	4	4	38.57809029	13.39119351
chrX	49174802	49186400	Xp11.23	PRICKLE3	1.497408752	1.109770837	1.34929546	4	4	64.97454751	68.16604604
chrX	102599348	102908110	Xq22.1	ARMCX5-GPRASP2	2.117582972	1.56949318	1.34921451	4	4	46.32504562	68.22110411
chr1	86704570	86748184	1p22.3	SH3GLB1	78.36347452	58.08287297	1.349166639	4	4	33.21754159	21.13324818
chr17	34036704	34040141	17q12	TLK2P1	11.75129845	8.711000877	1.349018168	4	4	78.09957885	40.75318417
chr15	42199570	42199666	15q15.1	MIR627	3.95685672	2.933194268	1.348992381	2	1	8.991674862	0
chr17	1494575	1516888	17p13.3	INPP5K	21.90349318	16.23693039	1.348992245	4	4	12.05371589	11.70664756
chr13	24420930	24512993	13q12.12	PARP4	58.89505195	43.6643443	1.348813383	4	4	29.52409794	24.59779771
chr1	208022242	208244320	1q32.2	PLXNA2	0.446075987	0.330718711	1.348807831	4	4	84.50617862	63.52976627
chr4	75479037	75515057	4q21.1	RCHY1	9.425584891	6.988152914	1.348794883	4	4	24.06422249	18.32522214
chr20	50731544	50753741	20q13.13	PARD6B	1.132994945	0.84003373	1.348749347	4	4	35.52305011	28.83188655
chr1	244969739	245125228	1q44	EFCAB2	4.737308329	3.51259378	1.348663872	4	4	67.82579807	45.84763813
chr13	21153595	21176602	13q12.11	SKA3	356.198407	264.1178209	1.348634506	4	4	70.66737768	16.35302276
chr7	100358003	100367831	7q22.1	PILRB	1.770403735	1.31273842	1.348634052	4	4	45.335702	50.33524232
chr1	153947672	153958656	1q21.3	CRTC2	13.89873717	10.30624488	1.348574318	4	4	57.2172989	17.17740343
chr3	113919144	113920395	3q13.31	VPS26AP1	0.371136586	0.27521009	1.348557336	2	1	29.46506692	0
chr18	47155019	47176374	18q21.1	IER3IP1	6.494240993	4.815802765	1.348527195	4	4	51.61511062	26.15241483
chr1	243056314	243101744	1q43	LINC01347	0.137728369	0.102142654	1.348392313	3	2	53.91779322	49.40216592
chr19	3572916	3579083	19p13.3	HMG20B	7.078897492	5.250016914	1.348357083	4	4	58.34085359	58.45831097
chr6	24947880	24948344	6p22.3	MTCO2P33	1.149603928	0.852613196	1.348329973	2	2	66.23401976	14.85792997
chr5	78002326	78294755	5q14.1	AP3B1	35.97056603	26.67829924	1.348308065	4	4	50.94727952	4.254545506
chr11	11962996	12009827	11p15.3	DKK3	0.171515636	0.127208087	1.348307646	2	4	38.97107002	45.82878801
chr10	37818406	37820987	10p11.21	TLK2P2	0.386848304	0.286959653	1.348093018	4	3	94.38175547	18.57910617

chr12	76764074	76853701	12q21.2	ZDHHC17	30.47505604	22.60722805	1.348022676	4	4	60.14785606	57.45549336
chr9	136940433	136944774	9q34.3	FBXW5	20.14130061	14.94218953	1.347948409	4	4	78.21736551	48.0253427
chr5	138187648	138213343	5q31.2	CDC23	10.84625128	8.047049632	1.347854403	4	4	9.478774583	26.38779984
chr9	33868540	33868665	9p13.3	RNU4ATAC11P	4.417788769	3.27810933	1.347663645	1	3	0	43.6401739
chr2	174431571	174494001	2q31.1	GPR155	12.8434389	9.530190275	1.347658182	4	4	20.35988234	39.53793261
chr3	122384176	122410114	3q21.1	FAM162A	3.646404576	2.706237876	1.347407265	4	4	44.76358917	28.18791565
chr6	136557046	136793098	6q23.3	MAP3K5	58.57477827	43.47662199	1.347270684	4	4	12.2191881	13.44970759
chr4	1009930	1026898	4p16.3	FGFRL1	1.214907326	0.90177608	1.347238358	4	4	75.84745772	31.6557797
chr14	59188657	59371405	14q23.1	DAAM1	9.312131632	6.912473297	1.347149021	4	4	53.06094874	36.76169629
chr9	5110861	5113421	9p24.1	TCF3P1	0.375270314	0.278567862	1.347141453	4	3	73.38718596	42.3463539
chr8	139727725	140458579	8q24.3	TRAPPC9	13.4697879	9.999129387	1.34709607	4	4	20.78930841	11.60033679
chr17	17303366	17347663	17p11.2	NT5M	15.67628994	11.63860635	1.34692157	4	4	89.93282796	40.52254963
chr6	142751467	142945756	6q24.2	HIVEP2	31.33900153	23.26716005	1.346919927	4	4	67.58750258	34.13833697
chr10	69269991	69401882	10q22.1	HK1	69.62698661	51.69360234	1.3469169	4	4	37.66548063	47.16847416
chr20	62238485	62296213	20q13.33	OSBPL2	15.47495373	11.48923248	1.346909269	4	4	45.65993544	23.01083719
chr20	41196686	41197157	20q12	RPL23AP81	0.76192889	0.56571024	1.34685363	1	1	0	0
chr20	3249302	3407662	20p13	C20orf194	2.15677837	1.601398946	1.34680891	4	4	34.94455365	35.40886771
chr17	48070052	48101521	17q21.32	CBX1	20.31373269	15.08292993	1.346802828	4	4	54.56312786	38.94559887
chr5	64718148	64768839	5q12.3	SREK1IP1	10.80367669	8.021731715	1.34680105	4	4	30.12747889	15.73728373
chr11	83157095	83185794	11q14.1	PCF11	48.19117956	35.7836651	1.346736826	4	4	41.24784092	24.70425731
chr1	40040065	40072649	1p34.2	CAP1	342.8648842	254.594511	1.346709647	4	4	53.72687903	32.14430352
chr7	154928517	154949490	7q36.2	PAXIP1-AS2	4.538304082	3.369934906	1.346703782	4	4	42.83708678	43.00035957
chr12	53064316	53079420	12q13.13	SPRYD3	9.170073003	6.80955819	1.346647278	4	4	56.31904624	35.40807436
chr17	6985123	7012334	17p13.1	ALOX12-AS1	1.860490201	1.381597316	1.346622623	4	4	28.88282059	22.32135741
chr1	92245786	92441936	1p22.1	GLMN	16.13425197	11.9814972	1.346597316	4	4	86.92524615	45.55755192
chr2	219249711	219254608	2q35	TUBA4A	62.13871377	46.14520574	1.346590892	4	4	75.84502323	32.08832734
chr3	138261437	138331785	3q22.3	NME9	0.090559048	0.067252278	1.346557331	1	1	0	0
chr8	90001352	90053633	8q21.3	DECR1	36.02734406	26.75645368	1.346491747	4	4	15.74811669	11.33047204
chr9	100427213	100451734	9q31.1	MSANTD3	6.932449844	5.149028041	1.346360864	4	4	57.88824634	11.44844522
chr12	12611833	12645108	12p13.1	CREBL2	33.86034246	25.14954736	1.346359916	4	4	10.9740362	12.7740873
chr1	211259277	211316385	1q32.2-q32.3	RCOR3	17.41467007	12.93485438	1.34633677	4	4	27.89826183	30.37981844
chr2	230864598	230879254	2q37.1	ITM2C	11.00584259	8.175651217	1.346173203	4	4	93.1219773	71.50332904
chr2	219434215	219493632	2q35	SPEG	0.073863366	0.054872483	1.346091196	3	3	11.9100422	103.5328608
chr3	101779177	101828231	3q12.3	NXPE3	12.14314149	9.021217124	1.346064652	4	4	62.94212017	37.28462433
chr15	43330341	43354555	15q15.3	ADAL	24.23194199	18.00313032	1.345984924	4	4	89.73034898	67.11632072
chr17	50634843	50691704	17q21.33	ABCC3	7.062433971	5.247249401	1.345930683	4	4	73.55425412	26.15959918
chr14	55576157	55580092	14q22.3	KTN1-AS1	0.259508655	0.192820479	1.345856294	3	4	113.1436201	40.59379577
chrX	45505388	45527239	Xp11.3	LINC01204	0.130998845	0.09734432	1.34572665	2	1	13.11220801	0
chr9	19049374	19051025	9p22.1	RRAGA	19.13033227	14.21569809	1.345718806	4	4	19.04512686	26.25653984
chr16	68245344	68261062	16q22.1	PLA2G15	1.506517798	1.119540614	1.34565712	4	4	47.14470334	40.14914084
chr18	12785478	12884351	18p11.21	PTPN2	16.953582	12.59906009	1.345622759	4	4	43.06935029	10.08665034
chr22	36281277	36388067	22q12.3	MYH9	315.0877906	234.1960258	1.345401954	4	4	75.30531069	40.21218937
chr12	105107714	105169134	12q23.3	WASHC4	67.97883586	50.53395769	1.345211002	4	4	25.06473874	34.18800629

chr14	74713118	74736691	14q24.3	FCF1	18.27179087	13.58360441	1.345135674	4	4	43.40811752	28.2393019
chr22	50583026	50595281	22q13.33	CHKB-DT	0.947735881	0.704612819	1.345044904	2	3	80.44362933	31.53530555
chr20	63895102	63936031	20q13.33	DNAJC5	31.57553716	23.47600215	1.345013387	4	4	45.08805153	9.689348752
chr16	57529339	57578182	16q21	ADGRG5	5.303238524	3.943021448	1.344968216	4	4	46.42262386	39.78768354
chr7	116952446	116954334	7q31.2	ST7-AS1	0.20648921	0.153533555	1.344912582	3	2	32.06071162	11.34395541
chr14	75522441	75546992	14q24.3	BATF	12.80514367	9.521595682	1.344852701	4	4	75.04863232	99.46828085
chr8	118131828	118621995	8q24.11-q24.12	SAMD12	0.457831729	0.340452196	1.344775376	4	4	48.30527502	57.72416869
chr12	100026251	100142864	12q23.1	UHRF1BP1L	34.03413303	25.30909217	1.344739385	4	4	41.80455545	24.36991249
chr14	50975261	51105173	14q22.1	TRIM9	0.348921991	0.25947852	1.344704725	3	4	81.62524593	92.05509341
chr15	49621022	49654448	15q21.2	DTWD1	3.793250489	2.820922761	1.344684279	4	4	56.33509991	32.76555648
chr4	105145875	105279803	4q24	TET2	45.19128766	33.608547	1.344636757	4	4	49.8692036	18.1182768
chr2	157736446	157875896	2q24.1	ACVR1	6.013367882	4.472167623	1.344620414	4	4	46.2761092	6.657957245
chrX	104166475	104195902	Xq22.2	FAM199X	14.74432014	10.9671331	1.344409702	4	4	19.8669334	27.00512629
chr7	12211222	12237264	7p21.3	TMEM106B	13.70995132	10.19805946	1.34436864	4	4	35.71675351	18.68972088
chr12	120095094	120117469	12q24.23	RAB35	15.96318959	11.87422101	1.344356786	4	4	55.95904565	13.73665159
chr15	78872781	78897739	15q25.1	MORF4L1	56.46418306	42.0009094	1.344356203	4	4	24.45465952	18.92480902
chrX	19533965	19887626	Xp22.12	SH3KBP1	52.14646968	38.79475531	1.344162871	4	4	62.00072199	37.52171274
chr8	23528795	23575463	8p21.2	SLC25A37	1146.924674	853.2819641	1.344133267	4	4	19.51329027	21.13480272
chr3	184812143	185052614	3q27.2	VPS8	29.55119756	21.98694413	1.344033868	4	4	12.57770065	25.77383761
chr1	115976498	116070054	1p13.1	SLC22A15	6.203151101	4.615473271	1.343990255	4	4	57.99807592	58.3604117
chr3	158666414	158672722	3q25.32	LXN	10.84440324	8.069209692	1.343923836	4	4	34.64569582	6.343726151
chr1	3857267	3885429	1p36.32	DFFB	2.159557016	1.606904439	1.343923735	4	4	68.01557368	17.71734639
chr1	169511954	169586630	1q24.2	F5	8.085662228	6.016519084	1.343910343	4	4	93.36748694	63.68639749
chr20	63743668	63832332	20q13.33	ZBTB46	0.383102303	0.285065394	1.343910241	4	4	76.66054439	38.12067261
chrY	13703567	13706024	Yq11.221	TMSB4Y	6.40353717	4.765687458	1.343675435	3	2	85.11722554	32.91994332
chr2	24076874	24169638	2p23.3	FAM228B	17.83366587	13.27480457	1.343422103	4	4	26.21717642	34.76034948
chr17	68126475	68152471	17q24.2	LRRC37A16P	0.257017609	0.191320438	1.343388147	2	2	47.47765818	38.21551628
chr3	38007496	38029663	3p22.2	PLCD1	2.733935427	2.035410091	1.343186535	4	4	79.68948439	36.37048736
chr15	74461265	74481292	15q24.1	UBL7-AS1	3.489980711	2.598451729	1.343100074	4	4	23.54010558	44.27504593
chr6	20401906	20493714	6p22.3	E2F3	15.02430593	11.18648153	1.343076989	4	4	35.79475407	28.4476719
chr15	31326855	31435665	15q13.3	KLF13	5.356103883	3.987951969	1.343071312	4	4	49.90435085	31.52519929
chr12	110491523	110502140	12q24.11	VPS29	31.16903402	23.20814201	1.343021514	4	4	36.71253737	15.96656348
chr11	59133972	59142808	11q12.1	FAM111A-DT	1.472445907	1.096451335	1.342919526	4	4	56.14123744	45.8053943
chr1	248810446	248826285	1q44	SH3BP5L	14.32544134	10.66775704	1.342872854	4	4	47.54778256	11.4067476
chr20	50123737	50153798	20q13.13	TMEM189	4.620522183	3.440786977	1.342867842	4	4	70.29558534	25.56872784
chr3	194394821	194399266	3q29	GP5	2.357658418	1.755788921	1.342791488	4	4	20.47774721	34.76817078
chr7	1567332	1570490	7p22.3	PSMG3	4.878361422	3.63335463	1.342660411	4	4	24.20264115	18.9525653
chr16	2047804	2088720	16p13.3	TSC2	5.441501631	4.052912129	1.34261525	4	4	67.29922689	29.35497012
chr8	42541155	42552997	8p11.21	SMIM19	5.103362297	3.801216658	1.342560227	4	4	32.54216533	26.02302396
chr9	108867517	108934332	9q31.3	ELP1	9.246784452	6.887507648	1.342544346	4	4	42.02763954	22.36327734
chr3	168008672	168095925	3q26.2	GOLIM4	36.5165105	27.19985067	1.342526139	4	4	48.93657935	32.14521178
chr14	31291788	31420582	14q12	HEATR5A	2.576523779	1.919256227	1.342459513	4	4	33.96711561	23.27166263
chr1	40531349	40548169	1p34.2	ZNF684	2.571425471	1.915475041	1.342447913	4	4	46.71810204	43.98469858

chr10	70052601	70112284	10q22.1	H2AFY2	1.048194192	0.780830999	1.342408529	4	4	95.34308786	27.70106751
chr7	23181298	23201011	7p15.3	NUPL2	34.39993553	25.62572698	1.342398425	4	4	77.32625792	44.48427022
chr2	86503430	86563497	2p11.2	CHMP3	17.36747786	12.93804936	1.342356748	4	4	39.11855257	21.25499782
chr12	71754863	71793476	12q21.1	RAB21	20.17715215	15.03161181	1.342314611	4	4	33.61160769	31.73054967
chr12	123260970	123272316	12q24.31	CDK2AP1	7.859062789	5.854963712	1.342290606	4	4	28.09451003	11.28960701
chr6	24704299	24720920	6p22.3	C6orf62	133.0524084	99.1241365	1.34228063	4	4	22.90125009	23.96607382
chr15	44534505	44536923	15q21.1	EIF3J-DT	1.645312997	1.225874387	1.34215464	4	4	32.93337745	44.74236391
chr12	26938372	26966648	12p11.23	FGFR1OP2	93.49465442	69.66066847	1.342144089	4	4	27.3438959	27.38133621
chrX	120426148	120469349	Xq24	LAMP2	94.84137334	70.6649427	1.342127648	4	4	45.039945	29.40545116
chr3	150541993	150546641	3q25.1	SERP1	49.75408405	37.07225208	1.342084207	4	4	30.0106981	16.28338625
chr19	55284101	55312562	19q13.42	BRSK1	0.253364802	0.18881916	1.341838414	4	4	49.9404946	50.49993308
chr2	106093303	106194339	2q12.2	UXS1	35.15818229	26.20347151	1.341737574	4	4	49.44229846	25.51300653
chr1	193012253	193060140	1q31.2	UCHL5	20.58859564	15.34571245	1.341651338	4	4	47.396098	42.94297839
chr9	34366149	34376952	9p13.3	MYORG	0.140899139	0.105034446	1.341456489	4	4	53.38970565	80.36659577
chr19	48872392	48876062	19q13.33	PPP1R15A	122.3640857	91.22175988	1.341391416	4	4	73.60857937	106.4462135
chr4	153344649	153415097	4q31.3	MND1	47.58362409	35.47569406	1.341302132	4	4	70.36119395	36.70902592
chr3	52287558	52295256	3p21.2	GLYCTK	3.331362829	2.483735801	1.341271011	4	4	51.91499397	16.03534756
chr8	98189826	98294393	8q22.2	NIPAL2	5.085137165	3.791338804	1.341251053	4	4	25.70937661	25.76809236
chr19	49453216	49471050	19q13.33	ALDH16A1	4.857962086	3.622395741	1.341090934	4	4	57.27702101	45.79612239
chr5	91380347	91420715	5q14.3	ARRDC3-AS1	1.22517786	0.913585147	1.341065871	4	4	78.55163604	26.64973742
chr8	98042086	98042217	8q22.2	SNORA72	21.25917377	15.85260053	1.341052765	4	3	63.88241523	35.25017942
chr7	21471058	21471146	7p15.3	MIR1183	5.46827752	4.077856461	1.340968612	2	1	56.12112453	0
chr9	34329506	34343713	9p13.3	NUDT2	12.82435282	9.564215145	1.340868291	4	4	45.55019028	40.20661302
chr11	87037844	87328834	11q14.2	TMEM135	10.71753175	7.99377476	1.340734768	4	4	48.86905379	25.77574285
chr12	24706788	24707076	12p12.1	RN7SL38P	1.78837266	1.33387937	1.34073043	2	2	19.91010394	60.95481321
chr20	11890723	11926595	20p12.2	BTBD3	1.59683829	1.191301484	1.340414925	4	4	53.98557129	40.46571937
chr1	149886975	149887411	1q21.2	HIST2H2AC	1057.260262	788.8156311	1.340313528	4	4	73.37465136	62.29912905
chr1	15847864	15940455	1p36.21-p36.13	SPEN	18.37207366	13.7073201	1.340311128	4	4	43.51893707	17.51412673
chr11	417930	442011	11p15.5	ANO9	0.879565402	0.656254194	1.340281571	4	4	78.06442895	37.86665873
chr2	95165417	95184317	2q11.1	ZNF2	1.732210382	1.292491857	1.3402099	4	4	63.34736557	63.36986624
chr16	1510427	1612108	16p13.3	IFT140	0.437869845	0.326728323	1.340164942	4	4	105.2585132	42.03238039
chr19	39328357	39336086	19q13.2	GMFG	176.1439603	131.4354596	1.340155547	4	4	63.44645297	27.41481084
chr4	108650585	108667822	4q25	OSTC	35.59769171	26.56238335	1.340154279	4	4	22.89773857	15.8508879
chr16	25100564	25111555	16p12.1	LCMT1-AS1	0.471951906	0.352168581	1.340130641	2	4	59.17058448	39.68055159
chr12	48904110	48921576	12q13.12	CCDC65	2.443319513	1.823297651	1.340055208	4	4	16.91158994	67.00924111
chr17	32007872	32053500	17q11.2	LRRC37B	9.713162265	7.248614819	1.340002539	4	4	16.35638093	17.42457881
chr19	48712441	48719898	19q13.33	MAMSTR	0.193824171	0.14465976	1.339862383	2	4	65.38773666	28.89461351
chr19	38730048	38744474	19q13.2	CAPN12	0.193469343	0.144405708	1.339762437	4	4	77.90926888	27.71707933
chr9	70384597	70414657	9q21.12	KLF9	12.7873075	9.545091329	1.339673667	4	4	54.76936217	8.971316159
chr4	118516340	118554204	4q26	CEP170P1	0.220487932	0.164591864	1.339604072	3	3	19.98921187	60.58694172
chr10	80153953	80205677	10q22.3	ANXA11	42.48366236	31.71416754	1.339579931	4	4	55.04781676	25.72097615
chr2	240559239	240565256	2q37.3	DUSP28	2.421563355	1.807994351	1.339364448	4	4	48.33082756	28.5813026
chr17	42833142	42843760	17q21.31	PSME3	25.42490523	18.98389136	1.339288386	4	4	21.03798917	10.61776212

chr3	153199700	153200017	3q25.2	RN7SL300P	1.075632539	0.803145027	1.339275601	1	1	0	0
chr7	66980120	66980409	7q11.21	RN7SL43P	1.399126358	1.044774123	1.339166359	2	2	42.1631833	22.87308853
chr17	61067208	61070947	17q23.2	RPL23AP74	0.652240631	0.487082329	1.339076767	2	1	20.08062915	0
chr1	20806292	21176888	1p36.12	EIF4G3	33.86268724	25.28861093	1.339048923	4	4	25.7426958	4.22871761
chr1	150965173	150975003	1q21.3	CERS2	35.0364913	26.16530373	1.339043936	4	4	42.13459931	13.07236859
chr19	43967006	43998326	19q13.31	ZNF155	3.562511096	2.660691081	1.338942022	4	4	87.23313374	59.33325525
chr1	117860227	117929679	1p12	GDAP2	12.74775408	9.521236562	1.33887589	4	4	33.34774313	19.17852239
chr1	212035553	212105006	1q32.3	DTL	61.26536584	45.76340539	1.33874141	4	4	91.07568994	68.15931563
chr1	45493863	45500111	1p34.1	CCDC163	1.243145857	0.928607869	1.338719926	4	4	103.5494257	34.95425719
chr5	32531778	32604079	5p13.3	SUB1	41.9933997	31.36852201	1.338711454	4	4	33.00893704	30.70405894
chr14	49620771	49623481	14q21.3	MGAT2	27.4825052	20.53085432	1.338595305	4	4	28.4710974	28.71378596
chr1	26432282	26471306	1p36.11	DHDDS	5.84009538	4.362982047	1.338555905	4	4	41.03739314	16.90406584
chr13	49495610	49528992	13q14.2	PHF11	16.38931546	12.24412522	1.338545233	4	4	34.77649694	20.06421556
chr5	140125936	140128806	5q31.3	IGIP	2.62879584	1.963950095	1.338524765	4	4	28.54894086	34.2972395
chr20	37693955	37872129	20q11.23	CTNNBL1	11.36139369	8.488055938	1.338515412	4	4	35.21821696	24.33014547
chr2	88885572	88886153	2p11.2	IGKV4-1	34.49547643	25.77241679	1.338464945	4	4	61.12094997	121.3921443
chr5	134196252	134226259	5q31.1	PPP2CA	34.70760185	25.9312324	1.338447834	4	4	30.8932335	7.460439819
chr17	22523111	22524665	17p11.2	MTRNR2L1	0.245479026	0.183412917	1.338395518	3	2	33.16179749	14.35867573
chr8	89757747	89791064	8q21.3	RIPK2	18.54741705	13.85942295	1.338253195	4	4	30.61045266	8.381167611
chrY	20756068	20781032	Yq11.223	RPS4Y2	1.412615506	1.05558399	1.338231273	2	2	32.29813854	40.86121605
chr2	74152598	74178967	2p13.1	MOB1A	172.4138906	128.8376494	1.338225987	4	4	30.88534036	20.36023917
chr1	11980181	12013515	1p36.22	MFN2	49.68860127	37.13059369	1.338211871	4	4	25.42456393	22.67149526
chr16	19501693	19522145	16p12.3	GDE1	45.49819501	34.00045654	1.338164238	4	4	15.31637556	19.00691862
chr9	96145974	96147965	9q22.32	EIF4BP3	2.943490298	2.199821896	1.33805846	4	4	84.76616774	40.93076876
chr5	119452443	119542335	5q23.1	HSD17B4	15.07474503	11.26623118	1.338046839	4	4	36.93891319	24.34264046
chr17	7189890	7220050	17p13.1	DLG4	1.225066646	0.915565161	1.338044193	4	4	80.0524439	43.22011724
chr3	182938158	182986364	3q26.33	DCUN1D1	174.2174011	130.2037239	1.338037008	4	4	61.57998248	53.55808832
chr2	233354507	233472098	2q37.1	DGKD	15.5347362	11.61020541	1.338024234	4	4	51.79623339	32.30088924
chr3	150603263	150630447	3q25.1	SELENOT	54.21663872	40.52213747	1.337951108	4	4	36.83435933	14.68081735
chr3	161344784	161373228	3q26.1	SPTSSB	0.498529757	0.372616228	1.337917459	4	3	72.7667007	29.39603634
chr18	21650897	21704805	18q11.2	ABHD3	14.88336711	11.12467184	1.337870215	4	4	27.2031996	49.98804022
chr17	19377721	19383544	17p11.2	MAPK7	3.890955209	2.908450191	1.337810502	4	4	83.91009254	31.18734807
chr1	45627304	45688110	1p34.1	GPBP1L1	100.8352503	75.37511076	1.337779132	4	4	33.54504103	15.234689
chr1	53921561	53946308	1p32.3	HSPB11	14.01406482	10.47577107	1.337759743	4	4	26.32721694	24.21282048
chr9	97238426	97297314	9q22.33	SUGT1P4-STRA6LP	0.456724561	0.341498611	1.337412647	4	4	45.43240021	40.54750421
chrMT	9207	9990	N/A	MT-CO3	5119.981747	3828.287385	1.337407888	4	4	76.35429183	56.98870932
chr2	106404680	106470935	2q12.2	RGPD3	0.927002887	0.693305779	1.337076534	4	4	59.83044484	62.4623402
chr8	143790920	143815379	8q24.3	SCRIB	2.517568482	1.883017513	1.336986228	4	4	59.45638033	35.42845623
chr12	71663897	71680648	12q21.1	THAP2	2.075921961	1.552861074	1.336836885	4	4	19.40644283	38.5389426
chr7	65960684	65982314	7q11.21	GUSB	11.17931317	8.362604833	1.336821887	4	4	52.0421976	20.12076032
chrX	23075586	23077412	Xp22.11	FAM3C2	2.119576999	1.585570147	1.336791691	4	4	41.47238791	64.02763788
chr19	58555712	58558976	19q13.43	UBE2M	22.80909719	17.06300211	1.336757567	4	4	48.91946565	22.21937501
chr5	141923820	141952031	5q31.3	DELE1	7.975429933	5.966792933	1.336635949	4	4	45.6135104	38.76907966

chr2	218450251	218568393	2q35	USP37	28.32420699	21.19105945	1.33661118	4	4	70.55863507	38.8721343
chr5	177456612	177480368	5q35.3	DBN1	2.979425489	2.229148355	1.336575685	4	4	77.8906598	35.75378009
chr12	10238383	10329607	12p13.2	KLRD1	19.90899752	14.89668105	1.336472028	4	4	36.37447983	36.4190337
chr7	157138952	157269372	7q36.3	UBE3C	28.41607213	21.26211081	1.336465245	4	4	31.80967697	16.33724273
chr7	6637322	6658279	7p22.1	ZNF316	4.862674764	3.638472633	1.336460448	4	4	60.7530982	21.00591611
chr15	88898683	88913469	15q26.1	MFGE8	0.470841349	0.35231714	1.336413406	4	4	81.27676431	54.12153465
chr12	51186936	51219875	12q13.13	POU6F1	0.612296484	0.458181573	1.336362091	4	4	62.3675082	58.27914219
chr11	1752752	1763992	11p15.5	CTSD	38.10814277	28.51758672	1.336303213	4	4	59.36640976	31.26713485
chr10	93306429	93482505	10q23.33	MYOF	7.506917425	5.617934052	1.336241643	4	4	56.91546188	57.2146419
chr6	36854829	36875024	6p21.2	PPIL1	8.579816264	6.42134687	1.336139666	4	4	62.528024	11.70727446
chr11	65422798	65445540	11q13.1	NEAT1	46.28302701	34.64021573	1.336106778	4	4	30.44675823	61.90850572
chr2	183607438	183608119	2q32.1	CACYBPP2	0.814705212	0.609799598	1.336021235	2	2	85.48009854	33.2520963
chr16	21158349	21180616	16p12.3	TMEM159	1.334833294	0.999112925	1.336018443	4	4	50.75251233	43.56448278
chr2	189674292	189763059	2q32.2	ANKAR	7.014043472	5.250127738	1.335975775	4	4	54.77427504	35.12727821
chr12	118032687	118062430	12q24.23	WSB2	15.00349417	11.2304846	1.335961422	4	4	25.76331482	34.762281
chr7	48171424	48647497	7p12.3	ABCA13	4.001862703	2.995706249	1.335866193	4	4	32.79413118	48.63210235
chr1	151131685	151148408	1q21.3	SEMA6C	0.227690219	0.170446952	1.335842129	4	3	29.13965093	65.3432215
chr8	39913809	39928790	8p11.21	IDO1	4.155427115	3.110762012	1.335822894	4	4	35.22591864	58.00008338
chr10	17589959	17617374	10p12.33	HACD1	13.4096185	10.03974989	1.335652645	4	4	103.4064597	76.28303728
chr3	132558138	132660131	3q22.1	ACAD11	0.271637132	0.203377039	1.335633231	4	4	64.47128366	26.15762412
chr16	57471922	57487139	16q21	DOK4	0.647484663	0.484777603	1.335632378	4	4	80.96774535	49.26066494
chr16	30053090	30070420	16p11.2	ALDOA	79.45397814	59.49845557	1.33539564	4	4	61.17906794	31.63494037
chr3	50088908	50118964	3p21.31	RBM5	18.59798864	13.92831885	1.335264423	4	4	36.93304722	25.50723515
chrX	48801377	48824982	Xp11.23	HDAC6	5.749815516	4.306204171	1.335239875	4	4	19.7470793	6.533155373
chr7	101217704	101224191	7q22.1	ZNHIT1	4.202214302	3.147307523	1.335177535	4	4	53.95044678	37.29273313
chr1	15681506	15734769	1p36.21	PLEKHM2	17.93838581	13.43611231	1.335087516	4	4	44.16439339	30.42738297
chr3	46016990	46086803	3p21.31	XCR1	0.15784791	0.118236623	1.335017067	3	3	113.7656942	100.7816825
chr15	68054179	68191464	15q23	PIAS1	58.4918143	43.81505822	1.334970594	4	4	24.76535266	4.759816627
chr6	95577497	95609452	6q16.1	MANEA	10.1262246	7.586055406	1.334847171	4	4	35.02922384	38.61455113
chr13	99200425	99386499	13q32.3	UBAC2	35.26429552	26.41880865	1.33481778	4	4	23.91206051	12.05044401
chr3	152834693	152841439	3q25.2	P2RY1	3.531544908	2.645713255	1.33481771	4	4	21.28083397	43.31743683
chr6	121079494	121335384	6q22.31	TBC1D32	13.56270516	10.16162273	1.33469875	4	4	61.68718793	50.74959175
chr1	77979158	78017964	1p31.1	DNAJB4	26.76380165	20.05294304	1.334657042	4	4	61.23908276	51.99589346
chrX	7898763	7928587	Xp22.31	PNPLA4	1.580004446	1.18385298	1.334628938	4	4	30.14407311	41.83943913
chr1	96678808	96679640	1p21.3	RPL7P9	4.500964899	3.373102674	1.334369373	4	4	43.58508354	61.05918318
chr1	35925683	36056470	1p34.3	AGO3	5.110769595	3.830398386	1.334265807	4	4	56.65540415	35.20823142
chr3	15601352	15653711	3p25.1	BTD	3.313540154	2.483471506	1.334237235	4	4	40.93579874	13.30815262
chr14	32934785	33804176	14q13.1	NPAS3	0.079154691	0.059327421	1.334200764	2	1	26.98111796	0
chr13	50172018	50173616	13q14.2	ST13P4	1.0915953	0.818207	1.334130972	4	4	55.87038085	71.69470707
chr2	60881495	60928171	2p16.1	REL	18.14238549	13.59884561	1.334112174	4	4	20.68037802	11.58592461
chr10	104309696	104338493	10q25.1	ITPRIP	20.89170774	15.65970012	1.334106501	4	4	65.61823567	9.189311208
chr2	74198563	74215298	2p13.1	MTHFD2	16.84482703	12.62644145	1.334091406	4	4	19.06718831	30.94059813
chr1	11273198	11299572	1p36.22	UBIAD1	3.920956592	2.939103103	1.334065684	4	4	79.0452859	41.4150763

chr14	105009573	105021088	14q32.33	CDCA4	3.4777838	2.606917418	1.334059827	4	4	26.19375787	42.57411342
chr5	55528842	55534542	5q11.2	RNF138P1	2.085431552	1.563390347	1.333916098	1	2	0	1.5377256
chr2	69520044	69520171	2p13.3	SNORA36C	2.175798213	1.631357981	1.333734373	1	1	0	0
chrMT	3230	3304	N/A	MT-TL1	4.834678108	3.625126424	1.333657794	2	2	2.386741862	9.562139825
chr6	24171755	24383292	6p22.3	DCDC2	0.067905165	0.050921541	1.333525328	1	1	0	0
chr6	30066864	30070333	6p22.1	PPP1R11	5.657698628	4.242813924	1.333477906	4	4	60.45369775	40.75616568
chr6	99398051	99426306	6q16.2	PNISR	30.91268187	23.18438275	1.333340732	4	4	54.40900868	19.71210474
chr8	42896890	43030539	8p11.21	HOOK3	22.44564101	16.8342861	1.33332895	4	4	18.80526694	22.50667464
chr3	105366713	105576913	3q13.11	ALCAM	6.96044226	5.220650677	1.333251866	4	4	39.59247135	54.94210964
chr16	31873758	31917951	16p11.2	ZNF267	60.96369636	45.74157397	1.332785277	4	4	43.75722695	21.42171041
chr5	115200247	115262887	5q22.3	PGGT1B	14.10560406	10.58405655	1.332721909	4	4	47.61906049	32.18398709
chr1	12230039	12512047	1p36.22-p36.21	VPS13D	12.95912657	9.724720407	1.332596314	4	4	42.34077153	17.5023782
chr1	28259452	28282491	1p35.3	SESN2	5.823049929	4.369834165	1.332556273	4	4	59.65182384	33.79497107
chr11	33698108	33703026	11p13	C11orf91	0.370505029	0.278052301	1.332501216	1	1	0	0
chr10	97614553	97634156	10q24.2	MORN4	0.268655502	0.201627654	1.332433801	2	3	63.28395461	39.04911422
chr1	154962298	154974492	1q21.3	SHC1	11.1579	8.374267159	1.332403157	4	4	40.55614703	33.16903837
chr17	39637080	39664201	17q12	STARD3	6.866693422	5.153785867	1.332359085	4	4	46.51788316	10.6675906
chr19	49640481	49658649	19q13.33	SCAF1	6.109471655	4.585884751	1.332234015	4	4	73.75147717	54.71049582
chr17	48874908	48875190	17q21.32	SUMO2P17	0.442921737	0.332483466	1.332161693	3	1	54.96502136	0
chr20	34560542	34677285	20q11.22	PIGU	12.00770773	9.014485817	1.332045773	4	4	54.17433167	21.59971343
chr17	4163818	4263995	17p13.2	ANKFY1	8.751493298	6.570362076	1.331965149	4	4	35.70390172	22.34770367
chr1	204398684	204412454	1q32.1	PPP1R15B	71.85384738	53.94941786	1.331874378	4	4	49.01824282	22.61122567
chr14	91965991	92040134	14q32.12	TRIP11	31.98451846	24.01538527	1.331834493	4	4	25.62741405	26.53643229
chr11	73305961	73369380	11q13.4	ARHGEF17	0.18779482	0.141009752	1.331786044	3	3	107.2882547	123.8545395
chr2	207074137	207173851	2q33.3	KLF7	12.15689296	9.128665138	1.331727342	4	4	68.05858198	40.76693231
chr9	75198071	75198177	9q21.13	RNU6-1228P	3.511266963	2.636841316	1.331618608	1	1	0	0
chrX	48786540	48794311	Xp11.23	GATA1	44.42285118	33.36145163	1.331562297	4	4	48.33836193	13.65415554
chr8	144314583	144326919	8q24.3	DGAT1	5.096231525	3.827481185	1.331484409	4	4	56.42707275	20.23316783
chr1	244408492	244452134	1q44	ADSS	36.65212835	27.52864621	1.331417756	4	4	7.005921137	4.906533463
chr12	51093756	51173143	12q13.12-q13.13	TFCP2	14.78090483	11.10182232	1.331394469	4	4	19.12838135	8.413879708
chr9	34810040	34982544	9p13.3	PHF24	0.223829766	0.168123974	1.331337587	3	3	115.9253619	8.176861853
chr13	49247875	49293486	13q14.2	CDADC1	5.525503817	4.15035156	1.331333921	4	4	30.44897584	29.65303809
chr1	120912238	120955811	1p11.2	LINC00623	6.547671975	4.918288757	1.331290678	4	4	68.56974557	71.47243702
chr8	123123110	123123684	8q24.13	HMGB1P19	1.984165299	1.490590803	1.331126755	2	4	0.522324763	58.76570212
chr4	25377226	25418498	4p15.2	ANAPC4	6.906274388	5.188503332	1.331072555	4	4	26.29071921	5.294433898
chr5	176392455	176416569	5q35.2	CLTB	17.21324896	12.93216449	1.331041604	4	4	32.84143959	10.63784827
chr12	75275979	75390937	12q21.1-q21.2	CAPS2	5.911608208	4.441508933	1.330990953	4	4	48.54811846	70.13836339
chr15	30919764	30991604	15q13.3	MTMR10	15.32337436	11.51542341	1.330682669	4	4	9.211290799	23.70439812
chr8	144973589	145002899	8q24.3	ZNF252P	4.408991789	3.313357904	1.330671758	4	4	43.73236706	41.54915969
chr20	54207963	54219953	20q13.2	PFDN4	21.54445046	16.19133881	1.330615752	4	4	14.38564908	14.57579512
chr1	28031884	28032180	1p35.3	RN7SL559P	1.186436427	0.891653564	1.330602461	3	2	17.09704672	5.255168937
chr2	27070472	27071699	2p23.3	OST4	883.3078122	663.8666424	1.330550077	4	4	26.08212129	25.33392852
chr17	21284656	21315240	17p11.2	MAP2K3	92.98948029	69.8905857	1.330500802	4	4	16.95775393	36.82637563

chr4	143559472	143561460	4q31.21	GUSBP5	0.595047127	0.447240289	1.330486411	3	3	51.2143796	83.78119635
chr5	65148736	65482663	5q12.3	ADAMTS6	3.360009169	2.525603075	1.330378951	4	4	24.65682935	38.50852463
chr12	88492793	88580473	12q21.32	KITLG	0.668940594	0.502832796	1.330344003	4	4	28.20013852	48.72003279
chrX	101882288	101932135	Xq22.1	ZMAT1	1.562941377	1.174851042	1.330331525	4	4	60.23879892	50.86188309
chr17	81683932	81702121	17q25.3	HGS	7.581432449	5.699024611	1.330303511	4	4	46.04621241	21.08514568
chr19	15673018	15698819	19p13.12	CYP4F12	0.702577556	0.528155939	1.330246435	4	3	93.97085463	51.90523881
chr4	67615761	67701171	4q13.2	UBA6	15.12569187	11.37065128	1.330239711	4	4	23.66952268	18.71507206
chr8	144148016	144261940	8q24.3	MROH1	1.289675244	0.969559104	1.330166712	4	4	79.94002394	18.07261704
chr10	68331693	68343196	10q21.3	HNRNPH3	31.14228917	23.41244728	1.3301595	4	4	44.05998925	18.76147278
chr17	6651739	6653298	17p13.1	C17orf100	0.475273076	0.357315328	1.330122271	4	4	43.29940428	50.36365383
chr17	58345175	58352238	17q22	SUPT4H1	63.07302679	47.42495371	1.32995442	4	4	37.95452553	10.61238935
chr5	50396192	50443299	5q11.1	EMB	133.2392716	100.1836158	1.329950716	4	4	12.84170962	19.68997485
chr5	140645363	140647785	5q31.3	NDUFA2	18.903371	14.21473114	1.329843725	4	4	39.1837873	19.23191989
chr19	39306817	39320867	19q13.2	LRFN1	2.631606035	1.978987834	1.329773731	4	4	94.5624938	45.00379739
chr1	65264694	65415869	1p31.3	DNAJC6	355.4889438	267.3384855	1.329733514	4	4	79.2422873	52.85369985
chr6	111259279	111280005	6q21	MFSD4B	11.59986133	8.723495346	1.329726316	4	4	66.02727423	54.8567022
chr8	119862665	119862860	8q24.12	RN7SL396P	74.37347777	55.93355568	1.329675485	4	4	67.445462	45.32917565
chr14	63761904	64226449	14q23.2	SYNE2	42.00854066	31.59371745	1.329648552	4	4	39.21468971	17.99481687
chr16	14833786	14896157	16p13.11	NOMO1	4.670622339	3.51298516	1.329530905	4	4	64.29706614	24.46661825
chr3	129430944	129440179	3q21.3	MBD4	28.47332088	21.4162604	1.329518802	4	4	55.73832754	28.98044737
chr2	175923862	176002901	2q31.1	LNPK	136.1329509	102.3956841	1.329479382	4	4	63.3734723	9.550043928
chr7	105110708	105399363	7q22.3	SRPK2	31.25884087	23.51268606	1.329445763	4	4	43.72078891	19.64836116
chr1	110871167	110899936	1p13.3	CD53	320.6446128	241.1941642	1.329404523	4	4	36.60608525	35.17533507
chr7	141790217	141791366	7q34	TAS2R5	0.420725904	0.316499727	1.3293089	2	2	57.6173475	37.29212321
chr1	228082660	228099212	1q42.13	ARF1	137.5176745	103.4526181	1.329281723	4	4	25.42350288	25.92091477
chr6	30184453	30213494	6p22.1	TRIM26	3.833937306	2.88426855	1.329258091	4	4	56.94211197	37.9499213
chrX	100887729	100888674	Xq22.1	HNRNPA1P27	0.414394128	0.311757759	1.329218331	1	1	0	0
chr1	35059786	35116458	1p34.3	ZMYM1	10.18833844	7.665680628	1.329084648	4	4	42.29413664	20.77157333
chr2	181123837	181399553	2q31.3	LINC01934	3.618799062	2.722786796	1.329079114	4	4	40.08964833	27.26221235
chr12	52006945	52015889	12q13.13	GRASP	2.294376915	1.726299384	1.329072428	4	4	78.361569	90.24886404
chr4	125314913	125492932	4q28.1	FAT4	0.350739289	0.263901067	1.329055971	4	4	63.50620168	68.59213999
chr12	6866834	6870946	12p13.31	TPI1	65.74193356	49.46673601	1.329012966	4	4	56.11145291	31.3892152
chr12	6618835	6636131	12p13.31	LPAR5	3.319998912	2.498141185	1.328987701	4	4	47.47554034	64.94937835
chr13	19552570	19568822	13q12.11	MRPS31P2	0.591403582	0.445011016	1.328963916	2	1	19.00688016	0
chr5	87311480	87413033	5q14.3	CCNH	27.04827278	20.35359672	1.328918576	4	4	26.26072474	13.19920153
chr19	48795062	48811063	19q13.33	BCAT2	1.247331765	0.938658187	1.32884556	4	4	56.70230214	36.86917872
chrX	47482716	47485227	Xp11.3	LINC01560	1.140150428	0.858080507	1.328721978	2	4	0.914372974	33.85228775
chr7	44748547	44769881	7p13	ZMIZ2	3.587468434	2.699940389	1.328721348	4	4	68.99891656	42.69355542
chr6	41905353	41921147	6p21.1	MED20	5.562328648	4.186543903	1.328620642	4	4	27.0283964	40.35912071
chr2	131104847	131149852	2q21.1	PLEKHB2	25.4846925	19.18217016	1.328561487	4	4	29.97976361	9.194828321
chr1	151281555	151291905	1q21.3	ZNF687	11.40856272	8.587318955	1.32853604	4	4	62.81640394	11.14487542
chr12	56470944	56488414	12q13.3	GLS2	0.083270711	0.062681076	1.32848249	3	1	16.53178616	0
chr1	29119425	29129138	1p35.3	TMEM200B	0.195762687	0.147358693	1.328477358	2	2	38.36099524	52.38015094

chr15	74782084	74803198	15q24.1	CSK	43.22893493	32.54062661	1.328460433	4	4	60.14213131	43.38000826
chr12	77571856	78213012	12q21.2	NAV3	0.217128319	0.163444823	1.328450273	3	4	76.11258644	31.4454145
chr5	36098550	36151961	5p13.2	LMBRD2	11.71211732	8.816843133	1.328379914	4	4	35.83319758	18.22264929
chr11	95790461	95832693	11q21	CEP57	37.29532468	28.07935475	1.328211599	4	4	41.40386047	14.93852584
chr10	124801785	124836670	10q26.13	ABRAXAS2	25.1565538	18.94181428	1.328096318	4	4	27.80619432	20.45553937
chr7	5620041	5781730	7p22.1	RNF216	19.15724017	14.42686856	1.327886235	4	4	29.87975321	12.64252011
chr17	7243587	7251940	17p13.1	CTDNEP1	11.97606281	9.019148282	1.327848533	4	4	37.19007119	35.59925273
chr17	49495293	49515020	17q21.33	NGFR	0.212752663	0.160225787	1.327830349	1	3	0	81.57075741
chr13	40931919	41061384	13q14.11	ELF1	192.6207866	145.0649973	1.327824011	4	4	26.81542699	12.28034891
chr1	148432959	148459920	1q21.2	LINC01138	1.646533143	1.240106402	1.327735378	4	4	13.40857893	39.28643766
chr12	93565628	93571398	12q22	SOCS2-AS1	0.294408461	0.221739113	1.327724537	4	4	67.82152066	54.48878426
chr15	64072559	64094008	15q22.31	CIAO2A	26.19469765	19.72962031	1.327683819	4	4	20.52453767	17.08413812
chr10	91409235	91514829	10q23.32	HECTD2	2.097421112	1.580017363	1.327467128	4	4	41.64593446	45.93670978
chr6	110814621	110895713	6q21	AMD1	81.46390036	61.37555561	1.327302043	4	4	9.921779205	18.58808152
chr9	19408927	19455173	9p22.1	ACER2	12.62248615	9.510084911	1.32727376	4	4	37.46677422	28.63094715
chr16	89873586	89911384	16q24.3	TCF25	11.15116857	8.40187134	1.327224391	4	4	72.62762346	21.04434146
chr17	59707465	59842255	17q23.1	VMP1	123.2331687	92.85182387	1.327202456	4	4	37.99848344	36.11352729
chr12	50400809	50480005	12q13.12	LARP4	90.78507116	68.40789447	1.327113952	4	4	60.22279906	49.37164927
chr20	49245897	49278219	20q13.13	ZNFX1	56.466177	42.54992558	1.32705701	4	4	45.19803552	12.84785066
chr17	82239019	82273742	17q25.3	CSNK1D	8.28331499	6.241982591	1.327032697	4	4	68.25554233	18.92938089
chr8	17246892	17333532	8p22	VPS37A	29.21205702	22.01524136	1.326901511	4	4	25.5718486	21.17502781
chr15	32615144	32639937	15q13.3	ARHGAP11A	6.050196439	4.559738113	1.326873669	4	4	72.61806522	50.500211
chr6	36196773	36232790	6p21.31	BRPF3	4.832543076	3.642503803	1.326709137	4	4	55.04320229	18.97114199
chr1	222618102	222668012	1q41	MIA3	27.6305022	20.82705656	1.32666381	4	4	33.8660493	17.83005861
chr17	48107533	48123601	17q21.32	SNX11	11.64099219	8.775313317	1.326561431	4	4	27.73539918	20.07250257
chr9	133654587	133657408	9q34.2	DBH-AS1	0.307856226	0.2320961	1.326417055	1	2	0	54.72762032
chr2	159712457	159768583	2q24.2	MARCH7	116.8241408	88.08033313	1.326336273	4	4	35.88462107	18.417819
chr1	26696033	26782110	1p36.11	ARID1A	11.76249014	8.868926125	1.326258667	4	4	68.15227121	43.26199071
chr8	58583504	58659845	8q12.1	NSMAF	18.94907347	14.2880669	1.326216737	4	4	34.76427094	20.22642861
chr17	3806748	3846251	17p13.2	NCBP3	9.165524624	6.912027621	1.326025463	4	4	31.48240247	11.733351
chr17	81244811	81295433	17q25.3	SLC38A10	13.53036117	10.20475419	1.325888004	4	4	65.81165153	40.06565321
chr17	76265205	76271298	17q25.1	UBALD2	61.5345809	46.41213532	1.325829559	4	4	41.91925589	39.56168987
chr9	98120975	98192674	9q22.33	CORO2A	2.266960356	1.709897444	1.325787324	4	4	80.47290296	33.94947568
chr19	17075781	17213295	19p13.11	MYO9B	36.256343	27.34780576	1.325749616	4	4	63.04890925	22.14598928
chr11	118401389	118409847	11q23.3	ATP5MG	67.39998579	50.84254329	1.325661177	4	4	11.26644121	14.91970627
chr22	30969211	30979395	22q12.2	TUG1	32.23562995	24.31886703	1.325539957	4	4	54.24458903	15.78566679
chr1	84298366	84350798	1p31.1	SAMD13	0.183358811	0.138363151	1.325199733	3	2	41.6360592	65.35451329
chr2	147877422	147877528	2q22.3	RNU6-1275P	3.036309017	2.291353585	1.325115878	1	2	0	2.46176704
chr17	4833340	4898061	17p13.2	MINK1	11.52211524	8.695370701	1.325086144	4	4	74.02104876	52.55995844
chr13	30713478	30764428	13q12.3	ALOX5AP	125.884136	95.01258892	1.324920596	4	4	42.57749666	41.03759904
chr17	28573115	28576963	17q11.2	ALDOC	5.397818687	4.074127562	1.324901738	4	4	58.87638433	36.58791895
chr3	14942779	14948441	3p25.1	FGD5-AS1	36.92665862	27.87173165	1.324878522	4	4	24.30115948	20.97894057
chr11	95066877	95071227	11q21	SRSF8	27.74001264	20.93862931	1.324824669	4	4	32.83025617	22.10618292

chr10	13586811	13645344	10p13	PRPF18	23.60194541	17.81563609	1.324788253	4	4	22.2695675	20.61555738
chr18	62715439	62980443	18q21.33	PHLPP1	2.604259899	1.965810204	1.324776875	4	4	44.57724741	17.58517123
chr7	64794388	64853800	7q11.21	ZNF138	10.71222981	8.087117848	1.324604144	4	4	46.26301299	27.56662984
chr4	184382741	184474580	4q35.1	IRF2	70.50973503	53.23210464	1.324571619	4	4	47.81024963	46.10167769
chr1	236518214	236552981	1q43	LGALS8	20.8539656	15.7441578	1.324552628	4	4	33.67157974	23.83711459
chr5	62412746	62628589	5q12.1	IPO11	11.0557411	8.34827676	1.324314156	4	4	81.19070411	52.93544485
chr8	124474767	124488618	8q24.13	RNF139	46.91453488	35.42565004	1.324309782	4	4	22.96816836	9.667623106
chr1	111117170	111140216	1p13.3	DRAM2	25.73063621	19.42959287	1.324301357	4	4	34.7935535	14.24582077
chr2	171922425	171983686	2q31.1	HAT1	82.52229045	62.32247976	1.32411757	4	4	79.93375572	64.54830575
chr12	14774336	14778002	12p12.3	H2AFJ	9.878467204	7.460643763	1.324077052	4	4	18.1523361	42.12878489
chr7	88205115	88226993	7q21.12	SRI	14.24422969	10.75834315	1.324017043	4	4	10.09993733	17.71905413
chr6	53794497	53924125	6p12.1	LRRC1	0.621458584	0.469380455	1.323997576	4	4	41.63045411	107.0610087
chr4	13568738	13627721	4p15.33	BOD1L1	42.06955069	31.7747436	1.323993396	4	4	61.36086628	39.52848583
chr8	100150611	100154002	8q22.2	POLR2K	30.98181175	23.40059026	1.323975652	4	4	32.90062052	14.92033285
chr19	57469586	57479014	19q13.43	ZNF772	3.010477593	2.273936692	1.323905632	4	4	93.7631966	34.64247776
chr19	58305374	58315657	19q13.43	ERVK3-1	5.065672869	3.826399749	1.323874451	4	4	53.56410348	13.61807931
chr6	35833034	35921180	6p21.31	SRPK1	47.00518856	35.50725131	1.323819412	4	4	42.85599857	28.23813828
chr1	35869808	35929610	1p34.3	AGO1	9.290897135	7.018984354	1.323681129	4	4	74.51628067	8.549916894
chr20	19212646	19722897	20p11.23	SLC24A3	3.78310919	2.858049816	1.323668037	4	4	15.43550273	10.74999837
chr18	45420997	45508330	18q12.3	SLC14A2-AS1	0.253452834	0.191482781	1.323632512	1	1	0	0
chr19	57279967	57294068	19q13.43	ZNF460	17.74252559	13.40479865	1.323595083	4	4	63.10411317	7.159956881
chr10	21623902	21626376	10p12.31	HNRNPRP1	0.301946941	0.228135387	1.323542763	2	2	61.45636428	26.51812661
chr5	108746085	109196841	5q21.3	FER	6.988731624	5.280401247	1.323522834	4	4	48.42709902	36.42477818
chr1	52020131	52056171	1p32.3	TXNDC12	27.3690681	20.67949171	1.323488434	4	4	55.45218614	25.73716879
chr4	7058895	7068210	4p16.1	GRPEL1	6.565259382	4.960590226	1.323483514	4	4	41.95707879	10.05259302
chr21	39405844	39445796	21q22.2	LCA5L	0.057517452	0.043460044	1.323455909	1	1	0	0
chr14	98135560	98162967	14q32.2	LINC02295	3.761293488	2.842212338	1.323368222	1	4	0	72.52950121
chr6	107867756	107958278	6q21	SEC63	23.54341607	17.79119829	1.323318176	4	4	34.77087603	24.48897119
chr13	18837767	18838262	13q11	RHOT1P3	57.50065823	43.45204755	1.323312973	4	4	94.69812652	54.50436892
chr16	67562407	67639185	16q22.1	CTCF	57.41787212	43.39118433	1.323261234	4	4	27.96615313	10.69830082
chr10	68482333	68527523	10q21.3	SLC25A16	7.479040425	5.652355376	1.323172364	4	4	74.13753121	45.28564185
chr4	184649613	184694959	4q35.1	PRIMPOL	30.42552503	22.99506158	1.323133009	4	4	77.46196342	57.70380411
chr10	67921899	68075346	10q21.3	HERC4	13.2584648	10.02098305	1.323070274	4	4	20.9208413	22.79211448
chr1	150574551	150579738	1q21.2	MCL1	616.2177683	465.7498439	1.323065968	4	4	42.94099908	36.00921327
chrX	153907376	153926260	Xq28	ARHGAP4	23.92564012	18.08449437	1.32299193	4	4	68.96773217	18.99147342
chr20	7977353	8019829	20p12.3	TMX4	56.93419758	43.03595112	1.322945029	4	4	50.51794472	30.70589188
chr4	87160103	87220608	4q22.1	KLHL8	46.08813591	34.839156	1.32288325	4	4	53.82795725	33.82794376
chr2	178431414	178451240	2q31.2	PRKRA	5.622332501	4.250361679	1.322789194	4	4	36.73190502	21.16935603
chr2	238244038	238290102	2q37.3	PER2	2.821756554	2.133278805	1.322732193	4	4	53.19493208	33.06535828
chr1	109656076	109665501	1p13.3	GSTM4	1.490973487	1.12719674	1.322726933	4	4	89.86907509	81.04983314
chr3	42489299	42537573	3p22.1	VIPR1	1.407083917	1.063871125	1.322607489	4	4	68.83771679	57.75360711
chrX	139726346	139933083	Xq27.1	ATP11C	17.81534416	13.47155563	1.322441494	4	4	22.35673805	26.21324589
chr2	47520775	47570331	2p16.3	KCNK12	0.050311432	0.038046649	1.322361722	1	2	0	2.773500801

chr19	58346806	58353499	19q13.43	A1BG	0.144274663	0.109107416	1.322317658	1	4	0	28.45939497
chr2	96208416	96242623	2q11.2	STARD7-AS1	0.995105287	0.752561796	1.322290465	4	4	45.82376608	39.95723677
chr1	27773183	27824452	1p35.3	STX12	26.47522828	20.02297255	1.32224265	4	4	20.52370315	17.66534856
chrX	132203024	132219480	Xq26.2	RAP2C	34.38722557	26.00736499	1.322211058	4	4	22.22649036	25.11511745
chr2	61800240	61854143	2p15	FAM161A	0.357173231	0.270135716	1.322199212	4	4	68.36548256	65.4505157
chr20	38420588	38435377	20q11.23	SNHG17	0.884356716	0.668980929	1.321946079	4	4	72.1727511	19.53652867
chr2	238170401	238203702	2q37.3	ILKAP	37.46982458	28.34491001	1.321924274	4	4	68.3101542	25.91153687
chr8	98433048	98944329	8q22.2	STK3	11.3034908	8.551058059	1.321882125	4	4	43.65981075	51.93626446
chrX	149924152	149938845	Xq28	CXorf40B	3.170796412	2.398867846	1.3217887	4	4	54.99095097	23.26275789
chr1	25819906	25832942	1p36.11	MTFR1L	4.780220395	3.616546302	1.321763914	4	4	61.43937336	25.28910961
chr2	183078559	183100008	2q32.1	DUSP19	1.282035837	0.969994578	1.321693818	4	4	33.15987565	39.85039721
chr5	33986986	34008115	5p13.2	AMACR	0.327833412	0.248084146	1.321460553	4	4	96.63799002	43.75107051
chr14	30874496	30890618	14q12	COCH	1.134742681	0.858793402	1.321322076	4	4	67.1742206	38.55323693
chr15	65611313	65660995	15q22.31	SLC24A1	0.713761539	0.540339536	1.320950054	4	4	43.95824118	72.39828779
chr17	1421353	1456267	17p13.3	CRK	3.741635192	2.832584246	1.320926358	4	4	45.13438548	41.03542245
chrX	136806941	136807944	Xq26.3	RANP4	0.517069134	0.391444413	1.320926079	1	1	0	0
chr14	29576479	29927805	14q12	PRKD1	0.385399009	0.291780763	1.320851331	1	3	0	99.65690836
chr18	49481178	49487274	18q21.1	C18orf32	1.798263505	1.361491406	1.320804154	4	4	59.26940105	67.17386705
chr16	2019520	2020755	16p13.3	NPW	0.924987222	0.700322191	1.320802386	2	3	67.63592	59.61489376
chr8	105318859	105804539	8q23.1	ZFPM2	1.109331395	0.839919629	1.320758983	4	4	53.56860345	13.44568703
chr14	106235062	106235515	14q32.33	IGHV3-21	6.636542427	5.025171244	1.320659955	2	4	68.86706231	87.67196056
chr5	150485783	150558211	5q33.1	NDST1	8.583859057	6.499750007	1.320644494	4	4	65.03715314	18.3361293
chr19	10350528	10380572	19p13.2	TYK2	18.43634746	13.96138369	1.320524374	4	4	55.84739905	24.45275607
chr20	32109506	32167258	20q11.21	TM9SF4	13.58532924	10.28801131	1.320501001	4	4	52.63987248	27.09608387
chr2	38875962	38882709	2p22.1	MORN2	10.3540055	7.841507557	1.320410064	4	4	74.0316046	67.12673444
chr3	4493348	4847840	3p26.1	ITPR1	12.02026437	9.104188648	1.32030045	4	4	43.41769006	21.72947255
chr21	29005759	29019371	21q21.3	RWDD2B	2.663448867	2.017486805	1.320181555	4	4	36.35422935	52.55710912
chr19	23739195	23758891	19p12	ZNF681	4.380565479	3.318203949	1.320161613	4	4	52.98253021	26.35242171
chr6	152121684	152637399	6q25.2	SYNE1	17.37841979	13.16425111	1.320122174	4	4	36.7449995	8.905923524
chr12	45215987	45440404	12q12	ANO6	46.55126663	35.26492729	1.32004431	4	4	48.46234355	1.437871248
chr21	46188446	46228824	21q22.3	LSS	1.891038421	1.432566974	1.320034913	4	4	59.54764009	52.81321769
chr2	239048168	239401647	2q37.3	HDAC4	6.194238552	4.692679272	1.319979098	4	4	57.34308859	36.97223921
chr3	142306607	142448062	3q23	XRN1	114.3310593	86.61638289	1.319970373	4	4	42.00991566	19.4391765
chr9	2015219	2193624	9p24.3	SMARCA2	20.93428003	15.85997201	1.319944324	4	4	31.83831146	18.60442843
chr9	83968083	83980782	9q21.32	HNRNPK	225.7728168	171.0518248	1.319908847	4	4	27.96379762	12.0487954
chr16	31201885	31203452	16p11.2	PYCARD-AS1	1.587705918	1.202919752	1.319876838	3	3	8.753247291	11.88261092
chr17	83226897	83240804	17q25.3	RPL23AP87	0.141933028	0.107541543	1.319797202	1	1	0	0
chr13	45954465	46052794	13q14.13	ZC3H13	31.48580402	23.85698959	1.319772719	4	4	56.65637839	27.33378273
chr1	44850522	44986722	1p34.1	EIF2B3	3.500544676	2.652446626	1.319741797	4	4	20.94692878	22.05319676
chr1	230642456	230693985	1q42.2	COG2	10.09751548	7.651354264	1.31970304	4	4	56.41997414	23.7619755
chr14	22920511	22929585	14q11.2	PRMT5	11.01256962	8.344837507	1.319686526	4	4	63.15346878	11.29439827
chr7	30028361	30130483	7p14.3	PLEKHA8	2.550426302	1.932680813	1.319631408	4	4	62.2722343	29.1504185
chr20	62877738	62937952	20q13.33	DIDO1	12.75846437	9.668526641	1.319587238	4	4	80.67441903	42.97871947

chr14	101803227	101803331	14q32.31	RNU6-790P	3.140156855	2.379676815	1.319572824	3	1	47.61303996	0
chr12	25052101	25108335	12p12.1	LRMP	41.45017936	31.413067	1.319520293	4	4	40.04528622	34.07640676
chr1	88979456	88992960	1p22.2	RBMXL1	13.90550848	10.53833254	1.319516956	4	4	63.69844806	14.62492304
chr2	165747803	165796352	2q24.3	GALNT3	12.35545175	9.364379282	1.319409581	4	4	33.85820401	51.92951339
chr15	45587123	45609716	15q21.1	BLOC1S6	42.80025263	32.44190105	1.319289291	4	4	15.8272034	36.28247776
chr8	96226676	96235634	8q22.1	UQCRB	55.10767357	41.77106624	1.319278595	4	4	31.62605946	8.524365073
chr1	36393420	36397959	1p34.3	LSM10	9.183534633	6.961726177	1.3191462	4	4	66.43435477	27.26977182
chr19	42230186	42243330	19q13.2	GSK3A	10.34198922	7.840313138	1.319078593	4	4	45.77038443	58.54588055
chr11	111879224	111884083	11q23.1	C11orf1	2.36181539	1.790513557	1.319071492	4	4	39.3397132	32.44776149
chr15	94231538	94483952	15q26.2	MCTP2	30.85639714	23.39397353	1.318989145	4	4	40.67093045	36.29214066
chr5	93740309	93741603	5q15	POU5F2	0.707755495	0.536601656	1.318958835	4	4	21.64052708	34.10676791
chr15	99136323	99251235	15q26.3	TTC23	0.8905346	0.675210716	1.318898795	4	4	48.3600022	61.43617441
chr16	11254417	11256182	16p13.13	SOCS1	3.490009364	2.646167678	1.318891993	4	4	36.62037562	80.10687844
chr12	80792520	80937915	12q21.31	LIN7A	14.13110498	10.71443066	1.318885289	4	4	51.76360529	67.94125882
chr6	149400359	149401046	6q25.1	SUMO4	3.799179482	2.880859227	1.318766098	4	4	58.63368852	46.08738147
chr9	113444032	113597743	9q32	RGS3	4.62423506	3.506610023	1.318719512	4	4	40.13980702	51.50921164
chr2	136114349	136118155	2q22.1	CXCR4	405.0949163	307.1977653	1.31867794	4	4	56.35454589	10.36853519
chr10	98134624	98244897	10q24.2	R3HCC1L	16.27784541	12.34467841	1.31861235	4	4	24.75096782	5.960765627
chr9	137277828	137282641	9q34.3	TOR4A	4.469602792	3.389764501	1.318558499	4	4	47.67547085	36.06426029
chrX	71043214	71045470	Xq13.1	SOCS5P4	0.767528308	0.582123174	1.318498115	1	2	0	95.88705626
chr1	114392777	114511192	1p13.2	TRIM33	42.6035397	32.31335697	1.31844982	4	4	26.27178375	18.89082226
chr2	72942036	73071836	2p13.2	SFXN5	2.14481623	1.62684921	1.31838662	4	4	32.49073386	32.57332314
chr15	34856351	34969794	15q14	AQR	19.01509691	14.42475429	1.318226746	4	4	31.84390573	14.56831944
chr2	61017677	61051990	2p15	PEX13	7.627363518	5.786251253	1.318187404	4	4	24.02592336	27.3110374
chr19	49500785	49500868	19q13.33	MIR150	7.667783927	5.81692417	1.318185299	3	2	68.2957219	58.45351971
chr7	100612102	100615380	7q22.1	MOSPD3	2.387763721	1.811482132	1.318127118	4	4	65.9479529	42.45622803
chr1	151510858	151538692	1q21.3	CGN	0.142216931	0.107894511	1.3181109	2	2	29.70476967	88.68251303
chr9	92325457	92615164	9q22.31	CENPP	2.644487889	2.006327589	1.31807383	4	4	74.79752695	40.13062631
chr19	3958453	3971123	19p13.3	DAPK3	5.179673767	3.929963647	1.317995338	4	4	65.94158491	28.45199181
chr10	17228210	17237597	10p13	VIM	222.7866958	169.0500355	1.317874292	4	4	56.31432159	48.42631305
chr12	63779803	63809558	12q14.2	RXYLT1	1.148967855	0.871848816	1.317852171	4	4	31.71314949	32.59136745
chr19	49664834	49673916	19q13.33	BCL2L12	1.014461272	0.769918868	1.317621003	4	4	14.33463941	50.29631626
chr15	39934101	40036153	15q15.1	EIF2AK4	4.858333471	3.687524365	1.317505456	4	4	40.72382592	16.13562708
chr22	21628089	21630326	22q11.21	YDJC	4.787500235	3.633908021	1.317452232	4	4	76.15581806	47.36289735
chr9	112040781	112175515	9q31.3-q32	SUSD1	72.02415902	54.66962526	1.317443803	4	4	40.59284113	21.45084188
chr3	119597848	119629811	3q13.33	PLA1A	0.144971722	0.110041636	1.31742609	1	1	0	0
chr10	12129641	12169958	10p14	SEC61A2	1.793666442	1.361538033	1.317382547	4	4	43.34534402	30.87804141
chr18	9546791	9615273	18p11.22	PPP4R1	39.37756856	29.89178938	1.317337282	4	4	27.06721202	40.86248353
chr18	75195108	75209506	18q22.3	ZADH2	5.639430057	4.28184703	1.317055471	4	4	47.68635821	36.54589307
chr3	52812990	52830701	3p21.1	ITIH4	0.17987993	0.136580404	1.317025896	3	4	121.7717591	54.52717136
chr17	43766121	43779000	17q21.31	DUSP3	10.8732615	8.255986566	1.317015406	4	4	46.44453391	36.74872865
chr16	30350766	30355361	16p11.2	CD2BP2	19.69803853	14.95660061	1.317013073	4	4	55.26836972	26.05506424
chr5	179732850	179777286	5q35.3	MAML1	1.239172131	0.940945764	1.316943206	4	4	88.14246607	56.58496458

chr4	118685370	118712270	4q26	METTL14	10.67865162	8.109118514	1.316869595	4	4	25.09705678	11.10998139
chr3	160514907	160515236	3q25.33	SCARNA7	5651.989052	4292.030726	1.316856615	4	4	34.57737439	38.53291907
chr19	51097606	51108390	19q13.41	CTU1	0.969154519	0.736007294	1.316772982	3	4	76.01205511	53.61836925
chr5	60945129	61153037	5q12.1	NDUFAF2	15.38680729	11.68530389	1.316765694	4	4	26.35026226	29.15607442
chr9	19230490	19374268	9p22.1	DENND4C	38.1496322	28.97362231	1.316702199	4	4	25.55453836	7.79403759
chr16	69355561	69386004	16q22.1	TERF2	91.0033639	69.12257076	1.316550627	4	4	51.00551394	28.79062444
chr11	9843817	9843875	11p15.4	RNU7-28P	7.32483052	5.563666939	1.31654727	2	2	27.17843954	22.70739606
chr17	68101555	68135929	17q24.2	LINC00674	12.73716392	9.674898992	1.316516476	4	4	37.24734683	16.78093168
chr8	26291491	26372680	8p21.2	PPP2R2A	21.38575668	16.24505709	1.316447001	4	4	26.67545533	41.48333165
chr2	44168803	44247330	2p21	PPM1B	40.35708622	30.65622199	1.316440305	4	4	18.89934422	16.06701913
chr12	106302026	106347455	12q23.3	TCP11L2	86.85562311	65.97893203	1.316414504	4	4	42.40306438	69.41932152
chr19	42247561	42255164	19q13.2	ERF	11.35339226	8.624651414	1.316388538	4	4	74.43799923	31.28622883
chr20	37179046	37241623	20q11.23	RPN2	55.38390281	42.07290833	1.316379233	4	4	43.07403714	19.78409888
chr3	185282938	185489094	3q27.2	MAP3K13	1.451091418	1.102339426	1.316374415	4	4	36.89056063	27.20511224
chr7	74191198	74191294	7q11.23	MIR590	7.438821725	5.651330418	1.316295664	2	4	33.92272561	39.02379459
chr2	96536719	96552638	2q11.2	ARID5A	19.70200088	14.96801778	1.316273215	4	4	60.83046849	29.89768978
chr4	169393270	169612629	4q33	NEK1	78.74184776	59.82347335	1.316236643	4	4	70.58891978	51.31218902
chr9	92947319	93036236	9q22.31	FGD3	44.21495407	33.59214492	1.316228963	4	4	57.376764	39.9399154
chr8	614737	731239	8p23.3	ERICH1	5.522338093	4.195785131	1.316163226	4	4	59.81934104	56.68287161
chr17	42980516	42995143	17q21.31	RUNDC1	6.613671802	5.025076614	1.316133526	4	4	53.48598084	35.14605811
chr9	211296	465260	9p24.3	DOCK8	68.97475434	52.40857753	1.316096669	4	4	45.86178801	38.11406306
chr5	83077409	83370333	5q14.2	XRCC4	15.38509794	11.69048355	1.316036063	4	4	17.35609812	13.79155173
chr8	8892225	9100262	8p23.1	ERI1	13.373588	10.16224387	1.316007386	4	4	20.50802247	27.43642429
chr6	159906942	159908076	6q25.3	MAS1	0.033903807	0.025764721	1.315900447	1	1	0	0
chr22	32359906	32371264	22q12.3	RFPL3S	0.40918407	0.310955903	1.315890989	2	4	56.07773918	64.64677561
chr8	141207166	141308321	8q24.3	SLC45A4	19.20516562	14.59482983	1.315888286	4	4	25.00320604	26.72583089
chr10	3775996	3785281	10p15.2	KLF6	67.00191974	50.9218617	1.315779068	4	4	58.30621552	71.04607024
chr12	118149801	118372950	12q24.23	TAOK3	29.48469825	22.40860442	1.315775748	4	4	33.40478231	16.88270507
chr10	70404105	70428618	10q22.1	EIF4EBP2	52.31393601	39.76061684	1.315722445	4	4	21.61968299	20.35582882
chr1	120155942	120176521	1p12	SEC22B	14.42824921	10.96651491	1.31566403	4	4	42.49551817	20.17136298
chr14	73851831	73885465	14q24.3	PTGR2	0.222656738	0.169235871	1.315659244	3	4	13.8295748	62.13737402
chr17	82458184	82490537	17q25.3	NARF	12.53837006	9.530784079	1.315565431	4	4	9.293150181	14.15203297
chr6	43511827	43516990	6p21.1	YIPF3	35.74337196	27.16997098	1.315546932	4	4	31.8453261	24.20579604
chr17	75205436	75235759	17q25.1	NUP85	9.536033715	7.24876089	1.315539836	4	4	53.22514674	42.78819208
chr17	8435852	8472744	17p13.1	NDEL1	39.33493752	29.90029905	1.315536592	4	4	61.04581544	32.1876115
chr10	118680982	118755246	10q26.11	CACUL1	22.48782177	17.09489947	1.31546967	4	4	17.4344613	32.10038722
chr8	24384285	24406013	8p21.2	ADAMDEC1	0.411680293	0.312967038	1.315411028	3	4	63.08954379	15.51588319
chr17	76752912	76753013	17q25.1	RNU6-97P	4.515905746	3.433140438	1.315386256	2	1	82.30205319	0
chr16	46622875	46626994	16q11.2	RAB43P1	0.707416434	0.537805828	1.315375172	2	1	52.14512434	0
chr12	6199707	6238271	12p13.31	CD9	26.9979873	20.52511795	1.31536332	4	4	86.96009993	40.94551595
chr22	39570753	39689737	22q13.1	CACNA1I	0.552796781	0.420266782	1.31534731	4	4	83.86930049	48.56086739
chr6	30326844	30343729	6p22.1	TRIM39	0.267132221	0.203096976	1.315293937	3	2	60.8921391	26.35460869
chr9	38392664	38398665	9p13.1	ALDH1B1	2.043031769	1.553378446	1.315218307	4	4	40.94545862	57.84094452

chr17	44299574	44308431	17q21.31	RUNDC3A-AS1	2.819058166	2.14341473	1.315218248	4	4	82.4671558	58.04814019
chr22	22712419	22713199	22q11.22	IGLV3-21	9.903009282	7.530069386	1.315128556	4	3	60.22394574	98.55436942
chr14	23953735	23969279	14q11.2	DHRS4	3.559378318	2.706819567	1.314966968	4	4	58.87612985	74.8152515
chr5	132656524	132661109	5q31.1	IL13	0.117708268	0.089514852	1.314957969	1	1	0	0
chr19	10602445	10644559	19p13.2	SLC44A2	83.58030466	63.56505692	1.314878153	4	4	44.48472618	27.01736213
chr17	41930594	41966517	17q21.2	TTC25	6.443047487	4.900208424	1.314851722	4	4	29.66975179	10.90819178
chr20	16272104	16573457	20p12.1	KIF16B	4.208656426	3.200918054	1.31482792	4	4	36.61253133	32.90767133
chr1	43389884	43454247	1p34.2	SZT2	2.840165281	2.160129391	1.314812572	4	4	68.00638579	18.90362572
chr13	32432488	32542710	13q13.1	N4BP2L2	142.7141456	108.5449772	1.314792718	4	4	74.69651513	36.79187217
chr9	98807699	98850081	9q22.33	GALNT12	1.552630633	1.180917005	1.314766937	4	4	82.36349396	51.53887689
chrX	121047608	121049942	Xq24	GLUD2	0.146066743	0.111102894	1.314697923	3	2	52.0086456	10.33038632
chr10	60869438	61001486	10q21.2	RHOBTB1	5.585514526	4.248516311	1.314697677	4	4	63.40991599	25.21664079
chr2	197499997	197503463	2q33.1	HSPE1	15.65000368	11.90420093	1.314662259	4	4	78.87979023	65.82427466
chr13	27435639	27450897	13q12.2	MTIF3	26.41885619	20.09569283	1.314652668	4	4	47.65486538	33.63939975
chr2	24819169	24920017	2p23.3	ADCY3	2.367446541	1.80115269	1.314406355	4	4	70.81339018	33.99524827
chr15	41282697	41313338	15q15.1	OIP5-AS1	28.10509322	21.38248624	1.314397816	4	4	52.41230523	16.82383254
chr12	104064458	104108954	12q23.3	HCFC2	6.641029759	5.052547701	1.314392293	4	4	28.66644161	16.84297748
chr1	68098459	68233018	1p31.3	WLS	10.66972202	8.117687762	1.314379455	4	4	88.82416015	100.2097483
chr7	143851368	143902198	7q35	TCAF1	1.938751961	1.475090518	1.314327451	4	4	51.9450537	40.33504342
chr6	37433131	37482844	6p21.2	CMTR1	13.58863989	10.33925837	1.314276073	4	4	30.49659127	19.65682858
chr17	35147793	35189345	17q12	UNC45B	0.392017903	0.298290507	1.314215151	4	3	18.54754905	71.01974366
chr4	20251905	20620561	4p15.31	SLIT2	0.069281706	0.05271804	1.314193509	1	2	0	0.37384921
chr18	12947984	12987665	18p11.21	SEH1L	3.155121991	2.401207553	1.313973042	4	4	34.7014214	5.441553476
chr17	59884990	59885649	17q23.1	NDUFB8P2	0.640327657	0.487339836	1.313924307	1	2	0	1.889336625
chr6	16141556	16141624	6p22.3	MIR4639	4.464156764	3.397802929	1.313836281	3	1	30.60991323	0
chr1	108692310	108702930	1p13.3	PRPF38B	22.75345217	17.31851463	1.313822384	4	4	51.3917911	32.80862616
chr11	126255151	126268982	11q24.2	SRPRA	25.30525736	19.2610715	1.313803199	4	4	49.04267322	21.26062696
chr17	30477402	30526814	17q11.2	GOSR1	30.53037726	23.23837295	1.313791517	4	4	60.53278108	35.55854113
chr6	33278207	33289527	6p21.32	WDR46	5.200823584	3.95864079	1.313790228	4	4	54.28789828	34.53980902
chr15	84601018	84623718	15q25.2	ZSCAN2	1.469537418	1.118555946	1.313780883	4	4	60.22399907	45.94252902
chr1	150211632	150211925	1q21.2	RN7SL480P	1.183799647	0.901092611	1.313738046	3	2	45.41615987	5.042990281
chr10	14897564	14954432	10p13	DCLRE1C	9.333663829	7.104733572	1.313724679	4	4	29.22401555	7.213350204
chr13	21492689	21604216	13q12.11	MICU2	42.42878732	32.30021748	1.313575902	4	4	42.34824989	28.39084761
chr3	181056680	181742228	3q26.33	SOX2-OT	0.055771179	0.042458039	1.313559937	2	2	16.09151895	72.95934384
chr19	40576851	40591399	19q13.2	SHKBP1	43.85233976	33.38501493	1.313533627	4	4	41.7721104	43.44862555
chr6	149217924	149411613	6q25.1	TAB2	57.70218213	43.93071398	1.313481547	4	4	30.7456998	8.742604248
chr19	12224686	12294899	19p13.2	ZNF44	10.22053706	7.781306114	1.313473203	4	4	51.00295504	27.82605133
chr1	114716913	114758050	1p13.2	CSDE1	310.052978	236.0624516	1.313436236	4	4	24.53044825	11.78453122
chr13	38349771	38363007	13q13.3	UFM1	26.93952299	20.51078247	1.313432242	4	4	14.89687815	22.016168
chr4	6716115	6717660	4p16.1	BLOC1S4	6.112375596	4.653759384	1.313427509	4	4	27.16572132	38.40315274
chr11	27494418	27506779	11p14.1	LIN7C	30.94750199	23.56400885	1.313337734	4	4	24.76352263	16.86291101
chr12	62466817	62598956	12q14.1	MON2	13.2681705	10.10285927	1.313308456	4	4	32.53472803	14.08802325
chr2	27377231	27380866	2p23.3	ZNF513	3.358108647	2.556990125	1.313305286	4	4	58.51122781	27.62772851

chr17	16415542	16437003	17p11.2	TRPV2	14.05837531	10.7047479	1.313284109	4	4	22.350131	24.18283221
chr1	74198212	74208702	1p31.1	FPGT	7.234206121	5.508853432	1.313196332	4	4	24.18995335	38.60291198
chr17	43044295	43125483	17q21.31	BRCA1	35.33077718	26.9049329	1.313170983	4	4	61.05677698	36.56890778
chr11	64823809	64844686	11q13.1	CDC42BPG	0.383741058	0.292250653	1.313054579	4	4	104.4386568	106.0863291
chr15	34229996	34338064	15q14	SLC12A6	50.36257318	38.35845245	1.312945908	4	4	40.13732908	26.32038385
chr12	123973215	124015439	12q24.31	ZNF664	13.67451907	10.41550859	1.312899793	4	4	31.9172917	21.07594797
chr17	20449863	20467535	17p11.2	LGALS9B	0.589051283	0.448672535	1.312875733	3	3	86.97667586	81.22576983
chr14	24094131	24104132	14q11.2-q12	PCK2	3.316639589	2.526250729	1.312870314	4	4	46.50702086	49.70340018
chr13	46490857	46491259	13q14.13	COX17P1	1.695801466	1.29206942	1.312469315	1	1	0	0
chr3	48440238	48444208	3p21.31	TMA7	118.8552232	90.56096154	1.312433318	4	4	7.474787144	24.51020883
chr15	44711487	44718159	15q21.1	B2M	2376.503378	1810.836925	1.312378462	4	4	59.43460688	34.15409595
chr14	21287977	21351316	14q11.2	RPGRIP1	1.645197033	1.253804067	1.312164378	4	4	82.26639841	79.27544864
chr3	31532501	31637622	3p23	STT3B	95.25292559	72.59278733	1.312154128	4	4	19.15789772	8.813824052
chr16	31713229	31761565	16p11.2	ZNF720	8.40534851	6.405940826	1.312117726	4	4	35.88125516	20.12169066
chr16	30472662	30523185	16p11.2	ITGAL	95.47967717	72.76923256	1.312088555	4	4	22.51713994	46.50189437
chr17	81189593	81222989	17q25.3	CEP131	1.61901619	1.233936648	1.312073997	4	4	87.63122016	55.96201982
chr12	106774556	106889316	12q23.3	RIC8B	7.303597594	5.566630738	1.312031988	4	4	36.87597702	21.47363934
chr3	174438534	175810548	3q26.31	NAALADL2	1.064775754	0.811555671	1.312018131	4	4	78.18416686	52.77669158
chr4	186191520	186213463	4q35.1-q35.2	CYP4V2	3.788660112	2.887923985	1.31189745	4	4	33.97485854	32.04402378
chr19	43543312	43575578	19q13.31	XRCC1	6.545495562	4.990167275	1.311678587	4	4	82.95332326	42.7207672
chr9	15422784	15466751	9p22.3	SNAPC3	9.590502847	7.312559124	1.311511153	4	4	26.9251798	12.63766269
chr10	86435256	86521816	10q23.2	WAPL	75.1665248	57.32084333	1.311329709	4	4	16.76901275	7.859782545
chr19	58551566	58555624	19q13.43	CHMP2A	50.2167837	38.29482682	1.311320298	4	4	75.67893357	65.84921756
chr4	16160505	16226538	4p15.32	TAPT1	9.884097788	7.537830746	1.311265551	4	4	32.63103407	27.37016892
chr14	22836533	22847600	14q11.2	MMP14	0.457710149	0.349090758	1.311149431	4	4	81.75901068	34.51936178
chrX	154675249	154751583	Xq28	GAB3	17.16821652	13.09449812	1.31110153	4	4	31.054503	36.82766028
chr7	64500253	64563135	7q11.21	ZNF680	14.36532568	10.95680769	1.311086777	4	4	28.52786322	10.68423461
chr17	76006845	76027452	17q25.1	EVPL	0.894052085	0.68192561	1.311069817	4	4	24.47047796	94.11331756
chr7	130164713	130205498	7q32.2	TMEM209	25.49408705	19.44710515	1.310945092	4	4	68.87530755	14.84635934
chr9	136830957	136831023	9q34.3	MIR4292	7.849112115	5.987462017	1.310924744	3	3	61.85369601	27.76972928
chr8	127415612	127417210	8q24.21	POU5F1B	0.127943303	0.097603569	1.310846568	4	4	89.61164978	39.26885184
chr19	15114974	15125799	19p13.12	ILVBL	1.339798496	1.022206508	1.310692591	4	4	51.26859376	33.15258504
chr16	46657979	46689232	16q11.2	VPS35	27.04070507	20.63100233	1.310683051	4	4	31.38522987	24.06129552
chr1	246839098	246931978	1q44	AHCTF1	45.9940044	35.09238409	1.310654878	4	4	49.63735358	29.30332215
chr3	128726136	128814798	3q21.3	RAB7A	68.61586236	52.35443624	1.31060264	4	4	56.73926146	32.97312561
chr19	12938600	12944490	19p13.13	CALR	133.2998822	101.7113627	1.310570213	4	4	58.6924097	57.17317676
chr5	131949973	132012068	5q31.1	ACSL6	3.658529194	2.791701242	1.310501689	4	4	48.93504571	23.66837062
chrX	24558087	24672862	Xp22.11	PCYT1B	3.654273999	2.788831814	1.310324266	4	4	70.44902726	31.88593819
chr1	34986165	35031968	1p34.3	ZMYM6	3.035633181	2.316816795	1.310260349	4	4	39.38349028	14.90637353
chr22	38704561	38738265	22q13.1	GTPBP1	17.8350327	13.61206811	1.31023681	4	4	46.99771237	7.46728483
chr11	119106752	119118347	11q23.3	C2CD2L	2.648121033	2.021655316	1.30987761	4	4	56.57013916	45.58242349
chr11	65717673	65720938	11q13.1	RNASEH2C	2.770176938	2.115037619	1.309753034	4	4	67.26423462	15.69020476
chr6	31580558	31582425	6p21.33	LTB	23.72275441	18.11376861	1.309653166	4	4	20.67647637	34.24729845

chr17	47522950	47623276	17q21.32	NPEPPS	13.38722705	10.22247346	1.309587851	4	4	29.05074407	28.17229061
chr4	2930556	2934859	4p16.3	MFSD10	5.523606171	4.217965243	1.309542837	4	4	31.48022776	31.52641501
chr9	128787207	128810432	9q34.11	TBC1D13	4.775534521	3.646799564	1.309513846	4	4	33.72530387	23.67996754
chr3	156711403	156715029	3q25.31	METTL15P1	0.716448101	0.547133222	1.30945823	4	2	43.45361599	78.21984734
chr7	84995192	85187176	7q21.11	SEMA3D	0.087596108	0.0668959	1.309439122	1	3	0	48.17821946
chr10	89829493	89840861	10q23.31	LINC00865	0.38172385	0.291538281	1.309343832	4	4	133.5771904	84.1929117
chr3	50606454	50611831	3p21.2	CISH	5.107233928	3.900839661	1.309265279	4	4	37.06191334	48.27577036
chr3	133573590	133590274	3q22.1	CDV3	127.6451906	97.50390982	1.309128945	4	4	34.70164127	19.11506158
chr12	47963547	47968878	12q13.11	TMEM106C	4.504779816	3.441372878	1.309006602	4	4	78.5463592	21.43564959
chr7	135361795	135510127	7q33	CNOT4	12.90661485	9.861146268	1.30883515	4	4	17.78928759	10.58405357
chr3	9962680	10011248	3p25.3	EMC3	194.9103215	148.9255969	1.3087765	4	4	48.82679142	56.07492178
chr1	234391313	234479174	1q42.2	TARBP1	5.537881394	4.231445656	1.308744539	4	4	46.90828432	16.14876191
chr14	57993536	58152305	14q23.1	ARMH4	0.090648954	0.069265288	1.308721236	1	2	0	71.06488256
chr21	25585656	25607858	21q21.3	MRPL39	20.59216429	15.7355874	1.308636517	4	4	32.75018286	23.38343864
chr1	5992298	6101193	1p36.31	KCNAB2	13.00502015	9.938387047	1.308564467	4	4	59.81855626	31.67819767
chr8	20197193	20226852	8p21.3	ATP6V1B2	50.85358736	38.86294707	1.308536568	4	4	45.74532844	30.60134757
chr6	85677007	85678733	6q14.3	SNHG5	25.98026585	19.85458833	1.308527047	4	4	46.93927169	58.25431969
chr3	148991408	149027669	3q24	GYG1	18.26220776	13.9574322	1.308421743	4	4	61.39607555	37.36130376
chr3	128052369	128071683	3q21.3	SEC61A1	36.13049107	27.61459198	1.308384028	4	4	32.2104609	33.7020056
chr17	2055099	2059687	17p13.3	HIC1	1.12599711	0.8606112	1.30836911	4	4	121.196385	66.44494035
chr6	149749695	149811421	6q25.1	PCMT1	87.61427838	66.9664526	1.308330888	4	4	59.88739425	48.73063692
chr16	2211602	2214821	16p13.3	PGP	3.46767377	2.650641466	1.308239464	4	4	98.49138709	48.31109649
chr3	153162212	153170624	3q25.2	RAP2B	27.80117818	21.25100547	1.308228838	4	4	22.74699222	11.81667675
chr12	70515871	70637440	12q15	PTPRB	0.092581089	0.070771461	1.308169817	3	4	43.32118638	57.10468729
chr3	191266465	191330536	3q28	UTS2B	0.094213304	0.072023232	1.308096035	2	2	39.62006678	2.978344003
chr9	130053706	130140169	9q34.11	GPR107	10.46003866	7.996453945	1.308084649	4	4	20.71704088	15.92620215
chr3	43286512	43351143	3p22.1	SNRK	110.3549938	84.36509709	1.308064562	4	4	35.99295039	15.05980156
chr2	32063551	32157637	2p22.3	SPAST	67.96146618	51.958198	1.308002756	4	4	29.84194335	5.670685225
chr3	122564338	122575203	3q21.1	DTX3L	81.12461145	62.02413251	1.307952375	4	4	40.3519345	51.94079752
chr1	154155304	154192135	1q21.3	TPM3	110.3546245	84.37591141	1.307892533	4	4	47.25928213	18.32009033
chr3	100260817	100325252	3q12.1-q12.2	TBC1D23	55.27430319	42.26369846	1.307843497	4	4	46.83561777	27.66929062
chr15	78167468	78234707	15q25.1	ACSBG1	0.665842447	0.509156959	1.307735139	4	4	34.16863406	32.41359132
chr7	158730997	158829628	7q36.3	ESYT2	88.56359486	67.7240719	1.307712197	4	4	41.70457583	25.68562179
chr12	7130371	7158945	12p13.31	CLSTN3	3.499834762	2.676504743	1.307613884	4	4	51.65769348	48.47773139
chr19	55308869	55334043	19q13.42	TMEM150B	2.158717903	1.650910391	1.307592413	4	4	26.96009462	56.15446086
chr19	4791716	4795559	19p13.3	FEM1A	7.997981476	6.116587393	1.30758885	4	4	42.06487855	9.007674709
chr2	238100310	238133287	2q37.3	ESPNL	0.065386317	0.050006708	1.307550921	2	2	22.11804547	4.81544865
chr1	93149742	93180689	1p22.1	TMED5	22.91018206	17.52156895	1.3075417	4	4	19.47249852	28.1608244
chr16	66720893	66751828	16q22.1	DYNC1LI2	15.1320222	11.57293897	1.307534952	4	4	10.69649634	37.55046277
chr6	25962689	25988869	6p22.2	TRIM38	19.71675392	15.07938513	1.307530363	4	4	41.80903595	20.47716328
chr17	30898983	30907629	17q11.2	TEFM	2.672363497	2.04384797	1.307515792	4	4	32.72496852	27.9971319
chr1	150067279	150145327	1q21.2	VPS45	5.487019661	4.196524476	1.307515229	4	4	16.22389396	17.02339393
chr3	28349149	28526366	3p24.1	ZCWPW2	0.467978543	0.357931185	1.307453955	4	4	45.35645845	95.91915448

chr5	137937909	138051961	5q31.2	FAM13B	255.028242	195.0697317	1.307369625	4	4	76.19435441	51.67313598
chr2	222199887	222298996	2q36.1	PAX3	0.072162681	0.055197002	1.307365936	1	1	0	0
chr19	11987617	12046275	19p13.2	ZNF433-AS1	1.306872071	0.999649293	1.307330561	4	4	42.68070243	23.31448782
chr7	141764097	141765197	7q34	TAS2R3	0.530458093	0.405795112	1.307206709	2	2	77.47901478	60.97261712
chr8	63167725	63168442	8q12.3	YTHDF3-AS1	1.436293152	1.098757341	1.307197775	4	3	45.3166808	33.92356415
chr19	21677550	21678221	19p12	MTDHP3	3.857225003	2.950879848	1.307144039	3	2	24.99264997	21.09207538
chr1	2391292	2405444	1p36.32	RER1	14.32067134	10.95636293	1.307064345	4	4	53.26329686	33.15914127
chr15	59399662	59523555	15q22.2	FAM81A	0.216730113	0.16581526	1.307057705	4	2	70.27391758	102.401422
chr19	305573	344841	19p13.3	MIER2	1.245661778	0.953092764	1.306968035	4	4	71.7180175	43.59057615
chr16	53491040	53504411	16q12.2	AKTIP	9.414231086	7.203149382	1.306960412	4	4	42.10839084	35.89194548
chr18	20949740	21111851	18q11.1	ROCK1	59.22402432	45.31537364	1.306930067	4	4	21.60705799	22.04745676
chr14	95086228	95158263	14q32.13	DICER1	38.5329727	29.48403692	1.306909661	4	4	47.81084518	24.11686126
chr17	64477785	64497066	17q23.3	POLG2	5.494027823	4.203901166	1.306887961	4	4	41.19083004	34.6491667
chr20	37101226	37179588	20q11.23	MROH8	1.093254289	0.836551012	1.306859084	4	4	34.9232968	21.73761703
chr11	17260337	17349980	11p15.1	NUCB2	32.94944839	25.21399552	1.30679203	4	4	42.94690306	23.82110973
chr14	24398786	24419288	14q12	NYNRIN	0.095368751	0.07298073	1.306766209	4	4	55.55609781	83.24890385
chr20	58689131	58715844	20q13.32	NPEPL1	0.938220296	0.717973498	1.306761737	4	4	87.56293227	36.190825
chr16	68022944	68079323	16q22.1	DUS2	8.266096348	6.325678414	1.306752542	4	4	30.6975	17.00238739
chr15	74955102	74957434	15q24.2	RPP25	0.974265329	0.745645152	1.306607207	4	4	75.74062185	80.01176721
chr3	131381671	131388830	3q22.1	NUDT16	8.611672474	6.591107741	1.306559203	4	4	53.11704161	44.40209271
chr14	54396868	54420218	14q22.2	CDKN3	78.17953471	59.83717213	1.306537925	4	4	84.67704845	49.72136901
chr7	99374254	99394805	7q22.1	ARPC1B	13.47016594	10.31057436	1.30644186	4	4	52.44243767	22.56063299
chr11	67583595	67586653	11q13.2	GSTP1	47.61227563	36.44510049	1.306410875	4	4	53.03370914	37.42472025
chr17	44186951	44191929	17q21.31	TMUB2	14.16897886	10.84604608	1.306372734	4	4	52.21020508	15.38512523
chr13	27236282	27237257	13q12.2	LINC00412	10.92645913	8.364062391	1.306357917	4	4	83.57967234	79.3356194
chr1	226631690	226739296	1q42.12	ITPKB	30.68864374	23.49218684	1.306334057	4	4	81.19445352	42.5502979
chr5	31908361	31909183	5p13.3	TPT1P5	0.656705892	0.502808874	1.306074587	2	2	31.8546338	16.80765362
chr14	91457481	91510554	14q32.12	PPP4R3A	64.63776022	49.49151934	1.306037096	4	4	25.98369856	2.291083336
chr11	45929319	46121434	11p11.2	PHF21A	20.45151537	15.66280738	1.305737527	4	4	39.76305072	19.60682923
chr8	144449582	144465648	8q24.3	CYHR1	2.977475214	2.280389078	1.305687368	4	4	70.2495824	33.37144437
chr17	50281579	50287855	17q21.33	TMEM92-AS1	0.979475353	0.750171131	1.305669216	2	3	86.54285224	86.79296941
chr11	33703010	33736479	11p13	CD59	17.8537603	13.67497171	1.305579322	4	4	21.8809283	30.51699828
chr8	100918576	100954068	8q22.3	YWHAZ	223.5512792	171.2474881	1.305428077	4	4	41.3915327	15.80664299
chr13	51598440	51599460	13q14.3	ATP5PBP1	0.784765508	0.601156903	1.305425429	2	3	52.13013699	27.31411257
chr15	43133115	43185147	15q15.2	TMEM62	4.782853101	3.663983513	1.305369711	4	4	31.91586375	30.30473836
chr1	119619377	119648173	1p12	ZNF697	1.183894912	0.906985981	1.305306739	4	4	84.5270984	40.56876683
chr12	26966524	27014427	12p11.23	TM7SF3	19.39665367	14.86065213	1.305235698	4	4	51.90224449	26.32916862
chr12	48930252	48957526	12q13.12	ARF3	26.0934588	19.99460298	1.305025103	4	4	50.83897199	21.7159698
chr7	139561570	139777894	7q34	HIPK2	10.0515236	7.70220858	1.305018358	4	4	63.11405195	18.59095773
chr9	126860665	126885878	9q33.3	ZBTB34	19.36818595	14.84185952	1.304970305	4	4	38.04177158	36.78989896
chr10	38010483	38067834	10p11.1	ZNF33A	29.89341205	22.90804418	1.304930784	4	4	34.83056134	27.58373336
chr11	63938970	63957322	11q13.1	NAA40	2.519346097	1.930644457	1.304924937	4	4	50.39906322	46.75460004
chr6	31142439	31158238	6p21.33	CCHCR1	0.754483014	0.578199368	1.304883843	4	4	101.0665232	39.36372404

chr7	1044573	1059269	7p22.3	GPR146	26.22925245	20.10083943	1.304883437	4	4	43.9691086	49.36030103
chr8	123416701	123467527	8q24.13	WDYHV1	2.645827117	2.027748499	1.304810295	4	4	24.58082738	30.02301863
chr10	88583277	88623161	10q23.31	LIPJ	0.148732475	0.114003824	1.304627075	1	3	0	53.56050861
chr1	52927229	53051617	1p32.3	SCP2	12.64730247	9.69420298	1.3046253	4	4	23.34438767	16.6241134
chr22	41433488	41448230	22q13.2	TOB2	10.10196869	7.743346244	1.304599894	4	4	44.55789381	38.73619237
chr10	110293040	110304969	10q25.2	SMNDC1	13.02915294	9.98733215	1.304567901	4	4	25.04054681	18.78163679
chrX	74028136	74293574	Xq13.2	FTX	2.490754674	1.909397474	1.304471546	4	4	42.8271951	34.8323244
chr5	53480338	53487134	5q11.2	FST	0.121990009	0.093520514	1.304419794	1	2	0	5.723582599
chr15	34084022	34101852	15q14	EMC7	21.28877773	16.32074443	1.30439992	4	4	43.19570827	34.70204124
chr3	183176116	183428269	3q27.1	MCF2L2	0.546263457	0.418806782	1.304332882	4	4	34.6810403	58.34514718
chr8	115408496	115669637	8q23.3	TRPS1	7.318458168	5.610954958	1.304315972	4	4	53.63355233	18.75625927
chr2	55547292	55618132	2p16.1	PPP4R3B	52.21719733	40.0341781	1.304315458	4	4	12.22696655	18.32161085
chr1	100266158	100292769	1p21.2	RTCA	14.73154011	11.29467087	1.304291225	4	4	37.71212506	11.58430881
chr12	123712318	123761755	12q24.31	ATP6V0A2	5.083241551	3.897829548	1.304121047	4	4	38.54219567	14.46728914
chr4	139495934	139556769	4q31.1	SETD7	3.745673534	2.872196082	1.30411484	4	4	57.75460902	32.67090948
chr14	24310151	24312053	14q12	LTB4R2	0.818863755	0.627926926	1.30407492	4	4	103.0805667	21.23025086
chr16	641804	648474	16p13.3	MCRIP2	0.52360366	0.401548602	1.303960857	4	4	71.08189281	62.52524457
chr5	35617367	35814611	5p13.2	SPEF2	0.480845699	0.368783086	1.303871349	4	4	40.17461287	56.4171158
chr2	36356088	36551135	2p22.2	CRIM1	2.780461582	2.132510224	1.303844432	4	4	45.84542097	24.1647833
chr19	7830159	7864976	19p13.2	EVI5L	1.554433493	1.192220892	1.303813331	4	4	89.32375982	54.70048983
chr5	69369297	69414801	5q13.2	RAD17	18.85010636	14.45769958	1.303810904	4	4	44.36413009	26.94123017
chr1	179882042	179920077	1q25.2	TOR1AIP1	28.75867317	22.06069958	1.303615648	4	4	21.97411982	19.6086561
chr10	88968429	89017061	10q23.31	FAS	36.87929154	28.29071622	1.303582817	4	4	26.94737985	55.06057149
chr9	129608884	129636742	9q34.11	NTMT1	2.330554736	1.787821288	1.303572539	4	4	75.26518847	36.38701847
chr2	63119559	63588799	2p15	WDPCP	2.210299481	1.695670755	1.303495667	4	4	42.50029723	14.22714152
chr12	56158261	56161582	12q13.2	MYL6	161.7723324	124.1077544	1.303482874	4	4	20.54468947	12.09193796
chr19	35154722	35169885	19q13.12	FXYD5	60.28126048	46.24981168	1.303383912	4	4	76.12803924	58.07020951
chr15	100566914	100603032	15q26.3	LINS1	32.23932336	24.73686093	1.303290803	4	4	85.89470663	39.78510092
chr9	131576770	131740068	9q34.13	RAPGEF1	24.18158815	18.55477502	1.303254182	4	4	55.67281008	31.6992353
chr6	32934629	32941070	6p21.32	HLA-DMB	6.434879454	4.937715913	1.303209736	4	4	87.04594855	52.61903393
chr17	28319041	28328685	17q11.2	TMEM97	1.835705277	1.40867353	1.303144581	4	4	46.11624785	38.74198103
chr7	55964577	56000182	7p11.2	NIPSNAP2	13.20392741	10.13240218	1.303138897	4	4	17.08622218	6.97555469
chr1	212791828	212816825	1q32.3	TATDN3	8.552572081	6.563280683	1.303094061	4	4	29.47342907	28.24902516
chr2	218874116	218893931	2q35	WNT10A	0.219787618	0.1686891	1.302915353	4	3	89.24902162	46.04267745
chr9	137205667	137219361	9q34.3	NDOR1	4.21688865	3.236727582	1.302824703	4	4	66.20340269	39.50271702
chr16	28111173	28111647	16p12.1	TPRKBP2	10.30838393	7.912409392	1.302812256	4	4	41.828781	75.95821132
chr11	2299480	2318201	11p15.5	TSPAN32	5.59921018	4.297795853	1.302809713	4	4	63.17087538	61.6554773
chr22	50185889	50199599	22q13.33	TRABD	11.23708017	8.625319474	1.302801618	4	4	63.01697349	22.81535622
chr3	141738209	141746803	3q23	RNF7	12.90150877	9.903509365	1.302720914	4	4	20.28225672	11.12268206
chr16	79202624	79601952	16q23.2	MAF	9.127480617	7.006719512	1.302675325	4	4	32.7813627	76.40340031
chr14	74019350	74078351	14q24.3	BBOF1	81.5582366	62.61249332	1.302587268	4	4	38.43117825	63.6526685
chr2	64454193	64461383	2p14	LGALSL	25.8379749	19.83809587	1.302442284	4	4	26.00231585	24.03794579
chr19	56382751	56393601	19q13.43	ZNF582	1.482858373	1.138525294	1.302437795	4	4	33.42992772	40.31949224

chr4	4418968	4542346	4p16.3-p16.2	STX18	12.00790513	9.219808785	1.302402838	4	4	19.2327282	5.491997934
chr1	235109341	235128941	1q42.3	TOMM20	41.76810099	32.07047072	1.30238503	4	4	24.67629402	15.94545512
chr12	123458101	123472362	12q24.31	SNRNP35	4.737913358	3.637981026	1.302346913	4	4	70.70349413	39.24705263
chr4	146214538	146232267	4q31.22	REELD1	0.151085607	0.116014172	1.30230303	1	1	0	0
chr5	179862071	179907856	5q35.3	TBC1D9B	3.372827602	2.589911824	1.302294376	4	4	76.87279131	41.85624616
chr1	45303910	45305619	1p34.1	LINC01144	0.625428457	0.480290032	1.302189128	1	2	0	53.50017875
chr4	127917805	127966032	4q28.2	MFSD8	14.76245291	11.33686303	1.302163823	4	4	71.5026664	45.84954091
chr2	148021011	148516923	2q23.1	MBD5	12.50015685	9.600074726	1.302089537	4	4	42.9549258	32.86046702
chr13	32031300	32299122	13q13.1	FRY	12.65635187	9.720116465	1.302078212	4	4	54.81245722	46.40194557
chr4	53996335	54064690	4q12	CHIC2	45.70294437	35.10248971	1.301985835	4	4	18.7713568	14.95104589
chr12	7729245	7751600	12p13.31	CLEC4C	8.071986193	6.199782888	1.30197885	4	4	47.83513101	82.6769707
chr11	64764604	64778844	11q13.1	SF1	33.82593392	25.98096068	1.301950853	4	4	66.493628	19.23755611
chr2	215939097	216013623	2q35	MREG	0.941056718	0.722832892	1.301900796	4	4	69.97802126	106.8211819
chr11	89870188	89878456	11q14.3	TRIM64B	0.3632221	0.278997401	1.301883456	2	3	15.94541691	33.71035651
chr15	59638062	59657541	15q22.2	GTF2A2	17.01674636	13.07137011	1.301833413	4	4	32.99783268	3.941155323
chr10	73248843	73252693	10q22.2	MRPS16	23.53174223	18.07609009	1.301815941	4	4	23.35417257	14.49529951
chr1	8011719	8026333	1p36.23	ERRFI1	0.345259423	0.265216658	1.301801426	4	4	44.8211529	43.79140313
chr18	23992773	24135970	18q11.2	TTC39C	17.18024565	13.19771375	1.301759227	4	4	22.55970595	17.61279053
chr14	22564907	22589237	14q11.2	DAD1	112.7505904	86.61617205	1.301726776	4	4	21.56706308	11.33566637
chr1	89522626	89597861	1p22.2	LRRC8B	8.967718194	6.889467855	1.301656149	4	4	20.58155773	15.81701334
chr2	230202743	230316571	2q37.1	SP140	24.04124265	18.46995384	1.301640646	4	4	53.67661657	53.76767647
chr19	17049761	17075606	19p13.11	HAUS8	1.195066579	0.918130424	1.301630517	4	4	62.292231	31.92123415
chr11	108155280	108222638	11q22.3	NPAT	92.62027519	71.15819785	1.301610749	4	4	68.79913076	21.06294133
chr20	33993652	34084884	20q11.22	RALY	23.86413698	18.3347672	1.301578401	4	4	36.16155555	14.45536269
chr16	1349240	1351872	16p13.3	TSR3	17.29891233	13.29127778	1.301523647	4	4	75.89107745	41.35737022
chr11	102711795	102724967	11q22.2	MMP8	4.990904882	3.834884167	1.301448666	4	4	45.12081898	102.0181663
chr5	68159175	68159962	5q13.1	EEF1B2P2	0.445101054	0.342017039	1.301400235	2	1	18.30080328	0
chr18	32091855	32131561	18q12.1	RNF138	66.79381978	51.33151713	1.301224345	4	4	6.533453942	11.5190537
chr11	72836743	73142682	11q13.4	FCHSD2	38.73768526	29.7716341	1.301160867	4	4	35.43297379	34.01071562
chr15	42359500	42412317	15q15.1	CAPN3	0.23659316	0.181836698	1.301129871	4	4	68.9298134	73.07502208
chr12	22625142	22690674	12p12.1	ETNK1	29.78752267	22.89417132	1.301096347	4	4	34.95553404	16.15088292
chr8	23020133	23069187	8p21.3	TNFRSF10B	10.65744764	8.191501075	1.301037202	4	4	60.571581	26.09398379
chr7	152025655	152122347	7q36.1	GALNT11	3.943298252	3.031022617	1.300979488	4	4	34.23332793	14.4680222
chr1	1373730	1375516	1p36.33	AURKAIP1	17.42801596	13.39809056	1.300783561	4	4	65.42353547	23.65793559
chr12	111452214	111599676	12q24.12	ATXN2	30.35575451	23.33736309	1.300736266	4	4	49.48776337	20.95932613
chr11	111602391	111726917	11q23.1	SIK2	5.297270352	4.073210313	1.300514814	4	4	27.96861044	27.75626641
chr21	25361795	25362924	21q21.2	RPL13AP7	0.555413118	0.427118345	1.300372894	1	2	0	2.35504781
chr6	106360808	106570449	6q21	CRYBG1	26.48673969	20.36950116	1.300313615	4	4	31.71056839	18.16148787
chrMT	7586	8269	N/A	MT-CO2	2475.540286	1903.831725	1.300293641	4	4	91.55579458	57.95749551
chr10	32009010	32056443	10p11.22	KIF5B	124.0849034	95.42946545	1.300278723	4	4	17.10456363	10.17623173
chr8	94719708	94793848	8q22.1	DPY19L4	3.685919133	2.834760559	1.300257661	4	4	34.94080428	20.39691772
chrX	116104366	116108497	Xq23	API5P1	0.219002283	0.168436376	1.300207754	1	1	0	0
chr18	74073343	74147865	18q22.3	FBXO15	0.173673741	0.133575957	1.30018714	2	3	9.632760633	35.76506985

chr19	47220822	47232998	19q13.32	BBC3	2.858542628	2.198658434	1.300130381	4	4	65.58942129	64.62207479
chr15	79843547	79923106	15q25.1	ST20-MTHFS	1.389800758	1.069012968	1.300078483	3	3	73.48550912	22.84685757
chr14	77320887	77331597	14q24.3	GSTZ1	0.764447525	0.588037685	1.299997508	4	4	81.95173253	37.56140213
chr22	32474682	32498831	22q12.3	FBXO7	561.7558185	432.1344672	1.299956058	4	4	39.84353606	59.53483637
chrX	134543377	134546630	Xq26.3	MIR503HG	0.256107403	0.197021793	1.299893779	2	3	32.0950492	25.06958744
chr15	85759326	85794958	15q25.3	KLHL25	0.3954231	0.304229769	1.299751505	4	4	113.5751242	28.98104753
chr3	194402674	194487006	3q29	ATP13A3	21.25666162	16.35546538	1.299667183	4	4	24.5939186	25.92161555
chr16	84122139	84145195	16q24.1	HSDL1	6.363015574	4.89588964	1.299664829	4	4	21.24976686	25.7583545
chr2	119366924	119372551	2q14.2	DBI	33.33590368	25.65055941	1.299617024	4	4	68.39293544	43.88341909
chrX	123859812	123913979	Xq25	XIAP	21.62108714	16.63736757	1.299549766	4	4	20.26237583	20.28602735
chr9	128255829	128276007	9q34.11	GOLGA2	8.475721015	6.522250491	1.299508663	4	4	55.61055772	32.45364858
chr8	23429152	23457731	8p21.3	ENTPD4	18.58440801	14.30128981	1.299491742	4	4	58.76524312	19.11985477
chr5	31532266	31555058	5p13.3	C5orf22	12.23587607	9.415976004	1.299480379	4	4	12.83460058	22.12690627
chr10	133308889	133313162	10q26.3	ZNF511	1.969824058	1.515945892	1.299402616	4	4	75.31548705	36.95154548
chrX	48903180	48911958	Xp11.23	SLC35A2	3.032862452	2.334266695	1.299278466	4	4	57.88220123	37.25273765
chr22	21383751	21389169	22q11.21	RIMBP3B	0.05509144	0.042405244	1.299165747	1	1	0	0
chr10	28050993	28303066	10p12.1	MPP7	22.45544274	17.28458616	1.299159988	4	4	25.48746913	34.30999953
chr7	102405742	102405855	7q22.1	MIR548O	4.513748422	3.474621392	1.299061944	1	2	0	65.58484423
chr10	119704395	119830482	10q26.11	INPP5F	3.059057196	2.35482326	1.299060209	4	4	19.5007373	25.71064914
chr18	34493290	34891844	18q12.1	DTNA	0.076661345	0.059016202	1.298988131	3	3	41.55294066	82.79676867
chr7	116210493	116258786	7q31.2	TES	44.6126861	34.34451671	1.29897551	4	4	38.79322325	25.13909117
chr3	156809271	156812021	3q25.31	PA2G4P4	0.834650966	0.642627277	1.298810362	4	4	72.0097646	45.35204977
chr10	80454265	80522631	10q23.1	TSPAN14	11.7860541	9.074866652	1.298757828	4	4	30.78144139	29.33767605
chr10	100909416	100965134	10q24.31	SLF2	22.36976523	17.22435176	1.298729006	4	4	26.25078043	15.60449025
chr20	62804800	62814000	20q13.33	OGFR	21.94625303	16.89964743	1.298621946	4	4	51.55900575	18.61843525
chr18	62523410	62587709	18q21.33	ZCCHC2	66.18553445	50.96750467	1.298582987	4	4	20.01100259	10.56747638
chr16	66932055	66934423	16q22.1	CIAO2B	11.82198463	9.104070778	1.298538304	4	4	16.47803398	31.54903073
chr2	113583653	113599037	2q14.1	WASH2P	1.719849624	1.324520492	1.298469623	4	4	81.91376614	34.06373535
chr16	29906335	29926232	16p11.2	KCTD13	1.466030252	1.1291523	1.298345894	4	4	68.9566012	60.5425866
chr8	132775358	132848807	8q24.22	PHF20L1	21.20761471	16.33666231	1.298160805	4	4	42.04499204	21.13599542
chr19	39406847	39409412	19q13.2	ZFP36	234.9157649	180.9635308	1.298138712	4	4	62.56289838	70.2581311
chr2	230327152	230407506	2q37.1	SP140L	18.9635805	14.60865939	1.298105459	4	4	16.50896237	19.09489599
chr12	53299686	53307181	12q13.13	C12orf10	11.39101073	8.775109706	1.298104651	4	4	22.59445292	16.93692287
chr10	17644125	17716824	10p12.33	STAM	168.2950447	129.6473674	1.298098435	4	4	56.38685881	21.85942118
chr20	32702691	32744008	20q11.21	COMMD7	9.440973103	7.273037838	1.29807837	4	4	40.5949932	20.650192
chr12	27710773	27756304	12p11.22	MRPS35	39.64667846	30.54437958	1.298002415	4	4	34.46154949	9.89195807
chr3	184174846	184184091	3q27.1	AP2M1	84.37509444	65.00762314	1.297926156	4	4	17.82731129	19.4056591
chr20	57487941	57489130	20q13.31	HMGB1P1	0.583994645	0.449949597	1.297911251	2	2	20.69459529	15.26669602
chr10	114120862	114174255	10q25.3	CCDC186	33.66207329	25.93763326	1.297808206	4	4	46.08733315	21.09752109
chr5	80556755	80570485	5q14.1	ANKRD34B	0.741489643	0.571355624	1.297772548	2	4	127.8271079	37.37277273
chr1	166839404	166856344	1q24.1	POGK	10.44415671	8.047778085	1.297768975	4	4	57.08044497	26.84674825
chr17	51177419	51260511	17q21.33	MBTD1	19.57462029	15.08419773	1.297690513	4	4	27.28937254	26.33257706
chr16	30370934	30377991	16p11.2	MYLPF	0.423146962	0.326115882	1.297535585	4	2	21.19866795	37.68614109

chr15	73566938	73569543	15q24.1	NPTN-IT1	1.286618997	0.991589828	1.29753146	4	4	71.09497767	47.78323431
chr12	57530050	57547331	12q13.3	DCTN2	13.09194804	10.09070213	1.297426866	4	4	28.65263861	15.2547705
chr3	47282840	47346816	3p21.31	KLHL18	4.528571839	3.490457189	1.297415093	4	4	54.16104182	26.49653992
chr9	134326463	134440586	9q34.2	RXRA	22.07463838	17.0144105	1.297408358	4	4	50.03972267	21.65364563
chr3	186147201	186362234	3q27.2-q27.3	DGKG	2.276470386	1.754771329	1.297303157	4	4	62.22918991	46.29331317
chr19	4007598	4039386	19p13.3	PIAS4	5.781535582	4.457664687	1.296987546	4	4	44.45295444	54.86227709
chr6	117675460	117710727	6q22.1	NUS1	15.44556423	11.90886306	1.296980589	4	4	31.31113117	18.01451069
chr9	113386312	113401338	9q32	ALAD	9.893313162	7.628249406	1.296931004	4	4	37.12667355	28.54300185
chr17	43398985	43401136	17q21.31	ARL4D	0.565999708	0.436428541	1.296889766	4	4	57.3904489	59.88981317
chr1	198638968	198757476	1q31.3-q32.1	PTPRC	576.2873945	444.3628536	1.296884719	4	4	46.48724423	41.69946626
chr4	119494395	119628826	4q26	PDE5A	32.67904397	25.20203165	1.296682919	4	4	31.0893982	35.09073474
chr5	69167010	69178245	5q13.2	CCNB1	97.85125562	75.46382214	1.296664453	4	4	118.3078059	80.05570454
chr2	196133583	196176503	2q32.3	STK17B	326.5627353	251.854732	1.296631327	4	4	29.02963995	13.16069432
chr12	10880961	11171623	12p13.2	PRH1	0.172280295	0.132868112	1.296626352	2	1	8.628151925	0
chr5	179837266	179858840	5q35.3	MRNIP	0.794877495	0.613051784	1.296591113	4	4	54.95748573	42.34224654
chr4	10078611	10078692	4p16.1	MIR3138	7.912771935	6.102777415	1.296585374	3	3	67.2789514	20.28424025
chr11	114400529	114410610	11q23.2	RBM7	7.154525441	5.518635042	1.296430256	4	4	53.02475941	4.478866678
chr19	1026275	1039068	19p13.3	CNN2	156.5838008	120.7887756	1.296343969	4	4	62.45209542	33.610636
chr11	74949247	74979031	11q13.4	SPCS2	16.45158542	12.69084023	1.296335399	4	4	18.45034116	28.56001594
chr17	29641611	29641702	17q11.2	RNU6-920P	11.5681356	8.924247068	1.296259003	3	4	38.0847316	85.65336062
chr3	45082274	45146422	3p21.31	CDCP1	0.407074061	0.314077977	1.296092343	4	4	76.14900712	109.4597295
chr3	153484495	153502697	3q25.2	C3orf79	0.708128394	0.546366386	1.296068742	1	1	0	0
chr3	198153206	198222513	3q29	FAM157A	0.451585361	0.348447971	1.295990791	4	4	74.39226451	91.89560202
chr2	38981549	39124959	2p22.1	SOS1	21.86579304	16.87333671	1.295878428	4	4	6.238864097	7.625664442
chr5	141639302	141651443	5q31.3	FCHSD1	6.993194623	5.396561141	1.295861279	4	4	69.63250866	16.40959154
chrX	23983716	24027186	Xp22.11	KLHL15	11.40287933	8.800567292	1.295698215	4	4	28.74507512	23.89164378
chr2	112645857	112663825	2q14.1	SLC20A1	22.5748791	17.42309368	1.295687179	4	4	24.02120795	9.158364629
chr7	75878992	75888926	7q11.23	RHBDD2	10.69979458	8.258251397	1.295648929	4	4	72.40097901	29.641207
chr8	100957883	100958180	8q22.3	RN7SL685P	1.518705244	1.172174704	1.295630455	1	1	0	0
chr1	203626787	203744081	1q32.1	ATP2B4	40.57080267	31.31473999	1.295581655	4	4	36.46569466	49.04735667
chr3	111732480	111976517	3q13.2	PHLDB2	1.274685086	0.98388765	1.295559595	4	4	46.54972773	44.50979757
chr7	73154924	73207296	7q11.23	GTF2IP4	22.78539213	17.58803846	1.295505021	4	4	58.33943991	26.66847285
chr2	86861825	86895042	2p11.2	ANAPC1P1	0.343675551	0.265283107	1.295504848	4	4	49.30670852	63.85563696
chr11	36272292	36289449	11p13	COMMD9	4.786019561	3.694638681	1.295395835	4	4	57.42990467	26.70221833
chr13	98671980	98672278	13q32.2	RN7SL60P	1.513131088	1.168084698	1.295395008	1	1	0	0
chr4	174283677	174334355	4q34.1	CEP44	9.843065042	7.598610602	1.295376952	4	4	52.4901289	57.01059975
chr12	9240003	9262877	12p13.31	LINC00987	0.836052759	0.645412979	1.295376427	4	4	75.19492729	36.24279002
chr18	31498004	31549008	18q12.1	DSG2	0.200458078	0.154752169	1.295349066	3	4	63.23554122	40.53732236
chr9	94374327	94460920	9q22.32	MFSD14B	38.28154646	29.55350104	1.295330337	4	4	23.82658848	28.60976765
chr3	50558025	50574835	3p21.31	C3orf18	0.888498545	0.685924612	1.295329734	4	4	26.78793523	54.85893202
chr1	232984109	233295730	1q42.2	PCNX2	2.923954598	2.257725222	1.295088778	4	4	27.9508314	28.92932455
chr6	82169987	82247754	6q14.1	IBTK	16.00369353	12.35785703	1.295021742	4	4	28.62823769	15.80067947
chr1	245749340	246507342	1q44	SMYD3	3.730020989	2.880366847	1.294981226	4	4	28.90341767	24.34735352

chr5	177331562	177351884	5q35.3	LMAN2	45.56554665	35.18693808	1.294956286	4	4	66.3652148	55.84534768
chr10	28898950	28899524	10p12.1	RPL21P93	0.861692022	0.665448407	1.294904326	1	1	0	0
chr3	52268278	52268361	3p21.2	MIRLET7G	4.416369748	3.410670518	1.29486848	1	1	0	0
chr7	26291895	26374330	7p15.2	SNX10	84.53151644	65.28238392	1.294859522	4	4	45.32750858	47.55235804
chr9	129827285	129835307	9q34.11	C9orf78	219.0212884	169.1500303	1.294834462	4	4	30.78773228	40.64240895
chr10	103389050	103396513	10q24.33	ATP5MD	63.88781329	49.34138415	1.294811939	4	4	7.320652949	22.7635017
chrX	9463295	9719740	Xp22.31-p22.2	TBL1X	15.35321841	11.85793661	1.29476307	4	4	65.39468466	38.06447612
chr5	157085832	157109237	5q33.3	HAVCR2	13.18305395	10.18195988	1.294746209	4	4	36.5211524	38.04636465
chr19	20167214	20200490	19p12	ZNF486	6.560082887	5.066991808	1.294670119	4	4	54.08258572	37.39812256
chr22	20111866	20117295	22q11.21	TRMT2A	4.666705177	3.604574036	1.294662041	4	4	96.84986606	34.79725239
chr20	17516793	17517342	20p12.1	RPS27AP2	0.736866883	0.569205981	1.294552248	1	1	0	0
chr16	55509001	55586670	16q12.2	LPCAT2	32.97119214	25.46989502	1.294516216	4	4	49.05048922	62.01643624
chr5	95713522	95796367	5q15	RHOBTB3	79.66484967	61.54143952	1.294491164	4	4	76.51297069	30.81898084
chr17	75272980	75289958	17q25.1	SLC25A19	1.715798625	1.325552859	1.294402266	4	4	38.1273005	57.12675154
chr6	41546426	41602384	6p21.1	FOXP4	0.822075818	0.635120459	1.294362048	4	4	78.37834924	38.26386643
chrX	48897862	48903145	Xp11.23	PQBP1	14.51117362	11.21250413	1.29419561	4	4	54.69473364	45.04126839
chr12	132084283	132126340	12q24.33	EP400P1	0.520516892	0.40221377	1.294129965	4	4	65.24946443	37.62929868
chr9	136375569	136400220	9q34.3	SNAPC4	2.485712154	1.920856236	1.294064651	4	4	55.56406651	34.76466289
chr18	46173553	46266992	18q21.1	C18orf25	10.67491671	8.249509562	1.294006223	4	4	19.67573842	21.47599253
chr14	106005095	106005532	14q32.33	IGHV1-3	8.170092721	6.313854746	1.293994406	3	3	78.80037251	132.5897037
chr1	6236240	6239444	1p36.31	LINC00337	0.197025516	0.152284337	1.293800266	1	1	0	0
chr15	30400562	30414260	15q13.2	GOLGA8R	0.360210499	0.278419468	1.293769081	3	4	52.41575532	126.2804837
chr5	14143350	14510204	5p15.2	TRIO	2.934993913	2.268578661	1.293758935	4	4	49.22011979	41.4704111
chr19	51571237	51589738	19q13.41	ZNF175	3.452284082	2.668582161	1.293677269	4	4	20.91110751	22.29866677
chr12	6870935	6873357	12p13.31	SPSB2	1.571979362	1.215142071	1.293658906	4	4	55.65183902	72.4883341
chr3	185586243	185631101	3q27.2	SENP2	19.05143583	14.72703531	1.293636868	4	4	26.62398896	16.48645386
chr2	171315746	171434803	2q31.1	METTL8	12.23146366	9.45528534	1.293611268	4	4	61.22143545	55.06635415
chr1	145168405	145216071	1q21.1	LINC01145	2.068498824	1.5990461	1.293582983	4	4	45.03552068	29.45727342
chr3	12557014	12583713	3p25.2	MKRN2	14.67968665	11.34830963	1.293557114	4	4	27.7545612	24.69739167
chr1	148102046	148152190	1q21.2	NBPF11	2.181650742	1.68669463	1.293447375	4	4	35.28660331	48.56951716
chr20	34516384	34540958	20q11.22	DYNLRB1	39.71618875	30.70841283	1.293332513	4	4	34.46703611	39.62660803
chr2	65227695	65271253	2p14	ACTR2	297.5173865	230.0506582	1.293269008	4	4	57.85213857	27.34057909
chr16	30658431	30670810	16p11.2	FBRS	15.47425472	11.96566101	1.293221888	4	4	62.34451068	19.73143167
chr11	43897303	43899888	11p11.2	SEC14L1P1	0.670907092	0.518787634	1.293221058	3	4	26.50486957	53.74277286
chr2	202206180	202238599	2q33.1	SUMO1	75.60782548	58.46592657	1.293194685	4	4	36.17979355	7.911615349
chr10	68721144	68792377	10q21.3	CCAR1	96.2483176	74.42772001	1.293178369	4	4	53.25550458	18.29382475
chr17	37609739	37643464	17q12	DDX52	6.463003759	4.998279665	1.293045646	4	4	51.74785902	22.02217688
chr1	100186922	100249864	1p21.2	DBT	3.693352407	2.856329359	1.293041503	4	4	41.93710333	20.65883557
chr5	96046983	96215614	5q15	MIR583HG	1.798417215	1.390871134	1.293014982	3	3	72.69878709	59.58935248
chr19	40191657	40215575	19q13.2	MAP3K10	1.488047119	1.150909335	1.292931662	4	4	66.4623633	40.26121654
chr5	146113026	146182787	5q32	LARS	20.80711773	16.09299739	1.292929913	4	4	52.35949037	17.01445179
chr3	57941776	57942514	3p14.3	PPIAP16	0.862762161	0.667362298	1.292794278	1	1	0	0
chr18	79679801	79754510	18q23	CTDP1	7.751549724	5.995986734	1.292789672	4	4	49.96117386	16.38199474

chr11	43576476	43856619	11p11.2	HSD17B12	6.556268735	5.071448879	1.292780208	4	4	29.9885135	40.99188583
chr22	35335640	35335758	22q12.3	MIR3909	7.570083112	5.855745716	1.292761585	2	2	0.0671191	39.22072027
chr19	58351970	58355183	19q13.43	A1BG-AS1	1.271659682	0.983699527	1.292731823	4	4	70.85762336	42.56134268
chr14	34982898	35029567	14q13.2	SRP54	38.01060498	29.40361994	1.292718551	4	4	21.54004228	6.740881187
chrX	103252995	103255193	Xq22.1	TCEAL8	31.26501908	24.18697588	1.292638618	4	4	38.68082457	23.38617791
chr12	40224890	40369285	12q12	LRRK2	148.5842621	114.9554334	1.292537967	4	4	48.49058572	60.9168531
chr10	125836340	125881315	10q26.2	DHX32	4.121693185	3.189004759	1.292470064	4	4	25.49725465	23.95632544
chr17	18780995	18806713	17p11.2	TVP23B	9.984939547	7.725570039	1.292453437	4	4	17.50823637	33.70926378
chr7	130876747	130876810	7q32.3	MIR29A	8.133674424	6.293306589	1.292432572	2	1	30.22708175	0
chr2	219498865	219506996	2q35	GMPPA	2.964767036	2.294056013	1.29236907	4	4	55.04368847	45.92348252
chr8	43073474	43073618	8p11.21	RNU1-124P	2.659793663	2.058371865	1.292183258	1	2	0	39.94948058
chr14	22540247	22540306	14q11.2	TRAJ5	21.58344436	16.70319674	1.292174468	4	4	38.10389478	27.92593122
chr2	9488486	9555820	2p25.1	ADAM17	27.19865533	21.0502187	1.292084216	4	4	29.75145998	12.89153082
chr1	206052723	206102701	1q32.1	RHEX	1.559866035	1.207395457	1.291926374	4	4	63.76871367	73.75024825
chr5	179806388	179838078	5q35.3	SQSTM1	17.21869989	13.32906934	1.291815613	4	4	37.84751406	26.69336745
chr20	46366045	46407051	20q13.12	ELMO2	12.39676085	9.596478471	1.291803122	4	4	42.43000684	31.93304584
chr12	27244145	27325959	12p11.23	STK38L	22.62672459	17.51691903	1.291706867	4	4	38.63099354	45.07863693
chr19	45406209	45410766	19q13.32	CD3EAP	0.693797575	0.537121886	1.291694853	4	4	85.71511899	64.77194315
chr14	30622254	30735828	14q12	SCFD1	124.6893849	96.5332677	1.291672683	4	4	72.16144379	38.11757412
chr2	102186973	102242910	2q12.1	IL1RL2	0.14339121	0.111015169	1.291636197	2	1	24.86642912	0
chr6	83859651	83960427	6q14.2	CYB5R4	28.52809095	22.08692657	1.291627917	4	4	51.84000204	37.8750426
chr11	9384622	9448127	11p15.4	IPO7	37.59233369	29.10540641	1.291592811	4	4	29.73499647	12.72539554
chr3	12733530	12769457	3p25.2	TMEM40	6.908218322	5.349496826	1.291377217	4	4	37.58662507	23.43942399
chr14	24136158	24138967	14q12	PSME1	168.5421036	130.5219446	1.291293231	4	4	44.18672637	36.86541053
chr1	1613730	1630610	1p36.33	MIB2	1.510934339	1.17013298	1.29125011	4	4	97.61007834	28.08706909
chr17	69148007	69244815	17q24.3	ABCA10	0.279151321	0.216189462	1.291234635	4	4	59.05976493	69.63095617
chr9	19108391	19127606	9p22.1	PLIN2	11.94077915	9.247568309	1.29123449	4	4	49.53199666	18.7184672
chr3	196932729	196942593	3q29	NCBP2	11.37409505	8.809185741	1.291163041	4	4	24.68009397	23.84792083
chr19	12668067	12671023	19p13.13	WDR83OS	10.937222	8.471194023	1.291107484	4	4	36.98577425	28.97940639
chr8	86342660	86468354	8q21.3	WWP1	21.23682936	16.44869876	1.291094795	4	4	32.33930689	27.27143708
chr16	27313668	27364778	16p12.1	IL4R	34.53746615	26.75208917	1.291019401	4	4	73.86342218	46.38816525
chr6	33410751	33416453	6p21.32	PHF1	6.100190223	4.725542813	1.29089725	4	4	46.01798249	32.31990551
chr18	31065974	31102432	18q12.1	DSC2	6.043342509	4.681576124	1.290877762	4	4	79.25326033	93.99716441
chr5	143277931	143435512	5q31.3	NR3C1	50.00611196	38.74664288	1.29059212	4	4	40.41829473	15.01249293
chr19	39391303	39400637	19q13.2	MED29	11.22197033	8.695458986	1.290555259	4	4	65.41045542	14.61520893
chr16	87392195	87404774	16q24.2	MAP1LC3B	46.57203359	36.08751142	1.290530484	4	4	20.21875044	45.50244386
chr14	106062149	106062604	14q32.33	IGHV3-7	18.72707037	14.51120748	1.29052461	4	3	42.55108861	108.5273819
chr17	4498843	4539037	17p13.2	SPNS2	0.458780695	0.355509361	1.290488367	3	4	59.65574179	49.88708439
chr5	138352596	138437027	5q31.2	KDM3B	35.14461829	27.23546671	1.290398974	4	4	55.96324103	27.55769339
chrX	15627316	15675624	Xp22.2	CLTRN	0.31519229	0.244263418	1.29037861	2	2	46.9322162	25.8794337
chr10	50021182	50067803	10q11.23	FAM21EP	0.444205443	0.344261378	1.290314483	3	3	81.16550221	65.63828884
chr19	36916329	36997932	19q13.12	ZNF568	2.023052737	1.567974111	1.290233508	4	4	32.25657275	34.55048812
chr11	74840910	74949187	11q13.4	XRRA1	3.384254148	2.623114276	1.290166494	4	4	44.44156374	67.3860802

chr8	56588392	56588781	8q12.1	RPL37P6	1.464776718	1.135379346	1.290120983	2	2	40.75195282	58.70085728
chr5	80407615	80483379	5q14.1	ZFYVE16	17.02172299	13.19446652	1.290065268	4	4	36.73132504	52.12986757
chr17	8474205	8631369	17p13.1	MYH10	7.910605099	6.132115023	1.290028819	4	4	82.50237987	43.22786316
chr12	56128202	56144676	12q13.2	ESYT1	48.48960607	37.58876808	1.290002534	4	4	37.83092779	41.52534787
chr1	2412481	2505530	1p36.32	PLCH2	0.294402123	0.228253798	1.289801638	4	4	128.2797221	54.05887331
chr11	47980504	48170842	11p11.2	PTPRJ	82.91201362	64.28682954	1.28972006	4	4	47.71692201	26.60920495
chr2	189766267	189784371	2q32.2	ORMDL1	16.64164865	12.90333538	1.289716819	4	4	22.23109904	18.13440906
chr1	25543585	25590400	1p36.11	LDLRAP1	19.55342085	15.16167424	1.289661058	4	4	94.92547644	33.92840483
chr9	128203355	128255248	9q34.11	DNM1	0.11251731	0.087249316	1.289606775	3	4	132.4552337	67.07723188
chr19	4043304	4066945	19p13.3	ZBTB7A	10.45807731	8.109984162	1.289531163	4	4	58.07945249	50.51331537
chr12	10505893	10523135	12p13.2	EIF2S3B	1.60359232	1.24363349	1.289441249	4	4	65.00712188	60.47234665
chr22	36467036	36482025	22q12.3	TXN2	21.61179665	16.76243204	1.289299583	4	4	16.79274387	18.19597919
chr1	3889133	3900293	1p36.32	C1orf174	4.719943501	3.661005663	1.289247801	4	4	44.5413421	40.64738555
chr21	14273799	14301386	21q11.2	ABCC13	15.82923204	12.27872131	1.289159649	4	4	84.29021178	124.7245702
chr6	24804281	25042288	6p22.3	RIPOR2	207.0340167	160.5974665	1.289148709	4	4	52.69584729	47.44587583
chr5	141850393	141878410	5q31.3	PCDH1	0.919319978	0.713122065	1.289148132	4	4	41.18006686	68.73416649
chr7	142587927	142588359	7q34	TRBV14	1.871108719	1.451532786	1.289057151	1	4	0	44.0076769
chr1	185118085	185157098	1q25.3	TRMT1L	37.11188859	28.79324165	1.288909704	4	4	41.20942414	26.27578
chr20	50934855	50958564	20q13.13	DPM1	69.62158616	54.01843392	1.28884866	4	4	31.68214016	24.4954888
chr19	6379569	6393280	19p13.3	GTF2F1	20.25128056	15.71294324	1.288827959	4	4	77.25021785	38.18330471
chr10	84139429	84153555	10q23.1	GHITM	145.1759486	112.6422998	1.288822661	4	4	23.52343684	14.36282056
chr5	65624731	65666233	5q12.3	TRAPPC13	6.861668684	5.324013809	1.288814967	4	4	25.78408999	28.64458085
chr13	114234845	114272723	13q34	CDC16	19.46887018	15.10606263	1.288811695	4	4	21.934852	20.19311216
chr6	27147626	27147688	6p22.1	MIR3143	11.68218355	9.064969566	1.288717349	3	1	59.32075253	0
chr16	67987390	68000644	16q22.1	DPEP2	19.42891448	15.07843857	1.288522972	4	4	52.95517494	43.36063439
chr7	80602188	80679277	7q21.11	CD36	90.25928233	70.05960958	1.288321229	4	4	50.00948208	45.01305542
chr1	38838197	38859823	1p34.3	RRAGC	2.962833166	2.299803791	1.288298236	4	4	35.58687664	28.44908865
chr2	26786014	26794589	2p23.3	CENPA	5.258522628	4.081932524	1.288243399	4	4	95.91361885	90.46157004
chr13	20403667	20525873	13q12.11	CRYL1	5.065020421	3.93206394	1.288132771	4	4	67.33266865	58.87385385
chr3	69084939	69106088	3p14.1	ARL6IP5	171.1356064	132.8574621	1.288114373	4	4	27.56628665	18.00611539
chr11	35662692	35811053	11p13	TRIM44	10.76868082	8.360579965	1.28803036	4	4	51.6680507	25.44037471
chrX	18675909	18827921	Xp22.13	PPEF1	0.509343369	0.395462516	1.287968769	4	4	58.13753546	76.03894992
chr19	21505427	21541157	19p12	ZNF429	5.931449427	4.605445919	1.287920764	4	4	56.6272414	53.95643145
chr1	85018082	85048902	1p22.3	MCOLN3	0.091795566	0.071278395	1.287845579	1	4	0	53.32325932
chr6	158168316	158199344	6q25.3	GTF2H5	2.050474845	1.592254904	1.287780519	4	4	53.07720174	10.36902323
chr17	74356416	74363655	17q25.1	BTBD17	0.180760426	0.140365924	1.287779977	1	1	0	0
chrY	12701231	12860843	Yq11.221	USP9Y	7.304278433	5.672486424	1.287667856	2	3	12.46638776	86.25196406
chr10	76869601	77637969	10q22.3	KCNMA1	0.040755235	0.031652139	1.287598114	4	3	117.7154563	38.54973922
chr22	50309032	50327060	22q13.33	DENND6B	0.676493591	0.525403233	1.287570285	4	4	86.29204012	27.32872744
chr1	169367879	169396544	1q24.2	BLZF1	15.7960006	12.26846489	1.287528696	4	4	18.56054916	27.91622727
chr15	72751367	72783785	15q24.1	ADPGK	16.20085647	12.58296128	1.287523351	4	4	33.575089	19.65995066
chr1	153728067	153774808	1q21.3	INTS3	4.207103653	3.268037424	1.287348676	4	4	57.17484705	27.71644673
chr8	95066808	95073182	8q22.1	MIR3150BHG	0.258399437	0.20073123	1.287290654	3	3	98.14270842	54.40507063

chr19	50376423	50383028	19q13.33	NR1H2	19.83324126	15.40863097	1.287151422	4	4	38.72302859	21.50377358
chr2	218217094	218254348	2q35	ARPC2	98.08509848	76.2095738	1.287044312	4	4	60.46212375	28.79167973
chr20	3917494	3917571	20p13	MIR103A2	6.600568867	5.129329294	1.28682884	2	2	23.73385769	1.144867336
chr14	22971174	22982642	14q11.2	AJUBA	0.106744975	0.082954326	1.286792142	3	3	87.54679709	46.83466082
chr2	157526767	157628887	2q24.1	ACVR1C	0.270602749	0.210298968	1.286752626	3	4	57.97317283	63.47961023
chr7	97946983	97947989	7q21.3	OR7E7P	0.551823479	0.428876694	1.286671638	3	3	75.06036084	76.08388725
chr15	82355136	82415611	15q25.2	UBE2Q2P2	18.53063015	14.40413301	1.286480077	4	4	63.25972329	31.8796314
chr6	57046532	57055739	6p12.1	KIAA1586	171.036881	132.954372	1.286432921	4	4	66.30099299	45.46065161
chr1	20499448	20508483	1p36.12	MUL1	8.05720707	6.26407345	1.2862568	4	4	56.48343419	17.11612359
chr6	44387514	44450424	6p21.1	CDC5L	26.12034004	20.30750834	1.286240518	4	4	38.71408234	29.45756839
chr17	18183553	18209954	17p11.2	ALKBH5	37.40195367	29.07856321	1.286238024	4	4	26.4550665	23.74865435
chr10	119892573	119944658	10q26.11-q26.12	SEC23IP	12.12015829	9.424292958	1.286054916	4	4	22.92249449	19.09103168
chr12	26335337	26833198	12p11.23	ITPR2	25.37065526	19.73057069	1.285855116	4	4	39.76500894	14.89827685
chrX	132023217	132075943	Xq26.2	STK26	55.8667505	43.45132822	1.285731709	4	4	38.07025814	13.61510163
chr15	63189529	63267776	15q22.2	RAB8B	128.6317421	100.0521703	1.285646696	4	4	28.40387334	28.1612069
chr9	133255176	133275214	9q34.2	ABO	0.347847104	0.27056231	1.285645083	4	4	64.01690184	64.26399592
chr11	64206662	64223891	11q13.1	FERMT3	82.11283288	63.87051439	1.285614084	4	4	70.45299721	31.38107158
chr14	77041049	77069503	14q24.3	LINC02288	0.28025122	0.218015895	1.28546233	2	2	55.29185054	38.10103845
chr7	27739373	27829767	7p15.2	TAX1BP1	72.22241634	56.18735408	1.285385609	4	4	6.410663196	20.53573455
chr19	10086119	10093252	19p13.2	C19orf66	8.843321718	6.881085989	1.285163669	4	4	50.81365744	25.0297019
chr2	102311502	102352367	2q12.1	IL1RL1	3.156284478	2.456090918	1.285084544	4	4	127.2436292	67.52847994
chr15	63153340	63157542	15q22.2	RPS27L	6.005442473	4.673287693	1.285057302	4	4	22.08037809	20.34715109
chrX	150562658	150673143	Xq28	MTM1	18.89780227	14.70580853	1.285057006	4	4	24.52061412	31.99367791
chr14	39215064	39217159	14q21.1	YTHDF2P1	0.269360761	0.209613529	1.285035186	1	1	0	0
chr1	95234155	95247225	1p21.3	RWDD3	9.217741362	7.173563848	1.284959827	4	4	29.58034894	45.58910493
chr9	136852367	136860799	9q34.3	MAMDC4	0.2279672	0.177416333	1.284927922	3	4	54.60485782	36.56516372
chr3	156539553	156555200	3q25.31	SSR3	36.14530812	28.13057357	1.284911878	4	4	23.43946087	10.63513982
chr3	75435308	75512177	3p12.3	LINC02018	0.431136684	0.335562944	1.284816133	3	2	54.32590268	19.34646
chr6	32439842	32445046	6p21.32	HLA-DRA	255.9990062	199.2549144	1.284781391	4	4	79.53645864	45.88924501
chrX	18892298	18984362	Xp22.13	PHKA2	5.154415364	4.012677123	1.284532796	4	4	52.65272457	37.20212634
chr2	61187455	61470714	2p15	USP34	48.53504004	37.78779987	1.284410318	4	4	18.75648992	16.45086258
chr2	240568526	240578732	2q37.3	RNPEPL1	11.49324718	8.948486326	1.284378917	4	4	79.82949304	29.05218194
chr2	74505043	74507694	2p13.1	PCGF1	3.331228159	2.593678052	1.284364555	4	4	37.32860493	47.7003452
chr17	39252644	39402659	17q12	FBXL20	12.64217397	9.843415733	1.284327952	4	4	16.5214671	28.10861949
chr1	77088981	77219447	1p31.1	PIGK	19.19465519	14.94584039	1.284280755	4	4	39.12164038	19.17798706
chr5	151181052	151224093	5q33.1	CCDC69	41.19560351	32.0794558	1.284174014	4	4	62.56328522	22.33450015
chr3	69019827	69052333	3p14.1	TMF1	41.12568615	32.02548095	1.284155146	4	4	14.8456367	14.09531654
chr15	58987588	59097419	15q22.1-q22.2	RNF111	17.61145619	13.71477831	1.284122557	4	4	24.24522987	9.378586138
chr11	66616582	66629934	11q13.2	RBM14	9.643148549	7.509750991	1.284083661	4	4	46.47221442	33.99976675
chr19	5586993	5622927	19p13.3	SAFB2	20.3497746	15.84800821	1.284058812	4	4	34.41761113	26.3040205
chr22	42573244	42582011	22q13.2	RRP7BP	0.960861916	0.748381907	1.283919223	4	4	72.0132273	41.14877234
chr1	205351221	205351607	1q32.1	SNRPGP10	13.71788882	10.68668609	1.283642909	4	4	14.36117923	20.8613941
chr5	169637247	170083382	5q35.1	DOCK2	57.98746953	45.17503734	1.283617523	4	4	52.14876377	29.48989589

chr20	50888918	50931213	20q13.13	ADNP	25.5074248	19.87160083	1.283611976	4	4	21.71690422	17.17542246
chr16	21953064	21983660	16p12.2	UQCRC2	25.11925119	19.57131328	1.28347295	4	4	57.64096549	18.01260395
chrX	130622330	130903317	Xq26.1	ENOX2	7.347419922	5.724764694	1.283444878	4	4	32.47132011	14.83363911
chr17	7036075	7040121	17p13.1	SLC16A13	0.866283062	0.675058	1.283272048	3	4	71.75522218	62.6549994
chr21	25562145	25575168	N/A	MIR155HG	1.150392594	0.896471218	1.28324543	4	4	47.03577481	39.08152974
chr5	170246233	170297842	5q35.1	LCP2	66.7294278	52.00143685	1.283222769	4	4	53.40675778	29.52970387
chr11	76860873	77026797	11q13.5	ACER3	8.974139135	6.995361067	1.282870041	4	4	31.77172596	39.71119774
chr6	3254325	3308224	6p25.2	PSMG4	0.92967009	0.724781453	1.282690231	4	4	47.87505285	21.98385796
chr19	7595902	7618310	19p13.2	CAMSAP3	0.122519042	0.095530161	1.282516868	2	4	70.54882867	31.56682711
chr1	43974648	43978300	1p34.1	ATP6V0B	28.0292394	21.85701371	1.282391079	4	4	37.63463361	34.40643153
chr17	42562074	42566281	17q21.2	COASY	7.909131062	6.167976387	1.282289452	4	4	66.93753399	40.44896587
chr1	179839967	179877806	1q25.2	TOR1AIP2	10.27522775	8.013199151	1.282287829	4	4	21.66680223	21.42951328
chr17	35809455	35847242	17q12	TAF15	17.79910138	13.88145828	1.282221293	4	4	30.37510662	33.37849323
chr12	103930334	103947930	12q23.3	HSP90B1	94.96086335	74.06099851	1.282197989	4	4	37.82015605	32.79990874
chrX	154487306	154490690	Xq28	SLC10A3	12.65558466	9.8708547	1.282116396	4	4	53.73260612	25.57240703
chr2	226800159	226999210	2q36.3	RHBDD1	129.5475228	101.0511996	1.28199886	4	4	67.64611082	28.55393584
chr6	31958804	31969852	6p21.33	SKIV2L	6.817701046	5.318153134	1.281967795	4	4	58.41628257	15.23155671
chr8	104489231	104589024	8q22.3	LRP12	1.425303122	1.111812967	1.281963032	4	4	38.38968401	20.29371479
chr20	58651253	58679526	20q13.32	STX16	7.064218714	5.511025722	1.28183374	4	4	31.84846706	15.43923952
chr22	44170228	44208469	22q13.31	PARVG	12.75112128	9.947652152	1.281822191	4	4	64.75647936	34.49036774
chr1	156934840	157046903	1q23.1	ARHGEF11	8.242510203	6.430479859	1.281787733	4	4	63.1380117	56.37274076
chrX	46545490	46548475	Xp11.3	ZNF674-AS1	2.199192798	1.715799048	1.281730982	4	4	63.05206251	42.12448478
chr1	241628864	241635930	1q43	CHML	7.528291527	5.874050965	1.281618354	4	4	36.13688898	22.64888175
chr5	69093949	69131072	5q13.1-q13.2	SLC30A5	13.5789462	10.59633034	1.2814763	4	4	22.00084755	22.49711345
chrX	1336574	1382689	Xp22.33 and Yp11	IL3RA	5.019048924	3.916771617	1.281424963	4	4	60.48444176	63.03405519
chr11	57376769	57381150	11q12.1	PRG3	0.375025018	0.292692712	1.281292642	2	1	45.59305401	0
chr9	37753204	37780459	9p13.2	TRMT10B	4.348445501	3.393939841	1.281238238	4	4	53.45701503	26.76400248
chr11	117144284	117178173	11q23.3	PAFAH1B2	29.67285558	23.1618459	1.281109274	4	4	16.64825972	21.54744975
chr4	105369077	105474070	4q24	PPA2	24.71331082	19.29071274	1.281098897	4	4	52.36593559	64.29355949
chr11	3000922	3057451	11p15.4	CARS	4.171184158	3.255989898	1.28108019	4	4	56.56922117	21.5643789
chr7	112762382	112790423	7q31.1	TMEM168	5.274119054	4.117241037	1.280983796	4	4	40.73286304	17.39702544
chr20	30278906	30286537	20q11.1	LINC01597	0.174376317	0.136133584	1.280920639	2	4	95.10996625	79.59322992
chr16	17102324	17470881	16p12.3	XYLT1	7.144195534	5.577725286	1.280843923	4	4	66.4452825	25.42734176
chr19	19516210	19528204	19p13.11	NDUFA13	0.441221191	0.344485951	1.280810407	4	4	124.5461341	51.93690311
chr1	16197854	16212700	1p36.13	ARHGEF19	0.841006757	0.656692497	1.280670575	4	4	66.17756648	38.76642515
chr1	23743366	23762059	1p36.11	ELOA	13.31605287	10.39854588	1.280568748	4	4	65.14589082	27.4777594
chr8	81799583	81842326	8q21.13	SNX16	9.818320442	7.667466425	1.280516914	4	4	61.89396532	53.57841131
chr3	93980139	94055678	3q11.1-q11.2	ARL13B	30.15226211	23.54862442	1.280425624	4	4	66.41797388	55.67343728
chr7	118184032	118204039	7q31.31	LSM8	29.64380256	23.15165761	1.280418148	4	4	40.09861095	29.67518714
chr7	76397524	76442071	7q11.23	ZP3	0.659219541	0.514848632	1.280414281	4	3	96.33992637	35.42742271
chr3	49020475	49023495	3p21.31	NDUFAF3	14.05943164	10.98045139	1.280405617	4	4	46.21773047	21.54479779
chr18	56602822	56638689	18q21.31	TXNL1	12.3116512	9.615449644	1.280403066	4	4	34.5184532	33.69712903
chrX	132369314	132490030	Xq26.2	MBNL3	188.8314849	147.4891654	1.280307502	4	4	25.5059103	44.44099779

chr9	4985086	5128183	9p24.1	JAK2	76.66195273	59.88311221	1.280193195	4	4	14.75082445	22.68011318
chr7	4973985	4998169	7p22.1	RNF216P1	3.407197355	2.661634523	1.280114653	4	4	28.76266494	19.4238637
chr17	4269217	4366674	17p13.2	UBE2G1	58.27690628	45.52872831	1.280002944	4	4	30.20405714	23.12663054
chr10	26697424	26747246	10p12.1	PDSS1	18.12079827	14.15703917	1.279985034	4	4	54.27728017	14.55339026
chr22	46267964	46294008	22q13.31	TTC38	8.111822186	6.33752411	1.279967073	4	4	7.603753594	32.02852606
chr1	16440672	16460090	1p36.13	NECAP2	22.56927099	17.63347078	1.279910874	4	4	59.67946035	29.80825186
chrX	75740538	75743079	Xq13.3	TTC3P1	0.298761705	0.233427817	1.279889041	3	4	33.49238141	85.79418162
chr3	49359136	49412097	3p21.31	RHOA	336.6408442	263.0314881	1.279849978	4	4	53.43786755	29.6513975
chr20	35278906	35284816	20q11.22	EIF6	14.99785838	11.71854667	1.279839455	4	4	30.49384845	28.39226235
chr3	122660587	122662120	3q21.1	EIF4BP8	0.219963857	0.171883178	1.279728821	1	1	0	0
chr15	64155815	64163155	15q22.31	PPIB	84.68606235	66.17693271	1.279691561	4	4	76.82742383	48.83761312
chr5	115578496	115626179	5q22.3	TMED7-TICAM2	0.159450662	0.124608085	1.279617308	2	3	69.21626952	40.74607751
chr3	57227737	57273471	3p14.3	APPL1	24.91289237	19.46935931	1.279594874	4	4	20.78648131	21.26120984
chr10	63167221	63522075	10q21.3	JMJD1C	98.49009577	76.97323822	1.279536863	4	4	34.456525	32.6692317
chr8	144876951	144902364	8q24.3	ZNF250	1.743711873	1.362773929	1.279531282	4	4	51.90420703	36.12675322
chr2	120013028	120179121	2q14.2	EPB41L5	2.01430003	1.574329863	1.279465046	4	4	23.7356494	35.23773438
chr1	210327873	210676298	1q32.2	HHAT	0.199536439	0.155955272	1.279446581	4	4	55.75828236	50.86962981
chr11	119044185	119057246	11q23.3	HYOU1	9.211456342	7.199710319	1.279420412	4	4	49.22488526	50.84077753
chr16	234546	269943	16p13.3	FAM234A	1.271995831	0.994305484	1.279280716	4	4	54.6298973	35.70158799
chr19	8390321	8404434	19p13.2	RAB11B	22.49824381	17.58696949	1.27925643	4	4	18.41616501	32.33431943
chr13	46553161	46753041	13q14.13-q14.2	LRCH1	6.194921717	4.842806985	1.279200624	4	4	23.15690022	14.89147053
chr17	51260536	51297933	17q21.33	UTP18	30.23663394	23.6384312	1.279130315	4	4	50.79433903	28.77483744
chrX	15243987	15270476	Xp22.2	ASB9	0.218682809	0.170971969	1.279056504	2	2	38.71961754	36.27048416
chr17	21123368	21192030	17p11.2	DHRS7B	1.468280138	1.147987123	1.279004014	4	4	64.30057066	44.60838146
chr5	139475528	139482790	5q31.2	TMEM173	10.25977336	8.021941792	1.278963825	4	4	46.57364994	32.27528424
chr8	41490401	41510980	8p11.21	GOLGA7	62.41183569	48.79944979	1.278945479	4	4	29.87380351	12.3903204
chrX	12867083	12890361	Xp22.2	TLR7	7.826630824	6.119892359	1.278883739	4	4	50.37435711	39.15062064
chr2	10878267	10885118	2p25.1	LINC01954	0.762976873	0.596618311	1.278835831	1	2	0	4.9967029
chr18	50375022	50394168	18q21.1	SKA1	3.431508051	2.683396752	1.278792653	4	4	80.15498074	29.36910265
chr7	100802565	100827521	7q22.1	EPHB4	4.487971446	3.509607129	1.278767475	4	4	46.9339058	57.56488931
chr14	24265538	24271627	14q12	RABGGTA	6.165782968	4.821887506	1.278707345	4	4	72.30459751	54.72310049
chr20	23374519	23421519	20p11.21	NAPB	6.176699946	4.830463354	1.278697196	4	4	17.58068172	31.53443948
chr8	129839593	130017129	8q24.21	FAM49B	59.76408894	46.73902039	1.278676541	4	4	29.60301039	27.79322743
chr3	42581840	42594998	3p22.1	SS18L2	11.35909587	8.884073494	1.278590939	4	4	29.53340713	21.57118473
chr9	127716064	127731600	9q34.11	TTC16	0.944731935	0.738891947	1.278579282	4	4	75.4506525	78.16989555
chr17	43483865	43544787	17q21.31	DHX8	16.65590559	13.027607	1.278508447	4	4	47.83813958	16.03524123
chr3	129314771	129316277	3q21.3	H1FX	23.13451729	18.0969137	1.278368106	4	4	66.10185442	18.22566039
chr7	102810966	103075216	7q22.1	FBXL13	11.32811691	8.862122049	1.278262345	4	4	22.24007591	63.3250379
chr2	73926826	73958961	2p13.1	DGUOK	20.98967661	16.42161857	1.278173435	4	4	19.62570362	17.59288465
chr15	64963021	64989946	15q22.31	SPG21	34.22470623	26.77697182	1.278139532	4	4	15.81668042	23.38067298
chr2	197453423	197475309	2q33.1	COQ10B	41.262933	32.2849201	1.278086886	4	4	28.94823799	35.32687872
chr14	23273635	23286100	14q11.2	HOMEZ	3.01997743	2.362970963	1.278042548	4	4	55.87415539	57.45696575
chr5	157731389	157741448	5q33.3	THG1L	5.168915573	4.044556782	1.277993079	4	4	36.55188464	11.73251988

chr3	157081667	157123002	3q25.31	LINC00880	0.181332873	0.141891804	1.277965804	2	2	29.78291967	10.35807638
chr4	168894484	169010317	4q32.3	CBR4	13.61103826	10.65068924	1.277949055	4	4	40.00765375	23.98212906
chr6	52577202	52583993	6p12.2	TRAM2-AS1	1.111270198	0.869647934	1.277839175	4	4	28.43013338	13.75441876
chrX	118346073	118449966	Xq24	WDR44	41.75961378	32.68213417	1.277750515	4	4	18.86862271	13.17272455
chr2	207109987	207110073	2q33.3	MIR2355	7.806045779	6.109422093	1.277706084	3	3	74.04308905	56.17211223
chr3	3148490	3179717	3p26.2	CRBN	30.49985058	23.87372045	1.277549121	4	4	41.7409205	20.57431182
chr5	34929540	34958964	5p13.2	DNAJC21	8.278377636	6.480119063	1.27750394	4	4	41.1191148	18.41895771
chr11	110174880	110296712	11q22.3	RDX	16.18644899	12.67079944	1.277460753	4	4	48.51692522	17.691625
chr16	88809469	88811934	16q24.3	APRT	16.0487556	12.5639445	1.277366006	4	4	38.4671382	15.60688445
chr10	102419222	102423138	10q24.32	FBXL15	1.885308487	1.47593872	1.277362307	4	4	101.8294242	44.31577181
chr10	24981979	25062279	10p12.1	ENKUR	14.66048554	11.47838408	1.277225561	4	4	81.72669752	35.54605595
chr2	203013784	203225194	2q33.2	NBEAL1	6.702927466	5.248041622	1.277224525	4	4	60.0309418	29.86436323
chrX	72181676	72263964	Xq13.1	PIN4	2.930024777	2.294060713	1.277221985	4	4	53.05292937	25.88831582
chr16	50666300	50681353	16q12.1	SNX20	23.67238491	18.53456324	1.277202198	4	4	46.0745729	24.47505699
chr20	33358839	33403030	20q11.21	CDK5RAP1	5.648102016	4.422287354	1.277190188	4	4	18.86795676	10.06708249
chr17	8878916	8965712	17p13.1	PIK3R5	32.63120942	25.54932787	1.277184652	4	4	42.29588376	20.05291235
chr22	19130279	19144726	22q11.2	ESS2	2.769529197	2.168474818	1.277178399	4	4	55.7871027	24.29774552
chr7	130070509	130135720	7q32.2	KLHDC10	21.45573498	16.80055663	1.277084769	4	4	51.26199537	20.57569461
chr3	155826511	155855358	3q25.31	SLC33A1	13.69489181	10.72398723	1.27703358	4	4	43.84139897	33.81934475
chr19	3178738	3180332	19p13.3	S1PR4	60.50899941	47.38706176	1.276909713	4	4	67.37147808	13.10756589
chr3	50341106	50345746	3p21.31	ZMYND10	0.2628651	0.205887498	1.276741438	4	3	47.22279872	31.17058398
chr19	44742620	44760044	19q13.32	BCL3	11.33423464	8.877928502	1.276675594	4	4	68.67349413	24.10568597
chr18	63389190	63422519	18q21.33	VPS4B	59.66396203	46.7370515	1.276588063	4	4	39.87071913	40.04225705
chr14	69191477	69244020	14q24.1	EXD2	1.409731004	1.104296872	1.276586976	4	4	18.74995409	28.77613493
chr1	161120977	161132783	1q23.3	DEDD	16.57076591	12.98182707	1.276458685	4	4	31.35438092	19.66966175
chrX	56563593	56567010	Xp11.21	UBQLN2	12.42850115	9.736900843	1.276432959	4	4	30.66546854	24.6527755
chr11	124622846	124635926	11q24.2	TBRG1	8.667495407	6.790424777	1.276429044	4	4	41.49140276	21.16690955
chr12	105173297	105236230	12q23.3	APPL2	14.27291147	11.18264917	1.276344384	4	4	29.43684884	27.21021015
chr19	55599772	55603138	19q13.42	ZNF524	5.521940437	4.326753388	1.27623184	4	4	62.61379795	23.87656311
chr10	48306639	48439360	10q11.22	MAPK8	22.77191469	17.84571453	1.276043873	4	4	75.51517015	61.67128398
chr19	57807082	57815989	19q13.43	ZNF552	9.832969883	7.705915971	1.276028693	4	4	72.71616204	33.95437885
chr14	21750212	21751630	14q11.2	RPL4P1	0.885111644	0.693648888	1.27602258	4	4	51.34749276	25.8805516
chr16	58463645	58515403	16q21	NDRG4	0.033903807	0.026570443	1.275997063	1	1	0	0
chr13	28700080	28719013	13q12.3	SLC46A3	11.44745008	8.971807616	1.27593575	4	4	24.99979394	31.0444675
chr1	224928422	225399294	1q42.12	DNAH14	5.593923704	4.384195924	1.275929224	4	4	70.37976223	52.40091498
chr15	80059568	80138393	15q25.1	ZFAND6	201.3840149	157.8349912	1.275914887	4	4	58.54338198	41.99935127
chr9	33025203	33039907	9p21.1	DNAJA1	131.5483809	103.1016292	1.275909817	4	4	40.66281761	16.59492159
chr15	42491244	42533061	15q15.1-q15.2	SNAP23	58.88247166	46.16061528	1.275599801	4	4	52.74258749	28.16505214
chr9	5510438	5571282	9p24.1	PDCD1LG2	0.775258023	0.607879858	1.275347443	4	4	24.91920995	83.1272698
chr4	36005186	36244833	4p14	ARAP2	41.32282128	32.40443644	1.275221106	4	4	24.67842035	38.88551352
chr6	44126628	44155519	6p21.1	TMEM63B	49.61202957	38.90705082	1.275142385	4	4	44.47107814	32.37812437
chr1	62194802	62212328	1p31.3	L1TD1	0.517870404	0.40616003	1.275040295	4	4	62.35616005	74.37957441
chr14	49893018	49897054	14q21.3	ARF6	95.40208872	74.82456477	1.275010272	4	4	39.34918646	35.54451355

chr17	3668723	3669669	17p13.2	EMC6	14.05307372	11.02285556	1.274903191	4	4	75.04261747	57.09935405
chr1	211940328	212035660	1q32.3	INTS7	13.7820535	10.81035611	1.274893571	4	4	52.45990027	23.81282922
chr19	14031740	14052919	19p13.12	IL27RA	21.4490322	16.82502718	1.274828978	4	4	33.15027073	40.71920452
chr1	34981526	34985347	1p34.3	TMEM35B	0.347954061	0.272954553	1.274769216	3	3	29.55490449	7.876830496
chr13	28300346	28495128	13q12.3	FLT1	0.390181515	0.306107874	1.274653637	4	4	34.8113028	51.59670341
chr1	153967487	153974364	1q21.3	CREB3L4	1.105800157	0.867586556	1.274570415	3	4	14.13082249	37.17760388
chr8	27284886	27311319	8p21.2	TRIM35	10.25493009	8.04651908	1.274455449	4	4	27.56447567	36.07248332
chr17	82752048	82943186	17q25.3	TBCD	5.580552439	4.378835741	1.274437492	4	4	43.20134453	36.50258239
chr1	202193990	202319761	1q32.1	LGR6	1.051581009	0.825144727	1.274420081	4	4	31.98286185	36.33063892
chr15	42313987	42314679	15q15.1	BNIP3P5	0.927074592	0.72754715	1.274246751	2	2	54.25093388	30.64479777
chr19	27790493	27793940	19q11	LINC00662	0.670713816	0.526420952	1.274101673	4	4	75.39459274	44.14877877
chr15	79843547	79897285	15q25.1	MTHFS	3.871176126	3.038416733	1.274076753	4	4	66.76250007	47.80362281
chr7	149126416	149183042	7q36.1	ZNF398	7.359808349	5.776876161	1.274011792	4	4	34.93982138	18.99180248
chr19	8444575	8489118	19p13.2	HNRNPM	166.1409302	130.4122097	1.273967603	4	4	8.497263362	12.77687149
chr1	150149434	150160065	1q21.2	PLEKHO1	19.39686949	15.2264692	1.273891487	4	4	48.07545943	27.20275236
chr2	73828911	73873661	2p13.1	STAMBP	6.357739641	4.990938407	1.273856562	4	4	38.84810237	19.61874609
chr1	247099962	247104400	1q44	ZNF669	7.257147695	5.697768496	1.273682442	4	4	50.17892973	28.26477564
chr16	4616493	4690974	16p13.3	MGRN1	6.028454344	4.733510666	1.273569401	4	4	56.19478425	14.09132111
chr7	6590021	6608726	7p22.1	C7orf26	7.50652448	5.894519438	1.273475227	4	4	48.0049232	29.79063134
chr2	89244781	89245566	2p11.2	IGKV2-30	2.49265558	1.957521832	1.273373068	3	2	48.48422954	86.23112461
chr7	66075789	66093343	7q11.21	ASL	2.447831105	1.922329495	1.273367084	4	4	67.17399498	48.25690898
chr2	98444538	98594068	2q11.2	INPP4A	14.65476962	11.50905653	1.273325018	4	4	41.97006735	20.07086391
chr19	42297033	42302800	19q13.2	PAFAH1B3	2.489972032	1.955497471	1.273318973	3	4	31.93372468	68.00299011
chr16	67518423	67546788	16q22.1	RIPOR1	2.301327995	1.807633311	1.273116612	4	4	85.83681814	29.14580064
chr2	65086854	65130301	2p14	RAB1A	53.46245186	41.99360594	1.273109338	4	4	16.61297244	9.459738994
chr11	6448613	6474459	11p15.4	TRIM3	0.690709806	0.542552959	1.27307352	4	4	57.35953158	16.99023281
chr15	99105069	99136586	15q26.3	SYNM	3.464432178	2.721368961	1.273047583	4	4	83.93323186	40.39463748
chr1	151291797	151327715	1q21.3	PI4KB	20.09696472	15.78675305	1.273027117	4	4	40.14075498	25.21705521
chr2	219229757	219236669	2q35	ANKZF1	6.660727338	5.232326543	1.272995346	4	4	47.50066176	36.40988441
chr14	65410592	65412617	14q23.3	FUT8-AS1	1.50679599	1.183688403	1.272966759	2	4	27.61461143	46.52786368
chr16	69187147	69309052	16q22.1	SNTB2	6.029874964	4.736923029	1.272951857	4	4	40.39589742	17.42464092
chr15	40323669	40326715	15q15.1	INAFM2	23.05330628	18.11079475	1.272904177	4	4	42.04687861	20.65648184
chr5	65926554	66081023	5q12.3	ERBIN	188.7663859	148.3032842	1.272840226	4	4	18.02672884	14.44460573
chr3	188152152	188890671	3q27.3-q28	LPP	9.509239402	7.47094137	1.272830147	4	4	40.76267248	27.26125221
chr17	5501399	5584512	17p13.2	NLRP1	14.19700507	11.15396201	1.272821716	4	4	59.66016451	37.08673828
chr4	83090048	83114758	4q21.22	PLAC8	57.98104482	45.55330349	1.272817565	4	4	51.3539899	38.36849824
chr17	32485028	32491256	17q11.2	CDK5R1	3.781009474	2.970633333	1.272795748	4	4	48.45965308	25.40532188
chr1	54806063	54842264	1p32.3	LEXM	0.765237844	0.601232787	1.272781293	4	4	69.93167281	57.01681021
chr11	58579114	58621550	11q12.1	ZFP91	22.32767779	17.54434262	1.272642599	4	4	29.78005591	21.83962068
chr5	132688808	132737578	5q31.1	KIF3A	3.991544394	3.13661155	1.272565739	4	4	64.32961731	37.38403128
chr17	19188016	19188232	17p11.2	SNORD3A	7803.701002	6132.4285	1.272530288	4	4	109.5906609	110.0770469
chr13	21671076	21704501	13q12.11	FGF9	0.203301027	0.159796674	1.272248179	4	4	66.56585552	104.0792951
chr3	151873643	151884619	3q25.1	SUCNR1	2.251165257	1.769497656	1.272205843	4	4	36.38755389	62.14276156

chr10	131900644	131971535	10q26.3	PPP2R2D	13.00750374	10.22459148	1.272178332	4	4	22.23056026	14.57107782
chr8	120395844	120445407	8q24.12	MRPL13	3.376255225	2.654063655	1.272107856	4	4	29.44193958	40.69428697
chr6	99425442	99579288	6q16.2	TSTD3	23.7224176	18.64829882	1.272095531	4	4	69.23525646	38.60330385
chr2	44317607	44361862	2p21	PREPL	8.325352354	6.545592448	1.271902035	4	4	32.51882837	42.87289081
chr20	2295967	2341079	20p13	TGM3	2.890379978	2.272929825	1.271653857	4	4	61.72792426	86.66951985
chr17	59619689	59696956	17q23.1	CLTC	49.68513528	39.07171785	1.271639386	4	4	24.00337691	6.941796219
chr2	3497919	3519579	2p25.3	ADI1	3.863033866	3.038128403	1.271517643	4	4	51.74796617	25.20975737
chr6	151654148	152129604	6q25.1-q25.2	ESR1	0.157785197	0.124097449	1.271462051	4	4	92.60965504	85.16556935
chr2	230416156	230545602	2q37.1	SP100	56.70607283	44.59924973	1.271457999	4	4	36.35192826	5.70236653
chr14	22478872	22478934	14q11.2	TRAJ57	5.799976272	4.562107862	1.271336945	1	4	0	29.28171677
chrX	20006713	20116996	Xp22.12	MAP7D2	0.191359895	0.150520325	1.271322629	3	3	78.21477338	36.44709504
chr17	43125557	43153671	17q21.31	NBR2	1.177641794	0.926408233	1.271190985	4	4	73.39699731	30.11840343
chr3	197509783	197573323	3q29	BDH1	0.230078782	0.181009965	1.271083508	4	4	38.34440384	104.8525022
chr5	98855204	98929772	5q15-q21.1	CHD1	57.28168181	45.06535963	1.2710801	4	4	38.93993195	11.95872141
chr19	7903780	7914483	19p13.2	MAP2K7	9.926585148	7.809603389	1.27107417	4	4	11.89124874	38.62387646
chr6	135283972	135497771	6q23.3	AHI1	3.557886854	2.799493576	1.270903739	4	4	32.34057916	18.01470312
chr1	240489573	240612372	1q43	GREM2	0.080580235	0.063403913	1.270903192	2	3	21.48334507	5.694021528
chr15	44288709	44415761	15q15.3	CASC4	62.76166466	49.3835739	1.270901632	4	4	11.68626306	13.62756723
chr7	129434433	129488399	7q32.1	STRIP2	1.569445418	1.234956956	1.2708503	4	4	53.3746458	79.77150175
chr6	2887265	2903318	6p25.2	SERPINB9	44.06939088	34.67945828	1.270763532	4	4	43.57397367	54.82281818
chr6	20099684	20212464	6p22.3	MBOAT1	15.35970504	12.08852457	1.270602127	4	4	19.8878352	44.25842493
chr14	73235841	73274640	14q24.2	PAPLN	0.157266424	0.123774121	1.270592126	3	2	46.95405171	57.37870392
chrX	13689121	13709825	Xp22.2	RAB9A	16.71753876	13.15736902	1.270583712	4	4	30.1911216	11.93873049
chr11	65120627	65122200	11q13.1	FAU	137.6748104	108.3636533	1.270488823	4	4	35.51358235	15.80317604
chr1	100345001	100520281	1p21.2	CDC14A	32.72639784	25.75902618	1.270482727	4	4	29.37374904	23.75799539
chr16	29962030	29973052	16p11.2	TMEM219	13.4872619	10.61672274	1.270379027	4	4	52.06043591	13.97019594
chr13	20747765	20748102	13q12.11	RANP8	0.477562714	0.375933961	1.270336718	1	1	0	0
chr3	169966136	169998373	3q26.2	SEC62	141.2986223	111.2478607	1.2701244	4	4	25.79103264	30.05397441
chr14	54938938	55027106	14q22.2-q22.3	WDHD1	27.80161746	21.88965256	1.270080344	4	4	79.53685611	43.25378851
chr12	89347825	89352859	12q21.33	DUSP6	133.3501609	104.9944671	1.270068457	4	4	75.40994582	53.79676015
chr20	34704308	34713439	20q11.22	TP53INP2	4.312713618	3.395666524	1.270063944	4	4	103.617111	39.13526568
chr1	53087179	53142632	1p32.3	SLC1A7	0.188609238	0.148513194	1.269983038	4	3	40.60901735	89.24679118
chr15	74899174	74907228	15q24.1-q24.2	FAM219B	4.613901008	3.633285032	1.269897893	4	4	27.69278489	30.24850384
chr11	86302211	86345943	11q14.2	HIKESHI	12.20506594	9.611468373	1.269844051	4	4	54.24608848	16.19968701
chr5	149063317	149109787	5q32	SH3TC2-DT	0.155556755	0.122504445	1.269804986	1	1	0	0
chr7	139228408	139329421	7q34	UBN2	15.64080609	12.31797418	1.26975474	4	4	34.25857259	32.52313196
chrX	103376323	103378164	Xq22.2	BEX3	54.97197761	43.29541566	1.269695111	4	4	36.4607869	47.98712927
chr10	126905409	127452517	10q26.2	DOCK1	0.756296936	0.595701165	1.269591165	4	4	64.0956944	19.44028484
chr20	64042350	64072347	20q13.33	TCEA2	0.882012135	0.69472572	1.269583248	4	4	87.01997366	40.3243914
chr18	79972867	80033935	18q23	TXNL4A	6.835305295	5.383927448	1.269576041	4	4	24.9302425	10.79837979
chr5	43526267	43557419	5p12	PAIP1	20.12060035	15.84854339	1.26955518	4	4	16.35002498	12.57508745
chr2	177230303	177265131	2q31.2	NFE2L2	98.11838538	77.29319385	1.269431117	4	4	38.80378791	53.56743357
chr4	139057258	139177218	4q31.1	ELF2	82.51693909	65.00792998	1.269336512	4	4	78.07226593	62.55368039

chr1	77881348	77889539	1p31.1	NEXN-AS1	0.212358537	0.167311252	1.269242413	3	2	28.36260017	30.18830108
chrX	115560850	115650861	Xq23	PLS3	0.977077913	0.769815852	1.269235897	4	4	58.5201274	50.31050686
chr21	13936993	13937525	21q11.2	RHOT1P2	8.681019251	6.840254184	1.269107699	4	4	120.8679672	50.76921173
chr16	31489475	31508419	16p11.2	C16orf58	6.353468279	5.006306433	1.269092966	4	4	43.50087555	22.52030361
chr9	93451696	93566115	9q22.31	FAM120A	73.70373723	58.0769194	1.269071052	4	4	14.93661075	18.89342992
chr4	25715910	25863595	4p15.2	SEL1L3	12.88921641	10.15734972	1.268954675	4	4	41.98148061	42.25558756
chr3	185482671	185499057	3q27.2	TMEM41A	1.948249693	1.535448128	1.268847614	4	4	50.81933259	24.34043219
chr8	96239398	96261613	8q22.1	MTERF3	8.068939222	6.359443289	1.268812199	4	4	64.34339418	20.64786329
chr2	119759631	119984899	2q14.2	PTPN4	76.29108384	60.12828624	1.268805226	4	4	46.01389181	19.09139043
chr22	32205115	32273615	22q12.3	SLC5A4-AS1	7.647672235	6.027917241	1.268708897	4	4	91.95620007	62.32565975
chr3	143001530	143060725	3q23	U2SURP	30.32584862	23.90729013	1.26847704	4	4	31.39335558	7.818914774
chr10	11920022	12043170	10p14	UPF2	66.21723492	52.21205631	1.268236488	4	4	9.59849261	16.54836598
chr11	126283087	126294933	11q24.2	TIRAP	2.96097033	2.33472158	1.268232733	4	4	66.03501227	34.78255926
chr12	14567732	14619755	12p13.1-p12.3	PLBD1-AS1	6.255182269	4.932239554	1.268223532	3	4	77.50294044	67.26759933
chr10	24583609	24725421	10p12.3	ARHGAP21	15.57064682	12.27759643	1.268216211	4	4	32.7695226	3.465231868
chr17	7646627	7657771	17p13.1	ATP1B2	2.675219043	2.109458898	1.268201549	4	4	42.99505797	45.97226646
chr15	65083036	65109132	15q22.31	UBAP1L	0.43648917	0.344212699	1.268079797	4	4	86.90334593	55.07320241
chr12	57744989	57748243	12q14.1	TSPAN31	2.782930776	2.194660218	1.268046303	4	4	50.51442785	31.87063778
chr3	180912446	180982753	3q26.33	FXR1	24.93386288	19.66387221	1.268003708	4	4	31.72902353	8.038693128
chr10	15778169	15860533	10p13	MINDY3	64.04707595	50.51205014	1.267956374	4	4	38.9408124	12.19192767
chr9	5629030	5778633	9p24.1	RIC1	17.29565413	13.64068103	1.267946526	4	4	49.68679035	16.27830487
chr17	7561992	7579006	17p13.1	SENP3-EIF4A1	0.136041685	0.107293399	1.267940862	2	2	55.56890982	36.84664972
chrX	56729219	56819179	Xp11.21	NBDY	8.384985523	6.613563542	1.267846822	4	4	57.48204459	55.41760186
chrX	139924148	139924231	Xq27.1	MIR505	3.833020725	3.023395852	1.267786592	1	1	0	0
chr2	24202864	24360538	2p23.3	ITSN2	28.80350178	22.71980762	1.267770496	4	4	29.93034135	17.55779131
chr19	36114949	36125948	19q13.12	TBCB	9.573146569	7.551632243	1.267692369	4	4	52.50052613	45.03693005
chr13	113165002	113209467	13q34	PCID2	5.424466865	4.279584286	1.267521914	4	4	39.81720202	24.09315274
chr8	37736579	37757801	8p11.23	ERLIN2	3.658960656	2.886786945	1.267485522	4	4	43.68035477	26.95870294
chr15	99257525	99260276	15q26.3	HSP90B2P	0.407092456	0.321182792	1.267479035	3	4	8.408547869	74.18523507
chr1	154548575	154558644	1q21.3	UBE2Q1	25.25766741	19.92804574	1.267443268	4	4	58.2864116	35.28947447
chr19	17281645	17287646	19p13.11	ANKLE1	0.75894108	0.598797123	1.267442763	4	4	66.19894891	78.89035632
chrX	47836864	47925626	Xp11.23	ZNF81	3.672582302	2.897929392	1.267312555	4	4	36.13517511	24.39993244
chr5	141966807	141990291	5q31.3	RNF14	74.99339048	59.17914077	1.267226754	4	4	45.57532315	114.7593447
chr7	815557	874920	7p22.3	SUN1	3.301690337	2.605515701	1.267192647	4	4	52.6794451	47.3664466
chr3	141876592	141926548	3q23	ATP1B3	28.41766908	22.42633531	1.267156167	4	4	22.07187042	26.3415421
chr2	70281362	70293771	2p13.3	SNRPG	24.2587658	19.14549111	1.267074616	4	4	25.92780377	30.12058244
chr1	1335278	1349142	1p36.33	DVL1	2.83342407	2.23624082	1.267047826	4	4	66.05789455	27.81001647
chr9	133761894	133992374	9q34.2	VAV2	2.388834609	1.885393463	1.267021794	4	4	62.22756035	42.51750585
chr1	22052627	22092943	1p36.12	CDC42	343.5424354	271.165426	1.266910906	4	4	19.95499614	14.1872252
chr12	95076749	95217464	12q22	FGD6	2.703685466	2.13409503	1.266900222	4	4	39.25564333	23.50005037
chr6	29926659	29929839	6p22.1	HLA-K	0.979559994	0.7732158	1.266864948	2	2	102.4693849	27.89793471
chr6	4115693	4135597	6p25.2	ECI2	3.640072196	2.873381372	1.266825292	4	4	45.60194902	41.50219412
chr1	93448067	93569671	1p22.1	FNBP1L	6.545168169	5.16670877	1.266796419	4	4	54.79714541	38.84865706

chr3	50350695	50368197	3p21.31	CYB561D2	2.480680846	1.958238608	1.26679192	4	4	79.0837875	69.38430766
chr14	54509869	54539310	14q22.2	CGRRF1	5.042468289	3.980915404	1.266660498	4	4	40.50794173	33.5220679
chr12	109850943	109880488	12q24.11	GLTP	21.76911101	17.18733546	1.266578584	4	4	60.56487916	19.43652534
chrX	109535781	109544698	Xq23	NXT2	16.57700856	13.0880241	1.266578395	4	4	21.44631517	19.73174165
chr20	46118242	46129745	20q13.12	CD40	3.404453946	2.6880784	1.266500987	4	4	36.8192125	29.99209402
chr19	10391128	10403595	19p13.2	CDC37	33.62040141	26.5461811	1.266487307	4	4	71.34649964	49.9418598
chr1	120850819	120850985	1p11.2	RNVU1-19	105.2433031	83.11130303	1.266293504	4	4	58.22691364	52.87461842
chr22	29508167	29553760	22q12.2	THOC5	9.850790003	7.779317141	1.266279524	4	4	58.00302388	35.95683555
chr6	110099819	110180004	6q21	WASF1	3.962912107	3.129642692	1.266250655	4	4	63.01113544	32.41910449
chr7	36852906	37449326	7p14.2-p14.1	ELMO1	12.06501445	9.528664275	1.266181083	4	4	43.27538518	13.16566081
chr1	19074506	19214332	1p36.13	UBR4	15.76443441	12.45100325	1.266117604	4	4	43.17060771	12.27257612
chr6	33112516	33129113	6p21.32	HLA-DPB2	0.198596542	0.15686603	1.26602644	2	2	40.24076113	8.957405987
chr1	203007367	203024848	1q32.1	TMEM183A	28.94893152	22.86911739	1.265852592	4	4	18.7828966	13.27772291
chr11	67119269	67258082	11q13.2	KDM2A	28.68415237	22.6602474	1.265835799	4	4	44.96854126	11.66612561
chr6	111555378	111606300	6q21	TRAF3IP2	2.329970921	1.840671708	1.265826443	4	4	19.07030125	63.77506506
chr2	27771717	27779741	2p23.2	MRPL33	18.47097	14.59274695	1.265763743	4	4	16.75517451	28.52101486
chr19	16517889	16542452	19p13.11	CHERP	3.127283346	2.470673486	1.265761488	4	4	83.39957743	50.6018066
chr4	82818509	82900571	4q21.22	SEC31A	28.45932511	22.48405478	1.265755906	4	4	16.70558618	15.30737832
chr2	130356007	130375409	2q21.1	PTPN18	30.7523174	24.29621272	1.265724735	4	4	71.83501049	47.44426447
chr9	135808487	135907551	9q34.3	CAMSAP1	9.577095346	7.566517604	1.265720355	4	4	26.73707528	23.3988231
chr16	68530249	68576072	16q22.1	ZFP90	8.386735183	6.626430662	1.265648976	4	4	43.98676497	23.42787018
chr1	228106679	228111746	1q42.13	MRPL55	4.926374168	3.892374091	1.265647662	4	4	60.32616797	31.00764642
chr9	40992337	41014139	9p11.2	FRG1HP	18.57555925	14.67675836	1.265644551	4	4	21.06833889	41.31868869
chr2	97113153	97264521	2q11.2	ANKRD36	15.87887408	12.5465162	1.265600253	4	4	40.92698218	22.58970445
chr7	151053812	151057965	7q36.1	CDK5	1.882257909	1.487440109	1.265434418	4	4	85.18853131	59.69635216
chr19	48637942	48646312	19q13.33	CA11	1.259376131	0.995217484	1.265428061	4	4	68.82094433	4.995966926
chr17	62966523	63427706	17q23.2-q23.3	TANC2	1.722494448	1.36124975	1.265377237	4	4	41.40836645	40.32563976
chr1	173608336	173705430	1q25.1	ANKRD45	0.252567647	0.199600682	1.265364652	4	4	53.37288613	80.68845837
chr16	1938229	1943326	16p13.3	MSRB1	53.77826132	42.50245641	1.265297723	4	4	74.70997059	44.3286799
chr11	3675010	3797792	11p15.4	NUP98	76.17276871	60.20259604	1.265273821	4	4	25.49282751	8.728488765
chr14	102139366	102224686	14q32.31	WDR20	45.80813268	36.20597296	1.265209272	4	4	59.22398251	52.59296174
chr11	17086575	17207996	11p15.1	PIK3C2A	22.95141885	18.14051858	1.265201915	4	4	21.8798485	9.284384008
chr22	23765834	23767972	22q11.23	CHCHD10	3.770572698	2.980301395	1.265164894	4	4	82.98489828	41.17684465
chr3	170357678	170396849	3q26.2	SKIL	20.11221386	15.89697105	1.265160124	4	4	20.4104558	16.33627034
chrX	13712242	13734635	Xp22.2	TRAPPC2	8.337435001	6.590049321	1.265155175	4	4	37.38092004	32.7457079
chr10	45614567	45672962	10q11.22	ZFAND4	56.86120668	44.94532586	1.265119467	4	4	72.17590579	88.13744432
chr20	32189146	32190360	20q11.21	TSPY26P	0.622410074	0.491986589	1.26509561	4	4	61.45814554	110.3889456
chr14	106301395	106301838	14q32.33	IGHV2-26	3.671712869	2.902329745	1.265091562	3	3	85.50272697	71.40383018
chr2	27370496	27377533	2p23.3	SNX17	15.97121062	12.62654379	1.264891714	4	4	19.29521462	9.019188255
chr4	70704204	70808622	4q13.3	RUFY3	3.784537251	2.991987163	1.264890872	4	4	47.71918044	21.85288524
chr12	55743278	55757194	12q13.2	GDF11	1.160228227	0.917267627	1.264874278	4	4	55.25816819	53.44048206
chrX	74242608	74242908	Xq13.2	RN7SL648P	3.966804431	3.13627296	1.264814792	3	4	79.30244533	81.82805459
chr16	30710537	30710665	16p11.2	SNORA30	3.960094947	3.131045578	1.264783552	3	3	20.01464023	41.19994656

chr4	6782732	6884172	4p16.1	KIAA0232	33.76635865	26.7001362	1.264651176	4	4	41.87942963	24.20021443
chr10	91906588	91909501	10q23.32	FGFBP3	0.33681001	0.266329019	1.264638797	3	4	67.15346247	32.5464223
chr11	73787872	73864611	11q13.4	MRPL48	2.998789416	2.371265838	1.264636536	4	4	37.85226562	13.34862548
chr2	173871572	173872567	2q31.1	RPL5P7	0.532691753	0.421225706	1.26462309	1	3	0	30.48319526
chr7	38335041	38335514	7p14.1	TRGV7	8.649549244	6.840215745	1.264514098	4	4	66.96339526	32.20092008
chr2	74549020	74557551	2p13.1	DOK1	10.51351931	8.314388237	1.26449704	4	4	47.73905857	42.36023419
chr3	52228610	52239167	3p21.2	TWF2	20.6907493	16.36304666	1.264480248	4	4	52.69105534	28.64960277
chr19	5205503	5340803	19p13.3	PTPRS	1.173267974	0.92791894	1.264407831	4	4	106.8232886	56.66746097
chr12	108782690	108857606	12q24.11	SSH1	4.82193608	3.814110643	1.264236026	4	4	55.16044971	21.73447621
chr6	125986430	126079719	6q22.32	TRMT11	11.08960948	8.77239709	1.264148142	4	4	36.98970634	47.59558233
chr7	140333752	140398550	7q34	SLC37A3	5.541335995	4.383678511	1.264083573	4	4	30.70254443	64.83114143
chr22	39447167	39492194	22q13.1	MGAT3	0.997794815	0.789342623	1.264083283	4	3	123.490422	61.49255307
chr8	22089154	22104380	8p21.3	FAM160B2	1.378897087	1.090844943	1.264063326	4	4	58.90251383	36.81875209
chr10	27154479	27187953	10p12.1	MASTL	74.98875797	59.32480037	1.264037258	4	4	59.59203905	20.86443655
chr11	118572387	118603033	11q23.3	ARCN1	64.18279659	50.77677399	1.264018793	4	4	28.84562299	19.04373673
chr6	36354221	36387800	6p21.31	ETV7	6.733655383	5.32725129	1.264001831	4	4	97.36570335	102.7471663
chrX	12975107	12977227	Xp22.2	TMSB4X	2524.595105	1997.508081	1.263872286	4	4	21.07901706	41.50748985
chr10	120851175	120909526	10q26.12	WDR11	8.945043664	7.07749983	1.263870559	4	4	30.62501059	26.69898262
chr7	144355396	144380632	7q35	ARHGEF5	0.437179434	0.345912714	1.263843207	4	4	79.63219014	81.26789702
chr2	61477934	61538522	2p15	XPO1	283.67467	224.4540908	1.263842726	4	4	68.95551786	39.38097658
chr6	75252922	75284916	6q14.1	TMEM30A	60.86849637	48.16563028	1.263732998	4	4	18.51133209	31.01296983
chr16	30761612	30800231	16p11.2	RNF40	23.33763426	18.4691252	1.26360258	4	4	27.00858854	35.69071722
chr14	21749190	21749705	14q11.2	TRAV5	5.641133197	4.464901291	1.263439621	4	3	56.37421162	25.15643467
chr18	79964582	79970822	18q23	HSBP1L1	1.408713088	1.115002429	1.263417057	4	4	60.07416219	36.6211351
chr14	105489855	105499248	14q32.33	TEDC1	0.773432143	0.612189356	1.263387114	4	4	69.01948755	47.76085171
chr12	42312086	42326130	12q12	ZCRB1	24.82265863	19.64900785	1.26330341	4	4	18.22109458	19.09118785
chr3	51975026	51981200	3p21.2	ABHD14A	0.861779838	0.682175915	1.263280951	4	4	89.17847379	48.07514704
chr1	40258050	40294184	1p34.2	ZMPSTE24	36.30163314	28.73619063	1.263272283	4	4	13.02756465	25.85278725
chr20	44885599	44908532	20q13.12	YWHAB	58.94358297	46.6595701	1.263268882	4	4	28.38726318	25.13774591
chr20	1160509	1162097	20p13	ACTG1P3	0.750305228	0.593975021	1.263193235	3	2	78.18034778	30.92576066
chr17	7906123	7906260	17p13.1	SCARNA21	10527.88774	8334.799488	1.263124296	4	4	12.17251895	18.72481009
chr14	57268888	57298742	14q22.3	AP5M1	11.55503183	9.148311532	1.263078087	4	4	10.86959363	18.69585701
chr12	113358566	113391582	12q24.13	PLBD2	1.691894327	1.339609951	1.262975336	4	4	54.68486638	17.89260353
chr19	10960922	11062282	19p13.2	SMARCA4	5.975740418	4.731766554	1.262898402	4	4	61.34051933	44.11919985
chr2	191678072	191688522	2q32.3	NABP1	32.19895252	25.49747512	1.262829059	4	4	47.82049285	59.85734696
chr17	17505561	17591987	17p11.2	PEMT	2.605968438	2.063917764	1.262631914	3	4	68.35897765	42.85210859
chrX	53049324	53080615	Xp11.22	GPR173	0.064419158	0.051026004	1.262477048	1	1	0	0
chr1	3487942	3624770	1p36.32	MEGF6	2.168817893	1.718044892	1.262375566	4	4	94.85308334	66.8012876
chr6	57186992	57222314	6p12.1-p11.2	RAB23	26.59500984	21.06947324	1.262253192	4	4	91.10559757	60.46644855
chr19	37411142	37469263	19q13.12	ZNF569	28.72544949	22.76373787	1.261895109	4	4	77.58529705	41.38134891
chr9	35732320	35737008	9p13.3	CREB3	8.464920324	6.70876427	1.261770422	4	4	54.2475991	36.45498567
chr14	75278778	75282234	14q24.3	FOS	286.8950154	227.3829878	1.261725946	4	4	66.69259836	116.7474515
chr7	99408620	99419616	7q22.1	BUD31	25.34833783	20.09194964	1.261616632	4	4	47.31881663	45.14481354

chr17	48830988	48865245	17q21.32	CALCOCO2	39.46252709	31.2796231	1.261604942	4	4	18.7916822	6.272387843
chr5	142108759	142154443	5q31.3	NDFIP1	28.07961274	22.25962097	1.261459608	4	4	28.55575676	37.14609385
chr20	59016026	59026654	20q13.32	TUBB1	460.9356995	365.4246337	1.26137008	4	4	68.59593175	16.51284953
chr6	73425133	73452320	6q13	CGAS	10.67835969	8.465871956	1.26134198	4	4	35.82343695	11.72315223
chr8	94487777	94553518	8q22.1	VIRMA	19.87438966	15.75749785	1.26126558	4	4	16.65446024	7.601422318
chr1	8861002	8878686	1p36.23	ENO1	113.4378062	89.94854412	1.261141104	4	4	61.85351202	41.19087963
chr16	66603996	66613892	16q22.1	CMTM3	15.49343445	12.28590336	1.261074094	4	4	38.04402223	24.69123621
chr18	76495521	76498088	18q23	C18orf65	0.160186455	0.127026642	1.261046121	3	3	3.0002076	15.97557452
chr16	88570381	88631964	16q24.2	ZC3H18	8.293540913	6.576730358	1.261043172	4	4	72.66556719	43.32835331
chr3	184276011	184293031	3q27.1	ECE2	0.227463927	0.180379465	1.261030055	4	3	113.7039286	100.6338077
chr2	151248214	151261875	2q23.3	RBM43	5.856621989	4.64449926	1.260980282	4	4	6.232977247	11.36590843
chr5	95852232	95860133	5q15	LINC01554	0.156447289	0.124071103	1.26094864	1	1	0	0
chr9	113150958	113164142	9q32	SLC31A2	34.11584759	27.05765816	1.260857365	4	4	39.78644967	29.36181472
chr12	92140278	92145897	12q21.33	BTG1	136.9602778	108.6340531	1.260749036	4	4	28.1280014	23.77764499
chr8	38974149	38988662	8p11.22	HTRA4	0.261098099	0.207100821	1.260729423	4	2	27.15552264	41.83737139
chr13	19422877	19561574	13q12.11	TPTE2	0.133682899	0.106038522	1.260701264	2	1	32.60930601	0
chr8	107249482	107498055	8q23.1	ANGPT1	6.143973319	4.873563843	1.260673609	4	4	68.9658931	49.61771098
chr13	51353171	51453333	13q14.3	INTS6	9.968709708	7.907652894	1.260640779	4	4	29.56444639	20.4987812
chr1	173931084	173993072	1q25.1	RC3H1	30.57158303	24.25372523	1.260490203	4	4	21.5370476	14.10514957
chr19	38335563	38370949	19q13.2	CATSPERG	0.627395407	0.497754724	1.260450936	4	4	33.10702752	18.38808754
chr7	32957404	33006931	7p14.3	FKBP9	0.354402296	0.281193296	1.260351158	4	4	63.03267555	48.93357994
chr10	13217269	13217375	10p13	RNU6-6P	20.55889037	16.31203554	1.260351004	4	4	55.64062986	35.76213386
chr3	101827990	101861025	3q12.3	NFKBIZ	40.74250848	32.32722328	1.260315745	4	4	46.928928	31.10788712
chr1	86862445	86914577	1p22.3	SELENOF	73.16595744	58.05446423	1.260298556	4	4	9.748037919	17.21882698
chr12	42326126	42448621	12q12	PPHLN1	12.95490944	10.27962494	1.260251178	4	4	45.5648027	27.03976703
chr19	38619072	38636959	19q13.2	EIF3K	41.72131287	33.10643114	1.260217772	4	4	21.4943872	18.93428061
chr1	157121191	157138591	1q23.1	ETV3	12.32468635	9.780054281	1.260185884	4	4	48.60624009	15.84701907
chr15	40693734	40695105	15q15.1	RAD51-AS1	4.208192564	3.339356936	1.260180521	4	4	39.44846143	30.14607302
chr20	34363235	34511773	20q11.22	ITCH	93.56116817	74.25436527	1.260008995	4	4	14.07847622	15.43092869
chr1	220266179	220267715	1q41	AURKAP1	0.361261376	0.286719451	1.259982102	1	4	0	55.41837577
chr11	77589630	77610356	11q14.1	AQP11	0.187509337	0.148830388	1.259886097	3	2	43.2129454	27.98464774
chr12	49096551	49110900	12q13.12	LMBR1L	6.247447086	4.959293904	1.25974528	4	4	40.12934699	23.4783444
chr17	60083566	60088467	17q23.1	WFDC21P	0.315528987	0.250473015	1.259732456	2	3	10.64748978	15.19049446
chr11	62787321	62790400	11q12.3	TMEM179B	19.59920676	15.5585179	1.259709111	4	4	67.80005129	59.00294793
chr14	104924301	104937789	14q32.33	PLD4	2.390785373	1.897935166	1.259677051	4	4	87.68355031	73.72561218
chr3	129591349	129591578	3q22.1	RN7SL752P	13.49117637	10.71113475	1.259546881	4	4	48.7273928	14.82888537
chr10	22321210	22331485	10p12.2	BMI1	9.384506241	7.450871609	1.259517911	4	4	45.45318364	18.33541898
chr18	50878702	50949795	18q21.2	ME2	13.18744506	10.47060144	1.259473502	4	4	25.22089389	31.85462538
chr1	175944826	176209087	1q25.1-q25.2	COP1	63.64584493	50.53651831	1.259403043	4	4	7.009970084	27.60775312
chr1	181033392	181061938	1q25.3	MR1	8.038030784	6.382471543	1.259391558	4	4	34.44809885	38.95414979
chr22	30331988	30356947	22q12.2	SF3A1	35.82740225	28.44988178	1.259316384	4	4	60.61778796	28.37215947
chr7	142656737	142657213	7q34	TRBV24-1	1.181898176	0.938546611	1.259285541	3	3	30.44137909	23.55842797
chr10	119029716	119033732	10q26.11	NANOS1	0.138957151	0.110353515	1.259200044	2	2	23.06936563	54.99494479

chr4	56435749	56461368	4q12	PAICS	9.272701342	7.363966666	1.259199255	4	4	65.22001817	37.89474994
chr9	99222288	99230619	9q22.33	SEC61B	29.38707087	23.33916933	1.259130968	4	4	30.93285995	9.190763961
chr3	27107336	27369460	3p24.1	NEK10	0.092365361	0.073356574	1.259128607	4	4	47.6541996	55.47483581
chr3	12484432	12539624	3p25.2	TSEN2	8.87048205	7.045018518	1.259114086	4	4	72.765328	39.73998313
chr10	100486646	100519838	10q24.31	SEC31B	0.798379158	0.634127162	1.259020597	4	4	63.11775884	24.62375265
chr3	33388336	33440405	3p22.3	UBP1	9.95262412	7.905337334	1.258975259	4	4	40.07800652	19.38508592
chr14	22271971	22272563	14q11.2	TRAV38-1	5.868140169	4.661102801	1.258959611	2	4	77.73769274	36.17414537
chr1	46675159	46719064	1p33	EFCAB14	49.2068163	39.08734661	1.258893749	4	4	41.81934594	18.46624126
chr6	151404548	151452181	6q25.1	RMND1	7.462229197	5.927981129	1.258814601	4	4	22.39283932	21.67233485
chr5	43601092	43705566	5p12	NNT	20.99738096	16.68069289	1.258783499	4	4	33.33788243	12.87565072
chr12	107732866	107761272	12q23.3	PRDM4	10.08309953	8.010377345	1.258754625	4	4	44.82087151	10.39034671
chr2	71130314	71150102	2p13.3	MPHOSPH10	11.81458207	9.386223788	1.258715149	4	4	61.51156597	50.13165355
chr3	196197443	196211437	3q29	ZDHHC19	0.235098735	0.186782243	1.258678186	4	3	53.41461848	40.66544512
chr13	26576858	26577721	13q12.13	RPS3AP44	0.363292323	0.288632182	1.258668804	1	1	0	0
chr1	183626193	183635941	1q25.3	ARPC5	64.46897956	51.22828234	1.258464595	4	4	45.69771686	25.84602134
chr3	129127416	129161230	3q21.3	ISY1	3.790900743	3.012499807	1.258390369	4	4	64.04676453	39.87719152
chr8	144529179	144700520	8q24.3	ARHGAP39	0.068819655	0.054689089	1.258379988	1	2	0	5.67058913
chr11	83193739	83256099	11q14.1	ANKRD42	2.854174914	2.268184856	1.258351984	4	4	22.07686336	24.23511724
chr3	9649433	9702394	3p25.3	MTMR14	16.5336709	13.13918969	1.258347835	4	4	52.68194581	11.76617701
chrMT	12337	14148	N/A	MT-ND5	132.5628207	105.3496101	1.258313349	4	4	65.59353556	28.53966282
chr16	67192160	67198918	16q22.1	E2F4	30.67933705	24.38227649	1.258263848	4	4	16.04663864	46.41416889
chr7	159028175	159144957	7q36.3	VIPR2	0.118791584	0.094413164	1.258209974	3	4	52.43682717	63.34502307
chr1	161225939	161230746	1q23.3	TOMM40L	2.826563652	2.246903168	1.25798196	4	4	74.8961899	45.81731597
chr4	88592423	88709303	4q22.1	HERC3	18.27198468	14.5251339	1.257956368	4	4	40.06076821	29.22586225
chr6	30234039	30326134	6p22.1	HCG17	0.441186918	0.350719653	1.257947521	1	2	0	11.65008368
chr1	7961654	7985282	1p36.23	PARK7	28.62493704	22.75576465	1.257920245	4	4	19.09247625	12.84638879
chr8	42391624	42405937	8p11.21	VDAC3	47.65368639	37.88359798	1.257897584	4	4	12.89874845	10.94109503
chrX	23664260	23686399	Xp22.11	PRDX4	3.738263622	2.972063447	1.257800746	4	4	64.1806234	35.64013443
chr3	127672928	127680926	3q21.3	ABTB1	26.83004943	21.33139312	1.257772959	4	4	36.38431943	21.57050518
chr12	64452060	64502119	12q14.2	TBK1	35.58666828	28.29350743	1.257768001	4	4	24.97299251	7.326758001
chr7	144183455	144186080	7q35	CTAGE4	0.183055893	0.145544253	1.257733573	2	2	56.30399961	49.31244784
chr1	65420652	65637493	1p31.3	LEPR	10.84275378	8.621595846	1.257627239	4	4	52.69601731	46.13679853
chr5	138561043	138561110	5q31.2	SNORD63	9.641605981	7.666791274	1.25758034	3	4	23.5971254	65.16455639
chr2	53849122	53849205	2p16.2	MIR3682	5.995305878	4.767470655	1.257544369	1	2	0	1.175672148
chr17	73207353	73232394	17q25.1	FAM104A	187.5967395	149.1770572	1.257544176	4	4	29.11002714	16.1518185
chr3	143265222	143848531	3q24	SLC9A9	20.61433368	16.39339104	1.257478311	4	4	24.51495693	12.77674266
chr5	119071002	119249432	5q23.1	DMXL1	17.5747056	13.97675919	1.25742351	4	4	29.24222632	10.76463696
chr3	52221080	52226163	3p21.2	TLR9	0.092019339	0.073190676	1.257254935	1	1	0	0
chr3	100492619	100577453	3q12.2	TMEM45A	0.658104397	0.523448694	1.257247184	4	4	52.10699145	20.26160326
chr2	27325849	27357376	2p23.3	GTF3C2	8.241507722	6.555280793	1.257231838	4	4	39.23344567	12.04228945
chr15	84527196	84570795	15q25.2	UBE2Q2P1	10.25139713	8.153990826	1.257224511	4	4	42.17690843	48.03779388
chr5	70415370	70450362	5q13.2	GTF2H2B	8.898278158	7.077989126	1.257176014	4	4	86.6286643	56.39525447
chr3	123201927	123274136	3q21.1	SEC22A	6.206809388	4.937735558	1.25701535	4	4	75.89621854	35.3804135

chr3	33798571	33869707	3p22.3	PDCD6IP	29.07508215	23.13025875	1.257014998	4	4	40.64538118	19.46044878
chr10	104129888	104232383	10q25.1	CFAP43	0.255408	0.203189782	1.256992345	3	4	21.81569691	64.72696096
chr10	97356701	97401370	10q24.1	RRP12	8.494198491	6.758362392	1.256842708	4	4	65.14711068	42.44833623
chr11	61402648	61429992	11q12.2	CPSF7	17.40186255	13.84610906	1.256805249	4	4	56.78781985	36.02946076
chr16	2475104	2509669	16p13.3	TBC1D24	0.595436177	0.473796771	1.256733295	4	4	57.68053794	36.51592929
chr11	18278670	18322498	11p15.1	HPS5	13.95750988	11.10684453	1.256658436	4	4	38.28972368	30.55891539
chr4	5897591	6018762	4p16.1	C4orf50	0.10221643	0.081339918	1.256657656	4	3	86.58457185	31.08499318
chr6	41066788	41097787	6p21.1	OARD1	8.103191917	6.448297799	1.256640461	4	4	25.28967682	21.39026358
chr1	182378093	182392206	1q25.3	GLUL	323.3521143	257.3166621	1.256631078	4	4	18.59045561	24.28260736
chr18	25061924	25352250	18q11.2	ZNF521	0.18559377	0.147696877	1.256585608	3	4	28.26186903	55.14083423
chrX	41333308	41364472	Xp11.4	DDX3X	166.7293582	132.6921189	1.256512893	4	4	34.79901318	18.2492805
chr1	46040140	46174901	1p34.1	PIK3R3	0.845073507	0.672558972	1.256504697	4	4	38.05502499	57.61214281
chr15	52192318	52295798	15q21.2	MYO5C	0.303418847	0.24149196	1.256434573	4	3	83.36788472	4.858303048
chr1	40450068	40466765	1p34.2	ZFP69B	1.590973391	1.266363182	1.256332633	4	4	82.57164356	59.65381422
chrX	107061885	107118827	Xq22.3	RBM41	15.08014581	12.00377317	1.256283803	4	4	53.46127841	49.71277119
chr9	35749280	35753267	9p13.3	RGP1	6.934008413	5.520377882	1.256074957	4	4	36.54355686	32.64325109
chr12	54497711	54543115	12q13.13-q13.2	NCKAP1L	41.89854121	33.35924349	1.255979957	4	4	40.93309964	37.78390709
chr2	227325151	227357836	2q36.3	MFF	19.15365121	15.25093467	1.255900155	4	4	31.52586769	24.66269877
chr7	2251770	2354477	7p22.3	SNX8	1.054119128	0.839393153	1.255810969	4	4	60.04318071	22.68516273
chr5	175959531	176034905	5q35.2	THOC3	3.492659352	2.781440484	1.255701631	4	4	52.05715455	52.20126465
chr7	103074881	103099773	7q22.1	ARMC10	3.015566816	2.401625973	1.255635494	4	4	23.65311824	26.02700675
chr7	32868166	32892144	7p14.3	KBTBD2	32.87703544	26.18423325	1.255604284	4	4	33.6638668	18.39207518
chr3	49908869	49930012	3p21.31	MON1A	1.379048557	1.098383109	1.255526005	4	4	70.70095363	77.25992736
chrX	69616067	70039472	Xq13.1	EDA	0.079749349	0.063518802	1.255523506	4	3	58.8634613	35.82230246
chr22	40857081	40932819	22q13.2	XPNPEP3	3.30051088	2.629158507	1.255348763	4	4	32.38169164	14.69760679
chr20	45948653	45972910	20q13.12	ZNF335	4.956617371	3.948448493	1.255332919	4	4	49.33862529	32.13090489
chr12	57931449	57936164	12q14.1	GIHCG	5.749356269	4.580250473	1.255249315	4	4	66.19363859	29.28337511
chr18	69401027	69849087	18q22.2	DOK6	0.165119994	0.131563189	1.255062258	3	4	27.3528919	56.56256955
chr7	91872876	91880733	7q21.2	MTERF1	5.704193893	4.545156804	1.255004863	4	4	36.34289287	15.48426078
chr8	102239451	102252174	8q22.3	UBR5-AS1	5.901194775	4.70257635	1.254885479	4	4	40.53306242	78.44392703
chr5	71220226	71259295	5q13.2	GUSBP9	6.146773984	4.898476851	1.254833731	4	4	46.71031253	69.23095776
chr22	37696988	37776556	22q13.1	TRIOBP	4.477067746	3.568495647	1.254609277	4	4	54.17605675	31.24799403
chr9	34398184	34458570	9p13.3	FAM219A	2.567663832	2.04700585	1.254350999	4	4	64.67551727	22.77129457
chr6	152987366	153003439	6q25.2	MTRF1L	7.168671403	5.715734311	1.25419955	4	4	56.49205862	8.651493004
chr9	19063656	19063786	9p22.1	SCARNA8	6.762086384	5.391763858	1.254151065	3	4	38.98962102	85.30202052
chr22	21954359	21982843	22q11.22	TOP3B	2.299607892	1.834076179	1.253823543	4	4	56.15668315	28.93583096
chr13	32315480	32399672	13q13.1	BRCA2	14.5869646	11.63470328	1.253746164	4	4	51.86773589	39.15371495
chr2	202291317	202291428	2q33.1	SNORD11B	9.88691687	7.886087487	1.253716356	3	3	34.48519456	75.39608762
chr18	80034346	80050652	18q23	RBFA	1.524348077	1.215886514	1.253692725	4	4	132.7450131	40.69495221
chr14	56118329	56301586	14q22.3	PELI2	28.15015217	22.45599545	1.253569552	4	4	55.62254595	30.74598002
chrX	135974596	136047269	Xq26.3	SLC9A6	5.835737073	4.655311642	1.253565287	4	4	26.11500432	23.00080804
chr11	47269376	47330031	11p11.2	MADD	13.558877	10.81641177	1.253546673	4	4	55.21136719	23.42490682
chr1	155262868	155273529	1q22	CLK2	6.490287003	5.177548992	1.253544295	4	4	68.59848609	40.06543046

chr13	26539139	26688945	13q12.13	WASF3	1.823410504	1.454604624	1.253543729	4	4	78.66036636	26.18288519
chr2	96266146	96274179	2q11.2	CIAO1	18.19300963	14.51335463	1.253535801	4	4	46.19418677	29.04353171
chr2	63892149	64019422	2p15-p14	VPS54	19.47674322	15.53757506	1.253525287	4	4	17.4069479	7.337606917
chr12	3613366	3753218	12p13.32	CRACR2A	2.530719714	2.01893952	1.253489611	4	4	51.54177049	25.12057653
chr3	9779964	9793011	3p25.3	TADA3	19.65717026	15.68276116	1.253425341	4	4	66.16456083	49.39330288
chr7	130877562	131109916	7q32.3	LINC-PINT	15.28167347	12.19249568	1.253367142	4	4	32.9224395	6.590091288
chr11	5719290	5720266	11p15.4	OR52U1P	0.819627674	0.653988268	1.253275806	3	2	65.69332505	17.223616
chr17	44850287	44899625	17q21.31	EFTUD2	9.180172754	7.325104667	1.253247997	4	4	59.53649337	37.82893633
chr13	110641410	110713601	13q34	CARS2	5.93742475	4.737649691	1.253242671	4	4	74.61827431	25.99025158
chr8	38176588	38213301	8p11.23	BAG4	12.80949346	10.22121233	1.253226432	4	4	21.82412822	7.194919918
chr4	38509767	38518056	N/A	LINC01259	1.151924972	0.919170804	1.253221889	3	4	105.0635458	96.60910515
chr8	22367230	22434129	8p21.3	SLC39A14	1.229581798	0.981202871	1.253137179	4	4	58.0137418	41.99527533
chr22	40769630	40819399	22q13.2	SLC25A17	3.543222086	2.827564714	1.253100262	4	4	49.51093256	18.86958839
chr19	45769709	45782557	19q13.32	DMPK	0.678591742	0.541577147	1.252991834	4	4	59.80349828	71.36347285
chr4	152779937	152911912	4q31.3	ARFIP1	26.10471535	20.83421813	1.252973123	4	4	13.49409193	55.48647573
chr8	85463902	85481492	8q21.2	CA2	80.54229004	64.28198467	1.252952759	4	4	57.51967073	69.05835075
chr1	23790789	23795539	1p36.11	LYPLA2	14.58796041	11.64294703	1.252943982	4	4	59.40807754	34.24327752
chr11	70398506	70436584	11q13.3	CTTN	15.12104728	12.06892809	1.25289066	4	4	71.41681861	9.935734733
chr17	68421281	68457524	17q24.2	WIPI1	51.72415735	41.28500332	1.252855836	4	4	39.16130857	36.08106101
chr1	9922113	9943427	1p36.22	LZIC	130.9254729	104.5046456	1.252819644	4	4	72.86658445	63.63033452
chr6	136256863	136289851	6q23.3	BCLAF1	70.48679659	56.26353168	1.252797229	4	4	37.10143706	12.83147464
chr1	33145399	33184774	1p35.1	TRIM62	2.37372051	1.894747194	1.252790091	4	4	84.08293977	32.58065026
chr10	125766453	125775821	10q26.2	MMP21	0.413345335	0.329955742	1.252729631	1	1	0	0
chr7	143288215	143307696	7q34	CASP2	5.359901892	4.278727078	1.25268609	4	4	40.39926924	27.29645978
chr12	22448546	22544546	12p12.1	C2CD5	24.4829323	19.54565348	1.252602392	4	4	28.63330453	15.14829745
chr9	94175957	94176036	9q22.32	MIRLET7A1	7.13915793	5.699908206	1.252504018	2	3	6.835515276	37.94476291
chr17	67377281	67697263	17q24.2	PITPNC1	11.51858203	9.197179336	1.252403765	4	4	27.01484966	23.93626637
chr7	139021498	139036472	7q34	ZC3HAV1L	1.617911344	1.291855926	1.252393019	4	4	38.43602284	48.96239758
chr10	122990806	123008850	10q26.13	IKZF5	10.48507918	8.372063349	1.252388897	4	4	32.22860834	27.96361374
chr15	56630176	56734086	15q21.3	ZNF280D	8.674277392	6.926545606	1.252323725	4	4	29.04208313	21.66691037
chr10	30434021	30461833	10p11.23	MAP3K8	22.82017265	18.22234108	1.25231838	4	4	15.55722545	24.26632309
chr1	3624992	3630131	1p36.32	TPRG1L	24.34386193	19.43950054	1.252288446	4	4	26.53860359	33.48969457
chr1	65057755	65057842	1p31.3	MIR3671	18.66335524	14.90423149	1.252218556	3	4	58.44872364	79.71554516
chr11	1792525	1792920	11p15.5	RPL36AP39	1.074882864	0.858520025	1.252018395	2	1	24.62614787	0
chr20	3471018	3651122	20p13	ATRN	5.169521976	4.129095754	1.251974351	4	4	43.76412012	29.15331494
chr16	56932090	56943881	16q13	HERPUD1	24.27370801	19.38918305	1.251920102	4	4	39.3736024	14.92163914
chr12	8082211	8097777	12p13.31	NECAP1	7.507948115	5.997417131	1.251863586	4	4	51.4081463	24.08737373
chr1	203861585	203871152	1q32.1	SNRPE	26.47603684	21.15076696	1.25177668	4	4	59.62779355	27.04693413
chr22	24540423	24555935	22q11.23	GUCD1	153.7092922	122.8047849	1.251655563	4	4	31.37209921	21.20406072
chr17	31391624	31538217	17q11.2	RAB11FIP4	10.88352795	8.696126734	1.251537412	4	4	66.38926992	23.16453398
chr15	58690473	58693607	15q21.3	HSP90AB4P	0.733199773	0.585841327	1.251533035	4	3	91.52809741	41.97572877
chr3	48625723	48635493	3p21.31	SLC26A6	1.22265446	0.97696699	1.251479806	4	4	60.20274162	36.8614247
chr9	133356545	133361165	9q34.2	SURF2	5.792338383	4.628500635	1.251450273	4	4	71.48669046	56.55654008

chr1	179025804	179076574 1q25.2	FAM20B	27.97916044	22.35849328	1.251388458	4	4	24.66318434	46.2475573
chr10	133362480	133373404 10q26.3	ECHS1	7.987788998	6.383657879	1.251287138	4	4	37.59630575	30.40729173
chr15	58771192	58861900 15q21.3-q22.1	MINDY2	8.219677267	6.569000151	1.251282855	4	4	23.5727323	37.19160859
chr1	112619453	112671619 1p13.2	CAPZA1	219.960867	175.7900973	1.251269954	4	4	17.15530881	20.45921029
chr20	35668688	35699365 20q11.22	NFS1	1.464875134	1.170743091	1.251235344	4	4	48.81480573	34.34121748
chr11	83259093	83286407 11q14.1	CCDC90B	23.12069648	18.47863256	1.251212524	4	4	48.03794708	19.21547396
chr14	75131468	75176646 14q24.3	TMED10	59.8958704	47.87453272	1.251100888	4	4	26.09293182	10.56139412
chr4	77157204	77170060 4q21.1	CCNG2	19.69412944	15.74195294	1.251060114	4	4	47.6531265	36.24377841
chr1	62783765	62865270 1p31.3	ATG4C	19.51407796	15.59831027	1.251037941	4	4	41.91745071	36.72132056
chr17	42810132	42824316 17q21.31	BECN1	24.64078299	19.69767843	1.250948587	4	4	56.53482846	6.938504875
chr12	28190427	28550166 12p11.22	CCDC91	95.15738771	76.07013678	1.25091648	4	4	71.14168621	51.92738551
chr17	74770529	74776425 17q25.1	NAT9	2.044490815	1.634448274	1.250875202	4	4	64.18188198	36.70393581
chrX	41085420	41236579 Xp11.4	USP9X	50.83206475	40.63923762	1.250812459	4	4	31.47795585	11.86033471
chr15	22664739	22685368 15q11.2	WHAMMP3	9.635977073	7.703787559	1.250810332	4	4	20.95354638	37.60538805
chrX	77826364	77895568 Xq21.1	MAGT1	31.32238966	25.04347817	1.250720425	4	4	23.36438613	11.09677685
chr2	235494089	236131800 2q37.2	AGAP1	0.880338738	0.703881678	1.250691367	4	4	31.85571833	61.842761
chr3	136336235	136752412 3q22.3	STAG1	51.4333402	41.12424296	1.250681751	4	4	54.13831845	26.90926692
chr19	22634324	22667670 19p12	ZNF492	3.824432082	3.058122559	1.250581692	4	4	98.49833274	54.30800175
chr13	48975241	49209779 13q14.2	FNDC3A	34.57462645	27.6485547	1.250503935	4	4	36.40958728	22.17497881
chr5	176526699	176544206 5q35.2	RNF44	12.76154879	10.20557377	1.250448929	4	4	56.70529211	29.47636438
chr19	32675407	32678300 19q13.11	RGS9BP	0.106593567	0.085247433	1.25040207	1	1	0	0
chr7	150567370	150573955 7q36.1	GIMAP4	329.1406736	263.2304226	1.250389945	4	4	62.9895091	47.33404049
chr19	52512747	52564464 19q13.41	ZNF808	11.77434062	9.417241191	1.250296173	4	4	40.92106498	19.38936142
chrMT	8366	8572 N/A	MT-ATP8	184.4211851	147.5028553	1.250288916	4	4	66.09013553	45.34730279
chr2	89117342	89117817 2p11.2	IGKV1-17	3.434673994	2.747155084	1.25026578	3	3	53.86309284	98.74746471
chr12	4321193	4360028 12p13.32	TIGAR	5.010625803	4.00773579	1.250238555	4	4	52.34129438	35.62133821
chr17	45434262	45490780 17q21.31	PLEKHM1	3.253639114	2.602433929	1.250229286	4	4	51.81165565	14.23520071
chr15	41621225	41769943 15q15.1	MGA	25.44611235	20.35449103	1.250147317	4	4	15.34018742	10.7774488
chr5	123512099	123617045 5q23.2	CSNK1G3	140.6371002	112.5120395	1.249973788	4	4	69.12444016	23.22251016
chr6	158109504	158168280 6q25.3	SERAC1	1.163686649	0.931006603	1.249923089	4	4	40.37768856	24.34654583
chr7	56051685	56063989 7p11.2	CCT6A	34.91962166	27.93996096	1.24980925	4	4	29.69489574	17.365765
chr10	45000825	45005329 10q11.21	ZNF22	26.8883468	21.51667405	1.24965163	4	4	24.52689051	26.42553723
chr17	74671117	74747335 17q25.1	RAB37	25.90976668	20.7337018	1.249644995	4	4	80.96498616	18.4178399
chr5	36885204	36886556 5p13.2	KRT18P31	4.353038695	3.483455545	1.249632337	4	4	64.32534223	29.59203715
chr1	116372986	116410259 1p13.1	ATP1A1	26.81425419	21.45808382	1.249610842	4	4	50.94948619	32.89756142
chr1	19682213	19800385 1p36.13	TMCO4	3.906698843	3.126368493	1.249596409	4	4	51.121332	62.51847165
chr18	51158202	51159443 18q21.2	SRSF10P1	0.373775789	0.299117325	1.249595921	1	1	0	0
chr7	32916815	32943170 7p14.3	RP9P	1.412681297	1.130543943	1.249558945	4	3	85.61223584	59.77728665
chr9	20658309	20995955 9p21.3	FOCAD	6.333608416	5.06879052	1.249530512	4	4	39.76249208	20.87434465
chr10	101845599	102056237 10q24.32	ARMH3	29.60972332	23.69816607	1.249452098	4	4	68.3458954	40.13479715
chr13	41961169	41981565 13q14.11	VWA8-AS1	0.28073165	0.224684248	1.249449628	3	3	89.02226131	82.45769248
chr13	28136843	28139174 13q12.2	PAN3-AS1	2.927806335	2.343416188	1.249375314	4	4	94.41774894	78.03567096
chr3	53071151	53130469 3p21.1	RFT1	4.444947487	3.557845677	1.249336787	4	4	52.48694663	16.07535994

chr14	105049389	105065550	14q32.33	GPR132	9.080488929	7.268371322	1.249315497	4	4	79.38311943	19.88823727
chr22	50568861	50582999	22q13.33	CHKB-CPT1B	0.208629629	0.167015212	1.249165427	4	4	63.30001086	37.29066993
chr9	27546546	27573866	9p21.2	C9orf72	41.52216045	33.24020752	1.24915467	4	4	34.18358102	39.66457816
chr14	51489100	51730727	14q22.1	FRMD6	0.187734902	0.150307629	1.249004485	3	4	72.86205778	53.42460387
chr6	89080710	89085160	6q15	PNRC1	410.3998829	328.5955917	1.248951274	4	4	35.26282038	27.17538247
chr5	140664799	140674119	5q31.3	WDR55	4.482791772	3.58930244	1.248931191	4	4	78.23889133	41.20566616
chr2	241227264	241315842	2q37.3	HDLBP	33.93057663	27.16853279	1.248892492	4	4	32.78957883	35.56370311
chr9	27325209	27529852	9p21.2	MOB3B	0.933520389	0.747480324	1.248889581	4	4	76.38477942	74.33219774
chr11	46677075	46700665	11p11.2	ARHGAP1	12.63261277	10.11558344	1.248826905	4	4	63.30893875	38.74099623
chr4	73995642	73998729	4q13.3	CXCL5	40.10245899	32.11450904	1.248733367	4	4	40.41039731	52.2220399
chr6	99432306	99515770	6q16.2	USP45	19.28106423	15.44051124	1.248732243	4	4	66.8249634	48.99234477
chr20	50510321	50584762	20q13.13	PTPN1	32.9815422	26.41237123	1.248715683	4	4	38.7899854	13.46898741
chr3	45224464	45226322	3p21.31	TMEM158	8.584333412	6.874531871	1.248715341	4	4	78.19693491	75.23936943
chr11	72576140	72674453	11q13.4	PDE2A	0.656366087	0.525640661	1.248697323	4	4	80.25103804	43.03915837
chr13	49996415	49996501	13q14.2	MIR3613	4.675935644	3.744766365	1.248658845	1	2	0	31.13179425
chr2	218398338	218405941	2q35	CTDSP1	35.35638316	28.31809724	1.248543744	4	4	34.59973233	15.84372459
chr2	177550152	177552796	2q31.2	TTC30B	1.732952481	1.388234925	1.24831356	4	4	46.9316908	29.44455792
chr8	103021020	103073057	8q22.3	ATP6V1C1	27.63553442	22.13904765	1.248271148	4	4	14.75404972	25.94947767
chr7	143642254	143647649	7q35	TCAF2C	0.127220254	0.101918608	1.248253452	1	1	0	0
chr7	100679506	100694280	7q22.1	GIGYF1	7.603947033	6.091806577	1.24822529	4	4	56.50471106	10.36338807
chr17	42017576	42025641	17q21.2	NKIRAS2	7.359320504	5.895852668	1.248219879	4	4	36.77598245	33.34363026
chr6	73055078	73056052	6q13	PGAM1P10	0.637689246	0.510928992	1.248097594	1	2	0	44.32809127
chr10	35079598	35079680	10p11.21	MIR3611	4.907770987	3.932324817	1.248058392	1	4	0	20.58706848
chr10	110936603	110937255	10q25.2	RPL13AP6	0.541107303	0.43356649	1.248037649	2	1	47.69462185	0
chr4	41990502	42087534	4p13	SLC30A9	29.96625232	24.01547195	1.247789441	4	4	45.52272398	27.19776568
chrX	9024232	9034127	Xp22.31	FAM9B	0.048238033	0.038661828	1.247691465	1	1	0	0
chr5	177592148	177597242	5q35.3	TMED9	11.11422537	8.908764782	1.247560761	4	4	30.6955091	15.4792221
chr6	85659871	85660653	6q14.3	PKMP3	0.589007763	0.472132676	1.247547125	3	3	46.29525359	31.03977072
chr15	41774434	41827855	15q15.1	MAPKBP1	1.976465043	1.584362733	1.247482664	4	4	60.49721782	55.21981063
chr6	149447630	149485012	6q25.1	ZC3H12D	1.277736919	1.024277623	1.247451756	4	4	64.9146197	53.13279265
chr15	43746392	43772606	15q15.3	PDIA3	84.84845915	68.0182996	1.247435758	4	4	31.95203905	26.61027386
chrX	154348532	154374638	Xq28	FLNA	301.1363774	241.4675797	1.24710894	4	4	59.14089171	61.30688252
chr7	5898710	5925974	7p22.1	CCZ1	16.6447656	13.34686244	1.247092017	4	4	59.77612783	69.08224582
chrX	13734713	13773978	Xp22.2	OFD1	31.81182418	25.50957675	1.247054175	4	4	36.73457902	21.04764432
chr19	39538806	39540198	19q13.2	EID2	6.446414706	5.169318286	1.247053161	4	4	38.59123278	21.90382943
chr17	42124976	42155044	17q21.2	RAB5C	17.35233081	13.91610429	1.246924459	4	4	47.1821807	20.14959059
chr19	49877425	49888750	19q13.33	TBC1D17	6.697309231	5.371391592	1.246848068	4	4	34.73003445	30.63393275
chr6	46159182	46171010	6p21.1	ENPP5	1.798396956	1.442357205	1.246845754	4	4	36.11387843	37.33583876
chrX	100671964	100732148	Xq22.1	SYTL4	2.842043054	2.279530666	1.246766756	4	4	12.74804081	27.497953
chrX	103675498	103688158	Xq22.2	MORF4L2	22.76149642	18.2568526	1.246737152	4	4	17.95760127	20.27678634
chr14	70366496	70417090	14q24.2	SYNJ2BP	4.257556329	3.414969351	1.246733394	4	4	37.47440292	33.35891193
chr2	222860934	222944651	2q36.1	ACSL3	20.33028111	16.3070154	1.246719931	4	4	17.51897321	25.36092133
chr17	4796144	4798503	17p13.2	PSMB6	19.76265291	15.85200324	1.246697506	4	4	52.29393451	34.65456331

chr8	94895768	95118496	8q22.1	NDUFAF6	3.73922926	2.999629227	1.246563817	4	4	46.89943606	21.24667834
chr19	6772668	6857366	19p13.3	VAV1	42.73176144	34.28055557	1.246530598	4	4	48.42911919	35.6520952
chr13	46211919	46276822	13q14.13	LRRC63	4.404581424	3.533690726	1.246453571	4	4	86.60421398	52.35538847
chr20	60047074	60072987	20q13.33	C20orf197	1.605678299	1.28822341	1.246428443	4	4	47.54426689	48.44633967
chr10	31307717	31320447	10p11.22	ZEB1-AS1	1.238556624	0.993729501	1.246371998	4	4	50.99417882	77.37930221
chr16	69762284	69941741	16q22.1	WWP2	16.53793449	13.26898598	1.246360085	4	4	47.68490164	18.22199256
chr6	131125698	131283535	6q23.2	AKAP7	207.5549393	166.5366848	1.246301616	4	4	73.10842034	24.44900317
chr11	114059576	114256770	11q23.2	ZBTB16	1.80582667	1.448960526	1.246291142	4	4	71.71931928	58.52658323
chr12	31671137	31729174	12p11.21	AMN1	10.96225681	8.796346233	1.246228436	4	4	18.41616088	29.17549077
chr1	148531385	148595717	1q21.2	NBPF14	3.170151248	2.543978051	1.246139387	4	4	76.02340684	57.210447
chr5	443219	467296	5p15.33	EXOC3	7.069820177	5.673950163	1.246013795	4	4	64.73279301	32.94750639
chr12	6883981	6884614	12p13.31	RPL13P5	0.787854808	0.632326475	1.245962077	3	4	109.6222271	56.65081151
chr2	105360826	105438773	2q12.2	FHL2	1.512789018	1.214163733	1.245951412	4	4	37.73271006	53.07016146
chr1	222668013	222712895	1q41	AIDA	44.69754204	35.87433725	1.245947534	4	4	35.36075808	35.05764169
chr17	80220287	80253509	17q25.3	SLC26A11	1.228452626	0.986020153	1.245869693	4	4	63.20780985	57.60011438
chr2	218270392	218346793	2q35	PNKD	6.155556605	4.941222085	1.245755908	4	4	55.31903746	43.70877956
chr5	103120248	103204911	5q21.1	PPIP5K2	16.6293553	13.34946559	1.245694458	4	4	19.47601536	20.19493309
chr18	9475532	9538108	18p11.22	RALBP1	79.99367718	64.21634434	1.245690299	4	4	17.20964951	15.90629233
chr20	35703609	35742336	20q11.22	RBM39	107.3619467	86.18930266	1.245652806	4	4	28.33370795	12.11709368
chr12	132136594	132144335	12q24.33	DDX51	1.602573576	1.286554612	1.245631986	4	4	66.30515911	45.26794502
chr15	98784426	98784502	15q26.3	MIR4714	3.819685242	3.066477951	1.245626188	1	1	0	0
chr17	75667116	75708062	17q25.1	SAP30BP	7.860877954	6.310874557	1.245608336	4	4	45.89908156	25.95478539
chr10	73495752	73507309	10q22.2	PPP3CB-AS1	2.978513087	2.391224228	1.245601751	4	4	65.72303197	31.3748742
chr5	140114123	140119416	5q31.3	PURA	5.416744304	4.348782677	1.245577143	4	4	33.01939432	17.04619561
chr1	32204807	32222371	1p35.2	TMEM234	3.787305477	3.040766491	1.245510133	4	4	69.84985688	32.04331701
chrX	77504878	77786269	Xq21.1	ATRX	34.67362804	27.84040686	1.245442576	4	4	24.8109664	14.1724508
chr19	36510687	36528488	19q13.12	ZNF260	6.539059622	5.250407777	1.245438431	4	4	41.40477081	29.88364061
chr11	130866260	130916488	11q24.3-q25	SNX19	10.57474293	8.490840636	1.245429443	4	4	41.12241281	21.03761264
chr20	36651766	36746138	20q11.23	NDRG3	15.02123976	12.0612896	1.245409094	4	4	35.43286568	28.217407
chr1	167936239	168075843	1q24.2	DCAF6	173.8015764	139.5545011	1.245402871	4	4	28.67121785	27.76099024
chr7	26201211	26213607	7p15.2	CBX3	50.35177528	40.43168119	1.245354479	4	4	28.6276521	7.902385027
chr5	179550551	179610026	5q35.3	RUFY1	39.76694189	31.93231865	1.245350904	4	4	47.91302913	26.73757499
chr1	51616874	51789219	1p32.3	OSBPL9	15.50270135	12.44911094	1.245285821	4	4	19.10571621	16.19074554
chr15	22479153	22482374	15q11.2	HERC2P7	0.909660503	0.73049312	1.245269091	4	4	64.88137527	35.30364956
chr9	83659963	83708253	9q21.2-q21.3	UBQLN1	232.4418321	186.6635702	1.245244757	4	4	62.53218075	39.01841677
chr22	27851669	27919306	22q12.1	PITPNB	20.24983965	16.26237595	1.245195642	4	4	45.85435887	11.5565845
chr5	41904313	41921636	5p13.1	C5orf51	10.34738471	8.310235468	1.245137367	4	4	17.15135036	22.67937553
chr1	113929374	113977869	1p13.2	HIPK1	65.98738004	52.99737142	1.245106659	4	4	11.79142632	7.691714975
chr5	149348116	149357642	5q32	GRPEL2-AS1	1.897338305	1.523841175	1.245102401	1	2	0	82.27296683
chr6	97142161	97283437	6q16.1	MMS22L	6.43684728	5.170054327	1.245025076	4	4	78.42090655	55.12925179
chr14	45135939	45200890	14q21.2	FANCM	8.785306539	7.056807215	1.244940704	4	4	59.7751079	26.77993317
chr19	35268978	35279821	19q13.12	USF2	19.98959276	16.05817783	1.244823228	4	4	65.31673539	32.57588962
chr19	49906825	49929731	19q13.33	NUP62	6.250227947	5.020999568	1.244817464	4	4	34.70127851	39.06669811

chr5	95620078	95622142	5q15	GPR150	0.185173352	0.148764116	1.244744753	2	1	22.22131655	0
chr16	56608533	56609497	16q13	MT2A	51.67079693	41.51172675	1.244727719	4	4	34.40231821	123.5374526
chr16	67029147	67101058	16q22.1	CBFB	28.52130161	22.91426949	1.244696089	4	4	27.44048201	23.82217666
chr5	93615130	94111699	5q15	FAM172A	27.87208645	22.39271893	1.244694159	4	4	37.90826947	21.2375002
chr3	19996803	19996925	3p24.3	RNU4-85P	6.658464582	5.349903139	1.244595352	1	4	0	97.62810224
chr8	116845934	116874866	8q24.11	RAD21	74.74995023	60.05983487	1.244591338	4	4	16.60074276	6.85321825
chr16	188969	229482	16p13.3	LUC7L	6.120057248	4.917488634	1.244549343	4	4	36.70482162	1.83691545
chr13	77044657	77327098	13q22.3	MYCBP2	41.65463809	33.47013184	1.244531641	4	4	33.9830405	15.70779665
chr10	22928024	23038527	10p12.2	ARMC3	1.497808058	1.203546998	1.244494865	4	4	52.52057662	71.8374583
chr13	50994511	51066157	13q14.3	GUCY1B2	0.086660849	0.069635918	1.244484908	1	1	0	0
chr15	42273658	42353666	15q15.1	GANC	3.297350187	2.649831317	1.244362298	4	4	41.1054993	28.83712582
chr10	47467993	47991879	10q11.22	ANXA8	0.064105773	0.051518308	1.244329956	1	1	0	0
chr3	19947080	19985175	3p24.3	RAB5A	67.52460527	54.266855	1.244306589	4	4	13.76239459	19.61232136
chr3	194640788	194672477	3q29	LSG1	10.95414246	8.803966668	1.24422807	4	4	17.3377275	20.39517722
chr2	99337371	99400265	2q11.2	EIF5B	7.79402094	6.26424815	1.244206927	4	4	36.79561503	25.63500431
chr17	64854134	64919491	17q24.1	LRRC37A3	0.578727193	0.465215805	1.243997272	4	4	42.59843846	51.65264859
chr16	2658389	2673439	16p13.3	ERVK13-1	2.725822941	2.191369816	1.243889973	4	4	63.67807869	22.87420192
chr5	132996985	133026621	5q31.1	ZCCHC10	29.54938535	23.75631681	1.243853817	4	4	41.86098729	25.81261053
chr22	42085526	42090955	22q13.2	NDUFA6	23.96732382	19.26926933	1.243810723	4	4	23.57828702	37.8276617
chr12	93677342	93894840	12q22	CRADD	2.88285795	2.317981919	1.243693028	4	4	21.85980241	38.83359874
chr12	55820960	55829636	12q13.2	DNAJC14	9.878348019	7.942890438	1.243671696	4	4	65.81137781	39.23945888
chr1	145933423	145958038	1q21.1	LIX1L	14.70584032	11.82519997	1.24360183	4	4	36.00979387	30.17576427
chr14	99481402	99511515	14q32.2	CCNK	6.854799259	5.512368658	1.243530628	4	4	32.85748199	9.684763122
chr8	42752620	42768786	8p11.21	CHRNA6	0.255129395	0.205165684	1.243528595	1	1	0	0
chr12	51281038	51324680	12q13.13	BIN2	137.5238981	110.6008909	1.243424867	4	4	58.17659383	32.49186704
chr6	73464073	73464177	6q13	RNU6-975P	3.574489896	2.874866242	1.24335868	1	2	0	42.14277974
chr1	40300487	40317653	1p34.2	COL9A2	1.473699082	1.185295187	1.243318202	4	4	92.43602029	76.17544091
chr16	71644924	71724701	16q22.2	PHLPP2	44.56383051	35.84372905	1.243281089	4	4	53.44860097	45.9891038
chr17	49700934	49708173	17q21.33	SLC35B1	3.777445287	3.038344406	1.243257769	4	4	46.21700254	5.283571443
chr10	120456954	120600123	10q26.12	PLPP4	0.302722017	0.243536399	1.24302576	2	2	65.57712631	46.93063324
chr12	46358188	46372862	12q13.11	SLC38A2	75.41517374	60.67084794	1.243021588	4	4	22.34237727	16.52896752
chr7	1815792	2232971	7p22.3	MAD1L1	7.819722155	6.291122681	1.242977216	4	4	10.78056497	19.02548328
chr2	219597851	219616451	2q35	STK11IP	3.249834767	2.61456213	1.242974772	4	4	48.88885527	26.19142193
chr17	60600183	60666280	17q23.2	PPM1D	12.30554931	9.90045026	1.242928249	4	4	50.40613361	24.58443522
chr4	185399540	185425985	4q35.1	UFSP2	13.31590566	10.71450887	1.242791977	4	4	33.85631587	24.91097278
chr11	65525077	65538711	11q13.1	SCYL1	15.00597247	12.0745112	1.242780948	4	4	72.55351605	43.0248867
chr17	30968642	30999911	17q11.2	RNF135	7.395180171	5.950611344	1.242759734	4	4	52.20047606	37.38372808
chr9	99216425	99221964	9q22.33	ALG2	8.755103729	7.045584214	1.242637014	4	4	41.23467193	39.35854865
chr17	50866679	50868371	17q21.33	TOB1-AS1	0.759524895	0.611251445	1.242573577	4	4	92.48404238	59.12402865
chr4	48497363	48780323	4p11	FRYL	40.43577515	32.54205713	1.24256973	4	4	46.15971113	30.04229994
chr12	132768909	132829131	12q24.33	GOLGA3	11.76119775	9.46574007	1.24250166	4	4	41.73220248	40.30751624
chr9	21802636	21865971	9p21.3	MTAP	2.93418724	2.361884514	1.242307667	4	4	30.39365953	9.365243608
chr20	35626031	35664956	20q11.22	CPNE1	17.80070111	14.32879419	1.242302798	4	4	58.61285307	35.6491851

chr7	100088960	100092200	7q22.1	COPS6	7.593579164	6.112586901	1.242285678	4	4	57.89947716	30.25004991
chrX	154428632	154436517	Xq28	ATP6AP1	14.63556941	11.78117863	1.242283974	4	4	60.47287301	39.21729987
chr14	100238765	100279034	14q32.2	YY1	27.00258058	21.73760303	1.242205984	4	4	32.42995743	17.91458579
chr10	124757831	124791941	10q26.13	EEF1AKMT2	2.274397301	1.831022622	1.242145932	4	4	52.57476524	32.71081001
chr12	38905683	38909592	12q12	CPNE8-AS1	0.659079993	0.530637505	1.242053166	1	1	0	0
chr7	38345030	38345499	7p14.1	TRGV5P	1.343777965	1.081936611	1.242011733	2	2	33.75863441	42.38705254
chr10	91798394	91865475	10q23.32	TNKS2	34.12090015	27.47239979	1.242006538	4	4	23.76577118	9.317462811
chr15	76216228	76311469	15q24.2-q24.3	ETFA	43.15455369	34.74640129	1.241986281	4	4	32.81487385	28.56026731
chr14	105248542	105251093	14q32.33	BTBD6	5.56078917	4.477967775	1.241810895	4	4	66.67140446	19.47954597
chr1	203305536	203309602	1q32.1	BTG2	246.171746	198.2412629	1.24177854	4	4	61.75326204	70.14211632
chr7	43926422	43956136	7p13	UBE2D4	1.388521836	1.118341892	1.241589756	4	4	81.09031139	38.82629799
chr1	112391085	112461164	1p13.2	CTTNBP2NL	0.64307539	0.517945568	1.241588751	3	4	58.56993874	56.36965781
chr2	74147981	74152389	2p13.1	BOLA3-AS1	0.937741379	0.755289943	1.241564763	3	4	15.68483819	71.67485438
chr17	28614440	28645159	17q11.2	KIAA0100	24.8965389	20.05484358	1.241422742	4	4	52.89598065	37.64790948
chr7	47987148	48035899	7p12.3	SUN3	0.190468294	0.153428163	1.241416766	1	1	0	0
chr17	82716706	82728017	17q25.3	FN3KRP	5.155415941	4.152933551	1.241391387	4	4	41.70550051	24.47686402
chr6	43629542	43640952	6p21.1	MAD2L1BP	9.451885123	7.614254175	1.241340899	4	4	9.522332031	34.01200467
chr12	106357658	106510198	12q23.3	POLR3B	3.8140336	3.072688477	1.24126921	4	4	37.12537763	31.87390181
chr19	48963941	48966879	19q13.33	FTL	7581.98778	6108.660708	1.241186594	4	4	71.25368136	31.65112908
chr16	47461131	47701523	16q12.1	PHKB	19.35946172	15.59870609	1.241094076	4	4	27.07352714	13.6816124
chr7	87503863	87713323	7q21.12	ABCB1	5.823939167	4.692602113	1.241089491	4	4	34.33803914	28.68802544
chr2	181891716	181930738	2q31.3	ITPRID2	24.69164977	19.89626445	1.24101938	4	4	40.74571311	29.76669657
chr10	35246981	35572670	10p11.21	CCNY	45.76967161	36.88232772	1.240964832	4	4	37.8517277	18.95994052
chr12	8359010	8359858	12p13.31	RPS3AP43	0.427109605	0.344205829	1.240855233	1	1	0	0
chr12	21501176	21518403	12p12.1	GOLT1B	20.56034696	16.56973078	1.240837719	4	4	42.35146985	35.81677063
chr19	4304594	4323846	19p13.3	FSD1	0.245449131	0.19781374	1.240809312	3	2	25.15957807	18.77344028
chr1	15735088	15741392	1p36.21	SLC25A34	0.312564104	0.251921269	1.240721378	4	4	95.58192211	61.09203393
chr4	672436	674338	4p16.3	ATP5ME	24.40789517	19.67246911	1.240713356	4	4	31.38021711	18.82464461
chr6	42183284	42194956	6p21.1	GUCA1B	0.49642974	0.400130056	1.240670959	3	4	39.00562159	44.14775755
chr12	56334159	56340410	12q13.3	IL23A	3.162390582	2.548947828	1.240665088	4	4	71.67671116	72.19207879
chr6	32115268	32128240	6p21.32	ATF6B	17.20476177	13.86757195	1.240647017	4	4	74.77231963	54.84394782
chr6	33416542	33418288	6p21.32	CUTA	12.4667682	10.04889868	1.240610398	4	4	47.06933569	18.54490481
chr10	59788747	59906656	10q21.2	CCDC6	10.53602533	8.493506289	1.240480076	4	4	34.48299892	16.90708483
chr11	47465933	47565569	11p11.2	CELF1	22.12978082	17.84008584	1.240452597	4	4	23.8647272	11.17436321
chr9	110243812	110256640	9q31.3	TXN	80.95065691	65.25933161	1.240445694	4	4	25.49993402	22.66530417
chr4	158666675	158672255	4q32.1	C4orf46	3.71890565	2.998221026	1.240370746	4	4	35.93706673	29.6693235
chr17	50719544	50756213	17q21.33	LUC7L3	30.23985238	24.38109053	1.240299417	4	4	39.71993849	39.24219587
chr10	124461772	124614141	10q26.13	LHPP	4.734088816	3.816892175	1.240299332	4	4	58.20682125	31.23717596
chr10	46772700	46776308	10q11.22	DUSP8P4	0.221499222	0.178588606	1.240276338	1	1	0	0
chr3	88051950	88149866	3p11.1	CGGBP1	50.81404402	40.97248981	1.240199076	4	4	27.66442072	19.02384382
chr2	84971108	85059472	2p11.2	KCMF1	15.19580302	12.25359756	1.240109523	4	4	30.82975113	9.192163872
chr12	109059986	109060100	12q24.11	RNA5SP372	3.087351123	2.489772123	1.240013532	1	3	0	33.62068722
chr12	56663341	56689575	12	PTGES3	195.6169272	157.7599209	1.239965932	4	4	10.33017733	15.91320625

chr13	76992597	77027196	13q22.3	FBXL3	40.62977814	32.76688683	1.239964552	4	4	8.064483989	13.67522005
chr19	19033578	19058178	19p13.11	ARMC6	3.365554108	2.714250569	1.239957042	4	4	44.88172576	34.69798061
chr10	119829404	119873628	10q26.11	MCMBP	27.4817833	22.16544709	1.239847913	4	4	45.85906502	39.61823209
chr14	70520817	70564742	14q24.2	ADAM20	0.107759639	0.086918541	1.23977735	2	2	30.13270831	1.255717296
chr11	62792125	62805492	11q12.3	NXF1	13.38687596	10.79862242	1.239683679	4	4	57.59248855	23.62176019
chr19	1905214	1913447	19p13.3	ADAT3	0.367073525	0.296103857	1.239678299	3	4	58.17992526	66.99021434
chr14	50637691	50637981	14q22.1	RN7SL452P	2.114552408	1.705761004	1.239653388	3	2	76.30710879	19.36252397
chr3	5122245	5180916	3p26.1	ARL8B	66.78446934	53.87402112	1.239641444	4	4	10.91086204	24.42067988
chr16	8852942	8869012	16p13.2	CARHSP1	10.1326393	8.174381954	1.239560294	4	4	8.981367407	20.8965321
chr5	127038454	127073539	5q23.2	C5orf63	1.135805489	0.916329772	1.239516083	4	4	35.72021555	29.65093307
chr1	43450607	43454241	1p34.2	HYI	1.084241621	0.874740631	1.239500696	4	4	39.17384744	10.23440925
chr7	87834499	87876377	7q21.12	SLC25A40	13.46305858	10.86192086	1.239473087	4	4	14.74063402	19.67909838
chr18	50266882	50281774	18q21.1	MBD1	8.805215239	7.10408275	1.239458428	4	4	54.44924964	27.19685763
chr2	38131105	38181855	2p22.2	CYP1B1-AS1	0.263670754	0.212733185	1.239443455	4	3	90.27508432	16.02085003
chr2	202199463	202202021	2q33.1	DAZAP2P1	0.943800778	0.761478535	1.239431888	2	2	15.92376464	21.4243308
chr14	39031919	39103528	14q21.1	SEC23A	12.3280832	9.946598106	1.239427095	4	4	30.86056314	23.2304538
chr20	60138492	60322256	20q13.33	MIR646HG	2.677866734	2.160692308	1.239355888	4	4	4.976270762	43.38251623
chr10	69630241	69633599	10q22.1	FAM241B	0.243974271	0.196866398	1.239288544	1	1	0	0
chr10	63222124	63223134	10q21.3	TATDN1P1	1.440549281	1.162419694	1.239267787	3	4	22.97214532	93.47675242
chr13	49308650	49444851	13q14.2	CAB39L	2.7706056	2.235694107	1.239259696	4	4	36.97242202	21.72515666
chr2	178522827	178620217	2q31.2	TTN-AS1	4.1125596	3.318610496	1.239241425	4	4	37.24118487	21.9675616
chr14	76151885	76214473	14q24.3	GPATCH2L	7.687479812	6.203663115	1.239183958	4	4	40.00387492	15.89446236
chr13	49890409	49893380	13q14.2	CTAGE10P	0.131552205	0.106166561	1.239111489	1	1	0	0
chr11	57597554	57614853	11q12.1	SERPING1	8.431643614	6.804845858	1.2390646	4	4	72.43608459	108.7320615
chr5	16451519	16465785	5p15.1	ZNF622	18.23558798	14.7182094	1.23898142	4	4	39.04019633	28.1532291
chr9	99906633	99974541	9q31.1	STX17	34.3528886	27.7274099	1.238950508	4	4	48.86938138	41.26688591
chr1	160367071	160372848	1q23.2	NHLH1	0.142477744	0.114999785	1.238939219	2	1	30.62322617	0
chr18	11851390	11854449	18p11.21	CHMP1B	65.24351524	52.66154919	1.238921305	4	4	39.67468523	32.52075301
chr13	21628398	21629059	13q12.11	RPS7P10	0.563780111	0.455075224	1.238872347	2	2	0.213001826	9.301243874
chr20	35954561	36030700	20q11.23	CNBD2	0.484440945	0.391042701	1.238844105	4	4	77.47786888	64.56371787
chr1	34862120	34929585	1p34.3	DLGAP3	0.214944321	0.173508006	1.238815006	3	2	63.3838197	32.87909431
chr4	40749874	40815729	4p14	NSUN7	1.50069152	1.211460251	1.238745984	4	3	80.58793149	18.4479937
chr6	110424587	110476674	6q21-q22.1	SLC22A16	5.380869362	4.34401861	1.238684694	4	4	74.42927274	58.73675404
chr15	49155759	49367883	15q21.1-q21.2	GALK2	11.15407144	9.005657417	1.238562709	4	4	43.37209711	30.23971874
chrX	18425605	18653629	Xp22.13	CDKL5	2.339443732	1.88884657	1.238556784	4	4	61.22874727	48.19757096
chr16	1771889	1773151	16p13.3	MRPS34	17.59933173	14.20971181	1.238542482	4	4	78.43740274	57.69757922
chr11	34150987	34358008	11p13	ABTB2	0.956610915	0.772392643	1.2385034	4	4	92.01822753	90.56570711
chr1	64833223	65067746	1p31.3	JAK1	194.8303761	157.312907	1.238489453	4	4	45.1896434	23.76805583
chr18	35332208	35344467	18q12.2	ZNF24	44.06775247	35.58313293	1.238444984	4	4	30.60905562	22.44221522
chr6	149658153	149718256	6q25.1	LATS1	15.15476002	12.23732174	1.238404967	4	4	16.98152052	4.150794058
chr1	45550779	45570051	1p34.1	AKR1A1	7.696482764	6.214867876	1.238398453	4	4	42.24662202	20.59634321
chr1	149475898	149556361	1q21.2	NBPF19	2.827343494	2.283080549	1.238389725	4	4	53.83548646	25.98142006
chr22	26524082	26590141	22q12.1	TPST2	15.53643616	12.54598961	1.238358762	4	4	34.26723961	17.91603397

chrX	155881280	155943769	Xq28 and Yq12	VAMP7	67.81053186	54.76252421	1.238265271	4	4	17.49121352	15.41450357
chr4	25234031	25279209	4p15.2	PI4K2B	8.120561522	6.558100407	1.238249038	4	4	6.336979342	25.09722342
chr14	50633104	50668353	14q22.1	SAV1	9.783742217	7.901472987	1.238217511	4	4	24.14563803	19.87246845
chr15	55202966	55291338	15q21.3	RAB27A	29.23854892	23.61399361	1.238187382	4	4	37.18972491	24.7605215
chrX	100886919	100957401	Xq22.1	XKRX	0.43237263	0.349220858	1.238106544	2	4	14.28953219	70.56981942
chr14	24249114	24263210	14q12	TGM1	0.169503879	0.136907346	1.238091921	3	4	7.507460131	55.0973913
chr13	47763559	47764572	13q14.2	NAP1L4P3	0.294755334	0.238077625	1.238063989	3	2	23.10319607	2.598323487
chr2	112482127	112532646	2q14.1	TTL	3.909670275	3.157986884	1.238026128	4	4	30.51922755	12.23370672
chr7	151238973	151277452	7q36.1	SMARCD3	2.013061083	1.626048195	1.238008252	4	4	71.79753066	19.76516186
chr12	62643982	62934885	12q14.1-q14.2	PPM1H	0.833189695	0.673038443	1.237952607	4	4	105.8435448	99.43016115
chr11	94706418	94876753	11q21	AMOTL1	0.669404985	0.540747374	1.237925539	4	4	98.47391489	29.20201421
chr1	160608100	160647311	1q23.3	SLAMF1	4.347593817	3.512190084	1.237858349	4	4	42.2965456	34.07280509
chr13	44400250	44405984	13q14.11	TUSC8	0.215042973	0.173735468	1.237760924	1	1	0	0
chr7	151341699	151378461	7q36.1	NUB1	46.8476925	37.85032497	1.237709122	4	4	31.45143862	28.9540961
chr20	38962338	39039723	20q11.23-q12	DHX35	2.937265185	2.373351259	1.237602388	4	4	28.47217289	26.91253749
chr14	51847131	51969800	14q22.1	GNG2	71.72368826	57.95842928	1.237502278	4	4	42.60258543	27.57379123
chr2	181456892	181538928	2q31.3	ITGA4	106.6280843	86.16568865	1.237477307	4	4	18.43941012	16.64818248
chr22	38734714	38756019	22q13.1	SUN2	65.91266576	53.26410168	1.237468833	4	4	47.7236596	32.79446682
chr16	31201486	31202776	16p11.2	PYCARD	29.62663844	23.94238357	1.237413909	4	4	66.81148255	46.08396484
chr8	143437655	143549729	8q24.3	ZC3H3	2.822016282	2.280616514	1.237391847	4	4	69.35648508	35.87488317
chr3	47585888	47781915	3p21.31	SMARCC1	49.89983034	40.3299535	1.237289558	4	4	13.38876035	9.896059032
chr10	92291163	92353964	10q23.32-q23.33	MARCH5	9.567151153	7.733677266	1.237076597	4	4	17.80517354	22.64355934
chr13	52652691	52700756	13q14.3	SUGT1	11.46661198	9.269403393	1.237038835	4	4	51.3270453	19.35829383
chr4	42561880	42562422	4p13	CCNL2P1	1.768355561	1.429557215	1.236995303	3	4	58.03440931	27.03994707
chr4	55948808	56033363	4q12	CEP135	43.23987657	34.95884164	1.236879557	4	4	74.91944755	38.89070424
chr7	155644494	155781485	7q36.3	RBM33	245.3352641	198.3509487	1.236874669	4	4	53.06267067	22.89146572
chr14	22519969	22520031	14q11.2	TRAJ24	15.13729721	12.24087407	1.23661898	3	4	76.18324141	52.81146601
chr1	109064376	109076002	1p13.3	TAF13	16.56150443	13.39258054	1.236617871	4	4	17.95030887	20.99023183
chrX	55717677	55760209	Xp11.21	RRAGB	6.274423182	5.073931348	1.236599936	4	4	31.00938599	9.077959087
chr1	5862808	5992473	1p36.31	NPHP4	0.441626836	0.357132392	1.236591376	4	4	73.09883149	38.42818623
chr9	93576627	93679587	9q22.31	PHF2	14.19361553	11.47804961	1.236587749	4	4	41.09000399	27.38734671
chr11	118359581	118399211	11q23.3	UBE4A	30.60495725	24.7505186	1.23653802	4	4	47.52998224	19.30258289
chr8	63168553	63212788	8q12.3	YTHDF3	40.10330912	32.43212639	1.236530366	4	4	35.34745917	15.77379017
chr1	15939933	15976132	1p36.13	ZBTB17	4.716094236	3.814080459	1.236495739	4	4	72.62138102	45.87875932
chr3	134157133	134250834	3q22.2	RYK	13.49260185	10.91218203	1.236471479	4	4	56.0875461	22.16542077
chr14	105838401	105845677	14q32.33	IGHD	19.40327048	15.69428441	1.236327186	4	4	118.3315887	103.3066983
chr19	15451624	15464581	19p13.12	RASAL3	18.19240996	14.71515392	1.236304429	4	4	53.74024335	52.43532002
chr3	64004022	64012243	3p14.1	PSMD6-AS2	10.40678479	8.417683065	1.236300383	4	4	50.42342182	25.58871869
chr9	35736862	35749236	9p13.3	GBA2	10.22135313	8.268228392	1.236220463	4	4	48.73751179	35.64976212
chr9	122816058	122828656	9q33.2	PDCL	14.73321494	11.91814164	1.236200691	4	4	17.8176823	12.31482624
chr7	155297772	155310235	7q36.3	INSIG1	28.30323458	22.89539675	1.236197603	4	4	28.13465622	16.22424277
chr6	34240673	34240736	6p21.31	MIR6835	9.863389741	7.979023088	1.236165083	3	1	42.91901345	0
chr12	39626167	39908300	12q12	C12orf40	0.212370154	0.171797751	1.236163755	3	1	60.6379892	0

chr22	43110748	43129712	22q13.2	BIK	1.569235761	1.269452202	1.236151908	4	3	83.89810861	43.22382453
chr2	177392743	177543836	2q31.2	AGPS	16.93547474	13.70144179	1.23603596	4	4	10.40712186	17.45239807
chr10	119499827	119542710	10q26.11	RGS10	364.8521913	295.1831148	1.236019857	4	4	38.9473162	41.81635651
chr19	4343527	4360086	19p13.3	MPND	3.67621425	2.974303027	1.235991833	4	4	34.65497645	33.68242199
chr17	77088643	77094986	17q25.2	SNHG20	2.576518088	2.084624755	1.235962531	4	4	12.81117576	33.69895233
chr3	49015399	49021251	3p21.31	DALRD3	2.405773497	1.946564526	1.235907398	4	4	48.92028334	10.16997948
chr14	20425852	20436518	14q11.2	KLHL33	0.260806957	0.211025574	1.235902133	3	3	52.06839253	70.75078945
chr13	60396457	60573879	13q21.2	TDRD3	25.13511613	20.33821266	1.235856688	4	4	63.12423653	29.54476069
chr12	6346843	6377357	12p13.31	SCNN1A	0.162091529	0.131158828	1.235841546	2	4	55.41016352	29.01187155
chr4	146253981	146522351	4q31.22	SLC10A7	12.82755595	10.3812426	1.23564745	4	4	59.36654962	30.08407998
chr7	99890407	99919600	7q22.1	TRIM4	10.08909298	8.165167927	1.235625901	4	4	41.87637572	19.24722413
chr5	133955507	133968715	5q31.1	C5orf15	32.84997453	26.58613107	1.235605679	4	4	20.18707405	15.35573868
chr2	20682489	20823139	2p24.1	LDAH	11.40205846	9.228684877	1.235501982	4	4	68.46125794	32.75801314
chr1	161766244	161964070	1q23.3	ATF6	46.16614406	37.36832462	1.235435212	4	4	27.3676021	24.86797627
chr1	20651767	20661544	1p36.12	DDOST	33.15254885	26.83480591	1.235430916	4	4	51.5120379	27.69937353
chr4	123396791	123403760	4q28.1	SPRY1	0.487310923	0.394457847	1.235394167	3	4	71.60787025	16.8337609
chr4	112231739	112273110	4q25	AP1AR	9.127444074	7.388981748	1.235277659	4	4	23.63643824	33.00237996
chr18	60016590	60017484	18q21.32	SINHCAFP2	0.498164609	0.40328407	1.235269741	2	1	47.77892117	0
chr8	102826302	102864200	8q22.3	AZIN1	65.15497062	52.74915928	1.235185006	4	4	13.21739428	15.72143466
chr5	139341832	139369720	5q31.2	PAIP2	205.8075947	166.6328352	1.235096279	4	4	37.98565265	9.76277951
chr5	43444252	43484659	5p12	TMEM267	4.943975113	4.002911577	1.23509476	4	4	26.36822329	20.02075907
chr19	46709293	46709345	19q13.32	MIR320E	13.18583922	10.67653554	1.235029769	3	1	48.9496522	0
chr12	103746315	103841239	12q23.3	NT5DC3	1.002895951	0.812071032	1.234985503	4	4	52.50132016	38.67858632
chr4	177430774	177442503	4q34.3	AGA	7.102675438	5.751566042	1.234911568	4	4	43.06481252	33.88550009
chr16	11927263	11976650	16p13.13	NPIPB2	1.22657417	0.993255191	1.234903357	4	4	50.4389298	76.34662517
chr3	9792495	9807105	3p25.3	ARPC4	26.09934652	21.13538382	1.234865037	4	4	38.92943655	15.33056845
chr11	62790315	62792014	11q12.3	TMEM223	2.894634273	2.34422283	1.234794848	4	4	80.33574722	42.85835362
chr12	15911172	16037381	12p12.3	DERA	10.56663125	8.557569357	1.234770156	4	4	11.19846134	3.711182319
chr18	57600656	57621945	18q21.31	NARS	68.17170015	55.21141436	1.234739246	4	4	16.751196	21.05388987
chr5	57173842	57264679	5q11.2	GPBP1	126.8833909	102.7650235	1.234694321	4	4	41.40860446	25.40341144
chr17	64542282	64662268	17q23.3-q24.1	SMURF2	10.36151562	8.39214613	1.23466816	4	4	40.81791801	17.5316196
chr13	52617484	52627573	13q14.3	MRPS31P4	2.431724319	1.969543662	1.234663829	4	4	73.33253901	12.59384144
chr2	152651593	152718012	2q23.3	PRPF40A	39.66068419	32.12298484	1.234651275	4	4	40.00728785	19.52465163
chr9	98072014	98119420	9q22.33	TRIM14	9.423919312	7.632971719	1.234633071	4	4	43.06085119	56.16776584
chr13	45464895	45536698	13q14.13	COG3	25.62664483	20.75678559	1.234615288	4	4	76.74150788	24.74945435
chr1	55066359	55215374	1p32.3	USP24	32.64386508	26.44069057	1.23460713	4	4	22.57949499	5.527642379
chr2	70124036	70125316	2p13.3	LINC01816	1.757419088	1.423660178	1.234437203	4	4	36.29521883	29.73070356
chr16	69105564	69118719	16q22.1	HAS3	0.119127638	0.096509226	1.234365283	2	4	13.13886122	97.25281857
chr6	35342558	35428191	6p21.31	PPARD	2.978585457	2.4131563	1.234311038	4	4	93.36871494	38.0318346
chr8	47997527	48063542	8q11.21	UBE2V2	14.28111312	11.57065131	1.234253175	4	4	27.01048821	18.78998355
chr7	5190204	5233855	7p22.1	WIPI2	13.83744688	11.21157672	1.234210605	4	4	14.96048625	15.51323779
chr19	1815246	1848683	19p13.3	REXO1	3.865478716	3.131984951	1.234194537	4	4	55.50791292	26.77726609
chr3	47011542	47013467	3p21.31	NRADDP	1.02495066	0.830473908	1.234175632	3	2	45.38271021	63.75844351

chr12	120469857	120498495	12q24.31	DYNLL1	31.39330486	25.43684905	1.234166417	4	4	19.93626746	20.73653784
chr20	18486540	18497250	20p11.23	RBBP9	6.121234523	4.960127091	1.234088242	4	4	43.25336004	34.42655775
chr1	150926246	150964744	1q21.3	SETDB1	10.99260943	8.907725553	1.234053448	4	4	36.64980231	35.28894201
chr3	186783572	186789897	3q27.3	EIF4A2	84.63624268	68.58575237	1.234020766	4	4	37.99118198	20.99498584
chr1	16231687	16237445	1p36.13	CPLANE2	0.869011114	0.704227106	1.233992708	4	4	54.04702969	88.39612745
chr10	22316383	22320317	10p12.2	COMMD3	4.000359064	3.242074643	1.233888637	4	4	21.45831287	31.1221527
chr20	24962925	24992930	20p11.21	APMAP	73.53605604	59.59880901	1.2338511	4	4	53.82821437	31.66969364
chr16	71758402	71758487	16q22.2	SNORD71	12.81685374	10.38909079	1.23368387	4	4	97.50749755	126.7211969
chr12	54549393	54579239	12q13.2	PDE1B	0.5162649	0.418486225	1.233648492	4	4	75.72811483	58.09369684
chr15	41332694	41381050	15q15.1	NUSAP1	476.9733258	386.6411017	1.233633268	4	4	72.87959637	11.48961165
chr2	27392623	27393576	2p23.3	FTH1P3	1.322110757	1.07182703	1.233511303	2	2	78.97710387	17.70370441
chr13	50909678	50970462	13q14.3	RNASEH2B	31.39740793	25.45419322	1.233486666	4	4	56.16877386	29.68942697
chr4	165076078	165112913	4q32.3	TMEM192	2.660418184	2.156974705	1.233402588	4	4	31.58452475	7.746795113
chr20	45890144	45898820	20q13.12	CTSA	38.84330362	31.49532608	1.233303746	4	4	53.82513374	33.54791112
chr15	76931621	76950260	15q24.3	RCN2	4.262659144	3.456370845	1.233275981	4	4	48.46151527	24.55294754
chr19	47331614	47342080	19q13.32	C5AR2	8.689833909	7.046590617	1.233196929	4	4	59.2496181	50.47280643
chr20	21125975	21246622	20p11.23	KIZ	10.46498358	8.486446239	1.233140856	4	4	19.24335075	24.18688041
chr14	75650890	75661195	14q24.3	ERG28	4.40215021	3.570218922	1.233019685	4	4	51.78797767	56.05150485
chr1	99708699	99766630	1p21.2	FRRS1	3.345752292	2.713529902	1.232988916	4	4	42.15568183	41.15740175
chr2	27130756	27134675	2p23.3	PREB	8.161172342	6.619319343	1.232932258	4	4	62.9876911	43.05744436
chr19	46466491	46471577	19q13.32	PNMA8A	0.08594674	0.069709571	1.232925962	1	1	0	0
chrX	87703273	87704306	Xq21.31	RPSAP15	0.386785596	0.313766062	1.232719669	1	1	0	0
chr3	65354231	66038834	3p14.1	MAGI1	0.027442818	0.022262836	1.232673936	2	2	16.48542023	4.411666795
chr5	112707505	112846239	5q22.2	APC	25.42199975	20.62433854	1.232621337	4	4	22.79025047	26.56728216
chr20	34844720	34872858	20q11.22	GGT7	1.058317189	0.858604375	1.232601673	3	4	79.65585094	88.81290021
chr19	34353330	34402413	19q13.11	GPI	9.510408994	7.716036857	1.232551006	4	4	73.54771521	45.0553027
chr16	50317562	50368969	16q12.1	BRD7	14.67569984	11.90716421	1.232510073	4	4	52.5083231	36.78355325
chr1	155320849	155331118	1q22	RUSC1	0.912016331	0.739981615	1.232485122	4	4	76.03319338	33.3781521
chr19	40799143	40808441	19q13.2	EGLN2	4.925634139	3.99660458	1.23245471	4	4	70.54335321	24.57251489
chr3	128619970	128650876	3q21.3	RPN1	55.25636997	44.83475748	1.23244494	4	4	62.5239856	26.87296362
chr16	23835979	24220611	16p12.2-p12.1	PRKCB	63.36571519	51.41499704	1.232436426	4	4	41.58488267	29.07855627
chr21	31668987	31732118	21q22.11	SCAF4	16.26380808	13.19717803	1.232370136	4	4	24.71171206	37.70767653
chrX	104099214	104157027	Xq22.2	SLC25A53	1.907579361	1.548037721	1.232256382	4	4	42.22469165	28.28305438
chr11	65386570	65413525	11q13.1	FRMD8	10.37747907	8.421702426	1.232230557	4	4	48.65944172	17.60501497
chr12	121803665	121832656	12q24.31	SETD1B	6.724128211	5.456982567	1.232206284	4	4	78.23172267	40.88119611
chr19	16197854	16235345	19p13.11	AP1M1	4.746945739	3.852483192	1.232178183	4	4	55.68517403	31.17313878
chr10	96832260	96986212	10q24.1	LCOR	16.77805047	13.61907583	1.231952203	4	4	28.15573182	22.60868707
chr6	25023009	25024591	6p22.3	ASS1P1	1.210687489	0.982765392	1.231919132	3	4	69.05177072	91.786438
chr19	1473201	1479229	19p13.3	C19orf25	2.433689735	1.975553942	1.231902447	4	4	52.5913446	37.00311096
chr12	3808861	3873471	12p13.32	PARP11	7.414112287	6.018575154	1.23187168	4	4	35.85227181	25.34974243
chr8	91102619	91219254	8q21.3	LRRC69	1.844656461	1.497530877	1.231798615	4	4	39.08538421	77.66553782
chr10	124939489	124987436	10q26.13	ZRANB1	72.43410193	58.80710237	1.231723704	4	4	26.89800893	35.42512132
chr15	68778535	68820922	15q23	ANP32A	37.0785672	30.10661916	1.231575256	4	4	52.59549748	27.83141211

chr22	24270817	24417740	22q11.23	SPECC1L	2.526330763	2.05149548	1.231458118	4	4	52.54216987	36.35541842
chr9	39443815	39464526	9p12	ZNF658B	0.35436912	0.287782737	1.231377269	4	3	123.8557849	39.36184311
chr1	1311585	1324687	1p36.33	INTS11	4.042349999	3.282878645	1.231343109	4	4	49.34705485	38.88535275
chr1	108648288	108661526	1p13.3	HENMT1	11.73686457	9.532350924	1.231266522	4	4	34.89326741	29.40607445
chr14	58265365	58298137	14q23.1	PSMA3-AS1	21.84672199	17.74336967	1.231261163	4	4	27.71198929	28.47934997
chr5	170331393	170335100	5q35.1	LINC01366	1.043852457	0.847795132	1.231255544	3	3	31.47597136	30.06370637
chr2	24077428	24085767	2p23.3	TP53I3	1.30102666	1.05668437	1.231234886	4	4	107.5513856	21.00395712
chr1	180913177	180946103	1q25.3	KIAA1614	0.062100843	0.050443096	1.231106885	2	1	69.39082914	0
chr10	72367326	72626191	10q22.1	MICU1	24.81215109	20.15480916	1.231078443	4	4	29.0939535	11.29225292
chr8	98117293	98159841	8q22.2	POP1	1.439092548	1.169081988	1.230959473	4	4	23.00309503	47.21579425
chrX	129779916	129795201	Xq26.1	SASH3	98.34278243	79.8946098	1.230906349	4	4	48.68574045	54.02117492
chr4	169729468	169757942	4q33	HPF1	17.60141027	14.30176806	1.230715685	4	4	24.56705015	19.10996045
chr2	39248941	39437312	2p22.1	MAP4K3	15.87159676	12.89625162	1.230713949	4	4	51.74072299	42.98902717
chr5	140700360	140706681	5q31.3	ZMAT2	175.1369145	142.3076053	1.230692584	4	4	20.91221101	26.71035522
chr22	20917407	20953749	22q11.21	CRKL	43.5612688	35.40352634	1.230421749	4	4	12.43824686	16.74791774
chr12	95020229	95073628	12q22	NR2C1	5.714479964	4.644532508	1.230367094	4	4	45.79173	48.75161269
chr3	160567779	160569472	3q25.33	KRT8P12	1.563363339	1.270653332	1.230361815	4	4	111.8489569	60.18356411
chr3	119468938	119494708	3q13.33	POGLUT1	3.417037197	2.777324019	1.23033437	4	4	23.38775185	25.75551183
chr18	59139857	59158837	18q21.32	SEC11C	6.078973872	4.941169323	1.230270302	4	4	44.25610629	14.7885026
chr10	80537902	80646560	10q23.1	SH2D4B	0.06566643	0.053376847	1.230241841	1	1	0	0
chr12	128267170	128707915	12q24.32-q24.33	TMEM132C	0.063356518	0.051499509	1.230235379	2	2	13.4910949	0.576526201
chr1	224385143	224434767	1q42.11-q42.12	WDR26	130.1553926	105.801218	1.230188037	4	4	35.47401686	75.66380422
chr15	89896532	89912990	15q26.1	ARPIN	1.456386067	1.18391532	1.23014378	4	3	81.02959467	42.39067359
chr12	79773563	79935764	12q21.2-q21.31	PPP1R12A	48.83316325	39.70058458	1.230036378	4	4	29.41605434	29.09252682
chr16	2026868	2039026	16p13.3	SLC9A3R2	0.310821363	0.252702543	1.229989061	4	3	51.71147462	49.14489837
chr6	39904258	39934551	6p21.2	MOCS1	0.120865123	0.09826541	1.22998645	1	2	0	4.688719138
chr8	102204501	102239118	8q22.3	RRM2B	28.10539093	22.8510017	1.229941308	4	4	38.40998859	44.43328875
chrX	74582976	74614626	Xq13.2	RLIM	30.81577774	25.05514233	1.229918287	4	4	27.18517625	24.55033205
chr1	116423724	116478826	1p13.1	LINC01762	0.515051422	0.418773345	1.229904979	4	3	45.58739725	70.06864029
chr1	235328438	235344532	1q42.3	GGPS1	22.21248259	18.06085692	1.229868698	4	4	20.93975121	20.6441945
chr22	37608474	37633564	22q13.1	GGA1	2.867043222	2.331190346	1.229862344	4	4	61.12638918	23.8524636
chr16	8797813	8849337	16p13.2	PMM2	2.555501918	2.07793059	1.22983026	4	4	52.08975695	14.28934824
chr12	101697639	101752038	12q23.2	CHPT1	61.36676657	49.90276607	1.229726755	4	4	58.18158246	38.31499326
chr16	3725054	3880727	16p13.3	CREBBP	23.19576541	18.86372863	1.229649019	4	4	42.161525	21.43313705
chr2	86913577	87013976	2p11.2	RGPD1	0.64471211	0.524336096	1.229577964	4	4	127.6517503	68.3860742
chr12	9595274	9607901	12p13.31	KLRB1	80.81656436	65.72893442	1.2295432	4	4	50.95564227	48.48652988
chr19	9291140	9309714	19p13.2	ZNF699	5.815756888	4.730068589	1.229529082	4	4	43.53326347	21.61066754
chr6	110243895	110358272	6q21	METTL24	0.17453118	0.141957353	1.229462066	3	2	33.92644241	7.898626733
chr14	94015600	94048930	14q32.12	OTUB2	0.073365792	0.059677422	1.229372672	2	2	11.92748219	1.094977896
chr11	67491760	67505372	11q13.2	PITPNM1	10.59425442	8.617655232	1.229366241	4	4	70.58121751	29.82686113
chr19	35382112	35384253	19q13.12	EEF1A1P7	0.205511075	0.16716964	1.229356451	1	1	0	0
chr11	94567308	94621421	11q21	PIWIL4	1.435907873	1.168107421	1.229260124	4	4	26.97552855	42.8550954
chr2	241356249	241494842	2q37.3	FARP2	5.287614766	4.301496639	1.229250005	4	4	60.26675331	26.10389737

chr11	114391448	114400550	11q23.2	C11orf71	7.187898386	5.848550504	1.229005098	4	4	23.60521836	34.66822751
chr14	77717599	77761156	14q24.3	SNW1	57.52219677	46.80519545	1.228970336	4	4	55.45490634	25.51778884
chr9	89311195	89316703	9q22.2	CKS2	17.20090611	13.99630054	1.228960899	4	4	54.17156153	60.7478252
chr16	20780193	20806473	16p12.3	ERI2	2.902040492	2.361439426	1.228928619	4	4	52.81773423	20.24646027
chr11	10797046	10809035	11p15.4	EIF4G2	99.67759119	81.11014609	1.22891643	4	4	50.13819667	20.04828416
chr17	62627401	62693601	17q23.2	MRC2	7.989712301	6.50163058	1.228878233	4	4	85.09492194	65.28252044
chr5	154559315	154560518	5q33.2	CIR1P1	0.373281993	0.303758951	1.228875696	1	1	0	0
chr10	97319265	97321915	10q24.1	FRAT1	24.68738978	20.09013953	1.228831176	4	4	59.29730508	47.76183111
chr6	24774931	24786099	6p22.3	GMNN	13.04116798	10.61317244	1.228771892	4	4	83.35373481	48.12492172
chr14	54426929	54441430	14q22.2	CNIH1	13.11015855	10.6702213	1.228667915	4	4	22.56482778	8.326146177
chr8	100509752	100559786	8q22.3	ANKRD46	4.750439182	3.866351421	1.228662029	4	4	28.15624668	41.02171946
chr13	49982549	50125541	13q14.2	DLEU2	121.4509134	98.85232441	1.228609586	4	4	84.61100908	58.71015601
chr19	49527579	49543638	19q13.33	RCN3	2.34081383	1.905281371	1.228592199	4	4	107.7988122	62.05482382
chr1	3630767	3650107	1p36.32	WRAP73	1.854313991	1.509313249	1.228581272	4	4	51.50643876	34.06391696
chr5	138946720	139198376	5q31.2	SIL1	4.87842312	3.970810825	1.228571024	4	4	32.70124745	18.80621222
chr11	62728073	62738636	11q12.3	TTC9C	9.274518936	7.549513395	1.228492282	4	4	10.90961318	21.68973548
chr16	16149565	16223617	16p13.11	ABCC6	0.108688049	0.088476288	1.22844268	3	3	78.23278342	54.61394168
chr8	140511298	140517153	8q24.3	CHRAC1	10.5791295	8.611942098	1.228425526	4	4	60.12849839	24.14194668
chr6	17759183	17987623	6p22.3	KIF13A	13.76171342	11.20285387	1.228411401	4	4	49.76047243	39.71592297
chr12	111841978	111902225	12q24.12-q24.13	MAPKAPK5	3.949673792	3.215277161	1.228408499	4	4	30.49360022	21.80645941
chr16	11750586	11797258	16p13.13	ZC3H7A	15.34300672	12.49074467	1.22835004	4	4	22.66439443	11.71948073
chr19	1609284	1652546	19p13.3	TCF3	11.76133266	9.575399465	1.228286371	4	4	24.48017418	52.70369846
chr7	6577413	6588996	7p22.1	ZDHHC4	9.532442184	7.761645368	1.228147092	4	4	41.06510677	15.01111212
chr4	121801317	121817021	4q27	EXOSC9	17.5082318	14.25621186	1.228112487	4	4	32.2593332	14.90997875
chr14	50703994	50705511	14q22.1	ZFP64P1	0.245082406	0.199564378	1.228086942	1	1	0	0
chr5	60873832	60945078	5q12.1	ERCC8	4.996056258	4.068318573	1.228039586	4	4	37.51601707	20.37267637
chr20	32853904	32961845	20q11.21	EFCAB8	0.244768732	0.199323737	1.227995904	3	4	15.94664247	65.80397177
chr2	64524305	64593005	2p14	AFTPH	56.83189764	46.28082794	1.227979277	4	4	22.45306863	17.63269041
chr5	141413176	141512979	5q31.3	PCDHGA10	0.084752653	0.069018999	1.227961206	2	3	19.55672296	74.08477122
chr3	172443291	172448456	3q26.31	GHSR	0.229884234	0.187216952	1.22790288	1	1	0	0
chr6	10396677	10419897	6p24.3	TFAP2A	0.039383338	0.032073876	1.227894536	1	2	0	0.831333447
chr10	124619294	124744361	10q26.13	FAM53B	23.18747309	18.88529346	1.227805813	4	4	41.91679096	37.21189056
chr6	89829900	89874436	6q15	CASP8AP2	39.53527364	32.20160932	1.227742168	4	4	34.35165227	12.72884943
chr1	54779696	54802379	1p32.3	TTC22	1.364276588	1.111232452	1.227714854	4	4	21.51091182	64.37501427
chr3	133784033	133821492	3q22.1	SRPRB	6.417094244	5.226919086	1.227701087	4	4	45.61168202	16.00190273
chr17	3636468	3663103	17p13.2	CTNS	3.583768157	2.919314876	1.227605897	4	4	37.68044339	34.91965637
chr6	31764135	31777331	6p21.33	VWA7	0.299233026	0.243757314	1.227585837	3	2	86.93367774	41.17594514
chr13	19736975	19737254	13q12.11	RN7SL166P	3.148212104	2.564612379	1.227558648	2	4	1.284806937	28.61991903
chr16	3020312	3022383	16p13.3	TNFRSF12A	1.886946822	1.537156042	1.227557106	4	4	83.82835782	44.13872853
chr12	122167719	122203471	12q24.31	LRRC43	0.522656304	0.425777857	1.227532844	4	4	85.24151034	62.3349372
chr7	30548357	30577779	7p14.3	GARS-DT	4.108073561	3.346941899	1.227411077	4	4	43.85603698	32.51426317
chr1	11674480	11691621	1p36.22	MAD2L2	2.604822112	2.122420832	1.227288233	4	4	44.06018763	42.58629072
chr7	107743697	107764997	7q22.3	CBLL1	19.20305714	15.64739027	1.227237054	4	4	23.20098336	9.507450444

chr3	10285750	10292947	3p25.3	GHRL	2.422759375	1.974338724	1.227124477	4	4	54.6192802	28.18231312
chr13	52012387	52029648	13q14.3	ALG11	2.624858926	2.139152268	1.227055673	4	4	32.55957138	4.198049595
chr6	30135998	30148773	6p22.1	TRIM40	0.66070457	0.538453203	1.227041767	3	4	53.12493247	51.07327782
chr6	167111807	167139141	6q27	CCR6	0.481074146	0.392069899	1.227011172	4	4	76.74300704	89.32383117
chr5	1801382	1816051	5p15.33	NDUFS6	14.89582757	12.14059033	1.226944256	4	4	64.81812655	39.82504721
chr1	227918656	227947930	1q42.13	WNT9A	0.077902573	0.063493965	1.226928777	2	1	18.2847505	0
chr20	17968594	17991122	20p11.23	MGME1	16.84377469	13.72866238	1.226905741	4	4	46.29907834	15.01350837
chr11	134253538	134265858	11q25	ACAD8	2.246468666	1.831027405	1.226889701	4	4	38.09423674	20.03943223
chr11	46617276	46676019	11p11.2	ATG13	16.18409879	13.19119072	1.226886878	4	4	41.13116383	11.1579696
chr10	133352773	133358031	10q26.3	FUOM	2.455522116	2.001563238	1.226802166	4	4	68.91893047	78.49735481
chr3	130678934	130746852	3q22.1	PIK3R4	16.32783768	13.30943297	1.226786875	4	4	38.82372527	13.24365753
chr17	20321174	20402191	17p11.2	CCDC144CP	5.008105941	4.082365508	1.22676569	4	4	31.32451136	41.54689723
chr5	157785743	157859175	5q33.3	CLINT1	26.41683229	21.53420806	1.226738044	4	4	39.15869751	36.44626532
chr17	42189087	42194532	17q21.2	GHDC	2.665702364	2.17309813	1.226682922	4	4	65.33988132	22.3312632
chr1	155745768	155750688	1q22	MSTO2P	0.573078993	0.46720652	1.226607438	3	4	12.08998757	46.05253606
chr6	52362128	52407777	6p12.2	PAQR8	5.043078138	4.111766297	1.226499216	4	4	67.50221216	46.18074226
chr4	102251041	102345498	4q24	SLC39A8	18.13935404	14.78973782	1.226482461	4	4	61.53546031	37.51360378
chr19	4969087	5153603	19p13.3	KDM4B	9.620139299	7.844305212	1.226385134	4	4	45.04813764	32.67584388
chr15	67839940	67840045	15q23	RNU6-1	17.62970433	14.37572574	1.2263523	4	4	60.65166005	49.11275136
chr16	8795180	8797648	16p13.2	TMEM186	1.800298577	1.468033527	1.22633342	4	4	48.31034476	39.23126439
chr4	109688629	109703473	4q25	CASP6	5.244264489	4.27647943	1.226304154	4	4	37.30698967	42.36261185
chr11	1309708	1310707	11p15.5	TOLLIP-AS1	0.959061712	0.782109344	1.226250165	2	4	8.336359268	31.98784392
chr6	63635802	63715522	6q12	PHF3	72.13645153	58.83042434	1.226175951	4	4	37.30967388	19.88383858
chr10	103967100	104029233	10q24.33-q25.1	SLK	77.39860117	63.12460325	1.226124161	4	4	14.42361072	24.53161172
chr5	115841606	115914081	5q22.3-q23.1	AP3S1	26.18810021	21.3587838	1.226104466	4	4	14.06512387	19.35579139
chr6	32968660	32981505	6p21.32	BRD2	19.48572501	15.89266706	1.226082754	4	4	67.05743043	38.64467479
chr5	176238359	176345991	5q35.2	SIMC1	3.186564036	2.599217703	1.225970427	4	4	38.32849201	34.64935651
chr2	227472169	227561222	2q36.3	AGFG1	21.82343082	17.80155539	1.225928316	4	4	10.21397109	17.37011196
chr12	56595596	56636816	12q13.3	BAZ2A	21.78329375	17.76935972	1.225890752	4	4	41.90846864	15.06953548
chr14	24078686	24115014	14q11.2-q12	NRL	0.461258983	0.376265058	1.225888434	4	4	87.07634788	54.44261478
chr16	50065941	50107272	16q12.1	HEATR3	8.009234221	6.533586389	1.225855716	4	4	14.47147592	29.99394523
chr2	27427790	27442259	2p23.3	NRBP1	23.75973756	19.3829324	1.225807173	4	4	36.88062349	17.24615274
chr3	179257260	179261567	3q26.32	LRRFIP1P1	0.731325679	0.596621957	1.225777345	3	3	59.1027576	34.92960715
chr5	160401641	160419161	5q33.3	SLU7	39.56276662	32.2780626	1.225685913	4	4	42.33147798	8.756092267
chr11	6748125	6750369	11p15.4	GVINP2	0.155771266	0.127093233	1.225645632	2	1	21.7025543	0
chr17	15999380	16045428	17p12	TTC19	5.163302293	4.212736112	1.225641045	4	4	45.07931154	14.75986917
chr16	668083	674174	16p13.3	RHOT2	5.049660472	4.12035804	1.225539243	4	4	105.4971302	12.58961558
chr10	12165328	12196144	10p14	NUDT5	11.39965851	9.30179729	1.225532889	4	4	60.46303688	33.83082688
chr11	59574398	59616144	11q12.1	OSBP	13.55267998	11.05935737	1.225449141	4	4	56.18551955	20.14732477
chr18	35240704	35258433	18q12.2	ZNF397	3.658697807	2.985657164	1.225424624	4	4	39.00671855	37.35290927
chr6	36594313	36604467	6p21.31-p21.2	SRSF3	49.18307898	40.13587809	1.225414301	4	4	23.4862269	20.63929504
chr1	70205682	70253052	1p31.1	SRSF11	21.59082636	17.62176377	1.225236397	4	4	35.99295833	4.917450241
chr2	130337933	130342681	2q21.1	CCDC115	7.943822965	6.483986058	1.225144979	4	4	63.76505837	23.91553107

chr6	85505496	85594156	6q14.3	SNX14	18.28429987	14.92500918	1.225077965	4	4	13.90472172	16.8040994
chr17	50478800	50504327	17q21.33	RSAD1	3.573276329	2.9172488	1.224878841	4	4	68.08137103	22.55834419
chr7	30167828	30168004	7p14.3	RPS27P16	2.858510876	2.333763204	1.224850436	1	4	0	50.96039319
chr6	15245975	15522042	6p22.3	JARID2	42.34145385	34.56925559	1.224829784	4	4	31.58969682	14.57886119
chr17	58968010	59106964	17q22	TRIM37	19.37132332	15.81735784	1.224687683	4	4	35.95892499	19.961456
chr2	32277889	32307398	2p22.3	YIPF4	10.95364786	8.944182107	1.224667357	4	4	17.50835031	53.51655216
chr1	108970211	109042228	1p13.3	WDR47	10.71896264	8.753239728	1.224570899	4	4	6.024628601	24.23621178
chr11	3855703	4093210	11p15.4	STIM1	15.00513409	12.25349238	1.224559793	4	4	50.01772391	29.5733075
chr18	76357682	76495255	18q23	ZNF516	10.21141398	8.338906052	1.224550788	4	4	54.02037735	38.46893741
chrX	40580964	40606637	Xp11.4	ATP6AP2	21.40962375	17.48450798	1.224491062	4	4	19.46770899	32.90891687
chr4	86934606	87141054	4q21.3-q22.1	AFF1	72.87323643	59.51330541	1.224486456	4	4	17.85505669	12.90649458
chr2	135905881	135985684	2q21.3	DARS	21.82313448	17.82424216	1.224351323	4	4	20.28779746	6.838102488
chr9	125234848	125241387	9q33.3	HSPA5	43.18410658	35.27326553	1.224272999	4	4	56.28305778	44.27095024
chr22	28687743	28741866	22q12.1	CHEK2	31.5675944	25.7854594	1.224240139	4	4	85.4166318	46.29842139
chr5	6599239	6633360	5p15.31	NSUN2	27.63890638	22.58121955	1.223977577	4	4	32.95813329	17.65686246
chr2	27032906	27041697	2p23.3	TMEM214	4.446622783	3.632935816	1.223975046	4	4	38.8643398	49.58602235
chr22	28794560	28800572	22q12	XBP1	45.99156431	37.57580473	1.223967514	4	4	35.67398424	22.66359783
chr17	1934677	2025345	17p13.3	RTN4RL1	0.23103029	0.188763156	1.223916229	2	3	43.75719353	57.10966807
chr2	38797729	38875934	2p22.1	DHX57	4.598353826	3.757090547	1.223913496	4	4	52.84819973	18.21156697
chr17	8072636	8087703	17p13.1	ALOX12B	0.213598746	0.174535637	1.22381165	1	1	0	0
chr3	126983259	127037392	3q21.3	PLXNA1	0.670987704	0.548313692	1.223729618	4	4	107.4833426	56.0277418
chr2	24972112	25039694	2p23.3	DNAJC27-AS1	0.222002153	0.181422946	1.223671853	4	4	51.65037939	63.2628883
chr3	196318332	196325570	3q29	TM4SF19-AS1	0.306023442	0.250139587	1.223410679	2	2	72.35568376	60.70418892
chr10	102854210	102864961	10q24.32	BORCS7	7.7015851	6.295314923	1.223383611	4	4	21.75534832	37.04999146
chrX	132218507	132432811	Xq26.2	RAP2C-AS1	1.084726225	0.886714859	1.223308953	4	4	40.76238248	50.36944993
chr19	12730640	12734775	19p13.13	TRIR	71.19780615	58.20416208	1.223242524	4	4	79.38065769	55.50748447
chr17	28042647	28205140	17q11.2	NLK	42.12645447	34.44107499	1.223145749	4	4	22.53441619	15.66105261
chr11	73675638	73761156	11q13.4	RAB6A	143.9644654	117.7044804	1.22310098	4	4	27.16088526	8.250661514
chr1	200969386	201023700	1q32.1	KIF21B	13.32846244	10.89742347	1.22308383	4	4	65.71648244	43.58157095
chr9	125747338	125967377	9q33.3	PBX3	23.34177387	19.08485122	1.223052441	4	4	59.63031245	17.30239044
chr5	6633322	6669562	5p15.31	SRD5A1	1.569929456	1.283787623	1.222888761	4	4	33.32196625	32.25749556
chrX	16786432	16844519	Xp22.2	TXLNG	14.42316575	11.79471193	1.222850192	4	4	46.74234658	25.35074675
chr22	39529093	39532855	22q13.1	RPS19BP1	5.602276905	4.581403844	1.222829747	4	4	52.55141501	22.45337852
chr22	50343317	50445090	22q13.33	PPP6R2	17.13413186	14.01306717	1.222725307	4	4	18.31077176	9.928293842
chr19	55675195	55695767	19q13.42	EPN1	3.239423401	2.649578376	1.222618448	4	4	55.76355064	36.28924076
chr20	21303284	21389825	20p11.22	XRN2	107.4791032	87.91463323	1.222539403	4	4	27.66431785	20.78800853
chr17	67337916	67366627	17q24.2	PSMD12	13.83683931	11.31890791	1.222453564	4	4	16.91698668	1.89358457
chr5	80111227	80256082	5q14.1	SERINC5	11.55567498	9.452931359	1.222443551	4	4	32.35290908	11.77350407
chr1	26317920	26320523	1p36.11	CD52	225.2758847	184.2859081	1.222425995	4	4	47.89146974	39.35763023
chr12	123384116	123409356	12q24.31	KMT5A	10.84882722	8.87501936	1.2224004	4	4	37.93790161	13.8055656
chr10	118357108	118359203	10q26.11	LINC00867	0.340355602	0.278453596	1.222306359	1	1	0	0
chr19	49976467	50025548	19q13.33	VRK3	5.476188437	4.480616611	1.222195272	4	4	50.78616672	23.61476151
chr1	113073170	113132260	1p13.2	LRIG2	6.786212201	5.552508252	1.222188585	4	4	29.10478181	26.52390396

chr10	97677422	97687432	10q24.2	AVPI1	1.352025263	1.106392667	1.222012133	4	4	74.97824374	47.50524783
chr17	63702832	63741970	17q23.3	STRADA	1.095601805	0.896604066	1.221946059	4	4	58.89301449	21.64535475
chr5	150401637	150412936	5q33.1	CD74	679.083799	555.7492537	1.221924806	4	4	74.80515971	58.99454542
chr22	32387575	32412287	22q12.3	RTCB	15.82068314	12.94774602	1.221887046	4	4	43.70615924	28.96096018
chr12	123602077	123620943	12q24.31	DDX55	3.429759581	2.806941748	1.221884844	4	4	62.94314316	25.93066114
chr7	5527146	5530709	7p22.1	ACTB	2645.606748	2165.250249	1.221848029	4	4	78.25136755	54.38106625
chr2	96335741	96377095	2q11.2	NCAPH	37.77713937	30.91978792	1.221778735	4	4	67.75463275	37.48597773
chr9	128128529	128130634	9q34.11	PTGES2-AS1	0.345488609	0.282779682	1.221758955	3	2	33.69778293	83.9928876
chr12	2676001	2691157	12p13.33	CACNA1C-AS1	0.402344574	0.329326246	1.22172034	3	4	101.6913028	119.2764633
chr12	76344486	76348442	12q21.2	BBS10	6.143427844	5.028702953	1.221672447	4	4	42.31593886	24.94441789
chr17	76527348	76557692	17q25.1	CYGB	0.110473732	0.090443609	1.221465322	2	1	15.65958613	0
chr19	32691363	32713796	19q13.11	NUDT19	4.372645866	3.580047914	1.221393113	4	4	18.82759695	20.90179001
chr19	6739682	6751526	19p13.3	TRIP10	0.779329158	0.638084187	1.221357893	4	4	90.16978962	75.96957747
chr5	134833603	134859735	5q31.1	C5orf24	5.888914768	4.821652538	1.22134781	4	4	43.31357767	10.28513978
chr8	131904088	132013642	8q24.22	EFR3A	33.10279837	27.10425815	1.221313573	4	4	27.96957413	32.23584236
chr12	120461668	120469755	12q24.31	SRSF9	21.00901841	17.2033874	1.22121405	4	4	58.71992598	7.414465924
chr11	7665095	7678569	11p15.4	CYB5R2	0.066944338	0.054821592	1.221130864	2	2	22.31428047	62.71665686
chr3	9809960	9836356	3p25.3	TTLL3	0.911650705	0.746620396	1.221036432	4	4	70.18140044	36.87218489
chr3	58332890	58426127	3p14.3	PXK	19.38774059	15.8781774	1.221030607	4	4	34.69322901	30.92914463
chr22	19175575	19178863	22q11.21	SLC25A1	8.706362292	7.130734243	1.220962946	4	4	43.81072396	43.49515725
chr6	5085486	5087221	6p25.1	PPP1R3G	0.121556247	0.099566346	1.22085677	3	1	39.41044008	0
chr10	134340	254637	10p15.3	ZMYND11	22.39144447	18.34144975	1.220811047	4	4	38.75448363	24.35592553
chr6	70413404	70561174	6q13	FAM135A	2.867290437	2.348708765	1.22079437	4	4	39.90497092	23.3563545
chr17	7351889	7354944	17p13.1	KCTD11	1.829329446	1.498564966	1.220720815	4	4	82.85945098	32.81974828
chrX	41514934	41923525	Xp11.4	CASK	2.672250486	2.189126284	1.220692705	4	4	54.28490776	45.70584275
chr9	133532164	133575519	9q34.2	ADAMTSL2	0.061454193	0.050344701	1.220668532	1	1	0	0
chr6	122443348	122471881	6q22.31	SERINC1	212.5892853	174.1590521	1.220661704	4	4	25.61652062	29.58707171
chr12	109999169	110039432	12q24.11	ANKRD13A	60.11335694	49.24992121	1.220577728	4	4	33.82953479	21.85642149
chr4	2035610	2043970	4p16.3	C4orf48	1.139614647	0.933690758	1.220548278	3	4	74.36658507	40.35911062
chr8	119557077	119673576	8q24.12	ENPP2	0.909621116	0.745276459	1.220515025	4	4	84.1416578	46.99115926
chr1	150568971	150569269	1q21.2	RN7SL600P	12.2526042	10.03899375	1.220501228	4	4	29.62814926	68.87834059
chr18	214348	268059	18p11.32	THOC1	7.994286629	6.550085365	1.220485869	4	4	38.7447349	26.76928899
chr2	48314640	48379295	2p16.3	FOXN2	66.77197715	54.71368036	1.220389064	4	4	10.81649357	13.18768023
chr1	168178845	168202114	1q24.2	TIPRL	31.17049618	25.54236912	1.220344755	4	4	19.15121196	29.89589785
chr6	113936156	113971195	6q21	HDAC2	5.199385673	4.261098483	1.220198428	4	4	35.93173333	25.00328226
chr11	94415570	94512701	11q21	MRE11	26.68909392	21.87428021	1.220113013	4	4	60.85950619	44.72941952
chr3	72725702	72848475	3p13	SHQ1	5.906876915	4.841632918	1.220017505	4	4	46.52287732	23.00843949
chrX	123960560	124102656	Xq25	STAG2	135.1352778	110.7737937	1.219921005	4	4	13.45815945	21.39389961
chr4	183639635	183659219	4q35.1	RWDD4	12.06559907	9.890769805	1.219884732	4	4	21.33855515	11.33489296
chr11	57703709	57712527	11q12.1	MED19	3.406250696	2.792436432	1.219813156	4	4	47.45501355	35.91205883
chr15	78437431	78501456	15q25.1	IREB2	19.21958655	15.75766756	1.219697425	4	4	22.28909798	8.422032554
chr7	101815904	102283958	7q22.1	CUX1	11.03980421	9.051997361	1.2195987	4	4	50.55695301	23.40243161
chr14	57202476	57268899	14q22.3	EXOC5	20.3374535	16.67554709	1.219597378	4	4	25.30569325	12.63082479

chr22	38483438	38506340	22q13.1	DDX17	94.44657757	77.44462215	1.219536941	4	4	28.9813819	19.11860011
chr5	147585439	147782848	5q32	JAKMIP2	1.51690293	1.243843205	1.219529057	4	4	48.27761366	55.80059405
chr18	9913958	9960021	18p11.22	VAPA	39.01672491	31.99450236	1.219482162	4	4	9.542231257	24.1727872
chr5	150846523	150902402	5q33.1	IRGM	0.270152583	0.221544099	1.219407716	3	3	55.61824087	6.775172792
chr16	75443060	75464757	16q23.1	TMEM170A	12.16084528	9.972840472	1.219396351	4	4	29.95625973	19.26258321
chr10	110868890	110870904	10q25.2	PDCD4-AS1	7.655479881	6.278418807	1.219332465	4	4	40.73839731	15.31685521
chr3	57571363	57597488	3p14.3	ARF4	59.25208269	48.5979572	1.219229904	4	4	13.07900763	33.55141228
chr13	40992795	40993478	13q14.11	RGS17P1	5.178670625	4.247522443	1.219221486	4	4	41.89616472	41.68006147
chr6	6144078	6320691	6p25.1	F13A1	186.6348605	153.0855912	1.219153671	4	4	66.25674915	9.240228018
chrX	54920563	54931436	Xp11.21	TRO	0.132917098	0.109028217	1.219107324	3	3	85.63073459	62.3414223
chr4	6260368	6303265	4p16.1	WFS1	0.224150913	0.183875639	1.219035398	4	4	61.78123782	77.95438203
chr13	98203835	98204192	13q32.2	RN7SKP8	1.009919653	0.828534542	1.218922811	1	2	0	19.86497546
chr11	30323099	30338458	11p14.1	ARL14EP	9.553734951	7.837934436	1.218909782	4	4	32.19744749	35.12436337
chr18	54406754	54407195	18q21.2	SNRPGP2	14.85861285	12.19110492	1.218807725	4	3	24.6099648	21.66289146
chr14	75081379	75127637	14q24.3	NEK9	10.43697913	8.563532514	1.218770304	4	4	31.62528928	17.62731624
chr11	46332905	46380554	11p11.2	DGKZ	13.04707502	10.70567449	1.218706494	4	4	63.07984137	48.97335048
chr20	62938089	62948475	20q13.33	GID8	31.55616041	25.89374725	1.218678784	4	4	20.36004985	23.07971747
chr7	1738630	1742310	7p22.3	ELFN1-AS1	0.332440627	0.272813276	1.218564693	1	1	0	0
chr9	34917045	34918408	9p13.3	GLULP4	0.316660241	0.259876857	1.218501117	2	2	27.119695	11.94409694
chr8	70573218	70608459	8q13.3	TRAM1	151.0564805	123.9720048	1.218472515	4	4	31.56525407	29.15353017
chrX	135344770	135363413	Xq26.3	ZNF449	2.127415031	1.746118632	1.218367981	4	4	40.06448373	44.77881222
chr17	28553388	28571869	17q11.2	PIGS	8.143470968	6.684134554	1.218328402	4	4	33.65785601	36.47576711
chr11	119096503	119102075	11q23.3	DPAGT1	2.112976599	1.734498047	1.218206387	4	4	54.56754079	18.77372391
chr2	108786733	108885477	2q13	CCDC138	13.39381171	10.99472026	1.218203955	4	4	75.9506764	64.52178839
chr13	44939249	44989483	13q14.12	NUFIP1	5.71136911	4.688689163	1.218116389	4	4	49.80801478	21.79868896
chr1	231332731	231337872	1q42.2	EXOC8	23.85369999	19.58413955	1.218011133	4	4	41.06628624	28.84183059
chr13	71437966	71868068	13q21.33	DACH1	2.103656822	1.727249777	1.217922764	4	4	87.0583068	34.7073376
chr6	138088505	138107523	6q23.3	PERP	1.184780171	0.972791483	1.217917911	4	4	46.23597548	80.76038758
chr13	102765888	102773821	13q33.1	TEX30	5.510830287	4.524833518	1.217907856	4	4	54.66519486	36.37238928
chr17	75167730	75183003	17q25.1	SUMO2	52.86237221	43.40621158	1.217852705	4	4	35.98206478	22.19548472
chr7	96481626	96709891	7q21.3	SEM1	3.007386384	2.469564569	1.217780017	4	4	27.32278977	35.32351239
chr11	118226690	118252368	11q23.3	MPZL3	37.58502717	30.86390173	1.217766551	4	4	25.42633616	69.42296988
chr10	99694793	99732666	10q24.2	COX15	11.79361856	9.685378782	1.217672414	4	4	45.37314164	35.71587393
chr17	64498254	64507038	17q23.3	DDX5	176.9501231	145.3223233	1.217638964	4	4	57.21530331	39.17149768
chr8	29067278	29263385	8p12	KIF13B	6.492856782	5.332984088	1.217490372	4	4	46.69386626	31.10088269
chr9	101533849	101563344	9q31.1	RNF20	24.52526611	20.14984998	1.217143857	4	4	43.28319976	30.82594984
chr17	31094927	31377677	17q11.2	NF1	5.869837589	4.823311581	1.216972507	4	4	35.92480602	23.54676937
chr11	2887344	2905504	11p15.4	SLC22A18AS	0.162602337	0.133627276	1.216834934	1	1	0	0
chr1	67407810	67430440	1p31.3	SERBP1	50.71343168	41.6773604	1.216810066	4	4	41.1357231	33.65427142
chr3	111978876	111993368	3q13.2	ABHD10	12.73649538	10.46714674	1.216806805	4	4	14.02499792	32.6554239
chr2	231961583	232343968	2q37.1	DIS3L2	2.529430137	2.07877456	1.216789057	4	4	56.00294053	27.268354
chr5	40759379	40798195	5p13.1	PRKAA1	43.36023237	35.64075866	1.216591173	4	4	15.74605344	16.11308492
chr12	122207662	122227534	12q24.31	DIABLO	1.68265957	1.383158777	1.216533921	4	4	47.46106872	23.78909805

chr17	5440912	5469060	17p13.2	DHX33	7.029238714	5.778151141	1.216520396	4	4	25.05453837	14.85324769
chr12	111642146	111686023	12q24.12	BRAP	63.26439151	52.00455677	1.21651631	4	4	41.24760119	11.94738017
chr1	11066613	11099910	1p36.22	EXOSC10	14.68867031	12.07461308	1.216492008	4	4	35.25712081	14.56868084
chr20	36605734	36612557	20q11.23	RAB5IF	11.10700289	9.130532121	1.216468301	4	4	64.18722644	31.26472811
chr2	182924851	183038858	2q32.1	NCKAP1	2.360271903	1.940277316	1.216461113	4	4	18.67400741	31.65322598
chr2	238238267	238240124	2q37.3	HES6	0.539715091	0.443723845	1.216331051	4	4	86.3732144	25.17261617
chr17	76557764	76565348	17q25.1	SNHG16	19.78404767	16.26565805	1.216307856	4	4	27.72223281	8.523658965
chr19	48296452	48322793	19q13.33	CCDC114	0.142210228	0.116940567	1.21608978	2	3	7.618046301	41.61506548
chr7	87345635	87399796	7q21.12	CROT	2.787778393	2.292571668	1.216004905	4	4	41.09996541	35.70056232
chrX	109623700	109625168	Xq23	KCNE5	0.418368394	0.344054957	1.215992927	3	1	51.6488999	0
chr3	179148114	179240093	3q26.32	PIK3CA	23.07599259	18.97906362	1.215865706	4	4	32.12715103	22.93643415
chr4	118640672	118643248	4q26	SEPT14P4	7.452463814	6.12962135	1.215811449	3	4	45.28764185	94.89042139
chr12	66136936	66170072	12q14.3	TMBIM4	11.39889679	9.375810606	1.215777203	4	4	28.12256619	43.03728761
chr15	78149362	78170542	15q25.1	IDH3A	6.293225331	5.176372805	1.215759677	4	4	54.99320115	31.35313551
chrX	47561100	47571921	Xp11.3	ARAF	19.53837422	16.07119567	1.215738681	4	4	41.21375594	33.72299332
chr3	121769761	121835079	3q21.1	IQCB1	10.02888294	8.249326771	1.215721382	4	4	40.3110435	5.540494872
chr3	69054730	69080373	3p14.1	UBA3	35.76827533	29.4238962	1.215619953	4	4	23.48491354	16.11085272
chr6	138773517	138793319	6q24.1	CCDC28A	22.60972246	18.60035777	1.215553095	4	4	25.58474188	30.79402876
chr3	50324909	50328238	3p21.31	TUSC2	7.64029634	6.286042683	1.21543819	4	4	56.09808976	23.7478226
chr17	47117697	47189339	17q21.32	CDC27	187.9740576	154.6610345	1.215393769	4	4	42.47281436	28.33893286
chr4	75949906	75990962	4q21.1	SDAD1	26.5041508	21.80755389	1.215365599	4	4	10.20051301	25.69334762
chr2	151800966	151828495	2q23.3	ARL5A	15.9777988	13.14686667	1.215331319	4	4	30.64952978	15.13171446
chr6	485138	693141	6p25.3	EXOC2	23.77498113	19.56301479	1.215302517	4	4	21.32798956	10.98137903
chr1	220253834	220254541	1q41	MORF4L1P1	14.5580143	11.97939711	1.215254338	4	4	22.63729281	34.50022669
chr17	80991841	81000133	17q25.3	CHMP6	5.699217826	4.690151064	1.215145898	4	4	62.64008832	16.76345085
chr3	150703564	150720146	3q25.1	ERICH6-AS1	2.078845461	1.710800264	1.21513043	3	4	71.89304649	64.4538357
chr12	121626550	121642040	12q24.31	ORAI1	17.21451257	14.1668686	1.215124743	4	4	55.08059554	52.45644146
chrX	135960588	135974063	Xq26.3	MMGT1	21.90510025	18.02735735	1.215103236	4	4	47.42448883	16.87715877
chr20	18567420	18569611	20p11.23	SMIM26	19.92412614	16.39734263	1.215082626	4	4	38.14469989	30.06084435
chrX	107820453	107931637	Xq22.3	MID2	0.742787201	0.611325007	1.215044687	4	4	43.00031449	56.02734782
chr11	10858217	10879276	11p15.4	ZBED5-AS1	6.121114804	5.037809909	1.215034889	4	4	3.842102721	7.656689108
chr1	213051233	213378753	1q32.3	RPS6KC1	26.43305396	21.75652453	1.214948367	4	4	48.42479173	50.48994236
chr8	89933336	89984724	8q21.3	NBN	50.78382582	41.80050181	1.214909478	4	4	14.7764462	32.65154886
chr20	59936832	59940297	20q13.33	PPP1R3D	11.51641968	9.480089436	1.214800742	4	4	37.14291425	48.04768341
chr16	67723066	67806652	16q22.1	RANBP10	33.98285134	27.97401699	1.214800554	4	4	31.96244287	17.93386651
chr17	38925168	38929384	17q12	LINC00672	0.759099	0.624882572	1.214786639	4	4	75.197434	51.49195038
chr22	50622754	50628173	22q13.33	ARSA	7.278140507	5.991534957	1.214737218	4	4	67.12739432	30.52008126
chr14	56311017	56345069	14q22.3	LINC02284	13.86826032	11.41738199	1.214662025	4	4	43.46403761	13.66234422
chr8	70109762	70405390	8q13.3	NCOA2	79.94840108	65.82080197	1.214637298	4	4	22.46481098	22.13670887
chr16	66598178	66696707	16q21-q22.1	CMTM4	0.336314096	0.276892442	1.214601932	4	4	88.0321135	39.98084096
chr9	128275364	128288990	9q34.11	SWI5	3.099261908	2.55187371	1.214504423	4	4	76.66030966	44.28281333
chr5	37379260	37752672	5p13.2	WDR70	4.704813599	3.873864008	1.214501487	4	4	55.70142063	16.19357557
chr14	101760573	101927992	14q32.31	PPP2R5C	42.97613778	35.38882996	1.21439838	4	4	6.784624786	9.257625753

chr6	136342281	136550819	6q23.3	MAP7	2.816994498	2.319822093	1.214314885	4	4	25.60233963	43.14007958
chr3	33496245	33718356	3p22.3	CLASP2	13.86632635	11.41923828	1.214295211	4	4	42.85806119	25.88272695
chr5	150777946	150796736	5q33.1	SMIM3	13.60487733	11.20493787	1.214185878	4	4	45.07473943	20.28018103
chr9	128504692	128542301	9q34.11	GLE1	30.59401173	25.19796341	1.214146208	4	4	33.60466043	7.95137607
chr19	5782960	5784765	19p13.3	PRR22	0.298248259	0.245649261	1.214122353	3	3	56.07643543	78.94487044
chr18	74389823	74390413	18q22.3	FAUP1	0.861722371	0.709750055	1.214120894	1	2	0	18.20828479
chr6	33208509	33212722	6p21.32	RING1	6.125049057	5.044932538	1.214099299	4	4	83.65009813	34.02567992
chr2	20248691	20352428	2p24.1	PUM2	55.46013266	45.68035942	1.214091425	4	4	17.63679721	10.64410676
chr5	154190735	154420984	5q33.2	GALNT10	13.69325405	11.27957239	1.213986983	4	4	12.38565826	17.15181969
chr1	151050971	151059649	1q21.3	CDC42SE1	183.2262595	150.9325574	1.21396114	4	4	35.44045876	30.19353461
chr1	6185020	6199619	1p36.31	RPL22	88.75848464	73.11837796	1.213901171	4	4	69.14121448	36.07688273
chr18	45825580	45842556	18q12.3	SIGLEC15	0.351391304	0.289473181	1.213899341	3	3	71.64971784	66.75148372
chr4	158768995	158908050	4q32.1	FNIP2	5.394735748	4.444570225	1.213781193	4	4	26.48394505	11.04129817
chr11	57712523	57740973	11q12.1	TMX2	12.34069948	10.16744064	1.213746893	4	4	33.2469955	28.49308061
chr18	158483	213739	18p11.32	USP14	34.53004696	28.44923494	1.21374255	4	4	83.85573048	56.91216772
chr17	32444463	32483319	17q11.2	PSMD11	7.454966562	6.142296333	1.213710013	4	4	22.49379465	18.77678787
chr1	53226892	53238610	1p32.3	MAGOH	21.63772925	17.82777921	1.213708617	4	4	15.75066822	7.968180794
chr7	6022247	6059229	7p22.1	EIF2AK1	224.8809097	185.2850007	1.213702722	4	4	21.21308984	25.4773845
chr3	184155311	184173614	3q27.1	DVL3	5.662759358	4.665693381	1.213701565	4	4	41.50431892	25.40528587
chr17	29255836	29294148	17q11.2	NUFIP2	43.92875594	36.19637408	1.213623106	4	4	35.96178541	16.93274937
chr9	37079896	37090401	9p13.2	EBLN3P	16.74224138	13.7955137	1.213600431	4	4	27.38914939	16.38744608
chr15	43777096	43796089	15q15.3	SERF2	160.3473776	132.1259285	1.213595087	4	4	16.91901392	13.53390112
chr6	32948614	32953122	6p21.32	HLA-DMA	16.21156893	13.36012431	1.213429498	4	4	73.46038253	52.09603016
chr4	147617371	147637273	4q31.23	TMEM184C	15.16042878	12.49441308	1.213376626	4	4	26.03524691	15.64969989
chr14	73886615	73932288	14q24.3	ZNF410	0.764793151	0.630309403	1.213361482	4	4	38.93600512	26.50341767
chr14	58298385	58373733	14q23.1	ARID4A	35.28105454	29.07742285	1.213348746	4	4	25.99936931	18.17743084
chr16	30834041	30894716	16p11.2	BCL7C	2.780283311	2.291446343	1.213331187	4	4	33.94734887	38.09547549
chr7	1470277	1504382	7p22.3	INTS1	6.074377066	5.006419351	1.213317671	4	4	73.43179781	25.83713198
chr1	51907956	51990764	1p32.3	RAB3B	0.021962543	0.018101277	1.213314599	1	1	0	0
chr8	86467055	86514348	8q21.3	RMDN1	7.202183601	5.935977257	1.213310511	4	4	32.12626876	28.21545488
chr2	177212694	177223959	2q31.2	HNRNPA3	36.3659388	29.9738198	1.213256737	4	4	32.33224768	14.86969476
chr13	95677139	95794989	13q32.1	DNAJC3	63.04343492	51.96427104	1.213207338	4	4	23.85686099	36.44185313
chr12	6534517	6538375	12p13.31	GAPDH	370.606149	305.5162139	1.213049037	4	4	57.32195175	47.36314985
chr6	159034468	159045152	6q25.3	TAGAP	172.3404959	142.0810313	1.212973289	4	4	38.60360116	53.9367825
chr3	9362862	9386791	3p25.3	THUMPD3	8.008717315	6.602589467	1.212966118	4	4	16.52616012	16.94454419
chr12	25204789	25251003	12p12.1	KRAS	29.52307007	24.34045347	1.212921941	4	4	44.16590785	22.75553522
chr10	17315414	17455966	10p12.33	ST8SIA6	7.684983409	6.336304998	1.212849352	4	4	51.15967787	35.77510619
chr15	60479178	60630639	15q22.2	RORA-AS1	3.528305877	2.90915283	1.21282933	4	4	93.54326237	64.73680225
chr10	14878783	14904315	10p13	SUV39H2	10.68958926	8.81401402	1.212794674	4	4	72.47763351	45.48743907
chr14	39114261	39136973	14q21.1	GEMIN2	3.524338836	2.90611211	1.212733268	4	4	62.7579977	20.38914318
chr12	96194382	96269835	12q23.1	ELK3	15.22426089	12.55454659	1.212649201	4	4	32.00946996	18.50935914
chr20	35953617	35955359	20q11.23	SCAND1	6.765528858	5.579512583	1.212566287	4	4	56.19888123	33.59970701
chr6	24976377	24977187	6p22.3	PPIAP29	5.493894291	4.531000122	1.212512501	4	4	44.69407891	109.2216175

chr19	44809059	44821421	19q13.32	BCAM	0.923698239	0.761824592	1.21248152	4	4	61.01454769	67.23179811
chr2	240586716	240599109	2q37.3	CAPN10	2.022748863	1.668366909	1.212412481	4	4	59.6054485	39.69839234
chr7	30424527	30478866	7p14.3	NOD1	2.79488507	2.305359586	1.212342355	4	4	41.42986831	27.39054733
chr22	37225261	37244299	22q13.1	RAC2	195.9426164	161.6332223	1.212266969	4	4	49.14991164	38.07319232
chr19	11089362	11133830	19p13.2	LDLR	5.153657994	4.251317546	1.2122496	4	4	52.54268671	85.3684878
chr2	74526648	74529897	2p13.1	AUP1	22.74323164	18.76119031	1.212248864	4	4	47.58563814	25.91663511
chr5	177131835	177300213	5q35.3	NSD1	23.9724996	19.77609745	1.212195665	4	4	36.97880683	18.55445341
chr14	100277986	100306547	14q32.2	SLC25A29	1.148652195	0.947662205	1.21209033	4	4	86.11512494	28.9700746
chr13	31311285	31312928	13q12.3	ANKRD26P4	0.347928037	0.287053955	1.212064945	1	3	0	15.72233962
chr20	63698642	63708013	20q13.33	ARFRP1	3.640573027	3.003849114	1.211969339	4	4	75.37336702	8.800761254
chr5	66596348	67169595	5q12.3	MAST4	2.354603492	1.942792069	1.211968861	4	4	39.41925239	43.31735466
chr3	47228026	47282847	3p21.31	KIF9	0.472815986	0.390122881	1.211966814	4	4	22.34378576	58.62229329
chr1	50441263	50960264	1p32.3	FAF1	32.96046738	27.1965527	1.211935489	4	4	66.59919348	24.62512562
chr22	46762617	47224975	22q13.31	TBC1D22A	12.89703283	10.64221817	1.211874501	4	4	25.88492892	13.94654229
chr15	22838641	22868384	15q11.2	NIPA2	24.58051692	20.28421724	1.211805052	4	4	21.95094964	24.63485975
chr7	5592805	5606656	7p22.1	FSCN1	0.711254007	0.586958179	1.211762666	4	4	66.32621288	63.20184258
chr2	10570766	10689987	2p25.1	NOL10	31.01430172	25.59453935	1.211754636	4	4	60.20730011	29.01356318
chr11	47248300	47269033	11p11.2	NR1H3	0.913471333	0.753850287	1.211741042	4	4	42.10808214	76.08965162
chr15	40894410	40903975	15q15.1	VPS18	11.33639574	9.355525996	1.21173259	4	4	63.73228505	12.42093272
chr15	40764087	40767713	15q15.1	GCHFR	2.196431621	1.812647694	1.211725604	4	4	47.23879749	5.788324035
chr16	53599239	53703938	16q12.2	RPGRIP1L	0.580929931	0.479482839	1.211576065	4	4	47.20935595	32.12532491
chr13	21140514	21149085	13q12.11	SAP18	36.84616938	30.41214388	1.211561064	4	4	7.735754018	8.721110256
chr13	97222289	97394120	13q32.1	MBNL2	25.29666962	20.87973272	1.211541832	4	4	8.816088325	17.2636334
chr12	6510222	6510551	12p13.31	SCARNA10	145.9199402	120.4441289	1.211515593	4	4	32.59215367	32.02439102
chr14	105917979	105932642	14q32.33	FAM30A	0.162220413	0.133913903	1.211378424	4	4	100.4081486	92.93262756
chr4	158893532	159082883	4q32.1	C4orf45	12.54814977	10.35989375	1.21122379	4	4	44.26436247	52.85376809
chr10	102502801	102633458	10q24.32	SUFU	1.752732457	1.447094501	1.211208015	4	4	75.40323047	38.80700124
chr2	111766150	111884196	2q13	ANAPC1	7.569606927	6.249650645	1.211204811	4	4	36.62567596	11.7074045
chr20	34928433	34956027	20q11.22	GSS	7.426375642	6.131481642	1.211187781	4	4	41.03396948	14.5550148
chr20	64255695	64276226	20q13.33	PCMTD2	16.57600384	13.68742282	1.211039072	4	4	48.58163342	58.28609727
chr19	19192199	19201869	19p13.11	RFXANK	11.45657095	9.460297615	1.211015913	4	4	55.55929245	38.50809702
chr4	140524146	140553770	4q31.1	ELMOD2	11.70231018	9.663673598	1.210958758	4	4	4.367422936	21.88082068
chr12	56712427	56726275	12q13.3	NACA	83.4843464	68.94094853	1.210954421	4	4	16.99732415	23.55503905
chr15	61852389	62060465	15q22.2	VPS13C	50.60541326	41.79154209	1.210900836	4	4	22.67218297	4.692629098
chr3	196712374	196736007	3q29	PIGX	13.02443875	10.757164	1.210768819	4	4	38.93812375	40.5135179
chr9	14588797	14693546	9p22.3	ZDHHC21	7.772586922	6.419735363	1.210733229	4	4	47.12052129	13.17501208
chr15	52547045	52569446	15q21.2	ARPP19	49.68732883	41.03930266	1.210725466	4	4	12.87066261	8.140266312
chr1	207752038	207795516	1q32.2	CD46	63.94622752	52.82110529	1.210618884	4	4	24.86743382	46.75037163
chr10	95237568	95291148	10q23.33	PDLIM1	37.25821122	30.77727253	1.210575472	4	4	53.93432843	21.84686164
chrX	55027927	55078903	Xp11.21	PAGE2B	52.49282478	43.36301686	1.210543652	4	4	58.77008192	57.11777627
chr19	16549837	16572382	19p13.11	SLC35E1	9.457579273	7.813559117	1.210406056	4	4	46.16260403	16.50948067
chr20	62143720	62182510	20q13.33	SS18L1	2.185373978	1.805490766	1.210404406	4	4	29.88742425	18.01276797
chr1	197504748	197782138	1q31.3	DENND1B	16.91105925	13.9722553	1.210331395	4	4	60.14347203	50.31774661

chr5	10488752	10489190	5p15.2	RPL30P7	1.289756841	1.065627544	1.210326111	2	1	25.09090317	0
chr6	108848410	108989090	6q21	ARMC2	3.991192404	3.29767548	1.210304782	4	4	35.45584056	17.15200478
chr7	90346665	90391455	7q21.13	GTPBP10	6.453780504	5.332440848	1.210286375	4	4	51.29174995	22.53938955
chr1	21678579	21784146	1p36.12	USP48	40.7455186	33.66645918	1.210270388	4	4	26.90765616	15.03789586
chr2	200910171	200963701	2q33.1	ORC2	23.75795932	19.63133383	1.210206067	4	4	58.8601805	38.85865565
chr11	71453171	71501538	11q13.4	NADSYN1	4.13375657	3.415793612	1.210189209	4	4	54.01132591	24.39729826
chr19	35758143	35769801	19q13.12	PROSER3	1.125465479	0.930015093	1.210158294	4	4	79.67012688	36.37076922
chr16	425621	522485	16p13.3	RAB11FIP3	0.822233208	0.679520021	1.210020578	4	4	54.93715439	59.17190367
chr4	113899929	113980464	4q26	ARSJ	0.214790579	0.177517	1.209971888	1	2	0	65.20847798
chr10	5889572	5937595	10p15.1	FBH1	12.88577183	10.65080486	1.209840195	4	4	31.14703316	26.59768113
chr19	33373669	33382686	19q13.11	CEBPG	9.534937244	7.881507111	1.209786036	4	4	25.26135795	16.1050827
chr5	110738136	110765174	5q22.1	SLC25A46	14.57709642	12.04940317	1.209777465	4	4	29.98705054	15.0419429
chr5	177086872	177098142	5q35.2	FGFR4	0.064222871	0.05308992	1.209699894	2	1	11.87002356	0
chr2	74361154	74392087	2p13.1	DCTN1	9.814875552	8.114417069	1.209560153	4	4	65.82042964	39.89164283
chr17	7306999	7312463	17p13.1	EIF5A	40.01224132	33.08167022	1.209498827	4	4	25.96073382	28.22599546
chr16	84053761	84116943	16q23.3-q24.1	MBTPS1	9.138743252	7.55591205	1.209482481	4	4	54.00032622	34.28879427
chr19	58354357	58362848	19q13.43	ZNF497	0.133885762	0.110698652	1.209461533	2	4	15.75276377	78.97715784
chr4	182890091	182917477	4q35.1	DCTD	11.35627945	9.3898672	1.209418536	4	4	20.70689409	17.43536819
chr4	13367723	13484365	4p15.33	RAB28	17.25991268	14.27147861	1.20939905	4	4	31.34988658	26.43371459
chr1	66924895	66988619	1p31.3	MIER1	66.49691937	54.98388602	1.209389226	4	4	18.09837583	9.549389798
chr17	7930345	7931999	17p13.1	TRAPPC1	46.5063918	38.4556734	1.209350602	4	4	59.41454388	35.95512134
chr9	112451803	112487187	9q32	C9orf147	0.466517836	0.385773878	1.209303851	1	3	0	12.9807765
chr10	100288145	100347030	10q24.31	PKD2L1	0.102691003	0.084919339	1.209277	1	1	0	0
chr12	122504643	122527013	12q24.31	RSRC2	31.52402677	26.06872359	1.209266217	4	4	18.66324612	16.44030586
chr1	26109131	26125548	1p36.11	PDIK1L	8.86783977	7.334520297	1.209055182	4	4	36.78891431	27.04305319
chr17	61942605	62065983	17q23.2	MED13	98.34461804	81.34398166	1.208996855	4	4	37.29248795	26.75292157
chr16	72093665	72112912	16q22.2	DHX38	10.79951077	8.933058496	1.208937653	4	4	57.11285425	51.63132702
chr18	47107402	47150520	18q21.1	HDHD2	4.001789251	3.310204343	1.208925141	4	4	48.60180116	47.85602143
chr16	28950992	28966465	16p11.2	NFATC2IP	5.963316556	4.933140645	1.208827598	4	4	51.6384988	9.498387174
chr2	201700263	201781172	2q33.1	ALS2	4.690194068	3.880397356	1.208689121	4	4	56.50994157	23.72052617
chr6	69705090	69706257	6q13	NPM1P37	3.717253657	3.075503056	1.208665246	4	4	44.54546807	34.7601365
chr19	2385254	2426261	19p13.3	TMPRSS9	1.26204815	1.044202261	1.208624226	4	3	41.26812079	17.31154511
chr19	10092337	10106407	19p13.2	ANGPTL6	0.302513392	0.250309938	1.208555259	1	3	0	49.58242767
chr1	11106531	11273497	1p36.22	MTOR	8.738442165	7.230660078	1.208526202	4	4	53.83185512	17.70595634
chr3	136841976	136862063	3q22.3	NCK1-DT	4.014891282	3.322176725	1.208512254	4	4	20.40559731	45.86682679
chr1	23084023	23177808	1p36.12	LUZP1	4.374625131	3.619938509	1.208480509	4	4	54.10874238	26.20596152
chr3	123491548	123585185	3q21.1	HACD2	10.82405212	8.957190152	1.208420491	4	4	35.34159009	16.62192342
chr6	160121808	160160590	6q25.3	SLC22A1	1.19563508	0.989426061	1.208412763	3	4	79.77549922	45.72411567
chr3	52705841	52708182	3p21.1	SPCS1	9.531684307	7.888456944	1.208307832	4	4	17.09712782	22.69074111
chr11	74012578	74171210	11q13.4	C2CD3	23.19447337	19.19604966	1.208294091	4	4	66.59525606	56.72927189
chr2	85577491	85582033	2p11.2	VAMP8	37.33644153	30.90275447	1.208191379	4	4	21.16405065	25.45896124
chr12	31280584	31326225	12p11.21	SINHCAF	4.222797646	3.495224738	1.208161982	4	4	58.52639059	18.91323888
chr17	76310736	76354202	17q25.1	PRPSAP1	3.054297798	2.528139647	1.208120684	4	4	16.66238192	18.03678585

chr19	55654050	55674716	19q13.42	U2AF2	25.69369486	21.26839766	1.20806914	4	4	47.50072328	39.62338143
chr1	156767482	156800819	1q23.1	PRCC	7.219304711	5.976324489	1.207984059	4	4	53.9567548	41.1294816
chr3	189596746	189897279	3q28	TP63	0.264559434	0.219017752	1.207936028	3	4	116.7862829	74.02920013
chr17	41976421	42021371	17q21.2	DNAJC7	10.53855843	8.724805253	1.207884661	4	4	41.52545196	19.46471284
chr5	179678646	179731641	5q35.3	CANX	95.65218701	79.19334771	1.207831084	4	4	22.87354983	26.10501758
chr12	752593	911452	12p13.33	WNK1	167.0773531	138.3434086	1.207700134	4	4	15.74901709	33.00391763
chr12	69470349	69579793	12q15	FRS2	10.18559701	8.434024171	1.207679371	4	4	21.59331458	12.17261905
chr16	1228336	1230184	16p13.3	TPSB2	0.180855453	0.149765348	1.207592115	3	1	12.30784925	0
chr4	183659267	183713594	4q35.1	TRAPPC11	20.30180962	16.81203579	1.207575922	4	4	24.39480211	9.137007736
chr14	35044907	35083383	14q13.2	FAM177A1	7.495584549	6.20746993	1.207510408	4	4	19.16945424	30.2252467
chr15	51829628	51912138	15q21.2	TMOD3	34.88960125	28.89449344	1.20748271	4	4	23.79392213	17.91616167
chr7	38178206	38230671	7p14.1	STARD3NL	12.08050309	10.00488276	1.207460735	4	4	19.32255674	52.46275706
chr1	32179669	32198285	1p35.2	TXLNA	12.84071282	10.63622092	1.207262704	4	4	44.94169619	42.19805092
chr3	170888415	170908637	3q26.2	EIF5A2	3.246463534	2.689159956	1.207240769	4	4	26.28511444	24.27738356
chr11	75150987	75206400	11q13.4	SLCO2B1	0.02769726	0.022943131	1.207213587	1	1	0	0
chr15	25672241	25865144	15q12	ATP10A	3.382584477	2.801994725	1.207205869	4	4	25.42395028	40.0285671
chr2	89027171	89027684	2p11.2	IGKV3-11	26.25450375	21.75057089	1.207071937	4	4	107.3365572	154.4305238
chr3	113828182	113947174	3q13.31	GRAMD1C	1.578072811	1.30742938	1.207004245	4	4	100.3193316	80.2043674
chr1	155897808	155911402	1q22	RIT1	52.93826997	43.85925867	1.207003301	4	4	8.553863902	40.33888665
chr17	59331692	59401734	17q22	YPEL2	22.46803345	18.6159146	1.206926113	4	4	22.18945663	32.50795452
chr17	2127405	2128275	17p13.3	MCUR1P1	1.45269219	1.203702533	1.206853147	2	3	96.51965738	38.32483765
chr16	69976297	70065948	16q22.1	PDXDC2P-NPIPB14	0.325279176	0.269529603	1.206840261	4	4	92.98070396	46.22413857
chr1	172532349	172611833	1q24.3	SUCO	150.3472826	124.5814911	1.206818776	4	4	68.30457103	51.99230323
chr8	28021964	28084068	8p21.1	NUGGC	1.582430584	1.311247472	1.206813068	4	4	45.68341597	92.98777734
chrX	111680741	111764164	Xq23	ALG13	6.679718658	5.535150163	1.206781833	4	4	26.72533543	15.79827184
chrX	154477769	154479257	Xq28	LAGE3	3.102942269	2.571463772	1.206683253	4	4	27.63203204	26.34721049
chr19	18001132	18014102	19p13.11	ARRDC2	12.16170973	10.07882376	1.206659629	4	4	67.28386247	25.49065884
chr9	136483495	136486067	9q34.3	C9orf163	0.117955718	0.097755286	1.206642855	2	2	31.36434788	3.009515256
chr19	2269486	2273488	19p13.3	OAZ1	495.7901004	410.9388423	1.206481474	4	4	14.22542638	29.73250117
chr14	23320188	23326185	14q11.2	PABPN1	5.337770945	4.424422928	1.206433253	4	4	25.54284912	21.0942808
chr6	31661229	31666283	6p21.33	GPANK1	1.306789041	1.083224611	1.206387879	4	4	85.62608571	68.97741215
chr11	85657564	85665148	11q14.1	CREBZF	14.90426682	12.35470407	1.206363724	4	4	30.56561092	10.31937733
chr10	87751512	87841359	10q23.31	ATAD1	19.36430406	16.05206685	1.206343348	4	4	28.13676756	14.54522261
chr18	35654569	35711834	18q12.2	GALNT1	33.54669997	27.81288362	1.206156845	4	4	30.45559826	11.70903763
chr2	99141613	99151487	2q11.2	C2orf15	0.590383315	0.489497061	1.206101859	2	3	51.471339	99.6132736
chr1	246566337	246668587	1q44	CNST	40.21524761	33.34355121	1.206087719	4	4	9.963575864	11.36350844
chr10	119573465	119598841	10q26.11	TIAL1	20.50997418	17.00637313	1.206016945	4	4	41.34153508	35.22032789
chr8	144373762	144393242	8q24.3	ADCK5	1.234704424	1.023831148	1.205964896	3	4	96.50157117	54.75075061
chr15	39581079	39598918	15q14	THBS1	28.65026006	23.75736079	1.205952981	4	4	47.51554894	22.48020143
chr1	18480930	18485999	1p36.13	KLHDC7A	0.201738991	0.167289555	1.205927	3	2	74.63475515	10.95861455
chr1	22023994	22026048	1p36.12	LINC01635	0.440711504	0.365461257	1.205904855	3	2	63.9997535	32.92328826
chr5	14704800	14871785	5p15.2	ANKH	15.15457066	12.5671721	1.205885504	4	4	28.58901866	13.95690004
chr4	1574055	1684313	4p16.3	FAM53A	0.163846657	0.135883407	1.205788554	3	3	46.00718712	57.50780092

chr17	8368001	8376711	17p13.1	KRBA2	0.62439797	0.517860694	1.205725744	4	3	75.96668521	30.50037774
chr5	176542511	176595768	5q35.2	CDHR2	0.321542482	0.266692496	1.205667526	4	4	72.79379178	65.66555506
chr8	101686542	102125081	8q22.3	NCALD	4.008907772	3.325604767	1.205467292	4	4	32.88543274	30.64197075
chr7	6009251	6023834	7p22.1	AIMP2	5.777749686	4.793187171	1.205408735	4	4	33.72526561	33.38556382
chr15	52019214	52066265	15q21.2	MAPK6	12.88393186	10.68922919	1.205319077	4	4	40.80128446	25.23840535
chr1	223220819	223365271	1q41	SUSD4	0.733132113	0.608247525	1.205318695	4	4	90.51971501	76.327939
chr1	173863248	173863941	1q25.1	GAS5-AS1	0.924497967	0.767032854	1.205291224	3	2	50.20294051	79.92591857
chr3	141487047	141615363	3q23	RASA2	59.6281749	49.47340819	1.205257068	4	4	21.52059806	12.38096107
chr6	170282200	170290609	6q27	DLL1	0.285720956	0.237064896	1.20524363	3	3	63.66233517	36.05650616
chr1	231626815	232041272	1q42.2	DISC1	2.368136667	1.9650316	1.205139229	4	4	33.71156319	49.5885198
chr2	17753687	17785920	2p24.2	GEN1	3.400567836	2.822375272	1.204860271	4	4	45.85546201	22.99823669
chr4	109709989	109730086	4q25	PLA2G12A	14.82288915	12.30405964	1.20471532	4	4	27.15589666	19.15110142
chr7	44606522	44709070	7p13	OGDH	36.38552581	30.20396848	1.204660435	4	4	25.63524192	35.32559115
chr7	1148850	1160219	7p22.3	ZFAND2A	6.536349981	5.42657958	1.204506427	4	4	26.60347376	32.18716135
chr5	116084976	116447435	5q23.1	COMMD10	17.50985516	14.53744238	1.204466006	4	4	40.63628792	33.62575271
chr6	41026901	41039199	6p21.1	UNC5CL	0.522263426	0.433609075	1.204456862	3	4	62.97959679	55.73418948
chr4	13627865	13627957	4p15.33	MIR5091	8.01726577	6.656706329	1.204389284	2	2	63.93775994	83.57408592
chr2	27364352	27370457	2p23.3	EIF2B4	2.137218173	1.774658567	1.204298231	4	4	60.66810812	33.15665527
chr8	8783354	8893621	8p23.1	MFHAS1	10.0618388	8.35496407	1.204294682	4	4	29.26707306	26.00723559
chr4	153466346	153637608	4q31.3	TMEM131L	53.87374703	44.74818251	1.203931512	4	4	23.81367456	14.06484028
chr10	110919370	111013667	10q25.2	SHOC2	127.6513006	106.0304595	1.203911604	4	4	11.23807657	16.87415281
chr13	113815610	113842841	13q34	GAS6-AS1	0.129914603	0.107911166	1.203903254	3	4	56.50900005	35.4003709
chr14	60971441	60981690	14q23.1	TRMT5	1.887627289	1.567998757	1.203844888	4	4	49.47908869	34.85373175
chr19	41956681	41959376	19q13.2	RABAC1	9.042260111	7.511374468	1.20380899	4	4	63.95499653	45.08935407
chrX	134990938	134992473	Xq26.3	SMIM10	1.477206514	1.227153645	1.203766553	4	4	43.59448716	55.18392711
chr17	81890790	81900533	17q25.3	ANAPC11	1.66397171	1.382341491	1.203734187	4	4	36.78866135	41.67710185
chr6	79914812	79947598	6q14.1	ELOVL4	0.897158371	0.745325672	1.20371323	4	4	93.96037748	71.60104378
chr1	193136503	193136583	1q31.2	MIR1278	6.27457863	5.21299095	1.203642724	2	3	59.77299526	34.89816296
chr17	2755686	3037741	17p13.3	RAP1GAP2	15.97525859	13.27248195	1.203637621	4	4	42.01027629	27.16125851
chr9	128061233	128067867	9q34.11	NAIF1	3.811496653	3.166712376	1.203613148	4	4	71.01457001	23.66372038
chr5	168555623	168579609	5q34	PANK3	35.0478204	29.11982101	1.203572659	4	4	27.6724909	21.20546372
chr12	76044745	76085033	12q21.2	NAP1L1	51.2143623	42.55370622	1.203522956	4	4	36.05934233	3.511418217
chr13	52024691	52033600	13q14.3	UTP14C	5.702193314	4.738024718	1.203495898	4	4	44.88831257	37.31516117
chr9	137447570	137459334	9q34.3	NSMF	3.409649036	2.833562308	1.203308297	4	4	77.82237873	25.00049056
chr5	1571957	1594531	5p15.33	SDHAP3	2.431884846	2.021175185	1.203203396	4	4	41.72785153	34.71955957
chr17	54900691	54961967	17q22	TOM1L1	0.609451196	0.506526396	1.203197308	4	4	20.07077096	21.6853224
chrX	120236452	120245267	Xq24	NKAPP1	3.90821448	3.248218191	1.203187179	4	4	39.0701893	37.96643153
chr6	44809057	45378051	6p21.1	SUPT3H	2.749138989	2.285039171	1.203103659	4	4	26.49438402	31.09922255
chrX	47444691	47485934	Xp11.3	ZNF41	6.413715928	5.331012955	1.203095168	4	4	56.72536835	19.13120629
chr6	8413066	8436169	6p24.3	SLC35B3	10.24719029	8.517507868	1.203073768	4	4	39.88181434	25.01635362
chr1	22428838	22531157	1p36.12	ZBTB40	5.151866883	4.28245325	1.203017659	4	4	38.00858201	21.47150062
chr17	64146078	64146157	17q23.3	SNORD104	23.77346244	19.76225513	1.202973157	4	4	54.29222838	51.52259958
chr16	67666814	67670523	16q22.1	C16orf86	0.678819982	0.564300426	1.202940758	4	4	96.38751415	36.2925994

chr17	75261668	75266376	17q25.1	MRPS7	26.04845944	21.65508916	1.202879344	4	4	36.78156339	27.31518788
chr14	100109655	100109753	14q32.2	MIR342	7.228439578	6.009461177	1.202843211	3	3	68.82819889	33.26763651
chr18	3603737	3610089	18p11.31	DLGAP1-AS2	1.131335345	0.940583009	1.202802235	4	4	67.94499139	40.02669564
chr1	168574128	168582077	1q24.2	XCL1	1.804992213	1.500714427	1.202755288	4	4	33.3266705	107.3390187
chr12	43718992	43758817	12q12	PUS7L	8.668357298	7.207618533	1.202665937	4	4	34.25308357	8.901194768
chr6	166364919	166383013	6q27	MPC1	15.73655632	13.08564933	1.202581234	4	4	37.89004959	15.46295319
chr17	14301050	14349406	17p12	HS3ST3B1	3.996373538	3.32333332	1.202519625	4	4	39.25554287	10.8353396
chr14	67470266	67533850	14q24.1	TMEM229B	4.623869035	3.845550121	1.202394687	4	4	27.89191526	54.88012835
chr2	186486158	186509361	2q32.1	ZC3H15	41.36875671	34.40571144	1.202380506	4	4	46.79825421	20.66340615
chr4	37826660	37862938	4p14	PGM2	26.78916942	22.28074657	1.202346131	4	4	47.2622857	41.93416647
chr12	7108308	7121851	12p13.31	C1RL-AS1	1.56417061	1.300938482	1.202340181	4	4	57.27033063	38.51448658
chr1	171700156	171742844	1q24.3	VAMP4	9.842324509	8.186158259	1.202313002	4	4	17.31731972	23.55296122
chr14	55580207	55684584	14q22.3	KTN1	20.15780188	16.76618895	1.202288841	4	4	17.22236928	7.584605015
chr3	124033341	124726325	3q21.1-q21.2	KALRN	0.275739534	0.229356688	1.202230189	4	4	61.50259161	24.28432473
chr4	1289851	1340148	4p16.3	MAEA	5.897221359	4.905311639	1.202211357	4	4	61.57475196	26.88640909
chr14	39233916	39388513	14q21.1	MIA2	1.25691079	1.045515411	1.202192505	4	4	66.99647774	56.99442291
chr17	42514188	42514307	17q21.2	MIR5010	2.820481255	2.346236865	1.202129801	2	1	21.20312837	0
chr16	69118009	69132590	16q22.1	CHTF8	16.49956593	13.72540471	1.202118719	4	4	54.31778289	34.67209559
chr1	39026295	39034636	1p34.3	NDUFS5	80.16722923	66.68935428	1.202099347	4	4	45.81328237	25.73152072
chr1	28553017	28578545	1p35.3	TRNAU1AP	8.017920609	6.670314848	1.202030308	4	4	56.3396927	33.67906315
chr15	82471477	82513999	15q25.2	GOLGA2P10	0.680179124	0.56594705	1.201842336	2	4	41.5104942	68.79197042
chr21	41167801	41282534	21q22.2-q22.3	BACE2	3.390003523	2.82067371	1.201841783	4	4	38.24324122	25.61011011
chr20	435477	462553	20p13	TBC1D20	10.77980153	8.969423329	1.201838862	4	4	37.69536845	18.56615613
chr10	113679162	113730909	10q25.3	CASP7	9.917056294	8.251712172	1.201818009	4	4	45.15509444	58.2514396
chr15	50424359	50501083	15q21.2	USP8	15.45032439	12.85643568	1.201757997	4	4	11.77492375	22.72691
chr3	195514422	195543401	3q29	PPP1R2	46.66504346	38.83104055	1.201745892	4	4	51.853004	24.87947229
chr15	55611540	55743124	15q21.3	PRTG	0.020779388	0.017291657	1.201700198	2	1	7.025910453	0
chr8	41929479	42052026	8p11.21	KAT6A	42.77025419	35.59426746	1.201605125	4	4	52.06038214	26.11872665
chr15	63277550	63309126	15q22.2	APH1B	6.357807562	5.291254904	1.201568943	4	4	38.87633631	43.93768941
chr1	21003511	21003964	1p36.12	RPS15AP6	0.882736478	0.734677156	1.201529774	4	1	31.84772725	0
chr8	144798876	144813137	8q24.3	ZNF517	0.370496516	0.308356673	1.201519372	3	4	93.29226994	39.91341267
chr20	59033145	59042846	20q13.32	PRELID3B	37.60070322	31.297194	1.201408127	4	4	11.10912287	16.70576903
chr8	54522490	54523381	8q11.23	SEC11B	0.699657561	0.582436433	1.201259951	1	2	0	16.3923667
chr20	46463660	46513572	20q13.12	ZNF334	0.087026074	0.072445802	1.201257644	2	1	75.08971118	0
chr20	63657810	63696253	20q13.33	RTEL1	0.283053455	0.235644855	1.201186655	3	3	112.5097672	11.08251651
chr22	41468756	41528989	22q13.2	ACO2	15.56621457	12.95933006	1.201158894	4	4	44.58850797	40.36318663
chr19	36054881	36111145	19q13.12	WDR62	1.563732767	1.301860399	1.201152419	4	4	66.31438997	30.0383631
chr9	14475	30487	9p24.3	WASHC1	2.715604935	2.260872503	1.201131391	4	4	78.08722026	76.75377506
chr3	182793503	182923010	3q26.33	ATP11B	71.26606171	59.33744356	1.2010302	4	4	9.972316255	10.04372409
chr12	56316223	56334053	12q13.3	PAN2	3.915105298	3.259798025	1.201026956	4	4	45.0682147	31.23883466
chr19	38930708	38933020	19q13.2	MRPS12	0.834079284	0.694483372	1.201006846	4	4	53.39012132	85.46742911
chr17	20840687	20844479	17p11.2	ABHD17AP6	0.322863658	0.268827679	1.201006008	2	3	3.302815802	11.98722167
chr14	67337864	67360003	14q23.3	ATP6V1D	23.25239568	19.36113217	1.200983263	4	4	31.93730827	30.43321371

chr7	2354540	2380745	7p22.3	EIF3B	20.58143726	17.13717199	1.200982127	4	4	12.41177277	37.68046753
chr2	32165841	32224379	2p22.3	SLC30A6	16.91556563	14.08551341	1.200919352	4	4	35.59227882	18.68748409
chr1	15526694	15571987	1p36.21	DNAJC16	19.82902747	16.51231089	1.200863259	4	4	45.88958759	25.70395724
chr2	60940218	61019693	2p16.1-p15	PUS10	2.067379588	1.721658109	1.200807279	4	4	18.22260707	35.12521363
chr1	27725979	27762915	1p35.3	FAM76A	6.641094254	5.530762063	1.200755733	4	4	30.44594217	7.223920987
chr5	76615482	76623434	5q13.3	F2RL2	0.090454749	0.075334411	1.200709577	3	1	14.75510328	0
chr20	3146519	3159886	20p13	FASTKD5	15.11320155	12.58736389	1.200664546	4	4	51.50291718	19.71812983
chr1	42682235	42703803	1p34.2	YBX1	1351.465929	1125.640943	1.200619023	4	4	56.41478973	32.77659014
chr7	1666075	1747954	7p22.3	ELFN1	0.079130311	0.065909189	1.20059603	1	1	0	0
chr11	114464276	114813702	11q23.2-q23.3	NXPE2	0.173869736	0.144820639	1.200586724	1	2	0	8.733387072
chr2	237858555	237912117	2q37.3	RAMP1	1.00441788	0.836928569	1.200123783	4	4	91.12088736	83.66012485
chr6	42963873	42980224	6p21.1	PEX6	5.420898925	4.516989875	1.200113145	4	4	57.49540553	42.51962992
chr4	39822863	39977956	4p14	PDS5A	58.68466138	48.90221145	1.200041054	4	4	69.20298925	23.92831059
chr14	49618688	49620685	14q21.3	RPL36AL	420.6144746	350.5001807	1.200040678	4	4	59.04036883	34.83281562
chr11	62615296	62622175	11q12.3	B3GAT3	4.745829481	3.954936609	1.199976119	4	4	33.36342564	42.13430428
chr1	112674312	112702377	1p13.2	MOV10	5.867647327	4.890029496	1.199920641	4	4	30.57522429	41.06556466
chr11	65027408	65040572	11q13.1	SNX15	0.128636454	0.107205838	1.199901576	1	1	0	0
chr7	44983023	44986696	7p13	SNHG15	3.161605707	2.635268274	1.199728217	4	4	71.17152758	16.50065164
chrX	150766336	150898816	Xq28	CD99L2	12.16598385	10.14096007	1.199687581	4	4	23.77481795	17.01543451
chr3	133824235	133895847	3q22.1	RAB6B	1.594138353	1.328798496	1.199684043	4	4	51.86146468	29.8620626
chr11	134224605	134247792	11q25	VPS26B	11.23985457	9.369384451	1.199636393	4	4	44.66492716	28.6125222
chr15	51938024	51971801	15q21.2	LEO1	15.60627296	13.00924449	1.199629461	4	4	47.88192508	38.93138876
chr19	48332356	48364237	19q13.33	TMEM143	0.827404635	0.689793244	1.199496577	4	4	54.61300863	31.04920786
chr4	55346221	55373100	4q12	SRD5A3	1.466181609	1.22234495	1.199482691	4	4	39.6190145	58.29918505
chr12	4273736	4305356	12p13.32	CCND2	29.61907545	24.69322478	1.199481871	4	4	38.71528769	29.67591233
chr11	64917553	64934478	11q13.1	PPP2R5B	18.28024471	15.24013813	1.199480251	4	4	53.13318355	48.39614163
chr1	3068227	3438621	1p36.32	PRDM16	0.063111692	0.052619455	1.199398426	2	1	75.35156123	0
chrX	54807599	54816015	Xp11.21	MAGED2	13.67982511	11.40730388	1.199216332	4	4	40.97884573	31.12093891
chr8	100702916	100722087	8q22.3	PABPC1	271.3500599	226.2973755	1.199086199	4	4	68.93868601	30.88371053
chr11	66546395	66563329	11q13.2	ACTN3	0.082647864	0.068928876	1.199031079	1	2	0	7.94779993
chr19	11483427	11505923	19p13.2	ZNF653	1.75263212	1.461792661	1.198960815	4	4	53.1986378	77.40522764
chr4	98871684	98930637	4q23	EIF4E	7.024726009	5.859460391	1.198869101	4	4	15.34237274	22.04571388
chr11	66520625	66546238	11q13.2	ZDHHC24	2.12292982	1.770846544	1.198822014	4	4	57.63909631	46.62636199
chrX	155489011	155612961	Xq28	TMLHE	13.58585284	11.33349224	1.198734914	4	4	15.4220986	8.702498864
chr11	130226677	130314712	11q24.3	ZBTB44	46.70015021	38.95937506	1.198688381	4	4	25.46653323	10.32267857
chr22	21549447	21624034	22q11.21	UBE2L3	39.72207225	33.13825369	1.198677294	4	4	44.88909089	56.74047745
chr17	15502264	15563631	17p12	TVP23C	0.719947569	0.600629045	1.198655934	4	4	67.83081254	38.08054082
chrX	107123427	107206440	Xq22.3	NUP62CL	69.00964838	57.57894554	1.198522268	4	4	79.70049455	66.78226348
chr14	22450089	22450139	14q11.2	TRDJ1	33.76917971	28.17646753	1.198488763	3	4	46.61554014	50.61023521
chr6	142147162	142220948	6q24.1-q24.2	VTA1	11.33105878	9.454769054	1.198449028	4	4	9.701270714	18.49146699
chr1	2404802	2412574	1p36.32	PEX10	1.466972314	1.224108892	1.198400178	4	4	72.61900912	38.09231808
chr16	71859680	71885353	16q22.2	ZNF821	1.04985719	0.876052259	1.19839562	4	4	102.7944027	47.88238706
chrX	19912860	19991040	Xp22.12	BCLAF3	6.259441912	5.223429119	1.198339591	4	4	11.39936183	29.94557629

chr1	160276809	160285151	1q23.2	PEX19	7.755593461	6.472000633	1.198330146	4	4	57.1959649	10.07614242
chr7	38358512	38358979	7p14.1	TRGV3	4.071749144	3.398116689	1.198236999	4	4	13.64706711	63.28637274
chr1	109483905	109492804	1p13.3	ATXN7L2	1.241878806	1.0364259	1.198232123	4	4	61.41173801	54.98406
chr17	46500213	46579780	17q21.31	ARL17A	0.297752531	0.248494933	1.198223756	4	4	47.86741634	49.54032921
chr11	112175485	112218929	11q23.1	BCO2	0.139255925	0.116223648	1.198172035	4	4	85.11014974	53.78843977
chr5	159263067	159286040	5q33.3	UBLCP1	35.54871421	29.66929905	1.19816495	4	4	16.82975897	22.76472634
chr17	30378924	30469657	17q11.2	CPD	58.00770743	48.41422271	1.198154265	4	4	50.71855868	43.35709127
chr17	31863171	31901710	17q11.2	UTP6	11.07313427	9.242371326	1.198083682	4	4	20.1405769	20.92079039
chr10	43138445	43185308	10q11.21	CSGALNACT2	50.80794849	42.40800292	1.198074538	4	4	14.87373791	8.396732843
chr19	49818279	49840384	19q13.33	MED25	16.63672603	13.88715154	1.19799413	4	4	7.425269673	26.80376221
chr15	81196878	81312763	15q25.1	IL16	39.14771098	32.68028063	1.197900086	4	4	66.40805345	50.75939679
chr1	144421386	144459464	1q21.1	NBPF15	2.1855728	1.824649542	1.197804154	4	4	70.33863896	24.02028102
chr12	56638175	56646068	12q13.3	ATP5F1B	138.4288098	115.5730264	1.197760533	4	4	17.83225818	41.26155918
chr6	43477523	43506556	6p21.1	TJAP1	2.162535882	1.805488735	1.197756508	4	4	42.25914611	22.92049183
chr16	11976734	12574289	16p13.13-p13.12	SNX29	9.699009499	8.097955927	1.197710828	4	4	42.50327957	25.62019423
chr22	37204237	37220451	22q13.1	SSTR3	0.755179106	0.630521186	1.197706157	4	4	64.0839144	75.85966336
chr3	63911518	63911772	3p21	SCAANT1	3.387757702	2.828573033	1.197691438	2	3	103.8815702	55.82187964
chr16	30075976	30085377	16p11.2	PPP4C	27.0245655	22.56551396	1.197604697	4	4	60.06609635	37.34377237
chr17	80101526	80119882	17q25.3	GAA	10.93610125	9.132039155	1.197553039	4	4	77.73869565	23.6700423
chr22	46150534	46243756	22q13.31	PPARA	2.914955547	2.434204037	1.197498444	4	4	24.62675974	19.44001105
chr8	91059906	91070189	8q21.3	OTUD6B-AS1	10.27121664	8.577287242	1.19749011	4	4	24.51850797	30.03257583
chr2	174799314	175005379	2q31.1	CHN1	0.420126775	0.350861095	1.197416244	4	4	56.71259108	49.38664371
chr1	220148268	220272501	1q41	RAB3GAP2	12.7453641	10.64510182	1.197298469	4	4	14.87641789	16.40310004
chr1	75786194	75795090	1p31.1	RABGGTB	16.61323119	13.87580971	1.197280125	4	4	31.12438313	14.24682694
chr2	37250502	37324785	2p22.2	PRKD3	12.67994856	10.59066221	1.197276271	4	4	16.77223829	27.12003848
chr10	124397303	124418976	10q26.13	OAT	54.63576387	45.63441018	1.197249261	4	4	45.97466408	32.33720242
chr15	90351439	90362823	15q26.1	ZNF774	0.069520581	0.058072672	1.197130744	3	2	39.64035073	69.71504514
chr11	65127279	65135178	11q13.1	SYVN1	5.3047296	4.431501333	1.19705021	4	4	47.51773503	28.49610992
chr2	32902129	33399509	2p22.3	LTBP1	31.79077519	26.55802486	1.197030854	4	4	15.05285904	12.26238724
chr5	141636950	141641077	5q31.3	RELL2	2.190321088	1.830013512	1.196887932	4	4	48.88018455	40.71869038
chr6	3063841	3115192	6p25.2	RIPK1	23.03106557	19.24302408	1.196852713	4	4	36.33881312	18.77241065
chr3	141944428	142149544	3q23	TFDP2	44.0851949	36.84009306	1.196663506	4	4	62.4131075	41.95767722
chr10	112308178	112356595	10q25.2	GUCY2GP	0.095323292	0.079659804	1.196629753	2	1	20.91265616	0
chr3	15877950	15880295	3p25.1	IMPDH1P8	0.24641777	0.205926813	1.196627899	1	1	0	0
chr6	166585518	166586685	6q27	RAMACL	1.144629725	0.956579326	1.196586309	1	1	0	0
chr1	54225431	54413479	1p32.3	SSBP3	21.14145035	17.66966681	1.196482683	4	4	23.70868946	57.62089504
chr8	28607736	28753690	8p21.1	EXTL3	6.248155705	5.222114588	1.196480008	4	4	56.7941533	28.81231265
chr5	133971875	134070987	5q31.1	VDAC1	28.48591705	23.80850971	1.196459476	4	4	41.95181165	40.96907729
chr10	92826831	93059494	10q23.33	EXOC6	44.56267889	37.25001464	1.196313057	4	4	32.02294702	37.37583285
chr7	27170591	27180261	7p15.2	HOXA10	0.308155952	0.257593376	1.196288338	3	4	41.10286852	78.91002684
chr17	75225464	75262393	17q25.1	GGA3	10.64676244	8.900227164	1.19623491	4	4	40.24038965	13.23349506
chr19	17302807	17306843	19p13.11	MRPL34	7.636163126	6.383529127	1.196229072	4	4	40.95524188	23.23035226
chr7	149003054	149028690	7q36.1	PDIA4	27.39697308	22.90387529	1.196171946	4	4	46.91908113	43.42143654

chr17	27878314	27894746	17q11.2	LYRM9	0.791667312	0.661850238	1.196142672	4	4	86.42065933	46.53783107
chr3	119821321	120095823	3q13.33	GSK3B	37.62833267	31.45889251	1.196111168	4	4	17.45355094	12.32510276
chr5	150357639	150400308	5q32-q33.1	TCOF1	4.050902863	3.386803132	1.196084539	4	4	44.13181135	47.48009014
chr19	11164267	11197567	19p13.2	KANK2	8.335041267	6.968646736	1.196077457	4	4	27.44931109	27.77969843
chr11	108008733	108107776	11q22.3	CUL5	24.5594115	20.53432257	1.196017615	4	4	44.56484401	25.89795547
chr8	123013164	123042423	8q24.13	DERL1	20.59121935	17.21758966	1.195940881	4	4	21.01145226	14.84554413
chr6	16299112	16761490	6p22.3	ATXN1	12.10569378	10.12261007	1.195906362	4	4	33.2000624	39.0116908
chr14	44924310	44961978	14q21.2	KLHL28	12.36916263	10.34370576	1.195815399	4	4	15.72497583	18.65045864
chr10	102102100	102120473	10q24.32	LDB1	25.50638875	21.33074728	1.195756924	4	4	55.00341909	25.60033805
chr14	23095474	23100456	14q11.2	C14orf119	14.19869851	11.87569363	1.195610037	4	4	30.6711916	15.76205429
chr10	123008913	123058290	10q26.13	ACADSB	5.7773391	4.832224995	1.195585699	4	4	35.33227762	24.10802534
chrX	16946691	17220724	Xp22.2	REPS2	12.61942491	10.55559507	1.19551999	4	4	38.22198779	35.60200823
chr8	86514422	86561498	8q21.3	CPNE3	30.15639299	25.22476563	1.195507361	4	4	15.98959889	10.40955063
chr11	85628573	85636540	11q14.1	TMEM126B	12.3218944	10.30738111	1.195443758	4	4	17.3192493	19.32083874
chr7	157337004	157417439	7q36.3	DNAJB6	28.11397838	23.51830742	1.195408235	4	4	24.4871491	30.64862491
chr5	10249921	10266412	5p15.2	CCT5	29.30667333	24.51708643	1.1953571	4	4	53.09859965	51.48156971
chr1	202331657	202341966	1q32.1	UBE2T	2.192668306	1.834413327	1.19529676	4	4	107.1341317	92.63170344
chr3	131026850	131350465	3q22.1	NEK11	1.304135614	1.091075897	1.195274882	4	4	45.34346013	29.24631351
chr7	142474096	142474567	7q34	TRBV7-5	1.707774654	1.428784377	1.195264087	1	3	0	53.53801799
chr13	41216369	41312038	13q14.11	MTRF1	50.11180838	41.92613125	1.195240459	4	4	56.53195154	23.91163671
chr10	50067888	50133509	10q11.23	WASHC2A	27.10682702	22.6795083	1.195212289	4	4	18.10007729	15.47969175
chr7	76326791	76359025	7q11.23	YWHAG	30.99167397	25.92986659	1.19521147	4	4	31.50413031	9.312734143
chr18	68673688	68715173	18q22.1	TMX3	14.79230337	12.37646675	1.195195985	4	4	9.549462196	20.78025934
chr22	32800816	32863041	22q12.3	TIMP3	0.06638664	0.055545309	1.195179956	4	3	14.8748129	0.585694395
chr7	102473100	102478934	7q22.1	POLR2J	25.10208583	21.004454	1.195083949	4	4	44.88326287	42.12501536
chr5	132875379	132963662	5q31.1	AFF4	25.2920968	21.16426475	1.19503782	4	4	28.59533282	5.077898478
chr19	4174109	4182604	19p13.3	SIRT6	2.994190346	2.50552932	1.195033051	4	4	47.14068081	44.45453249
chr9	101398830	101410660	9q31.1	ZNF189	10.25433311	8.581744539	1.194900764	4	4	9.484975716	27.91252637
chr1	121108441	121117251	1p11.2	HIST2H2BA	0.849604252	0.711086034	1.194798113	2	1	17.19865288	0
chr16	57186137	57240475	16q13	RSPRY1	10.16391849	8.506937206	1.194780007	4	4	58.21431819	12.11247361
chr12	124911645	124915041	12q24.31	UBC	205.3113757	171.8635937	1.194618192	4	4	43.63983603	18.61689706
chr1	1253912	1273854	1p36.33	UBE2J2	10.52785917	8.812761257	1.19461527	4	4	16.33235227	18.32272095
chr3	37141539	37142872	3p22.2	UBE2FP1	4.273257538	3.57719924	1.19458192	4	4	79.74264476	46.63832735
chr16	51645756	51647116	16q12.1	HNRNPA1P48	0.924953614	0.774295603	1.194574282	4	4	53.94083551	32.03570102
chr6	83067666	83171351	6q14.1	DOP1A	13.94021385	11.6697937	1.194555295	4	4	30.09935729	25.94544787
chr10	58385143	58399230	10q21.1	TFAM	18.01556764	15.08158915	1.194540407	4	4	19.39156672	17.56212651
chr14	76761457	76783020	14q24.3	VASH1	1.868406997	1.564129622	1.194534629	4	4	108.4443734	33.8799698
chr9	33441154	33447633	9p13.3	AQP3	10.92709597	9.147653104	1.194524524	4	4	33.90940351	45.24268289
chr16	80597899	80805043	16q23.2	CDYL2	4.817992734	4.033510316	1.194491239	4	4	29.52263792	55.33196596
chr14	61762357	61796428	14q23.2	SNAPC1	4.785294948	4.006170904	1.19448098	4	4	20.09133933	39.78304075
chr3	139355595	139389680	3q23	COPB2	24.17380442	20.23798516	1.194476833	4	4	36.17849306	31.6227085
chr3	97762581	97812585	3q11.2	ARL6	1.734845619	1.452405039	1.19446406	4	4	42.99684	20.29349804
chr1	100896076	100996913	1p21.2	SLC30A7	14.40035284	12.05604518	1.194450803	4	4	8.220713986	18.71161797

chr7	44876293	44885385	7p13	PURB	12.17376022	10.19278282	1.194350987	4	4	33.48330814	12.48004994
chr6	143843273	143863812	6q24.2	LTV1	9.100424229	7.619565616	1.19434948	4	4	54.15109155	24.46836901
chr7	30496621	30504841	7p14.3	GGCT	5.200402541	4.354289351	1.194317171	4	4	30.12930461	9.445311418
chr6	30914208	30926459	6p21.33	VARS2	0.96105608	0.804740705	1.194243156	4	4	74.79367038	56.81258864
chr17	78674047	78782342	17q25.3	CYTH1	44.48930426	37.2533054	1.194237767	4	4	45.59311004	25.15631804
chr2	112736349	112764677	2q14.1	CKAP2L	5.905565523	4.945115788	1.19422189	4	4	89.63062183	26.33224463
chr1	147928374	147993592	1q21.2	GPR89B	16.75390409	14.02961348	1.194181444	4	4	63.68031733	45.14339448
chr1	171485496	171593511	1q24.3	PRRC2C	44.969626	37.65980491	1.194101406	4	4	37.05637414	19.68600637
chr16	46796607	46831471	16q11.2	C16orf87	3.411799468	2.857289947	1.194068341	4	4	43.14735302	29.38897099
chr12	14365632	14502935	12p13.1	ATF7IP	47.97227102	40.1794184	1.193951354	4	4	19.22433853	27.0635174
chr12	53191318	53207375	12q13.13	ITGB7	23.5220508	19.7021793	1.193880659	4	4	39.4338982	45.47103422
chr10	70815920	70881189	10q22.1	SGPL1	9.176865765	7.68669084	1.193864298	4	4	46.39512238	26.1999075
chr5	131264009	131394690	5q31.1	CDC42SE2	126.4277241	105.900491	1.193835107	4	4	38.38598929	7.80157274
chr7	143316126	143352008	7q34	CLCN1	0.27774582	0.232650255	1.193834151	3	1	64.05388195	0
chr19	44905749	44909395	19q13.32	APOE	0.132092567	0.110654453	1.193739273	1	1	0	0
chr18	56651359	57029805	18q21.31	WDR7	9.15447689	7.669689081	1.193591656	4	4	37.83334278	22.88424387
chr19	37312837	37364455	19q13.12	HKR1	3.558783223	2.981970729	1.193433319	4	4	71.49877661	38.25570364
chr1	64982782	64986108	1p31.3	SLC2A3P2	0.483420647	0.405081463	1.193391186	2	4	28.69101473	87.19969757
chr5	151771915	151805354	5q33.1	G3BP1	27.39406808	22.95514345	1.193373857	4	4	29.10024656	20.39786496
chr17	1344539	1400262	17p13.3	YWHAE	42.76851603	35.83872593	1.193360392	4	4	27.49685898	16.51537903
chr12	100573661	100628288	12q23.1	GAS2L3	13.9247937	11.6687665	1.193338962	4	4	69.33796019	56.41347751
chr7	7968743	8089080	7p21.3	GLCCI1	12.44178733	10.42620253	1.193319168	4	4	49.83381835	17.11869641
chr1	109090741	109096934	1p13.3	TMEM167B	32.90559043	27.57765578	1.193197518	4	4	21.47542667	31.63627472
chr11	74988905	75007698	11q13.4	NEU3	2.40847303	2.018580101	1.193152072	4	4	53.50909014	25.43899305
chr11	123358299	123627771	11q24.1	GRAMD1B	1.605039371	1.345225894	1.193137434	4	4	41.78449994	39.67573902
chr5	78777209	78986087	5q14.1	ARSB	5.62032221	4.710871305	1.193053651	4	4	37.62017997	17.82249214
chr19	12131350	12140407	19p13.2	ZNF20	0.262407906	0.21994735	1.19304873	3	4	91.86978783	52.22330371
chr19	3750773	3761696	19p13.3	APBA3	2.653908754	2.224499351	1.193036426	4	4	72.971746	46.14960728
chr10	100150090	100186057	10q24.31	ERLIN1	5.879597681	4.928574023	1.19296122	4	4	34.24199373	28.47151409
chr1	110877252	110963962	1p13.3	LRIF1	12.7391452	10.67880766	1.19293704	4	4	38.08190405	50.74507601
chr4	153710160	153760983	4q31.3	RNF175	0.76459944	0.640997236	1.192827983	4	4	62.85836858	14.4429735
chr6	16238580	16295549	6p22.3	GMPR	113.7679592	95.37746098	1.192818073	4	4	14.66752728	59.05672589
chr2	134455759	134720934	2q21.3	TMEM163	0.142254055	0.119261002	1.192796069	2	3	21.5139971	116.0466793
chr3	179604787	179624500	3q26.33	NDUFB5	9.179700684	7.695994514	1.192789401	4	4	17.83137849	30.07151811
chr11	107502726	107565740	11q22.3	ALKBH8	4.544049743	3.809600827	1.192788943	4	4	44.67446449	18.56553575
chr2	85602842	85649284	2p11.2	USP39	17.47117983	14.64736849	1.192786257	4	4	18.94182179	9.491939125
chr8	28767658	28890226	8p21.1	INTS9	4.530896664	3.798611309	1.192777122	4	4	36.31803571	37.1016998
chr16	89644431	89657785	16q24.3	CHMP1A	15.96159524	13.38202322	1.192763978	4	4	51.04727328	34.33927502
chr19	12688916	12696900	19p13.13	FBXW9	0.529135655	0.443646212	1.192697335	3	4	59.15998236	79.40030216
chr1	113894194	113905124	1p13.2	AP4B1	3.779207794	3.168837071	1.192616632	4	4	32.19498843	12.55118362
chr9	136862114	136866336	9q34.3	EDF1	80.61375982	67.59437525	1.192610473	4	4	80.99258095	50.20898693
chr17	63477061	63498380	17q23.3	ACE	0.188397561	0.157971001	1.192608511	3	4	87.43409693	70.2546977
chr17	82657067	82698722	17q25.3	RAB40B	0.213908449	0.179365681	1.192582931	3	4	48.58872126	48.24126616

chr12	123289109	123365209	12q24.31	SBNO1	29.86762541	25.04493982	1.192561277	4	4	29.73043656	6.918947357
chr7	17790761	17940508	7p21.1	SNX13	28.94638286	24.27324305	1.192522268	4	4	41.05846859	45.69734835
chr13	79311827	79406221	13q31.1	RBM26	21.49735137	18.02780612	1.192455212	4	4	12.40225906	8.478782143
chr19	16574907	16628204	19p13.11	MED26	1.912208002	1.603593637	1.192451727	4	4	82.92044768	8.834477516
chr7	2234291	2242198	7p22.3	MRM2	5.480616843	4.596117962	1.192444774	4	4	65.85303858	41.93408381
chr14	104689606	104719610	14q32.33	INF2	2.500234705	2.096733901	1.192442543	4	4	61.58422285	39.86007879
chr19	35545600	35547527	19q13.12	TMEM147	5.900928186	4.948678704	1.192424996	4	4	52.55907115	12.36262817
chr3	12583601	12664201	3p25.2	RAF1	31.5624436	26.46918612	1.192422142	4	4	49.5428248	28.72372929
chr3	7519741	7535284	3p26.1	GRM7-AS1	0.540735259	0.453477106	1.192420194	2	1	4.888830405	0
chr17	40140254	40172178	17q21.1	CASC3	50.05726471	41.98049078	1.192393509	4	4	28.84444741	17.14179565
chr5	271621	314974	5p15.33	PDCD6	5.553249056	4.657275381	1.192381511	4	4	51.87851315	24.15758272
chr3	136148918	136197300	3q22.3	MSL2	42.49453345	35.63905494	1.192358594	4	4	52.9119678	23.87520405
chr1	244652760	244709034	1q44	DESI2	13.04185456	10.93789944	1.192354586	4	4	19.42431717	13.88904862
chr5	134926661	134928036	5q31.1	MTND4P12	0.318194345	0.266881884	1.192266561	1	3	0	53.13342957
chr12	115958576	116277219	12q24.21	MED13L	57.20024465	47.97645999	1.192256466	4	4	22.79160585	25.95404835
chrY	12662367	12692224	Yq11.221	TTTY15	5.669457551	4.755928277	1.192082223	2	2	39.86853347	25.59233964
chr13	49699307	49792921	13q14.2	KPNA3	22.31556322	18.72076378	1.192022051	4	4	22.31385571	20.56165354
chr13	23570248	23676105	13q12.12	TNFRSF19	0.189259452	0.158773413	1.192009728	1	3	0	66.4662081
chr2	74471958	74472815	2p13.1	MRPL53	1.075348257	0.902131455	1.192008383	4	4	96.90885025	34.05262741
chr19	34481933	34509147	19q13.11	WTIP	0.023388114	0.019621243	1.191979263	1	2	0	5.520896005
chrX	108084205	108091644	Xq22.3	PSMD10	11.52553325	9.669359908	1.191964448	4	4	6.446199634	12.73547533
chr5	135334381	135401998	5q31.1	H2AFY	14.49250342	12.15882069	1.191933312	4	4	37.07056675	16.97061094
chr17	42567060	42573203	17q21.2	MLX	13.35021203	11.20050026	1.191929978	4	4	53.33625722	20.73386721
chr6	157934168	157934257	6q25.3	RNU6-786P	4.524940061	3.796316468	1.191929097	1	2	0	23.3349908
chr8	56957929	56993874	8q12.1	IMPAD1	18.46539699	15.49297944	1.191855773	4	4	22.57335389	24.1296738
chr5	61157709	61162475	5q12.1	SMIM15	21.01152224	17.6304331	1.191775727	4	4	13.70622693	15.55816089
chr16	31033095	31040168	16p11.2	STX4	7.326963132	6.148086203	1.191746975	4	4	65.07866227	39.22828105
chr8	81478497	81483238	8q21.13	FABP4	0.213104007	0.178820248	1.191721906	2	2	15.2284483	9.504978122
chr20	34714774	34825649	20q11.22	NCOA6	8.370086651	7.023581856	1.191711982	4	4	17.97310204	18.45129383
chr17	46512710	47049128	17q21.31	LRRC37A2	4.761921496	3.99587205	1.191710204	4	4	87.5802326	52.87982333
chr21	34180677	34189919	21q22.11	LINC00310	0.123857447	0.103937151	1.191657133	2	3	35.62432208	43.7845473
chr17	78356777	78360079	17q25.3	SOCS3	79.37894421	66.61748377	1.191563231	4	4	69.517377	63.77516831
chr10	10462515	11336675	10p14	CELF2	44.6344966	37.46277572	1.191435918	4	4	37.25785813	15.78975965
chr1	25800176	25818222	1p36.11	SELENON	4.162091698	3.493356117	1.191430693	4	4	84.68981959	56.56618067
chr10	100977820	100987519	10q24.31	MRPL43	4.086044695	3.429871164	1.191311423	4	4	53.44376988	26.56565484
chr7	151058200	151076527	7q36.1	SLC4A2	3.063090339	2.57127101	1.191274793	4	4	50.44222518	42.89646638
chr4	56337687	56389418	4q12	AASDH	12.06575955	10.12966244	1.191131454	4	4	41.09393459	40.86123816
chr6	7541575	7586717	6p24.3	DSP	0.316412913	0.265643658	1.191117888	3	4	107.7970063	83.85435452
chr14	77708071	77717598	14q24.3	SLIRP	8.057346239	6.765259167	1.190988555	4	4	5.194304831	19.40273293
chr1	77695987	77759879	1p31.1	USP33	102.3526262	85.94286493	1.19093803	4	4	47.27244181	10.42403716
chr16	74999017	75110994	16q23.1	ZNRF1	2.967622494	2.491976006	1.190871215	4	4	84.95641485	56.69773125
chrMT	8527	9207	N/A	MT-ATP6	453.9598103	381.2415554	1.190740631	4	4	65.42519347	24.3131999
chr17	38765691	38799905	17q12	PIP4K2B	2.913218368	2.446645102	1.190699201	4	4	31.20016493	46.24395782

chr7	6409116	6448012	7p22.1	DAGLB	4.302261639	3.613432219	1.190630231	4	4	46.34597857	5.722044478
chr2	216081866	216102783	2q35	TMEM169	0.932809731	0.783480702	1.190596946	4	4	73.79380841	65.8915729
chr5	102754783	103031105	5q21.1	PAM	5.165201466	4.338390982	1.190579984	4	4	50.32202587	34.27481783
chr16	29742463	29746019	16p11.2	C16orf54	100.9952543	84.82938135	1.190569266	4	4	63.23214793	39.47284303
chr2	61888723	62136070	2p15	COMMD1	5.771034851	4.847805778	1.190442669	4	4	37.43076274	13.67011356
chr6	110440072	110440293	6q21	RN7SL617P	1.057122696	0.88805101	1.190385106	2	1	17.77203245	0
chr19	54236592	54242768	19q13.42	LILRA6	11.12062631	9.342172176	1.190368374	4	4	68.89580653	68.396368
chr10	74151185	74709303	10q11-q24	ADK	17.67503226	14.84846628	1.190360804	4	4	35.49299003	23.97501845
chr9	74980794	75028456	9q21.13	CARNMT1	39.44095287	33.13396568	1.190348093	4	4	62.26290062	28.91353101
chr14	21903077	21904598	14q11.2	TRAV8-5	0.841209723	0.706728901	1.190286292	3	2	26.10868578	8.359306937
chr8	132866943	133134902	8q24.22	TG	0.334545105	0.281070147	1.19025485	3	4	61.24536697	53.3840861
chr9	33290417	33371163	9p13.3	NFX1	12.99596574	10.91905927	1.190209286	4	4	11.19593922	10.18497148
chr17	81977546	82017669	17q25.3	ASPSCR1	0.887801979	0.745945412	1.190170171	4	4	82.42061073	50.61204866
chr5	218223	264816	5p15.33	SDHA	11.05063722	9.285867934	1.190048932	4	4	45.14337522	51.13698729
chr4	71062646	71572087	4q13.3	SLC4A4	1.579218561	1.327039481	1.190031332	4	4	56.25385005	31.14478465
chr22	26672843	26780893	22q12.1	MIATNB	6.287360121	5.283419431	1.190017224	4	4	26.1465183	24.60593771
chr12	10588478	10599835	12p13.2	KLRA1P	3.091958444	2.598284934	1.189999758	4	4	16.42455159	24.18248003
chr19	56595259	56624481	19q13.43	ZNF71	1.007286297	0.846464994	1.189991676	4	4	48.49107524	55.84557611
chr6	32041155	32109374	6p21.33-p21.32	TNXB	0.171007962	0.143733135	1.189760186	4	4	28.51165727	23.38537661
chr17	78696694	78696797	17q25.3	RNU6-638P	33.42318111	28.0937819	1.189700312	4	4	31.10974482	33.28191069
chr14	71320418	71741229	14q24.2	SIPA1L1	23.12776314	19.44051203	1.189668415	4	4	28.0721229	31.28107268
chr2	43222402	43226606	2p21	ZFP36L2	77.20784282	64.90069333	1.189630478	4	4	36.09092498	24.10297236
chr1	156009048	156020967	1q22	SSR2	24.68619845	20.75465356	1.18942956	4	4	63.37797947	28.71525184
chr8	27311482	27459391	8p21.2	PTK2B	47.39725314	39.85047259	1.189377442	4	4	21.39441471	28.13248178
chr12	63558913	63669201	12q14.2	DPY19L2	3.986615552	3.352185024	1.189258804	4	4	85.79116145	73.76379498
chr14	77800058	77933954	14q24.3	ADCK1	0.933598641	0.785037495	1.189240828	4	4	57.65393481	50.93429212
chr4	146175683	146204436	4q31.22	LSM6	8.247991152	6.935777438	1.189194899	4	4	41.72176792	27.67272627
chr17	65009289	65056802	17q24.1	GNA13	125.8412241	105.8221924	1.189176119	4	4	25.99211166	12.85958741
chr19	1086579	1095392	19p13.3	POLR2E	8.420063199	7.080842408	1.189132975	4	4	57.69330265	31.79983613
chr2	131047876	131093468	2q21.1	FAM168B	20.9565205	17.62395813	1.18909273	4	4	59.61684035	16.48611975
chr16	29858356	29863288	16p11.2	CDIPT	24.75179815	20.81573834	1.189090569	4	4	14.64695177	29.64016822
chr1	206160887	206161276	1q32.1	RPL22P4	2.431557212	2.044937554	1.18906184	3	3	75.67757815	86.72582186
chr11	68460718	68615334	11q13.2	PPP6R3	121.4515775	102.1415285	1.18905189	4	4	50.69874694	6.20925233
chr14	67819779	68683118	14q24.1	RAD51B	1.230677687	1.0350472	1.189006344	4	4	45.48794266	18.02047628
chr11	46936524	47164381	11p11.2	C11orf49	0.976098476	0.820939099	1.189002298	4	4	67.60168806	21.55928376
chr5	17130028	17217422	5p15.1	BASP1-AS1	0.796121897	0.669583872	1.188980095	2	2	12.65686777	35.05274552
chr10	96042848	96060870	10q24.1	CCNJ	3.542728128	2.979993622	1.188837487	4	4	47.31057766	18.39120436
chr6	151364115	151391705	6q25.1	ZBTB2	16.57855985	13.94579981	1.18878516	4	4	32.51392438	23.36253653
chr2	86106182	86142157	2p11.2	PTCD3	17.56107409	14.77317837	1.188713332	4	4	56.02257975	23.25377296
chr6	28994785	29005668	6p22.1	ZNF311	0.096565491	0.081240752	1.188633642	1	2	0	1.074213296
chr1	26279722	26281522	1p36.11	SH3BGRL3	385.215265	324.0857375	1.188621468	4	4	64.35601409	53.16500763
chr1	155316854	155324176	1q22	RUSC1-AS1	0.337324862	0.283801577	1.188594038	3	4	75.58387256	59.3513315
chr10	89213569	89252039	10q23.31	LIPA	39.80189348	33.4879324	1.188544369	4	4	56.44837592	15.34450969

chr17	28662183	28702684	17q11.2	SUPT6H	15.52558857	13.06294389	1.188521416	4	4	69.76186297	33.51562627
chr5	180233594	180292071	5q35.3	MAPK9	16.7273047	14.07475485	1.188461531	4	4	50.51286791	27.14231127
chr14	75053221	75069483	14q24.3	ACYP1	1.393718309	1.172743115	1.188425915	4	4	27.50486492	12.80945844
chr3	23945260	23980618	3p24.2	NR1D2	21.17128557	17.81460686	1.188422834	4	4	37.99936547	32.57465817
chr1	32072031	32102866	1p35.2	TMEM39B	2.913880976	2.451925329	1.188405267	4	4	86.67202958	28.70874478
chr17	18269958	18315092	17p11.2	TOP3A	7.22929936	6.083398115	1.188365322	4	4	27.1117137	16.95853016
chr20	1325343	1378735	20p13	SDCBP2-AS1	1.089999099	0.91726124	1.188319152	4	4	60.68776618	44.69965197
chr19	48469208	48482314	19q13.33	CYTH2	3.119750691	2.625389606	1.188300085	4	4	74.04840569	51.29464627
chr4	85475131	86002670	4q21.23-q21.3	ARHGAP24	2.368833699	1.993488144	1.188285822	4	4	52.20305738	52.27992749
chr1	203795537	203854128	1q32.1	ZC3H11A	0.465272148	0.391562629	1.188244518	4	4	76.16842914	66.78994968
chr19	10718053	10831910	19p13.2	DNM2	16.49268222	13.88039057	1.188200154	4	4	61.94392065	25.37568284
chr9	127691923	127715676	9q34.11	PTRH1	0.355035784	0.298802387	1.188195943	4	4	60.03662731	69.58928828
chr4	127623271	127716779	4q28.1	INTU	0.623778259	0.524985742	1.188181334	4	4	60.95577902	35.49815136
chr7	75416781	75486288	7q11.23	POM121C	3.854175549	3.243909487	1.188126723	4	4	63.53332042	30.37437097
chr13	41566825	41961746	13q14.11	VWA8	17.12460899	14.41382109	1.188068651	4	4	53.29038991	50.27310259
chr12	109929792	109996389	12q24.11	GIT2	18.34495932	15.44300356	1.187913947	4	4	43.65807329	16.69763717
chr2	170990823	171231314	2q31.1	TLK1	48.98594407	41.24442084	1.187698677	4	4	27.84711916	17.48027432
chr9	131502902	131523806	9q34.13	POMT1	5.048902243	4.251516222	1.187553329	4	4	44.04124354	67.10645832
chr16	67873023	67884514	16q22.1	EDC4	6.590427345	5.549619377	1.187545829	4	4	70.8770991	38.56616713
chr1	241497557	241519785	1q43	FH	11.17658626	9.411821532	1.187505121	4	4	25.30741839	18.26512168
chr3	120742711	120783134	3q13.33	GTF2E1	11.43756442	9.632229414	1.187426496	4	4	32.56031218	15.57208757
chr7	103484302	103484538	7q22.1	RN7SKP86	0.975124672	0.821327899	1.187253804	2	1	17.66843809	0
chr18	36108531	36129868	18q12.2	SLC39A6	17.79298354	14.98882646	1.187083164	4	4	56.55156992	35.95287553
chr1	1308563	1311677	1p36.33	PUSL1	0.798555486	0.67274015	1.187019217	4	4	104.6751803	8.99621685
chr6	26634383	26659752	6p22.2	ZNF322	2.976305031	2.507403083	1.187007008	4	4	55.86250326	42.15255937
chr1	22025191	22031224	1p36.12	LINC00339	3.422974728	2.883953946	1.186903395	4	4	35.61516609	48.61280803
chr3	114351811	114388978	3q13.31	ZBTB20-AS1	0.304685666	0.256724114	1.186821374	3	2	46.26112293	3.287192171
chr11	61391982	61398863	11q12.2	TMEM216	3.562060191	3.001373211	1.18681015	4	4	43.09681711	74.71952802
chr16	89948731	89968060	16q24.3	DEF8	8.823025303	7.434346284	1.186792351	4	4	52.85896641	53.40033399
chr14	94050922	94081212	14q32.12	DDX24	6.954448387	5.86018163	1.186729154	4	4	52.59769344	29.19563147
chr13	74231444	74259976	N/A	LINC00402	3.987730419	3.360289248	1.18672237	4	4	83.63405516	25.11638689
chr1	20642657	20652193	1p36.12	PINK1-AS	1.948655246	1.642103509	1.186682347	4	4	62.69258595	46.085161
chr10	14819245	14838073	10p13	CDNF	3.927945994	3.310119244	1.186647883	4	4	59.7016791	62.50483444
chr16	28823048	28837237	16p11.2	ATXN2L	8.222226743	6.929120426	1.186619114	4	4	53.96854353	38.8679217
chr11	6713139	6721879	11p15.4	GVINP1	33.34086437	28.0977887	1.186601007	4	4	54.03395634	37.24861813
chr1	21822232	21937297	1p36.12	HSPG2	0.161661511	0.136247031	1.186532357	4	4	84.06702953	63.16917162
chr14	24239641	24242674	14q12	TINF2	34.23551349	28.85481415	1.186474926	4	4	53.60455629	26.18583443
chr9	77177353	77421537	9q21.2	VPS13A	34.51097286	29.08759042	1.186450041	4	4	50.43549327	26.93372716
chr18	76822550	76970732	18q23	ZNF236	52.02785478	43.85183418	1.186446491	4	4	51.36987299	11.52157612
chr9	114329789	114333256	9q32	ORM2	1.363453381	1.149203659	1.186433205	2	1	25.09112528	0
chr11	47164298	47177125	11p11.2	ARFGAP2	11.75922007	9.911796328	1.18638637	4	4	45.67359769	12.72086942
chr16	57735602	57757250	16q21	KATNB1	3.859148533	3.253180731	1.186269332	4	4	74.46922091	42.1049464
chr15	30903852	30943108	15q13.3	FAN1	1.190225632	1.00336702	1.186231566	4	4	27.14202317	36.68752627

chr13	45119830	45284105	13q14.12-q14.13	GTF2F2	16.95823795	14.29594523	1.18622712	4	4	28.83021448	44.22893818
chr3	63864557	64003462	3p14.1	ATXN7	23.58849447	19.88560118	1.186209773	4	4	42.67595512	8.34526277
chr22	17734140	17774665	22q11.21	BID	20.9470556	17.65902868	1.18619523	4	4	54.82384953	35.3744901
chr9	78236062	78278437	9q21.2	CEP78	6.634205957	5.592949209	1.18617311	4	4	31.74960546	36.28287008
chr2	121755545	121767853	2q14.3	TSN	56.41689682	47.56711791	1.186048247	4	4	52.97968413	36.16571382
chr8	53715543	53843311	8q11.23	ATP6V1H	11.63203038	9.807786038	1.185999607	4	4	21.68330091	24.9449535
chr9	131125561	131234670	9q34.13	NUP214	10.86154033	9.15878621	1.185914824	4	4	48.3731656	23.69152821
chr10	95710901	95877266	10q24.1	ENTPD1	18.14787199	15.30339468	1.18587231	4	4	64.05681478	44.27452526
chr5	161288429	161548124	5q34	GABRB2	0.059587891	0.050248507	1.185863928	1	1	0	0
chr11	65572349	65574198	11q13.1	FAM89B	7.354036151	6.201570536	1.185834477	4	4	59.7415563	78.0679578
chr14	49767608	49786385	14q21.3	KLHDC2	14.89256441	12.55877724	1.185829172	4	4	31.04691617	35.89479558
chrX	30827442	30889394	Xp21.2	TAB3	12.73058115	10.73570841	1.185816592	4	4	20.93814556	41.35397094
chr15	38454127	38487710	15q14	FAM98B	5.385051386	4.541356298	1.185780422	4	4	48.44389138	14.86885508
chr7	26189920	26200793	7p15.2	HNRNPA2B1	117.7227675	99.28630293	1.185689909	4	4	26.75887062	15.25460794
chr15	88501924	88546703	15q26.1	DET1	1.059359093	0.893522233	1.18559903	4	4	69.12745777	37.77333076
chrMT	10059	10404	N/A	MT-ND3	1270.21374	1071.425537	1.185536181	4	4	56.00322702	33.81381701
chr6	109440721	109443922	6q21	SMPD2	4.416090646	3.724995635	1.185529079	4	4	96.74686201	25.70414714
chr9	134135382	134159972	9q34.2	WDR5	4.622380137	3.899129924	1.185490155	4	4	45.17432685	25.52915069
chr15	50702266	50765713	15q21.2	SPPL2A	14.94257616	12.60493675	1.185454276	4	4	24.05257298	14.4064398
chr20	53567065	53608969	20q13.2	ZNF217	62.82858794	53.00042251	1.185435605	4	4	64.10553449	49.81943142
chr13	40796985	40921750	13q14.11	TPTE2P5	22.1452732	18.68128089	1.185425846	4	4	81.67251943	57.77289547
chr7	73568944	73578683	7q11.23	TBL2	2.626757254	2.216016643	1.185350869	4	4	33.13598531	33.59028095
chr8	65602456	65634217	8q13.1	ARMC1	20.4002227	17.21091186	1.185307488	4	4	54.41995584	13.44423125
chr14	65410592	65744122	14q23.3	FUT8	16.2092968	13.67579568	1.185254385	4	4	35.26097116	49.16650981
chr1	150364111	150476566	1q21.2	RPRD2	15.4798642	13.06045225	1.18524718	4	4	26.80366676	17.08408567
chr15	42738974	42920995	15q15.2	TTBK2	11.02265282	9.299999981	1.185231489	4	4	21.16602497	29.10544818
chr6	7389829	7418037	6p24.3	RIOK1	15.39482333	12.98904895	1.185215592	4	4	30.32211332	25.58878869
chr22	37180166	37199401	22q12.3	C1QTNF6	0.736182918	0.621164047	1.185166659	4	4	54.03253037	27.67322926
chr2	89213423	89213897	2p11.2	IGKV1-27	5.632704486	4.752748891	1.185146663	3	1	69.87269308	0
chr15	76348185	76905457	15q24.3	SCAPER	4.259792979	3.594323384	1.185144608	4	4	27.38266425	24.44314092
chr1	149054033	149082311	1q21.2	NBPF9	0.783405112	0.661121803	1.184963359	4	4	82.67721175	27.80768842
chr11	78435961	78574864	11q14.1	NARS2	7.362403486	6.214293556	1.184753089	4	4	42.43440651	31.56518422
chr19	56221303	56368383	19q13.43	ZSCAN5A	0.535995555	0.452446608	1.184660346	4	4	87.40818203	37.08806052
chr1	1324757	1328896	1p36.33	CPTP	4.118087445	3.476246605	1.184636165	4	4	71.10606331	70.79595592
chrX	24054948	24078810	Xp22.11	EIF2S3	111.6784472	94.27478962	1.184605637	4	4	42.78347244	18.40452538
chr16	74296775	74306288	16q23.1	PSMD7	41.08367874	34.68193654	1.184584335	4	4	42.6591497	12.07838742
chr8	109334323	109345960	8q23.1	ENY2	7.600522709	6.416461536	1.184534913	4	4	37.75390522	18.11090782
chr9	130579573	130638352	9q34.11-q34.12	FUBP3	9.741729461	8.224351792	1.184498147	4	4	50.23323926	22.35604441
chr10	102479229	102502755	10q24.32	ACTR1A	24.55533188	20.73170072	1.184434032	4	4	39.15845826	32.14472763
chr9	104747568	104760124	9q31.1	NIPSNAP3A	16.97956556	14.33568186	1.184426784	4	4	28.41046901	12.07347756
chr9	136842506	136847345	9q34.3	AJM1	0.455434275	0.384518909	1.184426211	4	4	18.78746903	55.27265322
chr17	5078459	5096374	17p13.2	ZFP3	2.456086151	2.073694488	1.184401157	4	4	30.54912968	49.50227526
chr9	137220247	137221323	9q34.3	RNF208	1.018783615	0.860191333	1.184368612	2	3	73.1706171	86.74709038

chr2	46698960	46762788	2p21	SOCS5	6.398018468	5.402368928	1.184298694	4	4	44.30461464	30.89144213
chr1	247204570	247204661	1q44	RNA5SP82	5.117782387	4.321672485	1.184213382	3	1	26.12718507	0
chr16	30572939	30586038	16p11.2	ZNF785	0.95271091	0.804535953	1.184174438	4	4	39.03149378	39.17152158
chr6	38715329	39030798	6p21.2	DNAH8	0.063650718	0.053751331	1.184170073	3	3	117.0379986	34.36201517
chr1	40161369	40240921	1p34.2	RLF	32.49655356	27.44488656	1.184065873	4	4	2.239057665	21.72139832
chr18	46803224	46920167	18q21.1	PIAS2	6.161847508	5.204117388	1.184033151	4	4	64.91210586	27.59818779
chr17	63827410	63832027	17q23.3	PSMC5	8.243321338	6.962071819	1.184032792	4	4	57.08567393	38.00552275
chr11	118747763	118791696	11q23.3	DDX6	91.969392	77.67558684	1.184019275	4	4	19.33279136	15.71639302
chr9	34086383	34126773	9p13.3	DCAF12	1044.523203	882.2694926	1.183904932	4	4	36.3493084	25.73397255
chr3	121894324	121944187	3q13.33	SLC15A2	1.18348449	0.999670457	1.183874628	4	4	45.13141988	49.50533884
chr20	21166165	21166614	20p11.23	RPS15AP1	0.913114679	0.77132512	1.183825932	3	2	39.14372062	15.40710243
chr9	37485935	37503697	9p13.2	POLR1E	6.384241393	5.393013335	1.183798555	4	4	49.67464958	22.06328435
chr1	24356999	24415533	1p36.11	STPG1	0.485610402	0.410215336	1.183793876	4	4	40.58498844	33.34736985
chr1	151008391	151035713	1q21.3	PRUNE1	48.39786944	40.88429225	1.183776624	4	4	13.2965458	18.21031203
chr17	42609340	42615238	17q21.2	TUBG1	2.476379959	2.092243649	1.18360018	4	4	39.57487172	80.82035064
chr1	20588951	20618908	1p36.12	CDA	59.17813659	50.00406291	1.183466565	4	4	66.22363987	75.55755245
chr5	154857952	154876792	5q33.2	CNOT8	45.33077311	38.30861852	1.183304824	4	4	31.60094136	10.406666
chr11	93784239	93813330	11q21	MED17	0.933266718	0.788696616	1.183302552	4	4	55.39387582	26.2899141
chr1	38012712	38024825	1p34.3	UTP11	6.443103323	5.4460703	1.183073844	4	4	38.52749309	44.00048306
chr14	23969874	24006408	14q11.2	DHRS4L2	2.636075779	2.228255447	1.183022253	4	4	51.9986517	40.47351837
chr4	155342658	155376970	4q32.1	MAP9	1.25431577	1.060392187	1.182879113	4	4	35.0611705	50.97973743
chr19	14119509	14136628	19p13.12	ASF1B	6.620556933	5.596996477	1.182876737	4	4	54.45616862	18.5863734
chr17	36534762	36539303	17q12	PIGW	0.39049588	0.330155337	1.182764102	1	1	0	0
chr1	10474946	10630758	1p36.22	PEX14	3.06148201	2.589005318	1.182493519	4	4	63.98543347	43.52275743
chr4	155758973	155807642	4q32.1	GUCY1B1	18.28407859	15.46334346	1.18241431	4	4	17.45967247	25.5775915
chr8	26383007	26413129	8p21.2	BNIP3L	2584.134974	2185.706284	1.182288304	4	4	43.14039841	43.32657825
chr18	2537525	2571503	18p11.32	METTL4	8.005538645	6.771870684	1.182175357	4	4	25.64305527	41.84342207
chr19	58544469	58550722	19q13.43	TRIM28	23.40555033	19.79955077	1.182125322	4	4	49.98174434	38.49003537
chr1	28603096	28648707	1p35.3	TAF12	25.55103084	21.61484476	1.182105684	4	4	26.31951191	7.886205768
chr4	185396686	185400245	4q35.1	ANKRD37	0.889621594	0.752580542	1.182094864	4	4	35.56486559	30.92618059
chr20	31664452	31723999	20q11.21	BCL2L1	842.4262053	712.6677398	1.18207428	4	4	25.07474082	35.63968763
chr12	111686053	111757107	12q24.12	ACAD10	2.033884826	1.720713192	1.182001065	4	4	50.08714326	22.60293544
chr2	241881363	242078722	2q37.3	LINC01237	0.327806631	0.277337586	1.181976938	3	2	30.64967576	59.5146815
chr17	8140470	8156360	17p13.1	PER1	6.425163471	5.436093025	1.181945092	4	4	67.73517266	67.97081921
chr14	60091911	60134814	14q23.1	PCNX4	3.575429383	3.025080995	1.18192848	4	4	41.00887321	8.527935951
chr6	162726870	163315492	6q26	PACRG	0.119699808	0.101284753	1.181814681	1	1	0	0
chr14	39175183	39183218	14q21.1	PNN	100.7183362	85.22602576	1.181779103	4	4	27.66815413	12.85953176
chr6	78934419	79078298	6q14.1	PHIP	36.22004929	30.64967408	1.181743375	4	4	14.79117052	11.88547946
chr1	145607960	145670977	1q21.1	GPR89A	28.59981454	24.20181828	1.181721729	4	4	68.11540896	48.40302913
chr2	113702677	113756823	2q14.1	SLC35F5	12.14193342	10.27497724	1.181699301	4	4	4.205612042	33.62077281
chr19	49889654	49929539	19q13.33	IL4I1	0.595430247	0.503877928	1.181695436	4	4	67.69295961	61.13561388
chr7	64355090	64356399	7q11.21	TRIM60P18	3.051313794	2.582214308	1.18166559	3	4	29.33656689	65.38562495
chr2	87629755	87629834	2p11.2	MIR4435-1	3.675140213	3.110304522	1.181601412	1	2	0	23.11419596

chr7	152134925	152436642	7q36.1	KMT2C	38.31112991	32.4270226	1.18145691	4	4	8.066623222	10.66620436
chr1	53242364	53328149	1p32.3	LRP8	1.056618914	0.894359384	1.181425424	4	4	66.39189342	27.63947856
chr4	26583924	26858919	4p15.2	TBC1D19	4.277871188	3.621078841	1.181380295	4	4	68.80376448	35.79051057
chr9	129110949	129148946	9q34.11	PTPA	9.529061959	8.066232882	1.1813522	4	4	28.27216453	43.42914228
chr3	48856923	48898993	3p21.31	SLC25A20	19.66793576	16.65120297	1.181172063	4	4	49.84906185	42.09783513
chr13	42886388	42992312	13q14.11	EPSTI1	66.60865721	56.39613036	1.181085595	4	4	43.85185678	59.40974235
chr1	219173831	219445121	1q41	LYPLAL1	31.79135773	26.9178597	1.181050726	4	4	40.16966546	47.17640311
chr22	45604432	45605647	22q13.31	LINC01589	0.570642107	0.483168316	1.181042067	2	1	13.60569258	0
chr6	7107597	7251980	6p24.3	RREB1	5.651249887	4.78510351	1.181008911	4	4	48.89523702	10.26808022
chr6	31952087	31959087	6p21.33	NELFE	9.760443172	8.265894887	1.180809012	4	4	19.21803523	29.61800454
chr1	226223618	226227055	1q42.12	MIXL1	0.139804512	0.118400397	1.180777387	2	1	18.65810242	0
chr5	109334709	109409978	5q21.3	PJA2	111.0052113	94.0209972	1.180642778	4	4	36.88704631	24.41102989
chr2	90154098	90154574	2p11.2	IGKV1D-13	12.45491566	10.55024378	1.180533448	1	1	0	0
chrX	48590053	48605194	Xp11.23	WDR13	4.325640639	3.664257836	1.180495705	4	4	51.07578405	38.02616117
chr22	20507542	20587632	22q11.21	MED15	6.305178424	5.341170796	1.180486201	4	4	74.38882282	42.79364565
chr19	41431318	41447822	19q13.2	DMAC2	3.296254341	2.792391355	1.180441393	4	4	63.06846192	48.16490445
chr1	246540561	246566263	1q44	TFB2M	23.97413652	20.31066552	1.180371785	4	4	66.16767585	64.65137052
chr14	60245752	60299087	14q23.1	PPM1A	127.2732757	107.8254874	1.180363556	4	4	27.61238836	35.6610119
chr1	25959764	25998164	1p36.11	PAFAH2	7.400464987	6.269818966	1.180331526	4	4	27.54590278	14.74375375
chr17	50962174	51120865	17q21.33	SPAG9	26.78707013	22.69612103	1.180248823	4	4	18.13661664	28.9632232
chrX	16587999	16712979	Xp22.2	CTPS2	4.799378211	4.066678776	1.180171456	4	4	34.26588555	22.40022309
chrX	40626921	40647683	Xp11.4	CXorf38	14.1901578	12.02389498	1.180163152	4	4	36.31194543	5.634343704
chr7	30594735	30634033	7p14.3	GARS	28.64977432	24.27784295	1.18007907	4	4	9.847116105	41.776319
chr20	32277651	32335011	20q11.21	KIF3B	10.42868856	8.837621056	1.18003346	4	4	8.871858313	6.024605192
chr1	243124428	243255796	1q43	CEP170	2.617085923	2.217992134	1.179934718	4	4	38.4656047	22.99207854
chr15	72343435	72376473	15q23	HEXA	3.272393972	2.773667846	1.179807444	4	4	61.30572643	21.78146135
chr21	7816677	7829632	21p11.2	KCNE1B	0.163026948	0.138183083	1.179789484	2	2	69.08249031	7.679196502
chr11	31369830	31432835	11p13	DNAJC24	1.790258782	1.517490835	1.179749321	4	4	55.99306287	34.87121445
chr2	113157399	113203229	2q14.1	PSD4	20.27607478	17.1874714	1.179700859	4	4	40.63020711	42.71689404
chr11	448215	491400	11p15.5	PTDSS2	0.73185165	0.62037803	1.179686602	4	4	67.91805743	38.05668845
chr12	45919131	45992040	12q12	SCAF11	42.51063425	36.03556629	1.179685478	4	4	16.23150926	13.71275983
chr11	8335029	8594289	11p15.4	STK33	0.788500309	0.668404145	1.179675972	4	4	58.21958699	43.74806101
chr6	116571367	116593601	6q22.1	RWDD1	12.06132244	10.225367	1.179549101	4	4	37.07431148	30.9984595
chr1	151166022	151170297	1q21.3	SCNM1	44.9465757	38.10522222	1.179538475	4	4	44.36379546	8.101071134
chr7	135092303	135170826	7q33	CYREN	4.895132553	4.150325031	1.179457637	4	4	42.10963204	26.4912252
chr15	82988297	83012298	15q25.2	C15orf40	0.933294608	0.791300838	1.179443472	4	4	56.16795942	22.58973095
chr20	33811304	33854367	20q11.22	CHMP4B	74.17201979	62.88993321	1.179394157	4	4	58.84469838	43.53920202
chr1	225777813	225790466	1q42.12	SRP9	122.4878085	103.8701584	1.179239643	4	4	29.04319576	14.67724963
chrX	69160738	69165522	Xq13.1	PJA1	5.047744522	4.280569195	1.179222737	4	4	40.69229512	46.90382597
chr14	89794984	89954777	14q32.11	EFCAB11	3.881082911	3.291592731	1.179089647	4	4	73.43978364	31.51356129
chr6	37819531	38154624	6p21.2	ZFAND3	23.9856871	20.34422847	1.17899222	4	4	20.79366506	12.94873466
chr4	25914192	25929879	4p15.2	SMIM20	5.991075997	5.082012049	1.178878747	4	4	23.97657318	35.02570815
chr7	34928881	35038041	7p14.2	DPY19L1	9.064196367	7.689216115	1.178819301	4	4	29.37839048	17.25257472

chr17	35474906	35537861	17q12	SLFN12L	14.8071517	12.56107238	1.178812704	4	4	40.74532707	38.41540994
chr1	110362851	110391971	1p13.3	SLC16A4	0.325434596	0.276074881	1.178791038	4	4	31.51192551	51.84857449
chr7	100139565	100140698	7q22.1	RPL7P60	0.465945834	0.395275777	1.178786714	1	2	0	11.6046234
chr6	158869865	158918115	6q25.3	C6orf99	0.333077313	0.282590454	1.178657337	2	2	0.923747068	7.280912102
chr17	61679186	61864120	17q23.2	BRIP1	40.79523185	34.61340945	1.178596171	4	4	83.9133303	51.5375355
chr9	132375548	132406851	9q34.13	TTF1	12.01978768	10.1984599	1.178588511	4	4	57.46119765	23.05727024
chr12	109448655	109477544	12q24.11	KCTD10	5.745609591	4.87529883	1.178514342	4	4	47.89330927	27.1198848
chr4	158709127	158723400	4q32.1	PPID	24.75703573	21.00766735	1.178476188	4	4	43.07103378	39.80059761
chr5	160043538	160120701	5q33.3	PWWP2A	11.16816539	9.478734666	1.178233781	4	4	39.66243311	19.07861965
chr22	26443109	26483863	22q12.1	HPS4	6.010187707	5.101629152	1.178091846	4	4	43.29443717	28.75685262
chr10	12195962	12250590	10p14-p13	CDC123	53.99108109	45.82953537	1.178084845	4	4	17.70361236	8.683875068
chr13	32504506	32509395	13q13.1	N4BP2L2-IT2	3.859905059	3.276507285	1.178054777	4	4	63.4087876	18.35103554
chr16	11679080	11742878	16p13.13	TXNDC11	14.41442719	12.2358238	1.178051223	4	4	26.12677533	8.531530222
chr8	41578188	41625001	8p11.21	GPAT4	7.501032307	6.367704705	1.177980552	4	4	47.33587929	18.49871183
chr6	57114911	57172213	6p12.1	ZNF451-AS1	0.060770589	0.051590214	1.177947978	1	1	0	0
chr6	33250272	33271965	6p21.32	VPS52	4.800109173	4.074984272	1.177945448	4	4	47.63887007	15.11009582
chrX	6768840	7148190	Xp22.31	PUDP	3.877736016	3.29204106	1.177912409	4	4	22.33082923	42.53152093
chr19	10832067	10836307	19p13.2	TMED1	4.969444405	4.218976239	1.177879211	4	4	44.50757728	11.09531129
chr12	25195216	25205015	12p12.1	ETFRF1	12.82005918	10.88444317	1.17783326	4	4	9.899921269	38.19028792
chr19	38815422	38831857	19q13.2	ECH1	4.049976424	3.438503717	1.177831044	4	4	75.91102982	51.15480644
chr2	231708525	231713541	2q37.1	PTMA	229.3355384	194.7127447	1.177814728	4	4	69.24939265	32.03330459
chrX	123600561	123733054	Xq25	THOC2	29.1859734	24.78200063	1.177708524	4	4	27.53992607	7.595694302
chr20	63981078	64033100	20q13.33	PRPF6	33.13187648	28.13560373	1.177578302	4	4	53.08374033	40.14734561
chr5	138506093	138543300	5q31.2	ETF1	37.09875568	31.50604249	1.177512399	4	4	53.45764514	8.903908395
chr4	88709789	88730103	4q22.1	FAM13A-AS1	4.038123926	3.429431683	1.177490704	4	4	42.77904096	70.29951621
chrX	153764196	153779346	Xq28	PLXNB3	0.147435503	0.12521239	1.17748334	4	4	69.89597301	28.34181014
chr7	44826889	44848126	7p13	H2AFV	24.01462504	20.39521872	1.177463472	4	4	35.79515745	9.819385233
chr2	108719446	109842301	2q13	RANBP2	53.10964464	45.10596507	1.17744171	4	4	25.40187403	6.150678629
chr12	117038923	117099490	12q24.22	TESC	105.5201957	89.62142855	1.177399172	4	4	38.71757485	18.124311
chr10	109945559	110008381	10q25.1	ADD3-AS1	0.047616241	0.040450321	1.177153604	3	2	57.38063646	39.7089955
chr13	32303771	32311954	13q13.1	ZAR1L	0.404031331	0.343227821	1.177152044	3	1	31.87126445	0
chr12	2812621	2859907	12p13.33	ITFG2	4.404509916	3.74188151	1.177084283	4	4	43.54903073	17.95647639
chr16	89969775	89972832	16q24.3	CENPBD1	1.347908347	1.145196008	1.177011043	4	4	94.57166944	31.75482356
chr19	41262476	41307783	19q13.2	HNRNPUL1	50.25959001	42.70211899	1.176981171	4	4	66.42964824	38.96497398
chr16	176651	177522	16p13.3	HBA1	15656.11677	13302.03755	1.176971325	4	4	48.0554836	17.2512646
chr14	95157688	95179933	14q32.13	DICER1-AS1	0.29005347	0.246442176	1.176963602	3	4	103.1196178	74.67671423
chr4	439984	499171	4p16.3	ZNF721	9.190839289	7.809223819	1.176920972	4	4	42.93170125	23.17252394
chr19	24033405	24129968	19p12	ZNF254	8.782341477	7.462144273	1.176919282	4	4	35.41629461	19.38123064
chr4	82924603	83012951	4q21.22	LIN54	59.612531	50.6565292	1.176798568	4	4	20.53943453	6.762384287
chr5	151029943	151087660	5q33.1	TNIP1	68.80196414	58.46703025	1.176765159	4	4	41.70092298	28.40975528
chr10	110641933	110839471	10q25.2	RBM20	0.072621496	0.061715752	1.176709237	2	1	80.42943677	0
chr1	202348702	202588569	1q32.1	PPP1R12B	4.372759636	3.716289603	1.17664663	4	4	60.46545215	40.78160361
chr2	148644780	148788491	2q23.1	EPC2	30.68891489	26.08253406	1.176607872	4	4	15.99687646	11.71735942

chr8	38901235	38973912	8p11.22	PLEKHA2	39.57126684	33.63283319	1.17656656	4	4	30.1101446	27.84335049
chr12	54121274	54188994	12q13.13	SMUG1	1.321554921	1.123236996	1.176559289	4	4	48.34280793	53.87094547
chr22	24181476	24189106	22q11.23	SUSD2	0.143134178	0.121655217	1.176556019	3	2	54.9521561	28.67376009
chr12	56271699	56300391	12q13.3	CS	15.23770183	12.95149622	1.176520579	4	4	37.72753209	27.42741246
chr2	112829758	112836843	2q14.1	IL1B	41.27128118	35.08118341	1.176450654	4	4	74.93057782	67.60666801
chr16	67109881	67148544	16q22.1	C16orf70	4.085186354	3.472679521	1.176378738	4	4	39.11005954	20.28004736
chr19	1065923	1087831	19p13.3	ARHGAP45	63.18544684	53.71290048	1.176355145	4	4	56.55974071	27.68180165
chr18	68715221	69214350	18q22.1-q22.2	CCDC102B	1.187523427	1.009497555	1.176350968	4	4	22.67218048	32.53062676
chr3	45917899	45995824	3p21.31	FYCO1	5.429626373	4.616224651	1.176204969	4	4	59.90089823	37.90590944
chr3	42013802	42225890	3p22.1	TRAK1	6.492749908	5.520183685	1.17618367	4	4	51.55916999	24.82797656
chr11	2899721	2925246	11p15.4	SLC22A18	0.922393041	0.78423319	1.176171899	4	4	82.67548293	26.45725497
chr5	139276180	139276323	5q31.2	SNORA74D	43.88397537	37.31141141	1.176154257	4	4	67.14245619	30.13851311
chr3	196468783	196503768	3q29	RNF168	19.38146225	16.47882856	1.176143206	4	4	49.99168929	14.48999906
chr2	171434166	171491029	2q31.1	DCAF17	21.99083488	18.69854673	1.176071873	4	4	60.63536622	50.35757182
chr7	30010583	30026801	7p14.3	FKBP14	0.895300398	0.761282929	1.176041604	4	4	68.08705716	27.88614028
chr15	90868592	90883458	15q26.1	FURIN	75.65610001	64.33606548	1.175951614	4	4	23.98959466	24.45539433
chr4	141636596	141733987	4q31.21	IL15	1.585779489	1.348520494	1.175940222	4	4	25.01977893	44.38583669
chr5	33440696	33468091	5p13.3	TARS	20.97840276	17.84036507	1.175895374	4	4	33.6511988	37.62875087
chr12	49741510	49764934	12q13.12	TMBIM6	118.0591503	100.4031377	1.175851204	4	4	50.41384202	23.7989107
chr3	130081831	130103799	3q22.1	ALG1L2	0.43939546	0.373687631	1.175836241	1	3	0	97.85046261
chr11	45647689	45665656	11p11.2	CHST1	0.08662531	0.073674074	1.175790964	2	1	27.06782897	0
chr1	63593276	63660245	1p31.3	PGM1	8.231893338	7.001347739	1.175758389	4	4	58.99212141	26.09993658
chr1	207053063	207081023	1q32.1	PFKFB2	4.414274651	3.754585861	1.175702145	4	4	41.97611821	35.24929179
chr3	48636463	48662915	3p21.31	CELSR3	0.11572388	0.098433309	1.175657724	4	4	94.4029754	65.87115053
chrX	155197007	155239841	Xq28	VBP1	37.76409375	32.1222443	1.175636839	4	4	42.20004289	20.28295049
chr22	42500579	42512560	22q13.2	SERHL	0.188836344	0.160624959	1.175635131	1	1	0	0
chrX	130401949	130413656	Xq26.1	RBMX2	8.981841899	7.640951476	1.175487363	4	4	56.60727245	42.61325085
chr2	38778185	38781965	2p22.1	GEMIN6	2.659957364	2.262939237	1.175443565	4	4	51.73602757	45.65707336
chr3	52410508	52423641	3p21.1	PHF7	51.01793667	43.41331462	1.175167967	4	4	66.47611975	66.83569043
chr4	8366263	8440725	4p16.1	ACOX3	2.493211787	2.121754341	1.175070902	4	4	61.91621902	34.24405754
chr15	65442463	65517713	15q22.31	DPP8	24.60495556	20.94130515	1.174948523	4	4	21.51248816	15.0821694
chr10	32267716	32378798	10p11.22	EPC1	18.29136943	15.56807497	1.174928144	4	4	25.24984833	14.64086749
chr1	12019242	12032049	1p36.22	MIIP	9.039458291	7.693919259	1.174883435	4	4	42.34594669	34.19098291
chr6	24402908	24426194	6p22.3	MRS2	9.21300241	7.842059853	1.174819191	4	4	49.37660627	31.38664116
chr14	103334002	103345025	14q32.32	EIF5	59.37175856	50.53872732	1.174777477	4	4	28.54202755	13.53739479
chr4	173168753	173323967	4q34.1	GALNT7	8.21329644	6.991772252	1.174708807	4	4	35.49621143	43.16502947
chr7	101313367	101321823	7q22.1	IFT22	0.600705497	0.511367634	1.174703789	3	4	66.2374445	51.84394618
chr5	177511577	177517326	5q35.3	DDX41	8.020316161	6.828445354	1.174544973	4	4	60.82770368	38.9732788
chr22	31753951	31907034	22q12.2-q12.3	DEPDC5	7.691346498	6.54962126	1.174319276	4	4	20.55591735	23.80695546
chr11	65712002	65719606	11q13.1	KAT5	10.86938048	9.255951903	1.174312549	4	4	61.9326434	29.87618488
chr15	50500562	50546705	15q21.2	USP50	1.11978723	0.953647397	1.174215159	3	4	102.0340031	56.13822378
chrY	19705415	19745341	Yq11.223	KDM5D	10.27257539	8.748566599	1.17420097	2	2	6.127446265	1.094426917
chr7	74796144	74851576	7q11.23	GTF2IRD2	0.947584012	0.807041476	1.17414537	4	4	33.68091985	62.04211135

chr6	144284955	144853034	6q24.2	UTRN	60.94263026	51.9054045	1.174109533	4	4	13.76425832	13.57237369
chr13	42424892	42485956	13q14.11	LINC02341	0.365235862	0.311079988	1.174089868	2	4	70.15254052	46.49359809
chr5	10679230	10761275	5p15.2	DAP	19.55148899	16.65437456	1.173955162	4	4	36.74799622	15.64868943
chr2	180980385	181063427	2q31.3	UBE2E3	32.2902416	27.50640642	1.173917127	4	4	52.55391758	33.76269929
chr22	20080232	20111877	22q11.21	DGCR8	4.316210422	3.676857655	1.173885645	4	4	9.16636396	30.95874749
chr1	93310109	93345811	1p22.1	CCDC18-AS1	8.442455133	7.192828587	1.173732285	4	4	54.4909995	56.8263426
chr1	88852638	88891944	1p22.2	GTF2B	65.99950748	56.23053202	1.173730803	4	4	24.76851113	5.520145761
chr16	48244167	48362999	16q12.1	LONP2	15.3559379	13.08350049	1.173687265	4	4	21.60307337	9.186440088
chr4	99546707	99564057	4q23	TRMT10A	2.273525583	1.937220122	1.17360209	4	4	54.22532594	55.87941048
chr18	8609428	8639383	18p11.22	RAB12	25.55023417	21.77085017	1.173598365	4	4	19.52635942	29.90661649
chr13	111114619	111305734	13q34	ARHGEF7	5.653921332	4.817623103	1.17359146	4	4	30.11783852	16.51664474
chr9	96019732	96021811	9q22.32	LINC00092	0.215916008	0.183981879	1.173572142	3	3	50.68228903	53.37192349
chr11	129899706	130003097	11q24.3	PRDM10	4.028407244	3.43268381	1.173544511	4	4	32.17891071	29.38762529
chr18	45800586	45967339	18q12.3-q21.1	EPG5	10.96016163	9.339471423	1.173531256	4	4	25.50062579	18.71260271
chrX	101390890	101396154	Xq22.1	RPL36A	1.582937224	1.348902098	1.173500453	4	4	55.85762641	27.76446639
chr15	89316305	89334795	15q26.1	POLG	13.05736511	11.12812035	1.173366633	4	4	45.37988576	22.42178812
chr14	52707157	52727998	14q22.1	PSMC6	19.09364044	16.27318442	1.173319244	4	4	13.58913999	7.315902017
chr8	42270727	42332653	8p11.21	IKBKB	8.784035448	7.486574314	1.17330505	4	4	30.73910768	20.52191372
chr21	44939697	44976989	21q22.3	FAM207A	3.375576015	2.877071989	1.173267832	4	4	53.18073329	30.60521751
chr13	24928755	24968469	13q12.13	TPTE2P1	0.215587192	0.183749479	1.173266957	2	1	14.76883383	0
chr4	121192797	121192903	4q27	RNU6-948P	3.506453671	2.98873856	1.173221947	1	2	0	31.01331014
chr1	1399530	1402046	1p36.33	MRPL20-AS1	4.183814141	3.566130464	1.173208379	4	4	38.5537892	49.24383906
chr15	58879045	58934313	15q22.1	SLTM	182.8966047	155.9249471	1.172978463	4	4	57.25507381	33.07479318
chr10	72332830	72355149	10q22.1	DNAJB12	15.5300742	13.2402639	1.17294295	4	4	37.27759125	11.83609792
chr10	12068917	12123228	10p14	DHTKD1	12.3586725	10.53670667	1.172916062	4	4	42.52308893	30.65474231
chrX	49028726	49043517	Xp11.23	TFE3	26.92843971	22.95912365	1.172886218	4	4	36.52644198	15.40199362
chr12	9715860	9733435	12p13.31	CLECL1	1.491248096	1.271456897	1.172865631	4	4	64.53038976	41.31016742
chr2	98599323	98608518	2q11.2	COA5	7.948778329	6.777540064	1.172811707	4	4	42.27850436	41.23473147
chrX	49113385	49124547	Xp11.23	GPKOW	15.95841077	13.6072667	1.172785918	4	4	59.6937826	28.61561325
chr4	81426393	81471928	4q21.21	RASGEF1B	5.159726417	4.399727277	1.172737784	4	4	57.89924672	65.98190709
chr16	15865719	15888649	16p13.11	FOPNL	39.98849512	34.09844242	1.172736708	4	4	58.12008052	48.04047264
chr22	20707691	20858812	22q11.21	PI4KA	9.874130584	8.419762398	1.172732687	4	4	53.65149175	8.066219672
chr7	76461655	76505995	7q11.23	DTX2	2.340108191	1.995619666	1.172622334	4	4	70.22345773	46.9541488
chr1	76074684	76637316	1p31.1	ST6GALNAC3	1.427620599	1.217752034	1.172340969	4	4	32.30852557	69.61995184
chr2	189661399	189670831	2q32.2	ASNSD1	42.39761998	36.16533895	1.172327461	4	4	31.30662537	13.8593609
chr11	36483767	36510313	11p12	TRAF6	13.44105583	11.46600811	1.172252426	4	4	39.44758939	0.747434462
chr6	13357830	13487637	6p23	GFOD1	3.531545163	3.013170758	1.172036186	4	4	41.34485579	28.16290957
chr12	56731580	56752362	12q13.3	PRIM1	59.59234218	50.84573876	1.172022349	4	4	62.27442034	40.14277751
chr3	99817834	100178632	3q12.1	CMSS1	1.3313231	1.135981675	1.171958253	4	4	26.18822613	47.08525263
chr15	34224997	34230165	15q14	EMC4	7.792306189	6.649082541	1.171937052	4	4	39.98093472	19.89604792
chr6	87344853	87365463	6q15	C6orf163	7.50881188	6.407929852	1.17179995	4	4	55.13854459	49.60761139
chr17	18697998	18736119	17p11.2	TRIM16L	0.577318458	0.492690837	1.171766175	4	4	116.1969203	53.07462554
chr7	100400826	100429996	7q22.1	ZCWPW1	2.867845941	2.447476123	1.171756453	4	4	47.14202733	44.11702063

chr19	12317477	12347390	19p13.2	ZNF563	2.091987543	1.785378141	1.171733592	4	4	30.89177706	25.24306905
chr9	6532464	6645692	9p24.1	GLDC	0.237482407	0.202690348	1.171651284	3	3	49.30694764	51.08781177
chr16	56451480	56477495	16q13	OGFOD1	7.712877751	6.583202366	1.171599675	4	4	37.56107291	17.60507876
chr8	42152946	42171183	8p11.21	AP3M2	4.057526668	3.463255571	1.171593197	4	4	68.74544142	29.95288932
chr16	680111	682768	16p13.3	STUB1	9.923083576	8.469985762	1.171558472	4	4	76.56899527	44.93772549
chr18	3066807	3220108	18p11.31	MYOM1	1.945295827	1.660538458	1.171484959	4	4	87.32280419	14.68268986
chr3	14145145	14178672	3p25.1	XPC	17.30771769	14.77652749	1.17129804	4	4	59.87805895	57.74824651
chr13	23469512	23487464	13q12.12	LINC00327	0.093341628	0.079692636	1.171270434	1	1	0	0
chr5	65665928	65722821	5q12.3	SGTB	18.5419384	15.83199115	1.171169073	4	4	33.34805644	29.69913813
chr19	44954585	44993346	19q13.32	CLPTM1	11.96442901	10.21624341	1.17111824	4	4	62.35122394	33.93927308
chr5	35856849	35879603	5p13.2	IL7R	143.4183088	122.4633127	1.171112439	4	4	51.0560506	34.33884713
chr2	70985938	70994871	2p13.3	TEX261	7.56815048	6.462491366	1.171088679	4	4	28.2149159	14.99856176
chr3	112922685	112975090	3q13.2	CD200R1	8.184022967	6.988406386	1.171085726	4	4	39.70850193	36.12630876
chr17	57085192	57121349	17q22	AKAP1	3.147665019	2.688082431	1.170970422	4	4	79.45939577	47.08964105
chr2	200811546	200823851	2q33.1	BZW1	32.83783167	28.04391749	1.170943099	4	4	16.79383222	7.095864796
chr1	27970344	28088671	1p35.3	EYA3	36.08475369	30.81721203	1.17092856	4	4	56.55425218	19.96345446
chr14	50418492	50561126	14q22.1	MAP4K5	59.71091722	50.99516267	1.170913359	4	4	49.18397747	28.26087379
chr7	148987527	148989429	7q36.1	GHET1	0.301264589	0.257314906	1.170801152	1	3	0	85.52966607
chr5	69273087	69333070	5q13.2	CCDC125	9.686181127	8.273727733	1.170715479	4	4	22.02368582	36.62074406
chr14	105986582	105987019	14q32.33	IGHV1-2	8.916342832	7.616248199	1.17070014	4	3	99.58973967	115.4206309
chr11	33084584	33161491	11p13	CSTF3	18.9995924	16.23010414	1.17063897	4	4	58.72466429	43.46984223
chr5	863735	892824	5p15.33	BRD9	2.876103218	2.456912511	1.170616864	4	4	61.35653221	16.4965994
chr19	3506263	3536757	19p13.3	FZR1	7.912173466	6.75921863	1.170575165	4	4	16.02990688	34.9163007
chr7	129055223	129057239	7q32.1	TPI1P2	0.605754144	0.517484968	1.170573411	3	4	109.7764551	36.3525095
chr16	1997652	2009821	16p13.3	ZNF598	3.582280872	3.060331979	1.17055303	4	4	53.14510471	48.93796138
chr9	105558117	105655950	9q31.2	FKTN	2.44110441	2.085434976	1.17054928	4	4	17.1003323	28.65830769
chr5	159157409	159210053	5q33.3	RNF145	21.95230257	18.75400654	1.170539347	4	4	19.83580616	41.03460307
chr4	185204237	185368020	4q35.1	SNX25	5.22954599	4.46778186	1.170501639	4	4	28.85701449	19.1961728
chr5	140691426	140699318	5q31.3	HARS2	9.206356801	7.865579481	1.170461353	4	4	27.17431179	23.22656384
chr20	23350736	23354772	20p11.21	NXT1	7.575885075	6.473103943	1.170363576	4	4	76.94234349	32.91657375
chr17	11957433	12021522	17p12	ZNF18	6.913613265	5.90766964	1.170277569	4	4	65.78919031	21.87874631
chr6	57908553	57936929	6p11.2	GUSBP4	0.466870378	0.398943291	1.170267527	2	3	23.22224088	16.23334132
chrMT	10470	10766	N/A	MT-ND4L	651.3854448	556.6220352	1.17024732	4	4	44.05636112	20.34755826
chr19	21724041	21767628	19p12	ZNF100	1.494449867	1.277046576	1.170239125	4	4	39.64260431	28.94495882
chr2	112138385	112254558	2q13-q14.1	FBLN7	0.185629189	0.158627983	1.170217164	4	4	58.13813264	51.93815747
chr22	35648316	35668409	22q12.3	APOL6	81.17003971	69.36668997	1.170158757	4	4	46.68434047	50.68733265
chr17	81900964	81911477	17q25.3	PCYT2	1.265178328	1.081215255	1.170144726	4	4	64.70871107	49.33276561
chr17	75074306	75079549	17q25.1	TRIM80P	0.101199448	0.086486292	1.17012125	1	1	0	0
chr14	52730159	52774989	14q22.1	STYX	32.71494381	27.96113086	1.170015046	4	4	19.85592832	6.949061783
chr11	59636716	59669038	11q12.1	PATL1	24.21042543	20.69298398	1.169982321	4	4	20.20729928	10.99873985
chr18	12307669	12329826	18p11.21	TUBB6	1.756911737	1.501684404	1.169960701	4	4	33.89624438	66.81605883
chr12	110648686	110704945	12q24.11	HVCN1	17.29871485	14.78590635	1.169946194	4	4	62.56845103	53.06718282
chr7	21428034	21514822	7p15.3	SP4	26.31201692	22.49072244	1.169905369	4	4	29.88423693	36.31634488

chr11	85647919	85656553	11q14.1	TMEM126A	9.861672269	8.430198503	1.169803091	4	4	50.95199167	22.78610176
chr5	53560610	53683341	5q11.2	NDUFS4	98.10529855	83.86527898	1.169796366	4	4	87.25617757	46.32270705
chr2	178480454	178516324	2q31.2	PLEKHA3	5.205325	4.44998897	1.169738854	4	4	27.54896769	7.136396938
chr9	5860254	6008489	9p24.1	KIAA2026	36.84824015	31.50292643	1.169676736	4	4	23.00572547	6.101358261
chrX	129803288	129843934	Xq26.1	ZDHHC9	0.556555982	0.475838858	1.169631214	4	4	38.39918234	105.4337178
chr19	41829609	41831134	19q13.2	HNRNPA1P52	1.054168634	0.901331502	1.169568169	4	3	91.08671165	26.2220735
chr15	90902218	90922585	15q26.1	MAN2A2	13.97536622	11.94946567	1.169539007	4	4	21.23348459	11.92184538
chrX	73944324	74070384	Xq13.2	JPX	2.623942823	2.2436341	1.16950568	4	4	36.9600038	12.62832889
chr19	17511623	17521291	19p13.11	PGLS	9.429858459	8.063735562	1.169415637	4	4	55.75004768	19.73251658
chr9	21329671	21335432	9p21.3	KLHL9	12.05127412	10.3057002	1.169379458	4	4	17.11768286	17.32985797
chr17	1829971	1899554	17p13.3	RPA1	46.71512684	39.95392804	1.169224883	4	4	50.0443811	26.01711772
chr7	73302516	73308867	7q11.23	NSUN5	3.257908033	2.786384124	1.169224302	4	4	57.37988677	19.62869879
chr1	156054726	156058510	1q22	LAMTOR2	11.72675644	10.02965399	1.169208475	4	4	68.91485328	48.47118021
chr3	138187248	138298389	3q22.3	ARMC8	31.09463661	26.59570918	1.169159897	4	4	46.74335673	40.08924302
chr6	139135112	139180809	6q24.1	HECA	84.96853359	72.68394094	1.169013849	4	4	23.29708511	13.29846202
chr5	64768918	65018763	5q12.3	CWC27	19.72596049	16.87628529	1.168856781	4	4	28.4909763	9.226937757
chr1	23304688	23344364	1p36.12	HNRNPR	17.82226191	15.24845653	1.168791207	4	4	59.4471096	48.50573042
chrX	48508959	48520814	Xp11.23	PORCN	1.690554747	1.446453748	1.168758247	4	4	50.24504418	46.32380876
chr1	34850362	34859816	1p34.3	SMIM12	4.570900161	3.910939906	1.168747225	4	4	71.55548461	23.00911044
chr10	31805398	31928876	10p11.22	ARHGAP12	16.30394393	13.95014401	1.168729435	4	4	10.80418815	9.532122955
chrX	40649543	40736122	Xp11.4	MED14	8.450938774	7.230878042	1.168729264	4	4	22.85037116	8.602173618
chr8	67174298	67343824	8q13.2	ARFGEF1	34.41079241	29.44366606	1.168699317	4	4	19.93166372	15.78619651
chr10	806914	931900	10p15.3	LARP4B	11.37626921	9.734497758	1.168654972	4	4	15.06273064	13.1658996
chr17	29929856	30108452	17q11.2	EFCAB5	0.532159468	0.455426081	1.168487026	3	4	44.10995812	6.796424022
chr1	960103	965719	1p36.33	KLHL17	0.527651938	0.451573346	1.168474496	4	4	77.98134634	35.26397916
chr16	29992321	29996096	16p11.2	HIRIP3	2.229089395	1.907776815	1.16842252	4	4	77.63064764	57.71647524
chr11	62690262	62709619	11q12.3	BSCL2	1.216298718	1.041031498	1.168359191	4	4	64.71115182	74.37790918
chr9	136114581	136118885	9q34.3	TMEM250	8.270202775	7.078607826	1.168337472	4	4	48.88888944	42.82235794
chr17	47898386	47941404	17q21.32	SP2-AS1	0.213033825	0.182350278	1.168267069	2	4	66.2636577	81.11256831
chr1	35433490	35557603	1p34.3	KIAA0319L	10.46454128	8.958068349	1.168169394	4	4	28.30675171	25.06304816
chr1	231241173	231277973	1q42.2	GNPAT	17.35593875	14.85863007	1.16807126	4	4	15.51194743	7.593156405
chr9	36036905	36124455	9p13.3	RECK	2.205308077	1.888218219	1.167930727	4	4	40.91270789	44.79963317
chr7	75092556	75149817	7q11.23	GTF2IRD2B	1.783177798	1.526898194	1.167843282	4	4	48.317278	26.79825478
chr9	33041852	33076716	9p21.1	SMU1	17.78060537	15.22530011	1.167832834	4	4	34.22175874	12.77036909
chr7	151466012	151519924	7q36.1	RHEB	176.0721312	150.7784215	1.167754175	4	4	51.46955844	31.01310184
chr2	88992409	88992878	2p11.2	IGKV1-8	1.130090928	0.967771894	1.167724476	3	2	30.81605959	19.26241257
chr8	141391995	141432454	8q24.3	PTP4A3	2.296137566	1.966486657	1.16763445	4	4	91.5859288	31.29603852
chr10	24848607	24952644	10p12.1	PRTFDC1	4.587256897	3.929226524	1.167470715	4	4	39.08076263	40.56792746
chr2	196763032	196799768	2q33.1	GTF3C3	8.017875526	6.867794693	1.16745999	4	4	47.18938377	10.9157332
chr8	22245104	22251167	8p21.3	POLR3D	3.191076231	2.733511057	1.167391009	4	4	70.97601084	31.60485347
chr7	100539211	100568220	7q22.1	AGFG2	2.583115056	2.21279434	1.167354331	4	4	35.9621217	81.18774729
chr9	114603897	114646424	9q32	TMEM268	5.128937677	4.393751063	1.167325505	4	4	46.52616732	28.54369305
chr5	168292060	168472303	5q34	WWC1	0.12383865	0.106091589	1.16728057	3	4	56.90301613	53.73838746

chr11	5340883	5505652	11p15.4	OR51B5	0.112167933	0.096093623	1.167277595	1	2	0	13.94974721
chr10	114938193	114977680	10q25.3	TRUB1	13.17569008	11.28814134	1.167215193	4	4	62.76494066	10.31251316
chr19	51379909	51387956	19q13.41	LIM2	0.575598433	0.493151467	1.167183861	1	2	0	8.005968857
chr19	14433354	14471867	19p13.12	PKN1	31.11669714	26.66046238	1.167147692	4	4	50.04689192	43.46618469
chr17	5471251	5486230	17p13.2	DERL2	10.47005483	8.97227176	1.166934652	4	4	17.91832194	25.9800521
chr5	71455615	71578288	5q13.2	BDP1	16.50924416	14.14892959	1.166819303	4	4	24.1161291	5.497062443
chr1	226989865	227318502	1q42.13	CDC42BPA	41.850014	35.86690526	1.16681419	4	4	57.45738246	43.48079332
chr19	16496311	16521369	19p13.11	C19orf44	0.348938972	0.299061621	1.166779511	3	4	59.72086053	41.99529574
chr9	130893428	130896812	9q34.12	QRFP	0.187782859	0.160943574	1.166762082	2	1	9.791951559	0
chr2	210431249	210477618	2q34	LANCL1	10.78452796	9.243187201	1.166754251	4	4	38.1307069	23.5586496
chr2	88897295	88897784	2p11.2	IGKV5-2	4.091903622	3.507104698	1.166746925	2	1	29.06001475	0
chr10	73801911	73811849	10q22.2	NDST2	0.509299054	0.436536697	1.166680962	4	4	101.5510513	41.85384046
chrX	136869194	136880780	Xq26.3	RBMX	44.1327124	37.82798635	1.166668297	4	4	64.97392194	28.62872372
chr19	4657545	4670403	19p13.3	MYDGF	7.112348367	6.096349098	1.166657003	4	4	76.40847741	18.33336977
chr9	120952335	121075174	9q33.2	C5	11.75773187	10.07840386	1.166626386	4	4	71.21342303	43.57447473
chr6	130018369	130141449	6q23.1	L3MBTL3	11.13819587	9.547732965	1.166580162	4	4	46.81577353	42.00412175
chr4	174236658	174284251	4q34.1	FBXO8	11.38174543	9.7570191	1.16651872	4	4	22.73299331	20.20627446
chr1	30931506	31065717	1p35.2	PUM1	35.60930318	30.52786916	1.166452299	4	4	27.12677551	29.83274009
chr20	26187019	26209233	20p11.1	MIR663AHG	0.05678966	0.048687513	1.166411199	2	1	20.01224348	0
chr17	63832081	63842991	17q23.3	SMARCD2	24.85490761	21.30956538	1.166373277	4	4	53.3184182	35.15311311
chr5	38937920	39074421	5p13.1	RICTOR	60.37310206	51.76242931	1.166349858	4	4	20.37808373	34.18061731
chr5	113513662	113595287	5q22.2	YTHDC2	12.51984785	10.73426787	1.1663439	4	4	32.77722931	38.39465541
chr12	113185526	113192368	12q24.13	RITA1	3.313031546	2.840728778	1.166261127	4	4	72.70889136	45.05368711
chr16	726936	729746	16p13.3	HAGHL	0.247808366	0.212484644	1.166241292	3	3	100.9260403	54.35966816
chr10	23136910	23139778	10p12.2	YWHAZP3	1.780572983	1.526868487	1.166160019	3	1	34.92507105	0
chr1	36455761	36464439	1p34.3	MRPS15	6.520828772	5.592922556	1.165907217	4	4	29.82591777	27.62243941
chr11	72292425	72434684	11q13.4	CLPB	5.400673766	4.632522774	1.165816992	4	4	57.32343116	32.75309668
chr1	113697202	113759888	1p13.2	PHTF1	6.55683027	5.624521665	1.165757848	4	4	25.2503851	28.47585475
chr6	28123753	28130086	6p22.1	ZSCAN16	10.8568419	9.313754742	1.165678311	4	4	50.54601974	19.40359991
chr5	112976702	113022195	5q22.2	DCP2	77.09057592	66.13367615	1.165678069	4	4	22.29565123	21.14274925
chr3	88149743	88157966	3p11.1	C3orf38	23.18372783	19.888622	1.165677936	4	4	31.81935934	13.92343151
chr2	36552239	36598190	2p22.2	FEZ2	5.056263034	4.337616256	1.165677814	4	4	41.60985436	33.96099522
chr11	66682496	66729361	11q13.2	SPTBN2	0.071348497	0.061212724	1.165582782	2	4	7.948502407	65.76950969
chr16	56429136	56451349	16q13	NUDT21	41.71179629	35.78713579	1.165552799	4	4	35.83363293	33.30094806
chrX	44146254	44343677	Xp11.3	EFHC2	5.284740153	4.534380565	1.16548227	4	4	26.99547038	27.89888229
chr5	178859953	178902276	5q35.3	ZNF354B	3.695113333	3.170465124	1.165479887	4	4	83.92682518	39.45841681
chr10	17641284	17643918	10p12.33	STAM-AS1	0.344546086	0.29562666	1.165477043	4	4	76.29217946	60.62359684
chr2	135052265	135176667	2q21.3	RAB3GAP1	18.21492744	15.6288486	1.165468289	4	4	8.520284969	19.71578838
chr2	16549459	16665866	2p24.2	FAM49A	29.74853424	25.52566284	1.165436307	4	4	37.94771421	22.86948632
chr17	17206946	17237191	17p11.2	FLCN	2.827992533	2.426884257	1.165277052	4	4	30.24601364	28.42925408
chr12	15882354	15903478	12p12.3	STRAP	51.34671458	44.06407454	1.165273868	4	4	25.54583161	20.80759475
chr20	28580633	28602665	20q11.1	FRG1CP	41.25713377	35.40715422	1.165220269	4	4	75.18100838	21.69736379
chr1	160541094	160579516	1q23.3	CD84	12.22031147	10.4879095	1.165180865	4	4	39.86343255	32.12369951

chr16	2537964	2603190	16p13.3	PDPK1	8.057580299	6.915951214	1.165071882	4	4	35.53928832	25.15563319
chr5	73498425	73505623	5q13.2	BTF3	469.7178987	403.1894381	1.165005465	4	4	25.92038071	12.51872906
chr5	118974586	118974670	5q23.1	MIR1244-2	34.10604082	29.27637743	1.16496793	4	4	67.42669409	65.25648243
chr13	49912702	49936501	13q14.2	SPRYD7	2.910692279	2.498574858	1.164940994	4	4	30.89468758	50.38990686
chr4	7043429	7057952	4p16.1	TADA2B	12.40350687	10.64761102	1.164909842	4	4	48.72833702	22.45992752
chr19	52113091	52139938	19q13.41	ZNF616	3.21786259	2.762424333	1.164869043	4	4	53.62889242	60.44803595
chrX	155071403	155126766	Xq28	BRCC3	9.616369376	8.255396381	1.164858588	4	4	26.8606041	26.45201413
chr1	3780220	3796504	1p36.32	LRRC47	6.404384681	5.498515754	1.164747901	4	4	48.55884985	14.95737266
chr5	171861549	172009604	5q35.1	FBXW11	24.79970083	21.29510557	1.164572805	4	4	19.66591103	15.26405112
chr3	119669525	119677396	3q13.33	COX17	8.101604924	6.956759084	1.164565975	4	4	32.03389018	18.53041709
chr7	32485333	32495258	7p14.3	LSM5	5.604797043	4.812991679	1.164514177	4	4	46.06775125	30.02464379
chr3	161429072	161429620	3q26.1	RPL23AP42	12.93365193	11.10734344	1.164423519	4	4	31.80920655	32.83513654
chr12	71609599	71663969	12q21.1	ZFC3H1	31.76971118	27.28379077	1.164417051	4	4	24.78080408	33.03220416
chr3	45594105	45681263	3p21.31	LIMD1	4.196396245	3.603989022	1.164375424	4	4	48.54401694	12.78877554
chr12	68154770	68159741	12q15	IFNG	5.760190874	4.947050669	1.164368683	4	4	48.00618168	95.48462157
chr1	235440654	235504481	1q42.3	B3GALNT2	2.744042668	2.356717733	1.164349311	4	4	9.260256477	16.47620056
chr19	22052448	22091103	19p12	ZNF257	2.663104738	2.287216964	1.164342859	4	4	127.6198272	84.30291415
chr11	125592795	125622759	11q24.2	STT3A	12.53604169	10.76682064	1.164321587	4	4	32.63558324	34.525046
chr6	84124241	84227889	6q14.2-q14.3	CEP162	43.44609016	37.31476932	1.164313513	4	4	57.98926909	33.07827238
chr1	236213064	236282039	1q42.3	ERO1B	3.754357735	3.224560682	1.164300538	4	4	40.58150855	28.29689877
chr11	93478472	93553979	11q21	SMCO4	6.057605769	5.203005949	1.164251171	4	4	49.40351695	56.81443124
chr19	38303560	38315976	19q13.2	YIF1B	4.928383247	4.233249064	1.164208194	4	4	46.72539277	42.34147485
chr10	23439269	23442381	10p12.2	OTUD1	15.66767747	13.46007852	1.164010852	4	4	54.56319275	12.0273928
chr17	50367867	50373201	17q21.33	MRPL27	5.938291482	5.102244538	1.163858658	4	4	29.25049909	19.6147947
chr10	5412551	5459056	10p15.1	NET1	15.09949762	12.97405862	1.163822214	4	4	35.49821445	28.30149503
chr5	154445957	154461053	5q33.2	SAP30L	8.679095536	7.457596593	1.163792574	4	4	35.54139633	37.71416399
chr2	197486584	197500274	2q33.1	HSPD1	38.26245169	32.88124268	1.163655889	4	4	32.13763034	33.20454524
chr19	10780254	10780335	19p13.2	MIR4748	6.16265316	5.296246866	1.163588729	2	2	59.23786589	47.67289518
chr12	29336544	29381210	12p11.22	ERGIC2	34.35191601	29.52283262	1.16357114	4	4	14.97908002	23.78340574
chr18	12328944	12377309	18p11.21	AFG3L2	10.2125011	8.776943599	1.163560069	4	4	22.86686397	11.35444978
chr7	150685706	150693641	7q36.1	GIMAP2	68.99513841	59.29692487	1.163553398	4	4	20.0205536	28.36902554
chr9	131485665	131485750	9q34.13	SNORD62A	5.863868958	5.039703647	1.163534479	1	3	0	45.43655911
chr19	38374550	38383824	19q13.2	PSMD8	20.96566452	18.019441	1.163502493	4	4	67.47677981	59.50335222
chr9	123109494	123268576	9q33.3	STRBP	13.33072411	11.45770512	1.163472438	4	4	47.85673606	32.00373935
chr3	101574094	101594465	3q12.3	PCNP	67.08448013	57.66052749	1.163438544	4	4	9.62913895	9.930767393
chr6	106046729	106109939	6q21	PRDM1	17.63105178	15.15456359	1.163415341	4	4	50.48869321	59.22088758
chr3	88948198	88950993	3p11.1	ICE2P2	0.260681518	0.224073172	1.163376749	3	1	67.59737624	0
chr1	212726153	212798900	1q32.3	NSL1	14.6276348	12.57355654	1.163364936	4	4	20.17684221	29.52246427
chr1	203298758	203305325	1q32.1	LINC01136	3.227745467	2.774518307	1.163353458	4	4	12.94384552	91.08324813
chr11	95976593	96343308	11q21	MAML2	15.01572554	12.90734119	1.163347689	4	4	50.22863857	40.78824054
chr2	30447226	30644225	2p23.1	LCLAT1	14.45616349	12.42640314	1.163342548	4	4	48.47129913	48.67187603
chr6	90513573	90587301	6q15	MAP3K7	16.34551209	14.05085368	1.163310961	4	4	31.67075349	6.220660688
chr18	47833095	47931188	18q21.1	SMAD2	11.32426756	9.735123993	1.163238143	4	4	19.51086212	15.49863909

chrX	119925050	119943772	Xq24	NKAP	5.226030094	4.492865062	1.163184298	4	4	15.63344423	25.35492599
chr11	62662817	62665216	11q12.3	C11orf98	0.506771125	0.435700197	1.163118879	2	2	37.27076179	25.71521888
chr6	17615035	17706834	6p22.3	NUP153	120.4751177	103.5940356	1.16295419	4	4	48.87869308	19.15821969
chr19	40717103	40740860	19q13.2	ITPKC	3.316815613	2.852103477	1.162936633	4	4	44.08245283	16.57106676
chr5	32354346	32444761	5p13.3	ZFR	33.33095488	28.66263648	1.162871214	4	4	49.88233258	23.32396018
chr16	569968	584136	16p13.3	PIGQ	1.451318187	1.24804775	1.162870721	4	4	54.11022559	47.98024021
chr19	5891276	5904013	19p13.3	NDUFA11	0.862169097	0.741415179	1.162869498	4	4	49.35057179	24.01321246
chr2	207529892	207605989	2q33.3	CREB1	46.29425426	39.81096646	1.162851806	4	4	48.196475	28.14816129
chr1	186311652	186375325	1q31.1	TPR	54.28754653	46.68728689	1.162790776	4	4	47.30117588	27.16153382
chr11	62671654	62673690	11q12.3	UQCC3	1.463882028	1.259108951	1.162633327	4	4	50.93203524	61.18267235
chr9	72900662	72953317	9q21.13	ALDH1A1	11.60987523	9.986632786	1.162541517	4	4	10.9205148	34.92906387
chr17	49210227	49223225	17q21.32	ABI3	15.43075857	13.27493368	1.162398167	4	4	38.88303386	69.11033524
chr3	9385264	9397494	3p25.3	THUMPD3-AS1	6.68435331	5.750764354	1.162341716	4	4	29.10266151	22.27505561
chr10	72216000	72235860	10q22.1	ANAPC16	28.69384523	24.69013483	1.162158304	4	4	22.41605688	20.88361031
chr20	2655067	2655198	20p13	SNORA51	3.61641383	3.111923955	1.162115104	3	1	61.50010581	0
chrX	136665547	136781344	Xq26.3	ARHGEF6	45.65834869	39.29507297	1.161935715	4	4	48.8611369	28.25195135
chr7	141704338	141738230	7q34	WEE2-AS1	0.087332864	0.075162033	1.161927911	1	2	0	2.263190247
chr15	31001061	31161273	15q13.3	TRPM1	0.039080258	0.033634923	1.161895245	1	1	0	0
chrX	103707344	103728624	Xq22.2	GLRA4	0.163693313	0.14089075	1.161845713	1	2	0	45.54410996
chr3	33090416	33096801	3p22.3	TMPPE	4.800400655	4.131738736	1.161835479	4	4	45.73424805	31.57649333
chr11	47604309	47642654	11p11.2	MTCH2	19.99860332	17.21306283	1.161827126	4	4	42.55330367	17.59144728
chr3	184299098	184309054	3q27.1	PSMD2	18.95438109	16.31471141	1.161796897	4	4	32.75061474	31.14374599
chr17	18856299	18931287	17p11.2	PRPSAP2	42.32120454	36.42939006	1.161732449	4	4	53.7541369	45.44142608
chr22	31933521	31945362	22q12.3	C22orf24	0.305732474	0.263170513	1.161727698	2	1	33.74715503	0
chr16	29670588	29698699	16p11.2	QPRT	0.383124571	0.329794093	1.161708407	3	4	86.0821888	64.83517304
chr4	958884	973560	4p16.3	DGKQ	5.253532578	4.522327548	1.161687764	4	4	56.07607828	9.77962253
chr20	4852356	5010293	20p13	SLC23A2	4.897438073	4.215919604	1.161653573	4	4	55.51741761	13.54961412
chr2	174395729	174430074	2q31.1	SCRN3	6.724007873	5.788361471	1.161642704	4	4	17.30702656	8.825622989
chr4	112636964	112657592	4q25	LARP7	24.16443491	20.80290387	1.161589509	4	4	48.22458194	19.70890083
chr7	36324150	36390125	7p14.2	KIAA0895	0.107532383	0.092576807	1.161547767	3	4	53.7364565	67.87874691
chr7	964850	975599	7p22.3	COX19	4.56122099	3.927108078	1.161470705	4	4	61.631047	39.6746838
chr9	129634604	129642169	9q34.11	ASB6	4.639511069	3.994539609	1.161463278	4	4	48.53365533	24.57727278
chr2	206203376	206266243	2q33.3	GPR1-AS	0.06485934	0.055845929	1.161397823	1	1	0	0
chr7	75156578	75171998	7q11.23	NCF1C	49.55889131	42.67218397	1.161386334	4	4	51.48031539	15.43882227
chrX	101345656	101348969	Xq22.1	TIMM8A	1.61084158	1.387047211	1.161345892	4	4	47.36494082	23.04577211
chrX	103215092	103217200	Xq22.1	BEX4	22.19710206	19.11353989	1.16132868	4	4	56.22397353	36.31001135
chr10	70150204	70170529	10q22.1	SAR1A	27.19730657	23.42092063	1.161239859	4	4	27.66113562	13.58671383
chr8	19817149	19852083	8p21.3	INTS10	9.034145687	7.779895377	1.161216861	4	4	42.34739088	17.48919261
chr12	109112666	109268226	12q24.11	ACACB	0.835262042	0.719305693	1.161205938	4	4	84.39854717	36.25398697
chrX	77910656	78050395	Xq21.1	ATP7A	56.62922694	48.76915487	1.161168921	4	4	61.09917217	28.14438664
chr16	634427	636366	16p13.3	METTL26	4.633582607	3.990759283	1.16107795	4	4	42.2593869	53.18434518
chr15	48662534	48811904	15q21.1	CEP152	7.688241747	6.62193811	1.161025914	4	4	31.80688228	12.7439637
chr19	51167328	51173524	19q13.41	SIGLEC17P	1.659679204	1.429666449	1.160885607	4	3	44.50281581	58.69868213

chr8	58814412	58814552	8q12.1	RNU4-50P	2.040419423	1.757667368	1.160867784	1	1	0	0
chr2	57907651	58164001	2p16.1	VRK2	126.3779496	108.871037	1.160804131	4	4	68.44555747	30.97411722
chr16	50742026	50801935	16q12.1	CYLD	46.04870644	39.67144247	1.160752006	4	4	28.05477646	21.43870846
chr1	149940330	150010776	1q21.2	OTUD7B	3.139908365	2.705093015	1.160739519	4	4	12.67873605	28.41535383
chr1	9103390	9129170	1p36.22	GPR157	0.777887476	0.670170921	1.160729974	4	4	65.79989725	18.92546399
chr12	132490439	132585188	12q24.33	FBRSL1	1.737971652	1.497403482	1.160656879	4	4	105.1211769	34.68184456
chr1	43383908	43389812	1p34.2	MED8	9.589498672	8.262741449	1.160571068	4	4	47.36893756	12.90795865
chr18	36129874	36177931	18q12.2	ELP2	7.375069192	6.354832002	1.160545108	4	4	40.40959628	15.24893568
chr9	86287733	86354497	9q21.33	TUT7	134.5549969	115.9459156	1.160497946	4	4	13.36073994	11.8615182
chr6	26538344	26546937	6p22.2	HMGN4	59.90847223	51.62476186	1.160460021	4	4	29.42875673	11.39735117
chr12	26121951	26234777	12p12.1	SSPN	0.907145321	0.781725015	1.16044044	4	4	65.90685991	57.90255971
chr7	105010542	105014141	7q22.3	KMT2E-AS1	8.051315511	6.939035342	1.160293198	4	4	62.66921507	44.13637003
chr11	72097292	72103387	11q13.4	LAMTOR1	27.02727442	23.29381483	1.160276864	4	4	40.74852048	6.992152125
chr20	3190350	3204697	20p13	DDRGK1	11.92892603	10.2833193	1.160026804	4	4	63.82584487	58.11707775
chr15	50908569	51005900	15q21.2	AP4E1	13.37144116	11.5268509	1.160025515	4	4	44.25218829	32.6829522
chr9	17134991	17510020	9p22.2	CNTLN	35.76972101	30.83623023	1.159990075	4	4	41.35446607	30.9798371
chr1	28736621	28769775	1p35.3	YTHDF2	22.53707685	19.4288758	1.15997843	4	4	27.45913171	17.71619786
chr13	19674752	19783036	13q12.11	PSPC1	38.98290512	33.61434383	1.159710429	4	4	31.63762534	9.467383693
chr10	11849608	11894710	10p14	PROSER2-AS1	0.092170604	0.079482437	1.159634843	2	1	28.03755723	0
chr19	53479282	53480365	19q13.42	TPM3P6	1.57491002	1.358139821	1.159608161	3	3	74.37038852	61.08492015
chr1	169588849	169630139	1q24.2	SELP	15.5607594	13.42021928	1.159501128	4	4	35.95974005	23.55548419
chr17	49598884	49678163	17q21.33	SPOP	14.75111719	12.72294777	1.159410339	4	4	29.98668058	18.37532983
chrX	153444720	153446487	Xq28	TREX2	0.320364462	0.276322215	1.15938728	4	4	100.1856629	64.95735894
chr12	131894734	131923162	12q24.33	ULK1	5.991093716	5.167606848	1.159355557	4	4	46.30522928	22.47686501
chr15	74445977	74461829	15q24.1	UBL7	36.42689192	31.42052952	1.159334119	4	4	25.10873664	27.38776263
chr19	10221433	10231272	19p13.2	S1PR2	1.237704566	1.06763092	1.159300037	4	4	40.1561307	46.82149021
chr3	32525971	32570924	3p22.3	DYNC1LI1	28.28416684	24.39765247	1.159298702	4	4	20.54124517	32.59244152
chr3	157146502	157160760	3q25.31	CCNL1	51.03526596	44.02401648	1.15925965	4	4	25.04418451	34.78600905
chr3	179017223	179071868	3q26.32	ZMAT3	8.107807185	6.994471954	1.159173593	4	4	34.89369797	9.846319033
chr7	38722975	38909200	7p14.1	VPS41	18.66465228	16.10213551	1.159141424	4	4	35.09570842	18.66508451
chr5	77086798	77148351	5q13.3	ZBED3-AS1	0.169653877	0.146363996	1.159123024	4	4	67.1723586	83.48723241
chr2	197250858	197302519	2q33.1	ANKRD44-IT1	8.556555826	7.38289403	1.158970424	4	4	18.83344311	17.74094929
chr15	71822291	72118577	15q23	MYO9A	7.395526899	6.381934145	1.158822189	4	4	16.27559281	18.51645942
chr17	35587263	35726417	17q12	AP2B1	49.28846233	42.53841779	1.158681138	4	4	18.53371386	29.81788626
chr7	44200978	44214294	7p13	YKT6	12.52090653	10.80641012	1.158655501	4	4	45.18567329	20.25624056
chr16	71894408	71930639	16q22.2	IST1	22.83147897	19.70618292	1.158594694	4	4	20.57100777	22.96183514
chr16	89972580	90000787	16q24.3	AFG3L1P	2.146506344	1.852768061	1.158540235	4	4	17.55624307	18.54786403
chr16	50152918	50235310	16q12.1	TENT4B	11.48112178	9.910198277	1.15851585	4	4	34.50645801	17.57128671
chr3	52545352	52685907	3p21.1	PBRM1	32.65906737	28.19133485	1.158478928	4	4	24.82498022	5.174781913
chr11	60913899	60923443	11q12.2	TMEM109	17.92343994	15.47187344	1.158453113	4	4	47.49447352	46.27683452
chr3	179322910	179335535	3q26.33	ZNF639	9.355084123	8.075961329	1.158386444	4	4	18.1587723	18.31644596
chr5	151100706	151157882	5q33.1	ANXA6	66.78635738	57.65595516	1.158360089	4	4	59.58533642	50.35937235
chr2	102432289	102432366	2q12.1	MIR4772	14.04497204	12.12521878	1.158327309	3	2	63.61448345	21.91562178

chr9	6413148	6507056	9p24.1	UHRF2	25.21802938	21.77133983	1.158313158	4	4	42.64791486	33.41913345
chr4	17577304	17607967	4p15.32	LAP3	41.16770538	35.54113093	1.158311632	4	4	39.49912984	56.57528506
chr16	70250231	70251930	16q22.1	EXOSC6	7.008835793	6.051014949	1.15829094	4	4	36.02199451	7.377361625
chr12	104117080	104138262	12q23.3	NFYB	20.61096708	17.79507323	1.158240083	4	4	29.203977	16.26812704
chr7	99325873	99366262	7q22.1	ARPC1A	4.343757697	3.750516184	1.158175964	4	4	36.33339591	45.859536
chr22	29327680	29388583	22q12.2	AP1B1	13.38587425	11.55813539	1.158134404	4	4	37.79889801	54.54429065
chr8	144772224	144787371	8q24.3	ZNF34	1.862328468	1.608100435	1.158092136	4	4	86.40242657	64.9930878
chr22	49860163	49918469	22q13.33	ALG12	2.351779421	2.0307596	1.158078692	4	4	49.19563442	26.47710105
chr9	9441998	9442310	9p23	RN7SL5P	722.3157166	623.7373931	1.158044595	4	4	73.44329859	42.26386657
chr14	21918258	21918756	14q11.2	TRAV13-2	9.498552137	8.202479296	1.158009889	4	4	51.72289236	36.03906487
chr14	94127781	94129620	14q32.12	IFI27L2	3.333731083	2.878848046	1.1580087	4	4	62.95914418	89.4630058
chr7	39733568	39794623	7p14.1	LINC00265	0.706298153	0.609940249	1.157979251	4	4	43.93880307	58.72403734
chr22	24555650	24574964	22q11.23	SNRPD3	34.47820206	29.77742347	1.157863846	4	4	67.21348398	43.86280506
chr11	75434640	75525916	11q13.4-q13.5	GDPD5	2.0777191	1.794637874	1.15773724	4	4	10.07662084	42.43607525
chr12	110468413	110490387	12q24.11	FAM216A	6.140009273	5.303810285	1.157660049	4	4	62.55502039	55.45042552
chr9	35681993	35690056	9p13.3	TPM2	0.915776242	0.791065824	1.157648599	4	4	38.01716144	29.11589465
chrX	132626010	132961395	Xq26.2	HS6ST2	0.059539932	0.051432895	1.157623592	1	1	0	0
chr6	31652410	31658210	6p21.33	APOM	1.146820025	0.990725691	1.157555553	3	4	31.3202164	75.37212289
chr15	48988638	49046563	15q21.1	SECISBP2L	24.69424451	21.33345207	1.157536269	4	4	29.84943595	12.47668963
chr20	5291888	5292963	20p12.3	UBE2D3P1	1.988620831	1.718074288	1.157470806	3	4	66.41927188	69.30191521
chr19	2425624	2427916	19p13.3	TIMM13	4.12545985	3.564626086	1.157333126	4	4	85.84746193	46.3903076
chr1	197153682	197201390	1q31.3	ZBTB41	7.297454506	6.305461505	1.157322822	4	4	35.03838561	10.40443524
chrX	20124518	20141848	Xp22.12	EIF1AX	24.20207575	20.91304664	1.157271639	4	4	32.07522153	25.00593803
chr10	125032783	125032870	10q26.13	MIR4296	4.523162432	3.908471323	1.15727149	2	2	72.04943099	22.33923801
chr17	68205486	68257164	17q24.2	AMZ2	14.22730865	12.29403643	1.157252846	4	4	41.90724803	33.21553796
chr7	107201744	107564514	7q22.3	COG5	11.33525575	9.795134402	1.157233304	4	4	28.31941133	11.86656598
chr8	103140693	103230305	8q22.3	BAALC	0.112028265	0.096807736	1.157224301	2	4	63.84329797	35.70847431
chr16	56682470	56684196	16q13	MT1X	3.96041767	3.422360383	1.157218185	4	4	124.8419501	104.4533424
chr18	54357914	54382035	18q21.2	C18orf54	3.37808131	2.919338708	1.157139218	4	4	32.99869295	32.21811555
chr11	68030617	68036647	11q13.2	NDUFS8	4.04305615	3.494044211	1.157127931	4	4	82.31666606	57.67895388
chr9	69709522	69760050	9q21.12	PTAR1	16.55833984	14.31007989	1.157110231	4	4	16.57606267	21.1148879
chr11	102347182	102378670	11q22.2	BIRC2	223.6945846	193.3341782	1.157035898	4	4	34.81289501	66.17923724
chr7	66921208	66958551	7q11.21	TMEM248	31.08604817	26.86867351	1.156962519	4	4	23.81121857	15.75508555
chr20	32358062	32439319	20q11.21	ASXL1	17.77155527	15.36055519	1.156960478	4	4	42.50087837	20.34467445
chr3	188178543	188180808	3q27.3	FLJ42393	0.647183654	0.55939063	1.156944038	4	3	84.05203101	50.4864294
chr6	85607784	85643862	6q14.3	SYNCRIP	38.33277218	33.13703038	1.156795637	4	4	34.55644029	24.74071296
chr1	28508558	28508762	1p35.3	SNORA73B	3757.219198	3248.066767	1.156755531	4	4	49.53446844	41.75407487
chr14	35538352	35809323	14q13.2	RALGAPA1	7.442779593	6.434225572	1.156748316	4	4	24.91121382	13.29200988
chr16	24610209	24826227	16p12.1	TNRC6A	12.12006473	10.4793012	1.156571847	4	4	18.74178183	17.51804588
chr1	201380838	201399541	1q32.1	LAD1	0.082766431	0.071567739	1.156476823	2	1	1.509027321	0
chr1	84925583	84997122	1p22.3	MCOLN2	2.37472843	2.053719311	1.156306228	4	4	26.66810873	67.78680554
chr1	200640807	200669998	1q32.1	DDX59	11.77428467	10.18274106	1.156298152	4	4	51.57297975	19.75253726
chr13	112967484	112968638	13q34	MCF2L-AS1	0.39323957	0.340091082	1.156277216	2	2	30.57003188	7.285915319

chr19	50476477	50505905	19q13.33	EMC10	3.335173697	2.884515524	1.156233575	4	4	76.76316373	16.16601576
chr14	95407266	95516693	14q32.13	SYNE3	4.197183854	3.630310645	1.156150056	4	4	41.43570869	22.18316692
chr8	144393231	144409400	8q24.3	CPSF1	5.371212055	4.646522154	1.15596394	4	4	66.47592792	36.29686316
chr1	156308961	156338415	1q22	CCT3	62.75960764	54.2967099	1.155863914	4	4	47.38216839	7.688389476
chr6	14117256	14136918	6p23	CD83	12.21471913	10.56794631	1.155827138	4	4	59.01518233	40.88073
chr12	124993648	125051845	12q24.31	BRI3BP	7.940388472	6.870597717	1.155705631	4	4	65.07146297	40.5087777
chr17	76470893	76501440	17q25.1	RHBDF2	8.020719894	6.94061553	1.155620832	4	4	61.87491529	35.51793721
chr19	19320607	19358754	19p13.11	MAU2	6.352098094	5.496712575	1.155617655	4	4	47.74254409	19.12036499
chr20	56392371	56404526	20q13.2-q13.31	CSTF1	8.718416103	7.544511704	1.155597134	4	4	36.18726753	27.31395214
chrX	130202699	130268948	Xq26.1	ZNF280C	3.138922444	2.716285627	1.155593658	4	4	22.57032781	29.14513444
chr3	49724295	49786540	3p21.31	IP6K1	15.90574993	13.76537076	1.155490122	4	4	31.35012209	17.67650126
chr19	1985438	2015703	19p13.3	BTBD2	4.745173849	4.106685956	1.155475218	4	4	85.06230211	63.62848486
chr3	44915261	44976185	3p21.31	ZDHHC3	10.93226657	9.461811072	1.155409518	4	4	13.3104547	19.12647254
chr3	75729961	75785583	3p12.3	ZNF717	1.680923001	1.45484079	1.155399967	4	4	66.85932273	21.39717535
chr14	24216851	24232370	14q12	NEDD8	8.449838934	7.313841365	1.155321604	4	4	42.71024979	29.46297349
chr6	158609328	158611726	6q25.3	TATDN2P2	0.129660086	0.112228842	1.15531876	1	2	0	4.00704919
chr12	42157104	42238373	12q12	YAF2	9.799970813	8.482720276	1.155286334	4	4	43.63074894	33.02087083
chr1	10430102	10452003	1p36.22	CENPS-CORT	0.389445424	0.337102587	1.155272725	1	1	0	0
chr13	19957406	20091845	13q12.11	ZMYM2	21.10694679	18.27086443	1.155224312	4	4	14.89068528	9.927997139
chr1	230057789	230282130	1q42.13	GALNT2	7.659687453	6.630809157	1.155166326	4	4	42.8643136	29.24968763
chr8	144837978	144853578	8q24.3	COMMD5	4.272865823	3.698951297	1.15515601	4	4	27.89699058	37.48034314
chr17	76734115	76737411	17q25.1	SRSF2	42.17511363	36.51209878	1.15509968	4	4	36.42599537	21.28921798
chr1	16514122	16514285	1p36.13	RNU1-1	48.361563	41.87220737	1.154980022	4	4	50.28611628	61.21627166
chrX	153700492	153724746	Xq28	BCAP31	29.63887654	25.66284692	1.154933303	4	4	41.06323541	4.023645653
chr17	78108775	78132427	17q25.3	TMC6	10.30198413	8.920383366	1.154881322	4	4	43.30328573	21.14805458
chr2	134119922	134454621	2q21.2-q21.3	MGAT5	16.15356776	13.98930654	1.154708256	4	4	32.83165018	21.43663303
chr1	37489960	37514819	1p34.3	MEAF6	17.64858051	15.28520497	1.154618504	4	4	24.07291242	25.58928503
chr17	75909574	75938232	17q25.1	FBF1	0.528621113	0.457851414	1.154569139	4	4	58.02976351	16.04680949
chr3	196867856	196934714	3q29	SENP5	17.02513888	14.74650538	1.154520237	4	4	7.570844407	12.91927231
chr12	58872155	58920538	12q14.1	LRIG3	0.049991834	0.043301858	1.154496264	2	2	20.5448625	1.168169696
chrX	13378525	13379387	Xp22.2	GPX1P1	24.76619131	21.4530843	1.154434997	2	3	137.8837714	84.91959467
chr17	75626845	75667202	17q25.1	RECQL5	2.291356797	1.98484288	1.154427295	4	4	48.47394146	25.96165023
chr12	14942015	14961770	12p12.3	ARHGDIB	692.4979163	599.8723593	1.154408776	4	4	67.86199327	41.8040985
chr19	55226634	55229264	19q13.42	TMEM86B	2.426190334	2.101706821	1.154390475	4	4	12.22579149	57.95164579
chr10	130136375	130182877	10q26.3	GLRX3	18.21325633	15.77739287	1.154389479	4	4	54.75258751	39.03871687
chr1	156463721	156500842	1q22	MEF2D	13.70313172	11.87108158	1.15432883	4	4	69.57340663	26.71658361
chr12	53479669	53501205	12q13.13	MAP3K12	1.188887102	1.029950843	1.154314413	4	4	57.92714196	64.38997036
chr6	26382893	26394874	6p22.2	BTN2A2	4.020718996	3.483757201	1.154132956	4	4	42.79626371	33.2754251
chr5	60751789	60844292	5q12.1	ELOVL7	28.04695701	24.30437045	1.153988213	4	4	28.90144161	44.6094898
chr2	219245455	219250337	2q35	STK16	6.674375697	5.784246737	1.153888484	4	4	36.87363316	14.67558738
chr2	85593823	85597708	2p11.2	RNF181	24.99242339	21.66016848	1.15384252	4	4	32.53161665	27.89649371
chr2	170770042	170798971	2q31.1	ERICH2	1.051695247	0.911488085	1.153822264	1	1	0	0
chr13	49997007	50018467	13q14.2	TRIM13	4.500488945	3.900685945	1.153768596	4	4	26.26894111	40.71976781

chr11	67506490	67508728	11q13.2	CDK2AP2	13.78928038	11.95217603	1.153704593	4	4	68.17986604	68.16666261
chr16	287440	355226	16p13.3	AXIN1	10.02994332	8.693728712	1.153698678	4	4	40.95986797	31.63761861
chr6	43021602	43029599	6p21.1	RRP36	18.57864435	16.10602133	1.153521653	4	4	37.21269828	23.03528659
chr6	80004147	80042651	6q14.1	TTK	13.40122644	11.61803007	1.153485261	4	4	107.6012998	48.49872308
chr1	10032958	10181239	1p36.22	UBE4B	15.22366571	13.19810446	1.153473649	4	4	16.74481719	16.78707007
chr19	18588685	18592337	19p13.11	REX1BD	2.280023626	1.976852394	1.153360581	4	4	68.80820921	16.31322315
chr4	122923070	123319450	4q28.1	SPATA5	6.423136153	5.569237414	1.153324176	4	4	29.60543936	3.653570959
chr9	128111171	128118734	9q34.11	SLC25A25-AS1	0.393797218	0.341485499	1.153188698	4	4	91.69715137	28.03815091
chr20	56358927	56368663	20q13.2	FAM210B	389.6487896	337.9075036	1.153122631	4	4	23.45612958	37.66188366
chr1	161721544	161728143	1q23.3	FCRLB	0.653316542	0.566569325	1.153109626	3	3	71.68397132	38.79391006
chr22	38056311	38075704	22q13.1	PICK1	1.402724063	1.216542862	1.153041218	4	4	59.44727098	36.26987915
chr15	65696833	65696936	15q22.31	SNORD13E	9.609946777	8.334683777	1.153006765	4	2	31.31448942	75.20485242
chr19	35745577	35747519	19q13.12	PSENEN	6.235911093	5.408393604	1.153006151	4	4	61.03478184	63.3880274
chr14	66012433	66013933	14q23.3	YBX1P1	3.060179635	2.654092319	1.153004217	3	4	52.95977401	32.78538239
chr22	29603556	29698600	22q12.2	NF2	8.669202426	7.519233266	1.152937024	4	4	50.00907439	42.58186689
chr3	184361710	184368595	3q27.1	POLR2H	3.558597598	3.086604141	1.152916745	4	4	10.27878105	16.92333295
chr15	43826914	43868419	15q15.3	WDR76	18.51799192	16.06226931	1.15288765	4	4	72.56674352	31.01086873
chr2	171687409	171750158	2q31.1	DYNC1I2	16.24298672	14.08987446	1.152812736	4	4	28.86075516	20.32891296
chr19	52797408	52821686	19q13.41	ZNF28	6.625561787	5.747607962	1.152751167	4	4	51.46978681	59.24477544
chr20	47501857	47656877	20q13.12	NCOA3	37.81990898	32.8091739	1.152723598	4	4	38.44342121	23.6556859
chr1	92080305	92147840	1p22.1	BTBD8	0.048386696	0.04197637	1.152712731	1	1	0	0
chr6	31676684	31680373	6p21.33	LY6G5C	0.223149211	0.193601243	1.152622822	2	3	50.34243239	42.07076261
chr15	25337234	25439381	15q11.2	UBE3A	41.93464093	36.38249293	1.152604936	4	4	42.09532697	16.3330305
chr4	3074681	3243960	4p16.3	HTT	7.212204708	6.257338592	1.1525994	4	4	31.64247279	19.83183273
chr8	120635484	120638160	8q24.12	NCAPGP1	0.320258721	0.277871074	1.152544293	3	3	73.87050494	1.825007407
chr15	67403611	67521844	15q23	IQCH-AS1	0.547082607	0.474676718	1.152537265	4	4	85.46265647	43.22362573
chr9	34179005	34252523	9p13.3	UBAP1	82.99663849	72.01711838	1.152457087	4	4	31.81852071	24.40311066
chr13	99501374	99564048	13q32.3	TM9SF2	101.2033053	87.82029643	1.152390841	4	4	17.67704973	28.63276177
chr4	48135783	48269864	4p12-p11	TEC	6.96067802	6.040208144	1.152390423	4	4	21.8856217	42.98250486
chr2	167954020	168247595	2q24.3	STK39	28.24096807	24.50762516	1.152333932	4	4	46.10836266	20.26510497
chr6	144094881	144095617	6q24.2	SF3B5	33.68550751	29.23756518	1.152131079	4	4	32.49022792	25.07091979
chr1	243488233	243851079	1q43-q44	AKT3	11.60247204	10.07159257	1.151999742	4	4	21.79717371	35.30299774
chrX	47217881	47229997	Xp11.3	CDK16	5.512353326	4.785817602	1.151810157	4	4	21.6409717	17.72272589
chr3	136862208	136951610	3q22.3	NCK1	19.26499472	16.72601161	1.151798478	4	4	21.16906165	20.12717727
chr2	69829606	69881396	2p13.3	GMCL1	17.69891857	15.36715851	1.151736579	4	4	25.28709511	27.01057234
chr1	6785324	7769706	1p36.31-p36.23	CAMTA1	4.510560719	3.916517169	1.151676483	4	4	47.71659644	25.71077194
chr10	37758452	37858202	10p11.21	ZNF248	4.041804245	3.509508417	1.151672475	4	4	36.05292425	44.38032054
chr16	1773222	1776714	16p13.3	EME2	1.219492391	1.058928876	1.151628234	4	4	113.7526782	8.854731001
chr13	50082169	50528643	13q14.2-q14.3	DLEU1	0.765745952	0.664967882	1.15155329	4	4	42.54757717	21.87039458
chr2	170813210	170861151	2q31.1	GAD1	1.239924586	1.076788762	1.151502161	4	4	122.8282031	78.41048921
chr8	9555230	9782346	8p23.1	TNKS	5.467146083	4.74790773	1.151485327	4	4	24.45471785	9.942511574
chr16	1444933	1475084	16p13.3	CLCN7	4.025106832	3.495757666	1.151426162	4	4	72.7598255	25.03304037
chr4	8440774	8512531	4p16.1	TRMT44	1.274067704	1.106527368	1.151410928	4	4	45.18904479	35.87743257

chr17	48908369	48929060	17q21.32	UBE2Z	12.64727303	10.98417769	1.15140827	4	4	39.74235057	21.41885142
chr3	52254422	52278643	3p21.2	WDR82	41.62524361	36.15165145	1.151406421	4	4	35.41158036	7.058998403
chr16	67975663	67980549	16q22.1	DPEP3	1.390870159	1.208026039	1.151357764	3	4	47.98099328	57.33311209
chr5	176388526	176389771	5q35.2	HIGD2A	63.59232888	55.23633988	1.151277022	4	4	32.0964563	37.15955755
chr7	99572366	99611045	7q22.1	TMEM225B	0.291658093	0.253347101	1.151219382	1	2	0	40.82128226
chr19	35902365	35904271	19q13.12	HCST	66.35272896	57.63729411	1.151211728	4	4	10.62771568	29.1029139
chr19	34404398	34426168	19q13.11	PDCD2L	1.574748644	1.367982801	1.151146522	4	4	64.92698223	61.1035798
chr4	68310387	68350117	4q13.2	YTHDC1	16.1261695	14.00947194	1.15109046	4	4	40.47166933	20.41572134
chr5	17240611	17240715	5p15.1	RNU6-1003P	5.226295525	4.540414318	1.15106137	3	4	8.35681008	55.51455634
chr13	41431264	41432258	13q14.11	OR7E36P	0.552487252	0.479985546	1.15104977	1	1	0	0
chr1	155169408	155173328	1q22	KRTCAP2	0.245089715	0.212929606	1.151036343	4	3	63.20262345	15.28855126
chr12	12717270	12722383	12p13.1	CDKN1B	128.9314216	112.0150682	1.151018553	4	4	48.42673485	4.537723704
chr7	55434406	55572520	7p11.2	VOPP1	13.38604549	11.63062321	1.150931059	4	4	37.64287509	12.84192928
chr19	11435257	11450968	19p13.2	PRKCSH	25.58144885	22.22685416	1.150925303	4	4	67.75251485	48.39067438
chr7	73440398	73522285	7q11.23	BAZ1B	40.93955754	35.57707479	1.150728602	4	4	33.33922986	21.6421383
chr19	48900050	48923283	19q13.33	NUCB1	54.03146175	46.95637101	1.150673712	4	4	39.57511379	36.41902661
chr14	21990496	21990938	14q11.2	TRAV16	4.065613509	3.533651982	1.150541573	3	4	89.89277392	68.26716499
chr14	91271323	91417844	14q32.11-q32.12	CCDC88C	14.09610576	12.25180631	1.15053286	4	4	43.89762377	39.37524921
chr9	136992418	136993988	9q34.3	PAXX	9.187924224	7.986392355	1.150447388	4	4	61.67461307	19.72293208
chr13	23979626	24307074	13q12.12	SPATA13	17.41730886	15.13982392	1.150430081	4	4	34.19225609	62.26428179
chr11	3357927	3379222	11p15.4	ZNF195	3.6931435	3.210585021	1.150302352	4	4	33.10938079	25.38093365
chr6	32840717	32844935	6p21.32	PSMB8	38.73893335	33.67717676	1.150302284	4	4	41.68953871	29.57794179
chr15	100547752	100559283	15q26.3	PRKXP1	0.274567966	0.238693887	1.150293248	2	4	39.66501985	13.14151012
chr15	55318935	55355648	15q21.3	PIGB	7.570688643	6.581770894	1.150251014	4	4	23.97093276	22.92835479
chr3	16257061	16264989	3p25.1	DPH3	14.91035353	12.96358844	1.150171775	4	4	25.36565841	20.30598225
chr19	12884422	12887203	19p13.13	KLF1	31.81759235	27.66341221	1.150168754	4	4	42.27938721	59.87009122
chr1	161749768	161757164	1q23.3	DUSP12	9.418785462	8.189403225	1.15011866	4	4	28.47087119	24.29942538
chr7	24796537	24980218	7p15.3	OSBPL3	5.308230759	4.61545138	1.150100028	4	4	33.05839699	17.81384685
chr7	102433530	102456821	7q22.1	ORAI2	9.60963039	8.355598145	1.150082881	4	4	62.00741705	25.50627656
chr17	60041363	60078931	17q23.1	HEATR6	3.869631437	3.364965262	1.149976637	4	4	33.03593079	12.55385975
chr18	46077004	46077099	18q21.1	RNY4P37	3.875461507	3.370411254	1.149848257	1	2	0	31.21731147
chr1	113876814	113887547	1p13.2	BCL2L15	0.280875676	0.244286595	1.149779325	4	4	23.57754174	32.95756309
chr5	139391946	139395196	5q31.2	PROB1	0.166686844	0.144974093	1.149769874	4	4	55.35860048	82.78795058
chr2	113627218	113643398	2q14.1	RABL2A	0.68605194	0.596724977	1.149695364	3	4	52.05073499	57.1652405
chr10	90871952	90908555	10q23.31	RPP30	5.211296116	4.533110954	1.149607007	4	4	17.67159877	17.14724341
chr10	101610664	101695295	10q24.32	FBXW4	10.7931376	9.38881939	1.149573461	4	4	10.48183457	21.68497323
chr3	155024124	155183729	3q25.2	MME	74.40829501	64.72909901	1.149533921	4	4	62.77754722	59.7231379
chr6	37915573	37915742	6p21.2	RNU1-87P	2.814933804	2.448818742	1.149506803	2	3	26.57534531	52.85658933
chr1	81721612	81723258	1p31.1	ST13P20	0.354315395	0.308232542	1.149506773	3	3	45.10889825	35.34229189
chr3	185417408	185418843	3q27.2	RPL4P4	2.487149397	2.163724421	1.149476048	4	4	71.63886149	27.88319408
chr3	46407166	46409523	3p21.31	CCRL2	4.620162331	4.019610763	1.149405404	4	4	46.91753684	17.59574653
chr1	219968600	220046658	1q41	EPRS	49.71396116	43.25255396	1.149387877	4	4	14.27714803	14.24676855
chr17	12020818	12143831	17p12	MAP2K4	52.75675673	45.90361358	1.149294198	4	4	30.89018447	36.13280424

chr15	101632977	101652391	15q26.3	TM2D3	4.141304608	3.60352025	1.149238611	4	4	17.02801167	29.36683722
chr1	109279554	109283172	1p13.3	PSRC1	1.442914846	1.255551259	1.149228146	4	4	49.66429083	32.17468583
chr5	110946273	110951024	5q22.1	BCLAF1P1	0.110365684	0.09603519	1.149221278	2	1	31.39242971	0
chr7	77004561	77005876	7q11.23	UPK3BP1	0.75024394	0.652871904	1.149144167	2	4	14.84264681	37.16929535
chr8	25424357	25458468	8p21.2	KCTD9	13.40908174	11.66979419	1.149041836	4	4	43.97300832	21.24669321
chr2	10783391	10837977	2p25.1	PDIA6	31.35745134	27.29223173	1.148951528	4	4	50.59362701	34.89404904
chr11	57233577	57237453	11q12.1	APLNR	0.077331917	0.067312905	1.148842359	1	1	0	0
chr11	47214942	47239218	11p11.2	DDB2	4.084854183	3.555828748	1.148776972	4	4	84.44064274	66.2583419
chr22	19941740	19969975	22q11.21	COMT	4.348769711	3.78561434	1.14876195	4	4	54.5165457	30.65956071
chr3	49029707	49094373	3p21.31	QRICH1	19.69671148	17.14670388	1.148717073	4	4	43.80336698	27.76757249
chr2	47403067	47634501	2p21-p16.3	MSH2	17.81115459	15.50668141	1.148611629	4	4	47.52309688	23.40427698
chr5	178149463	178153960	5q35.3	NHP2	9.414643909	8.196720502	1.14858667	4	4	28.60642499	28.09345067
chr17	7259903	7263193	17p13.1	CLDN7	0.325466702	0.283379899	1.14851725	3	2	27.57577121	88.77520373
chr4	44678395	44700681	4p12	GUF1	12.06686209	10.50736792	1.148419108	4	4	23.57184393	15.30434893
chr2	190880821	190965552	2q32.2	GLS	33.55857872	29.22257728	1.148378475	4	4	44.66409566	15.29080175
chr5	142000669	142013055	5q31.3	GNPDA1	4.200497492	3.65790167	1.148335267	4	4	21.85365157	33.47821589
chr4	109429963	109433817	4q25	SEC24B-AS1	0.411013694	0.357922223	1.148332423	3	4	56.98095745	74.38085322
chr11	832952	838835	11p15.5	CD151	4.382080187	3.816109541	1.148310901	4	4	55.35793232	32.7925135
chr15	48206301	48304078	15q21.1	SLC12A1	0.022720377	0.019786186	1.148294937	1	1	0	0
chr16	68264510	68301823	16q22.1	SLC7A6	8.090119726	7.045406596	1.148282873	4	4	53.25068681	22.87268727
chr11	69048876	69090604	11q13.3	TPCN2	2.071448034	1.804038971	1.148227986	4	4	67.0826475	4.385728316
chr1	45688175	45694438	1p34.1	TMEM69	8.953992199	7.798241879	1.148206524	4	4	39.42795561	16.16291014
chr9	131258078	131281928	9q34.13	FAM78A	18.74051362	16.32172004	1.148194772	4	4	45.6736004	45.92300333
chr3	52455508	52493072	3p21.1	NISCH	5.74767978	5.005992758	1.148159827	4	4	50.38003773	18.87429111
chr11	34051663	34102610	11p13	CAPRIN1	65.29051321	56.86659331	1.148134773	4	4	12.03712737	22.2911548
chrX	46646399	46647338	Xp11.3	PGAM1P7	0.356049039	0.310119431	1.14810297	1	1	0	0
chr9	92844195	92878060	9q22.31	ZNF484	11.47925218	9.999034986	1.148036005	4	4	22.85360584	15.31779082
chr16	90186121	90225200	16q24.3	LINC02193	0.416025399	0.362380319	1.148035302	4	3	88.51359375	138.4653009
chr9	137877789	138124624	9q34.3	CACNA1B	0.05840862	0.050879929	1.147969761	1	1	0	0
chr12	9827303	9845005	12p13.31	KLRF1	44.34714479	38.63221818	1.14793162	4	4	62.01693719	51.65388808
chr15	72474326	72586555	15q24.1	ARIH1	10.65097943	9.278463207	1.147924952	4	4	21.72292764	31.46474592
chr7	64882493	64932239	7q11.21	ZNF273	3.919853676	3.414841392	1.147887479	4	4	30.76506521	10.28217846
chr14	102922430	102933597	14q32.32	AMN	0.228334626	0.198925373	1.147840635	1	4	0	70.6031059
chr1	74235387	74544432	1p31.1	TNNI3K	0.068095051	0.059325336	1.147824117	2	1	10.97547963	0
chr9	136006537	136095285	9q34.3	NACC2	7.746052054	6.748698332	1.147784606	4	4	58.63742383	16.38252123
chr4	48106081	48106188	4p12	RNU6-838P	4.477079459	3.901279266	1.147592662	3	3	63.44036185	64.20464116
chr2	111120914	111168445	2q13	BCL2L11	15.57327121	13.57077331	1.147559602	4	4	28.77654587	45.56596181
chr11	69641105	69654474	11q13.3	CCND1	0.267507794	0.233126689	1.147478198	4	3	56.62559947	58.95695421
chr9	85941142	86026355	9q21.33	NAA35	12.89413432	11.23694089	1.147477275	4	4	27.91088824	23.11853882
chr12	132725667	132761888	12q24.33	ANKLE2	3.913406052	3.410599888	1.147424553	4	4	52.61293589	25.5185535
chr12	123584529	123598144	12q24.31	TMED2	167.5311857	146.0077965	1.147412602	4	4	38.59731225	13.84367571
chr22	19875518	19941992	22q11.21	TXNRD2	2.531145679	2.206072186	1.147353969	4	4	18.69141619	24.12921843
chr20	38033462	38092366	20q11.23	RPRD1B	15.31162741	13.34632069	1.14725457	4	4	31.71885605	17.35339385

chr9	137618992	137836127	9q34.3	EHMT1	4.18766293	3.650191208	1.147244813	4	4	22.3008952	32.63597332
chr11	64185272	64204548	11q13.1	STIP1	16.59612446	14.46630167	1.147226488	4	4	48.45329524	44.56619266
chr12	21468910	21501669	12p12.1	RECQL	30.36970201	26.47258086	1.147213495	4	4	29.72358037	42.81307241
chr10	49981196	50010500	10q11.23	AGAP6	3.908856529	3.407377735	1.147174406	4	4	29.4772789	53.63967791
chr19	35937120	35945195	19q13.12	LRFN3	0.481181167	0.419470539	1.147115524	4	4	83.57842961	98.05299491
chrX	153802166	153830567	Xq28	PDZD4	2.367766955	2.064155882	1.14708728	4	4	55.78186305	54.88149983
chr3	50299889	50312951	3p21.31	HYAL1	0.093124583	0.081183634	1.147085667	1	2	0	3.749408959
chr3	32481312	32502911	3p22.3	CMTM6	169.2911087	147.6016422	1.14694597	4	4	35.93471793	40.87183036
chr12	56309842	56316348	12q13.3	CNPY2	1.167796149	1.018183136	1.146941162	4	4	55.43889157	9.135112899
chr14	77457877	77469472	14q24.3	AHSA1	24.49989383	21.36142341	1.146922345	4	4	36.94400798	36.83149923
chr11	9460965	9528524	11p15.4	ZNF143	19.93225052	17.38029962	1.146830087	4	4	30.74836721	19.31051362
chr1	54756898	54764553	1p32.3	PARS2	0.761532715	0.664041005	1.146815798	4	4	20.65457461	44.15355507
chr1	51789192	51878937	1p32.3	NRDC	54.0562471	47.13764004	1.146774575	4	4	14.07132702	9.046887826
chr11	124753123	124762327	11q24.2	ESAM	5.976221015	5.211686376	1.14669621	4	4	41.18701995	19.70004138
chr15	55181314	55197033	15q21.3	RSL24D1	50.28200861	43.85209053	1.146627401	4	4	17.04613809	12.94032193
chrX	120250752	120258396	Xq24	ZBTB33	22.67378983	19.77496084	1.146590884	4	4	38.01179396	18.12840899
chr13	51932669	52012130	13q14.3	ATP7B	1.532222415	1.336372982	1.146552972	4	4	48.15670773	16.56356823
chr3	119498521	119524281	3q13.33	TIMMDC1	64.79750152	56.52215582	1.146408883	4	4	32.9174829	24.87592361
chr8	106270178	106752694	8q23.1	OXR1	24.32486852	21.21836844	1.146406171	4	4	7.681897872	14.57566481
chr7	99438912	99457377	7q22.1	CPSF4	2.066251872	1.80250796	1.146320526	4	4	44.08894695	9.596100057
chr8	22620418	22669148	8p21.3	BIN3	6.98570051	6.094109709	1.1463037	4	4	54.8590395	19.89309801
chr17	2306762	2308048	17p13.3	HNRNPA1P16	0.448714281	0.391446882	1.14629673	2	2	32.40937453	54.06947212
chr2	36980999	37084384	2p22.2	HEATR5B	22.03713377	19.2263422	1.146194817	4	4	15.65372686	11.75322169
chr16	57245126	57253633	16q13	ARL2BP	28.82521246	25.14905875	1.146174604	4	4	40.15113664	9.826953561
chr16	71845991	71857333	16q22.2	ATXN1L	11.66974432	10.18150454	1.146170909	4	4	63.33797897	14.80766368
chr4	39761000	39761298	4p14	RN7SL558P	0.976379802	0.851872269	1.146157513	2	1	38.07365905	0
chr4	163494521	163520539	4q32.2	TMA16	4.557081324	3.976154615	1.146102646	4	4	45.03765923	20.7893262
chr1	173863899	173868882	1q25.1	GAS5	32.56559816	28.41893837	1.145911847	4	4	40.89945332	11.25996481
chr22	42269753	42274862	22q13.2	OGFRP1	0.509594328	0.44470889	1.14590542	2	4	62.99560917	66.68633758
chr3	23805893	23891640	3p24.2	UBE2E1	20.35389806	17.76273089	1.145876621	4	4	30.92259477	21.87660869
chr11	207511	215175	11p15.5	RIC8A	8.85008952	7.723631346	1.145845668	4	4	45.1116336	14.1138645
chr10	12349514	12835545	10p13	CAMK1D	10.38094359	9.059717346	1.145835261	4	4	76.74815948	51.38632339
chr3	53858992	53882202	3p21.1	ACTR8	11.09834119	9.686077011	1.145803526	4	4	27.6073295	20.98500173
chr11	61333182	61353426	11q12.2	TKFC	1.47224759	1.284973162	1.145741898	4	4	63.21511311	40.31655304
chr7	128455830	128458418	7q32.1	HILPDA	0.692058076	0.604081673	1.145636604	4	4	125.1652006	73.72697321
chr20	10435136	10628138	20p12.2	SLX4IP	14.08539513	12.29497023	1.145622548	4	4	51.54958157	33.400478
chr1	182789449	182830384	1q25.3	NPL	47.24586493	41.24350268	1.145534735	4	4	14.87935394	47.37123045
chr3	113211003	113287463	3q13.2	BOC	0.02769726	0.024179468	1.145486723	1	1	0	0
chr2	99306184	99336397	2q11.2	TXNDC9	16.20593268	14.14801965	1.145455907	4	4	10.02311493	27.17453324
chr22	38778508	38794156	22q13.1	DNAL4	2.141103914	1.869367492	1.145362762	4	4	52.83601808	58.41471506
chr3	20040032	20154404	3p24.3	KAT2B	226.7107864	197.9502868	1.145291528	4	4	49.26157823	42.64864175
chr14	55051644	55070194	14q22.3	MAPK1IP1L	22.35374584	19.5197018	1.1451889	4	4	29.17302688	20.23460374
chr4	88257609	88284831	4q22.1	PPM1K	7.968445158	6.958247033	1.145179974	4	4	13.18336779	31.61662704

chr10	72692131	72887694	10q22.1	MCU	19.74062899	17.23835937	1.145157063	4	4	18.71868671	32.31045799
chr17	42552558	42555214	17q21.2	HSD17B1	0.101761861	0.08886748	1.145096728	3	3	42.99205583	50.64045899
chr4	98995637	99063109	4q23	METAP1	11.00234979	9.608441634	1.145071199	4	4	19.75380349	21.94869831
chr1	40373672	40423326	1p34.2	SMAP2	180.526028	157.6601436	1.145032751	4	4	56.78040321	42.20381282
chr20	64080082	64100643	20q13.33	OPRL1	1.452796751	1.268805038	1.145011808	4	4	77.91361111	54.49558089
chr1	179293714	179358680	1q25.2	SOAT1	25.60945014	22.36649368	1.144991723	4	4	14.03752552	25.79507255
chr19	54200557	54207647	19q13.42	RPS9	49.0238762	42.82289574	1.144805258	4	4	31.161263	17.33267808
chr9	137086927	137109187	9q34.3	MAN1B1	2.848121074	2.487879233	1.144798765	4	4	55.05567696	32.18362494
chr2	26346086	26395891	2p23.3	SELENOI	9.891604364	8.640690083	1.144770183	4	4	45.06164403	37.35724564
chr18	51174550	51197681	18q21.2	MEX3C	16.7255915	14.61214	1.144636686	4	4	17.66847099	16.1384827
chr16	2682494	2709030	16p13.3	KCTD5	8.180614035	7.148080003	1.144449143	4	4	41.06019905	17.96818611
chr3	133661986	133779006	3q22.1	TF	0.036776818	0.032135427	1.144432214	1	1	0	0
chr3	10141635	10153670	3p25.3	VHL	25.69457332	22.45225171	1.144409641	4	4	36.55010275	15.03146028
chr7	127580628	127585600	7q32.1	GCC1	12.65948098	11.06236792	1.144373527	4	4	67.14747203	22.96699551
chr13	47942656	48001326	13q14.2	SUCLA2	7.502878995	6.557307795	1.144201131	4	4	31.35698772	28.10417194
chr11	106077565	106098692	11q22.3	AASDHPPT	9.006866724	7.872343742	1.144115021	4	4	15.49738112	14.74725648
chrX	71118556	71142454	Xq13.1	MED12	10.97645552	9.593956569	1.144101023	4	4	62.3984147	40.24231002
chr6	31620673	31637780	6p21.33	PRRC2A	10.84612058	9.480269365	1.144073038	4	4	71.00240535	36.25936337
chr7	108470422	108528161	7q31.1	PNPLA8	33.50936051	29.28984124	1.144060845	4	4	20.91834476	29.65088172
chr1	149923317	149928252	1q21.2	SF3B4	23.07572834	20.17005626	1.1440587	4	4	71.69381597	59.04496907
chr17	17843508	17972470	17p11.2	TOM1L2	3.580935045	3.13031389	1.143953984	4	4	54.34910221	25.60082881
chr1	23019443	23083691	1p36.12	KDM1A	53.67265694	46.91998302	1.143918934	4	4	46.02875674	29.52735075
chr9	133097720	133149220	9q34.13-q34.2	RALGDS	9.91302436	8.66593681	1.143906836	4	4	24.19368864	34.82977502
chr4	56809860	56822210	4q12	SPINK2	0.431495643	0.37723446	1.143839413	1	2	0	11.31183551
chr16	10928891	10942400	16p13.13	DEXI	2.779687775	2.430187728	1.143816069	4	4	46.66673758	50.39471304
chr13	72782023	73016454	13q21.33-q22.1	PIBF1	24.55291717	21.46604905	1.143802342	4	4	43.81406568	36.28032371
chr19	49289632	49332320	19q13.33	SLC6A16	1.026349156	0.897377528	1.143720591	4	4	67.65470695	25.36902363
chr4	1715952	1721373	4p16.3	TMEM129	2.996539896	2.620222257	1.143620503	4	4	79.93280891	14.65495537
chr4	176319939	176332245	4q34.2	SPCS3	51.80396252	45.30108387	1.143547971	4	4	16.08756169	22.86523438
chr19	10304803	10309557	19p13.2	ZGLP1	0.639907663	0.559609906	1.143488804	4	3	37.42664993	28.22664853
chr2	218637916	218659632	2q35	ZNF142	3.663523689	3.203863633	1.143470543	4	4	60.02549213	64.38002499
chr17	17972766	18016889	17p11.2	DRC3	0.058435789	0.051105716	1.143429608	2	3	58.59601592	54.98328837
chr1	77944051	77979435	1p31.1	FUBP1	40.053494	35.03131508	1.143362557	4	4	41.43135522	18.9865466
chr17	1464186	1492707	17p13.3	MYO1C	1.667396048	1.458379542	1.143321063	4	4	23.680317	48.12256053
chr2	86603393	86623877	2p11.2	RNF103	6.908213957	6.042549972	1.14326137	4	4	47.49660644	41.81906928
chr19	29811994	29824317	19q12	CCNE1	3.89696902	3.408659662	1.143255533	4	4	107.2935719	62.05767972
chr4	112275626	112285903	4q25	TIFA	10.43037931	9.123501599	1.143242997	4	4	39.73779296	71.70212168
chr11	67428464	67435408	11q13.2	RPS6KB2	5.337550467	4.668949393	1.143201611	4	4	60.75353833	33.99138487
chr8	122414327	122568644	8q24.13	SMILR	15.17466503	13.27391424	1.143194446	4	4	74.4672766	38.52917159
chr9	37885683	37887573	9p13.2	TMX2P1	0.371171559	0.324687647	1.143165017	1	1	0	0
chr17	2059839	2303836	17p13.3	SMG6	3.59992557	3.149160521	1.143138162	4	4	53.25333245	25.82669643
chr7	44577894	44582295	7p13	TMED4	13.03374907	11.40197037	1.143113746	4	4	28.99286166	10.66227683
chr10	130106009	130108838	10q26.3	CTAGE7P	0.263447656	0.230465034	1.143113343	4	4	30.04773902	48.94358057

chr10	112446988	112949403	10q25.2	VTI1A	8.443450801	7.386548073	1.143084796	4	4	29.78618038	11.47258232
chr1	39522280	39559698	1p34.3	PPIEL	1.207618504	1.056528332	1.143006266	4	3	40.5615488	24.32475703
chr2	9583967	9631055	2p25.1	YWHAQ	73.96916298	64.72116475	1.142889861	4	4	21.11869963	39.50874006
chr15	65231691	65286891	15q22.31	PARP16	4.778528611	4.181156444	1.142872475	4	4	32.87808969	18.65799422
chr22	31081296	31104624	22q12.2	SMTN	0.257475302	0.225301857	1.14280151	4	4	98.17120525	91.87428815
chr7	43940895	44019194	7p13	POLR2J4	1.280594472	1.120575462	1.142800744	4	4	156.4674276	149.5971827
chr2	23687624	23927114	2p24.1-p23.3	ATAD2B	68.44359493	59.89499483	1.142726452	4	4	65.52155815	23.27834352
chr4	97184113	98143486	4q22.3-q23	STPG2	0.614680816	0.537917663	1.142704281	4	4	117.4718266	50.24117454
chr9	36336398	36487384	9p13.2	RNF38	44.94313848	39.33107884	1.142687661	4	4	24.28114754	8.096712077
chr3	52198083	52214327	3p21.2	ALAS1	17.64804631	15.44496344	1.142640861	4	4	28.98332158	26.67784218
chr11	9573681	9589767	11p15.4	WEE1	3.005634032	2.630487343	1.1426149	4	4	47.0514408	17.60629826
chr9	35825817	35865515	9p13.3	TMEM8B	0.328813194	0.287773469	1.142611218	4	4	89.92917458	69.09338765
chr1	51354263	51519323	1p32.3	EPS15	88.9542479	77.85177749	1.142610365	4	4	28.58212331	18.01215934
chr17	77281410	77500596	17q25.3	SEPT9	36.94780904	32.33702911	1.142585143	4	4	66.10271725	42.71640642
chr9	33166948	33179983	9p21.1	B4GALT1-AS1	1.785346266	1.562598813	1.142549355	4	4	56.39769247	44.83261915
chr20	16729964	16741772	20p12.1	SNRPB2	23.57928649	20.63815487	1.142509427	4	4	38.88193949	23.62800423
chr5	62306162	62387184	5q12.1	KIF2A	88.35738616	77.3384852	1.142476297	4	4	20.45226934	4.995956739
chr2	25733753	25878516	2p23.3	ASXL2	19.07863788	16.70078808	1.142379497	4	4	19.64524192	7.232404209
chr7	74231502	74254458	7q11.23	RFC2	17.67474784	15.47238645	1.142341415	4	4	17.53519652	6.660633881
chr13	36227042	36297855	13q13.3	CCDC169	0.415075971	0.363358866	1.142330655	4	3	71.30957837	52.70875126
chr12	98599635	98599884	12q23.1	SNORA53	2771.782848	2426.508879	1.142292481	4	4	22.61181439	44.21212223
chrX	149540630	149555345	Xq28	CXorf40A	2.153919927	1.885821992	1.142165028	4	4	46.0548469	30.3088606
chr1	169794730	169854080	1q24.2	C1orf112	12.10347995	10.59801653	1.142051432	4	4	69.13428519	33.58847014
chr1	1541673	1574882	1p36.33	SSU72	11.51283941	10.08269644	1.141841321	4	4	47.56911444	15.90045921
chr8	85107088	85146080	8q21.2	LRRCC1	23.43140899	20.52429526	1.14164256	4	4	37.68350081	16.98337234
chr20	37517417	37527931	20q11.23	BLCAP	5.440678121	4.766063269	1.141545509	4	4	39.19812989	22.5839775
chr12	32679200	32745650	12p11.21	DNM1L	16.33270871	14.3078826	1.141518223	4	4	22.99610109	5.815673854
chr8	143553387	143563062	8q24.3	GSDMD	4.978768025	4.361569156	1.141508445	4	4	59.79022524	40.55804542
chr1	150622123	150629622	1q21.3	ENSA	24.04110711	21.06089756	1.141504394	4	4	35.26642057	8.846716688
chr19	10654261	10692419	19p13.2	ILF3	12.3659859	10.83331269	1.141477797	4	4	49.02523186	32.74114905
chr15	80404350	80597937	15q25.1	ARNT2	0.037886368	0.033191525	1.141447013	1	2	0	1.88921565
chr7	102973437	102978667	7q22.1	NFE4	5.932052865	5.197263265	1.1413801	4	4	72.84376434	90.80899497
chr19	53431974	53444672	19q13.42	TPM3P9	2.134417956	1.870097036	1.141340751	4	4	68.55032207	26.17832402
chr9	33750466	33799231	9p13.3	PRSS3	0.474651396	0.41592144	1.141204446	1	1	0	0
chr1	27206835	27207875	1p36.11	NPM1P39	0.81873036	0.717426569	1.141204404	1	2	0	45.55951773
chr17	41866916	41930542	17q21.2	ACLY	29.58182078	25.9234454	1.141122267	4	4	55.49377109	42.96933698
chr3	196588	409417	3p26.3	CHL1	0.127532347	0.111764903	1.141076878	3	4	69.63435788	72.41209538
chr15	34379068	34437466	15q14	GOLGA8A	1.340605939	1.174864446	1.141072865	4	4	23.0888876	25.052879
chr19	13097166	13103751	19p13.13	LYL1	39.01713212	34.19426689	1.141043095	4	4	19.6948202	36.92069144
chr21	44328944	44339417	21q22.3	CFAP410	1.01647363	0.890835925	1.141033496	4	4	54.56187631	37.58730625
chr10	62811996	62819167	10q21.3	EGR2	4.041067461	3.541745677	1.140981829	4	4	103.4832847	131.3281873
chr8	95244913	95270604	8q22.1	C8orf37	1.005277635	0.881071823	1.140971268	4	4	34.29214739	52.68630907
chr10	101784443	101818709	10q24.32	OGA	80.09802862	70.20192014	1.14096635	4	4	23.84085704	7.21606751

chr5	7851186	7901124	5p15.31	MTRR	6.680621768	5.855241646	1.140964314	4	4	35.68950584	32.00284475
chr6	143940300	144064599	6q24.2	PLAGL1	2.235269477	1.959489675	1.140740625	4	4	43.41956717	44.27961638
chr2	224378698	224401994	2q36.2	FAM124B	0.621227238	0.544593686	1.140716931	4	4	93.97316859	48.66148746
chr4	186069155	186085098	4q35.1	TLR3	1.041195196	0.912759562	1.140711354	4	4	23.44349044	17.30514971
chr3	113716460	113746299	3q13.31	NAA50	39.40779189	34.54847213	1.140652233	4	4	23.82137115	26.36899168
chr17	782329	792509	17p13.3	MRM3	4.399588304	3.857114185	1.140642484	4	4	36.11592413	16.96062501
chr17	37406877	37479725	17q12	TADA2A	17.71175187	15.52857411	1.140591	4	4	60.79804702	19.3978366
chr19	17794828	17813576	19p13.11	B3GNT3	0.12031576	0.105492281	1.140517195	1	1	0	0
chr17	45132600	45144181	17q21.31	ACBD4	1.140108953	0.999664946	1.140491078	4	4	11.16957667	55.0998074
chr3	64093850	64445476	3p14.1	PRICKLE2	0.04585717	0.040210006	1.140441763	3	4	94.73686328	44.71051068
chr20	25407470	25448568	20p11.21	GINS1	7.422929641	6.508927848	1.140422788	4	4	71.69262273	44.26053899
chrX	119871771	119876666	Xq24	NDUFA1	69.13524979	60.6264857	1.140347308	4	4	7.280789248	24.65540908
chr15	64165517	64356259	15q22.31	CSNK1G1	5.704608629	5.002623132	1.140323482	4	4	41.960288	34.93303967
chr4	6707701	6709879	4p16.1	MRFAP1L1	52.39373106	45.94760445	1.140292986	4	4	27.62580223	27.23139234
chr19	33194693	33208867	19q13.11	LRP3	0.445892233	0.391045556	1.140256489	3	4	102.1855467	85.40687442
chr7	66606703	66607157	7q11.21	RPL35P5	1.346609642	1.180997656	1.14023058	1	1	0	0
chr17	57971547	57988264	17q22	VEZF1	29.24985247	25.65306616	1.140208827	4	4	32.8249977	20.50999282
chr3	183697804	183812625	3q27.1	YEATS2	7.240819258	6.350537137	1.14019005	4	4	24.88980353	28.18796349
chr1	171683066	171684492	1q24.3	RPL4P3	0.226171015	0.198366658	1.140166485	2	2	14.98206567	3.570010089
chr20	35699310	35700982	20q11.22	ROMO1	8.175930388	7.171015442	1.140135655	4	4	28.33732682	20.29995877
chr16	67846732	67872567	16q22.1	NUTF2	15.24898868	13.37498121	1.140112905	4	4	29.53489599	58.6084023
chr9	130713881	130887675	9q34.12	ABL1	4.904263211	4.301643355	1.140090613	4	4	55.40206205	23.23068814
chrX	129906121	129929762	Xq26.1	UTP14A	6.135982265	5.382266335	1.140036907	4	4	48.6126714	29.18605744
chr22	42583721	42614962	22q13.2	POLDIP3	20.14224927	17.67006206	1.139908236	4	4	49.00021236	27.53132643
chr5	115289036	115289330	5q22.3	HMGN1P15	1.354383335	1.188252837	1.139810732	1	1	0	0
chr9	83832795	83921544	9q21.32	KIF27	6.541760641	5.73975343	1.139728513	4	4	52.42363649	54.74370795
chr16	28846600	28874213	16p11.2	SH2B1	2.759414594	2.42123002	1.139674699	4	4	76.44200989	16.51735231
chr15	68206992	68229742	15q23	CLN6	1.464520922	1.285138842	1.139581868	4	4	15.68477395	41.28304386
chr4	102885048	103019739	4q24	SLC9B1	1.674038253	1.469017205	1.139563408	4	4	69.5776308	41.18340875
chr11	72106376	72112780	11q13.4	ANAPC15	5.837560129	5.12263088	1.139562906	4	4	65.50449499	35.54513522
chr2	173906459	173965702	2q31.1	SP3	55.26416667	48.49644004	1.139550999	4	4	28.32821254	16.17266111
chr12	79584874	79691097	12q21.2	PAWR	1.050542373	0.921928345	1.139505449	4	4	52.18635093	50.28887309
chr2	95792212	95800191	2q11.1	GPAT2P1	0.128385906	0.112670958	1.139476472	1	2	0	17.2214627
chr2	64901840	64932447	2p14	LINC02245	0.194628666	0.170809869	1.139446252	2	2	0.670273361	9.659074228
chr12	124324411	124567464	12q24.31	NCOR2	4.232304693	3.714379685	1.13943782	4	4	75.05700857	27.93235966
chr8	144082621	144086216	8q24.3	GPAA1	6.663270621	5.848030709	1.139404178	4	4	69.23667863	32.69493376
chr19	10922185	10929012	19p13.2	YIPF2	7.178483015	6.300411898	1.139367256	4	4	20.52916826	21.91390747
chr6	113868013	113873347	6q21	LINC01268	0.323842991	0.284243574	1.139315084	4	2	72.79734166	97.18098825
chr16	31117664	31131393	16p11.2	KAT8	10.31190707	9.051056615	1.139304228	4	4	31.78633509	25.99320993
chr7	148807372	148884349	7q36.1	EZH2	124.3214271	109.122119	1.139287143	4	4	82.16138156	49.33554099
chr6	24667035	24705069	6p22.3	ACOT13	4.467652768	3.921473094	1.13927921	4	4	49.620925	23.05462468
chr5	40711576	40755970	5p13.1	TTC33	12.78690807	11.22383805	1.139263415	4	4	29.54368871	22.11788527
chr12	120490338	120495946	12q24.31	NRAV	0.628357271	0.551549158	1.139258872	4	4	91.26492451	70.59623046

chr5	43586363	43588822	5p12	AMD1P3	0.364905531	0.320314493	1.13921018	2	1	44.73201503	0
chr8	6935820	6938338	8p23.1	DEFA4	5.218802772	4.581114341	1.139199414	4	4	18.63995128	122.3254853
chr16	4461680	4476337	16p13.3	NMRAL1	2.545184916	2.234299729	1.139142114	4	4	114.9448082	30.368228
chr4	140257286	140382556	4q31.1	SCOC	9.541693387	8.376538338	1.139097441	4	4	50.21968196	55.404595
chr19	13764492	13774282	19p13.13	MRI1	2.427968169	2.131500257	1.139088847	4	4	83.46685911	45.29613865
chr8	133454848	133572534	8q24.22	ST3GAL1	40.66941228	35.70347545	1.139088332	4	4	11.72172905	16.78274749
chr2	241637612	241673857	2q37.3	ATG4B	4.428431059	3.887752004	1.139072414	4	4	60.4275425	17.73767336
chr1	110031577	110054641	1p13.3	STRIP1	5.887556493	5.168961073	1.139021248	4	4	35.46645527	16.89292654
chr2	90220908	90221382	2p11.2	IGKV1D-8	0.529429777	0.46484349	1.138942006	2	2	31.22119181	9.788372328
chr18	49814023	49851059	18q21.1	SNHG22	5.12531085	4.5002097	1.138904894	4	4	28.51235833	37.16863895
chr5	154817018	154850685	5q33.2	FAXDC2	52.95796932	46.50004151	1.138880044	4	4	50.71347688	9.707072048
chr17	74431350	74451658	17q25.1	GPRC5C	0.061399738	0.053915796	1.138807965	1	3	0	67.31253747
chr2	162318763	162377225	2q24.2	GCA	201.0516761	176.5521586	1.138766457	4	4	43.81913692	80.87117174
chr16	10385999	10483643	16p13.2-p13.13	ATF7IP2	27.87740007	24.48103001	1.13873477	4	4	54.320086	21.18099604
chr22	38084889	38110944	22q13.1	BAIAP2L2	0.272789899	0.239562942	1.138698235	2	2	71.50413527	68.62181336
chr11	62832321	62840426	11q12.3	WDR74	7.337315546	6.443749531	1.138671749	4	4	30.59072765	18.11637053
chr2	73762198	73780157	2p13.1	DUSP11	13.66723896	12.00351403	1.138603157	4	4	38.94230694	13.50798499
chr8	144423601	144428563	8q24.3	VPS28	19.54368356	17.16729405	1.138425398	4	4	30.87782369	37.19001877
chr9	35605284	35610041	9p13.3	TESK1	4.281538266	3.760965199	1.138414752	4	4	70.23029606	76.01778938
chr7	128954180	129055173	7q32.1	TNPO3	27.76866986	24.39470152	1.138307424	4	4	10.16902722	10.22031254
chr4	152927413	152928984	4q31.3	FAM192BP	0.615712353	0.540925419	1.138257385	4	4	38.94280305	51.30771589
chr2	218136	264866	2p25.3	SH3YL1	2.348756607	2.063594524	1.138187071	4	4	59.44600674	20.67156555
chr8	11795573	11839309	8p23.1	FDFT1	24.72406369	21.72428086	1.138084333	4	4	23.98289882	18.26516994
chr16	5024844	5033941	16p13.3	NAGPA	1.882933826	1.654504141	1.138065345	4	4	62.34900026	37.58989798
chr17	39156894	39166161	17q12	ARL5C	0.089399626	0.07856486	1.137908543	1	1	0	0
chr1	150265399	150282179	1q21.2	APH1A	23.59654196	20.73932283	1.137768198	4	4	37.21383222	7.820432776
chr1	9652610	9654586	1p36.22	PIK3CD-AS1	1.041814368	0.915683863	1.137744598	4	3	94.19839457	37.13510455
chr7	150716557	150724280	7q36.1	GIMAP1	17.46518155	15.3509833	1.137723963	4	4	71.67744993	46.22810121
chr16	74447427	74607144	16q23.1	GLG1	22.40611974	19.69388444	1.137719672	4	4	39.06963888	19.55093101
chr9	122239555	122264864	9q33.2	RBM18	11.11193634	9.766957813	1.137707007	4	4	20.18578473	12.99487263
chr2	663877	677468	2p25.3	TMEM18	3.260505839	2.865926446	1.137679525	4	4	57.45586491	19.47011164
chr14	101964528	102050798	14q32.31	DYNC1H1	26.68477293	23.45620881	1.137642197	4	4	32.41767767	19.54287341
chr16	81081947	81096375	16q23.2	GCSH	0.621690381	0.546482404	1.137621955	4	4	43.49942568	68.65766298
chr15	34140674	34210096	15q14	KATNBL1	17.43305604	15.3245194	1.13759235	4	4	32.93859487	46.16620442
chr16	4803203	4848032	16p13.3	GLYR1	21.95658355	19.30196607	1.137530937	4	4	35.27614113	19.29650256
chr16	66880361	66891101	16q22.1	PDP2	0.803967462	0.706817918	1.137446352	4	4	30.37473083	50.08584182
chr17	58475991	58477102	17q22	SETP3	0.352434695	0.30984905	1.137439975	1	1	0	0
chrX	11137543	11665701	Xp22.2	ARHGAP6	4.001321622	3.517929851	1.137408019	4	4	77.93863804	17.5121354
chr13	27424544	27435709	13q12.2	GTF3A	45.96474605	40.4120274	1.137402625	4	4	51.21581795	5.822146871
chr9	128322486	128334072	9q34.11	COQ4	1.708692313	1.502369484	1.137331616	4	4	45.35434424	60.17644589
chr14	61747039	61749089	14q23.2	HIF1A-AS2	1.138045262	1.000681723	1.137269959	3	4	42.98819976	77.63040767
chr1	77779624	77879540	1p31.1	MIGA1	8.050975633	7.079357562	1.137246645	4	4	35.22801226	28.31008046
chr7	100874971	100888664	7q22.1	SRRT	12.06882737	10.61281981	1.13719328	4	4	60.92181085	41.54784471

chr9	85546539	85742029	9q21.33	AGTPBP1	235.0387505	206.694093	1.13713337	4	4	15.31611163	23.50324962
chr9	113275634	113292905	9q32	PRPF4	9.741009055	8.566805924	1.137064285	4	4	29.45831012	33.87378833
chr4	70815782	70843274	4q13.3	GRSF1	11.16738335	9.821392832	1.137046806	4	4	19.43065908	8.347172663
chr8	70669354	71066635	8q13.3	XKR9	0.289297133	0.254432747	1.137027905	3	4	68.11495649	41.41827471
chr15	34978341	34988296	15q14	ZNF770	22.05103304	19.39512084	1.136937131	4	4	30.18353398	18.34691992
chr1	45583988	45618906	1p34.1	NASP	12.04892176	10.59787232	1.13691894	4	4	22.82306118	23.92699957
chr15	101653752	101724442	15q26.3	TARSL2	10.57775043	9.304122696	1.136888536	4	4	32.95302995	12.83722412
chr20	34088298	34112356	20q11.22	EIF2S2	100.4333888	88.34106517	1.136882249	4	4	36.11788908	15.00386323
chr1	89821014	89936432	1p22.2	LRRC8D	15.78033299	13.8815562	1.136784145	4	4	38.79415977	17.32195962
chr15	45479613	45523755	15q21.1	SLC30A4	1.665073378	1.464750073	1.136762789	4	4	33.00185247	63.23151517
chr1	169132530	169367963	1q24.2	NME7	4.692069617	4.127766516	1.136709065	4	4	50.59936583	27.09281641
chr4	83460939	83485178	4q21.23	ABRAXAS1	11.40376662	10.03253781	1.13667816	4	4	45.96040716	33.22259432
chr22	29268009	29300525	22q12.2	EWSR1	22.63690147	19.91521157	1.136663871	4	4	46.06712241	23.49302628
chr14	31561385	31861293	14q12	NUBPL	1.723666649	1.516511896	1.136599491	4	4	31.32263891	23.7820875
chr17	50381233	50397553	17q21.33	LRRC59	13.75507626	12.10214175	1.136581982	4	4	48.16561914	33.20098533
chr3	52401004	52410105	3p21.1	BAP1	10.64073974	9.362777529	1.13649392	4	4	43.10389239	13.6821958
chr2	56184123	56386174	2p16.1	CCDC85A	0.071964557	0.063322392	1.136478805	1	1	0	0
chr15	89687877	89688280	15q26.1	RPL36AP43	0.834856813	0.734605845	1.136469059	1	1	0	0
chr1	115856630	115857878	1p13.1	HNRNPA1P43	0.316662792	0.278651831	1.136410233	1	1	0	0
chr19	10572671	10587315	19p13.2	AP1M2	1.050802221	0.924782531	1.136269541	4	4	56.07779103	18.48542808
chr11	65653596	65662972	11q13.1	RELA	14.31346017	12.59695614	1.136263396	4	4	56.59370724	34.47102472
chr12	8914664	8941467	12p13.31	PHC1	1.652173214	1.454057709	1.136250098	4	4	52.03107256	48.40371671
chr1	100148448	100178273	1p21.2	LRRC39	1.390409395	1.223768253	1.136170506	4	4	100.9451183	51.62743306
chr9	105442183	105552433	9q31.2	FSD1L	3.995912589	3.517060782	1.136151132	4	4	55.35925941	46.67293814
chr1	159872364	159900116	1q23.2	CFAP45	3.290283427	2.896163333	1.136083518	4	4	87.6821012	69.83963403
chr9	129887187	130053846	9q34.11	FNBP1	44.23038341	38.93360218	1.136046524	4	4	47.30357238	34.19663497
chrX	152898132	152973481	Xq28	ZNF185	22.14525911	19.4951715	1.135935588	4	4	54.15554113	25.50072701
chr17	7014737	7017524	17p13.1	C17orf49	0.66285602	0.583538475	1.135925133	3	3	68.72335827	24.72452174
chr19	21020620	21060046	19p12	ZNF430	24.79412784	21.82733036	1.135921225	4	4	48.94981835	30.59951499
chr19	44025324	44033110	19q13.31	ZNF222	3.393748942	2.987718174	1.135899956	4	4	53.20698566	37.51545213
chr22	38861427	38872334	22q13.1	CBX6	11.26843748	9.92029864	1.135897001	4	4	60.83494948	37.33015766
chr7	18086942	19002416	7p21.1	HDAC9	3.326959847	2.929018596	1.135861633	4	4	34.51504545	47.11103402
chr9	134030305	134068533	9q34.2	BRD3	6.509129104	5.730798363	1.135815412	4	4	79.94504358	32.63156867
chr16	29973867	29992261	16p11.2	TAOK2	4.629732724	4.07639279	1.135742545	4	4	59.29714865	25.39274198
chr7	150368228	150374044	7q36.1	REPIN1	7.89681833	6.953064248	1.13573211	4	4	45.33034199	27.30592475
chr7	47962980	47979625	7p12.3	HUS1	7.307963768	6.434656068	1.135719406	4	4	28.10021461	19.70710287
chr1	32013694	32060859	1p35.2	KHDRBS1	39.59488108	34.86353388	1.135710488	4	4	40.10567729	20.96804669
chr19	10316212	10333638	19p13.2	RAVER1	3.087136382	2.718303078	1.135685129	4	4	85.64115555	43.37041388
chr10	1039419	1056716	10p15.3	IDI1	42.30360297	37.24965256	1.135677787	4	4	33.01827236	21.46907267
chr14	69050920	69153393	14q24.1	DCAF5	14.4932064	12.76251883	1.135607053	4	4	25.12111783	33.32384025
chr16	21446683	21502281	16p12.2	SMG1P3	11.11418066	9.787150805	1.135588986	4	4	51.03271761	43.58132599
chr22	37340644	37427470	22q13.1	ELFN2	0.034132909	0.030058075	1.135565369	1	1	0	0
chr17	32142454	32253374	17q11.2	RHOT1	177.7656617	156.5447933	1.135557804	4	4	56.77452252	37.26025783

chr9	125512902	125513207	9q33.3	RN7SL30P	1.305605332	1.149788296	1.135518022	1	2	0	31.54009018
chr20	20017290	20033629	20p11.23	NAA20	11.51862023	10.14397133	1.135513878	4	4	34.26802453	5.605557591
chrX	85861180	86047565	Xq21.2	CHM	5.096773126	4.488735975	1.135458435	4	4	42.32237024	27.64956249
chr19	15421507	15449951	19p13.12	WIZ	1.897249658	1.670947793	1.135433235	4	4	73.27190771	56.71949116
chr14	21385194	21437298	14q11.2	CHD8	23.48342251	20.68440446	1.135320214	4	4	45.6404014	34.83943462
chr15	81000954	81004004	15q25.1	TLNRD1	4.254136173	3.747098807	1.135314651	4	4	48.72923018	19.4085817
chr14	89156172	89619150	14q31.3-q32.11	FOXN3	31.18101409	27.4665818	1.135234603	4	4	49.45622417	16.21689842
chr6	130133203	130365425	6q23.1	SAMD3	16.46390797	14.50297632	1.135208912	4	4	35.32294959	38.56625496
chr20	45708860	45791908	20q13.12	WFDC3	0.152356862	0.134220402	1.135124465	2	1	30.31307388	0
chr21	32311019	32393003	21q22.11	URB1	2.700179724	2.37900506	1.135003775	4	4	66.89714282	51.50395337
chr6	22287244	22302897	6p22.3	PRL	0.20191366	0.177899533	1.134987012	2	2	27.10900541	6.390990887
chr6	11094033	11138738	6p24.2	SMIM13	2.764084591	2.435676553	1.134832368	4	4	42.56036674	43.28880031
chr1	161314376	161364751	1q23.3	SDHC	2.649092463	2.334396363	1.134808341	4	4	71.04105196	31.86801935
chr14	100333788	100376343	14q32.2	WARS	59.70618853	52.61698884	1.134732143	4	4	45.73141922	35.18236131
chr6	2948159	2972165	6p25.2	SERPINB6	8.231757525	7.254892574	1.134649127	4	4	47.86202845	27.72005866
chr17	39904595	39918650	17q21.1	GSDMB	3.087503963	2.72126931	1.134582289	4	4	16.05848924	17.52810161
chr7	23504780	23532041	7p15.3	TRA2A	57.26947714	50.47979194	1.134503035	4	4	5.581388255	23.69600322
chr7	128304173	128343915	7q32.1	RBM28	2.400517622	2.116036553	1.134440527	4	4	26.32764154	31.24370649
chr2	225010461	225010540	2q36.2	MIR4439	7.678893514	6.769113922	1.134401578	3	2	70.75129395	9.801749985
chr2	232550587	232583645	2q37.1	EIF4E2	10.09778562	8.901745647	1.13436016	4	4	55.5458391	28.71004817
chr1	147155106	147173366	1q21.1	PRKAB2	6.089877037	5.36866368	1.134337593	4	4	52.31867544	34.74715178
chrX	7147252	7354643	Xp22.31	STS	3.022560868	2.664794879	1.134256484	4	4	43.76570595	34.53209899
chr3	58492096	58502360	3p14.3	KCTD6	2.716140323	2.394713921	1.134223299	4	4	41.99998	45.25426132
chr7	143298517	143298812	7q34	RN7SL481P	1.435827948	1.265936424	1.134202256	2	4	19.2764762	67.35020995
chr3	126437348	126475938	3q21.3	ZXDC	6.069255564	5.351187147	1.13418862	4	4	58.76893217	18.18230609
chr10	109996350	110135565	10q25.1-q25.2	ADD3	97.44627353	85.91735698	1.134186117	4	4	45.72033342	25.74935564
chr16	2513726	2520223	16p13.3	ATP6V0C	7.56357434	6.668823451	1.134169227	4	4	25.48248309	56.48030434
chr1	155185824	155192915	1q22	MUC1	0.140070697	0.123507292	1.13410872	3	3	55.21118559	45.26208901
chr15	84641495	84654343	15q25.2	WDR73	1.767156802	1.558255174	1.134061244	4	4	90.85238657	52.88217377
chr16	85899168	85922606	16q24.1	IRF8	16.94867046	14.94634988	1.133967196	4	4	60.04252366	38.89337691
chr13	25301084	25349795	13q12.13	NUP58	23.33382485	20.57722369	1.133963707	4	4	5.669242235	30.05131889
chr11	108665025	108940930	11q22.3	DDX10	7.703866439	6.794826847	1.13378407	4	4	42.72110898	4.101467404
chr15	82659281	82709908	15q25.2	AP3B2	0.032374391	0.028554702	1.133767403	1	2	0	4.863865154
chr7	135557914	135648753	7q33	NUP205	15.83746982	13.96898942	1.133759167	4	4	22.97278701	12.69824837
chr2	218366665	218367902	2q35	CATIP-AS1	4.499759865	3.968916494	1.133750199	4	3	80.28249573	48.05017357
chr12	100203669	100268094	12q23.1	DEPDC4	2.242894631	1.978354307	1.133717364	4	4	106.7439845	73.12484388
chr10	99610518	99659507	10q24.2	SLC25A28	13.5594168	11.96051319	1.133681856	4	4	65.76014724	21.81351581
chr1	44782145	44792828	1p34.1	BEST4	0.155641922	0.13730151	1.133577638	3	2	25.70087221	12.84929129
chr22	17592136	17628822	22q11.21	ATP6V1E1	35.16407209	31.02098087	1.133557712	4	4	25.28022317	21.51565734
chrMT	4329	4400	N/A	MT-TQ	20.69718809	18.25891884	1.133538534	3	3	10.29670584	23.33696126
chr20	3208868	3227449	20p13	ITPA	3.977848994	3.509454205	1.133466563	4	4	42.50848056	26.35088066
chr5	82278447	82319932	5q14.2	ATP6AP1L	1.452955015	1.281871627	1.133463745	4	4	105.9382429	16.87766307
chr1	92508696	92792413	1p22.1	EVI5	15.46485474	13.64438034	1.133423018	4	4	12.32388482	49.03593816

chr1	70852353	71047808	1p31.1	PTGER3	0.373655395	0.329700811	1.133316579	4	4	47.24576426	64.10076817
chr1	224434640	224740549	1q42.12	CNIH3	2.37056167	2.091823993	1.133251018	4	4	59.42753352	25.93260883
chr19	7728957	7732181	19p13.2	CLEC4G	0.523513704	0.461972335	1.133214405	2	3	96.80435364	81.68211489
chr5	5140330	5320304	5p15.32	ADAMTS16	0.050050163	0.044166892	1.133205459	1	2	0	1.139564869
chr12	57755098	57760410	12q14.1	MARCH9	2.248857413	1.984721687	1.133084516	4	4	33.48421733	53.44874779
chr16	75647737	75657442	16q23.1	TERF2IP	64.8307392	57.21636129	1.133080429	4	4	36.22011369	30.17446805
chr2	64578892	64578997	2p14	RNU6-100P	5.194195479	4.58440681	1.133013647	1	2	0	27.7204711
chr1	165724291	165768922	1q24.1	TMCO1	17.04344808	15.04291522	1.132988376	4	4	12.64141881	16.64325361
chr6	53497341	53545129	6p12.1	GCLC	30.75368502	27.14405457	1.132980519	4	4	46.44728731	67.97939067
chr6	128883141	129516566	6q22.33	LAMA2	0.381865093	0.337059706	1.132930117	4	4	48.22162586	48.13092111
chr5	40832656	40832735	5p13.1	SNORD72	11.76676356	10.3862678	1.132915479	3	3	104.4143764	29.93903031
chr20	49219295	49244077	20q13.13	DDX27	11.94870444	10.54732867	1.132865468	4	4	61.91039074	30.13722992
chr12	96907222	96953691	12q23.1	NEDD1	40.1245943	35.42103735	1.132789927	4	4	44.23315825	12.23092214
chr6	166929509	166956589	6q27	RNASET2	15.4831896	13.66866158	1.132750965	4	4	40.52187498	44.73740039
chr17	2593210	2685617	17p13.3	PAFAH1B1	32.01135217	28.26072507	1.132715176	4	4	30.44333993	22.5587492
chr8	42338452	42371813	8p11.21	POLB	15.23470795	13.45018597	1.13267638	4	4	22.10365402	51.46408409
chr15	36579603	36810260	15q14	C15orf41	0.526425195	0.46478473	1.132621537	4	4	19.38239722	38.96420301
chr1	224227334	224330189	1q42.11	NVL	13.75647524	12.14592691	1.132599869	4	4	46.82685624	13.65254815
chr9	129488660	129513686	9q34.11	LINC00963	3.077073337	2.716831749	1.132596208	4	4	77.26636675	46.10675995
chrX	276324	303356	Xp22.33 and Yp11	PLCXD1	1.561430222	1.378732629	1.132511256	4	4	60.04231087	22.16783793
chr17	36948875	37056871	17q12	AATF	13.62444052	12.03041371	1.13249975	4	4	54.48840151	42.95699105
chr20	45886491	45890815	20q13.12	SPATA25	0.804468638	0.710451613	1.132334171	2	1	47.78226872	0
chr3	152243656	152465780	3q25.1-q25.2	MBNL1	182.9531868	161.5808866	1.132269977	4	4	23.67873259	7.981613724
chr19	16630741	16660157	19p13.11	SMIM7	3.244980606	2.866258635	1.132131123	4	4	38.91162435	18.85108875
chr16	9091377	9119698	16p13.2	C16orf72	18.3895988	16.24357073	1.132115537	4	4	27.0007591	35.8050832
chr6	36740773	36839444	6p21.2	CPNE5	3.547115981	3.133328875	1.132059902	4	4	74.0666021	42.10545009
chr1	154220172	154271510	1q21.3	UBAP2L	21.87002971	19.31910866	1.132041343	4	4	56.62935143	40.58207894
chr17	17811349	17837017	17p11.2	SREBF1	3.961779726	3.499793739	1.132003775	4	4	44.42385486	21.25907141
chr5	163503064	163519353	5q34	MAT2B	54.59549239	48.22933177	1.131997695	4	4	29.15588453	20.04227141
chr11	64270337	64289500	11q13.1	GPR137	2.853610145	2.520864229	1.131996762	4	4	72.07856382	68.36456231
chr3	160435503	160449838	3q25.33	TRIM59	3.774278265	3.334228024	1.131979648	4	4	43.65199017	35.91157515
chr3	171058414	171460408	3q26.2-q26.31	TNIK	16.26946664	14.37271203	1.131969151	4	4	28.13232363	34.4995544
chr13	95801580	96053482	13q32.1	UGGT2	19.89000754	17.57249676	1.13188284	4	4	77.13756433	61.42600416
chr17	7235028	7239656	17p13.1	PHF23	17.72287711	15.6581988	1.131859248	4	4	66.40708293	42.44877894
chr3	172750685	172829273	3q26.31	ECT2	35.83826822	31.66325868	1.131856597	4	4	62.24069033	43.79101546
chr1	67685177	67688338	1p31.3	GADD45A	17.37600557	15.35223313	1.131822675	4	4	15.27033313	35.26564535
chr18	49041474	49460733	18q21.1	DYM	15.56096617	13.7488815	1.131798698	4	4	27.57212746	25.65860725
chr17	44248390	44268161	17q21.31	SLC4A1	438.9951588	387.9206242	1.131662333	4	4	34.85207494	33.3593374
chr5	136132845	136182734	5q31.1	SMAD5	6.46921477	5.716565914	1.131661012	4	4	29.77519505	42.09700941
chr19	507497	519654	19p13.3	TPGS1	1.050885891	0.928657042	1.131618934	4	4	91.2543916	48.95867745
chr2	190648860	190692766	2q32.2	NAB1	19.19672241	16.96442059	1.131587272	4	4	33.23843562	26.90541708
chr12	49127782	49131521	12q13.12	TUBA1B	100.6728845	88.96618889	1.131585895	4	4	55.92968704	75.23108834
chr4	106041600	106316704	4q24	TBCK	13.85753456	12.24653557	1.131547324	4	4	46.71908527	38.50162017

chr3	100401193	100456326	3q12.2	LNP1	0.35898253	0.317288385	1.131407725	3	3	96.17102641	76.1979622
chr3	49357171	49358600	3p21.31	GPX1	293.3095474	259.2435208	1.131405508	4	4	36.80654358	51.99867441
chr11	65181215	65212006	11q13.1	CAPN1	18.58808485	16.4299475	1.131353879	4	4	42.03737287	32.2054742
chr7	101085424	101091742	7q22.1	TRIM56	8.742800835	7.728243777	1.131279122	4	4	61.35000397	31.30364394
chr7	123681927	123749071	7q31.32	WASL	8.789924975	7.769941479	1.131272996	4	4	26.38206847	17.60483472
chr7	142615797	142616415	7q34	TRBV18	3.124930861	2.762500101	1.13119665	2	4	17.40503396	38.2131067
chr1	19338773	19485641	1p36.13	CAPZB	59.90457438	52.96004824	1.13112764	4	4	58.22759356	31.8081809
chr10	125784980	125823280	10q26.2	UROS	8.931968894	7.896573662	1.131119556	4	4	73.69982243	37.70069507
chr4	4386256	4419058	4p16.3	NSG1	1.64726878	1.456348294	1.131095347	4	4	43.91062558	72.87189352
chr6	53057807	53100873	6p12.1	FBXO9	62.95306589	55.6579241	1.131071036	4	4	42.43265113	37.84156986
chr12	68294566	68332414	12q15	MDM1	13.84369446	12.24119454	1.13091042	4	4	22.93564319	18.43126407
chr6	116274859	116280117	6q22.1	TSPYL1	42.16313516	37.28343518	1.13088118	4	4	40.59629419	12.08176381
chr2	156435290	156586403	2q24.1	GPD2	13.65251927	12.07251882	1.130875791	4	4	26.39413761	26.96012178
chr15	52581321	52709817	15q21.2-q21.3	FAM214A	12.8756439	11.38568875	1.130862101	4	4	23.37439934	25.24802419
chr2	169827462	170084131	2q31.1	UBR3	23.37683503	20.67228292	1.130829871	4	4	21.15467138	21.73482725
chrX	119236245	119244466	Xq24	PGRMC1	90.5466647	80.07399614	1.130787385	4	4	39.36539762	28.56410439
chr5	115828196	115841851	5q22.3	ATG12	10.72305449	9.483245068	1.130736832	4	4	19.47698928	17.58502014
chr2	64607312	64616482	2p14	LINC02579	0.168669265	0.149168331	1.130731062	1	2	0	11.5016248
chr2	233475519	233566789	2q37.1	USP40	1.580673382	1.398000783	1.130667022	4	4	64.17033457	28.94487942
chr9	128120693	128128462	9q34.11	PTGES2	3.300597399	2.919187466	1.130656197	4	4	35.08341173	34.06176852
chr7	103344405	103369395	7q22.1	PSMC2	19.90179461	17.60204421	1.130652462	4	4	21.31382388	17.07536008
chr17	7572706	7579006	17p13.1	EIF4A1	3.618712512	3.200604211	1.130634178	4	4	51.72250492	33.27216471
chr17	35854946	35868891	17q12	HEATR9	0.23743081	0.210007241	1.130583921	2	2	11.00247196	18.60855516
chr9	137241259	137243707	9q34.3	TUBB4B	53.63814581	47.4430611	1.130579363	4	4	56.97166295	42.70918682
chr5	44495144	44510282	5p12	LINC02224	11.17518484	9.884680026	1.130556053	3	1	74.32460583	0
chr17	13494032	13601942	17p12	HS3ST3A1	0.16969014	0.150104201	1.130482285	1	2	0	40.37936948
chr17	79089345	79665600	17q25.3	RBFOX3	0.029126534	0.025764721	1.130481249	1	1	0	0
chr7	73736094	73738867	7q11.23	ABHD11	1.519265323	1.343967104	1.130433415	4	4	86.96757606	54.59173192
chrY	13231827	13480670	Yq11.221	UTY	24.91284826	22.0387619	1.130410518	2	2	19.3007837	10.74231715
chr3	4034714	4467282	3p26.1	SUMF1	7.222372871	6.389307794	1.130384246	4	4	31.08996664	30.12788868
chr19	44094329	44109831	19q13.31	ZNF224	5.784960796	5.118091645	1.130296446	4	4	29.99650291	14.94454257
chr17	7402974	7404138	17p13.1	TMEM256	2.475356248	2.19001281	1.130293045	4	4	19.6359218	48.01804949
chr2	39736060	39779276	2p22-p21	THUMPD2	5.515692082	4.88028388	1.130199025	4	4	27.17489401	33.67617537
chr5	76716043	76735780	5q13.3	F2R	19.00421582	16.81510928	1.130186875	4	4	13.80445822	53.32092043
chr22	40370562	40410289	22q13.1	SGSM3	0.223422708	0.197707808	1.130065172	3	3	37.98536653	61.73577922
chr4	39498755	39527598	4p14	UGDH	4.019813872	3.557213586	1.130045687	4	4	35.64280822	54.92816802
chr1	35176378	35193174	1p34.3	SFPQ	44.18210899	39.10082998	1.129953226	4	4	38.05505435	32.52103866
chr3	158110052	158544835	3q25.32	RSRC1	67.36748504	59.62151636	1.129919015	4	4	51.33591666	63.24277044
chr14	32203163	32203269	14q12	RNU6-8	9.997931555	8.84868554	1.129877597	2	4	51.13137783	55.46660704
chr13	41061257	41084012	13q14.11	WBP4	20.74484323	18.36056301	1.129858775	4	4	9.774296526	35.06039644
chr16	31060843	31074320	16p11.2	ZNF668	1.776840825	1.572710625	1.129795142	4	4	74.28524762	45.09386058
chr15	101976558	101979093	15q26.3	DDX11L9	1.851570552	1.63892265	1.12974859	4	4	25.63877183	60.884565
chr9	5299864	5340915	9p24.1	RLN1	0.944624092	0.836140797	1.129742856	3	4	46.56489503	105.1477261

chr5	139395269	139404204	5q31.2	SPATA24	0.966862981	0.855886642	1.129662427	4	4	58.57983182	54.6349318
chr3	23203007	23590805	3p24.3	UBE2E2	2.982675964	2.640343774	1.129654401	4	4	57.2014707	47.97268568
chr6	159789812	159798429	6q25.3	MRPL18	22.47082778	19.89213427	1.129633828	4	4	17.93970878	24.22300618
chr4	849275	932390	4p16.3	GAK	6.221255507	5.507690272	1.129557982	4	4	63.77513664	25.29063654
chr17	45254393	45262112	17q21.31	SPATA32	0.326326571	0.288901477	1.12954276	3	3	69.07656356	11.62791851
chr7	135926755	135977456	7q33	MTPN	253.128167	224.1144909	1.12945917	4	4	14.52468983	21.82780899
chr4	6997012	6997326	4p16.1	RN7SKP292	2.279561983	2.018303304	1.129444706	3	2	61.20720501	46.19500105
chr19	54155639	54159743	19q13.42	LENG1	2.540932356	2.24976136	1.129423059	4	4	74.88933287	49.66956405
chr16	24919384	25015601	16p12.1	ARHGAP17	3.701940324	3.277762042	1.129410945	4	4	32.92399488	43.36969042
chrMT	4263	4331	N/A	MT-TI	153.6948678	136.0846258	1.12940655	4	4	90.86346502	83.97730747
chr5	27472292	27496401	5p14.1	PURPL	0.118434314	0.104871613	1.129326707	1	1	0	0
chr1	145921556	145927536	1q21.1	RBM8A	29.38180989	26.0188712	1.129249984	4	4	28.05262475	23.66685054
chr11	66480013	66509659	11q13.2	DPP3	4.868759469	4.311554409	1.129235307	4	4	58.80968189	74.97958208
chr11	85957171	86069881	11q14.2	PICALM	837.4810399	741.6679465	1.12918597	4	4	32.83310463	20.45548746
chr17	42287547	42311943	17q21.2	STAT5A	23.74090041	21.02491202	1.129179536	4	4	58.19771386	29.58782452
chr3	126524288	126543291	3q21.3	CHST13	1.092790843	0.967789698	1.129161476	3	4	55.27347179	98.39603656
chr3	47016408	47164037	3p21.31	SETD2	99.42827207	88.05564646	1.129152713	4	4	29.02296226	10.48243534
chr19	10512742	10517992	19p13.2	S1PR5	21.90302629	19.39932017	1.129061539	4	4	32.79095243	54.76940792
chr7	108569745	108574850	14q24.2-q24.3	DNAJB9	19.06328779	16.8843062	1.129053664	4	4	24.75552434	27.67748644
chr1	114704464	114716894	1p13.2	NRAS	43.89565919	38.87834137	1.129051746	4	4	24.90184151	16.89886198
chr12	6581473	6581609	12p13.31	SCARNA11	5.969352185	5.287328214	1.128992176	4	3	62.11593122	104.757488
chr11	77874418	77994671	11q14.1	INTS4	10.20532833	9.039566491	1.128962139	4	4	13.7631044	16.17313176
chr2	53786931	53834524	2p16.2	ERLEC1	22.46298967	19.89816928	1.128897305	4	4	27.587524	18.64312445
chr8	93916855	93926068	8q22.1	PDP1	16.41798478	14.54351351	1.1288871	4	4	53.00446979	20.05380929
chr6	26421391	26430588	6p22.2	BTN2A3P	0.593262285	0.52555052	1.128839688	4	4	65.67756267	35.48074108
chr7	99756960	99784188	7q22.1	CYP3A4	0.097881858	0.08671591	1.128764697	1	3	0	12.03348002
chr19	2933218	2944971	19p13.3	ZNF77	1.344025759	1.190721741	1.128748819	3	4	24.42190457	42.29170916
chr3	131478998	131479233	3q22.1	SNORA58	2.862672069	2.53618387	1.12873207	1	1	0	0
chr17	77958853	78108835	17q25.3	TNRC6C	11.77429631	10.43178573	1.128694226	4	4	22.96237819	17.96670746
chr9	96450155	96491336	9q22.32	HABP4	4.215766118	3.735175579	1.128666117	4	4	52.15550669	57.55903615
chr3	38845764	39051945	3p22.2	SCN11A	0.038112008	0.033770821	1.128548445	1	1	0	0
chr22	24011192	24178628	22q11.23	CABIN1	8.63787205	7.654884351	1.128413135	4	4	76.2972686	39.15850767
chr5	149960737	149987400	5q32	SLC26A2	5.0602438	4.48444507	1.128399104	4	4	15.00656212	13.27268655
chr11	518967	554916	11p15.5	LRRC56	0.399798481	0.354306774	1.128396378	2	4	89.21104932	70.91829973
chr15	40250664	40252909	15q15.1	C15orf56	0.147671799	0.130878572	1.128311506	1	1	0	0
chr10	91923770	92030998	10q23.32	BTAF1	40.38964117	35.79768434	1.128275248	4	4	37.81995206	20.17204864
chr16	75293710	75433489	16q23.1	CFDP1	28.78450657	25.51304481	1.128227022	4	4	40.21321511	37.56718383
chr12	16347142	16377189	12p12.3	MGST1	1.046246061	0.927374239	1.12818107	4	4	100.341368	40.61476244
chr6	87155549	87265943	6q14.3	ZNF292	24.13752944	21.39540527	1.128164161	4	4	27.51866195	14.20269113
chr4	82422564	82430225	4q21.22	HNRNPDL	60.16383236	53.33358267	1.128066583	4	4	42.9323905	20.78872868
chr5	144158159	144170714	5q31.3	YIPF5	14.08818977	12.48988078	1.127968315	4	4	32.0119019	31.56838995
chr13	39655627	39791665	13q14.11	COG6	5.119692881	4.538973306	1.127940734	4	4	19.4475703	16.52563409
chr11	68754620	68844410	11q13.3	CPT1A	26.95002204	23.89324152	1.127934944	4	4	15.56174632	35.4688132

chr8	30578318	30658241	8p12	GTF2E2	12.77845552	11.32965136	1.127877206	4	4	37.37131706	42.67174821
chr2	201620184	201643529	2q33.1	TMEM237	0.471634574	0.41817639	1.127836448	4	4	57.72110592	33.28012569
chr11	129815781	129859511	11q24.3	TMEM45B	1.454685056	1.289809653	1.127829253	4	4	77.6753213	77.00204121
chr11	78139793	78173977	11q14.1	KCTD21-AS1	0.159745708	0.141641993	1.127813191	3	3	87.27691301	56.38915587
chr17	39921041	39927804	17q21.1	ORMDL3	31.79810844	28.19529867	1.127780514	4	4	28.23333448	15.80168831
chr1	237042208	237833988	1q43	RYR2	0.079437222	0.070439281	1.127740382	3	4	80.0903158	100.2421424
chr1	231528653	231566524	1q42.2	TSNAX	12.7368819	11.29547921	1.127608813	4	4	28.92935167	25.3954088
chr1	33472591	33504253	1p35.1	ZSCAN20	0.534716014	0.474209197	1.1275952	4	4	82.12566019	45.30827028
chr2	3379245	3479571	2p25.3	TRAPPC12	2.799637316	2.482955148	1.127542444	4	4	54.63885292	36.04553549
chr1	149782071	149792518	1q21.2	FCGR1A	19.23342046	17.05806747	1.127526344	4	4	103.0441222	90.84642713
chr11	64244479	64246941	11q13.1	PPP1R14B	5.000394962	4.435107383	1.127457473	4	4	61.98815323	39.64185398
chr12	105330636	105371518	12q23.3	C12orf75	18.99120382	16.84445997	1.127445098	4	4	18.93482466	66.66026262
chr3	196942623	196943545	3q29	NCBP2-AS2	8.420862624	7.469049024	1.127434376	4	4	83.18545979	29.85169132
chr2	223950846	223967714	2q36.1	MRPL44	23.79568263	21.1064241	1.127414219	4	4	37.95607369	30.38339849
chr19	39342459	39390748	19q13.2	SAMD4B	3.736638314	3.314377755	1.127402665	4	4	57.01589281	22.8805578
chr9	95905005	95905141	9q22.32	RNA5SP289	1.773655283	1.573262647	1.127373923	1	1	0	0
chr22	39994949	40030039	22q13.1	FAM83F	0.070831755	0.062829304	1.127368132	4	4	52.30495125	25.80418824
chr4	99948087	99950355	4q23	H2AFZ	40.07190901	35.54470335	1.127366534	4	4	14.0215924	31.88541532
chr15	75467121	75579291	15q24.2	PTPN9	6.019484827	5.339424488	1.12736585	4	4	18.24010625	12.01908231
chr17	7856681	7885466	17p13.1	NAA38	8.909255039	7.903434759	1.127263691	4	4	70.02648801	74.40957458
chr22	37906148	37942458	22q13.1	MICALL1	3.483612805	3.090428572	1.127226443	4	4	61.79923408	70.35389806
chr10	74120012	74151085	10q22.2	AP3M1	13.68731448	12.14274631	1.127200893	4	4	35.56686335	31.68303226
chr6	31879759	31897707	6p21.33	EHMT2	3.1939363	2.833594183	1.127167863	4	4	60.33985447	65.08506341
chr3	10300929	10321188	3p25.3	SEC13	9.467280092	8.399302448	1.127150755	4	4	63.86383663	35.3763103
chr19	41397789	41425005	19q13.2	BCKDHA	0.406012297	0.36021278	1.127145731	4	3	70.04530935	124.492549
chr1	53196429	53214197	1p32.3	CPT2	1.821960084	1.616439492	1.127144006	4	4	58.47412139	26.49392364
chr12	57055643	57088703	12q13.3	NEMP1	16.08482963	14.27090663	1.12710636	4	4	76.07466503	45.68663357
chr11	43358885	43494933	11p12-p11.2	TTC17	20.18098699	17.90558367	1.127077863	4	4	25.2011195	18.99885557
chr3	125225669	125375354	3q21.2	ZNF148	34.31439847	30.448244	1.126974628	4	4	23.18941583	25.80981166
chr7	121873105	122062036	7q31.32	PTPRZ1	0.028591451	0.025370493	1.126956857	1	1	0	0
chr6	42563905	42693505	6p21.1	UBR2	148.6258152	131.8861131	1.126925434	4	4	46.92055894	36.71839702
chr3	167735644	167825569	3q26.1	SERPINI1	3.521550257	3.125111146	1.126856004	4	4	23.71679817	21.35639705
chr15	72199029	72231624	15q23	PKM	78.13194771	69.33870743	1.126815751	4	4	65.81713624	53.34203477
chr19	10133344	10195135	19p13.2	DNMT1	39.52950388	35.08214828	1.126769763	4	4	28.75425	7.977984503
chr17	58325450	58353727	17q22	TSPOAP1-AS1	12.42511529	11.02845538	1.12664148	4	4	41.19228318	46.13015223
chr7	151207837	151227374	7q36.1	ABCF2	7.947189702	7.05422192	1.12658629	4	4	24.84760553	34.40536177
chr3	100709290	100748967	3q12.2	TFG	16.60473648	14.73980125	1.126523771	4	4	22.37676341	18.80625542
chr15	25080141	25080234	15q11.2	SNORD116-14	8.793106576	7.805638369	1.12650704	2	2	76.51382475	16.26510871
chr19	4867614	4915469	19p13.3	ARRDC5	2.382600756	2.115150791	1.126444869	3	4	48.74321864	34.92496327
chr12	120687124	120701864	12q24.31	MLEC	16.0650335	14.26210937	1.126413568	4	4	48.78508228	29.52157937
chr11	215030	236950	11p15.5	SIRT3	1.340027157	1.189748616	1.126311171	4	4	26.384306	52.35514372
chr20	58309715	58367507	20q13.32	RAB22A	12.84564709	11.40516353	1.126301	4	4	36.70721963	33.94535044
chr10	35008551	35126899	10p11.21	CUL2	32.71136902	29.04525436	1.12622078	4	4	54.59649197	30.62542155

chr17	56938199	56961050	17q22	COIL	9.660193364	8.57777792	1.126188327	4	4	54.43222618	27.36228034
chr16	4696510	4734377	16p13.3	ANKS3	0.42175558	0.374525944	1.126105113	4	4	69.53896532	47.11481381
chr9	95875618	96041092	9q22.32	ERCC6L2	15.91539709	14.13438974	1.126005252	4	4	51.29012452	22.18444887
chr12	52601813	52620133	12q13.13	KRT73	0.99118564	0.880272835	1.125998214	3	4	89.27294775	109.7628292
chr5	149141447	149260439	5q32	ABLIM3	4.519360554	4.013702934	1.125982822	4	4	72.73477154	17.23777728
chr10	31318495	31529814	10p11.22	ZEB1	41.83710795	37.15642075	1.1259725	4	4	35.69881269	11.16242006
chr4	82893452	82900916	4q21.22	THAP9-AS1	9.211828993	8.181225702	1.125971747	4	4	34.75593216	28.9708084
chr14	20289131	20305994	14q11.2	TTC5	1.576997165	1.400659531	1.125896144	4	4	27.81893939	20.55258145
chr1	162378841	162386818	1q23.3	C1orf226	0.121276701	0.107717868	1.125873574	3	1	76.42502183	0
chr19	16888860	16892600	19p13.11	F2RL3	0.813897442	0.722904608	1.125871149	4	4	77.78944777	23.8301775
chr12	57487953	57516655	12q13.3	MARS	8.251789366	7.329335776	1.125857734	4	4	39.41188019	14.38787775
chr17	42797618	42798725	17q21.2	COA3	5.780757447	5.134856131	1.125787617	4	4	39.59054017	55.55227552
chr19	1852399	1863565	19p13.3	KLF16	3.862546998	3.431004207	1.125777401	4	4	72.45660075	34.00613427
chr5	150690790	150701107	5q33.1	RBM22	26.81423287	23.81859563	1.125768844	4	4	57.69466268	21.1653783
chr9	92613184	92670265	9q22.31	IPPK	2.09041241	1.857048317	1.125663986	4	4	37.91677593	21.68168431
chr3	63833870	63864478	3p14.1	THOC7	46.03724141	40.90253626	1.125535129	4	4	20.20756147	16.51201294
chr10	79691500	79826594	10q22.3	NUTM2B-AS1	3.345930049	2.972760423	1.125529667	4	4	12.22122209	31.60686298
chr12	107316749	107659642	12q23.3	BTBD11	4.467205153	3.969270076	1.125447517	4	4	87.47507033	38.49478669
chr2	115144048	115161343	2q14.1	DPP10-AS1	0.329767868	0.293016728	1.125423348	1	1	0	0
chr2	238426928	238452250	2q37.3	ASB1	2.892712611	2.570389391	1.125398596	4	4	44.06245142	33.07538091
chr2	191029576	191172684	2q32.2-q32.3	STAT4	25.39069036	22.56156831	1.125395629	4	4	15.05697234	36.77802639
chr11	20387530	20510861	11p15.1	PRMT3	9.224370817	8.19656783	1.125394312	4	4	51.65422104	37.85749479
chr17	28335522	28347009	17q11.2	TNFAIP1	4.564677142	4.056160943	1.125368841	4	4	61.1049019	25.94963262
chr8	93754844	93832653	8q22.1	TMEM67	1.302624751	1.157629456	1.125251905	4	4	21.24037247	37.35084036
chr6	34587280	34696850	6p21.31	C6orf106	52.37604736	46.54612693	1.125250387	4	4	14.88677493	11.23834481
chr19	14147737	14206245	19p13.12	ADGRL1	0.701580113	0.623528997	1.1251764	4	4	64.44901176	68.41046164
chr17	44395277	44503602	17q21.31	GPATCH8	9.953297012	8.846306451	1.125135905	4	4	24.6810098	22.23183736
chr15	44427329	44527774	15q15.3-q21.1	CTDSPL2	20.8989995	18.57685658	1.12500193	4	4	20.87768927	7.157517254
chr6	132816802	132816877	6q23.2	SNORD100	11.72732864	10.42450098	1.12497746	4	3	74.63916828	90.52682055
chr1	150560895	150574552	1q21.2	ADAMTSL4-AS1	10.53812126	9.367788136	1.124931639	4	4	80.6502553	76.69671698
chr2	85605253	85612056	2p11.2	C2orf68	10.8870438	9.678162985	1.124908086	4	4	40.4923033	12.98030149
chr15	78507564	78537373	15q25.1	HYKK	0.166358558	0.147888051	1.124895191	3	4	22.28920633	88.78318907
chr3	38165051	38255488	3p22.2	OXSR1	27.01025901	24.01145353	1.124890627	4	4	23.22787718	30.25294626
chr12	55818022	55821175	12q13.2	ORMDL2	5.855217486	5.206025998	1.124700009	4	4	34.1953056	40.33293372
chr11	73870258	73876901	11q13.4	COA4	6.084940681	5.410500874	1.124653858	4	4	71.64936181	45.20766466
chr22	42079691	42084284	22q13.2	SMDT1	2.392263239	2.127165567	1.124624842	4	4	57.88200202	22.93768373
chr5	138139766	138178669	5q31.2	BRD8	7.627931909	6.782649761	1.124624178	4	4	55.03031174	34.12531376
chr20	45416067	45426245	20q13.12	PIGT	2.904141676	2.582356128	1.124609284	4	4	61.56763823	42.27434022
chr16	19523857	19553408	16p12.3	CCP110	7.040499077	6.260583157	1.124575603	4	4	25.33555361	15.60803257
chr14	77069180	77076191	14q24.3	LINC02289	5.052427795	4.492942106	1.124525461	4	3	115.3225575	26.38298977
chr16	2771414	2777296	16p13.3	ELOB	110.3657361	98.14473981	1.124520135	4	4	30.59762015	30.45257261
chr9	2621774	2656103	9p24.2	VLDLR	0.317074807	0.281966837	1.124510991	4	4	92.13575892	82.20870119
chr6	32637406	32654846	6p21.32	HLA-DQA1	4.266169109	3.794591422	1.124276275	3	3	91.26406187	167.7027186

chr1	16914082	16972979	1p36.13	CROCC	1.413960523	1.257700373	1.124242748	4	4	80.61581694	35.20040575
chr11	16777297	17014423	11p15.2-p15.1	PLEKHA7	0.20560528	0.182885475	1.124229689	4	4	94.71535275	85.53123408
chr15	75843286	75901047	15q24.2	UBE2Q2	73.50190501	65.38257324	1.124181894	4	4	45.7168592	12.20337383
chr6	70667771	70862015	6q13	SMAP1	15.26451612	13.57834745	1.124180699	4	4	20.43694277	18.24074911
chr22	32269381	32273194	22q12.3	CPSF1P1	0.796182932	0.708234176	1.124180333	4	4	52.37172692	80.30299852
chr20	25248039	25298012	20p11.21	PYGB	14.54339426	12.93783897	1.12409764	4	4	84.25950063	17.81791372
chr12	98515573	98550379	12q23.1	TMPO	42.45772811	37.77117555	1.124077487	4	4	26.2862471	33.92469096
chr16	70454590	70480274	16q22.1	FUK	1.299824418	1.156386104	1.124040157	4	4	65.90235822	24.99429223
chr1	108929508	108963499	1p13.3	CLCC1	5.561731158	4.947982965	1.124040078	4	4	42.97214576	26.16878432
chr22	50502947	50507781	22q13.33	LMF2	8.389153082	7.463432666	1.124034135	4	4	44.76517409	23.73181844
chr9	109015135	109119963	9q31.3	TMEM245	34.9864514	31.12894861	1.123920112	4	4	39.43168728	26.09145472
chr16	3579653	3611639	16p13.3	SLX4	1.955047045	1.739686489	1.12379274	4	4	55.3963246	34.95864808
chr3	42547967	42603127	3p22.1	SEC22C	4.547238827	4.046353277	1.123786905	4	4	22.25100551	7.684296471
chr22	50767492	50783705	22q13.33	RABL2B	1.080172294	0.961270435	1.123692412	4	4	52.46463829	58.46249731
chr19	51517819	51531856	19q13.41	SIGLEC6	0.676518073	0.602059848	1.123672465	4	4	82.34453322	86.69508026
chrX	134460145	134500668	Xq26.2-q26.3	HPRT1	44.95712424	40.010371	1.123636775	4	4	74.48886068	50.08732749
chr3	149129584	149173196	3q24	HPS3	10.55187627	9.391307303	1.123579064	4	4	34.42410125	14.35888113
chr9	26840685	26892828	9p21.2	CAAP1	15.09677545	13.43632975	1.123578814	4	4	42.825845	24.40017278
chr7	105604774	105877772	7q22.3	ATXN7L1	4.028304801	3.585393084	1.123532262	4	4	45.66485619	32.75592561
chr15	73983218	73994636	15q24.1	STOML1	0.267997828	0.238533539	1.123522623	4	4	40.96688985	36.79857044
chr8	30095406	30137706	8p12	LEPROTL1	51.24549961	45.61339391	1.123474822	4	4	27.28992339	12.39092631
chr17	7315632	7329319	17p13.1	NEURL4	1.991605347	1.772735857	1.123464243	4	4	51.166628	42.63789018
chr1	109493606	109500441	1p13.3	CYB561D1	4.212307883	3.749943695	1.123298968	4	4	52.00456476	25.94170776
chr19	19623655	19628395	19p13.11	LPAR2	5.10669758	4.546418531	1.123235255	4	4	50.6015107	41.2191193
chr3	49108046	49120938	3p21.31	USP19	12.14646641	10.81431967	1.123183592	4	4	52.61163573	19.31367061
chr3	196568854	196589059	3q29	FBXO45	4.13362088	3.680335349	1.123164193	4	4	32.68181652	22.66811882
chr6	28080704	28104244	6p22.1	ZNF165	0.496553739	0.442118709	1.12312311	3	4	99.81643017	83.78282206
chrX	135942487	135947151	Xq26.3	CT45A11P	0.99131294	0.882701214	1.123044722	4	4	64.27580861	73.78950941
chr10	21756548	22003763	10p12.31	DNAJC1	17.02093875	15.15638349	1.123021119	4	4	8.600228506	6.175529677
chr16	14937443	14952060	16p13.11	NPIPA1	0.279263333	0.248672021	1.123018712	4	4	76.35623094	53.11381617
chr4	7965310	8158832	4p16.1	ABLIM2	0.160622993	0.143031233	1.122992435	3	4	121.1808579	78.38055724
chr15	42942897	43106088	15q15.2	UBR1	22.62365115	20.14662274	1.122950057	4	4	25.03551393	19.6899709
chr11	60841956	60851088	11q12.2	CCDC86	2.339712423	2.083597928	1.122919346	4	4	77.07186238	63.92225232
chr1	40477220	40496343	1p34.2	ZFP69	2.814511588	2.506437852	1.122912976	4	4	25.62405521	62.72308655
chr15	100602550	100651701	15q26.3	ASB7	12.38629703	11.03161908	1.122799558	4	4	33.19066716	14.90575879
chrX	9786406	9949443	Xp22.2	SHROOM2	0.051117578	0.045527949	1.122773588	1	2	0	49.41151423
chr1	89995095	90035531	1p22.2	ZNF326	4.88484459	4.350801767	1.122745841	4	4	39.3693259	27.00063695
chr3	120596281	120602411	3q13.33	NDUFB4	21.09572319	18.78958554	1.122734886	4	4	19.89295386	6.148181216
chr3	10284419	10285746	3p25.3	LINC00852	2.698846533	2.403820733	1.122732031	4	4	83.90018556	61.47466026
chr11	925809	1012245	11p15.5	AP2A2	3.001727701	2.673717195	1.122679581	4	4	46.8602936	34.77388793
chr7	127588352	127591705	7q32.1	ARF5	29.32313951	26.12001504	1.122631034	4	4	55.67953263	24.98614607
chr11	134123230	134125221	11q25	PTP4A2P2	15.48161932	13.79050379	1.122628989	4	4	50.30776877	48.48926605
chr19	41363966	41384083	19q13.2	TMEM91	5.03488415	4.484993659	1.122606749	4	4	80.02197414	63.27074463

chr9	120843075	120854373	9q33.2	CUTALP	4.578363823	4.078382981	1.122592911	4	4	112.6758152	76.73164882
chr19	21289554	21289998	19p12	VN1R83P	3.166782501	2.820962682	1.122589292	3	4	20.59238933	21.7325693
chr9	104080024	104092474	9q31.1	SMC2-AS1	0.038423318	0.034227449	1.122587837	1	2	0	0.336651076
chr15	43735934	43746298	15q15.3	CATSPER2P1	0.196110607	0.174695408	1.12258593	3	3	49.34101775	57.89939344
chr2	190204634	190320045	2q32.2	HIBCH	2.487634952	2.215994126	1.122581925	4	4	28.43801366	16.16040333
chr8	10764961	10839899	8p23.1	PINX1	1.635967052	1.457415687	1.122512312	4	4	64.74726996	35.42649626
chr8	117520713	117540262	8q24.11	MED30	10.81832701	9.639095206	1.122338433	4	4	28.82055904	19.7232813
chr19	2252251	2260813	19p13.3	JSRP1	0.342940634	0.305562836	1.122324426	4	2	84.50218333	67.69311866
chr7	142544236	142544685	7q34	TRBV10-3	3.843693379	3.424832052	1.122301275	3	4	106.8397818	66.46622972
chr2	130416750	130428546	2q21.1	FAR2P2	0.081668852	0.072772096	1.122255044	4	2	66.27076969	47.82926512
chr16	75627724	75647687	16q23.1	KARS	38.97132022	34.72993813	1.122124666	4	4	57.56632975	25.25950644
chr10	92593068	92655395	10q23.33	KIF11	38.19874496	34.04335875	1.122061582	4	4	71.48915366	33.04848523
chr2	218264127	218270209	2q35	AAMP	13.1361592	11.70796009	1.121985308	4	4	52.79573043	35.19095762
chr22	23180365	23318037	22q11.23	BCR	7.260660329	6.472289339	1.12180713	4	4	49.11664504	33.94822625
chr2	202912218	202988261	2q33.2	CARF	10.08571635	8.99076989	1.121785617	4	4	49.69102908	20.92470288
chr2	131469724	131493080	2q21.1	MZT2A	2.776235405	2.474904523	1.121754548	4	4	24.86868563	23.94546088
chr16	20730589	20741964	16p12.3	THUMPD1	21.87885495	19.5049735	1.121706469	4	4	31.87531161	14.89196927
chr12	102120171	102197520	12q23.2	PARPBP	37.62197855	33.54034148	1.121693366	4	4	73.45509596	45.34373801
chr5	153990131	154038938	5q33.2	FAM114A2	40.32244168	35.94979242	1.121632114	4	4	80.99197226	55.42644526
chr2	112541915	112577153	2q14.1	POLR1B	5.299485547	4.724936075	1.121599417	4	4	35.15795048	29.21233911
chr12	111894880	112013219	12q24.12-q24.13	TMEM116	4.009391853	3.574802535	1.121570161	4	4	60.73103297	57.85376364
chr2	216672105	216695549	2q35	IGFBP5	0.045209388	0.040311493	1.12150121	1	1	0	0
chr2	42335561	42368957	2p21	COX7A2L	15.49947052	13.82102096	1.121441792	4	4	18.49647451	9.672543017
chr10	110871795	110900006	10q25.2	PDCD4	81.50392231	72.67984163	1.12141029	4	4	33.92116891	21.57559146
chr3	107522936	107811329	3q13.12	BBX	22.30517348	19.89083732	1.121379312	4	4	15.52570219	11.47708154
chr11	6610817	6612244	11p15.4	TAF10	12.3995957	11.05776046	1.12134783	4	4	48.39496539	23.93371565
chr5	103086000	103120149	5q21.1	GIN1	15.95738711	14.23118828	1.121296887	4	4	38.78141023	24.31952863
chr9	62838445	62847206	9q13	PTGER4P2-CDK2AI	0.098874495	0.088184659	1.121221036	1	1	0	0
chr20	32207889	32238664	20q11.21	POFUT1	5.659999781	5.048590983	1.121104839	4	4	59.2526437	44.98924125
chr15	65578757	65611289	15q22.31	INTS14	6.041955036	5.389530469	1.121054064	4	4	34.7691766	28.76784949
chr19	47064187	47114437	19q13.32	ZC3H4	15.33880245	13.68387348	1.120940096	4	4	57.07239099	41.38826075
chr10	13156683	13158243	10p13	BTBD7P1	0.227092826	0.20259955	1.120895017	1	1	0	0
chr8	12721894	12755486	8p23.1	LONRF1	5.12808265	4.575491953	1.120771865	4	4	22.65925845	29.08272227
chr2	121337776	121649476	2q14.2-q14.3	CLASP1	16.00003226	14.27630836	1.12074017	4	4	13.95206413	6.140293045
chr16	23388493	23453215	16p12.2	COG7	2.470409488	2.204267595	1.120739376	4	4	27.67134501	50.25575011
chr13	78614286	78659179	13q31.1	RNF219	39.12304754	34.91242016	1.120605428	4	4	38.56323032	26.85998149
chr1	162561440	162599843	1q23.3	UAP1	7.131283632	6.364378395	1.120499629	4	4	29.49856443	23.46593614
chr9	131009117	131093059	9q34.12	LAMC3	0.038611284	0.034459162	1.120493995	1	1	0	0
chr7	107168961	107202529	7q22.3	HBP1	27.21937637	24.29257846	1.120481155	4	4	37.81652341	17.75358981
chr1	153804907	153923953	1q21.3	GATAD2B	3.610541339	3.222416327	1.120445334	4	4	34.10427674	22.12693361
chr1	173868082	173903547	1q25.1	ZBTB37	6.567771915	5.861964109	1.120404662	4	4	35.59353388	10.81714047
chr16	1776712	1782580	16p13.3	SPSB3	12.4131724	11.07920443	1.120402867	4	4	19.17509538	14.22064786
chr10	70163652	70164741	10q22.1	CALM2P2	0.925638659	0.826233634	1.120311036	3	3	44.10452646	35.17699031

chr17	18658429	18682259	17p11.2	ZNF286B	0.543178284	0.484866286	1.12026408	4	4	50.25168112	20.2969831
chr16	1809103	1827194	16p13.3	HAGH	85.61020049	76.42110624	1.120242884	4	4	43.22276479	7.437199606
chr12	110533245	110583259	12q24.11	PPTC7	14.31925715	12.78388432	1.120102215	4	4	51.48664495	25.5333683
chr11	130002624	130005486	11q24.3	LINC00167	0.835265673	0.745785893	1.119980521	3	4	128.3298999	30.14399332
chr9	122264603	122325315	9q33.2	MRRF	2.095782636	1.871304465	1.119958123	4	4	25.67897709	20.12861987
chr5	181249959	181261355	5q35.3	TRIM52	5.417640444	4.837830424	1.119849182	4	4	42.15379148	26.36519572
chr2	197391974	197435093	2q33.1	SF3B1	93.29439089	83.31222911	1.119816285	4	4	31.15501415	8.858182914
chr20	2864195	3038669	20p13	PTPRA	371.0511037	331.3585833	1.119787211	4	4	42.95933664	19.16348294
chr4	105682627	105708725	4q24	INTS12	7.988527275	7.134358513	1.119726078	4	4	25.66592291	17.35616972
chr20	38472806	38578861	20q11.23	RALGAPB	14.37061189	12.83418926	1.119713259	4	4	23.46219057	16.60585176
chr10	6088986	6117459	10p15.1	RBM17	11.69033578	10.4406778	1.119691269	4	4	35.83718954	26.66733868
chr15	41417104	41483563	15q15.1	RTF1	17.88881111	15.97698558	1.119661216	4	4	55.37030704	25.96965648
chr8	52622458	52714466	8q11.23	RB1CC1	52.68251238	47.05652835	1.119557992	4	4	17.79707514	26.08023904
chr18	32018482	32090756	18q12.1	RNF125	12.818875	11.45088294	1.119466077	4	4	34.6170906	21.27186916
chrX	151396555	151409364	Xq28	VMA21	19.26182431	17.20663204	1.119441868	4	4	15.6253388	17.25766722
chr15	43403064	43510728	15q15.3	TP53BP1	4.236939788	3.784979285	1.119408977	4	4	50.9760466	36.02827866
chr8	140658382	141002079	8q24.3	PTK2	12.66998101	11.32167542	1.119090642	4	4	49.71634891	12.80364499
chr14	58244805	58272009	14q23.1	PSMA3	19.52572412	17.44843964	1.119052736	4	4	19.68933273	34.52341427
chr11	65014113	65022185	11q13.1	ARL2	1.71007224	1.52819217	1.119016491	4	4	66.54310243	67.01607827
chr1	28329002	28335967	1p35.3	MED18	4.645206905	4.15126935	1.118984704	4	4	56.54367889	25.30391552
chr18	74148511	74158969	18q22.3	TIMM21	2.050020397	1.832038981	1.118982957	4	4	23.15215382	23.6799312
chr1	13698874	13825079	1p36.21	PRDM2	16.78932092	15.0048867	1.118923538	4	4	18.6082565	10.98656784
chr19	46480788	46601200	19q13.32	PPP5D1	0.103784054	0.092767394	1.118755734	3	3	37.04957928	47.79700532
chr14	54842005	54902824	14q22.2	GCH1	30.49351289	27.26011047	1.118612961	4	4	21.30645048	53.15280185
chr1	149899582	149900795	1q21.2	BOLA1	2.062903297	1.844185004	1.11859889	4	4	28.52498944	71.14786676
chr18	30989365	31042815	18q12.1	DSC3	0.041440093	0.037046774	1.11858843	1	1	0	0
chr16	29074842	29116718	16p11.2	RRN3P2	6.329278864	5.658352569	1.118572727	4	4	48.14396324	26.63508027
chr11	108222484	108369099	11q22.3	ATM	57.85916178	51.72668856	1.118555303	4	4	45.40095518	11.24933178
chr4	55853648	55905078	4q12	EXOC1	18.22897587	16.29830502	1.118458383	4	4	11.97372058	15.31968826
chr10	70597348	70602788	10q22.1	PRF1	168.2991884	150.4780637	1.118430051	4	4	34.84065697	70.35326381
chr13	28135021	28295338	13q12.2	PAN3	90.85208786	81.24136001	1.118298461	4	4	36.44209245	21.87384803
chr20	20034361	20056046	20p11.23	CRNKL1	24.13137615	21.58089792	1.118182211	4	4	26.10004817	14.0428431
chr22	39077005	39087743	22q13.1	APOBEC3G	17.6381023	15.77509037	1.118098337	4	4	35.96675218	73.92438263
chr12	911028	991195	12p13.33	RAD52	1.728827397	1.546280978	1.118055141	4	4	23.08139334	28.23350599
chr9	93096123	93113295	9q22.31	CARD19	6.901108531	6.172877912	1.117972626	4	4	57.62026499	24.86998279
chr14	24131466	24132849	14q12	FITM1	0.561622188	0.502381473	1.117919785	3	4	59.77146523	56.46984436
chr17	63550435	63594279	17q23.3	DCAF7	19.85909946	17.76474559	1.117893829	4	4	21.46123546	42.4806052
chr3	47850690	48089279	3p21.31	MAP4	4.02338947	3.599423539	1.117787175	4	4	38.8749556	50.99788726
chr16	1959516	1961975	16p13.3	NDUFB10	23.52801265	21.04899489	1.117773688	4	4	63.51719764	36.45566212
chr6	112070657	112087548	6q21	TUBE1	1.885488032	1.686980731	1.117670165	4	4	54.58146932	25.14454503
chr12	101728648	101739721	12q23.2	SYCP3	0.234949597	0.210249521	1.117479821	2	3	19.89181449	13.04085259
chr17	69062044	69141927	17q24.2-q24.3	ABCA6	0.118426708	0.105982636	1.117416147	4	4	67.46592593	41.5128501
chr19	797392	812327	19p13.3	PTBP1	19.81868071	17.7363519	1.117404572	4	4	43.25339577	38.41713058

chr1	205086142	205122022	1q32.1	RBBP5	13.78761881	12.33991139	1.117319109	4	4	33.85862469	30.19866396
chr2	219372029	219400022	2q35	DNPEP	3.632325745	3.250958633	1.117309125	4	4	54.97111238	28.95175437
chrX	120603889	120621159	Xq24	MCTS1	7.134387138	6.385440342	1.117289765	4	4	18.33229125	17.36688807
chr2	48530169	48598515	2p16.3	STON1	0.409656107	0.366655959	1.117276554	4	4	30.90968001	26.90902404
chr17	60443149	60526240	17q23.2	APPBP2	12.13547657	10.86199293	1.117242172	4	4	18.24728994	22.74948867
chr19	14689787	14733746	19p13.12	ZNF333	2.486996604	2.226073007	1.117212507	4	4	44.38592527	55.95614845
chr22	30285118	30289627	22q12.2	CASTOR1	0.32277963	0.288936322	1.117130681	3	3	31.1442872	64.92404604
chr19	9523224	9538865	19p13.2	ZNF426	6.04102124	5.408115946	1.117028795	4	4	32.18327134	20.16995964
chrX	21839538	21885423	Xp22.12	MBTPS2	1.471945927	1.317741833	1.117021476	4	4	39.41240337	12.99571014
chr7	99027435	99144120	7q22.1	SMURF1	5.145918391	4.607073891	1.116960247	4	4	56.44136417	16.43519377
chr15	67819704	67834561	15q23	SKOR1	0.219271726	0.196311994	1.116955319	2	4	17.62001291	44.6146335
chr19	17816513	17821574	19p13.11	INSL3	1.601542909	1.433888375	1.116923003	4	4	61.93238061	54.6683243
chr12	122752821	122771064	12q24.31	DENR	22.55370738	20.19572163	1.116756697	4	4	29.86632514	23.32375811
chr21	43565189	43565648	21q22.3	H2BFS	0.710659631	0.636387459	1.116709044	1	2	0	16.6704535
chr11	236808	252984	11p15.5	PSMD13	17.20147628	15.40389113	1.116696823	4	4	29.76747762	14.36711573
chr11	112086824	112095801	11q23.1	SDHD	24.99142986	22.38096503	1.116637725	4	4	8.015769178	14.6838829
chr9	122761850	122762656	9q33.2	SKA2P1	1.270857677	1.138140671	1.116608613	1	3	0	25.35179609
chr19	17351448	17377349	19p13.11	PLVAP	27.05754445	24.23462978	1.116482682	4	4	50.33914403	62.17686213
chr17	47896032	47932271	17q21.32	SP2	4.486749383	4.018804105	1.116438937	4	4	57.34229519	41.14563993
chr2	27444373	27489819	2p23.3	IFT172	1.507967791	1.350740972	1.116400422	4	4	25.93327727	33.94615008
chr1	235166895	235328224	1q42.3	ARID4B	38.75125891	34.71267568	1.116343185	4	4	36.07148351	30.88308099
chr11	66435075	66438860	11q13.2	MRPL11	4.231606461	3.790724362	1.116305502	4	4	76.76312916	49.47424987
chr21	42653752	42775509	21q22.3	PDE9A	0.54494177	0.488201914	1.11622211	4	4	103.2968534	110.2151013
chrX	154886360	154888061	Xq28	F8A1	8.862719421	7.940248691	1.116176554	4	4	65.20139677	32.61951609
chr12	101393120	101407820	12q23.2	ARL1	11.17556019	10.01324137	1.116078178	4	4	12.3185277	13.83726058
chr5	36192589	36242279	5p13.2	NADK2	4.961374389	4.445552756	1.116030933	4	4	26.63373853	9.960885452
chr14	22545037	22545098	14q11.2	TRAJ1	10.75280308	9.635140835	1.115998537	3	2	9.808347967	46.01427103
chr7	140696681	140706646	7q34	NDUFB2	4.07441013	3.651219047	1.115904052	4	4	18.3089044	25.8538503
chr1	86914652	87109998	1p22.3	HS2ST1	6.595547104	5.910575801	1.115889099	4	4	19.66951073	21.92555885
chr8	232137	247340	8p23.3	ZNF596	0.678593199	0.608119857	1.115887257	4	4	64.79754039	17.49661198
chr14	96502376	96567116	14q32.2	PAPOLA	57.87032755	51.86203157	1.115851535	4	4	18.8279779	30.82640136
chr16	2223488	2235742	16p13.3	E4F1	2.3385219	2.095795707	1.11581577	4	4	73.87532109	11.7073342
chr18	12661956	12702777	18p11.21	CEP76	5.563504273	4.986090779	1.11580485	4	4	58.62895348	35.55242708
chr17	28357581	28363683	17q11.2	TMEM199	4.977356174	4.461421723	1.115643506	4	4	55.99680853	8.789716218
chr18	26016253	26091217	18q11.2	SS18	18.66860694	16.73401901	1.115608087	4	4	25.61089334	11.57313175
chr19	12922470	12933744	19p13.13	FARSA	29.5557342	26.49314998	1.115599097	4	4	11.92107705	25.10207056
chr2	216109297	216206293	2q35	XRCC5	104.2905342	93.48809103	1.115548868	4	4	38.48062185	23.3318398
chr19	52927135	52942594	19q13.41	ZNF321P	0.574511179	0.515012881	1.115527787	3	3	60.88804143	42.33059614
chr3	48744601	48847850	3p21.31	PRKAR2A	13.68517646	12.26797668	1.115520254	4	4	47.30489742	11.28581767
chr3	16315845	16513715	3p24.3	RFTN1	14.23424853	12.76093599	1.115454896	4	4	45.12881195	28.81133525
chr4	41359607	41700044	4p13	LIMCH1	0.083340781	0.074720354	1.115369183	3	4	62.04030306	59.99126239
chr1	207645113	207723691	1q32.2	CR1L	104.8796471	94.04113849	1.115252843	4	4	76.86223779	44.41508546
chr1	155247205	155255892	1q22	FAM189B	0.27987018	0.250959032	1.115202663	4	4	108.685388	76.46144881

chr8	144291569	144314726	8q24.3	HSF1	7.661056236	6.870045066	1.115139153	4	4	50.2753794	36.3871879
chr9	105694525	105776611	9q31.2	TMEM38B	5.724702092	5.133881429	1.115082647	4	4	64.16880892	22.77287702
chr3	50354749	50359508	3p21.31	TMEM115	11.55179932	10.35988846	1.11505055	4	4	55.70490002	14.70828145
chr16	2520345	2531422	16p13.3	AMDHD2	3.162425465	2.836303602	1.114981296	4	4	84.11748343	27.83174316
chr19	52702813	52735054	19q13.41	ZNF611	7.23481014	6.488974384	1.114938927	4	4	24.86155925	26.76367479
chr5	69560136	69592532	5q13.2	GTF2H2C	3.878629265	3.478808246	1.114930456	4	4	43.53382394	49.42755481
chr10	16823966	17130492	10p13	CUBN	0.477487667	0.428271155	1.114919046	4	4	71.84111798	37.296012
chr2	87455427	87521518	2p11.2	CYTOR	18.91043491	16.96159749	1.114897044	4	4	43.26839305	60.53638822
chr14	45203190	45257190	14q21.2	MIS18BP1	73.29410823	65.75541674	1.11464746	4	4	25.85859109	18.578184
chr12	6650538	6663143	12p13.31	ING4	8.959603152	8.038465791	1.11459119	4	4	27.47808986	19.99759909
chr18	23452823	23483140	18q11.2	RIOK3	436.9688768	392.0865881	1.114470349	4	4	27.58691186	71.99004819
chr20	34235012	34311976	20q11.22	AHCY	6.092646298	5.466914253	1.114457995	4	4	77.85660442	59.92793269
chr18	10525876	10552769	18p11.22	NAPG	14.81867014	13.29773333	1.114375644	4	4	8.701441495	8.311540727
chr14	55027126	55049489	14q22.3	SOCS4	10.82407014	9.713128296	1.114375288	4	4	27.59029188	12.46805184
chr10	132184983	132205776	10q26.3	DPYSL4	0.361244	0.324182998	1.114321238	3	3	65.48835276	36.90645827
chr22	45760524	45760598	22q13.31	MIR4762	5.430085802	4.873032311	1.114313523	1	2	0	12.55444362
chr19	36009116	36014243	19q13.12	ALKBH6	0.517899195	0.464782038	1.114284012	4	4	130.4592533	38.63310609
chr12	14786478	14803467	12p12.3	WBP11	19.99942658	17.95400889	1.113925402	4	4	43.15434955	18.20315428
chr6	13304951	13328583	6p24.1	TBC1D7	1.741004002	1.563030973	1.113864045	4	4	50.12665115	67.37848749
chr11	57406954	57427580	11q12.1	SLC43A3	3.246984296	2.915143183	1.113833555	4	4	42.73477641	33.1305754
chr2	159235793	159286799	2q24.2	WDSUB1	9.144773691	8.210756509	1.113755314	4	4	64.41603328	44.35508374
chr7	77122617	77295204	7q11.23	CCDC146	6.604613851	5.93118687	1.113540004	4	4	50.75265571	43.5457395
chr4	36281593	36347511	4p14	DTHD1	3.970958346	3.566132093	1.113519702	4	4	37.76890723	29.43960361
chr14	77394021	77426013	14q24.3	NOXRED1	0.572227363	0.513894799	1.113510711	2	4	54.55495262	41.19899086
chr16	14071072	14266779	16p13.12	MRTFB	0.793179034	0.712410184	1.113374081	4	4	51.86325184	16.70923772
chr7	102456220	102464874	7q22.1	ALKBH4	2.209049071	1.984110306	1.113370091	4	4	86.4965294	66.5862133
chr11	94962622	94973612	11q21	CWC15	15.76227621	14.15833209	1.113286234	4	4	25.56380138	17.78478739
chr5	37063928	37249499	5p13.2	CPLANE1	7.77081117	6.980298996	1.113249042	4	4	55.19996524	14.59778386
chr1	114567557	114581644	1p13.2	BCAS2	36.76927853	33.02951502	1.113224899	4	4	28.69876084	6.698074013
chr11	64226258	64230016	11q13.1	NUDT22	3.794026295	3.408197851	1.113206	4	4	54.21236297	24.02793752
chr17	49248243	49258665	17q21.32	FLJ40194	0.257175856	0.231031865	1.113161837	4	2	57.36825172	78.71088979
chr3	96617186	96618223	3q11.2	MTRNR2L12	1.351083183	1.213798593	1.11310327	4	4	45.31740019	53.92254668
chr16	67943474	67968694	16q22.1	SLC12A4	1.629093176	1.463678204	1.11301321	4	4	68.94931426	31.25415377
chr16	53054982	53327502	16q12.2	CHD9	37.84152642	34.00411172	1.112851491	4	4	17.68482502	6.163504408
chr4	6640091	6642745	4p16.1	MRFAP1	62.9867992	56.59999303	1.112841112	4	4	28.11107452	11.02527467
chr7	2537810	2555758	7p22.3	BRAT1	3.511182742	3.155366049	1.112765583	4	4	65.66925736	19.04829854
chr11	119067752	119081978	11q23.3	VPS11	5.588615215	5.022732774	1.112664254	4	4	46.74878888	53.00355293
chr14	88562909	88613509	14q31.3	ZC3H14	4.837042855	4.347919152	1.112496044	4	4	37.81340315	17.0470218
chr19	36573070	36594716	19q13.12	ZNF529-AS1	1.367221862	1.229009234	1.112458576	4	4	65.40835337	49.97923971
chr3	114234631	114237578	3q13.31	ZNF80	0.744862545	0.669569452	1.112450013	4	4	56.50640307	48.81695706
chr1	149843039	149843510	1q21.2	HIST2H2BD	0.714523531	0.642304883	1.11243671	2	4	27.36293042	28.3244182
chr6	41072968	41102407	6p21.1	NFYA	15.40646833	13.85048915	1.1123411	4	4	45.13044203	32.34927492
chr19	5691834	5720452	19p13.3	LONP1	4.50083593	4.046466379	1.112287984	4	4	51.00350908	38.53545219

chr2	101252883	101270316	2q11.2	CNOT11	34.44028498	30.96476111	1.112241262	4	4	22.37063011	12.72164866
chr8	123319850	123396465	8q24.13	ATAD2	124.6223988	112.0531994	1.112171713	4	4	72.31950612	28.15168906
chr2	53970838	54305300	2p16.2	ACYP2	11.59686762	10.42723879	1.112170523	4	4	76.28000028	60.6652301
chr19	21082147	21125094	19p12	ZNF714	2.776670964	2.496712102	1.112131015	4	4	46.19666459	7.943704015
chr9	133361449	133377949	9q34.2	SURF4	5.864088788	5.27309986	1.112076188	4	4	52.1186954	34.0653682
chr17	44034635	44067619	17q21.31	LSM12	5.999636689	5.395042834	1.1120647	4	4	52.89707767	31.85260999
chr6	155090289	155257723	6q25.2-q25.3	TIAM2	3.068738882	2.759557013	1.112040399	4	4	60.74872357	42.67218308
chr7	54752247	54759246	7p11.2	SEC61G	20.33818683	18.29033722	1.11196347	4	4	35.63310095	33.25128237
chrX	70133449	70166324	Xq13.1	IGBP1	34.4307026	30.96755636	1.111831434	4	4	26.8313672	13.20742795
chr7	44999746	45076470	7p13	CCM2	9.682146217	8.708751219	1.111772052	4	4	38.17140515	28.20705744
chr19	9648611	9675293	19p13.2	ZNF562	6.765435064	6.085306512	1.111765702	4	4	27.46058457	24.23910248
chr15	75674322	75712848	15q24.2	CSPG4	0.03544647	0.031883307	1.111756371	1	2	0	6.628315829
chr13	102845841	102876001	13q33.1	ERCC5	2.118368383	1.905664381	1.111616717	4	4	60.29906648	4.357216893
chr22	39514494	39522686	22q13.1	ATF4	83.06787219	74.73355071	1.11152048	4	4	64.4188013	26.98816449
chr3	194685893	194689037	3q29	FAM43A	2.839878242	2.554961234	1.111515198	4	4	72.2156843	72.55718526
chr4	14470465	14888169	4p15.33	LINC00504	0.455588333	0.409891733	1.111484559	4	4	33.94763374	49.11080206
chr11	77616151	77637806	11q14.1	CLNS1A	79.61186837	71.63139792	1.111410229	4	4	46.5785126	28.66525371
chr6	99542387	99568814	6q16.2	CCNC	12.66193006	11.39366386	1.111313289	4	4	15.90065978	9.422823114
chr8	66630253	66667217	8q13.1	VCPIP1	49.49485648	44.54324532	1.111164131	4	4	38.4049102	24.31972672
chr11	64305528	64316743	11q13.1	ESRRA	5.32551184	4.793178544	1.111060602	4	4	43.09878755	34.15291489
chr5	115520908	115544894	5q22.3	FEM1C	12.69437454	11.42708847	1.110901921	4	4	26.91135639	14.93809749
chr8	57994509	58149718	8q12.1	FAM110B	0.709071108	0.638300936	1.110872738	4	4	74.88778016	68.97968635
chr20	8132265	8884900	20p12.3	PLCB1	1.635129754	1.472012686	1.110812271	4	4	58.8927845	46.08346791
chr12	57229573	57234935	12q13.3	SHMT2	7.124408404	6.413792419	1.110794977	4	4	73.89104241	56.68361236
chr7	100706053	100707500	7q22.1	POP7	9.661392745	8.698317527	1.110719713	4	4	26.3831684	42.47939293
chr19	49446293	49452392	19q13.33	PIH1D1	7.219979741	6.500399761	1.110697804	4	4	50.59624855	42.7620998
chr19	57905411	57916616	19q13.43	ZNF417	6.273448141	5.648404063	1.110658528	4	4	38.09469606	23.29435649
chr4	38966009	39032668	4p14	TMEM156	9.86716184	8.884159098	1.110646684	4	4	40.84056253	22.54516569
chr17	4945650	4949088	17p13.2	PFN1	395.8727914	356.4435833	1.11061837	4	4	81.87092278	74.50291237
chr2	89319625	89320099	2p11.2	IGKV1-39	2.794669319	2.516319947	1.110617639	3	3	49.29743228	91.13251279
chr17	18225593	18244875	17p11.2	LLGL1	1.664639809	1.498841526	1.110617621	4	4	64.25317939	31.45136647
chr19	8308603	8321413	19p13.2	NDUFA7	0.550085494	0.495299217	1.110612484	2	4	81.66548038	75.48686954
chr16	31090842	31094999	16p11.2	VKORC1	5.366877516	4.832681284	1.110538271	4	4	64.71602312	32.84062326
chr20	35772001	35950372	20q11.22-q11.23	PHF20	27.7682008	25.00585406	1.110468002	4	4	20.96882166	20.69424605
chr21	16070488	16631727	21q21.1	MIR99AHG	0.047630055	0.042893684	1.110421181	3	2	14.18359165	3.024403104
chr16	30956618	30984664	16p11.2	SETD1A	3.332817672	3.001593931	1.110349284	4	4	85.20848888	61.32368193
chr3	138160988	138174949	3q22.3	DBR1	7.396814132	6.662231338	1.110260775	4	4	27.47602002	24.06905682
chr11	67468179	67491108	11q13.2	AIP	19.94535399	17.96458738	1.110259511	4	4	56.57674486	38.40858638
chr14	67652229	67652629	14q24.1	COX7A2P1	1.269418301	1.143518495	1.110098618	3	2	40.76923999	52.73795109
chr20	472498	543838	20p13	CSNK2A1	27.28334599	24.57745621	1.110096413	4	4	33.90457192	17.31553181
chr2	224470150	224585397	2q36.2	CUL3	24.25997633	21.85549251	1.11001737	4	4	28.1512029	22.54138566
chr20	5544434	5611046	20p12.3	GPCPD1	96.77517866	87.18543286	1.109992524	4	4	41.78191193	50.87973366
chr17	82614562	82648535	17q25.3	WDR45B	12.70419505	11.44550765	1.109972178	4	4	58.59522386	12.29594898

chr2	111098341	111115588	2q13	ACOXL-AS1	0.08921352	0.080379355	1.109905885	1	2	0	4.350144165
chr5	70924941	70953015	5q13.2	SMN1	1.576257503	1.420276127	1.109824683	4	4	51.59129141	33.20041174
chr11	65354811	65357613	11q13.1	TIGD3	4.148200056	3.737834385	1.109787013	4	4	64.33817569	87.93498254
chr1	155687902	155739010	1q22	DAP3	45.89070012	41.35119143	1.109779393	4	4	42.87638016	27.91636843
chr3	138494339	138594383	3q22.3	CEP70	53.65338655	48.34942399	1.109700636	4	4	66.06569194	30.40032544
chr5	176905005	177022640	5q35.2	UIMC1	125.4414441	113.04715	1.109638271	4	4	56.48953972	19.8551957
chr11	809936	812876	11p15.5	RPLP2	383.7689956	345.8560384	1.109620631	4	4	46.77728756	27.39767884
chr12	6666477	6689572	12p13.31	ZNF384	9.125159606	8.22416666	1.109554315	4	4	41.00850531	32.96681137
chr14	75985753	76084073	14q24.3	IFT43	0.634445037	0.571821034	1.109516788	3	4	54.40341385	44.68141133
chr2	218568578	218596435	2q35	CNOT9	17.52090321	15.79214608	1.109469424	4	4	34.31459175	10.41722441
chr12	100008987	100009894	12q23.1	RPS4XP1	0.556955224	0.502001613	1.109468994	3	2	18.72713239	50.70690305
chr7	127342871	127392798	7q31.33	ZNF800	25.99246003	23.42822496	1.109450677	4	4	31.285701	7.680987953
chr1	155913043	155934442	1q22	KHDC4	32.97828642	29.72554649	1.10942574	4	4	40.72238443	21.01147212
chr4	7040849	7043001	4p16.1	CCDC96	0.605332716	0.545638208	1.109403095	4	3	28.31919053	88.76206123
chr10	44292088	44385097	10q11.21	CXCL12	0.046821688	0.042205175	1.109382649	3	3	3.22936894	1.406494566
chr10	102714518	102739190	10q24.32	SFXN2	0.63842283	0.575489559	1.109356061	4	4	48.87092199	35.27833522
chr11	94543851	94549898	11q21	FUT4	2.826415108	2.547859857	1.10932911	4	4	61.93498066	7.969225374
chr10	67884669	67918390	10q21.3	SIRT1	21.80473673	19.65612796	1.109309869	4	4	35.05479016	24.19033244
chr22	22343224	22343734	22q11.22	IGLV9-49	1.599540802	1.441952706	1.109287978	3	2	67.00978343	61.65108586
chr16	11868128	11916662	16p13.13	GSPT1	294.452631	265.4441942	1.109282619	4	4	26.08367145	29.25861219
chr19	57191500	57222846	19q13.43	ZNF264	6.586448576	5.937722403	1.109255052	4	4	38.68427	22.67013591
chr3	170864875	170870256	3q26.2	RPL22L1	13.3858575	12.06819058	1.109185127	4	4	35.35491025	24.9231722
chr19	9835207	9849689	19p13.2	PIN1	3.900137328	3.516235388	1.109179818	4	4	46.60862587	42.94291019
chr17	75516508	75524739	17q25.1	TSEN54	4.750060259	4.282700063	1.109127464	4	4	42.56282058	31.1933344
chr5	133194827	133842066	5q31.1	FSTL4	0.178951713	0.161346222	1.109116223	4	4	85.98764733	40.04597101
chr22	44668547	44737681	22q13.31	PRR5	6.045761053	5.451130065	1.109083985	4	4	13.59571843	34.66272089
chr6	111299031	111483715	6q21	REV3L	21.0745949	19.00244477	1.109046502	4	4	30.0358698	13.54204767
chr11	18480311	18526956	11p15.1	TSG101	27.55292192	24.84421323	1.109027751	4	4	11.22218869	29.72226897
chrX	108439844	108697545	Xq22.3	COL4A5	0.033170813	0.029910451	1.109004111	1	1	0	0
chr10	15015072	15035425	10p13	DCLRE1CP1	0.658089585	0.593445688	1.108929761	3	1	20.12786505	0
chr1	92298965	92388175	1p22.1	RPAP2	7.870146361	7.097362165	1.108883297	4	4	54.52126941	32.50504018
chr3	98795970	98901692	3	DCBLD2	0.984811846	0.888133467	1.108855688	4	4	73.28961338	14.77874013
chr22	38111495	38192100	22q13.1	PLA2G6	2.247322209	2.026715923	1.108849141	4	4	47.94956026	8.494226518
chr15	79236332	79237205	15q25.1	HNRNPCP3	0.324384545	0.29255667	1.108792171	1	1	0	0
chr5	95885098	95962071	5q15	ELL2	20.74366459	18.70853402	1.108780868	4	4	16.53605757	48.06728293
chr2	143070973	143768352	2q22.2-q22.3	ARHGAP15	43.94615032	39.63579335	1.108749103	4	4	24.91596947	27.24709976
chr2	6917418	7076886	2p25.1	RNF144A	6.348039451	5.725533132	1.108724603	4	4	37.56818032	30.57719371
chr7	98106868	98209636	7q21.3	LMTK2	15.65515757	14.12024494	1.108702974	4	4	33.15392606	9.947339316
chr1	9848276	9910322	1p36.22	CTNNBIP1	4.073941209	3.674572009	1.108684549	4	4	39.90926676	18.94344409
chr16	53434420	53491648	16q12.2	RBL2	73.84383462	66.60937453	1.108610239	4	4	25.47922466	14.30054252
chr2	129979662	129982738	2q21.1	RAB6C	0.253209236	0.228407766	1.108584179	4	2	52.72696393	89.85017118
chr2	61145068	61164829	2p15	C2orf74	0.932699612	0.841387661	1.108525422	3	4	75.44512033	69.64967775
chr14	74285277	74303064	14q24.3	ABCD4	1.72613173	1.557179363	1.108498977	4	4	71.14124717	53.34162373

chr13	29936531	29950488	13q12.3	LINC00544	0.184982998	0.166881447	1.108469521	2	2	26.65690309	10.11275705
chr11	65261852	65306269	11q13.1	POLA2	2.680167555	2.418006914	1.108420137	4	4	34.87685663	29.30686385
chr9	110048598	110172512	9q31.3	AKAP2	0.205587662	0.185499342	1.108293216	3	4	56.60951214	91.08806404
chr3	156674416	156706770	3q25.31	TIPARP	28.14485121	25.39629522	1.108226652	4	4	13.2342826	23.96385936
chr12	57028517	57050765	12q13.3	MYO1A	0.21905206	0.197668936	1.108176451	4	3	32.34054207	41.51649184
chr6	57088970	57170305	6p12.1	ZNF451	49.36271005	44.54551528	1.10814096	4	4	60.89825705	36.42026673
chr9	111360693	111484745	9q31.3	ECPAS	30.84731759	27.83879518	1.108069418	4	4	13.01949536	11.31728152
chr17	32328441	32350049	17q11.2	C17orf75	1.654963196	1.493575781	1.108054387	4	4	37.98548435	28.090877
chr6	33771213	33794274	6p21.31	LEMD2	5.932364927	5.35390273	1.108044958	4	4	51.78238869	15.55696341
chr1	202878282	202889257	1q32.1	RABIF	8.369505906	7.554188362	1.107929205	4	4	16.73564713	21.84951734
chr12	74537771	74541452	12q21.1	ATXN7L3B	15.62161941	14.10029278	1.107893265	4	4	45.24500865	12.89581066
chr7	129830732	129952960	7q32.2	UBE2H	102.0352601	92.09915648	1.107884849	4	4	13.82697919	62.14346657
chr16	67657512	67660832	16q22.1	ACD	3.549694872	3.204162361	1.10783864	4	4	42.00220799	46.21640303
chr8	67062417	67198003	8q13.1-q13.2	CSPP1	61.63742917	55.64415298	1.107707205	4	4	57.30327059	32.65436925
chr1	150646225	150697196	1q21.3	GOLPH3L	10.51318537	9.491543301	1.107637087	4	4	36.42662464	12.34615581
chr14	35278558	35317479	14q13.2	PSMA6	2.495679407	2.253241044	1.107595396	4	4	30.5695196	26.26429558
chr2	61065870	61138034	2p15	KIAA1841	6.729606277	6.075912689	1.107587719	4	4	90.53583388	55.23568355
chr11	95089810	95132651	11q21	ENDOD1	36.54382357	32.99884498	1.107427354	4	4	38.78728002	15.87038941
chr7	150625375	150632648	7q36.1	GIMAP6	46.70574845	42.17790957	1.107350955	4	4	52.66634874	28.56666658
chr11	63759892	63768642	11q13.1	C11orf95	0.751252564	0.678503651	1.107219634	4	4	29.63340665	32.58123267
chr3	129249575	129277773	3q21.3	COPG1	24.23648548	21.89241575	1.107072228	4	4	35.21131898	30.40849133
chr16	14435700	14630286	16p13.12	PARN	83.64906009	75.56551569	1.106973986	4	4	61.51157328	34.21005427
chr2	63117851	63119542	2p15	DBIL5P2	0.171189203	0.154647694	1.106962528	1	2	0	2.759498762
chr12	15322257	15598331	12p13-p12	PTPRO	1.35850012	1.22738069	1.106828657	4	4	36.95040338	46.2045342
chr11	61481113	61490928	11q12.2	PPP1R32	1.150093641	1.039143421	1.106770843	4	3	78.44917277	10.03623448
chr22	49757557	49759053	22q13.33	RPL5P35	0.31053325	0.280586459	1.106729281	2	1	18.57912559	0
chr10	31992087	31992381	10p11.22	RN7SL825P	1.173692189	1.060515746	1.106718305	1	2	0	17.14229791
chr19	40447784	40465818	19q13.2	BLVRB	242.7234333	219.3250166	1.106683757	4	4	38.99680053	44.70334854
chr1	179954773	180114880	1q25.2	CEP350	56.74966196	51.28373854	1.106582	4	4	23.91140226	11.89441223
chr14	35825596	35871963	14q13.2	BRMS1L	1.518056776	1.371868387	1.106561526	4	4	16.474672	35.1512323
chr5	173144442	173164387	5q35.1	BNIP1	2.866486688	2.590577926	1.106504714	4	4	54.64559194	26.85618079
chr15	79922771	79924854	15q25.1	ST20-AS1	1.818962001	1.643951388	1.106457292	4	4	73.07400594	57.7661094
chr15	56090533	56247654	15q21.3	RFX7	8.51733504	7.698334793	1.106386676	4	4	30.51936932	31.73501201
chr10	43385617	43409248	10q11.21	HNRNPF	100.432682	90.78719734	1.106242785	4	4	50.27467709	46.19892133
chr3	30606472	30694142	3p24.1	TGFBR2	87.00571135	78.65202693	1.106210669	4	4	39.53181465	31.39420181
chr3	50329786	50340936	3p21.31	RASSF1	7.706006036	6.968158714	1.105888421	4	4	53.43434902	9.231099575
chr16	1782923	1789191	16p13.3	NUBP2	1.785784233	1.614797867	1.105887164	4	4	68.91723081	35.96768638
chr11	73400478	73598189	11q13.4	FAM168A	6.272288823	5.671853698	1.105862238	4	4	51.56788848	16.57928066
chr11	10750987	10779755	11p15.4	CTR9	22.68132177	20.5116916	1.105775292	4	4	42.61827925	39.07412264
chr19	12763002	12778484	19p13.13	HOOK2	0.607604793	0.549489946	1.105761438	4	4	84.61571819	22.60642526
chr9	65287480	65321282	9q21.11	CBWD4P	1.419053223	1.283564381	1.105556717	3	3	71.57400548	41.18016534
chr16	19167834	19268334	16p12.3	SYT17	0.173153981	0.15662326	1.105544481	4	4	95.39827057	37.00878159
chr6	154733378	154834244	6q25.2	SCAF8	57.04434945	51.59844849	1.10554389	4	4	5.28665638	13.3977648

chr12	54016852	54056030	12q13.13	HOXC4	0.333373021	0.301547911	1.10553915	4	4	42.78978949	78.90524479
chr17	39728500	39730563	17q12	MIEN1	7.226651672	6.537145317	1.105475146	4	4	27.92358576	27.24673535
chr9	124862118	124877733	9q33.3	ARPC5L	5.123063455	4.634308295	1.105464533	4	4	53.5705582	36.51625206
chr20	18467389	18484648	20p11.23	POLR3F	5.884968947	5.323936701	1.10537921	4	4	28.31824982	20.34238865
chr19	48630560	48637550	19q13.33	DBP	2.481615299	2.245085256	1.105354593	4	4	60.10953105	26.41125536
chr1	43699724	43707341	1p34.2-p34.1	KDM4A-AS1	0.303734446	0.274788826	1.105337688	2	2	62.91788998	71.97724908
chr1	114769479	114780687	1p13.2	SIKE1	15.01640884	13.58545797	1.105329601	4	4	5.28485502	26.0916577
chrX	100973359	101052118	Xq22.1	TRMT2B	13.62336125	12.32529814	1.105316974	4	4	37.26773993	13.96730784
chr17	82442586	82450831	17q25.3	CYBC1	15.39752372	13.9315212	1.105229178	4	4	59.77305555	32.52265568
chr10	112424411	112447572	10q25.2	ZDHHC6	12.16038455	11.00274839	1.105213363	4	4	30.40106498	18.98938543
chr12	19404101	19522239	12p12.3	AEBP2	13.08424601	11.83947129	1.105137695	4	4	30.17630091	17.2536347
chr2	32011649	32039840	2p22.3	DPY30	8.649532927	7.827197836	1.105061238	4	4	26.42552979	22.68437852
chr9	93446494	93453601	9q22.31	FAM120AOS	13.05916663	11.81938158	1.10489424	4	4	47.1023999	16.61340559
chr12	108561463	108569384	12q23.3	ISCU	53.49367806	48.4180882	1.104828382	4	4	28.72592953	26.28492669
chr17	60177231	60422470	17q23.1-q23.2	USP32	130.6873498	118.3014658	1.104697637	4	4	9.879813725	24.50378178
chr2	98997261	99154966	2q11.2	TSGA10	7.048438107	6.380879395	1.104618607	4	4	49.19430666	26.87242308
chr6	87513935	87590032	6q15	RARS2	24.26781027	21.97413522	1.104380674	4	4	27.0502372	15.12944983
chr9	122685740	122688631	9q33.2	TLK1P1	0.115007704	0.104138232	1.104375419	3	2	14.67287903	5.308820213
chr7	87152361	87196332	7q21.12	DMTF1	13.65795024	12.3672389	1.10436536	4	4	34.61636215	9.461463147
chr7	77657660	77697345	7q11.23	APTR	1.017329143	0.921191951	1.104361737	4	4	75.17704541	50.86144782
chr2	105249423	105329714	2q12.1-q12.2	TGFBRAP1	5.621435743	5.090326038	1.10433707	4	4	45.85259938	22.39829552
chr22	17628855	17730855	22q11.21	BCL2L13	21.78092771	19.72401076	1.104284924	4	4	15.27203036	43.36865889
chr5	34656303	34832612	5p13.2	RAI14	0.071202723	0.064481185	1.104240299	4	3	91.63987339	81.04936411
chr20	49046246	49096960	20q13.13	CSE1L	94.66762193	85.73650274	1.10416939	4	4	57.59954519	22.96116439
chr13	114281584	114305817	13q34	UPF3A	5.886246963	5.331237086	1.10410527	4	4	48.99611676	46.57548766
chr9	5764061	5867091	9p24.1	ERMP1	6.849442801	6.203861425	1.104061218	4	4	27.21411894	39.20577296
chr3	37052626	37176817	3p22.2	LRRFIP2	31.95542825	28.94861134	1.103867397	4	4	25.66963701	21.98466621
chr2	32628020	32821051	2p22.3	TTC27	17.89029853	16.20700062	1.103862396	4	4	63.97926979	19.88831322
chr3	133426402	133491146	3q22.1	BFSP2-AS1	0.609904082	0.55260221	1.103694613	3	2	49.64081661	75.43657642
chr7	128862803	128865849	7q32.1	ATP6V1F	43.92214534	39.79583534	1.10368698	4	4	43.16472296	21.69642834
chr5	112861188	112893079	5q22.2	SRP19	4.712521004	4.269959026	1.103645486	4	4	24.68231254	30.41569141
chr12	131711087	131799738	12q24.33	SFSWAP	6.72332318	6.092189246	1.103597231	4	4	59.22196709	16.26307849
chr2	95122987	95165187	2q11.1	ZNF514	1.565535133	1.418595616	1.103580975	4	4	52.21559434	28.29213162
chr5	139410203	139439525	5q31.2	DNAJC18	0.572763815	0.519039131	1.103507965	4	4	47.78154655	13.70084513
chr11	96352765	96389924	11q21	CCDC82	9.302371346	8.429947692	1.103490993	4	4	35.56509368	16.12005502
chr1	54053572	54112519	1p32.3	TCEANC2	2.448866655	2.219247165	1.10346729	4	4	51.33976687	37.95197578
chr13	113490738	113550229	13q34	TMCO3	4.599840355	4.168806709	1.10339497	4	4	31.53639312	27.32531646
chr9	91213815	91361969	9q22.31	AUH	11.48863418	10.41341334	1.103253449	4	4	18.02975355	8.020971826
chr3	113947901	113998002	3q13.31	ZDHHC23	0.581344714	0.526945808	1.103234345	4	4	52.78637719	53.90148642
chr2	102713867	102736888	2q12.1	MFSD9	3.511560688	3.183045904	1.10320768	4	4	55.2701539	43.10852494
chr3	48598999	48609665	3p21.31	UQCRC1	12.98056948	11.76649317	1.103180812	4	4	46.27170199	37.58550542
chr5	140245050	140303104	5q31.3	PFDN1	11.9294707	10.8143834	1.103111501	4	4	48.60383406	26.78980232
chr1	175320781	175743702	1q25.1	TNR	0.046493454	0.042148128	1.103096533	3	1	91.73285584	0

chr8	67043079	67062327	8q13.1	COPS5	5.187336405	4.702686753	1.103058034	4	4	15.51599226	18.61507761
chr4	82900650	82920283	4q21.22	THAP9	2.911244458	2.639299226	1.103036908	4	4	45.32094327	14.882303
chr2	182140035	182523375	2q32.1	PDE1A	0.045499787	0.041252353	1.102962218	1	1	0	0
chr8	54046367	54102017	8q11.23	LYPLA1	28.64700666	25.97294368	1.102955714	4	4	31.12756352	20.74985271
chr9	136440096	136484552	9q34.3	SEC16A	11.88571867	10.77698641	1.10287962	4	4	64.8090194	29.65261543
chr4	932387	958656	4p16.3	TMEM175	6.055626225	5.4909908	1.102829425	4	4	27.08511302	20.86389002
chr11	76186326	76210842	11q13.5	WNT11	0.342733019	0.310793315	1.102768312	3	2	103.6440988	11.71145453
chr8	125024260	125091819	8q24.13	WASHC5	18.40371797	16.68884579	1.102755589	4	4	21.63774309	33.46599223
chr13	21303512	21344859	13q12.11	LINC00539	0.671154412	0.608647398	1.102698235	4	3	70.50932059	50.76628171
chr7	39622955	39708124	7p14.1	RALA	8.968155704	8.133130142	1.102669642	4	4	24.50136668	39.45648344
chr19	49659569	49665875	19q13.33	IRF3	9.762615906	8.853635941	1.102667421	4	4	36.75825956	39.46781704
chr6	33289597	33290934	6p21.32	PFDN6	3.66522995	3.323974959	1.10266473	4	4	36.54137204	33.84765
chr7	134127352	134282475	7q33	LRGUK	0.80792052	0.732698428	1.102664465	4	4	51.22360488	17.31919983
chr2	62673851	63046487	2p15	EHBP1	8.08631183	7.333528666	1.102649515	4	4	13.5671792	23.19776696
chr1	36084075	36088275	1p34.3	TEKT2	0.140282081	0.127226121	1.102620126	2	2	22.97923823	7.283406387
chr19	17747718	17788568	19p13.11	FCHO1	9.405163036	8.530257212	1.102564999	4	4	67.10308518	37.44687187
chr1	43300895	43323110	1p34.2	TIE1	0.085076779	0.077169532	1.102465924	3	2	59.21928288	1.180444439
chr2	86020216	86106155	2p11.2	POLR1A	2.595015896	2.353957285	1.102405686	4	4	58.72215412	36.89800527
chr12	123152324	123244014	12q24.31	MPHOSPH9	34.47639334	31.27486862	1.102367328	4	4	59.75668552	35.09766429
chr12	98593625	98602000	12q23.1	SLC25A3	18.18063357	16.492385	1.102365339	4	4	23.88423723	8.752143921
chr10	97222173	97292673	10q24.1	ARHGAP19	8.073730663	7.324272997	1.102325195	4	4	39.75591703	38.28157116
chr16	15037853	15056079	16p13.11	NTAN1	16.22896167	14.72259937	1.102316328	4	4	33.40445078	8.052375062
chr17	62470908	62615481	17q23.2	TLK2	43.97737789	39.89813098	1.102241554	4	4	31.53071291	13.74466048
chr19	48619291	48630407	19q13.33	SPHK2	0.9992743	0.90673338	1.102059682	4	4	98.44136616	52.97294705
chr10	119207685	119455619	10q26.11	GRK5	5.918268258	5.370201646	1.102056989	4	4	58.82233436	18.04470718
chr11	46854715	46918622	11p11.2	LRP4	0.033826304	0.030693807	1.102056316	1	1	0	0
chr10	73168090	73242181	10q22.2	FAM149B1	5.311212144	4.819552855	1.102013466	4	4	19.76191592	37.62133622
chr10	112374158	112428380	10q25.2	ACSL5	18.37471192	16.6746138	1.101957271	4	4	20.89009875	43.61143919
chr2	37231631	37271365	2p22.2	NDUFAF7	4.26321441	3.868889132	1.101922093	4	4	69.9184892	10.35700899
chr1	173824647	173858544	1q25.1	DARS2	7.500243156	6.80661246	1.101905419	4	4	18.83489153	25.08749692
chr16	50806672	50807629	16q12.1	LINC02168	0.707105624	0.641717524	1.101895455	2	1	19.20144586	0
chr5	131170914	131205426	5q23.3-q31.1	LYRM7	7.932536037	7.199039919	1.101888047	4	4	39.41573671	17.93431277
chr18	23506184	23586617	18q11.2	NPC1	6.375350491	5.785844149	1.101887698	4	4	29.62153185	40.14204334
chrX	73524275	73563085	Xq13.2	MAP2K4P1	1.407618928	1.277488621	1.101864162	4	4	82.62570917	65.04972643
chr17	35435096	35522293	17q12	SLFN13	3.725187117	3.381052424	1.101783306	4	4	46.94819266	20.75483278
chr1	93885199	93910370	1p22.1	GCLM	39.04560478	35.43906974	1.101767204	4	4	71.36928305	87.59354181
chr11	18606401	18634823	11p15.1	SPTY2D1	17.50755296	15.8905581	1.101758217	4	4	23.76790811	10.53074266
chr5	52788302	52953655	5q11.2	ITGA1	1.008579216	0.915428166	1.101756811	4	4	68.80652005	52.84574346
chr19	42068477	42081567	19q13.2	ZNF574	2.178528197	1.977394624	1.101716456	4	4	71.24688217	34.88199307
chr5	157138440	157142910	5q33.3	MED7	7.945824653	7.212431813	1.101684544	4	4	3.693927869	27.13617317
chr21	42843075	42891695	21q22.3	WDR4	1.264337301	1.147756451	1.101572812	4	4	57.83885918	39.71311658
chr5	62578735	62581448	5q12.1	LRRC70	0.394379107	0.358026148	1.101537161	4	3	38.38675247	84.41617968
chr6	32180968	32184380	6p21.32	AGER	1.883237801	1.709684204	1.101512079	4	4	113.3900709	23.75754904

chr10	102423245	102432666	10q24.32	CUEDC2	7.042816152	6.393797753	1.101507496	4	4	66.88697582	40.42579711
chr1	206767603	206772494	1q32.1	IL10	0.416935332	0.378520956	1.101485466	3	4	65.96649749	65.63127799
chr16	527794	554636	16p13.3	CAPN15	4.115048163	3.73598707	1.1014621	4	4	38.16564177	55.42147683
chr14	73644875	73703728	14q24.3	DNAL1	1.80019794	1.634627758	1.101289227	4	4	25.06411353	40.9539187
chr16	30650702	30656440	16p11.2	PRR14	6.947573326	6.309031424	1.101210766	4	4	58.44375737	32.85108208
chr17	31851865	31859307	17q11.2	COPRS	1.917966237	1.741812047	1.10113272	4	4	40.53244798	47.19236585
chr11	86441108	86674746	11q14.2	ME3	0.284781995	0.258646647	1.101046534	4	4	37.42473721	73.40253951
chr2	70209444	70248793	2p13.3	TIA1	26.12666392	23.72992978	1.101000473	4	4	26.88989881	25.04934009
chr1	43707465	43931165	1p34.1	ST3GAL3	3.064189902	2.783100895	1.100998497	4	4	26.50823708	29.302098
chr12	121777754	121793688	12q24.31	RHOF	13.34757415	12.1232275	1.100991807	4	4	35.29207727	38.2895966
chr11	72814406	72845132	11q13.4	ATG16L2	16.23978414	14.75025362	1.100983383	4	4	61.78725244	50.76027852
chr22	41092586	41180077	22q13.2	EP300	48.83616588	44.35952046	1.100917354	4	4	4.094230823	15.05681662
chr9	127907886	127917052	9q34.11	ST6GALNAC4	28.78423386	26.14588436	1.100908788	4	4	58.65624637	27.50930201
chr4	15960240	15963236	4p15.32	FGFBP2	66.67541142	60.57032167	1.100793418	4	4	52.57137537	79.42935038
chr1	151399565	151401936	1q21.3	PSMB4	39.7402779	36.10253954	1.100761287	4	4	45.01534195	20.34684248
chr6	124962406	125092634	6q22.31	RNF217	2.860002115	2.598277302	1.100730131	4	4	9.006852698	23.91288071
chr12	119667864	119681624	12q24.23	PRKAB1	3.425730085	3.11235065	1.100688987	4	4	45.89807542	14.03524696
chr4	674199	682033	4p16.3	MYL5	0.866606226	0.787494528	1.100459997	4	4	44.20249958	25.85571819
chr17	47967904	47981786	17q21.32	CDK5RAP3	6.140649229	5.580109863	1.100453106	4	4	52.64772726	20.83401887
chr11	83111060	83111442	11q14.1	C1DP5	14.99008554	13.62182124	1.100446503	4	4	7.656865985	25.6064442
chr11	113898923	113949119	11q23.2	HTR3B	0.124493558	0.113138724	1.100362041	1	1	0	0
chr15	51907200	51907305	15q21.2	RNU6-90P	5.175462722	4.704198094	1.100179588	4	2	40.05131603	9.199471131
chrX	153056409	153060467	Xq28	PNMA3	0.27008927	0.245502301	1.100149647	3	4	98.66998147	72.70348838
chr16	84648511	84667686	16q24.1	KLHL36	5.57742517	5.070068661	1.100068962	4	4	66.92749018	29.19202281
chr9	95099054	95317730	9q22.32	FANCC	2.297485557	2.08850334	1.100063147	4	4	70.95987525	59.63774146
chr20	41028818	41124487	20q12	TOP1	202.887243	184.4438479	1.099994634	4	4	19.99145868	12.93089422
chr10	101007683	101031157	10q24.31	PDZD7	0.050459653	0.045873924	1.099963748	1	2	0	5.631264742
chr16	84699949	84779922	16q24.1	USP10	38.10165966	34.6432617	1.099828878	4	4	34.21776755	38.90992842
chr4	165277792	165280023	4q32.3	GK3P	0.185246386	0.168436376	1.099800349	2	1	23.10494079	0
chr6	42980344	43017086	6p21.1	MEA1	22.49035715	20.45172557	1.099680175	4	4	38.8458092	27.443854
chr5	1050374	1155887	5p15.33	SLC12A7	3.017355421	2.74399126	1.099622825	4	4	44.64396999	45.41385284
chr10	988409	1017771	10p15.3	GTPBP4	8.604615084	7.825238743	1.099597772	4	4	36.33054434	12.6515283
chr7	7338542	7542094	7p21.3	COL28A1	0.043140312	0.039234011	1.099564152	2	4	15.46311589	2.322367316
chr6	26440472	26453415	6p22.2	BTN3A3	19.81869625	18.02483573	1.099521602	4	4	38.50658196	50.63379178
chr14	23376432	23379772	14q11.2	CMTM5	6.808240629	6.192065853	1.099510372	4	4	75.45702569	12.70337456
chr6	43059594	43075099	6p21.1	KLC4	1.317819355	1.198606571	1.099459478	4	4	94.44792	28.74150945
chr16	3401190	3409364	16p13.3	ZNF174	2.592911297	2.358408695	1.099432555	4	4	49.92809515	37.37879541
chr3	100334718	100355634	3q12.2	NIT2	1.204164358	1.095273692	1.099418681	4	4	30.38432249	13.46179174
chr7	149195462	149226248	7q36.1	ZNF282	4.387886619	3.991108738	1.099415452	4	4	65.03561546	45.54391011
chrX	130339888	130373361	Xq26.1	SLC25A14	2.226744158	2.025595502	1.099303467	4	4	71.89893018	11.05468926
chr17	75038860	75046979	17q25.1	ATP5PD	15.42027232	14.0273877	1.099297507	4	4	19.05493081	12.74170395
chr20	5937832	5950558	20p12.3	TRMT6	6.185541815	5.626967647	1.09926735	4	4	91.0793455	37.2084874
chr1	202940825	202958572	1q32.1	ADIPOR1	1631.020893	1483.785542	1.099229536	4	4	32.73689482	17.15607494

chr3	161221290	161253532	3q26.1	NMD3	14.84274888	13.50502464	1.099053817	4	4	26.38003318	10.92898537
chr11	1469448	1486779	11p15.5	MOB2	8.632326108	7.854402667	1.099042979	4	4	35.9976172	34.73789539
chr3	196291219	196318294	3q29	TCTEX1D2	0.441089587	0.401346717	1.099023781	3	2	53.38915077	3.26890842
chr12	49323188	49331733	12q13.12	TROAP	9.212099264	8.382124857	1.099017185	4	4	76.82196181	52.84482881
chr3	197042560	197299272	3q29	DLG1	24.12554269	21.95495808	1.09886535	4	4	37.79713178	20.05695534
chrX	49876724	49879356	Xp11.23	USP27X-AS1	0.476312112	0.433466863	1.098843193	4	4	81.16069958	60.24475507
chr1	24643103	24673281	1p36.11	SRRM1	16.91618156	15.39760021	1.098624547	4	4	37.06513253	18.62302578
chr8	66870749	66926436	8q13.1	MCMDC2	0.31578309	0.287463856	1.09851407	4	4	36.46934872	30.0423433
chr3	150546678	150586016	3q25.1	EIF2A	44.76000411	40.74816702	1.098454419	4	4	22.12526764	14.22481636
chr2	70977183	70986677	2p13.3	ANKRD53	0.113742938	0.10354963	1.098438868	2	1	25.12329922	0
chr3	58008400	58172255	3p14.3	FLNB	6.798263743	6.189116411	1.098422342	4	4	70.06454923	55.39508455
chr11	68052859	68121391	11q13.2	CHKA	2.240507246	2.039816223	1.098386816	4	4	33.25300933	56.53363754
chr2	26190635	26244726	2p23.3	HADHA	55.95146629	50.94346128	1.098305158	4	4	40.2424589	24.17266325
chr9	132945545	132991697	9q34.13	GFI1B	9.124044825	8.307426499	1.098299795	4	4	37.43658087	33.17594967
chr7	37848597	37900401	7p14.1	NME8	5.397910925	4.914799171	1.098297354	4	4	25.85996884	62.3810714
chr3	179588465	179604646	3q26.33	MRPL47	65.76922985	59.88378918	1.098281033	4	4	56.12176741	38.69459118
chr1	167916675	167937069	1q24.2	MPC2	28.49625254	25.94786234	1.098211952	4	4	32.37128641	55.55487378
chr11	82901695	82934659	11q14.1	DDIAS	2.249108534	2.048006056	1.098194279	4	4	35.35617861	36.33245319
chr13	32586427	32778020	13q13.1	PDS5B	17.28023047	15.73526279	1.098185057	4	4	31.74830665	6.370099276
chr17	78146035	78166283	17q25.3	C17orf99	0.974339001	0.887336433	1.098049132	4	4	57.93549812	76.58858389
chr7	143263429	143269129	7q34	GSTK1	25.20078785	22.95486194	1.097840968	4	4	26.74782534	22.07444337
chr22	19330701	19431696	22q11.21	HIRA	2.491181985	2.269178384	1.097834354	4	4	71.05251613	28.98326909
chr4	113451032	113762177	4q26	CAMK2D	13.27872388	12.09597122	1.097780711	4	4	43.23918185	23.23609915
chrX	155466540	155494110	Xq28	TMLHE-AS1	1.763028141	1.606030012	1.097755414	4	4	72.27950725	55.5885594
chr1	161199315	161214395	1q23.3	NDUFS2	13.44265769	12.24680327	1.09764625	4	4	54.22287259	48.83769676
chr9	124353465	124415473	9q33.3	PSMB7	22.91405037	20.87616772	1.09761766	4	4	43.21887683	23.50610112
chr2	178463664	178480817	2q31.2	FKBP7	0.46706938	0.425537866	1.097597693	4	4	71.47037302	64.35020548
chr9	125146573	125189939	9q33.3	PPP6C	40.21671353	36.64096954	1.097588684	4	4	10.93626379	12.29728788
chrX	155026789	155056916	Xq28	FUNDC2	12.2227924	11.13700558	1.097493604	4	4	24.05417002	37.88002954
chr2	96274336	96305569	2q11.2	SNRNP200	19.75735977	18.00310697	1.097441669	4	4	54.52030167	36.75459333
chr7	35632659	35695811	7p14.2	HERPUD2	73.05513043	66.57140121	1.097395114	4	4	8.742169207	16.09503816
chr3	13549125	13638422	3p25.1	FBLN2	0.733269347	0.668197624	1.097383949	3	4	93.20590723	67.81226765
chr3	101649439	101677144	3q12.3	ZBTB11	33.69942639	30.71053205	1.097324733	4	4	33.55512171	31.27616448
chr4	39287449	39366381	4p14	RFC1	48.38488948	44.09354876	1.09732355	4	4	28.84199857	10.53283343
chr7	65038354	65074713	7q11.21	CCT6P3	3.553121288	3.238058346	1.097299958	4	4	11.76280524	62.09123473
chr6	32854161	32859851	6p21.32	PSMB9	33.62214791	30.64094358	1.097294795	4	4	49.41021719	61.34608405
chr4	77713387	77819663	4q21.1	CNOT6L	63.97346313	58.30118666	1.097292642	4	4	38.75764229	21.73425757
chr2	241584405	241637543	2q37.3	THAP4	5.509105843	5.020738939	1.097269926	4	4	30.75059605	44.93681985
chr15	44665732	44711406	15q21.1	PATL2	3.275951552	2.985728133	1.097203565	4	4	45.95036994	40.23363269
chr3	47413685	47477126	3p21.31	SCAP	11.97229645	10.91178535	1.097189512	4	4	54.1299637	22.36859254
chr19	53066614	53103434	19q13.41-q13.42	ZNF160	8.969404396	8.175070748	1.097165355	4	4	38.89979159	28.97276087
chrX	48922024	48960783	Xp11.23	OTUD5	21.3527332	19.46241153	1.097126796	4	4	58.82486562	25.84408121
chr13	27066150	27171896	13q12.13	USP12	176.168294	160.5751674	1.097107958	4	4	31.02676264	66.58329638

chr2	96859736	96870943	2q11.2	SEMA4C	1.868311466	1.703099799	1.097006451	4	4	47.11747697	56.26620673
chr16	67842310	67844195	16q22.1	THAP11	12.322313	11.23267352	1.097006245	4	4	23.69670768	19.10567336
chr1	180972712	181023121	1q25.3	STX6	19.2859929	17.58173491	1.096933436	4	4	33.57318885	2.719440131
chr12	122229561	122266823	12q24.31	VPS33A	3.04653577	2.777441016	1.096885857	4	4	59.53275343	27.2721175
chr12	103587250	103766724	12q23.3	STAB2	0.030913957	0.028183617	1.09687686	1	1	0	0
chr12	92772509	92975228	12q22	EEA1	21.85613639	19.9264527	1.096840302	4	4	15.4299343	21.53935762
chr3	149738470	149752499	3q25.1	COMMD2	8.800927239	8.024067459	1.096816207	4	4	32.54571524	12.82515211
chr7	152436895	152443016	7q36.1	FABP5P3	0.247907886	0.226046521	1.096711795	2	1	18.58429459	0
chr14	73949934	73963460	14q24.3	COQ6	1.086441511	0.990676976	1.096665753	4	4	26.74554055	10.25688808
chr20	45934674	45948023	20q13.12	PCIF1	11.01569511	10.04520333	1.096612458	4	4	50.61309994	17.36186612
chr5	62387953	62403960	5q12.1	DIMT1	8.004378559	7.299355404	1.096587043	4	4	22.17600302	7.745211577
chr1	93179802	93279037	1p22.1	CCDC18	117.8661843	107.484847	1.096584194	4	4	46.04052818	40.1933773
chr3	141324213	141449792	3q23	ZBTB38	12.70430605	11.58598494	1.096523612	4	4	29.27474185	53.65613751
chr15	63042639	63071915	15q22.2	TPM1	6.526771854	5.952892632	1.096403422	4	4	36.13759621	17.23643588
chrX	47145196	47186815	Xp11.3	RBM10	13.4811149	12.29581074	1.096399024	4	4	56.44344759	45.13077526
chr9	124942608	125143559	9q33.3	SCAI	2.695746134	2.458827391	1.096354361	4	4	32.38148282	12.33234354
chr3	154024401	154121210	3q25.2	ARHGEF26-AS1	0.04412958	0.040255105	1.096248033	1	2	0	6.650434074
chr2	111195866	111495161	2q13	MIR4435-2HG	10.55480315	9.628701722	1.096181339	4	4	36.52696093	60.38695251
chr4	88520978	88523801	4q22.1	PYURF	17.20751801	15.69772302	1.096179234	4	4	5.946375049	32.85459846
chr14	24423186	24441342	14q12	KHNYN	18.3947583	16.78101553	1.096164786	4	4	45.08810925	14.9436741
chr15	83016423	83067354	15q25.2	BTBD1	24.51003869	22.35982126	1.096164339	4	4	25.6926571	9.40961806
chr5	140673904	140691727	5q31.3	HARS	4.010267836	3.658565742	1.09613114	4	4	39.41588259	28.01279329
chr19	12092010	12114752	19p13.2	ZNF788P	3.056145692	2.788452822	1.096000502	4	4	68.01104624	55.68767295
chr19	29207260	29213229	19q12	UQCRFS1	14.26150044	13.01355296	1.095895984	4	4	19.21667065	27.58585524
chr2	161992241	162074542	2q24.2	DPP4	8.146543124	7.434701359	1.095745845	4	4	59.20516311	25.72389098
chr10	43554516	43574619	10q11.21	ZNF239	0.896812048	0.818485158	1.095697385	3	4	62.52839826	52.92079268
chr1	146938545	146996093	1q21.1	NBPF12	2.386442915	2.178019365	1.095694076	4	4	51.25111737	30.36358091
chr19	51964340	51986856	19q13.41	ZNF350	12.09039682	11.03463388	1.095677206	4	4	61.20144006	14.3101797
chrX	73212299	73214874	Xq13.2	NAP1L2	0.886526068	0.809122864	1.095663103	4	4	66.65001849	41.81138334
chr4	184584093	184624872	4q35.1	LINC02365	0.470086867	0.429050029	1.095645811	3	3	67.0344035	45.42131555
chr20	35171937	35215989	20q11.22	PROCR	0.669792894	0.611329748	1.095632751	4	4	85.48229587	29.32419845
chr5	151742822	151758649	5q33.1	ATOX1	2.562953775	2.339310789	1.095602084	4	4	30.12805165	15.81918536
chr1	103525956	103555239	1p21.1	RNPC3	10.71498524	9.78035187	1.095562346	4	4	4.614189723	28.93847648
chr11	64726911	64745481	11q13.1	RASGRP2	17.3905211	15.87599936	1.095396939	4	4	44.38079033	22.68640796
chr12	49622717	49645129	12q13.12	PRPF40B	0.212873771	0.19435086	1.095306557	3	4	91.29538972	66.29072484
chr5	94613476	94714918	5q15	SLF1	56.1833261	51.29477226	1.095303159	4	4	44.54207885	43.50082189
chr2	134918810	134959345	2q21.3	CCNT2	15.01067987	13.70470077	1.095294244	4	4	21.9245412	23.57851712
chr12	55973913	55996683	12q13.2	RAB5B	33.75166518	30.81719539	1.095221832	4	4	37.15983727	20.0479045
chr10	102869453	102901899	10q24.32	AS3MT	0.119727598	0.109318903	1.09521404	1	1	0	0
chr11	65111854	65116235	11q13.1	TM7SF2	1.626715131	1.485345023	1.095176612	4	4	23.13573831	25.60698851
chr14	52267256	52276724	14q22.1	PTGDR	4.805636147	4.388063947	1.09516092	4	4	52.17618876	61.62893983
chr18	74496265	74523454	18q22.3	CNDP2	6.587802077	6.0159262	1.095060321	4	4	40.85607073	37.21628972
chr14	54474491	54489026	14q22.2	GMFB	17.51491792	15.99472687	1.095043264	4	4	19.5918777	16.40842551

chr14	20891403	20892348	14q11.2	RNASE3	3.842837082	3.509607995	1.094947666	4	4	41.55655449	16.22496314
chr11	14878005	14898915	11p15.2	CYP2R1	3.716236873	3.394020406	1.094936514	4	4	27.71500666	31.71390222
chr11	112024814	112064278	11q23.1	DLAT	9.633222791	8.799320797	1.094768905	4	4	29.66716436	25.11692004
chr4	127891991	127892082	4q28.1	RNU6-583P	4.704373836	4.297157162	1.094764203	2	2	23.59681892	65.96578481
chr12	32725247	32756458	12p11.21	YARS2	8.020367754	7.326277142	1.094739879	4	4	20.74834735	25.38042498
chr19	41219199	41261767	19q13.2	AXL	0.244696602	0.223525855	1.094712741	4	4	36.4849981	63.55383106
chr17	10628526	10678347	17p13.1	MYH3	0.44496532	0.406483617	1.094669751	3	4	90.47203827	51.58257575
chr2	127941217	128028120	2q14.3	SAP130	14.63763723	13.37201958	1.094646709	4	4	19.35308049	25.61624238
chrX	153785766	153794523	Xq28	IDH3G	12.75497177	11.65231545	1.094629804	4	4	66.90417654	30.67074481
chr17	81902109	81902905	17q25.3	NPB	0.13038344	0.119116453	1.094588001	3	1	14.82431971	0
chr11	47418769	47426473	11p11.2	PSMC3	17.5625689	16.04627663	1.094494961	4	4	52.84350331	43.80837067
chr4	165327666	165343164	4q32.3	MSMO1	11.0138221	10.06335612	1.094448211	4	4	56.18505226	34.25097362
chr14	55766245	55767717	14q22.3	RPL13AP3	0.185060797	0.169120413	1.094254643	2	3	1.219148222	11.84618472
chr19	4542588	4581484	19p13.3	SEMA6B	0.34998019	0.319869005	1.094135989	3	3	75.77411936	38.49571513
chr1	78889764	79006810	1p31.1	ADGRL4	0.157448719	0.143903835	1.094124552	1	1	0	0
chr18	34976928	35143470	18q12.1-q12.2	MAPRE2	27.11010642	24.77881978	1.094083845	4	4	49.69154707	30.40573821
chr12	62965302	62966024	12q14.2	RPL14P1	1.885654559	1.723711525	1.093950195	4	4	47.11932581	25.72899345
chr5	55935095	55994993	5q11.2	IL6ST	44.63851158	40.80559151	1.093931246	4	4	17.91371339	14.94217951
chr4	109848182	109872315	4q25	LRIT3	0.075778574	0.069272906	1.093913596	3	2	17.57210656	7.205986824
chr10	50624951	50631636	10q11.23	SGMS1-AS1	0.807680818	0.73834447	1.093907859	4	4	75.38012683	42.56250061
chr6	149504495	149546102	6q25.1	PPIL4	59.99107584	54.84474701	1.093834489	4	4	32.35838097	21.59426291
chr4	76114659	76148515	4q21.1	NUP54	82.96568521	75.85169597	1.093788137	4	4	57.94878038	36.41432633
chr16	401858	412487	16p13.3	DECR2	0.132117649	0.120792693	1.09375531	3	3	40.26755511	44.64356487
chr11	57668002	57701187	11q12.1	ZDHHC5	23.5177867	21.50480056	1.093606362	4	4	30.19121465	17.85501417
chr2	110123336	110205062	2q13	NPHP1	0.047687398	0.043607041	1.093571047	3	2	85.51227789	1.946186537
chr13	110878540	110915107	13q34	ANKRD10	18.31099029	16.74434537	1.093562626	4	4	36.71393076	8.322008842
chr7	75027119	75073881	7q11.23	RCC1L	4.011013768	3.668024735	1.09350783	4	4	50.83391764	32.87389065
chr2	168455862	168775134	2q24.3	CERS6	14.46069108	13.22431089	1.093492977	4	4	32.4994912	24.55663771
chr2	28913662	28913750	2p23.2	SNORD92	5.716533626	5.227954509	1.09345512	2	3	33.44507844	68.09415209
chr19	55229779	55258670	19q13.42	PPP6R1	30.46039941	27.85765531	1.093430121	4	4	43.97013651	61.36302987
chr4	42408373	42657105	4p13	ATP8A1	23.0391864	21.07140479	1.093386351	4	4	20.95678758	12.1987994
chr10	46816582	46835825	10q11.22	AGAP13P	3.18871647	2.916399426	1.093374399	3	4	132.713942	63.79706307
chr15	65381484	65423072	15q22.31	IGDCC4	0.035733646	0.032684655	1.093285088	1	1	0	0
chr14	37197888	37579207	14q13.3-q21.1	MIPOL1	0.631587828	0.577719444	1.093243157	4	4	91.70169692	77.09783666
chr8	42849637	42897294	8p11.21	RNF170	3.796210027	3.472457386	1.093234446	4	4	36.29741409	27.84470949
chr8	43093506	43123180	8p11.21	POMK	5.437975552	4.974245027	1.093226313	4	4	32.71402018	18.23064406
chr8	22440970	22541466	8p21.3	PPP3CC	32.17109621	29.42778966	1.093221631	4	4	29.38620532	26.40809676
chr16	85707517	85751083	16q24.1	C16orf74	0.69151494	0.632548724	1.093220038	3	4	95.91605153	54.4656446
chr3	50569152	50589050	3p21.31	HEMK1	0.748616785	0.68478802	1.093209523	4	4	70.03980959	40.55806048
chr1	154957026	154961782	1q21.3	PYGO2	7.900245316	7.227028865	1.093152589	4	4	25.07307319	39.58958301
chr19	58575399	58584395	19q13.43	CENPBD1P1	3.403225906	3.113248659	1.093142977	4	4	80.88461965	44.13372051
chr4	108620558	108630484	4q25	RPL34	186.8348966	170.9209419	1.093107109	4	4	43.02338962	17.01037677
chr1	91829774	91830066	1p22.1	RN7SL653P	0.973911538	0.890971911	1.093088936	1	3	0	12.39319208

chr2	85354394	85391752	2p11.2	ELMOD3	2.862488672	2.618734914	1.09308073	4	4	25.63323856	17.08714005
chr20	38304150	38337505	20q11.23	BPI	2.440116013	2.232397251	1.093047401	4	4	35.18976626	78.46685888
chr2	200488952	200510094	2q33.1	KCTD18	6.924418513	6.334989608	1.093043389	4	4	48.62114735	22.29064293
chr1	37474518	37484377	1p34.3	ZC3H12A	8.985680429	8.220908079	1.093027722	4	4	59.01549046	49.947065
chr16	54283300	54286772	16q12.2	IRX3	0.211844166	0.193815682	1.093018706	2	2	56.3422241	68.77084897
chr1	184051651	184095846	1q25.3	TSEN15	15.60714153	14.27910482	1.0930056	4	4	15.59230436	20.1541879
chr6	31815514	31817942	6p21.33	HSPA1A	0.115764596	0.105914549	1.092999943	1	1	0	0
chr9	33524413	33575442	9p13.3	ANKRD18B	0.207533642	0.18989677	1.092876102	4	3	59.10662246	109.7474436
chr9	128455155	128501292	9q34.11	ODF2	11.35992633	10.39470799	1.092856706	4	4	17.86742404	21.01939577
chr15	101960813	101976605	15q26.3	WASH3P	4.144687247	3.792735386	1.092796313	4	4	91.9568577	54.57668234
chr1	17018722	17054170	1p36.13	SDHB	25.67321758	23.49362137	1.092773956	4	4	34.4871501	19.40720316
chr19	19645198	19663694	19p13.11	ATP13A1	4.126804645	3.776468211	1.092768273	4	4	61.27996689	34.42534709
chr12	131949920	132080466	12q24.33	EP400	2.658035649	2.432449599	1.092740277	4	4	66.11573024	36.61570492
chr3	154271828	154324497	3q25.2	DHX36	7.502840778	6.867020518	1.092590412	4	4	24.50310638	15.86622382
chr20	9514358	9530524	20p12.2	LAMP5	1.765169292	1.615746913	1.092478826	4	3	84.74522185	77.8872053
chr19	40422501	40426025	19q13.2	SERTAD1	2.446878793	2.240006699	1.092353337	4	4	75.77616983	49.56547799
chr3	108958239	109118146	3q13.13	MORC1	4.996466537	4.574322941	1.092285482	4	4	28.60749452	51.70287431
chr8	144517965	144525178	8q24.3	LRRC14	1.999386198	1.830464593	1.092283459	4	4	31.81230882	11.74361586
chr12	12357078	12471221	12p13.2	BORCS5	0.246992602	0.226130735	1.09225578	4	4	50.08896025	47.16025145
chr10	68898817	68956312	10q22.1	DDX50	31.17082526	28.54159685	1.092119177	4	4	13.07973601	8.88070472
chr6	32845209	32853971	6p21.32	TAP1	42.06362532	38.51673624	1.092086958	4	4	59.07388518	56.70689153
chr19	12014717	12035741	19p13.2	ZNF433	0.36818976	0.337149914	1.092065411	3	4	93.35260211	36.7497173
chr4	78887225	78939428	4q21.21	PAQR3	1.775005666	1.62543651	1.092017839	4	4	54.37508877	19.47649793
chr10	115093365	115948999	10q25.3	ATRNL1	0.04505334	0.041259159	1.091959726	3	2	62.24003312	52.60459235
chr17	79833156	79839414	17q25.3	CBX4	16.00163014	14.65409951	1.091955881	4	4	65.10943369	36.64423546
chr18	12911217	12912264	18p11.21	STK25P1	0.518749956	0.475108881	1.091854894	1	2	0	60.001212
chr17	76080993	76103787	17q25.1	EXOC7	13.16334287	12.05602402	1.091847764	4	4	32.44619184	17.77459642
chr19	21142009	21196053	19p12	ZNF431	4.508962456	4.129865266	1.091794082	4	4	28.39430799	11.76762777
chr14	73591706	73595766	14q24.3	ACOT4	0.327707779	0.300162415	1.091768197	3	4	66.75791741	43.24998688
chr10	69451464	69549508	10q22.1	TSPAN15	1.579674463	1.446938707	1.091735576	4	4	94.32097638	30.40547197
chr3	160579267	160585787	3q25.33	RPL6P8	0.409088395	0.374719211	1.091719837	1	1	0	0
chr9	113407235	113410749	9q32	POLE3	22.56608262	20.672205	1.091614688	4	4	64.89283186	30.14615657
chr12	70242993	70354993	12q15	CNOT2	13.4816591	12.35038809	1.091598014	4	4	21.98921189	6.931078997
chr1	228140084	228148955	1q42.13	GUK1	207.1634465	189.8012616	1.091475603	4	4	41.74877544	29.08644718
chr5	43486699	43515433	5p12	C5orf34	1.963283995	1.798802519	1.09143943	4	4	84.7913838	45.23884713
chr13	95433588	95579756	13q32.1	CLDN10	0.08586693	0.078676919	1.091386534	1	2	0	9.3193752
chr12	56104319	56113910	12q13.2	PA2G4	32.40523745	29.69377793	1.091314063	4	4	10.27892883	27.72181988
chr22	36510850	36529230	22q12.3	EIF3D	46.0228511	42.1728218	1.091291717	4	4	54.58072524	31.34523845
chr19	43584367	43596234	19q13.31	IRGQ	1.855750915	1.700520951	1.09128377	4	4	78.57705804	17.39979469
chr9	131523796	131531275	9q34.13	UCK1	4.513955155	4.136465257	1.091259052	4	4	83.58070942	36.3048869
chr11	64803514	64811294	11q13.1	MEN1	3.487562898	3.195933712	1.091250074	4	4	85.90836456	62.73463201
chr3	42905910	42919333	3p22.1	ZNF662	0.326376071	0.29909018	1.091229645	2	3	116.0973563	71.25360722
chr17	40627720	40647851	17q21.2	SMARCE1	7.492092503	6.865763716	1.091224926	4	4	25.85353107	14.48155165

chr14	99737699	99942060	14q32.2	EML1	0.032130849	0.029445178	1.091209193	2	1	0.337664436	0
chrX	119466033	119469120	Xq24	SLC25A5-AS1	0.481523849	0.441277428	1.09120435	4	4	81.41543409	63.77951837
chr1	228393672	228406816	1q42.13	TRIM11	3.893024639	3.567665875	1.091196534	4	4	61.62999965	29.06896662
chr4	151670656	151761023	4q31.3	GATB	0.591944398	0.542480278	1.091181416	4	4	68.23511441	56.3058445
chr1	150293842	150308979	1q21.2	MRPS21	13.0180822	11.93035454	1.091173121	4	4	25.63136931	14.33281961
chr14	92058552	92106621	14q32.12	ATXN3	6.170460054	5.655591036	1.091037173	4	4	32.87399412	26.14208149
chr1	116574398	116667755	1p13.1	IGSF3	0.112352235	0.102981813	1.090991041	2	2	21.12446108	95.04505765
chr17	38800434	38825350	17q12	CWC25	4.836308099	4.433444246	1.090869273	4	4	78.30101492	37.06189131
chr20	49812713	49892242	20q13.13	SLC9A8	7.517272238	6.891223202	1.090847302	4	4	19.48491936	25.22967589
chr15	41517176	41544277	15q15.1	RPAP1	1.180565185	1.082255017	1.090838265	4	4	60.7956003	60.14130192
chr9	107283236	107332194	9q31.2	RAD23B	90.29857905	82.78216771	1.090797469	4	4	16.60439157	6.391191091
chr5	163446528	163460154	5q34	NUDCD2	2.122387402	1.945737828	1.090787963	4	4	27.66324688	28.47400013
chr14	34710382	34714823	14q13.1	CFL2	2.473085962	2.267359741	1.090733825	4	4	26.23681943	18.05541529
chr11	124115404	124146912	11q24.2	VWA5A	2.037263173	1.867813627	1.0907208	4	4	70.89101857	40.68738903
chr4	2843857	2930076	4p16.3	ADD1	36.25010118	33.23810179	1.090618875	4	4	26.56767173	27.43183526
chr3	8877196	8969249	3p25.3	RAD18	19.04630046	17.4644375	1.090576233	4	4	57.17533301	28.26142955
chr19	19276513	19320512	19p13.11	SUGP1	4.915472625	4.507252963	1.090569503	4	4	50.84510992	26.07209677
chr12	118135884	118145588	12q24.23	PEBP1	29.89578907	27.41565838	1.090464021	4	4	80.67877306	47.03814215
chr7	44382366	44490875	7p13	NUDCD3	5.124982824	4.70035677	1.090339111	4	4	38.81869169	14.40692401
chr2	207239811	207245887	2q33.3	MYOSLID	1.465440466	1.344022742	1.09033904	4	3	107.7016406	50.31797069
chr10	75125616	75125871	10q22.2	RPS26P42	3.417689609	3.134690814	1.090279652	4	2	56.41766608	41.17946349
chr4	147635650	147684230	4q31.23	PRMT9	5.689525636	5.218577215	1.090244602	4	4	18.32198189	14.75850323
chr16	31180110	31194871	16p11.2	FUS	6.762127559	6.202493247	1.090227315	4	4	63.15436606	25.92774803
chr9	120756976	120793462	9q33.2	FBXW2	12.36493834	11.34267106	1.090125798	4	4	36.40428716	22.55257133
chr1	248272852	248273814	1q44	OR2T33	0.644541273	0.591321505	1.090001408	3	1	59.07874463	0
chr6	148745891	149076990	6q25.1	UST	1.768622726	1.62267834	1.089940429	4	4	49.2841235	67.79732968
chr8	100157869	100241904	8q22.2	SPAG1	2.507894155	2.300961238	1.089933248	4	4	60.06994184	58.35310788
chr8	63015079	63039051	8q12.3	GGH	1.07391858	0.985369555	1.089863772	4	4	33.21714744	42.59288784
chr17	439978	445940	17p13.3	RFLNB	1.428124676	1.310376785	1.089858041	4	4	102.0210187	59.46691424
chr8	38434348	38434851	8p11.23	RPS20P22	0.259697914	0.238286305	1.089856649	1	1	0	0
chr1	202891096	202928644	1q32.1	KLHL12	19.21736435	17.63300596	1.08985186	4	4	17.34652542	17.82025054
chr1	1702383	1724565	1p36.33	CDK11A	1.078288068	0.989435463	1.089801315	4	4	42.18641386	53.92030019
chr7	5045821	5069488	7p22.1	RBAK	8.754125364	8.03315582	1.089749229	4	4	31.1988489	9.324383392
chr15	41309268	41332621	15q15.1	OIP5	4.653252154	4.270348877	1.089665573	4	4	113.7072149	68.69753669
chr22	26491240	26513591	22q12.1	TFIP11	11.96125664	10.97715862	1.089649613	4	4	38.38410334	12.32097464
chr18	11689015	11885685	18p11.21	GNAL	0.385490929	0.353775604	1.08964814	4	4	38.76137045	61.62820461
chr19	12666804	12675832	19p13.13	WDR83	2.345004329	2.152256998	1.089555908	3	4	65.21085815	39.96349296
chr1	24319322	24364482	1p36.11	GRHL3	0.068293621	0.062683055	1.089506916	1	3	0	5.663257861
chr1	121184967	121392874	1p11.2	SRGAP2C	3.179008022	2.918377454	1.089306668	4	4	57.98353326	6.917778581
chr4	127965256	128039711	4q28.2	ABHD18	18.77222909	17.2338671	1.089263888	4	4	47.12142668	25.02054394
chr16	70687439	70801176	16q22.1-q22.2	VAC14	2.90229008	2.664480299	1.089251845	4	4	36.43819135	31.47441913
chr1	19881708	19912945	1p36.13	OTUD3	4.560181526	4.186575464	1.089239061	4	4	58.54932918	25.93993581
chr12	122865328	122896165	12q24.31	VPS37B	11.13832985	10.2265771	1.089155223	4	4	46.6682963	17.65543868

chr7	64313873	64356634	7q11.21	ZNF736	4.857343628	4.45978123	1.089143924	4	4	17.30147302	50.67630437
chr16	11834196	11851585	16p13.13	RSL1D1	32.91291903	30.21916836	1.089140463	4	4	34.18986346	21.33448992
chr2	26244748	26290465	2p23.3	HADHB	49.88453	45.80368648	1.089094216	4	4	26.89730587	19.70101544
chr5	151411344	151556318	5q33.1	SLC36A1	6.920341269	6.354312457	1.0890779	4	4	12.93889873	8.331692472
chr1	151059675	151068497	1q21.3	MLLT11	1.357392123	1.246392515	1.089056702	4	4	61.84816535	31.65119582
chr3	51391268	51397908	3p21.2	RBM15B	11.10397623	10.19597946	1.089054394	4	4	71.23458237	37.98731539
chr19	45710629	45733171	19q13.32	FBXO46	3.821049083	3.508868764	1.088968936	4	4	53.52171411	41.13702604
chr19	19063961	19113030	19p13.11	SLC25A42	2.676594763	2.457996893	1.088933338	4	4	50.10299131	30.54240993
chr10	1056836	1132297	10p15.3	WDR37	16.72296752	15.3579163	1.08888258	4	4	29.25953061	14.09718261
chr9	137110540	137117980	9q34.3	DPP7	12.36685455	11.35807331	1.088816229	4	4	65.08769132	49.85700283
chr4	139289917	139302551	4q31.1	NDUFC1	9.199699097	8.450221936	1.088693193	4	4	41.25665564	19.60762523
chr1	33007940	33036911	1p35.1	AK2	18.77495294	17.24569286	1.0886749	4	4	28.89742912	25.676001
chr17	28755978	28854985	17q11.2	FAM222B	3.430911092	3.151758549	1.088570409	4	4	38.88979262	16.86518569
chr5	55256055	55307722	5q11.2	DHX29	41.42753804	38.05772302	1.088544841	4	4	43.9606114	14.87664441
chr7	40132743	40134652	7p14.1	MPLKIP	1.865917792	1.714150456	1.088537932	4	4	21.34813602	14.34206756
chr7	43875894	43926411	7p13	URGCP	2.0175207	1.853531101	1.088474155	4	4	53.96518059	43.11616975
chr9	35658875	35665281	9p13.3	ARHGEF39	0.142912354	0.131304107	1.088407335	4	3	78.70410599	67.25547021
chr5	178204507	178211183	5q35.3	HNRNPAB	28.09092732	25.81064782	1.088346465	4	4	36.34541266	58.68253694
chr19	47778572	47784681	19q13.33	SELENOW	3.597222195	3.305510696	1.088250055	4	4	52.80684958	10.14934357
chr9	128729786	128772507	9q34.11	ZER1	60.61255768	55.69769758	1.08824171	4	4	35.50192766	10.76883056
chr7	6078743	6161564	7p22.1	USP42	8.303326332	7.630193945	1.088219565	4	4	27.99080491	17.06454766
chr20	26054614	26086917	20p11.1	FAM182A	0.046997762	0.043188314	1.088205529	2	1	11.14066453	0
chr2	169733846	169751886	2q31.1	KLHL23	0.405015305	0.372198023	1.088171563	4	4	62.85451017	49.76415133
chr7	120787320	120858337	7q31.31	TSPAN12	0.086332635	0.079338623	1.088153936	1	1	0	0
chr12	121402288	121582268	12q24.31	KDM2B	3.684893012	3.386537608	1.088100425	4	4	51.19637163	36.58050948
chr2	238060924	238099413	2q37.3	SCLY	0.13594262	0.124939389	1.08806855	3	4	0.758492441	59.15241899
chr12	48921959	48925547	12q13.12	FKBP11	2.143076812	1.969688099	1.088028512	4	4	58.94078273	22.42228456
chr1	156893697	156916434	1q23.1	PEAR1	2.431972352	2.235295447	1.087986984	4	4	66.77437699	38.17172955
chr17	16030094	16216475	17p12-p11.2	NCOR1	31.20912531	28.6879009	1.08788459	4	4	19.6061757	12.64026799
chr8	142212080	142403291	8q24.3	TSNARE1	1.420132519	1.30541368	1.087879299	4	4	71.5128937	51.50541336
chr1	32775238	32818032	1p35.1	YARS	7.263080536	6.676518603	1.087854459	4	4	23.71957547	46.59547072
chr12	116910949	117032498	12q24.22	FBXW8	2.094712956	1.925551144	1.087851113	4	4	20.70183215	39.0565996
chr6	21593741	21598619	6p22.3	SOX4	2.837305356	2.608384806	1.087763335	4	4	69.03988602	39.20467345
chr5	173056349	173139288	5q35.1	CREBRF	41.58859401	38.23952882	1.087581236	4	4	41.00113529	35.54497156
chr3	47380982	47413441	3p21.31	PTPN23	1.889184931	1.737126903	1.087534208	4	4	61.92027049	19.36445382
chr11	70270687	70384501	11q13.3	PPFIA1	9.534925493	8.767807063	1.087492622	4	4	37.11575887	22.29414762
chr14	22900645	22919187	14q11.2	RBM23	42.03709558	38.65643745	1.08745395	4	4	16.60129738	16.99395063
chr3	169772247	169790455	3q26.2	MYNN	15.535024	14.28580538	1.087444746	4	4	30.24587991	6.433851591
chr18	55222331	55635993	18q21.2	TCF4	2.259566284	2.077958481	1.087397224	4	4	51.47453386	42.21664089
chr20	35302566	35412142	20q11.22	UQCC1	4.288433345	3.943822815	1.087379821	4	4	56.63994586	32.34625584
chr7	92604921	92836627	7q21.2	CDK6	12.60015614	11.58814598	1.0873315	4	4	25.83097322	8.207926411
chr19	17405686	17425339	19p13.11	MVB12A	2.08044832	1.91335896	1.087327764	4	4	69.8267126	14.01781466
chr13	112485005	112606469	13q34	TUBGCP3	10.2929147	9.46631894	1.087319661	4	4	21.61536577	14.19393252

chr16	57152466	57186341	16q13	FAM192A	42.26313733	38.87046705	1.087281438	4	4	17.93692128	6.606035132
chr5	86617967	86620766	5q14.3	COX7C	67.88799999	62.44110121	1.087232587	4	4	35.97787845	16.77267132
chr12	110372900	110403730	12q24.11	ANAPC7	4.505985845	4.144724482	1.087161732	4	4	20.59475866	25.26262819
chrX	47190800	47215128	Xp11.3	UBA1	27.1388735	24.96395801	1.087122222	4	4	47.52480373	40.36002474
chr20	36612519	36646216	20q11.23	SLA2	21.91430975	20.15860243	1.087094694	4	4	42.15986042	33.32586822
chr11	11841423	11959325	11p15.3	USP47	20.61494216	18.96351785	1.087084281	4	4	37.79862641	11.03353504
chr2	90021634	90022185	2p11.2	IGKV6D-21	1.16429905	1.071080623	1.087032129	2	1	35.53715536	0
chr9	35056064	35072742	9p13.3	VCP	40.79966844	37.53458575	1.086988643	4	4	38.56147159	25.58398626
chr17	59859467	59892948	17q23.1	TUBD1	8.95203424	8.236091729	1.086927457	4	4	64.02566379	28.50556555
chr9	133030675	133058503	9q34.13	GTF3C5	8.505825151	7.825578672	1.086926029	4	4	64.04214883	30.78773101
chr22	27978014	28679865	22q12.1	TTC28	0.264868394	0.243695171	1.086884049	4	4	97.88178181	57.05757772
chr2	68462366	68467325	2p13.3	FBXO48	1.953882463	1.797693258	1.086883123	4	4	36.87181116	38.55610999
chr9	122931640	123104869	9q33.2-q33.3	RABGAP1	11.3195606	10.41499624	1.086852106	4	4	9.743223022	10.41589673
chr16	87302798	87317420	16q24.2	C16orf95	0.241427128	0.222151279	1.08676902	1	4	0	70.44087135
chr18	74590475	75065672	18q22.3	ZNF407	6.176569596	5.684186618	1.086623296	4	4	43.91986719	10.66722248
chrX	15674916	15703351	Xp22.2	CA5BP1	3.182565975	2.929086677	1.086538681	4	4	40.0248903	33.63524729
chr6	28349913	28369177	6p22.1	ZKSCAN3	1.221029108	1.123868701	1.086451742	4	4	37.36090585	22.6377502
chr8	17922784	18029948	8p22	PCM1	45.35086513	41.74463311	1.08638792	4	4	35.63052678	19.41478818
chr10	50305585	50623977	10q11.23	SGMS1	61.12596499	56.26610496	1.086372782	4	4	47.37401937	44.34273025
chr1	83865021	83999187	1p31.1	TTLL7	0.53652625	0.494004734	1.086075119	4	4	62.03816529	19.40520017
chr19	45527767	45584864	19q13.32	OPA3	1.685032587	1.551553383	1.086029398	4	4	72.23914927	38.10972503
chr2	196962617	197310797	2q33.1	ANKRD44	46.07783601	42.4281787	1.086019655	4	4	30.03673315	27.13243859
chr19	42220271	42227053	19q13.2	ZNF526	1.969582938	1.813581218	1.086018601	4	4	48.22727937	48.70953612
chr11	67682717	67683085	11q13.2	RPL37P2	2.719340813	2.504039655	1.085981529	1	2	0	57.06641333
chr3	6861115	7743038	3p26.1	GRM7	0.035238906	0.032449348	1.085966517	2	1	17.3569563	0
chr16	57758217	57863053	16q21	KIFC3	0.869413136	0.800596719	1.085956407	4	4	66.20687168	37.82252502
chr11	93741594	93763102	11q21	C11orf54	3.981810435	3.667000435	1.085849458	4	4	30.4942703	37.80319533
chr3	101723931	101770562	3q12.3	CEP97	5.089243817	4.68723127	1.085767594	4	4	38.89646907	29.87786428
chr19	47045907	47048624	19q13.32	TMEM160	1.999196203	1.841395265	1.085696396	3	4	108.4661049	58.876651
chr4	75724522	75814289	4q21.1	USO1	35.07030325	32.30240875	1.085686938	4	4	17.0075972	23.6110952
chr5	139471003	139473700	5q31.2	SMIM33	0.11653892	0.107346369	1.085634482	2	2	31.21089306	8.389767791
chr8	94823290	94894664	8q22.1	INTS8	20.78346974	19.14439888	1.085616209	4	4	14.39306266	17.69135727
chr19	44207522	44237268	19q13.31	ZNF227	8.482922721	7.814825605	1.085490982	4	4	23.64028985	11.23634102
chr14	21997686	21998168	14q11.2	TRAV17	10.70317062	9.860248318	1.085486925	4	4	57.7620144	56.90034848
chr1	184387016	184629021	1q25.3	C1orf21	3.532971796	3.254872733	1.085440841	4	4	26.78246527	48.66074752
chr7	16599776	16645817	7p21.1	ANKMY2	9.016559107	8.307212167	1.085389289	4	4	44.37226881	15.99692477
chr19	4472196	4502220	19p13.3	HDGFL2	6.092977598	5.613855795	1.085346297	4	4	62.70976058	49.50199829
chr16	30989256	31010638	16p11.2	STX1B	0.611542233	0.563474107	1.085306718	4	4	41.03026755	73.04072282
chr8	406808	469876	8p23.3	FBXO25	2.93278522	2.702421703	1.085243364	4	4	45.66444939	32.14977367
chr5	1798385	1801432	5p15.33	MRPL36	4.398158993	4.052805945	1.085213319	4	4	61.38651348	45.21847549
chr7	119619430	119907375	7q31.31	LINC02476	0.144276704	0.132964108	1.085080075	2	1	26.82670563	0
chr17	732546	742968	17p13.3	FAM57A	0.319750335	0.294684814	1.085058748	1	4	0	42.7738006
chr22	45163845	45188011	22q13.31	NUP50	56.27323031	51.86419981	1.085011058	4	4	26.50749478	19.34538738

chr17	38705620	38729803	17q12	MLLT6	4.64891489	4.284711499	1.08500068	4	4	72.51433047	43.57790713
chr3	185914568	185938136	3q27.2	TRA2B	33.13150333	30.5380123	1.084926648	4	4	22.04012503	12.58583858
chr1	145744438	145824123	1q21.1	RNF115	9.502383246	8.759467372	1.084812905	4	4	37.98197319	23.13673332
chr6	4021329	4064983	6p25.2	PRPF4B	26.26248133	24.20977378	1.084788382	4	4	34.50526452	11.80487264
chr9	129835417	129881838	9q34.11	USP20	7.564119951	6.973308584	1.084724684	4	4	43.03888404	28.71633684
chr7	27525440	27663001	7p15.2	HIBADH	42.17024886	38.87650699	1.084723195	4	4	91.37441388	20.19494335
chr4	88007647	88077779	4q22.1	PKD2	11.67696697	10.76612782	1.084602297	4	4	36.49520309	14.32170212
chr1	224356811	224379452	1q42.11	CNIH4	11.90668713	10.97802708	1.084592617	4	4	17.27748342	32.59085323
chr3	179347692	179393226	3q26.33	MFN1	21.50984056	19.83293458	1.084551582	4	4	18.25886572	10.89021804
chr14	21016763	21070872	14q11.2	NDRG2	0.572414829	0.527789987	1.084550375	4	4	72.62692067	56.47333481
chr2	119281472	119366880	2q14.2	C2orf76	5.696489668	5.252610139	1.084506468	4	4	41.21340323	44.67331237
chr19	15236836	15332543	19p13.12	BRD4	12.42077394	11.45490511	1.084319235	4	4	18.50905261	15.23567034
chr12	122773621	122827385	12q24.31	CCDC62	2.225634454	2.05266353	1.084266574	4	4	88.23018774	37.02920929
chrMT	1671	3229	N/A	MT-RNR2	8144.875353	7512.941824	1.084112661	4	4	65.95647291	36.07127959
chr9	123379654	123930158	9q33.3	DENND1A	23.42137385	21.60441174	1.084101439	4	4	38.63865291	29.96243368
chr12	2890867	2941140	12p13.33	TULP3	2.022697584	1.865854331	1.084059753	4	4	38.7162592	45.64116022
chr10	119651370	119677819	10q26.11	BAG3	2.761312848	2.547316854	1.084008393	4	4	49.88682178	63.84387345
chr10	72096032	72218777	10q22.1	ASCC1	4.006431289	3.695953415	1.084004813	4	4	38.76048039	31.46034312
chr22	29883165	30030868	22q12.2	MTMR3	53.36171029	49.2264991	1.084003764	4	4	7.076588799	26.66332986
chr15	75903859	75935268	15q24.2	FBXO22	3.145450316	2.901819038	1.083958123	4	4	47.60865847	9.573558513
chr3	51957454	51967466	3p21.2	PCBP4	0.763932776	0.704772925	1.083941718	4	4	58.91459799	37.90547044
chrX	74421493	74533929	Xq13.2	SLC16A2	0.129110249	0.11911349	1.083926339	1	3	0	48.96909917
chr14	22422546	22423042	14q11.2	TRDV2	21.49210444	19.82928246	1.083856891	4	4	69.08285222	49.29979978
chr11	71722143	71780314	11q13.4	ENPP7P8	0.396185458	0.365540687	1.083834091	4	2	82.12707354	11.95864422
chr7	104911873	104912145	7q22.3	RN7SL8P	1.728931579	1.595212971	1.083824926	2	2	24.57590091	46.3559759
chr17	63998351	64004305	17q23.3	PRR29	0.141158192	0.130246539	1.083776912	4	3	60.08763078	44.68128062
chrX	63426558	63561095	Xq11.1	LINC01278	0.867972145	0.800896105	1.083751238	4	4	64.50847132	30.52375057
chr4	159104069	159360169	4q32.1	RAPGEF2	36.92640688	34.07289929	1.083747132	4	4	20.58039873	37.21398817
chr6	116100833	116245690	6q22.1	NT5DC1	5.319294286	4.908481966	1.083694373	4	4	48.3804861	48.24759488
chr15	64460350	64686068	15q22.31	ZNF609	11.77335177	10.86426083	1.083677201	4	4	70.379855	39.52275794
chr3	101518867	101523887	3q12.3	FAM172BP	0.599486183	0.553216751	1.083637076	2	3	79.16154466	89.22316028
chr2	48440769	48515392	2p16.3	PPP1R21	16.79887213	15.50385066	1.083529022	4	4	24.47844077	19.82047767
chr7	2558972	2614734	7p22.3	IQCE	2.635051606	2.432006947	1.083488519	4	4	66.302586	41.38997228
chr7	74657665	74760692	7q11.23	GTF2I	10.56763342	9.754102081	1.083404021	4	4	18.8457473	14.82405809
chr4	39698044	39782792	4p14	UBE2K	174.8426292	161.3875786	1.083371042	4	4	58.56174192	22.24925658
chr1	100392836	100393658	1p21.2	BCAS2P2	1.152917359	1.064315476	1.083247764	2	3	79.60991303	107.2366183
chr15	67065857	67195195	15q22.33	SMAD3	5.116714744	4.723603965	1.083222637	4	4	34.63365188	29.87238166
chr10	69180204	69209098	10q22.1	SUPV3L1	6.903397735	6.373453567	1.083148667	4	4	32.6704324	40.50199848
chr1	151402724	151459465	1q21.3	POGZ	7.040934145	6.500667852	1.083109352	4	4	29.86638849	19.01566231
chr20	5064877	5113091	20p13-p12.3	TMEM230	28.93090471	26.71334602	1.083013138	4	4	16.13430678	16.88029693
chr20	3046029	3048250	20p13	MRPS26	4.14098858	3.82362996	1.082999303	4	4	52.52007796	14.8513771
chr12	48829756	48852174	12q13.12	DDX23	21.97173425	20.28817231	1.082982435	4	4	48.01663475	50.33836548
chr5	137751384	137754350	5q31.2	HNRNPA0	8.270863511	7.637277995	1.082959598	4	4	32.26666511	8.105242207

chr14	22052521	22053056	14q11.2	TRAV21	11.14018348	10.28689048	1.082949556	4	4	45.54628929	49.97205975
chr12	47661932	47706061	12q13.11	RPAP3	15.8147887	14.60581331	1.082773576	4	4	43.66756904	28.27278443
chrX	75053280	75156340	Xq13.3	ABCB7	24.38885087	22.52599227	1.082698181	4	4	23.56110845	32.00837518
chr15	80947326	80989878	15q25.1	MESD	6.832323883	6.31103438	1.082599693	4	4	37.1707867	44.34339874
chr11	47778083	47848544	11p11.2	NUP160	9.187555226	8.487208534	1.082517908	4	4	36.08553822	6.585656136
chr13	40911887	40921774	13q14.11	SUGT1P3	3.439923856	3.17773361	1.082508567	4	4	35.07128488	58.56717218
chr3	49721476	49723974	3p21.31	GMPPB	1.42611159	1.317426983	1.082497633	4	4	56.61332192	20.08260287
chr3	50669989	50672069	3p21.2	LINC02019	0.452872373	0.418363135	1.082486327	3	4	13.92008267	10.2853871
chr8	143267437	143276931	8q24.3	GLI4	1.899974051	1.755198737	1.082483716	4	4	77.50079717	56.77399306
chr1	109213893	109238182	1p13.3	SARS	26.24965668	24.25257466	1.082345155	4	4	50.50677192	33.80824254
chr5	82968810	82977347	5q14.2	ST13P12	0.313505063	0.289657889	1.082328754	1	1	0	0
chr8	123248450	123275541	8q24.13	ZHX1	10.40494585	9.614175198	1.082250493	4	4	21.81028094	11.43295781
chr17	61865367	61928032	17q23.2	INTS2	13.41709848	12.39808067	1.082191578	4	4	54.12840615	26.80247362
chr1	29147738	29182125	1p35.3	SRSF4	26.67005295	24.64524146	1.082158314	4	4	21.38998361	19.33045829
chr13	23328823	23433728	13q12.12	SACS	8.166731864	7.548690027	1.081874052	4	4	39.76362043	25.30768613
chr20	4193090	4195953	20p13	LINC01433	4.041375877	3.735982851	1.08174369	4	4	53.23558176	19.93519255
chr6	33200826	33204437	6p21.32	SLC39A7	4.930547232	4.558314669	1.081660129	4	4	63.24807653	35.30733576
chr12	48147788	48157595	12q13.11	ASB8	14.36594146	13.28205209	1.081605565	4	4	18.19443111	17.67173014
chr12	88142296	88199887	12q21.32	TMTC3	6.753472857	6.243962522	1.081600479	4	4	33.80758314	23.18324442
chr11	71928701	71997597	11q13.4	RNF121	3.210648284	2.968757702	1.081478721	4	4	30.0968969	21.89182433
chr6	152697895	152747175	6q25.2	MYCT1	4.432470733	4.098611497	1.081456668	4	4	26.05150546	57.14027314
chr14	67360317	67386516	14q23.3	EIF2S1	14.6675808	13.56296364	1.081443642	4	4	20.82415553	25.29044274
chr2	218200276	218201845	2q35	HMGB1P9	0.437344462	0.404416374	1.081421253	1	2	0	2.918756246
chr3	45742675	45742970	3p21.31	RN7SL145P	2.786777544	2.577013279	1.081398209	2	4	7.978232598	37.45743841
chrX	15738281	15787625	Xp22.2	CA5B	5.301370917	4.902375643	1.081388148	4	4	22.11534704	31.62302481
chr1	16460948	16468481	1p36.13	LINC01772	1.527206746	1.412353496	1.08132047	4	4	73.8694517	63.614351
chr1	40691570	40771603	1p34.2	NFYC	8.377889283	7.747886048	1.081312919	4	4	46.79181046	26.17628025
chr1	26900569	26946871	1p36.11	NUDC	21.10468364	19.51830621	1.081276388	4	4	27.24698031	38.8891989
chr14	106184899	106185335	14q32.33	IGHV1-18	8.380349509	7.750583763	1.081253976	4	4	71.30768127	113.4790005
chr14	69083345	69085714	14q24.1	DDX18P1	0.26066278	0.24107783	1.081239116	3	2	70.93242856	63.35847617
chr11	72002864	72080693	11q13.4	NUMA1	16.59858424	15.35149956	1.081235366	4	4	60.28426954	39.42863161
chr16	83899125	83916182	16q23.3	MLYCD	0.979413947	0.905875431	1.081179502	4	4	53.28055394	22.93451287
chr22	22819125	22819752	22q11.22	IGLV3-9	4.934497117	4.564227938	1.081124165	3	4	32.55025946	80.92233987
chr20	32819780	32850405	20q11.21	MAPRE1	57.61005008	53.29081729	1.081050226	4	4	32.98291994	9.178211781
chr1	169106709	169132722	1q24.2	ATP1B1	1.65543359	1.531339974	1.081035967	4	4	20.05285171	86.91007704
chr16	4474697	4510347	16p13.3	HMOX2	6.484881232	5.998784744	1.081032494	4	4	47.50882177	34.2126366
chr6	170535116	170553329	6q27	PSMB1	47.36113564	43.81327978	1.080976724	4	4	9.783589028	12.79502434
chr5	5422673	5490234	5p15.32	ICE1	21.00817382	19.43501287	1.080944683	4	4	28.77500253	6.473845713
chr10	103594027	103855406	10q24.33	SH3PXD2A	0.845265346	0.7819734	1.080938746	4	4	125.4676354	42.86444987
chr13	48075728	48095141	13q14.2	MED4	45.33411568	41.94001445	1.080927517	4	4	17.12483775	17.86112655
chr6	80106610	80469088	6q14.1	BCKDHB	3.710432202	3.432835989	1.08086498	4	4	27.95127228	20.07320934
chr1	28668728	28715603	1p35.3	GMEB1	8.690937906	8.041449836	1.080767534	4	4	34.2262725	12.35664293
chr17	75874164	75878599	17q25.1	TRIM47	0.383361966	0.354734648	1.080700654	2	3	45.32898721	72.45745262

chr17	35490032	35494381	17q12	E2F3P1	0.452379989	0.418598908	1.080700354	2	4	57.68778407	66.75310046
chr5	140564565	140569104	5q31.3	SLC35A4	7.599888882	7.032459123	1.080687246	4	4	41.63476276	27.05587941
chr9	135499989	135504673	9q34.3	MRPS2	5.096696452	4.716170782	1.080685303	4	4	56.08333617	67.43863347
chr18	63887705	63903890	18q21.33-q22.1	SERPINB2	0.751013968	0.694964608	1.080650668	4	2	52.54253003	23.52328236
chr11	124766492	124800972	11q24.2	MSANTD2	1.623216402	1.502196257	1.08056214	4	4	39.67203264	38.13138151
chr2	112275594	112340063	2q14.1	ZC3H6	27.11842751	25.09681089	1.08055273	4	4	11.59917249	23.43256187
chr1	156806238	156816853	1q23.1	SH2D2A	11.00971091	10.18920787	1.080526676	4	4	54.92799031	78.32257993
chr11	57551655	57568330	11q12.1	UBE2L6	76.90946578	71.18457863	1.080423137	4	4	55.16944621	53.13832176
chr6	35297818	35321771	6p21.31	DEF6	40.43485159	37.42548275	1.08040962	4	4	33.26402574	36.78715883
chr1	42456341	42473381	1p34.2	PPCS	16.50294572	15.27629066	1.080297966	4	4	33.85260466	17.42649772
chr1	2508531	2526628	1p36.32	PANK4	3.191524888	2.954340236	1.080283459	4	4	50.99704733	25.58862702
chrX	154295674	154330363	Xq28	TKTL1	1.669697094	1.545612638	1.08028173	4	4	75.48141552	24.5818716
chrX	77899464	77905384	Xq21.1	COX7B	21.31362953	19.7301367	1.08025757	4	4	11.41179512	18.29322992
chr19	37371057	37393066	19q13.12	ZNF527	1.484674064	1.374518218	1.080141423	4	4	38.58814404	27.06779421
chr6	31834915	31839766	6p21.33	C6orf48	23.54235788	21.79597654	1.080124024	4	4	25.06089066	19.35859775
chr20	17941596	17968991	20p11.23	SNX5	16.32329029	15.11375201	1.080028988	4	4	19.4543954	15.40877182
chr9	124658436	124698628	9q33.3	MIR181A2HG	1.406989128	1.302767826	1.079999905	3	4	47.2936426	46.31829901
chr2	87010464	87021846	2p11.2	PLGLB1	0.393364125	0.364273969	1.079857906	3	4	81.12781848	32.65657521
chr7	64043194	64085339	7q11.21	ZNF727	0.84995052	0.787186417	1.079732197	1	1	0	0
chr13	45333471	45341284	13q14.13	TPT1	841.4562881	779.4691084	1.07952487	4	4	8.480294147	22.13319053
chr6	152970519	152983619	6q25.2	FBXO5	5.198635707	4.815991377	1.079452869	4	4	38.2143018	48.46742199
chr2	42767087	42801002	2p21	HAAO	0.724073946	0.670778781	1.07945267	3	4	78.36390955	65.53818176
chr8	55738744	55773407	8q12.1	TMEM68	3.599593408	3.334739938	1.079422526	4	4	54.73794591	29.61548686
chr11	95768942	95790396	11q21	FAM76B	17.12298519	15.86383248	1.079372542	4	4	16.8979609	21.20490512
chr1	153974269	153977975	1q21.3	JTB	44.83903995	41.5458822	1.079265563	4	4	18.15157948	13.8220593
chrX	24148989	24216255	Xp22.11	ZFX	23.26210627	21.55439641	1.079227914	4	4	25.00871431	18.40318398
chr3	32951555	32955312	3p22.3	CCR4	8.487697003	7.865540092	1.079099071	4	4	36.6334443	32.27633315
chr8	85214053	85221079	8q21.2	C8orf59	14.95542901	13.8595101	1.079073423	4	4	61.69610009	27.03913163
chr22	43999164	44169233	22q13.31	PARVB	5.317973634	4.92838571	1.079049804	4	4	58.2727086	21.18930136
chr16	58392394	58406144	16q21	GINS3	0.711800326	0.659675946	1.079015128	4	4	46.47949891	62.60394109
chr3	122575926	122639047	3q21.1	PARP15	5.415635073	5.01955799	1.078906765	4	4	40.37577485	18.7721385
chr14	65006101	65102695	14q23.3	MAX	91.23215879	84.56180544	1.078881397	4	4	39.7158793	13.14599375
chr17	60677775	61392838	17q23.2	BCAS3	9.857445165	9.13780032	1.078754713	4	4	28.11160869	25.26905785
chr2	128091180	128195677	2q14.3	UGGT1	50.29888939	46.63363628	1.078596768	4	4	32.49913899	22.17623877
chr22	39130772	39152650	22q13.1	CBX7	3.982060799	3.692164529	1.078516618	4	4	73.07199883	38.9154138
chr14	21477176	21499177	14q11.2	TOX4	20.1435465	18.6777565	1.078477841	4	4	42.99241261	9.757228853
chr13	108218415	108235440	13q33.3	ABHD13	22.72465465	21.07383426	1.078335075	4	4	20.18878262	22.27133823
chr9	136401922	136410602	9q34.3	ENTR1	4.559332163	4.228172099	1.078322276	4	4	72.16357782	38.39568518
chr15	77994985	78077708	15q24.3-q25.1	TBC1D2B	8.133424087	7.542834039	1.078298163	4	4	34.62559793	34.78902034
chr13	95672835	95676925	13q32.1	DNAJC3-DT	1.365043676	1.265991106	1.078241126	4	4	35.49537875	73.88549512
chr1	156728471	156736960	1q23.1	RRNAD1	2.210814332	2.050428319	1.078220736	4	4	42.92317466	18.55749542
chr1	107056645	107059294	1p13.3	PRMT6	5.386749814	4.996303086	1.078147126	4	4	40.06992178	24.96159783
chr1	97545689	97547160	1p21.3	SEC63P1	0.23904914	0.221735278	1.078083478	3	3	28.76354858	11.72615288

chr17	81822361	81833294	17q25.3	MCRIP1	3.462087429	3.21140656	1.078059525	4	4	53.29290617	37.99795564
chr5	109689366	109869625	5q21.3	MAN2A1	50.78697984	47.1160271	1.077913037	4	4	66.0119937	47.77003133
chr17	42534238	42544449	17q21.2	NAGLU	1.935949674	1.796168387	1.077821928	4	4	73.35472408	40.93472552
chr1	159800506	159816257	1q23.2	FCRL6	16.22095723	15.05007478	1.077799112	4	4	30.54354906	80.05130187
chr2	47527537	47529164	2p16.3	HCG2040054	0.541501906	0.502437422	1.077749949	2	2	3.550376694	57.10844478
chr16	31458273	31467167	16p11.2	ARMC5	1.042083374	0.966915033	1.077740379	4	4	64.18196614	42.7915302
chr17	81911939	81918182	17q25.3	SIRT7	4.714341632	4.374314629	1.077732635	4	4	48.42017784	7.312379945
chr14	73957916	74019332	14q24.3	ENTPD5	6.900446547	6.404091811	1.077505874	4	4	20.77058452	41.55302824
chr14	22163349	22163870	14q11.2	TRAV29DV5	3.452470791	3.204140185	1.07750304	4	4	94.24454849	68.41273775
chr2	11481675	11642788	2p25.1	GREB1	0.022668888	0.021038659	1.077487296	3	3	19.9420602	7.464490944
chrX	75368427	75523502	Xq13.3	ZDHHC15	0.245384481	0.2277386	1.077483048	4	3	73.41961106	76.50675427
chr17	78129298	78142968	17q25.3	TMC8	19.65567569	18.24420631	1.077365349	4	4	61.44885108	24.55153269
chr6	38675925	38703176	6p21.2	GLO1	21.16024391	19.64143856	1.077326584	4	4	23.09463158	6.008716804
chr2	74461057	74465410	2p13.1	MOGS	10.19498824	9.463280671	1.077320709	4	4	35.55491883	29.29628086
chrX	147911951	147951127	Xq27.3	FMR1	16.42887953	15.25000374	1.077303312	4	4	19.15043144	10.09405684
chr1	35268694	35421944	1p34.3	ZMYM4	29.69065815	27.56120245	1.077262801	4	4	26.49523019	11.04580099
chr14	49782083	49853203	14q21.3	NEMF	53.36282677	49.53612764	1.077250672	4	4	31.08921125	24.76161174
chrX	24465227	24550466	Xp22.11	PDK3	16.16995987	15.01068551	1.077229941	4	4	28.56546073	20.61348501
chr19	18340573	18369953	19p13.11	PGPEP1	3.325892169	3.087509495	1.077208726	4	4	52.20719185	16.71691796
chr14	90540588	90816479	14q32.11	TTC7B	1.12281213	1.042394214	1.077147316	4	4	56.5383596	9.033253227
chr10	67796665	67838187	10q21.3	DNAJC12	0.136104696	0.126369341	1.077038903	1	2	0	5.951375869
chr15	50241945	50266049	15q21.2	HDC	2.089351328	1.939940959	1.077017998	4	4	74.16431415	46.70299961
chr8	28890370	29064945	8p21.1-p12	HMBOX1	6.788177292	6.303590124	1.07687479	4	4	20.8404529	14.75585847
chr14	74057662	74084493	14q24.3	ALDH6A1	2.999259389	2.785220119	1.076848242	4	4	55.45202732	29.98772268
chrX	134769566	134797232	Xq26.3	FAM122B	8.201363676	7.616526004	1.076785357	4	4	53.48570094	16.36097693
chr19	51491227	51501789	19q13.41	SIGLEC12	0.295542914	0.274471716	1.076770016	2	2	78.62331945	74.816443
chr17	34980498	35005828	17q12	LIG3	3.201360665	2.973161298	1.076753107	4	4	52.05079274	31.18008781
chr10	7750962	7788027	10p14	KIN	8.400837115	7.802140014	1.076734986	4	4	18.54873976	21.51474321
chr4	128809623	128875224	4q28.2	JADE1	12.13657861	11.27169277	1.076730785	4	4	30.23804509	24.75428519
chrX	120524589	120575829	Xq24	CUL4B	29.63445884	27.52498859	1.07663837	4	4	29.7042321	18.37124669
chr2	85538978	85545280	2p11.2	MAT2A	16.73704913	15.54593798	1.076618802	4	4	28.83889368	24.3334074
chr17	44677437	44689797	17q21.31	CCDC43	9.690185134	9.00078969	1.076592773	4	4	49.21075065	30.12520743
chr14	69380123	69398304	14q24.1	ERH	29.13418501	27.06267611	1.076544866	4	4	39.04835733	26.89460879
chr3	191329082	191398670	3q28	CCDC50	6.708762201	6.232043421	1.076494778	4	4	57.60085193	28.85695976
chr17	81867719	81871406	17q25.3	ARHGDIA	38.74301562	35.99015954	1.076489132	4	4	79.63451131	64.30802198
chr16	3022620	3024286	16p13.3	HCFC1R1	4.584476354	4.258815426	1.07646749	4	4	49.85475171	38.53640668
chr4	112788024	112788900	4q25	RPL7AP30	0.308213295	0.28633087	1.076423561	1	1	0	0
chr15	33851785	34074877	15q14	AVEN	5.096863732	4.735630171	1.076279935	4	4	29.53987472	33.50370784
chr16	30699141	30740129	16p11.2	SRCAP	0.584365328	0.542951215	1.076275937	4	4	81.16677595	29.7541029
chr9	136807922	136841187	9q34.3	RABL6	2.985793801	2.774453404	1.076173706	4	4	74.34543385	45.33537057
chr7	142645961	142646467	7q34	TRBV23-1	0.815099392	0.757422572	1.076148801	1	2	0	4.05903911
chr7	99458161	99466201	7q22.1	ATP5MF	12.74006275	11.8386432	1.076142133	4	4	27.93328293	42.21683042
chr20	46060985	46089941	20q13.12	NCOA5	10.72627942	9.96737022	1.076139361	4	4	36.89524484	23.84966397

chr20	58818918	58850903	20q13.32	GNAS-AS1	0.073657536	0.068450205	1.076074741	1	1	0	0
chr3	129001545	129040742	3q21.3	EFCC1	0.092762116	0.086204327	1.076072619	2	2	18.45955864	3.358604244
chr1	44819844	44842944	1p34.1	PTCH2	1.107762127	1.029487243	1.076032884	4	4	45.16551835	70.29006256
chr1	173183729	173462208	1q25.1	TNFSF4	9.692328974	9.007633241	1.076012834	4	4	33.49807732	54.18404279
chr11	2444991	2849110	11p15.5-p15.4	KCNQ1	4.41967355	4.108334366	1.075782338	4	4	63.72580251	39.44903635
chr8	143579722	143597675	8q24.3	EEF1D	22.33329614	20.76054849	1.075756555	4	4	68.04467032	34.97596527
chr3	58306245	58320193	3p14.3	RPP14	1.726828467	1.605249584	1.075738306	4	4	41.69600511	28.57567372
chr1	43969981	43973371	1p34.1	DPH2	2.986217687	2.775996422	1.075728219	4	4	56.7376609	36.24474005
chr3	52313010	52400497	3p21.1	DNAH1	2.698043539	2.508140065	1.07571486	4	4	73.75132151	26.66851106
chr16	1493345	1510459	16p13.3	TELO2	1.428283132	1.327772177	1.075698946	4	4	85.12397458	38.96660431
chr6	34877778	34888071	6p21.31	TAF11	12.86144997	11.95655139	1.075682238	4	4	28.58268247	11.05603179
chr17	81539885	81552403	17q25.3	FAAP100	2.090880176	1.943771401	1.075682138	4	4	58.24831013	24.29213785
chr5	134758734	134831138	5q31.1	DDX46	19.98443638	18.57880996	1.075657505	4	4	31.17157196	15.14698733
chr13	73686012	74168295	13q22.1	KLF12	22.40359036	20.8294449	1.075573087	4	4	47.68567928	16.85088761
chr19	5623035	5668478	19p13.3	SAFB	24.91003349	23.15990881	1.075566993	4	4	36.68886047	23.00254963
chr1	43979202	43991171	1p34.1	B4GALT2	0.388235409	0.360970516	1.075532186	4	4	53.69171758	98.6256849
chr14	75002909	75009591	14q24.3	EIF2B2	3.262733386	3.033651131	1.075513711	4	4	68.94244969	57.66241325
chr14	90061765	90185857	14q32.11	KCNK13	0.374900288	0.348587741	1.075483283	4	3	79.50556576	100.2517893
chr3	143971798	143992368	3q24	DIPK2A	54.75803207	50.91850646	1.075405307	4	4	54.56258391	62.02249691
chr8	11284416	11328146	8p23.1	MTMR9	5.915775893	5.500996296	1.075400814	4	4	39.95460991	17.10882707
chr6	36834886	36928964	6p21.2	C6orf89	29.6314276	27.55391521	1.075398083	4	4	16.72543923	30.29850672
chr7	121348851	121396369	7q31.31	FAM3C	8.666547853	8.059298141	1.075347716	4	4	24.11373726	31.32187774
chr19	43612101	43614498	19q13.31	SRRM5	0.149880398	0.139384893	1.075298727	2	2	59.01743796	49.17528873
chr3	37862152	37984469	3p22.2	CTDSPL	8.381384593	7.794649076	1.075274141	4	4	77.20952382	18.73943678
chr3	49805205	49813958	3p21.31	UBA7	15.69305378	14.59473741	1.075254274	4	4	26.96910453	25.57696872
chr3	42600007	42648741	3p22.1	NKTR	10.09244383	9.386390255	1.075220991	4	4	20.12975776	20.87137367
chr22	18110687	18131731	22q11.21	TUBA8	0.42675785	0.396921735	1.075168761	4	4	86.39840892	39.33491891
chr11	64230281	64234281	11q13.1	DNAJC4	6.375662869	5.929930065	1.075166621	4	4	72.56773438	40.71029666
chr7	74881990	74890612	7q11.23	STAG3L2	2.812142525	2.615628367	1.075130764	4	4	8.345570328	28.18003487
chr22	50546203	50551023	22q13.33	KLHDC7B	1.936192084	1.800904971	1.075121739	4	4	40.25469742	73.30311808
chr19	18831405	18868232	19p13.11	UPF1	10.67531355	9.929634981	1.075096272	4	4	52.96947127	16.66685734
chr4	103077618	103099867	4q24	BDH2	2.472848367	2.30014228	1.075084958	4	4	51.53544518	13.05822798
chr1	161159464	161165752	1q23.3	USP21	2.776425254	2.582560643	1.075066818	4	4	71.77139265	32.53921563
chr2	9423537	9473110	2p25.1	CPSF3	19.10716871	17.77469321	1.074964754	4	4	23.01688116	28.04505479
chr1	66812885	66924887	1p31.3	WDR78	16.87779663	15.70100746	1.074949915	4	4	90.26929051	63.51855977
chr5	68215737	68301821	5q13.1	PIK3R1	49.87862886	46.40105165	1.074946086	4	4	61.80074494	35.91622142
chr22	50217689	50245028	22q13.33	TUBGCP6	5.066193829	4.713076323	1.074922934	4	4	54.29962946	11.21262308
chr2	61868127	61888671	2p15	CCT4	47.3274866	44.03014393	1.074888301	4	4	42.08108881	26.23980388
chr8	123219956	123241398	8q24.13	C8orf76	1.661376365	1.545705519	1.074833689	4	4	51.04751571	43.39420728
chr12	47782710	47820612	12q13.11	HDAC7	6.476675473	6.025975526	1.074792861	4	4	65.02016437	42.52759916
chr1	201955491	201970661	1q32.1	TIMM17A	7.432328583	6.915376437	1.074754014	4	4	31.48627561	25.15920154
chr6	72621843	73198851	6q13	KCNQ5	0.64561032	0.600741338	1.074689353	4	4	49.83311576	49.60266305
chr11	6481447	6484681	11p15.4	TIMM10B	11.39950494	10.60772424	1.074641901	4	4	68.05696225	19.90036876

chr19	10651862	10653872	19p13.2	ILF3-DT	4.1098313	3.824471806	1.074614093	4	4	52.92591145	39.89939897
chr10	18512582	18659264	10p12.31	NSUN6	27.40407068	25.50180256	1.074593477	4	4	43.82093651	32.30800493
chr17	49788619	49829096	17q21.33	KAT7	12.07095211	11.23343174	1.074556057	4	4	32.70069546	12.91972918
chr12	51061205	51083671	12q13.12	CSRNP2	3.331238759	3.100976353	1.074254809	4	4	50.07172156	30.44739836
chr16	17932717	17933345	16p12.3	RPL7P47	0.615100957	0.572592104	1.074239328	1	1	0	0
chr3	32106511	32168715	3p22.3	GPD1L	6.231378474	5.800746681	1.074237304	4	4	30.97651477	17.18992147
chrX	154411518	154421726	Xq28	TAZ	2.748784638	2.558862516	1.074221308	4	4	64.75635856	14.59318476
chr1	202725185	202809470	1q32.1	KDM5B	9.550117636	8.890344194	1.074212362	4	4	23.93570432	20.74156349
chr19	4402596	4448322	19p13.3	CHAF1A	8.160100647	7.596647942	1.074171228	4	4	48.7700195	27.22547606
chr13	19633648	19673459	13q12.11	MPHOSPH8	23.64676341	22.01680298	1.074032566	4	4	29.58722297	41.5190041
chr3	57556247	57656480	3p14.3	PDE12	6.36803847	5.92958104	1.073944083	4	4	11.5527481	25.13359
chr4	17614628	17625628	4p15.32	MED28	12.52587641	11.66396143	1.073895561	4	4	31.91685752	19.15014077
chr19	9323772	9343845	19p13.2	ZNF559	2.605882682	2.426699335	1.073838297	4	4	48.33456405	27.50669089
chr2	86440647	86492716	2p11.2	KDM3A	12.68839993	11.81675442	1.07376353	4	4	27.41266251	10.85887984
chrX	15500777	15556529	Xp22.2	BMX	4.092323247	3.811260536	1.073745342	4	4	90.45003416	75.78863786
chr1	46278400	46303366	1p34.1-p33	LRRC41	2.981920277	2.777171557	1.073725629	4	4	30.71686815	32.15308959
chr12	1941591	2004535	12p13.33	DCP1B	4.605368004	4.289213323	1.073709246	4	4	40.47346236	14.03109639
chr7	25118651	25125361	7p15.3	CYCS	19.1778445	17.86181327	1.073678479	4	4	14.36296691	14.89922105
chr11	62750963	62754184	11q12.3	ZBTB3	2.245180992	2.091234603	1.073615073	4	4	54.96351721	10.21369217
chr5	160009100	160065545	5q33.3	TTC1	59.4735916	55.39814124	1.073566554	4	4	9.13675309	13.08162302
chr13	113977783	114132623	13q34	RASA3	27.00270982	25.15266381	1.073552687	4	4	48.43300701	37.74854595
chr6	169703905	169724010	6q27	PHF10	10.16137313	9.465734494	1.073490191	4	4	29.51289775	14.39170498
chr13	40863594	40863906	13q14.11	CYCSP34	1.207901293	1.125232733	1.073467965	2	2	31.8003756	36.35687599
chr5	129094749	129114028	5q23.3	ISOC1	15.14305415	14.1070368	1.073439756	4	4	21.19904873	15.9287574
chr6	42746958	42868560	6p21.1	BICRAL	22.96447113	21.39405813	1.073404167	4	4	29.11305866	9.743688618
chr11	110092425	110171841	11q22.3	ZC3H12C	1.65157708	1.5386592	1.073387194	4	4	34.92588101	17.8703899
chr1	88921044	88992960	1p22.2	KYAT3	17.75642011	16.54371753	1.073302907	4	4	13.41467534	22.90614528
chr12	57088894	57095476	12q13.3	NAB2	2.052357274	1.912472128	1.073143626	4	4	84.1448118	12.91294566
chr12	10372353	10390054	12p13.2	KLRK1	1.208477259	1.126133134	1.073121128	3	4	32.56926625	47.64894996
chr4	82353314	82373996	4q21.22	HNRNPD	24.76629577	23.07918133	1.073101139	4	4	16.29245711	14.76460091
chr1	156404252	156456649	1q22	C1orf61	0.05950851	0.055462147	1.072957197	2	3	86.18775376	107.7756649
chr16	18804853	18926428	16p12.3	SMG1	17.80543122	16.59593168	1.07287928	4	4	15.25312395	9.125359714
chr16	23603162	23641357	16p12.2	PALB2	7.091997425	6.610551704	1.072829885	4	4	41.00610528	30.50970205
chr8	103499934	104254430	8q22.3	RIMS2	0.022269733	0.0207587	1.072790377	1	1	0	0
chrMT	10760	12137	N/A	MT-ND4	333.1898346	310.5953545	1.072745712	4	4	58.1015267	39.63831477
chr3	170087580	170181752	3q26.2	PHC3	70.1488647	65.39321584	1.072723887	4	4	44.37434702	8.02190214
chr12	38316679	38329726	12q12	ALG10B	1.78652724	1.665436891	1.072707858	4	4	43.50374011	34.08559359
chr3	58427630	58433852	3p14.3	PDHB	11.11901267	10.36552533	1.07269167	4	4	29.84321954	8.053696019
chr17	7336506	7354944	17p13.1	ACAP1	14.54392965	13.55887185	1.072650425	4	4	49.6669762	33.91600258
chr13	25246201	25287566	13q12.13	MTMR6	27.98595384	26.09216601	1.072580706	4	4	32.0094413	17.28711962
chr9	137007234	137028922	9q34.3	ABCA2	2.75423351	2.567919042	1.07255465	4	4	48.09727889	34.08158486
chr18	670320	712664	18p11.32	ENOSF1	1.430883175	1.334095136	1.072549578	4	4	45.40723541	18.72425228
chr22	31496139	31618554	22q12.2	SFI1	3.201246845	2.984717896	1.072545868	4	4	45.40384803	29.42140736

chr3	9749944	9788246	3p25.3	OGG1	1.845924437	1.721088061	1.072533404	4	4	49.39634344	23.67597528
chr3	9397700	9478154	3p25.3	SETD5	9.999103345	9.323555175	1.07245607	4	4	19.38476293	2.690253466
chr12	42081845	42144871	12q12	GXYLT1	8.537979654	7.961534279	1.072403805	4	4	15.49531031	16.09397064
chr10	3141601	3148629	10p15.2	PITRM1-AS1	0.086910451	0.081042744	1.072402618	2	3	34.80075929	42.32800325
chr12	59595934	59789855	12q14.1	SLC16A7	7.803180461	7.276864867	1.072327246	4	4	33.6721149	24.66566867
chr22	41699503	41799477	22q13.2	MEI1	2.549559762	2.377655959	1.072299696	4	4	61.42977207	21.7451343
chr17	74203656	74209880	17q25.1	RPL38	206.4909524	192.5723208	1.072277426	4	4	32.51029669	19.23701411
chr19	8580240	8610735	19p13.2	ADAMTS10	1.187600336	1.107567879	1.07225964	4	4	77.17507649	46.0801151
chr1	33256307	33300719	1p35.1	ZNF362	8.07940408	7.535066459	1.072240587	4	4	46.36103389	36.50237007
chr20	36045618	36050960	20q11.23	NORAD	149.1971544	139.1620905	1.072110615	4	4	52.37425056	10.72388378
chr18	35982385	36067594	18q12.2	RPRD1A	16.16390919	15.07672437	1.072110147	4	4	41.77412055	30.84899094
chr12	30628981	30695995	12p11.21	IPO8	15.13624887	14.11851615	1.072084963	4	4	23.34269635	29.28969889
chr1	243255418	243503683	1q43-q44	SDCCAG8	25.48261563	23.76944874	1.072074321	4	4	29.82673461	27.20151388
chrX	54440375	54448032	Xp11.22	TSR2	14.78605968	13.79255842	1.072031688	4	4	57.42890711	33.24926621
chrX	102806741	102807824	Xq22.1	MTCYBP32	0.265411387	0.247583729	1.072006582	1	1	0	0
chr3	64010549	64024010	3p14.1	PSMD6	8.941107082	8.340785351	1.071974245	4	4	26.85015353	8.859444677
chr19	34172447	34229515	19q13.11	LSM14A	39.3330312	36.69278297	1.071955519	4	4	17.36844864	12.28774988
chr20	3783851	3786690	20p13	CENPB	13.55925064	12.64922415	1.071943266	4	4	54.22390484	36.69994409
chr6	89642775	89828021	6q15	MDN1	9.531459268	8.891782762	1.071940186	4	4	27.50083224	17.04084603
chr1	224114087	224162047	1q42.11	FBXO28	13.34005549	12.44500743	1.071920251	4	4	17.13431393	26.05820118
chr1	44405160	44651724	1p34.1	RNF220	5.330711477	4.973048817	1.071920199	4	4	64.62396454	33.62135177
chr2	236537131	236582358	2q37.3	ACKR3	1.124122937	1.048733271	1.071886407	4	4	84.18905616	76.48509008
chr15	75354326	75368680	15q24.2	MAN2C1	3.128368793	2.918596063	1.071874533	4	4	48.66401722	7.425534654
chr21	14816838	14918668	21q11.2	LINC02246	0.264755186	0.247016143	1.071813292	4	4	61.03981594	71.62906894
chr1	53765460	53838552	1p32.3	NDC1	23.53204088	21.9582279	1.071673042	4	4	56.21233622	25.59889965
chr12	49828543	49841154	12q13.12	BCDIN3D-AS1	0.151549645	0.141425249	1.071588323	1	1	0	0
chr14	23511760	23559581	14q11.2	THTPA	0.934978145	0.872555335	1.071540231	4	4	72.0619848	31.66221199
chr7	90595732	91210590	7q21.13	CDK14	8.557948964	7.98661462	1.071536486	4	4	16.4356566	42.6333587
chr16	69741854	69754968	16q22.1	NOB1	6.301836452	5.882051424	1.071367113	4	4	40.40896226	39.64771169
chr9	125200493	125234159	9q33.3	RABEPK	3.384595859	3.159655659	1.071191365	4	4	25.46939997	45.57896141
chr1	151769433	151790534	1q21.3	TDRKH	1.014087259	0.946701437	1.071179592	4	4	37.70420662	39.71295404
chr2	73214238	73227227	2p13.2	SMYD5	2.237163217	2.0885503	1.071156015	4	4	96.57590697	17.61618934
chr19	6952500	6990846	19p13.2	ADGRE4P	3.278846282	3.06118708	1.071102875	4	4	97.89036654	59.4488269
chr19	2841435	2860484	19p13.3	ZNF555	1.07003727	0.999033979	1.071071948	4	4	77.87434256	43.62922596
chr17	38351876	38405593	17q12	SOCS7	0.821759384	0.767268934	1.07101871	4	4	58.7758388	35.68617297
chr12	10212846	10223128	12p13.2	GABARAPL1	34.11500765	31.85298218	1.071014559	4	4	30.98885267	57.51128749
chr11	1919551	1938706	11p15.5	TNNT3	0.566315626	0.528770412	1.071004755	4	4	71.5486554	62.16505422
chr2	149569633	149587816	2q23.2	MMADHC	47.42242353	44.28255186	1.070905391	4	4	5.126329311	23.89264908
chr17	58519837	58556925	17q22	SEPT4-AS1	0.262106462	0.244757744	1.07088118	2	2	54.89243808	25.94336493
chr12	110124335	110218795	12q24.11	IFT81	3.999509361	3.734818282	1.070871207	4	4	69.61608524	63.15221859
chr16	2750903	2771412	16p13.3	SRRM2	18.84531418	17.59960123	1.070780748	4	4	66.91085452	24.4761081
chr16	1309153	1327018	16p13.3	UBE2I	11.11510533	10.38047211	1.070770694	4	4	37.66869631	25.92289332
chr15	77976009	77993241	15q24.3	ADAMTS7P3	0.145150518	0.135564861	1.070709013	1	1	0	0

chr12	6462237	6470899	12p13.31	VAMP1	3.04613226	2.845172026	1.070632015	4	4	79.61526294	9.907244555
chr12	50085200	50100711	12q13.12	SMARCD1	12.45923236	11.6374105	1.070618963	4	4	19.83162202	10.26608877
chr14	90396983	90408275	14q32.11	CALM1	81.13948479	75.78887639	1.070598862	4	4	25.68632648	20.64085759
chr11	287760	295688	11p15.5	PGGHG	14.34747071	13.40146343	1.070589849	4	4	67.41636677	45.28207967
chr18	44680072	45068510	18q12.3	SETBP1	2.412423942	2.253369935	1.070584951	4	4	53.65868043	45.23876089
chr2	62196127	62224731	2p15	B3GNT2	28.80916645	26.91057029	1.07055206	4	4	20.73456637	15.58543402
chr5	35048756	35230724	5p13.2	PRLR	0.274468036	0.256392851	1.070498006	4	4	49.41588411	50.38836607
chr22	21642253	21644299	22q11.21	SDF2L1	3.410578987	3.18605019	1.070472461	4	4	65.58235197	44.96755414
chr2	96184859	96208846	2q11.2	STARD7	34.44132297	32.17535501	1.070425577	4	4	43.09955102	29.5505227
chr4	169917761	169975902	4q33	LINC02275	0.101178358	0.094536175	1.070260758	3	2	45.68844418	54.68479635
chr7	6329409	6348959	7p22.1	FAM220A	4.926510254	4.603589009	1.070145542	4	4	23.01904229	36.35192242
chr20	52084011	52191985	20q13.2	ZFP64	1.50950654	1.410563819	1.070144094	4	4	69.22155359	60.31724991
chrX	71107404	71111631	Xq13.1	IL2RG	70.64497549	66.01665799	1.070108328	4	4	40.66598698	63.37549645
chr7	142722254	142722938	7q34	PGBD4P1	0.704370253	0.658241046	1.070079505	2	1	34.63383041	0
chr14	22946228	22957142	14q11.2	HAUS4	8.978568118	8.391171133	1.070001788	4	4	56.98392892	32.65014436
chr6	15522801	15663058	6p22.3	DTNBP1	3.910848477	3.655666527	1.069804494	4	4	23.46266613	33.71323204
chr5	179023752	179034387	5q35.3	ZNF879	2.119986888	1.981696864	1.069783642	4	4	33.68821846	13.13489174
chr1	160678746	160711851	1q23.3	CD48	95.76856566	89.53218023	1.069655239	4	4	43.91644788	35.22054237
chr1	120415027	120469670	1p11.2	NBPF8	3.614743969	3.379499781	1.069609174	4	4	78.03101359	29.09408347
chr6	34263270	34263723	6p21.31	RPL35P2	0.667528023	0.62415223	1.069495535	1	1	0	0
chr7	142407689	142408136	7q34	TRBV11-1	0.665579141	0.62239471	1.069384316	1	1	0	0
chr3	10280952	10293449	3p25.3	GHRLOS	1.435502058	1.342410313	1.069346715	4	4	66.51222286	56.02404017
chr7	142670777	142671244	7q34	TRBV25-1	2.055381442	1.922356924	1.069198657	1	4	0	67.64988154
chr1	25430858	25500209	1p36.11	MACO1	33.90071459	31.7070659	1.069184853	4	4	12.24349686	37.99935961
chr14	90256550	90272625	14q32.11	PSMC1	8.557528009	8.003843552	1.069177321	4	4	59.82069178	19.06446356
chr3	44748744	44761681	3p21.31	KIAA1143	2.988221793	2.794887958	1.069174092	4	4	28.84118016	27.65174383
chr3	48688003	48717278	3p21.31	IP6K2	7.773240888	7.270371616	1.069166928	4	4	42.20357431	29.20728081
chr12	75497639	75511638	12q21.2	KRR1	9.596834956	8.976051607	1.069159958	4	4	29.95615961	8.773827884
chr7	98215443	98252251	7q21.3	TECPR1	5.93241628	5.548790648	1.069136801	4	4	62.38991761	36.30364532
chr14	20455131	20457772	14q11.2	APEX1	23.61125285	22.08560409	1.069078878	4	4	59.71965228	49.47857748
chr6	154154483	154356766	6q25.2	IPCEF1	23.14674784	21.65121348	1.069073928	4	4	13.27803024	18.50981213
chr7	156472099	156603195	7q36.3	LINC01006	0.406344334	0.38010772	1.069024155	4	4	68.4899106	66.19675952
chr5	112452703	112452989	5q22.2	HMGB3P16	3.609127528	3.376120024	1.069016357	3	3	63.95237954	56.15302079
chr15	88604601	88632281	15q26.1	AEN	2.908612506	2.720950924	1.068969117	4	4	44.1932501	30.96891872
chr6	33420070	33453689	6p21.32	SYNGAP1	0.356946602	0.33392372	1.06894653	4	4	63.54212535	89.95458408
chr1	149903318	149917882	1q21.2	SV2A	0.344261899	0.322069766	1.068904739	3	4	67.7379342	62.61564724
chr6	8073360	8102595	6p24.3	EEF1E1	3.333357974	3.118484458	1.068903187	4	4	53.47500191	30.74971237
chr12	122271432	122422956	12q24.31	CLIP1	16.03894365	15.00625998	1.068816859	4	4	55.36250886	12.07757371
chr17	63773856	63819317	17q23.3	DDX42	14.85577126	13.90018187	1.068746538	4	4	21.87769869	13.16801827
chr4	128874390	129093607	4q28.2	SCLT1	202.8616098	189.8432963	1.068573996	4	4	49.73880899	34.36380885
chr3	101324189	101513883	3q12.3	SENP7	35.98215251	33.67417357	1.068538548	4	4	19.17735501	17.35847153
chr22	23894378	23895222	22q11.23	MIF	2.53164377	2.369283541	1.068527141	4	4	24.24548897	51.12503578
chr10	14518557	14774897	10p13	FAM107B	43.91430142	41.09972301	1.068481688	4	4	29.9788095	22.48592525

chrX	147981329	148026667	Xq27.3-q28	FMR1NB	0.408469994	0.382292462	1.068475146	1	3	0	31.89871366
chr14	36677318	37172660	14q13.3	SLC25A21	5.96356369	5.581491401	1.06845344	4	4	92.95341685	70.89243188
chr6	47477746	47627263	6p12.3	CD2AP	48.02200676	44.94984003	1.068346555	4	4	29.59342931	21.8064053
chr14	24290598	24299833	14q12	DHRS1	1.790320719	1.675826195	1.06832124	4	4	58.8499282	13.73330655
chr7	94509858	94633465	7q21.3	CASD1	8.497972422	7.955392829	1.068202741	4	4	21.53249383	20.93911573
chr20	18587893	18763917	20p11.23	DTD1	5.278704277	4.941872272	1.068158784	4	4	47.85249487	14.8226731
chr13	42562736	42608013	13q14.11	TNFSF11	0.492672092	0.461243328	1.068139228	3	4	75.26023546	68.86039501
chr2	216031656	216081843	2q35	PECR	3.371866285	3.157017828	1.068054243	4	4	70.86827096	34.29573779
chr16	88451782	88535166	16q24.2	ZFPM1	1.351826492	1.265870859	1.067902371	4	4	58.28372831	47.79856107
chr10	46005088	46030714	10q11.22	NCOA4	1290.446839	1208.395429	1.067901125	4	4	22.85099755	47.63656029
chr7	100101413	100107180	7q22.1	AP4M1	1.249546592	1.170269056	1.067742999	4	4	20.96657529	21.47384887
chr20	43457864	43463605	20q13.11	SRSF6	48.3926245	45.32429191	1.067697309	4	4	37.11905125	17.46119632
chr4	56978640	57031162	4q12	POLR2B	32.47333241	30.41527373	1.067665302	4	4	23.24744694	20.60135514
chr19	55635028	55645623	19q13.42	ZNF581	5.255361613	4.922429576	1.067635714	4	4	94.14056681	5.87143114
chr2	118088418	118110031	2q14.1-q14.2	INSIG2	7.007692293	6.563946796	1.067603457	4	4	11.07598348	27.2953047
chr9	134641789	134844843	9q34.3	COL5A1	0.084877702	0.079503184	1.067601294	4	3	49.16402696	79.30856564
chr11	116748170	116772998	11q23.3	BUD13	13.05643855	12.23066122	1.067516982	4	4	49.0196839	48.73442808
chr19	57849813	57865123	19q13.43	ZNF587	7.676783813	7.19130862	1.067508602	4	4	41.38138324	17.7683577
chr18	13217604	13652754	18p11.21	LDLRAD4	3.07026545	2.876122443	1.067501649	4	4	59.47592454	22.5823606
chr3	41199422	41240453	3p22.1	CTNNB1	34.92244277	32.71733191	1.067398859	4	4	25.98202009	19.94913784
chr3	108043094	108094200	3q13.12	CD47	41.08076954	38.48779484	1.06737135	4	4	16.48033685	13.92654815
chr14	74763316	74837310	14q24.3	YLPM1	7.286167186	6.827183431	1.067228859	4	4	57.71339445	29.45273312
chr15	44537068	44562803	15q21.1	EIF3J	65.67882795	61.54217437	1.067216565	4	4	9.10393683	26.33176518
chr8	91070196	91087095	8q21.3	OTUD6B	6.769476904	6.343270385	1.067190344	4	4	11.65273624	19.73899569
chr8	123497887	123541253	8q24.13	FBXO32	3.938415988	3.690516073	1.067172154	4	4	25.17165906	18.15912675
chr16	3298808	3305439	16p13.3	TIGD7	3.442973861	3.22625988	1.067171892	4	4	59.26307671	38.47557074
chr3	15206226	15252916	3p25.1	CAPN7	14.54276333	13.62749489	1.067163367	4	4	25.71813545	26.51889148
chr12	122527106	122626409	12q24.31	KNTC1	26.49420526	24.82844097	1.067090974	4	4	75.71736053	35.18385929
chr1	55217861	55234177	1p32.3	MIR4422HG	0.912648322	0.855307297	1.06704143	3	1	90.8276963	0
chr11	45909669	45918123	11p11.2	PEX16	4.580395527	4.292988574	1.066947989	4	4	32.09448664	16.51613255
chr19	17309528	17323301	19p13.11	DDA1	6.249835593	5.858058041	1.066878401	4	4	41.80487509	41.69851708
chr9	137174784	137188605	9q34.3	ANAPC2	2.658221621	2.49171619	1.066823594	4	4	39.80075757	9.855559036
chr22	46360834	46537502	22q13.31	CELSR1	0.276555475	0.259233696	1.066819164	3	4	42.25096657	70.54919799
chr10	46375590	46391784	10q11.22	ANXA8L1	0.038529051	0.036116782	1.066790813	1	1	0	0
chr16	4608883	4614926	16p13.3	UBALD1	5.114332791	4.794223973	1.066769684	4	4	45.17979154	37.24663826
chr2	219219380	219229688	2q35	ATG9A	10.38034006	9.730798857	1.066751067	4	4	6.90250969	40.61116804
chr3	5187674	5219965	3p26.1	EDEM1	18.5327493	17.37323166	1.066741621	4	4	31.60133294	20.4831615
chr8	28090231	28191153	8p21.1	ELP3	14.59349006	13.68046777	1.066739113	4	4	23.18672398	20.38718267
chr10	32958459	33082102	10p11.22	ITGB1-DT	0.246054659	0.230665066	1.066718351	3	1	11.13811303	0
chr4	705741	770980	4p16.3	PCGF3	5.342042985	5.008061359	1.066688805	4	4	60.01516289	32.02061819
chr3	28241593	28319776	3p24.1	CMC1	7.794036714	7.30785297	1.066528944	4	4	62.5829025	53.4894148
chr1	32108043	32176568	1p35.2	KPNA6	19.56393229	18.34390246	1.06650874	4	4	15.81049559	26.95191064
chr15	43804535	43824753	15q15.3	MFAP1	20.17380511	18.91664428	1.06645792	4	4	45.37505536	16.66141735

chr17	34961530	34963186	17q12	ZNF830	11.968077	11.22251885	1.066434119	4	4	63.25044687	38.47906319
chr8	30678061	30727999	8p12	GSR	41.81096082	39.21121937	1.066300959	4	4	29.24224652	28.04656497
chr17	28371662	28401045	17q11.2	SARM1	1.085440414	1.018026187	1.066220524	4	4	82.00121445	34.05737726
chrX	73563045	73687109	Xq13.2	CHIC1	6.146724556	5.765176345	1.066181534	4	4	37.78031909	2.195388731
chr6	95560096	95577451	6q16.1	MANEA-DT	3.369020229	3.160093398	1.066114132	4	4	40.353163	34.89526493
chr17	4671384	4704337	17p13.2	PELP1	2.560964026	2.402199789	1.066091188	4	4	66.70581491	66.62901667
chr19	20923227	20950697	19p12	ZNF85	1.172162469	1.099517653	1.066069714	4	4	73.39173949	39.11827695
chr7	66114818	66154568	7q11.21	CRCP	8.542122057	8.013034686	1.066028339	4	4	48.47054451	24.71341617
chr6	105302420	105302859	6q21	RPL35P3	1.201767001	1.127403117	1.065960332	2	2	15.61692737	26.7677352
chr12	132840352	132887618	12q24.33	CHFR	3.5942766	3.371877709	1.065956986	4	4	58.9060339	22.75201397
chr17	16442005	16492191	17p11.2	LRRC75A	2.752162773	2.581990708	1.065907311	4	4	31.15913512	22.06406237
chr17	58083419	58090257	17q22	DYNLL2	16.36125396	15.3497531	1.065896881	4	4	20.21290034	8.338758715
chr20	2835314	2841243	20p13	PCED1A	3.691376678	3.463221839	1.06587936	4	4	64.63226667	34.86670506
chr16	4958306	5019157	16p13.3	SEC14L5	2.080805096	1.952381353	1.065778001	4	4	77.17311622	25.62722263
chr1	148334479	148334554	1q21.2	MIR5087	5.806196412	5.447999902	1.065748259	2	3	47.15537849	43.51478629
chr1	9539469	9582773	1p36.22	SLC25A33	1.828551828	1.715855176	1.065679583	4	4	31.81938789	27.74375982
chr11	5225466	5227071	11p15.4	HBB	545020.9357	511454.6459	1.065629064	4	4	0	0
chr16	3443611	3486963	16p13.3	NAA60	4.428515579	4.155892652	1.065599127	4	4	66.66289276	29.77432532
chr1	23964347	23980463	1p36.11	SRSF10	19.23010073	18.04734135	1.065536488	4	4	24.52480601	18.14573817
chr16	89816758	89871319	16q24.3	SPIRE2	0.127735057	0.119890142	1.065434192	2	3	11.37134452	86.55694834
chr5	157743695	157760709	5q33.3	LSM11	1.110515277	1.042383764	1.065361257	4	4	38.21586847	16.38124093
chr14	70044217	70189626	14q24.2	SLC8A3	0.707010558	0.663685105	1.065280136	4	4	81.50187195	36.92120856
chr7	19695462	19709037	7p21.1	TWISTNB	7.583236823	7.118915966	1.065223534	4	4	18.64695511	19.33991711
chr7	7636563	7718607	7p21.3	RPA3	5.03978385	4.731459894	1.065164656	4	4	36.08869764	26.32221106
chr1	111449121	111461918	1p13.2	ATP5PB	36.90840365	34.65271508	1.065094137	4	4	6.546449729	21.51630861
chr2	179944876	180007296	2q31.3	CWC22	28.15561863	26.43606221	1.065045861	4	4	14.41174437	17.77848844
chr17	63819433	63827671	17q23.3	FTSJ3	7.951450183	7.467191129	1.064851568	4	4	54.54590772	50.78729943
chr16	30602558	30610775	16p11.2	ZNF689	3.841741063	3.607867682	1.064823159	4	4	68.80976579	28.83740199
chr10	95663396	95694143	10q24.1	TCTN3	4.39031962	4.123107989	1.064808303	4	4	30.17486848	19.57427006
chr7	129484504	129484656	7q32.1	RNU1-72P	3.141851785	2.950948025	1.064692349	2	4	27.35683272	71.13093424
chr20	45361937	45376802	20q13.12	SYS1	6.252222931	5.872405326	1.06467837	4	4	35.11622149	30.3379346
chr9	127885321	127906764	9q34.11	ST6GALNAC6	5.950206894	5.588841363	1.064658398	4	4	34.92980025	46.63622789
chr14	30893799	31026401	14q12	STRN3	180.7741215	169.8018334	1.064618195	4	4	45.62739644	24.6368911
chr14	22496887	22496952	14q11.2	TRAJ42	18.63495474	17.50393082	1.064615424	4	3	96.82012019	34.91220306
chr11	1274368	1309662	11p15.5	TOLLIP	7.326027491	6.882247485	1.064481844	4	4	31.459947	32.68335034
chr5	177519789	177554585	5q35.3	FAM193B	4.97392982	4.672647032	1.064477969	4	4	46.36509414	8.103400004
chr4	3463306	3501476	4p16.3	DOK7	0.293860121	0.276063844	1.06446435	3	2	49.064232	51.37198729
chr19	57769652	57780616	19q13.43	ZNF586	23.21102193	21.80582041	1.06444158	4	4	29.93727123	14.04613685
chr7	92246223	92401773	7q21.2	ANKIB1	34.2429114	32.1709749	1.064403908	4	4	30.36383118	7.751315577
chr1	52348437	52366291	1p32.3	CC2D1B	6.124904913	5.754398298	1.064386682	4	4	72.22676727	11.78254951
chr1	192636138	192660311	1q31.2	RGS13	0.239104177	0.224645537	1.064362018	4	2	79.36393108	52.73744463
chr11	119015712	119018347	11q23.3	RPS25	389.4395073	365.9023071	1.06432646	4	4	22.67550521	23.81028994
chr9	128818402	128822676	9q34.11	ENDOG	1.511589626	1.420364101	1.06422686	4	4	77.67603674	89.60480413

chr2	88627829	88631825	2p11.2	EIF2AK3-DT	0.686330389	0.644911672	1.06422386	4	4	82.1861273	65.27292473
chr1	113761832	113812537	1p13.2	RSBN1	20.01349213	18.80658032	1.064174975	4	4	23.76496647	20.73225342
chrX	53936680	54048935	Xp11.22	PHF8	9.990758864	9.38960604	1.064023221	4	4	23.75706041	23.6749239
chr5	53095679	53109772	5q11.2	MOCS2	3.494957001	3.284766889	1.063989354	4	4	12.05630963	19.12710827
chr22	22887780	22895834	22q11.22	IGLL5	1.141814616	1.073158219	1.063976024	3	4	82.19539041	75.22573651
chr17	15699577	15720787	17p12	ZNF286A	1.113400594	1.046501073	1.063926854	4	4	46.19926338	13.8356828
chr6	138904015	138988261	6q24.1	REPS1	14.82618893	13.93605933	1.063872403	4	4	47.10091637	24.50756542
chr16	69311384	69325043	16q22.1	VPS4A	3.340996909	3.140447631	1.063860093	4	4	59.85327477	19.54554373
chr3	160256986	160399532	3q25.33	IFT80	7.606890869	7.150341785	1.063849967	4	4	68.44606216	60.25647982
chr7	74773962	74789376	7q11.23	NCF1	65.49124821	61.56065365	1.063849136	4	4	73.46265392	58.98326964
chr2	46901870	46941855	2p21	MCFD2	12.13457778	11.40635697	1.063843418	4	4	31.89481098	28.51266616
chr15	65148219	65185420	15q22.31	CLPX	67.17274228	63.14211709	1.063834179	4	4	36.47026554	25.48307632
chr9	128947693	129007096	9q34.11	NUP188	5.08474937	4.779838458	1.06379105	4	4	64.75335746	52.03441696
chrX	71059247	71073426	Xq13.1	SNX12	10.33758911	9.718842468	1.063664644	4	4	27.58432342	17.48296807
chr3	59747278	61251474	3p14.2	FHIT	3.112139838	2.925893743	1.063654429	4	4	59.3904157	17.03552449
chr8	31033262	31175871	8p12	WRN	51.53234102	48.45267802	1.063560223	4	4	78.60059435	48.89750799
chr1	37956975	37990110	1p34.3	SF3A3	17.76846539	16.70717155	1.063523251	4	4	40.85136851	38.82094823
chr5	178602664	178627053	5q35.3	CLK4	9.530746016	8.961953628	1.063467455	4	4	24.68851559	24.32247154
chr9	35161969	35405338	9p13.3	UNC13B	0.205236292	0.193016424	1.06330999	4	4	48.11650182	28.26542915
chr5	81301583	81313147	5q14.1	ZCCHC9	4.884443216	4.59399542	1.063223354	4	4	59.15529992	36.34933102
chr12	53464468	53465057	12q13.13	PCBP2-OT1	1.297735406	1.220648717	1.063152231	3	3	56.91517436	68.26426582
chr12	57610180	57617245	12q13.3	ARHGEF25	0.144457299	0.135877331	1.063144954	2	1	42.22540305	0
chr3	184186075	184194012	3q27.1	ABCF3	5.025888756	4.72743379	1.063132553	4	4	55.34868888	35.91115613
chr2	27888709	28338901	2p23.2	BABAM2	8.49328675	7.989500665	1.063056016	4	4	28.14953027	25.15133225
chr2	46617172	46630176	2p21	CRIPT	9.928525873	9.339774769	1.06303697	4	4	25.91502919	37.01046776
chr17	35574795	35578637	17q12	PEX12	2.014423554	1.895018771	1.063009816	4	4	47.20536627	15.36844784
chr19	51345155	51366418	19q13.41	ETFB	1.100695054	1.035480359	1.062980137	4	4	58.0892777	18.6540841
chr14	23058564	23095614	14q11.2	ACIN1	18.49874437	17.40331106	1.062943959	4	4	46.96266659	30.3685734
chr12	57723765	57742201	12q14.1	AGAP2	9.407196398	8.850285228	1.062925788	4	4	52.25889376	41.51882708
chr19	29606263	29617255	19q12	POP4	3.492817482	3.286304933	1.062840349	4	4	55.08922025	38.89363554
chr15	99714798	99733458	15q26.3	LYSMD4	0.933503156	0.878327905	1.06281851	4	4	50.41505118	34.14241492
chr22	37469063	37486440	22q13.1	MFNG	20.65584414	19.43499781	1.062816901	4	4	47.37204274	22.95435948
chr6	146598965	146815462	6q24.3	ADGB	0.088378816	0.083163721	1.062708771	4	3	49.91226658	54.99479187
chr3	4896809	4979961	3p26.1	BHLHE40-AS1	0.263327462	0.247793229	1.062690305	4	3	64.3711107	45.43975266
chr16	53703963	54114467	16q12.2	FTO	2.524090461	2.37526856	1.062654768	4	4	36.42629131	13.40985207
chr4	70901970	70988174	4q13.3	MOB1B	49.68734567	46.75825372	1.062643314	4	4	43.56680799	53.30484384
chr16	3432422	3443537	16p13.3	ZNF597	2.706305044	2.546894069	1.062590344	4	4	98.94523189	11.48767486
chr15	64687574	64703281	15q22.31	OAZ2	18.61834919	17.52228947	1.062552312	4	4	33.91992087	51.37078055
chr2	175176258	175181762	2q31.1	ATP5MC3	6.373257532	5.998107274	1.062544773	4	4	25.86898281	19.41967044
chr12	121308245	121354209	12q24.31	ANAPC5	7.872717965	7.410118224	1.062428119	4	4	52.50746545	4.860278148
chr5	138473744	138474417	5q31.2	RPL7P19	0.547749655	0.515599082	1.062355761	4	1	31.04122508	0
chr16	67207139	67227062	16q22.1	LRRC29	0.519770928	0.489262761	1.062355383	4	4	57.37312392	42.58881152
chr2	88860568	88860605	2p11.2	IGKJ5	49.49481674	46.5922293	1.062297672	3	2	59.00006723	15.88605487

chr1	54641740	54710267	1p32.3	MROH7	0.030589964	0.02879617	1.062292799	1	1	0	0
chr5	134873770	134901633	5q31.1	TXNDC15	6.538096473	6.15540378	1.062171826	4	4	45.15237066	37.14301595
chr14	54567097	54793315	14q22.2	SAMD4A	0.6263057	0.58966913	1.062130724	4	4	42.34406652	57.29976635
chr1	28887091	29120046	1p35.3	EPB41	740.8465428	697.5116231	1.062127882	4	4	20.91490339	35.65228831
chr7	56079927	56093450	7p11.2	PHKG1	3.405521597	3.206384728	1.062106355	4	4	63.97867154	29.2002551
chr19	32581188	32587452	19q13.11	PDCD5	6.747031247	6.352638288	1.062083333	4	4	43.05083334	32.59780822
chr16	81006498	81033107	16q23.2	CENPN	11.68935054	11.00608206	1.062080991	4	4	97.22281087	35.15547769
chr14	96363452	96387290	14q32.2	GSKIP	10.22485305	9.627653267	1.062029631	4	4	8.126401768	18.98265548
chr2	55235583	55269314	2p16.1	MTIF2	13.63216515	12.83608387	1.062019015	4	4	37.01747081	21.05871152
chr4	101972422	101972802	4q24	MTND5P5	4.042110131	3.806439356	1.061913708	2	4	70.68138736	96.53288455
chr12	43793723	43806375	12q12	TWF1	18.58988065	17.50731879	1.061834817	4	4	9.853188138	15.88632473
chr13	67831564	67832843	13q21.32	NPM1P22	0.301580971	0.284023199	1.061818091	1	1	0	0
chr7	93885397	93890991	7q21.3	TFPI2	0.107324384	0.101081187	1.061764185	1	1	0	0
chr2	113437691	113496204	2q14.1	CBWD2	5.085873076	4.790085882	1.061749873	4	4	13.54360504	25.51394357
chr3	111542079	111665996	3q13.13-q13.2	CD96	23.29479511	21.94084914	1.061708914	4	4	28.54463846	32.96738354
chrX	150692962	150765103	Xq28	MTMR1	11.56427497	10.89264473	1.061659061	4	4	24.93964261	23.65402931
chr3	51968510	51974630	3p21.2	ABHD14B	9.476718309	8.926619786	1.061624505	4	4	51.09089818	37.85230331
chr13	52412602	52450678	13q14.3	VPS36	28.47105569	26.81853045	1.061618784	4	4	13.89180584	13.79731592
chr14	21941184	21941657	14q11.2	TRAV9-2	9.330984987	8.790247033	1.061515672	4	4	36.65798859	45.83151772
chr19	18418719	18434562	19p13.11	SSBP4	9.078726396	8.552728282	1.061500623	4	4	55.25442671	105.4002813
chr22	16783412	16825412	22q11.1	XKR3	1.212181903	1.141965042	1.06148775	3	3	123.082126	95.58727477
chr1	158747733	158840979	1q23.1	OR6N1	0.305745745	0.288043396	1.06145723	1	1	0	0
chr6	106642420	106642959	6q21	RPL21P65	0.602451235	0.567591226	1.061417455	2	1	31.16406404	0
chr1	219910395	219960104	1q41	SLC30A10	0.125790066	0.118513076	1.061402422	1	3	0	24.2013861
chr1	156282913	156292443	1q22	TMEM79	1.301516911	1.226247064	1.061382285	4	4	88.96847446	64.88168782
chr18	21740793	21870957	18q11.2	MIB1	31.39514159	29.57975381	1.061372647	4	4	12.98653807	11.10971733
chrX	48695554	48709016	Xp11.23	SUV39H1	2.577299429	2.428403753	1.061314218	4	4	56.04105178	58.64754469
chr15	22983026	23039673	15q11.2	TUBGCP5	9.158189833	8.62912441	1.0613116	4	4	58.42014218	32.23242547
chr13	99294273	99307495	13q32.3	GPR183	59.27661858	55.85700206	1.06122091	4	4	39.91119111	31.79198887
chr5	72955981	73090522	5q13.2	FCHO2	442.389787	416.927348	1.061071645	4	4	62.57261883	42.38372824
chr17	40443424	40457730	17q21.2	IGFBP4	1.26801377	1.195099299	1.061011224	4	4	51.17119832	90.01378159
chr7	76510342	76516521	7q11.23	UPK3B	0.107436394	0.101266084	1.060931652	1	2	0	12.78654067
chr5	95463895	95555005	5q15	TTC37	15.89602749	14.98335697	1.060912286	4	4	22.42011131	23.89597271
chr3	52694484	52706083	3p21.1	GLT8D1	5.98850011	5.644789942	1.060889807	4	4	34.59399201	28.5245777
chr6	26123467	26123904	6p22.2	HIST1H2BC	404.780564	381.5987712	1.060749128	4	4	29.60978889	46.8460879
chr1	150566562	150566860	1q21.2	RN7SL473P	9.966267278	9.396965726	1.060583551	4	4	60.89516755	73.57292635
chr3	22381721	22382915	3p24.3	HMGB1P5	3.186962817	3.00493251	1.06057717	4	4	52.82572596	46.3155436
chr4	39527839	39594707	4p14	UGDH-AS1	0.375747913	0.354287654	1.060572978	4	3	63.85891717	66.61653105
chr7	5121310	5144546	7p22.1	ZNF890P	0.432304024	0.407646701	1.060486993	4	3	113.3798506	75.22079056
chr11	9664077	9752991	11p15.4	SWAP70	9.98893532	9.420692955	1.060318532	4	4	38.14878071	53.94249508
chr11	65044818	65059020	11q13.1	NAALADL1	0.563906772	0.531845406	1.060283242	4	4	70.45235809	42.63045154
chr1	32364633	32394461	1p35.1	BSDC1	17.33284502	16.34780947	1.060254895	4	4	12.3117478	39.14992186
chr14	88385638	88470350	14q31.3	SPATA7	2.74108784	2.585345158	1.060240576	4	4	49.54887942	75.32826188

chr20	32443059	32585073	20q11.21	NOL4L	4.446657969	4.194136211	1.060208287	4	4	39.36093705	16.77679378
chr22	44492570	44498125	22q13.31	RTL6	5.506598238	5.194050464	1.060174189	4	4	46.77029208	45.19404177
chr7	140719327	140924928	7q34	BRAF	30.16787972	28.45653932	1.060138739	4	4	13.53922443	9.754670764
chr10	122374578	122432355	10q26.13	PLEKHA1	2.566050914	2.420523412	1.060122328	4	4	29.42289896	26.08449427
chr19	16067507	16103005	19p13.12-p13.11	TPM4	61.14745508	57.68035623	1.060108832	4	4	69.29801697	38.29062139
chr12	21437604	21471252	12p12.1	PYROXD1	9.421076414	8.887053041	1.06009004	4	4	54.29871687	21.0480006
chr3	195523087	195523221	3q29	RNU6ATAC24P	6.725423763	6.344466288	1.06004563	3	4	73.96758608	39.61332918
chr1	71063291	71081289	1p31.1	ZRANB2	21.02182923	19.83209339	1.059990431	4	4	43.02381468	19.19714174
chr11	799179	809867	11p15.5	PIDD1	0.653816741	0.61681763	1.059983874	3	4	57.27891705	65.14735576
chr3	40390951	40453308	3p22.1	ENTPD3-AS1	0.508419636	0.479654435	1.059970676	4	4	42.74424952	36.85917259
chr14	23469689	23478193	14q11.2	NGDN	6.523434241	6.154581064	1.059931484	4	4	48.352952	18.81477064
chr20	49903391	49915543	20q13.13	SPATA2	1.680017151	1.585244898	1.059783982	4	4	77.8489343	32.67859429
chr5	69234795	69277430	5q13.2	CDK7	30.24578771	28.54140934	1.059715985	4	4	60.53387229	43.97663059
chr6	159778498	159789703	6q25.3	TCP1	36.94612633	34.86544291	1.059677527	4	4	19.24132474	18.56517759
chr1	52404547	52418320	1p32.3	PRPF38A	14.20282725	13.40302802	1.059673025	4	4	45.06738213	26.97389318
chr9	133340901	133348156	9q34.2	MED22	0.293261985	0.276773914	1.059572345	4	4	55.16200009	75.04766731
chr17	82051465	82058061	17q25.3	GPS1	4.738536662	4.472197185	1.059554502	4	4	60.61944661	30.35183116
chr6	15314920	15315026	6p22.3	RNU6-522P	5.613547472	5.29882176	1.059395414	3	2	38.39670262	49.07409566
chr19	17871394	17895175	19p13.11	SLC5A5	0.191655514	0.180916855	1.059356879	2	3	79.94138914	53.65524881
chr8	37762546	37779768	8p11.23	PLPBP	118.3373475	111.7091873	1.059334065	4	4	69.01267639	54.03279219
chr4	67701278	67722505	4q13.2	UBA6-AS1	2.250113864	2.124135263	1.059308182	4	4	42.9662234	43.8628961
chr4	38612701	38664628	4p14	KLF3-AS1	0.359392377	0.339287177	1.059257175	4	4	115.0162803	39.39639569
chr10	80078646	80092552	10q22.3	TMEM254	2.554051609	2.411245678	1.059224961	4	4	33.84156806	36.8669929
chr22	20859004	20891214	22q11.21	SNAP29	11.06483893	10.44664725	1.059176085	4	4	65.86648219	29.15144093
chrX	46912276	47061242	Xp11.3	JADE3	0.536434036	0.506480781	1.059139964	4	4	59.44599585	65.12659464
chr22	49414524	49657542	22q13.33	C22orf34	0.681289042	0.643248452	1.059138253	4	4	87.97419029	32.83411044
chr22	31335349	31338021	22q12.2	PIK3IP1-AS1	1.725881711	1.629574617	1.05909953	3	4	41.03495343	28.51103693
chr2	27212031	27217178	2p23.3	ATRAID	12.37079524	11.68053188	1.059095199	4	4	37.4535551	9.706870979
chr20	25452697	25585531	20p11.21	NINL	0.209625759	0.197935775	1.059059481	3	4	113.6781178	47.0026339
chr17	62423876	62450093	17q23.2	METTL2A	3.094461908	2.921947262	1.059040986	4	4	56.55762334	60.52097214
chr3	14947584	15049279	3p25.1	NR2C2	14.48692684	13.67984152	1.058998148	4	4	21.05808913	4.247480696
chr2	38293957	38378584	2p22.2-p22.1	ATL2	20.18571652	19.06171797	1.058966277	4	4	62.68476998	52.10202744
chr7	142500069	142500534	7q34	TRBV5-6	2.704244589	2.553673619	1.058962496	2	4	14.171542	40.85821687
chr7	40134887	40860767	7p14.1	SUGCT	0.280192511	0.26460255	1.058918408	3	3	42.92974729	57.19503928
chr3	67654697	67947713	3p14.1	SUCLG2-AS1	0.757197932	0.715193496	1.058731569	4	4	98.03201429	41.88657053
chr2	32356907	32618899	2p22.3	BIRC6	38.43182244	36.30085306	1.058703011	4	4	19.8931559	11.7871073
chr1	10460531	10472556	1p36.22	DFFA	4.995105401	4.718351725	1.058654736	4	4	57.13122394	43.33814568
chr2	88016354	88016547	2p11.2	RNU2-63P	58.50252017	55.26247147	1.058630181	4	4	37.24351239	68.58311321
chr12	49002272	49018975	12q13.12	PRKAG1	10.86552153	10.26389767	1.058615535	4	4	33.72198865	14.78877761
chr19	40740856	40751372	19q13.2	C19orf54	0.941220754	0.889111539	1.058608187	4	4	110.9983042	49.99154966
chr11	94493987	94499578	11q21	ANKRD49	17.74982733	16.7671756	1.05860568	4	4	38.44807416	13.68898548
chr6	87590067	87677824	6q15	ORC3	25.29839771	23.89856823	1.05857378	4	4	45.60403213	23.85490355
chr22	19431902	19436078	22q11.21	MRPL40	3.629100363	3.428307366	1.058569135	4	4	51.81716421	48.12845591

chrX	149596556	149631912	Xq28	TMEM185A	1.825127626	1.724461143	1.058375617	4	4	60.48941484	24.07575054
chr10	131967683	131982013	10q26.3	BNIP3	2.16881798	2.049522258	1.0582066	4	4	50.68944599	31.76334084
chr9	113349532	113371233	9q32	BSPRY	0.852075248	0.805292078	1.05809466	4	4	55.34456524	32.47645705
chr19	39125780	39179406	19q13.2	PAK4	0.623285587	0.589105413	1.058020472	4	4	62.67308825	91.4569434
chr5	34905260	34915675	5p13.2	RAD1	2.731323869	2.581581853	1.058003978	4	4	31.90447359	24.66302983
chr1	150218417	150236156	1q21.2	ANP32E	81.23922639	76.7904262	1.057934308	4	4	33.14464744	18.3418376
chr14	73478484	73558947	14q24.3	HEATR4	0.063244931	0.059782352	1.057919744	1	1	0	0
chr12	14477	32015	12p13.33	WASH8P	4.333615199	4.096494007	1.057883935	4	4	67.71194909	43.27039481
chr18	21529281	21600736	18q11.2	ESCO1	27.8954258	26.37015583	1.057840764	4	4	13.06585418	18.02933311
chr12	6723984	6731875	12p13.31	COPS7A	6.078943032	5.746586466	1.057835476	4	4	43.33140097	30.78789368
chrX	47232915	47248328	Xp11.3	USP11	5.495933928	5.195765736	1.057771695	4	4	48.67847188	34.97673437
chrX	71239624	71255197	Xq13.1	ZMYM3	3.318617155	3.137465995	1.057738047	4	4	56.41946556	39.74739492
chr11	18599787	18610255	11p15.1	SPTY2D1OS	0.477492246	0.451436741	1.057716846	3	2	57.71891663	105.3899528
chr7	87876229	87909541	7q21.12	DBF4	45.59767997	43.11149006	1.057668847	4	4	51.08276309	13.34619358
chr16	11555	14090	16p13.3	DDX11L10	20.68729959	19.56152441	1.057550483	4	4	20.60700585	43.62069077
chr15	50557158	50686815	15q21.2	TRPM7	48.51093512	45.87121943	1.057546229	4	4	41.42051766	23.05391193
chr19	39445546	39476670	19q13.2	SUPT5H	13.45433032	12.72283932	1.057494321	4	4	35.73085823	29.0076198
chr13	114244505	114244561	13q34	MIR548AR	11.04856236	10.44798056	1.05748305	1	1	0	0
chr19	11721265	11739009	19p13.2	ZNF823	6.479496385	6.127349986	1.05747124	4	4	41.36146499	12.74378365
chr3	197950190	197955851	3q29	RPL35A	237.7604659	224.839945	1.057465416	4	4	42.12568405	27.52747346
chr6	96898083	97145030	6q16.1	KLHL32	0.359269001	0.339756013	1.057432356	4	4	58.73800645	55.17743965
chr4	2741660	2756376	4p16.3	TNIP2	7.177912479	6.788450615	1.057371245	4	4	72.37277645	22.92691425
chr2	218659656	218663443	2q35	BCS1L	1.365602808	1.291617177	1.057281392	4	4	78.25483149	28.31477266
chr4	173331376	173334444	4q34.1	HMGB2	56.93940186	53.85539682	1.05726455	4	4	39.10195729	46.56511265
chr10	87094478	87191483	10q23.2	SHLD2	15.43091425	14.59662284	1.057156468	4	4	46.35504628	20.71791693
chr4	56963344	56977660	4q12	NOA1	9.839301081	9.30750402	1.057136377	4	4	22.85745019	27.61070596
chr1	16124337	16156109	1p36.13	EPHA2	0.384146918	0.363408336	1.057066884	3	3	75.83217246	9.058851926
chr7	141073402	141511338	7q34	TMEM178B	0.050931819	0.048184968	1.05700639	4	3	96.48086201	51.39460103
chr5	55995167	56003649	5q11.2	FLJ31104	0.33489865	0.316897782	1.056803388	2	4	19.80981268	54.13596075
chr5	95555108	95605102	5q15	ARSK	1.959892594	1.85456962	1.05679106	4	4	29.85301688	16.75833639
chr16	28842411	28859562	16p11.2	TUFM	31.131702	29.46395249	1.056603048	4	4	78.3710399	48.11650356
chr4	1811479	1856247	4p16.3	LETM1	3.418005086	3.235186118	1.056509568	4	4	79.14520206	47.11121887
chr10	68956123	68985069	10q22.1	DDX21	69.61190434	65.89208372	1.056453225	4	4	37.03895951	13.94141376
chr6	57945834	57961446	6p11.2	LINC00680	1.211292649	1.146565644	1.056452942	4	4	51.50901565	64.28948483
chr15	92900321	93028007	15q26.1	CHD2	49.08775088	46.46546457	1.056435168	4	4	15.02017238	7.53692327
chr16	56657943	56659303	16q13	MT1F	0.976642911	0.924512638	1.05638676	4	4	20.18378574	118.4747143
chr7	74174376	74197101	7q11.23	EIF4H	55.01077684	52.08646653	1.05614338	4	4	27.49306263	20.78812786
chr2	74216242	74343407	2p13.1	SLC4A5	0.1314086	0.124425951	1.056118914	4	2	88.38516905	55.08433264
chr12	100200087	100224424	12q23.1	ACTR6	17.63414337	16.69715349	1.056116744	4	4	42.65482322	24.43057458
chr17	78971253	78979979	17q25.3	LGALS3BP	10.85186758	10.27556569	1.056084688	4	4	53.70440067	78.76739492
chr14	106511115	106511552	14q32.33	IGHV1-46	1.407806455	1.333048935	1.056080101	2	2	9.227324967	98.12556917
chr10	73247367	73276984	10q22.2	DNAJC9-AS1	0.802679483	0.760057804	1.056076891	3	3	74.34818391	88.42641694
chr12	120710435	120723640	12q24.31	UNC119B	4.28326024	4.055892376	1.056058653	4	4	24.1499476	17.45669314

chr11	62712625	62727385	11q12.3	HNRNPUL2	7.413068917	7.019708383	1.056036592	4	4	50.82416369	31.67289002
chr17	49404048	49414905	17q21.33	PHB	7.437952742	7.043474938	1.056006134	4	4	49.77682305	26.79288333
chr5	157460019	157474722	5q33.3	NIPAL4	0.209674235	0.198560762	1.055970137	1	2	0	52.44610833
chrX	11111286	11123086	Xp22.2	HCCS	8.171238788	7.73818568	1.055963132	4	4	38.7220609	8.105162017
chr7	64990355	65006746	7q11.21	ERV3-1	9.431854369	8.932529592	1.055899594	4	4	55.77014723	72.21910077
chr14	103562957	103592187	14q32.33	APOPT1	2.346445978	2.222389593	1.055821169	4	4	22.05097604	30.70520238
chr16	83953222	83966332	16q23.3	OSGIN1	0.551192967	0.522053226	1.055817566	4	4	43.35419933	70.52232389
chr19	20776249	20809995	19p12	ZNF66	6.170785907	5.844570874	1.055815053	4	4	66.26413951	39.23681335
chr6	30571393	30591532	6p21.33	ABCF1	11.31015586	10.71226279	1.055813891	4	4	51.11422389	44.23586625
chr22	22369648	22370087	22q11.22	IGLV7-46	7.510747237	7.114180644	1.055743115	4	3	109.2982177	100.8246516
chr9	5357966	5438539	9p24.1	PLGRKT	6.963454333	6.596089161	1.055694392	4	4	39.71674682	22.93629715
chr11	28108251	28527041	11p14.1	METTL15	8.430193719	7.98604091	1.055616145	4	4	67.43965535	41.81939573
chr6	149594873	149649018	6q25.1	KATNA1	36.19261772	34.2873858	1.055566555	4	4	63.33539627	40.43628439
chr20	35276333	35278178	20q11.22	MMP24OS	4.525496263	4.28730691	1.05555687	4	4	35.53324181	20.0943933
chr9	94259266	94303967	9q22.32	ZNF169	1.2656554	1.199045943	1.055552048	4	4	64.14470979	25.4252393
chr18	46084144	46104233	18q21.1	ATP5F1A	21.59459433	20.45963542	1.055473076	4	4	37.23687101	26.49240404
chr6	123803842	124825652	6q22.31	NKAIN2	0.196563661	0.18623672	1.055450617	4	4	49.87582414	88.68561791
chr12	280057	389455	12p13.33	KDM5A	36.38445708	34.47555587	1.055369701	4	4	37.49797751	16.59582875
chr20	1947210	2007517	20p13	PDYN-AS1	0.155497057	0.147340536	1.055358299	2	1	4.604438543	0
chr1	234373437	234384049	1q42.2	COA6	9.54521381	9.045029563	1.055299349	4	4	22.54460275	37.30455092
chr11	61873523	61892224	11q12.2	FADS3	0.374438947	0.354846793	1.055212993	4	4	73.0006265	32.54048663
chr14	56579792	56660629	14q22.3	TMEM260	5.802652415	5.49915431	1.05518996	4	4	29.46155888	40.64289962
chr19	43506719	43527256	19q13.31	ETHE1	6.093662771	5.775581096	1.055073536	4	4	64.66910078	31.48063247
chr1	200872955	200874178	1q32.1	GPR25	0.46374369	0.439544826	1.055054371	2	4	70.19566695	96.42551411
chr12	6439001	6451517	12p13.31	CD27-AS1	1.692520935	1.604373815	1.054941759	4	4	43.71026404	39.20672077
chr14	102503649	102509806	14q32.31	ANKRD9	2.332000703	2.210758164	1.054842063	4	4	29.0475339	46.38495875
chr3	49095932	49105129	3p21.31	QARS	17.42144965	16.51722711	1.05474421	4	4	56.81596555	30.77274929
chr11	113733183	113773763	11q23.2	ZW10	14.51733013	13.7643866	1.054702294	4	4	41.08440946	21.98817983
chr6	31777518	31795935	6p21.33	VARS	3.809328523	3.612023445	1.054624528	4	4	66.66993381	61.35881194
chr7	100336079	100341328	7q22.1	STAG3L5P	1.585200861	1.503100548	1.054620639	4	4	84.0889041	35.92949339
chr6	127288712	127344179	6q22.33	ECHDC1	12.76911972	12.10890863	1.054522757	4	4	25.50005875	29.33490144
chr19	17719452	17734515	19p13.11	MAP1S	4.556147097	4.320687266	1.054495921	4	4	50.34671078	32.34909782
chr2	202265716	202303661	2q33.1	NOP58	41.94455681	39.77763388	1.054475913	4	4	30.13545561	15.93845042
chr19	17292131	17303473	19p13.11	ABHD8	2.06371338	1.957229165	1.054405594	4	4	65.27587504	57.52782725
chrX	154762742	154777689	Xq28	DKC1	12.38987088	11.75230963	1.054249869	4	4	22.26730114	22.120853
chr2	64846130	64863631	2p14	LINC01800	0.138832856	0.131690313	1.054237418	1	2	0	2.833386126
chr3	100609589	100696174	3q12.2	ADGRG7	0.059693527	0.056626261	1.054166842	1	1	0	0
chr14	21209136	21269479	14q11.2	HNRNPC	108.4332683	102.866486	1.054116579	4	4	31.87219924	22.03648897
chr15	90998416	91022839	15q26.1	VPS33B	2.761171664	2.619443981	1.054106018	4	4	46.94724499	23.10629576
chr3	88059197	88144660	3p11.1	ZNF654	10.87111159	10.31333389	1.054083161	4	4	22.65674975	17.6416578
chr15	78264145	78282196	15q25.1	DNAJA4	19.80140511	18.78591439	1.054055965	4	4	43.02104745	58.90444491
chr19	35629016	35637686	19q13.12	RBM42	12.25476369	11.62778864	1.053920403	4	4	46.09201787	41.48848098
chr17	32492522	32877173	17q11.2	MYO1D	5.400834183	5.1245738	1.053908948	4	4	62.57936247	28.87361065

chr4	99896248	99946726	4q23	DNAJB14	15.01570189	14.24778067	1.053897602	4	4	7.362869159	13.5696189
chr14	105172937	105181323	14q32.33	NUDT14	0.905266316	0.858997732	1.053863453	4	4	94.16379507	24.44071649
chr8	66921690	66925541	8q13	SNHG6	45.59770419	43.26755652	1.053854386	4	4	51.95862059	22.5703132
chr17	81918269	81927711	17q25.3	MAFG	3.534992722	3.35467476	1.05375125	4	4	49.42254601	16.56372346
chr1	89103119	89104954	1p22.2	PTGES3P1	41.80367049	39.6714754	1.053746302	4	4	26.49875742	48.67910208
chr6	49676665	49714198	6p12.3	CRISP2	0.848701117	0.805430137	1.053724064	4	4	45.68135128	51.66350185
chr17	76389458	76453234	17q25.1	UBE2O	24.77990017	23.5168368	1.053708897	4	4	18.73522261	34.84576762
chr6	106969831	106976855	6q21	CD24	12.59819719	11.95613817	1.053701205	4	4	57.7457515	35.6062074
chr8	38988808	38997139	8p11.22	TM2D2	5.127254743	4.86656585	1.053567321	4	4	10.99031219	21.92722751
chr4	89237666	89308010	4q22.1	GPRIN3	12.68044438	12.03600539	1.053542597	4	4	28.91496399	53.11948323
chr19	18557762	18569387	19p13.11	KXD1	11.84676759	11.24486854	1.053526552	4	4	42.7323484	31.71271285
chr16	77019	85851	16p13.3	MPG	7.068418111	6.709641616	1.053471782	4	4	89.73110321	52.56861693
chr10	103877558	103918287	10q24.33	STN1	19.53553113	18.54493454	1.05341602	4	4	36.94008645	30.11215529
chr9	128159425	128163928	9q34.11	C9orf16	16.59514559	15.75517196	1.053314152	4	4	66.96351827	65.85614326
chr9	7796490	7799806	9p24.1	DMAC1	3.776353536	3.585471202	1.053237726	4	4	45.17118045	37.18796876
chr9	128342154	128342774	9q34.11	TMSB4XP4	0.641220123	0.608835049	1.053191868	3	3	48.33074117	44.7562261
chr12	113298766	113335168	12q24.13	SLC8B1	5.125521999	4.867319696	1.05304815	4	4	71.25552279	20.70293901
chr12	68808149	68845544	12q15	MDM2	32.25756934	30.63286911	1.053037808	4	4	16.01160779	21.46038627
chr1	151300665	151300963	1q21.3	RN7SL444P	1.748574296	1.660768987	1.052870273	1	3	0	53.58938833
chr14	104724197	104747325	14q32.33	ADSSL1	0.091049052	0.086477986	1.052858145	4	3	52.61741534	46.42067188
chr12	53711118	53727523	12q13.13	CALCOCO1	16.21228136	15.39898106	1.052815202	4	4	47.03836786	18.30968229
chr6	25031009	25031287	6p22.3	RN7SL334P	1.153973941	1.096111473	1.052788853	1	2	0	23.38380953
chr17	58520250	58540818	17q22	SEPT4	0.521684058	0.495532368	1.052774937	4	4	57.9042333	41.10746103
chr5	76952855	76981143	5q13.3	CRHBP	0.16688868	0.158522921	1.052773182	2	3	57.34589313	29.8073478
chr19	12796820	12801910	19p13.13	PRDX2	49.5985725	47.11516464	1.052709311	4	4	33.22550863	62.06510621
chr16	70480569	70523554	16q22.1	COG4	6.842817139	6.500386909	1.052678438	4	4	31.2293159	29.32143452
chr8	97775581	97852602	8q22.1	LAPTM4B	3.164703884	3.006366015	1.052667529	4	4	19.81282833	33.98564224
chr17	81665036	81666637	17q25.3	OXLD1	4.696335669	4.46178221	1.052569455	4	4	86.34997871	19.06180228
chr22	21009699	21028013	22q11.21	P2RX6	0.148569911	0.141172866	1.052397078	2	2	81.70384623	86.83966258
chr16	72008744	72025417	16q22.2	DHODH	0.711558228	0.676137442	1.052386962	4	4	50.48202951	62.85518375
chr9	121074773	121177610	9q33.2	CNTRL	28.81068296	27.3766859	1.052380228	4	4	18.92794512	26.2146853
chr17	76070824	76077541	17q25.1	GALR2	0.240647599	0.228685834	1.052306538	1	1	0	0
chr22	38683916	38701418	22q13.1	JOSD1	16.87740552	16.03868011	1.052293917	4	4	31.86151957	26.37351169
chr21	18857737	18858476	21q21.1	PPIAP22	97.20993216	92.38127409	1.052268797	4	4	69.47580616	57.40336633
chr6	132722787	132734765	6q23.2	VNN3	28.68710037	27.26421416	1.052188785	4	4	46.11059649	81.13305487
chr6	35573585	35728583	6p21.31	FKBP5	15.80223172	15.01938407	1.052122487	4	4	43.40202788	35.53586898
chr7	100187988	100272274	7q22.1	CASTOR3	2.610875479	2.481558151	1.052111343	4	4	55.40480156	15.29828073
chr1	3735984	3747373	1p36.32	TP73-AS1	1.203698985	1.144096297	1.052095866	4	4	84.12295517	41.33629938
chr11	111889143	111889827	11q23.1	RPL37AP8	1.365443957	1.297860361	1.052073087	1	1	0	0
chr15	43371059	43409771	15q15.3	TUBGCP4	4.50674042	4.283813026	1.052039478	4	4	54.49174612	16.48851505
chr3	36993350	37050846	3p22.2	MLH1	15.16285987	14.4134614	1.051992956	4	4	27.36435652	19.27292075
chr16	46979	53632	16p13.3	POLR3K	2.978445047	2.831360938	1.051948202	4	4	25.19303552	17.4666739
chr3	15042251	15065337	3p25.1	MRPS25	3.043274721	2.893165216	1.05188418	4	4	54.17739479	29.73923343

chr16	23061407	23149301	16p12.2	USP31	3.7568443	3.571874353	1.051785122	4	4	31.07952022	27.57755309
chr12	120446438	120463753	12q24.31	GATC	6.694655765	6.365550924	1.05170092	4	4	47.96771651	25.47619432
chr3	138608099	138633376	3q22.3	FAIM	2.144761144	2.039339016	1.051694263	4	4	61.95927639	25.71732413
chr3	120324729	120349339	3q13.33	LRRC58	14.60038447	13.88273149	1.051693933	4	4	29.56178457	13.07009311
chr12	1559641	1594190	12p13.33	FBXL14	5.339026525	5.076680775	1.051676629	4	4	71.97740235	59.95275624
chr2	219171897	219179272	2q35	CNPPD1	60.71868943	57.73560785	1.051667969	4	4	13.18919143	38.39542484
chr8	134477788	134713049	8q24.22	ZFAT	4.21238901	4.005562511	1.05163482	4	4	46.11583971	25.83908567
chr4	76949714	77040154	4q21.1	SEPT11	9.143793678	8.695054296	1.051608577	4	4	15.89829492	5.279663446
chr15	45361124	45402317	15q21.1	GATM	0.586001851	0.55726276	1.051571886	4	4	14.94008875	27.68029733
chr13	99486962	99499306	13q32.3	LINC01232	1.181998473	1.124051706	1.051551691	4	4	38.75876826	54.87530065
chr18	12658739	12725740	18p11.21	PSMG2	13.86381129	13.1846074	1.051514912	4	4	17.48576113	12.44781817
chr9	37422435	37438952	9p13.2	GRHPR	7.525149136	7.156652953	1.051490017	4	4	16.54823998	33.35309222
chr15	40767448	40807478	15q15.1	DNAJC17	1.928944054	1.834674593	1.051382115	4	4	55.00499029	54.01381244
chr5	146447310	146511508	5q32	TCERG1	10.30468112	9.801155	1.051374161	4	4	38.19594262	17.33190405
chr9	97501180	97601743	9q22.33	TMOD1	56.47945453	53.72041531	1.051359231	4	4	32.59731049	24.2718424
chr17	508668	714856	17p13.3	VPS53	1.527682302	1.453076309	1.051343479	4	4	45.63234486	14.9083656
chr16	56730105	56844950	16q13	NUP93	3.661598728	3.482826325	1.051329692	4	4	28.04746799	13.67076703
chr19	42325609	42378769	19q13.2	MEGF8	0.796619897	0.757835352	1.051178062	4	4	82.79861131	58.319789
chr17	5105734	5123116	17p13.2	ZNF232	0.890781188	0.847441844	1.051141379	4	4	61.66059622	38.45388918
chr9	128552558	128633665	9q34.11	SPTAN1	19.93248526	18.96308113	1.051120602	4	4	45.13632764	55.87988467
chr19	3610628	3626815	19p13.3	CACTIN	2.596304281	2.470107291	1.05108968	4	4	56.92937018	43.76519884
chr3	87089280	87157069	3p12.1-p11.2	LINC00506	0.053328923	0.050743031	1.050960536	2	1	12.0753581	0
chr10	42782506	42834937	10q11.21	BMS1	8.881751508	8.45113264	1.050953983	4	4	26.4501809	32.2517353
chr7	130913464	130921946	7q32.3	LINC00513	0.176857617	0.16828894	1.050916463	2	3	28.78619841	14.40950675
chr19	58367623	58381022	19q13.43	ZNF837	0.680234981	0.647298218	1.050883444	2	3	109.1806033	52.1843553
chr22	21472998	21517491	22q11.21	PI4KAP2	1.03593345	0.985849092	1.05080327	4	4	36.56507571	29.04471395
chr7	36919357	36919432	7p14.2	MIR1200	6.748967855	6.423120044	1.050730456	3	2	32.42456772	26.07863286
chrX	2219506	2500974	Xp22.33 and Yp11	DHRSX	4.910706237	4.673905743	1.050664371	4	4	24.90405778	45.38291581
chr19	56404014	56425031	19q13.43	ZNF583	1.067010045	1.015583135	1.050637814	4	4	38.14172392	24.91475635
chr1	150321468	150353228	1q21.2	PRPF3	14.29470298	13.60579731	1.050633245	4	4	30.68269779	7.483334782
chr1	153658654	153661852	1q21.3	SNAPIN	7.85832509	7.479983948	1.050580475	4	4	31.22598735	16.09755204
chr14	73058513	73121414	14q24.2	RBM25	17.90671554	17.04539515	1.050530972	4	4	40.84851349	6.862151094
chrX	15319451	15335554	Xp22.2	PIGA	4.883662116	4.649224489	1.050425104	4	4	32.6533021	13.57645952
chr2	98619106	98731126	2q11.2	MGAT4A	26.1139054	24.86105696	1.050394013	4	4	26.65760072	12.74830758
chr17	59912163	59912454	17q23.1	RPS29P21	2.758678111	2.626402697	1.050363722	1	2	0	52.51285096
chr1	23505696	23531250	1p36.12	E2F2	8.876861422	8.451523952	1.050326719	4	4	31.95187992	82.403242
chrMT	3307	4262	N/A	MT-ND1	325.0700578	309.4955182	1.050322343	4	4	44.8741908	34.37200757
chr6	36442767	36491143	6p21.31	KCTD20	39.10117789	37.22986065	1.05026388	4	4	32.71973747	21.22113186
chr15	41556935	41557663	15q15.1	ELOCP2	1.32309867	1.259843155	1.05020904	2	3	24.09821999	70.02831189
chr3	185643131	185825056	3q27.2	IGF2BP2	81.2847368	77.40152297	1.050169734	4	4	20.48111956	34.95832086
chr2	69457995	69643845	2p13.3	AAK1	5.901132259	5.619530869	1.050111192	4	4	33.80692049	21.28193984
chr17	28854957	28861067	17q11.2	ERAL1	4.393839892	4.184177236	1.050108455	4	4	44.0451414	18.66393921
chr16	85935277	85936851	16q24.1	LINC02132	0.483200223	0.460220964	1.049930926	3	3	52.18300006	28.41777341

chr6	117560269	117602542	6q22.1	GOPC	13.8762702	13.21640357	1.049927851	4	4	37.57943863	30.16259597
chr19	48954825	48961798	19q13.33	BAX	7.90087909	7.525270626	1.049912951	4	4	54.21563125	17.59645957
chr17	46027335	46225374	17q21.31	KANSL1	3.082370879	2.935888085	1.049893861	4	4	38.66578421	8.808629155
chr12	32106773	32383637	12p11.21	BICD1	0.800192742	0.762168061	1.049890153	4	4	47.53904515	21.19419948
chrX	16719584	16762684	Xp22.2	SYAP1	15.56889404	14.83006149	1.049819925	4	4	21.29828002	25.61959212
chr6	8013567	8064414	6p24.3	BLOC1S5	10.10836617	9.628673829	1.049819149	4	4	20.23577429	19.15069674
chrX	3604343	3713634	Xp22.33	PRKX	13.98440718	13.32217047	1.04970937	4	4	30.29806539	40.52896381
chr18	35468327	35497991	18q12.2	INO80C	2.082850128	1.984389294	1.0496177	4	4	57.91578632	41.91130889
chr12	93408312	93442336	12q22	UBE2N	13.93745375	13.27865077	1.049613699	4	4	21.21514895	23.24340275
chr12	82358417	82479239	12q21.31	METTL25	19.76279314	18.82886276	1.049600998	4	4	46.51028706	39.66148164
chr4	56907876	56935847	4q12	REST	26.71129607	25.4518405	1.04948387	4	4	36.65476804	35.92357122
chr12	69643508	69738364	12q15	BEST3	0.06793372	0.064732361	1.049455301	3	4	73.82844635	54.62489519
chr4	90127394	91602219	4q22.1	CCSER1	0.862634567	0.821990803	1.049445521	4	4	75.9068947	32.594362
chr18	35971878	35979287	18q12.2	C18orf21	9.222910539	8.788550479	1.049423402	4	4	5.69412148	21.24652987
chr6	87322588	87342328	6q15	SMIM8	2.357964984	2.247050564	1.049360002	4	4	39.89050118	35.76283185
chr19	52154050	52171683	19q13.41	ZNF836	4.106634887	3.913604529	1.049322908	4	4	43.58004239	57.20658346
chr5	146203550	146289223	5q32	RBM27	10.18242533	9.704923599	1.049202008	4	4	24.23030028	18.82168412
chr16	70523788	70577668	16q22.1	SF3B3	18.28133335	17.42493083	1.049148116	4	4	19.19156595	24.8547418
chr1	15491401	15524912	1p36.21	CASP9	3.196425428	3.046908009	1.049071852	4	4	64.9475878	36.47026939
chr4	1721490	1745178	4p16.3	TACC3	11.5871915	11.04616532	1.048978642	4	4	69.59337515	24.72528646
chr5	72320367	72368395	5q13.2	PTCD2	1.153849102	1.099993833	1.04895961	4	4	41.04247462	8.942393533
chr6	30061240	30064909	6p22.1	ZNRD1	2.128158108	2.028910868	1.048916511	4	4	52.87712767	22.75688957
chr5	141391916	141512979	5q31.3	PCDHGA8	0.031656421	0.030184383	1.048768211	2	2	8.250985154	0.591784837
chr1	9729021	9824526	1p36.22	CLSTN1	13.0061127	12.40141864	1.048760071	4	4	57.4260546	62.90504149
chr19	18899511	18919403	19p13.11	COPE	19.25152947	18.35716033	1.048720451	4	4	56.55764795	44.50995598
chr5	124636913	124748824	5q23.2	ZNF608	0.580025867	0.553113807	1.048655556	4	4	73.17936581	45.93552442
chr14	24606480	24609763	14q12	GZMH	80.99500612	77.23813321	1.048640131	4	4	48.32140337	111.7402906
chr3	132654446	132679786	3q22.1	UBA5	3.803444599	3.627039848	1.048636011	4	4	63.42054806	17.84716308
chr13	42271470	42323267	13q14.11	AKAP11	12.81182054	12.21856422	1.048553685	4	4	25.72150336	22.09666929
chr7	92112149	92134745	7q21.2	CYP51A1	0.184652599	0.176107072	1.048524606	3	4	54.66083555	58.91879351
chr11	45094013	45235124	11p11.2	PRDM11	0.319916418	0.305117034	1.048503958	4	4	70.82515966	56.13794815
chr5	21459480	21589372	5p14.3	GUSBP1	3.246818348	3.096622174	1.048503229	4	4	63.70772337	15.48928227
chr1	827591	859446	1p36.33	LINC01128	2.876372773	2.743327161	1.048497902	4	4	57.7318862	28.72798841
chr9	15167204	15307360	9p22.3	TTC39B	4.747526751	4.528724189	1.048314393	4	4	19.62489962	11.98283236
chr6	145798135	145864409	6q24.3	FBXO30	15.1863087	14.48653633	1.048305016	4	4	21.95594589	61.90141783
chr22	39019081	39055972	22q13.1	APOBEC3F	2.721815347	2.596405916	1.048301165	4	4	72.82589311	62.52542988
chr3	133600605	133661968	3q22.1	TOPBP1	22.55719039	21.51796677	1.048295623	4	4	22.45481049	21.90798579
chr3	56557125	56621837	3p14.3	CCDC66	118.8301868	113.3606191	1.048249275	4	4	48.64643343	33.51638795
chr4	114713439	114714951	4q26	CIR1P2	0.197796998	0.188696037	1.048230798	1	1	0	0
chr2	201141903	201141963	2q33.1	RNU7-45P	24.00105177	22.89687906	1.048223721	3	4	28.59481224	87.48750114
chr10	7818504	8016631	10p14	TAF3	5.887463366	5.616888523	1.04817166	4	4	50.23322275	51.16094733
chr12	57604327	57609804	12q13.3	DTX3	1.831552516	1.747453028	1.048126895	4	4	63.60281285	20.39927268
chr12	108522214	108561389	12q23.3	SART3	11.09846727	10.58897158	1.048115691	4	4	43.44480415	20.85589687

chr10	99396870	99430773	10q24.2	GOT1	3.364944667	3.210620088	1.048066907	4	4	36.42476052	34.94206059
chr5	139273752	139331677	5q31.2	MATR3	0.94660953	0.903270445	1.047980187	4	4	110.097386	75.44944063
chr7	132784862	133082158	7q32.3-q33	CHCHD3	11.44792418	10.92382221	1.047977892	4	4	23.6798254	27.042055
chr17	43640389	43661977	17q21.31	MEOX1	0.625104883	0.596501245	1.047952352	4	3	81.19828226	74.46606319
chr6	127263415	127289893	6q22.33	RNF146	13.18307695	12.58101902	1.047854464	4	4	17.46976302	47.51774092
chr9	137605640	137615360	9q34.3	ARRDC1	4.637656503	4.425985419	1.047824623	4	4	51.56862028	51.66354902
chr14	22766522	22771789	14q11.2	OXA1L	13.38199716	12.77170636	1.047784594	4	4	53.05349164	18.17657653
chr16	20854925	20900358	16p12.3	DCUN1D3	3.042808985	2.904147363	1.047746069	4	4	48.74733803	16.95395063
chr11	74592530	74669341	11q13.4	POLD3	7.231315322	6.902235614	1.047677264	4	4	58.12137553	24.17089757
chr7	100169606	100177426	7q22.1	GPC2	0.166252843	0.158691175	1.047650211	4	4	60.03084971	47.75267001
chr1	1724838	1745999	1p36.33	SLC35E2A	1.139651904	1.087855083	1.047613714	4	4	42.21476574	46.80133169
chr14	49981669	50007520	14q21.3	LINC01588	0.690609657	0.659222213	1.047612844	4	4	49.62558925	19.83979849
chrX	148500619	149000663	Xq28	AFF2	13.20786485	12.60766413	1.047606021	4	4	62.86732851	62.19761312
chrX	71366220	71530525	Xq13.1	TAF1	16.27974865	15.54074628	1.047552566	4	4	19.70703124	14.83389552
chr14	75661208	75955082	14q24.3	TTLL5	4.26101724	4.067705918	1.047523426	4	4	20.60688264	13.10267859
chr19	48615327	48619418	19q13.33	RPL18	68.10782491	65.0201729	1.047487601	4	4	58.55080334	34.2354776
chr1	223779893	223845972	1q41	TP53BP2	29.59417855	28.25302499	1.04746938	4	4	32.67478593	29.19535364
chr19	45178745	45202715	19q13.32	BLOC1S3	2.744072406	2.620053386	1.047334539	4	4	66.84325455	32.9737603
chr18	22914121	23026486	18q11.2	RBBP8	47.28773112	45.15198549	1.047301256	4	4	56.69370959	29.62223735
chr15	65655234	65792293	15q22.31	DENND4A	277.5478409	265.0271054	1.047243226	4	4	57.48834265	60.21244452
chr1	145848522	145859081	1q21.1	PIAS3	2.14075853	2.044244783	1.047212422	4	4	57.11443162	41.3436081
chr9	97155893	97238742	9q22.33	ANKRD18CP	0.540916637	0.51654379	1.047184475	3	3	25.09916696	69.44490193
chr1	160037467	160070261	1q23.2	KCNJ10	0.145062023	0.138528307	1.0471652	4	1	49.30455597	0
chr20	25006230	25058182	20p11.21	ACSS1	5.416799999	5.172979938	1.047133386	4	4	69.68215168	45.04333379
chr15	48878093	48880183	15q21.1	EID1	75.25330564	71.86745853	1.047112381	4	4	39.79489452	27.68998235
chr17	42700275	42745096	17q21.2	EZH1	45.37069179	43.33125115	1.047066276	4	4	45.16767657	28.59535377
chr10	97850238	97871578	10q24.2	GOLGA7B	0.581382564	0.555267793	1.047030949	4	4	80.48158697	55.85478602
chr5	127229519	127461222	5q23.2	MEGF10	0.030082526	0.028731691	1.047015504	2	3	7.009714543	2.174951748
chr7	142463117	142463581	7q34	TRBV5-4	3.605624618	3.443746236	1.047006478	4	4	51.14909185	55.35690632
chr1	160400574	160428678	1q23.2	VANGL2	0.050975448	0.048687513	1.046992244	2	1	12.4101527	0
chr1	100538172	100542027	1p21.2	GPR88	0.081577831	0.077917685	1.046974519	1	2	0	6.72661117
chr1	229441291	229508342	1q42.13	NUP133	19.02346363	18.17026969	1.046955491	4	4	20.30888177	23.57388183
chr6	107697297	107845959	6q21	SCML4	4.377335418	4.181021549	1.046953566	4	4	18.40366481	23.46134301
chr19	32597001	32675215	19q13.11	ANKRD27	8.939940648	8.539023894	1.046951122	4	4	14.11427998	17.27432574
chr3	123067009	123162106	3q21.1	PDIA5	1.516339995	1.44841491	1.046896151	4	4	42.08307067	66.28457504
chr1	156840873	156858920	1q23.1	INSRR	0.056626619	0.054090963	1.046877619	1	1	0	0
chr20	5126761	5197887	20p12.3	CDS2	13.19525523	12.6054256	1.046791727	4	4	31.56772085	21.19197895
chrX	47803147	47807533	Xp11.23	WASF4P	0.46072867	0.440134088	1.046791609	4	4	74.75662067	73.86915992
chr2	113969322	114028082	2q14.1	LINC01191	0.315966106	0.301854172	1.046750831	2	1	41.40608172	0
chr1	8352404	8826226	1p36.23	RERE	17.08537701	16.32602561	1.046511712	4	4	17.23266911	23.47893702
chr1	200551497	200620791	1q32.1	KIF14	53.98786883	51.58873984	1.046504896	4	4	57.68169389	38.79211421
chr1	228100727	228103462	1q42.13	C1orf35	0.900392338	0.860397297	1.046484387	4	4	80.32066453	57.12931164
chr11	554850	560780	11p15.5	LMNTD2	0.182865175	0.174742647	1.046482807	3	3	71.76631534	49.41116831

chr16	4796962	4802950	16p13.3	ROGDI	7.470593411	7.139014212	1.046446076	4	4	37.96182521	44.34436248
chr10	34109560	34815325	10p11.22-p11.21	PARD3	5.437099945	5.196411864	1.04631813	4	4	41.53786198	15.04058603
chrX	72329516	72573103	Xq13.1	HDAC8	2.510174988	2.399188983	1.046259801	4	4	30.18645352	18.18216974
chr10	102460896	102480197	10q24.32	MFSD13A	1.096945973	1.048529113	1.04617598	4	4	56.26040154	71.54707262
chr18	62044224	62187118	18q21.33	PIGN	5.753232309	5.499590108	1.046120201	4	4	57.14217257	40.12211964
chr8	144095035	144097527	8q24.3	CYC1	9.857855141	9.423525777	1.0460899	4	4	87.04628439	42.965216
chr10	118004916	118046921	10q26.11	RAB11FIP2	12.38806672	11.84369513	1.045962985	4	4	35.5705375	13.24630803
chr2	9206765	9405683	2p24	ASAP2	6.999482112	6.692207929	1.045915218	4	4	25.00730064	20.72955658
chr2	131209581	131265254	2q21.1	POTEE	0.064577412	0.061747768	1.045825853	2	1	14.39299452	0
chr3	183635619	183684519	3q27.1	KLHL24	27.7517734	26.53772458	1.045748037	4	4	12.08538213	9.328286551
chr20	63272785	63289793	20q13.33	ARFGAP1	4.479644611	4.283752705	1.045729042	4	4	62.11356869	20.11217381
chr16	28553915	28591790	16p11.2	SGF29	7.756478479	7.417539768	1.045694222	4	4	47.08064747	47.86213121
chr15	75598083	75626378	15q24.2	SNUPN	2.733896064	2.614471116	1.045678435	4	4	36.56197511	18.13821639
chr10	11823341	11872277	10p14	PROSER2	1.680438169	1.607128898	1.045615054	4	4	53.85882331	30.77662051
chr2	38563186	38603036	2p22.1	HNRNPLL	10.6860768	10.22033039	1.045570583	4	4	17.99295335	29.07539025
chr11	65084222	65088402	11q13.1	ZFPL1	6.274892995	6.0015972	1.045537177	4	4	69.70981744	32.51118265
chr10	97737121	97760907	10q24.2	ZFYVE27	6.226190644	5.955685976	1.045419565	4	4	48.48612835	28.97576572
chr11	67606852	67612541	11q13.2	NDUFV1	7.640390363	7.308450549	1.04541863	4	4	52.49879221	40.32746357
chr2	231455800	231455936	2q37.1	SNORA75	12.85455457	12.29734828	1.045311093	4	4	59.61482722	52.66300112
chr1	173714912	173786826	1q25.1	KLHL20	6.961628392	6.659926323	1.045301112	4	4	28.21964728	12.77950824
chr6	148193440	148552050	6q24.3-q25.1	SASH1	0.702148751	0.671719491	1.045300547	4	4	36.5231412	57.03883497
chr17	20868433	20905230	17p11.2	CCDC144NL-AS1	0.048155251	0.046068858	1.045288576	1	2	0	64.76799988
chr12	50129306	50167533	12q13.12	CERS5	2.82462998	2.702290608	1.045272471	4	4	54.40309583	35.0470205
chr20	36236136	36256941	20q11.23	AAR2	6.092269657	5.828682207	1.045222477	4	4	45.22533922	7.869152981
chr13	77697674	77764242	13q22.3	SLAIN1	19.52314691	18.6794191	1.045168847	4	4	13.28898156	25.26642183
chr3	45823316	45916037	3p21.31	LZTFL1	3.954564797	3.784392248	1.044966942	4	4	24.09164205	31.28155478
chr17	20111824	20112987	17p11.2	KCTD9P1	1.503802397	1.439201573	1.044886572	4	4	19.30690553	17.751193
chr17	17681376	17811453	17p11.2	RAI1	1.413299613	1.352655564	1.044833327	4	4	73.97320479	50.78171626
chr6	170575572	170584692	6q27	PDCD2	7.559336331	7.235051326	1.044821383	4	4	45.29834817	12.05835858
chr7	44039049	44044296	7p13	LINC00957	1.752111804	1.676976875	1.044803795	4	4	50.11135814	41.37780833
chr8	66667552	66681149	8q13.1	C8orf44	1.487206594	1.423433426	1.044802354	4	4	62.09512288	45.01655926
chr14	20469379	20478006	14q11.2	PNP	30.01302763	28.72605815	1.044801465	4	4	32.44165568	36.26012747
chr1	182203945	182314061	1q25.3	LINC01344	0.384149512	0.367688383	1.044769241	4	3	64.3581712	8.07889597
chr1	201134771	201171625	1q32.1	TMEM9	1.547292165	1.481068834	1.044713202	4	4	68.92394322	36.39249138
chr17	3662893	3668679	17p13.2	TAX1BP3	3.158648962	3.023465605	1.044711392	4	4	57.3001073	72.88521022
chr12	133079470	133107674	12q24.33	ZNF140	3.499883249	3.35024132	1.044666015	4	4	30.01484659	27.94012541
chr9	70258962	70354873	9q21.12	SMC5	30.265347	28.97412539	1.044564645	4	4	25.74822589	8.357784608
chr8	30785610	30812836	8p12	PPP2CB	4.10644308	3.931346225	1.04453865	4	4	29.86867749	23.67331331
chr19	57455174	57456739	19q13.43	VN1R1	0.464692743	0.44491474	1.044453469	2	3	104.212938	111.7818736
chr19	12671955	12681887	19p13.13	DHPS	9.133315685	8.744829722	1.044424646	4	4	19.57802435	14.55337334
chr1	209827967	209857565	1q32.2	UTP25	2.834018671	2.713503799	1.04441301	4	4	27.78426508	38.26627205
chrX	64185117	64205744	Xq11.2	AMER1	1.082468701	1.036631581	1.044217368	4	4	37.1225068	62.0804264
chr1	151254700	151267479	1q21.3	PSMD4	36.34876425	34.80975908	1.044211888	4	4	22.77107587	13.05334125

chr1	52423275	52553463	1p32.3	TUT4	10.86195735	10.40213316	1.044204797	4	4	42.32938903	17.20202505
chr7	726692	786479	7p22.3	DNAAF5	1.25778943	1.204602356	1.044153221	4	4	34.67099848	51.35204935
chr16	67184379	67190204	16q22.1	EXOC3L1	0.411299076	0.393979313	1.043961096	4	3	99.33705233	73.59717115
chr2	174072447	174248670	2q31.1	OLA1	14.17237165	13.57606644	1.043923269	4	4	23.8548553	10.85032781
chr1	93079219	93139081	1p22.1	MTF2	13.78932563	13.21001119	1.043854198	4	4	12.1149019	11.84144891
chr13	30457916	30617597	13q12.3	HMGB1	64.39631752	61.69210682	1.043833982	4	4	31.34321655	18.44913243
chr16	1827224	1840207	16p13.3	FAHD1	4.991571317	4.782278219	1.043764308	4	4	11.32648461	16.4534125
chr2	175072250	175168206	2q31.1	ATF2	51.1266016	48.98600762	1.04369807	4	4	16.58596374	8.948344122
chr11	133896435	133901740	11q25	MIR4697HG	0.211459123	0.20261524	1.043648656	3	4	69.84841444	80.66385539
chr12	121400083	121424352	12q24.31	RNF34	12.44474377	11.92473725	1.043607377	4	4	29.71188006	29.27177881
chr1	59814861	59876378	1p32.1	HOOK1	4.025078066	3.857119529	1.043545069	4	4	61.99735163	31.71317389
chr8	127794533	128101253	8q24.21	PVT1	2.747066183	2.632449341	1.043539999	4	4	75.48741122	48.73417771
chrX	46447189	46474639	Xp11.3	KRBOX4	2.856917431	2.738527506	1.043231234	4	4	48.70070533	33.47940569
chrX	131691525	131830643	Xq26.2	FIRRE	3.589232213	3.440512575	1.043226012	4	4	21.31289223	48.29539687
chr19	44141530	44160309	19q13.31	ZNF234	2.610913481	2.502753125	1.04321655	4	4	29.20276104	45.70679912
chr2	212999684	213155297	2q34	IKZF2	14.05897007	13.47672632	1.043203649	4	4	53.61182406	35.50776752
chr10	100188298	100229610	10q24.31	CHUK	12.76865941	12.23995424	1.043195028	4	4	17.17927737	23.83463755
chr1	149884460	149886682	1q21.2	HIST2H2BE	110.0732121	105.5232862	1.043117742	4	4	37.04689043	35.06728815
chr3	48989908	49007149	3p21.3	P4HTM	1.179186719	1.130528377	1.043040354	4	4	46.37411291	33.65960704
chr19	15226919	15233453	19p13.12	EPHX3	0.218199933	0.209201126	1.0430151	2	1	38.01445077	0
chr3	139344014	139357129	3q23	MRPS22	4.952137377	4.747924551	1.043010967	4	4	39.95355537	10.58383459
chr17	49710325	49764156	17q21.33	FAM117A	74.01114614	70.97842948	1.0427273	4	4	14.63733806	32.78467321
chr8	101197038	101206470	8q22.3	ZNF706	4.477777705	4.294429607	1.0426944	4	4	40.5375047	24.51749251
chrX	55484524	55489875	Xp11.21	USP51	0.478424684	0.458882785	1.042585819	3	4	101.5668812	91.16008184
chr12	28505394	28505483	12p11.22	RNA5SP355	3.109146216	2.982196477	1.042569207	3	3	35.10630488	41.01059707
chr10	17137336	17202071	10p13	TRDMT1	3.952428623	3.791114087	1.042550694	4	4	46.28285065	33.0603583
chr8	120535745	120812069	8q24.12	SNTB1	13.34818288	12.80382225	1.042515479	4	4	14.26899205	8.206917901
chr19	6467204	6481808	19p13.3	DENND1C	12.63482437	12.12014237	1.042465013	4	4	72.42031342	66.9736428
chr5	80608620	80622524	5q14.1	LINC01337	2.404456148	2.306642051	1.042405408	3	3	114.3976058	47.18591951
chr6	34757094	34773857	6p21.31	SNRPC	18.37266782	17.62567498	1.04238095	4	4	59.68765359	47.92738539
chr1	21596221	21669444	1p36.12	RAP1GAP	3.986370669	3.824302177	1.042378579	3	4	169.294911	109.4093549
chr14	22493403	22493465	14q11.2	TRAJ46	10.34615121	9.925939847	1.042334667	2	4	38.79469305	52.2508026
chr22	46335401	46357340	22q13.31	TRMU	2.346016189	2.250839626	1.042284916	4	4	52.83247571	51.63622191
chr6	35473597	35497084	6p21.31	TEAD3	0.159484095	0.153015574	1.042273611	2	1	51.79207074	0
chr16	58519946	58629886	16q21	CNOT1	53.79427981	51.61476785	1.042226519	4	4	20.13885521	16.93492307
chr5	141620876	141636856	5q31.3	HDAC3	6.74176329	6.468674508	1.042217116	4	4	44.98756783	18.61203126
chr10	93667883	93702959	10q23.33	FRA10AC1	17.74800912	17.02956274	1.042188187	4	4	52.66212058	55.53408739
chr14	57390553	57412748	14q22.3	NAA30	6.561575619	6.2959991	1.042181791	4	4	17.55965025	9.197048365
chrX	153688297	153696593	Xq28	SLC6A8	39.84470928	38.23260233	1.042165766	4	4	46.49111759	44.44052604
chr12	89708955	89711952	12q21.33	ATP2B1-AS1	3.657680707	3.509851124	1.042118477	4	4	71.42468284	64.20934677
chr2	210002565	210023363	2q34	RPE	15.81299522	15.17479964	1.042056277	4	4	18.97389416	20.66928458
chr13	40789411	40812460	13q14.11	SLC25A15	1.856105266	1.781411244	1.041929691	4	4	42.79954935	32.2510972
chr6	105277567	105403124	6q21	PREP	7.631743858	7.324647607	1.04192642	4	4	49.88668011	45.25992311

chr4	143184917	143224429	4q31.21	USP38	20.29931658	19.48278138	1.041910607	4	4	15.85972068	9.453941687
chr13	98640809	98641307	13q32.2	CALM2P4	1.011623976	0.971023824	1.041811694	1	3	0	69.29344014
chr9	127397138	127407898	9q33.3	SLC2A8	0.47271237	0.453807864	1.041657512	4	4	83.06749032	40.07735247
chr3	122421901	122514939	3q21.1	KPNA1	15.40416509	14.78836452	1.041640884	4	4	19.54076184	7.295988981
chr1	54172151	54200101	1p32.3	CYB5RL	0.254650056	0.244486905	1.041569305	4	4	85.58845646	84.1469589
chr5	34017858	34244724	5p13.2	C1QTNF3	0.506482266	0.486301828	1.041497763	3	4	32.58157327	64.88716004
chr11	44727139	44932427	11p11.2	TSPAN18	2.976824844	2.858328712	1.04145644	4	4	107.5472242	24.52841699
chr22	37019642	37029822	22q12.3	MPST	1.825587547	1.752927455	1.041450713	4	4	59.40467327	21.87855015
chr3	184335917	184346275	3q27.1	FAM131A	1.087929525	1.044700329	1.041379518	4	4	76.82958745	25.69222565
chr8	60516910	60623644	8q12.1-q12.2	RAB2A	28.68562752	27.54636353	1.041358054	4	4	24.30723835	15.85526798
chr14	67651149	67674885	14q24.1	VTI1B	38.21228006	36.69638434	1.04130913	4	4	15.45516535	23.79929617
chr14	91779751	91867536	14q32.12	TC2N	29.09742741	27.94451936	1.041257036	4	4	24.24559494	15.88760023
chr17	8224821	8248095	17p13.1	CTC1	5.76958437	5.54116968	1.041221385	4	4	51.0593723	31.56849992
chr12	4538784	4560047	12p13.32	RAD51AP1	3.100875704	2.978150471	1.04120854	4	4	65.06273265	28.54016443
chr6	89632224	89638755	6q15	LYRM2	3.85944715	3.706838713	1.04116943	4	4	42.07327333	19.61461605
chr12	56752449	56787790	12q13.3	HSD17B6	0.578187352	0.555331796	1.041156577	4	4	45.10258478	29.37736775
chr5	88179300	88268906	5q14.3	TMEM161B	6.473257571	6.217372895	1.041156398	4	4	45.32795041	17.04634931
chr11	2376177	2397419	11p15.5	CD81	6.457605806	6.203223311	1.041008115	4	4	27.04293932	34.29856467
chr2	232697305	232860577	2q37.1	GIGYF2	16.99233661	16.32310111	1.040999286	4	4	14.45609352	6.884716584
chr2	18986451	19026971	2p24.2-p24.1	LINC01376	0.240568831	0.23109527	1.040994177	4	2	62.23118288	11.92476342
chr19	4522531	4535196	19p13.3	PLIN5	0.765715566	0.735597012	1.040944367	4	4	68.59293348	46.98743383
chr17	17042438	17192648	17p11.2	MPRIP	5.93290079	5.699549478	1.040942063	4	4	44.53208531	31.64074874
chr8	27869882	27992852	8p21.1	SCARA5	0.288026738	0.276738079	1.040791853	2	3	77.58925933	69.44948869
chr1	202147012	202161588	1q32.1	PTPN7	8.02540113	7.711022761	1.040769996	4	4	41.24153668	48.61132847
chr1	45002547	45011355	1p34.1	HECTD3	5.892133353	5.661727511	1.040695325	4	4	12.90893021	49.73197554
chr3	196142636	196160890	3q29	LINC00885	0.454254097	0.436581867	1.040478616	2	4	43.50977252	42.82231615
chr5	72219409	72320257	5q13.2	MRPS27	7.627291162	7.331380493	1.040362203	4	4	66.24038691	50.14699314
chr19	10111521	10115389	19p13.2	P2RY11	1.153538429	1.108845756	1.040305581	3	4	24.33639529	21.5790847
chr13	24882274	24922889	13q12.12-q12.13	CENPJ	2.827282488	2.717961614	1.04022164	4	4	53.24638537	27.3235286
chr8	66429028	66430733	8q13.1	RRS1	4.528919835	4.353837445	1.04021335	4	4	37.21123792	54.39694604
chr4	1211440	1253919	4p16.3	CTBP1	10.48920621	10.08388974	1.040194457	4	4	44.18106513	28.81295266
chr6	139372255	139374650	6q24.1	CITED2	19.14463749	18.40601164	1.040129598	4	4	28.65268944	31.65809432
chr5	66144218	66183616	5q12.3	SREK1	6.600761327	6.346658819	1.040037209	4	4	16.44743061	5.198633032
chr1	218285287	218337983	1q41	RRP15	4.111928761	3.95390135	1.039967464	4	4	36.25533634	31.27131234
chr11	118208932	118209211	11q23.3	HSPE1P18	13.26955761	12.75964464	1.039962945	4	4	75.21100772	61.12467776
chr18	70288901	70330199	18q22.2	SOCS6	2.151402186	2.068762997	1.039946185	4	4	58.42857006	22.11036465
chr2	9474529	9512403	2p25.1	IAH1	2.905877153	2.794283015	1.039936591	4	4	56.20956414	23.83839392
chr9	136612028	136617181	9q34.3	LINC01451	0.161626773	0.155426392	1.039892713	3	3	45.97309984	44.75784899
chr10	72917318	72918630	10q22.1	NPM1P24	0.314215822	0.302169805	1.039865058	1	1	0	0
chr11	65833640	65855169	11q13.1	SNX32	0.182769198	0.175782326	1.039747292	2	1	72.55954108	0
chr14	64449106	64505213	14q23.3	ZBTB25	3.581741446	3.445322677	1.039595353	4	4	29.52469535	13.03600927
chr19	39971005	39981764	19q13.2	PSMC4	12.05750707	11.59949527	1.039485493	4	4	41.49927554	40.8865438
chr3	112990953	113001374	3q13.2	GTPBP8	5.183211175	4.986809801	1.039384172	4	4	48.02302755	10.88274875

chr3	194584268	194590832	3q29	TMEM44-AS1	3.874212165	3.727553101	1.039344594	4	4	37.93813698	19.96059399
chr8	10893764	11201410	8p23.1	XKR6	0.343424332	0.330427483	1.03933344	4	4	73.94440443	30.91689642
chr22	45671798	45845307	22q13.31	ATXN10	17.98789006	17.30723376	1.03932785	4	4	34.17344115	8.525794655
chr18	14071230	14132494	18p11.21	ZNF519	0.464297716	0.446729788	1.039325626	4	4	34.70185828	25.73379894
chr5	16473038	16617096	5p15.1	RETREG1	5.099361892	4.906445587	1.039318953	4	4	30.96217514	54.46430318
chr1	91680343	91906002	1p22.1	TGFBR3	20.29391565	19.52625809	1.039314115	4	4	20.66770394	87.38775448
chr19	6413104	6424811	19p13.3	KHSRP	12.95081215	12.46111043	1.039298402	4	4	58.98557198	62.12715766
chr4	4235542	4248242	4p16.3	TMEM128	9.536025615	9.176329036	1.039198309	4	4	55.33507212	32.19155196
chr14	34515929	34539737	14q13.1	EAPP	42.66752597	41.05921031	1.039170643	4	4	19.82615022	3.719291711
chr12	122727606	122730582	12q24.31	HCAR1	0.153639774	0.147863705	1.039063466	2	1	50.63572208	0
chr11	18394389	18408218	11p15.1	LDHA	42.01519952	40.43707438	1.039026689	4	4	47.77888185	60.10902483
chr17	45061299	45109016	17q21.31	NMT1	13.79280046	13.27641771	1.038894735	4	4	35.65541258	27.56491497
chr11	67403718	67410090	11q13.2	TBC1D10C	18.70965598	18.00933221	1.038886715	4	4	40.46395408	26.67075936
chr17	2689386	2711787	17p13.3	CLUH	2.329663635	2.242687159	1.03878226	4	4	67.52802014	56.7988852
chr8	11842524	11868150	8p23.1	CTSB	73.97226384	71.21085901	1.038777862	4	4	27.30806178	24.49564636
chr4	145098004	145129524	4q31.21	ABCE1	22.12915328	21.30470354	1.038698015	4	4	33.42347724	27.60762673
chr5	180829954	180831618	5q35.3	HEIH	10.24890696	9.867116037	1.038693264	4	4	71.87010381	23.826062
chr11	75815167	76144238	11q13.5	UVRAG	11.7484303	11.31095472	1.038677158	4	4	17.9789691	28.65947902
chr1	156249224	156291498	1q22	SMG5	11.9095108	11.46633688	1.03865	4	4	54.19429843	22.12700384
chr16	89696357	89701713	16q24.3	SPATA2L	3.667316038	3.530921949	1.038628463	4	4	66.25634525	36.20935329
chr19	53395133	53427321	19q13.42	ZNF765	3.915336651	3.769940454	1.03856724	4	4	36.24723128	21.11057686
chr7	148698157	148801110	7q36.1	CUL1	33.95701257	32.69832951	1.038493803	4	4	12.90563507	27.54811387
chr10	105686308	105689724	10q25.1	YWHAZP5	0.381131126	0.367014211	1.038464219	2	1	29.92757222	0
chr6	159726696	159756319	6q25.3	WTAP	30.68601085	29.55295357	1.0383399	4	4	32.76330884	17.59617489
chr17	80200668	80220400	17q25.3	SGSH	1.911642631	1.841196675	1.038260962	4	4	71.80315429	32.34695501
chr6	53001299	53061864	6p12.1	ICK	6.94900078	6.692974158	1.038253042	4	4	29.17344018	15.31640058
chr8	142449703	142545009	8q24.3	ADGRB1	0.069355739	0.066810311	1.03809934	3	2	74.2741844	69.47573524
chr19	55641026	55643470	19q13.42	ZNF580	2.164466456	2.08505318	1.038086931	4	4	71.33953118	61.28449582
chr19	18919675	18928633	19p13.11	DDX49	7.141587776	6.879945812	1.038029655	4	4	52.62656705	17.19723372
chr1	156721891	156728718	1q23.1	ISG20L2	6.666354521	6.422349682	1.037993079	4	4	53.02337975	24.8979093
chr4	122826708	122898235	4q28.1	FGF2	0.420648633	0.40527928	1.03792287	4	4	55.22693006	42.90676688
chr1	205767986	205775487	1q32.1	RAB29	21.10589914	20.33693961	1.037810976	4	4	25.2675231	51.48577537
chr1	45012133	45015669	1p34.1	UROD	13.78904787	13.2873893	1.037754487	4	4	23.13687711	16.7350545
chr11	66345372	66347690	11q13.2	B4GAT1	1.126566471	1.085597656	1.037738489	4	4	69.30242416	50.44724994
chr1	154272563	154275875	1q21.3	HAX1	16.96416904	16.34821239	1.037677309	4	4	49.73274089	31.78652557
chr22	40044817	40335808	22q13.1	TNRC6B	22.18072775	21.3760158	1.037645554	4	4	26.60158099	20.33476661
chr5	18731568	18746202	5p14.3	LINC02100	5.975887736	5.759164813	1.037630964	4	4	22.8579958	85.89402253
chr20	62136727	62143458	20q13.33	PSMA7	37.33653177	35.98473648	1.037565797	4	4	9.354615917	25.56913053
chr17	42679767	42699993	17q21.2	CNTNAP1	0.127461135	0.122848184	1.037550012	4	4	68.10518779	64.94775524
chr3	50296402	50299421	3p21.31	NAA80	2.359373002	2.27409914	1.037497865	4	4	58.53592805	62.52072236
chr6	42744496	42746146	6p21.1	TBCC	23.87754083	23.0166743	1.037401865	4	4	54.26371813	25.71321332
chr1	179365704	179554735	1q25.2	AXDND1	0.227741816	0.219537858	1.037369214	4	3	72.54648543	29.37077801
chr6	10762723	10838954	6p24.2	MAK	4.683579613	4.51501562	1.037334089	4	4	79.98506808	99.4813709

chr9	100174999	100176996	9q31.1	NANOGP5	0.497669772	0.479789133	1.037267702	1	1	0	0
chr17	30830830	30895869	17q11.2	ATAD5	46.76239015	45.08375766	1.037233642	4	4	66.51418841	40.22522488
chr10	104267626	104304948	10q25.1	GSTO2	0.099000295	0.095450192	1.037193249	4	4	86.82566119	21.217365
chr5	111491422	111512597	5q22.1	STARD4	6.361532834	6.133586907	1.03716356	4	4	25.60628566	8.634177283
chr11	65854811	65858333	11q13.1	CFL1	110.8267653	106.8564851	1.037155258	4	4	46.62821696	48.35293548
chr3	49024325	49029750	3p21.31	IMPDH2	17.16185067	16.54745887	1.037129072	4	4	49.44377939	52.24193724
chr1	167519548	167536087	1q24.2	AKR1D1P1	0.507963217	0.489799584	1.037083807	2	1	15.30349901	0
chr7	108562144	108569768	7q31.1	THAP5	19.85866094	19.14884115	1.037068551	4	4	30.11101398	36.71579842
chr5	150000606	150053143	5q32	HMGXB3	5.038084165	4.858022479	1.037064811	4	4	31.50843283	36.42981534
chr17	7884764	7912757	17p13.1	CHD3	21.89555135	21.11314506	1.03705778	4	4	71.86324789	39.49973152
chr18	58862600	58986480	18q21.32	ZNF532	0.845646369	0.815433	1.037051933	4	4	61.92975591	43.05552087
chr8	30063012	30083208	8p12	SARAF	240.3549404	231.7678993	1.037050175	4	4	44.79974501	26.78948557
chr8	11769625	11787355	8p23.1	NEIL2	3.532245788	3.40619361	1.037006757	4	4	28.88008561	28.90337329
chrX	155276207	155334681	Xq28	CLIC2	46.68278503	45.01784182	1.036984074	4	4	54.14164256	87.11941174
chr3	15381275	15427580	3p25.1	METTL6	3.956314245	3.81545157	1.036919005	4	4	16.31853477	11.88492196
chr4	105708778	105847726	4q24	GSTCD	1.421404816	1.370819284	1.036901678	4	4	55.89072688	25.39452397
chr8	28345585	28386460	8p21.1	ZNF395	4.313864599	4.160554937	1.036848369	4	4	75.52266432	36.7765271
chr13	39009865	39038110	13q13.3	PROSER1	3.619245807	3.490917349	1.036760669	4	4	24.26056136	26.64696782
chr14	89954923	90044768	14q32.11	TDP1	6.223933852	6.003546544	1.036709519	4	4	59.413257	27.43497081
chr3	57756229	57930013	3p14.3	SLMAP	17.80066992	17.17084469	1.036679921	4	4	19.12609589	14.28828288
chr1	100975218	101025806	1p21.2	DPH5	7.617877606	7.349219642	1.036555985	4	4	34.23046748	15.87068834
chr7	92144884	92165276	7q21.2	LRRD1	0.037800423	0.036467787	1.036542846	1	1	0	0
chr3	44439770	44477670	3p21.31	ZNF445	2.297438297	2.216652953	1.036444742	4	4	67.03386872	31.62933953
chr7	50303453	50405101	7p12.2	IKZF1	61.46367842	59.30508165	1.036398175	4	4	34.47628192	33.77277302
chr16	72044261	72094316	16q22.2	TXNL4B	43.86998296	42.33086695	1.036359189	4	4	82.23243179	56.9029334
chr22	29259852	29268213	22q12.2	RHBDD3	0.679113465	0.655288828	1.03635746	4	4	90.9581636	68.38030521
chr16	89508379	89557768	16q24.3	SPG7	2.743761254	2.64751998	1.036351481	4	4	53.20839652	43.96713581
chr10	73375101	73414085	10q22.2	ANXA7	42.34812207	40.87176626	1.036121654	4	4	25.63265251	16.24261328
chr16	1790413	1794908	16p13.3	IGFALS	0.115362439	0.111346204	1.036069804	1	1	0	0
chr2	27642568	27663840	2p23.3	SUPT7L	8.339253311	8.049134058	1.036043536	4	4	11.73498394	11.31338611
chr5	127940440	128083175	5q23.3	LINC01184	2.33315282	2.25205837	1.036009036	4	4	49.62731638	20.74913375
chr20	6001733	6002030	20p12.3	RN7SL498P	0.956377705	0.923171023	1.035970239	1	1	0	0
chr13	45341345	45391483	13q14.13	TPT1-AS1	0.99260694	0.958189528	1.035919211	4	4	45.48219021	28.4678047
chr15	66489748	66497815	15q22.31	SNAPC5	5.711215342	5.513269301	1.035903568	4	4	46.29698839	33.50414046
chr14	49625174	49635230	14q21.3	DNAAF2	3.220466045	3.10913325	1.035808306	4	4	44.13065983	42.82864238
chr17	44191805	44199976	17q21.31	ATXN7L3	15.31202104	14.78410389	1.035708431	4	4	36.81145642	16.33667453
chr16	3263768	3267567	16p13.3	LINC00921	3.242251616	3.130492078	1.03570031	4	4	78.3840303	40.43378908
chr16	89737549	89816658	16q24.3	FANCA	3.769807701	3.639885527	1.035694028	4	4	42.02634504	36.82755249
chr1	171781621	171797716	1q24.3	EEF1AKNMT	4.014949344	3.876757535	1.035646235	4	4	27.43135415	45.03686487
chr15	60488284	61229303	15q22.2	RORA	18.33042572	17.69955014	1.035643594	4	4	25.04362068	38.6808456
chr3	122739997	122793829	3q21.1	HSPBAP1	4.507181751	4.35208648	1.035636992	4	4	30.89355969	42.54027844
chr2	121649654	121728560	2q14.3	NIFK-AS1	2.200020498	2.124364235	1.035613603	4	4	57.75009328	14.90838675
chr4	73980825	73982124	4q13.3	PF4	730.3873916	705.3558602	1.035487805	4	4	41.06896223	17.72555126

chr1	75202131	75724261	1p31.1	SLC44A5	0.240014008	0.231796035	1.035453467	2	2	86.93193792	59.88833623
chr12	121132819	121188032	12q24.31	P2RX7	3.872872192	3.740400667	1.0354164	4	4	62.60754294	47.48231569
chr3	132243460	132244286	3q22.1	RPL7P16	0.32043834	0.309483867	1.035395941	2	1	29.23531372	0
chr3	142449235	142578826	3q23	ATR	9.711718628	9.379933256	1.035371826	4	4	32.71427105	18.47360861
chr6	83150728	83193936	6q14.1	PGM3	1.998407634	1.930190399	1.035342231	4	4	23.162594	22.88879695
chr17	35243036	35273655	17q12	SLFN5	67.83387808	65.52157581	1.0352907	4	4	9.046392836	74.38532059
chr7	149239651	149255609	7q36.1	ZNF212	1.657533824	1.601156954	1.035210084	4	4	79.89132871	15.65019709
chr8	122781349	122974515	8q24.13	ZHX2	11.41538588	11.02726223	1.035196736	4	4	56.41383525	57.15739716
chr19	23221584	23250456	19p12	ZNF724	1.153694153	1.114502365	1.03516528	4	4	41.47169153	25.79985161
chr3	40477113	40490237	3p22.1	ZNF619	2.53120576	2.445320744	1.035122189	4	4	53.39635937	33.20141962
chr10	95907440	96035768	10q24.1	CC2D2B	0.863119393	0.833850061	1.035101432	4	4	35.86874915	64.53614897
chr9	136410573	136423761	9q34.3	PMPCA	2.632534128	2.543285541	1.035091847	4	4	63.76561895	25.18139942
chr17	8039040	8049134	17p13.1	ALOX15B	0.314667803	0.304005125	1.035074007	4	3	48.60912939	36.26952238
chr1	26234153	26278810	1p36.11	CEP85	19.2279519	18.57656654	1.035064896	4	4	65.20285217	33.44636105
chr19	41883200	41907452	19q13.2	ARHGEF1	23.34199696	22.55151323	1.035052359	4	4	49.15794605	22.43506049
chr2	113215997	113278921	2q14.1	PAX8	0.088709035	0.085712181	1.034964151	3	2	67.54818429	85.54123661
chr3	108160812	108222570	3q13.12-q13.13	IFT57	6.300664439	6.088016634	1.034928913	4	4	42.48600454	28.77853646
chr16	19555073	19701163	16p12.3	VPS35L	8.225025267	7.947539517	1.034914674	4	4	31.41806504	13.14348254
chr3	130097782	130111433	3q22.1	FAM86HP	0.352141768	0.340263483	1.03490908	2	2	20.69361017	60.46039233
chr1	183471993	183554193	1q25.3	SMG7	11.64347778	11.25166253	1.034822876	4	4	18.360983	14.89577099
chrX	53159735	53225422	Xp11.22	KDM5C	14.00307484	13.53273478	1.034755729	4	4	30.60107944	34.75547268
chr12	103965815	103988878	12q23.3	TDG	17.85819151	17.25967878	1.034676933	4	4	21.07516274	4.062203463
chr1	155562042	155563944	1q22	ASH1L-AS1	1.896860897	1.83336045	1.034636095	4	4	25.86288624	49.40560389
chr6	89094818	89165569	6q15	PM20D2	4.407882497	4.260325556	1.034635133	4	4	46.99601228	37.83143192
chr12	118063593	118104402	12q24.23	VSIG10	0.382604217	0.369800417	1.034623541	4	4	65.49070049	79.7007858
chr15	66347206	66347342	15q22.31	SCARNA14	2.895910823	2.799040566	1.034608379	2	1	70.02269328	0
chrX	119805311	119853028	Xq24	UPF3B	5.560083794	5.374357473	1.034557865	4	4	63.568547	29.63876151
chr19	13774443	13778773	19p13.13	C19orf53	18.77745501	18.15186296	1.034464344	4	4	61.70321447	41.73157107
chr7	128870645	128910719	7q32.1	KCP	0.033186448	0.032086016	1.034296302	2	2	10.11200117	0.217780988
chr1	151176300	151190213	1q21.3	VPS72	6.401828845	6.189721612	1.034267653	4	4	54.70363388	60.68722748
chr11	76349956	76380965	11q13.5	THAP12	21.1578893	20.45693976	1.034264633	4	4	36.39773264	12.16219005
chr12	64759497	64881022	12q14.3	TBC1D30	0.62029341	0.599776834	1.034207017	4	4	50.844096	62.38324653
chr12	48857133	48865870	12q13.12	RND1	0.092094772	0.089049804	1.034193986	2	1	2.358267504	0
chr20	63735701	63739107	20q13.33	LIME1	0.543423127	0.525546829	1.034014663	3	4	25.84596468	77.87433073
chr17	9250471	9575958	17p13.1	STX8	6.751596235	6.532345917	1.033563795	4	4	68.54064927	31.03221787
chr4	37590800	37686403	4p14	RELL1	138.0044042	133.5236189	1.033557998	4	4	11.53874224	42.13893661
chr9	124517275	124771310	9q33.3	NR6A1	0.612178223	0.592313079	1.033538249	4	4	69.52247309	43.72834092
chr9	75890644	76362339	9q21.13	PCSK5	1.250841919	1.210362602	1.033443959	4	4	51.25350186	43.11618311
chr22	50481724	50486440	22q13.33	ADM2	0.176174288	0.170488057	1.033352667	3	3	46.8823315	35.35373894
chrX	75156379	75304897	Xq13.3	UPRT	11.39790065	11.03158669	1.033205918	4	4	31.80982984	15.03380535
chr16	2088708	2135898	16p13.3	PKD1	1.316003325	1.273824545	1.033111923	4	4	60.69035809	41.51826932
chr2	36837698	36966607	2p22.2	STRN	24.69043468	23.90370235	1.032912572	4	4	47.6162143	31.76854262
chr3	139493740	139495682	3q23	ACTG1P1	0.244391925	0.236611122	1.032884353	2	1	27.69526915	0

chr17	59893046	59950426	17q23.1	RPS6KB1	23.11202078	22.37636185	1.03287661	4	4	23.94588577	15.07654484
chrX	21940573	21994837	Xp22.11	SMS	29.95687352	29.00811988	1.032706485	4	4	36.42418262	39.44353173
chr6	27957241	27958182	6p22.1	OR2B6	1.641660355	1.589699498	1.032685961	4	3	45.74000087	31.35227345
chr5	36876759	37065819	5p13.2	NIPBL	48.91138067	47.36478151	1.032652936	4	4	20.8706976	3.424944149
chrX	53431258	53434376	Xp11.22	HSD17B10	11.98381714	11.60575326	1.032575557	4	4	78.32449786	74.51426383
chr12	48105253	48146404	12q13.11	PFKM	1.198527026	1.160740163	1.03255411	4	4	36.75071229	28.4829439
chr1	74733152	74766677	1p31.1	TYW3	5.939730101	5.753029364	1.032452596	4	4	24.20469094	38.92913375
chr20	58389119	58451101	20q13.32	VAPB	61.57008953	59.63548331	1.032440522	4	4	42.31045583	12.61201332
chr18	59327823	59359276	18q21.32	LMAN1	17.45291487	16.90588453	1.032357393	4	4	25.10887795	42.12541028
chr8	140520151	140642406	8q24.3	AGO2	39.72301677	38.47833938	1.032347482	4	4	23.19675212	18.31530694
chr12	54362445	54365224	12q13.13	GPR84	1.070296905	1.036846774	1.032261402	4	4	73.08335075	57.14048969
chr10	125719515	125764143	10q26.2	EDRF1	6.471511036	6.269321067	1.032250696	4	4	15.37607895	16.42916817
chr20	2658390	2664223	20p13	IDH3B	14.83855441	14.37559199	1.032204755	4	4	52.49526425	34.19022469
chr2	99154955	99163157	2q11.2	LIPT1	4.998893887	4.843145822	1.03215845	4	4	22.63686748	39.27031364
chr8	116644816	116755823	8q23.3-q24.11	EIF3H	43.59959063	42.2425818	1.032124192	4	4	22.41098236	8.219580893
chr11	66664994	66677921	11q13.2	RBM4B	2.423630966	2.348324911	1.032067988	4	4	29.62525264	29.18332501
chr1	84643707	84690757	1p22.3	SSX2IP	16.21161189	15.70819075	1.032048321	4	4	22.29365452	36.26129402
chr5	163437571	163445016	5q34	CCNG1	56.38225016	54.64241132	1.031840448	4	4	17.58719058	15.59460087
chr1	944203	959299	1p36.33	NOC2L	6.761769101	6.553121127	1.031839481	4	4	53.0157626	53.3794888
chr19	58451604	58457833	19q13.43	ZNF324B	1.056804763	1.024242089	1.031791971	4	4	76.3925757	49.0659423
chr1	1512143	1534687	1p36.33	ATAD3A	1.748675057	1.694794786	1.031791619	4	4	83.48835915	60.63413094
chr12	6556870	6568332	12p13.31	NOP2	4.18431096	4.055446182	1.031775734	4	4	49.35075011	32.48904285
chr22	46684410	46738255	22q13.31	CERK	21.34873722	20.6919498	1.031741205	4	4	44.36126634	22.91813105
chr19	9959561	10010532	19p13.2	COL5A3	0.666453649	0.645954317	1.031734956	4	4	71.13759761	165.4119345
chr16	89202112	89234453	16q24.3	ZNF778	1.95797958	1.897902483	1.03165447	4	4	24.14480307	14.4986618
chr2	86199433	86213798	2p11.2	MRPL35	9.013415309	8.736874603	1.031652132	4	4	34.99824072	11.62375691
chr11	119024351	119030906	11q23.3	SLC37A4	0.186086591	0.180385261	1.031606408	4	4	59.74501947	32.44247681
chr17	16786892	16804505	17p11.2	USP32P1	0.066475495	0.064445685	1.031496434	3	2	38.51975801	69.20973998
chr1	182839304	182887982	1q25.3	DHX9	56.46336341	54.73927491	1.031496371	4	4	30.33187693	18.47191438
chr19	45764785	45769239	19q13.32	SIX5	0.144158124	0.139760449	1.031465804	2	3	42.60652944	41.9549471
chr2	86143932	86195770	2	IMMT	19.00717037	18.42903579	1.031370853	4	4	14.73189696	13.93983984
chr18	41955198	42084606	18q12.3	PIK3C3	27.3081469	26.4802785	1.031263583	4	4	54.4928082	40.02179625
chr1	145911348	145918924	1q21.1	PEX11B	7.541957305	7.313775915	1.031198849	4	4	22.36748566	23.03597261
chr14	69398374	69462390	14q24.1	SLC39A9	14.42129872	13.98541482	1.031167034	4	4	24.2815397	10.52793893
chr19	1407569	1435687	19p13.3	DAZAP1	6.292516368	6.102326245	1.031166823	4	4	64.28065935	36.44979791
chr16	10866208	10941562	16p13.13	CIITA	4.864381181	4.717618312	1.031109526	4	4	65.11841691	34.41566925
chr1	168281040	168314426	1q24.2	TBX19	1.050278787	1.018613551	1.031086603	4	4	11.32603601	19.24030498
chr7	90245174	90320964	7q21.13	CFAP69	0.028003718	0.027161306	1.03101515	1	2	0	2.922556036
chr2	108534355	108687246	2q12.3	LIMS1	57.18518423	55.46664925	1.030983212	4	4	16.39162324	16.13394626
chr5	123344885	123423592	5q23.2	CEP120	18.06061223	17.51797646	1.030975939	4	4	29.73976306	26.30356449
chr5	55424842	55535078	5q11.2	PLPP1	1.442201571	1.398929064	1.030932595	4	4	117.2304488	59.50333149
chr2	25377243	25673674	2p23.3	DTNB	2.062264591	2.000605845	1.030820037	4	4	25.96945777	10.49072391
chr6	166409364	166862551	6q27	RPS6KA2	0.887798654	0.861376051	1.030674875	4	4	92.05635897	64.01457786

chr6	82880834	83065841	6q14.1	UBE3D	1.323822308	1.284702317	1.030450627	4	4	36.16065108	15.01734891
chr1	88537513	88685204	1p22.2	PKN2-AS1	0.367616269	0.356785778	1.030355725	4	4	13.40019948	55.68945187
chr18	6462110	6463029	18p11.31	RPL6P27	2.047862553	1.987539149	1.0303508	4	4	37.11999385	66.1074723
chr1	153981617	153990644	1q21.3	RAB13	3.613611925	3.507194138	1.030342714	4	4	6.620126797	74.3172409
chrX	135094985	135098634	Xq26.3	SMIM10L2B	0.104870243	0.101784943	1.030311949	1	2	0	7.781222255
chr5	55307748	55425581	5q11.2	MTREX	21.86636318	21.22311991	1.030308611	4	4	30.15299238	7.643987143
chr18	11883471	11908797	18p11.21	MPPE1	7.466003583	7.246432852	1.030300527	4	4	44.91226471	39.72687522
chr19	40393762	40414718	19q13.2	PRX	0.313470185	0.30425759	1.030278933	4	4	84.91529232	35.61098574
chr6	107065181	107115186	6q21	BEND3	0.398763981	0.387059573	1.030239293	4	4	23.66054001	53.26377291
chr18	67506582	67516730	18q22.1	DSEL	0.060514018	0.05874198	1.03016647	2	1	29.42710593	0
chr11	62624826	62646726	11q12.3	GANAB	25.6370326	24.88677574	1.030146808	4	4	52.8215373	33.20790813
chr10	38356380	38378505	10p11.1	HSD17B7P2	1.572840298	1.526885125	1.030097335	4	4	46.71968762	22.86709433
chr5	65589680	65624360	5q12.3	TRIM23	25.13455319	24.40425924	1.029924856	4	4	43.68946747	55.20554299
chr4	17841199	18023287	4p15.31	LCORL	5.2342563	5.082177506	1.029923944	4	4	32.23375616	19.30287924
chr19	44891220	44903689	19q13.32	TOMM40	2.859468123	2.776484476	1.029888029	4	4	66.17649623	44.78312401
chr2	200889327	200903932	2q33.1	NIF3L1	7.809797847	7.583321549	1.029865053	4	4	45.36432915	50.38829446
chr7	99504644	99534106	7q22.1	ZKSCAN5	4.452546995	4.323681952	1.029804469	4	4	37.79922963	15.9141683
chr6	109462516	109483237	6q21	ZBTB24	8.371237619	8.129833712	1.029693585	4	4	48.4437987	36.36952518
chr19	36214602	36238774	19q13.12	ZNF146	25.32058628	24.59404407	1.029541389	4	4	33.22008506	24.90102744
chr12	54325090	54351851	12q13.13	COPZ1	23.73641458	23.05597899	1.029512327	4	4	21.66466202	18.40494829
chr4	56049222	56330724	4q12	KIAA1211	1.295211252	1.258143507	1.029462255	4	4	44.89752275	54.58436143
chr2	175167633	175167709	2q31.1	MIR933	7.030582748	6.829711812	1.029411334	2	2	64.66655831	73.2608061
chr7	127651989	128092609	7q32.1	SND1	20.95308755	20.35457091	1.029404533	4	4	26.9641217	25.27317138
chr9	125437394	125707234	9q33.3	MAPKAP1	17.51076995	17.01226006	1.029302978	4	4	36.66579301	25.409186
chr11	68261335	68272001	11q13.2	C11orf24	2.478104023	2.407557302	1.029302198	4	4	38.71251221	56.010758
chr7	133253067	134067137	7q33	EXOC4	10.13715899	9.849201861	1.029236595	4	4	17.65682771	14.2838455
chr11	73876699	73927736	11q13.4	PAAF1	0.835268085	0.811555946	1.02921812	4	4	57.3631268	40.78411041
chr19	1205799	1228435	19p13.3	STK11	9.124421374	8.865898435	1.029159249	4	4	3.685935591	34.51569396
chr19	7739993	7747609	19p13.2	CD209	0.217420659	0.211264347	1.029140327	4	3	87.2208572	79.66771529
chr14	52775193	52791668	14q22.1	GNPNAT1	5.086552596	4.942621656	1.029120364	4	4	26.59692409	33.99603141
chr6	13621498	13711582	6p23	RANBP9	301.6130026	293.0930122	1.029069238	4	4	42.11540559	30.93307263
chr9	71683366	71769532	9q21.13	CEMIP2	26.53202087	25.78379645	1.029019172	4	4	41.81068488	6.314650693
chr2	241687012	241729481	2q37.3	ING5	2.317381405	2.252032693	1.029017657	4	4	54.94007216	36.9335329
chr13	45393316	45434016	13q14.13	SLC25A30	4.107960884	3.992253949	1.028982859	4	4	29.12694157	4.378712248
chr12	101280127	101386619	12q23.2	UTP20	4.692942318	4.560816337	1.02896981	4	4	33.21371008	17.22851971
chr1	229271062	229305894	1q42.13	RAB4A	14.82872739	14.41155468	1.0289471	4	4	31.47008164	22.6119021
chr20	35002404	35092835	20q11.22	TRPC4AP	31.74579401	30.85569279	1.028847228	4	4	45.96560753	11.89446996
chr19	39409623	39409678	19q13.2	MIR4530	5.328453105	5.179540454	1.028750167	1	1	0	0
chrY	20569690	20570564	Yq11.223	KDM5DP1	2.98526152	2.901923214	1.028718301	1	2	0	57.72080246
chr2	71182738	71227110	2p13.3	PAIP2B	1.168632748	1.136226178	1.028521232	4	4	60.28298447	37.72790786
chr11	126278202	126278618	11q24.2	RPL35AP26	0.801485835	0.779260635	1.028520881	1	1	0	0
chr12	82352304	82358805	12q21.31	CCDC59	15.29373195	14.86966527	1.028518912	4	4	43.58408358	16.11583624
chr5	168560896	168560973	5q34	MIR103A1	5.229239236	5.084334019	1.028500334	1	1	0	0

chr2	28810834	28850610	2p23.2	SPDYA	1.342265373	1.305099137	1.028477711	4	4	52.55757211	30.6019289
chr16	2155778	2178129	16p13.3	TRAF7	9.927187174	9.652914217	1.028413488	4	4	66.17147966	42.57857355
chr20	62302061	62308862	20q13.33	ADRM1	17.86771325	17.37451278	1.028386434	4	4	52.26067187	52.86353704
chr16	29545011	29613717	16p11.2	SMG1P2	4.189518753	4.074232486	1.028296438	4	4	56.015076	48.66107128
chr12	95474046	95515839	12q22	METAP2	29.06994474	28.27002975	1.028295513	4	4	24.46558419	35.13690417
chrX	64915802	65034744	Xq11.2	ZC4H2	0.8136579	0.791280308	1.028280235	4	4	38.24766227	62.94322129
chr3	113648385	113696657	3q13.2	USF3	15.47182801	15.04665846	1.028256743	4	4	47.30385731	25.23815438
chr4	138164094	138242349	4q28.3	SLC7A11	0.87249946	0.848530213	1.028247959	4	4	43.82283284	107.1411492
chr21	42403862	42447680	21q22.3	UBASH3A	8.166726015	7.942963199	1.028171201	4	4	42.79967627	53.66166009
chr16	87949216	88077318	16q24.2	BANP	12.85683727	12.50508563	1.028128687	4	4	16.09239947	14.38036396
chr12	118246061	118247011	12q24.23	RPS2P5	83.32135475	81.04193803	1.028126385	4	4	59.40918003	48.45398504
chr14	76781733	76786724	14q24.3	VASH1-AS1	1.546581302	1.504347354	1.028074598	4	4	119.3465883	42.78877388
chr19	42401507	42427426	19q13.2	LIPE	0.694108554	0.67519342	1.028014393	4	4	58.49210834	76.11519048
chr1	169792524	169794976	1q24.2	METTL18	9.391953767	9.136054156	1.028009861	4	4	22.34898581	33.42488818
chrX	136497079	136512346	Xq26.3	HTATSF1	24.07801771	23.42424457	1.027910106	4	4	55.71393667	40.0442207
chr1	2636988	2789737	1p36.32	TTC34	0.029337324	0.028541625	1.027878546	1	1	0	0
chr4	2469068	2515859	4p16.3	RNF4	14.35862293	13.96960228	1.027847654	4	4	37.79044032	13.10486358
chr10	123154244	123165370	10q26.13	BUB3	12.77701224	12.43113882	1.027823149	4	4	26.81500658	16.05605948
chr11	96389994	96393563	11q21	JRKL	4.543381186	4.420928829	1.027698333	4	4	40.17919027	67.66729656
chr10	47501854	47523638	10q11.22	AGAP9	1.507564994	1.466954893	1.027683265	4	4	71.66626223	76.38924769
chr9	123354065	123380335	9q33.3	CRB2	0.043312078	0.042148128	1.027615706	1	1	0	0
chr7	520391	525232	7p22.3	HRAT92	2.657856905	2.586477701	1.027597069	4	4	31.17720329	70.53572799
chr2	27309492	27323102	2p23.3	MPV17	3.272708205	3.185204107	1.027472054	4	4	73.37229314	47.75285481
chr19	51991332	52008230	19q13.41	ZNF615	5.535214648	5.38743123	1.027431147	4	4	27.92947341	14.48869046
chr1	175012958	175023455	1q25.1	MRPS14	7.623255536	7.419855096	1.027412994	4	4	22.62777219	23.10785935
chr8	144010992	144047114	8q24.3	SPATC1	0.122582585	0.119316449	1.027373723	1	1	0	0
chr3	183826488	183884908	3q27.1	PARL	1.816446855	1.768061195	1.027366507	4	4	24.69223274	78.55576395
chr7	103297426	103329902	7q22.1	PMPCB	25.27055954	24.59767293	1.027355702	4	4	24.8332114	15.68330891
chrX	119538149	119565434	Xq24	CXorf56	10.22674257	9.954738834	1.027324045	4	4	59.5189422	7.496795328
chr8	80613538	80613816	8q21.13	RN7SL107P	1.885636716	1.835610597	1.027253122	1	2	0	66.36812682
chr22	39014236	39020352	22q13.1	APOBEC3C	41.05629682	39.96923171	1.027197548	4	4	17.38927136	60.39118824
chr18	76794732	76822295	18q23	ZNF236-DT	1.415953717	1.378548723	1.027133603	4	4	64.19957667	62.74163026
chr2	169584340	169641406	2q31.1	PPIG	25.61607292	24.93980745	1.027115906	4	4	38.59552173	15.66544596
chr4	119334313	119378233	4q26	KLHL2P1	0.661160778	0.643711278	1.02710765	2	3	1.135318759	45.29003962
chr16	56361452	56425538	16q13	AMFR	36.44428569	35.4824436	1.027107549	4	4	18.04700785	21.26433392
chr12	120503279	120534613	12q24.31	COQ5	6.980634973	6.796418043	1.027105003	4	4	8.294867526	30.66329224
chr7	39949664	40097134	7p14.1	CDK13	23.71837942	23.09407608	1.027033051	4	4	16.0988965	14.74779057
chr2	102736906	102843838	2q12.1	TMEM182	0.37883677	0.368877857	1.026997858	4	4	56.31890072	46.10874516
chr6	158765741	158819424	6q25.3	EZR	64.60684503	62.91344985	1.026916267	4	4	51.00385036	38.28106737
chr10	94314907	94362976	10q23.33	NOC3L	11.29042337	10.99460912	1.02690539	4	4	52.56418573	25.81227081
chr18	31101588	31162789	18q12.1	DSCAS	0.511034613	0.497651862	1.026891795	1	2	0	22.73173482
chr17	8288497	8290092	17p13.1	RANGRF	3.372755502	3.284441199	1.026888684	4	4	43.16836573	36.70657533
chr7	66628881	66643229	7q11.21	KCTD7	1.77924354	1.732695995	1.026864231	4	4	36.09040091	42.8266116

chr3	101676430	101679217	3q12.3	ZBTB11-AS1	1.591009599	1.549431533	1.0268344	4	4	74.42493631	24.85803747
chr10	109864766	109923553	10q25.1	XPNPEP1	7.964863655	7.756782227	1.026825741	4	4	27.32201517	20.97811825
chr14	21768553	21769080	14q11.2	TRAV6	4.369978234	4.255862306	1.02681382	3	3	59.24843877	7.29236738
chr12	990030	1495933	12p13.33	ERC1	4.320015013	4.207438134	1.026756633	4	4	38.44255093	27.02516119
chr20	325571	330228	20p13	SOX12	0.67384337	0.656287622	1.026750082	4	4	83.64330199	50.8714309
chr2	97492663	97590337	2q11.2	ANKRD36B	4.444772869	4.329050949	1.026731476	4	4	41.5138976	24.00405488
chr3	44625019	44648471	3p21.31	ZNF197	1.196606188	1.165569686	1.026627753	4	4	40.26766495	6.2478452
chr1	11785730	11806103	1p36.22	MTHFR	3.668771667	3.573661544	1.026614194	4	4	52.63992829	22.78601569
chr2	24676309	24676415	2p23.3	RNU6-936P	7.962309905	7.755955873	1.02660588	3	2	64.65246713	5.226975643
chr17	45147317	45152101	17q21.31	HEXIM1	20.36724624	19.8402739	1.026560739	4	4	20.47483768	46.39210549
chr6	37257704	37332970	6p21.2	TBC1D22B	47.18414175	45.96445775	1.026535372	4	4	23.69440194	44.25318891
chr15	84980513	85139145	15q25.3	PDE8A	6.840167409	6.663401155	1.026527932	4	4	27.95042152	28.87596583
chr3	179562880	179588405	3q26.33	ACTL6A	6.257996285	6.096381495	1.026509954	4	4	30.50929999	29.17852106
chr8	142727203	142736932	8q24.3	THEM6	1.909166756	1.859921708	1.026476946	4	4	61.71874168	81.71840312
chr17	39461486	39567560	17q12	CDK12	19.02861258	18.53854215	1.026435219	4	4	17.5082896	27.10024991
chr14	93207056	93229215	14q32.12	UBR7	4.334170566	4.222752658	1.026385137	4	4	18.20450628	32.0601274
chr3	160399304	160434962	3q25.33	SMC4	73.7477739	71.85407055	1.026354851	4	4	34.98827665	16.76953683
chr1	45645772	45646685	1p34.1	RPS15AP10	4.40274225	4.28968819	1.026354843	3	4	62.88260975	78.12077555
chr12	57772600	57782541	12q14.1	EEF1AKMT3	0.501883525	0.48906021	1.026220319	4	4	51.43441464	70.90657034
chr2	169798812	169812064	2q31.1	SSB	18.33603118	17.8676431	1.026214318	4	4	35.4321003	24.64635075
chr19	29665420	29675477	19q12	PLEKHF1	4.545006195	4.428918658	1.02621126	4	4	57.77546957	50.21096421
chr1	58654739	58700091	1p32.1	MYSM1	16.1103965	15.69892813	1.026209966	4	4	13.22155136	11.46741972
chr2	127418143	127429246	2q14.3	PROC	0.290228055	0.282815476	1.026209947	2	2	29.60466248	88.77426374
chr6	43671197	43687812	6p21.1	MRPS18A	10.76907199	10.49478754	1.026135303	4	4	38.09179456	42.15339225
chr10	100232296	100267681	10q24.31	CWF19L1	13.08981739	12.75672412	1.026111191	4	4	24.52867673	15.21757442
chrMT	5587	5655	N/A	MT-TA	20.04323022	19.53446802	1.026044334	4	4	57.61622315	39.86464421
chrX	107522450	107605264	Xq22.3	FRMPD3	0.438033935	0.426924329	1.026022425	4	4	57.49982154	75.2920913
chr9	39803496	39810159	9p12	GLIDR	0.2720824	0.265206732	1.025925689	3	3	83.82423921	92.8462003
chr11	59806135	59810872	11q12.1	MRPL16	6.866696061	6.693494667	1.025876079	4	4	45.20730764	43.5954513
chr22	41301503	41360147	22q13.2	ZC3H7B	6.608752082	6.442491698	1.025806845	4	4	24.38319137	35.20745922
chr2	100391860	100417668	2q11.2	CHST10	0.863757936	0.842086689	1.025735173	4	4	53.67181493	49.95618785
chr5	18049544	18049915	5p15.1	RPL36AP21	0.408476309	0.398264744	1.025640143	1	1	0	0
chr22	26483867	26494620	22q12.1	SRRD	28.94164201	28.21939911	1.025593844	4	4	19.45437352	25.85004832
chr4	169620521	169723187	4q33	CLCN3	37.28771032	36.35730977	1.025590467	4	4	13.87107988	58.76476564
chr11	112074244	112085150	11q23.1	NKAPD1	9.736846002	9.494454783	1.025529767	4	4	48.24237348	24.84145489
chr2	117814679	117832377	2q14.1	DDX18	13.99184435	13.64358222	1.02552571	4	4	22.63936585	14.15321507
chr3	156673171	156675713	3q25.31	TIPARP-AS1	1.399428037	1.364644003	1.025489456	3	4	93.64187642	49.39866429
chr7	100428790	100434126	7q22.1	MEPCE	12.4228997	12.11434867	1.025469881	4	4	60.39344821	20.22005091
chr20	23356594	23358127	20p11.21	LINC01431	1.22081914	1.190552647	1.025422222	3	3	71.28029726	62.6484092
chr7	134289337	134320307	7q33	SLC35B4	3.156466793	3.078335283	1.025381092	4	4	40.99824525	22.42171961
chr16	27224994	27268792	16p12.1	NSMCE1	5.14188641	5.014927971	1.025316104	4	4	33.31234848	45.53658898
chr4	177309837	177362938	4q34.3	NEIL3	612.4744752	597.3868059	1.025256114	4	4	59.33077952	19.19203349
chr2	27199587	27212342	2p23.3	SLC5A6	1.732791208	1.690296859	1.025140169	4	4	54.33317016	33.60572365

chr16	2204628	2209454	16p13.3	MLST8	2.108605229	2.056915662	1.025129648	4	4	110.5932765	50.90571204
chr6	26499290	26510425	6p22.2	BTN1A1	0.164652232	0.160622203	1.02509011	2	1	55.59941524	0
chr16	3027710	3036985	16p13.3	BICDL2	0.142051474	0.138578928	1.025058254	2	3	41.6288544	52.60081525
chr1	158610498	158686715	1q23.1	SPTA1	30.83194537	30.07900667	1.025032033	4	4	59.17044537	33.55286376
chr2	88765902	88806596	2p11.2	ANKRD36BP2	376.2752714	367.090369	1.025020821	4	4	45.4976582	12.30263047
chr21	45981749	46005049	21q22.3	COL6A1	0.151780497	0.148078064	1.025003248	2	4	89.84319742	90.71094938
chr3	114056735	114088422	3q13.31	QTRT2	4.406109777	4.2988906	1.024941127	4	4	33.3871135	27.62797767
chr1	37692401	37709719	1p34.3	CDCA8	7.235518962	7.059531759	1.024929019	4	4	57.50093848	34.27297356
chr14	105643203	105644789	14q32.33	IGHG2	15.52129445	15.14559464	1.024805881	4	3	98.24384986	130.2771017
chr2	44275458	44320824	2p21	SLC3A1	0.080824528	0.078869077	1.02479364	3	3	63.32791706	46.8369979
chr1	161132703	161158856	1q23.3	UFC1	24.21520079	23.63008577	1.024761443	4	4	28.99826147	17.628025
chr13	113455819	113492047	13q34	DCUN1D2	2.137802933	2.086168827	1.024750684	4	4	21.64818956	29.73124136
chr4	5051517	5501001	4p16.2	STK32B	0.197859399	0.19308428	1.024730748	3	4	38.79348639	66.94165205
chr18	58671386	58753806	18q21.32	MALT1	15.17074957	14.8052669	1.02468599	4	4	32.56896705	16.78623571
chr20	33731657	33792269	20q11.22	ZNF341	0.840261494	0.820028621	1.024673375	4	4	79.55347479	49.68510557
chr9	15552874	16062431	9p22.3	CCDC171	0.86497446	0.844216484	1.024588452	4	4	26.58182558	55.40856385
chr6	26158008	26171349	6p22.2	HIST1H2BD	154.5738238	150.8679559	1.024563652	4	4	23.95388815	75.48271557
chr3	119428960	119463682	3q13.33	TMEM39A	5.56533166	5.432168149	1.024513879	4	4	19.95094578	6.790321496
chr15	98645863	98651221	15q26.3	IRAIN	0.834152935	0.814219029	1.024482241	2	1	32.73472033	0
chr15	44736362	44767829	15q21.1	TRIM69	0.80542693	0.786217534	1.024432673	4	4	82.33261213	87.64056056
chr6	38168451	38640541	6p21.2	BTBD9	4.010196904	3.914761534	1.024378336	4	4	47.80715078	21.42361524
chr17	21524606	21551678	17p11.2	C17orf51	0.256773226	0.250667884	1.024356301	4	4	60.0165504	46.78915973
chr3	49171120	49176487	3p21.31	KLHDC8B	3.304103275	3.225599456	1.024337746	4	4	19.31459016	62.17130509
chr7	151081085	151083533	7q36.1	TMUB1	7.33907775	7.164857842	1.024315892	4	4	80.15953804	78.44135049
chr12	46183055	46269425	12q13.11	SLC38A1	63.93825024	62.42392077	1.024258801	4	4	43.35837432	17.0941844
chr9	101389967	101398637	9q31.1	MRPL50	13.56798293	13.24705684	1.024226218	4	4	33.78757401	9.117240966
chr17	19533854	19579033	17p11.2	SLC47A1	0.207898256	0.202993157	1.024163868	4	4	51.57662171	95.43467838
chr4	145133650	145180019	4q31.21	OTUD4	25.3020548	24.70566981	1.0241396	4	4	33.17113539	19.07040917
chr17	17170083	17170189	17p11.2	RNU6-767P	2.704644189	2.640929603	1.024125818	1	1	0	0
chr14	103556544	103562814	14q32.33	BAG5	17.30502493	16.89763855	1.024109072	4	4	49.12872915	19.98494849
chr10	133278630	133312337	10q26.3	TUBGCP2	7.140815132	6.973046094	1.024059649	4	4	40.97310199	32.15788884
chr1	147173194	147295766	1q21.1	CHD1L	25.84858045	25.24278166	1.023998892	4	4	25.82692066	24.46415887
chr3	55508311	56468473	3p14.3	ERC2	0.111874599	0.10925684	1.023959675	3	4	88.02900324	49.40617512
chr14	24439765	24442905	14q12	SDR39U1	2.801297942	2.735785821	1.023946363	4	4	53.17673809	17.35277129
chr11	17115652	17115763	11p15.1	RNU6-593P	2.57247652	2.512340802	1.023936131	1	1	0	0
chr17	7312659	7315339	17p13.1	GPS2	1.014611817	0.990916861	1.023912153	4	4	83.90983879	46.45519375
chr8	10606349	10655107	8p23.1	RP1L1	0.029126534	0.028446571	1.023903163	1	1	0	0
chrX	333933	387327	Xp22.33 and Yp11	PPP2R3B	3.565192535	3.48213642	1.023852057	4	4	83.14618516	49.0042595
chr16	2239872	2251601	16p13.3	ECI1	1.316395883	1.285776879	1.023813621	4	4	69.3114211	12.06726199
chr11	107403370	107404637	11q22.3	SMARCE1P1	0.864889318	0.84480486	1.023774079	3	4	86.44025216	59.02963571
chr19	4090321	4124184	19p13.3	MAP2K2	18.10502043	17.6857537	1.023706466	4	4	47.2499074	37.03461857
chr11	64786914	64803254	11q13.1	MAP4K2	6.401723676	6.254181141	1.023591024	4	4	45.28323682	21.45769031
chr19	17933015	17943985	19p13.11	CCDC124	9.670868202	9.448053975	1.023583082	4	4	32.93685915	29.68889663

chr20	43507680	43541895	20q13.12	L3MBTL1	0.387850707	0.378927099	1.02354967	4	4	53.12275023	38.73407475
chr9	127224471	127393655	9q33.3	GARNL3	0.258670207	0.252730272	1.023503062	4	4	50.838708	35.80513357
chr16	25111702	25178231	16p12.1	LCMT1	10.87405086	10.62444116	1.023493913	4	4	38.6891511	26.30893256
chr10	119167699	119178865	10q26.11	PRDX3	20.0762564	19.61542275	1.023493434	4	4	14.94768272	16.54685016
chr7	140084746	140176941	7q34	KDM7A	66.30975703	64.79040286	1.023450297	4	4	14.967523	23.04856213
chr13	19823482	19863636	13q12.11	ZMYM5	35.71293341	34.89499962	1.023439857	4	4	36.66941797	38.5852929
chr3	122886513	122892416	3q21.1	LINC02035	2.398720677	2.343807746	1.02342894	4	4	83.42757592	38.98197955
chr1	111599007	111608318	1p13.2	LINC01160	0.185089719	0.180854363	1.023418601	2	2	17.92901882	9.631211006
chr14	31100115	31207744	14q12	HECTD1	36.22161686	35.3935589	1.023395725	4	4	15.14987349	4.118955195
chr6	31588883	31593024	6p21.33	NCR3	3.417535679	3.339486643	1.023371567	4	4	54.59376101	38.95297516
chr7	104981747	104991165	7q22.3	LINC01004	2.404617461	2.350093286	1.023200856	4	4	63.66451397	33.5406374
chr12	56162359	56189574	12q13.2	SMARCC2	13.80489701	13.49213943	1.023180726	4	4	60.90260037	39.53605302
chr16	1678277	1702072	16p13.3	JPT2	4.989135243	4.876127751	1.023175663	4	4	49.58455388	34.28674309
chr20	62388630	62407283	20q13.33	CABLES2	2.846624953	2.782220498	1.023148581	4	4	58.17548627	57.51185075
chr18	55774601	55781725	18q21.2	LINC01415	0.141646148	0.138445486	1.02311857	2	3	72.34595222	58.81580202
chr6	7590199	7611967	6p24.3	SNRNP48	14.11018216	13.79153676	1.023104416	4	4	23.27578731	28.00374435
chr3	6772837	6773370	3p26.1	MRPS36P1	2.52761415	2.471203834	1.022827059	1	1	0	0
chr2	127861630	127887575	2q14.3	AMMECR1L	4.891603284	4.782451864	1.022823318	4	4	54.15595384	16.85351963
chr2	208359845	208494506	2q34	PTH2R	3.472926031	3.395516653	1.022797526	4	4	78.33195337	34.6940186
chr19	1576671	1592761	19p13.3	MBD3	1.567868791	1.533063722	1.02270295	4	4	49.28211137	37.85892963
chr19	40818477	40826773	19q13.2	CYP2F2P	0.27216624	0.266130224	1.022680685	1	1	0	0
chr17	10672474	10697568	17p13.1	SCO1	3.094999171	3.026371452	1.022676568	4	4	33.13081302	4.568764394
chr2	132671788	133675182	2q21.2	NCKAP5	0.027426118	0.026819052	1.022635609	1	1	0	0
chr11	61362001	61378224	11q12.2	TMEM138	34.18679056	33.43304494	1.022544929	4	4	83.84832798	66.18757907
chr18	62187255	62307122	18q21.33	RELCH	17.16731664	16.79026958	1.022456284	4	4	16.250596	26.15513564
chr8	38104965	38140080	8p11.23	ASH2L	137.3928147	134.3804776	1.022416479	4	4	52.04990472	26.22621615
chr6	63211447	63213022	6q12	FKBP1C	0.338323705	0.330916193	1.022384856	3	3	24.33907675	49.49165107
chr12	48042893	48106308	12q13.11	SENP1	20.60177616	20.15117184	1.022361197	4	4	30.64244435	11.64280683
chr2	239943015	239943094	2q37.3	MIR4786	2.4251123	2.372221769	1.022295779	1	1	0	0
chr15	41848146	41894077	15q15.1	SPTBN5	0.13919159	0.136169122	1.02219643	4	2	73.41752391	38.53744447
chr6	160991521	161117385	6q26	MAP3K4	8.566986814	8.381016687	1.022189447	4	4	12.47635953	7.226691261
chr2	233251571	233295674	2q37.1	ATG16L1	3.944795385	3.859356153	1.022138209	4	4	25.33091866	2.393228402
chr1	209756032	209782323	1q32.2	TRAF3IP3	18.93129293	18.5232287	1.022029865	4	4	42.46283134	19.48772167
chr19	46601074	46610782	19q13.32	CALM3	85.00114875	83.17331636	1.021976187	4	4	61.68703432	47.50488215
chr1	220786697	220814399	1q41	MARC1	2.762832483	2.703538969	1.021931814	4	4	79.40446683	56.56049387
chr9	133351805	133356485	9q34.2	SURF1	5.7405598	5.617437943	1.021917796	4	4	55.95730225	23.13199541
chrX	53532096	53686729	Xp11.22	HUWE1	26.74660842	26.17297723	1.021916926	4	4	12.17999691	23.23047089
chr10	26746596	26861087	10p12.1	ABI1	66.43569241	65.01890461	1.021790398	4	4	24.19470367	8.157460744
chr5	81412807	81751253	5q14.1	SSBP2	5.881434442	5.756269198	1.021744161	4	4	25.45504124	26.49057418
chr16	4425805	4456775	16p13.3	DNAJA3	2.056194067	2.012492756	1.021715015	4	4	28.5710306	31.51445772
chr17	14029292	14069458	17p12	COX10-AS1	1.183594514	1.158524216	1.021639857	4	4	57.79399363	28.32288078
chrX	48913182	48919136	Xp11.23	PIM2	64.2550505	62.90071577	1.021531309	4	4	33.59733241	23.37961376
chr1	244835306	244845063	1q44	COX20	7.226426599	7.074749425	1.021439229	4	4	18.79153341	20.1721178

chr2	179101692	179264723	2q31.2	SESTD1	5.232285121	5.122647651	1.021402501	4	4	15.52927888	20.76504451
chr3	143342246	143347071	3q24	SLC9A9-AS1	1.893867014	1.854206618	1.021389416	1	1	0	0
chr11	66843412	66846532	11q13.2	RCE1	2.150529607	2.105559361	1.021357862	4	4	39.23297408	22.18207639
chr2	60451167	60553498	2p16.1	BCL11A	2.739486493	2.682256287	1.021336591	4	4	44.5317011	44.57204587
chr11	75562056	75572804	11q13.5	SERPINH1	0.723940344	0.708875589	1.02125162	4	4	61.15227388	81.51399796
chr22	29554808	29581337	22q12.2	NIPSNAP1	4.699403167	4.601805378	1.021208587	4	4	49.19041761	39.13134626
chr1	52142001	52348664	1p32.3	ZFYVE9	0.682662731	0.668543032	1.021120106	4	4	93.57148822	54.47546756
chr19	2476125	2478259	19p13.3	GADD45B	18.65884025	18.27293571	1.021118913	4	4	58.73036778	24.32218096
chr8	100257059	100336218	8q22.2	RNF19A	60.25428647	59.01161797	1.021058031	4	4	28.30543283	38.73814089
chr11	62676498	62679117	11q12.3	UBXN1	33.67471232	32.98039705	1.021052362	4	4	69.20784901	54.85579434
chr1	196044880	196088715	1q31.3	LINC01724	0.801784574	0.785345215	1.020932653	2	2	19.65935928	49.23338019
chr1	32292103	32333628	1p35.2-p35.1	HDAC1	25.80369225	25.27555479	1.020895187	4	4	63.51689165	45.02173208
chr11	93731503	93731573	11q21	SNORD6	25.24728505	24.73328996	1.020781509	4	3	59.73446868	70.55372881
chr19	17824782	17848071	19p13.11	JAK3	22.50812162	22.05092446	1.020733696	4	4	45.81908794	32.78004144
chr13	32400723	32429616	13q13.1	N4BP2L1	7.928154318	7.767376975	1.020699052	4	4	40.58781779	23.17316782
chr17	4143117	4157701	17p13.2	CYB5D2	3.549419684	3.477687183	1.020626496	4	4	74.33537649	26.88681169
chr1	148808181	149051277	1q21.2	PDE4DIP	1.286381686	1.260458293	1.020566641	4	4	63.46411879	77.96515771
chr8	78675870	78806830	8q21.13	IL7	1.038023181	1.01724462	1.020426317	4	4	56.92996761	54.94670506
chr11	65860244	65867653	11q13.1	MUS81	3.404735505	3.336593546	1.020422613	4	4	55.38430044	32.18128861
chr20	25294743	25390982	20p11.21	ABHD12	2.813005313	2.756720411	1.020417342	4	4	58.98816964	10.80580404
chr6	33318558	33323016	6p21.32	DAXX	7.645372953	7.492437332	1.020411999	4	4	40.5004242	54.78825933
chr15	34358888	34367196	15q14	LPCAT4	1.624237593	1.591754677	1.020406986	4	4	63.71368899	40.69327177
chr20	59957764	60037971	20q13.33	CDH26	0.417275895	0.408978531	1.020288017	4	3	39.37077953	52.52493043
chr11	64852727	64879713	11q13.1	EHD1	29.48111698	28.89595351	1.020250706	4	4	48.79421221	28.86772547
chr2	99400475	99490123	2q11.2	REV1	20.34135749	19.93874474	1.020192482	4	4	33.57427892	27.79518312
chr13	41311205	41377031	13q14.11	NAA16	26.84451853	26.31558828	1.020099503	4	4	55.40687699	44.76942491
chr15	40035690	40039202	15q15.1	SRP14	51.276519	50.26814476	1.020059906	4	4	65.43144197	44.77729314
chr11	2944430	2992377	11p15.4	NAP1L4	114.8021564	112.5454765	1.020051272	4	4	48.11569557	28.63671377
chr5	119138859	119138965	5q23.1	RNU6-701P	3.772284108	3.698222523	1.020026265	4	2	32.92046324	65.63303762
chrX	40051246	40177390	Xp11.4	BCOR	7.259698424	7.117534124	1.019973814	4	4	48.58429268	58.17211117
chr2	240126548	240136802	2q37.3	COPS9	4.050363906	3.971382077	1.019887744	4	4	40.9772333	33.07328261
chr1	52032105	52033800	1p32.3	KTI12	3.670188483	3.598636506	1.01988308	4	4	70.87892215	15.60495693
chr17	54968728	55164285	17q22	STXBP4	3.040550132	2.981428654	1.019829915	4	4	93.80418214	74.4482211
chr14	23295921	23302848	14q11.2	PPP1R3E	1.444057966	1.416061596	1.019770588	4	4	57.30987595	19.95344975
chr20	62796405	62801738	20q13.33	MRGBP	4.127217327	4.047363393	1.019729865	4	4	57.82301714	14.14084442
chr14	80478321	80959501	14q31.1	CEP128	12.20744246	11.97180774	1.019682467	4	4	32.38438034	13.24706626
chr7	106315225	106315743	7q22.3	LARP1BP2	3.602702041	3.533323457	1.019635503	4	2	74.12898549	14.90341777
chr19	29923644	30016612	19q12	URI1	23.43175541	22.98191941	1.019573474	4	4	36.60769982	10.75939086
chr6	158232236	158511828	6q25.3	TULP4	7.669823977	7.52268959	1.019558748	4	4	43.99362051	30.9394072
chr16	4538709	4600455	16p13.3	C16orf96	0.088280537	0.086589763	1.019526264	1	3	0	38.96070399
chr15	40439721	40468242	15q15.1	BAHD1	4.342571372	4.259446354	1.019515451	4	4	41.94863143	38.79026103
chr7	32495426	32588741	7p14.3	AVL9	7.993532906	7.840932674	1.019462	4	4	26.44798456	30.97951581
chr14	104986279	105000310	14q32.33	CLBA1	0.221883663	0.217650315	1.019450227	2	3	71.85906669	36.52598388

chr1	151197954	151249536	1q21.3	PIP5K1A	11.19551899	10.9822206	1.019422155	4	4	27.74350744	16.50243957
chr17	81637171	81648755	17q25.3	TSPAN10	0.060222057	0.059080679	1.01931896	1	1	0	0
chr19	41755520	41772211	19q13.2	CEACAM6	1.900402775	1.864495171	1.01925862	4	4	39.57477278	67.30901196
chr19	57719747	57727637	19q13.43	ZNF671	4.522605373	4.43721203	1.019244819	4	4	32.22643653	31.75460835
chr10	45454585	45594906	10q11.21-q11.22	MARCH8	106.7551988	104.7397594	1.019242353	4	4	20.64385324	57.30429023
chr14	77098235	77117287	14q24.3	CIPC	3.152876375	3.093531162	1.019183648	4	4	49.80722513	41.21944244
chr1	244850300	244864525	1q44	HNRNPU	31.50464505	30.91232424	1.019161316	4	4	36.60072761	16.14476256
chr10	122849094	122879641	10q26.13	FAM24B	0.772586545	0.758084391	1.019129999	3	4	21.75484294	51.59444802
chr16	70346829	70373383	16q22.1	DDX19A	1.191114215	1.168787618	1.019102356	4	4	43.76999186	41.2875756
chr14	23938731	23955112	14q11.2	DHRS4-AS1	3.096780057	3.038813258	1.019075472	4	4	61.12792653	24.07862955
chr7	77052733	77060881	7q11.23	SPDYE18	1.625191897	1.595084448	1.018875145	3	3	52.70213766	84.36711544
chr22	41688939	41698136	22q13.2	C22orf46	1.819300902	1.785688133	1.018823426	4	4	81.53429472	55.89665808
chr6	30163206	30172696	6p22.1	TRIM15	0.109575264	0.107552054	1.018811449	2	2	20.2778857	13.29364375
chr9	133330707	133336210	9q34.2	SURF6	1.114357062	1.093788264	1.0188051	4	4	91.83258199	41.11106578
chr9	96815628	96876698	9q22.33	ZNF782	3.872431191	3.801051335	1.018778977	4	4	51.1868333	31.93671324
chr17	82057872	82065821	17q25.3	DUS1L	3.712675368	3.644248291	1.018776733	4	4	56.33703118	11.94967462
chr3	152262616	152269626	3q25.1	MBNL1-AS1	0.818065342	0.803001102	1.018759924	4	4	65.07072236	27.42263344
chr19	7958573	8005645	19p13.2	ELAVL1	4.909333499	4.819058977	1.018732811	4	4	44.22420579	45.53086378
chr22	22792533	22793003	22q11.22	IGLV2-11	19.22922454	18.87588358	1.018719175	4	3	102.3878027	119.1227444
chr14	22148092	22148633	14q11.2	TRAV27	2.416525777	2.372367564	1.018613563	4	4	78.27203466	50.91367718
chr6	134917390	134950122	6q23.3	ALDH8A1	0.338460352	0.332293265	1.018559172	3	3	58.14812175	36.60841897
chr20	25624045	25678074	20p11.21	ZNF337-AS1	2.673846398	2.625256983	1.018508441	4	4	65.1274973	48.72044825
chr3	45678165	45688882	3p21.31	LIMD1-AS1	1.200916217	1.179186774	1.018427482	4	4	89.22017362	39.25011886
chr16	4354542	4416961	16p13.3	CORO7	0.536702361	0.526995303	1.018419628	4	4	86.3451148	36.93468321
chr3	131013873	131026854	3q22.1	ASTE1	5.486947305	5.388139892	1.018337945	4	4	31.16181208	8.596929346
chr19	21804949	21852095	19p12	ZNF43	1.745754348	1.714345534	1.018321169	4	4	62.8932372	21.09823235
chr14	70468876	70483756	14q24.2	ADAM20P1	0.121191771	0.119011949	1.018315995	3	2	21.95850107	40.80250199
chr2	96593155	96638379	2q11.2	KANSL3	7.53680389	7.40154878	1.018273893	4	4	42.16214855	16.1536323
chr5	138554882	138575629	5q31.2	HSPA9	40.30430012	39.58323098	1.018216531	4	4	44.23667969	34.01158542
chr22	31434648	31489950	22q12.2	EIF4ENIF1	10.26631014	10.08288507	1.018191724	4	4	16.77507456	3.960447026
chr3	25597905	25664891	3p24.2	TOP2B	70.40985149	69.15351179	1.018167403	4	4	22.89856408	20.40962485
chr11	61299447	61333212	11q12.2	DDB1	19.10823731	18.76888211	1.018080736	4	4	18.98937003	28.858312
chr20	1113229	1172246	20p13	PSMF1	132.3726217	130.0331111	1.017991653	4	4	32.48348804	32.05648791
chr16	68021274	68023867	16q22.1	DDX28	5.624584328	5.525232362	1.0179815	4	4	87.29048154	26.85234043
chr13	37000373	37009614	13q13.3	EXOSC8	4.680724571	4.59807164	1.017975564	4	4	33.88477002	14.34680226
chr19	45407333	45478866	19q13.32	ERCC1	5.294721826	5.201566003	1.017909188	4	4	51.31980003	22.89323853
chr4	143336830	143474565	4q31.21	GAB1	23.7115767	23.29530502	1.017869338	4	4	32.06714147	24.67157531
chr12	49636499	49707414	12q13.12	FMNL3	3.34795344	3.289686237	1.017712085	4	4	59.61716055	35.67858695
chr5	14660794	14716552	5p15.2	OTULIN	8.069005159	7.928812286	1.017681447	4	4	25.89524742	14.12804034
chr2	200881715	200881821	2q33.1	RNU6-312P	1.828952026	1.797235093	1.017647626	1	1	0	0
chr19	34956354	34964049	19q13.11	ZNF792	5.557193929	5.46094745	1.017624502	4	4	44.40156418	36.68502237
chr6	96521826	96555276	6q16.1	UFL1	20.11630427	19.76838963	1.017599544	4	4	26.07719576	17.40331215
chr10	102065367	102068038	10q24.32	HPS6	6.51270336	6.400224483	1.017574208	4	4	52.13901486	26.43709101

chr1	94418086	94518663	1p21.3	ABCD3	9.484493544	9.320984287	1.017542059	4	4	24.55979166	23.59639382
chrX	54192823	54358695	Xp11.22	WNK3	0.022720377	0.02232896	1.017529576	1	2	0	?
chr7	105532081	105567684	7q22.3	RINT1	9.328347176	9.168111426	1.017477509	4	4	37.2822757	7.853523085
chrY	2841582	2866956	Yp11.2	RPS4Y1	79.85482668	78.48451877	1.017459595	2	2	39.97054955	40.58166352
chr15	25083587	25083680	15q11.2	SNORD116-17	4.400507508	4.325935197	1.017238425	3	1	51.19651794	0
chr7	124822386	124929983	7q31.33	POT1	9.732399035	9.568148882	1.017166346	4	4	24.29715847	11.43821262
chr11	65333754	65354260	11q13.1	DPF2	6.624777519	6.513021442	1.017158868	4	4	60.54518958	48.97583886
chr7	92528762	92540481	7q21.2	RBM48	6.929099739	6.812444024	1.017123915	4	4	24.41072761	22.67150478
chr5	148982150	149063174	5q32	SH3TC2	0.680570998	0.669207097	1.016981142	4	4	43.40064504	4.172370617
chr10	15104588	15168710	10p13	NMT2	6.394411048	6.288044539	1.016915674	4	4	33.61859457	33.04933189
chr11	3797724	3826371	11p15.4	PGAP2	1.731982869	1.70326994	1.016857533	4	4	63.9140196	58.02049619
chr12	53251251	53254406	12q13.13	MFSD5	5.501888839	5.410988476	1.016799216	4	4	50.07900427	17.34050094
chr13	30617679	30660770	13q12.3	USPL1	13.3174036	13.09889134	1.016681737	4	4	15.18251265	29.76406307
chr15	65001512	65029639	15q22.31	MTFMT	3.442117965	3.386062887	1.016554648	4	4	32.75172656	47.04232325
chr1	24899511	24965158	1p36.11	RUNX3	29.18390501	28.70867971	1.016553367	4	4	37.19321687	68.17241397
chr15	42158701	42208331	15q15.1	VPS39	9.162210791	9.013517734	1.016496673	4	4	49.42543359	19.05934296
chr16	71729000	71809073	16q22.2	AP1G1	21.26620222	20.92127822	1.016486756	4	4	14.979612	5.773154924
chr6	1623800	2245634	6p25.3	GMDS	2.141900973	2.107234871	1.016450991	4	4	76.56674463	33.7732851
chr18	3594114	3597379	18p11.31	DLGAP1-AS1	5.686708596	5.59476355	1.016434125	4	4	51.3758482	17.22459161
chr10	88935074	88991397	10q23.31	ACTA2	3.774902301	3.714059583	1.016381729	4	4	23.74170057	128.7904224
chr17	29073521	29075609	17q11.2	TIAF1	0.345543672	0.339974481	1.016381203	4	4	75.9637321	48.50393927
chr11	122960253	122961284	11q24.1	ATP5PBP5	0.528861633	0.52034071	1.016375661	2	2	10.27146019	50.90492704
chr18	35591737	35591806	18q12.2	MIR3975	7.235667631	7.119264223	1.016350483	1	2	0	83.08742434
chr6	43336070	43369443	6p21.1	ZNF318	5.810456596	5.717044906	1.016339156	4	4	53.69890969	49.9780493
chr19	58467045	58473578	19q13.43	ZNF324	2.087518454	2.054324929	1.016157875	4	4	60.43728458	20.37237717
chr22	39500100	39518134	22q13.1	MIEF1	4.859153159	4.782196108	1.016092408	4	4	41.67016927	10.25997863
chr21	42221173	42221286	21q22.3	RNA5SP492	3.944096599	3.881632322	1.01609227	3	3	46.274716	49.85010125
chr1	225840556	225882720	1q42.12	TMEM63A	5.51196004	5.424839484	1.016059564	4	4	58.9170206	12.79230929
chr10	118046821	118210153	10q26.11	CASC2	0.025845776	0.025439511	1.01596986	1	1	0	0
chr1	179099327	179229693	1q25.2	ABL2	2.977686595	2.931127141	1.015884488	4	4	34.06300914	29.64603153
chr3	47802909	47850196	3p21.31	DHX30	4.702701817	4.629654767	1.015778077	4	4	38.63819939	38.68714072
chr2	24943636	24972094	2p23.3	DNAJC27	1.68096023	1.654873566	1.015763539	4	4	43.56048541	11.28180903
chr14	74493720	74495568	14q24.3	ISCA2	5.713036707	5.624653094	1.015713611	4	4	68.97052925	20.03897588
chr5	151020412	151028993	5q33.1	GPX3	0.781833474	0.769746332	1.015702759	4	4	123.4585157	57.02620101
chr2	15591621	15631111	2p24.3	DDX1	20.49443712	20.17825913	1.01566924	4	4	20.44659156	5.664139055
chr7	144451941	144836424	7q35	TPK1	3.786696409	3.728526599	1.015601286	4	4	33.47280551	27.6585916
chr12	94971328	95003713	12q22	NDUFA12	15.84925961	15.6059821	1.015588735	4	4	23.65749652	31.57596945
chr7	86876906	87059714	7q21.12	KIAA1324L	1.179415433	1.161338133	1.015565923	4	4	57.77672227	20.52278319
chr5	180843580	180861327	5q35.3	ZFP62	6.685836031	6.583367504	1.015564759	4	4	28.0553986	28.13367731
chr15	90620009	90717140	15q26.1	CRTC3-AS1	11.50867312	11.33273222	1.015525021	4	4	80.97128523	75.69114047
chr3	49673102	49683508	3p21.31	APEH	10.84928251	10.68366363	1.015502068	4	4	56.08120867	33.53105607
chr12	120438090	120440742	12q24.2	COX6A1	22.59015544	22.24819623	1.015370199	4	4	24.62153231	13.36138971
chr10	93893951	93914271	10q23.33	SLC35G1	0.764858972	0.753287995	1.015360628	4	4	42.48690928	31.21262112

chr10	68009947	68010883	10q21.3	POU5F1P5	1.188231329	1.170278797	1.015340389	2	3	64.07178602	35.74244274
chr17	7932054	7949919	17p13.1	CNTROB	2.896258531	2.852605878	1.015302729	4	4	28.91895302	18.38719148
chr3	114314500	115147280	3q13.31	ZBTB20	2.769627292	2.72793697	1.015282729	4	4	25.69517775	26.81604032
chr4	119212583	119295518	4q26	USP53	3.745665237	3.689552595	1.015208522	4	4	15.348689	43.95573383
chr6	5031029	5260950	6p25.1	LYRM4	1.2003506	1.182432864	1.01515328	4	4	40.30920308	38.232467
chr2	189441433	189475562	2q32.2	WDR75	9.777888334	9.632236464	1.015121293	4	4	32.60890111	17.46800895
chr15	74890005	74899457	15q24.1	MPI	1.843363732	1.81595284	1.015094495	4	4	63.87885772	39.35470682
chr1	47216290	47232389	1p33	TAL1	26.6797896	26.28841381	1.014887768	4	4	14.99461373	56.21021431
chr18	75210755	75289950	18q22.3	TSHZ1	5.12896378	5.054294588	1.014773415	4	4	45.59362643	29.20537026
chr2	191834305	191847280	2q32.3	CAVIN2	263.2013681	259.4235568	1.01456233	4	4	30.02921536	34.64857922
chr3	49828595	49856584	3p21.31	TRAIP	0.471575954	0.464828258	1.014516535	4	4	49.97905666	84.64060133
chr2	126555301	126558179	2q14.3	YWHAZP2	0.628482274	0.619496395	1.014505135	2	4	53.38685842	54.26924978
chr19	20619939	20661596	19p12	ZNF626	1.568001724	1.545877086	1.014312029	4	4	78.00775342	47.42183469
chr6	41921227	41933046	6p21.1	BYSL	1.93524006	1.908010092	1.014271396	4	4	16.69550765	53.56668594
chr7	45723787	45769018	7p12.3	SEPT7P2	7.064128997	6.965163957	1.014208573	4	4	32.66217613	60.88274
chr19	37667749	37692315	19q13.12	ZNF781	0.491512473	0.484648558	1.014162663	4	4	19.41318678	60.4654185
chr10	18659335	18681639	10p12.31	ARL5B	14.68994279	14.48538986	1.014121327	4	4	29.56658495	35.60240154
chr9	26903370	26947473	9p21.2	PLAA	15.33492925	15.12173747	1.014098365	4	4	5.819718535	17.97555728
chr5	96760273	96935983	5q15	ERAP1	27.51168239	27.12949254	1.014087615	4	4	45.15217545	28.639551
chr6	30287397	30327156	6p22.1	HCG18	0.272080513	0.268314393	1.014036222	4	4	64.11391156	37.3763711
chr8	96261886	96334552	8q22.1	PTDSS1	13.20777019	13.02554825	1.013989579	4	4	30.64080401	16.23370178
chr16	29995690	30005794	16p11.2	INO80E	3.059280177	3.017113813	1.013975729	4	4	45.38502145	33.16154419
chrMT	4470	5511	N/A	MT-ND2	222.2399513	219.1906055	1.013911845	4	4	35.04203198	29.91782588
chr19	36850350	36893218	19q13.12	ZNF345	1.689105989	1.66597373	1.013885128	4	4	53.81571591	36.47037081
chrX	119991393	119992543	Xq24	RHOXF1P3	0.107052051	0.105597466	1.013774812	2	1	25.1768343	0
chr11	13276552	13387268	11p15.3	ARNTL	9.031768826	8.909316412	1.013744311	4	4	50.81468874	19.34257354
chr5	74721204	74767371	5q13.3	GFM2	19.94749058	19.67798001	1.013696048	4	4	51.49130019	15.71842542
chr14	22531076	22531138	14q11.2	TRAJ13	14.31897969	14.12555662	1.013693129	4	3	51.80942104	18.93476299
chr3	39051986	39096671	3p22.2	WDR48	16.49415521	16.27482104	1.013476902	4	4	11.45646515	19.45794938
chr14	77335021	77377109	14q24.3	TMED8	11.39649725	11.24553812	1.013423913	4	4	37.74445074	28.05378369
chr13	20777329	20902774	13q12.11	XPO4	8.792851938	8.676741868	1.013381759	4	4	41.67931434	18.5357558
chr2	183117480	183161684	2q32.1	NUP35	25.93405933	25.5943218	1.013273942	4	4	69.24033329	51.86929299
chr3	44975291	45012668	3p21.31	EXOSC7	2.32624758	2.295842977	1.013243328	4	4	66.40825791	28.00490977
chr22	41621163	41664048	22q13.2	XRCC6	61.21113508	60.41119065	1.01324166	4	4	51.22893915	30.23261048
chr19	43596392	43600435	19q13.31	ZNF576	1.415971019	1.397487869	1.013225982	4	4	84.66864396	38.74591977
chr20	57648392	57711536	20q13.31	PMEPA1	0.736291664	0.726786693	1.013078076	3	4	94.64715157	104.0706475
chr18	63123346	63320280	18q21.33	BCL2	11.16035641	11.01775856	1.012942547	4	4	36.59973615	19.21619311
chr2	88861886	88861923	2p11.2	IGKJ1	20.32882846	20.06915482	1.012938943	4	4	85.44693692	71.50999456
chr17	3440011	3513852	17p13.2	SPATA22	0.536280677	0.529459959	1.012882407	2	1	119.6264762	0
chr8	130052104	130443848	8q24.21-q24.22	ASAP1	242.2973175	239.2186298	1.012869765	4	4	31.76538139	22.03602047
chr6	43576141	43620523	6p21.1	POLH	5.846841742	5.772905713	1.01280742	4	4	43.33248592	52.45155154
chr6	13615327	13632470	6p23	NOL7	14.12954638	13.95139162	1.012769677	4	4	46.04502177	38.32820359
chr16	48354581	48448435	16q12.1	SIAH1	4.337242324	4.282659178	1.012745153	4	4	44.58549871	33.11229398

chr11	73249316	73249869	11q13.4	OR8R1P	0.564297112	0.557340634	1.012481555	1	2	0	37.06215387
chr17	9643493	9729687	17p13.1	USP43	0.149713314	0.147874291	1.012436395	2	3	86.19773364	59.9375523
chr11	405716	417454	11p15.5	SIGIRR	7.191320053	7.103027297	1.012430299	4	4	33.89416374	24.96610063
chr5	150708747	150759095	5q33.1	DCTN4	20.69676448	20.44562162	1.012283454	4	4	18.09497444	44.24602714
chr1	239386565	239915450	1q43	CHRM3	0.07068664	0.069833531	1.012216321	2	4	89.14265073	79.3176893
chr5	119268692	119394604	5q23.1	TNFAIP8	27.78921456	27.45439593	1.012195447	4	4	27.06109105	27.41630749
chr14	50100456	50116579	14q21.3	VCPKMT	9.482872192	9.369176426	1.012135087	4	4	32.84944117	32.18098579
chrX	76172929	76178310	Xq13.3	PBDC1	10.46840693	10.3429264	1.012132014	4	4	39.69975078	22.16765761
chr2	110211526	110245420	2q13	MTLN	1.402038499	1.385302565	1.012081068	4	4	67.2881565	30.87818751
chr11	120336276	120489936	11q23.3	ARHGEF12	69.62250501	68.80175547	1.011929195	4	4	52.42642966	44.98696363
chr11	107326345	107457846	11q22.3	CWF19L2	40.6288442	40.15120182	1.011896092	4	4	23.67739715	11.84871797
chr11	68154863	68213899	11q13.2	KMT5B	20.60045647	20.35906089	1.011856912	4	4	16.59270059	28.34029436
chr10	80407829	80437115	10q23.1	PRXL2A	0.699569153	0.6914281	1.011774258	4	4	39.74498699	77.57097528
chr10	118305889	118342327	10q26.11	FAM204A	4.942289151	4.884974253	1.011732897	4	4	25.36817672	24.70303055
chr4	73054696	73069777	4q13.3	COX18	2.924772899	2.891034159	1.011670128	4	4	49.89344825	20.16353414
chr14	74661252	74713118	14q24.3	AREL1	20.0348842	19.8055385	1.011579877	4	4	17.76952047	10.47401549
chr15	79311062	79413886	15q25.1	TMED3	1.152502014	1.139398874	1.011500047	4	4	90.07729429	38.14324411
chr13	19304065	19305760	13q12.11	MRPL3P1	0.239825144	0.237123631	1.011392843	1	1	0	0
chr17	8159147	8162975	17p13.1	VAMP2	7.301411153	7.219248669	1.01138103	4	4	35.15553318	26.67102507
chr1	70180144	70180439	1p31.1	RN7SL242P	0.941303269	0.930711686	1.01138009	2	3	33.45263562	23.48019642
chr12	51048220	51068939	12q13.12	LETMD1	8.13811223	8.046635749	1.011368289	4	4	41.30888646	29.08144473
chr3	14673470	14773036	3p25.1	C3orf20	0.303607093	0.3001966	1.011360865	4	4	26.9471609	68.7177323
chr5	56919602	56952130	5q11.2	MIER3	9.727282468	9.618631137	1.011295924	4	4	24.24475333	11.5629083
chr19	39480412	39493785	19q13.2	TIMM50	1.318314562	1.303644498	1.011253117	4	4	69.54690504	55.28772857
chr6	134960378	135054898	6q23.3	HBS1L	12.73797593	12.59685184	1.011203123	4	4	31.15210554	25.20233606
chr8	144508081	144511228	8q24.3	MFSD3	0.750748684	0.742437134	1.011194954	4	4	59.83402109	42.70013776
chr6	28267010	28278224	6p22.1	ZSCAN26	4.549540212	4.499409209	1.011141686	4	4	34.79426367	14.0618111
chr9	15464066	15511019	9p22.3	PSIP1	32.90384492	32.54281401	1.011094029	4	4	36.18156458	30.80704772
chr1	37681570	37692336	1p34.3	C1orf109	2.51662134	2.489101736	1.011056038	4	4	36.3711864	44.23691307
chr11	790475	798281	11p15.5	SLC25A22	1.502201871	1.485893767	1.010975283	4	4	60.33912636	48.7266019
chr9	128309100	128322780	9q34.11	TRUB2	1.960693297	1.939424299	1.010966655	4	4	70.06180037	38.22090056
chr17	2416049	2511906	17p13.3	METTL16	5.350120464	5.292125804	1.01095867	4	4	20.96969309	17.29239979
chrX	43766610	43882475	Xp11.3	MAOB	0.45020294	0.445324783	1.010954156	2	4	36.66103789	61.67608808
chr12	69239537	69274358	12q15	CPSF6	30.25829125	29.93066608	1.010946137	4	4	19.09447135	18.99317124
chr11	63974607	63976543	11q13.1	COX8A	77.53039166	76.69102252	1.010944816	4	4	47.0307944	72.19583142
chr6	36968135	36987171	6p21.2	MTCH1	22.03894574	21.80275758	1.010832949	4	4	48.83841459	17.73862652
chr2	189746660	189763351	2q32.2	OSGEPL1	3.369412691	3.333468569	1.010782799	4	4	70.2111027	37.16409529
chr16	29618675	29636331	16p11.2	CA5AP1	0.74378996	0.735862163	1.010773481	2	4	5.798878375	68.29512942
chr12	95217746	95302790	12q22	VEZT	9.912844755	9.807960967	1.01069374	4	4	15.89050178	24.0027196
chr19	9751993	9786183	19p13.2	ZNF846	1.520402001	1.504399231	1.010637316	4	4	44.39181525	57.50560497
chr3	184135022	184145311	3q27.1	EIF2B5	6.115625954	6.051983356	1.01051599	4	4	41.42278001	21.52324674
chr8	38163321	38176730	8p11.23	LSM1	19.83281782	19.62669206	1.010502318	4	4	32.42659983	5.911488095
chr7	97852117	97928562	7q21.3	ASNS	1.983432885	1.962886777	1.010467291	4	4	34.45828565	33.87842011

chr8	78516101	78605267	8q21.13	PKIA	4.543083093	4.496383707	1.010385988	4	4	45.91217625	32.58721301
chr5	140401814	140539856	5q31.3	ANKHD1	0.669169201	0.662310031	1.010356434	4	4	23.03503347	30.31694818
chr8	127013154	127021014	8q24.21	PCAT1	0.292049357	0.289067303	1.010316121	3	4	45.97588554	92.47384611
chr2	170928438	170967135	2q31.1	GORASP2	7.500758961	7.424194925	1.010312773	4	4	52.9827841	44.65604724
chr2	231732425	231781327	2q37.1	PDE6D	5.860096936	5.800306807	1.010308098	4	4	70.92283971	50.51100122
chr17	4937130	4940251	17p13.2	SLC25A11	8.923578358	8.832606945	1.010299497	4	4	49.51273174	34.51652933
chr15	90529886	90645345	15q26.1	CRTC3	5.369565226	5.31494623	1.010276491	4	4	56.27966114	28.04571871
chr2	97712030	97744327	2q11.2	ZAP70	15.58782748	15.43025737	1.010211761	4	4	36.79466246	50.55987908
chr12	123621023	123633776	12q24.31	EIF2B1	11.17102398	11.0581903	1.010203629	4	4	46.03474169	24.50194459
chr4	139697626	139698635	4q31.1	H3F3AP6	2.013602839	1.993316432	1.010177214	3	4	50.5654676	73.28542318
chr7	1530732	1543043	7p22.3	MAFK	6.349677906	6.285737016	1.010172378	4	4	46.54543851	31.40449345
chr3	114291102	114329747	3q13.31	TIGIT	7.997783516	7.917301047	1.010165392	4	4	49.60178945	39.19939907
chr15	66293257	66333898	15q22.31	DIS3L	6.53340184	6.467736531	1.010152749	4	4	29.14157656	19.63231767
chr19	3094410	3124002	19p13.3	GNA11	0.356155325	0.352583353	1.010130857	4	4	86.28166619	52.27655621
chr6	116118909	116219937	6q22.1	COL10A1	0.160659149	0.159053583	1.010094496	2	4	63.23390076	52.15363972
chr1	85249953	85259677	1p22.3	C1orf52	7.332732334	7.261047867	1.009872469	4	4	12.90931349	24.82246954
chr6	156777847	157210779	6q25.3	ARID1B	3.057246176	3.027439501	1.009845507	4	4	31.75034998	11.70347919
chrX	134373312	134428792	Xq26.2	PHF6	9.248252869	9.159487028	1.009691137	4	4	14.70608243	14.35459764
chr2	195575944	195737702	2q32.3	SLC39A10	13.62849409	13.49857465	1.009624678	4	4	29.25325418	7.183827
chr6	96889311	96897891	6q16.1	NDUFAF4	4.801006652	4.75570154	1.009526483	4	4	36.46970939	38.62384079
chr10	97393174	97393582	10q24.1	RPL34P20	1.132574806	1.121908581	1.009507214	3	2	27.99424163	60.30025126
chr11	65890404	65891635	11q13.1	CCDC85B	3.655490729	3.621303437	1.009440604	4	4	90.73938728	57.73043223
chr2	135741619	135785063	2q21.3	UBXN4	73.26471322	72.58153394	1.009412577	4	4	20.46238595	9.67256483
chr5	137617500	137736090	5q31.2	KLHL3	0.693141761	0.686681828	1.009407462	4	4	66.50870712	33.01986544
chr3	188151206	188154088	3q27.3	LPP-AS2	0.871813094	0.863725439	1.009363688	4	4	106.7859404	62.88348048
chr1	6613696	6624033	1p36.31	PHF13	3.02398948	2.996364197	1.009219601	4	4	53.77704929	23.74405485
chr17	58000919	58007346	17q22	SRSF1	67.59923078	66.9837221	1.009188929	4	4	23.84412521	26.89339418
chr16	81035853	81047346	16q23.2	ATMIN	16.36288274	16.21483843	1.009130175	4	4	43.58847376	37.53368584
chr18	50227193	50266522	18q21.1	CFAP53	3.007747454	2.980865314	1.009018234	4	4	26.05705095	26.1030105
chr1	32651148	32686211	1p35.1	RBBP4	18.47363542	18.3125211	1.008798042	4	4	31.19179714	19.0692447
chr3	14651746	14672659	3p25.1	CCDC174	12.3287114	12.22169808	1.008756011	4	4	60.48167681	21.81907188
chr8	102252273	102412689	8q22.3	UBR5	34.62792471	34.32856117	1.008720538	4	4	21.51591676	13.6441111
chr1	174448074	174449545	1q25.1	GPR52	1.674656161	1.660188692	1.008714352	4	4	33.01852844	46.52902241
chrX	81113701	81201942	Xq21.1	HMGN5	1.407237074	1.395122387	1.008683601	4	4	29.57381174	59.27527441
chr2	216632828	216664436	2q35	IGFBP2	0.081005305	0.080319071	1.00854385	3	2	42.36610721	47.05291451
chr6	88609897	88963629	6q15	RNGTT	16.50616798	16.36713141	1.008494865	4	4	12.76912385	27.02848482
chr17	17476986	17493220	17p11.2	MED9	1.309174378	1.298224647	1.008434388	4	4	68.26331704	24.02870453
chr3	48847937	48851982	3p21.31	PRKAR2A-AS1	0.278583652	0.276289708	1.008302676	4	4	63.8720238	42.70518767
chr6	116635618	116668810	6q22.1	ZUP1	44.96922016	44.60195628	1.008234255	4	4	70.09508263	33.9106104
chr1	85649423	85708458	1p22.3	ZNHIT6	5.030397512	4.98976793	1.008142579	4	4	33.01953475	35.54831072
chr15	69414246	69448427	15q23	KIF23	22.96886955	22.78415734	1.008107046	4	4	92.55751535	67.88635246
chr15	39795049	39920939	15q14-q15.1	GPR176	0.250911327	0.248896019	1.008096987	4	4	55.59565712	83.92959346
chrX	154458281	154473646	Xq28	PLXNA3	0.435551086	0.432097023	1.007993721	4	4	72.94346797	18.031699

chr17	81556885	81637153	17q25.3	NPLOC4	14.50124209	14.38673719	1.00795906	4	4	12.95311278	6.880776497
chr8	18170419	18223689	8p22	NAT1	5.130995995	5.090601614	1.00793509	4	4	30.07685913	33.68744182
chr11	111293389	111305048	11q23.1	COLCA1	0.029631724	0.029398539	1.007931832	1	2	0	3.172689422
chr18	13663347	13726592	18p11.21	FAM210A	3.79562348	3.765933722	1.007883771	4	4	27.38033323	23.73109035
chr1	101786266	101787307	1p21.2	RPSAP19	0.314103726	0.311670182	1.007808072	2	4	28.37491006	10.63401362
chr7	75507747	75528123	7q11.23	PMS2P3	16.73292338	16.60363497	1.007786753	4	4	40.86025309	34.44667925
chr19	5904841	5910252	19p13.3	VMAC	2.376034362	2.357906219	1.007688238	4	4	63.798959	18.88784975
chr1	225982702	225999365	1q42.12	SDE2	30.3473684	30.11593061	1.007684896	4	4	24.16510049	32.92104088
chr4	77862651	77952790	4q21.1	MRPL1	17.62838446	17.49398839	1.007682414	4	4	24.42420798	11.14838636
chr20	35542029	35557634	20q11.22	ERGIC3	10.35531515	10.27655728	1.007663838	4	4	52.54687687	28.00004136
chr15	40942137	40956519	15q15.1	CHAC1	0.135071112	0.134045086	1.007654338	1	3	0	10.01655532
chr14	22462932	22466577	14q11.2	TRDC	70.81518109	70.28174041	1.007590032	4	4	38.3934018	43.25902919
chr6	118460772	118710101	6q22.31	CEP85L	16.56833105	16.44384837	1.007570167	4	4	39.96795581	14.66230311
chr12	48667457	48667593	12q13.11	SNORA2B	7.000562472	6.948032305	1.007560438	4	2	55.30424167	28.62630798
chr10	97435909	97446017	10q24.1	EXOSC1	40.11013455	39.81058962	1.007524253	4	4	40.73208529	34.21184693
chr16	16232532	16294811	16p13.11	NOMO3	0.703709203	0.698624855	1.007277651	4	4	67.71790259	45.38007976
chr3	124761948	124901411	3q21.2	ITGB5	4.467555554	4.435289743	1.007274792	4	4	79.13860399	7.784942417
chr22	45308960	45341955	22q13.31	FAM118A	3.890874937	3.862976149	1.007222097	4	4	37.56223143	26.95379599
chr17	12991612	13018064	17p12	ELAC2	5.092443122	5.05638467	1.007131271	4	4	46.1816677	31.50476673
chr11	66616582	66646473	11q13.2	RBM14-RBM4	0.448094587	0.444926174	1.00712121	4	4	51.3024227	52.94559194
chr7	6735305	6751601	7p22.1	PMS2CL	4.388709187	4.358476427	1.006936543	4	4	47.08107847	26.25356907
chr17	42968088	42980528	17q21.31	PTGES3L	0.495428377	0.492015831	1.006935845	4	4	74.34632644	64.47022591
chr12	112026689	112108831	12q24.13	NAA25	5.539759171	5.501666914	1.006923767	4	4	24.80377901	20.01493839
chr20	5911328	5925361	20p12.3	CHGB	0.073758353	0.073252113	1.006910927	1	3	0	39.82260756
chr5	96934859	97037513	5q15	LNPEP	43.98181262	43.68120287	1.006881902	4	4	2.946814068	15.0800196
chr11	66638617	66668385	11q13.2	RBM4	3.146381867	3.125057603	1.006823639	4	4	50.54920253	17.76429961
chr2	74654228	74699013	2p13.1	SEMA4F	0.70103458	0.696283903	1.006822902	4	4	58.41401642	31.36104749
chr1	22652981	22661637	1p36.12	C1QB	2.692641192	2.674594739	1.00674736	4	3	50.65833796	123.6356459
chr22	40824535	40857008	22q13.2	ST13	211.2632774	209.8591541	1.006690789	4	4	14.6138698	30.79518738
chr5	73552200	73565686	5q13.2	ANKRA2	12.0832717	12.00500534	1.006519477	4	4	26.46742052	19.89758645
chr13	75284664	75483144	13q22.2	TBC1D4	6.633047045	6.59016491	1.00650699	4	4	44.75396523	24.83756781
chr6	2854031	2854107	6p25.2	MIR4645	7.083385824	7.037663699	1.006496776	4	2	60.29669985	65.65989699
chr19	45162928	45179266	19q13.32	TRAPPC6A	5.886054	5.84816596	1.006478619	4	4	50.35037448	21.29014554
chr6	110958560	110967888	6q21	GTF3C6	25.49108221	25.33034593	1.006345601	4	4	26.84652592	18.130097
chr22	41673933	41690492	22q13.2	SNU13	15.63301605	15.53458978	1.006335943	4	4	30.05835475	18.81452062
chr9	126804072	126838210	9q33.3	ZBTB43	3.98291983	3.958534271	1.006160249	4	4	52.82153797	27.34807898
chr1	203765132	203778175	1q32.1	LAX1	9.928824819	9.868569263	1.006105805	4	4	36.43106509	49.55268882
chr2	86503430	86721122	2p11.2	RNF103-CHMP3	0.190409481	0.189275788	1.005989633	3	2	22.05346785	14.8001566
chr3	40523854	40539974	3p22.1	ZNF621	2.70915807	2.693199225	1.005925609	4	4	44.74985509	36.77532135
chr17	54951898	54968785	17q22	COX11	2.311155497	2.297710171	1.00585162	4	4	39.15229639	17.56560445
chr19	1446268	1473244	19p13.3	APC2	0.020504465	0.020390492	1.005589504	1	2	0	2.55375707
chr19	5916139	5978309	19p13.3	RANBP3	5.942384951	5.909400194	1.005581744	4	4	40.67129926	18.04802486
chr2	171783405	171894306	2q31.1	SLC25A12	4.05033562	4.028200046	1.005495152	4	4	34.73364665	29.50749466

chr4	7758713	7939926	4p16.1	AFAP1	3.182576567	3.165368453	1.00543637	4	4	78.25715159	58.37292383
chr16	11345438	11351763	16p13.13	RMI2	1.390956161	1.383763657	1.005197783	4	4	94.33303191	32.42431672
chr6	41149097	41158450	6p21.1	TREML1	32.83815386	32.67029313	1.005138023	4	4	87.37525471	40.07823122
chr17	47209017	47227650	17q21.32	MYL4	90.52852346	90.06975685	1.005093459	4	4	36.36922735	9.342254587
chr19	19792711	19821751	19p13.11	ZNF506	6.839253906	6.804792102	1.005064343	4	4	35.16271286	31.24989278
chr6	107152557	107459681	6q21	PDSS2	4.538191799	4.515520108	1.005020837	4	4	26.98299254	3.054947712
chr1	61681047	61725423	1p31.3	TM2D1	9.266275406	9.220154727	1.005002159	4	4	43.05307114	38.07013761
chr11	67398181	67401905	11q13.2	PPP1CA	45.63103842	45.40791014	1.004913864	4	4	50.49798851	46.38018612
chr7	156949722	156973182	7q36.3	NOM1	5.240644252	5.215040007	1.004909693	4	4	71.06606088	32.22396843
chr11	103050684	103092215	11q22.3	DCUN1D5	8.354205409	8.313820538	1.004857559	4	4	25.55646789	17.92894582
chr2	112584437	112589040	2q14.1	CHCHD5	1.051464263	1.046426183	1.004814559	4	4	86.26587964	70.79181823
chr22	31399553	31434186	22q12.2	DRG1	14.72974544	14.65938583	1.004799628	4	4	14.68935787	29.91048055
chr3	11556067	11720768	3p25.3-p25.2	VGLL4	4.510448189	4.48894999	1.004789137	4	4	55.09472027	30.95488529
chr1	44221070	44355298	1p34.1	ERI3	9.625621731	9.581562827	1.0045983	4	4	40.49836905	31.69729427
chr8	65643873	65771252	8q13.1	MTFR1	2.244648379	2.234389373	1.004591414	4	4	27.37974736	8.77414073
chr5	80626226	80654981	5q14.1	DHFR	6.029871651	6.002475796	1.004564092	4	4	74.53086243	36.12584649
chr22	50445000	50475071	22q13.33	SBF1	11.96975096	11.91557936	1.004546283	4	4	49.53821921	44.38763733
chr17	28719982	28724356	17q11.2	RPL23A	146.9644073	146.3038561	1.004514927	4	4	43.53238814	10.80207216
chr19	10871577	10923078	19p13.2	CARM1	30.14960229	30.01610375	1.004447564	4	4	32.39143028	29.17893125
chr12	122471599	122501184	12q24.31	ZCCHC8	15.46676691	15.39877601	1.004415344	4	4	48.43223998	32.88160877
chr10	84328552	84518521	10q23.1	CCSER2	26.43962608	26.32425407	1.004382726	4	4	21.09761962	19.03421965
chr8	74055858	74056690	8q21.11	RPS3AP32	0.704583903	0.701683706	1.004133197	2	1	84.04724381	0
chr7	67302638	67321526	7q11.21	STAG3L4	2.679966215	2.669189728	1.004037363	4	4	58.31177801	24.35565172
chr2	223875343	223945387	2q36.1	WDFY1	33.3602003	33.22629174	1.004030199	4	4	27.42374045	37.12917818
chr3	10026387	10101937	3p25.3	FANCD2	6.58492631	6.558624455	1.00401027	4	4	40.89874452	35.46833627
chr6	26124145	26124690	6p22.2	HIST1H2AC	311.5340024	310.3024495	1.003968879	4	4	26.36059988	17.1190415
chr16	84476355	84504752	16q24.1	MEAK7	0.904771952	0.901223517	1.003937352	4	4	82.43514293	25.05284642
chr8	43056299	43085788	8p11.21	FNTA	6.931535987	6.904457747	1.003921849	4	4	21.73682553	24.30528851
chr12	123671108	123708405	12q24.31	TCTN2	2.284808036	2.276022613	1.003859989	4	4	85.20465977	44.39138981
chr2	206765388	206796189	2q33.3	FASTKD2	5.841567893	5.819605895	1.003773795	4	4	25.7332746	13.44957543
chr1	84077975	84238498	1p31.1	PRKACB	69.99833911	69.7373433	1.003742555	4	4	31.59705602	35.1126429
chr1	212992182	213015875	1q32.3	ANGEL2	6.959559121	6.933625956	1.003740202	4	4	19.03582304	19.47448167
chr15	73683966	73714518	15q24.1	CD276	0.049140007	0.048956996	1.003738187	1	1	0	0
chr4	109815510	109824737	4q25	GAR1	3.734485742	3.720965355	1.00363357	4	4	37.05346173	32.82580391
chr10	97458324	97498794	10q24.1	MMS19	6.35755968	6.334661889	1.003614682	4	4	32.17663777	19.95944182
chrX	11758159	11775753	Xp22.2	MSL3	14.4678006	14.41636458	1.003567891	4	4	44.89770861	25.9809787
chr19	37906822	38208372	19q13.13-q13.2	SIPA1L3	3.221269682	3.209892718	1.003544344	4	4	73.9031829	66.89425297
chr14	67589306	67600302	14q24.1	PIGH	4.328377286	4.313223983	1.00351322	4	4	36.6635111	31.65468783
chr1	167430640	167518610	1q24.2	CD247	34.80893013	34.68868579	1.003466385	4	4	35.08041837	16.64671972
chr10	97426125	97433444	10q24.1	PGAM1	21.63256934	21.55795046	1.003461316	4	4	39.83450776	31.05915052
chr1	247121975	247172379	1q44	ZNF124	22.73549411	22.65756885	1.003439259	4	4	19.19694772	27.94815337
chr18	80109031	80140346	18q23	ADNP2	6.922534769	6.899503267	1.003338139	4	4	46.22076622	16.2139687
chr7	43608452	43729541	7p13	COA1	3.76542506	3.753017138	1.003306119	4	4	43.47800519	17.13618779

chr7	107891107	107921198	7q31.1	DLD	18.38883847	18.33015315	1.003201573	4	4	30.39952309	6.053327813
chr18	36187210	36268722	18q12.2	MOCOS	0.096986622	0.096678397	1.003188149	2	3	82.2499959	68.6170491
chr5	134738480	134752160	5q31.1	CAMLG	27.66802533	27.5807492	1.003164386	4	4	38.34494279	22.85968797
chr6	44246166	44253888	6p21.1	HSP90AB1	142.1555044	141.7242905	1.003042625	4	4	41.84701627	31.4997407
chr2	237966945	238099413	2q37.3	UBE2F-SCLY	0.12715102	0.126766917	1.003029986	2	3	34.58848376	62.76361343
chr1	43650126	43705518	1p34.2-p34.1	KDM4A	10.26081472	10.23033108	1.002979731	4	4	35.79925392	27.16473759
chr7	99394673	99408682	7q22.1	PDAP1	35.65678728	35.55480781	1.002868233	4	4	17.6875341	18.24132005
chr17	46590669	46757464	17q21.31	NSF	4.096828759	4.085402795	1.002796778	4	4	29.84517203	37.94858844
chr4	75642769	75724470	4q21.1	G3BP2	46.11444787	45.98644906	1.002783403	4	4	29.35366264	14.89687678
chr8	73290639	73294426	8q21.11	RPL7	341.082808	340.1472129	1.002750559	4	4	27.45531657	21.383685
chr10	114821744	114899827	10q25.3	FAM160B1	23.62287287	23.56054436	1.002645462	4	4	11.74822159	23.25600946
chr8	23288092	23296279	8p21.3	R3HCC1	4.999471391	4.986900771	1.002520728	4	4	53.51770186	39.73617094
chr6	43427538	43451994	6p21.1	ABCC10	1.870920583	1.866284485	1.002484133	4	4	48.23356319	50.41996117
chr8	144137691	144140843	8q24.3	HGH1	2.832806323	2.825908874	1.002440789	4	4	44.10818446	62.41965028
chr2	135839626	135876477	2q21.3	MCM6	11.45557187	11.42836231	1.00238088	4	4	27.99955302	70.75839358
chr3	123887383	123961412	3q21.1	CCDC14	17.64099422	17.59911088	1.002379855	4	4	31.0175639	28.13180528
chr2	165873362	165953838	2q24.3	TTC21B	7.266387841	7.250363785	1.002210104	4	4	3.86971609	14.51021994
chr3	108589490	108694846	3q13.13	DZIP3	18.19270562	18.15373356	1.002146779	4	4	76.49933252	31.65804488
chr19	1905214	1926017	19p13.3	SCAMP4	1.142893171	1.140454986	1.002137905	4	4	86.12715461	56.03634323
chr17	81666697	81673907	17q25.3	CCDC137	4.817829108	4.807729892	1.002100621	4	4	51.97413063	44.41158086
chr17	80135214	80147183	17q25.3	EIF4A3	5.961614398	5.949117878	1.002100567	4	4	36.40069006	27.2318359
chr7	107628389	107629732	7q22.3	WBP1LP2	0.384159699	0.383435135	1.001889667	2	2	20.50208242	42.20864358
chr2	99181079	99199557	2q11.2	MRPL30	7.158883289	7.145554574	1.001865316	4	4	46.96306636	22.70205181
chr7	44072062	44082540	7p13	POLM	3.551041622	3.544898726	1.001732883	4	4	59.25161244	14.97542162
chr13	52455360	52476631	13q14.3	CKAP2	17.37127397	17.34143242	1.001720824	4	4	16.41140305	25.69703141
chr14	24114697	24125242	14q12	DCAF11	27.85705841	27.81049752	1.00167422	4	4	10.79885777	15.64370395
chr1	6521347	6554598	1p36.31	NOL9	7.698942005	7.687192776	1.001528416	4	4	35.23799788	13.91051368
chr3	184242329	184258301	3q27.1	ALG3	2.71715652	2.713272317	1.001431556	4	4	42.87432769	27.00074156
chr16	74873568	74985146	16q23.1	WDR59	9.099943622	9.087888642	1.001326489	4	4	56.87667985	34.10468256
chr2	196639632	196733668	2q33.1	CCDC150	0.962849714	0.961716472	1.001178354	4	4	42.41928319	22.44332993
chr9	113373416	113377009	9q32	HDHD3	1.584315884	1.582580103	1.001096805	4	4	46.73693238	61.1213248
chr6	42050513	42083278	6p21.1	TAF8	5.755498441	5.749358831	1.001067877	4	4	36.37308561	25.89497827
chr4	13336387	13338931	4p15.33	HSP90AB2P	0.118551697	0.118439711	1.000945512	1	2	0	15.36420745
chr1	169463909	169485970	1q24.2	SLC19A2	2.661867878	2.659457662	1.000906281	4	4	29.01808476	27.67013932
chr1	201829160	201884294	1q32.1	IPO9	6.484219625	6.478369042	1.000903095	4	4	22.7244019	8.88391667
chr8	144930358	144950888	8q24.3	ZNF16	1.044163866	1.043358059	1.000772321	4	4	107.1118775	7.60440636
chr1	27891524	27914797	1p35.3	RPA2	29.506534	29.48696778	1.000663555	4	4	24.15129146	27.56907014
chr22	30922308	30926654	22q12.2	MORC2-AS1	0.375387021	0.375164648	1.000592735	2	3	97.9100442	31.1845483
chr8	109240919	109334121	8q23.1	NUDCD1	5.228760521	5.225790677	1.000568305	4	4	24.00267287	37.05258316
chr16	58113588	58129425	16q21	CFAP20	11.0171632	11.01272044	1.00040342	4	4	12.15632095	19.95138656
chr19	35742527	35745442	19q13.12	U2AF1L4	1.863482477	1.862740759	1.000398186	4	4	80.88667429	23.01938807
chr10	117241073	117279430	10q25.3	SLC18A2	2.873174647	2.872036688	1.00039622	4	4	58.1255782	42.83137212
chr6	100508194	100884455	6q16.3	ASCC3	16.98158285	16.9752427	1.000373494	4	4	20.33796826	26.54851759

chr5	80654648	80876815	5q14.1	MSH3	15.93739221	15.93182137	1.000349667	4	4	36.17185065	8.958475884
chr9	120816053	120843021	9q33.2	PSMD5	11.36142652	11.35827691	1.000277296	4	4	25.67691701	12.7059506
chr19	12954156	12957254	19p13.13	GADD45GIP1	4.053490842	4.052558018	1.000230182	4	4	66.18047322	52.48228802
chr13	23730189	23889448	13q12.12	MIPEP	3.065661806	3.065036405	1.000204044	4	4	73.3084359	35.59036743
chr4	23792021	24472829	4p15.2	PPARGC1A	0.056655568	0.056655557	1.000000195	3	2	50.72818107	82.94232672
chr10	99659377	99711245	10q24.2	ENTPD7	3.429256122	3.429315786	0.999982602	4	4	46.1940953	26.36651768
chr14	20311368	20333312	14q11.2	CCNB1IP1	2.771559933	2.771826434	0.999903854	4	4	43.44588347	13.85248534
chr2	10302124	10427617	2p25.1	HPCAL1	10.8131818	10.81457593	0.999871088	4	4	28.2379697	58.04892882
chr12	12355408	12357067	12p13.2	LOH12CR2	0.173715812	0.173741669	0.999851174	1	1	0	0
chr4	150264515	151015727	4q31.3	LRBA	32.57785295	32.58536102	0.999769588	4	4	30.68009358	13.92022552
chr20	35648925	35664956	20q11.22	RBM12	23.92729354	23.93411501	0.99971499	4	4	33.00156428	14.42842469
chr16	21796630	21819174	16p12.2	RRN3P1	2.47008115	2.470791967	0.999712312	4	4	63.14520657	34.71145867
chr7	22812632	22822852	7p15.3	TOMM7	70.01393744	70.04095616	0.999614244	4	4	11.9597098	25.51838831
chr7	2906075	3043945	7p22.2	CARD11	15.61219913	15.61875315	0.999580375	4	4	43.96599378	47.48768929
chr17	18087867	18107985	17p11.2	DRG2	2.671413997	2.672640331	0.999541153	4	4	64.99898852	15.93358993
chr10	73744164	73772175	10q22.2	SEC24C	21.02293632	21.03391036	0.999478269	4	4	36.58020143	42.10689089
chr20	18383367	18467185	20p11.23	DZANK1	0.380485176	0.380686188	0.999471974	4	4	23.96820001	32.08414496
chr4	78776378	78916365	4q21.21	BMP2K	64.88421092	64.93078844	0.999282659	4	4	32.90283795	50.62642728
chr11	47565430	47573461	11p11.2	PTPMT1	2.569801666	2.571861384	0.999199133	4	4	94.77257453	29.39550218
chr3	56727418	57079308	3p14.3	ARHGEF3	30.50070839	30.52652598	0.999154257	4	4	18.32927399	62.29560633
chr22	38290691	38318084	22q13.1	CSNK1E	1.075682593	1.076605817	0.999142468	4	4	72.44168341	34.59137911
chrMT	14747	15887	N/A	MT-CYB	240.489254	240.7099261	0.999083245	4	4	56.13409521	26.80870646
chr11	10852704	10858073	11p15.4	ZBED5	7.318090904	7.324918367	0.999067913	4	4	33.08859516	14.0676801
chr7	141551278	141662153	7q34	AGK	5.191733896	5.196767251	0.999031445	4	4	70.73055914	42.37530303
chr22	23638487	23717423	22q11.23	GUSBP11	0.099950526	0.10005969	0.998909014	3	2	45.88257812	89.36533102
chr8	143829776	143840974	8q24.3	NRBP2	0.840566819	0.841534975	0.998849536	4	4	91.35751529	19.64283477
chr12	79878305	79878419	12q21.2	RNA5SP363	2.296002932	2.298712082	0.998821449	2	2	36.19638662	8.741188408
chr5	176448328	176510074	5q35.2	FAF2	10.76354856	10.7764748	0.998800513	4	4	29.5397986	15.07228323
chr13	114314335	114327328	13q34	CHAMP1	3.670540723	3.675049422	0.99877316	4	4	54.33156742	26.82191963
chr1	202961873	202967276	1q32.1	CYB5R1	7.541727898	7.551512962	0.998704225	4	4	32.26806968	32.01563558
chr5	119154637	119154716	5q23.1	MIR5706	8.315796383	8.32758116	0.99858485	2	3	90.62364756	71.85337125
chr7	192939	260774	7p22.3	FAM20C	0.610262181	0.61113174	0.998577134	4	4	56.46478339	30.85908931
chr2	231781671	231809253	2q37.1	COPS7B	2.266333508	2.269723262	0.998506534	4	4	50.97397083	32.21744727
chr10	73005796	73006627	10q22.1	RPL17P50	1.462015722	1.46424209	0.998479508	3	2	71.80368932	84.72310269
chr16	66988589	67009758	16q22.1	CES4A	0.271781116	0.27223583	0.998329706	4	4	72.06394713	57.35706278
chr6	110982017	111028263	6q21	RPF2	4.774793708	4.784231207	0.998027374	4	4	25.84996999	9.247444315
chr19	57363435	57365405	19q13.43	TRAPPC2B	2.692700776	2.698096182	0.998000292	4	4	79.68631971	16.5468062
chrX	153794175	153798512	Xq28	SSR4	8.223013078	8.240255798	0.997907502	4	4	40.57240636	16.46710996
chr5	181261212	181272307	5q35.3	TRIM52-AS1	6.360570103	6.373979628	0.997896208	4	4	42.41015786	20.39905134
chr8	17221981	17246909	8p22	CNOT7	16.39702177	16.43195057	0.997874336	4	4	25.88427706	11.33318855
chr2	27582969	27623215	2p23.3	ZNF512	6.219717835	6.233135099	0.997847429	4	4	43.87366089	44.17056397
chr1	173799550	173824639	1q25.1	CENPL	3.620779779	3.628617302	0.99784008	4	4	74.11856383	20.95506229
chr12	89519412	89526262	12q21.33	POC1B-GALNT4	0.077173455	0.077343836	0.997797088	4	3	36.57544502	68.5258882

chr5	44808925	44815514	5p12	MRPS30	9.856145174	9.87820494	0.997766824	4	4	24.56492977	28.20779987
chr11	63985853	63998420	11q13.1	OTUB1	5.682401091	5.695218992	0.997749358	4	4	52.17360439	53.19541744
chr13	97953641	98024296	13q32.2	IPO5	9.126499884	9.147348364	0.997720817	4	4	22.39810809	16.5071347
chr11	123723927	123741683	11q24.1	ZNF202	1.677870123	1.681721032	0.997710138	4	4	37.51573462	41.91912566
chr5	1317744	1345070	5p15.33	CLPTM1L	6.919597188	6.935944438	0.997643111	4	4	37.54284624	26.60322123
chr11	62851978	62855885	11q12.3	SNHG1	15.75595401	15.79397356	0.997592781	4	4	56.28384976	14.97510986
chr1	52056185	52090716	1p32.3	BTF3L4	11.62114944	11.6499516	0.997527701	4	4	31.44705994	22.14288944
chr15	67521184	67527303	15q23	C15orf61	0.697118313	0.698987267	0.997326197	4	4	66.91769596	28.96833034
chr6	26956993	27023974	6p22.2	LINC00240	0.039291192	0.039397097	0.997311855	1	2	0	1.652165794
chr11	58526871	58578239	11q12.1	LPXN	37.20144042	37.30217229	0.997299571	4	4	25.69465063	59.53809339
chr19	12643212	12643942	19p13.13	RPL10P16	1.514952413	1.519224598	0.997187918	4	4	70.1928624	25.62756256
chr4	94207608	94291292	4q22.3	SMARCAD1	13.07454731	13.11393233	0.996996704	4	4	23.07345746	12.38328255
chr1	32361270	32362243	1p35.1	FAM229A	1.678024203	1.683182159	0.996935593	4	4	72.48707138	30.83439808
chr13	40729296	40771211	13q14.11	MRPS31	16.14384381	16.19348156	0.996934708	4	4	14.32703608	15.06193281
chr11	129863773	129895596	11q24.3	NFRKB	5.221114276	5.237178249	0.996932705	4	4	51.20399477	20.83834883
chr1	226656080	226675068	1q42.12	ITPKB-IT1	3.449617242	3.460259802	0.996924346	4	4	85.15208936	17.98087827
chr1	40072706	40097470	1p34.2	PPT1	65.32740888	65.53101214	0.996893024	4	4	24.93940025	42.03208831
chr1	1232249	1235041	1p36.33	B3GALT6	4.633310543	4.647899786	0.996861111	4	4	63.12999042	46.55878206
chr11	33740944	33774525	11p13	FBXO3	6.064567124	6.083885739	0.996824626	4	4	26.45690711	9.038137353
chr5	204760	218182	5p15.33	CCDC127	1.194545582	1.198395692	0.99678728	4	4	65.66031532	32.63619252
chr11	4094685	4138925	11p15.4	RRM1	20.37430184	20.44008763	0.996781531	4	4	53.75996837	35.58991354
chr19	7348901	7476990	19p13.2	ARHGEF18	1.602644212	1.608037807	0.996645853	4	4	65.97825288	13.46002586
chr5	78485215	78770256	5q14.1	LHFPL2	6.749072792	6.771821319	0.996640708	4	4	43.50704239	29.84531919
chr3	109326141	109339635	3q13.13	DPPA4	0.233010824	0.233798963	0.996628991	3	3	72.02029951	53.80328343
chr15	39597281	39782838	15q14	FSIP1	1.078951099	1.082626823	0.99660481	4	4	24.53404121	44.0550638
chr2	96497643	96508157	2q11.2	NEURL3	0.13106379	0.131520699	0.996525954	3	3	17.2097692	36.28210958
chr13	31134974	31162388	13q12.3	HSPH1	10.05332472	10.08868555	0.996495001	4	4	24.59096503	9.839552405
chr16	89195756	89201673	16q24.3	SLC22A31	0.105101228	0.105478338	0.996424764	2	2	19.92796063	9.5630649
chr6	34287194	34392680	6p21.31	NUDT3	9.255568362	9.291512154	0.996131545	4	4	66.89976402	27.44569131
chr20	31721507	31723409	20q11.21	ABALON	1.104831913	1.109160855	0.996097102	4	3	141.3102537	55.36521947
chr14	22455249	22455296	14q11.2	TRDJ4	7.50131346	7.531698012	0.995965777	2	2	23.68117025	62.03968643
chr15	77044017	77071228	15q24.3	TSPAN3	4.068423455	4.085431264	0.995836961	4	4	57.65783785	47.96778652
chr7	44795960	44803123	7p13	PPIA	92.94038581	93.32966144	0.995829026	4	4	34.24466563	47.80090947
chr14	45084099	45115601	14q21.2	PRPF39	8.190111478	8.224493621	0.995819543	4	4	26.05321215	5.541852139
chr17	29567180	29573157	17q11.2	TP53I13	1.680862003	1.687930555	0.995812297	4	4	70.50576076	42.12054307
chr9	34610485	34612113	9p13.3	RPP25L	5.517688919	5.540978653	0.99579682	4	4	36.03539285	47.41858997
chr1	46340177	46365152	1p33	NSUN4	1.519400219	1.525843305	0.99577736	4	4	48.49643265	27.56890329
chr12	109553715	109573580	12q24.11	MMAB	0.804499216	0.807921324	0.995764305	4	4	61.388713	46.14703492
chr16	3283183	3301649	16p13.3	ZNF263	4.583993518	4.60368571	0.995722516	4	4	66.56337379	39.1126441
chr1	23801877	23825459	1p36.11	HMGCL	3.409164435	3.423903499	0.995695245	4	4	38.25198193	19.20516598
chrX	108091667	108154671	Xq22.3	ATG4A	8.464487387	8.50145778	0.995651288	4	4	28.29683084	28.28103561
chr11	32602080	32794658	11p13	CCDC73	0.41606042	0.417882755	0.995639123	4	3	52.56460722	29.8651244
chr14	64986789	65062652	14q23.3	FNTB	1.191032505	1.196330461	0.995571495	4	4	75.57335237	13.5681991

chrX	2691133	2741309	Xp22.33 and Yp11.	CD99	42.47860838	42.66907958	0.995536084	4	4	55.56938365	54.26374561
chr1	32620818	32651008	1p35.1	ZBTB8OS	19.24688724	19.33405298	0.995491595	4	4	31.73444105	37.56399293
chr12	120443961	120446412	12q24.31	TRIAP1	6.350615871	6.37954088	0.995465973	4	4	19.10527725	14.85781685
chr8	81701331	81721304	8q21.13	ZFAND1	15.60949305	15.68097644	0.995441394	4	4	32.75471334	15.17368552
chr6	37353972	37394738	6p21.2	RNF8	2.806860773	2.819715236	0.995441219	4	4	34.01727475	20.8202199
chr2	157378487	157380509	2q24.1	FAM133DP	4.156621065	4.175833239	0.9953992	4	4	36.89225836	45.70658571
chr17	44070700	44076344	17q21.31	G6PC3	1.720863048	1.728821101	0.995396832	4	4	79.79152545	35.40194961
chr2	169811757	169824931	2q31.1	METTL5	9.108686572	9.150838245	0.995393682	4	4	41.05977354	21.44947926
chr20	63587594	63627101	20q13.33	GMEB2	5.898490717	5.926115121	0.995338531	4	4	56.24929554	23.40563307
chr10	119212857	119213146	10q26.11	RN7SL749P	3.323827314	3.339435622	0.995326064	3	3	53.06571746	32.19145226
chr11	106007920	106022287	11q22.3	MSANTD4	4.625376832	4.647393426	0.995262593	4	4	27.84444801	12.56259977
chr7	38260088	38265678	7p14.1	TRGC1	21.93009218	22.0345502	0.995259353	4	4	38.39979256	42.60373966
chr1	155135344	155138858	1q22	SLC50A1	9.517732394	9.563300841	0.995235071	4	4	39.49847434	78.09119642
chr5	154887416	154938216	5q33.2	GEMIN5	4.698447206	4.720976551	0.995227821	4	4	31.64640549	41.44289692
chr2	42169338	42332548	2p21	EML4	25.39063466	25.51352823	0.9951832	4	4	32.06366103	22.05928447
chr15	40807076	40814569	15q15.1	ZFYVE19	1.767120355	1.775973344	0.995015134	4	4	79.14150224	35.63169985
chr19	55485190	55487568	19q13.42	NAT14	0.531127346	0.533823908	0.994948594	2	4	33.16311175	40.93252935
chr6	33621333	33696574	6p21.31	ITPR3	3.867067059	3.886805462	0.99492169	4	4	59.57800969	52.5709385
chr7	149547154	149624790	7q36.1	ZNF767P	5.42691127	5.455021818	0.994846849	4	4	20.35411443	24.55285806
chr12	49227926	49273338	12q13.12	TUBA1C	9.296239846	9.345128064	0.994768588	4	4	38.77325244	33.90798732
chr3	131462201	131503016	3q22.1	MRPL3	25.06924659	25.20122717	0.99476293	4	4	46.86818913	16.35522466
chr2	206123079	206159519	2q33.3	NDUFS1	7.90750821	7.949441179	0.994725042	4	4	24.40778362	14.16576409
chr3	52535986	52540570	3p21.1	SMIM4	1.033661246	1.039156887	0.994711443	4	4	51.74184666	17.53144148
chr1	160215715	160262560	1q23.2	DCAF8	6.433804112	6.468141093	0.994691368	4	4	28.50994761	18.38289382
chr13	59665583	60163985	13q21.2	DIAPH3	14.89981255	14.97995187	0.994650228	4	4	72.00690931	61.47891114
chr1	148748774	148752423	1q21.2	NUDT4B	6.869841426	6.906914414	0.994632482	4	4	48.27059558	44.41402349
chr4	107931549	107953461	4q25	CYP2U1	1.393991016	1.401700693	0.99449977	4	4	48.8933384	43.05020383
chr2	65056361	65087008	2p14	CEP68	2.982375758	2.999043812	0.994442211	4	4	43.93185959	27.56468583
chr1	111755568	111767577	1p13.2	DDX20	5.193171353	5.222515303	0.994381261	4	4	25.83781934	16.65562492
chr2	230507101	230507386	2q37.1	RN7SL834P	24.07791209	24.2147954	0.994347121	4	4	57.09657199	30.7837909
chr2	100562956	100577472	2q11.2	PDCL3	8.636898165	8.686250811	0.994318303	4	4	48.82117285	38.26677514
chr12	94265652	94460616	12q22	CEP83	7.364635584	7.406966485	0.994284988	4	4	42.52717991	54.78676404
chr1	16367030	16398148	1p36.13	SZRD1	22.51557183	22.64726737	0.994184926	4	4	45.73893162	22.11576642
chr19	47608196	47703277	19q13.33	BICRA	0.514931825	0.51794794	0.994176798	4	4	86.16030081	70.32445378
chr3	36820550	36822500	3p22.2	LINC02033	1.512896051	1.521955761	0.994047324	4	4	71.25991647	59.75040851
chr11	64987945	64997045	11q13.1	BATF2	4.195973471	4.22137496	0.99398265	4	4	97.21290866	81.00036977
chr7	30911694	30925517	7p14.3	AQP1	1.318494007	1.326500669	0.993964072	4	4	83.44046032	20.51757519
chrX	71283192	71301168	Xq13.1	NONO	41.66710613	41.92037579	0.993958316	4	4	45.17950777	39.51984527
chr16	57619535	57665039	16q21	ADGRG1	8.818406255	8.87223358	0.993933058	4	4	28.98816863	95.28519426
chr22	36800701	36819473	22q12.3	PVALB	2.541025665	2.556870781	0.993802927	4	4	95.03816539	52.3169877
chr2	161409554	161409670	2q24.2	RNA5SP108	6.83903056	6.881817887	0.993782555	3	3	50.82956262	57.19096407
chr16	30443625	30445975	16p11.2	SEPHS2	8.791017797	8.846420274	0.9937373	4	4	52.7804824	28.79633354
chrX	55000323	55007873	Xp11.21	APEX2	3.817399611	3.842787054	0.993393482	4	4	43.06317982	56.80861497

chr15	78283233	78299726	15q25.1	WDR61	2.950701368	2.970440725	0.993354738	4	4	20.32696914	18.5168575
chr16	88706463	88715386	16q24.3	CTU2	0.723848515	0.728704872	0.993335634	4	4	82.89761155	31.38952764
chr7	142560485	142560931	7q34	TRBV12-3	2.804940527	2.823831518	0.993310156	4	4	40.53740383	54.09482362
chr12	63843911	64147863	12q14.2	SRGAP1	0.024124714	0.024287648	0.993291469	3	4	80.94437801	48.58379457
chr17	6640902	6644541	17p13.1	TXNDC17	3.372274191	3.395114544	0.993272583	4	4	12.66931112	25.14130643
chr8	27733316	27772656	8p21.1	CCDC25	10.46516788	10.53700782	0.993182131	4	4	4.853985305	14.65562763
chr19	14087840	14090575	19p13.12	SAMD1	5.55033812	5.588739648	0.993128768	4	4	71.35226583	36.46768061
chr12	8681677	8783098	12p13.31	RIMKLB	4.406135574	4.436949133	0.993055237	4	4	36.01602295	17.43407643
chr3	51671175	51704323	3p21.2	TEX264	2.875584301	2.895733927	0.993041617	4	4	57.42283227	43.7080275
chr20	33657087	33674458	20q11.22	NECAB3	0.690236548	0.695130112	0.992960219	4	4	65.25370844	34.97466319
chr1	67701466	67833472	1p31.3	GNG12	0.077888319	0.078448396	0.992860574	3	2	25.33253092	44.23681968
chr17	20999593	21043409	17p11.2	USP22	21.78430378	21.94385146	0.992729276	4	4	29.86642249	28.25905439
chr8	94427712	94436952	8q22.1	FSBP	0.517708826	0.521568185	0.992600471	3	4	54.4575932	85.01380011
chr16	86530176	86555840	16q24.1	MTHFSD	1.518800661	1.530239519	0.992524792	4	4	51.88374229	37.99538298
chr2	216216889	216220192	2q35	LINC01963	3.02125798	3.044032043	0.992518455	4	4	76.11511894	38.90634136
chr2	242088633	242160503	2q37.3	LINC01881	14.00161003	14.10792062	0.992464475	4	4	28.00087974	23.80086337
chr6	169702112	169706358	6q27	C6orf120	15.87230141	15.99509102	0.992323294	4	4	24.42627414	9.841021376
chr2	214725645	214809711	2q35	BARD1	31.68191445	31.92725523	0.992315632	4	4	59.16413614	37.78674832
chr6	43171295	43181506	6p21.1	SRF	8.629719803	8.697427787	0.992215172	4	4	64.53190045	50.41799863
chr10	87238667	87342558	10q23.2	NUTM2A-AS1	11.94464222	12.0385704	0.99219773	4	4	32.65406642	30.63441384
chr20	3846799	3876123	20p13	MAVS	7.128010471	7.185518913	0.99199662	4	4	51.48694501	28.66996045
chr1	205813811	205850148	1q32.1	PM20D1	0.33116775	0.333860081	0.991935751	1	3	0	71.7363098
chr7	25134697	25180742	7p15.3	C7orf31	1.960111023	1.976159545	0.991878934	4	4	51.91288365	55.01963056
chrMT	648	1601	N/A	MT-RNR1	535.5257599	539.9183265	0.991864387	4	4	58.45843055	28.51264313
chr20	41178449	41317640	20q12	ZHX3	3.698130322	3.728947807	0.991735608	4	4	77.53055137	34.17907545
chr19	56368099	56379828	19q13.43	ZNF542P	6.425331644	6.479023623	0.991712952	4	4	31.68755937	48.7358328
chr1	20742677	20787808	1p36.12	HP1BP3	272.4331482	274.7248072	0.991658347	4	4	71.86596299	61.57009426
chr22	28741992	28757518	22q12.1	HSCB	2.968796789	2.993781115	0.991654592	4	4	39.52800486	58.5734931
chr1	207801518	207822703	1q32.2	MIR29B2CHG	3.022494243	3.048033677	0.991621013	4	4	49.46939795	52.33126802
chr19	36666961	36723548	19q13.12	ZNF567	5.041164	5.083964199	0.991581334	4	4	19.31257865	34.13675706
chr1	28200278	28233031	1p35.3	DNAJC8	30.99065704	31.25445564	0.991559648	4	4	50.54212975	43.55405651
chr1	153633982	153646306	1q21.3	CHTOP	9.486483778	9.567281914	0.991554745	4	4	29.43456936	20.93938811
chr2	234493041	234497053	2q37.1	ARL4C	83.37357272	84.08383961	0.991552873	4	4	24.88657223	53.41583253
chr11	111690273	111766445	11q23.1	PPP2R1B	8.856775221	8.932289994	0.991545866	4	4	34.01781407	30.17232799
chr16	23581012	23596329	16p12.2	NDUFAB1	11.54713521	11.64622086	0.991492034	4	4	29.03618103	34.76427359
chrX	71533062	71575897	Xq13.1	OGT	31.09522041	31.36215496	0.991488641	4	4	37.15270602	3.642499233
chr6	118813449	118935162	6q22.31	MCM9	3.352614628	3.381754023	0.991383349	4	4	37.58460235	20.9000806
chr4	102728522	102730247	4q24	KRT8P46	3.823102835	3.856625324	0.991307818	4	4	57.20523666	17.19012584
chr2	17663812	17753833	2p24.2	SMC6	12.84419589	12.95691586	0.991300401	4	4	19.41835956	15.82726122
chr13	20566446	20691437	13q12.11	IFT88	13.78908823	13.91046387	0.991274508	4	4	57.27102858	49.95987811
chr5	139647299	139683885	5q31.2	CXXC5	5.181638016	5.227377223	0.991250066	4	4	61.62591454	57.2611258
chr16	58707131	58734357	16q21	GOT2	6.88315561	6.943929471	0.991247915	4	4	45.77958877	44.26528765
chr19	58561917	58574478	19q13.43	MZF1	1.423970283	1.436615965	0.99119759	4	4	71.75739704	31.90905796

chr7	141811910	141812257	7q34	MYL6P4	0.581055703	0.586240846	0.991155269	1	1	0	0
chr20	62182746	62203568	20q13.33	MTG2	3.468583887	3.499635625	0.991127151	4	4	56.22389342	30.92016453
chr19	6361452	6368904	19p13.3	CLPP	3.782017219	3.816016824	0.99109029	4	4	53.93663642	59.19917437
chr17	56791913	56833926	17q22	C17orf67	1.786925865	1.803056069	0.991053964	4	4	22.07127164	42.63497293
chr1	248838178	248849517	1q44	ZNF672	8.434042513	8.510621097	0.991001998	4	4	45.93719988	25.85017703
chr15	45402321	45421419	15q21.1	SPATA5L1	3.692347814	3.725877892	0.991000758	4	4	21.67374514	41.71792837
chr16	78099413	79212667	16q23.1-q23.2	WWOX	2.024737351	2.043513212	0.99081197	4	4	67.52721606	19.60953795
chr19	984328	994570	19p13.3	WDR18	2.252055439	2.272950786	0.990806951	4	4	75.18216977	81.91812082
chr4	53377572	53462611	4q12	FIP1L1	48.89927386	49.36163731	0.990633142	4	4	43.6970732	21.47281993
chr6	2854657	2876510	6p25.2	SERPINB9P1	3.961066881	3.998660617	0.990598418	4	4	48.87467026	35.68529813
chr4	95162504	95549210	4q22.3	UNC5C	0.024243822	0.024474976	0.990555476	1	2	0	5.174474227
chr19	53333719	53356119	19q13.42	ZNF845	13.44766709	13.57707453	0.99046868	4	4	18.91135987	25.11278129
chr20	37983007	38033468	20q11.23	TTI1	5.27356583	5.32438504	0.990455384	4	4	16.80677454	18.88006027
chr8	125091779	125367125	8q24.13	NSMCE2	23.72898693	23.95847986	0.990421223	4	4	57.00649592	47.72233451
chrX	53094157	53148008	Xp11.22	KANTR	0.484115594	0.48885005	0.990315115	4	4	68.48094696	34.75422549
chr6	158237110	158242815	6q25.3	SRP72P2	0.130010598	0.131286657	0.990280366	1	2	0	2.521303683
chr17	1779535	1829896	17p13.3	SMYD4	2.301181388	2.323834595	0.990251799	4	4	48.26061811	25.06197212
chr17	49223369	49230766	17q21.32	PHOSPHO1	235.3641226	237.6871771	0.99022642	4	4	21.31187831	52.07228892
chr1	47432133	47434641	1p33	FOXD2-AS1	0.471003993	0.475660009	0.990211462	4	4	83.86755135	35.99429433
chr1	152313459	152366692	1q21.3	FLG-AS1	0.078921319	0.079712235	0.990077858	4	3	45.93588054	64.00393146
chr2	227164565	227314792	2q36.3	COL4A3	0.064697929	0.065350363	0.990016368	2	3	80.06300551	48.74620211
chr19	52392752	52418408	19q13.41	ZNF528	1.441909721	1.456670832	0.989866543	4	4	68.23924104	32.36753763
chr1	116754430	116769229	1p13.1	CD2	73.13887326	73.89034325	0.98982993	4	4	3.975960303	63.23084815
chr11	124954121	125041489	11q24.2	CCDC15	25.11795931	25.37641448	0.989815142	4	4	40.29164746	53.57535822
chr3	20160593	20188143	3p24.3	SGO1	13.77342603	13.91588112	0.989763129	4	4	49.66006767	34.96244591
chr19	46346994	46390975	19q13.32	PPP5C	2.850139903	2.879707262	0.989732513	4	4	22.61226649	55.05831734
chr2	218663864	218672058	2q35	RNF25	3.448442965	3.484718356	0.989590151	4	4	61.44823308	23.03062195
chr20	35455137	35517531	20q11.22	CEP250	3.744481045	3.78389346	0.989584164	4	4	64.22185723	44.85964362
chr2	97646058	97648194	2q11.2	COX5B	21.17822988	21.40164278	0.989560946	4	4	40.77597684	16.66900841
chr21	33931122	33971186	21q22.11	LINC00649	1.987802937	2.008867941	0.989513993	4	4	54.03047288	69.22404978
chr18	63327736	63367273	18q21.33	KDSR	2.275311565	2.299577922	0.989447474	4	4	45.67090537	31.90413522
chr11	57741250	57743554	11q12.1	SELENOH	3.966120645	4.00846695	0.989435786	4	4	81.53692285	19.83481954
chr2	73700509	73701340	2p13.1	NAT8B	5.240608301	5.296610279	0.989426827	4	4	58.33196778	45.85953682
chr17	75441159	75500452	17q25.1	TMEM94	2.746672088	2.776526047	0.989247729	4	4	80.60705317	49.16385901
chr15	40735884	40755336	15q15.1	RMDN3	4.278575346	4.325106908	0.989241523	4	4	24.95549393	16.44507712
chr1	28236091	28238105	1p35.3	ATP5IF1	17.75983997	17.95602544	0.989074115	4	4	13.25595184	44.91550607
chr1	62454726	62688368	1p31.3	DOCK7	1.451543414	1.467624742	0.989042616	4	4	33.32160624	19.81204568
chr16	31527852	31528804	16p11.2	AHSP	385.5860262	389.8589499	0.989039821	4	4	39.96225246	45.15732809
chr22	49853849	49890078	22q13.33	ZBED4	5.539461055	5.601757406	0.988879142	4	4	62.33261432	41.90548917
chr16	2911873	2951208	16p13.3	FLYWCH1	1.094117311	1.10647576	0.9888308	4	4	60.10631899	28.35098997
chr1	1385711	1399342	1p36.33	CCNL2	6.307419254	6.378760424	0.988815825	4	4	29.01484968	34.23054004
chr4	139114733	139115539	4q31.1	PPP1R14BP3	1.399142197	1.415159348	0.988681733	3	3	75.33662922	68.42390958
chr7	103312474	103344873	7q22.1	DNAJC2	22.43203887	22.69013331	0.988625257	4	4	31.33145486	18.73196049

chr1	155749658	155859426	1q22	GON4L	23.34491389	23.61637238	0.988505496	4	4	28.94448608	19.79190957
chr1	32205635	32208687	1p35.2	IQCC	0.283305675	0.286613383	0.98845934	3	4	22.45251275	48.00524526
chr2	25232961	25342590	2p23.3	DNMT3A	4.550393103	4.603590538	0.98844436	4	4	73.1967292	38.6593382
chr14	24143365	24146646	14q12	PSME2	30.25462537	30.60889029	0.988426078	4	4	30.01163303	53.46703729
chr9	86422942	86423128	9q21.33	RNU2-36P	5.985392661	6.055849678	0.988365461	4	1	124.2893448	0
chr7	7566875	7609144	7p21.3	MIOS	7.290298391	7.377053666	0.988239848	4	4	27.01008811	14.88042983
chr3	158644524	158692572	3q25.32	GFM1	7.631907191	7.723049769	0.988198629	4	4	33.56940119	9.265583997
chr19	44165062	44198261	19q13.31	ZNF226	3.37531436	3.41562757	0.988197422	4	4	39.28748739	27.76055842
chrX	150361564	150514178	Xq28	MAMLD1	1.908297531	1.931100561	0.988191692	4	4	82.3330198	51.47692426
chr18	12991301	13125053	18p11.21	CEP192	72.80085266	73.67435545	0.988143733	4	4	66.17722082	39.32733264
chr19	44426251	44448578	19q13.31	ZNF229	0.409060918	0.413989738	0.988094342	4	4	96.41537729	61.79010119
chr16	396755	400754	16p13.3	NME4	5.315423342	5.379499271	0.988088867	4	4	43.17396579	38.74460521
chr2	127701023	127703833	2q14.3	SFT2D3	4.277897268	4.329562543	0.98806686	4	4	95.23753872	26.54778852
chr4	147506089	147507227	4q31.23	GTF2F2P1	0.605466021	0.612809901	0.988016055	1	2	0	33.33922478
chr2	189560590	189580811	2q32.2	SLC40A1	83.45266474	84.46669389	0.987994923	4	4	10.01471183	9.371288529
chr3	15427557	15442613	3p25.1	EAF1	11.89867921	12.04342973	0.987980955	4	4	33.47686944	20.01224386
chr3	42221840	42221965	3p22.1	RNU4-78P	1.817902955	1.840022995	0.987978389	2	2	36.89459783	10.53477405
chr15	82809628	82834861	15q25.2	WHAMM	17.42556934	17.63870981	0.987916323	4	4	32.91363965	44.65982824
chr11	13387996	13463297	11p15.3	BTBD10	20.31171575	20.5622339	0.987816589	4	4	26.54286256	17.72120364
chr16	75126285	75172234	16q23.1	ZFP1	4.62896137	4.686892018	0.987639859	4	4	26.75624252	25.07149712
chr2	113539514	113568938	2q13	PGM5P4	1.069108034	1.082513137	0.987616684	1	4	0	79.32574597
chr16	55566672	55567687	16q12.2	CAPNS2	1.00460305	1.017294972	0.987523852	2	4	52.39850441	64.27625487
chr16	68300615	68310965	16q22.1	SLC7A6OS	5.564788519	5.635104753	0.987521752	4	4	50.49058756	21.01140664
chr3	128879490	128913114	3q21.3	ACAD9	4.358593698	4.413719073	0.987510448	4	4	51.25071275	31.46221174
chr19	23652906	23687215	19p12	ZNF675	6.573273152	6.656978996	0.987425851	4	4	21.77531535	13.85820168
chr1	227728168	227781231	1q42.13	SNAP47	1.759150078	1.781617971	0.987389051	4	4	45.59615841	36.23748244
chr3	127689062	127823250	3q21.3	MGLL	2.196563075	2.224743954	0.987332979	4	4	71.84348827	16.8925066
chr1	16246840	16352491	1p36.13	FBXO42	5.186175535	5.253422795	0.987199344	4	4	25.48868228	13.80038608
chr3	11137093	11263253	3p25.3	HRH1	0.064342018	0.065178287	0.987169509	3	2	42.55590358	35.19633747
chr4	14909961	15002045	4p15.33-p15.32	CPEB2-DT	0.247359496	0.250578997	0.987151753	3	2	9.040401233	43.72987306
chr11	108505431	108607514	11q22.3	EXPH5	0.380863801	0.385836638	0.987111548	4	4	101.5348672	46.82698573
chr10	102245498	102382899	10q24.32	GBF1	14.16739259	14.35367101	0.987022245	4	4	27.43071704	27.99670119
chr8	23191457	23225167	8p21.3	TNFRSF10A	6.609444258	6.696492233	0.987000959	4	4	50.36944401	28.68263607
chr19	35995188	35996318	19q13.12	SDHAF1	5.419184603	5.490590789	0.986994808	4	4	33.10350626	48.32822352
chr17	35099792	35119869	17q12	RAD51D	0.797365311	0.80788373	0.986980282	4	4	55.15733427	41.73129153
chr6	34792017	34877514	6p21.31	UHRF1BP1	3.822973913	3.873539315	0.986945943	4	4	52.66588805	15.63797132
chr17	44319625	44324870	17q21.31	SLC25A39	884.4973415	896.2262832	0.986912968	4	4	31.70924758	19.80342059
chr22	41598028	41621077	22q13.2	DESI1	10.1646736	10.29994074	0.986867192	4	4	21.20610955	24.35994334
chr3	125446644	125520214	3q21.2	SNX4	24.00229959	24.32557092	0.986710638	4	4	28.13407867	28.04807962
chr15	48408306	48645788	15q21.1	FBN1	0.428075114	0.433853677	0.986680848	4	4	147.2334084	4.345468232
chr11	85694221	85811182	11q14.1	SYTL2	5.190409028	5.260664158	0.986645198	4	4	21.87520694	23.35073967
chr20	58839681	58911196	20q13.32	GNAS	80.8245677	81.92395909	0.986580344	4	4	27.33044744	25.40926879
chr10	100535884	100553924	10q24.31	HIF1AN	5.163925563	5.234512939	0.986515006	4	4	52.01504213	19.45335443

chr6	145857838	145964285	6q24.3	SHPRH	6.351644371	6.438589915	0.986496182	4	4	23.45550437	14.326844
chr9	137554444	137578935	9q34.3	DPH7	2.151925387	2.181753327	0.986328455	4	4	33.10702074	9.339452232
chr16	22437008	22492220	16p12.2	SMG1P1	1.437384313	1.45735349	0.986297644	4	4	54.92146193	23.94964099
chrX	110055329	110055426	Xq23	MIR652	3.97810377	4.033604709	0.986240362	2	1	5.899914851	0
chr16	8892094	8963912	16p13.2	USP7	79.51322976	80.62284556	0.986236956	4	4	21.37722284	23.24426447
chr14	24299854	24309126	14q12	NOP9	6.642002779	6.735222984	0.986159299	4	4	45.13447157	30.26299438
chr13	28003274	28100592	13q12.2	FLT3	2.159585273	2.190028345	0.986099234	4	4	59.91554926	46.18124086
chr4	20696282	20754828	4p15.31	PACRGL	7.320001208	7.423982357	0.985993885	4	4	55.68790497	61.01707381
chr12	26120026	26125070	12p12.1	BHLHE41	0.27400623	0.277925314	0.985898789	4	4	58.04838724	100.813496
chr10	27182838	27243542	10p12.1	ACBD5	15.19006508	15.40835213	0.985833199	4	4	26.6322033	30.00735296
chr1	117605982	117628389	1p12	TENT5C	981.3966972	995.5119845	0.985821078	4	4	34.79846848	70.72874974
chr14	105300718	105398147	14q32.33	PACS2	1.479500044	1.500965231	0.985699078	4	4	89.93494452	34.07921754
chrX	53556223	53556341	Xp11.22	MIR98	3.293710729	3.34149833	0.985698751	2	3	70.47818568	44.79587534
chr11	122655690	122814479	11q24.1	UBASH3B	11.52588153	11.69347959	0.985667392	4	4	31.73730129	36.64942472
chr16	68843606	69085182	16q22.1	TANGO6	5.714399011	5.797820348	0.985611604	4	4	34.44678921	13.88689279
chr19	49928702	49933936	19q13.33	ATF5	2.98279139	3.026942675	0.985413901	4	4	54.48487663	29.94595423
chr22	23772819	23784316	22q11.23	MMP11	0.052659106	0.053441448	0.985360767	2	1	12.74487386	0
chr19	37147438	37172741	19q13.12	ZNF585A	0.840221069	0.852726382	0.985334905	4	4	46.5356935	36.88003005
chr16	3539033	3577782	16p13.3	NLRC3	12.08603367	12.26607321	0.985322154	4	4	37.21800859	39.89359464
chr19	9607326	9621263	19p13.2	ZNF561	5.55167725	5.634987816	0.985215484	4	4	27.76084443	15.40913967
chrX	1462572	1537488	Xp22.33 and Yp11.	P2RY8	43.64357574	44.29918215	0.985200485	4	4	50.92773896	55.7063386
chr2	222571443	222656355	2q36.1	FARSB	19.77240301	20.0697789	0.985182901	4	4	54.47606368	30.48686556
chr8	65714334	65841734	8q13.1	PDE7A	25.12205259	25.50062727	0.985154299	4	4	18.07441021	25.8211759
chr4	17810813	17844865	4p15.31	NCAPG	157.8139302	160.1950119	0.985136356	4	4	68.55440634	34.12547238
chr11	62771303	62787342	11q12.3	TAF6L	1.165172749	1.182888265	0.985023509	4	4	50.14866004	34.11313607
chr20	13714322	13784933	20p12.1	ESF1	14.18933705	14.40550282	0.984994223	4	4	29.09966914	24.28081638
chr16	29806081	29811183	16p11.2	MAZ	27.21876138	27.63492791	0.98494056	4	4	55.63698808	60.81913153
chr12	34022281	34035296	12p11.1	ALG10	4.363296344	4.430308683	0.984874115	4	4	37.35866467	28.56925065
chr9	94029031	94109856	9q22.32	PTPDC1	0.837034177	0.849895758	0.984866872	4	4	59.65194999	35.9844894
chr12	117143473	117190495	12q24.22	FBXO21	4.921105938	4.996824119	0.984846739	4	4	33.8998007	26.6296495
chr11	65614311	65637439	11q13.1	PCNX3	4.972526729	5.049095219	0.984835206	4	4	80.29382396	35.26796893
chr5	176346061	176361865	5q35.2	KIAA1191	17.32503667	17.59306269	0.984765244	4	4	26.10150076	31.42484704
chr9	133348214	133351425	9q34.2	RPL7A	205.8933577	209.0855315	0.984732689	4	4	54.9670273	28.31597125
chr1	204516377	204558120	1q32.1	MDM4	12.99024088	13.19429649	0.984534559	4	4	32.53862995	18.6690699
chr6	135181315	135219173	6q23.3	MYB	5.020043363	5.098953201	0.984524306	4	4	24.44273188	14.08269009
chr14	32075419	32076699	14q12	ARHGAP5-AS1	0.426973888	0.433723352	0.984438322	1	3	0	59.78230367
chr20	45325288	45348424	20q13.12	SDC4	2.299264684	2.336080856	0.984240198	4	4	63.82650164	37.56257004
chr8	126552438	126558498	8q24.21	FAM84B	2.40336023	2.441883285	0.984224039	4	4	43.21647969	39.68252615
chr12	75391070	75432688	12q21.2	GLIPR1L2	0.239704591	0.243578968	0.98409396	4	1	83.8626844	0
chr16	89686612	89696364	16q24.3	CDK10	1.524922239	1.549681996	0.984022685	4	4	33.02053991	16.98410208
chr6	20534457	21232404	6p22.3	CDKAL1	37.90971304	38.5273383	0.983969169	4	4	60.36069908	45.14940202
chr4	52712394	52720697	4q12	DANCR	4.213313765	4.282141213	0.983926862	4	4	59.57126464	43.04920093
chr1	27534245	27604178	1p36.11-p35.3	AHDC1	0.975388432	0.991452333	0.983797606	4	4	80.82165322	84.14779309

chr17	64780759	64780824	17q24.1	MIR6080	4.02239477	4.088951298	0.983722837	2	2	15.86870641	9.854302742
chr10	104254194	104267464	10q25.1	GSTO1	25.14705116	25.56770311	0.983547527	4	4	26.14666664	24.52060545
chr1	35557792	35566779	1p34.3	NCDN	2.081189549	2.116209431	0.983451599	4	4	64.05167919	64.80377987
chr3	13316230	13420319	3p25.1	NUP210	19.12735859	19.44955748	0.983434127	4	4	42.6591164	58.07723211
chr6	30556709	30564696	6p21.33	PRR3	0.546897838	0.556145983	0.983371012	4	4	40.13038311	30.31042868
chrX	103576231	103587736	Xq22.2	TCEAL4	2.932049215	2.981732926	0.983337303	4	4	76.0788713	51.77821105
chr10	113854632	113912506	10q25.3	NHLRC2	13.27219499	13.49769192	0.983293668	4	4	29.1809121	20.0979197
chr17	64147433	64263323	17q23.3	TEX2	6.312402917	6.420110319	0.983223434	4	4	14.21821689	24.60648168
chr17	74748612	74769360	17q25.1	SLC9A3R1	58.47258513	59.47373128	0.983166582	4	4	55.12087228	68.12310715
chr6	110180421	110232220	6q21	CDC40	21.07748533	21.43886772	0.983143588	4	4	11.0308063	20.29072357
chrX	44873175	45112612	Xp11.3	KDM6A	22.50557979	22.8915554	0.983138952	4	4	19.05100587	35.42163635
chr12	43758909	43789543	12q12	IRAK4	15.01697422	15.27453085	0.983138164	4	4	14.0644729	23.68682298
chr9	88388382	88478708	9q22.1	SPIN1	11.22281166	11.41741465	0.982955599	4	4	35.50951422	24.33302944
chr8	208344	232318	8p23.3	RPL23AP53	2.908273428	2.959649163	0.982641275	4	4	56.12011684	47.26675287
chr19	8413272	8439021	19p13.2	MARCH2	54.01209235	54.97242299	0.982530684	4	4	26.19355632	15.77402065
chr11	33039417	33079454	11p13	TCP11L1	4.47077063	4.550455679	0.982488556	4	4	15.58488529	26.27543609
chr3	128064611	128153914	3q21.3	RUVBL1	3.633976439	3.69906155	0.982404967	4	4	29.52250806	38.44126953
chr17	29573469	29589592	17q11.2	GIT1	4.708274132	4.793030908	0.982316664	4	4	54.09486921	52.06172014
chrX	54530211	54567287	Xp11.22	GNL3L	18.61115196	18.95054027	0.982090837	4	4	22.58257946	18.90718059
chr10	62159131	62268879	10q21.2	RTKN2	1.80450591	1.837450258	0.982070617	4	4	87.79098129	59.27813227
chr9	98732009	98796542	9q22.33	ANKS6	0.544053488	0.55400209	0.982042302	4	4	91.61618811	44.75482786
chr11	117427772	117797261	11q23.3	DSCAML1	0.069400505	0.070684394	0.981836313	1	1	0	0
chr15	64094066	64144234	15q22.31	SNX1	15.87160764	16.16551219	0.981819038	4	4	26.40404034	26.46054612
chr12	54292107	54301037	12q13.13	NFE2	167.4415107	170.5430243	0.981813894	4	4	34.99982295	27.087144
chr20	10404780	10434239	20p12.2	MKKS	3.388246163	3.451045145	0.981802909	4	4	37.70225576	47.53469157
chr15	75369379	75455819	15q24.2	SIN3A	12.07953118	12.30348379	0.981797626	4	4	38.30053177	23.1012426
chr14	102777479	102911500	14q32.32	TRAF3	4.871485943	4.961895479	0.981779234	4	4	48.43252257	16.91763034
chr17	4004445	4143020	17p13.2	ZZEF1	11.34162747	11.55229241	0.98176423	4	4	36.83874626	9.350822732
chr7	977679	1138325	7p22.3	C7orf50	2.838425066	2.891218719	0.98174	4	4	75.93928102	26.9754605
chr9	128882112	128918042	9q34.11	LRRC8A	11.42608877	11.63956588	0.981659357	4	4	23.06939159	33.45093715
chr6	34466061	34536262	6p21.31	PACSIN1	0.847837578	0.863697724	0.981636925	4	4	98.69340458	104.7363543
chr1	88684144	88836255	1p22.2	PKN2	114.4247291	116.5690943	0.981604342	4	4	35.78136214	43.90689059
chr14	59505067	59577276	14q23.1	CCDC175	3.390710802	3.454628639	0.981497914	4	4	54.28539854	44.53255749
chr7	128476729	128502924	7q32.1	METTL2B	2.277845494	2.32079354	0.981494241	4	4	66.60719093	52.78632055
chr3	187120948	187139523	3q27.3	RPL39L	1.499013038	1.527350118	0.981446899	4	4	54.17115588	63.84671491
chr17	6578151	6640927	17p13.1	KIAA0753	3.977621017	4.052944113	0.981415215	4	4	60.72508473	22.69715281
chr15	50463381	50463498	15q21.2	RNA5SP395	2.053228503	2.092290541	0.98133049	2	2	26.7294159	19.28462958
chr7	38256337	38256396	7p14.1	TRGJP2	74.99531032	76.43998405	0.981100549	4	4	31.12897276	20.73706457
chr6	43037617	43053945	6p21.1	CUL7	1.784746894	1.819382989	0.980962725	4	4	84.97571474	46.90748195
chr22	30291990	30326966	22q12.2	TBC1D10A	1.878586912	1.915070652	0.980949141	4	4	66.91847226	53.90890139
chr22	28772674	28789301	22q12.1	CCDC117	20.3040402	20.7018658	0.980783104	4	4	20.11161016	22.70704793
chr3	48918820	48985541	3p21.31	ARIH2	15.79313769	16.10424015	0.980681954	4	4	24.72461461	13.23051162
chr10	62804756	62808479	10q21.3	ADO	5.922901227	6.039580212	0.980680945	4	4	36.47981352	9.312657085

chr17	42773436	42779600	17q21.2	VPS25	7.436315703	7.583854028	0.980545733	4	4	31.45483447	19.27430021
chr3	49007062	49015953	3p21.31	WDR6	10.50425139	10.71298669	0.980515677	4	4	62.45820664	44.11496924
chr1	89624849	89632894	1p22.2	LRRC8C-DT	0.640669978	0.653413665	0.980496754	3	4	55.13764258	47.94068839
chr16	81314941	81380198	16q23.2	GAN	1.95635817	1.995432551	0.98041809	4	4	45.88047159	21.32530672
chr1	42817105	42844989	1p34.2	ERMAP	28.13789148	28.7000012	0.980414296	4	4	44.68444048	88.20191775
chr8	119730773	119832858	8q24.12	TAF2	27.38540882	27.93376676	0.980369352	4	4	33.33278838	28.98243832
chr19	45349837	45370647	19q13.32	ERCC2	1.429702662	1.45834207	0.980361667	4	4	56.71327698	55.2532645
chr14	21200079	21206900	14q11.2	LINC00641	4.843670005	4.941058143	0.980290024	4	4	51.91798976	35.21381936
chrX	49250436	49264932	Xp11.23	FOXP3	0.619647713	0.632210465	0.980128844	4	4	120.4794192	59.68003657
chrX	110194186	110440233	Xq23	AMMECR1	15.8169708	16.13822576	0.98009354	4	4	68.63239966	54.59007622
chr19	55452978	55461682	19q13.42	ISOC2	0.743665097	0.758777068	0.980083781	4	4	69.77011631	70.16161749
chr2	43886224	43996005	2p21	LRPPRC	16.01138513	16.337503	0.980038696	4	4	24.40479644	9.834116579
chr15	72686179	72738476	15q24.1	BBS4	2.075957793	2.118290285	0.980015727	4	4	35.00302751	9.224752683
chrX	154021800	154097731	Xq28	MECP2	4.487728751	4.579861698	0.979883028	4	4	60.40229508	6.379496854
chr15	88459478	88467402	15q25.3	MRPL46	0.724173212	0.739133536	0.979759646	4	4	42.23829894	15.45118801
chr8	103398638	103415335	8q22.3	SLC25A32	9.037224576	9.224569067	0.979690705	4	4	30.08776859	6.02309598
chrX	154379236	154381523	Xq28	EMD	14.03502364	14.32684176	0.979631371	4	4	50.98575676	21.73248847
chr11	8682448	8689872	11p15.4	RPL27A	116.2484638	118.6801436	0.979510644	4	4	50.98556768	42.26948322
chr13	27172259	27183503	13q12.13	USP12-AS2	2.657043139	2.712904879	0.979408884	2	3	79.14687441	52.68993867
chr15	40161009	40221136	15q15.1	BUB1B	15.9754601	16.31455472	0.979215208	4	4	89.39891719	37.00367495
chr1	200342544	200373792	1q32.1	LINC00862	0.850440791	0.868508215	0.979197175	4	4	22.99174012	19.48822423
chr19	10553085	10566031	19p13.2	KRI1	6.187895508	6.320198757	0.9790666	4	4	61.16353084	53.85735702
chr12	20369245	20688579	12p12.2	PDE3A	2.041476403	2.085159602	0.979050429	4	4	45.19792179	18.59936914
chr9	32553526	32573184	9p21.1	NDUFB6	10.774424	11.00538152	0.979014129	4	4	21.74566379	54.5145633
chr9	122908056	122913328	9q33.2	ZBTB6	14.96875867	15.29118236	0.978914405	4	4	23.58091973	14.01343827
chr6	44254101	44257890	6p21.1	SLC35B2	4.36390939	4.457998601	0.978894293	4	4	36.54329207	30.1488343
chr10	16436943	16517962	10p13	PTER	10.75281584	10.98484869	0.97887701	4	4	28.43268901	20.3258871
chr22	39901073	39973343	22q13.1	GRAP2	45.20809094	46.18594923	0.978827797	4	4	7.834443312	4.897811226
chr2	64631621	64751086	2p14	SERTAD2	10.17973168	10.40025437	0.978796414	4	4	32.65095485	37.25315511
chr1	145289900	145405778	1q21.1	NBPF20	0.110868203	0.113271096	0.978786357	4	4	80.35723298	36.92707373
chr2	11677544	11827409	2p25.1	LPIN1	3.750316652	3.831628301	0.978778826	4	4	49.44253213	23.39000323
chr4	90838485	90839115	4q22.1	TMSB4XP8	74.20241921	75.81489273	0.978731441	4	4	45.06859925	105.5315913
chr5	128083791	128189688	5q23.3	SLC12A2	3.951222438	4.037101932	0.97872744	4	4	13.45714007	14.14503477
chr1	20988314	20988432	1p36.12	MIR1256	4.141139398	4.231170844	0.97872186	2	1	27.05648091	0
chr14	31111553	31112283	14q12	RPL21P5	0.79154546	0.808763535	0.978710619	3	2	65.57619526	20.71788132
chr12	53006258	53042215	12q13.13	EIF4B	140.8897726	143.9550629	0.978706617	4	4	55.64945442	37.14147455
chr14	22525650	22525715	14q11.2	TRAJ18	23.49950545	24.01168392	0.978669615	3	3	55.75087567	13.16182653
chrX	50365407	50470851	Xp11.22	DGKK	0.27619118	0.282214156	0.978658136	3	3	24.30756693	102.5610185
chr9	129007792	129028353	9q34.11	SH3GLB2	8.341760336	8.524058643	0.978613673	4	4	32.45727775	29.76288154
chr2	100820151	100996829	2q11.2	NPAS2	0.093084791	0.095119458	0.978609348	2	4	102.5130305	68.97967459
chr2	84905639	84906675	2p11.2	TMSB10	1049.013231	1072.030436	0.978529337	4	4	39.73217166	48.8772241
chr17	8377516	8383250	17p13.1	RPL26	125.6456104	128.4050833	0.978509628	4	4	29.5163839	18.36695232
chr1	85265776	85276904	1p22.3	BCL10	21.01554673	21.48147149	0.978310389	4	4	7.628410493	45.14208695

chr19	18683615	18782333	19p13.11	CRTC1	1.024799241	1.04751994	0.978310008	4	4	120.3990928	42.83044818
chr16	85778624	85799555	16q24.1	EMC8	2.477028494	2.532114629	0.978245007	4	4	44.10639929	16.34534146
chr4	106315610	106349225	4q24	AIMP1	13.73160798	14.03707915	0.978238267	4	4	42.05002636	10.26136598
chr2	14998067	15561348	2p24.3	NBAS	66.60039157	68.08885928	0.978139336	4	4	61.17257021	46.65004888
chr1	59893308	59926798	1p32.1	CYP2J2	0.252832176	0.258486499	0.97812527	3	3	99.06281759	63.057694
chr6	42206801	42217895	6p21.1	MRPS10	16.2518456	16.61662176	0.978047514	4	4	69.42131077	22.91693687
chr14	22320188	22320691	14q11.2	TRAV41	4.525328315	4.62692109	0.978043115	3	4	43.58698102	58.19812149
chr9	83622324	83644130	9q21.32	IDNK	2.069340659	2.116044793	0.97792857	4	4	49.96944308	34.84469078
chr19	14073009	14075062	19p13.12	MISP3	1.233633414	1.261585534	0.977843658	3	4	46.0337522	88.39514987
chr7	142812586	142813287	7q34	TRBV30	1.896839105	1.939838305	0.977833616	3	3	41.04562746	31.21575615
chr10	26938195	27100498	10p12.1	ANKRD26	16.50542904	16.88094778	0.977754878	4	4	38.41261583	45.60745304
chr3	52836756	52897581	3p21.1	STIMATE	0.212069202	0.216937116	0.977560718	4	4	72.43420626	54.49080989
chr16	87406246	87492948	16q24.2	ZCCHC14	1.620521292	1.657817462	0.977502849	4	4	58.50575863	30.77054459
chr8	19939071	19967259	8p21.3	LPL	0.126656212	0.129571947	0.977497175	4	2	54.48977002	71.08353966
chr7	56101562	56106630	7p11.2	CHCHD2	60.87462071	62.2847679	0.977359678	4	4	24.89195111	26.15019429
chr3	129055449	129062411	3q21.3	GP9	30.64108665	31.35102206	0.977355271	4	4	58.28219261	22.48031828
chr19	32345608	32387667	19q13.11	ZNF507	6.092147746	6.234336932	0.977192573	4	4	43.52835209	16.26754921
chr4	1982714	2041914	4p16.3	NELFA	1.977894551	2.024119744	0.977162817	4	4	92.11484735	64.79926944
chr2	222671738	222709930	2q36.1	MOGAT1	0.274808749	0.281237252	0.977142062	1	1	0	0
chr17	42113111	42121409	17q21.2	KAT2A	1.384160581	1.416607153	0.977095575	4	4	55.97224981	33.61807084
chr15	90717327	90815462	15q26.1	BLM	5.939475363	6.078940472	0.977057662	4	4	41.24112496	4.299142753
chr17	59697306	59707626	17q23.1	PTRH2	2.023403151	2.071050684	0.976993546	4	4	42.12753251	39.57331994
chr2	210021421	210171370	2q34	KANSL1L	7.491452478	7.668001835	0.976975833	4	4	22.0650817	16.88260706
chr3	50674969	51384198	3p21.2	DOCK3	0.120367353	0.123207421	0.976948887	3	3	51.58668945	28.38483374
chr6	117480351	117569858	6q22.1	DCBLD1	0.771687345	0.789968427	0.976858466	4	4	53.47110516	20.95176383
chr1	205302059	205321791	1q32.1	NUAK2	18.68351678	19.12696568	0.976815512	4	4	66.77802617	46.2491874
chr1	42767245	42775742	1p34.2	C1orf50	0.698227481	0.714828184	0.976776652	4	4	37.43527167	56.84408887
chr8	129351694	129680239	8q24.21	CCDC26	0.930530412	0.95270537	0.976724222	3	4	27.73608708	90.624745
chr1	6590724	6602898	1p36.31	KLHL21	5.969476157	6.111982255	0.976684144	4	4	69.44939639	29.53605288
chr5	154941073	154969411	5q33.2	MRPL22	8.257761498	8.455562538	0.976606992	4	4	44.06173682	59.77157099
chr19	50025893	50048776	19q13.33	ZNF473	1.815800004	1.859302944	0.976602554	4	4	69.35964143	35.15265942
chr3	47495640	47513725	3p21.31	ELP6	2.642392488	2.705723791	0.976593581	4	4	23.18887288	53.65528291
chr3	37452322	37819790	3p22.2	ITGA9	0.29413392	0.301184737	0.976589729	3	4	69.76875287	55.34900229
chr1	205789093	205813759	1q32.1	SLC41A1	2.422454531	2.480621658	0.976551391	4	4	42.35023291	55.28090376
chr1	31259568	31296797	1p35.2	SNRNP40	9.157938445	9.378184906	0.976515023	4	4	26.94804394	29.33435362
chr8	132570419	132685039	8q24.22	LRRC6	6.193254612	6.342264657	0.976505231	4	4	38.70690966	41.69165261
chr1	17406755	17439754	1p36.13	RCC2	6.295960112	6.447563826	0.976486667	4	4	54.64200033	55.82177202
chr22	22686769	22687281	22q11.22	IGLV3-25	5.449685754	5.581261667	0.976425418	4	3	80.65517896	75.38170704
chr12	120127210	120194710	12q24.23	GCN1	9.279380327	9.503442422	0.97642306	4	4	29.02447911	35.34168131
chr4	17793448	17810758	4p15.31	DCAF16	10.93154601	11.19619159	0.976362893	4	4	24.67332515	23.8787008
chr12	109573461	109597270	12q24.11	MVK	0.525183575	0.53790132	0.976356732	4	4	55.59976601	58.32862691
chr5	58969038	60524329	5q11.2-q12.1	PDE4D	16.14580867	16.5369888	0.976345142	4	4	42.90464477	47.01678881
chr17	44372181	44389601	17q21.31	ITGA2B	37.18032819	38.08445179	0.976260034	4	4	78.00872568	36.00944081

chr1	9234767	9271337	1p36.22	H6PD	6.537618515	6.697670012	0.976103407	4	4	53.10034819	30.34418358
chr3	136250325	136330171	3q22.3	PCCB	3.417327526	3.501302402	0.976016103	4	4	19.37739345	15.96729731
chr8	145052438	145065864	8q24.3	C8orf33	4.322726365	4.429160689	0.975969641	4	4	41.70622533	36.98023549
chr9	128720869	128724138	9q34.11	ZDHHC12	4.700800713	4.817161739	0.975844484	4	4	58.8234496	39.11256454
chr11	61348748	61362283	11q12.2	CYB561A3	3.790872746	3.884920154	0.975791675	4	4	43.99936034	20.46172681
chr12	107685732	107713162	12q23.3	PWP1	14.90575912	15.27577318	0.975777719	4	4	17.3520206	38.04486458
chr11	66002079	66004149	11q13.1	BANF1	9.845972097	10.09055029	0.97576166	4	4	60.04890537	49.3037324
chr16	1972063	1978750	16p13.3	TBL3	1.351259189	1.384845452	0.975747284	4	4	70.89006289	23.9397276
chr15	89575587	89628023	15q26.1	TICRR	0.836809793	0.857664566	0.975684232	4	4	69.21710056	54.82448637
chr12	132921433	132956306	12q24.33	ZNF605	1.01357124	1.038857429	0.975659616	4	4	52.85692319	79.61146532
chr22	38982399	38992779	22q13.1	APOBEC3B	1.69984948	1.742301086	0.975634747	3	3	118.0927407	28.37363528
chr17	50055968	50090485	17q21.33	ITGA3	0.286917924	0.294131914	0.975473625	4	3	89.20996316	32.14697112
chr7	5970925	6009106	7p22.1	PMS2	6.454201905	6.616910993	0.975410114	4	4	27.56857651	10.31929413
chr2	11444375	11466177	2p25.1	E2F6	2.571877348	2.636853869	0.975358316	4	4	56.65226376	22.18089521
chr6	170290703	170407748	6q27	FAM120B	3.153413809	3.233274847	0.975300263	4	4	36.91193088	37.21914824
chr16	720581	722590	16p13.3	FAM173A	1.133386523	1.162157435	0.975243534	4	4	65.52268914	32.47066412
chr2	127704234	127811187	2q14.3	WDR33	10.38950053	10.65403493	0.975170496	4	4	11.2399949	15.09040621
chr14	45115292	45134529	14q21.2	FKBP3	116.024488	118.9933135	0.975050484	4	4	46.05471832	29.88217352
chr12	71839707	71926849	12q21.1	TBC1D15	16.94605197	17.38073367	0.974990601	4	4	15.68584074	29.04460058
chr12	72253507	72273509	12q21.1	TRHDE-AS1	0.056806426	0.058265088	0.974965083	4	1	47.17891834	0
chr6	2765341	2785745	6p25.2	WRNIP1	11.13871671	11.42626156	0.974834739	4	4	22.65185698	23.64522429
chr2	205993721	206086182	2q33.3	INO80D	1.36625035	1.401600288	0.974778873	4	4	11.8150671	19.15006808
chr11	2398412	2403878	11p15.5	TSSC4	6.582690322	6.753440596	0.974716551	4	4	49.77952293	23.93998294
chr14	64914361	64935368	14q23.3	CHURC1	26.14945594	26.82889026	0.974675273	4	4	32.99054512	69.13691128
chr3	73061659	73063337	3p13	EBLN2	3.325692466	3.412243717	0.974635091	4	4	74.00354013	21.76442031
chr1	41506365	42035925	1p34.2	HIVEP3	1.127281467	1.156751461	0.974523486	4	4	48.74662718	62.57225476
chr11	66268526	66277492	11q13.2	RAB1B	68.5912408	70.38472088	0.9745189	4	4	61.80502985	59.37877428
chr10	73253758	73361064	10q22.2	CFAP70	1.050307793	1.077796641	0.97449533	4	4	31.05179273	40.10241911
chr22	39021113	39033258	22q13.1	APOBEC3D	0.684542555	0.702524815	0.974403381	4	4	49.79683729	47.29874117
chrX	53141688	53143984	Xp11.22	ACTG1P10	0.285550908	0.293059959	0.97437708	2	1	9.71836765	0
chr17	75880379	75897008	17q25.1	TRIM65	3.048653727	3.129135942	0.974279732	4	4	59.22488501	44.85952365
chr16	89159220	89163675	16q24.3	LINC00304	0.200947277	0.206258639	0.974249023	1	1	0	0
chr7	152464124	152465545	7q36.1	LINC01003	2.83223174	2.907190102	0.974216216	4	4	61.74819582	10.9350887
chr12	121712752	121782068	12q24.31	TMEM120B	0.517494471	0.531197011	0.97420441	4	4	56.15503095	38.06820928
chr9	114587714	114598872	9q32	ATP6V1G1	89.23442475	91.60437815	0.974128383	4	4	44.63576746	18.83655572
chr8	22599601	22604150	8p21.3	C8orf58	0.984704377	1.010876981	0.974109011	4	4	121.4735568	55.51187508
chr8	22987406	23020197	8p21.3	RHOBTB2	1.48827325	1.52832213	0.973795524	4	4	46.06049257	62.26440631
chr4	154534997	154550433	4q31.3	PLRG1	10.69804088	10.98608609	0.973780908	4	4	41.39559263	10.82599656
chr11	5291467	5292351	11p15.4	OR51AB1P	3.673588442	3.772577841	0.973760807	3	4	78.20953556	84.9516224
chr12	124946824	124989125	12q24.31	DHX37	2.549781211	2.618721027	0.973674242	4	4	83.34152506	45.26318548
chr7	20615622	20757014	7p21.1	ABCB5	1.379060625	1.416347998	0.97367358	4	4	70.51350623	59.91588757
chr17	7225341	7234544	17p13.1	DVL2	2.598090018	2.668403075	0.973649761	4	4	26.4230954	22.59413278
chr1	1280436	1292029	1p36.33	SCNN1D	0.220114238	0.226072333	0.973645184	4	4	82.4203622	65.46654314

chr9	3218297	3526529	9p24.2	RFX3	7.185689729	7.380370337	0.973621837	4	4	42.27411028	34.34586214
chr16	30923055	30948783	16p11.2	FBXL19	2.520525067	2.588827613	0.973616418	4	4	53.68235018	69.916162
chr7	92487023	92528531	7q21.2	PEX1	9.193137815	9.442590533	0.973582174	4	4	14.56712707	12.87567765
chr5	132630589	132656154	5q31.1	TH2LCRR	0.367993646	0.377984834	0.973567224	3	2	14.87642927	60.74029947
chr6	111660332	111873452	6q21	FYN	34.96674001	35.9172412	0.973536353	4	4	32.00584683	54.06992736
chr1	163068606	163076802	1q23.3	RGS4	0.051576688	0.05298291	0.973458945	2	1	21.83491985	0
chr11	118436490	118526832	11q23.3	KMT2A	9.114509313	9.363083965	0.973451626	4	4	32.98884294	31.74685306
chr7	149472793	149497832	7q36.1	ZNF746	7.609592236	7.817451742	0.973410836	4	4	59.65955023	43.96137353
chr1	19251581	19260128	1p36.13	MRTO4	3.482487481	3.577908717	0.973330444	4	4	61.60542045	26.41985614
chr1	31629862	31645237	1p35.2	PEF1	14.91508751	15.32414628	0.97330626	4	4	45.34709873	26.73062641
chr12	52233170	52252667	12q13.13	KRT7	0.1145011	0.117661975	0.973135972	1	1	0	0
chr11	46743534	46846308	11p11.2	CKAP5	33.99035258	34.92883088	0.9731317	4	4	33.23952718	17.39278907
chr2	201377207	201451596	2q33.1	TRAK2	210.9415935	216.7823619	0.973056994	4	4	57.70643739	98.56777739
chr3	194136142	194138612	3q29	HES1	0.857200594	0.88107982	0.972897772	4	4	76.78535988	83.86573712
chr16	70252295	70289509	16q22.1	AARS	20.64822972	21.2240615	0.972868917	4	4	18.4643698	50.08192211
chr7	36389803	36453791	7p14.2	ANLN	16.7553921	17.22327057	0.972834517	4	4	52.02141602	21.1623665
chr13	106543314	106568166	13q33.3	ARGLU1	21.46521995	22.06474247	0.972828936	4	4	61.33940548	17.9486659
chr1	231337936	231355029	1q42.2	SPRTN	4.398556379	4.521641835	0.972778592	4	4	43.56666635	8.45543166
chr19	39531533	39532867	19q13.2	EID2B	0.848212994	0.871971418	0.972753208	4	4	36.04153364	58.91057428
chr12	133181409	133207112	12q24.33	ZNF268	2.339334793	2.405007013	0.972693543	4	4	54.0647124	22.7126371
chr3	49688753	49721529	3p21.31	RNF123	12.94085006	13.30433514	0.9726792	4	4	47.39913385	38.45226226
chr3	129278806	129305293	3q21.3	HMCES	10.33817045	10.62861157	0.972673654	4	4	34.04851734	33.21097467
chr7	142482555	142483019	7q34	TRBV5-5	1.157932831	1.190471204	0.972667653	1	2	0	54.88568156
chr19	16661127	16689020	19p13.11	TMEM38A	1.190921601	1.224437416	0.97262758	4	3	58.44586774	28.06589268
chr1	155195588	155209180	1q22	THBS3	1.513155851	1.555844086	0.972562652	4	4	83.56621018	43.99803266
chr1	165662212	165698663	1q24.1	ALDH9A1	15.33638276	15.7700437	0.972500967	4	4	60.33850923	23.5124672
chr18	35366697	35377337	18q12.2	ZNF396	0.390946376	0.402014697	0.97246787	4	4	38.24829255	32.65278232
chr19	40222208	40227111	19q13.2	CNTD2	0.114735143	0.117997432	0.97235288	1	1	0	0
chr20	2098021	2148555	20p13	STK35	3.537290757	3.638126195	0.972283689	4	4	52.09508016	9.683264937
chr19	39995361	40021041	19q13.2	ZNF546	0.128912007	0.132589991	0.972260476	4	4	33.00220817	39.33908023
chr14	99397746	99486458	14q32.2	SETD3	79.07895162	81.33881149	0.972216709	4	4	29.17197402	8.28253478
chrX	149938548	150016787	Xq28	LINC00894	0.793250917	0.815987134	0.97213655	4	4	47.92729346	20.17460487
chr13	29509410	29595688	13q12.3	SLC7A1	5.524631955	5.683202526	0.972098377	4	4	12.39151245	27.37782052
chr19	58327000	58342346	19q13.43	ZSCAN22	1.170612809	1.204327379	0.972005477	4	4	67.72900133	54.4104244
chr10	96304328	96338564	10q24.1	DNTT	0.117757359	0.121164392	0.971880904	1	1	0	0
chr4	103105806	103198409	4q24	CENPE	24.50640911	25.2161443	0.971853937	4	4	50.00725459	35.1403586
chrX	87517712	87670050	Xq21.31	KLHL4	0.058832405	0.060541331	0.971772586	1	3	0	44.10726647
chr2	190454088	190607126	2q32.2	NEMP2	2.665963617	2.743408687	0.971770495	4	4	41.88936199	37.1920498
chr5	157266080	157395598	5q33.3	CYFIP2	27.5476586	28.35015116	0.971693535	4	4	33.45663934	31.09104956
chr1	20633455	20651511	1p36.12	PINK1	36.98371641	38.06269877	0.9716525	4	4	27.70398541	17.93243045
chr2	231395624	231401164	2q37.1	B3GNT7	3.897042776	4.010737528	0.971652408	4	4	67.89044293	54.79108192
chrX	153587925	153599177	Xq28	CCNQ	4.081638343	4.201193544	0.971542563	4	4	44.77198369	54.59487651
chr7	87196162	87220587	7q21.12	TMEM243	8.452086206	8.70022979	0.971478502	4	4	15.99791099	36.56635755

chr7	4775617	4794395	7p22.1	AP5Z1	3.605712025	3.71159374	0.971472709	4	4	59.60299821	7.521030818
chr11	59255377	59259500	11q12.1	WARSP1	1.328013662	1.367131556	0.971386884	3	2	54.90563677	26.49913847
chr2	231454748	231464494	2q37.1	NCL	54.24341034	55.84212958	0.971370733	4	4	57.8304537	52.59837383
chr14	36298558	36320676	14q13.3	MBIP	5.755841696	5.925713453	0.971333113	4	4	30.69797349	20.28716495
chr7	4799109	4883704	7p22.1	RADIL	0.038947483	0.040097426	0.971321279	1	1	0	0
chr7	104940949	105114085	7q22.3	KMT2E	92.87203086	95.64846003	0.970972568	4	4	6.568810798	11.82512771
chr6	12002096	12212034	6p24.1	HIVEP1	9.097870381	9.371400125	0.970812286	4	4	47.87998162	20.03878423
chr6	26596943	26600050	6p22.2	ABT1	9.516296393	9.802553792	0.970797671	4	4	46.94485307	9.953715385
chr6	143450781	143490616	6q24.2	PEX3	6.179930678	6.365869441	0.970791301	4	4	35.87225546	18.98863905
chrX	154778684	154805527	Xq28	MPP1	244.992891	252.3646595	0.97078922	4	4	36.02622252	36.54078766
chr16	4745961	4767219	16p13.3	ZNF500	1.059038004	1.090948256	0.970749986	4	4	83.69613112	29.89769975
chr19	18636965	18670499	19p13.11	KLHL26	0.808459378	0.832862143	0.970700115	4	4	38.13160396	28.03310119
chr12	53665160	53677546	12q13.13	ATP5MC2	21.22517939	21.86789093	0.97060935	4	4	33.05289819	29.60801373
chr8	38231491	38273922	8p11.23	DDHD2	4.654796673	4.796019741	0.97055411	4	4	13.29659027	17.00777447
chrX	135248589	135344629	Xq26.3	ZNF75D	3.435062964	3.539530183	0.970485569	4	4	30.78761601	22.34566799
chr5	69492292	69558104	5q13.2	OCLN	0.037646002	0.038792805	0.970437754	1	1	0	0
chr20	47209214	47357780	20q13.12	ZMYND8	58.80209047	60.59642818	0.970388722	4	4	36.63615365	13.39692547
chr17	50094737	50111369	17q21.33	PDK2	4.475207643	4.611951777	0.970350051	4	4	31.27103814	82.11668708
chr16	22345936	22374617	16p12.2	CDR2	6.148375309	6.336835831	0.970259523	4	4	25.23347063	13.59103171
chr17	80415117	80438086	17q25.3	ENDOV	0.387855022	0.399752989	0.970236703	4	4	128.9073135	18.26051515
chr10	100523729	100529923	10q24.31	NDUFB8	3.758954884	3.87433484	0.970219415	4	4	22.7861014	15.8359184
chr19	56477874	56495437	19q13.43	ZNF667-AS1	1.570745238	1.61898974	0.97020086	4	4	90.64895137	31.02890234
chr16	87693537	87765992	16q24.2	KLHDC4	2.587033769	2.666633249	0.970149821	4	4	23.01936898	31.42899644
chr15	24984860	24988232	15q11.2	PWAR5	8.147097818	8.398739955	0.970038108	4	4	36.69400901	39.47291717
chr1	40754355	40754446	1p34.2	MIR30E	5.241248109	5.403199432	0.970026773	2	3	15.05972029	54.20373946
chr1	26890488	26900471	1p36.11	GPATCH3	4.57401258	4.71609441	0.969872989	4	4	46.32865228	24.2824747
chr11	65996545	66002217	11q13.1	EIF1AD	6.887704026	7.101951176	0.969832635	4	4	37.21306526	20.18649642
chr2	222298147	222305217	2q36.1	CCDC140	0.120117214	0.12387179	0.969689821	1	1	0	0
chr9	109375694	109538395	9q31.3	PTPN3	0.050052027	0.051616571	0.969689116	2	3	3.010701475	49.10364667
chr20	46349528	46364458	20q13.12	SLC35C2	4.962147268	5.117340583	0.969673053	4	4	40.35169215	16.17002869
chr16	18374521	18402014	16p12.3	PKD1P5	0.124142501	0.128034512	0.969601859	3	4	81.18019551	95.87570223
chr1	96721605	96815049	1p21.3	PTBP2	3.274853201	3.377534997	0.969598599	4	4	27.02535071	42.3870451
chr11	123057492	123062366	11q24.1	HSPA8	268.8367468	277.2754523	0.969565624	4	4	50.75917442	59.13582257
chr17	7583647	7588212	17p13.1	MPDU1	5.304389834	5.471197874	0.969511605	4	4	52.51756445	27.8593161
chr13	46771256	46797700	13q14.2	ESD	13.31387113	13.73426216	0.969391073	4	4	14.82129775	20.72850519
chr22	37848868	37888782	22q13.1	EIF3L	32.69713137	33.73103788	0.969348512	4	4	47.55705422	23.72269534
chr17	15976560	15999692	17p12	ZSWIM7	3.245345934	3.348110245	0.969306772	4	4	42.02143052	28.30387749
chr14	31025106	31096450	14q12	AP4S1	2.674593542	2.75952085	0.969223893	4	4	33.56068869	53.38193241
chr5	41730065	41870689	5p13.1	OXCT1	17.30717807	17.85705704	0.96920663	4	4	15.50945367	31.71384871
chr1	43281883	43285840	1p34.2	C1orf210	0.148799144	0.153538669	0.969131391	1	1	0	0
chr11	60890547	60906589	11q12.2	PRPF19	10.31483211	10.64388444	0.969085316	4	4	50.20334743	37.34478578
chr11	76381303	76414634	11q13.5	GVQW3	0.142796103	0.147373141	0.968942523	4	4	76.11194756	69.04796484
chr22	37658730	37666932	22q13.1	PDXP	0.159034733	0.16413278	0.96893949	3	2	17.86364494	40.90693945

chr16	69709401	69726668	16q22.1	NQO1	0.582017654	0.600712268	0.968879253	4	4	74.06763307	59.48028704
chr6	37170146	37175428	6p21.2	PIM1	250.7645092	258.8198146	0.968876783	4	4	39.44042388	17.5170027
chr21	39445865	39515506	21q22.2	SH3BGR	0.601985188	0.621331076	0.968863801	4	3	53.76076162	81.03149696
chr6	13214040	13214255	6p24.1	RNU1-11P	2.337667196	2.413041882	0.968763623	1	2	0	64.17529577
chr19	43912624	43935315	19q13.31	ZNF45	8.129214285	8.391634357	0.968728372	4	4	29.37055325	39.46742914
chr17	78107401	78111799	17q25.3	TNRC6C-AS1	2.949205479	3.044444598	0.968717079	4	4	47.63180767	44.56597994
chr6	10671418	10694797	6p24.2	C6orf52	0.312535755	0.32267992	0.968562764	2	2	28.23775989	15.47416858
chr20	45841721	45857409	20q13.12	ACOT8	2.887980342	2.98188377	0.968508689	4	4	66.23806653	10.081075
chr14	49853616	49853914	14q21.3	RN7SL3	1327.748486	1371.150483	0.968346292	4	4	49.8366605	56.30100379
chrX	74732856	74925452	Xq13.3	NEXMIF	0.062736106	0.064793322	0.968249566	1	4	0	66.99573976
chr5	141387885	141512979	5q31.3	PCDHGB4	0.036808705	0.038017221	0.968211354	1	1	0	0
chr3	101561836	101566446	3q12.3	TRMT10C	11.44596559	11.82306207	0.968105007	4	4	27.05425005	21.68072836
chr6	157716046	157945077	6q25.3	SNX9	7.951627277	8.213739205	0.968088599	4	4	37.99483952	34.55309066
chr19	54447621	54462046	19q13.42	LENG8	3.639568415	3.759744988	0.968035978	4	4	80.11601758	12.80645063
chr7	141738321	141750488	7q34	SSBP1	7.655141784	7.90793871	0.968032513	4	4	32.96566711	39.47845324
chr2	71109676	71130288	2p13.3	MCEE	3.271145159	3.379212736	0.968019896	4	4	78.85864775	16.01538326
chr1	19302708	19312146	1p36.13	AKR7A2	8.794524782	9.086540346	0.967862844	4	4	68.54569673	20.11158071
chrX	46599251	46759172	Xp11.3	SLC9A7	3.164111547	3.269185961	0.96785915	4	4	64.10680391	35.97549887
chr3	15560704	15601852	3p25.1	HACL1	6.647729309	6.868689548	0.967830801	4	4	12.11768261	13.79470583
chr17	48542655	48545031	17q21.32	HOXB2	2.208779924	2.282614808	0.967653375	4	4	64.86115911	32.04381762
chr19	51450997	51458454	19q13.41	SIGLEC8	3.081556522	3.184654282	0.967626703	4	4	117.6607374	92.03542829
chr11	14457503	14499895	11p15.2	COPB1	30.91329206	31.94761303	0.967624468	4	4	5.807197415	17.67540396
chr3	133397882	133475212	3q22.1	BFSP2	0.112817202	0.116597906	0.967574849	1	1	0	0
chr2	74306670	74308058	2p13.1	KRT18P26	0.206448305	0.213388415	0.96747663	1	2	0	21.39136887
chr11	18322269	18367043	11p15.1	GTF2H1	9.72540948	10.05328275	0.967386447	4	4	14.13886712	10.51215265
chr2	206273572	206314438	2q33.3	ZDBF2	0.465889555	0.481618849	0.967340786	4	4	89.70934197	43.12067539
chr12	13044381	13083449	12p13.1	FAM234B	0.944727175	0.976677242	0.967286976	4	4	15.37092702	11.01577378
chr3	45755449	45796553	3p21.31	SLC6A20	0.038121275	0.039411876	0.967253503	1	2	0	1.598515449
chr11	62761539	62766715	11q12.3	POLR2G	12.68925963	13.11899637	0.96724317	4	4	19.68113951	12.15558947
chr19	52612364	52690581	19q13.41	ZNF83	6.273809562	6.487151931	0.967113092	4	4	37.43821545	20.11953789
chr2	230514924	230516086	2q37.1	HMGB1P3	1.202255258	1.243152134	0.967102276	3	3	7.420741324	42.54547076
chr19	49869033	49878356	19q13.33	AKT1S1	5.325002574	5.506146497	0.967101507	4	4	26.892211	34.86934605
chrX	65512582	65534806	Xq12	LAS1L	3.535237906	3.655749353	0.96703509	4	4	33.43877814	31.70506826
chr19	53235381	53254903	19q13.42	ZNF677	0.69323283	0.716915343	0.966966095	4	4	85.70038127	28.16344116
chr14	73490934	73493401	14q24.3	RIOX1	6.863248943	7.097998395	0.966927373	4	4	62.87182524	44.46827176
chr19	20077994	20127090	19p12	ZNF90	0.773084899	0.799528839	0.966925595	4	4	83.83866396	42.84253051
chr18	35290272	35310766	18q12.2	ZNF271P	11.35313697	11.74236371	0.966852778	4	4	22.34306703	22.90887665
chr3	119211742	119240907	3q13.32	B4GALT4	2.059046803	2.129666808	0.966839881	4	4	40.02464372	6.300014641
chr3	9731729	9748015	3p25.3	BRPF1	8.197607185	8.479080042	0.966803845	4	4	62.81861625	33.3169543
chr3	23916545	23924631	3p24.2	RPL15	89.62942571	92.71787877	0.966689779	4	4	49.43881694	21.83637716
chr3	198057531	198080671	3q29	ANKRD18DP	0.236602075	0.24475622	0.966684624	1	3	0	77.70056237
chr22	35256697	35295807	22q12.3	HMGXB4	4.937588815	5.107901367	0.96665704	4	4	11.78631317	13.24614921
chr2	96875882	96986600	2q11.2	FAM178B	0.289073106	0.299046445	0.96664953	2	3	49.27201388	43.09428374

chrX	136648177	136660390	Xq26.3	CD40LG	6.079474095	6.289284059	0.966640088	4	4	57.30172022	40.11998145
chr9	133406058	133418172	9q34.2	REXO4	4.351084426	4.501283807	0.966631879	4	4	44.09499058	42.55946509
chr19	8302127	8308356	19p13.2	CD320	1.624359741	1.680468613	0.966611175	4	4	43.10384529	33.43628359
chr6	32013390	32014184	6p21.33	STK19B	2.39917576	2.482059709	0.966606787	2	3	84.62963186	54.38372328
chr12	121918557	122003927	12q24.31	WDR66	0.344231773	0.356148703	0.966539453	4	4	42.25038518	39.39167714
chr5	149545270	149545844	5q32	RPL29P14	2.896837367	2.997204002	0.966513246	4	4	96.98343218	40.66890387
chr14	58395904	58417080	14q23.1	TOMM20L	0.936049151	0.968495607	0.966498087	2	2	36.81117398	35.00815022
chr16	47597693	47599023	16q12.1	NDUFA5P11	0.95442637	0.987521159	0.966487008	2	1	48.94630649	0
chr1	66999332	67054423	1p31.3	SLC35D1	5.540239431	5.732895768	0.966394586	4	4	35.45961201	6.579949258
chr19	38836378	38852339	19q13.2	HNRNPL	11.12848492	11.51588655	0.966359374	4	4	52.62443374	35.12041967
chr4	1166932	1208962	4p16.3	SPON2	7.266453793	7.519413634	0.9663591	4	4	38.85458445	44.8065979
chr2	237085441	237098846	2q37.3	COPS8	7.195218227	7.445847997	0.966339661	4	4	16.41186328	12.56309568
chr12	50952263	51028335	12q13.12	SLC11A2	7.882494618	8.15722082	0.966321103	4	4	64.61306742	32.8843412
chr19	57240685	57262738	19q13.43	ZNF805	7.160808089	7.410509634	0.966304403	4	4	37.69162698	35.68379915
chr12	64404348	64451128	12q14.2	XPOT	16.64326198	17.22369896	0.966300098	4	4	23.27513318	25.40914274
chr6	43054029	43060180	6p21.1	MRPL2	3.520845584	3.643886648	0.966233564	4	4	41.13265653	41.08873939
chr11	6474683	6481479	11p15.4	ARFIP2	2.868374796	2.968722581	0.966198329	4	4	64.32621785	18.651537
chr15	78104606	78131976	15q25.1	CIB2	0.34543962	0.357531505	0.966179527	4	4	19.96625345	49.36989595
chr1	88829019	88829443	1p22.2	ELOCP19	8.92039043	9.232752099	0.966168087	4	4	52.40425772	58.80166335
chr2	43230836	43596046	2p21	THADA	6.819608632	7.059776181	0.965980855	4	4	36.23648338	26.58266143
chr18	3496032	4455441	18p11.31	DLGAP1	0.054366762	0.056284303	0.965931154	3	3	55.92333811	78.53007391
chr1	235065474	235069818	1q42.3	LINC01348	0.116235683	0.120335791	0.965927776	4	2	23.45701817	51.51496884
chr1	27234516	27308633	1p36.11	WDTC1	27.19996371	28.15989145	0.965911525	4	4	34.31439303	19.01989298
chr3	51385237	51389397	3p21.2	MANF	8.942406664	9.258249062	0.965885299	4	4	57.50866129	50.44748977
chr8	143280155	143281700	8q24.3	MINCR	0.881956174	0.913124774	0.965866001	4	4	93.10175765	101.4893813
chr1	43946806	43968022	1p34.1	IPO13	2.391086168	2.475625454	0.965851342	4	4	32.83680392	43.71684444
chr14	23016543	23035220	14q11.2	PSMB5	6.519212951	6.749757299	0.965844054	4	4	9.466518443	18.87320257
chr17	59565525	59608352	17q23.1	DHX40	22.14660782	22.93136213	0.965778121	4	4	22.68061558	33.93251986
chr1	16562427	16613605	1p36.13	NBPF1	0.439737814	0.455340172	0.965734721	4	4	127.919858	47.33112057
chr18	46917550	47104795	18q21.1	KATNAL2	0.516907434	0.535314896	0.965613767	4	4	62.17071759	21.94374537
chr9	137255300	137273548	9q34.3	NELFB	7.452120376	7.718481696	0.965490451	4	4	38.35326572	36.71319172
chr16	1964996	1965504	16p13.3	SNHG9	3.167850227	3.281170069	0.965463588	4	4	32.358749	65.85338395
chr17	1650629	1684882	17p13.3	PRPF8	18.6876294	19.3576191	0.965388837	4	4	63.31835616	40.86062164
chr6	31560550	31574324	6p21.33	LTA	0.401525827	0.41596399	0.965289873	2	3	76.04216734	60.04774865
chr7	38341577	38378637	7p14.1	TRG-AS1	5.018762243	5.19933209	0.965270569	4	4	38.45340508	43.79549293
chrX	79170972	79172229	Xq21.1	GPR174	23.99653406	24.85994373	0.965269042	4	4	18.75240666	40.99613284
chr19	46918567	47005077	19q13.32	ARHGAP35	5.894263706	6.106434313	0.965254583	4	4	39.32318277	35.62856331
chr6	121435577	121449744	6q22.31	GJA1	0.221159883	0.229122324	0.965248083	2	1	76.60604162	0
chr17	3923869	3964464	17p13.2	ATP2A3	24.18218983	25.05758604	0.965064623	4	4	61.09780326	41.71585937
chr6	131573966	131628239	6q23.2	MED23	14.49346453	15.02050794	0.964911745	4	4	15.2909145	21.65542077
chr14	21642973	21643586	14q11.2	TRAV1-2	5.031730073	5.214764786	0.964900677	4	4	64.77131914	63.79936046
chr12	57797376	57818704	12q14.1	AVIL	0.564046939	0.584585773	0.964866004	4	4	89.2776257	60.18466356
chr1	92473043	92486876	1p22.1	GFI1	5.467035225	5.666477468	0.964803135	4	4	49.95618687	90.0544179

chr8	29748309	29798492	8p12	LINC02099	0.045209388	0.046866975	0.96463209	1	1	0	0
chr13	41189834	41194584	13q14.11	KBTBD7	7.515971595	7.791740183	0.964607574	4	4	40.38404289	55.20632005
chr9	35782086	35809731	9p13.3	NPR2	0.206625462	0.214241281	0.964452141	4	4	83.9499296	44.52912518
chr13	102596936	102679958	13q33.1	TPP2	27.67883615	28.70069408	0.964396055	4	4	29.32656759	16.93930992
chr12	132710807	132722737	12q24.33	PGAM5	3.447784226	3.575077908	0.964394151	4	4	42.31503017	34.03754635
chr1	84479237	84498350	1p22.3	RPF1	24.229392	25.12616307	0.964309271	4	4	46.78462933	23.29140267
chr11	47177521	47186459	11p11.2	PACSIN3	0.08587952	0.089066751	0.964215252	1	1	0	0
chr7	102748943	102913394	7q22.1	FAM185A	39.06781019	40.51804638	0.964207648	4	4	55.16372734	17.9027009
chr4	53276	88211	4p16.3	ZNF595	5.903821349	6.123464507	0.9641309	4	4	50.79675403	66.75411115
chr20	38210505	38260772	20q11.23	KIAA1755	0.031723865	0.03290682	0.964051372	1	1	0	0
chr14	105526603	105530202	14q32.33	TMEM121	0.343804314	0.356672983	0.963920259	1	3	0	108.6723502
chr12	48766191	48789096	12q13.12	ADCY6	0.19843014	0.205862832	0.96389493	4	4	57.4843867	90.11659821
chr7	139340359	139423457	7q34	LUC7L2	5.933710905	6.157596204	0.963640796	4	4	23.99921039	24.46954959
chr19	29698886	29715789	19q12	C19orf12	3.641428195	3.779076979	0.963576083	4	4	39.71365104	31.84056973
chr12	120196700	120201211	12q24.23	RPLP0	123.7346466	128.4184463	0.963527049	4	4	86.19578549	42.26724532
chr7	142787630	142787679	7q34	TRBJ1-3	7.477220541	7.760297008	0.96352247	2	2	28.30074498	49.07351333
chr3	13476987	13480053	3p25.1	HDAC11-AS1	0.480326286	0.498522486	0.963499741	1	1	0	0
chr2	162170684	162243557	2q24.2	FAP	0.036240738	0.0376143	0.963482996	1	3	0	5.443800125
chr12	95858931	95866460	12q23.1	SNRPF	9.064808513	9.408964437	0.96342255	4	4	64.40381618	55.55743082
chr13	72755402	72782128	13q21.33	DIS3	11.79840238	12.24733066	0.963344806	4	4	30.36110534	7.878796154
chr1	19215664	19251559	1p36.13	EMC1	3.003312303	3.118006704	0.963215473	4	4	28.32282164	19.69269736
chr17	4959226	4967837	17p13.2	SPAG7	8.868259618	9.20697616	0.963210881	4	4	71.63054524	23.97400081
chr11	134152442	134225454	11q25	NCAPD3	5.680575536	5.898397854	0.963070935	4	4	59.61871036	28.17404817
chr10	94545767	94613905	10q23.33	HELLS	5.573760786	5.787716363	0.963032816	4	4	62.82578868	25.82009741
chr20	3786772	3806121	20p13	CDC25B	16.74380743	17.38760111	0.962973979	4	4	24.71319604	87.83110504
chr9	32883872	33025131	9p21.1	APTX	3.128683663	3.24922724	0.962900848	4	4	30.68478871	20.73114788
chr12	113816738	113966371	12q24.13-q24.21	RBM19	5.370193951	5.577197941	0.962883872	4	4	63.38286904	25.44776535
chr2	232865678	232878704	2q37.1	SNORC	0.118462557	0.123036724	0.962822754	2	4	43.22536711	55.27479167
chr7	73536353	73557735	7q11.23	BCL7B	8.798573733	9.138802652	0.962770952	4	4	75.98586222	36.97289861
chr19	51927147	51946147	19q13.41	ZNF613	2.387833913	2.480239567	0.962743254	4	4	63.43547244	52.05577525
chr10	73782050	73783648	10q22.2	CHCHD1	5.87423044	6.101712765	0.962718284	4	4	26.04181531	17.88923016
chr16	67934504	67936877	16q22.1	PSMB10	19.10439625	19.84657876	0.962604007	4	4	53.39896629	41.83794248
chr3	14178728	14198369	3p25.1	LSM3	11.4639812	11.90939206	0.962600034	4	4	53.2291699	31.03781013
chr2	74425861	74441933	2p13.1	RTKN	0.141793344	0.147303659	0.962592134	3	3	43.26387627	36.64696826
chr14	35222127	35222765	14q13.2	DPRXP3	1.012018766	1.051430956	0.962515666	4	2	28.73085107	12.32141848
chr6	36130484	36144524	6p21.31	MAPK13	4.165849955	4.328175696	0.962495575	4	4	68.76695151	26.25433563
chr2	9405681	9423569	2p25.1	ITGB1BP1	3.310032067	3.439353756	0.962399422	4	4	14.34452076	22.28790434
chr11	65883740	65888539	11q13.1	FIBP	7.38490177	7.673433255	0.962398645	4	4	27.76043413	50.02917865
chr7	127593635	127601797	7q32.1	FSCN3	0.21771805	0.226225623	0.962393414	2	3	79.43497793	52.82429911
chr2	7720241	7731511	2p25.1	LINC01871	26.70996378	27.75583119	0.962319002	4	4	17.01244063	74.1926339
chr10	101353808	101557321	10q24.32	BTRC	16.77915981	17.43674358	0.962287467	4	4	62.57664779	51.55852837
chr9	77423079	77648316	9q21.2	GNA14	0.163773929	0.170198694	0.962251385	2	1	66.65576795	0
chr18	47085	73496	18p11.32	TUBB8P12	0.26956663	0.28014626	0.962235333	1	1	0	0

chr14	106657723	106658178	14q32.33	IGHV3-64	0.608032188	0.631926982	0.962187413	1	2	0	12.83082402
chr19	50418938	50431053	19q13.33	SPIB	2.389222583	2.483133563	0.962180456	4	4	82.67412944	105.3045459
chr3	37243191	37366879	3p22.2	GOLGA4	36.05031539	37.46820939	0.962157412	4	4	14.4794667	18.04321697
chr10	21524616	21743630	10p12.31	MLLT10	18.24074605	18.9603577	0.962046515	4	4	13.85327826	8.596967721
chr22	21697536	21735834	22q11.21-q11.22	YPEL1	3.847333662	3.999350184	0.961989695	4	4	50.4453033	24.08359475
chr19	11925068	11950773	19p13.2	ZNF700	5.57437512	5.794747399	0.961970339	4	4	22.13528285	27.97235085
chr13	102799049	102841538	13q33.1	BIVM	1.018532354	1.058842265	0.961930202	4	4	51.60192519	48.13298505
chr15	56918090	57292595	15q21.3	TCF12	12.71204105	13.21660639	0.961823381	4	4	17.27222918	13.90775846
chr16	5033702	5042971	16p13.3	NAGPA-AS1	0.089585148	0.09314537	0.961777798	1	1	0	0
chr11	32583767	32602873	11p13	EIF3M	22.10224718	22.98074746	0.961772336	4	4	32.0192165	15.78482915
chr20	20052514	20360714	20p11.23	CFAP61	0.034356885	0.035725037	0.961703251	1	2	0	0.20127999
chr11	14504876	14643634	11p15.2	PSMA1	7.221663933	7.509637591	0.961652789	4	4	19.43738155	15.95914448
chr9	122844565	122905351	9q33.2	RC3H2	22.21413328	23.10300873	0.961525554	4	4	5.52565306	10.79592661
chr17	10711084	10730329	17p13.1	TMEM220	0.274316987	0.285323478	0.961424518	4	4	55.13315102	71.45691653
chr20	10034999	10056764	20p12.2	ANKEF1	1.078159622	1.12143404	0.961411536	4	4	61.29422896	34.5961487
chr4	101790482	102074812	4q24	BANK1	12.37379627	12.87147466	0.961334781	4	4	33.77437998	56.932974
chr2	24009022	24026775	2p23.3	MFSD2B	11.20113546	11.65175776	0.96132581	4	4	48.64380538	34.30291195
chr6	170272474	170279469	6q27	LINC01624	0.099215871	0.103209879	0.961302079	1	3	0	59.5556592
chr16	3305406	3322999	16p13.3	ZNF75A	3.050218221	3.173655956	0.961105508	4	4	53.28914621	22.33246464
chr5	149358007	149375112	5q32	PCYOX1L	2.133166557	2.219683142	0.961023002	4	4	64.56200082	21.17650425
chr14	55271303	55353611	14q22.3	FBXO34	18.25470054	18.99634541	0.96095855	4	4	33.02098767	34.53733579
chr7	64433693	64435520	7q11.21	YWHAEP1	0.574605801	0.597960661	0.960942481	1	1	0	0
chrX	100820359	100843534	Xq22.1	CSTF2	3.72612805	3.877842962	0.960876468	4	4	21.4382032	56.23782555
chr7	72954845	73005932	7q11.23	TRIM74	0.152287869	0.158491224	0.960859951	1	1	0	0
chr9	83739421	83817837	9q21.32	GKAP1	52.38973931	54.52541988	0.960831469	4	4	55.41105646	29.72962719
chr19	19201409	19203429	19p13.11	NR2C2AP	1.570942293	1.635031197	0.960802641	4	4	24.08996846	18.45115235
chrX	49879867	49882565	Xp11.23	USP27X	1.179900024	1.228129727	0.960729147	4	4	62.32160706	77.49407801
chr19	45795710	45815347	19q13.32	RSPH6A	0.092657509	0.09644754	0.960703703	1	1	0	0
chr17	75784771	75825805	17q25.1	UNK	3.843393741	4.000655939	0.960690897	4	4	35.63873834	18.74844456
chr2	130151392	130182750	2q21.1	SMPD4	3.27596949	3.410020357	0.96068913	4	4	40.19638367	38.52771291
chr11	15111416	15269675	11p15.2	INSC	0.195368943	0.203374183	0.960637875	3	1	40.76901786	0
chr13	51335804	51365454	13q14.3	SERPINE3	0.090402223	0.094109512	0.960606651	1	1	0	0
chr17	42679402	42681827	17q21.2	CCR10	0.089916721	0.093607324	0.960573565	1	1	0	0
chr19	18193230	18196742	19p13.11	MPV17L2	1.228146318	1.278651341	0.960501333	3	4	70.58712426	40.29855269
chr6	35468401	35470781	6p21.31	RPL10A	190.0652329	197.894053	0.960439336	4	4	59.21877275	23.45127078
chr6	49727376	49744455	6p12.3	CRISP3	7.853164055	8.177128883	0.960381592	4	4	10.2240082	64.34580125
chr17	5432779	5439151	17p13.2	C1QBP	23.3175287	24.28864541	0.960017667	4	4	33.40003528	40.77970564
chr4	2937936	2963506	4p16.3	NOP14	19.5603175	20.3763072	0.959953995	4	4	49.25343786	37.18004531
chr3	48288402	48302904	3p21.31	NME6	2.696466119	2.808991198	0.959941107	4	4	49.25307487	31.01107767
chr1	145926831	145941203	1q21.1	LIX1L-AS1	0.270828373	0.282133696	0.9599292	4	4	62.40121256	62.07351445
chr6	43522330	43576075	6p21.1	XPO5	5.668185845	5.904866981	0.959917618	4	4	26.48757913	11.62379193
chr5	133051970	133105017	5q31.1	HSPA4	38.40591387	40.01074147	0.959890081	4	4	10.59481573	2.279552396
chr14	64744106	64879889	14q23.3	SPTB	20.47120514	21.32674071	0.959884373	4	4	76.44450935	46.10217718

chr16	28984826	28990783	16p11.2	LAT	0.308344945	0.321233618	0.959877571	4	4	63.32570337	36.479288
chr1	62436304	62451804	1p31.3	USP1	86.70527172	90.3314877	0.959856567	4	4	46.59602852	24.29173613
chr8	93728163	93741012	8q22.1	RBM12B	8.394792574	8.746178179	0.959824097	4	4	23.62470309	27.03621071
chr2	95849548	95992320	2q11.1	ANKRD36C	7.2982603	7.60381528	0.95981557	4	4	41.80632264	25.91512846
chr9	128819651	128833309	9q34.11	SPOUT1	4.985280671	5.194257998	0.959767627	4	4	35.70070864	8.762384212
chr9	5418172	5419109	9p24.1	RNF152P1	0.434866272	0.453121449	0.959712399	1	2	0	22.63011633
chr2	171999897	172082430	2q31.1	METAP1D	0.54144908	0.564191932	0.959689512	4	4	43.46318585	64.11917465
chr12	6492150	6493305	12p13.31	MRPL51	18.33168025	19.10257661	0.959644379	4	4	37.22519448	46.91692698
chr19	49806866	49817376	19q13.33	FUZ	2.161317124	2.252274064	0.959615509	4	4	66.7148951	26.14551936
chr1	166856510	166876417	1q24.1	TADA1	6.394558076	6.663827544	0.959592372	4	4	13.60134763	3.229922067
chr5	173707599	173746209	5q35.2	LINC01484	0.158716801	0.16540942	0.95953907	1	1	0	0
chr18	10610998	10626420	18p11.22	LINC01887	0.352502847	0.367372848	0.959523408	3	1	29.99196429	0
chr6	106559237	106630921	6q21	RTN4IP1	3.085392996	3.215617068	0.959502618	4	4	15.17024438	28.08838701
chr10	69404202	69416918	10q22.1	TACR2	0.082254536	0.085728196	0.959480541	1	1	0	0
chr17	57255851	57684689	17q22	MSI2	2.877704336	2.999461131	0.95940711	4	4	26.6697228	20.90691737
chr11	118162804	118176622	11q23.3	SCN2B	0.043137391	0.044962663	0.959404719	1	1	0	0
chr7	143437035	143437973	7q35	TAS2R62P	0.235522838	0.245493065	0.959386933	1	1	0	0
chr5	65722196	65829283	5q12.3	NLN	2.04051417	2.126929401	0.959370898	4	4	48.79215607	30.19422432
chr19	54906063	54938211	19q13.42	NCR1	6.146600825	6.407145066	0.959335361	4	4	71.721977	26.25804612
chr17	55392613	55421980	17q22	MMD	85.96309067	89.61831079	0.959213468	4	4	17.33727766	25.89003576
chr2	30231531	30260033	2p23.1	LBH	49.81556808	51.94460684	0.959013286	4	4	28.2457429	26.13475758
chr14	32072588	32159728	14q12	ARHGAP5	21.89911093	22.83516299	0.959008304	4	4	35.6236703	24.51447192
chr10	72059034	72095313	10q22.1	SPOCK2	42.2782821	44.08559526	0.959004452	4	4	52.6150823	34.49232008
chr9	120388869	120580170	9q33.2	CDK5RAP2	17.74034847	18.49953492	0.958961863	4	4	19.5177669	25.27233601
chr7	22510305	22512050	7p15.3	EEF1A1P6	0.705534537	0.735743022	0.958941527	3	4	60.28538956	78.58614697
chr3	126288121	126357393	3q21.3	KLF15	0.082868594	0.086418809	0.958918502	1	1	0	0
chr4	82274849	82276800	4q21.22	BIN2P1	0.227160087	0.2369201	0.958804623	2	2	24.63537011	36.81453473
chr8	66419587	66428977	8q13.1	RRS1-AS1	0.167341226	0.174535637	0.958779705	3	1	17.99693365	0
chr19	12610918	12631921	19p13.13	ZNF791	8.719234031	9.094837025	0.958701515	4	4	36.42449247	5.928394289
chr8	60678744	60868028	8q12.2	CHD7	6.376691294	6.651702969	0.958655449	4	4	49.99050486	40.56169735
chrX	145817829	145829856	Xq27.3	SLITRK2	0.032374391	0.033770821	0.958649801	1	1	0	0
chr19	52570273	52627269	19q13.41	ZNF701	6.83995241	7.135273076	0.958611161	4	4	44.20970873	28.17291322
chr5	59703606	59703703	5q12.1	MIR582	2.457845864	2.564092337	0.95856371	1	1	0	0
chr1	212364474	212415508	1q32.3	TMEM206	3.174966755	3.312377576	0.958515955	4	4	28.26378964	34.02507695
chr3	20174286	20186115	3p24.3	SGO1-AS1	0.574414668	0.599324632	0.958436609	4	4	54.73835669	20.43784654
chr20	18137855	18143169	20p11.23	PET117	1.9487117	2.033233554	0.958429835	4	4	45.18140272	62.9595224
chr20	61974798	62065810	20q13.33	TAF4	1.700753084	1.774543348	0.958417323	4	4	69.16752981	20.46053064
chr16	4340249	4351399	16p13.3	PAM16	0.108996332	0.113731644	0.95836417	1	2	0	14.68910048
chr19	18058994	18099027	19p13.11	IL12RB1	5.998159433	6.259210185	0.95829334	4	4	35.12164799	56.22195014
chr19	17267376	17279353	19p13.11	BABAM1	8.41148539	8.777881176	0.9582592	4	4	16.21744408	13.82324203
chr2	203433682	203535336	2q33.2	RAPH1	0.224534209	0.23433809	0.95816352	4	4	108.8200425	63.06468409
chr6	107028172	107051586	6q21	C6orf203	5.52580458	5.767332535	0.958121375	4	4	18.9036193	21.04795015
chr10	68414064	68472521	10q21.3	DNA2	77.34879096	80.73826811	0.958018951	4	4	93.76622331	80.42471155

chr14	93237550	93333092	14q32.12	BTBD7	3.884446274	4.054772638	0.957993609	4	4	54.86978892	43.67124652
chr2	120346142	120351808	2q14.2	INHBB	0.236309423	0.246671734	0.957991497	2	3	87.06970094	113.6490228
chr10	91162402	91284331	10q23.32	PCGF5	126.6376617	132.1918711	0.957983729	4	4	29.66032993	49.29730848
chrX	103710909	103714032	Xq22.2	TMEM31	0.301264589	0.314507684	0.957892617	1	1	0	0
chr8	55773232	55826445	8q12.1	TGS1	27.68140987	28.90037408	0.957821853	4	4	26.7401733	13.57861391
chr6	42984499	43012345	6p21.1	PPP2R5D	7.990998557	8.343352613	0.957768289	4	4	51.33667033	50.16215339
chr5	32227007	32313008	5p13.3	MTMR12	16.48678089	17.21529129	0.957682366	4	4	20.61899612	4.549440486
chr2	241675742	241686968	2q37.3	DTYMK	1.409803515	1.472112085	0.957674032	4	4	88.36698258	102.3728596
chr5	43287470	43313512	5p12	HMGCS1	11.90991012	12.43731984	0.957594584	4	4	15.24604907	24.22013169
chr20	31819375	31834697	20q11.21	MYLK2	0.079801099	0.083336001	0.957582535	1	1	0	0
chr19	43727992	43755036	19q13.31	SMG9	1.403432527	1.465611468	0.957574745	4	4	69.05083669	45.29050376
chr3	183919934	184018010	3q27.1	ABCC5	4.890433809	5.107133378	0.957569236	4	4	24.09227455	30.85591262
chr19	34428176	34469893	19q13.11	UBA2	44.3108446	46.27638743	0.95752601	4	4	31.36720215	23.56751
chr1	206572484	206612551	1q32.1	EIF2D	3.252938197	3.397585107	0.957426553	4	4	54.88304192	28.40091418
chr12	105304867	105327017	12q23.3	KCCAT198	1.078909617	1.126894414	0.957418551	2	3	4.320501185	87.62087238
chr19	45883607	45886170	19q13.32	IRF2BP1	5.627457135	5.877839879	0.957402252	4	4	51.88803231	43.5478639
chr16	20623235	20697788	16p12.3	ACSM1	0.359251101	0.375323886	0.957176227	4	3	48.98995127	110.3619071
chr1	215567379	215621821	1q41	KCTD3	2.535697004	2.649487611	0.957051844	4	4	38.54290018	45.37820273
chr16	180334	181179	16p13.3	HBQ1	98.93134556	103.3728594	0.957034043	4	4	44.46176531	42.38858091
chr11	17074653	17074744	11p15.1	SNORD14A	23.03021695	24.06572981	0.956971475	4	4	38.39645686	37.41266194
chr3	52899209	53046655	3p21.1	SFMBT1	4.989287133	5.214145059	0.956875399	4	4	51.09944157	39.0813211
chr11	125624910	125676256	11q24.2	CHEK1	2.857035331	2.985846412	0.956859442	4	4	63.23217877	48.15147301
chr15	75639085	75640323	15q24.2	IMP3	8.39267512	8.771087416	0.956856855	4	4	19.3883341	21.33299062
chr6	44297850	44313358	6p21.1	AARS2	2.749089272	2.873265251	0.956782278	4	4	67.37691184	31.37811077
chr22	37805107	37807436	22q13.1	H1F0	21.69291712	22.67280711	0.956781267	4	4	28.38320461	58.44791455
chr9	131490486	131490571	9q34.13	SNORD62B	8.648041642	9.039131164	0.956733726	2	1	63.91243593	0
chr12	69284217	69285684	12q15	C1GALT1P1	0.245709001	0.256842522	0.956652348	2	2	10.52411443	5.651817543
chr2	130342225	130347961	2q21.1	IMP4	5.502105777	5.751479751	0.956641771	4	4	43.21114756	34.019425
chr19	52031378	52049124	19q13.41	ZNF432	2.72421116	2.847738193	0.956622756	4	4	57.14948101	22.89502094
chr1	90915298	91022267	1p22.2	ZNF644	31.67708488	33.11494659	0.956579676	4	4	18.70667732	15.85492243
chr4	109433715	109540459	4q25	SEC24B	65.32658531	68.29357092	0.956555418	4	4	35.1713581	12.28281049
chr15	40594012	40664342	15q15.1	KNL1	45.75525631	47.83549814	0.956512592	4	4	57.25849553	37.33373326
chr17	44205033	44221626	17q21.31	UBTF	18.07380873	18.89562316	0.956507683	4	4	54.67527982	44.67784211
chr11	115755331	115760200	11q23.3	LINC00900	0.116479022	0.121776728	0.956496559	2	2	78.90137841	73.27050599
chr22	23070349	23125037	22q11.22-q11.23	GNAZ	13.781709	14.40877142	0.956480508	4	4	80.92086065	32.88132731
chr2	74421721	74426259	2p13.1	WDR54	1.126471176	1.177793193	0.956425273	4	4	46.4406311	70.1978782
chr11	125893485	125903278	11q24.2	PUS3	6.042526046	6.318044447	0.956391823	4	4	46.01056461	23.5825513
chr3	186930526	187078553	3q27.3	ST6GAL1	20.70902202	21.65355496	0.956379775	4	4	33.95646392	54.06392535
chr6	27357823	27375374	6p22.1	ZNF204P	1.244584159	1.301384802	0.956353691	4	4	47.01301728	58.33871889
chr9	36214441	36277056	9p13.3	GNE	4.303361673	4.499790444	0.956347129	4	4	36.79587376	6.754356688
chr1	27404226	27490187	1p36.11	WASF2	90.68996061	94.83127976	0.956329608	4	4	14.96399732	22.8220279
chr1	220829255	220832429	1q41	LINC01352	0.230344648	0.240872244	0.956293861	1	1	0	0
chr9	38566260	38620660	9p13.1	ANKRD18A	0.749223211	0.783557357	0.956181706	4	4	65.60343442	44.35628978

chr5	132866627	132868844	5q31.1	UQCRQ	9.320835431	9.749468011	0.956035285	4	4	26.78209058	36.07990045
chr7	98878490	99013243	7q22.1	TRRAP	7.823469306	8.18362449	0.955990749	4	4	50.0793363	28.81506944
chr3	180602130	180618347	3q26.33	TTC14	17.56437557	18.37461629	0.955904346	4	4	36.84148686	13.4914695
chr2	201063026	201064248	2q33.1	HNRNPA1P35	0.229884234	0.240490253	0.955898342	1	1	0	0
chr14	105209286	105315577	14q32.33	BRF1	1.242238173	1.299675816	0.955806177	4	4	58.85218428	39.35961482
chr1	228458107	228458558	1q42.13	HIST3H2BB	12.94529256	13.54392188	0.955800888	4	4	79.23438761	33.72431834
chr10	7295106	7295290	10p14	COX6CP17	3.486632189	3.647932829	0.955783002	2	3	16.60481695	60.74294658
chr2	232547970	232550590	2q37.1	TIGD1	2.837362949	2.968640789	0.955778469	4	4	26.12811832	24.66733167
chr19	55013705	55038264	19q13.42	GP6	1.023580676	1.070940414	0.955777429	4	4	56.53858832	27.2613768
chr4	10439880	10457423	4p16.1	ZNF518B	8.922809013	9.335941653	0.955748155	4	4	53.05896577	30.63457632
chr4	2935546	2951078	4p16.3	NOP14-AS1	1.818986633	1.903298414	0.95570228	4	4	75.39989447	38.00573048
chr1	46538696	46570255	1p33	MKNK1-AS1	0.116638087	0.12204624	0.955687672	2	1	24.45266973	0
chr22	41459717	41468704	22q13.2	PHF5A	23.04834458	24.11952635	0.955588607	4	4	11.08396499	14.06086552
chr12	31382223	31591018	12p11.21	DENND5B	1.512819514	1.583159161	0.955570073	4	4	35.66859407	55.67607109
chr14	106639119	106639556	14q32.33	IGHV4-61	2.591117302	2.711758116	0.955511956	2	1	38.403054	0
chr12	111034024	111350556	12q24.11-q24.12	CUX2	0.240899608	0.252136173	0.955434542	4	4	109.1398111	75.48518261
chr12	12813346	12829981	12p13.1	DDX47	0.600620388	0.628659315	0.955398851	4	4	67.60950164	30.22896862
chr7	44114680	44123668	7p13	POLD2	3.425973275	3.585913236	0.955397705	4	4	69.32998334	57.10313885
chr8	123041961	123165169	8q24.13	TBC1D31	63.91074109	66.89673494	0.955364132	4	4	61.87894116	36.94659783
chr7	91107391	91107788	7q21.13	PTP4A1P3	0.678417412	0.710212367	0.955231764	1	1	0	0
chr1	212856604	212858138	1q32.3	FLVCR1-DT	0.854297034	0.894444515	0.95511462	4	3	45.612213	67.98696774
chr17	73193034	73208507	17q25.1	COG1	3.562157852	3.729898136	0.955028186	4	4	36.01114924	21.25917467
chr19	54444816	54449038	19q13.42	LENG8-AS1	0.178484058	0.186889054	0.955026816	1	1	0	0
chr2	204545793	205620162	2q33.3	PARD3B	0.184876156	0.193589526	0.954990487	4	4	61.99318928	95.51958689
chr8	73294602	73325285	8q21.11	RDH10	1.292821599	1.35376194	0.954984448	4	4	54.73911751	16.93328628
chr3	4303304	4320649	3p26.1	SETMAR	1.601737183	1.677268238	0.954967814	4	4	59.18984497	14.73026545
chr8	56211638	56218798	8q12.1	CHCHD7	5.643259808	5.909758605	0.954905299	4	4	31.64492747	19.39943154
chr17	38752713	38764231	17q12	PSMB3	39.61548926	41.48707684	0.954887456	4	4	33.27627195	25.56361816
chr14	50311521	50326715	14q21.3	DMAC2L	4.64699741	4.867025343	0.954792113	4	4	52.95855294	75.41715482
chr1	30869466	30909735	1p35.2	SDC3	0.551628626	0.577778534	0.954740604	4	4	89.75443249	98.30731775
chr20	57351089	57378463	20q13.31	RAE1	3.899734684	4.084765707	0.95470217	4	4	37.89011677	9.781505238
chr1	110653560	110675033	1p13.3	KCNA3	18.63493335	19.51923187	0.954696039	4	4	57.90050932	28.34232425
chr14	92794231	92839963	14q32.12	GOLGA5	23.10223166	24.20097112	0.954599365	4	4	33.81442624	22.1200006
chr19	3777969	3801812	19p13.3	MATK	7.330514972	7.679251048	0.954587228	4	4	34.72138074	35.53130475
chr9	114402078	114505500	9q32	WHRN	0.319237586	0.33444169	0.954538848	4	4	45.24480873	55.74449938
chr2	28894643	28948220	2p23.2	WDR43	20.4282706	21.40235389	0.954487095	4	4	29.86011661	11.58805877
chr12	104986320	104994727	12q23.3	C12orf45	0.69382824	0.727021148	0.954343957	4	4	31.10478461	31.52609468
chr1	155127873	155134910	1q22	EFNA1	0.110924221	0.116233168	0.954325026	2	1	2.492805046	0
chr7	66996805	67239520	7q11.21	TYW1	9.269847708	9.713754282	0.954301235	4	4	27.20415796	10.36691041
chr1	155308748	155320666	1q22	FDPS	7.705945342	8.075081986	0.954286948	4	4	48.88413641	30.07668712
chr7	141652381	141702188	7q34	KIAA1147	1.005939254	1.054167662	0.954249775	4	4	48.7964271	37.50512497
chr22	19846138	19854848	22q11.21	RTL10	1.718411516	1.800908545	0.954191439	4	4	45.47988283	12.46374072
chr22	41205192	41231271	22q13.2	L3MBTL2	4.016017124	4.209182052	0.954108678	4	4	46.54392107	45.21487127

chr12	27780237	27803040	12p11.22	KLHL42	3.041833093	3.188305176	0.954059579	4	4	46.11881759	41.12927302
chr14	32203610	32833999	14q12	AKAP6	0.083160035	0.087165906	0.954043154	4	3	82.55775094	26.2357259
chr11	108116705	108148822	11q22.3	ACAT1	5.054672283	5.298225394	0.954031191	4	4	12.93451876	22.51606532
chr6	42879163	42889896	6p21.1	RPL7L1	11.82238146	12.3931967	0.953941243	4	4	49.42572893	11.8857215
chr10	121957088	121975228	10q26.13	NSMCE4A	5.867546037	6.150968355	0.953922325	4	4	27.75643883	10.33860558
chr4	189940819	189963204	4q35.2	FRG1	25.47397611	26.70448703	0.953921192	4	4	44.54133192	39.64914298
chr11	17788048	18013162	11p15.1	SERGEF	5.666852902	5.94071648	0.953900581	4	4	18.39883242	18.44023397
chr11	18261879	18263501	11p15.1	ST13P5	1.76034536	1.845530087	0.953842678	4	4	40.16500159	51.19379202
chr7	99416739	99438839	7q22.1	PTCD1	0.471080017	0.493880444	0.953834116	4	4	56.50650755	14.9538557
chr19	43575399	43582104	19q13.31	PINLYP	0.185112333	0.194099758	0.95369688	2	2	1.492258849	13.80450802
chr17	37084992	37406822	17q12	ACACA	1.39281911	1.461679026	0.952889852	4	4	37.26195538	15.71583689
chr4	109560199	109688719	4q25	MCUB	24.23924078	25.43876825	0.95284648	4	4	34.49193979	16.79949367
chr1	231528653	232041272	1q42.2	TSNAX-DISC1	0.123794599	0.129922367	0.952835152	2	3	68.62457941	44.85007527
chr9	3668086	3691756	9p24.2	RFX3-AS1	9.798950271	10.28494697	0.952746796	4	4	45.14587343	42.70150377
chr2	64988445	65023865	2p14	SLC1A4	2.586193164	2.714522717	0.952724819	4	4	36.95561145	65.93846561
chr9	122918029	122931551	9q33.2	ZBTB26	2.502377874	2.626570712	0.952716735	4	4	46.85378088	21.61934321
chr9	120855651	120902160	9q33.2	PHF19	3.357245398	3.524115818	0.952648996	4	4	13.55757403	33.73296521
chr5	169583634	169604778	5q35.1	SPDL1	3.773998108	3.961742481	0.952610657	4	4	40.149507	19.31126276
chr14	51989510	52004702	14q22.1	RTRAF	12.1854401	12.792074	0.952577362	4	4	62.44815785	46.9074895
chrX	153861514	153886174	Xq28	L1CAM	0.081310954	0.085362577	0.952536298	3	4	54.46393829	92.44967345
chr12	24810024	24949459	12p12.1	BCAT1	3.55330278	3.730608306	0.952472757	4	4	65.92420084	46.50975311
chr3	105655461	105869552	3q13.11	CBLB	8.902480209	9.34671422	0.952471639	4	4	45.47149353	46.78220007
chr16	85801	138698	16p13.3	NPRL3	56.83518287	59.67262424	0.952449864	4	4	80.65193464	58.30021822
chrX	18915243	18915535	Xp22.13	RN7SL48P	0.728932251	0.765369239	0.952392928	1	1	0	0
chr3	129440036	129520507	3q21.3-q22.1	IFT122	6.208064636	6.518492364	0.952377373	4	4	33.25933097	21.86209689
chr16	68310951	68360876	16q22.1	PRMT7	1.262303534	1.325504905	0.952319022	4	4	65.24870715	24.96703645
chr17	75633434	75641406	17q25.1	SMIM5	6.304359128	6.621189866	0.952148973	4	4	33.44980507	18.42741166
chr19	40292743	40348527	19q13.2	C19orf47	1.261028269	1.324410171	0.952143299	4	4	82.20091391	38.34459254
chr17	16840787	16845872	17p11.2	KRT17P1	0.186254304	0.195624246	0.952102344	1	1	0	0
chrX	153947556	153972360	Xq28	HCFC1	10.79859709	11.34224362	0.952068873	4	4	65.87817828	81.60906718
chr22	38578120	38656629	22q13.1	FAM227A	0.047366009	0.049752614	0.952030566	1	2	0	5.018968649
chr1	110407809	110416274	1p13.3	LAMTOR5-AS1	0.854657958	0.897792686	0.95195469	4	4	29.42741979	20.29363589
chr20	44195939	44217229	20q13.12	OSER1	41.77123255	43.88107915	0.95191899	4	4	39.56448175	40.40556488
chr3	151435051	151619926	3q25.1	IGSF10	0.042951058	0.045121208	0.951903983	1	2	0	1.97242213
chr14	22829862	22835037	14q11.2	MRPL52	1.946091594	2.044619729	0.951811022	4	4	64.10828291	17.63451843
chr5	75674198	75717488	5q13.3	POC5	6.486351963	6.814962189	0.951781064	4	4	21.64561191	20.57943691
chr16	89093809	89160556	16q24.3	ACSF3	2.623320539	2.756840142	0.951567883	4	4	49.38535809	39.1166323
chr15	65117379	65133836	15q22.31	PDCD7	7.454254645	7.834147601	0.951508068	4	4	39.04621076	25.71275453
chr8	17027238	17133069	8p22	MICU3	0.492106486	0.517228013	0.95143046	4	4	22.1066803	26.14356137
chr14	104661120	104665558	14q32.33	LINC02280	0.234556504	0.246547541	0.951364198	2	4	93.30681061	55.40584079
chr20	13104760	13638936	20p12.1	TASP1	20.67088529	21.72833192	0.951333281	4	4	73.43291395	49.68519513
chr17	45023338	45061129	17q21.31	DCAKD	3.103671249	3.262451241	0.951331076	4	4	50.67745285	26.91192417
chr16	21402129	21425378	16p12.2	NPIPB3	0.572225564	0.601506981	0.951319905	4	4	67.67291999	55.82594285

chr9	34613545	34620523	9p13.3	DCTN3	4.015921568	4.221464543	0.951310032	4	4	55.2245351	6.586492964
chr17	76038775	76072526	17q25.1	SRP68	3.996904435	4.201610412	0.951279163	4	4	23.08837013	18.05551445
chr7	92560758	92590394	7q21.2	FAM133B	28.30797796	29.75787298	0.951276927	4	4	17.03382965	20.97462311
chr15	72407779	72474239	15q24.1	TMEM202-AS1	0.767598775	0.806987539	0.951190369	4	4	62.28497366	19.49058328
chr16	3222325	3236221	16p13.3	ZNF200	5.244249864	5.51354493	0.951157546	4	4	46.04473129	52.00998694
chr11	116778189	116789272	11q23.3	ZPR1	5.113626022	5.376332908	0.951136418	4	4	55.52868211	11.80147536
chrX	48521776	48528716	Xp11.23	EBP	4.719822452	4.962506241	0.951096527	4	4	38.39940686	29.94147394
chr7	8113184	8262687	7p21.3	ICA1	2.30548111	2.424037543	0.951091338	4	4	11.68725891	22.59917235
chr1	228203506	228213664	1q42.13	OBSCN-AS1	0.295394375	0.310652145	0.950884711	2	4	65.59674134	57.41300788
chr9	129080988	129090438	9q34.11	DOLPP1	2.930972866	3.082844682	0.950736468	4	4	52.15577138	40.14521364
chr13	76991924	77002517	13q22.3	CLN5	0.781593001	0.822116482	0.950708347	4	4	45.78041583	46.41133741
chr2	88556740	88627576	2p11.2	EIF2AK3	8.217042885	8.643375979	0.950675165	4	4	28.21192151	20.34808819
chr3	40457292	40462372	3p22.1	RPL14	33.7260005	35.47693423	0.950645856	4	4	33.85160217	22.85398897
chr3	71289758	71305854	3p13	FOXP1-AS1	0.31340676	0.329686522	0.950620481	1	1	0	0
chr9	37120166	37358149	9p13.2	ZCCHC7	95.02720106	99.96403756	0.950613875	4	4	66.61018132	47.49433099
chr8	142651501	142669994	8q24.3	JRK	1.430105392	1.504442381	0.950588344	4	4	55.48798904	32.02885474
chr7	16646134	16706523	7p21.1	BZW2	7.644555703	8.042045996	0.950573487	4	4	31.59244617	19.38011585
chr20	3162617	3173597	20p13	LZTS3	0.120450647	0.126713664	0.950573463	3	4	89.12732626	61.72796819
chr1	161165824	161178277	1q23.3	PPOX	2.916071875	3.06772144	0.950566058	4	4	56.78631893	44.33444743
chr1	6634168	6701906	1p36.31	DNAJC11	4.926517488	5.183204553	0.950477149	4	4	39.54208718	21.57053585
chr7	77039480	77053038	7q11.23	PMS2P9	1.634627965	1.719829662	0.950459223	3	4	77.26359084	28.03340666
chr10	73731696	73735340	10q22.2	DUSP8P5	0.81138958	0.853761975	0.95036978	3	3	91.28473195	15.19717092
chr7	151028422	151047782	7q36.1	ABCB8	0.919694485	0.96777154	0.950321896	4	4	58.51425577	34.0164755
chr10	78033760	78056813	10q22.3	RPS24	295.1438888	310.5796501	0.950300153	4	4	14.48292708	29.49734298
chr7	91940867	92112908	7q21.2	AKAP9	29.72586799	31.28072236	0.950293527	4	4	13.45016044	20.91399402
chr17	48892786	48895871	17q21.32	ATP5MC1	2.590734056	2.726467471	0.950216382	4	4	54.82713007	12.69833688
chr10	52450877	52470533	10q21.1	LNCAROD	0.865242538	0.910577028	0.950213449	2	2	64.85793408	9.023866526
chr2	74909131	74938418	2p12	LINC01291	0.058434645	0.061496946	0.950204007	1	1	0	0
chr19	55613593	55618545	19q13.42	ZNF865	1.2269986	1.291337319	0.950176676	4	4	88.33108163	41.06060309
chr14	106324254	106324688	14q32.33	IGHV4-28	0.511614942	0.538525669	0.950028886	1	1	0	0
chr1	52895910	52927212	1p32.3	ECHDC2	0.530262355	0.558214209	0.949926295	4	4	39.59060902	35.01943594
chr1	111414315	111427777	1p13.2	OVGP1	0.380434128	0.400489011	0.94992401	4	4	87.58614258	60.9321436
chr7	76302558	76304301	7q11.23	HSPB1	15.44528438	16.25981035	0.949905568	4	4	44.80635148	39.38559665
chr19	47581023	47581321	19q13.33	RN7SL322P	0.714362177	0.752038216	0.94990143	1	1	0	0
chr5	154038959	154057454	5q33.2	MFAP3	12.60862559	13.27400349	0.949873608	4	4	16.62395049	39.0502003
chr5	132190147	132227863	5q31.1	P4HA2	0.198457806	0.208932074	0.949867593	4	3	89.31751764	35.09261761
chr15	88467453	88480767	15q25.3	MRPS11	2.189646358	2.30543059	0.949777611	4	4	69.88836273	34.40526966
chr5	56099680	56233359	5q11.2	ANKRD55	1.271157621	1.33851046	0.949680753	4	4	32.60206402	50.846146
chr9	101473158	101489195	9q31.1	TMEM246	0.238216838	0.250850191	0.949637855	4	3	74.3819492	75.99723673
chr8	74984473	75034558	8q21.13	CRISPLD1	0.053490182	0.056326975	0.949637047	1	1	0	0
chr5	113022099	113488830	5q22.2	MCC	0.441873627	0.465361312	0.949528068	4	4	71.65277312	98.91703571
chr2	18554723	18560693	2p24.2	RDH14	6.10582115	6.431270476	0.949395796	4	4	38.6246468	24.11271156
chr22	22880912	22881392	22q11.22	IGLV3-1	11.8167794	12.44822156	0.949274508	4	4	87.311967	143.8195005

chr8	73945138	73972287	8q21.11	ELOC	6.130600666	6.458687027	0.949202313	4	4	5.568792032	16.38936719
chr19	52904790	52929161	19q13.41	ZNF888	4.11753811	4.338215006	0.949131867	4	4	14.59105179	4.593215925
chr7	99923269	99929620	7q22.1	GJC3	0.198638447	0.209288133	0.949114719	1	1	0	0
chr17	32350138	32381885	17q11.2	ZNF207	16.84788622	17.75186491	0.949076973	4	4	19.22108511	16.39045175
chr1	10636604	10796676	1p36.22	CASZ1	1.36354737	1.436790872	0.949022852	4	4	57.71277344	48.23548932
chr19	57633167	57644046	19q13.43	ZNF211	10.92068042	11.50808751	0.948957019	4	4	33.08350574	17.3495222
chr20	63195429	63216186	20q13.33	YTHDF1	13.18663949	13.89634959	0.948928308	4	4	54.36933235	20.68049235
chr2	53852913	53860033	2p16.2	GPR75	0.96697627	1.019023252	0.948924637	3	4	60.8550074	11.3547529
chr9	133061978	133071863	9q34.13	CEL	0.392239473	0.413353662	0.948919799	2	3	54.74904172	48.39068473
chr5	179614178	179634784	5q35.3	HNRNPH1	23.74922997	25.02887393	0.948873291	4	4	63.37417991	45.42807782
chr14	96392088	96489427	14q32.2	AK7	0.786984266	0.829391065	0.948869959	4	4	65.0023564	47.74793915
chr9	136848816	136851043	9q34.3	PHPT1	2.611639219	2.752403419	0.948857715	4	4	68.51843885	27.72950417
chr4	145619388	145660035	4q31.21	MMAA	1.454812649	1.533331475	0.948792008	4	4	43.71150351	19.21440506
chr8	124998478	125022283	8q24.13	SQLE	4.100270574	4.321652703	0.948773734	4	4	18.74150358	11.33709322
chr15	40039311	40067509	15q15.1	SRP14-AS1	0.565234512	0.5957804	0.948729619	4	4	52.05620694	27.69890579
chr20	44619519	44651758	20q13.12	ADA	4.408954892	4.647508691	0.948670607	4	4	54.52439612	64.09875255
chr15	42575610	42720998	15q15.2	STARD9	0.459771906	0.484661409	0.948645585	4	4	52.90526024	31.80662606
chr8	124450807	124453026	8q24.13	TRMT12	6.352068529	6.696580465	0.948554051	4	4	42.84044275	19.97721855
chr19	896503	913225	19p13.3	R3HDM4	512.0058608	539.7754716	0.948553404	4	4	17.53874761	25.55929289
chr15	40405485	40435948	15q15.1	IVD	2.028563802	2.138745545	0.948483005	4	4	51.36307584	46.00470833
chr1	204422630	204494815	1q32.1	PIK3C2B	1.460578079	1.540203991	0.94830171	4	4	56.57083532	66.4390675
chr20	59146073	59259113	20q13.32	ZNF831	6.587147343	6.946596466	0.948255362	4	4	32.56699981	26.67245609
chr19	37696363	37720165	19q13.12	ZNF607	1.081392908	1.140465909	0.948202748	4	4	53.88136595	79.46604471
chr12	4487730	4538508	12p13.32	C12orf4	10.38702592	10.95443591	0.94820272	4	4	47.04587194	24.33625024
chr7	128081861	128081960	7q32.1	MIR593	3.740442586	3.945069704	0.948130925	1	1	0	0
chr17	35871491	35880373	17q12	CCL5	163.3205009	172.267943	0.948060899	4	4	41.47131988	93.11319343
chr19	11798576	11809622	19p13.2	ZNF491	0.417411845	0.440309265	0.947996961	4	4	62.89331891	43.19558474
chr8	28316979	28343355	8p21.1	PNOC	0.757191468	0.798783671	0.947930579	3	4	93.84184457	72.88851339
chr3	9890587	9894349	3p25.3	JAGN1	7.086301428	7.476958766	0.94775184	4	4	26.94486318	26.46520241
chr1	221701420	221742176	1q41	DUSP10	5.372399895	5.668783634	0.94771652	4	4	41.40132214	41.24731342
chr4	24563193	24563461	4p15.2	RN7SL16P	1.919125681	2.025057599	0.94768943	3	3	42.21285883	75.7161415
chr10	5275206	5285925	10p15.1	AKR1C7P	1.262703576	1.332444023	0.947659755	4	3	60.86924731	28.80106444
chr14	44962190	45074431	14q21.2	TOGARAM1	10.92866231	11.53244442	0.947644915	4	4	57.84848225	27.30539651
chr5	175478498	175529742	5q35.2	SFXN1	6.355753695	6.707907896	0.947501634	4	4	25.10579452	18.40280665
chr1	108875350	108934335	1p13.3	GPSM2	14.23574719	15.02471469	0.947488687	4	4	50.24409028	36.75294613
chr12	14770720	14771131	12p12.3	HIST4H4	21.74016118	22.94629094	0.947436831	4	4	14.77193739	35.1279489
chr22	38656653	38673850	22q13.1	CBY1	1.490795456	1.573586565	0.947387001	4	4	56.4821733	42.1920523
chr2	42048021	42058528	2p21	PKDCC	0.366737584	0.387136134	0.947309104	3	4	87.77325094	101.0185339
chr9	132670035	132694955	9q34.13	GTF3C4	3.361151129	3.548166046	0.947292513	4	4	40.08899734	9.942289292
chr12	57591159	57603428	12q13.3	PIP4K2C	6.065623196	6.403252622	0.9472722	4	4	34.00520951	36.58832214
chr9	33240164	33248567	9p13.3	SPINK4	0.653767626	0.690169641	0.947256425	1	1	0	0
chr19	617221	633568	19p13.3	POLRMT	2.408996889	2.543167254	0.947242807	4	4	66.14752333	63.46032667
chr2	27307400	27308445	2p23.3	UCN	0.609536771	0.643541161	0.947160504	2	1	44.17002026	0

chr19	50723348	50725724	19q13.33	CLEC11A	0.699622613	0.738714813	0.947080796	4	4	136.6375796	71.71031653
chr19	12875211	12881521	19p13.13	DNASE2	13.13196945	13.87185593	0.946662762	4	4	23.86192696	32.42887222
chr2	101002229	101019693	2q11.2	RPL31	95.13580925	100.497893	0.946644814	4	4	51.92470802	39.43007602
chr19	9565657	9584544	19p13.2	ZNF121	8.956315975	9.461413552	0.946614998	4	4	28.513316	13.47353168
chr1	155255979	155262385	1q22	SCAMP3	5.282121205	5.580591712	0.946516333	4	4	42.08446037	67.9695539
chr1	159204013	159206500	1q23.2	ACKR1	6.0387152	6.380064086	0.946497577	4	4	100.0015735	62.72487699
chr11	126269040	126278132	11q24.2	FOXRED1	1.038847536	1.097922508	0.946193861	4	4	61.73545191	42.64396467
chr8	56068180	56074581	8q12.1	RPS20	313.6912097	331.5430073	0.946155409	4	4	64.73727694	45.40777436
chrX	124375903	125204381	Xq25	TENM1	0.956651058	1.011104832	0.946144285	4	4	62.4654633	56.12103306
chr3	184710367	184712048	3q27.1	MAGEF1	4.642999816	4.90807719	0.945991604	4	4	52.36798254	60.59401668
chr5	141199775	141203045	5q31.3	PCDHB11	0.057882449	0.061189064	0.94596069	1	1	0	0
chr1	46303613	46316777	1p33	UQCRH	52.25780758	55.24551857	0.945919396	4	4	16.09042445	32.62740217
chr2	43227211	43228855	2p21	LINC01126	1.586790929	1.677564274	0.945889796	4	4	64.8119484	30.32874709
chr8	80558794	80559827	8q21.13	RPSAP47	1.527580641	1.615142952	0.945786649	3	3	101.2143556	71.03254867
chr14	66507407	67735831	14q23.3-q24.1	GPHN	1.182308631	1.250136336	0.945743754	4	4	33.62630952	24.3636855
chr15	35237326	35238063	15q14	ANP32AP1	0.205511075	0.21732117	0.94565603	1	1	0	0
chr12	10158300	10172191	12p13.2	OLR1	1.165087749	1.232089177	0.94561966	4	4	30.42771181	81.92574691
chr20	44910060	44959160	20q13.12	PABPC1L	1.17127062	1.238628737	0.9456188	4	4	53.56191574	57.51355335
chr11	65916810	65919060	11q13.1	C11orf68	8.671472196	9.171285124	0.945502411	4	4	30.17600019	45.09810836
chr15	101281510	101295282	15q26.3	SNRPA1	4.897477411	5.180668387	0.945336981	4	4	35.54991095	5.666520166
chr3	139584206	139584445	3q23	RN7SKP124	0.846539387	0.89552424	0.94530036	2	2	36.61632501	15.97109817
chr15	82262811	82284930	15q25.2	SAXO2	0.114620848	0.12125606	0.945279337	2	2	56.86841245	6.422879853
chr11	43311955	43344530	11p12	API5	39.4462351	41.73014153	0.945269622	4	4	24.21884177	14.36658313
chr17	6853576	6900349	17p13.1	ALOX12P2	0.161519638	0.170873695	0.945257475	3	4	6.235905621	36.52107759
chr14	95652097	95673452	14q32.13	TCL6	0.18227249	0.192857562	0.945114563	4	4	88.84261401	39.5467722
chr20	21755230	21755652	20p11.22	RPL41P1	706.4702203	747.5912911	0.944995252	4	4	35.2318362	22.24424869
chr9	37779714	37785092	9p13.2	EXOSC3	7.793260474	8.24735875	0.944940157	4	4	16.38358744	20.30798964
chr14	21351472	21384266	14q11.2	SUPT16H	26.35130943	27.89225634	0.944753594	4	4	35.03032402	38.00877326
chr11	93661650	93730381	11q21	CEP295	20.79432257	22.01076436	0.944734232	4	4	18.83344012	10.12554352
chr2	219206784	219209651	2q35	ZFAND2B	6.177641754	6.539120922	0.944720526	4	4	42.60919109	27.20389501
chr10	77975149	78029540	10q22.3	POLR3A	2.587077761	2.738975649	0.944542082	4	4	44.37673622	18.10118002
chr21	25607550	25717562	21q21.3	JAM2	0.086108004	0.09117077	0.944469421	3	2	65.88227716	66.39124207
chr3	181711924	181714436	3q26.33	SOX2	0.345998476	0.36634704	0.944455497	2	1	79.20862792	0
chr10	58269102	58289259	10q21.1	CISD1	4.578012497	4.847702408	0.944367478	4	4	63.45895435	60.97375544
chr8	20246165	20303981	8p21.3	LZTS1	0.118062608	0.12504144	0.944187848	3	3	55.33409902	43.10705795
chrX	16844650	16870411	Xp22.2	RBBP7	10.63389223	11.26427408	0.944037063	4	4	23.384733	15.9701587
chr20	48921721	49036693	20q13.13	ARFGEF2	14.01762851	14.84954483	0.943976982	4	4	16.54703153	7.007077639
chr10	102120634	102150333	10q24.32	PPRC1	2.676445038	2.835418075	0.943933123	4	4	30.8315864	38.50249933
chr16	15949577	16143074	16p13.11	ABCC1	5.985192981	6.340996178	0.943888438	4	4	36.27349506	32.00042198
chr3	151197754	151203674	3q25.1	GPR171	24.0394441	25.46975102	0.943842917	4	4	42.6563228	65.83963935
chr6	111483472	111602295	6q21	TRAF3IP2-AS1	1.075130159	1.139136386	0.943811621	4	4	22.80423792	44.4068646
chr1	205142497	205211599	1q32.1	DSTYK	5.669177123	6.007395789	0.94369962	4	4	17.54049947	28.00796825
chr22	43955381	43996532	22q13.31	SAMM50	3.423596201	3.62820675	0.943605598	4	4	46.03957673	18.04109322

chr19	37007815	37130367	19q13.12	ZNF420	4.838802166	5.12820088	0.943567204	4	4	36.05343648	19.09043089
chr13	75525214	75549439	13q22.2	COMMD6	25.93604418	27.4890354	0.943505067	4	4	45.94830137	26.14632634
chr7	154943690	155003419	7q36.2	PAXIP1	2.823116413	2.992383978	0.943433875	4	4	46.67899717	37.79941598
chr6	39104063	39115186	6p21.2	SAYSD1	3.894280296	4.12792785	0.943398344	4	4	34.62152453	19.6415933
chr9	63817572	63818501	9q13	DUX4L50	0.941113509	0.997673535	0.943308082	3	3	53.48348158	63.87221961
chr19	4247070	4269093	19p13.3	YJU2	13.11400369	13.90231728	0.943296245	4	4	54.05917454	42.86245837
chr15	64387804	64455303	15q22.31	TRIP4	7.80210171	8.271172076	0.943288525	4	4	42.65716919	13.75726462
chr7	100107070	100127208	7q22.1	TAF6	4.202269185	4.455094259	0.943250342	4	4	79.5846818	59.39201846
chr11	202924	207422	11p15.5	BET1L	6.950251057	7.368722597	0.943209758	4	4	45.86786864	11.04986674
chr9	6011019	6015660	9p24.1	RANBP6	16.8957715	17.9132967	0.943197212	4	4	15.57444918	12.45649892
chr20	31567587	31573263	20q11.21	HM13-AS1	0.88589933	0.939323204	0.943125141	2	3	27.36612068	74.71985505
chr7	99558422	99576453	7q22.1	ZNF655	13.04054275	13.82722452	0.943106314	4	4	16.12417128	9.489771046
chr11	34105564	34146911	11p13	NAT10	6.696068977	7.100196457	0.943082212	4	4	40.55081754	25.56613955
chr6	43014103	43021298	6p21.1	KLHDC3	20.81372752	22.07012678	0.943072404	4	4	52.17617597	25.08249016
chr12	31020763	31073847	12p11.21	DDX11-AS1	0.094679583	0.100405191	0.942974979	2	4	23.64684485	4.060868828
chr19	14514764	14530597	19p13.12	DNAJB1	35.96532846	38.14479962	0.942863216	4	4	61.882733	32.02912338
chr1	117060303	117107453	1p13.1	TTF2	1.807233319	1.9168142	0.942831767	4	4	41.81147334	45.31999501
chr3	51538683	51668660	3p21.2	RAD54L2	5.072174353	5.380082974	0.942768797	4	4	33.61348978	5.31074201
chr7	33094797	33109390	7p14.3	RP9	2.427235452	2.574676799	0.942734037	4	4	62.0871577	38.47228176
chr17	43778391	43784842	17q21.31	CFAP97D1	0.119020588	0.126281121	0.942505002	3	4	69.67676363	33.25387749
chr3	32238679	32370325	3p22.3	CMTM8	1.215708392	1.289909201	0.942475944	4	4	85.37762256	75.46515063
chr17	16988329	16990176	17p11.2	LINC02090	0.856730532	0.909072378	0.942422796	1	2	0	52.52285064
chr2	201451669	201480851	2q33.1	STRADB	800.7141843	849.6583246	0.942395503	4	4	41.68930205	48.22074495
chr7	142619057	142619532	7q34	TRBV19	4.985466915	5.290271152	0.942384005	3	4	54.60750556	26.37078059
chr2	199928911	199955736	2q33.1	TYW5	1.688957139	1.792240929	0.942371704	4	4	30.53179111	18.97916618
chr2	27381199	27409683	2p23.3	PPM1G	21.44746399	22.76015931	0.942324863	4	4	62.22092102	67.14676638
chr19	45093173	45147286	19q13.32	PPP1R37	0.740351748	0.78567563	0.942312221	4	4	77.5191137	41.238073
chr9	470291	746106	9p24.3	KANK1	0.439676815	0.466686229	0.94212511	4	4	64.31377506	17.26791675
chr17	47831627	47837713	17q21.32	LRRC46	0.169812936	0.180252673	0.942082763	3	1	21.96161099	0
chr15	32641613	32697098	15q13.3	SCG5	0.121384371	0.128856806	0.942009773	3	2	10.22230041	10.38729237
chr13	99606664	99909459	13q32.3	CLYBL	0.450692941	0.478474562	0.941937099	4	4	46.27426898	29.9019644
chr17	759309	789830	17p13.3	GLOD4	5.204882141	5.525870738	0.941911671	4	4	29.84856627	17.08861114
chrX	150980508	150990775	Xq28	HMGB3	1.214933073	1.289882895	0.941894089	4	4	83.55265805	113.5935625
chr9	35085496	35096601	9p13.3	PIGO	1.387568758	1.473225613	0.941857612	4	4	66.07197096	13.26665475
chr11	95832880	95924207	11q21	MTMR2	5.469514688	5.807634805	0.941780066	4	4	26.78657614	22.78485136
chr12	56118220	56122497	12q13.2	ZC3H10	1.510983589	1.604435042	0.941754293	4	4	84.35051895	11.79718156
chr5	141370264	141512979	5q31.3	PCDHGB3	0.113119717	0.120116029	0.94175372	3	1	22.20160327	0
chr19	37217243	37244935	19q13.12	ZNF383	2.180140361	2.315107196	0.941701691	4	4	21.0528313	17.24166142
chr17	63695889	63701227	17q23.3	LIMD2	44.03383805	46.76194561	0.941659665	4	4	62.37929398	28.51481097
chr4	337779	384864	4p16.3	ZNF141	9.320162893	9.897652154	0.941653914	4	4	37.79375731	15.99601961
chr14	24209581	24213830	14q12	CHMP4A	1.563364184	1.660390777	0.941564001	4	4	65.17644327	42.02439272
chr17	44011188	44026046	17q21.31	TMEM101	3.554065355	3.774870576	0.941506545	4	4	46.82399214	30.6359985
chr12	133130628	133159465	12q24.33	ZNF10	1.241371927	1.318759759	0.941317718	4	4	22.00398674	35.97038626

chr6	78867486	78979411	6q14.1	IRAK1BP1	1.187130663	1.261158901	0.941301419	4	4	20.33967088	35.65303769
chr5	131423921	131635236	5q31.1	RAPGEF6	5.022623752	5.336575471	0.941169815	4	4	26.68127417	19.80000425
chr3	62318973	62336213	3p14.2	C3orf14	0.396731453	0.421531517	0.94116676	4	4	24.50888382	35.10812955
chr19	40778219	40796944	19q13.2	RAB4B	1.737170156	1.84596061	0.941065669	4	4	73.40788513	46.87421355
chr15	77420651	77519899	15q24.3	HMG20A	7.575472961	8.049932143	0.941060474	4	4	22.24976868	14.94283726
chr20	25673195	25696904	20p11.21	ZNF337	5.616564614	5.968535103	0.941028999	4	4	49.61520132	38.67247141
chr19	1241750	1244825	19p13.3	ATP5F1D	4.852160158	5.156618492	0.940957755	4	4	62.13851768	51.96668517
chr15	38040393	38062340	15q14	LINC02345	0.295768863	0.314330181	0.940949617	4	3	68.57795318	67.26456159
chrX	57112142	57121905	Xp11.21	SPIN2B	1.401620452	1.489588713	0.940944598	4	4	52.53650456	85.47192
chr6	108986437	109094505	6q21	SESN1	6.442062846	6.846698112	0.940900671	4	4	39.61585231	25.81018654
chr17	42579508	42609427	17q21.2	RETREG3	20.3924858	21.67631135	0.940772878	4	4	27.82463459	8.445880984
chr11	36510366	36579762	11p12	RAG1	0.11527083	0.122528283	0.940769158	4	3	70.01840734	63.3278463
chr11	125569216	125584689	11q24.2	EI24	5.556247473	5.907348689	0.940565348	4	4	27.48220971	16.14901652
chr19	2754714	2783356	19p13.3	SGTA	6.81996239	7.251792905	0.940451896	4	4	65.79788044	60.60314282
chrX	47974851	48003995	Xp11.23	ZNF182	5.151231408	5.47774415	0.940392845	4	4	53.99006834	25.00151289
chr8	8236003	8244667	8p23.1	ALG1L13P	12.48829679	13.28273792	0.940189957	4	4	61.37836502	52.63595055
chr20	63657810	63698698	20q13.33	RTEL1-TNFRSF6B	0.072046535	0.076631062	0.940174044	4	2	61.14615037	41.6814509
chr3	15070069	15099163	3p25.1	RBSN	4.422004092	4.703555955	0.940140637	4	4	26.25526548	13.55985485
chr19	35748361	35754519	19q13.12	LIN37	0.978128654	1.040479009	0.940075336	4	4	73.64739237	47.23781228
chrX	136213220	136256482	Xq26.3	MAP7D3	3.686039455	3.921228069	0.940021695	4	4	42.58527869	38.95672385
chr2	158456880	158681429	2q24.1	PKP4	4.910595177	5.22415383	0.939979054	4	4	33.04428784	18.15445491
chr8	143816344	143830041	8q24.3	PUF60	4.428177791	4.711797571	0.939806459	4	4	63.20204909	45.36522476
chr3	67360460	67654614	3p14.1	SUCLG2	30.30753662	32.24888727	0.939801003	4	4	33.19248368	14.1565168
chr3	141763124	141818490	3q23	GRK7	0.074694046	0.079482437	0.93975535	1	1	0	0
chr3	61561569	62297609	3p14.2	PTPRG	0.066479989	0.070745202	0.939710213	3	3	70.33811185	64.80267936
chr19	37181579	37210549	19q13.12	ZNF585B	1.90807788	2.031292437	0.939341793	4	4	59.48797646	53.39069586
chr15	50244628	50244931	15q21.2	RN7SL494P	1.23469127	1.314435934	0.939331647	3	2	36.29160797	32.9699123
chr2	38743599	38751494	2p22.1	SRSF7	24.3905336	25.96661764	0.939303453	4	4	24.44432954	14.48087157
chr3	50347487	50351055	3p21.31	NPRL2	3.725059333	3.966126506	0.939218486	4	4	54.35124292	44.89684479
chr11	47572197	47579015	11p11.2	KBTBD4	6.994703623	7.447881173	0.939153493	4	4	39.26257139	22.60717949
chr16	589357	629273	16p13.3	RAB40C	2.213750344	2.357392291	0.93906744	4	4	71.01617808	40.81759007
chr17	27756766	27800529	17q11.2	NOS2	0.058443419	0.062237803	0.939034096	1	1	0	0
chr2	31234014	31269451	2p23.1	EHD3	5.69829839	6.06841214	0.939009787	4	4	61.93086277	12.92556729
chr4	139301467	139390781	4q31.1	NAA15	12.7085042	13.53437628	0.938979672	4	4	30.68636109	11.56224417
chr10	49954212	49954415	10q11.23	SNORA74C-2	1.2392859	1.319950983	0.938887819	1	2	0	8.568162702
chr12	49558294	49568233	12q13.12	MCRS1	7.373668201	7.853742201	0.938873216	4	4	43.37606348	9.623940824
chr12	12927726	13022498	12p13.1	GPRC5D-AS1	0.299246643	0.318754859	0.938798687	4	4	79.56749207	51.09988493
chr12	88049013	88142216	12q21.32	CEP290	9.012335281	9.600990165	0.938688107	4	4	14.41698219	8.14763383
chr19	7515286	7521025	19p13.2	ZNF358	1.085985782	1.157145931	0.938503739	4	4	91.50078623	46.68144716
chr12	389350	442642	12p13.33	CCDC77	16.33226897	17.40279966	0.938485145	4	4	44.64744637	20.35440679
chr1	26472411	26476642	1p36.11	HMGN2	41.43730943	44.15364087	0.938480012	4	4	27.52205995	22.68198805
chr17	81509971	81512866	17q25.3	ACTG1	429.0655927	457.193638	0.938476735	4	4	38.39110378	50.8751413
chr13	99499723	99501063	13q32.3	LINC00449	0.41861889	0.446083275	0.938432158	1	4	0	29.41533124

chr12	120534329	120577594	12q24.31	RNF10	280.9905626	299.4421929	0.938379992	4	4	31.41345745	31.07336513
chr16	30503919	30504001	16p11.2	MIR4518	7.089952731	7.55589332	0.938334149	3	3	45.92155498	23.98418095
chr14	75958061	75983011	14q24.3	TGFB3	0.231941593	0.247192296	0.938304297	3	4	66.08134013	62.93588974
chr11	44066118	44084237	11p11.2	ACCS	0.779688659	0.831010621	0.938241509	4	4	43.74671224	74.84335119
chr2	200510198	200584095	2q33.1	SGO2	5.133398806	5.471338953	0.938234471	4	4	38.15724303	30.33277058
chr11	117201156	117232525	11q23.3	PCSK7	3.06490162	3.266796249	0.938197973	4	4	63.77623994	20.38213903
chr2	85889280	85892252	2p11.2	ST3GAL5-AS1	1.610782871	1.717052318	0.938109371	3	4	92.50043548	42.869313
chrX	14834158	14835417	Xp22.2	NPM1P9	0.410411456	0.437555807	0.937963684	3	3	18.95597727	40.21881739
chr19	13206442	13506460	19p13.13	CACNA1A	0.023628153	0.025194651	0.937824166	4	4	47.50679421	94.82911767
chr17	36544888	36589848	17q12	GGNBP2	6.406568883	6.832186968	0.937703976	4	4	48.01965283	22.91791979
chr3	46916310	46982083	3p21.31	CCDC12	4.151101797	4.426930678	0.937692975	4	4	66.47865791	34.25220482
chr20	59933764	59948680	20q13.33	FAM217B	8.518720674	9.084877026	0.937681451	4	4	26.72332639	21.93749711
chr11	8964675	8976283	11p15.4	TMEM9B-AS1	2.965000392	3.162375831	0.937586344	3	4	51.17949642	46.60635571
chr19	52050028	52095780	19q13.41	ZNF841	4.959688111	5.290194568	0.937524707	4	4	36.07397726	20.94988755
chr3	159988836	159996019	3q25.33	IL12A	0.38127146	0.406706788	0.937460282	3	4	101.2644508	55.33659978
chr20	2652532	2658393	20p13	NOP56	7.888931796	8.415618535	0.937415564	4	4	35.28844021	43.66517904
chr17	19445929	19446875	17p11.2	RPS2P46	2.059920384	2.197516676	0.937385553	3	3	98.90850834	71.22158411
chrX	101418263	101535988	Xq22.1	ARMCX4	0.29634454	0.316147505	0.937361628	4	4	54.98519039	15.89633248
chr22	49918630	49927540	22q13.33	CRELD2	1.350189746	1.440502492	0.937304693	4	4	79.45153275	69.875694
chr6	61542697	62286227	6q11.1	KHDRBS2	0.554381724	0.591469336	0.9372958	4	4	66.0346836	68.33182917
chr19	57940833	57947709	19q13.43	ZNF256	4.254564905	4.53994629	0.93713992	4	4	79.92254745	21.50701561
chr11	47579010	47584563	11p11.2	NDUFS3	3.849207501	4.107587401	0.93709692	4	4	34.90821992	31.84300583
chr2	76445170	76445410	2p12	RN7SKP203	259.9042232	277.386494	0.936975047	4	4	33.27604887	47.77356994
chr2	27217390	27243792	2p23.3	CAD	2.401976817	2.56407878	0.936779648	4	4	70.8745765	55.18130753
chr9	137188602	137190370	9q34.3	SSNA1	11.16576938	11.91994698	0.936729786	4	4	58.22110553	27.75518406
chr19	45075717	45091524	19q13.32	GEMIN7	2.678378947	2.85951872	0.936653755	4	4	19.66703245	40.31049498
chr19	3762667	3767565	19p13.3	MRPL54	7.563376575	8.075226511	0.936614789	4	4	66.2948299	30.93877903
chr2	75655606	75711642	2p12	GCFC2	12.0548678	12.87084069	0.936602984	4	4	52.56240893	40.37905342
chr13	91347820	91354575	13q31.3	MIR17HG	2.408470415	2.571513249	0.936596541	4	4	30.16632171	35.94849148
chr18	21612269	21630247	18q11.2	SNRPD1	9.834516476	10.50066341	0.936561443	4	4	29.4106978	29.43883111
chrMT	7518	7585	N/A	MT-TD	2.820953227	3.012367995	0.936457044	1	1	0	0
chr17	76000906	76005999	17q25.1	CDK3	0.221721609	0.236776001	0.936419264	3	2	54.29617604	37.32593749
chr16	69132596	69169034	16q22.1	UTP4	6.754636432	7.213691696	0.936363337	4	4	46.3711105	21.55071138
chr1	100133080	100150498	1p21.2	TRMT13	6.689059068	7.143922345	0.936328636	4	4	33.86217441	37.51941885
chr14	90868122	91060649	14q32.11	RPS6KA5	3.669436687	3.919593427	0.936177886	4	4	29.23324025	19.08592439
chrX	33041291	33041898	Xp21.1	TBCAP1	1.579156596	1.686843767	0.936160554	1	4	0	53.17596763
chr1	100082632	100133095	1p21.2	SASS6	12.94422378	13.8283396	0.936064933	4	4	58.19740851	33.29015618
chr6	26204645	26205021	6p22.2	HIST1H4E	265.8378573	284.0051559	0.9360318	4	4	37.69814993	71.46051366
chr15	69452820	69455545	15q23	RPLP1	97.10705671	103.7490105	0.935980558	4	4	49.57542978	13.42507597
chr19	2229952	2236724	19p13.3	PLEKHJ1	2.482817708	2.652802807	0.935922452	4	4	48.31243497	23.01596083
chr10	45972453	46003742	10q11.22	TIMM23	89.55155919	95.68685653	0.935881504	4	4	43.50577319	60.49872007
chr17	1716523	1738599	17p13.3	WDR81	1.744825675	1.864386341	0.935871303	4	4	52.92562036	40.45170183
chr22	23856703	23886112	22q11.23	SLC2A11	0.106152132	0.113428257	0.93585263	2	4	91.55382533	59.25178941

chr1	220094113	220148041	1q41	IARS2	16.71687488	17.86603093	0.935679276	4	4	37.20195402	10.60446685
chr12	55966769	55972789	12q13.2	CDK2	3.490317483	3.7305135	0.935613149	4	4	30.88031562	54.70698966
chr1	236794304	236903981	1q43	MTR	12.31960793	13.16785125	0.935582252	4	4	12.31039638	9.690952484
chr1	170532122	170553834	1q24.2	GORAB	2.365309191	2.52845879	0.935474685	4	4	34.79382893	28.22293296
chr1	31500085	31508566	1p35.2	LINC01225	0.09951516	0.106381171	0.935458402	1	3	0	56.81431255
chr19	12043647	12052967	19p13.2	ZNF878	0.147243001	0.157409017	0.93541656	2	2	14.63976206	3.647695494
chr1	46394267	46413848	1p33	FAAH	1.628596546	1.74113775	0.935363412	4	4	87.61583553	114.5683119
chr1	24413360	24472983	1p36.11	NIPAL3	5.05752791	5.40726253	0.935321317	4	4	23.35292282	17.80936253
chr9	130172578	130237304	9q34.11	NCS1	0.238029479	0.254502668	0.93527302	4	4	56.96581867	34.49296072
chr2	89221698	89222431	2p11.2	IGKV2-28	1.04772367	1.120249457	0.935259253	1	1	0	0
chr3	14112077	14124871	3p25.1	CHCHD4	3.870827935	4.139307316	0.935139056	4	4	22.75364868	15.66573654
chr19	44259742	44278534	19q13.31	ZNF233	0.312789569	0.334500561	0.935094302	3	4	75.49461654	79.11329158
chr16	729753	741038	16p13.3	CIAO3	0.671694801	0.718378811	0.935014774	4	4	107.5340287	38.06753559
chr17	47733244	47746119	17q21.32	TBX21	26.99920843	28.88045365	0.934860953	4	4	40.47374991	74.7369238
chr12	110281227	110351093	12q24.11	ATP2A2	5.88844018	6.298858945	0.934842363	4	4	40.66326545	27.07005877
chr16	3135056	3142804	16p13.3	ZNF213	1.695117622	1.813469026	0.934737565	4	4	86.37953964	42.75344871
chr11	78188812	78226184	11q14.1	USP35	0.903161738	0.966257605	0.93470078	4	4	84.83679542	37.1225067
chr19	40230317	40285531	19q13.2	AKT2	7.15899425	7.659807978	0.934617979	4	4	54.50977238	23.17482785
chr6	49463327	49493107	6p12.3	CENPQ	6.393315588	6.841101912	0.934544708	4	4	41.28837106	25.81255625
chr17	5174021	5191883	17p13.2	ZNF594	1.353376696	1.448240289	0.934497339	4	4	59.74232939	52.43413261
chr7	129164849	129164958	7q32.1	RNY1P11	4.794244625	5.131936804	0.934197908	2	1	12.32962161	0
chr2	169694454	169701708	2q31.1	PHOSPHO2	0.840072266	0.899248031	0.934194168	4	4	55.00569522	10.75512698
chr15	72782835	72798199	15q24.1	ADPGK-AS1	0.515281355	0.551593979	0.934167838	4	4	109.3625516	59.39320393
chr14	72969445	73027212	14q24.2	ZFYVE1	10.39621636	11.12990535	0.934079494	4	4	16.84826346	25.07560469
chr13	110615460	110639996	13q34	NAXD	4.333735177	4.639767529	0.934041447	4	4	53.84866442	13.14957241
chr1	204218851	204378377	1q32.1	PLEKHA6	0.062985302	0.067440014	0.933945553	3	1	69.45708332	0
chr8	109362477	109537214	8q23.1-q23.2	PKHD1L1	6.633165664	7.10235545	0.933938847	4	4	30.54584599	9.605212789
chr7	142469581	142470013	7q34	TRBV6-6	2.018107554	2.160934132	0.933905168	4	4	54.35998369	53.61119169
chr17	2329125	2329210	17p13.3	SNORD91B	2.487469333	2.663514673	0.933904873	1	2	0	7.011630684
chr11	72080331	72110782	11q13.4	LRTOMT	0.302756065	0.324194769	0.933870915	4	4	79.52890784	48.611233
chr12	25803193	25806259	12p12.1	TDGP1	0.611157161	0.654642265	0.933574248	1	1	0	0
chr7	76048019	76067508	7q11.23	MDH2	17.18264602	18.40591492	0.933539359	4	4	61.19095272	62.82586103
chr4	87303789	87322906	4q22.1	HSD17B13	0.349125638	0.373993495	0.933507248	4	3	57.30456446	88.81522598
chr2	200585952	200677064	2q33.1	AOX1	0.056890947	0.060950777	0.93339166	2	4	12.19764225	48.49404252
chr10	129467184	129770983	10q26.3	MGMT	1.690900127	1.812225557	0.933051695	4	4	75.92660573	40.98335547
chr19	57614219	57624717	19q13.43	ZNF134	6.428982661	6.890619042	0.933005093	4	4	62.14153948	16.5103077
chr11	695386	705028	11p15.5	TMEM80	1.932122238	2.071058652	0.932915268	4	4	69.7442056	30.83578614
chr9	137048098	137054051	9q34.3	ENTPD2	0.104037684	0.111533687	0.932791575	2	1	19.79022206	0
chr20	41402101	41618494	20q12	CHD6	10.70475282	11.47621345	0.932777425	4	4	25.18657569	14.15336599
chr2	55519595	55545080	2p16.1	CFAP36	3.677016328	3.942630811	0.932630141	4	4	14.61306564	24.93450216
chr15	45023104	45075089	15q21.1	SORD	1.655324517	1.775061556	0.932544853	4	4	64.56847484	24.25470555
chr8	134800520	134800607	8q24.22	MIR30B	4.219413203	4.524968119	0.932473576	1	1	0	0
chr17	81239311	81241298	17q25.3	NDUFAF8	1.748182499	1.875119324	0.932304669	3	4	54.35976193	37.59510854

chr19	5690261	5691667	19p13.3	RPL36	60.68295656	65.09405924	0.932234942	4	4	18.94477311	19.65648382
chr7	135183838	135211564	7q33	WDR91	2.723995778	2.922513599	0.932072918	4	4	51.72060309	31.30348224
chr19	48029383	48048342	19q13.33	CABP5	5.367883008	5.759082637	0.932072579	4	4	94.3107928	54.92908871
chr8	6708357	6761503	8p23.1	AGPAT5	10.00273519	10.73213656	0.932035772	4	4	36.47860731	21.62632757
chr2	127048023	127107327	2q14.3	BIN1	6.562047691	7.041410854	0.931922285	4	4	51.32028444	35.94909156
chr19	9140380	9163419	19p13.2	ZNF317	9.624163963	10.32848518	0.931807888	4	4	56.45140317	22.77169025
chr17	67825517	67984378	17q24.2	BPTF	50.26970395	53.94981949	0.931786323	4	4	18.19604376	11.01295546
chr3	49416778	49422678	3p21.31	AMT	0.043619319	0.046816153	0.931715156	1	1	0	0
chr11	121292679	121313410	11q23.3-q24.1	SC5D	5.308509648	5.6976335	0.931704303	4	4	41.02689873	32.08758027
chr19	55428738	55442863	19q13.42	SHISA7	0.313199098	0.336161053	0.931693588	4	4	39.53886751	50.19359837
chrX	155258234	155264589	Xq28	RAB39B	3.847256898	4.129557487	0.931639022	4	4	37.76257679	24.8247302
chr2	69396113	69439649	2p13.3	NFU1	9.510084916	10.20836713	0.931597071	4	4	10.95103017	25.15777887
chr9	68705217	69009176	9q21.11	PIP5K1B	23.95397048	25.71384603	0.931559225	4	4	50.03493103	62.00703623
chr6	42915597	42925838	6p21.1	PTCRA	8.299334781	8.910795414	0.931379792	4	4	27.22163508	44.45320875
chrX	154483717	154486670	Xq28	UBL4A	10.56340066	11.34173714	0.931374139	4	4	50.48166079	21.89958409
chr5	181236928	181243906	5q35.3	RACK1	106.528553	114.3791813	0.931363136	4	4	53.68677667	33.86484883
chr6	73461731	73501456	6q13	MTO1	3.358294633	3.606143517	0.931270377	4	4	25.6446169	8.716596865
chr1	145844985	145848954	1q21.1	NUDT17	0.747046275	0.802357787	0.931063781	3	4	67.58009102	30.07704926
chr19	6463777	6467221	19p13.3	CRB3	0.199847727	0.214645082	0.931061292	2	3	40.33013018	30.1534858
chr10	31360913	31361214	10p11.22	SPTLC1P1	1.536383691	1.650163681	0.93104927	4	1	25.92260959	0
chr3	186546067	186570543	3q27.3	TBCCD1	4.259606099	4.575328602	0.930994573	4	4	33.61682411	11.78486599
chr14	22512852	22512908	14q11.2	TRAJ30	18.52386365	19.90084447	0.93080792	4	4	120.9420076	4.992898353
chr22	26657482	26676478	22q12.1	MIAT	1.888165724	2.028558638	0.930791789	4	4	24.33564489	45.63789503
chrX	102599168	102604159	Xq22.1	ARMCX5	2.007652577	2.157202434	0.930674166	4	4	70.81869246	35.0843109
chr4	73986439	73988190	4q13.3	PPBP	2331.631971	2505.347275	0.930662186	4	4	41.74946173	39.52249159
chr9	71862452	71911536	9q21.13	ABHD17B	12.11540488	13.01831877	0.930642819	4	4	23.4846554	9.884808794
chr19	55577078	55580845	19q13.42	ZNF579	0.375195181	0.403169961	0.930612936	4	4	98.75621566	38.50154576
chr16	87326987	87392107	16q24.2	FBXO31	2.285654237	2.456125554	0.930593403	4	4	48.3148278	35.21082472
chr12	39755021	40106085	12q12	SLC2A13	1.720679995	1.84908574	0.930557171	4	4	40.36335101	20.77600395
chr2	3188924	3377932	2p25.3	EIPR1	1.818965766	1.954906275	0.930461879	4	4	49.36756573	31.93407186
chr11	73974671	73982872	11q13.4	UCP2	27.56658819	29.62679029	0.930461516	4	4	23.29899286	55.16288205
chr3	148865256	148897091	3q24	CPA3	6.837166909	7.349732249	0.930260679	4	4	93.81069512	83.07073539
chr15	33310773	33866103	15q13.3-q14	RYR3	0.166558902	0.179047488	0.930249866	3	4	83.76430521	45.74108105
chr11	576446	612222	11p15.5	PHRF1	5.602741214	6.023042711	0.930217746	4	4	52.23176207	58.9826089
chr12	66233016	66235226	12q14.3	RBMS1P1	0.402491844	0.432686844	0.930215119	3	2	35.26625	51.93394314
chr2	68467559	68580162	2p13.3	APLF	40.04033015	43.04437234	0.93021057	4	4	50.13858468	17.90157493
chr2	75646783	75662208	2p12	MRPL19	6.803378023	7.31385951	0.930203542	4	4	22.23806345	27.98951436
chr8	38275042	38382272	8p11.23	NSD3	26.99777804	29.02467715	0.930166351	4	4	38.12538668	11.67212985
chr4	83035086	83075818	4q21.22	COPS4	9.911030852	10.65527324	0.93015267	4	4	31.18023055	17.27777821
chr2	179441982	179864550	2q31.2-q31.3	ZNF385B	0.136379462	0.146647242	0.929983131	4	3	71.80850369	85.89388818
chr19	14408798	14419383	19p13.12	DDX39A	5.344599244	5.747239867	0.929941914	4	4	50.09570484	31.33101707
chr19	647526	663233	19p13.3	RNF126	7.050704854	7.582135359	0.92991018	4	4	60.59879817	39.35731816
chr1	149842218	149842751	1q21.2	HIST2H2AA3	3.237487328	3.481540405	0.929900834	4	3	114.156964	62.30135967

chr1	36322030	36324154	1p34.3	EVA1B	1.652319356	1.776934968	0.929870472	4	4	84.80034272	47.48401975
chr2	169645425	169694421	2q31.1	CCDC173	0.18875837	0.203004991	0.929821329	4	4	38.12582326	52.56299468
chr2	202878919	202912226	2q33.2	WDR12	1.939672908	2.086124767	0.929797171	4	4	51.78660217	51.39328133
chr1	231223763	231241187	1q42.2	C1orf131	2.915845212	3.13614972	0.929753192	4	4	31.60725355	41.58761094
chr22	30554635	30574588	22q12.2	GAL3ST1	0.111981104	0.120449221	0.929695548	2	2	12.53926078	40.12237118
chr16	67674533	67719370	16q22.1	GFOD2	3.244914149	3.49035416	0.929680485	4	4	34.65456729	14.66833956
chr10	102152176	102163871	10q24.32	NOLC1	17.46835314	18.78974897	0.929674642	4	4	41.11692505	47.64764712
chr8	102864271	102977876	8q22.3	AZIN1-AS1	0.711858165	0.765711021	0.929669478	4	4	69.14075385	51.35488862
chr6	31546851	31558829	6p21.33	NFKBIL1	1.019735539	1.096924921	0.929631116	4	4	86.19500685	42.13633174
chr2	172464262	172556170	2q31.1	ITGA6-AS1	2.218641893	2.38675221	0.929565241	4	4	53.03649361	23.42221504
chr12	48998347	49019240	12q13.12	DDN-AS1	0.340233267	0.36601799	0.929553398	3	4	33.43722094	67.51439067
chrMT	7446	7514	N/A	MT-TS1	53.32979593	57.38126339	0.929393896	4	4	48.0189342	33.17865943
chr1	15834215	15848147	1p36.21	FLJ37453	1.725955595	1.857299431	0.929282358	4	4	47.32567504	5.889426576
chr4	83455932	83461723	4q21.23	MRPS18C	5.505882583	5.92703723	0.928943479	4	4	23.3187663	7.578914428
chr12	112405181	112418850	12q24.13	RPL6	141.0530038	151.8456115	0.928923809	4	4	54.24856058	29.85309076
chr1	42925375	42959176	1p34.2	SLC2A1	29.73405586	32.00919298	0.928922384	4	4	42.75330264	51.9438007
chr1	247297413	247333376	1q44	ZNF496	1.494348218	1.60882865	0.928842371	4	4	63.74543712	10.79340272
chr1	154402321	154406564	1q21.3	IL6R-AS1	0.211328053	0.227527026	0.928804184	3	2	16.68270589	38.50532952
chr4	56467596	56503681	4q12	SRP72	31.83414054	34.27521357	0.928780224	4	4	32.62456005	13.61593493
chr8	69466624	69660912	8q13.2-q13.3	SULF1	0.085882881	0.092469406	0.928770771	2	2	96.76300199	16.65406929
chr12	113157173	113185479	12q24.13	DDX54	7.542199979	8.121146406	0.928711244	4	4	66.96696411	56.62052648
chr12	4649098	4687554	12p13.32	NDUFA9	0.926217214	0.997401984	0.928629809	4	4	29.51860236	13.58509559
chr8	140094894	140100535	8q24.3	PEG13	0.393730492	0.424009773	0.928588249	4	4	84.36157222	109.3017569
chr3	93873037	93974090	3q11.1	PROS1	11.1370976	11.99366672	0.928581547	4	4	51.40895706	47.183006
chr2	24790267	24793382	2p23.3	PTRHD1	5.83060603	6.279634056	0.928494555	4	4	51.40574964	48.31977239
chr16	46578591	46621402	16q11.2	SHCBP1	1.806011014	1.945152942	0.928467359	4	4	35.02884608	40.6887916
chr1	2556365	2565622	1p36.32	TNFRSF14	4.20507774	4.529056611	0.928466588	4	4	49.11706022	16.15865329
chr1	121087345	121097161	1p11.2	FCGR1B	11.40217184	12.28130955	0.928416615	4	4	99.38319899	96.88538802
chr5	110387255	110738936	5q22.1	TMEM232	0.538203358	0.579879639	0.928129429	4	4	35.58185765	43.7286937
chr12	55716034	55719707	12q13.2	BLOC1S1	5.952506215	6.414912788	0.927916935	4	4	52.77015052	46.52677765
chr17	76725876	76733881	17q25.1	METTL23	6.328190094	6.819832812	0.927909858	4	4	41.1899515	19.63290586
chr12	49836043	49843129	12q13.12	BCDIN3D	1.351994136	1.457121512	0.927852705	4	4	31.67979396	47.25658487
chrX	12791355	12824227	Xp22.2	PRPS2	13.9479349	15.03326268	0.927804908	4	4	32.53687671	36.68962478
chr15	40531341	40565057	15q15.1	CCDC32	1.671070303	1.801161055	0.927773948	4	4	45.11179011	25.73272175
chr18	51030213	51085042	18q21.2	SMAD4	9.118438476	9.830575414	0.927558977	4	4	16.12040218	7.792593417
chr4	67468762	67545538	4q13.2	CENPC	16.5699411	17.86953244	0.927273344	4	4	34.28569111	33.33995267
chr2	216412414	216483053	2q35	SMARCAL1	3.690544294	3.98000465	0.927271352	4	4	34.35485045	28.98221296
chr6	27404010	27473118	6p22.1	ZNF184	9.359343888	10.09382321	0.927234774	4	4	41.83985904	12.39006896
chr3	108125064	108138610	3q13.12	LINC01215	4.652773101	5.019028286	0.927026674	4	4	69.48426753	31.25292188
chr3	113002444	113019733	3q13.2	NEPRO	7.777763119	8.390541903	0.926967913	4	4	21.9735056	16.48659092
chr16	4688930	4695859	16p13.3	NUDT16L1	2.687724866	2.899748442	0.92688208	4	4	52.54606315	41.93741082
chr11	6680385	6683401	11p15.4	MRPL17	3.512997206	3.790703973	0.926740054	4	4	49.84500708	16.16819206
chr10	38094327	38150293	10p11.1	ZNF37A	2.774754159	2.994181635	0.926715376	4	4	45.18123589	16.31148335

chr2	86515204	86515308	2p11.2	RNU6-640P	1.869622119	2.017513363	0.926696276	1	1	0	0
chr19	52269251	52292726	19q13.41	ZNF766	12.56316925	13.55734629	0.926668758	4	4	42.65481863	45.15550499
chr3	97941817	97972451	3q11.2	RIOX2	4.063246182	4.385263995	0.926568204	4	4	62.03231038	42.90756021
chr17	79932343	80035875	17q25.3	TBC1D16	0.175857814	0.189805029	0.926518202	4	4	108.1250092	31.67828171
chr16	23522013	23557375	16p12.2	EARS2	1.84777285	1.99454138	0.926414898	4	4	33.72337681	15.22629033
chr8	128008898	128008956	8q24.21	MIR1206	6.95663229	7.509584808	0.926367099	2	2	14.56462913	48.63648644
chr11	114439386	114450279	11q23.2	REXO2	16.06144218	17.34006221	0.926262086	4	4	33.01930716	48.4686949
chr10	19046931	19734478	10p12.31	MALRD1	0.198701288	0.214532676	0.926205233	4	4	70.79992103	46.95865957
chr12	31876969	31887203	12p11.21	LINC02422	1.836134271	1.982482707	0.926179212	4	4	71.79650487	43.82064076
chr5	180015303	180015397	5q35.3	MIR340	2.060769218	2.225096768	0.926148133	1	1	0	0
chr3	196431385	196433034	3q29	UBXN7-AS1	0.67732126	0.731376685	0.926090855	2	2	24.64490466	27.25495013
chr9	137204082	137205638	9q34.3	TMEM203	7.151108352	7.722463092	0.926013924	4	4	44.74829852	17.05230772
chr14	20447048	20455108	14q11.2	OSGEP	3.374269056	3.644146233	0.925942276	4	4	63.48167043	28.73843376
chr1	54200167	54225489	1p32.3	MRPL37	12.27469635	13.25775193	0.925850508	4	4	23.39769036	61.77438511
chr20	33662286	33663915	20q11.22	C20orf144	0.1345242	0.145316241	0.925734105	2	2	5.113500522	45.12295933
chr7	45100100	45111747	7p13	TBRG4	4.220857112	4.559820648	0.925662968	4	4	24.48303618	5.504458822
chr6	28425516	28443554	6p22.1	ZSCAN23	0.139125245	0.150306381	0.925611034	1	2	0	5.723850848
chr22	41525799	41544606	22q13.2	POLR3H	2.035900334	2.199704537	0.925533543	4	4	51.14055787	37.16211786
chr6	143422832	143450750	6q24.2	ADAT2	1.249651505	1.350201142	0.925529883	4	4	48.52472026	28.54774722
chr7	76980822	77023761	7q11.23	DTX2P1-UPK3BP1-	0.40543616	0.43808078	0.925482646	4	4	35.8251477	31.88667018
chr2	207821288	208025675	2q33.3	PLEKHM3	4.043188676	4.369009307	0.925424597	4	4	37.2437527	45.73382703
chr19	57977053	58003346	19q13.43	ZNF606	1.530821537	1.654303405	0.925357182	4	4	32.21816092	44.78046396
chr12	122917324	122975160	12q24.31	ABCB9	0.131835138	0.142480251	0.925287099	4	4	89.71885993	88.64049045
chr1	151759643	151763916	1q21.3	MRPL9	8.947943627	9.671427484	0.925193684	4	4	34.29475186	41.86562048
chr4	128060655	128222931	4q28.2	LARP1B	58.4722102	63.20611461	0.925103696	4	4	32.22975131	29.63387745
chr14	64465499	64474503	14q23.3	AKAP5	2.150197569	2.324423387	0.925045575	4	4	49.1173056	37.68412771
chr1	11012622	11030528	1p36.22	TARDBP	8.381707699	9.061494507	0.924980718	4	4	33.31274076	23.40291762
chr2	95402672	95416702	2q11.1	FAHD2A	1.084663745	1.172702582	0.924926543	4	4	82.62245899	50.82740611
chr15	50277192	50359306	15q21.2	GABPB1	16.73378556	18.09308956	0.924871648	4	4	37.44247108	32.28518639
chr1	172659008	172666873	1q24.3	FASLG	7.794990706	8.428947191	0.924788177	4	4	36.31818473	84.53551921
chr5	178711521	178730702	5q35.3	ZNF354A	7.247739846	7.837859128	0.924709124	4	4	37.68547838	33.42850678
chr12	55931091	55954023	12q13.2	DGKA	9.765111493	10.56048311	0.924684164	4	4	47.27661418	18.49610173
chr14	21090046	21104721	14q11.2	ZNF219	0.276230167	0.298765029	0.924573294	4	4	75.73819397	8.414447868
chr12	110708369	110742978	12q24.11	PPP1CC	29.44017489	31.8439107	0.924515056	4	4	9.814122237	25.58057901
chr17	46923108	46975519	17q21.32	GOSR2	2.577952249	2.788522331	0.924486858	4	4	31.53149172	9.867913446
chr7	38317017	38317492	7p14.1	TRGV9	5.540379208	5.992999066	0.924475233	4	4	39.30783171	28.65728371
chr10	49818274	49942027	10q11.23	PARG	18.98769569	20.54001482	0.924424634	4	4	12.71964809	7.815084362
chr5	74725100	74725204	5q13.3	RNU6-658P	2.934319971	3.174680788	0.924288194	3	2	33.97006205	22.81746794
chr14	68866659	68870128	14q24.1	BLZF2P	1.21095085	1.310191874	0.924254587	4	4	86.15877865	53.64154711
chr9	97633423	97673748	9q22.33	NCBP1	11.18526312	12.10206978	0.924243813	4	4	18.02633293	22.15405676
chr1	166475772	166490039	1q24.1	LINC01675	0.547599615	0.592523504	0.924182099	2	4	16.85268285	54.85169684
chr10	110567691	110604634	10q25.2	SMC3	73.03611558	79.03841631	0.924058439	4	4	38.21359326	37.24881411
chr13	78599092	78603560	13q31.1	POU4F1	0.103727035	0.112271752	0.923892552	1	1	0	0

chr7	142455174	142455635	7q34	TRBV7-4	3.573813718	3.86835843	0.923857957	2	4	42.89030567	59.98204323
chr11	65866441	65872934	11q13.1	EFEMP2	0.238501551	0.258166401	0.923828777	4	2	59.71752201	23.09592155
chr11	113797875	113875570	11q23.2	USP28	12.92328114	13.98894962	0.923820693	4	4	20.86434545	64.30530254
chr6	42224933	42452216	6p21.1	TRERF1	8.899592267	9.635645649	0.92361141	4	4	64.61258025	33.51816116
chr19	49474172	49487037	19q13.33	FLT3LG	0.555480561	0.601532079	0.923442955	3	4	81.57224297	86.0643043
chr22	42508335	42519823	22q13.2	RRP7A	6.131161408	6.639530466	0.923432981	4	4	68.5786766	36.15271946
chr4	78898858	78899154	4q21.21	RN7SL127P	2.545355224	2.756576433	0.92337553	3	4	87.86307932	27.70779757
chr3	128153453	128426193	3q21.3	EEFSEC	7.14089723	7.733952241	0.923317989	4	4	19.87776681	51.87766362
chr15	74920275	74938154	15q24.2	COX5A	43.30076834	46.89768911	0.923302814	4	4	46.12616571	54.47126861
chr7	120950749	120975657	7q31.31	ING3	8.29176094	8.981408772	0.923213847	4	4	18.48606438	20.83989862
chr1	234724042	234731643	1q42.3	LINC01132	0.146901202	0.159126316	0.923173525	2	2	23.87160727	74.93472516
chr8	144466246	144474116	8q24.3	KIFC2	1.111645688	1.204268827	0.923087655	4	4	94.0890573	33.05494081
chr12	55681546	55684611	12q13.2	METTL7B	0.149618024	0.162088303	0.923064904	1	2	0	5.818770602
chr19	52189802	52226425	19q13.41	PPP2R1A	9.657350194	10.46227238	0.923064306	4	4	45.43590913	31.47158445
chr8	42416462	42542213	8p11.21	SLC20A2	3.515020517	3.808139051	0.923028406	4	4	30.3088789	15.71771725
chr5	132481609	132490789	5q31.1	IRF1	92.08123868	99.75994988	0.923028117	4	4	61.29173217	25.2626086
chr12	10006138	10018800	12p13.2	CLEC12B	0.862115871	0.934024827	0.923011729	4	4	48.44765647	88.29560974
chr12	65169571	65248361	12q14.3	LEMD3	17.45651572	18.91292084	0.922994172	4	4	14.81733546	13.41992944
chr20	63101292	63104386	20q13.33	HAR1A	0.326371819	0.353602362	0.92299106	2	4	9.185413211	43.87242973
chr17	47941523	47949308	17q21.32	PNPO	3.880343063	4.204319746	0.92294195	4	4	59.61493818	64.92784643
chr20	31944955	31952080	20q11.21	PDRG1	1.697893057	1.83969845	0.922919219	4	4	28.53972912	19.07803473
chr14	77426675	77457727	14q24.3	VIPAS39	6.842946716	7.4155517	0.922783225	4	4	48.3566779	25.09373919
chr3	38346760	38453041	3p22.2	XYLB	0.298029533	0.323020734	0.922632827	4	4	45.98625565	113.3727196
chr2	87439523	87459684	2p11.2	LINC01943	0.725402029	0.786319564	0.922528273	4	4	101.8682737	71.26940799
chr19	45815442	45863290	19q13.32	SYMPK	2.925955223	3.171785401	0.9224947	4	4	64.79214092	34.28792597
chr15	28885479	29118315	15q13.1	APBA2	2.089753282	2.265531758	0.922411825	4	4	60.3627591	47.47407809
chr15	40978880	41117778	15q15.1	INO80	21.3459017	23.14258014	0.922364817	4	4	26.82432746	13.34498408
chr7	24573346	24694086	7p15.3	MPP6	47.99033638	52.04451441	0.922101723	4	4	70.78899909	45.1651828
chr14	89712511	89712750	14q32.11	RN7SKP255	15.96979173	17.32121217	0.921978876	4	4	58.21178821	79.52973264
chr11	115173625	115504523	11q23.3	CADM1	0.549774788	0.596345045	0.921907195	4	4	58.56594789	117.2548699
chr12	53157663	53181663	12q13.13	CSAD	2.236041238	2.42560918	0.921847285	4	4	48.50817769	58.07766139
chr9	137039457	137046224	9q34.3	NPDC1	0.973156633	1.055725078	0.921789823	4	4	62.59891356	68.9726297
chrX	13770922	13938824	Xp22.2	GPM6B	0.26586407	0.288428055	0.921769105	4	3	133.5315633	61.54998554
chr22	37830853	37849318	22q13.1	ANKRD54	1.170847199	1.270228288	0.921761237	4	4	69.04023757	49.7022878
chr11	17074392	17077673	11p15.1	RPS13	202.9455089	220.1810223	0.921721167	4	4	38.02903975	32.46740835
chr3	46370142	46376206	3p21.31	CCR5	11.73101193	12.72742873	0.921711068	4	4	15.55187697	120.3435817
chr8	74350383	74488867	8q21.11	GDAP1	1.899750972	2.061434123	0.921567636	4	4	30.94714104	44.8269875
chr1	211658256	211675630	1q32.3	NEK2	2.838925325	3.080696017	0.921520757	4	4	94.84980395	57.5763548
chr17	5486374	5490814	17p13.2	MIS12	7.38005	8.00930139	0.921434922	4	4	11.87227623	10.90205272
chr6	71164980	71167456	6q13	LYPLA1P3	0.378464307	0.410777956	0.921335485	2	2	40.60394189	14.80447281
chr20	33490070	33650031	20q11.21-q11.22	CBFA2T2	4.084012852	4.432978391	0.921279666	4	4	20.65904252	13.48012505
chr8	73008856	73047752	8q21.11	TERF1	5.621210153	6.10193387	0.921217809	4	4	19.53225895	7.335755092
chr10	73134524	73168095	10q22.2	ECD	17.56591437	19.07090243	0.921084592	4	4	12.07456652	14.69269976

chr12	47075707	47080826	12q13.11	AMIGO2	3.228386716	3.505299629	0.921001643	4	4	11.94859657	46.20750293
chr15	72114258	72143688	15q23	SENP8	0.295039488	0.320352363	0.92098427	4	4	42.40791394	60.737379
chr14	24426532	24429525	14q12	CBLN3	0.367760842	0.399430552	0.920712852	4	4	40.91783547	76.46147044
chr19	15353521	15379815	19p13.12	AKAP8	8.231570206	8.941097785	0.920644243	4	4	54.45438738	15.8583829
chr13	25372011	26025851	13q12.13	ATP8A2	0.296424291	0.322005545	0.92055648	4	4	65.45102295	66.9537284
chr19	38915264	38930896	19q13.2	SARS2	0.07287464	0.079169617	0.920487465	2	3	16.68186318	2.441935124
chr9	87966441	87974780	9q22.1	CDK20	0.188156716	0.204414625	0.92046602	3	3	59.96829062	45.68177874
chr12	66123069	66130753	12q14.3	LLPH	2.726214817	2.962139955	0.920353143	4	4	39.66547268	28.77402535
chr16	72116697	72173908	16q22.2	PMFBP1	0.909176461	0.987970761	0.920246324	4	4	113.7569214	28.30071469
chr7	100015552	100045374	7q22.1	ZKSCAN1	26.65580163	28.96602977	0.920243535	4	4	16.37300244	10.57919522
chr14	100376416	100530303	14q32.2	WDR25	1.281177109	1.392325565	0.920170642	4	4	65.17493347	51.65721354
chr9	127447674	127451432	9q33.3	RPL12	344.3241581	374.2252775	0.920098611	4	4	55.76354145	42.31923817
chr3	98531899	98534679	3q11.2	GPR15	5.751686956	6.251247569	0.920086254	4	4	58.77820218	78.75107935
chr19	1269266	1274810	19p13.3	CIRBP	23.47976883	25.52153682	0.919998235	4	4	45.29608783	21.19848662
chr7	43583093	43627379	7p13	STK17A	50.24956922	54.62284108	0.919936939	4	4	8.920388071	41.87864175
chr4	4542131	4710938	4p16.2	STX18-AS1	0.620982225	0.675058944	0.919893337	4	4	59.72347007	14.04478964
chr5	79236102	79327215	5q14.1	JMY	7.603859976	8.26665427	0.919823151	4	4	21.53182886	29.73579057
chr2	67397310	67410411	2p14	ETAA1	8.444816816	9.182657321	0.919648477	4	4	23.99440647	16.37963469
chr16	69339512	69343111	16q22.1	NIP7	7.042792632	7.658624386	0.919589769	4	4	36.32986144	12.1855629
chr19	58182989	58213562	19q13.43	ZNF274	4.472476075	4.86441115	0.919428053	4	4	72.81479702	33.2795049
chr1	165827495	165911618	1q24.1	UCK2	1.477123043	1.606725698	0.919337411	4	4	50.25987257	28.54070467
chr16	30530367	30534970	16p11.2	ZNF747	1.433009009	1.558863126	0.919265447	4	4	77.46559246	12.74993357
chr5	180988845	181006727	5q35.3	BTNL3	4.307036007	4.685847563	0.91915837	3	3	36.77184335	83.48951843
chr1	52602371	52609051	1p32.3	GPX7	3.796675042	4.130606725	0.919156748	4	4	50.79545101	40.6840068
chr5	74765429	74776912	5q13.3	NSA2	22.46367295	24.43962433	0.919149683	4	4	54.39933215	44.47367216
chr7	138460334	138585588	7q33-q34	TRIM24	18.16706161	19.76508766	0.919149053	4	4	13.49065776	5.299887554
chr6	30541377	30557594	6p21.33	GNL1	1.511879666	1.645116292	0.919010816	4	4	59.92933578	30.36954845
chr11	15966449	16476388	11p15.2	SOX6	75.30113825	81.9380585	0.919000763	4	4	68.86074276	26.46816247
chr12	80130377	80379090	12q21.31	OTOGL	0.045417358	0.049426656	0.918883889	3	3	24.17037623	32.25832639
chr19	35648223	35658784	19q13.12	COX6B1	103.8303896	113.0185214	0.918702424	4	4	33.6437007	13.46936958
chr19	36888124	36916387	19q13.12	ZNF829	1.428474976	1.555041434	0.918608948	4	4	63.58285948	24.98214042
chr16	85799567	85807003	16q24.1	COX4I1	20.8927746	22.74471665	0.918577044	4	4	56.91940484	19.35084264
chr5	58582188	58859394	5q11.2	RAB3C	0.288797355	0.314400217	0.918566016	3	3	125.1843622	24.17146271
chr12	111839767	111843005	12q24.12	MAPKAPK5-AS1	5.281997507	5.750297555	0.918560728	4	4	30.91389868	21.20715381
chr9	132891348	132945269	9q34.13	TSC1	6.508302262	7.085640722	0.918519936	4	4	35.96026506	14.04380481
chr22	20017000	20067164	22q11.21	TANGO2	21.35075388	23.24560068	0.918485789	4	4	31.04467975	17.49531436
chr11	4598672	4608259	11p15.4	TRIM68	2.0627039	2.246107634	0.918345973	4	4	62.03340821	32.40004478
chr9	136712572	136724718	9q34.3	DIPK1B	0.174049388	0.189537644	0.918284012	3	4	73.02389659	54.89402448
chr14	22476553	22476606	14q11.2	TRAJ59	5.542937199	6.036390008	0.918253657	1	2	0	32.09853606
chr7	38330343	38330821	7p14.1	TRGV8	7.58840862	8.264872425	0.918151936	4	4	68.25547344	49.86480246
chr19	7926718	7943823	19p13.2	TIMM44	2.144449233	2.335709279	0.91811479	4	4	73.18750001	50.8728797
chr9	106860154	107013634	9q31.2	ZNF462	0.108086612	0.117727616	0.918107534	4	3	51.21064069	96.67792363
chr19	43622368	43641984	19q13.31	CADM4	0.781685645	0.85157705	0.917927093	4	4	66.34669655	50.72769703

chr14	64504574	64533690	14q23.3	ZBTB1	26.37913085	28.74062442	0.917834298	4	4	28.9688404	27.4289922
chr9	129321016	129322603	9q34.11	C9orf106	0.475359492	0.517927304	0.917811224	4	3	76.66515695	62.43814739
chr12	53295451	53299450	12q13.13	PFDN5	168.4605526	183.5531359	0.9177754	4	4	36.57897906	9.840832125
chr19	54115410	54131719	19q13.42	PRPF31	6.850620038	7.464738792	0.917730711	4	4	64.94986863	50.24808434
chr19	9810267	9820528	19p13.2	FBXL12	3.304560085	3.600896371	0.917704856	4	4	93.99166281	39.13106316
chr11	66563463	66568576	11q13.2	CTSF	1.151870866	1.255354178	0.917566441	4	4	85.04346164	53.51434006
chr3	36985866	36993304	3p22.2	EPM2AIP1	7.353441587	8.014521217	0.91751477	4	4	47.75906426	12.40686791
chr19	18306230	18323191	19p13.11	LSM4	5.626373061	6.132511008	0.917466443	4	4	76.25312457	34.96072144
chr11	66593162	66606019	11q13.2	CCS	38.89995872	42.39972456	0.917457817	4	4	62.11963507	57.84686433
chr9	136791325	136793317	9q34.3	TMEM141	1.381597361	1.505974563	0.917410822	4	4	56.41555231	34.73871094
chr8	76980258	77001044	8q21.13	PEX2	11.00581456	11.99680046	0.917395817	4	4	9.304355871	16.96457235
chr7	142321075	142321544	7q34	TRBV5-1	4.303774064	4.691302716	0.917394234	4	4	78.80097237	55.50438701
chr1	220057482	220089916	1q41	BPNT1	4.385816075	4.780743525	0.917392044	4	4	42.27633275	47.27308594
chr15	49156298	49158625	15q21.1	NDUFAF4P1	1.348114356	1.469588236	0.917341554	1	4	0	64.32614895
chr10	125896223	126009599	10q26.2	FANK1	0.442282095	0.482139342	0.917332515	4	4	93.37134155	75.74737964
chr22	26429314	26445012	22q12.1	ASPHD2	2.737182562	2.983888504	0.917320657	4	4	47.69450591	26.84049251
chr4	74744759	74794718	4q13.3	BTC	0.083946291	0.091516273	0.917282665	1	1	0	0
chr7	149881359	150323669	7q36.1	ACTR3C	0.590400932	0.643643893	0.917278854	4	4	19.30579681	42.99802441
chr5	151253052	151270394	5q33.1	GM2A	15.73298578	17.15417385	0.917152054	4	4	47.93454019	53.68538572
chr1	47183589	47190099	1p33	PDZK1IP1	88.21769355	96.18808292	0.917137455	4	4	49.50763992	31.52333692
chrX	153972540	153983195	Xq28	TMEM187	1.793932133	1.956182465	0.91705767	4	4	49.56926052	62.03733637
chr15	52120926	52191368	15q21.2	GNB5	2.373557609	2.588353396	0.917014505	4	4	81.85422225	7.264728282
chr12	11512724	11514338	12p13.2	DDX55P1	0.202464741	0.220820589	0.91687438	1	1	0	0
chr12	10412315	10420595	12p13.2	KLRC3	3.858975091	4.209249105	0.916784679	4	4	35.83560558	37.31003447
chr20	3808494	3812434	20p13	LINC01730	0.708632856	0.773030824	0.916694179	2	2	89.16377229	24.76710111
chr16	22513523	22536521	16p12.2	NPIPB5	0.096629103	0.105422416	0.91658973	3	4	93.15259382	135.1452972
chr16	58157903	58197920	16q21	CSNK2A2	7.513857486	8.198516733	0.916489864	4	4	34.37440247	46.03257603
chr19	10543894	10553423	19p13.2	ATG4D	5.396262911	5.888222639	0.916450216	4	4	22.38489686	15.02352948
chr22	41244777	41302213	22q13.2	RANGAP1	5.423991469	5.918787555	0.916402459	4	4	44.11595655	17.62081219
chr20	18470615	18470691	20p11.23	MIR3192	9.284452535	10.13202068	0.91634757	3	1	67.6911066	0
chr11	90200429	90223364	11q14.3	CHORDC1	9.291396369	10.13966621	0.916341443	4	4	12.3681067	43.63376231
chr11	61430125	61446767	11q12.2	SDHAF2	2.511900742	2.741333239	0.916306236	4	4	28.1264375	43.92675962
chr14	23125295	23183660	14q11.2	SLC7A8	0.397827488	0.43421342	0.916202655	4	4	124.7430586	45.02602656
chr3	155869430	155943679	3q25.31	GMPS	32.47889552	35.45018623	0.916184059	4	4	54.93906328	33.79552805
chr18	36777647	36829216	18q12.2	TPGS2	11.41996794	12.46532469	0.916138827	4	4	41.08179662	20.87983336
chr4	142023160	142846535	4q31.21	INPP4B	9.516897044	10.38825357	0.91612098	4	4	38.24991697	16.57809804
chr5	149552057	149634968	5q32	ARHGEF37	1.52042136	1.659987717	0.91592326	4	4	21.20028665	26.52033063
chr1	205712819	205750244	1q32.1	NUCKS1	50.70472992	55.36450809	0.91583456	4	4	28.35935429	17.7801401
chr12	57747727	57752447	12q14.1	CDK4	3.491581395	3.812853144	0.915739805	4	4	57.676952	28.53076679
chr4	105171354	105352881	4q24	TET2-AS1	0.440733887	0.481400534	0.915524301	1	1	0	0
chr14	105472938	105480170	14q32.33	CRIP2	0.258443569	0.282301037	0.915489266	4	4	63.07150628	75.00544856
chr17	75032567	75065889	17q25.1	KCTD2	6.406783257	6.998353539	0.915470078	4	4	41.26743201	24.23143019
chr7	497245	520668	7p22.3	PDGFA	3.222828778	3.52045621	0.915457709	4	4	45.96475602	26.39870884

chr7	75185383	75237705	7q11.23	GTF2IP1	3.764929959	4.112740433	0.915430969	4	4	25.27923004	5.330611008
chr6	35214047	35253079	6p21.31	SCUBE3	0.095632199	0.104474174	0.91536688	3	1	103.4441743	0
chr19	55339853	55348121	19q13.42	KMT5C	0.830140583	0.906917669	0.915342827	4	4	75.87100796	16.31576213
chr6	138796107	138904070	6q24.1	ECT2L	0.088389579	0.096593856	0.915064187	2	3	21.74782534	68.97613009
chr20	63707442	63736142	20q13.33	ZGPAT	2.082557433	2.275938793	0.915032267	4	4	45.54170032	9.642593982
chr1	107139918	107484899	1p13.3	NTNG1	0.054935754	0.06003729	0.915027202	2	1	59.99612554	0
chr10	133481222	133481771	10q26.3	OR7M1P	0.604135866	0.660249106	0.915012017	2	2	44.58164365	37.73772291
chr19	2819874	2836735	19p13.3	ZNF554	0.513381623	0.561149183	0.91487547	4	4	63.68074686	61.82419653
chr9	97896855	97922570	9q22.33	TRMO	6.408720047	7.005446814	0.914819599	4	4	41.08424002	22.96863977
chr5	126600915	126627252	5q23.2	PHAX	10.42829342	11.40023345	0.914743849	4	4	46.89584259	33.60006951
chr9	113104723	113111677	9q32	FAM225B	0.033826304	0.036979602	0.914728724	1	1	0	0
chr7	77081655	77122289	7q11.23	FAM185BP	101.3785223	110.8311307	0.914711612	4	4	59.02015095	26.30254275
chr8	22138014	22141951	8p21.3	REEP4	8.343579246	9.122137436	0.91465178	4	4	11.19550039	105.4285947
chr16	84459259	84467361	16q24.1	ATP2C2-AS1	0.221035981	0.241666848	0.914630959	2	3	0.313853037	61.44790356
chr13	31199975	31332276	13q12.3	B3GLCT	6.730556619	7.359725338	0.914511929	4	4	53.47808963	81.61985416
chr3	37379111	37435497	3p22.2	C3orf35	1.46114466	1.597967706	0.914376839	4	4	35.84398144	31.28531693
chr22	21665984	21698283	22q11.21	PPIL2	5.233304963	5.724529788	0.914189489	4	4	27.46546718	39.08043999
chr2	3531813	3558371	2p25.3	RNASEH1	5.000758646	5.470439241	0.914142069	4	4	53.55564662	16.98881947
chr16	67660948	67662778	16q22.1	PARD6A	0.926795935	1.013842738	0.914141711	4	4	67.97250549	66.09753994
chr10	15097180	15104257	10p13	RPP38	4.366071958	4.777030385	0.913971988	4	4	37.58465835	38.41918356
chr19	48968125	48993353	19q13.33	GYS1	5.756053957	6.299073485	0.913793746	4	4	71.55021878	72.13623366
chr14	22986901	23010201	14q11.2	C14orf93	2.788199855	3.051309158	0.913771667	4	4	35.8419315	27.83527098
chr14	39396373	39432500	14q21.1	FBXO33	26.73963363	29.26368354	0.913748045	4	4	23.14372806	17.09667923
chr9	138216856	138272073	9q34.3	FAM157B	0.666977124	0.73006925	0.913580628	4	4	47.82677663	94.61565426
chr19	2236817	2248679	19p13.3	SF3A2	10.20813732	11.17387247	0.913572027	4	4	31.86488292	32.15217132
chr14	58427385	58548831	14q23.1	KIAA0586	13.57435708	14.86090402	0.913427411	4	4	33.55646286	17.09489776
chr16	20742440	20797811	16p12.3	ACSM3	1.31621114	1.440990754	0.913407068	4	4	51.76901826	29.08838472
chr7	10973872	11188565	7p21.3	PHF14	7.279613902	7.970701005	0.913296572	4	4	44.96040366	18.38690424
chr16	77722488	77742260	16q23.1	NUDT7	1.949541655	2.134661816	0.913278928	4	4	38.29880604	76.96187574
chr11	5514393	5516726	11p15.4	UBQLNL	0.13316803	0.14583354	0.913150917	4	3	28.58303366	43.74011786
chr7	5454448	5513798	7p22.1	FBXL18	0.90638958	0.992615568	0.913132545	4	4	88.60068119	25.55999805
chr12	119685790	119877318	12q24.23	CIT	3.387956874	3.710350112	0.913109753	4	4	51.61994523	21.00088982
chr17	28467778	28468454	17q11.2	RPS7P1	1.499530773	1.642310054	0.913061921	3	4	103.5429536	62.86768248
chr11	125094370	125111763	11q24.2	TMEM218	1.570944916	1.72068485	0.912976549	4	4	48.85015953	29.85660429
chr22	19714464	19723322	22q11.21	SEPT5	4.208786249	4.61084416	0.91280167	4	4	96.50752655	12.91944539
chrX	152830967	152869363	Xq28	NSDHL	3.140478673	3.440538131	0.912787057	4	4	51.30898389	50.62561026
chr1	9036946	9072227	1p36.23	SLC2A5	0.325338032	0.356433596	0.912759166	4	4	69.15100864	25.33695519
chr12	91984976	92142831	12q21.33	LINC01619	1.961563059	2.149382934	0.912616839	4	4	42.7292506	72.11733322
chr19	14305458	14363990	19p13.12	LINC01841	0.790362315	0.866124397	0.912527482	2	3	41.4722048	26.00393762
chr15	84631460	84642463	15q25.2	SCAND2P	0.969625827	1.062619884	0.912486056	4	4	54.38236596	9.233622309
chr22	23971365	23979828	22q11.23	DDT	3.408636415	3.736236182	0.912318239	4	4	62.78939855	51.99808027
chr2	96814946	96837109	2q11.2	CNNM3	4.04808314	4.437295936	0.91228604	4	4	43.65651339	11.93790161
chr17	82101454	82212892	17q25.3	CCDC57	1.000947882	1.097258169	0.912226412	4	4	72.56471426	58.35473888

chr1	178093729	178479514	1q25.2	RASAL2	0.191147879	0.209556501	0.912154376	4	4	87.79136162	52.82829403
chr2	215311956	215359745	2q35	ATIC	2.461538452	2.698872857	0.912061658	4	4	65.02995142	46.92177334
chr4	2231836	2242164	4p16.3	HAUS3	7.447313308	8.165566131	0.912038821	4	4	11.10578058	9.658611176
chr11	113687546	113709811	11q23.2	TMPRSS5	0.307558793	0.337234715	0.912002171	4	3	55.41988203	62.1781318
chr2	3558386	3561750	2p25.3	RNASEH1-AS1	1.553177604	1.703075301	0.911984105	4	4	63.12608354	32.48239561
chr10	87357720	87370695	10q23.2	NUTM2D	0.160550337	0.176053411	0.911941076	4	4	44.14514174	71.74495004
chr3	179653145	179789401	3q26.33	USP13	1.190185718	1.305131817	0.911927594	4	4	37.54143071	25.09133567
chr2	176269395	176338025	2q31.1	MTX2	7.963273828	8.732724308	0.911888839	4	4	36.97492475	24.99877237
chr16	3382081	3402007	16p13.3	ZSCAN32	4.445167157	4.874723814	0.911880822	4	4	36.61647488	26.07027294
chr14	49570984	49598710	14q21.3	RPS29	28.38809854	31.13311366	0.911829727	4	4	9.026414019	11.97792437
chr16	28688558	28735730	16p11.2	EIF3C	0.488271556	0.535568828	0.91168778	4	4	88.29803419	60.95657034
chr3	130111707	130120569	3q22.1	LINC02021	0.203116896	0.222818983	0.911578059	1	4	0	46.51821818
chr15	25065025	25065122	15q11.2	SNORD116-6	5.110535575	5.606883301	0.911475289	3	4	20.65368779	62.73623044
chr22	38681949	38684760	22q13.1	TOMM22	13.30143225	14.59349104	0.911463352	4	4	42.19206056	25.33084504
chr2	128517289	128519258	2q14.3	ISCA1P6	0.534044834	0.586008566	0.911325985	1	1	0	0
chr7	20002725	20003292	7p21.1	RPL21P75	3.253784536	3.570871951	0.911201684	4	4	54.09519041	36.32060511
chr6	391739	411443	6p25.3	IRF4	8.388762984	9.206987253	0.911130075	4	4	36.71868974	57.02552687
chr19	3630181	3700492	19p13.3	PIP5K1C	4.753134575	5.216904205	0.911102522	4	4	70.34184064	37.88393885
chr1	21436775	21484900	1p36.12	NBPF3	1.484862008	1.629780672	0.91108088	4	4	72.75463428	63.45588961
chr17	59155355	59206709	17q22	PRR11	6.515335319	7.151314077	0.911068266	4	4	21.94228515	39.15911932
chr10	85599555	86366493	10q23.1-q23.2	GRID1	0.080065772	0.087897934	0.910894809	1	1	0	0
chr10	60778331	60794852	10q21.2	CDK1	3.536591889	3.882658912	0.910868549	4	4	66.96420172	52.14034968
chr19	19710472	19733112	19p13.11	ZNF14	7.566593033	8.307167121	0.910851187	4	4	39.89716338	21.81177604
chr9	2804152	2844130	9p24.2	PUM3	18.91272214	20.76426136	0.91083048	4	4	42.26769985	18.67665538
chr2	12716872	12742734	2p24.3	TRIB2	13.20058352	14.49626455	0.910619661	4	4	27.00785728	37.34759592
chr11	48096782	48096858	11p11.2	MIR3161	5.429924746	5.963289875	0.910558577	2	2	42.03120294	63.29785293
chr11	76444928	76553031	11q13.5	EMSY	18.24526751	20.04037169	0.910425605	4	4	13.40534465	13.30515828
chr2	84423523	84459462	2p11.2	SUCLG1	8.877631828	9.751285962	0.910406265	4	4	51.33548711	45.2415608
chr12	48653211	48682252	12q13.11	KANSL2	8.875775363	9.751855456	0.910162728	4	4	32.43788314	24.09049594
chr1	169853074	169894267	1q24.2	SCYL3	4.887491717	5.369978361	0.910151101	4	4	37.95239851	20.43299496
chr2	234444590	234454595	2q37.1	LINC01891	5.589968493	6.142156856	0.910098622	4	4	43.06979346	14.4514724
chr7	22854240	22861436	7p15.3	SNHG26	1.102898476	1.211941361	0.91002627	3	4	63.90305724	41.93417213
chr15	43358172	43371032	15q15.3	ZSCAN29	5.479813193	6.022441345	0.909898973	4	4	42.71226048	15.79559199
chr1	110338929	110373005	1p13.3	RBM15	15.32772093	16.84553891	0.909897927	4	4	24.49547872	11.72850441
chr22	39399754	39437127	22q13.1	TAB1	3.752503691	4.124597664	0.909786601	4	4	65.08194456	57.09261151
chr5	132073792	132076170	5q31.1	CSF2	0.574344322	0.631297733	0.909783597	3	2	55.45408521	76.49415088
chr9	92383271	92404699	9q22.31	OGN	0.087596108	0.096287286	0.909737016	1	4	0	35.76932149
chr22	39312882	39319665	22q13.1	RPL3	113.0232734	124.2460378	0.90967306	4	4	59.36697749	17.64882579
chr8	26379259	26382953	8p21.2	SDAD1P1	0.925877595	1.017997161	0.909509014	4	4	45.04830678	20.82626824
chr6	35259473	35295987	6p21.31	ZNF76	1.652679085	1.817313152	0.909407981	4	4	62.38435855	31.41626549
chr1	32816767	32858879	1p35.1	S100PBP	4.626117879	5.087103759	0.909381467	4	4	31.78144624	43.2847767
chr17	5282263	5386340	17p13.2	RABEP1	34.55140065	37.99538517	0.909357821	4	4	10.35640066	15.88537048
chrX	17737449	17754988	Xp22.13	SCML1	1.322578854	1.454434397	0.909342393	4	4	52.0963961	15.5860547

chr1	166747323	166748749	1q24.1	RPL4P2	0.206108631	0.226657987	0.909337601	1	2	0	11.75198898
chr15	89243954	89317132	15q26.1	FANCI	10.10962356	11.11792738	0.909308293	4	4	74.22667562	43.28601971
chr18	79395772	79529323	18q23	NFATC1	2.85665977	3.141596983	0.909301793	4	4	85.85246036	57.12314927
chr8	144827509	144848070	8q24.3	ZNF7	1.211398917	1.332390558	0.909192061	4	4	74.79607805	27.79876942
chr14	53990931	53992023	14q22.2	ATP5F1CP1	0.548878925	0.603706864	0.909181191	1	2	0	58.70044719
chr20	38748454	38772446	20q11.23	ACTR5	2.407953061	2.648627341	0.909132449	4	4	9.436522782	45.64499321
chr7	99616608	99738689	7q22.1	ZSCAN25	3.755668106	4.131784447	0.908969999	4	4	43.30798372	21.54291255
chr1	28505943	28510892	1p35.3	SNHG3	5.692399597	6.262943862	0.908901584	4	4	42.60212086	33.91916927
chr7	100093560	100093643	7q22.1	MIR25	8.581031884	9.441740049	0.908840091	1	1	0	0
chr10	42574169	42638837	10q11.21	ZNF33B	5.383836297	5.924256768	0.908778351	4	4	27.3947366	26.64586667
chr4	176065834	176182828	4q34.2	WDR17	0.188141765	0.207032917	0.908752907	4	4	95.90212866	28.64681252
chr20	36751791	36773827	20q11.23	DSN1	5.607727554	6.171197815	0.908693534	4	4	28.9649802	33.93421333
chr5	115962454	116027602	5q23.1	LVRN	0.068093031	0.07494213	0.90860816	2	1	49.05782301	0
chr2	134866633	134918606	2q21.3	CCNT2-AS1	4.1736163	4.593824291	0.908527631	3	4	15.56632288	55.03473911
chr19	4675226	4723843	19p13.3	DPP9	3.470684048	3.820159094	0.908518196	4	4	59.86440366	41.79232307
chr17	3276113	3386311	17p13.3	OR3A2	0.442189221	0.486728098	0.908493309	1	2	0	16.67454111
chr7	6047721	6048023	7p22.1	RN7SL851P	1.374377609	1.512830222	0.908481063	3	3	24.32912653	56.19797888
chr4	37960435	37961010	4p14	PTTG2	1.346567601	1.482311997	0.90842387	4	3	79.53768766	66.93849863
chr11	818890	825573	11p15.5	PNPLA2	34.34437327	37.81123296	0.908311382	4	4	33.04333706	67.12370469
chr3	108545752	108589644	3q13.13	CIP2A	7.542771954	8.304355392	0.908291083	4	4	60.01829886	38.99859611
chr10	14838160	14871741	10p13	HSPA14	3.240342278	3.568015816	0.908163653	4	4	89.57507089	92.23766496
chr1	23691779	23696835	1p36.11	RPL11	531.7337418	585.5055501	0.908161745	4	4	65.31327565	57.87600335
chr7	6688433	6706935	7p22.1	ZNF12	10.21679702	11.25001789	0.908158291	4	4	25.80097548	10.31778177
chr17	58301227	58329030	17q22	TSPOAP1	1.932372326	2.127995866	0.908071466	4	4	53.00204615	18.86162122
chr11	1947272	2013176	11p15.5	MRPL23	1.136895972	1.252036396	0.908037479	4	4	108.8511309	75.48625517
chr11	111352251	111379671	11q23.1	POU2AF1	0.789736303	0.869772526	0.907980281	4	4	76.63059009	59.40758013
chr17	38850068	38853800	17q12	RPL23	87.22652336	96.09824799	0.907680683	4	4	54.04449885	27.83967776
chr1	228149714	228159826	1q42.13	GJC2	0.248231005	0.273479211	0.907677785	4	2	57.34447519	33.67386956
chr1	110593580	110631536	1p13.3	KCNA2	0.086105539	0.094864997	0.907663962	4	3	42.97967505	60.44928467
chr16	69424595	69466264	16q22.1	CYB5B	8.002466548	8.816628952	0.907656043	4	4	32.86991137	40.44748786
chr22	37125838	37175118	22q12.3	IL2RB	31.70895852	34.93625866	0.90762319	4	4	39.95390654	43.55832064
chr3	121431420	121546006	3q13.33	POLQ	10.38455596	11.44236666	0.907553154	4	4	71.10151544	60.35144821
chr1	46188681	46220305	1p34.1	POMGNT1	1.853361887	2.042202513	0.907530901	4	4	59.43554922	41.60271595
chr9	32550976	32566860	9p21.1	SMIM27	6.502056242	7.164600217	0.907525339	4	4	19.20780935	31.32063637
chr16	26031717	26032931	16p12.1	HMGN2P3	1.637191237	1.80406821	0.907499633	4	3	30.99072742	49.3789532
chr4	9078958	9141608	4p16.1	ENPP7P10	0.411131269	0.453065078	0.907444181	2	2	19.9233439	35.49156566
chr5	79976716	79991265	5q14.1	MTX3	3.476410532	3.831557376	0.907310054	4	4	30.03881091	15.07755898
chr11	113314529	113385952	11q23.2	TTC12	1.962881877	2.163460803	0.907287931	4	4	72.99669044	19.47280894
chr2	53864067	53970840	2p16.2	PSME4	46.65195058	51.42231854	0.907231566	4	4	44.73621844	60.76523868
chr7	7640636	7879223	7p21.3	UMAD1	9.502845381	10.47509941	0.907184267	4	4	43.32305061	23.67487003
chr6	31706863	31710679	6p21.33	LY6G6F	2.060195692	2.271004083	0.907173927	3	4	77.71416444	59.16022167
chr6	143864436	143938346	6q24.2	ZC2HC1B	0.203888827	0.224755805	0.907157112	1	2	0	6.250992852
chr11	43880807	43920275	11p11.2	ALKBH3	5.318323269	5.864234982	0.906908281	4	4	49.24513579	27.55106813

chr19	55385932	55403248	19q13.42	RPL28	39.76980305	43.85431958	0.906861706	4	4	49.99906876	54.83497842
chr8	80034868	80171659	8q21.13	TPD52	2.037989053	2.247353533	0.906839544	4	4	54.52177424	49.78207845
chr10	73241954	73247331	10q22.2	DNAJC9	7.95591634	8.773604332	0.90680136	4	4	22.59435703	19.95457564
chr17	82035881	82037697	17q25.3	DCXR	5.296125274	5.840497465	0.906793523	4	4	52.94752406	67.71099348
chr12	14553691	14553970	12p13.1	RN7SKP134	1.040502664	1.14763176	0.906652029	2	2	50.39403581	24.3873953
chr7	111953653	111953765	7q31.1	RNA5SP237	6.404339431	7.063946271	0.906623463	2	1	77.749711	0
chr22	49773278	49827927	22q13.33	BRD1	9.94102195	10.96708572	0.906441529	4	4	60.78695384	26.89572239
chr17	212149	386267	17p13.3	RPH3AL	0.173100286	0.190989991	0.906331716	4	3	89.45269375	54.27526236
chr1	116775078	116775567	1p13.1	FTH1P22	3.082765718	3.401425293	0.906315868	4	3	29.71784065	71.06926821
chr20	51386957	51562857	20q13.2	NFATC2	13.53496789	14.93442842	0.906292997	4	4	33.7307174	69.06092043
chr1	27830741	27851672	1p35.3	PPP1R8	17.10597931	18.88072639	0.906002182	4	4	33.61110847	19.63887382
chr5	173607145	173617072	5q35.2	BOD1	6.397685272	7.062194229	0.905906162	4	4	25.77089118	27.20103625
chr9	96492743	96619830	9q22.32-q22.33	CDC14B	4.889826266	5.398719947	0.905738085	4	4	25.95064383	27.56668658
chr4	164956948	164959131	4q32.3	FAM218A	0.375678578	0.414809605	0.90566509	2	4	5.061950522	42.73724961
chr7	76609986	76627303	7q11.23	POMZP3	2.108817168	2.328698472	0.905577598	4	4	62.59845958	33.89682587
chrX	22032327	22251310	Xp22.11	PHEX	0.689455913	0.761393115	0.905518975	4	4	40.837809	66.31555632
chr5	470510	472965	5p15.33	PP7080	1.812958691	2.002186524	0.905489408	4	4	72.90239207	69.63672898
chr11	62371968	62373372	11q12.3	RCC2P6	0.145538743	0.160733664	0.905465222	1	2	0	9.228045537
chr17	37514797	37609496	17q12	SYNRG	5.128522665	5.663995223	0.905460274	4	4	27.39368551	24.43613084
chr1	197084127	197146694	1q31.3	ASPM	15.96095947	17.62757508	0.905454062	4	4	82.37605504	48.59016481
chr17	39671122	39688070	17q12	PGAP3	2.075205392	2.291933017	0.905438936	4	4	36.97338236	11.66055417
chr3	101681090	101686719	3q12.3	RPL24	115.1332602	127.1669057	0.905371249	4	4	19.69417552	18.12139563
chr7	116953848	116959817	7q31.2	ST7-OT4	0.198589911	0.219397293	0.905161173	4	2	47.0822697	9.951927336
chr1	28369554	28500369	1p35.3	PHACTR4	10.34225145	11.42600647	0.905150148	4	4	6.23085153	25.39350756
chr19	58126328	58155169	19q13.43	ZNF329	3.132554721	3.460845296	0.905141505	4	4	69.00091877	27.78435147
chr1	44721786	44721901	1p34.1	RNU5F-1	104.6526325	115.6238118	0.905113151	4	4	25.49427531	23.99002412
chr19	7629785	7631624	19p13.2	PET100	3.531077156	3.901333373	0.905094956	4	4	39.43355151	62.4831979
chr19	9538666	9539730	19p13.2	ZNF426-DT	0.889879831	0.983288211	0.905004069	2	3	10.7172375	64.50067706
chr7	55365448	55433742	7p11.2	LANCL2	1.986854904	2.195564803	0.904940224	4	4	61.4009255	26.40778015
chr12	112160188	112382092	12q24.13	HECTD4	21.23805062	23.47172479	0.904835534	4	4	35.02461994	31.38767525
chr3	194587673	194633819	3q29	TMEM44	0.150925831	0.166801678	0.90482202	4	1	55.47685847	0
chr10	89853906	89914956	10q23.31	LINC01374	0.839677089	0.928030117	0.904795086	2	2	25.77733734	19.62311644
chr16	715173	719655	16p13.3	METRN	0.385495322	0.426100014	0.904706195	3	4	59.7041452	48.9356019
chr2	37084451	37099244	2p22.2	GPATCH11	10.98410507	12.14176552	0.904654686	4	4	39.87991444	39.4257953
chr1	159780946	159782546	1q23.2	DUSP23	10.95434108	12.10994501	0.904573974	4	4	75.89237694	43.56665673
chr5	71587288	71658706	5q13.2	MCCC2	3.917795006	4.331173279	0.904557438	4	4	51.5888481	28.0635525
chr1	30718772	30726746	1p35.2	MATN1-AS1	0.670837947	0.741659872	0.904508888	4	4	51.83969026	23.89603581
chr16	67806573	67828068	16q22.1	TSNAXIP1	0.274089502	0.303031958	0.904490417	4	4	46.84735197	64.56295161
chr12	2876258	2889525	12p13.33	RHNO1	8.620897621	9.53213409	0.904403729	4	4	52.0765144	36.5214843
chr17	82023252	82031151	17q25.3	LRRC45	1.614319832	1.785163956	0.904297797	4	4	70.38347396	53.94970613
chr3	9986893	10007004	3p25.3	EMC3-AS1	0.991578949	1.096541559	0.904278493	4	4	63.3828112	70.96178016
chr9	128683432	128696396	9q34.11	SET	79.43199313	87.84380762	0.904241235	4	4	40.86108121	24.01610865
chr11	70203163	70207402	11q13.3	FADD	6.743593741	7.457937595	0.904216971	4	4	46.08487352	34.92374779

chr20	3107573	3159910	20p13	UBOX5	2.473627305	2.735801662	0.904169092	4	4	42.87522662	15.67330104
chr15	63608618	63833948	15q22.31	HERC1	63.74194254	70.50010748	0.904139651	4	4	13.99585498	20.65575049
chr6	139028682	139043302	6q24.1	ABRACL	42.54114162	47.05361957	0.904099238	4	4	39.63059393	32.42647483
chr16	23463542	23510494	16p12.2	GGA2	7.91829729	8.760810636	0.903831577	4	4	54.9573294	29.13789082
chr2	19896631	19901986	2p24.1	TTC32	7.524208423	8.326332503	0.903664179	4	4	40.05364886	23.96854803
chr17	59109859	59155439	17q22	SKA2	17.2670897	19.10817973	0.903649115	4	4	22.9938962	52.68568078
chr9	112878920	112895538	9q32	SLC46A2	2.199901067	2.434468932	0.903647213	4	4	103.1337302	31.93789923
chr14	21723889	21724321	14q11.2	TRAV3	3.384186554	3.745235681	0.903597755	4	4	67.91002757	32.72752698
chr2	95025655	95053992	2q11.1	MAL	10.39175116	11.50114557	0.903540529	4	4	54.06095317	30.9580099
chr5	118836074	118988605	5q23.1	DTWD2	2.395073331	2.650864083	0.903506651	4	4	43.50942308	36.28722219
chr17	69593987	69903000	17q24.3	LINC01483	0.060809246	0.067312905	0.90338169	1	1	0	0
chr19	57411147	57421939	19q13.43	ZNF17	3.219513156	3.563862371	0.903377522	4	4	66.17613748	31.43621348
chr7	142391938	142392412	7q34	TRBV9	2.47100056	2.735419785	0.903335047	1	4	0	68.70532401
chr7	118224658	118242731	7q31.31	ANKRD7	0.124712043	0.138069994	0.903252328	2	4	26.66288682	84.98403213
chr9	123485529	123485622	9q33.3	MIR7150	3.804688901	4.212212215	0.903251951	2	2	20.11185953	39.40144975
chr15	24823608	24978723	15q11.2	SNRPN	3.543727278	3.923613237	0.90317956	4	4	22.20424176	40.95218034
chr14	106737110	106737547	14q32.33	IGHV1-69-2	1.636304528	1.811849939	0.90311261	1	1	0	0
chr1	145823183	145844402	1q21.1	POLR3C	50.88359916	56.34434911	0.903082562	4	4	61.43692019	40.05989316
chr9	89318473	89367117	9q22.2	SECISBP2	15.17876699	16.81132207	0.902889548	4	4	6.437575885	51.48008786
chr1	241593150	241640399	1q43	OPN3	3.660575577	4.054316882	0.902883441	4	4	44.56230545	37.35053183
chr16	85918347	85918416	16q24.1	MIR6774	5.02259779	5.564579003	0.902601578	2	2	22.86748107	66.80044866
chr8	24294040	24359018	8p21.2	ADAM28	2.536302455	2.809997202	0.902599637	4	4	81.3631149	60.30239778
chr15	76259289	76260152	15q24.2	TYRO3P	0.227581814	0.252177547	0.902466602	3	3	45.74197617	105.8674715
chr16	1419744	1420800	16p13.3	C16orf91	4.121324634	4.56782001	0.902251977	4	4	50.9130376	37.3578718
chr8	99877995	99894014	8q22.2	COX6C	18.30997595	20.29376514	0.902246371	4	4	15.82545282	20.22736757
chr19	9853718	9936552	19p13.2	OLFM2	0.971018386	1.076301938	0.902180282	4	4	95.28828211	93.73816285
chr8	33485167	33501260	8p12	MAK16	7.56718543	8.387682014	0.902178387	4	4	15.51176493	22.55318284
chr12	9669708	9699555	12p13.31	CLEC2D	24.88257028	27.58067454	0.902174102	4	4	33.45844952	31.71804543
chr1	155335261	155563160	1q22	ASH1L	33.66099747	37.31570657	0.902059764	4	4	3.391890066	17.78241455
chr1	115641953	115698224	1p13.1	VANGL1	1.875170818	2.078927758	0.901989408	4	4	52.23935477	58.20751788
chr1	153661788	153671028	1q21.3	ILF2	40.7063971	45.12966029	0.901987669	4	4	39.48410382	48.28631145
chr14	73715122	73790285	14q24.3	ELMSAN1	5.285964191	5.862050515	0.901726141	4	4	62.6696224	18.82325084
chr15	80152823	80186582	15q25.1	FAH	1.169647197	1.297345456	0.901569579	4	4	13.02281459	22.82611891
chr12	29500813	29784759	12p11.22	TMTC1	0.399949242	0.443670638	0.901455288	4	3	110.6400567	158.7919334
chr16	2253116	2268412	16p13.3	RNPS1	7.99822809	8.872771137	0.901435185	4	4	59.98029019	44.1778922
chr19	33621955	33773509	19q13.11	CHST8	0.550399512	0.610595334	0.901414539	3	4	94.77648465	90.65716048
chr10	8045420	8075201	10p14	GATA3	4.672091524	5.183152353	0.901399613	4	4	63.4382628	62.32032055
chr2	202635178	202769757	2q33.2	FAM117B	8.410814572	9.330879201	0.90139572	4	4	38.28618195	26.7484025
chr1	116560024	116560111	1p13.1	MIR548AC	4.424180642	4.908235551	0.901379038	2	3	6.591606905	74.34959499
chr1	101236749	101241520	1p21.2	S1PR1	47.76978062	52.99781025	0.901353856	4	4	41.57884549	33.77673818
chr15	82130220	82262763	15q25.2	EFL1	11.42928637	12.68049149	0.901328342	4	4	24.54887297	16.8371792
chr16	23557541	23574393	16p12.2	UBFD1	3.457129866	3.835715379	0.901299894	4	4	10.26344397	18.01049817
chr15	71163472	71165512	15q23	HMGB1P6	8.099450757	8.986499686	0.901290941	4	4	25.38062685	14.11340811

chr11	102317373	102339403	11q22.2	BIRC3	35.81785595	39.7434207	0.901227306	4	4	46.3018868	17.50513161
chr2	200712305	200735177	2q33.1	LINC01792	0.114231636	0.126763445	0.901140204	1	2	0	2.25565869
chr12	66302545	66343643	12q	HELB	7.435364589	8.251876031	0.901051417	4	4	14.74101691	27.85499464
chr7	50437320	50450406	7p12.2	FIGNL1	3.390656461	3.763602519	0.900907161	4	4	53.33408829	33.30718617
chr5	95384796	95450441	5q15	FAM81B	1.085228738	1.204690589	0.900836072	4	4	74.75770349	73.91167703
chr7	4642284	4771443	7p22.1	FOXK1	4.141847555	4.597852138	0.90082226	4	4	63.07764849	28.74544136
chr16	89560657	89566829	17p11.2	RPL13	89.93287933	99.83481334	0.900816823	4	4	59.90708899	23.96449789
chr16	18782955	18790334	16p12.3	RPS15A	74.91593277	83.17056982	0.900750505	4	4	24.90150256	12.52423806
chr1	119711796	119744226	1p12	PHGDH	0.478475065	0.531208836	0.900728739	3	4	96.79241921	100.5607195
chr14	22112423	22113031	14q11.2	TRAV25	3.809926759	4.229840044	0.900725966	4	4	18.7182438	66.82982393
chr14	34568652	34569134	14q13.1	RPS19P3	1.427198538	1.584539146	0.900702606	3	4	51.9066247	58.99761058
chr19	52764813	52786825	19q13.41	ZNF600	5.460622448	6.062840508	0.900670641	4	4	31.15899489	55.05925772
chr21	45593654	45603056	21q22.3	LINC01694	0.054607071	0.060633415	0.900610186	3	2	48.3774155	52.9515022
chr14	85934678	86129778	14q31.3	LINC02328	8.876918476	9.856742148	0.900593557	4	4	62.81720457	20.23844872
chr19	8322584	8343262	19p13.2	KANK3	0.448705121	0.498235852	0.900587783	4	3	74.62367124	50.77535085
chr3	196018694	196082161	3q29	TFRC	187.7013022	208.4229793	0.900578731	4	4	68.22465039	41.8911662
chr18	26454910	26657512	18q11.2	KCTD1	0.407397326	0.45240744	0.900509783	3	3	64.61449854	2.926714815
chr9	105519824	105527319	9q31.2	RALGAPA1P1	0.400074873	0.444345277	0.90036936	4	4	39.46602221	35.41861925
chr19	3594506	3608749	19p13.3	TBXA2R	1.251051023	1.389611419	0.900288387	4	4	100.5504152	47.41042859
chr7	73093644	73096950	7q11.23	PMS2P6	4.232464936	4.701325488	0.900270561	4	4	55.16199529	54.63677856
chr12	79935281	79941395	N/A	PPP1R12A-AS1	0.397817675	0.441891358	0.900261268	4	4	52.68837282	31.87583984
chr15	57591904	57685364	15q21.3	MYZAP	1.41930157	1.576760098	0.90013793	4	4	49.95584198	95.54570496
chr3	183451859	183451990	3q27.1	SNORA63D	5.708769108	6.343276141	0.899971715	3	2	87.3788644	6.043750994
chr9	128833113	128882558	9q34.11	KYAT1	0.813622893	0.904100971	0.899924808	4	4	63.73947646	35.97492983
chr12	67269281	67314692	12q14.3-q15	CAND1	11.23781689	12.48815585	0.899878015	4	4	13.38192569	20.04609046
chr5	150586026	150659230	5q33.1	SYNPO	0.199797953	0.222029474	0.89987131	3	4	73.30145334	104.6879838
chr17	2336492	2381074	17p13.3	SGSM2	1.920011766	2.133673042	0.899862222	4	4	98.92936929	15.22667954
chr1	27323874	27326525	1p36.11	ACTG1P20	0.955543584	1.062003298	0.89975576	2	2	8.678061132	16.81198269
chr6	34417454	34426125	6p21.31	RPS10	3.712241335	4.1259571	0.899728535	4	4	12.31076399	23.72429642
chr14	106276546	106276983	14q32.33	IGHV1-24	3.303493033	3.67182835	0.899686129	3	2	88.20170736	116.2582335
chr13	43023226	43109170	13q14.11	DNAJC15	4.738057303	5.266500976	0.899659437	4	4	23.56533971	15.34604831
chr19	47130794	47210636	19q13.32	SAE1	9.839913619	10.93782371	0.899622619	4	4	35.40383401	45.12036762
chr17	62136923	62140639	17q23.2	POLRMTP1	0.268514158	0.298495976	0.899557046	2	3	63.24282385	44.62763432
chr6	43076268	43161720	6p21.1	PTK7	0.31494814	0.350141314	0.899488655	4	4	80.29444789	43.80602841
chr18	9136753	9285985	18p11.22	ANKRD12	84.2378076	93.65091776	0.899487262	4	4	4.072599058	20.02792334
chr19	35248656	35267964	19q13.12	LSR	1.196532732	1.330335449	0.899421821	4	4	120.5776385	53.0821843
chr16	67326844	67385206	16q22.1	LRRC36	0.099964489	0.111154068	0.899332707	2	2	23.12406775	53.68456223
chr9	122369906	122395703	9q33.2	PTGS1	34.64175851	38.52708261	0.899153431	4	4	70.68137866	26.2430441
chr19	11585274	11587311	19p13.2	GAPDHP76	0.253919619	0.282406562	0.899127901	1	2	0	22.31903124
chr1	236819556	236820590	1q43	RPSAP21	0.502937296	0.559402274	0.899061945	3	2	26.64739049	51.01411386
chr6	79484991	79537430	6q14.1	LCA5	1.87281868	2.083271357	0.89897971	4	4	40.44658501	22.06485864
chr5	37291633	37371126	5p13.2	NUP155	8.660421043	9.63534473	0.898817975	4	4	38.93425032	21.00680762
chr22	38201932	38216511	22q13.1	MAFF	2.150710613	2.392970432	0.898761884	4	4	80.71536367	8.806353288

chr16	84177847	84187070	16q24.1	TAF1C	1.624190015	1.807170343	0.898747603	4	4	49.95146719	12.72026837
chr9	127802858	127814520	9q34.11	FPGS	2.708977844	3.014293249	0.898710782	4	4	52.72548756	51.33960853
chr11	6390414	6394998	11p15.4	SMPD1	7.397575752	8.231876296	0.898650015	4	4	50.07404992	40.7428989
chr20	13995500	16053197	20p12.1	MACROD2	0.495141577	0.550996753	0.898628847	4	4	58.59801061	71.42701511
chr14	92703807	92748702	14q32.12	LGMN	1.494088788	1.662715382	0.898583609	4	4	94.08627276	31.52324624
chr8	127735434	127742951	8q24.21	MYC	16.85356635	18.7560174	0.898568496	4	4	70.65956517	57.13930571
chr20	3820524	3825307	20p13	AP5S1	0.976641891	1.087016735	0.898460768	4	4	52.8195557	43.76482656
chr19	40750854	40765392	19q13.2	SNRPA	13.73478756	15.28708597	0.89845688	4	4	43.18879289	68.83788643
chr14	72926332	72962098	14q24.2	DCAF4	0.994928437	1.107453788	0.898392735	4	4	92.71678014	17.7392374
chr10	46286997	46330419	10q11.22	ANTXRL	0.174578285	0.194354274	0.89824773	2	2	65.64971792	21.93258895
chr16	13920137	13952348	16p13.12	ERCC4	3.35518301	3.735825735	0.89811015	4	4	27.38840036	18.36394259
chr14	22513939	22513998	14q11.2	TRAJ29	13.18997673	14.68662318	0.898094583	3	4	77.91664629	73.83028304
chr22	40346500	40387408	22q13.1	ADSL	1.165362163	1.297676423	0.898037556	4	4	29.84340692	8.788102539
chr1	167220829	167427345	1q24.2	POU2F1	8.127351848	9.050343203	0.898015872	4	4	24.26798123	36.57970179
chr19	40028260	40064448	19q13.2	ZNF780B	0.480974627	0.535659151	0.897911715	4	4	20.6691923	41.01409393
chr3	140941820	140979943	3q23	SLC25A36	7.627540573	8.495140106	0.897871074	4	4	36.46227507	24.38940214
chr1	43996483	44031492	1p34.1	SLC6A9	5.006317801	5.576278615	0.897788318	4	4	61.99169936	63.69860906
chr3	58306245	58320193	3p14.3	HTD2	0.146194271	0.162846553	0.897742494	2	3	14.44684862	31.22166552
chr6	34246380	34249108	6p21.31	SMIM29	4.164308687	4.638665963	0.897738427	4	4	65.42697732	25.14886838
chr11	65213840	65242757	11q13.1	SLC22A20P	0.160267955	0.178543803	0.897639415	3	2	46.90302492	85.20625013
chr12	56237807	56258391	12q13.3	ANKRD52	1.856855526	2.068862931	0.897524673	4	4	58.60680263	51.9834825
chr19	12163047	12189881	19p13.2	ZNF136	5.810373566	6.474247025	0.897459356	4	4	44.8098297	28.59686354
chr11	134248265	134253407	11q25	THYN1	8.451880303	9.418432884	0.897376496	4	4	42.90275445	38.87021634
chr11	32892826	32980270	11p13	QSER1	9.272943958	10.33581816	0.897165934	4	4	12.62724793	9.449387739
chr14	106537810	106538265	14q32.33	IGHV3-48	5.946700612	6.628786124	0.897102501	4	3	57.07678935	133.8120914
chrX	49269822	49301465	Xp11.23	PPP1R3F	0.907845701	1.012006405	0.897075055	3	4	56.09089654	44.54132522
chr17	752333	755336	17p13.3	DBIL5P	0.125790066	0.140249106	0.89690458	1	2	0	49.14015891
chr12	52285913	52291515	12q13.13	KRT81	0.120370291	0.134220402	0.896810686	1	1	0	0
chr6	116598184	116633051	6q22.1	RSPH4A	0.288093263	0.321259476	0.896761917	3	4	33.43147467	46.90965928
chr2	42494569	42802118	2p21	MTA3	1.837321009	2.049252481	0.896581083	4	4	30.88106636	24.80974458
chr15	43533462	43590272	15q15.3	PPIP5K1	0.401182914	0.447498463	0.896501211	4	4	83.95385242	34.92251198
chr1	39738845	39765698	1p34.2	PPIE	2.112884637	2.356836542	0.896491802	4	4	35.79858804	12.05317457
chr13	33818069	33973946	13q13.2	RFC3	5.539156512	6.179270696	0.896409428	4	4	44.95471145	25.45124808
chr7	30134936	30162765	7p14.3	MTURN	38.46849448	42.92280767	0.896225027	4	4	32.28831331	35.81350025
chr12	47208420	47216443	12q13.11	PCED1B-AS1	13.86911389	15.47617998	0.896158736	4	4	61.63428115	30.24732163
chr9	125261794	125367207	9q33.3	GAPVD1	29.00630049	32.36772682	0.896148829	4	4	22.89319675	42.58301196
chr5	149345414	149354583	5q32	GRPEL2	6.798958097	7.587392139	0.896086293	4	4	37.48047474	19.85237334
chr4	48063504	48135322	4p12	TXK	17.45569805	19.48032984	0.89606789	4	4	39.37145597	27.13387171
chr2	217799789	218002995	2q35	TNS1	69.68797462	77.78039687	0.895958075	4	4	47.58579832	35.14587825
chr1	40979299	41012565	1p34.2	CTPS1	5.25836209	5.869040645	0.895949169	4	4	42.32115705	33.00445924
chr7	27106190	27153238	7p15.2	HOXA3	0.109841527	0.122617966	0.895802876	1	1	0	0
chr14	90274276	90332137	14q32.11	NRDE2	1.633330357	1.823331767	0.895794384	4	4	53.50743705	30.95877172
chr17	82519718	82604607	17q25.3	FOXK2	5.380939843	6.007117294	0.895760742	4	4	30.02601031	36.68353159

chr2	186694062	186763787	2q32.1	FAM171B	0.209905496	0.234332737	0.895758309	3	4	75.96409743	51.24318862
chr17	66302640	66810744	17q24.2	PRKCA	4.214896076	4.705888106	0.895664321	4	4	55.81853281	29.32240067
chr18	5238100	5246508	18p11.31	LINC00667	5.159261566	5.760614563	0.895609576	4	4	52.40522346	26.96361559
chr19	52222020	52222098	19q13.41	MIR6801	5.53090048	6.175770003	0.895580709	2	2	21.96271067	26.5564062
chr5	111092172	111130502	5q22.1	WDR36	8.890862736	9.927543101	0.895575335	4	4	19.77631508	16.01660857
chr14	22531939	22531998	14q11.2	TRAJ12	23.08051878	25.77211929	0.895561538	3	4	45.19784974	57.85492587
chr17	58335976	58336022	17q22	MIR4736	5.171120471	5.77519896	0.895401268	2	2	33.47195789	9.515221172
chr7	93921704	93926514	7q21.3	GNG11	78.29361274	87.44266046	0.89537089	4	4	24.99640215	42.86449667
chr7	99486519	99500308	7q22.1	ZNF394	32.27845419	36.05189043	0.895333193	4	4	27.07784742	13.27671925
chr6	31639028	31660900	6p21.33	BAG6	85.71426018	95.7458818	0.895226599	4	4	26.46696475	29.14516606
chr6	31665880	31670070	6p21.33	CSNK2B	1.65788467	1.852266176	0.895057466	4	4	53.06785446	42.00040857
chr11	69665563	69675397	11q13.3	LTO1	1.568327086	1.752239594	0.895041461	4	4	36.78464048	18.0850789
chr2	231056864	231172827	2q37.1	PSMD1	23.74534121	26.53117617	0.894997683	4	4	13.16432691	18.88554754
chr1	37534449	37554344	1p34.3	SNIP1	5.705588194	6.37514576	0.894973764	4	4	51.82090114	26.64749228
chr7	73680918	73683451	7q11.23	DNAJC30	1.424370962	1.59173957	0.894851764	3	4	55.86527549	37.27519298
chr1	150487364	150507609	1q21.2	TARS2	4.309064784	4.816375582	0.894669593	4	4	47.81569542	35.32945674
chr5	177600102	177610347	5q35.3	B4GALT7	1.26863902	1.418071137	0.894622975	4	4	59.33459253	38.05161766
chr12	52585589	52602900	12q13.13	KRT72	0.63902572	0.714367232	0.894533919	3	4	69.1721175	95.47613758
chr11	9758293	9811319	11p15.4	SBF2-AS1	1.057128576	1.181792322	0.894512984	4	4	62.55826098	62.09299149
chr1	11054592	11060053	1p36.22	SRM	4.905762987	5.48490035	0.894412418	4	4	99.40291838	60.71638061
chr6	75237675	75244659	6q14.1	COX7A2	22.56133555	25.22612537	0.894363887	4	4	20.94566503	12.83792181
chr3	189956728	190122437	3q28	P3H2	0.161756361	0.180923244	0.894060695	2	3	35.53015464	78.6790454
chr22	22720663	22721155	22q11.22	IGLV3-19	10.84245679	12.13025742	0.893835672	4	4	70.82103798	149.3191672
chr6	166999317	167042418	6q27	FGFR1OP	1.204335163	1.347522794	0.893740105	4	4	21.85188951	51.60320433
chr19	13952474	13961449	19p13.12	DCAF15	6.533166895	7.31037265	0.893684523	4	4	52.38556359	9.553767222
chr11	62593203	62601840	11q12.3	MTA2	27.3603045	30.61565847	0.893670294	4	4	44.52563241	57.53535534
chr13	48771096	48771870	13q14.2	PSME2P2	0.572380296	0.640493754	0.893654766	2	3	41.42022912	74.67369785
chr9	128371319	128392016	9q34.11	URM1	3.325382498	3.721128645	0.893648894	4	4	31.56441316	39.04106633
chr2	190968989	191014250	2q32.2	STAT1	149.3169102	167.1044405	0.893554413	4	4	52.5378481	54.85250271
chr7	137227341	137343990	7q33	PTN	0.133449062	0.149351878	0.893521151	1	1	0	0
chr10	30309801	30349338	10p11.23	MTPAP	6.130026821	6.860957704	0.893465182	4	4	26.5993912	43.45156033
chr12	96303375	96303513	12q23.1	RNU4-24P	5.061139345	5.664802172	0.893436203	3	3	54.26279475	65.75057416
chrX	47651792	47659180	Xp11.23	UXT	37.19904922	41.6404102	0.893340124	4	4	37.07948352	25.53876014
chr6	52805446	52813462	6p12.2	GSTA6P	1.117487943	1.250994012	0.89328001	3	3	69.2840587	62.16389891
chr7	128627172	128627275	7q32.1	RNU6-177P	2.898511391	3.244938807	0.893240694	2	2	49.35919542	22.89104016
chr2	138287130	138288880	2q22.1	YWHAEP5	0.365419217	0.409118394	0.893186964	3	3	20.7259037	29.34381324
chr3	149517229	149724788	3q25.1	WWTR1	0.133801466	0.149802515	0.893185712	3	4	48.43355351	85.64863816
chr19	21290385	21329439	19p12	ZNF708	12.97105993	14.52255969	0.893166233	4	4	48.74392968	12.48464253
chr4	112539333	112636995	4q25	ZGRF1	10.03327926	11.23704018	0.892875624	4	4	73.14837042	51.34032644
chr14	21852558	21853006	14q11.2	TRAV8-3	15.30621431	17.14390559	0.892807898	4	4	30.06610742	62.22439977
chr9	129036511	129072082	9q34.11	MIGA2	2.294193105	2.569734854	0.89277425	4	4	62.25958798	30.1968027
chr9	97654398	97697409	9q22.33	XPA	9.426765199	10.56092787	0.892607668	4	4	43.59110963	7.200978246
chr14	99511266	99604390	14q32.2	CCDC85C	0.109129608	0.122264969	0.89256644	4	4	90.98487057	26.53410306

chr14	34546714	34546877	14q13.1	RNU1-27P	2.052969706	2.300288223	0.892483683	2	1	56.83751483	0
chr11	6497296	6572025	11p15.4	DNHD1	0.507048368	0.568166393	0.892429354	4	4	70.50278942	20.01732992
chr5	112160526	112162501	5q22.1	EPB41L4A-AS1	5.776020546	6.47254208	0.892388257	4	4	47.45070643	21.76859297
chr17	40388521	40417950	17q21.2	TOP2A	40.2721199	45.13353781	0.892288127	4	4	53.15522784	34.5232544
chr2	37201629	37231597	2p22.2	CEBPZ	23.20753468	26.00981203	0.892260761	4	4	36.22685373	11.11489747
chr7	100837314	100852614	7q22.1	SLC12A9-AS1	0.582931444	0.653382482	0.892174891	1	2	0	8.422519438
chr1	21423108	21427960	1p36.12	NBPF2P	0.426385074	0.477954073	0.892104698	1	1	0	0
chr6	73515750	73521032	6q13	EEF1A1	1185.551944	1328.967596	0.892084914	4	4	67.42262632	40.69182939
chr19	54353624	54370556	19q13.42	LAIR1	1.553308133	1.741510553	0.891931508	4	4	24.78601531	46.5373718
chr16	66408524	66410901	16q21	LINC00920	1.252307715	1.404097799	0.891894935	4	4	64.04789301	38.51833062
chr19	48445773	48453907	19q13.33	GRWD1	2.12715425	2.385043176	0.891872429	4	4	53.56537325	35.74333148
chr1	160091339	160099442	1q23.2	IGSF8	4.728630591	5.301948055	0.891866639	4	4	58.30855401	59.34168366
chr12	7060661	7071032	12p13.31	C1S	0.087710419	0.098359518	0.891732904	1	4	0	48.84008119
chr19	55262222	55280423	19q13.42	HSPBP1	2.514831634	2.820220368	0.891714585	4	4	62.68210189	82.26335102
chr9	6720863	7175648	9p24.1	KDM4C	19.29946606	21.64401176	0.891676935	4	4	34.47077662	48.60308038
chr6	159761957	159779112	6q25.3	ACAT2	3.376781256	3.787613393	0.891532716	4	4	58.02739094	37.62540557
chr2	170751691	170753082	2q31.1	EIF2S2P4	3.661499015	4.107392095	0.891441316	4	4	33.51185623	28.3793766
chr22	19788411	19854939	22q11.21	GNB1L	0.279053146	0.313122623	0.891194456	4	4	90.10563028	54.46135293
chr9	109046229	109046298	9q31.3	MIR32	4.210105193	4.724880428	0.891050103	1	1	0	0
chr16	788622	798074	16p13.3	CHTF18	0.477673075	0.536092413	0.891027485	4	4	75.85031975	46.3799871
chr15	25097662	25097755	15q11.2	SNORD116-25	3.856744362	4.328496857	0.891012397	1	1	0	0
chr14	31446036	31457474	14q12	DTD2	4.126992821	4.631843253	0.891004422	4	4	15.05352642	14.05836945
chr1	180949690	180954887	1q25.3	KIAA1614-AS1	0.726751015	0.815682011	0.890973449	2	4	102.8149613	93.15302592
chr17	82389210	82418637	17q25.3	OGFOD3	1.723567508	1.934488504	0.89096808	4	4	32.94754767	18.80484667
chr1	172441457	172444090	1q24.3	PIGC	46.23707976	51.90443138	0.890811796	4	4	121.9277005	102.6175181
chr17	47837681	47841333	17q21.32	SCRN2	2.26317306	2.540604815	0.890800901	4	4	85.54230308	14.81995851
chr1	84364958	84384801	1p31.1	UOX	0.097448712	0.109408951	0.890683174	1	1	0	0
chr3	141231825	141294903	3q23	PXYLP1	0.843046036	0.946530011	0.89067016	4	4	32.21935823	31.84537658
chr3	122411853	122416035	3q21.1	WDR5B	2.922474571	3.281504948	0.890589719	4	4	38.90435605	14.91346698
chr15	68277803	68295865	15q23	FEM1B	31.15913525	34.98825897	0.890559752	4	4	61.23844121	66.53924263
chr6	24797321	24798896	6p22.3	ARMH2	1.039640885	1.167420785	0.890545121	2	3	26.72109488	44.75014579
chr14	96279202	96363401	14q32.2	ATG2B	9.560767762	10.73799611	0.890367966	4	4	32.43915352	16.90093047
chr13	49488970	49489364	13q14.2	SNRPGP14	2.284340956	2.565696092	0.890339648	2	1	60.20799263	0
chr9	34646589	34650598	9p13.3	GALT	2.107303451	2.366887853	0.8903267	4	4	42.68646666	16.69652355
chr19	3474407	3480542	19p13.3	SMIM24	19.64399969	22.06558472	0.890255116	4	4	38.36635881	40.2609527
chr9	97600080	97633680	9q22.33	TSTD2	2.455696728	2.758726965	0.890155771	4	4	21.89166461	32.66172563
chr1	119558552	119559845	1p12	GAPDHP27	0.233125449	0.261897847	0.890138855	1	1	0	0
chr11	62646848	62653302	11q12.3	INTS5	3.369837947	3.785949314	0.890090613	4	4	33.58613642	21.78253016
chrX	72272603	72277291	Xq13.1	RPS4X	307.0671176	344.9854687	0.890087107	4	4	45.34772239	44.21382372
chr7	100435277	100436615	7q22.1	PPP1R35	6.86680528	7.715232077	0.890032239	4	4	41.2057189	8.568557436
chr6	30345129	30346858	6p22.1	RPP21	0.157453242	0.176912748	0.89000507	1	2	0	14.67344639
chr17	7591230	7614897	17p13.1	FXR2	6.703743709	7.533818793	0.889820142	4	4	32.38609428	25.61284201
chr10	45854835	45972428	10q11.22	PARGP1	14.97367962	16.82959331	0.8897232	4	4	30.65712251	38.96855253

chr2	47783082	47806954	2p16.3	MSH6	5.643367533	6.342956753	0.889706134	4	4	23.45729868	45.28039253
chr15	45167214	45201175	15q21.1	SHF	0.291639418	0.327810968	0.889657291	4	4	54.02203422	90.03190947
chr10	5265989	5271236	10p15.1	LINC02561	0.959924852	1.078991112	0.889650379	3	2	50.7623166	93.15235679
chr19	34677639	34686397	19q13.11	ZNF302	5.495708598	6.178906626	0.889430595	4	4	65.09667454	13.19860191
chr1	155139891	155140573	1q22	DPM3	6.005289967	6.75226688	0.889373906	4	4	109.7439571	43.80709081
chr14	64945814	64972203	14q23.3	RAB15	0.633948734	0.712957546	0.889181604	4	4	81.69021252	45.98274496
chr1	42846563	42852477	1p34.2	ZNF691	1.461979606	1.644376286	0.889078503	4	4	72.5289271	38.46125475
chr2	190408355	190502315	2q32.2	MFSD6	12.34458911	13.88583975	0.889005587	4	4	35.06007884	52.75628133
chr19	42397148	42652355	19q13.2	LIPE-AS1	0.241083759	0.271192641	0.888976037	3	4	79.33691522	36.50660401
chr2	241494707	241509730	2q37.3	STK25	2.940845756	3.308538954	0.888865386	4	4	70.84162942	28.71562553
chr5	123023383	123036730	5q23.2	PPIC	0.162641731	0.183008697	0.888710395	1	2	0	47.10920032
chr9	93058688	93085138	9q22.31	SUSD3	9.351662183	10.52322838	0.888668557	4	4	44.03742686	16.51738397
chr11	14643691	14874139	11p15.2	PDE3B	24.79152236	27.89746217	0.888665866	4	4	25.62903072	30.19535527
chr16	681666	684446	16p13.3	JMJD8	4.1112964	4.626817967	0.888579674	4	4	43.4841744	38.22818414
chr6	136231030	136253659	6q23.3	MTFR2	0.772080584	0.868982923	0.888487637	4	4	44.66534681	56.97682223
chr20	55074556	55075178	20q13.2	RPL12P4	5.056474525	5.69111129	0.88848632	4	4	57.83725249	49.54776018
chr3	180983709	180989774	3q26.33	DNAJC19	4.263552545	4.798787409	0.88846456	4	4	24.8959539	19.88779392
chr16	30875460	30895220	16p11.2	MIR762HG	1.166418554	1.312991628	0.888367091	4	4	82.73507416	38.78422766
chr17	40019097	40054742	17q21.1	MED24	4.415743757	4.970732386	0.888348721	4	4	31.4908228	24.70253123
chr8	122670385	122694106	8q24.13	LINC01151	8.507337528	9.577481618	0.888264563	4	4	66.96120511	84.03289255
chr9	26946990	27066134	9p21.2	IFT74	38.88941143	43.78184456	0.888254294	4	4	53.08305955	40.9714164
chr10	95605929	95656901	10q24.1	ALDH18A1	6.548045986	7.371995607	0.888232486	4	4	26.37066315	53.37515482
chr10	102402617	102420164	10q24.32	PSD	0.080852316	0.091035105	0.888144377	3	3	67.18470675	58.69840168
chr6	73368555	73369792	6q13	OOEP	0.30684732	0.345496809	0.888133584	2	1	37.7176409	0
chr22	19440886	19448232	22q11.21	C22orf39	2.903308033	3.269131478	0.888097665	4	4	46.63035909	39.725299
chr20	56369389	56392337	20q13.2	AURKA	102.3044626	115.2029406	0.888036903	4	4	85.17694639	63.48334304
chr19	13104900	13117567	19p13.13	TRMT1	2.656124054	2.991213058	0.887975548	4	4	35.44624078	43.59785056
chr7	151076621	151080883	7q36.1	FASTK	3.219966458	3.626420023	0.887918784	4	4	56.83179859	17.73434839
chr11	5619944	5644398	11p15.4	TRIM34	0.175484477	0.197640829	0.887895876	2	1	63.67476858	0
chr6	132814569	132817564	6q23.2	RPS12	1698.942565	1913.492576	0.88787518	4	4	28.03593797	30.53495321
chr3	184561799	184582408	3q27.1	EPHB3	0.258566026	0.291291552	0.887653706	4	2	127.82556	17.96736718
chr19	47745536	47757066	19q13.33	NOP53	22.39348877	25.22794155	0.887646292	4	4	70.62896729	37.74991917
chr1	58575423	58577494	1p32.1	TACSTD2	0.883516319	0.995501932	0.887508392	3	3	129.6630121	143.9961192
chr15	57591904	57717557	15q21.3	GCOM1	0.536205016	0.604184064	0.887486195	4	4	59.14254816	67.55148585
chr8	116766503	116774684	8q24.11	UTP23	7.515277729	8.468440013	0.887445352	4	4	25.09127387	19.85583655
chr8	30384501	30572261	8p12	RBPMS	0.054194564	0.061072871	0.887375416	1	1	0	0
chr6	31969811	31972290	6p21.33	DXO	0.835539702	0.941718195	0.887250248	4	4	56.00818257	64.82042939
chr16	75596879	75623323	16q23.1	ADAT1	8.703382486	9.809671492	0.887224663	4	4	41.30414339	16.21615933
chr11	22622533	22625841	11p14.3	FANCF	4.176701529	4.707812135	0.887185259	4	4	37.53053425	31.71952157
chr12	12473282	12562514	12p13.2	DUSP16	1.645871129	1.855169204	0.88718114	4	4	73.94819105	21.74766854
chr12	52948871	52952901	12q13.13	KRT18	0.718489946	0.81002532	0.886996897	3	4	70.45596609	122.6246373
chrX	136639543	136642429	Xq26.3	LINC00892	0.842158472	0.949629623	0.88682835	4	4	34.0431314	51.45678766
chr1	163321933	163355764	1q23.3	NUF2	4.88095531	5.503991464	0.886802849	4	4	53.1011686	39.90503076

chr1	222814514	223005995	1q41	DISP1	0.690717875	0.778953919	0.88672495	4	4	74.33314333	58.05586962
chr14	22007629	22008181	14q11.2	TRAV19	11.54894352	13.02533239	0.886652499	4	4	17.95952219	56.91927111
chr14	22537622	22537680	14q11.2	TRAJ7	17.54189378	19.78596048	0.886582878	2	4	0.409342095	50.11306681
chr14	20989806	20997035	14q11.2	METTL17	3.195118536	3.603872908	0.886579138	4	4	60.25065919	16.0876635
chr6	26156331	26157115	6p22.2	HIST1H1E	273.2839606	308.2463004	0.886576612	4	4	53.64276746	72.2450143
chr7	65373799	65401135	7q11.21	ZNF92	21.72747714	24.5077699	0.886554641	4	4	20.94800192	24.59945005
chr12	6970781	6997428	12p13.31	EMG1	1.601156988	1.806158795	0.886498459	4	4	48.91448154	22.64178387
chr12	67989445	68021327	12q15	IFNG-AS1	2.060888153	2.324949935	0.886422594	4	4	79.88654109	93.59801872
chr7	98617285	98629869	7q22.1	NPTX2	0.149452073	0.168604232	0.886407599	1	1	0	0
chr6	151411262	151413957	6q25.1	HSPA8P15	0.364418073	0.411139423	0.8863613	1	3	0	26.87566093
chr17	50693190	50707909	17q21.33	ANKRD40	7.171127957	8.091298896	0.886276486	4	4	33.45448812	27.24570306
chr17	8247596	8270491	17p13.1	PFAS	1.675576648	1.890590158	0.88627175	4	4	68.97012033	64.81854846
chr6	27132316	27132796	6p22.1	HIST1H2BJ	64.36530363	72.62953534	0.886213898	4	4	50.80583685	83.77283702
chr1	154206701	154220797	1q21.3	C1orf43	29.1058679	32.84634189	0.886122053	4	4	26.62546793	8.755557738
chr7	129502479	129512932	7q32.1	SMKR1	0.567106165	0.640013698	0.886084418	2	2	62.3647797	31.6160422
chr1	156737302	156741267	1q23.1	MRPL24	5.785530151	6.529595107	0.886047306	4	4	67.27928534	35.41517271
chr7	100119642	100125508	7q22.1	CNPY4	3.351934414	3.783607544	0.885909645	4	4	54.38347619	37.47280424
chr19	57746796	57758159	19q13.43	ZNF776	11.42093348	12.89238563	0.885866573	4	4	57.11732269	31.15276739
chr7	41685101	41710532	7p14.1	INHBA	0.146704429	0.165617863	0.885800758	3	4	85.99362773	41.18939056
chr14	59452773	59465636	14q23.1	GPR135	0.277907287	0.313742334	0.885781922	4	3	47.57697548	29.71485847
chrX	10015755	10144478	Xp22.2	WWC3	23.15566356	26.14313215	0.885726447	4	4	46.75081575	29.42387982
chr17	70169535	70180044	17q24.3	KCNJ2	17.53789332	19.80113367	0.885701476	4	4	108.3041443	86.13454354
chr2	199955317	199964125	2q33.1	MAIP1	6.142005291	6.934778381	0.885681554	4	4	39.14251698	22.55330747
chr19	46746015	46758575	19q13.32	FKRP	1.215190771	1.372053683	0.885672905	4	4	57.65221512	41.63347109
chr7	100582490	100601117	7q22.1	FBXO24	0.117593656	0.132774115	0.885667026	4	3	30.38667946	69.6991173
chr11	14277920	14364506	11p15.2	RRAS2	8.360274771	9.4399108	0.885630696	4	4	23.47605715	31.340258
chrX	41919254	41919349	Xp11.4	RNU6-202P	4.667247821	5.270771338	0.885496168	2	1	79.67253581	0
chr11	19117099	19176420	11p15.1	ZDHHC13	2.293647569	2.590649337	0.885356245	4	4	10.97940107	27.97664766
chr16	1962061	1964826	16p13.3	RPS2	259.2410502	292.8336866	0.885284248	4	4	81.17554694	51.74147361
chr5	73565741	73583377	5q13.2	UTP15	3.699735858	4.179543681	0.885200907	4	4	21.08224967	13.84630517
chr10	35126830	35212958	10p11.21	CREM	12.08437389	13.65249979	0.885140016	4	4	32.5042103	63.90049577
chr1	98661284	98760500	1p21.3	SNX7	0.112976109	0.127638551	0.885125286	1	1	0	0
chrX	65034815	65507887	Xq11.2-q12	ZC3H12B	0.303362267	0.342776613	0.885014484	4	4	89.61182677	70.46584517
chr7	42932340	42937857	7p14.1	MRPL32	5.766027431	6.515421128	0.884981541	4	4	27.72367672	20.33003134
chr15	90930180	90954093	15q26.1	UNC45A	4.016365272	4.538953118	0.884865996	4	4	41.87561209	26.89970863
chr13	48037567	48052742	13q14.2	NUDT15	4.330571624	4.894184035	0.884840372	4	4	46.53486023	5.570541453
chr8	11339637	11368452	8p23.1	TDH	0.15217567	0.172023906	0.884619317	2	2	21.30883712	45.39738899
chr1	151870866	151909885	1q21.3	THEM4	3.187911388	3.603934456	0.884564197	4	4	71.41495027	36.28220879
chr5	97175950	97176113	5q15	RNU1-73P	2.032998352	2.299220581	0.884211967	2	1	68.26233573	0
chr20	38805693	38923024	20q11.23	PPP1R16B	15.68166548	17.73952851	0.883995619	4	4	48.86274918	55.53316901
chr1	42657770	42681654	1p34.2	PPIH	11.31422164	12.8011501	0.883844151	4	4	33.79169565	41.6282214
chr3	37789783	37790532	3p22.2	NDUFAF4P3	1.496257689	1.692955281	0.883814065	4	4	85.94648735	61.86903964
chrX	1403139	1453794	Xp22.33 and Yp11	ASMTL	4.319393357	4.88801241	0.883670702	4	4	88.28372962	30.79211613

chr7	149867694	149873862	7q36.1	ATP6V0E2-AS1	0.353818743	0.400409159	0.883642982	3	4	22.69380267	60.46117074
chr1	11654375	11663327	1p36.22	FBXO44	1.58047006	1.788926912	0.883473802	4	4	31.25193549	27.92077278
chr5	82273320	82278416	5q14.2	RPS23	204.9899576	232.0435571	0.883411547	4	4	67.24052939	27.13350285
chr19	40069008	40091706	19q13.2	ZNF780A	0.272685505	0.308715223	0.883291409	4	4	63.20670774	38.35095241
chr3	10248493	10281222	3p25.3	TATDN2	2.746610743	3.10953852	0.883285647	4	4	52.06930811	9.915334136
chr16	67939884	67944112	16q22.1	LCAT	1.367360833	1.5481978	0.883195179	4	4	44.79296702	71.71381426
chr8	144428780	144444488	8q24.3	TONSL	0.484280022	0.548366297	0.883132361	3	3	35.53312989	44.58827125
chr19	13836587	13836659	19p13.12	MIR23A	18.81166268	21.30121185	0.883126407	4	2	75.56267783	41.96096141
chr11	61258286	61295316	11q12.2	VWCE	1.42192125	1.610204534	0.883068716	4	4	27.80133622	47.6184034
chr3	98657539	98660967	3q12.1	WWP1P1	0.190098272	0.215306994	0.882917314	3	2	54.64295527	83.45454838
chr15	89361579	89398487	15q26.1	MIR9-3HG	0.32381556	0.366845788	0.882702136	4	4	53.37176562	40.60803786
chr9	35099776	35103195	9p13.3	STOML2	15.39919577	17.44587687	0.882683965	4	4	21.14036209	26.36419201
chr20	44942130	44960473	20q13.12	TOMM34	4.196182404	4.754153132	0.882635096	4	4	76.63440348	55.52435173
chr2	64390956	64455286	2p14	LGALSL-DT	0.303070203	0.343399049	0.882559823	1	2	0	11.01565335
chr6	137866317	137883314	6q23.3	TNFAIP3	54.57806879	61.84682839	0.882471587	4	4	56.21938321	96.6961415
chr15	89681531	89690784	15q26.1	PEX11A	0.596314608	0.675735295	0.882467755	4	4	45.06711285	62.40608071
chr6	116249964	116254098	6q22.1	TSPYL4	3.612176646	4.093329364	0.882454434	4	4	74.26911339	38.77927375
chrX	130129362	130165887	Xq26.1	AIFM1	5.692949145	6.451712494	0.882393496	4	4	43.21627791	52.14468858
chr16	58025566	58046901	16q21	MMP15	0.14443148	0.163741439	0.882070417	3	2	91.95787855	41.00709177
chr12	45729706	45908040	12q12	ARID2	13.62537651	15.44874058	0.881973287	4	4	16.37542094	16.10129024
chr3	52075233	52154690	3p21.2	POC1A	1.031923101	1.170059938	0.881940375	4	4	46.64824464	52.00034235
chr19	14477759	14496149	19p13.12	GIPC1	1.740833074	1.973918949	0.881917201	4	4	73.54340492	68.20210537
chr1	117929749	117960426	1p12	WDR3	3.630807364	4.117933388	0.881706192	4	4	34.81458615	35.41086851
chr17	27928924	27991707	17q11.2	LINC01992	0.190115058	0.215634423	0.881654495	2	2	17.62597974	11.51831732
chr11	107790991	107859188	11q22.3	SLC35F2	1.716134474	1.946503239	0.881649945	4	4	44.80336075	83.43567863
chr12	10699077	10723442	12p13.2	YBX3	198.5883562	225.2862002	0.881493656	4	4	16.53475184	17.11097953
chr6	136821683	136913934	6q23.3	PEX7	4.083358911	4.633170406	0.881331476	4	4	43.02971064	49.91166648
chr17	5419641	5433020	17p13.2	RPAIN	2.178981642	2.472697244	0.881216513	4	4	53.26657853	10.0870828
chr13	40347132	40481006	13q14.11	LINC00598	0.125841528	0.142829472	0.881061352	4	4	31.33555651	74.72964267
chr17	16939084	16972088	17p11.2	TNFRSF13B	0.220565152	0.250344422	0.881046801	2	4	36.77551864	120.3719663
chr6	72630495	72678558	6q13	KCNQ5-IT1	1.817164008	2.062606957	0.881003529	4	4	54.28989555	98.92412034
chr10	133641889	133643091	10q26.3	AGGF1P2	0.161403448	0.183204796	0.881000123	2	2	1.768133613	29.40989671
chr9	136428615	136439861	9q34.3	INPP5E	0.653139422	0.741405049	0.88094817	4	4	72.15656286	54.42562209
chr11	2129112	2149603	11p15.5	IGF2	0.178673903	0.202833592	0.880889115	4	4	104.8227036	108.3849132
chr15	40331452	40340967	15q15.1	CCDC9B	0.325808571	0.369912336	0.880772385	4	4	73.58085289	52.70064389
chr17	2322678	2337384	17p13.3	TSR1	4.702381811	5.338935685	0.880771391	4	4	63.81502628	19.69875319
chr6	30699807	30717988	6p21.33	MDC1	0.523747357	0.594689306	0.880707542	4	4	40.3966223	77.65668884
chr17	75130225	75131965	17q25.1	NT5C	4.725637062	5.365858459	0.880686119	4	4	58.89439691	34.04746667
chr7	56064007	56080672	7p11.2	SUMF2	8.58495213	9.748069506	0.880682285	4	4	45.45746393	10.34410506
chr2	226970293	227164559	2q36.3	COL4A4	0.137044583	0.155659263	0.880413926	3	2	28.29300713	30.2747042
chr12	119989806	120094495	12q24.23	BICDL1	2.939092921	3.338390999	0.880392058	4	4	76.61995656	79.97396774
chr11	83455009	85628535	11q14.1	DLG2	0.128962992	0.146507431	0.880248809	4	4	40.77933292	46.65597985
chr4	145757627	145938960	4q31.21-q31.22	ZNF827	1.93536587	2.198766853	0.880205133	4	4	35.57248938	33.95139927

chr20	36620402	36621439	20q11.23	HNRNPA3P2	1.03892306	1.180323213	0.880202175	2	2	96.43600767	59.49634053
chr14	61187305	61550980	14q23.1	PRKCH	30.26564394	34.38593814	0.880175024	4	4	33.99921845	61.79451823
chr13	46341739	46390042	13q14.13	RUBCNL	10.53439799	11.96864518	0.880166287	4	4	61.26587408	57.21646499
chr1	96446930	96448623	1p21.3	EEF1A1P11	0.315968812	0.358995742	0.880146407	3	2	40.85079132	10.09771264
chr10	14954228	14988050	10p13	MEIG1	0.158925946	0.180588808	0.880043167	1	1	0	0
chr1	217426992	217631112	1q41	GPATCH2	3.008165098	3.418283047	0.880022238	4	4	43.18285011	23.93693535
chr14	50329404	50397404	14q21.3	CDKL1	17.66019085	20.07110354	0.879881408	4	4	34.07369443	50.30033582
chr2	88861221	88861258	2p11.2	IGKJ3	27.66626349	31.44331613	0.879877408	3	3	84.61828671	96.92337559
chr19	19865830	19893484	19p13.11	ZNF253	7.250793174	8.240871693	0.87985755	4	4	19.37374384	26.7224367
chr5	178130532	178148570	5q35.3	RMND5B	1.455695057	1.654487766	0.879846371	4	4	63.54780077	23.31960094
chr19	12559485	12561278	19p13.2	PGK1P2	0.183329549	0.208392565	0.879731718	1	1	0	0
chr22	41833079	41907308	22q13.2	SREBF2	5.103922146	5.804491937	0.879305579	4	4	50.99538679	45.25690884
chr2	241970683	241977276	2q37.3	LINC01238	0.08278947	0.094158062	0.879260554	1	2	0	11.98134607
chr12	132144421	132152473	12q24.33	NOC4L	2.976247972	3.384993498	0.879247766	4	4	69.63130537	55.24632309
chr8	91954967	92103451	8q21.3	RUNX1T1	0.364452444	0.414505259	0.879246854	4	4	114.2985199	98.00062602
chr1	226360691	226408100	1q42.12	PARP1	18.71055527	21.28112495	0.879208939	4	4	46.27277259	62.95943121
chr1	236523069	236524508	1q43	LGALS8-AS1	0.352510142	0.400986561	0.879107123	3	4	59.51433919	43.74500432
chr5	97160867	97183301	5q15	RIOK2	5.742031135	6.531880165	0.879077844	4	4	49.22227488	12.59783419
chr2	97913054	97913117	2q11.2	RNU7-96P	7.175600433	8.163064694	0.879032655	4	3	55.95973883	19.99179718
chr11	65122183	65127369	11q13.1	MRPL49	8.721159626	9.921503943	0.879015891	4	4	49.84667064	32.55712205
chr9	33921693	34048949	9p13.3	UBAP2	132.1200547	150.3152123	0.878953319	4	4	49.1901468	18.0891471
chr16	2969245	2973489	16p13.3	PAQR4	1.558382401	1.773043428	0.878930756	4	4	83.21256265	87.77587693
chr16	28878957	28879921	16p11.2	ATP2A1-AS1	0.623297856	0.709241864	0.878822709	2	2	31.69206472	33.07520677
chr6	29887573	29891079	6p22.1	HLA-H	11.88678601	13.52929231	0.878596289	4	4	63.48112389	29.62252433
chr4	16360691	16361826	4p15.32	ZEB2P1	0.119727598	0.136277464	0.878557571	1	1	0	0
chr7	149714945	149734575	7q36.1	KRBA1	0.604236067	0.687886763	0.878394672	4	4	70.22477958	67.79897765
chr2	127846266	127858155	2q14.3	POLR2D	3.745726626	4.264788357	0.878291327	4	4	46.15361696	2.765119644
chr1	226083575	226090289	1q42.12	LINC01703	0.336969454	0.383698103	0.878215065	2	1	29.07591266	0
chr12	6494132	6531966	12p13.31	NCAPD2	13.0493233	14.85946229	0.87818274	4	4	19.73900574	54.21207232
chr2	28927983	28928060	2p23.2	SNORD53B	5.224319324	5.949316104	0.878137795	1	2	0	20.59549821
chr4	48341475	48426198	4p11	SLAIN2	42.25708731	48.13361852	0.877912125	4	4	10.34794841	7.533178411
chr17	28328325	28335489	17q11.2	IFT20	5.752450865	6.552505738	0.877900928	4	4	24.82982347	38.26143136
chrX	104112445	104121330	Xq22.2	ZCCHC18	0.373874845	0.425931773	0.877781064	3	4	88.35341355	40.38474204
chr6	136922241	136925660	6q23.3	SLC35D3	0.672102077	0.765688995	0.877774243	3	3	65.03560487	73.90643606
chrX	19360058	19515261	Xp22.12	MAP3K15	0.137053003	0.156137301	0.877772329	4	3	72.11534831	60.19847409
chr1	11664093	11674354	1p36.22	FBXO6	7.458828164	8.497632811	0.877753644	4	4	51.56508214	69.61545099
chr14	22096050	22096619	14q11.2	TRDV1	10.98333863	12.51651089	0.877508015	4	4	53.5892651	26.6140863
chr14	70722526	70809262	14q24.2	MAP3K9	0.484515511	0.552289731	0.877285027	4	4	81.76014989	62.58030167
chr9	121455041	121500027	9q33.2	GGTA1P	5.096342031	5.809326248	0.877269035	4	4	82.80999382	55.91393004
chr12	47079603	47236663	12q13.11	PCED1B	15.71302354	17.91286436	0.877192125	4	4	45.19825083	44.08546123
chr17	57838926	57850072	17q22	MRPS23	2.754891689	3.140669687	0.877166962	4	4	36.94907483	27.74615746
chr10	13317428	13348298	10p13	SEPHS1	5.331717535	6.079685566	0.87697258	4	4	53.99362242	11.3780633
chr4	48885380	48906828	4p11	OCIAD2	3.470779246	3.958101088	0.87687989	4	4	44.66057193	13.51362568

chr1	178542796	178548889	1q25.2	C1orf220	0.196273195	0.223834417	0.876867806	3	4	40.69945924	59.58379568
chr19	14566078	14572077	19p13.12	NDUFB7	21.87117296	24.94509346	0.87677254	4	4	65.02450558	53.12550056
chr3	53283423	53347627	3p21.1	DCP1A	10.56711855	12.05367461	0.87667196	4	4	21.90485768	11.54563066
chr13	75549480	75606020	13q22.2	UCHL3	2.034946459	2.321234437	0.876665634	4	4	38.33295983	50.91997683
chr14	22168429	22168988	14q11.2	TRAV30	3.330259964	3.798902429	0.876637404	4	4	45.99781878	110.5133584
chr16	23641366	23676207	16p12.2	DCTN5	4.160404313	4.746237492	0.876568928	4	4	42.2028618	35.8233738
chr5	154682663	154817607	5q33.2	LARP1	19.21469119	21.92080453	0.876550455	4	4	27.73234139	41.19353571
chr19	7619525	7629553	19p13.2	XAB2	5.46765995	6.237729202	0.87654654	4	4	58.66145693	31.5493742
chr9	35507534	35507931	9p13.3	RPL36AP33	0.800428168	0.913171196	0.876536811	2	1	20.34582506	0
chr2	216498797	216501467	2q35	RPL37A	58.46365645	66.70305705	0.876476417	4	4	2.81631873	9.869850844
chr14	102317377	102343174	14q32.31	ZNF839	1.880666559	2.146055709	0.876336318	4	4	39.97100611	31.22258562
chr11	34915829	34996128	11p13	PDHX	31.31053583	35.72897057	0.876334676	4	4	20.82099553	12.9026696
chr7	141778489	141779388	7q34	TAS2R4	0.259572789	0.296232108	0.876247989	3	2	59.15198259	42.59522644
chr22	20424584	20437859	22q11.21	SCARF2	0.201227491	0.229689107	0.876086346	2	4	80.35795994	60.07841302
chr11	66278201	66284214	11q13.2	CNIH2	0.112879459	0.128860595	0.875981202	1	4	0	10.4820014
chr19	43766533	43782139	19q13.31	KCNN4	1.926466671	2.199381532	0.875912907	4	4	76.00975687	32.06048904
chr17	19237377	19336715	17p11.2	EPN2	2.299103169	2.624839545	0.875902366	4	4	37.18944356	30.45275458
chr12	52609851	52615305	12q13.13	KRT73-AS1	0.229372349	0.261873153	0.875891041	3	3	113.2854834	62.60773123
chr1	109539872	109545833	1p13.3	GPR61	0.077993067	0.08904474	0.87588629	3	4	35.42544977	83.25693259
chr16	56470403	56520096	16q13	BBS2	2.872478664	3.279532543	0.875880518	4	4	49.00520917	31.09183157
chr2	130181922	130190729	2q21.1	MZT2B	10.11812175	11.55207423	0.875870562	4	4	49.23073663	43.35501726
chr3	18345388	18445588	3p24.3	SATB1	18.57060017	21.20259622	0.875864445	4	4	67.46414906	21.16354074
chr22	17137520	17165287	22q11.1	HDHD5	1.668397378	1.905009634	0.87579472	4	4	55.15867368	39.23079075
chr19	12576106	12610809	19p13.2-p13.13	ZNF490	1.835626322	2.096080747	0.87574218	4	4	40.40133925	29.45156119
chr16	30378312	30400108	16p11.2	ZNF48	1.396778478	1.595003324	0.875721359	4	4	59.28944711	54.38299765
chr9	20341665	20622543	9p21.3	MLLT3	7.947747157	9.07771042	0.875523319	4	4	43.81116627	20.92333236
chrX	130110633	130184873	Xq26.1	RAB33A	2.380534703	2.719548862	0.875341766	4	4	47.52994595	67.90279075
chr13	98793487	99086406	13q32.3	DOCK9	3.711811244	4.240579815	0.875307483	4	4	46.92261219	30.8948975
chr10	49942033	49988221	10q11.23	TIMM23B	9.462994909	10.8113782	0.875281091	4	4	28.6925514	56.46592697
chr1	154983294	154993111	1q21.3	FLAD1	3.306369965	3.777982876	0.875168065	4	4	52.97296315	17.84883707
chr7	142720680	142721160	7q34	TRBV28	9.939410603	11.35752297	0.875138939	4	4	60.83691842	69.46711353
chr11	46396412	46594069	11p11.2	AMBRA1	8.170577093	9.336324761	0.875138484	4	4	21.28801317	21.15291369
chr3	149964904	149970954	3q25.1	PFN2	0.637898883	0.728945028	0.87509875	3	4	40.48346781	61.94898981
chr11	116835751	116837950	11q23.3	APOA1	0.156728403	0.179129049	0.874946883	1	1	0	0
chr1	61742466	62163920	1p31.3	PATJ	2.65386846	3.033498548	0.87485404	4	4	53.80534569	28.64259461
chr15	20408396	20506180	15q11.1-q11.2	HERC2P3	1.226166532	1.401684733	0.874780543	4	4	92.82154142	50.87903177
chr11	45847118	45883248	11p11.2	CRY2	4.208136395	4.810524408	0.874777059	4	4	43.82772928	12.26424345
chr17	34927859	34981222	17q12	CCT6B	0.812201126	0.928714268	0.874543607	4	4	37.1592193	40.88010205
chr15	38488097	38564806	15q14	RASGRP1	30.11272809	34.43332942	0.874522696	4	4	24.9125607	50.19953877
chr2	202073253	202205203	2q33.1	KIAA2012	0.02929225	0.033495229	0.874520082	1	1	0	0
chr2	241074394	241102332	2q37.3	MTERF4	4.870095848	5.56898702	0.874502999	4	4	78.44621106	32.9110046
chr17	5384833	5419739	17p13.2	NUP88	27.22821006	31.13681728	0.874469918	4	4	54.28747512	39.06650107
chr11	867859	915058	11p15.5	CHID1	2.68910407	3.07532773	0.874412195	4	4	17.5313375	22.16882715

chrX	55452089	55453568	Xp11.21	MAGEH1	7.297835146	8.346233194	0.874386682	4	4	60.39033646	42.55563345
chr3	113286930	113441514	3q13.2	CFAP44	2.196546745	2.512207775	0.874349155	4	4	30.93430988	48.41865628
chr5	441835	443160	5p15.33	EXOC3-AS1	0.489055413	0.559340605	0.87434277	3	3	49.9562314	45.88204427
chr4	2071958	2242164	4p16.3	POLN	2.360658775	2.700172525	0.874262201	4	4	71.32080232	31.96337343
chr1	151762969	151771330	1q21.3	OAZ3	0.137022455	0.156729468	0.874260957	1	2	0	42.08882286
chr6	43506965	43510343	6p21.1	LRRC73	0.136028965	0.155601455	0.874213964	2	1	25.76668321	0
chr19	3052910	3063107	19p13.3	AES	29.79940405	34.08861326	0.874174723	4	4	30.7473946	41.55470959
chr3	58716417	59050045	3p14.2	C3orf67	0.099889109	0.114272834	0.874128219	4	3	46.40443979	65.48662558
chr16	19067595	19080095	16p12.3	COQ7	3.374193064	3.860072927	0.874126766	4	4	39.65847619	9.268308897
chr4	73836556	73838760	4q13.3	CXCL6	1.370137687	1.567498704	0.874091751	4	4	61.32481578	77.29892735
chr9	137306894	137423262	9q34.3	EXD3	0.228641713	0.261579456	0.874081307	4	4	64.47657227	39.36548563
chr19	2163963	2232578	19p13.3	DOT1L	3.5898381	4.107449834	0.873982214	4	4	35.68301704	24.61972938
chr20	49278178	49289260	20q13.13	ZFAS1	15.47492273	17.70761692	0.873913345	4	4	25.00683763	43.06188286
chr7	130018286	130051451	7q32.2	ZC3HC1	3.255407845	3.725975716	0.873706136	4	4	38.48820327	14.41443375
chr11	119121523	119135055	11q23.3	HINFP	1.457566848	1.668531275	0.873562797	4	4	38.4533046	4.418557089
chr11	32090931	32105726	11p13	RCN1	2.791124002	3.195122859	0.873557646	4	4	51.0736596	44.62686583
chr4	24527464	24584561	4p15.2	DHX15	18.70031309	21.407193	0.873552786	4	4	25.19797395	13.79971961
chr14	76886320	76886775	14q24.3	RPL22P2	1.74890035	2.002118006	0.873525109	2	3	80.46766222	61.48089031
chr4	212600	255985	4p16.3	ZNF876P	3.697770326	4.233193823	0.87351784	4	4	69.09876759	36.01112216
chr22	45133758	45163781	22q13.31	NUP50-DT	3.420907844	3.916766397	0.87340104	4	4	64.95705994	20.99839191
chr19	44286348	44305707	19q13.31	ZNF235	1.273142367	1.457734355	0.873370626	4	4	43.9921215	35.71624289
chr2	53669980	53787009	2p16.2	ASB3	0.784265738	0.898063771	0.873285131	4	4	37.30492705	24.44404334
chr13	98577255	98578841	13q32.2	STK24-AS1	1.119606742	1.282087927	0.873268298	3	3	91.88580579	48.80930134
chr6	5261001	5771592	6p25.1	FARS2	6.763532014	7.747187756	0.873030605	4	4	21.1357321	19.70850626
chr10	73730562	73731771	10q22.2	GLUD1P3	1.281035838	1.467426537	0.87298124	4	4	79.40855657	39.22892875
chr19	34758074	34773229	19q13.11	ZNF599	0.224319986	0.256964091	0.872962388	4	4	38.99247944	31.50378008
chr1	235131183	235161616	1q42.3	RBM34	0.885614256	1.014743747	0.8727467	4	4	47.44762992	33.79440176
chr12	9752486	9760901	12p13.31	CD69	55.74424807	63.88122414	0.872623354	4	4	37.1287075	64.90734735
chr12	10441673	10454685	12p13.2	KLRC1	6.100244342	6.990795763	0.872610866	4	4	74.37905609	13.62225322
chr12	48688458	48716998	12q13.11-q13.12	CCNT1	14.75760251	16.91349774	0.87253404	4	4	25.4497922	17.58816508
chr11	36594246	36659291	11p12	C11orf74	8.381652853	9.606391292	0.872507958	4	4	39.47628886	55.93575246
chr19	37564253	37594830	19q13.12	ZNF571	1.53843618	1.763317653	0.872466839	4	4	22.92528128	53.65797627
chr11	66744736	66843516	11q13.2	C11orf80	5.241505476	6.008027406	0.872417038	4	4	64.6432801	24.06013451
chr9	96754549	96778549	9q22.33	ZNF510	5.163645245	5.919074427	0.872373765	4	4	27.14386261	12.54476235
chr20	380629	397559	20p13	TRIB3	1.331903104	1.526801492	0.872348574	4	4	86.64608674	47.67509582
chr15	100344457	100350718	15q26.3	SPATA41	0.27976813	0.320788658	0.872126002	2	3	71.70637575	110.7346021
chr17	73232090	73248959	17q25.1	C17orf80	6.462160955	7.410166157	0.872066944	4	4	7.833183297	20.96286903
chr5	85464339	85465372	5q14.3	RPL5P17	0.454402057	0.521069141	0.872057126	1	1	0	0
chr13	91398619	92867237	13q31.3	GPC5	0.247933955	0.284324604	0.872010202	4	4	74.1734732	21.4262578
chr20	33666498	33668525	20q11.22	ACTL10	0.254445715	0.291799572	0.871987963	3	3	61.33863039	44.95386726
chr7	141006161	141014981	7q34	MRPS33	2.459635924	2.820902833	0.871932168	4	4	36.59926971	32.69241194
chr2	73254682	73284449	2p13.2	FBXO41	0.768787037	0.881726001	0.871911496	4	4	70.00256721	28.75621565
chr17	79794377	79797116	17q25.3	CBX8	0.810724841	0.929926478	0.871816064	4	4	77.3326225	57.11551343

chr1	26169897	26170873	1p36.11	ZNF593	3.107367937	3.56457397	0.871736135	4	4	69.05736062	62.15895317
chr5	111719769	111720061	5q22.1	RN7SKP57	1.274631459	1.46227394	0.871677614	1	1	0	0
chrX	107777733	107795777	Xq22.3	NCBP2L	0.49070112	0.562978615	0.871615914	4	4	72.22476083	47.66702607
chr16	1614640	1677908	16p13.3	CRAMP1	2.745549483	3.149970354	0.871611214	4	4	16.7869256	19.75101181
chr4	6024925	6200591	4p16.1	JAKMIP1	3.315663857	3.804073611	0.871608753	4	4	89.66612225	135.613646
chr12	5560241	5948790	12p13.31	ANO2	0.694546024	0.796944233	0.871511451	4	4	72.36591831	22.30997841
chr11	62815966	62816067	11q12.3	RNU6-118P	8.091294497	9.287045019	0.871245319	2	4	38.68731066	29.74075928
chr18	74591774	74597835	18q22.3	LINC00909	3.846713427	4.415416664	0.87120055	4	4	31.48135382	28.92973315
chr4	2059512	2069089	4p16.3	NAT8L	0.138069327	0.158491224	0.87114809	3	1	110.7756347	0
chr20	50958814	50961863	20q13.13	MOCS3	1.465139096	1.681881452	0.871131015	4	4	28.30037956	15.12612849
chr19	44072137	44089613	19q13.31	ZNF284	0.400469653	0.459714842	0.871126222	4	4	62.10734528	36.90877765
chr3	32752830	32753920	3p22.3	SUGT1P2	1.052235383	1.208053913	0.871016907	4	3	28.46351039	41.39993268
chr16	3658037	3717597	16p13.3	TRAP1	3.239448388	3.719219293	0.871002254	4	4	50.90296917	48.52377465
chr22	39223359	39245055	22q13.1	PDGFB	0.531955484	0.610841301	0.870857101	4	4	71.82413517	45.38137131
chr15	78540405	78549262	15q25.1	PSMA4	16.71263182	19.19147472	0.870836247	4	4	32.39602758	32.3586444
chr3	66998303	67011210	3p14.1	KBTBD8	5.460122078	6.270475855	0.870766781	4	4	36.14157529	16.87292891
chr11	68903851	68940601	11q13.3	IGHMBP2	1.78873084	2.054287892	0.870730362	4	4	73.7873093	30.42402868
chr1	109399031	109426486	1p13.3	PSMA5	8.086105762	9.28758304	0.870636174	4	4	34.11485568	43.86665605
chr2	74529405	74533556	2p13.1	HTRA2	5.121664578	5.882820114	0.870613835	4	4	31.83077082	18.14525489
chr11	119305759	119317130	11q23.3	MCAM	0.058603897	0.067317362	0.870561396	2	2	46.53417887	66.26282653
chr1	209779208	209784730	1q32.2	C1orf74	0.572299012	0.657398503	0.870551133	4	4	58.25639609	25.35549799
chr5	54977867	54985586	5q11.2	ESM1	0.386475207	0.443993479	0.870452439	3	3	5.43901204	86.20454855
chrX	103628720	103630953	Xq22.2	TCEAL1	1.858794018	2.135507779	0.870422499	4	4	66.56057938	50.20395056
chr9	123109494	123115477	9q33.3	MIR600HG	0.671669799	0.771666565	0.870414541	4	4	76.80600032	55.48050481
chr16	25236001	25257937	16p12.1	ZKSCAN2	1.14863468	1.319743055	0.870347206	4	4	60.72289015	28.05103913
chr2	223755330	223837602	2q36.1	AP1S3	0.839343536	0.964386557	0.870339316	4	4	34.19144983	45.34325276
chr20	297563	300321	20p13	ZCCHC3	5.33814639	6.134019748	0.87025256	4	4	36.15410086	40.53782943
chr7	1570073	1589626	7p22.3	PSMG3-AS1	0.37824014	0.434633908	0.870249957	4	4	90.19748117	68.53997015
chr10	35158306	35158637	10p11.21	ATP6V1G1P4	0.770760005	0.885762253	0.870165784	1	2	0	21.58144023
chr11	124673844	124694794	11q24.2	SPA17	0.295832436	0.340033192	0.870010465	4	4	55.81533495	17.7148947
chr3	160404588	160404685	3q25.33	MIR15B	3.599036768	4.137123979	0.86993689	1	1	0	0
chr7	44565417	44574538	7p13	DDX56	4.894330391	5.626164743	0.86992305	4	4	62.81287926	14.80126167
chr12	133119969	133130473	12q24.33	ZNF891	0.636305116	0.731465162	0.869904883	4	4	48.26601296	26.86665472
chr9	137582079	137590485	9q34.3	ZMYND19	1.588355374	1.826062118	0.869825489	4	4	57.90938552	63.23668023
chr19	49085140	49108613	19q13.33	SNRNP70	11.79517958	13.56100347	0.869786635	4	4	64.66472058	27.78000986
chr19	49591177	49626439	19q13.33	PRR12	2.165747886	2.490411806	0.869634444	4	4	93.09306967	61.44019491
chr6	149998019	150005144	6q25.1	RAET1K	0.122144238	0.140475369	0.869506444	1	1	0	0
chr10	102918293	103087169	10q24.32	CNNM2	1.300249719	1.495465843	0.869461328	4	4	43.85929396	22.16122365
chr19	32875925	32972029	19q13.11	CEP89	3.79254634	4.362232907	0.869404826	4	4	25.99975593	19.33037992
chr7	158631169	158704831	7q36.3	NCAPG2	7.766036712	8.934400081	0.869228671	4	4	91.67503937	73.41845033
chr11	57657744	57661865	11q12.1	CLP1	5.089361987	5.85503477	0.869228312	4	4	50.82743953	44.60942846
chr19	11814284	11835617	19p13.2	ZNF440	1.755298769	2.019474496	0.869185906	4	4	77.48835337	29.257739
chr13	49660676	49691487	13q14.2	EBPL	3.486140387	4.011053948	0.869133258	4	4	59.18378926	21.44615898

chr22	26560428	26561616	22q12.1	HMGB1P10	2.033246878	2.339843341	0.868967098	3	4	96.1262859	71.70969226
chr12	56998833	57006513	12q13.3	ZBTB39	2.119430706	2.439272551	0.868878184	4	4	56.80235654	21.68867463
chr14	22489488	22489543	14q11.2	TRAJ49	11.97618431	13.78394193	0.868850462	3	3	16.8916581	66.2524135
chr14	102080738	102139749	14q32.31	HSP90AA1	89.58206609	103.1085814	0.868812904	4	4	51.94080971	29.07311995
chr6	104682670	104859919	6q16.3	HACE1	3.129868145	3.602716994	0.868752153	4	4	41.73834248	12.13861396
chr11	7987320	7996171	11p15.4	EIF3F	14.99017587	17.25487704	0.868750084	4	4	53.57148719	33.67996951
chr22	37807894	37817177	22q13.1	GCAT	8.390244751	9.660809549	0.868482575	4	4	76.76607639	44.17379183
chr1	225919877	225924340	1q42.12	PYCR2	5.979860092	6.885659824	0.868451281	4	4	36.59280761	13.52349369
chr3	120686681	120742537	3q13.33	RABL3	5.795810885	6.673774519	0.868445715	4	4	20.06305057	19.50813407
chr5	38474963	38606290	5p13.1	LIFR	0.038213575	0.044015791	0.868178778	2	1	41.1996956	0
chr9	92210207	92293756	9q22.31	IARS	12.91463721	14.88482682	0.867637721	4	4	15.65606413	34.72880878
chr5	90458203	90474768	5q14.3	MBLAC2	2.426664019	2.797493661	0.867442187	4	4	32.55440601	51.62294686
chr11	126202094	126211692	11q24.2	RPUSD4	2.47051157	2.848131118	0.867414971	4	4	57.55991026	18.70299889
chr12	130871879	130877678	12q24.33	RAN	24.23416699	27.93869823	0.867405016	4	4	20.30142872	40.04893924
chr15	48329991	48343373	15q21.1	DUT	5.519871327	6.364238952	0.867326222	4	4	25.96350462	29.57161245
chr5	40831328	40835285	5p13.1	RPL37	90.99356656	104.9495885	0.867021661	4	4	14.95765098	22.60288383
chr11	108472106	108498432	11q22.3	KDELC2	3.15907514	3.643787908	0.866975581	4	4	36.59226906	43.9593938
chr11	3714772	3714875	11p15.4	RNU6-1143P	6.544612752	7.548855711	0.866967525	1	2	0	37.30077586
chr11	124739933	124747206	11q24.2	NRGN	124.8523204	144.0176704	0.866923622	4	4	77.24640224	54.05619588
chr7	1542235	1560821	7p22.3	TMEM184A	0.063988438	0.073824488	0.866764395	2	3	58.59300174	61.25975233
chr16	10944488	11193272	16p13.13	CLEC16A	14.82979277	17.10964469	0.866750481	4	4	26.2186795	6.614460326
chr4	587325	670892	4p16.3	PDE6B	0.685702389	0.791220833	0.86663844	4	4	84.81787361	67.03789315
chr5	178007692	178063588	5q35.3	FAM153C	0.427149177	0.493020823	0.866391757	3	4	62.86678406	102.1807412
chr5	179797597	179806977	5q35.3	MGAT4B	4.861210868	5.611312621	0.8663233	4	4	64.48576225	35.16434758
chr19	49676166	49688450	19q13.33	PRMT1	5.188990545	5.98987714	0.866293319	4	4	69.98352809	54.52819765
chr19	19668070	19683506	19p13.11	ZNF101	10.73058151	12.38683483	0.866289222	4	4	60.50289518	9.871209697
chr9	136803927	136808848	9q34.3	CCDC183-AS1	0.389501638	0.449629349	0.866272718	4	4	100.3997705	37.09702392
chrX	149527591	149539752	Xq28	LINC00893	0.432631187	0.499473806	0.866173925	4	4	29.24978	63.69339868
chr6	132817219	132817351	6q23.2	SNORA33	15.85445826	18.30413929	0.86616792	4	4	55.61749374	46.05609328
chr6	44192365	44218236	6p21.1	MYMX	0.261797405	0.302275194	0.866089611	1	1	0	0
chr19	43607224	43619862	19q13.31	ZNF428	4.720606916	5.45079956	0.866039351	4	4	16.5867909	23.35153861
chr4	114598402	114687914	4q26	UGT8	0.249306054	0.287898442	0.865951382	3	4	65.44693217	35.10631405
chr14	106470263	106470720	14q32.33	IGHV3-43	0.815028612	0.941259053	0.865891923	2	2	0.444793135	52.53796756
chr15	66499315	66504855	15q22.31	RPL4	166.9707812	192.8384402	0.865858389	4	4	53.60310016	31.61688078
chr12	53180715	53190870	12q13.13	ZNF740	3.405069846	3.933133835	0.865739634	4	4	55.38541375	14.04691563
chr19	19119616	19138501	19p13.11	TMEM161A	1.09580789	1.266094682	0.865502324	4	4	71.68049789	40.65972559
chr9	131430090	131500191	9q34.13	PRRC2B	12.33954677	14.25756349	0.865473738	4	4	42.95597709	34.23156809
chr3	111071759	111201444	3q13.13	NECTIN3	0.280610521	0.324229589	0.865468577	4	3	58.03498538	89.4650638
chr7	2679522	2765187	7p22.3	AMZ1	0.130883268	0.151240406	0.865398813	1	3	0	34.1597382
chr20	18288228	18316996	20p11.23	ZNF133	1.415881622	1.636301456	0.865293872	4	4	48.8373152	45.21698376
chrX	119434519	119434820	Xq24	RN7SL118P	1.637533573	1.892508912	0.865271261	2	3	80.49417571	93.0288091
chr17	78787381	78840840	17q25.3	USP36	3.552675241	4.106057628	0.865227808	4	4	49.08567291	38.60270311
chr19	57389850	57403497	19q13.43	ZNF548	3.241571146	3.746894799	0.865135351	4	4	66.55062022	18.58507247

chr12	120579045	120581452	12q24.31	POP5	2.317252287	2.678696787	0.865067035	4	4	76.6685995	30.91763477
chr7	100336065	100367831	7q22.1	STAG3L5P-PVRIG2	0.077065417	0.089087207	0.865055939	3	3	3.032808406	43.0487643
chr12	123633739	123662604	12q24.31	GTF2H3	5.730503145	6.624486332	0.865048678	4	4	38.56252817	15.21707222
chr13	20138252	20161327	13q12.11	GJA3	0.056280628	0.065063068	0.865016517	2	3	21.50080848	38.91370874
chr12	116713320	116738100	12q24.22	C12orf49	4.724579119	5.461992218	0.864991917	4	4	41.41064229	33.44146485
chr8	143213193	143217170	8q24.3	GPIHBP1	0.103170076	0.119279493	0.864943955	1	2	0	15.46518366
chr14	21521080	21537216	14q11.2	SALL2	0.36965006	0.427371272	0.864938952	4	4	110.3216201	38.69838261
chr12	53307456	53321628	12q13.13	AAAS	4.041347776	4.672783243	0.864869515	4	4	12.72628197	45.91804754
chr6	31463180	31465809	6p21.33	HCP5	2.115350625	2.446150451	0.864767179	4	4	50.6830503	61.07953452
chr12	14914027	14938549	12p12.3	ERP27	2.669507301	3.087141819	0.864718065	4	4	36.36007863	28.7402708
chr19	20531230	20565821	19p12	ZNF737	9.885857448	11.43281278	0.864691624	4	4	42.60548937	22.92002689
chr8	108201743	108248734	8q23.1	EIF3E	65.15920591	75.35953676	0.864644459	4	4	44.75452727	20.68532184
chr1	8893411	8894603	1p36.23	HMGN2P17	0.295153242	0.341434358	0.864450913	1	1	0	0
chr9	135932969	135961380	9q34.3	UBAC1	12.26792034	14.19346595	0.864335771	4	4	13.40365755	24.10870444
chr4	40191011	40244764	4p14	RHOH	8.998467214	10.41085457	0.864335118	4	4	15.70926339	12.83645792
chr18	2916994	3013315	18p11.31	LPIN2	50.39672526	58.30733145	0.864329133	4	4	18.55345793	54.85059077
chr15	57706521	57717557	15q21.3	POLR2M	5.988934012	6.929449103	0.864272747	4	4	35.25922152	18.04670208
chr5	121961961	122075624	5q23.1	SRFBP1	20.26888506	23.45204607	0.864269369	4	4	25.00693256	11.94356106
chr4	74123273	74303099	4q13.3	MTHFD2L	3.665359029	4.241087459	0.86424981	4	4	35.71108259	20.49644915
chr19	55591371	55599703	19q13.42	FIZ1	0.556527833	0.643954829	0.864234273	4	4	91.61715523	58.05287048
chr10	17752110	17800916	10p12.33	TMEM236	0.690528334	0.799092138	0.864141069	4	4	66.58030956	87.11108446
chr3	9933788	9945413	3p25.3	CRELD1	1.592024723	1.842558716	0.864029303	4	4	96.35184549	31.9661038
chrX	23833348	23907940	Xp22.11	APOO	5.173756854	5.988108857	0.864005144	4	4	71.66406703	73.17571695
chr8	28494205	28574268	8p21.1	FZD3	0.507032564	0.586883628	0.863940549	4	4	45.76462584	55.45753951
chrX	57905430	57910633	Xp11.21	ZXDA	2.221808562	2.571844683	0.863896866	4	4	45.52002179	19.67444641
chr3	108296490	108378285	3q13.13	HHLA2	0.104685004	0.121188607	0.86381886	2	2	10.78782177	61.00034704
chr17	7686071	7703502	17p13.1	WRAP53	1.059791554	1.226872469	0.86381558	4	4	61.21135887	68.28387746
chr2	164687643	164687777	2q24.3	SNORA70F	1.79974482	2.083738311	0.863709618	1	1	0	0
chr9	33252471	33264761	9p13.3	BAG1	78.738871	91.166581	0.863681298	4	4	40.28274842	41.49983633
chr1	32231660	32241710	1p35.2	MTMR9LP	0.080171818	0.092826206	0.863676563	1	2	0	7.359373112
chr7	143390813	143408892	7q34-q35	EPHA1	0.510668943	0.591294497	0.863645688	3	4	81.58374164	41.97988055
chr11	27338514	27363248	11p14.1	CCDC34	2.448696861	2.837382221	0.863012689	4	4	45.96146884	17.19839556
chr18	5289019	5297053	18p11.31	ZBTB14	8.451714575	9.794190155	0.862931436	4	4	41.51029979	10.40645185
chr17	47823272	47831541	17q21.32	MRPL10	9.171057217	10.63021377	0.862734976	4	4	32.78205946	85.70720083
chr16	675666	678271	16p13.3	RHBDL1	0.193388051	0.224189527	0.862609658	2	2	14.93418547	52.20866751
chr5	77784801	77786597	5q14.1	ACTBP2	0.515713395	0.597933982	0.8624922	4	3	19.94089102	59.46951489
chr12	26905171	26938338	12p11.23	INTS13	12.23247187	14.18755719	0.862197184	4	4	9.083212253	24.28812823
chr10	121740421	121928801	10q26.13	ATE1	16.42901773	19.06100961	0.861917499	4	4	19.35306992	12.49103758
chr15	59768330	59769188	15q22.2	RPS3AP6	1.567679598	1.819030238	0.861821627	4	4	59.27614883	25.49882251
chr16	2235816	2238711	16p13.3	DNASE1L2	0.186262822	0.21615588	0.861706018	2	2	41.28972191	63.18395032
chr11	62707255	62709206	11q12.3	GNG3	0.657703322	0.763261357	0.861701324	2	1	60.59485488	0
chr17	39980768	39997960	17q21.1	PSMD3	6.790913345	7.881141657	0.861666195	4	4	27.12618605	28.72004739
chr16	142422	154511	16p13.3	HBZ	7.800011061	9.052801693	0.861612938	4	4	67.47102701	115.6425371

chr1	92831986	92841924	1p22.1	RPL5	172.6319805	200.3795966	0.861524743	4	4	59.66046752	17.44428865
chr13	21176233	21179084	13q12.11	MRPL57	6.909076847	8.020047221	0.861475831	4	4	33.59169375	37.20315698
chrX	106940300	107000244	Xq22.3	MORC4	0.844821874	0.980706399	0.861442196	4	4	53.64259766	40.34833161
chr22	19046162	19048375	22q11.21	DGCR11	1.747900817	2.029161972	0.861390486	4	4	111.7939141	17.9662985
chr17	7251737	7259940	17p13.1	ELP5	3.401445661	3.949112671	0.861318971	4	4	92.37564105	48.18467069
chr11	71434411	71448431	11q13.4	DHCR7	1.183583926	1.374154843	0.861317728	4	4	40.34625345	30.98209826
chr11	787110	790126	11p15.5	CEND1	0.266563463	0.309483867	0.861316184	1	1	0	0
chr3	48223347	48225491	3p21.31	CAMP	12.83054061	14.89730029	0.861266159	4	4	53.34079728	85.37451635
chr2	127257290	127294176	2q14.3	ERCC3	3.515493983	4.082121461	0.861192891	4	4	53.40650582	18.38820422
chrX	154506159	154516257	Xq28	FAM3A	1.655031408	1.921846412	0.861167364	4	4	39.18800405	40.31969445
chr7	95772506	96110322	7q21.3	DYNC1I1	0.311702044	0.362021473	0.861004297	3	4	104.804376	69.92635797
chr4	175632937	176002691	4q34.2	GPM6A	0.158274045	0.18383622	0.860951363	2	4	24.82257606	145.510224
chrX	119786504	119791659	Xq24	RPL39	141.5178221	164.3739091	0.860950639	4	4	64.86402994	71.26605871
chr3	130212820	130273806	3q22.1	COL6A4P2	0.186981883	0.217206731	0.86084755	3	2	103.8442768	14.56594876
chr4	127880861	127899224	4q28.1	PLK4	4.967141848	5.770224043	0.860823048	4	4	49.11686776	51.718254
chr14	52553082	52637713	14q22.1	GPR137C	0.116083969	0.134868239	0.860721324	4	3	48.96434568	72.10828004
chr1	43336875	43354464	1p34.2	MPL	1.830206488	2.126382085	0.860713839	4	4	66.92998538	43.4812843
chrX	52891599	52908560	Xp11.22	FAM156B	0.054329122	0.063126962	0.860632614	1	1	0	0
chr11	47589664	47596336	11p11.2	C1QTNF4	0.162641731	0.18898194	0.860620499	1	1	0	0
chr11	65116403	65117738	11q13.1	ZNHIT2	1.445454368	1.679707533	0.860539314	3	4	12.68520665	33.70148959
chr2	172555373	172672698	2q31.1	PDK1	6.611151394	7.683798272	0.860401478	4	4	19.66535336	22.86692226
chr7	142778642	142779537	7q34	WBP1LP1	0.246335552	0.28633649	0.860300942	1	1	0	0
chr19	708953	748330	19p13.3	PALM	0.085614016	0.099516911	0.860296155	2	1	51.95960644	0
chr12	56416373	56449465	12q13.3	TIMELESS	1.844401077	2.143948231	0.860282469	4	4	58.49292552	88.19808322
chrX	47142216	47145210	Xp11.3	NDUFB11	12.26155348	14.2561406	0.860089264	4	4	39.76854676	26.2269647
chr12	100156391	100173343	12q23.1	GOLGA2P5	0.368341189	0.428406654	0.859793343	4	4	64.22962862	69.21355847
chr2	36696690	36814794	2p22.2	VIT	0.126187227	0.146768182	0.859772365	2	2	33.59663544	9.517736676
chr17	76140556	76154648	17q25.1	RNF157-AS1	0.518770752	0.603423383	0.85971271	2	4	62.67088266	51.83739388
chr11	111927144	112022650	11q23.1	DIXDC1	0.328490932	0.382094775	0.85971061	4	4	72.76908978	95.52491042
chr22	20482748	20484155	22q11.21	KRT18P5	0.349229092	0.406260047	0.859619583	2	2	71.69356382	16.87134301
chr20	2461635	2470853	20p13	SNRPB	18.58474541	21.62165963	0.859542964	4	4	38.15408303	18.28855264
chr19	9412426	9435578	19p13.2	ZNF266	9.55024087	11.11337155	0.859346853	4	4	25.82458797	21.28964168
chr20	36573473	36593952	20q11.23	TGIF2	6.228496082	7.248021484	0.85933742	4	4	42.74480519	20.55564226
chr1	178725147	178921842	1q25.2	RALGPS2	12.89048472	15.0007407	0.859323214	4	4	42.56947065	54.95176774
chr3	121653994	121654296	3q13.33	RN7SL172P	3.538104988	4.118044365	0.859171168	3	4	114.1202116	54.0602653
chr12	54280690	54287088	12q13.13	HNRNPA1	118.6764396	138.1317839	0.859153746	4	4	49.58289985	33.37444282
chr11	12110576	12263790	11p15.3	MICAL2	47.50630952	55.29551791	0.859134905	4	4	15.41144687	17.90701633
chr18	2571511	2616635	18p11.32	NDC80	12.98500075	15.11529745	0.859063528	4	4	41.64131481	23.41734212
chr7	149069641	149090777	7q36.1	ZNF786	2.492051656	2.901222994	0.858965912	4	4	43.54807908	35.55497514
chr9	113313222	113340340	9q32	WDR31	3.48053594	4.052372216	0.858888512	4	4	94.59047391	68.59875607
chr19	35612744	35625349	19q13.12	HAUS5	0.998587064	1.162718598	0.858838128	4	4	78.4540987	23.83880746
chr9	130693760	130704894	9q34.12	EXOSC2	3.326839457	3.87387397	0.858788769	4	4	41.69983738	23.42031936
chr16	34265919	34268850	16p11.2	BCLAF1P2	0.193332732	0.225126579	0.858773463	3	4	50.41912697	55.87579204

chr19	1009651	1021142	19p13.3	TMEM259	9.277556162	10.80548321	0.858597064	4	4	56.35101245	11.47276883
chr17	36486627	36499310	17q12	ZNHIT3	2.422795157	2.821874432	0.85857653	4	4	42.65892041	1.231570221
chr14	69879388	70032366	14q24.2	SMOC1	0.102333282	0.119200648	0.858496024	2	2	75.22856609	9.711369627
chr3	100363459	100401398	3q12.2	TOMM70	13.19547281	15.37142832	0.858441554	4	4	24.21041347	9.045423401
chr2	174557967	174558072	2q31.1	RNU6-5P	12.57955808	14.65426568	0.858422958	4	4	52.25716359	45.16597603
chr11	46700767	46705916	11p11.2	ZNF408	2.24505296	2.6153644	0.858409237	4	4	44.70547076	39.03917733
chr19	54102338	54106974	19q13.42	NDUFA3	9.283946547	10.81741336	0.858240897	4	4	34.31005432	29.73974425
chr12	109088188	109093631	12q24.11	ALKBH2	1.430724076	1.667355355	0.858079876	4	4	61.70892899	41.76401395
chr8	124539102	124549986	8q24.13	NDUFB9	11.41439812	13.30229673	0.858077244	4	4	47.20719076	54.84928262
chr17	59210010	59215250	17q22	SMG8	4.902817271	5.713962172	0.858041605	4	4	59.54043048	23.98508907
chr18	36828817	37232127	18q12.2	KIAA1328	4.804809643	5.601556809	0.857763262	4	4	25.73222397	38.2198168
chr19	12525370	12551542	19p13.2	ZNF564	0.795077038	0.926924503	0.857758141	4	4	82.18636411	41.22940476
chr22	20131841	20148007	22q11.21	ZDHHC8	1.11074524	1.29504724	0.857687044	4	4	72.09697492	58.70332214
chr1	89615810	89719535	1p22.2	LRRC8C	7.918977009	9.233531114	0.857632569	4	4	28.23643453	21.33779616
chr17	19406979	19417276	17p11.2	RNF112	0.118362954	0.138023147	0.857558729	2	2	47.71926665	7.630216455
chr16	14632811	14669236	16p13.12	BFAR	4.908071462	5.723864382	0.857475149	4	4	17.28487514	22.09072602
chr11	58884906	58893648	11q12.1	GLYATL1P2	0.894258727	1.042970845	0.857414885	4	4	55.53511997	81.61750835
chr1	154199085	154206365	1q21.3	C1orf189	0.743535615	0.867218865	0.857379429	3	1	32.14428932	0
chr14	22543179	22543240	14q11.2	TRAJ3	10.44834314	12.18648898	0.857371074	3	4	10.09008108	36.91181695
chr13	99372173	99372686	13q32.3	HMGB3P4	1.360871688	1.587322264	0.857337996	3	4	81.79594571	63.86283651
chr22	22758741	22759211	22q11.22	IGLV2-14	51.53062617	60.11122145	0.857254684	4	4	66.13463274	140.8790099
chr3	48564073	48595302	3p21.31	COL7A1	0.075759966	0.08838561	0.85715271	2	3	4.994702716	126.602619
chr5	176647387	176659058	5q35.2	TSPAN17	3.421797272	3.992054437	0.857151957	4	4	20.41676208	45.94518642
chr6	89433178	89633834	6q15	ANKRD6	0.710861703	0.829425126	0.857053495	4	4	76.69404628	57.32912283
chr6	31494881	31511124	6p21.33	MICB	3.086659325	3.602265232	0.856866201	4	4	59.53147255	47.72952287
chr17	40177010	40195656	17q21.1	RAPGEFL1	0.536119487	0.625674937	0.856865851	4	4	77.92107101	65.3765212
chr10	73423579	73433561	10q22.2	MSS51	0.834242269	0.973764668	0.856718565	4	4	46.98256013	30.79347209
chr19	19900913	19935573	19p12	ZNF93	2.892163814	3.3763581	0.856592733	4	4	41.48748446	27.6840697
chr9	5107937	5109290	9p24.1	MTND4P14	0.643246262	0.750943323	0.856584302	3	4	63.28460495	66.81099181
chr5	176595802	176610130	5q35.2	GPRIN1	0.254022547	0.296563075	0.856554872	3	4	62.86265293	76.1314117
chr6	53647901	53665854	6p12.1	KLHL31	0.050700076	0.059194568	0.856498792	1	3	0	38.86237201
chr22	46039907	46044868	22q13.31	LINC00899	0.116324401	0.1358202	0.856458763	4	4	86.32912648	70.45520345
chr10	43643859	43648878	10q11.21	ZNF32	6.358703406	7.424638649	0.856432711	4	4	57.54421095	34.14725634
chr10	46109495	46131678	10q11.22	AGAP7P	2.111522461	2.465556778	0.856407964	4	4	65.12266846	46.27076868
chr12	6852121	6866632	12p13.31	USP5	8.26184398	9.647680156	0.856355502	4	4	51.20662646	53.15957302
chr5	67182275	67196789	5q12.3	CD180	5.092088739	5.947123998	0.856227101	4	4	44.24437779	97.7453562
chr19	39498917	39508481	19q13.2	DLL3	0.089957672	0.105082984	0.856063168	1	2	0	9.67468997
chr19	54532692	54545768	19q13.42	KIR3DX1	0.13924742	0.162679105	0.855963771	3	4	47.21282144	73.47804813
chr14	67241295	67336061	14q23.3	MPP5	4.422205578	5.167405111	0.855788444	4	4	30.59302365	19.92298421
chr19	49856465	49859289	19q13.33	PTOV1-AS2	2.048951953	2.394234	0.855786006	4	4	99.10048008	25.90383109
chr12	9936579	9943495	12p13.31	LINC02470	1.647184017	1.925061239	0.855652789	4	2	136.5502873	122.269495
chr7	156640202	156678333	7q36.3	RNF32	1.669314394	1.95094242	0.855645137	4	4	44.57994424	78.17627613
chr20	45405993	45410610	20q13.12	DBNDD2	0.23512861	0.274801059	0.855632111	2	2	61.32672688	93.56427899

chr1	51195095	51235096	1p32.3	LINC01562	0.215066221	0.251367673	0.855584245	2	2	17.74247297	83.63034844
chr7	66847450	66884273	7q11.21	GTF2IP23	4.360583603	5.096665116	0.855575853	4	4	58.74751326	127.5657481
chr7	13891229	13991425	7p21.2	ETV1	0.030464485	0.035619396	0.855277978	2	3	17.02945091	42.47701864
chr12	101873133	101873662	12q23.2	HSPE1P4	3.16303506	3.698881211	0.855132912	3	2	53.25860885	38.74994621
chr1	151629326	151629426	1q21.3	RNU6-1062P	3.110019347	3.637537413	0.854979343	1	1	0	0
chr4	55078481	55125595	4q12	KDR	0.098582605	0.1153056	0.854968061	1	1	0	0
chr5	171387116	171410884	5q35.1	NPM1	133.0948748	155.7553492	0.854512385	4	4	45.03603667	32.73709566
chr14	55148116	55191678	14q22.3	DLGAP5	54.93437297	64.29238856	0.85444598	4	4	58.22063965	32.49848812
chr19	55476332	55484487	19q13.42	ZNF628	0.765991405	0.89652839	0.854397266	4	4	106.2977618	81.25167743
chr1	248826377	248826443	1q44	MIR3124	14.11289189	16.52029254	0.854276149	4	3	43.95434429	25.87985095
chr17	81228277	81239092	17q25.3	TEPSIN	2.826389442	3.308667507	0.854237978	4	4	62.7438249	36.34297254
chr22	25448087	25461678	22q11.23	CRYBB2P1	1.17972726	1.381103771	0.854191614	4	4	48.21554594	31.46567972
chr2	229763837	229923234	2q36.3	TRIP12	76.96345114	90.12595958	0.853954305	4	4	13.02267882	32.85328185
chr3	188562237	188568666	3q28	LPP-AS1	0.44466171	0.520733397	0.85391433	1	1	0	0
chr1	153990755	153992155	1q21.3	RPS27	1105.094339	1294.292861	0.853820933	4	4	43.57169935	27.75611647
chr22	39097203	39104067	22q13.1	APOBEC3H	1.978620432	2.317408894	0.853807214	4	4	57.65045008	113.3884075
chr14	70583221	70600690	14q24.2	MED6	6.226968036	7.293526704	0.8537664	4	4	12.39955057	18.17484836
chr3	128489203	128503207	3q21.3	GATA2-AS1	0.171890167	0.20137253	0.853592926	2	4	18.58220702	83.1896831
chr14	35122303	35274078	14q13.2	KIAA0391	1.567141854	1.835938617	0.85359164	4	4	45.20100374	49.30327246
chr12	50175780	50283570	12q13.12	LIMA1	3.268392023	3.829270845	0.853528558	4	4	43.53493502	59.37337161
chr19	55401282	55408439	19q13.42	UBE2S	3.253695498	3.812056563	0.853527602	4	4	23.11071789	49.42401771
chr19	36313061	36331718	19q13.12	LINC00665	0.27327447	0.320205756	0.853433971	3	4	76.76503845	66.51172494
chr6	43225629	43229473	6p21.1	DNPH1	2.386632988	2.797360533	0.853173182	4	4	49.23834845	37.68977828
chr1	151070180	151125545	1q21.3	GABPB2	9.512103039	11.14910042	0.853172245	4	4	32.16030127	23.19756903
chr19	41859918	41871416	19q13.2	RPS19	51.38647789	60.23635163	0.853080847	4	4	28.43122574	10.20195852
chr1	247034638	247078813	1q44	ZNF670	1.044614509	1.224579942	0.853039049	4	4	88.74979267	11.35594118
chr4	106921802	107036296	4q25	DKK2	0.097713153	0.114551775	0.853004275	2	3	45.59815157	49.78945179
chr20	45898620	45912364	20q13.12	PLTP	0.661403969	0.775465988	0.852911642	4	4	48.02450145	75.57767254
chr16	58515479	58521990	16q21	SETD6	0.880093799	1.032091891	0.852728141	4	4	49.66298454	57.43644888
chr2	3575263	3580919	2p25.3	RPS7	58.61102792	68.74843629	0.852543433	4	4	32.50059432	14.92287725
chr3	112120841	112133956	3q13.2	GCSAM	3.131479012	3.673323918	0.852491934	4	4	59.90098281	67.09732241
chr19	10486120	10503378	19p13.2	KEAP1	5.780346827	6.780814282	0.852456149	4	4	19.86874494	58.67729491
chr1	36088852	36093932	1p34.3	ADPRHL2	7.968506182	9.348646923	0.85237	4	4	66.95495561	12.6571679
chr12	122021886	122062044	12q24.31	BCL7A	0.59295293	0.695747117	0.852253522	4	4	64.70938044	50.51258665
chr10	119060011	119060138	10q26.11	SNORA19	5.786782758	6.790338063	0.852208344	4	4	49.06677132	40.87375743
chr3	12834945	12841582	3p25.2	RPL32	176.7890624	207.4822625	0.852068318	4	4	10.82775186	12.77042201
chr19	52428971	52452263	19q13.41	ZNF534	0.073755724	0.086563702	0.85203985	1	1	0	0
chr10	112149864	112216306	10q25.2	GPAM	1.041320829	1.222333183	0.851912428	4	4	57.88101906	29.81673284
chr2	28981298	29052230	2p23.2	TOGARAM2	0.186374104	0.218791767	0.851833259	4	4	45.56802215	100.8340817
chr2	68157830	68176238	2p14	PNO1	4.813785097	5.651203533	0.851815913	4	4	20.29403847	30.49271208
chr16	68085366	68229259	16q22.1	NFATC3	48.40188121	56.82528902	0.851766565	4	4	22.56588713	25.53422223
chr2	219178190	219185475	2q35	RETREG2	36.27945212	42.59509285	0.851728443	4	4	40.76742913	19.93814576
chr18	79902420	79951665	18q23	PQLC1	10.2296121	12.01134066	0.851662807	4	4	21.78641282	30.00855341

chr17	50373220	50381479	17q21.33	EME1	0.246504023	0.289459973	0.851599691	2	4	90.58550408	129.2610038
chr4	52872630	53366106	4q12	SCFD2	10.26917852	12.05907371	0.851572747	4	4	33.06991257	34.46045867
chrX	1386152	1392146	Xp22.33 and Yp11.	SLC25A6	68.19247225	80.07919044	0.851562957	4	4	60.18106825	45.43327326
chr9	128945530	128947733	9q34.11	DOLK	2.277183979	2.674341253	0.85149342	4	4	80.52763769	16.48117515
chr8	61202255	61203545	8q12.2	NPM1P6	0.389691622	0.457687095	0.851436771	2	2	2.661808212	59.62863352
chr5	134524153	134583230	5q31.1	JADE2	11.33056193	13.30989392	0.851288672	4	4	27.76741907	35.62088409
chr9	5299864	5339438	9p24.1	RLN2	0.396695995	0.466043901	0.85119877	4	4	31.88435675	38.11622152
chr9	137615332	137618895	9q34.3	ARRDC1-AS1	1.542954188	1.812836762	0.85112693	4	4	66.92738556	28.55887488
chr2	38814	47077	2p25.3	FAM110C	0.147914662	0.173802765	0.85104896	4	2	59.91133537	14.11863736
chr9	19376256	19380237	9p22.1	RPS6	351.9759342	413.6365742	0.850930397	4	4	68.82898499	43.31191448
chr8	97273486	97277948	8q22.1	TSPYL5	0.84259562	0.990311947	0.85083859	4	4	72.74595711	17.46541345
chr9	37588413	37592639	9p13.2	TOMM5	0.828608802	0.973896025	0.850818548	4	4	81.23519152	53.32327969
chr20	5950652	6000941	20p12.3	MCM8	6.219234521	7.310218473	0.85075905	4	4	40.47953028	31.790805
chr10	4963262	4979967	10p15.1	AKR1C1	0.187435628	0.220328564	0.850709614	4	4	66.72959095	87.06843697
chr4	107989714	108035175	4q25	HADH	0.894835078	1.051885533	0.850696249	4	4	45.57638382	11.42560321
chr7	150322650	150338156	7q36.1	LRRC61	1.619665705	1.904123699	0.850609499	4	4	54.57234687	47.18734338
chr22	24429206	24495074	22q11.23	ADORA2A-AS1	0.146729755	0.172507164	0.850571951	3	1	74.7708522	0
chr1	63523301	63585401	1p31.3	EFCAB7	1.610756098	1.893956696	0.850471451	4	4	37.08972979	22.87909176
chr22	36140323	36166177	22q12.3	APOL3	13.69120166	16.10100579	0.850332075	4	4	37.40758346	50.07975449
chr19	11575255	11619159	19p13.2	ZNF627	3.790845699	4.458131794	0.850321586	4	4	23.57375517	27.60637232
chr12	9417691	9448172	12p13.31	DDX12P	0.425959021	0.500994495	0.850226949	4	4	53.18469241	46.99311186
chr4	39459022	39477653	4p14	LIAS	0.51609339	0.6071108	0.850081056	4	4	30.04838859	48.81513496
chr17	64506736	64537951	17q23.3	CEP95	5.668909072	6.668684163	0.850079106	4	4	21.97994035	36.70131357
chr17	10697610	10712689	17p13.1	ADPRM	9.733038968	11.44965702	0.850072535	4	4	37.88662094	55.33398724
chr2	217284019	217756593	2q35	DIRC3	0.268441168	0.315811795	0.850003616	3	4	46.91603022	57.80656525
chr22	31346759	31351155	22q12.2	LINC01521	0.592047523	0.69654624	0.849975908	2	4	16.3100639	56.74512267
chr14	89290198	89291673	14q31.3	CAP2P1	0.744695069	0.87626238	0.849853977	4	3	58.93835928	73.54760254
chr5	157624796	157672703	5q33.3	SOX30	0.130139553	0.153135053	0.849835162	2	1	64.86948049	0
chr12	109023089	109088026	12q24.11	USP30	2.594578995	3.053081022	0.849823171	4	4	29.10227131	23.204437
chr19	57830294	57846238	19q13.43	ZNF587B	2.936041622	3.455172742	0.849752484	4	4	47.70717007	29.46203043
chr3	193241128	193281639	3q29	HRASLS	0.90956886	1.070465302	0.849694855	4	4	90.91731175	69.84254094
chr11	67415678	67425607	11q13.2	CARNS1	0.519897603	0.611888411	0.84966081	4	4	50.96919342	21.34915438
chr3	126006357	126101550	3q21.2-q21.3	SLC41A3	1.571643816	1.850257739	0.849418858	4	4	81.31690316	11.71244898
chr1	40861056	40862346	1p35-p34	CITED4	2.099151286	2.471870726	0.849215642	4	4	88.49414343	50.90034177
chr19	52838532	52857649	19q13.41	ZNF468	6.025156041	7.095237479	0.84918314	4	4	20.97275786	19.03267468
chr12	109713835	109770509	12q24.11	FAM222A	0.180887775	0.213118135	0.848767634	2	4	38.78409466	53.62054039
chr2	177614298	177618966	2q31.2	TTC30A	0.692961841	0.816461517	0.848737909	4	4	81.96826893	51.38972139
chr20	18485225	18486067	20p11.23	RPL21P3	0.45864243	0.540384778	0.848733067	1	1	0	0
chr12	64186312	64222296	12q14.2	C12orf66	1.420385176	1.673557668	0.848721979	4	4	82.17664098	22.19994886
chrX	48636228	48637159	Xp11.23	VN1R110P	0.945408143	1.113974729	0.848680063	3	3	28.42438294	66.87246057
chr16	46689646	46698394	16q11.2	ORC6	1.420072717	1.673579252	0.848524332	4	4	72.29175256	57.56972872
chr10	70137981	70146740	10q22.1	TYSND1	1.811458338	2.134845003	0.848519839	4	4	57.16558436	39.06413759
chr15	65530489	65578355	15q22.31	HACD3	3.990592497	4.70380594	0.848375241	4	4	21.28045163	33.63942506

chr17	48133438	48430250	17q21.32	SKAP1	14.80750891	17.45484176	0.848332463	4	4	32.5043543	29.99746434
chr9	100302075	100352977	9q31.1	TEX10	5.97644694	7.045044848	0.848319219	4	4	35.91433479	6.138059285
chr17	67717833	67744208	17q24.2	NOL11	11.76443631	13.86908176	0.848249114	4	4	22.72685341	2.311962693
chrX	44523639	44542975	Xp11.3	FUNDC1	47.55732723	56.06620137	0.84823523	4	4	69.4685204	57.82770167
chrX	119468400	119471396	Xq24	SLC25A5	57.22848222	67.470877	0.848195322	4	4	44.0145971	53.92203763
chr16	30378133	30382850	16p11.2	SEPT1	7.17029849	8.454690109	0.848085311	4	4	23.98997353	56.83262974
chr17	68198660	68200302	17q24.2	ARHGAP27P2	0.465440502	0.548879135	0.847983595	2	2	57.06322034	53.25219486
chr4	41981696	41986467	4p13	DCAF4L1	0.142963892	0.168605673	0.847918633	1	2	0	96.16021373
chr1	101800028	101997234	1p21.1	OLFM3	0.105126021	0.123984922	0.847893588	1	1	0	0
chr6	28241697	28259258	6p22.1	ZKSCAN4	3.208055909	3.784045769	0.847784648	4	4	37.70882412	36.464722
chr14	53036740	53153328	14q22.1	DDHD1	5.536474282	6.531738082	0.847626499	4	4	23.98996297	42.07055382
chr12	4590072	4613888	12p13.32	DYRK4	0.791721496	0.934094311	0.84758197	4	4	39.71513019	37.80795227
chr2	135787840	135837195	2q21.3	LCT	0.101066097	0.119243333	0.847561824	2	4	12.01867678	34.26175921
chr14	24809654	25050297	14q12	STXBP6	0.052807109	0.062317692	0.847385505	2	1	48.53709878	0
chr7	99546295	99559395	7q22.1	FAM200A	3.197876045	3.773935577	0.847358409	4	4	17.656344	22.21014469
chr11	74171063	74254703	11q13.4	PPME1	4.94738924	5.838676768	0.847347684	4	4	42.69511584	40.11707751
chr19	2428165	2456968	19p13.3	LMNB2	3.434696916	4.054091319	0.847217452	4	4	67.86406879	77.90271076
chr7	32826302	32838744	7p14.3	DPY19L1P2	51.94389194	61.31372495	0.847182127	4	4	73.65101466	36.53164511
chr8	6406592	6648505	8p23.1	MCPH1	5.485163178	6.474913453	0.847140771	4	4	26.11652258	26.47699663
chr14	103928343	104052667	14q32.33	TDRD9	0.891923958	1.052982936	0.847045026	4	4	42.33497456	63.21207344
chr1	158931177	159010083	1q23.1	PYHIN1	24.68551793	29.14688092	0.846935149	4	4	20.79294725	88.1031634
chr4	99070978	99088788	4q23	ADH5	9.901403231	11.69303502	0.846777865	4	4	15.21573426	25.74937131
chr7	116499601	116508541	7q31.2	CAV2	0.612066796	0.722903509	0.846678414	4	4	99.16415664	55.62347188
chr1	6264272	6393766	1p36.31	ACOT7	1.375823418	1.624991124	0.846665189	4	4	42.7522152	122.3682532
chr19	53991149	54012666	19q13.42	CACNG6	1.021313036	1.206433132	0.846555859	4	3	98.40233028	39.86760625
chr19	3976056	3985463	19p13.3	EEF2	400.7503925	473.4365195	0.846471229	4	4	61.95044652	42.79041135
chr11	18080343	18106091	11p15.1	SAAL1	4.896195759	5.784538235	0.846428109	4	4	28.65968742	33.27804576
chr9	96917928	97013704	9q22.33	MFSD14C	1.745975788	2.062838474	0.846394815	4	4	40.77098406	20.93915981
chr12	6914450	6923696	12p13.31	ENO2	1.966439846	2.323638646	0.846276098	4	4	56.74500018	58.57152997
chrX	24693833	24996986	Xp22.11-p21.3	POLA1	25.15957982	29.73911292	0.846009761	4	4	49.32236446	20.43843173
chr19	6372433	6375250	19p13.3	ALKBH7	3.842779455	4.542440173	0.845972497	4	4	53.52059977	21.30199628
chr3	52708449	52770949	3p21.1	NEK4	29.21077698	34.53420665	0.845850529	4	4	48.84522609	41.61594509
chr2	6828554	6840464	2p25.2	NRIR	9.435291843	11.15517915	0.845821633	4	4	62.59830468	95.07227047
chr7	152759749	153070979	7q36.1-q36.2	ACTR3B	2.223297408	2.62864578	0.845795742	4	4	23.84133381	26.28039965
chr12	50111981	50120457	12q13.12	COX14	5.204637806	6.15504002	0.845589596	4	4	30.40675793	26.18505246
chr1	179900262	179900564	1q25.2	RN7SL230P	2.006504404	2.372907385	0.845589009	4	4	39.57998107	87.14165039
chr1	43164174	43172570	1p34.2	EBNA1BP2	3.20007729	3.785253763	0.845406277	4	4	75.09910108	54.21655669
chr12	8231049	8242946	12p13.31	FAM86FP	0.771819017	0.913140296	0.845235962	3	2	111.3732377	31.47420721
chr11	47407132	47416501	11p11.2	SLC39A13	1.674041624	1.980808953	0.845130279	4	4	65.53352883	8.028923985
chr10	99732201	99756137	10q24.2	CUTC	10.30491789	12.19555362	0.844973357	4	4	54.27360316	9.292050118
chr17	82018703	82022930	17q25.3	CENPX	2.07597594	2.457020262	0.844916085	3	4	75.76313805	32.41983435
chr13	44989530	45033611	13q14.12	GPALPP1	6.355588215	7.523259105	0.844791882	4	4	22.74075496	22.4926706
chr8	99013085	99877586	8q22.2	VPS13B	45.33212129	53.67856001	0.844510756	4	4	26.75713733	32.21004408

chr17	28744004	28750958	17q11.2	TRAF4	1.028927193	1.218696336	0.844285129	4	4	82.96824953	41.6345554
chr3	49412206	49416476	3p21.31	TCTA	4.852505327	5.748543456	0.844127798	4	4	56.80523398	35.87151717
chr12	54455952	54473602	12q13.13	GTSF1	0.693410095	0.821485807	0.844092606	4	4	24.06941697	33.58461393
chr22	41995103	42059402	22q13.2	WBP2NL	0.186249288	0.22066062	0.844053135	4	4	32.72629876	47.53899944
chr15	20669468	20756151	15q11.2	NBEAP1	0.392721245	0.465331647	0.843959889	4	4	136.9259244	75.12052566
chr12	27524112	27695564	12p11.23-p11.22	PPFIBP1	0.36257331	0.429630978	0.843917988	4	4	40.67664638	41.35429442
chr9	120793496	120801728	9q33.2	B3GNT10	1.610990669	1.908943217	0.843917543	4	4	31.45456184	21.81965567
chr17	48930096	48944842	17q21.32	SNF8	4.308605922	5.105968116	0.843837216	4	4	60.24477129	32.11794207
chr17	39200283	39204730	17q12	RPL19	235.3880453	278.9530409	0.843826777	4	4	45.48961768	25.24730642
chr4	145622597	145626132	4q31.21	NCOA4P3	0.130271501	0.154402707	0.843712544	1	2	0	24.72370386
chr14	99971454	100144236	14q32.2	EVL	15.57500375	18.46055855	0.843690819	4	4	34.28261355	28.68691031
chr4	151099573	151104652	4q31.3	RPS3A	313.7711222	371.9104538	0.843673844	4	4	61.50483676	19.8759211
chr2	121726945	121736927	2q14.3	NIFK	12.84032128	15.22076898	0.843605293	4	4	29.68801499	28.96850399
chr19	7069460	7087968	19p13.2	ZNF557	5.054178858	5.991552955	0.843550728	4	4	48.74520999	29.26343609
chr12	21764955	21775593	12p12.1	KCNJ8	0.094976126	0.112607367	0.84342729	1	2	0	8.670775219
chr14	20457853	20461612	14q11.2	PIP4P1	5.679737719	6.734175725	0.843419886	4	4	52.30370907	34.05303792
chr19	41386370	41397351	19q13.2	EXOSC5	3.097555747	3.673258662	0.843271883	4	4	42.44977762	53.01731972
chrX	153334155	153352926	Xq28	ZNF275	1.516463492	1.798453477	0.843204182	4	4	72.32339258	25.65228044
chr17	40819092	40836274	17q21.2	TMEM99	0.482346403	0.572123452	0.843080984	3	4	51.7558201	55.90948945
chr7	29684772	29685821	7p14.3	ZNRF2P2	0.473520133	0.561654931	0.843080167	3	3	45.778073	40.58763731
chr4	40056223	40187039	4p14	N4BP2	11.4452207	13.57556924	0.843074828	4	4	36.63024981	38.17599252
chr4	2535388	2732575	4p16.3	FAM193A	12.27133159	14.55824711	0.842912715	4	4	26.35403196	19.26016713
chr5	178207144	178232822	5q35.3	PHYKPL	3.760801395	4.461859541	0.842877585	4	4	64.53997144	21.73940902
chr19	48993448	49015995	19q13.33	RUVBL2	3.660856834	4.343615633	0.842813256	4	4	66.63668256	56.61087143
chr11	64234584	64239264	11q13.1	VEGFB	3.70121095	4.391845929	0.842746082	4	4	62.2953972	50.51777316
chr20	46684365	46689637	20q13.12	TP53RK	8.316596095	9.869781989	0.842632198	4	4	23.27025565	24.06757417
chr22	30925130	30968287	22q12.2	MORC2	5.368400705	6.371852165	0.842518088	4	4	22.5622895	22.97009344
chr11	64269828	64284704	11q13.1	BAD	4.958595111	5.88567161	0.84248586	4	4	57.97562695	54.94811507
chr3	113964137	114056613	3q13.31	CCDC191	1.80576882	2.143623785	0.842390737	4	4	69.59252492	60.29240861
chr8	21919669	22006585	8p21.3	XPO7	157.7686532	187.2972984	0.842343454	4	4	51.04447907	60.99359146
chr9	7476951	7478376	9p24.1	RPL4P5	0.451913877	0.536497291	0.842341395	1	1	0	0
chr10	88932684	88939974	10q23.31	ACTA2-AS1	0.672150225	0.798277537	0.842000675	3	3	32.69455376	26.8333679
chr1	32221584	32231604	1p35.2	EIF3I	28.90282674	34.33077739	0.84189258	4	4	48.81097519	45.57184359
chr3	111674676	111846447	3q13.2	PLCXD2	3.429035547	4.073976933	0.841692431	4	4	34.32278526	57.92758007
chr17	74862497	74873031	17q25.1	FDXR	0.650117967	0.772553756	0.841518098	4	4	128.5282355	100.7718458
chr6	157289022	157324259	6q25.3	TMEM242	2.226425121	2.646279812	0.841341536	4	4	66.58939223	29.95901934
chr6	135851696	136195571	6q23.3	PDE7B	0.490104114	0.582635214	0.841185191	4	4	39.92818829	67.30027849
chr1	59020476	59088247	1p32.1	LINC01358	0.04339226	0.051585439	0.841172634	3	3	13.42401342	43.21003668
chr6	37212178	37259034	6p21.2	TMEM217	0.987576245	1.1741894	0.841070653	4	4	36.70422443	88.53251553
chr2	238320489	238400900	2q37.3	TRAF3IP1	2.033882612	2.418210061	0.841069453	4	4	60.13790807	34.05780854
chr5	98928562	99004021	5q21.1	LINC02062	5.304772137	6.307495299	0.841026729	4	4	56.10589286	57.50390186
chr10	5948897	5978741	10p15.1	IL15RA	16.88937653	20.08316638	0.840971798	4	4	24.79716668	15.35908655
chr19	53365693	53386590	19q13.42	ZNF525	11.47562207	13.64611036	0.840944545	4	4	40.47994982	40.11605432

chr7	101154398	101161277	7q22.1	AP1S1	2.889290537	3.435856705	0.840922886	4	4	30.24281287	45.66137871
chr4	152536264	152539263	4q31.3	MIR4453HG	1.60779526	1.911955566	0.840916645	4	4	47.65478798	27.09164493
chr11	65174128	65175491	11q13.1	PGAM1P8	0.987273634	1.174326744	0.840714596	3	4	42.14258431	43.89303599
chr6	106283413	106283711	6q21	RN7SL47P	0.877193144	1.043400806	0.840705833	2	2	29.79226636	16.88924015
chr1	204131061	204152182	1q32.1	ETNK2	0.095803794	0.11396701	0.840627418	2	2	31.00930927	70.83668064
chr14	49862551	49862849	14q21.3	RN7SL2	776003.6698	923210.422	0.840549079	4	4	?	0
chr10	13161554	13211110	10p13	MCM10	22.5226116	26.79576686	0.840528719	4	4	65.42485018	49.36724812
chr1	56494747	56579584	1p32.2	PLPP3	0.188048859	0.22374386	0.840464893	3	4	20.07166768	80.80416041
chr9	104093760	104141419	9q31.1	SMC2	7.881861861	9.378572154	0.84041171	4	4	19.5570385	30.19783164
chrX	92113098	92114477	Xq21.31	EIF4A1P10	0.41456909	0.493304895	0.840391195	2	2	49.00276654	20.60497712
chr8	103414714	103443453	8q22.3	DCAF13	3.194367295	3.801450675	0.840302182	4	4	25.85703303	19.51946532
chr22	17787649	18024559	22q11.21	MICAL3	1.035881705	1.232815526	0.840256861	4	4	47.89503167	24.77592177
chr8	23243637	23262000	8p21.3	CHMP7	18.70368482	22.26001507	0.840236844	4	4	49.18711953	44.98817346
chr1	149782689	149812373	1q21.2	HIST2H2BF	54.55801391	64.93257545	0.840225627	4	4	50.54948787	58.30884156
chr6	122399551	122433119	6q22.31	HSF2	7.82349525	9.311239606	0.840220592	4	4	15.44157355	17.25828507
chr16	29425411	29447026	16p11.2	SMG1P6	3.597137431	4.281520695	0.840154162	4	4	51.69881243	39.87464546
chr17	2680956	2681234	17p13.3	RN7SL608P	5.150963576	6.13210669	0.839999014	3	3	51.41903924	34.82257243
chr11	65040904	65044833	11q13.1	SAC3D1	1.174011596	1.397722257	0.83994627	4	4	41.58558483	67.36010639
chr17	51435427	51445802	17q21.33	LINC02073	0.687732088	0.818831373	0.839894648	3	2	84.27704373	97.03371006
chr1	226939339	226987545	1q42.13	COQ8A	4.793276642	5.707453965	0.839827473	4	4	61.06312445	30.15811895
chr17	68424451	68424548	17q24.2	MIR635	5.588568597	6.655621904	0.839676394	4	4	86.61786028	70.81607983
chr9	124857879	124861961	9q33.3	RPL35	108.9064727	129.7604882	0.839288401	4	4	61.57396084	39.73635029
chr10	93061264	93068697	10q23.33	CYP26C1	0.113700948	0.135480178	0.83924416	3	3	25.34192622	25.36283045
chr16	57462639	57472009	16q21	POLR2C	8.886784228	10.58915048	0.83923486	4	4	38.7763441	8.457600673
chr4	81086874	81217837	4q21.21	PRKG2	0.311081337	0.370686639	0.839202994	4	4	34.53418633	37.90325885
chr14	104752420	104759659	14q32.33	SIVA1	1.824711179	2.174537987	0.839125915	4	4	70.58199851	29.52668239
chr1	155659442	155689032	1q22	YY1AP1	193.3997314	230.4958219	0.839059597	4	4	46.57176514	30.76915841
chr9	98943392	99070792	9q22.33	COL15A1	0.073641049	0.087788886	0.838842498	4	4	38.17296457	66.02457675
chr12	132623758	132687524	12q24.33	POLE	1.504299069	1.793315282	0.83883692	4	4	50.9979088	37.82458619
chr3	51394261	51505620	3p21.2	DCAF1	6.286490168	7.496342408	0.83860766	4	4	43.58114365	39.10674249
chr19	8209329	8262433	19p13.2	CERS4	2.336477156	2.786629899	0.838459803	4	4	69.11385844	50.63854071
chr2	110637698	110678107	2q13	BUB1	13.50955303	16.1164452	0.838246453	4	4	63.77846067	37.90371036
chr14	69885282	69886162	14q24.2	RPL7AP6	0.579792306	0.691729994	0.838177195	3	3	33.70157597	81.92065616
chr6	18224169	18264568	6p22.3	DEK	178.2045089	212.6560013	0.837994262	4	4	13.75292828	7.784796791
chr16	57428169	57447528	16q21	CIAPIN1	3.968747359	4.736091552	0.837979442	4	4	56.03155101	12.36090286
chr7	134986139	135135782	7q33	AGBL3	0.431204836	0.514586131	0.837964356	4	4	18.02950107	37.66466327
chr15	74630558	74696434	15q24.1	EDC3	2.614483973	3.120300152	0.837895025	4	4	23.33709579	24.51670074
chr11	65375192	65383701	11q13.1	SLC25A45	1.204258964	1.437324353	0.837847742	4	4	79.65425808	26.74467167
chr11	75742129	75742426	11q13.5	RN7SL786P	0.716618683	0.855356878	0.837800808	1	1	0	0
chr22	30378812	30387313	22q12.2	RNF215	0.186867117	0.223065277	0.837723913	4	4	53.89206815	30.40973398
chr9	120902393	120929173	9q33.2	TRAF1	3.814031132	4.552951366	0.837705222	4	4	65.33389858	14.56058003
chr15	63046034	63049387	15q22.2	TPM1-AS	0.128527453	0.153428163	0.837704434	3	1	53.70462344	0
chr17	49288346	49288454	17q21.32	MIR6129	2.645939914	3.158560452	0.837704376	1	2	0	25.86886837

chr1	23557930	23559794	1p36.12	ID3	0.68057561	0.812550786	0.837579167	3	4	104.979606	135.3363213
chr22	41367333	41399328	22q13.2	TEF	0.872278814	1.041446168	0.837564956	4	4	78.86162097	39.48472119
chr4	39182404	39285810	4p14	WDR19	1.857103644	2.217633768	0.83742576	4	4	26.25152995	19.94624177
chr2	231508426	231514339	2q37.1	LINC00471	0.587117154	0.701132533	0.837383984	2	3	59.37631958	80.89693078
chr19	4445006	4458759	19p13.3	UBXN6	177.3869182	211.8810868	0.837200341	4	4	28.89492967	33.68896929
chr16	5071809	5087379	16p13.3	ALG1	2.117260845	2.529314121	0.837088928	4	4	36.15766736	27.62295959
chr19	14070325	14071304	19p13.12	EEF1DP1	0.344268675	0.411269218	0.837088358	2	3	54.88575043	39.39503324
chr1	54715822	54742657	1p32.3	TTC4	0.938831781	1.121707999	0.836966289	4	4	93.92825188	48.16753442
chr10	103367953	103389065	10q24.33	TAF5	5.076340404	6.065944793	0.836858985	4	4	35.12526297	7.222690037
chr16	88651935	88663153	16q24.2	MVD	1.972468506	2.35722063	0.836777211	4	4	64.54793377	25.14857114
chr6	32008420	32013023	6p21.33	TNXA	0.141801877	0.169468809	0.836743221	1	3	0	30.44964397
chr2	26308173	26346817	2p23.3	ADGRF3	0.1068014	0.127652329	0.836658454	3	4	52.64412737	23.12411018
chr2	161308275	161411717	2q24.2	PSMD14	6.029405206	7.207537459	0.83654164	4	4	33.21275522	20.86744729
chr19	45687454	45692316	19q13.32	SNRPD2	16.2273753	19.40288297	0.836338359	4	4	7.742193683	29.77778788
chr15	38696598	38700038	15q14	C15orf53	0.208840492	0.249710571	0.836330202	2	1	33.80083372	0
chrX	2609265	2657229	Xp22.33 and Yp11	CD99P1	1.221735942	1.460887682	0.83629697	4	4	42.46704758	51.9827792
chrX	107628424	107651026	Xq22.3	PRPS1	13.13903893	15.71170344	0.836258078	4	4	41.34428894	15.44842463
chr7	103099576	103149560	7q22.1	NAPEPLD	1.936589191	2.316332108	0.836058519	4	4	60.71154501	29.51241503
chr3	132680609	132722459	3q22.1	NPHP3	0.492743217	0.589377771	0.836039703	4	4	51.18442224	44.78639702
chr14	22528527	22528586	14q11.2	TRAJ16	18.69459522	22.36443284	0.835907414	3	4	75.65436087	49.98453117
chr1	156525405	156572605	1q22	IQGAP3	0.28428997	0.340104089	0.835891066	4	4	27.39067416	83.25185617
chr21	46300426	46324046	21q22.3	C21orf58	1.903214019	2.276973384	0.835852555	4	4	30.2779897	26.91029502
chr20	63739858	63744050	20q13.33	SLC2A4RG	3.704364438	4.432932707	0.835646441	4	4	60.46691886	30.81047343
chr14	63597039	63641923	14q23.2	WDR89	10.76759238	12.88583097	0.835614902	4	4	24.71201828	22.74531356
chr12	56670450	56670746	12q13.3	RN7SL809P	1.831918024	2.192514331	0.835532976	4	4	20.71094755	61.13073687
chr10	49454480	49539538	10q11.23	ERCC6	10.5747351	12.6571076	0.835478012	4	4	45.14925041	51.5301462
chr13	99086723	99088625	13q32.3	DOCK9-DT	0.694779953	0.831614413	0.835459248	3	3	65.0485859	105.7486222
chr10	4987775	5018033	10p15.1	AKR1C2	0.053719092	0.064299466	0.835451598	1	2	0	53.23038422
chr8	54135221	54148514	8q11.23	MRPL15	8.352271265	9.998003111	0.835393945	4	4	39.59800792	50.16670276
chr4	158672101	158708713	4q32.1	ETFDH	9.574336668	11.46283131	0.835250594	4	4	13.63219097	46.73248717
chr7	129611702	129757082	7q32.2	NRF1	8.412686155	10.07236124	0.835224825	4	4	17.08613155	14.5050361
chr1	206470243	206496890	1q32.1	IKBKE	6.652483905	7.96536033	0.835176769	4	4	58.68291178	39.79450542
chr5	43120843	43176324	5p12	ZNF131	24.84948956	29.75537041	0.835126205	4	4	26.0572206	25.42209613
chr14	77547566	77547858	14q24.3	RN7SL587P	2.409539402	2.885544416	0.835038057	4	3	55.76392403	84.37869089
chr17	58489532	58518109	17q22	MTMR4	10.15207362	12.15851078	0.834976734	4	4	24.7451525	39.33979518
chrX	78945332	78963727	Xq21.1	P2RY10	42.19599134	50.54261436	0.83485969	4	4	52.84276866	19.86676032
chr1	35599541	35641844	1p34.3	PSMB2	8.96392617	10.73710803	0.834854799	4	4	38.8781771	47.25100619
chr7	2403489	2448484	7p22.3	CHST12	1.85221199	2.218818155	0.834774128	4	4	37.28105497	39.93486457
chr8	98041710	98045590	8q22.2	RPL30	321.7905809	385.5079145	0.834718481	4	4	28.11388163	19.16576678
chr1	149607012	149679523	1q21.2	LINC00869	2.216539249	2.655449397	0.83471342	4	4	43.45473752	14.50968212
chr14	105398499	105413859	14q32.33	TEX22	0.24652335	0.295351891	0.834676726	3	4	49.06759983	38.46199467
chr7	144238573	144355718	7q35	OR2A1-AS1	0.361312062	0.432946108	0.834542809	4	4	83.71660468	68.139994
chr12	8645943	8662888	12p13.31	MFAP5	0.054451905	0.065256249	0.834432049	1	1	0	0

chr2	58159243	58241681	2p16.1	FANCL	178.9850641	214.540516	0.834271621	4	4	85.17072995	73.57718117
chr19	6752160	6767513	19p13.3	SH2D3A	1.018936096	1.221978668	0.833841148	4	4	73.52375297	49.45630392
chr2	201790324	201895550	2q33.1	CDK15	0.13164264	0.15788786	0.833773034	2	3	90.72805226	76.74086688
chr17	58352500	58418894	17q22	RNF43	0.133854021	0.16054236	0.833761391	3	4	48.34864488	50.12149455
chr4	120059422	120066858	4q27	MAD2L1	1.88970594	2.266629978	0.833707292	4	4	52.42973707	54.66350853
chr4	16501534	16898809	4p15.32	LDB2	0.176669829	0.211911494	0.833696301	4	3	50.40116263	17.8685168
chr17	19335372	19378193	17p11.2	B9D1	0.201995347	0.242314482	0.833608231	3	4	33.37117057	34.21965079
chr2	73228006	73233284	2p13.2	PRADC1	2.023934978	2.428229278	0.833502419	4	4	79.92879097	36.59977448
chr19	56567521	56582894	19q13.43	ZNF470	0.669153735	0.802895483	0.833425707	4	4	55.58594054	22.63750056
chr2	112215035	112255087	2q14.1	ZC3H8	3.006298577	3.608162131	0.833193872	4	4	15.77229662	24.98911229
chr22	29788608	29838304	22q12.2	ASCC2	92.35178484	110.8445607	0.833164787	4	4	20.96192436	38.83595213
chr14	74881891	74903747	14q24.3	DLST	9.94720151	11.94162867	0.832985331	4	4	17.82635572	3.701394485
chr1	174999435	175012027	1q25.1	CACYBP	13.51001106	16.22412992	0.832710976	4	4	29.36113353	25.86319049
chrX	65270912	65273021	Xq11.2	CCT4P2	0.146009686	0.175361169	0.832622676	1	3	0	14.83748072
chr20	49280313	49280415	20q13.13	SNORD12B	28.5902571	34.34483305	0.832447112	4	4	36.04300525	61.61602624
chr11	78068347	78080219	11q14.1	NDUFC2	6.189960513	7.437762604	0.832234214	4	4	25.75208359	20.75356921
chr8	125922523	125951197	8q24.13	LINC00861	6.354394128	7.635398677	0.832228204	4	4	33.67782333	12.72740609
chr19	18563766	18577460	19p13.11	UBA52	506.9100622	609.1109081	0.832213076	4	4	45.08886616	35.04276246
chr5	33986986	34124528	5p13.2	C1QTNF3-AMACR	0.139410268	0.167519805	0.832201708	3	2	69.8915289	13.35764465
chr3	38051278	38052400	3p22.2	PPP2R2DP1	0.413464855	0.496932761	0.832033803	2	1	40.94069638	0
chr16	21834563	21857756	16p12.2	NPIPB4	0.432303511	0.519601854	0.831989932	4	4	79.68988328	14.45909963
chr2	221418027	221574202	2q36.1	EPHA4	3.383674896	4.068792934	0.831616391	4	4	73.45166702	45.8524835
chr1	113924001	113929525	1p13.2	HIPK1-AS1	0.706861382	0.849994911	0.831606605	4	4	75.16011484	107.0721142
chr8	19313443	19396218	8p21.3	SH2D4A	0.074357618	0.089444891	0.831323251	2	2	10.60890531	42.65757282
chr5	172641266	172691540	5q35.1	NEURL1B	0.115724124	0.139214912	0.831262411	3	4	71.10670602	64.95119763
chr3	4414560	4414963	3p26.1	MRPS10P2	1.225148122	1.473934892	0.831209118	2	1	38.14850139	0
chr19	57351253	57359898	19q13.43	ZNF304	3.252811652	3.913371586	0.831204393	4	4	76.16086133	30.80605416
chr1	89006677	89022870	1p22.2	GBP3	13.69283106	16.47352962	0.831202018	4	4	89.93088868	79.76373719
chr2	27628648	27650846	2p23.3	GPN1	5.272001604	6.34278041	0.831181479	4	4	12.73284228	12.46781175
chr11	119018469	119023674	11q23.3	TRAPPC4	1.63087649	1.962201803	0.831146158	4	4	33.82254306	23.1922882
chr8	80628451	80884416	8q21.13	ZNF704	0.086039881	0.10352397	0.831110715	3	4	92.96033201	74.85141734
chr3	186900073	186900622	3q27.3	RPS20P14	1.347283734	1.621244094	0.831018437	4	3	57.23228707	95.37718978
chr11	123072006	123195305	11q24.1	CLMP	0.055250648	0.066491155	0.830947328	3	3	11.43835193	39.05336496
chr1	93869458	93879206	1p22.1	DNTTIP2	14.09981254	16.97255641	0.830741828	4	4	33.90694331	14.41890838
chr9	128920895	128942041	9q34.11	PHYHD1	0.180447858	0.217214297	0.830736561	3	2	47.64292576	14.00748343
chr19	4639505	4655568	19p13.3	TNFAIP8L1	1.536759776	1.85054861	0.830434698	4	4	59.71759401	30.98212372
chr2	43637300	43767987	2p21	PLEKHH2	0.083289091	0.100299498	0.83040387	2	2	43.60336928	29.41930604
chr16	53829	57669	16p13.3	SNRNP25	1.012220808	1.219360032	0.83012464	4	4	56.80729037	62.69225777
chr8	144262046	144291438	8q24.3	BOP1	2.241271124	2.700014649	0.830095912	4	4	57.0043031	55.92134495
chr17	8188333	8190246	17p13.1	BORCS6	4.571679394	5.507412523	0.830095689	4	4	63.50478721	30.73284414
chr7	30771417	30892387	7p14.3	MINDY4	0.058595419	0.070595915	0.830011462	2	2	47.37291259	68.6238949
chr2	73233425	73253022	2p13.2	CCT7	25.63433686	30.88786149	0.829916207	4	4	47.06584696	52.32750838
chr19	41966582	41994276	19q13.2	ATP1A3	0.426310905	0.513732931	0.82982982	3	4	53.96024681	100.6702576

chr7	100049774	100065041	7q22.1	ZSCAN21	3.253383723	3.92105897	0.829720682	4	4	44.94236218	42.27116784
chr11	106046801	106077765	11q22.3	KBTBD3	1.079800728	1.301528309	0.829640601	4	4	36.64238949	40.38056573
chr15	101764629	101767030	15q26.3	DNM1P47	0.070307185	0.084756449	0.829520174	4	4	56.69359085	71.85220608
chr1	171314131	171342084	1q24.3	FMO4	1.227073527	1.479312736	0.829488922	4	4	45.87354842	52.20638413
chr6	3268973	3456574	6p25.2	SLC22A23	6.680281263	8.055276486	0.829305024	4	4	88.05322441	80.4617308
chr16	30553764	30558321	16p11.2	ZNF764	1.821589035	2.196657842	0.829254789	4	4	59.86185869	26.03320518
chr1	44213444	44220679	1p34.1	DMAP1	2.453444938	2.958943348	0.829162525	4	4	58.20290209	36.90384538
chr3	25788283	25794534	3p24.2	OXSM	2.041965031	2.462795854	0.829124764	4	4	67.84284734	17.66453678
chr19	2289775	2308157	19p13.3	LINGO3	0.247601278	0.298668622	0.829016709	3	4	50.52823823	48.26586451
chr22	37686337	37693478	22q13.1	NOL12	0.353844821	0.426831948	0.829002662	4	4	127.2449507	65.1761753
chr13	102784281	102799054	13q33.1	KDELC1	0.534078936	0.644312282	0.828913169	4	4	47.92226995	61.78932635
chr11	1554051	1572839	11p15.5	DUSP8	0.196765771	0.237385882	0.828885734	2	4	96.87810419	79.09757874
chr1	193057281	193057415	1q31.2	SCARNA18B	1.471339538	1.775605809	0.828640867	1	3	0	8.710445421
chr11	118896140	118926072	11q23.3	BCL9L	4.124952332	4.978240437	0.828596446	4	4	78.38051337	71.66319147
chr17	46372813	46423097	17q21.31	NSFP1	1.496795364	1.806424908	0.828595397	4	4	146.6642533	41.85341905
chr14	22919456	22923407	14q11.2	PRMT5-AS1	0.292593299	0.353122942	0.828587621	4	4	17.91778535	71.60899116
chr14	55366391	55411858	14q22.3	ATG14	16.16972913	19.51549449	0.828558515	4	4	19.83835171	59.56775456
chr10	6393038	6580646	10p15.1	PRKCQ	21.72981827	26.22890329	0.828468428	4	4	24.5875074	47.82555892
chr12	132186735	132189695	12q24.33	LINC02361	2.043716966	2.466914245	0.828450754	3	4	42.1307365	29.83074659
chrX	303352	318819	Xp22.33 and Yp11.	GTPBP6	4.203676327	5.074407186	0.828407373	4	4	51.61016576	23.53117647
chr19	2732524	2740076	19p13.3	SLC39A3	1.305201236	1.575573894	0.828397348	4	4	90.22300451	51.0959791
chr9	69035563	69079077	9q21.11	FXN	3.557293943	4.294745255	0.828289859	4	4	28.82087138	24.99854025
chrX	108018886	108079184	Xq22.3	VSIG1	2.094311742	2.528849617	0.828167768	4	4	65.18543231	59.37359229
chr1	23959164	23963462	1p36.11	PNRC2	100.2063801	121.007362	0.828101517	4	4	15.94091418	17.20085365
chr1	236549005	236604541	1q43	HEATR1	8.523687511	10.29376716	0.828043551	4	4	14.16382822	15.79960268
chr4	89112817	89114901	4q22.1	TIGD2	2.624658463	3.169867791	0.828002502	4	4	37.74281204	9.158411694
chr22	27919376	28002679	22q12.1	TTC28-AS1	0.954052473	1.152290161	0.827962006	4	4	61.46352026	40.74188379
chr8	89900721	89900824	8q21.3	RNU6-925P	8.804373267	10.63415889	0.827933206	2	1	42.92366822	0
chr3	27715949	27722715	3p24.1	EOMES	11.16308821	13.48621213	0.827740814	4	4	37.31762664	97.19162713
chr10	94402429	94536332	10q23.33	TBC1D12	5.576917071	6.740569128	0.827365904	4	4	41.73148387	86.06678054
chr17	4538896	4555386	17p13.2	MYBBP1A	2.570535349	3.107259847	0.827267585	4	4	68.10063071	53.75670906
chrY	7273972	7381547	Yp11.2	PRKY	3.776518659	4.565063054	0.827265388	3	2	97.86322214	19.85533367
chr17	38297023	38323218	17q12	MRPL45	3.569816603	4.315794854	0.827151596	4	4	46.69143696	21.56999866
chr17	60810115	60811518	17q23.2	KRT18P61	0.429952883	0.519818275	0.827121522	2	3	30.46080073	54.83410691
chr11	57325985	57335877	11q12.1	SSRP1	9.803751773	11.85305319	0.827107718	4	4	58.8920292	59.66562596
chr22	40813883	40813958	22q13.2	MIR4766	3.875461507	4.686239448	0.826987513	1	2	0	59.37265764
chr1	48324976	48326158	1p33	PPP1R8P1	0.211537002	0.255812652	0.826921579	1	1	0	0
chr20	62309060	62367318	20q13.33	LAMA5	0.067089045	0.081146983	0.826759566	4	4	62.33233043	84.22647951
chr9	135462718	135472249	9q34.3	PPP1R26-AS1	0.091446314	0.110632284	0.826578922	1	3	0	33.1522479
chr6	28161761	28169595	6p22.1	ZNF192P1	0.241765997	0.29258412	0.826312777	2	4	89.97015152	38.13136127
chr14	22086452	22086961	14q11.2	TRAV23DV6	5.615092343	6.797181144	0.82609132	4	4	38.25840438	39.33404791
chr22	19449941	19479215	22q11.21	UFD1	4.057471629	4.913417832	0.825794135	4	4	53.78749454	11.06937441
chr2	27051423	27070622	2p23.3	AGBL5	2.215606788	2.68301901	0.825788703	4	4	58.80078491	28.22816354

chr11	6514830	6603678	11p15.4	RRP8	2.452677503	2.970159729	0.82577293	4	4	51.59519176	10.98463823
chr14	22516273	22516331	14q11.2	TRAJ27	9.468871484	11.4673621	0.825723597	3	4	54.34775797	35.35353934
chr11	134068925	134151757	11q25	JAM3	10.46474762	12.67477685	0.825635649	4	4	75.01526383	23.45915572
chr2	206737763	206765549	2q33.3	MDH1B	0.117757359	0.142639085	0.825561655	1	3	0	30.63574649
chr20	1266291	1309327	20p13	SNPH	1.22195062	1.480344172	0.825450354	4	4	58.64134454	43.0971671
chrX	56976370	56995801	Xp11.21	SPIN3	0.295291078	0.35781328	0.825265841	4	4	80.8468158	47.3909956
chr4	2247432	2262143	4p16.3	MXD4	9.460777405	11.46607458	0.825110402	4	4	29.13611993	24.49627371
chr22	22856590	22857060	22q11.22	IGLV2-5	2.511007047	3.043686895	0.824988619	3	2	20.01931624	94.89341503
chr4	15968226	16084059	4p15.32	PROM1	0.071770022	0.087013041	0.824819148	2	3	11.66718492	71.44863048
chr10	101578882	101588319	10q24.32	POLL	15.09763951	18.30894443	0.824604584	4	4	44.71508158	15.68676819
chr1	214281222	214337136	1q32.3	SMYD2	3.450651128	4.184879552	0.824552077	4	4	33.72322098	18.34533845
chr1	45329242	45340470	1p34.1	MUTYH	2.146513618	2.603608317	0.824437994	4	4	38.38453405	21.61194358
chr1	151340640	151347319	1q21.3	RFX5	11.94547071	14.4919676	0.824282184	4	4	22.58032929	48.86431145
chr14	22518446	22518505	14q11.2	TRAJ26	18.57889195	22.5401855	0.824256391	4	4	26.15316563	28.92176758
chr19	45340740	45351548	19q13.32	KLC3	4.48547693	5.443471745	0.824010327	4	4	46.35394927	72.09878272
chr5	139299852	139299968	5q31.2	RNA5SP195	4.123833903	5.005136612	0.823920349	2	3	30.78460648	50.21061798
chr20	51596775	51768411	20q13.2	ATP9A	3.025260285	3.672562518	0.823746436	4	4	16.0350818	21.88618033
chr19	12737459	12748324	19p13.13	ASNA1	19.73941376	23.96842706	0.823558998	4	4	37.13066568	42.86756162
chr2	202773150	202871985	2q33.2	ICA1L	0.443791546	0.53888074	0.823543157	4	4	70.16511142	44.93881142
chr1	10430102	10442815	1p36.22	CENPS	0.183865151	0.223281224	0.82346893	4	2	20.65549563	44.63526393
chr1	185323846	185335039	1q25.3	GS1-279B7.1	0.203116896	0.246675473	0.82341748	1	1	0	0
chr18	54901509	54959539	18q21.2	CCDC68	0.691820393	0.840360229	0.823242663	4	4	46.61153994	26.83212081
chr19	37467465	37488652	19q13.12	ZNF570	2.430821369	2.953146823	0.823129196	4	4	41.05095803	23.32222813
chr2	61177418	61188925	2p15	AHSA2P	10.60719117	12.88944379	0.822936299	4	4	12.64749268	25.24410753
chr7	73293495	73303235	7q11.23	POM121B	0.180959643	0.219906433	0.822893814	3	2	46.70339565	2.863871799
chr7	53655509	53811931	7p12.1	LINC01446	0.059718343	0.072574172	0.822859448	1	2	0	2.01520295
chr1	233904712	234324518	1q42.2	SLC35F3	0.277231007	0.337066518	0.822481592	4	2	58.71271207	103.9470342
chr15	56428731	56465137	15q21.3	MNS1	1.155640061	1.405092623	0.822465397	4	4	58.85034546	52.92140809
chr1	94657533	94820281	1p21.3	SLC44A3-AS1	0.041022369	0.04988008	0.822419866	3	1	64.07573237	0
chr9	111549722	111599855	9q31.3	PTGR1	0.264278672	0.321343045	0.822419142	4	4	59.26083408	40.45048121
chr5	36152043	36184041	5p13.2	SKP2	3.465644349	4.21521885	0.822174239	4	4	35.57677359	14.14111973
chr2	62452778	62511894	2p15	TMEM17	0.246993185	0.300420775	0.822157473	4	3	67.69598366	63.02474647
chr6	125275350	125302136	6q22.31	HDDC2	2.88465054	3.509098657	0.822048857	4	4	10.06412813	19.76731712
chr9	33460811	33473943	9p13.3	NOL6	4.133900575	5.029117537	0.821993231	4	4	46.02968173	22.12529918
chr5	150444229	150449756	5q33.1	RPS14	104.1414038	126.7020891	0.821939121	4	4	23.16378528	21.88830518
chr17	7668402	7687550	17p13.1	TP53	8.316119211	10.11770309	0.821937463	4	4	48.3112881	72.26993485
chr17	4932275	4935030	17p13.2	GP1BA	5.800891302	7.057999727	0.821888853	4	4	70.16908877	32.55484609
chr3	49554489	49671549	3p21.31	BSN	0.025949878	0.031575216	0.821843258	2	2	30.64810582	73.06559552
chr15	90234255	90242080	15q26.1	GDPGP1	0.400519366	0.487390404	0.821762928	4	4	97.16297476	46.00369316
chr1	28505943	28539196	1p35.3	RCC1	3.426509155	4.169979742	0.821708825	4	4	50.68345212	47.26646319
chr19	58498333	58512065	19q13.43	SLC27A5	0.17424577	0.212088404	0.821571418	3	4	35.6505171	52.64064459
chr8	143291389	143299952	8q24.3	ZNF696	0.670928025	0.816687847	0.821523215	4	4	69.71103109	39.07542186
chr11	18455827	18479600	11p15.1	LDHAL6A	0.147483623	0.179527778	0.821508653	1	1	0	0

chr4	986997	1004557	4p16.3	IDUA	0.687714195	0.837215989	0.821429839	4	4	68.14011198	13.97954108
chr5	55102646	55110252	5q11.2	GZMA	98.65747843	120.1101778	0.821391495	4	4	11.07502836	83.41763178
chr16	55478830	55506691	16q12.2	MMP2	0.058549954	0.071305471	0.82111446	1	2	0	39.41361993
chr4	185445182	185471759	4q35.1	CCDC110	0.089009361	0.108401558	0.821107767	2	2	27.39311634	41.61692109
chr6	157381525	157678114	6q25.3	ZDHHC14	0.60977429	0.742854775	0.820852622	4	4	49.15122816	29.37867001
chr1	86346824	86396346	1p22.3	ODF2L	5.962738332	7.264544858	0.820799988	4	4	33.56883328	18.15402789
chr12	40156239	40167847	12q12	LINC02471	1.01094046	1.231922107	0.820620438	2	2	80.76572212	53.03410817
chr19	34923795	34945172	19q13.11	ZNF30	1.303513112	1.588674422	0.820503619	4	4	35.15929954	81.44200924
chr2	224765090	225042689	2q36.2	DOCK10	23.01805012	28.05848444	0.820359709	4	4	21.48310802	30.92430305
chr22	22318666	22319226	22q11.22	IGLV5-52	2.8075265	3.422320219	0.820357629	4	3	36.53851111	48.35490905
chr18	12446512	12661814	18p11.21	SPIRE1	0.960057769	1.170394868	0.820285354	4	4	47.62975379	27.09227365
chr14	96039324	96093971	14q32.2	C14orf132	0.045292622	0.055218324	0.820246235	3	3	36.84573471	33.22530061
chr1	160485030	160523263	1q23.2-q23.3	SLAMF6	33.90896983	41.34054098	0.820235271	4	4	27.36579922	69.27212426
chr3	113532296	113629579	3q13.2	SIDT1	3.472337807	4.23438042	0.820034447	4	4	34.97417404	21.88705567
chr19	57487711	57494680	19q13.43	ZNF419	1.189717716	1.451056172	0.819897768	4	4	55.45490234	26.09133291
chr1	111439890	111449308	1p13.2	WDR77	3.250579156	3.964666789	0.8198871	4	4	53.80677772	17.19158419
chr15	25082767	25082860	15q11.2	SNORD116-16	2.127342607	2.595157659	0.819735402	1	2	0	28.00373659
chr22	22822821	22823289	22q11.22	IGLV2-8	11.98156029	14.6164911	0.819728908	4	3	70.77483863	117.1818828
chr5	16373361	16441102	5p15.1	LINC02150	0.245294922	0.299259236	0.819673688	3	2	71.95064848	37.35102335
chr9	128633653	128656850	9q34.11	WDR34	0.89174679	1.088192891	0.819474927	3	4	72.59942798	93.6211495
chr19	36797550	36828111	19q13.12	ZNF790-AS1	1.513354912	1.846781855	0.819455155	4	4	60.37705319	44.81883436
chr16	28605587	28623586	16p11.2	SULT1A1	3.078477524	3.757625608	0.819261375	4	4	66.19263164	29.18243183
chr12	55829608	55836246	12q13.2	TMEM198B	1.050868642	1.28273072	0.819243373	4	4	100.4077843	58.78534936
chr22	20701114	20704603	22q11.21	TMEM191A	0.374816865	0.457520873	0.819234459	3	3	17.86206217	48.90587058
chr16	1966639	1968976	16p13.3	RNF151	0.225897016	0.275856143	0.818894272	1	1	0	0
chr1	236394380	236484708	1q42.3-q43	EDARADD	0.255855437	0.312454125	0.818857608	3	4	59.21668165	26.16799369
chr1	65058434	65058508	1p31.3	MIR101-1	5.622357268	6.867096447	0.818738649	4	4	32.71992715	50.69569454
chr2	68130149	68157560	2p14	WDR92	0.547516598	0.668798738	0.818656745	4	4	47.76663265	61.08087078
chr9	34652185	34661902	9p13.3	IL11RA	1.256364513	1.53491848	0.818521979	4	4	71.99072612	32.901782
chr20	13785026	13821580	20p12.1	NDUFAF5	1.36320726	1.665554672	0.818470437	4	4	61.53227923	28.50526223
chr11	57299629	57324952	11q12.1	TNKS1BP1	0.472430481	0.57739759	0.818206534	4	4	123.0811583	49.98504022
chr22	31305404	31305512	22q12.2	RNA5SP496	4.543563941	5.553149941	0.818195797	1	1	0	0
chr2	63588609	63607197	2p15	MDH1	7.969883927	9.741002981	0.818178984	4	4	15.72480084	19.12866517
chr9	66093946	66154762	9q21.11	ANKRD20A3	0.068283679	0.083458675	0.818173533	1	2	0	83.56075006
chr1	53946326	53968168	1p32.3	LRRC42	2.382203944	2.911650702	0.818162681	4	4	42.12307847	50.15424381
chr22	30576625	30607013	22q12.2	PES1	5.137826802	6.280806633	0.818020216	4	4	64.33083183	33.21585636
chr1	110286375	110339171	1p13.3	RBM15-AS1	0.452861064	0.553788338	0.817751174	3	3	65.88388664	21.64855614
chr11	3626460	3637559	11p15.4	TRPC2	0.317067202	0.387751143	0.817707973	4	3	52.00175694	86.3624127
chr3	46515385	46580099	3p21.31	LRRC2	0.734276775	0.89803183	0.817651169	4	4	36.15136169	42.46748616
chr19	10928736	10930254	19p13.2	TIMM29	3.875362101	4.74064931	0.817474959	4	4	50.32284773	37.14428115
chr1	70259002	70354977	1p31.1	ANKRD13C	51.23584919	62.67955713	0.817425195	4	4	56.17860202	50.16007513
chr6	33572546	33580296	6p21.31	BAK1	9.564365708	11.7008121	0.817410418	4	4	50.97455268	57.38910346
chr12	133037286	133063304	12q24.33	ZNF84	3.215932792	3.935300284	0.817201372	4	4	32.32146433	28.1865977

chr6	26225155	26225616	6p22.2	HIST1H3E	3.788436328	4.636464559	0.817095932	4	4	25.77999965	4.342980351
chr19	36817391	36850787	19q13.12	ZNF790	2.843964334	3.480706785	0.817065185	4	4	32.66338862	26.47199006
chr16	86196181	86199720	16q24.1	LINC01082	1.574868793	1.927533088	0.817038526	3	1	44.09769191	0
chr1	116392865	116418622	1p13.1	ATP1A1-AS1	0.509251769	0.623334268	0.816980222	3	4	88.82624852	55.77027419
chr19	10701436	10713367	19p13.2	QTRT1	0.9076657	1.111183845	0.816845659	4	4	72.17032011	61.75645557
chr5	140102922	140108012	5q31.3	MALINC1	0.326648752	0.399929326	0.816766189	3	4	20.20235058	38.97876862
chr11	122028329	122422871	11q24.1	MIR100HG	0.039753392	0.048674241	0.81672341	2	2	45.81146071	4.763343476
chr1	145919013	145925341	1q21.1	GNRHR2	0.318888871	0.390490686	0.816636306	4	4	51.89153589	49.58925073
chr3	70954714	71583989	3p13	FOXP1	17.92396791	21.95067854	0.816556439	4	4	26.25764756	13.38698594
chr17	42998273	43002959	17q21.31	RPL27	129.2357493	158.2695043	0.816554963	4	4	47.40311004	17.34730322
chr6	35023522	35023831	6p21.31	HSPE1P11	0.946457659	1.159231107	0.816452952	3	2	23.05904626	52.55363336
chr2	64185078	64205485	2p14	LINC00309	0.162938604	0.199579135	0.816411016	2	3	72.60573444	57.78361858
chr19	38587640	38618035	19q13.2	MAP4K1	9.92945112	12.16332814	0.816343274	4	4	58.7011304	97.26368456
chr12	121002422	121016502	12q24.31	C12orf43	1.399542185	1.714410115	0.816340369	4	4	73.40409037	44.84992881
chr19	36334453	36379203	19q13.12	ZFP14	1.955283634	2.395655988	0.816178802	4	4	40.9770466	41.38033842
chr11	65570459	65571888	11q13.1	SSSCA1	2.580325934	3.16225153	0.815977448	4	4	43.88703202	33.796254
chrX	102153707	102156014	Xq22.1	BEX5	0.728005486	0.892272851	0.815900074	3	2	52.11143998	21.44922158
chr2	172427354	172506455	2q31.1	ITGA6	22.77201438	27.9106195	0.815890682	4	4	67.38029289	34.65700334
chr19	37738274	37779626	19q13.12	ZNF573	2.417498625	2.963029894	0.815887356	4	4	46.19658212	40.14301713
chr19	54965279	55001142	19q13.42	NLRP2	0.619711546	0.759633935	0.815802873	4	4	32.52870375	102.1935899
chr18	13726645	13764556	18p11.21	RNMT	9.047913973	11.09216254	0.815703335	4	4	36.08556982	11.4280898
chr2	101348354	101387970	2q11.2	CREG2	0.089911657	0.11023148	0.815662246	2	1	13.79878954	0
chr7	27141052	27143668	7p15.2	HOXA5	0.272848389	0.334600585	0.815445045	3	2	26.66670399	14.83604144
chr14	76786473	76812940	14q24.3	ANGEL1	1.605194422	1.968524582	0.815430214	4	4	63.7270881	48.38989486
chr8	489790	545781	8p23.3	TDRP	3.171570611	3.891019193	0.815100223	4	4	42.06451682	56.46724928
chr19	37497159	37507087	19q13.12	ZNF793-AS1	0.440449526	0.540378929	0.815075317	4	3	42.77471072	74.16415145
chr7	102537919	102572621	7q22.1	POLR2J3	0.395701485	0.48548964	0.815056498	4	4	49.1716553	18.69618877
chr20	62642058	62690722	20q13.33	SLCO4A1	0.16461269	0.201965497	0.815053523	3	4	84.16544458	88.88179635
chr1	244943902	244944215	1q44	RN7SKP55	3.98010807	4.883602831	0.814994218	3	4	93.15502326	99.11070048
chr7	142580981	142581427	7q34	TRBV12-5	1.094137074	1.342604588	0.814936195	2	2	12.0285291	75.89834378
chr9	37510892	37576459	9p13.2	FBXO10	0.217838949	0.267314588	0.814916052	2	3	30.32486558	69.90541458
chr14	22539073	22539134	14q11.2	TRAJ6	18.67411458	22.91555777	0.814909887	3	4	91.80419015	7.918527559
chr12	6828373	6839854	12p13.31	P3H3	0.093428108	0.114698876	0.814551209	3	4	49.83474589	36.46318688
chr8	124462493	124474563	8q24.13	RNF139-AS1	0.45811231	0.562414188	0.814546147	4	4	75.82108343	39.67498308
chr11	128458761	128587593	11q24.3	ETS1	134.1730898	164.7754027	0.814278634	4	4	45.64588894	42.18246487
chr12	122983480	123150015	12q24.31	PITPNM2	2.145467084	2.63490056	0.814249735	4	4	81.53263172	38.42656641
chr5	52787940	52802617	5q11.2	PELO	3.102615809	3.811783829	0.813953768	4	4	55.13515584	9.578857333
chr16	71282300	71290178	16q22.2	CMTR2	0.702460791	0.86306437	0.813914715	4	4	27.48748616	21.38395419
chr12	2742626	2812924	12p13.33	ITFG2-AS1	0.720946827	0.885901774	0.813799959	4	1	110.7305584	0
chr9	136881933	136926615	9q34.3	TRAF2	2.104946824	2.587149181	0.813616331	4	4	67.02569632	43.27253837
chr18	50282343	50288322	18q21.1	CXXC1	5.292791499	6.505924979	0.81353405	4	4	43.65594907	48.83769366
chr17	45247925	45268630	17q21.31	MAP3K14-AS1	0.388696257	0.477850137	0.813427113	4	4	119.1149025	54.36179041
chr15	82536750	82540564	15q25.2	RPS17	146.9979597	180.7300632	0.813356434	4	4	51.31544649	40.97550107

chr13	87671232	87679619	13q31.2	SLITRK5	0.012556367	0.015441044	0.813181271	2	4	34.38406253	45.02025109
chr2	219209766	219219017	2q35	ABCB6	0.161739981	0.19893286	0.813038032	4	4	49.94255629	65.14523072
chr2	90159860	90160335	2p11.2	IGKV1D-12	1.096176943	1.348443695	0.81292007	1	2	0	17.92194945
chrX	49053432	49069858	Xp11.23	CCDC120	0.299181125	0.368085516	0.812803309	4	4	36.68354331	60.40722827
chr1	182407621	182414813	1q25.3	LINC00272	0.811144592	0.998367411	0.812471023	2	3	38.20999925	89.6012826
chr13	21483834	21484174	13q12.11	HIST1H2BPS3	1.186986708	1.461034182	0.812429115	1	1	0	0
chr10	51695486	51699595	10q21.1	CSTF2T	14.68107041	18.07357453	0.812294789	4	4	36.53138626	24.59344889
chr14	22123527	22124285	14q11.2	TRAV26-1	5.125050705	6.311625697	0.812001685	4	4	47.31043264	90.35587376
chrX	2486414	2500967	Xp22.33 and Yp11	ZBED1	10.29557785	12.68068424	0.811910277	4	4	61.63456155	25.55481601
chr4	52713249	52713370	4q12	SNORA26	7.793427155	9.59901654	0.811898503	3	4	103.888517	54.51325088
chr19	53467735	53494292	19q13.42	ZNF813	2.549420751	3.140705723	0.811734997	4	4	37.98554947	26.05463602
chr14	22533497	22533560	14q11.2	TRAJ10	29.16982532	35.93794209	0.811672111	4	4	60.396835	35.84664959
chr1	1401896	1407313	1p36.33	MRPL20	8.078020918	9.953380414	0.811585671	4	4	46.29354256	19.0814941
chr15	50354174	50358312	15q21.2	GABPB1-AS1	1.287234075	1.586286286	0.81147652	4	4	44.31747542	23.432857
chr22	20115938	20127357	22q11.21	RANBP1	3.616471535	4.456736227	0.811461875	4	4	99.21371807	79.46848267
chr20	45857581	45879131	20q13.12	ZSWIM3	1.192498189	1.469706674	0.811385163	4	4	75.44449045	43.62004478
chr14	22508602	22508658	14q11.2	TRAJ33	7.802742805	9.617940522	0.811269605	4	3	38.24192419	50.2457437
chr4	73073966	73259766	4q13.3	ANKRD17	32.12434379	39.60215781	0.811176602	4	4	29.2168085	33.67553066
chr3	72928843	72929707	3p13	FTH1P23	0.609538841	0.751439333	0.811161745	4	3	25.3419775	63.66786985
chr2	96760902	96811891	2q11.2	CNNM4	2.165260077	2.669412552	0.811137295	4	4	56.6140935	44.28070108
chr10	120761812	120851179	10q26.12	WDR11-AS1	3.447139439	4.249851909	0.811119896	4	4	50.79458585	50.28205758
chr18	3411927	3458412	18p11.31	TGIF1	2.128696346	2.624704667	0.811023188	4	4	66.83897785	16.09098639
chr19	11848733	11869491	19p13.2	ZNF439	1.603594795	1.97744982	0.810940828	4	4	86.09930845	34.10696859
chr19	55647393	55653161	19q13.42	CCDC106	0.671659843	0.828284472	0.810904787	3	4	47.07532605	86.21302097
chr1	45339670	45343978	1p34.1	TOE1	2.770866202	3.417471324	0.81079428	4	4	75.22348014	35.86681725
chr8	22604632	22620471	8p21.3	CCAR2	8.292398956	10.22805998	0.810749934	4	4	46.96726859	41.70188175
chr9	121038	179083	9p24.3	CBWD1	2.493430044	3.076537689	0.810466276	4	4	38.64861283	37.48656409
chr5	79373824	79513836	5q14.1	HOMER1	1.075847018	1.3276539	0.810336954	4	4	39.84342281	40.27650353
chr2	96010551	96023136	2q11.2	FAHD2CP	0.94280559	1.163869727	0.810061099	3	3	46.26883447	15.934168
chr18	70003804	70205726	18q22.2	RTTN	2.028582917	2.504443543	0.809993471	4	4	28.16235838	14.32308626
chr16	75538117	75556286	16q23.1	TMEM231	0.170351296	0.210317883	0.809970573	3	1	67.7849124	0
chr1	63440770	63529395	1p31.3	ITGB3BP	44.21130625	54.58761418	0.80991461	4	4	66.37716758	39.9798581
chr16	74621396	74666892	16q23.1	RFWD3	6.923055353	8.548072308	0.809896677	4	4	23.09696766	25.95417866
chr6	88139864	88172849	6q15	CNR1	0.183425029	0.226526892	0.809727392	4	3	61.74251612	82.63990373
chr19	3708337	3750813	19p13.3	TJP3	0.345278069	0.426441499	0.809672768	4	3	67.2839496	34.52033456
chr12	43795763	44389762	12q12	TMEM117	0.894592436	1.104988265	0.809594512	4	4	79.02977667	31.36600193
chr4	25120005	25160582	4p15.2	SEPSECS	11.17574594	13.80455437	0.809569483	4	4	48.12084159	50.13582502
chr12	122078728	122147347	12q24.31	MLXIP	1.654535482	2.043732157	0.809565714	4	4	68.83586106	36.33621237
chr1	1659798	1692804	1p36.33	SLC35E2B	5.066698304	6.258654098	0.80955078	4	4	51.02342817	20.64819512
chr19	13731760	13763298	19p13.13	CCDC130	4.080252893	5.040503617	0.809493099	4	4	42.27441717	10.65740916
chr16	2882804	2899382	16p13.3	FLYWCH2	2.526460676	3.121255174	0.809437401	4	4	94.38509745	55.19480595
chr20	44475870	44494603	20q13.12	TTPAL	11.61159858	14.34988447	0.809177147	4	4	28.95522099	20.46957952
chr5	176063709	176114798	5q35.2	FAM153B	0.204698433	0.253017753	0.809027947	4	4	45.10798617	73.14508775

chrX	130494941	130497448	Xq26.1	FAM45BP	0.480941882	0.594478984	0.809014103	3	1	77.49432678	0
chr3	148824782	148825387	3q24	HMGB1P30	1.745527894	2.157841668	0.808923064	3	4	7.399743913	83.03550151
chr5	17404019	17441694	5p15.1	LINC02217	1.14851136	1.420068899	0.808771575	4	4	26.65863883	115.795512
chr4	148078764	148444698	4q31.23	NR3C2	2.544598481	3.146480465	0.808712626	4	4	57.18157751	32.95027582
chr22	29073078	29168333	22q12.1	KREMEN1	5.494261168	6.794764902	0.8086021	4	4	61.88054102	50.42180609
chr15	90083978	90102554	15q26.1	IDH2	20.50649859	25.36334121	0.808509353	4	4	51.02378597	98.22129137
chr4	109488698	109488795	4q25	MIR576	3.70002031	4.576705988	0.808446144	3	3	58.37276591	79.73437651
chr5	16661907	16936276	5p15.1	MYO10	0.201043892	0.248696432	0.808390735	4	3	100.5430974	128.2313437
chr2	73385758	73609919	2p13.1	ALMS1	13.29681942	16.45722617	0.807962368	4	4	7.355509403	24.1465485
chr7	39566376	39612089	7p14.1	YAE1	2.083719568	2.579364292	0.807842295	4	4	43.56458279	19.53496741
chr11	122838419	122872643	11q24.1	CRTAM	4.945887253	6.123388043	0.807704365	4	4	32.9887299	34.6382831
chr19	12390014	12401274	19p13.2	ZNF799	1.260930973	1.561173394	0.807681567	4	4	49.96382667	28.94151469
chr16	3052125	3059370	16p13.3	MMP25-AS1	0.662729849	0.821080238	0.807143831	4	4	76.18248087	29.29382925
chr2	84459572	84819589	2p11.2	DNAH6	0.165324894	0.204843408	0.807079397	4	4	49.37029559	76.78977387
chr13	52617470	52643784	13q14.3	HNRNPA1L2	1.242714475	1.539873408	0.807023791	4	4	76.11756454	39.44335575
chr19	36383102	36418674	19q13.12	ZFP82	1.066902571	1.322113746	0.806967308	4	4	50.23395228	44.35845233
chr3	183015218	183099587	3q27.1	MCCC1	9.598496923	11.89787928	0.80674015	4	4	42.64570577	42.14978321
chr2	240435698	240468078	2q37.3	GPC1	0.076418349	0.094735508	0.806649488	3	3	42.62071555	62.28315233
chr6	34236800	34246231	6p21.31	HMGA1	19.58196959	24.27752683	0.80658832	4	4	44.12285905	98.41305015
chr14	97458816	97581383	14q32.2	LINC02325	1.079760633	1.338967786	0.806412704	4	4	100.0946455	20.03365345
chr10	68988721	69016983	10q22.1	KIF1BP	1.035435264	1.284110327	0.806344472	4	4	19.02271417	9.298619395
chr2	97827152	97828350	2q11.2	HMGN1P36	0.608176691	0.754415409	0.806156243	1	1	0	0
chr7	150379317	150398631	7q36.1	ZNF775	0.476837762	0.591553677	0.806076914	4	3	48.91373538	66.56009486
chr19	51889235	51905052	19q13.41	ZNF649	2.832972382	3.51469495	0.80603649	4	4	68.21852893	30.45346002
chr16	30085793	30091927	16p11.2	TBX6	0.25880439	0.321128874	0.805920648	2	2	90.61833319	53.03622903
chr12	6965352	6970753	12p13.31	PHB2	20.86186691	25.88645064	0.805899086	4	4	44.86725008	51.64923402
chrX	100627108	100637104	Xq22.1	TSPAN6	0.252597744	0.313448441	0.805866965	3	4	63.07175285	53.20013571
chr1	17369	17436	1p36.33	MIR6859-1	5.37978088	6.677829331	0.805618205	1	4	0	35.46841793
chr17	82418354	82442645	17q25.3	HEXD	1.985149371	2.464792759	0.805402143	4	4	76.95659472	13.53132532
chr1	32251239	32286167	1p35.2	LCK	48.37256144	60.06190607	0.805378394	4	4	46.54153812	69.06618692
chrX	14690863	14873255	Xp22.2	FANCB	16.44370374	20.41884533	0.805319962	4	4	53.86288518	80.38288805
chr1	94981565	95072974	1p21.3	ALG14	0.269021277	0.334161279	0.805064184	4	4	56.03574256	40.84082474
chr12	9951316	9995209	12p13.31	CLEC12A	43.15934086	53.61218267	0.805028609	4	4	56.83651123	71.83368731
chr2	164680185	164842168	2q24.3	COBLL1	3.683456242	4.57632171	0.804894515	4	4	38.37964684	40.87786997
chr1	100264742	100266174	1p21.2	RTCA-AS1	23.19535827	28.82278171	0.804757795	4	4	44.0959903	130.8640485
chr1	47179250	47180339	1p33	LINC00853	2.492605256	3.097663389	0.80467273	4	4	28.12302912	70.90326846
chr6	26020490	26020958	6p22.2	HIST1H3A	52.97174988	65.83640867	0.80459659	4	4	23.43547425	81.54776331
chr19	44474428	44500539	19q13.31	ZNF180	4.237998981	5.267520705	0.804552885	4	4	48.1765523	26.11994325
chr12	2794942	2805423	12p13.33	FKBP4	6.195744755	7.702743152	0.804355621	4	4	43.87068492	22.46027226
chr14	82642632	82706825	14q31.1	LINC02301	0.356176429	0.442861589	0.804261282	4	2	43.60638595	98.12399052
chr16	19858971	19886045	16p12.3	GPRC5B	0.072749025	0.090471654	0.804108497	1	3	0	64.8922544
chr11	8092965	8169094	11p15.4	RIC3	0.535263638	0.66567202	0.804095143	4	4	22.39273455	46.75997336
chr13	111305838	111344248	13q34	TEX29	0.394287824	0.490395049	0.80402081	3	4	14.20923189	85.73009562

chr15	35217356	35546203	15q14	DPH6	1.282381776	1.595124002	0.803938612	4	4	57.63059877	24.15988291
chr12	57762334	57767193	12q14.1	CYP27B1	0.08575422	0.106671115	0.803912288	2	2	4.708386335	42.06533338
chr7	158839245	158958695	7q36.3	WDR60	6.216751294	7.733425756	0.803880646	4	4	43.51691262	36.90599511
chr11	8911117	8920079	11p15.4	AKIP1	1.608525568	2.001016844	0.803854087	4	4	51.46506732	24.36682409
chrX	55908123	56291531	Xp11.21	KLF8	0.461374841	0.573976771	0.803821451	4	4	64.21057752	82.23101672
chr2	241106099	241150264	2q37.3	PASK	3.689825873	4.59077467	0.803747981	4	4	58.75418036	33.05079801
chr12	66130751	66134449	12q14.3	LLPH-DT	1.484002883	1.846487432	0.803689674	4	4	61.83711327	75.70924879
chr9	21967752	21995043	9p21.3	CDKN2A	0.206601949	0.257084193	0.803635364	4	3	48.14454656	94.40802573
chr16	3500931	3539048	16p13.3	CLUAP1	1.478042186	1.83919961	0.803633373	4	4	53.8150821	16.43905566
chr13	19818182	19818611	13q12.11	ST6GALNAC4P1	0.43998214	0.547514948	0.803598406	1	3	0	17.46435834
chr4	183796331	184023530	4q35.1	STOX2	0.1281167	0.159538237	0.803046985	4	4	96.99690704	35.6883366
chr4	184694065	184734132	4q35.1	CENPU	19.85896703	24.73057483	0.803012755	4	4	70.74785116	37.24868569
chr1	19424384	19424599	1p36.13	RN7SL277P	1.572158507	1.957849258	0.803002836	2	1	24.09942206	0
chr17	16217195	16351800	17p11.2	PIGL	0.987831425	1.230300384	0.802918895	4	4	57.96203568	24.02874811
chr4	119449570	119454628	4q26	GTF2IP12	0.590900382	0.736036897	0.802813535	4	4	31.26740047	20.62864927
chr22	20999772	21002115	22q11.21	THAP7	2.780512836	3.463492137	0.802806164	4	4	52.05618914	23.60235868
chr12	53500921	53506431	12q13.13	TARBP2	1.009599999	1.257690746	0.802741057	4	4	99.73692498	19.25487245
chr22	22697951	22698421	22q11.22	IGLV2-23	13.34441558	16.62377729	0.802730652	4	3	94.22997279	118.5598087
chr7	42909273	42914356	7p14.1	C7orf25	0.380223713	0.473668942	0.802720378	4	4	57.69367649	42.5041651
chr8	119873655	120056202	8q24.12	DEPTOR	0.80665238	1.004992169	0.802645438	4	4	47.17631719	51.67643641
chr3	35639174	35798933	3p22.3	ARPP21	0.042552441	0.05301837	0.802598055	2	4	46.39879887	45.07024729
chr20	62386275	62388520	20q13.33	RPS21	107.0551197	133.4104561	0.802449244	4	4	24.7096643	10.75463033
chr6	106629566	106668417	6q21	QRSL1	3.301222959	4.114186523	0.802399925	4	4	24.3111332	19.10658169
chr4	48574453	48574520	4p11	RNU5E-3P	9.608114905	11.97848882	0.80211411	2	2	53.25669586	3.732153633
chr2	24029336	24047426	2p23.3	WDCP	2.43396343	3.034622373	0.802064682	4	4	35.79722508	30.71158682
chr16	28904421	28925211	16p11.2	RABEP2	1.263004406	1.575423173	0.801692159	4	4	75.64874725	48.49968149
chr17	36495636	36535457	17q12	MYO19	0.465289735	0.580412427	0.801653641	4	4	71.44737132	23.41096318
chr15	48134632	48178517	15q21.1	MYEF2	0.436889377	0.545040235	0.801572707	4	4	50.28884073	52.02787432
chr22	24952688	25197448	22q11.23	KIAA1671	0.831070584	1.036812086	0.801563364	4	4	65.86266797	100.5383543
chr16	30778455	30787219	16p11.2	ZNF629	0.974544622	1.215875761	0.801516613	4	4	87.31121945	47.01254782
chr17	49206234	49210574	17q21.32	GNGT2	3.260319825	4.067906935	0.801473553	4	4	76.56232365	38.28834377
chr11	18490058	18491092	11p15.1	YWHABP2	1.098874506	1.371072365	0.801470829	3	2	109.2713343	75.95642917
chr1	89052304	89065360	1p22.2	GBP1	83.81894364	104.5911711	0.801395976	4	4	89.36641904	85.05022906
chr15	81312666	81324183	15q25.1	STARD5	0.318585532	0.397605696	0.801259978	4	4	77.11763938	46.24525508
chr11	46256355	46274663	11p11.2	LINC02489	0.60718291	0.757909008	0.801129032	1	1	0	0
chr1	72274535	72275148	1p31.1	GDI2P2	0.62603247	0.781474528	0.801091331	3	2	11.09377919	65.15883076
chr2	237324012	237434822	2q37.3	COL6A3	0.34081042	0.425451896	0.80105512	4	4	111.0568021	64.91845143
chr8	60402171	60413818	8q12.1	LINC01301	0.054737087	0.068341479	0.800935065	1	1	0	0
chr1	154974642	154979249	1q21.3	CKS1B	1.99696704	2.493758763	0.800785974	4	4	27.4074405	87.67054441
chr17	58420167	58488401	17q22	HSF5	2.772386507	3.46308405	0.800554207	4	4	52.22319467	54.60751042
chr1	203216079	203229732	1q32.1	CHIT1	0.602729312	0.752913024	0.800529799	4	4	34.65557349	56.76390445
chr3	48290279	48291678	3p21.31	FCF1P2	6.680757449	8.346328809	0.800442638	4	4	66.37422591	58.04913126
chr19	44111426	44134816	19q13.31	ZNF225	1.329149375	1.660750998	0.800330319	4	4	43.44023383	33.45049368

chr7	66493632	66495474	7q11.21	GS1-124K5.4	2.003561602	2.504004808	0.800142873	4	4	72.49537581	57.47641182
chr19	58216147	58216615	19q13.43	RPS15AP36	1.067117127	1.333786169	0.800066121	1	1	0	0
chr14	64388041	64460007	14q23.3	MTHFD1	3.446583754	4.308533294	0.799943628	4	4	23.83384996	56.37808373
chr6	30880958	30900156	6p21.33	DDR1	0.146819512	0.183537998	0.799940685	3	2	55.77873342	16.06815897
chr16	70113626	70163631	16q22.1	PDPR	4.304685702	5.383721142	0.799574419	4	4	43.13291739	38.28470103
chr7	67276131	67302442	7q11.21	PMS2P4	4.894532622	6.123103785	0.799354836	4	4	55.0223189	58.00623303
chr17	30248195	30292166	17q11.2	BLMH	5.125338094	6.412347824	0.799291965	4	4	39.61477954	49.14035865
chr6	24494969	24537207	6p22.3	ALDH5A1	17.13935837	21.44497451	0.799224935	4	4	35.57584798	90.41772024
chr21	28439661	28885373	21q21.3	N6AMT1	1.279016935	1.600467172	0.799152246	4	4	53.40623109	36.61547906
chr4	143424974	143425984	4q31.21	RPSAP36	0.176381466	0.220732947	0.799071767	1	3	0	11.64341662
chr11	128965060	129279167	11q24.3	ARHGAP32	1.714933201	2.146315101	0.799012783	4	4	57.46421986	29.57096592
chr8	93957305	93957720	8q22.1	RPL34P18	0.764487797	0.957119987	0.798737679	2	1	6.037071676	0
chr19	57571015	57578927	19q13.43	ZNF416	1.742843307	2.182108101	0.798697052	4	4	56.71155704	23.12707216
chr8	144511284	144517828	8q24.3	RECQL4	0.289945181	0.363047298	0.798642994	4	4	36.34530335	122.157417
chr6	26285126	26285499	6p22.2	HIST1H4H	40.66738558	50.92717531	0.79853998	4	4	53.04556543	93.58930663
chr19	48600600	48615239	19q13.33	FAM83E	0.093124583	0.116623011	0.798509501	1	1	0	0
chr11	86294788	86295578	11q14.2	SETP17	0.440320788	0.551510428	0.798390684	2	3	28.83877327	61.03959182
chr15	90966038	90994651	15q26.1	PRC1	9.70420726	12.1579302	0.798179221	4	4	59.86749264	38.10111155
chr22	21417403	21451463	22q11.21	HIC2	1.186138885	1.486155789	0.798125536	4	4	77.59282063	30.3476681
chr18	32411506	32413237	18q12.1	HNRNPA1P7	0.720902416	0.903407764	0.797981204	3	3	54.75017913	100.0346516
chr3	169911572	169939175	3q26.2	SAMD7	0.093951575	0.117745326	0.797921909	1	1	0	0
chr5	78358764	78360541	5q14.1	SCAMP1-AS1	2.283766479	2.862373912	0.797857495	4	4	44.49087946	19.17310012
chr15	24823608	25419462	15q11.2	SNHG14	0.722875799	0.906063853	0.797819929	4	4	49.46933075	33.75190677
chr1	35720218	35769985	1p34.3	CLSPN	10.34739221	12.96972549	0.797811197	4	4	49.88120464	41.44505183
chr6	30005971	30009956	6p22.1	HLA-J	0.317282512	0.397773265	0.797646651	2	4	9.698110087	84.37317568
chr1	227731189	227735499	1q42.13	JMJD4	1.953616091	2.44931091	0.797618662	4	4	58.20585554	33.46903082
chr10	74824927	75032623	10q22.2	KAT6B	1.608565585	2.016860787	0.797559056	4	4	22.50226297	28.27705818
chr3	53333061	53333127	3p21.1	SNORD38C	6.320993423	7.92816083	0.797283703	2	3	22.68935723	71.59023795
chr2	203325013	203432173	2q33.2	ABI2	1.867147522	2.34210005	0.797210829	4	4	38.46583037	11.73915548
chr1	151047740	151050796	1q21.3	C1orf56	0.539809708	0.677384678	0.796902743	4	2	66.13372859	93.24418069
chr10	73110375	73131828	10q22.2	NUDT13	0.512529496	0.643199034	0.796844319	4	4	51.24307358	54.4956126
chr12	56116590	56117832	12q13.2	RPL41	428.6171859	538.1593844	0.796450268	4	4	16.40373242	15.29189582
chr22	41800610	41832164	22q13.2	CCDC134	9.267165982	11.63853491	0.796248501	4	4	54.94136803	37.59757094
chr6	41053201	41064891	6p21.1	APOBEC2	0.640861178	0.804853268	0.796245979	4	3	105.3194694	95.02703015
chr10	68560360	68700794	10q21.3	TET1	1.887577844	2.370638303	0.796231901	4	4	20.19485207	49.37699857
chr1	92832729	92961522	1p22.1	DIPK1A	4.281801354	5.377948337	0.79617748	4	4	36.25412309	29.01354004
chr2	206159594	206162929	2q33.3	EEF1B2	123.07379	154.5833685	0.796164498	4	4	64.0553472	28.67197306
chr2	105337359	105359940	2q12.2	C2orf49	10.76685053	13.52879592	0.795846918	4	4	24.27536557	18.70480173
chr13	66302834	67230336	13q21.32	PCDH9	0.628107635	0.789242186	0.795836368	4	4	63.60828637	70.51931842
chr17	21504518	21505094	17p11.2	RPL21P120	0.559824016	0.703463971	0.795810502	2	2	30.89688697	35.17696472
chr20	31547175	31548177	20q11.21	MCTS2P	2.186126935	2.747217269	0.795760481	3	4	68.2627436	47.21396725
chr1	91260766	91408008	1p22.2	HFM1	1.037009069	1.303986143	0.795260804	4	4	69.98618481	110.164998
chr1	193304745	193365953	1q31.2	LINC01031	0.783829064	0.985856066	0.795074546	4	3	61.53541931	41.23497235

chr19	11887773	11980222	19p13.2	ZNF69	0.879654925	1.106541939	0.794958504	4	4	97.4424377	33.7804164
chr1	998962	1001052	1p36.33	HES4	0.512584291	0.644939058	0.79477942	4	2	56.93777106	16.70037629
chrX	123874459	123874565	Xq25	RNU6-122P	4.649004744	5.849759466	0.794734343	1	2	0	53.15911952
chr5	55073015	55096553	5q11.2	GZMAP1	0.944903464	1.189065474	0.794660583	2	1	36.70534552	0
chr8	143723933	143741626	8q24.3	FAM83H	0.055661699	0.070053981	0.794554392	2	2	11.06015526	42.58950312
chr19	11505916	11529172	19p13.2	ECSIT	9.741180092	12.2604668	0.794519512	4	4	29.53925999	22.93258339
chr3	183807064	183810451	N/A	YEATS2-AS1	0.140236325	0.176536232	0.794377013	2	3	36.86942553	54.97966574
chr2	241734579	241768816	2q37.3	D2HGDH	0.720000614	0.906491678	0.794271621	4	4	83.32364599	63.78069047
chr15	39250669	39254845	15q14	C15orf54	5.772088173	7.269122766	0.794055673	4	4	63.96306634	86.37575948
chr11	118077012	118125505	11q23.3	TMPRSS4	0.049983732	0.062956848	0.793936368	3	3	41.49516863	31.3247099
chr7	152646498	152676165	7q36.1	XRCC2	1.099694965	1.385736226	0.79358174	4	4	43.93472296	11.03205827
chr3	177010419	177011046	3q26.32	MTND5P15	1.102805512	1.390040534	0.793362125	3	3	47.5577347	17.33321362
chr22	42090933	42125349	22q13.2	NDUFA6-DT	0.100450668	0.126646591	0.793157298	4	4	50.42329707	91.49474103
chr10	27812164	27999500	10p12.1	ARMC4	0.060102257	0.07577992	0.79311586	1	3	0	40.1873678
chr6	28141643	28159472	6p22.1	ZKSCAN8	5.44595288	6.866845816	0.793079243	4	4	35.66082674	44.28317208
chr10	6580425	6585361	10p15.1	PRKCQ-AS1	8.207909914	10.34947096	0.793075313	4	4	36.06625808	11.38540285
chr8	93700252	93732370	8q22.1	FAM92A	0.19452325	0.245337517	0.792880162	4	4	45.69299303	60.57917782
chr7	100063847	100082548	7q22.1	ZNF3	4.331578606	5.463302044	0.792849923	4	4	26.41376144	26.17365928
chr4	44701795	44726634	4p12	GNPDA2	2.938613154	3.706444448	0.79283885	4	4	31.5476062	3.169916212
chrX	70444850	70505490	Xq13.1	DLG3	1.614300034	2.03621801	0.792793319	4	4	31.32857441	53.29109287
chr14	105851966	105856217	14q32.33	IGHM	76.98393692	97.12568417	0.792621824	4	4	92.8398443	127.1553822
chrX	49071156	49074045	Xp11.23	PRAF2	0.893679284	1.127555517	0.792581182	4	4	51.68832467	66.39179442
chr15	50944129	50946573	15q21.2	DCAF13P3	0.997871984	1.259090553	0.79253393	3	4	73.5679459	36.44420371
chr16	46884380	46931297	16q11.2	GPT2	0.358706767	0.452739092	0.792303499	4	2	86.36467721	91.14768015
chr9	71910977	71973459	9q21.13	C9orf85	3.516486893	4.438690512	0.792235206	4	4	29.49122564	16.72459273
chr7	140672895	140695114	7q34	ADCK2	3.075392284	3.88287534	0.792039923	4	4	44.49063871	46.67922847
chr14	105419849	105470729	14q32.33	MTA1	2.109863245	2.66383826	0.792038795	4	4	59.73864257	28.05427399
chr17	17494437	17496395	17p11.2	RASD1	0.559159949	0.706089557	0.791910804	3	2	93.80410408	82.77226241
chr16	70661204	70686051	16q22.1	MTSS1L	0.445977234	0.563344674	0.791659626	3	3	75.87960544	72.95042207
chr10	84194635	84219621	10q23.1	CDHR1	0.222013165	0.280454282	0.791619808	3	4	51.99940858	58.08131922
chr14	60734735	60969881	14q23.1	MNAT1	5.526739817	6.982280969	0.791537872	4	4	20.28763273	24.83221224
chr17	81681165	81683924	17q25.3	ARL16	1.307162546	1.651894595	0.79131111	4	4	70.41208255	41.25120134
chr22	41576886	41589867	22q13.2	PMM1	0.963488707	1.217655978	0.791265123	4	4	40.45273604	12.51177969
chr22	22762079	22762574	22q11.22	IGLV3-13	1.59472985	2.016410296	0.790875673	3	2	30.01580665	53.86482011
chr1	47250139	47314787	1p33	STIL	79.35779203	100.3545488	0.79077424	4	4	46.76701815	39.10998578
chr1	826206	827522	1p36.33	LINC00115	0.91817887	1.161313819	0.79063803	4	4	58.47598205	53.92326174
chr19	57320497	57330776	19q13.43	ZNF543	2.571679047	3.253022108	0.790550744	4	4	63.50098417	14.14842451
chrX	70286361	70289958	Xq13.1	PDZD11	5.367210517	6.789312452	0.790538152	4	4	40.79523544	65.68469409
chr16	15104312	15123498	16p13.11	NPIPP1	0.155616438	0.196862555	0.790482669	1	2	0	14.35255256
chr17	28346628	28357589	17q11.2	POLDIP2	15.47219823	19.57574999	0.790375758	4	4	37.71702136	30.73053795
chr13	38050817	38143232	13q13.3	LINC00571	0.522873879	0.661652699	0.790254283	4	2	47.82578468	95.58110733
chrX	154398065	154402339	Xq28	RPL10	144.6929953	183.1013818	0.790234316	4	4	66.39652536	40.15272293
chr4	25312774	25373176	4p15.2	ZCCHC4	4.015022987	5.08126709	0.790161768	4	4	57.91641629	58.43049569

chr2	88860886	88860923	2p11.2	IGKJ4	29.81240705	37.73401403	0.790067206	3	3	40.10472858	7.821104704
chr17	81125276	81125373	17q25.3	MIR657	2.00070709	2.532406243	0.79004192	1	2	0	20.35364407
chr9	112272596	112272837	9q32	RN7SL430P	1.897766235	2.402156291	0.790026128	2	3	76.77079345	86.38825068
chr14	88612148	88792752	14q31.3	EML5	1.756482721	2.224012095	0.789781101	4	4	50.50188071	39.40913866
chr1	214603189	214664571	1q41	CENPF	9.219376717	11.67370189	0.789756052	4	4	41.58066962	20.53917543
chr3	39334915	39336077	3p22.1	HNRNPA1P21	0.58676411	0.743067538	0.789651115	2	2	69.34717945	7.343267337
chr5	43573185	43603230	5p12	NNT-AS1	1.326122167	1.679431094	0.789625827	4	4	46.19413908	33.52361317
chr6	150865679	151101887	6q25.1	MTHFD1L	1.457090271	1.845446527	0.789559735	4	4	23.04589352	27.27141636
chr15	75335996	75341927	15q24.2	COMMD4	0.835987183	1.05900466	0.789408409	4	4	89.57633806	53.02489774
chr12	47753619	47782753	12q13.11	SLC48A1	8.293947225	10.51420991	0.78883219	4	4	17.21604296	43.36832366
chr2	38665910	38734767	2p22.1	GALM	5.94655622	7.540694556	0.788595291	4	4	34.35176399	102.9871253
chr4	155666710	155737062	4q32.1	GUCY1A1	8.641395012	10.96114584	0.788366028	4	4	58.053569	30.87573091
chr11	767095	777502	11p15.5	GATD1	1.721832588	2.18439678	0.78824168	4	4	82.66229022	23.96655266
chrX	45747015	45747124	Xp11.3	MIR222	1.782140999	2.261114311	0.788169351	1	1	0	0
chr22	20441519	20497305	22q11.21	KLHL22	2.366343706	3.002339118	0.788166697	4	4	67.09556902	36.84703564
chr16	2039815	2047872	16p13.3	NTHL1	0.57106239	0.724621712	0.788083465	4	4	60.28364992	45.58379461
chr3	68144992	68145773	3p14.1	COPS8P2	0.540860691	0.686427807	0.787935286	1	2	0	37.31688379
chr2	6912276	6918682	2p25.1	RNF144A-AS1	0.260669799	0.330829518	0.787927876	2	3	12.42423981	59.28845829
chr8	39934955	40016391	8p11.21	IDO2	0.123775727	0.1571007	0.787875081	2	1	51.32250685	0
chr16	56989485	57083524	16q13	NLRC5	13.5516954	17.20102694	0.787842229	4	4	23.98287831	47.86339496
chr16	79770471	79801343	16q23.2	LINC01229	0.097608597	0.123919525	0.787677301	1	1	0	0
chr19	58083834	58118433	19q13.43	ZSCAN18	1.282027121	1.628054146	0.787459756	4	4	92.97704266	39.41363592
chr7	73683568	73698221	7q11.23	BUD23	2.27121325	2.88428322	0.787444601	4	4	69.3175997	30.2987955
chr21	10413532	10491615	21p11.2	BAGE2	0.076179384	0.096742703	0.78744321	1	2	0	48.3375256
chrX	48458537	48470256	Xp11.23	SLC38A5	15.63698909	19.85939797	0.78738485	4	4	62.40726413	48.79468199
chr16	20806429	20849682	16p12.3	REXO5	3.107048927	3.946444837	0.78730327	4	4	77.41456738	46.35331712
chr22	46085997	46113928	22q13.31	MIRLET7BHG	0.442618271	0.562436183	0.78696621	4	4	70.94409202	21.34974514
chr14	98972823	98973301	14q32.2	RPL3P4	1.322961347	1.681324133	0.786856812	2	4	94.79241882	123.7452425
chr1	39841031	39883511	1p34.2	TRIT1	8.024272272	10.19888861	0.786779088	4	4	33.53607866	31.4620849
chr17	19063920	19064136	17p11.2	SNORD3B-2	13.53771465	17.20726018	0.786744346	4	4	69.27487715	62.80602538
chr10	98416193	98446963	10q24.2	HPS1	36.07154961	45.85388894	0.786662821	4	4	25.09811744	58.94239193
chr8	25458557	25511308	8p21.2	CDCA2	5.767630373	7.333571478	0.786469511	4	4	63.69462702	42.55478004
chr1	212432887	212446379	1q32.3	NENF	4.894026081	6.224525561	0.786248853	4	4	69.56001094	8.072896793
chr6	115941031	116100739	6q22.1	FRK	0.044262384	0.056300118	0.786186352	3	3	53.6134408	31.31955097
chr15	30572555	30600708	15q13.2	ULK4P2	0.246798494	0.31393895	0.786135309	2	1	19.61358702	0
chr7	142300998	142301432	7q34	TRBV2	6.577053712	8.366647949	0.786103796	4	4	30.46244054	39.20420498
chr22	43203223	43343388	22q13.2	SCUBE1	0.03590295	0.045672548	0.786094749	2	1	46.56330216	0
chr19	12891129	12915345	19p13.13	GCDH	1.535213289	1.953769398	0.785769954	4	4	63.78089958	18.09724974
chr3	51993607	51995942	3p21.2	RPL29	84.33261775	107.3250066	0.785768578	4	4	42.81225295	21.64550646
chr11	71814045	71821547	11q13.4	ZNF705E	0.312272394	0.397419905	0.785749255	3	4	102.8404188	66.61518982
chr20	18142224	18188387	20p11.23	KAT14	3.740103837	4.759928003	0.785747985	4	4	54.1810563	34.57559135
chr16	1363205	1414749	16p13.3	UNKL	2.327214358	2.962756117	0.785489681	4	4	89.489718	24.17273476
chr6	116461370	116463782	6q22.1	CALHM6	10.28990987	13.10035804	0.785467835	4	4	69.81887572	87.74430212

chr19	4903080	4962154	19p13.3	UHRF1	2.056098504	2.617700058	0.78545993	4	4	102.1534103	100.2311678
chr12	67648732	67665405	12q15	DYRK2	19.3320619	24.6178582	0.785286102	4	4	31.21760554	29.69046311
chr9	112280988	112281408	9q32	HSPE1P28	1.85960588	2.368289667	0.785210486	1	1	0	0
chr2	55231903	55235853	2p16.1	RPS27A	163.0273829	207.6262221	0.7851965	4	4	22.61378395	33.83718453
chr11	90251232	90915052	11q14.3	DISC1FP1	0.110383938	0.140615562	0.785005135	3	2	13.17714585	42.20062438
chr20	30377164	30419843	20q11.1	FRG1BP	18.20821315	23.19687888	0.784942373	4	4	93.24711031	19.25612305
chr19	1876976	1885519	19p13.3	ABHD17A	9.41733095	12.00024605	0.784761488	4	4	52.16918759	39.12009078
chr19	39834453	39846414	19q13.2	FBL	13.49673354	17.20301476	0.784556296	4	4	70.51357614	24.23712914
chr2	28849821	28870360	2p23.2	TRMT61B	5.912404608	7.536749227	0.784476759	4	4	36.93007425	36.6334314
chr16	19716456	19858467	16p12.3	IQCK	1.224614275	1.561175122	0.784418261	4	4	77.70675085	36.92314032
chr13	52064762	52129092	13q14.3	NEK5	0.098688677	0.125813647	0.784403597	4	3	53.47843034	97.28936332
chr20	58981208	58995133	20q13.32	NELFCD	4.212171192	5.370958427	0.78424945	4	4	49.05714884	42.88985202
chr19	57527325	57540876	19q13.43	ZNF549	1.340915206	1.710210385	0.784064474	4	4	61.72150908	16.39407925
chr3	15254184	15264498	3p25.1	SH3BP5-AS1	1.473756853	1.879913072	0.783949468	4	4	62.26626328	22.90431866
chr12	12725917	12791466	12p13.1	APOLD1	0.413356751	0.52738758	0.783781733	4	4	70.23824543	28.86110509
chr14	106129540	106129992	14q32.33	IGHV3-13	2.184179645	2.787230278	0.78363803	3	1	24.32442311	0
chr7	125033433	125035389	7q31.33	EEF1GP1	0.171156446	0.218420176	0.78361097	1	2	0	14.98999392
chr16	31700613	31707437	16p11.2	CLUHP3	1.359731171	1.735456314	0.783500662	4	4	54.22812306	26.96496731
chr12	39530965	39651333	12q12	ABCD2	5.550757451	7.085783069	0.783365423	4	4	31.42341887	63.31383949
chr19	8510578	8514164	19p13.2	ZNF414	0.806100862	1.029136647	0.783278746	4	4	104.8134354	25.25275281
chr3	124730366	124749273	3q21.2	UMPS	1.989128245	2.539852057	0.783166972	4	4	51.76881159	21.38576411
chr15	43197227	43225856	15q15.2	EPB42	41.7195366	53.27241909	0.783135764	4	4	37.40979497	34.82592485
chr19	49496365	49499712	19q13.33	RPS11	1167.716801	1491.33907	0.782998866	4	4	38.501866	57.40682551
chr3	49940041	50077252	3p21.31	RBM6	26.25983492	33.53779637	0.782992258	4	4	24.52636545	13.81427782
chr1	164559360	164886047	1q23.3	PBX1	2.862823159	3.656306952	0.782982172	4	4	22.23390799	29.27638929
chr3	94057922	94063389	3q11.2	DHFR2	2.334295207	2.981808948	0.782845329	4	4	46.33772887	34.48504164
chr13	41127573	41132800	13q14.11	KBTBD6	1.699981038	2.1715996	0.782824347	4	4	44.51916698	40.85436947
chr2	168865045	168890443	2q24.3	SPC25	10.04854767	12.84270573	0.782432292	4	4	104.4155407	56.41373515
chr7	2242222	2251145	7p22.3	NUDT1	3.809739226	4.86998723	0.782289367	4	4	26.02825327	26.93699221
chr17	38730257	38735605	17q12	CISD3	0.716447793	0.915867939	0.782261025	4	3	95.84496659	97.16032609
chr10	3067475	3136805	10p15.2	PFKP	8.06644536	10.3123632	0.782211138	4	4	77.26270161	74.28456375
chr19	17523301	17556755	19p13.11	FAM129C	1.865423583	2.385373569	0.782025762	4	4	76.78716746	84.11492258
chr1	67307351	67397730	1p31.3	IL12RB2	2.318966005	2.965597264	0.781955808	4	4	41.81673483	47.83752643
chr12	122974703	122980041	12q24.31	OGFOD2	0.269074551	0.344187134	0.781768185	4	4	92.69167801	42.44802521
[truncated: 537,437 more chars]
