# Supplementary material for: The transcriptome profile of human trisomy 21 blood cells
Source: Hum Genomics. 2021 May 1;15:25. doi: 10.1186/s40246-021-00325-4 (PMC8088681; doi:10.1186/s40246-021-00325-4)
Supplement: Supplementary file 5 — Additional file 5: Supplementary Table 4. A) Segment map of the T21 blood cell transcriptome map. B) Segment map of the normal control blood cell transcriptome map. "Genes" column list only over-/under-expressed genes, in red over-expressed genes, in blue under-expressed genes. [file 40246_2021_325_MOESM5_ESM.pdf]

"The transcriptome profile of human trisomy 21 blood cells"

Francesca Antonaros, Rossella Zenatelli, Giulia Guerri, Matteo Bertelli, Chiara Locatelli, Beatrice Vione, Francesca Catapano, Alice Gori, Lorenza Vitale, Maria Chiara Pelleri, Giuseppe Ramacieri, Guido Cocchi, Pierluigi Strippoli, Maria Caracausi, Allison Piovesan

Supplementary Table 4. A) Segment map of the T21 blood cell transcriptome map. **B**) Segment map of the normal control blood cell transcriptome map.
"Genes" column list only over-/under-expressed genes, in red over-expressed genes, in blue under-expressed genes.

A

Chromosome	Location	Segment start	Segment End	Expression Value	Over-/Under-expression	p-value	q-value	Genes	Number of Over-expressed genes	Number of Under-expressed genes
chr11	11p15.4	5,000,001	5,500,000	78,960.77	Over	0.000447481	0.000447481	*HBB HBG1 HBG2*	3	0
chr14	14q21.3	49,500,001	50,000,000	55,597.69	Over	0.00021233	0.00021233	*RPL36AL POLE2 RN7SL3 RN7SL2*	4	0
chr1	1q21.3	153,000,001	153,500,000	4,444.81	Over	1.47826E-05	1.47826E-05	*S100A9 S100A12 S100A8*	3	0
chr1	1q21.3	153,250,001	153,750,000	1,170.54	Over	5.48849E-06	1.0977E-05	*S100A9 S100A12 S100A8 S100A6 S100A4*	5	0
chrMT	4q23	1	500,000	784.78				*MT-RNR1 MT-RNR2 MT-ND1 MT-ND2 MT-TW MT-CO1 MT-CO2 MT-ATP8 MT-ATP6 MT-CO3 MT-ND3 MT-ND4L MT-ND4 MT-CYB MT-TT MT-TP*	16	0
chr4	4q13.3	73,500,001	74,000,000	612.28	Over	1.69931E-05	3.39863E-05	*RASSF6 CXCL8 PF4V1 PF4 PPBP*	4	1
chr4	4q13.3	73,750,001	74,250,000	375.58	Over	0.000972727	0.000972727	*PF4V1 PF4 PPBP*	3	0
chr12	12q24.23	120,250,001	120,750,000	236.90	Over	0.00593083	0.00593083	*RNU4-2 RNU4-1 RNF10*	3	0
chr10	10q23.31	89,000,001	89,500,000	209.22	Over	0.000410394	0.000820788	*IFIT2 IFIT3 IFIT1B*	3	0
chr2	2q33.1	201,250,001	201,750,000	162.35	Over	0.000687302	0.000687302	*ALS2CR12 TRAK2 STRADB*	3	0
chr15	15q22.31	65,250,001	65,750,000	154.45	Over	0.000888465	0.000888465	*RNU5A-1 RNU5B-1 DENND4A*	3	0
chr1	1q23.3	161,500,001	162,000,000	139.70				*FCGR2AFCGR3A FCGR3B*	2	0

B

Chromosome	Location	Segment start	Segment End	Expression Value	Over-/Under-expression	p-value	q-value	Genes	Number of Over-expressed genes	Number of Under-expressed genes
chr11	11p15.4	4,750,001	5,250,000	102,528.26	Over	0.000128605	0.000128605	*HBB HBD HBG1*	3	0
chr14	14q21.3	49,500,001	50,000,000	66,099.56	Over	0.000208834	0.000208834	*RPL36AL POLE2 RN7SL3 RN7SL2*	4	0

chr11	11p15.4	5,000,001	5,500,000	57,175.03	Over	3.31376E-05	6.62752E-05	*HBB HBD HBG1 HBG2*	4	0
chr16	16p13.3	1	500,000	6,263.58	Over	0.001098081	0.001098081	*HBM HBA2 HBA1*	3	0
chr1	1q21.3	153,000,001	153,500,000	1,902.25	Over	1.45772E-05	2.91544E-05	*S100A9 S100A12 S100A8*	3	0
chrMT	4q23	1	500,000	655.24				*MT-RNR1 MT-RNR2 MT-ND1 MT-TI MT-ND2 MT-TW MT-CO1 MT-CO2 MT-ATP8 MT-ATP6 MT-CO3 MT-ND3 MT-ND4L MT-ND4 MT-CYB MT-TT MT-TP*	17	0
chr1	1q21.3	153,250,001	153,750,000	563.08	Over	9.85701E-05	9.85701E-05	*S100A9 S100A12 S100A8 S100A6 S100A4*	5	0
chr4	4q13.3	73,500,001	74,000,000	459.53	Over	0.000692632	0.000692632	*CXCL8 PF4 PPBP*	3	0
chr2	2q33.1	201,250,001	201,750,000	202.85	Over	0.000482119	0.000964237	*ALS2CR12 TRAK2 STRADB*	3	0
chr6	6p22.2	25,750,001	26,250,000	169.43	Over	1.07E-10	2.14E-10	*HIST1H4B HIST1H3B HIST1H2BB HIST1H3C HIST1H1C HIST1H4C HIST1H2BC HIST1H2AC HIST1H1E HIST1H2BD HIST1H4D HIST1H4E HIST1H2AE HIST1H1D HIST1H4F*	15	0
chr6	6p22.2	26,000,001	26,500,000	139.88	Over	3.38555E-09	3.38555E-09	*HIST1H4B HIST1H3B HIST1H2BB HIST1H3C HIST1H1C HIST1H4C HIST1H2BC HIST1H2AC HIST1H1E HIST1H2BD HIST1H4D HIST1H4E HIST1H2AE HIST1H1D HIST1H4F HIST1H2BI*	16	0
chr12	12q24.23	120,250,001	120,750,000	128.58	Over	0.007064632	0.007064632	*RNU4-2 RNU4-1 RNF10*	3	0
chr2	2q33.1	201,000,001	201,500,000	127.79	Over	0.002780481	0.002780481	*ALS2CR12 TRAK2 STRADB*	3	0
chr1	1q21.3	153,500,001	154,000,000	103.47				*S100A6 S100A4 RPS27*	3	0
chr15	15q22.31	65,250,001	65,750,000	102.47	Over	0.000913335	0.000913335	*RNU5A1 RNU5B1 DENND4A*	3	0
chr6	6p22.1	27,500,001	28,000,000	100.76				*HIST1H2AI HIST1H3H HIST1H2AJ HIST1H2BMHIST1H2AL HIST1H1B HIST1H3I HIST1H4L*	8	0
