# Supplementary material for: The transcriptome profile of human trisomy 21 blood cells
Source: Hum Genomics. 2021 May 1;15:25. doi: 10.1186/s40246-021-00325-4 (PMC8088681; doi:10.1186/s40246-021-00325-4)
Supplement: Supplementary file 6 — Additional file 6: Supplementary Table 5. Expression values from RNA sequencing performed here for genes implicated in one-carbon metabolic process (Gene Ontology, GO:0006730) and folic acid-containing compound metabolic process (GO:0006760). Trisomy 21 (T21) vs normal control (N) differential expression ratios between 0.58 and 0.76 are highlighted in blue (under-expression), ratios between 1.30 and 1.70 in red (over-expression). Extreme expression ratios (< 0.58 or > 0.76) are highlighted in green and orange respectively. Sample count represents the number of samples with an available expression value for that gene. [file 40246_2021_325_MOESM6_ESM.pdf]

"The transcriptome profile of human trisomy 21 blood cells"

Francesca Antonaros, Rossella Zenatelli, Giulia Guerri, Matteo Bertelli, Chiara Locatelli, Beatrice Vione, Francesca Catapano, Alice Gori, Lorenza Vitale, Maria Chiara Pelleri, Giuseppe Ramacieri, Guido Cocchi, Pierluigi Strippoli, Maria Caracausi, Allison Piovesan

Supplementary Table 5. Expression values from RNA sequencing performed here for genes implicated in one-carbon metabolic process (Gene Ontology, GO:0006730) and folic acid-containing compound metabolic process (GO:0006760). Trisomy 21 (T21) vs normal control (N) differential expression ratios between 0.58 and 0.76 are highlighted in blue (under-expression), ratios between 1.30 and 1.70 in red (over-expression). Extreme expression ratios (< 0.58 or > 0.76) are highlighted in green and orange respectively. Sample count represents the number of samples with an available expression value for that gene.

Gene symbol	T21 expression value	N expression value	T21/N Ratio	SD as % of T21 expression	SD as % of N expression	T21 sample count	N sample count	Chromosome location	Description
AHCY	6.093	5.467	1.114	77.857	59.928	4	4	20q11.22	adenosylhomocysteinase
AHCYL1	17.196	12.498	1.376	51.857	23.727	4	4	1p13.3	adenosylhomocysteinase like 1
AHCYL2	3.173	2.289	1.386	58.050	25.102	4	4	7q32.1	adenosylhomocysteinase like 2
ALDH1L2	0.076	0.097	0.778	66.265	97.029	2	2	12q23.3	aldehyde dehydrogenase 1 family member L2
ATIC	2.462	2.699	0.912	65.030	46.922	4	4	2q35	5-aminoimidazole-4-carboxamide ribonucleotide formyltransferase/IMP cyclohydrolase
CA1	297.645	192.838	1.543	84.200	46.868	4	4	8q21.2	carbonic anhydrase 1
DHFR	6.030	6.002	1.005	74.531	36.126	4	4	5q14.1	dihydrofolate reductase
DHFR2	2.334	2.982	0.783	46.338	34.485	4	4	3q11.2	dihydrofolate reductase 2
DHFRP1	1.216	1.837	0.662	95.825	27.043	2	3	18q11.2	dihydrofolate reductase pseudogene 1
DMGDH	0.092	0.201	0.458	41.266	72.780	2	2	5q14.1	dimethylglycine dehydrogenase
FOLR2	3.578	0.994	3.601	51.865	35.759	3	4	11q13.4	folate receptor beta
FPGS	2.709	3.014	0.899	52.725	51.340	4	4	9q34.11	folylpolyglutamate synthase
GART	10.931	6.910	1.582	40.052	53.577	4	4	21q22.11	phosphoribosylglycinamide formyltransferase, phosphoribosylglycinamide synthetase, phosphoribosylaminoimidazole synthetase
GCH1	30.494	27.260	1.119	21.306	53.153	4	4	14q22.2	GTP cyclohydrolase 1
GGH	1.074	0.985	1.090	33.217	42.593	4	4	8q12.3	gamma-glutamyl hydrolase
GNMT	0.632	0.352	1.794	43.700	44.983	3	3	6p21.1	glycine N-methyltransferase
MAT2A	16.737	15.546	1.077	28.839	24.333	4	4	2p11.2	methionine adenosyltransferase 2A
MAT2B	54.595	48.229	1.132	29.156	20.042	4	4	5q34	methionine adenosyltransferase 2B
MTHFD1	3.447	4.309	0.800	23.834	56.378	4	4	14q23.3	methylenetetrahydrofolate dehydrogenase, cyclohydrolase and formyltetrahydrofolate synthetase 1
MTHFD1L	1.457	1.845	0.790	23.046	27.271	4	4	6q25.1	methylenetetrahydrofolate dehydrogenase (NADP+ dependent) 1 like
MTHFD2	16.845	12.626	1.334	19.067	30.941	4	4	2p13.1	methylenetetrahydrofolate dehydrogenase (NADP+ dependent) 2, methenyltetrahydrofolate cyclohydrolase
MTHFD2L	3.665	4.241	0.864	35.711	20.496	4	4	4q13.3	methylenetetrahydrofolate dehydrogenase (NADP+ dependent) 2 like
MTHFR	3.669	3.574	1.027	52.640	22.786	4	4	1p36.22	methylenetetrahydrofolate reductase
MTHFS	3.871	3.038	1.274	66.763	47.804	4	4	15q25.1	methenyltetrahydrofolate synthetase
MTR	12.320	13.168	0.936	12.310	9.691	4	4	1q43	5-methyltetrahydrofolate-homocysteine methyltransferase
MTRR	6.681	5.855	1.141	35.690	32.003	4	4	5p15.31	5-methyltetrahydrofolate-homocysteine methyltransferase reductase
PIPOX	0.775	0.441	1.755	53.819	52.484	4	4	17q11.2	pipecolic acid and sarcosine oxidase
PM20D2	4.408	4.260	1.035	46.996	37.831	4	4	6q15	peptidase M20 domain containing 2
SARDH	0.156	0.359	0.433	65.005	74.376	4	4	9q34.2	sarcosine dehydrogenase
SFXN1	6.356	6.708	0.948	25.106	18.403	4	4	5q35.2	sideroflexin 1

SFXN3	9.067	4.187	2.166	80.854	29.500	4	4	10q24.31	sideroflexin 3
SHMT1	0.377	0.575	0.655	82.536	73.687	4	4	17p11.2	serine hydroxymethyltransferase 1
SHMT2	7.124	6.414	1.111	73.891	56.684	4	4	12q13.3	serine hydroxymethyltransferase 2
SLC19A1	10.555	3.017	3.498	88.960	40.740	4	4	21q22.3	solute carrier family 19 member 1
SLC25A32	9.037	9.225	0.980	30.088	6.023	4	4	8q22.3	solute carrier family 25 member 32
SLC46A1	0.677	0.406	1.667	71.601	40.673	4	4	17q11.2	solute carrier family 46 member 1
TYMS	2.402	4.766	0.504	109.371	105.712	4	4	18p11.32	thymidylate synthetase
