# Supplementary material for: The transcriptome profile of human trisomy 21 blood cells
Source: Hum Genomics. 2021 May 1;15:25. doi: 10.1186/s40246-021-00325-4 (PMC8088681; doi:10.1186/s40246-021-00325-4)
Supplement: Supplementary file 12 — Additional file 12: Supplementary Table 11. Details of transcriptome maps used in the comparisons. [file 40246_2021_325_MOESM12_ESM.docx]

**"The transcriptome profile of human trisomy 21 blood cells"**
Francesca Antonaros, Rossella Zenatelli, Giulia Guerri, Matteo Bertelli, Chiara Locatelli, Beatrice Vione, Francesca Catapano, Alice Gori, Lorenza Vitale, Maria Chiara Pelleri, Giuseppe Ramacieri, Guido Cocchi, Pierluigi Strippoli, Maria Caracausi, Allison Piovesan

**Supplementary Table 11.** Details of transcriptome maps used in the comparisons.

| **Study** | **DS samples** | **n samples** | **Source** | **Method** | **T21 expression values** | **n expression values** |
| --- | --- | --- | --- | --- | --- | --- |
| This study | 2 F and 2 M, mean age: 11.52 ± 0.54 | 2 F and 2 M, mean age: 7.86 ± 4.33 | Blood cells | RNA-Seq analysed by TRAM | 19,378 | 19,357 |
| (Pelleri et al., 2018) | 2 M and 4 F, mean age: 49.16 ± 6.65 | 2 M and 2 F,  mean age: 47.80 ± 5.54 | Peripheral blood leucocytes | Microarray analysed by TRAM | 24,699 | 24,699 |
| (Powers et al., 2019) | 5 F and 5 M, mean age: 25.56 ± 9.55 | 4 F and 5 M, mean age: 25.90 ± 9.66 | Peripheral blood leucocytes | RNA-Seq analysed by TRAM | 22,614 | 21,099 |

DS: Down syndrome; n: healthy control; T21: trisomy 21.
