# Supplementary material for: The transcriptome profile of human trisomy 21 blood cells
Source: Hum Genomics. 2021 May 1;15:25. doi: 10.1186/s40246-021-00325-4 (PMC8088681; doi:10.1186/s40246-021-00325-4)
Supplement: Supplementary file 3 — Additional file 3: Supplementary Table 2. Fragments per kilobase million values (FPKM). ID: Ensembl gene accession number identifier. A: trisomy 21 samples, B: normal control samples. FPKM values for each sample are accessible through NCBI's Gene Expression Omnibus (GEO) Series accession number GSE151282 (https://www.ncbi.nlm.nih.gov/geo/query/acc.cgi?acc=GSE151282). [file 40246_2021_325_MOESM3_ESM.pdf]

"The transcriptome profile of human trisomy 21 blood cells"

Francesca Antonaros, Rossella Zenatelli, Giulia Guerri, Matteo Bertelli, Chiara Locatelli, Beatrice Vione, Francesca Catapano, Alice Gori, Lorenza Vitale, Maria Chiara Pelleri, Giuseppe Ramacieri, Guido Cocchi, Pierluigi Strippoli, Maria Caracausi, Allison Piovesan

Supplementary Table 2. Fragments per kilobase million values (FPKM). ID: Ensembl gene accession number identifier. A: trisomy 21 samples, B: normal control samples. FPKM values for each sample are accessible through NCBI's Gene Expression Omnibus (GEO) Series accession number GSE151282 (https://www.ncbi.nlm.nih.gov/geo/query/acc.cgi?acc=GSE151282).

ID	Gene Symbol	A1	A2	A3	A4	B1	B2	B3	B4
ENSG00000223972	DDX11L1	0.04225	0	0	0.0627	0.15409	0	0.07042	0.15561
ENSG00000227232	WASH7P	3.03836	1.08372	0.07973	0.08052	1.38523	0.57429	0.27131	0.73277
ENSG00000278267	MIR6859-1	2.15589	0	0	0	3.93162	1.90164	1.79677	2.6470
ENSG00000243485	MIR1302-2HG	0	0	0	0	0	0	0	0
ENSG00000284332	MIR1302-2	0	0	0	0	0	0	0	0
ENSG00000237613	FAM138A	0	0	0	0	0	0.10608	0	0
ENSG00000268020	OR4G4P	0	0	0	0	0	0	0	0
ENSG00000240361	OR4G11P	0	0	0	0	0	0	0	0
ENSG00000186092	OR4F5	0	0	0	0	0	0	0	0
ENSG00000238009	AL627309.1	0	0.13098	0	0	0.09567	0	0	0.02415
ENSG00000239945	AL627309.3	0	0	0	0	0	0	0	0
ENSG00000233750	CICP27	0	0	0	0	0	0	0	0
ENSG00000268903	AL627309.6	1.26213	0.9696	0.57067	0.14409	0.94428	0.85637	0.16183	0.47681
ENSG00000269981	AL627309.7	1.5486	0.42961	0	0.38305	0	0.45532	0	0.95067
ENSG00000239906	AL627309.2	0	0	0	0	0	0.80069	0	0
ENSG00000241860	AL627309.5	0.14199	0.27573	0.08694	0.0878	0.17262	0.06262	0.05917	0.14527
ENSG00000222623	RNU6-1100P	0.70481	0	0	0	0	0	0	0
ENSG00000241599	AL627309.4	0	0	0	0	0	0	0	0
ENSG00000279928	FO538757.1	0.38579	0.64215	0	0	2.50152	0	0.64305	2.52623
ENSG00000279457	WASH9P	1.0494	0.61135	0.15421	0.46723	1.59479	0.46282	0.52475	1.09517
ENSG00000273874	MIR6859-2	0	0	0	0	0	0	0	0
ENSG00000228463	AP006222.1	0.14519	0.24166	0.10667	0.35014	0.44128	0.03202	0.121	0.20054
ENSG00000241670		0	0	0	0	0	0	0	0
ENSG00000236679	RPL23AP24	0	0	0	0.28256	0	0	0	0
ENSG00000236743		0	0	0	0	0	0	0	0
ENSG00000236601	AL732372.1	0	0	0	0	0	0	0	0
ENSG00000237094	AL732372.2	0.10707	0.22915	0.06743	0.09081	0.14878	0.08095	0.10199	0.05634
ENSG00000269732	WBP1LP7	0	0	0	0	0	0	0	0
ENSG00000278566		0	0	0	0	0	0	0	0
ENSG00000224813		0	0	0	0	0	0	0	0
ENSG00000233653	CICP7	0	0	0	0	0	0	0	0
ENSG00000250575	AL732372.3	0	0	0	0	0	0	0	0
ENSG00000278757	RF00026	0	2.34632	0	0	0	1.24338	0	5.19214
ENSG00000230021	AL669831.3	0	0.05448	0.02405	0.04858	0.03979	0	0.05456	0.04019
ENSG00000235146	AC114498.1	0	0	0	0	0	0	0	0
ENSG00000225972	MTND1P23	0	0	0	0.29243	0	0	0	0
ENSG00000225630	MTND2P28	2.94886	2.68793	0.61905	1.56301	2.30474	1.98179	0.58515	12.4134
ENSG00000237973	MTCO1P12	0.99761	0.31629	0.20942	0.21151	0.69307	1.08947	0.63347	0.58326
ENSG00000278791	MIR6723	0	0	0	0	0	0	0	0
ENSG00000229344	MTCO2P12	0	0.1789	0	0	0.13067	0.18961	0.3583	0.26392
ENSG00000240409	MTATP8P1	0	0	0	0	0	0	0	0
ENSG00000248527	MTATP6P1	37.0269	26.5158	2.37255	5.4313	26.0414	22.4064	7.89418	9.11864
ENSG00000198744	MTCO3P12	0	0.22305	0	0	0.65168	0.70921	0.22336	0.16453
ENSG00000268663	WBP1LP6	0	0	0	0	0	0	0	0
ENSG00000273547		0	0	0	0	0	0	0	0
ENSG00000283574		0	0	0	0	0	0	0	0
ENSG00000229376	CICP3	0	0	0	0	0	0	0	0
ENSG00000228327	AL669831.1	0.26211	0.36041	0.06699	0.32135	0.38795	0.16084	0.07598	0.1819

ENSG00000223181	RNU6-1199P	0	0	0	0	0	0	1.17481	0
ENSG00000229905	AL669831.2	0	0	0	0	0	0	0	0
ENSG00000237491	AL669831.5	0.11958	0.13269	0.01952	0.05915	0.06461	0.02344	0.04429	0.03263
ENSG00000230092	AL669831.4	37.1545	89.5475	82.1442	135.982	74.0936	32.0314	106.487	86.1784
ENSG00000177757	FAM87B	0	0	0	0	0	0	0	0
ENSG00000228794	LINC01128	0.78177	0.69572	0.47772	1.41295	1.36451	0.90122	0.87732	1.1309
ENSG00000225880	LINC00115	0.61223	0.46321	0.08179	0.1652	0.74433	0.29456	0.27832	0.47834
ENSG00000230368	FAM41C	0	0	0	0	0	0	0	0
ENSG00000234711	TUBB8P11	0	0	0	0	0	0	0	0
ENSG00000283040	AL669831.6	0	0	0	0	0	0	0	0
ENSG00000272438	AL645608.6	0	0	0	0	0	0	0	0
ENSG00000230699	AL645608.2	0	0	0	0	0	0	0	0
ENSG00000241180	AL645608.4	0	0	0	0	0	0	0	0
ENSG00000223764	LINC02593	0	0	0	0	0	0	0	0
ENSG00000187634	SAMD11	0	0	0	0	0	0	0	0
ENSG00000188976	NOC2L	4.40675	3.5684	0.64173	1.09984	4.24748	4.83256	1.01468	1.20234
ENSG00000187961	KLHL17	0.34545	0.25156	0.03173	0.09613	0.23624	0.15236	0.14395	0.15905
ENSG00000187583	PLEKHN1	0.05175	0.08613	0	0.1536	0.03146	0.09129	0	0
ENSG00000187642	PERM1	0.02141	0.03563	0	0	0	0	0	0
ENSG00000272512	AL645608.7	0.03514	0.17547	0	0.05215	0	0.06199	0	0.04314
ENSG00000188290	HES4	0.32782	0.21826	0.09635	0.0973	0	0	0.21857	0.24149
ENSG00000187608	ISG15	10.2714	74.3449	31.5595	3.34903	4.25717	19.7674	4.92872	10.9869
ENSG00000231702	AL645608.3	0	0	0	0	0	0	0	0
ENSG00000224969	AL645608.1	0.15563	0	1.14346	0.46194	0.56762	0	0.51881	0
ENSG00000188157	AGRN	0.30661	0.29487	0.13016	0.06067	0.15739	0.1803	0.03407	0.05856
ENSG00000242590	AL645608.5	0	0	0	0	0	0	0	0
ENSG00000217801	AL390719.1	0.03376	0	0	0	0	0.05956	0	0.08291
ENSG00000273443	AL645608.8	0	0	0	0	0	0	0	0
ENSG00000237330	RNF223	0	0	0	0	0	0	0	0
ENSG00000131591	C1orf159	0.35943	0.40528	0.01704	0.06883	0.19735	0.24545	0.17394	0.09965
ENSG00000223823	LINC01342	0	0	0	0	0.05501	0	0	0
ENSG00000207730	MIR200B	0	0	0	0	0	0	0	0
ENSG00000207607	MIR200A	0	0	0	0	0	0	0	0
ENSG00000198976	MIR429	0	0	0	0	0	0	0	0
ENSG00000272141	AL390719.2	0	0	0	0	0	0	0	0
ENSG00000205231	TTLL10-AS1	0	0	0	0	0	0	0	0
ENSG00000162571	TTLL10	0	0	0	0	0	0	0	0
ENSG00000186891	TNFRSF18	0.74905	0.44529	0	0.31762	0.19515	0.09439	0.26755	0.26277
ENSG00000186827	TNFRSF4	0.72406	0.56715	0.12518	0.18963	0.36247	0.90165	0.14199	0.10459
ENSG00000078808	SDF4	13.0443	12.3848	5.51265	12.1977	12.2065	10.5723	8.27682	6.45976
ENSG00000176022	B3GALT6	2.82432	1.97709	0.34909	0.86182	3.14492	2.84048	0.65996	1.2315
ENSG00000184163	C1QTNF12	0.04086	0	0	0	0	0	0.13621	0
ENSG00000260179	AL162741.1	0	0.07831	0.13827	0	0.2288	0	0.07842	0
ENSG00000160087	UBE2J2	4.52683	3.80629	1.53159	3.32452	4.33101	3.32061	3.29306	2.31105
ENSG00000230415	LINC01786	0.14289	0	0	0	0.34743	0	0	0
ENSG00000162572	SCNN1D	0.1639	0.06063	0.02676	0.05406	0.13284	0.03213	0.15178	0.04472
ENSG00000131584	ACAP3	1.27312	0.77562	0.15896	0.32108	0.76886	0.55782	0.38836	0.28606
ENSG00000278073	MIR6726	0	0	0	0	0	0	0	0
ENSG00000169972	PUSL1	0.61844	0.14706	0.12983	0.08741	0.28643	0.36367	0.24544	0.21695
ENSG00000127054	INTS11	2.48793	1.61787	0.31449	0.82052	1.61538	2.21812	0.68373	0.91968
ENSG00000283712	MIR6727	0	0	0	0	0	0	0	0
ENSG00000240731	AL139287.1	0.56969	0.94825	0.5581	0.56366	0	0	0	0.46631
ENSG00000224051	CPTP	3.29794	1.71843	0.25285	0.85122	1.95248	2.9849	0.33461	0.66899
ENSG00000169962	TAS1R3	0.19217	0.03554	0	0	0.05192	0.03767	0	0.05243
ENSG00000107404	DVL1	2.07563	1.05566	0.14121	0.68455	1.14492	1.42399	0.48052	0.6607
ENSG00000284372	MIR6808	0	0	0	0	0	0	0	0
ENSG00000162576	MXRA8	0.14166	0.03369	0	0.03003	0.04921	0	0.03373	0.02485
ENSG00000175756	AURKAIP1	13.2635	7.9682	1.41984	2.41167	7.31515	6.5082	2.78182	4.20597

ENSG00000223663	NDUFB4P8	0	0	0	0	0	0	0	0
ENSG00000221978	CCNL2	3.00692	2.27987	0.69189	2.00102	2.44583	1.90664	2.3901	2.66707
ENSG00000224870	MRPL20-AS1	2.09015	2.31937	0.42659	0.81859	2.64703	1.89487	0.72582	0.81978
ENSG00000242485	MRPL20	5.38973	3.41344	0.8886	1.56077	5.24216	5.38026	2.23501	3.16345
ENSG00000264293	RN7SL657P	0	0	0	0	0	0	0	0
ENSG00000272455	AL391244.2	0.52942	0.16022	0	0.14286	0.23406	0.33962	0.16045	0.29546
ENSG00000235098	ANKRD65	0	0	0	0	0.03371	0	0.04621	0
ENSG00000225905	AL391244.1	0.13499	0	0.19837	0	0	0	0	0
ENSG00000205116	TMEM88B	0	0	0	0	0	0	0	0
ENSG00000225285	LINC01770	0	0	0	0	0	0	0	0
ENSG00000179403	VWA1	0	0	0	0.04296	0	0	0	0
ENSG00000215915	ATAD3C	0.05531	0.06137	0	0.05472	0.02241	0	0	0
ENSG00000160072	ATAD3B	0.83014	0.65118	0.33652	0.53812	0.73084	0.99315	0.23857	0.21088
ENSG00000197785	ATAD3A	1.5047	0.59633	0.10529	0.38991	0.75498	1.55899	0.23887	0.41054
ENSG00000205090	TMEM240	0	0.08841	0	0	0.12915	0	0	0.06522
ENSG00000160075	SSU72	7.2824	5.74365	1.11991	1.99506	5.1218	5.26584	2.38185	3.36596
ENSG00000215014	AL645728.1	0.31399	0.17421	0	0	0.08483	0.24619	0.11631	0.38552
ENSG00000274481	AL691432.3	0	0	0	0	0	0	0	0
ENSG00000228594	FNDC10	1.01953	0.64369	0.10332	0.2087	0.29919	0.80626	0.0586	0.21582
ENSG00000272106	AL691432.2	0.82724	0.898	0	0.48041	0.65591	1.01521	0.35971	0.35328
ENSG00000197530	MIB2	1.04512	0.69584	0.086	0.12409	0.59974	0.70799	0.26479	0.33876
ENSG00000189409	MMP23B	0	0	0	0	0.11762	0	0	0
ENSG00000248333	CDK11B	9.62487	6.61113	1.4823	5.05255	7.61375	7.71125	4.65883	4.28312
ENSG00000272004	FO704657.1	0	0.91506	0	0.81589	0.22279	0	0.30545	0.89997
ENSG00000189339	SLC35E2B	3.15205	2.60149	0.26945	1.55501	3.45002	2.63399	1.60093	1.96182
ENSG00000269737	AL691432.1	0.0403	0	0	0	0	0	0	0
ENSG00000215914	MMP23A	0	0	0	0	0	0	0	0
ENSG00000008128	CDK11A	0.54574	0.62289	0.13748	0.23141	0.1327	0.71519	0.28589	0.38288
ENSG00000268575	AL031282.2	0.41853	0.55251	0.06362	0.19277	0.42111	0.33098	0.16839	0.19491
ENSG00000227775	AL031282.1	0.05314	0.08844	0	0	0	0.04687	0.13285	0
ENSG00000215790	SLC35E2A	0.44914	0.7476	0.165	0.26663	0.38224	0.63388	0.56149	0.16544
ENSG00000008130	NADK	37.624	41.3777	9.01162	10.0583	23.1991	14.1616	6.3174	21.7479
ENSG00000078369	GNB1	43.8066	37.3612	11.2464	20.9197	36.6494	29.0785	16.4316	21.1915
ENSG00000231050	AL109917.1	0.24272	0	0	0	0.39345	0.85637	0	0.09933
ENSG00000169885	CALML6	0.06316	0	0	0	0	0	0	0
ENSG00000178821	TMEM52	0	0	0	0	0.05591	0	0	0
ENSG00000142609	CFAP74	0	0	0	0	0	0	0	0
ENSG00000233542	AL391845.2	0	0	0	0	0	0	0	0
ENSG00000187730	GABRD	0.02639	0	0	0	0	0	0	0
ENSG00000226969	AL391845.1	0	0	0	0	0	0	0	0
ENSG00000067606	PRKCZ	1.3823	0.7989	0.14106	0.25644	0.92198	0.57577	0.22401	0.28286
ENSG00000271806	AL590822.2	0.05241	0.05815	0.02567	0	0.10619	0	0	0.02145
ENSG00000182873	PRKCZ-AS1	0.04227	0.07036	0	0	0.15418	0	0	0
ENSG00000162585	FAAP20	0.32232	0.1453	0.01974	0.08969	0.34288	0.36723	0.19028	0.06596
ENSG00000234396	AL590822.1	0	0	0	0	0	0	0	0
ENSG00000157933	SKI	6.70312	4.21411	0.62006	1.48507	4.13337	3.91338	2.39136	1.56903
ENSG00000116151	MORN1	0.18104	0.10762	0.019	0.04798	0.08647	0.11406	0.05389	0.05557
ENSG00000272161	AL589739.1	0	0	0	0	0	0	0	0
ENSG00000269896	AL513477.1	0.27408	0.20276	0	0	0.33322	0.16117	0	0.07478
ENSG00000272420	AL513477.2	0.12905	0	0	0	0	0	0	0
ENSG00000157916	RER1	8.39779	6.71036	0.98421	2.19827	6.57688	4.70349	2.01809	4.28557
ENSG00000157911	PEX10	0.98662	0.74062	0.14214	0.17227	0.7056	0.54605	0.54818	0.21377
ENSG00000149527	PLCH2	0.24477	0.05658	0.01998	0.04036	0.14052	0.03598	0.07933	0.09183
ENSG00000224387	AL139246.1	0	0	0	0	0	0	0	0
ENSG00000229393	AL139246.4	0	0	0	0	0	0	0	0
ENSG00000157881	PANK4	1.69364	1.67233	0.2531	0.61774	1.60539	1.29135	0.6938	0.96923
ENSG00000197921	HES5	0	0	0	0	0	0	0	0
ENSG00000272449	AL139246.5	0	0	0	0	0	0	0	0

ENSG00000238164	TNFRSF14-AS1	0.04778	0	0	0.03545	0	0.02107	0.03982	0
ENSG00000157873	TNFRSF14	2.61661	2.21691	0.48496	0.68221	2.00616	2.55754	1.13949	1.44713
ENSG00000225931	AL139246.2	0	0	0	0	0	0	0	0
ENSG00000228037	AL139246.3	0.23419	0.1949	0.17207	0	0.14236	0	0	0.28753
ENSG00000157870	PRXL2B	0.30442	0.18097	0	0.06454	0.07931	0.23016	0	0.13349
ENSG00000142606	MMEL1	0.07628	0.03174	0	0.0283	0.02318	0	0	0.02341
ENSG00000237058	AL831784.1	0	0	0	0	0	0	0	0
ENSG00000215912	TTC34	0.00832	0	0	0	0	0	0	0.01021
ENSG00000283259	AC242022.1	0	0	0	0	0	0	0	0
ENSG00000233234	AL592464.2	0	0	0	0	0	0	0	0
ENSG00000231630	AL592464.1	0	0	0	0	0	0	0	0
ENSG00000169717	ACTRT2	0	0	0	0	0	0	0	0
ENSG00000177133	LINC00982	0	0	0	0	0	0	0	0
ENSG00000142611	PRDM16	0.03002	0	0	0.01114	0.01825	0	0	0
ENSG00000283572	MIR4251	0	0	0	0	0	0	0	0
ENSG00000226286	AL008733.1	0	0	0	0	0	0	0	0
ENSG00000279839	AL512383.1	0	0	0	0	0	0	0	0
ENSG00000272235	AL590438.1	0	0	0	0	0	0	0	0
ENSG00000130762	ARHGEF16	0	0	0	0	0.06408	0	0	0
ENSG00000272088	AL512413.1	0	0	0	0	0	0	0	0
ENSG00000162591	MEGF6	1.72863	0.83199	0.09181	0.38637	0.96219	0.20208	0.41658	0.94613
ENSG00000207776	MIR551A	0	0	0	0	0	0	0	0
ENSG00000238260	AL513320.1	0.47138	0.19616	0	0	0.28655	0.2079	0.19643	0
ENSG00000158109	TPRG1L	13.2086	10.0579	2.85571	6.2189	9.37682	5.89242	6.07359	5.96501
ENSG00000116213	WRAP73	0.99289	0.75413	0.22665	0.37197	0.85554	0.66322	0.30529	0.57994
ENSG00000078900	TP73	0	0	0	0	0	0.03824	0	0
ENSG00000227589	AL136528.1	0	0	0	0	0	0	0	0
ENSG00000235131	AL136528.2	0	0	0	0	0	0	0	0
ENSG00000227372	TP73-AS1	0.98629	0.32156	0.07471	0.36217	0.74171	0.574	0.37286	0.21223
ENSG00000276189	RF02197	0	0	0	0	0	0	0	0
ENSG00000162592	CCDC27	0	0	0	0	0	0	0	0
ENSG00000235169	SMIM1	1.15252	5.62723	2.48397	2.39465	17.1881	36.8688	1.28072	12.7354
ENSG00000130764	LRRC47	3.52218	2.62064	0.71555	1.22854	2.86136	3.17839	1.32569	1.75371
ENSG00000266075	RN7SL574P	0	0	0	0	0.30006	0.43539	0	0
ENSG00000272153	AL365330.1	0	0	0	0	0	0.73058	0	0
ENSG00000116198	CEP104	2.94095	2.19975	0.99836	1.32598	2.35351	2.33142	1.10142	1.01698
ENSG00000169598	DFFB	1.6289	0.97733	0.167	0.50598	0.59872	0.969	0.442	0.58138
ENSG00000198912	C1orf174	2.70617	2.53026	0.44185	0.84292	1.60443	2.91747	0.72395	1.046
ENSG00000236423	LINC01134	0.01804	0.09007	0	0	0.06578	0.22273	0	0.04429
ENSG00000233304	LINC01346	0	0	0	0	0	0	0	0
ENSG00000226374	LINC01345	0	0	0	0	0	0	0	0
ENSG00000229280	EEF1DP6	0	0	0	0	0	0	0	0
ENSG00000235054	LINC01777	0	0	0	0	0	0	0	0
ENSG00000227169	Z98747.1	0	0	0	0	0	0	0	0
ENSG00000232596	LINC01646	0	0	0	0	0	0	0	0
ENSG00000196581	AJAP1	0.10045	0	0	0.00877	0.02874	0	0.0591	0.04353
ENSG00000231510	AL691497.1	0	0	0	0	0	0	0	0
ENSG00000260972	Z98259.1	0	0	0	0	0	0	0	0
ENSG00000283356	Z97988.1	0	0	0	0	0	0	0	0
ENSG00000236948	AL365255.1	0	0.11142	0	0	0	0	0	0
ENSG00000264341	MIR4417	0	0	0	0	0	0	0	0
ENSG00000264101	MIR4689	0	0	0	0	0	0	0	0
ENSG00000131697	NPHP4	0.30618	0.19459	0.03272	0.09088	0.18274	0.21606	0.11135	0.06835
ENSG00000069424	KCNAB2	7.81132	5.29804	0.75696	2.29347	5.45879	5.42529	2.37082	2.2183
ENSG00000116254	CHD5	0.00741	0.02467	0.01089	0	0.05406	0	0	0
ENSG00000116251	RPL22	43.7409	28.3358	6.66068	14.564	51.0875	32.0113	16.0331	21.8197
ENSG00000226944	AL031847.1	0	0	0	0	0.70728	0	0	0
ENSG00000158286	RNF207	0.10018	0.03706	0	0	0.0406	0.01964	0	0.02733

ENSG00000116237	ICMT	4.03077	3.52856	0.96526	1.99404	3.43036	3.95046	0.94559	1.52134
ENSG00000225077	LINC00337	0	0	0	0.06456	0	0.07674	0	0
ENSG00000173673	HES3	0	0	0	0	0	0	0	0
ENSG00000158292	GPR153	0.35914	0.53801	0.13194	0.1066	0.10916	0.06336	0.23945	0.04409
ENSG00000097021	ACOT7	0.56877	0.79879	0.13059	0.44844	0.41058	2.35169	0.11851	0.1964
ENSG00000271746	AL031848.2	0.3503	0.14577	0.12869	0	0.21294	0.15449	0.14597	0.21505
ENSG00000069812	HES2	0	0	0	0	0	0.02392	0	0
ENSG00000187017	ESPN	0.08446	1.01923	1.21009	1.17513	1.4119	0.52149	0.59832	0.75181
ENSG00000265392	MIR4252	0	0	0	0	0	0	0	0
ENSG00000231868	AL031848.1	0	0	0	0	0	0	0	0
ENSG00000215788	TNFRSF25	1.81474	0.43597	0.08248	0.49978	1.38747	1.22117	0.71724	0.71207
ENSG00000171680	PLEKHG5	0.13511	0.0173	0	0.10797	0.01264	0.055	0.12126	0.02552
ENSG00000162408	NOL9	3.65848	2.19157	1.12248	2.83413	3.22594	3.58165	2.83127	2.23358
ENSG00000253022	RNU6-731P	0	0	0	0	0	0	0	0
ENSG00000229519	AL591866.1	0	0	0	0	0	0	0	0
ENSG00000173662	TAS1R1	0	0	0	0	0	0	0	0
ENSG00000204859	ZBTB48	3.32074	1.70698	0.35881	0.91802	1.78115	1.26354	0.75973	0.87939
ENSG00000162413	KLHL21	3.60076	3.12933	0.49947	0.72513	3.17674	1.79886	1.91211	2.33437
ENSG00000116273	PHF13	2.15062	1.0938	0.38041	0.70928	1.54943	1.29979	0.66384	1.10021
ENSG00000041988	THAP3	0.79205	0.65918	0.15871	0.29387	0.7441	0.5081	0.15002	0.19891
ENSG00000007923	DNAJC11	3.08603	1.82425	0.63573	1.28411	2.8227	2.92561	1.17782	1.50497
ENSG00000228750	LINC01672	0	0	0	0	0	0	0	0
ENSG00000237436	CAMTA1-DT	0.88314	0	0	0.52427	1.07369	0.31159	0	0.43372
ENSG00000171735	CAMTA1	1.63653	1.0823	0.70857	1.97339	1.46559	1.31464	1.29085	1.61462
ENSG00000227950	AL590128.1	0	0	0	0	0	0	0	0
ENSG00000237365	AL512330.1	0	0	0	0	0	0	0	0
ENSG00000207056	RNU1-8P	0	0	0	0	0	0	0	0
ENSG00000237402	CAMTA1-IT1	0	0	0	0	0	0	0	0
ENSG00000237728	Z97987.1	0	0	0	0	0	0	0	0
ENSG00000225126	AL365194.1	0	0	0	0	0	0	0	0
ENSG00000270171	AL359881.3	0	0	0	0	0	0	0	0
ENSG00000270035	AL359881.2	0.1286	0	0	0	0	0	0	0
ENSG00000269978	AL359881.1	0	0	0	0	0	0	0	0
ENSG00000049245	VAMP3	43.7677	54.1965	20.9574	36.2619	36.0341	33.9678	26.1625	34.6619
ENSG00000269925	Z98884.2	1.67863	1.55227	0	0.55362	1.36056	0.32904	0.31089	0.687
ENSG00000049246	PER3	1.23087	0.52726	0.30589	0.64474	0.57218	0.86219	0.36206	0.46671
ENSG00000236266	Z98884.1	0.17663	0	0	0	0	0	0	0
ENSG00000049247	UTS2	0.69576	0.06812	0	0.36444	0.04976	0.361	0.13644	0.15075
ENSG00000049249	TNFRSF9	0.9494	1.34901	0.13611	0.22338	0.50674	0.44935	0.15439	0.81027
ENSG00000270330	AL009183.1	0	0.34369	0.30342	0	0	0	0	0.25351
ENSG00000116288	PARK7	14.0668	10.4855	3.99939	6.35378	7.43568	11.5987	6.7793	8.56042
ENSG00000200344	RF00019	0	0	0	0	0	0	0	0
ENSG00000116285	ERRFI1	0.19262	0.08744	0.0772	0.10395	0.12774	0.06178	0.08756	0.129
ENSG00000238290	AL034417.2	0	0	0	0	0	0	0	0
ENSG00000232848	AL034417.1	0	0	0	0	0	0	0	0
ENSG00000229305	AL358876.1	0	0	0	0	0	0	0	0
ENSG00000227634	LINC01714	0	0	0	0	0	0	0	0
ENSG00000200975	RNU1-7P	0	0	0	0	0	0	0	0
ENSG00000233645	AL358876.2	0	0	0	0	0	0	0	0
ENSG00000251977	RNU6-991P	0	0	0	0	0	0	0	0
ENSG00000162426	SLC45A1	0	0.12674	0	0	0.06172	0	0	0
ENSG00000252254	RF00019	0	0	0	0	0	0	0	0
ENSG00000142599	RERE	5.73505	6.75645	2.8092	5.59864	6.71687	6.18286	6.65975	5.63313
ENSG00000232912	RERE-AS1	0.73092	0.69521	0	0.15497	0	0.55261	0.69618	1.02561
ENSG00000221083	RF00599	0	0	0	0	0	0	0	0
ENSG00000270282	AL096855.1	0	0	0	0	0	0	0	0
ENSG00000234619	RPL7P11	0	0	0	0.16533	0	0	0	0
ENSG00000224315	RPL7P7	0	0	0.30644	0	0.3803	0	0.3476	0.12802

ENSG00000222511	RF00019	0	0	0	1.05617	0	0	0	0
ENSG00000226545	AL357552.1	0	0	0	0	0	0	0	0
ENSG00000228423	AL357552.2	0.20765	0.34563	0	0.30817	0.50491	0	0	0
ENSG00000229716	RPL23AP19	0	0	0	0	0	0	0	0
ENSG00000074800	ENO1	66.1888	44.42	10.287	19.5144	46.3146	62.8263	18.8611	22.9199
ENSG00000274258	MIR6728	0	0	0	0	0	0	0	0
ENSG00000236269		0.78818	0.49197	0.14478	0	0.35934	0	0	0.72578
ENSG00000230679	ENO1-AS1	0.48224	0	0	0	0.19543	0.56716	0	0.19736
ENSG00000201725	RNU6-304P	0	0	0	0	0	0	0	0
ENSG00000238249	HMGN2P17	0.09865	0	0	0	0	0.17404	0	0
ENSG00000232208	AL139415.1	1.45768	0	0.30601	0	1.51903	0.36736	0.3471	0
ENSG00000232663	AL139415.2	0	0	0	0	0	0	0	0
ENSG00000131686	CA6	0.02754	0	0	0	0.03348	0.09715	0	0
ENSG00000265141	RN7SL451P	0	0	0	0	0	0	0	0
ENSG00000197241	SLC2A7	0	0	0	0	0	0	0	0
ENSG00000142583	SLC2A5	0.21904	0.1276	0.03219	0.09752	0.10652	0.1932	0.18255	0.10757
ENSG00000252404	RF00424	0	0	0	0	0	0	0	0
ENSG00000180758	GPR157	0.46393	0.3861	0.0401	0.20251	0.28201	0.24071	0.27293	0.25129
ENSG00000228526	MIR34AHG	0.03207	0.02669	0	0	0	0	0	0
ENSG00000284357	MIR34A	0	0	0	0	0	0	0	0
ENSG00000234546	LNCTAM34A	0	0	0	0	0	0.0778	0	0
ENSG00000281937		0	0	0	0	0	0	0	0
ENSG00000049239	H6PD	3.78258	3.08915	0.46224	1.34217	3.8339	2.62203	1.44189	2.07589
ENSG00000171621	SPSB1	0.49542	0.43024	0.09496	0.12787	0.6285	1.29198	0.28723	0.3967
ENSG00000252956	RNA5SP40	0	0	0	0	0	0	0	0
ENSG00000235263	AL928921.1	0	0	0	0	0	0	0	0
ENSG00000171612	SLC25A33	0.64666	0.44321	0.44718	0.64921	0.64745	0.57039	0.69745	0.65384
ENSG00000231181	AL954705.1	0.26226	0	0	0	0.15942	0.23133	0.21857	0
ENSG00000188807	TMEM201	0.07366	0.10509	0.03093	0.03123	0.15352	0.37127	0.05262	0.01292
ENSG00000171608	PIK3CD	46.6867	39.6354	5.91984	11.2875	34.2139	21.0755	10.7477	21.6891
ENSG00000179840	PIK3CD-AS1	0.76396	0.60235	0.05909	0.05967	0.53773	0.35467	0	0.24684
ENSG00000233268	AL691449.1	0	0	0	0	0	0	0	0
ENSG00000231789	PIK3CD-AS2	0.07806	0.25987	0	0	0.37962	0.41314	0	0.19169
ENSG00000171603	CLSTN1	7.80604	6.38684	1.14321	2.21133	7.00557	10.3049	1.5605	2.20176
ENSG00000271264	MZT1P1	0	0	0	0	0	0	0	0
ENSG00000280113	AL357140.3	0	0	0	0	0	0	0	0
ENSG00000178585	CTNNBIP1	2.56848	1.24237	0.48389	0.91225	1.70814	1.35547	1.24412	1.29376
ENSG00000223989	AL357140.1	0	0	0	0	0	0	0	0
ENSG00000162441	LZIC	17.9971	22.8384	29.3765	58.8704	27.2071	27.0749	62.0522	34.1757
ENSG00000228150	AL357140.2	0.33318	0	0	0.24724	0	0	0	0.20454
ENSG00000173614	NMNAT1	2.36914	1.79969	0.74769	0.58994	1.44984	1.29031	0.92762	1.0542
ENSG00000202415	RN7SKP269	0	0.38247	0	0	0	0	1.53204	0
ENSG00000283611	AL357140.4	0	0	0	0.26404	0	0	0	0
ENSG00000265521	MIR5697	0.93975	0	0	0	0	0	0	0
ENSG00000241326	AL603962.1	0	0	0	0	0	0	0	0
ENSG00000162444	RBP7	14.9836	16.2961	1.12593	3.53775	10.5574	5.10639	4.11525	7.94399
ENSG00000130939	UBE4B	6.96225	5.68072	2.10047	4.6837	5.34036	5.05744	4.72498	4.43675
ENSG00000224340	AL590639.1	0	0	0	0	0	0	0	0
ENSG00000233623	PGAM1P11	0.43118	0	0	0	0.13105	0	0	0
ENSG00000201746	RNU6-828P	0	0	0	0	0	0	0	0
ENSG00000054523	KIF1B	4.42201	4.89549	0.77807	1.1296	3.17843	1.91924	0.93773	2.08232
ENSG00000264881	MIR1273D	0	0	0	0	0	0	0	0
ENSG00000199562	RNU6-37P	0.68505	0	0	0	0	0	1.14187	0
ENSG00000264501	RN7SL731P	0	0	0	0	0	0	0	0
ENSG00000142657	PGD	110.84	88.7708	8.57063	12.6171	71.089	31.8266	16.807	27.7031
ENSG00000271989	AL139424.1	0	0	0	0	0	0	0	0
ENSG00000251503	CENPS-CORT	0.13066	0	0	0	0.11914	0	0	0
ENSG00000175279	CENPS	0.04215	0.07016	0.06194	0.06256	0	0.14872	0	0.05175

ENSG00000241563	CORT	0	0	0	0	0	0	0	0
ENSG00000160049	DFFA	3.36504	2.11884	0.40665	1.01853	3.16255	2.90961	0.84873	1.07366
ENSG00000203469	AL354956.1	0	0	0	0	0	0	0	0
ENSG00000142655	PEX14	1.98241	1.75171	0.14386	0.69013	1.07119	2.15879	0.53033	0.72118
ENSG00000243267	RN7SL614P	0.78536	0	0	0.38852	0	0	0	0
ENSG00000130940	CASZ1	0.70481	0.91485	0.12353	0.2783	0.51099	1.23198	0.26945	0.44459
ENSG00000272078	AL139423.1	0.00925	0	0	0.01373	0	0	0	0
ENSG00000225158	HSPE1P24	0	0	0	0	0	0	0	0
ENSG00000175262	C1orf127	0.06155	0	0	0	0.02494	0	0	0
ENSG00000215785	CFL1P6	0	0	0	0	0	0	0	0
ENSG00000120948	TARDBP	4.44318	3.73803	0.81615	2.34738	4.62391	4.98418	1.93202	2.68316
ENSG00000277726	AL109811.3	0	0	0	0	0	0	0	0
ENSG00000009724	MASP2	0	0.0287	0	0	0	0	0	0
ENSG00000271895	AL109811.2	0.58977	0.42072	0	0.12504	0.3073	0.14863	0.56175	0
ENSG00000116649	SRM	3.97784	1.22137	0.34051	0.91706	2.91108	4.83727	0.83685	1.04317
ENSG00000171824	EXOSC10	5.42151	3.39421	2.22498	5.90774	5.32948	5.29913	4.45735	3.58309
ENSG00000226849	AL109811.1	0.05466	0	0	0.04056	0.03323	0.04821	0.04556	0.16778
ENSG00000230337	EXOSC10-AS1	0	0	0	0	0	0	0	0
ENSG00000198793	MTOR	4.98992	3.38269	0.85325	1.92096	3.92675	4.28941	1.79455	2.00505
ENSG00000225602	MTOR-AS1	0	0	0	0	0	0	0	0
ENSG00000253086	RNU6-537P	0	0	0	0	0	0	0	0
ENSG00000171819	ANGPTL7	0	0	0	0	0	0	0	0
ENSG00000207451	RNU6-291P	0	0	0	0	0	0	0	0
ENSG00000238173	RPL39P6	0	0	0	0	0	0	0	0
ENSG00000120942	UBIAD1	2.79816	1.08036	0.4239	0.68499	1.70097	1.98471	0.48084	0.86774
ENSG00000238199	UBE2V2P3	0	0	0	0	0	0	0	0
ENSG00000224338	MTCYBP45	0	0	0	0	0	0	0	0
ENSG00000204624	DISP3	0.01193	0	0	0	0.08706	0.02105	0.01989	0
ENSG00000235643	LINC01647	0	0	0	0	0	0	0	0
ENSG00000116661	FBXO2	0.06284	0.26148	0	0.04663	0.11459	0.16628	0.15711	0.23145
ENSG00000132879	FBXO44	0.81052	0.76104	0.24432	0.33928	0.65694	1.28322	0.45034	0.56137
ENSG00000116663	FBXO6	3.68323	4.91807	0.89216	1.02118	2.46043	8.0686	0.94452	2.63382
ENSG00000116670	MAD2L2	1.60978	1.17457	0.29164	0.55636	1.17964	1.36159	0.29406	0.73102
ENSG00000162490	DRAXIN	0.06985	0.03322	0.01466	0.02962	0.06066	0.05281	0.03326	0.08576
ENSG00000177674	AGTRAP	16.8576	15.082	2.03484	4.0655	11.1259	6.8506	3.71308	5.98748
ENSG00000215910	C1orf167	0	0	0	0	0	0	0	0
ENSG00000177553	AL953897.1	0	0	0	0	0	0	0	0
ENSG00000177000	MTHFR	2.49741	1.66039	0.35855	0.7562	1.79734	1.81042	0.71772	1.32167
ENSG00000011021	CLCN6	1.19385	1.0903	0.23288	0.31359	0.79637	0.46595	0.31699	0.42806
ENSG00000242349		0.51367	0.684	0.37741	0.15247	0.18735	0.36247	0.25686	0.31534
ENSG00000276470	RF02156	0	0	0	0	0	0	0	0
ENSG00000278852	RF02157	0	0	0	0	0	0	0	0
ENSG00000175206	NPPA	0.06545	0	0	0.19426	0	0	0	0
ENSG00000275915	RF02158	0	0	0	0	0	0	0	0
ENSG00000120937	NPPB	0	0	0	0	0	0	0	0
ENSG00000224904	AL021155.1	0	0	0	0	0	0	0	0.03413
ENSG00000199347	RNU5E-1	95.2904	116.925	65.526	116.038	115.852	89.4407	38.6904	129.746
ENSG00000201801	RNU5E-4P	9.77338	12.2008	7.18093	32.6357	13.3675	12.9312	4.07268	16.4995
ENSG00000116685	KIAA2013	12.4439	11.3418	1.54299	2.68566	9.61515	8.04011	2.495	4.6082
ENSG00000083444	PLOD1	6.80646	7.20125	0.70864	1.45184	3.21624	2.69805	1.17128	1.48867
ENSG00000116688	MFN2	19.2353	21.4429	7.27768	8.99074	19.0311	13.9895	12.7266	10.5868
ENSG00000270914	AL096840.1	0	0	0	0	0	0	0	0
ENSG00000116691	MIIP	4.46922	5.44951	0.95457	1.73533	3.72767	5.13397	1.25602	2.5203
ENSG00000202461	RF00019	0	0	0	0	0	0	0	0
ENSG00000276869	MIR6729	0	0	0	0	0	0	0	0
ENSG00000243539	RN7SL649P	0	0	0	0	0	0	0	0
ENSG00000120949	TNFRSF8	3.01986	3.06816	0.23053	0.61116	0.92979	0.34594	0.4576	0.67414
ENSG00000201135	RNU6-777P	0	0	0	0	0	0	0	0

ENSG00000227875	RPL23AP89	0	0	0	0	0	0	0	0
ENSG00000283789	MIR7846	0	0	0	0	0	0	0	0
ENSG00000028137	TNFRSF1B	118.573	100.043	17.0466	28.3374	54.8993	50.7496	34.5842	34.8422
ENSG00000263676	MIR4632	0	0	1.7658	0	0	0	0	1.47536
ENSG00000225196	RPL10P17	0	0	0	0	0	0	0	0
ENSG00000252969	RF00156	0	0	0	0	0	0	0	0
ENSG00000048707	VPS13D	6.63338	4.74677	1.26419	2.27101	5.40317	4.71446	2.41029	3.02492
ENSG00000239149	SNORA59A	1.44672	8.02687	2.12593	5.00987	1.17259	5.10441	3.21527	2.96043
ENSG00000229484	AC243836.1	0	0	0	0	0	0	0.27895	0
ENSG00000162496	DHRS3	2.51095	1.76824	0.42575	1.7916	1.87861	0.97963	1.40853	1.21538
ENSG00000221340	RNU6ATAC18P	0	0	0	0	1.76469	0	0	0.89106
ENSG00000276830	MIR6730	0	0	0	1.62367	0	0	0	0
ENSG00000272482	AC254633.1	1.27479	0.35365	0	0	0	0	0	0.26086
ENSG00000204518	AADACL4	0	0	0	0	0	0	0	0
ENSG00000226166	AC244670.1	0	0	0	0	0	0	0	0
ENSG00000188984	AADACL3	0	0	0	0	0	0	0	0
ENSG00000157330	C1orf158	0	0	0	0	0	0	0	0
ENSG00000116726	PRAMEF12	0	0	0	0	0	0	0	0
ENSG00000116721	PRAMEF1	0	0	0	0	0	0	0	0
ENSG00000228338	LINC01784	0	0	0	0	0	0	0	0
ENSG00000239810	PRAMEF11	0	0	0	0	0	0	0	0
ENSG00000231103	PRAMEF30P	0	0	0	0	0	0	0	0
ENSG00000179172	HNRNPCL1	0	0	0	0	0	0	0	0
ENSG00000120952	PRAMEF2	0	0	0	0	0	0	0	0
ENSG00000243073	PRAMEF4	0	0	0	0	0	0	0	0
ENSG00000187545	PRAMEF10	0	0	0	0	0	0	0	0
ENSG00000204510	PRAMEF7	0	0	0	0	0	0	0	0
ENSG00000207434	RNU6-1072P	0	0	0	0	0	0	0	0
ENSG00000234064	PRAMEF29P	0	0	0	0	0	0	0	0
ENSG00000232423	PRAMEF6	0	0	0	0	0	0	0	0
ENSG00000274178	PRAMEF28P	0	0	0	0	0	0	0	0
ENSG00000274764	PRAMEF27	0	0	0	0	0	0	0	0
ENSG00000277058	HNRNPCL3	0	0	0	0	0	0	0	0
ENSG00000229571	PRAMEF25	0	0	0	0	0	0	0	0
ENSG00000277862	PRAMEF34P	0	0	0	0	0	0	0	0
ENSG00000236179	PRAMEF35P	0	0	0	0	0	0	0	0
ENSG00000275774	HNRNPCL2	0	0	0	0	0	0	0	0
ENSG00000229978	PRAMEF36P	0	0	0	0	0	0	0	0
ENSG00000233336	AC245056.1	0	0	0	0	0	0	0	0
ENSG00000280267	PRAMEF26	0	0	0	0	0	0	0	0
ENSG00000179412	HNRNPCL4	0	0	0	0	0	0	0	0
ENSG00000204505	PRAMEF9	0	0	0	0	0	0	0	0
ENSG00000279169	PRAMEF13	0	0	0	0	0	0	0	0
ENSG00000279804	PRAMEF18	0	0	0	0	0	0	0	0
ENSG00000250894	PRAMEF31P	0	0	0	0	0	0	0	0
ENSG00000270601	PRAMEF5	0	0	0	0	0	0	0	0
ENSG00000231219	PRAMEF32P	0	0	0	0	0	0	0	0
ENSG00000207511	RNU6-771P	0	0	0	0	0	0	0	0
ENSG00000182330	PRAMEF8	0	0	0	0	0	0	0	0
ENSG00000237700	PRAMEF33	0	0	0	0	0	0	0	0
ENSG00000204501	PRAMEF15	0	0	0	0	0	0	0	0
ENSG00000228229	AC243961.1	0	0	0	0	0	0	0	0
ENSG00000204481	PRAMEF14	0	0	0	0	0	0	0	0
ENSG00000204480	PRAMEF19	0	0	0	0	0	0	0	0
ENSG00000204479	PRAMEF17	0	0	0	0	0	0	0	0
ENSG00000204478	PRAMEF20	0	0	0	0	0	0	0	0
ENSG00000162494	LRRC38	0	0	0	0	0	0	0	0
ENSG00000259961	AL354712.1	0	0	0	0	0	0	0	0

ENSG00000215909	BRWD1P1	0	0	0	0	0	0	0	0
ENSG00000162493	PDPN	0	0	0	0	0	0.03488	0	0
ENSG00000222952	RNA5SP41	0	0	0	0	0	0	0	0
ENSG00000237445	AL359771.1	0	0	0	0	0	0	0	0
ENSG00000253085	RF00564	0	0	0	0	0	0	0	0
ENSG00000116731	PRDM2	7.75079	7.88593	2.20891	4.163	7.56305	7.67134	4.41831	4.22672
ENSG00000189337	KAZN	0.12181	0.02645	0.00778	0.02358	0.13521	0.02803	0.04414	0.19507
ENSG00000252151	RNU6-1265P	0	0	0	0	0	0	0	0
ENSG00000231606	AL357873.1	0	0	0	0	0	0	0	0
ENSG00000234593	KAZN-AS1	0	0	0	0	0	0	0	0
ENSG00000235829	TBCAP2	0	0	0	0	0	0	0	0
ENSG00000175147	TMEM51-AS1	0.02065	0	0	0	0	0	0	0
ENSG00000171729	TMEM51	0.58902	0.43574	0	0.09713	0.15914	0.17319	0.10909	0
ENSG00000204464	C1orf195	0	0	0	0	0	0	0	0
ENSG00000215720	AL035405.1	0	0	0	0	0	0	0	0
ENSG00000278480	AL391094.1	0.07588	0	0	0	0	0.13386	0	0
ENSG00000142621	FHAD1	0.0229	0	0	0	0.01392	0	0	0
ENSG00000233485	AL031283.2	0	0	0	0	0	0	0	0
ENSG00000236045	AL031283.3	0	0	0	0	0	0	0	0
ENSG00000228140	AL031283.1	0.04247	0.11781	0.0416	0	0.05163	0	0.09438	0.10428
ENSG00000142634	EFHD2	85.0875	90.9158	11.4906	17.8372	55.4911	35.2018	21.4335	30.1531
ENSG00000162438	CTRC	0.04205	0	0	0	0.02556	0	0	0.02582
ENSG00000142615	CELA2A	0	0	0	0	0	0	0	0
ENSG00000215704	CELA2B	0	0	0	0	0	0	0	0
ENSG00000132906	CASP9	2.35106	1.59776	0.30664	0.39228	2.04652	1.15347	0.76522	0.93943
ENSG00000116138	DNAJC16	4.50263	5.5711	3.96238	8.04173	7.48276	5.25523	6.16395	6.22192
ENSG00000251866	SCARNA21B	0	0	0	0	0	0	0	1.28567
ENSG00000272510	AL121992.3	0.21185	0.35263	0.31131	0	0.25756	0	0.35312	0.26011
ENSG00000116771	AGMAT	0.90892	0.0488	0.3016	0.43514	1.63975	1.29312	0.53759	0.86397
ENSG00000237301	AL121992.1	0	0	0	0	0	0	0	0
ENSG00000235084	CHCHD2P6	0	0	0	0	0.39873	0	0	0
ENSG00000252417	RNU7-179P	0	0	0	0	0	0	0	0
ENSG00000236500	CD24P1	0	0	0	0	0	0	0	0
ENSG00000197312	DDI2	13.7227	16.5781	17.334	47.419	17.7264	20.799	27.7866	23.3076
ENSG00000215695	RSC1A1	0	0	0	0	0	0	0	0
ENSG00000271742	AL121992.2	0	0	0	0	0	0	0	0
ENSG00000116786	PLEKHM2	11.5943	7.84383	1.93373	3.27254	7.56692	8.25061	3.49764	2.9475
ENSG00000237938	AL450998.2	0	0	0	0	0	0	0	0
ENSG00000162461	SLC25A34	0.25236	0.03819	0.03371	0.10215	0.16736	0.16189	0.03824	0.05634
ENSG00000224459	SLC25A34-AS1	0	0	0	0	0	0	0	0
ENSG00000162460	TMEM82	0	0	0	0	0	0	0	0
ENSG00000162458	FBLIM1	0	0	0	0	0.07559	0	0	0
ENSG00000224321	RPL12P14	0	0	0.21982	0	0.18187	0.2639	0	0
ENSG00000233954	UQCRHL	0.67248	0.39177	0.24705	0.44912	0.32703	0.53386	0.33628	0.4954
ENSG00000178715	AL450998.1	0.16735	0	0	0	0	0	0	0
ENSG00000179743	FLJ37453	0.91885	0.98672	0.30489	0.21995	0.72072	0.94121	0.64227	0.61866
ENSG00000065526	SPEN	11.9594	7.42477	1.90408	3.80513	7.70235	6.44905	4.07145	3.60828
ENSG00000270620	AL034555.1	0	0	0	0	0	0	0	0
ENSG00000116809	ZBTB17	3.50943	2.30205	0.35566	0.48748	2.2069	2.83632	0.60514	0.91271
ENSG00000234607	AL355994.4	0	0	0	0	0	0	0	0
ENSG00000231353	AL355994.1	0	0	0	0	0	0	0	0
ENSG00000275503	AL355994.5	0	0	0	0	0	0	0	0
ENSG00000224966	TBC1D3P6	0	0	0	0	0	0	0	0
ENSG00000183888	SRARP	0	0	0	0	0	0	0	0
ENSG00000233078	AL355994.3	0	0	0	0	0	0	0	0
ENSG00000173641	HSPB7	0	0	0	0	0	0	0	0
ENSG00000186510	CLCNKA	0	0	0	0	0	0.0372	0	0
ENSG00000232456	AL355994.2	0	0	0	0	0	0	0	0

ENSG00000184908	CLCNKB	0	0	0	0	0	0	0	0
ENSG00000185519	FAM131C	0	0	0	0	0	0	0	0
ENSG00000142627	EPHA2	0.22912	0	0.0259	0.13078	0.14999	0	0.14689	0.1082
ENSG00000227959	AL451042.2	0	0	0	0	0	0	0	0
ENSG00000224621	AL451042.1	0	0	0	0	0	0	0	0
ENSG00000234166	ARHGEF19-AS1	0	0	0	0	0	0	0	0
ENSG00000142632	ARHGEF19	0.48932	0.09774	0.11505	0.37763	0.42833	0.34529	0.1305	0.21628
ENSG00000237276	ANO7L1	1.21553	1.28753	0.5954	1.03866	0.22391	0.77977	0.12279	0.58792
ENSG00000132881	CPLANE2	0.28466	0.39485	0.06972	0.35206	0.17304	0.16739	0.15816	0.52425
ENSG00000233929	MT1XP1	0.39409	0	0	0	0	0.69522	1.31377	0.48386
ENSG00000037637	FBXO42	2.89644	2.105	0.70738	1.33197	2.63858	2.37494	1.56397	1.80315
ENSG00000055070	SZRD1	14.9782	9.48412	2.29179	4.17084	9.93106	13.9745	5.91984	6.50284
ENSG00000226457	RPL22P3	0	0	0	0.28553	0	0	0	0
ENSG00000187144	SPATA21	0	0	0	0	0	0	0	0
ENSG00000157191	NECAP2	13.8979	10.7106	1.74388	3.22893	10.5164	9.90948	3.60453	4.88921
ENSG00000226029	LINC01772	1.17461	0.52638	0.09958	0.30172	0.87881	0.2391	0.64008	0.27734
ENSG00000080947	CROCCP3	0.17214	0.25071	0.09486	0.11177	0.11772	0.13286	0.17933	0.09247
ENSG00000206652	RNU1-1	19.219	12.6472	5.25434	22.5531	17.3886	7.88487	25.3301	12.0728
ENSG00000261135	AL137802.2	0.06604	0	0	0.19601	0.16057	0.1165	0.22014	0
ENSG00000224174	AL137802.1	0.22416	0	0	0	0	0	0	0
ENSG00000233421	LINC01783	0	0	0	0	0	0	0	0
ENSG00000277344	RNU1-6P	0	0	0	0	0	0	0	0
ENSG00000268991		0	0	0	0	0	0	0	0
ENSG00000225710	AL355149.1	0	0	0	0	0	0	0	0
ENSG00000219481	NBPF1	0.55221	0.04995	0.03528	0.08017	0.2919	0.12707	0.2101	0.11054
ENSG00000271732	AL137798.1	0	0	0	0	0	0	0	0
ENSG00000215908	CROCCP2	1.17678	0.7835	0.32664	0.40751	0.89021	0.59973	1.1987	1.13981
ENSG00000283773	AL137798.3	0	0	0	0	0	0	0	0
ENSG00000186301	MST1P2	0.03199	0	0	0.04748	0.0389	0	0.15999	0.2357
ENSG00000282143	AL137798.2	0	0	0	0	0	0	0	0
ENSG00000207513	RNU1-3	1.34086	0	0	1.32666	0	0	2.23501	3.29258
ENSG00000237847		0	0	0	0	0	0	0	0
ENSG00000280114	AL021920.2	0.16277	0	0	0	0	0	0	0
ENSG00000236698	EIF1AXP1	0.33701	0.28048	0.24762	0	0	0.29727	0.28087	0
ENSG00000268869	ESPNP	0	0	0	0	0	0	0	0
ENSG00000279151	AL021920.1	0	0	0	0	0	0	0	0
ENSG00000279132		0	0	0	0	0	0	0	0
ENSG00000282740	AL021920.3	0	0	0	0	0	0	0	0
ENSG00000058453	CROCC	1.04113	0.85273	0.10928	0.18395	0.7233	0.78715	0.28923	0.32464
ENSG00000227684	CROCCP4	0	0	0	0	0	0	0	0
ENSG00000207389	RNU1-4	1.78781	5.20768	2.62717	3.31664	5.43394	2.36546	0	2.74381
ENSG00000186715	MST1L	0.01415	0	0	0.021	0	0	0	0
ENSG00000223643	BX284668.1	0	0	0	0	0	0	0	0
ENSG00000230239	BX284668.3	0	0	0	0	0	0	0	0
ENSG00000263811	MIR3675	0	0	0	0	0	0	0	0
ENSG00000228549	BX284668.2	0.02474	0	0	0	0.03008	0	0	0
ENSG00000277234	RNU1-5P	0	0	0	0	0	0	0	0
ENSG00000238142	BX284668.5	2.88584	1.68122	1.27221	1.92731	1.75427	1.27275	0.48102	2.30307
ENSG00000235241	BX284668.4	0	0	0	0	0	0	0	0
ENSG00000207005	RNU1-2	24.5824	18.5989	13.7926	29.1864	33.1471	14.1928	18.625	14.2678
ENSG00000272426	BX284668.6	0.16735	1.39279	0.24592	0.74511	1.01731	0.59046	1.1158	1.02736
ENSG00000117122	MFAP2	0.02515	0	0	0	0	0	0	0
ENSG00000226526	AL049569.1	0	0	0	0	0	0	0	0
ENSG00000159363	ATP13A2	3.18151	2.39232	0.51263	0.84908	2.00186	2.14166	0.58148	0.87376
ENSG00000117118	SDHB	14.142	12.7856	3.5438	4.10929	11.4744	10.9773	5.11152	8.44408
ENSG00000117115	PADI2	24.1925	43.7143	5.04009	3.2723	9.53119	5.8562	6.61538	19.9726
ENSG00000204362	AL590644.1	0	0	0	0	0	0	0	0
ENSG00000282843	AL590644.3	0	0	0	0	0	0	0	0

ENSG00000142623	PADI1	0	0	0	0	0	0	0	0
ENSG00000142619	PADI3	0	0	0	0	0	0	0	0
ENSG00000266634	MIR3972	0	0	0	0	0	0	0	0
ENSG00000159339	PADI4	12.0399	14.0447	2.06654	2.81942	10.4384	4.83124	3.00207	6.60362
ENSG00000276747	PADI6	0	0.15277	0	0.0454	0	0	0.05099	0
ENSG00000179051	RCC2	4.28148	3.07535	0.54823	1.13373	3.30462	5.42194	0.91798	1.48323
ENSG00000227751	AC004824.1	0	0	0	0	0	0	0	0
ENSG00000074964	ARHGEF10L	1.80039	1.07696	0.17913	0.36183	0.64982	0.49627	0.35949	0.51808
ENSG00000236648	AL136529.1	0	0	0	0	0	0	0	0
ENSG00000117148	ACTL8	0	0	0	0	0	0	0	0
ENSG00000261781	LINC01654	0	0	0	0	0	0	0	0
ENSG00000117154	IGSF21	0	0	0	0	0	0	0	0
ENSG00000280222	AL365209.1	0	0	0	0	0	0	0	0
ENSG00000230035	IGSF21-AS1	0	0	0	0	0	0	0	0
ENSG00000225387	AL591896.1	0	0	0	0	0	0	0	0
ENSG00000179023	KLHDC7A	0.02849	0.14228	0	0.04229	0.06928	0	0	0.05248
ENSG00000235282	DYNLL1P3	0	0	0	0	0	0	0	0
ENSG00000225478	AL031737.1	0	0	0	0	0	0	0	0
ENSG00000009709	PAX7	0	0	0	0	0	0	0	0
ENSG00000179002	TAS1R2	0	0	0	0	0	0	0	0
ENSG00000255275	AL080251.1	0	0	0	0	0	0	0	0
ENSG00000159423	ALDH4A1	0.05961	0.19844	0	0.11796	0.09663	0.07011	0	0.09758
ENSG00000265606	MIR4695	0	0	0	0	0	0	0	0
ENSG00000221662	MIR1290	0	0	0	0	0	0	0	0
ENSG00000169991	IFFO2	3.43521	2.54349	0.60924	1.51189	2.53467	3.28086	0.5726	1.17805
ENSG00000272084	AL137127.1	0.08619	0.14346	0.03166	0.03198	0.05239	0.19005	0.10774	0.02645
ENSG00000127481	UBR4	7.90782	6.59612	1.39253	2.56148	5.83276	6.75513	4.16428	3.55611
ENSG00000230424	EMC1-AS1	0.86746	1.44389	0	0	1.23041	0.51011	0.48197	0.71004
ENSG00000127463	EMC1	1.67937	1.08834	0.47536	0.67417	1.64845	1.54203	0.71128	1.06475
ENSG00000053372	MRTO4	2.12992	0.95116	0.34352	0.80953	2.02107	2.10785	0.9958	0.79728
ENSG00000211454	AKR7L	0	0	0	0	0.0322	0.09343	0	0
ENSG00000162482	AKR7A3	0.08573	0.07135	0	0	0.20846	0	0	0.05263
ENSG00000270728	AL035413.1	0	0	0	0	0	0	0	0
ENSG00000053371	AKR7A2	5.96774	3.90482	0.60479	1.46595	4.60344	5.44547	2.33247	2.32446
ENSG00000200403	RNU6-1099P	0	0	0	0	0	0	0	0
ENSG00000040487	PQLC2	1.17346	0.68136	0.28071	0.28351	0.56403	0.674	0.31841	0.5696
ENSG00000077549	CAPZB	38.099	33.8745	4.62392	8.63432	27.7698	32.4677	10.5276	15.0922
ENSG00000240490	RN7SL277P	0	0	0.37401	0.37773	0	0	0	0.62498
ENSG00000201609	RNU4-28P	0	0	0	0	0	0	0	0
ENSG00000235185	AL031727.2	0	0	0	0.52553	0	0.62469	0	0
ENSG00000173436	MINOS1	1.19199	1.02705	0.20607	0.37462	1.58558	0.6927	0.42075	1.17081
ENSG00000270136	MINOS1-NBL1	0	0	0	0	0	0	0	0
ENSG00000226396	AL031727.1	0.6444	0.26815	0	0.23909	0.19586	0	0.26853	0.1978
ENSG00000158747	NBL1	0.21155	0	0	0.0314	0.10288	0.03732	0.03526	0.02597
ENSG00000158748	HTR6	0.14778	0	0	0	0	0.06518	0.06158	0
ENSG00000162542	TMCO4	2.29629	1.83585	0.31884	0.75136	1.69265	2.3604	0.33152	0.66599
ENSG00000178828	RNF186	0	0	0	0	0	0	0	0
ENSG00000235434	AL391883.1	0	0	0	0	0	0	0	0
ENSG00000169914	OTUD3	1.39733	0.77529	0.97097	2.12202	1.93589	1.43319	1.19165	1.56933
ENSG00000188784	PLA2G2E	0	0	0	0	0	0	0	0
ENSG00000242688	RN7SL304P	0	0	0	0	0	0	0	0
ENSG00000188257	PLA2G2A	0	0.07241	0	0	0	0	0	0
ENSG00000127472	PLA2G5	0	0	0	0	0	0	0	0
ENSG00000117215	PLA2G2D	0.05468	0.09102	0	0	0.03324	0	0	0
ENSG00000158786	PLA2G2F	0	0	0	0	0	0	0	0
ENSG00000227066	Z98257.1	0.08333	0	0	0	0	0	0	0
ENSG00000187980	PLA2G2C	0.04445	0	0	0	0.02702	0	0	0
ENSG00000225986	UBXN10-AS1	0.14373	0	0	0.85322	0.34948	0	0	0.17646

ENSG00000162543	UBXN10	0.56577	0.45991	0.21268	0.58581	0.39991	0.27854	2.87302	0.21001
ENSG00000228105	LINC01757	0	0	0	0	0	0	0	0
ENSG00000158816	VWA5B1	0	0	0	0	0	0	0	0
ENSG00000226664	AL020998.1	0	0	0	0	0	0	0	0
ENSG00000236963	LINC01141	0	0	0	0	0	0	0	0
ENSG00000226487	AL139254.1	0	0	0	0	0	0	0	0
ENSG00000162545	CAMK2N1	0	0.18663	0	0	0.20447	0	0	0.03442
ENSG00000090432	MUL1	4.50987	3.85343	0.97199	0.98166	3.28979	3.55369	1.65379	1.8088
ENSG00000215520	RPS4XP4	0	0	0	0	0	0	0	0
ENSG00000183114	FAM43B	0	0	0	0	0	0	0	0
ENSG00000158825	CDA	38.1344	42.8233	3.80298	7.56868	41.6433	13.5623	6.85123	15.1397
ENSG00000158828	PINK1	15.233	18.7958	8.17432	5.98366	21.3025	17.0597	9.44902	13.0455
ENSG00000284005	MIR6084	0	0	0	0	0	0	0	0
ENSG00000117242	PINK1-AS	1.45182	0.57668	0.21819	0.41624	1.10318	0.90224	0.275	0.48614
ENSG00000244038	DDOST	19.8808	16.0077	2.53351	6.59671	15.0329	15.2551	5.61284	7.50802
ENSG00000117245	KIF17	0	0	0	0.02434	0.07975	0.05786	0	0
ENSG00000235432	AL663074.2	0	0	0	0	0	0	0	0
ENSG00000189410	SH2D5	0	0	0	0	0	0	0	0
ENSG00000233069	AL663074.1	0	0	0	0	0	0	0	0
ENSG00000127483	HP1BP3	43.0228	44.1342	63.1636	153.209	66.2447	59.7559	162.272	97.8455
ENSG00000075151	EIF4G3	11.71	13.4774	3.53584	7.85624	11.7386	12.2224	7.71585	8.08435
ENSG00000251914	RNU7-200P	0	0	0	0	0	0	0	0
ENSG00000221808	MIR1256	0	0	0.90516	0.91417	0	2.17331	0	0
ENSG00000233072	RPS15AP6	0.19039	0.31691	0.27978	0.28256	0	0.33587	0	0
ENSG00000236073	AL627311.1	0.63739	0	1.87329	1.89193	1.54986	0	3.18731	2.34775
ENSG00000235112	HSPE1P27	0	0	0	0	0	0	0	0
ENSG00000117298	ECE1	29.9457	29.7254	4.01796	4.61198	14.4132	10.11	6.30662	11.2233
ENSG00000236936	AL031005.1	1.31799	0.50626	0.74491	0.75232	0.36978	0.71542	1.01394	1.74268
ENSG00000231105	AL031728.1	0.14784	0.39373	0.30415	0.08777	0.25164	0.20865	0	0.21782
ENSG00000236216	PPP1R11P1	0	0	0	0	0	0	0	0
ENSG00000228823	AL592309.1	0	0	0	0	0	0	0	0
ENSG00000236009	AL592309.2	0	0	0	0	0	0	0	0
ENSG00000227001	NBPF2P	0	0.18458	0	0	0	0	0.18484	0
ENSG00000187952	HS6ST1P1	0	0	0	0	0	0	0	0
ENSG00000186543	CROCCP5	0	0	0	0	0	0	0	0
ENSG00000142794	NBPF3	0.9254	1.00301	0.18975	0.11179	1.17741	0.43661	0.35872	0.43598
ENSG00000176378	PFN1P10	0	0.37311	0	0	0	0	0	0
ENSG00000162551	ALPL	68.9126	124.686	3.54629	4.52219	44.2768	1.80615	4.67268	24.7215
ENSG00000233431	LINC02596	1.33923	0.89166	0	0	1.08546	0.15751	0.44646	0.43848
ENSG00000076864	RAP1GAP	0.03821	4.13408	0	0.0189	3.23638	3.28054	0.06369	0.1251
ENSG00000090686	USP48	10.8828	14.2859	7.08868	14.9484	14.3664	13.2212	11.3471	12.0918
ENSG00000231978	AL590103.1	0	0	0	0	0	0	0	0
ENSG00000187942	LDLRAD2	0.04898	0.02717	0	0	0.01985	0	0.02721	0.02004
ENSG00000142798	HSPG2	0.11606	0.08279	0.00609	0.03691	0.09575	0.08044	0.03455	0.02036
ENSG00000232037	AL590556.1	0	0	0	0	0	0	0	0.18633
ENSG00000283234	AL590556.2	0.12616	0	0	0.09362	0	0	0	0.07745
ENSG00000219073	CELA3B	0	0	0	0	0	0	0	0
ENSG00000273610	RN7SL386P	0	0	0	0	0	0	0	0
ENSG00000201919	RNU6-1022P	0	0	0	0	0	0	0	0
ENSG00000142789	CELA3A	0	0	0	0	0	0	0	0
ENSG00000278124	RN7SL186P	0	0	0	0	0	0	0	0
ENSG00000201273	RNU6-776P	0	0	0	0	0	0	0	0
ENSG00000228397	LINC01635	0.0651	0.32507	0.09566	0	0.15829	0	0.10851	0
ENSG00000218510	LINC00339	1.73654	1.87627	0.49246	0.633	1.51861	0.75244	0.76172	0.93513
ENSG00000070831	CDC42	144.441	137.743	48.4982	88.6189	111.78	103.148	87.2717	105.73
ENSG00000230068	CDC42-IT1	1.2711	0.70525	0.15566	0.47161	0.90147	0.18687	0.35312	0.13005
ENSG00000271428	AL031281.1	0	0	0	0	0	0	0	0
ENSG00000271840		0.97995	0.32623	0.144	0.29087	0.47656	0.51863	0.16334	0.7219

ENSG00000162552	WNT4	0	0	0	0	0	0	0	0
ENSG00000266564	MIR4418	0	0	0	0	0	0	0	0
ENSG00000234397	PPIAP34	0	0	0	0	0	0	0	0
ENSG00000279625	AL591122.1	0	0	0	0	0	0	0	0
ENSG00000184677	ZBTB40	2.27433	2.10313	0.51509	1.2219	2.44793	1.8695	1.11419	1.37117
ENSG00000237200	ZBTB40-IT1	2.68499	0.44692	0.39456	0.39848	2.28504	0	1.34264	0.98898
ENSG00000070886	EPHA8	0	0	0	0	0	0	0	0
ENSG00000276835	MIR6127	0	0	0	0	0	0	0	0
ENSG00000173372	C1QA	1.67116	1.24694	0.4234	0.25657	0.14012	2.0332	0.48027	0.28301
ENSG00000159189	C1QC	0.21963	0.54835	0.08068	0	0.20026	0.96863	0	0
ENSG00000173369	C1QB	0.91568	1.44795	0.74007	0.27179	0.38964	3.31154	0	0.16864
ENSG00000133216	EPHB2	0.35139	0.21446	0.04303	0.01738	0.12104	0.08265	0.08786	0.02876
ENSG00000265422	MIR4684	0	0	0	0	0	0	0	0
ENSG00000225952	AL512444.1	0	0	0	0	0	0	0	0
ENSG00000264014	MIR4253	0	0	0	0	0	0	0	0
ENSG00000215906	LACTBL1	0	0	0	0	0	0	0	0
ENSG00000227868	TEX46	0	0	0	0	0.27087	0	0	0
ENSG00000004487	KDM1A	10.5466	12.2345	10.7218	22.818	17.0035	18.8436	22.3679	14.224
ENSG00000240553	AL031428.1	0	0	0	0	0	0	0	0
ENSG00000263793	MIR3115	0	0	0	0	1.31054	0	0	0
ENSG00000266802	MIR4419A	0	0	0	0	0	0	0	0
ENSG00000169641	LUZP1	3.18004	1.66754	0.49072	0.86731	1.87313	2.35648	0.8476	0.94116
ENSG00000229010	AL161672.1	0	0	0	0	0	0	0	0
ENSG00000206935	RNU6-514P	0	0	0	0	0	0	0	0
ENSG00000252578	RNU6-135P	0	0	0	0	0	0	0	0
ENSG00000179546	HTR1D	0	0	0	0	0	0	0	0
ENSG00000215381	AL109936.1	0	0	0	0	0	0	0	0
ENSG00000261326	LINC01355	0	0	0	0.05995	0	0.07126	0.04489	0
ENSG00000125944	HNRNPR	9.89335	6.45481	1.16703	2.53282	8.85415	10.9548	2.61936	2.98745
ENSG00000125945	ZNF436	0.11683	0.22224	0.02453	0.02477	0.28407	0	0.05564	0
ENSG00000249087	ZNF436-AS1	0	0	0	0	0	0.09741	0	0
ENSG00000201405	RF00019	0	0	0	0	0	0	0	0
ENSG00000271420	AL109936.2	0	0	0	0	0	0	0	0
ENSG00000204219	TCEA3	1.04268	0.41653	0.09193	0.1238	2.28179	1.14045	0.59091	0.58889
ENSG00000232482	AL357134.1	0	0	0	0	0	0	0	0
ENSG00000088280	ASAP3	0.04409	0.04892	0	0.02181	0.12506	0.05185	0.07348	0
ENSG00000007968	E2F2	4.37895	1.72158	1.77648	2.85072	2.23731	3.3886	5.66458	1.07198
ENSG00000235052	AL021154.1	0	0	0	0	0	0	0	0
ENSG00000117318	ID3	0.44869	0.13579	0	0.06054	1.04143	0.07196	0.13598	0.10016
ENSG00000197880	MDS2	0.03422	0	0	0.05079	0.20802	0	0.17112	0
ENSG00000229239	AL451000.1	0.0618	0	0	0	0	0	0	0
ENSG00000142676	RPL11	344.146	251.324	49.1679	64.1655	379.161	294.222	94.0795	138.279
ENSG00000011007	ELOA	8.15808	5.79376	0.89911	2.34078	5.7196	6.33246	2.42502	2.4874
ENSG00000236810	ELOA-AS1	0.81626	0.36231	0.71969	0.72685	1.05855	0.672	2.44905	0.73494
ENSG00000057757	PITHD1	41.6464	45.6555	32.9153	102.018	47.6159	87.2529	34.206	30.0127
ENSG00000011009	LYPLA2	9.41289	7.72276	1.2753	1.53568	6.57418	7.47841	2.39242	2.95072
ENSG00000117308	GALE	0.77391	0.15335	0	0.32816	0.33604	0.22755	0.18428	0.40723
ENSG00000117305	HMGCL	1.16597	1.7898	0.51401	0.73062	1.18129	1.73696	1.23087	1.17706
ENSG00000179163	FUCA1	10.9575	6.6756	0.89455	3.9858	5.09363	2.84271	2.14875	3.03361
ENSG00000188822	CNR2	3.20881	0.83599	0.38953	1.34585	2.64602	0.83681	0.5116	1.33608
ENSG00000229106	BTBD6P1	0.46283	0.0963	0.08501	0.08586	0	0	0.19287	0.07103
ENSG00000232557	AL590609.3	0	0	0	0	0	0	0	0
ENSG00000264926	MIR378F	0	0	0	0	0	0	0	0
ENSG00000230228	AL590609.2	0	0	0	0	0.24216	0	0	0
ENSG00000189266	PNRC2	54.9467	42.6364	15.1236	29.0208	51.5445	54.2666	40.552	33.6611
ENSG00000188529	SRSF10	9.22228	7.57433	2.27439	4.68931	8.81888	9.04692	4.62471	5.18422
ENSG00000215905	AL590609.1	0	0	0	0	0	0	0	0
ENSG00000225315	AL591178.1	0	0	0	0	0	0	0	0.19823

ENSG00000142661	MYOM3	0	0	0	0	0	0	0	0
ENSG00000230703	AL591178.2	0	0	0	0	0	0	0	0
ENSG00000142677	IL22RA1	0	0	0	0	0	0	0	0
ENSG00000185436	IFNLR1	0.47646	0.52045	0.04376	0.19888	0.57927	1.78615	0.19855	0.3839
ENSG00000230023	AL590683.1	0	0	0	0	0	0	0	0
ENSG00000232298	AL138902.1	0	0	0	0	0	0.15107	0	0.10514
ENSG00000158055	GRHL3	0.0186	0	0	0	0.02262	0.03282	0	0.02284
ENSG00000001460	STPG1	0.21531	0.2007	0.07594	0.1406	0.178	0.28868	0.10049	0.11632
ENSG00000001461	NIPAL3	2.57222	2.04991	0.58416	1.42287	2.55865	2.7914	1.32522	1.4068
ENSG00000117602	RCAN3	11.849	3.74111	2.54179	7.59363	15.1235	7.80219	9.61628	7.64282
ENSG00000264443	AL445686.2	0	0	0	0	0	0	0	0
ENSG00000184454	NCMAP	0	0	0	0	0.02162	0	0	0
ENSG00000227312	AL445686.1	0	0	0	0	0	0	0	0
ENSG00000239106	RF00019	0	0	0	0	0	0	0	0
ENSG00000133226	SRRM1	10.3651	6.94588	1.91216	4.12874	8.53198	8.64401	4.18836	3.96657
ENSG00000169504	CLIC4	17.5627	15.9602	5.79906	9.43858	11.3998	8.94682	8.53495	10.7116
ENSG00000238482	RNU6-1208P	0	0	0	0	0	0	0	0
ENSG00000020633	RUNX3	13.9373	9.33339	2.5958	8.44214	8.68902	25.8166	7.56367	3.89994
ENSG00000278034	MIR6731	0	1.69456	0	0	0	0	3.3939	0
ENSG00000229162	AL445471.1	0	0	0	0	0	0.1577	0	0
ENSG00000261025	AL445471.2	0	0	0	0	0	0	0	0
ENSG00000264371	MIR4425	0	0	0	0	0	0	0	0
ENSG00000233755	AL050344.1	0	0	0	0	0	0.44952	0	0
ENSG00000233419	AL121994.1	0	0	0	0	0	0	0	0
ENSG00000231953	AL031432.1	0	0	0	0	0	0	0	0
ENSG00000117614	SYF2	31.3212	28.4204	7.25551	15.1719	26.8672	17.766	11.8192	15.4891
ENSG00000117616	RSRP1	15.9873	25.4812	6.61735	15.253	16.0578	7.69713	12.9112	13.3726
ENSG00000272432	AL031432.3	0.30227	0	0	0.2243	0	0	0	0
ENSG00000261349	AL031432.2	5.58935	3.72139	0.70401	0.94802	2.32985	1.40863	0.79856	0.19607
ENSG00000187010	RHD	1.593	2.61473	2.89361	3.31644	1.58705	2.18577	6.56447	0.08149
ENSG00000224183	SDHDP6	0.45813	1.52511	0.2244	0	0.18566	0.2694	0.25454	0
ENSG00000259984	AL928711.1	2.23574	2.65814	0.23467	0.94802	0.97077	1.40863	0.53238	1.76465
ENSG00000183726	TMEM50A	53.3523	49.1254	26.4073	48.81	32.4061	29.6166	35.2567	22.1958
ENSG00000188672	RHCE	0.81344	1.20354	1.01826	2.10149	1.64827	2.7106	4.01744	1.62757
ENSG00000238084	AL031284.1	0	0	0	0	0	0	0	0
ENSG00000204178	MACO1	10.611	9.87352	5.95553	10.0884	13.1245	10.1396	16.3357	8.59484
ENSG00000157978	LDLRAP1	11.8161	4.47613	1.21744	2.94288	10.0725	7.76302	3.37315	4.00209
ENSG00000225643	AL606491.1	0.91056	0	0	0.22523	0	0	0.25296	0
ENSG00000117643	MAN1C1	1.1207	0.47311	0.19094	0.28926	1.80682	0.71633	0.37902	0.41878
ENSG00000233478	AL031280.1	0	0	0	0	0	0	0	0
ENSG00000162430	SELENON	3.74455	1.51683	0.26782	0.71311	2.51799	2.3676	0.63521	0.54926
ENSG00000255054	AL020996.2	0	0	0	0	0	0	0	0
ENSG00000228172	AL020996.1	0.18371	0.04368	0	0	0	0.0463	0.08749	0
ENSG00000117640	MTFR1L	2.94865	1.71991	0.68513	0.65455	1.17964	1.75617	1.0712	1.5162
ENSG00000127423	AUNIP	0.12176	0.10134	0	0.09035	0.25906	0.48331	0.10148	0.11212
ENSG00000272478	AL020996.3	0	0	0	0	0	0	0	0
ENSG00000236528	AL033528.2	0.86337	0.71854	0.50749	0.64067	0.41987	0.15231	0.57564	0.42401
ENSG00000182749	PAQR7	1.6452	1.03617	0.22869	0.32995	1.10821	0.70598	0.48175	0.68242
ENSG00000207302	RF00019	0	0	0	0	0	0	0	0
ENSG00000117632	STMN1	1.63904	1.09128	0.73977	1.04251	2.16351	8.26141	1.11233	1.19306
ENSG00000229786	SNRPFP2	0	0	0	0	0	0	0	0
ENSG00000223624	AL033528.1	0	0	0	0	0	0	0	0
ENSG00000283938	MIR3917	0	0	0	0	0	0	0	0
ENSG00000158006	PAFAH2	3.49777	3.02237	0.92687	2.09912	3.41595	3.47304	1.52925	1.94779
ENSG00000207237	RNU6-110P	0	0	0	0	0	0	0	0
ENSG00000252190	RF00492	0	0	0	0	0	0	0	0
ENSG00000252691	RF00283	0	0	0	0	0	0	0	0
ENSG00000158008	EXTL1	0	0	0	0.02398	0	0	0	0

ENSG00000158014	SLC30A2	0	0	0	0	0	0	0	0
ENSG00000284309	AL391650.2	0	0	0	0	0	0	0	0
ENSG00000158022	TRIM63	0	0	0	0	0	0	0	0
ENSG00000175087	PDIK1L	2.58612	2.25991	1.16383	2.42279	3.37995	2.4237	2.82887	2.14326
ENSG00000197245	FAM110D	0	0	0	0	0	0	0	0
ENSG00000282872	C1orf232	0	0	0	0	0	0	0	0
ENSG00000236782	AL391650.1	0	0	0	0	0	0	0	0
ENSG00000142684	ZNF593	1.90638	1.42793	0.42021	0.28293	1.62241	3.36312	0.47665	0.81922
ENSG00000142675	CNKSR1	0.10878	0.06036	0	0.02691	0.08817	0	0	0.02226
ENSG00000188782	CATSPER4	0.03611	0	0	0	0	0	0	0
ENSG00000236155	AL355877.1	0	0	0	0	0	0	0	0
ENSG00000278572	AL355877.3	0	0	0	0	0	0	0	0
ENSG00000130695	CEP85	4.23147	3.53289	5.6221	7.50385	6.73881	6.34396	8.36018	6.37382
ENSG00000270733	AL355877.2	0	0	0	0	0	0	0	0
ENSG00000142669	SH3BGRL3	256.264	197.427	27.9839	61.2148	186.367	254.207	56.7803	64.0965
ENSG00000158062	UBXN11	5.45389	3.68308	0.29768	0.71691	1.79976	1.8693	1.03895	1.16705
ENSG00000169442	CD52	131.078	123.966	23.0404	38.9767	97.5041	126.546	37.1115	46.9717
ENSG00000176092	CRYBG2	0.13325	0.12098	0	0.01798	0.07364	0.04274	0	0
ENSG00000225854	AL451139.1	0.63555	0	0	0	0	0	0	0
ENSG00000176083	ZNF683	2.06436	5.23113	0.09055	1.006	1.79807	3.53311	0.15407	0.41613
ENSG00000131914	LIN28A	0	0	0	0	0	0	0	0
ENSG00000117682	DHDDS	2.43636	1.19915	1.03941	2.25503	1.57658	2.44944	1.59385	1.20618
ENSG00000223583	AL513365.1	0.27402	0.68416	0.20133	0.40668	0.83287	0	0.22837	0.50466
ENSG00000225891	AL513365.2	4.01836	4.15152	2.64703	5.55239	4.71695	1.46667	6.69798	4.59342
ENSG00000198830	HMGN2	22.4371	17.5062	6.05327	7.76112	17.6173	26.6973	13.0994	12.949
ENSG00000224129	DPPA2P2	0	0	0	0	0	0	0	0
ENSG00000117676	RPS6KA1	18.5742	18.828	2.39812	5.08431	12.1013	10.2852	3.88303	8.73357
ENSG00000238705	MIR1976	0	0	0	0	0	0	0	0
ENSG00000243905	RN7SL679P	0	0	0	0	0	0	0	0
ENSG00000252802	RF00019	0	0	0	0	0	0	0	0
ENSG00000252429	RNU7-29P	0	0	0	0	0	0	0	0
ENSG00000229247	AL627313.1	0	0	0	0	0	0	0	0
ENSG00000238316	RF01210	0	0	0	0	0	0	0	0
ENSG00000235069	AL627082.1	0	0	0	0	0	0	0	0
ENSG00000260063	AL512408.1	0.18325	0.12201	0.05386	0	0.04456	0.06466	0.12218	0.045
ENSG00000117713	ARID1A	7.9322	5.80847	0.8524	1.54413	5.82656	5.59577	1.61147	1.85963
ENSG00000060642	PIGV	2.16737	2.30885	0.60513	0.86848	2.16072	1.07059	0.65028	1.30392
ENSG00000241188	RN7SL165P	0	0	0	0.37383	0	0	0	0
ENSG00000204160	ZDHHC18	33.0499	31.7815	5.07262	8.14492	18.9184	8.39718	8.31617	17.2676
ENSG00000175793	SFN	0.55531	0.18486	0	0	0.13503	0	0.18512	0.06818
ENSG00000142751	GPN2	1.70774	0.85766	0.1947	0.56807	1.28869	0.98691	1.17788	0.88569
ENSG00000226698	AL034380.1	0	0	0	0	0	0	0	0
ENSG00000198746	GPATCH3	3.09673	1.80956	0.58093	0.78228	2.56336	2.03412	1.3179	1.05165
ENSG00000090273	NUDC	11.0618	9.31297	2.33833	4.38038	10.5779	12.8135	5.39031	3.37174
ENSG00000131910	NR0B2	0	0	0	0	0.07709	0	0	0
ENSG00000175707	KDF1	0.04088	0	0.06007	0	0.19881	0	0.06814	0
ENSG00000172746	RPL12P13	0	0	0	0	0	0	0	0
ENSG00000224064	AL356390.1	0	0	0	0	0	0	0	0
ENSG00000225294	OSTCP2	0	0	0	0	0	0	0	0
ENSG00000253368	TRNP1	0.89029	0.24698	0	0	0.1353	0.13088	0.12366	0
ENSG00000158246	TENT5B	0.09286	0	0	0.04594	0.03763	0	0	0
ENSG00000090020	SLC9A1	2.38036	2.47121	0.30652	0.43704	1.32765	1.29875	0.49085	0.73817
ENSG00000229985	AL590640.2	0	0	0	0	0	0	0	0
ENSG00000214973	CHCHD3P3	0	0.19064	0	0	0	0	0	0
ENSG00000225159	NPM1P39	0	0	0	0.25095	0.20557	0	0	0.31141
ENSG00000225990	SNRPEP7	0	0	0	0	0	0	0	0
ENSG00000224311	AL590640.1	0	0	0	0	0	0	0	0
ENSG00000142784	WDTC1	12.3871	11.5446	4.5596	3.99098	11.5094	15.1121	10.344	7.38024

ENSG00000252098	RF00019	0	0	0	0	0	0	0	0
ENSG00000243659	FO393419.3	0.77979	0	0	0	0.94805	0	0.43326	0
ENSG00000186501	TMEM222	1.81777	0.92678	0.19252	0.63191	1.57288	2.10897	0.92809	0.94501
ENSG00000206888	RNU6-48P	1.3701	0	0	0	0	0	0	0
ENSG00000241547	ACTG1P20	0.31325	0	0.23016	0	0.38084	0	0	0.3846
ENSG00000142765	SYTL1	5.27699	3.45225	0.81017	2.47417	3.33549	3.19574	1.13778	2.27249
ENSG00000142733	MAP3K6	0.93273	0.69001	0.02176	0.06592	0.306	0.05224	0.04936	0.12724
ENSG00000142748	FCN3	0	0.0563	0	0	0	0	0	0
ENSG00000174950	CD164L2	0	0	0	0	0	0	0	0
ENSG00000231207	FO393419.1	0	0	0	0	0	0	0	0
ENSG00000181773	GPR3	0	0	0	0	0	0	0	0
ENSG00000158195	WASF2	38.8456	39.3368	20.1726	19.1084	42.3917	43.0584	39.6935	24.7191
ENSG00000241169	FO393419.2	0	0	0	0	0	0	0	0
ENSG00000237429	BX293535.1	0.25901	0.43113	0	0	0.3149	0	0	0.31801
ENSG00000126705	AHDC1	0.62475	0.59666	0.0301	0.1672	0.386	0.84919	0.13657	0.16347
ENSG00000000938	FGR	100.835	91.3483	11.7822	17.1915	38.9404	28.2148	14.9472	33.9108
ENSG00000235912	AL031729.1	0	0	0	0	0	0	0	0
ENSG00000233975	LINC02574	0.33243	0.55333	0.24425	0.24668	0	0	0	0
ENSG00000126709	IFI6	15.5075	103.249	29.3879	6.66548	5.46033	23.321	4.37872	15.6064
ENSG00000225886	AL445490.1	0	0	0	0	0	0	0.074	0
ENSG00000206767	RNU6-949P	0	0	0	0	0	0	0	0
ENSG00000226662	CHMP1AP1	0	0	0	0	0	0	0	0
ENSG00000207095	RNU6-424P	0	0	0	0	0	0	0	0
ENSG00000009780	FAM76A	2.43487	2.02642	0.96125	2.42705	2.23123	2.43622	1.7264	1.87401
ENSG00000231344	AL020997.1	0	0	0	0	0	0	0	0
ENSG00000230833	RPEP3	0	0.54146	0	0.16093	0	0	0	0
ENSG00000117758	STX12	9.85343	9.77083	2.99672	8.68362	7.15992	7.69953	6.98899	7.81566
ENSG00000269971	AL020997.2	2.7886	4.64163	0	0	0	0	1.99207	1.46734
ENSG00000270031	AL020997.3	0	0	0.30004	0.90907	0.24824	0	0	0
ENSG00000223062	RNU6-1245P	0	0	0	0	0	0	0	0
ENSG00000117751	PPP1R8	9.56883	6.7518	1.71945	4.09056	9.38902	10.8257	4.59422	5.87423
ENSG00000252947	SCARNA1	3.53255	7.34991	7.13767	13.762	3.22108	4.67392	8.83231	9.75872
ENSG00000130775	THEMIS2	68.6368	60.4366	8.55465	15.9847	38.8981	22.079	22.6064	25.8247
ENSG00000117748	RPA2	14.4858	14.4428	3.41441	7.11229	15.6694	15.7557	5.32534	10.8317
ENSG00000130768	SMPDL3B	0.60397	0.22848	0.12103	0.04074	0.16689	0.04843	0	0.06741
ENSG00000227050	AL512288.1	0	0	0	0	0	0	0	0.18329
ENSG00000158156	XKR8	15.4457	10.4083	1.92416	3.25207	7.47242	4.33711	4.23155	5.77451
ENSG00000158161	EYA3	7.58669	10.2003	5.91488	17.3003	11.918	10.6314	10.5351	11.175
ENSG00000238231	AL512288.2	0.19973	0.6649	0	0.59284	0	0	0	0
ENSG00000240750	RN7SL559P	0.49361	0.4108	0	0.36628	0	0.43539	0	0.30302
ENSG00000228589	SPCS2P4	0.75345	0.17916	0.31634	0.63898	0.13086	0	0.71765	0.26431
ENSG00000228943	AL137792.2	0	0	0	0	0	0	0	0
ENSG00000214812	AL137792.1	0.3948	0.43809	0	0	0.31999	0.23216	0	0.48472
ENSG00000253005	RNU6-176P	0	1.16199	0	0	3.39492	0	0	0
ENSG00000169403	PTAFR	50.5128	37.2559	7.2557	10.4024	50.4477	15.8274	10.9822	23.771
ENSG00000126698	DNAJC8	21.3962	11.1109	2.75998	5.23588	16.7557	16.3879	5.41519	7.57247
ENSG00000130770	ATP5IF1	7.57711	6.5254	2.66259	5.52485	7.77017	12.7278	3.78896	4.12571
ENSG00000270605	AL353622.1	0.37687	0.06273	0.05538	0.11186	0.09164	0.46539	0.18845	0.3239
ENSG00000271398	AL353622.2	0.17247	0.57416	0.25344	0.51193	0	0	0	0
ENSG00000130766	SESN2	4.0121	3.07406	0.5303	0.97665	1.60013	3.18317	1.48612	0.88616
ENSG00000130772	MED18	2.35984	3.14237	0.48015	0.70045	1.98626	1.72928	1.02876	1.87215
ENSG00000204138	PHACTR4	4.44487	4.28246	1.52994	3.29834	6.91318	6.55213	2.63651	2.92533
ENSG00000206779	RF00019	0	0	0	0	0	0	0	0
ENSG00000229820	CR391992.1	0	0.61933	0	0	0	0	0	0
ENSG00000221216	RNU6ATAC27P	0	0	0	0	0	0	0	0
ENSG00000180198	RCC1	2.01727	1.31799	0.36015	0.81142	1.99412	3.45896	0.72277	0.8796
ENSG00000242125	SNHG3	3.03027	1.6415	0.57967	1.75633	2.46335	2.87852	2.86921	1.51903
ENSG00000274266	SNORA73A	0	0	0	0	0	0	0	0

ENSG00000200087	SNORA73B	581.732	861.834	1701.77	4094.4	1101.29	578.1	1957.28	2028.02
ENSG00000231468	PRDX3P2	0	0	0	0	0	0	0	0
ENSG00000279443	AL513497.1	0.17595	0	0	0	0.04278	0.18624	0	0.08641
ENSG00000180098	TRNAU1AP	1.88734	2.09432	1.12436	3.86087	2.5013	2.60963	3.06088	1.8371
ENSG00000197989	SNHG12	5.07915	4.91358	4.11463	8.98763	4.24868	5.28428	4.48634	3.22465
ENSG00000221539	SNORD99	1.98109	1.64876	1.45559	8.82046	2.40856	3.49491	3.30217	2.43235
ENSG00000278274	SNORA61	0	0	0	0	0	0	0	0
ENSG00000273544	SNORA44	0	0	0	0	0	0	0	0
ENSG00000274582	SNORA16A	0	0	0	0	0	0	0	0
ENSG00000280498	SNORA16A	0	0	0	0	0	0	0	0
ENSG00000120656	TAF12	10.7269	11.7466	4.42471	4.18944	8.92311	10.6792	7.11019	6.93174
ENSG00000188060	RAB42	0.03332	0	0	0	0	0	0	0
ENSG00000229388	LINC01715	0.52464	0	0.22027	0.3337	0.63785	0.26444	0	0.09202
ENSG00000270103	AL360012.1	0	0	0	0	0	0	0	0
ENSG00000274978	RNU11	0	0.91051	0.80384	1.62367	0	0	0	0
ENSG00000162419	GMEB1	5.29978	3.3057	1.26742	1.56635	3.50451	4.34443	2.11862	2.32691
ENSG00000252777	RF00422	0.56385	0	0	0	0	0	0	0
ENSG00000198492	YTHDF2	11.4538	8.91588	2.65168	5.1306	10.3458	10.1198	4.52754	6.2268
ENSG00000116329	OPRD1	0	0	0	0	0	0	0	0
ENSG00000233427	AL009181.1	0	0	0.09928	0	0.16427	0.35754	0	0
ENSG00000199756	RF00019	0	0	0	0	0	0	0	0
ENSG00000159023	EPB41	278.458	223.851	181.125	268.829	261.466	230.724	400.331	218.225
ENSG00000225616	AL138785.1	0	0	0	0	0	0	0	0.27189
ENSG00000206704	RF00019	0	0	0	0	0	0	0	0
ENSG00000277958	RF00017	0	0	0	0	0	0	0.43326	0.31914
ENSG00000253304	TMEM200B	0	0	0.04082	0.08244	0.03377	0	0	0.06821
ENSG00000225750	AL357500.1	0.52545	0	0	0.38991	0	0.92697	1.31377	0.64514
ENSG00000116350	SRSF4	11.0779	11.5382	3.1734	8.47729	11.7947	11.6472	8.29831	6.04163
ENSG00000277916	RF02271	0	0	0	0	0	0	0	0
ENSG00000116353	MECR	0.44855	0.15554	0.19225	0.19416	0.409	0.5605	0.43614	0.39009
ENSG00000237934	AL590729.1	0	0	0	0	0	0	0	0
ENSG00000060656	PTPRU	0	0	0.03224	0	0	0.01935	0	0.01347
ENSG00000230523	LINC01756	0	0	0	0	0	0	0	0
ENSG00000225011	AL671862.1	0	0	0	0	0	0	0	0
ENSG00000228176	AC092265.1	0	0	0	0	0	0	0	0
ENSG00000270927	AL645944.1	0	0	0	0	0	0	0	0
ENSG00000233399	LINC01648	0	0	0	0	0	0	0	0
ENSG00000233372	AL137076.1	0	0	0	0	0	0	0	0
ENSG00000231251	AL021921.1	0	0	0	0	0	0	0	0
ENSG00000236335	AL161638.2	0	0	0	0	0	0	0	0
ENSG00000231949	AL161638.1	0	0	0	0	0	0	0	0
ENSG00000162510	MATN1	0.01591	0	0	0.02362	0	0	0	0
ENSG00000186056	MATN1-AS1	0.27324	0.27288	0.05354	0.21627	0.39863	0.35349	0.21254	0.22365
ENSG00000162511	LAPTM5	377.405	294.363	52.1791	80.1771	244.785	163.2	96.0948	146.238
ENSG00000264773	MIR4420	15.2312	9.50715	4.19665	1.4128	5.7868	3.35875	4.76027	4.67517
ENSG00000229607	AL137027.1	0	0	0	0	0	0	0	0
ENSG00000223382	LINC01778	0	0	0	0	0	0	0	0
ENSG00000222784	RN7SKP91	0	0	0	0	0	0	0	0
ENSG00000235143	AL445235.1	0	0	0	0	0	0	0	0
ENSG00000162512	SDC3	0.26968	0.48341	0.03048	0.06157	0.13871	0.73192	0.05187	0.14008
ENSG00000266210	RN7SL371P	0	0	0	0	0	0	0	0
ENSG00000134644	PUM1	19.3076	15.0553	4.06713	8.74098	17.4034	17.5384	6.27417	8.5226
ENSG00000237329	AL356320.2	0	0	0	0	0	0	0	0
ENSG00000232768	AL356320.1	0	0	0	0	0	0	0	0
ENSG00000215900	SELENOWP1	0	0	0	0	0	0	0	0
ENSG00000225142	AC114495.1	0	0	0	0	0	0	0	0
ENSG00000084628	NKAIN1	0	0	0	0	0	0	0	0
ENSG00000060688	SNRNP40	4.70286	4.18117	1.05466	1.99015	4.08729	6.39729	2.01483	2.71313

ENSG00000229447	AC114495.2	0.33547	0.55839	0	0.24894	0.20393	0	0.27959	0.82377
ENSG00000121766	ZCCHC17	20.8721	16.958	7.90613	19.1409	18.6491	18.0404	10.8124	11.6654
ENSG00000229044	AL451070.1	0.50091	0.27792	0	0.49561	0.812	0.58912	0.83495	0.82002
ENSG00000121769	FABP3	0	0	0	0	0	0	0	0
ENSG00000168528	SERINC2	0.68158	0.41254	0.0607	0.15326	0.30132	0.32793	0.24099	0.07608
ENSG00000215899	AC209007.1	0	0	0	0	0	0	0	0
ENSG00000206981	RNU6-40P	0	0	0	0	0	0	0	0
ENSG00000260386	LINC01225	0	0	0.0303	0	0.02507	0	0.06874	0.02532
ENSG00000265961	RF00019	0	0	0	0	0	0	0	0
ENSG00000284543	LINC01226	0.02233	0.02478	0	0	0.00905	0.02626	0.02481	0
ENSG00000229167	AC114488.1	0	0	0	0	0	0	0	0
ENSG00000142910	TINAGL1	0	0	0	0	0.08437	0	0	0
ENSG00000121764	HCRTR1	0	0	0	0	0	0	0	0
ENSG00000162517	PEF1	8.65648	8.05355	1.45102	2.34473	7.64315	8.94208	2.85289	5.41518
ENSG00000235790	AC114488.2	0.06616	0.05506	0	0	0.08043	0.05835	0	0
ENSG00000264078	AC114488.3	0	0	0	0	0	0	0	0
ENSG00000084636	COL16A1	0	0	0	0	0	0	0	0
ENSG00000121753	ADGRB2	0.0567	0.01573	0.01389	0.09818	0.02298	0.01667	0	0.04641
ENSG00000266580	MIR4254	0	0	0	0	0	0	0	0
ENSG00000254545	AL354919.2	0	0	0	0	0	0	0	0
ENSG00000134668	SPOCD1	0.20692	0.29275	0.12162	0.07677	0.27672	0.62055	0.22418	0.17783
ENSG00000203620	AL354919.1	0	0	0	0	0	0	0	0
ENSG00000269967	AL136115.2	1.87309	2.90991	0	0.92662	1.36635	1.10146	0.83257	0.9199
ENSG00000184007	PTP4A2	75.6396	59.0671	17.5656	30.0649	51.9314	62.4378	27.0407	30.9755
ENSG00000228634	AL136115.1	0	0	0	0.21042	0	0	0	0
ENSG00000121774	KHDRBS1	19.8164	14.3499	4.50309	10.1873	17.5849	19.1012	10.6584	8.52815
ENSG00000203325	AL445248.1	0.11747	0	0.17262	0	0	0	0	0
ENSG00000121775	TMEM39B	2.28739	1.03837	0.30557	0.34719	1.16926	1.51322	0.47659	0.86167
ENSG00000266203	MIR5585	0	0	0	0	0	0	0	0
ENSG00000276493	RF00017	0	0	0	0	0	0	0	0
ENSG00000025800	KPNA6	7.82001	6.9323	2.98085	5.23568	6.62418	8.95501	7.72611	4.83674
ENSG00000250135	AL049795.2	0	0	0	0	0	0	0	0
ENSG00000084652	TXLNA	6.80862	5.88166	1.11872	2.49421	5.71059	7.52608	1.60417	2.85705
ENSG00000160050	CCDC28B	0.28613	0.25978	0.11467	0.42464	0.63248	1.05542	0.13007	0.38324
ENSG00000224066	AL049795.1	0.43213	0.44955	0	0	0.19702	0.28588	0.18007	0.19896
ENSG00000160051	IQCC	0.11832	0.09847	0	0.0878	0.14385	0.20874	0.09861	0.03632
ENSG00000222046	DCDC2B	0.03922	0	0	0	0	0	0	0
ENSG00000160055	TMEM234	2.3195	1.63999	0.3318	0.57881	1.34761	1.19498	0.82115	1.38612
ENSG00000084623	EIF3I	19.2068	13.085	2.74749	6.11723	16.6868	25.2627	7.2954	7.46063
ENSG00000220785	MTMR9LP	0.02476	0	0	0	0	0	0.04126	0.03039
ENSG00000183615	FAM167B	0	0	0	0	0.09521	0.27631	0	0
ENSG00000182866	LCK	24.5871	17.3932	4.22597	11.338	31.1468	51.3529	7.61111	10.6735
ENSG00000277617	RF00017	0	0	0	0	0	0	0	0
ENSG00000252450	RF00019	0	0	0	0	0	0	0	0
ENSG00000116478	HDAC1	12.3739	7.26571	2.17439	4.72146	13.7846	15.2381	3.76129	7.17615
ENSG00000175130	MARCKSL1	18.1633	10.1255	1.64836	3.13743	10.0184	7.07828	3.95522	3.49606
ENSG00000233775	AL109945.1	0	0	0	0	0	0	0	0
ENSG00000162526	TSSK3	0.10037	0	0	0	0	0.11804	0	0
ENSG00000225828	FAM229A	1.18517	1.11502	0.03786	0.38238	0.87707	0.9545	0.34357	0.53777
ENSG00000160058	BSDC1	6.70936	5.89516	2.54212	5.29097	6.13912	5.36136	7.85181	5.42479
ENSG00000224333	GAPDHP20	0	0	0	0	0	0	0	0
ENSG00000270850	AL356986.1	0	0	0	0	0	0	0	0
ENSG00000229259	LRRC37A12P	0	0	0	0	0	0	0	0
ENSG00000263529	RN7SL122P	0	0	0	0	0	0	0	0.29899
ENSG00000254553	AL033529.1	0	0	0	0	0	0	0	0
ENSG00000273274	ZBTB8B	0	0	0	0	0	0	0	0
ENSG00000160062	ZBTB8A	0.01036	0	0	0.03075	0	0	0	0.0636
ENSG00000176261	ZBTB8OS	6.89202	7.32535	2.71496	7.36329	7.14246	6.59193	9.34259	5.98961

ENSG00000200591	RF00019	0	0	0	0	0	0	0	0
ENSG00000162521	RBBP4	10.1552	6.17128	2.10423	5.69775	8.43174	10.7327	4.51564	5.607
ENSG00000162520	SYNC	0	0	0.03089	0	0	0.07417	0	0
ENSG00000224409	AC114489.1	0	0	0	0	0	0	0.30932	0
ENSG00000162522	KIAA1522	0.13517	0.06136	0	0.03647	0.01494	0.04336	0.04097	0.03018
ENSG00000134684	YARS	3.73107	3.04576	0.91817	1.92084	2.89749	5.41686	1.11587	1.77539
ENSG00000116497	S100PBP	2.26423	2.00384	0.56235	1.19506	2.56862	3.68498	0.66446	1.62492
ENSG00000160097	FNDC5	0	0	0.03431	0	0.05678	0	0	0
ENSG00000121905	HPCA	0	0	0	0	0	0	0	0
ENSG00000121900	TMEM54	0	0	0	0	0.18867	0	0	0
ENSG00000116514	RNF19B	48.221	41.1837	8.46029	14.3797	25.4375	20.8088	14.7927	20.2752
ENSG00000278966	AL031602.1	0	0	0	0	0	0	0	0
ENSG00000217644	AL355864.1	0	0	0	0	0	0	0	0
ENSG00000239670	AL355864.2	0	0	0	0	0	0	0	0
ENSG00000236065	AL020995.1	0	0	0	0	0	0	0	0
ENSG00000004455	AK2	10.6104	7.49904	1.95604	3.60909	9.16531	9.43816	3.7548	4.93436
ENSG00000276645	RF00017	1.06232	0	0	0	0	0	0	0
ENSG00000142920	AZIN2	0.06705	0.0186	0.01642	0.03317	0.04076	0.07886	0.07451	0.05488
ENSG00000278997	AL662907.1	0.59287	0.44856	0	0.15998	0.19658	0.38033	0.08984	0.2647
ENSG00000116525	TRIM62	1.50782	1.37201	0.14772	0.29837	1.00213	1.2768	0.43564	0.46899
ENSG00000279179	AL662907.2	0.04252	0	0	0	0	0	0	0
ENSG00000160094	ZNF362	4.68717	2.93025	1.03478	1.76356	4.2806	5.08551	1.72395	1.62108
ENSG00000270115	AL513327.3	1.09403	0.26015	0	0.23195	0.19001	0	1.04205	0.19189
ENSG00000184389	A3GALT2	0.57322	0.23853	0	0.21268	0	0.1264	0	0
ENSG00000225313	AL513327.1	0.01826	0.09116	0	0.05419	0.04439	0	0.06086	0.04483
ENSG00000134686	PHC2	25.4894	27.8596	3.1394	5.96955	15.5484	6.52407	7.78516	12.2468
ENSG00000284154	MIR3605	10.262	3.66025	1.07714	2.17571	0.89117	0	3.66541	2.69991
ENSG00000222112	RN7SKP16	4.90303	2.44833	1.08074	0.72766	1.49025	0.43248	1.22589	1.20397
ENSG00000233246	AL513327.2	0	0	0	0	0	0	0.24935	0.18367
ENSG00000234512	TLR12P	0	0	0	0	0	0	0	0
ENSG00000121903	ZSCAN20	0.30381	0.25284	0.02029	0.12297	0.26863	0.3167	0.09207	0.11868
ENSG00000235907	AC115285.1	0	0	0	0	0	0	0	0
ENSG00000121904	CSMD2	0	0	0	0	0	0	0	0
ENSG00000227000	HSPD1P14	0	0	0	0	0	0	0	0
ENSG00000176256	HMGB4	0	0	0	0	0	0	0	0
ENSG00000231163	CSMD2-AS1	0	0	0	0	0	0	0	0
ENSG00000201148	RNA5SP42	0	0	0	0	0	0	0	0
ENSG00000142698	C1orf94	0	0	0	0	0	0	0	0
ENSG00000270241	AC115286.1	0	0	0	0	0	0	0	0
ENSG00000207941	MIR552	0	0	0	0	0	0	0	0
ENSG00000163866	SMIM12	2.92622	2.09605	0.34613	0.7126	1.9275	2.52518	0.95135	1.0567
ENSG00000189280	GJB5	0	0	0	0	0	0	0	0
ENSG00000189433	GJB4	0	0	0	0	0	0	0	0
ENSG00000255811	AL121988.1	0	0	0	0	0	0	0	0.32142
ENSG00000188910	GJB3	0	0	0	0	0	0	0	0
ENSG00000187513	GJA4	0	0	0	0	0	0.07531	0	0
ENSG00000230163	AL122010.1	0	0	0	0	0	0	0	0
ENSG00000116544	DLGAP3	0.07372	0.12271	0	0.02735	0.08963	0	0	0.04526
ENSG00000241014	AC114490.1	0.37242	0.32818	0.03219	0.06502	0.34624	0.46376	0.10955	0.24207
ENSG00000271741	AC114490.2	0	0	0	0	0	0	0	0
ENSG00000243749	TMEM35B	0.0795	0.13233	0.11683	0	0.09666	0.14025	0	0.09761
ENSG00000163867	ZMYM6	1.57438	1.39933	0.40431	0.56712	0.96634	0.95727	0.67517	0.89143
ENSG00000233940	RPL12P45	0	0	0	0	0	0	0	0
ENSG00000197056	ZMYM1	2.67209	2.62819	1.80713	4.28113	3.4517	2.91943	2.32821	3.03843
ENSG00000228348	EFCAB14P1	0	0	0	0	0	0	0	0
ENSG00000116560	SFPQ	25.2001	17.9086	3.85832	9.5367	18.145	25.4932	9.82247	9.57416
ENSG00000201868	RF00019	0	0	0	0	0	0	0	0
ENSG00000146463	ZMYM4	8.75156	9.23799	5.02862	10.5841	12.5628	10.907	9.65044	8.41479

ENSG00000240374	RN7SL503P	0	0	0	0	0	0	0	0
ENSG00000201542	RF00091	0	0	0	0	0	0	0	0
ENSG00000229994	RPL5P4	0	0.55083	0	0.49113	0	0.14595	0	0.50788
ENSG00000227409	ZMYM4-AS1	0	0	0	0	0	0	0	0
ENSG00000278702	RF00017	0	0	0	0	0	0	0	0
ENSG00000142687	KIAA0319L	4.61315	5.16974	1.27525	2.23695	5.15505	3.97511	1.95407	3.1778
ENSG00000236274	AC004865.1	0	0	0	0	0	0	0	0
ENSG00000020129	NCDN	1.25223	0.81885	0.13144	0.48674	1.03308	1.91982	0.34788	0.29285
ENSG00000239636	AC004865.2	1.20981	1.53991	0.10458	0.31685	1.12477	1.00436	0.47449	0.96113
ENSG00000116819	TFAP2E	0.20091	0.11147	0.04921	0	0.12213	0.11815	0.05582	0.12334
ENSG00000126067	PSMB2	6.09953	4.13161	0.88965	2.31363	4.25066	8.62435	2.24531	2.21137
ENSG00000142686	C1orf216	1.13112	0.70603	0.29681	0.53958	0.51569	0.89082	0.16834	0.39679
ENSG00000092853	CLSPN	2.23518	2.23228	1.71196	3.73945	3.80446	7.28887	5.44178	2.77758
ENSG00000232335	AL354864.1	0	0	0.39968	0	0	0	0	0.16697
ENSG00000134698	AGO4	34.3958	25.9564	3.72041	7.26037	23.5453	9.00396	7.6163	14.8612
ENSG00000092847	AGO1	6.75149	3.52336	0.59729	1.51984	3.81213	3.32452	2.02373	2.19062
ENSG00000271164	AL139286.1	0	0	0	0	0	0	0	0
ENSG00000271914	AL139286.2	0.3702	0	0	0	0	0	0	0.22727
ENSG00000126070	AGO3	2.9449	1.82808	0.40729	1.0695	2.45569	1.71136	0.82582	1.38248
ENSG00000271554	AL138787.2	0.22246	0.18514	0.16345	0	0	0	0	0.13657
ENSG00000232862	AL138787.1	0	0	0	0	0	0	0	0
ENSG00000092850	TEKT2	0.03706	0.06168	0	0	0.04505	0	0	0.0455
ENSG00000116863	ADPRHL2	3.82322	5.99802	0.64577	1.10873	4.54132	4.3414	2.34399	2.75171
ENSG00000171812	COL8A2	0.4081	0.36576	0.11533	0.04659	0.15266	0	0.10465	0.05781
ENSG00000054116	TRAPPC3	6.38027	4.7392	1.83239	1.66556	3.86585	4.47293	2.56347	3.13854
ENSG00000116871	MAP7D1	15.6446	15.2077	1.7983	3.57047	9.07905	10.3773	4.56641	5.13927
ENSG00000264592	RN7SL131P	0	0	0	0	0	0	0	0
ENSG00000054118	THRAP3	59.9744	49.4887	8.14223	16.6073	52.8566	41.6659	16.8204	22.8413
ENSG00000231849	UBE2V2P4	0	0	0	0	0	0	0	0
ENSG00000214193	SH3D21	0.62258	0.68176	0.16853	0.21884	0.25894	0.11561	0.13654	0.16092
ENSG00000142694	EVA1B	1.39216	1.10825	0.08895	0.17966	0.6623	1.49493	0.60535	0.29727
ENSG00000116883	AL591845.1	0.1994	0.06638	0	0	0	0	0	0.04896
ENSG00000196182	STK40	21.4918	21.1059	4.70165	6.55828	14.7306	10.2941	8.37121	11.1571
ENSG00000181817	LSM10	6.36968	5.38273	0.93602	0.94533	4.34861	3.19822	1.63343	2.04539
ENSG00000222821	RNU4-27P	0	0	0	0	0	0	0	0
ENSG00000116885	OSCP1	0.10517	0	0	0	0.12786	0.04638	0.04382	0
ENSG00000201448	SNORA63C	0	0	0	0	0	0	0.94713	0
ENSG00000116898	MRPS15	3.11702	2.76151	1.07123	1.08189	2.8422	3.19288	1.1313	2.00611
ENSG00000119535	CSF3R	122.712	113.121	11.6672	18.3138	74.1247	16.1084	23.3953	40.9679
ENSG00000230214	FTLP18	0	0	0	0	0	0	0	0
ENSG00000234481	AC117945.1	0	0	0	0	0	0	0	0
ENSG00000163873	GRIK3	0	0	0	0	0	0	0	0
ENSG00000264698	MIR4255	0	0	0	0	0	0	0	0
ENSG00000207328	RNU6-636P	0	0	0	0	0	0	0	0
ENSG00000223944	AL353604.1	0	0	0	0	0	0	0	0
ENSG00000252368	RNA5SP43	0	0	0	0	0	0	0	0
ENSG00000230451	RPS29P6	0	0	0	0	0	0	0	0
ENSG00000233621	LINC01137	1.0509	0.87461	0.15443	0.38991	0.57495	0.92697	0.26275	0.4516
ENSG00000163874	ZC3H12A	5.58971	3.36198	0.79379	1.46395	3.08382	6.04919	2.03569	1.55714
ENSG00000283724	MIR6732	0	0	0	0	0	0	0	0
ENSG00000163875	MEAF6	9.7958	6.12317	2.25498	3.55652	8.38264	8.7889	3.11845	4.67148
ENSG00000263675	MIR5581	1.22167	0	0	1.8131	4.45583	0	4.07268	1.49995
ENSG00000163877	SNIP1	3.60848	3.04545	0.50412	1.03713	3.02771	2.06217	1.90609	2.29322
ENSG00000232273	FTH1P1	0	0	0	0	0	0	0	0
ENSG00000237749	AL034379.1	0	0	0	0	0	0	0	0
ENSG00000163879	DNALI1	0	0	0	0.02441	0	0	0	0
ENSG00000134697	GNL2	6.00948	3.95014	1.51868	4.09012	4.21927	4.63677	3.25389	2.94507
ENSG00000169218	RSPO1	0	0.0395	0	0	0	0	0	0

ENSG00000116922	C1orf109	1.12414	0.52281	0.3401	0.80964	1.68827	1.16655	0.85422	0.38564
ENSG00000134690	CDCA8	1.72192	2.02606	1.00341	2.81988	1.98518	4.39943	2.87017	1.71319
ENSG00000183317	EPHA10	0	0	0	0	0	0.01586	0	0
ENSG00000200421	RF00019	0	0	0	0	0	0	0	0
ENSG00000214141	ACTN4P2	0	0	0	0	0	0	0	0
ENSG00000227416	AL929472.1	0	0	0	0	0	0	0	0
ENSG00000185090	MANEAL	0.14573	0.28299	0.07138	0.07209	0.08859	0.12854	0.12145	0.05964
ENSG00000233728	AL929472.3	0	0	0	0	0	0.18473	0	0.25713
ENSG00000196449	YRDC	2.89027	1.00226	0.5309	0.83406	2.78185	1.84124	1.7397	1.33073
ENSG00000197982	C1orf122	1.87276	1.3672	0.53109	0.19504	0.719	0.69554	0.54765	0.36305
ENSG00000188786	MTF1	5.69119	5.47396	0.88598	1.39643	3.74281	3.4004	1.55314	2.62454
ENSG00000235673	AL929472.4	0	0	0	0	0	0	0	0
ENSG00000204084	INPP5B	3.38183	2.14505	0.79609	2.21713	2.61462	2.02728	1.27243	1.6528
ENSG00000230955	AL929472.2	0	0	0	0	0	0	0	0
ENSG00000252448	RF00092	0	0	0	0	0	0	0	0
ENSG00000222282	RNU6-584P	0	0	0	0	0	0	0	0
ENSG00000277168	RF00017	0	0	0	0	0	0	0	0
ENSG00000183431	SF3A3	11.3353	6.97559	1.93158	3.07538	10.3217	10.2304	3.09317	3.66444
ENSG00000212541	RNU6-510P	0	0	0	0	0	0	0	0
ENSG00000183386	FHL3	10.461	6.69707	1.02124	1.79138	3.11286	4.90404	1.3413	1.21254
ENSG00000183520	UTP11	3.07493	3.24395	0.70006	1.57474	3.44883	3.82014	0.75799	1.48887
ENSG00000185668	POU3F1	0	0	0	0	0	0	0	0
ENSG00000224592	AL139158.2	0	0	0	0	0	0	0	0
ENSG00000223589	AL139158.1	0	0	0	0	0	0	0	0
ENSG00000265596	MIR3659	0	0	0	0	0	0	0	0
ENSG00000237290	LINC01343	0	0	0	0	0	0	0	0
ENSG00000275350	AL513479.1	0	0	0	0	0	0	0	0
ENSG00000200796	RNU6-753P	0	0	0	0	0	0	0	0
ENSG00000238063	LINC01685	0	0.40751	0.07195	0.21801	0.11906	0.08638	0	0
ENSG00000215895	AL354702.1	0	0	0	0	0	0	0	0
ENSG00000116954	RRAGC	1.78747	0.92091	0.47947	0.75794	1.00034	1.17624	0.49657	1.06248
ENSG00000273637	AL139260.2	0	0	0	0	0	0	0	0
ENSG00000228436	AL139260.1	0.22531	0.12501	0	0.11146	0.27392	0.26498	0.12518	0.09221
ENSG00000214114	MYCBP	5.66817	4.1352	1.66586	2.75633	3.95879	2.89345	1.80919	3.46484
ENSG00000274944	AL139260.3	0	0	0	0	0	0	0	0
ENSG00000131233	GJA9	0	0	0	0	0	0	0	0
ENSG00000158315	RHBDL2	0	0	0	0	0	0	0	0
ENSG00000199963	RNU6-605P	0	0	0	0	0	0	0	0
ENSG00000207466	RF00019	0	0	0	0	0	0	0	0
ENSG00000235549	EIF1P2	0	0	0	0	0	0	0	0
ENSG00000174574	AKIRIN1	30.8605	27.9538	6.71984	11.5493	21.9459	15.6155	11.6787	16.1542
ENSG00000168653	NDUFS5	42.0006	29.3889	7.0761	17.8663	34.3132	38.2272	16.7218	18.722
ENSG00000127603	MACF1	11.2717	10.8529	2.29662	5.29517	10.1208	10.3445	4.28036	5.59793
ENSG00000206654	RNU6-608P	0	0	0	0	0	0	0	0
ENSG00000222378	RNA5SP44	0	0	0	0	0	0	0	0
ENSG00000270040	AL356055.1	0	0	0	0	0	0	0	0
ENSG00000226438	AL442071.1	0	0	0	0	0	0	0	0
ENSG00000217897	HSPE1P8	0	0	0	0	0	0	0	0
ENSG00000183682	BMP8A	0.08664	0	0	0	0.05267	0	0	0
ENSG00000237624	OXCT2P1	0	0	0	0	0	0	0	0
ENSG00000182109		0.16774	0.11966	0	0	0.23306	0.16909	0.11982	0.02942
ENSG00000243970	PPIEL	0.63112	0.45022	0.13249	0.40142	0.54807	0	0.30057	0.33209
ENSG00000090621	PABPC4	9.12201	5.12716	1.20706	2.96747	5.91308	4.53792	2.70231	2.72035
ENSG00000228060	PABPC4-AS1	0	0	0	0	0	0	0	0
ENSG00000201457	SNORA55	1.6289	0	0.79788	1.61164	0.66012	0.95787	0	1.99993
ENSG00000163909	HEYL	0	0	0	0	0	0	0.03337	0
ENSG00000225903	AL035404.2	0	0	0	0	0	0	0	0
ENSG00000116981	NT5C1A	0	0	0	0	0	0	0	0

ENSG00000116983	HPCAL4	1.1227	0.39342	0	0.26309	0.88003	0.20848	0.14774	0.09069
ENSG00000084072	PPIE	0.98085	0.72561	0.28026	0.59306	1.18145	1.23368	0.71149	0.66904
ENSG00000225333	AL035404.1	0	0	0	0	0	0	0	0
ENSG00000252413	RNU7-121P	0	0	0	0	0	0	0	0
ENSG00000116985	BMP8B	0.50164	0.22772	0.11169	0.20304	0.33266	0	0.05068	0.09332
ENSG00000198754	OXCT2	0	0	0	0	0	0	0	0
ENSG00000261798	AL033527.3	0	0	0.09006	0	0	0	0	0
ENSG00000229213	AL033527.1	0	0	0	0	0	0	0	0
ENSG00000043514	TRIT1	3.46821	1.87846	1.61793	2.81871	4.91932	3.69046	4.08338	2.36568
ENSG00000202222	RF00019	0	1.07972	0	0	0	0	2.16248	0
ENSG00000116990	MYCL	3.61941	3.08455	0.53187	0.77351	2.3058	1.12378	0.72396	1.1554
ENSG00000236546	AL033527.2	0	0	0	0	0	0	0	0
ENSG00000207356	RF00019	0	0	0	0	0	0	0	0
ENSG00000168389	MFSD2A	0.8049	0.63943	0.13441	0.29864	0.40032	0.6777	0.15246	0.24706
ENSG00000228477	AL663070.1	0.30331	0	0	0	0	0	0.16852	0
ENSG00000131236	CAP1	191.073	181.445	33.2123	52.8241	143.792	108.325	52.0243	100.356
ENSG00000131238	PPT1	26.2959	37.1635	8.66664	11.3195	41.9436	16.8583	15.4111	30.4618
ENSG00000226745	AL512599.1	0	0	0	0	0	0	0	0
ENSG00000117000	RLF	10.6207	11.1734	4.70796	8.49943	11.186	10.6207	11.4434	8.97665
ENSG00000207508	RNU6-1237P	0	0	0	0	0	0	2.28374	0
ENSG00000188800	TMCO2	0	0	0	0	0	0	0	0
ENSG00000259943	AL050341.2	2.04537	0.9501	0.48929	1.97664	2.08189	1.00697	1.58573	1.69365
ENSG00000084073	ZMPSTE24	17.3811	13.936	4.58357	9.53246	13.622	11.6569	6.841	11.7913
ENSG00000231296	AL050341.1	0	0	0	0	0	0	0	0
ENSG00000049089	COL9A2	1.1478	0.47028	0.07785	0.31449	0.85875	0.21806	0.50038	0.15177
ENSG00000227311	AL031985.2	0	0	0	0	0	0	0	0
ENSG00000084070	SMAP2	122.194	110.483	11.866	31.3684	108.849	45.9411	52.7956	42.4084
ENSG00000213172	AL031985.1	0.17536	0.29189	0	0	0	0	0	0.2153
ENSG00000187801	ZFP69B	0.82947	0.56249	0.09029	0.31915	0.93375	0.32518	0.51207	0.22631
ENSG00000260920	AL031985.3	0.59861	0.34874	0.04398	0.17768	0.40028	0.47522	0.14967	0.25724
ENSG00000279667	AL603839.4	0	0	0	0	0	0	0	0
ENSG00000187815	ZFP69	1.30893	0.9391	0.39796	0.56938	1.61881	1.03513	0.2257	0.88667
ENSG00000238287	AL603839.3	0.12116	0.20167	0	0.53943	0.2946	0.42748	0	0
ENSG00000164002	EXO5	0.80602	0.94703	0.34836	0.49256	1.64284	1.92378	1.10642	0.69855
ENSG00000227278	AL603839.1	0	0	0	0	0	0	0	0
ENSG00000238186	AL603839.2	0	0	0	0	0	0	0	0
ENSG00000117010	ZNF684	0.9648	0.7628	0.63799	0.39376	0.87973	1.48928	0.32163	0.56267
ENSG00000236505	AL356379.1	0	0	0	0	0	0	0	0
ENSG00000233910	GTF2F2P2	0	0	0	0	0	0	0	0
ENSG00000117016	RIMS3	0.57749	0.15177	0.05955	0.1654	0.25867	0.08937	0.2702	0.07463
ENSG00000237899	AL031289.1	0	0	0	0	0	0	0	0
ENSG00000272145	NFYC-AS1	0.869	0.28929	0.19155	0.25794	0.89803	0.99647	0.79667	0.53347
ENSG00000066136	NFYC	5.06617	5.05144	0.75526	1.85244	4.0467	4.27441	1.50941	2.16354
ENSG00000198974	MIR30E	1.59349	0	1.1708	0	1.93732	0	2.65609	0.97823
ENSG00000207962	MIR30C1	0	0	0	1.22231	0	1.45294	0	0
ENSG00000117013	KCNQ4	0.01752	0	0	0.05201	0.0213	0.03091	0	0.02151
ENSG00000264582	RN7SL326P	0	0	0	0.36752	0	0	0	0.30404
ENSG00000179862	CITED4	1.44818	1.01983	0.08185	0.33066	1.69295	0.68783	1.02126	0.47871
ENSG00000229528	AC119677.1	0	0	0	0	0	0	0	0
ENSG00000228940	AL391730.1	0	0	0	0	0	0	0	0
ENSG00000233118	UBE2V1P8	0	0	0	0	0	0	0	0
ENSG00000171793	CTPS1	1.98617	1.22857	0.88743	2.15102	1.99052	2.69893	2.66193	1.53236
ENSG00000281207	SLFNL1-AS1	0.1069	0.07626	0	0.04533	0.1114	0.08082	0.02545	0.01875
ENSG00000171790	SLFNL1	0	0	0	0	0.02068	0	0	0
ENSG00000010803	SCMH1	3.08234	3.78619	1.59863	1.19867	9.03792	9.68312	5.02788	4.31063
ENSG00000215893	RPL23AP17	0	0	0	0	0	0	0	0
ENSG00000235358	AC093151.3	0	0	0	0	0	0	0	0
ENSG00000213987	AC093151.1	0	0	0	0	0	0	0	0

ENSG00000204060	FOXO6	0	0	0	0	0	0	0	0
ENSG00000229901	AC093151.2	0	0	0	0	0	0	0	0
ENSG00000252563	RNA5SP45	0	0	0	0	0	0	0	0
ENSG00000127129	EDN2	0.04472	0	0	0	0	0	0	0
ENSG00000127124	HIVEP3	0.63928	0.62643	0.14394	0.22954	0.47636	1.13687	0.24061	0.21521
ENSG00000230881	AL445933.2	0	0	0	0	0	0	0	0
ENSG00000230638	AL445933.1	0	0	0	0	0	0	0	0
ENSG00000227538	HNRNPFP1	0	0	0	0	0	0	0	0
ENSG00000228776	AC114492.1	0	0	0	0	0	0	0	0
ENSG00000044012	GUCA2B	0	0	0	0	0	0	0	0
ENSG00000197273	GUCA2A	0	0	0	0	0	0	0	0
ENSG00000198815	FOXJ3	10.6447	10.2131	7.62577	15.9241	11.9072	11.4623	14.0561	8.93051
ENSG00000227527	AC096540.1	0	0	0	0	0	0	0	0
ENSG00000177181	RIMKLA	0.00816	0	0.01199	0	0.00992	0.0288	0	0
ENSG00000235002	AL513331.1	0	0	0	0	0	0	0	0
ENSG00000066185	ZMYND12	1.00183	0.22234	0.29443	0.2478	0.4466	0.17674	0.33398	0.41001
ENSG00000127125	PPCS	9.76395	7.16034	1.99624	2.81415	7.63857	7.83859	3.49087	5.52492
ENSG00000186409	CCDC30	0.27683	0.32526	0.18346	0.30612	0.33656	0.22024	0.15381	0.19327
ENSG00000230116	AL445669.1	0	0	0	0.14409	0.11804	0	0	0
ENSG00000236876	TMSB4XP1	0	0	0	0.80582	0	0	0.90504	0.66664
ENSG00000200254	RNU6-536P	0	0	0	0	0	0	0	0
ENSG00000236180	AL445669.2	0.34822	0	0	0	0.63504	0	0	0
ENSG00000171960	PPIH	5.12696	4.81612	0.96972	2.78745	5.98637	8.86556	3.04605	2.67997
ENSG00000234917	AC098484.2	0	0.20301	0	0	0	0	0	0
ENSG00000065978	YBX1	520.432	572.428	210.126	194.743	557.418	524.014	201.059	461.456
ENSG00000164007	CLDN19	0	0	0	0	0	0	0	0
ENSG00000117385	P3H1	1.43004	0.55901	0.1592	0.3698	0.7771	0.64981	0.30699	0.42564
ENSG00000164008	C1orf50	0.38528	0.24049	0.14154	0.14295	0.35131	0.59473	0.24083	0.05913
ENSG00000283580	AC098484.3	0	0	0	0	0	0	0	0
ENSG00000228452	AC098484.1	0	0	0	0	0	0	0	0
ENSG00000274386	TMEM269	0.10057	0.0279	0.02463	0	0	0.02957	0.02794	0.04116
ENSG00000177868	SVBP	12.7426	20.0991	10.5217	8.46511	8.26247	12.2033	12.744	8.4931
ENSG00000164010	ERMAP	6.72698	5.16382	7.07084	10.4534	5.23423	7.31585	21.9772	6.62686
ENSG00000228192	AL512353.1	0	0.15091	0.06661	0.06728	0.05511	0.15994	0	0.05566
ENSG00000164011	ZNF691	0.93606	0.95881	0.10581	0.21372	0.96295	1.0162	0.30005	0.48623
ENSG00000233708	AL512353.2	0.03736	0	0	0	0	0	0	0
ENSG00000237090	AL512353.3	0	0	0	0	0	0	0	0
ENSG00000225099	ATP6V1E1P1	0	0	0	0	0	0	0	0
ENSG00000117394	SLC2A1	7.49927	7.72729	7.52925	10.8763	8.87203	13.3667	18.2973	7.98745
ENSG00000284138	ATP6V0CP4	0.33318	0.27729	0	0.24724	0.20254	0.58778	32.4889	0.20454
ENSG00000227533	SLC2A1-AS1	0.65369	0.32642	0.19212	0.54975	0.50333	0.49972	4.06783	0.53506
ENSG00000283973	AC099795.1	0	0	0	0	0.1046	0	0.57362	0
ENSG00000207256	RNU6-880P	0	0	0	0	0	0	0	0
ENSG00000283136	RF00026	0	0	0	0	0	0	0	0
ENSG00000277513	AL161637.1	0	0	0	0	0	0	0	0
ENSG00000186973	FAM183A	0	0	0	0	0	0	0.14494	0
ENSG00000117395	EBNA1BP2	2.51127	1.43902	0.27223	0.42769	2.10216	2.97769	0.6519	0.6571
ENSG00000284410	MIR6733	0	0	0	0	0	0	0	0
ENSG00000243710	CFAP57	0	0	0.01869	0.05662	0	0	0	0.01561
ENSG00000199240	RNA5SP46	0	0	0	0	0	0	0	0
ENSG00000179178	TMEM125	0	0	0	0	0	0	0	0
ENSG00000253313	C1orf210	0.04835	0	0	0	0.05878	0	0	0
ENSG00000066056	TIE1	0.0219	0.01823	0	0.04875	0	0.03864	0	0.02689
ENSG00000117400	MPL	1.22987	0.5264	0.25818	0.36505	0.38449	0.99184	0.82	0.86287
ENSG00000234694	AL139289.2	0.33268	0.55375	0.16296	0	0.13482	0	0.36968	0.27231
ENSG00000117399	CDC20	0.46757	0.11973	0.31712	0.16014	0.52473	4.94913	0.1199	0.2208
ENSG00000066322	ELOVL1	10.4965	10.0254	2.14826	4.12231	7.10943	7.73704	2.8754	4.12831
ENSG00000283836	MIR6734	0	0	0	0	0	0	0	0

ENSG00000159479	MED8	4.79814	3.60972	0.71703	2.29319	4.11967	3.96926	2.1237	2.86233
ENSG00000229431	AL139289.1	0	0	0	0	0	0.1769	0	0
ENSG00000198198	SZT2	1.78274	1.49587	0.2624	0.3519	1.18158	1.02768	0.59529	0.6721
ENSG00000229372	SZT2-AS1	0	0	0	0	0.12749	0	0	0
ENSG00000274975	MIR6735	0	0	0	0	0	0	0	0
ENSG00000178922	HYI	0.40128	0.40076	0.11793	0.39703	0.35777	0.42475	0.26755	0.29561
ENSG00000229348	HYI-AS1	0	0	0	0.19961	0	0	0	0
ENSG00000142949	PTPRF	0.49953	0.21641	0.14078	0.21327	0.2995	0.25351	0.26234	0.22684
ENSG00000066135	KDM4A	5.85326	4.97753	1.1038	2.16646	5.42764	6.35058	2.2206	2.67973
ENSG00000236200	KDM4A-AS1	0	0.18955	0	0.05634	0.04615	0	0	0.13982
ENSG00000126091	ST3GAL3	1.18785	1.04351	0.43638	1.27321	0.86248	1.07687	1.20998	0.89126
ENSG00000229444	AL451062.1	0	0	0.22967	0	0	0	0	0.38378
ENSG00000283414	RF00026	0.81445	0	0	0	0	0	0	0.99997
ENSG00000233674	AL451062.2	0.27048	0.45022	0	0	0	0	0	0
ENSG00000283477	MIR6079	0	0	0	0	0	0	0	0
ENSG00000224751	SHMT1P1	0	0	0	0	0	0	0	0
ENSG00000117407	ARTN	0	0	0	0	0	0	0	0
ENSG00000237950	AL357079.1	0.1316	0	0	0.19531	0	0	0.21935	0
ENSG00000117408	IPO13	1.54301	1.05614	0.31787	0.62065	1.27986	1.5264	0.31248	0.9738
ENSG00000132768	DPH2	1.7512	1.04103	0.14705	1.03959	1.70325	1.32401	0.5004	0.92147
ENSG00000117410	ATP6V0B	16.8166	12.8739	3.10801	4.46221	11.8486	5.96261	6.08309	9.9035
ENSG00000117411	B4GALT2	0.19433	0.19408	0.02856	0.1442	0.0945	0.44567	0.09717	0.02386
ENSG00000159214	CCDC24	0.10312	0.17165	0	0.06122	0.12538	0.07277	0	0.02532
ENSG00000196517	SLC6A9	0.85249	1.20613	1.19009	1.89779	1.53739	5.23864	1.75254	0.8199
ENSG00000230615	AL139220.2	1.14546	2.62351	1.58898	0.6528	1.06953	1.55194	0.91647	1.06885
ENSG00000227163	AL139220.1	0	0	0	0	0	0	0	0
ENSG00000271647	KRT8P47	0	0	0	0	0	0	0	0
ENSG00000239560	RN7SL479P	0	0	0	0	0	0	0	0
ENSG00000171872	KLF17	0	0	0	0	0	0	0	0
ENSG00000233514	AL356653.1	0	0	0	0	0	0	0	0.24999
ENSG00000283039	KLF18	0	0	0	0	0	0	0	0
ENSG00000226804	AL359839.1	0	0	0	0	0	0	0	0
ENSG00000200171	RF00019	0	0	0	0	0	0	0	0
ENSG00000238037	OOSP1P1	0	0	0	0	0	0	0	0
ENSG00000227994	AL035417.1	0	0	0	0	0	0	0	0
ENSG00000271329	AL035417.2	0	0	0	0	0	0	0.15349	0
ENSG00000178028	DMAP1	1.67574	1.2203	0.21162	0.46631	1.70307	1.24716	1.09109	0.56258
ENSG00000117419	ERI3	2.74668	2.817	1.67156	3.95285	3.33935	4.30713	4.30082	2.41854
ENSG00000233602	ERI3-IT1	0.11842	0	0	0	0.28794	0.2089	0.39477	0.29078
ENSG00000199385	RNU6-369P	0	0	0	0	0	0	0	0
ENSG00000187147	RNF220	4.02558	2.41825	0.41142	0.9321	3.10947	2.92343	1.16038	1.08699
ENSG00000263381	MIR5584	0	0	0	0	0	0	0	0
ENSG00000126106	TMEM53	0.36297	0.13426	0	0.14964	0.09807	0.42689	0	0.09903
ENSG00000198520	ARMH1	1.41229	0.38537	0.10207	0.41233	0.98518	0.73519	0.23155	0.22741
ENSG00000199377	RNU5F-1	37.5899	35.4555	16.5714	39.9811	54.0793	51.9458	25.0626	46.9215
ENSG00000200169	RNU5D-1	125.748	68.3668	27.8571	64.7087	106.786	66.8854	33.7049	48.8777
ENSG00000142945	KIF2C	0.44229	0.13385	0.08863	0.44756	0.19554	2.41174	0.80426	0.51836
ENSG00000225721	AL592166.1	0.09582	0.15949	0	0.1422	0.11649	0.16904	0.15971	0.23529
ENSG00000142937	RPS8	87.2807	58.8829	19.8251	28.4669	102.013	78.6681	45.5762	63.5701
ENSG00000264294	SNORD55	0	0	0	0	0	0	0	0
ENSG00000200913	SNORD46	1.40962	3.51948	5.17856	9.41415	5.99824	3.73015	1.17481	6.05749
ENSG00000202031	SNORD38A	0	0	1.53877	0	0	0	0	0
ENSG00000207421	SNORD38B	0	0	0	0	0	0	0	0
ENSG00000281859	SNORD38B	0	0	0	0	0	0	0	0
ENSG00000234093	RPS15AP11	0	0	0	0	0	0	0	0
ENSG00000142959	BEST4	0.03581	0	0.05262	0.05314	0	0.06317	0.05969	0
ENSG00000173846	PLK3	13.4212	5.50599	1.39325	1.00062	7.60783	2.71335	2.66907	3.59574
ENSG00000188396	TCTEX1D4	0.09607	0	0	0	0.2336	0	0	0

ENSG00000222009	BTBD19	1.10367	1.14383	0.0918	0.09272	1.13928	0.25715	0.17355	0.25567
ENSG00000117425	PTCH2	0.44131	0.47391	0.12552	0.25353	0.89999	0.62785	0.09492	0.26218
ENSG00000202444	RNU5E-6P	0	0	0	0	0	0	0	0
ENSG00000226499	AL136380.1	0	0	0	0	0	0	0	0
ENSG00000070785	EIF2B3	1.39681	1.46653	0.4421	0.95678	1.43694	1.51641	0.68057	0.71238
ENSG00000253039	RNA5SP47	0	0	0	0	0	0	0	0
ENSG00000229815	CCNB1IP1P1	0	0	0	0	0	0	0	0
ENSG00000232369	PPIAP35	0	0	0	0	0	0	0	0
ENSG00000268949	MRPS17P1	0	0	0	0	0	0	0	0
ENSG00000126107	HECTD3	2.87311	2.76364	0.93351	1.45705	2.98401	4.25351	0.74603	1.38266
ENSG00000126088	UROD	4.96567	3.39845	2.07427	4.67614	5.57745	7.24822	4.66369	3.00197
ENSG00000162415	ZSWIM5	0.15761	0.08072	0.01782	0.16194	0.1474	0.10694	0.04042	0.05954
ENSG00000234315	OSTCP5	0	0	0	0	0	0	0	0
ENSG00000281912	LINC01144	0.21433	0	0	0	0.26058	0.15124	0	0
ENSG00000186603	HPDL	0	0	0	0	0.10941	0.63505	0	0.11049
ENSG00000132781	MUTYH	1.20011	0.96758	0.248	0.52876	1.41346	1.3563	0.84392	0.5986
ENSG00000132773	TOE1	2.16533	1.42388	0.31426	0.23804	2.24254	1.65059	0.89118	0.72208
ENSG00000070759	TESK2	5.33976	5.03166	4.3773	8.80896	6.6529	6.4617	7.24549	4.68678
ENSG00000236872	PPIAP36	0	0	0	0	0	0	0	0
ENSG00000280670	CCDC163	0.53174	0.08851	0.07814	0.82862	0.48486	0.51593	0.22158	0.26114
ENSG00000132763	MMACHC	0.27681	0.20734	0.06102	0.16433	0.70674	0.39067	0.09228	0.11895
ENSG00000117450	PRDX1	18.7826	14.0286	6.1571	14.6525	15.048	18.5217	8.66983	11.7079
ENSG00000234379	HMGB1P48	0	0	0	0	0	0	0	0
ENSG00000117448	AKR1A1	4.83815	3.51835	0.93184	1.56852	3.31225	3.14889	1.37017	2.10503
ENSG00000281133	AL355480.3	0	0	0	0	0	0	0	0
ENSG00000280836	AL355480.1	0	0	0	0	0	1.30618	0	0
ENSG00000132780	NASP	4.66954	5.42162	1.41388	3.2489	5.32296	6.50919	2.35415	2.59023
ENSG00000281112	AL355480.2	0	0	0	0	0	0	0	0
ENSG00000281825	AL355480.4	0	0	0	0	0	0	0	0
ENSG00000159588	CCDC17	0.92646	0.80776	0	0.22916	0.42909	0.38914	0.33091	0.40625
ENSG00000159592	GPBP1L1	31.2017	30.0056	18.8532	40.7306	28.0191	32.6541	29.5876	27.1577
ENSG00000225447	RPS15AP10	0.57566	2.87455	0.84592	0	0.93316	0.33851	2.23891	1.88476
ENSG00000234329	AL604028.2	2.13948	1.70316	0.41008	1.10442	0.79165	0.4923	0.93031	1.48472
ENSG00000159596	TMEM69	5.46973	3.7518	0.88326	2.49775	3.10575	3.60525	2.95557	2.32465
ENSG00000197429	IPP	1.56295	1.38309	0.61052	1.90852	1.99641	1.78	1.08825	0.99592
ENSG00000230896	AL604028.1	0.73545	0	0.18012	0.54575	0.74512	1.51368	0.40863	0.60199
ENSG00000236111	AL603882.1	0.10764	0	0	0	0	0	0	0
ENSG00000086015	MAST2	0.80028	0.8674	0.23247	0.70434	0.76932	0.98498	0.49635	0.43416
ENSG00000232467	TMA16P2	0	0	0	0	0	0	0	0
ENSG00000117461	PIK3R3	0.41547	0.39517	0.11629	0.27894	0.16837	0.62824	0.1484	0.23076
ENSG00000278139	AL358075.4	0	0	0	0	0	0	0	0
ENSG00000226957	AL358075.1	0	0	0	0	0	0	0	0
ENSG00000233114	AL358075.3	0	0	0	0	0	0	0	0
ENSG00000227857	AL358075.2	0	0	0	0	0	0	0	0
ENSG00000117472	TSPAN1	0	0	0	0	0.113	0	0	0
ENSG00000250719	AL672043.1	0	0	0	0	0	0	0	0
ENSG00000085998	POMGNT1	1.2536	0.90552	0.15641	0.40369	1.25091	1.35613	0.41397	0.46465
ENSG00000171357	LURAP1	0.07907	0	0	0	0	0	0	0
ENSG00000085999	RAD54L	0.15975	0.07597	0.06707	0	0.16647	0.76492	0	0.05604
ENSG00000132128	LRRC41	1.69495	1.02075	0.40052	0.66996	1.43937	1.9233	0.66726	0.70065
ENSG00000173660	UQCRH	24.5202	18.4573	7.57364	17.5386	18.6715	32.4198	16.3138	17.4497
ENSG00000117481	NSUN4	0.89158	0.93653	0.15264	0.35971	0.83139	0.68718	0.34628	0.56328
ENSG00000225779	AL122001.1	0	0.32975	0	0	0	0	0	0.48647
ENSG00000117480	FAAH	1.21463	0.70322	0.11641	0.23513	2.08667	0.32607	0.30809	0.22694
ENSG00000232022	FAAHP1	0.23683	0.09855	0.0435	0.26362	0.03599	0	0.04935	0
ENSG00000224863	LINC01398	0	0	0	0	0	0	0	0
ENSG00000197587	DMBX1	0	0	0	0	0	0	0	0
ENSG00000282881	AL136373.1	0	0	0	0	0	0	0	0

ENSG00000269956	MKNK1-AS1	0.02998	0	0.04405	0	0	0	0.04997	0
ENSG00000162456	KNCN	0	0	0	0	0.12686	0	0	0.06405
ENSG00000079277	MKNK1	5.06835	6.09287	0.89428	1.9816	3.83191	2.01899	2.02877	2.19695
ENSG00000142961	MOB3C	5.01803	3.27913	0.87395	1.43429	3.6906	2.40438	1.81742	1.84071
ENSG00000123472	ATPAF1	3.11175	2.72945	1.0815	2.49113	2.44886	2.41449	1.72176	1.53773
ENSG00000233647	NENFP1	0.1573	0	0	0	0.38247	0.27749	0	0.57938
ENSG00000186118	TEX38	0	0	0	0	0	0	0	0.0604
ENSG00000228237	EFCAB14-AS1	0	0	0	0	0	0	0	0
ENSG00000159658	EFCAB14	25.1915	16.5396	5.80987	10.9565	22.1197	18.5172	10.2643	11.8851
ENSG00000236476	AL593856.1	0	0	0	0	0	0	0	0
ENSG00000142973	CYP4B1	0	0	0	0	0	0	0	0
ENSG00000154198	CYP4Z2P	0	0	0	0	0	0	0	0
ENSG00000238145	AL731892.1	0	0	0	0	0	0	0	0
ENSG00000187048	CYP4A11	0	0	0	0	0	0	0	0
ENSG00000259832	CYP4A26P	0	0	0	0	0	0	0	0
ENSG00000261196	CYP4A43P	0	0	0	0	0	0	0	0
ENSG00000261593	CYP4A27P	0	0	0	0	0	0	0	0
ENSG00000261290	CYP4A44P	0	0	0	0	0	0	0	0
ENSG00000186377	CYP4X1	0	0	0	0	0	0	0	0
ENSG00000186160	CYP4Z1	0	0	0	0	0	0	0	0
ENSG00000225154	AL450996.1	0	0	0	0	0	0	0	0
ENSG00000225506	CYP4A22-AS1	1.75093	1.05979	2.68992	7.44137	3.58015	4.49292	14.46	4.59268
ENSG00000162365	CYP4A22	0	0	0	0	0	0	0	0
ENSG00000225514	MTND1P34	0	0	0	0	0	0	0	0
ENSG00000224805	LINC00853	0.88714	1.29207	0.32591	0.82289	1.07857	0.39126	1.84842	0.95307
ENSG00000162366	PDZK1IP1	29.6158	14.5935	26.0671	22.317	25.1609	44.0631	42.9076	35.423
ENSG00000162367	TAL1	10.2492	9.48241	6.12269	6.75617	7.00426	8.03094	15.2404	8.68393
ENSG00000226252	AL135960.1	0.03632	0.06046	0.02669	0	0	0.03204	0.03027	0.0223
ENSG00000123473	STIL	14.5877	20.9177	19.0136	36.2719	28.6137	43.5946	46.955	30.2171
ENSG00000162368	CMPK1	69.315	47.61	25.4994	46.3556	53.8085	48.7106	41.7651	41.674
ENSG00000225762	LINC01389	0.45528	0	0.22301	0.22523	0.73803	0	0	0.55899
ENSG00000186790	FOXE3	0	0	0	0	0	0	0	0
ENSG00000237424	FOXD2-AS1	0.26293	0.29177	0.04293	0.04336	0.24863	0.2577	0.09739	0.17935
ENSG00000186564	FOXD2	0.01568	0.07829	0.02304	0	0.01906	0.1383	0.0784	0.0385
ENSG00000271355	AL356458.1	0	0	0	0	0	0	0	0
ENSG00000229554	RPL21P24	0	0	0	0	0	0	0	0
ENSG00000227883	ATP6V0E1P4	0	0	0	0	0	0	0	0
ENSG00000227947	LINC01738	0	0	0	0	0	0	0	0
ENSG00000269113	TRABD2B	0	0	0	0	0	0	0	0
ENSG00000223814	AL691459.1	0	0	0	0	0	0	0	0
ENSG00000225028	AC096541.1	0	0	0	0	0	0	0	0
ENSG00000226133	AL356289.1	0	0	0	0	0	0	0	0
ENSG00000231413	AL356289.2	0	0	0	0	0	0	0	0
ENSG00000237180	CYP46A4P	0	0	0	0	0	0	0	0
ENSG00000242267	SKINT1L	0	0	0.05359	0	0	0	0	0
ENSG00000279096	AL356289.3	0	0	0	0	0	0	0	0
ENSG00000223720	AL109659.1	0	0	0	0	0	0	0	0
ENSG00000117834	SLC5A9	0.11314	0.18833	0.0475	0.02399	0.05895	0.05703	0	0.05953
ENSG00000272491	AL109659.2	0.04379	0.14577	0	0.06499	0.05324	0	0.07299	0.05376
ENSG00000279214	AL109659.3	0	0	0	0	0	0	0	0.09473
ENSG00000132122	SPATA6	0.98738	0.69848	0.32646	0.40298	0.81029	0.26128	0.39088	0.30307
ENSG00000224986	PPP1R8P1	0.07021	0	0	0	0	0	0	0.0862
ENSG00000206700	RNU6-723P	0	0	0	0	0	0	0	0
ENSG00000235105	AL356968.2	0.93302	0.48532	2.05659	1.3847	1.06344	0.92586	2.8188	1.21714
ENSG00000223175	RNU4-61P	0	0	0	0	0	0	0	0
ENSG00000232514	AL356968.1	0	0	0	0	0	0	0	0
ENSG00000186094	AGBL4	0	0	0	0	0.02389	0	0	0
ENSG00000162373	BEND5	0.10285	0.11413	0.05038	0.05088	0.37514	0.30241	0.11429	0.08419

ENSG00000229846	AC099788.1	0	0	0	0	0	0	0	0
ENSG00000230114	AL590432.1	0	0	0	0	0	0	0	0
ENSG00000225623	AGBL4-IT1	0	0	0	0	0	0	0	0
ENSG00000237478	AL390023.1	0	0	0	0	0	0	0	0
ENSG00000215887	ZNF859P	0	0	0	0	0	0	0	0
ENSG00000279324	AL645730.1	0	0	0	0	0	0	0	0
ENSG00000233595	MTND2P29	0	0	0	0	0	0	0	0
ENSG00000162374	ELAVL4	0	0	0	0	0	0	0	0
ENSG00000230828	AL583843.1	0	0	0	0	0	0	0	0
ENSG00000237337	AL592182.2	0	0	0	0	0	0	0	0
ENSG00000233407	AL592182.1	0	0	0	0	0	0.51725	0	0
ENSG00000234080	AL596275.1	0	0	0	0	0	0	0	0
ENSG00000229316	HMGB1P45	0	0	0	0	0	0	0	0
ENSG00000225671	FCF1P6	0	0	0	0	0	0	0	0
ENSG00000142700	DMRTA2	0	0	0	0	0	0	0	0
ENSG00000185104	FAF1	5.88734	6.35511	6.8383	15.7303	11.3389	9.8205	11.5622	8.99374
ENSG00000225767	AL049637.1	0	0	0	0	0	0	0	0.17892
ENSG00000207194	RNU6-1026P	0	0	0	0	0	0	0	0
ENSG00000230585	PHBP12	0	0	0	0	0	0	0	0
ENSG00000237130	MRPS6P2	0	0	0	0	0	0	0	0
ENSG00000123080	CDKN2C	1.65688	1.58118	0.84405	1.63933	0.88633	2.68912	0.99423	0.94934
ENSG00000265538	MIR4421	0	0	0	0	0	0	0	0
ENSG00000275816	MIR6500	0	0	0	0	0	0	0	0
ENSG00000204006	C1orf185	0	0	0	0	0	0	0	0
ENSG00000200262	RF00019	0	0	0	0	0	0	0	0
ENSG00000213478	CFL1P2	0	0	0	0	0	0	0	0
ENSG00000203356	LINC01562	0.06196	0.10313	0	0	0.03767	0	0.15492	0
ENSG00000123091	RNF11	110.232	138.774	173.405	291.896	99.7068	76.5019	276.882	156.558
ENSG00000233406	AL162430.1	0	0	0	0	0	0	0	0
ENSG00000236434	AL162430.2	0	0	0	0	0	0	0	0
ENSG00000085831	TTC39A	0.17225	0.10426	0.12656	0.1162	0.14278	0.02762	0.48287	0.21149
ENSG00000261664	TTC39A-AS1	0	0	0	0	0	0	0	0
ENSG00000085832	EPS15	26.1674	24.4017	16.5914	33.1475	30.7117	32.6408	32.4993	25.475
ENSG00000232027	AL671986.1	0	0	0	0	0	0	0	0
ENSG00000206595	RNU6-877P	0	0	0	0	0	0	0	0
ENSG00000238140	AC104170.2	0	0	0	0	0	0	0	0
ENSG00000227070	AC104170.1	0.46198	0.25632	0.67887	0.68562	0.56166	0.27166	0.77004	0.56721
ENSG00000252032	RNU6-1281P	0	0	0	0	0	0	0	0
ENSG00000227742	CALR4P	0	0	0	0	0	0	0	0
ENSG00000117859	OSBPL9	7.6587	6.201	1.73057	3.39277	6.55999	4.96987	4.13127	3.77075
ENSG00000236973	GAPDHP51	0	0	0	0	0	0	0	0
ENSG00000232846	SLC25A6P3	0	0.27761	0	0	0	0	0	0.10239
ENSG00000212624	RF00568	0	0	0	0	0	0	0	0
ENSG00000078618	NRDC	24.7076	24.2828	7.33148	13.4757	23.0522	19.1113	14.702	17.6991
ENSG00000266993	AL050343.1	0.28208	0.2683	0	0.23922	0.04899	0	0.06717	0.09895
ENSG00000272175	AL050343.2	0.26179	0	0	0	0	0	0	0
ENSG00000283899	MIR761	0	0	0	0	0	0	0	0
ENSG00000230604	TSEN15P2	0	0	0	0	0	0	0	0
ENSG00000169213	RAB3B	0.00571	0	0	0	0.00694	0	0	0
ENSG00000200839	RNA5SP48	0	0	0	0	0	0	0	0
ENSG00000229032	AL445685.2	0	0	0	0	0	0	0	0
ENSG00000264613	RN7SL290P	0	0	0	0	0	0	0	0
ENSG00000117862	TXNDC12	15.1649	14.4192	2.64092	3.50632	12.4469	9.61823	4.34195	6.99151
ENSG00000198841	KTI12	2.22381	1.63722	0.43991	0.25388	1.87176	1.81067	0.92669	1.15515
ENSG00000223390	AL445685.1	0	0	0	0	0.11695	0	0	0
ENSG00000228369	TXNDC12-AS1	0	0	0	0	0	0	0	0
ENSG00000134717	BTF3L4	6.74334	4.4573	1.53826	2.98068	5.9786	4.93884	2.96219	3.76607
ENSG00000157077	ZFYVE9	0.60568	0.12602	0.07417	0.16854	0.38353	0.08904	0.23136	0.30985

ENSG00000241745	RN7SL788P	0	0	0	0	0	0	0	0
ENSG00000228407	AL139156.2	0	0	0	0	0	0	0	0
ENSG00000223429	AL139156.1	0	0	0	0	0	0	0	0
ENSG00000224255	PDCL3P6	0	0	0	0	0	0	0	0
ENSG00000236004	AL139156.3	0	0	0	0	0	0	0	0
ENSG00000237663	DNAJC19P7	0	0	0	0	0	0	0	0
ENSG00000213735	ANAPC10P1	0	0	0	0	0	0	0	0
ENSG00000154222	CC2D1B	4.04888	2.40924	0.53174	1.03053	2.6515	2.69148	1.58125	2.04129
ENSG00000272100	AL513218.1	0	0	0	0	0	0	0	0
ENSG00000224680	PLA2G12AP1	0.2581	0	0	0	0	0	0.21511	0
ENSG00000085840	ORC1	0.41793	0.46376	0.20471	0.86146	0.56457	3.11299	0.54182	0.17104
ENSG00000134748	PRPF38A	9.1593	6.29666	1.59915	2.76867	7.19801	7.61061	2.50493	4.51735
ENSG00000134744	TUT4	4.42544	3.3818	1.29664	3.06902	5.84795	4.75769	2.72283	3.22787
ENSG00000272371	AL591167.1	0	0	0.23621	0	0	0	0	0
ENSG00000236290	EEF1GP7	0	0	0	0	0	0	0	0
ENSG00000116157	GPX7	1.86394	1.01012	0.44589	1.35098	1.52832	3.28824	0.79479	1.11765
ENSG00000182183	SHISAL2A	1.40288	0.82701	0.17179	0.73738	1.88325	2.01083	0.43845	1.00475
ENSG00000162377	COA7	0.96795	0.84952	0.18103	0.49626	0.77028	0.83828	0.23468	0.36733
ENSG00000233664	NDUFS5P3	0	0	0	0	0	0	0	0
ENSG00000239640	RN7SL62P	0	0	0	0	0	0	0	0
ENSG00000162378	ZYG11B	7.913	7.54554	1.51099	2.91935	5.3483	3.15472	2.23555	4.72051
ENSG00000252018	RNU2-30P	0	0	0	0	0	0	0	0
ENSG00000228929	RPS13P2	0	0	0.7102	2.15181	0	0.2842	0.26853	0.59339
ENSG00000206627	RNU6-969P	0	0	0	0	0	0	0	0
ENSG00000203995	ZYG11A	0	0.02599	0.02295	0.02318	0	0	0	0
ENSG00000242391	AC099677.4	0	0	0	0	0	0	0	0
ENSG00000121310	ECHDC2	0.29138	0.18978	0.10239	0.13161	0.31574	0.16762	0.19005	0.20998
ENSG00000230953	AC099677.1	0	0	0	0	0	0	0	0
ENSG00000231866	AC099677.2	0	0	0	0	0	0	0	0
ENSG00000116171	SCP2	5.57806	5.39159	1.53043	3.3009	3.80932	4.53097	2.51569	3.73859
ENSG00000264834	MIR1273F	0.74041	0	0	0	0	0	0	0
ENSG00000266188	MIR5095	0	0	0	0	0	0	0	0
ENSG00000265815	MIR1273G	0	0	0	0	0	0	0	0
ENSG00000237279	AC099677.3	0	0	0	0	0	0	0	0
ENSG00000236360	AL445183.3	0	0	0	0	0	0.25759	0	0
ENSG00000226147	TUBBP10	0	0	0	0	0	0	0	0
ENSG00000174348	PODN	0.01496	0.0498	0.04396	0.0888	0	0.02639	0.02493	0.0551
ENSG00000232993	AL445183.1	0	0	0	0	0	0	0	0
ENSG00000227644	HIGD1AP11	0	0	0	0	0	0	0	0
ENSG00000162383	SLC1A7	0.04067	0.11282	0.03984	0.08048	0	0.04783	0.0226	0.09987
ENSG00000235563	AL445183.2	0	0	0	0	0	0	0	0
ENSG00000277397	AL606760.4	0	0	0	0	0	0	0	0
ENSG00000157184	CPT2	1.03092	0.98055	0.19581	0.36428	0.94642	0.72995	0.44421	0.48219
ENSG00000236723	AL606760.2	0.17922	0.19887	0.08779	0	0.07263	0.21078	0.09958	0.29339
ENSG00000162384	C1orf123	2.46676	1.9306	0.48012	0.99403	1.50944	2.16144	0.70798	1.10315
ENSG00000259818	AL606760.3	0.02041	0.10193	0	0	0	0	0	0.10025
ENSG00000162385	MAGOH	10.0206	8.75659	2.87139	6.24607	8.89339	7.86654	5.17784	6.09003
ENSG00000226754	AL606760.1	0	0.20662	0	0.18423	0.22638	0.21899	0	0.30482
ENSG00000157193	LRP8	0.7503	0.44008	0.084	0.2227	0.46044	0.55466	0.23821	0.24565
ENSG00000234578	AL355483.4	0	0	0	0	0	0	0	0
ENSG00000228838	AL355483.2	0	0	0	0	0	0	0	0
ENSG00000225030	AL355483.1	0.61857	0	0	0	0	0	0.51553	0
ENSG00000232762	AL355483.3	0	0	0	0	0	0	0	0
ENSG00000225675	LINC01771	0.38783	0.21518	0	0	0.15717	0	0	0
ENSG00000226938	AC119428.1	0	0	0	0	0	0	0	0
ENSG00000230138	AC119428.2	0.68989	0.43062	0.12672	0.12798	0.7339	0.30426	0	0.10588
ENSG00000236253	SLC25A3P1	0	0	0	0.03438	0	0	0	0
ENSG00000143006	DMRTB1	0	0	0	0	0	0	0	0

ENSG00000174332	GLIS1	0	0	0	0.03495	0	0	0	0
ENSG00000239007	RNU7-95P	0	0	0	0	0	0	0	0
ENSG00000058804	NDC1	4.15392	5.41732	4.447	10.6138	7.32371	9.26713	9.18434	6.5548
ENSG00000201003	RF00418	0	0	0	0	0	0	0	0
ENSG00000229687	AL049745.1	0	0	0	0	0	0	0	0
ENSG00000058799	YIPF1	11.8512	15.5455	2.23538	3.99021	10.5374	6.17851	3.30217	7.16675
ENSG00000211452	DIO1	0	0	0	0	0	0	0	0
ENSG00000081870	HSPB11	4.68887	4.2982	2.6296	4.97537	5.0672	5.9541	2.22764	3.17048
ENSG00000116212	LRRC42	1.15529	1.16391	0.26806	0.54145	1.03495	1.60902	1.46961	0.4106
ENSG00000219102	HNRNPA3P12	0	0	0	0	0	0	0	0
ENSG00000203985	LDLRAD1	0	0	0	0	0	0	0	0
ENSG00000116209	TMEM59	11.3368	11.1753	5.16848	9.38156	9.54578	7.67475	10.0159	10.732
ENSG00000280378	AL353898.3	0.58473	0.06952	0	0	0.15234	0.07368	0	0.10256
ENSG00000116205	TCEANC2	1.48228	1.31386	0.22136	0.54549	1.46513	1.15865	0.46201	0.61404
ENSG00000283749	MIR4781	0	0	0	0	1.17259	0	1.60764	0
ENSG00000225183	AL353898.1	0	0	0	0	0.37444	0	0	0
ENSG00000279049	AL353898.2	0	0	0	0	0	0	0	0
ENSG00000256407	AL357673.1	0	0	0	0	0	0	0	0
ENSG00000157211	CDCP2	0	0	0	0	0	0	0	0
ENSG00000280425	AL357673.2	0	0	0	0	0	0	0	0
ENSG00000215883	CYB5RL	0.15603	0.10935	0.02413	0.03656	0.12979	0.24628	0.02738	0.03025
ENSG00000116221	MRPL37	6.55648	5.02585	1.98609	2.00586	6.15321	10.0953	2.54043	1.97718
ENSG00000157216	SSBP3	9.42373	7.77288	2.59651	6.16043	8.45645	14.7198	3.10885	3.23693
ENSG00000198711	SSBP3-AS1	0.05587	0	0	0	0	0	0	0.0686
ENSG00000225632	AL161644.1	0.06598	0	0	0	0.24064	0.11639	0.21995	0.16201
ENSG00000232245	AL035415.1	0	0	0	0	0	0	0	0
ENSG00000235741	AC099796.1	0	0	0	0	0	0	0	0
ENSG00000237173	AC099796.2	0	0	0	0	0	0	0	0
ENSG00000227453	HNRNPA1P63	0	0	0	0	0	0	0	0
ENSG00000162390	ACOT11	0.14258	0.03747	0.02205	0.01114	0.03649	0.01324	0.02502	0.06449
ENSG00000162391	FAM151A	0	0	0	0	0	0	0	0
ENSG00000230728	AL590093.1	0	0	0	0	0	0	0	0
ENSG00000184313	MROH7	0.00807	0	0	0	0.00981	0	0	0
ENSG00000271723	MROH7-TTC4	0	0	0	0	0	0	0	0
ENSG00000243725	TTC4	0.68583	0.4843	0.06108	0.09253	0.68221	0.54995	0.24249	0.28068
ENSG00000162396	PARS2	0.24985	0.36389	0.13768	0.27811	0.30376	0.33058	0.10412	0.34511
ENSG00000006555	TTC22	0.54476	0.7254	0.21347	0.35933	0.67702	0.83289	0.04036	0.40131
ENSG00000237453	AC096536.1	0	0	0	0	0	0	0	0
ENSG00000162398	LEXM	0.20625	0.3433	0.06062	0.42853	0.4012	0.21831	0.20627	0.10129
ENSG00000116133	DHCR24	0.51741	0.94736	0.17741	0.12798	0.69196	2.34283	0.14374	0.38116
ENSG00000242396	AC096536.2	0	0	0	0	0	0	0	0
ENSG00000233203	DHCR24-DT	0	0	0	0	0	0	0	0
ENSG00000143001	TMEM61	0	0	0	0	0	0	0	0
ENSG00000233271	AL590440.1	0	0	0	0	0	0	0	0
ENSG00000162399	BSND	0	0	0	0	0	0	0	0
ENSG00000169174	PCSK9	0	0	0	0	0	0	0	0
ENSG00000162402	USP24	12.7522	10.043	3.81556	8.25226	11.9307	12.9729	8.42766	8.28719
ENSG00000231090	MIR4422HG	0.11489	0	0.33766	0.17051	0	0	0	0.28212
ENSG00000231095	GYG1P3	0	0	0	0	0	0	0	0
ENSG00000265822	MIR4422	0	0	0	0	0	0	0	0
ENSG00000231900	GOT2P1	0	0	0	0	0	0	0	0
ENSG00000232198	MTCO2P34	0	0	0	0	0	0	0	0
ENSG00000199831	RN7SKP291	0	0	0	0.33268	0	0.39545	0	0
ENSG00000252162	RNU6-830P	0	0	0	0	0	0	0	0
ENSG00000272051	RF00019	0	0	0	0	0	0	0	0
ENSG00000234810	AL603840.1	0.05968	0	0.0877	0.155	0.12697	0.05264	0.19895	0.31141
ENSG00000230250	LINC01753	0	0	0	0	0	0	0	0
ENSG00000227104	PIGQP1	0	0	0	0	0	0	0	0

ENSG00000233079	LINC01755	0	0	0	0	0	0	0	0
ENSG00000235612	AL353681.1	0	0	0	0	0	0	0	0
ENSG00000229871	RPSAP20	0	0	0	0	0	0	0	0
ENSG00000260971	AC119674.1	0	0	0	0	0	0	0	0
ENSG00000223956	LINC01767	0	0	0	0	0	0	0	0
ENSG00000162407	PLPP3	0.05547	0.06924	0.06113	0	0.18545	0.09785	0.06934	0.01703
ENSG00000236776	RPL21P23	0	0	0	0	0	0	0	0
ENSG00000224980	RPL23AP85	0	0	0	0	0	0	0	0
ENSG00000225475	AC099789.1	0.51986	0	0	0	0	0	0	0
ENSG00000162409	PRKAA2	0	0	0	0	0.00953	0	0	0
ENSG00000187889	FYB2	0	0	0	0	0	0	0	0
ENSG00000229913	AL360295.1	0	0	0	0	0	0	0	0
ENSG00000157131	C8A	0	0	0	0	0	0	0	0
ENSG00000021852	C8B	0	0	0	0	0	0	0	0
ENSG00000236341	AL161740.1	0	0	0	0	0	0	0	0
ENSG00000173406	DAB1	0.06798	0	0	0.04324	0.01181	0.01713	0.01619	0.03577
ENSG00000227935	AL390243.1	0	0	0	0	0	0	0	0
ENSG00000236888	RPS20P5	0	0	0	0	0	0	0	0
ENSG00000226759	DAB1-AS1	0	0	0	0	0	0	0	0
ENSG00000229887	HNRNPA1P6	0.05066	0	0	0.07518	0	0	0	0
ENSG00000223416	RPS26P15	0	0	0	0	0	0	0	0
ENSG00000235038	AL445193.2	0	0	0	0	0	0	0	0
ENSG00000230546	AL445193.1	0	0	0	0	0	0	0	0
ENSG00000233216	AL357373.1	0	0	0	0	0	0	0	0
ENSG00000162600	OMA1	15.8706	14.6686	9.58938	18.4626	14.2376	12.2078	14.3935	12.7806
ENSG00000270209	AL035411.2	0	0	0	0	0	0	0	0
ENSG00000264128	RN7SL713P	0	0	0	0	0	0	0	0
ENSG00000184292	TACSTD2	0.09354	0.93414	0.04582	0	1.02346	0.055	0	0.07656
ENSG00000185839	AL035411.1	0	0	0	0	0.38746	0	0	0
ENSG00000162601	MYSM1	6.28195	5.96528	2.8923	4.68589	7.15885	6.42363	4.93478	5.82997
ENSG00000283445	AL136985.3	0.01547	0.12873	0	0	0	0	0	0
ENSG00000177606	JUN	8.22041	2.44706	0.36513	0.3073	5.23623	0.94975	0.62126	1.04234
ENSG00000234807	LINC01135	0	0.1225	0	0.21845	0.3579	0	0	0
ENSG00000272226	AL136985.2	0.13701	0	0	0	0	0	0	0
ENSG00000231740	AL136985.1	0	0	0	0	0	0	0	0
ENSG00000232453	AC105277.1	0.25189	0.20964	0	0.06231	0.05104	0.07406	0.13995	0.05154
ENSG00000237352	LINC01358	0.01089	0	0.01601	0.01616	0.01324	0.01921	0	0.02675
ENSG00000228121	PHBP3	0	0	0	0	0	0	0	0
ENSG00000241042	AL592431.2	0	0	0	0	0	0	0	0
ENSG00000235215	AL592431.1	0	0	0	0	0	0	0	0
ENSG00000224609	HSD52	0.02775	0	0	0.08238	0	0	0	0
ENSG00000270457	AC093424.1	0	0.36097	0	0	0	0	0	0.26626
ENSG00000172456	FGGY	1.84244	2.19483	0.57511	3.17225	1.82274	1.32775	1.40506	0.97582
ENSG00000266150	MIR4711	0	0	0	0	0	0	0	0
ENSG00000226883	AL035416.1	0	0	0	0	0	0	0	0
ENSG00000134709	HOOK1	1.2148	0.55967	0.81288	1.89948	2.18902	1.10981	1.35595	1.22517
ENSG00000134716	CYP2J2	0.02452	0	0.03602	0.14553	0.14902	0.12974	0.04086	0
ENSG00000265535	RN7SL475P	0	0	0	0	0	0	0	0
ENSG00000162598	C1orf87	0.08288	0	0	0	0	0	0.02763	0.02035
ENSG00000229332	PGBD4P8	0	0	0	0	0	0	0	0
ENSG00000238242	AC097066.1	0	0	0	0	0	0	0	0
ENSG00000226476	LINC01748	0.04831	0	0	0.01024	0.00839	0	0.0115	0.00847
ENSG00000231252	AC099792.1	0	0.02847	0	0	0	0	0	0
ENSG00000162599	NFIA	2.21142	2.49134	2.42733	6.39386	2.93449	3.47305	3.75352	2.73998
ENSG00000237928	NFIA-AS2	0.07954	0	0	0	0	0	0	0
ENSG00000270742	AC096947.1	0	0	0.45836	0	0	0	0.51992	0
ENSG00000237853	NFIA-AS1	0	0	0	0	0	0	0	0
ENSG00000223920	AC099791.1	0	0	0	0	0	0	0	0

ENSG00000162604	TM2D1	3.08297	2.74276	1.50362	3.98381	3.3443	2.60218	3.69908	3.39365
ENSG00000271200	AC099791.2	0.17166	0	0	0	0.2087	0	0	0
ENSG00000132849	PATJ	1.8654	0.68885	0.35399	0.82504	1.93748	1.02429	0.96781	0.93281
ENSG00000200575	RNU6-414P	0	0	0	0	0	0	0	0
ENSG00000212360	RNU6-1177P	0	0	0	0	0	0	0	0
ENSG00000236646	LAMTOR5P1	0.27148	0	0	0.40291	0	0	0	0
ENSG00000242860	RN7SL180P	0	0	0	0	0	0	0.41558	0
ENSG00000263908	MIR3116-1	0	0	0	0	0	0	0	0
ENSG00000283690	MIR3116-2	0	0	0	0	0	0	0	0
ENSG00000234204	PIGPP2	0	0	0	0	0	0	0	0
ENSG00000234088	RPS15AP7	0	0	0	0	0	0	0	0
ENSG00000240563	L1TD1	0.3047	0.22189	0.02798	0.16958	0.13892	0.10079	0.09523	0.28058
ENSG00000237227	AL162739.1	0	0	0	0	0	0	0	0
ENSG00000200174	RF00019	0	0	0	0	0	0	0	0
ENSG00000132854	KANK4	0	0	0	0	0	0	0	0
ENSG00000201153	RNU6-371P	0	0	0	0	0	0	0	0
ENSG00000162607	USP1	22.5249	23.3256	21.376	33.8632	32.2559	28.5742	36.4277	36.8602
ENSG00000116641	DOCK7	0.35955	0.49055	0.41575	0.51611	0.7596	0.5667	0.49124	0.47401
ENSG00000270549	AL451044.1	0	0	0	0	0	0	0	0
ENSG00000132855	ANGPTL3	0	0	0	0	0	0	0	0
ENSG00000278967	AL138847.2	0	0	0	0	0	0	0	0
ENSG00000213703	AL138847.1	0.13425	0	0	0	0	0	0	0
ENSG00000235545	AC103923.1	0	0	0	0	0	0	0	0
ENSG00000125703	ATG4C	6.24585	3.9231	3.35957	8.01027	6.79121	3.99162	6.52152	6.0191
ENSG00000234318	AC099794.1	0	0	0	0	0	0	0	0
ENSG00000237163	AC099794.2	0	0	0	0	0	0	0	0
ENSG00000229537	LINC01739	0	0	0	0	0	0	0	0
ENSG00000227485	AL162400.1	0	0	0	0	0	0	0	0
ENSG00000229225	AL162400.2	0	0	0	0	0	0	0	0
ENSG00000203605	AC096543.1	0	0	0	0	0	0	0	0
ENSG00000224209	LINC00466	0	0	0	0	0	0	0	0
ENSG00000252259	RNA5SP49	0	0	0	0	0	0	0	0
ENSG00000228734	AC096543.2	0	0	0	0	0	0	0	0
ENSG00000223683	RPSAP65	0	0	0	0	0	0	0	0
ENSG00000230798	FOXD3-AS1	0	0	0	0	0	0	0	0
ENSG00000187140	FOXD3	0	0	0	0	0	0	0	0
ENSG00000275836	MIR6068	0	0	0	0	0	0	0	0
ENSG00000252784	RF00019	0	0	0	0	0	0	0	0
ENSG00000236674	AL049636.1	0	0	0	0	0	0	0	0
ENSG00000088035	ALG6	3.02086	3.22891	3.41638	8.32925	3.40264	3.18708	3.50497	4.69076
ENSG00000142856	ITGB3BP	5.41125	10.6833	8.70157	17.4966	16.1125	16.5578	24.7763	25.3225
ENSG00000203965	EFCAB7	0.82244	0.57753	0.20772	0.57215	1.0624	0.95216	0.49266	0.568
ENSG00000264271	RN7SL488P	0	0	0	0	0	0	0	0
ENSG00000251720	RNU7-123P	0	0	0	0	0	0	0	0
ENSG00000116652	DLEU2L	0.10309	0.20592	0.1515	0.21421	0.12534	0.07275	0.06874	0.12658
ENSG00000276884	RF02110	0	0	0	0	0	0	0	0
ENSG00000278287	RF02109	0	0	0	0	0	0	0	0
ENSG00000277565	RF02108	0	0	0	0	0	0	0	0
ENSG00000275823	RF02107	0	0	0	0	0	0	0	0
ENSG00000277689	RF02106	0	0	0	0	0	0	0	0
ENSG00000274199	RF02105	0	0	0	0	0	0	0	0
ENSG00000079739	PGM1	3.8532	5.10109	0.73741	1.06392	4.18347	2.97196	1.49365	2.22242
ENSG00000244256	RN7SL130P	0	0	0	0	0.58437	0.84795	0	0.59014
ENSG00000185483	ROR1	0.03275	0	0	0.0486	0.15926	0	0.05459	0.04021
ENSG00000177452	AL161742.1	0	0	0	0	0	0	0	0
ENSG00000228056	CFL1P3	0	0	0	0	0	0	0	0
ENSG00000207190	RNU6-809P	0	0	0	0	0	0	0	0
ENSG00000200508	RF00019	0	0	0	0	0	0	0	0

ENSG00000223949	ROR1-AS1	0	0	0	0	0	0	0	0
ENSG00000177414	UBE2U	0	0	0	0	0	0	0	0
ENSG00000238653	RNU7-62P	0	0	0	0	0	0	0	0
ENSG00000158966	CACHD1	0.28881	0.03846	0.01698	0.17145	0.40731	0.3057	0.50066	0.28368
ENSG00000264470	MIR4794	0	0	0	0	0	0	0	0
ENSG00000162437	RAVER2	0.88154	0.64321	0.33716	0.89609	0.73407	3.94113	0.58373	0.74133
ENSG00000162434	JAK1	112.811	86.1418	15.1587	37.232	89.6386	55.741	39.1666	50.0348
ENSG00000234784	AL606517.2	0	0	0	0	0	0	0	0
ENSG00000233877	AL606517.1	0	0	0	0	0	0	0	0
ENSG00000226891	LINC01359	0.23263	0.22126	0.14651	0.19728	0.17508	0.15634	0.59087	0.42163
ENSG00000185031	SLC2A3P2	0.19811	0.16488	0	0	0.12043	0.08737	0.08255	0.30404
ENSG00000272506	AL357078.3	0	0.39485	0	0	0.1442	0.20924	0.1977	0.43688
ENSG00000212257	RNU6-1176P	0.68505	1.14027	1.00667	0	1.66573	0	0	1.68219
ENSG00000265996	MIR3671	13.3273	8.31876	1.22402	0	3.03807	1.46945	6.94206	9.20424
ENSG00000199135	MIR101-1	1.95468	1.62678	1.43619	1.45048	1.18822	3.44831	1.62907	3.59988
ENSG00000231485	AL357078.1	0.94581	0.78715	0	1.40369	0.57495	0.41713	0	2.03219
ENSG00000235804	AL357078.2	0	0	0	0	0	0	0	0
ENSG00000234496	MRPS21P1	0	0	0	0	0	0	0	0
ENSG00000162433	AK4	0.12575	0.0644	0	0	0.08232	0.08532	0.06449	0.07126
ENSG00000231622	RPS29P7	0	0	0	0	0	0	0	0
ENSG00000116675	DNAJC6	40.3394	62.5658	98.5906	217.243	75.8786	55.9719	117.12	121.911
ENSG00000229294	AL139294.1	0.28633	0	0.63114	1.69978	0	1.01025	0.95453	0.17578
ENSG00000234144	COX6CP13	0	0	0	0	0	0	0	0
ENSG00000222624	RNU2-15P	0	0	0	0	0	0	0	0
ENSG00000213625	LEPROT	17.7757	13.2562	6.29377	12.2973	11.146	9.5064	9.79869	10.1734
ENSG00000116678	LEPR	1.66328	3.06899	1.72418	5.05179	3.13515	2.195	2.90138	4.4959
ENSG00000237852	AC119800.1	1.90616	3.74967	2.80107	7.45812	3.58152	3.97412	6.64337	4.25518
ENSG00000224570	AC097063.1	0	0	0	0	0	0	0	0
ENSG00000239319	RN7SL854P	0	0	0.36513	0	0	0	0	0
ENSG00000184588	PDE4B	24.1593	24.4186	4.23678	9.69054	14.3304	13.4937	11.6753	12.702
ENSG00000227466	AL590783.1	0	0	0	0	0	0	0	0
ENSG00000223152	RNU4-88P	0	0	0	0	0	0	0	0
ENSG00000118473	SGIP1	0.83256	0.88505	0.29815	0.4984	0.62093	0.78992	1.46938	0.91054
ENSG00000264720	MIR3117	0	0	0	0	0	0	0	0
ENSG00000248458	AL139147.1	0	0	0	0	0	0	0	0
ENSG00000152760	TCTEX1D1	0.11823	0	0.06949	0	0.08624	0.04171	0	0.02903
ENSG00000172410	INSL5	0	0	0	0	0	0	0	0
ENSG00000152763	WDR78	1.77128	3.05749	3.29376	7.98368	3.4429	3.99277	9.20362	5.49057
ENSG00000231080	AL592161.1	0	0	0	0	0	0	0	0
ENSG00000198160	MIER1	20.6708	26.5755	11.4966	19.4744	25.3788	21.1482	18.505	21.8221
ENSG00000116704	SLC35D1	3.10474	1.98001	0.64677	1.76543	2.68999	3.04288	1.60607	1.76723
ENSG00000203963	C1orf141	0	0	0	0.04332	0	0	0	0.01792
ENSG00000252433	RF00322	0	0	0	0	0	0	0	0
ENSG00000275678	AL133320.1	0	0	0	0	0	0	0	0
ENSG00000162594	IL23R	0.40386	0.14938	0.19782	0.29969	0.35461	0.03958	0.0748	0.22038
ENSG00000253074	RNU6-586P	0	0	0	0	0	0	0	0
ENSG00000252116	RNU4ATAC4P	0	0	0	0	0	0	0	0
ENSG00000228523	AL389925.1	0.10069	0	0	0	0	0	0	0
ENSG00000081985	IL12RB2	1.22946	0.7494	0.22902	0.66819	0.82106	2.26059	0.51955	1.0843
ENSG00000142864	SERBP1	25.468	18.9625	5.41657	11.3236	21.9773	26.1959	8.43611	11.3376
ENSG00000223263	RNU6-387P	0	0	0	0	0	0	0	0
ENSG00000235200	LINC01702	0	0	0	0	0	0	0	0
ENSG00000207504	RNU6-1031P	0	0	0	0	0	0	0	0
ENSG00000238139	AL590559.1	0	0	0	0	0	0	0	0
ENSG00000242482	RN7SL392P	0	0	0	0	0	0	0	0
ENSG00000232048	HNRNPCP9	0	0	0	0	0	0	0	0.10477
ENSG00000116717	GADD45A	6.05817	6.54565	2.96747	4.46921	5.34097	6.31247	7.14539	4.21929
ENSG00000172380	GNG12	0.03103	0.02583	0.0228	0	0.03773	0.02737	0	0

ENSG00000238778	RNU7-80P	0	0	0	0	0	0	0	0
ENSG00000232284	GNG12-AS1	0	0	0	0	0	0	0	0
ENSG00000235055	AL157407.1	0	0	0	0	0	0	0	0
ENSG00000162595	DIRAS3	0	0	0	0	0	0	0	0
ENSG00000228431	ARL5AP3	0	0	0	0	0	0	0	0
ENSG00000116729	WLS	6.42957	8.76364	0.43384	0.90066	3.84863	0.05787	1.23028	6.26294
ENSG00000234383	CTBP2P8	0.27557	0.27521	0	0.08179	0.20102	0	0	0.27067
ENSG00000221203	MIR1262	0	1.31192	0	0	2.87473	0	0	0
ENSG00000229133	RPS7P4	0	0	0	0	0	0	0	0
ENSG00000225242	COX6B1P7	0	0	0	0	0	0	0	0
ENSG00000237622	ELOCP18	0	0	0	0	0	0	0	0
ENSG00000229440	AL139413.1	0	0	0	0	0	0	0	0
ENSG00000116745	RPE65	0	0	0	0	0	0	0	0
ENSG00000024526	DEPDC1	1.84989	2.45918	2.77312	7.46244	3.09433	3.6796	4.05612	2.92673
ENSG00000233589	AL138789.1	0	0	0	0	0.09283	0	0	0
ENSG00000234264	DEPDC1-AS1	0	0	0	0.08724	0	0	0	0.07217
ENSG00000225167	TXNP2	0	0	0	0	0	0	0	0
ENSG00000223883	LINC01707	0	0	0	0	0	0	0	0
ENSG00000235446	AL512271.1	0	0	0	0	0	0	0	0
ENSG00000229639	LINC01758	0	0	0	0	0	0	0	0
ENSG00000033122	LRRC7	0.03002	0	0.00735	0.03712	0.03649	0.01765	0.00834	0.02457
ENSG00000270615	SGO1P1	0	0	0	0	0	0	0	0
ENSG00000240692	RN7SL538P	0	0	0	0	0	0	0	0
ENSG00000229359	PIN1P1	0	0	0	0	0	0	0	0
ENSG00000237919	AL158840.1	0	0	0	0	0	0	0	0
ENSG00000066557	LRRC40	6.5828	6.76885	6.61074	13.8057	8.7139	7.66724	9.87102	8.33191
ENSG00000244389	RN7SL242P	0.2468	0.4108	0	0	0.30006	0.43539	0.41138	0
ENSG00000116754	SRSF11	10.0352	5.52948	2.33912	5.36675	7.54121	8.31759	5.17677	5.73568
ENSG00000228988	AL353771.1	0	0	0	0	0	0	0	0
ENSG00000118454	ANKRD13C	10.9751	11.0989	11.2519	22.8699	19.3749	18.0389	36.9333	20.0755
ENSG00000197568	HHLA3	0.3599	0.09984	0	0.17805	0.58342	0.42328	0.09998	0
ENSG00000226088	AL158839.1	0	0	0	0	0	0	0	0
ENSG00000116761	CTH	0.48583	1.87006	0.66931	0.6309	0.40608	1.44632	0.10123	0.33553
ENSG00000278636	RF00017	0	0	0	0	0	0	0	0
ENSG00000271992	AL354872.2	0	0	0	0	0	0	0	0
ENSG00000233020	AL354872.1	0	0	0	0	0	0	0	0
ENSG00000237033	CASP3P1	0	0	0	0	0	0	0	0
ENSG00000229051	LINC01788	0	0	0	0	0	0	0	0
ENSG00000226208	AL513285.1	0	0	0	0	0	0	0	0
ENSG00000050628	PTGER3	0.25059	0.08848	0.07811	0.10143	0.24003	0.06698	0.07594	0.11188
ENSG00000235782	AL031429.1	0.78291	0.65158	0.14381	0.14524	0.23796	0.34529	0.32625	0
ENSG00000269933	AL031429.2	0	0	0	0	0	0.19925	0	0
ENSG00000235079	ZRANB2-AS1	0	0	0	0	0	0	0	0
ENSG00000132485	ZRANB2	11.7298	6.42664	2.28674	5.62121	10.0308	10.7738	4.72277	6.56097
ENSG00000207721	MIR186	2.55699	0	0	0	0	4.51088	4.2621	1.04648
ENSG00000229956	ZRANB2-AS2	0.0197	0.0164	0	0.01462	0.02395	0.01738	0.04926	0.03628
ENSG00000226324	AL358453.1	0	0	0	0	0	0	0	0
ENSG00000172260	NEGR1	0.27339	0.10921	0.0241	0.04869	0.1396	0	0.05468	0.03357
ENSG00000231985	AL354949.1	0	0	0	0	0	0	0	0
ENSG00000271618	AL359821.1	0	0	0	0	0	0	0	0
ENSG00000228853	NEGR1-IT1	0	0	0	0	0	0	0	0
ENSG00000233994	GDI2P2	0.24112	0	0.17716	0.17892	0	0.21268	0.40191	0
ENSG00000227207	RPL31P12	0	0	0	0	0	0	0	0
ENSG00000212366	RNU6-1246P	0	0	0	0	0	0	0	0
ENSG00000225087	AL583808.1	0	0	0	0	0	0	0	0
ENSG00000227016	AL583808.2	0	0	0	0	0	0	0	0
ENSG00000280317	AL732618.1	0	0	0	0	0	0	0	0
ENSG00000229636	KRT8P21	0	0	0	0	0	0	0	0

ENSG00000251825	RN7SKP19	0	0	0	0	0	0	0	0
ENSG00000233973	LINC01360	0	0	0	0	0	0	0	0
ENSG00000223479	LINC02238	0	0	0	0	0	0	0	0
ENSG00000200601	RNA5SP50	0	0	0	0	0	0	0	0
ENSG00000252005	RNU4ATAC8P	0	0	0	0	0	0	0	0
ENSG00000162620	LRRIQ3	0.02511	0.02786	0.0123	0.14904	0.19331	0.1624	0.06975	0.0822
ENSG00000254685	FPGT	1.77113	2.59779	1.26268	2.31625	2.11066	1.48492	1.98762	2.43293
ENSG00000259030	FPGT-TNNI3K	0.06266	0	0.01151	0.01162	0.02857	0.02764	0.01306	0.00962
ENSG00000116783	TNNI3K	0	0	0.02283	0.02305	0	0	0.02589	0
ENSG00000237324	AC093158.1	0	0	0	0	0	0	0	0
ENSG00000233894	AC105271.1	0	0	0	0	0	0	0	0
ENSG00000162621	LRRC53	0	0	0	0	0	0	0	0
ENSG00000178965	ERICH3	0.02505	0.0278	0.01227	0	0.01015	0.08838	0	0
ENSG00000234497	ERICH3-AS1	0	0	0	0	0	0	0	0
ENSG00000272864	AC135803.1	0	0	0	0.19254	0	0	0	0
ENSG00000116791	CRYZ	1.05868	0.98563	0.68557	1.9174	1.24349	1.7727	0.41873	0.81515
ENSG00000162623	TYW3	3.00125	2.69603	0.72338	1.53186	3.49439	2.99748	0.847	1.7937
ENSG00000229943	AC133865.1	0	0	0	0	0	0	0	0
ENSG00000261213	AC099786.3	0	0	0	0	0	0	0	0
ENSG00000224127	AC099786.1	0	0	0	0	0	0	0	0
ENSG00000162624	LHX8	0	0	0	0	0	0	0	0
ENSG00000224149	AC099786.2	0	0	0	0	0	0	0	0
ENSG00000206999	RNU6-622P	0	0	0	0	0	0	0	0
ENSG00000137968	SLC44A5	0.11637	0.03874	0	0	0.12733	0	0.05819	0
ENSG00000224493	AC093156.1	0	0	0	0	0	0	0	0
ENSG00000252338	RNU6-503P	0	0	0	0	0	0	0	0
ENSG00000213579	AL096829.1	0	0	0	0	0	0	0	0
ENSG00000230863	AL137803.1	0	0	0	0	0	0	0	0
ENSG00000178193		0.36377	0	0	0.13497	0.11057	0	0	0
ENSG00000117054	ACADM	9.11848	9.18316	6.19305	15.8072	13.5454	10.1114	8.09767	9.03169
ENSG00000181227	DLSTP1	0.05414	0	0	0	0	0	0	0
ENSG00000137955	RABGGTB	7.62383	5.30292	1.96738	4.74661	6.54095	6.7138	4.83514	3.59197
ENSG00000206620	SNORD45C	0	0	0	0	0	0	0	0
ENSG00000207241	SNORD45A	0	0	0	0	0	0	0	0
ENSG00000201487	SNORD45B	0	0	0	0	0	0	0	0
ENSG00000057468	MSH4	0.85285	1.046	0.39576	2.53145	3.90192	1.42536	3.47911	2.56268
ENSG00000154007	ASB17	0	0	0	0	0	0	0	0
ENSG00000225605	AC092813.1	0	0	0	0	0	0	0	0.08028
ENSG00000230027	AC092813.2	0	0	0.53992	0.54529	1.7868	0	0	0
ENSG00000184005	ST6GALNAC3	0.64823	0.80923	0.23814	0.25554	0.45562	0.37523	0.21948	0.78345
ENSG00000223905	AC103592.1	0	0	0	0	0	0	0	0
ENSG00000272855	AC104458.1	0	0.26352	0	0	0	0	0.26389	0
ENSG00000226415	TPI1P1	0	0	0	0	0	0	0	0
ENSG00000199921	RNU6-161P	0	0	0	0	0	0	0	0
ENSG00000237552	LINC02567	0	0	0	0	0	0	0	0
ENSG00000117069	ST6GALNAC5	0	0	0	0	0	0	0.02866	0
ENSG00000276081	MIR7156	0	0	0	0	0	0	0	0
ENSG00000230498	AL035409.1	0	0	0	0	0	0	0	0
ENSG00000142892	PIGK	6.63466	4.97949	2.53996	7.9325	6.25579	5.60593	5.03082	5.90948
ENSG00000226084	AC113935.1	0.92451	0.65951	0.77632	1.56808	0.64228	0.46599	0.44029	1.1351
ENSG00000228187	AC093433.1	0	0	0	0	0	0	0	0
ENSG00000154027	AK5	0.76277	0.12344	0.09341	0.28301	1.36528	0.50461	0.72401	0.40322
ENSG00000233099	AC095030.1	0	0.64726	0.57143	0	0	0	0.32409	0.23872
ENSG00000251767	RNU7-8P	0	0	0	0	0	0	0	0
ENSG00000036549	AC118549.1	5.29735	3.84663	2.02083	3.79204	5.95562	4.85206	2.91616	3.70658
ENSG00000251785	RNA5SP20	0	0	0	0	0	0	0	0
ENSG00000243437	RN7SL370P	0	0	0	0	0	0	0	0
ENSG00000077254	USP33	23.1832	29.0471	21.3622	46.263	33.2738	32.8233	31.2074	32.5358

ENSG00000232672	ACTG1P21	0	0	0	0	0	0	0	0
ENSG00000180488	MIGA1	2.75048	2.04746	1.22377	3.20892	3.3161	2.22394	2.78898	2.29824
ENSG00000222849	RNA5SP21	0	0	0	0	0	0	0	0
ENSG00000219201	AC138392.1	0.07959	0.26495	0.70172	0.94494	0.19352	0.56161	1.32661	0.5863
ENSG00000235613	NSRP1P1	0.38967	0.6486	0	0.06426	0.42111	0.45828	0.2165	0.05316
ENSG00000232015	HSPE1P25	0	0.97218	0	0.86682	0	0	0	0
ENSG00000235927	NEXN-AS1	0.06396	0.10646	0	0.04746	0.07776	0	0.05331	0
ENSG00000162614	NEXN	7.51841	4.15313	1.3355	1.27522	3.67636	1.89478	1.45977	2.57656
ENSG00000162613	FUBP1	19.9357	14.2551	3.93519	8.27509	17.3641	18.9882	10.6956	8.94252
ENSG00000162616	DNAJB4	4.78593	5.77307	5.837	11.8764	8.7397	3.21314	9.78605	7.99339
ENSG00000137960	GIPC2	0.05535	0.09213	0.16267	0.10953	0.15701	0	0.21527	0.20387
ENSG00000273338	AC103591.3	6.66367	7.00527	2.06151	3.64354	9.8071	4.33102	2.92297	2.15304
ENSG00000213561	AC103591.2	0	0	0	0	0	0	0	0
ENSG00000251958	RNU6-1102P	0	0	0	0	0	0	0	0
ENSG00000213560	AC103591.1	0	0	0	0	0	0	0	0
ENSG00000202263	RNA5SP22	0	0	0	0	0	0	0	0
ENSG00000233333	RNFT1P2	0	0	0	0	0	0	0	0
ENSG00000237413	MGC27382	0	0	0	0	0	0	0	0.03627
ENSG00000122420	PTGFR	0	0.05176	0.0457	0.02308	0	0	0	0.01909
ENSG00000227556	AC096531.1	0	0	0	0	0	0	0	0
ENSG00000212308	RNA5SP23	0	0	0	0	0	0	0	0.85711
ENSG00000137959	IFI44L	3.53617	25.903	18.1347	2.2947	1.44466	5.68265	1.09771	3.22548
ENSG00000137965	IFI44	18.451	66.5719	34.4072	6.93923	7.56486	16.0529	5.21574	13.4245
ENSG00000238015	AC104837.2	0	0.86327	0.50808	0.76971	0	0	0	0
ENSG00000235400	AC104837.1	0.04619	0	0	0	0	0	0	0
ENSG00000162618	ADGRL4	0	0.06636	0	0	0	0	0	0.04895
ENSG00000230787	PSAT1P3	0	0	0	0	0	0	0	0
ENSG00000282898	AL353651.2	0	0	0	0	0	0	0	0
ENSG00000235011	AL353651.1	0	0	0	0	0	0	0	0
ENSG00000232676	ADH5P2	0	0	0	0	0	0	0	0
ENSG00000235756	AC098657.1	0	0	0	0	0	0	0	0
ENSG00000260322	AC098657.2	0	0	0	0	0	0	0	0
ENSG00000277670	AC098657.3	0	0	0	0	0	0	0	0
ENSG00000236407	HMGB1P18	0	0	0	0	0	0	0	0
ENSG00000225598	AL606519.1	0	0	0	0	0	0	0	0
ENSG00000213559	HNRNPA1P64	0	0	0	0	0	0	0	0
ENSG00000227062	AL596276.1	0	0	0	0	0	0	0	0
ENSG00000234108	AL596276.2	0	0	0	0	0	0	0	0
ENSG00000224326	AL136234.1	0	0	0	0	0	0	0	0
ENSG00000234184	LINC01781	0	0.17209	0	0.15344	1.00555	0	0.68931	0.76161
ENSG00000227937	MTND2P30	0	0	0	0	0	0	0	0
ENSG00000229752	RPL7P10	0	0	0	0	0	0	0	0
ENSG00000235089	AL357632.1	0	0.18975	0.16752	0	0	0.40221	0	0.13996
ENSG00000223026	RN7SKP247	0	0	0	0	0	0	0	0
ENSG00000117114	ADGRL2	0	0	0	0	0.01149	0	0	0
ENSG00000224494	HNRNPA3P14	0	0.10609	0	0	0	0	0	0
ENSG00000227288	AL138799.1	0.08752	0	0	0	0	0	0	0
ENSG00000227960	AL138799.2	0	0	0	0	0	0	0	0
ENSG00000234953	AL138799.4	0	0	0	0	0	0	0	0
ENSG00000242598	MED28P8	0	0	0	0	0	0	0	0
ENSG00000236676	AC117944.1	0	0	0	0	0	0	0	0
ENSG00000234683	AL138799.3	0	0	0	0	0	0	0	0
ENSG00000215875	ST13P20	0.06883	0.22912	0	0.10215	0.16736	0.12142	0	0.0845
ENSG00000233290	AL157944.1	0	0	0	0	0	0	0	0
ENSG00000230817	LINC01362	0	0.06497	0.05736	0	0	0	0	0
ENSG00000236268	LINC01361	0	0	0	0	0	0	0.07704	0
ENSG00000231364	LINC01712	0	0	0	0	0	0	0	0
ENSG00000233008	LINC01725	0.01351	0	0	0	0.03285	0	0	0.04977

ENSG00000237076	AL035706.1	0	0	0	0	0	0	0	0
ENSG00000137941	TTLL7	0.2781	0.26617	0.07152	0.09286	0.21977	0.22077	0.15065	0.2134
ENSG00000233061	TTLL7-IT1	0	0	0	0	0	0	0	0
ENSG00000229486	AC104454.1	0	0	0	0	0	0	0	0
ENSG00000271576	AL359504.2	0	0	0.06728	0	0	0	0.07632	0.16864
ENSG00000142875	PRKACB	26.2051	19.4706	9.26692	22.1936	33.7001	39.7542	14.6144	20.1256
ENSG00000213540	AL359504.1	0.29736	0.49496	0	0	0	0.2623	0	0.18255
ENSG00000226970	AL450063.1	0	0	0	0	0	0	0	0
ENSG00000199959	RF00410	0	0	0	0	0	0	0	0
ENSG00000203943	SAMD13	0.08486	0	0.03118	0.06297	0.07738	0.03743	0	0
ENSG00000249237	AL359273.1	0	0	0	0	0	0	0	0
ENSG00000240520	UOX	0.03039	0	0	0	0	0	0	0.03731
ENSG00000137976	DNASE2B	0	0	0	0	0	0	0	0
ENSG00000117133	RPF1	14.974	10.7322	3.05439	3.08478	12.8415	14.5925	7.07062	6.35396
ENSG00000174021	GNG5	31.3768	43.4491	7.84607	9.68506	23.1607	21.3969	7.58133	12.9492
ENSG00000122432	SPATA1	0.47953	1.33981	0.40267	0.71168	0.54136	0.33234	0.28547	0.6939
ENSG00000117151	CTBS	13.4368	15.895	2.56129	4.42104	9.34961	4.28617	5.14147	9.07882
ENSG00000180869	LINC01555	0	0	0	0	0	0	0	0
ENSG00000232622	AC114482.1	0	0	0	0	0	0	0	0
ENSG00000117155	SSX2IP	5.54059	4.22277	3.04227	5.45251	4.8174	4.70007	6.35015	6.78181
ENSG00000171517	LPAR3	0.08276	0.17218	0.0608	0.24564	0.05031	0.25548	0.03448	0.0254
ENSG00000153898	MCOLN2	0.72406	1.1343	0.40682	0.63211	0.44015	2.10385	0.56795	0.41835
ENSG00000162643	WDR63	0.09342	0.0622	0.05491	0	0.02272	1.15369	0	0
ENSG00000055732	MCOLN3	0	0	0	0.03285	0.01346	0.01952	0.0369	0.04077
ENSG00000266110	MIR4423	0	0	0	0	0	1.6164	0	0
ENSG00000280099	AL603750.1	0.12807	0	0	0.12672	0	0	0	0
ENSG00000097096	SYDE2	0.12499	0.03783	0.10018	0.20236	0.34536	0.14032	0.58713	0.37667
ENSG00000162642	C1orf52	3.23636	3.06805	1.07084	2.35384	2.81421	2.64673	2.71512	2.71044
ENSG00000223254	RF00019	0	0	0	0	0	0	0	0
ENSG00000142867	BCL10	10.0629	8.91584	3.13322	4.708	8.15612	5.91742	6.41458	10.4076
ENSG00000223653	AL078459.1	0.09657	0.05358	0	0	0	0	0	0
ENSG00000153904	DDAH1	0.33668	0	0.07068	0.11897	0.15593	0.02828	0	0.05905
ENSG00000199459	RF00019	0	0	0	0	0	0	0	0
ENSG00000273264	AL360219.1	0	0	0	0	0	0	0	0
ENSG00000282057	AC092807.3	0.06097	0	0	0	0	0	0	0
ENSG00000272691	AC092807.2	0	0	0	0	0	0	0	0
ENSG00000142871	CYR61	0	0.0994	0	0	0.1452	0	0	0
ENSG00000199934	RF00136	0	0	0	0	0	0	0	0
ENSG00000230285	AC092807.1	0	0	0	0	0	0	0	0
ENSG00000117174	ZNHIT6	2.41299	1.63413	0.73003	1.28148	3.25002	2.71269	1.08438	1.32156
ENSG00000171502	COL24A1	0.46732	0.3953	0.11258	0.17055	0.90346	0.86496	0.38309	0.41387
ENSG00000201620	RNA5SP51	0	0	0	0	0	0	0	0
ENSG00000229505	AC104455.1	0	0	0	0	0	0	0	0
ENSG00000122417	ODF2L	2.4281	1.69052	0.9192	2.25597	2.97655	2.90708	2.62344	2.5435
ENSG00000278281	MIR7856	0	0	0	1.9426	0	0	0	0
ENSG00000231349	AC119749.1	0	0	0	0	0	0	0	0
ENSG00000137975	CLCA2	0	0	0	0	0	0	0	0
ENSG00000016490	CLCA1	0	0	0	0	0	0	0	0
ENSG00000016602	CLCA4	0	0	0	0	0	0	0	0
ENSG00000224466	CDCA4P2	0	0	0	0	0	0	0	0
ENSG00000236915	CLCA4-AS1	0	0.46747	0	0.2084	0.34144	0	0	0.17241
ENSG00000153923	CLCA3P	0	0	0	0	0	0	0	0
ENSG00000261737	AL049597.2	0.6102	0.6348	0	0.3396	0.46367	0.13456	0.12714	0.1873
ENSG00000097033	SH3GLB1	33.8965	40.0725	10.4881	15.6515	29.5545	19.1309	17.845	24.2878
ENSG00000230721	AL049597.1	0	0	0	0	0	0	0	0
ENSG00000183291	SELENOF	31.9273	30.8067	10.756	19.7604	27.502	20.107	18.7076	24.179
ENSG00000153936	HS2ST1	3.07844	2.92334	0.75395	1.83042	2.80701	2.22801	1.52952	2.0352
ENSG00000267734	AL121989.1	0	0	0	0	0	0	0	0

ENSG00000267561	AC093155.3	0	0	0.05351	0.05404	0	0.12848	0	0
ENSG00000235251	AC093155.2	0	0	0.11496	0	0	0	0	0
ENSG00000225568	AC093155.1	0.08874	0	0	0.2634	0.32367	0.15655	0	0.10896
ENSG00000267272	LINC01140	0.04365	0	0	0	0.01327	0	0	0
ENSG00000284240	AC099062.1	0	0	0	0	0	0	0	0
ENSG00000143013	LMO4	2.63896	2.30086	0.79405	1.62255	1.8792	1.52966	1.31962	2.3452
ENSG00000227290	LINC01364	0	0	0	0	0	0	0	0
ENSG00000199318	RNA5SP52	0	0	0	0	0	0	0	0
ENSG00000279778	AL445437.1	0	0	0	0	0	0	0	0
ENSG00000224235		0	0	0	0	0	0	0	0
ENSG00000270507	AL049861.1	0	0	0	0	0	0	0	0
ENSG00000239504	RN7SL583P	0	0	0	0	0	0	0	0
ENSG00000230053	AC093578.1	0	0	0	0	0	0	0	0
ENSG00000274321	RF02271	0	0	0	0	0	0	0	0
ENSG00000237505	PKN2-AS1	0.12241	0.16979	0.08994	0.12111	0.09921	0.07198	0.17003	0.20038
ENSG00000237959	RPL36AP10	0	0	0	0	0	0	0	0
ENSG00000065243	PKN2	29.3648	38.3521	23.2545	46.7351	36.6313	28.7984	49.478	58.7323
ENSG00000207234	RNU6-125P	5.4804	0	0	0	1.66573	0	1.14187	0.84109
ENSG00000241975	ELOCP19	5.07109	5.37144	0.67745	1.71047	4.20362	0.81328	4.22636	3.96213
ENSG00000137947	GTF2B	24.8398	26.5951	9.68215	19.1658	23.6309	22.2009	19.6038	19.9005
ENSG00000235308	AL445991.1	0.15432	0	0	0	0	0	0	0
ENSG00000137944	KYAT3	6.96131	7.19564	2.24209	5.56666	7.18807	5.6637	6.51699	6.20535
ENSG00000213516	RBMXL1	7.76664	6.14	1.20694	1.60388	5.60592	4.39767	3.0503	3.50293
ENSG00000117226	GBP3	1.99584	13.1748	1.65444	1.77217	6.74026	14.5051	2.53061	2.47141
ENSG00000202385	RF00019	0	2.03347	0	0	0	0	0	0
ENSG00000117228	GBP1	17.2385	71.0739	11.587	7.93229	19.6728	103.641	7.7097	46.8735
ENSG00000234518	PTGES3P1	18.6665	21.2189	4.23719	11.7119	25.0929	25.1663	5.81811	9.68912
ENSG00000162645	GBP2	45.5966	106.573	13.3012	16.0856	48.9318	67.6229	21.9013	56.2697
ENSG00000213512	GBP7	0.02987	0.09944	0	0	0	0	0	0
ENSG00000162654	GBP4	15.1219	29.1729	6.38162	6.81797	13.454	57.8283	6.62051	14.5564
ENSG00000154451	GBP5	26.8969	100.238	14.7887	18.5562	35.4913	161.723	24.0369	78.5803
ENSG00000237568	AC099063.1	0	0	0	0	0	0	0	0
ENSG00000238081	AC099063.2	0	0	0	0	0	0	0	0
ENSG00000233235	BX119321.1	0	0	0	0	0	0	0	0
ENSG00000183347	GBP6	0.22086	0.73524	0.07212	0.07284	0.35802	1.55849	0.20452	0.48207
ENSG00000215874	CAPNS1P1	0	0	0	0	0	0.20428	0	0.14218
ENSG00000225492	GBP1P1	0.0935	2.0231	0.59536	0.18501	0.11367	5.88281	0.10389	1.07139
ENSG00000197147	LRRC8B	4.15901	2.57001	1.58692	2.66236	2.69097	3.52683	2.51412	2.11487
ENSG00000231999	LRRC8C-DT	0.28084	0.11687	0	0.2084	0.08536	0.43352	0.29258	0.21551
ENSG00000230735	AC093423.2	2.51603	0.55839	0.24648	0.99575	1.01964	0.88772	2.5163	0.41189
ENSG00000171488	LRRC8C	4.09517	2.42477	1.08412	2.49602	4.54553	5.70234	2.33439	2.37198
ENSG00000271949	AC093423.3	0	0	0	0	0	0	0	0
ENSG00000226394	AC093423.1	0	0	0	0	0	0	0	0
ENSG00000231613	AC099568.1	0.28802	0	0	0	0	0	0	0
ENSG00000272931	AC099568.2	0.94921	0	0.15498	0.15653	0.76935	0.55818	0	0.25898
ENSG00000171492	LRRC8D	9.78949	4.35026	1.97579	3.56491	7.3468	6.42295	3.32395	4.35888
ENSG00000272672	AL391497.1	0.26801	0.22305	0	0	0	0	0	0.16453
ENSG00000252797	RN7SKP272	0	0	0	0	0	0	0	0
ENSG00000228175	GEMIN8P4	0	0	0	0	0	0	0	0
ENSG00000162664	ZNF326	2.33568	1.22186	0.61781	1.52522	1.9472	1.69527	1.82425	1.14709
ENSG00000212459	RNU6-695P	0	0	0	0	0	0	0	0
ENSG00000229201	AC092783.1	0	0	0	0	0	0	0	0
ENSG00000199666	SNORD3G	0	0	0	0	0	0	0	0
ENSG00000143032	BARHL2	0	0	0	0	0	0	0	0
ENSG00000233593	LINC02609	0	0	0	0	0	0	0	0
ENSG00000225446	LINC01763	0	0	0	0	0	0	0	0
ENSG00000272094	AC091614.1	0.08339	0	0	0	0	0	0	0
ENSG00000232882	PHKA1P1	0.59018	0.78588	0	0	0.14351	0.20823	0.19675	0

ENSG00000122482	ZNF644	15.6067	11.4563	4.20713	7.3964	13.8058	18.5023	10.4281	9.55124
ENSG00000232393	RPL5P6	0	0	0	0	0	0	0	0
ENSG00000162669	HFM1	0.27675	0.06282	0.33274	0.52274	0.15294	0.02219	1.02743	0.49423
ENSG00000206817	RF00019	0	0	0	0	0	0	0	0
ENSG00000215873	FEN1P1	0	0	0	0	0	0	0	0
ENSG00000097046	CDC7	2.61713	4.21902	3.5733	6.26963	4.40948	8.14333	12.4688	5.59161
ENSG00000241318	WDR82P2	0.31595	0	0	0	0	0	0.13166	0
ENSG00000229067	AL714022.1	0	0	0	0	0	0	0	0
ENSG00000203914	HSP90B3P	0	0	0	0.06942	0	0	0	0
ENSG00000069702	TGFBR3	7.25004	8.14324	2.89547	5.59862	3.81855	20.1432	2.89134	5.06589
ENSG00000239794	RN7SL653P	0	0.41641	0	0	0.30415	0.44134	0	0.30716
ENSG00000137948	BRDT	0	0	0	0.0502	0	0	0	0
ENSG00000172031	EPHX4	0.22794	0.21681	0.19141	0.33829	0.07918	0	0.05428	0.11994
ENSG00000233228	LPCAT2BP	0	0	0	0	0	0	0	0
ENSG00000230667	SETSIP	0	0.13422	0	0	0	0	0	0
ENSG00000284413		0	0.06228	0	0	0	0	0	0.04594
ENSG00000189195	BTBD8	0.01213	0	0	0	0	0	0	0.0149
ENSG00000224678	GAPDHP46	0	0	0	0	0	0	0	0
ENSG00000233401	PRKAR1AP1	0	0.105	0	0	0	0.22257	0	0.1549
ENSG00000069712		0	0	0	0	0	0	0	0
ENSG00000273487	AC104836.1	0	0	0	0.08413	0	0	0	0
ENSG00000226773	AL663109.1	0	0	0	0	0	0	0	0
ENSG00000203910	C1orf146	0	0	0	0	0	0	0	0
ENSG00000233125	ACTBP12	0	0	0	0	0	0	0	0
ENSG00000174842	GLMN	2.11589	2.18022	2.88718	7.47668	3.21555	4.13264	5.79412	3.77307
ENSG00000122484	RPAP2	1.62491	1.85767	1.23159	3.70615	2.63056	2.36114	3.01495	2.50429
ENSG00000242764	RN7SL824P	0	0	0	0	0	0	0	0
ENSG00000162676	GFI1	1.70982	2.99385	0.45683	1.97732	1.53882	6.42446	0.66624	1.1996
ENSG00000067208	EVI5	5.82789	4.74707	2.19957	4.91613	5.10407	3.40623	6.37771	4.91526
ENSG00000223787	AL354890.1	0	0	0	0	0.25756	0	0.70624	0.26011
ENSG00000229992	HMGB3P9	0	1.04638	0	0	0.15286	0.2218	0	0.15437
ENSG00000201317	RNU4-59P	0	0	0.92857	0	0	0	2.10656	0
ENSG00000225505	AC104332.1	0	0	0	0	0	0	0	0
ENSG00000234202	AC104332.2	0	0	0	0	0.57126	0	0.78321	0
ENSG00000223896	CCNJP2	0	0	0	0	0	0	0	0
ENSG00000122406	RPL5	109.215	61.8064	15.3558	36.4376	105.152	93.3372	53.3112	60.7869
ENSG00000154511	DIPK1A	2.41884	1.22364	0.55756	1.33739	2.47947	3.63964	1.06725	1.6596
ENSG00000206680	SNORD21	0	0	0	0	0	0	0	0
ENSG00000207523	SNORA66	2.20452	2.75207	1.61976	2.45381	1.3401	6.80588	2.75595	4.73669
ENSG00000207022	RF00432	0	0	0	0	0	0	0	0
ENSG00000229052	AL449283.1	0	0	0.03165	0	0	0	0	0
ENSG00000252121	RNU6-970P	0	0	0	0	0	0	0	0
ENSG00000239710	RN7SL692P	0	0	0	0	0	0	0	0
ENSG00000252752	RNU6-210P	0	0	0	0	0	0	0	0
ENSG00000222664	RN7SKP123	0	0	0	0	0	0	0	0
ENSG00000251837	RF00019	0	0	0	0	0	0	0	0
ENSG00000143033	MTF2	6.35129	6.13243	1.96871	3.88455	6.62079	5.79923	3.65983	4.72146
ENSG00000117500	TMED5	11.6761	9.59443	3.08636	5.56455	9.68433	6.93011	4.07138	7.44006
ENSG00000122483	CCDC18	26.1815	34.0705	25.8911	51.5443	34.7101	29.6363	53.7264	45.4059
ENSG00000237003	AC126124.2	0	0	0	0	0	0	0	0
ENSG00000225297	AC126124.1	0	0	0	0	0	0	0	0
ENSG00000223745	CCDC18-AS1	2.23607	1.759	1.64294	3.79591	2.10409	2.13448	4.31435	2.08727
ENSG00000229567	AL139421.1	0	0	0	0.6437	0	0	0	0
ENSG00000117505	DR1	11.5952	10.0736	3.84658	6.02102	9.98686	9.57821	3.91682	6.33736
ENSG00000229635	AL137159.1	0	0	0	0	0	0	0	0
ENSG00000207379	RF00019	0	0	0	0	0	0	0	0
ENSG00000137942	FNBP1L	2.84453	1.64137	0.70596	3.00202	1.76758	2.52022	2.46552	1.13311
ENSG00000212601	RNA5SP53	0	0	0	0	0	0	0	0

ENSG00000137936	BCAR3	0.33437	0.26618	0	0.06473	0.12372	0.10259	0.04847	0.0714
ENSG00000224093	AL109613.1	0.11254	0	0	0	0	0	0.06253	0
ENSG00000230439		0.04387	0.07302	0	0	0	0	0.07312	0
ENSG00000211575	MIR760	0	0	0	0	4.45583	0	0	1.12496
ENSG00000260464	AL049796.1	0.08301	0	0	0	0	0	0.06918	0.05096
ENSG00000067334	DNTTIP2	8.24739	6.24728	1.77637	2.69107	6.7439	8.35823	5.4111	4.94332
ENSG00000023909	GCLM	6.39687	7.54112	7.69101	20.1916	7.77716	6.41787	26.4423	10.5107
ENSG00000223490	CHCHD2P5	0	0	0	0	0	0	0	0
ENSG00000234099	MTND4P11	0	0	0	0	0	0	0	0
ENSG00000235794	MTND3P21	0	0	0	0	0	0	0	0
ENSG00000232752	MTCO3P21	0	0	0	0	0	0	0	0
ENSG00000229842	MTATP6P13	0	0	0	0	0	0	0	0
ENSG00000250890	AL117351.2	0	0	0	0	0	0	0	0
ENSG00000236689	MTCO2P21	0	0	0	0	0	0	0	0
ENSG00000226960	MTCO1P21	0	0	0	0	0	0	0	0
ENSG00000233129	AL117351.1	0	0	0	0	0	0	0	0
ENSG00000198691	ABCA4	0	0	0	0	0	0	0	0
ENSG00000233482	AC093579.1	0	0	0	0	0	0	0	0
ENSG00000137962	ARHGAP29	0.08499	0.04716	0.00694	0.01402	0.06315	0.01666	0.03148	0.0058
ENSG00000264963	RN7SL440P	0	0	0	0	0	0	0	0
ENSG00000226835	AC097059.1	0	0	0	0	0	0	0	0
ENSG00000178596	GAPDHP29	0	0	0	0	0	0	0	0
ENSG00000236098	AC097059.2	0	0	0	0	0	0	0	0
ENSG00000232918	AC118469.1	0	0	0	0	0	0	0	0
ENSG00000117528	ABCD3	3.7045	2.9208	1.26065	3.3132	3.98234	3.92114	3.86739	2.76491
ENSG00000117525	F3	0.10198	0.04244	0	0	0.06199	0.13493	0.0425	0.0313
ENSG00000223675	AC093117.1	0	0	0	0	0	0	0	0
ENSG00000224081	SLC44A3-AS1	0.02146	0.01191	0	0.01062	0	0	0	0.01757
ENSG00000232857	KATNBL1P2	0	0	0	0	0	0	0.14632	0
ENSG00000263526	MIR378G	0	0	0	0	0	0	0	0
ENSG00000143036	SLC44A3	0	0.04253	0	0.03792	0	0	0	0
ENSG00000117519	CNN3	0.07748	0	0	0.15333	0.40822	0.45564	0.12915	0.22198
ENSG00000235501	AC105942.1	0.07243	0	0.21287	0.1075	0	0	0	0
ENSG00000172339	ALG14	0.14458	0.038	0.05591	0.11293	0.18502	0.21478	0.0761	0.07474
ENSG00000230427	AC095033.1	0	0	0	0	0	0	0	0
ENSG00000152078	TMEM56	3.72344	7.08777	12.6166	32.5975	6.36892	4.2277	12.5284	7.72063
ENSG00000271092	TMEM56-RWDD3	0.07476	0.06222	0.43942	0.4438	0.18178	0.06594	0.24922	0.45893
ENSG00000231992	AC092802.3	2.44334	5.22894	19.2346	32.1177	9.97258	8.92867	41.8904	22.4993
ENSG00000206679	RF00019	0	0	0	0	0	0	0	0
ENSG00000226026	AC092802.1	0.05852	0.14612	0.215	0.17371	0.24903	0.25811	0.92672	0.17963
ENSG00000122481	RWDD3	1.81217	2.83168	1.68473	3.3481	3.37223	1.63108	3.45212	2.08873
ENSG00000228852	AC092802.2	0	0	0	0	0	0	0	0
ENSG00000228504	LINC01760	0	0	0	0	0	0	0	0
ENSG00000230434	LINC01650	0	0	0	0	0	0	0	0
ENSG00000237954	AL445218.1	0	0	0	0	0	0	0	0
ENSG00000233907	LINC01761	0	0	0	0	0	0	0	0
ENSG00000228971	LINC02607	0	0	0	0	0	0	0	0
ENSG00000271252	AL683887.1	0	0	0	0	0	0	0	0
ENSG00000237435	AC099566.1	0.03272	0	0	0	0.03978	0	0	0
ENSG00000200800	RNU1-130P	0	0	0	0	0	0	0	0
ENSG00000231987	LINC01787	0	0	0	0	0	0	0	0
ENSG00000225923	AC092393.1	0	0	0	0	0	0	0	0
ENSG00000234422	UBE2WP1	0	0	0	0	0	0	0	0
ENSG00000228502	EEF1A1P11	0.10554	0.08784	0	0.07832	0.12832	0	0	0.12959
ENSG00000241992	RN7SL831P	0	0	0	0	0	0	0	0
ENSG00000234226	NDUFS5P2	0	0	0	0	0	0	0	0
ENSG00000137970	RPL7P9	0.98126	1.79664	0.72098	1.89319	0.7158	0.86554	1.79917	1.32526
ENSG00000223229	RN7SKP270	0	0	0	0	0	0	0	0

ENSG00000117569	PTBP2	1.18985	1.01855	0.52454	1.20247	0.98506	1.19946	1.6433	1.09914
ENSG00000188641	DPYD	26.1551	22.7429	5.34975	11.4982	18.3821	14.2096	10.6763	18.6615
ENSG00000232878	DPYD-AS1	0	0	0	0	0	0	0	0
ENSG00000232542	DPYD-IT1	0.73118	1.5213	0.53723	0.27129	0.44447	0.32247	0.91407	0.89773
ENSG00000228057	SEC63P1	0.05827	0.09699	0	0.08648	0	0.10279	0.09712	0.07154
ENSG00000226128	RPL26P9	0	0	0	0	0	0	0	0
ENSG00000230718	AC114878.1	0	0	0	0	0	0	0	0
ENSG00000235777	DPYD-AS2	0	0	0	0	0	0	0	0
ENSG00000270911	AC114878.2	0	0	0	0	0	0	0	0
ENSG00000269998		0	0	0	0	0	0	0	0
ENSG00000259946	BX005019.1	0	0	0	0	0	0	0	0
ENSG00000225206	MIR137HG	0	0	0	0	0	0	0	0
ENSG00000284247	MIR2682	0	0	0	0	0	0	0	0
ENSG00000284202	MIR137	0	0	0	0	0	0	0	0
ENSG00000233557	NFU1P2	0	0	0	0	0	0	0	0
ENSG00000226053	LINC01776	0	0	0	0	0	0	0	0
ENSG00000280040	AC095031.1	0	0	0	0	0	0	0	0
ENSG00000162627	SNX7	0.03546	0	0	0	0	0	0	0.04354
ENSG00000117598	PLPPR5	0	0	0	0	0	0	0	0
ENSG00000232825	AL445433.2	0	0	0	0	0	0	0	0
ENSG00000227034	AL445433.1	0.28522	0	0	0.84658	0	0	0	0
ENSG00000117600	PLPPR4	0.03969	0	0	0	0	0	0	0
ENSG00000233983	AL365220.1	0	0	0	0	0	0	0	0
ENSG00000224445	LINC01708	0	0.11675	0.10308	0.4164	0.08528	0	0.23384	0
ENSG00000099260	PALMD	0	0	0	0	0	0	0	0
ENSG00000223656	HMGB3P10	0	0	0	0	0	0	0	0
ENSG00000156869	FRRS1	1.17493	0.53623	0.80758	1.06873	0.64511	0.90264	1.16874	1.11682
ENSG00000201491	RNU4-75P	0	0	0	0	0	0	0	0
ENSG00000202254	RF00019	0	0	0	0	0	0	0	0
ENSG00000231996	AC096949.1	0	0	0	0	0	0	0	0
ENSG00000162688	AGL	3.7893	2.63286	0.88123	2.83766	3.74049	2.7138	1.47764	2.11281
ENSG00000228084	AC118553.1	0.55954	0	0	0.16609	0.54422	0.39485	0	0.1374
ENSG00000117620	SLC35A3	1.00076	0.56863	0.31302	0.77542	0.7134	0.70902	0.34166	0.54774
ENSG00000283761	AC118553.2	0.13986	0.2328	0.37368	0.69819	0.23187	0.26917	0.72058	0.31222
ENSG00000212248	RNU6-750P	0	0	1.00667	1.01669	1.66573	0	0	0.84109
ENSG00000202259	RNU6-1318P	0	0	0	0	0	0	0	0
ENSG00000156875	MFSD14A	1.94272	1.69974	0.2562	0.36964	1.09011	1.18635	0.37364	0.9174
ENSG00000241073	AC093019.2	0	0	0	0	0	0	0	0
ENSG00000156876	SASS6	3.56167	2.94885	2.03387	6.32666	4.73404	4.81826	5.96752	4.44094
ENSG00000226952	AC093019.1	0	0	0	0	0.35364	0	0	0
ENSG00000122435	TRMT13	2.5539	1.8598	1.22491	2.52684	2.99715	2.19013	1.83285	2.89611
ENSG00000122477	LRRC39	0.47861	0.12256	0.1082	0.92886	0.4476	0.32474	0.3682	0.67803
ENSG00000137992	DBT	1.00518	0.9499	0.52413	1.22231	1.159	1.13261	0.95124	1.11471
ENSG00000271415	RPL23AP90	0	0	0	0	0	0	0	0
ENSG00000225169	BRI3P1	0	0	0	0	0	0	0	0
ENSG00000230287	AL445928.1	0	0	0	0	0	0	0	0
ENSG00000224616	RTCA-AS1	9.87735	3.46123	5.09286	8.74401	2.73881	4.27982	27.7289	5.31898
ENSG00000137996	RTCA	5.50296	5.42349	1.87976	2.50741	5.22317	5.74814	3.4196	3.31896
ENSG00000207750	MIR553	2.15589	3.58848	0	0	1.31054	0	0	0
ENSG00000215871	AC104457.1	0	0	0	0	0	0	0	0
ENSG00000223906	AC104457.2	0	0	0	0	0	0	0	0
ENSG00000079335	CDC14A	11.6014	9.16321	5.11636	12.2455	13.4178	10.0826	8.30012	6.9365
ENSG00000234332	BCAS2P2	0.54377	0	0	0.1614	0.13222	0	0.18128	0.66763
ENSG00000228086	AL589990.1	0	0	0	0	0	0	0	0
ENSG00000181656	GPR88	0.021	0	0	0	0	0	0.035	0.02578
ENSG00000213421	AC099670.1	0	0	0	0	0	0	0	0
ENSG00000230402	LINC01349	0	0	0	0	0	0	0	0
ENSG00000224685	AC099670.2	0	0	0	0	0	0	0	0

ENSG00000162692	VCAM1	0.16965	0.02017	0.01781	0.1079	0.01473	0.76959	0.0606	0
ENSG00000162694	EXTL2	0.43974	0.22716	0.33424	0.38257	0.3134	0.32101	0.25275	0.35373
ENSG00000273204	AC104506.1	0	0	0	0	0.41546	0	0	0
ENSG00000162695	SLC30A7	6.3709	5.30218	2.37907	4.59768	5.01122	5.54237	3.17996	5.07147
ENSG00000230946	HNRNPA1P68	0	0	0	0	0.18206	0	0	0
ENSG00000117543	DPH5	3.03567	2.25942	0.79788	2.38083	4.17078	3.83146	1.76894	2.33326
ENSG00000235795	AC093157.2	0.98316	0	0	1.45912	0.66406	0.77086	0.54626	0.13412
ENSG00000233184	AC093157.1	0.69687	0.47257	0.11378	0.65118	0.97275	0.68298	0.51625	0.85561
ENSG00000252765	RF00424	0	0	0	0	0	0	0	0
ENSG00000271578	AC119501.1	0	0	0	0	0	0	0	0
ENSG00000225938	AL109741.1	1.09132	0.6055	0	0.53988	1.10567	3.20873	1.21271	0.44664
ENSG00000170989	S1PR1	30.0983	17.8384	5.15596	10.4145	29.8313	26.4434	13.0495	12.6893
ENSG00000260940		2.724	0.82438	0.7278	1.10256	2.70963	1.31059	1.85747	0.60809
ENSG00000228399	AL109741.2	0	0	0	0	0.25982	0.377	0	0
ENSG00000173810	PPIAP7	0	0	0	0	0	0	0	0
ENSG00000231671	LINC01307	0	0	0	0	0	0	0	0
ENSG00000226715	LINC01709	0	0	0	0	0	0	0	0
ENSG00000252530	RNU6-965P	0	0	0	0	0	0	0	0
ENSG00000183298	RPSAP19	0.0833	0	0	0.12362	0.10127	0.14695	0.13884	0.10227
ENSG00000118733	OLFM3	0.0327	0	0	0	0	0	0.05451	0
ENSG00000252717	RNU6-352P	0	0	0	0	0	0	0	0
ENSG00000271277	AL356280.1	0	0	0	0	0	0	0	0
ENSG00000162699	DNAJA1P5	0	0	0	0	0	0	0	0
ENSG00000233359	AC114485.1	0	0	0	0	0	0	0	0
ENSG00000230864	AC099567.1	0	0	0	0	0	0	0	0
ENSG00000060718	COL11A1	0	0.01433	0.01265	0	0	0	0	0
ENSG00000234257	SOD2P1	0	0	0	0	0	0	0	0
ENSG00000230759	AC095032.2	0	0	0	0	0	0	0	0
ENSG00000224613	AC095032.1	0	0	0	0	0	0	0	0
ENSG00000222069	RN7SKP285	0	0	0	0	0	0	0	0
ENSG00000185946	RNPC3	4.4405	4.08649	1.85092	2.91489	4.26961	3.03177	3.71861	4.02346
ENSG00000240038	AMY2B	0.69474	0.66778	0.05752	0.43567	0.90413	0.60418	0.76658	0.63674
ENSG00000236085	ACTG1P4	0	0	0	0	0	0	0	0
ENSG00000243480	AMY2A	0	0	0	0	0	0	0	0
ENSG00000237763	AMY1A	0	0	0	0	0	0	0	0
ENSG00000234441	AC105272.1	0	0	0	0	0	0	0	0
ENSG00000174876	AMY1B	0	0	0	0	0	0	0	0
ENSG00000227408	AMYP1	0	0	0	0	0	0	0	0
ENSG00000187733	AMY1C	0	0	0	0	0	0	0	0
ENSG00000225191	AL136455.1	0	0	0	0	0	0	0	0
ENSG00000215869	AC092506.1	0	0	0	0	0	0	0	0
ENSG00000228347	FTLP17	0	0	0	0.20721	0.16975	0	0	0
ENSG00000225036	CDK4P1	0	0	0	0	0	0	0	0
ENSG00000230768	LINC01676	0	0	0	0	0	0	0	0
ENSG00000227850	SEPT2P1	0	0	0	0	0	0	0	0
ENSG00000237897	AL512844.2	0	0	0	0	0	0	0	0
ENSG00000232952	AL512844.1	0	0	0.06055	0.1223	0	0	0.13736	0.05059
ENSG00000233047	LINC01677	0	0	0	0	0	0	0	0
ENSG00000237480	AL355306.2	0	0	0	0	0	0	0	0
ENSG00000230932	AL355306.1	0	0	0	0	0	0	0	0
ENSG00000270342	AL499605.1	0	0	0	0	0	0	0	0
ENSG00000270976	AL596327.1	0	0	0	0	0	0	0	0
ENSG00000233449	MTATP6P14	0	0	0	0	0	0	0	0
ENSG00000232551	MTCO1P14	0	0	0	0	0	0	0	0
ENSG00000237460	LINC01661	0	0	0	0	0	0	0	0
ENSG00000198890	PRMT6	2.91408	1.63238	0.4941	1.82973	2.55495	3.16359	1.07422	1.44491
ENSG00000162631	NTNG1	0.02406	0	0	0.0119	0.0195	0	0	0
ENSG00000224550	AC114491.1	0	0	0	0	0	0	0	0

ENSG00000134215	VAV3	9.69811	10.7896	2.63693	4.45855	8.27311	4.12603	4.15053	6.721
ENSG00000275455	MIR7852	0	0	0	0	0	0	0	0
ENSG00000230489	VAV3-AS1	0.94734	0.59132	0	0.35149	0.28794	0	0	0.14539
ENSG00000226822	AL390036.1	0.09876	0.13151	0	0	0.02401	0	0.03292	0.07275
ENSG00000085491	SLC25A24	4.35459	4.34147	0.5264	1.32909	2.87166	1.63911	1.34346	1.60807
ENSG00000260879	AL359258.2	0	0	0	0	0	0	0	0
ENSG00000280186	AL359258.3	0	0	0	0	0	0	0	0
ENSG00000196427	NBPF4	0	0	0	0	0	0	0	0
ENSG00000238122	AL359258.1	0	0	0	0	0	0	0	0
ENSG00000241361	SLC25A24P1	0	0	0	0	0.04735	0.06871	0	0
ENSG00000243967	NBPF5P	0	0	0	0	0	0	0	0
ENSG00000238118	SLC25A24P2	0	0	0	0	0	0	0	0
ENSG00000224698	AL390038.1	0	0	0	0	0	0	0	0
ENSG00000186086	NBPF6	0	0	0	0	0	0	0	0
ENSG00000283354	AL392088.2	0	0	0	0	0	0	0	0
ENSG00000227832	ST13P21	0	0	0	0	0	0	0	0
ENSG00000226483	AL392088.1	0	0	0	0	0	0	0	0
ENSG00000162636	FAM102B	4.71692	2.99967	1.12838	1.86058	2.39105	2.41898	1.58031	1.87594
ENSG00000162639	HENMT1	4.26024	4.52628	1.37639	4.66353	3.96727	6.02318	1.91379	3.6355
ENSG00000134186	PRPF38B	12.5002	12.354	2.11095	3.45975	11.0917	7.91387	3.42365	4.9199
ENSG00000143107	FNDC7	0.022	0	0	0	0	0	0	0
ENSG00000232971	AL591719.2	0	0	0	0	0	0	0	0
ENSG00000116266	STXBP3	18.0049	17.6131	7.32711	11.3861	15.4499	10.6608	11.8968	13.8469
ENSG00000228076	AL591719.1	0	0	0	0	0	0	0	0
ENSG00000162641	AKNAD1	0	0	0	0	0	0	0	0
ENSG00000274068	AL449266.1	0	0	0	0	0	0	0	0
ENSG00000203897	SPATA42	0	0	0	0	0	0	0	0
ENSG00000121957	GPSM2	2.5794	3.07319	2.93922	6.07126	3.8732	6.11513	7.16578	4.92267
ENSG00000121940	CLCC1	3.17477	1.64677	0.58587	1.92851	2.81856	1.64114	1.59986	1.70422
ENSG00000085433	WDR47	4.42487	4.52394	1.9239	2.53333	3.50583	3.50835	2.59664	3.78464
ENSG00000237349	BX679664.2	0.25901	0	0.38061	0	0	0	0	0.31801
ENSG00000244716	BX679664.3	0.66036	0.65951	0.19408	0.19601	0.64228	0.23299	0	0.64863
ENSG00000228665	BX679664.1	0	0	0	0	0	0	0	0
ENSG00000228054	RANP5	0	0	0	0	0	0	0	0
ENSG00000197780	TAF13	6.59703	4.2703	2.61591	4.66225	4.13756	6.28086	4.27631	4.6927
ENSG00000273382	AL356488.3	0.68529	0.21123	0.0373	0	0.52458	0.31343	0.29614	0.18697
ENSG00000215717	TMEM167B	16.2979	17.1249	5.03952	6.49289	12.1381	7.54837	10.4978	11.9629
ENSG00000270066	AL356488.2	0	0	0	0	0	0	0	0
ENSG00000278249	SCARNA2	0	0	0	0	0	0	0	0
ENSG00000251484	AL356488.1	0	0	0	0	0	0	0	0
ENSG00000179902	C1orf194	0	0.09879	0	0	0	0	0	0
ENSG00000116299	KIAA1324	5.20984	11.7417	0.56753	1.47387	0.69952	2.55843	1.2218	0.64837
ENSG00000031698	SARS	15.0193	12.5952	2.3587	4.73598	13.0794	13.4503	4.45914	6.87412
ENSG00000143126	CELSR2	0.23149	0.12844	0.02617	0.04405	0.1732	0.3665	0.09894	0.06559
ENSG00000134222	PSRC1	0.8986	0.42735	0.18864	0.38104	0.28093	0.72469	0.42795	0.53588
ENSG00000221986	MYBPHL	0	0	0	0	0	0.07228	0	0
ENSG00000134243	SORT1	6.65785	5.67419	1.81091	1.78143	4.38773	3.96688	2.24086	3.58613
ENSG00000143106	PSMA5	4.02102	3.66622	0.80917	1.99555	3.26948	6.98067	1.64359	3.41184
ENSG00000143028	SYPL2	0	0	0	0	0	0	0	0
ENSG00000162650	ATXN7L2	0.81258	0.58299	0.08235	0.33268	0.54506	0.84033	0.18682	0.20642
ENSG00000174151	CYB561D1	2.20287	1.26511	0.32182	1.01329	1.67583	1.54538	0.90186	1.29697
ENSG00000181754	AMIGO1	0.88348	0.28462	0.3141	0.69789	1.5592	1.08095	0.80757	1.10222
ENSG00000156097	GPR61	0.02899	0.02413	0.0213	0	0.01762	0.10228	0.02416	0.0178
ENSG00000254942	AL355310.3	0.37784	0	0	0	0	0	0	0
ENSG00000065135	GNAI3	7.45843	6.97281	1.92644	3.1506	4.81756	3.97348	3.74086	4.39818
ENSG00000206832	RNU6V	0	0	0	0	0	0	0	0
ENSG00000225113	AL355310.1	0	0	0	0	0	0	0	0
ENSG00000284443	MIR197	0	0	0	0	0	0	0	0

ENSG00000134183	GNAT2	0.10616	0.1767	0	0	0.12906	0	0	0.06517
ENSG00000116337	AMPD2	26.0521	20.9934	2.38382	5.67605	13.7012	4.71913	7.86123	9.00894
ENSG00000228703	AL355310.2	0	0	0	0	0	0	0	0
ENSG00000235045	RPL7P8	0	0	0	0	0	0	0	0.12328
ENSG00000168765	GSTM4	1.04624	0.63712	0.09375	0.22723	0.43434	1.26048	0.14887	0.26631
ENSG00000213366	GSTM2	0.12061	0.05354	0.01182	0.02387	0.00978	0.22696	0.10722	0.08885
ENSG00000134184	GSTM1	1.66326	0	0	0	0.03548	2.21354	0	0
ENSG00000260246	AC000032.1	0	0	0	0	0	0	0	0
ENSG00000134201	GSTM5	0.09752	0.10145	0.01791	0.03618	0.0741	0.08602	0	0.01497
ENSG00000241720	AL158847.1	0	0	0	0	0	0	0	0
ENSG00000134202	GSTM3	0.24691	0.02569	0.06803	0	0.16885	0.51725	0.28294	0.35999
ENSG00000198758	EPS8L3	0.02108	0	0	0	0	0	0	0
ENSG00000224927	NDUFA5P10	0	0	0	0	0	0	0	0
ENSG00000228420	LINC01768	0	0	0	0	0	0	0	0
ENSG00000235005	AL450468.1	0.16289	0	0.23936	0	0.19804	0	0.27151	0
ENSG00000261055	AL450468.2	0.50445	0.12918	0	0.17277	0.18871	0.06846	0.06468	0.19057
ENSG00000184371	CSF1	5.47926	1.71145	0.47714	0.66259	2.53303	1.14562	0.38336	1.06309
ENSG00000168710	AHCYL1	9.94923	8.06549	1.3904	3.74463	7.25064	6.92968	2.55687	3.99557
ENSG00000143093	STRIP1	3.29014	2.00803	0.57139	1.3761	2.59398	2.48	1.29625	1.94635
ENSG00000258634	AL160006.1	0.74761	0.28939	0.25549	0.23223	0.63413	0.55209	0.31878	0.34154
ENSG00000156150	ALX3	0	0	0	0	0	0	0	0
ENSG00000258673	LINC01397	0	0	0	0	0	0	0	0
ENSG00000186150	UBL4B	0	0	0	0	0	0	0	0
ENSG00000197106	SLC6A17	0	0	0	0	0	0	0	0
ENSG00000227091	AL355990.1	0	0	0	0	0	0	0	0
ENSG00000235526	AL355990.2	0	0	0	0	0	0	0	0
ENSG00000224965	LINC02586	0.098	0.16311	0	0	0	0.34575	0	0
ENSG00000116396	KCNC4	0.05561	0.02923	0.01721	0.0391	0.03559	0.04131	0.0244	0.01797
ENSG00000282852	AL137790.1	0	0	0	0	0	0	0	0
ENSG00000200536	RF00402	0	0	0	0	0	0	0	0
ENSG00000227963	RBM15-AS1	0.19652	0.23364	0	0.04166	0.20479	0.34668	0	0.13787
ENSG00000162775	RBM15	7.98927	6.76664	1.86827	3.65725	7.25146	9.38687	4.84012	5.14544
ENSG00000224699	LAMTOR5-AS1	0.29307	0.34033	0.17026	0.35402	0.36459	0.43284	0.26129	0.36819
ENSG00000168679	SLC16A4	0.13887	0.1541	0.10203	0.0687	0.1407	0.20416	0.03858	0.08525
ENSG00000273373	AL355488.1	0.48867	0.81339	0.30235	0.45805	0.28142	0.77133	0.55731	0.78945
ENSG00000134248	LAMTOR5	9.94527	11.6491	2.21021	5.55773	9.29232	6.82298	4.75828	6.29376
ENSG00000143125	PROK1	0	0	0	0	0	0	0	0
ENSG00000270380	AL358215.1	0	0	0	0	0	0	0	0
ENSG00000282887	AL358215.2	0	0	0	0	0	0	0	0
ENSG00000283999	AL358215.3	0	0	0	0	0	0	0	0
ENSG00000240194	CYMP	0	0	0	0	0	0	0	0
ENSG00000235407	CYMP-AS1	0	0	0	0	0	0	0	0
ENSG00000143105	KCNA10	0	0	0	0	0	0	0	0
ENSG00000177301	KCNA2	0.03796	0.04914	0.01859	0.01878	0.04615	0.06697	0	0.01036
ENSG00000259834	AL365361.1	9.91797	8.69236	3.03245	6.37526	11.4948	10.1785	6.24766	6.59272
ENSG00000177272	KCNA3	11.0136	6.07905	1.59328	3.64172	10.8577	11.6275	4.47059	5.21976
ENSG00000199710	RF00019	0	0	0	0	0	0	0	0
ENSG00000237415	NRBF2P3	0	0	0	0	0	0	0	0
ENSG00000232791	OR11I1P	0	0	0	0	0	0	0	0
ENSG00000143119	CD53	159.781	173.158	44.2249	56.2757	123.607	90.0209	49.6945	106.525
ENSG00000261654	AL360270.2	0.11023	0.06116	0.03599	0.14541	0.01489	0.04321	0.02041	0.09022
ENSG00000232811	AL360270.1	0.21751	0.05172	0.04566	0	0.11333	0.43853	0.25897	0.03815
ENSG00000121931	LRIF1	4.25106	7.81717	1.39811	2.76396	6.98954	4.67822	1.21472	4.69744
ENSG00000273010	AL360270.3	0.05438	0.09051	0.07991	0	0.06611	0.09593	0	0.06676
ENSG00000225672	CCNT2P1	0	0	0	0	0	0	0	0
ENSG00000252760	RNA5SP54	0	0	0	0	0	0	0	0
ENSG00000156171	DRAM2	12.6372	9.91465	3.39824	5.20008	10.2805	8.32409	6.46333	5.59255
ENSG00000134255	CEPT1	4.26053	4.93081	2.94475	4.93952	5.44467	5.31818	3.67427	4.43941

ENSG00000272982	AL355816.1	11.1814	13.4416	3.65132	5.53148	9.06271	13.1504	4.14171	5.33881
ENSG00000273221	AL355816.2	2.94561	3.20579	0.49945	1.51325	3.30572	7.79468	1.32189	3.61657
ENSG00000162777	DENND2D	9.67591	8.98195	1.48436	5.50339	13.2827	29.567	3.27882	7.4576
ENSG00000064886	CHI3L2	1.69069	0.74057	0.35959	1.51871	4.84124	8.35915	0.51913	1.4476
ENSG00000236040	CHIAP1	0	0	0	0	0.08956	0	0.12279	0.09045
ENSG00000203878	CHIAP2	0	0	0	0	0	0	0	0
ENSG00000134216	CHIA	0	0	0	0	0	0	0	0
ENSG00000229283	AL356387.1	0	0	0	0	0	0	0	0
ENSG00000232240	AL356387.2	0	0	0	0	0	0	0	0
ENSG00000173947	PIFO	0	0	0	0	0	0.03035	0	0
ENSG00000234020	CHIAP3	0	0	0	0	0	0	0	0
ENSG00000236012	HIGD1AP12	0	0	0	0	0	0	0	0
ENSG00000227179	PGCP1	0	0	0	0	0	0	0	0
ENSG00000085465	OVGP1	0.14277	0.04753	0.04196	0.25427	0.27773	0.10075	0.19039	0.07012
ENSG00000260948	AL390195.2	0.6522	0.24124	0	0.05377	0.61672	0.06392	0.42277	0.3559
ENSG00000233337	UBE2FP3	0	0	0	0.20761	0	0	0	0
ENSG00000243960	AL390195.1	0.59594	0.28341	0	0.12635	0.20701	0.45056	0.42572	0.31358
ENSG00000116455	WDR77	2.11669	1.08742	0.2688	1.04714	1.58853	2.53553	1.08895	1.18713
ENSG00000199890	RF00019	0	0	0	0	0	0	0	0
ENSG00000116459	ATP5PB	17.7238	14.0654	5.06252	8.26424	13.0659	19.8765	8.84841	12.7959
ENSG00000143110	C1orf162	48.6727	30.5462	4.82534	10.1397	27.2372	19.9013	6.93003	9.49391
ENSG00000121933	TMIGD3	0.12843	0.26721	0	0.1906	0.07807	0.05664	0.05352	0.07884
ENSG00000200360	RNU6-792P	0	0	0	1.01669	0	0	0	0
ENSG00000282608	ADORA3	1.41854	0.59029	0.24052	0.36437	1.12764	0.14437	0.18188	0.66987
ENSG00000116473	RAP1A	16.8808	16.4751	7.08747	16.2431	14.3856	9.84097	13.8701	13.4865
ENSG00000231346	LINC01160	0.05323	0.0886	0	0	0.06472	0	0	0.06536
ENSG00000215867	KRT18P57	0	0	0	0	0	0	0	0
ENSG00000201028	RNU6-151P	0	0	0	0	0	0	0	0
ENSG00000197852	INKA2	6.10154	5.77098	0.64187	1.11419	4.02436	1.08362	1.08074	2.74013
ENSG00000227811	INKA2-AS1	3.30213	0.85881	0.30328	1.32728	0.75275	1.69908	0.97469	1.3092
ENSG00000064703	DDX20	2.74009	1.60137	0.68003	1.50012	2.88715	2.44917	1.40064	1.56998
ENSG00000171385	KCND3	0.26102	0.06207	0.0137	0.02767	0.11334	0.11512	0.35739	0.20602
ENSG00000232558	KCND3-IT1	0	0	0	0	0	0	0	0
ENSG00000237556	KCND3-AS1	0	0	0	0	0	0	0	0
ENSG00000231437	LINC01750	0	0.03399	0	0	0	0	0.13613	0
ENSG00000231246	AL445426.1	0	0	0	0	0	0	0	0
ENSG00000234388	TXNP3	0	0	0	0	0	0	0	0
ENSG00000238761	RF01210	0	0	0	0	0	0	0	0
ENSG00000143079	CTTNBP2NL	0.3762	0.24718	0	0.11754	0.38516	0.22705	0.13202	0.1094
ENSG00000283296	MIR4256	0	0	0	0	0	0	0	0
ENSG00000134245	WNT2B	0.01167	0.03885	0.00858	0.03464	0.00709	0	0	0.02149
ENSG00000273483	AL354760.1	0	0	0	0	0	0	0	0
ENSG00000007341	ST7L	3.74138	4.76842	3.59888	9.75061	5.2079	4.59152	6.13086	4.23855
ENSG00000116489	CAPZA1	84.7835	104.017	34.2442	53.9451	75.6521	71.4189	47.9255	76.443
ENSG00000235299	MRPL53P1	0	0	0	0	0	0	0	0
ENSG00000252750	RNU7-70P	0	0	0	0	0	0	0	0
ENSG00000155363	MOV10	2.4212	3.2803	1.026	1.00279	1.34175	3.77466	0.97609	1.71449
ENSG00000225075	AL603832.1	0	0	0	0	0	0	0	0
ENSG00000155366	RHOC	4.38565	2.36263	0.56157	1.18832	2.10181	1.34834	1.03131	0.87137
ENSG00000271810	AL603832.3	0	0	0	0	0	0	0	0
ENSG00000155367	PPM1J	0.28055	0.08491	0	0.15141	0.15504	0	0.04251	0.03131
ENSG00000261595	AL603832.2	0	0	0	0	0	0	0	0
ENSG00000184599	FAM19A3	0	0	0	0	0	0	0	0
ENSG00000234790	NUTF2P4	0	0	0	0	0	0	0	0
ENSG00000215866	LINC01356	0	0	0	0	0	0	0	0
ENSG00000224167	AL390729.1	0	0	0	0	0	0	0	0
ENSG00000228040	AL390729.2	0	0	0	0	0	0	0	0
ENSG00000155380	SLC16A1	0.34498	0.27562	0.08111	0.16383	0.41941	0.68161	0.62102	0.18636

ENSG00000229020	AKR7A2P1	0	0	0	0	0	0	0	0
ENSG00000226419	SLC16A1-AS1	0.00723	0.01203	0.01062	0.01073	0.01758	0	0	0.03551
ENSG00000238198	AL357055.3	0.08943	0	0.0438	0	0	0	0	0
ENSG00000198799	LRIG2	1.86546	1.84149	1.47008	2.2096	2.1392	2.00363	2.21682	1.93635
ENSG00000237278	RLIMP2	0	0	0	0	0	0	0	0
ENSG00000233839	AL357055.1	0	0	0	0	0	0	0	0
ENSG00000236887	AL357055.2	0	0	0	0	0	0	0	0
ENSG00000081026	MAGI3	0.2061	0.13424	0.13168	0.10639	0.18521	0.44263	0.29873	0.17603
ENSG00000232499	AL391058.1	0	0.23714	0	0	0.17321	0	0	0
ENSG00000238256	MTND5P20	0	0	0	0	0	0	0	0
ENSG00000116793	PHTF1	3.21008	3.26717	0.87133	1.48689	2.59768	2.0019	1.55068	2.39741
ENSG00000232450	AL133517.1	0.19599	0.81556	0	0.72718	0	0.17288	0	0.24063
ENSG00000081019	RSBN1	9.12626	10.0913	2.36942	5.12102	9.94542	7.1113	4.9811	6.5198
ENSG00000231128	AL137856.1	0.13574	0	0.29921	0.40291	0.24755	0	0.45252	0.49998
ENSG00000134242	PTPN22	36.6583	38.827	17.9882	32.8314	35.0886	38.7677	16.6869	24.243
ENSG00000226167	AP4B1-AS1	0	0	0	0	0	0	0	0.0519
ENSG00000188761	BCL2L15	0.08622	0.14351	0.05068	0.10236	0.06289	0.1521	0.11497	0.06351
ENSG00000134262	AP4B1	1.66561	1.24985	0.50155	1.15492	1.34447	1.63777	0.93302	1.1566
ENSG00000118655	DCLRE1B	4.11152	5.48109	2.9799	9.52565	7.03434	6.89225	4.34143	4.45417
ENSG00000235527	HIPK1-AS1	0.18996	0.11857	0.10468	0.42288	0.20208	0.12567	0.71242	0.14577
ENSG00000163349	HIPK1	25.1733	25.6639	10.4116	16.3061	23.9597	22.5042	19.1828	17.4854
ENSG00000116774	OLFML3	0.12992	0	0	0	0.12636	0.18336	0	0.0319
ENSG00000270780	AL591742.1	0	0	0	0	0	0	0	0
ENSG00000282048	AL591742.2	0	0	0	0	0	0	0	0
ENSG00000134207	SYT6	0	0	0	0	0	0	0	0
ENSG00000232895	AL121999.1	0	0	0	0	0	0	0	0
ENSG00000273698	MRPL57P1	0	0	0	0	0	0	0	0
ENSG00000271419	AL035410.2	0	0	0	0	0	0	0	0
ENSG00000197323	TRIM33	13.2911	13.177	5.9291	15.26	13.8971	11.359	11.8556	12.5962
ENSG00000226984	AL035410.1	0	0	0	0	0	0	0	0
ENSG00000225650	EIF2S2P5	0	0	0	0	0	0	0	0
ENSG00000201114	RF00019	0	0	0	0	0	0	0	0
ENSG00000236480	PKMP1	0	0	0	0	0	0	0	0
ENSG00000116752	BCAS2	14.6714	10.2378	4.97524	10.6357	13.858	15.0316	11.0047	12.3322
ENSG00000175984	DENND2C	1.13713	0.36209	0.69746	0.85115	0.45682	0.31399	0.62631	0.93481
ENSG00000116748	AMPD1	0	0	0	0	0	0	0	0
ENSG00000242769	RN7SL432P	0	0	0	0	0	0	0	0
ENSG00000213281	NRAS	19.2601	14.0958	5.71375	10.1475	15.3035	21.7409	11.6166	12.5013
ENSG00000009307	CSDE1	149.1	131.215	40.677	62.8841	111.743	98.3602	82.6166	74.2966
ENSG00000201900	RNY1P13	0	0	0	0	0	0	0	0
ENSG00000052723	SIKE1	6.60375	5.98243	2.15647	3.76523	5.98748	7.56915	2.86069	4.45863
ENSG00000227970	NR1H5P	0	0	0	0	0	0	0	0
ENSG00000198765	SYCP1	0	0	0	0	0	0	0	0.02075
ENSG00000134200	TSHB	0	0	0	0	0	0	0	0
ENSG00000134198	TSPAN2	8.36183	8.05041	2.90607	3.89204	6.19374	2.80618	3.26053	4.75058
ENSG00000233730	LINC01765	1.04502	3.72735	1.31626	0.44312	1.089	0.79009	0.49768	2.19952
ENSG00000228035	NGF-AS1	0	0	0	0	0	0	0	0
ENSG00000134259	NGF	0	0	0	0	0	0	0	0
ENSG00000226973	AL512638.1	0	0	0	0	0	0	0	0
ENSG00000274468	AL512638.2	0	0	0	0	0	0	0	0
ENSG00000229101	ELOCP20	0	0	0	0	0	0	0	0
ENSG00000232185	CNOT7P2	0	0	0	0	0	0	0	0
ENSG00000270631	AL592436.1	0	0	0	0	0	0	0	0
ENSG00000239984	RN7SL420P	0	0	0	0	0	0	0	0
ENSG00000207502	RF00406	0	0	0	0	0	0	0	0
ENSG00000173218	VANGL1	0.84123	0.79436	0.15452	0.7923	0.63922	1.99775	0.48538	0.50649
ENSG00000118729	CASQ2	0	0	0	0	0	0	0	0
ENSG00000177551	NHLH2	0	0	0	0	0	0	0	0

ENSG00000214204	HNRNPA1P43	0	0.13408	0	0	0.09793	0	0	0
ENSG00000271143	AL357137.1	0	0	0	0	0	0	0	0
ENSG00000228127	LINC01649	0	0	0	0	0	0	0	0.04828
ENSG00000163393	SLC22A15	2.89489	4.44967	0.61062	0.96616	2.50914	0.85524	1.06204	2.09177
ENSG00000237993	AL365318.1	0	0	0	0	0	0	0	0
ENSG00000173212	MAB21L3	0.02251	0.11242	0.03308	0	0.02737	0.03971	0	0.02764
ENSG00000235933	LINC01779	0	0	0	0	0	0	0	0
ENSG00000221040	RF00012	0	0	0	0	0	0	0	0
ENSG00000163399	ATP1A1	14.7792	8.63688	2.37353	4.52462	12.6415	12.7157	4.27404	5.61475
ENSG00000203865	ATP1A1-AS1	0.35785	0.04964	0	0.13277	0.21753	0.21043	0.14912	0.36614
ENSG00000212385	RNU6-817P	0	0	0	0	0	0	1.17481	0
ENSG00000233154	LINC01762	0.17237	0.28691	0.12665	0.06395	0.15717	0.07602	0	0.26454
ENSG00000230381	AL136376.1	0.28466	0	0	0	0	0	0	0
ENSG00000200547	RF00019	0	0	0	0	0	0	0	0
ENSG00000224950	AL390066.1	0.50947	0.63601	0.09358	0.09451	0.03871	0.67408	0	0.27367
ENSG00000116815	CD58	25.2008	27.7137	15.2841	27.3337	19.6427	13.127	13.9791	18.7263
ENSG00000177173	NAP1L4P1	3.61117	3.47993	0.93098	1.22231	2.15667	1.22941	1.47841	2.64468
ENSG00000264419	MIR548AC	1.66592	0	0	1.2362	1.01269	0	2.77683	1.02269
ENSG00000143061	IGSF3	0.02972	0.04946	0	0	0	0.08737	0	0.01216
ENSG00000211543	MIR320B1	0	0	0	0	0	0	0	0
ENSG00000203864	C1orf137	0	0	0	0	0	0	0	0
ENSG00000231072	GAPDHP64	0	0	0	0	0	0	0	0
ENSG00000223489	NEFHP1	0	0	0	0	0	0	0	0
ENSG00000116824	CD2	29.1193	31.247	11.6118	20.3447	31.5279	63.3081	9.82391	19.2966
ENSG00000225079	FTH1P22	1.26866	1.40779	0.41428	1.04602	1.02827	2.73544	0	0.51921
ENSG00000272715	AL157904.1	0	0	0	0	0	0	0	0
ENSG00000134247	PTGFRN	0.25387	0.07683	0.06783	0.03425	0.11224	0.10179	0.0577	0.0425
ENSG00000252510	RNA5SP55	0	0	0	0	0	0	0	0
ENSG00000134256	CD101	9.57423	5.64146	1.29437	2.98393	4.5862	3.64829	1.53204	1.57518
ENSG00000236137	AL445231.1	0	0	0	0	0	0.06645	0.06279	0
ENSG00000116830	TTF2	1.06556	0.79094	0.22218	0.45946	0.86657	1.48605	0.37203	0.43315
ENSG00000215930	MIR942	1.70466	0	0	0	0	0	0	0
ENSG00000134253	TRIM45	0	0	0.02466	0	0	0	0.05594	0.0206
ENSG00000228453	RPS15AP9	0	0	0	0	0	0	0	0
ENSG00000134258	VTCN1	0	0	0	0	0	0	0	0
ENSG00000277141	RF00017	0	0	0	0	0	0	0	0
ENSG00000235202	LINC01525	0	0	0	0	0	0	0	0
ENSG00000271427	AL358072.1	0.12748	0.21219	0.18733	0	0	0	0	0.46955
ENSG00000198162	MAN1A2	55.6406	69.4036	43.6672	68.0037	132.601	101.93	99.1775	97.2959
ENSG00000279513	AL157902.2	0.09826	0.16355	0	0.51039	0.29865	0.08667	0.16378	0.18096
ENSG00000226755	VPS25P1	0	0	0	0	0	0	0	0
ENSG00000236866	AL157902.1	0	0	0	0.09971	0.08168	0	0	0.08249
ENSG00000183508	TENT5C	218.626	309.678	331.514	366.478	239.008	290.238	828.43	245.375
ENSG00000213262	VDAC2P3	0	0	0	0	0	0	0	0
ENSG00000212266	RF00561	0	0	0	0	0	0	0	0
ENSG00000270719	AL390877.1	0	0	0	0	0	0	0	0
ENSG00000228217	PNRC2P1	0.17452	0	1.02585	0.25901	0.21218	0	0.58181	0.21428
ENSG00000196505	GDAP2	6.38305	5.60291	1.55988	2.76732	4.44904	4.10259	2.47946	3.84134
ENSG00000065183	WDR3	2.09022	1.40117	0.42981	1.01639	2.4892	2.35341	0.70157	1.27879
ENSG00000155761	SPAG17	0.04604	0.01277	0	0.01139	0	0	0	0.00942
ENSG00000222209	RNA5SP56	0	0	0	0	0	0	0	0
ENSG00000226126	PSMC1P12	0	0	0	0	0	0	0	0
ENSG00000229911	AL139148.1	0	0	0	0	0	0	0	0
ENSG00000092607	TBX15	0	0	0	0	0	0	0	0
ENSG00000226172	AL139420.1	0	0	0	0	0	0	0	0
ENSG00000239216	AL139420.2	0	0	0	0	0	0	0	0
ENSG00000116874	WARS2	0.97175	0.66602	0.30799	0.39589	1.15826	1.479	0.66696	0.53806
ENSG00000224238	WARS2-IT1	0	0	0	0	0	0	0	0

ENSG00000223575	RBMX2P3	0	0	0.11255	0	0	0	0	0
ENSG00000236804	RPS3AP12	0	0	0	0	0	0.17173	0.16226	0
ENSG00000231365	WARS2-AS1	0.36234	0.3574	0.29581	0.25892	0.48947	0.42615	0.71581	0.56022
ENSG00000227056	RPL6P2	0	0	0	0	0	0	0	0.11494
ENSG00000227712	AL359915.1	0.08163	0	0	0	0	0	0	0
ENSG00000232650	LINC01780	0	0	0	0	0	0	0	0
ENSG00000194297	RNU1-75P	0	0	0	0	0	0	0	0
ENSG00000116882	HAO2	0	0	0	0	0	0	0	0
ENSG00000230921	HAO2-IT1	0	0	0	0	0	0	0	0
ENSG00000203859	HSD3B2	0	0	0	0	0	0	0	0
ENSG00000232780	GAPDHP74	0	0	0	0	0	0	0	0
ENSG00000203858	HSD3BP2	0	0	0	0	0	0	0	0
ENSG00000235595	GAPDHP23	0	0	0	0	0	0	0	0
ENSG00000187481	HSD3BP1	0	0	0	0	0	0	0	0
ENSG00000229570	GAPDHP58	0	0	0	0	0	0	0	0
ENSG00000203857	HSD3B1	0	0	0	0	0	0	0	0
ENSG00000226443	GAPDHP32	0.07345	0	0	0	0	0	0	0
ENSG00000249798	HSD3BP3	0	0	0	0	0	0	0	0
ENSG00000239873	GAPDHP27	0.07186	0	0	0	0	0	0	0.08823
ENSG00000203855	HSD3BP4	0	0	0	0	0	0	0	0
ENSG00000240244	GAPDHP33	0	0	0	0	0	0	0.12061	0
ENSG00000260941	LINC00622	0	0	0	0	0	0	0	0
ENSG00000198857	HSD3BP5	0	0	0	0	0	0	0	0
ENSG00000143067	ZNF697	0.78521	0.84711	0.0641	0.12948	0.45964	0.20522	0.31509	0.35706
ENSG00000092621	PHGDH	0.29988	0.15191	0.01916	0	0.23777	0.57503	0.0652	0.03202
ENSG00000134240	HMGCS2	0	0.04746	0	0	0	0	0	0
ENSG00000134193	REG4	0.04197	0.03493	0	0.09343	0.30616	0.03702	0	0.10306
ENSG00000215864	NBPF7	0	0	0	0	0	0	0	0
ENSG00000227205	PFN1P9	0	0	0	0	0	0	0	0
ENSG00000226446	NOTCH2P1	0	0	0	0	0	0	0	0
ENSG00000134249	ADAM30	0	0	0	0	0	0	0	0
ENSG00000261662	AL359752.1	5.07008	2.42401	0.3963	1.12068	2.49186	1.80789	1.25866	1.78802
ENSG00000134250	NOTCH2	33.5297	26.6274	5.01174	6.22668	19.9634	14.503	7.22599	14.1633
ENSG00000273406	AC245008.1	0	0	0	0	0	0	0	0
ENSG00000207149	RNU6-465P	0	0	0	0	0	0	0	0
ENSG00000265808	SEC22B	7.34302	6.75274	1.47847	2.99841	5.40579	4.23691	3.04301	4.43311
ENSG00000274642	AC244669.2	0	0.14303	0	0	0	0	0	0
ENSG00000277313	RF00100	0	0	0	0	0	0	0	0
ENSG00000223804	AC244669.1	2.51618	2.31452	0.77842	0.49135	2.41509	1.63538	0.66222	1.78856
ENSG00000273727	RF00003	0	0	0	2.01455	1.65031	1.59644	0	3.33322
ENSG00000270392	PFN1P2	0	0	0	0	0.0482	0	0	0
ENSG00000270231	NBPF8	2.06286	2.74691	0.18188	0.51025	2.05654	1.45567	0.77939	1.11441
ENSG00000275131	AC241952.1	0.71465	0.51366	0.1074	0.26515	0.76025	0.40114	0.32488	0.35895
ENSG00000273694	RF00004	0	0	0	0	0	0	0	0
ENSG00000275933	RF00100	0	0	0	0	0	0	0	0
ENSG00000273136	NBPF26	4.98228	4.63195	0.5	0.75746	3.04344	1.39345	1.1343	2.35735
ENSG00000275538	RNVU1-19	59.2548	64.2919	3.86996	23.4508	51.7624	28.6499	9.51104	44.1902
ENSG00000263353	PPIAL4A	0	0	0	0	0	0	0	0
ENSG00000281741	AC241377.3	0.20211	0	0	0.09999	0.16382	0	0	0.33087
ENSG00000226067	LINC00623	3.8858	3.93955	0.57101	0.68155	4.29478	1.49565	0.52994	1.73488
ENSG00000277610	RNVU1-4	1.78781	2.23186	3.28396	8.62326	2.17358	5.51941	5.21501	3.84134
ENSG00000275585	AC241377.2	0	0	0	0	0	0	0	0
ENSG00000273516	RF00003	0	0	0	0	0	0	0	0
ENSG00000275296	AC241377.1	0	0	0	0	0	0	0	0
ENSG00000198019	FCGR1B	4.03011	9.66547	1.11439	0.51451	4.42554	2.52278	1.40852	10.109
ENSG00000234998	AC244453.3	0.1597	0.26581	0.70401	0	0	0	0	1.56858
ENSG00000233029	AC244453.2	0.12413	0.03444	0	0	0.10061	0.0365	0	0.12701
ENSG00000223345	HIST2H2BA	0	0.32277	0	0.28779	0	0	0	0.23809

ENSG00000227193	AC244453.1	0	0	0	0	0	0	0	0
ENSG00000213244	HIST2H3DP1	0	0	0	0	0	0	0	0.21634
ENSG00000268993	RPL22P6	0	0	0	0	0	0	0	0
ENSG00000188610	FAM72B	0.36251	0.6034	0.46168	0.68148	0.23506	0.72479	0.44312	0.29673
ENSG00000171943	SRGAP2C	1.95508	1.55202	0.3536	0.41663	1.2677	1.45036	0.90244	0.78783
ENSG00000275129	AC243994.1	0	0	0	0	0	0	0	0
ENSG00000230806	SRGAP2-AS1	0	0	0	0	0	0	0	0.06795
ENSG00000227082	AC244021.1	0	0	0	0	0.02434	0.03531	0.03336	0.02458
ENSG00000231429		0	0	0	0	0	0	0	0
ENSG00000228826	AL592494.1	0	0	0	0	0	0	0	0
ENSG00000233432	AL592494.2	0	0	0	0	0	0	0	0
ENSG00000272583	AL592494.3	0.31595	0	0.23214	0	0.19206	0.83607	0.78996	1.16376
ENSG00000231752	EMBP1	2.04693	1.23895	0.24859	0.30128	1.41912	1.46233	0.31017	0.60233
ENSG00000224857	LINC01691	0	0	0	0	0	0	0	0
ENSG00000276118	AC242852.1	0	0	0	0	0	0	0	0
ENSG00000277095	AC242852.2	0	0	0	0	0	0	0	0
ENSG00000277702	AC239859.6	0	0	0	0	0	0	0	0
ENSG00000252830	RNA5SP533	0	0	0	0	0	0	0	0
ENSG00000264145	AC239859.4	0	0	0	0	0	0	0	0
ENSG00000274927	AC239859.5	0	0	0	0	0	0	0	0
ENSG00000242943	NKAIN1P1	0	0	0	0	0	0	0	0
ENSG00000223495	AC239859.2	0	0	0	0	0	0	0	0
ENSG00000235887	AC239859.3	0	0	0	0	0	0	0	0
ENSG00000203825	AC239859.1	0	0	0	0	0	0	0	0
ENSG00000179571	NBPF17P	0	0	0	0	0	0	0	0
ENSG00000226028	PFN1P12	0	0	0	0	0	0	0	0
ENSG00000202167	RNU1-114P	0	0	0	0	0	0	0	0
ENSG00000207349	RNVU1-17	0	0	0	0	0	0	0	0
ENSG00000252826	RNU1-92P	0	0	0	0	0	0	0	0
ENSG00000206737	RNVU1-18	3.12867	10.4154	3.94075	12.6032	3.80376	0.78849	2.98001	12.0728
ENSG00000232721	AC239800.2	0	0	0	0	0	0	0	0
ENSG00000223779	AC239800.1	0.30584	2.54538	0	0	0	0	0	0.12517
ENSG00000242352	FAM91A3P	0	0	0	0	0	0	0	0
ENSG00000270962	AC239800.3	0	0	0	0	0	0	0	0
ENSG00000263590	LINC02591	0	0	0	0	0	0	0	0
ENSG00000252105	RNU1-143P	0	0	0	0	0	0	0	0
ENSG00000233430	AC239798.1	0	0	0	0	0	0	0	0
ENSG00000237503	AC239798.3	0	0	0	0	0	0	0	0
ENSG00000265531	FCGR1CP	1.95121	9.6351	1.33806	0.77221	1.97686	2.18006	0.8673	3.11436
ENSG00000240929	HIST2H2BB	0.12928	0	0	0	0	0.91225	0	0
ENSG00000234571	AC239798.2	0.04141	0.06893	0	0.03073	0.02517	0.03653	0	0.02542
ENSG00000273213	AC239798.4	0	0	0	0	0	0	0	0
ENSG00000230186	AC246680.1	0	0	0	0	0	0	0	0
ENSG00000203818	HIST2H3PS2	0	0	0	0	0	0	0	0
ENSG00000268074	RPL22P5	0	0	0	0	0	0	0	0
ENSG00000263513	FAM72C	0.06382	0.15935	0.37515	0.28416	0.0388	0.0563	0.37234	0.23508
ENSG00000270872	SRGAP2D	1.07689	0.71699	0	0.63929	1.22197	0.50661	0.11967	0.08815
ENSG00000276674	IGKV1OR1-1	0	0	0	0	0	0	0	0
ENSG00000275075	AC244015.1	0	0	0	0	0	0	0	0
ENSG00000275767	AC244015.2	0	0	0	0	0	0	0	0
ENSG00000232527	LSP1P5	0	0	0	0	0	0	0	0
ENSG00000276442	RNA5SP529	0	0	0	0	0	0	0	0
ENSG00000175658	DRD5P2	0	0	0	0	0	0	0	0
ENSG00000276756	AC245595.1	0	0	0	0	0	0	0	0
ENSG00000271567	PPIAL4E	0	0	0	0	0	0	0	0
ENSG00000271439	AC246785.4	0	0	0	0	0	0	0	0
ENSG00000207205	RNVU1-15	23.545	27.3595	15.6675	48.7887	21.604	52.5084	5.92389	12.545
ENSG00000237343	AC246785.3	0.23344	0	0	0	0	0	0	0.57323

ENSG00000233586	AC246785.2	0	0	0	0	0	0.25207	0	0
ENSG00000266338	NBPF15	1.31239	0.89678	0.1015	0.59457	0.87336	0.60927	0.6908	0.54276
ENSG00000227212	PFN1P6	0	0	0	0	0	0	0	0.22556
ENSG00000229002	AC246785.1	0	0	0	0	0	0	0	0
ENSG00000275291	RF00003	0	0	0	0	0	0	0	0
ENSG00000278099	RF00003	0	0	0	0	0	0	0	0
ENSG00000277918	RF00003	0	0	0	0.66333	0	0	0	0
ENSG00000279782	PPIAL4F	0	0	0	0	0	0	0	0
ENSG00000277199	LINC01632	0	0	0	0	0	0	0	0
ENSG00000224363	FP700111.1	0	0	0	0	0	0	0	0
ENSG00000278273	FP700107.1	0	0	0	0	0	0	0	0
ENSG00000222854	RNA5SP59	0	0	0	0	0	0	0	0
ENSG00000273825	FP700111.2	0	0	0	0	0	0	0	0
ENSG00000196369	SRGAP2B	1.07467	1.0969	0.25327	0.39121	0.57932	0.67965	0.60837	0.21161
ENSG00000271644	AC242498.1	0	0	0	0	0	0	0	0
ENSG00000215784	FAM72D	0.27522	0.2036	0.31456	0.58999	0.18589	0.64737	0.20389	0.26282
ENSG00000283752	AC241585.3	0	0	0	0	0	0	0	0
ENSG00000272419	LINC01145	0.84726	0.96704	0.19565	0.62872	0.67691	1.13169	0.36315	0.50527
ENSG00000277678	RF00003	0	0	0	0	0	0	0	0
ENSG00000281571	AC241585.2	0.40423	0.11214	0	0	0.24573	0.11885	0.1123	0
ENSG00000223612	AC241585.1	0.4237	0.47017	0.20754	0	0.85854	0.24916	0.23541	0.1734
ENSG00000256374	PPIAL4D	0	0	0	0	0	0	0	0
ENSG00000271291	AC245014.2	0	0	0	0	0	0	0	0
ENSG00000276216	AC245014.3	3.37984	6.68058	2.48332	3.13504	8.73189	5.9625	3.16894	5.18715
ENSG00000207501	RNVU1-14	0	0	0	0	0	0	0	0
ENSG00000236140	AC245014.1	0.57717	0	0	0	0	0	0	0.23621
ENSG00000162825	NBPF20	0.07332	0.05139	0.01701	0.01145	0.06099	0.03404	0.03859	0.04264
ENSG00000234367	PFN1P3	0	0	0	0	0	0	0	0
ENSG00000270384	RF00003	0	0	0	0	0	0	0	0
ENSG00000270722	RF00003	4.02258	2.23186	3.28396	13.2666	7.60752	18.1352	4.47001	8.23144
ENSG00000278182	AC239860.2	0	0	0	0	0	0	0	0
ENSG00000275064	AC239860.1	0	0	0	0	0	0	0	0
ENSG00000272150	NBPF25P	0.01915	0.06376	0.02815	0	0.04657	0.06758	0.19156	0
ENSG00000117262	GPR89A	4.9333	6.87217	4.20621	11.7067	9.91076	7.28353	13.28	6.64006
ENSG00000174827	PDZK1	0.08867	0	0	0	0	0.20857	0	0
ENSG00000117281	CD160	1.99356	1.53624	0.35263	1.56152	1.90756	4.81948	1.75378	1.35981
ENSG00000265491	RNF115	3.67796	2.89523	1.06338	2.44352	3.6725	3.98207	3.6186	2.24157
ENSG00000186141	POLR3C	11.8099	12.1441	9.14007	20.1368	16.9427	24.1678	29.178	13.6945
ENSG00000186364	NUDT17	0.4866	0.2209	0	0.1313	0.37647	0.3902	0.36868	0.16294
ENSG00000131788	PIAS3	1.31193	0.73637	0.24659	0.4528	0.85315	1.45324	0.38142	0.61809
ENSG00000278549	MIR6736	0	0	0	0	0	0	0	1.52537
ENSG00000198483	ANKRD35	0.10898	0.03628	0	0.03235	0	0	0	0
ENSG00000143127	ITGA10	0.06959	0.07722	0	0.05164	0.0282	0	0	0.04272
ENSG00000244619	AC243547.1	0	0	0	0	0	0	0	0
ENSG00000131779	PEX11B	2.79163	3.05353	1.17208	1.71642	4.02431	3.79915	1.52892	2.44823
ENSG00000265241	RBM8A	15.8281	13.1948	3.27867	7.05111	13.8023	11.7851	6.69421	7.38024
ENSG00000211451	GNRHR2	0.17515	0.04859	0.08579	0.08665	0.28392	0.103	0.14597	0.10752
ENSG00000234222	LIX1L-AS1	0.15797	0.07513	0.03316	0.06699	0.05487	0.23888	0.11285	0.05542
ENSG00000280778	AC243547.3	0	0	0	0	0	0	0	0
ENSG00000271601	LIX1L	7.06265	5.31005	1.54458	3.22936	7.37595	6.3436	2.61266	2.96594
ENSG00000272031	ANKRD34A	0.33889	0.23227	0.05859	0.05917	0.04847	0.17584	0.03323	0.02448
ENSG00000278431	AC243547.2	0.6265	0	0	0	0	0	0	0
ENSG00000121851	POLR3GL	9.37136	7.25676	1.19749	1.93504	7.08376	6.68482	1.49415	3.40178
ENSG00000265972	TXNIP	685.909	737.133	209.96	466.083	512.058	259.658	356.834	472.404
ENSG00000168509	HJV	0	0	0	0	0	0	0	0
ENSG00000201558	RNVU1-6	0.89391	2.97582	6.56792	29.8497	2.17358	9.46184	3.72501	7.13391
ENSG00000233396	LINC01719	0	0	0.04382	0	0	0	0	0.03661
ENSG00000271425	NBPF10	0.60304	0.52491	0.10569	0.09853	0.67936	0.18544	0.11988	0.23775

ENSG00000213240	AC239799.1	0	0.03577	0	0	0	0	0	0
ENSG00000264343	NOTCH2NLA	0.54608	0.39767	0.21733	0.27015	0.40111	0.2007	0.22756	0.23746
ENSG00000276509	AC239799.2	0	0	0	0	0	0	0	0
ENSG00000201789	RNU6-1071P	0	0	0	0	0	0	0	0
ENSG00000271121	NUDT4P2	0	0	0	0	0	0	0	0
ENSG00000277406	SEC22B4P	1.26653	1.84462	0.93057	1.87967	3.84953	1.95504	2.375	2.72129
ENSG00000270339	PPIAL4H	0	0.18486	0	0.16483	0	0	0	0
ENSG00000273768	RF00003	58.9978	40.1735	24.9581	81.5893	61.947	109.6	27.5651	31.8282
ENSG00000274428	RF00003	0.45813	0	0	0	0	4.04099	0.76363	2.24993
ENSG00000277655	AC245407.1	0	0	0	0	0	0	0	0
ENSG00000277420		0	0	0	0	0	0	0	0
ENSG00000274408	RNA5SP536	0	0	0	0	0	0	0	0
ENSG00000276975	HYDIN2	0	0	0	0	0	0	0	0
ENSG00000274450	RF00619	0	0	0	0	0	0	0	0
ENSG00000268043	NBPF12	1.60269	0.95634	0.25181	0.59838	1.32351	0.74685	0.70566	0.65592
ENSG00000244371	PFN1P8	0	0	0	0	0	0	0	0
ENSG00000234225	AC244394.2	0	0	0	0	0	0	0	0
ENSG00000227242	NBPF13P	0.20555	0.61586	0.06041	0.06101	0.09996	0.07252	0.06853	0.05048
ENSG00000225603	AC244394.1	0	0.07328	0	0.06534	0	0	0	0
ENSG00000201142	RNU1-151P	0	0	0	0	0	0	0	0
ENSG00000230832	AC241584.1	0.11813	0.09831	0	0.08766	0.21543	0	0.09845	0
ENSG00000283342	AC241584.2	0	0	0	0	0	0	0	0
ENSG00000131791	PRKAB2	3.97379	1.98878	0.67075	0.87666	1.77907	2.34466	2.48389	1.46699
ENSG00000237188	AC242426.2	0.24312	0.16861	0.02977	0.03007	0.19705	0.21445	0.06754	0.199
ENSG00000273071		0	0	0	0	0	0	0	0
ENSG00000131781	FMO5	1.10208	1.01912	0.12596	0.34529	0.4764	0.34564	0.28575	0.52621
ENSG00000180867	PDIA3P1	0.63106	0.7272	0.07133	0.07204	0.70821	0.51382	0.32366	0.4172
ENSG00000226015	CCT8P1	0.57508	0.07363	0	0.1313	0.05378	0	0.14747	0.21725
ENSG00000236806	RPL7AP15	0	0.15347	0	0	0	0	0	0
ENSG00000131778	CHD1L	8.66316	6.61361	5.20064	7.4758	9.10701	13.2912	8.64961	6.77111
ENSG00000278811	LINC00624	0	0	0	0	0	0	0	0
ENSG00000213226	AC242426.1	0	0	0	0	0	0	0	0
ENSG00000226653	OR13Z1P	0	0	0	0	0	0	0	0
ENSG00000207209	RF00019	0	0	0	0	0	0	0	0
ENSG00000272443	OR13Z2P	0	0	0	0	0	0	0	0
ENSG00000272480	OR13Z3P	0	0	0	0	0	0	0	0
ENSG00000116128	BCL9	0.59371	0.26606	0.11745	0.13556	0.9578	0.7654	0.19031	0.32242
ENSG00000162836	ACP6	0.41676	0.29584	0.06304	0.13644	0.15648	0.10812	0.15324	0.26337
ENSG00000277762	RN7SL261P	0	0	0	0	0	0	0	0
ENSG00000227139	AC241644.1	0	0	0	0	0	0	0	0
ENSG00000265107	GJA5	0	0	0	0	0	0	0	0
ENSG00000274415	AC241644.3	0	0	0	0	0	0	0	0
ENSG00000234190	AC241644.2	0	0	0	0	0	0	0	0
ENSG00000223728	AC239801.1	0	0	0	0	0	0	0	0
ENSG00000121634	GJA8	0	0	0	0	0	0	0	0
ENSG00000188092	GPR89B	3.09278	4.27018	2.95308	8.2282	4.74781	5.07894	7.22201	3.2548
ENSG00000215859	PDZK1P1	0	0	0	0	0	0	0	0
ENSG00000244211		0	0	0	0	0	0	0	0
ENSG00000273059	AC239803.2	0	0	0	0	0.4145	0	0	0
ENSG00000206791	RNU1-129P	0	0	0	0	0	0	0	0
ENSG00000274372	AC239803.3	0	0	0	0	0	0	0	0
ENSG00000206585	RNVU1-7	6.25735	1.48791	7.88151	17.9098	4.34715	13.4043	5.96002	8.7802
ENSG00000229828	PDE4DIPP1	0	0	0	0	0	0	0	0
ENSG00000263956	NBPF11	1.29419	0.67703	0.30791	0.51219	1.2138	0.43488	0.53417	0.46913
ENSG00000225080	PFN1P4	0	0	0	0	0	0	0	0
ENSG00000198658	ABHD17AP1	0	0	0	0.2332	0	0	0	0
ENSG00000227733	AC239809.3	0	0	0	0	0	0	0.01182	0
ENSG00000232265	AC239803.1	0.05212	0	0	0.03867	0	0	0	0

ENSG00000238765	RNA5SP57	0	0	0	0	0	0	0	0
ENSG00000224335	AC239809.1	0	0	0	0	0	0	0	0
ENSG00000227700	AC239809.2	0	0	0	0	0	0	0	0
ENSG00000199879	RNU1-120P	3.26817	2.33137	2.7443	6.92903	1.70287	0	0.77822	2.29292
ENSG00000234283	LINC01731	0	0	0	0	0	0	0.29091	0
ENSG00000228626	AC245100.3	0	0	0	0	0	0	0	0
ENSG00000274020	LINC01138	0.49877	0.79765	0.3018	0.4064	0.46372	0.46583	0.34233	0.6244
ENSG00000238107	AC245100.6	0	0	0	0	0	0	0	0
ENSG00000235988	AC245100.5	0.22145	0	0	0	0	0	0	0
ENSG00000224481	AC245100.1	0	0	0	0	0.06695	0	0	0
ENSG00000252744	RF00019	0	0	0	0	0	0	0	0
ENSG00000283676	MIR5087	1.92896	1.60537	0	0	1.17259	3.40294	0	2.36834
ENSG00000202408	RNU1-122P	0.46987	2.34632	3.45237	4.18407	0.57126	1.65784	0.78321	2.30762
ENSG00000272824	AC245100.7	0	0	0.2437	0.24612	0.60486	0	0	0
ENSG00000207340	RNVU1-1	0.44695	0	0	0	0.54339	0	0	1.64629
ENSG00000252135	RNVU1-2	0	0	0	0	0	0	0	0
ENSG00000278596	MIR6077	0	0	0	0	0	0	0	0
ENSG00000238825	RNU1-13P	0.90494	0	4.65431	2.01455	4.95093	4.78933	0.7542	3.88876
ENSG00000231551	AC245100.4	0.24213	0.25647	0.06469	0.19601	0.16057	0.19416	0.18345	0.08108
ENSG00000201183	RNVU1-3	1.36584	0.75782	1.33806	2.70275	0	2.40954	0	3.35393
ENSG00000225871	AC245100.2	0	0	0	0	0.10615	0	0	0
ENSG00000280649	AC245100.8	0.7621	1.1532	0.10181	0.30847	0.84231	1.5889	0.34645	0.42532
ENSG00000236334	PPIAL4G	0	0	0	0	0	0	0	0
ENSG00000274210	RF00003	61.3057	75.4234	44.3912	135.817	52.3898	124.61	31.1004	50.7256
ENSG00000270629	NBPF14	2.27475	1.58099	0.14224	0.40404	1.73585	0.43759	0.59497	1.10676
ENSG00000252515	RNU6-1171P	0	0	0	0	0	0	0	0
ENSG00000177144	NUDT4B	1.76261	2.63037	0.89315	3.06693	2.95578	5.57563	1.41835	1.56711
ENSG00000275140	SEC22B3	0	0.21182	0	0	0	0	0	0
ENSG00000271546	AC245389.1	0	0	0	0	0	0	0	0
ENSG00000178104	PDE4DIP	0.79873	0.49028	0.13258	0.30324	1.16142	0.41196	0.19019	0.33884
ENSG00000252656	RN7SKP88	0	0	0	0	0	0	0	0
ENSG00000222788	RNU2-38P	0	0	0	0	0	0	0	0
ENSG00000254913	AC239802.1	0	0	0	0	0	0	0	0
ENSG00000255148	AC239802.2	0	0	0	0	0	0	0	0
ENSG00000254539	AC239804.1	0	0	0	0	0	0	0	0
ENSG00000269713	NBPF9	0.60773	0.33719	0.07144	0.10823	0.3448	0.24301	0.2026	0.25867
ENSG00000274019	AC239804.2	0	0	0	0	0	0	0	0
ENSG00000201699	RNU1-59P	0	0	0	0	1.10021	0	0	0
ENSG00000274265	AC245297.3	1.54201	1.00833	0.64742	1.06252	0.40173	1.263	1.92771	0.74378
ENSG00000215861	AC245297.1	0.54995	0.26154	0.11545	0.1166	0.09552	0.2772	0	0
ENSG00000272755	AC245297.2	0	0	0	0	0.15773	0	0	0
ENSG00000273591	RF00100	0	0	0	0	0	0	0	0
ENSG00000274423	SEC22B2	0	0	0	0	0	0	0	0
ENSG00000271383	NBPF19	1.52181	1.47436	0.27523	0.51541	1.26666	0.81229	0.67643	0.82403
ENSG00000263464	PPIAL4C	0	0	0	0	0	0	0	0
ENSG00000277147	LINC00869	0.97435	1.40652	0.35478	0.31992	1.32087	1.30818	0.70425	0.91045
ENSG00000275557	AC242842.1	1.47585	0.5459	0	0	2.79113	1.44644	2.18667	1.20801
ENSG00000206828	RF00003	4.46953	6.69559	3.94075	19.8998	3.80376	12.6158	3.72501	21.9505
ENSG00000234232	AC243772.3	0	0	0.03225	0	0	0	0	0
ENSG00000275229	RNU1-68P	0	0	0	0	0	0	0	0
ENSG00000226500	AC243772.1	0	0	0	0	0	0.31539	0	0
ENSG00000150337	FCGR1A	3.98531	20.2262	2.22757	1.48773	3.6265	7.46195	1.46714	13.5386
ENSG00000203814	HIST2H2BF	34.5631	27.1809	4.68468	9.5647	26.155	44.6684	7.22531	22.2177
ENSG00000233030	AC243772.2	0.3724	0.86091	0.0608	0.09211	0.15092	0.0365	0.06897	0.20321
ENSG00000183598	HIST2H3D	10.3441	13.3586	2.3587	5.29371	8.45633	67.3302	2.97276	8.10193
ENSG00000270882	HIST2H4A	0.19536	0.45526	0.17225	0.28994	0.0475	0.41358	0.06513	0.33581
ENSG00000203811	HIST2H3C	0	0	0	0	0	0	0	0
ENSG00000203812	HIST2H2AA3	0.53406	3.5558	0.3924	0.19815	0	2.59095	1.3353	0.32786

ENSG00000220323	HIST2H2BD	0.29736	0.24748	0	0	0.36153	0.2623	0.24783	0.18255
ENSG00000261716	AC239868.1	2.34781	2.61428	0.28552	0.40852	0.76774	1.02832	0.5128	0.93436
ENSG00000272993		2.18481	2.00014	0.16053	0.48637	0.79687	0.19272	0.72835	1.74361
ENSG00000203819		1.01104	0.84144	0.18571	0.37512	0.9219	0	0	1.39651
ENSG00000272196	HIST2H2AA4	0.25993	0.64898	0.19098	1.15729	0.15801	0.9171	0.6499	0.31914
ENSG00000203852	HIST2H3A	0	0	0.06504	0	0	0.23426	0	0
ENSG00000270276	HIST2H4B	0	0	0	0	0.0924	0.06704	0	0.04665
ENSG00000264207	AC239868.2	0	0	0	0	0	0	0	0
ENSG00000184678	HIST2H2BE	53.1544	64.5633	16.054	19.5358	45.4519	61.3553	18.3772	47.0085
ENSG00000184260	HIST2H2AC	503.517	584.389	85.3426	104.881	514.135	526.2	93.3583	174.456
ENSG00000184270	HIST2H2AB	38.7951	49.0518	12.6077	20.7606	47.3928	165.835	13.3683	28.396
ENSG00000178096	BOLA1	0.7024	0.58457	0.36863	0.52122	0.97595	0.79658	0.08363	1.04719
ENSG00000159164	SV2A	0.16714	0.05058	0	0.11275	0.22168	0.05361	0.15197	0.07462
ENSG00000143368	SF3B4	15.3784	9.56503	1.39141	3.24661	11.3926	16.0128	3.2654	3.56781
ENSG00000014914	MTMR11	2.29767	2.11282	0.51944	0.62	1.0158	1.04878	0.61599	1.3612
ENSG00000264522	OTUD7B	0.98721	1.21237	0.61035	0.8755	0.93676	1.96458	0.70236	0.83515
ENSG00000223945	AC244033.1	0	0	0	0	0	0	0	0
ENSG00000136631	VPS45	2.38794	2.08478	0.82566	1.71986	2.1347	2.16827	1.01459	1.40845
ENSG00000023902	PLEKHO1	13.1193	7.84274	1.7884	3.2644	7.561	7.16976	2.99635	6.53901
ENSG00000266187	RN7SL480P	0.24932	0.41499	0.36637	0	0	0.43984	0	0.30611
ENSG00000143401	ANP32E	25.5992	22.0664	12.6687	31.7153	30.1835	35.906	31.35	22.8925
ENSG00000222222	RNU2-17P	0	0	0	0	0	0	0	0
ENSG00000276110	AC242988.1	0	0	0	0	0	0	0	0
ENSG00000118298	CA14	0	0	0	0	0	0	0	0
ENSG00000117362	APH1A	13.9087	9.48526	2.57422	4.76114	10.1356	9.0105	6.05097	7.35939
ENSG00000118292	C1orf54	1.41067	0.72248	0.15946	0.40261	0.26385	0.38286	0.36175	0.13323
ENSG00000159208	CIART	0.05438	0	0	0.04035	0	0	0	0
ENSG00000266472	MRPS21	7.19156	4.0149	2.23173	3.04945	6.13661	5.91005	3.79719	3.29057
ENSG00000117360	PRPF3	6.04356	5.06382	1.70402	4.29231	5.39046	6.35368	4.02492	4.97471
ENSG00000163125	RPRD2	7.3401	5.26404	2.26818	5.04214	6.26203	7.60658	3.64947	3.65632
ENSG00000143374	TARS2	2.78674	1.79556	0.42272	1.12068	2.71044	2.63254	0.98895	1.23616
ENSG00000283933	MIR6878	0	0	0	0	0	0	0	0
ENSG00000143369	ECM1	0.21816	0	0.03206	0	0	0.11546	0.03636	0.16071
ENSG00000228126	FALEC	0	0	0.19031	0	0	0	0	0
ENSG00000237781	AL356356.1	8.92831	6.78844	0.32395	1.79946	4.69035	1.16672	0.91865	3.38335
ENSG00000143382	ADAMTSL4	2.67811	1.36577	0.2512	0.30444	1.17769	0.66345	0.34192	0.36379
ENSG00000264553	MIR4257	0	0	0	0	0	1.50363	0	0
ENSG00000203804	ADAMTSL4-AS1	5.39549	6.93336	0.78054	0.91277	3.9766	1.28227	2.05032	5.59478
ENSG00000277452	RN7SL473P	5.63849	6.52888	0.36025	2.54682	3.57659	1.29744	2.45178	6.32086
ENSG00000274963	RN7SL600P	5.39334	5.71277	2.52173	1.81916	5.96098	1.72992	1.63452	6.01987
ENSG00000143384	MCL1	281.312	312.348	84.4264	108.139	260.173	153.038	118.798	207.625
ENSG00000253047	RF00561	1.21158	0	0	0	0	0	0	0.74378
ENSG00000143420	ENSA	11.2626	11.2835	2.68648	4.95111	9.84777	9.13398	6.09456	7.20894
ENSG00000276103	RF00015	0	0	0	0.92979	1.52336	0	0	1.53841
ENSG00000143457	GOLPH3L	2.64225	3.62837	1.65017	4.08477	4.22963	5.47701	3.00955	2.62232
ENSG00000143452	HORMAD1	0.701	3.44972	0.26873	1.58316	1.03753	0.26884	0.50803	1.12262
ENSG00000206931	RNU6-1042P	0	0	0	0	0	0	0	0
ENSG00000163131	CTSS	460.641	486.562	87.3329	120.647	268.362	127.966	149.951	292.065
ENSG00000236713	AL356292.1	0	0	0	0	0	0	0	0
ENSG00000143387	CTSK	1.63149	0.9052	0.22833	0.5765	0.75563	0.41117	0.32374	0.81078
ENSG00000224690	UBE2D3P3	0	0	0	0	0	0	0	0
ENSG00000143437	ARNT	12.003	8.01406	2.46307	4.12371	8.50684	7.26337	4.35019	5.40038
ENSG00000200175	RNU6-1309P	0.67871	0	0	0	0	0	0	0
ENSG00000224800	RPS27AP6	0.16362	0	0.24043	0	0	0	0	0
ENSG00000283324	CTXND2	0.11928	0	0	0	0	0	0	0.07323
ENSG00000229357	CYCSP51	0	0	0	0	0.28024	0	0	0
ENSG00000143379	SETDB1	6.57254	4.93676	1.21488	2.28522	5.35228	4.61242	1.37804	3.17204
ENSG00000143418	CERS2	18.8144	17.1033	3.03844	7.19148	13.6246	11.3329	7.28767	9.36016

ENSG00000259357	AL590133.2	0.09422	0	0	0	0	0	0.15704	0.11568
ENSG00000231073	AL590133.1	0	0	0	0	0.48433	0	0	0
ENSG00000143412	ANXA9	0.24086	0	0	0	0	0.07082	0	0
ENSG00000143409	MINDY1	16.624	16.892	2.78375	4.30323	12.5262	7.33176	3.35094	6.83522
ENSG00000143363	PRUNE1	17.8525	16.451	8.08822	13.1765	16.8749	20.5599	15.5169	11.077
ENSG00000200759	RNU6-884P	0	0	0	0	0	0	0	0
ENSG00000163141	BNIPL	0.07604	0.08438	0	0.11285	0.03081	0.08943	0.04225	0.03112
ENSG00000143443	C1orf56	0.3459	0.05234	0.13863	0.14001	0.45877	0	0	0.07722
ENSG00000197622	CDC42SE1	100.718	91.2148	20.6984	34.3985	76.8482	54.4877	34.2245	61.8407
ENSG00000213190	MLLT11	0.82744	0.50899	0.13216	0.34705	0.6342	0.82506	0.26985	0.37545
ENSG00000143458	GABPB2	4.58676	3.22647	1.33631	2.16648	4.98487	5.75563	4.29473	2.41912
ENSG00000233717	RPS29P29	0	0	0	0	0	0	0	0
ENSG00000261168	AL592424.1	0.04772	0	0	0	0	0	0	0
ENSG00000143434	SEMA6C	0.07571	0.06301	0.07417	0.09364	0.07671	0.13356	0.02103	0
ENSG00000163154	TNFAIP8L2	15.4317	24.236	4.38902	4.70974	12.9363	7.24497	3.73386	12.9113
ENSG00000163156	SCNM1	10.417	12.4437	9.63322	17.4923	16.7588	15.961	14.0704	13.4238
ENSG00000163155	LYSMD1	0.32149	0.58377	0	0.13013	0.4264	0.5156	0.04872	0.07177
ENSG00000163157	TMOD4	0	0	0	0	0	0	0	0
ENSG00000163159	VPS72	4.30609	3.1945	0.54509	1.14889	4.17642	3.89785	0.72582	1.28709
ENSG00000143398	PIP5K1A	5.69586	5.07898	1.19571	3.05928	5.5564	6.19644	2.78042	3.08038
ENSG00000159352	PSMD4	18.6944	16.0421	4.51922	6.90018	17.841	17.3014	10.4138	10.5249
ENSG00000232671	ZNF687-AS1	1.29631	0.39231	0.17317	0.34979	0.71637	0	0.19643	0.14469
ENSG00000143373	ZNF687	6.67311	5.68822	0.96703	1.35361	3.91614	3.60501	2.34777	3.1185
ENSG00000143393	PI4KB	9.98207	7.62553	2.03346	3.38958	8.13418	9.59884	3.78454	4.07428
ENSG00000265753	RN7SL444P	0	0	0.35905	0	0.29706	0	0.81454	0.59998
ENSG00000273481	AL391069.3	0.30478	0.25366	0	0	0.18527	0	0	0
ENSG00000143390	RFX5	7.38438	4.6558	1.52669	2.15864	6.78188	10.3483	2.39778	3.78749
ENSG00000224645	AL391069.1	0	0	0	0	0	0	0	0
ENSG00000237976	AL391069.2	0	0	0	0.35206	0.43261	0.20924	0.1977	0
ENSG00000143416	SELENBP1	25.6677	37.3616	45.9567	59.0187	51.3998	78.1259	20.6772	30.5384
ENSG00000159377	PSMB4	20.8896	14.1344	4.14284	7.15828	14.8665	20.3735	8.66246	13.0533
ENSG00000143442	POGZ	4.07462	2.85067	0.99619	1.51444	3.4269	3.77663	1.62955	1.73476
ENSG00000238711	RNY4P25	0	0	0	1.13318	0	0	0	0
ENSG00000143375	CGN	0.03324	0	0	0.06577	0.06735	0	0.01847	0
ENSG00000252840	RF00405	0	0	0	0	0	0	0	0
ENSG00000143367	TUFT1	0.69936	0.56631	0.19443	0.14026	0.43662	0.36679	0.22054	0.16245
ENSG00000232536	AL365436.2	0	0	0	0	0	0	0	0
ENSG00000207606	MIR554	0.76355	0	0	0	0	0	0	0
ENSG00000223861	AL365436.1	0.29616	0	0	0	0	0	0	0
ENSG00000250734	AL391335.1	0	0	0	0	0	0	0	0
ENSG00000143376	SNX27	12.3885	13.6763	3.3034	5.31764	8.73623	5.88804	4.27067	6.89411
ENSG00000206635	RNU6-1062P	0	1.14027	0	0	0	0	1.14187	0
ENSG00000227045	AL589765.1	0.1899	0	0	0	0.23087	0.335	0	0
ENSG00000159409	CELF3	0.04954	0.02749	0	0	0.02008	0	0	0.02027
ENSG00000178796	RIIAD1	0	0	0	0	0	0	0	0
ENSG00000206980	RNU6-662P	0	0	0	0	0	0	0	0
ENSG00000269621	AL589765.7	0	0	0	0	0	0	0	0
ENSG00000236940	AL589765.3	0	0	0	0	0	0	0	0
ENSG00000143436	MRPL9	5.46112	2.69334	1.12945	2.04123	4.08206	7.49323	2.36	1.73835
ENSG00000143450	OAZ3	0	0	0	0.04696	0	0.05582	0.07912	0
ENSG00000249602	AL589765.4	0	0	0	0	0.69987	0	0	0
ENSG00000232937	AL589765.2	0	0	0	0	0	0	0	0
ENSG00000268288	AL589765.5	0.71165	0	0	0	0	0	0	0
ENSG00000182134	TDRKH	0.50383	0.33545	0.14807	0.32046	0.56004	0.58409	0.19196	0.26511
ENSG00000203288	TDRKH-AS1	0	0	0.0944	0	0.0781	0.11333	0	0
ENSG00000269489	AL589765.6	0	0	0	0	0	0	0	0
ENSG00000213171	LINGO4	0	0	0	0	0	0	0	0
ENSG00000143365	RORC	0.58046	0.3285	0.10236	0.60303	0.25405	0.08192	0.0774	0.25656

ENSG00000225556	C2CD4D	0	0	0	0	0	0	0	0
ENSG00000201134	RF00019	0	0	0	0	0	0	0	0
ENSG00000234614	C2CD4D-AS1	4.92532	2.41123	0.63862	1.71993	1.23284	0.76667	0.72439	1.95646
ENSG00000196407	THEM5	0.74492	0.24798	1.20412	1.2161	0.72453	1.8398	0.745	0.36584
ENSG00000159445	THEM4	2.58805	0.97273	0.24536	0.81775	2.39539	1.35498	0.83495	1.02502
ENSG00000223810	KRT8P28	0	0	0	0	0	0	0	0
ENSG00000197747	S100A10	45.3674	27.9125	5.86388	11.0689	22.6401	18.0182	12.0359	10.4404
ENSG00000229021	AL450992.1	0.03568	0	0	0.02647	0	0.03147	0	0
ENSG00000240667	NBPF18P	0	0	0	0	0	0	0	0
ENSG00000163191	S100A11	573.144	747.302	65.8956	97.7472	417.276	139.581	95.4085	192.038
ENSG00000232678	SPTLC1P4	0	0	0	0	0	0	0	0
ENSG00000182898	TCHHL1	0	0	0	0	0	0	0	0
ENSG00000159450	TCHH	0	0	0	0	0.01274	0	0	0
ENSG00000226716	AL589986.1	0	0	0	0	0	0	0	0
ENSG00000233792	PUDPP2	0	0	0	0	0	0	0	0
ENSG00000215853	RPTN	0	0	0	0	0	0	0	0
ENSG00000237975	FLG-AS1	0.01906	0.01586	0.02801	0.04243	0.04635	0.01681	0.03177	0
ENSG00000236427	AL589986.2	0	0	0	0	0	0	0	0
ENSG00000197915	HRNR	0	0	0	0	0	0	0	0
ENSG00000143631	FLG	0	0	0	0	0	0	0	0
ENSG00000143520	FLG2	0	0	0	0	0	0	0	0
ENSG00000183586	HMGN3P1	0	0	0	0	0.92349	0	0	0
ENSG00000143536	CRNN	0	0	0	0	0	0	0	0
ENSG00000186207	LCE5A	0	0	0	0	0	0	0	0
ENSG00000169509	CRCT1	0	0	0	0	0	0	0	0
ENSG00000185966	LCE3E	0	0	0	0	0	0	0	0
ENSG00000163202	LCE3D	0	0	0	0	0	0	0	0
ENSG00000244057	LCE3C	0	0	0	0	0	0	0	0
ENSG00000187238	LCE3B	0	0	0	0	0	0	0	0
ENSG00000185962	LCE3A	0	0	0	0	0	0	0	0
ENSG00000226947	LCEP4	0	0	0	0	0	0	0	0
ENSG00000176075		0	0	0	0	0	0	0	0
ENSG00000187223	LCE2D	0	0	0	0	0	0	0	0
ENSG00000187180	LCE2C	0	0	0	0	0	0	0	0
ENSG00000159455	LCE2B	0	0	0	0	0	0	0	0
ENSG00000187173	LCE2A	0	0	0	0	0	0	0	0
ENSG00000187170	LCE4A	0	0	0	0	0	0	0	0
ENSG00000198854	C1orf68	0	0	0	0	0	0	0	0
ENSG00000229713	LCEP2	0	0	0	0	0	0	0	0
ENSG00000233819	LCEP1	0	0	0	0	0	0	0	0
ENSG00000203786	KPRP	0	0	0	0	0	0	0	0
ENSG00000240386	LCE1F	0	0	0	0	0	0	0	0
ENSG00000186226	LCE1E	0	0	0	0	0	0	0	0
ENSG00000172155	LCE1D	0	0	0	0	0	0	0	0
ENSG00000197084	LCE1C	0	0	0	0	0	0	0	0
ENSG00000196734	LCE1B	0	0	0	0	0	0	0	0
ENSG00000186844	LCE1A	0	0	0	0	0	0	0	0
ENSG00000235942	LCE6A	0	0	0	0	0	0	0	0
ENSG00000163206	SMCP	0	0	0	0	0	0	0	0
ENSG00000163207	IVL	0	0	0	0	0	0	0	0
ENSG00000224308	LINC01527	0	0	0	0	0	0	0	0
ENSG00000283227	SPRR5	0	0	0	0	0	0	0	0
ENSG00000184148	SPRR4	0	0	0	0	0	0	0	0
ENSG00000169474	SPRR1A	0	0	0	0	0	0	0	0
ENSG00000163209	SPRR3	0	0	0	0	0	0	0	0
ENSG00000252920	RF00322	0	0	0	0	0	0	0	0
ENSG00000234262	AL356867.1	0	0	0	0	0	0	0	0
ENSG00000169469	SPRR1B	0	0	0	0	0	0	0	0

ENSG00000163216	SPRR2D	0	0	0	0	0	0	0	0
ENSG00000241794	SPRR2A	0	0	0	0	0	0	0	0
ENSG00000196805	SPRR2B	0	0	0	0	0	0	0	0
ENSG00000203785	SPRR2E	0	0	0	0	0	0	0	0
ENSG00000244094	SPRR2F	0	0	0	0	0	0	0	0
ENSG00000229035	SPRR2C	0	0	0	0	0	0	0	0
ENSG00000159516	SPRR2G	0	0	0	0	0.1569	0	0	0
ENSG00000229699	AL161636.1	0	0	0	0	0	0	0	0
ENSG00000203784	LELP1	0	0	0	0	0	0	0	0
ENSG00000203783	PRR9	0	0	0	0	0	0	0	0
ENSG00000230779	AL161636.2	0	0	0	0	0	0	0	0
ENSG00000203782	LOR	0	0	0	0	0	0	0	0
ENSG00000159527	PGLYRP3	0	0	0	0	0	0	0	0
ENSG00000163218	PGLYRP4	0	0	0	0	0	0	0	0
ENSG00000207321	RNU6-160P	0	0	0	0	0	0	0	0
ENSG00000163220	S100A9	3924.93	6228.57	634.15	542.797	2559.67	815.987	711.695	2509.94
ENSG00000163221	S100A12	268.579	380.393	16.2195	28.3515	114.234	47.9302	40.3337	161.578
ENSG00000237008	LAPTM4BP1	0	0	0	0	0	0	0	0
ENSG00000143546	S100A8	842.371	1294.19	219.282	124.249	570.134	266.979	257.842	1028.7
ENSG00000224784	S100A15A	0	0	0	0	0	0	0	0
ENSG00000184330	S100A7A	0	0	0	0	0	0	0	0
ENSG00000203781	S100A7P1	0	0	0	0	0	0	0	0
ENSG00000197364	S100A7L2	0	0	0	0	0	0	0	0
ENSG00000143556	S100A7	0	0	0	0	0	0	0	0
ENSG00000263841	RN7SL44P	0	0.404	0	0	0	0	0	0
ENSG00000238279	BX470102.1	0	0	0	0	0	0	0	0
ENSG00000197956	S100A6	180.345	218.51	49.3643	44.8139	128.673	58.7297	54.4841	101.49
ENSG00000196420	S100A5	0.0935	0.15562	0	0.13876	0.22734	0.32988	0.15584	0
ENSG00000196154	S100A4	124.789	126.294	51.3874	57.0992	70.0761	49.7062	58.885	101.587
ENSG00000188015	S100A3	0.07675	0	0	0.11391	0	0	0	0
ENSG00000196754	S100A2	0	0	0	0	0	0	0	0
ENSG00000188643	S100A16	0.04309	0	0	0	0.10478	0	0	0
ENSG00000189334	S100A14	0	0	0	0	0.13957	0	0	0
ENSG00000189171	S100A13	0.06616	0.38541	0	0.09818	0.04022	0.05835	0	0.20306
ENSG00000271853	AL162258.1	0.07367	0	0	0	0	0.25992	0	0
ENSG00000160678	S100A1	0	0	0	0	0	0	0	0
ENSG00000272030	AL162258.2	0.27818	0.34727	0	0.10321	0.1691	0	0.23184	0.17077
ENSG00000160679	CHTOP	5.18641	3.75244	1.1298	2.12737	4.68952	4.18395	2.32414	3.83987
ENSG00000143553	SNAPIN	5.26965	2.44965	1.11621	2.04326	3.69396	4.10381	1.89918	2.44811
ENSG00000143621	ILF2	23.78	16.5281	4.01389	8.67997	22.3866	33.5043	8.51673	9.90323
ENSG00000169418	NPR1	0	0	0	0	0	0	0	0
ENSG00000274940	MIR8083	0	0	0	0	0	0	0	0
ENSG00000242565	RN7SL372P	0	0	0	0	0	0	0	0
ENSG00000235015	GEMIN2P1	0	0	0	0	0	0	0	0
ENSG00000199565	RF00019	0	0	0	0	0	0	0	0
ENSG00000143624	INTS3	2.61637	1.83305	0.42261	0.77035	1.71411	2.10364	0.70151	0.9215
ENSG00000243613	AL513523.4	0	0	0	0	0	0	0	0
ENSG00000233222	AL513523.3	0	0	0	0	0	0	0	0
ENSG00000279767		0	0	0	0	0	0	0	0
ENSG00000143554	SLC27A3	1.60805	2.05206	0.25599	0.13921	0.61909	0.66193	0.22336	0.36196
ENSG00000207039	RF00019	0	0	0	0	0	0	0	0
ENSG00000143614	GATAD2B	1.92624	1.57013	0.3844	0.87057	1.57089	2.06966	0.88527	0.94406
ENSG00000231827	AL513523.2	0	0	0	0	0	0	0	0
ENSG00000223599	AL513523.1	0	0	0	0	0	0	0	0
ENSG00000236327	AL831737.1	0	0	0	0	0	0	0	0
ENSG00000198837	DENND4B	6.28233	5.48588	1.15043	2.05121	3.70144	3.69998	1.85268	2.72934
ENSG00000160741	CRTC2	8.76682	6.24905	0.83499	2.13837	5.10718	5.47749	2.57079	2.91519
ENSG00000143570	SLC39A1	9.10845	9.75924	1.58964	1.22015	6.28657	4.84728	1.29826	3.18762

ENSG00000276584	MIR6737	1.04715	1.74298	0	0	0	0	0	0
ENSG00000282386	AL358472.4	0	0.51553	0	0	0.12552	0	0.17208	0
ENSG00000273026	AL358472.3	0	0	0	0	0	0.62319	0	0.21686
ENSG00000143578	CREB3L4	0.45434	0.33611	0	0.33715	0.491	0.48982	0.16829	0.30991
ENSG00000143543	JTB	19.403	18.4318	8.71216	8.79884	17.9901	19.5351	11.0256	16.9045
ENSG00000272654	AL358472.2	0.15508	0.25813	0	0	0.25139	0.09119	0	0.1904
ENSG00000143545	RAB13	1.51876	1.46357	0.70478	0.90951	1.49014	3.66642	0.57737	0.52343
ENSG00000177954	RPS27	477.227	433.994	170.111	209.062	625.324	490.319	359.437	610.001
ENSG00000143552	NUP210L	0.08713	0.12431	0.07316	0.12931	0.10593	0.21958	0.04149	3.5302
ENSG00000231416	AL358472.1	0.66839	0	0	0	0.81261	0.39304	0.37137	0.27355
ENSG00000200220	RNU6-179P	0	0	0	0	0	0	0	0
ENSG00000231837	RPS7P2	0	0	0	0	0	0	0	0
ENSG00000263987	MIR5698	0	0	0	0	0	0	0	1.24996
ENSG00000143549	TPM3	66.7296	54.6704	11.4532	17.9732	42.864	43.0525	21.06	29.3196
ENSG00000264384	RN7SL431P	1.6823	2.40017	0.70632	0.35667	2.0453	2.11987	0	3.2458
ENSG00000215938	MIR190B	0	0	0	0	0	0	0	0
ENSG00000163263	C1orf189	0.18941	0.31527	0	0.2811	0	0	0.31571	0
ENSG00000143612	C1orf43	16.3581	14.886	4.74108	6.13232	13.7632	16.7756	9.62347	11.6058
ENSG00000143569	UBAP2L	11.8015	8.9925	1.50739	3.94372	11.248	12.702	3.46853	4.64199
ENSG00000201129	SNORA58B	0	0	0	0	0	0	0	0
ENSG00000143575	HAX1	10.9865	8.15277	1.39953	2.62499	9.18046	9.9009	3.0616	4.67734
ENSG00000252682	RF00273	0	0	0	0	0	0	0	0
ENSG00000212292	RNU6-239P	0	0	0	0	0	0	0	0
ENSG00000222457	RNU6-121P	0	0	0	0	0	0	0	0
ENSG00000278694	AL354980.1	0	0	0	0	0	0	0	0
ENSG00000143595	AQP10	2.04678	1.22434	0.23498	0.56956	0.34993	1.07196	0.47977	0.82458
ENSG00000143515	ATP8B2	7.41522	4.04439	1.2376	2.9452	6.77908	5.77224	1.79107	3.00703
ENSG00000238365	RNU7-57P	1.22167	2.03347	0	0	2.97056	0	0	0
ENSG00000237920	AL162591.1	0	0	0	0	0	0	0	0
ENSG00000226855	RPSAP17	0	0	0	0	0	0	0	0
ENSG00000228013	IL6R-AS1	0.08349	0.06948	0	0.06195	0.1015	0	0	0.05125
ENSG00000160712	IL6R	57.1818	53.0537	7.86197	11.9019	40.8503	15.8709	10.7923	30.3337
ENSG00000228264	PSMD8P1	0	0	0	0	0	0	0	0
ENSG00000169291	SHE	0.13026	0.24572	0.01276	0.03866	0	0.10724	0.01447	0.01066
ENSG00000273110	AL162591.2	0.32291	0.53748	0.31634	0.15974	0.13086	0.37977	0.53824	0.26431
ENSG00000163239	TDRD10	0.02709	0	0	0	0	0.14336	0	0.03326
ENSG00000160714	UBE2Q1	15.326	12.4618	1.57908	3.66018	12.6575	10.5041	3.40613	6.14255
ENSG00000229780	UBE2Q1-AS1	0	0	0	0	0	0	0	0.20833
ENSG00000160716	CHRNB2	0	0	0	0	0	0	0	0
ENSG00000233875	AL592078.1	1.3701	0.38009	0.67111	0	0	0.40284	0	0.84109
ENSG00000160710	ADAR	56.87	85.6804	19.3746	19.0987	46.2007	44.832	17.8679	46.7649
ENSG00000143603	KCNN3	0.03773	0.06281	0	0.024	0.02621	0	0.01797	0.01986
ENSG00000163344	PMVK	2.97783	2.19234	0.58906	1.01987	2.01905	3.53587	0.47727	0.42186
ENSG00000270361	AL451085.1	0.3187	0.53047	0	0.15766	0.25831	0.56223	0.17707	0
ENSG00000163346	PBXIP1	56.4742	30.7635	7.04854	16.1865	36.2159	21.7255	15.2289	16.0784
ENSG00000268154	RF00017	1.29506	0.43113	0.38061	0	0	0.45693	0	0
ENSG00000163348	PYGO2	4.03859	3.26028	0.94954	2.1877	3.95256	4.73787	1.17805	1.85944
ENSG00000271380	AL451085.2	0.64218	0.61081	0	0	0.22307	0	0.61167	0.22527
ENSG00000160691	SHC1	6.7358	5.39512	1.36441	2.07951	4.90532	4.88419	1.40696	2.77743
ENSG00000173207	CKS1B	0.8465	0.52185	0.3225	0.55835	0.64798	2.93136	0.36581	0.46192
ENSG00000264349	MIR4258	0	0	0	0	0	0	0	0
ENSG00000160688	FLAD1	2.09742	1.60897	0.24121	0.8391	1.75173	2.05921	1.09442	0.96289
ENSG00000163352	LENEP	0	0	0	0	0	0	0	0
ENSG00000160685	ZBTB7B	23.6612	19.6212	3.57962	5.53662	13.1097	10.0372	4.82702	7.94768
ENSG00000163354	DCST2	0.07161	0	0	0	0.02902	0	0	0
ENSG00000163357	DCST1	0	0.03821	0	0	0	0	0	0
ENSG00000232093	DCST1-AS1	0	0	0	0	0.21683	0	0	0.10949
ENSG00000143537	ADAM15	2.93914	1.24158	0.26176	0.727	1.28586	0.70705	0.74229	0.87482

ENSG00000243364	EFNA4	1.7411	1.06262	0.25585	0	1.62287	0.71669	0	0.21377
ENSG00000251246	AL691442.1	0	0	0	0	0	0	0	0
ENSG00000143590	EFNA3	0.07679	0	0	0.05699	0	0	0	0.04714
ENSG00000202027	RF00019	0	0	0	0	0	0	0	0
ENSG00000169242	EFNA1	0.02887	0.04805	0	0	0	0	0.04812	0
ENSG00000169241	SLC50A1	4.82404	4.69263	0.82857	1.99905	3.69415	9.67075	1.35756	1.57687
ENSG00000179085	DPM3	6.24585	2.55002	0.17317	0.52469	5.0146	3.95004	1.57145	1.15752
ENSG00000223452	HMGN2P18	0	0	0	0	0	0	0	0
ENSG00000163463	KRTCAP2	0.15327	0.09567	0.02815	0.05687	0.06988	0	0.0958	0.07057
ENSG00000273088	AL713999.1	0	0	0	0	0	0	0	0
ENSG00000163462	TRIM46	0.03234	0.05383	0	0	0	0.02853	0	0.01985
ENSG00000185499	MUC1	0.06216	0.05173	0	0.02306	0.05668	0.08224	0.0259	0
ENSG00000231064	AC234582.1	0.02972	0	0	0	0	0	0.04955	0
ENSG00000284586	MIR92B	0	0	0	0	0	0	0	0
ENSG00000169231	THBS3	1.23674	0.81814	0.0233	0.25885	1.0795	0.47551	0.47572	0.42828
ENSG00000173171	MTX1	3.8685	3.72204	0.55861	0.79648	2.25646	1.02566	0.85727	1.48256
ENSG00000236263	AC234582.2	0.38715	0	0	0	0	0	0	0
ENSG00000160766	GBAP1	0.20049	0.13349	0.02946	0.05951	0.0975	0.14148	0	0.02462
ENSG00000236675	MTX1P1	0	0	0	0	0	0	0	0
ENSG00000177628	GBA	5.78687	5.71915	0.97439	1.64014	4.10406	3.8992	1.03826	2.02296
ENSG00000160767	FAM189B	0.24608	0.10923	0.02411	0.02435	0.13962	0.23153	0.02735	0.04029
ENSG00000116521	SCAMP3	3.06169	2.24831	0.44109	1.55917	2.48155	4.55398	0.60039	1.10561
ENSG00000176444	CLK2	4.13438	2.79256	0.587	1.03747	3.03531	2.46644	0.93217	1.37325
ENSG00000143630	HCN3	0	0	0	0	0	0	0	0
ENSG00000143627	PKLR	0	0.13981	0	0.16621	0	0	0	0
ENSG00000160752	FDPS	4.79074	3.83943	0.57942	1.75555	4.4822	4.41705	1.57737	2.17852
ENSG00000225855	RUSC1-AS1	0.16678	0.1388	0	0.03094	0.17742	0.11033	0.03475	0.12798
ENSG00000160753	RUSC1	0.67533	0.40467	0.0397	0.24054	0.42695	0.40507	0.18011	0.21558
ENSG00000116539	ASH1L	14.9622	14.104	5.61415	8.90184	16.0324	17.1074	15.0428	10.8435
ENSG00000283701	MIR555	0	0	0	0	0	0	0	0
ENSG00000207134	RNU6-106P	0.68505	0	0	0	0	0	0	0
ENSG00000227773	ASH1L-IT1	1.14532	1.63404	1.20216	2.42825	1.98921	0.57728	0.81817	1.80798
ENSG00000207144	RNU6-1297P	0	0	0	0	0	0	0	0
ENSG00000237872	POU5F1P4	0	0	0	0	0	0	0	0
ENSG00000235919	ASH1L-AS1	0.72755	0.50458	0.44547	0.62986	1.10567	0.53479	0.70741	0.3722
ENSG00000271267	AL353807.4	0	0	0	0	0	0	0	0
ENSG00000225082	DAP3P1	0	0.43888	0	0.39132	0	0	1.31849	0.64746
ENSG00000223503	AL353807.1	0	0	0	0	0	0	0	0
ENSG00000232519	AL353807.2	0.33318	0	0.48961	0	0	0	0	0
ENSG00000125459	MSTO1	0.33097	0.23196	0.0256	0.15511	0.55063	0.27657	0.37746	0.1711
ENSG00000246203	AL353807.3	0.05852	0.38965	0.043	0.08685	0.10673	0.05162	0	0.14371
ENSG00000163374	YY1AP1	36.0712	51.5749	47.5354	74.0248	81.9531	82.7947	108.352	83.3026
ENSG00000132676	DAP3	12.4777	10.9563	7.48927	19.6326	15.9081	15.558	19.094	12.1029
ENSG00000227673	AL162734.1	0	0	0	0	0	0	0	0
ENSG00000203761	MSTO2P	0.21445	0.21418	0	0.19096	0.20858	0.07567	0.21448	0.21064
ENSG00000116580	GON4L	7.63813	8.19711	4.06841	8.39051	10.649	10.3951	9.51551	6.9564
ENSG00000234937	AL139128.1	0	0	0	0	0	0	0	0
ENSG00000132718	SYT11	4.71416	4.6801	1.19225	2.01378	2.82316	6.07074	1.11921	1.94078
ENSG00000283442	RF00015	0	0	0	0	0	0	0	0
ENSG00000143622	RIT1	24.1092	19.8626	8.79522	15.4615	18.4646	9.46989	18.4253	20.4153
ENSG00000132680	KHDC4	7.77881	8.89893	4.64498	13.8419	11.1653	13.1262	9.84381	8.52856
ENSG00000207475	SNORA80E	0.53504	0.89057	0	3.17623	0.65049	0	0.89183	0.65691
ENSG00000280466	SCARNA4	0	0	0	0	0	0	0	0
ENSG00000281394	SCARNA4	0	0	0	0.8433	0	0	0	0
ENSG00000173080	RXFP4	0	0	0	0	0	0	0	0
ENSG00000116584	ARHGEF2	18.5036	18.0308	3.14829	4.72774	14.9552	11.2254	3.26349	7.20174
ENSG00000277817	MIR6738	3.43595	3.81277	0	0	0	0	0	1.4062
ENSG00000273002	AL355388.2	0.2039	0.05656	0	0.10087	0.16526	0.3597	0.16993	0.04172

ENSG00000224276	AL355388.1	0.36559	0	0	0	0.22224	0	0.30469	0
ENSG00000163479	SSR2	13.5558	11.2451	1.83005	3.20583	11.1614	13.007	4.44559	5.76436
ENSG00000160803	UBQLN4	2.30599	2.10793	0.63884	0.98182	1.88436	1.93401	0.44108	0.90508
ENSG00000116586	LAMTOR2	7.90094	7.29675	0.97377	1.28606	6.81699	5.21564	1.52938	2.56598
ENSG00000132698	RAB25	0.12943	0	0	0	0.05245	0	0.07191	0
ENSG00000254726	MEX3A	0	0	0	0	0.01455	0.02112	0	0.0147
ENSG00000160789	LMNA	3.484	2.44541	0.46262	0.87215	1.23755	1.38846	1.067	0.83748
ENSG00000196189	SEMA4A	16.0633	16.1855	1.67366	2.80655	5.76084	2.63476	2.47155	3.41677
ENSG00000252236	RF00568	0	0	0	0	0.63203	0	0	0
ENSG00000160785	SLC25A44	6.96519	8.69936	0.75324	1.52148	5.31547	2.92561	1.42401	3.04803
ENSG00000260238	PMF1-BGLAP	0	0.12116	0	0	0	0	0	0
ENSG00000160783	PMF1	2.58878	2.7113	0.29921	0.73387	1.90964	1.796	0.53333	1.14282
ENSG00000242252	BGLAP	0.0616	0	0	0	0	0	0	0.07563
ENSG00000160781	PAQR6	0.72128	0.50024	0.04416	0.0446	0.14615	0.05302	0.05009	0.1845
ENSG00000198952	SMG5	7.91876	6.07282	1.05602	1.90744	5.6454	5.89997	2.53391	3.56323
ENSG00000163472	TMEM79	0.59749	1.03431	0.10536	0.14188	0.72642	0.42162	0.11951	0.64556
ENSG00000198715	GLMP	4.2447	1.79269	0.74478	0.3761	1.56615	2.01177	0.63361	0.64821
ENSG00000189030	VHLL	0	0	0	0	0	0	0	0
ENSG00000163468	CCT3	15.8714	19.9805	9.5704	28.5637	22.9778	25.5212	15.5994	16.5749
ENSG00000163467	TSACC	0.06574	0	0	0	0.15985	0	0	0
ENSG00000132677	RHBG	0	0	0	0	0	0	0	0
ENSG00000237390	AL139130.1	0	0	0	0	0	0	0	0
ENSG00000125462	C1orf61	0.02976	0	0	0.00883	0.00724	0.06301	0.00992	0
ENSG00000207933	MIR9-1	0	0	0	0	0	0	0	0
ENSG00000236947	AL139412.1	0	0	0	0	0	0	0	0
ENSG00000116604	MEF2D	10.766	6.10042	0.99299	1.78477	5.84828	7.45563	3.4745	2.46086
ENSG00000206651	RF00019	2.5947	5.39861	8.57899	5.77623	1.57729	0	8.64993	3.98217
ENSG00000260460	AL365181.1	0	0	0	0	0	0	0	0
ENSG00000183856	IQGAP3	0.05436	0.12668	0.06391	0.11295	0.02644	0.32606	0.07249	0.12014
ENSG00000187862	TTC24	0.23319	0.21564	0.11422	0.15382	0.37801	0.68564	0.12957	0.38175
ENSG00000163382	NAXE	3.07156	1.26237	0.50151	1.18184	3.50381	5.28486	1.01132	1.30363
ENSG00000160818	GPATCH4	1.89983	1.04492	0.21849	0.56391	2.20934	2.38981	0.74349	0.81133
ENSG00000272971	AL365181.4	0	0	0	0	0	0	0	0
ENSG00000132702	HAPLN2	0	0	0	0	0	0	0	0
ENSG00000272068	AL365181.2	0	0	0	0	0	0.08027	0	0
ENSG00000132692	BCAN	0	0	0	0	0	0	0	0
ENSG00000272405	AL365181.3	0	0	0	0	0	0	0.03792	0
ENSG00000229953	AL590666.2	0	0	0.1421	0	0	0	0	0
ENSG00000132688	NES	0	0	0	0	0	0	0	0
ENSG00000237588	AL590666.3	0	0	0	0	0	0	0	0
ENSG00000143320	CRABP2	0.13327	0.11092	0	0.19779	0.08102	0.11756	0	0
ENSG00000223356	AL590666.1	0.50785	0	0	0.25124	0	0	0	0
ENSG00000143319	ISG20L2	4.24869	3.64031	0.65504	1.17841	3.40411	4.00567	1.4396	1.69322
ENSG00000143303	RRNAD1	1.31843	1.07802	0.27192	0.48059	1.01237	1.22416	0.57832	0.53958
ENSG00000143314	MRPL24	3.67583	2.37539	0.57193	0.96271	3.62776	3.81451	1.15332	1.75216
ENSG00000143321	HDGF	64.8253	74.6712	32.875	37.1818	64.5352	85.218	17.6175	34.8213
ENSG00000143294	PRCC	3.97906	3.44523	0.60307	1.00629	3.27571	3.77736	0.95175	1.55545
ENSG00000027869	SH2D2A	5.89437	4.61417	0.60032	3.07476	2.58978	10.3985	2.04282	2.04213
ENSG00000198400	NTRK1	0.25241	0.11204	0	0.12487	0.08183	0.11874	0.0561	0.04132
ENSG00000027644	INSRR	0	0	0.02112	0	0	0	0.02395	0
ENSG00000187800	PEAR1	1.47131	1.03696	0.21426	0.53114	0.82187	1.77716	0.50816	0.6347
ENSG00000160838	LRRC71	0	0	0	0	0.03387	0.04915	0	0
ENSG00000132694	ARHGEF11	5.58557	4.75765	0.60175	1.19117	4.19197	2.0372	0.86004	3.07699
ENSG00000211581	MIR765	0	0	0	0	0	1.13431	0	0
ENSG00000266160	RN7SL612P	0	0	0	0	0	0	0	0
ENSG00000237842	AL157713.1	0.04788	0	0	0	0	0	0	0.05878
ENSG00000224520	KRT8P45	0	0	0	0	0	0	0	0.0622
ENSG00000253831	ETV3L	0	0	0	0	0	0	0.06183	0

ENSG00000117036	ETV3	7.26774	5.54832	1.05996	2.2221	5.24841	5.03211	2.64144	3.20699
ENSG00000235700	CYCSP52	0	0	0	0	0	0	0	0
ENSG00000229961	AL357143.1	0.34023	0.25169	0.01852	0.14961	0.12256	0	0.18904	0.15471
ENSG00000284592	AL357143.2	0	0	0	0	0	0	0	0
ENSG00000228239	AL138900.1	0	0	0	0	0.22335	0	0	0
ENSG00000271736	AL138900.3	18.529	17.8454	9.07606	30.7851	21.677	15.4187	209.202	23.1789
ENSG00000237189	AL138900.2	0	0	0	0	0	0	0	0
ENSG00000143297	FCRL5	0.2597	0.04912	0.03469	0.21899	1.01179	0.10412	0.05903	0.16667
ENSG00000163518	FCRL4	0	0	0	0	0.09199	0	0	0.02323
ENSG00000236731	AL135929.2	0	0	0	0	0	0	0	0
ENSG00000233712	AL135929.1	0	0	0	0	0	0	0	0
ENSG00000160856	FCRL3	1.90594	1.41827	0.18123	1.81365	4.7707	3.97548	1.85008	2.10608
ENSG00000227217	AL356276.1	0	0	0	0	0	0	0	0
ENSG00000231700	AL356276.2	0	0	0	0	0	0	0	0
ENSG00000232366	VDAC1P9	0	0	0	0	0.10597	0	0	0
ENSG00000132704	FCRL2	0.10144	0.14473	0.04259	0.38714	0.72238	0.02557	0.33818	0.32027
ENSG00000163534	FCRL1	1.69513	0.62012	0.27373	1.35464	5.93356	1.21589	1.45933	2.24135
ENSG00000073754	CD5L	0	0	0	0.04429	0	0	0	0
ENSG00000227425	MRPS21P2	0	0	0	0	0	0	0	0
ENSG00000236957	AL139010.1	0	0	0	0	0	0	0	0
ENSG00000183853	KIRREL1	0.01862	0.0155	0	0	0.04528	0	0	0
ENSG00000226520	KIRREL1-IT1	0	0	0	0	0	0	0	0
ENSG00000231666	LINC01704	0	0	0	0	0	0	0	0
ENSG00000227295	ELL2P1	0.42303	0.12803	0	0.2283	0.09351	0	0.12821	0.14165
ENSG00000158473	CD1D	20.0738	10.3434	2.00798	2.75224	5.73543	3.27154	2.06074	5.07307
ENSG00000229914	AL138899.2	0.88137	0.48901	0	0	0.17859	0	0	0
ENSG00000176320	AL138899.1	0.44002	0.12207	0.05388	0	0.04458	0.06469	0	0
ENSG00000158477	CD1A	0.69943	0.29105	0.05139	0	0.38266	0.12339	0	0.08588
ENSG00000230942	HMGN1P5	0	0	0	0	0	0	0	0
ENSG00000158481	CD1C	8.78583	3.74103	0.75062	1.44037	6.14812	0.27034	0.93657	1.44246
ENSG00000158485	CD1B	0.63054	0	0	0	0.06388	0.18539	0	0
ENSG00000158488	CD1E	0.56564	0.49845	0.04889	0.19752	0.24271	0.1174	0	0.20426
ENSG00000186306	OR10T2	0	0	0	0	0	0	0	0
ENSG00000180708	OR10K2	0	0	0	0	0	0	0	0
ENSG00000203758	OR10T1P	0	0	0	0	0	0	0	0
ENSG00000236257	EI24P2	0	0	0	0	0	0	0	0
ENSG00000173285	OR10K1	0	0	0	0	0	0	0	0
ENSG00000236656	AL365440.2	0	0	0	0	0	0	0	0
ENSG00000198965	OR10R2	0	0	0	0	0	0	0	0
ENSG00000198703	OR10R3P	0	0	0	0	0	0	0	0
ENSG00000227445	OR10R1P	0	0	0	0	0	0	0	0
ENSG00000231434	AL365440.1	0	0	0	0	0	0	0	0
ENSG00000197532	OR6Y1	0	0	0	0	0	0	0	0
ENSG00000186440	OR6P1	0	0	0	0	0	0	0	0
ENSG00000279111	OR10X1	0	0	0	0	0	0	0	0
ENSG00000198967	OR10Z1	0	0	0	0	0	0	0	0
ENSG00000163554	SPTA1	7.39218	5.73227	6.13513	11.2638	10.2851	12.8731	14.4545	7.84613
ENSG00000231237	OR6K1P	0	0	0	0	0	0	0	0
ENSG00000196171	OR6K2	0	0	0	0	0	0	0	0
ENSG00000203757	OR6K3	0.22078	0.1225	0	0.54611	0.3579	0.38949	0.73603	0
ENSG00000180437	OR6K4P	0.07675	0	0.11279	0.56956	0	0.67703	0.12794	0.09424
ENSG00000226949	OR6K5P	0	0.12911	0.22797	0.23023	0	0	0.12929	0.19047
ENSG00000180433	OR6K6	0	0	0	0	0	0	0	0
ENSG00000197403	OR6N1	0	0.12993	0	0	0	0	0	0.09584
ENSG00000188340	OR6N2	0.07683	0	0	0.11403	0	0	0	0
ENSG00000229724	OR2AQ1P	0	0	0	0	0	0	0	0
ENSG00000180409	OR10AA1P	0.5476	0.26042	0	0	0.09511	0.13801	0.26079	0.28814
ENSG00000163563	MNDA	863.477	1398.45	147.682	148.2	731.891	176.289	140.694	554.8

ENSG00000229849	PYHIN5P	0	0	0	0	0	0	0	0
ENSG00000163564	PYHIN1	11.2089	10.5911	2.91035	7.37954	9.04231	30.1071	3.37146	6.9328
ENSG00000163565	IFI16	33.5487	92.048	13.6728	10.5621	35.1147	33.8546	8.58539	34.9516
ENSG00000163568	AIM2	2.62699	12.3891	2.19824	2.11182	3.63742	5.98606	1.15551	4.03172
ENSG00000233473	RAD1P2	0	0	0	0	0	0	0	0
ENSG00000162706	CADM3	0	0	0	0	0	0	0	0
ENSG00000222552	RNA5SP60	0	0	0	0	0	0	0	0
ENSG00000225670	CADM3-AS1	0	0	0	0	0	0	0	0
ENSG00000213088	ACKR1	1.14858	0.86901	0.76719	3.92579	2.79283	5.89455	1.04428	1.19654
ENSG00000215846	MPTX1	0	0	0	0	0	0	0	0
ENSG00000248642	OR10J2P	0	0.25392	0	0	0	0	0	0
ENSG00000179639	FCER1A	48.1958	23.9828	5.91733	18.9558	22.872	4.55089	9.1242	13.2871
ENSG00000196266	OR10J3	0	0	0	0	0.09002	0	0	0
ENSG00000228560	AL513323.1	0	0	0	0	0	0	0	0
ENSG00000248442	OR10J7P	0	0	0	0	0	0	0	0
ENSG00000251686	OR10J8P	0	0	0	0	0	0	0	0
ENSG00000249197	OR10J9P	0	0	0	0	0	0	0	0
ENSG00000249730	OR10J4	0	0	0	0	0	0	0	0
ENSG00000196184	OR10J1	0	0	0	0	0	0	0	0
ENSG00000224943	AL663023.1	0	0	0	0	0	0	0	0
ENSG00000184155	OR10J5	0	0	0	0	0	0	0	0
ENSG00000231100	AL606752.1	0	0	0	0	0	0	0	0
ENSG00000271890	OR10AE1P	0	0	0	0	0	0	0	0
ENSG00000132703	APCS	0	0	0	0	0.09624	0	0	0
ENSG00000158731	OR10J6P	0	0	0	0	0	0	0	0
ENSG00000276632	AL445528.2	0	0	0	0	0	0	0	0
ENSG00000223603	CRPP1	0	0	0	0	0	0	0	0
ENSG00000132693	CRP	0	0	0	0	0	0	0	0
ENSG00000235226	AL445528.1	0	0	0	0	0	0	0	0
ENSG00000158716	DUSP23	8.08525	5.61978	0.91394	0.79117	7.45339	7.05337	1.92527	3.05445
ENSG00000181036	FCRL6	4.08901	9.04443	2.01636	3.8692	4.43748	15.0565	1.60102	4.07699
ENSG00000158714	SLAMF8	0.39146	0.77375	0.14381	0.50834	0.17847	2.71917	0.08156	0.36047
ENSG00000188004	SNHG28	0.08793	0	0	0.13049	0.14253	0.10341	0.04885	0.07197
ENSG00000212161	RF00570	0	0	0	0	0	0	0	0
ENSG00000243284	VSIG8	0	0	0	0	0	0.14226	0	0
ENSG00000256029	AL590560.1	0	0	0	0	0	0	0	0
ENSG00000272668	AL590560.2	0	0	0	0	0	0.17666	0	0
ENSG00000213085	CFAP45	2.34561	1.90566	0.1231	0.33154	2.003	0.34483	1.44289	0.51427
ENSG00000266458	MIR4259	0	0	0	0	0	0	0	0
ENSG00000279430	AL590560.3	3.81607	4.49923	0	0.47196	1.7398	0.56101	0	0.97611
ENSG00000158710	TAGLN2	174.217	146.734	25.8999	34.7309	102.912	66.7097	56.7424	75.7032
ENSG00000085552	IGSF9	0.02212	0	0	0	0	0	0	0
ENSG00000162723	SLAMF9	0.06341	0	0	0	0	0	0	0
ENSG00000224259	LINC01133	0.04647	0.07734	0.03414	0.20688	0.28246	0.32789	0.1549	0.05705
ENSG00000273933	AL513485.1	0	0	0	0	0	0	0	0
ENSG00000177807	KCNJ10	0.01115	0.05567	0.04915	0.06618	0	0	0.05575	0
ENSG00000237409	AL513302.1	0	0	0	0	0	0	0	0
ENSG00000260766		0.6367	0.13247	0.05848	0.23623	0.43542	0.28081	0.33165	0.14658
ENSG00000143315	PIGM	1.6123	0.93223	0.39903	1.13345	1.09358	0.92815	0.7638	0.85434
ENSG00000225279	AL121987.1	0	0	0	0	0	0	0	0
ENSG00000162728	KCNJ9	0	0	0	0	0	0	0	0
ENSG00000162729	IGSF8	3.39353	2.07113	0.41556	1.00728	3.67881	3.94122	0.7542	0.90275
ENSG00000018625	ATP1A2	0	0	0	0	0	0	0	0
ENSG00000132681	ATP1A4	0	0	0	0.03462	0	0	0	0
ENSG00000143318	CASQ1	0	0	0	0	0	0	0	0
ENSG00000227741	AL121987.2	0.04645	0.23196	0.13652	0.06894	0	0.40973	0.07743	0
ENSG00000162734	PEA15	14.6548	14.9576	1.30516	3.14029	8.44798	7.60386	1.95941	2.88658
ENSG00000132716	DCAF8	3.5457	2.33178	0.89442	1.53421	3.12443	2.55658	2.38338	2.06399

ENSG00000258465	AL139011.2	0	0	0	0	0	0.08996	0	0
ENSG00000228606	AL139011.1	1.57918	0.43809	0.19338	0.78123	0.63998	2.08942	0.87742	0.6463
ENSG00000224261	RPSAP18	0	0.13771	0	0	0	0	0	0
ENSG00000162735	PEX19	4.5022	2.6256	0.65193	1.53631	3.37609	2.86973	1.9172	1.89637
ENSG00000122218	COPA	27.4053	22.9255	5.99755	11.1195	19.3763	24.6298	9.46937	11.8128
ENSG00000235082	SUMO1P3	0.72336	0	0	0.35785	0.29315	0	0.40191	0.29604
ENSG00000202078	RF00019	9.73013	5.39861	3.81288	5.77623	4.73186	4.57741	2.16248	0.79643
ENSG00000162736	NCSTN	14.9937	15.5762	1.75438	3.12678	11.2917	9.32284	4.30192	5.02259
ENSG00000171786	NHLH1	0.0572	0.0476	0	0	0	0	0.04767	0
ENSG00000201608	RNU4-42P	0	0	0	0	0	0	0	0
ENSG00000162738	VANGL2	0.01373	0	0	0.02037	0.01669	0	0	0
ENSG00000162739	SLAMF6	13.3201	14.7512	3.56954	10.4586	19.2448	35.177	3.91547	9.24223
ENSG00000234425	AL138930.1	0.3463	0.38428	0	0.85658	0	0.40728	0	0.56691
ENSG00000066294	CD84	7.63496	3.62532	1.39662	2.52717	5.75816	6.70661	2.03304	2.72276
ENSG00000117090	SLAMF1	2.19781	1.08604	0.58033	1.45251	1.92053	2.09007	0.71551	0.88542
ENSG00000235101	SETP9	0	0	0	0	0	0.15542	0	0
ENSG00000228863	AL121985.1	0	0.02961	0	0.07919	0.12975	0	0	0.02184
ENSG00000117091	CD48	52.8371	46.4706	11.5807	13.4238	46.7631	57.191	18.7338	24.7671
ENSG00000026751	SLAMF7	5.73299	7.85116	1.33723	2.11584	5.49488	17.1774	1.2893	3.85462
ENSG00000275801	AL121985.3	0	0	0	0	0.1704	0	0	0
ENSG00000274562	AL121985.2	0	0	0	0	0	0	0	0
ENSG00000122224	LY9	2.01781	1.21768	0.56704	0.95447	3.10804	3.04914	1.03179	1.3621
ENSG00000122223	CD244	16.1635	10.7617	2.0736	4.34077	7.20544	7.69444	3.20739	5.41809
ENSG00000226003	PPIAP37	0	0	0	0	0	0	0	0
ENSG00000179914	ITLN1	3.31161	0.90499	2.4695	0.29342	3.78581	9.41718	2.47162	1.75989
ENSG00000213080	AL354714.2	0	0	0	0	0.08124	0.23576	0	0.08204
ENSG00000232188	AL354714.3	0	0	0	0	0	0	0	0
ENSG00000198358	AL354714.1	0	0	0	0	0	0	0	0
ENSG00000233691	AL354714.4	0	0	0	0	0	0	0	0
ENSG00000158764	ITLN2	0	0	0	0	0	0	0.07468	0
ENSG00000158769	F11R	10.2152	9.97069	2.46641	3.52169	7.3355	2.39939	2.96648	7.46126
ENSG00000270149	AL591806.3	0.06935	0	0	0	0	0	0	0
ENSG00000232879	AL591806.2	0	0	0	0	0	0	0	0
ENSG00000215845	TSTD1	5.08862	5.04167	1.60236	1.70821	4.86091	1.70991	2.42341	2.23133
ENSG00000158773	USF1	23.2829	22.0931	3.0894	3.72554	16.2134	11.348	5.70105	11.5578
ENSG00000186517	ARHGAP30	77.3243	75.9662	11.7839	20.5155	54.4133	48.9005	16.6478	31.794
ENSG00000143217	NECTIN4	0	0.03484	0	0	0	0	0	0
ENSG00000228917	AL591806.1	0	0	0	0	0	0	0	0
ENSG00000162755	KLHDC9	0	0	0	0	0	0	0.07104	0
ENSG00000143256	PFDN2	5.63016	9.19119	4.77314	7.07027	6.4501	7.44928	4.15088	6.77969
ENSG00000158793	NIT1	2.86012	2.41251	0.56796	1.26195	2.25552	1.97735	0.86973	1.35245
ENSG00000158796	DEDD	8.60064	8.52027	1.98551	2.62227	5.43356	7.2884	4.59097	3.09455
ENSG00000238934	RF01225	0	0	0	0	0	0	0	0
ENSG00000143222	UFC1	12.3779	8.36998	2.74732	6.31474	10.8163	12.9653	7.73701	5.93648
ENSG00000224985	AL590714.1	0.1934	0	0.56841	0.57407	0.94055	0	0.96713	0.47492
ENSG00000143258	USP21	1.77214	1.19934	0.22894	0.49133	1.56262	1.51161	0.6492	0.57384
ENSG00000143224	PPOX	0.82735	0.39725	0.65465	1.20427	0.96719	1.12274	1.56471	0.87907
ENSG00000158850	B4GALT3	7.33003	7.99799	4.98419	5.00582	5.65856	10.8369	3.04666	3.63227
ENSG00000158859	ADAMTS4	0.00709	0	0	0.02105	0.03449	0.07508	0	0.01742
ENSG00000158864	NDUFS2	9.13749	6.47065	1.5312	1.72487	7.99078	7.95384	1.63664	3.41979
ENSG00000158869	FCER1G	315.632	281.733	37.8787	59.2453	135.154	126.897	29.5311	85.6092
ENSG00000158874	APOA2	0	0	0	0	0	0	0	0
ENSG00000158882	TOMM40L	1.58234	1.8548	0.262	0.23153	1.00253	1.65129	0.29719	0.82089
ENSG00000284035	MIR5187	0	0	0	0	0	0	0	2.36834
ENSG00000143257	NR1I3	0	0	0	0	0	0	0	0
ENSG00000248485	PCP4L1	0	0	0	0	0	0	0	0
ENSG00000158887	MPZ	1.80358	1.86921	0.20003	0.25252	0.4551	0.66037	0.34034	0.41781
ENSG00000143252	SDHC	1.70827	0.93057	0.26624	0.50705	1.49157	1.01367	0.58674	0.69277

ENSG00000188931	CFAP126	0	0.15763	0	0	0	0	0	0
ENSG00000236484	RRM2P2	0	0	0	0	0	0	0	0
ENSG00000283696	AL592295.4	0.66169	0.12238	0.21608	0.21823	0.53631	1.03761	0.12255	0.63188
ENSG00000206921	RNU6-481P	0.68505	2.28053	0	1.01669	0	1.20852	1.14187	0
ENSG00000283360	AL592295.3	0	0	0	0	0	0	0	0
ENSG00000215840	AL592295.1	0.24876	0	0	0	0.10081	0	0	0.10181
ENSG00000277882	AL592295.2	0	0	0	0	0	0	0	0
ENSG00000283317	AL831711.1	0	0.21443	0	0	0.15662	0.45452	0	0
ENSG00000143226	FCGR2A	168.836	249.619	20.0671	30.1341	115.58	25.9159	26.372	85.8817
ENSG00000273112	AL590385.2	3.97651	4.85386	0.19478	0.9836	0.48345	0.46767	0.66282	0.32549
ENSG00000173110	HSPA6	27.02	41.6815	4.2704	3.71642	18.3044	7.58091	5.05005	17.1947
ENSG00000224203	RPS23P10	0	0	0	0	0	0	0	0
ENSG00000203747	FCGR3A	82.5186	129.763	20.0644	27.4723	28.4402	38.3389	15.6133	36.0536
ENSG00000224515	AL590385.1	0	0	0	0	0	0	0	0
ENSG00000244682	FCGR2C	12.7354	9.61072	1.35395	3.40335	1.14507	2.81741	1.05799	2.21222
ENSG00000225217	HSPA7	4.8309	2.40598	0.39128	0.90325	0.69369	0.53684	0.19021	0.88736
ENSG00000231586	RPS23P9	0	0	0	0	0	0	0	0.20547
ENSG00000162747	FCGR3B	412.92	920.523	106.803	48.3217	170.2	120.307	95.5814	385.555
ENSG00000072694	FCGR2B	1.20128	3.06351	0.51824	0.34348	0.92453	0.15554	0.38577	0.50066
ENSG00000234211	AL451067.1	0	0	0	0	0	0	0	0
ENSG00000213075	RPL31P11	0	0	0	0	0	0	0	0
ENSG00000199595	RF00019	0	0	0	0	0	0	1.19785	0
ENSG00000132185	FCRLA	1.49958	0.8196	0.13156	0.76399	4.78917	0.67124	0.44768	1.374
ENSG00000162746	FCRLB	0.42419	0.09414	0	0.16788	0.27505	0.34922	0	0.10416
ENSG00000241347	RN7SL466P	0	0	0	0	0	0	0	0
ENSG00000081721	DUSP12	4.11846	3.53146	1.03924	2.40786	3.48981	3.88963	3.05104	1.94091
ENSG00000226889	AL359541.1	0.38244	0.21219	0	0.18919	0	0	0.21249	0
ENSG00000118217	ATF6	19.9555	21.8811	5.88399	10.7683	18.6253	11.7675	11.4283	14.2011
ENSG00000229808	AL391825.1	1.07479	1.52063	0	0.23926	0.52268	0.47402	0	0.72578
ENSG00000162745	OLFML2B	0.34343	0.28582	0.03154	0.03186	0.18267	0.22719	0.03578	0
ENSG00000227818	AL590408.1	0	0	0	0	0	0	0	0
ENSG00000198929	NOS1AP	0.06603	0.0314	0.04158	0.028	0.02294	0.03328	0.03145	0.03474
ENSG00000266144	MIR4654	0	0	0	0	0	0	0	0
ENSG00000227094	AL512785.1	0	0	0	0	0	0	0	0
ENSG00000252262	RNA5SP61	0	0	0	0	0	0	0	0
ENSG00000207729	MIR556	0	0	0	0	0	0	0	0
ENSG00000254706	AL512785.2	0	0	0	0	0	0	0	0
ENSG00000239887	C1orf226	0.01631	0.08143	0.02396	0	0.03965	0	0	0
ENSG00000171722	SPATA46	0	0	0	0	0	0	0	0
ENSG00000198574	SH2D1B	6.26342	5.27816	0.77021	7.66206	4.46061	2.91264	4.32458	4.37597
ENSG00000227243	SLAMF6P1	0	0	0	0	0	0	0	0
ENSG00000152332	UHMK1	27.2161	22.0106	11.2187	21.1194	22.0912	25.1043	14.7076	19.9734
ENSG00000227941	UQCRBP2	0	0	0	0	0	0	0	0
ENSG00000259788	AL596325.1	0	0.56381	0	0.30162	0.32945	0.11951	0.11292	0.08318
ENSG00000117143	UAP1	2.68718	2.3429	1.1909	2.65871	2.20393	4.28907	2.02627	1.64964
ENSG00000272574	AL596325.2	0.43545	0	0	0.21542	0	0.51213	0	0.17821
ENSG00000162733	DDR2	0.04782	0.03411	0.01004	0.04055	0.04983	0.01205	0.03416	0.00839
ENSG00000243173	RN7SL861P	0	0	0	0	0	0	0	0
ENSG00000132196	HSD17B7	0.60432	0.76921	0.23507	0.31654	0.75632	1.25424	0.38515	0.65468
ENSG00000185860	CCDC190	0	0	0	0	0	0	0	0
ENSG00000230739	AL392003.2	0	0	0	0	0	0	0	0
ENSG00000227667	AL392003.1	0	0	0	0	0	0	0	0
ENSG00000117152	RGS4	0.01516	0.02523	0	0	0.01843	0	0	0
ENSG00000143248	RGS5	0.04299	0.03577	0	0.01595	0.02613	0.15166	0.07165	0.02639
ENSG00000232892	AL499616.1	0	0	0	0	0	0	0	0
ENSG00000225755	AL451063.1	0	0	0	0	0	0	0	0
ENSG00000232995	RGS5	0.06065	0.20189	0.02971	0.12001	0.09831	0.07132	0.26956	0.22338
ENSG00000237756	AL592435.1	0	0	0	0	0	0	0	0

ENSG00000143228	NUF2	1.19914	1.01613	1.08931	2.36209	1.35186	3.57704	2.32586	1.41875
ENSG00000225122	AL603841.1	0	0	0	0	0	0	0	0
ENSG00000200327	RNA5SP62	0	0	0	0	0	0	0	0
ENSG00000212527	RNA5SP63	0	0	0	0	0	0	0	0
ENSG00000228289	AL355862.1	0	0	0	0	0	0	0	0
ENSG00000212538	RF00012	0	0	0	0	0	0	0	0
ENSG00000225719	NMNAT1P2	0	0	0	0	0	0	0	0
ENSG00000199849	RNU5F-6P	0	0	0	0	0	0	0	0
ENSG00000213070	HMGB3P6	0	0	0	0	0	0	0	0
ENSG00000185630	PBX1	0.93484	0.99908	0.69447	0.76282	0.99815	1.59441	1.58123	1.01225
ENSG00000207082	RNU6-171P	0	0	0	0	0	0	0	0
ENSG00000269887	AL391001.1	1.74281	3.07154	3.01298	3.65155	2.86669	2.89369	13.6705	2.89501
ENSG00000276359	RF00017	0	0	0	0	0	0	0	0
ENSG00000233693	AL357568.1	0	0	0	0	0	0	0	0
ENSG00000271917	AL357568.2	0	0	0	0	0	0	0.08054	0
ENSG00000201987	RF00019	0	0	0	0	0	0	0	0
ENSG00000225487	AL390119.1	0	0	0	0	0	0	0	0
ENSG00000201270	RNU6-755P	0	0	0	0	0	0	0	0
ENSG00000162761	LMX1A	0	0	0	0	0	0	0	0
ENSG00000224702	AL390730.1	0	0	0	0	0	0	0	0
ENSG00000238022	AL390730.2	0	0	0	0	0	0	0	0
ENSG00000143171	RXRG	0	0	0	0	0	0	0	0
ENSG00000237463	LRRC52-AS1	0	0	0	0	0	0	0	0
ENSG00000230748	AL157714.1	0	0	0	0	0	0	0	0
ENSG00000162763	LRRC52	0	0	0	0	0	0	0	0
ENSG00000237783	AL356441.2	0	0	0	0	0	0	0	0
ENSG00000236206	AL356441.1	0	0	0	0	0	0	0	0
ENSG00000143198	MGST3	1.43093	1.55397	0.43593	0.51797	1.18808	0.67727	0.5672	0.65347
ENSG00000143149	ALDH9A1	8.95204	7.37657	0.65123	3.68319	8.91706	6.6845	3.58268	5.98529
ENSG00000206990	RF00019	0	0	0	0	0	0	0	0
ENSG00000230175	AL451074.3	0	0	0	0	0	0	0	0
ENSG00000225272	AL451074.2	0	0	0	0	0	0	0	0
ENSG00000215838	AL451074.1	0.03954	0	0	0	0	0	0	0
ENSG00000273365	AL451074.5	0	0	0	0	0	0	0.1676	0.12345
ENSG00000143183	TMCO1	8.33555	6.4675	2.50767	5.24057	7.06997	6.97042	3.89472	6.14056
ENSG00000224358	TMCO1-AS1	0.06819	0.22699	0.0501	0.0506	0.58029	0.12029	0.17048	0.25115
ENSG00000230659	AL451074.4	0	0	0	1.00033	0.20487	0	0	0
ENSG00000143179	UCK2	0.94392	0.61402	0.17538	0.41865	0.79144	0.93787	0.4702	0.31971
ENSG00000236364	AL358115.1	0	0	0	0	0	0	0	0
ENSG00000283936	MIR3658	0	0	0	0	0	0	0	0
ENSG00000271527	AL606495.2	0	0	0	0	0	0	0	0
ENSG00000226292	AL606495.1	0	0	0	0	0	0	0	0
ENSG00000207341	RNA5SP64	0	0	0	0	0	0	0	0
ENSG00000188859	FAM78B	0.02744	0	0	0	0.01668	0	0	0
ENSG00000203307	AL626787.1	0	0	0	0	0	0	0	0
ENSG00000230898	AL596087.2	0.30289	0	0	0	0	0	0	0
ENSG00000215952	MIR921	0	0	0	0	0	0	0	0
ENSG00000215835	AL596087.1	0.03723	0	0.0547	0	0.04526	0	0	0
ENSG00000229588	AL390115.1	0	0	0	0	0	0	0	0
ENSG00000225325	AL583804.1	0	0	0	0	0	0	0	0
ENSG00000230231	FMO7P	0	0	0	0	0	0	0	0
ENSG00000234142	LINC01675	0.20913	0	0	0.15519	0.12713	0.5534	0.17429	0.12838
ENSG00000238087	FMO8P	0	0	0	0	0	0	0	0
ENSG00000215834	FMO9P	0	0	0	0	0	0	0	0
ENSG00000234984	FMO10P	0	0	0	0	0	0	0	0
ENSG00000232341	RPL4P2	0.05718	0	0	0	0	0	0.0953	0.0702
ENSG00000232148	FMO11P	0	0	0	0	0	0	0	0
ENSG00000232223	CNN2P10	0	0	0	0	0	0	0	0

ENSG00000143157	POGK	6.03895	3.87053	0.94352	1.73841	4.91576	4.11753	1.63428	2.23714
ENSG00000152382	TADA1	2.77072	2.59417	1.01788	1.98259	3.09788	3.49137	2.26794	2.06541
ENSG00000225171	DUTP6	0	0	0	0	0.19289	0.2799	0	0.1948
ENSG00000143195	ILDR2	0.10559	0.07909	0	0.02351	0.01926	0.05588	0.0352	0.0713
ENSG00000143194	MAEL	0.06985	0.15503	0	0	0	0.12323	0.07762	0
ENSG00000200036	RNA5SP65	0	0	0	0	0	0	0	0
ENSG00000227907	AL158837.1	0	0	0	0	0	0	0	0
ENSG00000143167	GPA33	1.55763	0.87694	0.06732	0.50993	2.22792	1.41435	0.68726	0.30936
ENSG00000198842	DUSP27	0.03406	0	0	0	0	0	0	0
ENSG00000213068	AL451050.1	0	0	0	0	0	0	0	0
ENSG00000231605	LINC01363	0	0	0	0	0	0	0	0
ENSG00000272205	AL451050.2	0	0	0	0	0	0	0	0.13196
ENSG00000143190	POU2F1	2.45684	2.74951	1.66156	3.01089	3.4961	2.53649	3.78493	3.54207
ENSG00000272033	AL136984.1	0.50338	0.51562	0.1707	0.22987	0.47077	0.20493	0.19363	0.47542
ENSG00000198821	CD247	16.8078	14.1849	3.01727	11.8925	14.3373	19.5859	10.0252	10.8871
ENSG00000233411	AL359962.1	2.66107	1.208	0	0.35903	0.88234	0.85354	0.80647	1.78212
ENSG00000273160	AL359962.2	1.93532	3.22135	1.06647	0.71806	1.47057	1.28031	2.82265	2.37616
ENSG00000227776	AKR1D1P1	0.15662	0	0.11508	0	0.19042	0	0	0
ENSG00000143162	CREG1	38.0682	34.7234	27.9704	44.3156	27.5174	34.6852	47.2267	28.6933
ENSG00000231029	AL031733.1	0	0	0	0	0	0	0	0
ENSG00000241666	AL031733.2	0.40674	0.04836	0	0.08624	0.10597	0.25627	0.14528	0.03567
ENSG00000198771	RCSD1	41.3292	29.3179	4.87896	9.47452	26.9937	24.3788	7.28637	12.8118
ENSG00000197965	MPZL1	8.30578	4.98804	0.68569	1.26191	6.18988	1.75611	2.81729	3.29739
ENSG00000143199	ADCY10	0.08358	0.11925	0.03509	0.23037	0.13065	0.25277	0.25873	0.11728
ENSG00000250762	Z99943.2	0	0	0	0	0	0	0	0
ENSG00000232194	Z99943.1	0.10209	0.33986	0	0	0	0	0	0
ENSG00000143158	MPC2	8.24342	8.55781	5.65583	8.014	8.45072	8.96873	15.3204	6.27722
ENSG00000143164	DCAF6	44.0081	50.537	34.7602	66.0719	53.0093	51.1013	65.8952	48.9803
ENSG00000221545	MIR1255B2	0	1.82102	0	0	0	0	0	2.68648
ENSG00000224837	GCSHP5	0	0	0.20635	0.4168	0	0	0.23406	0
ENSG00000143147	GPR161	0.00653	0.04348	0	0.00969	0.03176	0	0	0.01604
ENSG00000143155	TIPRL	13.4536	13.4891	4.71667	6.55417	14.8897	13.895	4.74302	8.10533
ENSG00000237049	RPL34P1	0	0	0	0	0	0	0	0
ENSG00000237131	AL021397.1	0	0	0	0	0	0	0	0
ENSG00000213064	SFT2D2	10.8373	6.49132	2.8702	6.51493	7.83871	9.16165	4.67118	5.52606
ENSG00000214262	ANKRD36BP1	0	0	0	0	0	0	0	0
ENSG00000206880	RNU6-1310P	0	0	0	0	0	0	0	0
ENSG00000143178	TBX19	0.38188	0.40145	0.26581	0.29829	0.46428	0.53186	0.36852	0.24677
ENSG00000227722	AL022100.1	0	0	0	0	0	0	0.07281	0.05363
ENSG00000207974	MIR557	0	0	0	0	0	0	0	0
ENSG00000228697	AL023755.1	0	0	0	0	0	0	0	0
ENSG00000283255	AL022100.2	0	0	0	0	0	0	0	0
ENSG00000215833	QRSL1P1	0	0	0	0	0	0	0	0
ENSG00000143185	XCL2	2.85417	3.887	0.95322	5.39115	3.62776	1.60209	0.64875	3.98217
ENSG00000227777	AL031736.1	0.23722	0	0	0	0	0	0	0
ENSG00000143184	XCL1	0.48259	0.53552	0.47278	0.63664	0.52153	0.0946	0.26814	1.18504
ENSG00000229757	AL031736.2	0	0	0	0	0	0	0	0
ENSG00000143196	DPT	0	0	0	0	0	0	0	0
ENSG00000235736	AL031275.1	0	0	0	0	0	0	0	0
ENSG00000225826	LINC00626	0	0	0	0	0	0	0	0
ENSG00000229612	SUMO1P2	0	0	0	0	0	0	0	0
ENSG00000203601	LINC00970	0	0	0	0	0	0	0	0
ENSG00000213063	RPL29P7	0	0	0	0	0	0	0	0
ENSG00000252987	RNA5SP66	0	0	0	0	0	0	0	0
ENSG00000237707	AL031726.1	0	0	0	0	0	0	0	0
ENSG00000143153	ATP1B1	0.54443	0.86502	0.36365	0.51418	1.53442	0.43657	0.28874	0.33422
ENSG00000143156	NME7	1.20102	1.16968	0.90121	2.06687	1.4757	1.53272	1.66116	1.4589
ENSG00000235575	Z99758.1	0	0	0	0	0	0	0	0

ENSG00000117475	BLZF1	6.19791	7.98485	2.70695	3.27496	6.20551	5.24694	2.59073	5.34799
ENSG00000117477	CCDC181	0.01851	0.0308	0	0.02746	0	0.06529	0	0
ENSG00000117479	SLC19A2	0.8586	1.13002	0.3521	1.00755	1.3109	0.81018	0.96519	0.95611
ENSG00000234604	AL021068.2	1.46601	1.22008	0	1.08786	0	0	0	0.89997
ENSG00000213062	AL021068.1	0.24138	0.26786	0	0.47765	0.19565	0.14194	0	0.29637
ENSG00000198734	F5	3.88109	7.34642	0.46649	0.63831	4.94263	1.58975	0.81931	2.1374
ENSG00000174175	SELP	7.75009	5.90815	1.72836	3.64697	4.59628	5.89128	4.23605	5.85368
ENSG00000000460	C1orf112	1.53554	2.69903	2.70774	5.85222	3.31555	3.96582	4.77091	4.01194
ENSG00000188404	SELL	437.166	717.723	84.8272	127.389	374.486	183.694	132.807	297.04
ENSG00000007908	SELE	0	0	0	0	0	0	0	0
ENSG00000230704	AL021940.1	0	0	0	0	0	0	0	0
ENSG00000171806	METTL18	3.53031	2.55151	1.97953	1.93029	5.25212	3.11397	2.16797	4.0493
ENSG00000000457	SCYL3	2.79016	2.41074	0.61032	1.05893	2.69305	1.87871	1.31358	2.01359
ENSG00000239494	RN7SL333P	0	0	0	0	0	0	0	0.60604
ENSG00000075945	KIFAP3	6.18444	6.66659	3.2457	7.58309	5.56755	5.8967	5.22788	6.21916
ENSG00000243051	RN7SL269P	0	0	0	0	0	0	0	0
ENSG00000235378	MRPS10P1	0	0	0	0	0	0	0	0.69229
ENSG00000232959	AL356475.1	0	0	0	0	0	0	0	0
ENSG00000224896	SIGLEC30P	0	0	0	0	0	0	0	0
ENSG00000203740	METTL11B	0	0	0	0	0	0	0	0
ENSG00000263390	MIR3119-2	0	0	0	0	0	0	0	0
ENSG00000283340	MIR3119-1	0	0	0	0	0	0	0	0
ENSG00000233985	LINC01681	0	0	0	0	0	0	0	0
ENSG00000238051	ISCUP1	0	0	0	0	0	0	0	0
ENSG00000224286	LINC01142	0	0	0	0	0	0	0	0
ENSG00000226967	HAUS4P1	0	0	0	0	0	0	0	0
ENSG00000231407	GORAB-AS1	0.1611	0	0.06764	0.06831	0	0	0	0.02826
ENSG00000120370	GORAB	1.37936	0.74376	0.37113	0.66315	1.51165	1.37092	0.61527	0.66788
ENSG00000225545	AL162399.1	0	0	0	0	0	0	0	0
ENSG00000235303	AL023495.1	0	0	0	0	0	0	0	0
ENSG00000116132	PRRX1	0	0.0148	0	0	0.04323	0	0	0
ENSG00000271811	Z97200.1	0	0	0	0	0	0	0	0
ENSG00000117501	MROH9	0	0	0	0	0	0	0	0
ENSG00000226552	BX284613.1	0	0	0	0	0	0	0	0
ENSG00000007933	FMO3	0	0	0	0	0	0	0	0
ENSG00000221390	MIR1295A	0	0	0	0	0	0	0	0
ENSG00000283598	MIR1295B	0	0	0	0	0	0	0	0
ENSG00000117507	FMO6P	0	0	0	0	0	0	0	0
ENSG00000094963	FMO2	0	0	0.02251	0	0	0	0	0
ENSG00000225243	AL021026.1	0	0	0	0	0	0	0	0
ENSG00000231424	AL445673.1	0	0	0	0	0	0	0	0
ENSG00000010932	FMO1	0	0	0	0	0	0	0	0
ENSG00000206692	RF00019	0	0	0	0	0	0	0	0
ENSG00000230547	HMGB1P11	0	0	0	0	0	0	0	0
ENSG00000076258	FMO4	0.61156	0.43626	0.12838	0.38898	1.02677	0.46238	0.29125	0.57209
ENSG00000225704		0	0.5259	0	0	0.38412	0.55738	0	0
ENSG00000268062	SRP14P4	0	0	0	0	0	0	0	0
ENSG00000239428	GM2AP2	0	0	0	0	0	0	0	0
ENSG00000234333	CYCSP53	0	0	0	0	0	0	0	0
ENSG00000117523	PRRC2C	26.1588	19.9038	4.62703	9.72906	20.2099	20.2592	10.7514	10.3308
ENSG00000283683	MYOCOS	0	0	0	0	0	0	0	0
ENSG00000034971	MYOC	0	0	0	0	0	0	0	0
ENSG00000233328	PFN1P1	0.69315	0	0	0	0	0	0	0
ENSG00000230364	RPL4P3	0.05591	0	0	0.08298	0	0.09864	0	0.06865
ENSG00000117533	VAMP4	3.92148	3.64204	1.41976	2.69908	4.00757	3.05796	2.2262	2.91326
ENSG00000232261	Z98751.1	0	0	0	0	0	0	0	0
ENSG00000271459	Z98751.3	0	0	0	0	0	0	0	0
ENSG00000236741	Z98751.2	0	0	0	0	0	0	0	0

ENSG00000253060	RF00601	0	0	0	0	0	0	0	0
ENSG00000010165	EEF1AKNMT	2.2218	1.24336	0.56292	1.05176	2.07248	2.63557	0.51081	1.03472
ENSG00000213060	AL031864.1	0	0	0	0	0	0	0.28884	0
ENSG00000224600	AL031864.2	0	0	0	0	0	0.36946	0	0
ENSG00000197959	DNM3	6.33428	4.62015	2.56711	4.02341	4.20057	3.77811	4.03349	6.40283
ENSG00000233540	DNM3-IT1	0.92551	0.92431	0.272	2.4724	0.45008	0	0	0.90906
ENSG00000230630	DNM3OS	0	0	0	0.02496	0	0.05934	0	0
ENSG00000283844	MIR214	0	0	0	0	0	0	0	0
ENSG00000283152	MIR3120	0.90494	0	0	0	0	0	0	0
ENSG00000208024	MIR199A2	0	0	0	0	0	0	0	0
ENSG00000206684	RNU6-157P	0	0	0	0	0	0	0	0
ENSG00000135845	PIGC	42.0021	20.6189	0.80399	1.68644	3.17238	48.1609	28.5048	1.75688
ENSG00000180999	C1orf105	0.21246	0	0	0	0	0.14993	0.07083	0.05217
ENSG00000094975	SUCO	17.8654	27.5578	42.8727	78.275	40.4491	24.5879	67.6681	52.8764
ENSG00000251943	RNU6-693P	0	0	0	0	0	0	0	0
ENSG00000117560	FASLG	2.17416	3.94201	1.08399	1.38287	1.88807	8.97238	1.03543	2.28806
ENSG00000229785	SLC25A38P1	0	0	0	0	0	0	0.12957	0
ENSG00000279061	Z98043.1	0	0	0	0	0	0	0	0
ENSG00000224228	AL031599.1	0.63503	0.906	0.66655	0.94245	0.99264	0.8002	1.05849	1.33659
ENSG00000227177	AIMP1P2	0	0	0	0	0	0	0	0
ENSG00000224000	Z97198.1	0.1114	0	0.3274	0.49598	0.27087	0	1.11411	1.09419
ENSG00000120337	TNFSF18	0.07301	0	0.16093	0.2167	0	0	0	0
ENSG00000230849	GOT2P2	0	0	0	0	0	0	0.09767	0
ENSG00000226375	AL022310.1	0.21246	0.17682	0	0.47298	0.25831	0	0.17707	0
ENSG00000117586	TNFSF4	5.6156	2.35227	1.31159	2.81485	2.93891	1.70579	3.78133	4.54323
ENSG00000232751	AL645568.3	0	0	0	0	0	0	0	0
ENSG00000231615	AL645568.2	0.4386	0.09126	0	0.08137	0.06665	0	0.09138	0.26925
ENSG00000203739	AL645568.1	0.54606	1.33664	0.80243	2.71726	0.58578	0.62333	0.96374	1.49864
ENSG00000117592	PRDX6	99.9325	35.6696	45.552	105.936	68.3341	54.0848	30.0934	132.885
ENSG00000162753	SLC9C2	0	0	0.02191	0.02213	0	0	0	0
ENSG00000238272	AL139142.1	0	0	0	0	0	0	0	0
ENSG00000183831	ANKRD45	0.07554	0.04191	0.11101	0.07474	0.15307	0.08884	0.04197	0.03092
ENSG00000232113	TEX50	0	0	0	0	0	0	0	0
ENSG00000076321	KLHL20	3.10157	2.32315	0.93686	1.96909	3.33088	3.52613	2.06793	1.69247
ENSG00000225591	BX248409.1	0.86236	0.47846	0.84482	0	0	0	0	0
ENSG00000200674	RN7SKP160	0	0.35885	0.31681	0	0.26211	0	0	0.2647
ENSG00000120334	CENPL	0.55192	0.57106	0.87679	1.81531	1.19693	1.63153	1.29291	1.35527
ENSG00000252357	RF00019	0	0	0	0	0	0	0	0
ENSG00000117593	DARS2	2.60297	2.249	1.45993	2.24118	2.24664	4.27651	1.92097	2.41521
ENSG00000270084	GAS5-AS1	0.5281	0.35161	0	0.15675	0.12841	0	0	0.38904
ENSG00000234741	GAS5	9.89181	6.88839	4.3311	10.9954	12.2471	13.7467	10.2294	8.50151
ENSG00000185278	ZBTB37	3.49103	2.97123	0.64748	1.39168	2.62808	2.72389	1.67602	2.07607
ENSG00000117601	SERPINC1	0	0	0	0	0	0	0	0
ENSG00000252231	RNA5SP67	0	0	0	0	0	0	0	0
ENSG00000135870	RC3H1	15.2104	14.2598	3.82821	7.25994	12.336	9.14666	7.69732	8.75102
ENSG00000200755	RNA5SP68	0.61597	1.02528	0	0	0	0	0	0
ENSG00000236535	RC3H1-IT1	0.81808	1.63404	0.48087	0.72848	0.79568	0.86593	0.27272	0.40177
ENSG00000224977	AL121983.1	1.96926	1.09261	0	0	2.39418	0	0.72943	0.5373
ENSG00000225713	RPL30P1	0	0	0	0	0	0	0	0
ENSG00000227373	AL121983.2	1.07608	0.42145	0.27905	0	0.30783	0.335	0.21102	1.24348
ENSG00000152061	RABGAP1L	24.5566	18.6152	10.0603	28.8092	20.5726	14.081	26.4188	17.8975
ENSG00000237317	AL022400.1	0	0	0	0	0	0	0	0
ENSG00000203737	GPR52	1.01057	0.44856	0.39601	0.39995	0.40954	1.30738	0.67379	0.33087
ENSG00000223828	BANF1P4	0	0	0	0	0	0	0	0
ENSG00000231378	NDUFAF4P4	0	0	0	0	0	0	0	0
ENSG00000237249	Z99127.3	0.18557	0	0	0.82622	0	0.32737	0.61863	0
ENSG00000223525	RABGAP1L-IT1	0.29837	0.16555	0.14615	0	0	0	0	0
ENSG00000235869	Z99127.2	0	0	0	0	0	0	0	0

ENSG00000229531	Z99127.1	0	0.50261	0.11093	0.22407	0.18356	0.13317	0.50332	0.18537
ENSG00000252552	RNU6-307P	0	0	0	0	0	0	0	0.84903
ENSG00000116161	CACYBP	6.23971	5.62575	1.69534	2.99034	6.75635	10.1477	4.71279	3.67091
ENSG00000120333	MRPS14	4.43645	2.9632	1.2042	1.50975	4.39743	4.23728	1.60144	2.15105
ENSG00000206659	RF00019	0	0	0	0	0	0	0	0
ENSG00000181101	SDCCAG3P2	0	0	0	0	0	0	0	0
ENSG00000120332	TNN	0	0	0	0.02172	0.03559	0	0	0
ENSG00000235750	KIAA0040	29.3784	41.8089	4.95444	7.05072	31.6184	14.9507	5.95192	19.7568
ENSG00000230687	AL008626.1	0	0	0	0	0	0	0	0
ENSG00000233946	RPS29P4	0	0	0	0	0	0	0	0
ENSG00000260990	Z94057.1	0	0	0	0	0	0	0	0
ENSG00000116147	TNR	0.02828	0.00941	0	0.00839	0.01375	0	0	0
ENSG00000235628	TNR-IT1	0	0	0	0	0	0	0	0
ENSG00000224718	LINC01657	0	0	0	0	0	0	0	0
ENSG00000227740	AL513329.1	0.44627	1.11423	0.16395	0	0	0.39364	0	0.41095
ENSG00000230777	RPS29P5	0	0	0	0	0	0	0	0
ENSG00000143207	COP1	25.644	29.2888	9.6464	16.271	21.9319	14.9495	18.5587	23.0069
ENSG00000252906	SCARNA3	4.10072	7.67886	5.27271	8.36813	1.24639	3.61711	2.56322	3.7761
ENSG00000228686	AL590723.1	0	0	0	0	0	0	0	0
ENSG00000236021	AL359265.3	1.39258	2.31795	2.04638	1.87886	1.38523	0.67001	1.0551	2.17609
ENSG00000273629	RF00066	0	0	0	0	0	0	0	0
ENSG00000232463	AL359265.2	0	0	0	0	0	0	0	0
ENSG00000253025	RNU2-12P	0	0	0	0	0	0	0	0
ENSG00000227815	AL359265.1	0.0922	0	0.27098	0.54735	0	0	0.15369	0.22641
ENSG00000231020	AL591043.1	0	0	0	0	0	0	0	0
ENSG00000225904	MORF4L1P7	0	0	0	0	0	0	0	0
ENSG00000116183	PAPPA2	0	0	0	0	0	0.01183	0.01118	0.00823
ENSG00000237514	PTP4A1P7	0	0	0	0	0	0	0	0
ENSG00000152092	ASTN1	0	0	0	0	0	0	0	0
ENSG00000202609	MIR488	0	0	0	0	0	0	0	0
ENSG00000198797	BRINP2	0	0	0	0	0	0	0	0
ENSG00000224968	LINC01645	0	0	0	0	0	0	0	0
ENSG00000227579	AL122019.1	0	0	0	0	0	0	0	0
ENSG00000236720	LINC01741	0	0	0	0	0	0	0	0
ENSG00000120341	SEC16B	0	0	0	0	0	0	0	0
ENSG00000254154	CRYZL2P-SEC16B	0	0.01556	0	0	0	0	0	0
ENSG00000242193	CRYZL2P	0.55591	0.24234	0.0389	0.19644	0.22529	0.2335	0.11031	0.14626
ENSG00000276563	AL359075.1	0	0	0	0	0	0	0	0
ENSG00000224687	RASAL2-AS1	0	0.05179	0	0	0.11348	0	0.05186	0
ENSG00000075391	RASAL2	0.11879	0.10646	0.00671	0.0339	0.06665	0.0806	0.14469	0.04488
ENSG00000270575	AL160281.1	0	0	0	0	0	0	0	0
ENSG00000213058	AL365357.1	0	0	0	0.60944	0.24963	0.36222	0.68448	1.76465
ENSG00000188585	CLEC20A	0.02832	0	0	0.04203	0.06887	0	0	0.03477
ENSG00000264809	RF00017	0	0	0	0	0	0	0	0
ENSG00000240021	TEX35	0	0	0	0	0	0	0	0
ENSG00000213057	C1orf220	0.10566	0.08793	0	0.0392	0.16057	0.0932	0.04403	0.06486
ENSG00000201347	RNA5SP69	0	0	0	0	0	0	0	0
ENSG00000273384	AL137796.1	0	0	0	0	0	0	0	0
ENSG00000266417	MIR4424	0	0	0	0	0	0	0	0
ENSG00000273062	AL449106.1	0.14808	0.06162	0	0.16483	0.22504	0	0.24683	0.09091
ENSG00000116191	RALGPS2	6.33332	3.58927	1.38486	4.08924	11.778	3.05738	3.98038	5.00093
ENSG00000228196	PTPN2P1	0	0	0	0	0	0	0	0
ENSG00000201791	RF00092	0	0	0	0	0	0	0	0
ENSG00000116194	ANGPTL1	0	0	0	0	0	0	0	0
ENSG00000116199	FAM20B	8.19303	11.8998	5.13688	5.63834	8.16054	8.41522	11.957	5.30534
ENSG00000232750	AL139132.1	0	0	0	0	0	0	0	0
ENSG00000186283	TOR3A	3.16256	2.24269	0.46748	0.80541	2.22962	2.14584	0.65504	1.21773
ENSG00000143322	ABL2	1.40994	1.17803	0.43875	0.6893	1.66039	0.94616	0.86633	0.94362

ENSG00000234041	AL512326.3	0	0	0	0	0	0	0	0
ENSG00000231905	SETP10	0	0	0	0	0	0	0	0
ENSG00000212338	RF00272	0	0	0	0	0	0	0	0
ENSG00000201619	RF00272	0	0	0	0	0	0	0	0
ENSG00000231564	EIF4A1P11	0	0	0	0	0	0	0	0
ENSG00000225711	AL512326.1	0	0	0	0	0	0	0	0
ENSG00000223794	COX5BP8	0	0	0	0	0	0	0	0
ENSG00000228191	AL512326.2	0	0	0	0	0	0	0	0
ENSG00000057252	SOAT1	10.6524	10.2588	3.72117	7.40768	11.9586	8.91614	4.97093	9.50718
ENSG00000162779	AXDND1	0.03611	0.04008	0.07076	0.12506	0	0.14866	0.08026	0.05912
ENSG00000224620	MEF2AP1	0	0	0	0	0	0	0	0
ENSG00000236539	HNRNPA1P54	0	0	0	0	0	0	0	0
ENSG00000261250	AL160286.3	0	0	0	0	0	0	0	0
ENSG00000116218	NPHS2	0	0	0	0	0	0	0	0
ENSG00000251875	RNU5F-2P	0	0	0	0	0	0	0	0
ENSG00000227141	AL160286.1	0	0	0	0	0	0	0	0
ENSG00000261060	AL160286.2	0	0	0	0	0	0	0	0
ENSG00000162782	TDRD5	0	0	0	0	0	0	0	0
ENSG00000243062	AL359853.3	0	0	0	0	0	0	0	0
ENSG00000143340	FAM163A	0	0	0	0.0374	0	0	0	0
ENSG00000229407	AL359853.1	0	0	0	0	0	0	0	0
ENSG00000231966	AL359853.2	0.8306	0	0	0	0	0	0	0.25495
ENSG00000169905	TOR1AIP2	4.34536	4.33713	1.4266	2.33985	3.37091	3.06221	2.26548	3.16105
ENSG00000272906	AL353708.3	1.11173	0.86356	0.21782	0.54998	1.1714	0.91525	0.49416	0.36399
ENSG00000143337	TOR1AIP1	14.4466	13.4761	4.06658	6.75456	10.9646	9.66981	5.13373	9.08863
ENSG00000264916	RN7SL230P	0.72575	1.208	0.35549	0.35903	0.29411	1.28031	1.61294	0.29702
ENSG00000261831	AL353708.2	0	0	0	0	0	0	0	0
ENSG00000260360	AL353708.1	0.11663	0	0	0.17309	0.49627	0.20575	0.1944	0.21479
ENSG00000135837	CEP350	21.1223	18.1294	8.93285	20.3788	20.377	18.7625	18.7529	19.5714
ENSG00000235145	RPSAP16	0.08928	0	0	0.1325	0.32564	0.63002	0.14882	0.10962
ENSG00000223450	AL590632.1	0	0	0	0	0	0	0	0
ENSG00000261817	AL390718.1	0	0	0	0	0	0	0	0
ENSG00000116260	QSOX1	4.07793	2.30625	0.55529	0.81785	1.70366	1.49991	0.85294	1.2662
ENSG00000121454	LHX4	0	0.02013	0	0	0	0.02134	0.06048	0
ENSG00000230124	ACBD6	2.20526	2.59869	1.77803	2.70806	2.63364	2.30668	1.64273	2.92323
ENSG00000235060	VDAC1P4	0	0	0	0	0	0	0	0
ENSG00000265435	MIR3121	0	0	0	0	0	0	0	0
ENSG00000206905	RF00019	0	0	1.03571	0	0	0	0	0
ENSG00000236719	OVAAL	0	0	0	0	0	0	0	0
ENSG00000143324	XPR1	5.91968	5.38575	2.29874	3.81233	4.04393	4.80762	3.29364	3.34595
ENSG00000283575	RF00026	0	0	0	0	0.9283	0	0	0
ENSG00000225857	AL162431.1	0.19599	0	0	0	0	0	0	0
ENSG00000135835	KIAA1614	0.02876	0	0.01057	0	0.01748	0	0	0
ENSG00000243155	AL162431.2	0.1512	0.10067	0	0	0.18382	0	0.15121	0.07426
ENSG00000232586	KIAA1614-AS1	0.45813	0.08473	0	0	0.30943	0.9878	0.08485	0.125
ENSG00000251520	AL162431.3	0	0	0	0	0	0	0	0
ENSG00000270711	AL162431.4	0.17835	0	0	0	0	0	0	0
ENSG00000135823	STX6	9.61947	10.1909	1.84595	3.76224	8.18657	8.46506	5.39502	5.61353
ENSG00000153029	MR1	4.35976	4.13743	1.07161	1.43139	2.36424	3.31994	1.21553	2.66679
ENSG00000162783	IER5	12.0242	8.36114	2.18617	2.51963	6.44755	2.71715	2.24639	3.28786
ENSG00000237292	LINC01732	0.21979	0	0	0	0	0	0	0
ENSG00000225359	AL358393.1	0	0	0	0	0	0	0	0
ENSG00000179452	LINC01699	0.28301	0	0	0	0	0	0	0
ENSG00000198216	CACNA1E	0.11962	0.48746	0.03637	0.04285	0.13038	0.07277	0.03438	0.16712
ENSG00000273573	RF00017	0	0	0	0	0	0	0	0
ENSG00000252977	RNA5SP70	0	0	0	0	0	0	0	0
ENSG00000272880	AL161734.1	0	0	0	0	0	0	0	0
ENSG00000222397	RN7SKP229	0	0	0	0	0	0	0	0

ENSG00000179930	ZNF648	0	0	0	0	0	0	0	0
ENSG00000224810	AL355482.1	0.02976	0	0	0	0	0	0	0
ENSG00000225982	AL355482.2	0	0	0	0	0	0	0	0
ENSG00000232309	AL390856.1	0	0.19428	0	0	0	0	0	0
ENSG00000228918	LINC01344	0.11594	0.15439	0.03408	0.20649	0.14096	0	0.15461	0.11388
ENSG00000232096	YPEL5P1	0	0	0	0	0	0	0	0
ENSG00000206764	RNU6-152P	0	0	0	0	0	0	0	0
ENSG00000228664	AL513480.1	0	0	0	0	0	0	0	0
ENSG00000231684	EIF1P3	0	0	0	0	0	0	0	0
ENSG00000135821	GLUL	152.574	137.935	47.8344	81.228	117.79	75.2362	96.9786	92.4283
ENSG00000203730	TEDDM1	0	0	0	0	0	0	0	0
ENSG00000203729	LINC00272	0.29716	0	0.14556	0	0.24086	0	0.66043	0.12162
ENSG00000121446	RGSL1	0	0.01346	0	0	0	0	0	0
ENSG00000270443	AL139344.1	0	0	0	0	0	0	0	0
ENSG00000233537	TEDDM2P	0	0	0	0	0	0	0	0
ENSG00000135828	RNASEL	7.09667	11.2148	2.03997	2.84175	7.52217	2.9557	1.95489	7.25854
ENSG00000276839	RF00017	0	0	0	0	0	0	0	0
ENSG00000143333	RGS16	0.09061	0.05027	0	0.08965	0.25703	0.31968	0.15103	0.07416
ENSG00000261504	LINC01686	0	0	0	0	0	0.18795	0	0
ENSG00000135824	RGS8	0	0	0	0	0	0	0	0
ENSG00000227181	LINC01688	0	0	0	0	0	0	0	0
ENSG00000271269	AL353778.1	0	0	0	0	0	0	0	0
ENSG00000135838	NPL	17.8131	16.3577	7.05824	11.633	18.8981	8.73342	21.7837	13.4408
ENSG00000135829	DHX9	31.2939	21.6568	6.39224	14.569	27.0113	29.5275	14.1552	17.0907
ENSG00000157060	SHCBP1L	0	0	0	0	0	0	0	0
ENSG00000224040	HMGN1P4	0	0	0	0.37512	0	0	0	0
ENSG00000199837	RNA5SP71	0	0	0	0	0	0	0	0
ENSG00000226570	AL450304.1	0	0	0	0	0	0	0	0
ENSG00000176855	KRT18P28	0	0	0	0	0	0	0	0
ENSG00000207472	RNU6-41P	0	0	0	0	0	0	0	0
ENSG00000135862	LAMC1	0.63656	0.31932	0.07688	0.18118	0.83752	0.07692	0.3343	0.56743
ENSG00000224468	LAMC1-AS1	0	0	0	0	0	0	0	0
ENSG00000058085	LAMC2	0	0	0	0	0.02724	0	0	0
ENSG00000157064	NMNAT2	0.01023	0.01703	0	0	0	0	0	0
ENSG00000233562	AL356981.1	0	0	0	0	0	0	0	0
ENSG00000232860	SMG7-AS1	0.17334	0.14426	0.03184	0.06431	0.07903	0.07645	0.14446	0.0266
ENSG00000116698	SMG7	5.45544	5.83857	1.63183	2.87758	5.07891	5.37957	4.12801	2.96491
ENSG00000116701	NCF2	308.022	337.44	49.3321	54.8759	198.382	73.312	54.1633	149.934
ENSG00000162704	ARPC5	39.3824	30.8352	8.57737	11.3865	24.9047	20.5458	11.5234	23.8462
ENSG00000143344	RGL1	1.31264	1.14994	0.32487	0.3281	0.43676	0.46313	0.16122	0.15268
ENSG00000173627	APOBEC4	0	0	0	0	0	0	0	0
ENSG00000270994	AL157899.1	0	0	0	0	0	0	0	0
ENSG00000227554	AL590422.1	0	0	0	0	0	0	0	0
ENSG00000198756	COLGALT2	0.0923	0.42678	0.16578	0.12177	0.04988	0.05428	0.22224	0.05037
ENSG00000198860	TSEN15	7.50106	5.19398	2.51318	3.47331	6.82146	7.72801	3.55088	3.94175
ENSG00000199840	RF00019	0	0	0	0	0	0	0	0
ENSG00000207080	RF00019	0	0	0	0	0	0	0	0
ENSG00000231791	AL445228.1	0	0	0	0	0	0	0	0
ENSG00000244568	RN7SL654P	0	0	0	0	0	0	0	0
ENSG00000271387	AL445228.2	0	0	0	0	0	0.27166	0	0.09453
ENSG00000116667	C1orf21	1.50329	1.66815	0.42218	1.11056	0.90165	2.84059	0.81298	0.95157
ENSG00000230470	AL078645.1	0	0	0	0.25718	0	0.6114	0	1.0638
ENSG00000232036	AL078645.2	0.05532	0	0	0	0	0	0.09221	0.13584
ENSG00000116406	EDEM3	16.362	14.4615	4.08551	7.73655	14.4797	9.51114	6.74779	9.19114
ENSG00000135842	FAM129A	93.6129	147.935	12.4671	17.0871	86.3278	17.083	21.3217	59.1667
ENSG00000252222	RNU7-13P	14.1872	27.5503	3.47464	0	12.9363	2.08567	1.97065	4.3547
ENSG00000260976	LINC01633	0	0	0	0	0	0	0	0
ENSG00000201312	RNA5SP72	0	0	0	0	0	0	0	0

ENSG00000121481	RNF2	7.99051	8.41748	3.75531	7.74561	10.0866	7.71489	7.5594	7.2254
ENSG00000232792	FTH1P25	0	0	0	0	0	0	0	0
ENSG00000121486	TRMT1L	8.44918	11.5964	7.51493	14.0574	10.7589	9.07405	12.1563	9.209
ENSG00000116668	SWT1	145.462	161.554	166.486	432.379	173.287	125.197	160.667	189.385
ENSG00000231084	RPL22P24	0	0	0	0	0	0	0	0
ENSG00000213051	RPL5P5	0	0	0	0	0	0.14481	0	0
ENSG00000201421	RF00019	0	0	0	0	0	0	0	0
ENSG00000206640	RF00019	0	0	0	0	0	0	0	0
ENSG00000233583	AL356273.1	0	0	0	0.09443	0	0	0	0
ENSG00000252612	RF00019	0	0	0	3.50922	0	0	0	0
ENSG00000238061	AL356273.2	0.226	0.37618	0	0.22361	0.18318	0	0	0
ENSG00000279838	AL356273.3	8.21819	9.1399	1.13725	3.33631	4.48048	1.82038	2.33426	3.61979
ENSG00000116679	IVNS1ABP	49.0518	45.6089	9.22408	25.0664	28.5142	14.3843	14.6697	27.8311
ENSG00000273004	AL078644.1	2.14442	1.62245	0.42971	1.30196	0.82954	0.51587	0.32495	0.95742
ENSG00000261024	GS1-279B7.1	0.06737	0	0	0	0	0	0	0.08272
ENSG00000252407	RNU7-183P	1.18226	0	0	0	0	0	0	0
ENSG00000270920	MCRIP2P2	0	0	0	0	0	0	0	0
ENSG00000279401	AL133383.2	0.06276	0	0	0	0	0	0	0
ENSG00000228309	LINC01350	0	0	0	0	0	0	0	0
ENSG00000207108	RF00019	0	0	0	0	0	0	0	0
ENSG00000201596	RF00019	0	0	0	0	0	0	0	0
ENSG00000261729	AL133383.1	0	0	0.11955	0	0	0	0	0.09989
ENSG00000143341	HMCN1	0.03945	0.0197	0.09276	0.15809	0.01919	0	0.00658	0.00969
ENSG00000224691	AL133553.1	0	0	0	0	0	0	0	0
ENSG00000116690	PRG4	0	0	0	0	0.053	0.02564	0.02422	0
ENSG00000047410	TPR	33.7122	26.6443	4.93653	9.89201	27.4566	19.6742	9.86346	17.6496
ENSG00000202025	RNU6-1240P	0	0	0	0	0	0	0	0
ENSG00000157181	ODR4	7.03669	7.27917	7.27261	16.9869	8.64254	7.33391	10.4092	11.5562
ENSG00000262180	OCLM	0.16584	0.13802	0	0.12306	0	0	0.41464	0.20361
ENSG00000229739	AL596220.1	0.38579	0.32107	1.13383	1.28825	0.46904	0.85074	1.12535	0.7105
ENSG00000116703	PDC	0	0	0	0	0	0	0	0
ENSG00000273198	AL096803.3	0	0	0	0	0	0	0	0
ENSG00000228238	AL096803.1	0	0	0	0	0	0	0	0
ENSG00000233196	AL096803.2	0	0	0	0	0	0	0	0
ENSG00000073756	PTGS2	87.9632	156.766	27.8433	50.1087	67.8227	15.0688	4.35944	75.8203
ENSG00000273129	PACERR	1.06619	1.62678	0.26112	1.05489	0.86416	0.31348	0.1481	2.07266
ENSG00000116711	PLA2G4A	4.02833	4.32865	1.76089	3.6325	2.47977	2.51877	2.08238	2.91121
ENSG00000236030		13.4128	31.9952	31.3852	170.025	48.2975	22.607	90.5672	26.7474
ENSG00000231599		0.14868	0.24748	0.21849	2.20661	0.36153	0	0.99132	1.0953
ENSG00000272827		0	0	0	0	0	0	0	0
ENSG00000271558	AL391813.1	0	0	0	0	0	0	0	0
ENSG00000225462	FDPSP1	0	0	0	0	0	0	0	0
ENSG00000274702	AL357559.1	0	0	0	0	0	0	0	0
ENSG00000230426	ERVMER61-1	0	0	0	0	0	0	0	0
ENSG00000236401	SLC4A1APP2	0	0	0	0	0	0	0	0
ENSG00000223847	AL645474.1	0	0	0	0	0	0	0	0
ENSG00000150732	AL136372.1	0	0	0	0	0	0	0	0
ENSG00000222240	RN7SKP156	0	0	0	0	0	0	0.35725	0.5263
ENSG00000261182	AL596211.1	0	0	0	0	0	0	0	0
ENSG00000224278	AL592447.1	0	0	0	0	0	0	0	0
ENSG00000225006	AL929288.1	0	0	0	0	0	0	0	0
ENSG00000235083	AL929288.2	0	0	0	0	0	0	0	0
ENSG00000231022	RPS3AP9	0	0	0	0	0	0	0	0
ENSG00000237283	AL691515.1	0	0	0	0	0	0	0	0
ENSG00000238054	AL691515.2	0	0	0	0	0	0	0	0
ENSG00000226486	LINC01035	0	0	0	0	0	0	0	0
ENSG00000271036	CLPTM1LP1	0	0	0	0	0	0	0	0
ENSG00000226196	GAPDHP75	0	0	0	0	0	0	0	0

ENSG00000252553	RNA5SP73	0	0	0	0	0	0	0	0
ENSG00000232212	LINC01701	0	0	0	0	0	0	0	0
ENSG00000238270	AL591504.1	0	0	0	0	0	0	0	0
ENSG00000230987	AL359976.1	0	0	0	0	0	0	0	0
ENSG00000162670	BRINP3	0	0	0	0	0	0	0	0
ENSG00000225811	AL354771.1	0	0	0	0	0	0	0	0
ENSG00000237457	LINC01351	0	0	0	0	0	0	0	0
ENSG00000241505	AL391645.1	0	0	0	0	0	0	0	0
ENSG00000231175	LINC01720	0	0	0	0	0	0	0	0
ENSG00000236025	AL139135.1	0	0	0	0	0	0	0	0
ENSG00000238108	AL139135.2	0	0	0	0	0	0	0	0
ENSG00000228020	HNRNPA1P46	0	0	0	0	0	0	0	0
ENSG00000261642	AL713866.1	0	0	0	0	0	0	0	0
ENSG00000271187	AL713866.2	0	0	0	0	0	0	0	0
ENSG00000233882	LINC01680	0	0	0	0	0	0	0	0
ENSG00000223344	AL359081.1	0	0	0	0	0	0	0	0
ENSG00000228215	AL954650.1	0.10722	0.29744	0.21007	0.2652	0.3476	0.06305	0.417	0.26328
ENSG00000150681	RGS18	225.753	210.623	138.308	219.859	151.841	91.7072	140.991	213.029
ENSG00000226723	AL513175.1	0	0	0.27619	0.13947	0	0	0.31328	0
ENSG00000236792	AL513175.2	0	0	0	0	0	0	0	0.20315
ENSG00000253148	RGS21	0	0	0	0	0	0	0	0
ENSG00000232498		0	0.3081	0	0	0	0.32654	0	0
ENSG00000236069	AL136987.1	0	0	0	0	0	0	0	0
ENSG00000090104	RGS1	5.67957	1.48176	2.30236	6.52661	6.38558	74.1258	2.43352	3.01666
ENSG00000127074	RGS13	0.14851	0.0206	0.09093	0.03673	0.12037	0.0655	0	0
ENSG00000231767	AL136454.1	0.1573	0	0	0	0	0	0	0.19313
ENSG00000283795	MIR4426	0	0	0	0	0	0	0	0
ENSG00000228687	AL035407.2	0	0	0	0	0	0	0	0
ENSG00000226814	AL035407.1	0	0	0	0	0	0	0	0
ENSG00000116741	RGS2	853.935	1030.61	168.2	140.03	692.355	223.704	144.04	493.567
ENSG00000223075	RN7SKP126	0	0	0	0	0	0	0	0
ENSG00000234871		0	0	0	0	0.64577	0.46852	0.44268	0.32608
ENSG00000234473	ZNF101P2	0.11928	0.09927	0	0	0.07251	0.21043	0.29824	0.14646
ENSG00000116750	UCHL5	5.27111	4.92723	2.87841	8.93346	5.56561	4.60094	7.55717	5.03962
ENSG00000238754	SCARNA18B	0.54297	0	0	0	0.66012	0.95787	0	0.66664
ENSG00000116747	TROVE2	18.7076	16.6351	9.08859	18.4417	17.0949	16.6932	12.8999	14.6534
ENSG00000272579		0.84863	0.70627	0.31176	0.62973	0.12897	0.56141	1.0609	0.91169
ENSG00000023572	GLRX2	3.44213	2.81775	2.98514	3.18234	2.05812	3.68325	2.6336	2.84055
ENSG00000134371	CDC73	15.1203	15.2967	9.17742	19.9603	13.6706	15.0525	15.6835	13.2675
ENSG00000221680	MIR1278	3.61977	1.50628	0	0	0	3.19288	1.5084	1.11107
ENSG00000162630	B3GALT2	0.62688	0.67079	0.1974	0.53163	0.62605	0.27648	0.11196	0.63223
ENSG00000276735	RF00017	0	0	0	0	0	0	0	0
ENSG00000232077	LINC01031	0.0941	0.15662	0.27654	0.2793	0	0.33199	0.31369	0.46212
ENSG00000226640	AL357793.1	0	0	0	0	0	0	0	0
ENSG00000237011	AL357793.2	0	0	0	0	0	0	0	0
ENSG00000237213	RPL23AP22	0	0	0	0	0	0	0	0
ENSG00000227240	AL356272.1	0	0	0	0.13075	0.10711	0.15542	0	0
ENSG00000233057	EEF1A1P14	0	0	0	0.15812	0	0.09398	0	0
ENSG00000231714	AL513348.1	0	0	0	0	0	0	0	0
ENSG00000251813	RNU6-983P	0	0	0	0	0	0	0	0
ENSG00000228167	AL353072.1	0	0	0	0	0	0	0	0
ENSG00000227421	LINC01724	0	0.39294	0.17345	0	0.43052	0	0.19675	0
ENSG00000162687	KCNT2	0.13421	0.05155	0.01517	0.09193	0.0251	0.12749	0	0.02535
ENSG00000265986	MIR4735	0	0	0	0	0	0	0	0
ENSG00000000971	CFH	0.19799	0.16478	0.06612	0.14692	0.15318	0.03175	0.06	0.14364
ENSG00000116785	CFHR3	0	0	0	0	0	0	0	0
ENSG00000276911		0	0	0	0	0	0	0	0
ENSG00000244414	CFHR1	0	0	0	0	0	0	0	0

ENSG00000134365	CFHR4	0	0	0	0	0	0	0	0
ENSG00000080910	CFHR2	0	0	0	0	0	0	0	0
ENSG00000273844	AL139418.1	0	0	0	0	0	0	0	0
ENSG00000134389	CFHR5	0	0	0	0	0	0	0	0
ENSG00000143278	F13B	0	0	0	0	0	0	0	0
ENSG00000066279	ASPM	2.44402	2.79049	2.81974	7.24439	3.51162	8.7063	8.86585	5.21614
ENSG00000235748	SEPT14P12	0.39268	0.21787	0	0.38852	0.47741	0.23091	1.30907	0.16071
ENSG00000177888	ZBTB41	2.99639	1.98075	0.85547	2.55383	3.01843	2.77895	1.86938	1.94458
ENSG00000237861	AL513325.1	0	0	0.10417	0	0	0	0	0
ENSG00000134376	CRB1	0.01729	0	0.01693	0.02565	0.00701	0.0305	0.06723	0.06367
ENSG00000221879	MRPS21P3	0	0	0	0	0	0	0	0
ENSG00000230260	AL136322.1	0	0	0	0	0	0	0.22296	0
ENSG00000213047	DENND1B	4.84672	3.43764	2.7004	6.64101	3.86759	5.51409	7.87514	3.77629
ENSG00000207139	RF00019	0	0	0	0	1.76469	1.28031	0	0.89106
ENSG00000213046	EEF1A1P32	0	0	0	0	0	0	0	0
ENSG00000235582	AL365258.2	0	0	0	0	0	0	0	0
ENSG00000233040	FAM204BP	0.3137	0	0	0	0.25426	0.5534	0.17429	0.12838
ENSG00000224901	AL365258.1	0	0	0	0	0	0	0	0
ENSG00000203724	C1orf53	0	0	0	0	0	0.21164	0	0
ENSG00000143355	LHX9	0	0	0	0	0	0	0	0
ENSG00000151414	NEK7	32.9284	35.9686	16.378	29.4525	30.2363	20.4636	25.1266	30.3053
ENSG00000232824	PRR13P1	0	0	0	0	0	0	0	0
ENSG00000151418	ATP6V1G3	0	0	0	0	0	0	0	0
ENSG00000223881	AL157402.1	0	0	0	0	0	0	0	0
ENSG00000081237	PTPRC	300.846	297.537	54.0436	91.9175	223.063	169.97	85.2971	202.711
ENSG00000261573	AL157402.2	0	0.29329	0	0	0	0	0	0
ENSG00000236278	PEBP1P3	0	0	0	0	0	0	0	0
ENSG00000229989	MIR181A1HG	1.24047	0.86344	0.09943	1.17154	0.49357	1.71089	0.52632	0.69229
ENSG00000207975	MIR181B1	0	0	0	1.97792	1.6203	0	1.11073	0.81816
ENSG00000207759	MIR181A1	0	0	0.97922	0	0	1.17556	1.11073	0
ENSG00000227747	AC096631.2	0	0	0	0	0	0	0	0
ENSG00000225172	AC096631.1	0	0	0	0	0	0	0	0
ENSG00000233410	LINC01222	0.04106	0	0	0	0	0	0	0
ENSG00000235492	LINC01221	0	0	0	0	0.13462	0	0	0
ENSG00000231718	AC099673.1	0	0	0	0	0	0	0	0
ENSG00000228190	RPL23AP16	0	0	0	0	0	0	0	0
ENSG00000236468	AC099335.1	0	0	0	0	0	0	0	0
ENSG00000231984	AL596266.1	0	0	0	0	0	0	0	0
ENSG00000228530	AL445687.1	0	0	0	0	0	0	0	0
ENSG00000200139	RNU6-778P	0	0	0	0	0	0	0	0
ENSG00000229747	AL445687.2	0	0	0	0	0	0	0	0
ENSG00000202491	RNU6-716P	0	0	0	0	0	0	0	0
ENSG00000202329	RNU6-609P	0	0	0	0	0	0	0	0
ENSG00000116833	NR5A2	0	0	0	0	0	0	0.03233	0.01191
ENSG00000252860	RNU6-570P	0	0	0	0	0	0	0	0
ENSG00000229220	AC096633.1	0	0	0	0	0	0	0	0
ENSG00000233827	CCNQP1	0	0	0	0	0	0	0	0
ENSG00000203721	LINC00862	0.32332	0.34596	0.20362	0.17137	0.42116	0.32593	0.30795	0.34025
ENSG00000273093	AC097065.2	0	0	0	0	0	0	0	0
ENSG00000213045	AC097065.1	0	0	0	0	0	0	0	0
ENSG00000230623	AC104461.1	0.05304	0	0	0	0.03224	0.04678	0.0442	0
ENSG00000162702	ZNF281	15.3189	12.8614	2.50856	4.22253	14.0548	7.21187	4.99205	7.62999
ENSG00000282849	AL359834.1	0	0	0	0	0	0	0	0
ENSG00000118193	KIF14	7.20211	16.0118	12.9517	22.8126	16.7897	15.8863	25.3192	20.3937
ENSG00000118197	DDX59	5.29665	5.02227	1.30124	1.80093	5.08384	4.9179	2.29601	3.88578
ENSG00000260088	AL445483.1	0.09959	0	0	0	0	0.1757	0	0
ENSG00000118200	CAMSAP2	1.62115	0.83148	0.15235	0.25179	0.93964	0.19953	0.37705	0.32402
ENSG00000231333	RPL34P6	0	0	0	0	0	0	0	0

ENSG00000170128	GPR25	0.23954	0.09968	0	0	0.43685	0.10565	0.09982	0.07353
ENSG00000163362	INAVA	0.01354	0.02254	0	0.0201	0.03293	0	0	0
ENSG00000233217	MROH3P	0.02395	0	0	0	0	0	0	0
ENSG00000201032	RNU6-704P	0	0	0	0	0	0	0	0
ENSG00000116852	KIF21B	8.09019	6.18578	0.91313	1.76385	6.01446	6.89663	1.87041	2.42214
ENSG00000229191	AL358473.1	0.18806	0.10434	0	0.03101	0.0254	0.07372	0.03483	0.12827
ENSG00000234132	AL358473.2	0	0	0	0	0	0	0	0
ENSG00000081248	CACNA1S	0	0	0	0	0	0	0	0
ENSG00000232237	ASCL5	0	0	0	0	0	0	0	0
ENSG00000116857	TMEM9	0.95487	0.46747	0.27513	0.2223	0.88775	0.62757	0.31208	0.62067
ENSG00000163395	IGFN1	0	0	0	0	0	0	0	0
ENSG00000229821	AC103925.1	0	0	0	0	0	0	0	0
ENSG00000081277	PKP1	0	0	0	0	0	0	0	0
ENSG00000118194	TNNT2	0	0.01681	0	0	0	0	0	0
ENSG00000159166	LAD1	0.02105	0.03504	0	0	0.02559	0	0	0
ENSG00000282221	AC119427.1	0	0	0	0	0	0	0	0
ENSG00000159173	TNNI1	0	0	0	0	0	0	0	0
ENSG00000227048	AC096677.3	0	0	0	0	0	0	0	0
ENSG00000174307	PHLDA3	0	0	0	0	0	0.04121	0	0
ENSG00000224818	AC096677.2	0	0	0	0	0	0	0	0
ENSG00000159176	CSRP1	2.71378	1.5727	0.48021	0.80129	1.90014	1.69191	0.72233	0.64545
ENSG00000224536	AC096677.1	0.1934	0	0.07105	0.21528	0.3527	0.51179	0.24178	0.11873
ENSG00000223396	RPS10P7	0.44062	0.15716	0	0	0.07653	0.22209	0.05246	0.19321
ENSG00000242150		0	0	0	0	0	0	0	0
ENSG00000134369	NAV1	0.22199	0.17389	0.00959	0.0533	0.11907	0.06335	0.04353	0.01202
ENSG00000236390	AC092800.1	0	0	0	0	0	0	0	0
ENSG00000231871	IPO9-AS1	0.05754	0.2873	0	0	0.1399	0.1015	0.0959	0.07064
ENSG00000264802	MIR5191	0	0	0	0	0	0	0	0
ENSG00000235121	AL645504.1	0	0	0	0	0	0	0	0
ENSG00000200942	RNU6-501P	0	0	0	0	0	0	0	0
ENSG00000221028	MIR1231	0	0	0	0	0	0	0	0
ENSG00000198700	IPO9	2.74376	2.32446	0.82266	1.74386	2.73745	3.49462	2.16366	1.81278
ENSG00000277681	MIR6739	0	0	0	0	1.18822	0	0	0
ENSG00000198892	SHISA4	10.7893	7.82643	4.5652	10.5297	14.6997	3.62902	2.09932	5.97919
ENSG00000223774	AL513217.1	0.21848	0.18183	0	0.16212	0.13281	0	0.18209	0
ENSG00000163431	LMOD1	5.53767	0.70667	0.97651	2.219	2.98477	0.19538	0.18461	0.43061
ENSG00000134375	TIMM17A	3.75578	2.46587	0.7972	1.91993	2.20699	4.2699	1.80853	2.58745
ENSG00000206637	SNORA70H	0	0	0	0	0	0	0	0
ENSG00000238003	RPL10P4	0	0	0	0	0	0	0	0
ENSG00000176393	RNPEP	9.96581	4.87473	1.6719	2.53281	3.99603	3.8656	2.21252	3.13011
ENSG00000273478		0	0	0	0	0	0	0	0
ENSG00000234678	ELF3-AS1	0	0	0	0	0	0	0	0
ENSG00000275207	MIR6740	0	0	0	0	0	0	1.08124	0
ENSG00000163435	ELF3	0.10193	0	0	0	0.04957	0	0.11894	0.01252
ENSG00000249007	AL691482.3	0	0	0	0	0	0	0	0
ENSG00000232296	AL691482.1	0	0	0	0	0	0	0	0
ENSG00000235811	AL691482.2	0	0	0	0	0	0	0	0
ENSG00000212512	RF00019	0	0	0	0	0	0	0	0
ENSG00000227109	CRIP1P3	0	0	0	0	0	0	0	0
ENSG00000170075	GPR37L1	0	0	0	0	0	0	0	0
ENSG00000143862	ARL8A	18.557	17.2974	2.12701	2.80915	11.5513	9.82115	3.77367	5.24034
ENSG00000143851	PTPN7	4.46802	3.30534	0.63833	2.00773	4.14952	5.99923	1.30331	1.58478
ENSG00000243323	PTPRVP	0.03349	0	0	0.07454	0	0	0	0.02056
ENSG00000133067	LGR6	0.30389	0.53244	0.23503	0.23737	0.17501	0.5361	0.26659	0.35347
ENSG00000077152	UBE2T	0.48179	0.45825	0.20228	1.73649	0.33471	2.18555	0.57362	0.16901
ENSG00000077157	PPP1R12B	2.51188	1.96891	0.33742	0.75384	1.82725	1.13544	0.92785	1.7556
ENSG00000229120	CYCSP4	0	0	0	0	0	0	0	0
ENSG00000272262	RNU6-89P	0	0	0	0	0	0	0	0

ENSG00000232626	AC099336.1	0.23464	0.07811	0	0	0	0.16557	0	0.11523
ENSG00000236439	AC099336.2	0	0	0	0.42829	0	0	0	0.35432
ENSG00000253042	RF00156	0	0	0	0	0	0	0	0
ENSG00000143858	SYT2	0.01886	0	0	0	0	0	0	0
ENSG00000226862	AC104463.2	0.46688	0	0	0	0.28381	0	0	0.28661
ENSG00000225620	AC104463.1	0	0	0	0	0	0	0	0
ENSG00000117139	KDM5B	4.63128	3.72322	1.14912	2.33009	4.20821	3.21889	3.07169	2.91009
ENSG00000235449	AC098934.3	1.15737	0.64215	0	0	0.46904	0	0.64305	1.89467
ENSG00000226148	SLC25A39P1	0	0	0	0	0	0	0	0
ENSG00000260021	AC098934.4	0	0	0.3639	0	0	0.43686	0	0
ENSG00000228288	PCAT6	0.32944	0	0	0	0	0.14529	0	0
ENSG00000184774	MGAT4EP	0	0	0	0	0	0	0	0
ENSG00000214796	AC098934.1	0	0	0	0	0	0	0	0
ENSG00000234996	AC098934.2	0	0	0	0.04687	0	0	0	0
ENSG00000183155	RABIF	4.49455	3.76569	1.15249	2.10409	3.48399	4.04432	1.60896	2.88879
ENSG00000117153	KLHL12	10.9992	7.79728	2.69891	3.46081	9.30808	8.04558	4.16211	6.61296
ENSG00000230280	HNRNPA1P59	0	0	0	0	0	0	0	0
ENSG00000199471	RF00019	1.45149	0	0	0	0	0	0	0
ENSG00000159346	ADIPOR1	512.483	684.975	407.512	299.987	555.744	650.163	752.788	421.433
ENSG00000159348	CYB5R1	4.47549	2.87143	1.05227	1.42503	3.83851	2.89975	1.68188	2.87735
ENSG00000234761	AC096632.2	0	0	0	0	0	0	0	0
ENSG00000231547	AC096632.1	0	0	0	0	0	0	0	0
ENSG00000163444	TMEM183A	11.6523	9.66734	5.16363	4.51247	10.1158	12.5586	6.03922	7.51093
ENSG00000143847	PPFIA4	0.09062	0.08798	0.0111	0.03362	0	0.02664	0.01259	0.02781
ENSG00000122180	MYOG	0	0	0	0	0	0	0	0
ENSG00000163485	ADORA1	0.06996	0.02911	0	0.05191	0.04253	0.03085	0.02915	0
ENSG00000234775	AC105940.2	0	0	0	0	0	0	0	0
ENSG00000224671	AC105940.1	0	0	0	0	0	0	0	0
ENSG00000133055	MYBPH	0.24366	0.33797	0	0.06027	0.19749	0	0	0.44874
ENSG00000133048	CHI3L1	23.8312	9.09336	3.79274	3.79856	8.00167	1.2142	9.53637	13.5999
ENSG00000133063	CHIT1	0.29396	0.28411	0.12541	0.11259	0.25363	0.3513	0.12645	0.43078
ENSG00000236523	NPM1P40	0	0	0	0	0	0	0	0
ENSG00000231507	LINC01353	1.7515	0.72884	0.64345	0.51988	0.7453	0.15449	0.14597	0.96771
ENSG00000233791	LINC01136	1.37186	1.47753	0.51386	0.87827	1.53706	0.80672	0.04484	1.84948
ENSG00000159388	BTG2	162.553	137.188	23.715	29.0805	145.748	51.2182	25.0831	72.7941
ENSG00000202300	RNU6-487P	0	0	0	0	0	0	0	0
ENSG00000122176	FMOD	0	0	0	0	0.05385	0	0	0
ENSG00000229652	AL359837.1	0	0	0	0	0	0	0	0
ENSG00000271588	LARP7P1	0	0	0	0	0	0	0	0
ENSG00000188783	PRELP	0	0	0	0	0	0	0	0
ENSG00000188770	OPTC	0	0	0	0	0	0	0	0
ENSG00000058668	ATP2B4	19.311	18.6759	4.26601	9.22122	12.9719	20.5122	4.56244	8.11001
ENSG00000236035	AL513343.1	0	0	0	0	0	0	0	0
ENSG00000221643	SNORA77	1.17281	0	0.86171	1.74057	0	0	0	0
ENSG00000122188	LAX1	3.30058	3.7007	1.11149	2.82339	5.48968	7.48051	1.56641	2.22319
ENSG00000058673	ZC3H11A	0.15464	0.06673	0.05892	0.28051	0.14623	0.07073	0.12411	0.25316
ENSG00000257315	ZBED6	0.59904	0.5181	0.04315	0.0523	0.24991	0.37298	0.02937	0.09374
ENSG00000227417	AC114402.2	0	0	0	0	0	0	0	0
ENSG00000223505	AC114402.1	0	0	0	0	0	0	0	0
ENSG00000182004	SNRPE	8.55336	6.2908	2.84996	10.48	7.07371	11.2292	5.22209	9.34162
ENSG00000236430	KRT8P29	0	0	0	0	0	0	0	0
ENSG00000232917	HSPE1P6	0	0	0	0	0	0	0	0
ENSG00000237379	CBX1P3	0	0	0	0	0.1691	0	0	0
ENSG00000176754	LINC00303	0	0	0	0	0	0	0	0
ENSG00000143842	SOX13	0.30624	0.57551	0.13065	0.33721	0.31227	0.08714	0.0494	0.09703
ENSG00000143845	ETNK2	0.03278	0.02728	0	0	0	0.08675	0	0.02012
ENSG00000261065	AL592146.1	0	0	0	0	0	0	0	0
ENSG00000230550	ERLNC1	0	0	0	0	0	0	0	0

ENSG00000143839	REN	0	0	0	0	0	0	0	0
ENSG00000225522	AL592114.2	0	0	0	0	0	0	0	0
ENSG00000170498	KISS1	0	0	0	0	0	0	0	0
ENSG00000174567	GOLT1A	0	0	0	0	0	0	0	0
ENSG00000143850	PLEKHA6	0.03554	0.01479	0	0.01318	0	0	0	0.02181
ENSG00000231691	AL592114.3	0	0	0	0	0	0	0	0
ENSG00000219133	AL592114.1	0	0.25261	0	0	0	0.26773	0	0.18633
ENSG00000280924	LINC00628	0	0	0.08356	0	0	0	0	0
ENSG00000226330	AL606489.1	0	0	0	0	0	0	0	0
ENSG00000237848	AL606489.2	0	0	0	0	0	0	0	0
ENSG00000158615	PPP1R15B	37.4986	27.7769	7.89257	11.4676	27.3641	21.9189	13.2769	19.852
ENSG00000133056	PIK3C2B	0.92657	0.6869	0.11442	0.38134	0.97504	1.1401	0.20766	0.26768
ENSG00000198625	MDM4	7.41793	5.17605	1.57539	3.44897	5.99076	4.82805	4.45079	4.98712
ENSG00000236779	AL512306.1	0	0	0	0	0	0	0	0
ENSG00000200408	RNA5SP74	0.67248	0	0	0	0	0	0	1.65132
ENSG00000240710	AL512306.3	0.42928	0.71454	0	0	0.39143	0	0	0.7906
ENSG00000170382	LRRN2	0.0129	0	0.01896	0.0383	0	0.31867	0.04301	0.01584
ENSG00000240219	AL512306.2	0.03303	0.10997	0.04854	0.04902	0.08032	0.05827	0.05506	0.32446
ENSG00000228153	AL161793.1	0	0	0	0	0	0	0	0
ENSG00000252650	RNA5SP75	0	0	0	0	0.80285	0	0	0
ENSG00000251861	RF00601	0	0	0	0	0	0	0	0
ENSG00000163531	NFASC	0	0.01147	0	0.00511	0.01257	0	0	0
ENSG00000229657	AL391822.1	0	0	0	0	0	0	0	0.16014
ENSG00000184144	CNTN2	0	0	0	0	0	0	0	0
ENSG00000174529	TMEM81	1.43079	1.83196	0.16173	0.16334	1.20428	0.38832	0.09173	0.40539
ENSG00000117222	RBBP5	7.15694	6.28881	1.66316	2.56896	7.32521	6.19546	2.21944	4.65925
ENSG00000271580	AL583832.1	0	0	0	0	0	0	0	0
ENSG00000236108	AC093422.3	0	0	0	0	0	0	0	0
ENSG00000133059	DSTYK	2.90072	2.07761	0.98168	1.35672	2.70374	1.95387	2.41753	1.6836
ENSG00000213041	AC093422.1	0	0	0	0.19708	0	0	0	0
ENSG00000133069	TMCC2	5.68506	4.79284	4.96908	4.2515	8.85063	14.4838	20.3306	4.93135
ENSG00000225063	AC093422.2	0	0	0	0	0	0	0	0
ENSG00000163545	NUAK2	12.0144	12.0909	1.31473	2.40271	10.4889	2.93122	5.82318	8.99709
ENSG00000162873	KLHDC8A	0.42161	0.10025	0.32453	0.26817	0.43936	1.62924	0.26772	0.3451
ENSG00000235363	SNRPGP10	6.51559	4.88034	1.91491	2.90095	3.96074	5.74719	2.71512	4.39986
ENSG00000226235	LEMD1-AS1	0	0	0	0	0	0	0	0.06472
ENSG00000186007	LEMD1	0	0	0	0	0	0	0	0
ENSG00000281406	BLACAT1	0	0	0	0	0	0	0	0
ENSG00000199059	MIR135B	0	0	0	0	0	0	0	0
ENSG00000224717	AC098936.1	0	0	0	0	0.03089	0.04482	0	0
ENSG00000117266	CDK18	0.21461	0.05358	0.04731	0.01593	0.19569	0.03786	0.01789	0.01317
ENSG00000253097	RNU2-19P	0	0	0	0	0	0	0	0
ENSG00000174514	MFSD4A	0.02532	0	0	0	0.12313	0	0.0422	0.09326
ENSG00000206762	RNU6-418P	0	0	0	0	0	0	0	0
ENSG00000158711	ELK4	10.2266	7.62679	3.16701	5.80573	11.9928	18.1369	6.17845	6.79685
ENSG00000236942	AC096533.1	0	0	0	0	0	0	0	0.25495
ENSG00000158715	SLC45A3	2.21762	1.35126	0.23277	0.47017	1.13141	0.52395	0.16502	0.53483
ENSG00000275213	RF00017	0	0	0	0	0	0	0	0
ENSG00000069275	NUCKS1	20.4247	13.6994	8.29328	21.5544	22.8441	27.9524	16.9104	17.7904
ENSG00000201944	RF00139	1.11061	0	0	1.64827	0	0	0	0.6818
ENSG00000117280	RAB29	11.1027	9.52307	2.94118	4.25951	9.25142	14.8898	2.98999	5.54078
ENSG00000133065	SLC41A1	1.18766	0.84448	0.28805	0.49627	1.7243	1.72904	0.44206	0.42471
ENSG00000162877	PM20D1	0	0.14003	0	0	0.20455	0.14841	0	0.03443
ENSG00000174502	SLC26A9	0	0	0	0	0	0	0	0
ENSG00000227687	AL713965.1	0	0	0	0	0	0	0	0
ENSG00000276600	RAB7B	0.01989	0	0	0	0.04835	0	0.06629	0
ENSG00000196188	CTSE	1.39959	1.14197	3.42781	6.96457	3.80356	4.35719	4.4371	2.15643
ENSG00000263961	RHEX	1.07158	0.48021	0.12113	0.51992	0.92699	0.14542	0.34349	0.37952

ENSG00000252692	RF00271	0	0	0	0	0	0	0	0
ENSG00000198049	AVPR1B	0	0	0	0	0	0	0	0
ENSG00000226780	AC244035.1	0	0	0	0	0	0	0	0
ENSG00000225483	RPL22P4	0	0.31284	0.82857	0.55788	0	0.33157	1.25313	0.46152
ENSG00000236889	AC244035.2	0	0	0.79494	1.60569	0	0	0	0
ENSG00000196550	FAM72A	0.56191	0.48378	0.3132	1.03523	0.4947	1.1622	0.48446	0.26169
ENSG00000266028	SRGAP2	2.13237	2.00818	0.46728	0.53439	1.21096	1.72416	0.66254	0.57989
ENSG00000229509	AC244023.1	0	0	0	0	0	0	0	0
ENSG00000263528	IKBKE	4.60182	3.37929	0.56826	1.06174	3.59664	5.93518	1.25693	2.23153
ENSG00000278465	MIR6769B	0	0	0	0	0	0	0	0
ENSG00000162888	C1orf147	0	0	0	0	0	0	0	0.04294
ENSG00000261000	AC244034.2	1.58409	0.2397	0	0.21372	1.22557	0.25405	0.24004	0
ENSG00000266094	RASSF5	49.9947	35.8015	6.93285	14.6742	29.8163	27.0821	17.602	22.5638
ENSG00000234981	AC244034.1	0.1851	0	0	0	0.11252	0.16327	0	0
ENSG00000279946	AC244034.3	0.04676	0	0	0	0	0	0	0
ENSG00000143486	EIF2D	2.16338	0.9078	0.17365	0.93083	1.88975	1.94032	0.84847	0.85935
ENSG00000237605	AL591846.2	0	0	0	0	0	0	0	0
ENSG00000143479	DYRK3	0.76463	1.38843	0.89379	1.98589	1.05639	1.25694	4.72153	0.91747
ENSG00000162889	MAPKAPK2	25.1266	20.3008	4.39072	6.06814	15.2316	16.7073	8.62107	7.03672
ENSG00000224114	AL591846.1	0	0	0	1.25619	1.02906	2.09049	3.66823	6.23536
ENSG00000199349	RF00019	0	0	0	0	0	0	0	0
ENSG00000136634	IL10	0.22235	0.12337	0.05446	0	0.09011	0.32688	0.06177	0.1365
ENSG00000284486		0.0535	0	0.07862	0.07941	0	0.09439	0	0
ENSG00000142224	IL19	0	0	0	0	0.03445	0	0	0
ENSG00000162891	IL20	0	0	0	0	0	0	0	0
ENSG00000162892	IL24	0	0	0	0	0	0	0	0
ENSG00000162894	FCMR	26.7852	12.6789	2.79836	10.7488	41.484	35.0816	13.5293	10.9622
ENSG00000271680	AC098935.2	0	0	0	0	0.38247	0	0	0
ENSG00000226945	AC098935.1	0	0	0	0	0	0	0	0
ENSG00000162896	PIGR	0.0335	0	0	0	0.26474	0	0	0.04113
ENSG00000162897	FCAMR	0	0	0	0	0	0	0	0
ENSG00000182795	C1orf116	0.85264	0.77614	0.29366	0.47453	0.53451	0.9166	0.53296	0.3435
ENSG00000123836	PFKFB2	2.32186	1.3042	0.46479	0.99216	1.17983	1.17937	1.61757	1.35918
ENSG00000180667	YOD1	107.067	109.342	93.7232	177.217	50.4143	37.8159	217.249	107.193
ENSG00000123843	C4BPB	0	0.0379	0	0	0	0	0	0
ENSG00000123838	C4BPA	0.06006	0.49983	0	0.04457	7.30165	1.64222	0.05005	7.33692
ENSG00000275392	AL445493.3	0	0	0	0	0	0	0	0
ENSG00000226565	AL445493.1	0	0	0	0	0	0	0	0
ENSG00000224462	C4BPAP1	0	0	0	0	0	0	0	0
ENSG00000243636	AL445493.2	0	0	0	0	0	0	0	0
ENSG00000232621	C4BPAP2	0	0	0	0	0	0	0	0
ENSG00000237074	AL596218.1	0	0	0	0	0	0	0	0
ENSG00000196352	CD55	62.6897	79.827	9.34372	21.5696	42.5417	21.6216	21.2192	27.7685
ENSG00000274245		0.63372	0.35161	0.31041	0.3135	1.02728	0.74531	0.52816	0.51872
ENSG00000283044	AL391597.1	0	0	0	0	0.18605	0	0	0
ENSG00000117322	CR2	1.06537	0.22479	0.1764	0.42312	2.59049	0.79414	0.47522	0.82904
ENSG00000203710	CR1	16.3991	17.3462	2.4521	5.56026	11.0298	2.80843	5.50957	8.14016
ENSG00000236911	AL137789.1	0	0	0.14305	0.14447	0	0	0.08113	0
ENSG00000197721	CR1L	13.9292	17.0142	30.5086	50.8558	28.4929	28.4037	53.2773	34.7893
ENSG00000244703	CD46P1	9.99113	30.2223	41.3633	155.551	32.5866	55.2499	85.9906	36.8009
ENSG00000226289	CDCA4P3	0	0	0	0	0.25868	0	0	0
ENSG00000274059	RNA5SP534	0	0	0	0	0	0	0	0
ENSG00000117335	CD46	28.5609	31.7351	6.6177	14.1042	22.8011	11.9541	16.0232	30.8706
ENSG00000234219	CDCA4P4	2.15589	1.43539	0.31681	0.31996	0.65527	0.95082	0.89838	0.2647
ENSG00000203709	MIR29B2CHG	1.74046	1.64164	0.27543	0.73517	0.92236	0.59834	1.23482	1.46845
ENSG00000284214	MIR29C	0	0	0	0	0	0	0	0
ENSG00000284203	MIR29B2	0	0	0	0	0	0	0	0
ENSG00000174059	CD34	0.00795	0.02647	0	0	0.058	0.01403	0	0.01952

ENSG00000284237	AL356275.1	0	0	0	0	0	0	0	0
ENSG00000076356	PLXNA2	0.36701	0.27445	0.05471	0.02368	0.2328	0.0563	0.08866	0.13061
ENSG00000226843	AL606753.1	0	0	0	0	0	0	0	0
ENSG00000261453	LINC01735	0	0	0	0	0	0	0	0
ENSG00000259815	AC099793.1	0	0	0	0	0	0	0	0
ENSG00000227887	RPS26P13	0	0	0	0	0	0	0	0
ENSG00000232812	LINC01717	0	0	0	0	0	0	0	0
ENSG00000236950	LINC01774	0	0	0	0	0	0	0	0
ENSG00000278684	AC104465.1	0	0	0	0	0	0	0	0
ENSG00000232537	AC092810.3	0	0	0	0	0	0	0	0
ENSG00000228081	AC092810.2	0	0	0	0	0	0	0	0
ENSG00000224540	AC092810.1	0	0	0	0.35551	0	0.10565	0	0
ENSG00000225712	ATP5MC2P1	0	0	0	0	0	0	0	0
ENSG00000227940	LINC01696	0	0	0	0	0	0	0	0
ENSG00000231648	LINC01698	0	0	0	0	0	0	0	0
ENSG00000230937	MIR205HG	0	0	0	0	0	0	0	0
ENSG00000284485	MIR205	0	0	0	0	0	0	0	0
ENSG00000224260	AL023754.1	0	0	0	0	0	0	0	0
ENSG00000008118	CAMK1G	0	0	0	0	0	0	0	0
ENSG00000196878	LAMB3	0.53162	0.31231	0.11488	0.04641	0.32316	0.05517	0.07819	0.13438
ENSG00000283876	MIR4260	0	0	0	0	0	0	0	0
ENSG00000227591	HSD11B1-AS1	1.53669	1.27891	0	0.45612	0.56048	0.54219	0.25614	0.18867
ENSG00000123689	G0S2	184.774	170.333	24.1299	30.5253	177.822	23.4434	6.06669	59.0281
ENSG00000117594	HSD11B1	0.09689	0	0	0.0719	0	0	0	0
ENSG00000236136	ADORA2BP1	0	0	0	0	0	0	0	0
ENSG00000009790	TRAF3IP3	11.6146	9.16244	2.07089	3.27059	10.602	8.87663	4.16201	6.66559
ENSG00000162757	C1orf74	0.34428	0.31258	0.06899	0.11612	0.36149	0.22086	0.23476	0.24978
ENSG00000117595	IRF6	0.2237	0.04965	0.04383	0.08853	0.01813	0.02631	0.12429	0.07324
ENSG00000117597	UTP25	1.50472	0.98773	0.41524	0.70245	1.72633	1.64506	0.57699	0.68521
ENSG00000143469	SYT14	0	0	0	0	0	0	0	0
ENSG00000203706	SERTAD4-AS1	0	0	0	0	0	0	0	0
ENSG00000082497	SERTAD4	0	0	0	0	0	0	0	0
ENSG00000228110	ST13P19	0	0	0	0	0	0	0	0
ENSG00000233455	AL035414.1	0	0	0	0	0	0	0	0
ENSG00000054392	HHAT	0.0883	0.09186	0.01622	0.06552	0.08051	0.01947	0.07359	0.06776
ENSG00000200972	RNU5A-8P	0	0	0	0	0	0	1.07176	0
ENSG00000270936	AL034351.1	0	0	0	0	0	0	0	0
ENSG00000143473	KCNH1	0	0	0	0	0	0	0	0
ENSG00000279333	AC096636.1	0	0	0	0.06014	0.02463	0	0.03377	0
ENSG00000284299	AL590132.1	0	0	0	0	0	0	0	0
ENSG00000224668	IPO8P1	0	0	0	0	0	0	0	0
ENSG00000283952	AC092017.3	0	0	0	0	0	0	0	0
ENSG00000284376	AC092017.4	0	0	0	0	0	0	0	0
ENSG00000234233	KCNH1-IT1	0	0	0	0	0	0	0	0
ENSG00000234004	AC092017.2	0	0	0.29755	0	0	0	0	0.24861
ENSG00000226986	AC092017.1	0	0.74057	0.16345	0	0	0	0	0
ENSG00000117625	RCOR3	5.18391	5.60928	3.24611	4.40312	5.53794	3.86854	4.97214	5.07792
ENSG00000082512	TRAF5	1.87679	1.31177	0.5492	1.55546	3.50659	2.63726	0.92089	1.63595
ENSG00000153363	LINC00467	0.62622	0.21944	0.1453	0.29349	0.44077	0.2035	0.10987	0.28326
ENSG00000236809	SNX25P1	0.08789	0.14629	0	0	0	0	0	0
ENSG00000238137	ARPC3P2	0	0	0	0	0	0	0	0
ENSG00000198570	RD3	0.01718	0	0	0	0	0	0	0
ENSG00000223649	AC105275.1	0	0	0	0	0	0	0	0
ENSG00000170385	SLC30A1	11.6231	9.56186	6.8674	11.2482	7.8144	5.95297	8.16916	6.7243
ENSG00000261314	AC105275.2	0	0	0	0	0	0	0	0
ENSG00000228792	AL356310.1	0	0	0	0	0	0	0	0
ENSG00000227764	LINC01693	0	0	0	0	0	0	0	0
ENSG00000117650	NEK2	0.30789	0.19711	0.62645	1.33566	0.80623	2.67398	1.1843	0.43617

ENSG00000224763	FDPSP8	0.06731	0	0	0	0	0	0	0
ENSG00000231057	AC096637.2	0	0.04976	0	0	0	0	0	0
ENSG00000226868	AC096637.1	0	0	0	0	0	0	0	0
ENSG00000123684	LPGAT1	36.1378	26.5815	6.24322	11.8565	24.8644	17.4885	10.2477	19.955
ENSG00000241395	RN7SL344P	0	0	0	0	0	0	0	0
ENSG00000212205	RF00019	0	0	0	0	0	0	0	0
ENSG00000229258	LPGAT1-AS1	0.05394	0	0	0	0	0	0	0
ENSG00000233626	AC092814.1	0	0	0	0	0	0	0	0
ENSG00000143493	INTS7	3.22958	2.80268	2.23094	5.22319	3.47339	6.50089	3.81885	3.03323
ENSG00000212187	RF00568	0	0	0	0	0	0	0	0
ENSG00000143476	DTL	4.8012	6.70104	13.7381	33.813	8.03065	14.1254	29.3024	15.9454
ENSG00000220749	RPL21P28	2.27641	1.51563	1.11505	0	1.10704	0.53545	1.01185	2.23595
ENSG00000264358	MIR3122	0	0	0	0	0	0	0	0
ENSG00000252879	RN7SKP98	0	0	0	0	0	0	0	0
ENSG00000229983	AL606468.1	0	0	0	0.21331	0	0	0	0
ENSG00000226251	LINC02608	0.14344	0.23876	0	0.21289	0	0.75917	0	0.17612
ENSG00000066027	PPP2R5A	25.9282	27.3044	11.3487	16.7516	20.8987	17.1063	11.8826	23.9286
ENSG00000230063	AL360091.2	0.78939	0	0.16571	0.33473	1.50813	0.59682	0	0.9692
ENSG00000234915	AL360091.3	0	0	0	0	0.23028	0	0	0
ENSG00000225338	RPL23AP18	0	0	0	0	0	0	0	0
ENSG00000201544	SNORA16B	3.80076	8.1339	0	0	0.66012	0.95787	0.90504	1.99993
ENSG00000229832	AL360091.1	1.62561	2.70583	0.21717	0.21933	0.35934	0.26071	1.23166	1.27012
ENSG00000065600	TMEM206	1.73566	1.29796	0.44357	0.9333	1.77377	2.0413	0.54507	0.9883
ENSG00000117691	NENF	3.78653	2.35174	0.41524	0.671	2.47355	3.19042	2.07245	1.94288
ENSG00000224535	AC092803.1	0	0	0	0	0	0	0	0
ENSG00000228067	LINC01740	0	0	0	0	0	0	0	0
ENSG00000260805	AC092803.2	0.1544	0.1285	0.11344	0.05729	0.23464	0.06809	0	0.04739
ENSG00000162772	ATF3	0.92533	0.4832	0.31994	0.21542	0.39705	0.96024	0.09073	0.20049
ENSG00000235862	AL590648.2	0	0	0	0	0	0	0	0
ENSG00000162771	FAM71A	0	0	0	0	0	0.0561	0	0
ENSG00000226036	AL590648.1	0	0	0	0	0	0	0	0
ENSG00000237980	AL590648.3	0.92981	0.25795	0.22773	0	0	0.54677	0	0
ENSG00000123685	BATF3	0.51335	0.58463	0.11911	0.0401	0.0657	0.14299	0	0.09952
ENSG00000117697	NSL1	6.55779	5.4279	2.29067	3.80258	7.18859	5.87925	2.32952	4.88373
ENSG00000203705	TATDN3	3.67663	2.19023	1.45021	3.01544	2.04677	2.86753	2.70938	2.20954
ENSG00000236905	AC104333.3	0	0	0	0	0	0	0	0
ENSG00000185523	SPATA45	0.14486	0.24112	0.21287	0.64497	0	0	0.24146	0
ENSG00000198468	FLVCR1-DT	0.5212	0.27604	0.17407	0.1758	0.60486	0.12538	0	0.31996
ENSG00000235182	AC104333.1	0	0	0	0	0	0	0	0
ENSG00000236317	AC104333.2	0	0	0	0	0.46415	0	0	0
ENSG00000162769	FLVCR1	3.44769	6.07959	5.09978	12.0404	5.98999	5.56029	9.42621	7.02709
ENSG00000282718	AC104333.4	0	0	0	0	0	0	0	0
ENSG00000143494	VASH2	0.03227	0.01343	0	0	0.01962	0	0.01345	0
ENSG00000174606	ANGEL2	3.03235	2.59914	0.91785	1.92872	3.3927	3.44784	1.57847	2.58513
ENSG00000136643	RPS6KC1	6.21691	6.80015	4.50693	11.0192	7.32801	7.39521	12.0799	6.52806
ENSG00000228646	RPL31P13	0	0	0	0	0	0	0	0
ENSG00000225233	AL592402.1	0	0	0	0	0	0	0	0
ENSG00000228255	AC096639.1	0	0	0	0	0	0	0	0
ENSG00000230461	PROX1-AS1	0	0	0	0	0	0	0	0
ENSG00000281664	LINC00538	0	0	0	0	0	0	0	0
ENSG00000117707	PROX1	0	0	0	0	0.02995	0.01448	0	0.01008
ENSG00000274895	AC011700.1	0	0	0	0	0	0	0	0
ENSG00000272167	AL606537.1	0	0	0	0	0	0	0	0
ENSG00000283518	AL592287.1	0	0	0	0	0	0	0	0
ENSG00000143499	SMYD2	1.79915	1.42248	0.37454	0.89004	1.64052	1.90437	1.22455	1.43583
ENSG00000228470	AL929236.1	0	0	0	0	0	0	0	0
ENSG00000152104	PTPN14	0	0	0.00574	0	0.01425	0.02068	0	0.0096
ENSG00000213036	AL445305.1	0	0	0	0.37839	0.30997	0	0	0

ENSG00000215819	KRT18P12	0	0	0	0	0	0	0	0
ENSG00000117724	CENPF	2.30749	2.02478	2.06398	3.39665	3.97175	6.02865	4.003	3.3951
ENSG00000250536	ABHD17AP3	0	0	0	0	0	0	0	0
ENSG00000224584	UBE2V1P13	0	0	0	0	0	0	0	0
ENSG00000185221	GAPDHP24	0	0	0	0	0	0	0	0
ENSG00000223869	AC099563.1	0	0	0	0	0	0	0	0
ENSG00000082482	KCNK2	0	0	0	0	0	0	0	0
ENSG00000232809	VDAC1P10	0	0	0	0	0	0	0	0
ENSG00000282265	AL450990.1	0	0	0	0	0	0	0	0
ENSG00000136636	KCTD3	1.17483	0.76735	0.26224	0.72833	1.28369	0.57717	1.28898	0.73035
ENSG00000042781	USH2A	0	0	0	0	0	0	0	0
ENSG00000202498	RF00108	0	0	0	0	0	0	0	0
ENSG00000229242	AL358452.1	0	0	0	0	0	0	0	0
ENSG00000233620	AC093581.1	0	0	0	0	0	0	0	0
ENSG00000236292	AC138024.1	0	0	0	0	0	0	0	0
ENSG00000226013	MRPS18BP1	0	0	0	0	0	0	0	0
ENSG00000196482	ESRRG	0	0.01118	0	0	0	0	0	0
ENSG00000092978	GPATCH2	1.77587	1.51941	0.34145	0.64042	1.82612	1.90315	0.91293	0.82529
ENSG00000162814	SPATA17	0.01125	0	0	0	0.02736	0	0	0
ENSG00000234070	SPATA17-AS1	0	0	0	0	0	0	0	0
ENSG00000228247	UBBP2	0	0	0	0	0	0	1.05784	0
ENSG00000231814	LINC00210	0	0	0	0	0	0	0	0
ENSG00000230714	AL355526.1	0	0	0	0	0	0	0	0
ENSG00000232100	LINC01653	0	0	0	0	0	0	0	0
ENSG00000201493	RNU1-141P	0	0	0	0	0	0	0	0
ENSG00000067533	RRP15	1.57145	0.92865	0.83351	1.5456	1.98967	1.21389	1.16244	1.62116
ENSG00000229016	AC096638.2	0	0	0	0	0	0	0	0
ENSG00000223375	AC096638.1	0	0	0	0	0.41258	0	0	0
ENSG00000232480	TGFB2-AS1	0	0	0	0	0	0	0	0
ENSG00000092969	TGFB2	0.19922	0.3553	0.04182	0.04224	0.10381	0.0251	0.04744	0.06989
ENSG00000281453	TGFB2-OT1	0	0	0	0	0	0	0	0
ENSG00000228208	C1orf143	0	0	0	0	0	0	0	0
ENSG00000212610	RF00012	0	0	0	0	0	0	0	0
ENSG00000226693	NXNP1	0	0	0	0	0	0	0	0
ENSG00000225561	LINC01710	0	0	0	0	0.45701	0	0	0.46152
ENSG00000228063	LYPLAL1-DT	5.66964	5.0556	3.57063	7.51283	2.83105	0.71443	3.03763	2.85902
ENSG00000143353	LYPLAL1	8.69916	6.41717	5.48375	12.6171	9.40442	6.32172	13.3879	11.0448
ENSG00000225920	RIMKLBP2	0	0	0	0	0	0	0	0
ENSG00000223842	AL360093.1	0	0	0	0	0	0	0	0
ENSG00000277007	AC096642.1	0.16825	0	0	0	0.03409	0	0	0
ENSG00000228536	LYPLAL1-AS1	0	0	0	0	0	0	0	0
ENSG00000238232	AL356364.1	0	0	0	0	0	0	0	0
ENSG00000215817	ZC3H11B	0	0	0.02327	0	0.05777	0.02794	0	0.03889
ENSG00000196660	SLC30A10	0	0	0	0.03771	0.03089	0.06723	0	0.04679
ENSG00000252086	RNA5SP76	0	0	0	0	0	0	0	0
ENSG00000221673	RF00012	0	0	0	0	0	0	0	0
ENSG00000136628	EPRS	18.3587	17.3722	9.07169	17.6253	21.5842	22.066	11.2765	14.1188
ENSG00000162813	BPNT1	2.61366	2.07724	0.41522	1.04837	2.57645	3.40622	0.51023	1.59005
ENSG00000067704	IARS2	9.38033	6.47392	1.47412	5.19769	8.81538	9.0037	4.6056	5.9854
ENSG00000207590	MIR215	0	0	0	0	0	0	0	0
ENSG00000207624	MIR194-1	0	0	0	0	0	0	0	0
ENSG00000232134	RPS15AP12	0	0	0	0	0.22968	0	0	0
ENSG00000118873	RAB3GAP2	5.58398	5.83857	1.74941	3.31279	5.47933	4.56292	2.78756	3.91519
ENSG00000281696	MIR664A	0	0	0	0	0	0	0	0
ENSG00000222370	SNORA36B	0	0	0	0	0	0	0	0
ENSG00000270585	AL445435.1	0	0	0	0	0	0	0	0
ENSG00000270708	KF455155.1	0	0	0	0	0	0	0	0
ENSG00000218283	MORF4L1P1	5.38193	6.18244	2.33919	2.69996	4.88437	3.61057	5.18034	4.18807

ENSG00000213033	AURKAP1	0.12096	0	0	0	0.07353	0.10669	0.20162	0.07426
ENSG00000230318	XRCC6P3	0	0	0	0	0	0	0	0
ENSG00000232883	AL451081.2	0	0	0	0	0	0	0	0
ENSG00000226927	AL451081.1	0	0	0	0	0	0	0	0
ENSG00000260505	AC096644.4	0	0	0	0	0	0	0	0
ENSG00000235163	AC096644.3	0	0	0	0	0	0	0	0
ENSG00000234863	AC096644.2	0	0	0	0	0	0	0	0
ENSG00000226226	PRELID3BP1	0	0	0	0	0	0	0	0
ENSG00000231529	AC096644.1	0	0	0	0	0	0	0	0
ENSG00000116141	MARK1	0	0	0	0	0.01645	0	0.02255	0
ENSG00000243872	RN7SL464P	0	0	0	0	0	0	0	0
ENSG00000233012	HDAC1P2	0	0	0	0	0	0	0	0
ENSG00000162817	C1orf115	0.06443	0.50047	0.06312	0.15937	0.20889	0.07578	0.21479	0.13184
ENSG00000117791	MARC2	0.31048	0.04698	0	0	0.06863	0	0	0.06931
ENSG00000186205	MARC1	1.66268	2.06906	0.17452	0.35251	1.93481	0.90789	0.39592	1.10819
ENSG00000221571	RNU6ATAC35P	0	0	0	0	0	0	0	0
ENSG00000276642	RF00619	0	0	0	0	0	0	0	0
ENSG00000272823	AL445423.1	0	0	0	0	0	0.24125	0	0.1679
ENSG00000238078	LINC01352	0.0762	0	0	0	0.09264	0	0	0
ENSG00000257551	HLX-AS1	0	0	0	0	0.63316	0.22968	0	0
ENSG00000136630	HLX	5.75006	6.38066	0.41868	1.13399	2.89708	1.41649	1.2952	2.67129
ENSG00000226211	AL445466.1	0	0	0	0	0	0	0	0
ENSG00000234754	C1orf140	0	0	0	0	0	0	0	0
ENSG00000232436	AL360013.2	0	0	0	0	0	0	0	0
ENSG00000227585	AL360013.1	0	0	0	0	0	0	0	0
ENSG00000143507	DUSP10	3.06885	2.7634	0.5175	1.19463	2.04901	4.52636	1.21593	1.17361
ENSG00000227925	LINC01655	0.17329	0	0	0	0	0	0	0
ENSG00000200033	RNU6-403P	0	0	0	0	1.71378	0	0	0
ENSG00000238042	LINC02257	0.14689	0	0.10793	0	0	0.12957	0	0.09018
ENSG00000228437	LINC02474	0.05366	0	0	0	0	0	0	0
ENSG00000232679	LINC01705	0	0	0	0	0	0	0	0
ENSG00000236230	AL356108.1	0.10859	0	0	0	0	0	0	0
ENSG00000227613	QRSL1P2	0	0	0	0	0	0	0	0
ENSG00000226643	AL513314.1	0	0	0	0	0	0	0	0
ENSG00000234419	CICP13	0	0	0	0	0	0	0	0
ENSG00000276997	AL513314.2	0	0	0.13566	0.0685	0	0.16286	0.07694	0.05667
ENSG00000222399	RNU6-791P	0	0	0	0	0	0	0	0
ENSG00000267305	AL592148.2	0	0	0	0	0	0	0	0
ENSG00000143512	HHIPL2	0	0	0	0.03308	0	0	0	0
ENSG00000143498	TAF1A	0.70895	0.96932	0.33486	1.27762	1.0774	1.38469	1.56154	1.08805
ENSG00000225265	TAF1A-AS1	0.1011	0.08414	0	0.15005	0	0.17836	0	0.24827
ENSG00000154305	MIA3	12.7488	12.8354	3.13473	5.72219	11.6007	8.78522	4.74498	7.48575
ENSG00000229399	AL592148.1	0	0	0	0	0	0	0	0
ENSG00000272750	AL592148.3	0.41553	0.36888	0.04071	0.32891	0.60624	0.43984	0.1847	0.44216
ENSG00000186063	AIDA	11.6221	17.1827	6.89756	13.9324	12.2556	14.0251	17.6688	11.0157
ENSG00000162819	BROX	22.1814	27.9787	14.2105	30.7571	20.4539	19.968	19.9463	25.2281
ENSG00000197520	FAM177B	0.30976	0.09374	0.04138	0.16717	0.65049	0.09936	0.14081	0.24202
ENSG00000228106	AL392172.1	8.57113	9.01653	7.75863	15.1628	14.9222	4.11282	9.94358	9.68163
ENSG00000278610	AC093152.1	0.12997	0	0	0	0	0	0	0
ENSG00000154309	DISP1	0.40859	0.11335	0.06004	0.28298	0.57954	0.28832	0.27242	0.11705
ENSG00000234523	NDUFB1P2	0.41648	0	0	0	1.01269	0.73473	0	0
ENSG00000187554	TLR5	6.93554	13.6095	1.19215	1.7706	5.80187	1.59956	0.47727	4.43345
ENSG00000236846	AL359979.2	0	0	0	0	0	0	0	0.29363
ENSG00000226601	AL359979.1	0	0	0.22118	0	0	0	0	0
ENSG00000143502	SUSD4	0.60695	0.12952	0.04574	0.25406	0.51086	0.32946	0.10376	0.09554
ENSG00000251789	RNU4-57P	0	0	0	0	0	0	0	0
ENSG00000178395	CCDC185	0	0	0	0	0	0	0	0
ENSG00000203697	CAPN8	0.01412	0.0235	0	0.02095	0.01716	0.07472	0	0

ENSG00000227002	SNRPEP10	0	0	0	0	0	0	0	0
ENSG00000212398	RNU6-1248P	0	0	0	0	0	0	0	0
ENSG00000162909	CAPN2	20.0259	13.7801	3.76117	6.03815	6.65942	15.1785	3.93278	7.04254
ENSG00000143514	TP53BP2	8.00174	9.34767	5.19602	10.6407	10.1002	9.54028	12.9298	9.31367
ENSG00000227621	PHBP11	0	0	0	0	0	0	0	0
ENSG00000188460	ACTBP11	0	0	0	0	0	0	0	0
ENSG00000233771	CICP5	0	0	0	0	0	0	0	0
ENSG00000272645	GTF2IP20	0.15916	0.38266	0.05197	0.41992	0.516	0.46796	0.26529	0.58623
ENSG00000212157	RNU6-1319P	0	0	0	0	0	0	0	0
ENSG00000185495	AC138393.1	0.62362	0.42082	0.37151	0.3502	0.53277	0.62441	0.4776	0.53804
ENSG00000278467	AC138393.3	0.89547	0.63879	0	0.18985	1.24421	2.03108	0.42646	0.94238
ENSG00000229930	AC138393.2	0	0	0	0	0	0.52566	0	0
ENSG00000252484	RN7SKP49	0	0	0	0	0	0	0	0
ENSG00000143756	FBXO28	5.91836	4.07772	2.2277	3.63579	4.45288	4.21483	4.4069	5.13716
ENSG00000236773	AC092809.3	0	0	0	0	0.38579	0	0	0
ENSG00000143753	DEGS1	16.9043	18.5802	5.22509	7.87237	15.839	7.81527	6.21832	10.8784
ENSG00000201898	RF00139	0	0	0	0	0	0	0	0
ENSG00000232628	AC092809.2	0	0	0	0	0	0	0	0
ENSG00000237101	AC092809.4	0.16621	0.36888	0	0	0	0	0	0
ENSG00000143748	NVL	3.80741	4.03061	1.85752	6.14787	6.23983	5.18537	4.03629	3.55276
ENSG00000221406	MIR320B2	0	0	0.78054	0	1.29155	0.93704	0	0
ENSG00000229742	AC092809.1	0	0	0	0	0	0	0	0
ENSG00000206887	RNU6-1008P	0	0	0	0	0	0	0	0
ENSG00000143771	CNIH4	3.65208	5.5763	2.21851	3.15816	5.01696	4.31208	2.4925	5.26071
ENSG00000162923	WDR26	34.1989	37.5653	25.2713	54.6472	27.3531	27.4885	76.2622	27.1339
ENSG00000266618	MIR4742	5.17414	7.17697	1.26722	2.55966	0	1.52132	0	0
ENSG00000143786	CNIH3	0.74609	0.3248	0.45541	1.14135	1.08849	0.68847	0.7653	0.67646
ENSG00000229991	AKR1B1P1	0	0	0	0	0	0	0	0
ENSG00000233384	AC096537.1	0	0	0	0	0	0	0	0
ENSG00000234247	AC096537.2	0	0	0	0	0	0	0	0
ENSG00000229400	AL596330.1	0	0	0	0	0	0	0	0
ENSG00000225334	AL391811.1	0	0	0	0	0	0	0	0
ENSG00000185842	DNAH14	0.67743	1.09041	1.31272	2.63499	1.1268	1.22791	2.1901	1.67262
ENSG00000143815	LBR	69.201	65.7969	14.2608	34.8892	50.5339	41.6198	22.6604	44.7332
ENSG00000234476	AC092811.1	0.14778	0	0	0	0	0.13035	0	0.09072
ENSG00000282418	AC092811.2	0.39338	0.21826	0	0	0	0.23133	0	0
ENSG00000154380	ENAH	0.00487	0.00811	0.00716	0.01446	0.05331	0.0086	0	0.02991
ENSG00000227496	AC099066.2	0	0	0	0	0	0	0	0
ENSG00000226349	AC099066.1	0.13727	0.45696	0	0	0	0	0	0
ENSG00000223306	RNU6-1304P	0	0	0	0	0	0	0	0
ENSG00000143742	SRP9	43.0979	33.2558	22.5651	44.6978	36.4099	52.9819	35.0703	38.6967
ENSG00000234112	AC099066.3	0	0	0	0	0	0	0	0
ENSG00000143819	EPHX1	1.19829	0.63242	0.04295	0.1735	0.53299	0.30936	0.19486	0.10765
ENSG00000242861	AL591895.1	5.6871	2.10359	0.61905	2.81342	5.37773	5.2022	2.10656	3.36196
ENSG00000196187	TMEM63A	3.27544	2.12414	0.43757	1.2521	2.76685	2.37637	1.58353	1.76704
ENSG00000243709	LEFTY1	0.19381	0.06452	0.05696	0.17258	0.09425	0.20515	0.25845	0
ENSG00000255835	AL117348.2	0	0	0	0	0	0	0	0
ENSG00000143811	PYCR2	3.47983	2.74367	0.56817	1.38927	3.58743	3.9131	1.72993	2.09877
ENSG00000284519	MIR6741	0	0	0	0	0	0	0	0
ENSG00000248322	AL117348.1	0	0	0	0	0	0	0	0
ENSG00000143768	LEFTY2	0.03064	0	0	0	0	0	0	0.07525
ENSG00000213032	NDUFA3P3	0	0	0	0	0	0	0	0
ENSG00000143751	SDE2	13.1295	10.4973	3.8208	9.086	10.7164	12.5896	14.3543	9.39592
ENSG00000272562	AL512343.2	2.39348	2.24097	0.21982	1.77609	3.0918	0.7917	1.24674	1.83667
ENSG00000163041	H3F3A	70.6209	77.9908	9.38132	15.9755	49.4022	32.3074	19.0688	35.9016
ENSG00000225518	LINC01703	0.08928	0	0	0.1325	0	0	0.14882	0
ENSG00000270598	AL512343.1	0	0	0	0	0	0	0	0
ENSG00000182827	ACBD3	14.9756	11.0483	2.40836	4.65182	12.4036	9.07134	3.75624	6.087

ENSG00000234478	ACBD3-AS1	0	0	0	0	0	0	0	0
ENSG00000223570	AL592045.1	0	0	0	0	0	0	0	0
ENSG00000185155	MIXL1	0.03613	0.06013	0	0	0.04392	0	0	0
ENSG00000183814	LIN9	4.70297	7.85557	6.42598	18.9555	10.0713	9.92691	13.0102	10.9001
ENSG00000236994	YBX1P9	0	0	0	0	0	0	0	0
ENSG00000275406	AL359742.1	0	0	0	0	0	0	0	0
ENSG00000143799	PARP1	12.6581	8.101	1.59265	3.59635	14.8921	14.9171	2.01106	3.94184
ENSG00000270287	AL359704.2	0	0	0	0	0	0	0	0
ENSG00000229965	AL359704.1	0	0	0	0.13929	0	0	0	0
ENSG00000223282	RN7SKP165	0	0	0	0	0	0	0	0
ENSG00000177770	CDKN2AIPNLP1	0	0	0	0	0	0	0	0
ENSG00000203685	STUM	0	0	0	0	0	0	0	0
ENSG00000143772	ITPKB	16.7937	8.32349	1.82177	4.56108	16.1742	12.9018	5.01849	4.66869
ENSG00000228382	ITPKB-IT1	1.85357	2.52431	0.12381	0.37512	1.53649	1.48634	0.98306	1.34478
ENSG00000228548	ITPKB-AS1	0	0	0	0	0.16564	0	0	0
ENSG00000238183	RPS27P5	0	0	0	0	0	0	0	0
ENSG00000143801	PSEN2	1.13311	0.29266	0.08613	0.14497	0.23752	0.48251	0.22795	0.3598
ENSG00000163050	COQ8A	3.65703	1.54917	0.45589	1.10223	3.06314	1.6419	1.80207	2.11228
ENSG00000228525	AL353689.1	0	0	0	0	0	0	0	0
ENSG00000143776	CDC42BPA	5.73678	9.64436	9.77128	17.0435	10.7219	11.8685	18.6987	12.7744
ENSG00000233706	AL353689.2	0	0	0	0	0	0	0	0
ENSG00000228625	AL451047.1	0	0	0	0	0	0	0	0
ENSG00000228729	AL627308.1	0	0	0	0	0	0	0	0
ENSG00000236636	AL627308.2	0	0	0	0	0	0	0	0
ENSG00000234277	LINC01641	0	0	0	0	0	0	0	0
ENSG00000232864	NUCKS1P1	0	0.17355	0	0	0.12677	0	0	0.12802
ENSG00000242757	AL606462.1	0	0	0	0	0	0	0	0
ENSG00000224834	BTF3P9	0	0	0	0.3844	0	0	0	0
ENSG00000237193	AL451054.2	0	0	0	0	0	0	0	0
ENSG00000278180	AL451054.3	0	0	0	0	0	0	0	0
ENSG00000237469	TUBB8P10	0	0	0	0	0	0	0	0
ENSG00000235827	TUBB8P9	0	0	0	0	0	0	0	0
ENSG00000227711	AL451054.1	0	0	0	0	0	0	0	0
ENSG00000202264	RNA5SP77	0	0	0	0	0	0	0	0
ENSG00000181450	ZNF678	2.45842	1.75178	0.86993	1.54972	1.98926	2.39332	1.20604	1.11045
ENSG00000225934	AL592310.1	0.10924	0.72732	0	0	0.26562	0.19272	0	0.13412
ENSG00000215812	ZNF847P	0	0	0	0	0	0	0	0
ENSG00000143740	SNAP47	0.82435	0.74169	0.18007	0.42985	0.77198	1.3167	0.3528	0.56077
ENSG00000081692	JMJD4	1.35899	0.50896	0.22466	0.50422	0.92937	1.85801	0.7362	0.54228
ENSG00000230005	SNAP47-AS1	0	0	0	0	0	0	0	0
ENSG00000185888	PRSS38	0	0	0	0	0	0	0	0
ENSG00000143816	WNT9A	0.01846	0	0	0.0274	0.02244	0	0	0
ENSG00000264483	MIR5008	0	0	0	0	0	0	0	0
ENSG00000233003	CICP26	0	0	0	0	0	0	0	0
ENSG00000274886	SEPT14P17	0	0	0	0	0	0	0	0
ENSG00000154342	WNT3A	0	0	0	0	0	0	0	0
ENSG00000276255	AL136379.1	0.08323	0.04618	0.04077	0	0.03373	0	0.04625	0.13626
ENSG00000143761	ARF1	70.7535	58.1595	20.617	29.1775	52.8833	57.8179	22.4177	30.9133
ENSG00000284163	MIR3620	0	0	0	0	0	0	0	0
ENSG00000143793	C1orf35	0.61254	0.59475	0.07501	0.07576	0.57922	0.51028	0.08508	0.27158
ENSG00000162910	MRPL55	2.60399	2.52298	0.22845	1.21129	2.07907	1.30272	1.10131	1.2884
ENSG00000224566	FAM96AP2	0	0	0	0	0.18683	0.81328	0	0
ENSG00000280157	AL359510.2	0.03259	0	0	0	0.07925	0	0.10865	0.04002
ENSG00000227625	AL359510.1	0	0	0	0	0	0	0	0
ENSG00000143774	GUK1	56.0806	68.2731	61.5736	42.3171	85.552	116.093	36.305	71.7881
ENSG00000198835	GJC2	0.06536	0.16319	0.04802	0.0485	0.07946	0.17295	0	0
ENSG00000203684	IBA57-DT	0.80047	0.38068	0	0	0.13903	0	0.38122	0.1404
ENSG00000181873	IBA57	0.4519	0.25073	0.15811	0.26614	0.39243	0.56943	0.9565	0.32585

ENSG00000162913	OBSCN-AS1	0.12059	0.06691	0	0	0.19548	0.21274	0.067	0.04935
ENSG00000154358	OBSCN	0.78544	0.20404	0.05337	0.14824	0.9356	0.5126	0.29136	0.28429
ENSG00000269890	AL353593.1	0.07413	0.0617	0.02723	0	0.04507	0.06539	0	0.04551
ENSG00000269934	AL353593.2	0.09885	0	0	0	0.12018	0	0	0.06069
ENSG00000270110	AL353593.3	0	0	0	0	0	0	0	0
ENSG00000270104	AL670729.3	0.1277	0	0	0	0	0	0	0.15679
ENSG00000154370	TRIM11	2.53742	2.05431	0.26634	0.78134	1.71035	2.28385	0.6186	1.19742
ENSG00000270094	AL670729.2	0	0	0	0	0	0	0	0
ENSG00000284311	MIR6742	0	0	0	0	0	0	0	0
ENSG00000231563	AL670729.1	1.03605	0.43113	0.38061	1.15321	0.3149	0.45693	2.15866	0.31801
ENSG00000162931	TRIM17	0	0.10316	0	0	0.05023	0	0	0.02537
ENSG00000168148	HIST3H3	0	0	0	0	0	0	0	0
ENSG00000230841	RPL23AP15	0	0	0	0	0	0	0	0
ENSG00000181218	HIST3H2A	1.4742	2.31748	1.32386	2.30942	3.78372	10.5472	3.68589	1.81
ENSG00000196890	HIST3H2BB	2.13948	3.66438	4.64756	2.43894	3.92053	9.35377	3.87628	4.30189
ENSG00000266174	MIR4666A	0	0	0	0	0	0	0	0
ENSG00000181201	HIST3H2BA	0.27977	0.23284	0.41112	0.20761	0.17007	3.94845	0	0
ENSG00000279306	AL139288.1	0.07856	0	0.2309	0.1166	0	0	0.13095	0.19292
ENSG00000168159	RNF187	9.46099	11.9966	3.93476	5.56347	14.8935	27.1614	4.31443	7.17785
ENSG00000215811	BTNL10	0.70192	0.33381	0.2947	0.59527	0.60955	1.23828	3.67711	0.49246
ENSG00000202056	RNA5SP19	0	0	0	0	0	0	0	0
ENSG00000251823	RNA5SP162	0	0	0	0	0	0	0	0
ENSG00000199352	RNA5S1	0	0	0	0	0	0	0	0
ENSG00000201588	RNA5S2	0	0	0	0	0	0	0	0
ENSG00000199337	RNA5S3	0	0	0	0	0	0	0	0
ENSG00000200381	RNA5S4	0	0	0	0	0	0	0	0
ENSG00000199396	RNA5S5	0	0	0	0	0	0	0	0
ENSG00000200624	RNA5S6	0	0	0	0	0	0	1.02673	0
ENSG00000202521	RNA5S7	0	0	0	0	0	0	0	0
ENSG00000200343	RNA5S8	0	0	0	0	0	0	0	0
ENSG00000201321	RNA5S9	5.54372	8.20225	66.9818	106.957	11.9821	5.43327	7.18708	56.7208
ENSG00000199910	RNA5S10	0	0	0	0	0	0	0	0
ENSG00000199334	RNA5S11	0	0	0	0	0	0	0	0
ENSG00000199270	RNA5S12	0	0	0	0	0	0	0	0
ENSG00000202526	RNA5S13	0	0	0	0	0	0	0	0
ENSG00000201355	RNA5S14	0	0	0	0	0	0	0	0
ENSG00000201925	RNA5S15	0	0	0	0	0	0	0	0
ENSG00000202257	RNA5S16	0	0	0	0	0	0	0	0
ENSG00000200370	RNA5S17	0	0	0	0	0	0	0	0
ENSG00000212237	RNA5SP18	0	0	0	0	0	0	0	0
ENSG00000183929	DUSP5P1	0	0	0	0	0	0	0	0
ENSG00000206878	RF00432	0	0	0	0	0	0	0	0
ENSG00000234975	FTH1P2	0.88957	1.48069	0	2.11234	0.64891	0.94159	0.59311	0.21844
ENSG00000116574	RHOU	5.75069	5.21857	1.33041	1.89106	3.4652	3.22392	1.64882	2.182
ENSG00000271475	AL078624.2	0	0	0	0	0	0	0	0
ENSG00000271399	AL078624.1	0	0	0	0	0	0	0	0
ENSG00000282111	AL137793.2	0	0	0	0	0	0	0	0
ENSG00000229840	ISCA1P2	0	0	0	0.2584	0.21168	0	0	0
ENSG00000236975	AL137793.1	0	0	0	0	0.5951	0.21588	0	0
ENSG00000233920	AL162595.2	0	0	0	0	0	0	0	0
ENSG00000177800	TMEM78	0	0	0	0	0	0	0	0
ENSG00000177788	AL162595.1	0	0	0	0	0.07716	0	0	0
ENSG00000240524		0	0	0	0	0	0	0	0
ENSG00000168118	RAB4A	8.23798	4.38641	2.11523	3.91103	4.71161	7.1102	4.31876	5.30194
ENSG00000213029	SPHAR	0	0	0	0	0	0	0	0
ENSG00000237481	AL117350.1	0	0.19064	0	0.16998	0.41773	0	0.38181	0.28124
ENSG00000154429	CCSAP	6.89349	6.44816	2.2014	2.62281	5.20784	2.95251	2.88722	4.18157
ENSG00000252506	RNU6-180P	0	0	0	0	0	0	0	0

ENSG00000252051	RN7SKP276	0	0	0	0	0	0	0	0
ENSG00000230331	AL160004.2	0	0	0	0	0	0	0	0
ENSG00000143632	ACTA1	0	0	0	0.14553	0.11922	0	0	0
ENSG00000069248	NUP133	6.89602	5.1974	3.50779	7.08539	6.71307	7.76296	7.53579	5.71363
ENSG00000226920	AL160004.1	0	0	0	0	0	0	0	0
ENSG00000223635	AL121990.1	0.12403	0.20644	0.72903	0.36814	0.15079	0.2188	0	0.15228
ENSG00000135776	ABCB10	10.9537	14.1617	26.8273	53.6772	21.8604	19.1719	34.7651	20.7653
ENSG00000199672	RNU4-21P	0	0	1.70975	0.86338	0	0	0	0
ENSG00000201492	RNA5SP78	0	0	0	0	0	0	0	0
ENSG00000229367	HMGN2P19	0	0	0	0	0	0	0	0
ENSG00000135801	TAF5L	2.14917	1.4842	0.36957	0.91616	1.62612	2.11755	0.70503	0.81407
ENSG00000135763	URB2	0.90389	0.75226	0.19744	0.59822	1.18802	1.22826	0.57008	0.74985
ENSG00000213028	AL354983.1	0	0	0	0	0	0	0	0
ENSG00000225984	HMGB1P26	0	0	0	0	0	0	0	0
ENSG00000227043	LINC01682	0	0	0	0	0	0	0	0
ENSG00000228058	LINC01736	0	0	0	0	0	0	0.18682	0
ENSG00000143641	GALNT2	4.12746	3.08842	0.73729	1.86859	3.5794	4.00811	1.19924	1.81319
ENSG00000227006	AL136988.2	0	0	0	0	0	0	0.14191	0
ENSG00000224407	AL136988.1	0	0	0	0	0	0	0	0.08746
ENSG00000177614	PGBD5	0	0.01079	0	0.01925	0.00788	0.01144	0.01081	0
ENSG00000282564	AL133516.1	0	0	0	0	0	0	0	0
ENSG00000232964	LINC01737	0	0	0	0	0	0	0	0
ENSG00000235817	AL158214.1	0	0.604	0	0	0	0	0.90728	0.22277
ENSG00000135775	COG2	2.43262	2.37592	1.81197	3.7893	3.16121	2.73094	2.85959	2.37787
ENSG00000135744	AGT	0	0	0	0	0	0	0	0
ENSG00000244137	AL512328.1	0	0	0	0	0	0	0	0
ENSG00000135773	CAPN9	0	0	0	0	0	0	0	0
ENSG00000202063	RNA5SP79	0	0	0	0	0	0	0	0
ENSG00000225656	AL118511.2	0	0	0	0	0	0	0	0
ENSG00000119280	C1orf198	3.20982	2.76594	0.58438	1.5598	1.39865	1.87917	1.08899	1.27297
ENSG00000223393	AL118511.1	0	0	0	0	0	0	0.15388	0.11335
ENSG00000240502	RN7SL837P	0	0	0	0	0	0	0	0
ENSG00000143643	TTC13	9.81515	10.3412	9.97351	24.3812	13.1602	11.5128	20.1313	11.6022
ENSG00000173409	ARV1	4.57435	3.31578	4.44517	12.3732	6.14443	5.01108	5.4726	4.34812
ENSG00000182118	FAM89A	0.58175	0.45868	0.49493	0.45441	0.48393	0.27007	0.40829	0.45111
ENSG00000283799	MIR1182	0	0	0	0	0	0	0	0
ENSG00000227934	AL732414.1	0	0	0	0	0	0	0	0
ENSG00000229595	AL109810.1	0	0	0	0	0	0	0	0
ENSG00000119283	TRIM67	0	0	0	0	0	0	0	0
ENSG00000235710	AL109810.2	0	0	0	0	0	0	0	0
ENSG00000143633	C1orf131	1.51665	1.35239	0.43778	0.60291	2.025	1.83946	0.49658	0.91444
ENSG00000116906	GNPAT	7.64652	6.65221	2.41024	5.38272	5.95422	7.29493	4.69776	5.1905
ENSG00000222407	RNA5SP80	0	0	0	0	0	0	0	0
ENSG00000116903	EXOC8	11.0094	11.7463	2.21764	4.18078	10.1698	6.08526	5.48614	7.42917
ENSG00000010072	SPRTN	2.1099	1.87991	0.383	1.04991	2.05213	2.03623	1.4895	1.43239
ENSG00000135766	EGLN1	18.0602	29.2552	4.34414	5.86979	17.4553	6.53234	5.54983	17.3181
ENSG00000223982	SNRPD2P2	0	0	0	0	0	0	0	0
ENSG00000233461	AL445524.1	2.72812	4.77996	0.73849	0.95893	2.35666	1.13987	1.07701	3.96657
ENSG00000270106	TSNAX-DISC1	0.02061	0	0	0.06117	0.02505	0	0.0687	0.0506
ENSG00000116918	TSNAX	7.28188	5.18062	1.62235	2.89352	6.45421	4.3097	2.77994	4.28283
ENSG00000229228	LINC00582	0	0	0	0	0	0	0	0
ENSG00000162946	DISC1	1.2779	0.92646	0.50911	0.33716	0.87694	0.57111	0.46388	1.03902
ENSG00000222986	RNU5A-5P	0	0	0.92857	0	0	0	0	0
ENSG00000274121	RF02102	0	0	0	0	0	0	0	0
ENSG00000226758	DISC1-IT1	1.05814	0.48035	0	0.14276	0.35085	0.3394	0	0.23621
ENSG00000235152	AL353052.1	0	0	0	0	0	0	0	0
ENSG00000236372	AL353052.2	0	0	0	0	0	0	0	0
ENSG00000224436	BX323014.1	0	0	0	0	0	0	0	0

ENSG00000242794	RN7SL299P	0	0	0	0	0	0	0	0
ENSG00000116991	SIPA1L2	13.4111	15.7281	1.25462	1.56608	5.97144	1.03233	1.75891	2.81498
ENSG00000238382	RNU6-1211P	0	0	0	0	0	0	0	0
ENSG00000225919	LINC01745	0	0	0	0	0	0	0	0
ENSG00000236497	LINC01744	0	0	0	0	0	0	0	0
ENSG00000212916	MAP10	0.62543	0.48581	0.15318	0.52598	0.659	1.10334	0.45175	0.56312
ENSG00000206835	RNU1-74P	0	0	0	0	0	0	0	0
ENSG00000232175	AL122003.1	0	0	0	0	0	0	0	0
ENSG00000135778	NTPCR	0.39299	0.23857	0.07474	0.18527	0.59583	0.51385	0.17725	0.3406
ENSG00000135749	PCNX2	1.23626	1.0333	0.48341	1.05518	1.3869	1.15131	0.57486	0.56676
ENSG00000231940	RPS7P3	0	0.21036	0	0.18756	0	0	0	0
ENSG00000251508	AL133380.1	0	0	0	0	0	0	0	0
ENSG00000143674	MAP3K21	0.39817	0.41615	0.10886	0.12368	0.25893	0.09801	0.09261	0.19327
ENSG00000252501	RNU4-77P	0	0	0	0	0	0	0	0
ENSG00000135750	KCNK1	0.02021	0.06728	0.08909	0.62986	0.19656	0.03565	0.33686	0.02481
ENSG00000265744	MIR4427	0	0	0	0	0	0	0	0
ENSG00000225486	AL663058.1	0	0.34563	0.61028	0	0	0	0	0
ENSG00000183780	SLC35F3	0.11471	0.11456	0.03371	0.0681	0.25103	0	0.03824	0
ENSG00000236101	RAC1P7	0	0	0	0	0	0	0	0
ENSG00000233332	AL122008.3	0	0	0	0	0	0	0	0
ENSG00000231272	AL122008.1	0.16217	0	0	0.24068	0	0	0	0
ENSG00000236244	AL122008.4	0	0	0	0	0	0	0	0
ENSG00000232166	AL122008.2	0	0.21405	0	0	0	0	0	0
ENSG00000264377	MIR4671	0	0	0	0	0	0	0	0
ENSG00000235605	AL355472.1	0	0	0	0	0	0	0	0
ENSG00000236358	AL355472.2	0	0.29758	0	0.26533	0	0.31539	0	0
ENSG00000273367	AL355472.3	0.11709	0	0	0	0	0	0	0.14377
ENSG00000231663	COA6-AS1	2.03966	0.42438	0.18733	0.75677	3.40968	1.57423	1.27493	0.9391
ENSG00000168275	COA6	3.73208	3.30015	2.22797	1.90397	2.76496	6.48102	1.7496	3.15026
ENSG00000059588	TARBP1	2.1584	1.24482	0.72338	2.23388	1.6113	1.85375	1.45171	1.52262
ENSG00000231768	LINC01354	0	0	0	0	0	0	0	0
ENSG00000230404	AL161640.1	0	0	0	0	0	0	0	0
ENSG00000212144	RF00096	0	0	0	0	0	0	0	0
ENSG00000168264	IRF2BP2	29.1401	15.109	4.40068	9.78703	17.6951	14.809	8.61263	9.83033
ENSG00000228830	AL160408.2	0.31732	0	0	0	0	0	0	0
ENSG00000224939	LINC00184	0	0	0	0	0	0.05697	0	0
ENSG00000272362	AL160408.5	0	0	0	0	0	0	0	0
ENSG00000228044	AL160408.1	0	0	0	0	0	0	0	0
ENSG00000241475	AL160408.4	0	0	0	0	0	0	0	0
ENSG00000230628	AL160408.3	0	0	0	0	0	0	0	0
ENSG00000282097	AL160408.6	0	0	0	0	0	0	0	0
ENSG00000227630	LINC01132	0.03865	0	0	0.05736	0.09398	0	0.03221	0
ENSG00000279261	PP2672	0	0	0	0	0	0	0	0
ENSG00000201638	RNY4P16	0	0	0	0	0	0	0	0
ENSG00000239690	RN7SL668P	0	0	0	0	0	0	0	0
ENSG00000237520	AL391832.1	0.69315	0	0	0.51435	0.63203	0	0	0
ENSG00000238005	AL391832.2	1.9359	2.5632	0.51723	0.71827	1.44427	0.38809	0.66004	1.0804
ENSG00000258082	AL391832.3	8.05784	5.48029	0.76393	2.31459	4.00288	2.29276	2.0219	3.40414
ENSG00000280587	LINC01348	0.04494	0.0374	0.03302	0.03335	0.02732	0	0	0.05518
ENSG00000233018	AL732292.1	0	0	0	0.48135	0	0	0	0
ENSG00000273416	AL732292.2	0	0	0	0.25299	0	0	0.28414	0
ENSG00000173726	TOMM20	18.9149	9.9894	5.07855	13.9437	13.8883	12.9969	13.246	10.3159
ENSG00000207181	SNORA14B	5.42966	9.94143	6.38305	10.4757	7.26136	0	5.43024	5.9998
ENSG00000188739	RBM34	0.42384	0.39193	0.1038	0.24462	0.57255	0.33231	0.31399	0.43365
ENSG00000054267	ARID4B	19.9916	21.96	4.35797	7.1227	20.8124	12.7759	7.99971	12.9961
ENSG00000263439	MIR4753	0	0	1.29776	0	0	0	0	0
ENSG00000236863	RPL23AP23	0	0	0	0	0	0	0	0
ENSG00000152904	GGPS1	8.29416	8.36026	3.16723	7.59701	7.55702	6.82375	5.67758	7.6553

ENSG00000227962	AL391994.1	0.25993	0	0	0	0	0	0	0
ENSG00000230026	AL357556.1	0	0	0	0	0	0	0	0
ENSG00000116957		3.82486	3.46831	2.51668	6.6932	6.2812	5.99225	5.23358	3.67979
ENSG00000229795	RPS21P1	0	0	0	0	0	0	0	0
ENSG00000162885	B3GALNT2	0.7456	0.98428	0.51004	0.83945	0.98463	1.40606	0.87853	0.58399
ENSG00000238236	MTCYBP14	0	0	0	0	0	0	0	0
ENSG00000235638	MTND6P14	0	0	0	0	0	0	0	0
ENSG00000231199	MTND5P19	0	0	0	0	0	0	0	0
ENSG00000226663	MTND4P10	0	0	0	0	0	0	0	0
ENSG00000231582	MTND4LP21	0	0	0	0	0	0	0	0
ENSG00000232714	MTND3P8	0	0	0	0	0	0	0	0
ENSG00000235761	MTCO3P46	0	0	0	0	0	0	0	0
ENSG00000168243	GNG4	0	0	0	0	0	0	0	0
ENSG00000270710	FO393422.1	0	0	0	0	0	0	0	0
ENSG00000227236	AL583844.1	0	0	0	0	0	0	0	0
ENSG00000143669	LYST	42.8613	33.18	8.98989	15.7183	23.1811	18.7434	14.4768	24.5113
ENSG00000235674	LDHAP2	0	0	0	0	0	0	0	0
ENSG00000229463	LYST-AS1	0	0	0	0	0	0	0	0
ENSG00000222831	MIR1537	16.823	10.0007	3.5316	1.78337	2.92186	6.3596	10.0148	2.95072
ENSG00000206803	RNU6-968P	0	0	0	0	0	0	0	0
ENSG00000237845	AL139161.2	0	0	0	0	0	0	0	0
ENSG00000229291	AL139161.1	0.38579	0	0	0	0.46904	0	0	0
ENSG00000116962	NID1	5.01252	2.22628	0.25716	1.55832	0.95743	1.54363	0.72925	0.67528
ENSG00000252822	RF00019	0	0	0	0	0	0	0	0
ENSG00000235371	AL122018.1	0	0	0	0	0	0	0	0
ENSG00000077585	GPR137B	5.46022	5.91813	1.67939	1.60187	2.89465	4.14425	1.53453	1.90986
ENSG00000086619	ERO1B	2.24235	1.75241	0.40184	0.99431	1.32985	1.2784	0.98	1.41014
ENSG00000222650	RNU2-70P	0	0	0	0	0	0	0	0
ENSG00000237922	AL450309.1	0	0	0	0	0	0	0	0
ENSG00000186197	EDARADD	0.11788	0	0.08661	0.02916	0.1672	0.13863	0.09824	0.09649
ENSG00000244457	ENO1P1	0	0	0	0	0	0	0	0
ENSG00000116977	LGALS8	11.2026	10.5297	2.18534	4.63824	8.79236	7.31124	3.57355	5.92257
ENSG00000223776	LGALS8-AS1	0.20124	0.08374	0	0.07466	0.24466	0.1775	0.16771	0.06177
ENSG00000273058	AL359921.2	0.40497	0.44939	0	0.20034	0.65648	0.95257	0.22501	0.33148
ENSG00000230325	AL359921.1	0	0	0	0	0.1804	0.26176	0	0.36436
ENSG00000119285	HEATR1	4.05829	2.791	1.23795	2.21202	4.35292	5.35882	2.48438	3.17262
ENSG00000077522	ACTN2	0	0	0	0	0	0	0	0
ENSG00000116984	MTR	5.43276	4.17279	2.10238	3.53884	6.21325	6.04834	4.44722	3.79801
ENSG00000226498	RPSAP21	0.16565	0.27573	0	0.12292	0.30209	0	0.13806	0
ENSG00000237991	RPL35P1	0	0	0	0	0	0.69898	0	0.24324
ENSG00000244020	MT1HL1	0	0	0	0	0	0	0	0
ENSG00000198626	RYR2	0	0.02059	0.01212	0.04896	0.06016	0.01455	0.02749	0.00506
ENSG00000252396	RN7SKP195	0	0	0	0	0	0	0	0
ENSG00000266262	MIR4428	0	0	0	0	0	0	0	0
ENSG00000252290	RF00402	0	0	0	0	0	0	0	0
ENSG00000237250	AL359924.1	0	0	0	0	0	0	0	0
ENSG00000116996	ZP4	0	0	0	0	0	0	0	0
ENSG00000243781	AL590396.1	0	0	0	0	0	0	0	0
ENSG00000283174	AL590396.3	0	0	0	0	0	0	0	0
ENSG00000283166	AL590396.2	0	0	0	0	0	0	0	0
ENSG00000283377	AL590396.4	0	0	0	0	0	0	0	0
ENSG00000270188	MTRNR2L11	0	0	0	0	0	0	0	0
ENSG00000232154	MTCYBP15	0	0	0	0	0	0	0	0
ENSG00000225723	MTND6P15	0	0	0	0	0	0	0	0
ENSG00000227860	MTND5P18	0	0	0	0	0	0	0	0
ENSG00000230019	YWHAQP9	0	0	0	0	0	0	0	0
ENSG00000232989	AL356010.1	0	0	0	0	0	0	0	0
ENSG00000234464	AL356010.2	0	0	0	0	0	0	0	0

ENSG00000252371	RNU6-725P	0	0	0	0	0	0	0	0
ENSG00000215808	LINC01139	0	0	0	0	0	0	0	0
ENSG00000231877	AL359551.1	0	0	0	0	0	0	0	0
ENSG00000215149	KRT18P32	0	0	0	0	0	0	0	0
ENSG00000224783	MIPEPP2	0	0	0	0	0	0	0	0
ENSG00000227854	AL590135.1	0	0	0	0	0	0	0	0
ENSG00000259776	AC093426.1	0	0	0	0	0	0	0	0
ENSG00000133019	CHRM3	0.03614	0	0.00885	0	0.01465	0.07438	0.03012	0.00739
ENSG00000233355	CHRM3-AS2	1.82874	0.53347	0.19393	0.8394	2.33794	1.69622	1.0056	0.60183
ENSG00000234601	CHRM3-AS1	0	0	0	0	0	0	0	0
ENSG00000231979	AL356361.2	0	0	0	0	0	0	0	0
ENSG00000215805	AL356361.1	0	0	0	0	0	0	0	0
ENSG00000217327	RPS7P5	0	0	0	0	0	0	0	0
ENSG00000155816	FMN2	0	0	0	0	0	0	0	0
ENSG00000228818	AL359918.1	0	0.08971	0	0	0.03276	0	0	0
ENSG00000202041	RF00019	0	0	0	0	0	0	0	0
ENSG00000223694	ADH5P3	0	0	0	0	0	0	0	0
ENSG00000233735	AL359918.2	0	0	0	0	0	0	0	0
ENSG00000252317	RF00019	0	0	0	0	0	0	0	0
ENSG00000233519	AL590490.1	0	0	0	0	0	0	0	0
ENSG00000180875	GREM2	0	0.02926	0.02583	0	0.02137	0	0.0293	0.02158
ENSG00000224359	AL358176.1	0	0	0	0	0	0	0	0
ENSG00000226014	AL358176.2	0	0	0	0	0	0	0	0
ENSG00000231440	AL358176.4	0	0	0	0	0	0	0	0
ENSG00000228844	AL358176.3	0	0	0	0	0	0	0	0
ENSG00000251750	RNU5F-8P	0	0	0	0	0	0	0	0
ENSG00000234872	AL358176.5	0	0	0	0	0	0	0	0
ENSG00000199241	RF00019	0	0	0	0	0	0	0	0
ENSG00000230015	AL365184.2	0	0	0	0	0	0	0	0
ENSG00000226919	AL365184.1	0	0	0	0	0	0	0	0
ENSG00000223963	THAP12P8	0	0	0	0.05144	0	0	0	0
ENSG00000182901	RGS7	0.14233	0	0	0	0.06922	0	0	0
ENSG00000215802	RFKP1	0	0	0	0	0	0	0	0
ENSG00000233615	HNRNPA1P42	0	0	0	0	0	0	0	0
ENSG00000238085	AL590682.1	0	0	0	0	0	0	0	0
ENSG00000265831	MIR3123	0	0	0	0	0	0	0	0
ENSG00000224348	AL592076.1	0	0	0	0	0	0	0	0
ENSG00000225554	AL359764.1	0	0	0	0	0	0	0	0
ENSG00000091483	FH	4.72243	3.47456	1.55889	2.53935	4.61809	5.49364	2.39569	2.85705
ENSG00000117009	KMO	1.72587	1.45688	0.39854	0.60376	1.34889	0.47845	0.36987	1.01409
ENSG00000054277	OPN3	2.40652	1.31333	0.4348	0.90752	2.73393	1.49634	0.95351	1.2836
ENSG00000203668	CHML	4.30559	2.40481	0.85766	2.31459	3.15241	2.07615	1.78622	1.92658
ENSG00000162843	WDR64	0.04452	0	0	0	0.01804	0	0	0
ENSG00000229022	AL365366.1	0	0	0	0	0	0	0	0
ENSG00000174371	EXO1	0.54887	0.97253	0.91063	2.33863	1.91579	3.46706	2.50853	1.34778
ENSG00000235990	RPL23AP20	0	0	0	0	0	0	0	0
ENSG00000196289	BECN2	0	0	0	0	0	0	0	0
ENSG00000213026	CFL1P4	0	0	0	0	0	0	0	0.18072
ENSG00000197769	MAP1LC3C	0	0	0	0	0	0	0	0
ENSG00000224625	TUBB8P6	0	0	0	0	0	0	0	0
ENSG00000277099	BX571673.1	0	0	0	0	0	0	0	0
ENSG00000180287	PLD5	0	0.01278	0	0	0	0	0	0
ENSG00000272865	AL591686.2	0	0	0	0	0	0	0	0
ENSG00000252084	RN7SKP12	0	0	0	0	0	0	0	0
ENSG00000224525	AL591686.1	0	0	0	0	0	0	0	0
ENSG00000226750	AL360271.2	0	0	0	0	0	0	0	0
ENSG00000235467	RPL10AP5	0	0	0	0	0	0	0	0
ENSG00000213690	AL360271.1	0	0	0	0	0	0	0	0

ENSG00000270859	AC099785.1	0	0	0	0	0	0	0	0
ENSG00000215800	RSL24D1P4	0	0	0	0	0	0	0	0
ENSG00000270818	AL445675.1	0	0	0	0	0	0	0	0
ENSG00000277704	AL445675.2	0	0	0	0	0	0	0	0
ENSG00000230199	AL603825.1	0	0	0	1.35982	0	0	0	0
ENSG00000234116	AL606534.3	0	0	0	0	0	0	0	0
ENSG00000231512	SEPT14P21	0	0	0	0	0	0	0	0.06164
ENSG00000282831		0	0	0	0	0	0	0	0
ENSG00000278455	AL606534.5	0	0	0	0	0	0	0	0
ENSG00000253326	AL606534.4	0	0	0	0	0	0	0	0
ENSG00000214837	LINC01347	0.01908	0.09529	0.02804	0	0	0.03367	0	0.04686
ENSG00000212230	RNU6-747P	0	0	0	0	0	0	0	0
ENSG00000143702	CEP170	1.80252	1.19343	0.37418	0.52707	1.18127	0.92205	0.59197	0.82272
ENSG00000227230	AL606534.1	0	0	0	0	0	0	0	0
ENSG00000232085	AL606534.2	0	0	0	0	0	0	0	0
ENSG00000054282	SDCCAG8	7.09314	6.69419	5.30272	6.64325	8.94181	9.54895	10.5605	7.28838
ENSG00000224727	FCF1P7	0	0	0	0	0	0	0	0
ENSG00000265201	MIR4677	0	0	0	0	0	0	0	0
ENSG00000117020	AKT3	4.55661	4.13348	1.45513	3.74292	6.51798	3.04343	2.77243	3.42891
ENSG00000236031	AL591721.1	0	0	0	0	0	0	0	0
ENSG00000226766	FABP7P1	0	0	0	0	0	0	0	0
ENSG00000232184	AL662889.1	0	0	0	0	0	0	0.31409	0
ENSG00000228939	AKT3-IT1	0	0	0	0	0	0	0	0
ENSG00000226828	AL591885.1	0	0	0	0	0	0	0	0
ENSG00000179456	ZBTB18	21.8437	18.0857	2.95069	6.04365	18.526	6.65429	10.7604	14.3313
ENSG00000279774	AL590483.3	0	0.03408	0	0	0	0	0.03413	0.02514
ENSG00000229960	AL590483.1	0	0	0	0	0	0	0.26107	0
ENSG00000244066	RN7SL148P	0	0	0	0	0	0	0	0
ENSG00000237759	AL590483.2	0	0	0	0	0	0	0	0
ENSG00000173728	C1orf100	0.06368	0	0	0	0	0	0	0
ENSG00000240963	AL645465.1	0	0.57013	0	0	0	0	0	0
ENSG00000225401	TGIF2P1	0.10701	0	0	0.15881	0	0.56633	0	0.13138
ENSG00000035687	ADSS	15.4758	12.7799	4.7011	9.49575	11.6683	13.3686	8.76525	9.27957
ENSG00000179397	CATSPERE	0.23683	0.30974	0.12429	0.37659	0.0617	0.14922	0.14099	0.33232
ENSG00000227735	CYCSP5	0	0	0	0	0	0	0	0
ENSG00000121644	DESI2	4.92594	4.85261	1.96266	3.96437	3.946	5.59912	3.78223	3.91445
ENSG00000232059	AL451007.1	0	0	0	0	0	0	0	0
ENSG00000284188	AL451007.3	0	0	0	0	0	0	0	0
ENSG00000282317	AL451007.2	0	0	0	0	0	0	0	0
ENSG00000203667	COX20	3.39074	2.40806	1.10726	2.19182	3.86588	2.65855	2.13514	2.33134
ENSG00000153187	HNRNPU	18.3251	14.6766	3.134	7.93954	16.5787	15.3426	7.14781	9.65054
ENSG00000273175	BX323046.1	0.27402	0.68416	0	1.42336	1.99888	0.48341	0.22837	0.33644
ENSG00000201758	RN7SKP55	0.23876	0	1.40344	0.7087	0.29028	0.42121	2.38789	3.5178
ENSG00000252073	RNU6-947P	0	0	0	0	0	0	0	0
ENSG00000272195	AL356512.1	0.42151	0.3508	0	0	0.15374	0.07436	0.14052	0.36227
ENSG00000203666	EFCAB2	1.37965	0.70877	0.60071	2.40145	1.90513	0.60096	1.46213	1.18156
ENSG00000252282	RNU6-1089P	0	0	0	0	0	0	0	0
ENSG00000251754	RNU6-999P	0	0	0	0	0	0	0	0
ENSG00000223353	AL589763.1	0	0	0	0	0	0	0	0
ENSG00000201170	RNU1-132P	0	0	0	0	0	0	0	0
ENSG00000162849	KIF26B	0	0.00847	0	0	0	0	0	0
ENSG00000232192	KIF26B-AS1	0	0	0	0	0	0	0	0
ENSG00000232793	DNAJC19P8	0	0	0	0	0	0	0	0
ENSG00000238224	AC104462.2	0	0	0	0	0	0	0	0
ENSG00000231612	AC104462.1	0	0	0	0	0	0	0	0
ENSG00000185420	SMYD3	0.96218	1.16476	0.71613	1.39088	0.85076	1.36675	1.14557	0.99724
ENSG00000235096	AC118555.1	0	0	0	0	0	0	0	0
ENSG00000226876	AC092801.1	0	0	0	0	0	0	0	0

ENSG00000225222	CHCHD4P5	0	0	0	0	0	0	0	0
ENSG00000202184	RNU6-1283P	0	0	0	0	0	0	0	0.85711
ENSG00000230184	SMYD3-IT1	0.20305	1.01392	0	0.30135	0	0	0	0.2493
ENSG00000207326	RF00019	0	0	0	0	0	0	0	0.78945
ENSG00000227728	AL356583.1	0	0	0	0	0	0	0	0
ENSG00000242042	LINC01743	0	0	0	0	0	0	0	0
ENSG00000230813	AL356583.3	0.35156	0	0	0	0	0	0	0
ENSG00000229112	AL356583.2	0	0	0	0	0	0	0	0
ENSG00000162851	TFB2M	4.32345	4.09336	5.82868	8.30021	4.967	4.05849	11.4379	7.59715
ENSG00000162852	CNST	16.9697	15.0399	6.14781	9.36858	15.0334	13.1015	11.3474	12.5224
ENSG00000225300	AL591623.1	0.29919	0	0.05496	0.0555	0	0.06598	0.06234	0.09183
ENSG00000228955	AL591623.2	0	0	0	0	0	0	0	0
ENSG00000260698	AL591848.3	0	0	0	0	0.06005	0.04357	0.1235	0.03032
ENSG00000235021	AL591848.2	0	0	0	0	0	0	0	0
ENSG00000143653	SCCPDH	6.99911	7.55546	5.25014	10.909	6.55113	4.28801	6.39458	7.22709
ENSG00000228879	AL591848.1	0	0	0	0	0	0	0	0
ENSG00000223519	KIF28P	1.67019	1.07726	0.70562	0.46476	0.93914	0.99442	0.41759	0.89715
ENSG00000260855	AL591848.4	0.10556	0.10542	0.09307	0	0.10267	0	0.07038	0.10368
ENSG00000227953	LINC01341	0.0368	0.03062	0.02704	0	0.04474	0.03246	0.092	0.02259
ENSG00000235386	AC113174.1	0	0	0	0	0	0	0	0
ENSG00000252011	RF00402	0	0	0	0	0	0	0	0
ENSG00000153207	AHCTF1	23.7316	17.1795	3.94581	8.68339	17.5655	9.5009	9.29481	14.5453
ENSG00000197472	ZNF695	0.05855	0	0.02868	0.14482	0.04745	0.03443	0	0.02396
ENSG00000135747	ZNF670-ZNF695	0.22928	0.03816	0	0.06805	0.02788	0.04045	0.03822	0.0563
ENSG00000236775	AL512637.2	0	0	0	0	0	0	0	0
ENSG00000277462	ZNF670	0.91901	0.24475	0.16206	0.16367	0.40223	0.58365	0.42892	0.45134
ENSG00000215796	AL512637.1	0	0	0	0	0	0	0	0
ENSG00000228982	AL627095.1	0	0	0	0	0	0	0	0
ENSG00000188295	ZNF669	5.18285	2.21834	1.08802	1.48344	3.10558	1.76334	1.7278	2.09084
ENSG00000221953	C1orf229	0	0	0	0	0	0	0	0
ENSG00000226164	FGFR3P6	0	0	0	0	0	0	0	0
ENSG00000196418	ZNF124	6.71825	6.67171	3.98234	6.35808	8.26652	7.9299	8.1147	10.0617
ENSG00000224014	AL390728.3	0	0	0	0	0	0.47893	0	0
ENSG00000215795	AL390728.2	0	0	0	0	0.05336	0.07743	0	0.05389
ENSG00000259865	AL390728.6	1.70603	2.15424	1.03737	3.14309	1.43044	0.83025	1.47087	3.7559
ENSG00000227671	AL390728.4	10.0449	6.60879	3.44086	5.64074	7.09633	6.70506	5.54337	7.29143
ENSG00000283881	MIR3916	0	0	0	0	0	0	0	0
ENSG00000252516	RNA5SP82	1.59349	2.65236	0	1.18245	0	0	1.32805	0
ENSG00000232347	AL390728.5	0	0	0	0	0	0	0	0
ENSG00000214144	AL390728.1	0.03776	0	0	0.05605	0.04591	0	0	0
ENSG00000235818	VN1R17P	0	0	0	0	0	0	0	0
ENSG00000197617	VN1R5	0	0	0	0	0	0	0	0
ENSG00000202079	RF00019	0	0	0	0	0	0	0	0
ENSG00000162714	ZNF496	0.89094	0.4055	0.24548	0.22726	0.75315	0.81042	0.48728	0.47003
ENSG00000162711	NLRP3	15.7633	9.64372	1.72089	3.75044	8.89855	4.53964	3.23622	3.21621
ENSG00000177535	OR2B11	0	0	0	0	0	0	0	0
ENSG00000203664	OR2W5	0	0	0	0	0	0	0	0
ENSG00000169224	GCSAML	4.88669	1.92919	1.10476	1.11575	1.44719	1.13286	0.80931	1.2115
ENSG00000227135		0.0895	0.14897	0.06576	0	0	0	0	0
ENSG00000196242	OR2C3	0.02604	0	0	0	0	0	0	0
ENSG00000236817	AL606804.1	0	0	0	0	0	0	0	0
ENSG00000177489	OR2G2	0	0	0	0	0	0	0	0
ENSG00000177476	OR2G3	0	0	0	0	0	0	0	0
ENSG00000283369	RF00026	0	0	0	0	0	0	0	0
ENSG00000198452	OR14L1P	0.55291	0	0.11607	0.11723	0.09603	0	0.13166	0.09698
ENSG00000235749	AL390860.1	0	0	0	0	0	0	0.12984	0.09564
ENSG00000230411	OR3D1P	0	0	0	0	0	0	0	0
ENSG00000197437	OR13G1	0	0	0	0	0	0	0	0

ENSG00000239395	AC118470.1	0	0	0	0	0	0	0	0
ENSG00000169214	OR6F1	0	0	0	0	0	0	0	0
ENSG00000241128	OR14A2	0	0	0	0	0	0	0	0
ENSG00000153230	OR14K1	0	0	0	0	0	0	0	0
ENSG00000221888	OR1C1	0	0	0	0	0	0	0	0
ENSG00000228336	OR9H1P	0	0	0	0	0	0	0	0
ENSG00000196772	OR14A16	0	0	0	0	0	0	0	0
ENSG00000232908	HSD17B7P1	0	0	0	0	0	0	0	0
ENSG00000230576	OR6R1P	0	0	0	0	0	0	0.12998	0
ENSG00000197591	OR11L1	0	0	0	0	0	0	0	0
ENSG00000162722	TRIM58	78.0961	116.372	89.4109	88.4116	82.0979	144.789	91.872	52.2681
ENSG00000238243	OR2W3	30.5612	33.1811	32.5991	34.6503	28.9511	74.5766	8.14535	9.71397
ENSG00000177462	OR2T8	0.46837	0.3898	0.34413	0.57926	0.56944	0.55085	0.26023	0.38337
ENSG00000177275	OR2AJ1	0	0	0	0	0	0	0	0
ENSG00000226191	CLK3P2	0.20179	0.08397	0	0.07487	0	0.089	0.08409	0
ENSG00000224830	OR2X1P	0	0	0	0	0	0	0	0.131
ENSG00000279263	OR2L8	0	0	0	0	0.09491	0	0	0
ENSG00000187080	OR2AK2	0	0	0	0	0.15445	0	0	0
ENSG00000237492	OR2L9P	0	0	0	0	0	0	0	0
ENSG00000224227	OR2L1P	0.46887	0	0	0.11598	0.09501	0.13786	0.26051	0.19189
ENSG00000199442	RF00019	0	0	0	0	0	0	0	0
ENSG00000232215	OR2L6P	0	0	0	0	0	0	0	0
ENSG00000197454	OR2L5	0	0	0	0	0	0	0	0
ENSG00000200085	RF00019	0	0	0	0	0	0	0	0.88232
ENSG00000203663	OR2L2	0	0	0	0	0	0	0	0
ENSG00000200982	RF00019	0	0	0	0	0	0	0	0
ENSG00000198128	OR2L3	0	0	0	0	0	0	0	0
ENSG00000197067	OR2T32P	0	0	0	0.11297	0	0	0	0
ENSG00000196071	OR2L13	0.43322	0.07211	0.06366	0	0.15801	0.15285	0.14442	0.10638
ENSG00000177233	OR2M1P	0	0	0	0	0	0	0	0
ENSG00000162727	OR2M5	0	0	0	0	0	0	0	0
ENSG00000198601	OR2M2	0	0	0	0	0	0	0	0
ENSG00000228198	OR2M3	0	0	0	0	0	0	0	0
ENSG00000171180	OR2M4	0	0	0	0	0	0	0	0
ENSG00000177212	OR2T33	0.07612	0	0.2237	0.22593	0	0.26856	0	0
ENSG00000177201	OR2T12	0	0	0	0	0	0	0	0
ENSG00000177186	OR2M7	0	0	0	0	0	0	0	0
ENSG00000177174	OR14C36	0	0	0	0	0	0	0	0
ENSG00000196944	OR2T4	0	0	0	0	0	0	0	0
ENSG00000198104	OR2T6	0	0	0	0	0	0	0	0
ENSG00000175143	OR2T1	0	0	0	0	0	0	0	0
ENSG00000227152	OR2T7	0	0	0	0	0	0	0	0
ENSG00000196240	OR2T2	0	0	0	0	0	0	0	0
ENSG00000196539	OR2T3	0	0	0	0	0	0	0	0
ENSG00000229255	AC138089.1	0	0	0	0	0	0	0	0
ENSG00000203661	OR2T5	0	0	0	0	0	0	0	0
ENSG00000271934	OR2AS2P	0	0	0	0	0	0	0	0
ENSG00000188558	OR2G6	0.07708	0	0	0	0	0	0	0
ENSG00000224521	AC098483.1	0	0	0	0	0	0	0	0.06762
ENSG00000227102	OR2AS1P	0	0	0	0	0	0	0	0
ENSG00000182783	OR2T29	0	0	0	0	0	0	0	0
ENSG00000183310	OR2T34	0	0	0	0	0	0	0	0
ENSG00000184022	OR2T10	0	0	0	0	0	0	0	0
ENSG00000201602	RF00019	0	0	0	0	0	0	0	0
ENSG00000271701	AC098483.2	0	0	0	0	0	0	0	0
ENSG00000279301	OR2T11	0	0	0	0	0	0	0	0
ENSG00000177151	OR2T35	0	0	0	0	0	0	0	0
ENSG00000187701	OR2T27	0	0	0	0	0	0	0	0

ENSG00000189181	OR14I1	0	0	0	0	0	0	0	0
ENSG00000229703	CR589904.1	0	0	0	0	0	0	0	0
ENSG00000276520	CR589904.2	0	0	0	0	0	0	0	0
ENSG00000259823	LYPD8	0.03277	0	0	0	0	0	0	0
ENSG00000242529	AHCYP8	0	0	0	0	0	0	0	0
ENSG00000229663	DPY19L4P1	0	0	0	0	0	0	0	0
ENSG00000175137	SH3BP5L	6.92951	6.68566	1.35437	2.39795	5.53348	4.75736	3.35702	3.1573
ENSG00000264500	MIR3124	5.47017	1.82102	3.21534	3.24733	7.9806	5.79008	0	6.7162
ENSG00000171161	ZNF672	5.44707	3.94628	0.92137	1.51212	3.81146	5.70341	2.02491	2.26135
ENSG00000171163	ZNF692	1.30594	1.08687	0.19191	0.55376	0.83923	0.72407	0.40426	0.48102
ENSG00000227237	AL672291.1	0	0	0	0	0	0	0	0
ENSG00000185220	PGBD2	2.18837	3.04102	4.04185	7.09148	3.56369	1.70007	3.37995	3.5496
ENSG00000200495	RNU6-1205P	0	0	0	0	0	0	0	0
ENSG00000233084	RPL23AP25	0	0	0	0.23496	0	0.27929	0.26389	1.16627
ENSG00000184731	FAM110C	0.07389	0.03075	0.02715	0.05483	0	0.09777	0.06158	0
ENSG00000227061	AC079779.1	0	0	0	0	0	0	0	0
ENSG00000035115	SH3YL1	1.06356	0.53546	0.29907	0.59435	0.88598	0.81073	0.83168	0.63679
ENSG00000143727	ACP1	11.8761	14.7231	8.44104	27.4841	12.7463	12.9623	20.7942	10.3194
ENSG00000189292	ALKAL2	0.03346	0.05569	0	0	0	0	0	0
ENSG00000228643	AC079779.2	0.159	0.26466	0.35048	0.23598	0	0.14025	0	0
ENSG00000235779	AC079779.3	0	0	0	0	0	0	0	0
ENSG00000277997	AC079779.4	0	0	0	0	0	0	0	0
ENSG00000233684	LINC01865	0.19482	0	0	0.14457	0	0	0.08118	0.0598
ENSG00000236856	AC105393.2	0	0	0	0	0	0	0	0
ENSG00000226277	AC105393.1	0	0	0	0	0	0	0	0
ENSG00000223985	LINC01874	0	0	0	0	0	0	0	0
ENSG00000284452	AC093326.2	0	0	0	0	0	0	0	0
ENSG00000225942	LINC01875	0	0	0	0	0	0	0	0
ENSG00000233633	AC093326.1	0	0	0	0	0	0	0	0
ENSG00000151353	TMEM18	1.48228	1.18525	0.38439	0.53918	1.51944	1.56384	0.70246	0.87428
ENSG00000233296	AC092159.2	0.15949	0.05309	0.14062	0	0.07756	0.11254	0	0.03916
ENSG00000280360	AC092159.4	0.13327	0	0	0	0	0	0	0
ENSG00000233970	AC092159.3	0	0	0	0	0	0	0	0
ENSG00000227713	AC092159.1	0	0	0	0	0	0	0	0
ENSG00000272342	LINC01115	0	0	0	0	0	0	0	0
ENSG00000231173	AC116609.2	0	0	0	0	0	0	0	0
ENSG00000223751	AC116609.1	0	0	0	0	0	0	0	0
ENSG00000237667	LINC01115	0	0	0	0	0	0	0	0.1185
ENSG00000228799	LINC01939	0	0	0	0	0.23483	0	0	0
ENSG00000234796	AC113607.1	0	0	0	0	0	0	0	0
ENSG00000235688	SNTG2-AS1	0.27199	0	0	0	0	0	0	0
ENSG00000172554	SNTG2	0.08201	0	0.0482	0	0.03988	0.08681	0.05468	0
ENSG00000235403	AC114808.1	0	0	0	0	0	0	0	0
ENSG00000236665	AC114808.2	0	0	0	0	0	0	0	0
ENSG00000233553	AC108462.1	0	0	0	0	0	0	0	0
ENSG00000115705	TPO	0.01071	0	0	0	0	0	0	0
ENSG00000228613	AC141930.1	0	0	0	0	0	0	0	0
ENSG00000283766	AC141930.2	0	0	0	0	0	0	0	0
ENSG00000231482	AC105450.1	0	0	0	0	0	0	0	0
ENSG00000203635	AC144450.1	0	0	0	0	0	0	0	0
ENSG00000130508	PXDN	0.03907	0.03252	0	0	0.20583	0.12636	0.02171	0
ENSG00000186487	MYT1L	0	0.00933	0	0	0.04088	0	0.00934	0
ENSG00000284600	AC093390.2	0	0	0	0	0	0	0.07095	0
ENSG00000232057	AC093390.1	0	0	0	0	0	0	0	0
ENSG00000225619	MYT1L-AS1	0	0	0	0	0	0	0	0
ENSG00000234929	AC018685.2	0	0	0	0	0	0	0	0
ENSG00000227364	AC018685.1	0	0	0	0	0	0	0	0
ENSG00000237720	AC011995.2	0	0	0	0	0	0	0	0

ENSG00000228391	AC011995.1	0	0	0	0	0	0	0	0
ENSG00000234423	LINC01250	0.03127	0.1041	0	0	0	0	0.05212	0
ENSG00000236760	AC019118.2	0	0	0.11314	0	0	0	0	0
ENSG00000226649	AC019118.1	0	0	0	0	0	0	0	0
ENSG00000032389	EIPR1	1.15504	0.63381	0.20517	0.54628	1.1265	1.18676	0.42314	0.59219
ENSG00000224885	EIPR1-IT1	0.20418	0.50978	0	0	0	0.1801	0	0
ENSG00000171853	TRAPPC12	1.58673	1.10157	0.23434	0.65084	1.58981	1.18157	0.49175	0.71465
ENSG00000225234	TRAPPC12-AS1	0	0	0	0	0	0	0	0
ENSG00000271868	AC114810.1	0	0	0	0	0	0	0	0
ENSG00000182551	ADI1	2.54442	1.6458	0.27122	0.84133	1.82721	1.37219	0.7911	0.90645
ENSG00000235078	AC231981.1	0.15051	0.12527	0	0.22338	0.54897	0	0	0
ENSG00000242282	AC108488.1	0.622	0.23892	0.07031	0.14202	0.29085	0.33763	0	0.17623
ENSG00000255767	AC108488.2	0	0	0	0	0	0	0	0
ENSG00000171865	RNASEH1	3.63304	1.78861	0.63914	0.91129	2.51953	3.24972	1.74848	1.41357
ENSG00000234171	RNASEH1-AS1	0.72217	0.40068	0.17687	0.80384	0.80483	0.95551	0.70219	0.29556
ENSG00000171863	RPS7	33.3219	24.0826	8.14173	11.0091	32.2595	35.0668	16.3038	26.227
ENSG00000118004	COLEC11	0	0	0	0	0	0	0	0.0324
ENSG00000237370	AC010907.2	0	0	0	0	0	0	0	0
ENSG00000188765	TMSB4XP2	0	0	0	0	0	0.61577	0	0.42856
ENSG00000151360	ALLC	0	0	0	0	0	0	0	0
ENSG00000232718	GAPDHP48	0	0	0	0	0	0	0	0
ENSG00000224661	AC010907.1	0	0	0	0	0	0	0	0
ENSG00000214866	DCDC2C	0	0	0	0	0	0	0	0
ENSG00000237401	LINC01304	0	0	0	0	0	0	0	0
ENSG00000229550	AC012445.1	0	0	0	0	0	0	0	0
ENSG00000231821	NPM1P48	0	0	0	0	0	0	0	0
ENSG00000231532	LINC01249	0	0	0	0	0	0	0	0
ENSG00000252238	RF00322	0	0	0	0	0	0	0	0
ENSG00000207192	RNU6-649P	0	0	0	0	0	0	0	0
ENSG00000228585	AC073143.1	0	0	0	0	0	0	0	0
ENSG00000232835	AC107057.1	0	0	0	0	0	0	0	0
ENSG00000224128	LINC01248	0	0	0	0	0	0	0	0
ENSG00000230090	AC108025.1	0	0	0	0	0	0	0	0
ENSG00000176887	SOX11	0	0	0	0	0.01022	0	0	0
ENSG00000242540	AC108025.2	0	0	0	0	0	0	0	0
ENSG00000236106	AC010729.1	0	0	0	0	0	0	0	0
ENSG00000237266	LINC01810	0	0	0	0	0	0	0	0
ENSG00000232044	LINC01105	0	0	0	0	0	0	0	0
ENSG00000276733	MIR7158	0	0	0	0	0	0	0	0
ENSG00000234275	AC017053.1	0	0	0	0	0	0	0	0
ENSG00000227007	LINC01247	0	0	0	0	0	0	0	0
ENSG00000231610	PIK3CDP1	0	0	0	0	0	0	0	0
ENSG00000226488	LINC01824	0	0	0	0	0	0	0	0
ENSG00000236172	MIR7515HG	0	0	0	0	0	0	0	0
ENSG00000283394	MIR7515	0	0	0	0	0	0	0	0
ENSG00000205837	LINC00487	0	0	0.04892	0	0	0	0	0.04087
ENSG00000225964	NRIR	1.13002	2.38249	3.21038	3.01872	1.92338	3.987	8.66438	1.38742
ENSG00000134326	CMPK2	5.48645	22.3484	11.1169	3.38849	3.21085	6.162	5.42456	4.87432
ENSG00000134321	RSAD2	5.85311	76.9305	44.2532	7.13386	2.98653	15.3013	5.38362	10.2396
ENSG00000271947	AC017076.1	0	0	0	0	0	0	0	0.1557
ENSG00000228203	RNF144A-AS1	0.07885	0	0	0.09362	0.09587	0.27821	0	0.05809
ENSG00000151692	RNF144A	2.95557	2.50689	0.58242	1.6638	3.52447	1.87785	1.94416	1.69622
ENSG00000223884	AC068481.1	0	0	0	0	0	0	0	0
ENSG00000223145	RN7SKP112	0	0	0	0	0	0	0	0
ENSG00000272002	AC010904.2	0	0	0	0	0	0	0	0.14195
ENSG00000213774	AC010904.1	0	0	0	0	0	0	0	0
ENSG00000229727	AC013460.1	0.02263	0.03767	0	0.16793	0	0.03992	0.03772	0
ENSG00000221255	RNU6ATAC37P	0	0	0	0	0	0	0	0

ENSG00000230515	AC092580.2	0	0	0	0	0	0	0	0
ENSG00000235576	LINC01871	9.04943	10.0419	5.31921	5.81981	3.66735	25.5431	7.0392	7.77752
ENSG00000232979	AC092580.3	0	0	0	0	0	0	0	0
ENSG00000229405	AC092580.1	0	0	0	0	0	0	0	0
ENSG00000226506	AC007463.1	0	0	0	0	0	0	0	0
ENSG00000235665	LINC00298	0.02808	0	0.04127	0.12504	0.10243	0.19818	0	0.10344
ENSG00000236790	LINC00299	1.37708	1.21511	0.68266	4.82617	1.97678	1.60981	1.24448	1.97594
ENSG00000229740	U91324.1	0	0	0	0	0	0	0	0
ENSG00000231435		0.14151	0.11777	0	0	0	0	0	0
ENSG00000236008	LINC01814	0.08184	0.04087	0	0	0.0398	0.04332	0.01364	0.03015
ENSG00000231083	AC011747.1	0	0	0	0	0	0	0	0
ENSG00000225642	SNRPEP5	0	0	0	0	0	0	0	0
ENSG00000235092	ID2-AS1	0.14233	0.05923	0.05229	0.10562	0.04326	0	0	0.04369
ENSG00000115738	ID2	56.1138	34.8042	10.2585	21.4118	28.4931	31.7855	18.895	18.979
ENSG00000134313	KIDINS220	14.1571	13.8354	5.45511	7.49929	12.31	9.98332	8.7408	8.07876
ENSG00000143797	MBOAT2	23.0532	59.7284	121.818	313.053	49.785	49.62	137.692	140.253
ENSG00000230886	HMGB1P25	0	0	0	0	0	0	0	0
ENSG00000223640	RPL30P3	0	0	0	0	0	0	0	0
ENSG00000242136	AC093904.2	0	0	0	0	0	0	0	0
ENSG00000261104	AC093904.4	0	0	0	0	0	0	0	0
ENSG00000244310	AC093904.3	0	0	0	0	0	0	0	0
ENSG00000240980	AC093904.1	0	0	0	0	0	0	0	0
ENSG00000151693	ASAP2	3.87335	2.23884	1.02542	1.60597	3.12302	2.51559	1.90485	2.47095
ENSG00000213772	EIF1P7	0	1.08613	0	0.32281	0.26444	0	0	0
ENSG00000119185	ITGB1BP1	1.5806	1.18756	0.56454	0.96112	1.78821	1.76211	0.82332	1.10509
ENSG00000119203	CPSF3	6.63184	5.30894	3.1151	6.11902	5.69836	10.0526	4.86258	5.13382
ENSG00000134330	IAH1	1.99752	1.25974	0.23701	0.71812	1.26706	1.79478	0.72382	0.79212
ENSG00000151694	ADAM17	11.1733	11.1765	3.66828	5.03216	9.44562	7.79497	7.43184	7.24435
ENSG00000239300	AC080162.1	0.14929	0	0	0.22156	0.363	0.26336	0.24884	0.54988
ENSG00000271855	AC073195.1	0.58507	0.97384	0.12282	0.24809	0.40646	0.14745	0.41795	0.20524
ENSG00000134308	YWHAQ	35.9342	26.7582	9.76691	18.7257	25.2601	45.2329	15.0417	19.041
ENSG00000207086	RF00019	0	0	0	0	0	0	0	0
ENSG00000240687	AC082651.1	0	0	0	0	0	0	0	0
ENSG00000244260	AC082651.4	0	0	0	0	0	0	0	0
ENSG00000200034	RNU4-73P	0	0	0	0	0	0	0	0
ENSG00000240960	AC082651.2	0	0	0	0	0	0	0	0
ENSG00000243491	AC082651.3	0.15059	0	0.05532	0.16762	0.09154	0	0	0
ENSG00000115750	TAF1B	2.2726	1.86135	0.66261	1.92731	1.66655	2.89551	0.87186	1.68302
ENSG00000269973	AC010969.2	0.72597	0.52867	0.20003	0.16835	0.6068	0.60033	0.26471	0.38996
ENSG00000134317	GRHL1	1.02318	0.42961	0.50118	0.54721	0.91896	0.4228	0.23047	0.24899
ENSG00000188525	AC010969.1	0	0.05058	0	0	0	0	0	0
ENSG00000260476	AC104794.3	0	0.09776	0	0	0	0	0	0
ENSG00000260077	AC104794.2	0.9792	0.38807	0.20556	0.41521	1.30387	0.24678	0.23317	1.374
ENSG00000172059	KLF11	9.61578	4.7591	0.96236	1.61199	4.56361	3.49415	2.23647	1.72581
ENSG00000271787	AC104794.4	0	0	0	0	0	0	0	0
ENSG00000205795	CYS1	0	0	0	0	0	0	0	0
ENSG00000233502	AC104794.1	0	0	0	0	0	0	0	0
ENSG00000212558	RF00568	0	0	0	0	0	0	0	0
ENSG00000272524		0.22078	0.73499	0	0	0.53685	0	0	0
ENSG00000171848	RRM2	1.15387	0.64021	0.60868	1.77833	1.69061	12.4223	0.937	0.65386
ENSG00000163009	C2orf48	0	0.19295	0	0.22938	0.23489	1.09066	0.25763	0.42698
ENSG00000206647	RF00410	0	0	0	0	0	0	0	0
ENSG00000265418	MIR4261	0	0	0	0	0	0	0	0
ENSG00000264030	RN7SL66P	0	0	0	0	0	0	0	0
ENSG00000206898	RF00432	0	0	0	0	0	0	0	0
ENSG00000115756	HPCAL1	5.19461	4.63621	1.41615	2.16281	4.20079	9.41279	1.82183	2.69832
ENSG00000115758	ODC1	88.7606	53.5637	32.59	46.2926	58.6405	44.5707	35.5262	41.845
ENSG00000206633	SNORA80B	0.53897	0	3.16806	2.39968	0	0	0.89838	1.32349

ENSG00000257135	AC007249.2	0.25089	0.4176	0.06145	0.06206	0.25418	0	0.27879	0.15402
ENSG00000217258	AC007249.1	0.14838	0.24698	0	0	0.1804	0	0	0.54654
ENSG00000115761	NOL10	6.65147	6.89169	6.39411	13.968	10.557	8.55541	11.5662	7.437
ENSG00000234818	AC007314.1	0	0	0	0	0	0	0	0
ENSG00000243819	RN7SL832P	0.12523	0.06948	0	0	0.203	0.22092	0	0.05125
ENSG00000277446	RF00017	0	0	0	0	0	0	0	0
ENSG00000236162	AC092687.2	0	0	0	0	0	0	0	0
ENSG00000143882	ATP6V1C2	0	0	0	0.03194	0.05233	0	0.07174	0
ENSG00000239053	RNU7-138P	0	0	0	0	0	0	0	0
ENSG00000272275	AC092687.3	0.37303	0.62091	0	0.27681	0.68028	0	0	0
ENSG00000143870	PDIA6	20.8229	13.5962	2.574	7.48051	14.1465	17.7209	5.27333	7.32968
ENSG00000238962	RNU7-176P	0	0	0	0	0	0	0	0
ENSG00000232056	AC092687.1	0	0	0	0	0	0	0	0
ENSG00000270488	AC079587.1	0	0	0	0	0	0	0	0
ENSG00000271952	LINC01954	0	0	0	0.23598	0	0.2805	0	0.19522
ENSG00000162975	KCNF1	0	0	0	0	0	0	0	0
ENSG00000226080	AC062028.2	0	0	0	0	0	0	0	0
ENSG00000145063	AC062028.1	0	0	0	0	0.09884	0	0	0
ENSG00000150873	C2orf50	0.0499	0.04153	0	0.03703	0	0	0	0
ENSG00000162976	PQLC3	9.6982	7.1328	2.79346	3.63416	3.95639	6.36612	2.8464	5.38004
ENSG00000134318	ROCK2	8.61974	7.22791	4.69291	8.78221	9.23229	7.27817	5.91331	7.95735
ENSG00000207267	RNU6-1081P	0	0	0	0	0	0	0	0
ENSG00000230154	AC018463.1	0	0.13305	0	0	0	0	0	0
ENSG00000226961	PPIAP60	0	0	0	0	0	0	0	0
ENSG00000230952	AC099344.1	0	0	0	0	0	0	0	0
ENSG00000231403	AC099344.2	0	0	0	0	0	0	0	0
ENSG00000224177	LINC00570	4.93111	5.54584	11.2284	28.2184	4.75289	4.85899	14.2914	6.98159
ENSG00000169016	E2F6	0.99128	0.89999	0.26485	0.98969	1.11752	0.95386	1.02142	0.90727
ENSG00000196208	GREB1	0.00593	0.00987	0	0.0088	0.00721	0	0.00988	0.00728
ENSG00000201610	RNA5SP84	0	0	0	0	0	0	0	0
ENSG00000264010	MIR4429	0	0	0	0	0	0	0	0
ENSG00000251718	RNU2-13P	0	0	0	0	0	0	0	0
ENSG00000202248	RNA5SP85	0	0	0	0	0	0	0	0
ENSG00000239899	RN7SL674P	18.1252	23.0707	36.4269	34.8114	20.0918	38.0882	24.4361	24.2174
ENSG00000169006	NTSR2	0	0	0	0	0	0	0	0
ENSG00000230044	AC110754.1	0	0	0	0	0	0	0	0
ENSG00000276411	AC106875.2	0	0	0	0	0	0	0	0
ENSG00000134324	LPIN1	2.05792	1.39897	0.40866	0.99972	1.79571	2.24587	0.85498	1.16091
ENSG00000228496	AC106875.1	0	0	0	0	0	0	0	0
ENSG00000203643	AC012456.1	0	0	0	0	0	0	0	0
ENSG00000230790	AC012456.2	0	0	0	0	0	0	0	0
ENSG00000265056	MIR548S	0	1.48791	0	0	0	0	0	0
ENSG00000265172	MIR4262	0	0	0	0	0	0	0	0
ENSG00000224184	MIR3681HG	0.03782	0	0	0	0	0	0	0
ENSG00000238503	RF00093	0	0	0	0	0	0	0	0
ENSG00000264089	MIR3681	0	0	0	0	0	0	0	0
ENSG00000207183	RNU6-843P	0	0	0	0	0	0	0	0
ENSG00000223360	AC096559.1	0	0	0	0	0	0	0	0
ENSG00000261117	AC009486.1	0	0	0	0.13381	0.32884	0	0	0
ENSG00000071575	TRIB2	6.34268	4.17509	1.50255	3.20097	7.10913	9.94923	2.47663	4.27623
ENSG00000264370	MIR3125	0	0	0	0	0	0	0	0
ENSG00000225649	AC064875.1	0	0	0	0	0	0	0	0
ENSG00000229370	AC073062.1	0	0	0	0	0	0	0	0
ENSG00000230448	LINC00276	0	0	0	0	0.02316	0	0.03175	0
ENSG00000227718	AC016730.1	0	0	0	0	0	0	0	0
ENSG00000251859	RNU6-1288P	0	0	0	0	0	0	0	0
ENSG00000237261	RF00017	0	0	0	0	0	0	0	0
ENSG00000228505	AC011897.1	0	0	0	0	0	0	0	0

ENSG00000162981	FAM84A	0	0	0	0	0	0	0	0
ENSG00000235127	AC068286.1	0	0	0	0	0	0	0	0.19271
ENSG00000151779	NBAS	12.6891	13.608	15.9139	35.2326	18.0863	20.9691	35.9249	27.4578
ENSG00000229087	AC007738.1	0.21063	0	0	0	0	0	0	0
ENSG00000234022	AC008278.2	0.71687	2.38647	3.68703	4.78764	0.21789	1.26466	1.49365	2.6405
ENSG00000079785	DDX1	7.09482	6.62086	3.96234	6.47095	9.69519	9.34499	6.43893	6.24556
ENSG00000224194	AC008278.1	0	0	0	0	0	0	0	0
ENSG00000231031	LINC01804	0	0	0	0	0	0	0	0
ENSG00000237326	AC113608.2	0	0	0	0	0	0	0	0
ENSG00000202160	RNU5E-7P	0	0	0	0	0	0	0	0
ENSG00000214657	AC113608.1	0	0	0	0	0	0	0	0
ENSG00000238371	RF01210	0	0	0	0	0	0	0	0
ENSG00000223850	MYCNUT	0	0	0	0	0	0	0	0
ENSG00000233718	MYCNOS	0.0952	0	0	0	0	0	0	0
ENSG00000134323	MYCN	0	0	0	0.04181	0	0	0	0
ENSG00000243541	RN7SL104P	0	0	0	0	0	0	0	0
ENSG00000226764	AC010145.1	0	0	0	0	0	0	0	0
ENSG00000236289	GACAT3	0	0	0	0	0	0	0	0
ENSG00000236989	AC130710.1	0	0	0	0	0	0	0	0
ENSG00000271315	AC174048.1	0	0	0	0	0	0	0	0
ENSG00000251704	RF00561	0	0	0	0	0	0	0	0
ENSG00000226041	AC010745.1	0	0	0	0	0	0	0	0
ENSG00000228876	AC010745.2	0	0	0	0	0	0	0	0
ENSG00000231266	AC010745.3	0	0	0	0	0	0	0	0
ENSG00000232444	AC010745.4	0	0	0	0	0	0	0	0
ENSG00000224400	AC010880.1	0	0	0	0	0	0	0	0
ENSG00000237633	AC104623.1	0	0	0	0	0	0	0	0
ENSG00000279663	AC104623.2	0.0183	0.06091	0.02689	0	0	0.06456	0.122	0.11233
ENSG00000197872	FAM49A	17.0469	14.7136	2.88661	5.19168	11.4177	7.66661	8.14087	9.51511
ENSG00000223536	AC008164.1	0	0	0	0	0	0	0	0
ENSG00000224604	AC008069.1	0	0	0	0	0	0	0	0
ENSG00000237670	LINC01866	0	0	0	0	0	0	0	0
ENSG00000222842	RN7SKP168	0	0	0	0	0	0	0	0
ENSG00000214843	ZFYVE9P2	0	0	0	0	0.29509	0	0	0
ENSG00000236348	PSMC1P10	0	0	0	0	0	0	0	0
ENSG00000214842	RAD51AP2	0	0	0	0	0	0	0	0
ENSG00000163032	VSNL1	0	0	0	0	0	0	0	0
ENSG00000163029	SMC6	5.30502	4.19777	1.76526	3.18916	7.16634	6.10756	3.84301	3.5178
ENSG00000178295	GEN1	0.8243	0.88718	0.7377	1.4257	0.92681	1.59631	1.12604	0.76095
ENSG00000151379	MSGN1	0	0	0	0	0	0	0	0
ENSG00000170745	KCNS3	0	0	0	0	0.02846	0	0	0
ENSG00000212455	RF00561	0	0	0	0	0	0	0	0
ENSG00000260331	AC079148.1	0.0895	0	0	0	0.10881	0	0.14918	0
ENSG00000240857	RDH14	3.55648	1.922	0.61085	1.8508	2.6954	2.28149	2.46362	2.21165
ENSG00000250741	NT5C1B-RDH14	0	0	0	0	0	0	0	0
ENSG00000185013	NT5C1B	0	0	0	0	0	0	0	0
ENSG00000207170	RNU6-1215P	0	0	0	0	0	0	0	0
ENSG00000224626	AC106053.1	0	0.33154	0.2927	0.29561	0.24216	0.35139	0	0
ENSG00000236204	LINC01376	0.04948	0.08235	0.03635	0.14686	0	0	0.08247	0.09112
ENSG00000266738	MIR4757	0	0	0	0	0	0	0	0
ENSG00000143867	OSR1	0	0	0	0	0	0	0	0
ENSG00000234597	AC010096.1	0	0	0	0	0	0	0	0
ENSG00000237992	LINC01808	0	0	0	0	0	0	0	0
ENSG00000235911	AC019055.1	0	0	0	0	0	0	0	0
ENSG00000213403	CISD1P1	0	0	0	0	0	0	0	0
ENSG00000228784	LINC00954	0.31305	0.18237	0	0.13938	0.32351	0.4142	0.28699	0.30749
ENSG00000183891	TTC32	4.42902	3.13315	0.75931	1.64329	3.41031	2.7998	2.89148	3.12679
ENSG00000271991	AC013400.1	0.94147	0.44774	0.19764	0.19961	0.49055	0	0.44837	0.4954

ENSG00000118965	WDR35	0.9822	0.65395	0.16495	0.47202	1.42159	1.12215	0.3898	0.63168
ENSG00000227210	AC079145.1	0.13549	0	0	0	0	0	0	0
ENSG00000132031	MATN3	0	0	0	0	0	0	0	0
ENSG00000068697	LAPTM4A	25.011	20.4392	5.14769	10.7891	18.6843	9.70171	9.92009	11.7468
ENSG00000223734	AC098828.2	0.25585	0.21293	0	0	0	0	0	0
ENSG00000234378	AC098828.3	0	0	0	0	0	0	0	0
ENSG00000213729	AC098828.1	0	0	0	0	0	0	0	0
ENSG00000266059	RN7SL140P	0	0	0	0	0	0	0	0
ENSG00000200763	RNU6-961P	0	0	0	0	0	0	0	0
ENSG00000115884	SDC1	0.04078	0.06788	0	0	0.4462	0	0	0
ENSG00000235411	AC007041.1	0.06346	0.10564	0	0	0	0.11196	0	0
ENSG00000238735	RNU7-113P	0	0	0	0	5.74946	0	0	0
ENSG00000055917	PUM2	23.5301	19.7752	6.66845	16.3069	21.813	18.346	16.6164	15.0769
ENSG00000232800	SLC7A15P	0	0	0	0	0	0	0	0
ENSG00000212171	RNA5SP86	0	0	0	0	0	0	0	0
ENSG00000143878	RHOB	32.7565	57.4037	7.44734	16.6022	22.6549	10.8487	4.79038	20.4504
ENSG00000228950	AC023137.1	0	1.98066	0	0	0	0	0.79338	0
ENSG00000200829	RF00019	0	0	0	0	0	0	0	0
ENSG00000227047	AC012065.1	0	0	0	0	0	0	0	0
ENSG00000279307	AC012065.5	0	0	0.42241	0.21331	0.17474	0	0.23957	0.35293
ENSG00000118960	HS1BP3	2.55689	1.84514	0.26159	0.43231	1.33788	1.14195	0.44508	0.71529
ENSG00000269976	AC012065.3	0	0	0	0	0	0	0	0
ENSG00000231948	HS1BP3-IT1	0.24433	0	0.17952	0	0	0	0	0.15
ENSG00000233416	AC012065.2	0.19547	0	0	0.58019	0.23764	0	0	0
ENSG00000143869	GDF7	0	0.02503	0	0	0.14626	0.01326	0.02507	0.01846
ENSG00000270100	AC012065.4	0.64772	0.53907	0	0	0	0.19044	0	0.26509
ENSG00000118961	LDAH	2.18264	2.31191	2.13824	5.86505	3.05566	3.79213	4.16179	3.08585
ENSG00000236436	AC012361.1	0	0	0	0	0	0	0	0
ENSG00000261012	AC115619.1	0	0	0	0	0	0	0	0
ENSG00000084674	APOB	0	0	0	0.00734	0	0	0	0
ENSG00000280390	AC010872.1	0	0	0	0	0	0	0	0
ENSG00000218819	TDRD15	0.03584	0.01989	0.03511	0.01773	0.01453	0	0	0.01467
ENSG00000271629	AC067959.1	0	0	0	0	0	0	0	0
ENSG00000233005	AC018742.1	0	0	0	0.12334	0	0	0	0
ENSG00000231204	AC011752.1	0	0	0	0	0	0	0	0
ENSG00000270654	NUTF2P8	0	0	0	0	0	0	0	0
ENSG00000235537	AC009411.2	0	0	0	0	0	0	0	0
ENSG00000228538	AC009411.1	0	0	0	0	0	0	0	0
ENSG00000229621	LINC01822	0	0	0	0	0	0	0	0
ENSG00000264192	RN7SL117P	0	0	0	0	0	0	0	0
ENSG00000231200		0	0	0	0	0	0	0	0
ENSG00000228999	AC096570.1	0	0	0	0	0	0	0	0
ENSG00000223691	AC068044.1	0	0	0	0	0	0	0	0
ENSG00000234189	AC099799.1	0	0	0	0	0	0	0	0
ENSG00000206882	RNA5SP87	0	0	0	0	0	0	0	0
ENSG00000234207	AC096570.2	0	0	0	0	0	0	0	0
ENSG00000233587	LINC01884	0	0	0	0	0	0	0	0
ENSG00000222616	RN7SKP27	0	0	0	0	0	0	0	0
ENSG00000232451	AC018467.1	0	0	0	0	0	0	0	0
ENSG00000223530	AC012506.1	0	0	0	0	0	0	0	0
ENSG00000233714	AC012506.3	0	0	0	0	0	0	0	0
ENSG00000223634	AC012506.2	0	0	0	0	0	0	0	0
ENSG00000235497	AC012506.4	0	0	0	0	0	0	0	0
ENSG00000119771	KLHL29	0.00662	0	0	0	0.08855	0.01168	0.02207	0.00813
ENSG00000224361	AC011239.1	0	0	0	0	0	0	0	0
ENSG00000279526	AC011239.2	0	0	0	0	0	0	0	0
ENSG00000283031	AC009242.1	0	0	0	0	0	0	0.04515	0
ENSG00000119778	ATAD2B	13.1832	15.1871	14.4549	34.3299	22.6699	22.7393	26.3878	18.159

ENSG00000224464	PGAM1P6	0	0	0.1442	0	0.3579	0	0	0
ENSG00000238111	AC066692.1	0	0	0	0	0	0	0	0
ENSG00000173960	UBXN2A	5.92197	6.71915	9.45353	26.0662	10.4889	9.84588	19.5822	11.4033
ENSG00000234946	SDHCP3	0	0.48225	0	0.21499	0.17612	0	0	0
ENSG00000243847	RN7SL610P	0	0	0	0	0	0	0.41986	0
ENSG00000205639	MFSD2B	2.74174	2.55673	2.54844	4.46124	4.23696	5.50697	5.31338	2.69707
ENSG00000163026	WDCP	1.14323	0.91974	0.33599	0.6504	1.83005	1.14286	0.76224	1.07613
ENSG00000222940	RNU6-370P	0	0	0	0	0	0	0	0
ENSG00000119782	FKBP1B	3.05964	1.7461	0.96345	2.07582	1.80678	2.00483	1.09285	2.14662
ENSG00000115128	SF3B6	25.5357	27.113	9.89852	16.5405	23.2284	24.1987	12.1466	19.0219
ENSG00000219626	FAM228B	4.3688	7.07332	2.38829	6.43951	6.7617	3.18282	4.32453	5.21749
ENSG00000115129	TP53I3	1.29988	0.42217	0.04659	0.18821	0.46254	0.44745	0.31708	0.42819
ENSG00000176732	PFN4	0	0	0	0	0	0	0.1157	0.08522
ENSG00000276087	AC008073.3	0.03214	0	0.04722	0	0	0	0	0
ENSG00000232642	AC008073.2	0	0	0	0	0	0	0	0
ENSG00000186453	FAM228A	0.06472	0	0	0.09606	0	0	0.05394	0
ENSG00000223754	AC008073.1	0.53997	0	0	0	0	0	0	0
ENSG00000198399	ITSN2	14.5967	15.1014	3.53014	5.72795	12.4757	9.40097	5.76246	8.42178
ENSG00000242628	AC009228.1	0	0	0	0	0	0	0	0
ENSG00000251805	RF00602	0.57266	0.95319	0	0.84989	0.69622	2.0205	0.95453	0
ENSG00000232963	HMGN2P20	0	0	0	0	0	0	0	0
ENSG00000233747	RPL36AP13	0.23123	0	0	0	0	0	0	0
ENSG00000084676	NCOA1	40.3564	43.7128	14.3735	21.0187	35.7788	26.9768	25.5598	24.3001
ENSG00000202430	RNA5SP88	0	0	0	0	0	0	0	0
ENSG00000206732	RNU6-936P	2.05515	4.56107	1.00667	0	3.33146	0	0	2.52328
ENSG00000184924	PTRHD1	3.90017	2.16395	0.56189	1.24845	2.78925	5.1266	1.2747	1.17367
ENSG00000138092	CENPO	0.65091	0.55277	0.15616	0.27601	0.79136	1.49981	0.37642	0.3425
ENSG00000138031	ADCY3	1.54877	1.04728	0.12446	0.55668	0.95619	1.08863	0.34286	0.57938
ENSG00000271936	AC012073.1	0.39693	0.22023	0.09721	0.09818	0.08043	0.70024	0.22054	0.08122
ENSG00000115137	DNAJC27	0.72988	0.67252	0.13407	0.59964	0.53876	0.82774	0.63002	0.52808
ENSG00000237953	AC013267.1	0	0	0	0.40744	0	0	0	0.33707
ENSG00000202479	RF00016	0	0	0	0	0.99019	0	0	0
ENSG00000224165	DNAJC27-AS1	0.10377	0.04318	0.03812	0.09625	0.12616	0.02288	0.06486	0.0637
ENSG00000207069	RF00019	0	0	0	0	0	0	0	0
ENSG00000084710	EFR3B	0.02768	0.01152	0	0	0	0	0	0
ENSG00000276653	RN7SL856P	0	0	0	0	0	0	0	0
ENSG00000229593	SUCLA2P3	0	0	0	0	0	0	0	0
ENSG00000115138	POMC	0.95319	0.07933	0.14007	0.14146	0.4056	0.58855	0.07944	0.05852
ENSG00000230452	LINC01381	0	0	0	0	0	0	0	0
ENSG00000119772	DNMT3A	3.54915	2.2534	0.28676	0.77833	3.12872	2.30222	0.95549	1.2953
ENSG00000221445	MIR1301	0.89391	0	0	0	0	0	0	0
ENSG00000235072	AC012074.1	0.28364	0	0	0.06014	0.04926	0.21445	0.13508	0.0995
ENSG00000138101	DTNB	0.80009	0.68295	0.30147	0.6546	0.96026	1.04955	0.58132	0.61711
ENSG00000224220	AC104699.1	0	0	0.26018	0.26277	0	0	0	0
ENSG00000201160	RF00019	0	0	0	0	0	0	0	0
ENSG00000143970	ASXL2	8.03754	6.56745	2.79562	5.52157	7.51364	8.62873	5.20695	4.96183
ENSG00000187536	TPM3P7	0	0.33336	0	0	0.85221	0	0	0.12295
ENSG00000217643	PTGES3P2	0.6083	0	0.44695	0.67709	2.21867	3.75594	0.76046	0.18672
ENSG00000218682	AC064847.1	0.14486	0	0	0.21499	0	0.76667	0.24146	0.53358
ENSG00000217718	NDUFB4P4	0	0	0	0	0	0	0	0
ENSG00000084731	KIF3C	1.45387	1.1814	0.23551	0.32281	1.2387	0.74723	0.32439	0.46383
ENSG00000237629	UQCRHP2	0.50903	0	0	0	0	0	0	0.31249
ENSG00000268412	TRMT112P6	0	0.32798	0	0	0	0	0	0
ENSG00000084733	RAB10	45.9868	46.4378	17.888	36.7912	35.416	29.7015	21.1317	28.8754
ENSG00000199872	RNU6-942P	0.68505	2.28053	0	0	0	1.20852	0	0.84109
ENSG00000235440	AC011742.3	0	0	0	0	0	0	0	0
ENSG00000231655	AC011742.2	0	0	0	0	0	0	0	0
ENSG00000214465	SMARCE1P6	0.18084	0.30101	0.08858	0	0.14657	0	0	0.07401

ENSG00000227133	AC011742.1	0	0	0.24819	0	0.20534	0	0	0
ENSG00000237951	PPIL1P1	0	0	0	0	0	0	0	0
ENSG00000157833	GAREM2	0.0864	0.02054	0	0.03663	0.24009	0.34837	0.08229	0.01515
ENSG00000084754	HADHA	32.6591	26.6683	6.02022	11.8463	26.1902	28.8415	12.2469	14.7358
ENSG00000138029	HADHB	15.1567	13.9968	8.58188	16.3941	16.8447	20.3938	19.0693	14.6981
ENSG00000173567	ADGRF3	0.01272	0.06354	0	0.03777	0.04641	0.06734	0.06362	0.03124
ENSG00000138018	SELENOI	2.75133	2.21133	2.20416	3.80343	2.44883	3.79528	4.02886	2.27308
ENSG00000157856	DRC1	0.14429	0.20015	0	0.10707	0.02924	0	0	0.11811
ENSG00000115155	OTOF	0.17663	0.12379	0.19125	0.04139	0.0113	0.082	0	0.01141
ENSG00000173557	C2orf70	0	0	0	0	0	0	0	0
ENSG00000157884	CIB4	0	0	0	0	0	0	0	0
ENSG00000225378	AC015977.2	0	0	0	0	0	0	0	0
ENSG00000225024	AC015977.1	0	0	0	0	0	0	0	0
ENSG00000171303	KCNK3	0.01178	0	0	0	0	0	0	0
ENSG00000213699	SLC35F6	5.29746	3.97106	0.69012	1.81217	3.2431	3.74481	1.25249	2.19111
ENSG00000115163	CENPA	0.62839	0.43069	1.14069	3.12697	0.62916	1.23899	2.6494	0.90769
ENSG00000145835	CDKN2AIPNLP2	0	0	0	0	0	0	0	0
ENSG00000157851	DPYSL5	0	0	0	0	0	0	0	0
ENSG00000230286	AC013472.2	0	0	0	0	0	0	0	0
ENSG00000084764	MAPRE3	0.29668	0.42327	0.03114	0.0629	0.23187	0.03738	0.14129	0.13009
ENSG00000205500	AC013472.1	0	0.0476	0	0	0	0	0	0.03511
ENSG00000119777	TMEM214	2.68718	1.66488	0.54844	0.7533	1.8513	2.97601	0.54745	0.89814
ENSG00000084693	AGBL5	1.5401	0.87759	0.22428	0.41183	1.24827	1.24833	1.13323	0.5792
ENSG00000231636	AGBL5-AS1	0	0	0	0	0	0	0	0
ENSG00000272056	AC013472.3	0.33369	0.92571	0	0	0.40569	0	0	0.13657
ENSG00000229122	AGBL5-IT1	0	0	0	0	0	0	0	0
ENSG00000272148	AC013403.2	1.06896	0	0	0	0.55698	0.2694	0	0.37499
ENSG00000228474	OST4	386.993	308.33	167.938	305.931	246.155	421.567	189.473	202.525
ENSG00000138080	EMILIN1	0.1852	0.15413	0	0.10994	0.06755	0.09801	0.09261	0.09095
ENSG00000138030	KHK	0.35932	0.23923	0.0352	0.17775	0.05825	0.25355	0.07986	0.05882
ENSG00000138028	CGREF1	0	0	0.05334	0	0	0	0	0
ENSG00000143994	ABHD1	0.03166	0	0	0.0235	0	0.02793	0.02639	0
ENSG00000138073	PREB	5.1886	3.71215	0.5685	1.11454	4.12244	4.37597	1.02418	1.6485
ENSG00000186143	PRR30	0	0	0	0	0	0	0	0
ENSG00000163792	TCF23	0	0	0	0	0	0	0	0
ENSG00000236099	AC013403.1	0	0	0	0	0	0	0	0
ENSG00000138074	SLC5A6	1.12448	0.60939	0.15371	0.54335	0.89021	1.06107	0.56666	0.28897
ENSG00000138085	ATRAID	7.73302	4.89484	1.1737	3.3945	5.38496	6.2126	3.08628	3.83346
ENSG00000084774	CAD	1.85383	1.02857	0.14069	0.55542	1.73535	1.82713	0.36268	0.6091
ENSG00000115194	SLC30A3	0	0	0	0	0	0	0	0
ENSG00000163793	DNAJC5G	0	0	0	0	0	0	0	0
ENSG00000138100	TRIM54	0	0	0	0	0	0	0	0
ENSG00000163794	UCN	0.27661	0	0	0.13684	0	0.32531	0	0
ENSG00000115204	MPV17	2.12408	1.75692	0.19149	0.54151	1.5843	2.29888	0.62991	0.65598
ENSG00000115207	GTF3C2	4.04225	2.7582	0.71945	2.36612	3.06773	3.52125	1.65306	2.18866
ENSG00000234945	GTF3C2-AS1	0	0	0	0	0	0	0	0
ENSG00000234072	AC074117.1	0.49411	0.10728	0.09471	0.15942	0.2873	0.26529	0.14324	0.15826
ENSG00000115211	EIF2B4	1.24889	1.11934	0.20167	0.38699	1.10124	0.92002	0.41177	0.50551
ENSG00000115234	SNX17	7.61233	5.86548	1.97404	3.52506	5.5624	6.04485	3.56968	4.68511
ENSG00000163795	ZNF513	1.95261	1.85721	0.13664	0.78197	1.20581	1.2029	0.56828	1.0655
ENSG00000115241	PPM1G	15.8531	9.69031	1.60571	3.5358	13.9816	18.8343	2.8664	3.51895
ENSG00000213453	FTH1P3	0.62757	0	0	0.18628	0	0	0.41843	0.30821
ENSG00000115216	NRBP1	13.645	11.6994	2.56702	3.75616	10.1676	9.77907	4.44792	6.65394
ENSG00000157992	KRTCAP3	0.36572	0	0.07677	0.15508	0.12704	0.09217	0.17417	0
ENSG00000138002	IFT172	0.75037	0.47926	0.21797	0.37553	0.6789	0.84658	0.36359	0.29995
ENSG00000235267	AC074117.2	0	0	0	0	0	0	0	0
ENSG00000200003	RNU6-986P	0	0	0	0	0	0	0	0
ENSG00000115226	FNDC4	0	0	0	0	0	0	0	0

ENSG00000084734	GCKR	0.0466	0	0.03424	0.03458	0	0	0	0
ENSG00000221843	C2orf16	0	0	0	0	0	0	0	0
ENSG00000243943	ZNF512	3.63335	2.57999	0.56331	1.36044	4.61996	2.32279	1.36126	1.7803
ENSG00000259080	AC074091.1	0	0	0	0	0	0	0	0
ENSG00000176714	CCDC121	0.56702	0.5148	0.18937	0.34426	0.43869	0.22734	0.12888	0.41138
ENSG00000198522	GPN1	2.50466	2.05193	0.94889	1.24874	2.47414	3.7972	1.76128	1.60966
ENSG00000206731	RF00340	0.57717	0	0	0	0.70171	0	0	0
ENSG00000119760	SUPT7L	3.93494	2.73479	1.0956	2.4589	3.1054	4.62785	2.39344	1.91555
ENSG00000163798	SLC4A1AP	9.03761	7.75587	3.78561	8.48224	8.69431	6.95358	5.39686	6.56787
ENSG00000205334	LINC01460	0	0	0	0	0	0	0	0
ENSG00000243147	MRPL33	7.3348	7.8485	2.6596	4.38253	6.13799	5.71358	3.41375	6.66645
ENSG00000171174	RBKS	1.49634	2.01945	0.53486	0.78026	1.45045	1.07017	0.43816	1.26617
ENSG00000158019	BABAM2	4.77576	4.02858	0.9209	2.27706	3.75698	4.80344	1.72897	2.17564
ENSG00000270846	AC021171.2	0	0	0	0	0	0	0	0
ENSG00000265321	MIR4263	0	5.87993	1.29776	0	2.14739	1.55797	0	1.0843
ENSG00000270528	AC021171.1	0.10382	0	0	0	0	0	0.17306	0
ENSG00000277035	RF02271	0	0	0	0	0	0	0	0
ENSG00000223522	AC093690.1	0	0	0	0	0	0	0	0
ENSG00000225991	RPL23AP34	0.35756	0.29758	0	0	0	0.94618	0	0
ENSG00000229951	FLJ31356	0.02443	0.20328	0	0	0.0297	0	0	0.05998
ENSG00000075426	FOSL2	35.2931	29.3898	3.40133	9.62154	17.4946	16.8393	12.4622	11.531
ENSG00000270640	AC104695.3	7.42909	24.3193	1.0917	4.77775	3.91391	2.18432	0.41277	4.86471
ENSG00000270210	AC104695.2	0.37832	0.78715	0.13899	0	0.68994	0.16685	0	0.11613
ENSG00000227938	AC104695.1	0	0	0	0	0.10497	0	0	0
ENSG00000163803	PLB1	4.31545	4.50238	0.31381	0.60745	1.69839	0.28255	0.50426	0.83027
ENSG00000242915	SNRPGP7	0	0	0	0	0	0	0	0
ENSG00000230730	AC074011.1	0	0	0	0	0	0	0	0
ENSG00000222232	RNA5SP89	0	0	0	0	0	0	0	0
ENSG00000226833	AC092164.1	0.25487	0.25454	0.14981	0.22695	0	0.17985	0.08497	0.43809
ENSG00000213639	PPP1CB	50.7503	54.692	34.0533	50.2756	46.0746	36.5485	45.8388	43.1392
ENSG00000163806	SPDYA	0.77867	0.52545	0.1237	0.4685	0.61407	0.44552	0.38587	0.56846
ENSG00000273233	AC097724.1	0	0	0	0	0	0	0	0
ENSG00000171103	TRMT61B	2.24607	1.2462	1.20497	2.11645	4.16109	2.01263	2.37705	2.45128
ENSG00000163811	WDR43	9.53082	5.70161	2.86889	6.11097	10.9184	9.54966	7.07048	6.12329
ENSG00000264994	SNORD92	1.72471	2.87079	0	0	4.19373	1.52132	0	1.05879
ENSG00000265145	SNORD53	9.39748	4.69263	1.38095	5.57875	1.14252	8.28922	1.56641	0
ENSG00000222224	RF00019	0	0	0	0	0	0	0	0
ENSG00000265706	SNORD53B	0	0	0	1.39469	0	0	1.56641	2.30762
ENSG00000189350	TOGARAM2	0.08508	0.06773	0.02174	0.06588	0.03598	0.26755	0.0185	0.06812
ENSG00000200283	RF00019	0	0	0	0	0	0	0	0
ENSG00000179270	PCARE	0	0	0	0	0	0	0	0
ENSG00000229224	AC105398.1	0	0	0	0	0	0	0	0
ENSG00000115295	CLIP4	4.40273	3.27971	1.06675	3.02221	3.14057	3.22656	1.90146	2.56969
ENSG00000171094	ALK	0	0	0	0	0	0	0	0
ENSG00000242699	RN7SL516P	0	0	0	0	0	0	0	0
ENSG00000230737	AC106870.2	0	0	0	0	0	0	0	0
ENSG00000197644	AC106870.1	0	0	0	0	0	0	0	0
ENSG00000233862	AC016907.2	0.01738	0.02893	0.02554	0	0	0	0	0.02134
ENSG00000225943	AC016907.1	0	0	0	0	0	0	0	0
ENSG00000119801	YPEL5	77.1786	67.9053	29.0932	52.6916	49.8595	39.2736	52.1079	42.4599
ENSG00000207187	SNORA10B	0	0	0	0.81794	0	0.97227	0	0
ENSG00000231712	AC104698.1	0	0	0	0	0	0	0	0
ENSG00000213626	LBH	20.4142	15.658	5.27288	17.1035	20.2993	27.7728	20.5295	11.4109
ENSG00000235997	LINC01936	0	0	0	0	0.06038	0.0438	0	0
ENSG00000280154	AC073255.1	0	0	0	0	0	0	0	0
ENSG00000172954	LCLAT1	4.46608	2.48334	2.5363	6.15058	3.22466	5.41881	6.48529	3.28046
ENSG00000278908	AC132154.1	0	0	0	0	0	0	0	0
ENSG00000162949	CAPN13	0	0	0	0	0	0	0	0

ENSG00000230118	AC092569.1	0	0	0	0	0	0	0	0
ENSG00000158089	GALNT14	0.91485	1.33576	0.04717	0.26202	0.42929	0.05663	0.05351	0.23647
ENSG00000234579	AC009305.1	0	0	0	0	0	0	0	0
ENSG00000214711	CAPN14	0.01826	0.0304	0	0.0271	0.08881	0	0	0.04484
ENSG00000201671	RNA5SP90	0	0	0	0	0	0	0	0
ENSG00000013016	EHD3	3.60493	2.78967	0.34851	0.93862	3.19098	2.81719	1.6867	1.90244
ENSG00000213620	AL121657.1	0	0	0	0	0	0	0	0
ENSG00000158125	XDH	0	0	0	0.01802	0	0	0	0
ENSG00000277893	SRD5A2	0	0	0	0	0	0	0	0
ENSG00000228563	AL133247.1	0	0	0	0	0	0	0	0
ENSG00000270422	AL133247.2	0	0	0	0	0	0	0	0
ENSG00000223647	LINC01946	0	0	0	0	0	0	0	0
ENSG00000237007	KRT18P52	0	0.14422	0	0	0	0	0	0
ENSG00000242272	AK2P2	0	0	0.26596	0	0	0	0	0
ENSG00000273165	AL121652.1	0.65447	0	0.24043	0.9713	0.39784	0	0.54545	0.60266
ENSG00000162959	MEMO1	0.75828	0.32723	0.10317	0.18756	0.39266	0.37159	0.09362	0.27585
ENSG00000162961	DPY30	2.67032	3.15578	1.45188	3.13081	2.56474	3.86287	2.13649	3.08187
ENSG00000271228	AL121655.1	0	0	0.34972	0	0	0	0	0
ENSG00000021574	SPAST	21.4994	22.2344	11.5917	22.8715	22.1039	22.9452	18.0704	18.507
ENSG00000272716	AL121658.1	0.2059	0.68544	0.15128	0	0.62582	0	0	0
ENSG00000152683	SLC30A6	4.78281	5.18705	3.08607	6.5754	5.45242	5.68876	5.05885	4.59135
ENSG00000229816	DDX50P1	0.45813	0.20335	0.04488	0.13598	0.18566	0.10776	0.25454	0.1125
ENSG00000201113	RNU6-647P	0	0	0	0	0	0	0	0
ENSG00000091106	NLRC4	11.347	21.5804	2.64529	3.45209	9.56578	3.1757	1.48343	8.1206
ENSG00000119820	YIPF4	4.63782	4.55799	1.46166	2.88127	3.64975	2.26214	2.29719	4.49507
ENSG00000272754	AL133245.1	0.4833	0.44692	0.07891	0	0	0.09473	0.26853	0.1978
ENSG00000115760	BIRC6	18.5329	13.8716	4.98974	11.5458	16.6789	19.1892	10.5683	11.268
ENSG00000230046	BIRC6-AS1	0	0	0	0	0	0	0	0
ENSG00000276334	AL133243.2	1.67311	1.27955	0.19935	1.00665	1.42939	0.71796	0.67836	0.77727
ENSG00000276517	AL133243.3	0.75643	0.28431	0.502	0.86913	0.38566	0.90398	0.56942	0.5093
ENSG00000207653	MIR558	0.77979	0	0	0	3.7922	0	0	0
ENSG00000274159	AL133243.1	0.60082	0.66671	0.2943	0.89169	0.48698	0.70662	0.33383	0.49179
ENSG00000279897	BIRC6-AS2	0	0	0	0	0	0	0	0
ENSG00000279544	AL133243.4	0.40081	0.15697	0.06929	0.24493	0.28664	0.20796	0.27509	0.26053
ENSG00000018699	TTC27	5.0171	3.83072	2.23206	7.30931	5.3442	8.28245	5.98434	5.34048
ENSG00000265057	MIR4765	0	0	0	0	0	0	0	0
ENSG00000230876	LINC00486	0	0	0	0	0	0	0.05815	0.04284
ENSG00000236854	AL121656.1	0	0	0	0	0	0	0	0
ENSG00000206951	RF00019	0	0	0	0	0	0	0	0
ENSG00000049323	LTBP1	12.7018	9.62217	4.15924	7.288	10.0072	12.8624	9.20228	9.20969
ENSG00000223951	AL133244.1	0	0	0	0	0	0	0	0.69765
ENSG00000212204	RNA5SP91	0	0	0	0	0	0	0	0
ENSG00000252246	RNA5SP92	0	0	0	0	0	0	0	0
ENSG00000283591	MIR4430	0	0	0	0	0	0	0	0
ENSG00000152689	RASGRP3	1.69439	0.73526	0.41659	4.09977	2.54894	1.40733	4.32983	2.906
ENSG00000237133	AC020594.1	0	0	0.31868	0	0	0	0.36148	0.26626
ENSG00000119812	FAM98A	1.50444	1.23967	0.26266	0.88426	1.61174	2.60148	0.74485	0.951
ENSG00000238038	ATP6V0E1P3	0	0	0	0	0	0	0	0
ENSG00000187999	HNRNPA1P61	0	0	0	0.11773	0	0	0	0
ENSG00000203386		0	0	0	0	0	0	0	0.08264
ENSG00000239649	MYADML	0	0	0	0	0	0	0	0
ENSG00000235064	SLC25A5P2	0	0	0	0	0	0	0	0
ENSG00000237790	LINC01318	0	0	0	0	0	0	0	0
ENSG00000226785		0	0	0	0	0	0	0	0
ENSG00000228262	LINC01320	0	0	0	0	0	0	0	0
ENSG00000272027	AC073218.2	0	0	0	0	0	0	0	0
ENSG00000232153	AC073218.1	0	0	0	0	0	0	0	0
ENSG00000226994	AC012593.1	0	0	0	0	0	0	0	0

ENSG00000264352	RN7SL602P	0	0	0	0	0	0	0	0
ENSG00000237320	AC019064.1	0	0	0	0	0	0	0	0
ENSG00000196085	AC013442.1	0	0	0	0	0	0	0	0
ENSG00000265301	MIR548AD	0	0	0	0	0	0	0	0
ENSG00000207255	RNU6-1117P	0	0	0	0	0	0	0	0
ENSG00000229013	AC083939.1	0	0	0	0	0	0	0	0
ENSG00000234587	MRPL50P1	0	0	0	0	0	0	0	0
ENSG00000233752	AC009414.1	0	0	0	0	0	0	0	0
ENSG00000260025	CRIM1-DT	0	0	0	0	0	0	0	0
ENSG00000150938	CRIM1	1.04979	0.97648	0.27223	1.11505	0.82585	1.41622	0.51466	0.72028
ENSG00000273090	AC007378.1	0	0	0	0.22759	0.18644	0	0	0.18828
ENSG00000171055	FEZ2	3.00976	2.539	0.56641	0.98586	2.19352	1.54804	1.02523	1.90305
ENSG00000270677	AC007363.1	0	0	0	0	0	0	0	0
ENSG00000205221	VIT	0	0.06577	0	0.02932	0	0.06971	0	0.04852
ENSG00000279519	AC007382.1	1.59592	0.97868	0.12343	0.41553	1.39564	1.23483	1.12006	0.75628
ENSG00000115808	STRN	15.0993	13.2316	2.42663	4.58868	12.4518	13.6672	4.68517	6.55701
ENSG00000252756	RNU6-577P	0	0	0	0	1.81871	0	1.24674	0
ENSG00000008869	HEATR5B	8.39658	7.19621	3.46533	6.31026	8.54677	10.3059	6.01521	5.83456
ENSG00000152133	GPATCH11	6.65872	4.53002	1.01315	2.28881	7.41168	5.76142	2.35893	3.27465
ENSG00000055332	EIF2AK2	13.1972	39.168	8.09382	4.83582	8.79315	10.6547	3.95413	9.87599
ENSG00000224891	AC007899.1	0	0	0	0	0	0	0	0
ENSG00000138068	SULT6B1	0	0	0	0	0	0	0.09221	0
ENSG00000233159	AC007390.1	0	0	0	0	0	0	0	0
ENSG00000218739	CEBPZOS	4.29068	3.5839	2.1552	5.55738	3.69897	3.13783	4.26513	2.49034
ENSG00000115816	CEBPZ	13.4724	9.11113	2.34483	5.33587	12.4503	13.5049	6.80089	8.80379
ENSG00000272054	AC007390.2	0.40476	0.03208	0.02832	0.05721	0.09373	0.13601	0.38553	0.16565
ENSG00000003509	NDUFAF7	2.75052	1.83798	0.14751	1.10246	1.73303	2.12509	1.03741	1.2818
ENSG00000115825	PRKD3	5.70289	4.25196	1.66134	2.99469	5.47192	3.55973	2.96983	4.44538
ENSG00000232028	AC007391.1	0	0	0	0	0.17682	0	0	0
ENSG00000207364	RNU6-939P	0	0	0	0	0	0	0	0
ENSG00000115828	QPCT	28.4289	46.8036	5.67788	5.65066	26.9167	7.3139	7.80375	28.1176
ENSG00000253078	RNU6-1116P	0	0	0	0	0	0	0	0
ENSG00000236213	AC006369.1	2.40768	4.00758	0.78623	2.7792	0.48786	3.06762	0.22296	2.13497
ENSG00000163171	CDC42EP3	26.9998	26.2753	4.23362	3.26169	13.0087	8.0649	3.68678	8.46695
ENSG00000236572	AC006369.2	0.19089	0	0	0	0	0	0.31818	0.23437
ENSG00000225402	AC010878.1	0	0	0	0	0	0	0	0.08596
ENSG00000237803	LINC00211	3.0224	4.13747	1.24525	1.1738	2.16353	1.19595	1.36541	2.28894
ENSG00000115841	RMDN2	1.46625	1.58637	1.54416	3.26412	1.88662	1.18556	2.87172	1.59021
ENSG00000235848	RMDN2-AS1	0.04467	0	0	0	0	0	0	0
ENSG00000138061	CYP1B1	12.3467	5.45731	0.41194	1.10341	4.8011	0.36553	1.84875	2.75349
ENSG00000232973	CYP1B1-AS1	0.19686	0.13107	0.01446	0.04382	0.09573	0	0.08203	0.06043
ENSG00000280276	AC009229.4	0	0	0	0	0	0	0	0
ENSG00000229160	AC009229.2	0	0	0	0	0.25389	0	0	0
ENSG00000199603	RNU6-951P	0	0	0	0	0	0	0	0
ENSG00000273006	AC009229.3	0	0	0	0	0	0	0	0
ENSG00000227292	AC009229.1	0	0	0	0	0	0	0	0
ENSG00000227770	RPL7P12	0	0	0	0	0	0	0	0
ENSG00000213594	GAPDHP25	0	0	0	0	0	0	0	0
ENSG00000119787	ATL2	4.12727	3.72221	4.43294	9.02093	6.44107	4.9776	10.2567	6.78111
ENSG00000231367	LINC02613	0	0	0	0	0	0	0	0
ENSG00000235009	LINC01883	0	0	0	0	0	0	0	0
ENSG00000213553	RPLP0P6	0.38417	1.40681	0.45163	0.45612	0.84072	0.13555	0.12807	0.47168
ENSG00000271443	AC011247.2	0	0	0	0	0	0	0	0
ENSG00000143889	HNRNPLL	4.15818	4.26401	1.5276	2.79173	2.99413	6.81163	2.99108	3.3124
ENSG00000235586	AC011247.1	0	0	0	0	0	0	0	0
ENSG00000143891	GALM	2.60098	2.66757	0.96518	0.85781	2.26784	9.03792	0.61309	1.41931
ENSG00000232518	AC074366.1	0	0.11235	0	0	0	0	0	0
ENSG00000115875	SRSF7	13.2104	9.80291	3.08895	6.02423	12.3155	14.3538	7.09824	7.72039

ENSG00000152147	GEMIN6	1.27742	1.14706	0.19759	0.7733	1.45088	1.21573	0.3362	0.70165
ENSG00000227238	TTC39DP	0	0	0	0	0	0	0	0
ENSG00000232114	AC018693.2	0	0	0	0	0	0	0	0
ENSG00000163214	DHX57	2.68967	1.69605	0.40469	0.96729	2.07585	1.87854	0.97928	1.11581
ENSG00000223922	ASS1P2	0	0	0	0	0	0	0.0999	0
ENSG00000225284	AC018693.1	0	0	0	0	0	0	0	0
ENSG00000188010	MORN2	1.55844	2.68347	1.65835	5.6626	2.41739	1.42205	4.6579	2.30931
ENSG00000207295	RNU6-851P	0	0	0	0	0	0	0	0
ENSG00000214694	ARHGEF33	0.01335	0.02222	0.03924	0	0	0	0	0
ENSG00000265905	RN7SL96P	0	0	0	0	0	0	0	0
ENSG00000269210	AC019171.1	0.06941	0	0	0	0	0	0	0
ENSG00000115904	SOS1	8.20603	8.04784	3.10478	6.08345	7.78316	8.78086	4.62974	5.56713
ENSG00000229692	SOS1-IT1	0.70144	1.07025	0.25769	0.52051	0.92386	1.03119	0	0.78945
ENSG00000206985	RNU6-198P	0	0	0	0	0	0	0	0
ENSG00000225900	HSPE1P13	0.49361	1.23241	0.72535	0.73256	0	1.30618	0	1.21208
ENSG00000202309	RF00019	0	0	0	0	0	0	0	0
ENSG00000205111	CDKL4	0.10603	0.17648	0.46742	1.04904	0.17187	0.1247	0.47128	0.52072
ENSG00000011566	MAP4K3	3.41915	3.70027	3.40878	6.99298	4.52414	3.53812	6.16238	4.40566
ENSG00000252473	RF00272	1.6289	0	0	0	1.32025	0.95787	2.71512	0.66664
ENSG00000273035	AC007684.1	0.30734	0	0	0.45612	0.37365	0	0.51229	0
ENSG00000231312	MAP4K3-DT	0.11961	0.0181	0.01598	0.01614	0.1322	0.05755	0.05437	0.0267
ENSG00000152154	TMEM178A	0	0	0	0	0	0	0	0
ENSG00000138050	THUMPD2	1.9557	1.5638	1.26789	1.84961	2.58748	1.52211	2.01343	1.27121
ENSG00000227028	SLC8A1-AS1	0	0.0144	0	0	0	0	0	0.01062
ENSG00000235653	AC007253.1	0	0.13724	0	0	0	0	0	0
ENSG00000183023	SLC8A1	3.76499	4.55264	0.85986	1.31504	3.47572	2.57774	1.06452	2.63973
ENSG00000233128	LINC01794	0	0	0	0	0	0	0	0
ENSG00000237442	HNRNPA1P57	0	0	0	0	0	0	0	0
ENSG00000236090	LDHAP3	0.07272	0	0	0	0	0	0	0
ENSG00000236533	AC009413.1	0	0	0	0	0	0	0	0
ENSG00000207218	RF00019	0	0	0	0	0	0	0	0
ENSG00000223245	RNU4-63P	0	0	0.91283	0.92191	0	0	0	0
ENSG00000214691	LINC01913	0	0	0	0	0	0	0	0
ENSG00000234362	LINC01914	0	0	0	0	0	0	0	0
ENSG00000205086	C2orf91	0	0	0	0	0	0	0	0
ENSG00000226398	AC013480.1	0	0	0	0	0	0	0	0
ENSG00000162878	PKDCC	0.01868	0	0.08233	0.22173	0.0227	0.09884	0.34242	0.11465
ENSG00000224875	AC083949.1	0.23221	0	0.11374	0	0.56463	0.5462	0.12902	0.47517
ENSG00000143924	EML4	14.7706	11.1103	3.1836	5.19391	13.415	12.5799	6.44747	6.77374
ENSG00000115944	COX7A2L	5.45339	5.05013	2.78653	3.68765	5.13554	6.59825	5.10084	4.15865
ENSG00000221300	RF00612	0	0	0	0	0	0	0	0
ENSG00000171126	KCNG3	0	0	0	0	0.0233	0.03382	0	0.02353
ENSG00000232526	VDAC1P13	0	0	0	0	0	0	0	0
ENSG00000236913	AC025750.2	0	0	0	0	0	0	0	0.19999
ENSG00000057935	MTA3	0.80367	0.50741	0.29525	0.43185	1.18766	1.08778	0.55432	0.5359
ENSG00000273913	RF00017	0	0	0	0	0	0	0	0
ENSG00000215263	AC025750.1	0	0	0	0	0	0	0	0
ENSG00000226523	AC074375.1	0	0	0	0	0.46415	0	0	0
ENSG00000200550	RNU6-137P	0	0	0.99735	0	1.65031	0	0	0.83331
ENSG00000162881	OXER1	3.41602	2.4662	0.18144	1.40487	2.50187	1.30691	0.61742	1.81914
ENSG00000162882	HAAO	0.59157	0.22379	0	0.07981	0.52306	0.28462	0.08964	0.19809
ENSG00000226491	FTOP1	0	0	0	0	0	0	0	0
ENSG00000232202	AC098824.1	0	0	0	0	0	0	0	0
ENSG00000224739	AC016735.1	0	0	0	0	0.37602	0	0	0
ENSG00000231826	LINC01819	0.0797	0.04422	0	0	0	0	0.04428	0
ENSG00000233978	LINC02590	0	0	0	0	0	0	0	0
ENSG00000207087	RNU6-242P	2.05515	0	0	1.01669	0	1.20852	1.14187	0
ENSG00000230587	LINC02580	0.53897	0.6408	0.11314	0.34281	0.37444	0.4075	0	0.18907

ENSG00000152518	ZFP36L2	41.1124	24.4611	8.16015	26.0191	26.2335	36.0716	24.5947	16.6066
ENSG00000279873	LINC01126	1.02425	0.44475	0.06544	0.52873	1.02869	0.70705	0.5196	0.43741
ENSG00000234936	AC010883.1	0.55252	0.30655	0	0.27333	1.56738	0.97471	0	0.22612
ENSG00000115970	THADA	2.60314	2.01631	1.00745	2.70817	2.36266	3.1464	2.97288	1.98728
ENSG00000252804	RNU6-958P	0	0	0	0	0	0	0	0
ENSG00000264071	RN7SL531P	0	0	0	0	0	0	0	0
ENSG00000207411	RF00019	0	0	0	0	0	0	0	0
ENSG00000152527	PLEKHH2	0.01491	0	0	0.03318	0	0	0.04969	0.02745
ENSG00000223658	C1GALT1C1L	0	0	0	0	0	0	0	0
ENSG00000229695	AC011242.1	0	0	0	0	0	0	0	0
ENSG00000252490	RN7SKP66	0	0	0	0	0	0	0	0
ENSG00000138036	DYNC2LI1	0.5238	0.63408	1.11959	2.2379	1.21575	0.75605	1.50807	0.97442
ENSG00000138075	ABCG5	0	0	0	0	0	0	0	0
ENSG00000143921	ABCG8	0	0	0	0	0	0	0	0
ENSG00000138095	LRPPRC	7.78816	4.70033	2.06378	5.21636	7.38682	8.90343	4.7445	4.89636
ENSG00000200924	RNU6-1048P	0	0	0	0	0	0	0	0
ENSG00000264047	RN7SL455P	0	0	0	0	0	0	0	0
ENSG00000219391	AC019129.1	0	0	0	0	0	0	0	0
ENSG00000252599	RNU6-566P	0	0	0	0	0	0	0	0
ENSG00000224637	PDSS1P2	0	0	0	0	0	0	0	0
ENSG00000273106	AC019129.2	0.35612	0	0	0	0.07216	0.10471	0.09893	0.14574
ENSG00000138032	PPM1B	12.0636	12.711	6.0355	13.5082	13.5626	11.1416	11.4216	10.1616
ENSG00000226972	RPL12P19	0	0	0	0	0.36674	0	0.2514	0
ENSG00000138079	SLC3A1	0.04483	0	0.01647	0.01663	0.04087	0	0.03736	0.01376
ENSG00000138078	PREPL	3.74009	2.88822	0.83233	1.9214	4.56896	2.74389	1.12395	2.18563
ENSG00000143919	CAMKMT	5.56502	6.66446	10.3099	22.3514	10.2269	5.57295	11.4802	7.9376
ENSG00000259439	LINC01833	0	0	0	0	0	0	0	0
ENSG00000278957	AC012354.3	0	0	0	0	0	0	0	0
ENSG00000274520	RF02246	0	0	0	0	0	0	0	0
ENSG00000277311	RF02247	0	0	0	0	0	0	0	0
ENSG00000276229	RF02248	0	0	0	0	0	0	0	0
ENSG00000277809	RF02249	0	0	0	0	0	0	0	0
ENSG00000276048	RF02250	0	0	0	0	0	0	0	0
ENSG00000274547	RF02251	0	0	0	0	0	0	0	0
ENSG00000276670	RF02252	0	0	0	0	0	0	0	0
ENSG00000236502	SIX3-AS1	0	0	0	0	0	0	0	0
ENSG00000138083	SIX3	0	0.04836	0	0	0	0	0	0
ENSG00000225156	AC012354.1	0	0	0	0	0	0	0	0
ENSG00000231848	AC012354.2	0	0.26466	0	0	0	0	0	0
ENSG00000170577	SIX2	0	0	0	0	0	0	0	0
ENSG00000231156	AC093702.1	0	0	0	0	0	0	0	0
ENSG00000205054	LINC01121	0	0	0	0	0	0	0	0
ENSG00000231054	AC093833.1	0	0	0	0	0	0	0	0
ENSG00000068784	SRBD1	11.3764	11.0763	6.25574	11.9244	9.29145	13.2259	8.23415	9.1335
ENSG00000239396	RN7SL414P	0.24932	0	0	0	0	0	0	0.30611
ENSG00000171132	PRKCE	1.67937	1.08364	0.3805	0.6478	1.07933	0.82223	0.45626	0.654
ENSG00000228481	U51244.1	0	0	0	0	0	0	0	0
ENSG00000233829	AC017078.1	0	0	0	0	0	0	0	0
ENSG00000232696	AC017006.2	0	0	0	0	0	0	0	0
ENSG00000231336	AC017006.1	0	0	0	0	0	0	0	0
ENSG00000233335	AC016696.1	0	0	0	0	0	0	0	0
ENSG00000278764	RF00017	0	0	0	0	0	0	0	0
ENSG00000116016	EPAS1	1.18453	0.50127	0.14751	0.17878	0.58581	0.35418	0.20079	0.32045
ENSG00000228100	LINC01820	0	0	0	0	0	0	0	0
ENSG00000187600	LINC02583	0	0	0	0	0	0	0	0
ENSG00000241791	RN7SL817P	0	0	0	0	0	0	0	0
ENSG00000250565	ATP6V1E2	0.63824	0.4157	0.04078	0.26769	0.50606	0.3182	0.13876	0.25553
ENSG00000253515	AC018682.2	0	0	0	0	0	0	0	0

ENSG00000119729	RHOQ	20.2968	16.3241	2.86833	5.39336	13.3704	7.75479	6.53357	9.60566
ENSG00000250116	AC018682.1	0	0	0	0	0	0	0	0
ENSG00000151665	PIGF	0.86877	0.78431	0.82224	1.90124	0.76979	0.90918	1.37447	1.17513
ENSG00000119878	CRIPT	4.465	3.81128	1.09354	2.88849	3.37537	2.7771	4.00748	3.61956
ENSG00000279254	AC020604.1	0.0384	0	0	0	0.04668	0	0.19201	0.04714
ENSG00000171150	SOCS5	3.40729	1.89048	0.70611	1.53432	3.43437	1.74676	1.79606	1.48386
ENSG00000260977		0.02976	0	0	0	0.03618	0	0	0.03654
ENSG00000222005	LINC01118	0	0	0	0	0.03931	0	0	0
ENSG00000239332	LINC01119	0.0259	0	0	0	0.06298	0	0	0
ENSG00000226548	AC016722.1	0	0	0	0	0	0	0	0
ENSG00000228925	AC016722.2	0	0	0	0	0	0	0	0
ENSG00000180398	MCFD2	6.86044	4.33564	1.34777	2.81311	5.76865	6.47206	2.30337	3.18308
ENSG00000068724	TTC7A	6.31845	4.72302	0.75953	1.51852	3.75755	3.34956	1.33626	1.26921
ENSG00000272814	AC093732.2	0	0	0	0	0	0	0	0
ENSG00000233845	AC093732.1	0	0.24208	0	0	0	0	0	0
ENSG00000239605	STPG4	0.06911	0.03834	0	0	0	0	0	0
ENSG00000273269	AC073283.3	0.11014	0	0	0.08173	0.06695	0.09715	0	0.06762
ENSG00000225187	AC073283.1	1.27849	0.28374	0	0	0.93262	0.75181	0.14207	0
ENSG00000143933	CALM2	53.0538	42.4369	18.0745	32.2353	33.753	26.9983	29.0618	35.9933
ENSG00000234690	EPCAM-DT	0.02064	0	0	0	0	0	0	0
ENSG00000226087	AC073283.2	0	0	0	0	0	0	0	0
ENSG00000236824	BCYRN1	0	0.61004	0	0	0	0	0	0
ENSG00000119888	EPCAM	2.38612	4.02013	3.97674	15.1583	4.03307	2.20739	5.09287	4.25155
ENSG00000222685	RN7SKP119	0	0	0	0	0	0	0.44755	0
ENSG00000207923	MIR559	0	0	0	0	0	0	0	0
ENSG00000277396	RF00017	0	0	0	0	0	0	0	0
ENSG00000095002	MSH2	3.41381	3.27	3.99905	6.78716	5.99071	6.22128	6.36132	4.32982
ENSG00000184261	KCNK12	0	0	0	0.01627	0.01333	0	0	0.01346
ENSG00000235760	HCG2040054	0	0.16012	0	0.14276	0.11695	0	0	0.23621
ENSG00000230979	AC079250.1	0.27608	0.45954	0.20285	0.20487	0	0.24353	0.23009	0
ENSG00000116062	MSH6	2.84053	2.6304	0.77407	1.31616	4.06144	4.3053	1.11144	1.30988
ENSG00000224443	AC006509.2	0	0	0	0	0	0	0	0
ENSG00000224058	AC006509.1	0	0	0	0	0	0	0	0
ENSG00000283502	RF00026	0	0	0	0	0	0	0	0
ENSG00000138081	FBXO11	16.289	14.1947	5.49685	9.53451	13.1269	11.2787	9.93125	8.76562
ENSG00000235369	RPL36AP15	1.10226	0	0	0.40897	0.67005	1.4584	1.37797	1.015
ENSG00000236549	RPS27AP7	0	0	0	0	0	0	0	0
ENSG00000233230	AC079807.1	0.85631	0.4276	0.12583	0.25417	0.72876	0.60426	0.4282	0.52568
ENSG00000226473	PPIAP62	0	0	0	0	0	0	0	0
ENSG00000230773	AC092650.1	0.58518	0.2541	0.26171	0.5664	0.64958	0.49373	0.59373	0.68724
ENSG00000275423	AC092650.2	0	0	0	0	0	0	0	0
ENSG00000201010	RN7SKP224	0	0	0	0	0	0	0.37594	0
ENSG00000170802	FOXN2	24.3265	26.1335	11.3197	16.1759	24.5514	26.707	14.3335	21.4936
ENSG00000251889	RNU4-49P	0	0	0	0	0	0	0	0
ENSG00000272663	AC093635.1	1.32073	0.21984	0	0.19601	0.16057	0	0.22014	0.16216
ENSG00000162869	PPP1R21	6.80712	6.96319	1.96824	4.68364	5.91137	6.27947	5.90258	5.51922
ENSG00000202227	RNU6-282P	0	0	0	0	0	0	0	0
ENSG00000243244	STON1	0.19017	0.18992	0.09781	0.08467	0.1734	0.16774	0.09509	0.15177
ENSG00000068781	STON1-GTF2A1L	0	0	0	0	0	0	0	0
ENSG00000242441	GTF2A1L	0	0	0	0	0	0	0	0
ENSG00000279956	AC073082.1	0	0	0	0	0	0	0	0
ENSG00000239995	TPT1P11	0	0	0	0	0	0	0	0
ENSG00000138039	LHCGR	0	0	0	0	0	0	0	0
ENSG00000232285	ELOBP3	0	0	0	0	0	0	0	0
ENSG00000282890	AC009975.1	0	0	0	0	0	0	0	0
ENSG00000214602	CTBP2P5	0	0	0	0	0	0	0	0
ENSG00000170820	FSHR	0	0	0	0	0	0	0	0
ENSG00000212036	MIR548BA	0	0	0	0	0	0	0	0

ENSG00000282998	AC009975.2	0	0	0	0	0	0	0	0
ENSG00000206915	RNU6-439P	0	0	0	0	0	0	0	0
ENSG00000282828	AC009971.1	0.10892	0.18129	0	0.96986	0.66209	0.19214	0.36309	0.26745
ENSG00000234253	RPL7P13	0	0	0	0	0	0	0	0
ENSG00000212580	RF00072	0	0	0	0	0	0	0	0
ENSG00000179915	NRXN1	0.1085	0.08529	0.06201	0.07604	0.05863	0	0.06531	0.08141
ENSG00000230327	MTCO1P42	0	0	0	0	0	0	0	0
ENSG00000283058	AC009234.1	0	0	0	0	0	0	0	0
ENSG00000216191	MIR8485	0	0	0	0	0	0	0	0
ENSG00000238165	AC007560.1	0	0	0	0.45901	0.37602	0	0	0
ENSG00000231918	AC007402.1	0	0	0	0	0	0	0	0
ENSG00000224344	KNOP1P3	0	0	0	0	0	0	0	0
ENSG00000223710	CRYGGP	0	0	0	0	0	0	0	0
ENSG00000230840	AC097463.2	0	0	0	0	0	0	0	0
ENSG00000226366	AC097463.1	0	0	0	0	0	0	0	0
ENSG00000236538	ZNF863P	0	0	0	0	0	0	0	0
ENSG00000202195	RF00019	0	0	0	0	0	0	0	0
ENSG00000225444	LINC01867	0	0	0	0	0	0	0	0
ENSG00000225842	AC139712.2	0	0	0	0	0	0	0	0
ENSG00000236837	AC139712.3	0	0	0	0	0	0	0	0
ENSG00000204993	AC139712.1	0	0	0	0	0	0	0	0
ENSG00000233083	FTH1P6	0	0	0	0	0	0	0	0
ENSG00000264975	MIR4431	0	0	0	0	0	0	0	0
ENSG00000228033	AC010967.1	0.1499	0	0	0	0	0	0	0
ENSG00000232604	AC010967.2	0	0	0	0	0	0	0	0
ENSG00000232668	AC010967.3	0	0	0	0	0	0	0	0
ENSG00000251942	RF00424	0	0	0	0	0	0	0	0
ENSG00000223897	AC069157.1	0	0	0	0	0	0	0	0
ENSG00000115239	ASB3	0.38402	0.27117	0.1026	0.24178	0.3254	0.61587	0.25216	0.30004
ENSG00000207456	RNU6-997P	0	0	0	0	0	0	0	0
ENSG00000270898	GPR75-ASB3	0	0	0	0	0	0.05526	0	0.07692
ENSG00000143942	CHAC2	0.4994	1.10833	1.38617	4.52931	1.88892	1.37045	2.58974	1.7032
ENSG00000068912	ERLEC1	10.2372	8.47281	2.74272	5.62395	9.11108	10.8259	4.47806	6.2498
ENSG00000265452	MIR3682	0	0	0	1.29507	2.12183	0	1.45453	0
ENSG00000119737	GPR75	0.51986	0.34612	0	0.15431	0.42136	0.55026	0.34661	0.29786
ENSG00000068878	PSME4	13.4776	13.8199	8.15138	15.8276	12.9398	17.7913	32.1646	12.2855
ENSG00000170634	ACYP2	1.37327	2.28582	2.2133	6.24576	2.65698	1.74534	4.40578	5.62028
ENSG00000235937	AC008280.1	0	0	0	0	0	0	0	0
ENSG00000233266	HMGB1P31	1.54497	0.28573	0	0	0.83482	0.90851	0.28614	0.21077
ENSG00000241114	AC008280.2	0	0	0.35086	0	0	0	0	0
ENSG00000272156	AC008280.3	0.40055	0	0.2943	0	0.60872	0.17666	0.33383	0.12295
ENSG00000252934	RNU7-172P	0	0	0	0	0	0	0	0
ENSG00000178021	TSPYL6	0	0	0	0	0	0	0	0
ENSG00000177994	C2orf73	0	0	0	0	0	0	0	0
ENSG00000115306	SPTBN1	10.5094	5.2749	1.1584	3.46275	15.291	11.7543	4.0872	5.23816
ENSG00000228108	AC092839.1	0	0	0	0	0	0	0	0
ENSG00000237887	RPL23AP32	0	0	0	0	0	0	0	0
ENSG00000234943	AC092839.2	0	0	0	0	0	0	0	0
ENSG00000238018	AC093110.1	0	0	0	0	0	0	0	0
ENSG00000214595	EML6	0.07622	0.07612	0.01493	0.0377	0.1112	0.03586	0.07623	0.08734
ENSG00000231334	AC104781.1	0	0	0	0	0	0	0	0
ENSG00000252507	RNU7-81P	0	0	0	0	0	0	0	0
ENSG00000115310	RTN4	22.2306	19.7594	6.67943	13.9339	13.7082	9.86771	8.90055	11.4325
ENSG00000200086	RNU6-433P	0	0	1.03571	0	0	0	0	0.86536
ENSG00000162994	CLHC1	1.23334	3.60644	2.76755	3.24032	4.33628	1.05849	2.38916	1.71891
ENSG00000203327	AC012358.1	0.08002	0.1332	0	0	0.09729	0.14117	0	0
ENSG00000227799	AC012358.2	0.20446	0.08508	0	0	0	0.18035	0.1704	0
ENSG00000143947	RPS27A	79.7639	56.7944	23.6764	39.6911	89.4014	65.4409	57.6551	101.067

ENSG00000085760	MTIF2	6.40889	3.32685	1.62816	4.28823	6.15417	6.97532	3.00562	3.94771
ENSG00000162997	PRORSD1P	0.59864	0.22143	0.04887	0.24679	0.64694	0.52804	0.44349	0.245
ENSG00000240401	AC012358.3	0.10823	0.10294	0.06816	0.09178	0.0188	0.05455	0.07731	0.07593
ENSG00000206964	RF00019	0	0	0	0	0	0	0	0
ENSG00000115355	CCDC88A	9.29301	6.04293	2.6897	4.273	5.25983	3.00663	3.68129	4.55031
ENSG00000233594	BTF3P5	0	0	0	0	0	0.2769	0	0.19271
ENSG00000238619	RNU6-775P	0	0	0	0	0	0	0	0
ENSG00000238493	RNU6-221P	0	0	0	0	0	0	0	0
ENSG00000239189	RNU6-634P	0	0	0	0	0	0	0	0
ENSG00000163001	CFAP36	1.61031	1.39239	0.67609	1.11734	2.23745	1.36506	1.32464	1.28384
ENSG00000275052	PPP4R3B	19.6699	18.9019	7.32775	13.1729	20.0531	17.5626	9.01074	15.0269
ENSG00000212175	RF00586	0	0	0	0	0	0	0	0
ENSG00000272606	AC015982.1	4.88669	3.41099	2.54807	6.55054	6.32441	4.72753	2.36478	3.87084
ENSG00000138035	PNPT1	6.38201	9.17321	6.62984	12.5652	8.24511	6.47315	7.87723	7.60785
ENSG00000115380	EFEMP1	0	0.03079	0	0	0.17994	0.03264	0	0
ENSG00000202344	RN7SKP208	0	0	0	0	0	0	0	0
ENSG00000272180	AC011306.1	0	0	0	0	0	0	0	0
ENSG00000226702	MIR217HG	0	0	0	0	0	0	0	0
ENSG00000207548	MIR217	0	0	0	0	0	0	0	0
ENSG00000207798	MIR216A	0	0	0	0	0	0	0	0
ENSG00000211520	MIR216B	0	0	0	0	0	0	0	0
ENSG00000229805	LINC01813	0	0	0	0	0	0	0	0
ENSG00000271894	AC007744.1	0	0	0	0	0	0	0	0
ENSG00000233251	AC007743.1	0	0	0	0	0	0	0	0
ENSG00000055813	CCDC85A	0	0.03064	0	0	0.02238	0	0	0
ENSG00000199395	RNA5SP93	0	0	0	0	0	0	0	0
ENSG00000231570	PPIAP63	0	0	0	0	0	0	0	0
ENSG00000271115	AC010738.1	0	0	0	0	0	0	0	0
ENSG00000270569	AC068276.1	0	0	0	0	0	0	0	0
ENSG00000212168	RF00592	0	0	0	0	0	0	0	0
ENSG00000271615	ACTG1P22	0	0	0	0	0	0	0	0.05291
ENSG00000028116	VRK2	21.9128	26.3906	32.3541	66.1891	36.114	31.508	47.7718	45.909
ENSG00000115392	FANCL	16.0458	23.3192	53.2764	106.666	41.7873	37.2066	135.908	78.878
ENSG00000273063	AC007250.1	0	0	0	0	0	0	0	0
ENSG00000233426	EIF3FP3	0.0675	0.89877	0.09918	0.20034	0.16412	0.23814	0.22501	0.33148
ENSG00000225226	LINC01795	0	0	0	0	0	0	0	0
ENSG00000233723	LINC01122	0	0	0	0.01965	0	0	0	0
ENSG00000231043	AC007238.1	0	0.07008	0	0.24994	0.10237	0.07427	0.14036	0.20677
ENSG00000222030	LINC01793	0	0	0	0	0	0	0	0
ENSG00000271955	AC007100.1	0	0	0	0	0	0	0	0
ENSG00000233891	AC007179.2	0	0	0	0	0	0	0	0
ENSG00000231815	AC007179.1	0	0	0	0	0	0	0	0
ENSG00000270447	AC007179.3	0	0	0	0	0	0	0	0
ENSG00000200101	RNU6-508P	0	0	0	0	0	0	0	0
ENSG00000252726	RNA5SP94	0	0	0	0	0	0	0	0
ENSG00000228590	MIR4432HG	0.25591	0.24341	0.05372	0.16277	0.13334	0	0.06094	0
ENSG00000223929		0.42127	0	0	0	0.25608	0	0.23406	0
ENSG00000200807	RNU1-32P	0	0	0	0	0	0	0	0
ENSG00000266078	MIR4432	0	0	0	0	0	0	0	0
ENSG00000119866	BCL11A	1.7242	0.9178	0.32153	0.80534	1.71317	0.98817	0.94827	0.5158
ENSG00000233953	AC009970.1	0	0	0	0	0	0	0	0
ENSG00000242158	RN7SL361P	0	0	0	0	0	0	0	0
ENSG00000271134	AC012498.2	0	0	0	0	0	0	0	0
ENSG00000213495	AC012498.1	0	0	0	0	0	0	0	0
ENSG00000252718	RNU6-612P	0	0	0	0	0	0	0	0
ENSG00000271707	ATP1B3P1	0	0	0	0	0	0	0	0
ENSG00000115421	PAPOLG	7.89826	9.41508	4.09265	8.67624	9.18318	7.81864	6.03644	7.73884
ENSG00000228414	LINC01185	0	0.06013	0	0	0	0	0	0

ENSG00000241524	RN7SL632P	0	0	0	0	0	0	0	0
ENSG00000237024	RPL21P33	0	0	0	0	0	0	0	0
ENSG00000162924	REL	8.58373	6.8186	2.2586	4.58147	5.81178	6.5374	4.78734	4.27796
ENSG00000201076	RNU4-51P	0.52357	0	0.76939	0.77704	0	0	0	0
ENSG00000267520	AC010733.2	0	0	0	0	0	0	0	0
ENSG00000237522	NONOP2	0.47636	0	0	0.08837	0.36197	0.10505	0.19851	0.07311
ENSG00000232713	AC010733.1	0.54838	0	0	0.54257	0	0.32247	0	0
ENSG00000162927	PUS10	0.76644	0.89302	0.3942	0.48343	0.72216	1.03098	0.31938	0.72929
ENSG00000222251	RNA5SP95	0	0	0	0	0	0	0	0
ENSG00000162928	PEX13	2.54257	2.68377	0.92951	2.44814	2.48803	1.9911	1.61253	2.3451
ENSG00000162929	KIAA1841	0.99972	0.85491	1.23995	3.98827	1.77297	1.34295	3.04228	2.39857
ENSG00000274769	AC016747.4	0	0	0	0.15732	0.06444	0	0	0
ENSG00000212978	AC016747.1	0.87234	0.15697	0.17323	0.34991	0.51595	0.45752	0.19649	0.40526
ENSG00000237651	C2orf74	0.33868	0.56374	0	0.07181	0.17647	0.34142	0.56453	0.17821
ENSG00000271889	AC016747.2	0	0	0	0	0	0	0	0
ENSG00000173209	AHSA2P	4.63483	3.62445	1.77339	3.11484	4.67804	5.67725	5.5828	3.34993
ENSG00000115464	USP34	17.5597	15.0247	7.65438	16.2893	15.7435	14.5685	14.756	13.5207
ENSG00000273302	AC016747.3	0	0	0	0.13753	0	0	0	0
ENSG00000271146	AC018889.1	0	0	0	0	0	0	0	0
ENSG00000234624	AC016894.1	0.94277	0.78462	0	0	0	0.41579	0.39286	0.57876
ENSG00000206937	SNORA70B	0.54297	0	0	0.80582	0	0	0	0
ENSG00000270820	AC016727.1	0.89618	0.99447	0.27436	0.6096	1.36195	0.85637	0.7469	0.82524
ENSG00000082898	XPO1	45.0082	60.3894	63.1674	155.011	67.8879	70.4654	114.802	83.0713
ENSG00000271243	AC016727.2	0	0	0	0	0	0	0	0
ENSG00000226884	RPS29P10	0	0	0	0	0	0	0	0
ENSG00000206973	RNU6-1145P	0	0	0	0	0	0	0	0
ENSG00000213486	AC108039.1	0	0	0	0	0	0	0	0
ENSG00000227361	RPS24P7	0	0	0	0	0	0	0	0
ENSG00000229831	AC108039.2	0	0	0	0	0	0	0	0
ENSG00000170264	FAM161A	0.19539	0.16262	0.04102	0.08285	0.20362	0.12311	0.04653	0.06854
ENSG00000230702	AC107081.1	0	0	0	0	0	0	0	0
ENSG00000277498	AC107081.3	0	0	0	0	0	0	0	0
ENSG00000115484	CCT4	18.882	15.0894	5.25248	10.7248	21.7596	25.9081	13.0815	10.2717
ENSG00000236498	AC107081.2	0	0	0	0	0	0	0	0
ENSG00000173163	COMMD1	1.93057	1.37719	1.1708	1.77368	2.04909	2.27052	1.78776	1.3921
ENSG00000278523	RF00019	0	0	0	0	0	0	0	0
ENSG00000229839	AC018462.1	0	0	0	0	0.09708	0	0	0
ENSG00000242735	RPSAP26	0	0	0	0	0	0	0	0
ENSG00000271657	AC018462.2	0.18557	0	0	0	0	0	0	0
ENSG00000170340	B3GNT2	12.426	13.9877	3.8402	8.06102	12.7399	14.2374	6.49123	9.94026
ENSG00000266097	MIR5192	0.79674	5.30472	0	0	0	4.21669	1.32805	0
ENSG00000239958	RN7SL51P	0	0	0	0	0	0	0	0
ENSG00000270335	AC093159.2	0	0	0	0	0	0	0	0
ENSG00000228541	AC093159.1	0.17288	0.28776	0	0.51314	0.21018	0	0.28816	1.06129
ENSG00000241625	RN7SL18P	0	0	1.11045	0.74767	0	0.88874	0	0
ENSG00000186889	TMEM17	0.1229	0.04091	0.03612	0.14592	0.02988	0.21682	0.16389	0
ENSG00000233702	AC107083.1	0	0	0	0	0	0	0	0.32257
ENSG00000229503	AC092155.2	0	0	0	0	0	0	0	0
ENSG00000232144	PSAT1P2	0	0	0	0	0	0	0	0
ENSG00000230595	RSL24D1P2	0	0	0	0	0	0	0	0
ENSG00000226622	AC092155.1	0	0	0	0	0	0	0	0
ENSG00000115504	EHBP1	2.6666	2.18217	1.4416	2.40893	3.00345	3.25679	3.00285	2.15713
ENSG00000252436	RF00019	0	0	0	0	0	0	0	0
ENSG00000226605	AC092567.1	0	0	0	0	0	0	0	0
ENSG00000237162	AC007098.2	0.19973	0	0	0	0.24282	0.35235	0.33292	0.24522
ENSG00000231609	AC007098.1	0	0	0	0	0	0	0	0
ENSG00000115507	OTX1	0.10617	0.05049	0	0.02251	0.14751	0	0.05056	0.11173
ENSG00000270437	AC009501.2	0	0	0	0	0	0	0	0

ENSG00000232403	AC009501.1	0.23494	0	0	0	0	0	0	0
ENSG00000242412	DBIL5P2	0	0.07211	0	0	0	0.07643	0	0.05319
ENSG00000143951	WDPCP	0.50419	0.56984	0.49393	0.92379	0.67352	0.73572	0.65365	0.5579
ENSG00000283636	RF00017	0	0	0	0	0	0	0	0
ENSG00000233716	AC074367.1	0.32578	0.10845	0.09575	0	0.39607	0	0	0
ENSG00000279485	AC016734.2	0	0	0	0	0	0	0	0
ENSG00000014641	MDH1	3.46671	2.56731	1.12247	2.39808	3.62539	5.33832	2.66887	3.06603
ENSG00000228305	AC016734.1	0.11208	0.74623	0	0	0	0.19772	0	0
ENSG00000229920	RPS4XP5	0	0	0	0.06868	0.05626	0	0	0
ENSG00000234488	AC096664.2	0	0	0	0	0	0	0	0
ENSG00000228872	AC096664.1	0	0	0	0	0	0	0	0
ENSG00000229221	HNRNPA1P66	0	0	0	0	0	0	0	0
ENSG00000169764	UGP2	17.6007	16.4405	7.62166	14.7256	17.8891	14.9581	13.231	13.1099
ENSG00000251775	RF01293	0	0	0	0	0.62319	0	0	0
ENSG00000143952	VPS54	6.85346	5.99138	3.06787	6.45321	6.2667	7.84862	5.01585	5.40937
ENSG00000228079	AC012368.2	1.57072	3.48596	0.19235	0.58278	0.79568	0	0.21818	2.08922
ENSG00000197329	PELI1	60.5772	48.1527	8.51267	14.5048	26.7242	13.1349	32.3148	31.0899
ENSG00000225889	AC012368.1	10.5782	12.0865	1.93209	4.03567	5.59477	2.89937	3.73564	7.26434
ENSG00000230923	LINC00309	0	0	0.06514	0.0329	0.08084	0.15641	0	0.02721
ENSG00000270354	AC012368.3	0	0	0	0	0.81385	0	0	0.41095
ENSG00000238012	AC114752.1	0	0	0	0	0	0	0	0
ENSG00000238201	AC114752.2	23.2239	6.34203	13.5976	41.7371	5.07347	2.88071	25.1014	25.8407
ENSG00000264297	MIR4433B	0	0	0	0	0	0	0	0
ENSG00000283222	MIR4433A	0	0	1.3298	0	0	0	0	0
ENSG00000236148	RPL23AP37	0.13352	0.66671	0	0.19815	0	0.23554	0	0
ENSG00000223935	LGALSL-DT	0.10138	0	0	0	0.12326	0	0	0.12448
ENSG00000237217	AC008074.1	0	0	0	0	0.20773	0	0	0
ENSG00000119862	LGALSL	14.3561	10.5458	3.229	5.38086	9.26119	6.29923	6.95906	7.93629
ENSG00000223863	LINC01805	0	0	0	0	0	0	0	0
ENSG00000260101	AC008074.2	1.19156	0.57581	0.11297	0.22818	0.7477	0.47466	0.06407	0.37754
ENSG00000119844	AFTPH	28.3313	26.1492	6.87337	13.7138	24.6628	16.1892	15.5082	17.603
ENSG00000283204	MIR4434	12.4472	13.8123	2.03234	2.05256	5.04434	2.43985	2.30529	3.39612
ENSG00000252414	RNU6-100P	2.07454	0	0	0	0	0	1.15264	1.69806
ENSG00000233694	LINC02579	0	0	0	0.05639	0	0	0.06334	0.04665
ENSG00000179833	SERTAD2	6.21546	3.61708	1.39063	2.27143	6.51969	3.52444	3.97271	2.26642
ENSG00000226756	AC007365.1	1.28597	0.58377	0.17179	0	0.71066	1.44367	0.38973	0.86122
ENSG00000227791	AC007365.2	0	0	0	0	0	0	0	0
ENSG00000275588	RN7SL341P	0	0	0	0	0	0	0	0
ENSG00000199964	RF00019	0	0	0	0	0	0	0	0
ENSG00000234572	LINC01800	0.03708	0	0	0	0	0.06541	0	0.04552
ENSG00000237638	LINC02245	0.0751	0.08334	0	0	0.06087	0	0	0.06147
ENSG00000244534	RN7SL211P	0	0	0	0	0	0	0	0
ENSG00000227394	AC007386.1	0.12595	0	0	0	0	0	0.20993	0
ENSG00000115902	SLC1A4	1.17255	0.84398	0.32598	0.79955	0.61646	2.73942	0.72192	0.57067
ENSG00000252892	RNU6-548P	0	0	0	0	0	1.12445	0	0
ENSG00000232613	LINC02576	0	0	0	0.28805	0.15731	0.11413	0.21568	0.15887
ENSG00000011523	CEP68	1.5046	1.08949	0.27481	0.89572	1.77758	1.1847	1.03435	0.77233
ENSG00000138069	RAB1A	24.5659	24.8069	6.89866	13.3816	18.9347	14.7672	14.4911	15.6145
ENSG00000272025	RF00090	0	0	0	0	0	0	0	0
ENSG00000273763	AC007318.2	0	0	0	0	0	0	0	0
ENSG00000172974	AC007318.1	0	0.27698	0.24453	0.24696	0.10115	0.44034	0.13868	0.10215
ENSG00000138071	ACTR2	192.584	152.995	23.6639	59.7858	123.23	95.6973	51.9133	89.7072
ENSG00000198369	SPRED2	0.73955	0.49712	0.35528	0.16886	0.39769	0.22581	0.26077	0.17462
ENSG00000232693	AC012370.1	0.41491	0	0	0	0.16814	0	0	0
ENSG00000204929	AC007389.1	0	0	0	0	0	0	0	0
ENSG00000234255	AC012370.2	0	0	0	0	0	0	0	0
ENSG00000237979	AC007389.4	0	0	0	0	0	0	0	0
ENSG00000223739	AC007389.2	0	0	0	0	0	0	0	0

ENSG00000243029	RN7SL635P	0	0	0	0	0	0	0	0
ENSG00000251900	VTRNA2-2P	0	0	0	0	0	0	0	0
ENSG00000235725	AC007389.3	0	0	0	0	0	0	0	0
ENSG00000281920	AC007389.5	0	0	0	0	0	0	0	0
ENSG00000214533	KRT18P33	0	0	0	0	0	0	0	0
ENSG00000280257	AC007741.1	0	0	0	0	0	0	0	0
ENSG00000227293	AC118345.1	0	0	0	0	0	0	0	0
ENSG00000225815	AC092669.1	0	0	0	0	0	0	0	0
ENSG00000264525	MIR4778	0	0	0	0	0	0	0	0
ENSG00000232164	LINC01873	0	0	0	0	0	0	0	0
ENSG00000226819	MEIS1-AS3	0	0.34514	0	0	0.12605	0	0	0
ENSG00000143995	MEIS1	2.5877	1.06007	0.65018	1.15412	1.04325	1.13535	1.03921	0.97948
ENSG00000230749	MEIS1-AS2	0	0	0	0	0	0.12567	0.11874	0.08746
ENSG00000232046	LINC01798	0.22807	0	0	0	0.05546	0	0.07603	0
ENSG00000237179	LINC01797	0	0	0	0	0	0	0	0
ENSG00000224071	AC009474.1	0	0	0	0	0	0	0	0
ENSG00000230355	AC009474.2	0	0	0	0	0	0	0	0
ENSG00000230525	LINC01799	0	0	0	0	0	0	0	0
ENSG00000232688	LINC01628	0	0	0	0	0	0	0	0
ENSG00000223859	AC007403.1	0	0	0	0	0	0	0	0
ENSG00000235885	LINC01828	0	0	0	0	0	0	0	0
ENSG00000236780	LINC01829	0	0	0	0	0	0	0.04382	0
ENSG00000236605	AC023115.1	0	0	0	0	0	0	0	0
ENSG00000143971	ETAA1	4.18439	2.30997	1.45225	2.46531	3.32334	4.97068	3.32964	2.65912
ENSG00000224173	AC007422.1	0	0	0	0	0	0	0	0
ENSG00000235495	AC007422.2	0	0	0	0	0	0	0	0
ENSG00000237013	LINC01812	0	0	0	0	0	0	0	0
ENSG00000235968	AC079112.1	0	0	0	0	0	0	0	0
ENSG00000197223	C1D	4.87168	4.53145	2.31611	6.71367	3.48404	3.06939	4.36724	3.56872
ENSG00000243667	WDR92	0.28752	0.20938	0.05281	0.18669	0.37141	0.38042	0.02995	0.30889
ENSG00000236259	PPIAP64	0	0	0	0	0	0	0	0
ENSG00000273398	AC017083.3	0	0	0	0	0	0	0	0
ENSG00000115946	PNO1	1.67215	1.55134	0.80564	1.58663	2.49953	2.7081	2.42167	1.27894
ENSG00000221823	PPP3R1	36.988	37.2592	28.5059	45.8692	37.7348	25.0703	47.1936	30.8018
ENSG00000273275	AC017083.2	0.31414	0	0.15388	0.62163	0.12731	0	0	0.3857
ENSG00000273064	AC017083.1	0.22462	0.62313	0.22005	0	0.18206	0.13209	0	0.27578
ENSG00000119865	CNRIP1	0.08264	0.06878	0.18215	0.24529	0.22606	0	0.17218	0.0761
ENSG00000203395	AC015969.1	0.1553	0	0.07607	0.38413	0.12587	0.18264	0	0
ENSG00000115956	PLEK	111.632	133.385	29.545	29.3137	70.535	79.8346	38.9694	82.7734
ENSG00000229462	AC127383.1	0	0	0	0	0	0	0	0
ENSG00000204923	FBXO48	0.4916	0.49527	0.55131	0.74879	0.51903	0.63903	0.7116	0.85772
ENSG00000169621	APLF	6.8215	10.7559	7.75963	16.8477	14.1513	17.9967	17.3467	13.1811
ENSG00000214525	AC130709.1	0	0	0	0	0	0	0	0
ENSG00000169618	PROKR1	0	0	0.02521	0	0.02086	0	0	0
ENSG00000163219	ARHGAP25	46.2362	53.4626	6.73706	11.4732	42.8911	23.5285	9.81566	28.9866
ENSG00000237576	LINC01888	1.60982	1.42909	0.63083	0.31855	1.30478	0	0.53666	1.05414
ENSG00000228329	LINC01890	0.21623	0.11997	0	0	0.43814	0	0	0
ENSG00000163217	BMP10	0	0	0	0	0	0	0	0
ENSG00000283586	GKN3P	0	0	0	0	0.16322	0	0	0
ENSG00000183607	GKN2	0	0	0	0	0	0	0	0
ENSG00000169605	GKN1	0	0	0	0	0	0	0	0
ENSG00000169604	ANTXR1	0.00896	0	0	0	0.05444	0.0158	0	0
ENSG00000266649	MIR3126	0	0	0	0	0	0	0	0
ENSG00000251850	RNA5SP96	0	0	0	0	0	0	0	0
ENSG00000199460	RNU6-1216P	0	0	0	0	0	0	0	0
ENSG00000271597	AC112230.1	0	0	0	0	0	0	0	0
ENSG00000198380	GFPT1	3.78655	3.64677	1.66443	3.33744	3.34715	3.26707	2.74237	2.11137
ENSG00000252250	RF00019	0	0	0	0	0	0	0	0

ENSG00000169599	NFU1	4.47075	3.72079	1.4218	2.42626	4.2591	6.18013	2.11327	3.76865
ENSG00000115977	AAK1	3.17631	2.22228	0.5977	1.27642	2.76319	3.54939	1.49051	1.37237
ENSG00000244236	RN7SL604P	0	0	0	0	0	0	0	0
ENSG00000207016	SNORA36C	0	0.92431	0	0	0.67513	0	0	0
ENSG00000232228	AC092431.2	1.15252	0	0	0.34209	0.84072	1.62656	0.38421	0.56602
ENSG00000229948	B3GALNT1P1	0	0	0	0	0	0	0	0
ENSG00000196975	ANXA4	1.47954	1.04913	0.57523	0.64988	1.17768	0.99489	0.44236	0.71685
ENSG00000231024	AC092431.1	0	0	0	0	0	0	0	0
ENSG00000087338	GMCL1	8.59291	6.69287	1.94769	5.7686	9.16153	5.70107	3.74832	5.85109
ENSG00000275381	AC019206.1	0	0	0	0	0	0	0	0
ENSG00000270723	RPL23AP92	0	0	0	0	0	0	0	0
ENSG00000124380	SNRNP27	8.86048	6.97191	1.75183	4.25579	6.81596	7.50293	3.32975	3.99547
ENSG00000059728	MXD1	107.833	137.954	16.9093	26.0188	72.4748	28.0369	34.6861	68.6421
ENSG00000244617	ASPRV1	1.07745	2.0176	0.69269	0.64962	0.77778	0.35639	0.22449	1.52958
ENSG00000179818	PCBP1-AS1	2.16699	2.97885	0.88943	1.39177	1.94867	0.90299	1.12098	1.88077
ENSG00000263669	RN7SL470P	0	0	0	0	0	0	0	0
ENSG00000169564	PCBP1	77.4052	87.5672	17.1111	27.7248	53.0117	60.2223	31.0687	32.3475
ENSG00000233060	AC016700.3	0.16362	0.54468	0.24043	0	0	0.28864	0.27272	0.80355
ENSG00000225392	AC016700.1	0	0	0	0	0	0	0	0
ENSG00000231327	LINC01816	0.99653	0.73721	0.32542	0.32866	0.40385	0.97668	0.55369	0.40784
ENSG00000115998	C2orf42	2.14045	2.28556	0.74183	1.31861	3.26516	2.63611	1.38	2.05778
ENSG00000116001	TIA1	9.57442	7.36815	4.64636	9.00648	8.6999	8.74866	8.54356	8.86758
ENSG00000221238	MIR1285-2	0	0	0	0	0	0	0	0
ENSG00000231414	AC016700.2	0	0	0	0	0	0.82892	0	0
ENSG00000116005	PCYOX1	3.52141	2.12008	0.5872	1.26021	2.88453	1.38784	1.0199	1.16521
ENSG00000143977	SNRPG	7.18711	6.79713	4.50058	7.03016	7.49672	12.4629	3.74369	6.61817
ENSG00000035141	FAM136A	3.3016	1.8582	0.76789	1.2338	2.05032	2.97509	1.22735	0.99154
ENSG00000233849	AC022201.2	0	0	0	0.37904	0	0	0	0.31358
ENSG00000235289	BRD7P6	0	0	0	0	0	0	0	0
ENSG00000229229	AC022201.1	0	0	0	0	0	0	0	0
ENSG00000163235	TGFA	4.99204	7.27675	0.75962	1.03021	5.42278	0.49505	0.71393	3.89872
ENSG00000224606	TGFA-IT1	0	0	0	0	0	0	0	0
ENSG00000075340	ADD2	0.27185	0.14441	0.18699	0.53221	0.5274	0.67345	0.4242	0.37638
ENSG00000235035	AC005234.1	0	0	0	0	0	0	0	0
ENSG00000183733	FIGLA	0	0	0	0	0	0	0	0
ENSG00000228034	HMGN2P21	0	0	0	0	0	0	0	0
ENSG00000152672	CLEC4F	0.11847	0.19719	0.13056	0	0.86416	0.20899	0.04937	0.07272
ENSG00000231386	AC007395.1	0	0	0	0	0	0	0	0
ENSG00000116031	CD207	0	0	0	0	0.09536	0.13838	0	0
ENSG00000237751	LINC01143	0	0	0	0	0	0	0	0
ENSG00000277764	RF02272	0	0	0	0	0	0	0	0
ENSG00000116035	VAX2	0	0	0	0	0	0	0	0
ENSG00000116039	ATP6V1B1	0	0	0	0	0	0	0	0
ENSG00000258881	AC007040.2	0	0	0	0	0	0	0	0
ENSG00000239322	ATP6V1B1-AS1	0	0	0	0	0	0	0	0
ENSG00000226186	ELOCP21	0	0	0	0	0	0	0	0
ENSG00000241159	RN7SL160P	0	0	0	0	0	0	0	0
ENSG00000144031	ANKRD53	0.02902	0.0483	0	0	0	0	0	0.03563
ENSG00000144043	TEX261	3.47574	2.57764	0.8344	1.96631	3.13791	3.79436	1.66349	1.83797
ENSG00000228384	AC007040.1	0	0	0	0	0	0	0	0
ENSG00000236469	AC007881.2	1.67176	0.8562	0	0.19085	0.15635	0.22686	0	0.15789
ENSG00000205847	OR7E91P	0	0	0	0	0	0	0	0
ENSG00000234485	OR7E46P	0	0	0	0	0	0	0	0
ENSG00000234896	OR7E62P	0	0	0	0	0	0	0	0
ENSG00000124357	NAGK	5.67843	5.26774	1.19897	1.55338	2.7254	2.38443	1.41495	2.2565
ENSG00000272735	AC007881.3	3.50199	2.21103	0.53236	0.71687	2.34904	1.06517	0.60386	1.33439
ENSG00000124370	MCEE	2.88868	0.97667	0.46428	0.4689	1.317	1.75176	0.90281	1.16376
ENSG00000124383	MPHOSPH10	5.69399	4.25581	1.15251	2.04861	7.30402	4.53823	1.96095	1.8296

ENSG00000233870	AC007881.1	0	0	0	0	0	0	0	0
ENSG00000124374	PAIP2B	0.47863	0.25261	0.13724	0.55441	0.5961	0.3501	0.31134	0.50166
ENSG00000075292	ZNF638	17.6758	20.5108	11.6487	20.599	21.9684	14.2125	20.3928	23.3398
ENSG00000281195	AC007878.1	1.7701	2.19086	0.40018	0.87567	0.99325	0.64055	1.21046	1.39314
ENSG00000200779	RNU6-105P	0	0	0	0	0	0	0	0
ENSG00000135636	DYSF	20.873	42.4396	2.67166	4.99434	20.4354	7.17028	5.53643	16.5699
ENSG00000233971	RPS20P10	0	0	0	0	0	0	0	0
ENSG00000003137	CYP26B1	0	0	0	0	0.19547	0	0	0
ENSG00000144036	EXOC6B	2.37937	2.16569	2.21829	4.6941	2.71798	1.95293	2.7918	2.06525
ENSG00000228568	AC006461.1	0	0	0	0	0	0	0	0
ENSG00000222536	RNU2-39P	0	0	0	0	0	0	0	0
ENSG00000212378	RF00592	0	0	0	0	0	0	0	0
ENSG00000116096	SPR	0.24764	0.24731	0	0	0.18064	0	0	0.12162
ENSG00000135638	EMX1	0	0	0	0	0	0	0	0
ENSG00000278060	AC012366.1	0.46491	0	0	0	0.18841	0	0	0.19027
ENSG00000144040	SFXN5	1.14377	0.97941	0.28174	0.5691	0.77164	0.52485	0.45183	0.57633
ENSG00000135631	RAB11FIP5	0.4985	0.67418	0.28996	0.30826	0.21465	0.12825	0.08655	0.20402
ENSG00000272702	AC010913.1	0	0	0	0	0	0.04474	0	0
ENSG00000214513	NOTO	0	0	0	0	0	0	0	0
ENSG00000135632	SMYD5	1.32288	0.57755	0.12747	0.48278	1.081	0.95645	0.72296	0.55915
ENSG00000135617	PRADC1	1.35452	0.86715	0.15311	0.38659	1.26676	1.74621	0.52102	0.57567
ENSG00000135624	CCT7	18.8813	12.0276	2.12968	5.48321	15.5352	21.0658	4.42313	6.14015
ENSG00000163013	FBXO41	0.5673	0.21322	0.08067	0.23085	0.4561	0.54882	0.27453	0.20222
ENSG00000135625	EGR4	0	0	0	0	0	0.0544	0	0
ENSG00000252988	RNU6-111P	0	0	0	0	0	0	0	0
ENSG00000214484	RPSAP28	0	0	0	0	0	0	0	0
ENSG00000116127	ALMS1	5.55985	4.34938	2.36827	3.71547	6.62002	6.72598	6.89405	4.22001
ENSG00000230002	ALMS1-IT1	0.45226	0.23163	0.4601	0.25815	0.38066	0.30686	0.40591	0.21357
ENSG00000144035	NAT8	0	0	0	0	0.18821	0	0	0
ENSG00000163016	ALMS1P1	0	0	0.32562	0	0.05388	0.07818	0	0.05441
ENSG00000204872	NAT8B	1.72471	0.79744	1.54883	1.70644	1.5144	1.52132	2.07627	2.47051
ENSG00000144034	TPRKB	6.26652	5.84115	2.5784	4.62353	4.35353	4.422	3.10375	4.00085
ENSG00000273245	AC092653.1	0.13804	0.68931	0.20285	0.40974	0.33566	0.24353	0.69028	1.1864
ENSG00000280037	AC092653.2	1.0002	0.47567	0.41994	0.21206	0.17372	0.25207	1.66718	0.35087
ENSG00000144048	DUSP11	7.42518	5.92141	1.79321	1.90314	6.63849	6.27585	3.2062	3.45361
ENSG00000187833	C2orf78	0	0	0	0	0	0	0	0
ENSG00000124356	STAMBP	3.1613	2.69439	0.81156	1.44143	2.53528	2.48612	1.14276	1.54321
ENSG00000271536	AC073046.2	0	0	0	0	0	0	0	0
ENSG00000163017	ACTG2	0	0	0	0	0	0.03393	0	0
ENSG00000114956	DGUOK	10.8817	6.54066	3.25733	4.63554	8.39105	8.97628	3.86275	5.25756
ENSG00000237883	DGUOK-AS1	0.50844	0.2821	0.12452	0.25153	0.10303	0	0.14125	0.10404
ENSG00000201876	RNA5SP97	0	0	0	0	0	0	0	0
ENSG00000235499	AC073046.1	7.70593	3.12842	0.55238	1.67363	2.05654	3.64726	1.25313	3.23066
ENSG00000187605	TET3	11.7379	7.79518	2.05403	4.37058	5.62108	5.81698	3.99269	3.59372
ENSG00000279181	AC110801.1	0	0	0	0	0	0	0	0
ENSG00000257800	FNBP1P1	0.66169	0.18356	0.21608	0.05456	0.17877	0.3891	0.2451	0.36107
ENSG00000217702	AC073263.1	0	0	0	0	0	0.1023	0	0
ENSG00000163170	BOLA3	0.69677	1.12112	1.53585	5.51512	0.90359	1.43407	2.12925	1.73949
ENSG00000225439	BOLA3-AS1	0.35971	0	0.23493	0.23726	0.43732	0.07051	0.19986	0.44164
ENSG00000114978	MOB1A	92.8775	79.3434	22.3122	38.4297	61.413	51.6645	29.9	53.1737
ENSG00000279201	AC073263.3	0	0	0	0	0	0	0	0
ENSG00000279070	AC073263.2	0	0.06549	0	0	0	0	0	0
ENSG00000065911	MTHFD2	6.57746	6.31338	2.14543	3.68576	3.34874	7.67374	3.58764	5.10045
ENSG00000264324	AC006030.1	0.04347	0.02412	0	0.0215	0.03523	0.02556	0.02415	0.01779
ENSG00000188687	SLC4A5	0.10047	0.04561	0.01342	0.02711	0.06663	0	0.03045	0
ENSG00000229798	KRT18P26	0.05722	0	0	0	0.06957	0	0.09538	0
ENSG00000252214	RNU6-542P	0	0	0	0	0	0	0	0
ENSG00000234632	NECAP1P2	0	0	0	0	0	0	0	0

ENSG00000225658	TAF13P2	0	0	0	0	0	0	0	0
ENSG00000204843	DCTN1	6.24236	4.99067	0.71197	1.60485	4.87457	5.32657	1.33428	2.18979
ENSG00000237737	DCTN1-AS1	0	0	0	0	0	0	0	0
ENSG00000159239	AC005041.1	0.01042	0	0	0	0	0	0	0
ENSG00000234521	AC005041.2	0.07464	0	0	0.11078	0	0	0	0
ENSG00000284308	C2orf81	0	0	0	0	0.11902	0	0	0
ENSG00000233155	HMGA1P8	0.4567	0	0	0	0.27762	0	0	0
ENSG00000005448	WDR54	0.71763	0.59724	0.11299	0.3043	0.62319	0.90428	0.25632	0.0944
ENSG00000114993	RTKN	0.07062	0.04702	0	0.04192	0.05151	0.04983	0	0.06936
ENSG00000115274	INO80B	0.62609	0.24049	0.28309	0.2859	0.52697	0.76466	0.64221	0.59131
ENSG00000274049	INO80B-WBP1	0	0	0	0	0	0	0	0
ENSG00000239779	WBP1	0.48105	0.35031	0.08836	0.31235	0.58485	0.47736	0.40092	0.59063
ENSG00000115275	MOGS	6.25569	4.26313	1.33227	1.95101	4.93256	5.31802	1.92678	2.56021
ENSG00000204822	MRPL53	0.81808	0.54468	0.08014	0.08094	0.39784	0.6735	0.27272	0.20089
ENSG00000135637	CCDC142	0.61371	0.6727	0.10998	0.17772	0.80072	0.60735	0.5489	0.53296
ENSG00000115282	TTC31	1.56846	1.31733	0.42291	0.81151	1.38206	1.29464	0.52767	0.77736
ENSG00000179528	LBX2	0.09205	0	0.04509	0	0.07461	0.05413	0.05114	0
ENSG00000272183	AC005041.3	0.22589	0.37599	0	0	0.13731	0.19925	0	0
ENSG00000257702	LBX2-AS1	0.49242	0.29273	0	0.0522	0.29934	0.06205	0.05863	0.12955
ENSG00000115289	PCGF1	1.9517	1.34585	0.49166	0.62069	1.69488	1.72153	0.41827	0.61618
ENSG00000115297	TLX2	0	0	0	0	0	0	0	0
ENSG00000144045	DQX1	0	0	0.07327	0	0.06062	0	0	0.03061
ENSG00000115307	AUP1	16.4467	11.5802	2.04468	4.13006	10.3191	9.86776	3.85003	6.62849
ENSG00000115317	HTRA2	3.05316	1.87017	0.71785	1.23249	2.34596	3.79188	1.66924	1.6494
ENSG00000115318	LOXL3	1.57924	1.70583	0.37032	0.34907	0.51064	0.44457	0.19603	0.37129
ENSG00000115325	DOK1	6.10636	5.92235	1.27179	1.39149	4.93956	3.18084	1.40253	3.30589
ENSG00000159374	M1AP	0.19547	0	0	0.10879	0.02971	0.08621	0.08145	0.09
ENSG00000233872	AC005033.1	0	0	0	0	0	0	0	0
ENSG00000108442	TVP23BP2	0	0	0	0	0	0	0	0
ENSG00000135622	SEMA4F	0.412	0.41146	0.08649	0.12229	0.40072	0.35303	0.17659	0.21679
ENSG00000224646	AC007387.1	0	0	0	0	0	0	0	0
ENSG00000272711	AC019069.1	0.87982	0.18306	0	0.08161	0.53483	0.48504	0	0.33757
ENSG00000159399	HK2	11.6164	6.67988	1.27893	2.15275	6.11354	3.96636	1.47084	4.60077
ENSG00000204792	LINC01291	0.01755	0	0	0	0	0.03097	0	0
ENSG00000236209	AC104135.1	0.48034	0	0	0.14258	0	0	0	0.11795
ENSG00000230836	LINC01293	0.1737	0	0	0	0	0	0	0
ENSG00000115350	POLE4	2.62395	1.70165	0.05008	0.60689	1.20148	1.74339	0.39761	0.92047
ENSG00000115353	TACR1	0	0	0	0	0	0	0	0
ENSG00000263909	MIR5000	0.71165	0	0	0	0	0	0	0
ENSG00000270571	AC007681.1	0	0	0.03133	0.03164	0	0.11284	0	0.02618
ENSG00000236167	GAPDHP57	0	0	0	0	0	0	0	0
ENSG00000115363	EVA1A	0	0	0	0	0	0	0	0
ENSG00000237293	AC007099.2	0	0	0	0	0	0	0	0
ENSG00000238410	RF01233	0	0	0	0	0	0	0	0
ENSG00000231172	AC007099.1	0	0	0	0	0	0	0	0
ENSG00000230477	AC005034.1	0	0	0	0	0	0	0	0
ENSG00000221638	RF00012	0	0	0	0	0	0	0	0
ENSG00000115364	MRPL19	3.47304	2.86392	0.88962	2.00973	3.48642	4.55303	1.59331	1.85823
ENSG00000226247	SUPT4H1P1	0	0	0	0	0	0	0	0
ENSG00000005436	GCFC2	2.95079	2.47812	2.73966	5.0561	4.305	3.99885	5.92457	4.79216
ENSG00000270696	AC005034.3	2.01398	1.39678	0.30828	1.05859	0.96921	1.55441	0.41962	0.30909
ENSG00000271452	AC005034.5	0.1573	0.26182	0	0.93378	0.38247	1.10997	0.78657	0
ENSG00000270462	AC005034.2	0	0	0	0	0	0	0	0
ENSG00000270996	AC005034.4	0	0	0	0	0	0	0	0
ENSG00000233107	SUCLA2P2	0	0	0	0	0	0	0	0
ENSG00000228209	AC073091.1	0	0	0	0	0	0	0	0
ENSG00000271356	AC073091.2	0	0	0	0	0	0	0	0
ENSG00000200488	RN7SKP203	64.6898	123.111	39.5816	84.866	74.0849	97.7628	150.149	89.1838

ENSG00000251947	RN7SKP164	0	0	0	0	0	0	0	0
ENSG00000176204	LRRTM4	0	0	0	0	0.01647	0	0.02258	0
ENSG00000200028	RNA5SP98	0	0	0	0.79406	0	0	0	0
ENSG00000234653	AC079117.1	0	0	0	0	0	0	0	0
ENSG00000227088	AC084149.1	0	0	0	0	0	0	0	0
ENSG00000230968	AC084149.2	0	0	0	0	0	0	0	0
ENSG00000237077	AC105399.1	0	0	0.51538	0	0.2132	0	0.87689	0
ENSG00000233605	LINC01851	0	0	0	0	0	0	0	0
ENSG00000229494	AC012494.1	0	0	0	0	0	0	0	0
ENSG00000214429	CYCSP6	0	0	0	0	0	0	0	0
ENSG00000270470	AC064872.1	0	0	0	0	0	0	0	0
ENSG00000234877	AC092660.1	0	0	0	0	0	0	0	0
ENSG00000202423	RNU6-827P	0	0	0	0	0	0	0	0
ENSG00000200048	RNU6-812P	0	0	0	0	0	0	0	0
ENSG00000143954	REG3G	0	0	0	0	0	0	0	0
ENSG00000172023	REG1B	0	0	0	0	0	0	0.05125	0
ENSG00000115386	REG1A	0	0	0	0	0	0	0	0
ENSG00000204787	REG1CP	0	0	0	0	0	0	0	0
ENSG00000172016	REG3A	0	0	0	0	0	0	0	0
ENSG00000224879	AC011754.1	0	0	0	0	0	0	0	0
ENSG00000066032	CTNNA2	0.00973	0.01619	0	0	0	0	0	0.02389
ENSG00000229385	AC010975.1	0	0	0	0	0	0	0	0
ENSG00000277171	AC010975.3	0	0	0	0	0	0	0	0
ENSG00000230237	AC010975.2	0	0	0	0	0	0	0	0
ENSG00000252031	RNU6-561P	0	0	0	0	0	0	0	0
ENSG00000266436	MIR4264	0	0	0	0	0	0	0	0
ENSG00000276917	MIR8080	0	0	0	0	0	0	0	0
ENSG00000224731	AC016716.2	0	0	0	0	0	0	0	0
ENSG00000223427	AC016716.1	0	0	0	0	0	0	0	0
ENSG00000162951	LRRTM1	0	0	0	0	0	0	0	0
ENSG00000237031	AC008067.1	0	0	0	0	0	0	0	0
ENSG00000230975	AC012355.1	0	0	0	0	0	0	0	0
ENSG00000234429	AC105342.1	0	0	0	0	0	0	0	0
ENSG00000231722	AC012075.1	0	0	0	0	0	0	0	0
ENSG00000235519	LINC01815	0	0	0	0	0	0	0	0
ENSG00000200890	RNA5SP99	0	0	0	0	0	0	0	0.78945
ENSG00000233444	AC013262.1	0	0	0	0	0	0	0	0
ENSG00000242118	RN7SL201P	0	0	0	0	0	0	0	0
ENSG00000231781	AC079896.1	0	0	0	0	0	0	0	0
ENSG00000229560	LYARP1	0	0	0	0	0	0	0	0
ENSG00000252897	RNU6-685P	0	0	0	0	0	0	0	0
ENSG00000201311	RF00019	0	0	0	0	0	0	0	0
ENSG00000237498	AC010105.1	0	0	0	0	0	0	0	0
ENSG00000226860	AC109638.1	0	0	0	0	0	0	0	0
ENSG00000226700	MTND4P25	0	0	0	0	0	0	0	0
ENSG00000225045	MTND5P27	0	0	0	0	0	0	0	0
ENSG00000224695	MTND6P7	0	0	0	0	0	0	0	0
ENSG00000227789	MTCYBP7	0	0	0	0	0	0	0	0
ENSG00000168129	DHFRP3	0	0	0	0	0	0	0	0
ENSG00000223977	AC138623.1	0	0	0.49868	0.50364	0	0	0	0
ENSG00000232548	LINC01809	0	0	0	0	0	0	0	0
ENSG00000224627	RPL37P10	0	0	0	0	0	0	0	0
ENSG00000199295	RNU6-1312P	0	0	0	0	0	0	0	0
ENSG00000228225	AC016908.1	0	0	0	0	0	0	0	0
ENSG00000228902	ST6GALNAC2P1	0	0	0	0	0	0	0	0
ENSG00000182814	FUNDC2P2	0	0	0.08807	0.08895	0.2186	0	0	0.29435
ENSG00000228272	AC106874.1	0	0	0	0	0	0	0	0
ENSG00000163541	SUCLG1	5.84898	4.46928	0.80421	1.59904	5.40605	6.15483	1.2543	2.68773

ENSG00000115423	DNAH6	0.06509	0.04514	0.02391	0.07245	0.09232	0.00957	0.06329	0.13319
ENSG00000259056	DUXAP1	0.15832	0	0	0	0.19248	0	0.52778	0.19438
ENSG00000235847	LDHAP7	0.07596	0.25287	0	0	0	0	0	0
ENSG00000186854	TRABD2A	1.99735	0.5286	0.42982	0.8434	3.75934	1.38585	1.37907	1.43651
ENSG00000276362	AC010087.1	0	0	0	0	0	0	0	0
ENSG00000213400	RPL12P18	0	0	0	0	0	0	0	0
ENSG00000034510	TMSB10	552.708	456.302	138.551	201.999	468.402	718.254	193.863	290.495
ENSG00000213399	AC022210.1	0	0	0	0	0.11238	0	0.15407	0.11349
ENSG00000176407	KCMF1	6.73392	7.0896	1.7709	3.24937	5.68164	5.27439	3.68013	4.11131
ENSG00000260840	LINC01964	0	0	0	0	0	0	0	0
ENSG00000235463	AC078974.1	0	0	0	0	0	0	0	0
ENSG00000225787	LSM3P3	0	0	0	0	0	0.42259	0	0
ENSG00000152284	TCF7L1	0	0.02764	0	0	0	0	0	0
ENSG00000231134	TCF7L1-IT1	0	0	0	0	0	0	0	0
ENSG00000200701	RNU6-674P	0	0	0	0	0	0	0	0
ENSG00000230299	AC093162.1	0	0	0	0	0	0	0	0
ENSG00000246575	AC093162.2	0	0	0	0	0	0	0	0
ENSG00000152291	TGOLN2	31.0551	23.5367	4.73083	8.8952	21.3699	14.3355	8.74812	13.5739
ENSG00000239143	RF00019	0	0	0	0	0	0	0	0
ENSG00000270532	PEBP1P2	0	0.43731	0	0.19496	0	0	0.43792	0
ENSG00000042445	RETSAT	2.34268	1.55367	0.34963	0.78771	0.7788	1.51752	0.51862	0.96626
ENSG00000115459	ELMOD3	1.32282	1.29421	0.41548	0.67438	1.41182	1.19354	0.84158	0.73148
ENSG00000271014	AC062037.1	0	0	0	0	0	0	0	0
ENSG00000202382	RF00019	0	0	0	0	0	0	0	1.76465
ENSG00000278590	RN7SL113P	0.4903	0.40806	0	0	0	0	0.81726	0
ENSG00000273196	AC062037.2	0	3.3427	0	0	2.44155	0	0	1.23284
ENSG00000042493	CAPG	12.8212	11.9341	1.36347	2.29506	10.5969	4.61296	2.57764	3.34857
ENSG00000152292	SH2D6	0	0	0	0.0282	0	0.06704	0.03167	0
ENSG00000207207	RF00019	0	0	0	0	0	0	0	0
ENSG00000244399	RN7SL251P	0	0	0	0	0	0	0	0
ENSG00000199936	RF00019	0	0	0	0	0	0	0	0
ENSG00000229648	RPSAP22	0	0	0	0	0	0	0	0
ENSG00000168906	MAT2A	7.9067	5.95495	1.99248	4.65497	8.5204	7.92528	3.07697	5.61604
ENSG00000115486	GGCX	1.74832	1.26058	0.22495	0.60981	0.67587	0.69645	0.4566	0.60341
ENSG00000118640	VAMP8	19.2506	16.1446	5.3313	6.3733	13.2325	18.9396	6.91121	10.9996
ENSG00000276281	RN7SL126P	0	0	0	0	0	0	0.40863	0
ENSG00000168899	VAMP5	1.5215	2.68151	0.92063	0.39848	0.65287	5.84192	1.19346	2.08784
ENSG00000168894	RNF181	11.9564	9.40065	3.09015	4.63677	10.2265	13.4611	4.30636	7.1555
ENSG00000168890	TMEM150A	0.63966	0.47321	0.06963	0.21096	0.25923	0.12538	0.03949	0.37814
ENSG00000168883	USP39	7.14085	6.29801	2.453	5.53288	5.95314	6.8386	5.28665	4.28124
ENSG00000168887	C2orf68	6.44472	4.83832	0.92752	2.26791	4.92737	4.89351	2.43641	3.14062
ENSG00000168878	SFTPB	0	0.02969	0	0	0	0	0	0
ENSG00000115523	GNLY	32.1455	30.3122	9.95586	25.7078	24.8569	15.3852	8.93026	15.6042
ENSG00000203363	AC012454.1	0	0.12262	0	0	0.26869	0	0	0.09045
ENSG00000199687	RNU1-38P	0	0	0	0	0	0	0	0
ENSG00000168874	ATOH8	0.09651	0.16064	0	0.08952	0.19067	0.02128	0	0.02962
ENSG00000278783	MIR6071	0	0	0	0	0	0	0	0
ENSG00000229498	AC105053.1	0	0	0	0.06358	0	0	0	0
ENSG00000252321	RN7SKP83	0	0	0	0	0	0	0	0
ENSG00000115525	ST3GAL5	1.123	0.972	0.42246	0.62666	0.87926	1.09358	0.48668	0.56255
ENSG00000232504	ST3GAL5-AS1	1.02976	0.31164	0	0.27787	0.91052	0.99089	0.62417	0.22988
ENSG00000272564	AC012511.1	0	0	0	0	0	0	0	0
ENSG00000068654	POLR1A	1.59408	1.18227	0.28683	0.53109	1.40409	1.5113	0.52419	0.52591
ENSG00000132300	PTCD3	4.26215	4.48949	2.91434	6.65729	5.93585	5.61064	5.91428	4.97622
ENSG00000208772	SNORD94	0.53504	2.67172	25.1595	65.9067	4.55341	4.71941	16.0529	22.9919
ENSG00000132305	IMMT	8.22008	6.18307	3.56704	5.81319	10.0832	9.60268	4.81242	5.5091
ENSG00000200241	RF00019	1.32073	0	0	0.98005	0	0	1.10072	0
ENSG00000265420	MIR4779	0	0	0	0	0	0	0	0

ENSG00000273080	AC009309.1	0.47805	0	0	0.47298	0	0.56223	0	0.19565
ENSG00000132313	MRPL35	2.89066	2.74033	1.18151	3.15362	4.11952	4.22146	2.36128	2.74998
ENSG00000068615	REEP1	0.04087	0.09071	0	0	0.03313	0.19229	0	0.26765
ENSG00000202537	RF00096	0	0	0	0	0	0	0	0
ENSG00000251974	RF00413	0	0	0	0	0	0	0	0
ENSG00000115548	KDM3A	6.70586	5.89	1.52467	2.86897	5.58349	4.9324	3.9231	3.94906
ENSG00000115561	CHMP3	8.20947	6.7628	1.90318	2.57316	6.37455	4.42218	4.52649	4.88585
ENSG00000249884	RNF103-CHMP3	0.04877	0	0.07167	0.07238	0	0.08604	0.08129	0
ENSG00000200563	RNU6-640P	0.6981	0	0	0	0.84873	0	0	0
ENSG00000228363	AC015971.1	0.23067	0.25596	0.15065	0.15215	0.1558	0.04521	0.04272	0.15734
ENSG00000239305	RNF103	3.87771	2.64922	0.63786	1.48884	2.09921	1.70169	2.7655	2.16732
ENSG00000153561	RMND5A	34.1077	36.9038	13.8046	27.82	27.7147	25.0109	24.1883	19.9464
ENSG00000153563	CD8A	13.5144	18.3315	4.06076	5.8973	12.5803	68.1075	4.20268	7.67724
ENSG00000172116	CD8B	9.79173	11.7165	3.69833	2.80135	11.4742	46.272	3.99839	5.0213
ENSG00000233673	ANAPC1P1	0.05733	0.09543	0.12638	0.12763	0.03485	0.25286	0.09557	0.07039
ENSG00000213605	AC111200.1	0	0	0	0	0	0	0	0
ENSG00000187627	RGPD1	0.04267	0.01776	0.14109	0.42748	0.15564	0.05646	0.37342	0.20957
ENSG00000231351	AC111200.2	0	0	0	0	0	0	0	0
ENSG00000226114	NDUFB4P5	0	0	0	0	0	0	0	0
ENSG00000183281	PLGLB1	0.2504	0.15156	0	0.03378	0.16606	0.12048	0.11383	0.1677
ENSG00000231259	AC125232.1	4.06067	3.20619	1.45353	2.08609	6.96224	3.85731	2.8636	3.06808
ENSG00000266931	AC092651.2	12.3022	3.41282	1.50649	2.28222	13.7103	9.04278	0.85441	3.7761
ENSG00000230395	AC092651.1	0	0	0	0.34867	0	0	0	0
ENSG00000204745	AC083899.1	0.17274	0.31948	0.05641	0.22788	0.07001	0.30474	0.22395	0.21209
ENSG00000264793	MIR4771-1	0	0	0	0	0	0	0	0
ENSG00000233671	AC083899.2	0	0	0	0	0	0	0	0
ENSG00000281477	LINC01955	0	0	0	0	0	0	0	0
ENSG00000271097	AC083899.3	0	0	0	0	0	0	0	0
ENSG00000233999	IGKV3OR2-268	0.20943	0.69719	0.30775	0	0.50924	0	0	0
ENSG00000224881	AC068279.1	0	0	0	0	0	0	0	0
ENSG00000280721	LINC01943	0.19878	0.08272	0.07303	0.51627	0.06042	0.78902	0.2485	0.24406
ENSG00000222041	CYTOR	3.9874	4.97778	3.74007	6.86206	2.93961	8.00715	9.75746	4.45298
ENSG00000273445	AC133644.2	0.27148	0.90377	0.59841	0.60437	0.66012	1.19733	0	0.49998
ENSG00000214354	AC133644.1	0.07823	0	0	0	0	0	0	0
ENSG00000265507	MIR4435-1	0	1.52511	0	0	1.11396	0	0	1.12496
ENSG00000225933	AC133965.1	0	0.34466	0	0	0	0	0	0
ENSG00000234231	AC093616.1	1.99433	0.69886	0.41132	0.83082	2.33958	1.35793	0.69984	0.77324
ENSG00000125551	PLGLB2	0.22764	0	0.04779	0.04826	0	0	0.32524	0.15971
ENSG00000185304	RGPD2	0.09393	0.13898	0.09202	4.69345	1.47195	0.38666	3.63601	2.78077
ENSG00000229604	MTATP8P2	0	0	0	0	0	0	0	0
ENSG00000225428	NDUFB4P7	0	0	0	0	0	0	0	0
ENSG00000233205	AC108479.1	0	0	0	0	0	0	0	0
ENSG00000275597	AC108479.2	0	0	0	0	0	0	0	0
ENSG00000222724	RNU2-63P	15.1135	14.4649	8.88362	24.673	26.1838	46.6589	4.40857	14.8449
ENSG00000172086	KRCC1	27.3883	31.3199	6.02166	7.26066	28.316	17.9989	3.97278	14.4776
ENSG00000115593	SMYD1	0	0	0	0	0	0	0	0
ENSG00000265003	MIR4780	0	0	0	0	0	0	0	0
ENSG00000163586	FABP1	0	0	0	0	0	0	0	0
ENSG00000144115	THNSL2	0.23936	0.07589	0	0.11841	0.05543	0.02011	0	0.09796
ENSG00000232508	MRPL45P1	0	0	0	0	0	0	0	0
ENSG00000283262	RF00026	0	0	0	0	0	0	0	0
ENSG00000212133	RNU6-1168P	0	0	0	0	0	0	0	0
ENSG00000252395	RNU6-1007P	0	0	0	0	0	0	0	0
ENSG00000224291	AC012671.1	0	0	0	0	0	0	0	0
ENSG00000214336	FOXI3	0	0	0	0	0	0	0	0
ENSG00000172073	TEX37	0	0	0	0	0	0	0	0
ENSG00000225420	AC104134.1	0	0	0	0	0	0	0	0
ENSG00000172071	EIF2AK3	4.43795	3.00867	0.892	1.86181	5.05115	4.09305	2.38335	2.31866

ENSG00000234028	EIF2AK3-DT	0.44497	0.25638	0.0503	0.1524	0.49937	0.15096	0.14263	0.23114
ENSG00000153574	RPIA	46.5663	18.9483	21.1581	42.9727	26.571	29.1791	29.7517	24.8369
ENSG00000230006	ANKRD36BP2	88.3578	123.04	133.698	104.958	185.824	170.427	117.349	117.694
ENSG00000283270	AC244205.2	0	0	0	0	0	0	0	0
ENSG00000240040	AC244205.1	0	0	0	0	0	0	0	0
ENSG00000265510	MIR4436A	0	0	0	0	0	0	0	0
ENSG00000211592	IGKC	460.825	203.425	40.367	112.114	343.005	30.4118	34.3413	55.2372
ENSG00000211593	IGKJ5	25.0764	6.4215	11.3383	0	23.4518	0	0	14.2101
ENSG00000211594	IGKJ4	15.8487	6.59505	0	8.82046	14.4514	0	13.2087	12.1618
ENSG00000211595	IGKJ3	21.2185	3.21075	0	5.72556	30.4873	0	6.43054	2.36834
ENSG00000211596	IGKJ2	0	0	0	0	0	0	0	0
ENSG00000211597	IGKJ1	21.2185	3.21075	2.83458	2.86278	18.7614	6.80588	3.21527	4.73669
ENSG00000211598	IGKV4-1	25.478	12.2462	2.80297	8.08816	27.9939	0.96143	3.40651	6.18939
ENSG00000211599	IGKV5-2	1.07795	0	0	1.06653	1.52896	0	0	0
ENSG00000197794	IGKV7-3	0	0	0	0	0	0	0	0
ENSG00000225136	PGBD4P5	0	0	0	0	0	0	0	0
ENSG00000253435	IGKV2-4	0	0	0	0	0	0	0	0
ENSG00000243466	IGKV1-5	10.6093	11.0083	2.83458	5.52108	10.0508	0.7292	0.68899	3.89085
ENSG00000239855	IGKV1-6	1.15737	0	0.85037	0.57256	0.70355	0	0.32153	0.47367
ENSG00000243063	IGKV3-7	0	0	0	0	0	0	0	0
ENSG00000240671	IGKV1-8	0.55252	0.30655	0	0.27333	0.44782	0	0.30699	0
ENSG00000241755	IGKV1-9	1.9714	2.08816	0.26336	1.59588	1.52522	0	0.59746	0.22004
ENSG00000253278	IGKV2-10	0	0	0	0	0	0	0	0
ENSG00000241351	IGKV3-11	22.8129	4.35745	0.82434	6.10532	32.0547	0.98963	1.55842	2.52543
ENSG00000243290	IGKV1-12	5.15681	3.06554	0.81191	1.36665	2.23911	0	0	0.67837
ENSG00000253497	IGKV1-13	0	0	0	0	0.25682	0	0	0
ENSG00000253265	IGKV2-14	0	0	0	0	0	0	0	0
ENSG00000244437	IGKV3-15	6.30184	2.76037	0.73109	1.96897	5.84702	0.29256	0	1.01807
ENSG00000240864	IGKV1-16	2.52091	1.93664	0.28496	2.59014	4.00789	0	0.64646	0.23809
ENSG00000240382	IGKV1-17	1.35741	1.93664	0.28496	0	2.59334	0	0.32323	0.47617
ENSG00000254157	IGKV2-18	0	0	0	0	0	0	0	0
ENSG00000253732	IGKV2-19	0	0	0	0	0	0	0	0
ENSG00000239951	IGKV3-20	20.1576	10.6757	2.42356	12.2384	16.2638	1.29312	1.52725	4.94984
ENSG00000211611	IGKV6-21	0.90271	0.60103	0.26531	0.80384	0.6585	0	0	0
ENSG00000253578	IGKV1-22	0	0	0	0	0	0	0	0
ENSG00000253625	IGKV2-23	0	0	0	0	0	0	0	0
ENSG00000241294	IGKV2-24	1.69155	1.56421	0	0.83681	0.91402	0	0	0.92305
ENSG00000253202	IGKV3-25	0	0	0	0	0	0	0	0
ENSG00000254098	IGKV2-26	0	0.35571	0	0	0	0	0	0
ENSG00000277852	AC244255.1	0	0	0	0	0	0	0	0
ENSG00000244575	IGKV1-27	3.64583	0	0.28197	1.4239	2.09961	0	0	0
ENSG00000244116	IGKV2-28	0.3759	0	0	0	0.45701	0	0	0
ENSG00000253998	IGKV2-29	0	0	0	0	0	0	0	0
ENSG00000243238	IGKV2-30	1.31565	1.25137	0	0.27894	1.37103	0	0	0.23076
ENSG00000253158	IGKV3-31	0	0	0	0	0	0	0	0
ENSG00000253870	IGKV1-32	0	0	0	0	0	0	0	0
ENSG00000242076	IGKV1-33	0	1.44903	1.02341	0.5168	0.21168	0	0	0.21377
ENSG00000253860	IGKV3-34	0	0	0	0	0	0	0	0
ENSG00000253461	IGKV1-35	0	0	0	0	0	0	0	0
ENSG00000253487	IGKV2-36	0	0	0	0	0	0	0	0
ENSG00000239862	IGKV1-37	0	0	0	0	0	0	0	0.22612
ENSG00000253592	IGKV2-38	0	0	0	0	0	0	0	0
ENSG00000242371	IGKV1-39	0.92086	0	0.81191	0.54666	2.23911	0	0.61397	0.22612
ENSG00000273962	IGKV2-40	0	0	0	0.33996	0.27849	0	0	0
ENSG00000283427	AC006453.3	0	0	0	0	0	0	0	0
ENSG00000283196	LSP1P5	0	0.05054	0	0	0.11075	0	0	0.03728
ENSG00000276861	RF00001	0	0	0	0	0	0	0	0
ENSG00000283132	AC006453.1	0.05831	0	0	0	0	0	0	0

ENSG00000283214	AC006453.2	0	0	0	0	0	0	0	0
ENSG00000283659	AC006453.4	0	0	0	0	0	0	0	0
ENSG00000251039	IGKV2D-40	0	0.36312	0.32058	0	0.79568	0	0	0
ENSG00000251546	IGKV1D-39	0.97474	0.64898	0.28647	0.86797	0.23701	0	0	0
ENSG00000254009	IGKV2D-38	0	0	0	0	0	0	0	0
ENSG00000250036	IGKV1D-37	0	0	0	0	0	0	0	0
ENSG00000253127	IGKV2D-36	0	0	0	0	0	0	0	0
ENSG00000232747	IGKV1D-35	0	0	0	0	0	0	0	0
ENSG00000253501	IGKV3D-34	0	0	0	0	0	0	0	0
ENSG00000239975	IGKV1D-33	0.15303	0	0	0.68133	0.37209	0.53992	0	0.18789
ENSG00000253191	IGKV1D-32	0	0	0	0	0	0	0	0
ENSG00000253999	IGKV3D-31	0	0	0	0	0	0	0	0
ENSG00000239571	IGKV2D-30	0.18557	0.30888	0.27269	0	0.22561	0	0	0.68352
ENSG00000243264	IGKV2D-29	2.93201	0	0	0.54393	4.67863	0	0	0.44999
ENSG00000242534	IGKV2D-28	0.72936	0	0.53589	0.27061	0.44337	0	0.30393	0.22387
ENSG00000224607	IGKV1D-27	0	0	0	0	0.26602	0	0	0
ENSG00000211623	IGKV2D-26	0.18795	0	0	0	0	0	0	0
ENSG00000254097	IGKV3D-25	0	0	0	0	0	0	0	0
ENSG00000241566	IGKV2D-24	0.7518	0	0	0	0.2285	0	0	0
ENSG00000254345	IGKV2D-23	0	0	0	0	0	0	0	0
ENSG00000253365	IGKV1D-22	0	0	0	0	0	0	0	0
ENSG00000225523	IGKV6D-21	0.53766	0	0	0.26598	0.43578	0	0	0
ENSG00000211625	IGKV3D-20	1.48248	0.54835	0	0.24446	2.60341	0	0.27456	0.40448
ENSG00000253765	IGKV2D-19	0	0	0	0	0	0	0	0
ENSG00000254220	IGKV2D-18	0	0	0	0	0	0	0	0
ENSG00000211626	IGKV6D-41	0	0	0	0.29322	0	0	0	0
ENSG00000242766	IGKV1D-17	0	0	0	0	0.16751	0	0	0
ENSG00000241244	IGKV1D-16	0.38783	0.64555	0	0.86338	0.23576	0	0	0.23809
ENSG00000224041	IGKV3D-15	1.29571	0.92431	0	0.54942	0.22504	0	0	0
ENSG00000254292	IGKV2D-14	0	0	0	0	0	0	0	0
ENSG00000276566	IGKV1D-13	5.06864	0	0	0	4.26622	0	0	0
ENSG00000278857	IGKV1D-12	0.39515	0	0	0	0.48041	0	0	0.48516
ENSG00000211632	IGKV3D-11	0.66036	0	0	0.24501	0.20071	0	0.27518	0
ENSG00000253906	IGKV2D-10	0	0	0	0	0.31714	0	0	0
ENSG00000211633	IGKV1D-42	0	0	0	0	0	0	0	0
ENSG00000242580	IGKV1D-43	0.68891	0	0	0	0	0	0	0
ENSG00000239819	IGKV1D-8	0.13727	0	0	0.20372	0.16689	0	0	0.16853
ENSG00000228325	IGKV3D-7	0	0	0	0	0	0	0	0
ENSG00000275490	AC233263.2	0	0	0	0	0	0	0	0
ENSG00000270999	IGKV1OR2-118	0	0	0	0	0	0	0	0
ENSG00000265897	AC233263.1	0	0	0	0	0	0	0	0
ENSG00000281904	AC233263.6	0	0	0	0	0	0	0	0
ENSG00000230964	AC233266.1	0	0	0	0	0	0	0	0
ENSG00000261600	AC233266.2	0	0	0	0	0	0	0	0
ENSG00000278131	AC116050.1	0	0	0	0	0	0	0	0
ENSG00000143429	LSP1P4	0	0.07982	0	0	0	0	0	0.02944
ENSG00000222835	RNA5SP100	0	0	0	0	0	0	0	0
ENSG00000184303	DRD5P1	0	0	0	0	0	0	0	0
ENSG00000230572	AC027612.2	0	0	0	0	0	0	0	0
ENSG00000271627	AC027612.4	0	0	0	0	0	0	0	0
ENSG00000223703	AC027612.1	0	0	0	0	0	0	0.05101	0
ENSG00000232531	AC027612.3	0	0	0	0.1039	0	0.37052	0	0
ENSG00000236969	GGT8P	0	0	0	0	0.16751	0	0	0
ENSG00000235235	IGKV1OR2-1	0	0	0	0	0	0	0	0
ENSG00000214330	ABCD1P5	0	0	0	0	0	0	0	0
ENSG00000237085	AC127391.1	0	1.24498	0	0	0	0	0	0
ENSG00000227497	PABPC1P6	0	0	0	0	0	0	0	0
ENSG00000214329	SLC9B1P2	0	0	0	0	0	0	0	0

ENSG00000226481	ACTR3BP2	0	0	0	0	0	0	0	0
ENSG00000234898	CHEK2P3	0	0	0	0	0	0	0	0
ENSG00000235147	AC128677.1	0	0	0	0	0	0	0	0
ENSG00000223816	IGKV1OR2-2	0	0	0	0	0	0	0	0
ENSG00000261522	AL845331.3	0	0	0	0	0	0	0	0
ENSG00000259916	AL845331.2	0	0	0	0	0	0	0	0
ENSG00000237383	CNN2P11	0	0	0	0	0	0	0	0
ENSG00000227265	AL845331.1	0	0	0	0	0	0	0	0
ENSG00000213355	CNN2P8	0	0	0	0	0	0	0	0
ENSG00000234837	AC073464.2	0	0	0	0	0	0	0	0
ENSG00000223564	CYP4F32P	0	0	0	0	0	0	0	0
ENSG00000232502	AC073464.1	0	0	0	0	0	0	0	0
ENSG00000235571	SNX18P14	0	0	0	0	0	0	0	0
ENSG00000204717		0	0	0	0	0	0	0	0
ENSG00000229089	ANKRD20A8P	0	0	0.0153	0.01545	0	0	0	0
ENSG00000212140	RNU6-1320P	0	0	0	0	0	0	0	0
ENSG00000278631	AC097374.2	0	0	0	0	0	0	0	0
ENSG00000259848	AC097374.1	0	0	0	0	0	0.03624	0	0
ENSG00000163060	TEKT4	0.03305	0	0	0	0	0	0	0
ENSG00000237055	MTCO1P48	0	0	0	0	0	0	0	0
ENSG00000232128	MTCO3P45	0	0	0	0	0	0	0	0
ENSG00000233158	RPS24P6	0	0.30502	0	0	0.22279	0	0.6109	0
ENSG00000231331	AC103563.3	0	0	0	0	0	0	0	0
ENSG00000232594	AC103563.4	0	0	0	0	0	0	0	0
ENSG00000233105	AC103563.5	0	0	0	0	0	0	0	0
ENSG00000224585	AC103563.1	0	0	0	0	0	0	0	0
ENSG00000233447	AC103563.6	0	0	0	0	0	0	0	0
ENSG00000235186	AC103563.8	0	0	0	0	0	0	0	0
ENSG00000277591	RN7SL575P	0	0	0.36025	0	0	0	0	0
ENSG00000233850	AC103563.7	0.92009	0.5105	0	0.22759	2.23724	0.54105	0.25561	0.37656
ENSG00000172005	MAL	5.63462	0.92113	2.43964	2.98657	7.09508	5.41388	3.27044	2.22367
ENSG00000231062	AC103563.2	0	0	0	0	0	0	0	0
ENSG00000144029	MRPS5	3.09827	2.66933	0.64125	1.14954	2.90468	3.06006	0.67282	1.25907
ENSG00000144026	ZNF514	0.82843	0.55515	0.18972	0.41515	0.58862	0.58838	0.39454	0.63406
ENSG00000275111	ZNF2	0.78877	1.09918	0.08087	0.40835	0.98126	0.42068	0.18345	0.49548
ENSG00000275768	SLC2AXP1	0	0	0	0	0	0	0	0
ENSG00000233757	AC092835.1	0.4454	0.58251	0.187	0.2833	0.58019	0.61737	0.10606	0.42967
ENSG00000155066	PROM2	0.02418	0	0	0	0.0147	0	0	0
ENSG00000115041	KCNIP3	0	0	0	0	0	0	0	0
ENSG00000235129	FABP7P2	0	0	0	0	0	0	0	0
ENSG00000115042	FAHD2A	0.85398	0.45228	0.09507	0.15363	0.72363	0.84458	0.19411	0.25418
ENSG00000248821	AC009238.3	0	0	0	0	0	0	0	0
ENSG00000233275	AC009238.2	0	0	0	0	0	0	0	0
ENSG00000227120	AC009238.1	0	0	0	0	0	0	0	0
ENSG00000204705	UBTFL5	0	0	0	0	0	0	0	0
ENSG00000277747	AC133104.2	0	0	0	0	0	0	0	0
ENSG00000234903	AC133104.1	0	0	0	0	0	0	0	0
ENSG00000144010	TRIM43B	0	0	0	0	0	0	0	0
ENSG00000238162	AC009237.11	0	0	0	0	0	0	0	0
ENSG00000223917	AC009237.1	0	0	0	0	0	0	0	0
ENSG00000237308	AC009237.10	0	0	0	0	0	0	0	0
ENSG00000224719	AC009237.2	0	0	0	0	0	0	0	0
ENSG00000226185	TRIM64FP	0	0	0	0	0	0	0	0
ENSG00000272913	AC009237.14	0	0	0	0.13649	0.16772	0.48674	0	0
ENSG00000229689	AC009237.3	0	0	0	0.02185	0.05371	0.20781	0.04909	0
ENSG00000236431	AC009237.8	0	0	0	0	0	0	0	0
ENSG00000273305	AC009237.15	0	0	0	0	0	0	0	0
ENSG00000233037	AC009237.5	0	0	0	0	0	0	0	0

ENSG00000168992	OR7E102P	0	0	0	0	0	0	0	0
ENSG00000232717	TRIM51JP	0	0	0	0	0	0	0	0
ENSG00000274028	AC009237.16	0	0	0	0	0	0	0	0
ENSG00000230083	AC009237.4	0	0	0	0	0	0	0	0
ENSG00000144015	TRIM43	0	0	0	0	0	0	0	0
ENSG00000236026	AC009237.7	0	0	0	0	0	0	0	0
ENSG00000271003	AC009237.13	0	0	0	0	0	0	0	0
ENSG00000270193	AC009237.12	0	0	0	0	0	0	0	0
ENSG00000204699	UBTFL3	0	0	0	0	0	0	0	0
ENSG00000275497	AC009237.17	0	0	0	0	0	0	0	0
ENSG00000235959	AC009237.6	0	0	0	0	0	0	0	0
ENSG00000236750	AC009237.9	0	0	0	0	0	0	0	0
ENSG00000235584	AC008268.1	0	0	0	0	0	0	0	0
ENSG00000237510	GPAT2P1	0	0.05435	0	0	0.0397	0	0	0.04009
ENSG00000232931	LINC00342	0.53197	0.27962	0.20572	0.62329	0.44252	0.39515	0.37335	0.27501
ENSG00000174501	ANKRD36C	2.23988	2.13451	1.16835	2.46145	2.92067	2.72981	2.86427	2.86555
ENSG00000275961	RN7SL210P	0	0	0	0	0	0	0	0
ENSG00000231584	FAHD2CP	0.23977	0.39909	0	0.1525	0.49972	0.48341	0	0.42055
ENSG00000186281	GPAT2	0	0.02147	0	0.01914	0.06271	0.182	0.0215	0
ENSG00000274286	ADRA2B	0	0	0	0	0	0	0	0
ENSG00000188886	ASTL	0.35454	0.07377	0.06512	0.26309	0.21552	1.17272	0.14774	0.16324
ENSG00000158050	DUSP2	17.2248	5.43102	4.40445	9.62855	13.1922	73.357	6.95644	4.89114
ENSG00000228873	AC012307.1	0	0	0	0	0	0	0	0
ENSG00000084090	STARD7	18.2836	11.7199	3.92885	7.68943	15.4046	20.1555	7.36293	9.19544
ENSG00000204685	STARD7-AS1	0.53048	0.46747	0.09171	0.32418	0.41732	0.16515	0.31208	0.26819
ENSG00000135956	TMEM127	22.7258	17.5721	3.02824	4.39107	11.9251	9.07843	6.42852	7.59045
ENSG00000144021	CIAO1	10.7832	7.80903	1.65673	3.26546	8.29044	7.08953	2.63699	4.86712
ENSG00000144028	SNRNP200	12.6528	8.26343	1.31118	3.35537	10.5462	11.7997	3.56723	4.39854
ENSG00000230747	AC021188.1	0.69338	0.49463	0.14556	0.58803	0	1.39797	0.33022	0.24324
ENSG00000198885	ITPRIPL1	1.15024	1.62608	0.46309	0.63139	0.61301	1.72342	0.26264	0.54168
ENSG00000121152	NCAPH	5.21018	6.58801	8.70781	20.6421	9.37927	10.0988	13.8282	11.7507
ENSG00000163121	NEURL3	0.02936	0	0.04314	0.04357	0.07138	0.05179	0	0.03604
ENSG00000235480	AC013270.1	0	0.27729	0	0	0	0	0	0
ENSG00000196843	ARID5A	11.8638	9.85651	1.20857	2.77686	7.79927	5.6948	5.58637	3.48376
ENSG00000114982	KANSL3	3.9435	3.01807	0.89612	1.5446	3.35114	3.77249	2.00584	1.91674
ENSG00000249715	FER1L5	0	0	0	0	0	0	0	0
ENSG00000114988	LMAN2L	1.81289	1.93664	0.23857	0.68267	1.57903	1.00242	0.18041	0.86376
ENSG00000158158	CNNM4	1.47893	1.00379	0.211	0.38357	1.27434	1.87445	0.69407	0.45836
ENSG00000264157	MIR3127	0	0	0	0	0	0	0	0
ENSG00000273265	CNNM3-DT	2.11953	0.294	0.25955	1.04854	0.64422	1.86957	2.35528	0.86744
ENSG00000168763	CNNM3	2.7554	1.6301	0.43905	0.93611	2.17948	2.48902	1.24504	1.22279
ENSG00000163126	ANKRD23	0.03562	0.11857	0.05234	0.05286	0.02165	0.03142	0.02968	0
ENSG00000213337	ANKRD39	0.11002	0.18313	0.08084	0.06123	0.1672	0.41244	0.06877	0.10131
ENSG00000168758	SEMA4C	1.08337	1.01063	0.13996	0.53006	1.27372	0.37804	0.69454	0.42389
ENSG00000168754	FAM178B	0.06203	0.13767	0	0	0.10055	0.21886	0.06893	0
ENSG00000274206	RF00017	0	0	0	0	0	0	0	0
ENSG00000252845	RNA5SP101	0	0	0	0	0	0	0	0
ENSG00000275094	AC079395.3	0	0	0	0	0	0	0	0
ENSG00000273634	AC079395.2	0	0	0	0	0	0	0	0
ENSG00000248134	AC079395.1	0	0	0	0	0	0	0	0
ENSG00000144188	TRIM43CP	0	0	0	0	0.17338	0	0	0
ENSG00000230343	AC018892.1	0	0	0	0	0	0	0	0
ENSG00000236847	AC018892.2	0	0	0	0	0	0	0	0
ENSG00000270748	IGKV2OR2-1	0	0	0	0	0	0	0	0
ENSG00000271402	IGKV2OR2-2	0	0	0	0	0	0	0	0
ENSG00000204670	IGKV1OR2-3	0	0	0	0	0	0	0	0
ENSG00000188383	GPAT2P2	0	0	0	0	0	0	0	0
ENSG00000144199	FAHD2B	0.46811	0.04583	0	0.1226	0.23434	0.29146	0.0918	0.10142

ENSG00000279791	AC018892.3	0	0	0	0	0	0	0	0
ENSG00000275655	RN7SL313P	0	0	0	0	0	0	0	0
ENSG00000135976	ANKRD36	4.14554	4.47955	3.11751	6.31932	5.61422	3.78228	4.3734	4.88734
ENSG00000277701	AC159540.2	0.0478	0.11934	0	0	0.02906	0	0	0
ENSG00000237837	AC159540.1	0	0	0	0	0	0	0	0
ENSG00000271351	IGKV1OR2-9	0	0	0	0	0	0	0	0
ENSG00000276050	IGKV2OR2-10	0	0	0	0	0	0	0	0
ENSG00000271015	IGKV2OR2-7D	0	0	0	0	0	0	0	0
ENSG00000270252	IGKV3OR2-5	0	0	0	0	0	0	0	0
ENSG00000271569	IGKV1OR2-6	0	0	0	0	0	0	0	0
ENSG00000270450	IGKV2OR2-7	0	0	0	0	0	0	0	0
ENSG00000278537	IGKV2OR2-8	0	0	0	0	0	0	0	0
ENSG00000270187	IGKV1OR2-11	0	0	0	0	0	0	0	0
ENSG00000230606	AC092683.1	0.22221	0.11558	0.04082	0.14428	0.05065	0.0245	0.13889	0.05115
ENSG00000278766	AC092683.2	0	0	0	0	0	0	0	0
ENSG00000196912	ANKRD36B	1.72128	1.74787	0.31816	1.54238	2.02687	1.47054	1.56988	1.32915
ENSG00000235833	AC017099.1	0.0713	0.11869	0.10478	0.10582	0	0	0.35656	0
ENSG00000228970	UBTFL6	0	0	0	0	0	0	0	0
ENSG00000135940	COX5B	12.0048	11.1764	2.69098	2.94424	8.47258	12.1146	5.59605	7.49455
ENSG00000115073	ACTR1B	5.70283	3.54382	1.11736	2.55789	3.66697	3.30881	1.90115	2.52066
ENSG00000228486	C2orf92	0.14658	0.15014	0.06628	0.13387	0.06854	0.07956	0.13156	0.08306
ENSG00000201806	RNU4-8P	0	0	0	0	0	0	0	0
ENSG00000115085	ZAP70	7.08479	5.58599	1.2645	3.76738	5.99807	12.5238	3.08379	3.62735
ENSG00000075568	TMEM131	6.8869	6.39875	3.1439	7.81649	5.77869	5.27803	5.5421	5.21893
ENSG00000235734	HMGN1P36	0.24597	0	0	0	0.29905	0	0	0
ENSG00000238719	RNU7-96P	1.14532	1.90638	1.68303	3.39955	4.17734	0	1.90907	2.81241
ENSG00000168658	VWA3B	0	0	0.02593	0	0	0	0	0.01083
ENSG00000231635	ATP5F1BP1	0	0	0	0	0	0	0	0
ENSG00000222000	AC092675.1	0	0	0	0	0	0	0	0
ENSG00000144191	CNGA3	0	0	0	0	0	0	0	0
ENSG00000227987	AC092675.2	0	0	0	0	0	0	0	0
ENSG00000040933	INPP4A	7.3984	5.83813	1.2579	3.24204	5.90642	6.32487	2.50161	3.35092
ENSG00000183513	COA5	2.33711	2.34292	1.28788	3.35028	2.64767	1.87408	2.16914	3.29337
ENSG00000115446	UNC50	4.83191	2.95447	1.59397	3.51236	3.9563	3.53723	1.75326	4.15681
ENSG00000071073	MGAT4A	12.8333	8.17191	3.60165	9.18409	10.8735	9.97702	8.84317	8.8128
ENSG00000236564	YWHAQP5	0	0	0	0	0	0	0	0
ENSG00000226791	LINC02611	0.66288	0.34843	0.05127	0.05178	0.89074	0.80012	0.11631	0.04284
ENSG00000201070	RNU4-84P	0	0	0	0	0	0	0	0
ENSG00000196872	KIAA1211L	0.49854	0.36304	0.09157	0.11561	0.26517	0.10994	0.02597	0.11477
ENSG00000238830	RNU7-46P	0	0	0	0	0	0	0	0
ENSG00000135951	TSGA10	2.32573	1.63781	1.13921	3.15663	2.59798	1.99885	2.58442	2.19654
ENSG00000231822	AC019097.1	0	0.166	0	0	0	0	0	0
ENSG00000273045	C2orf15	0.24457	0	0	0.1037	0.42477	0.06164	0.11647	0
ENSG00000241962	AC079447.1	0	0.12244	0	0.10917	0.04471	0.12977	0.0613	0.09031
ENSG00000144182	LIPT1	1.60817	1.82022	1.13433	0.90695	1.72055	1.36177	1.82279	1.85602
ENSG00000273155	AC092587.1	0	0.18237	0	0.16261	0	0.19329	0	0
ENSG00000158411	MITD1	11.5442	20.5473	12.5354	42.453	18.4444	14.7785	23.6698	16.9751
ENSG00000185414	MRPL30	3.26285	2.73656	0.52036	1.87691	3.50562	3.83741	1.51777	2.39123
ENSG00000185674	LYG2	0	0	0	0	0	0	0	0
ENSG00000144214	LYG1	0.20119	0.11163	0	0.69671	0.32614	0.47324	0.22357	0.32936
ENSG00000115514	TXNDC9	7.00206	6.62901	2.9453	3.63134	6.48754	7.80646	2.64666	5.75265
ENSG00000158417	EIF5B	3.31958	2.5383	0.73786	2.05621	3.54974	3.60885	1.33293	1.75816
ENSG00000135945	REV1	5.00997	5.51229	4.33961	6.90325	7.46749	8.30565	8.69684	5.88474
ENSG00000273306	AC018690.1	0.11709	0.58471	0.17207	0	1.13887	0	0.7807	0.28753
ENSG00000144218	AFF3	1.12221	0.25863	0.04228	0.3331	1.4833	0.57869	0.54678	0.33209
ENSG00000230393	AC092667.1	0.05669	0	0	0	0	0	0	0
ENSG00000232084	LINC01104	0	0	0	0	0	0	0	0
ENSG00000170500	LONRF2	0.01578	0.00876	0	0.01562	0.01919	0	0.03508	0

ENSG00000269383	AC012493.2	0	0	0	0	0	0	0	0
ENSG00000270699	AC012493.3	0	0	0	0	0	0	0	0
ENSG00000115526	CHST10	0.39207	0.43506	0.04801	0.29094	0.49653	0.46111	0.2723	0.12034
ENSG00000238029	AC012493.1	0	0	0	0	0	0	0	0
ENSG00000204640	NMS	0	0	0	0	0	0	0	0
ENSG00000204637	AC068538.1	0.21816	0	0	0	0	0	0	0
ENSG00000115539	PDCL3	5.60461	4.26463	1.00007	1.48533	4.0397	6.35612	2.26878	1.62201
ENSG00000227259	HMGN2P22	0	0	0	0	0	0	0	0
ENSG00000234988	LINC01849	0	0	0	0	0	0	0	0
ENSG00000226034	NANOGNBP1	0	0	0	0	0	0	0	0
ENSG00000234169	LINC01868	0	0	0	0	0	0	0	0
ENSG00000228488	AC092168.1	0	0	0	0	0	0	0	0
ENSG00000170485	NPAS2	0.05281	0	0.00862	0	0.07134	0.06211	0.02934	0.0072
ENSG00000232034	AC092168.2	0	0.17017	0	0	0	0	0	0
ENSG00000230140	AC016738.2	0	0	0	0	0	0	0	0
ENSG00000223947	AC016738.1	0	0	0.03091	0.03122	0.05114	0	0	0
ENSG00000071082	RPL31	22.5559	20.5841	23.5293	29.3427	30.1775	29.8773	40.3611	50.5699
ENSG00000204634	TBC1D8	4.50986	5.20003	0.60734	0.9742	2.55675	1.99436	0.62812	1.13429
ENSG00000271532	BBIP1P1	0	0	0	0	0	0	0	0
ENSG00000272902	TBC1D8-AS1	0.08874	0.14771	0	0	0.07193	0	0	0.03632
ENSG00000158435	CNOT11	13.1814	11.9118	4.4462	10.8551	15.4819	15.8739	8.33247	9.49718
ENSG00000163162	RNF149	93.414	124.203	22.1843	31.1331	62.0094	22.9959	27.1343	74.1415
ENSG00000212283	SNORD89	28.9343	20.3347	31.1804	102.106	15.6345	5.67157	31.081	41.8407
ENSG00000264857	MIR5696	0	0	0	0	0	0	0	1.05879
ENSG00000175874	CREG2	0.03076	0.03413	0	0	0.03739	0	0	0
ENSG00000226058	AC092570.1	0	0	0	0	0	0	0	0
ENSG00000196460	RFX8	0.02873	0	0	0.04264	0	0	0	0
ENSG00000223826	LINC01870	0	0	0	0	0	0	0	0
ENSG00000237549	AC092570.3	0	0	0	0	0	0	0	0
ENSG00000232556	AC092570.2	0	0	0	0	0	0	0	0
ENSG00000071054	MAP4K4	30.4514	30.4282	3.90836	5.70259	23.4247	15.0383	6.15958	10.8273
ENSG00000281162	LINC01127	2.53813	1.77883	0.05609	0.2549	2.22734	0.40399	1.52686	1.54642
ENSG00000115590	IL1R2	13.954	13.9756	1.19176	5.62868	9.04797	0.79952	4.65184	8.31733
ENSG00000115594	IL1R1	3.03219	2.15319	0.68433	1.04439	1.33367	0.5477	2.01823	1.24519
ENSG00000226925	IL1R1-AS1	0	0	0	0	0	0	0	0
ENSG00000115598	IL1RL2	0.05545	0	0	0.04114	0	0	0.04621	0
ENSG00000205716	FAM183DP	0	0	0	0	0	0	0	0
ENSG00000115602	IL1RL1	3.50811	0.61578	0.22495	0.22719	1.56644	0.24755	1.25455	0.59518
ENSG00000115604	IL18R1	10.2045	7.70786	1.31147	2.84897	4.87245	2.40622	1.65602	4.94126
ENSG00000236785	SDR42E1P5	0.08464	0.14089	0	0	0	0	0	0
ENSG00000115607	IL18RAP	32.4736	38.8732	5.91955	6.712	15.3235	9.02479	8.36231	35.4405
ENSG00000264764	MIR4772	5.63849	9.38527	0	1.39469	0	0	3.13283	4.61523
ENSG00000236525	AC007278.2	5.92511	8.33725	1.07714	0.81589	2.82203	0.96984	1.32362	5.69981
ENSG00000234389	AC007278.1	16.9027	14.4351	0.32468	0.24594	2.6191	1.26681	1.19694	6.91763
ENSG00000180251	SLC9A4	0	0.02725	0	0	0	0	0	0.0201
ENSG00000115616	SLC9A2	0	0	0	0	0	0	0	0
ENSG00000135953	MFSD9	2.08135	1.70426	0.29599	0.54804	1.75499	1.36211	0.50361	1.23651
ENSG00000170417	TMEM182	0.09659	0.10048	0.08871	0.21503	0.19083	0.1491	0.06038	0.17789
ENSG00000277527	LINC01796	0	0	0	0	0	0	0	0
ENSG00000224095	LINC01935	0	0	0	0	0	0	0	0
ENSG00000227680	AC108051.1	0	0	0	0	0	0	0	0
ENSG00000229209	AC073987.1	0	0	0	0	0	0	0	0
ENSG00000227623	AC010881.1	0	0	0	0	0	0	0	0
ENSG00000236596	AC092568.1	0	0	0	0	0	0	0	0
ENSG00000221849	AC018880.1	0	0	0	0	0	0	0	0
ENSG00000231723	AC018880.2	0	0	0	0	0	0	0	0
ENSG00000232597	AC013727.1	0	0	0	0	0	0	0	0
ENSG00000236141	AC013727.2	0	0	0	0	0	0	0	0

ENSG00000256637	LINC01965	0	0	0	0	0	0	0	0
ENSG00000273515	RF02091	0	0	0	0	0	0	0	0
ENSG00000228968	AC096554.1	0	0	0	0	0	0	0	0
ENSG00000233955	AHCYP3	0	0	0	0	0	0	0	0
ENSG00000227157	AC068535.1	0	0	0	0	0	0	0	0
ENSG00000225765	LINC01831	0	0	0	0	0	0	0	0
ENSG00000235597	LINC01102	0	0	0	0	0	0	0	0
ENSG00000234781	LINC01103	0	0	0	0	0	0	0	0
ENSG00000231626	AC013402.2	0	0	0	0	0	0	0	0
ENSG00000236109	AC013402.3	0	0	0	0	0	0	0	0
ENSG00000230690	AC013402.1	0	0	0	0	0	0	0	0
ENSG00000225669	RPL23AP27	0	0	0	0	0	0	0	0
ENSG00000228528	AC068057.1	0	0	0	0	0	0	0	0
ENSG00000279904	AC068057.2	0	0	0	0	0	0	0	0
ENSG00000207249	RF00139	0	0	0	0	0	0	0	0
ENSG00000234177	LINC01114	0	0	0	0	0	0	0	0
ENSG00000213301	HMGB3P11	0	0	0	0	0	0	0	0
ENSG00000233639	PANTR1	0	0	0	0	0	0	0	0
ENSG00000269707	AC018730.2	0	0	0	0	0	0	0	0
ENSG00000198914	POU3F3	0	0.1991	0	0	0	0	0	0
ENSG00000239587	AC018730.1	0	0	0	0	0	0	0	0
ENSG00000229743	LINC01159	0	0	0	0	0.02659	0	0	0
ENSG00000224509	AC010884.1	0	0	0	0	0	0	0	0
ENSG00000135972	MRPS9	7.54056	6.68976	5.73724	16.2468	8.51611	10.3314	8.86844	6.7674
ENSG00000231851	UTAT33	0	0	0	0	0	0	0	0
ENSG00000226508	LINC01918	0.07265	0.48368	0.10675	0	0	0	0	0
ENSG00000135973	GPR45	0	0	0	0	0	0	0	0
ENSG00000272861	AC012360.2	0	0	0	0	0.22792	0	0	0
ENSG00000135966	TGFBRAP1	3.64976	2.33655	0.53812	1.32246	2.9384	2.71329	1.32252	1.40878
ENSG00000235319	AC012360.1	0	0	0.15981	0	0	0	0	0
ENSG00000272994	AC012360.3	0.34004	0.31838	0.09369	0.22079	0.46509	0.33743	0.2834	0.15656
ENSG00000135974	C2orf49	5.85726	4.09424	1.40064	3.19421	5.62586	5.26143	4.97126	4.15255
ENSG00000115641	FHL2	0.36506	0.36827	0.37389	0.60747	0.39004	0.99531	0.33191	0.3124
ENSG00000238273	AC108058.1	0.05619	0	0	0	0.03416	0.24782	0	0
ENSG00000071051	NCK2	24.9319	13.9147	2.73788	6.14876	15.4389	18.1642	7.02843	7.25394
ENSG00000235522	AC010978.1	0	0	0	0	0	0	0	0
ENSG00000234162	AC009505.1	0	0	0	0	0	0	0	0
ENSG00000119147	C2orf40	0	0	0	0	0	0	0	0
ENSG00000115652	UXS1	7.01379	8.11305	7.11651	17.3332	10.8239	8.94853	10.9602	9.40593
ENSG00000233339	AC018878.1	0	0	0	0	0	0	0	0
ENSG00000273595	AC092106.2	0	0	0	0	0	0	0	0
ENSG00000213295	RPL22P10	0	0	0	0	0	0	0	0
ENSG00000224061	AC092106.1	0	0	0	0	0	0	0	0
ENSG00000230353	AC114755.3	0	0	0	0	0	0	0	0
ENSG00000233939	AC114755.4	0	0	0	0	0	0	0	0
ENSG00000227513	AC114755.1	0	0	0	0	0.10267	0	0	0
ENSG00000229682	AC114755.2	0	0	0	0	0	0	0	0
ENSG00000235486	AC114755.5	0.44104	0.73411	0.38886	1.57091	0.21448	0.15561	0.44108	0.4332
ENSG00000240935	PLGLA	0	0	0	0.10977	0	0	0	0
ENSG00000153165	RGPD3	0.18898	0.20971	0.22217	0.48615	0.10722	0.17781	0.33601	0.41766
ENSG00000237666	AC097527.1	0.27661	0	0	0	0	0	0	0.16981
ENSG00000254126	CD8B2	0.92639	0.19275	0.17016	0.17186	0.28157	0	0.38604	0.85305
ENSG00000231505	AC108868.1	0	0	0	0	0	0	0	0
ENSG00000232001	AC108868.2	0	0	0	0	0	0	0	0
ENSG00000214199	EEF1A1P12	0.05446	0	0	0	0.06621	0.28821	0	0.13373
ENSG00000144057	ST6GAL2	0.56107	0.63315	0.46115	0.81857	0.79775	0.50329	0.36458	0.44368
ENSG00000238250	ST6GAL2-IT1	0	0	0	0	0	0	0	0
ENSG00000227294	AC016994.1	0	0	0	0	0	0	0	0

ENSG00000229360	PPP1R2P5	0	0	0	0	0	0	0	0
ENSG00000226905	AC005040.1	0	0	0	0	0	0	0	0
ENSG00000229457	LINC01789	0	0	0	0	0	0	0	0
ENSG00000225588	AC096669.1	0	0	0	0	0	0	0	0
ENSG00000237880	LINC01885	0	0	0	0	0	0	0	0
ENSG00000224568	LINC01886	0	0	0	0	0	0	0	0
ENSG00000232991	GACAT1	0	0	0	0	0	0	0	0
ENSG00000230651	RGPD4-AS1	0	0	0	0	0	0	0	0
ENSG00000196862	RGPD4	0.02683	0.08932	0.01971	0.07964	0.11417	0.11833	0.02236	0.06588
ENSG00000233494	AC009963.2	0	0	0	0	0	0	0	0
ENSG00000227270	AC009963.1	0	0	0	0	0	0	0	0
ENSG00000237745	RPL22P8	0	0	0	0.3209	0	0	0	0
ENSG00000235325	AC009963.3	0	0	0	0	0	0	0	0
ENSG00000115665	SLC5A7	0	0	0	0	0	0	0	0
ENSG00000231221	LINC01593	0	0	0	0	0	0	0	0
ENSG00000229890	ACTP1	0	0	0	0	0	0	0	0
ENSG00000225328	LINC01594	0	0	0	0	0	0	0	0
ENSG00000236877	SETD6P1	0	0	0	0	0	0	0	0
ENSG00000196228	SULT1C3	0	0	0	0	0	0	0	0
ENSG00000227597	WASF1P1	0	0	0	0	0	0	0	0
ENSG00000198203	SULT1C2	0	0	0	0	0	0	0	0
ENSG00000261209	SULT1C2P2	0	0	0	0	0	0	0	0
ENSG00000237223	SULT1C2P1	0	0	0	0	0	0.06453	0	0
ENSG00000271070	GMCL1P2	0	0	0	0	0	0	0	0
ENSG00000198075	SULT1C4	0.05059	0.25261	0	0.07508	0.09225	0	0.04216	0.09316
ENSG00000271284	SMIM12P1	0	0	0	0	0	0	0	0
ENSG00000135968	GCC2	11.893	6.87113	2.3275	4.08704	7.39396	6.55224	5.73094	4.716
ENSG00000214184	GCC2-AS1	0	0	0	0.2723	0.11154	0.32368	0	0
ENSG00000169756	LIMS1	30.4671	22.2476	7.63742	16.7007	20.8944	28.6712	16.0056	22.2705
ENSG00000228763	LIMS1-AS1	0	0	0	0	0	0	0	0
ENSG00000233648	AC010095.1	0	0	0	0	0	0	0	0
ENSG00000235644	RPL10P5	0	0	0	0	0	0	0	0
ENSG00000153201	RANBP2	22.6598	18.943	6.38298	14.9711	22.0292	19.2359	14.9473	14.9898
ENSG00000163006	CCDC138	1.89701	2.06295	3.66108	6.02488	3.19811	2.36492	6.23971	4.00608
ENSG00000227470	AC073415.1	0	0	0	0	1.74739	0	0	0
ENSG00000135960	EDAR	0.43059	0.02757	0.07301	0.04916	0.76512	0.26295	0.38647	0.16267
ENSG00000277628	RF00017	0	0	0	0	0	0	0	0
ENSG00000259863	SH3RF3-AS1	0.0914	0	0	0	0.05556	0	0	0.05611
ENSG00000172985	SH3RF3	0.94736	0.81998	0.09281	0.01875	0.72178	0.22284	0.16844	0.1861
ENSG00000264934	MIR4265	0	0	0	0	0	0	0	0
ENSG00000228551	SNRPGP9	0	0	0	0	0	0	0	0
ENSG00000265965	MIR4266	0	0	0	0	0	0	0	0
ENSG00000186522	SEPT10	1.02915	0.64977	0.13037	0.26334	0.53932	0.15651	0.32534	0.06536
ENSG00000237916	AC011753.4	0	0	0	0	0	0	0	0
ENSG00000230696	AC011753.2	0	0	0	0	0	0	0	0
ENSG00000198142	SOWAHC	0.80273	0.49778	0.25442	0.07008	0.15309	0.27767	0.10494	0.1546
ENSG00000230603	AC011753.1	0	0	0	0	0	0	0	0
ENSG00000231099	BMS1P19	0	0	0	0	0	0	0	0.229
ENSG00000233307	AC011753.3	0	0	0	0	0	0	0	0
ENSG00000233707	RPL22P11	0	0	0	0	0	0	0	0
ENSG00000282033	AC074387.1	0	0	0	0	0	0	0	0
ENSG00000015568	RGPD5	0.00517	0	0.0152	0.12279	0.02515	0.03649	0.03448	0.03174
ENSG00000256977	LIMS3	0	0	0	0	0	0	0	0
ENSG00000284337	AC013271.1	0	0	0	0	0	0	0	0
ENSG00000186148	AC013268.1	0	0	0	0	0	0	0	0
ENSG00000283684	AC013268.5	0	0	0	0	0	0	0	0
ENSG00000240069	GPAA1P1	0	0	0	0	0	0	0	0
ENSG00000225108	ZBTB45P1	0	0	0	0	0	0	0	0

ENSG00000204588	LINC01123	0	0	0	0	0	0	0	0
ENSG00000235721	AC013268.3	0	0	0	0	0	0	0	0
ENSG00000229730	AC013268.2	0	0	0	0	0	0	0	0
ENSG00000283331		0	0	0	0	0	0	0	0
ENSG00000283283	AC013268.4	0	0	0	0	0	0	0	0
ENSG00000265682	MIR4267	0	0	0	0	0	0	0	0
ENSG00000144063	MALL	0	0.07705	0	0.0687	0.02814	0	0	0
ENSG00000264979	MIR4436B1	0	0	0	0	0	0	0	0
ENSG00000144061	NPHP1	0.02493	0.01037	0	0.00925	0.01515	0	0	0.0153
ENSG00000233370	AC140479.3	0	0	0	0	0	0	0	0
ENSG00000175701	MTLN	0.71478	0.35692	0.10504	0.68952	0.39105	0.88267	0.65528	0.35104
ENSG00000261760	AC140479.4	0	0	0	0	0	0	0	0
ENSG00000282304	AC140479.5	0	0	0	0	0	0	0	0
ENSG00000282785	AC140479.6	0	0	0	0	0	0	0	0
ENSG00000263881	MIR4436B2	0	0	0	0	0	0	0	0
ENSG00000283167	AC140479.7	0	0	0	0	0	0	0	0
ENSG00000226991	AC140479.1	0	0	0	0	0	0	0	0
ENSG00000230650	AC140479.2	0	0	0	0	0	0	0	0
ENSG00000175772	LINC01106	0	0	0	0	0	0	0	0
ENSG00000226065	ZBTB45P2	0	0	0	0	0	0	0	0
ENSG00000230596	GPAA1P2	0	0	0	0	0	0	0	0
ENSG00000273471	AC112229.4	0	0	0	0	0	0	0	0
ENSG00000184115	AC112229.1	0	0	0	0	0	0	0	0
ENSG00000257207	AC112229.3	0	0	0	0	0	0	0	0
ENSG00000256671	LIMS4	0	0	0	0	0	0	0	0
ENSG00000227574	AC112229.2	0	0	0	0	0	0	0	0
ENSG00000231583	AC108938.1	0	0	0	0	0	0	0	0
ENSG00000183054	RGPD6	0.54525	0.22002	0.45323	1.83094	0.70977	0.38864	0.99147	0.63564
ENSG00000231536	AC226101.1	0	0	0	0	0	0	0	0
ENSG00000216867	RPL22P12	0	0	0	0.2811	0	0	0	0
ENSG00000169679	BUB1	2.07798	2.58904	3.14285	6.09575	4.28446	8.61793	7.29193	4.35662
ENSG00000235881	AC114776.1	0	0	0	0	0	0	0	0
ENSG00000153093	ACOXL	0.06377	0.02123	0	0	0.01551	0	0.02126	0.01566
ENSG00000236330	RPL5P9	0.08273	0	0	0.12278	0	0	0	0
ENSG00000204581	ACOXL-AS1	0	0	0.03139	0	0.02597	0	0.0356	0
ENSG00000153094	BCL2L11	6.4625	8.7594	1.84431	3.74165	4.28317	10.6433	3.52337	3.63611
ENSG00000230499	AC108463.2	0	0	0	0	0.43261	0	0	0.21844
ENSG00000172965	MIR4435-2HG	2.83715	2.85643	1.8248	3.32756	2.04444	3.72722	5.46218	2.67874
ENSG00000227992	AC108463.1	2.1124	0.70322	0	0	0.51364	2.98125	0	0.51872
ENSG00000271590	AC108463.3	0.19784	0.24698	0.07268	0.0734	0	0	0.16489	0.36436
ENSG00000229118	AC068491.2	0	0	0	0	0	0	0	0
ENSG00000270190	AC068491.3	0.24192	0.20133	0	0.17951	0	0.42677	0	0.14851
ENSG00000226928	RPS14P4	0	0	0	0	0.4264	0.30936	0.2923	0
ENSG00000266139	MIR4435-2	0	0	0	0	0	0	1.65109	0
ENSG00000279191	AC068491.4	0.23855	0.10829	0.06374	0.12874	0.18456	0.11477	0	0.15976
ENSG00000223973	AC068491.1	0	0.29494	0	0	0	0	0	0
ENSG00000280878	AC017002.5	0	0	0	0	0	0	0	0
ENSG00000240350	AC017002.3	0.08245	0	0.12116	0	0.50122	0.72729	0.13744	0
ENSG00000224959	AC017002.1	0.99842	0.41547	0.24453	0.49392	0.10115	1.90812	0	1.02153
ENSG00000271635	AC017002.4	0	0	0	0	0	0	0	0
ENSG00000225744	AC017002.2	0	0	0	0	0	0	0	0
ENSG00000237583	AC093166.4	0	0	0	0	0	0	0	0
ENSG00000227842	AC093166.1	0	0	0	0	0	0	0	0
ENSG00000228615	AC093166.2	0	0	0	0	0	0	0	0
ENSG00000153107	ANAPC1	2.74457	2.12396	1.0103	2.31007	3.20972	3.26991	1.81524	1.70175
ENSG00000266063	MIR4771-2	0	0	0	0	0	0	3.30217	0
ENSG00000230958	AC093166.3	0	0	0	0	0	0	0	0
ENSG00000236307	EEF1E1P1	0	0.28374	0	0	0	0	0	0

ENSG00000153208	MERTK	1.38803	0.8307	0.16043	0.23146	0.47402	0.22011	0.25996	0.24893
ENSG00000240183	RN7SL297P	0	0	0	0	0	0	0	0
ENSG00000225496	AC104651.1	0	0	0	0	0	0	0.16623	0
ENSG00000232277	AC104651.2	0	0	0	0	0	0	0	0
ENSG00000153214	TMEM87B	10.2364	7.68356	2.68465	5.96086	8.08764	5.78163	3.92855	4.77724
ENSG00000144152	FBLN7	0.12258	0.07419	0.01637	0.06615	0.06774	0.11795	0.01857	0.06841
ENSG00000278962	AC092645.1	0.12905	0	0	0.04788	0.07845	0.28458	0.10755	0.11883
ENSG00000144161	ZC3H8	1.39937	1.03522	0.55489	0.8571	2.07939	1.29312	1.14775	1.10451
ENSG00000188177	ZC3H6	8.78138	13.1074	4.11582	6.95867	14.7911	8.88519	7.84654	7.96805
ENSG00000235258	NDUFB4P6	0	0	0	0	0	0	0	0
ENSG00000236555	AC115115.1	0	0	0	0	0	0	0	0
ENSG00000169629	RGPD8	1.7564	0.40752	1.83017	8.68896	2.01891	1.59621	3.37123	2.19569
ENSG00000283698	AC012442.3	0	0	0	0	0	0	0	0
ENSG00000114999	TTL	2.18988	1.427	0.51351	1.25852	1.62576	1.5864	0.93196	0.9153
ENSG00000125630	POLR1B	2.28159	1.94705	0.45952	1.22038	2.60493	3.00345	1.00385	1.23712
ENSG00000207383	RF00019	0	0	0	0	0.88234	0	0	0
ENSG00000125611	CHCHD5	0.897	0.32458	0.08596	0.23152	0.49786	1.03202	0.09751	0.26336
ENSG00000243389	AC012442.2	0.17247	0	0.25344	0.25597	0.20969	0	0	0
ENSG00000228251	AC012442.1	0.13856	0	0.20362	0	0.16846	0.24445	0.23096	0.17013
ENSG00000231747	AC079922.1	0	0	0	0	0.74471	0.3602	0	0
ENSG00000237753	AC079922.2	0.39877	0.12068	0.3729	0.26901	0.26444	0.19186	0.36255	0.13353
ENSG00000144136	SLC20A1	7.978	9.38527	2.72327	6.37851	8.61285	8.6718	4.92928	5.82552
ENSG00000144130	NT5DC4	0	0.11551	0.02549	0.02575	0	0	0	0.0639
ENSG00000169607	CKAP2L	0.82865	0.86498	0.94939	2.79312	1.75877	2.18039	2.08355	1.58646
ENSG00000115008	IL1A	0	0	0	0	0	0	0	0
ENSG00000125538	IL1B	19.2484	34.9553	3.40955	3.23265	22.8549	11.6531	2.20998	17.9355
ENSG00000280228	AC079753.1	0.0379	0	0	0.05625	0	0	0.06317	0
ENSG00000180152	XIAPP3	0	0	0	0	0.1121	0	0	0
ENSG00000125571	IL37	0	0	0	0	0	0.15413	0	0
ENSG00000227368	CDK8P2	0	0	0	0	0	0	0	0
ENSG00000136688	IL36G	0	0	0	0	0	0	0	0
ENSG00000236124	HMGN2P23	0	0	0	0	0	0	0	0
ENSG00000232090	AC016724.1	0	0	0	0	0	0	0	0
ENSG00000136694	IL36A	0	0	0	0	0.10036	0	0	0.10135
ENSG00000136696	IL36B	0	0	0	0	0	0	0	0
ENSG00000136695	IL36RN	0	0	0	0	0	0	0	0
ENSG00000136697	IL1F10	0	0	0	0	0	0	0	0
ENSG00000201805	RNU6-1180P	0	0	0	0	0	0	0	0
ENSG00000136689	IL1RN	22.5574	38.0714	7.27797	3.7086	12.5628	3.69349	3.26465	10.7245
ENSG00000125637	PSD4	11.3151	10.892	1.89914	4.11932	11.4296	9.17746	2.27236	5.62398
ENSG00000234174	AC016683.1	0	0	0	0	0	0.1204	0	0
ENSG00000189223	PAX8-AS1	0.59407	0.36022	0.11224	0	0.01548	0.4267	0.25463	0.3126
ENSG00000125618	PAX8	0.01981	0.06595	0.01456	0	0	0.0699	0	0.01216
ENSG00000231292	IGKV1OR2-108	0	0	0	0	0.25246	0	0	0
ENSG00000234997	AC016745.1	0.28858	0.24017	0	0	0	0	0	0.35432
ENSG00000272563	AC016745.2	0.10645	0.07087	0.09385	0	0.02588	0.07512	0	0.07842
ENSG00000136682	CBWD2	2.0691	2.07804	0.78258	1.34751	2.72784	1.63257	1.45522	1.37204
ENSG00000184492	FOXD4L1	0	0.04898	0	0	0	0	0	0
ENSG00000234148	LINC01961	0	0	0	0	0	0	0	0
ENSG00000231943	PGM5P4-AS1	0	0	0	0	0	0.18023	0	0.06272
ENSG00000225398	PGM5P4	0	0	0.24205	0	1.00131	0.29059	0.27456	0.20224
ENSG00000226516	FAM138B	0	0	0	0	0	0	0	0
ENSG00000221055	MIR1302-3	0	0	0	0	0	0	0	0
ENSG00000146556	WASH2P	1.43726	0.74245	0.10924	0.25744	0.78331	0.74317	0.28914	0.39552
ENSG00000236397	DDX11L2	0.1573	0.87274	0	0.23345	1.5299	1.94245	0.43698	1.48064
ENSG00000279267	AL078621.3	0.25127	1.01574	0	0.63929	1.3529	0.31663	0	1.36626
ENSG00000240356	RPL23AP7	1.2056	4.07077	0.35432	1.9426	3.97842	3.15987	0.51674	6.51295
ENSG00000144134	RABL2A	0.39041	0.22338	0	0.10864	0.38566	0.25828	0.10168	0.19473

ENSG00000144158	AL078621.1	0	0	0	0	0	0	0	0
ENSG00000175509	AL078621.2	0	0	0	0	0	0	0	0
ENSG00000233479	AC017074.2	0	0	0	0	0	0	0	0
ENSG00000227359	AC017074.1	0	0.21748	0.192	0.19391	0.15885	0.461	0	0.64169
ENSG00000202427	RNU6-744P	0	0	0	0	0	0	0	0
ENSG00000115084	SLC35F5	4.6865	4.39198	2.15573	3.12695	4.52466	2.7964	3.84019	4.26715
ENSG00000265429	MIR4782	0	0	0	0	0	0	0	2.27841
ENSG00000244063	AC104653.2	0.1762	0.29329	0.25893	1.30752	0.85689	0	1.46851	0.86536
ENSG00000228857	AC104653.1	0.76468	0.54549	0.32105	1.297	1.19531	1.15629	1.82087	1.07299
ENSG00000270019	AC110769.2	0.66292	0.78817	0.20875	0.35138	0.51812	0.50121	0.31571	0.4651
ENSG00000115091	ACTR3	48.3153	49.739	12.3188	23.3028	40.0652	37.4767	17.3196	33.6191
ENSG00000279957	AC110769.3	0.81237	1.04014	0	0.18548	0.30389	0.33072	0.5208	0.07672
ENSG00000234199	LINC01191	0.08685	0.14456	0	0	0	0.15321	0	0
ENSG00000243179	AC110769.1	0.26982	0.1497	0	0	0.21869	0.31733	0	0.22085
ENSG00000212182	RF00012	0	0	0	0	0	0	0	0
ENSG00000235242	AC010982.1	0	0	0	0	0	0	0	0
ENSG00000231926	SEPHS1P7	0	0	0	0	0	0	0	0
ENSG00000222923	RNU2-41P	0	0	0	0	0	0	0	0
ENSG00000175497	DPP10	0.02931	0.04878	0.17226	0.91334	0.02672	0.0517	0.15876	0
ENSG00000231538	DPP10-AS3	0	0	0	0	0	0	0	0
ENSG00000235717	DPP10-AS2	0	0	0	0	0	0	0	0
ENSG00000235026	DPP10-AS1	0	0	0	0.10933	0	0	0.12279	0
ENSG00000230391	RPSAP23	0	0	0	0	0	0	0	0
ENSG00000271667	AC104408.1	0	0	0	0	0	0	0	0
ENSG00000224869	AC062016.1	0	0	0	0	0	0	0	0
ENSG00000226341	MTCYBP39	0	0	0	0	0	0	0	0
ENSG00000235871	AC062016.2	0	0	0	0	0	0	0	0
ENSG00000230158	MTND1P28	0	0	0	0	0	0	0	0
ENSG00000230950	MTND2P21	0	0	0	0	0	0	0	0
ENSG00000228898	MTCO1P43	0	0	0	0	0	0	0	0
ENSG00000239185	RNU7-190P	0	0	0	0	0	0	0	0
ENSG00000227291	AC092170.1	0	0	0	0	0	0	0	0
ENSG00000238207	AC009312.1	0	0	0	0	0	0	0	0
ENSG00000088205	DDX18	7.4318	5.53242	2.07169	3.53335	7.35545	7.01654	3.34591	4.94061
ENSG00000236255	AC009404.1	0.12644	0.15033	0.05309	0.02681	0.15373	0.28679	0.24087	0.06653
ENSG00000125631	HTR5BP	0.03218	0	0	0.09551	0	0.1703	0	0
ENSG00000125633	CCDC93	5.79859	4.38716	1.09517	2.49539	4.19893	3.52741	2.95414	2.67815
ENSG00000224967	AC009303.1	1.87416	1.73308	0	0.6181	0.25317	1.46945	1.04131	0.51135
ENSG00000235066	AC009303.2	0	0	0	0	0	0	0	0
ENSG00000272895	AC009303.3	0	0	0	0	0	0	0	0
ENSG00000279227	AC009303.4	0.2932	0.40669	0	0.21757	0.23764	0.68966	0.16291	0
ENSG00000243510	RN7SL111P	0	0	0	0	0	0	0	0
ENSG00000125629	INSIG2	3.09903	2.53283	1.14531	2.01047	3.11344	1.99697	2.25802	2.27841
ENSG00000226856	THORLNC	0	0	0	0.22224	0	0.13209	0	0
ENSG00000258910	LINC01956	0	0	0	0	0	0	0	0
ENSG00000163064	EN1	0	0	0	0	0	0	0	0
ENSG00000019169	MARCO	10.6478	4.75358	0.46629	0.41861	1.54315	1.61717	0.52892	1.60168
ENSG00000259094	AC013457.1	0.0725	0	0	0	0	0	0	0
ENSG00000144119	C1QL2	0	0	0	0	0	0	0	0
ENSG00000264833	RN7SL468P	0	0	0	0	0	0	0	0
ENSG00000115107	STEAP3	0.86765	0.40117	0.14167	0.31	0.64464	0.14173	0.10713	0.19728
ENSG00000229867	STEAP3-AS1	0.04556	0.03791	0	0	0	0	0	0
ENSG00000186132	C2orf76	2.65169	2.20687	0.55666	1.89743	2.70574	1.50363	1.18392	2.55806
ENSG00000155368	DBI	19.0774	19.4513	2.23241	3.90224	13.9944	17.7295	4.18793	6.42068
ENSG00000171227	TMEM37	0	0	0	0	0	0	0	0
ENSG00000080293	SCTR	0	0	0	0	0	0	0	0
ENSG00000231013	AC013275.1	0	0	0	0	0	0	0	0
ENSG00000163075	CFAP221	0	0	0	0	0	0	0	0

ENSG00000144120	TMEM177	0.22659	0.23572	0.04162	0.08407	0.48208	0.69952	0.37768	0.2782
ENSG00000229326	AC069154.1	0.13524	0	0.19873	0	0.49327	0	0	0
ENSG00000088179	PTPN4	16.6843	20.0318	15.6231	32.9075	22.3541	23.5818	21.1951	26.705
ENSG00000233741	AC016691.1	0	0	0	0	0	0	0	0
ENSG00000115109	EPB41L5	0.63183	0.52584	0.37139	0.73844	0.96981	0.52945	0.43443	0.54303
ENSG00000224789	AC012363.1	0.25496	0.84875	0.18733	0	0	2.02401	0	1.09562
ENSG00000202046	RF00019	0	0	0	0	0	0	0	0
ENSG00000236878	MTATP6P26	0	0	0	0	0	0	0	0
ENSG00000227788	MTCO3P43	0	0	0	0	0	0	0	0
ENSG00000233286	MTND3P10	0	0	0	0	0	0	0	0
ENSG00000243104	MTND4LP14	0	0	0	0	0	0	0	0
ENSG00000234065	MTND4P26	0	0	0	0	0	0	0	0
ENSG00000223549	MTND5P28	0	0	0	0	0	0	0	0
ENSG00000226479	TMEM185B	6.19149	8.75988	0.70765	1.58252	4.72557	2.24521	1.54804	3.84314
ENSG00000144118	RALB	52.0269	78.9393	9.79218	12.39	46.5608	14.2842	12.6162	30.812
ENSG00000235840	AC012363.2	0	0.21984	0	0	0.16057	0	0	0
ENSG00000163083	INHBB	0.11439	0.03808	0	0	0.22251	0	0.03813	0.02809
ENSG00000280409	LINC01101	0	0	0	0	0	0	0	0
ENSG00000237614	AC073257.2	0	0	0	0	0	0	0	0
ENSG00000232140	AC073257.1	0	0	0	0	0	0	0	0
ENSG00000201006	RF00019	0	0	0	0	0	0	0	0
ENSG00000229774	AC018866.1	0	0	0	0	0	0	0	0
ENSG00000074047	GLI2	0	0	0	0	0	0	0	0
ENSG00000271709	AC017033.1	0	0	0	0	0	0	0	0
ENSG00000201584	RF00019	0	0	0	0	0	0	0	0
ENSG00000234455	AC067960.1	0	0	0	0	0	0	0	0
ENSG00000236145	AC079988.1	0	0	0	0	0	0	0	0
ENSG00000115112	TFCP2L1	0.2581	0.13018	0.03448	0.13929	0.08558	0	0.02607	0.04801
ENSG00000232723	AC013399.1	0	0	0	0	0	0	0	0
ENSG00000074054	CLASP1	6.25427	5.6191	2.2611	4.9117	6.52329	6.59897	4.68734	4.79626
ENSG00000265451	AC012447.1	0.32244	0.1789	0.15794	0.15951	0	0.18961	0	0.52784
ENSG00000264229	RNU4ATAC	22.5096	25.9388	35.6219	79.662	20.3495	27.4915	23.0892	43.9356
ENSG00000222467	RF00019	3.18697	0	0	2.36491	0	1.40556	0	0
ENSG00000236859	NIFK-AS1	1.3505	1.05221	0.12667	0.68231	0.90828	1.0645	0.81422	0.56447
ENSG00000230993	RPL12P15	0	0	0	0	0	0	0	0
ENSG00000279721	AC018737.2	0	0	0	0	0	0	0	0
ENSG00000213239	NPM1P32	0	0	0	0	0	0	0	0
ENSG00000155438	NIFK	6.68001	5.43479	1.4086	3.73437	9.50529	7.82106	3.19556	4.41342
ENSG00000211460	TSN	13.9881	10.9356	11.0476	25.5981	17.1277	15.0526	23.316	16.9906
ENSG00000270798	AC018737.1	0	0	0	0	0	0	0	0
ENSG00000224655	LINC01823	0	0	0	0	0	0	0	0
ENSG00000237856	AC062020.1	0	0	0	0	0	0	0	0
ENSG00000232740	LINC01826	0	0	0	0	0.30519	0	0	0
ENSG00000226708	AC073409.1	0.27249	0	0.80085	0.40441	0	0	0	0
ENSG00000235640	AC092646.2	0	0	0	0	0	0	0	0
ENSG00000227738	AC092646.1	0	0	0	0	0	0	0	0
ENSG00000279874	AC064859.1	0	0	0	0	0	0	0	0
ENSG00000223118	RN7SKP102	0	0	0	0.34535	0	0	0.77575	0
ENSG00000228400	AC079154.1	0	0	0	0	0	0	0	0
ENSG00000155052	CNTNAP5	0	0	0	0	0	0	0	0
ENSG00000238046	MTND5P22	0	0	0	0	0	0	0	0
ENSG00000270815	AC019159.1	0	0	0	0	0	0	0	0
ENSG00000252674	RNA5SP102	0	0	0	0	0	0	0	0
ENSG00000206726	RNU6-259P	0	0	0	0	0	0	0	0
ENSG00000235491	LINC01889	0	0	0	0	0	0	0	0
ENSG00000224410	AC097499.1	0	0	0	0	0	0	0	0
ENSG00000226813	LINC01941	0	0	0	0.17434	0	0	0	0
ENSG00000235774	AC023347.1	0	0	0	0	0	0	0	0

ENSG00000236655	AC023347.2	0	0	0	0	0	0	0	0
ENSG00000213236	YWHAZP2	0.30206	0.16759	0	0	0.12241	0.35525	0.33566	0.12362
ENSG00000136732	GYPC	213.638	283.006	145.756	117.946	387.198	629.248	80.9887	217.026
ENSG00000235128	AC013474.1	0.64218	0.61081	0	0.13615	0.44614	0.48553	1.07042	0.56319
ENSG00000206963	RNU6-675P	0	0	0	0	0	0	0	0
ENSG00000237524	TEX51	0	0	0	0	0	0	0	0
ENSG00000238788	RNU7-182P	0	0	0	0	0	0	0	0
ENSG00000260634	AC012508.2	0	0	0	0	0	0	0	0
ENSG00000260163	AC012508.1	0	0	0	0	0	0	0	0
ENSG00000136717	BIN1	4.30556	2.99343	0.48191	1.55431	3.67836	4.94554	1.34012	1.50666
ENSG00000237630	NIFKP9	0	0	0	0	0	0	0	0
ENSG00000186684	CYP27C1	0	0	0	0	0	0	0	0
ENSG00000226789	AC110926.1	0	0	0.03813	0	0	0	0	0
ENSG00000163161	ERCC3	2.25814	1.86285	0.33474	0.74964	2.20353	2.21897	1.02353	1.17953
ENSG00000169967	MAP3K2	30.1915	29.6964	6.40218	11.5422	22.7593	10.4614	12.39	17.4942
ENSG00000200250	RNU6-1147P	0	0	0	0	0	0	0	0
ENSG00000236682	AC068282.1	0.35424	0.13607	0.04004	0.04044	0.33129	0	0.04542	0.03346
ENSG00000115718	PROC	0.11752	0.0978	0	0	0.17859	0	0	0.03607
ENSG00000264075	MIR4783	0	0	0	0	0	0	0	0
ENSG00000163166	IWS1	10.9156	10.4369	1.68709	3.87959	9.14788	7.04204	2.88522	5.16128
ENSG00000231731	AC010976.1	0	0	0	0	0	0	0	0
ENSG00000202429	RNU4-48P	0	0	0	0	0	0	0	0
ENSG00000169994	MYO7B	1.74088	1.48351	0.15912	0.22252	0.20254	0.05878	0.12496	0.16363
ENSG00000272789	AC010976.2	0	0	0	0	0	0	0	0
ENSG00000072163	LIMS2	0.06909	0.01438	0	0.05127	0.0945	0.10666	0.08638	0.06362
ENSG00000144230	GPR17	0.10977	0.09136	0	0	0	0	0	0.06739
ENSG00000136709	WDR33	4.09738	3.40586	1.59576	3.5665	4.35804	4.87802	4.01402	3.18508
ENSG00000173349	SFT2D3	2.57091	1.30665	0.25955	0.61165	2.60073	2.28503	1.17764	1.01202
ENSG00000201470	RNY4P7	2.29064	0	0	0	1.8566	5.38799	0	0.93747
ENSG00000200390	RF00019	0	0	0	0	0	2.43985	0	0
ENSG00000274010	AC006011.2	0.0633	0	0	0	0.07696	0.22334	0	0
ENSG00000144231	POLR2D	1.86547	1.35409	0.35039	1.12408	1.84168	2.12798	1.356	1.34324
ENSG00000202532	RNU6-395P	0	0	0	0	0	0	0	0
ENSG00000244563	AC006011.1	0	0	0	0	0	0	0	0
ENSG00000273950	RF00017	0	0	0	0	0	0	0	0
ENSG00000144233	AMMECR1L	2.80207	2.38328	0.22624	1.25671	2.58309	2.00989	1.5141	1.51224
ENSG00000272667	AC012306.2	1.74525	1.16199	0.51292	0.86338	0.70728	2.46308	1.16362	0.57141
ENSG00000204399	AC012306.1	0	0	0	0	0	0	0	0
ENSG00000136715	SAP130	6.973	5.58008	1.89663	4.42805	6.82691	7.89532	4.10714	2.696
ENSG00000234044	AC108059.3	0	0	0	0	0	0	0	0
ENSG00000200432	RF00019	0	0	0	0	0	0	0	0
ENSG00000136731	UGGT1	15.9273	14.1263	9.59545	18.6246	16.0267	17.3304	19.8305	14.5594
ENSG00000229173	AC108059.1	0	0.16829	0	0	0	0	0	0
ENSG00000232101	AC108059.2	0	0	0	0	0	0	0	0
ENSG00000229758	DYNLT3P2	0.24848	0	0	0	0	0	0	0.61015
ENSG00000185631	AC017079.1	0	0	0	0	0	0	0.29441	0
ENSG00000238180	AC017079.2	0	0	0	0	0	0	0	0
ENSG00000136720	HS6ST1	1.89394	1.64137	0.20701	0.62721	1.10373	1.18736	0.60007	0.65341
ENSG00000252742	RF00019	0	0	0	0	0	0	0	0
ENSG00000238379	RNA5SP103	0	0	0	0	0	0	0	0
ENSG00000227653	ISCA1P6	0.18795	0	0	0	0.2285	0	0	0
ENSG00000237532	AC012451.1	0	0	0	0	0	0	0	0
ENSG00000238277	AC068483.1	0	0	0	0	0	0	0	0
ENSG00000204460	LINC01854	0	0	0	0	0	0	0	0
ENSG00000233428	PPIAP65	0	0	0	0	0	0	0	0
ENSG00000229536	LINC02572	0	0	0	0	0	0	0	0.15985
ENSG00000214100	PLAC9P1	0	0	0	0	0	0	0	0
ENSG00000237574	LINC01856	0	0	0	0	0	0	0	0

ENSG00000234559	RPL22P7	0	0	0	0	0	0	0	0
ENSG00000225449	RAB6C-AS1	0	0	0	0.0551	0	0	0	0
ENSG00000222014	RAB6C	0.04768	0.07936	0.07006	0.14151	0.14491	0.04205	0	0
ENSG00000180178	FAR2P1	0	0	0	0	0	0	0	0
ENSG00000250207	AC079776.2	0	0	0	0	0	0	0	0
ENSG00000217289	AC079776.1	0	0.10591	0	0.09443	0	0	0	0.07812
ENSG00000231147	ARHGAP42P2	0	0	0	0	0	0	0	0
ENSG00000231240	KLF2P1	0	0	0	0	0	0	0	0
ENSG00000248313	CYP4F27P	0	0	0	0	0	0	0	0
ENSG00000196604	POTEF	0	0	0	0	0.02117	0	0	0
ENSG00000228471	AC018865.2	0	0	0	0	0	0	0	0
ENSG00000224087	AC018865.1	0	0	0	0	0	0	0	0
ENSG00000252223	RNU6-1049P	0	0	0	0	0	0	0	0
ENSG00000223760	MED15P9	0	0	0	0	0	0	0	0
ENSG00000152076	CCDC74B	0	0	0	0	0	0	0	0
ENSG00000136699	SMPD4	2.07726	1.24076	0.39434	0.75228	1.43795	2.55994	0.62954	0.92743
ENSG00000152082	MZT2B	3.91743	1.54611	1.30562	2.69717	4.27171	4.34602	5.78926	2.82635
ENSG00000152086	TUBA3E	0	0	0	0	0	0	0	0
ENSG00000227632	AC018804.1	0.23722	0.19742	0	0	0.43261	0	0	0
ENSG00000230756	RHOQP3	0	0	0	0	0	0	0	0
ENSG00000213225	NOC2LP1	0	0	0	0	0.03267	0	0	0
ENSG00000234398	AC134915.1	0	0	0	0	0	0	0	0
ENSG00000236595	MED15P5	0	0	0	0	0	0	0	0
ENSG00000237722	MTND1P29	0	0	0	0	0	0	0	0
ENSG00000231954	MTND2P22	0	0	0	0	0	0	0	0
ENSG00000236211	MTCO1P7	0	0	0	0	0	0	0	0
ENSG00000230496	MTCO2P7	0	0	0	0	0	0	0	0
ENSG00000232990	MTATP6P7	0	0	0	0	0	0	0	0
ENSG00000249997	MTCO3P7	0	0	0	0	0	0	0	0
ENSG00000283940	AC068137.3	0	0	0	0	0	0	0	0
ENSG00000284171	AC068137.4	0	0	0	0	0	0	0	0
ENSG00000230548	MTND4P27	0	0	0	0	0	0	0	0
ENSG00000234934	MTND5P29	0	0	0	0	0	0	0	0
ENSG00000248136	MTND6P8	0	0	0	0	0	0	0	0
ENSG00000233550	MTCYBP8	0	0	0	0	0	0	0	0
ENSG00000226915	AC068137.1	0	0	0	0	0	0	0	0
ENSG00000271435	AC068137.2	0	0	0	0	0	0	0	0
ENSG00000224352	AC132479.1	0	0	0	0	0	0	0	0
ENSG00000136710	CCDC115	3.77937	2.61309	0.65506	1.40944	3.62876	3.31656	1.35684	2.11786
ENSG00000136718	IMP4	3.13249	2.52802	0.41847	1.60601	3.85457	2.74633	1.42401	1.49179
ENSG00000072135	PTPN18	23.0412	14.7915	2.12667	3.90001	13.7981	17.1998	4.04164	5.36179
ENSG00000178162	FAR2P2	0.01367	0.06824	0.02008	0.02028	0.01661	0	0	0.03356
ENSG00000230646	KLF2P2	0	0	0	0	0	0	0	0
ENSG00000239402	CYP4F62P	0	0	0	0	0	0	0	0
ENSG00000196834	POTEI	0	0	0	0	0	0	0	0
ENSG00000251973	RNU6-473P	0	0	0	0	0	0	0	0
ENSG00000225819	AC013269.2	0	0	0	0	0	0	0	0
ENSG00000274711	RF00017	0	0	0	0	0	0	0	0
ENSG00000152093	CFC1B	0	0	0	0	0	0	0	0
ENSG00000225341	AC013269.1	0	0	0	0	0	0	0	0
ENSG00000184761	PRSS40B	0	0	0	0	0	0	0	0
ENSG00000183292	PRSS40A	0	0	0	0	0	0.03168	0	0
ENSG00000232408	AC140481.2	0	0	0	0	0	0	0	0
ENSG00000136698	CFC1	0	0	0	0	0	0	0	0
ENSG00000277668	RF00017	0	0	0	0	0	0	0	0
ENSG00000222038	POTEJ	0	0.03607	0	0	0	0	0	0
ENSG00000251757	RNU6-848P	0	0	0	0	0	0	0	0
ENSG00000214081	CYP4F30P	0	0	0	0	0	0	0	0

ENSG00000282944	AC140481.3	0	0	0	0	0	0	0	0
ENSG00000240253	FAR2P3	0	0	0	0	0	0	0	0
ENSG00000223349	KLF2P3	0	0	0	0	0	0	0	0
ENSG00000173302	GPR148	0	0	0	0	0	0	0	0
ENSG00000229797	AC140481.1	0	0	0	0	0	0	0	0
ENSG00000178171	AMER3	0	0	0	0	0	0	0	0
ENSG00000232503	CYCSP8	0	0	0	0	0	0	0	0
ENSG00000233221	AC133785.1	0	0	0	0	0	0	0	0
ENSG00000136002	ARHGEF4	0.02072	0	0.0203	0.01025	0.05879	0.03656	0	0.02544
ENSG00000281516	RF00423	0	0	0	0	0	0	0	0
ENSG00000281159	RF00426	0	0	0	0	0	0	0	0
ENSG00000284479	SMIM39	0	0	0	0	0	0	0	0
ENSG00000152102	FAM168B	12.8124	6.59991	1.98186	4.38345	9.85448	8.39872	5.10302	4.80205
ENSG00000115762	PLEKHB2	13.466	10.0827	2.83373	4.98008	9.09976	8.30302	5.90199	7.08954
ENSG00000230450	NEK2P4	0.05515	0	0	0.08186	0.06706	0	0.36774	0
ENSG00000225448	AC009477.1	0	0	0	0	0	0	1.68913	0
ENSG00000236956	NF1P8	0.60206	0.41755	1.10589	0	0.48798	0.35404	8.53004	1.29359
ENSG00000248796	MED15P8	0	0	0	0	0	0	0	0
ENSG00000188219	POTEE	0.01749	0	0	0.02595	0	0	0	0.02147
ENSG00000212199	RNU6-127P	0	0	0	0	0	0	0	0
ENSG00000231431	FAR2P4	0	0	0	0	0	0	0	0
ENSG00000236516	KLF2P4	0	0	0	0	0	0	0	0
ENSG00000227817	ARHGAP42P1	0	0	0	0	0	0	0	0
ENSG00000224425	AC073869.2	0	0	0	0	0	0	0	0
ENSG00000233087	RAB6D	0.12088	0.24144	0.03553	0.07176	0.14696	0.04265	0.0403	0
ENSG00000283303	AC073869.5	0	0	0	0	0	0	0	0
ENSG00000233285	MTCYBP10	0	0	0	0	0	0	0	0
ENSG00000227275	MTND6P10	0	0	0	0	0	0	0	0
ENSG00000235080	MTND5P23	0	0	0	0	0	0	0	0
ENSG00000231777	MTND4P21	0	0	0	0	0	0	0	0
ENSG00000284003	AC073869.6	0	0	0	0	0	0	0	0
ENSG00000283865	MTND3P18	0	0	0	0	0	0	0	0
ENSG00000227661	MTCO3P18	0	0	0	0	0	0	0	0
ENSG00000236590	MTATP6P4	0	0	0	0	0	0	0	0
ENSG00000234480	MTCO2P18	0	0	0	0	0	0	0	0
ENSG00000237910	MTCO1P18	0	0	0	0	0	0	0	0
ENSG00000234939	MTND2P18	0	0	0	0	0	0	0	0
ENSG00000234576	MTND1P26	0	0	0	0	0	0	0	0
ENSG00000226831	MED15P3	0	0	0	0	0	0	0	0
ENSG00000276460	AC073869.4	0	0	0	0	0	0	0	0
ENSG00000223631	LINC01120	0	0	0	0	0	0	0	0
ENSG00000214077	GNAQP1	0	0.11435	0.10095	0	0.33408	0.12119	0	0
ENSG00000217950	NOC2LP2	0	0	0	0	0	0	0	0
ENSG00000232742	RHOQP2	0.1266	0	0	0.18789	0	0	0	0
ENSG00000273073	AC073869.3	0	0	0	0	0	0	0	0
ENSG00000173272	MZT2A	1.39688	1.07313	0.39475	0.69104	1.13219	1.35852	0.53732	0.94549
ENSG00000075886	TUBA3D	0	0	0	0	0	0	0	0
ENSG00000284149	MIR4784	0	0	0	0	0	0	0	0
ENSG00000152117	AC073869.1	1.6855	1.18455	0.22016	0.63926	1.29782	1.35457	1.27987	0.78178
ENSG00000163040	CCDC74A	0	0	0	0	0	0	0	0
ENSG00000224679	MED15P4	0	0	0	0	0	0	0	0
ENSG00000204434	POTEKP	0	0	0	0	0	0	0	0
ENSG00000251956	RNU6-617P	0	0	0	0	0	0	0	0
ENSG00000273588	AC093838.1	0	0	0	0	0	0	0	0
ENSG00000224559	LINC01087	0	0	0	0	0	0	0	0
ENSG00000270930	GRAMD4P8	0	0	0	0	0	0	0	0
ENSG00000197927	C2orf27A	0.45706	0.04755	0.04198	0	0.17365	0.10079	0.04762	0
ENSG00000213222	AC093724.1	0	0	0	0	0	0	0	0

ENSG00000186825	C2orf27B	0	0	0	0	0	0	0	0
ENSG00000274353	AC103564.3	0	0	0	0	0	0	0	0
ENSG00000229203	AC103564.1	0	0	0	0	0	0	0	0
ENSG00000232760	AC103564.2	0	0	0	0	0	0	0	0
ENSG00000244337	AJ239322.2	0	0	0	0	0	0	0	0
ENSG00000235615	AJ239322.1	0	0	0	0	0	0	0	0
ENSG00000236485	LINC01945	0	0	0	0	0	0	0	0
ENSG00000226886	AC093787.1	0	0	0	0	0	0	0	0
ENSG00000271179	AC098826.3	0	0	0	0	0	0	0	0
ENSG00000225594	AC098826.1	0	0	0	0	0	0	0	0
ENSG00000227745	AC098826.2	0	0	0	0	0	0	0	0
ENSG00000163046	ANKRD30BL	0	0	0	0	0	0	0	0.01819
ENSG00000239108	RNU6-1132P	0	0	0	0	0	0	0	0
ENSG00000202174	RNA5-8SP5	0	0	0	0	0	0	0	0
ENSG00000221288	MIR663B	0	0	0	0	0	0	0	0
ENSG00000233786	CDC27P1	0.10252	0.51192	0.3013	0.15215	0.24928	0.18086	0.17088	0
ENSG00000230803	AC097532.1	0	0	0	0	0	0	0	0
ENSG00000018607	ZNF806	0	0	0	0	0	0	0	0
ENSG00000272769	AC097532.2	0	0	0	0	0	0	0	0
ENSG00000230992	FAM201B	0	0	0	0	0	0	0	0
ENSG00000252705	RNU6-175P	0	0	0	0	0	0	0	0
ENSG00000183840	GPR39	0.06493	0	0	0	0	0	0.03607	0
ENSG00000232349	YBX1P7	0	0	0	0	0	0	0	0
ENSG00000200718	RN7SKP103	0	0	0	0	0	0	0	0
ENSG00000150551	LYPD1	0	0	0	0	0.07389	0	0	0
ENSG00000230065	AC010974.1	0	0	0	0	0	0	0	0
ENSG00000176771	NCKAP5	0.00767	0	0	0	0	0	0	0.00942
ENSG00000228721	AC016909.1	0	0	0	0	0	0	0	0
ENSG00000233729	NCKAP5-AS1	0	0	0	0	0	0	0	0
ENSG00000279512	AC011755.1	0	0	0	0	0	0	0	0
ENSG00000226953	NCKAP5-AS2	0	0	0	0	0	0	0	0
ENSG00000252688	RNU6-579P	0	0	0	0	0	0	0	0
ENSG00000222068	RN7SKP154	0	0	0	0	0	0	0	0
ENSG00000232474	NCKAP5-IT1	0	0	0	0	0	0	0	0
ENSG00000223430	AC011243.1	0	0	0	0	0	0	0	0
ENSG00000200708	RN7SKP93	0	0	0	0	0	0	0	0
ENSG00000152127	MGAT5	8.28026	5.70359	1.46917	3.84263	8.07224	5.27619	4.04514	4.04976
ENSG00000263813	MIR3679	6.46768	12.5597	0	0	0	1.90164	0	0
ENSG00000222921	RNA5SP104	0	0	0	0	0	1.09586	0	0
ENSG00000234060	EDDM3CP	0	0	0	0	0	0	0	0
ENSG00000152128	TMEM163	0.05387	0	0	0.04797	0.0131	0.01901	0.10774	0
ENSG00000224043	CCNT2-AS1	1.69155	1.6089	0	1.31499	1.37103	0.85261	2.28249	1.78016
ENSG00000234682	VDAC2P4	0	0	0	0	0	0	0	0
ENSG00000228098	AC110620.1	0	0	0	0	0	0	0	0
ENSG00000153086	ACMSD	0	0	0	0	0	0	0	0
ENSG00000263783	MIR5590	0	0	0	0	0	0	0	0
ENSG00000082258	CCNT2	6.69155	5.73699	1.76736	4.1449	5.46614	4.49978	4.91089	5.51023
ENSG00000176601	MAP3K19	0	0	0	0	0	0	0	0
ENSG00000115839	RAB3GAP1	8.63593	7.21486	2.79481	5.36626	8.46938	5.579	5.05014	5.25401
ENSG00000208308	SNORA40B	4.58127	1.90638	1.68303	3.39955	2.7849	1.01025	0.95453	1.4062
ENSG00000121988	ZRANB3	0.80417	0.93698	1.35898	4.96488	1.42743	1.33355	3.1098	1.76736
ENSG00000235651	AC064850.1	0	0	0	0	0	0	0.0874	0
ENSG00000048991	R3HDM1	10.0883	10.8291	6.88726	18.2916	13.3935	13.573	11.2001	10.6884
ENSG00000201308	RNU6-512P	0	0	0	0	0	0	0	0
ENSG00000207654	MIR128-1	0	2.97582	0	1.32666	0	0	0	1.09753
ENSG00000144224	UBXN4	24.8636	23.6523	10.9147	20.5569	31.6232	28.8877	27.199	24.1365
ENSG00000115850	LCT	0	0	0.03271	0.03304	0.02706	0.0589	0.07421	0.04099
ENSG00000200664	RF00019	0	0	0	0	0	0	0	0

ENSG00000226806	AC011893.1	0.39366	0.06553	0	0.11685	0.09572	0	0.13124	0.24167
ENSG00000076003	MCM6	4.5924	3.94054	1.20322	3.35497	2.72674	10.8964	2.64061	2.01062
ENSG00000214070	AC011999.1	0.07158	0	0	0	0	0.12628	0	0
ENSG00000115866	DARS	8.27231	8.26764	2.81763	5.71843	8.59197	8.66589	4.87017	5.24647
ENSG00000231890	DARS-AS1	0.39073	0.18582	0.13124	0.13254	0.1493	0.31511	0.01861	0.19189
ENSG00000237262	AC068492.1	0	0	0	0	0	0	0	0
ENSG00000121966	CXCR4	274.481	139.18	41.7973	89.171	120.692	163.442	115.038	82.0252
ENSG00000227347	HNRNPKP2	0	0	0	0	0	0	0.1758	0
ENSG00000279024	AC112255.1	0.67626	0.19576	0	0.13091	0.32172	0	0.24505	0.0361
ENSG00000230037	UBBP1	0	1.28973	0	0	0.37681	0	0	0
ENSG00000251976	RN7SKP141	0	0	0	0	0	0	0	0
ENSG00000229568	SMC4P1	0	0	0	0	0	0	0	0
ENSG00000144229	THSD7B	0	0	0	0	0	0	0	0
ENSG00000212425	RNA5SP105	0	0	0	0	0	0	0	0
ENSG00000222394	RF00019	0	0	0	0	0	0	0	0
ENSG00000150540	HNMT	4.94731	3.25801	0.5662	1.34953	2.34222	1.38665	0.97621	1.87336
ENSG00000224231	LINC01832	0	0	0	0	0	0	0	0
ENSG00000233045	AC097523.1	0	0	0	0	0	0	0	0
ENSG00000234645	YWHAEP5	0.09582	0.15949	0	0.1422	0.23298	0.16904	0	0.11764
ENSG00000237939	AC097523.2	0	0	0	0	0	0	0	0
ENSG00000232915	AC097721.1	0.2925	0.09737	0	0.08682	0	0	0	0
ENSG00000228043	AC114763.1	0.33496	0.37169	0.16407	0.08285	0.27149	0.29546	0.27916	0.34272
ENSG00000230569	AC114763.2	0	0	0	0	0	0	0	0
ENSG00000144228	SPOPL	24.9661	24.8054	5.41234	7.1481	18.1457	8.95666	8.40604	16.5857
ENSG00000241772	AC092620.2	2.51076	4.75961	0.10249	0.82805	2.28939	0.73822	1.74377	1.79823
ENSG00000260059	AC092620.3	0	0	0	0	0	0	0	0
ENSG00000237772	AC092620.1	0.53897	0.7177	0.1584	0.15998	0.39316	0.19016	0.35935	0.66174
ENSG00000144227	NXPH2	0	0	0	0	0	0	0	0
ENSG00000222826	RN7SKP286	0	0	0	0	0	0	0	0
ENSG00000229104	YY1P2	0	0	0	0	0	0	0	0
ENSG00000232992	AHCYP4	0	0	0	0	0	0	0	0
ENSG00000226939	AC062021.1	0	0	0	0	0	0	0	0
ENSG00000229131	AC016710.1	0	0	0	0	0	0	0	0
ENSG00000206901	RF00139	0	0	0	0	0	0	0	0
ENSG00000235232	MRPS18BP2	0	0	0	0	0	0	0	0
ENSG00000228374	AC012497.1	0	0	0	0	0	0	0	0
ENSG00000223554	AC078851.1	0	0	0	0	0	0	0	0
ENSG00000240977	RN7SL283P	0	0	0	0	0	0	0	0
ENSG00000224028	LINC01853	0	0	0	0	0	0	0	0
ENSG00000227149	MTCO1P44	0	0	0	0	0	0	0	0
ENSG00000231567	MTND2P19	0	0	0	0	0	0	0	0
ENSG00000223921	MTND1P27	0	0	0	0	0	0	0	0
ENSG00000168702	LRP1B	0.01721	0.01432	0.00632	0	0	0	0.00717	0.00528
ENSG00000277306	RF02271	0	0	0	0	0	0	0	0
ENSG00000283506	MIR7157	0	0	0	0	0	0	0	0
ENSG00000236440	AC117947.1	0	0	0	0	0	0	0	0
ENSG00000238065	AC010740.1	0	0	0	0	0	0	0	0
ENSG00000201892	RF00019	0	0	0	0	0	0	0	0
ENSG00000252015	RNU6-904P	0	0	0	0	0	0	0	0
ENSG00000233329	AC068287.1	0	0	0	0	0	0	0	0
ENSG00000244125	AC078882.1	0	0	0	0	0	0	0	0
ENSG00000228134	UBE2V1P14	0	0	0	0	0	0	0	0
ENSG00000229781	AC013444.1	0	0	0	0	0	0	0	0
ENSG00000115919	KYNU	2.21567	2.56495	0.28153	0.55011	0.95699	0.74942	0.74974	0.78747
ENSG00000270390	AC013444.2	0	0	0	0	0	0	0	0
ENSG00000075884	ARHGAP15	20.2853	23.2416	6.50505	9.12081	22.3579	12.7526	10.7148	16.8401
ENSG00000224288	MTCYBP11	0	0	0	0	0	0	0	0
ENSG00000226839	MTND6P11	0	0.50521	0.22301	0.45046	0.36901	0	0.25296	0.18633

ENSG00000224852	MTND5P24	0	0	0	0	0	0	0	0
ENSG00000232772	MTND4P22	0	0	0	0	0	0	0	0
ENSG00000236300	MTND4LP12	0	0	0	0	0	0	0	0
ENSG00000227557	MTND3P9	0	0	0	0	0	0	0	0
ENSG00000225476	MTCO3P5	0	0	0	0	0	0	0	0
ENSG00000284167	AC013437.2	0	0	0	0	0	0	0	0
ENSG00000231766	MTCO2P5	0	0	0	0	0	0	0	0
ENSG00000257284	AC013437.1	0	0	0	0	0	0	0	0
ENSG00000228655	AC096558.1	0	0	0	0	0	0	0	0
ENSG00000257640	AC096558.2	0	0	0	0	0	0	0	0
ENSG00000258268	AC079793.1	0	0	0	0	0	0	0	0
ENSG00000283994	AC092652.3	2.17437	1.84983	0.63904	1.00396	1.2924	0.42621	0.88595	1.77977
ENSG00000231758	AC092652.1	0	0	0	0	0	0	0	0
ENSG00000257277	AC092652.2	0	0	0	0	0	0	0	0
ENSG00000257226	AC079584.2	0	0	0	0	0	0	0	0
ENSG00000236356	AC079584.1	0	0	0	0	0	0	0	0
ENSG00000200287	RF00019	0	0	0	0	0	0	0	0
ENSG00000232377	AC016910.1	0	0	0	0	0	0	0	0
ENSG00000121964	GTDC1	5.23278	6.39248	5.2024	11.2381	6.25491	7.85257	8.71361	5.78289
ENSG00000169554	ZEB2	16.3216	11.5266	2.59255	4.50478	7.26281	11.217	4.75119	4.75755
ENSG00000283118	AC009951.2	0	0	0	0	0	0	0	0
ENSG00000279166	AC009951.1	5.38396	5.69435	1.8543	1.62305	1.87506	3.61123	2.61748	2.0313
ENSG00000238057	ZEB2-AS1	0.51909	0.90949	0.20073	0.40546	0.29894	0.09639	0.09108	0.26835
ENSG00000275372	RF01984	0	0	0	0	0	0	0	0
ENSG00000273537	RF01986	0	0	0	0	0	0	0	0
ENSG00000277444	RF01987	0	0	0	0	0	0	0	0
ENSG00000232606	LINC01412	0	0	0	0	0.24618	0	0	0
ENSG00000226674	TEX41	0	0	0	0	0.01775	0	0.00811	0
ENSG00000234940	AC023128.1	0	0	0	0	0	0	0	0
ENSG00000276706	RF01992	0	0	0	0	0	0	0	0
ENSG00000235352	AC023128.2	0	0	0	0	0	0	0	0
ENSG00000268580	LINC01966	0	0	0	0	0	0	0	0
ENSG00000235435	AC096666.1	0	0	0	0	0	0	0	0
ENSG00000214064	RPL6P5	0	0	0	0	0	0	0	0
ENSG00000225107	AC092484.1	0	0	0	0	0	0	0	0
ENSG00000226218	AC079163.1	0	0	0	0	0	0	0	0
ENSG00000234119	AC079248.1	0	0	0	0	0	0	0	0
ENSG00000279876	AC079248.2	0	0	0	0	0	0	0	0
ENSG00000233842	AC062031.1	0	0	0	0	0	0	0	0
ENSG00000251905	RNU7-2P	0	0	0	0	0	0	0	0
ENSG00000230559	RPL17P12	0	0	0	0	0	0	0	0
ENSG00000227134	AC093084.1	0	0	0	0	0	0	0	0
ENSG00000198526	PABPC1P2	0	0	0	0	0	0	0	0
ENSG00000271583	AC103881.1	0	0	0	0	0	0	0	0
ENSG00000231040	LINC01911	0	0	0	0	0	0	0	0
ENSG00000227902	AC062032.1	0	0	0	0	0	0	0	0
ENSG00000207225	RNU6-692P	0	0	0	0	0	0	0	0
ENSG00000207161	RF00019	0	0	0	0	0	0	0	0
ENSG00000202074	RNU6-715P	0	0	0	0	0	0	0	0
ENSG00000253083	RNA5SP106	0	0	0	0	0	0	0	0
ENSG00000229143	AC009480.2	0	0	0	0	0	0	0	0
ENSG00000121989	ACVR2A	5.40279	3.55032	2.30702	5.0456	4.69936	3.82014	3.24852	2.69858
ENSG00000212389	RNU6-1275P	1.3701	0	0	0	0.83287	1.20852	0	0
ENSG00000223911	AC009480.1	0	0	0	0	0	0	0	0
ENSG00000115947	ORC4	6.43793	8.48517	10.7533	33.0445	10.676	7.16545	21.4073	13.0024
ENSG00000204406	MBD5	2.83459	3.97373	2.04273	5.39982	2.89879	3.77444	4.05488	3.09439
ENSG00000281469	AC019226.1	1.13865	0.71073	0.20915	0.6337	0.51913	0.25109	1.6607	0.69901
ENSG00000281772	AC019226.2	0.51863	0	0.25404	0	0	0	0	0

ENSG00000212181	RF00554	0	0	0	0	0	0	0	0
ENSG00000224007	AC019070.1	0	0	0	0	0	0	0	0
ENSG00000232238	RPS29P8	0	0	0	0	0	0	0	0
ENSG00000135999	EPC2	17.4124	12.5025	4.20667	7.737	13.6022	10.6331	7.70467	8.99648
ENSG00000222126	RNU2-9P	0	0	0	0	0	0	0	0
ENSG00000223962	UBBP3	0	0	0	0	0.36978	0	0	0
ENSG00000231079	AC105402.3	0	0	0	0	0	0	0	0
ENSG00000228848	AC105402.2	0	0	0	0	0	0	0	0
ENSG00000226340	AC105402.1	0.26899	0	0	0	0	0	0	0
ENSG00000168280	KIF5C	0.61249	0.27804	0.07013	0.37777	0.27078	0.0842	0.14585	0.07813
ENSG00000150556	LYPD6B	0.09719	0	0	0.02885	0	0	0	0
ENSG00000228236	TXNP5	0	0.38733	0	0	0	0	0	0
ENSG00000224337	FAM8A3P	0	0	0	0	0	0	0	0
ENSG00000187123	LYPD6	0	0	0	0	0	0	0	0
ENSG00000223604	AC007394.1	0	0	0	0	0	0	0	0
ENSG00000168288	MMADHC	18.668	18.4878	8.30838	12.6114	16.961	17.7059	13.8296	18.7716
ENSG00000231969	AC007364.1	0.07172	0	0	0	0	0.12653	0	0
ENSG00000207270	RNU6-601P	0	0	0	0	0	0	0	0
ENSG00000162947	LINC01931	0	0	0	0	0	0	0	0
ENSG00000230645	LINC01818	0	0	0	0	0	0	0	0
ENSG00000231420	LINC01817	0	0	0	0	0	0	0	0
ENSG00000115963	RND3	0.04079	0	0	0	0.14878	0	0	0
ENSG00000236049	LINC01920	0	0	0	0	0	0	0	0
ENSG00000237220	AC104777.2	0	0	0	0	0	0	0	0
ENSG00000232359	AC104777.1	0	0	0	0	0	0	0	0
ENSG00000224048	LINC02612	0	0	0	0	0	0	0	0
ENSG00000228064	AC023469.2	0	0	0	0	0	0	0	0
ENSG00000222031	AC023469.1	0	0.52029	3.90434	1.85562	0.57004	0.55144	5.47076	0.76757
ENSG00000236885	AC018731.1	0	0.21182	0.37401	1.32205	0.30943	0.6735	5.93932	0
ENSG00000213201	FABP5P10	0	0	0	0	0	0	0	0
ENSG00000184898	RBM43	2.34648	1.86233	1.00475	1.49906	1.85148	2.7414	1.45052	1.33555
ENSG00000123609	NMI	25.4234	45.1572	9.81187	11.0036	22.9962	21.329	9.37418	22.4993
ENSG00000123610	TNFAIP6	4.59578	22.949	15.3487	3.37587	4.45866	7.28863	3.63678	22.7415
ENSG00000264684	MIR4773-1	0	0	0	0	0	0	0	0
ENSG00000283569	MIR4773-2	0	0	0	0	0	0	0	0
ENSG00000242113	RN7SL124P	0.51439	0.4281	0	0	0.31269	0.45373	0.4287	0
ENSG00000080345	RIF1	8.81654	9.16105	4.31069	9.94677	9.36005	7.75899	7.53959	8.17434
ENSG00000183091	NEB	0.10311	0.03193	0.03524	0.01779	0.06122	0.00846	0.03198	0.04416
ENSG00000162980	ARL5A	8.88423	5.66805	2.03688	4.68122	6.0508	5.77629	3.56572	4.54273
ENSG00000283228	AC068547.1	0	0	0	0	0	0	0	0
ENSG00000182389	CACNB4	0.50325	0.11774	0.03267	0.05399	0.09829	0.01783	0.1078	0.07196
ENSG00000225214	AC079790.1	0	0	0	0	0	0	0	0
ENSG00000115145	STAM2	7.11552	8.2384	3.49052	4.61233	7.18785	5.53798	3.62791	5.79424
ENSG00000234272	RPL30P2	0	0	0	0	0	0	0	0
ENSG00000199884	RF00019	0	0	0	0	0	0	0	0
ENSG00000157827	FMNL2	0.88828	1.4224	0.39655	0.43387	0.42378	0.65459	0.26239	0.59363
ENSG00000213197	AC012066.1	0	0	0	0.15675	0	0.37266	0	0
ENSG00000270776	AC012443.1	0	0	0	0	0	0	0	0
ENSG00000196504	PRPF40A	20.1545	18.5009	4.19063	6.95882	16.9897	16.5708	7.27752	10.6834
ENSG00000177917	ARL6IP6	5.20916	5.13682	4.50282	10.1347	5.37521	5.52152	5.28998	6.127
ENSG00000234932	AC009969.1	0	0	0	0	0	0	0	0
ENSG00000226213	UBQLN4P2	0	0	0	0	0	0	0	0
ENSG00000214025	ATP5PBP4	0	0	0	0	0	0	0	0
ENSG00000224612	AC079150.1	0	0	0	0	0	0	0	0
ENSG00000226338	AC079150.2	0	0	0	0	0	0	0	0
ENSG00000227708	LINC01850	0	0	0	0.30472	0	0	0	0
ENSG00000214024	RPL23AP29	0	0	0	0	0	0	0	0
ENSG00000242575	AC012501.2	0	0	0	0	0	0	0	0

ENSG00000227400	AC012501.1	0	0	0	0	0	0	0	0
ENSG00000200377	RF00275	0	0	0	0	0	0	0	0
ENSG00000177519	RPRM	0	0	0	0	0	0	0	0.06118
ENSG00000236917	DNAJA1P2	0	0	0	0	0	0	0	0
ENSG00000144278	GALNT13	0	0	0	0	0	0	0	0.0138
ENSG00000230251	PHBP4	0	0	0	0	0	0	0	0
ENSG00000224675	AC009227.1	0	0	0	0	0	0	0	0
ENSG00000238004	AC009227.2	0	0.23876	0	0	0	0	0	0
ENSG00000223290	RNA5SP107	0	0	0	0	0	0	0	0
ENSG00000235949	AC061961.1	0	0	0	0	0	0	0	0
ENSG00000162989	KCNJ3	0	0	0	0	0	0	0	0
ENSG00000228837	CBX3P6	0	0	0	0	0	0	0	0
ENSG00000230991	AC092625.1	0	0	0	0	0	0	0	0
ENSG00000223197	RNU6-1001P	0	0	0	0	0	0	0	0
ENSG00000232982	MTCO1P45	0	0	0	0	0	0	0	0
ENSG00000229701	MTND2P20	0	0	0	0	0	0	0	0
ENSG00000227682	ATP5F1AP2	0	0	0	0	0	0	0	0
ENSG00000233709	MTND4P28	0	0	0	0	0	0	0	0
ENSG00000233039	MTND5P30	0	0	0	0	0	0	0	0
ENSG00000233248	MTND6P9	0	0	0	0	0	0	0	0
ENSG00000229935	MTCYBP9	0	0	0	0	0	0	0	0
ENSG00000206718	RNU6-546P	0	0	0	0	0	0	0	0
ENSG00000226383	LINC01876	0.02699	0	0	0.40054	0.03281	0.04761	0	0.16568
ENSG00000235548	AC073551.1	0	0	0	0	0	0	0	0
ENSG00000282440	AC074099.1	0	0	0	0	0	0	0	0
ENSG00000153234	NR4A2	4.23135	2.61425	0.62451	1.4534	3.43707	13.0713	1.90955	0.9982
ENSG00000115159	GPD2	6.27996	5.0645	1.98718	2.9192	4.8326	6.86771	2.47605	5.03128
ENSG00000283436	LINC01958	0	0	0	0	0	0	0	0
ENSG00000238069	RPLP0P7	0	0	0	0	0	0	0	0
ENSG00000213194	CDK7P1	0	0	0	0	0.1286	0	0.35261	0
ENSG00000136542	GALNT5	0.05376	0.28632	0.31597	0.92543	0.8496	0.77762	4.76679	1.34639
ENSG00000222404	RN7SKP281	0	0	0	0	0	0	0	0
ENSG00000136541	ERMN	1.11794	0.38883	0.14712	0.42098	0.44629	0.50041	1.72438	0.59411
ENSG00000230562	FAM133DP	2.86412	1.58911	0.62353	0.62973	1.80555	1.4971	0.88408	1.95363
ENSG00000115165	CYTIP	121.497	97.6637	24.5404	45.3803	78.614	67.3342	46.5126	55.2637
ENSG00000123612	ACVR1C	0.13552	0.05308	0	0.08282	0.05815	0.05625	0.06644	0.13703
ENSG00000234584	AC019186.1	0.1345	0.44774	0	0.19961	0	0	0	0.16513
ENSG00000115170	ACVR1	4.00488	1.83471	0.6749	1.33596	1.94315	2.13899	1.50046	1.37589
ENSG00000007001	UPP2	0	0	0	0	0	0	0	0.03044
ENSG00000270557	AC013731.1	0.16603	0	0	0.1232	0.20185	0	0	0.20384
ENSG00000251980	RNU6-436P	0	0	0	0	0	0	0	0
ENSG00000271320	AC091488.1	0	0	0	0	0	0	0	0
ENSG00000228937	AC005539.1	0	0	0	0	0	0	0	0
ENSG00000212410	RNU6-932P	0	0	0	0	0	0	0	0
ENSG00000231158	PTP4A1P1	0	0	0	0	0	0	0	0
ENSG00000237327	UPP2-IT1	0	0	0	0	0	0	0	0
ENSG00000227480	CCDC148-AS1	0	0	0	0	0	0	0	0
ENSG00000153237	CCDC148	0.23209	0.22535	0.17052	0.25833	0.18811	0	0.16119	0.21371
ENSG00000234890	HNRNPDLP2	0	0	0	0	0	0	0	0
ENSG00000243723	RN7SL393P	0	0	0	0	0	0	0	0
ENSG00000144283	PKP4	2.17015	1.70364	0.58804	1.53042	2.47001	2.11786	1.9241	1.50231
ENSG00000227331	AC005042.1	0.27488	0	0.26928	0	0	0	0.15273	0.1125
ENSG00000204380	PKP4-AS1	0	0	0	0	0	0	0	0
ENSG00000228586	AC005042.2	0	0	0	0	0	0	0	0
ENSG00000231675	AC005042.3	0	0	0	0	0	0	0	0
ENSG00000163331	DAPL1	0	0	0	0	0	0	0	0
ENSG00000243792	OR7E89P	0	0	0	0	0	0	0	0
ENSG00000251491	OR7E28P	0	0	0	0	0	0	0	0

ENSG00000188668	OR7E90P	0	0	0	0	0	0	0	0
ENSG00000222300	RNU2-21P	0	0	0	0	0	0	0	0
ENSG00000115183	TANC1	0.06895	0.09017	0.17369	0.22658	0.15567	0.04344	0.04104	0.04837
ENSG00000213189	BTF3L4P2	0	0	0	0.31441	0.25756	0	0	0
ENSG00000202029	RNU6-580P	0	0	0	0	0	0	0	0
ENSG00000226878	GSTM3P2	0	0	0	0	0	0	0	0
ENSG00000275141	MIR6888	0	0	0	0	0	0	0	0
ENSG00000196151	WDSUB1	2.60929	1.48582	1.21084	3.71961	4.13234	1.51419	3.777	2.90857
ENSG00000123636	BAZ2B	13.469	12.3865	2.38483	3.48734	9.07791	4.43101	3.86874	7.01155
ENSG00000223642	AC008277.1	0	0	0	0	0	0	0	0
ENSG00000270893	AC008277.2	0	0	0	0	0	0	0	0
ENSG00000225369	AC009506.2	0	0	0	0	0	0	0	0
ENSG00000207117	RF00019	0.71165	2.3691	1.04577	3.16852	0	0	2.37243	1.74752
ENSG00000224152	AC009506.1	5.93664	9.06489	1.44195	1.31067	2.38599	1.90419	1.14493	2.53004
ENSG00000226266	AC009961.1	0.56509	0.40311	0.31634	0.51917	0.55616	0.23736	0.26912	0.36342
ENSG00000230783	AC009961.3	0.19163	0.15949	0	0.1422	0	0	0.15971	0
ENSG00000136536	MARCH7	51.5131	47.6159	14.5948	21.2623	47.081	32.7392	24.8519	31.5305
ENSG00000241399	CD302	57.4351	35.6594	4.54645	8.16894	31.2728	13.5182	12.0531	21.2459
ENSG00000248672	LY75-CD302	0.09081	0.04319	0	0	0	0	0.04325	0
ENSG00000054219	LY75	3.15763	3.55014	0.73458	1.45905	2.73488	1.89602	1.0971	1.88218
ENSG00000227055	AC009961.2	0	1.45827	0	0	0.35505	1.03037	0	0.71711
ENSG00000153246	PLA2R1	0	0	0	0	0.01161	0	0	0
ENSG00000115221	ITGB6	0.12684	0.11516	0	0.08556	0.09813	0	0.03844	0.05663
ENSG00000153250	RBMS1	13.5822	11.1379	2.73748	5.60341	8.79426	6.12211	4.20595	7.8561
ENSG00000283734	MIR4785	0	0	0	0	0	0	0	0
ENSG00000279844	AC096656.1	0	0	0	0	0	0	0	0
ENSG00000244372	RN7SL423P	0	0	0	0	0	0	0	0
ENSG00000232337	AC009313.2	0	0	0	0	0	0	0	0
ENSG00000224467	AC009313.1	0.14573	0	0.21414	0.21627	0	0	0	0
ENSG00000136560	TANK	33.0042	44.482	23.4774	60.7593	38.7679	28.4398	46.3843	38.3443
ENSG00000235724	AC009299.3	0	0	0	0	0	0.04243	0.04009	0
ENSG00000227403	LINC01806	0.89428	0.87259	0.31721	1.55604	1.23721	1.63204	2.31305	0.60579
ENSG00000225813	AC009299.1	0	0	0	0	0	0	0	0
ENSG00000115233	PSMD14	2.30432	1.77513	0.93749	2.28933	2.33847	4.13233	2.34901	2.09268
ENSG00000232320	AC009299.2	0.14344	0	0	0.21289	0	0	0	0
ENSG00000271924	RNA5SP108	1.253	0	0.92063	2.78938	3.8084	4.42092	0	0.76921
ENSG00000136535	TBR1	0.01527	0	0	0	0.00928	0	0.01273	0
ENSG00000224076	AC009487.1	0	0	0	0	0	0	0	0
ENSG00000251621	AC009487.3	0	0	0	0	0	0	0	0
ENSG00000144290	SLC4A10	2.42716	0.54944	0.58494	1.90195	1.08592	0.25691	0.38839	1.54962
ENSG00000280105	AC009487.4	0	0	0	0	0	0	0	0
ENSG00000226121	AC009487.2	0.16119	0.16098	0.04737	0.09569	0.20904	0.17062	0.08956	0.15833
ENSG00000238570	RF01210	0	0	0	0	0	0	0	0
ENSG00000226946	AC062022.1	0	0	0	0	0	0	0	0
ENSG00000235716	KRT18P46	0	0	0	0	0	0	0	0
ENSG00000234732	RPEP5	0	0	0	0	0	0	0	0
ENSG00000197635	DPP4	5.63399	1.75377	0.75264	2.73647	4.28771	4.18217	1.73184	1.83264
ENSG00000230918	AC008063.1	0.22906	0	0	0	0.13924	0	0.19091	0.14062
ENSG00000231445	TIMM8AP1	0	0	0	0	0	0	0	0
ENSG00000224674	EIF3EP2	0	0	0	0	0	0	0	0
ENSG00000233397	AC008063.2	0	0	0	0	0	0	0	0
ENSG00000115263	GCG	0	0	0	0	0	0	0	0
ENSG00000236841	AC007750.1	0	0	0	0	0	0	0	0
ENSG00000078098	FAP	0.01047	0	0	0	0.01273	0.01847	0	0.01285
ENSG00000115267	IFIH1	9.56725	26.4873	7.25547	4.44465	6.57354	10.8521	2.67135	9.97759
ENSG00000115271	GCA	72.24	137.293	31.4852	35.7176	73.5504	13.3759	40.0154	120.528
ENSG00000184611	KCNH7	0	0	0	0	0	0	0	0
ENSG00000212312	RNA5SP109	0	0	0	0	0	0	0	0

ENSG00000237750	AC011900.1	0	0	0	0	0	0	0	0
ENSG00000230282	RPL7P61	0	0	0	0	0	0	0	0
ENSG00000200902	RNU6-627P	0	0	0	0	0	0	0	0
ENSG00000182263	FIGN	0	0	0	0	0	0	0	0
ENSG00000237844	AC016766.1	0	0	0	0	0	0	0	0
ENSG00000226847	PRPS1P1	0	0	0	0	0	0	0	0
ENSG00000229641	CYP2C56P	0	0	0	0	0	0	0	0
ENSG00000115290	GRB14	1.11512	0.90743	0.61905	0.9562	0.90382	0.87432	0.70219	0.91275
ENSG00000277998	AC107075.1	0.46688	0.38856	0	0.34645	0.28381	0	0.38911	0
ENSG00000082438	COBLL1	1.64284	0.88783	0.67721	1.43756	1.88318	1.18939	2.17646	1.33597
ENSG00000224331	AC019181.1	0	0	0	0	0.86242	0	0	0
ENSG00000206869	SNORA70F	0.54297	0	0	0	0	0	0	0.66664
ENSG00000236283	AC019197.1	0.05949	0.03301	0	0.05886	0.12054	0.01749	0.09916	0.07304
ENSG00000199508	RNA5SP110	0	0	0	0	0	0	0	0
ENSG00000223318	RNA5SP111	0	0	0	0	0	0	0	0
ENSG00000169507	SLC38A11	0.0132	0.04395	0.0194	0	0	0.02329	0.08803	0.03242
ENSG00000153253	SCN3A	0.13409	0.11159	0	0.0995	0.44377	0.09199	0.09933	0.10061
ENSG00000136531	SCN2A	0.00309	0	0	0	0	0	0.02063	0
ENSG00000233204	MAPRE1P3	0	0	0	0	0	0	0	0
ENSG00000178662	CSRNP3	0.01167	0	0	0	0	0	0	0
ENSG00000115339	GALNT3	3.87932	7.52464	1.86189	2.18221	4.24093	3.42182	1.59048	5.33913
ENSG00000235192	AC009495.3	1.88191	3.75893	1.79754	2.37401	0.91519	0.49799	0.47053	3.5814
ENSG00000229195	AC009495.1	0	0	0	0	0	0.34761	0.32844	0
ENSG00000232411	AC009495.2	0.28972	0.48225	0.63862	0.64497	0.52836	0.25556	0	0.17786
ENSG00000123607	TTC21B	3.06309	2.92963	1.07171	1.93384	3.08563	3.01922	2.43129	2.57885
ENSG00000224490	TTC21B-AS1	0	0	0	0	0	0	0	0
ENSG00000236107	SCN1A-AS1	0	0	0	0	0	0	0	0.03301
ENSG00000144285	SCN1A	0	0	0	0	0	0	0	0
ENSG00000238813	RF00019	0	0	0	0	0	0	0	0
ENSG00000222376	RN7SKP152	0	0	0	0	0	0	0	0
ENSG00000169432	SCN9A	0.48094	0.67102	0.20786	0.28341	0.27516	0.04991	0.15326	0.14762
ENSG00000136546	SCN7A	0.15204	0.01489	0	0	0.02175	0	0.01491	0.03294
ENSG00000283657	AC074101.1	0	0	0	0	0	0	0	0
ENSG00000163092	XIRP2	0.11813	0.15917	0.01653	0.06679	0.03419	0	0.04688	0.02763
ENSG00000225182	AC092601.1	0	0	0	0	0	0	0	0
ENSG00000254552	XIRP2-AS1	0	0	0	0	0	0	0	0
ENSG00000228222	AC073050.1	0	0	0	0	0	0	0	0
ENSG00000238357	RNU7-148P	0	0	0	0	0	0	0	0
ENSG00000214211	CTAGE14P	0	0	0	0	0	0	0	0
ENSG00000235335	AC016723.1	0	0.26466	0	0	0	0	0	0
ENSG00000172318	B3GALT1	0	0	0	0	0	0	0	0
ENSG00000198648	STK39	7.11084	8.84827	5.02175	11.8596	9.1278	14.6149	6.8757	8.20082
ENSG00000238259	AC067940.1	0.24433	0.40669	0	0	0	0	0	0
ENSG00000241074	RN7SL813P	0	0	0	0	0	0	0	0
ENSG00000172292	CERS6	7.22779	4.49886	1.81482	4.14651	5.64902	6.19682	5.38262	3.13206
ENSG00000265694	MIR4774	0	0	0	0	1.17259	0	1.60764	0
ENSG00000199348	RNU6-766P	0	0	0	0	0	0	0	0
ENSG00000227617	CERS6-AS1	0	0	0	0	0	0	0.02841	0
ENSG00000163072	NOSTRIN	0.01397	0	0	0	0	0	0	0
ENSG00000152253	SPC25	0.59723	1.06037	2.34034	6.38178	2.66237	3.65248	5.5084	6.64834
ENSG00000152254	G6PC2	0	0	0	0	0	0	0	0
ENSG00000073734	ABCB11	0	0	0	0	0	0	0	0
ENSG00000073737	DHRS9	2.07196	3.90427	1.43619	1.10236	1.68728	3.86211	0.3584	1.70394
ENSG00000235321	AC007556.1	1.43258	0.79484	0	0.35435	0	1.26363	0.79596	0
ENSG00000235995	UBE2V1P6	0	0	0	0	0	0	0	0
ENSG00000081479	LRP2	0	0	0	0	0.00548	0	0	0
ENSG00000163093	BBS5	0.08044	0	0	0.04775	0.07824	0.02838	0	0
ENSG00000251569	AC093899.2	0	0	0	0	0	0	0	0

ENSG00000239474	KLHL41	0.02798	0	0	0	0.03401	0.09871	0	0.03435
ENSG00000138399	FASTKD1	4.89108	6.71581	6.99378	19.6259	6.0866	7.98938	9.88203	9.05834
ENSG00000229827	AC093899.1	0	0	0	0	0	0	0	0
ENSG00000138398	PPIG	15.3604	11.9889	2.64607	5.37432	13.8005	10.0915	7.26316	8.23263
ENSG00000154479	CCDC173	0.09835	0.05457	0.04817	0.04865	0.11957	0.05783	0.10928	0.04025
ENSG00000226072	AC016772.1	0	0	0	0	0	0	0	0
ENSG00000144362	PHOSPHO2	0.29557	0.08199	0.21717	0.36554	0.41923	0.43452	0.32844	0.24193
ENSG00000213160	KLHL23	0.15967	0.06644	0.05866	0.17772	0.16177	0.30515	0.06653	0.09802
ENSG00000206961	RF00432	0	0	0	0	0	0	0	0
ENSG00000231550	PTCHD3P2	0	0	0	0	0	0	0	0
ENSG00000214369	AC009967.1	0	0	0	0	0	0	0	0
ENSG00000138385	SSB	10.9671	9.25861	1.6432	4.68079	10.754	9.20585	3.65609	5.91414
ENSG00000138382	METTL5	3.09576	2.29964	1.54146	3.75909	2.83058	4.60377	3.11315	3.45538
ENSG00000144357	UBR3	8.27479	7.49292	3.38245	7.66462	7.69576	7.20897	7.69774	6.54654
ENSG00000227176	AC092641.1	0	0	0	0	0	0	0	0
ENSG00000252807	RNU6-1006P	0	0	0	0	0	0	0	0
ENSG00000071909	MYO3B	0.1602	0.25184	0.03924	0.27738	0.02164	0.50243	0.0445	0.14205
ENSG00000231898	MYO3B-AS1	0	0	0	0	0	0	0	0
ENSG00000214821	HMGB1P4	0	0	0	0	0	0	0	0
ENSG00000213981	AC007277.1	0	0	0	0	0.04071	0	0.05582	0
ENSG00000275414	RF02271	0	0	0	0	0	0	0	0
ENSG00000222033	LINC01124	0	0	0	0	0	0	0	0
ENSG00000204335	SP5	0	0	0	0	0	0	0	0
ENSG00000234350	AC007405.1	0.20955	0	0.12317	0.46649	0.89168	0.07393	0.83829	0.48883
ENSG00000128692	EIF2S2P4	0.81941	1.36392	0.76626	1.54776	2.08301	1.57697	0.99334	1.55483
ENSG00000239467	AC007405.3	0	0	0	0.225	0	0	0	0.09307
ENSG00000222509	RF00019	0	0	0	0	0	0	0	0
ENSG00000204334	ERICH2	0	0	0	0.36202	0	0	0	0.29949
ENSG00000128683	GAD1	0.06225	0.04441	0.24831	0.92393	0.98394	0.28241	0.23719	0.25114
ENSG00000235934	AC007405.2	0	0	0	0	0	0	0	0
ENSG00000115806	GORASP2	5.05141	3.58714	0.62531	1.46677	4.5059	5.15794	1.14401	1.75275
ENSG00000198586	TLK1	17.2008	14.0105	9.04774	17.3271	17.2832	15.4129	16.2907	14.8895
ENSG00000123600	METTL8	1.97185	1.8288	2.66238	5.58291	2.93694	2.70844	5.14239	2.84088
ENSG00000224553	AC008065.1	0.6319	0	0	0	0	0	0	0.25861
ENSG00000115827	DCAF17	3.13037	4.07917	4.67037	9.8457	5.72619	5.0833	9.7974	6.89934
ENSG00000228507	DAP3P2	0.43118	0	0	0	0	0	0	0
ENSG00000234061	AC007969.2	0.38377	0	0	0	0	0.67703	0.63969	0
ENSG00000233762	AC007969.1	0.16398	0.27295	0	0.73011	0.39873	0.86786	0	0.60401
ENSG00000071967	CYBRD1	10.253	9.71165	2.78858	7.272	5.35458	6.79536	6.44421	4.19036
ENSG00000233934	RPL21P38	0	0	0	0	0	0	0	0
ENSG00000077380	DYNC1I2	8.86786	7.27633	1.90421	3.68412	7.47859	7.87714	3.27896	4.15961
ENSG00000115840	SLC25A12	2.02785	1.2038	0.58348	0.96811	2.10335	2.8269	0.89821	1.07948
ENSG00000252779	RNU6-182P	0	0	0	0	0	0	0	0
ENSG00000128708	HAT1	9.33797	12.2188	18.4335	46.7186	15.8284	13.2549	37.8777	25.74
ENSG00000172878	METAP1D	0.28716	0.22641	0.06663	0.17944	0.44099	0.18664	0.20153	0.09278
ENSG00000144355	DLX1	0	0	0	0	0	0	0	0
ENSG00000115844	DLX2	0.02468	0	0	0	0.03001	0.04354	0.08228	0.0303
ENSG00000236651	DLX2-DT	0	0	0	0	0	0	0	0
ENSG00000232555	AC104088.1	0	0	0	0	0.17474	0.25355	0	0
ENSG00000278924	AC078883.3	0	0	0	0	0	0	0	0
ENSG00000091409	ITGA6	18.2662	4.25806	2.37422	5.22864	19.3839	9.91661	8.3037	6.7639
ENSG00000226963	AC078883.2	0	0	0	0	0	0.23174	0	0
ENSG00000232788	ITGA6-AS1	1.36119	0.90628	0.40005	0.20202	0.9102	0.96053	0.90756	0.91919
ENSG00000225205	AC078883.1	1.43785	1.79498	0.39617	0.62239	1.38391	0.52845	2.89598	0.88268
ENSG00000152256	PDK1	3.24564	2.62331	1.12179	1.66654	3.47293	2.89329	3.03747	2.27971
ENSG00000199866	RF00019	0	0	0	0	0	0	0	0
ENSG00000230104	AC018712.1	0	0	0	0	0	0	0	0
ENSG00000228016	RAPGEF4-AS1	0	0	0	0	0	0	0	0

ENSG00000239041	RF01210	0	0	0	0	0	0	0	0
ENSG00000091428	RAPGEF4	0.01028	0.01711	0.01511	0	0.0375	0.01814	0.05142	0
ENSG00000232292	ALDH7A1P2	0	0	0	0	0	0	0	0
ENSG00000091436	MAP3K20	1.87514	2.77634	0.32785	0.81201	1.73726	1.6212	0.66407	0.99134
ENSG00000238133	MAP3K20-AS1	0	0	0	0	0	0	0	0
ENSG00000231735	AC092573.1	0	0	0	0	0	0	0	0
ENSG00000236391	AC092573.2	0	0.26698	0	0	0	0	0	0
ENSG00000144354	CDCA7	0.44718	0.46521	0.1369	0.58071	0.58898	4.93055	1.18019	0.5948
ENSG00000213148	AC073465.1	0	0	0	0	0.19206	0	0	0
ENSG00000236832	AC073465.2	0	0	0	0	0	0	0	0
ENSG00000226131	PPIAP66	0	0	0	0	0	0	0	0
ENSG00000237016	AC013410.1	0	0	0	0	0	0	0	0
ENSG00000237617	AC013410.2	0	0	0	0	0	0	0	0
ENSG00000270460	AC106900.2	0	0	0	0	0	0	0	0
ENSG00000234415	RPL5P7	0.18032	0	0	0	0.21923	0.15906	0.15028	0
ENSG00000224638	AC106900.1	0.48224	0.80269	0	0	0.58629	0	0	0.19736
ENSG00000172845	SP3	29.1859	25.521	6.03778	10.6103	23.4364	18.3747	14.8502	17.0762
ENSG00000271151	AC016737.1	0.2059	0.22848	0	0	0.25033	0	0.3432	0
ENSG00000273258	AC016737.2	0	0.34081	0	0.60774	0	0	0	0
ENSG00000260868	LINC01960	0	0	0	0	0	0.06864	0	0.09554
ENSG00000235414	RPSAP24	0	0	0	0	0	0	0	0
ENSG00000138430	OLA1	6.4288	5.16036	2.01301	4.81511	7.10012	6.84717	4.34641	3.64414
ENSG00000270799	AC013467.2	0	0	0	0	0	0	0	0
ENSG00000243770	RN7SL65P	0	0	0	0	0	0	0	0.2857
ENSG00000201088	RF00019	0	0	0	0	0	0	0	0
ENSG00000227180	AC013467.1	0	0	0	0	0	0	0	0
ENSG00000237804	HNRNPA1P39	0	0	0	0.11332	0	0	0	0
ENSG00000231453	LINC01305	0	0.03552	0	0	0	0	0	0
ENSG00000280414	AC018470.1	0	0	0	0	0	0	0	0
ENSG00000217236	SP9	0	0	0	0	0	0	0	0
ENSG00000138433	CIR1	28.4353	36.591	8.32997	9.95179	23.6729	14.228	14.4419	18.1352
ENSG00000144306	SCRN3	3.16621	2.70868	0.93574	1.91635	2.90317	2.746	1.71005	1.78083
ENSG00000163328	GPR155	4.43444	5.79497	2.07969	3.06656	4.27861	3.11254	2.37472	4.72632
ENSG00000226853	AC010894.2	0.18534	0	0	0.41259	0	0	0.30893	0.34133
ENSG00000179997	AC010894.1	0	0	0	0	0	0	0	0
ENSG00000279884	AC010894.5	0.61662	0.76977	0.22653	0.34317	0.93708	1.0878	0.5139	1.70341
ENSG00000236449	AC010894.3	0.01981	0	0	0	0	0.03494	0	0
ENSG00000206965	RNU6-5P	6.16545	7.98186	2.01334	1.01669	5.83006	10.8767	2.28374	4.20547
ENSG00000115935	WIPF1	61.7439	59.1783	11.0654	21.4208	43.6546	45.722	16.7042	28.7426
ENSG00000237798	AC010894.4	0	0	0	0	0	0	0	0
ENSG00000235655	H3F3AP4	6.59881	13.6555	2.09662	4.23497	5.20389	2.83165	4.45914	5.47427
ENSG00000138435	CHRNA1	0	0	0	0	0	0	0	0
ENSG00000128656	CHN1	0.19169	0.24544	0.02167	0.15319	0.10756	0.18209	0.22121	0.05431
ENSG00000212167	RNU6-763P	0	0	0	0	0	0	0	0
ENSG00000199827	RNU6-1290P	0	0	0	0	0	0	0	0
ENSG00000227187	AC007435.1	0	0	0	0	0.21474	0	0	0
ENSG00000115966	ATF2	17.3978	19.8362	8.50324	16.5515	22.6859	24.8685	14.6166	16.4314
ENSG00000277948	RF02271	0.78396	0	0	1.16348	0	0	0.65337	0.48127
ENSG00000215973	MIR933	4.75976	1.58453	0	0	0	1.67937	3.17351	0
ENSG00000229750	AC096649.1	0.14749	0	0	0	0	0	0.24584	0
ENSG00000154518	ATP5MC3	2.94066	2.42937	0.82612	1.76496	2.54995	3.8908	1.67592	1.57959
ENSG00000200121	RF00019	0	0	0	0	0	0	0	0
ENSG00000233131	AC096649.2	0	0	0	0	0	0	0	0
ENSG00000229066	AC093459.1	0	0	0	0	0	0	0	0
ENSG00000223976	EXTL2P1	0	0	0	0	0	0	0	0
ENSG00000235047	AC016751.2	0	0	0	0	0	0	0	0
ENSG00000229779	AC016751.1	0	0	0	0	0	0	0	0
ENSG00000144320	LNPK	20.0648	29.4789	26.1551	56.3248	40.6115	43.5198	38.2935	38.4771

ENSG00000174279	EVX2	0	0	0	0	0	0	0	0
ENSG00000128714	HOXD13	0	0	0	0	0	0	0	0
ENSG00000170178	HOXD12	0	0	0	0	0	0	0	0
ENSG00000128713	HOXD11	0	0	0	0	0	0	0	0
ENSG00000128710	HOXD10	0	0	0	0	0	0	0	0
ENSG00000237380	HOXD-AS2	0	0	0	0	0	0	0	0
ENSG00000128709	HOXD9	0	0	0	0	0	0	0	0
ENSG00000175879	HOXD8	0	0	0	0	0	0	0	0
ENSG00000128652	HOXD3	0	0	0	0	0	0	0	0
ENSG00000207744	MIR10B	0	0	0	0	0	0	0	0
ENSG00000278500	AC009336.2	0	0	0	0	0	0	0	0
ENSG00000170166	HOXD4	0	0	0	0	0	0	0	0
ENSG00000272729	AC009336.1	0	0	0	0	0	0	0	0
ENSG00000224189	HAGLR	0	0	0	0	0	0	0	0
ENSG00000226363	HAGLROS	0	0	0	0	0	0	0	0
ENSG00000128645	HOXD1	0	0	0	0	0	0	0	0
ENSG00000283880	MIR7704	0	0	0	0	0	0	0	0
ENSG00000279160	AC009336.3	0	0	0	0	0	0	0	0
ENSG00000218175	AC016739.1	0.21246	0.35365	0	0	0.51662	0	0.35415	1.30431
ENSG00000230384	RPSAP25	0	0	0	0	0	0	0	0
ENSG00000128654	MTX2	2.73222	2.62919	1.25468	3.23126	3.9965	4.7447	3.34448	1.93937
ENSG00000237260	PPIAP67	0	0	0	0	0.20208	0	0	0
ENSG00000229434	AC017048.1	0	0	0	0	0	0	0	0
ENSG00000236231	AC017048.2	0	0	0	0	0	0	0	0
ENSG00000283203	MIR1246	0	0	0	0	0	0	0	0
ENSG00000272551	AC017048.3	0	0	0	0	0	0	0	0
ENSG00000163364	LINC01116	0	0	0	0	0	0	0	0
ENSG00000224577	LINC01117	0	0	0	0	0	0	0	0
ENSG00000252027	RNU6ATAC14P	0	0	0	0	0	0	0	0
ENSG00000230552	AC092162.2	0	0	0	0	0.03369	0	0	0
ENSG00000237053	FUCA1P1	0	0	0	0	0	0	0	0
ENSG00000223777	AC092162.1	0	0	0	0	0	0	0	0
ENSG00000279205	AC092162.3	0	0	0	0	0	0	0	0
ENSG00000227098	AC073636.1	0	0	0	0	0	0	0	0
ENSG00000206866	RNU6-187P	0	0	0	0	0	0	0	0
ENSG00000236501	AC074286.1	0	0	0	0	0	0	0	0
ENSG00000251746	RNA5SP112	0	0	0	0	0	0	0	0
ENSG00000229337	AC079305.3	0	0	0.4941	0.99803	0.81758	0	0	1.65132
ENSG00000226273	AC079305.2	0	0	0	0	0	0	0	0
ENSG00000201102	RF00019	0	0	0	0	0	0	0	0
ENSG00000213069	KRT8P40	0	0	0	0	0	0	0	0
ENSG00000170144	HNRNPA3	17.1314	15.4912	3.32087	9.39456	15.4658	16.0327	7.99954	8.98031
ENSG00000284252	MIR4444-1	0	0	0	0	0	0	0	0
ENSG00000116044	NFE2L2	23.0358	25.3853	18.5919	37.7721	25.3831	19.6379	44.8546	25.6188
ENSG00000225808	DNAJC19P5	9.74245	6.94985	0.68173	2.40981	2.53813	1.63686	5.41305	2.27841
ENSG00000265396	MIR3128	1.11061	0	1.63203	1.64827	0	0	5.55365	6.81796
ENSG00000222043	AC079305.1	0.73447	0.24451	0.21586	0.43601	0.71436	1.29571	0	0
ENSG00000213963	AC019080.1	0.18399	0.03403	0	0.21238	0.06214	0.1082	0.10223	0.1757
ENSG00000271825	AC019080.3	0	0	0	0	0	0	0	0
ENSG00000271996	AC019080.4	0	0	0	0	0	0	0	0
ENSG00000278418	MIR6512	0	0	0	0	0	1.67937	0	0
ENSG00000280374	AC019080.5	0	0.16117	0	0	0.23545	0.17082	0.4842	0.59443
ENSG00000237178	AC019080.2	0	0	0	0	0	0	0	0
ENSG00000238295	RF01210	0	0	0	0	0.85689	0	0	0
ENSG00000018510	AGPS	8.39874	6.32017	2.58661	5.05054	5.28891	8.05888	4.14952	4.46641
ENSG00000196659	TTC30B	0.83939	0.57709	0.1877	0.4333	0.77647	0.54725	0.36499	0.51529
ENSG00000237655	AC073834.1	0	0	0	0	0	0	0	0
ENSG00000197557	TTC30A	0.55205	0.1021	0.09014	0.18207	0.56677	0.43284	0.14314	0.22593

ENSG00000128655	PDE11A	0.01759	0	0	0.03482	0	0	0.01955	0
ENSG00000229941	AC012499.1	0.19784	0	0	0	0	0	0	0.48581
ENSG00000236664	AC011998.3	0	0	0	0	0	0	0	0
ENSG00000213962	API5P2	0	0	0	0	0	0	0	0
ENSG00000229744	AC011998.2	0	0	0	0	0	0	0	0
ENSG00000206788	RNU6-629P	0	0	0	0	0	0	0	0
ENSG00000249909	CYCTP	0	0	0	0	0	0	0	0
ENSG00000227241	AC011998.1	0	0	0	0	0	0	0	0
ENSG00000155636	RBM45	1.31769	0.7396	0.24767	0.59123	1.26671	1.18932	0.91942	1.07229
ENSG00000223096	RNU5E-9P	0	0	0	0	0	0	0	0
ENSG00000079156	OSBPL6	0.07658	0.04249	0.03751	0.00947	0.03103	0.01126	0.07446	0.00784
ENSG00000223960	PRKRA-AS1	0.91603	0.60989	0.26922	0.5166	0.57911	0.87264	0.09161	0.31491
ENSG00000180228	PRKRA	2.65972	2.16711	0.49197	1.46299	2.44217	2.13277	1.08508	1.21031
ENSG00000204311	PJVK	0.05969	0.04968	0	0.08859	0.03629	0	0.04975	0
ENSG00000238082	AC009948.1	0.20561	0	0	0	0	0	0	0
ENSG00000079150	FKBP7	0.0476	0.15845	0.10492	0.28256	0.26041	0.33587	0.07934	0.05844
ENSG00000116095	PLEKHA3	2.77336	1.83103	0.69062	1.27235	2.10972	2.08641	1.41179	1.29989
ENSG00000237298	TTN-AS1	2.03001	1.68357	0.57367	0.85326	1.49722	1.11443	1.01748	1.31157
ENSG00000155657	TTN	0.57913	0.13844	0.11045	0.28071	0.71982	0.38149	0.4498	0.27458
ENSG00000270956	AC009948.3	2.60623	2.34979	1.11703	0.32233	2.64049	2.1073	1.08605	1.86661
ENSG00000270277	AC009948.2	1.79378	1.90002	0.29954	0.66554	1.09042	1.22264	0.74749	1.40151
ENSG00000279598	AC009948.4	0.69873	0.71998	0.39115	0.49381	0.8495	0.35219	0.3605	0.5515
ENSG00000271011	AC010680.3	1.97347	1.64242	0.62143	0	1.37103	0.49735	0.23496	0.69229
ENSG00000270574	AC010680.2	1.17724	0.80686	0.10176	0.51387	1.26287	0.42758	0.23086	0.68019
ENSG00000271141	AC010680.4	1.6199	0	0.5951	0.80137	2.13355	1.19072	0.22501	0.33148
ENSG00000271401	AC010680.5	0.15832	0.26352	0	0	0	0	0	0.19438
ENSG00000267784	AC010680.1	0	0	0	0.0606	0.04965	0	0	0
ENSG00000163492	CCDC141	0.66062	0.13504	0.17883	0.43002	1.02158	0.44982	0.59888	0.32017
ENSG00000238542	RNU7-104P	0	0	0	0	0	0	0	0
ENSG00000231541	RPS6P2	0	0	0	0	0	0	0	0
ENSG00000187231	SESTD1	2.79664	2.03186	0.78776	1.52409	1.80605	3.00805	1.87324	1.34016
ENSG00000237477	AC093911.1	0	0	0	0	0	0	0	0
ENSG00000237964	AC068042.1	0	0	0	0	0	0	0	0
ENSG00000144331	ZNF385B	0.0138	0.02298	0.06086	0.08195	0	0.14612	0.02301	0.0339
ENSG00000213126	AC092642.1	0	0	0	0	0	0	0	0
ENSG00000221240	MIR1258	0	0	0	0	0	0	0	0
ENSG00000280636	RF00416	0	0	0	0	0	0	0	0
ENSG00000202216	RF00560	0	0	0	0	0	0	0	0
ENSG00000163510	CWC22	15.1276	12.4798	3.85619	7.10184	13.1369	10.3092	7.0941	9.34085
ENSG00000270289	AC012669.1	0	0	0	0	0	0	0	0
ENSG00000225258	AC009478.1	0	0	0	0	0	0	0	0
ENSG00000281131	SCHLAP1	0	0	0	0	0	0	0	0
ENSG00000279559	AC104820.1	0	0	0	0	0	0	0	0
ENSG00000226564	FTH1P20	0.13376	0.89057	0.19656	0.39703	0.32524	0.47194	0	0
ENSG00000236667	AC104076.2	0	0.26015	0	0	0	0	0	0
ENSG00000170035	UBE2E3	7.64316	6.68933	8.07945	11.819	9.02258	9.52549	13.0687	10.6556
ENSG00000236153	AC104076.1	0.27661	0	0	0.41051	0	0	0	0.33961
ENSG00000238171	AC068196.1	0	0	0	0	0	0	0	0
ENSG00000234663	LINC01934	1.60746	0.57629	0.72681	1.32128	1.62358	1.04706	0.90687	0.81981
ENSG00000266705	MIR4437	1.22167	0	0	0	0	0	2.03634	0
ENSG00000226681	AC020595.1	0	0	0	0	0	0	0	0
ENSG00000115232	ITGA4	41.5996	36.0883	16.564	33.6505	38.3379	46.9555	26.3041	27.0413
ENSG00000188452	CERKL	0	0	0	0	0	0	0	0.01889
ENSG00000162992	NEUROD1	0	0	0	0	0	0	0	0
ENSG00000234595	AC013733.2	0	0	0	0	0	0	0	0
ENSG00000225570	AC013733.1	0	0	0	0	0	0	0	0
ENSG00000223873	SAP18P2	0	0	0	0.47401	0	0	0	0.19607
ENSG00000221023	RNU6ATAC19P	0	0	0	0	0	0	0	0

ENSG00000260742	AC009962.1	0.34603	0.22399	0.0565	0.08559	0.39732	0.20348	0.06409	0.14162
ENSG00000138434	ITPRID2	13.9303	13.166	3.21501	3.49676	11.4116	6.53172	4.9474	8.43429
ENSG00000150722	PPP1R1C	0	0	0	0	0	0	0	0
ENSG00000213958	KRT18P29	0	0	0	0	0	0	0	0
ENSG00000222418	RNA5SP113	0	0	0	0	0	0	0	0
ENSG00000115252	PDE1A	0	0	0	0.01721	0.0141	0	0	0
ENSG00000242121	RN7SL267P	0	0	0	0	0	0	0	0
ENSG00000077232	DNAJC10	4.58232	3.17943	0.77449	1.75372	3.61201	2.3452	1.4101	1.99902
ENSG00000232430	RPL31P15	0	0	0	0	0	0	0	0
ENSG00000212404	RF00019	0	0	0	0	0	0	0	0
ENSG00000162998	FRZB	0	0	0	0	0	0	0	0
ENSG00000207178	RNU6-1122P	0	0	0	0	0	0	0	0
ENSG00000061676	NCKAP1	0.6998	0.65555	0.4783	0.80671	0.62524	0.60866	0.78126	0.70335
ENSG00000202141	RF00019	0.64868	0	0	0	0.78864	0	1.08124	0
ENSG00000221498	RF00599	0	0	0	0	0	0	0	0
ENSG00000231203	KRT8P10	0	0	0	0	0	0	0	0
ENSG00000162999	DUSP19	0.55902	0.31773	0.2204	0.50589	0.46415	0.26459	0.29545	0.45199
ENSG00000224643	AC064871.1	0.12278	0	0	0.18222	0	0	0	0
ENSG00000163002	NUP35	3.55088	5.42796	4.89851	12.9865	7.73108	6.48796	13.7702	9.85386
ENSG00000272800	AC021851.1	0	0	0	0	0.16473	0.23902	0	0.24953
ENSG00000213120	LIN28AP1	0	0	0	0	0.22004	0	0	0
ENSG00000177855	CACYBPP2	0.10811	0	0	0.3209	0	0.38145	0.18021	0
ENSG00000234172	AC093639.1	0	0	0	0	0	0	0	0
ENSG00000266808	MIR548AE1	0	0	0	0	0	0	0	0
ENSG00000283839	AC096667.1	1.37504	0.77466	0.27978	0.25116	0.69442	0.70907	0.52892	0.83114
ENSG00000170396	ZNF804A	0.21862	0.12996	0	0.02318	0.17087	0.02755	0.05206	0.07669
ENSG00000237824	RPL23AP33	0	0	0	0	0	0	0	0
ENSG00000225406	AC080125.1	0	0	0	0	0	0	0	0
ENSG00000212581	RF00096	0	0	0	0	0	0	0	0
ENSG00000226747	FSIP2-AS2	0	0	0	0	0	0	0	0
ENSG00000188738	FSIP2	0.01872	0	0.00458	0.02778	0.00379	0	0.0208	0.00766
ENSG00000231646	FSIP2-AS1	0.0833	0	0	0.6181	0	0	0.27768	0.20454
ENSG00000224019	RPL21P32	0	0	0.22582	0	0	0	0.51229	0
ENSG00000237877	LINC01473	5.61127	6.39493	5.42284	14.1046	9.71064	5.79674	9.69016	6.20669
ENSG00000226410	AC104058.1	0.49361	0	0	0	0.30006	0	0	0.30302
ENSG00000232568	RPL23AP35	0	0	0	0	0	0	0.24387	0.17963
ENSG00000259915	AC017071.1	0	0	0.09774	0.44422	0.36391	0.05867	0	0.04083
ENSG00000227692	MED28P3	0	0	0	0	0	0	0	0
ENSG00000065548	ZC3H15	23.4092	17.0617	5.23921	7.24078	17.4526	18.7037	7.62405	11.4908
ENSG00000224323	DPRXP1	0	0	0	0	0	0	0	0
ENSG00000138448	ITGAV	3.16019	2.11	0.80021	1.17914	1.96445	1.7166	0.90768	1.27142
ENSG00000227227	AC017101.1	0	0	0	0	0	0	0	0
ENSG00000144369	FAM171B	0.12582	0.08377	0.01849	0	0.13767	0.15537	0.04194	0.06179
ENSG00000163012	ZSWIM2	0	0	0	0	0	0	0	0
ENSG00000226553	AC018735.1	0	0	0	0	0	0	0	0
ENSG00000224063	AC007319.1	0	0	0.14635	0.14781	0	0.1757	0.99604	0.12228
ENSG00000222845	RN7SKP42	0	0	0	0	0	0	0	0
ENSG00000199626	RNU6-989P	0	0	0	0	0	0	0	0
ENSG00000064989	CALCRL	0.73968	0.57589	0.17532	0.46036	0.81226	0.21047	0.1392	0.3369
ENSG00000231739	GAPDHP59	0	0	0	0	0	0	0	0
ENSG00000003436	TFPI	1.70204	1.9998	1.43447	3.09251	1.23245	1.67794	2.63886	1.51352
ENSG00000231689	LINC01090	0	0	0	0	0	0	0	0
ENSG00000235616	ST13P2	0	0	0	0	0	0	0	0
ENSG00000213115	AC104131.1	0	0	0	0	0	0	0	0
ENSG00000200650	RNA5SP114	0	0	0	0	0	0	0	0
ENSG00000144366	GULP1	0	0	0.03597	0.00908	0	0.01079	0.0306	0
ENSG00000207951	MIR561	0	0	0	0	0	0	0	0
ENSG00000223523	AC092598.1	0	0	0	0	0	0	0	0.11292

ENSG00000174325	DIRC1	0	0	0	0	0	0	0	0
ENSG00000168542	COL3A1	0.11838	0.16122	0	0	0.68038	0.20884	0	0
ENSG00000221502	MIR1245A	0	0	0	0	0	0	0	0
ENSG00000283238	MIR1245B	0	0	0	0	0	0	0	0
ENSG00000284180	MIR3606	0	0	0	0	0	0	0	0
ENSG00000204262	COL5A2	0.1434	0.08525	0.01505	0.0152	0.3736	0.14456	0	0.01258
ENSG00000228073	AC133106.1	0	0	0	0	0	0	0	0
ENSG00000264725	MIR3129	0	0	0	0	0	0	0	0
ENSG00000213601	KRT18P19	0	0	0	0	0	0	0	0
ENSG00000115368	WDR75	3.92112	2.84189	1.16942	2.98484	5.22457	5.10508	2.67707	2.43379
ENSG00000233996	AC013439.1	0	0	0	0	0	0	0	0
ENSG00000138449	SLC40A1	27.5596	33.4185	13.3088	22.6114	33.074	37.4198	30.531	31.0937
ENSG00000276828	AC012488.1	0	0	0	0	0	0	0	0
ENSG00000138381	ASNSD1	19.6424	17.4437	6.10838	7.68973	18.7558	14.4081	12.6377	11.2856
ENSG00000151687	ANKAR	1.68038	1.50445	1.27206	3.2307	2.16678	1.4373	2.14314	1.95374
ENSG00000128694	OSGEPL1	0.75694	0.64699	0.60125	1.45736	1.39284	1.19098	0.7843	1.53219
ENSG00000253559	OSGEPL1-AS1	0.2557	0	0	0.37949	0.10362	0	0.28414	0.10465
ENSG00000273240	AC013468.1	0.12258	0	0.36025	0.36383	0	0	0	0.1505
ENSG00000128699	ORMDL1	7.64857	6.47342	1.98664	4.28766	5.60638	5.19469	3.88952	5.6845
ENSG00000064933	PMS1	4.3276	4.22802	3.02168	7.24041	4.75485	4.45739	4.41319	3.61374
ENSG00000187699	C2orf88	34.664	19.8566	11.585	14.6934	14.5927	19.5358	16.5841	20.1844
ENSG00000223824	AC019178.2	0	0	0	0	0	0	0	0
ENSG00000204253	HNRNPCP2	1.08162	1.2464	0.85584	0.86436	1.315	0.58711	0.41605	0.91938
ENSG00000213110	AC019178.1	0	0	0	0	0.20254	0	0	0
ENSG00000138379	MSTN	0	0	0	0	0	0	0	0
ENSG00000198130	HIBCH	0.69969	1.18104	0.44893	0.78979	0.97048	1.14743	0.57493	0.83487
ENSG00000151689	INPP1	1.89432	1.65953	0.32232	0.53268	0.94547	1.44227	0.46532	0.51413
ENSG00000151690	MFSD6	7.13436	4.97664	1.21448	3.31895	3.69411	11.1709	4.4164	2.37266
ENSG00000272979	AC093388.1	1.53419	1.98618	0.75149	1.01196	0.82899	0.90218	0.56828	1.25577
ENSG00000189362	NEMP2	1.29268	0.71098	0.37991	0.83412	1.32561	1.92352	0.44967	0.84187
ENSG00000233654	AC108047.1	3.9916	0.604	3.99928	4.57762	2.64703	6.40157	8.16552	3.78701
ENSG00000251935	RN7SKP179	0	0	0	0	0	0	0	0
ENSG00000284052	AC006460.2	0.14076	0.06248	0.02758	0.02785	0.07986	0.149	0.09385	0.05761
ENSG00000138386	NAB1	7.09455	9.33726	2.55675	3.29451	6.3277	8.91714	3.85017	6.53754
ENSG00000228509	AC006460.1	0	0.10845	0	0	0	0	0	0
ENSG00000235852	AC005540.1	0.07976	0.13276	0	0	0	0.28142	0.13295	0.19586
ENSG00000115419	GLS	9.8236	9.78352	5.78742	14.6014	13.1494	11.5929	7.9945	11.4019
ENSG00000115415	STAT1	46.1624	88.0646	23.9878	18.7809	39.7661	116.665	28.461	88.3616
ENSG00000229023	AC067945.1	0.83026	1.18455	0.34859	0	0.43261	0.83697	0.59311	2.91253
ENSG00000230686	AC067945.2	0	0	0	0	0	0	0	0
ENSG00000231858	AC067945.3	0	0	0	0	0.17138	0.49735	0.23496	0.17307
ENSG00000138378	STAT4	8.98576	9.73197	3.01599	6.24558	7.61161	14.057	5.26094	7.19976
ENSG00000207402	RNU6-959P	0	0	0	0	0	0	0	0
ENSG00000280083	AC079777.1	0	0	0	0	0	0	0	0
ENSG00000230611	HMGB1P27	0	0	0	0	0	0	0	0
ENSG00000227542	AC092614.1	0	0	0	0	0	0	0	0
ENSG00000128641	MYO1B	0.0692	0	0.0113	0.05706	0.26175	0	0.0769	0.07552
ENSG00000252130	RNU6-1045P	0	0	0	0	0	0	0	0
ENSG00000173559	NABP1	16.5013	20.3966	3.90098	4.09739	12.9421	4.58951	5.89397	16.3228
ENSG00000274845	RF02271	0	0	0	0	0	0	0	0
ENSG00000225884	AC098872.1	0.13499	0.22469	0	0	0	0.23814	0	0
ENSG00000168497	CAVIN2	143.309	76.3639	43.2991	61.6868	96.5684	79.253	90.1312	128.32
ENSG00000233766	AC098617.1	0.02844	0	0	0.04221	0.01729	0	0	0.03492
ENSG00000213946	DNAJB1P1	0	0	0	0	0	0	0	0
ENSG00000144339	TMEFF2	0	0	0	0	0	0	0	0
ENSG00000229395	AC062039.1	0	0	0	0	0	0	0	0
ENSG00000232227	AC013401.1	0	0	0	0	0	0	0	0
ENSG00000227418	PCGEM1	0	0	0	0	0	0	0	0

ENSG00000226242	RPS17P8	0	0	0	0	0	0	0	0
ENSG00000278406	RF01981	0	0	0	0	0	0	0	0
ENSG00000232658	AC092638.1	0	0	0	0	0	0	0	0
ENSG00000235218	AC092638.2	0	0	0	0	0	0	0	0
ENSG00000224219	AC074290.1	0	0	0	0	0	0	0	0
ENSG00000260142	AC068135.2	0	0	0	0	0	0	0	0
ENSG00000224670	AC068135.1	0	0	0	0	0	0	0	0
ENSG00000215043	GLULP6	0	0	0.11798	0	0	0	0	0
ENSG00000236695	HNRNPA1P47	0	0	0	0	0	0	0	0
ENSG00000225539	LINC01821	0	0	0	0	0	0	0	0
ENSG00000225394	AC106883.1	0	0	0	0	0	0	0	0
ENSG00000276123	RF00017	0	0	0	0	0	0	0	0
ENSG00000252517	RF00273	0	0	0	0	0	0	0	0
ENSG00000230173	LINC01790	0	0	0	0	0	0	0	0
ENSG00000235056	AC010983.1	0	0	0	0	0	0	0	0
ENSG00000224099	AC104823.1	0	0	0	0	0	0	0	0
ENSG00000271893	AC064834.1	0.29145	0	0	0	0	0	0	0
ENSG00000202206	RNU6-169P	0	0	0	0	0	0	0	0
ENSG00000223466	LINC01825	0.60365	0	0.25344	0.25597	0.31453	0.30426	0.14374	0.10588
ENSG00000234919	LINC01827	0	0	0	0	0	0	0	0
ENSG00000196950	SLC39A10	5.98738	3.79237	1.61952	4.0891	6.27437	6.18797	4.45127	4.09846
ENSG00000213107	AHCYP5	0	0	0	0	0	0	0	0
ENSG00000201813	RNU6-915P	0	0	0	0	0	0	0	0
ENSG00000118997	DNAH7	0.055	0.02289	0.0101	0.03061	0.01672	0.00606	0.03438	0.02954
ENSG00000203435	E2F3P2	0	0	0	0	0	0	0	0
ENSG00000227878	AC114760.1	0	0	0	0	0	0	0	0
ENSG00000081320	STK17B	178.572	122.565	45.2791	81.457	133.445	100.03	85.749	90.8998
ENSG00000272211	AC114760.2	0	0	0	0	0	0	0	0
ENSG00000138411	HECW2	1.00491	1.27853	0.2546	0.37713	0.72321	0.22415	0.15402	0.84381
ENSG00000229056	HECW2-AS1	3.64905	1.13885	1.07245	1.76007	1.6082	1.85076	0.53221	1.7921
ENSG00000276200	RN7SL820P	0	0	0	0	0	0	0	0
ENSG00000282836	AC068544.1	0	0	0	0	0	0	0	0
ENSG00000144395	CCDC150	0.13626	0.28981	0.24473	0.41568	0.33132	0.38728	0.42901	0.3253
ENSG00000252923	RF00424	0	0	0	0	0	0	0	0
ENSG00000119041	GTF3C3	2.81647	1.82025	0.89446	1.9292	2.45839	3.43982	1.85721	2.24201
ENSG00000187944	C2orf66	0.13176	0.0731	0	0.06518	0	0	0	0.05392
ENSG00000280176	AC012486.1	0	0	0	0	0	0	0	0
ENSG00000197121	PGAP1	0.31018	0.22947	0.21946	0.28132	0.32124	0.74987	0.38298	0.33852
ENSG00000065413	ANKRD44	23.9601	22.6817	4.88931	10.5885	21.3193	17.6329	8.92765	17.096
ENSG00000225636	HNRNPA3P15	0	0	0	0	0	0	0	0
ENSG00000224442	AC017035.1	0.46018	0.21885	0	0	0.23978	0.23195	0.21916	0.16143
ENSG00000231621	AC013264.1	0.6444	0	0.47347	0.71727	0.58758	1.98941	0.26853	0.59339
ENSG00000236977	ANKRD44-IT1	3.7713	3.65227	1.10838	2.5441	2.83439	3.02413	2.28588	2.86239
ENSG00000230482	ATP5MC2P3	0	0	0	0	0	0	0	0
ENSG00000225979	AC010746.1	0.32434	0	0	0.24068	0.19716	0	0	0
ENSG00000213104	NPM1P46	0	0	0	0	0	0.14949	0	0
ENSG00000115524	SF3B1	52.3997	34.23	9.462	19.7296	38.0817	33.8943	24.7587	32.0155
ENSG00000202434	RF00394	0	0	0	0	0	0	0	0
ENSG00000206836	RNU6-1029P	0	0	0	0	0	0	0	0
ENSG00000115520	COQ10B	11.9026	15.7777	7.47904	15.1868	11.1969	9.9763	12.6579	15.9032
ENSG00000144381	HSPD1	13.7967	13.3649	4.41513	10.2251	14.8213	21.4149	6.82137	9.66753
ENSG00000115541	HSPE1	1.7426	2.59199	3.21453	8.25385	2.56937	3.00877	7.0453	4.82534
ENSG00000270757	HSPE1-MOB4	0	0	0	0	0	0.14529	0	0
ENSG00000115540	MOB4	9.11136	7.18532	2.50731	5.19112	7.17746	6.38131	3.5835	4.92302
ENSG00000231699	AC020550.1	0.4497	0.74852	0	0	1.09346	0	0.74957	0
ENSG00000162944	RFTN2	0.0244	0.02031	0	0.01811	0.01484	0	0.02034	0
ENSG00000222017	AC011997.1	0.21308	0	0.10437	0.10541	0	0.50121	0	0.08721
ENSG00000247626	MARS2	1.62674	1.41447	0.46382	0.4324	2.33197	1.49914	1.21411	0.77507

ENSG00000152430	BOLL	0	0	0	0	0	0	0	0
ENSG00000115896	PLCL1	1.21664	0.62428	0.26885	0.65166	1.43467	0.87144	1.17408	0.58403
ENSG00000237035	AC020719.1	0	0	0	0	0	0	0	0
ENSG00000236653	LINC01923	0.29769	0	0	0.06797	0.08352	0.08079	0.11451	0
ENSG00000225421	AC019330.1	0	0	0	0	0	0	0	0
ENSG00000231557	AC020718.1	0	0	0	0	0	0	0	0
ENSG00000230407	AC018717.1	0	0	0	0	0	0	0	0
ENSG00000238698	RNU7-147P	0	0	0	0	0	0	0	0
ENSG00000119042	SATB2	0.0602	0.20042	0.02949	0	0.0244	0.0118	0.01115	0.04106
ENSG00000257045	AC016746.1	0	0	0	0	0	0	0	0
ENSG00000225953	SATB2-AS1	0	0	0	0	0	0	0.02733	0
ENSG00000238217	LINC01877	0	0	0	0	0	0	0	0
ENSG00000213938	SEPHS1P6	0	0	0	0.09227	0	0	0	0
ENSG00000226124	FTCDNL1	0.35188	0.04505	0.07955	0.04017	0.26327	0.04775	0.04512	0.03323
ENSG00000232732	AC097717.1	0.01101	0	0	0.01634	0.04017	0	0.01836	0.01352
ENSG00000227004	AC108032.1	0	0	0	0	0	0	0	0
ENSG00000240302	RN7SL717P	0	0	0	0	0	0	0	0
ENSG00000178074	C2orf69	5.06755	3.77726	1.78019	3.06403	3.92064	3.85287	2.21836	2.82812
ENSG00000162971	TYW5	0.88692	0.48822	0.30787	0.51822	0.68773	0.72689	0.71008	0.54019
ENSG00000162972	MAIP1	2.39219	1.42744	0.92857	2.41151	2.35962	4.22015	2.40749	2.05043
ENSG00000196141	SPATS2L	0.63208	0.99365	0.88755	0.18762	0.26469	1.38765	0.21072	0.29318
ENSG00000201649	RNY4P34	0	0	1.13383	0	0	0	0	0
ENSG00000155729	KCTD18	4.62322	3.05332	0.70129	1.35014	3.55379	3.52549	1.46666	1.77614
ENSG00000163535	SGO2	1.25373	1.47901	1.05532	2.04131	1.33187	2.89889	2.0289	2.00259
ENSG00000138356	AOX1	0.01319	0	0	0.01958	0.01604	0.02327	0.04398	0.0162
ENSG00000244301	AOX3P	0	0	0	0	0	0	0	0.02088
ENSG00000284084	AOX3P-AOX2P	0	0	0	0	0	0	0	0
ENSG00000237166	LINC01792	0.03576	0	0	0	0	0.06308	0	0.0439
ENSG00000243478	AOX2P	0	0	0	0	0	0	0	0
ENSG00000201737	RNU1-133P	0	0	0	0	0	0	0	0
ENSG00000230408	AC007163.1	0.08358	0	0	0	0	0	0	0
ENSG00000082153	BZW1	16.4461	13.2647	3.99681	8.00588	12.8632	14.1547	7.84312	9.47279
ENSG00000207116	RNU6-31P	0	0	0	0	0	0	0	0
ENSG00000224346	BICD1P1	0	0	0	0	0	0	0	0
ENSG00000223092	RNA5SP115	0	0	0	0	0	0	0	0
ENSG00000013441	CLK1	37.43	42.2192	31.8767	92.4573	38.7666	28.3135	54.5439	53.7197
ENSG00000252759	RF00019	12.2167	20.3347	3.36606	3.39955	10.2113	14.817	3.81814	8.43722
ENSG00000240344	PPIL3	5.13153	5.31746	3.99214	11.125	7.73731	7.01141	6.45702	5.03415
ENSG00000201499	RNU6-312P	0.68505	0	0	0	0.83287	0	0	0
ENSG00000196290	NIF3L1	4.83745	3.13473	0.81396	1.4249	4.66909	5.14641	0.73862	2.26693
ENSG00000252916	RNU6-762P	0	0	0	1.06653	0	0	0	0
ENSG00000115942	ORC2	5.22587	4.7047	4.98419	10.5523	6.35348	5.93926	9.35983	6.70929
ENSG00000183308	AC005037.1	0.15067	0	0.1107	0	0	0.2658	0	0
ENSG00000155744	FAM126B	13.9276	22.7444	3.1364	6.00402	13.6284	4.93626	6.53345	13.3907
ENSG00000225695	HNRNPA1P35	0.07612	0	0	0	0.09254	0	0	0
ENSG00000226658	RPL23AP30	0	0	0	0	0	0	0	0
ENSG00000119013	NDUFB3	18.0759	27.4326	5.17574	8.67919	14.6239	18.4061	6.20317	12.9733
ENSG00000252148	RNU6-1206P	0	0	0	0	0.86521	0	0	0
ENSG00000273975	RF00017	0	0	0	0	0	0	0	0
ENSG00000232719	AC007272.1	0	0	0	0	0	0	0	0
ENSG00000224672	RPL17P10	0.79244	0.43967	0	0	0.16057	0.46599	0.22014	0
ENSG00000003402	CFLAR	20.8035	28.2154	4.21217	8.80272	17.207	9.54528	6.12827	14.7365
ENSG00000232133	IMPDH1P10	0	0	0	0	0	0	0	0.05836
ENSG00000226312	CFLAR-AS1	0.03383	0	0	0.1506	0	0	0.05638	0.0623
ENSG00000238829	RNU7-45P	10.4715	9.68321	0	3.45351	8.4873	4.10514	3.87874	18.5708
ENSG00000235579	AC007283.2	0	0	0	0	0	0	0	0
ENSG00000234431	AC007283.1	0	0	0	0	0	0	0	0
ENSG00000003400	CASP10	4.12905	3.14736	1.13413	2.36242	2.92051	2.1274	1.81711	2.99674

ENSG00000227348	MTND5P25	0	0	0	0	0	0	0	0
ENSG00000225796	MTND4P23	0	0	0	0	0	0	0	0
ENSG00000235318	MTND4LP13	0	0	0	0	0	0	0	0
ENSG00000064012	CASP8	24.1771	27.3563	4.44656	6.71368	19.1571	9.56939	7.20295	13.1833
ENSG00000155749	ALS2CR12	79.4203	101.496	39.6518	80.134	114.82	167.786	128.765	99.0822
ENSG00000115993	TRAK2	42.243	47.3255	42.7168	88.332	49.2928	48.2855	175.685	40.2054
ENSG00000082146	STRADB	202.492	200.964	261.145	289.581	285.96	249.29	598.314	257.277
ENSG00000213090	AC007256.1	1.93524	1.05902	1.36347	2.20326	2.28835	1.44979	8.39575	1.98547
ENSG00000202008	RF00019	0	0	0	0	0	0	0	0
ENSG00000155754	C2CD6	0.03227	0.14322	0.03161	0.17559	0.03923	0.0759	0.05378	0.07923
ENSG00000234842	MTCO2P16	0	0	0	0.16069	0	0	0	0
ENSG00000224299	MTATP6P16	0	0	0	0	0	0	0	0
ENSG00000227736	MTCO3P16	0	0	0	0	0	0	0	0
ENSG00000224590	MTND3P16	0	0	0	0	0	0	0	0
ENSG00000234731	MTND4LP16	0	0	0	0	0	0	0	0
ENSG00000249177	MTND4P29	0	0	0	0	0	0	0	0
ENSG00000227841	MTND5P31	0	0	0	0	0	0	0	0
ENSG00000155755	TMEM237	0.30674	0.12764	0.08451	0.11381	0.13985	0.27056	0.09587	0.17653
ENSG00000241790	ENO1P4	0	0	0	0	0	0	0	0
ENSG00000082126	MPP4	0	0.07433	0.02188	0	0.0181	0	0	0
ENSG00000222972	RNU6-651P	0	0	0	0	0	0	0	0
ENSG00000003393	ALS2	2.76006	1.90345	0.52903	0.81715	2.17127	1.40719	1.43548	1.11803
ENSG00000230799	AC007279.1	0	0	0	0	0	0	0	0
ENSG00000138395	CDK15	0.07162	0.01987	0	0	0.11609	0.04211	0.03979	0
ENSG00000202137	RF00019	0	0	0	0	0	0	0	0
ENSG00000231124	UBE2V1P11	0	0	0	0	0	0	0	0
ENSG00000212184	RNU6-440P	0	0	0	0	0	0	0	0
ENSG00000273209	AC069148.1	0	0	0	0	0	0	0	0
ENSG00000155760	FZD7	0.07598	0.09485	0	0	0	0	0	0
ENSG00000182329	KIAA2012	0.00923	0	0	0	0.01122	0	0	0
ENSG00000222035	KIAA2012-AS1	0	0	0	0.03517	0	0	0	0
ENSG00000231903	AC079354.2	0	0	0	0	0	0	0	0
ENSG00000227890	PSMA2P3	0	0	0	0	0	0	0	0
ENSG00000223825	DAZAP2P1	0.29145	0	0.21414	0	0.35434	0	0.2429	0
ENSG00000116030	SUMO1	34.0759	28.9235	7.46631	13.3217	26.1502	29.0414	17.4463	20.2535
ENSG00000224463	AC079354.1	0	0	0	0.7064	0	0	0	0.5844
ENSG00000055044	NOP58	17.9085	15.1262	5.38733	15.3335	18.8823	21.4017	11.3699	11.594
ENSG00000212534	SNORD70	0	1.38646	0	0	2.02538	0	0	0
ENSG00000212309	SNORD70B	0	0	0	3.8395	0	0	0	0
ENSG00000271852	SNORD11B	3.25779	0	1.19682	3.62619	1.98037	7.18399	1.35756	0
ENSG00000238317	SNORD11	9.59885	2.90496	2.56462	1.29507	6.36548	4.61828	2.90906	2.14279
ENSG00000264275	RN7SL753P	0	0	0	0.37512	0	0	0	0
ENSG00000226261	AC064836.1	0	0	0	0	0	0	0	0
ENSG00000272966	AC064836.2	0	0.26409	0	0	0	0	0	0
ENSG00000242696	RN7SL40P	0	0	0	0	0	0	0	0
ENSG00000273456	AC064836.3	0.32675	0	0	0	0.26483	0	0	0.13373
ENSG00000204217	BMPR2	4.4165	4.9111	4.19994	7.01165	5.03996	4.78128	4.86668	3.19149
ENSG00000270915	AC073410.2	0	0	0	0	0	0	0	0
ENSG00000236047	AC073410.1	0.12445	0.20715	0	0.1847	0.1513	0	0	0
ENSG00000223619	MTCO1P17	0	0	0	0	0	0	0	0
ENSG00000233888	MTCO2P17	0	0	0	0	0	0	0	0
ENSG00000235433	MTATP6P17	0	0	0	0	0	0	0	0
ENSG00000237542	MTCO3P17	0	0	0	0	0	0	0	0
ENSG00000233134	MTND3P17	0	0	0	0	0	0	0	0
ENSG00000241221	MTND4LP17	0	0	0	0	0	0	0	0
ENSG00000227454	MTND4P30	0	0	0	0	0	0	0	0
ENSG00000233311	MTCO1P54	0	0	0	0	0	0	0	0
ENSG00000138439	FAM117B	5.17976	2.71085	0.97169	2.30802	5.82102	3.71561	3.00042	2.25519

ENSG00000163596	ICA1L	0.23516	0.06907	0.05082	0.26688	0.31113	0.26843	0.0807	0.2123
ENSG00000240761	AC098831.1	0	0	0	0	0	0	0	0
ENSG00000233579	KRT8P15	0	0.08409	0	0	0	0	0	0
ENSG00000138442	WDR12	1.28908	0.57665	0.23679	0.47828	1.23419	1.37868	0.34916	0.45503
ENSG00000138380	CARF	1.88428	2.47238	1.68381	3.62787	3.82843	3.83324	3.62183	2.54276
ENSG00000232479	AC010900.1	0.16968	0	0.08311	0	0	0.09978	0	0
ENSG00000270664	AC010900.2	0	0	0	0	0	0	0	0
ENSG00000144426	NBEAL1	1.92472	1.31873	1.13792	3.23514	1.74685	1.99779	2.15621	1.89601
ENSG00000232994	RPL7P14	0	0.49396	0	0	0	0	0.16489	0
ENSG00000202059	RF00408	0	0	0	0	0	0	0	0
ENSG00000228513	AC023271.1	0	0	0	0	0	0	0	0
ENSG00000225124	RPL23AP36	0	0	0	0	0	0	0	0
ENSG00000204196	RPL12P16	0	0	0	0	0	0	0	0
ENSG00000119004	CYP20A1	5.40409	4.27961	4.93266	10.1401	6.57015	6.42569	6.82527	7.16118
ENSG00000264041	RN7SL670P	0	0	0	0	0	0	0	0
ENSG00000238285	MRPL50P2	0	0	0	0	0	0	0	0
ENSG00000138443	ABI2	0.7446	0.59093	0.27239	0.65278	1.11152	1.09187	0.82218	0.74448
ENSG00000256458	AC080075.1	0	0	0	0	0	0	0	0
ENSG00000173166	RAPH1	0.19426	0.09388	0.00921	0.0279	0.14476	0.12161	0.01045	0.10002
ENSG00000233459	AC125238.2	0	0.08473	0	0	0	0	0	0
ENSG00000233031	AC125238.1	0	0	0	0	0	0	0	0
ENSG00000178562	CD28	9.8434	3.79637	1.764	4.1421	10.3802	13.3944	7.9536	4.56894
ENSG00000224791	KRT18P39	0	0	0	0	0	0	0	0
ENSG00000213925	NPM1P33	0	0	0	0.12475	0	0	0	0
ENSG00000206970	RNU6-474P	0	0	0	0	0	0	1.14187	0
ENSG00000163599	CTLA4	5.46295	1.84164	0.45728	1.12891	2.48013	14.8831	1.15264	1.06129
ENSG00000163600	ICOS	7.64873	3.18283	1.46605	3.29031	9.50129	16.8668	3.88021	3.09631
ENSG00000235951	AC009965.2	0	0	0	0	0	0	0	0
ENSG00000234714	AC009965.1	0	0	0	0	0	0	0	0
ENSG00000237271	AC009498.1	0	0	0	0	0	0	0	0
ENSG00000236681	DSTNP5	0	0	0	0	0	0	0	0
ENSG00000225304	AC106901.1	0	0	0	0	0	0	0	0
ENSG00000236634	AC016903.1	0	0	0	0	0	0	0	0
ENSG00000237843	AC016903.2	0	0	0	0	0	0	0	0
ENSG00000116117	PARD3B	0.0706	0.12927	0.01038	0.07335	0.06009	0.01246	0.05884	0.15603
ENSG00000223890	AC008171.1	0	0	0	0	0	0	0	0
ENSG00000118257	NRP2	0.15297	0.06146	0.0155	0.00783	0.04489	0	0.03517	0.00648
ENSG00000225216	AC007362.1	0	0	0	0	0	0	0	0
ENSG00000201875	RN7SKP178	0	0	0	0	0	0	0	0
ENSG00000213739	PPIAP68	0	0	0	0	0	0	0	0
ENSG00000225610	AC007679.1	0	0	0	0	0	0	0	0
ENSG00000114933	INO80D	0.50489	0.49235	0.2623	0.45413	0.67583	0.47683	0.57805	0.42578
ENSG00000252485	RF00006	0	0	0	0	0	0	0	0
ENSG00000207386	RF00019	0	0	0	0	0	0	0	0
ENSG00000237041	AC007679.2	0	0	0	0	0	0	0	0
ENSG00000227946	AC007383.2	0	0	0	0.10521	0	0	0	0
ENSG00000023228	NDUFS1	3.28023	2.29736	1.15019	2.3144	3.06547	3.59434	2.91807	2.25946
ENSG00000231955	AC007383.3	0	0	0	0	0	0	0	0
ENSG00000237580	GCSHP3	0	0	0	0	0	0	0	0
ENSG00000114942	EEF1B2	84.5155	50.0139	8.60587	18.5759	92.7	73.4005	37.643	45.9149
ENSG00000277502	RF02271	0	0	0	0	0	0	0	0
ENSG00000281780	RF00134	0	0	0	0	0	0	0	0
ENSG00000207047	SNORD51	0	0	0	0	0	0	0	0
ENSG00000207406	SNORA41	0	0	0	0	0	0	0	0
ENSG00000183671	GPR1	0.10346	0	0.03801	0.03839	0	0	0	0
ENSG00000279220	GPR1-AS	0.01775	0	0	0	0.02158	0	0	0
ENSG00000222496	RN7SKP200	0	0	0	0	0	0	0	0
ENSG00000159186	AC007383.1	0	0	0	0	0	0	0	0

ENSG00000224070	HMGN1P6	0	0	0	0	0	0	0	0
ENSG00000252716	RN7SKP260	0	0	0	0	0	0	0	0
ENSG00000204186	ZDBF2	0.07089	0.1062	0.07292	0.32615	0.25856	0.16258	0.24814	0.08704
ENSG00000180105	AC017081.1	0	0	0	0	0	0	0	0
ENSG00000234491	HNRNPA1P51	0	0	0	0	0	0	0	0
ENSG00000114948	ADAM23	0.02283	0	0	0.03388	0.24982	0.02014	0.09514	0.02803
ENSG00000228577	AC010731.2	0	0	0	0	0	0	0	0
ENSG00000231653	AC010731.3	0	0	0	0	0	0	0	0
ENSG00000235118	FAM237A	0	0	0	0	0	0	0	0
ENSG00000232125	DYTN	0.37678	0.57013	0.302	0.96585	0.2915	0.2417	0.79931	0.42055
ENSG00000213083	AC010731.1	0	0	0	0	0	0	0	0
ENSG00000207319	RF00019	0	0	0	0	0	0	0	0
ENSG00000138400	MDH1B	0	0	0.03463	0	0	0.08316	0.03929	0.05788
ENSG00000118246	FASTKD2	2.37542	1.74613	0.90367	1.48531	2.68274	2.7228	1.6682	1.71733
ENSG00000263468	MIR3130-2	0	0	0	0	0	0	0	0
ENSG00000283469	MIR3130-1	0	0	0	0	0	0	0	0
ENSG00000260171	AC008269.2	0	0	0	0	0	0	0	0
ENSG00000229321	AC008269.1	0.56734	0.18887	0.16674	0	0.13795	1.00087	0.5674	0
ENSG00000144410	CPO	0	0.10092	0.17819	0	0	0	0	0
ENSG00000118263	KLF7	9.19618	5.99372	0.98852	1.29199	5.59024	2.09423	2.63831	3.13853
ENSG00000253008	MIR2355	1.68507	0	2.47618	1.25041	1.02433	0	2.80874	2.0689
ENSG00000237892	KLF7-IT1	2.35009	4.65681	0.16445	1.99302	3.12929	0.78969	2.05188	1.9236
ENSG00000277590	MIR7845	2.96163	7.39445	1.08802	1.09885	5.40101	1.30618	1.23414	0.90906
ENSG00000224342	AC007879.1	0.13231	0	0	0	0	0	0	0
ENSG00000234902	AC007879.3	0.73635	0.22285	0	0	0.24416	0	0.22316	0.16438
ENSG00000225916	AC007879.2	0.26606	0	0	0	0	0	0	0
ENSG00000240440	AC007879.4	0.20305	0	0.29838	0	0.49372	0	0	0
ENSG00000229647	MYOSLID	0.36528	0.20267	0.71571	0.18071	0.4441	0	0.30444	0.67274
ENSG00000223725	AC009226.1	1.48427	0.38009	0.16778	0.42362	0.83287	0	0.76125	0.14018
ENSG00000225064	LINC01802	0.42046	0	0	0	0	0	0.23361	0
ENSG00000221628	MIR1302-4	0	0	0	0	0	0	0	0
ENSG00000118260	CREB1	23.9614	20.6554	4.66854	10.6002	22.6259	14.464	7.3372	13.281
ENSG00000144401	METTL21A	1.74679	1.13071	0.68133	1.74428	1.4158	1.08426	0.93459	0.71489
ENSG00000223433	RPS29P9	0	0	0	0	0	0	0	0
ENSG00000224137	LINC01857	0.59594	0	0.21893	0.66333	1.99245	0.26283	1.49	1.28045
ENSG00000199251	RNU6-664P	0	0	0	0	0	0	0	0
ENSG00000213082	PPP1R14BP2	0	0	0	0	0	0	0	0
ENSG00000200764	RF00019	0	0	0	0	0	0	0	0
ENSG00000163249	CCNYL1	9.00098	10.783	9.70272	30.4609	9.69359	8.73617	15.756	12.6382
ENSG00000272851	AC096772.1	0.25993	0.21633	0.38196	0	0	0	0	0
ENSG00000284079	MIR4775	0	0	0	0	0	0	0	0
ENSG00000163251	FZD5	0.33875	0.65479	0.49778	1.65417	0.66426	0.80964	0.71035	0.34883
ENSG00000178385	PLEKHM3	2.7132	1.90263	0.5999	0.81093	1.90893	1.52901	2.2089	1.01016
ENSG00000225111	AC083900.1	1.25033	0.78044	0.689	0.23195	0.57004	1.37859	0.78154	1.15135
ENSG00000212246	RNU6-360P	0	0	0	0	0	0	0	0
ENSG00000185078	AC093698.1	0	0	0	0	0	0	0	0
ENSG00000251941	RNA5SP116	0	0	0	0	0	0	0	0
ENSG00000224839	RPL12P17	0	0	0	0	0	0.25966	0	0
ENSG00000229150	CRYGEP	0	0	0	0	0	0	0	0
ENSG00000118231	CRYGD	0	0	0	0	0	0	0	0
ENSG00000163254	CRYGC	0	0	0	0	0	0	0	0
ENSG00000224529	AC093698.2	0	0	0	0	0	0	0	0
ENSG00000182187	CRYGB	0	0	0	0	0	0	0	0
ENSG00000168582	CRYGA	0	0	0	0	0	0	0	0
ENSG00000188674	C2orf80	0	0	0	0	0.0621	0	0	0
ENSG00000233869	RPSAP27	0	0	0	0	0	0	0	0
ENSG00000228111	TPT1P2	0	0	0	0	0	0	0	0
ENSG00000138413	IDH1	5.35662	6.53848	1.25488	1.93561	3.37892	3.8621	1.19049	2.36383

ENSG00000231908	IDH1-AS1	0.11191	0.18627	0	0	0	0	0	0
ENSG00000115020	PIKFYVE	12.3227	9.68679	3.29148	6.89954	10.4814	7.24633	5.84282	6.82954
ENSG00000228380	MYL6BP1	0	0	0	0	0	0	0	0
ENSG00000144407	PTH2R	0.21706	0.66702	1.12867	1.3877	1.54279	1.06042	1.5029	0.98402
ENSG00000213081	AC012362.1	0	0	0	0	0	0	0	0
ENSG00000227430	AC019185.1	0	0	0	0	0	0	0	0
ENSG00000231896	AC019185.2	0	0	0	0	0.34276	0	0	0.17307
ENSG00000232409	AC019185.3	0	0	0	0	0	0	0	0
ENSG00000202164	RNA5SP117	0	0	0	0	0	0	0	0
ENSG00000235864	HSPA8P6	0	0	0	0	0	0	0	0
ENSG00000232799	CRYGFP	0	0	0	0	0	0	0	0
ENSG00000240286	MEAF6P1	0	0	0	0	0	0	0	0
ENSG00000229670	PKP4P1	0	0	0	0	0	0	0	0
ENSG00000078018	MAP2	0.19658	0.095	0.00932	0.02823	0.04626	0	0	0.03114
ENSG00000222317	RNA5SP118	0	0	0	0	0	0	0	0
ENSG00000144406	UNC80	0.00381	0	0	0.00565	0.00926	0	0.00634	0
ENSG00000237194	SNAI1P1	0	0	0	0	0	0	0	0
ENSG00000197713	RPE	6.07714	4.642	2.14082	4.91111	6.04738	8.29769	4.16287	4.5484
ENSG00000144445	KANSL1L	2.46896	3.13248	1.63645	2.20364	3.01219	3.59411	2.93544	2.48019
ENSG00000272807	AC007038.2	1.28771	0.98926	0.43668	0.58803	1.44514	0.87373	0.99065	0.36485
ENSG00000229127	AC007038.1	0.9	0.44387	0.93068	0.89047	1.17525	1.1761	2.05579	1.47335
ENSG00000231063	AC006994.1	0	0	0	0	0	0	0	0
ENSG00000279317	AC006994.2	0	0	0	0	0.01998	0	0	0
ENSG00000115361	ACADL	0	0	0	0	0	0	0	0
ENSG00000199203	RF00019	0	0	0	0	0	0	0	0
ENSG00000168530	MYL1	0	0	0	0	0	0	0	0
ENSG00000234281	LANCL1-AS1	0	0	0	0	0	0	0	0
ENSG00000115365	LANCL1	4.2133	2.9492	1.33259	3.5199	4.24042	5.73461	2.30222	2.50087
ENSG00000021826	CPS1	0.01404	0	0.01031	0	0.00853	0	0	0.00862
ENSG00000280837	CPS1-IT1	0	0	0	0	0	0	0	0
ENSG00000271074	AC012491.1	0	0	0	0	0	0	0	0
ENSG00000231017	AC013404.1	0	0	0	0	0	0	0	0
ENSG00000178568	ERBB4	0.00563	0	0.00828	0	0	0	0	0
ENSG00000199585	RNA5SP119	0	0	0	0	0	0	0	0
ENSG00000232631	MTND2P23	0	0	0	0	0	0	0	0
ENSG00000236007	MTCO1P46	0	0	0	0	0	0	0	0
ENSG00000221782	MIR548F2	0	0	0	0	0	0	0	0
ENSG00000273118	AC093865.1	0.16928	0.28177	0.24876	0.75371	0.20581	0	0	0.83138
ENSG00000234308	LINC01878	0	0	0	0	0	0	0	0
ENSG00000231254	PCED1CP	0	0	0	0	0	0	0	0
ENSG00000223373	AC108066.1	0	0	0	0	0	0	0	0
ENSG00000266354	MIR4776-1	0	0	0	0	0	0	0	0
ENSG00000283637	MIR4776-2	0	0	0	0	0	0	0	0
ENSG00000030419	IKZF2	7.43783	3.02031	1.12642	4.80826	9.07184	5.3246	2.76503	3.99987
ENSG00000270659	AC079610.2	0.10623	0	0.15611	0.15766	1.29155	0.74963	0.53122	0.39129
ENSG00000236193	LINC01953	0	0	0	0	0	0	0	0
ENSG00000272519	AC079610.3	0	0	0	0	0	0	0	0
ENSG00000196096	AC079610.1	0.04244	0	0	0.12598	0	0	0.14149	0
ENSG00000144451	SPAG16	0.69243	0.47284	0.67835	1.35702	1.31673	1.48778	2.36755	1.08995
ENSG00000265734	MIR4438	0	0	0	0	0	0	0	0
ENSG00000197585	AC068051.1	0.20165	0	0.14816	0	0.24516	0	0.16806	0
ENSG00000213917	RPL5P8	0	0	0	0	0	0	0	0
ENSG00000174453	VWC2L	0	0	0	0	0	0	0	0
ENSG00000224257	VWC2L-IT1	0	0	0	0	0	0	0	0
ENSG00000177261	ENSAP3	0	0	0	0	0	0	0	0
ENSG00000138376	BARD1	6.10131	5.18843	7.58099	10.3819	10.3409	11.8932	15.8893	10.2947
ENSG00000229267	AC016708.1	0	0	0	0.25803	0.12683	0	0	0
ENSG00000207274	SNORA70I	0	0	0	2.41746	0	0	0	0.66664

ENSG00000230076	RPL10P6	0	0	0	0	0.28024	0	0	0
ENSG00000144452	ABCA12	0	0	0	0	0	0	0	0
ENSG00000227769	AC072062.1	0	0	0	0	0	0	0	0
ENSG00000237571	AC073284.1	0	0	0	0	0	0	0	0
ENSG00000138363	ATIC	1.47422	0.74547	0.19195	0.63698	1.40663	2.13983	0.68431	0.41241
ENSG00000115414	FN1	0.08467	0.28186	0.00622	0	0.61761	0.17177	0	0.0052
ENSG00000230695	AC012462.3	0	0	0	0	0	0	0	0
ENSG00000225166	AC012462.1	0	0	0	0	0	0	0	0
ENSG00000228618	AC012462.2	0	0	0	0	0	0	0	0
ENSG00000234938	AC012668.2	0	0	0	0	0	0	0	0
ENSG00000237525	AC012668.3	0	0	0.10623	0.21457	0	0.25505	0	0.04438
ENSG00000227981	AC012668.1	0	0	0	0	0	0	0	0
ENSG00000235770	LINC00607	0	0	0	0	0	0.02298	0	0
ENSG00000230838	LINC01614	0	0	0	0	0	0	0	0
ENSG00000216721	AC093850.1	0	0	0	0	0	0	0	0
ENSG00000227824	AC122136.1	0	0	0	0	0	0	0	0
ENSG00000226276	AC093382.1	0.1934	0.32192	0.28421	0	0.47027	0	0.32238	0.23746
ENSG00000118242	MREG	0.64761	0.23955	0.07049	0.35597	0.64155	0.04231	0.31984	0.0589
ENSG00000115425	PECR	1.77377	1.57208	0.1354	0.68376	1.82042	2.03193	0.61436	0.8485
ENSG00000163449	TMEM169	0.60612	0.43719	0.11876	0.11994	0.61409	0.28514	0.10103	0.27287
ENSG00000079246	XRCC5	56.8369	44.3099	10.4894	20.9288	48.1879	50.0394	21.0008	32.6184
ENSG00000232970	POLHP1	0	0	0	0	0	0	0	0
ENSG00000236478	AC012513.2	0	0	0	0	0	0	0	0
ENSG00000279348	AC012513.3	2.1131	1.21086	0.10181	0.82258	3.2429	2.38335	1.03934	1.36101
ENSG00000260804	LINC01963	1.58336	1.08521	0.30795	0.4838	1.95332	1.76633	0.54337	0.7433
ENSG00000144583	MARCH4	0	0.02744	0	0	0	0	0	0
ENSG00000231092	AC012513.1	0	0	0	0	0	0	0	0
ENSG00000233581	AC069155.1	0	0	0	0	0	0	0	0
ENSG00000207303	RF00019	0	0	0	0	0	0	0	0
ENSG00000235042	AC098820.2	0.11006	0	0	0	0	0	0	0
ENSG00000138375	SMARCAL1	1.74182	1.55745	0.38076	1.26048	2.17016	2.00621	0.76783	1.46696
ENSG00000232485	AC098820.1	0.05108	0	0.07506	0.15162	0	0.09011	0.08514	0.12543
ENSG00000241520	AC098820.3	0	0	0	0	0	0	0	0
ENSG00000197756	RPL37A	27.4515	20.0464	9.6519	15.6476	31.3817	26.9171	21.2891	27.0609
ENSG00000224391	LINC01280	0	0	0	0	0	0	0	0
ENSG00000225794	AC073321.1	0	0	0	0	0	0	0	0
ENSG00000235444	PSMB3P2	0	0	0	0	0	0	0	0
ENSG00000115457	IGFBP2	0.01538	0.05119	0	0.02282	0	0.05425	0	0.01888
ENSG00000115461	IGFBP5	0	0.01894	0	0	0.01383	0	0	0
ENSG00000236886	AC007563.2	0	0	0.23831	0	0	0.14304	0	0
ENSG00000237930	AC007563.3	0	0	0	0	0	0	0	0
ENSG00000222832	RNA5SP120	0	0	0	0	0	0	0	0
ENSG00000229352	AC007563.1	0	0	0	0	0	0	0	0
ENSG00000234588	FABP5P14	0	0	0	0	0	0	0	0
ENSG00000231597	AC007557.2	0	0	0	0	0	0	0	0
ENSG00000118245	TNP1	0	0	0	0	0	0	0	0
ENSG00000237479	AC007557.4	0	0	0	0	0	0	0	0
ENSG00000227021	AC007557.1	0	0	0	0	0	0	0	0
ENSG00000236295	AC007557.3	0	0	0	0	0	0	0	0
ENSG00000223874	LINC01921	0	0	0	0	0	0	0	0
ENSG00000259855	AC007749.1	0	0	0	0.37192	0.45701	0	0.20886	0.30768
ENSG00000251982	RN7SKP43	0	0	0	0	0	0	0	0
ENSG00000233143	DIRC3-AS1	0	0	0	0	0	0	0.21935	0
ENSG00000278004	RF01983	0	0	0	0	0	0	0	0
ENSG00000231672	DIRC3	0.10999	0.05231	0	0.1166	0.11462	0.1386	0.05238	0.19292
ENSG00000224414	AC010886.1	0	0	0	0	0	0	0	0
ENSG00000231035	RPL7L1P9	0.19678	0.16377	0	0.14602	0.11962	0	0	0
ENSG00000079308	TNS1	16.4039	22.2815	20.3479	18.7088	30.2751	51.4013	25.5486	15.3448

ENSG00000275458	MIR6809	0	0	0	0	0	0	0	0
ENSG00000223923	AC010136.1	0	0	0	0	0	0	0	0
ENSG00000188282	RUFY4	0.25235	0.19602	0.02472	0.02497	0.04091	0.08904	0	0
ENSG00000229754	CXCR2P1	66.0084	31.4405	12.4798	18.6887	22.5017	34.5107	18.1098	22.6521
ENSG00000180871	CXCR2	365.345	491.357	38.8717	53.0293	234.5	42.7037	57.4788	170.524
ENSG00000163464	CXCR1	136.23	193.85	23.3784	32.3996	70.491	18.9186	44.2953	83.269
ENSG00000224159	HMGB1P9	0	0.18887	0	0	0	0.20017	0	0.13931
ENSG00000163466	ARPC2	60.5325	58.2525	8.07608	11.7207	42.3311	42.4274	15.1562	25.4411
ENSG00000261338	AC021016.2	2.00431	1.37021	0.05259	0.53118	1.65353	1.13653	1.07385	1.31832
ENSG00000179921	GPBAR1	4.62892	4.07904	0.6402	0.92945	1.98626	1.68125	0.31771	0.80235
ENSG00000127837	AAMP	7.49344	5.24559	1.26924	2.4598	6.10194	7.33041	2.21792	3.00946
ENSG00000127838	PNKD	4.14294	2.54184	0.58179	0.98629	2.30356	3.94122	1.27271	0.85067
ENSG00000135926	TMBIM1	26.7579	21.8265	6.0428	8.59958	16.4843	15.3459	7.0461	10.6627
ENSG00000284173	MIR6513	0	0	0	0	0	0	0	0
ENSG00000237281	CATIP-AS2	0.13701	0	0	0	0	0	0	0
ENSG00000278524	MIR6810	0	0	0	0	0	0	0	0
ENSG00000230580	AC021016.1	0	0	0	0	0	0	0	0
ENSG00000158428	CATIP	0.05755	0.06386	0	0	0.04665	0	0.03198	0.02355
ENSG00000225062	CATIP-AS1	1.79704	3.77833	0.13899	0.84221	2.64475	1.00112	0	1.16125
ENSG00000018280	SLC11A1	24.1124	23.977	2.12299	4.06254	10.3024	3.30881	3.42206	8.72378
ENSG00000144579	CTDSP1	23.0895	15.8996	3.44083	6.76603	14.4412	14.6902	6.90124	9.17677
ENSG00000273361	AC021016.3	2.15137	3.32518	0.45163	0.45612	1.30779	1.89766	0.25614	0
ENSG00000199121	MIR26B	1.90391	0	1.39888	5.65121	0	3.35875	0	1.16879
ENSG00000127831	VIL1	1.75836	1.19893	0.10896	0.23581	0.75981	0.52323	0.37078	0.42918
ENSG00000135913	USP37	4.56845	5.12199	5.18279	13.7475	6.56144	6.89013	10.2584	8.47658
ENSG00000222714	RN7SKP38	0	0	0	0	0	0	0	0
ENSG00000144580	CNOT9	5.90394	5.19038	2.85119	6.89038	6.92512	8.38607	5.03598	4.50921
ENSG00000207393	RNU6-136P	0	0	0	0	0	0	0	0
ENSG00000115556	PLCD4	0.11244	0.06239	0.07344	0.03708	0.07595	0	0.06248	0.03068
ENSG00000273466	AC012510.1	0.67602	1.12524	0	0.28666	1.17413	1.36297	0.16098	0.35572
ENSG00000115568	ZNF142	2.2009	1.30836	0.33882	0.85548	2.08967	2.23716	0.33192	0.66912
ENSG00000074582	BCS1L	1.10634	0.39719	0.09563	0.32195	0.68572	0.80365	0.32543	0.34624
ENSG00000163481	RNF25	2.24035	1.208	0.55642	0.51513	1.72632	2.22663	0.99932	0.81358
ENSG00000163482	STK36	0.78317	0.20782	0.11675	0.13476	0.28979	0.40047	0.13243	0.12542
ENSG00000135912	TTLL4	2.86143	2.12741	0.46253	0.94842	1.5191	1.29564	0.90622	0.93686
ENSG00000135929	CYP27A1	11.5737	5.23536	0.3972	1.2764	1.61324	3.59801	2.86712	1.68952
ENSG00000272555	AC009974.1	2.2554	1.0726	0	0	0.39172	0	0	0
ENSG00000115592	PRKAG3	0	0	0	0	0	0	0	0
ENSG00000283639	MIR9500	0	0	0	0	0	0	0	0
ENSG00000232625	RPL23AP31	0	0	0	0	0	0	0	0
ENSG00000115596	WNT6	0	0.07164	0	0	0	0	0	0
ENSG00000135925	WNT10A	0.17186	0.07152	0.03157	0.03188	0.07836	0	0.03581	0.07913
ENSG00000228135	LINC01494	0	0	0	0	0	0	0	0
ENSG00000200029	RNU6-642P	0	0	0	0	0	0	0	0
ENSG00000235024	AC097468.2	0	0	0	0	0	0	0	0
ENSG00000224928	KRT8P30	0	0	0	0	0	0	0	0
ENSG00000171450	CDK5R2	0	0	0	0	0	0	0	0
ENSG00000236445	LINC00608	0	0	0	0	0	0	0	0
ENSG00000163497	FEV	0	0	0	0	0	0	0	0
ENSG00000163499	CRYBA2	0	0	0	0	0	0	0	0
ENSG00000198973	MIR375	0	0	0	0	0	0	0	0
ENSG00000224090	AC097468.1	0	0	0	0	0	0	0	0
ENSG00000181378	CFAP65	0.00697	0	0	0	0	0	0	0
ENSG00000163501	IHH	0.03623	0	0	0	0	0	0	0
ENSG00000264755	MIR3131	0	0	0	0	0	0	0	0
ENSG00000272644	AC097468.3	0.80373	0.53512	0.23621	0.47713	0.78173	2.26863	0	0.39472
ENSG00000187736	NHEJ1	0.07073	0.11773	0	0.06998	0	0.08319	0.0393	0
ENSG00000280537	AC068946.1	0.01576	0.02624	0	0	0	0.05562	0	0

ENSG00000240317	RN7SL764P	0	0	0	0	0	0	0	0
ENSG00000213901	SLC23A3	0.02036	0	0.02991	0.06042	0.07424	0	0	0.04998
ENSG00000115649	CNPPD1	22.0167	22.1472	13.3081	18.2886	24.8623	38.5124	19.7436	11.4127
ENSG00000144567	RETREG2	19.6103	15.5486	4.50623	6.27205	17.8891	20.3207	17.7611	11.2318
ENSG00000158552	ZFAND2B	3.86359	2.69453	0.60264	1.24931	4.01497	2.81775	1.51106	2.09357
ENSG00000115657	ABCB6	0.04225	0.07032	0.02069	0.0836	0.06849	0.02484	0.14084	0.06916
ENSG00000198925	ATG9A	4.31549	4.61881	1.68138	2.58059	3.94322	6.16679	3.25874	1.55968
ENSG00000163516	ANKZF1	3.82134	3.38968	0.44008	1.77783	3.46621	1.79629	1.37775	1.72081
ENSG00000163521	GLB1L	0.66617	0.42923	0.03158	0.15946	0.23514	0.41701	0.25074	0.05277
ENSG00000115661	STK16	3.62474	3.26808	0.61649	1.36978	2.14223	3.34529	1.62236	2.18399
ENSG00000127824	TUBA4A	39.5883	28.0868	3.69697	8.77433	24.2072	24.9493	10.963	9.70794
ENSG00000243910	TUBA4B	0.04368	0	0	0	0	0	0	0
ENSG00000135924	DNAJB2	6.36366	5.16142	3.3489	3.105	10.9163	9.66653	6.68401	6.26891
ENSG00000054356	PTPRN	0.1331	0.05908	0.09127	0	0.06473	0.12523	0.05916	0.04358
ENSG00000207647	MIR153-1	0	0	0	0	0	0	0	0
ENSG00000230432	AC114803.1	0	0	0	0	0	0	0	0
ENSG00000182698	RESP18	0	0	0	0	0	0	0	0
ENSG00000232789	AC053503.3	0	0	0	0	0	0	0	0
ENSG00000123992	DNPEP	1.91999	1.34019	0.29579	0.91919	1.46834	2.02135	0.61942	1.17867
ENSG00000229525	AC053503.2	0	0	0	0	0	0	0	0
ENSG00000175084	DES	0.53547	0	0	0.04415	0.1085	0.05248	0	0
ENSG00000234638	AC053503.4	0	0	0	0	0	0	0	0
ENSG00000280323	AC053503.6	0	0	0	0	0	0	0	0
ENSG00000072195	SPEG	0.02456	0.02725	0	0.0243	0.04478	0.01444	0.00682	0
ENSG00000227432	AC053503.1	0	0	0	0	0	0	0	0
ENSG00000268603	AC053503.5	0	0	0	0	0	0	0	0
ENSG00000144591	GMPPA	1.55344	1.25853	0.24242	0.59167	1.22009	1.84315	0.38955	0.60763
ENSG00000072182	ASIC4	0	0	0	0	0	0.04926	0	0
ENSG00000123989	CHPF	0.39338	0.21826	0.09635	0.06487	0.37198	0.88675	0.10928	0.0805
ENSG00000188760	TMEM198	0.02843	0	0.04178	0.0422	0	0	0	0
ENSG00000268896	AC009955.3	0	0	0	0	0	0	0	0
ENSG00000265252	MIR3132	0	0	0	0	0	0	0	0
ENSG00000124006	OBSL1	0.04576	0	0	0.03396	0.00927	0	0.01271	0.00936
ENSG00000269068	AC009955.4	0	0	0	0	0	0	0	0
ENSG00000123999	INHA	0	0	0	0	0	0	0	0
ENSG00000222678	RN7SKP213	0	0	0	0	0	0	0	0
ENSG00000144589	STK11IP	1.84088	1.2428	0.32159	0.85974	1.36164	0.86299	0.83685	0.96414
ENSG00000228973	AC009955.1	0	0	0	0	0	0	0	0
ENSG00000114923	SLC4A3	0.08639	0	0	0.02137	0.03501	0	0.048	0
ENSG00000267919	AC009955.2	0	0	0	0	0	0	0	0
ENSG00000231802	AC009502.2	0	0	0	0	0	0	0	0
ENSG00000227308	AC009502.1	0	0	0	0	0	0	0	0
ENSG00000223903	RPL23P4	0	0	0	0	0	0	0	0
ENSG00000228909	LINC01803	0	0	0	0	0	0	0	0
ENSG00000266518	MIR4268	0	0	0	0	0	0	0	0
ENSG00000225911	AC009310.1	0	0	0	0	0	0	0	0
ENSG00000239498	AC019211.1	0	0	0	0	0	0	0	0
ENSG00000235337	AC093083.1	0	0	0	0	0	0	0	0
ENSG00000236451	AC067956.1	0	0	0	0	0	0	0	0
ENSG00000224819	AC093843.1	0	0	0	0	0	0	0	0
ENSG00000232089	AC011233.1	0	0	0	0	0	0	0	0
ENSG00000116106	EPHA4	2.23181	1.25982	0.27092	0.72005	2.99651	2.01996	0.80871	0.97693
ENSG00000226726	TMEM256P2	0	0	0	0	0	0	0	0
ENSG00000272944	AC079834.2	0	0	0	0	0.04572	0.26539	0.06269	0
ENSG00000230406	AC079834.1	0	0	0	0	0	0	0	0
ENSG00000234446	AC068489.1	0	0	0	0	0	0	0	0
ENSG00000233868	AC009302.1	0	0.31284	0	0	0	0.33157	0	0
ENSG00000226666	HSPA9P1	0	0	0	0	0	0.06373	0	0

ENSG00000226331	RPL23AP28	0	0	0	0	0	0	0	0
ENSG00000135903	PAX3	0	0.02633	0	0	0	0.02791	0	0
ENSG00000163081	CCDC140	0.03794	0	0	0	0.04613	0	0	0
ENSG00000267034	AC010980.2	0.10382	0	0.07628	0	0	0	0	0
ENSG00000237732	AC010980.1	0	0.04753	0	0	0	0	0	0
ENSG00000200822	RF00019	0	0	0	0	0	0	0	0
ENSG00000163082	SGPP2	0.30762	0.25601	0.06458	0.09783	0.26714	0.77525	0.07325	0.08093
ENSG00000228670	NANOGP2	0	0	0	0	0	0	0	0
ENSG00000202016	RNU6-619P	0	0	0	0	0	0	0	0
ENSG00000234285	GAPDHP49	0	0	0	0	0	0	0	0
ENSG00000261428	AC097461.1	0.49369	0.04325	0.15273	0.23138	0.50545	0.04584	0.25987	0.25522
ENSG00000116120	FARSB	4.24267	4.49067	4.25229	8.33087	6.85106	8.25231	8.95771	6.30434
ENSG00000124003	MOGAT1	0	0.11642	0	0	0	0	0	0.08588
ENSG00000233751	AC104772.2	0	0	0	0	0	0	0	0
ENSG00000203531	AC104772.1	0	0	0	0	0	0	0	0
ENSG00000235225	AC016712.1	0.19392	0	0	0	0	0.34209	0	0
ENSG00000123983	ACSL3	7.77551	10.2123	2.81187	6.28072	9.29324	7.44793	3.78016	5.09652
ENSG00000224121	ATG12P2	0	0	0	0	0	0	0	0
ENSG00000234193	AC013476.1	0	0	0	0	0	0	0	0
ENSG00000278083	AC013476.2	0	0	0	0	0	0	0	0
ENSG00000152049	KCNE4	0	0	0	0	0	0	0	0
ENSG00000243125	RN7SL807P	0	0	0	0	0	0	0	0
ENSG00000233299	HIGD1AP4	0	0	0	0	0	0	0	0
ENSG00000231189	AC013448.1	0	0	0	0	0	0	0	0
ENSG00000235610	AC013448.2	0	0	0	0	0	0	0	0
ENSG00000171951	SCG2	0	0	0	0	0.06555	0	0	0
ENSG00000226539	AC012512.1	0	0	0	0	0	0	0	0
ENSG00000152056	AP1S3	0.35932	0.34176	0.12572	0.35551	0.58246	0.3924	0.17112	0.39915
ENSG00000085449	WDFY1	15.973	17.6067	4.88189	6.64626	12.7148	22.7632	6.933	9.31623
ENSG00000135900	MRPL44	10.1899	13.192	2.71133	3.36066	9.3807	13.9077	4.40352	6.53869
ENSG00000228802	AC073641.1	0	0	0	0	0	0	0	0
ENSG00000135919	SERPINE2	0.15664	0.09606	0.19384	0.41601	0.79184	0.26179	0.2611	0.4555
ENSG00000224826	AC019109.1	0	0	0	0	0	0	0	0
ENSG00000228681	AC008072.1	0	0	0	0	0	0	0	0
ENSG00000124019	FAM124B	0.53821	0.20674	0.03042	0.12289	0.30201	0.10956	0.17252	0.25416
ENSG00000274629	AC073052.2	0	0	0	0	0	0	0	0
ENSG00000036257	CUL3	8.32075	8.26116	3.66068	7.26184	9.90702	7.18211	7.81614	7.4626
ENSG00000228446	AC073052.1	2.15589	3.07584	0.7543	1.06653	1.37295	0	1.19785	1.6386
ENSG00000283428	CCDC195	0	0	0	0	0	0	0	0
ENSG00000135905	DOCK10	11.9506	7.9391	3.15403	6.42981	12.5561	17.6818	5.80812	7.78375
ENSG00000263828	MIR4439	1.83251	4.57532	0	1.35982	0	3.23279	0	2.24993
ENSG00000144460	NYAP2	0	0	0	0.01992	0	0	0	0
ENSG00000228206	AC016717.1	0	0	0	0	0	0	0	0
ENSG00000273301	AC016717.2	0	0	0	0	0	0	0	0
ENSG00000279015	AC016717.3	0	0	0	0	0	0	0	0
ENSG00000230621	AC068138.1	0	0	0	0	0	0	0	0
ENSG00000235070	AC062015.1	0.12928	0	0	0	0	0	0	0
ENSG00000263363	MIR5702	0	0	0	0	0	0	0	0
ENSG00000169047	IRS1	0.51553	0.26969	0.05411	0.08744	0.68944	0.41575	0.22096	0.20797
ENSG00000272622	AC010735.2	0.12551	0	0	0	0	0	0	0
ENSG00000261379	AC010735.1	0.14289	0	0	0	0	0	0	0
ENSG00000144468	RHBDD1	17.6319	22.6718	33.7636	45.7855	27.6254	53.2482	41.7735	34.4863
ENSG00000212391	RF00554	0	0	0	0	0	0	0	0
ENSG00000081052	COL4A4	0.05926	0.03699	0	0.04398	0.07205	0	0	0.04548
ENSG00000169031	COL4A3	0.00803	0	0	0.03577	0.0293	0	0.01339	0.02959
ENSG00000236432	AC097662.1	0.08029	0.10023	0	0.02979	0.14641	0	0	0.02464
ENSG00000168958	MFF	5.94746	5.59454	2.87237	7.35851	6.04255	4.94118	5.62228	5.61933
ENSG00000168955	TM4SF20	0	0	0	0	0	0	0	0

ENSG00000284092	MIR5703	0	0	0	0	0	0	0	0
ENSG00000173744	AGFG1	9.51544	9.35909	3.12013	5.97557	9.18798	6.96433	4.8762	6.86492
ENSG00000042304	C2orf83	0	0	0	0	0	0	0	0
ENSG00000236116	AC064853.1	0	0	0	0	0	0	0	0
ENSG00000250599		0	0	0	0	0.15648	0	0	0
ENSG00000135917	SLC19A3	0	0	0	0	0	0	0	0
ENSG00000252346	RNA5SP121	0	0	0	1.07709	0	0	0	0
ENSG00000226718	SNRPGP8	0	0	0	0	0	0	0	0
ENSG00000229172	AC073065.1	0	0	0	0	0	0	0	0
ENSG00000115009	CCL20	0.10916	0.09085	0	0.24301	0.26543	0	0	0
ENSG00000183514	TDGF1P2	0	0	0	0	0	0	0	0
ENSG00000123977	DAW1	0	0	0	0	0	0	0	0
ENSG00000272237	RF00402	0	0	0	0	0	0	0	0
ENSG00000153820	SPHKAP	0	0	0	0	0	0	0	0
ENSG00000200281	RNU6-624P	0	0	0	0	0	0	0	0
ENSG00000203387	AC074019.1	0	0	0	0	0	0	0	0
ENSG00000232023	LINC01807	0	0	0	0	0	0	0	0
ENSG00000228226	AC012070.1	0	0	0	0	0	0	0	0
ENSG00000237322	RPL7L1P10	0	0	0	0	0	0	0	0
ENSG00000153823	PID1	7.25595	4.71495	0.28818	1.61691	2.54316	0.99944	1.0896	1.79245
ENSG00000226297	AC007677.1	0	0	0	0	0	0	0	0
ENSG00000253006	RN7SKP283	1.35741	0.45188	0.39894	0.80582	0.33006	0.95787	0	0
ENSG00000187957	DNER	0.01863	0	0	0.02765	0.04529	0	0	0.02287
ENSG00000238782	RNU7-9P	0	0	0	0	0	0	0	0
ENSG00000235993	AC007559.1	0	0	0	0	0	0	0	0
ENSG00000153827	TRIP12	31.8601	29.7998	10.8107	16.9562	34.8924	29.9202	44.4327	23.2603
ENSG00000222344	RNU6-613P	0	0	0	0	0	0	0	0
ENSG00000153832	FBXO36	0.22297	0.09278	0.19113	0.27576	0.20331	0.19667	0.2168	0.15969
ENSG00000252333	RNU6-964P	0	0	0	0	0	0	0	0
ENSG00000231534	FBXO36-IT1	0	0	0	0	0	0	0	0
ENSG00000206725	RNU6-1027P	0	1.14027	0	0	0	0	0	0
ENSG00000163053	SLC16A14	0.62245	0.02467	0	0.08798	0.07207	0.02614	0.24703	0.09098
ENSG00000199400	RNY4P19	0	0	1.12202	0	0	0	0	0
ENSG00000225963	AC009950.1	0.09815	0.06535	0	0	0	0	0	0.0241
ENSG00000283451	AC009950.2	0	0	0	0	0	0	0	0
ENSG00000135899	SP110	20.2764	30.4444	6.11922	4.92824	17.8753	12.432	5.49945	13.9879
ENSG00000079263	SP140	8.96885	15.1254	2.54716	3.43	6.41793	14.7103	2.53904	4.74008
ENSG00000185404	SP140L	7.04462	6.80224	3.05031	6.25759	8.1388	7.25519	3.43835	4.76268
ENSG00000279964	AC009949.1	0.04005	0.13334	0	0	0.0487	0.07066	0	0
ENSG00000067066	SP100	17.0151	29.0179	9.72311	10.6734	18.5112	20.2095	14.9506	16.105
ENSG00000243650	RN7SL834P	5.38219	8.95867	7.15582	4.56444	8.41311	9.49492	11.1073	7.86688
ENSG00000250011	HMGB1P3	0.47598	0.39613	0	0.3532	0.43401	0.41984	0	0.5844
ENSG00000235419	AC010149.1	0	0	0	0	0	0	0	0.13005
ENSG00000183022	TPM3P8	0	0	0	0	0	0.40221	0	0
ENSG00000283202	AC010149.2	0	0	0	0	0	0	0	0
ENSG00000199791	RNU6-451P	0	0	0	0	0	0	0	0
ENSG00000226125	LINC01907	0.11238	0.04676	0	0	0.03416	0	0.04683	0.06899
ENSG00000135932	CAB39	57.7787	53.7884	7.13404	16.3382	42.4801	28.3473	11.2606	26.5626
ENSG00000237758	BANF1P3	0.28084	0	0	0	0	0	0	0
ENSG00000201044	RNU6-268P	1.3701	0	0	0	0	0	0	0
ENSG00000270891	AC012507.3	0	0	0	0	0	0	0	0
ENSG00000135916	ITM2C	10.5838	2.19625	0.74257	2.20821	7.543	1.68388	2.05896	1.8613
ENSG00000232520	AC012507.2	0	0	0	0	0	0	0.05394	0
ENSG00000135898	GPR55	0.24848	0.14771	0.02608	0.21072	0.60418	0.75145	0.38459	0.06537
ENSG00000230385	AC012507.1	2.19901	1.42343	0.71809	1.08786	2.37644	0.64656	1.2218	1.94994
ENSG00000283164	AC012507.4	0.14544	0.24208	0	0	0.35364	0.25657	0	0
ENSG00000235013	COX20P2	0	0.34081	0	0	0	0	0	0
ENSG00000238062	SPATA3-AS1	0	0	0	0	0	0	0	0

ENSG00000173699	SPATA3	0	0	0	0	0	0.18777	0	0
ENSG00000204128	C2orf72	0	0	0	0	0	0	0	0
ENSG00000261829	AC009407.1	0	0	0	0	0	0	0	0
ENSG00000173692	PSMD1	11.2204	8.60904	3.59254	6.52253	10.0851	12.8888	9.35138	7.47807
ENSG00000135914	HTR2B	0.19582	0	0	0	0	0.05757	0	0.04007
ENSG00000201574	RNU1-93P	0	0	0	0	0	0	0	0
ENSG00000135931	ARMC9	0.03794	0.02526	0.03345	0.04504	0.04612	0.01338	0.03794	0.01863
ENSG00000237039	AC018738.1	0.34905	0	0	0	0.42437	0	0.58181	0.42856
ENSG00000263641	MIR4777	0	0	0	0	0	0	0	0
ENSG00000224376	AC017104.1	0.1836	0.0764	0	0.47683	0.39062	0.64777	0	0.33812
ENSG00000156966	B3GNT7	2.88887	0.89759	0.25471	1.3148	0.93659	2.61613	1.99033	0.6148
ENSG00000172799	ZBTB8OSP2	0	0	0	0.21584	0.17682	0.25657	0	0
ENSG00000230047	AC017104.2	0	0	0	0	0	0	0	0
ENSG00000233538	AC017104.3	0	0	0	0.19152	0	0	0	0
ENSG00000115053	NCL	37.671	24.0354	4.2128	9.81254	34.6486	37.7167	8.78132	11.1961
ENSG00000206885	SNORA75	4.28031	1.78115	3.93117	3.17623	1.30097	6.60717	5.35096	4.59839
ENSG00000207280	SNORD20	0	1.52511	1.34642	4.07946	3.34188	6.46559	9.16352	1.12496
ENSG00000202400	SNORD82	1.04715	3.48596	1.53877	1.55408	1.2731	0	5.2363	0
ENSG00000283312	AC017104.4	0	0	0	0	0	0.16245	0	0.05653
ENSG00000283491	AC017104.5	0.45884	1.14562	1.17996	3.40487	1.1157	1.82129	0.57362	1.69009
ENSG00000223198	RNU2-22P	0	0.57551	0	0	0	0	0	0
ENSG00000181798	LINC00471	0.11435	0	0	0.25457	0.20854	0.10087	0	0.4212
ENSG00000171596	NMUR1	0.91125	0.96186	0.09798	0.79165	0.83766	0.98023	0.29637	0.62763
ENSG00000231494	AC104634.1	0.15866	0.26409	0.46629	0	0.19289	0.83969	0	0.1948
ENSG00000228884	RPL23AP26	0	0	0	0	0	0	0	0
ENSG00000177673	TEX44	0	0	0	0	0	0.0935	0	0
ENSG00000239202	RN7SL499P	0	0	0	0	0	0	0	0
ENSG00000187514	PTMA	143.953	90.3686	18.5901	39.7078	107.742	118.172	43.0416	52.3518
ENSG00000277986	RF00015	0	0	0	0	0	0	0	0
ENSG00000284378	MIR1244-1	0	0	0	0	0	0	0	0
ENSG00000156973	PDE6D	4.57806	2.78265	0.41574	0.87792	3.6272	4.1289	0.68592	1.48416
ENSG00000281072	AC073476.4	0	0	0	0	0	0	0	0
ENSG00000144524	COPS7B	1.2688	0.86488	0.21308	0.48421	1.05776	1.44959	0.4834	0.66763
ENSG00000237641	AC073476.1	0	0	0	0	0	0	0	0
ENSG00000261096	AC073476.3	0.11695	0	0.1289	0.04339	0.10664	0.10316	0.04874	0.0718
ENSG00000260622	AC073476.2	0	0	0	0	0	0	0	0
ENSG00000222246	MIR1471	0	0	0	0	0	0	0	0
ENSG00000163273	NPPC	0	0	0	0	0	0	0	0
ENSG00000144535	DIS3L2	1.4095	0.8502	0.24703	0.71967	1.02975	0.78703	0.74362	0.55568
ENSG00000227033	AC019130.1	0	0	0	0	0	0	0	0
ENSG00000207626	MIR562	0	0	0	1.14511	0	0	0	0
ENSG00000251485	AC068134.3	0.4651	0	0	0	0.11309	0.1641	0	0.11421
ENSG00000230122	ECEL1P3	0	0	0	0	0	0	0	0
ENSG00000275157	AC068134.4	0	0	0	0	0	0	0	0
ENSG00000163283	ALPP	0	0	0	0	0	0	0	0
ENSG00000224516	AC068134.1	0	0	0	0	0	0	0	0
ENSG00000244280	ECEL1P2	0	0	0	0	0	0	0	0
ENSG00000163286	ALPG	0	0	0	0	0	0	0	0
ENSG00000204121	ECEL1P1	0	0	0	0	0	0	0	0
ENSG00000237087	AC068134.2	0	0	0	0	0	0	0	0
ENSG00000223788	DIS3L2P1	0	0	0	0	0	0	0	0
ENSG00000163295	ALPI	0	0	0	0	0	0	0	0
ENSG00000171551	ECEL1	0	0	0	0	0	0	0	0
ENSG00000237412	PRSS56	0	0	0	0	0	0	0	0
ENSG00000135902	CHRND	0	0	0	0	0	0	0	0
ENSG00000196811	CHRNG	0	0	0	0	0	0	0	0
ENSG00000221944	TIGD1	1.53582	0.92959	0.53344	0.70452	1.22217	1.82268	0.65163	1.09711
ENSG00000135930	EIF4E2	6.71009	5.37198	0.82545	1.63702	4.7557	5.47733	1.70239	2.62079

ENSG00000284407	MIR5001	0	0	0	0	0	0	0	0
ENSG00000237126	AC073254.1	0	0	0	0	0	0	0	0
ENSG00000115468	EFHD1	0.04803	0	0	0.03564	0	0.16948	0	0
ENSG00000244384	RN7SL359P	0	0	0	0	0	0	0.42424	0
ENSG00000204120	GIGYF2	7.62726	7.66645	2.3444	4.42733	8.01758	7.99042	4.84496	4.94394
ENSG00000213055	EEF1B2P7	0	0	0	0	0	0	0	0
ENSG00000241409	AC064852.1	0	0	0	0	0	0	0	0.10575
ENSG00000115474	KCNJ13	0	0	0	0	0	0	0	0
ENSG00000252452	RNU6-107P	2.87452	1.19616	1.05602	1.06653	1.74739	0	1.19785	0.88232
ENSG00000199781	RF00019	2.22122	0	0	0	0	0	0	0.90906
ENSG00000182600	SNORC	0.05946	0	0	0.02942	0.0482	0.03497	0.03304	0.07301
ENSG00000066248	NGEF	0	0	0	0	0	0	0	0
ENSG00000222001	AC106876.1	0	0	0	0	0	0	0	0
ENSG00000115488	NEU2	0	0	0	0	0	0	0	0
ENSG00000168918	INPP5D	17.4702	13.9282	2.71735	5.6141	11.3641	13.8086	3.78603	5.68119
ENSG00000263941	RN7SL32P	0	0	0	0	0	0	0	0
ENSG00000085978	ATG16L1	2.18192	1.45272	0.64126	0.93548	1.70953	1.96737	1.1719	1.23528
ENSG00000252010	SCARNA5	0	0	0	0	0	0	0	0
ENSG00000251791	SCARNA6	0	0	0	0	0	0	0	0
ENSG00000130561	SAG	0.01233	0	0	0	0.0075	0	0	0
ENSG00000259793	AC013726.1	0	0	0	0	0	0	0	0
ENSG00000077044	DGKD	8.75463	5.59408	1.36696	3.57386	7.78893	4.76433	2.95103	3.45399
ENSG00000201013	RF00019	0	0	0	0	0	0	0	0
ENSG00000085982	USP40	1.08163	0.49576	0.11518	0.53509	0.81951	0.74667	0.37887	0.36569
ENSG00000271639	AC019072.1	0	0	0	0	0	0	0	0
ENSG00000228949	UGT1A12P	0	0	0	0	0	0	0	0
ENSG00000227846	UGT1A11P	0	0	0	0	0	0	0	0
ENSG00000242366	UGT1A8	0	0	0	0	0	0	0	0
ENSG00000242515	UGT1A10	0	0	0	0	0	0	0	0
ENSG00000234143	UGT1A13P	0	0	0	0	0	0	0	0
ENSG00000241119	UGT1A9	0	0	0	0	0	0	0	0
ENSG00000244122	UGT1A7	0	0	0	0	0	0	0	0
ENSG00000167165	UGT1A6	0	0	0	0	0	0	0	0
ENSG00000224814	AC114812.2	0	0	0	0	0	0	0	0
ENSG00000240224	UGT1A5	0	0	0	0	0	0	0	0
ENSG00000244474	UGT1A4	0	0	0	0	0	0	0	0
ENSG00000233445	RPL17P11	0	0	0	0	0	0	0	0
ENSG00000243135	UGT1A3	0	0	0	0	0	0	0	0
ENSG00000248114	AC114812.3	0	0	0	0	0	0	0	0
ENSG00000227802	DNAJB3	0	0	0	0	0	0	0	0
ENSG00000228445	UGT1A2P	0	0	0	0	0	0	0	0
ENSG00000248705	AC114812.4	0	0	0	0	0	0	0	0
ENSG00000178836	AC114812.1	0	0	0	0	0	0	0	0
ENSG00000241635	UGT1A1	0	0	0	0	0	0	0	0
ENSG00000185038	MROH2A	0	0	0	0	0	0	0	0
ENSG00000123485	HJURP	0.3453	0.28738	0.19733	0.17082	0.39649	1.89517	0.22383	0.21198
ENSG00000224287	MSL3P1	0	0	0	0	0	0.05677	0	0
ENSG00000279809	AC005538.2	0	0	0	0	0	0	0	0
ENSG00000144481	TRPM8	0	0	0	0	0	0.01636	0	0
ENSG00000237581	AC005538.1	0	0	0	0	0	0	0	0
ENSG00000072080	SPP2	0	0	0	0	0	0	0	0
ENSG00000279218	AC006037.2	0	0	0	0	0	0	0	0
ENSG00000226442	AC006037.1	0	0	0	0	0	0	0	0
ENSG00000261298	AC122134.1	0	0	0	0	0	0	0	0
ENSG00000237784	AC097713.2	0	0	0	0.30558	0	0	0	0
ENSG00000228162	AC097713.1	0	0	0	0	0	0	0	0
ENSG00000231682	LINC01891	2.58867	1.13391	0.80085	1.81984	2.48467	2.64392	2.04391	2.17465
ENSG00000232690	HSPE1P9	0	0	0	0	0	0	0	0

ENSG00000188042	ARL4C	41.4632	27.8494	8.05238	24.3704	31.0676	68.571	17.7501	21.776
ENSG00000280744	LINC01173	0	0	0	0	0	0	0	0
ENSG00000278593	AC104394.1	0	0	0	0	0	0	0	0
ENSG00000235726	AC010148.1	0.02466	0	0	0	0	0.08699	0	0
ENSG00000130147	SH3BP4	0.401	0.17114	0.01511	0.04578	0.26251	0.05442	0.10283	0.10099
ENSG00000226542	AC114814.1	0	0	0	0	0	0	0	0
ENSG00000231032	AC114814.2	0	0	0	0	0	0	0	0
ENSG00000235293	AC114814.3	0	0	0	0	0	0	0	0
ENSG00000157985	AGAP1	0.42157	0.41225	0.1239	0.27372	0.24986	0.62285	0.06149	0.27174
ENSG00000235529	AGAP1-IT1	0	0	0	0	0	0	0	0
ENSG00000222007	AC064874.1	0	0	0	0	0	0	0	0
ENSG00000228499	TMSB10P1	0	0	0	0	0	0	0	0.6818
ENSG00000238812	RNU7-127P	0	0	0	0	0	0	0	0
ENSG00000264676	RN7SL204P	0.27977	0	0.41112	0	0	0	0	0
ENSG00000207049	RF00019	0	0	0	1.06653	0	0	0	0
ENSG00000168505	GBX2	0	0	0	0	0	0	0	0
ENSG00000233611	AC019068.1	0	0	0	0	0	0	0	0
ENSG00000182177	ASB18	0	0	0	0	0	0	0	0
ENSG00000200745	RNU1-31P	0	0	0	0	0	0	0	0
ENSG00000132321	IQCA1	0.04907	0	0	0.03641	0.01491	0	0	0.01506
ENSG00000270540	AC093915.1	0	0	0	0	0	0	0	0
ENSG00000232893	IQCA1-AS1	0	0	0	0	0	0	0	0
ENSG00000231546	RPL3P5	0	0	0	0	0	0	0	0
ENSG00000144476	ACKR3	0.71418	0.59437	0.09541	0.14453	0.4736	0.1718	0.21644	0.59785
ENSG00000232328	AC084030.1	0	0	0	0	0	0	0	0
ENSG00000202341	RNU6-1051P	0	0	0	0	0	0	0	0
ENSG00000124835	AC105760.1	0	0	0	0.04876	0.03994	0	0	0
ENSG00000227252	AC105760.2	0.03131	0	0	0.09294	0.03807	0.16571	0.10438	0
ENSG00000198612	COPS8	3.63179	2.41805	1.02195	2.20186	3.17536	4.14408	1.90625	2.40979
ENSG00000224844	AC107079.1	0	0	0	0	0	0	0	0
ENSG00000224132	AC112715.1	0	0	0	0	0	0	0	0
ENSG00000163359	COL6A3	0.30988	0.11186	0.01646	0.03879	0.31774	0.23711	0.06846	0.0871
ENSG00000222022	AC112721.1	0.49864	0.2075	0.91593	0.18501	0	0	0.20779	0
ENSG00000222032	AC112721.2	0.54196	0	0.3982	0	0.16473	0.71707	0.22584	0.33271
ENSG00000115648	MLPH	0	0	0	0	0.08302	0	0	0
ENSG00000277842	MIR6811	0	0	0	0	0	0	0	0
ENSG00000222449	RNU6-1140P	0	0	0	0	0	0	0	0
ENSG00000071677	PRLH	0	0	0	0	0	0	0	0
ENSG00000124839	RAB17	0.01533	0	0	0	0.0559	0	0	0
ENSG00000234949	AC104667.2	0	0	0	0	0	0	0	0
ENSG00000227107	AC104667.1	0.14254	0	0	0	0	0.06286	0	0
ENSG00000124831	LRRFIP1	30.6973	24.3381	3.34881	6.10611	22.2399	13.3647	6.50889	8.6284
ENSG00000177483	RBM44	0.37565	0.26221	0.12465	0.14387	0.47144	0.44893	0.38377	0.22317
ENSG00000132329	RAMP1	0.71281	0.29662	0.17458	0.08816	0.36109	0.20958	0.59407	0.07293
ENSG00000263723	RF00157	0	0	0	0	0	0	0	0
ENSG00000184182	UBE2F	4.01267	4.66385	3.65994	5.33918	3.42756	3.72252	4.81456	4.62939
ENSG00000258984	UBE2F-SCLY	0.04588	0	0	0.03405	0.08368	0.04047	0	0.02817
ENSG00000251971	RNU6-1333P	0	0	0	0	0	0	0	0
ENSG00000132330	SCLY	0.04373	0.05199	0	0.04636	0.01519	0.07715	0.08331	0.03068
ENSG00000144488	ESPNL	0.02734	0	0	0.02029	0.01662	0	0.02279	0
ENSG00000168427	KLHL30	0	0	0	0	0	0.06941	0.03279	0
ENSG00000279484	KLHL30-AS1	0	0.11784	0	0	0	0	0	0
ENSG00000178752	ERFE	0.02052	0.06831	0.03016	0.12182	0	0.97744	0.03421	0.0252
ENSG00000132323	ILKAP	4.54766	8.10662	9.39195	16.189	9.11527	13.9584	12.5228	8.84691
ENSG00000186235	LINC02610	0.02522	0.04199	0.1112	0.07487	0.24533	0.0445	0.08409	0.15485
ENSG00000225057	AC012485.1	0	0	0	0	0	0	0	0
ENSG00000144485	HES6	0.35984	0.17969	0.05288	0.10681	0.21874	0.25393	0.11996	0.13254
ENSG00000132326	PER2	1.69474	1.22417	0.25061	0.60111	1.37362	0.86496	0.63959	0.54964

ENSG00000283635	AC012485.3	0	0.05033	0	0	0.03676	0	0	0
ENSG00000232306	AC012485.2	0	0	0	0	0	0	0	0
ENSG00000204104	TRAF3IP1	1.34948	0.89848	0.18664	0.37699	1.19672	1.17633	0.44987	0.81869
ENSG00000202099	RNU6-234P	0	0	0	0	0	0	0	0
ENSG00000065802	ASB1	1.42446	1.02085	0.29073	0.80745	1.45521	1.64039	0.69252	0.5101
ENSG00000229915	AC016999.1	0	0.26993	0	0	0.19716	0	0	0
ENSG00000274346	AC016999.2	0.26367	0	0	0	0	0	0.21975	0.16187
ENSG00000225493	LINC01107	0	0	0	0	0	0	0	0
ENSG00000234279	LINC01937	0	0	0	0	0	0	0	0
ENSG00000224252	AC113618.1	0	0	0	0	0	0	0	0
ENSG00000226992	AC145625.1	0	0	0	0	0	0	0	0
ENSG00000233608	TWIST2	0.19516	0	0	0	0	0	0	0
ENSG00000227744	LINC01940	0	0	0	0	0	0	0	0
ENSG00000068024	HDAC4	3.42704	3.10315	0.45123	1.07419	2.19325	1.84759	0.88656	1.74366
ENSG00000266109	MIR4440	0	0	0	0	0	0	0	0
ENSG00000264810	MIR4441	2.19901	0	0	0	0	0	0	0
ENSG00000265215	MIR4269	0	0	0	0	0	0	0	0
ENSG00000264292	MIR2467	0	1.50628	0	0	0	0	0	0
ENSG00000222020	HDAC4-AS1	2.27195	1.19421	0	0.53239	0.72689	1.05475	0.79726	0.73407
ENSG00000196758	AC079612.1	0	0	0	0	0	0	0	0
ENSG00000227459	AC079612.2	0	0	0	0	0	0	0	0
ENSG00000259889	AC093802.2	0	0	0	0	0	0	0	0
ENSG00000220256	AC093802.1	0	0	0	0	0	0	0	0
ENSG00000130414	NDUFA10	4.00506	4.49984	4.52722	10.0361	6.05847	7.52745	6.52176	4.6904
ENSG00000264279	MIR4786	0.91625	0	0	0	1.11396	0	0	0
ENSG00000182083	OR6B2	0	0	0	0	0	0	0	0
ENSG00000221961		0	0	0	0	0	0	0	0
ENSG00000178586	OR6B3	0	0	0	0	0	0	0	0
ENSG00000231278	OR9S24P	0	0	0	0	0	0	0	0
ENSG00000213048	OR5S1P	0	0	0	0	0	0	0	0
ENSG00000172428	COPS9	2.08505	1.49594	0.63392	0.74693	1.4423	2.02942	0.89883	1.80965
ENSG00000178602	OTOS	0	0	0	0	0	0	0	0
ENSG00000227479	AC124861.1	0	0	0	0	0	0	0	0
ENSG00000276366	RF00012	0	0	0	0	0	0	0	0
ENSG00000063660	GPC1	0.0225	0.01872	0	0.03339	0.01368	0.07938	0.0375	0
ENSG00000218416	AC110619.1	0	0	0	0	0	0	0	0
ENSG00000207611	MIR149	0	0	0	0	0	0	0	0
ENSG00000144504	ANKMY1	0.99633	0.7973	0.09385	0.38862	0.84636	0.60842	0.36195	0.31366
ENSG00000188542	DUSP28	1.60079	1.05265	0.31945	0.46928	0.76887	0.73215	0.82354	0.50955
ENSG00000142327	RNPEPL1	6.29859	4.07884	0.86259	1.86679	4.41789	5.96672	2.2209	2.01341
ENSG00000260942	CAPN10-DT	0.2199	0.09151	0.05386	0.08159	0.35647	0.35561	0.15273	0.1125
ENSG00000142330	CAPN10	1.12785	0.93049	0.18735	0.4221	1.08504	0.83047	0.37599	0.40941
ENSG00000178623	GPR35	0.96505	0.2877	0.127	0.08551	0.26267	0.27951	0.24009	0.10611
ENSG00000276949	AC011298.3	0	0	0	0	0	0	0	0
ENSG00000185176	AQP12B	0	0	0	0	0	0	0	0
ENSG00000219159	AC011298.1	0	0	0	0	0	0	0	0
ENSG00000184945	AQP12A	0	0	0	0	0	0	0	0
ENSG00000274977	AC011298.2	0	0	0	0	0	0	0	0
ENSG00000130294	KIF1A	0	0	0	0	0	0	0	0
ENSG00000172482	AGXT	0	0	0	0	0	0	0	0
ENSG00000172478	MAB21L4	0	0	0	0	0	0	0	0
ENSG00000226321	CROCC2	0.06131	0.06123	0	0	0.01491	0	0	0.01505
ENSG00000233392	AC104809.2	0.49676	0.32156	0.04055	0.08192	0.13421	0.14606	0.138	0
ENSG00000223991	AC104809.1	0	0	0	0	0	0	0	0
ENSG00000162804	SNED1	0.38829	0.13465	0.02377	0.04002	0.17047	0.04757	0.17978	0.07283
ENSG00000229996	AC093585.1	0	0	0	0	0	0	0	0
ENSG00000225521	AC005237.1	0	0	0	0	0	0	0	0
ENSG00000122085	MTERF4	3.3781	1.21542	0.34684	1.00707	3.63174	2.82357	1.52449	1.26782

ENSG00000115687	PASK	2.69577	0.89057	0.41328	1.04856	2.35176	2.3476	1.37204	0.81693
ENSG00000235861	AC005237.2	0	0	0	0	0	0	0	0
ENSG00000115685	PPP1R7	7.52196	7.29276	5.61218	12.3431	10.4178	7.55829	8.53097	7.37876
ENSG00000146205	ANO7	0.01287	0	0	0	0	0	0	0
ENSG00000115677	HDLBP	17.2871	14.2019	4.29724	5.54555	16.5914	14.5107	4.2726	9.10166
ENSG00000168385	SEPT2	26.8046	17.7605	6.01493	11.2206	17.2605	16.9308	9.17429	11.8077
ENSG00000223374	AC005104.1	0	0	0	0	0	0	0	0
ENSG00000006607	FARP2	1.22057	1.12587	1.15838	2.39264	1.44066	1.90204	1.65303	1.58602
ENSG00000263752	MIR3133	0	0	0	0	0	0	0	0
ENSG00000224232	AC005104.2	0	0	0	0.16045	0	0	0	0.13274
ENSG00000115694	STK25	2.26164	1.4318	0.21304	0.58811	2.00936	1.80736	0.80551	0.84254
ENSG00000234235	BOK-AS1	0	0	0	0	0	0	0	0
ENSG00000176720	BOK	0.08276	0.64287	0	0.36849	0.20124	0	0.04598	0.27097
ENSG00000273113	AC133528.1	0	0	0	0	0	0	0	0
ENSG00000176946	THAP4	2.84164	1.97726	0.65032	1.79754	2.97339	3.61596	1.20356	0.77214
ENSG00000207186	RNA5SP122	0	0	0	0	0	0	0	0
ENSG00000168397	ATG4B	3.14712	2.18726	0.34076	0.8358	2.00036	1.98701	0.93872	1.31511
ENSG00000168393	DTYMK	0.957	0.34845	0.04395	0.39946	0.58175	1.84656	0.04985	0.25703
ENSG00000168395	ING5	1.32897	0.77303	0.35698	0.42416	1.5028	1.09661	0.51211	0.61407
ENSG00000235351	AC114730.3	0	0	0	0	0	0	0	0
ENSG00000180902	D2HGDH	0.58318	0.30046	0.06121	0.14425	0.28698	0.80835	0.18516	0.22163
ENSG00000215692	AC114730.1	0	0	0	0	0	0	0	0
ENSG00000234793	AC114730.2	0	0	0	0	0	0	0	0
ENSG00000154252	GAL3ST2	0	0	0	0	0	0	0	0
ENSG00000215023	AC131097.1	0	0	0	0	0	0	0	0
ENSG00000224272	AC131097.3	0	0	0	0	0	0	0	0
ENSG00000204099	NEU4	0	0	0	0	0	0	0	0
ENSG00000235151	AC131097.4	0	0	0	0	0	0	0	0
ENSG00000188389	PDCD1	0.34674	0.51943	0	0.15438	0.12647	2.20209	0	0.25543
ENSG00000188011	RTP5	0	0	0	0	0	0	0	0
ENSG00000233806	LINC01237	0.13735	0	0.06728	0.1359	0.05566	0	0.15263	0
ENSG00000216921	AC131097.2	0	0	0	0	0	0	0	0
ENSG00000237940	LINC01238	0.02568	0	0	0	0.03123	0	0.04281	0
ENSG00000280119	AC093642.2	0	0	0	0	0	0	0	0
ENSG00000226423	AC093642.1	0.51894	0	0	0	0.31546	0	0	0
ENSG00000232002	LINC01880	0	0	0	0	0	0	0	0
ENSG00000261186	LINC01238	0	0	0	0	0	0	0	0
ENSG00000220804	LINC01881	3.26454	5.1811	2.95642	4.05623	5.76882	4.88853	5.75785	3.5887
ENSG00000224160	CICP10	0	0	0	0	0	0	0	0
ENSG00000244528	SEPT14P2	0	0	0	0	0	0	0	0
ENSG00000271153	RPL23AP88	0	0	0	0	0	0	0	0
ENSG00000223587	LINC01986	0	0	0	0	0	0	0	0
ENSG00000224918	AC066595.1	0	0	0	0	0	0	0	0
ENSG00000224318	CHL1-AS2	0	0	0	0	0	0	0	0
ENSG00000134121	CHL1	0.04326	0	0.00908	0.0642	0.06762	0.03271	0.0103	0.0607
ENSG00000252017	RNU6-1194P	0	0	0	0	0	0	0	0
ENSG00000231660	RPS8P6	0	0	0	0	0	0	0	0
ENSG00000234661	CHL1-AS1	0	0	0	0	0	0	0	0
ENSG00000224957	LINC01266	0	0	0	0.0267	0	0	0	0
ENSG00000238075	RPSAP32	0	0	0	0	0	0	0	0
ENSG00000224239	AC090044.1	0	0	0	0	0	0	0	0
ENSG00000235158	AC087430.1	0	0	0	0	0	0	0	0
ENSG00000244169	RN7SL120P	0	0	0	0	0	0	0	0
ENSG00000134115	CNTN6	0	0	0	0	0	0	0	0
ENSG00000232563	CRB3P1	0	0	0	0	0	0	0	0
ENSG00000184423	RPL23AP38	0	0	0	0	0	0	0	0
ENSG00000214074	RPL23AP39	0	0	0	0	0	0	0	0
ENSG00000214073	RPL21P17	0	0	0	0	0	0	0	0

ENSG00000225044	AC018814.1	0	0	0	0.30472	0	0	0	0
ENSG00000223040	RN7SKP144	0	0	0	0	0	0	0	0
ENSG00000144619	CNTN4	0	0	0	0.01193	0	0	0	0
ENSG00000227588	CNTN4-AS2	0	0	0	0	0	0	0	0
ENSG00000230398	HINT2P1	0	0	0	0	0	0	0	0
ENSG00000225310	DNAJC19P4	0	0	0	0	0	0	0	0
ENSG00000237990	CNTN4-AS1	0.07055	0.23486	0	0.05235	0	0	0	0.17324
ENSG00000091181	IL5RA	6.34159	2.18136	1.2893	1.41751	5.04994	0.50941	0.66644	1.84085
ENSG00000253049	RF00416	0	0	0	0	0	0	0	0
ENSG00000072756	TRNT1	7.86405	9.75095	8.88181	22.9085	11.7735	9.67846	15.7873	10.6829
ENSG00000113851	CRBN	9.44873	8.13592	4.96648	10.1936	9.79753	9.00759	9.44974	8.4442
ENSG00000271870	AC024060.1	1.63219	1.97585	0	0.88086	1.26279	0.78529	1.7313	2.55052
ENSG00000223727	AC034195.1	0	0	0	0.32546	0	0	0.09138	0
ENSG00000144455	SUMF1	3.1315	3.15902	0.79019	2.15944	3.85847	3.40392	1.93323	1.21688
ENSG00000175928	LRRN1	0.29224	0.08584	0.05052	0.05103	0.64789	0.33359	0.20058	0.31659
ENSG00000229241	PNPT1P1	0.15832	0	0.13959	0.04699	0	0	0.21111	0
ENSG00000170364	SETMAR	0.90418	0.51307	0.18118	0.30498	0.79948	0.94256	0.54805	0.42892
ENSG00000234325	MRPS10P2	0.57717	0.32023	0	0	0	0	0	0.47243
ENSG00000231249	ITPR1-DT	0.34173	0.5688	0.25108	0	0	0.15071	0.1424	0.10489
ENSG00000150995	ITPR1	5.9743	4.82546	0.96897	2.23561	5.0519	4.34215	2.0087	3.12669
ENSG00000235947	EGOT	0	0	0	0.07431	0	0	0	0
ENSG00000275981	AC018816.2	0	0	0	0	0.75737	0	0.34612	0.25495
ENSG00000235978	AC018816.1	0.64453	0.19506	0	0.08696	0.14247	0.3101	0	0.28776
ENSG00000235831	BHLHE40-AS1	0.13489	0.17962	0.03964	0.04004	0.0656	0.19037	0	0.06625
ENSG00000134107	BHLHE40	33.0683	13.8957	3.14411	5.18837	13.796	20.1197	6.14564	5.74649
ENSG00000230182	RNF10P1	0	0	0	0	0	0	0	0
ENSG00000224082	UBTFL8	0	0	0	0	0	0	0	0
ENSG00000207283	RF00019	0	0	0	0	0	0	0	0
ENSG00000237566	AC090955.1	0	0	0	0	0	0	0	0
ENSG00000134108	ARL8B	26.2783	24.1219	11.657	23.5763	20.6842	16.5979	19.9876	20.5614
ENSG00000233912	AC026202.2	0.21816	0	0	0	0.26523	0.57728	0	0.13392
ENSG00000268509	AC026202.3	0.26016	0.21652	0.09558	0	0.15815	0.11474	0.10841	0
ENSG00000134109	EDEM1	12.2673	6.26472	2.15173	4.24969	9.92969	7.19462	4.61024	6.65856
ENSG00000265180	MIR4790	0	0	0	0	0	0	0	0
ENSG00000241227	RN7SL553P	0	0	0	0	0	0	0	0
ENSG00000230944	AC026202.1	0	0	0	0	0	0	0	0
ENSG00000229642	AC087857.1	0	0	0	0	0	0	0	0
ENSG00000226022	AC026167.1	0	0	0	0	0	0	0	0
ENSG00000232958	AC027119.1	0	0	0	0	0	0	0	0
ENSG00000189229	AC069277.1	0.19398	1.13008	3.77694	2.26712	0.1474	0	0.36375	0
ENSG00000226258	GRM7-AS3	0	0	0	0	0	0	0	0
ENSG00000223719	AC069277.2	0	0	0	0	0	0	0	0
ENSG00000196277	GRM7	0.00892	0	0.01311	0	0.01085	0	0	0
ENSG00000236152	MRPS36P1	0	0	0	0.7087	0	0	0.79596	0
ENSG00000237665	GRM7-AS2	0	0	0	0	0	0	0	0
ENSG00000236202	GRM7-AS1	0.14458	0.24065	0	0	0.17577	0	0	0
ENSG00000270207	AC068313.1	0.07272	0	0	0	0	0	0	0
ENSG00000227110	LMCD1-AS1	0.03405	0	0	0	0	0	0	0
ENSG00000228351	AC018832.1	0	0	0	0	0	0	0	0
ENSG00000253081	RNU4ATAC17P	0	0	0	0	0	0	0	0
ENSG00000231401	AC023481.1	0	0	0	0	0	0	0	0
ENSG00000071282	LMCD1	0.01606	0	0	0.01192	0.01952	0	0	0.02957
ENSG00000224884	AC034187.1	0	0	0	0	0	0	0	0
ENSG00000125046	SSUH2	0	0	0	0	0	0.04119	0	0
ENSG00000215160	OR7E122P	0	0	0	0	0	0	0	0
ENSG00000182533	CAV3	0	0	0	0	0	0.13222	0	0
ENSG00000180914	OXTR	1.75375	2.2434	0.47724	0.53019	0.23691	0.88805	0.1624	0.1595
ENSG00000070950	RAD18	3.13859	4.36805	4.61213	6.26262	5.47487	8.01833	7.99605	5.12943

ENSG00000281863	RF00416	0	0	0	0	0	0	0	0
ENSG00000199815	RF00560	0	0	0	0	0	0	0	0
ENSG00000196220	SRGAP3	0.24402	0.09026	0.03984	0.06323	0.14128	0.03417	0.04519	0.06182
ENSG00000224808	SRGAP3-AS1	0	0	0	0	0	0	0	0
ENSG00000228723	SRGAP3-AS2	0	0	0	0	0	0	0	0
ENSG00000227929	SRGAP3-AS3	0	0	0	0	0	0	0	0
ENSG00000235830	SRGAP3-AS4	0	0	0	0	0	0	0	0
ENSG00000254485	AC026191.1	0	0	0	0	0	0	0	0
ENSG00000214041	PGAM1P4	0	0	0	0	0	0	0	0
ENSG00000206573	THUMPD3-AS1	2.82688	1.9638	0.80678	1.68163	2.18709	2.24621	2.10286	2.09396
ENSG00000134077	THUMPD3	3.64794	2.75173	1.22471	2.41295	3.12282	3.68774	2.20904	1.52651
ENSG00000168137	SETD5	4.67283	3.88332	1.30556	2.40561	4.37008	4.65417	2.9392	2.55636
ENSG00000156959	LHFPL4	0	0	0	0	0	0	0	0
ENSG00000235338	DUSP5P2	0	0	0	0	0	0	0	0
ENSG00000163719	MTMR14	11.4962	7.05415	1.48534	3.10868	6.97358	6.16589	3.79593	3.90251
ENSG00000144550	CPNE9	0.03139	0.1045	0	0	0.03817	0	0	0
ENSG00000156983	BRPF1	6.47752	4.49986	0.55066	1.33077	4.52336	5.05253	1.76232	1.82393
ENSG00000114026	OGG1	1.20027	0.7684	0.22201	0.42353	0.83678	1.09574	0.41972	0.50496
ENSG00000134072	CAMK1	7.78816	3.3642	0.99002	1.31983	3.44017	0.85574	0.89838	2.01832
ENSG00000171148	TADA3	12.5168	7.95332	1.88258	2.26102	7.59828	10.3535	2.42398	3.59223
ENSG00000241553	ARPC4	13.3477	12.5796	3.16454	5.12571	9.5588	11.6839	5.62138	7.43324
ENSG00000250151	ARPC4-TTLL3	0.02651	0	0	0	0	0	0	0
ENSG00000214021	TTLL3	0.69305	0.48262	0.05196	0.14694	0.36971	0.22456	0.22397	0.33863
ENSG00000269886	AC022382.1	0	0	0.32058	0.32377	0.79568	0	0	0.26785
ENSG00000156990	RPUSD3	0.95943	0.68442	0.14099	0.30512	0.94982	2.0069	0.36554	0.28608
ENSG00000187288	CIDEC	0	0	0	0	0	0	0	0
ENSG00000171135	JAGN1	2.85215	3.11973	1.01786	1.8141	2.4273	4.45655	1.90164	2.85149
ENSG00000163701	IL17RE	0.07838	0.07828	0.02304	0.11632	0.01906	0	0.02613	0.01925
ENSG00000163702	IL17RC	0.24218	0.13437	0.04745	0.09585	0.09815	0.08545	0.08074	0.05947
ENSG00000212327	RNU6-882P	0	0	0	0	0	0	0	0
ENSG00000163703	CRELD1	0.9895	1.09801	0.05702	0.11518	0.94353	1.26642	0.45276	0.42878
ENSG00000269894	AC018809.1	0.2651	0	0.19478	0.19672	0.3223	0	0.22094	0.32549
ENSG00000163704	PRRT3	0.22331	0.06195	0.08204	0.02762	0.20362	0.49243	0.15509	0.15993
ENSG00000230082	PRRT3-AS1	0.47753	0.39742	0	0.53153	0	0	0.39798	0.14658
ENSG00000269982	AC018809.2	0.09587	0.03191	0	0.02846	0	0.03382	0.03196	0
ENSG00000125037	EMC3	37.148	56.1182	52.9741	69.0929	45.0384	36.9765	93.2233	50.9843
ENSG00000180385	EMC3-AS1	0.51314	0.26281	0.08701	0.3222	0.35992	0.38299	0.72374	0.16962
ENSG00000232901	CYCSP10	0	0	0	0	0.60624	0	0.41558	0
ENSG00000206567	AC022007.1	0.14469	0.09634	0.04252	0.08589	0.21109	0.1021	0.14471	0.14212
ENSG00000186162	CIDECP	1.55493	0.97057	0.32132	0.54086	0.94522	1.07153	1.13392	1.13354
ENSG00000144554	FANCD2	1.80232	1.2961	1.13139	2.49306	1.95721	3.0252	3.01878	1.86912
ENSG00000201182	RNU6-670P	0	0	0	0	0	0	0	0
ENSG00000229217	CYCSP11	0	0	0	0	0	0	0	0
ENSG00000163705	FANCD2OS	0	0	0	0	0	0	0	0
ENSG00000254999	BRK1	54.574	35.1282	20.9662	55.8486	35.7045	37.4406	41.3213	27.2984
ENSG00000134086	VHL	14.0755	9.49888	2.3266	6.58447	11.5706	9.88331	5.36515	8.11747
ENSG00000134070	IRAK2	2.3781	2.41705	0.77314	1.06193	1.79103	1.37368	0.70158	1.42114
ENSG00000157014	TATDN2	1.64439	1.00725	0.32863	0.72237	1.48741	1.64773	0.98674	0.85604
ENSG00000272410	AC022384.1	0	0	0	0	0	0	0	0
ENSG00000231177	LINC00852	1.82147	1.19436	0.40555	0.08192	0.93948	2.33696	0.46002	0.47438
ENSG00000157017	GHRL	1.42055	1.4187	0.36531	0.26353	0.77718	0.93977	0.88794	0.52324
ENSG00000240288	GHRLOS	0.77068	0.71267	0.12583	0.25417	0.88492	0.83086	0.4282	0.10514
ENSG00000276678	RF02126	0	0	0	0	0	0	0	0
ENSG00000157020	SEC13	7.14699	4.30144	0.73181	1.55808	4.72911	4.91508	1.79479	1.81779
ENSG00000157087	ATP2B2	0	0	0	0	0.00877	0.01272	0	0
ENSG00000264534	MIR378B	0	0	0	0	0	0	0	0
ENSG00000216135	MIR885	0	0	0	0	0	0	0	0
ENSG00000236999	ATP2B2-IT1	0	0	0	0	0	0	0	0

ENSG00000224771	ATP2B2-IT2	0	0	0	0	0	0	0	0
ENSG00000226567	LINC00606	0	0	0	0	0	0	0	0
ENSG00000230599	AC018495.1	0	0	0	0	0	0	0	0
ENSG00000132164	SLC6A11	0	0.01821	0	0	0	0	0	0
ENSG00000157103	SLC6A1	0	0	0	0	0	0	0	0
ENSG00000232287	SLC6A1-AS1	0	0	0	0	0	0	0	0
ENSG00000196639	HRH1	0.01322	0.022	0	0.03923	0.03214	0	0	0.01623
ENSG00000213964	CHCHD4P4	0	0	0	0	0	0	0	0
ENSG00000197548	ATG7	3.89198	4.36485	1.14628	1.97053	2.50209	2.76689	1.53538	2.55737
ENSG00000226621	AC083855.1	0	0	0	0	0	0	0	0
ENSG00000235240	AC026185.1	0	0	0	0	0	0	0	0
ENSG00000270531	AC022001.1	0	0	0	0	0	0	0	0
ENSG00000144560	VGLL4	2.71483	2.05708	0.44658	0.82687	2.51241	2.80569	0.94556	1.26862
ENSG00000271716	AC022001.2	0.25276	0	0	0	0.15365	0	0.21066	0
ENSG00000272483	AC022001.3	0	0	0	0	0.29905	0	0.205	0
ENSG00000144559	TAMM41	0.433	0.47362	0.56357	1.23015	1.09798	1.06941	1.50534	0.98731
ENSG00000230342	FANCD2P2	0	0	0	0	0	0	0	0
ENSG00000224263	CYCSP12	0	0	0	0	0	0	0	0
ENSG00000225275	NUP210P2	0	0	0	0	0	0	0	0
ENSG00000231897	MARK2P14	0	0	0	0	0	0	0	0
ENSG00000251038	AC090958.1	0	0	0	0	0	0	0	0
ENSG00000263988	RN7SL147P	0	0	0.36146	0	0	0	0	0
ENSG00000157152	SYN2	0.05159	0.04294	0.03791	0	0.04704	0	0	0.01584
ENSG00000226642	ACTG1P12	0	0	0	0	0	0	0	0
ENSG00000157150	TIMP4	0	0	0	0	0	0	0	0
ENSG00000233026	MTCO1P5	0	0	0	0	0	0	0	0
ENSG00000236062	GSTM5P1	0	0	0	0	0	0	0	0
ENSG00000132170	PPARG	0.07247	0.03016	0	0.05377	0	0	0	0.04449
ENSG00000225026	AC091492.1	0	0	0	0	0.32763	0	0	0
ENSG00000154743	TSEN2	1.73324	1.29178	1.88172	3.58972	2.46891	2.39593	3.40647	2.12804
ENSG00000200114	RNA5SP123	0	0	0	0	0	0	0	0
ENSG00000251774	RNU6-377P	0	0	0	0	0	0	1.19785	0
ENSG00000225526	MKRN2OS	0	0	0	0	0	0	0	0
ENSG00000075975	MKRN2	6.94912	4.88377	1.8154	4.04891	6.9153	5.03989	2.70272	3.82361
ENSG00000274514	RF00017	0	0	0	0	0	0	0	0
ENSG00000132155	RAF1	18.4395	20.7571	2.89635	5.35125	13.8326	8.3714	7.17792	11.8704
ENSG00000233252	CRIP1P1	0	0	0	0	0	0	0	0
ENSG00000088726	TMEM40	3.1485	2.91149	0.63503	1.61865	1.82637	2.3234	1.78365	1.86968
ENSG00000213943	KRT18P17	0	0	0	0	0	0	0	0
ENSG00000144712	CAND2	0.08029	0.06682	0.01966	0.01986	0.13014	0	0.04461	0.06572
ENSG00000272263	AC034198.2	0.14373	0	0	0.63992	0.34948	0	0	0
ENSG00000144713	RPL32	71.7437	59.6595	36.1652	42.1106	93.9079	95.0025	52.6492	73.6428
ENSG00000207496	SNORA7A	0	0	0	0	0	0	0	0
ENSG00000250939	AC034198.1	0	0	0	0	0	0	0	0
ENSG00000232746	LINC02022	0	0	0	0	0.14034	0	0	0
ENSG00000144711	IQSEC1	18.3198	16.1862	2.67641	4.90026	10.7479	8.8876	5.73442	6.52551
ENSG00000231362	AC069271.1	0	0	0.69047	0	0	0	0	0
ENSG00000132182	NUP210	12.0853	7.29188	1.72458	4.9992	9.06197	16.1837	3.47935	3.6408
ENSG00000238138	AC027124.2	0	0	0	0	0	0	0	0
ENSG00000223398	AC027124.1	0	0	0	0	0	0	0	0
ENSG00000244502	HDAC11-AS1	0.14233	0	0	0	0	0.25109	0	0
ENSG00000163517	HDAC11	0.40252	0.39276	0.08159	0.1236	0.15187	0.12243	0.04627	0.05113
ENSG00000163520	FBLN2	0.56361	0.12678	0	0.11304	0.50003	0.2956	0.05078	0.22443
ENSG00000224514	LINC00620	0	0	0	0	0	0	0	0
ENSG00000154764	WNT7A	0.30415	0.10658	0	0.07127	0.1557	0.19768	0.10673	0.01965
ENSG00000233121	VN1R20P	0	0	0	0	0.09225	0	0	0
ENSG00000275340	FGD5P1	0	0	0	0	0.04607	0	0	0
ENSG00000180438	TPRXL	0	0	0	0	0	0	0	0.02401

ENSG00000224752	VN1R21P	0	0	0	0	0	0	0	0
ENSG00000163528	CHCHD4	1.90283	1.44592	0.54708	1.16644	2.11225	1.7514	1.17216	1.42208
ENSG00000170876	TMEM43	26.6976	26.779	4.25187	6.97422	20.408	9.41239	6.36213	13.303
ENSG00000268279	AC090004.1	0.32944	0.09139	0.08068	0.08149	0.20026	0	0	0.40448
ENSG00000228242	AC093495.1	1.02237	2.0664	0.40242	0.37933	1.0876	0.41869	0.42603	1.70356
ENSG00000154767	XPC	11.7177	9.15445	0.89799	2.53939	10.8656	5.47109	2.44463	4.14534
ENSG00000170860	LSM3	7.20515	4.92511	1.58112	1.25467	5.72309	7.86378	2.46602	3.58573
ENSG00000255021	AC093496.1	0	0	0	0	0	0	0	0
ENSG00000251576	LINC01267	0	0	0	0	0	0	0	0
ENSG00000199609	RNA5SP124	0	0	0	0	0	0	0	0
ENSG00000131389	SLC6A6	30.9666	29.1939	3.84972	5.58822	19.1319	7.78628	8.52627	13.7165
ENSG00000144596	GRIP2	0.00617	0	0	0	0	0	0	0
ENSG00000207163	RNU6-905P	0	0	0	0	0	0	0	0
ENSG00000234123	RHBDF1P1	0	0	0	0	0	0	0	0
ENSG00000283392	AC090952.2	0	0	0	0	0	0	0	0
ENSG00000235629	AC090952.1	0	0.27356	0	0	0	0.57987	0.82184	0
ENSG00000154781	CCDC174	8.26587	5.69556	0.69669	2.29441	7.16743	5.05465	3.88256	3.54319
ENSG00000131379	C3orf20	0.12595	0.06988	0.09254	0.09346	0.07656	0.03703	0.20993	0.12886
ENSG00000230172	AC090957.1	0	0	0	0	0	0	0	0
ENSG00000260902	LINC02011	0	0	0	0	0	0	0	0
ENSG00000154783	FGD5	0.01811	0	0	0	0	0	0	0
ENSG00000225733	FGD5-AS1	19.0715	12.4363	4.74437	8.69268	12.0536	10.4589	7.38178	11.9797
ENSG00000177463	NR2C2	7.2116	5.26388	1.84975	3.51545	6.016	6.31189	4.36174	4.6289
ENSG00000131368	MRPS25	1.4434	0.95195	0.22011	0.94983	1.53963	1.00893	1.15757	0.76906
ENSG00000131381	RBSN	2.41527	1.70975	0.61193	0.94763	2.07013	1.99167	1.66588	1.49976
ENSG00000228874	AC090954.1	0	0	0	0	0	0	0	0
ENSG00000227578	RPS3AP53	0	0	0	0	0	0	0	0
ENSG00000230524	COL6A4P1	0	0	0	0.02383	0	0	0	0
ENSG00000131375	CAPN7	6.02325	3.90131	2.42443	4.31412	5.85833	4.71233	5.5219	4.56577
ENSG00000224660	SH3BP5-AS1	0.8055	0.72358	0.13152	0.30361	0.68396	0.72178	0.70329	0.73781
ENSG00000131370	SH3BP5	20.5149	13.1719	2.46041	7.45466	13.9493	6.56042	2.67334	5.84256
ENSG00000201162	RNU6-454P	0	0	0	0	0	0	0	0
ENSG00000226427	HMGN2P7	0	0	0	0	0	0	0	0
ENSG00000206562	METTL6	1.34696	1.49468	0.54668	1.25654	1.38806	1.94624	1.43264	1.26002
ENSG00000144597	EAF1	6.31703	4.94949	1.65167	1.96372	4.23789	6.77682	4.36358	3.35393
ENSG00000206926	RNU6-1024P	0	0	0	0	0	0	0	0
ENSG00000249786	EAF1-AS1	0.01696	0	0.02493	0	0	0	0	0.04166
ENSG00000206561	COLQ	1.22899	0.32146	0.1548	0.70352	0.34152	0.27876	0.23412	0.49579
ENSG00000263573	MIR4270	0	0	0	0	0	0	0	0
ENSG00000241064	RN7SL110P	0	0	0	0	0	0	0	0
ENSG00000131373	HACL1	3.16715	2.11668	1.05775	1.74484	3.23795	3.25925	2.43961	1.82645
ENSG00000169814	BTD	1.95916	1.25171	0.34897	0.67551	1.08268	1.11716	0.69271	0.9233
ENSG00000206560	ANKRD28	8.0412	4.45792	3.96429	7.52509	4.79725	3.72706	4.55088	5.85827
ENSG00000264354	MIR3134	0	3.29753	1.45559	1.47008	1.20428	0	0	1.21618
ENSG00000279465	AC090950.2	0	0	0	0	0	0	0	0
ENSG00000270409	AC090950.1	0	0	0	0	0	0	0	0
ENSG00000263740	RN7SL4P	821.212	1338.78	2896.23	4987.18	872.437	1728.39	2062.57	2186.17
ENSG00000207815	MIR563	0	0	0	0	0	0	0	1.1392
ENSG00000224728	IMPDH1P8	0.09613	0	0	0	0	0	0.08012	0
ENSG00000131386	GALNT15	0	0	0	0	0	0	0	0
ENSG00000154813	DPH3	5.90601	6.61079	2.47966	3.17623	5.4791	5.15505	5.04225	4.11834
ENSG00000154814	OXNAD1	7.56455	3.6032	3.2164	4.78336	10.9513	9.46236	8.80019	6.09824
ENSG00000270739	AC090953.1	0	0	0	0	0	0	0	0
ENSG00000131378	RFTN1	8.13425	6.48873	1.08255	3.21164	7.20248	7.68939	3.06986	2.88307
ENSG00000271964	AC090948.1	1.46868	0.66671	0.7848	1.58522	0.97395	2.35541	1.78041	0.98357
ENSG00000272498	AC090948.2	1.81951	0.86531	0.19098	0.38576	3.47618	1.60493	1.08316	1.59569
ENSG00000272529	AC090948.3	1.46335	0.88572	0	0.19743	2.74952	1.40811	0.44349	0.32667
ENSG00000269391	AC010139.1	0	0	0	0	0	0	0	0

ENSG00000233570	LINC00690	0	0	0	0	0	0	0	0
ENSG00000092345	DAZL	0.02019	0	0	0	0.04909	0	0	0.04957
ENSG00000229271	AC091493.1	0	0	0	0	0.21683	0	0	0
ENSG00000224039	CDYLP1	0	0	0	0	0	0	0	0
ENSG00000154822	PLCL2	5.8401	3.50899	1.13031	1.86031	4.84894	7.33755	4.98598	3.16546
ENSG00000283885	MIR3714	0	0	0	0	0	0	0	0
ENSG00000226441	PLCL2-AS1	0	0	0	0	0	0	0	0.28938
ENSG00000131374	TBC1D5	9.71137	7.90365	6.0696	11.2078	8.69894	6.8391	8.9231	8.16195
ENSG00000231780	AC090960.1	0	0	0	0	0	0	0	0
ENSG00000227309	AC140076.1	0	0	0	0	0	0	0	0
ENSG00000271841	RNU7-10P	0	0	0	0	0	0	0	0
ENSG00000233133	AC104451.1	0.83296	0	0	0	0.50634	1.46945	0.69421	0
ENSG00000213383	AC104297.1	0.08425	0	0	0	0	0	0	0
ENSG00000214286	PDCL3P3	0	0	0	0	0	0	0	0
ENSG00000226238	AC132807.1	0	0	0	0	0	0	0	0
ENSG00000274840	AC132807.2	0	0.71981	0	0.3209	0	0	0	0.26548
ENSG00000182568	SATB1	9.34772	4.91346	1.71905	3.82117	9.66998	12.6814	6.11403	4.79215
ENSG00000272477	AC144521.1	1.91685	0.63812	1.01404	2.16206	0.65253	3.65211	1.91706	1.41209
ENSG00000228956	SATB1-AS1	0.80872	0.2891	0.12761	0.5397	1.39894	1.03411	0.69662	0.71305
ENSG00000236570	RAD23BP1	0.11948	0.09944	0.08779	0.08866	0.14526	0	0.29873	0
ENSG00000251761	RNU6-138P	0	0	0	0	0	0	0	0
ENSG00000183960	KCNH8	0.11923	0.03969	0	0.05308	0.11596	0.04207	0.01987	0.05855
ENSG00000271898	MIR4791	0	0	0	0	0	0	0	0
ENSG00000163576	EFHB	0.01761	0.23446	0.02587	0.31358	0.10703	0.06212	0.14675	0.02162
ENSG00000232953	HSPA8P18	0	0	0	0.16927	0.04622	0	0	0
ENSG00000144566	RAB5A	28.4293	25.1431	9.96034	20.9811	25.0986	18.0046	21.3049	20.2107
ENSG00000183977	PP2D1	0	0	0.04269	0.04312	0	0	0	0
ENSG00000201545	RNU4-85P	0	0	0	1.76887	0.72453	1.05132	3.97334	1.46337
ENSG00000230697	SAP18P3	0	0	0	0	0	0	0	0
ENSG00000229468	RPL39P18	0	0	0.69493	0	0	0	0	0
ENSG00000114166	KAT2B	34.1262	55.7636	53.3599	101.101	66.798	51.7568	103.4	75.5247
ENSG00000266745	MIR3135A	0.95195	0	0	0	0	0	0	0
ENSG00000129810	SGO1	2.64249	3.98408	2.58874	6.10996	4.49308	4.86439	6.0005	5.38384
ENSG00000231304	SGO1-AS1	0.15617	0.27852	0.04918	0.26489	0.21699	0.39358	0.20453	0.17805
ENSG00000199577	RNU6-822P	0	0	0	0	0	0	0	0
ENSG00000261734	AC116096.1	0.22174	0	0	0	0	0.05588	0.0528	0
ENSG00000206807	RNU6-815P	0	0	0	0	0	0	0	0
ENSG00000282987	AC099753.1	0	0	0	0	0	0	0	0
ENSG00000236380	VENTXP7	0	0	0	0	0	0	0	0
ENSG00000151789	ZNF385D	0.06458	0.0627	0.00791	0.03195	0.13085	0.02848	0.17043	0.21143
ENSG00000225542	ZNF385D-AS1	0	0.34612	0	0.15431	0.12641	0	0	0.12766
ENSG00000223351	ZNF385D-AS2	0	0	0	0	0	0	0	0
ENSG00000132967	HMGB1P5	1.84735	0.76873	0.38781	0.97917	0.72192	1.16392	1.53963	0.81005
ENSG00000234723	AC092421.1	0	0	0	0	0	0	0	0
ENSG00000271235	AC114477.1	0	0	0	0	0	0	0	0
ENSG00000226183	RANP7	0	0	0	0	0	0	0	0
ENSG00000231915	SALL4P5	0	0	0	0	0	0	0	0
ENSG00000215016	RPL24P7	0	0	0	0	0	0	0	0
ENSG00000233153	UBE2E2-AS1	0	0	0	0	0	0	0	0
ENSG00000182247	UBE2E2	2.21647	1.00618	0.4935	0.53163	1.9598	0.82943	0.821	0.63223
ENSG00000206728	RF00019	0	0	1.03571	0	0	0	0	0
ENSG00000228171	AC121251.1	0	0	0	0	0	0	0	0
ENSG00000252562	RNU6-922P	0	0	0	0	0	0	0	0
ENSG00000212269	RNU6-788P	0	0	0	0	0	0	0	0
ENSG00000223791	UBE2E1-AS1	0	0	0	0	0	0	0	0
ENSG00000170142	UBE2E1	9.68118	6.19171	2.6981	4.4944	6.92872	7.78226	4.84906	7.96331
ENSG00000231395	ARL4AP4	0	0	0	0	0	0	0	0.17374
ENSG00000197885	NKIRAS1	0.82313	0.91339	0.31015	1.03367	0.92375	0.96807	0.45734	0.59601

ENSG00000174748	RPL15	51.0452	33.1561	8.26665	17.7643	49.8278	48.4325	24.6307	27.1315
ENSG00000174738	NR1D2	11.8767	6.47777	2.18389	5.68228	7.99294	6.66556	8.04039	4.56171
ENSG00000224074	LINC00691	0	0	0	0	0	0	0	0
ENSG00000242109	NPM1P23	0	0	0	0	0	0	0	0
ENSG00000151090	THRB	0.47975	0.2942	0.12368	0.21235	0.31721	0.04454	0.32267	0.08267
ENSG00000229756	RPL31P20	0	0	0	0	0	0	0	0
ENSG00000224822	THRB-IT1	0	0	0	0	0	0	0	0
ENSG00000228791	THRB-AS1	0	0	0	0	0	0	0	0
ENSG00000272554	AC012087.1	0	0	0	0	0	0	0	0
ENSG00000265028	MIR4792	0	0	0	0	0	0	0	0
ENSG00000233544	EIF3KP2	0	0	0	0	0	0	0	0
ENSG00000237838	AC092422.1	0	0	0	0	0	0	0	0
ENSG00000244404	RN7SL216P	0	0	0	0	0	0	0	0
ENSG00000201713	RNA5SP125	0	0	0	0	0	0	0	0
ENSG00000077092	RARB	0	0.066	0.02914	0	0	0	0	0
ENSG00000213877	CFL1P7	0	0	0	0	0	0	0	0
ENSG00000201962	RNA5SP126	0	0	0	0	0	0	0	0
ENSG00000229421	AC133141.1	0	0	0	0	0	0	0	0
ENSG00000272166	RF01161	0	0	0	0	0	0	0	0
ENSG00000077097	TOP2B	32.3206	26.2427	8.64349	18.179	37.9232	34.1251	17.9106	22.1568
ENSG00000275227	RF02271	1.6199	1.34816	0.5951	0	0.98471	4.28658	5.40024	2.98333
ENSG00000283971	MIR4442	0	0	0	0	0	1.93003	0	0
ENSG00000238225	CRIP1P2	0.64299	0	0	0	0	0	0	0
ENSG00000151092	NGLY1	3.66238	4.14706	1.09965	2.39104	3.20032	2.68688	2.49467	2.68067
ENSG00000213872	AC092798.1	0	0	0	0	0	0	0	0
ENSG00000213871	TAF9BP1	0	0	0	0	0	0	0.16378	0.12064
ENSG00000151093	OXSM	0.73713	0.73617	0.08666	1.00646	1.07542	0.88427	0.78636	0.94124
ENSG00000230891	LINC00692	0	0	0	0	0	0	0	0
ENSG00000234158	RPEP2	0	0	0	0	0	0	0	0
ENSG00000237603	HMGB3P12	0	0	0	0	0	0	0	0.16187
ENSG00000230465	VENTXP4	0	0	0	0	0	0	0	0
ENSG00000225386	AC099754.1	0	0	0	0	0	0	0	0
ENSG00000179796	LRRC3B	0	0	0	0	0	0	0	0
ENSG00000234165		0	0	0	0	0	0	0	0
ENSG00000163491	NEK10	0.02295	0.05093	0.01124	0.04541	0.0465	0.02699	0.01275	0.02818
ENSG00000224237	MINOS1P3	0	0	0	0	0	0	0	0
ENSG00000207251	RNU6-342P	0	0	0	0	0	0	0	0
ENSG00000033867	SLC4A7	5.092	4.88491	2.12829	4.42338	5.2547	6.01106	2.3506	3.18209
ENSG00000227523	RPS20P15	0	0	0	0	0	0	0	0
ENSG00000230807	AC099535.1	0	0	0	0	0	0.34855	0	0
ENSG00000201033	RNU1-96P	0	0	0	0	0	0	0	0
ENSG00000232167	AC137675.1	0	0	0	0	0	0	0	0
ENSG00000238912	RF00019	0	0	0	0	0	0	0	0
ENSG00000223968	AC098614.2	0	0	0	0	0	0	0	0
ENSG00000213846	AC098614.1	0.26905	0.29855	0	0.1331	0.3271	0.21095	0	0.11011
ENSG00000271943	AC098614.4	0	0	0	0.23547	0	0	0	0
ENSG00000232766	AC098614.3	0	0	0	0	0	0	0	0
ENSG00000272282	LINC02084	2.27392	1.33586	0.09828	0.79406	0.81311	2.12373	0.22296	1.06748
ENSG00000163508	EOMES	3.66903	6.87466	1.14903	2.29117	5.2651	14.9968	0.96915	2.09238
ENSG00000225548	LINC01980	0	0	0	0	0	0	0	0
ENSG00000229243	LINC01981	0	0	0	0	0	0	0	0
ENSG00000235493	LINC01967	0	0	0	0	0	0	0	0
ENSG00000187118	CMC1	1.45404	1.79492	1.73798	3.55185	1.7847	1.98828	3.81524	2.60527
ENSG00000163512	AZI2	4.80279	5.49605	2.4091	2.82716	3.60734	4.74557	2.63643	4.29502
ENSG00000206559	ZCWPW2	0.17724	0.08429	0.11162	0.18789	0.03078	0.04467	0.29543	0.15544
ENSG00000283563	AC098650.1	0	0	0	0	0.05042	0	0.03456	0
ENSG00000234048	FAM96AP1	0	0	0	0	0	0	0	0
ENSG00000230480	AC093142.1	0	0	0	0	0	0	0	0

ENSG00000144642	RBMS3	0.03198	0.01331	0.00587	0.02373	0.06803	0.04231	0.01332	0.01963
ENSG00000215004	MESTP4	0	0	0	0	0	0	0	0
ENSG00000235904	RBMS3-AS3	0	0	0	0	0	0	0	0
ENSG00000226216	RPS12P5	0	0	0	0	0	0	0	0
ENSG00000203506	RBMS3-AS2	0	0	0	0	0	0	0	0
ENSG00000227795	MTND4LP9	0	0	0	0	0	0	0	0
ENSG00000235593	RBMS3-AS1	0	0	0	0	0	0	0	0
ENSG00000199927	RF01848	0	0	0	0	0	0	0	0
ENSG00000281710	RF00012	0	0	0	0	0	0	0	0
ENSG00000227260	LINC01985	0	0	0	0	0	0	0	0
ENSG00000227549	AC116035.1	0	0	0	0	0	0	0	0
ENSG00000163513	TGFBR2	48.0254	45.7532	8.65558	14.4076	49.5408	28.067	18.6231	30.5156
ENSG00000224618	AC096921.1	0	0	0	0	0	0	0	0
ENSG00000261468	AC096921.2	2.90753	2.9929	0.33731	0.68133	2.27908	2.09221	1.72175	1.92582
ENSG00000144644	GADL1	0	0	0	0	0	0	0	0
ENSG00000265376	MIR466	0	0	0	0	0	0	0	0
ENSG00000213854	CNN2P6	0	0	0	0	0	0	0	0
ENSG00000222983	RNA5SP127	0	0	0	0	0	0	0	0
ENSG00000230111	AC115283.1	0	0	0	0	0	0	0	0
ENSG00000227339	THRAP3P1	0	0	0	0.03905	0	0	0	0
ENSG00000163527	STT3B	35.7142	24.611	12.4004	32.5793	33.9433	28.8191	25.6696	22.4274
ENSG00000144645	OSBPL10	0.26931	0.03056	0.01799	0.15442	0.53577	0.09718	0.20404	0.21793
ENSG00000232490	OSBPL10-AS1	0	0	0	0	0	0	0	0
ENSG00000223965	ZNF587P1	0	0	0	0	0	0	0	0
ENSG00000197385	ZNF860	0.47175	0.14957	0.03301	0.10002	1.17438	0.03963	0.18722	0.49646
ENSG00000235670	RPL21P40	0	0	0	0	0	0	0	0
ENSG00000201701	RF00402	0	0	0	0	0	0	0	0
ENSG00000252398	RF00019	0	0	0	0	0	0	0	0
ENSG00000251590	NIFKP7	0	0	0	0	0	0	0	0
ENSG00000152642	GPD1L	3.40399	2.33752	0.71779	1.83499	2.4868	2.04658	1.83194	1.8929
ENSG00000236732	AC094019.1	0	0	0	0	0	0	0	0
ENSG00000237433	RPSAP11	0	0	0	0	0	0	0	0
ENSG00000261572	AC097639.1	0	0	0	0	0.04713	0	0	0
ENSG00000170293	CMTM8	0.43705	0.10393	0.1835	0.7413	1.06272	0.44059	0.52036	0.07666
ENSG00000270377	AC097639.2	0	0	0	0	0	0	0	0
ENSG00000234737	KRT18P15	0.11356	0.18901	0	0	0	0.10016	0	0
ENSG00000153551	CMTM7	7.69937	5.98495	1.00506	2.58116	6.22462	5.37794	1.82407	2.56723
ENSG00000091317	CMTM6	102.152	92.8957	23.6029	28.62	75.2029	32.8774	42.7479	72.1322
ENSG00000276147	MIR548AY	0	0	0	0	0	0	0	0
ENSG00000213849	AC104306.1	0	0.32886	0	0	0	0	0	0
ENSG00000144635	DYNC1LI1	10.5312	10.247	3.58344	8.94129	11.4956	6.30532	8.82679	9.07015
ENSG00000226955	AC104306.2	0	0	0	0	0	0	0	0
ENSG00000231552	IGBP1P3	0	0	0	0	0	0	0	0
ENSG00000237676	RPL30P4	0.22012	1.09918	0.9704	0.98005	0	0	0.36691	0.54052
ENSG00000182973	CNOT10	8.71255	8.56023	9.17991	17.1731	11.0155	11.8478	12.5811	9.74999
ENSG00000251224	CNOT10-AS1	0	0	0	0	0	0	0	0
ENSG00000213842	SUGT1P2	0.51724	0.49197	0.21717	0.21933	0.26951	0.91248	0	0.36289
ENSG00000227337	RPL23AP43	0	0	0	0	0	0	0	0
ENSG00000206557	TRIM71	0.07596	0.08429	0.03721	0.0501	0	0.02978	0	0.01036
ENSG00000183813	CCR4	4.40513	2.0499	1.11368	3.0228	4.80856	2.13082	2.44755	2.20994
ENSG00000170266	GLB1	8.71743	7.80027	1.77713	4.53689	5.37069	5.09661	3.49967	3.67082
ENSG00000223899	SEC13P1	0	0	0	0	0	0	0	0
ENSG00000235424	SUMO2P10	0	0	0	0	0	0	0	0
ENSG00000188167	TMPPE	3.14197	2.12269	0.51603	0.98746	2.5616	2.08673	0.83179	1.27076
ENSG00000275090	RN7SL296P	0	1.22417	0.72049	0.72766	0.29805	0.86496	0.40863	0.30099
ENSG00000170275	CRTAP	9.51426	5.13912	0.72399	2.42107	4.56565	2.64611	2.59143	2.27177
ENSG00000272149	AC112211.1	0	0	0	0	0	0	0	0
ENSG00000173705	SUSD5	0	0	0	0.04344	0	0	0	0.01797

ENSG00000230119	AC123900.1	0	0	0	0	0	0	0	0
ENSG00000153558	FBXL2	0	0	0	0	0.02326	0	0	0
ENSG00000153560	UBP1	4.93425	3.80026	0.98223	2.10661	4.19104	4.54446	1.91533	2.12083
ENSG00000252700	RNU7-110P	0	0	0	0	0	0	0	0
ENSG00000202175	RNA5SP128	0	0	0	0	0	0	0	0
ENSG00000163539	CLASP2	3.75393	3.30468	2.37932	5.95028	4.32372	4.03807	4.62664	3.65644
ENSG00000238008	COX6CP10	0	0	0	0	0	0	0	0
ENSG00000228112	AC112220.1	0	0	0	0	0	0	0	0
ENSG00000227707	SDAD1P3	0	0	0	0	0	0	0	0
ENSG00000271324		0	0.48609	0	0.2167	0	0	0	0
ENSG00000271643	AC112220.2	0.6384	0.28981	0	0.08613	0.14112	0.30715	0.19348	0.21377
ENSG00000271020		0.27505	1.37345	0	0.2041	0.1672	0	0	0.3377
ENSG00000170248	PDCD6IP	15.4338	9.74664	3.18208	8.18481	11.6449	9.80068	5.81296	7.83665
ENSG00000226320	LINC01811	0	0	0	0	0	0	0	0
ENSG00000236452	AC123023.1	0.13207	0	0	0	0	0	0	0
ENSG00000227498	AC018359.1	0	0	0	0	0	0	0	0
ENSG00000235534	FECHP1	0	0	0	0	0	0	0	0
ENSG00000228767	KRT8P18	0	0	0	0	0	0	0	0
ENSG00000212442	RNU6-243P	0	0	0	0	0	0	0	0
ENSG00000172995	ARPP21	0.01453	0	0	0.01078	0.01766	0.01281	0.03632	0.01784
ENSG00000230830	ARPP21-AS1	0	0	0	0	0	0	0	0
ENSG00000207625	MIR128-2	0	0	0	0	0	0	0	0
ENSG00000226489	AC104308.1	0	0	0	0	0	0	0	0
ENSG00000236158	RFC3P1	0	0	0	0	0	0	0	0
ENSG00000144681	STAC	0.25011	0.13877	0.19602	0.04949	0.04054	0.20591	0.02779	0.10236
ENSG00000231382	NBPF21P	0	0	0	0	0	0	0	0
ENSG00000201315	RN7SKP227	0.44424	0.36972	0.32641	0	0	0.39185	1.11073	0.54544
ENSG00000163673	DCLK3	0	0	0	0	0.01599	0	0.02192	0
ENSG00000230067	HSPD1P6	0.06728	0	0	0	0	0	0	0
ENSG00000279727	LINC02033	0.70901	1.18014	0.24043	0.08094	1.06091	0.38486	0.27272	0.66962
ENSG00000281100	AC105749.1	1.38716	1.94436	0.16093	0.16253	0.75447	1.03037	0.12169	0.67229
ENSG00000168016	TRANK1	17.6611	34.5492	4.89565	8.10759	15.6435	19.0202	3.17011	14.4211
ENSG00000234073	AC011816.1	0.40275	1.11729	0.59183	0.59772	0.48965	0.23683	0.22377	1.6483
ENSG00000210181	RNU6ATAC4P	0	0	0	0	0	0	0	0
ENSG00000272334	AC011816.2	0.26367	0	0	0	0.64113	0	0.21975	0.32373
ENSG00000236165	PRADC1P1	0	0	0	0	0	0	0	0
ENSG00000178567	EPM2AIP1	5.06048	2.58251	0.80858	1.87423	3.94807	3.67598	2.7365	2.19289
ENSG00000076242	MLH1	4.69023	5.49245	2.47022	5.60561	5.65179	5.67478	5.18886	4.71389
ENSG00000224858	RPL29P11	0	0	0	0	0	0	0	0
ENSG00000093167	LRRFIP2	9.68936	11.3251	5.63667	11.0993	12.4164	11.6021	12.1905	9.04706
ENSG00000206645	RF00019	1.43726	0	0	0	0	0	0	0
ENSG00000225558	UBE2D3P2	0	0	0.24205	0.24446	0	0	0	0
ENSG00000199594	RNU6-1301P	0	0	0	0	0	0	0	0
ENSG00000224080	UBE2FP1	2.62373	0.81885	1.20485	0.24337	1.1962	0.86786	1.64	1.40935
ENSG00000271993	AC126118.1	0.11672	0	0	0	0	0.20591	0.19455	0
ENSG00000231449	AC097359.1	0.31663	0	0	0	0	0	0	0
ENSG00000271653	AC097359.3	0	0	0	0	0.07365	0	0	0
ENSG00000270194	AC097359.2	0.39887	0.20428	0.04509	0.22768	0.26112	0.05413	0.10229	0.2637
ENSG00000144674	GOLGA4	17.1423	14.8406	4.84547	9.40236	17.0232	13.7417	13.917	11.8269
ENSG00000230409	TCEA1P2	4.85433	13.0627	15.5745	28.4572	13.1806	8.99188	19.2845	13.0128
ENSG00000222208	RNA5SP129	0	2.06794	0	0	0	0	0	0
ENSG00000198590	C3orf35	0.50108	0.67518	0.17532	0.46036	0.37712	1.09444	0.59658	0.49803
ENSG00000144668	ITGA9	0.15941	0.11793	0	0.02629	0.1615	0.21872	0.07381	0.04349
ENSG00000239105	RNU7-73P	0	0	0	0	0	0	0	0
ENSG00000235257	ITGA9-AS1	0.48492	0.18344	0.11337	0.11449	0.12059	0.11665	0.1837	0.27063
ENSG00000235482	RPL21P135	0	0	0	0	0	0	0	0
ENSG00000236411	NDUFAF4P3	0.13962	0.69719	0.61551	0.20721	1.35797	0.73892	0.23272	0.51427
ENSG00000144677	CTDSPL	5.85543	2.61026	0.68761	1.23876	3.38262	3.50275	2.27665	3.13654

ENSG00000199075	MIR26A1	0	0	0	0	0	0	0	0
ENSG00000136059	VILL	0.95078	0.77244	0.06653	0.45355	0.5642	0.619	0.3396	0.40301
ENSG00000187091	PLCD1	1.88603	0.93331	0.07491	0.68086	1.15682	1.3189	0.59476	0.35465
ENSG00000200579	RF00019	0	0	0	0	0	0	0	0
ENSG00000008226	DLEC1	0.18461	0.07316	0.10335	0.14352	0.13894	0.09305	0.05861	0.10794
ENSG00000231668	PPP2R2DP1	0.11507	0	0	0.17078	0	0	0.19181	0
ENSG00000060971	ACAA1	3.66192	3.76018	0.4699	0.64298	2.09435	1.78336	1.18638	1.46913
ENSG00000201965	RF00019	0	0	0	0	0	0	0	0
ENSG00000172936	MYD88	27.6921	24.7137	2.80829	3.59983	14.1416	11.346	4.37999	7.62572
ENSG00000172939	OXSR1	13.2242	11.4075	3.38656	6.10633	9.59379	9.57865	11.0616	6.63682
ENSG00000172940	SLC22A13	0.05738	0	0	0	0.10464	0	0	0
ENSG00000237982	AP006193.1	0	0	0.22209	0	0	0	0	0
ENSG00000144671	SLC22A14	0	0.03334	0	0	0	0	0	0
ENSG00000199514	RNU6-235P	0	0	0	0	0	0	0	0
ENSG00000230123	DLEC1P1	0	0	0	0	0	0	0	0
ENSG00000093217	XYLB	0.15091	0.09134	0.04032	0.12216	0.01668	0.43564	0.09147	0.05053
ENSG00000229589	ACVR2B-AS1	0	0	0	0	0	0	0	0
ENSG00000114739	ACVR2B	0.2421	0.09299	0.11631	0.17274	0.26038	0.29568	0.24833	0.24008
ENSG00000157036	EXOG	0.5333	0.7008	0.18561	0.33325	0.80193	0.69323	0.79535	0.56862
ENSG00000207512	RF00019	0	0	0	0	0	0	0	0
ENSG00000230683	DDTP1	0	0	0	0	0	0	0	0
ENSG00000232439	RPL18AP7	0	0	0	0	0	0	0	0
ENSG00000183873	SCN5A	0.01464	0	0	0	0	0.01291	0	0
ENSG00000185313	SCN10A	0	0	0	0	0	0	0	0
ENSG00000231243	AC116038.1	0	0	0	0	0	0	0	0
ENSG00000168356	SCN11A	0.00952	0	0	0	0	0	0.01586	0
ENSG00000206708	RNU6-1227P	0	0	0	0	0	0	0	0
ENSG00000114742	WDR48	7.5963	7.07154	2.50297	3.98067	7.4859	5.6643	6.31461	4.87965
ENSG00000114745	GORASP1	2.29649	2.1843	0.46488	0.57384	1.68091	1.01283	0.52731	1.02139
ENSG00000168026	TTC21A	0.19096	0.15893	0.04209	0.05668	0.10447	0.01684	0.04775	0.08206
ENSG00000284158	MIR6822	0	0	0	0	0	0	0	0
ENSG00000144655	CSRNP1	35.7379	40.2393	8.11038	7.37875	26.1749	10.2999	5.51218	14.0288
ENSG00000283849	AC092053.2	0	0	0	0	0	0	0	0
ENSG00000214305	RF00017	0	0	0	0	0	0	0	0
ENSG00000168334	XIRP1	0	0	0	0	0.02756	0.03999	0	0.01392
ENSG00000215034	DSTNP4	0	0	0	0	0	0	0	0
ENSG00000168329	CX3CR1	40.6912	69.8336	12.8491	20.7154	25.967	58.5176	3.57682	23.6135
ENSG00000270367	AC092053.1	0	0	0	0	0	0	0	0
ENSG00000179934	CCR8	0.3893	0.27771	0.08173	0.33015	0.06762	0.09811	0	0.13657
ENSG00000228168	HNRNPA1P21	0.3051	0.12696	0	0	0.2782	0	0	0.28095
ENSG00000238267	AC104850.1	0	0	0	0	0	0	0	0
ENSG00000223668	EEF1A1P24	0	0.08854	0	0	0	0	0	0
ENSG00000144659	SLC25A38	8.4171	7.0335	4.85739	8.95168	13.2163	15.0894	20.2782	9.24656
ENSG00000168028	RPSA	58.1784	29.3242	8.67513	15.5606	57.9168	69.3868	32.687	31.9426
ENSG00000206760	SNORA6	0	0	0	0	0	0	0	0
ENSG00000202363	SNORA62	0	0	0	0	0	0	0	0
ENSG00000168314	MOBP	0.00794	0	0	0	0	0	0	0
ENSG00000230757	NFU1P1	0	0	0	0	0	0	0	0
ENSG00000170011	MYRIP	0	0.02078	0	0.05558	0	0	0	0.01533
ENSG00000201570	RNU4-56P	0	0	0	0	0	0	0	0
ENSG00000280739	EIF1B-AS1	0.33729	0.35931	0.15861	0.18021	0.27885	0.11901	0.11244	0.16565
ENSG00000265327	RN7SL411P	0	0	0	0	0	0	0	0
ENSG00000202517	RF00264	0	0	0	0	0	0	0	0
ENSG00000114784	EIF1B	28.101	48.4971	13.3516	36.3567	21.3004	16.5698	37.1909	19.8634
ENSG00000223797	ENTPD3-AS1	0.12378	0.24723	0.07276	0.1837	0.09029	0.21836	0.20632	0.21276
ENSG00000230274	PGAM1P3	0	0	0	0	0	0	0	0
ENSG00000168032	ENTPD3	0.04357	0	0	0	0.02648	0	0	0.02675
ENSG00000188846	RPL14	20.1818	13.0269	3.73365	7.12543	21.3752	15.9354	8.68265	11.0175

ENSG00000177873	ZNF619	1.52641	1.10819	0.26248	0.53019	1.55964	0.8594	0.73081	0.73768
ENSG00000199906	RNU5B-2P	0.64868	0	0	0	0	0	0	0
ENSG00000177842	ZNF620	0.44527	0.45881	0.31158	0.25175	0.43824	0.44887	0.21206	0.41653
ENSG00000172888	ZNF621	1.71098	1.24141	0.24498	0.62506	1.48279	1.82653	0.52651	0.76488
ENSG00000234067	RPL5P10	0	0	0	0	0	0	0	0
ENSG00000226845	EEF1GP3	0	0	0	0	0	0.1004	0	0
ENSG00000226302	AC122683.1	0	0	0	0	0	0	0	0
ENSG00000229072	HMGN2P24	0	0	0	0	0	0	0	0
ENSG00000234287	AC099560.2	0	0.47846	0.84482	0.42661	0.34948	1.01421	0	1.41172
ENSG00000231873	AC099560.1	0	0	0	0	0.17823	0	0	0
ENSG00000233096	AC099541.1	0	0	0	0	0.26602	0	0	0
ENSG00000233919	AC009743.1	0	0	0	0	0	0	0	0
ENSG00000275256	MRPS31P1	0	0	0	0	0	0	0	0
ENSG00000168036	CTNNB1	16.8012	14.5178	4.16915	7.63178	15.2795	13.6859	8.16506	12.4777
ENSG00000168038	ULK4	1.45954	1.1498	0.49117	1.15747	2.23503	2.16208	1.26285	1.61417
ENSG00000252409	RF00602	0	0	0	0	0	0	0	0
ENSG00000222942	RN7SKP58	0	0	0	0	0	0	0	0
ENSG00000223513	ATP6V0E1P2	0.59837	0	0	0	0	0	0	0
ENSG00000251986	RF00019	0	0	0	0	0	0	0	0
ENSG00000212145	RF00096	1.02518	0	0	0.76074	0	0	0	0
ENSG00000234270	RPL36P20	0	0	0	0	0	0	0	0
ENSG00000235339	GEMIN2P2	0	0	0	0	0	0	0	0
ENSG00000182606	TRAK1	4.40122	2.93033	0.56836	1.33607	3.22675	2.95281	1.71177	1.23631
ENSG00000229339	AC093414.1	0	0	0	0	0	0	0	0
ENSG00000234562	TPMTP2	0	0	0	0	0	0	0	0
ENSG00000222872	RNU4-78P	0.58175	0.96832	0	0	0.70728	1.02628	0	0
ENSG00000187094	CCK	0	0	0	0	0.04274	0	0	0
ENSG00000281160	AC018358.1	0	0	0	0	0	0	0	0
ENSG00000231280	SALL4P6	0	0	0	0	0	0	0	0
ENSG00000229572	EIF4BP4	0	0	0	0	0	0.07265	0	0
ENSG00000157093	LYZL4	0	0	0	0	0	0	0	0
ENSG00000235787	AC092047.1	0	0	0	0	0	0	0	0
ENSG00000114812	VIPR1	1.02247	0.42961	0.08752	0.39778	0.82068	0.2802	0.29784	0.3047
ENSG00000232354	VIPR1-AS1	0.02821	0	0	0	0	0	0	0
ENSG00000093183	SEC22C	2.3538	1.57015	0.6997	1.29332	1.91144	1.91773	1.31779	1.19128
ENSG00000008324	SS18L2	2.65417	3.3839	2.40655	3.01717	3.0209	3.98495	3.01215	3.53609
ENSG00000114857	NKTR	5.04267	3.77498	1.35294	2.8707	4.11284	3.53901	3.60431	3.0801
ENSG00000230084	AC006059.1	0.28013	0.46627	0.13722	0	0.22705	0	0.15564	0.22929
ENSG00000114853	ZBTB47	2.09374	2.03294	0.19942	0.23017	0.91921	0.3078	0.4524	0.52365
ENSG00000240203	RN7SL567P	0	0	0	0	0	0	0	0
ENSG00000157119	KLHL40	0	0	0	0	0	0	0	0
ENSG00000010282	HHATL	0	0	0	0	0	0	0	0
ENSG00000244607	CCDC13	0.17713	0.09828	0.04338	0.06572	0.17945	0.02604	0.04921	0.03625
ENSG00000230970	HHATL-AS1	0	0	0	0	0	0	0	0
ENSG00000173811	CCDC13-AS1	0	0	0	0	0	0	0	0
ENSG00000280571	AC006059.2	0	0	0	0	0	0	0	0
ENSG00000225611	LINC02158	3.35041	3.94453	0.48033	0.60639	2.98049	1.29744	0.27242	1.10364
ENSG00000181061	HIGD1A	12.7346	9.83352	8.4242	22.0169	10.0556	10.1905	15.9746	14.6144
ENSG00000235288	AC099329.1	0.03747	0.18713	0.05507	0.27808	0.09112	0.13222	1.12436	0.23005
ENSG00000144648	ACKR2	0.14776	0.04472	0.05922	0.17942	0.22863	0.37914	0.55974	0.26387
ENSG00000273328	AC099329.2	0.01864	0	0	0	0	0	0.12426	0
ENSG00000273291	AC092042.3	0	0	0	0	0	0	0	0
ENSG00000240747	KRBOX1	0	0	0	0	0	0	0	0
ENSG00000180432	CYP8B1	0	0	0	0	0	0	0	0
ENSG00000236459	HNRNPA1P22	0	0	0	0	0	0	0	0
ENSG00000182983	ZNF662	0.20241	0	0	0.02146	0.1582	0.02551	0.16869	0
ENSG00000206552	KRBOX1-AS1	0	0	0	0	0	0	0	0
ENSG00000144649	FAM198A	0	0	0	0.02466	0	0	0.0831	0

ENSG00000232375	AC092042.2	0	0	0	0	0	0	0	0
ENSG00000144647	POMGNT2	1.45612	0.64022	0.8882	0.36697	0.83505	1.01782	0.45795	0.77584
ENSG00000227245	AC092042.1	0	0	0	0	0	0	0	0
ENSG00000163788	SNRK	54.9149	44.9514	11.3562	22.9228	41.1052	30.6377	29.5256	29.4447
ENSG00000241939	RN7SL517P	4.56625	2.00014	0.70632	1.78337	1.16874	0.42397	1.20177	1.47536
ENSG00000271941		0.20333	0.16922	0	0	0.1236	0	0	0.12482
ENSG00000234617	SNRK-AS1	0.68414	0.32536	0	0.14505	0	0	0	0.12
ENSG00000160746	ANO10	4.11769	4.4308	2.5263	5.63785	3.38802	3.32635	4.41393	3.45553
ENSG00000251811	RF00019	0	0	0	0	0	0	0	0
ENSG00000223916	AC097638.1	0	0.23691	0	0	0	0.25109	0	0
ENSG00000011198	ABHD5	16.5457	24.5017	3.19203	8.74538	10.5917	5.1366	8.55108	10.895
ENSG00000235886	AC006055.1	0	0	0	0	0	0	0	0
ENSG00000271192	AC006058.2	0	0	0	0	0	0	0	0
ENSG00000272121	AC006058.3	0	0	0	0	0	0	0	0
ENSG00000252980	RNU6-367P	0	0	0	0	0	0	0	0
ENSG00000207954	MIR138-1	0	0	0	0	0	0	0	0
ENSG00000280435	AC006058.4	0	0	0	0	0	0	0	0
ENSG00000261786	AC006058.1	0.0434	0	0	0.02147	0	0	0	0
ENSG00000173769	TOPAZ1	0	0	0	0	0	0	0	0
ENSG00000271937	AC104187.1	0	0	0	0	0	0.42259	0.19964	0.29411
ENSG00000179152	TCAIM	2.38102	1.49318	0.59136	1.33136	1.31489	1.58256	0.74066	0.99848
ENSG00000225873	C3orf86	11.3847	28.6781	1.34195	1.98778	7.69779	1.82583	0.50739	4.70915
ENSG00000283036	LINC01988	0	0	0	0	0	0	0	0
ENSG00000185219	ZNF445	1.27793	1.69393	0.11987	0.59376	1.11921	0.76062	0.59565	1.03012
ENSG00000178917	ZNF852	0.17349	0.2599	0.15297	0.02575	0.25311	0.12243	0.05784	0.0639
ENSG00000214825	EI24P3	0	0	0	0	0	0	0	0
ENSG00000277509	AC099669.1	0	0	0	0	0	0	0	0
ENSG00000196345	ZKSCAN7	0.29613	0.14497	0.05119	0.10341	0.14825	0.15365	0.02904	0.10694
ENSG00000236869	ZKSCAN7-AS1	0.16075	0	0	0	0	0	0.26794	0
ENSG00000144792	ZNF660	0.01125	0	0	0	0.01368	0	0	0.02764
ENSG00000214820	MPRIPP1	0	0	0	0	0	0	0	0
ENSG00000186448	ZNF197	0.37912	0.64908	0.12734	0.4662	0.50043	0.6306	0.37916	0.37238
ENSG00000233509	ZNF197-AS1	0	0	0.1729	0	0	0	0	0
ENSG00000169981	ZNF35	0.30278	0.04582	0	0	0.26772	0.04856	0.09176	0
ENSG00000272077	AC124045.1	0	0	0	0	0	0	0	0
ENSG00000235805	SOCS5P3	0	0	0	0	0	0	0	0
ENSG00000196653	ZNF502	0.0224	0	0	0.03324	0.05446	0.27656	0.11199	0
ENSG00000186446	ZNF501	0.01875	0.09361	0	0	0.09117	0	0	0.06905
ENSG00000163807	KIAA1143	1.83983	1.2992	0.38915	0.91017	1.54205	1.72121	0.65051	0.80431
ENSG00000163808	KIF15	4.64628	5.86379	8.29509	11.177	14.2317	16.5646	17.0151	14.0317
ENSG00000169964	TMEM42	0.1652	0	0	0.03502	0.02869	0.04163	0.11801	0.05795
ENSG00000284498	MIR564	0	0	0	0	0	0	0	0
ENSG00000163810	TGM4	0	0	0	0	0	0	0	0
ENSG00000235845	AC098649.1	0	0	0	0	0	0	0	0
ENSG00000163812	ZDHHC3	5.08642	4.69818	1.8403	2.3447	4.4857	5.21734	2.37649	2.55479
ENSG00000227123	RPL12P44	0	0	0	0	0	0	0	0
ENSG00000075914	EXOSC7	1.66667	0.76908	0.16974	0.44082	1.3843	1.16445	0.55011	0.6686
ENSG00000163815	CLEC3B	0	0	0	0	0	0	0	0
ENSG00000252410	RNU5B-3P	0	0	0	0	0	0	0	0
ENSG00000163814	CDCP1	0.1915	0.28126	0.01655	0.06687	0.06848	0.37758	0.03755	0.05532
ENSG00000224094	RPS24P8	0.54702	0.3035	0.80384	1.08244	0.22168	0.96501	0.30393	0.44775
ENSG00000249992	TMEM158	1.25334	1.54782	3.03001	2.28012	0.44239	5.27803	3.63913	1.241
ENSG00000202268	RF00012	0	0	0	0	0	0	0	0
ENSG00000011376	LARS2	2.05236	1.28954	0.35951	0.58498	2.04904	2.58959	0.81559	0.98458
ENSG00000232455	LARS2-AS1	0	0	0	0	0	0	0	0
ENSG00000144791	LIMD1	2.29624	1.59815	0.45897	0.96141	1.63143	2.03054	0.93518	1.17885
ENSG00000230530	LIMD1-AS1	0.57717	0.0739	0.13048	0.59302	0.59375	0.39162	0.29602	0.54511
ENSG00000211456	SACM1L	8.91329	9.45931	3.56019	7.69431	7.95165	7.00028	4.68646	6.53678

ENSG00000244357	RN7SL145P	0	1.23657	0	0.73504	0.90321	0.87373	0.82554	1.21618
ENSG00000279017	AC098476.1	0	0.11903	0	0	0	0	0.1192	0.0878
ENSG00000163817	SLC6A20	0.01102	0	0	0	0.0134	0	0.01837	0
ENSG00000163818	LZTFL1	1.62867	1.74516	0.50858	1.2992	1.96772	1.11336	1.52704	1.08731
ENSG00000236930	AC099782.1	0.15464	0	0	0	0	0	0	0
ENSG00000173585	CCR9	0.75675	0.1303	0.07669	0.23237	1.11039	0.36828	0.47846	0.16019
ENSG00000201635	RF00019	0	0	0	0	0.79568	0	0	0
ENSG00000163820	FYCO1	3.29625	3.42913	0.49649	0.74603	2.42453	3.34364	0.82415	1.20401
ENSG00000172215	CXCR6	1.63365	0.42083	0.4001	1.12567	1.22952	5.66104	0.42142	1.19391
ENSG00000173578	XCR1	0.1221	0.02258	0	0.02013	0.09896	0	0.02261	0.01666
ENSG00000226933	NRBF2P2	0	0	0	0	0	0	0	0
ENSG00000229515	FLT1P1	0	0	0	0	0	0	0	0
ENSG00000183625	CCR3	15.0381	5.43704	2.14341	2.95746	7.51797	1.1235	1.67792	7.16344
ENSG00000163823	CCR1	130.792	87.117	22.4026	9.63975	43.8241	21.3073	10.7819	40.5992
ENSG00000236736	UQCRC2P1	0	0	0	0	0	0	0	0
ENSG00000121807	CCR2	22.7186	24.4475	3.77994	8.39279	14.0849	10.3228	2.422	11.2587
ENSG00000223552	AC098613.1	4.80475	3.53378	4.92594	5.63828	4.21131	1.77409	3.35251	3.56696
ENSG00000160791	CCR5	5.01045	3.95398	1.93603	2.10343	3.44623	16.2696	0.89838	1.59309
ENSG00000121797	CCRL2	2.63412	2.4314	0.35189	1.13726	1.54302	2.45021	0.95796	1.38186
ENSG00000283646	LINC02009	0.04968	0.16538	0	0	0.21139	0.2191	0	0.21348
ENSG00000012223	LTF	5.0052	2.12588	2.78986	2.12602	1.72064	25.2412	1.06444	2.22503
ENSG00000163825	RTP3	0	0	0	0	0	0	0	0
ENSG00000163827	LRRC2	0.19557	0.32553	0.0958	0.29025	0.17437	0.59803	0.43465	0.24012
ENSG00000268324	LRRC2-AS1	0	0	0	0	0	0	0	0
ENSG00000241186	TDGF1	0	0	0	0	0	0	0	0
ENSG00000283877	AC104304.1	0	0	0	0	0	0	0	0
ENSG00000283473	FAM240A	0	0	0	0	0.0488	0	0	0
ENSG00000251967	RF00591	0	0	0	0	0	0	0	0
ENSG00000178038	ALS2CL	0.22661	0.06736	0.02379	0.08408	0.23615	0.07139	0.14839	0.1888
ENSG00000181585	TMIE	0	0	0	0	0.14657	0	0.06698	0.04934
ENSG00000206549	PRSS50	0	0	0	0	0	0	0	0
ENSG00000283706	PRSS50	0	0	0	0	0	0	0	0
ENSG00000261603	PRSS46P	0	0	0	0	0	0	0	0
ENSG00000188086	PRSS45	0	0	0	0	0	0	0	0
ENSG00000238013	AC109583.1	0	0	0	0	0	0	0	0
ENSG00000226074	PRSS44	0	0	0	0	0	0.11284	0	0
ENSG00000178055	PRSS42	0.03947	0	0	0	0	0	0	0
ENSG00000160808	MYL3	0	0	0	0	0	0	0	0
ENSG00000160801	PTH1R	0	0	0	0	0	0	0	0
ENSG00000273682	AC109583.2	2.8411	1.8916	0.83499	0	0	5.01208	0.94713	0.69765
ENSG00000160799	CCDC12	3.31501	2.38679	0.36252	0.52631	2.73686	2.61126	0.89952	1.11692
ENSG00000160796	NBEAL2	20.6188	17.4955	2.41339	4.40948	10.2377	10.0747	4.79065	8.55151
ENSG00000236409	NRADDP	0.42431	0.5297	0	0.15743	0	0	0.17682	0.39073
ENSG00000181555	SETD2	30.8037	31.073	19.8064	36.2691	38.0945	34.2902	28.806	32.0011
ENSG00000275940	MRPL57P3	0	0	0	0	0	0	0	0
ENSG00000227398	KIF9-AS1	0.80813	0.67257	0.85767	2.13218	0.74597	0.39601	1.04769	1.52506
ENSG00000088727	KIF9	0.20048	0.14599	0.11048	0.16736	0.25897	0.19894	0.04177	0.13846
ENSG00000239128	SNORD13P3	0	0	0	1.04602	0	0	0	0.86536
ENSG00000114648	KLHL18	2.34511	2.43533	0.48795	0.6776	1.89234	1.50107	0.79562	1.32499
ENSG00000280173	AC104447.1	0.51766	0.74676	0.15214	0.15365	1.04893	0.66969	0.34514	0.55083
ENSG00000260236	AC099778.1	0.19179	0.38307	0.1691	0.05693	0.1399	0.33834	0.25574	0.18838
ENSG00000076201	PTPN23	1.38488	0.77518	0.21611	0.36377	0.9089	0.73509	0.5107	0.58684
ENSG00000114650	SCAP	7.11947	5.69283	0.90602	1.70922	5.95431	5.31531	2.36566	3.31365
ENSG00000276925	AC099778.2	0	0	0	0	0	0	0	0
ENSG00000163832	ELP6	1.28325	1.03116	0.39015	0.59105	1.21046	2.45899	0.55319	0.57046
ENSG00000271161	BOLA2P2	0	0	0	0	0	0	0	0
ENSG00000244732	RN7SL870P	0	0	0	0	0	0	0	0
ENSG00000114646	CSPG5	0.05037	0	0	0.02492	0.02041	0	0	0

ENSG00000173473	SMARCC1	17.7858	15.4486	8.25643	15.5815	18.9074	18.5045	14.6393	12.1723
ENSG00000214773	AC112512.1	0	0	0	0.07618	0.12481	0	0.17112	0.12605
ENSG00000235695	AC130472.1	0	0	0	0	0	0	0	0
ENSG00000132153	DHX30	2.98161	1.95365	0.54246	1.09572	2.47418	3.38976	0.88354	1.10404
ENSG00000274420	RF02271	0.59113	0	0	0	0	0	0	0
ENSG00000221585	MIR1226	0	0	0	0	0	0	0	0
ENSG00000047849	MAP4	2.39871	1.89426	0.44692	0.81537	1.9919	2.92494	0.61323	0.72875
ENSG00000199591	RF00019	0	0	0	0	0.83287	0	0	0
ENSG00000224895	VPS26BP1	0	0	0	0	0	0	0	0.1192
ENSG00000270285	AC124916.2	0	0	0	0	0	0	0	0
ENSG00000265078	RN7SL664P	0	1.20008	1.05948	1.07002	0	0.84795	0.40059	1.18029
ENSG00000236768	AC124916.1	0	0	0	0	0	0	0	0
ENSG00000164045	CDC25A	0.24926	0.14818	0.23547	0.31708	0.25975	0.94228	0.56386	0.17488
ENSG00000235627	SNRPFP4	0	0	0	0	0	0	0	0
ENSG00000253056	RNU7-128P	0	0	0	0	0	0	0	0
ENSG00000231884	NDUFB1P1	0	0	0	0	0	0	0	0
ENSG00000265483	MIR4443	0	0	0	0	0	0	0	0
ENSG00000164047	CAMP	5.80499	3.43917	3.18081	1.46021	3.82783	15.6216	2.62401	2.89923
ENSG00000164048	ZNF589	2.46946	1.14915	0.37069	0.68964	1.45273	1.2882	0.64177	0.68464
ENSG00000229759	MRPS18AP1	0	0	0	0	0	0	0.20744	0
ENSG00000199476	RF00019	0	0	0	0	0	1.19733	0	0
ENSG00000228638	FCF1P2	4.65199	3.76699	0.36952	0.93298	6.72579	3.99247	1.04786	2.4699
ENSG00000172113	NME6	1.36778	1.57814	0.29692	0.46135	1.43615	1.48067	0.51815	1.08775
ENSG00000229453	SPINK8	0	0	0	0	0.40143	0.29124	0	0.2027
ENSG00000252466	MIR2115	0	0	0	0	0	0	0	0
ENSG00000164049	FBXW12	0	0	0	0	0.02555	0	0	0
ENSG00000277483	RN7SL321P	0.24515	0	0	0.36383	1.78829	3.89233	0.40863	1.80596
ENSG00000164050	PLXNB1	0.03766	0.05014	0	0	0.00916	0	0.02511	0.01849
ENSG00000164051	CCDC51	0.54747	0.35438	0.08939	0.31598	0.51769	1.12678	0.20279	0.44812
ENSG00000232112	TMA7	40.3506	43.0618	22.5074	28.5574	36.3039	40.7576	22.4194	43.2997
ENSG00000244380	AC134772.1	0.20923	0.11609	0	0	0	0.12304	0.11625	0
ENSG00000164053	ATRIP	0.17161	0.42848	0.25218	0.89143	0.23472	0.37844	0.35757	0.28972
ENSG00000282827		0	0	0	0.0239	0	0.05682	0	0
ENSG00000213689	TREX1	0	0	0	0	0	0	0	0
ENSG00000164054	SHISA5	37.0902	57.9436	11.8014	11.2191	24.4956	28.3077	9.84987	16.5175
ENSG00000114268	PFKFB4	5.88131	7.53188	1.42866	2.0129	5.55976	1.24929	1.88062	4.14101
ENSG00000278799	MIR6823	1.20164	2.00014	0	0	0	0	0	0
ENSG00000145040	UCN2	0	0	0	0	0.06017	0	0	0
ENSG00000114270	COL7A1	0.01986	0.03306	0	0	0.00805	0	0.01104	0.07316
ENSG00000284251	MIR711	0	0	0	0	0	0	0	0
ENSG00000010256	UQCRC1	8.38107	5.95715	1.23159	2.48768	5.56291	7.83223	1.88783	3.30953
ENSG00000183396	TMEM89	0	0	0	0	0	0	0	0
ENSG00000225697	SLC26A6	0.80512	0.63177	0.15211	0.1707	0.2517	0.75075	0.36426	0.28243
ENSG00000284364	MIR6824	0	0	0	0	0	0	0	0
ENSG00000008300	CELSR3	0.0634	0.09674	0.00776	0.00784	0.07066	0.05593	0.02642	0.01297
ENSG00000284575	MIR4793	0	0	0	0	0	0	0	0
ENSG00000228350	LINC02585	0	0	0	0	0.90935	0	0	0
ENSG00000271973	AC141002.1	0	0	0	0	0	0	0	0
ENSG00000213672	NCKIPSD	1.85868	1.28018	0.18837	0.50731	1.06493	1.16837	0.28489	0.31477
ENSG00000068745	IP6K2	4.28802	4.36055	0.88102	1.35402	4.16744	4.13871	1.45556	2.27233
ENSG00000114302	PRKAR2A	8.72676	6.13348	1.07001	3.17646	6.0359	5.64425	3.31012	3.76568
ENSG00000224424	PRKAR2A-AS1	0.16842	0.09345	0.0825	0.02777	0.0455	0.16506	0.09358	0.13786
ENSG00000178537	SLC25A20	11.8715	9.41842	1.68473	3.18344	8.27319	11.0913	2.15757	5.81212
ENSG00000221883	ARIH2OS	0.55044	0.1527	0.20222	0.2723	0.72498	1.05197	0.15292	0.28159
ENSG00000177479	ARIH2	8.59453	6.28533	2.01535	3.84013	6.70293	8.33905	5.15117	4.20967
ENSG00000235236	AC137630.2	0.11106	0	0	0	0.13503	0	0.18512	0
ENSG00000223343	AC137630.1	0.09373	0.15602	0	0	0	0.33072	0	0
ENSG00000273211	AC137630.4	0.30606	0	0	0	0	0.26996	0	0

ENSG00000178467	P4HTM	0.5564	0.57483	0.14097	0.28474	0.66479	0.49078	0.3198	0.31801
ENSG00000178252	WDR6	7.82202	4.07155	0.79201	1.8254	6.25026	6.55823	1.86588	2.23975
ENSG00000178149	DALRD3	1.31391	1.25919	0.29254	0.41363	0.94393	0.98336	0.5973	0.58662
ENSG00000199032	MIR425	2.5276	0	0	1.25041	0	0	0	3.10335
ENSG00000178057	NDUFAF3	7.70949	6.46635	1.28336	3.26268	4.6865	6.32215	2.40947	3.51262
ENSG00000207605	MIR191	0	2.65236	0	1.18245	0	1.40556	0	0
ENSG00000178035	IMPDH2	10.4941	6.33582	1.00994	3.96226	12.5335	9.18659	2.4674	3.63493
ENSG00000272434	AC137630.3	0.18747	0.31204	0	0	0	1.6536	0	0
ENSG00000198218	QRICH1	9.57077	8.03184	1.95879	3.52134	8.91329	10.5585	4.39929	3.89513
ENSG00000241461	RN7SL182P	0	0	0	0	0	0	0	0
ENSG00000172053	QARS	11.8015	6.68611	1.02955	3.74326	8.27187	9.58577	3.14015	4.09078
ENSG00000284488	MIR6890	0	0	0	0	0	0	0	0
ENSG00000172046	USP19	6.59972	5.85453	1.18408	1.8982	6.04892	4.76091	2.7715	3.48619
ENSG00000172037	LAMB2	0.82216	0.37322	0.04707	0.11092	0.14279	0.18836	0.08899	0.03933
ENSG00000270441	AC135506.1	0.8149	0.06782	0.05987	0.18141	0.29722	0.14376	0.61124	0.15008
ENSG00000177352	CCDC71	3.29255	2.32919	0.3024	0.79406	3.6027	4.06595	1.23484	1.97074
ENSG00000185909	KLHDC8B	1.15487	1.6396	0.49914	0.60493	0.82592	2.27704	0.39632	1.66816
ENSG00000236980	C3orf84	0	0	0	0	0	0	0	0
ENSG00000173421	CCDC36	0	0	0	0	0	0	0	0
ENSG00000270538	AC121247.2	0	0.89712	0	0	0	0	0	0
ENSG00000225399	AC121247.1	0	0	0	0	0	0	0	0
ENSG00000188315	C3orf62	3.07639	3.09267	0.55949	0.65547	3.18472	1.37023	0.76156	1.90727
ENSG00000199546	RF00019	0	0	0	0	0	0	0	0
ENSG00000264633	MIR4271	0	0	0	0	0	0	0	0
ENSG00000114316	USP4	18.515	19.9897	13.3288	30.8926	19.7531	14.0206	14.8253	15.7181
ENSG00000233276	GPX1	115.223	64.9856	84.8861	87.8683	105.939	207.988	60.2756	55.9561
ENSG00000067560	RHOA	215.87	169.735	32.2057	57.3	141.475	108.177	65.065	86.6509
ENSG00000235908	RHOA-IT1	1.27602	0.23599	0.20834	0	0	0.50024	0.23633	0.6963
ENSG00000145022	TCTA	3.33547	2.30959	0.3529	1.02964	3.95786	2.58906	1.28985	1.53981
ENSG00000145020	AMT	0.01276	0	0	0	0	0	0	0.01566
ENSG00000283189	AC104452.1	0	0	0	0	0	0	0.01795	0.02644
ENSG00000145029	NICN1	0	0	0	0	0	0	0	0
ENSG00000201301	RNA5SP130	0	0	0	0	0	0	0	0
ENSG00000173402	DAG1	1.96936	1.21603	0.28006	0.67569	1.54471	1.60636	0.42356	0.41599
ENSG00000226913	BSN-DT	0	0	0	0	0.03424	0	0	0
ENSG00000164061	BSN	0.00904	0.00752	0	0	0.01648	0.00797	0	0
ENSG00000235120	BSN-AS1	0	0	0	0	0	0	0	0
ENSG00000164062	APEH	7.26881	5.64443	0.99201	1.81737	6.35593	6.28695	1.75328	2.94914
ENSG00000173531	MST1	0.05767	0.072	0.02119	0.08559	0.05259	0.05087	0.04806	0.05311
ENSG00000259970	AC099668.1	0	0	0	0	0	0	0	0
ENSG00000164068	RNF123	2.96818	3.72995	3.49514	3.7633	7.32482	8.80804	2.26078	3.8253
ENSG00000176020	AMIGO3	0	0	0	0	0	0	0	0
ENSG00000173540	GMPPB	0.9785	0.561	0.17574	0.32271	0.63447	0.70966	0.34432	0.41381
ENSG00000176095	IP6K1	8.68327	7.5786	1.84357	3.32633	6.94082	5.27194	4.84022	4.63831
ENSG00000239576	COX6CP14	0	0	0	0	0	0	0	0
ENSG00000244730	AC139451.1	0	0	0	0	0	0	0	0
ENSG00000187492	CDHR4	0	0	0	0	0	0	0.03863	0
ENSG00000185614	INKA1	0.77529	0.93853	0	0.3138	0.94258	0.74603	0.11748	0.17307
ENSG00000182179	UBA7	8.0961	7.22118	1.75376	3.90145	6.50973	8.33627	2.84953	4.91073
ENSG00000283726	MIR5193	0	0	0	0	0	0	0	0
ENSG00000183763	TRAIP	0.08986	0.33652	0.13204	0.10002	0.19118	0.51519	0.07489	0.05516
ENSG00000164076	CAMKV	0	0	0	0	0	0	0	0
ENSG00000264706	RN7SL217P	0	0	0	0	0	0	0	0
ENSG00000234667	ACTBP13	0	0	0	0	0	0	0	0
ENSG00000164078	MST1R	0	0	0	0	0	0	0	0.01714
ENSG00000230698	AC105935.2	0	0	0	0	0	0	0	0
ENSG00000228008	AC105935.1	0.23876	0	0	0	0	0.70202	0	0.09772
ENSG00000164077	MON1A	0.744	0.83457	0.1426	0.16803	0.47193	1.11279	0.02696	0.33759

ENSG00000004534	RBM6	11.504	9.49066	3.26004	6.73393	16.3539	11.4224	12.198	9.26839
ENSG00000003756	RBM5	8.24346	8.75524	1.76614	3.78777	7.29746	5.27574	3.82992	6.07619
ENSG00000281691	RBM5-AS1	0	0	0	0	0	0	0	0
ENSG00000235016	SEMA3F-AS1	0	0	0	0	0	0	0	0
ENSG00000001617	SEMA3F	0.04557	0	0	0	0.01847	0	0	0
ENSG00000207922	MIR566	0	0	0	0	0	0	0	0
ENSG00000114349	GNAT1	0	0	0	0	0	0	0	0
ENSG00000188338	SLC38A3	0	0.0615	0	0	0	0	0	0
ENSG00000114353	GNAI2	68.3466	56.9213	7.05421	13.5947	43.7895	34.4493	15.8435	20.8817
ENSG00000275334	MIR5787	0	0	0	0	0	0	0	0
ENSG00000213600	U73169.1	4.99321	11.3032	0.8805	1.77851	2.42825	0	2.33041	1.96179
ENSG00000230454	U73166.1	0.36301	0.41831	0.08207	0.08288	0.27159	0.09852	0.13963	0.23999
ENSG00000232352	SEMA3B-AS1	0	0	0	0	0	0	0	0
ENSG00000012171	SEMA3B	0.05083	0.12692	0	0	0.0618	0	0	0.04681
ENSG00000283848	MIR6872	0	0	0	0	0	0	0	0
ENSG00000179564	LSMEM2	0	0	0	0	0.1193	0	0.08178	0
ENSG00000214706	IFRD2	1.73772	1.80778	0.87053	1.14295	1.32042	2.0205	0.78996	0.82433
ENSG00000186792	HYAL3	1.55267	0.66113	0.42449	0.37512	0.4829	0.5096	0.30094	0.399
ENSG00000243477	NAA80	1.70855	0.8922	0.24615	0.54691	1.71065	1.53661	0.44673	0.28792
ENSG00000114378	HYAL1	0	0.03896	0	0	0.02845	0	0	0.02873
ENSG00000068001	HYAL2	0.54834	0.77849	0.1185	0.26329	0.29411	0.34142	0.24194	0.13861
ENSG00000114383	TUSC2	4.38705	4.05681	0.89538	1.17557	3.6669	2.52604	2.03126	1.64583
ENSG00000068028	RASSF1	5.53285	2.84896	0.73116	1.68362	3.33915	3.82704	1.9241	2.19922
ENSG00000281358	RASSF1-AS1	1.67013	1.38997	0.13635	0.41311	0.78964	0.16369	0.30932	0.79744
ENSG00000235058	ZMYND10-AS1	0	0	0	0	0	0	0	0.35999
ENSG00000004838	ZMYND10	0.12799	0.12173	0.02687	0.08141	0.08892	0	0.09143	0.0449
ENSG00000114388	NPRL2	1.81407	2.05725	0.26365	0.94673	1.38147	2.91892	0.66457	1.59092
ENSG00000114395	CYB561D2	2.03807	0.87734	0.41309	0.73011	1.7943	1.23981	1.11286	0.69029
ENSG00000272104	Z84492.1	0	0	0	0	0	0	0	0
ENSG00000126062	TMEM115	7.40677	5.22021	1.12764	1.23789	5.19205	6.00355	2.94745	2.74456
ENSG00000007402	CACNA2D2	0.75364	0.77196	0.27261	0.2409	0.28193	0.18409	0.09663	0.42707
ENSG00000271858	CYB561D2	0.54884	0.30451	0	0	0.59312	0.10758	0.40659	0.22462
ENSG00000202322	RNA5SP131	0	0	0	0	0	0	0	0
ENSG00000088543	C3orf18	0.51906	0.22539	0.19898	0.26795	0.439	0.3185	0.07523	0.27708
ENSG00000114735	HEMK1	0.45282	0.27008	0.08872	0.16241	0.39454	0.19971	0.19499	0.28725
ENSG00000280382	AC096920.1	0	0.35991	0.31774	0	0	0.38145	0	0
ENSG00000114737	CISH	2.69571	2.62538	0.5057	1.25555	2.16167	2.42839	0.38241	1.51404
ENSG00000114738	MAPKAPK3	25.5177	17.3942	3.324	5.16045	14.0005	9.2342	5.79585	9.13513
ENSG00000273356	LINC02019	0.17791	0	0.10458	0.15843	0.17304	0.18832	0.17793	0.13106
ENSG00000272543	MIR4787	0	0	0	0	0	0	0	0
ENSG00000088538	DOCK3	0.03349	0.02787	0	0.06213	0.04072	0.07385	0	0.03084
ENSG00000228337	PPIAP69	0	0	0	0	0	0	0	0
ENSG00000213587	AC126120.1	0	0	0	0	0	0	0	0
ENSG00000235278	ZNF652P1	0	0	0	0	0	0	0	0
ENSG00000240959	ST13P14	0	0	0	0	0	0	0	0
ENSG00000145050	MANF	6.42376	4.10355	0.66333	1.80365	5.53022	6.67692	1.09968	2.34478
ENSG00000259956	RBM15B	7.59383	3.72952	0.77854	1.8019	5.42134	6.19201	1.82139	2.20893
ENSG00000145041	DCAF1	3.55318	3.40249	0.5819	1.52481	4.31986	4.51241	1.46281	1.61624
ENSG00000164080	RAD54L2	2.17614	1.85202	0.58717	1.61482	2.55601	2.75454	1.59847	1.60008
ENSG00000221015	RNU6ATAC29P	0	0	0	0	0	0	0	0
ENSG00000164081	TEX264	1.93303	1.30846	0.34087	0.40164	1.39441	2.34162	0.66589	0.5696
ENSG00000201595	RNA5SP132	0	0	0	0	0	0	0	0
ENSG00000164082	GRM2	0.01033	0.03439	0	0	0	0.01822	0	0
ENSG00000214686	IQCF6	0	0	0	0	0	0	0	0
ENSG00000224792	IQCF4	0	0	0	0	0	0	0	0
ENSG00000229972	IQCF3	0	0	0	0	0	0	0	0
ENSG00000184345	IQCF2	0	0	0	0	0	0	0	0
ENSG00000241789	RN7SL504P	0	0	0	0	0	0	0	0

ENSG00000235455	IQCF5-AS1	0	0	0	0	0	0	0	0
ENSG00000214681	IQCF5	0	0	0	0	0	0	0	0
ENSG00000173389	IQCF1	0	0	0	0	0	0	0	0
ENSG00000114767	RRP9	1.16129	0.61855	0.06826	0.68939	1.69423	2.21256	0.38714	0.74142
ENSG00000041880	PARP3	1.22668	0.75005	0.14715	0.22292	0.91308	2.20819	0.25037	0.52252
ENSG00000280422	AC115284.2	0	0	0	0	0	0	0	0
ENSG00000180929	GPR62	0	0	0	0	0	0	0	0
ENSG00000090097	PCBP4	0.47063	0.39168	0.05763	0.20372	0.33377	0.51891	0.19612	0.16853
ENSG00000272762	AC115284.1	0	0	0	0	0	0	0	0
ENSG00000114779	ABHD14B	4.17402	2.89486	1.05635	2.16814	4.45442	5.44083	1.85532	1.87909
ENSG00000248487	ABHD14A	0.58566	0.35915	0.13589	0.04575	0.44971	0.38065	0.20552	0.11354
ENSG00000114786	ABHD14A-ACY1	0	0	0	0	0	0	0	0
ENSG00000243989	ACY1	0.01529	0	0.02247	0.02269	0.01859	0.05395	0	0
ENSG00000162244	RPL29	53.0967	33.5013	8.95738	18.093	56.6301	57.1488	30.2256	29.7009
ENSG00000164086	DUSP7	3.04985	2.18181	0.41957	0.57783	1.97231	1.94609	0.56244	0.49396
ENSG00000164087	POC1A	0.19573	0.38009	0.19175	0.48414	0.27762	1.03588	0.435	0.24031
ENSG00000242849	ALDOAP1	0	0	0	0	0	0	0	0
ENSG00000023330	ALAS1	9.36487	8.51558	2.46349	3.21724	7.8715	9.38224	3.13159	4.71988
ENSG00000280003	AC097637.2	0.11012	0.30548	0.10788	0	0.13388	0	0	0.1352
ENSG00000239732	TLR9	0	0	0	0.02811	0	0	0.03157	0
ENSG00000173366	AC097637.1	0	0	0	0	0	0	0	0
ENSG00000247596	TWF2	12.4578	9.63225	1.94877	2.92242	9.62499	9.35809	3.75115	4.3913
ENSG00000243224	AC006252.1	0.2663	0.11082	0.09783	0.19761	0.32377	0.58725	0.11097	0.08174
ENSG00000164088	PPM1M	14.5328	10.7283	1.35736	3.50332	9.40828	5.43175	2.77138	5.01524
ENSG00000164091	WDR82	23.9001	18.3673	4.32662	9.1383	17.8669	16.7568	10.6049	12.0394
ENSG00000199150	MIRLET7G	1.74525	0	0	0	0	0	0	1.07139
ENSG00000168237	GLYCTK	1.89759	1.3768	0.286	0.86653	1.27183	1.37337	0.72992	0.65713
ENSG00000242797	GLYCTK-AS1	0	0	0	0	0	0	0	0
ENSG00000207926	MIR135A1	0	0	0	0	0	0	0	0
ENSG00000114841	DNAH1	2.10834	1.3105	0.16306	0.49405	1.36836	0.98811	0.75746	0.74608
ENSG00000239557	AC092045.1	0.53591	0.59468	0.0875	0	0.72394	0.21009	0.29776	0.07311
ENSG00000163930	BAP1	6.65576	4.85138	1.00149	1.87228	4.75994	4.42551	2.39285	3.22245
ENSG00000010318	PHF7	6.22628	11.0884	10.1412	25.3952	11.434	11.8673	28.1955	12.6963
ENSG00000010319	SEMA3G	0.11702	0	0.04299	0.04342	0.03557	0.05161	0.02438	0.01796
ENSG00000114854	TNNC1	0	0	0	0	0	0.22142	0	0
ENSG00000010322	NISCH	3.95267	2.06433	0.56438	0.98561	2.64597	2.72429	1.26702	1.54236
ENSG00000010327	STAB1	4.62454	2.18942	0.33572	0.56509	1.5571	1.09917	0.98086	1.20699
ENSG00000168268	NT5DC2	0.29513	0.07756	0	0.04611	0.491	0.43844	0	0.01907
ENSG00000168273	SMIM4	0.62019	0.54073	0.17359	0.13149	0.50267	0.5731	0.29536	0.32634
ENSG00000163939	PBRM1	13.8036	10.5781	4.54108	9.04857	13.0804	13.984	8.39088	9.06123
ENSG00000252768	RNU6-856P	2.24389	1.24498	0	0	0	1.31951	0	0.91834
ENSG00000221518	RNU6ATAC16P	1.06232	0	0	0	0	0	0	0
ENSG00000163938	GNL3	7.35654	4.22875	1.331	3.04915	8.72903	6.35257	4.01376	5.01792
ENSG00000280904	RF01183	0	0	0	0	0	0	0	0
ENSG00000252787	RF00569	0	0	0	0	0	0	0	0
ENSG00000212493	SNORD19	1.07795	0	0	0	2.62108	0	1.79677	0
ENSG00000238862	SNORD19B	0	0	0	0	0	0	0	0
ENSG00000222345	SNORD19C	2.89343	0	1.41729	1.43139	1.17259	1.70147	1.60764	0
ENSG00000212452	SNORD69	0.95195	0	0	0	0	0	0	2.33759
ENSG00000016864	GLT8D1	3.29396	2.16087	0.59794	1.75414	2.75619	2.52949	1.195	2.3552
ENSG00000114902	SPCS1	4.4195	3.62084	1.19367	2.18634	4.05076	3.44896	1.95066	2.72155
ENSG00000114904	NEK4	7.27154	6.41447	5.2753	12.4769	12.5776	11.8568	18.1808	9.87137
ENSG00000275956	AC104446.1	0	0	0	0	0	0	0	0
ENSG00000270941	AC006254.2	0	0	0	0	0	0	0	0
ENSG00000271137	AC006254.3	0	0	0	0	0	0	0	0
ENSG00000055957	ITIH1	0.06452	0	0	0	0	0	0.02689	0
ENSG00000162267	ITIH3	0	0	0	0	0	0	0	0
ENSG00000055955	ITIH4	0.12078	0.02234	0	0.01992	0.08158	0.02367	0.04474	0.06591

ENSG00000243696	AC006254.1	0.05804	0.05796	0.03411	0.05168	0.07056	0	0	0
ENSG00000239799	ITIH4-AS1	0	0	0	0	0	0	0	0
ENSG00000272573	MUSTN1	0	0	0	0.12061	0	0	0	0
ENSG00000248592	TMEM110-MUSTN	0.04676	0.01946	0	0.01735	0	0.02062	0.01948	0.01435
ENSG00000213533	STIMATE	0.11513	0.04259	0.0188	0.11391	0.12442	0.13541	0.02132	0.07853
ENSG00000275789	MIR8064	0.81445	0	0	0	1.98037	0	0	0
ENSG00000279144	AC099667.1	0	0	0	0	0	0	0	0.38378
ENSG00000163935	SFMBT1	1.60135	1.28973	0.75908	2.26158	1.80557	2.41491	2.47546	1.04648
ENSG00000272305	AC096887.1	0.12494	0.06932	0	0	0.05063	0	0.20826	0.05113
ENSG00000280417	AC096887.2	0	0	0	0.05559	0.18215	0.13215	0	0.04599
ENSG00000242142	SERBP1P3	0	0	0.13314	0	0.11016	0	0	0
ENSG00000163933	RFT1	2.45759	1.44507	0.39254	0.93166	1.86742	1.9557	0.95732	1.00033
ENSG00000163932	PRKCD	22.3182	24.7897	2.50571	4.51672	13.0946	8.49132	2.91419	7.89727
ENSG00000163931	TKT	40.0619	29.9022	3.03643	6.0303	29.1619	12.1192	7.73667	10.6213
ENSG00000272886	DCP1A	5.21608	4.58005	1.28336	2.85858	5.19253	6.22604	3.68914	3.49589
ENSG00000274967	RF00019	0	1.08936	0	0	0	0	0	0
ENSG00000157388	CACNA1D	0.27415	0.17186	0.06278	0.09511	0.25539	0.04397	0.02374	0.153
ENSG00000207109	SNORD38C	2.12465	0	0	1.5766	6.45773	1.87408	1.77073	0
ENSG00000212608	RF00568	0	0	0	0	0	0	0	0
ENSG00000227224	AC092035.1	0	0	0	0	0	0	0	0
ENSG00000251987	RF00154	0	0	0	0	0	0	0	0
ENSG00000271916	AC012467.1	0	0	0	0	0	0	0	0
ENSG00000016391	CHDH	0.02835	0.03145	0	0	0	0	0.01575	0
ENSG00000056736	IL17RB	0.16889	0	0	0	0.02933	0	0	0
ENSG00000271976	AC012467.2	1.36214	0.28341	0.0834	0.33693	0.41401	1.10138	0.42572	0.52263
ENSG00000113812	ACTR8	4.6331	4.35102	1.19211	3.61192	4.66843	5.21572	2.1936	2.87743
ENSG00000113811	SELENOK	21.1268	30.2063	59.1556	61.3521	23.833	14.9321	25.1697	36.6224
ENSG00000242983	CABYRP1	0	0	0	0	0	0	0	0
ENSG00000157445	CACNA2D3	0.81619	1.01891	0.34598	0.20965	0.62973	0.13845	0.10465	0.32761
ENSG00000229806	RPS15P5	0	0	0.24934	0	0	0	0	0.20833
ENSG00000265992	ESRG	0.07003	0	0.0343	0	0.25543	0.12355	0.15564	0
ENSG00000243715	CACNA2D3-AS1	0	0	0	0	0	0	0	0
ENSG00000144771	LRTM1	0	0	0	0.06868	0	0	0	0
ENSG00000239991	AC092059.1	0	0	0	0	0	0	0	0
ENSG00000243572	LINC02017	0	0	0	0	0	0	0	0
ENSG00000240708	LINC02030	0	0	0	0	0	0	0	0
ENSG00000242568	AC121764.2	0	0	0	0	0	0	0	0
ENSG00000242775	AC121764.3	0	0	0	0	0	0	0	0
ENSG00000114251	WNT5A	0.01982	0	0	0	0.01205	0	0	0
ENSG00000244586	WNT5A-AS1	0	0	0	0	0	0	0	0
ENSG00000242317	AC121764.1	0	0	0	0	0.15829	0	0	0
ENSG00000187672	ERC2	0.07392	0.01538	0	0.02743	0.05617	0.0163	0.0462	0.04538
ENSG00000240777	AC025572.1	0	0	0	0	0	0	0	0
ENSG00000281708	ERC2-IT1	0	0	0	0	0	0	0	0
ENSG00000264013	MIR3938	0	0	0	0	0	0	0	0
ENSG00000200379	RN7SKP45	0	0	0	0	0	0	0	0
ENSG00000200238	RNA5SP133	0	0	0	0	0	0	0	0
ENSG00000180376	CCDC66	24.908	31.5719	23.9442	63.5317	46.2653	32.1361	54.5309	42.6726
ENSG00000163946	FAM208A	27.0922	24.7768	20.1415	38.2035	27.2412	21.666	25.3216	28.3439
ENSG00000163947	ARHGEF3	13.9064	11.8419	3.96944	7.37446	10.8929	26.4742	5.13254	7.61577
ENSG00000240198	ARHGEF3-AS1	0	0	0	0	0	0	0	0
ENSG00000186451	SPATA12	0	0.05056	0	0	0	0	0	0
ENSG00000272202	AC097358.2	0	0	0	0	0	0	0	0
ENSG00000144730	IL17RD	0	0	0	0	0	0	0	0
ENSG00000229863	AC097358.1	0	0	0	0	0	0	0	0
ENSG00000163666	HESX1	0.1762	0.39105	0.17262	0	0	0	0	0.07211
ENSG00000157500	APPL1	12.9654	9.68018	3.24689	6.48105	11.4801	8.47615	4.7253	6.51337
ENSG00000239388	ASB14	0	0	0	0.03366	0	0	0	0

ENSG00000174844	DNAH12	0.07178	0.03676	0.08114	0.08195	0.16111	0.09741	0.13806	0.04746
ENSG00000212392	RF00019	0	0	0	0	0	0	0	0
ENSG00000206918	RNU6-1181P	0	0	0	0	0	0	0	0
ENSG00000239831	RNF7P1	0	0	0	0	0	0	0	0
ENSG00000237186	AC092418.1	0	0	0	0	0	0	0	0
ENSG00000202428	RNU6-108P	0	0	0	0	0	0	0	0
ENSG00000206815	RNU6-483P	0	0	0	0	0	0	0	0
ENSG00000174840	PDE12	2.89203	2.01825	0.99925	1.79952	2.66942	3.07851	1.32463	2.11237
ENSG00000168374	ARF4	25.5916	21.5807	8.96579	17.6908	16.9305	18.0887	24.0594	14.7394
ENSG00000272146	ARF4-AS1	0.61391	0.40874	0.90213	1.27554	0.74637	1.08301	3.06986	0.75374
ENSG00000210841	RNU6ATAC26P	0	0	0	0	0	0	0	0
ENSG00000174839	DENND6A	14.3339	11.5484	8.55573	17.027	14.1919	13.1987	17.0013	12.2244
ENSG00000239801	DENND6A-AS1	0	0.33519	0	0.29886	0	0.35525	0.33566	0.24724
ENSG00000241933	DENND6A-DT	0.10045	0.0418	0.0369	0	0.03053	0.0443	0	0
ENSG00000163681	SLMAP	7.9378	7.03746	2.55827	5.59808	8.05685	6.57208	5.5878	6.25978
ENSG00000270772	PDHA1P1	0	0	0	0	0	0	0	0
ENSG00000213509	PPIAP16	0.30103	0	0	0	0	0	0.25088	0
ENSG00000136068	FLNB	5.6005	1.8714	0.57902	1.57502	5.08435	2.36343	1.41866	1.3159
ENSG00000244161	FLNB-AS1	0	0	0	0	0	0	0	0
ENSG00000283148	AC137936.1	0	0	0	0	0	0	0	0
ENSG00000283511	AC137936.2	0	0	0	0	0	0	0	0
ENSG00000163687	DNASE1L3	0.58212	0.19379	0.06843	0.20734	0.16985	0	0.19406	0.11435
ENSG00000163686	ABHD6	1.57262	1.05385	0.2401	0.75777	1.06771	0.64854	0.27234	0.42629
ENSG00000163684	RPP14	0.96287	0.69936	0.20581	0.41571	0.91522	0.54047	0.52526	0.51587
ENSG00000255154	HTD2	0.03459	0.05758	0	0	0.08411	0	0.05766	0.04247
ENSG00000273493	AC098479.1	0	0	0	0	0	0	0	0
ENSG00000168297	PXK	9.09067	8.61397	2.13255	4.9171	8.88836	5.6275	3.97075	5.56388
ENSG00000275607	AC135507.2	0	0	0	0	0	0	0	0
ENSG00000168291	PDHB	6.41786	4.3713	1.34728	2.69831	5.08213	4.66038	2.87514	3.14808
ENSG00000272182	AC135507.1	2.35188	0.65245	0.192	0.38783	2.3828	0.69151	0.65337	1.28338
ENSG00000272360	AC116036.2	0.15866	0	0	0	0.19289	0	0	0
ENSG00000168301	KCTD6	1.57687	1.27258	0.24576	0.60279	0.95856	0.80082	0.59736	1.23203
ENSG00000168306	ACOX2	0.05698	0.03162	0	0	0.02309	0	0	0.02332
ENSG00000236722	AC116036.1	0	0	0	0	0	0	0	0
ENSG00000168309	FAM107A	0	0	0	0	0	0	0	0
ENSG00000243384	AC119424.1	0	0	0	0	0	0	0	0
ENSG00000244383	FAM3D-AS1	0	0	0	0	0	0	0	0
ENSG00000198643	FAM3D	0.08939	0.0744	0	0.06633	0.05434	0	0	0.05488
ENSG00000163689	C3orf67	0.03876	0.0129	0.03418	0.04602	0.05655	0	0.06461	0.00952
ENSG00000242428	C3orf67-AS1	0	0	0	0	0	0	0	0
ENSG00000243295	AC114400.1	0	0	0	0	0	0	0	0
ENSG00000243903	AC138057.1	0	0	0	0	0	0	0	0
ENSG00000242545	AC126121.2	0	0	0	0	0	0	0	0
ENSG00000241804	AC126121.1	0	0	0	0	0	0	0	0
ENSG00000277592	RF00017	0	0	0	0	0	0	0	0
ENSG00000189283	FHIT	0.68732	0.49351	0.75255	1.40007	1.11416	1.0461	1.14565	0.86042
ENSG00000240097	AC104164.2	0	0	0	0	0	0	0	0
ENSG00000281426	MIR548BB	0	0	0	0	0	0	0	0
ENSG00000232407	PPIAP70	0.14808	0	0	0	0	0	0.49366	0.18181
ENSG00000225673	AC104164.1	0	0.20679	0	0.18438	0.30209	0	0	0
ENSG00000244183	PPIAP71	0.14868	0	0	0	0	0	0	0
ENSG00000212211	RF00012	0	0	0	0	0	0	0	0
ENSG00000144724	PTPRG	0.00501	0.03336	0	0.03718	0.03046	0	0.00835	0.03691
ENSG00000252420	RF00019	0	0	0	0	0	0	0	0
ENSG00000226360	RPL10AP6	0.11242	0.18713	0	0	0.27336	0	0.56218	0.13803
ENSG00000252184	RNU2-10P	0	0	0	0	0	0	0	0
ENSG00000237456	AC104849.1	0	0	0	0.26861	0	0	0	0
ENSG00000241472	PTPRG-AS1	0	0	0	0	0	0	0	0

ENSG00000114405	C3orf14	0.16546	0.19279	0.09726	0.07367	0.20117	0.2919	0.11032	0.10158
ENSG00000153266	FEZF2	0	0	0	0	0.04064	0.05897	0	0
ENSG00000163618	CADPS	0.00501	0	0	0	0	0	0	0
ENSG00000225662	AC012519.1	0	0	0	0	0	0	0	0
ENSG00000244632	RN7SL863P	0	0	0	0	0	0	0	0
ENSG00000252449	RNU6-139P	0	0	0	0	0	0	0	0
ENSG00000226082	AC096915.1	0	0	0	0	0	0	0	0
ENSG00000244342	LINC00698	0	0	0	0	0	0	0	0
ENSG00000242841	KRT8P35	0	0	0	0	0	0	0	0
ENSG00000240359	UBL5P3	0	0	0	0	0	0	0	0
ENSG00000163630	SYNPR	0	0	0	0	0	0	0	0
ENSG00000241359	SYNPR-AS1	0	0	0	0	0	0	0	0
ENSG00000252301	RNA5SP134	0	1.27092	0	0	0	0	0	1.87494
ENSG00000188817	SNTN	0	0	0	0	0	0	0	0
ENSG00000224479	AC104162.1	0	0	0.02987	0	0	0	0.03388	0
ENSG00000163632	C3orf49	0	0	0	0	0	0	0	0
ENSG00000163634	THOC7	15.9009	15.2511	9.2378	13.4763	13.2376	17.3962	15.615	15.3358
ENSG00000240549	THOC7-AS1	0	0	0	0	0	0	0	0
ENSG00000163635	ATXN7	11.4042	14.6059	2.23132	4.76797	8.64739	9.14995	6.11434	7.46705
ENSG00000280620	SCAANT1	0.28745	2.39232	0	0	0	2.02842	0.47914	0.70586
ENSG00000239653	PSMD6-AS2	4.80511	5.61358	1.14029	1.63887	3.33825	3.00113	2.93511	3.40787
ENSG00000271843	AC012557.1	0.23994	0	0.35258	0.35609	0	0.21164	0.79987	0.58918
ENSG00000163636	PSMD6	4.85294	3.98537	1.15707	1.96752	3.37985	4.3515	2.41066	2.90027
ENSG00000243410	PSMD6-AS1	0.49864	2.21331	0	0.24668	0.80831	0	0.83116	0.40815
ENSG00000272181	AC012557.2	0.8768	2.0432	0	0	1.27919	0	0.2923	0.43061
ENSG00000241111	PRICKLE2-AS1	0	0	0	0	0	0	0	0
ENSG00000200222	RF00012	0	0	0	0	0	0	0	0
ENSG00000189196	LINC00994	0	0	0	0	0	0	0	0.04227
ENSG00000163637	PRICKLE2	0.02982	0.00827	0	0.00738	0.01209	0.00877	0.02486	0.01831
ENSG00000241572	PRICKLE2-AS1	0	0	0	0	0	0	0	0
ENSG00000241101	PRICKLE2-AS2	0	0	0	0	0	0	0	0
ENSG00000239926	PRDX3P4	0	0	0	0	0	0	0	0
ENSG00000226017	PRICKLE2-AS3	0	0	0	0	0	0	0	0
ENSG00000244564	AC096888.1	0	0	0	0	0	0	0	0
ENSG00000212340	RNU6-739P	0	0	0	0	0	0	0	0
ENSG00000163638	ADAMTS9	0.00686	0	0	0	0	0	0	0
ENSG00000241158	ADAMTS9-AS1	0	0	0	0	0	0	0	0
ENSG00000241684	ADAMTS9-AS2	0	0	0	0	0	0	0	0
ENSG00000243149	LINC02040	0	0	0	0	0	0	0	0
ENSG00000151276	MAGI1	0.00629	0	0	0.00934	0.00765	0	0	0.00773
ENSG00000270059	AC121493.1	0	0	0	0	0	0	0	0
ENSG00000244226	ILF2P1	0	0	0	0	0	0	0	0
ENSG00000223409	RPL17P17	0	0	0	0	0	0	0	0
ENSG00000272610	MAGI1-IT1	0	0	0	0	0	0	0	0
ENSG00000240175	MAGI1-AS1	0	0	0	0	0	0	0	0
ENSG00000202071	RF00019	0	0	0	0	0	0	0	0
ENSG00000203647	AC106827.1	0	0	0	0	0	0	0	0
ENSG00000144741	SLC25A26	0.66409	1.23051	1.41777	3.51462	1.03589	1.83468	1.90058	1.67687
ENSG00000206759	RNU6-787P	0	0	0	0	0	0	0	0
ENSG00000241587	RN7SL482P	0	0	0	0	0	0	0	0
ENSG00000144749	LRIG1	1.28213	0.60727	0.15318	0.49504	1.66017	1.91246	0.39962	0.47353
ENSG00000271174	AC104440.1	0	0	0	0	0	0	0	0
ENSG00000214560	RPL21P41	0	0	0	0.41207	0	0	0	0
ENSG00000163376	KBTBD8	2.07395	1.75181	0.86426	2.15918	3.36822	2.7031	2.27024	1.86227
ENSG00000283298	MIR4272	0	0	0	0	0	0	0	0
ENSG00000241884	AC114401.1	0	0	0	0	0	0	0	0
ENSG00000172340	SUCLG2	9.74966	8.60772	4.60137	11.7235	15.3719	16.028	11.0318	8.6502
ENSG00000242510	NDUFB4P1	0	0	0	0	0	0	0	0

ENSG00000241316	SUCLG2-AS1	0.31428	0.04024	0.07105	0.50231	0.3821	0.21325	0.16119	0.32651
ENSG00000183662	FAM19A1	0.04998	0.0416	0.03672	0	0.06077	0.08818	0.08331	0.18411
ENSG00000214552	COPS8P2	0	0.19459	0	0	0	0	0.19486	0.28707
ENSG00000240568	AC107626.1	0	0	0	0.59446	0	0	0	0
ENSG00000244640	AC096922.1	0	0	0	0	0	0	0	0
ENSG00000241506	PSMC1P1	0.11106	0.27729	0.0816	0	0.06751	0.09796	0	0
ENSG00000163377	FAM19A4	0	0	0	0	0	0	0.03706	0
ENSG00000252828	RNA5SP135	0	0	0	0	0	0	0	0
ENSG00000163378	EOGT	1.80492	1.34004	0.95407	2.11983	1.4366	2.22201	1.19042	0.74931
ENSG00000244513	AC109587.1	0	0	0.02875	0.05808	0	0.06904	0.03262	0
ENSG00000144747	TMF1	18.2963	17.7984	5.24796	9.13931	16.5144	12.3972	9.39177	11.9842
ENSG00000265355	MIR3136	1.8795	1.56421	0	1.39469	5.71261	0	1.56641	3.46143
ENSG00000144744	UBA3	17.8877	16.0459	4.85316	7.55088	14.889	14.172	6.93327	11.4853
ENSG00000144746	ARL6IP5	92.4247	68.3577	25.2842	40.3309	61.4488	64.6559	32.4102	52.9206
ENSG00000163380	LMOD3	0	0	0	0	0	0	0	0
ENSG00000114541	FRMD4B	3.57223	3.21107	0.94855	2.11192	2.49701	2.04456	1.89512	1.81921
ENSG00000239263	RBM43P1	0	0	0	0	0.08244	0	0	0
ENSG00000240265	AC099328.1	0	0	0	0	0	0	0	0
ENSG00000240842	AC099328.2	0	0	0	0	0	0	0	0
ENSG00000241667	AC104445.1	0	0	0	0	0	0	0	0
ENSG00000187098	MITF	1.48781	0.78026	0.14975	0.27223	0.40885	0.84494	0.47561	0.28777
ENSG00000241665	RN7SL418P	0	0	0	0	0	0	0	0
ENSG00000240405	SAMMSON	0	0	0	0	0	0	0	0
ENSG00000271034	AC139700.1	0	0	0	0	0	0	0.07934	0
ENSG00000242728	UQCRHP4	0	0	0	0	0	0	0	0
ENSG00000242120	MDFIC2	0	0	0	0	0	0	0	0
ENSG00000240156	COX6CP6	0	0	0	0	0	0	0	0
ENSG00000243873	HMGB1P36	0	0	0	0	0	0	0	0
ENSG00000206939	RNU6-281P	0	0	0	0	0	0	0	0
ENSG00000114861	FOXP1	9.71386	7.34128	2.11824	4.64582	11.4894	9.60255	7.2261	5.48118
ENSG00000277539		1.14277	1.08694	0	0.72685	1.19087	0.576	1.90482	0.80176
ENSG00000244203	FOXP1-AS1	0.10471	0	0	0	0.12731	0	0	0
ENSG00000221264	MIR1284	0	0	0.89762	0	1.48528	0	0	0
ENSG00000274387	AC097634.2	0	0	0	0	0	0	0	0
ENSG00000242094	FOXP1-IT1	0.53852	0.89636	0.27393	0.30739	0.65471	0.47501	0.31072	0.48317
ENSG00000277855	AC097634.3	0.62573	0.59516	0	0	0	0	0	0
ENSG00000270562	AC097634.1	0.77252	0.54402	0.17465	0.13229	0.46961	0.10483	0.44573	0.29184
ENSG00000163412	EIF4E3	13.1518	19.899	5.49612	6.78208	9.49949	12.0206	4.00127	10.7121
ENSG00000170837	GPR27	11.9521	15.4069	1.93683	2.00055	8.84976	1.58535	1.54785	6.21557
ENSG00000163421	PROK2	46.2447	101.119	19.3778	15.9939	51.8557	3.04829	10.8386	56.3319
ENSG00000239250	RN7SL271P	0	0	0	0	0	0	0	0
ENSG00000244130	UBE2Q2P9	0	0	0	0	0	0	0	0
ENSG00000241163	LINC00877	2.94524	5.45261	0.37539	0.60213	1.73556	1.32546	0.60113	1.23612
ENSG00000273461	AC105265.3	0	0	0	0	0	0	0	0
ENSG00000276471	AC105265.4	0.47909	0.39872	0	0	0.87369	0	0.39928	0.88232
ENSG00000239350	AC105265.1	0	0	0	0	0	0	0	0
ENSG00000242609	AC105265.2	0.08747	0.14559	0	0	0	0	0	0
ENSG00000243083	LINC00870	0	0	0	0	0	0	0	0
ENSG00000272774	AC112219.2	0	0	0	0	0	0	0	0
ENSG00000242618	AC112219.1	0	0	0	0	0	0	0	0
ENSG00000260670	AC134508.1	0	0	0	0	0	0	0	0
ENSG00000163602	RYBP	3.97234	3.46663	0.44788	0.6785	2.76675	1.64888	1.21926	1.04778
ENSG00000244345	AC104435.2	0	0	0	0	0	0	0	0
ENSG00000199469	RNU1-62P	0	0	0	0	0	0	0	0
ENSG00000239568	AC104435.1	0	0	0	0	0	0	0	0
ENSG00000222838	RNA5SP136	0	0	0	0	0	0	0	0
ENSG00000144736	SHQ1	1.782	1.50396	1.0327	2.55156	1.60199	2.45739	1.92443	1.41752
ENSG00000241537	AC134050.1	0	0	0	0	0	0	0	0

ENSG00000240135	PSMD12P	0	0	0	0	0	0	0.08944	0
ENSG00000271266	LAPTM4BP2	0	0	0	0	0	0	0	0
ENSG00000172986	GXYLT2	0.01999	0	0	0	0	0	0	0
ENSG00000276890	RF00017	0	0	0	0	0	0	0	0
ENSG00000242960	FTH1P23	0.13327	0.22183	0.19584	0.19779	0	0.23511	0.44429	0.16363
ENSG00000163605	PPP4R2	20.3161	25.3264	7.69385	13.6438	17.0344	13.3128	11.6772	17.7912
ENSG00000238959	RNU7-19P	0	0	0	0	0	0	0	0
ENSG00000255423	EBLN2	2.00823	2.32536	0.12831	0.58313	1.80463	1.23227	1.16432	1.07203
ENSG00000252651	RNU6-557P	0	0	0	0	0	0	0	0
ENSG00000223247	RNU2-64P	0	0	2.25579	0	0	0	0	0
ENSG00000182921	CCDC75P1	0	0	0	0	0	0	0	0
ENSG00000252937	RNU6-1270P	0	0	0	0	0	0	0	0
ENSG00000121440	PDZRN3	0.01195	0	0	0	0	0	0	0
ENSG00000239119	RNU7-119P	0	0	0	0	0	0	0	0
ENSG00000239677	PDZRN3-AS1	0	0	0	0	0	0	0	0
ENSG00000278934	AC117489.1	0	0	0	0	0	0	0	0
ENSG00000242364	AC108725.1	0	0	0	0	0	0	0	0.22004
ENSG00000242741	LINC02005	0	0	0	0	0	0	0	0
ENSG00000278554	RF00017	0	0	0	0	0	0	0	0
ENSG00000239454	LINC02047	0	0	0	0	0	0	0	0
ENSG00000223738	AC016930.1	0	0	0	0	0	0	0	0
ENSG00000213452	AKR1B1P2	0	0	0	0	0	0	0	0
ENSG00000113805	CNTN3	0	0	0	0	0	0	0	0
ENSG00000241022	NIPA2P2	0	0	0	0	0	0	0	0
ENSG00000242638	RN7SL294P	0	0	0	0	0	0	0	0
ENSG00000266780	MIR4444-2	0	0	0	0	0	0	0	0
ENSG00000213300	HNRNPA3P6	0.14573	0.24256	0	0.10814	0.44293	0.12854	0.2429	0.08946
ENSG00000228868	MYLKP1	0	0	0	0	0	0	0	0
ENSG00000243674	OR7E66P	0	0	0	0	0	0	0	0
ENSG00000179799	OR7E22P	0	0	0	0	0	0	0	0
ENSG00000230655	OR7E55P	0	0	0	0	0	0	0	0
ENSG00000244699	AC139453.2	0	0	0	0	0	0	0	0
ENSG00000240665	LSP1P2	0	0	0	0	0	0	0	0
ENSG00000242953	AC139453.1	0	0	0	0	0	0	0	0
ENSG00000238278	ALG1L6P	0	0	0	0	0	0	0	0
ENSG00000244026	FAM86DP	0.30323	0.20189	0.02971	0.03	0.5407	0.49927	0.30326	0.1241
ENSG00000272690	LINC02018	0.19683	0.06553	0.1157	0	0.14358	0	0	0.09667
ENSG00000239959	ENPP7P2	0	0	0	0	0	0	0	0
ENSG00000243794	SNRPCP10	0	0	0	0	0	0	0	0
ENSG00000272710	AC133041.1	0	0	0	0	0	0	0	0
ENSG00000244674	RPS3AP15	0	0	0	0	0	0	0	0
ENSG00000239383	AC108724.1	0	0	0	0	0	0	0	0
ENSG00000244222	OR7E121P	0	0	0	0	0	0	0	0
ENSG00000172971	UNC93B3	0	0	0	0	0	0	0	0
ENSG00000243422	RPL23AP49	0	0	0	0.09559	0.07831	0	0.32209	0
ENSG00000282912	AC108724.2	0	0	0	0	0	0	0	0
ENSG00000282511	CLUHP10	0	0	0	0	0	0	0	0
ENSG00000283894	MIR1324	0	0	0	0	0	0	0	0
ENSG00000241532	AGGF1P3	0	0.30175	0	0	0	0	0	0
ENSG00000241622	RARRES2P1	0	0	0	0	0	0	0	0
ENSG00000172969	FRG2C	0	0	0	0	0	0	0	0
ENSG00000236138	DUX4L26	0	0	0	0	0	0	0	0
ENSG00000242516	LINC00960	0	0	0	0	0	0	0	0
ENSG00000227124	ZNF717	1.05614	0.30187	0.21947	0.53831	0.73929	0.63988	0.4979	0.39294
ENSG00000266396	MIR4273	0	0	0	0	0	0	0	0
ENSG00000185008	ROBO2	0	0	0	0	0	0	0	0
ENSG00000222883	RF00019	0	0	0	0	0	0	0	0
ENSG00000240809	AC026877.1	0.36364	0.25941	0	0.1542	0.31579	0.36658	0.08659	0.25513

ENSG00000199520	RNU6-386P	0	0	0	0	0	0	0	0
ENSG00000213450	VDAC1P7	0	0	0	0	0	0	0	0
ENSG00000206891	RNU6-217P	0	0	0	0	0	0	0	0
ENSG00000244461	LINC02077	0	0	0	0	0	0	0	0
ENSG00000240573	AC117462.1	0	0	0	0	0	0	0	0
ENSG00000243957	RN7SL647P	0	0	0	0	0	0	0	0
ENSG00000222574	RN7SKP61	0	0	0	0	0	0	0	0
ENSG00000240241	AC108752.1	0	0	0	0	0	0	0	0
ENSG00000239226	MRPS17P3	0	0	0	0	0	0	0	0
ENSG00000169855	ROBO1	0.04044	0	0.01981	0.01	0.13931	0.04756	0.08988	0.01655
ENSG00000240752	AC055731.1	0	0	0	0	0	0	0	0
ENSG00000240964	RN7SL751P	0	0	0	0	0	0	0	0
ENSG00000265193	MIR3923	0	0	0	0	0	0	0	0
ENSG00000243877	HMGB1P38	0	0	0	0	0	0	0	0
ENSG00000271389	OSBPL9P1	0	0	0	0	0	0	0	0
ENSG00000239699	HNRNPA3P8	0	0	0	0	0	0	0	0
ENSG00000242586	AC108740.1	0	0	0	0	0	0	0	0
ENSG00000242828	AC068756.1	0.18189	0.30275	0	0.26994	0	0	0	0
ENSG00000242781	LINC02050	0	0	0	0.15279	0	0	0	0
ENSG00000243694	LINC02027	0	0	0	0	0	0	0	0
ENSG00000242145	AC099542.2	0	0	0.19478	0	0	0	0	0
ENSG00000241593	AC099542.1	0.05813	0	0	0	0.07067	0	0	0
ENSG00000244281	AC107302.1	0	0	0	0	0	0.1253	0	0
ENSG00000114480	GBE1	5.51418	4.83693	0.62491	2.1248	3.61913	1.95055	1.48856	2.78467
ENSG00000222389	RNU2-28P	0	0	0	0	0	0	0	0
ENSG00000241319	SETP6	0	0	0	0	0	0	0	0.15845
ENSG00000240404	AC017015.1	0	0	0	0	0	0	0	0
ENSG00000242190	AC017015.2	0	0	0	0	0	0	0	0
ENSG00000242009	AC129807.1	0	0	0	0.18407	0	0	0	0
ENSG00000239440	LINC02008	0	0	0	0	0.04418	0	0	0
ENSG00000241367	RPL7AP23	0	0	0	0	0	0	0	0
ENSG00000241095	CYP51A1P1	0	0	0	0	0	0	0	0
ENSG00000242195	SRRM1P2	0	0	0	0	0	0	0	0
ENSG00000242641	LINC00971	0	0	0	0	0	0	0	0
ENSG00000241776	AC117482.1	0	0	0	0	0	0	0	0
ENSG00000242339	LINC02025	0	0	0	0	0	0	0	0
ENSG00000175161	CADM2	0.13941	0.06107	0	0.09801	0.08029	0.16827	0.04892	0
ENSG00000264084	MIR5688	0	0	0	0	0	0	0	0
ENSG00000241648	CADM2-AS2	0	0	0	0	0	0	0	0
ENSG00000239519	CADM2-AS1	0	0	0	0	0	0	0	0
ENSG00000243915	THAP12P2	0	0	0	0.04799	0	0	0	0
ENSG00000201410	RF00342	0	0	0	0	0	0	0	0
ENSG00000251773	RNU6-1129P	0	0	0	0	0	0	0	0
ENSG00000239767	AC108733.1	0	0	0	0	0	0	0	0
ENSG00000222934	RN7SKP284	0	0	0	0	0	0	0	0
ENSG00000241328	LINC02070	0	0	0	0	0	0	0	0
ENSG00000277746	AC108706.1	0	0	0	0	0	0	0	0
ENSG00000206538	VGLL3	0	0	0	0	0.00701	0	0	0
ENSG00000241293	PPATP1	0	0	0	0	0	0	0	0
ENSG00000281392	LINC00506	0.01463	0.02435	0	0	0	0.02581	0	0
ENSG00000264119	MIR4795	0	0	0	0	0	0	0	0
ENSG00000083937	CHMP2B	20.862	17.716	5.8608	7.14497	14.2311	10.2417	7.67069	12.2855
ENSG00000064835	POU1F1	0.04672	0	0	0.06933	0	0	0	0
ENSG00000239829	KRT8P25	0	0	0	0	0	0	0	0
ENSG00000240902	APOOP2	0	0	0	0	0	0	0	0
ENSG00000240255	PSMC1P6	0	0	0	0	0	0	0	0
ENSG00000239572	AC108749.1	0	0	0	0	0	0	0	0
ENSG00000200703	RNU6-873P	0	0	0	0	0	0	0	0

ENSG00000221059	RNU6ATAC6P	0	0	0	0	0	0	0	0
ENSG00000179097	HTR1F	0.07003	0	0	0	0	0	0	0.02866
ENSG00000163320	CGGBP1	26.8241	17.5963	5.72593	12.1357	20.446	22.1364	9.95892	13.6174
ENSG00000175105	ZNF654	4.48271	4.71061	1.36508	3.12603	5.72574	4.70675	2.59269	3.66036
ENSG00000241535	CBX5P1	0	0	0	0	0	0	0	0
ENSG00000179021	C3orf38	10.7063	9.89258	2.91119	5.06707	8.55807	11.0796	6.04227	6.02913
ENSG00000242159	ABCF2P1	0	0	0.05769	0	0	0	0	0
ENSG00000283434	CSNKA2IP	0	0	0	0	0	0	0	0
ENSG00000207316	RF00019	0	0	0	0	0	0	0	0
ENSG00000242365	NDUFA5P5	0	0	0	0	0	0	0	0
ENSG00000242705	ICE2P2	0	0.04378	0.11595	0.07807	0	0	0.08768	0
ENSG00000241861	GAPDHP50	0	0	0	0	0	0	0	0
ENSG00000044524	EPHA3	0	0	0	0	0	0	0	0
ENSG00000240951	MTCO2P6	0	0	0	0	0	0	0	0
ENSG00000240309	MTCO1P6	0	0	0	0	0	0	0	0
ENSG00000212598	RF00012	0	0	0	0	0	0	0	0
ENSG00000222490	RNU6-712P	0	0	0	0	0	0	0	0
ENSG00000189002	PROS2P	0	0	0	0	0	0	0	0
ENSG00000271024	HSPE1P19	0	0	0	0	0	0	0	0
ENSG00000283367	RF01699	0	0	0	0	0	0	0	0
ENSG00000283544	ABBA01000935.2	0	0.29119	0	0.7789	0.42538	0.61724	0.2916	0.42958
ENSG00000251727	RNU6-488P	0	0	0	0	0	0	0	0
ENSG00000184500	PROS1	6.23938	6.83181	1.21601	1.69479	4.8492	2.94886	3.22761	6.66494
ENSG00000201339	RF00019	0	0	0	0	0	0	0	0
ENSG00000200366	RNU6-511P	0	0	0	0	0	0	0	0
ENSG00000169379	ARL13B	5.01139	5.96238	6.48254	14.0631	8.79582	6.31928	13.7651	6.75953
ENSG00000239614	HMGN1P7	0.23419	0	0	0	0	0	0	0
ENSG00000178750	STX19	0	0	0	0.18789	0.15391	0	0	0
ENSG00000178700	DHFR2	1.24291	1.30938	0.20808	0.60709	1.37721	2.13716	0.55072	0.92721
ENSG00000178694	NSUN3	21.8145	15.611	11.1441	33.0122	15.4353	9.40867	103.772	16.8176
ENSG00000242457	RBBP4P2	0	0.19305	0	0	0.0705	0.1023	0	0
ENSG00000178660	ARMC10P1	0.08573	0	0	0.12723	0.10423	0	0.2858	0.10526
ENSG00000242911	AC140059.1	0	0	0	0	0	0	0	0
ENSG00000234629	WDR82P1	0	0	0	0	0	0	0	0
ENSG00000239589	LINC00879	0	0	0	0	0	0	0	0
ENSG00000244048	RPS18P6	0	0	0	0	0	0	0	0
ENSG00000244681	MTHFD2P1	0	0	0	0	0	0	0	0
ENSG00000271671	AC107304.1	0	0	0	0	0	0	0	0
ENSG00000243547	HNRNPKP4	0	0.08803	0.31086	0.07849	0	0.0933	0.08815	0.12987
ENSG00000278103	MIR8060	0	0	0	0	0	0	0	0
ENSG00000241815	AC107015.1	0	0	0	0	0	0	0	0
ENSG00000199286	RNU6-1094P	0	0	0	0	0	0	0	0
ENSG00000269028	MTRNR2L12	0.27951	0.34893	0.30805	0.62223	0.25486	0.49309	0.34942	0.68635
ENSG00000218754	RPL18AP8	0	0	0	0	0	0	0	0
ENSG00000241018	RCC2P5	0	0	0	0	0	0	0	0
ENSG00000176243	CDV3P1	0	0	0	0	0	0	0	0
ENSG00000080224	EPHA6	0	0	0	0	0	0	0	0
ENSG00000251172	AC117470.1	0	0	0	0	0	0	0	0
ENSG00000261292	AC110491.1	0	0	0	0	0	0	0	0
ENSG00000113966	ARL6	0.51281	0.90693	0.21194	0.5708	0.72088	0.53715	0.48081	0.51157
ENSG00000282527	AC110491.3	0	0	0	0	0	0	0	0
ENSG00000080200	CRYBG3	4.66134	3.39378	0.69976	1.44331	2.91103	1.38434	0.67077	2.10808
ENSG00000279658	AC110491.2	0	0	0	0	0	0	0	0
ENSG00000170854	RIOX2	2.52503	1.33003	0.37575	0.86965	2.5906	1.74796	0.78139	1.34734
ENSG00000183185	GABRR3	0	0	0	0	0	0	0	0
ENSG00000250750	OR5BM1P	0	0	0	0	0	0	0	0
ENSG00000213439	OR5AC1	0	0	0	0	0	0	0	0
ENSG00000196578	OR5AC2	0	0	0	0	0	0	0	0

ENSG00000249225	AC117460.1	0	0	0	0	0	0	0	0
ENSG00000251090	OR5AC4P	0	0	0	0	0	0	0	0
ENSG00000248365	POU5F1P7	0	0	0	0	0	0	0	0
ENSG00000231192	OR5H1	0	0	0	0	0	0	0	0
ENSG00000236032	OR5H14	0	0	0	0	0	0	0	0
ENSG00000233412	OR5H15	0	0	0	0	0	0	0	0
ENSG00000249321	OR5H5P	0	0	0	0	0	0	0	0
ENSG00000249975	OR5H3P	0	0	0	0	0	0	0	0
ENSG00000248507	OR5H4P	0	0	0	0	0	0	0	0
ENSG00000251088	AC117473.1	0	0	0	0	0	0	0	0
ENSG00000187900	OR5H7P	0	0	0	0	0	0	0	0
ENSG00000230301	OR5H6	0	0	0	0	0	0	0	0
ENSG00000197938	OR5H2	0	0	0	0	0	0	0	0
ENSG00000232535	OR5H8	0	0	0	0	0	0	0	0
ENSG00000196098	OR5K4	0	0	0	0	0	0	0	0
ENSG00000206536	OR5K3	0	0	0	0	0	0	0	0
ENSG00000244653	UBFD1P1	0	0	0	0	0	0	0	0
ENSG00000232382	OR5K1	0	0	0	0	0	0	0	0
ENSG00000231861	OR5K2	0	0	0	0	0	0	0	0
ENSG00000080822	CLDND1	8.73928	6.85231	3.28283	8.98728	8.26768	9.56763	4.02818	5.24422
ENSG00000080819	CPOX	3.27912	2.39624	3.70212	9.73315	3.33033	3.73897	5.33247	3.63327
ENSG00000248839	AC021660.2	0	0	0	0	0	0	0	0
ENSG00000250562	RPL38P4	0	0	0	0	0	0	0	0
ENSG00000154165	GPR15	3.62989	0.3898	1.0324	1.56401	5.83671	0.61971	1.85417	1.29389
ENSG00000240827	AC021660.1	0	0	0	0	0	0	0	0
ENSG00000244153	WWP1P1	0.05294	0.04406	0	0.11786	0.03218	0	0.13237	0
ENSG00000239445	ST3GAL6-AS1	0.1739	0	0	0	0.21143	0.30679	0.28987	0.53379
ENSG00000064225	ST3GAL6	2.82104	2.65826	1.16485	1.19374	1.61566	0.55525	1.12698	2.14688
ENSG00000249274	PDLIM1P4	0.58291	0.72768	0	0	0.26576	0.38562	0	0.26838
ENSG00000057019	DCBLD2	0.22106	0.07575	0.30573	0.50176	0.39523	0.42439	0.3468	0.2315
ENSG00000206712	RNU6-26P	0	0	0	0	0	0	0	0
ENSG00000239462	AC091212.1	0.37114	0	0	0	0.22561	0	0	0
ENSG00000207331	RNU6-1263P	0	0	0	0	0	0	0	0
ENSG00000240476	LINC00973	0	0	0	0	0	0	0	0
ENSG00000275108	RF00066	0	0	0	0	0	0	0	0
ENSG00000241305	ACTG1P13	0	0	0	0	0	0	0	0
ENSG00000240572	AC107297.1	0	0	0	0	0	0	0	0
ENSG00000243296	AC078828.1	0	0	0	0	0	0	0	0
ENSG00000243089	AC107029.1	0	0	0	0	0	0.27809	0	0
ENSG00000244464	AC107029.2	0	0	0	0	0	0	0	0
ENSG00000144810	COL8A1	0	0.01297	0	0	0	0	0	0
ENSG00000273374	AC069222.1	0	0	0	0	0	0.03849	0	0
ENSG00000184220	CMSS1	0.54899	0.50766	0.28385	0.30176	0.70453	0.77121	0.27114	0.2122
ENSG00000168386	FILIP1L	0.19868	0.22736	0	0	0.19626	0.02191	0.0414	0.04574
ENSG00000264897	MIR3921	0	0	0	0	0	0	0	0
ENSG00000235156	TMEM30CP	0	0	0	0	0	0	0	0.06971
ENSG00000277000	DUSP12P1	0	0	0	0.21083	0.86353	0	0.23678	0.17441
ENSG00000243974	VTI1BP1	0	0	0	0	0	0	0.17454	0
ENSG00000036054	TBC1D23	12.5098	15.7468	10.8172	23.8916	15.1402	14.8378	18.4169	16.3346
ENSG00000114021	NIT2	0.45892	0.36876	0.18603	0.42274	0.50022	0.50251	0.42204	0.31087
ENSG00000154174	TOMM70	6.40885	4.91477	1.59961	4.20038	7.32851	7.99347	4.15055	4.97849
ENSG00000206535	LNP1	0.26074	0.05425	0	0.04837	0	0.11499	0.05433	0.20008
ENSG00000181458	TMEM45A	0.1953	0.20318	0.10762	0.28985	0.16324	0.23687	0.20346	0.22481
ENSG00000144820	ADGRG7	0.01792	0	0	0	0.02178	0	0	0
ENSG00000252989	RF00322	0	0	0	0	0	0	0	0
ENSG00000243495	GMFBP1	0	0	0	0	0	0	0	0
ENSG00000114354	TFG	6.16463	5.25267	2.86863	5.729	5.88877	6.73229	5.72491	4.30703
ENSG00000154175	ABI3BP	0.00668	0	0	0	0.01624	0	0	0

ENSG00000213431	AC080014.1	0	0	0	0	0	0	0	0
ENSG00000201642	RNU6-865P	0	0	0	0	0	0	0	0
ENSG00000242573	ACTR3P3	0	0	0	0	0	0	0	0
ENSG00000081148	IMPG2	0.17584	0.11708	0.11628	0.36536	0.47033	0.27919	0.61552	0.41021
ENSG00000138468	SENP7	14.1994	11.0639	6.41636	11.2255	18.0156	12.7169	10.0289	12.706
ENSG00000241738	ZNF90P1	0	0	0	0	0	0	0	0
ENSG00000232830	AC110994.1	0	0	0	0	0	0	0	0
ENSG00000282978	AC110994.2	0.59594	0.24798	0.87572	1.10555	0.90566	1.05132	1.98667	0.73168
ENSG00000201554	RF00019	0	0	0	0	0	0	0	0
ENSG00000175841	FAM172BP	0.33655	0.11204	0	0	0.40917	0	0.11219	0.08264
ENSG00000174173	TRMT10C	6.52249	3.81277	1.88271	2.47764	5.23938	4.31496	4.52999	3.90877
ENSG00000081154	PCNP	28.2143	25.3693	12.7784	23.2511	26.3156	22.1356	19.9301	21.8755
ENSG00000242299	AC073861.1	1.11787	0.26581	0.93868	0.47401	0.77662	0	0.79856	0.39214
ENSG00000243396	AC084198.1	0	0	0	0	0	0	0	0
ENSG00000252348	RNU6-1256P	0	0	0	0	0	0	0	0
ENSG00000212319	RF00019	0	0	0	0	0	0	0	0
ENSG00000201511	RF00019	0	0	0	0	0	0	0	0
ENSG00000066422	ZBTB11	10.4178	9.9864	5.85339	12.7621	12.2932	10.0262	14.1522	10.5458
ENSG00000201121	RNY1P12	0	3.48596	1.02585	0	0.84873	0	0	1.71423
ENSG00000256628	ZBTB11-AS1	1.06891	0.71168	0.19634	0.1983	0.77973	0.89571	0.35634	0.52496
ENSG00000114391	RPL24	55.1802	42.1707	18.876	20.4024	58.6136	68.8988	31.5245	46.173
ENSG00000244119	PDCL3P4	0.40722	0.50837	0	0	0.12377	0.1796	0.16969	0.125
ENSG00000182504	CEP97	1.80373	1.59697	0.73313	1.72007	1.773	2.50499	1.03629	2.02611
ENSG00000144815	NXPE3	7.52952	4.70513	1.03535	2.1543	6.13032	3.32452	2.00922	2.9391
ENSG00000249474	AC020651.1	0	0	0	0	0	0	0	0
ENSG00000144802	NFKBIZ	23.6096	22.8017	4.76336	5.41901	20.2483	13.5841	6.35536	11.9701
ENSG00000214407	LINC02085	0.32165	0.17846	0.10504	0.05304	0.13035	0.06305	0	0.08776
ENSG00000202177	RF00019	0	0	0	0	0	0	0	0
ENSG00000241280	AC106712.1	0.16991	0.33938	0	0	0	0.2398	0	0
ENSG00000170044	ZPLD1	0	0.04757	0	0	0	0	0.02382	0
ENSG00000241754	AC108748.1	0	0	0	0	0	0	0	0
ENSG00000201065	RNU6-461P	0	0	0	0	0	0	0	0
ENSG00000201922	RNU1-43P	0	0	0	0	0	0	0	0
ENSG00000236691	NDUFA4P2	0	0	0	0	0	0	0	0
ENSG00000265328	MIR548AB	0	0	0	0	0	0	0	0
ENSG00000240107	AC046144.1	0	0	0	0	0	0	0	0
ENSG00000243195	TUBBP11	0	0	0	0	0	0	0	0
ENSG00000214405	RAP1BP2	0	0	0	0	0	0	0	0
ENSG00000243635	AC091804.1	0	0	0	0	0	0	0	0
ENSG00000170017	ALCAM	4.11478	2.70224	0.7352	1.46987	4.19577	1.47703	1.1573	1.52941
ENSG00000114423	CBLB	3.39262	2.8132	0.83697	2.89421	3.40209	7.65604	2.31152	1.97629
ENSG00000244652	AC079382.1	0	0	0	0	0	0	0	0
ENSG00000200610	RF00019	0	0	0	0	0	0	0	0
ENSG00000200361	RF00019	0	0	0	0	0	0	0	0
ENSG00000242759	LINC00882	0	0	0	0	0	0	0	0
ENSG00000239997	FCF1P3	0	0	0	0	0	0	0	0
ENSG00000241787	MTND4P16	0	0	0	0	0	0	0	0
ENSG00000239632	MTND4LP3	0	0	0	0	0	0	0	0
ENSG00000242214	MTND3P6	0	0	0	0	0	0	0	0
ENSG00000242836	MTCO3P35	0	0	0	0	0	0	0	0
ENSG00000249580	MTATP6P22	0	0	0	0	0	0	0	0
ENSG00000243658	MTND5P16	0	0	0	0	0	0	0	0
ENSG00000240034	MTND6P6	0	0	0	0	0	0	0	0
ENSG00000250630	MTCO1P35	0	0	0	0	0	0	0	0
ENSG00000239586	MTND1P16	0	0	0	0	0	0	0	0
ENSG00000240796	MTND2P14	0	0	0	0	0	0	0	0
ENSG00000252626	RNU6-1308P	0	0	0	0	0	0	0	0
ENSG00000239455	AC074043.1	0	0	0	0	0	0	0	0

ENSG00000243701	DUBR	0.40083	0.29008	0.12805	0.10346	0.04238	0.03074	0.02905	0.12838
ENSG00000239828	AC063944.1	0.13287	0.07372	0.19525	0.29579	0	0.15627	0.40604	0.08157
ENSG00000241218	AC063944.2	0	0	0	0	0	0	0	0
ENSG00000272597	AC063944.3	0	0	0	0	0	0	0	0
ENSG00000138483	CCDC54	0	0	0	0	0	0	0	0
ENSG00000273125	LINC01990	0	0	0	0	0	0	0	0
ENSG00000114439	BBX	10.4918	9.02968	2.95653	5.05597	9.85933	9.68995	5.3596	6.96365
ENSG00000241469	LINC00635	0.09672	0	0	0.07177	0	0	0	0.01979
ENSG00000240423	LINC00636	0	0	0	0	0	0	0	0
ENSG00000279277	AC012020.1	0.33935	0.63546	0.12467	0.25182	0.61887	1.04766	0.98989	0.31249
ENSG00000196776	CD47	16.8698	13.7231	5.62104	12.9385	18.2748	15.8221	11.0806	13.3143
ENSG00000271856	LINC01215	3.05418	0.94142	0.54023	1.13318	3.16309	1.74611	1.60267	1.35412
ENSG00000114446	IFT57	3.13366	1.84665	0.60064	1.50209	3.83348	2.8156	1.36261	1.57722
ENSG00000114455	HHLA2	0	0.03364	0.0297	0	0.02457	0	0.06737	0
ENSG00000243188	HNRNPA1P17	0	0	0	0	0	0	0	0
ENSG00000144821	MYH15	0.07622	0	0.014	0.08483	0.08108	0.05042	0.06352	0.02339
ENSG00000241634	AC069499.1	0.14401	0.2397	0	0	0.35016	0.25405	0	0.35362
ENSG00000163507	CIP2A	1.2006	1.62536	1.34084	3.4686	2.04351	3.8689	3.84232	2.65333
ENSG00000202379	RF00156	0	0	0	0	0	0	0	0
ENSG00000276763	AC069499.2	0	0	0	0	0	0	0	0
ENSG00000198919	DZIP3	4.46825	4.09495	2.45647	10.1889	7.95045	5.71257	7.88599	5.0988
ENSG00000241777	AC092185.1	0	0	0	0	0	0	0	0
ENSG00000163515	RETNLB	0	0	0	0	0	0	0	0
ENSG00000163519	TRAT1	18.557	12.2588	3.91997	10.672	32.608	35.5505	9.42451	9.39843
ENSG00000138472	GUCA1C	0	0	0	0	0	0	0	0
ENSG00000114487	MORC1	1.73319	1.19934	0.82989	1.8208	2.32025	0.54968	2.04499	1.81716
ENSG00000221633	RF00012	0	0	0	0	0	0	0	0
ENSG00000239314	MORC1-AS1	0	0	0	0	0	0	0	0
ENSG00000241224	C3orf85	0	0	0	0	0	0	0	0
ENSG00000261364	AC063923.2	0	0	0	0	0	0	0	0
ENSG00000214381	LINC00488	0	0	0	0	0	0	0	0
ENSG00000252889	RNU6-1236P	0	0	0	0	0	0	0	0
ENSG00000239288	AC063923.1	0	0	0	0	0	0	0	0
ENSG00000163530	DPPA2	0	0	0	0	0	0	0	0
ENSG00000277635	RF00017	0	0	0	0	0	0	0	0
ENSG00000121570	DPPA4	0.13205	0	0.06468	0.02177	0.1427	0	0.04891	0.07206
ENSG00000241257	AC124945.1	0	0	0	0	0	0	0	0
ENSG00000244561	AC092905.1	0	0	0	0	0	0	0	0
ENSG00000230618	H3F3AP3	0	0	0	0	0	0	0	0
ENSG00000228980	LINC01205	0	0	0	0	0	0	0	0
ENSG00000244196	PPIAP15	0	0	0	0	0	0	0	0
ENSG00000265956	MIR4445	0	0	0	0	0	0	0	0
ENSG00000242029	AC078980.1	0	0	0	0	0	0	0	0
ENSG00000271249	DIMT1P1	0	0	0	0	0	0	0	0
ENSG00000214380	AC068781.1	0	0	0	0	0	0	0	0
ENSG00000243945	AC078918.1	0	0	0	0	0	0	0	0
ENSG00000240895	AC117430.1	0	0	0	0	0	0	0	0
ENSG00000221206	RNU6ATAC15P	0	0	0	0	0	0	0	0
ENSG00000244722	RPSAP29	0	0	0	0	0	0	0	0
ENSG00000201426	RF00019	0	0	0	0	0	0	0	0
ENSG00000206532	AC117402.1	0	0	0	0	0	0	0	0
ENSG00000242242	NECTIN3-AS1	0	0	0	0	0	0	0	0
ENSG00000177707	NECTIN3	0.07112	0.21703	0.03484	0.08796	0.24498	0	0.01976	0.10187
ENSG00000153283	CD96	12.1998	7.36879	2.39281	6.32475	10.8728	14.565	5.36474	4.95124
ENSG00000239311	AC092916.1	0	0	0	0	0	0	0	0
ENSG00000244142	ATP6V0CP2	0	0	0	0	0	0	0	0
ENSG00000240787	AC092916.2	0	0	0	0	0	0	0	0
ENSG00000177494	ZBED2	0	0	0	0.04707	0.03856	0	0	0

ENSG00000244720	AC055748.1	1.37581	0.38167	0.33696	0.90749	0.74341	1.07872	0.38221	0.93845
ENSG00000240891	PLCXD2	1.46209	1.86262	0.4081	1.00615	1.44986	3.80414	0.76244	1.04298
ENSG00000240766	PLCXD2-AS1	0	0	0	0	0	0	0	0
ENSG00000144824	PHLDB2	0.39899	0.51543	0.16627	0.60098	0.40544	0.43072	0.17867	0.48987
ENSG00000144827	ABHD10	6.13869	3.91752	1.85406	2.77276	6.72579	4.32323	2.18396	3.57486
ENSG00000144834	TAGLN3	0	0	0	0	0	0	0	0
ENSG00000239439	RFKP2	0	0	0	0	0	0	0	0
ENSG00000176040	TMPRSS7	0	0	0	0	0	0	0	0.02951
ENSG00000114529	C3orf52	0.14609	0.10132	0.05367	0.30715	0.0296	0.21477	0.06088	0.07474
ENSG00000207940	MIR567	0	0	0	0	0	0	0	0
ENSG00000174500	GCSAM	2.01513	1.03206	0.19931	1.1215	3.1331	1.19638	0.48446	1.21328
ENSG00000261488	TBILA	0.07568	0	0	0	0	0	0.06308	0.04646
ENSG00000172139	SLC9C1	0.43118	0.31579	0.50689	2.3037	0.25162	0.51725	0.40248	0.02118
ENSG00000244144	AC128688.1	0	0	0	0	0	0	0	0
ENSG00000239482	AC112487.1	0.05808	0	0	0	0	0	0	0
ENSG00000283669	AC112487.2	0	0	0	0	0	0	0	0
ENSG00000091972	CD200	0.19606	0.16317	0.03601	0.36371	1.72811	0.56204	0.32679	0.30089
ENSG00000243081	AC092894.1	0.14069	0	0	0	0	0	0	0
ENSG00000186265	BTLA	2.77578	1.78211	0.84493	2.47173	8.55732	3.8825	1.42109	2.92119
ENSG00000242525	OR7E100P	0.07222	0.12021	0	0.10718	0.2634	0.2548	0.36112	0
ENSG00000279349	AC092692.1	0.08474	0.37613	0.04151	0.04192	0.03434	0.24916	0.18833	0.06936
ENSG00000144848	ATG3	18.448	25.0366	10.2056	14.7931	16.6392	13.3311	11.4763	18.3646
ENSG00000138459	SLC35A5	8.23175	9.35239	3.83979	6.50069	6.50058	5.14263	3.67573	6.3979
ENSG00000091986	CCDC80	0.03365	0.028	0	0	0.02045	0.00989	0	0
ENSG00000242308	AC048334.1	0	0	0	0	0	0	0	0
ENSG00000240893	LINC02042	0.10875	0	0	0	0.08815	0	0	0
ENSG00000242770	CD200R1L-AS1	0.0952	0	0.13989	0	0	0	0	0
ENSG00000206531	CD200R1L	0.04287	0.07135	0	0	0.15635	0.07562	0	0
ENSG00000163606	CD200R1	4.63641	2.03087	1.18609	2.73009	4.24474	3.57636	1.34539	1.91287
ENSG00000272844	AC074044.1	0.51839	0.17257	0	0.46161	0.37815	0.3658	0	1.01835
ENSG00000163607	GTPBP8	3.39105	1.69013	0.56306	1.25106	2.4457	2.67006	1.3093	1.52896
ENSG00000163608	NEPRO	3.90544	2.42364	1.19424	2.27823	3.97965	4.75909	1.93788	2.75784
ENSG00000240057	AC078785.1	0	0	0	0	0.22257	0.12918	0.06103	0
ENSG00000243483	AC078785.3	0	0	0	0	0	0	0	0
ENSG00000241219	AC078785.2	0	0	0	0	0	0	0	0
ENSG00000243795	LINC02044	0	0	0	0	0	0	0	0
ENSG00000144857	BOC	0	0	0	0.01053	0.00863	0	0	0
ENSG00000206530	CFAP44	0.61572	0.67319	0.50562	0.81524	0.74123	0.55375	1.17723	0.91161
ENSG00000240751	AC026348.1	0	0	0	0	0	0	0	0
ENSG00000243849	CFAP44-AS1	0	0	0	0	0	0	0	0
ENSG00000275670	MIR8076	0	0	0	0	0	0	0	1.0843
ENSG00000163611	SPICE1	1.0246	0.56848	0.35267	0.84935	1.24568	1.40043	1.20011	1.01999
ENSG00000072858	SIDT1	1.70974	1.66513	0.34746	1.18773	2.31084	2.47072	1.06112	1.10542
ENSG00000239453	SIDT1-AS1	0	0	0	0	0	0.27809	0	0
ENSG00000265253	MIR4446	2.18807	0	0	0	0	0	0	0
ENSG00000241529	RN7SL767P	2.98171	4.13588	9.1283	3.31889	2.11463	3.94511	2.89919	6.1015
ENSG00000176542	USF3	8.99581	7.48676	1.48971	2.53395	7.8556	7.2572	4.43791	3.4196
ENSG00000121579	NAA50	20.4776	14.6918	4.82001	10.767	13.4035	21.5988	10.0045	10.2778
ENSG00000239280	AC108693.1	2.2058	1.97699	0.49868	1.51091	1.65031	2.99333	1.1313	1.04163
ENSG00000242659	AC108693.2	0	0.40267	0	0	1.17646	0.42677	0	0.89106
ENSG00000114573	ATP6V1A	46.7522	31.8459	6.29906	14.8441	26.1096	15.7292	14.8617	26.8412
ENSG00000178075	GRAMD1C	1.454	0.64018	0.08271	0.16706	0.85536	0.08274	0.29709	0.7947
ENSG00000240776	AC079944.1	0	0	0	0	0	0	0	0
ENSG00000241889	AC079944.2	0.39946	0.33245	0	0	0.12141	0	0	0.36784
ENSG00000240375	VPS26AP1	0.14975	0.12463	0	0	0	0	0	0.09193
ENSG00000273394	AC128687.2	0	0	0	0	0	0	0	0
ENSG00000184307	ZDHHC23	0.28753	0.18802	0.06036	0.27433	0.34958	0.09058	0.22252	0.16391
ENSG00000163617	CCDC191	1.3546	0.87428	0.1625	0.30771	1.54606	0.92662	0.29952	0.66187

ENSG00000270880	AC128687.1	0	0	0	0	0.14397	0	0	0
ENSG00000151576	QTRT2	2.4573	2.22687	0.45471	0.98601	2.66664	1.91062	1.07708	1.26269
ENSG00000240912	AC092896.1	0	0	0	0	0	0	0	0
ENSG00000151577	DRD3	0	0	0	0	0	0	0	0
ENSG00000241490	AC093010.2	0.21274	0.3541	0.10421	0.24557	0.28738	0.6255	0.0788	0.17413
ENSG00000240487	AC093010.1	0	0	0	0	0	0	0	0
ENSG00000174255	ZNF80	0.29837	0.24832	0.07308	0.36902	0.33252	0.21932	0.37301	0.12211
ENSG00000181847	TIGIT	3.97129	2.5239	0.56589	2.07177	2.73597	6.13546	1.9658	1.99468
ENSG00000259976	AC093010.3	0	0	0	0	0	0	0	0
ENSG00000181722	ZBTB20	1.3477	0.88429	0.44272	1.02738	1.138	0.92505	1.10787	0.94029
ENSG00000284134	MIR568	0	0	0	0	0	0	0	0
ENSG00000241560	ZBTB20-AS1	0.10806	0.05996	0	0.10691	0	0.12709	0	0.08845
ENSG00000242290	ZBTB20-AS5	0	0	0	0	0	0	0	0
ENSG00000241295	ZBTB20-AS2	0	0	0	0	0	0	0	0
ENSG00000264623	MIR4796	0	0	0	0	0	0	0	0
ENSG00000239946	ZBTB20-AS3	0	0	0	0	0	0	0	0
ENSG00000240002	YBX1P3	0	0	0	0	0	0	0	0
ENSG00000242767	ZBTB20-AS4	0	0	0	0	0	0.38315	0	0
ENSG00000242880	AC046136.1	0.27505	0.45782	0.60627	0.8164	0.3344	0.48522	0.22923	0.16885
ENSG00000244157	EIF4E2P2	0	0	0	0	0	0	0	0
ENSG00000172020	GAP43	0	0	0	0	0	0	0	0
ENSG00000241596	AC092468.1	0	0	0	0	0	0	0	0.16014
ENSG00000185565	LSAMP	0	0.02362	0	0	0	0	0	0
ENSG00000243359	RN7SL815P	0	0	0	0	0	0	0	0
ENSG00000240922	LSAMP-AS1	0	0	0	0	0	0	0	0
ENSG00000241397	LINC00903	0	0	0	0	0	0	0	0
ENSG00000241156	RN7SL582P	0	0	0	0	0	0.46348	0	0
ENSG00000198406	BZW1P2	0.62119	0.29542	0.26081	0.39511	0.64734	0.15655	0.59167	0.87164
ENSG00000243197	TUSC7	0	0	0	0	0	0	0	0
ENSG00000278072	RF01879	0	0	0	0	0	0	0	0
ENSG00000265433	MIR4447	0	0	0	0	0	0	0	0
ENSG00000274732	RF01884	0	0	0	0	0	0	0	0
ENSG00000242385	LINC00901	0	0	0	0	0	0	0	0
ENSG00000200372	RNU5E-8P	0	0	0	0	0	0	0	0
ENSG00000243014	PTMAP8	0	0	0	0	0.26058	0	0	0
ENSG00000206889	RNU6-1200P	0	0	0	0	0	0	0	0
ENSG00000239268	AC092691.1	0	0	0	0	0	0	0	0
ENSG00000240393	AC092691.2	0	0	0	0	0	0	0	0
ENSG00000241213	LINC02024	0	0	0	0	0	0	0	0
ENSG00000242816	AC092691.3	0	0	0	0	0	0	0	0
ENSG00000243276	AC068633.1	0	0	0	0	0	0	0	0
ENSG00000144847	IGSF11	0.01496	0	0	0	0.07276	0.0264	0	0.03674
ENSG00000239877	IGSF11-AS1	0	0	0	0	0	0	0	0
ENSG00000163424	C3orf30	0	0	0	0	0	0	0	0
ENSG00000251012	AC083800.1	0	0	0	0	0	0	0	0
ENSG00000114638	UPK1B	0	0	0	0	0	0	0	0
ENSG00000121578	B4GALT4	1.08745	0.7855	0.22613	0.7156	0.93544	1.0497	0.7353	0.70537
ENSG00000240254	B4GALT4-AS1	0	0	0	0	0	0	0	0
ENSG00000031081	ARHGAP31	3.86655	2.32731	0.30992	0.85786	2.0038	1.75006	0.49476	0.64256
ENSG00000242829	RPS26P21	0.42127	2.10359	0	0	0.51216	0.74317	0	0
ENSG00000241155	ARHGAP31-AS1	0	0	0.34524	0	0.14282	0	0	0
ENSG00000207310	RNU6-1127P	0	0	0	0	0	0	0	0
ENSG00000176142	TMEM39A	2.19915	2.21917	0.8887	1.87667	2.60683	2.57023	1.60372	1.56942
ENSG00000163389	POGLUT1	1.71451	1.15183	0.4705	0.96569	1.60729	1.27544	0.6542	0.93839
ENSG00000272662	AC073352.1	0	0.24208	0	0	0.17682	0	0.72726	0
ENSG00000113845	TIMMDC1	20.3791	19.9772	10.9687	25.6224	27.5511	22.3572	24.5703	14.538
ENSG00000121594	CD80	0.1145	0.03812	0	0.16992	0.30624	0	0	0.16869
ENSG00000272967	AC073352.2	0.16697	0	0	0	0.203	0	0	0.205

ENSG00000144843	ADPRH	2.76365	1.61268	0.5835	0.56573	1.58344	0.89664	0.47654	0.81904
ENSG00000144837	PLA1A	0	0	0	0.0494	0.04047	0	0	0
ENSG00000242052	RPL10P7	0.11728	0	0	0	0	0	0	0
ENSG00000121577	POPDC2	0.17988	0.0499	0	0.13348	0.29159	0.21155	0	0
ENSG00000138495	COX17	4.01599	3.31423	0.94225	2.05351	2.46179	4.34611	2.08134	2.07176
ENSG00000183833	MAATS1	0	0	0.01516	0	0.02508	0	0	0
ENSG00000239994	AC069444.1	0	0	0	0	0	0	0	0
ENSG00000144852	NR1I2	0.05449	0	0	0	0	0	0	0
ENSG00000240471	PHBP8	0	0	0	0	0	0	0	0
ENSG00000082701	GSK3B	15.7295	13.4109	5.17985	8.60122	13.569	14.6982	11.5832	9.28523
ENSG00000239835	AC092910.1	0	0	0	0	0	0	0	0
ENSG00000241546	AC092910.2	0	0	0	0	0	0	0	0
ENSG00000242622	AC092910.3	0.60137	0.38927	0.09819	0.34708	0.32495	0.11788	0.16707	0.12306
ENSG00000244308	RN7SL762P	0	0	0	0	0	0	0	0
ENSG00000244139	RN7SL397P	0	0	0	0.36505	0	0	0	0
ENSG00000175697	GPR156	0	0	0	0	0	0	0	0
ENSG00000206721	RF00019	0	0	0	0	0	0	0	0
ENSG00000240882	AC063952.2	0	0	0	0	0	0	0	0
ENSG00000163428	LRRC58	6.72438	4.53885	1.8945	4.22589	5.87496	7.00309	4.90082	3.83766
ENSG00000240661	AC063952.1	0	0.02861	1.187	0	0.02089	0	0.02865	0
ENSG00000282950	AC063952.4	0	0	0	0	0	0	0	0
ENSG00000242613	AC063952.3	0	0	0	0	0	0	0	0
ENSG00000163430	FSTL1	0.79793	0.25165	0.12343	0.31164	0.83735	0.62233	1.02202	1.27875
ENSG00000284121	MIR198	0	0	0	0	0	0	0	0
ENSG00000240774	AC126182.1	0	0	0	0	0	0	0	0
ENSG00000244441	AC126182.2	0	0	0	0	0	0	0	0
ENSG00000065518	NDUFB4	9.62334	8.39656	2.623	5.29819	7.83132	8.96763	5.75651	6.71762
ENSG00000113924	HGD	1.79381	1.02253	0.43331	1.8599	1.37424	0.52019	3.39959	3.22819
ENSG00000144840	RABL3	2.97333	2.31827	0.89685	1.37027	3.23438	3.39568	1.51291	1.92137
ENSG00000240452	MTCO1P29	0	0	0	0	0	0	0	0
ENSG00000243257	MTCO2P29	0	0	0	0	0	0.68419	0	0
ENSG00000153767	GTF2E1	5.1332	6.08232	1.11869	3.09894	4.54839	5.60223	2.39285	2.91088
ENSG00000213371	NAP1L1P3	0	0	0	0.12362	0.10127	0	0	0
ENSG00000243593	LINC02049	0	0	0	0	0	0	0	0
ENSG00000145087	STXBP5L	0	0	0	0	0	0	0	0
ENSG00000270782	AC072026.1	0	0	0	0	0	0	0	0
ENSG00000264477	MIR5682	0	0	0	0	0	0	0	0
ENSG00000243813	AC078857.1	0	0	0	0	0	0	0	0
ENSG00000242103	AC079841.1	0	0	0	0	0	0	0	0
ENSG00000051341	POLQ	1.45041	1.90801	2.1199	5.35828	2.85363	4.51216	6.64193	2.95841
ENSG00000242445	RPL7AP11	0	0	0	0	0	0	0	0
ENSG00000186103	ARGFX	0	0	0	0	0	0	0	0
ENSG00000163833	FBXO40	0.18545	0.02058	0.01817	0	0.03006	0	0.06182	0.03036
ENSG00000180353	HCLS1	112.678	86.6014	9.24229	20.6763	80.0703	39.38	22.2146	35.6419
ENSG00000243544	RN7SL172P	2.66107	0.40267	0	0.35903	2.0588	0.42677	1.61294	1.78212
ENSG00000222057	RNU4-62P	2.2554	0	0	0	0	0.99471	0.93985	0
ENSG00000173230	GOLGB1	15.3087	11.0646	2.36036	4.30008	10.4552	9.99401	4.32497	6.72039
ENSG00000173226	IQCB1	4.3033	2.28091	1.51909	3.99606	3.65352	4.07148	2.6047	2.80411
ENSG00000145088	EAF2	9.81479	10.5465	5.05099	11.9234	6.2432	4.52957	7.93827	6.81334
ENSG00000163406	SLC15A2	0.53116	0.56977	0.17345	0.22773	0.60272	0.29152	0.21642	0.44926
ENSG00000145103	ILDR1	0.12411	0.04132	0.03648	0.03684	0.06036	0	0	0.03048
ENSG00000202388	RF00019	0	0	0	0	0	0	0	0
ENSG00000114013	CD86	23.0812	16.917	3.0121	4.43635	9.44901	5.9891	5.08936	5.74115
ENSG00000036828	CASR	0	0	0	0	0	0	0	0
ENSG00000240236	HNRNPA1P23	0	0	0	0	0	0	0	0
ENSG00000121552	CSTA	37.9048	40.0771	6.51148	7.52705	23.3014	9.8891	7.83093	27.5956
ENSG00000160124	CCDC58	2.47693	2.16624	0.98707	1.86917	2.24578	2.22185	1.60948	1.28862
ENSG00000114023	FAM162A	1.77791	1.22234	0.36918	0.94646	1.55067	1.60233	0.70867	0.75927

ENSG00000196981	WDR5B	1.59606	1.24632	0.37642	0.6726	1.50923	1.32093	1.08386	1.25802
ENSG00000272758	AC083798.2	0.63387	0.67525	0.33533	0.33866	0.36991	0.80512	0.46489	0.49808
ENSG00000242531	AC083798.1	0	0	0	0.83043	0	0	0	0
ENSG00000114030	KPNA1	7.05161	7.44958	1.95009	4.42815	7.03358	6.68669	4.81435	4.60049
ENSG00000275348	AC096861.1	0	0	0	0	0	0	0	0
ENSG00000138496	PARP9	15.221	38.7812	10.6533	6.28031	13.2808	20.8479	5.19451	19.4836
ENSG00000163840	DTX3L	27.6937	53.2072	13.6386	12.5322	21.3376	32.9009	8.32858	31.9315
ENSG00000173200	PARP15	1.69042	1.52871	0.8215	2.25197	2.16845	2.32465	1.19806	1.97742
ENSG00000243918	EIF4BP8	0	0	0.05899	0	0	0	0.06691	0
ENSG00000173193	PARP14	23.7327	52.4305	13.1884	12.4593	15.8788	25.4927	7.89312	32.1914
ENSG00000169087	HSPBAP1	1.90198	2.38	0.51538	1.10107	2.37798	0.83289	1.66385	1.55681
ENSG00000138463	DIRC2	6.82859	4.64827	1.22811	1.39159	6.14598	2.01375	3.26177	2.10227
ENSG00000273033	LINC02035	2.00786	1.20315	0.15736	0.31785	1.39957	0.54313	0.82554	0.80531
ENSG00000082684	SEMA5B	0	0	0	0	0	0	0	0
ENSG00000065485	PDIA5	0.81849	0.7266	0.16037	0.4319	1.23835	0.60966	0.36381	0.24565
ENSG00000284421	MIR7110	0	0	0	0	0	0	0	0
ENSG00000121542	SEC22A	1.23259	1.01284	1.14638	3.4039	2.01072	1.51387	2.21058	1.55167
ENSG00000273454	AC112503.2	0.13279	0	0	0	0.48433	0.46852	0	0.16304
ENSG00000173175	ADCY5	0.00826	0	0	0	0	0	0	0
ENSG00000272678	AC112503.1	0.18747	0.31204	0	0	0	0	0	0
ENSG00000206527	HACD2	5.34239	3.99019	1.06687	3.21214	4.61322	5.36483	2.39748	2.38826
ENSG00000239523	MYLK-AS1	0.22439	0.1245	0.10991	0.11101	0.36374	0.39585	0.12467	0.18367
ENSG00000065534	MYLK	2.89339	1.94844	0.48592	0.84901	1.52769	1.59839	0.9811	1.38851
ENSG00000250174	MYLK-AS2	0	0	0	0	0	0	0	0
ENSG00000273123	AC020634.1	0.66758	0.44448	0	0.3963	0.81163	0.94216	0	0.16393
ENSG00000207002	RF00392	0	0	0	0	0	0	0	0
ENSG00000175455	CCDC14	4.58809	5.12686	3.24377	5.21882	5.07098	8.99964	6.54595	5.27865
ENSG00000065371	ROPN1	0	0	0	0	0	0	0	0
ENSG00000160145	KALRN	0.16761	0.06477	0.03079	0.10217	0.09461	0.13728	0.05987	0.07717
ENSG00000266383	MIR5002	0	0	0	0	0	0	0	0
ENSG00000240622	RPL7P15	0	0	0	0	0	0	0	0
ENSG00000276656	MIR6083	0	0	0	0	0	0	0	0
ENSG00000252751	RNU6-143P	0	0	0	0	0	0	0	0
ENSG00000260391	AC022336.2	0	0	0	0	0	0	0	0
ENSG00000114491	UMPS	1.27664	0.85321	0.18476	0.54544	1.42276	1.17727	0.66096	0.83122
ENSG00000265981	MIR544B	2.81924	1.56421	1.38095	0	0	0	3.13283	0
ENSG00000242199	AC022336.1	0	0	0	0	0	0	0	0
ENSG00000082781	ITGB5	2.99023	1.76001	0.28403	0.62433	1.65876	2.42698	1.44031	1.2424
ENSG00000244286	ITGB5-AS1	0	0	0	0	0	0	0	0
ENSG00000280042	AC022336.3	0	0	0	0	0	0	0	0
ENSG00000276626	RF00100	0	0	0	0	0	0	0	0
ENSG00000243986	ENO1P3	0	0	0	0	0	0	0	0
ENSG00000173702	MUC13	0	0	0	0	0	0	0	0
ENSG00000173706	HEG1	2.2304	2.10211	0.43411	1.46874	1.54439	1.56347	0.87404	0.68008
ENSG00000252642	RNA5SP137	0	0	0	0	0	0	0	0
ENSG00000221955	SLC12A8	0	0	0	0	0	0.0926	0	0.01611
ENSG00000199327	RNU6-230P	0	0	0	0	0	0	0	0
ENSG00000264986	MIR5092	0	0	0	0	0	0	0	0
ENSG00000163848	ZNF148	18.7722	17.2155	4.15617	9.0329	15.9287	12.376	6.84043	11.2007
ENSG00000229048	DUTP1	0	0	0	0	0	0	0	0
ENSG00000243508	AC108688.1	0.41122	0.34224	0.15107	0.15257	0.24998	0.18136	0	0.50489
ENSG00000252953	RNU6-232P	0	0	0	0	0	0	0	0
ENSG00000114520	SNX4	8.76682	5.9	4.21472	8.03143	10.2637	8.92628	10.8695	7.34195
ENSG00000201800	RF00019	0	0	0	0	0	0	0	0
ENSG00000252965	RF00019	7.60664	6.90614	0	3.07884	5.04434	4.87969	3.45793	0
ENSG00000144909	OSBPL11	19.921	18.5152	3.70098	5.32118	13.4175	9.19468	4.72279	10.9517
ENSG00000243072	AF186996.3	0	0	0	0	0	0	0	0
ENSG00000242001	AF186996.2	0	0	0	0	0	0	0	0

ENSG00000234458	OR7E130P	0	0	0	0	0	0	0	0
ENSG00000243429	OR7E29P	0	0	0	0	0	0	0	0
ENSG00000228963	OR7E93P	0	0	0	0	0	0	0	0
ENSG00000239978	OR7E53P	0	0	0	0	0	0	0	0
ENSG00000179170	OR7E97P	0	0	0	0	0	0	0	0
ENSG00000239432	AF186996.1	0	0	0	0	0	0	0	0
ENSG00000272840	AC092902.2	0	0	0	0	0	0	0	0
ENSG00000239804	AC092902.1	0	0	0	0	0	0	0	0
ENSG00000221737	MIR548I1	0	0	0	0	0	0	0	0
ENSG00000242229	RPS3AP14	0	0	0	0	0	0	0	0
ENSG00000241346	SNRPCP11	0	0	0	0	0	0	0	0
ENSG00000241288	LINC02614	0.04355	0	0	0.01616	0.03971	0.01921	0.01815	0.01337
ENSG00000241278	ENPP7P4	0	0	0	0	0	0	0	0
ENSG00000248787	AC092903.2	0	0	0	0	0	0	0	0
ENSG00000171084	FAM86JP	0.0582	0	0	0	0	0.20534	0	0
ENSG00000189366	ALG1L	0	0.43419	0	0	0.10571	0	0.28987	0
ENSG00000241439	AC092903.1	0	0	0	0	0	0	0	0
ENSG00000114547	ROPN1B	0	0	0	0	0.06604	0	0	0
ENSG00000114544	SLC41A3	1.05636	0.53675	0.11438	0.34656	0.86521	0.96121	0.68578	0.50514
ENSG00000248607	AC117422.1	0	0	0	0	0	0	0	0
ENSG00000250012	AC079848.1	0.11364	0	0	0	0	0	0	0.13953
ENSG00000144908	ALDH1L1	0	0	0	0	0	0	0	0
ENSG00000250218	ALDH1L1-AS1	0	0	0	0	0	0	0	0
ENSG00000272020	RNU1-30P	0	0	0	0	0	0	0	0
ENSG00000246022	ALDH1L1-AS2	0	0	0	0	0	0	0	0
ENSG00000250934	AC016924.1	0	0	0	0	0	0	0	0
ENSG00000251448	AC063919.1	0	0	0	0	0	0	0	0
ENSG00000163884	KLF15	0.02574	0	0	0	0.03129	0	0	0
ENSG00000249833	CCDC37-DT	0	0	0	0	0	0	0	0
ENSG00000163885	CFAP100	0	0	0	0	0	0	0	0
ENSG00000279328	AC073439.1	0.24792	0	0.12144	0.06132	0	0.07289	0.06887	0.05073
ENSG00000070476	ZXDC	3.72629	2.53735	0.43923	1.09422	2.58011	2.37285	1.42823	1.68813
ENSG00000159650	UROC1	0	0	0	0	0.05354	0	0	0
ENSG00000180767	CHST13	0.65556	0.341	0	0.18242	0.94646	0.07228	0.13659	0.40245
ENSG00000180697	C3orf22	0	0.05579	0	0	0	0	0	0
ENSG00000201968	RNA5SP138	0	0	0	0	0	0	0	0
ENSG00000283374		0	0	0	0	0	0	0	0
ENSG00000206483		0	0	0	0	0	0	0	0
ENSG00000197763	TXNRD3	0.57585	0	0	0.14244	0.14585	0.25397	0.07999	0.23567
ENSG00000248557	AC024558.1	0	0	0	0	0	0	0	0
ENSG00000198284	NUP210P1	0	0	0	0	0	0	0	0
ENSG00000248850	AC078867.1	0	0	0	0	0	0	0	0
ENSG00000159685	CHCHD6	0.1857	0.22079	0.03898	0.03937	0.35479	0.1872	0.2211	0.13029
ENSG00000249558	RCC2P4	0	0	0	0	0	0	0	0
ENSG00000280053	AC011199.1	0	0	0	0	0.05301	0	0	0
ENSG00000114554	PLXNA1	0.55591	0.13378	0.03937	0.18886	0.39901	0.25995	0.10048	0.14802
ENSG00000214324	C3orf56	0	0	0	0	0	0	0	0
ENSG00000212556	RF00019	0	0	0	0	0	0	0	0
ENSG00000243016	AC112482.1	0	0	0	0	0	0	0	0
ENSG00000201288	RNU6-1047P	0	0	0	0	0	0	0	0
ENSG00000239620	PRR20G	0	0	0	0	0	0	0	0
ENSG00000244215	LINC02016	0	0	0	0	0	0	0	0
ENSG00000239921	LINC01471	0	0	0	0	0	0	0	0
ENSG00000240562	AC016968.1	0	0	0	0	0	0	0	0
ENSG00000282975	LINC02034	0	0	0	0	0	0	0	0
ENSG00000282860	AC084035.1	0.24152	0.201	0	0	0	0.42607	0.60386	0.14827
ENSG00000163870	TPRA1	1.79378	1.49287	0.71889	1.54285	1.01607	1.33052	0.95135	1.50162
ENSG00000275067	MIR6825	0	0	0	0	0	0	0	0

ENSG00000276083	MIR7976	0	1.84861	0	0	0	0	0	0
ENSG00000073111	MCM2	1.42055	0.54757	0.4175	0.53261	1.34529	6.2783	0.39879	0.47734
ENSG00000114631	PODXL2	0.26825	0.16744	0.04927	0.09953	0.52997	0.11831	0.05589	0.20585
ENSG00000114626	ABTB1	13.8995	11.8258	4.08153	4.15753	9.69573	7.23423	7.61019	8.284
ENSG00000074416	MGLL	1.77773	0.59181	0.22392	0.54921	1.01449	0.93444	0.84663	0.65925
ENSG00000187715	KBTBD12	0.0378	0	0.01851	0.0187	0.12254	0.06668	0.021	0.06187
ENSG00000201210	RNA5SP139	0	0	0	0	0	0	0	0
ENSG00000058262	SEC61A1	16.6358	11.5592	3.78386	8.15256	13.7365	15.9678	6.19096	6.74451
ENSG00000175792	RUVBL1	1.61346	1.45841	0.43965	0.9039	1.64983	2.90292	0.8371	0.86586
ENSG00000222627	RNU2-37P	0	0	0	0	0	0.73058	0	0
ENSG00000239608	RUVBL1-AS1	0	0	0	0	0	0	0	0
ENSG00000212344	RNU6-823P	0	0	0	0	0	0	0	0
ENSG00000132394	EEFSEC	2.72492	2.4694	1.15678	1.93217	3.93866	6.24927	1.76634	1.11521
ENSG00000179407	DNAJB8	0	0	0	0	0	0	0	0
ENSG00000242049	DNAJB8-AS1	0	0	0	0	0	0	0	0
ENSG00000179348	GATA2	9.45228	3.80336	0.89379	2.04934	3.61743	1.62401	0.73982	1.41283
ENSG00000244300	GATA2-AS1	0.06421	0.05344	0	0	0.03903	0.22656	0.05352	0.03942
ENSG00000239405	TMED10P2	0.11977	0	0	0	0	0	0	0.44116
ENSG00000198685	LINC01565	0	0	0	0	0	0	0	0
ENSG00000163902	RPN1	32.225	23.5643	4.1553	8.50229	25.9849	22.4158	9.27371	14.4509
ENSG00000277250	RF00017	0	0	0	0	0	0	0	0
ENSG00000242551	POU5F1P6	0	0	0	0	0	0	0	0.09091
ENSG00000207130	RF00399	0	0	0	0	0	0	0	0
ENSG00000075785	RAB7A	41.8165	41.2682	5.352	9.80953	32.4717	26.7	10.9274	14.7732
ENSG00000239503	MARK2P8	0	0	0	0	0	0	0	0
ENSG00000242992	FTH1P4	0.13255	0	0	0	0	0	0	0
ENSG00000244232	RN7SL698P	0.97087	0	0	0	0.29509	0	0	0
ENSG00000239483	RPS15AP16	0	0	0	0	0	0	0	0
ENSG00000244065	MARK2P17	0	0	0	0	0	0	0	0
ENSG00000243022	MARK3P3	0	0	0	0	0	0	0	0
ENSG00000242062	MARK2P6	0	0	0	0	0	0	0	0
ENSG00000261159	AC112484.3	0	0	0	0	0	0.15945	0	0
ENSG00000231305	AC112484.1	0.10069	0	0	0	0.06121	0.08881	0	0
ENSG00000250796	AC112484.2	0	0.37085	0	0	0	0	0.37137	0
ENSG00000177646	ACAD9	2.79514	1.9174	0.3734	0.90508	2.51265	2.33102	0.96005	1.18554
ENSG00000114656	KIAA1257	0.4786	0.53108	0.02758	0.11142	0.34227	0.149	0.14078	0.20739
ENSG00000187695		0.38579	0.50455	0.04049	0.0409	0.30152	0.14584	0.1378	0.1015
ENSG00000279507	AC112484.4	0	0.53512	0	0.95426	0	0	0	0
ENSG00000114654	EFCC1	0.02489	0	0	0.03694	0.03026	0	0	0.03056
ENSG00000249505	AC108673.1	0	0	0	0	0	0	0	0
ENSG00000169704	GP9	17.9747	10.9794	2.18093	7.34212	10.5256	18.9095	9.48306	9.71847
ENSG00000172780	RAB43	0.82244	1.05117	0.06474	0.17437	0.55352	0.36273	0.41616	0.16229
ENSG00000261796	ISY1-RAB43	0	0	0	0	0	0	0	0
ENSG00000273174	AC108673.2	1.42709	1.51161	0.38129	0.38508	0.63091	0.22887	0.4325	0
ENSG00000240682	ISY1	2.93058	2.20103	0.31511	0.55692	1.93354	1.79687	0.53614	0.81177
ENSG00000273437	AC108673.3	0.65642	0.3642	0.32153	0.64947	0	0.38601	0.36472	0.80594
ENSG00000169714	CNBP	211.837	172.091	31.4701	43.1304	152.15	83.7473	65.4334	77.8075
ENSG00000241673	RPS27P12	0	0	0	0	0	0	0	0
ENSG00000181789	COPG1	13.8981	11.5805	2.62895	5.16271	10.9155	12.5367	4.05887	7.46416
ENSG00000278658	MIR6826	0	0	0	0	0	0	0	0
ENSG00000183624	HMCES	6.21591	3.85798	1.04501	2.11083	6.98075	5.43639	2.54634	2.39302
ENSG00000184897	H1FX	11.3817	5.26248	2.28722	5.55839	8.04238	9.95366	6.08064	4.47895
ENSG00000206417	H1FX-AS1	0.17146	0.07135	0.0315	0	0.05212	0.07562	0.07145	0.05263
ENSG00000248916	NUP210P3	0	0	0	0	0	0	0	0
ENSG00000270492	AC137695.1	0	0	0	0	0	0	0	0
ENSG00000270773	AC137695.2	0	0	0	0	0	0	0	0
ENSG00000244932	AL449212.1	0	0	0	0	0	0	0	0
ENSG00000251474	RPL32P3	1.54608	0.9752	0.38264	0.38645	0.81123	0.60292	0.48829	0.43959

ENSG00000207088	SNORA7B	4.21872	3.51104	25.5724	40.6968	3.84676	2.7909	9.66895	18.7764
ENSG00000172771	EFCAB12	0.0847	0.04699	0	0.02793	0.01144	0.06641	0.03137	0.03466
ENSG00000129071	MBD4	16.4904	9.96136	2.59977	4.92712	12.0292	7.62924	4.95129	9.17133
ENSG00000163913	IFT122	1.66592	1.67135	1.46436	2.23532	3.43112	2.48264	2.37108	1.8119
ENSG00000163914	RHO	0	0	0	0	0	0	0	0
ENSG00000178804	H1FOO	0	0	0	0	0	0	0	0
ENSG00000004399	PLXND1	5.44973	3.29516	0.57518	1.66452	1.6198	1.58021	1.07902	0.80404
ENSG00000239437	RN7SL752P	2.04322	4.25117	3.00248	4.92758	3.72613	4.9562	3.83144	3.44936
ENSG00000248659	AC023162.1	0	0	0	0	0.18801	0	0	0
ENSG00000172765	TMCC1	9.14965	9.97102	2.81029	3.32521	7.10061	2.71226	5.23474	6.03126
ENSG00000202412	RNY3P13	0	0	0	0	0	0	0	0
ENSG00000283509	RF00026	0	0	0	0	0	0	0	0
ENSG00000203644	AC083799.1	29.0785	27.2173	3.31428	3.82543	19.684	8.38395	13.0236	14.2413
ENSG00000271270	TMCC1-AS1	0.30126	0.15835	0.1398	0.09413	0.21204	0.16783	0.05286	0.03893
ENSG00000250643	AC083906.3	0.1573	0	0	0	0	0.27749	0	0
ENSG00000170893	TRH	0	0	0	0	0	0	0	0
ENSG00000248459	AC083906.1	0	0	0	0	0	0	0	0
ENSG00000249869	AC083906.2	0	0	0	0	0	0	0	0
ENSG00000180770	OR7E129P	0	0	0	0	0	0	0	0
ENSG00000251178	OR7E21P	0	0	0	0	0	0	0	0
ENSG00000251610	AC083906.4	0	0	0	0	0	0	0	0
ENSG00000251287	ALG1L2	0.1467	0	0	0	0.05945	0.08627	0	0.24015
ENSG00000248243	LINC02014	0.10281	0	0	0	0	0	0	0
ENSG00000253540	FAM86HP	0.11268	0	0	0.08362	0	0.09939	0.18783	0
ENSG00000249846	LINC02021	0	0	0.05457	0	0.13544	0.13101	0.06189	0.04559
ENSG00000248618	ENPP7P3	0	0	0	0	0	0	0	0
ENSG00000250580	SNRPCP8	0	0	0	0	0	0	0	0
ENSG00000228252	COL6A4P2	0.0933	0.03106	0	0.02769	0.06806	0	0.09331	0
ENSG00000249253	AC093004.1	0	0	0	0	0	0	0	0
ENSG00000172752	COL6A5	0.00765	0	0.02247	0	0.0093	0	0	0.00939
ENSG00000206384	COL6A6	0.02262	0	0	0	0.00917	0.03991	0.02514	0
ENSG00000196455	PIK3R4	6.53104	4.81135	2.36426	3.68285	5.96763	5.94122	3.93177	5.25652
ENSG00000222783	RN7SKP212	0	0	0	0	0	0	0	0
ENSG00000232829	GSTO3P	0	0	0	0	0	0	0	0
ENSG00000017260	ATP2C1	6.13342	5.11884	4.27927	7.53456	5.89029	5.72833	6.21426	5.5266
ENSG00000284095	AC055733.4	0	0	0	0	0.06582	0	0	0
ENSG00000250592	AC055733.2	0	0	0	0	0	0	0	0
ENSG00000252894	RF00019	0	0	0	0	0	0	0	0
ENSG00000249098	AC055733.1	0	0	0	0	0	0	0	0
ENSG00000251579	AC055733.3	0	0	0	0	0	0	0	0
ENSG00000034533	ASTE1	2.5453	1.88691	0.75434	1.17452	2.15836	2.79226	1.60435	1.86454
ENSG00000114670	NEK11	0.34221	0.38733	0.26149	0.50787	0.58246	0.62784	0.38787	0.21848
ENSG00000250129	AC116424.1	0	0	0	0	0	0	0	0
ENSG00000201550	RNU6-726P	0	0	0	0	0	0	0	0
ENSG00000250608	AC010210.1	0.3316	0.08715	0.10258	0.28491	0.10609	0.03079	0.17454	0.10714
ENSG00000246082	NUDT16P1	1.74374	2.21642	0.55907	0.65874	1.77308	0.7271	0.31708	1.28456
ENSG00000198585	NUDT16	5.31842	4.8602	0.72024	1.20718	4.09513	2.11564	1.40797	2.22784
ENSG00000261167	AC107027.3	0.67538	0.38644	0.12406	0.46985	0.5132	0.5585	0.38698	0.36279
ENSG00000114686	MRPL3	12.5272	6.29141	2.53911	6.61129	12.0463	14.1933	6.9303	7.12684
ENSG00000249020	SNORA58	0	0	0	0.79406	0	0	0.89183	0
ENSG00000248468	AC107027.1	0	0	0	0	0	0	0	0
ENSG00000248530	BCL2L12P1	0	0	0	0	0	0	0	0
ENSG00000196353	CPNE4	0.11405	0.01898	0.0838	0.03385	0.0416	0.02012	0	0.014
ENSG00000251447	AC107027.2	0	0	0	0	0	0	0	0
ENSG00000265859	MIR5704	0	0	0	0	0	0	0	0
ENSG00000240854	AC117409.1	0	0	0	0	0.06803	0	0	0
ENSG00000242899	RPL7P16	0.09932	0.16532	0	0	0.12075	0	0	0
ENSG00000014257	ACPP	10.2267	6.60528	0.66791	1.4529	5.67473	2.46719	1.31126	2.31807

ENSG00000240890	AC020633.1	0.13049	0.27149	0	0.04841	0.03966	0.05755	0.05437	0.12016
ENSG00000213264	NIP7P2	0	0	0	0	0	0	0	0
ENSG00000138246	DNAJC13	14.3504	12.798	2.97391	5.73395	10.1493	9.19599	4.43386	7.70839
ENSG00000240303	ACAD11	0.16794	0.15052	0.03797	0.03835	0.10994	0.09116	0.0646	0.06345
ENSG00000274810	NPHP3-ACAD11	0	0	0	0	0	0	0.01569	0
ENSG00000129048	ACKR4	0	0	0	0	0.03921	0	0	0
ENSG00000251614	HSPA8P19	0	0	0	0	0	0	0	0
ENSG00000081307	UBA5	1.66421	1.09016	0.26822	1.08355	1.33146	2.23507	1.10959	1.0546
ENSG00000113971	NPHP3	0.29468	0.20123	0.06662	0.11214	0.33989	0.11997	0.21411	0.2412
ENSG00000248724	NPHP3-AS1	0	0	0.02065	0	0	0	0	0
ENSG00000249725	AC079942.1	0	0	0	0	0	0	0	0
ENSG00000144868	TMEM108	0.0261	0	0	0	0.01058	0	0.0145	0
ENSG00000251011	TMEM108-AS1	0	0	0	0	0	0	0	0
ENSG00000170819	BFSP2	0.03541	0	0	0	0.04305	0	0	0
ENSG00000249820	AC055753.1	0	0	0	0	0	0	0	0
ENSG00000249993	BFSP2-AS1	0.16398	0.13647	0	0.24337	0.09968	0	0	0.302
ENSG00000214301	AC022296.1	0.10748	0	0	0	0	0	0	0
ENSG00000272832	AC022296.3	0	0	0	0	0	0	0	0
ENSG00000250983	AC022296.2	0	0	0	0	0	0	0	0
ENSG00000201827	RF00438	0	0	0	0	0	0	0	0
ENSG00000091527	CDV3	68.1305	51.2676	13.2947	24.861	48.8744	48.2365	22.8301	32.4377
ENSG00000163781	TOPBP1	12.1398	8.46475	2.71145	5.57704	9.23312	12.7625	5.3073	5.96757
ENSG00000242337	TFP1	0.22136	0	0.04647	0.04693	0	0.05579	0	0.07765
ENSG00000252641	RNU6-678P	0	0	0	0	0	0	1.12092	0
ENSG00000201394	RNA5SP140	0	0	0	0	0	0	0	0
ENSG00000091513	TF	0	0.01425	0	0	0	0.0151	0	0
ENSG00000244062	AC080128.1	0	0	0	0	0	0	0	0
ENSG00000144867	SRPRB	2.71807	2.15996	0.85037	1.32729	2.87817	2.38206	1.34457	1.61478
ENSG00000154917	RAB6B	0.9277	0.5689	0.25112	0.32609	0.32649	0.64602	0.44762	0.56951
ENSG00000221972		0	0	0	0	0	0	0	0
ENSG00000174640	SLCO2A1	0.0555	0	0.04078	0	0.03374	0	0.02313	0
ENSG00000243832	LINC02000	0	0	0	0	0	0	0	0
ENSG00000163785	RYK	3.88045	3.27499	2.00784	5.27233	4.70667	4.16202	4.32725	3.44465
ENSG00000231845	HMGB3P14	0	0	0	0	0	0	0	0
ENSG00000240006	LINC02004	0	0	0	0	0	0	0	0
ENSG00000260633	AC010207.1	0	0	0	0	0	0	0	0
ENSG00000214289	RPL39P5	0	0	0	0	0	0	0	0
ENSG00000114019	AMOTL2	0	0	0	0	0.01489	0	0	0
ENSG00000277723	MIR6827	0	0	0	0	0	0	0	0
ENSG00000248377	HMGN1P9	0	0	0	0	0	0	0	0
ENSG00000214288	HMGB3P13	0	0	0	0	0	0	0	0
ENSG00000284561	MIR4788	0	0	0	0	0	0	0	0
ENSG00000129055	ANAPC13	12.6466	11.1716	3.24192	6.22555	8.53767	7.50984	5.95623	7.85901
ENSG00000182923	CEP63	10.5646	9.49196	1.17127	3.18313	6.64235	3.42582	2.02909	4.20805
ENSG00000249691	AC026117.1	0.24904	0	0	0	0	0	0	0
ENSG00000154928	EPHB1	3.61444	3.84127	0.6194	1.03218	2.24201	0.9295	1.31736	1.87602
ENSG00000174611	KY	0.08868	0.06326	0.01862	0	0	0	0	0.03111
ENSG00000253087	RNU6-1174P	0	0	0	0	0	0	0	0
ENSG00000201413	RNA5SP141	0	0	1.79523	0	0.74264	1.0776	1.01817	1.49995
ENSG00000240086	AC092969.1	0	0	0	0	0	0	0	0
ENSG00000242222	AC092989.1	0	0	0	0	0	0	0	0
ENSG00000241905	NDUFS6P1	0	0	0	0	0	0	0	0
ENSG00000073711	PPP2R3A	0.18706	0.07784	0.01374	0.04164	0.03411	0.099	0.06236	0.01148
ENSG00000227267	AC072039.1	0	0	0	0	0	0.22257	0	0
ENSG00000273455	AC072039.2	0	0	0	0	0	0	0.27832	0.41001
ENSG00000174579	MSL2	20.4435	19.7321	3.06546	7.51572	20.7793	17.9438	8.22529	10.6513
ENSG00000241438	TDGF1P6	0	0	0	0	0	0	0	0
ENSG00000244740	AC092991.1	0	0	0	0	0	0	0	0

ENSG00000114054	PCCB	1.51817	1.2473	0.52913	1.09768	1.86942	1.78551	1.15173	1.00368
ENSG00000118007	STAG1	12.5281	12.5647	7.04273	19.5914	15.9697	14.5859	18.3909	14.2922
ENSG00000200571	RNU6-1284P	0	1.22008	1.07714	1.08786	0	0	0	0.89997
ENSG00000244101	HMGN1P10	0	0	0	0	0	0	0	0
ENSG00000252914	RNU6-789P	1.43726	0	0	0	0.87369	0	0	0
ENSG00000240695	AC117382.1	0.60247	1.33708	0.14755	0.74511	1.22078	1.23998	0.33474	0.36985
ENSG00000261758	AC117382.2	0.06979	0.11616	0	0	0.14141	0.04104	0.03878	0.05712
ENSG00000242968	AC096992.1	0	0	0	0	0	0	0	0
ENSG00000168917	SLC35G2	0.06839	0.02846	0	0	0	0	0	0
ENSG00000273486	AC096992.2	0.13058	0	0	0.0646	0	0.38394	0	0.16033
ENSG00000239213	NCK1-DT	1.33367	1.36609	0.60302	1.37029	1.37199	2.0813	0.4275	1.32256
ENSG00000158092	NCK1	8.75199	8.87156	2.25428	4.40747	8.48844	8.46581	3.96668	4.9502
ENSG00000214283	RAD51AP1P1	0	0	0	0	0	0	0	0
ENSG00000174564	IL20RB	0.1482	0.19734	0.13067	0.08798	0.07207	0.15687	0.04941	0.14557
ENSG00000249407	IL20RB-AS1	0	0	0	0	0	0	0	0
ENSG00000201325	RNA5SP142	0	0	0	0	0	0	0	0
ENSG00000243886	AC117440.1	0	0	0	0	0	0	0	0
ENSG00000242070	NPM1P17	0	0	0	0	0	0	0	0
ENSG00000168875	SOX14	0	0	0	0	0	0	0	0
ENSG00000239513	LINC01210	0	0	0	0	0	0	0	0
ENSG00000261146	AC007159.1	0	0	0	0	0	0	0	0
ENSG00000241478	HSPA8P9	0	0	0.05544	0	0	0	0	0
ENSG00000066405	CLDN18	0	0	0	0	0	0	0	0
ENSG00000272609	AC016252.1	0	0	0	0	0	0	0	0
ENSG00000158163	DZIP1L	0.07265	0	0	0	0	0.05126	0.02422	0.05352
ENSG00000240668	KRT8P36	0	0	0	0	0	0	0	0
ENSG00000118017	A4GNT	0	0	0	0	0	0	0	0
ENSG00000138231	DBR1	3.52584	2.74133	0.88625	1.96227	4.00461	2.65989	2.01056	1.85121
ENSG00000114098	ARMC8	8.66385	7.64411	6.32196	13.2889	9.61027	7.65524	13.213	9.01067
ENSG00000181322	NME9	0	0.03264	0	0	0.02384	0	0	0
ENSG00000158186	MRAS	0.84068	0.53672	0.11846	0.27346	0.40603	0.26411	0.21115	0.19795
ENSG00000158220	ESYT3	0	0	0	0	0	0.02635	0	0
ENSG00000280399	AC022497.1	0	0	0	0	0	0	0	0
ENSG00000114107	CEP70	7.9768	12.0904	14.0857	25.7689	19.517	15.4765	22.5564	15.6579
ENSG00000158234	FAIM	0.77405	0.41948	0.31743	1.06862	1.20369	1.0162	0.6001	0.50833
ENSG00000174715	PPIAP72	0.43631	0.72624	0.42744	0.21584	0.17682	0	0	0.5357
ENSG00000051382	PIK3CB	6.84448	5.61084	2.27777	3.92183	5.21077	3.98643	4.20238	3.71453
ENSG00000239207	GAPDHP39	0.14975	0	0	0	0	0.13209	0	0.09193
ENSG00000242321	RPL23AP40	0	0	0	0	0	0	0	0
ENSG00000241429	EEF1A1P25	0	0	0	0	0	0	0	0
ENSG00000242244	ATP5MC1P3	0	0	0	0	0	0	0	0
ENSG00000244578	LINC01391	0	0	0	0	0	0	0	0
ENSG00000183770	FOXL2	0	0	0	0	0	0	0	0
ENSG00000206262	FOXL2NB	0	0	0	0	0	0	0	0
ENSG00000206260	PRR23A	0	0	0	0	0	0	0	0
ENSG00000175110	MRPS22	2.94182	2.38032	0.54037	0.86915	2.03667	2.57086	1.27129	1.62202
ENSG00000184814	PRR23B	0	0	0	0	0	0	0	0
ENSG00000233701	PRR23C	0	0	0	0	0	0	0	0
ENSG00000251137	RPL7L1P7	0	0	0	0	0	0	0	0
ENSG00000232416	BPESC1	0	0	0	0	0	0	0	0
ENSG00000281473	PISRT1	0	0	0	0	0	0	0	0
ENSG00000276304	RF01873	0	0	0	0	0	0	0	0
ENSG00000272656	AC024933.1	0	0	0	0	0	0	0	0
ENSG00000184432	COPB2	13.8049	11.7726	2.516	5.18851	11.803	10.5638	3.43665	7.12096
ENSG00000283418	RF00026	0	0	0	0	0	0	0	0
ENSG00000248932	AC097103.2	0.58779	0.34531	0.30485	2.66833	0.71461	0.12199	0.23053	0.38206
ENSG00000114113	RBP2	0	0	0	0	0	0	0	0
ENSG00000248790	AC097103.1	0	0	0	0.39848	0	0	0	0

ENSG00000178631	ACTG1P1	0.06493	0	0	0.09636	0	0	0	0.07971
ENSG00000114115	RBP1	0	0.03124	0	0	0	0	0	0
ENSG00000163864	NMNAT3	0.65845	0.70709	0.37455	0.72503	0.15494	0.29977	0.53106	0.78236
ENSG00000214280	AC046134.1	0.33018	0.73278	1.13213	3.1035	0.93666	0.38832	0.18345	1.08105
ENSG00000201600	RN7SKP124	0.21496	0	0	0.31902	0	0	0.3583	0.26392
ENSG00000244692	RN7SL724P	0	0.45022	0	0.80285	0	0	0.45085	0
ENSG00000261763	AC110716.2	0	0	0	0	0	0	0	0
ENSG00000250543	AC110716.1	0	0	0	0	0	0	0	0
ENSG00000251471	AC016933.1	0	0	0	0	0	0	0	0
ENSG00000250161	TRMT112P5	0	0	0	0	0	0	0	0
ENSG00000158258	CLSTN2	0	0.00836	0	0	0	0	0	0
ENSG00000249290	AC010181.1	0	0	0	0	0	0	0	0
ENSG00000251058	AC010181.2	0	0	0	0	0	0	0	0
ENSG00000250433	CLSTN2-AS1	0	0	0	0	0	0	0	0
ENSG00000249305	AC048346.1	0	0	0	0	0	0	0	0
ENSG00000155890	TRIM42	0	0	0	0	0	0	0	0
ENSG00000261826	AC121333.1	0	0	0	0	0	0	0	0
ENSG00000224085	RPL23AP41	0	0	0	0	0	0	0	0
ENSG00000114120	SLC25A36	4.70917	2.38046	0.88243	2.21629	3.85208	3.61019	3.51645	2.12454
ENSG00000248773	AC108727.1	2.23818	0.62091	0.27408	0.55362	1.36056	0.98711	0	0.229
ENSG00000175093	SPSB4	0	0	0.0576	0	0	0.03458	0	0
ENSG00000251270	AC108727.2	0	0	0	0	0	0	0	0
ENSG00000155893	PXYLP1	0.35206	0.43035	0.15359	0.21226	0.56847	0.36877	0.27508	0.31068
ENSG00000241400	AC022215.1	0	0	0	0	0	0	0	0
ENSG00000249417	AC117383.1	0	0	0	0	0	0	0	0
ENSG00000249540	AC117383.2	0	0	0	0	0	0	0	0
ENSG00000177311	ZBTB38	5.81545	6.83583	1.44445	2.99403	4.52372	9.75082	2.04163	2.78654
ENSG00000215606	KRT18P35	0	0	0	0	0	0	0	0
ENSG00000155903	RASA2	17.845	20.8267	9.41801	20.1489	21.8293	19.0742	16.8398	19.9426
ENSG00000250170	RASA2-IT1	0.8384	0.79744	0	0.17775	0.29123	1.26776	0.39928	0.29411
ENSG00000249986	YWHAQP6	0	0	0	0	0	0	0	0
ENSG00000242104	AC092978.1	0	0.09194	0	0	0	0	0	0
ENSG00000241899	TPT1P3	0	0	0	0.21247	0	0	0	0
ENSG00000241526	AC112771.1	0	0	0	0	0	0	0	0
ENSG00000114125	RNF7	6.45192	4.80745	1.88251	3.18026	4.67247	4.35559	3.45537	2.94557
ENSG00000114124	GRK7	0.02263	0	0	0	0	0.03992	0	0
ENSG00000228398	HMGN2P25	0	0	0	0	0	0	0	0
ENSG00000069849	ATP1B3	10.2405	9.44338	4.00176	9.72504	6.75962	13.4116	8.18003	6.93089
ENSG00000200389	RNU6-509P	0	0	0	0	0	0	0	0
ENSG00000244124	ATP1B3-AS1	0	0	0	0	0	0	0	0
ENSG00000279147	AC112504.1	0	0	0	0	0	0	0	0
ENSG00000114126	TFDP2	6.82666	9.52727	9.1082	20.894	12.5169	11.5758	19.2457	12.5347
ENSG00000233597	AC133435.1	0	0.21182	0	0	0	0	0	0.15624
ENSG00000206604	RNU6-425P	0	0	0	0	0	0	0	0
ENSG00000175066	GK5	1.99559	1.78627	0.7885	2.05796	1.09948	3.00997	1.19588	1.45825
ENSG00000114127	XRN1	25.9506	36.5557	19.966	38.8676	31.6616	33.0456	32.508	36.5036
ENSG00000251804	RNU6-1294P	0	0	0	0	0	0	1.5084	2.22215
ENSG00000202125	RNU1-100P	0.44424	1.47889	0	0	1.0802	0.78371	4.44292	1.63631
ENSG00000175054	ATR	4.23518	2.86079	1.37918	3.13403	4.10068	5.04995	2.28795	2.90964
ENSG00000242479	AC109992.1	0.07158	0	0	0.21247	0	0	0.17898	0.04394
ENSG00000244327	AC109992.2	0.47909	0	0	0.1422	0	0	0	0.11764
ENSG00000242390	RPL6P9	0	0	0	0.12664	0	0	0	0
ENSG00000252745	RNA5SP143	0	0	0	0	0	0	0	0
ENSG00000120756	PLS1	0.73383	0.55312	1.07836	1.58226	0.87534	0.73278	1.01548	0.84999
ENSG00000239641	PLS1-AS1	0	0	0	0	0	0	0	0
ENSG00000199319	RN7SKP25	0	0	0	0	0	0	0	0
ENSG00000144935	TRPC1	0.70718	0.61414	1.80728	2.46411	0.35512	0.46105	1.20438	0.79276
ENSG00000251787	RNU7-47P	0	0	0	0	0	0	0	0

ENSG00000163710	PCOLCE2	0	0	0	0	0	0.18579	0	0.05172
ENSG00000240950	AC021074.1	0	0	0	0	0	0	0	0
ENSG00000243818	AC021074.3	0	0	0	0	0	0	0.05931	0
ENSG00000188582	PAQR9	1.05493	0.73164	0.93013	2.89643	1.83834	1.02358	9.17305	3.77776
ENSG00000241592	AC021074.2	0	0	0	0	0	0	0	0
ENSG00000241570	PAQR9-AS1	0	0	0	0	0	0	0.02764	0
ENSG00000163714	U2SURP	14.1287	8.7243	3.53621	6.6927	11.6145	11.2936	6.51672	8.37803
ENSG00000268129	AC026304.1	0.6533	0.43497	0.192	0.38783	0.31771	0.2305	0.21779	0.16042
ENSG00000243733	AC018450.2	0	0	0	0	0	0	0	0
ENSG00000175040	CHST2	6.59095	5.35218	1.36347	5.00956	4.72618	4.34614	2.69319	3.86936
ENSG00000241679	AC018450.1	0.58955	0.81775	0.43317	0.5833	0.35838	0.34668	0.8189	0.48256
ENSG00000244171	PBX2P1	0.05678	0.09451	0	0.16853	0	0	0	0
ENSG00000181804	SLC9A9	6.02616	6.23924	3.42618	7.37305	7.65352	7.43535	4.81307	5.24084
ENSG00000240888	AC131210.1	0.33243	0.27666	0.24425	0.24668	0.20208	0	0.55411	0.20407
ENSG00000240012	SLC9A9-AS1	0.70753	0	0	0	0.8602	0	0	0
ENSG00000244493	SLC9A9-AS2	0.22416	0	0	0	0.54506	0	0	0.55044
ENSG00000243033	GAPDHP47	0	0	0	0	0	0.38832	0	0
ENSG00000243759	ST13P15	0.46351	0.22043	0.19461	0.19654	0.16101	0.23363	0.22074	0
ENSG00000248374	AC073358.1	0	0	0	0	0	0	0	0
ENSG00000235110	AC107421.1	0	0	0	0.26927	0	0	0	0
ENSG00000181744	DIPK2A	11.1013	12.5411	16.1757	22.4233	16.1602	13.0797	32.2207	14.7453
ENSG00000222778	RNA5SP144	0	0	0	0	0	0	0	0
ENSG00000280397	AC011592.1	0	0	0	0	0	0	0	0
ENSG00000244024	LARP7P4	0	0	0	0	0	0	0	0
ENSG00000241695	GM2AP1	0	0	0	0	0	0	0	0
ENSG00000239797	RPL21P39	0	0	0	0	0	0	0	0
ENSG00000244358	AC055758.2	0	0	0	0	0	0	0	0
ENSG00000240095	AC055758.1	0	0	0	0	0	0	0	0
ENSG00000261051	AC107021.2	0	0.11648	0	0	0	0	0	0
ENSG00000243415	AC107021.1	0	0	0	0	0	0	0	0
ENSG00000240032	LNCSRLR	0.20677	0.17209	0	0	0	0	0	0
ENSG00000152952	PLOD2	1.24628	0.84573	0.87343	1.77847	0.9907	0.91327	1.58198	0.96518
ENSG00000114698	PLSCR4	0.79769	1.21229	1.68184	4.96703	1.41254	2.32498	2.34128	2.00135
ENSG00000243596	AC130509.1	0	0	0	0	0	0	0	0
ENSG00000241358	AC069528.1	0	0	0	0	0	0	0	0
ENSG00000163746	PLSCR2	0.05733	0.04772	0.04213	0.04254	0.0697	0	0	0.17598
ENSG00000279320	AC069528.2	0.36816	0.6128	0.0541	0.27319	0	0.1299	0.30683	0.31641
ENSG00000188313	PLSCR1	16.5517	46.7231	15.8344	6.61738	8.00818	9.77287	4.5325	15.988
ENSG00000199812	RNU6-428P	0	0	0	0	0	0	0	0
ENSG00000231213	PLSCR5	0	0	0	0	0	0	0	0
ENSG00000241457	PLSCR5-AS1	0	0	0	0	0	0	0	0
ENSG00000243620	AC092957.1	0	0	0	0	0	0	0	0
ENSG00000242671	LINC02010	0	0	0	0	0	0	0	0
ENSG00000207156	RNU6-505P	0	0	0	0	0	0	0	0
ENSG00000239412	RPL21P71	0	0	0	0	0	0	0	0
ENSG00000174963	ZIC4	0	0	0	0	0	0	0	0
ENSG00000241202	ZIC4-AS1	0	0	0	0	0	0	0	0
ENSG00000152977	ZIC1	0	0	0	0	0.02619	0	0	0
ENSG00000240804	NPM1P28	0	0	0	0	0	0	0	0
ENSG00000239661	AC092925.1	0	0	0	0	0	0	0	0
ENSG00000239922	AC092958.1	0	0	0	0	0	0	0	0
ENSG00000241131	LINC02032	0	0	0	0	0	0	0	0
ENSG00000241792	AC092958.2	0	0	0	0	0	0	0	0
ENSG00000244650	AC025566.1	0	0	0	0	0	0	0	0
ENSG00000205412	HNRNPA1P20	0	0	0	0	0	0	0	0
ENSG00000243347	LINC02045	0	0	0	0	0	0	0.35988	0
ENSG00000242440	LINC02046	0	0	0	0	0	0	0	0
ENSG00000241993	RPL38P1	0	0	0	0	0	0	0.57905	0

ENSG00000144891	AGTR1	0	0	0	0	0	0	0	0
ENSG00000153002	CPB1	0	0	0	0.01908	0	0	0	0
ENSG00000244089	HMGB1P30	0.60479	0	0.35549	0.53854	0.73529	0.42677	1.61294	0.44553
ENSG00000240521	AC092979.1	0.05184	0	0	0.07693	0	0	0	0
ENSG00000163751	CPA3	6.10836	3.69723	0.17486	0.76527	6.12436	0.62977	0.99172	3.65248
ENSG00000241627	UBQLN4P1	0	0	0	0	0	0	0	0
ENSG00000163754	GYG1	6.9104	12.1626	1.68734	2.78858	6.32015	8.9498	2.22715	5.40854
ENSG00000071794	HLTF	7.02097	5.06343	4.72053	13.219	7.78144	8.37178	9.93831	7.60433
ENSG00000239718	HLTF-AS1	0	0	0	0	0.42948	0	0	0
ENSG00000201031	RF00019	0.66637	0	3.91687	1.97792	2.43045	1.17556	3.33219	1.63631
ENSG00000240511	MED28P2	0	0	0	0	0	0	0	0
ENSG00000163755	HPS3	5.62407	4.4661	1.25689	1.99974	4.30199	4.65076	2.36314	3.51012
ENSG00000047457	CP	0	0	0	0	0	0	0	0
ENSG00000240216	CPHL1P	0	0.03101	0	0.02765	0.02265	0.06574	0.06212	0
ENSG00000244468	AC093001.1	0	0	0	0	0	0	0	0
ENSG00000163762	TM4SF18	0	0	0	0	0.03977	0	0	0
ENSG00000243822	AC073522.1	0	0	0	0	0	0	0	0
ENSG00000169908	TM4SF1	0.39967	0.16631	0	0.02966	0.07289	0.28203	0.03331	0.02454
ENSG00000240541	TM4SF1-AS1	0	0	0	0	0	0	0	0
ENSG00000243885	AC108751.4	0	0	0	0	0	0	0	0
ENSG00000240246	AC108751.2	0	0	0	0	0	0	0	0
ENSG00000241929	AC108751.3	0	0	0	0	0	0	0	0
ENSG00000282502	FKBP1AP4	0	0	0	0	0	0	0	0
ENSG00000239408	AC108751.1	0	0	0	0	0	0	0	0
ENSG00000169903	TM4SF4	0	0	0.05033	0	0.24986	0	0	0
ENSG00000244503	AC108751.5	0	0	0.08062	0	0	0	0	0
ENSG00000018408	WWTR1	0.04356	0.029	0	0.06465	0.04237	0.16908	0.01452	0.04279
ENSG00000252581	RNU6-1098P	0	0	0	0	0	0	0	0
ENSG00000241985	WWTR1-IT1	0	0	0	0	0	0	0	0
ENSG00000241313	WWTR1-AS1	0	0	0	0	0	0	0	0
ENSG00000114744	COMMD2	4.2326	3.46035	1.20879	1.82012	3.58212	3.95772	2.09409	2.95644
ENSG00000206199	ANKUB1	0.11201	0.01864	0.01646	0	0.12256	0.05928	0.05601	0.08252
ENSG00000251854	RNU6-507P	0	0	0	0	0	0	0	0
ENSG00000082996	RNF13	70.6587	110.63	41.5447	72.6934	77.7902	44.4676	57.6785	61.5743
ENSG00000252172	RNU6-720P	0	0	0	0	0	0	0	0
ENSG00000239351	NPM1P29	0	0	0	0	0	0	0	0
ENSG00000070087	PFN2	0.21308	0.39014	0	0.1265	0.4145	0.07518	0.39069	0.18313
ENSG00000242791	AC117395.1	0.20333	0.33844	0	0.15088	0.49441	0	0.33892	0.37447
ENSG00000224831	TMEM183B	0	0.21633	0.09549	0.28932	0.15801	0	0.10832	0
ENSG00000182000	PPIAP73	0.36377	0	0	0.26994	0	0	0	0.22332
ENSG00000241947	HNRNPA1P24	0	0.11664	0	0	0.0852	0	0	0.25812
ENSG00000243944	AC117386.2	0	0	0	0	0	0	0	0
ENSG00000240477	AC022494.1	0	0.16759	0	0.14943	0.61207	0	0.16783	0.12362
ENSG00000243321	LINC01998	0	0	0	0	0	0	0	0
ENSG00000241143	RPL32P9	0	0	0	0	0	0	0	0
ENSG00000241048	AC117386.1	0	0	0	0	0	0	0	0
ENSG00000244541	LINC01213	0	0	0	0	0	0	0	0
ENSG00000278234	RF00004	0	0	0	0	0	0	0	0
ENSG00000243550	LINC01214	0	0	0	0	0	0	0	0
ENSG00000196428	TSC22D2	2.94432	2.39294	0.60887	1.09941	2.43476	1.82738	1.13014	1.06369
ENSG00000120742	SERP1	23.3025	17.7907	5.86645	10.5814	14.6073	15.548	12.7346	15.3936
ENSG00000144895	EIF2A	21.8606	14.4425	6.44041	12.0083	20.5156	20.6911	11.9489	11.7425
ENSG00000198843	SELENOT	23.6653	22.8969	6.17369	9.05013	18.8578	19.2326	10.0758	16.0406
ENSG00000271711	AC069236.1	0	0	0	0	0	0	0	0
ENSG00000163645	ERICH6	0.07065	0.0588	0	0.47184	0.0859	0.12464	0.29441	0.04337
ENSG00000240137	ERICH6-AS1	0.97497	0	0.13025	0.78926	1.40087	0.31273	0.44322	0.54412
ENSG00000244668	SNRPCP3	0	0	0	0	0	0.54333	0	0
ENSG00000181788	SIAH2	48.6241	101.635	37.3315	31.7586	38.6501	113.895	28.946	25.9984

ENSG00000244265	SIAH2-AS1	0	0	0	0	0	0.58645	0.27705	0.40815
ENSG00000239265	CLRN1-AS1	0	0.07964	0	0	0	0	0	0
ENSG00000214237	MINDY4B	0	0	0	0	0	0	0	0
ENSG00000260234	AC020636.2	0	0	0	0	0	0	0	0
ENSG00000243273	AC020636.1	0	1.07972	0.19064	0	0.47319	0	0	0.95572
ENSG00000163646	CLRN1	0	0	0	0	0	0	0	0
ENSG00000276055	RF02089	0	0	0	0	0	0	0	0
ENSG00000144893	MED12L	1.12689	1.21825	1.22063	2.76727	1.00282	1.78305	1.51043	1.33366
ENSG00000199994	RNA5SP145	0	0.97607	4.30856	14.7949	1.42587	0	0.97744	2.15993
ENSG00000174946	GPR171	10.3315	6.3893	2.88037	9.21194	11.6671	21.9002	3.06302	6.16693
ENSG00000174944	P2RY14	11.952	16.9035	7.53806	5.95133	8.95915	12.357	1.38891	11.0938
ENSG00000239570	SETP11	0.42493	0	0	0	0	0	0	0
ENSG00000138271	GPR87	0	0	0	0	0	0	0	0
ENSG00000181631	P2RY13	86.6928	126.643	18.4719	18.8525	50.4264	21.6611	15.8251	49.8826
ENSG00000169313	P2RY12	13.4851	10.501	7.82215	7.78295	6.80719	6.39951	7.95257	10.118
ENSG00000152580	IGSF10	0.0125	0	0	0	0.0152	0	0.02084	0
ENSG00000242810	MRPL42P6	0	0	0	0	0	0	0	0
ENSG00000242119	AC117454.1	0	0	0	0	0	0	0	0
ENSG00000264330	MIR5186	0	0	0	0	0	0	0	0
ENSG00000241151	LINC02066	0	0	0	0	0	0	0	0
ENSG00000197953	AADACL2	0	0	0	0	0	0	0	0
ENSG00000242908	AADACL2-AS1	0	0	0	0	0	0	0	0
ENSG00000242097	AC068647.1	0	0	0	0	0	0	0	0
ENSG00000240602	AADACP1	0	0	0	0	0	0	0	0
ENSG00000250271	AC068647.2	0	0	0	0	0	0	0	0
ENSG00000114771	AADAC	0	0	0	0	0	0	0	0
ENSG00000198829	SUCNR1	1.1045	0.81709	0.56678	0.36427	0.29841	1.70106	0.52601	0.45203
ENSG00000239941	AC108718.1	2.11698	0.88093	0.19443	0.19636	0.64344	0.70024	0	0.81225
ENSG00000152601	MBNL1	86.2594	67.8748	24.0682	47.4392	65.4301	81.1678	56.2771	49.0484
ENSG00000229619	MBNL1-AS1	0.58996	0.31498	0.09814	0.18172	0.3654	0.43202	0.35253	0.164
ENSG00000221962	TMEM14EP	0	0	0	0	0	0	0	0
ENSG00000201217	RF00019	6.48676	7.55805	4.7661	5.77623	2.36593	4.57741	5.40621	6.37147
ENSG00000243305	AC026347.1	0	0	0	0	0	0	0	0
ENSG00000244545	AC092924.1	0	0	0	0	0	0	0	0
ENSG00000242561	ATP5MGP5	0	0	0	0	0	0	0	0
ENSG00000169860	P2RY1	1.43407	1.04884	0.70245	0.93517	0.93779	0.6708	0.99599	1.22717
ENSG00000241810	HMGN2P13	0	0	0	0	0	0	0	0
ENSG00000241671	AC117394.1	0	0	0	0	0	0	0	0
ENSG00000244268	AC117394.2	0	0.37774	0	0	0.2759	0	0	0
ENSG00000181467	RAP2B	14.8339	11.6734	3.65023	7.43823	9.46551	10.0185	5.54501	7.18813
ENSG00000265813	RN7SL300P	0	0.38367	0	0	0	0.40664	0	0
ENSG00000241220	AC078788.1	0	0	0.1699	0	0	0	0.19271	0
ENSG00000241912	AC078788.2	0	0	0	0	0	0	0	0
ENSG00000238755	LINC02006	0.02603	0	0	0	0	0	0	0
ENSG00000243012		0	0.47382	0	0	0.17304	0	0	0
ENSG00000207323	RNU6-901P	0	0	0	0	0	0	0	0
ENSG00000237787	C3orf79	0	0	0	0.18756	0	0	0.21066	0
ENSG00000200162	RF00019	0	0	0	0	0	0	0	0
ENSG00000243486	AC068985.1	0	0	0	0	0	0	0	0
ENSG00000201398	RF00096	0	0	0	0	0	0	0	0
ENSG00000240068	RPL21P42	0	0	0	0	0	0	0	0
ENSG00000243069	ARHGEF26-AS1	0	0.01854	0	0	0	0.01965	0	0.01368
ENSG00000114790	ARHGEF26	0.01335	0.02223	0	0	0.03247	0	0	0
ENSG00000174953	DHX36	2.76906	1.97533	1.20339	2.27624	2.94468	2.91386	2.54494	2.09613
ENSG00000271922	RF01210	0	0	0	0	0	0	0	0
ENSG00000279305	AC134026.1	0	0	0	0	0	0	0	0
ENSG00000174948	GPR149	0	0	0	0	0	0	0	0
ENSG00000240048	DDX50P2	0	0	0	0	0	0	0	0.04104

ENSG00000243861	AC092994.1	0	0	0	0	0	0	0	0
ENSG00000243154	SYPL1P1	0	0	0	0	0	0	0	0
ENSG00000242151	DYNLL1P5	0	0	0	0	0	0	0	0
ENSG00000240601	RPL9P15	0	0	0	0	0	0	0	0
ENSG00000243969	AC073359.2	0.41767	0.3476	0	0.30993	0.25389	0	0	0
ENSG00000243953	AC073359.1	0.42207	0.23418	0	0	0.6842	0	0.23451	0.34548
ENSG00000196549	MME	38.3874	53.4326	3.79574	9.38588	38.7685	10.0949	14.3009	37.9541
ENSG00000240666	MME-AS1	0	0	0	0	0	0	0	0
ENSG00000241336	LINC01487	0	0	0	0	0	0.27959	0	0
ENSG00000240045	DWORF	0	0	0	0	0	0	0	0
ENSG00000242790	AC104831.1	0	0	0	0	0	0	0	0
ENSG00000239443	PABPC1P10	0.19239	0	0	0	0	0	0	0
ENSG00000114805	PLCH1	0.33722	0.23813	0.21023	0.19716	0.23605	0.10817	0.11923	0.27603
ENSG00000248772	AC108729.3	0	0	0	0	0	0	0	0
ENSG00000243518	AC108729.2	0	0	0	0	0	0	0	0
ENSG00000242152	AC108729.1	0	0	0	0	0.0941	0	0	0
ENSG00000272096	RN7SL715P	0	0	0	0	0	0	0	0
ENSG00000239508	PLCH1-AS1	0	0	0	0	0	0	0	0
ENSG00000242925	PLCH1-AS2	0	0	0	0	0	0	0	0
ENSG00000239212	RPL6P7	0	0	0	0	0	0	0	0
ENSG00000214919	AC104472.1	0	0	0	0	0	0	0	0
ENSG00000241307	AC104472.2	0	0	0	0	0	0	0	0
ENSG00000174928	C3orf33	0.79002	0.45739	0.8076	0.86662	0.4176	0.30298	0.80156	0.71694
ENSG00000169359	SLC33A1	3.56337	3.57395	2.4839	5.96643	3.86015	3.29089	4.74659	3.59913
ENSG00000163655	GMPS	7.46616	7.79975	6.78895	14.717	10.7522	16.3441	16.7491	10.5849
ENSG00000240489	SETP14	0.25189	0.27952	0	0.37383	0	0	0	0
ENSG00000174930	VN2R1P	0	0	0	0	0	0	0	0
ENSG00000169282	KCNAB1	0	0	0	0	0	0	0.03566	0.0394
ENSG00000242017	ALG1L15P	0	0	0	0.13701	0	0	0	0
ENSG00000242537	MRE11P1	0.03446	0	0	0	0	0	0	0
ENSG00000227709	AC091607.1	0	0	0	0	0	0	0	0
ENSG00000240596	KCNAB1-AS2	0	0	0	0	0	0	0	0
ENSG00000242370	KCNAB1-AS1	0	0	0	0	0	0	0	0
ENSG00000272990	AC084036.1	0.28858	0.48035	0.21204	0	1.05256	0.5091	0	0.17716
ENSG00000114850	SSR3	19.0895	11.4704	4.56277	9.73358	14.6377	12.0173	8.15944	9.43345
ENSG00000243926	TIPARP-AS1	1.11061	0.19459	0	0.26025	0.28426	0.92808	0.68203	0.28707
ENSG00000163659	TIPARP	11.7327	9.32949	4.11822	8.29685	12.5695	15.2162	6.65602	6.45383
ENSG00000174912	METTL15P1	0.21043	0.11675	0.20615	0.3123	0.34112	0.12374	0	0
ENSG00000240875	LINC00886	0.09589	0.09577	0.02818	0.05693	0.20985	0.06767	0.0959	0.04709
ENSG00000230457	PA2G4P4	0.49611	0.20644	0.09113	0.27611	0.30158	0.3282	0.3101	0.07614
ENSG00000197980	LEKR1	0.02617	0	0.11537	0.33012	0.1909	0.09233	0.10905	0.12852
ENSG00000241879	KLF3P2	0	0	0	0	0	0	0	0
ENSG00000239706	AC117392.1	0	0	0	0	0	0	0	0
ENSG00000222499	RN7SKP177	0	0	0	0	0	0	0	0
ENSG00000243629	LINC00880	0.07265	0.06046	0	0	0	0	0.06055	0.0446
ENSG00000241544	LINC02029	0	0	0	0	0	0.08638	0	0
ENSG00000241135	LINC00881	0.05776	0	0	0	0	0.2038	0	0
ENSG00000163660	CCNL1	29.5082	23.7615	5.60226	11.0668	22.052	14.6511	9.42002	20.8986
ENSG00000201778	RF00019	0	0	0	0	0	0	0	0
ENSG00000244515	KRT18P34	0	0	0	0	0	0	0	0
ENSG00000241770	AC104411.1	0	0	0	0	0	0.4041	0.38181	0.56248
ENSG00000222675	RNA5SP146	0	0	0	0	0	0	0	0
ENSG00000243176	AC092944.1	0.47474	0.71119	0.34881	0.91594	0.86577	0.67001	0.55393	0.58288
ENSG00000270981	AC092944.2	0	0	0	0	0	0	0	0
ENSG00000197415	VEPH1	3.09799	0.93079	1.01896	0.74693	0.47591	0.59191	0.68976	0.57673
ENSG00000163661	PTX3	1.09573	0.18867	0.16657	0.2243	0.64311	0.19997	0.25192	0.09278
ENSG00000174899	PQLC2L	0	0	0	0	0	0	0	0.0566
ENSG00000251751	RN7SKP46	0	0	0	0	0	0	0	0

ENSG00000239716	AC079943.1	0	0	0	0	0	0	0	0
ENSG00000242536	AC079943.2	0	0	0	0	0	0	0	0
ENSG00000168779	SHOX2	0.01453	0.04837	0	0	0	0	0	0
ENSG00000174891	RSRC1	12.8064	15.4031	10.4409	25.0226	18.7742	13.2167	37.5829	17.9748
ENSG00000241723	AC074276.1	0	0	0	0	0	0	0	0
ENSG00000277143	AC074276.2	0	0	0	0	0	0	0	0
ENSG00000243314	AC106707.2	0.19599	0.32623	0	0	0	0.34575	0	0
ENSG00000243150	AC106707.1	0	0	0	0	0	0	0	0
ENSG00000178053	MLF1	0.04108	0.13674	0.1509	0.06096	0.02497	0.07246	0.03423	0.05043
ENSG00000243025	MTAPP1	0	0	0	0	0	0	0	0
ENSG00000243165	AC025033.1	0	0	0	0	0	0	0	0
ENSG00000168827	GFM1	3.76565	2.78274	0.7394	2.07165	3.95657	3.67949	2.24556	2.3416
ENSG00000079257	LXN	4.0939	5.3541	1.07427	2.60391	3.25892	4.21295	2.51834	2.63289
ENSG00000272087	AC080013.4	0	0	0	0	0	0	0	0
ENSG00000272440	AC080013.6	0	1.13496	0	0.50598	0	0.60145	0.56828	0
ENSG00000118849	RARRES1	0.03278	0.10913	0.04817	0.19461	0	0	0.10928	0.161
ENSG00000118855	MFSD1	30.3847	23.4188	6.4775	11.685	18.9761	13.2051	11.2522	17.2063
ENSG00000240207	AC080013.1	0.07019	0	0.02579	0.05209	0.23469	0.09287	0	0.04309
ENSG00000243675	AC080013.2	0	0	0	0	0	0	0	0
ENSG00000271778	AC080013.3	0	0	0.37271	0	0	0.22372	0	0
ENSG00000272247	AC080013.5	0.53977	0.35938	0	0	0.65623	0.19044	0	0.13254
ENSG00000232374	GPR79	0	0	0	0	0	0	0	0.10368
ENSG00000279311	AC092999.1	0.30478	0.19024	0.05598	0	0.04632	0	0	0.04678
ENSG00000283154	IQCJ-SCHIP1	0	0.05535	0	0	0	0	0	0
ENSG00000214216	IQCJ	0	0	0	0	0	0	0	0
ENSG00000263634	MIR3919	0	0	0	0	0	0	0	0
ENSG00000244249	AC063955.1	0	0	0	0	0	0	0	0
ENSG00000243116	AC092943.1	0	0	0	0	0	0	0	0
ENSG00000241211	IQCJ-SCHIP1-AS1	0	0	0	0	0	0	0	0
ENSG00000252763	RNU2-31P	0	0	0	0	0	0	0	0
ENSG00000151967	SCHIP1	0	0	0	0	0	0	0	0.01177
ENSG00000244040	IL12A-AS1	0	0	0	0	0	0	0	0
ENSG00000168811	IL12A	0.28745	0.05981	0	0.05333	0.21842	0.31694	0.05989	0.08823
ENSG00000242107	LINC01100	0	0	0	0	0	0	0	0
ENSG00000184100	BRD7P2	0.11312	0	0	0	0.04584	0.19956	0	0.04629
ENSG00000180044	C3orf80	0.02697	0.13467	0	0.12007	0	0	0	0
ENSG00000248710	AC079594.2	0	0	0	0.03238	0	0	0	0
ENSG00000068885	IFT80	1.82868	1.24134	1.42618	3.85109	2.23568	1.51389	3.9847	2.37065
ENSG00000243687	AC079594.1	0	0	0	0	0	0	0	0
ENSG00000113810	SMC4	20.4464	21.7702	14.9146	25.2508	26.7972	28.3639	27.0308	29.7862
ENSG00000207779	MIR15B	0	0	0	1.11006	1.81871	0	0	0
ENSG00000198987	MIR16-2	0	0	0	0	1.10021	0	0	0
ENSG00000213186	TRIM59	1.00654	0.76013	0.80803	1.72895	1.3257	1.28243	1.569	0.8582
ENSG00000244009	B3GAT3P1	0	0	0	0	0	0	0	0
ENSG00000238427	RNU7-136P	0	0	0	0	0	0	0	0
ENSG00000186432	KPNA4	15.0292	15.0226	3.63891	6.48346	10.8591	8.47608	6.11356	8.32757
ENSG00000238741	SCARNA7	2020.42	2127.01	1069.96	3450.16	2441.53	2112.09	1391.74	1346.14
ENSG00000229320	KRT8P12	1.57558	0.71524	0.10524	0.05314	0.91424	0.63171	0.11938	0.43965
ENSG00000242285	RPL6P8	0	0.14473	0	0	0	0	0.14494	0
ENSG00000179674	ARL14	0	0	0	0	0	0	0	0
ENSG00000207084	RF00139	0	0	0	0	0	0	0	0
ENSG00000260572	AC069224.1	0.47015	0.21343	0.06281	0.12686	0.15589	0.3016	0.35621	0.10495
ENSG00000163590	PPM1L	3.28606	2.51918	0.85673	2.2549	1.89732	1.74535	1.49204	2.10405
ENSG00000169255	B3GALNT1	0.2979	0.26251	0.72101	1.22231	0.44739	0.30914	0.46734	0.12909
ENSG00000169251	NMD3	6.69925	5.0367	1.81404	5.3622	6.46091	5.57722	4.2659	4.06641
ENSG00000271052	AC108738.1	0	0	0	0	0	0	0	0
ENSG00000240138	EEF1GP4	0	0	0	0	0	0	0	0
ENSG00000243838	PSMC1P7	0	0	0	0	0	0	0	0

ENSG00000196542	SPTSSB	0.09786	0.13032	0.05752	0.26143	0.19037	0.13812	0	0.12016
ENSG00000240567	LINC02067	0	0	0	0	0	0	0	0
ENSG00000269888	AC112491.1	0	0	0	0	0	0	0	0
ENSG00000234851	RPL23AP42	5.7582	4.4037	1.14346	3.46451	7.37909	5.2164	2.59406	2.67507
ENSG00000182447	OTOL1	0	0	0	0	0	0	0	0
ENSG00000240354	AC131211.1	0	0	0	0	0	0	0	0
ENSG00000241874	TOMM22P6	0	0	0	0	0	0	0	0
ENSG00000243044	AC128716.1	0	0	0	0	0	0	0	0
ENSG00000241168	AC128685.1	0	0	0	0	0	0	0	0
ENSG00000244571	RPS6P4	0	0	0	0	0	0	0	0
ENSG00000241369	LINC01192	0	0	0	0	0	0	0	0
ENSG00000238924	RNU7-82P	0	0	0	0	0	0	0	0
ENSG00000240792	AC079910.1	0	0	0	0	0	0	0	0
ENSG00000214210	AC084017.1	0	0	0	0	0	0	0	0
ENSG00000221251	MIR1263	0	0	0	0	0	0	0	0
ENSG00000241636	LINC01323	0	0	0	0	0	0	0	0
ENSG00000241767	LINC01324	0	0	0	0	0	0	0	0
ENSG00000090402	SI	0	0	0	0	0	0	0	0
ENSG00000200052	RF00019	0	0	0	0	0	0	0	0
ENSG00000239205	LINC02023	0	0	0	0	0	0	0	0
ENSG00000121871	SLITRK3	0	0	0	0	0	0	0	0
ENSG00000244128	LINC01322	0	0	0	0	0	0	0	0
ENSG00000114200	BCHE	0	0	0	0	0	0	0	0
ENSG00000242178	MTND4P17	0	0	0	0	0	0	0	0
ENSG00000240084	MTND4LP10	0	0	0	0	0	0	0	0
ENSG00000240824	MTND3P7	0	0	0	0	0	0	0	0
ENSG00000242456	MTCO3P38	0	0	0	0	0	0	0	0
ENSG00000242326	MCUR1P2	0	0	0	0	0	0	0	0
ENSG00000244429	AC104629.1	0	0	0	0	0	0	0	0
ENSG00000244321	LINC01326	0	0	0	0	0	0	0	0
ENSG00000274149	AC072046.2	0	0	0	0	0	0	0	0
ENSG00000251759	RN7SKP298	0	0	0	0	0	0	0	0
ENSG00000224962	PSAT1P4	0	0	0	0	0	0	0	0
ENSG00000240458	AC072046.1	0	0	0	0	0	0	0	0
ENSG00000231516	CBX1P5	0	0	0	0	0	0	0	0
ENSG00000240033	AC069439.2	0	0	0	0	0	0	0	0
ENSG00000239227	AC069439.1	0	0	0	0	0	0	0	0
ENSG00000220343	PPIAP74	0	0	0	0	0	0	0	0
ENSG00000169064	ZBBX	0.01845	0	0	0	0	0	0	0
ENSG00000241669	LINC01327	0	0	0	0	0	0	0	0
ENSG00000114204	SERPINI2	0.10342	0.30126	0	0.03837	0.03143	0	0.0431	0.03174
ENSG00000174776	WDR49	1.43172	1.4424	0.52597	1.53771	1.00774	0.79761	0.75362	0.83266
ENSG00000114209	PDCD10	29.1411	45.227	30.127	63.3781	37.5242	29.8108	57.975	43.5009
ENSG00000241120	HMGN1P8	4.07224	1.69456	0.37401	2.6441	1.54716	0.898	0.84847	0.62498
ENSG00000163536	SERPINI1	1.24289	1.86696	0.71275	0.71984	1.25309	1.06958	1.11165	0.93049
ENSG00000244227	LRRC77P	0	0	0	0	0.01654	0	0	0
ENSG00000244706	AC026353.1	0	0	0	0	0	0	0	0
ENSG00000239238	MEMO1P3	0	0	0	0	0	0	0	0
ENSG00000242036	AC128713.1	0	0	0	0	0	0	0	0
ENSG00000241203	HMGN2P26	0	0	0	0	0	0	0	0
ENSG00000173905	GOLIM4	8.17077	7.56153	8.99355	12.8754	10.279	9.93422	13.3828	8.98615
ENSG00000206120	EGFEM1P	0	0	0	0	0	0	0	0
ENSG00000241882	AC124893.1	0	0	0	0	0	0	0	0
ENSG00000222357	RNU2-20P	0	0	0	0	0	0	0	0
ENSG00000207717	MIR551B	0	0	0	0	0	0	0	0
ENSG00000226765	RPSAP33	0	0	0	0	0	0	0	0
ENSG00000231688	RPL21P43	0	0	0	0	0	0	0	0
ENSG00000242268	LINC02082	0	0	0	0	0	0	0	0

ENSG00000272797	AC092954.1	0	0	0	0	0	0	0	0
ENSG00000273177	AC092954.2	0	0	0	0	0	0	0	0
ENSG00000241383	LINC01997	0	0	0	0	0	0	0	0
ENSG00000085276	MECOM	0.05637	0.0134	0	0.0239	0.00979	0.01421	0.01342	0
ENSG00000241479	AC074033.1	0	0	0	0	0	0	0	0
ENSG00000213178	RPL22P1	0.53634	2.08307	0.52543	4.2453	1.95622	1.89237	1.788	1.31703
ENSG00000242795	AC007849.1	0	0	0	0	0	0	0	0
ENSG00000244381	AC007849.2	0	0	0	0	0	0	0	0
ENSG00000206777	RNU6-637P	0	0	0	0	0	0	0	0
ENSG00000270141	TERC	0.27098	0	0	0	0.16473	0	0	0.16635
ENSG00000277925	RF00024	0	0	0	0	0	0	0	0
ENSG00000184378	ACTRT3	0	0	0	0	0.04445	0	0.06094	0
ENSG00000269889	AC078802.1	0.26086	0	0.12777	0.38714	0	0.30679	0.28987	0.10676
ENSG00000085274	MYNN	7.64984	5.15289	1.82343	3.66416	6.01887	6.81539	4.43517	4.33494
ENSG00000269984	AC078795.1	0	0	0	0	0	0	0	0
ENSG00000171757	LRRC34	0.18132	0.1006	0.15543	0.40366	0.20208	0.15994	0.15112	0.07421
ENSG00000270135	AC078795.3	0.93178	0	0.45642	0.69143	1.32165	0.27397	1.03543	0.38134
ENSG00000270096	AC078795.2	0.70256	0.09745	0.17207	0.43445	0.3559	0.30985	0.29276	0.64694
ENSG00000188306	LRRIQ4	0	0	0	0	0	0	0.06687	0.04926
ENSG00000114248	LRRC31	0	0	0	0	0.02863	0	0	0
ENSG00000240128	KRT18P43	0	0	0	0	0	0	0	0
ENSG00000187033	SAMD7	0.02918	0	0	0	0	0	0.04864	0
ENSG00000244039	AC008040.3	0	0	0.08743	0	0	0.10496	0	0
ENSG00000239219	AC008040.1	0.02757	0.07648	0.0135	0.15001	0.03352	0.08105	7.19879	0.06769
ENSG00000244193	AC008040.4	0	0	0	0	0	0	0	0
ENSG00000008952	SEC62	38.2127	52.6939	29.5099	50.345	45.6374	32.6215	49.9324	43.9655
ENSG00000240373	SEC62-AS1	0	0	0	0	0	0	0	0
ENSG00000173890	GPR160	6.34678	9.49685	1.69134	1.58615	6.77671	2.00146	2.60366	5.14788
ENSG00000201342	RNU4-38P	0	1.8916	0	0	0	0	0	0
ENSG00000242583	AC008040.2	0	0	0	0	0	0	0.17257	0
ENSG00000173889	PHC3	17.9709	21.5979	12.1154	30.7023	29.5335	27.8434	23.8887	21.8197
ENSG00000268220	AC008040.5	0.86429	0.54804	0.18144	0.18324	0.60045	0.07261	0.1372	0.25266
ENSG00000199536	RNU6-315P	0	0	0	0	0	0	0	0
ENSG00000163558	PRKCI	4.25421	3.13324	0.70076	1.65758	3.21925	2.54594	0.66937	1.75649
ENSG00000200118	RF00019	0	0	0	0	0	0	0	0
ENSG00000239628	AC073288.1	0	0	0	0	0	0	0	0.19189
ENSG00000136603	SKIL	10.4742	7.99593	2.7406	4.30558	8.16502	5.98204	4.83572	5.80552
ENSG00000242578	AC073288.2	0	0	0	0	0	0	0	0
ENSG00000013297	CLDN11	0	0	0	0	0.01477	0	0	0
ENSG00000283474	MIR6828	0	0	0	0	0	0	0	0
ENSG00000013293	SLC7A14	0	0	0	0	0	0	0	0
ENSG00000241416	KRT8P13	0	0	0	0	0	0	0	0
ENSG00000213174	AC026316.1	0	0	0	0	0	0	0	0
ENSG00000244738	AC026316.3	0	0	0	0	0	0	0	0
ENSG00000239739	AC026316.2	0	0	0	0	0	0	0	0
ENSG00000163584	RPL22L1	8.22502	5.14051	1.85235	2.94647	6.81976	5.83738	2.52135	3.59833
ENSG00000163577	EIF5A2	1.1402	1.5052	0.53924	0.71966	1.17909	1.8265	0.65536	0.78846
ENSG00000240704	KLF7P1	0	0	0	0	0	0	0	0
ENSG00000199488	RNU1-70P	0	0	0	0	0	0	0	0
ENSG00000163581	SLC2A2	0	0	0	0	0	0	0	0
ENSG00000154310	TNIK	7.61126	5.68314	1.80724	4.41627	8.03022	9.23981	3.11077	3.18891
ENSG00000207963	MIR569	1.52709	0	0	2.26637	0	0	1.27271	0
ENSG00000222506	RF00019	0	0	0	0	0	0	0	0
ENSG00000279673	AC092919.2	0.98031	0.74169	0.39288	0.92584	0.48757	0.55026	0.22282	0.6018
ENSG00000240497	AC092919.1	1.92053	3.19673	2.35183	2.37523	1.94578	1.12936	3.20123	0.786
ENSG00000207114	RNU6-348P	0	0	0	0	0	0	0	0
ENSG00000234594	AC008134.1	0	0	0	0	0	0	0	0
ENSG00000075651	PLD1	1.63599	1.78378	0.46071	1.18438	1.36527	0.52292	0.75062	0.77686

ENSG00000186329	TMEM212	0	0	0	0	0	0	0	0
ENSG00000234717	TMEM212-AS1	0	0	0	0	0	0	0	0
ENSG00000235943	TMEM212-IT1	0	0	0	0	0	0	0	0
ENSG00000075420	FNDC3B	17.5435	22.4617	3.25496	4.97611	12.0747	6.16838	4.77857	8.57244
ENSG00000237483	RPS27AP8	0	0	0	0	0	0	0	0
ENSG00000243398	RN7SL141P	4.40904	3.21075	0.80988	0.40897	0.67005	0.97227	0.91865	1.35334
ENSG00000279862	AC092964.1	0	0	0	0	0	0	0	0
ENSG00000236686	BZW1P1	0	0	0	0	0	0	0	0
ENSG00000121853	GHSR	0	0.09792	0	0	0.07152	0	0	0
ENSG00000121858	TNFSF10	56.8378	155.874	29.681	12.31	48.6767	40.6962	10.2022	51.3862
ENSG00000224611	AC007919.1	0	0	0	0.50132	0	0.59591	0	0
ENSG00000223387	LINC02068	0	0	0.14687	0	0	0.14105	0.03332	0
ENSG00000224426	SLC31A1P1	0	0	0	0	0	0	0	0
ENSG00000144959	NCEH1	4.04006	2.8675	2.32992	4.50257	2.87294	3.06604	3.73555	1.77823
ENSG00000231384	AC007919.2	0	0	0	0	0	0	0	0
ENSG00000252082	RNU6-547P	0	0	0	0	0	0	0	0
ENSG00000114346	ECT2	4.87869	7.65465	7.60011	16.1823	7.73025	11.5696	15.7086	10.7198
ENSG00000200355	RF00139	0	0	0	0	0	0	0	0
ENSG00000201458	RNU4-4P	0	0	0	0	0	0	0	0
ENSG00000227440	ATP5MC1P4	0	0	0	0	0	0	0	0
ENSG00000237501	AC108667.1	0	0	0	0	0	0	0	0
ENSG00000144962	SPATA16	0	0	0	0	0	0	0	0
ENSG00000237473	AC068759.1	2.96321	1.55756	1.83343	0.46292	0.75844	1.92592	1.03983	1.53186
ENSG00000169760	NLGN1	0.00722	0.03607	0.04246	0.01072	0.04391	0.01274	0	0.05321
ENSG00000237645	AC092967.1	0	0	0	0	0	0	0	0
ENSG00000228213	NLGN1-AS1	0	0	0	0	0	0	0	0
ENSG00000252982	RN7SKP234	0	0	0	0	0	0	0	0
ENSG00000232601	AC069218.2	0	0	0	0	0	0	0	0
ENSG00000213169	AC069218.1	0	0	0	0	0.11695	0	0	0
ENSG00000177694	NAALADL2	0.14561	0.24237	0.20604	0.52824	0.47207	0.09514	0.35058	0.30458
ENSG00000253020	RN7SKP40	0	0	0	0	0	0	0	0
ENSG00000230497	EEF1B2P8	0	0	0	0	0	0	0	0
ENSG00000230292	NAALADL2-AS3	0	0	0	0	0	0	0	0
ENSG00000226779	NAALADL2-AS2	0	0	0	0	0	0	0	0
ENSG00000264974	MIR4789	0	0	0	0	0	0	0	0
ENSG00000199792	RNU6-1233P	0	0	0	0	0	0	0	0
ENSG00000201648	RNU4-91P	0	0	0	0	0	0	0	0
ENSG00000236241	AC119744.1	0	0	0	0	0	0	0	0
ENSG00000214192	UBE2V1P2	0	0	0	0.24724	0	0	0.55537	0
ENSG00000201452	RNU6-1317P	0	0	0	0	0	0	0	0
ENSG00000225552	NAALADL2-AS1	0	0	0	0	0	0	0	0
ENSG00000260217	AC104640.1	0	0	0	0	0	0	0	0
ENSG00000230362	ACTG1P23	0	0	0	0	0	0	0	0
ENSG00000236357	EI24P1	0	0	0	0	0	0	0	0
ENSG00000271413	AC104640.2	0	0	0	0	0	0	0	0
ENSG00000238026	AC117434.1	0	0	0	0	0	0	0	0
ENSG00000283333	MIR7977	0	0	0	0	0	0	0	0
ENSG00000223715	LINC01208	0	0	0	0.2092	0	0	0	0
ENSG00000232461	AC092920.1	0	0	0	0	0	0.38145	0	0
ENSG00000252302	RNA5SP147	0	0	0	0	0	0	0	0
ENSG00000228308	LINC01209	0	0	0	0	0	0	0	0
ENSG00000231888	MTND5P15	0.21003	0.69919	0	0.31171	0.5107	0.74104	0	0.51574
ENSG00000177565	TBL1XR1	25.2189	19.5794	8.23427	17.4106	19.7312	17.486	15.5108	16.4357
ENSG00000231310	TBL1XR1-AS1	0	0	0	0	0	0	0	0
ENSG00000201343	RF00019	1.9991	1.10917	1.95844	0.98896	4.05076	0	3.33219	1.63631
ENSG00000200882	RNU6-681P	0.70481	0	0	1.04602	0.85689	1.24338	3.52443	1.73071
ENSG00000203645	LINC00501	0	0	0	0	0	0	0	0
ENSG00000236893	ASS1P7	0	0	0	0	0	0	0	0

ENSG00000226782	AC117465.1	0	0	0	0	0	0	0	0
ENSG00000228221	LINC00578	0	0	0	0.08816	0.07222	0	0	0
ENSG00000252028	RN7SKP52	0	0	0	0	0	0	0	0
ENSG00000236385	AC026355.2	0	0	0	0	0	0	0	0
ENSG00000200288	RF00425	0	0	0	0	0	0	0	0
ENSG00000228561	AC026355.1	0	0	0	0	0	0	0	0
ENSG00000277241	AC026355.3	0	0	0	0	0	0	0	0
ENSG00000231574	LINC02015	0	0	0	0	0	0	0	0
ENSG00000225790	AC007953.1	0	0	0	0	0	0	0	0
ENSG00000270321	AC007953.2	0	0	0	0	0	0	0	0
ENSG00000199858	RNU6-1120P	0	0	0	0	0	0	0	0
ENSG00000223930	AC109779.1	0.02633	0	0	0	0	0.04645	0.04389	0.03233
ENSG00000197584	KCNMB2	0	0	0	0	0	0	0	0
ENSG00000275163	AC117457.1	0	0	0	0	0	0	0	0
ENSG00000223941	LINC01014	0	0	0	0	0	0	0	0
ENSG00000252336	RNA5SP148	0	0	0	0	0	0	0	0
ENSG00000271131	PPIAP75	0	0	0	0	0	0	0	0
ENSG00000237978	KCNMB2-AS1	0	0	0	0	0	0	0.07225	0
ENSG00000172667	ZMAT3	4.87858	2.80799	0.9715	2.43601	3.03954	3.12354	2.27996	2.36982
ENSG00000200616	RF00019	0	0	0	0	0	0	0	0
ENSG00000229102	AC076966.1	0	0.3476	0	0	0	0	0	0.2564
ENSG00000121879	PIK3CA	10.1644	8.21946	2.76982	5.6988	8.32362	6.18322	5.98503	7.53562
ENSG00000201957	RF00402	0	0	0	0	0	0	0	0
ENSG00000171121	KCNMB3	0.07347	0.04076	0	0.02423	0.0794	0.0288	0.02721	0.03007
ENSG00000240429	LRRFIP1P1	0.52218	0.21729	0.09592	0	0.27775	0	0.272	0.12021
ENSG00000121864	ZNF639	3.7083	2.77494	1.42907	3.06104	3.45605	3.12958	3.06386	2.88662
ENSG00000260743	AC007823.1	0	0.23373	0	0.06947	0	0.08257	0	0.17241
ENSG00000171109	MFN1	7.70287	9.05284	3.3994	6.15424	7.80912	7.85813	6.62485	7.22152
ENSG00000114450	GNB4	20.0052	20.6556	6.30819	6.83487	13.073	12.15	7.19014	12.7672
ENSG00000242539	AC007620.2	0.26435	0.37715	0.11099	0.28023	0.41321	0.19986	0.06295	0.23183
ENSG00000272699	AC007620.3	0.14749	0.49098	0	0.21888	0.17931	0.26018	0	0
ENSG00000239255	AC007620.1	0	0	0	0	0	0	0	0
ENSG00000181260	MTHFD2P7	0	0	0	0	0	0	0	0
ENSG00000136518	ACTL6A	3.05169	2.32397	0.76206	1.8353	3.32217	3.69462	1.13038	1.88565
ENSG00000272910	AC090425.2	1.02416	0	0	0.43427	0.71151	0	0	0.17963
ENSG00000272660	AC090425.1	0	0	0	0.77704	0	0.92366	0.87272	0
ENSG00000136522	MRPL47	12.9479	13.2695	13.6018	30.1741	18.734	22.8419	30.5897	18.9848
ENSG00000136521	NDUFB5	4.71939	3.40817	1.43105	2.20499	3.23315	3.94257	1.56081	2.92786
ENSG00000239096	RF01210	0	0	0	0	0	0	0	0
ENSG00000243977	AC125604.1	0	0	0	0	0.12605	0.1829	0	0
ENSG00000058056	USP13	0.64268	0.3614	0.16591	0.45113	0.73912	0.67414	0.39086	0.34122
ENSG00000114757	PEX5L	0	0	0	0	0	0	0	0
ENSG00000270178	AC007687.1	0	0	0	0	0	0	0	0
ENSG00000243799	PEX5L-AS1	0	0	0	0	0	0	0	0
ENSG00000244302	PEX5L-AS2	0	0	0	0	0	0	0	0
ENSG00000201822	RNA5SP149	0	0	7.02482	43.7508	0	0	0	1.95646
ENSG00000282327	AC134503.1	0	0	0	0	0	0	0	0
ENSG00000213158	GAPDHP36	0	0	0	0	0	0	0	0
ENSG00000239880	AC068760.1	0.03753	0	0	0.1114	0	0	0	0
ENSG00000241696	LINC02053	0	0	0	0	0	0	0	0
ENSG00000201810	RF00096	0	0	0	0	0	0	0	0
ENSG00000239774	AC125618.1	0	0.21671	0	0	0.31658	0.91873	0.65105	0
ENSG00000199986	RNU6-486P	0	0	0	0	0	0	0	0
ENSG00000241946	AC068298.1	0	0	0	0	0	0	0	0
ENSG00000163728	TTC14	7.44022	5.83158	1.93755	5.59094	8.46168	7.67597	5.27465	7.23862
ENSG00000145075	CCDC39	0	0	0	0	0	0	0	0
ENSG00000243187	CCDC39-AS1	0	0	0	0	0	0	0	0
ENSG00000242811	RN7SL229P	0	0	0	0	0	0	0	0

ENSG00000242068	AC108734.3	0.06292	0	0	0.09338	0	0	0	0
ENSG00000241473	AC108734.2	0	0	0	0	0	0	0	0
ENSG00000239516	FLYWCH1P1	0	0	0	0	0	0	0	0
ENSG00000114416	FXR1	8.51948	7.561	3.23481	7.49064	8.42076	9.67639	6.57832	6.01772
ENSG00000213157	AC108734.1	0	0	0	0	0	0	0	0
ENSG00000205981	DNAJC19	1.98275	1.32692	0.7209	1.51681	2.11236	1.80301	1.56729	1.80697
ENSG00000242808	SOX2-OT	0.01852	0	0	0.01832	0	0.03267	0	0.00758
ENSG00000242140	AC083904.1	0	0	0	0	0	0	0	0
ENSG00000206932	RNU6-4P	10.9608	29.6469	8.05338	26.4339	14.1587	12.0852	12.5606	10.9342
ENSG00000237806	FAUP2	0	0	0	0	0	0	0	0
ENSG00000241231	AC022294.1	0	0	0	0	0	0	0	0
ENSG00000235294	RPL7AP25	0	0	0	0	0	0	0	0
ENSG00000239381	AC125613.1	0	0	0	0	0	0	0	0
ENSG00000276074	RF01951	0	0	0	0	0	0	0	0
ENSG00000276690	RF01953	0	0	0	0	0	0	0	0
ENSG00000181449	SOX2	0.05834	0.1942	0	0	0.14185	0	0	0
ENSG00000278026	RF01954	0	0	0	0	0	0	0	0
ENSG00000266317	RN7SL703P	0	0	0	0	0	0	0	0
ENSG00000199839	RNA5SP150	0	0	0	0	0	0	0	0
ENSG00000239805	AC007547.2	0	0	0	0	0	0	0	0
ENSG00000213155	AC007547.1	0	0	0	0.22201	0	0	0	0.18367
ENSG00000242512	LINC01206	0	0	0	0	0	0	0	0
ENSG00000252257	RN7SKP265	0	0	0	0	0	0	0	0
ENSG00000240478	AC012081.1	0	0	0	0	0	0	0	0
ENSG00000242012	AC084211.1	0	0	0	0	0	0	0	0
ENSG00000241098	LINC01994	0	0	0	0	0	0	0	0
ENSG00000244199	EIF4EP3	0	0	0	0	0	0	0	0
ENSG00000244247	LINC01995	0	0	0	0	0.14351	0	0	0
ENSG00000228766	RPL7L1P8	0	0	0	0	0	0.17451	0	0
ENSG00000238837	LINC02031	0	0	0	0	0	0	0	0
ENSG00000243378	AC083801.1	0	0	0	0	0	0	0	0
ENSG00000260377	AC083801.2	0	0	0	0	0	0	0	0
ENSG00000240063	AC069431.1	0.0781	0	0.05739	0.11591	0	0	0	0.09589
ENSG00000058063	ATP11B	25.3315	30.7542	10.3895	21.3762	28.9561	22.8785	19.3846	22.0002
ENSG00000270554	AC069431.2	0	0	0	0	0	0	0	0
ENSG00000273261	AC092953.2	0.10486	0	0.30819	0	0.12749	0	0.17479	0
ENSG00000043093	DCUN1D1	37.6087	45.4558	36.1607	95.0905	43.446	28.3773	75.7454	49.9306
ENSG00000244346	AC092953.1	0.80995	0	0	0.40068	0	0	0	0.16574
ENSG00000078070	MCCC1	2.88384	2.40007	1.54554	4.7331	3.07299	5.74586	5.51381	2.85341
ENSG00000243368	MCCC1-AS1	0	0	0	0	0	0	0	0
ENSG00000078081	LAMP3	0.38417	1.31089	0.70567	0.17105	0.39701	0.54219	0.22413	0.25943
ENSG00000053524	MCF2L2	0.18888	0.12457	0.17282	0.15338	0.2513	0.07544	0.23167	0.09189
ENSG00000176597	B3GNT5	10.0315	5.59081	2.47451	1.65718	3.19681	0.63544	2.16142	2.71981
ENSG00000202502	RNA5SP151	0	0	0	0	0	0	0	0
ENSG00000240024	LINC00888	0.72234	0.22902	0.15164	0.35734	0.46001	0.42477	0.05733	0.1267
ENSG00000201229	SNORA63D	0.55531	0	0.81601	2.4724	0	0	1.85122	2.04539
ENSG00000253092	RF01241	0	0	0	0	0	0	0	0
ENSG00000199363	SNORA63E	0	0	0	0	0	0	0	0
ENSG00000251730	RF00394	0	0	0	0	0	0	0	0
ENSG00000271682	AC092960.1	0	0	0	0	0	0	0.34809	0
ENSG00000172578	KLHL6	6.76982	4.90533	1.18479	1.87739	5.28988	2.90602	1.56404	3.00388
ENSG00000242522	KLHL6-AS1	0	0	0	0	0	0	0	0
ENSG00000243584	AC068769.1	0	0	0	0	0	0	0	0
ENSG00000114796	KLHL24	11.9569	11.0019	3.94744	8.1495	12.6309	10.8383	9.11056	9.239
ENSG00000163872	YEATS2	3.10801	2.95105	0.9282	1.87488	3.4034	4.01409	1.26823	1.78901
ENSG00000233885	YEATS2-AS1	0	0.0746	0.03293	0	0.10898	0	0.03735	0.05503
ENSG00000283765	AC131160.1	0.02397	0	0	0	0	0	0	0
ENSG00000180834	MAP6D1	0.09132	0.10134	0	0	0	0	0	0

ENSG00000175193	PARL	0.48479	0.80693	0.33245	0.57559	0.39293	0.11403	0.91581	0.95235
ENSG00000234371	RPSAP31	0	0	0	0	0	0	0	0
ENSG00000263932	MIR4448	0	0	0	0	0	0	0	0
ENSG00000233441	CYP2AB1P	0	0	0	0	0	0	0	0
ENSG00000114770	ABCC5	2.55632	1.56763	0.86616	1.42626	2.11867	1.57105	1.77274	2.09241
ENSG00000223882	ABCC5-AS1	0	0	0	0	0	0	0	0
ENSG00000223529	EEF1A1P8	0	0	0	0	0	0	0	0.06484
ENSG00000186090	HTR3D	0	0	0	0	0	0	0	0
ENSG00000178084	HTR3C	0	0	0	0	0	0	0	0
ENSG00000233576	HTR3C2P	0	0	0	0	0	0	0	0
ENSG00000207351	RF00019	0	0	0	0	0	0	0	0
ENSG00000238020	HTR3E-AS1	0	0	0	0	0	0	0	0
ENSG00000186038	HTR3E	0	0	0	0	0	0	0	0
ENSG00000205955	HSP90AA5P	0	0	0	0	0	0	0	0
ENSG00000273181	AC131235.3	0	0	0	0	0.14374	0.20857	0	0.14516
ENSG00000272721	AC131235.2	0.37979	0.63217	0	0	0	0.335	0	0
ENSG00000145191	EIF2B5	3.76657	2.22534	0.68006	1.52628	3.28209	3.15229	1.94991	1.21532
ENSG00000228205	AC131235.1	0.10014	0	0	0	0	0.17666	0	0
ENSG00000161202	DVL3	3.50253	2.64452	0.70748	1.01819	2.63399	2.71788	1.06331	1.25612
ENSG00000161203	AP2M1	34.4771	26.5515	15.7857	15.5539	35.5267	31.621	16.6726	20.6827
ENSG00000161204	ABCF3	3.13923	2.27287	0.35167	1.02372	3.20046	1.9619	1.07937	1.39999
ENSG00000145198	VWA5B2	0	0	0	0	0	0	0	0
ENSG00000221120	MIR1224	0	0	0	0	0	0	0	0
ENSG00000214160	ALG3	1.67169	1.16232	0.34205	0.6909	1.33778	1.82918	0.70543	0.64952
ENSG00000145194	ECE2	0.13425	0.09311	0.01644	0.0166	0.14962	0.05921	0	0.01374
ENSG00000163888	CAMK2N2	0.05382	0	0	0	0	0	0	0
ENSG00000175166	PSMD2	10.5656	7.76397	2.23211	4.38893	8.97214	10.4341	3.33851	4.48913
ENSG00000114867	EIF4G1	21.0216	13.0771	3.14389	5.29634	14.8294	17.4619	4.1876	5.82033
ENSG00000212158	SNORD66	0	1.60537	1.41729	0	0	0	0	4.73669
ENSG00000175182	FAM131A	0.77201	0.35543	0.13276	0.20721	0.55916	0.3912	0.34224	0.38318
ENSG00000114859	CLCN2	0.0516	0	0.01264	0.06382	0.05228	0.04552	0.02867	0.01056
ENSG00000163882	POLR2H	1.39797	1.55128	0.58205	1.03737	1.44468	1.97297	0.85441	0.88681
ENSG00000090534	THPO	0	0	0	0	0	0	0	0
ENSG00000090539	CHRD	0	0	0	0	0	0	0	0
ENSG00000224153	LINC02054	0	0	0	0	0	0	0	0
ENSG00000283625	AC128714.1	0	0	0	0	0	0	0	0
ENSG00000227509	LINC01839	0	0	0	0	0	0	0	0
ENSG00000230215	LINC01840	0	0	0	0	0	0	0	0
ENSG00000182580	EPHB3	0.25878	0.05384	0.02377	0.024	0.09832	0	0	0.09929
ENSG00000177383	MAGEF1	3.27074	1.26781	0.52672	1.46289	4.08542	1.42275	0.97087	1.54029
ENSG00000272970	AC107294.2	0	0	0	0	0	0	0	0
ENSG00000273403	AC107294.3	0	0	0	0	0	0	0	0
ENSG00000272922	AC107294.1	0	0	0	0	0	0	0	0
ENSG00000229433	LINC02069	0	0	0	0	0	0	0	0
ENSG00000156931	VPS8	11.222	10.7108	4.26034	8.90981	9.37075	7.11053	8.26244	8.87295
ENSG00000187068	C3orf70	0.03727	0	0	0	0	0	0	0
ENSG00000223358	EHHADH-AS1	0	0	0	0	0	0	0	0
ENSG00000113790	EHHADH	0.3899	0.38939	0.09167	0.18517	0.36026	0.49524	0.10398	0.11489
ENSG00000229704	EIF2S2P2	0	0	0	0.22156	0	0	0	0
ENSG00000264614	MIR5588	0	0	0	0	0	0	0	0
ENSG00000073803	MAP3K13	0.73167	0.77325	0.19626	0.35334	0.37417	0.39952	0.513	0.34935
ENSG00000229638	RPL4P4	0.79922	0.47511	0.33556	1.10141	0.90227	0.80568	0.95156	0.63082
ENSG00000163900	TMEM41A	0.90484	0.5208	0.22368	0.61496	0.81221	0.8951	0.38058	0.45684
ENSG00000163898	LIPH	1.97388	1.18397	0.39197	1.02927	0.86479	0.34508	0.82995	0.655
ENSG00000163904	SENP2	8.29217	6.82458	2.32887	4.3399	7.02342	6.51159	5.50485	4.1427
ENSG00000236489	AC133473.1	0.73545	0	0	0	2.98049	0.43248	0.40863	0.90298
ENSG00000073792	IGF2BP2	30.9301	29.9159	15.5827	17.9893	25.4834	44.5969	32.8997	18.0374
ENSG00000163915	IGF2BP2-AS1	0	0	0	0	0	0	0	0

ENSG00000265470	MIR548AQ	0	0	0	0	0	0	0	1.55167
ENSG00000136527	TRA2B	13.6897	13.6636	4.48465	10.3064	14.4931	15.3056	8.89698	9.11518
ENSG00000171658	NMRAL2P	0	0	0	0	0.03391	0	0	0
ENSG00000244405	ETV5	0.13897	0.08411	0	0	0.06144	0.06686	0.08423	0.03102
ENSG00000277118	RF00017	0	0	0	0	0	0	0	0
ENSG00000234197	ETV5-AS1	0	0	0	0	0	0	0	0
ENSG00000058866	DGKG	1.32205	0.96691	0.17661	0.51529	1.15272	0.42405	0.46744	0.68043
ENSG00000234548	LINC02020	0	0	0	0	0	0	0	0
ENSG00000224406	LINC02052	0	0	0	0	0	0	0	0
ENSG00000226859	LINC02051	0	0	0	0	0	0	0	0
ENSG00000213139	CRYGS	0.34077	0.11344	0.10015	0.15172	0.58002	0.18035	0.28401	0.33472
ENSG00000113838	TBCCD1	1.39927	1.21829	1.10719	0.95846	1.77972	2.20267	1.4353	1.63871
ENSG00000090520	DNAJB11	4.16959	1.89474	0.67662	1.78431	2.50354	2.95583	1.06596	1.69598
ENSG00000197099	AC068631.1	0	0	0	0	0.01606	0	0.01101	0
ENSG00000283149	AC068631.2	0	0	0	0	0	0	0	0
ENSG00000283390	AC068631.3	0	0	0	0	0	0	0.54302	0
ENSG00000145192	AHSG	0	0	0	0	0	0	0	0
ENSG00000275696	RF00017	0	0	0	0	0	0	0	0
ENSG00000090512	FETUB	0	0	0	0	0	0	0	0
ENSG00000113905	HRG	0	0	0	0	0	0	0	0
ENSG00000225175	AC109780.1	0	0	0	0	0	0	0	0
ENSG00000226724	AC109780.2	0	0	0	0	0	0	0	0
ENSG00000113889	KNG1	0	0	0	0	0	0	0	0
ENSG00000226652	PSMD10P2	0	0	0	0	0	0	0	0
ENSG00000207505	RNU6-1105P	0	0	0	0	0	0	0	0
ENSG00000230951	GPS2P2	0	0	0	0	0.10036	0	0	0
ENSG00000263826	AC112907.3	0.42759	0.15251	0.13464	0.13598	0.22279	0.70044	0.15273	0.22499
ENSG00000156976	EIF4A2	39.8831	33.9189	8.34243	21.6046	41.7031	26.0395	21.5155	22.6277
ENSG00000238942	SNORD2	0	0	0	0	0	0	0	0
ENSG00000200418	SNORA63B	0	0	0	0	0	0	0	0
ENSG00000283958	MIR1248	0	0	0	0	0	0	0	0
ENSG00000221420	SNORA81	0	0	0	0	0	0	0	0
ENSG00000200320	SNORA63	0	0	0	0	0	0	0	0
ENSG00000263776	SNORA4	0	0	0	0	0	0	0	0
ENSG00000163918	RFC4	2.17675	0.97704	0.9704	2.61347	2.05174	3.53806	3.70985	2.28221
ENSG00000231724	AC112907.1	0	0	0	0	0	0	0	0
ENSG00000232233	LINC02043	0	0	0	0	0	0	0	0
ENSG00000231982	AC112907.2	0	0	0	0	0	0	0	0
ENSG00000181092	ADIPOQ	0	0	0	0	0	0	0	0
ENSG00000226482	ADIPOQ-AS1	0	0	0	0	0	0	0	0
ENSG00000223803	RPS20P14	0.20361	1.01674	0.29921	0.30218	1.48528	0.3592	0	0.24999
ENSG00000233565	AC007690.1	0	0	0	0	0	0	0	0
ENSG00000073849	ST6GAL1	12.7681	8.30634	2.09519	4.75514	13.7118	14.9505	2.34988	5.44841
ENSG00000163923	RPL39L	0.34576	0.80572	0.40647	0.20526	1.00887	1.21992	0.23053	0.25471
ENSG00000198491	AC007920.1	0	0	0	0	0	0	0	0
ENSG00000175077	RTP1	0	0	0	0	0	0	0	0
ENSG00000127241	MASP1	0.01114	0	0	0	0	0	0.00928	0
ENSG00000283175	AC007920.2	0	0	0	0.05514	0	0	0	0
ENSG00000136514	RTP4	1.65091	5.26034	3.67364	0.98005	2.46591	6.74019	0.94348	2.31653
ENSG00000280270	AC068299.1	0.08382	0	0	0	0	0.07393	0.06986	0
ENSG00000239093	RF01210	0	0	0	0	0	0	0	0
ENSG00000228952	LINC02041	0	0	0	0	0	0	0	0
ENSG00000157005	SST	0	0	0	0	0	0	0	0
ENSG00000198471	RTP2	0	0	0	0	0	0	0	0
ENSG00000228804	AC072022.1	0	0	0	0	0	0	0	0
ENSG00000113916	BCL6	39.2795	57.2441	4.7889	7.63955	30.9012	12.1951	8.10695	22.4917
ENSG00000223401		8.39366	7.89216	1.22402	6.18101	4.90765	2.2607	2.24282	8.65356
ENSG00000224049	AC108681.1	0	0	0	0	0	0	0	0

ENSG00000236864	AC068295.1	0	0	0	0	0	0	0	0
ENSG00000224187	LINC01991	0.04279	0.07123	0	0	0	0.22647	0	0.21015
ENSG00000225058	AC092941.1	0	0	0	0	0	0	0	0
ENSG00000236412	AC092941.2	0	0.24208	0	0	0	0	0	0.08928
ENSG00000234238	AC022498.1	0	0	0	0	0	0	0	0
ENSG00000270959	LPP-AS2	0.84815	0.25668	0.07554	0.11443	0.59369	0.27204	0.17136	0.31556
ENSG00000145012	LPP	5.67514	3.91803	0.91115	2.07049	3.15494	2.48647	2.53595	3.05218
ENSG00000279891	FLJ42393	0.19375	0.59123	0.0949	0.04792	0.19629	0	0.10765	0.27752
ENSG00000224563	LPP-AS1	0.14838	0	0	0	0	0.26176	0	0
ENSG00000207651	MIR28	0	0	1.25249	0	0	0	0	0
ENSG00000234076	TPRG1-AS1	0.21033	0.70019	0	0	0.38357	0.7421	0	0
ENSG00000188001	TPRG1	0.36131	0.37108	0.12426	0.09127	0.07477	0.20343	0.03844	0.10382
ENSG00000236182	AC009319.1	0	0	0	0	0	0	0	0
ENSG00000230115	TPRG1-AS2	0	0	0	0	0	0	0	0
ENSG00000073282	TP63	0.18684	0.03274	0	0.02919	0.14347	0.17348	0.01639	0.04829
ENSG00000227971	AC078809.1	0	0	0	0	0	0	0	0
ENSG00000216058	MIR944	0	0	0	0	0	0	0	0
ENSG00000090530	P3H2	0.07847	0.04354	0	0	0.1272	0	0.0654	0.01606
ENSG00000230077	MTAPP2	0	0	0	0.13075	0	0	0	0
ENSG00000225764	P3H2-AS1	0	0	0	0	0	0	0	0
ENSG00000212489	RNU6-1109P	0	0	0	0	0	0	0	0
ENSG00000238077	NMNAT1P3	0	0	0	0	0	0	0	0
ENSG00000163347	CLDN1	0	0	0	0	0.07299	0	0	0
ENSG00000113946	CLDN16	0	0	0	0.03036	0	0.10827	0	0
ENSG00000198398	TMEM207	0	0	0	0	0	0	0	0
ENSG00000196083	IL1RAP	15.4046	17.1749	1.94776	3.80251	10.0698	1.59989	5.32781	5.54403
ENSG00000231047	GCNT1P3	2.4221	1.27313	0	0.18919	0.30997	0.44978	0.42498	0.9391
ENSG00000223117	RN7SKP296	3.86923	2.50457	0	1.5951	0.52268	1.13764	2.1498	1.84745
ENSG00000273370	AC108747.1	0	0	0	0	0	0	0	0
ENSG00000228730	LINC02013	0	0	0	0	0	0	0	0
ENSG00000232610	CCT6P4	0	0	0	0	0	0	0	0
ENSG00000214147	AC092952.1	0	0	0	0	0	0	0	0
ENSG00000205835	GMNC	0	0	0	0	0	0	0	0
ENSG00000188729	OSTN	0.06831	0	0	0	0	0	0	0
ENSG00000233308	OSTN-AS1	0	0	0	0	0	0	0	0.17475
ENSG00000188958	UTS2B	0.03808	0	0	0.02826	0.02315	0	0.03174	0
ENSG00000152492	CCDC50	4.97059	2.14794	0.60869	1.85605	3.29273	2.33273	2.0713	1.57461
ENSG00000253548	PYDC2	0	0	0	0	0	0	0	0
ENSG00000223812	AC073365.1	0	0	0	0	0	0	0	0
ENSG00000201860	RF00019	0	0	0	0	0	0	0	0
ENSG00000237653	AC026320.2	0	0	0	0	0	0	0	0
ENSG00000232353	AC026320.1	0	0	0	0	0	0	0	0
ENSG00000222583	RN7SKP222	0	0	0	0	0	0	0	0
ENSG00000114279	FGF12	0	0	0	0	0	0	0	0
ENSG00000231383	FGF12-AS1	0	0	0	0	0	0	0	0
ENSG00000230126	FGF12-AS2	0	0	0	0	0	0	0	0
ENSG00000226709	FGF12-AS3	0	0	0	0	0	0	0	0
ENSG00000238902	RF01210	0	0	0	0	0	0	0	0
ENSG00000200184	RNU1-20P	0	0	0	0	0	0	0	0
ENSG00000180611	MB21D2	0.65291	0.25358	0.38378	0.3876	0.23814	0.19197	0.10883	0.29393
ENSG00000232130	AC092966.1	0	0	0	0	0	0	0	0
ENSG00000236229	VEZF1P1	0	0	0	0.07059	0	0	0	0
ENSG00000127252	HRASLS	0.68974	0.20874	0.18428	0.09306	0.2287	0.33185	0.31355	0.69288
ENSG00000187527	ATP13A5	0.01177	0	0	0.01747	0	0	0	0
ENSG00000236508	ATP13A5-AS1	0.15399	0	0	0	0	0	0.25668	0
ENSG00000127249	ATP13A4	0.21943	0.24349	0.10748	0.25328	0.13339	0.17204	0.30479	0.0898
ENSG00000225473	ATP13A4-AS1	0	0	0	0	0	0	0	0
ENSG00000236297	AC048351.1	0.15866	0	0	0.23547	0.86802	0.13995	0	0

ENSG00000198836	OPA1	26.7848	17.8218	17.7228	62.0032	24.3362	18.7049	45.5428	31.8527
ENSG00000224855	OPA1-AS1	0	0	0	0	0	0	0	0.54324
ENSG00000207370	RF00019	0.64868	0	0	1.92541	0.78864	0	4.32497	3.18574
ENSG00000243991	RN7SL447P	0	0	0	0	0	0	0	0
ENSG00000223986	AC069421.1	0	0	0	0	0	0	0	0
ENSG00000229155	LINC02038	0	0	0	0	0	0	0	0
ENSG00000214146	LINC02026	0.03139	0.05225	0	0	0	0	0	0
ENSG00000238043	AC024559.1	0	0	0	0	0	0	0	0
ENSG00000224403	DPPA2P3	0	0	0	0	0	0	0	0
ENSG00000230102	LINC02028	0	0	0	0	0	0	0	0
ENSG00000276407	AC024559.2	0	0	0	0	0	0	0	0
ENSG00000229964	AC080129.1	0	0	0	0	0	0	0	0
ENSG00000232874	AC080129.2	0	0	0	0	0	0	0	0
ENSG00000114315	HES1	0.24884	0.76921	0.10448	0.15827	0.82115	0.37627	0.11851	0.17458
ENSG00000242201	RN7SL215P	0	0	0	0	0	0	0	0
ENSG00000225742	LINC02036	0	0	0	0	0	0	0	0
ENSG00000238097	LINC02037	0	0	0	0	0	0	0	0
ENSG00000228271	LINC02048	0	0	0	0	0	0	0	0
ENSG00000214145	LINC00887	0.01765	0	0	0	0	0	0	0
ENSG00000178772	CPN2	0	0	0	0	0	0	0	0
ENSG00000172061	LRRC15	0	0	0	0	0	0	0	0
ENSG00000178732	GP5	1.14078	0.62144	0.45719	0.76957	0.65564	0.95136	0.3803	0.81491
ENSG00000133657	ATP13A3	6.27106	5.2448	3.63162	8.48169	6.20452	6.44161	6.77635	5.71964
ENSG00000271483	AC108676.2	0	0	0	0	0	0	0	0
ENSG00000233058	LINC00884	0.15051	0.12527	0	0.18615	0.15249	0.13276	0.04181	0.0308
ENSG00000244675	AC108676.1	0	0	0	0	0.06003	0	0	0
ENSG00000252053	RNU6-1101P	0	0	0	0	0	0	0	0
ENSG00000232873	RPL23AP93	0	0	0	0	0	0	0	0
ENSG00000231770	TMEM44-AS1	1.96516	2.07164	0.38504	0.97217	1.75207	1.61784	0.98268	1.44767
ENSG00000145014	TMEM44	0.03214	0.02675	0.07085	0.0477	0	0.08505	0	0
ENSG00000229334	AC046143.1	0	0	0	0	0	0	0	0.09259
ENSG00000272707	AC046143.2	0.45813	0	0	0	1.11396	0	0	0
ENSG00000041802	LSG1	4.24466	3.46932	1.47553	2.61917	4.56866	4.509	2.13017	2.52171
ENSG00000185112	FAM43A	1.17563	2.29928	0.25914	0.47981	1.75089	0.31109	0.4899	1.33516
ENSG00000233987	AC106706.1	0	0	0	0	0	0	0	0
ENSG00000237222	LINC01968	0	0	0	0	0	0	0	0
ENSG00000234805	AC090505.1	0	0	0	0	0	0	0	0
ENSG00000230401	LINC01972	0	0	0	0	0	0	0	0
ENSG00000238031	AC090505.2	0	0	0	0	0	0	0	0
ENSG00000173950	XXYLT1	1.24674	1.01505	0.29871	0.30168	0.56017	0.64548	0.36141	0.44924
ENSG00000233303	XXYLT1-AS1	0	0	0	0	0	0	0	0
ENSG00000265333	MIR3137	0	0	0	0	0	0	0	0
ENSG00000244314	RN7SL36P	0	0	0	0	0	0	0	0
ENSG00000230266	XXYLT1-AS2	0.84146	0	0.13739	0.13876	0.22734	0.32988	0.15584	0.11479
ENSG00000206600	RNU6-25P	0	0	0	0	0	0	0	0
ENSG00000114331	ACAP2	47.3731	47.121	14.1375	26.945	35.8899	22.7535	19.4475	32.1562
ENSG00000229325	ACAP2-IT1	4.78046	2.12189	0.31221	1.10362	2.58309	2.06149	0.53122	2.73904
ENSG00000227433	AC090018.1	0	0	0	0	0	0	0	0
ENSG00000207368	RF00019	1.32073	3.29753	0	0.98005	0.80285	0	1.10072	3.24314
ENSG00000184203	PPP1R2	25.8682	16.7565	4.83305	8.93461	23.6787	15.363	10.7974	10.464
ENSG00000252620	RNU6ATAC24P	4.88669	0.90377	0	1.61164	3.30062	3.83146	0.90504	2.66658
ENSG00000223711	AC069213.1	1.19703	0.23441	0	0.209	0.51364	0.37266	0.46947	0.60517
ENSG00000265882	RN7SL73P	0	0	0	0	0	0	0	0
ENSG00000236844	AC069213.3	0.30928	0	0	0	0	0	0	0.37973
ENSG00000236425	AC069213.2	0	0	0	0	0	0	0	0
ENSG00000189058	APOD	0	0	0	0	0	0	0	0
ENSG00000224769	MUC20P1	0	0	0	0	0	0.07697	0	0
ENSG00000282206	AC233280.19	0	0	0	0	0	0	0	0

ENSG00000271662	AC233280.2	0	0	0	0	0	0	0	0
ENSG00000229178	AC233280.1	0	0	0	0	0	0	0	0
ENSG00000242086	MUC20-OT1	0.11246	0.39516	0.52024	0.5316	0.75956	0.71272	1.2427	0.9307
ENSG00000215837	SDHAP2	0	0	0	0	0	0	0	0
ENSG00000207650	MIR570	0	0	0	0	0	0	0	0
ENSG00000283426	SMBD1P	0	0	0	0	0	0	0	0
ENSG00000176945	MUC20	0	0	0	0	0	0	0	0
ENSG00000145113	MUC4	0	0	0	0	0	0	0	0
ENSG00000223783	LINC01983	0	0.12938	0	0	0	0	0	0
ENSG00000235836	KIF3AP1	0	0	0	0	0	0	0	0
ENSG00000061938	TNK2	3.28317	2.88362	0.92487	1.11703	2.37445	2.57548	0.96256	1.14717
ENSG00000276489	MIR6829	0	0	0	0	0	0	0	0
ENSG00000224614	TNK2-AS1	1.53875	1.10599	1.33615	0.98613	1.19049	1.1722	0.81609	0.47231
ENSG00000226155	AC124944.1	0.4946	0.49396	0.29073	0.29362	0.24053	1.04706	0.82443	0.12145
ENSG00000273009		0	0	0	0	0	0	0	0
ENSG00000239122	RNU2-11P	0	0	0	0	0	0	0	0
ENSG00000231443	AC124944.2	0	0	0	0.08759	0	0	0	0
ENSG00000260261	AC124944.3	0.04179	0.03478	0.03071	0.03101	0.05081	0	0.06966	0.05131
ENSG00000185485	SDHAP1	1.79261	1.16441	0.96374	1.3951	2.0731	2.1597	2.84225	2.71092
ENSG00000231464	AC024937.2	0	0	0	0	0	0	0.09837	0
ENSG00000072274	TFRC	24.9632	39.1689	49.4525	90.703	54.1231	72.4356	107.758	78.4836
ENSG00000228413	AC024937.1	0	0	0	0	0	0	0	0
ENSG00000252174	RNU7-18P	0	0	0	0	0	0	0	0
ENSG00000224652	LINC00885	0.24059	0.13349	0	0	0.195	0.28296	0.20051	0.04923
ENSG00000163958	ZDHHC19	0.03014	0.1505	0.04429	0.08946	0.07329	0	0.10048	0.03701
ENSG00000222335	RF00019	0	0	0	0	0	0	0	0
ENSG00000163959	SLC51A	0.04155	0	0	0	0	0	0	0
ENSG00000161217	PCYT1A	7.61669	8.25828	1.60666	2.84986	5.73037	4.84636	1.73056	4.8958
ENSG00000272741	AC069257.3	0	0	0	0	0	0	0	0
ENSG00000228028	AC069257.1	0	0	0	0	0	0	0	0
ENSG00000213123	TCTEX1D2	0.11766	0	0.1729	0.08731	0	0.20756	0	0.14446
ENSG00000237418	AC069257.2	0	0.78715	0	0	0	0	0	0
ENSG00000273331	TM4SF19-TCTEX1D	0	0	0	0	0	0	0	0
ENSG00000235897	TM4SF19-AS1	0.13727	0	0	0.05093	0	0.18162	0.0572	0
ENSG00000145107	TM4SF19	0.0386	0.1285	0	0	0	0	0	0.04739
ENSG00000212146	RNU6-910P	0	0	0	0	0	0	0	0
ENSG00000163960	UBXN7	26.2469	41.4187	22.1419	61.4599	44.8385	35.1596	36.1639	31.4125
ENSG00000206644	RNU6-1279P	0	0	0	0	0	0	0	0
ENSG00000241868	RN7SL434P	1.08192	0.90043	0	0	1.31538	0	0.45085	0.33209
ENSG00000243339	RN7SL738P	0	0	0	0	0	0	0	0
ENSG00000226345	AC083822.1	0	0	0	0	0	0	0	0
ENSG00000225822	UBXN7-AS1	0.1899	0	0	0.28183	0.23087	0	0.31653	0
ENSG00000225240	AC117490.1	0	0	0	0	0	0	0	0
ENSG00000163961	RNF168	10.7202	6.77698	2.15549	3.23488	6.55	9.40757	4.98136	4.94841
ENSG00000273013	AC117490.2	0	0	0	0	0	0	0	0
ENSG00000214097	SMCO1	0	0	0	0	0	0	0	0
ENSG00000213358	AC092933.1	0	0	0	0	0	0	0	0
ENSG00000225770	RPS29P3	0	0	0	0	0	0	0	0
ENSG00000185798	WDR53	3.34481	2.51214	0.71929	0.84752	2.43	1.94292	1.08786	1.35221
ENSG00000174013	FBXO45	1.7908	1.59248	0.54073	1.25606	1.93862	1.99074	0.79736	1.2349
ENSG00000232065	LINC01063	0.42127	0	0.61905	0.93781	0	0.37159	0.35109	0.51722
ENSG00000174004	NRROS	5.23934	5.07321	1.11045	0.97197	2.75619	3.46609	1.42754	1.29893
ENSG00000163964	PIGX	6.94095	6.06475	1.01754	3.05853	5.63243	2.99575	2.55573	4.95935
ENSG00000174007	CEP19	17.6507	19.6234	2.83844	2.02645	12.2277	4.40635	1.11023	9.32273
ENSG00000201441	RNU6-646P	1.38303	0	0	0	0	1.21992	0	0
ENSG00000180370	PAK2	46.3183	36.0188	9.43141	17.5931	31.4822	22.3563	16.7779	23.5543
ENSG00000272359	RNU4-89P	0	0	0	0	0	0	0	0
ENSG00000206892	RNU6-42P	0	2.28053	2.01334	4.06676	0	1.20852	5.70936	0.84109

ENSG00000119231	SENP5	6.53295	6.45162	2.95505	5.65964	6.13113	6.1577	5.00184	5.37296
ENSG00000230732	AC016949.1	1.86752	1.55425	0.22869	0.23097	0.31535	0.45758	0.69175	0.25477
ENSG00000114503	NCBP2	5.58998	4.15125	1.28482	2.46757	5.24523	4.52617	2.15022	2.32296
ENSG00000225578	NCBP2-AS1	0	0	0	0.19357	0.63428	0	0	0
ENSG00000270170	NCBP2-AS2	6.14829	1.9936	0.58668	1.54054	3.98016	4.36674	1.59713	2.05876
ENSG00000119227	PIGZ	0.13697	0.07599	0	0.03388	0.05551	0	0.0761	0
ENSG00000233487	RPSAP69	0	0	0	0	0	0	0	0
ENSG00000163975	MELTF	0.0786	0.05815	0.02567	0.02592	0.01062	0.09244	0.01456	0.03217
ENSG00000228109	MELTF-AS1	0	0	0.09457	0.09551	0	0	0.10727	0.07901
ENSG00000075711	DLG1	7.72915	6.95385	4.3865	9.70083	9.3321	9.16855	9.3515	6.88006
ENSG00000265850	MIR4797	0	0	0	0	0	0	0	0
ENSG00000227375	DLG1-AS1	0	0	0	0	0	0	0	0
ENSG00000235126	AC128709.2	0	0	0	0	0	0	0	0
ENSG00000229912	AC128709.1	0	0	0	0	0	0	0	0
ENSG00000237167	AC128709.3	0	0	0	0	0	0	0	0
ENSG00000272989	LINC02012	0	0	0	0	0	0	0	0
ENSG00000161267	BDH1	0.09651	0.08032	0.05909	0.04774	0.02933	0.22701	0.01341	0.04937
ENSG00000214135	AC132008.2	2.07217	1.53295	0.5844	0.62128	1.24692	1.29238	1.04666	1.07935
ENSG00000213519	AC132008.1	0	0	0	0.38037	0	0	0.4272	0
ENSG00000249626	AC024560.2	0	0	0	0	0	0	0	0
ENSG00000273308	AC024560.3	0	0	0	0	0	0	0	0
ENSG00000236833	AC024560.1	0	0	0	0	0	0	0	0
ENSG00000145016	RUBCN	4.29777	3.39975	1.23809	1.32619	2.65912	3.13783	1.06392	1.34792
ENSG00000284146	MIR922	0	0	0	0	0	0	0	0
ENSG00000122068	FYTTD1	9.70031	7.693	1.89254	4.32432	7.86226	6.44545	3.45607	4.69891
ENSG00000273375	AC055764.2	0.76582	0.1821	0	0.16237	0.26602	0	0	0.13432
ENSG00000186001	LRCH3	10.039	12.53	11.241	24.2377	17.1831	14.5487	18.1158	15.4873
ENSG00000234136	AC055764.1	0	0	0	0	0	0	0	0
ENSG00000234742	AC144530.1	0	0	0	0	0	0	0	0
ENSG00000114473	IQCG	1.24554	0.96314	0.21618	0.36388	0.70349	0.64016	0.39234	0.4937
ENSG00000199306	RNU6-858P	0	0	0	0	0	0	1.14187	0
ENSG00000182899	RPL35A	57.3698	73.5658	72.9792	62.4869	94.6846	79.4027	69.5652	101.908
ENSG00000185621	LMLN	0.37106	0.39303	0.26024	0.30037	0.27682	0.3273	0.25302	0.20708
ENSG00000201622	RNU6-621P	0	0	0	0	0	0	0	0
ENSG00000232832	LMLN-AS1	0	0	0	0	0	0	0	0
ENSG00000226435	ANKRD18DP	0	0	0.06246	0	0.02584	0.22496	0	0.07828
ENSG00000212297	RNU6-821P	0	0	0	0	0	0	0	0
ENSG00000232783	FRG2FP	0	0	0	0	0	0	0	0
ENSG00000213113	TUBB8P8	0	0	0	0	0.08423	0	0	0
ENSG00000236246	AC073135.1	0	0	0	0	0	0	0	0
ENSG00000236438	FAM157A	0.1964	0.30971	0.06076	0.04602	0.20108	0.01824	0.05169	0.21576
ENSG00000222792	RNU6-860P	0	0	0	0	0	0	0	0
ENSG00000230304	CICP6	0	0	0	0	0	0	0	0
ENSG00000226008	SEPT14P3	0	0	0	0	0	0	0	0
ENSG00000248302	BNIP3P41	0	0	0	0	0	0	0	0
ENSG00000272602	ZNF595	3.22425	1.67517	0.57513	2.01916	1.94865	2.33439	1.05622	3.9358
ENSG00000250238	AC253576.1	0	0	0	0	0	0	0	0
ENSG00000250312	ZNF718	1.5308	1.36289	2.77263	2.06054	1.25516	2.29232	0.56373	1.50796
ENSG00000275426	AC253576.2	0.37914	0.21036	0	0.37512	0	0	0.21066	0
ENSG00000250892	AC108475.1	0	0	0	0	0	0	0	0
ENSG00000198155	ZNF876P	1.49006	0.3481	0.69146	1.51307	1.14415	1.47574	1.83009	1.54061
ENSG00000251188	AC079140.3	0	0	0	0	0	0	0	0
ENSG00000251689	AC079140.5	0	0	0	0	0	0	0	0
ENSG00000186777	ZNF732	1.63781	0.05564	2.16116	3.91887	0.12191	12.8545	4.28996	2.62645
ENSG00000251535	AC079140.4	0	0	0	0.20564	0.16846	0.24445	0	0
ENSG00000131127	ZNF141	3.89217	2.40024	1.1814	2.82191	5.22847	4.1423	3.31827	2.83591
ENSG00000248564	AC079140.1	0	0	0	0	0	0	0	0
ENSG00000250321	AC079140.2	0.5162	0	0	0	0	0	0	0

ENSG00000207642	MIR571	0	0	0	1.13318	0	0	3.81814	0
ENSG00000281016	AC092574.2	0	0	0	0	0	0	0	0
ENSG00000249025	ZNF519P4	0	0.97607	0	0.87029	0	0	0	0
ENSG00000272885	AC092574.1	0.17452	0.2905	0	0	0	0	0	0
ENSG00000251595	ABCA11P	0.02627	0	0.03861	0.03899	0.06388	0.13904	0.13138	0.06451
ENSG00000182903	ZNF721	4.97846	3.07408	0.96083	2.21317	4.8533	3.37951	1.85469	2.66188
ENSG00000174227	PIGG	1.70216	0.98547	0.31637	0.62905	1.19422	0.90204	0.785	0.66083
ENSG00000273238	TMEM271	0	0	0	0	0	0	0	0
ENSG00000283183	AC116565.1	0	0	0	0	0	0	0	0
ENSG00000133256	PDE6B	0.57649	0.16792	0.08471	0.12834	0.26283	0.7119	0.12011	0.21234
ENSG00000242686	AC107464.1	0	0	0	0	0.07575	0	0	0
ENSG00000272927	AC107464.3	0	0.33109	0	0	0.12092	0	0.33156	0
ENSG00000169020	ATP5ME	8.42174	8.17716	4.01063	9.14263	8.24803	8.11638	5.5891	8.521
ENSG00000215375	MYL5	0.5108	0.19839	0.15012	0.27797	0.41401	0.2403	0.31219	0.25086
ENSG00000169026	SLC49A3	0.99804	0.69218	0.16296	0.32915	0.40446	0.44017	0.23105	0.57865
ENSG00000185619	PCGF3	3.25779	1.77467	0.49324	1.05489	1.90836	1.93315	1.56325	2.25447
ENSG00000248416	AC107464.2	0	0	0	0	0	0	0	0
ENSG00000272588	AC139887.4	0	0.16969	0	0	0.61973	0.7194	0	0.25034
ENSG00000249592	AC139887.2	1.95468	1.62678	0.06839	0.48349	1.64088	1.23154	0.3103	0.85711
ENSG00000233799	AC139887.1	0.51259	0.4266	0	0	0	0	1.28161	0.31468
ENSG00000168993	CPLX1	0.03031	0	0	0	0	0	0	0
ENSG00000260262	AC139887.3	0	0	0	0	0	0	0	0
ENSG00000178950	GAK	4.57069	2.96266	0.42645	1.1198	2.88137	3.5325	1.30605	1.36584
ENSG00000127419	TMEM175	2.83247	2.49203	0.6838	1.74153	2.11538	2.56982	1.51756	2.23564
ENSG00000145214	DGKQ	3.56915	2.67584	0.49847	0.85365	2.15171	2.44574	1.20459	1.41243
ENSG00000145217	SLC26A1	0.03415	0	0	0	0.08303	0	0	0
ENSG00000127415	IDUA	0.53143	0.30502	0.02693	0.19038	0.26735	0.42026	0.336	0.26999
ENSG00000127418	FGFRL1	0.65431	0.85362	0.12993	0.10498	0.4515	0.46796	0.3832	0.15199
ENSG00000250259	AC019103.1	0	0.44856	0	0	0	0	0	0
ENSG00000178222	RNF212	0.01465	0.02439	0.02153	0.02174	0.10688	0	0.04884	0.03598
ENSG00000251639	AC092535.2	0.06088	0	0	0	0	0	0	0
ENSG00000251652	AC092535.3	0	0	0	0	0	0.50218	0	0
ENSG00000215367	TMED11P	0	0	0	0	0	0	0	0.11904
ENSG00000212458	RF00554	0	0	0	0	0	0	0	0
ENSG00000227189	AC092535.1	0.03386	0	0	0	0	0	0	0
ENSG00000159674	SPON2	2.65749	2.82042	0.80611	2.96705	1.91187	5.93548	1.80842	2.39473
ENSG00000273179	AC092535.4	0	0.30733	0.27132	0.27402	0	0.32572	0	0
ENSG00000280927	CTBP1-AS	0	0	0	0	0	0	0	0
ENSG00000159692	CTBP1	6.82944	4.58207	1.10793	2.04274	4.61518	6.65041	2.19197	3.11077
ENSG00000196810	CTBP1-DT	0.99359	0.32764	0.08264	0.33387	0.69516	1.12445	0.42185	0.4028
ENSG00000090316	MAEA	3.17533	3.65147	0.40432	0.9712	2.58571	1.86287	1.21474	1.93562
ENSG00000163945	UVSSA	1.71315	0.8726	0.20635	0.47238	0.81946	0.61105	0.43692	0.58619
ENSG00000254094	AC078852.2	0.97363	0.23152	0	0	0	0.98149	0.46368	0.17077
ENSG00000253399	AC078852.1	0.16621	0	0	0	0	0.29322	0.55411	0.20407
ENSG00000179979		0.2973	0.13746	0.09708	0.07354	0.16064	0.20396	0.2753	0.32446
ENSG00000235608	NKX1-1	0	0	0	0	0	0	0	0
ENSG00000272783	AC147067.2	0	0	0	0	0	0	0	0
ENSG00000244459	AC147067.1	1.89897	2.52867	0.18603	0.37577	1.53915	0.44667	0	1.08805
ENSG00000174137	FAM53A	0.05188	0.08635	0.02541	0	0.08409	0.06101	0.02882	0
ENSG00000207009	RF00019	0	0	0	0	0	0	0	0
ENSG00000163950	SLBP	20.7672	16.862	11.6965	23.8405	18.9142	22.9136	19.1774	18.2571
ENSG00000270195	AC016773.1	0.36559	0.30426	0.13431	0	0.88894	1.45113	0.30469	0.33665
ENSG00000168936	TMEM129	2.32125	1.63056	0.06259	0.47408	1.34633	1.27734	0.70994	0.86284
ENSG00000013810	TACC3	7.68037	7.3078	0.69301	1.64978	6.64826	4.19946	3.0882	3.50172
ENSG00000068078	FGFR3	0	0	0	0.0225	0	0	0	0
ENSG00000168924	LETM1	2.40035	1.14793	0.32904	0.55828	1.51359	2.55969	0.56731	0.71479
ENSG00000109685	NSD2	1.29729	0.98712	0.62637	1.05617	1.88814	3.83175	1.51984	1.22872
ENSG00000249784	SCARNA22	0.5864	0.97607	15.5108	26.9789	1.42587	2.06899	17.594	19.4394

ENSG00000185049	NELFA	1.44501	0.8223	0.12704	0.27494	1.48653	1.43801	0.22645	0.40942
ENSG00000284587	MIR943	0	0	0	0	0	0	0	0
ENSG00000243449	C4orf48	0.27148	0.22594	0	0.60437	0.16503	0.47893	0.45252	0.33332
ENSG00000185818	NAT8L	0.0874	0.02078	0	0.01853	0	0	0.06243	0
ENSG00000130997	POLN	0.26582	0.60048	0.51618	1.22579	1.15421	0.97139	1.20266	0.81593
ENSG00000249006	AL136360.1	0	0	0	0	0	0	0.06431	0
ENSG00000250623	AL136360.2	0	0	0	0	0	0	0	0
ENSG00000214367	HAUS3	2.58268	2.62421	1.24983	2.46296	3.87135	3.87917	2.36857	2.54697
ENSG00000249047	COX6B1P5	0	0	0	0	0	0	0	0
ENSG00000123933	MXD4	5.50428	3.5956	1.43456	1.91123	5.35357	7.38349	2.68319	3.13676
ENSG00000284276	MIR4800	0	0	0	0	0	0	0	0
ENSG00000159733	ZFYVE28	0.43414	0.59584	0.11192	0.23738	0.44447	0.36278	0.15234	0.16832
ENSG00000251148	AL158068.2	0	0	0	0	0	0	0	0
ENSG00000239247	RN7SL589P	0.23645	0	0	0	0.86242	0	0	0.29031
ENSG00000249077	AL158068.1	0	0	0	0	0	0	0	0
ENSG00000206113	CFAP99	0	0	0	0	0.01714	0	0	0
ENSG00000063978	RNF4	7.08805	5.53821	1.48162	3.05258	6.59488	7.64843	3.56289	4.56794
ENSG00000248399	AL645924.1	0	0	0	0	0	0	0	0
ENSG00000251229	AL645924.2	0	0	0	0	0	0	0	0
ENSG00000125386	FAM193A	6.56217	5.41753	1.47044	2.95452	6.74874	8.80784	3.79235	3.49177
ENSG00000248155	CR545473.1	0.26462	0	0	0	0	0	0	0
ENSG00000206738	RF00019	0	0	0	0	0	0	0	0
ENSG00000168884	TNIP2	5.84001	3.17967	0.52133	1.05303	3.31782	4.28472	1.36464	1.94335
ENSG00000087266	SH3BP2	8.58529	7.00536	1.1413	2.07945	4.46604	3.63828	2.44046	3.31819
ENSG00000087274	ADD1	19.933	15.2394	4.89833	8.60757	18.6039	18.7564	7.33679	9.593
ENSG00000109736	MFSD10	3.01648	2.12424	0.75014	1.27416	1.69262	2.98821	0.85089	1.39597
ENSG00000249673	NOP14-AS1	1.35496	0.96657	0.07485	0.36287	0.76793	1.32996	0.5434	0.36274
ENSG00000087269	NOP14	9.48885	6.65564	1.64402	3.4745	9.7983	10.4897	5.31819	3.99365
ENSG00000125388	GRK4	0.82905	0.65092	0.34479	3.69119	0.68464	0.77267	1.12116	0.61458
ENSG00000199335	RNU6-204P	0	0	0	1.01669	0	0	0	0
ENSG00000251075	HTT-AS	0	0	0	0	0	0	0	0
ENSG00000278778	RF02134	0.3187	0	0	0	0	0	0	0
ENSG00000274693	RF02135	0	0.69719	0	0	0	0	0	0
ENSG00000197386	HTT	3.29949	2.69889	0.84374	1.98032	3.12656	3.84483	1.55691	1.66808
ENSG00000188981	MSANTD1	0.04852	0	0	0.02881	0	0.03424	0.01618	0.03575
ENSG00000159788	RGS12	0.30325	0.28844	0.01592	0.05626	0.25018	0.17196	0.07221	0.09973
ENSG00000248840	AL645949.1	0.134	0	0	0	0	0	0	0.16453
ENSG00000248843	AL645949.2	0	0	0	0	0	0	0	0
ENSG00000109758	HGFAC	0	0	0	0	0	0	0	0
ENSG00000175920	DOK7	0.04802	0.15985	0.07056	0	0.14595	0	0	0.05896
ENSG00000261643		0.17027	0.42512	0	0.12635	0.93153	0.30038	0	0.4181
ENSG00000163956	LRPAP1	6.73012	5.70604	0.66673	1.09733	3.8511	2.38643	1.49854	2.04257
ENSG00000270090	AL590235.1	0	0	0	0	0	0	0	0
ENSG00000216560	LINC00955	0	0	0	0	0	0	0	0
ENSG00000250681	AL121796.1	0	0	0	0	0	0	0	0
ENSG00000250632	LINC02171	0	0	0	0	0	0	0	0
ENSG00000250986	LINC02600	0	0	0	0	0	0	0	0
ENSG00000184160	ADRA2C	0	0.05599	0	0	0	0	0	0
ENSG00000251166	OR7E163P	0	0	0	0	0	0	0	0
ENSG00000234386	OR7E162P	0	0	0	0	0	0	0	0
ENSG00000249522	AC147876.1	0	0	0	0	0	0	0	0
ENSG00000253917	AC226119.1	0	0	0	0	0	0	0	0
ENSG00000271977	AC226119.2	0	0	0	0	0	0	0	0
ENSG00000251271	ALG1L7P	0	0	0	0	0	0	0	0
ENSG00000251669	FAM86EP	0.25305	0	0.06198	0	0.35893	0.0744	0	0.02589
ENSG00000250476	ENPP7P9	0	0	0	0	0	0	0	0
ENSG00000243894	AC116562.1	0	0	0	0	0	0	0	0
ENSG00000250940	AC116562.3	0	0	0	0	0	0	0	0

ENSG00000248669	AC116562.2	0	0	0	0	0	0	0	0
ENSG00000198787	OR4D12P	0	0	0	0	0	0	0	0
ENSG00000250381	UNC93B4	0	0	0	0	0	0	0	0
ENSG00000250710	OR7E99P	0	0	0	0	0	0	0	0
ENSG00000249844	OR7E43P	0	0	0	0	0	0	0	0
ENSG00000163982	OTOP1	0	0	0	0	0	0	0	0
ENSG00000132406	TMEM128	2.78516	2.24771	1.48828	3.63245	3.18091	2.97782	3.16529	4.09313
ENSG00000145220	LYAR	13.5917	12.4166	3.66943	6.56749	11.7208	18.6245	4.95254	5.66605
ENSG00000168826	ZBTB49	1.97903	1.77375	0.52198	0.64014	1.94335	2.95416	1.56479	1.52643
ENSG00000248516	AC105415.1	1.03532	0.25849	0.38035	0.38413	0.12587	0.45661	0.51771	0.31779
ENSG00000168824	NSG1	1.14453	0.72841	0.19787	0.42465	1.20732	0.17816	0.33666	0.55796
ENSG00000168818	STX18	4.96276	3.88999	1.93934	3.93767	3.97783	4.94741	2.77266	2.83562
ENSG00000248221	STX18-IT1	0.54096	0	0	0.20071	0.32884	0.23858	0.22542	0
ENSG00000237170	RPS7P15	0	0	0	0	0	0	0	0
ENSG00000247708	STX18-AS1	0.26406	0.15983	0.07055	0.32064	0.27726	0.31762	0.22007	0.26526
ENSG00000280310	AC092437.1	0	0	0	0	0	0	0	0
ENSG00000273396	LINC01396	0	0	0	0.20642	0	0	0	0
ENSG00000163132	MSX1	0	0	0	0	0	0	0	0
ENSG00000251587	LDHAP1	0	0	0	0	0	0	0	0
ENSG00000200761	RN7SKP113	0	0	0	0	0	0	0	0
ENSG00000170891	CYTL1	0.29232	0.24329	0	0.43384	0.1777	0	0	0
ENSG00000152953	STK32B	0.06884	0.11458	0	0.04087	0.08369	0.02429	0.13769	0.05071
ENSG00000252266	RF00019	0	0	0	0	0	0	0	0
ENSG00000201496	RN7SKP275	0	0	0	0	0	0	0	0
ENSG00000082929	LINC01587	0	0	0	0	0	0	0	0
ENSG00000173040	EVC2	0.22619	0.44294	0	0.03949	0.04853	0.30515	0	0.04901
ENSG00000072840	EVC	0.20509	0.25603	0	0.03044	0.06234	0.12663	0	0
ENSG00000072832	CRMP1	0	0	0	0	0	0	0	0
ENSG00000181215	C4orf50	0.03342	0.09536	0.00702	0.02126	0.04063	0.03369	0	0.02345
ENSG00000263631	MIR378D1	0	0	0	0	0	0	0	0
ENSG00000152969	JAKMIP1	1.05759	3.10462	0.36734	0.31391	1.05201	5.15619	0.0641	0.37774
ENSG00000249896	LINC02495	0.02598	0	0	0	0.07896	0.06875	0	0
ENSG00000251408	AC113615.1	0.03557	0	0	0	0.02163	0.06276	0.02965	0
ENSG00000109501	WFS1	0.16242	0.0811	0.02387	0.07231	0.13823	0.08596	0.02707	0.03988
ENSG00000074211	PPP2R2C	0	0	0	0	0	0	0	0
ENSG00000013288	MAN2B2	6.62323	5.40249	0.62948	1.21036	5.04774	3.83663	1.19462	2.08355
ENSG00000179010	MRFAP1	34.3449	24.08	7.78948	13.3985	29.2356	25.0831	17.5332	15.6944
ENSG00000251580	LINC02482	1.38958	0	0.25525	0.51557	0.63353	0.61285	0.86858	0.21326
ENSG00000170846	AC093323.1	4.12085	1.73828	0.871	1.17289	2.95111	3.73446	2.02301	1.6634
ENSG00000246526	LINC002481	0.96607	1.13901	0.17745	0.41818	0.53832	0.28405	0.20129	0.64248
ENSG00000279859	AC093323.2	0	0	0	0	0	0	0	0.09221
ENSG00000163993	S100P	20.0431	2.19234	2.44039	4.50441	13.3675	1.91947	10.7862	26.7179
ENSG00000282742	AC093323.3	0	0	0	0	0	0	0	0.09483
ENSG00000178988	MRFAP1L1	25.487	21.2675	5.18806	11.2778	27.5935	20.2272	9.97619	15.6463
ENSG00000186222	BLOC1S4	2.0399	2.03725	1.26566	1.27825	1.87382	1.19955	2.11568	1.78102
ENSG00000170871	KIAA0232	20.216	14.3556	3.86548	6.34871	14.1555	8.38343	8.76927	10.6103
ENSG00000132405	TBC1D14	33.203	28.9919	6.57571	13.4566	26.6879	12.9705	12.4706	18.9627
ENSG00000187904	AC097382.1	0	0.41289	0	0.36814	0	0	0.41347	0.15228
ENSG00000202392	RN7SKP292	0.23123	1.15465	0	0.68635	0.56225	0	0	0.85171
ENSG00000245748	AC097382.2	0.01705	0	0	0	0	0	0	0.02093
ENSG00000173013	CCDC96	0.24539	0.2334	0.10303	0.2081	0.17048	0.55658	0.05843	0
ENSG00000173011	TADA2B	7.00516	4.58377	1.2308	1.92108	6.03266	4.29845	2.66529	3.27205
ENSG00000109519	GRPEL1	4.17656	2.30756	0.8252	1.38033	2.26152	2.8791	1.55029	1.40048
ENSG00000245468	LINC02447	0.01948	0	0	0	0	0	0	0.07175
ENSG00000200867	RN7SKP36	0	0	0	0	0	0	0	0.5769
ENSG00000184985	SORCS2	0.08123	0.07726	0	0.01722	0.01411	0	0.03868	0.01425
ENSG00000264658	MIR4798	0	0	0	0	0	0	0	0
ENSG00000178597	PSAPL1	0	0	0	0	0	0	0	0

ENSG00000266690	MIR4274	0	0	0	0	0	0	0	0
ENSG00000272620	AFAP1-AS1	0.11866	0.01796	0.11096	0.04803	0.03935	0.01903	0.01798	0
ENSG00000196526	AFAP1	2.63665	1.47101	0.35418	0.28183	2.43304	0.51539	0.97394	1.05816
ENSG00000228919	AC097381.1	0.05656	0	0	0	0	0	0	0
ENSG00000163995	ABLIM2	0.08482	0.02017	0	0.01798	0.11786	0.02138	0.0404	0.04463
ENSG00000207807	MIR95	0	0	0	0	0	0	0	0
ENSG00000273267	AC097381.3	0	0	0	0	0	0	0	0
ENSG00000251460	AC097381.2	0	0	0	0	0	0	0	0
ENSG00000250471	GMPSP1	0	0	0	0	0.15391	0	0	0
ENSG00000125089	SH3TC1	2.92011	2.15384	0.32539	0.64698	1.69938	1.61136	1.03807	0.90906
ENSG00000170801	HTRA3	0.08829	0	0	0.03276	0	0.03894	0	0.0542
ENSG00000249145	LINC02517	0	0	0	0	0	0	0	0
ENSG00000248986		0	0	0	0	0	0	0	0
ENSG00000251615	AC104825.1	0.22561	0.60084	0.03315	0.23438	0.54858	0.398	0.07521	0.1385
ENSG00000087008	ACOX3	1.37224	1.26894	0.19605	0.45257	1.13539	1.14316	0.38122	0.77221
ENSG00000202054	RNA5SP152	0	0	0	0	0	0	0	0
ENSG00000155275	TRMT44	0.65481	0.61604	0.11156	0.3521	0.49612	0.75337	0.20564	0.39615
ENSG00000251186	AC105345.2	0	0	0	0	0	0	0	0
ENSG00000205959	AC105345.1	0.02173	0	0	0	0.05283	0	0.03621	0.05335
ENSG00000155269	GPR78	0	0	0	0	0	0	0	0
ENSG00000109625	CPZ	0	0	0	0	0	0	0	0
ENSG00000250915	AC209005.1	0	0	0	0	0	0	0	0
ENSG00000215612	HMX1	0	0	0	0	0	0	0	0
ENSG00000258507	AC116612.1	0	0	0	0	0	0	0	0
ENSG00000197468	AC073648.1	0	0	0	0	0	0	0	0
ENSG00000249272	UNC93B8	0	0	0	0	0	0	0	0
ENSG00000250804	AC073648.3	0	0	0	0	0	0	0	0
ENSG00000249347	AC073648.2	0	0	0	0	0	0	0	0
ENSG00000271057	AC073648.5	0	0	0	0	0	0	0	0
ENSG00000251313	AC073648.4	0	0	0	0	0	0	0	0
ENSG00000250342	SNRPCP16	0	0	0	0	0	0	0	0
ENSG00000249767	ENPP7P10	0.15713	0	0	0.1166	0	0	0.13095	0.19292
ENSG00000163612	FAM86KP	0.07427	0	0	0	0	0	0	0
ENSG00000250268	ALG1L14P	0	0	0	0	0	0	0	0
ENSG00000229924	FAM90A26	0	0	0	0	0	0	0	0
ENSG00000231396	USP17L10	0	0	0	0	0	0	0	0
ENSG00000233136	USP17L11	0	0	0	0	0	0	0	0
ENSG00000227551	USP17L12	0	0	0	0	0	0	0	0
ENSG00000232399	USP17L13	0	0	0	0	0	0	0	0
ENSG00000249482	USP17L14P	0	0	0	0	0	0	0	0
ENSG00000223569	USP17L15	0	0	0	0	0	0	0	0
ENSG00000250231	USP17L16P	0	0	0	0	0	0	0	0
ENSG00000249104	USP17L17	0	0	0	0	0	0	0	0
ENSG00000250844	USP17L18	0	0	0	0	0	0	0	0
ENSG00000248920	USP17L19	0	0	0	0	0	0	0	0
ENSG00000250745	USP17L20	0	0	0	0	0	0	0	0
ENSG00000249811	USP17L21	0	0	0	0	0	0	0	0
ENSG00000248933	USP17L22	0	0	0	0	0	0	0	0
ENSG00000250913	USP17L23	0	0	0	0	0	0	0	0
ENSG00000232264	USP17L24	0	0	0	0	0	0	0	0
ENSG00000230430	USP17L25	0	0	0	0	0	0	0	0
ENSG00000229579	USP17L26	0	0	0	0	0	0	0	0
ENSG00000227140	USP17L5	0	0	0	0	0	0	0	0
ENSG00000235780	USP17L27	0	0	0	0	0	0	0	0
ENSG00000231051	USP17L28	0	0	0	0	0	0	0	0
ENSG00000231637	USP17L29	0	0	0	0	0	0	0	0
ENSG00000251694	USP17L9P	0	0	0	0	0	0	0	0
ENSG00000228856	USP17L30	0	0	0	0	0	0	0	0

ENSG00000205946	USP17L6P	0	0	0	0	0	0	0	0
ENSG00000219492	AC116655.1	0	0	0	0	0	0	0	0
ENSG00000188438	DEFB108F	0	0	0	0	0	0	0	0
ENSG00000252307	RNA5SP153	0	0	0	0	0	0	0	0
ENSG00000232773	DEFB130D	0	0	0	0	0	0	0	0
ENSG00000186146	DEFB131A	0	0	0	0	0	0	0	0
ENSG00000248364	OR7E86P	0	0	0	0	0	0	0	0
ENSG00000250884	OR7E85P	0	0	0	0	0	0	0	0
ENSG00000251624	UNC93B7	0	0	0	0	0	0	0	0
ENSG00000249866	OR7E83P	0	0	0	0	0	0	0	0
ENSG00000249443	AC097493.3	0	0	0	0	0	0	0	0
ENSG00000242034	AC097493.1	0	0	0	0	0	0	0	0
ENSG00000221275	MIR548I2	0	0	0	0	0	0	0	0
ENSG00000244004	AC097493.2	0	0	0	0	0	0	0	0
ENSG00000249799	SNRPCP13	0	0	0	0	0	0	0	0
ENSG00000250942	ENPP7P11	0	0	0.34359	0.1735	0	0	0	0
ENSG00000186234	FAM86MP	0	0	0	0	0	0	0	0
ENSG00000251087	ALG1L3P	0	0	0	0	0	0	0	0
ENSG00000203436	RF00017	0	0	0	0	0	0	0	0
ENSG00000249563	AC098976.1	0	0	0	0	0	0	0	0
ENSG00000250425	OR7E35P	0	0	0	0	0	0	0	0
ENSG00000109667	SLC2A9	0.88661	0.55341	0.13029	0.26316	0.43117	0.13686	0.2032	0.17689
ENSG00000169676	DRD5	0	0	0	0	0	0	0	0
ENSG00000252002	RNA5SP154	0	1.09918	0	0	0	0	0	0
ENSG00000249219	AC108199.1	0	0	0	0	0	0	0	0
ENSG00000250413	AC005674.1	0.1597	0	0	0	0	0	0	0
ENSG00000261490	AC005674.2	1.67066	1.70325	0.42963	0.37192	1.52336	1.25259	0.69618	1.69225
ENSG00000071127	WDR1	47.6494	37.8948	6.24788	11.0912	32.2446	36.3137	12.1291	17.029
ENSG00000264931	MIR3138	0.89391	2.97582	2.62717	0	3.26037	3.15395	1.49	0
ENSG00000223086	RNA5SP155	3.40932	3.78321	0.83499	0.8433	1.38165	1.00242	0	1.3953
ENSG00000250393	AC093664.1	0	0	0	0	0	0	0	0
ENSG00000251296	AC006499.6	0.12076	0	0	0	0.29363	0	0	0
ENSG00000248777	AC006499.1	0	0	0	0	0	0	0	0
ENSG00000271544	AC006499.8	0	0	0	0	0	0	0	0
ENSG00000250040	RAF1P1	0	0	0	0	0	0	0	0
ENSG00000250573	AC006499.4	0	0	0	0	0	0	0	0
ENSG00000250074	AC006499.2	0	0	0	0	0	0	0	0
ENSG00000250505	AC006499.3	0	0	0	0	0	0	0	0
ENSG00000251278	AC006499.5	0	0	0	0	0	0	0	0
ENSG00000251338	AC006499.7	0	0	0	0	0	0	0	0
ENSG00000250613	AC110768.2	0	0	0	0	0	0	0	0
ENSG00000178163	ZNF518B	6.08316	3.3453	0.7013	1.82557	5.44261	3.40325	2.40887	3.02879
ENSG00000248419	AC110768.1	0	0	0	0	0	0	0	0
ENSG00000109684	CLNK	0.07087	0.0983	0.03471	0	0.08616	0.04167	0.01969	0.05801
ENSG00000249334	AC084048.1	0	0	0	0	0	0	0	0
ENSG00000283083	LINC02498	0	0	0	0	0	0	0	0
ENSG00000207716	MIR572	0	0	0	0	0	0	0	0
ENSG00000250896	RNPS1P1	0	0	0	0	0	0.14086	0	0
ENSG00000002587	HS3ST1	0.05386	0.02988	0	0	0.17461	0	0	0.0551
ENSG00000222182	RNA5SP156	0	0	0	0	0	0	0	0
ENSG00000251152	AC025539.1	0.03648	0	0	0	0.02217	0	0	0.02239
ENSG00000249631	AC005699.1	0	0	0.05343	0	0	0	0	0
ENSG00000248300	LINC02360	0	0	0	0	0	0	0	0
ENSG00000248262	AC108037.1	0	0	0	0	0	0	0	0
ENSG00000251210	LINC02270	0	0	0	0	0	0	0	0
ENSG00000212282	RNU6-578P	0	0	0	0	0	0	0	0
ENSG00000249995	ECM1P2	0	0	0	0	0	0	0	0
ENSG00000249780	AC093809.1	0	0	0	0	0	0	0	0

ENSG00000250371	AC007370.2	0	0	0	0	0	0	0	0
ENSG00000250098	AC007370.1	0	0	0	0	0	0	0	0
ENSG00000283271	RF00026	0	0	0	0	0	0	0	0
ENSG00000205940	HSP90AB2P	0	0	0	0.04732	0.03876	0	0.05314	0
ENSG00000157869	RAB28	5.64357	6.09768	2.52189	6.66135	5.57732	6.22979	5.94123	3.64689
ENSG00000281202	LINC01097	0	0	0	0	0	0	0	0
ENSG00000109705	NKX3-2	0	0	0	0	0	0	0	0
ENSG00000246095	LINC01096	0	0	0	0	0	0	0	0
ENSG00000038219	BOD1L1	26.4454	26.1787	3.30069	5.31582	20.0878	8.88713	7.30309	13.9251
ENSG00000266240	MIR5091	4.72905	0	0	1.16974	0	1.39045	0	2.90313
ENSG00000249148	AC006445.2	0	0	0	0	0	0	0	0
ENSG00000242209	AC006445.1	0	0	0	0	0	0	0	0
ENSG00000250634	LINC01182	0.05346	0.08899	0	0	0	0	0	0
ENSG00000250497	AC007126.1	0	0	0	0	0	0	0	0
ENSG00000224569	AC095052.1	0.16987	0	0	0	0.10326	0	0	0
ENSG00000248698	LINC01085	0.0626	0.0521	0	0	0.03805	0	0	0.03843
ENSG00000251317		0	0	0	0	0	0	0	0
ENSG00000251679	AC073848.1	0	0	0	0	0	0	0	0
ENSG00000249988	AC092546.1	0.22589	0.37599	0	0.16762	0	0	0	0
ENSG00000251412	AC006296.2	0	0	0	0	0	0	0	0
ENSG00000248425	AC006296.1	0	0	0	0	0	0	0.82694	0
ENSG00000248360	LINC00504	0.18703	0.09579	0.12685	0.17081	0.24487	0.07614	0.19184	0.12365
ENSG00000251252	MTND2P31	0	0	0	0	0	0	0	0
ENSG00000202449	RF00092	0	0	0	0	0	0	0	0
ENSG00000247624	CPEB2-DT	0	0.079	0.06974	0.07043	0	0.16745	0	0.05827
ENSG00000137449	CPEB2	6.62839	5.18498	1.95107	2.63741	3.50159	2.27021	2.62168	3.12237
ENSG00000249252	AC098829.1	0.08082	0	0	0	0.39302	0	0	0.3969
ENSG00000199420	RN7SKP170	0	0	0	0	0	0	0	0
ENSG00000163145	C1QTNF7	0	0	0	0	0	0	0	0
ENSG00000251379	AC099550.1	0.84157	0.14008	0.12367	0.1249	0.61389	0.29693	0	0.72328
ENSG00000048342	CC2D2A	0.12528	0.09625	0.02832	0.05721	0.03515	0	0.01606	0
ENSG00000249930	AC007016.1	0	0	0	0	0	0.52997	0	0.73768
ENSG00000273133	AC116651.1	0.79101	2.41384	0	0.39132	0.16028	0.23258	0	1.13306
ENSG00000118564	FBXL5	122.734	127.691	38.3229	34.5412	80.2693	42.84	35.027	88.6195
ENSG00000237765	FAM200B	6.72592	7.26935	1.83362	2.66488	4.64361	3.03347	1.82624	3.97953
ENSG00000109743	BST1	17.3412	26.9401	3.54227	4.1616	13.6366	5.29397	4.96101	11.9593
ENSG00000214846	AC114744.1	0	0	0	0	0	0	0	0
ENSG00000248188	AC005798.1	1.13644	0.31527	0	0	0.69083	0	0.31571	0.23255
ENSG00000004468	CD38	3.61423	2.04682	0.5971	2.33282	2.97706	15.6004	1.26547	1.89053
ENSG00000251644	HPRT1P1	0	0	0	0	0	0	0	0
ENSG00000137440	FGFBP1	0	0	0	0	0	0	0	0
ENSG00000137441	FGFBP2	20.2895	39.4331	4.99793	13.1413	16.2549	59.0696	8.21052	16.9194
ENSG00000007062	PROM1	0.01972	0.03282	0	0	0.05994	0.03479	0.01644	0
ENSG00000169762	TAPT1	3.40845	2.54054	1.52737	3.09903	3.54053	2.42831	2.87189	2.72474
ENSG00000249234	AC108063.1	0	0	0	0	0	0	0	0
ENSG00000263327	TAPT1-AS1	0.58364	0.61821	0.07797	0.23623	0.4193	0.37441	0.26532	0.39087
ENSG00000242358	RPS21P4	0	0	0	0	0	0	0	0
ENSG00000248851	AC006427.1	0	0	0	0	0.12516	0	0	0
ENSG00000249506	ZEB2P1	0.03782	0	0	0	0	0	0	0.04644
ENSG00000248138	AC097515.1	0	0	0	0	0	0	0	0
ENSG00000169744	LDB2	0.09186	0.06553	0.03857	0.03895	0	0.11575	0.06562	0.08056
ENSG00000249998	AC106894.1	0	0	0	0	0	0	0	0
ENSG00000251212	MTND5P4	0	0	0	0	0	0	0	0
ENSG00000250819	LINC02493	0	0	0	0	0	0	0	0
ENSG00000206780	SNORA75B	0	0	0	0	0	0	0	0
ENSG00000215278	RPS7P6	0	0	0	0	0	0	0	0
ENSG00000151552	QDPR	1.03037	0.39885	0.07042	0.2845	0.87398	0.25364	0.11982	0.23536
ENSG00000249581	CLRN2	0	0	0	0	0	0	0	0

ENSG00000249488	NACAP5	0	0	0	0	0	0	0	0
ENSG00000283219	AC006160.2	0	0	0	0	0	0	0	0
ENSG00000002549	LAP3	17.8748	21.4039	8.62281	4.62735	7.39303	19.3711	6.94023	21.4947
ENSG00000249502	AC006160.1	1.27054	0.81339	0.57447	0.72524	1.18822	0.51725	0.65163	0.95997
ENSG00000118579	MED28	5.03932	4.88834	1.39277	3.72459	4.95814	4.45091	4.35007	3.45827
ENSG00000047662	FAM184B	0.01107	0.03685	0	0	0	0	0	0.01359
ENSG00000163257	DCAF16	5.96631	3.39475	1.65507	2.95908	5.97689	4.24237	4.21136	2.95256
ENSG00000109805	NCAPG	23.8774	32.1571	30.3595	104.232	49.5837	52.6865	75.5278	65.6004
ENSG00000178177	LCORL	2.05722	1.96258	0.84095	2.00614	1.97331	2.07136	1.75977	1.89589
ENSG00000235413	KRT18P63	0	0	0	0	0	0	0	0
ENSG00000251048	AC093871.1	0	0	0	0	0	0	0	0
ENSG00000279918	AC006840.1	0	0	0	0	0	0	0	0
ENSG00000248238	LINC02438	0	0	0	0	0	0	0	0
ENSG00000251816	RNA5SP157	0	0	0	0	0	0	0	0
ENSG00000279379	AC024595.1	0	0	0	0	0	0	0	0
ENSG00000248515	AC024230.1	0	0	0	0	0	0	0	0
ENSG00000249441	AC110767.1	0	0	0	0	0	0	0	0
ENSG00000241612	AC114728.1	0	0.25261	0	0	0	0.26773	0	0
ENSG00000250611	AC098862.1	0	0	0	0	0	0	0	0
ENSG00000145147	SLIT2	0	0.02513	0	0	0.01835	0.02663	0	0
ENSG00000248228	SLIT2-IT1	0	0	0	0	0	0	0	0.14975
ENSG00000207732	MIR218-1	0	0	0	0	0	0	0	0
ENSG00000163138	PACRGL	1.49073	1.72482	1.57617	3.21069	1.79029	2.245	4.5757	2.03118
ENSG00000185774	KCNIP4	0	0	0	0	0	0	0	0
ENSG00000272995	AC097505.1	0	0	0	0	0	0	0	0
ENSG00000250243	AC110296.1	0	0	0	0	0	0	0	0
ENSG00000283281	MIR7978	0	0	0	0	0	0	0	0
ENSG00000250092	AC096576.3	0	0	0	0	0	0	0	0
ENSG00000242768	AC096576.1	0	0	0	0	0	0	0	0
ENSG00000248343	AC096576.2	0	0	0	0	0	0	0	0
ENSG00000280650	KCNIP4-IT1	0	0	0	0	0	0.01313	0	0.00914
ENSG00000250039	AC096719.1	0	0	0	0	0	0	0	0
ENSG00000239001	RNU6-420P	0	0	0	0	0	0	0	0
ENSG00000152990	ADGRA3	0.24566	0.2638	0.26783	0.85853	0.6744	0.99256	0.63402	0.68106
ENSG00000244538	AC098583.1	0	0	0	0	0	0	0	0
ENSG00000251516	AC098583.2	0	0	0	0	0	0	0	0
ENSG00000249948	GBA3	0	0	0	0.11131	0	0	0	0
ENSG00000237350	CDC42P6	0.50903	1.0591	0.56101	0.56659	0.77358	0.2245	0.21212	0.31249
ENSG00000248837	AC097512.1	0	0	0	0	0	0	0	0
ENSG00000249645	AC114912.1	0	0	0	0	0	0	0	0
ENSG00000249547	AC092440.1	0	0	0	0	0	0	0	0
ENSG00000251220	RFPL4AP3	0	0	0	0	0	0	0	0
ENSG00000250137	AC093607.1	0	0	0	0	0	0	0	0
ENSG00000251292	ERVH-1	0	0	0	0	0	0	0	0
ENSG00000109819	PPARGC1A	0.02697	0.02245	0.00991	0	0.03279	0	0	0.00828
ENSG00000249453	AC092834.1	0	0	0	0	0	0	0	0
ENSG00000109606	DHX15	9.74404	7.87778	2.13791	5.10475	9.60827	10.6941	5.59856	7.03481
ENSG00000207697	MIR573	3.70204	4.92964	1.08802	5.49423	3.60067	3.91854	3.70243	2.72718
ENSG00000243005	RN7SL16P	0.81748	0.45356	0.40042	0	1.65644	0.48071	0.4542	0
ENSG00000249256	ATP5MGP3	0	0	0	0	0	0	0	0
ENSG00000261121	LINC02473	0	0	0	0	0	0	0	0
ENSG00000251638	AC006390.1	0	0	0	0	0	0	0	0
ENSG00000248444	HNRNPA1P65	0	0	0	0	0	0	0	0
ENSG00000109610	SOD3	0	0	0	0	0	0	0	0
ENSG00000181982	CCDC149	1.01742	0.56026	0.06745	0.18165	0.66033	0.32389	0.06376	0.26298
ENSG00000153012	LGI2	0.02281	0	0	0	0	0	0	0
ENSG00000109618	SEPSECS	2.88167	2.4668	2.54075	5.09715	3.44626	5.74765	7.33239	3.87022
ENSG00000281501	SEPSECS-AS1	0.97599	0.84028	0.04946	0.64932	0.2455	0.02969	0.36463	0.2066

ENSG00000281028	AC104662.2	0	0	0	0	0	0	0	0
ENSG00000271172	AC104662.1	0	0	0	0.21289	0	0	0	0.17612
ENSG00000038210	PI4K2B	3.33372	2.99851	1.46053	2.21259	2.94538	3.47023	1.48411	2.64398
ENSG00000278135	RF00004	0	0	1.13383	1.14511	0	0	0	0
ENSG00000168228	ZCCHC4	0.99955	1.08144	0.66097	1.9532	1.27599	1.44006	3.02674	1.67722
ENSG00000053900	ANAPC4	2.61158	1.77461	0.99176	2.29359	2.34267	2.51928	1.54886	1.71732
ENSG00000249320	AC133963.2	0	0	0	0	0	0	0	0
ENSG00000248608	AC133963.1	0	0	0	0	0	0	0	0
ENSG00000238632	RNU7-126P	0	0	0	0	0	0	0	0
ENSG00000248545	AC092436.2	0	0	0	0	0	0	0	0
ENSG00000228154	AC092436.1	0	0	0	0	0	0	0	0
ENSG00000157765	SLC34A2	0	0	0	0	0	0	0	0
ENSG00000242640	RPS29P11	0	0	0	0	0	0	0	0
ENSG00000249277	AC092436.3	0	0	0	0	0	0	0	0
ENSG00000250050	MTND4P9	0	0	0	0	0	0	0	0
ENSG00000248133	MTND4LP22	0	0	0	0	0	0	0	0
ENSG00000251353	MTND3P5	0	0	0	0	0	0	0	0.27026
ENSG00000248654	MTCO3P44	0	0	0	0	0.32945	0	0	0
ENSG00000091490	SEL1L3	8.15318	4.07292	1.16502	3.64604	7.3302	3.83325	2.03933	3.2086
ENSG00000250541	AC092436.4	0	0	0	0	0	0	0	0
ENSG00000250317	SMIM20	3.18302	2.04357	0.80184	1.61964	1.87963	2.56698	0.98533	2.23318
ENSG00000251009	AC133961.1	0	0	0	0	0	0	0	0
ENSG00000283314	LINC02357	0	0	0	0	0	0	0	0.05549
ENSG00000248452	AC097714.1	0	0	0	0	0	0	0	0
ENSG00000168214	RBPJ	19.8679	22.0467	3.20127	5.45516	15.2075	5.82763	4.39707	11.3992
ENSG00000163394	CCKAR	0	0	0	0	0	0	0	0
ENSG00000109680	TBC1D19	1.06727	1.208	0.53324	2.24921	1.34947	1.09203	1.5655	1.31038
ENSG00000200999	RF00284	0	0	0	0	0	0	0	0
ENSG00000109689	STIM2	8.79519	7.52289	2.19824	5.33773	6.91367	7.58015	4.73937	4.18137
ENSG00000240005	AC106047.1	0.99559	1.45002	1.28013	1.10817	0.4539	1.53681	3.11155	1.06957
ENSG00000248340	AC106047.2	0	0	0	0	0	0	0	0
ENSG00000251080	AC024132.2	0	0	0	0	0	0	0	0
ENSG00000205830	AC024132.1	0	0	0	0	0	0	0	0
ENSG00000249699	LINC02261	0	0	0	0	0	0	0	0
ENSG00000222206	RF00019	0	0	0	0	0	0	0	0
ENSG00000251325	AC024132.3	0	0	0	0	0	0	0	0
ENSG00000250858	IGBP1P5	0	0	0	0	0	0	0	0
ENSG00000251410	AC007106.1	0	0	0	0	0	0	0	0
ENSG00000250038	AC109588.1	0	0	0	0	0	0	0	0
ENSG00000250064	AC097480.1	0	0	0	0	0	0	0	0
ENSG00000243548	RN7SL101P	0	0	0	0	0	0	0	0
ENSG00000283275	MIR4275	0	0	0	0	0	0	0	0
ENSG00000250927	MESTP3	0	0	0	0	0	0	0	0
ENSG00000250190	LINC02364	0	0	0	0	0	0	0	0
ENSG00000276507	AC068944.2	0	0	0	0	0	0	0	0
ENSG00000249228	AC068944.1	0	0	0	0	0	0	0	0
ENSG00000248176	AC109349.1	0	0	0	0	0	0	0	0
ENSG00000248338	LINC02472	0	0	0	0	0	0	0	0
ENSG00000251373	AC092593.2	0	0	0	0	0	0	0	0
ENSG00000251113	AC092593.1	0	0	0	0	0	0	0	0
ENSG00000283666	RF00026	0	0	0	0	0	0	0	0
ENSG00000249542	EEF1A1P21	0	0	0	0	0	0	0	0
ENSG00000249564	AC097510.1	0	0	0	0	0	0	0	0
ENSG00000244031	RPS3AP17	0	0	0	0	0	0	0	0
ENSG00000248281	AC106868.1	0	0	0	0	0	0	0	0
ENSG00000169851	PCDH7	0	0.01992	0	0	0.00728	0	0	0
ENSG00000249678	AC097716.1	0	0	0	0	0	0	0	0
ENSG00000250624	MTCYBP43	0	0	0	0	0	0	0	0

ENSG00000251182	LINC02497	0	0	0	0	0	0	0	0
ENSG00000251434	AC104071.1	0	0.40534	0.17893	0.36141	0.14803	1.07402	0.40591	0.44849
ENSG00000249882	LINC02501	0	0	0	0	0	0	0	0
ENSG00000251129	LINC02506	0	0.33245	0	1.77851	0	3.52348	8.82228	0.12261
ENSG00000251329	LINC02353	0	0	0	0	0	0	0	0
ENSG00000250076	AC017013.1	0	0	0	0	0	0	0	0
ENSG00000249452	AC093878.1	0	0	0	0	0.20629	1.19733	0	0
ENSG00000249766	AC097535.2	0	0	0	0	0	0	0	0
ENSG00000248417	AC097535.1	0	0	0	0	0	0	0	0
ENSG00000250954	AC016687.3	0	0	0	0	0	0	0	0
ENSG00000250723	AC016687.2	0	0	0	0	0	0	0	0
ENSG00000239983	AC016687.1	0	0	0	0	0	0	0	0
ENSG00000248685	LINC02484	0	0	0	0	0	0	0	0
ENSG00000250597	AC093689.1	0	0	0	0	0	0	0	0
ENSG00000275250	AC093913.1	0	0	0	0	0	0	0	0
ENSG00000238694	RF01210	0	0	0	0	0	0	0	0
ENSG00000250416	SEC63P2	0	0	0	0	0	0	0	0
ENSG00000206665	RNU6-573P	0	0	0	0	0	0	0	0
ENSG00000047365	ARAP2	11.4539	15.9923	5.72891	10.7578	11.2651	22.6514	7.04886	10.7826
ENSG00000248466	AC098827.1	0.32969	0.18292	0	0	0.40082	0.19387	0	0.40478
ENSG00000247193	AC104078.1	0.04106	0.1367	0	0	0	0.36222	0	0.10084
ENSG00000197057	DTHD1	1.43521	1.62881	0.46015	1.58783	1.33247	2.62398	0.95691	0.96116
ENSG00000251438	AC104078.2	0	0	0	0	0	0	0	0
ENSG00000283207	MIR1255B1	0	0	0	0	0	0	0	0
ENSG00000248215	LINC02505	0	0	0	0	0	0	0	0
ENSG00000251588	AC125336.1	0	0	0	0	0	0	0	0
ENSG00000261761	LINC02616	0	0	0	0	0	0	0	0
ENSG00000247810	AL136537.1	0	0	0	0	0	0	0	0
ENSG00000264319	MIR4801	0	0	0	0	0	0	0	0
ENSG00000174145	NWD2	0	0	0	0	0	0	0	0
ENSG00000154274	C4orf19	0	0	0	0	0	0	0	0
ENSG00000248936	AC027607.1	0.68633	0	0	0	0.16689	0	0.2288	0.33707
ENSG00000181826	RELL1	53.1587	67.1767	21.4653	45.0899	92.8241	55.258	35.33	31.7093
ENSG00000207075	RF00019	0	0	0	0	0	0	0	0
ENSG00000239532	AC108022.1	0	0.25525	0	0	0	0	0	0
ENSG00000169299	PGM2	16.4847	14.1641	2.76264	4.87539	15.326	9.11187	3.59548	8.23677
ENSG00000279386	AC021106.3	0	0	0	0	0	0	0	0
ENSG00000249863	AC021106.1	2.21702	1.03788	0.50905	1.13104	2.10578	0.85556	0.11548	1.27595
ENSG00000065882	TBC1D1	11.5972	10.7257	3.14841	5.03459	9.43263	8.94817	3.46304	6.8554
ENSG00000250254	PTTG2	0.31824	1.23598	0.15588	0.31486	0.64484	0	0.17682	0.78145
ENSG00000251332	AC021106.2	0	0	0	0	0	0	0	0
ENSG00000249649	MRPS33P2	0	0	0	0	0	0	0	0
ENSG00000251642	AC098680.1	0	0	0	0	0	0	0	0
ENSG00000248227	LINC02513	0	0	0	0	0	0	0	0
ENSG00000280015	AC024023.1	0	0	0	0	0	0	0	0
ENSG00000249534	LINC01258	0	0	0	0	0.04328	0	0	0
ENSG00000249667	LINC01259	0.77464	0.16117	0	0.14371	0.94179	0.34164	0.1614	0.11889
ENSG00000251635	LINC02278	0	0	0	0	0	0	0	0
ENSG00000231160	KLF3-AS1	0.07588	0.05052	0.0223	0.2928	0.18451	0.08032	0.15178	0.1118
ENSG00000271958	AC021860.1	0	0	0.28647	0	0	0	0	0
ENSG00000109787	KLF3	55.7814	42.2389	13.2568	26.9675	35.8618	38.0522	31.9178	21.4667
ENSG00000222230	RNA5SP158	0	0	0	0	0	0	0	0
ENSG00000174123	TLR10	2.6247	2.3146	0.38314	1.62521	6.27642	0.88927	0.78228	2.07012
ENSG00000174125	TLR1	75.8218	86.6301	16.3887	16.096	48.2036	23.8093	23.0082	54.4944
ENSG00000174130	TLR6	17.6844	23.9771	4.17648	5.31761	13.1866	4.07113	3.84661	10.4686
ENSG00000197712	FAM114A1	0.42135	0.40077	0.04423	0.335	0.4025	0.21238	0.12542	0.33257
ENSG00000207944	MIR574	0	0	0	0	0	0	0	0
ENSG00000121895	TMEM156	3.46188	3.47643	1.1772	4.1612	4.8002	3.28074	2.47985	3.26687

ENSG00000109790	KLHL5	8.17932	10.5311	0.90041	4.27191	6.23678	5.631	3.68156	4.21643
ENSG00000249207	AC079921.1	0	0	0	0	0	0	0	0
ENSG00000249685	AC079921.2	0.4833	1.87705	0	0.47818	0.58758	0.5684	0.26853	0
ENSG00000157796	WDR19	0.7848	0.48127	0.4006	0.62528	0.9441	0.93271	0.8675	0.60857
ENSG00000035928	RFC1	12.884	13.9822	10.0837	15.4622	17.9056	18.5913	15.6203	15.0285
ENSG00000206675	RNU6-32P	0.68505	1.14027	0	0	3.33146	1.20852	0	0
ENSG00000222592	RNU6-887P	0	0	0	0	0	0	0	0
ENSG00000134962	KLB	0	0	0	0	0	0	0	0
ENSG00000264621	MIR5591	0	0	0	0	0	0	0	0
ENSG00000238797	RF00019	0	0	0	0	0	0	0	0
ENSG00000163682	RPL9	54.3399	41.8925	24.3918	29.5748	82.636	64.7551	54.1357	67.1595
ENSG00000121897	LIAS	0.24361	0.16635	0.10097	0.17613	0.41008	0.16529	0.177	0.21474
ENSG00000224097	AC021148.1	0.0444	0	0	0.06589	0.10795	0	0	0
ENSG00000109814	UGDH	2.21079	1.62879	0.47932	1.21022	2.22516	1.95007	0.36246	1.1347
ENSG00000249348	UGDH-AS1	0.28931	0.13133	0.03865	0.1171	0.22383	0	0.04384	0.12917
ENSG00000163683	SMIM14	8.08147	5.60938	1.47444	2.91347	7.1601	2.77055	1.96332	4.31172
ENSG00000252796	RNU7-11P	0	0	0	0	0	0	0	0
ENSG00000255458	AC108471.2	0.06371	0	0.04681	0.09456	0.07746	0.33719	0	0.15645
ENSG00000078140	UBE2K	35.9304	42.1738	38.3006	93.5619	62.8829	60.6003	71.4213	53.8781
ENSG00000252975	RF00019	0	0	0	0	0	0	0	0
ENSG00000249019	AC108471.1	0	0	0	0	0	0	0	0
ENSG00000243260	RN7SL558P	0.24515	0	0	0.36383	0	0.43248	0	0
ENSG00000180610	ZBTB12BP	0	0	0	0	0	0	0	0
ENSG00000121892	PDS5A	15.0757	12.0869	9.24095	31.5999	17.4013	19.4328	21.0041	18.5392
ENSG00000271278	ELOCP33	0	0	0	0	0	0	0	0
ENSG00000252970	RNA5SP159	0	0	0	0	0	0	0	0
ENSG00000250568	AC098591.2	1.8616	1.54931	0	1.03605	2.54619	1.23154	1.5515	0.57141
ENSG00000231707	PABPC1P1	0	0	0	0	0	0	0	0
ENSG00000249064	KRT18P25	0	0	0	0	0	0	0	0
ENSG00000205794	AC098591.1	0	0	0	0	0	0	0	0
ENSG00000078177	N4BP2	5.43324	3.10139	1.21568	3.32939	5.05614	7.54702	3.55298	3.2485
ENSG00000200455	RNU6-1112P	0	0	0	0	0	0	0	0
ENSG00000201863	RF00432	0.59113	0	0	0	0	0	0	0
ENSG00000248977	AC095057.1	0	0	0	0	0	0	0	0
ENSG00000260296	AC095057.3	0.69689	0.52726	0	0.28207	0.46214	0.55882	0.1056	0.38892
ENSG00000168421	RHOH	3.9379	2.41875	1.46705	2.74928	5.56979	4.67699	3.10628	3.09161
ENSG00000250338	AC095057.2	0.13255	0.22063	0	0	0	0.23384	0.22094	0
ENSG00000249241	LINC02265	0.07659	0	0	0	0	0	0	0
ENSG00000174343	CHRNA9	0.04628	0	0	0	0	0	0	0
ENSG00000239010	RNU7-74P	0	0	0	0	0	0	0	0
ENSG00000163694	RBM47	16.0087	18.9639	2.63486	2.76293	11.5359	3.28425	3.76093	8.45829
ENSG00000250893	AC098869.2	0	0	0	0	0	0	0	0
ENSG00000242197	AC098869.1	0	0	0	0	0	0	0	0
ENSG00000263642	MIR4802	0	0	0	1.35982	1.11396	0	0	0
ENSG00000179299	NSUN7	0.65115	1.21286	0.13669	0.13805	0.60316	0	0.36179	0.3807
ENSG00000248780	ARL4AP2	0	0.40602	0	0.18101	0.44484	0	0.2033	0.44924
ENSG00000163697	APBB2	0.09052	0.06592	0	0.04198	0.07566	0.00998	0.11316	0.00695
ENSG00000250906	AC131953.1	0	0	0	0	0	0	0	0
ENSG00000207195	RF00019	0	0	0	0	0	0	0	0
ENSG00000201736	RNA5SP160	0	0	0	0	0	0	0	0
ENSG00000199790	RNU6-836P	0	0	0	0	0	0	0	0
ENSG00000207198	RNU6-1195P	0	0	0	0	0	0	0	0
ENSG00000251173	UCHL1-AS1	0	0	0	0	0	0	0	0
ENSG00000154277	UCHL1	0.03212	0	0.0472	0	0.11716	0	0	0
ENSG00000207481	RF00019	0	0	0	1.07709	0	0	1.20971	0
ENSG00000064042	LIMCH1	0.04049	0.01123	0.02975	0	0.04102	0.0119	0.0225	0.04143
ENSG00000239627	RPL12P20	0	0	0	0	0	0	0	0
ENSG00000249216	AC105389.2	0	0	0	0	0	0	0	0

ENSG00000249081	OR5M14P	0	0	0	0	0	0	0	0
ENSG00000109132	PHOX2B	0	0	0	0	0	0	0	0
ENSG00000250467	AC105389.3	0	0	0	0	0	0	0	0
ENSG00000249122	AC105389.1	0	0	0	0	0	0	0	0
ENSG00000200338	RNU1-49P	0	0	0	0	0	0	0	0
ENSG00000249745	HMGB1P28	0	0	0	0	0	0	0	0
ENSG00000245870	LINC00682	0	0	0	0	0	0	0	0
ENSG00000249771	AC108210.1	0	0	0	0	0	0	0	0
ENSG00000249887	AC108210.2	0	0	0	0	0	0	0	0
ENSG00000109133	TMEM33	15.6564	13.8888	3.83012	7.24798	10.2311	9.08635	4.46314	8.89049
ENSG00000182308	DCAF4L1	0.04608	0	0	0	0.01867	0	0	0.0943
ENSG00000272862	AC106052.1	0.14749	0	0	0	0.17931	0.52037	0	0
ENSG00000014824	SLC30A9	8.24819	6.55215	6.64919	12.8134	8.83149	9.28897	10.5523	8.03434
ENSG00000249212	ATP1B1P1	0.48066	0	0	0	0.38958	0.84795	0.13353	0.19671
ENSG00000188848	BEND4	0.13381	0.0696	0.02458	0.09929	0.25418	0.51636	0.04182	0.09241
ENSG00000250781	AC024022.1	0	0	0	0	0	0	0	0
ENSG00000178343	SHISA3	0	0	0	0	0	0	0	0
ENSG00000124406	ATP8A1	9.35972	6.92896	3.44331	7.73225	10.6313	8.35983	6.46959	7.52185
ENSG00000240167	RPS7P7	0	0	0	0	0	0	0	0
ENSG00000248283	CCNL2P1	1.05903	0.62956	0	0.22453	0.45984	0.93414	0.37827	0.55726
ENSG00000260519	AC096734.2	0.16928	0.56355	0	0.75371	1.23487	0.89592	0.28217	1.03923
ENSG00000251517	AC096734.1	0	0.10816	0	0	0	0.22928	0	0
ENSG00000252595	RN7SKP82	0	0	0	0	0	0	0	0
ENSG00000215203	GRXCR1	0	0	0	0	0	0	0	0
ENSG00000248939	AC111198.1	0	0	0	0	0	0	0	0
ENSG00000250657	AC097451.1	0	0	0	0.15974	0	0	0	0
ENSG00000277096	AC098590.2	0	0	0	0	0	0	0	0
ENSG00000213851	AC098590.1	0	0	0	0.12128	0	0	0	0
ENSG00000248143	LINC02383	0	0	0	0	0	0	0	0
ENSG00000251501	AC114774.1	0	0	0	0	0	0	0	0
ENSG00000239464	RN7SL691P	0	0	0	0	0	0	0	0
ENSG00000239679	RN7SL193P	0	0	0	0	0	0	0	0
ENSG00000124399	NDUFB4P12	0	0	0.27619	0	0.2285	0	0	0
ENSG00000250863	AC110799.1	0	0	0	0	0	0	0	0
ENSG00000251350	LINC02475	0	0	0	0	0	0	0	0
ENSG00000183783	KCTD8	0	0	0	0	0	0	0	0
ENSG00000251159	AC093852.1	0	0	0	0	0	0	0	0
ENSG00000177752	YIPF7	0	0	0.05941	0	0.04915	0	0	0
ENSG00000151806	GUF1	6.36893	4.10895	1.64449	2.75994	4.10169	4.76137	3.92272	3.37438
ENSG00000163281	GNPDA2	1.35324	0.80201	0.48208	1.04982	1.65769	1.82664	1.14491	1.18318
ENSG00000273369	AC096586.2	0.16621	0.82999	0.4885	0.49336	0.20208	0.58645	0	0.40815
ENSG00000272936	AC096586.1	1.56792	0	1.34402	0.96957	0.95312	2.76603	0.65337	0.96254
ENSG00000249729	AC093755.1	0	0	0	0	0	0	0	0
ENSG00000248987	PRDX4P1	0	0	0	0	0	0	0	0
ENSG00000275959	AC108467.2	0	0	0	0	0	0	0	0
ENSG00000248744	AC108467.1	0	0	0	0	0	0	0	0
ENSG00000248320	THAP12P9	0	0	0	0	0	0	0	0
ENSG00000224560	AC108043.1	0	0	0	0	0	0	0	0
ENSG00000200720	RNU6-931P	0	0	0	0	0	0	0	0
ENSG00000222257	RN7SKP199	0	0	0	0	0	0	0	0
ENSG00000163285	GABRG1	0	0	0.01588	0.01603	0	0	0	0
ENSG00000260878	AC104072.1	0	0	0	0	0	0	0	0
ENSG00000151834	GABRA2	0	0	0	0	0	0	0	0
ENSG00000249330	AC095060.1	0	0	0	0	0	0	0	0
ENSG00000252243	RNU6-412P	0	0	0	0	0	0	0	0
ENSG00000249936	RAC1P2	1.13938	0.42145	0.37207	2.06674	0.46174	0.89335	1.0551	0.62174
ENSG00000170516	COX7B2	0	0	0	0	0	0	0	0
ENSG00000109158	GABRA4	0	0	0	0	0	0	0	0

ENSG00000163288	GABRB1	0	0	0	0	0.02838	0	0	0
ENSG00000260918	AC107398.3	0	0	0	0	0	0	0	0
ENSG00000169019	COMMD8	6.05994	6.14879	3.65959	5.60561	4.037	6.29718	2.83658	7.64414
ENSG00000282904	AC107398.4	0	0	0	0.11672	0	0	0	0
ENSG00000145246	ATP10D	5.18118	3.79888	1.12244	1.47506	2.88664	2.50019	0.7363	2.12422
ENSG00000242431	AC107398.1	0	0	0	0	0	0	0	0
ENSG00000248254	AC107398.2	0	0	0	0	0	0	0	0
ENSG00000145244	CORIN	0.02907	0.14515	0.01068	0.05392	0.00883	0.07692	0	0.06245
ENSG00000277388	MIR8053	0	0	0	0	0	0	0	0
ENSG00000242262	AC092597.1	0	0	0	0	0	0	0	0
ENSG00000282917	AC107068.2	0	0	0	0	0	0	0	0
ENSG00000259959	AC107068.1	0.64299	0.69422	0.07661	0.69635	0.84511	0.49051	0.5214	0.51207
ENSG00000170448	NFXL1	1.99434	5.33311	1.05696	2.13496	2.22593	2.10521	1.38965	3.07082
ENSG00000163293	NIPAL1	0.01225	0.12236	0	0	0.08937	0	0	0.10529
ENSG00000198515	CNGA1	0	0	0	0	0	0	0	0
ENSG00000074966	TXK	8.78253	4.98998	1.89244	6.57978	9.06054	5.69836	7.00285	7.33559
ENSG00000200269	RNU6-838P	1.35741	1.12971	0	2.01455	3.30062	1.19733	0	0.83331
ENSG00000283499	RF00026	0	0	0	1.87562	1.53649	0	0	1.55167
ENSG00000135605	TEC	3.51041	2.27063	0.90875	1.91657	2.94107	1.47602	2.78923	1.69722
ENSG00000202014	RF00019	0	0	0	0	0	0	0	0
ENSG00000109171	SLAIN2	20.9542	18.0095	5.8612	11.6223	22.047	20.6732	14.0646	16.871
ENSG00000145248	SLC10A4	0	0.13632	0.18053	0	0.04979	0.07224	0.20477	0.15083
ENSG00000182223	ZAR1	0	0	0	0	0.06104	0	0	0
ENSG00000075539	FRYL	11.7268	9.67222	7.08519	17.4697	11.4734	11.8612	14.8081	11.5669
ENSG00000251722	RNU5E-3P	0	5.38273	0	1.59979	0	0	3.59354	3.97046
ENSG00000109180	OCIAD1	11.1956	10.6117	6.35224	14.1694	12.0045	8.33916	13.2185	10.4239
ENSG00000248256	OCIAD1-AS1	0	0	0	0.41051	0	0	0	0
ENSG00000145247	OCIAD2	1.83029	1.03189	0.43381	0.87624	1.97399	2.13523	1.23017	1.01487
ENSG00000252913	RNU6-158P	0	0	0	0	0	0	0	0
ENSG00000251334	AC020593.1	0	0	0	0	0	0	0	0
ENSG00000109182	CWH43	0	0	0	0	0	0	0	0
ENSG00000225455	TPI1P4	0	0	0	0	0	0	0	0
ENSG00000249828	AC118282.3	0	0	0	0	0	0	0	0
ENSG00000249079	AC118282.2	0	0	0	0	0	0	0	0
ENSG00000280043	AC118282.4	0	0	0	0	0	0	0	0
ENSG00000250016	SNX18P23	0	0	0	0	0	0	0	0
ENSG00000237961	AC118282.1	0	0	0	0	0	0	0.15971	0
ENSG00000268736	MTCO3P39	0	0	0	0	0	0	0	0
ENSG00000248946	MTND3P22	0	0	0	0	0	0	0	0
ENSG00000248583	AC119751.3	0	0	0	0	0	0	0	0
ENSG00000251056	ANKRD20A17P	0	0	0	0	0	0	0	0
ENSG00000250769	AC119751.5	0	0	0	0	0	0	0	0
ENSG00000248532	AC119751.2	0	0	0	0	0	0	0	0
ENSG00000269848	AC119751.8	0	0	0	0	0	0	0	0
ENSG00000268967	AC119751.7	0	0	0	0	0	0	0	0
ENSG00000268391	MTCO3P42	0	0	0	0	0	0	0	0
ENSG00000232471	AC119751.1	0	0	0	0	0	0	0	0
ENSG00000251630	AC119751.6	0	0	0	0	0	0	0	0
ENSG00000251539	SNX18P24	0	0	0	0	0	0	0	0
ENSG00000250753	AC119751.4	0	0	0	0	0	0	0	0
ENSG00000249337	SNX18P25	0	0	0	0	0	0	0	0
ENSG00000109184	DCUN1D4	8.68068	12.4489	16.8104	24.6676	13.9548	16.0431	19.8213	15.1077
ENSG00000250473	DUTP7	0	0	0	0	0	0	0	0
ENSG00000272576	AC027271.1	0.24033	0	0	0	0.58437	0	0	0.14754
ENSG00000243929	AC104784.1	0	0	0	0	0	0	0	0
ENSG00000188993	LRRC66	0.0661	0.03667	0	0.0327	0	0.03887	0	0
ENSG00000163069	SGCB	0.62953	0.40302	0.16604	0.11978	0.56912	0.34172	0.05381	0.09909
ENSG00000251040	LINC02480	0	0	0	0	0	0	0	0

ENSG00000163071	SPATA18	0.01484	0	0	0	0	0	0	0
ENSG00000251286	AC097522.1	0	0	0	0	0	0	0	0
ENSG00000199924	RNU6-1252P	0	0	0	0	0	0	0	0
ENSG00000109189	USP46	1.12618	0.67045	0.26575	0.48799	0.46972	0.8411	0.31514	0.57529
ENSG00000248866	USP46-AS1	0.69943	0.46568	0	0.31141	0.17007	0.61695	0.34975	0.17175
ENSG00000260120	AC104066.2	0	0	0	0	0	0	0	0
ENSG00000226950	DANCR	1.92474	0.80093	0.51854	1.14261	2.57405	1.3582	1.818	0.86649
ENSG00000264585	MIR4449	0	0	0	0	0	0	0	0
ENSG00000212588	SNORA26	0.60082	0	0.8829	3.56674	2.19139	3.1798	5.00739	2.95072
ENSG00000248375	AC104066.1	0	0	0	0	0	0.17219	0.16269	0
ENSG00000226887	ERVMER34-1	0	0	0	0	0	0	0	0
ENSG00000212490	RF00568	0	0	0	0	0	0	0	0
ENSG00000199901	RF00019	0	0	0	0	0	0	0	0
ENSG00000250302	LINC01618	0.10083	0	0	0	0	0	0	0
ENSG00000128045	RASL11B	0	0	0	0	0	0	0	0
ENSG00000184178	SCFD2	5.73054	2.94966	1.31895	3.21063	6.57533	3.69462	4.94859	3.16473
ENSG00000248115	AC023154.1	0	0	0	0	0	0	0	0
ENSG00000207385	RNU6-310P	0	0	0	0	0	0	1.14187	0
ENSG00000145216	FIP1L1	14.3843	15.6083	6.80379	17.3138	22.1527	22.072	18.9687	11.8644
ENSG00000282278	AC058822.1	0	0	0	0	0	0	0	0
ENSG00000072201	LNX1	0.02561	0	0	0	0	0	0	0
ENSG00000250930	LNX1-AS1	0	0	0	0	0	0	0	0
ENSG00000173966	AC095040.1	0	0	0	0	0	0	0	0
ENSG00000248494	LNX1-AS2	0	0	0	0.12264	0	0	0	0
ENSG00000249341	AC124017.1	0.02368	0	0	0	0	0	0	0
ENSG00000249706	AC105384.1	0	0	0	0	0	0	0	0
ENSG00000250815	AC105384.2	0	0	0	0	0	0	0	0.14062
ENSG00000229585	RPL21P44	0.62119	1.03397	0.68462	0.46096	0.56642	0.27397	1.03543	0.19067
ENSG00000272650	AC110792.3	1.22987	1.22827	1.80728	1.09516	0	0.43393	2.05001	1.51002
ENSG00000109220	CHIC2	19.5208	19.5325	6.04767	10.3998	14.4696	13.9319	13.349	12.6324
ENSG00000269506	AC110792.2	0	0	0	0	0	0	0	0
ENSG00000251105	AC110792.1	0	0	0	0	0	0	0	0
ENSG00000250147	MORF4L2P1	0	0	0	0	0	0	0	0
ENSG00000180613	GSX2	0	0	0	0	0.04627	0	0	0
ENSG00000242670	RPL22P13	0	0	0	0	0	0	0	0
ENSG00000134853	PDGFRA	0.23349	0.11283	0.01107	0.02236	0.04579	0.03986	0.03766	0.04624
ENSG00000248184	LINC02283	0	0	0	0	0	0	0	0
ENSG00000248518	AC098587.1	0	0	0	0	0	0	0	0
ENSG00000251264	AC098868.1	0	0	0	0	0	0	0	0
ENSG00000250456	LINC02260	0	0	0	0	0	0	0	0
ENSG00000157404	KIT	0.55341	0.24125	0.03873	0.33244	0.91311	0.41841	0.32945	0.19413
ENSG00000249727	AC097494.1	0	0	0	0	0	0	0	0
ENSG00000251256	LINC02358	0	0	0	0	0	0	0	0
ENSG00000248317	AC111194.1	0	0	0	0	0	0	0	0
ENSG00000244422	RPL38P3	0	0	0	0	0	0	0	0
ENSG00000252815	RNU6-410P	0	0	0	0	0	0	0	0
ENSG00000250646	AC111194.2	0	0	0	0	0	0	0	0
ENSG00000244034	RN7SL424P	0	0	0	0	0	0	0	0
ENSG00000128052	KDR	0	0	0	0.03476	0.04271	0	0	0
ENSG00000239545	RN7SL822P	0	0	0	0	0	0	0	0
ENSG00000250812	AC021220.1	0	0	0	0	0	0	0	0
ENSG00000206839	RNU6-746P	0	0	0	0	0	0	0	0
ENSG00000250464	LRRC34P2	0	0	0	0	0	0	0	0
ENSG00000128039	SRD5A3	0.76285	0.7453	0.17059	0.39379	0.54438	1.11173	0.16586	0.32578
ENSG00000251111	FCF1P8	0	0	0	0	0	0	0	0
ENSG00000249700	SRD5A3-AS1	0	0	0.04185	0.04226	0.03462	0.02512	0.02373	0.03496
ENSG00000273257	AC069200.1	0.22694	0	0	0	0	0	0	0
ENSG00000134851	TMEM165	7.54333	8.95364	5.93229	10.6083	8.12106	8.49842	8.87954	7.31985

ENSG00000239040	RF00019	1.42331	3.55365	2.09153	0	2.59563	1.25545	3.55865	0.87376
ENSG00000134852	CLOCK	2.24974	1.6597	0.7601	1.38734	1.66687	1.82501	1.09071	1.13242
ENSG00000238585	RF00019	0	0	0	0	0	0	0	0
ENSG00000223305	RN7SKP30	0.43892	0.73059	0	0	0	0.38716	0	0
ENSG00000272969	AC024243.1	0	0	0	0	0	0	0	0.11568
ENSG00000163440	PDCL2	0	0.42512	0.50041	0.88444	0.62102	0	0.70953	0.31358
ENSG00000109255	NMU	0	0	0.33143	0.78103	0.0914	0	0.50125	0.27691
ENSG00000251923	RNU6-276P	0	0	0	0	0	0	0	0
ENSG00000250821	EXOC1L	0	0	0	0	0	0	0	0
ENSG00000090989	EXOC1	7.11481	7.43871	3.062	5.61527	7.76667	6.48131	4.70715	6.37907
ENSG00000202358	RNU6-652P	2.05515	2.28053	0	2.03338	0.83287	1.20852	0	0.84109
ENSG00000174799	CEP135	7.61	8.30336	8.20321	18.6472	11.9441	10.297	16.7432	13.7808
ENSG00000109265	KIAA1211	0.53092	0.18802	0.26559	0.53647	0.48067	0.47827	0.73432	0.24965
ENSG00000200741	RNA5SP161	0	0	0	0	0	0	0	0
ENSG00000252489	RNU6-197P	0	0	0	0	0	0	0	0
ENSG00000250324	MRPL22P1	0	0	0	0	0	0	0	0
ENSG00000157426	AASDH	3.10274	2.70023	2.49959	4.74506	4.09776	2.88965	4.75175	2.74594
ENSG00000242083	RPL7AP31	0	0	0	0	0	0	0	0
ENSG00000269921	AC068620.1	1.10851	0.69192	0.40724	0.41129	0.67385	0.48889	0.69289	0.68051
ENSG00000128059	PPAT	1.13205	0.47108	0.41588	0.78004	1.60571	2.21107	0.89855	1.17481
ENSG00000270147	AC068620.2	0	0	0	0	0.15914	0	0.43636	0.32142
ENSG00000283156	AC068620.3	0	0	0.02349	0	0	0	0	0
ENSG00000128050	PAICS	2.94489	2.12626	1.13202	3.83507	3.72258	5.38441	1.67415	1.49656
ENSG00000174780	SRP72	18.8206	12.7282	3.96732	6.80924	17.664	17.2893	9.83269	8.69889
ENSG00000196503	ARL9	0.0416	0	0	0	0	0	0.20803	0
ENSG00000249693	THEGL	1.63526	2.72188	3.18396	5.88523	4.42353	3.8945	8.79044	9.13523
ENSG00000248590	GLDCP1	0	0	0	0	0	0	0	0
ENSG00000171476	HOPX	1.58835	1.08585	0.65298	1.34702	0.90806	1.11749	0.97706	1.06794
ENSG00000280285	AC108215.1	0	0	0	0	0	0	0	0
ENSG00000241250	RPL17P20	0	0	0	0	0	0	0	0
ENSG00000227040	AC022483.1	0	0	0	0.19186	0	0	0	0
ENSG00000240545	RN7SL492P	0	0	0	0	0	0	0	0
ENSG00000265894	RN7SL357P	0	0.40942	0.36146	0.73011	0	0	0	0
ENSG00000128040	SPINK2	0	0	0	0.14091	0	0	0.15826	0.11658
ENSG00000278226	RF00017	0	0	0	0	0	0	0	0
ENSG00000251799	RF00019	0	0	0	0	0	0	0	0
ENSG00000084093	REST	16.3441	10.4387	2.92796	6.29575	14.9923	12.0192	5.00476	7.2151
ENSG00000269949	AC069307.1	0.49195	0.27295	0	0	0	1.44644	0	0.20134
ENSG00000084092	NOA1	5.02263	3.57272	1.26166	2.67584	4.93208	5.8692	2.28976	2.29275
ENSG00000047315	POLR2B	16.6451	13.9976	3.61419	8.11146	15.1473	15.7777	6.45997	11.2401
ENSG00000251703	RNU6-998P	2.17724	1.208	0	0	1.76469	1.28031	1.20971	0.89106
ENSG00000163453	IGFBP7	4.0638	2.02294	0.44648	2.25463	1.9855	0.40201	0.88628	1.25903
ENSG00000250384	UBE2CP3	0	0	0	0	0	0	0	0
ENSG00000245067	IGFBP7-AS1	0	0	0	0	0	0	0	0
ENSG00000250192	AC105390.1	0	0	0	0	0	0	0	0
ENSG00000243920	RPS26P24	0	0	0	0	0	0	0	0
ENSG00000248505	LINC02380	0	0	0	0	0	0	0	0
ENSG00000251459	AC013724.1	0	0	0	0	0	0	0	0
ENSG00000251049	AC107396.1	0	0	0	0	0	0	0	0
ENSG00000250333	AC093725.2	0	0	0	0	0	0	0	0
ENSG00000249831	AC093725.1	0	0	0	0	0	0	0	0
ENSG00000248660	SRIP1	0	0	0	0	0	0	0	0
ENSG00000249105	AC096725.1	0	0	0	0	0	0	0	0
ENSG00000250340	LINC02494	0	0	0	0	0	0	0	0
ENSG00000250078	AC019133.1	0	0	0	0	0	0	0	0
ENSG00000251339	AC017091.1	0	0	0	0	0	0	0	0
ENSG00000251266	LINC02429	0	0	0	0	0	0	0	0
ENSG00000249382	AC097501.1	0	0	0	0	0	0	0	0

ENSG00000250375	AC097501.2	0	0	0	0	0	0	0	0
ENSG00000249111	AC108517.1	0	0	0	0	0	0	0	0
ENSG00000199780	RF00019	0	0	0	0	0	0	0	0
ENSG00000249392	AC096588.1	0	0	0	0	0	0	0	0
ENSG00000249892	AC093857.1	0	0	0	0	0	0	0	0
ENSG00000201676	RF00019	0	0	0	0	0	0	0	0
ENSG00000201775	RNU6-1325P	0	0	0	0	0	0	0	0
ENSG00000283043	AC097655.1	0	0	0	0	0	0	0	0
ENSG00000249066	LINC02496	0	0	0	0	0	0	0	0
ENSG00000248237	AC105420.1	0	0	0	0	0	0	0	0
ENSG00000265829	MIR548AG1	0	0	0	0	0	0	0	0
ENSG00000240152	LINC02271	0	0	0	0	0	0	0	0
ENSG00000150471	ADGRL3	0.00524	0	0.0077	0	0	0	0.01747	0.0193
ENSG00000241261	RPL17P19	0	0	0	0	0	0	0	0
ENSG00000205682	AC020741.1	0	0	0	0	0	0	0	0
ENSG00000226439	AC108161.1	0	0	0	0	0	0	0	0
ENSG00000202169	RF00019	0	0	0	0	0	0	0	0
ENSG00000248692	ADGRL3-AS1	0	0	0	0	0	0	0	0
ENSG00000225405	RPS15AP17	0	0	0	0	0	0	0	0.23076
ENSG00000241344	RPL21P47	0	0	0	0	0	0	0	0
ENSG00000250249	AC105313.1	0	0	0	0	0	0	0	0
ENSG00000248847	AC074087.1	0	0	0	0	0	0	0	0
ENSG00000248336	HMGN1P11	0	0	0	0	0	0	0	0
ENSG00000180673	EXOC5P1	0.14891	0	0.21882	0.3315	0	0.13135	0.37231	0.09141
ENSG00000251055	AC097491.1	0	0	0	0	0	0	0	0
ENSG00000231442	LARP1BP1	0	0	0	0	0	0	0	0
ENSG00000250775	AC093730.1	0	0	0	0	0	0	0	0
ENSG00000205678	TECRL	0	0	0	0	0	0	0	0
ENSG00000252104	RNU6-191P	0	0	0	0	0	0	0	0
ENSG00000250768	DPP3P1	0	0	0	0	0	0	0	0
ENSG00000251430	MTCO3P27	0	0	0	0	0	0	0	0
ENSG00000251385	MTCYBP16	0	0	0	0	0	0	0	0
ENSG00000249893	MTND6P16	0	0	0	0	0	0	0	0
ENSG00000250169	MTND5P13	0	0	0	0	0	0	0	0
ENSG00000248259	MTND4LP31	0	0	0	0	0	0	0	0
ENSG00000249045	MTND3P24	0	0	0	0	0	0	0	0
ENSG00000248328	MTCO3P28	0	0	0	0	0	0	0	0
ENSG00000244669	AC104689.1	0	0	0	0	0	0	0	0
ENSG00000250125	LINC02232	0	0	0	0	0	0	0	0
ENSG00000248447	AC107058.1	0	0	0	0	0	0	0	0
ENSG00000248998	EFL1P2	0	0	0	0	0	0	0	0
ENSG00000249351	AC096754.1	0	0	0	0	0	0	0	0
ENSG00000145242	EPHA5	0	0	0	0	0	0	0	0
ENSG00000243199	AC115223.1	0.33701	0	0	0.25008	0.10243	0.29727	0.14044	0.31033
ENSG00000250846	EPHA5-AS1	0	0	0	0	0	0	0	0
ENSG00000248479	AC104137.1	0	0	0	0	0	0	0	0
ENSG00000222765	RNU2-40P	0	0	0	0	0	0	0	0
ENSG00000251527	AC097110.1	0	0	0	0	0	0	0	0
ENSG00000249413	AC116049.2	0	0	0	0	0	0	0	0
ENSG00000272304	AC096721.1	0	0	0	0	0	0	0	0
ENSG00000236562	AC116049.1	0	0	0	0	0	0	0	0
ENSG00000221563	MIR1269A	0	0	0	0	0	0	0	0
ENSG00000244099	RPS23P3	0	0	0	0	0	0	0	0
ENSG00000252890	RNU6-699P	0	0	0	0	0	0	0	0
ENSG00000250075	AC104806.2	0.03206	0.05337	0	0.28553	0	0.22627	0.26724	0.27558
ENSG00000206629	RNU1-63P	0.89391	0	0.65679	0	0	0	0	0
ENSG00000274759	RNA5SP527	0	0	0	0	0	0	0	0
ENSG00000250030	AC104806.1	0.95195	0.39613	0	0.3532	0.28934	0	0.39669	0

ENSG00000145241	CENPC	6.48495	4.05137	2.85136	6.30257	6.8806	5.44992	7.60347	6.82318
ENSG00000035720	STAP1	3.14461	1.99047	0.78101	1.64329	6.62317	1.64081	1.40267	3.20835
ENSG00000279464	AC096720.2	0.05964	0.14891	0.04382	0.04426	0.10877	0.15783	0.09941	0
ENSG00000033178	UBA6	5.79632	4.96273	2.11056	4.14893	5.02022	6.40225	2.5757	4.10151
ENSG00000270257	AC096720.1	0	0.66671	0	0	0	0	0	0
ENSG00000248049	UBA6-AS1	1.28851	0.94105	0.25117	0.42929	1.23085	1.48448	0.35065	0.565
ENSG00000250656	ST3GAL1P1	0	0	0	0	0	0	0	0
ENSG00000270228	AC079880.1	0	0	0	0	0	0	0	0
ENSG00000270292	AC079880.2	0	0	0	0	0	0	0	0
ENSG00000109163	GNRHR	0	0	0	0	0	0	0	0
ENSG00000202374	RF00091	0.48543	0	0	0.72044	0	0.85637	0	0
ENSG00000232398	TMPRSS11CP	0	0	0	0	0	0	0	0
ENSG00000153802	TMPRSS11D	0	0	0	0	0	0	0	0
ENSG00000187054	TMPRSS11A	0	0	0	0	0	0	0	0
ENSG00000229009	TMPRSS11GP	0	0	0	0	0	0	0	0
ENSG00000207428	RNU6-95P	0	0	0	0	0	0	0	0
ENSG00000227725	GCOM2	0	0	0	0	0	0	0	0
ENSG00000283131	MTND2P41	0	0	0	0	0	0	0	0
ENSG00000198092	TMPRSS11F	0.03511	0	0.15476	0.1042	0	0	0.05852	0
ENSG00000215127	SYT14P1	0	0	0	0	0	0	0	0
ENSG00000250426	FTLP10	0	0	0	0	0	0	0	0
ENSG00000250026	TMPRSS11BNL	0	0	0	0	0	0	0	0
ENSG00000185873	TMPRSS11B	0	0	0	0	0	0	0	0
ENSG00000250642	AC098799.3	0	0	0	0	0	0	0	0
ENSG00000248639	AC098799.1	0	0	0	0	0	0	0	0
ENSG00000249531	AC098799.2	0	0	0	0	0	0	0	0
ENSG00000248122	APOOP4	0	0	0	0	0	0	0	0
ENSG00000083896	YTHDC1	9.42314	7.02694	1.66963	3.04761	8.41488	5.05699	3.7356	4.40259
ENSG00000162840	MT2P1	0	0	0	0	0	0	0	0
ENSG00000087128	TMPRSS11E	0	0	0	0	0	0	0	0
ENSG00000250566	UGT2B29P	0	0	0	0	0	0	0	0
ENSG00000197888	UGT2B17	0.07058	0.11749	0	0.26188	0.08581	0	0	0
ENSG00000249472	AC147055.2	0	0	0	0	0	0	0	0
ENSG00000251101	AC147055.4	0	0	0	0	0	0	0	0
ENSG00000251074	AC147055.3	0	0	0	0	0	0	0	0
ENSG00000196620	UGT2B15	0	0.05874	0	0	0	0	0	0
ENSG00000248635	AC147055.1	0	0	0	0	0	0	0	0
ENSG00000250376	AC093720.1	0	0	0	0	0	0	0	0
ENSG00000251236	AC021146.8	0	0	0	0	0	0	0	0
ENSG00000109181	UGT2B10	0	0	0	0	0	0	0	0
ENSG00000251424	AC021146.9	0	0	0	0	0	0	0	0
ENSG00000249235	AC021146.4	0	0	0	0	0	0	0	0
ENSG00000251529	AC021146.11	0	0	0	0	0	0	0	0
ENSG00000248547	AC021146.2	0	0	0	0	0	0	0	0
ENSG00000248613	AC021146.3	0	0	0	0	0	0	0	0
ENSG00000272626	AC021146.12	0	0	0	0	0	0	0	0
ENSG00000249985	AC021146.6	0	0	0	0	0	0	0	0
ENSG00000198277	AC021146.1	0	0	0	0	0	0	0	0
ENSG00000135220	UGT2A3	0	0	0	0	0	0	0	0
ENSG00000251498	AC021146.10	0	0	0	0	0	0	0	0
ENSG00000249735	AC021146.5	0	0	0	0	0	0	0	0
ENSG00000250277	AC021146.7	0	0	0	0	0	0	0	0
ENSG00000251685	UGT2B27P	0	0	0	0	0	0	0	0
ENSG00000249890	AC107401.1	0	0	0	0	0	0	0	0
ENSG00000250919	UGT2B26P	0	0	0	0	0	0	0	0
ENSG00000171234	UGT2B7	0	0	0	0	0	0	0	0
ENSG00000248763	AC111000.2	0	0	0	0	0	0	0	0
ENSG00000251284	AC111000.5	0	0	0	0	0	0	0	0

ENSG00000250100	AC111000.3	0	0	0	0	0	0	0	0
ENSG00000251427	AC111000.6	0	0	0	0	0	0	0	0
ENSG00000196472	AC111000.1	0	0	0	0	0.12499	0	0	0
ENSG00000250696	AC111000.4	0.70271	0.16133	0.17804	0.25174	0.55974	0.25649	0.72702	0.59502
ENSG00000213759	UGT2B11	0.03132	0	0	0.04649	0	0	0	0
ENSG00000268803	AC114797.1	0	0	0	0	0	0	0	0
ENSG00000249763	AC114786.1	0.77704	0	0.07612	0.15376	0.3149	0	0.17269	0.25441
ENSG00000135226	UGT2B28	0	0	0	0	0	0	0	0
ENSG00000251691	AC114786.3	0.20794	0	0	0	0	0	0	0.12766
ENSG00000250612	AC114786.2	0	0	0	0	0	0	0	0
ENSG00000268209	AC114786.4	0	0	0	0	0	0	0	0
ENSG00000215110	UGT2B25P	0	0	0	0	0	0	0	0
ENSG00000248824	AC108078.1	0	0	0	0	0	0	0	0
ENSG00000249956	UGT2B24P	0	0	0	0	0	0	0	0
ENSG00000250828	AC108078.3	0	0	0	0	0	0	0	0
ENSG00000249686	AC108078.2	0	0	0	0	0	0	0	0
ENSG00000156096	UGT2B4	0	0	0	0	0	0	0	0
ENSG00000248886	UGT2A3P7	0	0	0	0	0	0	0	0
ENSG00000251177	AC093829.1	0	0	0	0	0	0	0	0
ENSG00000173610	UGT2A1	0	0.03113	0	0.02776	0	0	0	0
ENSG00000271271	UGT2A2	0	0	0	0	0	0	0	0
ENSG00000173597	SULT1B1	14.9371	16.5365	3.71932	3.99295	16.7669	3.93772	3.08939	12.8585
ENSG00000236959	SULT1D1P	0	0	0	0	0	0	0	0
ENSG00000109193	SULT1E1	0	0	0	0	0	0	0	0
ENSG00000251489	AC108941.1	0	0	0	0	0	0	0	0
ENSG00000126545	CSN1S1	0	0	0	0.11044	0	0	0	0
ENSG00000135222	CSN2	0	0	0	0	0	0	0	0
ENSG00000126549	STATH	0	0	0	0	0	0	0	0
ENSG00000205649	HTN3	0	0	0	0	0	0	0	0
ENSG00000126550	HTN1	0	0	0	0	0	0	0	0
ENSG00000234124	CSN1S2AP	0	0	0	0	0	0	0	0
ENSG00000187533	PRR27	0	0	0	0	0	0	0	0
ENSG00000109205	ODAM	0	0	0	0	0	0	0	0
ENSG00000181617	FDCSP	0	0	0	0	0	0	0	0
ENSG00000171209	CSN3	0	0	0	0	0	0	0	0
ENSG00000145309	CABS1	0	0	0	0	0	0	0	0
ENSG00000109208	SMR3A	0	0	0	0	0	0	0	0
ENSG00000171201	SMR3B	0	0	0	0	0	0	0	0
ENSG00000171199	OPRPN	0	0	0	0	0	0	0	0
ENSG00000171195	MUC7	0	0	0	0	0	0	0	0
ENSG00000187689	AMTN	0	0	0	0	0	0	0	0
ENSG00000178522	AMBN	0	0	0	0	0	0	0	0
ENSG00000132464	ENAM	0.01124	0	0	0.03336	0	0	0	0
ENSG00000132465	JCHAIN	76.6163	41.541	9.48909	21.3686	69.5959	1.53943	12.3635	21.6422
ENSG00000132467	UTP3	10.0421	8.71069	2.39018	3.20112	8.98475	7.04883	3.88997	4.64529
ENSG00000207448	RNU6-520P	0	0	0	0	0	0	0	0
ENSG00000207058	RNU6-784P	0	0	0	0	0	0	0	0
ENSG00000272986	AC009570.1	0.19678	0.32754	0	0.29204	0.11962	0	0	0.1208
ENSG00000018189	RUFY3	2.46151	1.7689	0.48051	0.64705	1.63436	1.55431	0.69644	0.95908
ENSG00000132463	GRSF1	5.47046	4.27853	1.48046	3.21396	3.98364	5.29871	3.15456	2.98256
ENSG00000283313	RF00026	0	0	0	0	0	0	0	0
ENSG00000207007	RNU6-891P	0	0	0	0	0	0	0	0
ENSG00000173542	MOB1B	12.7511	12.754	12.2021	20.1107	13.7103	13.488	27.071	15.8704
ENSG00000156136	DCK	25.236	29.7554	19.8939	53.6006	29.5215	23.3619	21.2784	21.5859
ENSG00000080493	SLC4A4	0.55925	0.51715	0.18262	0.78388	0.44384	0.49329	0.45314	0.5722
ENSG00000249170	AC096713.1	0	0	0	0	0	0	0	0
ENSG00000270098	LDHAL6EP	0	0	0	0	0	0	0	0
ENSG00000145321	GC	0	0	0	0	0.14971	0	0	0

ENSG00000222236	RNA5SP163	0	0	0	0	0	0	0	0
ENSG00000248567	AC068721.1	0	0	0	0	0	0	0	0
ENSG00000056291	NPFFR2	0	0	0	0	0	0	0	0
ENSG00000156140	ADAMTS3	0.11242	0.08506	0.21026	0.28819	0.22366	0.37863	0.7155	0.22587
ENSG00000250877	AC095056.1	0	0	0	0	0	0	0	0
ENSG00000270665	HNRNPA1P67	0	0	0	0	0	0	0	0
ENSG00000252955	RNU4ATAC9P	0	0	0	0	0	0	0	0
ENSG00000210839	RNU6ATAC5P	0	0	0	3.45351	0	0	0.96969	0
ENSG00000163626	COX18	1.98592	1.22428	0.35513	0.59258	1.4691	1.4644	0.68306	0.89017
ENSG00000132466	ANKRD17	16.7333	15.9679	4.13252	6.02558	20.6696	25.0097	9.5577	9.50642
ENSG00000248641	HMGA1P2	0	0	0	0	0	0	0	0
ENSG00000250220	AC053527.1	0.96307	0.89057	0	0.47643	0.78058	0.56633	1.07019	0.26277
ENSG00000221639	RF00334	0.5864	0	0.86171	0	0.71293	0	0	0
ENSG00000249976	AC108157.1	0	0	0	0	0	0	0	0
ENSG00000163631	ALB	0	0	0	0	0.02639	0	0	0
ENSG00000081051	AFP	0	0	0	0	0	0	0	0
ENSG00000079557	AFM	0	0	0	0	0	0	0	0
ENSG00000250436	LINC02499	0	0	0	0	0	0	0	0
ENSG00000249970	AC074250.1	0	0	0	0	0	0	0	0
ENSG00000169435	RASSF6	0.02211	0.03679	0	0.0164	0.22844	0.039	0.11054	0.04071
ENSG00000228277	AC112518.1	0.12748	0.42438	0	0	0	0	0.21249	0.31303
ENSG00000169429	CXCL8	1008.7	416.621	155.224	221.255	208.997	23.2579	93.4352	199.901
ENSG00000249051	AC112518.2	0	0	0	0	0	0	0	0
ENSG00000124875	CXCL6	0.53724	0.83835	0.09868	0.299	0.48988	0.11847	0.55969	0.98943
ENSG00000250550	PPBPP1	0	0	0	0	0	0	0	0.24128
ENSG00000109272	PF4V1	157.273	99.8039	18.365	24.5914	10.7555	37.9017	29.2577	21.3786
ENSG00000163739	CXCL1	31.9558	35.9624	5.56854	7.72244	22.623	4.19066	4.61947	23.6798
ENSG00000249920	HNRNPA1P55	0	0	0	0	0	0	0	0
ENSG00000250339	CXCL1P1	0	0	0	0	0	0	0	0
ENSG00000163737	PF4	376.696	184.427	131.967	206.225	242.12	353.466	226.357	312.606
ENSG00000163736	PPBP	1028.67	700.824	418.503	854.31	865.117	501.151	1491.11	1487.28
ENSG00000163735	CXCL5	18.4839	9.37417	4.83822	14.3162	10.6392	11.0053	18.5822	7.05651
ENSG00000244194	RN7SL218P	0	0	0	0	0	0.43248	0	0
ENSG00000163734	CXCL3	0.23123	0.10997	0.19417	0.04902	0.12048	0.05827	0.11012	0.04056
ENSG00000248848	PPBPP2	0	0	0	0	0	0	0	0
ENSG00000251017	AC093677.1	0.10811	0	0.15887	0	0.26288	0	0.18021	0
ENSG00000081041	CXCL2	0.48867	0.48803	0.14362	0	0.23764	0.17242	0.08145	0.35999
ENSG00000163738	MTHFD2L	1.31387	0.96921	0.59238	1.4292	1.57921	1.75155	1.61762	1.26485
ENSG00000269559	AC093677.2	0	0.06549	0	0	0.04784	0	0	0
ENSG00000182585	EPGN	0	0.06726	0	0.11994	0.04913	0	0.33677	0
ENSG00000124882	EREG	0.57697	0.72561	0.24493	0.74211	1.05998	0.31666	0.17097	0.34632
ENSG00000250532	AC021180.1	0	0	0	0	0	0	0	0
ENSG00000109321	AREG	2.40183	0.96245	0.3268	4.55474	1.18966	1.49085	2.96554	0.65532
ENSG00000249942	AC239584.1	0	0	0	0	0	0	0	0
ENSG00000174808	BTC	0.02603	0	0	0	0	0.04592	0	0
ENSG00000250356	HSPE1P23	0	0	0	0	0	0	0	0
ENSG00000169116	PARM1	0.32034	0.24237	0.04279	0.10805	0.79663	0.07706	0.04854	0.1609
ENSG00000249717	AC110760.2	0	0	0	0	0	0	0	0
ENSG00000248165	AC110760.1	0	0	0	0	0	0.21917	0	0
ENSG00000260265	LINC02562	0	0	0	0	0	0	0	0
ENSG00000250315	AC110760.3	0	0	0	0	0	0	0	0
ENSG00000250560	AC093870.1	0	0	0	0	0	0	0	0
ENSG00000251185	AC025244.1	0	0	0	0	0	0	0	0
ENSG00000251454	AC025244.2	0	0	0	0	0.21118	0	0	0
ENSG00000251383	LINC02483	0	0	0	0	0	0	0	0
ENSG00000248646	AC096759.1	0	0	0	0	0	0	0.25831	0
ENSG00000250735	AC096759.2	0	0	0	0	0	0	0	0
ENSG00000163743	RCHY1	3.93019	2.62044	1.31258	3.2644	3.28503	2.69851	2.54969	2.35788

ENSG00000174796	THAP6	1.60444	2.15702	0.66499	1.03794	1.76308	1.34265	0.75431	1.71736
ENSG00000174792	ODAPH	0	0	0	0.10593	0	0	0	0
ENSG00000138769	CDKL2	0.01939	0.03227	0.02849	0.02877	0.07071	0.0684	0	0.09521
ENSG00000138757	G3BP2	29.6957	19.4656	5.50341	10.3591	20.8555	22.9712	13.0634	13.5816
ENSG00000201644	RF00019	0	0	0	1.15729	0	0	0	1.91483
ENSG00000138768	USO1	17.3186	13.9879	4.57808	10.9307	16.9376	17.4746	6.55108	11.7891
ENSG00000222644	RNU2-16P	0	0	0	0	0	0	0	0
ENSG00000229717	AC110615.1	0	0	0	0	0	0	0	0
ENSG00000156194	PPEF2	0.01472	0	0	0	0	0	0	0
ENSG00000138744	NAAA	8.8276	5.36301	1.90837	1.68339	4.15705	2.43601	3.04149	4.01646
ENSG00000198301	SDAD1	10.2885	8.21312	4.13454	6.48165	11.5895	10.742	4.33983	7.45034
ENSG00000245928	AC112719.2	0	0	0	0	0	0	0	0
ENSG00000138755	CXCL9	0.32102	0.3117	0	0.11911	0.32524	1.27424	0.04459	0.13138
ENSG00000156219	ART3	0	0	0	0	0	0.03945	0.03727	0
ENSG00000169245	CXCL10	1.05961	1.45248	1.00753	0.83254	0.90935	4.94816	0.41558	5.20391
ENSG00000169248	CXCL11	0.03842	0.19184	0.11291	0.05702	0.04671	0.54219	0	0.1415
ENSG00000225846	AC112719.1	0	0	0	0	0	0	0	0
ENSG00000138750	NUP54	14.3461	22.167	18.4296	33.2356	27.5116	26.1998	39.8545	23.596
ENSG00000138760	SCARB2	3.0395	3.14783	0.82424	1.54677	1.46063	1.37729	0.93494	1.46575
ENSG00000189157	FAM47E	0	0	0	0	0	0.0136	0	0
ENSG00000272414	FAM47E-STBD1	0	0	0	0	0	0	0	0
ENSG00000118804	STBD1	0.04287	0.1427	0.0315	0	0.02606	0.11343	0.03573	0.05263
ENSG00000163749	CCDC158	0.05003	0	0	0	0.04562	0	0	0.03072
ENSG00000249858	SNX5P1	0.14083	0	0	0.209	0	0	0	0
ENSG00000212368	RNU6-1000P	0	0	0	0	0	0	0	0
ENSG00000240426	AC096743.1	0	0	0	0	0	0	0	0
ENSG00000199857	RF00278	0	0	0	0	0	0	0	0
ENSG00000138771	SHROOM3	0	0	0	0.01653	0	0	0	0
ENSG00000249278	AC112249.1	0	0	0	0	0	0	0	0
ENSG00000207307	RNU6-145P	0	0	0	0	0	0	0	0
ENSG00000263445	MIR4450	0	0	0	0	0	0	0	0
ENSG00000283682	MIR548AH	0	0	0	0	0	0	0	0
ENSG00000231335		0	0	0	0	0	0	0	0
ENSG00000233860	AC107072.2	0	0	0	0	0	0	0	0
ENSG00000224218	AC107072.1	0	0	0	0	0	0	0	0
ENSG00000270244	AC104687.2	0	0	0	0	0	0	0	0
ENSG00000242727	AC104687.1	0	0	0	0	0	0	0	0
ENSG00000186212	SOWAHB	0.02276	0	0	0	0	0	0	0
ENSG00000138758	SEPT11	4.33646	3.1166	1.23265	2.11191	3.67867	4.62441	2.57169	2.77706
ENSG00000234036	TXNP6	0	0	0.34746	0	0.28747	0	0	0.29031
ENSG00000118816	CCNI	101.927	94.4498	53.1803	60.5431	71.0361	97.9258	71.9822	62.5721
ENSG00000214062	RPL7P17	0	0	0	0	0	0	0	0
ENSG00000271676	AC104771.1	0	0	0	0	0	0	0	0
ENSG00000201641	RNU6-1187P	0	0	0	0	0	0	0	0
ENSG00000138764	CCNG2	13.1668	8.60641	2.26356	3.15975	9.98403	4.53483	4.05578	6.26402
ENSG00000270669	AC008638.3	0	0	0	0	0	0	0	0
ENSG00000250006	AC008638.2	0	0	0	0	0	0	0	0
ENSG00000248831	AC008638.1	0	0	0	0	0	0	0	0
ENSG00000249036	AC092674.1	0	0	0	0	0	0	0	0
ENSG00000156234	CXCL13	0	0	0	0	0	0	0	0
ENSG00000221711	RF00612	0	0	0	0	0	0	0	0
ENSG00000138767	CNOT6L	24.2352	19.7464	7.73903	21.6712	23.1473	26.2296	24.9917	16.7465
ENSG00000169288	MRPL1	5.01747	3.76763	3.04903	5.59885	7.15502	7.65355	6.09958	6.48461
ENSG00000250214	AC112225.1	0	0	0	0	0	0	0	0
ENSG00000248926	AC114801.2	0	0	0	0	0	0	0	0
ENSG00000248956	HMGB1P44	0	0	0	0	0	0	0	0
ENSG00000248640	HNRNPA1P56	0	0	0	0	0	0	0	0
ENSG00000248128	AC114801.1	0	0	0	0	0	0	0	0

ENSG00000249072	AC114801.3	0.41413	0	0	0	0	0	0	0
ENSG00000138759	FRAS1	0	0	0	0	0.0094	0	0.00644	0
ENSG00000249565	SERBP1P5	0.1862	0.20662	0	0	0	0	0	0.0762
ENSG00000248117	MINOS1P4	0	0	0	0	0	0	0	0
ENSG00000138772	ANXA3	6.8965	19.3861	5.15126	1.16791	7.89751	1.74166	1.02552	10.2066
ENSG00000200998	RF00019	0	0	0	0	0	0	0	0
ENSG00000251442	LINC01094	1.41583	2.31531	0.80302	1.69574	1.87233	0.48202	17.4721	2.43977
ENSG00000251019	HIGD1AP13	0	0	0	0	0	0	0	0
ENSG00000239793	AC098818.1	1.74525	0	0	0	0	0	0	0.71426
ENSG00000260278	AC098818.2	0	0	0.09286	0.04689	0	0.05574	0	0.03879
ENSG00000138756	BMP2K	19.6933	19.2135	10.5785	24.7061	24.5075	14.9246	37.2393	20.747
ENSG00000266270	MIR5096	0	3.48596	0	0	0	0	0	0
ENSG00000163291	PAQR3	0.63236	0.58038	0.24317	0.72799	0.60355	0.72981	0.65016	0.57323
ENSG00000242175	RN7SL127P	0.24433	0.81339	0	1.08786	0.89117	1.29312	0.81454	1.19996
ENSG00000249307	LINC01088	0.44035	0.42756	0.35051	1.14368	0.24538	0.25895	0.58108	0.60824
ENSG00000156269	NAA11	0	0	0	0	0	0	0	0
ENSG00000196475	GK2	0	0	0	0	0	0	0	0
ENSG00000250334	LINC00989	22.1774	11.7032	13.8082	22.1375	15.2589	5.8541	18.7843	20.2907
ENSG00000249646	OR7E94P	0	0	0	0	0	0	0	0
ENSG00000251399	AC092542.1	0.82592	0	0.30342	1.22575	0.25103	0.36426	1.03251	1.26756
ENSG00000248408	LINC02469	0	0	0	0	0	0	0	0
ENSG00000251321	PCAT4	0	0	0	0	0	0	0	0
ENSG00000212620	RF00072	0	0	0	0	0	0	0	0
ENSG00000163297	ANTXR2	15.5087	13.0494	2.29318	4.46657	10.9137	4.32612	3.88936	5.84835
ENSG00000236530	KPNA2P1	0	0	0	0.06838	0	0	0	0
ENSG00000244002	RPSAP39	0	0	0	0	0	0.14645	0	0
ENSG00000248719	AC021127.1	0	0.21556	0	0	0.6298	0	0	0
ENSG00000152784	PRDM8	1.46984	1.35624	0.14086	0.14227	0.95177	0.78917	0.02663	0.45116
ENSG00000138675	FGF5	0	0	0	0	0	0	0	0
ENSG00000197826	CFAP299	0	0.05449	0	0.04859	0	0	0	0.0201
ENSG00000232327	AC105917.1	0	0	0	0	0	0	0	0
ENSG00000152785	BMP3	0.03835	0	0	0.03794	0.07771	0.02255	0	0.06278
ENSG00000138669	PRKG2	0.14328	0.07155	0.08422	0.10632	0.20903	0.15166	0.11941	0.10555
ENSG00000251059	AC139722.1	0	0	0	0	0	0	0	0
ENSG00000207065	RNU5A-2P	0	0	0	0	0	0	0	0
ENSG00000138670	RASGEF1B	3.05208	1.30599	1.38569	0.59825	1.81156	4.30489	0.8159	0.75123
ENSG00000248282	MTCYBP44	0	0	0	0	0	0	0	0
ENSG00000248448	COX5BP1	0	0	0	0	0	0	0	0
ENSG00000252762	RF00322	0	0	0	0	0	0	0	0
ENSG00000248543	NPM1P41	0	0	0	0	0	0	0	0
ENSG00000250826	HNRNPA3P13	0	0	0	0	0	0	0	0
ENSG00000202485	RNU6-499P	0	0	0	0	0	0	0	0
ENSG00000234568	BIN2P1	0	0.07974	0.0704	0	0.11649	0	0	0.05882
ENSG00000284516	VAMP9P	0	0	0	0	0	0	0	0
ENSG00000273156	AC124016.2	0.66136	0.18347	0.16198	0.16359	0.13401	0.38891	0.55119	0.406
ENSG00000138668	HNRNPD	12.0015	9.26293	3.39451	6.9189	11.5273	11.6327	7.42078	5.96886
ENSG00000272677	AC124016.1	0.16181	0.808	0	0	0.09836	0.57091	0.40457	0.39734
ENSG00000250325	IGBP1P4	0	0	0	0	0	0	0	0
ENSG00000202440	RF00150	0	0	0	0	0	0	0	0
ENSG00000152795	HNRNPDL	37.3328	21.1666	6.01908	12.6745	30.9532	21.4182	16.8902	15.3337
ENSG00000145293	ENOPH1	7.8911	7.53139	4.94686	14.6122	8.80165	7.34363	8.32636	7.46642
ENSG00000249242	TMEM150C	0.09984	0.03324	0.08803	0	0	0	0	0.04903
ENSG00000214988	RPL7AP26	0	0	0	0	0	0	0	0
ENSG00000227304	AC067942.1	0	0	0	0	0	0	0	0
ENSG00000249960	AC067942.3	0	0	0	0	0	0	0	0
ENSG00000248113	AC067942.2	0	0	0.08495	0	0	0	0	0
ENSG00000231782	LINC00575	0	0	0	0	0	0	0	0
ENSG00000145284	SCD5	0.3928	0.36955	0.10039	0.22812	0.16611	0.33141	0.05693	0.23065

ENSG00000270480	AC073413.1	0	0	0	0	0	0	0	0
ENSG00000207746	MIR575	0	0	0	0	0	0	0	0
ENSG00000270842	AC073413.2	0	0	0	0	0	0	0	0
ENSG00000138674	SEC31A	11.502	9.45916	3.71021	8.31506	10.1249	10.2799	8.36357	6.23927
ENSG00000251022	THAP9-AS1	4.00093	4.16222	0.8329	2.20194	3.99272	2.61741	2.41749	2.96784
ENSG00000168152	THAP9	1.4721	0.90126	0.34811	0.87892	1.06973	1.13431	0.98714	0.95565
ENSG00000189308	LIN54	19.863	21.6868	10.2335	24.271	23.8329	20.7304	17.4389	17.6191
ENSG00000199530	RF00019	0	1.12971	0	0	0	0	0	0
ENSG00000251874	RNU6-615P	0	0	0	0	0	0	0	0
ENSG00000138663	COPS4	4.99495	3.5583	1.32906	2.28798	3.97351	6.05582	2.84379	3.88658
ENSG00000145287	PLAC8	31.9036	22.4511	4.69659	9.40193	28.3564	25.4059	8.14958	12.2161
ENSG00000250057	AC114781.2	0.37208	1.23866	0	0	0	0	0.3101	0.22842
ENSG00000250677	AC114781.3	0	0	0	0	0	0	0	0
ENSG00000248401	AC114781.1	0	0.11869	0	0	0	0	0	0
ENSG00000173085	COQ2	6.28893	5.84972	1.35904	2.15689	4.07996	3.30971	2.99505	2.72522
ENSG00000173083	HPSE	12.7783	14.6534	2.55538	3.31818	4.87018	4.57426	2.40686	3.29792
ENSG00000274154	AC114781.5	0	1.12971	0	0	0	0	0	0
ENSG00000251647	AC114781.4	0	0	0	0	0	0.20237	0	0
ENSG00000163312	HELQ	6.06065	6.14479	5.2508	13.7996	6.74435	6.05986	10.3654	7.15032
ENSG00000163319	MRPS18C	2.50059	2.30678	0.88544	1.31902	2.71049	3.29524	1.75763	1.68305
ENSG00000163322	ABRAXAS1	3.69471	2.10224	2.54846	4.3643	3.72418	3.54165	4.57175	2.75419
ENSG00000213608	SLC25A14P1	0	0	0	0	0	0	0	0
ENSG00000239525	RPL30P5	0	0	0	0	0	0	0	0
ENSG00000138678	GPAT3	14.1824	17.8017	3.26124	3.13684	9.17377	3.84058	2.21954	9.2644
ENSG00000201633	RF00019	0	0	0	0	0	0	0	0
ENSG00000249171	AC021192.1	0	0	0	0	0	0	0	0
ENSG00000250546	AC079160.1	0	0	0	0	0	0	0	0
ENSG00000200108	RNU6-774P	0	0	0	0	0	0	0	0
ENSG00000214980	AC025538.1	0	0	0	0	0	0	0	0
ENSG00000248749	AC104063.1	0	0	0	0	0	0	0	0
ENSG00000163623	NKX6-1	0	0	0	0	0	0	0	0
ENSG00000258013	RPL3P13	0	0	0	0	0	0	0	0
ENSG00000163624	CDS1	0.03286	0	0.02415	0.02439	0.11986	0.02899	0.02739	0.0807
ENSG00000163625	WDFY3	14.8505	21.0562	2.28771	3.32372	9.46362	2.10201	2.42099	9.40762
ENSG00000239466	RN7SL552P	0.26558	1.32618	0	0	0.32289	0	0	0
ENSG00000251260	WDFY3-AS1	0	0	0	0	0	0	0	0
ENSG00000252062	RNU6-469P	1.45149	0	3.19942	0	0	0	0	1.78212
ENSG00000180769	WDFY3-AS2	0.05908	0.21636	0	0	0.01437	0	0.0197	0.18861
ENSG00000201901	RN7SKP48	0	0.37198	0	1.65832	0.81509	0.39424	5.96002	1.64629
ENSG00000248725	AC097488.1	0	0	0	0	0	0	0	0
ENSG00000138639	ARHGAP24	1.12157	1.24971	0.21793	0.49523	1.4988	0.50691	0.52531	0.62593
ENSG00000266421	MIR4451	0	1.84861	0	0	4.05076	3.91854	0	0
ENSG00000273389		0	0	0	0	0	0	0	0
ENSG00000109339	MAPK10	0.12712	0.07508	0.03013	0.05477	0.02493	0.00723	0.0205	0.05035
ENSG00000250062	AC104827.1	0	0	0	0	0	0	0	0
ENSG00000252757	RN7SKP96	0	0	0	0	0	0	0	0
ENSG00000266515	MIR4452	0	0	0	0	0	0	0	0
ENSG00000163629	PTPN13	1.02664	0.51394	0.21552	0.779	0.34723	0.35406	0.10293	0.16112
ENSG00000145283	SLC10A6	0	0	0	0	0	0	0	0
ENSG00000243312	AC093827.1	0	0	0	0	0.1035	0	0	0
ENSG00000163633	C4orf36	0.27818	0.18521	0.1022	0.1445	0.21983	0.09815	0.16229	0.11954
ENSG00000250202	AC093827.2	0	0	0	0	0	0	0	0
ENSG00000251411	AC093827.3	0.06604	0	0	0	0.08029	0.1165	0	0
ENSG00000172493	AFF1	20.3654	27.1913	14.7225	22.6025	22.7587	24.7106	23.1429	20.5434
ENSG00000235043	TECRP1	0.16472	0.27418	0	0	0	0	0	0
ENSG00000145332	KLHL8	9.59862	10.8504	10.3115	22.9731	14.3236	9.2667	14.6703	14.7588
ENSG00000248196	AC092658.1	0.08227	0	0	0	0	0	0.13713	0
ENSG00000248180	GAPDHP60	0	0	0	0	0	0	0	0

ENSG00000250572	AC108516.1	0	0	0	0	0	0	0	0
ENSG00000263981	MIR5705	0	0	0	0	0	0	0	0
ENSG00000170509	HSD17B13	0.21406	0.1018	0.08987	0.04538	0.29743	0	0.05097	0.07509
ENSG00000255723	AC108516.2	0	0	0	0	0	0	0	0.51574
ENSG00000198189	HSD17B11	73.9024	86.2327	17.7828	25.778	53.3236	28.199	31.8622	61.7967
ENSG00000240966	RN7SL681P	2.00823	0.83567	0	0.37255	1.22078	0.8857	0.41843	0
ENSG00000249262	AC112250.1	0	0	0	0	0	0	0	0
ENSG00000170502	NUDT9	1.8187	1.57109	0.50745	0.75166	1.34347	1.13716	0.65234	0.70664
ENSG00000272856	AC112250.2	0	0	0	0	0	0	0	0
ENSG00000152583	SPARCL1	0.02108	0	0.03098	0	0.07689	0.03719	0	0
ENSG00000249001	AC093895.1	0	0	0	0	0	0	0	0
ENSG00000152591	DSPP	0	0	0	0	0	0	0	0
ENSG00000152592	DMP1	0	0	0	0	0	0	0	0
ENSG00000200024	RF00019	0	0	0	0	0	0	0	0
ENSG00000249973	CHCHD2P7	0	0	0	0	0	0	0	0
ENSG00000029559	IBSP	0	0	0	0	0	0	0	0
ENSG00000152595	MEPE	0	0	0	0	0	0	0.04933	0
ENSG00000183199	HSP90AB3P	0	0	0	0	0	0.05951	0	0
ENSG00000118785	SPP1	5.65306	1.31418	0.09282	0.23435	1.72781	0	0.31585	0.81428
ENSG00000202000	RNU1-36P	0	0	0	0	0	0	0	0
ENSG00000118762	PKD2	2.53311	3.68378	2.17465	3.6407	4.31157	4.93916	3.88897	2.9628
ENSG00000118777	ABCG2	4.86855	8.22953	9.17613	15.2812	8.7499	6.42825	13.2564	7.44406
ENSG00000241853	AC097484.1	0	0	0	0	0	0	0	0
ENSG00000201707	RNU6-818P	0	0	0	0	0	0	0	0
ENSG00000221440	RNU6ATAC31P	0	0	0	0	0	0	0	0
ENSG00000251285	AC097484.2	0	0	0	0	0	0	0	0
ENSG00000199326	RNU6-1298P	0	0	0	0	0	0	0	0
ENSG00000163644	PPM1K	2.90726	2.67172	1.36407	1.82729	3.61294	2.3199	1.77291	2.21609
ENSG00000200469	RNU6-112P	0	0	0	0	0	0	0	0
ENSG00000246375	AC107067.1	0.13295	0.07377	0.06512	0	0	0	0	0.05441
ENSG00000207480	RF00019	0	0	0	0	0	0	0	0
ENSG00000138642	HERC6	2.36616	4.48607	2.23126	1.76521	1.7691	2.29917	1.16001	1.91086
ENSG00000138646	HERC5	5.99814	42.0348	14.0123	2.57927	2.71127	5.18224	2.81994	7.89315
ENSG00000249934	NCOA4P2	0	0	0	0	0	0	0	0
ENSG00000145337	PYURF	7.0015	7.26134	2.69087	4.79584	4.51804	6.84089	5.29658	7.4722
ENSG00000138641	HERC3	10.1792	9.39723	1.98361	3.32632	7.61734	4.79265	3.87739	6.33753
ENSG00000255072	PIGY	0	0	0	0	0	0	0	0
ENSG00000249755	AC083829.1	0	0	0	0	0	0	0	0
ENSG00000252322	RN7SKP244	0	0	0	0	0	0	0	0
ENSG00000207524	RNU6-33P	6.16545	5.70133	3.02002	10.1669	5.83006	6.04261	2.28374	10.9342
ENSG00000177432	NAP1L5	1.64223	0.3154	0.74253	3.42152	1.49744	1.44856	0.73698	1.27958
ENSG00000248019	FAM13A-AS1	2.22664	1.81254	0.40602	0.86836	1.24487	0.37274	1.11073	2.03541
ENSG00000278151	RF02114	0	0	0	0	0	0	0	0
ENSG00000276003	RF02115	0	0	0	0	0	0	0	0
ENSG00000138640	FAM13A	5.31379	5.23487	5.78713	16.6615	7.76863	5.35252	9.939	9.29595
ENSG00000180346	TIGD2	1.50383	1.09986	0.30134	0.74395	1.5513	1.64805	0.94949	1.00712
ENSG00000270720	AC104785.1	0.12466	0.2075	0	0	0	0.21992	0	0.15306
ENSG00000212226	RNU6-907P	0	0	0	0	0	0	0	0
ENSG00000185477	GPRIN3	5.59695	3.65905	1.57267	3.72755	3.8038	10.1715	2.82851	2.99497
ENSG00000251095	AC097478.1	0	0	0.12424	0.10755	0	0	0	0.02966
ENSG00000145335	SNCA	345.574	274.23	303.096	439.479	260.534	393.251	430.371	265.283
ENSG00000274238	AC097478.2	0	0	0	0	0	0	0	0
ENSG00000277695	AC097478.4	0	0	0	0	0	0	0	0
ENSG00000276542	AC097478.3	0	0	0	0	0	0	0	0
ENSG00000247775	SNCA-AS1	0.04903	0	0.03602	0.32745	0.05961	0.21624	0.16345	0.0301
ENSG00000138722	MMRN1	12.0393	6.59864	3.06916	4.2843	5.79016	4.88146	5.36618	5.14502
ENSG00000184305	CCSER1	0.2179	0.1521	0.15494	0.5216	0.29056	0.32241	0.4218	0.23302
ENSG00000252087	RN7SKP248	0	0	0	0	0	0	0	0

ENSG00000251401	AC093729.1	0	0	0	0	0	0	0	0
ENSG00000187653	TMSB4XP8	16.8319	21.6904	15.9576	30.6212	5.28099	10.5365	27.1512	65.3312
ENSG00000248984	AC004054.1	0	0	0	0	0	0	0	0
ENSG00000249049	AC074124.1	0	0	0	0	0	0	0	0
ENSG00000279013	AC110774.1	0	0	0	0	0.04416	0	0	0
ENSG00000249891	KRT19P6	0	0	0	0	0	0	0	0
ENSG00000249052	AC093810.1	0	0	0	0	0	0	0	0
ENSG00000248817	PMPCAP1	0	0	0	0	0	0	0	0
ENSG00000280056	AC097520.1	0	0	0	0	0	0	0	0
ENSG00000249152	LNCPRESS2	0	0	0	0	0	0	0	0
ENSG00000248511	AC112695.1	0	0	0	0	0	0	0	0
ENSG00000152208	GRID2	0	0	0	0	0	0	0	0
ENSG00000251449	MTND1P19	0	0	0	0	0	0	0	0
ENSG00000249239	AC095059.2	0	0	0	0	0	0	0	0
ENSG00000248627	AC095059.1	0	0	0	0	0	0	0	0
ENSG00000248750	AC020699.1	0	0	0	0	0	0	0	0
ENSG00000250908	AC110800.1	0	0	0	0	0	0	0	0
ENSG00000252342	RNA5SP164	0	0	0	0	0	0	0	0
ENSG00000172238	ATOH1	0	0	0	0	0	0	0	0
ENSG00000246541	AC096746.1	0	0	0	0	0	0	0	0
ENSG00000250897	HMGB3P15	0	0	0	0	0	0	0	0
ENSG00000163104	SMARCAD1	5.24818	3.63339	1.72329	4.01506	5.88653	7.27161	3.40626	4.41931
ENSG00000163106	HPGDS	0.17362	0.34678	0	0	0.16886	0	0.11576	0.04263
ENSG00000241103	AC109925.1	0	0	0	0	0	0	0	0
ENSG00000163110	PDLIM5	6.49723	6.35896	1.64145	2.64853	4.65271	3.90617	2.00512	3.17303
ENSG00000249951	AC108067.1	1.36373	0.56748	0	0	0	0	0.56828	0
ENSG00000249599	BMPR1B-DT	0	0	0	0	0	0	0	0
ENSG00000138696	BMPR1B	0.01161	0	0	0	0	0.06146	0	0
ENSG00000182168	UNC5C	0.00686	0	0	0	0.00834	0	0.01143	0
ENSG00000271474	AC106881.1	0	0	0	0	0	0	0	0
ENSG00000242936	RPL30P6	0	0	0	0	0	0	0	0
ENSG00000163114	PDHA2	0	0	0	0	0	0	0	0
ENSG00000200622	RNU6-1059P	0	0	0	0	0	0	0	0
ENSG00000201744	RNU6-34P	0	0	0	0	0	0	0	0
ENSG00000250655	AC096661.1	0	0	0	0	0	0	0	0
ENSG00000248510	LINC02267	0.03025	0.15106	0.26673	0.08979	0.07356	0.05337	0	0.03714
ENSG00000201640	RN7SKP28	0	0	0	0	0	0	0	0
ENSG00000236764	COX7A2P2	0	0	0	0	0	0	0	0
ENSG00000253170	AC108515.1	0	0	0	0	0	0	0	0
ENSG00000163116	STPG2	0.11304	0.06272	0.07383	0.48465	0.21378	0.17726	0.33497	0.12337
ENSG00000254044	AC034154.1	0	0	0	0	0	0	0	0
ENSG00000251620	STPG2-AS1	0	0	0	0	0	0	0	0
ENSG00000250293	CRYZP2	0	0	0	0	0	0	0	0.17159
ENSG00000242697	RPL5P12	0.08613	0	0	0	0	0	0	0
ENSG00000214904	DUTP8	0	0	0	0	0	0	0	0
ENSG00000214559	AC019077.1	0	0	0	0	0.44895	0	0	0.45339
ENSG00000138698	RAP1GDS1	5.59483	4.04895	1.23124	3.24913	4.23896	3.86219	2.02733	2.68797
ENSG00000242318	AC058823.1	0.77321	0	0	0	0	0.27281	0	0.37973
ENSG00000168785	TSPAN5	21.2502	22.8417	19.7451	36.4571	21.6135	23.7913	27.8565	23.3048
ENSG00000251523	AC108159.1	0	0	0	0	0	0	0	0
ENSG00000260641	AC114811.2	0.50624	0.79942	0.24797	0.38529	0.22094	0.1374	0.32454	0.25499
ENSG00000249764	AC114811.1	0	0	0	0	0	0	0	0
ENSG00000227118	BTF3P13	0	0	0	0	0	0	0	0
ENSG00000151247	EIF4E	2.80862	2.54997	1.12561	2.21949	2.61642	2.06833	1.99769	2.34797
ENSG00000249055	TBCAP3	0	0	0	0	0	0	0	0
ENSG00000263923	AC019131.1	0.23569	0	0.17317	0	0.28655	0	0	0
ENSG00000214886	FAM177A1P1	0	0	0	0	0	0	0	0
ENSG00000252548	RNU7-149P	0	0	0	0	0	0	0	0

ENSG00000251437	NDUFS5P4	0	0	0	0	0	0	0	0
ENSG00000164024	METAP1	4.66473	3.82295	1.56979	2.72162	4.82706	5.46521	1.8103	2.73248
ENSG00000265213	MIR3684	0	0	0	1.47008	0	0	0	0
ENSG00000250496	ABT1P1	0	0	0	0	0	0	0	0
ENSG00000272777	AC019131.2	0.33701	0.56096	0	0.37512	0.20487	0.4459	0.42131	0.31033
ENSG00000197894	ADH5	5.58866	3.96164	1.63807	2.5039	5.16459	6.67021	2.81221	3.51407
ENSG00000246090	AP002026.1	0.2088	0.10427	0.03068	0.04648	0.11424	0.07367	0.08701	0.10255
ENSG00000198099	ADH4	0	0	0	0	0	0	0	0
ENSG00000249065	PCNAP1	0	0	0	0	0	0	0	0
ENSG00000172955	ADH6	0	0	0	0	0	0	0	0
ENSG00000187758	ADH1A	0	0	0	0	0	0	0	0
ENSG00000196616	ADH1B	0	0	0	0	0	0	0	0
ENSG00000248144	ADH1C	0	0	0	0	0	0	0	0
ENSG00000196344	ADH7	0	0	0	0	0	0	0	0
ENSG00000250300	AP001960.1	0	0	0	0	0	0	0	0
ENSG00000138813	C4orf17	0	0	0	0	0	0	0	0
ENSG00000145331	TRMT10A	1.22195	0.808	0.24598	0.54654	0.50877	1.7718	0.39062	0.61656
ENSG00000138823	MTTP	0	0	0	0	0	0.07187	0	0.01667
ENSG00000248676	AC083902.1	0	0	0	0	0	0	0	0
ENSG00000248713	C4orf54	0	0	0	0	0	0	0	0.00892
ENSG00000070190	DAPP1	33.9909	40.1061	15.5839	13.6102	23.9055	12.0839	14.5069	31.1863
ENSG00000279913	AP001962.1	2.22917	1.7461	0.38538	0.58382	0.31884	0	0.65571	2.57594
ENSG00000109270	LAMTOR3	11.0662	10.1626	3.21154	6.15234	8.49835	5.53844	4.59694	8.28416
ENSG00000164031	DNAJB14	6.39514	6.56782	2.38852	4.30884	6.7461	6.51602	3.70895	5.4846
ENSG00000279098	AC097460.3	0.99503	0.27604	0	0.24612	0.20162	1.4628	0.82928	0.61084
ENSG00000164032	H2AFZ	18.8741	16.847	6.45442	10.0141	12.8083	18.7539	8.32927	17.1162
ENSG00000245322	AC097460.1	0	0.07118	0	0.25388	0	0.15089	0.14257	0.26254
ENSG00000250403	AC097460.2	0	0	0	0	0	0	0	0
ENSG00000248820	DYNLL1P6	0	0	0	0	0	0	0	0
ENSG00000145358	DDIT4L	0.02448	0.04075	0.03598	0.03633	0	0	0	0
ENSG00000249710	AP001961.1	0	0	0	0	0	0	0	0
ENSG00000164035	EMCN	0.41942	0.02327	0.02054	0	0	0	0	0.0515
ENSG00000250223	LINC01216	0.26687	0	0	0	0	0	0	0
ENSG00000251219	LINC01217	0	0	0	0	0	0	0	0
ENSG00000251636	LINC01218	12.8009	1.01462	0.22394	0.22617	0.18527	0	0	1.49683
ENSG00000138814	PPP3CA	20.1495	18.6067	2.97541	6.28132	16.9412	6.69752	6.09378	9.84046
ENSG00000273882	MIR8066	0	0	0	0	0	0	0	1.15381
ENSG00000221265	MIR1255A	0	2.15944	0	0.96271	0	1.14435	0	0
ENSG00000254531	FLJ20021	1.67013	0.92665	0.40904	0.1377	1.01525	0.32737	0	0.22784
ENSG00000153064	BANK1	7.3014	3.37589	1.43759	3.41726	10.3853	2.56771	3.91757	3.54481
ENSG00000199529	RNU6-462P	0	0	0	0	0	0	0	0
ENSG00000251149	MTND5P5	1.94948	0	0	0.57865	4.02921	0.68783	0.32495	1.19677
ENSG00000251309	AP002075.1	0	0	0	0	0	0	0	0
ENSG00000138821	SLC39A8	3.43878	3.84101	3.87305	8.22608	5.47352	5.80706	6.52383	3.88876
ENSG00000241144	RN7SL728P	0	0	0	0	0	0	0	0
ENSG00000248161	AC098487.1	0	0.36898	0.10858	0	0	0.13035	0	0.09072
ENSG00000251572	AF213884.2	0	0	0	0	0	0	0	0
ENSG00000260651	AF213884.3	0.30606	0	0	0	0	0	0	0
ENSG00000109320	NFKB1	13.8939	15.7946	2.27237	6.13734	12.9039	11.8007	4.21783	6.88966
ENSG00000109323	MANBA	11.6419	12.5378	6.68983	11.9268	9.85491	5.77818	8.41375	11.4776
ENSG00000241981	AF213884.1	0	0	0	0	0	0	0	0
ENSG00000230069	LRRC37A15P	4.00312	2.97142	0.71544	1.12399	3.55151	2.48126	1.35255	1.0627
ENSG00000248971	KRT8P46	2.46733	1.7112	0.30214	0.6103	1.49986	1.99499	1.45657	1.13601
ENSG00000224207	AC018797.1	0	0	0	0	0	0	0	0
ENSG00000251288	AC018797.3	0	0	0	0	0	0	0	0
ENSG00000109332	UBE2D3	67.1052	69.0562	26.4016	48.3372	56.0951	43.0354	43.8048	46.8085
ENSG00000246560	AC018797.2	1.57724	0.69087	0.24397	0.616	1.00925	1.31801	1.93717	1.01922
ENSG00000252739	RNU7-151P	0	0	0	0	0	0	0	0

ENSG00000145354	CISD2	28.8197	48.1576	36.4005	67.1826	28.4066	25.3465	21.5116	18.0892
ENSG00000164037	SLC9B1	0.36933	0.28689	0.32564	0.91355	0.47896	0.39093	0.6977	0.57439
ENSG00000250651	PABPC1P7	0	0	0	0	0	0.08547	0	0
ENSG00000249283	ACTR3BP4	0	0	0	0	0	0	0	0
ENSG00000164038	SLC9B2	0.94115	1.45302	1.50326	3.09717	1.75779	1.20312	2.61458	1.49046
ENSG00000164039	BDH2	0.88836	0.68524	0.25472	0.93254	1.0537	1.29962	0.61397	0.74488
ENSG00000138778	CENPE	5.20026	6.09784	5.89667	10.3556	7.52075	9.33827	11.4884	9.52002
ENSG00000248740	LINC02428	0	0	0	0	0	0	0	0
ENSG00000251577	AC105460.2	0	0	0	0	0	0	0	0
ENSG00000250920	AC105460.1	0	0	0	0	0	0	0	0
ENSG00000251571	DDX3P3	0	0	0	0	0	0	0	0
ENSG00000169836	TACR3	0	0	0	0	0	0	0	0
ENSG00000252460	RNU6-635P	0	0	0	0	0	0	0	0
ENSG00000249614	LINC02503	0	0	0	0	0	0	0	0
ENSG00000251170	AC004052.1	0	0	0	0	0	0	0	0
ENSG00000168772	CXXC4	0.07558	0.04193	0.03702	0.09347	0.16846	0.06667	0.20997	0.20106
ENSG00000245384	CXXC4-AS1	0	0	0	0	0	0	0	0
ENSG00000250670	AC004063.1	0	0	0	0	0	0	0	0
ENSG00000248242	AC004053.1	2.80453	1.9097	3.55924	3.78385	2.63475	1.57423	3.3998	6.26067
ENSG00000244470	AC105918.1	0	0	0	0	0	0	0	0.1074
ENSG00000248373	AC096577.1	0.14123	0.23508	0.10377	0.1048	0	0	0	0
ENSG00000251906	RNU6-351P	0	0	0	0	0	0	0	0
ENSG00000276992	AC096577.2	0	0	0	0	0	0	0	0
ENSG00000251473	AC004069.2	0	0	0	0	0	0	0	0
ENSG00000252136	RF00322	0	0	0	0	0	0	0	0
ENSG00000251259	AC004069.1	1.86923	2.44461	0.3924	0.3963	1.46093	0	0.4451	0.98357
ENSG00000168769	TET2	23.0482	25.7733	4.82535	6.12803	18.2085	12.6612	9.34486	12.7233
ENSG00000251586	TET2-AS1	0.1365	0	0	0	0	0.2408	0	0
ENSG00000243383	RN7SL89P	0	0	0	0	0	0	0	0.31801
ENSG00000138777	PPA2	4.63049	5.53154	5.11491	10.4485	4.95944	3.97327	11.1312	7.83172
ENSG00000200917	RNU6-553P	0	0	0	0	0	0	0	0
ENSG00000249264	EEF1A1P9	0.106	0	0	0	0	0.0935	0.26503	0.19522
ENSG00000216425	AC004066.1	0	0	0	0	0	0	0	0
ENSG00000250922	ATP5F1EP1	0	0	0	0	0	0	0	0
ENSG00000250522	AC004066.2	0	0	0	0	0	0	0	0
ENSG00000236699	ARHGEF38	0	0	0	0.07177	0	0	0.04836	0.01187
ENSG00000249885	ARHGEF38-IT1	0	0	0	0	0	0	0.26794	0
ENSG00000248778	AC105391.1	0	0	0	0.1041	0	0	0	0
ENSG00000138785	INTS12	3.25403	2.81775	1.24381	2.26114	2.72129	3.08596	2.72766	2.10155
ENSG00000138780	GSTCD	0.45412	0.23258	0.27378	0.69125	0.75031	0.65734	0.34936	0.4146
ENSG00000251175	AC008243.1	0	0	0	0	0	0	0	0
ENSG00000168743	NPNT	0	0	0	0	0	0	0	0.01471
ENSG00000250740	AC109361.2	0	0	0	0	0	0	0	0
ENSG00000249635	AC109361.1	0	0	0	0	0	0.03667	0	0
ENSG00000145348	TBCK	3.23888	2.98653	3.06314	5.49215	3.78537	4.44538	5.61862	4.25202
ENSG00000164022	AIMP1	7.20229	3.99607	1.48883	3.20343	7.0158	5.90607	4.40554	4.65129
ENSG00000250298	GIMD1	0	0	0	0	0	0	0	0
ENSG00000249613	LINC02173	0	0	0	0	0	0	0	0
ENSG00000248915	ACTR6P1	0	0	0	0	0	0	0	0
ENSG00000155011	DKK2	0.03394	0.02825	0	0	0.04127	0.08982	0	0.02084
ENSG00000213522	RAC1P5	0	0	0	0	0	0	0	0
ENSG00000251081	AC104663.1	0	0	0	0	0	0	0	0
ENSG00000252470	RNU6-551P	0	0	0	0	0	0	0	0
ENSG00000138801	PAPSS1	10.896	7.64746	3.0041	6.09926	8.12248	5.98597	2.81036	4.19193
ENSG00000164023	SGMS2	4.44506	3.22799	0.63486	1.35359	2.24105	1.25332	1.04017	1.14338
ENSG00000245293	AC096564.1	0	0	0.03476	0	0.02876	0	0	0
ENSG00000222691	RNU6-733P	0	0	0	0	0	0	0	0
ENSG00000155016	CYP2U1	0.73348	0.62045	0.10602	0.49967	0.96485	0.48789	0.38081	0.41337

ENSG00000249604	AC096564.2	0	0	0	0	0	0	0	0
ENSG00000138796	HADH	0.47306	0.4038	0.09804	0.23403	0.48665	0.56706	0.33361	0.32019
ENSG00000138795	LEF1	15.1906	2.59447	2.0494	4.36279	28.3923	20.7439	8.09066	7.72219
ENSG00000232021	LEF1-AS1	0.18329	0.07627	0.06734	0.11334	0.38997	0.35029	0.76379	0.31881
ENSG00000239939	RPSAP34	0	0	0	0	0	0	0	0
ENSG00000278589	ZACNP1	0	0	0	0	0	0	0	0
ENSG00000250485	EXOC7P1	0	0	0	0	0	0	0	0
ENSG00000234492	RPL34-AS1	0.06416	0.10679	0.04714	0.11902	0.1365	0.31125	0.08021	0.07877
ENSG00000109475	RPL34	73.0195	54.0462	25.365	39.6284	84.9143	64.9036	48.8289	67.7166
ENSG00000198856	OSTC	12.8287	9.73352	5.4623	12.3788	9.64466	12.4753	8.31159	10.7418
ENSG00000206601	RNU6-431P	2.05515	0	0	1.01669	0	0	0	0.84109
ENSG00000164089	ETNPPL	0	0	0	0	0	0	0	0
ENSG00000249257	AC097473.1	0	0	0	0	0	0	0	0
ENSG00000250185	RCC2P8	0	0	0	0	0	0	0	0
ENSG00000188517	COL25A1	0.06611	0.01834	0.12952	0	0	0.01944	0	0.04058
ENSG00000246774	ZCCHC23	0	0	0	0	0	0	0	0
ENSG00000249465	RBMXP4	0	0	0	0	0	0	0	0
ENSG00000247950	SEC24B-AS1	0.11347	0.09443	0	0.2105	0.10346	0.05004	0.14185	0.2438
ENSG00000138802	SEC24B	18.9185	21.0166	11.2482	26.4304	28.9941	31.6404	26.1263	20.9516
ENSG00000243227	RN7SL55P	0	0	0	0	0	0	0.41138	0
ENSG00000207988	MIR576	0.74796	1.24498	1.09912	0	0.90935	1.31951	0	2.75501
ENSG00000225094	SETP20	0	0	0	0	0	0	0	0
ENSG00000249965	CDC42P4	0.39059	0	0	0.38645	0.15829	0	0.21702	0.15985
ENSG00000005059	MCUB	7.56437	6.05595	3.92676	9.15191	9.17157	12.6918	9.42221	8.25267
ENSG00000248785	HIGD1AP14	0.77979	2.59593	0	0.38576	0.63203	1.83421	0.43326	0.95742
ENSG00000138794	CASP6	3.16893	1.38147	0.77612	1.23175	2.6602	1.86347	0.75459	1.52851
ENSG00000273447	AC004067.1	1.04715	0.87149	0.46163	0.77704	1.2731	1.84731	0.34909	0.64284
ENSG00000123739	PLA2G12A	8.39841	5.06424	2.28696	3.2669	4.22742	5.0953	3.97296	4.95776
ENSG00000205403	CFI	0	0.06297	0	0.02807	0	0	0	0
ENSG00000272795	AC126283.1	0	0	0	0.29886	0.24483	0	0	0
ENSG00000109534	GAR1	1.82713	1.90825	0.31588	1.0634	1.74226	2.7809	0.83603	1.09967
ENSG00000180245	RRH	0.15113	0.16771	0.07403	0.2243	0.06125	0	0.16795	0.06185
ENSG00000183423	LRIT3	0.01881	0.03131	0.02764	0	0	0.03318	0.03135	0
ENSG00000249681	KRT19P3	0	0	0	0	0	0	0	0
ENSG00000138798	EGF	5.01271	3.56447	1.82834	4.22572	2.66172	2.68037	2.97125	3.95123
ENSG00000207260	RNU6-35P	0	0	0	0	0	0	0	0
ENSG00000170522	ELOVL6	0.59267	0.70226	0.22142	0.99885	0.32975	0.67341	0.82045	0.37
ENSG00000263940	RN7SL275P	0	0	0	0	0	0	0	0
ENSG00000248200	AC093770.1	0	0	0	0	0	0	0	0
ENSG00000166530	HSBP1P2	0	0	0	0	0	0	0	0
ENSG00000250594	AC108145.1	0	0	0	0	0	0	0	0
ENSG00000212160	RNU6-205P	0	0	0	0	0	0	0	0
ENSG00000250304	ZBED1P1	0	0	0	0	0	0	0	0
ENSG00000138792	ENPEP	0.03758	0.01564	0.05522	0.11155	0.02284	0.03315	0.03132	0.01154
ENSG00000251464	RPL7L1P13	0	0	0	0	0	0	0	0
ENSG00000251046	ZNF969P	0.21918	0	0.09202	0.27882	0.07614	0.05524	0.05219	0.19222
ENSG00000199894	RF00494	0	0	0	0	0	0	0	0
ENSG00000250511	AC098798.1	0	0	0	0	0	0	0	0
ENSG00000250103	PANCR	0	0	0	0	0	0	0	0
ENSG00000164093	PITX2	0	0	0	0	0	0	0	0
ENSG00000249519	LINC01438	0	0	0	0	0	0	0	0
ENSG00000215961	MIR297	0	0	0	0	0	0	0	0
ENSG00000251483	LYPLA1P2	0	0	0	0	0	0	0	0
ENSG00000251312	AC004062.1	0	0	0	0	0	0	0	0
ENSG00000200963	RNU6-289P	0	0	0	0	0	0	0	0
ENSG00000241722	AC083795.1	0	0	0	0.33996	0	0	0	0
ENSG00000248656	AC083795.2	0	0	0	0	0	0	0	0
ENSG00000250855	AC139718.2	0	0	0	0	0	0	0	0

ENSG00000248432	AC139718.1	0	0	0	0	0	0	0	0
ENSG00000249815	AC004704.1	0	0	0	0	0	0	0	0
ENSG00000250556	CCDC34P1	0	0	0	0	0	0	0	0
ENSG00000250307	TUBB8P3	0	0	0	0	0	0	0	0
ENSG00000240459	AC093663.1	0	0	0	0	0	0	0	0
ENSG00000174749	FAM241A	1.66348	1.75453	0.41145	0.52553	1.2515	0.71186	0.48043	0.97065
ENSG00000243280	AC093663.2	0.46837	0	0	0	0.28472	0	0.39035	0.57506
ENSG00000260526	AC109347.1	0.25201	0.11985	0	0.16029	0.08754	0.12703	0.36006	0.08841
ENSG00000138660	AP1AR	3.74589	3.04667	1.40741	2.90601	3.75201	2.74093	1.66739	3.29258
ENSG00000272567	AC109347.2	0	0.69919	0	0	0.25535	0.37052	0	0.77361
ENSG00000145365	TIFA	2.44909	6.28703	1.74877	2.15012	4.04695	2.58624	1.37992	6.16215
ENSG00000073331	ALPK1	5.00762	10.028	1.31092	1.49167	5.38677	2.14033	1.44733	5.11139
ENSG00000251441	RTEL1P1	0.29837	1.19194	0.14615	0.05904	0.09673	0.10527	0.03316	0.26865
ENSG00000248697	TOX4P1	0.27557	0.2621	0	0	0.14358	0	0.06562	0.09667
ENSG00000178403	NEUROG2	0	0	0	0	0	0	0	0
ENSG00000249509	AC023886.1	0	0	0	0	0	0	0	0
ENSG00000248439	RPL23AP94	0	0	0	0	0	0	0	0
ENSG00000138658	ZGRF1	1.5541	1.63518	2.33106	5.01922	2.68241	4.41788	5.93252	3.37131
ENSG00000250966	AC023886.2	0	0.29686	0	0	0	0	0.29728	0
ENSG00000174720	LARP7	13.1054	10.2495	2.95468	3.65017	9.51625	9.91295	4.5784	7.80283
ENSG00000249532	AC106864.1	0	0	0.14497	0	0	0	0	0
ENSG00000199169	MIR367	0	0	0	0	0	0	0	0
ENSG00000199145	MIR302D	0	0	0	0	0	0	0	0
ENSG00000207927	MIR302A	0	0	0	0	0	0	0	0
ENSG00000199102	MIR302C	0	0	0	0	0	0	0	0
ENSG00000284463	MIR302B	0	0	0	0	0	0	0	0
ENSG00000220924	OSTCP4	0	0	0	0.24228	0	0	0	0
ENSG00000250046	AC106864.2	0	0	0	0	0	0	0	0
ENSG00000248633	WRBP1	0	0	0	0	0	0	0	0
ENSG00000241002	AC017007.3	0	0	0	0	0	0	0	0
ENSG00000202536	RF00019	0	0	0	0	0	0	0	0
ENSG00000241741	RPL7AP30	0.09209	0	0	0	0.11196	0	0	0
ENSG00000250500	AC017007.4	0	0	0	0	0	0	0	0
ENSG00000145362	ANK2	0	0	0	0.01791	0	0.0071	0	0
ENSG00000234841	AC017007.2	0	0.09378	0	0	0	0	0	0
ENSG00000251126	AC017007.5	0	0	0	0	0	0	0	0
ENSG00000228814	AC017007.1	0	0	0	0	0	0	0	0
ENSG00000248152	AC093879.1	0	0	0	0	0	0	0	0
ENSG00000249373	AC093879.2	0	0	0	0	0	0	0	0
ENSG00000283193	MIR1243	0	0	0	0	0	0	0	0
ENSG00000275810	MIR8082	0	0	0	0	0	0	0	0
ENSG00000196656	AC004057.1	1.03033	0.245	0	0	0.3579	0	0	0.36143
ENSG00000239279	RN7SL184P	0	0	0	0	0	0	0	0
ENSG00000206820	RNU1-138P	0	0	0	0	0	0	0	0
ENSG00000145349	CAMK2D	6.55054	3.51294	1.37513	3.39817	5.89431	7.42889	3.4847	2.7257
ENSG00000250229	AC111193.1	0	0	0	0	0	0	0	0
ENSG00000180801	ARSJ	0.07122	0	0	0	0.03464	0	0	0.08744
ENSG00000249304	AC104779.1	0	0	0	0	0	0	0	0
ENSG00000248716	AC093815.1	0	0	0	0	0	0	0	0
ENSG00000174607	UGT8	0.09359	0.02596	0	0.11575	0.11379	0.08256	0.15601	0.09576
ENSG00000207931	MIR577	0	0	0	0	0	0	0	0
ENSG00000250215	CIR1P2	0.05462	0	0	0	0	0.09636	0	0
ENSG00000240637	RN7SL808P	0	0	0	0	0	0	0	0
ENSG00000138653	NDST4	0	0	0	0	0	0	0	0
ENSG00000251308	MRPS33P3	0	0	0	0	0	0	0	0
ENSG00000250693	RPF2P2	0	0	0	0	0	0	0	0
ENSG00000248488	PGAM4P2	0	0	0	0	0	0	0	0
ENSG00000250363	KRT18P21	0	0	0	0	0	0	0	0

ENSG00000248173		0	0	0	0	0	0	0	0
ENSG00000250442	EIF3KP3	0	0	0	0	0	0	0	0
ENSG00000249259	AC027613.1	0.07491	0	0	0	0	0	0	0
ENSG00000250627	TTC39CP1	0	0	0	0	0	0	0	0
ENSG00000270394	MTRNR2L13	0	0	0	0	0	0	0	0
ENSG00000284253	MIR1973	0	0	0	0	0	0	0	0
ENSG00000250272	TRMT112P1	0	0	0	0	0	0	0	0
ENSG00000248139	CUL4AP1	0	0	0	0	0	0	0	0
ENSG00000250791	AC106892.1	0	0	0	0	0	0	0	0
ENSG00000213493	ACTN4P1	0	0	0	0	0	0	0	0
ENSG00000201752	RNU6-119P	0	0	0	0	0	0	0	0
ENSG00000271053	AC093765.1	0	0	0	0	0	0	0	0
ENSG00000174599	TRAM1L1	0	0	0	0	0	0	0	0
ENSG00000223605	AC107399.1	0	0	0	0	0	0	0	0
ENSG00000224932	LINC02262	0	0	0	0	0	0	0	0
ENSG00000228358	LINC02263	0	0	0	0	0	0	0	0
ENSG00000236922	LINC01378	0	0	0	0	0	0	0	0
ENSG00000237684	RPSAP35	0	0	0	0	0	0	0	0
ENSG00000213492	NT5C3AP1	0.88154	1.3206	1.29542	0.32708	0.91099	0.85534	0.66123	0.37882
ENSG00000229565	LINC02264	0	0	0	0	0	0	0	0
ENSG00000164100	NDST3	0.30328	0.36556	0.39957	0.4501	0.178	0.18449	0.17432	0.0642
ENSG00000251463	FKBP4P1	0	0	0	0	0	0	0	0
ENSG00000269893	SNHG8	18.2655	7.27028	5.01809	15.6755	26.2619	15.9713	11.9136	25.3513
ENSG00000275994	SNORA24	0	0	0	0	0	0	0	0
ENSG00000164099	PRSS12	0	0	0	0.038	0.03113	0	0	0.01572
ENSG00000230671	NDUFS5P5	0	0	0	0	0	0	0	0
ENSG00000273716	AC092670.1	0	0	0	0	0	0	0	0
ENSG00000154608	CEP170P1	0.0799	0.1064	0.04696	0	0.09714	0	0.07991	0.01962
ENSG00000260404	AC110079.1	0.48071	0.46905	0.09743	0.46742	0.3426	1.0235	0.66312	0.42739
ENSG00000223225	RNU6-1054P	0	0	0	0	0	0	1.17481	0
ENSG00000248213	CICP16	0	0	0	0	0	0	0	0
ENSG00000251155	SEPT14P4	1.57635	0	1.15821	2.92435	0.47912	0.69522	3.9413	2.90313
ENSG00000281731	METTL14-DT	0	0	0	0.19119	0.03915	0.11363	0	0
ENSG00000145388	METTL14	3.42894	3.59162	1.33426	2.17191	4.11687	3.65586	2.3147	2.67552
ENSG00000150961	SEC24D	3.65763	3.55448	0.92295	1.22823	2.72201	2.60709	0.92374	2.01405
ENSG00000172403	SYNPO2	0.05745	0.09563	0.06908	0.03876	0.0381	0.0645	0.06094	0.0513
ENSG00000249002	AC096745.1	0	0	0	0	0	0	0	0
ENSG00000280551	AC096745.2	0.07659	0	0	0	0	0	0	0
ENSG00000172399	MYOZ2	0	0	0	0	0	0	0	0
ENSG00000178636	AC092656.1	0.17864	0	0	0.04419	0.10859	0	0.04963	0.10966
ENSG00000145390	USP53	1.32493	1.59275	0.53181	0.88303	1.74505	1.19032	0.83839	1.83007
ENSG00000164096	C4orf3	42.4015	46.3152	9.71752	13.5809	34.9333	16.2256	13.3513	31.0473
ENSG00000145384	FABP2	0	0	0	0	0	0	0	0
ENSG00000250412	KLHL2P1	0	0.23418	0	0.2088	0.17105	0.4964	0	0.17274
ENSG00000201186	RNU4-33P	0	0	0	1.27983	0	0	0	0
ENSG00000249244	AC110373.1	0	0	0	0	0	0	0	0
ENSG00000283050	GTF2IP12	0.21208	0.28682	0.11687	0.15738	0.3223	0.25722	0.30932	0.26039
ENSG00000260091	AC093752.3	0	0	0	0	0	0	0	0
ENSG00000223208	RNU6-1217P	0	0	0	0	0	0	0	0
ENSG00000245958	AC093752.1	1.74806	1.13761	0.44422	1.17037	1.78968	1.11296	1.18303	1.04892
ENSG00000250950	AC093752.2	0.38377	1.06465	0	0.56956	0.93316	0.22568	0.85292	0.31413
ENSG00000138735	PDE5A	16.6022	9.44864	4.24244	7.4119	9.81879	6.32338	9.43695	11.3797
ENSG00000221245	RF00614	0	0	0	0	0.70171	0	0	0.70864
ENSG00000250772	LINC01365	0	0	0	0	0	0	0	0
ENSG00000250183	AC097173.1	0	0	0	0	0	0	0	0
ENSG00000250392	LINC02502	0	0	0	0	0	0	0	0
ENSG00000200553	RF00019	0	0	0	0	0	0	0	0
ENSG00000253825	AC097173.2	0	0	0	0	0.18801	0	0	0

ENSG00000164109	MAD2L1	0.63762	0.4033	0.33731	1.022	0.74419	2.1147	0.5314	0.50103
ENSG00000250938	AC108866.1	0.10471	0.03486	0.03078	0.12433	0.10185	0.07389	0.10473	0.10285
ENSG00000250219	LTV1P1	0	0	0	0	0	0	0	0
ENSG00000225665	SAR1AP3	0	0	0	0	0	0	0	0
ENSG00000249409	AC025741.1	0	0	0	0	0	0	0	0
ENSG00000138738	PRDM5	2.03334	1.82045	0.47536	0.78872	1.32968	0.25816	0.61623	0.83217
ENSG00000212359	RNU6-550P	0	0	0	0	0	0	0	0
ENSG00000251609	SETP12	0	0	0	0	0	0.16064	0	0
ENSG00000173376	NDNF	0	0.03431	0	0	0.07518	0	0	0
ENSG00000223006	RN7SKP137	0	0	0	0	0	0	0	0
ENSG00000250344	AC105254.1	0	0	0	0	0	0	0	0
ENSG00000271302	AC105254.2	0	0	0	0	0	0	0	0
ENSG00000050730	TNIP3	0.60305	0.25904	0.14293	0.23097	0.14191	0.9266	0.06485	0.07165
ENSG00000252183	RNU6-948P	1.3701	0	0	0	0	1.20852	1.14187	0
ENSG00000207148	RF00019	0	0	0	0	0	0	0	0
ENSG00000186867	QRFPR	0	0	0	0	0	0	0	0
ENSG00000213480	AC093816.1	0	0	0	0	0	0	0	0
ENSG00000224062	TUBB4BP5	0	0	0	0	0	0	0	0
ENSG00000164111	ANXA5	71.73	60.3608	8.27203	10.7567	33.8079	42.4078	9.18165	23.4336
ENSG00000164112	TMEM155	0.04848	0	0	0.03597	0.05894	0.47038	0.08081	0
ENSG00000226757	PP12613	0	0	0	0	0	0.05799	0	0
ENSG00000123737	EXOSC9	4.73006	4.53226	3.61257	6.07316	5.14535	7.85039	4.51271	5.08156
ENSG00000145386	CCNA2	1.10029	1.35177	0.57745	1.98287	0.79625	12.1085	1.22268	0.6433
ENSG00000138686	BBS7	3.03584	2.17286	1.9629	3.24397	2.82354	2.27614	2.42893	1.45367
ENSG00000261501	AC079341.1	0	0	0	0	0	0	0	0
ENSG00000138741	TRPC3	0.01682	0.11199	0.04943	0.02496	0	0	0	0.02065
ENSG00000237868	AC097533.1	0	0	0	0	0	0	0	0
ENSG00000241037	RN7SL335P	0	0	0	0	0	0	0	0
ENSG00000138688	KIAA1109	15.7379	15.7532	4.74636	7.19038	11.5717	9.32409	6.76856	9.33384
ENSG00000164113	ADAD1	0	0	0	0	0	0	0	0
ENSG00000109471	IL2	0.39303	0	0	0.19443	0.15928	0	0	0.08043
ENSG00000138684	IL21	0	0	0	0	0.24789	0.89925	0	0
ENSG00000227145	IL21-AS1	0.01956	0	0	0.02903	0.07133	0.37952	0.0326	0
ENSG00000224786	CETN4P	1.09403	0	0	0	0	0.27572	0.52102	0
ENSG00000181004	BBS12	0.70481	0.39105	0.31385	0.19018	0.44143	1.05499	1.17481	0.47201
ENSG00000244083	AC021205.2	0	0	0	0	0	0	0	0
ENSG00000138685	FGF2	0.21638	0.14407	0.06359	0.1124	0.171	0.22904	0.21641	0.05313
ENSG00000240775	AC021205.1	0	0	0	0	0	0	0	0
ENSG00000273007	AC021205.3	0.04289	0	0	0.19096	0	0	0.07149	0.10532
ENSG00000170917	NUDT6	0.1557	0.14809	0.04903	0.21457	0.05408	0.13734	0.11122	0.04096
ENSG00000145375	SPATA5	1.78651	1.93805	1.12324	2.36118	2.49617	2.74398	1.67411	1.81151
ENSG00000250322	AC026402.1	0	0	0	0	0	0	0	0
ENSG00000164056	SPRY1	0.20346	0.03079	0	0.2196	0.15741	0.16315	0.18498	0.13626
ENSG00000249125	AC093821.1	0	0	0	0	0	0	0	0
ENSG00000251526	LINC02435	0	0	0	0	0	0	0	0
ENSG00000249464	LINC01091	0	0	0	0	0	0	0	0.01157
ENSG00000226655	AC096732.1	0	0	0	0	0	0	0	0
ENSG00000228489	RPL21P50	0	0	0	0	0	0	0	0
ENSG00000250458	AC093767.1	0	0	0	0	0	0	0	0
ENSG00000250484	PPIAP76	0	0	0	0	0	0	0	0
ENSG00000249837	AC121154.1	0	0	0	0	0	0	0	0
ENSG00000248506	AC097474.1	0	0	0	0	0	0	0	0
ENSG00000248565	TECRP2	0	0	0	0	0	0	0	0
ENSG00000261083	LINC02516	0	0	0	0	0	0	0	0
ENSG00000151458	ANKRD50	2.884	2.55283	0.4532	0.85356	1.82408	0.49995	0.66689	0.93129
ENSG00000196159	FAT4	0.19619	0.15586	0.00655	0.17206	0.16263	0.01573	0.08176	0.13139
ENSG00000201297	RF00019	0	0	0	0	0	0	0	0
ENSG00000283550	MIR2054	0	0	0	0	0	0	0	0

ENSG00000250149	AC105919.1	0	0	0	0	0	0	0	0
ENSG00000251436	NUP58P1	0	0	0	0	0	0	0	0
ENSG00000249555	TMEM248P1	0	0	0	0	0	0	0	0
ENSG00000251649	LINC02379	0	0	0	0	0	0	0	0
ENSG00000251005	AC105758.1	0	0	0	0	0	0	0	0
ENSG00000223620	RBM48P1	0	0	0	0	0	0	0	0
ENSG00000199862	RF00019	0	0	0	0	0	0	0	0
ENSG00000250945	AC096773.1	0	0	0	0	0	0	0	0
ENSG00000248491	AC093772.1	0	0	0	0	0	0	0	0
ENSG00000248209	AC097462.1	0	0	0	0	0	0	0	0
ENSG00000249795	AC097462.2	0	0	0	0	0	0	0	0
ENSG00000164066	INTU	0.14144	0.11391	0.16091	0.38596	0.13313	0.19317	0.25096	0.23527
ENSG00000151475	SLC25A31	0	0	0	0	0	0	0	0
ENSG00000164070	HSPA4L	0.05677	0.09449	0.00927	0.06553	0.00767	0.03338	0.03154	0.03098
ENSG00000282855	AC093591.3	0	0	0	0	0	0	0	0
ENSG00000244021	AC093591.1	0.15239	0	0	0.22617	0.18527	0	0	0
ENSG00000261668	AC093591.2	0	0	0	0.02831	0.02319	0	0.03179	0.04684
ENSG00000142731	PLK4	1.17752	0.92554	1.12953	1.6262	1.51113	5.04899	1.49931	1.24494
ENSG00000251821	RNU6-583P	0	1.32618	0	1.18245	0.96866	0	0	1.95646
ENSG00000164073	MFSD8	2.37865	2.98767	3.13085	7.32024	3.33544	3.3982	5.35132	4.49717
ENSG00000164074	ABHD18	5.0478	4.71794	3.41844	7.97751	6.93328	6.35502	7.13393	5.75393
ENSG00000138709	LARP1B	14.0565	14.7931	11.6633	21.4305	26.5059	16.4875	25.4311	25.4268
ENSG00000241651	AC099340.1	0	0	0	0	0	0	0	0
ENSG00000248394	FOSL1P1	0	0	0	0	0	0	0	0
ENSG00000164040	PGRMC2	2.82046	2.32096	2.35173	5.85552	3.14008	3.07486	3.88251	2.56801
ENSG00000251432	LINC02615	1.45127	1.37506	2.78882	4.53964	3.06737	3.97822	12.2441	4.63281
ENSG00000273077	AC110609.1	0	0	0	0	0	0	0	0
ENSG00000248187	AC078850.1	0	0	0	0	0	0	0.07528	0
ENSG00000248802	AC078850.2	0	0	0	0	0	0	0	0
ENSG00000280711	JADRR	0	0	0	0	0	0	0	0
ENSG00000077684	JADE1	6.67243	4.86484	1.2979	2.68121	6.52097	3.92369	3.35932	3.83491
ENSG00000151466	SCLT1	36.0402	62.5345	44.7769	93.2524	71.2332	53.7863	92.5488	74.9433
ENSG00000275647	AC093783.1	0.2228	0.37085	0	0	0.27087	0	0.37137	0
ENSG00000281369	AC093826.2	0	0	0	0	0	0	0	0
ENSG00000151470	C4orf33	1.95398	1.49576	0.52206	0.75987	1.07982	0.94011	0.9231	1.10331
ENSG00000251345	AC093826.1	0	0	0	0	0	0	0	0
ENSG00000248958	ZSWIM5P3	0	0	0	0	0	0	0	0
ENSG00000251195	AC105417.1	0	0	0	0	0	0	0	0
ENSG00000250193	AC082650.1	0	0	0	0	0	0	0	0
ENSG00000246876	LINC02466	0	0	0	0	0	0	0	0
ENSG00000249618	LINC02465	0	0	0	0	0	0	0	0
ENSG00000234125	EEF1GP8	0	0	0	0	0.07722	0	0	0
ENSG00000251326	LINC02479	0	0	0	0	0	0	0	0
ENSG00000248877	PGBD4P4	0	0	0	0	0	0	0	0
ENSG00000249018	GAPDHP56	0	0	0	0	0	0	0	0
ENSG00000280059	AC093831.2	0	0	0	0	0	0	0	0
ENSG00000250503	AC093831.1	0	0	0	0	0	0	0	0
ENSG00000250213	AC025554.1	0	0	0	0	0	0	0	0
ENSG00000206782	RNU6-224P	0	0	0	0	0	0	0	0
ENSG00000250102	LINC02377	0	0	0	0	0	0.14148	0	0
ENSG00000248715	AC105424.1	0	0	0	0	0	0	0	0
ENSG00000241392	RN7SL205P	0	0	0	0	0	0	0	0
ENSG00000252014	RF00156	0	0	0	0	0	0	0	0
ENSG00000249416	AC107393.1	0	0	0	0	0	0	0	0
ENSG00000251676	SNHG27	0	0	0	0	0	0	0	0
ENSG00000184139	RPL7AP28	0	0	0	0	0.62428	0	0	0
ENSG00000251555	AC096711.2	0.16145	0	0	0	0.19629	0	0	0
ENSG00000251598	AC096711.3	0	0	0	0	0	0	0	0

ENSG00000249463	AC096711.1	0	0	0	0	0	0	0	0
ENSG00000251051	ELL2P2	0	0	0	0	0	0	0	0
ENSG00000248369	AC114741.1	0	0	0	0	0	0	0	0
ENSG00000251398	LINC01256	0	0	0	0	0	0	0	0
ENSG00000249038	AARSP1	0	0	0	0	0	0	0	0
ENSG00000251488	AC115622.1	0	0	0	0	0	0	0	0
ENSG00000249513	AC110751.1	0	0	0	0	0	0	0	0
ENSG00000250241	AC105383.1	0	0	0	0	0	0	0	0
ENSG00000248735	R3HDM2P1	0	0	0	0	0	0	0	0
ENSG00000138650	PCDH10	0	0	0	0	0	0	0	0
ENSG00000250191	AC105252.1	0	0	0	0	0	0	0	0
ENSG00000251388	AC079380.1	0	0	0	0	0	0	0	0
ENSG00000254535	PABPC4L	0	0	0	0	0	0	0	0
ENSG00000251199	AC109830.1	0	0	0	0	0	0	0	0
ENSG00000248191	PES1P1	0	0	0	0	0	0	0	0
ENSG00000251291	LINC02462	0	0	0	0	0	0	0	0
ENSG00000249000	AC093722.1	0	0	0	0	0	0	0	0
ENSG00000249847	AC104619.2	0	0	0	0	0	0	0	0
ENSG00000251228	AC104619.4	0	0	0	0	0	0	0	0
ENSG00000241411	AC104619.1	0	0	0	0	0	0	0.19363	0
ENSG00000250144	AC104619.3	0	0	0	0	0	0	0	0
ENSG00000248434	AC107463.1	0	0	0	0	0	0	0	0
ENSG00000248456	LINC02485	0	0	0	0	0	0	0	0
ENSG00000283432	RF00026	0	0	0	0	0	0	0	0
ENSG00000250834	KRT18P54	0	0	0	0	0	0	0	0
ENSG00000248104	TARS2P1	0	0	0	0	0	0	0	0
ENSG00000250865	AC105362.1	0	0	0	0	0	0	0	0
ENSG00000248330	LINC00613	0	0	0	0	0	0	0	0
ENSG00000207322	RNU1-89P	0	0	0	0	0	0	0	1.64629
ENSG00000251492	AC104136.1	0	0	0	0	0	0	0	0
ENSG00000261166	AC073429.2	0	0	0	0	0	0	0	0
ENSG00000251567	AC018680.1	0	0	0	0	0	0	0	0
ENSG00000250665	AC073429.1	0	0	0	0	0	0	0	0
ENSG00000249311	TERF1P3	0	0	0	0	0	0	0	0.07335
ENSG00000279749	AC093875.1	0	0	0	0	0	0	0	0
ENSG00000248869	LINC02511	0	0	0	0	0	0	0	0
ENSG00000250341	LINC02510	0	0	0	0	0	0	0	0
ENSG00000250813	SERF1AP1	0	0	0	0	0	0	0	0
ENSG00000251440	STMN1P2	0	0	0	0	0	0	0	0
ENSG00000213448	RPS23P2	0	0	0	0	0	0	0	0
ENSG00000189184	PCDH18	0	0	0	0	0	0.01934	0	0
ENSG00000251632	LINC02172	0	0	0	0	0	0	0	0
ENSG00000280352	AC116563.2	0	0	0	0	0	0	0	0
ENSG00000250126	AC116563.1	0	0	0	0	0	0	0	0
ENSG00000250777	AC131956.2	0	0	0	0	0	0	0	0
ENSG00000250034	AC131956.1	0	0	0	0	0	0	0	0
ENSG00000248307	LINC00616	0	0	0	0	0	0	0	0
ENSG00000250033	SLC7A11-AS1	0	0	0	0	0	0	0	0
ENSG00000151012	SLC7A11	0.33439	0.56925	0.18985	0.15791	0.18479	1.15301	0.11401	0.13996
ENSG00000249091	AC093903.1	0	0	0	0	0	0	0	0
ENSG00000248397	LINC00498	0	0	0.47347	0	0	0	0	0
ENSG00000251372	LINC00499	0	0	0	0	0	0	0	0
ENSG00000249381	LINC00500	0	0	0	0	0	0	0	0
ENSG00000248305	AC105416.1	0	0	0	0	0	0	0	0
ENSG00000248795	AC098859.1	0	0	0	0	0	0	0	0
ENSG00000250977	AC098859.2	0.1302	0	0	0	0	0	0	0
ENSG00000250501	AC093766.1	0.20629	0	0.70731	0	0	0	0	0.16885
ENSG00000250195	AC109927.1	0.22078	0	0	0.32767	0	0	0	0.27108

ENSG00000250577	AC109927.2	0	0	0	0	0	0	0	0
ENSG00000151014	NOCT	2.10768	0.48725	0.08603	0.52134	0.85415	0.46478	0.63432	0.17971
ENSG00000109381	ELF2	13.093	14.852	16.0942	37.4396	18.271	14.5778	39.9655	23.6347
ENSG00000252503	RNU6-531P	0	1.17316	0	0	0	0	0	0
ENSG00000240723	RN7SL382P	0	0	0	0	0	0	0	0
ENSG00000179967	PPP1R14BP3	0.98832	0.27418	0	0.24446	0.40052	0	0.27456	0.80896
ENSG00000276396	RF00017	0	0	0	0	0	0	0	0
ENSG00000265892	RN7SL311P	0	0	0	0	0	0	0	0
ENSG00000207384	RF00019	0	0	0	0	0	0	0	0
ENSG00000252362	RNU6-506P	0	0	0	0	0	0	0	0
ENSG00000206722	RNU6-1074P	0	0	0	0	0	0	0	0
ENSG00000137463	MGARP	0.10949	0	0.08044	0	0	0	0	0
ENSG00000109390	NDUFC1	5.51358	3.39436	0.83241	2.1858	4.26989	4.59686	1.82547	2.87471
ENSG00000164134	NAA15	4.84543	3.89442	1.89653	4.92851	5.1844	6.47092	4.27732	4.36456
ENSG00000200520	RNU6-1214P	0	0	0	0	0	0	0	0
ENSG00000273247	AC097376.2	0.86626	1.33452	0.2573	0.3556	0.88512	0.5365	0.47619	0.97306
ENSG00000248863	AC097376.1	0.35468	0.19679	0.08687	0.08773	0	0	0.09853	0.36289
ENSG00000239005	RF01225	0.57717	0	0	0	0	0	0	0.70864
ENSG00000172007	RAB33B	6.00631	12.7395	3.42765	3.74302	6.85922	4.32068	2.6973	9.50446
ENSG00000145391	SETD7	2.63367	1.70089	0.30187	0.67229	1.61378	1.21729	0.67605	0.80193
ENSG00000249302	FTH1P24	0	0	0	0	0	0	0	0
ENSG00000272717	AC112236.2	0	0	0	0	0	0	0.14459	0
ENSG00000261268	AC112236.1	0	0	0	0	0	0	0	0
ENSG00000085871	MGST2	3.22503	3.41256	1.7941	3.14528	2.52058	2.35703	2.64942	1.83841
ENSG00000201533	RN7SKP237	0	0	0	0	0	0	0.40591	0
ENSG00000178458	H3F3AP6	0.89173	1.18743	0	0.26469	1.30097	0.31463	0.29728	1.09485
ENSG00000196782	MAML3	6.77391	4.78499	0.76807	0.9774	3.73649	3.52233	1.5682	1.86103
ENSG00000252233	RN7SKP253	0.23722	0	0.34859	0	0.2884	0.41848	0	0
ENSG00000250698	AC131182.1	0	0	0	0	0	0	0	0
ENSG00000153130	SCOC	2.29428	2.36496	2.37881	4.04446	2.93091	1.35599	3.86303	3.7749
ENSG00000196951	SCOC-AS1	0.18249	0	0.13408	0.13542	0.07396	0	0.15209	0.03734
ENSG00000249754	NDUFB4P9	0	0	0	0	0	0	0	0
ENSG00000241923	RPL14P3	0	0	0	0	0.14327	0	0.39286	0
ENSG00000153132	CLGN	0.04688	0	0	0.06958	0.0855	0	0.19536	0.20146
ENSG00000205301	MGAT4D	0	0	0	0	0	0	0	0
ENSG00000280262	AC093671.1	0	0	0	0	0	0	0	0
ENSG00000179387	ELMOD2	4.97739	3.90309	1.6904	3.1682	4.16873	3.06352	3.31861	3.77534
ENSG00000109424	UCP1	0	0	0	0	0	0	0	0
ENSG00000242102	RN7SL152P	0	0	0	0	0	0	0	0
ENSG00000248621	AC108019.1	0	0	0	0	0	0	0	0
ENSG00000109436	TBC1D9	8.41194	4.65997	0.6344	1.90273	5.5509	2.79256	1.83172	2.23266
ENSG00000249661	TNRC18P1	0.32132	0.10028	0	0.05961	0.19532	0	0.03347	0.04931
ENSG00000207155	RNY1P14	0	0	0	0	0	0	0	0
ENSG00000248335	AC096733.1	0	0	0	0	0	0	0	0
ENSG00000273472	AC096733.2	0.36808	0.08752	0	0	0.31964	0	0.08765	0.12912
ENSG00000222613	RF00019	0	0	0	0	0	0	0	0
ENSG00000170153	RNF150	0.01998	0	0.03914	0.04942	0.14573	0	0	0
ENSG00000109445	ZNF330	10.232	10.0551	2.03875	3.90359	8.996	7.18965	4.14334	5.07476
ENSG00000248747	AC107220.1	0	0	0	0	0	0	0	0
ENSG00000248810	LINC02432	1.95468	2.8306	0.37341	0.2901	1.8061	0.37931	0.19549	0.38399
ENSG00000250189	AC097504.1	0.0607	0.10104	0.0446	0.09009	0.0738	0.05355	0	0.03727
ENSG00000272632	AC097504.2	2.26036	1.61245	0.47451	0.47923	0.98146	0.85448	0.80736	0.39646
ENSG00000250141	LINC02276	0	0	0	0	0	0	0	0
ENSG00000164136	IL15	0.59685	0.74509	0.23297	0.45673	0.3288	0.74033	0.32644	0.60685
ENSG00000109452	INPP4B	5.2879	2.81896	1.17966	3.21292	5.45291	5.56425	2.48625	3.17308
ENSG00000242134	RPL5P13	0.10339	0	0	0.15344	0	0.54716	0	0.38081
ENSG00000250835	LSM3P4	0	0	0	0	0	0	0	0
ENSG00000251248	AC139720.2	0	0	0	0	0	0	0	0

ENSG00000249806	AC139720.1	1.28848	0.71489	0.21038	0.21247	1.74056	1.26281	0.7159	0.7031
ENSG00000250326	AC104596.1	0.50502	0.84061	0.10602	0.21415	0.43857	0.25455	0.12026	0.26574
ENSG00000251300	AC104596.2	0	0	0	0	0	0	0	0
ENSG00000170185	USP38	8.5754	7.55209	2.891	5.25278	7.94234	8.20342	6.74683	6.25252
ENSG00000250969	AC097658.2	0	0	0	0	0	0	0	0
ENSG00000200049	RF00019	0	0	0	0	0	0	0	0
ENSG00000109458	GAB1	6.59189	7.57203	4.55594	9.69708	8.64834	10.3383	9.98549	6.8216
ENSG00000265623	MIR3139	5.78687	3.21075	0	1.43139	0	0	0	1.18417
ENSG00000228981	AC097658.1	0	0	0	0	0.21143	0	0.28987	0.21352
ENSG00000243175	RPSAP36	0.05813	0	0	0	0	0.10255	0.09689	0.07137
ENSG00000250540	AC104685.1	0	0	0	0	0	0	0	0
ENSG00000153147	SMARCA5	90.041	121.63	91.7849	176.133	124.933	109.371	113.948	110.154
ENSG00000245112	SMARCA5-AS1	0.07757	0	0	0	0	0	0	0
ENSG00000248924	AC139713.1	0	0	0	0	0	0	0	0
ENSG00000236296	GUSBP5	0.33707	0.21579	0	0.11544	0.31523	0.09148	0	0.0955
ENSG00000183090	FREM3	0	0	0	0	0.02595	0	0	0
ENSG00000246448		4.79172	4.95794	3.61584	3.07521	5.6682	3.65546	10.5774	7.95027
ENSG00000250345	AC104090.1	0	0	0	0	0	0	0	0
ENSG00000197465	GYPE	4.57213	11.385	7.04118	25.6765	8.09343	4.77499	25.3628	9.7901
ENSG00000249741	AC093890.1	0.27148	0.22594	0	0.60437	0.99019	0.23947	0.45252	0.83331
ENSG00000251600	AC139713.2	0.30526	0.31757	0.33643	0.62293	0.83503	0.20194	0.63602	1.17123
ENSG00000248828		0.2643	0.14664	0.64732	0.65376	0.10711	0.46627	0.88111	0.75719
ENSG00000250361	GYPB	24.4334	20.2568	40.9258	143.728	44.9567	39.9661	113.442	45.056
ENSG00000170180	GYPA	23.7308	35.6785	56.1266	135.734	45.0405	28.6091	71.2487	38.2334
ENSG00000261129	AC106871.1	0	0	0	0	0	0	0	0
ENSG00000250504	KRT18P51	0	0	0	0	0	0	0	0
ENSG00000248890	HHIP-AS1	0	0	0	0.06987	0.05724	0	0	0
ENSG00000164161	HHIP	0	0	0	0	0.01476	0	0	0
ENSG00000273621	RF02271	0	0	0	0	0	0	0	0
ENSG00000249193	HSPD1P5	0	0	0	0	0	0	0	0
ENSG00000250406	AC109811.1	0	0	0	0	0	0	0	0
ENSG00000251452	AC109811.2	0	0	0	0	0	0	0	0
ENSG00000164162	ANAPC10	3.10958	2.64149	2.86775	6.49277	4.1195	3.93459	3.61036	3.2123
ENSG00000222594	RN7SKP235	0	0	0	0	0	0	0	0
ENSG00000164163	ABCE1	9.35998	5.53079	2.6821	6.00796	10.8675	13.2717	4.97302	5.88967
ENSG00000164164	OTUD4	8.71374	7.48032	3.24861	9.4716	9.32856	9.67881	9.26607	6.97391
ENSG00000238713	RF00019	2.5947	0	0.95322	0	2.36593	4.57741	1.08124	2.3893
ENSG00000240992	RPS23P4	0	0	0	0	0	0	0	0
ENSG00000272727	LINC02266	0	0	0	0	0	0	0	0
ENSG00000251333	RTN3P1	0	0	0.1515	0	0	0	0	0
ENSG00000278943	AC079228.1	0	0	0	0	0	0	0	0
ENSG00000248745	NMNAT1P4	0	0	0	0	0	0	0	0
ENSG00000170365	SMAD1	0.39929	0.26182	0.05334	0.2514	0.47074	0.4696	0.60505	0.28226
ENSG00000250582	SMAD1-AS2	0	0	0	0	0	0	0	0
ENSG00000250902	SMAD1-AS1	0	0	0	0	0	0	0	0
ENSG00000248686		0	0	0	0	0	0	0	0
ENSG00000151611	MMAA	0.72565	0.60392	0.14031	0.48179	0.82419	0.67376	0.47745	0.46892
ENSG00000249245	NCOA4P3	0.03905	0	0	0	0.04748	0	0.06509	0
ENSG00000248356	AC093864.1	0	0	0	0	0	0	0	0
ENSG00000237136	C4orf51	0	0.10874	0	0	0	0	0	0
ENSG00000151612	ZNF827	1.12997	0.7668	0.281	0.5031	1.0779	1.38006	0.56505	0.46957
ENSG00000279481	AC104791.2	0.08709	0.19328	0.04266	0.04308	0.07059	0.10243	0.04839	0.03564
ENSG00000251687	AC104791.1	0	0	0	0	0	0.16286	0	0
ENSG00000201314	RF00019	0	0	0	0	0	0	0	0
ENSG00000260303	AC108206.1	0.01762	0.02934	0	0	0	0	0	0
ENSG00000251024	AC097372.2	0	0	0	0	0	0	0	0
ENSG00000248809	LINC01095	0.03248	0	0	0.0482	0	0	0	0
ENSG00000164167	LSM6	4.09982	3.70279	0.81049	1.85539	3.01749	3.53524	1.25643	2.48299

ENSG00000279845	AC097372.3	0.47189	0.52364	0.23115	0.23345	0.19124	1.10997	0.78657	0.57938
ENSG00000250673	REELD1	0	0.05399	0	0	0	0	0	0.03982
ENSG00000280250	AC097372.4	0.3899	0	0	0	0	0	0	0
ENSG00000251010	AC097372.1	0	0	0	0	0	0	0	0
ENSG00000120519	SLC10A7	2.70752	2.60912	2.77057	6.63744	3.34504	3.98358	4.09278	4.15868
ENSG00000278438	MIR7849	0	0	0	0	0	0	0	0
ENSG00000207182	RNU1-44P	0	0	0	0	0	0	0	0
ENSG00000151615	POU4F2	0	0	0	0	0	0	0	0
ENSG00000137473	TTC29	0	0	0	0	0	0	0	0
ENSG00000248764	AC092435.2	0	0	0	0	0	0	0	0
ENSG00000248346	AC092435.1	0	0	0	0	0	0	0	0
ENSG00000260090	AC092435.3	0	0	0	0	0	0	0	0
ENSG00000279376	AC092435.4	0	0	0	0	0	0	0	0
ENSG00000221369	MIR548G	0	0	0	0	0	0	0	0
ENSG00000241281	AC010683.1	0	0	0	0	0	0	0	0
ENSG00000249004	PRMT5P1	0	0	0	0	0	0	0	0
ENSG00000151617	EDNRA	0	0	0	0	0	0	0	0
ENSG00000280219	AC093908.1	0	0	0	0	0	0	0	0
ENSG00000251254	GTF2F2P1	0	0	0	0.16116	0	0.38315	0.18101	0
ENSG00000251688	LINC02507	0	0	0	0	0	0	0	0
ENSG00000251298	AC093835.1	0	0	0.18964	0	0	0	0.21511	0
ENSG00000164168	TMEM184C	6.7669	6.00721	1.94306	3.60156	6.52488	5.76305	3.37085	4.46929
ENSG00000164169	PRMT9	2.49867	2.21613	0.80404	1.46166	2.54999	2.76706	1.15523	1.74665
ENSG00000071205	ARHGAP10	2.47991	2.82369	1.47169	2.15367	2.0459	2.40379	1.34002	1.98246
ENSG00000252951	RNA5SP165	0	0	0	0	0	0	0	0
ENSG00000264274	MIR4799	0	0	0	0	0	0	0	0
ENSG00000240014	RN7SL254P	0	0	0	0	0	0	0	0
ENSG00000270387	AC115621.1	0	0	0	0	0	0	0	0
ENSG00000151623	NR3C2	1.59759	0.68326	0.30976	0.62568	1.68603	0.84159	1.14654	1.04885
ENSG00000250354	AC069272.1	0	0	0	0	0	0	0	0
ENSG00000251418	ASS1P8	0	0	0	0	0	0	0	0
ENSG00000201846	RNA5SP166	0	0	0	0	0	0	0	0
ENSG00000250357	AC108935.1	0	0	0	0	0	0	0	0.17374
ENSG00000225176	ATP5MGP4	0	0	0	0	0	0	0	0
ENSG00000279016	AC093648.1	0	0	0	0	0	0	0	0
ENSG00000249317	AC002460.1	0	0	0	0	0	0	0	0
ENSG00000239168	RNU7-197P	0	0	0	0	0	0	0	0
ENSG00000249752	AC108156.1	0	0	0	0	0	0	0	0
ENSG00000250252	LINC02430	0	0	0	0	0	0	0	0
ENSG00000248210	LINC02355	0	0	0	0	0	0	0	0
ENSG00000234828	IQCM	0	0	0	0	0	0	0	0
ENSG00000250905	AC093893.1	0	0	0	0	0	0	0	0
ENSG00000251482	AKIRIN2P1	0	0	0	0	0	0	0	0
ENSG00000202331	RNA5SP167	0	0	0	0	0	0	0	0
ENSG00000207121	RNU6-1230P	0	0	0	0	0	0	0	0
ENSG00000250704	AC105343.1	0	0	0	0	0	0	0	0
ENSG00000170390	DCLK2	0.1114	0.02649	0.02339	0.21256	0.01935	0.1123	0.10611	0.05862
ENSG00000238721	RNU7-194P	0	0	0	0	0	0	0	0
ENSG00000198589	LRBA	12.319	8.44228	5.62776	11.7974	13.8251	17.3149	11.1222	9.70562
ENSG00000248521	AC092612.1	0.07345	0.12225	0	0	0.0893	0.25914	0.36728	0
ENSG00000249690	AC110813.1	0	0	0	0	0	0	0	0
ENSG00000181541	MAB21L2	0	0	0	0	0	0	0	0
ENSG00000183432	ZBTB8OSP1	0	0	0	0	0	0	0	0
ENSG00000252451	RNA5SP168	0	0	0	0	0	0	0	0
ENSG00000235606	AK4P6	0	0	0	0	0	0	0	0
ENSG00000145425	RPS3A	186.241	120.914	35.3372	52.2785	176.14	132.239	108.297	150.354
ENSG00000201264	SNORD73B	6.10836	1.69456	4.48808	4.53274	3.71319	1.796	1.69695	6.2498
ENSG00000109686	SH3D19	0.02823	0.01566	0	0	0.06865	0	0.04706	0.01155

ENSG00000208797	SNORD73A	0	0	0	0	0	0	0	0
ENSG00000270681	AC095055.1	0.85616	0.43849	0.09678	0.09774	0.32028	0.34855	0.21955	0.16172
ENSG00000249012	AC104819.1	0	0	0	0	0	0	0	0
ENSG00000189099	PRSS48	0	0	0	0	0	0	0	0
ENSG00000250350	AC104819.2	0	0	0	0	0	0	0	0
ENSG00000252544	RNU6-1282P	0	0	0	0	0	0	0	0
ENSG00000270265	AC104819.3	0.40722	0.45188	0.39894	0	0.66012	0	0.67878	0.33332
ENSG00000251611	FAM160A1-DT	0.4095	0	0	0	0	0	0	0
ENSG00000164142	FAM160A1	0.31211	0.34633	0.41496	0.13234	0.16262	0.02622	0.22296	0.25547
ENSG00000253019	RN7SKP35	0	0	0	0	0	0	0	0
ENSG00000251603	AC092611.2	0.48224	0	0	0.35785	0.29315	0	0	0.29604
ENSG00000278978	AC092611.3	0	0	0.45006	0	0	0	0.34034	0.12534
ENSG00000059691	GATB	0.37408	0.29057	0.05497	0.08328	0.35625	0.36296	0.09353	0.12248
ENSG00000251455	AC092611.1	0	0	0	0.04241	0	0	0	0
ENSG00000249184	AC107074.1	0	0	0	0	0	0	0	0
ENSG00000249708	AC112242.1	0	0	0	0	0	0	0	0
ENSG00000249818	AC097375.2	0	0	0	0	0	0	0	0
ENSG00000248991	AC097375.1	1.20164	0.50003	0	0	0.36523	0	0	0.18442
ENSG00000251249	AC097375.3	0	0	0	0	0	0	0	0
ENSG00000253077	RNA5SP169	0	0	0	0	0	0	0	0
ENSG00000245954	LINC02273	0.72899	0.60671	0.16069	0.43276	1.94984	2.12197	0.24302	0.31327
ENSG00000250706	AC079340.1	0	0	0	0	0	0	0	0
ENSG00000251377	AC079340.2	0	0	0	0	0	0	0.15525	0
ENSG00000244544	RN7SL446P	0	0	0	0	0	0.43686	0	0
ENSG00000270302	AC080078.1	0.35756	0	0	0	0	0	0	0.43901
ENSG00000270883	AC080078.2	0.9499	0	0	0.46992	1.15486	0	0.26389	0.38876
ENSG00000270750		1.902	1.21765	0	0.43427	2.31241	0.77432	0.48775	0.71854
ENSG00000109670	FBXW7	25.9939	24.4918	14.9001	29.6208	60.468	47.6041	66.9753	37.969
ENSG00000270751	FBXW7-AS1	0	0	0	0	0	0	0	0
ENSG00000264678	MIR3140	8.95893	10.8452	1.19682	4.83492	5.94111	4.31039	4.07268	4.99984
ENSG00000268471	MIR4453HG	1.00177	0.73205	0.25133	0.32636	0.68323	1.33622	0.65163	0.44999
ENSG00000283792	MIR4453	0	0	0	0	0	0	0	0
ENSG00000243417	AC023424.2	0.47169	0.47108	0.27726	0	0	0	0.47174	0.23165
ENSG00000240669	AC023424.1	1.11787	0.79744	0.23467	0.23701	0.58246	0	0.79856	0.19607
ENSG00000170006	TMEM154	59.0224	54.5126	10.235	13.7919	33.9792	14.5416	14.9944	32.3033
ENSG00000248571	AC106882.1	0.12366	0.10292	0	0.18353	0.03759	0	0.05153	0
ENSG00000169989	TIGD4	0.0593	0.09871	0.04357	0.08801	0.18025	0	0	0.21844
ENSG00000164144	ARFIP1	12.0708	10.6604	5.0211	5.86102	8.68413	5.3009	5.23753	12.8387
ENSG00000250980	AC113155.1	0.09532	0	0	0	0	0	0	0
ENSG00000231841	FAM192BP	0.19239	0.16012	0.14136	0.28553	0.11695	0.1697	0.32068	0.23621
ENSG00000137460	FHDC1	1.14514	1.03632	0.99659	2.27703	2.01401	1.82406	6.41201	1.55615
ENSG00000249022	AC093599.1	0	0.43419	0.19166	0.19357	0.31714	0	0.43481	0.16014
ENSG00000109654	TRIM2	0.27998	0.15534	0.04571	0.16159	0.16074	0.05488	0.06482	0.06684
ENSG00000252030	RNU6-1196P	0	0	0	0	0	0	0	0
ENSG00000201786	RF00019	0	0	0	0	0	0	0	0
ENSG00000213406	ANXA2P1	0	0.13176	0	0	0	0	0	0
ENSG00000121211	MND1	7.51923	8.9629	10.194	24.1906	12.1496	9.15709	15.6061	15.5455
ENSG00000243883	RN7SL419P	0	0	0	0	0	0	0	0
ENSG00000121210	TMEM131L	17.8177	17.5198	8.55916	14.498	17.7313	22.088	15.0026	13.824
ENSG00000250771	AC106865.1	0.24605	0.26062	0	0.16598	0.13597	0.15784	0.07457	0.21971
ENSG00000248208	WDR45P1	0	0	0	0	0	0	0	0.08318
ENSG00000137462	TLR2	45.9523	48.8324	11.233	15.2041	28.3052	8.46684	14.8071	27.5348
ENSG00000145428	RNF175	0.20037	0.33351	0.07361	0.34692	0.2639	0.2651	0.27832	0.22551
ENSG00000249309	AC020703.1	1.12553	3.7469	0.41349	0.4176	1.0263	0.4964	1.17256	0.69096
ENSG00000145423	SFRP2	1.37077	0.84061	0.53009	1.98084	1.79812	2.67278	4.62986	1.24012
ENSG00000280241	AC079298.3	0	0.08867	0.07828	0.07906	0	0	0	0
ENSG00000197410	DCHS2	0	0	0	0	0	0	0	0
ENSG00000278981	AC079298.1	0	0	0	0	0	0	0	0

ENSG00000280005	AC079298.2	0	0	0	0	0	0	0	0
ENSG00000249200	AC110775.1	0	0	0	0	0	0	0	0
ENSG00000250609	AC110753.1	0	0	0	0	0	0	0	0
ENSG00000171566	PLRG1	4.9796	3.04478	1.13069	2.88716	4.8362	6.17234	3.02486	3.35105
ENSG00000200350	RNU6-1285P	1.3962	0	1.02585	1.03605	0	0	4.65449	0
ENSG00000248611	AC107385.1	0	0	0	0	0	0	0	0
ENSG00000171564	FGB	0	0	0	0	0	0	0	0
ENSG00000171560	FGA	0	0	0	0	0	0	0	0
ENSG00000171557	FGG	0	0	0	0	0	0	0	0
ENSG00000121207	LRAT	0	0	0	0	0	0	0	0
ENSG00000249074	NDUFB2P1	0	0	0	0	0	0	0	0
ENSG00000249041	AC009567.1	0	0	0	0	0	0	0	0
ENSG00000249692	AC009567.2	0	0	0	0	0	0	0	0
ENSG00000151962	RBM46	0	0	0	0	0.02213	0	0	0
ENSG00000222582	RNU2-66P	0	0	0	0	0	0	0	0
ENSG00000252604	RNU2-44P	0	0	0	0	0	0	0	0
ENSG00000250910	AC097467.3	0	0	0	0	0	0	0	0
ENSG00000250538	AC104407.1	0	0	0	0	0	0	0	0
ENSG00000185149	NPY2R	0	0	0	0	0	0	0	0
ENSG00000249924	AC097467.1	0	0	0	0	0	0	0	0
ENSG00000164114	MAP9	0.69518	0.42704	0.19458	0.35619	0.77475	0.3212	0.30349	0.30483
ENSG00000250205	YWHAEP4	0	0	0	0	0	0	0	0
ENSG00000250398	AC097467.2	0	0	0	0	0	0	0	0
ENSG00000271606	AC097467.4	0	0	0	0	0	0	0	0
ENSG00000249247	MTCYBP17	0	0	0	0	0	0	0	0
ENSG00000249770	MTND6P17	0	0	0	0	0	0	0	0
ENSG00000248748	MTND5P9	0	0	0	0	0	0	0	0
ENSG00000248548	MTND4P8	0	0	0	0	0	0	0	0
ENSG00000249127	MTND3P3	0	0	0	0	0	0	0	0
ENSG00000249698	MTCO3P9	0	0	0	0	0	0	0	0
ENSG00000248420	MTATP6P9	0	0	0	0	0	0	0	0
ENSG00000249386	MTCO2P9	0	0	0	0	0	0	0	0
ENSG00000251223	MTCO1P9	0	0	0	0	0	0	0	0
ENSG00000248907	MTND2P33	0	0	0	0	0	0	0	0
ENSG00000251407	MTND1P22	0	0	0	0	0	0	0	0
ENSG00000251244	AC107208.1	0	0	0	0	0	0	0	0
ENSG00000164116	GUCY1A1	6.44524	3.0682	0.72855	1.58481	5.76492	3.00516	3.92011	4.02692
ENSG00000260244	AC104083.1	1.822	1.02646	0.37072	0.45761	2.59001	0.8901	1.07463	1.7552
ENSG00000061918	GUCY1B1	9.34953	6.61808	3.00812	4.23575	6.93981	5.10441	4.95414	6.6217
ENSG00000256394	ASIC5	0	0	0	0	0	0	0	0
ENSG00000151790	TDO2	0	0	0	0	0	0	0	0
ENSG00000256043	CTSO	5.13648	3.05641	0.4011	1.14162	3.89169	1.61968	0.78586	1.31005
ENSG00000249013	FTH1P21	0	0	0	0	0	0	0	0
ENSG00000251511	AC096736.3	0	0	0	0	0	0	0	0
ENSG00000251283	LINC02272	0	0	0	0	0	0	0	0
ENSG00000249479	AC096736.2	0	0	0	0	0	0	0	0
ENSG00000248912	AC096736.1	0	0	0	0	0	0	0	0
ENSG00000145431	PDGFC	2.52941	2.09419	0.78961	1.32262	1.38622	1.01729	1.04857	0.80455
ENSG00000248629	AC092608.1	0	0	0	0	0	0	0	0
ENSG00000280284	AC092608.3	0	0	0	0	0	0	0	0
ENSG00000278880	AC092608.2	0	0	0	0	0	0	0	0
ENSG00000250622	AC093325.1	0	0	0	0	0	0	0	0
ENSG00000252350	RPPH1-3P	0	0	0	0	0	0	0	0
ENSG00000252524	RF00019	0	0	0	0	0	0	0	0
ENSG00000109738	GLRB	0	0	0	0	0	0	0	0
ENSG00000199859	RNU6-582P	0	0	0	0	0	0	0	0
ENSG00000120251	GRIA2	0	0.01365	0	0	0	0	0	0
ENSG00000249819	AC112240.1	0	0	0	0	0	0	0	0

ENSG00000234111	LINC02433	0	0	0	0	0	0	0	0
ENSG00000249275	AC093817.1	0	0	0	0	0	0	0	0
ENSG00000200521	RF00019	0	0	0	0	0	0	1.20971	0
ENSG00000249627	AC017037.1	0	0	0	0	0	0	0	0
ENSG00000279703	AC017037.3	0	0	0	0	0	0	0	0
ENSG00000279994	AC017037.4	0	0	0	0	0	0	0	0
ENSG00000250475	AC017037.2	0	0	0	0	0	0	0	0
ENSG00000279139	AC098679.3	0	0	0	0	0	0	0	0
ENSG00000280140	AC098679.4	0	0	0	0	0	0	0	0
ENSG00000164125	FAM198B	7.92537	6.92104	1.44894	1.73947	2.92909	3.61023	2.31026	3.06081
ENSG00000248429	FAM198B-AS1	0.45496	0.12982	0.0573	0.03858	0.11063	0.11466	0.10834	0.09576
ENSG00000251073	NUDT19P5	0	0	0	0	0	0	0	0.15817
ENSG00000250604	AC098679.1	0	0	0	0	0	0	0	0
ENSG00000164124	TMEM144	0.17447	0.132	0.19811	0.25893	0.26032	0.02798	0.18506	0.21421
ENSG00000251429	AC098679.2	0.04054	0.03374	0.08936	0.09025	0	0.07152	0	0.04978
ENSG00000171509	RXFP1	0.0129	0.08586	0.01895	0.01914	0	0	0	0
ENSG00000199634	RNU4-79P	0	0	0	0	0	0	0	0
ENSG00000248387	AC121161.1	0	0	0	0	0	0	0.18739	0
ENSG00000249901	AC121161.2	0	0	0	0	0	0	0	0
ENSG00000205208	C4orf46	1.8276	1.37382	0.57755	0.90412	1.43351	2.18409	0.75339	0.77209
ENSG00000171503	ETFDH	4.53539	3.23147	1.65419	2.49386	3.7884	4.46101	5.90099	2.42369
ENSG00000271817	RF00012	0	0	0	0	1.22078	0	0	1.23284
ENSG00000171497	PPID	11.0433	13.0324	2.40414	4.68269	12.1829	10.0501	3.40878	7.28155
ENSG00000052795	FNIP2	2.71807	1.91971	0.70369	1.71166	1.82858	1.54679	1.45024	1.6065
ENSG00000206703	RNU6-128P	0	0	0	0	0	0	0	0
ENSG00000164123	C4orf45	3.46622	2.54538	2.39697	5.2199	4.524	1.4388	4.6731	4.31836
ENSG00000274535	RF00157	0	0	0	0	0	0	0	0
ENSG00000248988	AC093700.1	0	0	0	0	0	0	0	0
ENSG00000249367	FABP5P12	0	0	0	0	0	0	0	0
ENSG00000206978	RF00019	0	0	0	0	0	0	0	0
ENSG00000109756	RAPGEF2	11.9125	12.9885	6.91774	11.7077	12.7087	9.19886	16.8233	11.9346
ENSG00000264105	MIR3688-1	0	0	0	0	0	0	0	0
ENSG00000283379	MIR3688-2	0	0	0	1.25041	0	0	0	1.03445
ENSG00000248552	AC074344.1	0	0	0	0	0	0	0	0
ENSG00000250180	AC074344.2	0	0	0	0	0	0	0	0
ENSG00000250488	LINC02233	0	0	0	0	0	0	0	0
ENSG00000250997	AC093853.1	0	0	0	0	0	0	0	0
ENSG00000249425	LINC02477	0	0	0	0	0	0	0	0
ENSG00000242635	RPS14P7	0	0	0	0	0	0	0	0
ENSG00000249568	AC104793.1	0	0	0	0	0	0	0	0
ENSG00000168843	FSTL5	0	0	0	0	0	0	0	0
ENSG00000249419	AC023136.1	0	0	0	0	0	0	0	0
ENSG00000251253	MTHFD2P4	0	0	0	0	0	0	0	0
ENSG00000248396	TOMM22P4	0	0	0	0	0	0	0	0
ENSG00000248431	AC005150.1	0	0	0	0	0	0	0	0
ENSG00000251123	AC084752.1	0	0	0	0	0	0	0	0
ENSG00000283468	MIR4454	0	0	0	0	0	0	0	0
ENSG00000250027	AC022272.1	0	0	0	0	0	0	0	0
ENSG00000145414	NAF1	0.80801	0.50752	0.49287	1.2218	0.88968	0.83375	2.61742	0.52411
ENSG00000250547	AC079238.1	0	0	0	0	0	0	0	0
ENSG00000164128	NPY1R	0	0	0	0	0	0	0	0
ENSG00000164129	NPY5R	0	0	0	0	0	0	0	0
ENSG00000227283	RPL35AP12	0	0	0	0	0	0	0	0
ENSG00000151005	TKTL2	0.05524	0	0	0	0	0	0	0
ENSG00000198498	TMA16	2.71251	1.63234	0.3986	1.4864	2.20699	1.546	1.25206	1.30653
ENSG00000145416	MARCH1	14.7923	12.5626	2.69776	3.47622	10.4501	6.55183	3.6463	5.67393
ENSG00000273449	AC093788.1	0.07907	1.31616	0	0.46941	0.2884	0.69747	0.79081	0.19417
ENSG00000253001	RN7SKP105	0	0	0	0	0	0	0	0

ENSG00000177803	AC112247.1	0	0	0.16395	0.16558	0	0	0.18597	0
ENSG00000250746	AC105250.1	0	0	0	0	0	0	0	0
ENSG00000248546	ANP32C	0	0	0	0.15431	0	0	0	0
ENSG00000251162	AC074198.2	0	0	0	0	0	0	0	0
ENSG00000223037	RNU6-284P	0	0	0	0	0	0	0	0
ENSG00000248162	AC074198.1	0	0	0	0	0	0	0	0
ENSG00000248771	SMIM31	0	0	0	0	0.02794	0	0	0
ENSG00000201050	RNU6-668P	0	0	0	0	0	0	0	0
ENSG00000248329	APELA	0	0	0	0	0	0	0	0
ENSG00000250227	TRIM60P14	0	0	0	0	0	0	0	0
ENSG00000244146	AC106872.3	0	0	0	0	0	0	0	0.20179
ENSG00000221891	FAM218BP	0	0.26639	0	0	0	0	0	0
ENSG00000270960	AC106872.11	0	0	0	0	0	0	0	0
ENSG00000248645	AC106872.6	0	0	0	0	0	0	0	0
ENSG00000121089	NACA3P	0.11312	0.18828	0.16623	0.67152	0	0	1.1313	0.13888
ENSG00000236941	AC106872.1	0	0	0	0	0	0	0	0
ENSG00000183439	TRIM61	0.139	0.07712	0	0	0.16899	0.16348	0	0
ENSG00000250486	FAM218A	0.1011	0	0.09905	0	0.20487	0.11891	0.11235	0.20689
ENSG00000248632	AC106872.5	0.20706	0.51699	0.07607	0	0.25174	0	0.08629	0.38134
ENSG00000248229	AC106872.4	0	0	0	0	0	0	0	0
ENSG00000250887	AC106872.10	0	0	0	0	0	0	0	0
ENSG00000250712	AC106872.9	0	0	0	0	0	0	0	0
ENSG00000240674	AC106872.2	0	0	0	0	0	0	0	0
ENSG00000276623	AC106872.12	0	0	0	0	0	0	0	0
ENSG00000250430	AC106872.8	0	0	0	0	0	0	0	0
ENSG00000176979	TRIM60	0	0	0	0	0	0	0	0
ENSG00000249617	AC106872.7	0.23494	0	0	0	0	0.41446	0.3916	0
ENSG00000250374	TRIM75P	0	0	0	0	0	0	0	0
ENSG00000170088	TMEM192	1.43867	0.82205	0.39968	0.69047	1.01813	1.06066	0.68004	0.66788
ENSG00000109466	KLHL2	13.971	19.9776	3.11569	5.73148	11.5309	4.10779	5.04877	12.0631
ENSG00000200974	RNU4-87P	3.57563	4.46373	1.31358	1.32666	0	0	0	1.09753
ENSG00000229894	GK3P	0	0.06532	0.05766	0	0	0	0.06541	0
ENSG00000052802	MSMO1	2.20509	2.99537	3.57557	3.46068	2.77334	4.29251	4.64725	3.57872
ENSG00000109472	CPE	0.02478	0	0	0	0	0	0	0
ENSG00000207559	MIR578	0	0	0	0	0	0	0	0
ENSG00000251596	HADHAP1	0	0	0	0	0	0	0	0
ENSG00000248287	SCGB1D5P	0	0	0	0	0	0	0	0
ENSG00000248327	NOL8P1	0	0	0	0	0	0	0	0
ENSG00000250725	AC080079.1	0	0	0	0	0	0	0	0
ENSG00000249500	LINC01179	0	0	0	0	0	0	0	0
ENSG00000038295	TLL1	0	0	0	0	0	0	0	0
ENSG00000252959	RNA5SP170	0	0	0	0	0	0	0	0
ENSG00000206711	RF00019	0	0	0	0	0	0	0	0
ENSG00000249675	AC097487.1	0	0	0	0	0	0	0	0
ENSG00000196104	SPOCK3	0.09813	0	0	0	0.01988	0	0.02726	0
ENSG00000212373	RNA5SP171	0	0	0	0	0	0	0	0
ENSG00000250150	AC079349.1	0	0	0	0	0	0	0	0
ENSG00000240772	RN7SL776P	0	0	0	0	0	0	0	0
ENSG00000248913	PHBP14	0	0	0	0	0	0	0	0
ENSG00000222721	RN7SKP188	0	0	0	0	0	0	0	0
ENSG00000270336	AC105148.1	0	0	0	0	0	0	0	0
ENSG00000248601	AC116634.1	0	0	0	0	0	0	0	0
ENSG00000109511	ANXA10	0	0	0	0	0.04918	0	0	0
ENSG00000137628	DDX60	8.1119	27.9445	12.8299	8.18713	7.05012	14.1764	4.85101	11.693
ENSG00000181381	DDX60L	16.8598	40.9907	12.875	7.82155	13.2546	8.2974	6.18138	20.7742
ENSG00000207171	RF00432	0	0	0	0	0	0	0	0
ENSG00000129116	PALLD	0.64502	0.74793	0.13845	0.2689	0.47581	0.33242	0.25369	0.16907
ENSG00000229226	BTF3L4P4	0	0	0	0	0	0	0	0

ENSG00000249623	AC079858.1	0	0	0	0	0	0	0	0
ENSG00000206613	RNU6-1336P	0	0	0	0	0	0	0	0
ENSG00000214676	RPL9P16	0	0	0	0	0	0	0	0
ENSG00000249609	AC080188.1	0	0	0	0	0	0	0	0
ENSG00000279384	AC080188.2	0.098	0	0.144	0	0.23828	0	0	0.12032
ENSG00000145439	CBR4	4.18859	4.34551	1.64417	4.59838	5.52837	3.66932	4.08862	3.36388
ENSG00000201176	RNU6-853P	0	0	0	0	0	0	0	0
ENSG00000201818	RNY4P17	0	0	0	0	0	0	0	0
ENSG00000251445	AC021151.1	0	0	0	0	0	0	0.66765	0
ENSG00000154447	SH3RF1	0.61257	0.84608	0.38305	0.29015	0.31692	0.82774	0.45622	0.25604
ENSG00000214671	RPL6P12	0	0	0	0	0	0	0	0
ENSG00000251171	AC096741.1	0	0	0	0	0	0	0	0
ENSG00000137601	NEK1	11.4256	12.3653	16.4008	33.0626	14.2664	20.0318	33.5305	19.152
ENSG00000109572	CLCN3	16.0353	13.3529	6.33845	11.3835	11.5304	9.21723	22.216	9.90488
ENSG00000056050	HPF1	6.94209	5.35274	2.85037	5.07566	8.19178	6.93385	3.57352	4.38704
ENSG00000229204	PTGES3P3	0.15208	0.75939	0.67042	1.12848	0.36978	0.53656	0.50697	0.56015
ENSG00000248625	AC079768.1	0	0	0	0.30601	0	0	0.34369	0
ENSG00000248319	LINC02275	0.05038	0	0.02468	0.02492	0.02042	0	0	0.04124
ENSG00000251200	AC084866.2	0	0	0	0	0	0	0	0
ENSG00000249955	AC084866.1	0	0	0	0	0	0	0	0
ENSG00000198948	MFAP3L	14.9854	7.73283	4.92914	6.82011	7.18765	4.05348	5.45136	10.4504
ENSG00000109576	AADAT	0	0	0	0	0	0	0	0
ENSG00000248822	APOBEC3AP1	0	0	0	0	0	0	0	0
ENSG00000248739	AC069306.1	0	0	0	0	0	0	0	0
ENSG00000250266	LINC01612	0	0	0	0	0	0	0	0
ENSG00000251061	LINC02512	0	0	0	0	0	0	0	0
ENSG00000250082	AC074255.1	0	0	0	0	0	0	0	0
ENSG00000181359	HSP90AA6P	0	0	0	0	0	0	0	0
ENSG00000250968	LINC02382	0	0	0	0	0	0	0	0
ENSG00000249943	AC097486.1	0	0	0	0	0	0	0	0
ENSG00000251961	RNU6ATAC13P	0	0	0	0	0	0	0	0
ENSG00000248872	LINC02431	0	0	0	0	0	0	0	0
ENSG00000249525	AC034159.1	0	0	0	0	0	0	0	0
ENSG00000248755	AC092639.1	0	0	0	0	0	0	0	0
ENSG00000278502	MIR6082	0	0	0	0	0	0	0	0
ENSG00000248339	LINC02504	0	0	0	0	0	0	0	0
ENSG00000251176	AC074254.1	0	0	0	0	0	0	0	0
ENSG00000249297	LINC02174	0	0	0	0	0	0	0	0
ENSG00000174473	GALNTL6	0.00793	0	0	0	0.01929	0	0	0
ENSG00000248637	AC108064.1	0	0	0	0	0	0	0	0
ENSG00000245213	AC105285.1	0.28301	0.14495	0.03199	0.09693	0.18527	0.19203	0.03629	0.08019
ENSG00000241652	RN7SL253P	0	0	0	0	0	0.43686	0	0
ENSG00000109586	GALNT7	4.04673	4.38982	0.89435	2.04419	4.34874	2.50524	1.21023	2.58258
ENSG00000221296	MIR548T	0	0	0	0	1.20428	0	0	0
ENSG00000248774	AC097534.1	2.81383	2.81018	1.24047	3.54963	1.19734	2.97839	1.87609	1.20917
ENSG00000164104	HMGB2	30.0862	31.9201	5.66711	10.7414	22.0945	40.2148	10.0826	16.1833
ENSG00000272870	AC097534.2	0.14689	0.0978	0.04317	0.17441	0.17859	0.10366	0.14691	0.18035
ENSG00000164105	SAP30	2.99865	4.99126	1.79523	3.37895	2.90304	3.52669	1.20329	2.79536
ENSG00000164106	SCRG1	0	0.02261	0	0.02016	0	0	0	0
ENSG00000242882	RPL5P11	0	0	0	0	0	0	0	0
ENSG00000252898	RNU6-1096P	0	0	0	0	0	0	0	0
ENSG00000261646	AC093849.1	0	0	0	0	0	0	0	0
ENSG00000164107	HAND2	0	0	0	0	0	0	0	0
ENSG00000237125	HAND2-AS1	0	0	0	0	0	0	0	0
ENSG00000261672		0	0	0	0	0	0	0	0
ENSG00000234801	MORF4	0.30196	0.62826	0	0.44814	0.18356	0.26635	0.25166	0.09268
ENSG00000213370	RANP6	0	0.18945	0	0	0	0	0	0
ENSG00000250043	AC106895.1	0	0	0	0	0	0	0	0

ENSG00000250708	LINC02269	0	0	0	0	0	0	0	0
ENSG00000251216	AC106895.2	0	0	0	0	0	0	0	0
ENSG00000248174	LINC02268	0	0	0	0	0.05372	0	0	0
ENSG00000250957	AC012055.1	0	0	0	0	0	0	0	0
ENSG00000251213	AC012055.2	0	0	0	0	0	0	0	0
ENSG00000164117	FBXO8	3.62589	4.42878	1.83995	3.17453	4.47169	3.63546	3.04364	3.84329
ENSG00000164118	CEP44	2.23025	2.29236	2.0842	4.25564	2.04923	1.59555	3.56331	3.05374
ENSG00000249875	AC116616.1	0	0	0	0	0	0	0	0
ENSG00000265846	MIR4276	0	0	0	0	0	0	0	0
ENSG00000164120	HPGD	3.34087	1.52262	0.70134	1.27891	2.62724	1.02907	0.79553	0.91153
ENSG00000250596	AC096751.1	0	0	0	0	0	0	0	0
ENSG00000251584	AC096751.2	0	0	0	0.13736	0.11252	0	0	0
ENSG00000250760		0	0	0	0	0	0	0	0
ENSG00000145451	GLRA3	0	0.01271	0.01122	0	0.02785	0	0.01273	0
ENSG00000168594	ADAM29	0	0	0.01808	0	0	0	0	0
ENSG00000250200	AC105914.1	0	0	0	0	0	0	0	0
ENSG00000250431	AC105914.2	0	0	0	0	0	0	0	0
ENSG00000249411	AC022325.1	0	0	0	0	0	0	0	0
ENSG00000249284	AC095050.2	0	0	0	0	0	0	0	0
ENSG00000248551	AC095050.1	0	0	0	0	0	0	0	0
ENSG00000249945	AC131094.1	0	0	0	0	0	0	0	0
ENSG00000251174	TSEN2P1	0	0	0	0	0	0	0	0.06489
ENSG00000248574	ADAM20P2	0	0	0	0	0	0	0	0
ENSG00000150625	GPM6A	0.04165	0	0	0.06181	0.22786	0.01837	0.01736	0.02557
ENSG00000249106	AC097537.1	0	0	0	0	0	0	0	0
ENSG00000248249	AC110794.1	0	0	0	0	0	0	0	0
ENSG00000250372	MARK2P4	0	0	0	0	0	0	0	0
ENSG00000150627	WDR17	0.15021	0.01563	0.04139	0.0418	0.11414	0.08281	0.07824	0.05763
ENSG00000201516	RF00432	0	0	0	0	0	0	0	0
ENSG00000150628	SPATA4	0	0	0	0	0	0	0	0
ENSG00000164122	ASB5	0	0	0	0	0	0	0	0
ENSG00000248980	AC019163.1	0.72099	0.20001	0	0.17834	0.43828	0.21199	0.2003	0.29507
ENSG00000129128	SPCS3	24.9485	17.4502	7.06424	15.9253	19.321	17.4536	12.9853	18.9585
ENSG00000248480	AC093801.2	0	0	0	0	0	0	0	0
ENSG00000248388	AC093801.1	0	0	0	0	0	0	0	0
ENSG00000150630	VEGFC	0.51492	0.25208	0.17804	0.44953	0.18413	0.21374	0.20195	0.18594
ENSG00000249458	AC092673.1	0	0	0	0	0	0	0	0
ENSG00000250263	LINC02509	0	0	0	0	0	0	0	0
ENSG00000222859	RN7SKP136	0	0	0	0	0	0	0	0
ENSG00000249008	AC097518.1	0	0	0	0	0	0	0	0
ENSG00000109674	NEIL3	115.338	160.932	114.23	298.857	233.98	212.388	277.146	202.014
ENSG00000249084	AC027627.1	0	0	0	0	0	0	0	0
ENSG00000038002	AGA	3.69927	2.05248	1.28854	1.26069	2.56523	1.64359	1.6443	2.42235
ENSG00000250131	AC078881.1	0.13568	0	0.09969	0.05034	0.08248	0.05984	0.05654	0
ENSG00000201931	RNA5SP172	0	0	0	0	0	0	0	0
ENSG00000251416	AC103879.1	0	0	0	0	0	0	0	0
ENSG00000231171	LINC01098	0	0	0	0	0	0	0	0
ENSG00000251504	LINC01099	0	0	0	0	0	0	0	0
ENSG00000253000	RNU1-45P	0	0	0	0	0	0	0	0
ENSG00000241877	AC095041.1	0	0	0	0	0	0	0	0
ENSG00000201727	RNA5SP173	0	0	0	0	0	0	0	0
ENSG00000212191	RF00571	0	0	0	0	0	0	0	0
ENSG00000250839	AC018710.1	0	0	0	0	0	0	0	0
ENSG00000250993	AC020551.1	0	0	0	0	0	0	0	0
ENSG00000248921	AC021193.1	0	0	0	0	0	0	0	0
ENSG00000251025	NDUFB5P1	0	0	0	0	0	0	0	0
ENSG00000249883	AC104803.1	0	0	0	0	0	0	0	0
ENSG00000248197	LINC00290	0	0	0	0	0	0	0	0

ENSG00000248770	AC019235.1	0	0	0	0	0	0	0	0
ENSG00000249460	LINC02500	0	0	0	0	0	0	0	0
ENSG00000225356	AC093840.1	0	0.36312	0	0	0	0	0	0
ENSG00000251336	TEMN3-AS1	0	0	0	0	0	0	0	0
ENSG00000251742	RN7SKP13	0	0	0	0	0	0	0	0
ENSG00000177822	TENM3-AS1	0	0	0	0	0	0	0	0
ENSG00000251433	CCNHP1	0	0	0	0	0	0	0	0
ENSG00000218336	TENM3	0.00612	0.02036	0	0	0	0	0	0
ENSG00000248266	AC108142.1	0	0	0	0	0	0	0	0
ENSG00000221227	MIR1305	0	0	0	0	0	0	0	0
ENSG00000252343	RNU2-34P	0	0	0	0	0	0	0	0
ENSG00000251714	RN7SKP67	0	0	0	0	0	0	0	0
ENSG00000248816	AC079226.2	0	0	0	0	0	0	0	0
ENSG00000248694	AC079226.1	0	0	0	0	0	0	0	0
ENSG00000272646	AC079766.1	0	0	0	0	0	0	0	0
ENSG00000129187	DCTD	5.49858	3.73136	1.61602	3.2642	4.01102	5.78284	2.57333	3.11588
ENSG00000250636	AC019193.1	0	0	0	0	0	0	0	0
ENSG00000230219	FAM92A1P2	0	0	0	0	0	0	0	0
ENSG00000273180	AC019193.3	0	0	0	0	0	0	0	0
ENSG00000272784	AC019193.2	0	0	0	0	0	0	0	0
ENSG00000251359	WWC2-AS2	0.06716	0.05589	0	0	0	0	0	0
ENSG00000151718	WWC2	0.9488	0.808	0.14051	0.30564	0.28615	0.24653	0.14712	0.32509
ENSG00000251128	WWC2-AS1	0	0	0	0	0	0	0	0
ENSG00000177300	CLDN22	0	0	0	0	0.03453	0	0	0
ENSG00000185758	CLDN24	0	0	0	0	0	0	0	0
ENSG00000250292	AC093844.1	0	0	0	0	0	0	0	0
ENSG00000248376	AC112698.1	0	0	0	0	0	0	0	0
ENSG00000168564	CDKN2AIP	4.47653	3.32438	0.94456	2.18048	4.63306	5.02182	3.74998	3.3541
ENSG00000213434	VTI1BP2	0	0	0	0	0	0	0	0
ENSG00000232648	AC107214.1	0.13113	0.21826	0	0	0	0	0	0.48299
ENSG00000168556	ING2	4.78046	4.19244	0.75536	2.51748	4.81205	1.36022	0.77112	1.13601
ENSG00000272744	AC107214.2	0	0	0	0	0	0	0	0
ENSG00000182552	RWDD4	4.70009	3.51609	2.0953	3.64448	5.23272	4.79795	2.99289	2.62599
ENSG00000252157	RNU6-479P	0	0	0	0	0	0	1.16362	0
ENSG00000168538	TRAPPC11	10.579	9.06552	2.50798	4.89824	8.19306	7.81494	4.53914	5.97823
ENSG00000201433	RNU6-335P	0	0	0	0	0	0	0	0
ENSG00000251739	RNU6-1053P	0	3.62401	0	0	0	0	1.20971	1.78212
ENSG00000283383	AC108477.1	0	0	0	0	0	0	0	0
ENSG00000173320	STOX2	0.03239	0.12938	0.01904	0.00961	0.063	0.06856	0.04319	0.07953
ENSG00000248206	AC074194.1	0	0	0	0	0	0	0	0
ENSG00000283355	AC074194.2	0.03122	0	0.04587	0	0	0	0	0
ENSG00000250726	AC107222.1	0	0	0	0	0	0	0	0
ENSG00000164303	ENPP6	0.03117	0	0	0	0	0	0	0
ENSG00000240159	AC079080.1	0	0	0	0	0	0	0	0
ENSG00000244512	RN7SL28P	0	0	0	0	0	0	0	0
ENSG00000227765	MYL12BP2	0	0	0	0	0	0	0	0
ENSG00000180712	LINC02363	0.37382	0.20741	0	0.09247	0.11362	0.05496	0.05193	0.0765
ENSG00000249096	LINC02362	0.60929	1.4992	0.11678	0.23589	0.934	0.23367	0.26494	0.42283
ENSG00000168310	IRF2	33.1922	46.83	8.22719	11.1718	31.2309	21.2061	6.78344	22.8459
ENSG00000251878	RF00152	0	0	0	0	0	0	0	0
ENSG00000270426	AC099343.2	2.24389	2.84568	0.47105	0.63432	0.90935	2.26201	1.78105	1.04953
ENSG00000271646	AC099343.3	1.58139	1.65733	0.21517	0.30423	1.53097	1.54988	0.92746	1.79778
ENSG00000271538	LINC02427	0.23419	0.09745	0.08603	0.17378	0.21354	0.41314	0.09759	0.28753
ENSG00000249056	AC099343.1	0	0	0	0	0	0	0	0
ENSG00000254233	LINC02365	0.28904	0.09622	0.08495	0	0	0.20396	0.09636	0.21293
ENSG00000164305	CASP3	15.0191	14.4777	6.13067	9.30626	9.71399	14.6753	9.01918	10.2135
ENSG00000164306	PRIMPOL	4.22837	3.99113	7.46382	14.8083	5.04671	8.73298	13.4514	6.83762
ENSG00000151725	CENPU	3.47513	3.67719	4.55951	9.94654	11.5583	7.53188	11.8332	7.2534

ENSG00000151726	ACSL1	108.807	184.818	26.7571	29.0315	80.5369	10.9269	26.5592	100.738
ENSG00000249138	SLED1	3.02571	6.33599	1.14742	1.30369	2.37328	0.34437	0.81345	2.51656
ENSG00000251139	AC084871.1	2.10097	2.91422	0.34304	1.90548	0.99334	0	0.19455	2.14961
ENSG00000251230	MIR3945HG	4.53481	8.53812	1.42016	0.33099	2.98261	0.78689	0.24783	1.91677
ENSG00000266698	MIR3945	28.4226	90.8839	17.586	19.9811	46.377	5.27803	3.74021	41.3252
ENSG00000249173	LINC01093	0.1844	3.37634	0	0	0.56048	0.16266	0	0.33961
ENSG00000251147	AC084871.2	0	0	0	0	0	0	0	0
ENSG00000263458	MIR4455	0	0	0	0	0	0	0	0
ENSG00000279460	AC112243.1	0	0	0	0	0	0	0	0
ENSG00000248517	LINC02437	0	0	0	0	0	0	0	0
ENSG00000187821	HELT	0	0	0	0	0	0	0	0
ENSG00000250754	LINC02436	0	0	0	0	0	0	0	0
ENSG00000249520	AC093824.1	0	0	0	0	0	0	0	0
ENSG00000151729	SLC25A4	0.25725	0.25188	0.08895	0.06737	0.22077	0.66738	0.02522	0.27869
ENSG00000164323	CFAP97	9.60164	6.87472	2.11173	8.13602	7.05324	8.99511	2.94539	5.20169
ENSG00000109762	SNX25	1.62105	1.81664	0.91983	1.92942	1.95132	2.26515	1.09687	1.73412
ENSG00000109771	LRP2BP	0.15935	0.2155	0	0.11825	0.06054	0.05271	0.0664	0.04891
ENSG00000250410	AC112722.1	0	0	0	0	0	0.22029	0	0
ENSG00000186352	ANKRD37	0.39775	0.33103	0.12525	0.29516	0.34541	0.45109	0.3315	0.13953
ENSG00000109775	UFSP2	3.55827	3.33428	2.40139	4.45939	4.55037	3.67337	4.08586	3.91573
ENSG00000207231	RF00019	0	0	0	0	0	0	0	0.78945
ENSG00000205129	C4orf47	0	0	0	0	0.13048	0.28399	0	0
ENSG00000168491	CCDC110	0.02164	0	0	0.03211	0.05261	0	0	0.02656
ENSG00000249679	AC106897.1	0	0	0	0	0	0	0	0.22842
ENSG00000154553	PDLIM3	0	0	0	0	0	0	0	0
ENSG00000154556	SORBS2	0.20154	0.02188	0.0837	0.03901	0.02131	0.00773	0.0073	0.01614
ENSG00000233110	AC093797.1	0	0	0	0	0	0	0	0
ENSG00000235902	AC108472.1	0	0	0	0	0	0	0	0
ENSG00000207497	RF00019	0	0	0	0	0	0	0	0
ENSG00000235149	AC096659.1	0	0	0	0	0	0	0	0
ENSG00000222727	RNU4-64P	0	0	0	0	0	0	0	0
ENSG00000164342	TLR3	0.29434	0.3629	0.24029	0.33975	0.31808	0.46155	0.39976	0.26769
ENSG00000109794	FAM149A	0.01405	0	0	0	0.03416	0.02478	0.02342	0
ENSG00000253013	RF00322	0	0	0	0	0	0	0	0
ENSG00000250327	RPSAP70	0	0	0	0	0	0	0	0
ENSG00000251008	ORAOV1P1	0	0	0	0.27541	0	0	0	0
ENSG00000279943	FLJ38576	0.17885	0.19847	0.0438	0.08848	0.32617	0	0.14906	0.0732
ENSG00000145476	CYP4V2	2.02553	1.33118	0.45032	1.23129	1.49936	2.00422	0.68522	0.66991
ENSG00000164344	KLKB1	0.02482	0.12395	0	0.03684	0	0.17516	0	0.06095
ENSG00000088926	F11	0	0	0	0	0	0	0	0
ENSG00000251165	F11-AS1	0	0	0	0	0	0	0	0
ENSG00000213332	SLC25A5P6	0	0	0	0	0	0	0	0
ENSG00000272297	AC018709.1	0	0	0	0	0	0	0	0
ENSG00000212387	RNU6-1055P	0	0	0	0	0	0	0	0
ENSG00000168412	MTNR1A	0	0	0	0	0	0	0	0
ENSG00000083857	FAT1	0.00906	0.05279	0.17312	0.10087	0.18179	0.55955	0.35498	0.22253
ENSG00000250829	AC108865.1	0	0	0	0	0	0	0	0
ENSG00000272218	AC108865.2	0	0	0	0	0	0	0	0
ENSG00000249539	MRPS36P2	0	0	0	0	0	0	0	0
ENSG00000249742	AC110772.1	0	0	0	0	0	0	0	0
ENSG00000250971	AC108474.1	0	0	0	0	0	0	0	0
ENSG00000249747	LINC02374	0	0	0	0	0	0	0	0
ENSG00000250658	AC097652.1	0	0	0	0.36022	0.25293	0	0.11559	0.04257
ENSG00000250042	LINC02514	0	0	0	0	0	0	0	0
ENSG00000250620	LINC02515	0	0	0	0	0	0	0	0
ENSG00000251643	AC093763.2	0	0	0	0.08895	0	0	0	0
ENSG00000251490	AC093763.1	0	0	0	0	0	0	0	0
ENSG00000250590	LINC02492	0	0	0	0	0	0	0	0

ENSG00000249642	AC097521.1	0	0	0	0	0	0	0	0
ENSG00000249162	ADAM20P3	0	0	0	0	0	0	0	0
ENSG00000213331	AC108073.1	0	0	0	0	0	0	0	0
ENSG00000179059	ZFP42	0	0	0	0	0	0	0	0
ENSG00000179046	TRIML2	0	0	0	0	0	0	0	0
ENSG00000241011	AC108073.2	0	0	0	0	0	0	0	0
ENSG00000201085	RNU6-173P	0	0	0	0	0	0	0	0
ENSG00000184108	TRIML1	0	0	0	0	0	0	0	0
ENSG00000247130	AC138781.1	0	0	0	0	0	0	0	0
ENSG00000248370	LINC02434	0	0	0	0	0	0	0	0
ENSG00000242169	AC093789.1	0	0	0	0	0	0	0	0
ENSG00000248880	ICE2P1	0	0	0	0	0	0	0	0
ENSG00000249378	LINC01060	0	0	0	0	0	0	0	0
ENSG00000250523	AC093909.3	0	0	0	0	0	0	0	0
ENSG00000275826	AC093909.5	0	0	0	0	0	0	0	0
ENSG00000252275	RNU7-192P	0	0	0	0	0	0	0	0
ENSG00000180015	AC093909.1	0	0.10874	0	0.19391	0	0.11525	0	0
ENSG00000278974	AC093909.6	0	0	0	0	0	0	0	0
ENSG00000249269	AC093909.2	0	0	0	0	0	0	0	0
ENSG00000251619	LINC02508	0	0	0	0	0	0	0	0
ENSG00000250626	AC093909.4	0	0	0	0	0	0	0	0
ENSG00000250024	AC122138.1	0	0	0	0	0	0	0	0
ENSG00000251310	AC107391.1	0	0	0	0	0	0	0	0
ENSG00000249877	AC105924.1	0	0	0	0	0	0	0	0
ENSG00000205100	HSP90AA4P	0	0	0	0	0	0	0	0
ENSG00000250739	LINC01262	0.16678	0.1388	0	0	0	0	0	0
ENSG00000272566	AF250324.1	0	0	0	0	0	0	0	0
ENSG00000202215	RNU1-51P	0	0	0	0	0	0	0	0
ENSG00000245685	FRG1-DT	0	0	0	0	0.03868	0	0.15909	0
ENSG00000250666	LINC01596	0	0	0	0	0	0	0	0
ENSG00000109536	FRG1	16.747	14.0807	2.14544	5.22579	16.0796	16.5143	4.58087	6.80118
ENSG00000249462	MLLT10P2	0	0	0	0	0	0	0	0
ENSG00000271307	AF146191.1	0	0	0	0	0	0	0	0
ENSG00000251297	TUBB7P	0	0	0	0	0	0	0	0
ENSG00000199572	RNA5SP174	0	0	0	0	0	0	0	0
ENSG00000201145	RNA5SP175	0	0	0	0	0	0	0	0
ENSG00000224807	DUX4L9	0	0	0	0	0	0	0	0
ENSG00000205097	FRG2	0	0	0	0	0	0	0	0
ENSG00000250053	RARRES2P4	0	0	0	0	0	0	0	0
ENSG00000214273	AGGF1P1	0.07072	0	0	0	0	0	0	0
ENSG00000249003	CLUHP4	0	0	0	0	0	0	0	0
ENSG00000281591	DBET	0	0	0	0	0	0	0	0
ENSG00000277162	U85056.1	0	0	0	0	0	0	0	0
ENSG00000281720	DUX4L8	0	0	0	0	0	0	0	0
ENSG00000281652	DUX4L7	0	0	0	0	0	0	0	0
ENSG00000281058	DUX4L6	0	0	0	0	0	0	0	0
ENSG00000280799	DUX4L5	0	0	0	0	0	0	0	0
ENSG00000258834	DUX4L4	0	0	0	0	0	0	0	0
ENSG00000280757	DUX4L1	0	0	0	0	0	0	0	0
ENSG00000281627	DUX4L3	0	0	0	0	0	0	0	0
ENSG00000280457	DUX4L2	0	0	0	0	0	0	0	0
ENSG00000260596	DUX4	0	0	0	0	0	0	0	0
ENSG00000261914	RPL23AP84	0.15797	0	0	0	0	0	0	0
ENSG00000282994	AC215524.1	0	0	0	0	0	0	0	0
ENSG00000250020	AC113430.1	0	0	0	0	0	0	0	0
ENSG00000153404	PLEKHG4B	0.01653	0	0	0	0.0067	0	0.00918	0
ENSG00000199540	RF00019	0	0	0	0	0	0	0	0
ENSG00000185028	LRRC14B	0	0	0	0	0	0	0	0

ENSG00000261434	AC021087.4	0	0	0	0	0	0	0	0
ENSG00000164366	CCDC127	0.55914	0.40246	0.19986	0.20185	0.69816	0.37323	0.40303	0.39891
ENSG00000260774	AC021087.3	0.15266	0.04235	0.03739	0.03776	0.12373	0	0.08482	0.09371
ENSG00000073578	SDHA	5.78848	3.97332	0.97653	2.15417	4.93262	7.31168	1.3263	2.08272
ENSG00000248925	AC021087.1	0.28084	0.7012	0.4127	0	0	0	0.23406	0
ENSG00000249915	PDCD6	3.12721	2.30059	0.55599	0.91677	2.47833	2.11138	1.05539	1.73491
ENSG00000250848	AC021087.2	0.30466	0.10142	0	0.09043	0	0	0.10156	0
ENSG00000063438	AHRR	0.04773	0.01589	0	0	0.06964	0.03368	0.04774	0
ENSG00000214278	AC010442.1	0	0	0	0	0	0	0	0
ENSG00000250645	AC010442.2	0	0	0	0	0	0	0	0
ENSG00000221990	EXOC3-AS1	0.22039	0.07337	0	0.19625	0.21435	0.15552	0	0.27059
ENSG00000180104	EXOC3	4.41665	3.5341	0.52659	1.10355	3.51805	2.60773	1.0005	1.92489
ENSG00000188242	PP7080	1.24552	0.57343	0.15577	0.3933	1.70759	1.12201	0.44172	0.22776
ENSG00000225138	SLC9A3-AS1	0.10223	0.17017	0.10015	0.15172	0.06215	0.03006	0.0568	0.16736
ENSG00000066230	SLC9A3	0	0	0	0	0	0	0	0
ENSG00000250385	AC106772.2	0	0	0	0	0	0	0	0
ENSG00000264233	MIR4456	0	0	0	0	0	0	0	0
ENSG00000249650	AC106772.1	0	0	0	0	0	0	0	0
ENSG00000112877	CEP72	0.44084	0.30018	0.05889	0.1487	0.5116	0.70701	0.167	0.31984
ENSG00000171368	TPPP	0.25561	0.30391	0	0.01806	0.13319	0.02147	0.02029	0
ENSG00000271781	AC026740.1	0.278	0	0	0	0.22533	0	0	0.11378
ENSG00000206077	ZDHHC11B	0.34833	0.17646	0	0.02248	0.07365	0.13359	0.05049	0.05578
ENSG00000249908	BRD9P2	0	0	0	0	0	0	0	0
ENSG00000188818	ZDHHC11	0	0.01755	0.01549	0	0.03845	0	0	0
ENSG00000247872	SPCS2P3	0	0	0	0	0	0	0	0
ENSG00000028310	BRD9	1.99868	1.28739	0.23631	0.44323	1.26618	1.25637	0.81692	0.54532
ENSG00000071539	TRIP13	0.47357	0.27418	0.27231	0.85562	0.32543	2.14309	0.65209	0.35392
ENSG00000248949	AC122719.1	0	0	0	0	0	0	0	0
ENSG00000250173	AC122719.2	0	0	0	0	0	0	0	0
ENSG00000215246	AC116351.1	0	0	0	0	0	0	0	0
ENSG00000272347	AC116351.2	0	0	0	0	0	0	0	0
ENSG00000145506	NKD2	0	0	0	0	0	0	0	0
ENSG00000113504	SLC12A7	1.56792	0.9379	0.68401	0.54538	1.05737	1.96647	0.87797	0.43615
ENSG00000263834	MIR4635	0	1.54441	0	0	0	0	0	0
ENSG00000249201	CTD-3080P12.3	0	0	0	0	0	0	0	0
ENSG00000174358	SLC6A19	0.05557	0.90188	0.26541	0.02062	0.28715	0.56372	0.20842	0.08529
ENSG00000164363	SLC6A18	0	0	0	0	0	0	0	0
ENSG00000164362	TERT	0.04764	0	0	0	0	0	0	0
ENSG00000263670	MIR4457	0	0	0	0	0	0	0	0
ENSG00000049656	CLPTM1L	4.13621	2.60503	0.7228	1.89135	3.32981	4.35838	1.49069	1.85292
ENSG00000250584	LINC01511	0	0	0	0	0	0	0	0
ENSG00000248951	MTCO2P32	0	0	0	0	0	0	0	0
ENSG00000279908	AC026748.1	0	0	0	0	0	0	0	0
ENSG00000142319	SLC6A3	0	0	0	0	0	0	0	0
ENSG00000153395	LPCAT1	4.89873	4.32121	0.71323	1.64167	2.77203	3.52451	1.24175	2.00948
ENSG00000273742	MIR6075	3.85791	2.5686	0	0	0	1.36118	0	0
ENSG00000251532	AC091849.2	0	0	0	0	0.25756	0.37373	0	0.13005
ENSG00000185986	SDHAP3	1.37477	1.24817	0.27548	0.55645	1.29155	1.1024	0.5208	0.46034
ENSG00000271119	AC026412.3	0	0	0	0	0	0	0	0
ENSG00000248126	AC091849.1	0	0	0	0	0	0	0	0
ENSG00000188002	AC026412.1	0.67548	0.4715	0.06404	0.16169	0.50333	0.1922	0.21792	0.32104
ENSG00000232615	AC026412.2	0	0	0	0	0	0	0	0
ENSG00000263746	MIR4277	0	0	0	0	0	0	0	0
ENSG00000260066	AC112176.1	0	0	0	0	0	0	0	0
ENSG00000171421	MRPL36	1.89298	2.71324	0.46362	0.78039	2.42929	2.5046	0.52588	1.16209
ENSG00000145494	NDUFS6	8.8405	7.31546	1.26198	1.87432	7.61576	7.57512	2.18931	2.79109
ENSG00000249966	AC025183.2	0	0	0	0	0	0	0	0
ENSG00000250417	LINC02116	0	0	0	0	0	0	0	0

ENSG00000279222	AC025183.3	0	0	0	0	0	0	0	0
ENSG00000113430	IRX4	0	0	0	0	0	0	0	0
ENSG00000249116	AC025183.1	0	0	0	0	0	0	0	0
ENSG00000249326	CTD-2194D22.4	0	0	0	0	0	0	0	0
ENSG00000248994	AC126768.2	0	0	0	0	0	0	0	0
ENSG00000248597	AC126768.1	0	0	0	0	0	0	0	0
ENSG00000249731	AC126768.3	0	0	0	0	0	0	0	0
ENSG00000201026	RF00019	0	0	0	0	0	0	0	0
ENSG00000275733	AC092266.1	0	0	0	0	0	0	0	0
ENSG00000281560	LSINCT5	0	0	0	0	0	0	0	0
ENSG00000259757	AC091891.1	0	0	0	0	0	0	0	0
ENSG00000170561	IRX2	0	0	0	0	0	0	0	0
ENSG00000186493	C5orf38	0	0	0	0	0	0	0	0
ENSG00000249541	AC116359.1	0	0	0	0	0	0	0	0
ENSG00000248137	AC094105.1	0	0	0	0	0	0	0	0
ENSG00000248736	AC112192.1	0	0	0	0	0	0	0	0
ENSG00000249808	LINC01377	0	0	0	0	0	0	0	0
ENSG00000248118	LINC01019	0	0	0	0	0	0	0	0
ENSG00000249830	LINC02162	0	0	0	0	0	0	0	0
ENSG00000250716	LINC01017	0	0	0	0	0	0	0	0
ENSG00000170549	IRX1	0	0	0	0	0	0	0	0
ENSG00000259603	AC016595.1	0	0	0	0	0	0	0	0
ENSG00000248962	AC025773.1	0	0	0	0	0	0	0	0
ENSG00000249994	AC025187.1	0	0	0	0	0	0	0	0
ENSG00000250921	LINC02063	0	0	0	0	0	0	0	0
ENSG00000260763	AC106799.3	0	0	0	0	0	0	0	0
ENSG00000248973	AC106799.2	0	0	0	0	0	0	0	0
ENSG00000231291	AC106799.1	0	0	0	0	0	0	0	0
ENSG00000251371	AC010634.1	0	0	0	0	0	0	0	0
ENSG00000249521	LINC02114	0	0	0	0	0	0	0	0
ENSG00000248311	AC026719.1	0	0	0	0	0	0	0	0
ENSG00000248467	AC026719.2	0	0	0	0	0	0	0	0
ENSG00000249941	AC026415.1	0	0	0	0	0	0	0	0
ENSG00000251506	AC010451.3	0	0	0	0	0	0	0	0
ENSG00000250481	AC010451.1	0	0	0	0	0	0	0	0
ENSG00000215231	LINC01020	0	0	0	0	0	0	0	0
ENSG00000251426	AC010451.2	0	0	0	0	0	0	0	0
ENSG00000250529	LINC02121	0	0	0	0	0	0	0	0
ENSG00000223007	RN7SKP73	0	0	0	0	0	0	0	0
ENSG00000250579	AC022424.1	0	0	0	0	0	0	0	0
ENSG00000145536	ADAMTS16	0	0.02081	0	0	0.0152	0.02206	0	0
ENSG00000250866	AC022424.2	0	0	0	0	0	0	0	0
ENSG00000251568	ALG3P1	0	0	0	0	0	0	0	0.08079
ENSG00000248729	MTCO1P30	0	0	0	0	0	0	0	0
ENSG00000250808	MTCO2P30	0	0	0	0	0	0	0	0
ENSG00000251476	MTCO1P31	0	0	0	0	0	0	0	0
ENSG00000164151	ICE1	10.3472	6.36896	2.69347	5.72886	9.47853	9.64101	6.1245	5.84908
ENSG00000261037	AC010266.2	0	0	0	0	0	0	0	0
ENSG00000259861	AC010266.1	0	0	0	0	0	0	0	0
ENSG00000278913	AC026797.1	0	0	0	0	0	0	0	0
ENSG00000250730	HMGB3P3	0	0	0	0	0	0	0	0
ENSG00000250490	LINC02145	0.05015	0	0	0.03722	0.12195	0.13272	0.0418	0.03079
ENSG00000133398	MED10	3.70401	4.02368	0.74483	1.79381	2.89155	2.75131	2.40461	1.38825
ENSG00000215218	UBE2QL1	0.01698	0	0	0	0	0	0	0
ENSG00000271230	AC027334.1	0	0	0	0	0	0	0	0
ENSG00000250056	LINC01018	0	0	0	0	0	0	0	0
ENSG00000037474	NSUN2	9.07934	8.1989	4.55818	10.6099	9.58174	9.08893	6.74588	8.35243
ENSG00000145545	SRD5A1	0.76008	0.74328	0.19546	0.39482	0.79703	0.58664	0.36424	0.34996

ENSG00000248677	LINC02102	0.02179	0	0	0	0	0	0	0
ENSG00000112941	TENT4A	2.84127	1.96005	1.63516	2.85392	3.06031	2.32513	1.9448	1.89677
ENSG00000251365	LINC02236	0	0	0	0	0	0	0	0
ENSG00000272071	AC122710.2	0	0	0	0	0	0	0	0
ENSG00000249734		0	0	0	0	0	0	0	0
ENSG00000283420	MIR4278	0	0	0	0	0	0	0	0
ENSG00000200243	RN7SKP79	0	0	0	0.32377	0	0.76971	1.45453	0.80355
ENSG00000250060	AC122710.1	0	0	0	0	0	0	0	0
ENSG00000250974	LINC02196	0	0	0	0	0	0	0	0
ENSG00000212258	RNA5SP176	0	0	0	0	0	0	0	0
ENSG00000237611	RF00017	0	0	0	0	0	0	0	0
ENSG00000248775	AC091951.2	0	0	0	0	0	0	0	0
ENSG00000247732		0	0	0	0	0	1.27401	0	0
ENSG00000205976	AC091951.1	0	0	0	0	0	0	0	0
ENSG00000251250	AC091951.3	0	0	0	0	0	0	0	0
ENSG00000251678	AC027343.3	0	0	0	0	0	0	0	0
ENSG00000250668	LINC02123	0	0	0	0	0	0	0	0
ENSG00000249638	AC027343.1	0	0	0	0	0	0	0	0
ENSG00000249865	AC027343.2	0	0	0	0	0	0	0	0
ENSG00000223597	LINC02142	0	0	0	0	0	0	0	0
ENSG00000078295	ADCY2	0	0	0	0	0	0	0	0
ENSG00000250761	AC093305.1	0	0	0	0	0	0	0	0
ENSG00000215217	C5orf49	0	0	0	0	0	0	0	0
ENSG00000124275	MTRR	3.94816	2.42214	0.79567	1.43976	2.49603	3.70145	1.20338	1.48195
ENSG00000124279	FASTKD3	1.61635	1.11033	0.67863	0.95192	1.74677	2.21781	1.62508	1.41752
ENSG00000251168	AC025174.1	0	0	0	0.36877	0	0	0	0
ENSG00000199773	RNU1-76P	0	0	0	0	0	0	0	0
ENSG00000280338	AC091941.1	0	0	0	0	0	0	0	0
ENSG00000280251	AC091912.2	0	0	0	0	0	0	0	0
ENSG00000249486	AC091912.1	0	0	0	0	0	0	0	0
ENSG00000245729	LINC02226	0	0	0	0	0	0	0	0
ENSG00000249159	AC091965.1	0	0.17555	0	0	0.12823	0	0	0
ENSG00000251549	AC091965.3	0	0	0	0	0	0	0	0
ENSG00000272049	AC091965.4	0	0	0.1835	0	0	0	0	0
ENSG00000247516	MIR4458HG	0.15204	0.02812	0.02482	0.05014	0.16431	0.08941	0.14079	0.08297
ENSG00000250037	AC091965.2	0	0	0	0	0	0	0	0
ENSG00000279075	AC091965.5	0	0.35675	0	0	0	0	0	0
ENSG00000284000	MIR4458	0	0	0	0	0	0	0	0
ENSG00000249782	AC027328.1	0	0	0	0	0	0	0	0
ENSG00000279097	AC091953.2	0	0	0	0	0	0	0	0
ENSG00000279264	AC091953.4	0	0	0	0	0	0	0	0
ENSG00000250389	MTND6P2	0	0	0	0	0	0	0	0
ENSG00000248765	MTCYBP37	0	0	0	0	0	0	0	0
ENSG00000279002	AC091953.1	0	0	0	0	0	0	0	0
ENSG00000280373	AC091953.5	0	0	0	0	0	0	0	0
ENSG00000279125	AC091953.3	0	0	0	0	0	0	0	0
ENSG00000280438	AC091932.4	0	0	0	0	0	0	0	0
ENSG00000278877	AC091932.2	0	0	0	0	0	0	0	0
ENSG00000260981	AC091932.1	0	0	0	0	0	0	0	0
ENSG00000280185	AC091932.3	0	0	0	0	0	0	0	0
ENSG00000250198	LINC02199	0	0	0	0	0	0	0	0
ENSG00000279635	AC091895.1	0	0	0	0	0	0	0	0
ENSG00000250619	AC021088.1	0	0	0	0	0	0	0	0
ENSG00000112902	SEMA5A	0.06059	0.01009	0.0089	0.00899	0.07367	0.01069	0.0808	0.01488
ENSG00000266415	MIR4636	0	0	0	0	0	0	0	0
ENSG00000248537	AC091906.1	0	0	0	0	0	0	0	0
ENSG00000279698	AC027335.2	0	0	0	0	0	0	0	0
ENSG00000251370	SEMA5A-AS1	0	0	0	0	0	0	0	0

ENSG00000250001	AC027335.1	0	0	0	0	0	0	0	0
ENSG00000250786	SNHG18	0	0	0	0.05576	0	0	0	0
ENSG00000239112	SNORD123	0	0	0	0	0	0	0	0
ENSG00000248525	AC026787.1	0	0	0	0	0	0	0	0
ENSG00000169777	TAS2R1	0	0	0	0	0	0	0	0
ENSG00000249781	LINC02112	0	0	0	0	0	0	0	0
ENSG00000222054	RNA5SP177	0	0	0	0	0	0	0	0
ENSG00000250814	LINC02221	0	0	0	0	0	0	0	0
ENSG00000279011	AC091905.2	0	0	0	0	0	0	0	0
ENSG00000279755	AC091905.3	0	0	0	0	0	0	0	0
ENSG00000278874	AC091905.1	0	0	0	0	0	0	0	0
ENSG00000280016	AC091905.4	0	0	0	0	0	0	0	0
ENSG00000249807	AC034229.1	0	0	0	0	0	0	0	0
ENSG00000280449	AC034229.6	0	0	0	0	0	0	0	0
ENSG00000280368	AC034229.5	0	0	0	0	0	0	0	0
ENSG00000260515	AC034229.2	0	0	0	0	0	0	0	0
ENSG00000271998	AC034229.3	0	0	0	0	0	0	0	0
ENSG00000272417	AC034229.4	0	0	0	0	0.20208	0	0	0.20407
ENSG00000150756	FAM173B	1.60772	1.33803	0.70001	1.59069	1.88224	0.99794	1.43917	1.38907
ENSG00000248968	AC012640.1	0	0	0	0	0	0	0	0
ENSG00000150753	CCT5	16.056	14.8776	2.19523	4.15469	13.5835	15.5688	2.82486	6.75094
ENSG00000271980	AC012640.4	0	0	0	0	0	0	0	0
ENSG00000271715	AC012640.3	0	0	0	0	0	0	0	0
ENSG00000164237	CMBL	0.79387	1.32139	1.65265	0.75273	0.10724	0.89476	3.05083	0.18952
ENSG00000252341	RF00019	0	0	0	0	0	0	0	0
ENSG00000252671	RF00019	0	0	0	0.98896	0	0	0	0
ENSG00000280369	AC012640.5	0	0	0	0	0	0	0	0
ENSG00000259802	AC012640.2	0.73219	0.13541	0.11955	0.12074	0.49454	0.14352	0	0.29966
ENSG00000145495	MARCH6	10.4752	9.99875	6.26178	8.03226	8.16798	8.1105	8.71947	7.09751
ENSG00000250600	ROPN1L-AS1	0.77567	0	0	0	0	0.68419	0.64646	0
ENSG00000145491	ROPN1L	4.51824	2.68593	0.53353	0.83819	2.35421	0.71168	0.33621	1.58498
ENSG00000277073	MIR6131	0	0	0	0	0	0	0	0
ENSG00000246016	LINC01513	1.36584	2.27345	0	0.45046	0	0.26773	0.50592	0.18633
ENSG00000244361	RPL30P7	0.45387	0.37774	0	0	0	0	0.37827	0
ENSG00000249166	AC092336.1	0	0	0	0	0	0	0	0
ENSG00000249396	LINC02212	0	0	0	0	0	0	0	0
ENSG00000249160	LINC02213	0	0	0	0	0	0	0	0
ENSG00000280017	AC112200.2	0.04215	0	0	0	0	0	0	0.05175
ENSG00000279450	AC112200.1	0	0	0	0	0	0	0	0
ENSG00000164236	ANKRD33B	1.94059	1.55894	0.33031	0.68943	1.28442	0.97813	0.48707	0.70835
ENSG00000250106	ANKRD33B-AS1	0	0	0	0	0	0	0	0
ENSG00000251196	AC106760.1	0	0	0	0	0	0	0	0
ENSG00000251548	AC106760.2	0.11842	0	0	0	0	0	0	0
ENSG00000279979	AC012629.4	0	0.47108	0	0	0	0	0	0
ENSG00000278865	AC012629.3	0	0.74395	0	0.33166	0	0	0	0
ENSG00000112977	DAP	9.72685	7.76847	2.75614	3.46328	6.89388	9.04144	5.63462	4.49851
ENSG00000272324	AC012629.2	0	0	0	0	0	0	0	0
ENSG00000250576	AC012629.1	0	0	0	0	0	0	0	0
ENSG00000169862	CTNND2	0	0	0	0	0	0	0	0.02623
ENSG00000207312	RNU6-429P	0	0	0	0	0	0	0	0
ENSG00000212305	RNU6-679P	0	0	0	0	0	0	0	0
ENSG00000248783	AC106771.1	0	0	0	0	0	0	0	0
ENSG00000248131	LINC01194	0	0	0	0	0	0	0	0
ENSG00000251112	AC106794.2	0	0	0	0	0	0	0	0
ENSG00000249053	AC106794.1	0	0	0	0	0	0	0	0
ENSG00000248457	LINC02220	0	0	0	0	0	0	0	0
ENSG00000248145	AC016553.1	0	0	0	0	0	0	0	0
ENSG00000242608	RPS23P5	0	0	0	0	0	0	0	0

ENSG00000248462	NENFP3	0	0	0	0	0	0	0	0
ENSG00000243744	AC016546.1	0	0	0	0	0	0	0	0
ENSG00000039139	DNAH5	0	0	0	0	0.01082	0	0	0
ENSG00000251423	AC016576.1	0	0	0	0	0	0	0	0
ENSG00000038382	TRIO	2.06916	1.48502	0.25851	0.69623	1.56845	0.76109	0.57948	0.71998
ENSG00000145569	OTULINL	10.1476	8.62505	2.06227	3.20429	7.17483	5.68159	3.29893	5.21338
ENSG00000248791	AC010627.1	0.50982	0.18858	0.08324	0.08407	0	0.09993	0.56652	0.34775
ENSG00000250526	CCT6P2	0	0.15318	0	0	0	0	0	0
ENSG00000250182	EEF1A1P13	0.05277	0	0	0.07832	0	0	0.08796	0.12959
ENSG00000261360	AC010491.1	0.05241	0	0.03851	0	0	0	0.04368	0
ENSG00000154124	OTULIN	4.06751	3.79595	0.96285	2.008	3.21747	4.72872	2.14177	2.51791
ENSG00000154122	ANKH	3.6296	4.33459	3.41036	4.37546	4.74906	5.74851	4.4419	4.41495
ENSG00000249485	RBBP4P1	0.11489	0.28685	0	0.25577	0.06984	0.30402	0	0.14106
ENSG00000264792	MIR4637	0	0	0	0	0	0	0	0
ENSG00000272057	AC016575.1	0	0.31939	0	0	0	0.33851	0	0
ENSG00000249928	UQCRBP3	0	0	0	0	0	0	0	0
ENSG00000250662	HNRNPKP5	0	0	0	0	0	0	0	0
ENSG00000250247	SEPHS2P1	0	0	0	0	0	0	0	0
ENSG00000202269	RF00096	0	0	0	0	0	0	0	0
ENSG00000249332	AC016651.1	0	0	0	0	0	0	0	0
ENSG00000248486	LINC02149	0	0	0	0	0	0	0	0
ENSG00000248834	MARK2P5	0	0	0	0	0	0	0	0
ENSG00000248699	AC114964.1	0	0	0	0	0	0	0	0
ENSG00000183580	FBXL7	0	0	0	0	0	0	0	0
ENSG00000250250	CTD-2350J17.1	0	0	0	0	0	0	0	0
ENSG00000216077	MIR887	0	0	0	0	0	0	0	0
ENSG00000222312	RNA5SP178	0	0	0	0	0	0	0	0
ENSG00000183654	MARCH11	0	0	0	0	0	0	0	0
ENSG00000250981	AC016650.1	0	0	0	0	0	0	0	0
ENSG00000250448	AC092335.1	0	0	0	0	0	0	0	0
ENSG00000248403	NACAP6	0	0	0	0	0	0	0	0
ENSG00000248150	LINC02150	0	0.05755	0.10162	0.05131	0.08407	0	0	0.12735
ENSG00000249737	AC020980.1	0	0	0	0	0	0	0	0
ENSG00000173545	ZNF622	9.44837	10.3409	1.77516	3.00938	9.07427	8.44827	3.37991	3.9728
ENSG00000154153	RETREG1	2.56744	2.02991	0.63666	1.11929	3.68718	0.99079	1.68506	1.2412
ENSG00000246214	AC022113.1	0	0.07288	0.12869	0.06499	0.10647	0	0	0.10752
ENSG00000250415	AC022113.2	0	0.17331	0	0	0	0.18368	0.3471	0
ENSG00000201370	RF00019	0	0	0	0	0	0	0	0
ENSG00000145555	MYO10	0.11024	0.05243	0.03086	0.02337	0.24253	0.02778	0	0.02578
ENSG00000253093	RNA5SP179	0	0	0	0	0	0	0	0
ENSG00000243538	RPS26P28	0	0	0	0	0	0	0	0
ENSG00000201436	RNU6-660P	0	0	0	0	0	0	0	0
ENSG00000176788	BASP1	88.5423	137.363	9.71709	13.1505	61.4612	7.58256	15.431	41.0406
ENSG00000271892	AC026790.1	0	0	0	0	0	0	0	0
ENSG00000206814	RF00019	0	0	0	0	0	0	0	0
ENSG00000215196	BASP1-AS1	0.24924	0.37337	0	0	0.21211	0	0	0.27541
ENSG00000251990	RNA5SP180	0.72575	2.41601	0	0	0	0	0	0
ENSG00000250816	DCAF13P2	0	0	0	0	0	0	0	0
ENSG00000252908	RNU6-1003P	2.0943	0	1.02585	1.03605	1.69746	1.23154	1.16362	2.57134
ENSG00000185296	AC026785.1	0	0	0	0	0	0	0	0
ENSG00000201715	RN7SKP133	0	0	0	0.31996	0	0	0	0
ENSG00000223361	FTH1P10	0.31527	0.13119	0.11582	0.35092	0.09582	0.13904	0.13138	0.09677
ENSG00000248223	AC026785.2	0	0	0	0	0	0	0	0
ENSG00000249199	AC026785.3	0	0	0	0	0	0	0	0
ENSG00000250822	LINC02111	0	0	0	0	0	0	0	0
ENSG00000248455	LINC02217	0.52411	0.37388	0.22005	0.44448	0.36411	0.13209	0.2496	1.19506
ENSG00000249662	LINC02218	6.784	4.81975	2.06675	1.4734	4.92857	0	0.5516	3.65677
ENSG00000284552		1.22371	1.42581	1.07894	0.54484	1.33898	0	0.40795	0.90147

ENSG00000268799	AC106774.4	0	0	0	0	0	0	0	0
ENSG00000250667	AC106774.3	0	0	0	0	0	0	0	0
ENSG00000284373	TAF11L2	0.36834	0	0.18043	0	0.14927	0	0	0.3015
ENSG00000248205	AC106774.1	0	0	0	0	0	0	0	0
ENSG00000249620	AC106774.2	0	0	0	0	0	0	0	0
ENSG00000284439	TAF11L3	0	0	0	0	0	0	0	0
ENSG00000284283	TAF11L4	0	0	0	0	0	0	0	0
ENSG00000284234	TAF11L5	0	0	0	0	0	0	0	0
ENSG00000284042	TAF11L6	0	0	0	0	0	0	0	0
ENSG00000284465	TAF11L7	0	0	0	0	0	0	0	0
ENSG00000250088	AC233724.7	0	0	0	0	0	0	0	0
ENSG00000283967	TAF11L8	0	0	0	0	0	0	0	0
ENSG00000283988	TAF11L9	0	0	0	0	0	0	0	0
ENSG00000284356	TAF11L10	0	0	0	0	0	0	0	0
ENSG00000248422	AC233724.3	0	0	0	0	0	0	0	0
ENSG00000249282	AC233724.6	0	0	0	0	0	0	0	0
ENSG00000283740	TAF11L11	0	0	0	0	0	0	0	0
ENSG00000249156	TAF11L12	0	0	0	0	0	0	0	0
ENSG00000271296	AC233724.11	0	0	0	0	0	0	0	0
ENSG00000250351	AC233724.8	0	0	0	0	0	0	0	0
ENSG00000250386	AC233724.9	0	0	0	0	0	0	0	0
ENSG00000283776	TAF11L13	0	0	0	0	0	0	0	0
ENSG00000250782	TAF11L14	0	0	0	0	0	0	0	0
ENSG00000248471	AC233724.4	0	0	0	0	0	0	0	0
ENSG00000248160	AC233724.1	0	0	0	0	0	0	0	0
ENSG00000248542	AC233724.5	0	0	0	0	0	0	0	0
ENSG00000248337	AC233724.2	0	0	0	0	0	0	0	0
ENSG00000269466	H3.Y	0	0	0	0	0	0	0	0
ENSG00000274832	AC233724.12	0	0	0	0	0	0	0	0
ENSG00000250715	AC233724.10	0	0	0	0	0	0	0	0
ENSG00000249937	LINC02223	0	0.19742	0	0.08801	0	0	0	0
ENSG00000240785	RPL36AP21	0.11435	0	0	0	0	0.20173	0	0
ENSG00000212278	RF00136	0	0	0	0	0	0	0	0
ENSG00000241552	RN7SL58P	0	0	0	0	0	0	0	0
ENSG00000248693	LINC02100	2.15589	2.5632	1.35774	1.37125	0.74888	4.34661	3.08018	0.37814
ENSG00000249191	UBE2V1P12	0	0	0	0	0	0	0	0
ENSG00000249927	AC025768.1	0	0	0	0	0	0	0	0
ENSG00000249285	AC114981.1	0	0	0	0	0	0	0	0
ENSG00000276764	RF00017	0	0	0	0	0	0	0	0
ENSG00000249174	AC106744.1	0	0	0	0	0	0	0	0
ENSG00000251487	AC106744.2	0	0	0	0	0	0	0	0
ENSG00000242654	RPL32P14	0	0	0	0	0	0	0	0
ENSG00000249557	HSPD1P15	0	0	0	0	0	0	0	0
ENSG00000145526	CDH18	0	0	0	0	0	0	0	0
ENSG00000248766	AC093303.1	0	0	0	0	0	0	0	0
ENSG00000214132	AC094103.1	0.10442	0	0	0	0	0	0	0
ENSG00000249854	CDH18-AS1	0	0	0	0	0	0	0	0
ENSG00000251629	LINC02241	0	0	0	0	0	0	0	0
ENSG00000249490	LINC02146	0	0	0	0	0	0	0	0
ENSG00000248308	AC138938.1	0	0	0	0	0	0	0	0
ENSG00000248286	AC140172.1	0	0	0	0	0	0	0	0
ENSG00000249359	AC093274.1	0	0	0	0	0	0	0	0
ENSG00000183666	GUSBP1	0.84623	0.55549	0.4904	1.66274	1.28966	1.30363	1.15227	1.00972
ENSG00000233974	AC138951.1	0	0	0	0	0	0	0	0
ENSG00000279107	AC138951.2	0	0	0	0	0	0	0	0
ENSG00000272130	AC091946.2	0	0	0	0	0	0	0	0
ENSG00000253766	AC091946.1	0	0	0	0	0	0	0	0
ENSG00000154162	CDH12	0	0	0	0	0.01503	0	0	0

ENSG00000213430	HSPD1P1	0.04247	0	0	0.18908	0	0.07492	0	0.05214
ENSG00000253104	AC139497.1	0	0	0	0	0	0	0	0
ENSG00000168967	PMCHL1	0	0	0	0	0	0	0	0
ENSG00000249206	GCNT1P2	0	0	0	0	0	0	0	0
ENSG00000249404	AC010445.1	0	0	0	0	0	0	0	0
ENSG00000250319	AC010460.2	0	0	0	0	0	0	0	0
ENSG00000206912	RF00019	0	0	0	0	0	0	0	0
ENSG00000250332	AC010460.3	0	0	0	0	0	0	0	0
ENSG00000249289	AC010460.1	0	0	0	0	0	0	0	0
ENSG00000164256	PRDM9	0.01752	0	0	0	0	0	0	0
ENSG00000248591	AC109445.1	0	0	0	0	0	0	0	0
ENSG00000249607	AC108103.1	0	0	0	0	0	0	0	0
ENSG00000248874	C5orf17	1.6995	0	0	0	0.7636	1.10801	0	0
ENSG00000252083	RF00561	0	0	0	0	0	0	0	0
ENSG00000234865	AC026784.1	0.41648	0	0	0	0	0.36736	0	0
ENSG00000200600	RF00019	0	0	0	0	0	0	0	0
ENSG00000226432	AC010342.1	0.33432	0	0	0.49617	0	0.2949	0	0
ENSG00000250694	AC010387.1	0	0	0	0	0	0	0	0
ENSG00000277455	RF00017	0	0	0	0	0	0	0	0
ENSG00000040731	CDH10	0.02003	0	0	0.05945	0	0	0	0
ENSG00000251294	AC091885.2	0	0	0	0	0	0	0	0
ENSG00000249562	AC091885.1	0	0	0	0	0	0	0	0
ENSG00000250099	AC091893.1	0	0	0	0	0	0	0	0
ENSG00000248156	BTG4P1	0	0	0	0	0	0	0	0
ENSG00000223004	RF00070	0	0	0	0	0	0	0	0
ENSG00000248908	LINC02239	0	0	0	0	0	0	0	0
ENSG00000250524	AC106821.1	0	0	0	0	0	0	0	0
ENSG00000199911	RF00019	0	0	0	0	0	0	0	0
ENSG00000251273	LINC02228	0	0	0	0	0	0	0	0
ENSG00000245662	LINC02211	0.07823	0	0	0.3483	0	0	0	0
ENSG00000251654	AC099499.1	0	0	0	0	0	0	0	0
ENSG00000260761	AC106754.1	0	0	0	0	0	0	0	0
ENSG00000279671	AC106754.2	0	0	0	0	0	0	0	0
ENSG00000248605	AC022140.1	0	0	0	0	0	0	0	0
ENSG00000279531	AC022140.2	0	0	0	0	0	0	0	0
ENSG00000201016	RNU6-374P	0	0	0	0	0	0	0	0
ENSG00000279869	AC022418.1	0	0	0	0	0	0	0	0
ENSG00000251593	MSNP1	0.04232	0.28177	0	0	0	0	0	0.10392
ENSG00000222560	RNU4-43P	0	0	0	0	0	0	0	0
ENSG00000249099	AC025475.1	0	0	0	0	0	0	0	0
ENSG00000230571	AC113347.1	0	0	0	0	0	0	0	0
ENSG00000248426	AC113347.2	0	0	0	0	0	0	0	0
ENSG00000250703	AC113347.3	0	0	0	0	0	0	0	0
ENSG00000251033	AC113347.4	0	0	0	0	0	0	0	0
ENSG00000249616	CCNB3P1	0	0	0	0	0	0	0	0
ENSG00000113100	CDH9	0	0	0	0	0	0	0	0
ENSG00000207261	RNU6-738P	0	0	0	0	0	0	0	0
ENSG00000248490	AC113355.1	0	0	0	0	0	0	0	0
ENSG00000250337	PURPL	0	0	0	0.04124	0	0.04902	0	0
ENSG00000250079	AC093300.1	0	0	0	0	0	0	0	0
ENSG00000249785	LINC02103	0	0	0	0	0	0	0	0
ENSG00000252867	RNU6-909P	0	0	0	0	0	0	0	0
ENSG00000250453	AC008825.1	0	0	0	0	0	0	0	0
ENSG00000270495	AC008825.2	0	0	0	0	0	0	0	0
ENSG00000271035	AC010385.2	0	0	0	0	0	0	0	0
ENSG00000162685	LSP1P3	0	0	0	0	0	0	0	0
ENSG00000226400	AC010385.1	0	0	0	0	0	0	0	0
ENSG00000277878	AC010385.3	0	0	0	0	0	0	0	0

ENSG00000249226	SUCLG2P4	0	0	0	0	0	0	0	0
ENSG00000252601	RF00425	0	0	0	0	0	0	0	0
ENSG00000248687	AC024589.1	0	0	0	0	0	0	0	0
ENSG00000270430	AC024589.2	0	0	0	0	0	0	0	0
ENSG00000259786	LINC02109	0	0	0	0	0	0	0	0
ENSG00000248391	LINC02064	0	0	0	0	0	0	0	0
ENSG00000276098	AC027345.1	0	0	0	0	0	0	0	0
ENSG00000248418	UBL5P1	0	0	0	0	0	0	0	0
ENSG00000250984	AC108082.1	0	0	0	0	0	0	0	0
ENSG00000249744	AC010374.2	0	0	0	0	0	0	0	0
ENSG00000233787	AC010374.1	0	0	0	0	0	0	0	0
ENSG00000223136	RN7SKP207	0	0	0	0	0	0	0	0
ENSG00000280305	AC018765.1	0	0	0	0	0	0	0	0
ENSG00000250587	HPRT1P2	0	0	0	0	0	0	0	0
ENSG00000250118	AC114300.1	0	0	0	0	0	0	0	0
ENSG00000241668	RPL19P11	0	0	0	0	0	0	0	0
ENSG00000253940	AC026421.1	0	0	0	0	0	0	0	0
ENSG00000254138	AC113386.1	0.96195	1.28093	0	0	0.2339	0	1.60342	0.94485
ENSG00000113361	CDH6	0.10658	0.00986	0	0	0.0144	0	0.00987	0
ENSG00000250482	DUX4L51	0	0	0	0	0	0	0	0
ENSG00000113360	DROSHA	5.86026	7.4491	3.95966	9.91376	7.81203	8.82578	4.56998	3.89833
ENSG00000206682	RF00019	0	0	0	0	0	0	0	0
ENSG00000082213	C5orf22	5.56956	4.24047	1.72983	3.23332	4.59254	5.7651	2.07929	2.78275
ENSG00000279923	AC022417.1	0.39379	0.18727	0.08267	0	0	0.19848	0.09377	0.06907
ENSG00000199765	RNU6-363P	0	0	0	0	0	0	0	0
ENSG00000133401	PDZD2	0.04686	0.0312	0	0.01391	0.09116	0.05787	0.03906	0.04027
ENSG00000248624	AC022447.2	0	0	0	0	0	0	0	0
ENSG00000248878	AC022447.4	0	0	0	0	0	0	0	0
ENSG00000249114	AC022447.5	0	0	0	0	0	0	0	0
ENSG00000248813	AC022447.3	0	0	0	0	0	0	0	0
ENSG00000248378	AC022447.1	0	0	0	0	0	0	0	0
ENSG00000250164	AC022447.7	0	0	0	0	0	0	0	0
ENSG00000249688	AC022447.6	0	0	0	0	0	0	0	0
ENSG00000252373	RNU6-358P	0	0	0	0	0	0	0	0
ENSG00000224282	AC010469.1	0	0	0	0	0	0	0	0
ENSG00000251887	RNU6-760P	0	0	0	0	0	0	0	0
ENSG00000241739	AC010469.2	0	0	0	0	0	0	0	0.18749
ENSG00000243914	RPL5P14	0	0	0	0	0	0	0	0
ENSG00000214108	TPT1P5	0.14069	0	0	0.2088	0.17105	0	0	0.17274
ENSG00000249774	AC025458.1	0	0	0	0	0	0	0	0
ENSG00000266243	MIR4279	0	0	0	0	0	0	0	0
ENSG00000250764	AC025178.1	0.52734	0.29259	0	0.26088	0.32056	0.6202	0.1465	0.10791
ENSG00000113384	GOLPH3	27.4514	23.2627	10.636	19.0619	22.7512	24.6551	13.1174	19.5182
ENSG00000200065	RF00019	0	0	0	0	0	0	0	0
ENSG00000272086	AC025181.2	1.64514	0.76065	0	0.40693	1.2223	0.32247	0.15234	0.11222
ENSG00000230829	AC025181.1	0	0	0	0	0	0	0	0
ENSG00000150712	MTMR12	8.9626	6.88704	1.98139	4.80658	8.03577	8.32539	5.37637	5.30731
ENSG00000199731	RNU6-1079P	2.66547	1.10917	0	0	0.81015	0	0	0
ENSG00000207052	RNU6-378P	0	0	0	0	0	0	0	0
ENSG00000056097	ZFR	19.3503	14.7122	3.33038	6.55085	15.5351	15.6309	6.36806	8.67905
ENSG00000207956	MIR579	0	0	0	0	0.90935	0	0	0.91834
ENSG00000249142	AC074134.1	0	0	0	0	0	0	0	0.16245
ENSG00000113387	SUB1	20.3219	14.4856	4.34368	7.80541	15.4071	19.5826	6.42089	9.14063
ENSG00000250885	LINC02061	0	0	0	0	0	0	0	0
ENSG00000113389	NPR3	0.02229	0	0.02184	0.05513	0.12646	0.05243	0.01238	0.04561
ENSG00000251062	AC025459.1	0	0	0	0	0	0	0	0
ENSG00000250697	AC010343.3	0	0	0.11002	0	0	0	0	0.09193
ENSG00000248279	LINC02120	0	0	0	0	0	0	0	0

ENSG00000249102	AC034223.1	0	0	0	0	0	0	0	0
ENSG00000251281	AC034223.2	0	0	0	0	0	0	0	0
ENSG00000250672	AC010343.2	0	0	0	0	0	0	0	0
ENSG00000240376	AC010343.1	0	0	0	0.17295	0	0	0	0
ENSG00000251443	LINC02160	0	0	0	0	0	0	0	0
ENSG00000249572	AC034231.1	0.546	0.45441	0.60175	0.60774	0	0.48161	0	0
ENSG00000113407	TARS	10.1656	11.501	3.71103	3.1011	7.8394	11.805	2.7778	5.65039
ENSG00000249360	AC034232.1	0	0	0	0	0	0	0	0
ENSG00000250583	AC034232.2	0	0	0	0	0	0	0	0
ENSG00000151388	ADAMTS12	0	0	0	0	0.00911	0	0	0
ENSG00000201623	RNU6-923P	0	0	0	0	0	0	0	0
ENSG00000182631	RXFP3	0	0	0	0	0	0	0	0
ENSG00000164175	SLC45A2	0	0	0	0	0	0	0.0374	0
ENSG00000250914	AC139783.2	0	0	0	0	0	0	0	0
ENSG00000242110	AMACR	0.24242	0.15131	0.02226	0.04497	0.14736	0.10691	0.05051	0.09301
ENSG00000273294	C1QTNF3-AMACR	0	0.1069	0.03146	0.03177	0.05205	0	0.07137	0
ENSG00000250767	AC139783.1	0.21527	0	0	0.15974	0	0	0	0
ENSG00000082196	C1QTNF3	0.22548	0.20471	0	0.09126	0.27413	0.03616	0.3075	0.12583
ENSG00000278900	AC139792.2	0.12487	0.20785	0	0.18532	0	0	0	0
ENSG00000279995	AC139792.3	0.32723	0	0	0	0.39784	1.15457	0	0.80355
ENSG00000271771	AC139792.1	0	0	0	0	0	0	0	0
ENSG00000215158	AC138409.2	0.85894	0.85412	0.34424	1.00988	0.56961	0.72813	1.02266	0.49306
ENSG00000215156	AC138409.1	0	0	0	0	0	0	0	0
ENSG00000249380	AC138853.1	0	0	0	0	0	0	0	0
ENSG00000271874	AC025754.2	0	0	0	0	0	0	0	0
ENSG00000039560	RAI14	0.00954	0.01587	0.01401	0.05661	0.04637	0	0.01589	0.01171
ENSG00000250234	AC025754.1	0	0	0	0	0	0	0	0
ENSG00000272323	AC026801.2	0	0	0	0	0	0.08263	0	0
ENSG00000205838	TTC23L	0	0	0	0	0	0	0	0
ENSG00000250140	AC026801.1	0	0	0	0	0	0	0	0
ENSG00000241829	RPL21P54	0	0	0	0	0	0	0	0
ENSG00000113456	RAD1	1.11586	1.11802	0.4776	0.54666	1.1459	1.41429	0.61397	1.0109
ENSG00000113460	BRIX1	4.22746	3.74961	4.85798	8.07589	4.87287	5.0837	5.85178	4.83121
ENSG00000168724	DNAJC21	4.51659	2.63059	0.76627	2.1669	3.12108	3.45322	1.45755	1.9995
ENSG00000113492	AGXT2	0	0	0	0	0	0	0	0
ENSG00000113494	PRLR	0.12302	0.10239	0.03287	0.09129	0.11558	0.03946	0.14914	0.08926
ENSG00000250886	AC010368.1	0	0	0	0	0	0	0	0
ENSG00000201368	RF00012	0	0	0	0	0	0	0	0
ENSG00000152582	SPEF2	0.19074	0.12095	0.09343	0.18872	0.24295	0.05608	0.13626	0.14498
ENSG00000238441	RNU7-130P	0	0	0	0	0	0	0	0
ENSG00000248969	AC137810.1	0.27148	0.22594	0	0.20146	0.16503	0	0.22626	0.16666
ENSG00000168685	IL7R	72.1754	28.1062	11.358	46.3327	79.0727	35.9768	41.876	34.8404
ENSG00000279737	AC112204.3	0	0	0	0	0	0	0	0
ENSG00000248349	AC112204.1	0	0	0	0	0	0	0	0
ENSG00000152611	CAPSL	0.06458	0	0	0	0	0	0	0
ENSG00000249748	AC112204.2	0	0	0	0	0	0	0	0
ENSG00000145626	UGT3A1	0	0	0	0	0	0	0	0
ENSG00000168671	UGT3A2	0	0	0	0	0	0.048	0	0.03341
ENSG00000251591	AC016612.1	0	0	0	0	0	0	0	0
ENSG00000164187	LMBRD2	4.14333	3.62215	1.50935	4.22431	4.36006	4.315	2.21988	3.27029
ENSG00000207756	MIR580	0	0	0	0	0	0	0	0
ENSG00000145604	SKP2	2.04408	1.14472	0.42109	1.21913	1.9045	2.39279	1.14634	1.4073
ENSG00000201223	RNU6-1305P	0	1.17316	0	0	0	0	1.17481	0
ENSG00000152620	NADK2	1.99569	1.39866	0.77175	1.80382	2.06145	2.09122	1.52569	1.2712
ENSG00000245711	NADK2-AS1	0	0	0	0	0	0	0	0
ENSG00000164188	RANBP3L	0	0	0	0	0.0795	0	0	0
ENSG00000222178	RNA5SP181	0	0	0	0	0	0	0	0
ENSG00000242292	AC010631.1	0	0	0	0	0	0	0	0

ENSG00000079215	SLC1A3	0.0234	0.21422	0.05158	0	0.02845	0.08256	0	0.0431
ENSG00000274441	AC008957.2	0	0	0	0	0	0	0	0
ENSG00000250155	AC008957.1	1.77085	0.31581	0.09294	0.28159	1.15337	0.22314	0.31626	0.23295
ENSG00000279400	AC008957.3	0	0	0	0	0	0	0	0
ENSG00000272103	AC026741.1	0.30415	0.25313	0	0	0	0.26828	0	0.18672
ENSG00000164190	NIPBL	23.9576	22.6411	7.17004	11.2875	22.4694	21.2922	14.7252	16.3559
ENSG00000249850	KRT18P31	2.04242	2.36085	0.33348	0.5894	1.58644	1.10095	1.4185	1.11451
ENSG00000244073	RPS4XP6	0	0	0.13515	0.27299	0	0	0	0
ENSG00000197603	CPLANE1	1.83904	2.11054	1.28722	4.19456	2.64773	4.22615	1.83117	2.04998
ENSG00000271378	C8orf59P2	0	0	0	0	0	0	0	0
ENSG00000228212	OFD1P17	0	0	0.03569	0	0.02953	0	0.04048	0
ENSG00000242493	RN7SL37P	0	0	0	0.35435	0	0	0	0
ENSG00000270558	AC025449.1	0.69282	0.46128	0.20362	0.41129	1.17924	0.48889	0.46193	0.17013
ENSG00000113569	NUP155	3.1994	2.37859	1.48161	3.5229	3.66775	5.92428	3.18915	2.65127
ENSG00000251880	RNU7-75P	0	0	0	0	0	0	0	0
ENSG00000250455	AC117532.1	0	0	0	0	0	0	0	0.27863
ENSG00000082068	WDR70	3.43644	1.85465	0.50498	1.02001	1.86108	2.14938	0.95467	1.38083
ENSG00000248216	KCTD9P5	0	0	0	0	0	0	0	0
ENSG00000222743	RNU6-1190P	0	0	0	0	0	0	0	0
ENSG00000206743	RNU6-484P	0	0	0	0	0	0	0	0
ENSG00000248587	GDNF-AS1	0	0	0	0	0	0	0	0
ENSG00000168621	GDNF	0	0	0	0	0	0	0	0
ENSG00000248539	LINC02117	0	0	0	0	0	0	0	0
ENSG00000250032	AC008869.1	0	0	0	0	0	0	0	0
ENSG00000251518	LINC02110	0	0	0	0	0	0	0	0
ENSG00000248533	AC034226.1	0	0	0	0	0	0	0	0
ENSG00000250003	LINC02107	0	0	0	0	0	0	0	0
ENSG00000248461	LINC02119	0	0	0	0	0	0	0	0
ENSG00000276332	AC010338.1	0	0	0	0	0	0	0	0
ENSG00000164318	EGFLAM	0.02561	0	0	0	0	0	0	0
ENSG00000248730	EGFLAM-AS4	0	0	0	0	0	0	0	0
ENSG00000249071	EGFLAM-AS3	0	0	0	0	0	0	0	0
ENSG00000248572	EGFLAM-AS2	0	0	0	0	0	0	0	0
ENSG00000249491	EGFLAM-AS1	0	0	0	0	0	0	0	0
ENSG00000248234	AC091839.1	0	0	0	0	0	0	0	0
ENSG00000251257	AC010457.1	0	0	0	0	0	0	0	0
ENSG00000113594	LIFR	0	0.02057	0.00908	0	0.01502	0	0	0
ENSG00000244968	LIFR-AS1	0	0	0	0	0	0	0	0
ENSG00000265304	MIR3650	0	0	0	0	0	0	0	0
ENSG00000251189	AC010425.1	0	0	0	0	0	0	0	0
ENSG00000249740	OSMR-AS1	0	0	0	0	0	0	0	0
ENSG00000248577	AC091435.1	0	0	0	0	0	0	0	0
ENSG00000250629	AC091435.2	0	0	0	0	0	0	0	0
ENSG00000145623	OSMR	0	0	0	0	0	0	0	0
ENSG00000164327	RICTOR	24.4623	25.7613	8.1322	14.3629	20.1187	15.1452	24.4361	19.8193
ENSG00000249089	AIG1P1	0	0	0	0	0	0	0	0
ENSG00000082074	FYB1	155.132	188.793	30.0186	41.2316	119.066	93.3165	36.9345	83.6383
ENSG00000251215	GOLGA5P1	1.49593	1.74298	0.21982	0.44402	0.27281	0.65975	0.74804	0.8265
ENSG00000113600	C9	0	0	0	0	0	0	0	0
ENSG00000153071	DAB2	6.68698	4.75892	1.11664	2.59383	3.6723	3.63621	1.88408	2.89222
ENSG00000271334	LINC02104	0	0	0	0	0	0	0	0
ENSG00000251515	CCDC11P1	0	0	0	0	0	0	0	0
ENSG00000250492	INTS6P1	0	0	0.04257	0	0	0	0.14488	0
ENSG00000248651	GCSHP1	0	0	0	0	0	0	0	0
ENSG00000250048	LINC00603	0	0	0	0	0	0	0	0
ENSG00000249668	KRT18P56	0	0	0	0	0	0	0	0
ENSG00000250585	LINC00604	0	0	0	0	0	0	0	0
ENSG00000199361	RNU1-150P	0	0	0	0	0	0	0	0

ENSG00000283286	AC093277.1	0.18077	0	0.06641	0	0	0	0	0
ENSG00000113638	TTC33	3.91164	3.68476	2.10027	4.81649	4.2592	3.88637	3.85116	4.63113
ENSG00000199552	RF00092	0	0	0	0	0	0	0	0
ENSG00000171522	PTGER4	21.855	17.311	3.58956	8.76108	13.3416	17.9554	6.32339	8.95199
ENSG00000132356	PRKAA1	18.9868	16.7499	5.77713	10.6747	19.2138	15.3685	9.0104	12.9312
ENSG00000212567	RF00191	2.29064	0.95319	0	0	0	0	0.95453	0
ENSG00000145592	RPL37	43.5235	34.8232	12.7756	22.8833	50.8926	56.0198	25.6259	33.7227
ENSG00000212296	SNORD72	1.83251	1.52511	0	6.79911	3.34188	0	3.05451	4.49985
ENSG00000132357	CARD6	8.59809	9.62422	1.73851	2.49769	4.37581	4.23298	1.08321	3.39612
ENSG00000112936	C7	0	0	0	0	0	0.02476	0	0
ENSG00000253098	RNU7-161P	0	0	0	0	0	0	0	0
ENSG00000248120	AC114967.1	0	0	0	0	0	0	0	0
ENSG00000171495	MROH2B	0	0	0	0	0	0	0	0
ENSG00000039537	C6	0	0	0	0	0	0	0	0
ENSG00000182836	PLCXD3	0	0	0	0	0.02305	0	0	0
ENSG00000251478	AC008817.1	0	0	0	0	0	0	0	0.05762
ENSG00000083720	OXCT1	5.17332	4.16004	2.51692	4.79861	7.24578	11.0997	5.15639	4.07712
ENSG00000248668	OXCT1-AS1	0	0	0	0.15804	0.12947	0.06262	0	0
ENSG00000240281	AC034222.1	0	0	0	0	0	0	0	0
ENSG00000205765	C5orf51	4.38066	3.3064	1.73982	3.24092	4.33428	3.80602	1.88577	3.35955
ENSG00000151876	FBXO4	1.75104	1.21442	1.25083	2.05733	1.68535	2.23099	1.6215	1.46312
ENSG00000239694	RPSAP38	0	0	0	0	0	0	0	0
ENSG00000199487	RF00019	0	0	0	0	0	0	0	0
ENSG00000249266	MTHFD2P6	0	0	0	0	0	0	0	0
ENSG00000260786	AC108099.1	0	0	0	0	0	0	0	0
ENSG00000112964	GHR	0	0.07383	0	0	0.04045	0.01956	0	0.01362
ENSG00000248873	SERBP1P6	0	0	0	0	0.09562	0	0	0.19313
ENSG00000250860	AC093225.1	0	0	0	0	0	0	0	0
ENSG00000270513	AC113368.1	0	0	0	0	0	0	0	0
ENSG00000198865	CCDC152	0.70502	0.1778	0.21976	0.28536	0.80519	0.94223	0.64099	0.86561
ENSG00000250722	SELENOP	0.01289	0.0858	0.34087	0.28688	0.06267	0.34101	0.06444	0.1424
ENSG00000272234	AC008945.1	0.24272	0	0	0	0	0	0.20229	0
ENSG00000249092	PPIAP77	0	0	0	0	0	0	0	0
ENSG00000251163	PRELID3BP4	0	0	0	0	0	0	0	0
ENSG00000251656	PRELID3BP5	0	0	0	0	0	0	0	0
ENSG00000249516	PRELID3BP6	0	0	0	0	0	0	0	0
ENSG00000250711	PRELID3BP7	0	0	0	0	0	0	0	0
ENSG00000271788	AC008875.1	0	0	0	0.26828	0.10988	0.15945	0	0.11097
ENSG00000177738	AC025171.1	1.10582	0.65737	0.15476	0.3126	1.37644	1.30055	0.39498	1.03445
ENSG00000251131	AC025171.3	1.3255	1.10315	0.19478	0.9836	1.77266	1.87069	0.66282	0.81372
ENSG00000272382	AC025171.5	0.15935	0	0.11708	0	0	0	0	0.19565
ENSG00000177721	ANXA2R	2.30408	1.43178	0.67716	1.23102	2.80124	3.68533	1.84346	2.30085
ENSG00000215068	AC025171.2	1.18951	0.83598	0.23306	0.62769	0.89984	0.88602	0.92527	1.39555
ENSG00000272144	AC025171.4	0.28004	0	0	0	0.34046	0	0.35009	0.25787
ENSG00000172262	ZNF131	7.62613	8.22368	3.64274	7.1025	12.7668	11.8591	13.1276	8.39099
ENSG00000278487	AC106800.4	0	0	0	0	0	0	0	0
ENSG00000177453	NIM1K	0.02025	0	0	0	0.02462	0.07146	0	0
ENSG00000249779	AC106800.2	0	0	0	0	0.08079	0	0	0
ENSG00000249888	AC106800.3	0	0	0	0	0	0	0	0
ENSG00000247763	AC106800.1	0	0	0	0	0	0	0	0
ENSG00000261604	AC114947.2	0	0	0	0	0	0	0	0
ENSG00000112972	HMGCS1	5.92389	4.25551	1.85556	3.1465	5.06043	8.03042	3.71582	3.06243
ENSG00000188850	AC114947.1	0	0	0	0	0	0	0	0
ENSG00000151882	CCL28	0.81013	0.24272	0.04762	0.3607	1.14252	0.94326	0.29708	0.35808
ENSG00000151881	TMEM267	1.78725	1.42949	1.02325	1.41235	1.91892	2.17021	1.00592	0.99743
ENSG00000249492	AC114956.3	1.06878	0.59299	0.52352	0.66091	0.86626	0.94273	1.18766	0.98417
ENSG00000172244	C5orf34	0.46254	0.56729	0.14309	1.33679	0.56234	0.60125	0.93329	0.59779
ENSG00000249855	EEF1A1P19	0	0.08828	0	0.07872	0.25794	0	0	0.13024

ENSG00000248554	AC114956.2	0	0.2133	0	0	0	0	0	0
ENSG00000248240	AC114956.1	0.28633	0	0.21038	0.42494	0.34811	0.25256	0.7159	0.17578
ENSG00000172239	PAIP1	8.40868	6.83622	2.28706	5.00459	9.01416	7.5505	4.28766	4.96298
ENSG00000248092	NNT-AS1	0.56813	0.22251	0.27829	0.46292	0.59592	0.68783	0.48278	0.80696
ENSG00000279557	AC010435.1	0	0	0	0	0	0	0.10381	0
ENSG00000249286	AMD1P3	0.07374	0	0.10836	0	0	0	0.12292	0
ENSG00000112992	NNT	7.66342	5.81557	3.30523	8.07482	6.57161	9.34746	4.93463	5.33761
ENSG00000224993	RPL29P12	0	0	0	0	0	0	0	0
ENSG00000249276	AC104119.1	0	0	0	0	0	0	0	0
ENSG00000212561	RNU6-381P	0	0	0	0	0	0	0	0
ENSG00000070193	FGF10	0	0	0	0	0	0	0	0
ENSG00000248464	FGF10-AS1	0	0	0	0	0	0	0	0
ENSG00000249203	LINC02224	6.45698	0	2.13824	0.94479	0	0	0	3.12645
ENSG00000250418	AC093292.1	0	0	0	0	0	0	0	0
ENSG00000263556	RN7SL383P	0	0	0	0	0	0	0	0
ENSG00000251141	MRPS30-DT	0.03315	0.16555	0.04872	0.0984	0.04031	0.05849	0	0.08141
ENSG00000248779	AC093297.1	0	0	0	0	0	0	0	0
ENSG00000112996	MRPS30	5.25938	4.11154	1.2331	2.45566	5.53207	5.838	2.10792	2.84415
ENSG00000272335	AC093297.2	0.96103	0.58169	0.17118	0.73475	1.41624	1.13026	0.67959	1.39447
ENSG00000248148	AC114954.1	0	0	0	0	0	0	0	0
ENSG00000164588	HCN1	0	0	0	0	0	0	0	0
ENSG00000250422	AC117529.2	0	0	0	0	0	0	0	0
ENSG00000250017	AC117529.1	0	0	0	0	0	0	0	0
ENSG00000250122	AC122694.1	0	0	0	0	0	0	0	0
ENSG00000170571	EMB	53.7936	52.1733	20.1003	33.7511	46.0416	43.1203	25.2402	44.853
ENSG00000271455	AC112187.2	0	0	0	0	0	0	0	0
ENSG00000213956	AC112187.1	0	0	0	0	0	0	0	0
ENSG00000271752	AC112187.3	0	0.29904	0.52801	0	0	0	0	0
ENSG00000151883	PARP8	29.7692	29.4223	6.8553	13.609	22.7666	19.1987	9.69569	16.1988
ENSG00000249405	AC022441.1	0	0	0	0	0	0	0	0
ENSG00000270830	AC022441.3	0	0	0	0	0	0	0	0
ENSG00000251601	AC022441.2	0	0	0	0	0	0	0	0
ENSG00000250360	AC008808.2	0	0	0	0	0	0	0	0
ENSG00000251573	LINC02106	0	0	0	0	0	0	0	0
ENSG00000248918	AC008808.1	0	0	0	0	0	0	0	0
ENSG00000207060	RNU6-480P	0	0	0	0	0	0	0	0
ENSG00000278734	AC091834.1	0	0	0	0	0	0	0	0
ENSG00000206606	RNU6-1296P	0	0	0	0	0	0	0	0
ENSG00000240627	AC026798.1	0	0	0	0	0	0	0	0
ENSG00000259663	AC010478.1	0	0	0	0	0	0	0	0
ENSG00000016082	ISL1	0	0	0	0	0	0	0	0
ENSG00000249338	HMGB1P47	0	0	0	0	0	0	0	0
ENSG00000248891	AC091860.1	0	0	0	0	0	0	0	0
ENSG00000251125	AC091860.2	0	0	0	0	0	0	0	0
ENSG00000251873	RNA5SP182	0	0	0	0	0	0	0	0
ENSG00000249270	KATNBL1P4	0	0	0	0	0	0	0	0
ENSG00000279470	AC091903.1	0	0	0	0	0	0	0	0
ENSG00000248789	LINC02118	0	0	0	0	0	0	0	0
ENSG00000244621	RPS17P11	0	0	0	0	0	0	0	0
ENSG00000250827	MFSD4BP1	0	0	0	0	0	0	0	0
ENSG00000248898	AC022126.1	0	1.02874	0	0	0.15028	0	0.20604	0
ENSG00000213949	ITGA1	0.71855	0.47973	0.08122	0.21094	0.6288	0.43183	0.12504	0.29569
ENSG00000152684	PELO	1.65174	1.5991	0.34675	0.57532	1.55734	1.99216	1.20804	1.09677
ENSG00000251237	B3GNTL1P1	0	0	0	0.37642	0	0	0	0
ENSG00000241809	AC022133.1	0	0	0	0	0	0	0	0
ENSG00000249899	AC025180.1	0	0	0	0.17805	0	0	0	0
ENSG00000164171	ITGA2	1.62169	0.33934	0.35405	0.22005	0.29292	0.19617	0.35527	0.21617
ENSG00000272123	AC008966.2	0.64724	0	0	0.48029	0	0.28546	0	0.39734

ENSG00000164172	MOCS2	1.39039	1.13685	0.62729	0.88694	1.33453	2.04403	1.11812	0.80863
ENSG00000247796	AC008966.1	0.16065	0.1146	0.03372	0.10218	0.25111	0.36437	0	0.08453
ENSG00000213940	AC026477.1	0	0	0	0	0	0	0	0
ENSG00000240052	AC108114.1	0	0	0	0	0	0	0	0
ENSG00000134363	FST	0	0	0	0.03664	0.03002	0	0.04115	0
ENSG00000164258	NDUFS4	15.9565	18.571	17.6775	50.2301	26.5986	22.6516	46.441	28.0858
ENSG00000250447	LINC02105	0	0	0	0	0	0	0	0
ENSG00000217791	ASS1P9	0	0	0	0	0	0	0	0
ENSG00000272416	AC025175.1	0.96575	0	0	0.14333	0.11741	0	0.16098	0
ENSG00000185305	ARL15	4.24706	3.21329	2.92192	3.3521	3.02767	3.67808	3.41088	3.69754
ENSG00000222960	RNU6-272P	0	0	0	0	0	0	0	0
ENSG00000207627	MIR581	0.76355	0	2.24404	1.13318	0.9283	0	0	0
ENSG00000241230	RN7SL801P	0.24272	0	0.35667	0	0	0	0	0
ENSG00000265421	MIR4459	5.55306	5.54584	1.63203	0	0	0	0	1.36359
ENSG00000279638	AC008873.1	1.06232	1.76824	0	0	0	1.87408	1.77073	0
ENSG00000249069	LINC01033	0.11626	0	0	0	0.07067	0	0.19378	0.07137
ENSG00000251597	RPL37P25	0	0	0	0	0	0	0	0
ENSG00000169271	HSPB3	1.12194	0.46687	0.27478	0	0	0.32988	0.15584	0
ENSG00000178996	SNX18	30.1002	31.6529	3.3352	6.36069	24.7807	14.2665	6.70311	12.648
ENSG00000280159	AC016583.1	0.0603	0	0	0.04475	0.14663	0	0	0.1851
ENSG00000232517	AC112198.2	0.16764	0.01744	0.03079	0.01555	0.05095	0.01848	0.06986	0.09005
ENSG00000226810	AC112198.1	0	0	0	0	0	0	0	0
ENSG00000249843	AC112198.4	0	0	0	0	0	0	0	0
ENSG00000249016	AC112198.3	0	0	0	0	0	0	0	0
ENSG00000164283	ESM1	0.13599	0.13582	0	0.1211	0.16534	0.43184	0	0.03339
ENSG00000240535	AC034238.1	0.03541	0.11788	0.02602	0.02628	0	0.03123	0.02951	0
ENSG00000113088	GZMK	12.7925	8.93693	2.95013	5.88967	15.0987	43.0765	3.96891	5.67497
ENSG00000207229	RF00019	0	0	0	0	0	0	0	0
ENSG00000251376	AC034238.3	0	0	0	0	0	0	0	0
ENSG00000248029	AC034238.2	0	0	0	0	0	0	0	0
ENSG00000249454	GZMAP1	0.3485	0	0.1707	0	0	0.61479	0	0
ENSG00000145649	GZMA	32.2226	45.3096	17.9523	28.1091	23.4255	126.997	17.7667	38.9585
ENSG00000164287	CDC20B	0	0	0	0	0	0	0	0
ENSG00000164294	GPX8	0	0	0	0	0	0	0	0
ENSG00000198983	MIR449A	0	0	0	0	0	0	0	0
ENSG00000207728	MIR449B	0	0	0	0	0	0	0	0
ENSG00000251856	MIR449C	0	0	0	0	0	0	0	0
ENSG00000234602	MCIDAS	0	0	0	0	0	0	0	0
ENSG00000152669	CCNO	0	0	0	0	0	0	0	0
ENSG00000251307	AC026704.1	0	0	0	0	0	0	0	0
ENSG00000067248	DHX29	9.98706	12.6545	8.66899	17.4875	15.6622	14.0402	14.0918	13.3836
ENSG00000039123	MTREX	9.02604	6.49179	3.42235	8.16968	9.46983	9.41629	7.26247	6.86812
ENSG00000271410	AC020728.1	0	0	0	0	0	0	0	0
ENSG00000067113	PLPP1	0.94534	0.3026	0.05343	0.26981	1.10512	0.57728	0.24242	0.40177
ENSG00000279883	AC010480.1	0	0	0	0	0	0	0	0
ENSG00000265135	MIR5687	0	0	0	0	0	0	0	1.16879
ENSG00000250853	RNF138P1	0	0	0	0.59772	0.65287	0	0	0.49449
ENSG00000249410	AK4P2	0	0	0	0	0	0	0	0
ENSG00000177058	SLC38A9	2.59206	3.92728	4.2607	8.34727	4.89874	4.63136	5.88537	4.69213
ENSG00000152670	DDX4	0.03829	0	0	0	0	0	0	0
ENSG00000251368	AC016632.1	0	0	0	0	0	0	0	0
ENSG00000199322	RNA5SP183	0	0	0	0	0	0	0	0
ENSG00000248854	HNRNPH1P3	0	0	0.08793	0	0	0	0	0
ENSG00000164509	IL31RA	0.28833	0.39994	0.11769	0.07132	0.1558	0.22607	0	0.118
ENSG00000134352	IL6ST	19.4689	16.5707	6.3293	11.4967	22.3367	15.99	12.3206	13.9083
ENSG00000262211	AC008914.1	0	0	0	0.13649	0	0	0	0
ENSG00000264281	AC016596.1	0	0	0	0	0	0	0	0
ENSG00000227908	FLJ31104	0.12809	0	0	0.09505	0.19466	0.05649	0.16013	0.07863

ENSG00000238326	RF01210	0	0	0	0	0	0	0	0
ENSG00000249236	AC008892.1	0	0	0	0	0	0	0	0.23437
ENSG00000164512	ANKRD55	0.68896	0.36861	0.2531	0.36518	0.77779	0.43408	0.24608	0.63442
ENSG00000253032	RNU6-299P	0	0	0	1.08786	0	0	0	0
ENSG00000243592	RPL17P22	0.13255	0.22063	0	0	0	0	0	0
ENSG00000223003	RNA5SP184	0	0	0	0	0	0	0	0
ENSG00000251435	C1GALT1P2	0.19919	0.22103	0.09757	0.19708	0	0	0.11067	0.16304
ENSG00000212265	RNA5SP185	0	0	0	0	0	0	0	0
ENSG00000248781	PSMC1P4	0	0	0	0.16396	0	0	0	0
ENSG00000210678	RNU6ATAC2P	0	0	0.85487	0	0	0	0	0
ENSG00000249697	AC034245.1	0	0	0	0	0	0	0	0
ENSG00000250787	HMGN1P17	0	0	0	0	0	0	0	0
ENSG00000248727	LINC01948	0.59802	0.99541	0	0	0.51933	0.15071	0.1424	0.41957
ENSG00000226221	RPL26P19	1.00411	0.27856	1.47553	0.99348	1.22078	0.29523	0.83685	0.41095
ENSG00000225940	C5orf67	0	0	0	0	0.23238	0	0	0.35201
ENSG00000234553	AC022431.1	0	0.45526	0.20096	0.20296	0.16626	0	0	0.33581
ENSG00000228650	AC008940.1	0	0	0	0	0	0	0	0
ENSG00000095015	MAP3K1	66.4795	45.0495	13.8202	24.6023	46.7528	26.4121	19.54	26.7565
ENSG00000237705	AC008937.2	0	0	0	0	0	0	0	0
ENSG00000225230	AC008937.1	0	0	0	0	0	0	0	0.09847
ENSG00000155542	SETD9	1.24375	0.54006	0.55625	0.88281	1.01904	0.85858	0.45068	0.39836
ENSG00000155545	MIER3	4.31178	3.4297	1.17751	2.45396	4.80918	5.1608	2.6077	2.7641
ENSG00000271828	AC008937.3	0.12935	0.07177	0	0	0.10484	0.30426	0	0.05294
ENSG00000235635	AC016644.1	0	0	0	0	0	0	0	0
ENSG00000233943	AC114973.1	0	0	0	0	0	0	0	0
ENSG00000248652	AC034244.1	0	0	0	0	0	0	0	0
ENSG00000250480	AC034244.2	0	0	0	0	0	0	0	0
ENSG00000207293	RF00019	0	0	0	0	0	0	0	0
ENSG00000062194	GPBP1	32.4483	38.5141	24.5818	44.8119	39.1056	33.8464	40.6827	41.5221
ENSG00000248586	AC025470.1	0	0	0	0	0	0	0	0
ENSG00000250961	AC025470.2	0.89889	0	0.12008	0.24255	0	0.28832	0.13621	0.40132
ENSG00000250308	SALL4P1	0	0	0	0	0	0	0	0
ENSG00000169067	ACTBL2	0	0	0	0	0	0	0	0
ENSG00000248294	AC008780.1	0	0	0	0	0	0	0	0
ENSG00000249436	AC008780.2	0	0	0	0	0	0	0	0
ENSG00000249198	AC106822.1	0	0	0	0	0	0	0	0
ENSG00000249584	LINC02225	0	0	0	0	0	0	0	0
ENSG00000248132	LINC02101	0	0	0	0	0	0	0	0
ENSG00000248271	PGAM1P1	0	0	0	0	0	0	0	0
ENSG00000145632	PLK2	0.50732	0.48619	0.13555	0.34224	0.54203	0.08136	0.23063	0.26425
ENSG00000175857	GAPT	43.6264	28.6289	6.74652	5.84977	31.666	11.339	4.25559	13.171
ENSG00000271257	AC008814.1	0	0	0	0	0	0	0	0.22167
ENSG00000265699	MIR548AE2	0	0	0	0	0	0	0	0
ENSG00000250127	LINC02108	0	0	0	0	0	0	0	0
ENSG00000152932	RAB3C	0.24292	0.02527	0	0.0338	0.13844	0	0.08857	0.13048
ENSG00000248475	AC016642.1	0.3155	0	0	0	0.12786	0	0.52588	0.6456
ENSG00000240412	RPL5P15	0	0	0	0	0	0	0	0
ENSG00000248733	AC008852.1	0	0	0	0	0	0	0	0
ENSG00000113448	PDE4D	9.98283	3.87542	1.61822	5.09641	4.06566	11.4896	6.68291	3.43256
ENSG00000247345	AC092343.1	0	0.33473	0	0.44768	0.12225	0.17738	0	0.61726
ENSG00000276214	AC008833.1	0	0	0	0	0	0	0	0
ENSG00000251306	NDUFB4P2	0	0	0	0	0	0	0	0
ENSG00000202601	MIR582	0.74796	0	0	0	0	1.31951	0	0
ENSG00000248935	AC034234.1	0	0	0	0	0	0	0	0
ENSG00000202017	RNU6-806P	0	0	0	0	0	0	0	0
ENSG00000213896	AC109486.1	0	0	0	0.29087	0	0	0	0
ENSG00000214244	SETP21	0	0	0	0.12196	0.09991	0	0	0
ENSG00000273701	RF02159	0	0	0	0	0	0	0	0

ENSG00000152931	PART1	0.00752	0	0	0	0	0	0	0
ENSG00000275634	RF02160	0	0	0	0	0	0	0	0
ENSG00000276233	RF02161	0	0	0	0	0	0	0	0
ENSG00000035499	DEPDC1B	1.09777	0.96171	1.24525	3.68717	2.64587	6.65925	6.45251	2.22273
ENSG00000251303	CAB39P1	0	0	0	0	0	0	0	0
ENSG00000250148	KRT8P31	0.15618	0	0	0	0	0	0	0
ENSG00000164181	ELOVL7	9.53097	7.06561	4.54301	8.27304	8.06257	6.1604	9.87909	12.1543
ENSG00000272308	AC104113.1	1.22422	0	0	0.22711	0.74419	0	0	0.18789
ENSG00000049167	ERCC8	1.37618	1.20825	0.7778	1.66085	1.45249	2.0009	1.61327	1.16976
ENSG00000215032	GNL3LP1	0.04461	0	0	0	0	0	0	0
ENSG00000233847	AC022445.1	0	0	0	0	0	0	0	0
ENSG00000164182	NDUFAF2	4.46865	4.31888	2.96558	4.17172	5.87101	4.19596	4.80552	3.00875
ENSG00000279985	AC008498.2	0	0	0	0	0	0	0	0
ENSG00000276945	AC008498.1	0.41413	0.45954	0.20285	0	0.50348	0.9741	0	0.16949
ENSG00000188725	SMIM15	9.79368	6.92606	3.16915	6.36372	8.82221	9.71293	4.69436	5.35808
ENSG00000251279	SMIM15-AS1	0.36834	0	0	0.27333	0	0	0	0.56531
ENSG00000249279	LINC02057	0	0	0	0	0	0	0	0
ENSG00000130449	ZSWIM6	8.1282	8.71648	1.37066	2.82791	5.47563	3.17344	2.70969	3.59923
ENSG00000251682	AC122718.2	2.97666	4.74822	0.54677	1.47256	1.5079	0.4376	1.44714	1.37051
ENSG00000250461	AC122718.1	2.56157	1.31192	0.57911	0	1.31759	0.52142	0.8211	1.08867
ENSG00000213891	RPL3P6	0	0	0	0	0	0	0	0.07475
ENSG00000178722	C5orf64	0.01813	0	0	0.02691	0	0	0.12088	0.02226
ENSG00000248529	AC026746.1	0	0	0	0	0	0	0	0
ENSG00000212215	RNU6-913P	0	0	0	0	0	0	0	0
ENSG00000251575	C5orf64-AS1	0	0	0	0	0	0	0	0
ENSG00000249878	AC026434.2	0	0	0	0	0	0	0	0
ENSG00000248199	AC026434.1	0	0	0	0	0	0	0	0
ENSG00000251983	RN7SKP157	0	0	0	0	0	0	0	0
ENSG00000068796	KIF2A	45.141	26.9369	12.2741	23.7898	32.4061	35.8898	26.8213	27.0896
ENSG00000086189	DIMT1	3.68856	1.89414	1.26858	2.59152	3.48261	3.9112	2.02762	1.97531
ENSG00000086200	IPO11	2.304	1.87705	1.69999	5.17959	2.36384	2.93267	4.521	2.32751
ENSG00000199279	RNU6-661P	0	0	0	0	0	0	1.14187	0
ENSG00000268942	CKS1BP3	0	0	0	0	0	0	0	0
ENSG00000241991	AC114982.2	0	0	0	0	0	0	0	0
ENSG00000240436	AC114982.1	0	0	0	0	0	0	0	0
ENSG00000186105	LRRC70	0.09778	0.16275	0.04789	0.19348	0.27738	0	0.05433	0.08003
ENSG00000217416	ISCA1P1	0.37881	0	0.55666	0.5622	0.23028	0	0	0
ENSG00000249579	AC113420.1	0	0	0	0	0	0	0	0
ENSG00000248185	AC025445.1	0	0	0	0	0	0	0	0
ENSG00000248285	AC122707.1	0	0	0	0	0	0	0	0
ENSG00000178394	HTR1A	0	0	0	0	0	0	0	0
ENSG00000164197	RNF180	0	0	0	0.02002	0	0	0	0
ENSG00000236068	AC091862.1	0	0	0	0	0	0	0	0
ENSG00000186479	RGS7BP	0	0	0	0	0	0	0	0
ENSG00000242547	RN7SL169P	0	0	0	0	0	0	0	0
ENSG00000201869	RNU6-294P	0	0	0	0	0	0	0	0
ENSG00000224296	MRPL49P1	0	0	0	0	0	0	0	0
ENSG00000145642	SHISAL2B	0	0	0	0	0	0	0	0
ENSG00000153006	SREK1IP1	4.25	3.33472	1.41484	3.11765	3.961	3.29413	2.82067	2.36426
ENSG00000153015	CWC27	7.80041	6.13305	2.85125	6.22464	7.4105	9.02416	5.1943	5.31801
ENSG00000272370	AC092354.2	0.28522	0.23737	0	0.21165	0.17338	0.75474	0	0
ENSG00000272354	AC092354.1	0.19495	0	0	0	0	0.68783	0	0
ENSG00000207439	RF00019	0	0	0	0	0	0	0	0
ENSG00000049192	ADAMTS6	0.81283	1.46689	0.69152	1.09205	0.53052	1.63017	1.05537	0.70384
ENSG00000248288	AC008868.1	0	0	0	0	0	0	0	0
ENSG00000250732	RPEP1	0	0.18542	0	0	0	0	0	0
ENSG00000205644	AC025186.1	0	0	0	0	0	0	0	0
ENSG00000250081	AC025176.1	0.40165	0	0.29511	0	0	0	0	0.24657

ENSG00000123219	CENPK	10.9475	12.8743	40.4677	88.4262	23.6019	16.5838	52.108	43.8506
ENSG00000113593	PPWD1	9.91699	7.462	5.96034	6.16366	9.27266	15.0641	11.1279	14.0345
ENSG00000113595	TRIM23	5.77924	5.7362	5.17618	9.61804	8.97285	3.793	11.4631	10.2035
ENSG00000207352	RNU6-540P	0.72575	0	0	0	0	1.28031	1.20971	0.89106
ENSG00000113597	TRAPPC13	2.09014	1.95587	1.37527	2.28405	1.82051	1.99957	2.09729	1.97894
ENSG00000253251	SHLD3	1.71681	1.24245	1.09688	2.27098	1.452	1.64603	1.67967	1.46635
ENSG00000197860	SGTB	10.8395	6.17684	2.11755	3.34987	8.60469	4.61185	5.95172	5.16685
ENSG00000123213	NLN	1.2855	1.00299	0.25581	0.38753	1.12332	1.19296	0.41292	0.77272
ENSG00000251648	AC008958.1	0	0	0	0	0	0	0	0
ENSG00000269961	AC010359.1	0.72288	0	0.21245	0	0.17577	0.76516	0	0
ENSG00000112851	ERBIN	66.0966	62.9509	29.7015	50.8149	59.7455	57.3017	46.595	54.5744
ENSG00000252904	RF00598	0	0	0	0	0	0	0	0
ENSG00000153914	SREK1	3.49623	2.53515	0.95667	1.93238	2.80932	3.21153	2.06313	1.87
ENSG00000253744	AC025442.1	0	0.25366	0	0	0	0	0	0
ENSG00000248846	LINC02065	0	0	0	0	0	0	0	0
ENSG00000251391	AC113378.1	0	0	0	0	0	0.29059	0	0
ENSG00000250313	LINC02229	0	0	0	0	0	0	0	0
ENSG00000234838	PPIAP78	0	0	0	0	0	0	0	0
ENSG00000069020	MAST4	1.04893	0.89792	0.26424	0.6301	1.18417	0.76662	0.62443	0.40476
ENSG00000248803	AC092349.1	0	0	0	0	0	0	0	0
ENSG00000249057	MAST4-IT1	0	0	0	0	0	0	0	0
ENSG00000229666	MAST4-AS1	1.13205	1.2562	0.41588	0.28001	1.03224	0.16642	0.31449	0.11583
ENSG00000134061	CD180	2.74165	2.90599	0.74115	0.69095	5.50297	1.02665	0.45268	1.69896
ENSG00000251206	AC010420.1	0	0	0	0	0	0	0	0
ENSG00000249364	AC112206.2	0	0	0	0	0	0	0	0
ENSG00000250978	AC079467.1	0	0	0	0	0	0	0	0
ENSG00000239870	AC112206.1	0	0	0	0	0	0	0	0
ENSG00000249736	LINC02242	0	0	0	0	0	0	0	0
ENSG00000249238	BCL9P1	0	0	0	0	0	0	0	0
ENSG00000250669	AC112206.4	0	0	0	0	0	0	0	0
ENSG00000249721	AC112206.3	0	0	0	0	0	0	0	0.23135
ENSG00000250421	AC106798.1	0	0	0	0	0	0	0	0.12211
ENSG00000249894	AC024581.1	0	0	0	0	0	0	0	0.15901
ENSG00000273860	AC024581.2	0	0	0	0	0	0	0	0
ENSG00000253801	AC010376.1	0	0	0	0	0	0	0	0
ENSG00000252108	RNU6-1232P	0	0	0	0	0	0	0	0
ENSG00000213864	EEF1B2P2	0.10843	0	0	0.16093	0.13183	0	0	0
ENSG00000253787	LINC02219	0	0	0	0	0	0	0	0
ENSG00000145675	PIK3R1	29.3215	12.1136	4.18171	11.6798	17.9856	26.8784	19.6995	8.79325
ENSG00000251044	AC024579.1	0	0	0	0	0	0	0	0
ENSG00000248884	AC010280.2	0.71513	0.59516	0.26272	0	0	0.31539	0.596	0.65852
ENSG00000249588	AC010280.3	0	0	0	0	0	0	0	0
ENSG00000248359	AC010280.1	0	0	0	0	0	0	0	0
ENSG00000249335	AC093523.1	0	0	0	0	0	0	0	0
ENSG00000250289	VWA8P1	0	0	0	0	0	0	0	0
ENSG00000212249	RF00096	0	0	0	0	0	0	0	0
ENSG00000250066	AC093248.1	0	0	0	0	0	0	0	0
ENSG00000276309	RN7SKP251	0	0	0	0	0	0	0	0
ENSG00000249352	LINC02198	0	0	0	0	0	0	0	0
ENSG00000250237	AC010273.2	0	0	0	0	0	0	0	0
ENSG00000249183	SUMO2P4	0	0	0	0	0	0	0	0
ENSG00000145740	SLC30A5	5.22126	3.86023	2.32784	4.36347	4.94579	4.69493	2.51374	4.5123
ENSG00000248664	AC010273.1	0.07748	0.12897	0.34159	1.03496	0.28261	0.13669	1.29155	0.95134
ENSG00000220986	RF00407	0	0	0	0	0	0	0	0
ENSG00000280894	SNORA50D	0	0	0	0	0	0	0	0
ENSG00000134057	CCNB1	6.27552	4.86508	18.4857	72.3395	13.0992	17.9965	54.9764	23.4668
ENSG00000280187	AC022107.1	0.04738	0	0.03481	0	0.05761	0.04179	0.03949	0.02909
ENSG00000153044	CENPH	10.0608	16.8006	26.9285	52.3568	16.9576	10.3726	38.3311	32.4455

ENSG00000134056	MRPS36	3.25779	3.82772	3.02725	7.60789	3.0288	3.29618	4.23239	4.99984
ENSG00000134058	CDK7	6.73538	7.52453	4.85961	14.7239	11.1396	8.13551	15.1714	7.78534
ENSG00000183323	CCDC125	2.9017	3.86083	1.72462	2.89382	4.31427	1.67916	2.84964	2.97267
ENSG00000213830	CFL1P5	0	0	0	0	0	0	0	0
ENSG00000215006	CHCHD2P2	0	0	0	0	0	0	0	0
ENSG00000271045	NDUFB9P1	0	0	0	0	0	0	0	0
ENSG00000085231	AK6	1.49177	0.90293	1.69393	5.98774	1.56634	1.01679	2.93866	1.95646
ENSG00000273841	TAF9	0.68266	0.37876	0.29259	0.591	0.82996	1.35484	0.71118	0.94293
ENSG00000152942	RAD17	5.78084	4.48841	3.39277	6.35608	6.62108	4.53944	5.43481	4.67405
ENSG00000152939	MARVELD2	0.08979	0.02491	0.04398	0.19989	0.05458	0	0.04989	0.01837
ENSG00000244717	RPS27P14	0	0	0	0	0	0	0	0
ENSG00000249295	AC145146.1	0	0	0	0	0	0	0	0
ENSG00000266477	RN7SL616P	0	0	0	0	0	0	0	0
ENSG00000197822	OCLN	0.01084	0	0	0	0	0.01913	0	0
ENSG00000212260	RNU6-724P	0	0	0	0	0	0	0	0
ENSG00000183474	GTF2H2C	0.86001	1.13472	0.86307	1.77444	1.33885	0.75859	1.17128	1.75127
ENSG00000248477	AC139495.1	0.06831	0.11371	0.10039	0	0	0	0	0
ENSG00000250138	AC139495.3	0.2478	0.13749	0.29132	0.04904	0.84357	0.11658	0.02754	0.1217
ENSG00000253203	GUSBP3	0.22624	0	0	0.25182	0.48134	0.09978	0.09427	0.06944
ENSG00000248769	AC139495.2	0	0	0	0	0	0	0	0
ENSG00000198237	AC131392.1	0.2316	0	0	0.51557	1.68942	0.40857	0	0.5687
ENSG00000251158	AC131392.2	0	0	0	0	0	0	0	0
ENSG00000249230	CDH12P2	0	0	0	0	0	0	0	0
ENSG00000205572	SERF1B	0	0	0	0	0	0	0	0
ENSG00000205571	SMN2	0.12172	0.24313	0.10732	0.10839	0.65113	0.04295	0.12173	0.269
ENSG00000179978	AC140134.1	0.08273	0.03443	0.03039	0	0.17602	0	0.03448	0.02539
ENSG00000253492	CDH12P3	0	0	0	0	0	0	0	0
ENSG00000254701	AC138866.2	1.38066	1.46243	0.36888	1.67649	2.59415	3.32136	0.83685	0.92463
ENSG00000253816	AC138866.1	2.50726	2.4549	0.86691	4.59658	2.86895	3.64259	0.73751	2.89729
ENSG00000226259	GTF2H2B	1.56585	0.42928	2.24686	4.64779	3.22513	0.64997	3.4391	2.64631
ENSG00000269983	AC146944.4	0.20418	0	0	0	0	0	0	0
ENSG00000250687	AC146944.2	1.06232	0.88412	0.48783	0	0.48433	0.23426	0.11067	0.08152
ENSG00000253333	AC146944.3	0.39515	0	0.871	0.29322	0.48041	0	1.31731	0
ENSG00000196302	AC146944.1	0	0.29189	0	0	0	0	0	0
ENSG00000253366	AC139272.1	0	0	0	0	0	0	0	0
ENSG00000254335	CDH12P1	0	0	0	0	0	0	0	0
ENSG00000172058	SERF1A	0	0	0	0	0.02713	0	0	0
ENSG00000172062	SMN1	0.78105	0.74852	0.10434	0.38639	0.71938	0.91859	0.27616	0.46495
ENSG00000249437	NAIP	3.52902	4.42478	0.97376	1.14355	2.13588	0.48935	0.46237	1.49475
ENSG00000145736	GTF2H2	1.63903	1.08283	2.56994	6.10634	2.44465	1.25198	3.3094	2.90447
ENSG00000230847	OCLNP1	0.10795	0.17969	0	0.16021	0.65623	0.19044	0.17994	0
ENSG00000251634	AC145138.1	0.61705	0.34236	0.56672	0.95393	0.65642	0.45357	0.21428	0.3788
ENSG00000253900	CDH12P4	0	0	0	0	0	0	0	0
ENSG00000215630	GUSBP9	1.12963	2.92486	1.65998	1.30394	1.67857	4.87133	1.25528	0.61642
ENSG00000235558	AC146949.1	0.69315	0.28844	0	0.77153	0.63203	0.9171	0.28884	0.21276
ENSG00000250387	LINC02197	0	0	0	0	0	0	0	0
ENSG00000253985	AC145141.2	0	0	0	0	0	0	0	0
ENSG00000169040	PMCHL2	0	0	0	0	0	0	0	0
ENSG00000249981	AC145141.1	0	0	0	0	0	0	0	0
ENSG00000145734	BDP1	5.87063	4.9447	2.16537	4.56627	6.85788	6.21811	4.75517	4.25525
ENSG00000253536	AC138832.1	0	0	0	0	0	0	0	0
ENSG00000247911	HMGN1P12	0	0	0	0	0	0	0	0
ENSG00000131844	MCCC2	2.38099	1.33453	0.30347	1.37019	2.12675	2.85026	1.29591	0.86507
ENSG00000164326	CARTPT	0	0	0	0	0	0	0	0
ENSG00000278824	AC143336.1	0	0	0	0	0	0	0	0
ENSG00000131711	MAP1B	1.53217	0.12558	0	0.00861	0.19051	0.03072	0.04837	0.00713
ENSG00000264099	MIR4803	0	0	0	0	0	0	0	0
ENSG00000113048	MRPS27	3.78266	2.08001	0.69482	1.6708	2.95641	5.46164	1.46369	1.61721

ENSG00000244748	RN7SL153P	0	0	0	0	0	0	0	0.30821
ENSG00000049883	PTCD2	0.39327	0.34241	0.20449	0.46693	0.50756	0.55503	0.36306	0.312
ENSG00000244061	AC093278.1	0	0	0	0	0	0	0	0
ENSG00000251108	YBX1P5	0	0	0	0	0	0	0	0
ENSG00000261269	AC093278.2	0.67843	1.17835	0.04335	0.26266	0.32276	0.26018	0.09833	0.25351
ENSG00000178175	ZNF366	0.60853	0.58319	0.08129	0.1642	0.29145	0.16266	0.03074	0.09056
ENSG00000248371	LINC02056	0	0	0	0	0	0	0	0
ENSG00000251613	AC063979.2	0	0	0	0	0	0	0	0
ENSG00000226544	RPL7P22	0	0	0	0	0	0	0	0
ENSG00000223497	AC063979.1	0	0	0	0	0	0	0	0
ENSG00000206770	RF00019	0	0	0	0	0	0	0	0
ENSG00000249085	AC035140.1	0	0	0	0	0	0	0	0
ENSG00000083312	TNPO1	15.9367	15.7489	7.28465	14.0876	12.7235	11.099	11.7314	13.4128
ENSG00000263593	MIR4804	0	0	0	0	0	0	0	2.46567
ENSG00000242602	AC008953.1	0	0	0	0	0	0	0	0
ENSG00000271926	AC008972.1	0	0	0.1683	0	0	0	0	0
ENSG00000272081	AC008972.2	0	0.24698	0.21804	0.22021	0	0.26176	0.98931	0
ENSG00000157107	FCHO2	61.5402	96.7784	102.598	215.796	128.853	130.874	250.862	145.035
ENSG00000251467	AC020893.2	0	0.33751	0.14898	0.15046	0.12326	0	1.01394	0.12448
ENSG00000248930	AC020893.1	0	0	0	0	0	0	0	0
ENSG00000157111	TMEM171	0.19101	0	0.14034	0.07087	0.05806	0.08424	0	0.05863
ENSG00000251599	AC116345.3	0	0	0	0	0	0	0	0
ENSG00000250747	AC116345.2	0	0	0	0	0	0	0	0
ENSG00000164325	TMEM174	0	0	0	0	0	0	0	0
ENSG00000249743	AC116345.1	0	0	0	0	0	0	0	0
ENSG00000251543	LINC02230	0	0	0	0	0	0	0	0
ENSG00000249149	AC099522.1	0	0	0	0	0	0	0	0
ENSG00000251493	FOXD1	0.02795	0	0	0	0	0	0	0
ENSG00000247993	FOXD1-AS1	0	0	0	0	0	0	0	0
ENSG00000251604	LINC01385	0	0	0	0	0	0	0	0
ENSG00000251324	LINC01386	0	0	0	0	0	0	0	0
ENSG00000272525	AC099522.2	0.59113	0	0.14478	0.43865	0.5989	0.17381	0.32844	0
ENSG00000145741	BTF3	148.324	166.865	87.9788	116.326	202.364	185.607	116.336	134.613
ENSG00000255883	FUNDC2P1	0.25719	0.21405	0.37794	0	0.15635	0.22686	0	0.31578
ENSG00000164331	ANKRA2	5.92688	4.03789	1.53935	3.19116	4.25642	5.64128	4.64092	3.28308
ENSG00000164338	UTP15	1.41475	1.08092	0.56234	1.13587	1.49444	2.29124	1.46902	1.26716
ENSG00000214944	ARHGEF28	0.05632	0.11719	0.02069	0.09752	0.03994	0.03312	0.00782	0.06339
ENSG00000249293	AC091868.2	0	0	0	0	0	0	0	0
ENSG00000184084	AC091868.1	0	0	0	0	0	0	0	0
ENSG00000238913	RNU7-196P	0	0	0	0	0	0	0	0
ENSG00000259968	AC093283.1	0	0	0	0	0	0	0	0
ENSG00000248474	LINC02122	0	0	0	0	0	0	0	0
ENSG00000248942	LINC01335	0	0	0	0	0	0	0	0.23135
ENSG00000244326	RN7SL814P	0	0	0	0	0	0	0	0
ENSG00000251041	AC106732.1	0	0	0	0	0	0	0	0
ENSG00000249343	LINC01333	0	0	0	0	0	0	0	0
ENSG00000250446	LINC01332	0	0	0	0	0	0	0	0
ENSG00000248673	LINC01331	0	0.42961	0	0	0	0	0	0.15845
ENSG00000171617	ENC1	3.04063	3.95726	0.60679	1.37421	1.91681	9.64651	5.42282	1.1676
ENSG00000049860	HEXB	15.2619	11.2449	3.91146	8.5019	8.53001	6.02281	6.8963	7.97487
ENSG00000164347	GFM2	4.3313	5.2036	3.64436	8.9942	7.85637	8.31884	7.36517	6.71172
ENSG00000207336	RNU6-658P	0.69151	1.15102	0	1.02628	0	0	1.15264	0.84903
ENSG00000164346	NSA2	12.7338	9.83543	2.36615	3.33241	14.4397	16.2356	4.16132	5.58627
ENSG00000198780	FAM169A	2.21183	1.70741	0.76939	3.56804	1.55889	1.43261	1.62076	1.73172
ENSG00000199645	RNU6-1330P	0	0	0	0	0	0	0	0
ENSG00000250071	AC093214.1	0	0	0	0	0	0.11681	0	0
ENSG00000271714	AC010501.1	0	0	0	0	0.04542	0	0	0
ENSG00000212363	RF00561	0	0	0	0	0	0	0	0

ENSG00000249856	AC116337.3	0.17329	0	0	0	0	0	0	0
ENSG00000196390	AC116337.1	0	0	0	0	0	0	0.17785	0
ENSG00000239517	AC116337.2	0	0	0	0	0	0	0	0
ENSG00000182383	RPL27AP5	0	0	0	0	0	0	0	0.20044
ENSG00000249157	AC093259.1	0	0	0	0	0	0	0	0
ENSG00000176928	GCNT4	1.94007	0.50526	0.34909	1.11645	3.33746	1.67635	2.79382	1.34493
ENSG00000250889	LINC01336	2.03355	0.15386	0	0.68591	3.03424	1.30453	1.38666	0.90791
ENSG00000145700	ANKRD31	0.2337	0.01945	0.03434	0.19076	0.34095	0.1443	0.19477	0.15781
ENSG00000251663	SUMO2P5	0	0	0	0	0	0	0	0
ENSG00000247372	AC008897.2	0.27818	0.46303	0.20439	0.20642	0.1691	0	0.46368	0
ENSG00000113161	HMGCR	12.2541	11.8377	2.66902	4.56848	9.79351	4.8687	3.59759	6.82037
ENSG00000271815	AC008897.3	0.30352	0.25261	0	0	0.18451	0.53545	0	0.18633
ENSG00000113163	COL4A3BP	10.6196	10.2971	2.36557	4.22785	7.60315	4.99583	4.01789	6.43649
ENSG00000242198	AC008897.1	0	0	0	0	0.25682	0	0	0
ENSG00000122008	POLK	4.43586	5.89607	3.4071	6.95368	5.76493	5.5673	4.40144	5.29801
ENSG00000251724	RNU7-175P	0	0	0	0	0	0	0	0
ENSG00000248881	AC010245.1	0	0	0	0	0	0	0	0
ENSG00000272040	AC010245.2	0.12562	0.2091	0	0.27965	0.07636	0.11081	0.1047	0.15424
ENSG00000189045	ANKDD1B	0.0892	0.22271	0.19662	0.23167	0.10845	0.07868	0.22302	0.08214
ENSG00000152359	POC5	1.93455	2.44159	1.21834	2.42938	2.84305	3.82535	1.45284	2.37521
ENSG00000207333	RNU6-680P	0.6981	0	0	0	0	0	0	0
ENSG00000251078	SLC25A5P9	0	0	0	0	0	0	0	0
ENSG00000251419	AC108120.3	0	0	0	0	0	0	0	0
ENSG00000249301	AC108120.2	0	0	0	0	0	0	0	0
ENSG00000248684	BIN2P2	0	0	0	0	0	0	0	0
ENSG00000228367	AC108120.1	0	0	0	0	0	0	0	0
ENSG00000249777	SAP18P1	0	0	0	0	0	0	0	0
ENSG00000251342	AC026774.2	0	0	0	0	0	0	0	0
ENSG00000248127	AC026774.1	0	0	0	0	0	0	0	0
ENSG00000122012	SV2C	0	0	0	0.01809	0.00741	0	0	0
ENSG00000252833	RNA5SP186	0	0	0	0	0	0	0	0
ENSG00000254893	AC113404.3	1.72067	1.52749	2.13517	4.82357	1.81302	0.87692	1.78458	1.87787
ENSG00000251668	AC113404.2	0	0	0	0	0	0	0	0
ENSG00000249014	HMGN2P4	0.19239	0	0	0	0	0	0	0
ENSG00000251107	PDCD5P2	0	0	0	0	0	0	0	0
ENSG00000250348	AC113404.1	0.77567	0.48416	0.71239	0.28779	0.11788	0.17105	1.61614	1.30948
ENSG00000251235	SNRPCP2	0	0	0	0	0	0	0	0
ENSG00000145703	IQGAP2	52.5826	39.4706	9.11698	23.1225	27.5784	28.0743	18.2559	16.3735
ENSG00000250574	AC112173.1	0	0.37311	0	0.33268	0.27253	0	0	0.27522
ENSG00000249713	AC026725.1	0	0	0	0	0	0	0	0
ENSG00000164220	F2RL2	0.02138	0	0.03141	0.03173	0	0	0	0.02625
ENSG00000225407	AC025188.1	0	0	0	0	0	0	0	0
ENSG00000277188	RF02154	0	0	0.80988	0	0	0	0	0
ENSG00000278237	RF02155	0	0	0	0	0	0	0	0
ENSG00000181104	F2R	8.65326	8.43197	2.64914	4.94967	4.71227	12.117	3.54581	5.0687
ENSG00000164251	F2RL1	3.29579	4.7434	1.16526	1.87562	6.89916	3.67214	1.32176	9.52302
ENSG00000264391	RN7SL208P	0	0	0	0.73504	0	0	0	0
ENSG00000171643	S100Z	2.85099	1.37369	0.05512	0.44539	0.45607	0.33089	0.4377	0.64481
ENSG00000252684	RNU6ATAC36P	0	0	0	0	0	0	0	0
ENSG00000145708	CRHBP	0.02615	0	0	0.07762	0	0.09227	0.04359	0.06421
ENSG00000164252	AGGF1	23.0596	29.856	23.7564	42.9242	26.3592	28.0333	32.0025	28.0985
ENSG00000132846	ZBED3	0.15754	0.11238	0.03307	0.2004	0.13681	0.05955	0.01876	0.01382
ENSG00000250615	AC008581.1	0.19039	0	0	0	0	0	0	0
ENSG00000238961	SNORA47	10.0921	4.4206	7.02482	27.5906	3.22886	5.62225	7.08292	13.0431
ENSG00000250802	ZBED3-AS1	0.03557	0.01973	0.05226	0.10557	0.01441	0.02091	0.11856	0.07278
ENSG00000251566	HMGB1P35	0	0	0	0	0	0	0	0
ENSG00000113231	PDE8B	0.27445	0.10542	0.03102	0.03133	0.0385	0.01862	0.0176	0
ENSG00000251400	ALDH7A1P1	0	0	0	0	0	0	0	0

ENSG00000164253	WDR41	3.89313	3.05443	1.6686	2.91901	1.78726	2.2178	1.19983	2.26549
ENSG00000244019	RPS2P24	0	0	0	0.12504	0.10243	0	0	0
ENSG00000244363	RPL7P23	0	0	0.28839	0.14563	0.2386	0	0	0
ENSG00000171540	OTP	0	0	0	0	0	0	0	0
ENSG00000171530	TBCA	10.403	11.9694	2.81789	5.79858	9.87919	8.83786	4.43493	5.53285
ENSG00000213763	ACTBP2	0.19582	0.21729	0.09592	0.19374	0.07936	0.34545	0	0.32056
ENSG00000253572	AC108482.1	0	0	0	0	0	0	0	0
ENSG00000132842	AP3B1	15.8433	12.1341	4.47949	6.42892	12.7519	12.5066	7.72193	9.03664
ENSG00000253558	AC024568.1	0.16217	0.21594	0.04766	0.04814	0.03943	0.17165	0.05406	0
ENSG00000249372	ATP6V1G1P6	0	0	0	0	0	0	0	0
ENSG00000251605	AC104108.1	0	0	0	0	0	0	0	0
ENSG00000200331	RNU6-183P	0	0	0	0	0	0	0	0
ENSG00000206714	RF00019	0	0	0	0	0	0	0	0
ENSG00000245556	SCAMP1-AS1	1.47255	0.74894	0.48087	0.42494	1.09407	1.15457	0.88635	1.10487
ENSG00000085365	SCAMP1	9.25944	8.8173	2.40489	4.06934	7.69692	5.82486	3.30217	4.31831
ENSG00000145685	LHFPL2	2.4994	3.60963	1.01273	1.13191	1.91036	3.08001	2.38939	2.82052
ENSG00000248909	HMGB1P21	0.12616	0.41999	0	0	0	0	0	0
ENSG00000254170	AC008802.1	0	0	0	0	0	0	0	0
ENSG00000254310	AC025755.1	0	0	0	0	0	0	0	0
ENSG00000113273	ARSB	3.54827	2.05356	0.74916	1.08952	2.3305	2.14051	1.24067	1.82773
ENSG00000132837	DMGDH	0.01395	0	0	0.04141	0.11873	0.04922	0	0
ENSG00000214890	AC020937.1	0	0	0	0	0	0	0	0
ENSG00000132840	BHMT2	0.02304	0	0	0	0	0	0	0
ENSG00000145692	BHMT	0	0	0	0	0	0	0	0
ENSG00000201955	RNY3P1	0	0	1.05602	0	0	0	0	0
ENSG00000249829	AC016559.1	0	0	0	0	0	0	0	0
ENSG00000206592	RF00425	0	0	0	0	0	0	0	0
ENSG00000250330	AC016559.2	0	0	0	0	0	0	0	0
ENSG00000152409	JMY	3.51018	2.53678	1.10787	2.43029	3.68609	3.28928	3.62136	2.05036
ENSG00000213757	AC020898.1	0	0	0	0	0	0	0	0
ENSG00000152413	HOMER1	0.4288	0.37669	0.14002	0.40658	0.88335	0.60937	0.31766	0.40948
ENSG00000213755	AC093270.1	0	0	0	0	0	0	0	0
ENSG00000243160	AC093270.2	0	0	0	0	0	0	0	0
ENSG00000207391	RF00019	0	0	0	0	0	0	0	0
ENSG00000277818	RF00019	0	0	0	0	0	0	0	0
ENSG00000164329	TENT2	35.8517	37.7677	26.3418	74.4791	39.931	36.366	55.6555	38.0216
ENSG00000164309	CMYA5	0.01034	0.02581	0	0.00767	0.00629	0.0456	0	0.03174
ENSG00000250258	AC008496.2	0	0	0	0	0.19373	0	0	0
ENSG00000238254	AC008496.1	0	0	0	0	0	0	0	0
ENSG00000250888	LINC01455	0	0	0	0	0	0	0	0
ENSG00000177034	MTX3	1.85195	0.92477	0.61232	1.03069	1.59861	1.96026	1.41998	1.02321
ENSG00000113296	THBS4	0.49344	0.05299	0	0.09449	0.13547	0	0.0796	0.03909
ENSG00000251050	AC112184.1	0	0	0	0	0	0	0	0
ENSG00000249778	TRMT112P2	0	0	0	0	0	0	0	0
ENSG00000249825	CTD-2201I18.1	0.34009	0.18869	0.06663	0	0.16539	0.07999	0	0.08351
ENSG00000164300	SERINC5	5.41519	3.34025	1.53073	3.11466	4.57223	4.21581	3.39603	2.84944
ENSG00000251675	AC010260.1	0	0	0	0	0	0	0	0
ENSG00000249438	KRT18P45	0	0	0	0	0	0	0	0
ENSG00000184188	AC026410.1	0	0	0	0	0	0	0	0
ENSG00000251828	RF00322	0	0	0	0	0	0	0	0
ENSG00000244076	AC026410.2	0	0	0	0	0	0	0	0
ENSG00000164299	SPZ1	0	0	0	0	0	0	0	0
ENSG00000227087	RBMX2P5	0	0	0	0	0	0	0	0
ENSG00000248569	AC026410.3	0.15662	0.2607	0	0.4649	0.09521	0	0.26107	0
ENSG00000220157	HNRNPA1P12	0	0.12696	0	0.1132	0	0	0	0
ENSG00000206774	RNU6-211P	0	0	0	0	0	0	0	0
ENSG00000039319	ZFYVE16	9.51031	8.44191	1.96425	2.52482	7.38686	3.77973	2.44126	7.3499
ENSG00000249042	AC008771.1	0.23251	0	0	0.25881	0	0	0.09689	0.07137

ENSG00000152380	FAM151B	1.08453	0.58865	0.55433	0.31492	0.97458	0.5823	0.31439	0.43421
ENSG00000242706	RPS27AP9	0	0	0	0	0	0	0	0
ENSG00000240003	RPL7P24	0.77979	0	0	0	0.23701	0	0.32495	0
ENSG00000189127	ANKRD34B	0.41896	0.03032	0	0	0.31005	0.19281	0.15181	0.24602
ENSG00000251366	DBIP2	0.27977	0	0	0	0	0	0	0
ENSG00000251221	LINC01337	0.16659	0	0.2448	1.48344	0.60761	0	1.11073	0.61362
ENSG00000228716	DHFR	2.27765	0.66414	0.53747	1.97388	2.02124	4.60466	1.49642	1.65338
ENSG00000249655	AC008434.1	0.26703	0	0	0	0	0	0	0.16393
ENSG00000271043	MTRNR2L2	0	0	0	0	0	0	0	0
ENSG00000113318	MSH3	5.15235	4.301	2.88213	6.26055	8.17549	7.46927	4.7222	4.18547
ENSG00000248967	AC022493.2	0	0	0	0	0	0	0	0
ENSG00000244630	AC022493.1	0	0	0	0	0	0	0	0
ENSG00000251450	RASGRF2-AS1	0	0	0	0	0.07643	0.1109	0.10479	0
ENSG00000113319	RASGRF2	1.57996	0.47815	0.21107	0.56844	2.11212	1.17041	0.77524	1.10009
ENSG00000251001	AC026427.2	0	0	0	0	0	0	0	0
ENSG00000249772	AC026427.1	0	0	0	0	0	0	0	0
ENSG00000247572	CKMT2-AS1	0.60392	0.12565	0.0832	0.25208	0.73423	0.59928	0.18874	0.30123
ENSG00000131730	CKMT2	0	0	0	0	0	0	0	0
ENSG00000248794	AC026436.1	0.54297	0	0	0	0.33006	0	0	0
ENSG00000131732	ZCCHC9	2.61634	2.18935	0.56725	0.65776	2.6768	2.47173	0.71492	1.63248
ENSG00000172497	ACOT12	0.02999	0.04992	0	0	0	0	0.09998	0
ENSG00000145687	SSBP2	2.7348	1.83813	0.73502	1.90886	3.09586	1.71896	2.15472	1.61108
ENSG00000249483	AC026726.1	0	0	0	0	0	0.21516	0.2033	0
ENSG00000214857	SEM1P1	0	0	0	0	0	0	0	0
ENSG00000152348	ATG10	0.53808	0.60456	0.47442	1.19786	0.89951	1.16283	1.74896	1.02401
ENSG00000236447	ATG10-IT1	0	0	0	0	0	0	0	0.1848
ENSG00000251495	PPIAP11	0.29736	0.24748	0.21849	0	0	0	0	0
ENSG00000248192	ATG10-AS1	0	0	0	0	0	0	0	0
ENSG00000265684	RN7SL378P	0	0	0	0	0	0	0	0
ENSG00000251374	AC010598.1	0	0	1.04577	0	0.86521	0	0	0
ENSG00000186468	RPS23	126.863	62.049	17.3459	36.5638	132.827	108.149	54.3476	69.3817
ENSG00000205464	ATP6AP1L	0.23464	0.11887	0.25486	0.83274	0.49613	0.64791	0.52715	0.35072
ENSG00000248393	AC026782.1	0	0	0	0	0	0	0	0
ENSG00000248870	AC026782.2	0	0	0	0	0	0	0	0
ENSG00000249100	AC008885.2	0	0	0	0	0	0	0	0
ENSG00000240411	RPL5P16	0	0	0	0	0	0	0	0
ENSG00000248105	AC008885.1	0	0	0	0	0	0	0	0
ENSG00000283359	MIR3977	0	0	0	0	0	0	0	0
ENSG00000281327	LINC01338	0	0	0	0	0	0.20461	0	0
ENSG00000248112	AC108174.1	0	0	0	0	0	0	0	0
ENSG00000249857	AC027338.2	0	0	0	0	0	0	0	0
ENSG00000248400	ST13P12	0	0.11153	0	0	0	0	0.11168	0
ENSG00000249664	AC027338.1	0.08885	0	0	0.13186	0	0	0	0.10909
ENSG00000271862	AC104118.1	1.89132	0.76783	0.13557	0.13692	0.95342	0.97655	0.46135	0.39646
ENSG00000174695	TMEM167A	32.3191	26.3507	8.25192	15.981	17.9395	13.7532	10.1568	18.3918
ENSG00000238835	SCARNA18	4.37614	1.82102	3.21534	3.24733	0	0.96501	2.73538	3.3581
ENSG00000152422	XRCC4	5.83444	6.88463	2.01259	3.17088	5.76128	4.73563	3.56131	4.23753
ENSG00000243385	AC094085.1	0.12551	0.20892	0	0	0.1526	0	0	0
ENSG00000251552	COQ10BP2	0	0	0	0.15026	0	0	0	0
ENSG00000251395	FTH1P9	0	0	0	0	0	0	0	0
ENSG00000038427	VCAN	110.852	79.0739	8.44659	17.5674	41.7011	20.0712	17.6956	26.166
ENSG00000249835	VCAN-AS1	0	0	0	0	0	0	0	0
ENSG00000145681	HAPLN1	0	0	0	0	0	0	0	0
ENSG00000253073	RN7SKP295	0	0	0	0	0	0	0	0
ENSG00000206744	RNU6-620P	0	0	0	0	0	0	0	0
ENSG00000242858	AC020899.1	0	0	0	0.18071	0	0	0	0
ENSG00000202157	RNU4-11P	0	0	0	0	0	0	0	0
ENSG00000250013	AC093286.1	0	0	0	0	0	0	0	0

ENSG00000164176	EDIL3	0.01277	0.29769	0.56316	0.68252	0.18637	0.06761	0	0.18821
ENSG00000252861	RNU6-448P	0	0	0	0	0	0	0	0
ENSG00000250320	AC113383.1	0.0535	0	0	0	0	0	0	0
ENSG00000214853	PPIAP79	0	0	0	0	0	0	0	0
ENSG00000249349	AC114928.1	0	0	0	0	0	0	0	0
ENSG00000248674	RBBP4P6	0	0	0	0	0	0	0	0
ENSG00000250253	AC117522.3	0	0	0	0	0	0	0	0
ENSG00000249792	AC117522.2	0	0	0	0	0	0	0	0
ENSG00000248170	AC117522.1	0	0	0	0	0	0	0	0
ENSG00000248667	AC010486.1	0	0	0	0	0	0	0	0
ENSG00000249023	AC010486.2	0	0	0	0	0	0	0	0
ENSG00000249842	AC010486.3	0	0	0	0	0	0	0	0
ENSG00000243859	RPL5P17	0	0	0	0.12404	0.20323	0	0	0
ENSG00000251066	AC026700.1	0	0	0	0	0	0	0	0
ENSG00000250874	AC010595.1	0	0	0	0	0	0	0	0
ENSG00000241965	RPS2P25	0	0	0	0	0	0	0	0
ENSG00000249153	AC026414.1	0	0	0	0	0	0	0	0
ENSG00000250359	PTP4A1P4	0	0	0	0	0	0	0	0
ENSG00000205449	NBPF22P	0	0	0	0	0	0	0	0
ENSG00000280009	AC020923.1	0	0	0	0	0	0	0	0
ENSG00000248195	AC016550.1	0	0	0	0	0	0	0	0
ENSG00000248363	AC016550.2	0	0	0	0	0	0	0	0
ENSG00000250124	AC016550.3	0	0	0	0	0	0	0	0
ENSG00000251585	AC026444.1	0	0	0	0	0	0	0	0
ENSG00000127184	COX7C	22.2512	17.5697	15.0248	27.1282	22.581	26.8616	24.4606	23.4363
ENSG00000284078	SNORD138	0	0	0	0	0	0	0	0
ENSG00000207013	RNU6-804P	0	0	0	0	0	0	0	0
ENSG00000250544	LINC02059	0	0	0	0	0	0	0	0
ENSG00000248701	AC008539.1	0	0	0	0	0	0	0	0
ENSG00000242477	AC091429.1	0	0	0	0	0	0	0	0
ENSG00000249061	AC109492.1	0	0	0	0.16762	0	0	0	0
ENSG00000251951	RN7SKP34	0	0	0	0	0	0	0	0
ENSG00000265919	MIR4280	0	0	0	0	0	0	0	0
ENSG00000233828	LINC01949	0	0	0	0	0	0	0	0
ENSG00000145715	RASA1	11.3761	11.2401	5.68216	14.6794	12.6555	11.5669	9.738	9.4435
ENSG00000241243	RN7SL629P	0	0	0	0	0	0	0	0
ENSG00000207452	RNU6-606P	0	0	0	0	0	0	0	0
ENSG00000272367		2.13958	2.50612	0.69869	0.58803	1.05977	1.67756	1.45296	1.16753
ENSG00000134480	CCNH	10.3344	10.1586	3.0049	8.82425	7.70687	10.6557	5.89926	6.41181
ENSG00000200462	RNU6-727P	0	0	0	0	0	0	0	0
ENSG00000249362	LINC02488	0	0	0	0	0	0	0	0
ENSG00000248708	LINC02144	0	0	0	0	0	0	0	0
ENSG00000164180	TMEM161B	2.13074	1.63983	1.06614	2.78821	2.53478	2.68108	2.36774	1.92221
ENSG00000247828	TMEM161B-AS1	1.50267	1.64913	0.92208	3.50447	1.90721	1.83524	1.89918	1.27727
ENSG00000207129	RNA5SP187	0	0	0	0	0	0	0	0
ENSG00000240083	RPS3AP22	0	0	0	0	0	0	0	0
ENSG00000206958	RF00156	0	0	0	0	0	0	0	0
ENSG00000250306	AC091826.1	0	0	0	0	0	0	0	0
ENSG00000250156	LINC02060	0	0	0	0	0	0	0	0
ENSG00000271904	AC091826.2	0	0	0	0	0	0	0	0
ENSG00000245526	LINC00461	0	0.07905	0	0	0.01443	0	0	0
ENSG00000250705	AC008526.1	0	0	0	0	0	0	0	0
ENSG00000284447	MIR9-2	0	0	0	0	0	0	0	0
ENSG00000245864	MEF2C-AS2	0	0	0	0	0	0	0	0
ENSG00000250377	AC008525.1	0.21686	0	0	0	0	0	0	0
ENSG00000250555	AC008525.2	0	0	0	0	0	0	0	0
ENSG00000081189	MEF2C	17.2416	7.82598	1.70436	3.48892	9.88585	3.97121	2.92069	4.50942
ENSG00000248309	MEF2C-AS1	0.55919	0.44987	0.43825	1.02354	0.48722	0.32883	0.54371	0.69801

ENSG00000250831	AC074131.1	0	0	0	0	0	0	0	0
ENSG00000250437	LINC02161	0	0	0	0	0	0	0	0.21428
ENSG00000214942	AC008448.1	0	0	0	0	0	0	0	0
ENSG00000249265	AC113167.1	0	0	0	0	0	0	0	0
ENSG00000264342	MIR3660	0	0	0	0	0	0	0	0
ENSG00000248555	LINC01339	0	0	0	0	0	0	0	0
ENSG00000253321	AC099554.1	0	0	0	0	0	0	0	0
ENSG00000260871	AC093510.2	0	0.13617	0	0	0.09946	0	0	0.10044
ENSG00000153140	CETN3	1.55757	1.0606	0.97102	3.53746	2.00842	2.08165	1.18011	1.99929
ENSG00000255647	AC093510.1	0.32821	0.1821	0.16077	1.13657	1.3301	0.57901	0.54708	0.26865
ENSG00000176055	MBLAC2	1.05895	0.70935	0.37954	0.91996	1.85267	1.38971	0.51662	0.66594
ENSG00000113356	POLR3G	2.38924	2.91436	1.42046	2.43611	1.97347	2.73488	1.82404	2.01536
ENSG00000176018	LYSMD3	13.4798	14.9945	5.96666	11.4177	11.7117	9.51206	11.2617	11.5852
ENSG00000164199	ADGRV1	0.01517	0.01082	0.00318	0.01608	0.03162	0	0.02167	0.00266
ENSG00000249514	TMEM251P1	0	0	0	0	0	0	0	0
ENSG00000212930	AC093281.1	0	0	0	0	0	0	0	0
ENSG00000251093	AC093281.2	0.36834	0	0	0	0.29855	0	0.40931	0.45225
ENSG00000199643	RNU4-90P	0	0	0	0	0	0	0	0
ENSG00000234292	AC123595.1	1.12123	0.93314	0.41191	0.208	0.0852	0	0.35042	0.51624
ENSG00000248323	LUCAT1	14.0088	9.45314	1.59494	1.64827	6.29095	1.42492	3.49207	7.06588
ENSG00000271762		0	0	0	0	0	0	0	0
ENSG00000277001	RF00017	0	0	0	0	0	0	0	0
ENSG00000240388	AC008799.1	0	0	0	0	0	0	0	0
ENSG00000241059	AC008799.2	0	0	0	0	0	0	0	0
ENSG00000113369	ARRDC3	70.9679	67.1615	14.3648	20.1961	44.3416	22.5916	37.813	48.7195
ENSG00000281357	ARRDC3-AS1	0.88002	0.43082	0.15214	0.23048	0.47202	0.36529	0.25886	0.31779
ENSG00000251356	RAB5CP2	0	0	0	0	0	0	0	0
ENSG00000248826	PCBP2P3	0	0.14473	0	0	0	0	0	0
ENSG00000276426	RF02271	0	0	0	0	0	0	0	0
ENSG00000250049	AC114316.2	0	0	0	0	0	0	0	0
ENSG00000248864	AC114316.1	0	0	0	0	0	0	0	0
ENSG00000249776	AC124854.1	0	0	0	0	0	0	0	0
ENSG00000249169	AC026780.1	0	0	0	0	0	0	0	0
ENSG00000249984	AC026780.2	0	0	0	0	0	0	0	0
ENSG00000248588	AC008517.1	0	0	0	0	0	0	0	0
ENSG00000248950	LDHBP3	0	0	0	0	0	0	0	0
ENSG00000249958	CCT7P2	0	0	0	0	0	0	0	0
ENSG00000248528	LINC02058	0	0	0	0	0	0	0	0
ENSG00000251361	AC012625.1	0	0	0	0	0	0	0	0
ENSG00000213730	POLD2P1	0	0	0	0	0	0	0	0
ENSG00000237187	NR2F1-AS1	0	0	0	0	0.01154	0.05025	0	0
ENSG00000175745	NR2F1	0	0.11617	0	0	0	0	0	0
ENSG00000278905	AC106818.1	0	0	0	0	0	0	0	0
ENSG00000113391	FAM172A	9.03426	7.33831	4.0783	8.9028	9.34925	8.82309	9.1316	7.13445
ENSG00000284336	MIR2277	0	0	0	0	0	0	0	0
ENSG00000249353	NPM1P27	0.97145	0.73499	0.90843	0.65534	0.75159	1.40218	2.64969	1.0843
ENSG00000248483	POU5F2	0.2351	0.21741	0.19194	0.25846	0.13762	0.23042	0.18868	0.28866
ENSG00000272406	AC108102.1	0	0	0	0	0	0	0	0
ENSG00000251023	AC114980.1	0.39471	0.54106	0.20471	0.13783	0.22583	0.32768	0.30961	0.19955
ENSG00000185261	KIAA0825	1.14851	2.287	0.53841	0.66926	0.92518	0.95713	0.4463	1.15924
ENSG00000251544	MTND5P12	0	0	0	0	0	0	0	0
ENSG00000254132	MTND6P3	0.27048	0	0	0	0	0	0	0
ENSG00000251340	MTCYBP35	0	0	0	0	0	0	0	0
ENSG00000232578	AC093311.1	0.07254	0.06037	0.0533	0.21531	0	0.25594	0	0
ENSG00000270133	AC025766.1	0.71687	0.29831	0.79008	0.26598	0.21789	0	0.59746	0
ENSG00000133302	SLF1	10.2427	16.947	13.8671	18.9864	16.3474	12.0403	25.2863	22.1705
ENSG00000175471	MCTP1	7.95517	9.64845	2.05823	3.23954	5.45507	3.8294	2.50647	5.14418
ENSG00000249175	AC008534.1	0	0	0	0	0	0	0	0

ENSG00000243806	RPL7P18	0.69905	0.99734	0	0.14821	0.24282	0	0	0.24522
ENSG00000276514	RF02271	1.2285	0.68161	0	0	0	0	0	0
ENSG00000249545	AC012312.1	0	0	0	0	0	0	0	0
ENSG00000153347	FAM81B	0.79323	0.34231	0.04317	0.34881	0.21431	0.15549	0.68558	0.57714
ENSG00000225165	AC090071.1	0	0	0	0	0	0	0	0
ENSG00000198677	TTC37	8.0076	5.54858	2.08985	4.8397	8.02843	8.21382	2.94429	5.50526
ENSG00000212259	RNU6-308P	0	0	0	0	0	0	0	0
ENSG00000164291	ARSK	1.05346	0.53736	0.34956	0.70607	0.9709	0.89925	0.59476	0.56326
ENSG00000178015	GPR150	0.07099	0	0	0.05268	0	0	0.05917	0
ENSG00000175449	RFESD	1.81006	2.31387	3.34079	8.12347	2.13021	2.65674	6.49279	2.82685
ENSG00000145757	SPATA9	0.13038	0	0	0.0774	0.0317	0	0	0.12806
ENSG00000250240	AC008840.1	0.08064	0	0.1185	0.23935	0.49019	0.14226	0	0.19801
ENSG00000164292	RHOBTB3	11.1199	14.7659	19.9014	44.6326	20.9146	22.0034	28.7988	22.5171
ENSG00000173221	GLRX	6.26229	8.58634	3.67432	3.37657	4.54621	5.92116	2.68466	5.87719
ENSG00000251348	HSPD1P11	0.04307	0	0	0.06392	0.05236	0	0.21536	0.26439
ENSG00000251000	AC008592.3	1.22752	0.58377	0	0	0.4264	0	0	0
ENSG00000251409	AC008592.4	0	0	0	0	0	0	0	0
ENSG00000272021	AC008592.5	0	0	0	0	0	0	0	0
ENSG00000236882	LINC01554	0	0	0	0.05278	0	0.06274	0	0
ENSG00000250362	AC008592.1	0	0	0	0	0	0	0	0.10588
ENSG00000118985	ELL2	8.06224	5.47773	2.8369	6.56158	6.26298	7.67612	10.0705	4.18791
ENSG00000251314	AC104123.1	0.09852	0.08199	0	0.07311	0	0	0.08211	0
ENSG00000250955	AC008592.2	0.12837	0	0	0	0	0	0	0
ENSG00000213716	FABP5P5	0	0	0	0	0	0	0	0
ENSG00000250551	MIR583HG	0.10412	0	0.45901	0.77263	0.63293	0	0.17355	0.63918
ENSG00000207578	MIR583	0	0	0	0	0	0	0	0
ENSG00000206997	RNU6-524P	0	0	0	0	0	0	0	0
ENSG00000249746	AC099509.1	0	0	0	0	0	0	0	0
ENSG00000250158	AC099509.2	0	0	0	0	0	0	0	0
ENSG00000175426	PCSK1	0	0	0	0	0	0	0	0
ENSG00000153113	CAST	19.3026	16.6233	5.41685	12.1125	12.6502	10.5128	7.89147	8.67581
ENSG00000249180	AC020900.1	0	0	0	0	0	0	0	0.15929
ENSG00000164307	ERAP1	15.9056	12.9764	2.39967	5.3674	16.9709	13.867	5.22861	8.82413
ENSG00000248734	AC008906.1	1.68422	1.09698	0.32282	0.32603	1.06833	0.25837	0	0.17981
ENSG00000272109	AC008906.2	0	0	0	0	0	0	0	0
ENSG00000247121	AC009126.1	0.66479	0.62544	0.08495	0.21448	0.42169	0.66288	0.33725	0.35488
ENSG00000164308	ERAP2	2.69617	2.58089	3.26538	1.74421	14.1564	22.9806	1.90958	9.44593
ENSG00000113441	LNPEP	20.6466	18.2941	6.89352	13.7675	22.2762	20.8884	10.8286	15.7403
ENSG00000227836	AC008850.1	0.60746	0.33704	0	0.30051	0.49236	0	0	0.49722
ENSG00000200884	RF00019	0	0	0	0	0	0	0	0
ENSG00000213701	SETP22	0	0	0	0	0	0	0	0
ENSG00000251513	LIX1-AS1	1.59437	0.95266	0.12015	2.66959	5.31817	2.66845	3.20272	1.90736
ENSG00000145721	LIX1	0	0	0	0	0	0	0	0
ENSG00000058729	RIOK2	3.26401	1.90708	0.80267	1.24564	3.14225	2.82032	2.24286	1.93014
ENSG00000206698	RNU1-73P	0.44695	0	0.65679	0	0	0	0.745	0
ENSG00000248758	AC008883.1	0	0	0	0	0	0	0	0
ENSG00000251054	AC008883.2	0	0	0	0	0	0	0	0
ENSG00000249761	AC074133.1	0	0	0	0	0	0	0	0
ENSG00000251389	YTHDF1P1	0	0	0	0	0	0	0	0
ENSG00000249977	AC106748.1	0	0	0	0	0	0	0	0
ENSG00000250331	LINC01340	0.10124	0	0	0.15026	0.12309	0	0	0.24861
ENSG00000248931	MTCYBP40	0	0	0	0	0	0	0	0
ENSG00000251193	AC112203.1	0	0	0	0	0	0	0	0
ENSG00000248202	LINC02234	0	0	0	0	0	0	0	0
ENSG00000249101	AC008834.1	0	0	0	0	0	0	0	0
ENSG00000248901	LINC01846	0	0	0	0	0	0	0	0
ENSG00000249135	AC116347.2	0	0	0	0	0	0	0	0
ENSG00000238000	AC116347.1	0.71264	0.67782	0	0.15109	0.37132	0.3592	0	0.24999

ENSG00000176183	AC233964.1	0	0	0	0	0	0	0	0
ENSG00000250221	KRT8P32	0	0	0	0	0	0	0	0
ENSG00000250830	MRPS35P2	0	0	0	0	0	0	0	0
ENSG00000249990	MTCO2P24	0	0	0	0	0	0	0	0
ENSG00000249448	MTCO1P24	0	0	0	0	0	0	0	0
ENSG00000251102	CTBP2P4	0	0	0	0	0	0	0	0.09782
ENSG00000251553	DDX18P4	0	0	0	0	0	0	0	0
ENSG00000174136	RGMB	0.32964	0.09145	0.03229	0.11415	0.33397	0.07754	0.18315	0.22934
ENSG00000246763	RGMB-AS1	0.03127	0	0	0.09282	0	0.05517	0.05212	0.03839
ENSG00000279232	AC008522.1	0.05045	0	0.07413	0.03743	0	0	0.04204	0
ENSG00000250162	CSNK1A1P3	0	0	0	0	0	0	0	0
ENSG00000153922	CHD1	29.5252	26.6883	6.55345	10.7879	20.3046	17.7917	16.6583	16.3231
ENSG00000222610	RNU6-402P	0	0	0	0	0	0	0	0
ENSG00000248489	LINC02062	2.60692	1.27624	1.35206	0.68276	2.79655	2.70527	0.76682	3.76557
ENSG00000200351	RF00019	0	0	0	0	0	0	0	0
ENSG00000241597	AC022121.1	0	0	0	0	0	0	0	0
ENSG00000249444	AC114324.2	0	0	0	0	0	0	0	0
ENSG00000206356	AC114324.1	0	0	0	0	0	0	0	0
ENSG00000227762	GUSBP8	0	0	0	0	0	0	0	0
ENSG00000249515	LINC02113	0	0	0	0	0	0	0	0
ENSG00000248236	AC008837.1	0	0	0	0	0	0	0	0
ENSG00000248928	AC008837.2	0	0	0	0	0	0	0	0
ENSG00000250391	AC092284.1	0	0	0	0	0	0	0	0
ENSG00000249963	EEF1A1P20	0	0	0	0	0	0	0	0
ENSG00000199378	RF00019	0	0	0	0	0	0	0	0
ENSG00000270232	MTCYBP22	0	0	0	0	0	0	0	0
ENSG00000270230	MTND6P22	0	0	0	0	0	0	0	0
ENSG00000271687	MTND5P10	0	0	0	0	0	0	0	0
ENSG00000270906	MTND4P35	0.15993	0	0	0	0	0	0	0
ENSG00000270350	MTND4LP5	0	0	0	0	0	0	0	0
ENSG00000271480	MTND3P19	0	0	0	0	0	0	0	0
ENSG00000270388	MTCO3P22	0	0	0	0	0	0	0	0
ENSG00000270307	MTATP6P2	0	0	0	0	0	0	0	0
ENSG00000270225	MTCO2P22	0.10764	0.35832	0	0	0	0	0	0
ENSG00000271207	MTCO1P22	0	0	0	0	0	0	0	0
ENSG00000274455	AC092278.1	0	0	0	0	0	0	0	0
ENSG00000207077	RNU6-1119P	0	0	0	0	0	0	0	0
ENSG00000275374	AC113385.3	0	0	0	0	0.1193	0.51932	0	0.12048
ENSG00000273957	AC113385.2	0	0	0	0	0	0	0	0
ENSG00000249787	AC113385.1	0	0	0	0	0	0	0	0
ENSG00000247877	AC021086.1	0	0	0	0	0.10405	0	0	0
ENSG00000174132	FAM174A	16.8239	13.9586	1.74959	2.53526	10.5732	2.28305	2.24342	5.14814
ENSG00000250806	AC027315.1	0	0	0	0	0	0	0	0
ENSG00000207269	RN7SKP62	0	0	0	0	0	0	0	0
ENSG00000113532	ST8SIA4	34.7222	39.4798	7.33848	10.8311	24.7318	10.9004	7.52458	23.3249
ENSG00000221263	MIR548P	2.61787	2.90496	1.28231	1.29507	2.12183	0	0	1.07139
ENSG00000277506	RN7SL802P	0	0	0	0	0	0	0	0
ENSG00000251261	OR7H2P	0	0	0	0	0	0	0	0
ENSG00000249495	AC117525.1	0	0	0	0	0	0	0	0
ENSG00000199786	RNA5SP188	0	0	0	0	0	0	0	0
ENSG00000248261	AC008948.1	0	0	0	0	0	0	0	0
ENSG00000173930	SLCO4C1	6.23891	6.49614	1.67609	4.48685	5.81414	3.41825	1.46598	4.5724
ENSG00000222987	RN7SKP68	0	0	0	0	0	0	0	0
ENSG00000205359	SLCO6A1	0	0	0	0	0	0	0	0
ENSG00000249017	AC094108.1	0	0	0	0	0	0	0	0
ENSG00000250958	LINC00492	0	0	0	0	0	0	0	0
ENSG00000247402	AC099487.1	0	0	0	0	0	0	0	0
ENSG00000250682	LINC00491	0	0	0	0	0	0	0	0

ENSG00000145730	PAM	1.74012	1.50338	0.59665	2.33657	2.68982	2.52893	0.85634	1.0072
ENSG00000175749	EIF3KP1	0	0	0	0	0.13585	0	0	0
ENSG00000145723	GIN1	3.43541	4.843	3.915	4.57826	5.3274	5.50395	4.17785	6.36995
ENSG00000145725	PPIP5K2	7.32155	6.13928	2.7443	3.64719	6.1716	4.93436	3.46661	5.6385
ENSG00000248203	AC011362.1	0	0	0	0	0	0	0	0
ENSG00000181751	C5orf30	2.342	1.77815	0.8151	1.95131	1.97315	1.8121	1.267	1.16028
ENSG00000250567	AC010406.1	0	0	0	0	0	0	0	0
ENSG00000226926	PDZPH1P	0	0	0	0	0	0	0	0
ENSG00000248757	LINC02115	0.16968	0	0	0	0	0	0	0
ENSG00000112874	NUDT12	0.35765	0	0.32534	0.35386	0.35199	0.18027	0.14194	0.4182
ENSG00000283462	AC008505.1	0	0	0	0	0	0	0	0
ENSG00000251026	LINC02163	0	0	0	0	0	0	0	0
ENSG00000201910	RNU1-140P	0	0	0	0	0	0	0	0
ENSG00000239808	RN7SL255P	0	0	0	0	0	0	0	0
ENSG00000251574	AC099520.1	0.03043	0	0	0	0	0	0	0
ENSG00000278958	AC091931.1	0	0	0	0	0	0	0	0
ENSG00000253776	AC099520.2	0	0	0	0	0	0	0	0
ENSG00000252881	RNU6-334P	0	0	0	0	0	0	0	0
ENSG00000253584	AC091987.1	0	0	0	0	0	0	0	0
ENSG00000232159	RAB9BP1	0.11709	0	0	0	0	0	0	0
ENSG00000201790	RNA5SP189	0	0	0	0	0	0	0	0
ENSG00000251204	AC027313.1	0	0	0	0	0	0	0	0
ENSG00000250145	AC114940.1	0	0	0	0	0	0	0	0
ENSG00000252337	RF00322	0	0	0	0	0	0	0	0
ENSG00000251027	LINC01950	0	0	0	0	0	0	0	0
ENSG00000250273	PSMC1P5	0	0	0	0	0	0	0	0
ENSG00000184349	EFNA5	0.08997	0.29951	0.03777	0.01908	0.04688	0.04535	0.02142	0.03156
ENSG00000249959	AC024587.2	0	0	0	0	0	0	0	0
ENSG00000248827	AC024587.1	0.03398	0	0	0.05043	0	0.05995	0	0.04172
ENSG00000239708	RN7SL782P	0	0	0	0	0	0	0	0
ENSG00000251732	RN7SKP122	0	0	0	0	0	0	0	0
ENSG00000145743	FBXL17	3.48233	2.94322	1.2992	3.78909	3.26853	2.59419	2.45113	2.5033
ENSG00000244245	AC133134.1	0.20418	0.33986	0.60008	1.81815	0.24824	1.0806	0	0
ENSG00000272523	LINC01023	0	0	0	0	0.2044	0	0	0
ENSG00000151422	FER	1.93292	1.55042	1.02856	2.49311	2.12744	1.55774	2.25263	1.95675
ENSG00000249921	AC034207.1	0	0	0	0	0	0	0	0
ENSG00000250383	AC008871.1	0	0	0	0	0	0	0	0
ENSG00000207404	RF00019	0	1.10917	0	0	0	0	0	0
ENSG00000250441	AC109481.1	0	0	0	0	0	0	0	0
ENSG00000206593	RNU6-47P	0	0	0	0	0	0	0	0
ENSG00000176857	GJA1P1	0	0	0	0	0	0	0	0
ENSG00000249476	AC008467.1	0.33495	0.55752	0.01969	0.27838	0.60269	0.18909	0.35732	0.2632
ENSG00000198961	PJA2	59.3063	54.1169	11.9957	21.2901	50.6822	29.1986	28.2441	34.8465
ENSG00000248867	AC010625.1	0	0	0	0	0	0	0	0
ENSG00000251367	AC091917.3	0	0	0	0	0	0	0	0
ENSG00000248876	AC091917.2	0	0	0	0	0	0	0	0
ENSG00000248440	AC091917.1	0	0	0	0	0	0	0	0
ENSG00000214794	KRT18P42	0	0	0	0	0	0	0	0
ENSG00000271849	AC012603.1	0.13883	0	0.204	0	0.16878	0.24491	0.2314	0.17045
ENSG00000112893	MAN2A1	10.6026	9.84123	7.7492	21.4717	13.8559	14.6002	26.4009	14.4041
ENSG00000202512	RN7SKP230	0	0.72841	1.28614	2.27313	0.26602	1.15802	1.09415	1.34324
ENSG00000249068	AC008417.1	0	0	0	0	0	0	0	0
ENSG00000254106	LINC01848	0	0	0	0	0	0	0	0
ENSG00000253224	PGAM5P1	0	0	0	0	0	0	0	0
ENSG00000186952	TMEM232	0.1303	0.12758	0.19148	0.22751	0.18638	0.14874	0.2683	0.29174
ENSG00000221436	MIR548F3	0	0	0	0	0	0	0	0
ENSG00000207177	RF00432	0	0	0	0	0	0	0	0
ENSG00000164209	SLC25A46	7.69021	4.38954	1.8596	4.74123	5.45481	7.10333	3.24516	3.78112

ENSG00000270779	AC008782.1	0	0	0	0	0	0	0	0
ENSG00000248966	BCLAF1P1	0.02664	0	0.03914	0	0.03238	0	0	0
ENSG00000248428	AC010395.1	0	0	0	0	0	0	0	0
ENSG00000251627	AC010395.2	0	0	0	0	0	0	0	0
ENSG00000145777	TSLP	0	0	0	0	0	0.0604	0	0
ENSG00000253613	AC008572.1	0	0	0	0	0	0	0	0
ENSG00000134987	WDR36	3.93501	3.29882	1.20995	2.84184	4.88885	4.22256	2.74491	3.27967
ENSG00000250728	AC010468.3	0	0	0	0	0	0	0	0
ENSG00000214784	AC010468.1	0.08972	0	0.13184	0.13315	0	0	0	0.11016
ENSG00000152495	CAMK4	5.00613	1.82443	0.91375	2.04902	6.2465	7.85539	4.25128	2.15449
ENSG00000249318	AC010468.2	0	0	0	0	0	0	0	0
ENSG00000248268	AC010275.1	0	0	0	0	0	0	0	0
ENSG00000164211	STARD4	3.1595	1.69575	1.11807	1.91389	2.30474	2.91202	2.10656	1.85251
ENSG00000246859	STARD4-AS1	0.01298	0	0	0	0	0.0229	0.04327	0.01594
ENSG00000249619	HMGN1P13	0	0	0	0	0	0	0	0
ENSG00000251099	AC008967.1	0	0	0	0	0	0	0	0
ENSG00000134986	NREP	1.4916	0.7579	0.42684	0.53595	1.18351	0.63707	0.30097	0.44338
ENSG00000253057	RN7SKP57	0	0	0	0.37128	0.6083	0	0	0
ENSG00000249439	HMGN1P14	0	0	0	0	0	0	0	0
ENSG00000250095	NREP-AS1	0	0	0	0	0	0	0	0
ENSG00000129595	EPB41L4A	1.13598	4.00913	3.97871	8.65773	3.10486	5.09291	3.1321	6.10327
ENSG00000224032	EPB41L4A-AS1	3.98943	1.82429	0.70864	1.36633	3.09137	2.28152	2.5576	2.09921
ENSG00000238363	SNORA13	0	0	3.23952	4.90763	0.67005	3.88908	1.8373	4.73669
ENSG00000250882	AC010261.1	0	0	0	0	0	0	0	0
ENSG00000251187	AC010261.2	0	0	0	0	0	0	0	0
ENSG00000251076	AC104126.1	0	0	0	0	0	0	0	0
ENSG00000248350	AC010265.1	0	0	0	0	0	0	0	0.42653
ENSG00000278921	EPB41L4A-DT	0.15752	0	0	0	0	0	0	0
ENSG00000248430	HMGB3P16	0.2554	0	0.75062	1.51618	0	1.35169	1.70286	0.62716
ENSG00000251014	AC137549.1	0	0	0	0	0	0	0	0
ENSG00000250358	LINC02200	0	0	0	0	0	0	0	0
ENSG00000134982	APC	12.474	12.3382	3.16908	5.96398	11.3545	6.75664	5.56883	8.58011
ENSG00000250045	CBX3P3	0	0	0	0	0	0	0	0
ENSG00000212370	RNU6-482P	0	0	0	0	0	0	0	0.84109
ENSG00000258864	AC008575.1	0	0	0	0	0	0	0	0
ENSG00000272869		0	0.05612	0	0	0	0	0.0562	0.0414
ENSG00000153037	SRP19	2.4447	2.13634	0.56881	1.42105	2.00624	1.47354	1.15457	1.85097
ENSG00000129625	REEP5	21.3663	17.162	4.04882	6.94377	15.5902	12.2892	5.40135	8.61674
ENSG00000249947	XBP1P1	0.11242	0.56139	0	0	0.13668	0.19833	0	0
ENSG00000212643	ZRSR2P1	0	0	0	0	0	0	0	0
ENSG00000270067		0.76697	2.00612	0.16101	0.32522	0.66604	0	0	0.40357
ENSG00000279522		0.18337	0.53412	0.13473	0.06803	0.44586	0.16174	0	0.11257
ENSG00000272389		0	0.69919	0	0.31171	0	0.37052	0	0.25787
ENSG00000172795	DCP2	24.1314	25.3924	13.8336	21.7222	26.5174	23.8326	23.9302	30.5055
ENSG00000171444	MCC	0.31377	0.10218	0.05012	0.15185	0.46441	0.04813	0.12507	0.1005
ENSG00000232633	AC079465.1	0	0	0	0	0	0	0	0
ENSG00000212122	TSSK1B	0	0	0	0	0	0	0	0
ENSG00000251741	RNU4ATAC13P	0	0	0	0	0	0	0	0
ENSG00000047188	YTHDC2	3.44951	3.59844	2.14113	4.90823	3.91369	3.47908	4.95906	3.56127
ENSG00000251628		0	0	0	0	0	0	0	0
ENSG00000222706	RN7SKP89	0	0	0	0	0	0	0	0
ENSG00000080709	KCNN2	0	0	0	0	0	0	0	0
ENSG00000246316	AC010230.1	0	0	0	0	0	0	0	0
ENSG00000251542	LINC01957	0	0	0	0	0	0	0	0
ENSG00000251132	AC094104.2	0	0	0	0	0	0	0	0
ENSG00000250242	AC094104.1	0	0	0	0	0	0	0	0
ENSG00000152503	TRIM36	0.27579	0.43909	0.12334	0.10677	0.27699	0.04231	0.05996	0.25028
ENSG00000250472	TRIM36-IT1	0	0	0	0	0	0	0	0

ENSG00000249791	AC008494.2	0	0	0	0	0	0	0	0
ENSG00000164219	PGGT1B	8.48518	6.52231	1.23083	2.56183	5.96827	3.89323	2.33094	3.86315
ENSG00000271797	AC008494.3	0.46589	0	0.22821	0	0.0944	0.27397	0.12943	0.09534
ENSG00000243304	AC008494.1	0.09209	0	0	0	0.11196	0	0	0
ENSG00000164221	CCDC112	4.01157	3.15315	1.04799	0.92612	3.35983	1.69061	1.18874	2.18904
ENSG00000250197	HMGN1P15	0.49695	0	0	0	0	0	0.41417	0
ENSG00000249026	CTNNA1P1	0	0	0	0	0	0	0	0
ENSG00000248722	AK3P4	0	0	0	0	0	0	0	0
ENSG00000249944	AC008628.2	0	0	0	0	0	0	0	0
ENSG00000249433	AC008628.1	0	0	0	0	0	0	0	0
ENSG00000250444	CCT5P1	0	0	0	0	0	0	0	0
ENSG00000145780	FEM1C	5.8605	5.72701	1.5557	2.8805	4.16777	5.49175	4.09649	3.83756
ENSG00000251201	TMED7-TICAM2	0.07865	0	0	0.02918	0	0.06937	0.06555	0.02414
ENSG00000243414	TICAM2	0.27115	0.03761	0	0.06707	0.05494	0.19931	0	0
ENSG00000249249	AC010226.1	0.06018	0	0	0.08932	0.14633	0	0.10031	0.44334
ENSG00000134970	TMED7	22.5887	18.8935	6.86006	12.0687	17.5533	16.1391	9.35021	11.787
ENSG00000249021	AC008549.2	0	0	0	0	0	0	0	0
ENSG00000248709	AC008549.1	0	0	0	0	0	0	0	0
ENSG00000251118	AC008549.3	0	0	0	0	0	0	0	0
ENSG00000222598	RNU2-49P	0	0	0	0	0	0	0	0
ENSG00000129596	CDO1	0	0	0	0	0	0.11815	0	0
ENSG00000145782	ATG12	5.50907	4.33784	1.67243	2.68048	3.52937	4.26287	2.99677	3.34148
ENSG00000177879	AP3S1	10.6352	10.9113	3.60394	7.95993	9.9483	7.64225	6.34207	8.13294
ENSG00000172901	LVRN	0.01105	0	0	0.03279	0.02686	0	0	0
ENSG00000248295	DDX43P1	0	0	0	0	0	0	0	0
ENSG00000268714		0	0	0	0	0	0	0	0
ENSG00000268223	ARL14EPL	0.07659	0	0	0	0	0	0	0
ENSG00000185641	AC034236.1	0.19652	0	0	1.45825	2.38919	2.08008	1.63781	0.96512
ENSG00000272265	AC034236.3	0.159	0	0	0.23598	0	0	0	0
ENSG00000271918	AC034236.2	0.54634	0.37891	0	0.40541	0.55352	0.48191	0.22767	0.1118
ENSG00000145781	COMMD10	5.65222	5.05068	3.06006	8.21191	5.09961	5.353	6.94206	4.78475
ENSG00000212457	RNU6-644P	0	0	0	0	0	0	0	0
ENSG00000232916	HMGN2P27	0	0	0	0	0	0	0	0
ENSG00000250015	AC018752.1	0	0	0	0	0	0	0	0
ENSG00000092421	SEMA6A	0.01338	0	0	0	0	0	0	0
ENSG00000248445	SEMA6A-AS1	0.15546	0.04313	0.03807	0.07691	0.15751	0.13713	0.21594	0.09544
ENSG00000239528	RPS14P8	0	0	0	0	0	0.35044	0.33111	0.48779
ENSG00000249167	SEMA6A-AS2	0	0	0	0	0	0	0	0
ENSG00000197575	RPS17P2	0.17966	0	0	0	0	0	0	0
ENSG00000251311	LINC02214	0	0	0	0	0	0	0	0
ENSG00000249150	AC010267.1	0	0	0	0	0	0	0	0
ENSG00000250650	AC093534.1	0	0	0	0	0	0	0	0
ENSG00000250949	AC093534.2	0	0	0	0	0	0	0	0
ENSG00000243758	RPL35AP15	0	0	0	0	0	0	0	0
ENSG00000248663	LINC00992	0	0	0	0	0	0	0	0
ENSG00000251477	AC114322.1	0	0	0	0	0	0	0	0
ENSG00000249582	RPL7L1P4	0	0	0	0	0	0	0	0
ENSG00000249797	LINC02147	0	0	0	0	0	0	0	0
ENSG00000250427	LINC02148	0	0	0	0	0	0	0	0
ENSG00000250891	LINC02208	0	0	0	0	0	0	0	0
ENSG00000249551	LINC02216	0	0	0	0	0	0	0	0
ENSG00000249128	LINC02215	0	0	0	0	0	0	0	0
ENSG00000238904	RNU7-34P	0	0	0	0	0	0	0	0
ENSG00000249426	AC093206.1	0	0	0	0	0	0	0	0
ENSG00000169570	DTWD2	0.80969	0.6078	0.41994	1.03673	1.64066	1.20433	0.5028	0.95513
ENSG00000197744	PTMAP2	0.89664	1.49246	0	0.33268	0.81758	18.1906	0	0
ENSG00000283498	MIR1244-2	6.89886	8.61236	5.06889	17.9176	6.29059	7.60658	10.0619	16.9406
ENSG00000249494	AC008629.1	0	0	0	0	0.06675	0	0	0

ENSG00000223179	RNU6-373P	0	0	0	0	0	0	0	0
ENSG00000250678	AC008629.2	0	0	0	0	0	0	0	0
ENSG00000250928	AC008629.3	0	0	0	0	0	0	0	0
ENSG00000172869	DMXL1	6.89602	5.04801	2.41726	6.07546	6.9313	6.05913	3.98336	4.9801
ENSG00000248885	AC118465.1	0	0	0	0	0	0	0	0
ENSG00000248979	LAMTOR3P2	0	0	0	0	0	0	0	0
ENSG00000206786	RNU6-701P	0.68505	1.14027	1.00667	1.01669	0.83287	0	0	1.68219
ENSG00000264536	MIR5706	0.91625	0	0	4.07946	0	3.23279	4.58176	1.12496
ENSG00000145779	TNFAIP8	15.7973	10.4441	3.3243	6.24971	16.3069	13.3343	5.36314	9.34596
ENSG00000243333	RN7SL174P	0	0	0	0	0	0	0.42277	0
ENSG00000202092	RNA5SP190	0	0	0	0	0	0	0	0
ENSG00000133835	HSD17B4	7.43601	4.58032	1.84518	3.2645	6.53948	4.06898	3.71098	3.30204
ENSG00000213663	AC010409.1	0	0	0	0	0	0	0	0
ENSG00000249919	FABP5P6	0	0	0	0	0	0	0	0
ENSG00000164334	FAM170A	0	0	0	0	0	0	0	0
ENSG00000250895	AC010409.2	0	0	0	0	0	0	0	0
ENSG00000234259	AC008550.1	0	0	0	0	0	0	0	0
ENSG00000251293	AC008574.1	0	0	0	0	0	0	0	0
ENSG00000251975	RNU6-718P	0	0	0	0	0	0	0	0
ENSG00000261036	AC113418.1	0	0	0	0	0	0	0	0
ENSG00000184838	PRR16	0.32234	0.10731	0	0.09568	0	0.11373	0	0.07915
ENSG00000270741	PRELID3BP8	0	0	0	0	0	0	0	0
ENSG00000222609	RNU4-69P	0	0	0	0	0	0	0	0
ENSG00000248927	AC114284.1	0	0	0	0	0	0	0	0
ENSG00000251135	AC008565.1	0	0	0	0	0	0	0	0
ENSG00000248853	AC113352.1	0	0	0	0	0	0	0	0
ENSG00000250847	AC010350.1	0	0	0	0	0	0	0	0
ENSG00000229855	AC008568.1	0	0	0	0	0	0	0	0
ENSG00000279860	AC008568.2	0	0.1055	0	0	0	0	0	0
ENSG00000244088	RPL23AP44	0	0	0	0	0	0	0	0
ENSG00000213661	AC106806.1	0	0	0	0	0	0	0	0
ENSG00000249600	AC106806.2	0	0	0	0	0	0	0	0
ENSG00000181867	FTMT	0	0	0	0	0	0	0	0
ENSG00000151304	SRFBP1	6.50302	6.66713	4.43182	5.83967	10.7708	10.1421	6.48015	9.0836
ENSG00000113083	LOX	0.48893	0.28594	0.01942	0.11767	0	0.09325	0.11013	0.21092
ENSG00000247311	AC010255.1	0	0	0	0	0	0	0	0
ENSG00000164185	ZNF474	0	0	0	0	0	0.06105	0.05769	0
ENSG00000250803	AC010255.3	0	0	0	0	0	0	0	0
ENSG00000249610	AC010255.2	0	0	0	0	0	0	0	0
ENSG00000272139	AC113349.2	0	0	0	0	0	0	0	0
ENSG00000064692	SNCAIP	0.01001	0	0	0.0297	0	0	0	0
ENSG00000249621	AC113349.1	0	0	0	0	0	0	0	0
ENSG00000249916	AC119150.1	0	0	0	0	0	0	0	0
ENSG00000250328	MGC32805	0	0	0	0	0	0	0	0
ENSG00000251538	LINC02201	0	0	0	0	0	0	0	0.03786
ENSG00000240058	ARGFXP1	0	0	0	0	0	0	0	0
ENSG00000205302	SNX2	24.4334	20.937	8.33049	13.579	21.6193	14.0133	16.2562	13.2224
ENSG00000260686	AC008669.1	0.70675	0.50417	0.24728	0.69927	1.473	0.47497	0.56097	1.11567
ENSG00000064652	SNX24	0.70203	0.68737	0.30342	0.7661	0.55227	0.07285	0.34417	0.35492
ENSG00000263432	RN7SL689P	0	0	0	0	0	0	0	0
ENSG00000168938	PPIC	0	0.05815	0	0	0.08495	0.06164	0	0
ENSG00000249996	AC106786.2	0	0	0	0	0	0	0	0
ENSG00000276047	RN7SL711P	0	0	0.72049	0	0	0	0.81726	0.30099
ENSG00000223652	AC106786.1	0	0	0	0	0	0	0	0
ENSG00000061455	PRDM6	0	0	0	0	0	0	0	0
ENSG00000213655	AC010493.1	0	0	0	0	0	0	0	0
ENSG00000168944	CEP120	8.08397	6.34517	1.79612	4.05903	9.48989	8.28347	3.97384	5.30443
ENSG00000250539	KRT8P33	0.61813	0.25722	0.22708	0.22934	0.43838	0.72698	0.60103	0.50596

ENSG00000249400	HMGB3P17	0	0	0	0	0	0	0	0
ENSG00000279772	AC026422.1	0	0	0	0	0	0	0	0
ENSG00000151292	CSNK1G3	21.5943	28.1352	34.6408	69.971	43.8276	34.2676	44.2781	41.0747
ENSG00000235275	KRT18P16	0	0	0	0	0	0	0	0
ENSG00000253807	LINC01170	0	0	0	0	0	0	0	0
ENSG00000250862	HMGB1P29	0	0	0	0	0	0	0	0
ENSG00000251421	AC016556.1	0	0	0	0	0	0	0	0
ENSG00000248600	AC025465.2	0	0	0	0	0	0	0	0
ENSG00000251214	AC025465.4	0	0	0	0	0	0	0	0
ENSG00000250438	AC025465.3	0	0	0	0	0	0	0	0
ENSG00000248296	AC025465.1	0	0	0	0	0	0	0	0
ENSG00000168916	ZNF608	0.41299	0.24677	0.03112	0.15716	0.34761	0.18681	0.12356	0.22103
ENSG00000249112	AC112196.1	0	0	0	0	0.32133	0	0	0
ENSG00000251456	AC113398.2	0	0	0	0	0	0	0	0
ENSG00000242814	AC113398.1	0	0	0	0	0	0	0	0
ENSG00000248443	AC109464.1	0	0	0	0	0	0	0	0
ENSG00000249261	AC109464.2	0	0	0	0	0	0	0	0
ENSG00000249423		0	0	0	0	0	0	0	0
ENSG00000250411		0	0	0	0	0	0	0	0
ENSG00000250194	AC109458.1	0	0	0	0	0	0	0	0
ENSG00000249365		0	0	0	0	0	0	0	0
ENSG00000250721	HMGB1P22	0	0	0	0	0	0	0	0
ENSG00000281608		0	0	0	0	0	0	0	0
ENSG00000222107	RN7SKP117	0	0	0	0	0	0	0	0
ENSG00000250402	AC109471.1	0	0	0	0	0	0	0	0
ENSG00000260192	LINC02240	0	0	0	0	0	0	0	0
ENSG00000249950	AC010587.2	0	0	0	0	0	0	0	0
ENSG00000249904	AC010587.1	0	0	0	0	0	0	0	0
ENSG00000241888	RPSAP37	0	0	0	0	0	0	0	0
ENSG00000248752	AC010235.1	0	0	0	0	0	0	0	0
ENSG00000248107	LINC02039	0	0	0	0	0	0	0	0
ENSG00000155324	GRAMD2B	0.26122	0.28986	0.19193	0.21537	0.5293	0.92164	0.43541	0.40981
ENSG00000250602	AC093535.1	0.0434	0	0	0	0.21105	0	0.07234	0.10657
ENSG00000279118	AC093535.2	0	0	0	0	0	0	0	0
ENSG00000164904	ALDH7A1	0.00408	0.02037	0.01199	0.00605	0.01488	0	0	0.02003
ENSG00000202082	RNU6-290P	0	0	0	0	0	0	0	0
ENSG00000164902	PHAX	6.17766	4.54813	0.84794	2.14096	7.40748	5.98804	2.51772	3.22981
ENSG00000230929	AC099513.1	0	0	0	0	0	0	0	0
ENSG00000196900	TEX43	0	0	0	0	0	0	0	0
ENSG00000213630	BOLA3P3	0	0	0.46428	0	0	0	0	0
ENSG00000249768	HSPE1P10	0	0	0	0	0	0	0	0
ENSG00000251072	LMNB1-DT	0.32079	0.26698	0.35355	0.11902	0	0.14148	0	0.19693
ENSG00000252185	RNU6-752P	0	0	0	0	0	0	0	0
ENSG00000113368	LMNB1	23.9896	61.5248	7.73833	6.0187	25.6581	21.0715	8.37404	27.5714
ENSG00000173926	MARCH3	1.14642	1.24859	0.87352	1.30232	1.60028	1.37327	3.8926	0.97313
ENSG00000164241	C5orf63	0.44857	0.32241	0.20974	0.39338	0.34705	0.32373	0.44182	0.28789
ENSG00000279446	AC005915.1	0	0	0.05338	0	0	0	0	0
ENSG00000214743	MRPS5P3	0	0	0	0	0	0	0	0
ENSG00000250080	AC011416.1	0	0	0	0	0	0	0	0
ENSG00000271585	SELENOTP2	0	0	0	0	0	0	0	0
ENSG00000250956	AC011416.2	0	0.84144	0	0	0	0	0	0
ENSG00000283897	AC011416.3	0.06316	0	0	0	0.11519	0.05571	0	0.11633
ENSG00000145794	MEGF10	0.00814	0.01355	0	0	0.00989	0.01436	0	0.00999
ENSG00000249577	AC010424.1	0	0	0	0	0	0	0	0
ENSG00000271766	AC010424.2	0	0	0	0	0	0	0	0
ENSG00000250859	HNRNPKP1	0	0	0.07811	0.15777	0.06462	0	0	0
ENSG00000164244	PRRC1	4.8441	3.17674	0.92693	2.55642	4.22773	3.43823	1.50973	2.07517
ENSG00000282925	AC010424.3	0	0	0	0	0	0	0	0

ENSG00000205279	CTXN3	0	0	0	0	0	0	0	0
ENSG00000248799	AC022118.1	0	0	0	0	0	0	0	0
ENSG00000230561	CCDC192	0	0	0	0	0	0	0	0
ENSG00000251032	CUL1P1	0	0	0	0	0	0	0	0
ENSG00000250603	AC068658.1	0	0	0	0	0	0	0	0
ENSG00000245937	LINC01184	1.34943	0.906	0.21663	0.67318	1.14429	1.28031	0.52925	0.72399
ENSG00000064651	SLC12A2	1.56031	1.28141	0.72725	1.25475	1.80506	2.30394	1.21447	1.01272
ENSG00000250329	KDELC1P1	0.04858	0.08085	0	0	0	0.08569	0.16194	0
ENSG00000138829	FBN2	1.5831	0.96157	0.28569	0.25556	0.68208	0.44097	0.24999	0.32054
ENSG00000207290	RF00019	0	0	0	0	0	0	0	0
ENSG00000113396	SLC27A6	0	0	0	0	0.05103	0.03702	0.03498	0.02576
ENSG00000248634	AC008588.1	0	0	0	0	0	0	0	0
ENSG00000066583	ISOC1	4.68518	5.5973	2.77613	5.77574	5.69612	7.93201	4.72347	3.52566
ENSG00000264563	MIR4633	0	0	0	0	0	0	0	0
ENSG00000265691	MIR4460	0	0	0	0	0	0	0	0
ENSG00000249421	ADAMTS19-AS1	0	0	0	0	0	0	0	0
ENSG00000145808	ADAMTS19	0	0	0	0	0	0	0	0
ENSG00000251680	AC008591.1	0	0	0	0	0	0	0	0
ENSG00000186367	MINAR2	0	0	0	0	0	0	0	0
ENSG00000198108	CHSY3	0	0	0	0	0.02263	0.03284	0.09308	0
ENSG00000221562	RNU6ATAC10P	0	0	0	0	0	0	0	0
ENSG00000199455	RNA5SP191	0	0	0	0	0	0	0	0
ENSG00000248610	HSPA8P4	0.03818	0.06355	0	0	0	0	0	0
ENSG00000252514	RNU7-53P	0	0	0	0	0	0	0	0
ENSG00000249312	ARL2BPP4	0	0	0	0	0	0	0	0
ENSG00000249418	AC005741.1	0	0	0	0	0	0	0	0
ENSG00000244192	AC113367.1	0	0	0.20247	0	0	0	0	0
ENSG00000250405	AC113367.3	0	0	0	0	0	0	0	0
ENSG00000248955	AC113367.2	0	0	0	0	0	0	0	0
ENSG00000169567	HINT1	21.0133	13.4865	2.89314	6.57434	19.4713	25.1143	8.20426	10.9707
ENSG00000186687	LYRM7	2.71226	2.05557	1.17025	3.22024	3.08702	2.97268	2.17389	2.81994
ENSG00000158985	CDC42SE2	64.4055	45.1861	13.6788	27.5576	47.8425	52.3129	32.1879	36.3335
ENSG00000279370	AC004777.1	0	0	0	0	0	0	0	0
ENSG00000158987	RAPGEF6	2.51692	1.59022	0.72616	1.63301	2.50728	2.16198	1.48264	2.05476
ENSG00000273217	AC008695.1	0	0	0	0	0	0	0	0
ENSG00000275939	RF02271	1.3247	0	0	1.31067	0.53685	0.77899	1.47205	0.54215
ENSG00000217128	FNIP1	17.1852	22.6596	8.35454	10.7799	16.1581	10.567	12.3073	15.3772
ENSG00000279584	AC005593.1	1.13865	1.65837	0.62746	0.42247	0.51913	1.00436	0.47449	0.52425
ENSG00000239642	MEIKIN	0.08773	0	0.06446	0.1302	0	0	0	0
ENSG00000281938	AC026398.1	0.0469	0	0.06891	0.2088	0.08552	0.20683	0.15634	0.08637
ENSG00000234758	AC034228.3	0	0	0	0	0	0	0	0
ENSG00000164398	ACSL6	0.86746	0.53443	0.91052	1.4128	0.93821	1.2322	1.15486	0.80225
ENSG00000223548	AC034228.1	0.07942	0	0	0.23572	0.1931	0	0	0
ENSG00000231585	AC034228.2	0	0	0	0	0	0	0	0
ENSG00000164399	IL3	0	0	0	0	0	0	0	0
ENSG00000164400	CSF2	0.09314	0	0.13687	0.27646	0.11324	0.49293	0	0
ENSG00000253067	RF00285	0	0	0	0	0	0	0	0
ENSG00000227616	AC063976.3	0	0	0	0	0	0	0	0
ENSG00000235775	AC063976.4	0	0	0	0	0	0	0	0
ENSG00000224015	AC063976.1	0	0	0	0	0	0	0	0
ENSG00000237714	P4HA2-AS1	0	0	0	0	0	0	0	0
ENSG00000072682	P4HA2	0.13794	0.1148	0.01267	0.02559	0.05241	0	0.08622	0.09527
ENSG00000224431	AC063976.2	0	0	0	0	0	0	0	0
ENSG00000283678	MIR6830	0	0	0	0	0	0	0	0
ENSG00000131435	PDLIM4	0	0	0	0	0	0	0	0
ENSG00000197208	SLC22A4	3.32688	7.67092	3.56642	4.24944	4.54203	2.54967	3.9545	3.91728
ENSG00000233006	MIR3936HG	0.20525	0.12812	0	0.45692	0	0.22631	0.29936	0.1575
ENSG00000263597	MIR3936	0	0	0	0	1.6203	0	0	0

ENSG00000197375	SLC22A5	0.16623	0.09541	0.06738	0.14462	0.08363	0.11123	0.09554	0.07038
ENSG00000197536	C5orf56	1.45425	1.94401	0.29896	0.53677	0.69622	1.31598	0.54007	0.95289
ENSG00000283782	AC116366.3	0.03272	0.01362	0.02404	0.07284	0.05967	0.04329	0.0409	0
ENSG00000238160	AC116366.2	7.41075	5.25162	0.9704	1.74231	5.97679	2.71827	4.28059	5.85566
ENSG00000202533	RF00019	0.66036	1.09918	1.94079	5.88031	0	0	1.10072	4.86471
ENSG00000234290	AC116366.1	1.15944	1.24298	0.20214	0.14583	1.19459	0.38135	0.8189	0.62733
ENSG00000125347	IRF1	46.3731	53.7376	7.45808	9.31772	36.5425	50.6039	24.7055	46.0198
ENSG00000113525	IL5	0	0	0	0	0	0	0	0
ENSG00000113522	RAD50	0.31508	0.14984	0.03307	0.1002	0.28274	0.33086	0.20007	0.14737
ENSG00000223442	TH2LCRR	0.13562	0	0.09964	0.10063	0.08244	0	0	0.16651
ENSG00000169194	IL13	0	0.04226	0	0	0	0.04479	0	0
ENSG00000113520	IL4	1.34414	0.89493	1.05344	0.66495	0.87156	0.47425	0	0.33006
ENSG00000230612	AC004039.1	0.04097	0	0	0	0	0	0	0
ENSG00000131437	KIF3A	2.36143	1.68962	0.34544	0.61846	2.19544	1.65881	0.55213	0.97081
ENSG00000205089	CCNI2	0.13909	0	0	0.0344	0.1691	0.08179	0.07728	0.02846
ENSG00000164402	SEPT8	0.69393	0.42721	0.1816	0.19751	0.38138	0.35217	0.19014	0.21008
ENSG00000198944	SOWAHA	0	0	0	0	0	0	0	0
ENSG00000272203	AC004775.1	0	0	0	0	0	0	0	0
ENSG00000164403	SHROOM1	0.89552	0.27604	0.04874	0.34457	0.1613	0.02926	0	0.04072
ENSG00000201274	RNA5SP192	0	0	0	0	0	0	0	0
ENSG00000164404	GDF9	0	0	0	0	0.02718	0.11831	0	0.02745
ENSG00000164405	UQCRQ	4.97964	3.89565	1.09763	2.43881	2.72435	4.48023	3.15411	4.64659
ENSG00000164406	LEAP2	0.09143	0.05073	0.08958	0	0.11116	0.32261	0	0.07484
ENSG00000072364	AFF4	11.6857	8.92087	3.02251	4.94962	9.86855	9.85549	6.86771	6.58029
ENSG00000235253	AC010240.2	0	0	0	0	0	0	0	0
ENSG00000202417	RF00019	0	0	0	0	0	0	0	0
ENSG00000225364	ATP6V0E1P1	0	0	0	0	0	0	0	0
ENSG00000272023	AC010240.3	0.37114	0.30888	0	0.27541	0	0	0.30932	0.22784
ENSG00000229738	AC010240.1	0	0	0	0	0	0	0	0
ENSG00000199677	RF00019	0	0	0	0	0	0	0	0
ENSG00000155329	ZCCHC10	7.64798	10.1555	5.13525	12.2857	10.7957	7.93355	7.20955	10.7968
ENSG00000248648	AC113410.1	0	0	0	0	0	0	0	0
ENSG00000170606	HSPA4	13.2433	14.8693	5.56024	11.2311	17.7092	18.871	12.7326	11.9635
ENSG00000279691	AC113410.3	2.5276	3.00514	0	0	0.878	0	0	0
ENSG00000251616	AC113410.2	0	0	0	0	0	0	0	0
ENSG00000248245	AC010307.2	0.08983	0	0	0	0	0	0	0
ENSG00000240611	AC010307.1	0	0	0	0	0	0	0	0
ENSG00000053108	FSTL4	0.10885	0.12683	0.016	0.01615	0.02647	0.11522	0.07258	0.05346
ENSG00000249478	AC010307.3	0	0	0	0	0	0	0	0
ENSG00000250244	AC010307.4	0	0	0	0	0	0	0	0
ENSG00000250409	AC010608.1	0	0	0	0	0	0	0	0
ENSG00000221287	MIR1289-2	0	0	0	0	0	0	0	0
ENSG00000280104	AC010608.3	0	0	0	0	0	0	0	0
ENSG00000279197	AC010608.2	0	0	0	0	0	0	0	0
ENSG00000251243	AC005178.1	0	0	0	0	0	0	0	0
ENSG00000249073	WSPAR	0	0	0	0	0	0	0	0
ENSG00000113583	C5orf15	14.9506	13.5509	4.49179	10.0611	12.9149	14.4154	6.55799	8.73217
ENSG00000213585	VDAC1	18.5163	11.2635	3.33733	6.29396	13.1576	16.0669	4.48085	5.5199
ENSG00000271737	AC008608.2	0.35241	0	0	0	0	0.93254	0	0
ENSG00000271128	AC008608.1	0	0	0.84814	0	0	0	0.96205	0
ENSG00000081059	TCF7	8.46418	2.06116	0.58872	2.11885	10.0603	7.35033	3.50891	2.32527
ENSG00000113558	SKP1	12.0187	12.6298	12.314	24.7538	14.8632	9.6394	18.8949	15.0383
ENSG00000272772	AC104109.3	0	0	0	0	0	0	0	0
ENSG00000113575	PPP2CA	13.6216	12.5435	3.91964	6.82193	11.5233	11.2842	7.87773	9.66582
ENSG00000273345	AC104109.4	0.04201	0.27968	0.06173	0.24937	0.5107	0	0.07002	0.10315
ENSG00000266751	MIR3661	0	0	0	0	1.8566	0	0	0
ENSG00000270177	AC104109.2	0.20677	0.34417	0	0.07672	0.12569	0.27358	0.25849	0.1904
ENSG00000239615	AC104109.1	0	0	0	0	0	0	0	0

ENSG00000006837	CDKL3	0.14387	0.03991	0.31712	1.53019	0.69964	0.2961	0.55954	0.47103
ENSG00000119048	UBE2B	25.1016	35.9983	20.8816	62.9047	24.3169	12.2593	39.7244	29.7802
ENSG00000279469	AC109454.4	0	0	0	0	0.13731	0	0	0.13867
ENSG00000248559	AC109454.2	2.2554	3.05021	0.41428	0	1.5424	0.74603	0.23496	1.38457
ENSG00000237190	CDKN2AIPNL	1.5436	0.76386	0.18392	0.30958	1.57235	2.20794	0.62585	1.07566
ENSG00000243648	AC109454.1	0	0	0.21629	0	0	0	0	0
ENSG00000250564	AC109454.3	0	0	0	0	0	0	0	0
ENSG00000250994	AC005355.1	0	0	0	0	0	0	0	0
ENSG00000207222	RNU6-456P	0	0	0	0	0	0	0	0
ENSG00000251169	LINC01843	0	0	0	0	0	0	0	0
ENSG00000240250	RN7SL541P	0	0	0	0	0	0	0	0
ENSG00000280420	AC005355.2	0.30289	0	0	0.22476	0	0	0	0
ENSG00000043143	JADE2	6.05579	4.59524	1.47225	2.98481	7.38955	7.5673	2.67196	3.43514
ENSG00000152700	SAR1B	2.81487	2.07002	1.27728	3.78066	2.21918	2.11136	2.52987	2.63514
ENSG00000207459	RNU6-1311P	0	0	0	0	0	0	0	0
ENSG00000113615	SEC24A	7.14579	6.21818	2.96172	6.55645	6.47244	6.80588	4.73383	4.27427
ENSG00000201298	RNU6-1164P	0	0	0	0	0	0	0	0
ENSG00000222266	RNU6-757P	0	2.44017	0	0	0	0	0	0
ENSG00000164615	CAMLG	8.77888	7.41609	4.7043	9.3063	12.6392	9.54846	11.2773	9.0767
ENSG00000145833	DDX46	10.794	8.91712	2.00898	5.26116	10.0887	8.89001	5.57774	4.99658
ENSG00000241179	AC010301.1	0	0	0	0	0	0	0	0
ENSG00000181904	C5orf24	2.69402	2.12521	0.50658	1.95174	1.89379	2.79301	1.51103	1.42653
ENSG00000270892	AC006077.1	0	0	0	0	0	0	0	0
ENSG00000113621	TXNDC15	4.17666	2.73447	0.5933	1.61164	3.18213	3.09464	0.81221	2.0854
ENSG00000132570	PCBD2	0.75641	0.45938	0.7961	2.24519	0.7705	0.63114	1.36305	1.14206
ENSG00000279799	AC006077.2	0.64158	0.26698	0.2357	0	0	0.56592	0.26735	0.19693
ENSG00000244921	MTCYBP18	0.09813	0	0	0	0	0	0	0
ENSG00000249119	MTND6P4	0.14042	0.23373	0	0	0	0	0	0
ENSG00000248923	MTND5P11	0	0	0	0	0	0	0	0
ENSG00000247627	MTND4P12	0.10646	0	0	0	0.06472	0.09391	0	0.13071
ENSG00000198868	MTND4LP30	0	0	0	0	0	0	0	0
ENSG00000263963	MIR4461	0	0	0	0	1.20428	0	0	0
ENSG00000249192	MTND3P25	0	0	0	0	0	0	0	0
ENSG00000152705	CATSPER3	0	0	0	0	0.11273	0.08179	0	0
ENSG00000069011	PITX1	0	0	0	0	0	0.03683	0	0
ENSG00000224186	C5orf66	0.03307	0	0.12958	0.13087	0.16081	0	0.05512	0.05413
ENSG00000277619	AC008406.3	0	0	0	0	0	0	0	0
ENSG00000249082	C5orf66-AS1	0	0	0	0	0	0	0	0
ENSG00000248753	AC008406.2	0	0	0	0	0	0	0	0
ENSG00000248482	AC008406.1	0	0	0	0	0	0	0	0
ENSG00000249647	C5orf66-AS2	0	0	0	0	0	0	0	0
ENSG00000113648	H2AFY	8.46695	6.92276	1.42768	2.74879	5.62948	4.79176	3.01833	3.74099
ENSG00000270021	AC026691.1	0.18171	0.06049	0	0	0.13255	0.12822	0.06058	0
ENSG00000251380	DCANP1	0	0	0	0	0	0	0	0
ENSG00000255833	TIFAB	0.21038	0.16479	0.05456	0.01837	0.03009	0.04366	0.06188	0.12156
ENSG00000249639	AC022092.1	0	0	0	0	0	0	0	0
ENSG00000181965	NEUROG1	0	0	0	0	0	0	0	0
ENSG00000250167	AC034206.1	0.08264	0.82531	0.72862	0.24529	1.10517	0	1.10196	0.50731
ENSG00000145824	CXCL14	0	0	0	0	0	0	0	0
ENSG00000251045	LINC01959	0	0	0	0	0	0	0	0
ENSG00000223539	AC011431.1	0	0	0	0	0	0	0	0
ENSG00000265924	MIR5692C1	0	0	0	0	0	0	0	0
ENSG00000250378	AC114296.1	0	0	0	0	0	0	0	0
ENSG00000145832	SLC25A48	0	0	0	0	0	0	0	0
ENSG00000145839	IL9	0	0.20644	0	0	0	0	0	0
ENSG00000254239	AC002428.2	0	0	0	0	0	0	0	0
ENSG00000253927	AC002428.1	0	0	0	0	0	0	0	0
ENSG00000145826	LECT2	0	0	0	0	0	0	0	0

ENSG00000164616	FBXL21	0	0	0	0	0	0	0	0.02324
ENSG00000120708	TGFBI	20.8826	12.0915	1.71018	4.69408	10.395	4.28832	4.94353	3.53766
ENSG00000270123	VTRNA2-1	0	0	0	0	0	0	0	0
ENSG00000278815	RF00006	0	0	0	0	0	0	0	0
ENSG00000164621	SMAD5-AS1	0	0	0	0	0	0	0	0
ENSG00000277859	RF02173	0	0	0	0	0	0	0	0
ENSG00000113658	SMAD5	3.19786	2.07077	0.8466	2.18092	3.02503	2.34202	1.1273	2.77813
ENSG00000275646	RF02174	0	0	0	0	0	0	0	0
ENSG00000277524	RF02175	0	0	0	0	0	0	0	0
ENSG00000274934	RF02176	0	0	0	0	0	0	0	0
ENSG00000271824	SMIM32	0	0	0	0	0	0	0	0
ENSG00000069018	TRPC7	0	0	0	0	0	0	0	0
ENSG00000248211	TRPC7-AS1	0	0	0	0	0	0	0	0
ENSG00000250947	TRPC7-AS2	0	0	0	0	0	0	0	0
ENSG00000279240	AC063980.1	0	0	0	0	0	0	0	0
ENSG00000248351	HSPD1P18	0	0	0	0	0	0	0	0
ENSG00000213568	HNRNPA1P13	0	0	0	0	0	0	0	0
ENSG00000249803	AC112178.1	0	0	0	0	0	0	0	0
ENSG00000250284	AC109439.2	0	0	0	0	0	0	0	0
ENSG00000248616	AC109439.1	0	0	0	0	0	0	0	0
ENSG00000222285	RNA5SP193	0	0	0	0	0	0	0	0
ENSG00000152377	SPOCK1	0.11781	0.07844	0	0.01748	0.04297	0.04157	0.01964	0.04339
ENSG00000146021	KLHL3	0.43285	0.16468	0.05452	0.28449	0.39093	0.20727	0.26798	0.22017
ENSG00000216009	MIR874	0	0	0	0	0	1.65784	0	0
ENSG00000177733	HNRNPA0	4.15811	2.83838	0.93815	2.79257	3.47234	3.4825	2.35231	2.60936
ENSG00000270697	AC106791.2	0	0	0	0	0	0	0	0
ENSG00000226306	NPY6R	0	0	0	0	0	0	0	0
ENSG00000250159	AC106791.1	0.52514	0.14568	0.19292	0.71441	0.47884	0.3088	0.43766	0.32238
ENSG00000120729	MYOT	0	0	0	0	0	0	0	0
ENSG00000078795	PKD2L2	0	0.02622	0	0	0	0	0	0
ENSG00000031003	FAM13B	35.3259	45.8069	57.0371	146.273	54.1271	44.4652	104.611	80.6378
ENSG00000246323	AC113382.1	0	0	0	0.08682	0	0	0.09751	0.07183
ENSG00000250260	AC113382.2	0.3404	0	0	0	0	0	0	0
ENSG00000222924	RNU6-1148P	0	0	0	0	0	0	0	0
ENSG00000061492	WNT8A	0	0	0	0	0	0	0	0
ENSG00000112981	NME5	0	0	0	0	0	0	0	0
ENSG00000212460	RNU6-460P	0	0	0	0	0	0	0	0
ENSG00000199880	RNU6-888P	0	0	0	0	0	0	0	0
ENSG00000112983	BRD8	4.16125	3.68831	0.58092	1.5285	4.45204	2.86299	1.40457	2.18415
ENSG00000112984	KIF20A	0.1852	0	0.02721	0.1924	0.27019	1.60088	0.24695	0.06821
ENSG00000094880	CDC23	4.4285	3.98945	1.60941	2.49703	3.70515	4.9283	1.82556	2.37758
ENSG00000146013	GFRA3	0	0	0	0	0	0	0	0
ENSG00000249851	RPS27AP18	0	0	0	0	0	0	0	0
ENSG00000158402	CDC25C	1.46745	1.64173	4.77236	8.71143	4.4456	6.15366	17.3627	5.46421
ENSG00000120709	FAM53C	17.2985	15.4349	2.94835	5.09138	11.236	6.12539	5.493	8.21984
ENSG00000249971	AC104116.1	0	0	0	0	0	0	0	0
ENSG00000120733	KDM3B	22.4293	19.571	3.13901	5.52059	16.9368	12.2473	5.35623	8.88553
ENSG00000132563	REEP2	0.15624	0	0	0	0	0	0.0372	0.0274
ENSG00000271196	AC113403.1	0	0	0	0	0	0.67001	0	0
ENSG00000120738	EGR1	29.7126	1.51636	3.08931	0.13867	7.58258	7.99442	0.15574	0.48756
ENSG00000241458	RPL7P19	0.11861	0.19742	0.17429	0.17603	0	0	0.1977	0
ENSG00000120705	ETF1	22.3517	14.4522	4.42468	6.43115	13.9457	15.2049	11.0469	9.81928
ENSG00000113013	HSPA9	24.7626	17.5655	4.01292	7.83092	20.8395	25.0132	8.48653	9.90706
ENSG00000222937	SNORD63B	4.18859	3.48596	0	1.55408	5.09238	1.84731	6.98173	2.57134
ENSG00000206989	SNORD63	3.23384	3.58848	0	3.19958	1.31054	1.90164	3.59354	3.97046
ENSG00000044115	CTNNA1	12.4187	8.93992	3.87917	6.59121	9.10342	5.25741	5.2027	5.7484
ENSG00000232174	AC113340.1	0	0	0	0	0	0	0	0
ENSG00000238745	RF01210	0	0	0	0	0	0	0	0

ENSG00000253404	AC034243.1	0	0	0	0	0	0	0	0
ENSG00000146006	LRRTM2	0.03608	0	0.01767	0	0.01462	0.02122	0	0.02953
ENSG00000120725	SIL1	2.22953	2.25097	0.56395	1.30185	2.06628	2.32123	1.00522	1.09944
ENSG00000201532	RNA5SP194	0	0	0	0	0	0	0	0
ENSG00000249593	AC011405.1	0	0	0	0	0	0	0	0
ENSG00000242683	RPL12P21	0	0	0	0	0	0	0	0
ENSG00000252533	RNU6-572P	0	0	0	0	0	0	0	0
ENSG00000280987	MATR3	0.06921	0.0384	0.0339	0.01712	0.01402	0	0.01923	0
ENSG00000281398	SNHG4	0.26641	0	0	0.28241	0.27762	0.20142	0.12687	0
ENSG00000252213	SNORA74D	4.67875	11.249	12.2229	20.0598	13.2727	21.0934	11.2649	9.57416
ENSG00000200959	SNORA74A	48.4967	58.5394	45.1528	158.783	83.2656	94.0449	43.1951	68.6341
ENSG00000015479	MATR3	0.8003	0.4844	0.22273	0.66585	0.49386	0.68453	0.42445	0.50619
ENSG00000199545	RNA5SP195	1.253	0	0.92063	0	2.28504	1.10523	0	2.30762
ENSG00000253015	RN7SKP64	0	0	0	0	0	0	0	0
ENSG00000120727	PAIP2	60.8592	61.8156	37.5191	70.5144	64.3401	57.9815	61.2062	59.2476
ENSG00000272742	AC135457.1	0	0	0	0.16383	0	0	0	0.13554
ENSG00000170482	SLC23A1	0	0	0	0	0	0	0.03815	0.0281
ENSG00000170476	MZB1	3.09583	1.12722	0.23694	0.67004	2.19557	0.17067	0.59128	0.31675
ENSG00000228672	PROB1	0.08587	0.04764	0.02103	0.06372	0.0696	0.15148	0.02385	0.01757
ENSG00000170469	SPATA24	0.66969	0.20267	0.17893	0.27106	0.37009	0.42961	0.10148	0.44849
ENSG00000170464	DNAJC18	0.23685	0.2816	0.08287	0.11717	0.23311	0.27856	0.1692	0.16617
ENSG00000279686	AC142391.1	0	0	0	0	0	0	0	0
ENSG00000200378	RNU5B-4P	0	0	0	0	0	0	0	0
ENSG00000249751	ECSCR	0	0	0	0	0	0	0	0
ENSG00000283288	SMIM33	0.02826	0	0	0.04194	0	0	0.0471	0.03469
ENSG00000184584	TMEM173	6.4352	5.10217	1.03517	2.03443	4.62944	4.73583	1.36461	2.45447
ENSG00000249689	AC138517.1	0	0	0	0	0	0	0	0
ENSG00000249215	NCOA4P4	0	0	0	0.05846	0	0	0.13131	0
ENSG00000131508	UBE2D2	30.8373	39.0798	48.0411	106.981	39.8218	40.1099	65.385	58.6651
ENSG00000207020	RF00019	0	0	0	0	0	0	0	0
ENSG00000275506	AC010378.1	0	0	0	0	0	0	0	0
ENSG00000272255	AC113361.1	1.21492	0	0.19837	0	0	0	0	0
ENSG00000171604	CXXC5	3.31107	1.23529	0.39148	2.00514	4.18747	1.17495	1.3639	1.63546
ENSG00000250635	CXXC5-AS1	0	0	0	0	0	0	0	0
ENSG00000249526	AC008667.1	0	0	0	0	0	0.22766	0	0
ENSG00000200756	RNU6-236P	0	0	0	0	0	0	0	0
ENSG00000249131	PSD2-AS1	0	0	0	0	0	0	0	0
ENSG00000251387	AC008667.3	0	0	0	0	0	0	0	0
ENSG00000271792	AC008667.4	0	0	0	0	0	0	0	0
ENSG00000146005	PSD2	0.01624	0	0.02387	0.0241	0.03949	0	0	0
ENSG00000158458	NRG2	0	0	0	0	0	0	0	0
ENSG00000250692	AC008667.2	0	0	0	0	0	0	0	0
ENSG00000207317	RF00019	0	0	0	0	0	0	0	0
ENSG00000245146	MALINC1	0.08526	0.14191	0	0.12653	0.23322	0.1128	0.17764	0.13085
ENSG00000185129	PURA	2.05133	1.58206	0.75139	1.60701	2.15752	2.06941	1.09295	1.55842
ENSG00000182700	IGIP	0.91148	0.81151	0.40494	1.00669	1.21124	0.7105	0.49466	0.7027
ENSG00000254363	AC011379.2	0.07163	0.03974	0.03509	0.01772	0.08708	0	0.0398	0.01466
ENSG00000120306	CYSTM1	9.25899	17.7568	8.1833	10.7541	8.93206	7.75279	9.17051	8.36128
ENSG00000250069	AC011379.1	0.48463	0.484	0.14243	0.10789	0.41244	0.21374	0.04039	0.20826
ENSG00000113068	PFDN1	7.88761	5.48925	1.4988	1.66509	5.24945	7.07736	2.60682	2.67153
ENSG00000113070	HBEGF	9.83653	2.95117	0.7495	1.44183	1.7717	2.05665	0.20242	0.2982
ENSG00000253965	AC008438.2	0	0	0	0	0	0	0	0
ENSG00000113073	SLC4A9	0.02017	0.03356	0	0	0	0	0	0
ENSG00000249637	AC008438.1	0.8011	0	0	0.19815	0.48698	0.23554	0	0
ENSG00000222790	RNU4-14P	0	0	0	0	0	0	0	0
ENSG00000131503	ANKHD1	0.3756	0.26323	0.13556	0.17603	0.32045	0.18599	0.2636	0.25889
ENSG00000254996	ANKHD1-EIF4EBP3	0.2189	0.18218	0.03784	0.14333	0.21134	0.07951	0.07512	0.11067
ENSG00000200051	RF00279	0	0	0	0	0	0	0	0

ENSG00000213523	SRA1	6.38896	4.56274	0.98325	1.44151	2.99155	3.54122	1.58302	2.8886
ENSG00000243056	EIF4EBP3	1.16687	0.88284	0	0	0.51587	0.37427	0	0.13024
ENSG00000113108	APBB3	1.86255	1.16675	0.23544	0.53501	0.80351	0.4593	0.46736	0.59014
ENSG00000283155	AC116353.4	0.06616	0.11012	0	0.09818	0	0.11671	0	0
ENSG00000283717	MIR6831	0	0	0	0	0	0	0	0
ENSG00000176087	SLC35A4	4.90127	3.12376	0.96388	1.43317	3.27847	4.30718	1.51852	2.14758
ENSG00000283602	AC116353.5	2.10532	1.57694	0.10312	0.2083	0.4266	0.30951	0.05849	0.25849
ENSG00000200235	RF00443	0	0	0	0	0	0	0	0
ENSG00000251453	HAUS1P1	0	0	0	0	0	0	0	0
ENSG00000240518	AC116353.1	0	0	0	0	0	0	0	0
ENSG00000170458	CD14	185.466	123.108	16.3913	20.3613	60.3404	24.7516	15.8718	33.2121
ENSG00000131495	NDUFA2	4.87684	4.50972	4.12614	6.726	6.16869	5.5618	4.26974	5.92723
ENSG00000113119	TMCO6	2.67917	0.76647	0.2153	0.71447	1.22147	0.8862	0.52333	0.66817
ENSG00000113141	IK	41.5637	35.0979	5.02402	10.0683	32.6217	26.715	9.72669	13.8457
ENSG00000284325	MIR3655	0	0	0	0	0	0	0	0
ENSG00000120314	WDR55	3.65785	2.22556	0.35086	0.58114	1.91586	2.59466	0.55717	1.02016
ENSG00000256453	DND1	0.18268	0	0	0	0	0	0.07612	0.05607
ENSG00000170445	HARS	2.5285	1.56199	0.51073	0.76083	2.05995	2.23797	0.81106	0.97081
ENSG00000112855	HARS2	3.91451	4.08127	1.20104	2.29829	3.47785	2.61811	2.65298	2.71998
ENSG00000146007	ZMAT2	86.2857	58.1762	26.6907	43.7064	67.3785	91.4888	36.4788	41.2735
ENSG00000199990	VTRNA1-1	4.44245	1.23241	8.70416	7.69192	0	5.22472	1.23414	7.27249
ENSG00000202111	VTRNA1-2	0	0	1.21027	0	0	0	0	0
ENSG00000202515	VTRNA1-3	6.58879	0	4.84108	9.77849	4.00524	2.90588	2.74563	2.02241
ENSG00000250515	AC116353.3	0	0	0	0	0	0	0	0
ENSG00000248106	AC116353.2	0	0	0	0	0	0	0	0
ENSG00000204970	PCDHA1	0	0	0	0	0	0	0	0
ENSG00000279726	AC005609.5	0	0	0	0	0	0	0	0
ENSG00000204969	PCDHA2	0	0	0	0	0	0	0	0
ENSG00000255408	PCDHA3	0	0	0	0	0	0	0	0
ENSG00000204967	PCDHA4	0	0	0	0	0	0	0	0
ENSG00000204965	PCDHA5	0	0	0	0	0	0	0	0
ENSG00000081842	PCDHA6	0	0	0	0	0	0	0	0
ENSG00000204963	PCDHA7	0	0	0	0	0	0	0	0
ENSG00000204962	PCDHA8	0	0	0	0	0	0	0	0
ENSG00000204961	PCDHA9	0	0	0	0	0	0	0	0
ENSG00000278907	AC005609.2	0	0	0	0	0	0	0	0
ENSG00000250120	PCDHA10	0	0	0	0	0	0	0	0
ENSG00000249504	PCDHA14	0	0	0	0	0	0	0	0
ENSG00000278946	AC005609.4	0	0	0	0	0	0	0	0
ENSG00000249158	PCDHA11	0	0	0	0	0	0	0	0
ENSG00000251664	PCDHA12	0	0	0	0	0	0	0	0
ENSG00000278915	AC005609.3	0	0	0	0	0	0	0	0
ENSG00000278901	AC005609.1	0	0	0	0	0	0	0	0
ENSG00000239389	PCDHA13	0	0	0	0	0	0	0	0
ENSG00000248383	PCDHAC1	0	0	0	0	0	0	0	0
ENSG00000279028	AC010223.1	0	0	0	0	0	0	0	0
ENSG00000243232	PCDHAC2	0	0	0	0	0	0	0	0
ENSG00000279047	AC244517.5	0	0	0	0	0	0	0	0
ENSG00000171815	PCDHB1	0	0	0	0	0	0	0	0
ENSG00000279558	AC244517.9	0	0	0	0	0	0	0	0
ENSG00000112852	PCDHB2	0.01777	0	0	0	0	0	0	0
ENSG00000272154	AC244517.2	0	0	0	0	0	0	0	0
ENSG00000113205	PCDHB3	0	0	0	0	0	0	0	0
ENSG00000272108	AC244517.1	0	0	0	0	0	0	0	0
ENSG00000081818	PCDHB4	0	0	0	0	0	0	0	0
ENSG00000113209	PCDHB5	0	0	0	0	0	0	0	0
ENSG00000280029	AC244517.11	0	0	0	0.07119	0	0	0.07996	0
ENSG00000113211	PCDHB6	0	0	0	0	0	0	0	0

ENSG00000255622	PCDHB17P	0	0	0	0	0	0	0	0
ENSG00000280336	AC244517.12	0	0	0	0	0	0	0	0
ENSG00000113212	PCDHB7	0	0	0	0	0	0	0	0
ENSG00000120322	PCDHB8	0.02675	0	0	0	0	0	0	0
ENSG00000279472	AC244517.8	0	0	0	0	0	0	0	0
ENSG00000272674	PCDHB16	0	0	0	0	0	0	0	0
ENSG00000279068	AC244517.6	0	0	0	0	0	0	0	0
ENSG00000278936	AC244517.4	0	0	0	0	0	0	0	0
ENSG00000177839	PCDHB9	0	0.02763	0	0	0	0	0	0
ENSG00000279375	AC244517.7	0	0	0	0	0	0	0	0
ENSG00000120324	PCDHB10	0	0	0	0	0	0	0	0
ENSG00000197479	PCDHB11	0.01746	0	0	0	0	0.0308	0	0
ENSG00000120328	PCDHB12	0	0	0	0	0	0	0	0
ENSG00000187372	PCDHB13	0	0	0	0	0	0	0	0
ENSG00000120327	PCDHB14	0.01518	0	0	0	0	0	0	0
ENSG00000279983	AC244517.10	0	0	0	0	0	0	0	0
ENSG00000146001	PCDHB18P	0	0	0	0	0	0	0	0
ENSG00000262096	PCDHB19P	0	0	0	0	0	0	0	0
ENSG00000113248	PCDHB15	0.01825	0	0	0	0	0	0	0
ENSG00000277795	AC244517.3	0	0	0	0	0	0	0	0
ENSG00000178913	TAF7	47.0544	40.9981	5.73578	12.8242	36.0983	21.4649	14.0891	23.1024
ENSG00000120329	SLC25A2	0.20692	0.17221	0	0.23032	0.06289	0.18251	0	0
ENSG00000272070	AC005618.1	0	0	0	0	0.02831	0	0	0
ENSG00000204956	PCDHGA1	0	0	0	0	0	0	0	0
ENSG00000081853	PCDHGA2	0	0	0.01602	0	0	0	0	0
ENSG00000254245	PCDHGA3	0	0	0.02209	0	0	0	0	0
ENSG00000254221	PCDHGB1	0	0	0	0	0	0	0	0
ENSG00000280026	AC005618.3	0	0	0	0	0	0	0	0
ENSG00000262576	PCDHGA4	0	0	0	0	0.01824	0	0	0
ENSG00000253910	PCDHGB2	0.01537	0.02558	0	0	0.03737	0	0	0
ENSG00000253485	PCDHGA5	0.02557	0	0	0	0	0	0	0.0157
ENSG00000262209	PCDHGB3	0.03082	0.0513	0	0.04574	0	0	0	0.03784
ENSG00000253731	PCDHGA6	0	0	0	0	0.05633	0	0.01545	0.03413
ENSG00000253537	PCDHGA7	0.05387	0.04483	0	0	0	0	0	0.03307
ENSG00000253953	PCDHGB4	0.01061	0	0	0	0	0	0.01769	0
ENSG00000253767	PCDHGA8	0.00846	0.01409	0	0	0.01029	0.01493	0	0
ENSG00000276547	PCDHGB5	0.03022	0	0	0	0	0	0.02519	0.01855
ENSG00000261934	PCDHGA9	0	0	0	0	0.01877	0	0	0
ENSG00000253305	PCDHGB6	0.06485	0.01799	0.01588	0.01604	0	0.01907	0	0.02654
ENSG00000253846	PCDHGA10	0.02207	0.03673	0	0	0.01342	0	0.01839	0.04064
ENSG00000254122	PCDHGB7	0	0.04914	0	0	0	0	0	0
ENSG00000253873	PCDHGA11	0	0	0	0	0	0.02224	0	0
ENSG00000248449	PCDHGB8P	0	0.04247	0	0	0	0	0	0
ENSG00000279855	AC005618.2	0	0	0	0	0	0	0	0
ENSG00000253159	PCDHGA12	0.01522	0	0	0	0	0	0	0
ENSG00000276545	PCDHGB9P	0	0	0	0	0	0	0	0
ENSG00000273557	AC008781.3	0	0	0	0	0	0	0	0
ENSG00000240184	PCDHGC3	0.02699	0	0.01983	0.02003	0.06562	0	0.02249	0
ENSG00000242020	RN7SL68P	0	0	0	0	0	0	0	0
ENSG00000242419	PCDHGC4	0	0	0	0	0	0	0	0
ENSG00000240764	PCDHGC5	0	0	0	0	0	0	0	0.01804
ENSG00000131504	DIAPH1	28.5563	22.2758	5.5819	10.1535	19.882	21.9658	8.93666	10.2807
ENSG00000246422	AC008781.2	0	0	0	0	0	0	0	0
ENSG00000240974	RPS27AP10	0	0	0	0	0	0	0	0
ENSG00000228737	AC008781.1	0	0.19647	0	0	0	0	0	0.14492
ENSG00000171720	HDAC3	4.08367	2.9771	0.7443	1.47992	3.0982	3.267	1.55661	2.00166
ENSG00000164620	RELL2	1.44003	1.08097	0.20746	0.50286	0.78955	1.19549	0.28239	0.76269
ENSG00000197948	FCHSD1	3.48819	2.9131	0.54983	1.00313	2.65604	2.85325	1.26747	1.83758

ENSG00000120318	ARAP3	7.00299	5.56198	0.48755	1.26618	3.86086	1.27514	1.44183	2.86606
ENSG00000278925	AC005753.1	0	0	0	0	0	0	0	0
ENSG00000156453	PCDH1	0.19868	0.47538	0.29196	0.16586	0.27174	0.28478	0.08279	0.44213
ENSG00000226040	AC005740.1	0	0	0	0	0	0	0	0
ENSG00000081791	DELE1	4.98205	3.73269	0.67689	1.83498	3.40432	3.40013	1.05067	1.59247
ENSG00000113555	PCDH12	0.31169	0.26835	0.03159	0.0638	0.1176	0.05688	0.01792	0
ENSG00000271871	AC005740.4	0	0	0	0	0	0	0.12767	0.28212
ENSG00000013561	RNF14	18.5198	14.459	14.2553	25.9585	11.0971	8.9717	54.2915	11.2609
ENSG00000254099	AC005740.3	0	0	0	0	0	0	0	0
ENSG00000113552	GNPDA1	2.20216	1.75574	0.76142	0.96125	2.15986	1.3385	0.83284	1.40869
ENSG00000250347	AC005740.2	0	0	0	0	0	0	0	0
ENSG00000131507	NDFIP1	9.41807	7.12931	4.61325	10.3657	9.55667	7.19114	10.8104	6.81253
ENSG00000280047	AC091825.1	0.27967	0.1862	0	0.16602	0.136	0.04934	0.18646	0.06867
ENSG00000187678	SPRY4	0	0	0	0	0	0	0	0
ENSG00000281881		0	0	0	0	0	0	0	0
ENSG00000277968	RF02038	0	0	0	0	0	0	0	0
ENSG00000274721	RF02039	0	0	0	0	0	0	0	0
ENSG00000231185	SPRY4-AS1	0	0	0	0	0	0	0	0
ENSG00000225147	RPS12P10	0	0	0	0	0	0	0	0
ENSG00000113578	FGF1	0	0	0	0	0	0	0	0
ENSG00000261757	AC005592.1	0	0	0	0	0	0	0	0
ENSG00000236714	LINC01844	0	0	0	0	0	0	0	0
ENSG00000145819	ARHGAP26	17.1841	21.1255	4.43058	7.75935	11.0102	6.966	5.95935	9.94573
ENSG00000226272	ARHGAP26-AS1	0	0	0	0	0	0	0	0
ENSG00000230789	ARHGAP26-IT1	4.34512	3.77346	0	1.1215	1.60777	1.33311	1.57449	2.31951
ENSG00000113580	NR3C1	24.0312	23.5982	4.95587	8.62268	21.2881	14.9304	11.3895	14.4435
ENSG00000231579	RPL7P21	0	0	0	0	0	1.22184	0	0
ENSG00000279679	AC091925.2	2.00431	0.95319	0.42076	0.84989	1.74056	1.01025	2.38633	1.05465
ENSG00000279130	AC091925.1	0.31732	0	0	0.23547	0	0.2799	0.26446	0
ENSG00000249429	AC016598.1	0	0	0	0	0	0	0	0
ENSG00000253023	RNU7-156P	0	0	0	0	0	0	0	0
ENSG00000251205	AC016598.2	0	0	0	0	0	0	0	0
ENSG00000249881	AC008696.2	0.08885	0.14789	0	0.13186	0.43208	0	0.1481	0
ENSG00000266478	MIR5197	0	0	0	0	0	0	0	0
ENSG00000225869	AC008696.1	0	0	0	0	0	0	0	0
ENSG00000158497	HMHB1	0	0	0	0	0	0	0	0
ENSG00000239390	RN7SL87P	0	0	0	0	0	0	0	0
ENSG00000145817	YIPF5	7.07058	7.1537	1.39699	3.49784	6.54951	6.14939	2.11282	5.22815
ENSG00000183775	KCTD16	0.00513	0	0	0.00761	0	0	0	0
ENSG00000249605	AC008652.1	0	0	0	0	0	0	0	0
ENSG00000250972	CKS1BP5	0	0	0	0	0	0	0	0
ENSG00000213488	AC008716.1	0	0	0	0	0	0	0	0
ENSG00000201423	RN7SKP246	0	0	0	0	0	0	0	0
ENSG00000249229	NAMPTP2	0	0	0	0	0	0	0	0
ENSG00000251031	AC132803.1	0	0	0	0	0	0	0	0
ENSG00000215325	ASS1P10	0	0	0	0	0	0	0	0
ENSG00000250842	AC137770.1	0	0	0	0	0	0	0	0
ENSG00000248125	AC008700.1	0	0	0	0	0	0	0	0
ENSG00000186314	PRELID2	1.77285	3.94967	4.44884	8.80405	6.4164	8.90146	14.798	6.53002
ENSG00000249363	AC011411.1	0	0	0	0	0	0	0	0
ENSG00000204928	GRXCR2	0.23419	0.44549	0.34413	0.64547	0.32539	0.17706	0.39035	0.1643
ENSG00000156463	SH3RF2	0.01014	0.10122	0.02979	0	0.01232	0.03576	0	0.03733
ENSG00000248493	AC005351.1	0	0	0	0	0	0	0	0
ENSG00000173261	PLAC8L1	0.11174	0.18599	0	0	0.13585	0	0.3725	0.13719
ENSG00000251556	AC091887.1	0	0	0	0	0	0	0	0
ENSG00000133706	LARS	9.26412	5.41058	2.06989	5.15921	8.85897	7.26363	4.18405	5.25482
ENSG00000239602	AC091959.2	0	0	0	0	0	0	0	0
ENSG00000239317	AC091959.1	0	0	0	0	0	0	0	0

ENSG00000275740	AC091959.3	0	0	0	0	0.05383	0	0	0
ENSG00000091009	RBM27	5.30419	3.65395	1.49011	2.96027	4.403	4.04959	2.67467	3.94028
ENSG00000238374	RNU7-180P	0	0	0	0	0	0	0	0
ENSG00000250025	AC011396.2	0	0	0	0	0	0	0	0
ENSG00000091010	POU4F3	0	0	0	0	0	0	0	0
ENSG00000271724	AC011396.3	0	0	0	0	0	0	0	0
ENSG00000248842	AC011396.1	0	0	0	0	0	0	0	0
ENSG00000113649	TCERG1	5.14802	3.38835	1.1743	2.19719	4.56117	5.14932	2.90238	2.37541
ENSG00000277568	RF02271	0	0	0	0	0.57126	0	0	0.5769
ENSG00000173250	GPR151	0	0	0	0	0	0	0	0
ENSG00000250407	AC008728.1	0.31824	0	0	0	0	0	0	0
ENSG00000156475	PPP2R2B	0.29418	0.67444	0.15497	0.36245	0.26993	0.95961	0.18504	0.21808
ENSG00000280646	RNA5SP196	0	0	0	0	0	0	0	0
ENSG00000241291	RN7SL791P	0	0	0	0	0	0	0.41417	0
ENSG00000248568	KRT8P48	0	0.20001	0	0.08917	0	0	0	0
ENSG00000249553	PPP2R2B-IT1	0	0	0	0	0	0	0	0
ENSG00000250343	STK32A-AS1	0	0	0	0	0	0	0	0
ENSG00000169302	STK32A	0	0	0	0	0	0	0	0
ENSG00000113657	DPYSL3	0.04093	0.01703	0	0	0.14927	0.0361	0	0
ENSG00000272239	AC011373.1	0	0	0	0	0	0	0	0
ENSG00000280780	JAKMIP2-AS1	0.03481	0	0	0	0	0	0	0
ENSG00000176049	JAKMIP2	0.82982	0.82874	0.13936	0.32841	0.75905	0.6971	0.09221	0.56278
ENSG00000164266	SPINK1	0	0	0	0	0	0	0	0
ENSG00000249518	AC011352.2	0	0	0	0	0	0	0	0
ENSG00000164265	SCGB3A2	0.13637	0	0.1002	0	0.0829	0	0	0
ENSG00000178776	C5orf46	0	0	0	0	0	0	0	0
ENSG00000248362	AC011352.1	0	0	0	0	0	0	0	0
ENSG00000251320	AC011352.3	0	0	0	0	0	0	0	0
ENSG00000250346	EEF1GP2	0	0	0	0	0	0.10403	0	0
ENSG00000133710	SPINK5	0	0	0	0	0	0	0	0
ENSG00000196800	SPINK14	0	0	0	0	0	0	0	0
ENSG00000178172	SPINK6	0	0	0	0	0	0	0	0
ENSG00000249503	HMGN1P16	0	0	0	0	0	0	0	0
ENSG00000248109	MARCOL	0	0	0	0	0	0	0	0
ENSG00000214510	SPINK13	0	0	0	0	0	0	0	0
ENSG00000247199	AC091948.1	0.39661	0.12003	0.10597	0.32106	0.17534	0.06361	0	0.08854
ENSG00000249834	PGBD4P3	0	0	0	0	0	0	0	0
ENSG00000145879	SPINK7	0	0	0	0	0	0	0	0
ENSG00000204909	SPINK9	0	0	0	0	0	0	0	0
ENSG00000283653	AC091948.2	0	0	0	0	0	0	0	0
ENSG00000145868	FBXO38	7.65611	8.01546	1.66721	3.15556	6.70265	5.5449	3.69819	4.84965
ENSG00000274799	RF00017	1.03972	0	0.76393	0	0	0	0	0.31914
ENSG00000251330	AC114939.1	0	0	0	0	0	0	0	0
ENSG00000164270	HTR4	0.02081	0	0	0.01544	0	0.01836	0	0
ENSG00000253297	AC008627.1	0	0	0	0	0	0	0	0
ENSG00000169252	ADRB2	4.5347	8.29218	1.22011	2.11695	4.76255	4.13137	1.70336	3.11056
ENSG00000169247	SH3TC2	0.341	0.42164	0.07874	0.1952	0.27836	0.32657	0.2436	0.22728
ENSG00000272411	AC116312.1	0.23344	0	0	0	0	0	0	0
ENSG00000251842	RNU6-732P	0	0	0	0	0	0	0	0
ENSG00000207714	MIR584	0	0	0	0	0	0	0	0
ENSG00000250072	SH3TC2-DT	0.05099	0	0	0	0	0	0	0.04173
ENSG00000200161	RN7SKP145	0	0	0	0	0	0	0	0
ENSG00000173210	ABLIM3	3.74364	1.72247	0.32799	0.79801	1.7515	2.52359	1.03156	1.20827
ENSG00000248647	AC012613.1	0	0	0	0	0	0	0	0
ENSG00000253406	AC012613.2	0	0	0	0	0	0	0	0
ENSG00000157510	AFAP1L1	0.01553	0.05169	0	0.04609	0.05663	0	0	0
ENSG00000164284	GRPEL2	3.41703	1.96031	0.82876	1.96942	3.06534	4.06751	2.90313	2.01623
ENSG00000253618	GRPEL2-AS1	0	0	0	0.54257	0.88894	0.32247	0	0

ENSG00000145882	PCYOX1L	1.66674	0.96497	0.15973	0.50189	1.20408	1.17188	0.54356	0.69696
ENSG00000127743	IL17B	0.12106	0	0	0	0	0	0	0
ENSG00000253865	AC131025.1	0.37398	0	0	0.18501	0	0	0	0
ENSG00000249669	CARMN	0	0	0	0	0	0	0	0
ENSG00000275871	AC131025.2	0	0	0	0	0	0	0	0
ENSG00000284182	MIR143	0	0	0	0	0	0	0	0
ENSG00000276365	MIR145	0	0	0	0	0	0	0	0
ENSG00000113712	CSNK1A1	17.7787	15.7216	4.17507	8.05246	12.8003	11.7343	7.52342	8.89004
ENSG00000230551	AC021078.1	1.92672	1.8225	0.56127	0.781	1.0732	1.28773	1.31574	1.05254
ENSG00000241112	RPL29P14	1.02757	2.28053	0.25167	0.25417	1.87395	1.51065	0.57094	0.84109
ENSG00000183111	ARHGEF37	0.24782	0.64167	0.3844	0.53126	0.70302	0.70437	0.80321	0.4226
ENSG00000222213	RNU6-588P	0	0	0	0	0	0	0	0
ENSG00000221043	RF00012	0	0	0	0	0	0	0	0
ENSG00000242928	RN7SL868P	0	0	0	0	0	0	0	0
ENSG00000155846	PPARGC1B	0.77344	0.46384	0.08357	0.16036	0.31805	0.62203	0.12323	0.18853
ENSG00000199047	MIR378A	0	0	0	0	0	1.95927	0	0
ENSG00000132915	PDE6A	0	0	0	0	0	0.01744	0.03295	0
ENSG00000251583	AC008427.1	0	0	0	0	0	0	0	0
ENSG00000200334	RF00019	0	0	0	0	0	0	0	0
ENSG00000155850	SLC26A2	2.23155	2.06986	0.65084	1.50424	1.99855	2.29894	1.16416	1.61045
ENSG00000164296	TIGD6	0.47392	0.18406	0.02321	0.09378	0.55698	0.39016	0.15799	0.15517
ENSG00000113716	HMGXB3	2.58652	1.88964	0.53315	1.14639	2.23396	3.6958	1.13148	1.09209
ENSG00000248696	AC011406.1	0	0	0	0	0	0	0	0
ENSG00000241907	RPS20P4	0	0	0	0	0	0	0	0
ENSG00000182578	CSF1R	28.2472	17.9543	3.534	4.64625	10.9687	8.03335	2.84637	4.38541
ENSG00000214485	RPL7P1	0.19625	0.16333	0	1.16504	0	0	0.16356	0.36143
ENSG00000199409	RF00019	0	0	0	0	0	0	0	0
ENSG00000113721	PDGFRB	0.66758	0.92314	0.25657	0.65543	0.09989	0.10871	0.03424	0.35308
ENSG00000113722	CDX1	0	0	0	0	0	0.07356	0	0
ENSG00000011083	SLC6A7	0	0	0	0	0	0	0	0
ENSG00000070808	CAMK2A	0	0	0	0	0	0	0	0
ENSG00000183876	ARSI	0	0	0	0	0	0	0	0
ENSG00000070814	TCOF1	1.96282	1.66601	0.46799	0.76202	1.7305	2.71741	0.57418	0.86183
ENSG00000019582	CD74	482.576	285.823	46.2791	106.599	354.675	399.063	87.6061	101.558
ENSG00000164587	RPS14	39.1828	33.8584	24.961	26.6697	43.4012	48.9757	50.9264	51.4156
ENSG00000254333	NDST1-AS1	0.39837	0	0	0	0	0.35139	0	0
ENSG00000070614	NDST1	6.96157	3.99482	0.56519	1.3357	3.62864	3.12118	1.94894	1.6528
ENSG00000171992	SYNPO	0.10004	0	0.0105	0.07423	0.21719	0.07564	0.01191	0.05264
ENSG00000253852	AC011383.1	0	0	0	0	0	0	0	0
ENSG00000164591	MYOZ3	0	0	0	0	0	0	0	0.01491
ENSG00000250309	AC008453.1	0	0	0	0	0	0	0	0
ENSG00000086589	RBM22	17.1338	15.6265	2.35391	4.57412	13.2134	12.1979	5.30631	7.54332
ENSG00000132912	DCTN4	8.49859	8.34685	2.75286	6.64547	6.63827	6.12604	10.5863	5.41357
ENSG00000256235	SMIM3	9.0211	5.59816	1.38569	2.3791	4.01254	5.98871	3.03879	4.12937
ENSG00000237693	IRGM	0.06455	0	0.04743	0.14371	0.07848	0.11388	0	0.07926
ENSG00000145908	ZNF300	0.19941	0.11064	0.02442	0.14797	0.14142	0.02932	0.58168	0.20403
ENSG00000197083	ZNF300P1	0.04688	0	0	0.03479	0	0.04135	0	0
ENSG00000274235	AC022106.1	0	0	0	0	0	0	0	0
ENSG00000277866	AC022106.2	0	0	0	0	0	0	0	0
ENSG00000211445	GPX3	0.7184	0.23144	0.06811	0.03439	0.36627	0.20441	0.15451	0.4268
ENSG00000145901	TNIP1	33.1262	34.1615	8.44159	8.13123	31.2439	31.8536	11.0517	18.2542
ENSG00000197043	ANXA6	39.9795	29.2937	4.70393	11.3055	33.9093	39.3154	8.19488	13.7972
ENSG00000254298	AC008641.1	0	0	0	0	0	0	0	0
ENSG00000198624	CCDC69	25.1185	20.5262	3.27909	5.77373	16.4026	15.6169	7.85497	9.26467
ENSG00000196743	GM2A	10.0483	7.65447	1.58721	2.98742	13.7684	7.44864	2.83696	4.44058
ENSG00000186334	SLC36A3	0	0	0	0	0	0	0	0
ENSG00000186335	SLC36A2	0	0	0	0	0	0	0	0
ENSG00000270904	AC008385.1	0	0	0	0	0	0	0	0

ENSG00000253897	AC034205.2	0	0	0	0	0	0	0	0
ENSG00000253472	AC034205.1	0	0	0	0	0	0	0	0
ENSG00000271494	AC034205.3	0	0	0	0	0	0	0	0
ENSG00000123643	SLC36A1	2.60437	2.44716	1.52775	1.49619	2.80883	2.89006	2.11803	2.0114
ENSG00000200227	RNA5SP197	0	0	0	0	0	0	0	0
ENSG00000086570	FAT2	0.00504	0	0	0.01497	0	0	0	0.01857
ENSG00000271795	AC011337.1	0.0442	0.03678	0.03247	0	0.05373	0	0	0.02713
ENSG00000276622	MIR6499	0	0	0	0	0	0	0	0
ENSG00000260581	AC011374.1	0	0	0	0	0	0	0	0
ENSG00000113140	SPARC	29.3292	24.1188	6.26529	7.61117	25.6411	43.4965	17.906	23.0069
ENSG00000249035	CLMAT3	0	0	0	0	0	0	0	0
ENSG00000222102	RN7SKP232	0	0	0	0	0	0	0	0
ENSG00000272112	AC011374.2	1.5329	0.46391	0.20478	1.2409	1.18596	1.2292	0.23228	0.68439
ENSG00000177556	ATOX1	1.42711	1.11785	0.3084	0.68523	0.95257	1.11071	0.74628	0.91045
ENSG00000253921	AC091982.1	0	0	0	0	0	0	0	0
ENSG00000213433	RPLP1P6	0.14096	0	0.20714	0.2092	0.68551	0	0	0.34614
ENSG00000275765	AC091982.3	2.32219	0.84028	0.29673	0.74921	1.7185	0.71246	1.17805	0.86774
ENSG00000145907	G3BP1	11.4742	8.05457	2.93465	7.26933	10.2435	13.8005	5.59394	7.52273
ENSG00000145888	GLRA1	0	0	0	0	0	0	0	0
ENSG00000270978	AC091982.2	0	0	0	0	0	0	0.10958	0.08071
ENSG00000222308	RNA5SP198	0	0	0	0	0	0	0	0
ENSG00000254226	LINC01933	0	0	0	0.02905	0	0.03453	0.09787	0.02403
ENSG00000132911	NMUR2	0	0	0	0	0	0	0	0
ENSG00000242080	AC008571.1	0	0	0	0	0	0	0	0
ENSG00000249484	LINC01470	0	0	0	0	0	0	0	0
ENSG00000155511	GRIA1	0	0	0	0	0	0	0	0
ENSG00000242976	RN7SL177P	0	0	0	0	0	0	0	0
ENSG00000251183	LINC01861	0	0	0	0.20258	0	0	0.22752	0.16759
ENSG00000261382	AC091962.1	0	0	0	0	0	0	0	0
ENSG00000055147	FAM114A2	4.87325	8.24205	7.21056	23.044	12.9936	10.7315	21.6425	9.44949
ENSG00000037749	MFAP3	5.12429	4.27743	2.00053	2.83769	5.05838	4.58744	6.70564	3.26784
ENSG00000164574	GALNT10	6.37607	5.13793	2.10379	2.57039	5.1113	6.45573	3.63188	2.71809
ENSG00000245275	SAP30L-AS1	0.22302	0.37122	0	0.22066	0	0	0.24783	0.04564
ENSG00000253119	HNRNPA3P7	0.07464	0.24849	0	0	0	0	0	0
ENSG00000221430	MIR1294	0	0	0	0	0	0	0	0
ENSG00000241963	RN7SL655P	0	0	0	0.36505	0	0.86786	0.82	0
ENSG00000164576	SAP30L	4.52498	3.05247	1.06837	1.52992	3.99739	3.84777	1.12142	2.27824
ENSG00000113196	HAND1	0	0	0	0	0	0	0	0
ENSG00000254293	AC026688.2	0	0	0	0	0	0	0	0
ENSG00000253886	AC026688.1	0	0.20892	0	0.18628	0	0	0	0.07705
ENSG00000253146	CIR1P1	0	0	0.09507	0	0	0	0.10784	0
ENSG00000264760	MIR3141	0	0	0	0	0	0	0	0
ENSG00000221552	MIR1303	0	0	0	0	0	0	0	0
ENSG00000274472	RF00019	0	0	0	0	0	0	1.19785	0
ENSG00000155506	LARP1	9.9955	8.51991	2.39731	3.36913	11.1973	15.2427	5.51049	3.81523
ENSG00000272197	RN7SL803P	0	0	0	0	0	0	0	0
ENSG00000241187	AC008379.1	0	0	0	0	0	0	0	0
ENSG00000170271	FAXDC2	20.9792	10.8291	5.80182	24.6884	22.6679	21.5937	15.4459	13.7326
ENSG00000277039	RF00017	0	0	0	0	0.28201	0	0	0
ENSG00000263361	MIR378H	0	0	0	0	0	0	0	0
ENSG00000155508	CNOT8	21.1694	16.7645	5.97497	9.8267	17.2564	16.1557	11.1921	13.511
ENSG00000082516	GEMIN5	2.49666	1.83976	0.51593	1.2737	3.27251	2.43162	0.78029	1.2932
ENSG00000082515	MRPL22	2.07362	2.15432	1.73831	3.26337	2.41953	2.74975	4.84824	2.17004
ENSG00000253261	AC008410.1	0	0	0	0	0	0	0	0
ENSG00000226650	KIF4B	0	0	0	0	0.02036	0.02954	0	0.02056
ENSG00000253646	AC010476.1	0	0	0	0	0	0	0	0
ENSG00000271477	AC010591.1	0	0	0	0	0	0	0	0
ENSG00000270442	AC008725.1	0	0	0	0	0	0	0	0

ENSG00000200275	RNA5SP199	0	0	0	0	0	0	0	0
ENSG00000170624	SGCD	0.09696	0.03458	0.10177	0.02056	0.01684	0	0.06926	0.24659
ENSG00000253370	AC027308.1	0	0	0	0	0	0	0	0
ENSG00000254163	AC025434.1	0	0	0	0	0	0.28111	0	0
ENSG00000199468	RNU6-556P	0	0	0	0	0	0	0	0
ENSG00000231989	PPP1R2B	0.07363	0	0	0	0	0	0	0
ENSG00000145850	TIMD4	0.28283	0.15692	0.13854	0.13992	0.05731	2.07897	0.23572	0.05788
ENSG00000270560	APOOP1	0	0	0	0	0	0	0	0
ENSG00000113249	HAVCR1	0.64747	0.22689	0.05008	0.15172	0.45573	0.48094	0.39761	0
ENSG00000135077	HAVCR2	6.57082	5.35631	1.07601	4.03228	3.44378	7.64857	2.60163	2.93366
ENSG00000254246	AC011377.1	0	0	0	0	0	0	0	0
ENSG00000155868	MED7	3.12023	3.22508	1.36815	2.166	3.02868	3.46252	1.63578	3.15129
ENSG00000113263	ITK	10.6236	6.31029	2.15328	6.6379	11.4348	18.1704	8.57705	7.15458
ENSG00000170613	FAM71B	0	0	0	0	0	0	0	0
ENSG00000253980	AC010609.1	0	0.64328	0	0	0	0	0	0
ENSG00000253653	AC009185.1	0	0.26786	0	0.23883	0	0	0	0
ENSG00000055163	CYFIP2	15.2005	11.808	2.56511	6.18642	15.5262	17.0724	5.56317	7.96411
ENSG00000172568	FNDC9	0	0	0	0	0	0	0	0
ENSG00000251405	AC016571.1	0.02763	0	0	0	0	0	0	0
ENSG00000248544	AC008676.1	0.1411	0	0	0	0	0	0	0
ENSG00000135074	ADAM19	15.0045	10.7533	1.24436	3.44545	12.759	4.1459	2.4582	5.50216
ENSG00000253155	AC008676.2	0	0	0	0	0	0	0	0
ENSG00000172548	NIPAL4	0.06968	0	0	0	0.10589	0	0	0.04277
ENSG00000253519	AC106801.1	0.03973	0.39678	0	0.11792	0.14491	0	0	0
ENSG00000252068	RNU6-390P	0	0	0	0	0	0	0	0
ENSG00000254336	AC008694.2	0	0	0	0	0	0	0	0
ENSG00000039600	SOX30	0.02112	0	0	0.06268	0	0	0	0.05186
ENSG00000187658	C5orf52	0	0	0	0	0	0	0	0
ENSG00000253798	AC008694.1	0	0	0	0	0	0	0	0
ENSG00000113272	THG1L	2.73169	1.81876	0.73593	1.01353	1.88197	1.88747	1.13833	1.42542
ENSG00000155858	LSM11	0.65685	0.51887	0.14724	0.29741	0.4196	0.45173	0.40826	0.41007
ENSG00000253630	AC026407.1	0	0	0	0	0	0	0	0
ENSG00000113282	CLINT1	16.9093	9.48401	3.06159	5.96203	12.2871	11.2295	4.04837	6.27492
ENSG00000206819	RNU6-260P	0	0	0	0	0	0	0	0
ENSG00000244331	AC008677.2	0	0	0	0	0	0	0	0
ENSG00000222626	RNU2-48P	0	0	0	0	0	0	0	0
ENSG00000253743	MARK2P11	0	0	0	0	0	0	0	0
ENSG00000213414	AC008677.1	0	0.07816	0	0	0	0	0	0
ENSG00000253422	AC008677.3	0	0	0	0	0	0	0	0
ENSG00000253792	AC025437.5	0	0	0	0	0	0	0	0
ENSG00000253673	AC025437.4	0	0	0	0	0	0	0	0
ENSG00000254047	AC025437.6	0	0	0	0	0	0	0	0
ENSG00000253449	AC025437.3	0.09507	0	0	0.28219	0	0	0	0
ENSG00000253424	AC025437.2	0	0	0	0	0	0	0	0
ENSG00000253134	AC025437.1	0	0	0	0	0	0	0	0
ENSG00000276778	LINC02227	0.03911	0	0	0	0	0	0	0
ENSG00000254350	AC091979.1	0	0	0	0	0	0	0	0
ENSG00000272085	AC091979.2	0	0	0	0	0	0	0	0
ENSG00000254135	AC091939.1	0	0	0	0	0	0	0	0
ENSG00000164330	EBF1	0.4034	0.11849	0	0.2113	1.05301	0.18838	0.17799	0.24764
ENSG00000253811	AC136424.2	0	0	0	0	0	0	0	0
ENSG00000253456	AC136424.1	0	0	0	0	0	0	0	0
ENSG00000245812	LINC02202	0.04561	0.03796	0	0.06769	0	0.1207	0	0
ENSG00000279204	AC134043.2	0.03275	0	0	0.09722	0	0.05778	0	0
ENSG00000145860	RNF145	9.30949	11.397	2.96482	5.28132	9.03914	5.63322	4.47518	9.83063
ENSG00000253256	AC134043.1	0	0	0	0	0	0	0	0
ENSG00000253315	LINC01932	0	0	0	0	0	0	0	0
ENSG00000252458	RF00263	0	0	0	0	0	0	0	0

ENSG00000164332	UBLCP1	12.8193	12.1735	5.3535	11.3827	11.5559	10.8243	7.67051	11.8382
ENSG00000249738	AC008691.1	0.01731	0.01441	0	0.05139	0.0421	0	0.02886	0.02126
ENSG00000113302	IL12B	0	0	0	0	0	0	0	0
ENSG00000221601	RNU4ATAC2P	0	0	0	0	0	0	0	0
ENSG00000253298	LINC01845	0	0	0	0	0	0	0	0
ENSG00000283413	AC008703.1	0	0	0	0	0	0	0	0
ENSG00000254199		0	0	0	0	0	0	0	0
ENSG00000253311	LINC01847	0	0	0	0	0	0	0	0
ENSG00000170214	ADRA1B	0	0	0	0	0	0	0	0
ENSG00000248626	GAPDHP40	0.07286	0	0	0.10814	0	0	0	0
ENSG00000199398	RF00019	0	0	0	0	0	0	0	0
ENSG00000113312	TTC1	22.391	24.1121	9.39301	17.9626	25.705	25.5554	20.4895	15.7425
ENSG00000223908	AC068657.1	0	0	0	0	0	0	0	0
ENSG00000170234	PWWP2A	4.4926	4.31059	1.12512	2.25593	5.09238	3.63503	2.75891	3.29022
ENSG00000170231	FABP6	0	0	0	0	0	0	0	0
ENSG00000247699	AC008609.1	0.02536	0	0	0	0	0	0	0
ENSG00000253772	AC112191.1	0	0	0	0	0	0	0	0
ENSG00000135083	CCNJL	13.3724	9.14548	0.77328	1.99839	6.56709	0.51878	1.83167	4.61767
ENSG00000254373	AC112191.2	0.88048	1.09918	0.32347	0.98005	0.53524	0.77665	0	0.27026
ENSG00000270612	AC112191.3	0	0	0	0.78546	0	0	0	0
ENSG00000145861	C1QTNF2	0	0	0	0	0.03692	0	0	0
ENSG00000221886	ZBED8	0.10024	0.29199	0.03683	0.11158	0.12187	0.08842	0.04177	0.0923
ENSG00000164609	SLU7	21.2026	23.1677	4.13306	6.63589	16.1329	13.4095	10.386	10.9796
ENSG00000164611	PTTG1	1.42013	1.69709	1.28422	2.91825	2.34634	10.5993	2.67061	1.38595
ENSG00000253522	MIR3142HG	0.38227	0.15907	0.04681	0.14183	0.3873	0	0.47789	0.39112
ENSG00000265237	MIR3142	0	0	0	0	0	0	0	0
ENSG00000283733	MIR146A	0	0	0	0	0	0	0	0
ENSG00000118322	ATP10B	0	0	0	0	0	0	0	0
ENSG00000253687	AC008456.1	0	0	0	0	0	0	0	0
ENSG00000254391	AC011363.1	0	0	0	0	0	0	0	0
ENSG00000253417	LINC02159	0	0	0	0	0	0	0	0
ENSG00000145864	GABRB2	0	0	0	0.02175	0.01782	0	0	0
ENSG00000145863	GABRA6	0	0	0	0	0	0	0	0
ENSG00000253403	AC091944.1	0	0	0	0	0	0	0	0
ENSG00000118990	GLRXP3	0	0	0	0	0	0	0	0
ENSG00000022355	GABRA1	0	0	0	0	0	0	0	0
ENSG00000280776	LINC01202	0	0	0	0	0	0	0	0
ENSG00000113327	GABRG2	0	0	0	0	0	0	0	0
ENSG00000254173		0	0	0	0	0	0	0	0
ENSG00000201474	RNU6-164P	0	0	0	0	0	0	0	0
ENSG00000254186	AC113414.1	0	0	0	0	0	0	0	0
ENSG00000254351	ARL2BPP5	0	0	0	0	0	0	0	0
ENSG00000250061		0.05964	0	0	0.26555	0.07251	0	0.09941	0.14646
ENSG00000249449	MRPL57P6	0	0	0	0	0	0	0	0
ENSG00000250114	AC008723.1	0	0	0	0	0	0	0	0
ENSG00000113328	CCNG1	22.8708	19.783	8.38471	18.7283	21.6459	29.8219	17.8377	17.3541
ENSG00000170584	NUDCD2	1.12681	0.77229	0.42613	0.45496	1.13826	0.96469	0.64909	0.41708
ENSG00000072571	HMMR	6.05249	6.4166	6.92369	13.1007	8.35469	8.73906	11.889	8.11711
ENSG00000251018	HMMR-AS1	0	0	0	0	0	0	0	0.09404
ENSG00000038274	MAT2B	19.4026	18.4445	9.13468	20.5834	15.876	21.2299	17.4509	20.1721
ENSG00000224012	AC116917.1	0	0	0	0	0	0	0	0
ENSG00000251998	RNU6-168P	0	0	0	0	0	0	0	0
ENSG00000273569	AC008432.1	0	0	0	0	0	0	0	0
ENSG00000271679	LSM1P2	0	0	0	0	0	0	0	0
ENSG00000253331	AC008662.1	0	0	0	0	0	0	0	0
ENSG00000253538	AC008662.2	0	0	0	0	0	0	0	0
ENSG00000241956	AC109466.1	0	0	0	0	0	0	0	0
ENSG00000253236	LINC02143	0	0	0	0	0	0	0	0

ENSG00000253600	AC008446.1	0	0	0	0	0	0	0	0
ENSG00000206845	RNU6-209P	0	0	0	0	0	0	0	0
ENSG00000254066	LINC01938	0	0	0	0	0	0	0	0
ENSG00000253693	AC008415.1	0	0	0	0	0	0	0	0
ENSG00000252794	RN7SKP60	0	0	0	0	0	0	0	0
ENSG00000253512	AC008562.1	0	0	0	0	0	0	0	0
ENSG00000253946	AC008489.1	0	0	0	0	0	0	0	0
ENSG00000254252	RPL7P20	0	0	0	0	0	0	0	0
ENSG00000254171	AC114321.1	0	0	0	0	0	0	0	0
ENSG00000229119	AC026403.1	0	0	0	0.73753	0	0	0	0
ENSG00000254130	LINC01947	0	0	0	0	0	0	0	0
ENSG00000253713	AC091819.2	0	0	0	0	0	0	0	0
ENSG00000253469	AC091819.1	0	0	0	0	0	0	0	0
ENSG00000145934	TENM2	0	0.01105	0	0	0	0	0	0
ENSG00000254365	AC091820.2	0	0	0	0	0	0	0	0
ENSG00000254297	AC091820.1	0	0	0	0	0	0	0	0
ENSG00000253527	AC008601.1	0	0	0	0	0	0	0	0
ENSG00000254187	AC008708.2	0	0	0	0	0	0	0	0
ENSG00000253357	AC008708.1	0	0	0	0	0	0	0	0
ENSG00000253660	AC008464.1	0	0	0	0	0	0	0	0
ENSG00000253947	AC008705.1	0	0	0	0	0	0	0	0
ENSG00000253065	RF00561	0	0	0	0	0	0	0	0
ENSG00000253925	AC011369.1	0	0	0	0	0	0	0	0
ENSG00000279739	AC011369.2	0.04678	0	0	0	0	0	0	0
ENSG00000253978	CTB-178M22.2	0	0	0	0	0	0	0	0
ENSG00000113645	WWC1	0.03637	0.02422	0	0.06478	0.07076	0.02567	0.03638	0.03573
ENSG00000113643	RARS	20.141	23.2533	32.8085	61.182	67.0264	57.0771	136.931	66.5836
ENSG00000188573	FBLL1	0	0	0	0	0	0	0	0
ENSG00000120137	PANK3	10.4893	8.17618	5.3857	12.0713	11.6492	10.8534	11.8138	8.93166
ENSG00000253861	SLC2A3P1	0	0	0	0	0	0	0	0
ENSG00000199035	MIR103A1	0	0	0	1.39469	0	0	1.56641	0
ENSG00000283612	MIR103B1	0	0	0	0	0	0	0	0
ENSG00000233913	RPL10P9	0.22729	0.18916	0	0	0.13817	0	0.18943	0
ENSG00000254192	AC011365.2	0	0	0	0	0	0	0	0
ENSG00000184347	SLIT3	0.00514	0	0	0.00763	0.0125	0.00907	0	0
ENSG00000254042	AC011365.1	0	0	0	0	0	0	0	0
ENSG00000207739	MIR218-2	0	0	0	0	0	0	0	0
ENSG00000202345	RF00019	0	0	0	0	0	0	0	0
ENSG00000248965	AC011389.2	0	0	0	0	0	0	0	0
ENSG00000279752	AC011389.3	0	0	0	0	0	0	0	0
ENSG00000248222	AC011389.1	0	0	0	0	0	0	0	0
ENSG00000207619	MIR585	0	0	0	0	0	0	0	0
ENSG00000206614	RNU6-477P	0	0	0	0	0	0	0	0
ENSG00000040275	SPDL1	1.56513	1.27704	0.45097	1.54855	1.71629	1.81367	1.56018	1.05502
ENSG00000134516	DOCK2	36.3147	30.4406	4.32026	9.30038	24.654	18.7798	9.58296	14.7039
ENSG00000253269	AC008680.1	0	0	0	0	0	0	0	0
ENSG00000204767	INSYN2B	0	0	0	0	0.08915	0	0	0.03001
ENSG00000263831	MIR378E	4.63926	1.54441	0	2.75407	2.25612	1.63686	1.54659	0
ENSG00000168269	FOXI1	0.0317	0	0	0	0	0	0	0
ENSG00000253219	KRT18P41	0	0	0	0	0	0	0	0
ENSG00000249601	LINC01187	0	0.05273	0	0	0	0	0	0
ENSG00000234511	C5orf58	0	0	0	0	0	0	0	0
ENSG00000043462	LCP2	33.9383	39.2435	5.84367	9.79132	25.7853	23.8488	9.56326	20.4121
ENSG00000250274	AC034199.1	0.36953	0.3355	0.09873	0.04986	0.12253	0.11853	0.16798	0.20623
ENSG00000235172	LINC01366	0.29767	0.49547	0	0.22088	0.27142	0.39384	0	0.36547
ENSG00000182132	KCNIP1	0	0	0	0	0	0	0	0
ENSG00000145936	KCNMB1	0.49438	0.30106	0	0.0179	0.10262	0.04254	0.0402	0.04441
ENSG00000253647	CTD-2270F17.1	0	0	0	0	0	0	0	0

ENSG00000253858	AC008619.1	0	0	0	0	0	0	0	0
ENSG00000253591	AC027312.1	0	0	0	0	0	0	0	0
ENSG00000253348	AC008514.1	0	0	0	0	0	0	0	0
ENSG00000094755	GABRP	0	0	0	0	0	0	0	0
ENSG00000204764	RANBP17	0.82225	3.00431	3.81149	10.705	4.06369	3.0898	8.88078	5.44989
ENSG00000244501	RN7SL623P	0	0	0	0	0	0	0	0
ENSG00000253966	AC008514.2	0	0	0	0	0	0	0	0
ENSG00000253213	AC010306.1	0.13125	0.10923	0	0.29217	0	0	0.32815	0
ENSG00000275038	AC091980.2	0	0	0	0	0	0	0	0
ENSG00000164438	TLX3	0	0	0	0	0	0	0	0
ENSG00000213393	AC091980.1	0	0	0	0	0	0	0	0
ENSG00000265740	RN7SL339P	0	0	0	0	0	0	0	0
ENSG00000254256	RPSAP71	0	0	0	0	0	0	0	0
ENSG00000206909	SNORA70J	0	0	0	0	0	0	0	0
ENSG00000254012	RPL10P8	0	0	0	0	0	0	0	0
ENSG00000264249	MIR3912	0	0	0	0	0	0	0	0
ENSG00000181163	NPM1	80.8257	46.6297	13.1125	28.2479	87.1379	93.9436	35.4923	41.2839
ENSG00000156427	FGF18	0	0	0	0	0	0	0	0
ENSG00000253110	AC011410.1	0	0	0	0	0	0	0	0
ENSG00000185662	SMIM23	0	0	0	0	0	0	0	0
ENSG00000072803	FBXW11	9.59463	7.76636	4.22976	8.07554	10.4345	8.1385	7.55822	6.9874
ENSG00000072786	STK10	40.0759	34.7502	5.66853	10.5995	26.785	26.5372	9.76582	16.2199
ENSG00000214360	EFCAB9	0	0	0	0	0	0	0	0
ENSG00000168246	UBTD2	2.24276	1.58933	0.48946	0.98866	1.80879	1.44942	0.37013	0.98149
ENSG00000212529	RF00191	0	0	0	0	0	0	0	0
ENSG00000253218	KLF3P1	0.07172	0.23876	0	0.10644	0.0872	0.12653	0	0
ENSG00000174705	SH3PXD2B	0.26681	0.22205	0.06534	0.0396	0.14056	0.04707	0.0593	0.0546
ENSG00000254299	LINC01944	0	0	0	0	0	0	0	0
ENSG00000280318	AC027309.3	0	0	0	0	0	0	0	0
ENSG00000214357	NEURL1B	0.06846	0.01899	0	0.03387	0.01387	0.12078	0.03804	0.07005
ENSG00000253683	AC027309.2	0.38177	0.63546	1.12202	1.69978	0	0	0	1.4062
ENSG00000265160	MIR5003	0	0	0	0	0	1.30618	0	0
ENSG00000253445	AC027309.1	0	0	0	0	0	0	0	0
ENSG00000253295	AC022217.2	0	0	0	0	0	0	0	0
ENSG00000213386	AC022217.1	0.09932	0.49597	0	0	0	0	0	0.12195
ENSG00000253736	AC022217.3	0.41648	0.69323	0.61201	0.92715	1.26586	0.73473	1.38841	1.02269
ENSG00000120129	DUSP1	178.114	398.235	43.6404	109.325	283.549	51.8144	28.2001	216.947
ENSG00000206741	RF00019	0	0	0	0	0	0	0	0
ENSG00000270243	AC022217.4	0	0	0	0	0	0	0	0
ENSG00000253628	AC110011.1	0	0	0	0	0	0	0	0
ENSG00000113719	ERGIC1	10.2908	9.75964	1.14273	1.96967	8.17486	3.74976	3.34421	4.44925
ENSG00000204758	AC008429.1	0.02637	0	0	0	0.03206	0.04652	0.04395	0
ENSG00000254295	AC008429.3	0.1499	1.24753	0.22027	0	0.54673	0	0	0.36809
ENSG00000037241	RPL26L1	3.21161	2.83009	1.11045	1.68225	2.44994	2.55513	0.83973	2.24219
ENSG00000253785	AC008429.2	0.16968	0	0.12467	0.12591	0	0	0.42424	0.10416
ENSG00000113732	ATP6V0E1	76.9134	69.6869	15.3972	22.4542	56.0617	43.7413	20.7774	39.2593
ENSG00000254328	AC008429.4	2.41649	2.01113	0.59183	1.19545	0.48965	0	4.69924	0.98898
ENSG00000212402	SNORA74B	36.4678	44.9185	51.9814	125.564	46.1101	39.8872	43.1582	59.5503
ENSG00000207210	RF00019	0	0	0	0	0	0	0	0
ENSG00000164463	CREBRF	16.6654	22.4172	5.04814	7.45349	15.2683	9.84544	12.2646	17.3602
ENSG00000253439	CDC42P5	0	0	0	0.19672	0	0	0	0
ENSG00000253172	AC008378.1	0	0	0	0	0	0	0	0
ENSG00000113734	BNIP1	1.59218	1.15008	0.26487	0.71335	1.38788	1.5899	0.75111	0.55326
ENSG00000254200	RPL7AP33	0	0	0	0	0	0	0	0
ENSG00000183072	NKX2-5	0	0	0	0	0	0	0	0
ENSG00000201277	RF00019	0	0	0	0	0	0	0	0
ENSG00000252169	RNA5SP200	0	0	0	0	0	0	0	0
ENSG00000113739	STC2	0	0	0	0	0	0	0	0

ENSG00000274994	MIR8056	0	0	0	0	0	0	0	0
ENSG00000199219	RNU6-500P	0	0	0	0	0	0	0	0
ENSG00000253968	AC016573.1	0	0	0	0	0	0	0	0
ENSG00000253141	AC008632.1	0	0	0	0	0	0	0	0
ENSG00000253768	AC008663.1	0	0	0	0	0	0	0	0
ENSG00000254164	AC008663.3	0	0	0	0	0	0	0	0
ENSG00000253955	AC008663.2	0	0	0	0	0	0	0	0
ENSG00000145919	BOD1	3.34174	2.47886	0.80065	1.50942	4.15112	2.49909	1.8769	2.49744
ENSG00000253959	LINC01863	0	0	0	0	0	0	0	0
ENSG00000253428	LINC01942	0	0	0	0	0	0	0	0
ENSG00000253686	LINC01484	0.05208	0	0	0	0.06332	0	0	0
ENSG00000254211	LINC01485	0	0	0	0	0	0	0	0
ENSG00000113742	CPEB4	20.1019	19.2776	8.8713	13.5347	13.2825	10.3228	15.6056	13.5013
ENSG00000185056	C5orf47	0.07973	0.13271	0	0	0.03231	0.04689	0.0443	0.13053
ENSG00000253109	RPL12P22	0	0	0	0	0	0	0	0
ENSG00000170091	NSG2	0	0	0	0	0	0	0	0
ENSG00000253244	AC113423.1	0	0	0	0	0	0	0	0
ENSG00000253447	AC113423.2	0	0	0	0	0	0	0	0
ENSG00000254011	AC011333.1	0	0	0	0	0	0	0	0
ENSG00000249722	AC025752.1	0	0	0	0	0	0	0	0
ENSG00000249306	LINC01411	0	0	0	0	0	0	0	0
ENSG00000213376	GAPDHP71	0	0	0	0	0	0	0	0
ENSG00000249031	SUMO2P6	0	0	0	0	0	0	0	0
ENSG00000250256	AC108112.1	0	0	0	0	0	0	0	0
ENSG00000248929	HIGD1AP3	0	0	0	0	0	0	0	0
ENSG00000120149	MSX2	0	0	0	0	0	0	0	0
ENSG00000251670	AC113346.2	0	0	0	0	0	0	0	0
ENSG00000266890	MIR4634	0	0	0	0	0	0	0	0
ENSG00000251144	AC113346.1	0	0	0	0	0	0	0	0
ENSG00000204754	LINC01951	0	0	0	0	0	0	0	0
ENSG00000251122	NIFKP2	0	0	0	0	0	0	0	0
ENSG00000248293	AC008413.1	0	0	0	0	0	0	0	0
ENSG00000249403	AC008413.2	0	0	0	0	0	0	0	0
ENSG00000248355	ARL2BPP6	0	0	0	0	0	0	0	0
ENSG00000184845	DRD1	0	0	0	0	0	0	0	0
ENSG00000164466	SFXN1	3.27199	1.83689	1.03845	1.89642	3.13062	4.11571	1.96857	1.60454
ENSG00000270839	AC091393.1	0	0	0	0	0	0	0	0
ENSG00000222111	RN7SKP148	0	0	0	0	0	0	0	0
ENSG00000113749	HRH2	14.2109	16.328	1.59323	1.85811	8.53662	2.41364	1.39844	5.37225
ENSG00000200648	RNU6-226P	0.68505	4.56107	0	1.01669	0	0	0	3.36438
ENSG00000145920	CPLX2	0.23749	0	0	0	0	0.01822	0	0
ENSG00000280068	AC138965.3	0	0	0	0	0	0	0	0
ENSG00000250820	AC138965.2	0.12905	0	0	0	0	0	0	0.15845
ENSG00000051596	THOC3	1.18877	1.09536	0.31194	1.60674	1.85821	1.94735	0.42461	0.59946
ENSG00000250801	AC138965.1	0	0	0	0	0	0	0	0
ENSG00000272494	OR1X1P	0	0	0	0	0	0	0	0
ENSG00000250992	AC139491.3	0.10716	0	0	0	0	0	0.35725	0
ENSG00000248469	AC139491.1	0	0	0	0	0	0	0	0
ENSG00000182230	FAM153B	0.05445	0.03399	0.08002	0.06061	0.08275	0.01201	0.18153	0.10028
ENSG00000251458	AC139491.4	0.62119	0.34466	0.60855	2.45843	0	0	0.34514	0.50846
ENSG00000251623	AC139491.5	0.07967	0.26524	0.11708	0.47298	0	0	0	0
ENSG00000248596	AC139491.2	0	0	0.02561	0.07759	0	0	0.02905	0.04279
ENSG00000283559	AC139491.6	0	0	0	0	0	0	0	0
ENSG00000283235	AC139493.2	0	0	0.04422	0	0	0	0	0
ENSG00000270989	AC139493.1	0	0	0	0	0	0	0	0
ENSG00000170085	SIMC1	1.52268	0.9036	0.4086	0.94323	1.23952	1.47158	0.4414	1.05669
ENSG00000251667	BRCC3P1	0.38539	0	0	0.11439	0.18742	0	0	0.09463
ENSG00000122203	KIAA1191	7.58279	3.99222	2.68404	6.90008	7.08806	6.93265	7.59591	4.82491

ENSG00000250909	AC138956.1	0	0	0	0	0	0	0	0
ENSG00000251414	AC138956.2	0.13883	0	0	0	0	0	0	0
ENSG00000175414	ARL10	0.06397	0.09583	0.047	0.09493	0.12443	0.18056	0.10662	0.08639
ENSG00000221464	MIR1271	0	0	0	0	0	0	0	0
ENSG00000048162	NOP16	0.64029	0.38367	0.15054	0.3801	0.96527	1.58138	0.42691	0.3459
ENSG00000146066	HIGD2A	32.4804	26.1186	6.28869	14.8196	24.5471	37.7482	12.4375	14.8199
ENSG00000175416	CLTB	9.85007	7.12065	2.00742	4.48161	6.03144	6.08825	4.4342	3.6193
ENSG00000280139	AC010297.1	0	0	0	0	0	0	0	0
ENSG00000113194	FAF2	5.88471	4.65165	1.26637	2.35696	5.19373	5.82055	3.48852	2.63008
ENSG00000146083	RNF44	8.76755	6.16509	0.93251	2.34485	6.42396	5.20903	2.46088	2.71899
ENSG00000074276	CDHR2	0.08224	0.25095	0.08056	0.04068	0.16664	0.19344	0.02285	0.06731
ENSG00000243959	RN7SL684P	0	0	0	0	0	0	0	0
ENSG00000207420	RF00019	0	0	0	1.06653	0	0	0	0
ENSG00000169258	GPRIN1	0.12185	0.1159	0	0.02583	0.19047	0.24566	0.05803	0.02137
ENSG00000074317	SNCB	0	0	0	0	0	0	0	0
ENSG00000266329	MIR4281	0	0	0	0	0	0	0	0
ENSG00000175766	EIF4E1B	0	0	0	0	0	0	0	0
ENSG00000048140	TSPAN17	1.57424	1.68449	0.53702	0.79269	1.36708	3.39707	0.79657	1.15624
ENSG00000248484	AC113391.1	0	0	0	0	0	0	0	0
ENSG00000251446	AC113391.2	0	0	0	0	0	0	0	0
ENSG00000248859	LINC01574	0	0	0	0	0	0	0	0
ENSG00000113763	UNC5A	0	0	0	0	0	0	0	0
ENSG00000160883	HK3	29.6098	22.77	2.12809	3.57108	9.99515	5.42402	2.89668	6.20952
ENSG00000087206	UIMC1	24.1757	31.8988	27.3433	48.2752	37.5535	47.1787	44.319	40.2983
ENSG00000113761	ZNF346	3.42569	2.86405	1.95384	2.6233	5.22985	4.66361	6.02297	3.24573
ENSG00000251666	ZNF346-IT1	0	0.81885	0	0	0.71772	0	0.164	0
ENSG00000160867	FGFR4	0.01518	0.02527	0	0	0.01845	0	0	0
ENSG00000165671	NSD1	14.2109	9.44561	2.62889	4.95324	10.6996	10.1892	4.53707	6.37691
ENSG00000240729	AC146507.2	0.76996	0.51264	0	0.22854	1.12332	0	0.25668	1.32349
ENSG00000270706	PRMT1P1	0	0	0	0	0	0	0	0
ENSG00000169228	RAB24	4.36607	4.01771	0.26081	0.31608	1.63992	1.94124	0.88751	1.43821
ENSG00000213347	MXD3	2.47463	2.05951	0.62697	0.42214	1.02016	1.10394	0.59265	1.11754
ENSG00000169230	PRELID1	53.6363	42.3613	5.22772	6.84625	29.0402	26.2072	9.05763	14.3995
ENSG00000169223	LMAN2	29.8239	28.4704	2.51348	5.3539	23.3662	20.409	3.78412	9.01116
ENSG00000239393	AC146507.1	0	0	0	0	0	0	0	0
ENSG00000169220	RGS14	15.6369	15.3595	1.70209	3.15155	13.0964	6.02797	1.73762	6.80252
ENSG00000131183	SLC34A1	0.04855	0	0	0	0	0	0	0
ENSG00000196570	PFN3	0	0	0	0	0	0	0	0
ENSG00000131187	F12	0.15883	0.08812	0.0778	0.03929	0.09655	0.2335	0	0.19501
ENSG00000198055	GRK6	33.6963	25.8865	4.52621	5.99508	25.497	19.419	6.84546	12.0272
ENSG00000246334	PRR7-AS1	0.17237	0	0	0	0.05239	0.30408	0	0.05291
ENSG00000131188	PRR7	1.8201	0.82624	0.30393	0.30696	1.70991	0.65678	0.20685	0.40631
ENSG00000113758	DBN1	2.07629	0.90571	0.31563	0.53128	1.13159	1.59145	0.45349	0.58017
ENSG00000279821	AC145098.2	0.4118	0.32256	0.0356	0	0.20615	0.08547	0.16151	0.32715
ENSG00000196923	PDLIM7	9.7634	9.30221	1.12431	2.34505	5.78338	3.08095	2.05159	3.02237
ENSG00000248996	AC145098.1	0	0	0	0	0	0	0	0.6221
ENSG00000146094	DOK3	10.3694	12.7804	1.25119	1.51185	7.20919	2.65544	2.73708	3.73354
ENSG00000183258	DDX41	5.4135	4.19606	0.45118	1.75074	4.14542	4.50425	1.48146	1.32932
ENSG00000146067	FAM193B	3.39728	1.87091	0.68667	0.91842	2.08819	2.58445	1.28411	1.47307
ENSG00000272459	AC139795.3	0	0	0.1991	0	0	0	0	0
ENSG00000184840	TMED9	5.88806	4.40031	1.14777	2.67506	3.50623	4.7167	2.80414	3.31956
ENSG00000027847	B4GALT7	1.05629	0.39621	0.1749	0.26495	0.88628	0.65614	0.49596	0.29226
ENSG00000247679	AC139795.2	0.37676	0.3484	0	0.18638	0.50895	0	0	0.1028
ENSG00000246596	AC139795.1	3.2077	1.37088	0.50959	0.51466	2.47693	2.6	2.96239	1.38375
ENSG00000249849	AC138819.1	0	0	0	0	0	0	0.28348	0.20881
ENSG00000170074	FAM153A	0.15471	0.05723	0.02526	0.07654	0.05225	0.07582	0.44414	0.03166
ENSG00000249109	AC140125.2	0	0.08043	0	0.07171	0	0	0.24162	0
ENSG00000248943	AC140125.1	0	0	0	0	0.26058	0	0.35725	0

ENSG00000170089	AC106795.1	0.09515	0.06335	0.25167	0.4801	0.11568	0	0.41234	0.16355
ENSG00000214351	OR1X5P	0	0	0	0	0	0	0	0
ENSG00000249186	AC140125.3	0	0	0	0	0	0	0	0
ENSG00000250101	AC106795.3	0	0	0	0	0.16292	0	0	0
ENSG00000249684	AC106795.2	0.15475	0.17172	0.2274	0.15311	0.06271	0.182	0.60187	0.19
ENSG00000280207	AC106795.5	0	0	0	0	0	0	0	0
ENSG00000249129	SUDS3P1	0	0	0	0	0	0	0	0
ENSG00000175325	PROP1	0	0	0	0	0	0	0	0
ENSG00000204677	FAM153C	0.11974	0.08542	0.17596	0	0.12478	0.12071	0.4277	0.06301
ENSG00000218227	AC136632.1	0.12403	0.41289	0	0	0	0.4376	0	0
ENSG00000145911	N4BP3	0.13262	0	0.01772	0.05368	0.24917	0.08507	0.18086	0.05921
ENSG00000145916	RMND5B	1.2469	0.49114	0.15386	0.36728	0.87948	0.80599	0.4125	0.57263
ENSG00000145912	NHP2	4.85433	3.55521	1.07001	2.30539	4.60338	4.96694	1.6992	2.38403
ENSG00000251211	AC136632.2	0	0	0	0	0	0	0	0
ENSG00000244234	GMCL2	0	0	0	0.06881	0	0	0	0
ENSG00000197451	HNRNPAB	17.0943	10.3002	3.01438	5.78432	13.0932	20.7478	4.16006	4.91122
ENSG00000175309	PHYKPL	2.70449	1.96038	0.29486	0.63443	2.17435	1.81609	1.17789	1.74596
ENSG00000050767	COL23A1	0.04088	0.06805	0.03004	0	0	0	0.03407	0
ENSG00000271681	AC136601.2	0	0	0	0	0	0	0	0
ENSG00000253698	AC136601.1	0	0	0	0	0	0	0	0
ENSG00000242341	RN7SL646P	0	0	0	0	0	0	0	0
ENSG00000245688	AC008659.1	0	0	0	0	0	0	0	0
ENSG00000113240	CLK4	4.3477	4.46523	1.10106	2.52607	4.03747	3.03533	3.02213	3.53219
ENSG00000252464	RN7SKP70	1.89337	0.90043	0.39747	0.40142	0	0.95433	3.15595	1.32837
ENSG00000169131	ZNF354A	4.30965	4.05585	0.85508	1.37635	4.39946	2.50219	1.93985	3.25963
ENSG00000250420	AACSP1	0	0	0	0	0	0	0	0
ENSG00000274813	AC126915.2	0	0	0	0	0	0	0	0
ENSG00000253725	AC126915.1	0	0	0	0	0	0	0	0
ENSG00000278441	AC126915.3	0	0	0	0	0	0	0	0
ENSG00000178338	ZNF354B	1.7983	1.13875	0.22979	0.66722	1.97245	1.68967	0.48872	1.07996
ENSG00000206624	RNU1-39P	0	0	0	0	0	0	0	0
ENSG00000198939	ZFP2	0.02775	0	0.08157	0.16476	0.5399	0.04896	0.04626	0.13631
ENSG00000213328	AC104117.1	0	0	0	0	0	0	0	0
ENSG00000253869	PIGFP1	0	0	0	0	0	0	0	0
ENSG00000272431	AC104117.5	0	0	0	0	0	0	0	0
ENSG00000178187	ZNF454	0	0	0	0	0	0	0	0
ENSG00000254035	AC104117.3	0	0	0	0	0	0	0	0
ENSG00000113262	GRM6	0	0	0	0	0	0	0	0
ENSG00000254158	AC104117.4	0	0	0	0	0	0	0	0
ENSG00000234284	ZNF879	1.03825	0.62842	0.30514	0.61635	0.98687	1.03236	0.56638	0.60261
ENSG00000177932	ZNF354C	1.165	0.90193	0.27869	0.68355	1.43285	1.50557	0.92578	1.11436
ENSG00000253144	AC104117.2	0	0.47382	0.20915	0	0.17304	0.25109	0	0
ENSG00000087116	ADAMTS2	0	0	0	0	0	0	0	0
ENSG00000253979	AC109479.3	0	0	0	0	0	0	0	0
ENSG00000253163	AC109479.1	0	0	0	0	0	0	0	0
ENSG00000253617	AC109479.2	0	0	0	0	0	0	0	0
ENSG00000250674	AC136628.1	0	0	0	0	0	0	0	0
ENSG00000274716	RF00015	0	0	0	0	0	0	0	0
ENSG00000253652	AC136628.4	0	0	0	0	0	0	0	0
ENSG00000251545	AC136628.2	0	0	0	0	0	0	0	0
ENSG00000253520	AC136628.3	0	0	0	0	0	0	0	0
ENSG00000176783	RUFY1	21.8004	8.99944	4.48277	12.1282	13.2522	9.97567	12.8981	12.8197
ENSG00000249140	PRDX2P3	0	0	0	0	0	0	0	0
ENSG00000244945	AC136604.2	0.07457	0	0	0	0	0	0.06215	0
ENSG00000169045	HNRNPH1	18.0622	9.6397	1.46573	4.47536	16.4509	15.3455	4.56243	5.10743
ENSG00000273549	RF00017	0	0	0	0	0	0	0	0
ENSG00000204661	C5orf60	0	0	0	0	0	0	0	0
ENSG00000228259	AC136604.1	0	0	0	0	0	0	0	0

ENSG00000250999	AC136604.3	0	0	0	0	0	0	0	0
ENSG00000204659	CBY3	0	0	0	0	0	0	0	0
ENSG00000127022	CANX	50.2228	36.221	11.7647	25.2641	41.7498	44.0718	21.9474	20.2223
ENSG00000225051	HMGB3P22	0	0	0	0	0	0	0	0.10909
ENSG00000161021	MAML1	0.86397	0.87883	0.06046	0.10176	0.6169	0.67741	0.14858	0.20205
ENSG00000213316	LTC4S	0	0	0	0	0	0	0	0
ENSG00000161013	MGAT4B	3.62833	1.97612	0.37244	0.85128	2.51376	4.21234	1.33409	1.37575
ENSG00000221394	MIR1229	0	0	0	0	0	0	0	0
ENSG00000161011	SQSTM1	9.21282	8.79915	1.80133	2.71467	7.25368	7.02818	2.85737	3.60976
ENSG00000161010	MRNIP	0.22432	0.20105	0.12678	0.34572	0.35663	0.16742	0.28762	0.15889
ENSG00000222448	RN7SKP150	0	0	0	0	0	0	0	0
ENSG00000252202	RF00019	0	0	0	0	0	0	0	0
ENSG00000245317	AC008393.1	0.05041	0	0	0	0	0	0	0
ENSG00000197226	TBC1D9B	2.86864	1.11661	0.20655	0.64477	1.78653	1.34124	0.55377	0.69029
ENSG00000113269	RNF130	12.4168	10.3624	1.73895	2.49438	7.62447	2.75325	3.53524	5.56603
ENSG00000242979	RPS15AP18	0	0	0	0	0	0	0	0
ENSG00000249876	AC010285.2	0	0.18049	0	0	0	0.38258	0	0
ENSG00000249412	AC010285.1	0	0	0	0	0	0	0	0
ENSG00000198995	MIR340	0.77158	0	0	0	0.93807	0	0	0
ENSG00000278965	AC122713.2	0.19781	0.18292	0	0.06524	0.02672	0	0.03664	0.05397
ENSG00000253480	AC122713.1	0	0	0	0.10718	0	0	0	0
ENSG00000146090	RASGEF1C	0.02476	0	0	0	0	0	0	0
ENSG00000229721	AC104115.1	0.11766	0.19584	0	0	0.14304	0	0	0
ENSG00000050748	MAPK9	4.10089	3.36528	3.29577	7.14305	5.41368	5.70387	6.03056	4.22097
ENSG00000253908	AC104115.2	0	0	0	0	0	0	0	0
ENSG00000248367	AC008610.1	0.18651	0.31045	0	0	0	0	0.31089	0.458
ENSG00000131459	GFPT2	0.21469	0.02552	0.02253	0.11379	0.01864	0.21642	0	0.03766
ENSG00000250509	AC034213.1	0	0	0	0	0	0	0	0
ENSG00000200555	RNU6-525P	0	0	0	0	0	0	0	0
ENSG00000113300	CNOT6	9.41574	12.3009	7.50694	16.8316	11.5827	13.1157	12.9119	9.83873
ENSG00000250749	AC122714.1	0	0	0	0	0	0	0	0
ENSG00000161055	SCGB3A1	0.08664	0.14422	0	0.12859	0.31602	0	0	0
ENSG00000037280	FLT4	0.16561	0.05513	0.01217	0.14747	0.08054	0	0.02761	0.05083
ENSG00000251352	AC122714.2	0	0	0	0	0	0	0	0
ENSG00000248717	LINC02222	0	0	0	0	0	0	0	0
ENSG00000236791	OR2AI1P	0	0	0	0	0	0	0	0
ENSG00000199892	RNU1-17P	0	0	0	0	0	0	0	0
ENSG00000174339	OR2Y1	0	0	0	0	0	0	0	0
ENSG00000131446	MGAT1	10.5113	7.69404	1.35852	2.80986	5.37348	4.89276	2.91308	4.01162
ENSG00000280161	AC022413.1	0.74492	0.37198	0.10947	0.11055	0.09057	0.26283	0.24833	0.27438
ENSG00000278970	HEIH	8.9185	5.096	0.39121	1.58042	5.93392	4.14862	3.03232	2.56045
ENSG00000245060	LINC00847	2.51008	2.28797	0.21956	0.88696	3.19701	2.95208	0.94636	2.34807
ENSG00000196670	ZFP62	2.63721	2.87797	0.7956	1.86623	4.05558	2.40322	1.77579	2.31586
ENSG00000113303	BTNL8	2.40972	3.5135	0.19215	1.13665	0.81758	0.16477	0.65387	0.9174
ENSG00000222852	RF00019	1.6289	1.35565	0	1.20873	0.99019	0	1.35756	2.9999
ENSG00000243664	RPS29P12	0.48224	0	0	0	0	0	0	1.18417
ENSG00000249287	AC091874.3	0	0	0	0	0	0	0	0
ENSG00000248761	AC091874.2	0	0	0	0	0	0	0	0
ENSG00000231228	AC091874.1	0	0	0	0	0	0	0	0
ENSG00000168903	BTNL3	1.23169	2.20015	0.4856	0	2.44706	0.21199	0	2.545
ENSG00000206686	RNU6-1036P	0	0	0	0	0	0	0	0
ENSG00000165810	BTNL9	0.02634	0	0	0	0	0	0	0.01617
ENSG00000277912	MIR8089	0	0	0	0	0	0	0	0
ENSG00000241870	AC008620.1	0	0	0	0	0	0	0	0
ENSG00000214295	FOXO1B	0	0	0	0	0	0	0	0
ENSG00000248560	AC008620.2	0	0	0	0	0	0	0	0
ENSG00000185372	OR2V1	0	0	0	0	0	0	0	0
ENSG00000182613	OR2V2	0	0	0	0	0	0	0	0

ENSG00000248473	LINC01962	0	0	0	0.26469	0	0.31463	0	0
ENSG00000250222	AC008443.5	0.30165	0	0	0.22384	0.36674	0	0	0
ENSG00000146054	TRIM7	0.12986	0.11639	0	0.01482	0.02429	0.03524	0.01665	0.02453
ENSG00000250900	AC008443.6	0.04939	0	0	0	0.06005	0	0.08233	0
ENSG00000248514	AC008443.4	0	0	0	0	0	0	0	0
ENSG00000146063	TRIM41	1.37978	0.87673	0.14302	0.32288	0.57773	0.67671	0.24812	0.41473
ENSG00000264732	MIR4638	0	0	0	0	0	0	0	0
ENSG00000247049	AC008443.2	0	0	0	0	0	0	0	0
ENSG00000204628	RACK1	71.2445	41.0633	8.91922	24.2634	70.5197	60.5974	26.639	30.1993
ENSG00000272296	SNORD96A	0	0	0	0	0	0	0	0
ENSG00000264549	SNORD95	0	0	0	0	0	0	0	0
ENSG00000233937	AC008443.1	0.19919	0.18419	0.09757	0.16423	0.13454	0.19522	0.07378	0.2989
ENSG00000183718	TRIM52	3.32879	2.56586	0.59093	1.09416	2.82479	2.26621	1.0613	1.61836
ENSG00000248275	TRIM52-AS1	3.34196	3.61575	0.6548	1.15729	2.57328	3.63567	1.48548	2.39354
ENSG00000248103	AC008443.3	0	0	0	0	0	0	0	0.16393
ENSG00000274525	AC008443.7	0.29797	0	0	0	0	1.05132	0	0
ENSG00000222533	RNU6-705P	0	0	0	1.04602	0	0	0	0
ENSG00000238035	AC138035.1	0.07976	0.53105	0	0.05919	0.09697	0.63319	0.13295	0.04896
ENSG00000274553	AC138035.3	0	0	0	0	0	0	0	0
ENSG00000250765	AC138035.2	0	0	0	0	0	0	0	0
ENSG00000230178	OR4F3	0	0	0	0	0	0	0	0
ENSG00000251035	WBP1LP4	0	0	0	0	0	0	0	0
ENSG00000213285	RPL23AP45	0	0	0	0	0	0	0	0
ENSG00000271530	WBP1LP12	0	0	0	0	0	0	0	0
ENSG00000220212	OR4F1P	0	0	0	0	0	0	0	0
ENSG00000170590		0.10083	0	0.14816	0	0.12258	0	0	0
ENSG00000217929	CICP18	0	0	0	0	0	0	0	0
ENSG00000218577	AL035696.2	0	0	0	0	0	0	0	0
ENSG00000263667	AL035696.3	0	0	0	0	0	0	0	0
ENSG00000205653	AL035696.1	0	0	0	0	0	0	0	0
ENSG00000112679	DUSP22	6.52303	4.69924	1.32606	1.7793	3.43239	2.52438	1.86945	3.57709
ENSG00000137265	IRF4	4.93392	2.93747	0.89488	1.95512	3.52055	8.11213	2.1337	1.57167
ENSG00000112685	EXOC2	8.72308	7.12512	4.75558	5.78531	9.65744	8.14849	6.8164	6.35741
ENSG00000230433	AL031770.1	0	0	0	0	0	0	0	0
ENSG00000188996	HUS1B	0.28605	0.11903	0	0.21226	0.26083	0.12616	0	0.0878
ENSG00000219712	AL357054.1	0.05974	0	0	0	0.21789	0.10539	0.19915	0.07335
ENSG00000272463	AL357054.4	0.44282	0.04336	0	0.15463	0.25335	0.36762	0.08684	0.09595
ENSG00000271727	AL357054.2	0.34657	0.28844	0.25464	0.51435	0.21068	0.3057	0	0.21276
ENSG00000271911	AL357054.3	0	0	0	0	0	0	0	0
ENSG00000272485	AL392183.1	0	0	0	0	0	0	0	0
ENSG00000276687	AL356130.1	0	0	0	0	0	0	0	0
ENSG00000278343	AL356130.2	0	0	0	0	0	0	0	0
ENSG00000270937	AL033381.2	0	0	0	0	0	0	0	0
ENSG00000176515	AL033381.1	0	0	0	0	0	0	0	0
ENSG00000164379	FOXQ1	0	0	0	0	0	0	0	0
ENSG00000281809	LINC01394	0	0	0	0	0	0	0	0
ENSG00000261730	AL034346.1	0	0	0	0	0	0	0	0
ENSG00000137273	FOXF2	0	0	0	0	0	0	0	0
ENSG00000275859	MIR6720	0	0	0	0	0	0	0	0
ENSG00000243439	RN7SL352P	0	0	0	0	0	0	0	0
ENSG00000218027	AL512329.1	0	0	0.06766	0	0	0	0	0
ENSG00000272279	AL512329.2	0.40128	0	0	0	0.48786	0	0	0
ENSG00000280916	FOXCUT	0	0	0	0	0	0	0	0
ENSG00000054598	FOXC1	0.1867	0.06215	0	0.13855	0.0227	0	0	0
ENSG00000112699	GMDS	1.63495	0.96382	0.15016	0.30331	1.07669	1.44214	0.42581	0.58548
ENSG00000250903	GMDS-DT	0.47283	0.49074	0.13896	0.27244	0.39902	0.28459	0.31526	0.36881
ENSG00000236086	HMGN2P28	1.11909	0.93136	0.41112	0.41521	0.34014	0.49356	0	0
ENSG00000272465	AL031768.1	0.10457	0	0	0	0.12713	0	0	0

ENSG00000280423	AL359852.1	0	0	0	0	0	0	0	0
ENSG00000164385	LINC01600	0	0	0	0	0	0	0	0
ENSG00000228718	LINC02521	0	0	0	0	0	0	0	0
ENSG00000145949	MYLK4	0.11362	0.39927	0.50091	0.07495	0.62931	0.62362	0.21044	0.62003
ENSG00000124535	WRNIP1	5.63412	5.19252	1.30579	2.4131	6.84982	4.53609	3.02536	3.71409
ENSG00000021355	SERPINB1	72.3053	91.5655	15.8751	15.7166	41.6204	26.2051	22.351	42.4388
ENSG00000266750	MIR4645	0.95195	3.16905	1.39888	1.4128	4.62944	0	0	1.16879
ENSG00000230438	SERPINB9P1	1.7572	1.67135	0.29511	1.11766	2.25844	2.39138	0.83685	0.92463
ENSG00000170542	SERPINB9	20.5768	19.8805	3.92353	6.89487	12.3136	28.073	5.28123	9.68157
ENSG00000232999	AL133351.3	0	0	0	0	0	0	0	0
ENSG00000228365	AL133351.2	0	0	0	0	0	0	0	0
ENSG00000124570	SERPINB6	4.83606	4.64401	0.79721	1.44925	2.99631	3.03523	1.86022	2.79754
ENSG00000244041	LINC01011	1.32989	1.56254	0.11496	0.7353	1.77536	1.56407	0.91277	1.69685
ENSG00000124588	NQO2	1.98503	2.14291	0.17707	0.47061	1.41871	0.90623	0.44398	0.87988
ENSG00000224846	AL133351.1	3.26384	1.81089	0.9992	1.21097	1.3227	1.43946	2.04012	1.6697
ENSG00000271361	HTATSF1P2	9.64172	12.3749	0.85352	1.37922	9.46247	2.86904	1.74267	4.99191
ENSG00000274855	AL133351.4	0	0	0	0	0	0	0	0
ENSG00000232654	FAM136BP	0	0	0	0	0	0	0	0
ENSG00000217707	SERPINB8P1	0	0	0	0	0	0	0	0
ENSG00000229282	AL031963.2	0	0	0	0	0	0	0	0
ENSG00000137275	RIPK1	9.96164	14.1012	2.54583	4.98805	10.2997	10.208	4.27386	6.65778
ENSG00000272277	AL031963.3	0.34494	0.43062	0	0	0.31453	0.15213	0	0
ENSG00000222248	RNA5SP201	0	0	0	0	0	0	0	0
ENSG00000137274	BPHL	0.19023	0.12665	0.01864	0.01882	0.15418	0.15661	0.10569	0.12456
ENSG00000228170	AL031963.1	0	0	0	0	0.12823	0.18606	0.1758	0
ENSG00000137267	TUBB2A	20.5852	17.8739	28.8152	21.48	19.0927	0.67389	13.3005	14.4871
ENSG00000216819	TUBB2BP1	0	0	0	0	0	0	0	0
ENSG00000230269	LINC02525	0	0	0	0	0	0	0	0
ENSG00000137285	TUBB2B	1.00228	0.05561	0.9819	0	0.04062	0	0	0.32816
ENSG00000180822	PSMG4	0.3518	0.30449	0.12407	0.36547	0.36783	0.26066	0.26974	0.25052
ENSG00000137266	SLC22A23	0.85674	1.12438	0.94422	3.96118	1.61247	2.77574	5.38264	1.45646
ENSG00000272320	AL445309.1	0	0	0	0	0	0	0	0
ENSG00000228793	AL138881.1	0	0	0	0	0	0	0	0
ENSG00000270477	AL033523.1	0	0	0	0	0	0	0	0
ENSG00000168994	PXDC1	0.17777	0.34521	0.17415	0	0.21613	0.10454	0.29631	0.14551
ENSG00000270504	AL391422.4	0.0531	0.04419	0	0	0	0	0.13276	0
ENSG00000219992	AL391422.1	0	0	0	0	0	0	0	0
ENSG00000238158	AL391422.3	0	0	0	0	0	0	0	0
ENSG00000145945	FAM50B	0.30352	0.63151	0	0.22523	1.3838	0.73625	0.3162	0.27949
ENSG00000233068	AL391422.2	0	0	0	0	0	0	0	0
ENSG00000260604	AL590004.3	0	0	0	0	0	0	0	0
ENSG00000218472	AL590004.2	0	0	0	0.29322	0	0	0	0
ENSG00000217135	AL590004.1	0	0	0	0	0	0	0	0
ENSG00000217566	TDGF1P4	0	0	0	0	0	0	0	0
ENSG00000216657	GLRX3P2	0	0	0	0.1048	0.17171	0.37373	0.70624	0
ENSG00000279926	AL138831.3	0	0	0	0	0	0	0	0
ENSG00000272248	AL138831.2	0	0	0	0.22201	0	0.2639	0	0
ENSG00000230648	AL138831.1	0.35072	0	0	0.52051	0	0.61872	1.16919	0
ENSG00000112739	PRPF4B	15.0537	12.249	2.72746	5.99367	12.6548	11.6001	6.40518	7.91296
ENSG00000145975	FAM217A	0	0	0	0	0	0	0	0
ENSG00000185689	C6orf201	0	0	0	0.06555	0	0	0.03681	0
ENSG00000198721	ECI2	2.017	1.70529	0.44695	0.6177	1.61535	1.94857	0.45361	0.96306
ENSG00000254821	AL136309.4	0.3436	0	0.1683	0	0.13924	0.20205	0	0
ENSG00000234817	AL136309.2	0	0.05358	0	0.04778	0	0	0	0.03952
ENSG00000237716	AL136309.3	0	0	0	0	0	0	0	0
ENSG00000216307	AL136309.1	0.10608	0	0	0	0	0	0	0
ENSG00000231811	AL159166.1	0	0	0	0	0	0	0.37137	0
ENSG00000201185	RNA5SP202	0	0	3.62064	11.8842	0	1.08665	0	3.78139

ENSG00000260239	LINC02533	0	0	0	0	0	0	0	0
ENSG00000270695	AL133393.1	0	0	0	0	0	0	0	0
ENSG00000260673	AL034376.1	0	0	0	0	0	0	0	0
ENSG00000205444	KU-MEL-3	0	0	0	0	0	0	0	0
ENSG00000217385	PSMC1P11	0	0	0	0	0	0	0	0
ENSG00000153046	CDYL	44.6176	55.7399	24.7473	19.1834	114.862	111.275	50.9916	43.7968
ENSG00000236336	AL022725.1	0.19865	0	0	0	0	0	0	0
ENSG00000217030	AL359643.1	0	0	0	0	0	0	0	0
ENSG00000124787	RPP40	0.59776	0.74623	0.2745	0.44357	1.04469	1.51589	0.68501	1.00914
ENSG00000272142	AL359643.3	0.09846	0.16388	0.14468	0.21918	0.29925	0.52107	0.24617	0.06044
ENSG00000271978	AL359643.2	0	0	0	0	0	0.11853	0	0.0275
ENSG00000220685	AL139094.1	0	0	0	0	0	0	0	0.07576
ENSG00000219607	PPP1R3G	0.04481	0	0.02195	0.04434	0.03632	0	0	0
ENSG00000214113	LYRM4	0.68309	0.41346	0.1711	0.38017	0.69836	0.32865	0.38816	0.40028
ENSG00000265083	MIR3691	0	0	0	0	0	0	0	0
ENSG00000145982	FARS2	2.96194	2.59482	0.91632	2.17479	3.9043	3.19017	2.85832	2.06714
ENSG00000220446	AL121978.1	0	0	0	0	0	0	0	0
ENSG00000269985	AL021328.1	0	0	0	0	0	0	0	0
ENSG00000218574	HNRNPA1P37	0	0	0	0	0	0	0	0
ENSG00000270174	AL022097.1	0	0	0	0	0	0	0	0
ENSG00000217239	AL136968.2	0	0	0	0	0	0	0	0
ENSG00000216360	AL136968.1	0	0	0	0	0	0	0	0
ENSG00000233064	AL136307.1	0	0	0	0	0.21683	0	0	0
ENSG00000239472	RN7SL221P	0	0	0	0	0	0	0	0
ENSG00000215057	PKMP5	0	0	0	0	0	0	0	0
ENSG00000124785	NRN1	0.12595	0.20964	0	0.04673	0.11484	0.05555	0.15745	0.03866
ENSG00000124491	F13A1	140.505	78.7722	16.6049	28.275	69.4122	63.3049	47.4914	59.448
ENSG00000263572	MIR7853	0	0.92431	0	2.4724	0	0.97963	0	2.04539
ENSG00000283616	MIR5683	0	0	0	0	0	0	0	0
ENSG00000216863	LY86-AS1	0.27473	0.19598	0.28837	0.05825	0.15905	0.13847	0.21806	0.16062
ENSG00000241216	SNAPC5P1	0	0	0	0	0	0	0	0
ENSG00000218868	CNN3P1	0	0	0	0	0	0	0	0
ENSG00000112799	LY86	15.3147	8.85389	1.20255	3.55679	10.3756	4.33102	2.63068	4.95199
ENSG00000261211	AL031123.2	0.11023	0.11008	0	0.13087	0	0	0	0.0812
ENSG00000279368	AL031123.3	0.0399	0	0.05864	0	0	0	0	0
ENSG00000226281	AL031123.1	0.38715	1.07402	0.18964	0.19152	0.31379	0.91065	0	0.79223
ENSG00000224532	AL136361.1	0.14486	0	0	0	0	0	0	0
ENSG00000219986	BTF3P7	0.30041	0	0	0	0	0	0	0
ENSG00000223342	AL158817.1	0	0	0	0	0	0	0	0
ENSG00000240936	RN7SL554P	0	0	0	0	0	0	0	0
ENSG00000203498	AL139390.1	0	0	0	0	0	0	0	0
ENSG00000124782	RREB1	3.4029	2.53153	0.63053	0.88269	2.28809	2.64559	1.42334	1.12666
ENSG00000225092	AL355336.1	0.30615	0.21839	0.06427	0.12982	0.10634	0.07716	0.0729	0.21479
ENSG00000201483	RF00019	0	0	0	0	0	0	0	0
ENSG00000124783	SSR1	13.4148	10.9119	3.77744	9.1158	10.2395	11.1662	6.37331	8.62779
ENSG00000238221	AL139095.4	0.4398	0	0	0.43514	0	0	0	0
ENSG00000164304	CAGE1	0.06261	0	0.09201	0.06195	0.05075	0	0.06958	0
ENSG00000219375	AL139095.1	0	0	0	0	0	0	0	0
ENSG00000220472	AL139095.2	0.10041	0	0	0.29804	0	0	0	0.24657
ENSG00000230570	AL139095.3	0	0	0	0	0	0	0	0
ENSG00000124784	RIOK1	5.86493	3.65611	2.42914	4.97383	5.28588	5.31309	5.43527	3.72555
ENSG00000233503	HNRNPLP1	0	0	0	0	0	0	0	0.05996
ENSG00000219993	AL138878.1	0	0	0	0	0	0	0	0
ENSG00000234115	AL138878.2	0	0	0	0	0	0	0	0
ENSG00000261189	AL031058.1	0	0	0	0	0	0	0	0
ENSG00000096696	DSP	0.21061	0	0.01067	0.08623	0.03532	0.02562	0.18158	0.13375
ENSG00000168566	SNRNP48	4.30809	4.18124	2.21482	3.80265	5.00861	4.94115	5.48513	5.25857
ENSG00000236512	AL390026.1	0	0	0	0	0	0	0	0

ENSG00000153162	BMP6	6.90816	6.97818	1.62733	2.34789	3.91088	4.41893	2.90068	3.49629
ENSG00000239264	TXNDC5	0.5848	0.30593	0.02455	0.12399	0.22345	0.23581	0.08355	0.08206
ENSG00000259040	BLOC1S5-TXNDC5	0	0	0	0	0	0	0	0
ENSG00000217746	AL096800.1	0	0	0	0	0	0	0	0
ENSG00000219294	PIP5K1P1	0	0	0	0	0.05381	0	0	0
ENSG00000188428	BLOC1S5	3.71477	2.68676	1.49467	3.21599	4.81203	3.51073	3.20654	3.31211
ENSG00000265818	EEF1E1-BLOC1S5	0	0	0	0	0.11604	0	0	0
ENSG00000124802	EEF1E1	0.90451	1.15813	0.35785	1.54892	1.31116	2.08666	0.63787	1.15326
ENSG00000232234	AL355499.1	0	0	0	0	0	0	0	0
ENSG00000234763	AL359378.1	0	0	0	0	0	0	0	0
ENSG00000124786	SLC35B3	3.67016	4.05363	1.00809	3.05435	3.33614	3.08606	3.08738	3.32699
ENSG00000251164		0	0.43888	0	0	0	0	0	0
ENSG00000276019	RF02101	0	0	0	0	0	0	0	0
ENSG00000230939		0	0	0	0	0	0	0	0
ENSG00000225775	AL137220.1	0	0	0	0	0	0	0	0
ENSG00000181355	OFCC1	0	0	0	0	0	0	0	0
ENSG00000216368	AL136226.1	0	0	0	0	0	0	0	0
ENSG00000229765	AL354868.1	0	0	0.22347	0	0	0	0	0
ENSG00000221583	RNU6ATAC21P	0	0	0	0	0	0	0	0
ENSG00000216781	AL138885.1	0	0	0	0	0	0	0	0
ENSG00000137203	TFAP2A	0	0	0.01306	0	0.01081	0	0.01482	0
ENSG00000229950	TFAP2A-AS1	0	0	0	0	0	0	0	0
ENSG00000228478	AL138885.2	0	0	0	0	0	0	0	0
ENSG00000183674	LINC00518	0	0	0	0	0	0	0	0
ENSG00000229401	MIR5689HG	0	0	0	0	0	0	0	0
ENSG00000263705	MIR5689	0	0	0	0	0	0	0	0
ENSG00000238076	MRPL48P1	0	0	0	0	0	0	0	0
ENSG00000231056	LINC02522	0	0	0	0	0	0	0	0
ENSG00000111846	GCNT2	2.1654	1.2373	0.72427	2.29035	0.78586	1.18308	2.35687	0.85315
ENSG00000237685	AL139039.3	0	0	0	0	0	0	0	0
ENSG00000216359	AL139039.1	0	0.17631	0	0	0	0	0	0
ENSG00000217181	AL139039.2	0	0	0	0	0	0	0	0
ENSG00000205318	GCNT2P	0	0.13063	0	0.11647	0	0	0	0
ENSG00000137434	C6orf52	0.08292	0	0	0.12306	0.10081	0	0.13821	0
ENSG00000201048	RF00019	0	0	0	0	0	0	0	0
ENSG00000111845	PAK1IP1	7.00425	8.94729	14.7807	43.4539	13.0209	7.97423	28.0336	20.3993
ENSG00000111843	TMEM14C	8.54523	9.05135	7.25703	15.0702	10.0518	7.63537	10.174	7.28969
ENSG00000272097	AL024498.1	0	0	0	0.07395	0.06058	0	0.08306	0.06118
ENSG00000137210	TMEM14B	4.53655	4.35835	5.50314	12.4036	5.20079	4.53867	6.21686	3.21485
ENSG00000272162	AL024498.2	0	0	0	0	0	0	0	0
ENSG00000222383	RNA5SP203	0	0	0	0	0	0	0	0
ENSG00000111837	MAK	2.22017	3.14693	0.33135	0.46336	4.6393	0.153	0.57823	1.76757
ENSG00000124827	GCM2	0	0	0	0	0	0	0	0
ENSG00000235051	AL357497.1	0	0	0	0	0	0	0	0
ENSG00000153157	SYCP2L	0.16651	0.12318	0.05437	0.21966	0.58481	0.06528	0.37006	0.11358
ENSG00000197977	ELOVL2	0	0	0	0	0	0	0	0
ENSG00000274541	AL121955.1	0	0	0	0	0	0	0	0
ENSG00000230314	ELOVL2-AS1	0	0	0	0	0	0	0	0
ENSG00000224531	SMIM13	1.36031	0.85885	0.32167	1.06744	1.31166	1.18609	0.36487	1.07505
ENSG00000244476	ERVFRD-1	0	0	0.03322	0	0	0	0.07537	0
ENSG00000271245	AL136139.1	0	0.54959	0	0.49003	0	0	0	0
ENSG00000247925	AL139807.1	0.10412	0	0	0	0	0	0.17355	0.25567
ENSG00000111859	NEDD9	18.7302	17.7377	3.3066	4.79922	11.5278	7.43989	4.1635	9.32202
ENSG00000233656	AL445430.1	0	0	0	0	0	0	0	0
ENSG00000242753	AL445430.2	0	0	0	0	0	0.377	0	0
ENSG00000202313	RNU1-64P	0	0	0	0	0	0	0	0
ENSG00000218020	THAP12P5	0	0	0	0	0	0	0	0
ENSG00000205269	TMEM170B	16.5851	10.5638	2.66644	4.47528	7.27896	4.43823	7.08652	7.26477

ENSG00000271897	AL357518.1	0	0	0	0	0	0	0	0
ENSG00000277473	RF00017	0	0	0	0	0	0	0	0
ENSG00000207419	RF00272	0	0	0	0	0	0	0	0
ENSG00000111863	ADTRP	0.26494	0.0294	0.02596	0.05243	0.21474	0.9971	0.38273	0.54215
ENSG00000234427	AL022724.1	0	0	0	0	0	0	0	0
ENSG00000215124	AMD1P4	0	0	0	0	0	0	0	0
ENSG00000225102	AL157373.1	0	0	0	0	0	0	0	0
ENSG00000229896	AL157373.2	0	0	0	0	0	0	0	0
ENSG00000095951	HIVEP1	5.21428	4.54517	0.91241	1.73399	4.79715	3.7101	2.64859	2.65589
ENSG00000078401	EDN1	0.21633	0	0	0	0.13151	0.06361	0	0.04427
ENSG00000218893	SUMO2P12	0	0	0	0	0	0	0	0
ENSG00000223321	RN7SKP293	0	0	0	0	0	0	0	0
ENSG00000212802	RPL15P3	0.11919	0.19839	0.17514	0	0.28981	0.63079	0	0
ENSG00000234015	LINC02530	0	0	0	0	0	0	0	0
ENSG00000112137	PHACTR1	7.09569	1.64523	0.5579	1.14634	1.78265	0.25405	0.65465	0.90817
ENSG00000206702	RNU1-11P	0	0	0	0.66333	0.54339	0	0	1.09753
ENSG00000215022	AL008729.1	1.2707	0.31334	0.55327	0.66354	0.28609	0.37361	0.35301	0.60672
ENSG00000145979	TBC1D7	1.15691	0.66149	0.2336	0.3932	0.63348	0.38949	0.32385	0.96503
ENSG00000272379	AL008729.2	0.92981	0.25795	0	0.22999	0.18841	0	0	0.57081
ENSG00000145990	GFOD1	1.86578	1.27877	0.34272	1.13	1.08414	2.20239	0.83466	0.83377
ENSG00000237786	GFOD1-AS1	0	0	0	0	0	0	0	0
ENSG00000218265	RPS4XP7	0	0	0.13809	0	0.11425	0	0	0
ENSG00000202351	RN7SKP204	0	0	0	0	0	0	0	0
ENSG00000124523	SIRT5	9.72156	14.5503	8.98858	21.9176	11.7918	9.67801	10.008	11.936
ENSG00000261071	AL441883.1	0.07014	0.23351	0	0	0	0	0.11692	0
ENSG00000225921	NOL7	8.17505	5.36445	1.38613	3.61647	9.17448	8.04299	2.94805	2.9436
ENSG00000010017	RANBP9	66.2484	66.0895	55.7396	113.856	113.186	84.5337	123.969	114.6
ENSG00000050393	MCUR1	4.32033	4.23011	2.09844	6.39388	4.50218	4.07769	3.22748	4.45751
ENSG00000272209	AL023583.1	0	0	0.18848	0	0	0	0	0
ENSG00000180537	RNF182	1.34414	14.0802	0.97443	1.38309	4.11811	15.5554	35.6982	0.35207
ENSG00000220868	MRPL35P1	0	0	0	0	0	0	0	0
ENSG00000233981	AL022396.1	0	0	0	0	0.23087	0	0.31653	0
ENSG00000112149	CD83	9.09332	3.34735	2.05577	1.3409	6.62617	6.0157	3.2549	1.43136
ENSG00000238987	RNU7-133P	0	0	0	0	0	0	0	0
ENSG00000230631	AL353152.1	0	0	0	0	0	0	0	0
ENSG00000226673	LINC01108	0	0	0	0	0	0	0	0
ENSG00000237346	AL080313.2	0	0.24951	0	0.22247	0	0	0	0.36809
ENSG00000234540	AL080313.1	0	0.30126	0	0	0	0	0	0
ENSG00000229646	AL109914.1	1.50617	0.83567	0	1.49022	2.44155	2.65709	0	2.46567
ENSG00000206960	RNU6-793P	0	0	0	0	0	0	0	0
ENSG00000234261	AL138720.1	0.0845	0	0.12417	0.0627	0.20546	0.37266	0.42253	0.05187
ENSG00000216754	AL050335.1	0	0	0	0	0	0	0	0
ENSG00000242989	RN7SL332P	0	0	0	0	0	0	0	0
ENSG00000271888	AL136162.1	0.20399	0.22636	0	0	0.41334	0	0	0
ENSG00000008083	JARID2	25.8958	16.3692	4.27991	9.85673	16.3566	15.8442	12.8782	9.38124
ENSG00000235488	JARID2-AS1	0.1611	0.26815	0	0	0.39172	0	0.26853	0
ENSG00000201367	RNU6-522P	1.3701	0	1.00667	2.03338	1.66573	3.62556	0	0
ENSG00000201519	RNU6-645P	0	0	0	0	0	0	0	0
ENSG00000277411	RF00001	0	1.15102	0	0	0	0	0	0
ENSG00000047579	DTNBP1	1.63317	2.07878	0.56469	0.78417	2.21916	2.24558	0.88072	0.79617
ENSG00000214027	ARPC3P5	0	0	0.20171	0	0	0	0	0
ENSG00000232947	LINC02543	0	0	0	0	0	0	0	0
ENSG00000274212	AL365265.1	0	0	0	0	0	0	0	0
ENSG00000217585	AL021407.2	0	0	0	0	0	0	0	0
ENSG00000007944	MYLIP	20.76	15.8897	3.51589	6.09745	11.5179	7.54639	8.66099	9.34688
ENSG00000263712	MIR4639	1.06232	1.76824	0	1.5766	1.29155	0	0	0
ENSG00000218073	AL021407.3	0.45247	0.50209	0.22163	0.22384	0	0	0.2514	0.37036
ENSG00000217078	AL021407.1	0	0	0	0	0	0	0	0

ENSG00000216364	MRPL42P2	0.17207	0	0	0	0	0	0	0
ENSG00000251831	RNU6-1114P	0	0	0	0	0	0	0	0
ENSG00000207193	RF00019	0	0	0	0	0	0	0	0
ENSG00000137198	GMPR	42.9227	46.9558	24.8534	26.3679	53.9811	74.0994	17.4074	15.5237
ENSG00000282024	AL009031.1	0.35177	0	0	0.06526	0.21384	0.15514	0.65964	0.05399
ENSG00000124788	ATXN1	6.29991	6.27544	1.50918	1.93329	4.54043	7.33345	1.68223	3.09506
ENSG00000229931	AL137003.1	0.52507	0.34959	0.30864	0	0.12767	2.40839	0	0
ENSG00000272341	AL137003.2	0.17329	0.38458	0.12732	0.04286	0.31602	0.86615	0	0.10638
ENSG00000230873	STMND1	0	0	0	0	0	0	0	0
ENSG00000112183	RBM24	0.04257	0	0	0	0.02588	0.03755	0	0
ENSG00000219314	AL034372.1	0	0	0	0	0	0	0	0
ENSG00000112186	CAP2	0	0	0	0	0	0	0	0
ENSG00000184612	RPL7P26	0	0	0	0	0	0	0	0
ENSG00000218359	SUMO2P13	0	0	0	0	0	0	0	0
ENSG00000137414	FAM8A1	36.8618	49.3564	12.8741	12.1881	20.1594	17.5291	11.3899	29.4987
ENSG00000124789	NUP153	29.3201	34.4173	22.8111	57.4237	38.6763	39.6513	42.8746	39.2375
ENSG00000206881	RNU6-190P	0.71863	0	0	0	0	0	0	0.88232
ENSG00000272269	AL138724.1	0.26949	0	0.198	0.29996	0.32763	0.23771	0	0.16544
ENSG00000201415	RNA5SP204	0	0	0	0	0	0	0	0
ENSG00000201442	RF00019	0	0	0	0	0	0	0	0.90906
ENSG00000137177	KIF13A	8.79525	6.79896	1.25857	2.1413	7.27287	2.7894	2.70145	4.48125
ENSG00000220920	AL023807.1	0	0	0	0	0	0	0	0
ENSG00000187566	NHLRC1	0.13043	0.10855	0.04792	0.09678	0.43607	0.11505	0.27175	0.1201
ENSG00000137364	TPMT	10.669	7.17246	1.86239	2.5307	5.26688	7.15463	2.80389	3.39505
ENSG00000165097	KDM1B	15.7767	14.4445	4.68831	6.79467	8.00983	6.92289	5.34456	7.26636
ENSG00000124795	DEK	65.5854	71.5097	27.194	56.4259	100.172	91.5946	69.1449	74.8497
ENSG00000199715	RF00019	0	0	0	0	0	0	0	0
ENSG00000278102	AL138825.1	0	0	0	0	0	0	0	0
ENSG00000200681	RNU6-263P	0	0	0	0	0	0	1.14187	0
ENSG00000217414	DDX18P3	0	0	0	0	0	0	0	0
ENSG00000218976	AL138725.1	0	0	0	0	0.06782	0	0	0
ENSG00000137393	RNF144B	13.2153	15.6784	2.28957	6.01212	9.72965	5.39736	8.31062	10.1562
ENSG00000283408	MIR548A1HG	0	0	0	0	0	0	0.11955	0
ENSG00000207775	MIR548A1	0	0	0	0	0	0	0	0
ENSG00000217379	AL589647.1	0	0	0	0	0	0	0	0
ENSG00000231662	AL357052.1	0	0	0	0	0	0	0	0
ENSG00000228412	AL022068.1	0	0	0	0	0	0	0	0
ENSG00000218643	RPL5P20	0	0	0	0	0	0	0	0
ENSG00000201523	RNA5SP205	0	0	0	0	0	0	0	0
ENSG00000214012	KRT18P38	0	0	0	0	0	0	0	0
ENSG00000217314	UQCRFS1P3	0	0	0	0	0	0	0	0
ENSG00000200957	RNU6-801P	0	0	0	0	0	0	0	0
ENSG00000172201	ID4	0	0.05205	0	0	0	0	0	0
ENSG00000229700	AL008627.1	0.45247	0	0	0	0	0	0	0
ENSG00000172197	MBOAT1	6.9051	6.52888	1.86128	4.66918	5.76228	2.88321	3.47335	5.36772
ENSG00000227803	AL158198.1	0.1929	0	0.28346	0	0.23452	0	0	0.23683
ENSG00000112242	E2F3	7.39533	6.07207	1.96071	2.865	4.40061	5.03324	2.7209	5.24576
ENSG00000240869	RN7SL128P	29.3201	40.4028	49.4425	63.4881	28.342	52.9966	30.8455	37.4742
ENSG00000224707	E2F3-IT1	2.38226	0.91506	0	0	0	0.32328	0	0.89997
ENSG00000222431	RNU6-141P	0	0	0	0	0	0	0	0
ENSG00000201683	RF00019	0	1.19616	0	0	3.49477	0	0	0.88232
ENSG00000145996	CDKAL1	8.48959	7.10523	7.79416	17.54	14.6591	10.8462	19.4076	11.9488
ENSG00000225832	AL035090.1	0	0	0	0	0	0	0	0
ENSG00000233848	AL513188.1	0	0	0	0	0	0	0	0
ENSG00000252046	RNU6-150P	0	0	0	0	0	0	0	0
ENSG00000227089	AL031767.1	0	0	0	0	0	0	0	0
ENSG00000280989	LINC00581	0	0	0	0	0	0	0	0
ENSG00000231754	AL512380.1	0.06961	0	0	0	0	0	0	0

ENSG00000283480	AL512380.2	0	0	0	0	0	0	0	0
ENSG00000124766	SOX4	1.95401	1.50113	0.07363	0.81794	1.43147	0.7292	1.00216	0.61515
ENSG00000220771	BOLA2P3	0	0	0	0	0	0	0	0
ENSG00000272168	CASC15	0.22063	0.10099	0.03242	0.04912	0.114	0.13623	0.04597	0.02709
ENSG00000280443	AL136313.1	0	0	0	0	0	0	0	0
ENSG00000222515	RN7SKP240	0	0	0	0	0	0	0	0
ENSG00000260455	NBAT1	0	0	0	0	0.04594	0	0	0
ENSG00000219404	AL359694.1	0	0	0	0	0	0	0	0
ENSG00000261568	AL359694.2	0	0	0	0	0	0	0	0
ENSG00000172179	PRL	0.05374	0	0	0.07975	0.06533	0.0948	0	0
ENSG00000112273	HDGFL1	0	0	0	0	0	0	0	0
ENSG00000228772	AL033539.1	0	0	0	0	0	0	0	0
ENSG00000233358	AL035401.1	0	0	0	0	0	0	0	0
ENSG00000218476	AL591416.1	0	0	0	0	0	0	0	0
ENSG00000207394	RNU6-1060P	0	0	0	0	0	0	0	0
ENSG00000235743	AL139231.1	0.23494	0	0	0	0	0	0	0
ENSG00000219453	AL133270.1	0	0	0	0	0	0	0	0
ENSG00000278128	AL139093.1	0	0	0	0	0	0	0	0
ENSG00000218512	SPTLC1P2	0	0	0	0	0	0	0	0
ENSG00000220748	AL032822.1	0	0	0	0	0	0	0	0
ENSG00000219582	HNRNPA1P58	0	0	0	0	0	0	0	0
ENSG00000152954	NRSN1	0	0	0	0	0	0	0	0
ENSG00000251830	RF00218	0	0	0	0	0	0	0	0
ENSG00000146038	DCDC2	0	0	0.02156	0	0	0.02588	0	0
ENSG00000146049	KAAG1	0	0	0	0	0	0	0	0
ENSG00000202050	RNU6-391P	0	0	0	0	0	0	0	0
ENSG00000124532	MRS2	3.02741	2.11548	1.48991	4.13271	2.84729	2.9223	3.47522	2.50722
ENSG00000112293	GPLD1	0.68547	1.51301	1.16057	1.79135	0.90584	0.92009	4.91801	1.51855
ENSG00000112294	ALDH5A1	5.16087	3.84244	2.59293	6.79295	4.64536	4.37671	16.1654	5.5057
ENSG00000137261	KIAA0319	0.63366	0.51035	0.03004	0.0455	0.41005	0.03606	0.08518	0.08784
ENSG00000218186	KRT8P43	0	0	0	0	0	0	0	0
ENSG00000111802	TDP2	26.5898	36.7767	7.44421	9.38903	23.3924	12.5871	13.5183	18.5425
ENSG00000112304	ACOT13	1.90174	2.22137	0.49028	0.84176	1.98758	2.26605	0.97322	1.02409
ENSG00000272345	AL031775.1	1.57013	1.23797	0.24287	0.61322	1.60752	1.60364	1.23971	0.8117
ENSG00000112308	C6orf62	59.1509	40.5514	24.4662	47.2878	34.793	42.7286	44.2438	33.1992
ENSG00000272402	AL031775.2	0.18099	0	0	0.53721	0.22004	0	0	0.22221
ENSG00000224164	AL133264.2	0	0	0	0	0	0	0	0.16157
ENSG00000218806	AL133264.1	0.13043	0.2171	0	0	0	0	0	0
ENSG00000112312	GMNN	1.27299	1.82323	3.61077	5.22839	2.87938	3.34247	5.18131	3.45304
ENSG00000260286	ARMH2	0.45025	0.29978	0	0	0.43792	0.31772	0	0.55281
ENSG00000282804	AL512428.1	0	0	0	0	0	0	0	0
ENSG00000111913	RIPOR2	108.861	127.762	22.8625	36.5983	103.524	48.533	26.9579	75.8592
ENSG00000217083	MTCO2P33	0.63054	0.26238	0	0	0.3833	0	0.26275	0
ENSG00000214975	PPIAP29	2.05652	3.66759	0.86344	0.87203	5.17913	0.25914	1.22425	0.54106
ENSG00000272053		32.3802	49.3957	31.0593	51.2243	12.9782	43.6898	18.2677	14.6791
ENSG00000220517	ASS1P1	0.47405	0.69043	0.08708	0	0.14409	1.1499	0.09877	0.36377
ENSG00000244618	RN7SL334P	0.26273	0	0	0	0	0.46348	0.43792	0
ENSG00000229313	AL133268.3	0	0	0	0	0.3089	0	0	0
ENSG00000223623	AL133268.2	0	0	0	0	0	0	0	0
ENSG00000230372		0	0	0	0	0	0	0	0
ENSG00000168405	CMAHP	2.63047	2.69647	2.33341	3.92771	2.26204	3.28231	2.64679	2.28439
ENSG00000219682	AL133268.1	0	0.08709	0.07688	0	0.06361	0.1846	0	0.06424
ENSG00000262400	AL590084.3	0	0	0	0	0	0	0	0
ENSG00000217805	AL590084.1	0	0	0	0	0	0	0	0
ENSG00000219681	AL590084.2	0	0	0	0	0	0	0	0
ENSG00000235733		0.39356	0.16377	0.14458	0.29204	0	0	0.164	0.3624
ENSG00000271108	KATNBL1P5	0	0	0	0	0	0	0	0
ENSG00000216718	AL024509.2	0	0	0	0	0	0	0	0

ENSG00000213972	AL024509.1	0.23722	0.13162	0.23239	0	0	0	0	0
ENSG00000079691	CARMIL1	0.99186	0.28712	0.15843	0.56001	1.40248	1.17919	0.95243	0.79421
ENSG00000207286	RF00019	0	0	0	0	0	0	0	0
ENSG00000207490	RNU6-987P	0	0	0	0	0	0	0	0
ENSG00000079689	SCGN	0	0	0.43609	0.44043	0.06013	0	0.32977	0.06073
ENSG00000217408	PRELID1P2	0	0	0	0	0	0	0	0
ENSG00000164508	HIST1H2AA	0	0	0	0	0	0	0	0
ENSG00000146047	HIST1H2BA	0	0	0	0	0	0	0	0
ENSG00000178762	HIST1H2BPS1	0	0	0	0	0	0	0	0
ENSG00000216436	HIST1H2APS1	0	0	0	0	0	0	0	0
ENSG00000146039	SLC17A4	0	0	0	0	0	0	0	0
ENSG00000124568	SLC17A1	0	0	0	0	0	0	0	0.04403
ENSG00000124564	SLC17A3	0	0	0	0.03027	0	0	0	0
ENSG00000242387	HIST1H2APS2	0	0	0	0	0	0	0	0
ENSG00000277410	AL138726.1	0	0	0	0	0	0	0	0
ENSG00000112337	SLC17A2	0	0	0	0	0	0	0	0
ENSG00000112343	TRIM38	8.70602	12.0414	3.20407	2.38073	6.96801	7.00616	3.71227	6.44407
ENSG00000272558	U91328.2	0	0	0	0	0	0	0	0
ENSG00000272462	U91328.1	0.40821	0.26588	0.15649	0.18438	0.25893	0.25048	0.14792	0.26149
ENSG00000272810	U91328.3	0	0.23599	0	0	0	0	0	0.17408
ENSG00000224447	HIST1H1PS2	0	0	0	0	0	0	0	0
ENSG00000124610	HIST1H1A	0.33935	0.75314	0.16623	0.16788	0.27505	1.796	0.3771	0
ENSG00000275714	HIST1H3A	17.1212	15.4366	7.60025	17.9986	23.4175	65.4425	8.91827	12.9193
ENSG00000278637	HIST1H4A	8.69267	9.77632	4.48808	22.6637	11.1396	70.0439	21.1466	8.65356
ENSG00000278705	HIST1H4B	28.1152	27.2987	15.9849	57.87	23.8051	147.321	28.453	42.1219
ENSG00000274267	HIST1H3B	18.1913	13.3586	16.5109	27.2626	25.1522	389.509	17.8365	17.5177
ENSG00000278463	HIST1H2AB	5.59545	7.14045	5.20754	12.1796	11.1112	88.8402	4.35248	4.351
ENSG00000276410	HIST1H2BB	10.0042	18.8937	18.0937	32.5501	24.0919	208.392	9.29981	8.73987
ENSG00000234816	HIST1H2APS5	0.39729	0.99194	0.29191	1.47406	0.72453	2.80351	0	0.97558
ENSG00000278272	HIST1H3C	19.0831	14.8429	9.95895	16.6752	27.9709	451.175	5.35096	5.69324
ENSG00000187837	HIST1H1C	102.301	86.8503	30.7035	60.3235	122.431	242.913	45.104	104.997
ENSG00000010704	HFE	0.85248	0.62348	0.0949	0.23003	0.51821	0.20508	0.12918	0.26959
ENSG00000197061	HIST1H4C	49.2039	56.6568	82.4569	93.0305	76.8247	297.268	83.1388	111.307
ENSG00000187475	HIST1H1T	0.61254	0.16993	0	0.15151	0.37235	0	0	0.62672
ENSG00000180596	HIST1H2BC	130.077	208.175	101.127	92.8722	144.681	295.06	57.7207	133.377
ENSG00000180573	HIST1H2AC	151.391	140.222	55.2777	56.5451	135.492	143.964	87.2403	126.309
ENSG00000168298	HIST1H1E	149.323	136.572	18.1428	56.557	143.603	301.156	35.9735	70.3027
ENSG00000158373	HIST1H2BD	44.7713	71.3	23.6204	41.7158	52.3855	161.231	22.3039	36.2675
ENSG00000283064	AL353759.1	0.23226	0.46391	0.13652	0.27576	0.2259	0.32779	0.15485	0.39923
ENSG00000217159	LARP1P1	0.50206	0	0	0	0	0	0	0
ENSG00000274290	HIST1H2BE	2.8356	4.38947	3.00016	3.78751	4.72301	30.4145	3.35582	3.93411
ENSG00000277157	HIST1H4D	37.8249	36.759	34.5237	94.8388	53.6985	490.307	27.4123	38.6526
ENSG00000216331	HIST1H1PS1	1.02161	0.63768	0	0.18952	0.46577	0.22528	0.42572	0.62716
ENSG00000282988	AL031777.3	2.05975	1.86329	0.329	0.46518	1.41541	0.71094	0.22391	1.15451
ENSG00000197409	HIST1H3D	0	0	0	0	0	0	0	0
ENSG00000196866	HIST1H2AD	0	0	0.27408	0.27681	0	0	0	0.229
ENSG00000277224	HIST1H2BF	20.1024	27.5437	12.0683	18.0097	23.2478	118.824	11.646	13.7705
ENSG00000217275	AL031777.1	1.46894	2.68957	0.43172	0.21801	2.14309	1.55485	0.73455	1.62319
ENSG00000276966	HIST1H4E	96.715	100.183	23.035	82.4489	134.604	274.798	39.5217	49.3259
ENSG00000273802	HIST1H2BG	8.60108	10.8169	4.70459	17.8709	15.8017	62.6328	6.6108	12.8483
ENSG00000277075	HIST1H2AE	53.1391	69.274	23.7013	44.241	96.8203	360.752	29.2849	38.7217
ENSG00000277720	AL031777.2	0	0	0	0	0	0	0	0
ENSG00000274750	HIST1H3E	1.28597	1.13856	0.56289	0.93396	2.06242	2.26863	1.50502	1.41093
ENSG00000218281	HIST1H2APS3	0	0	0	0	0	0	0	0
ENSG00000124575	HIST1H1D	42.5934	44.883	11.8065	37.732	64.7635	149.31	32.2879	48.9173
ENSG00000274618	HIST1H4F	94.4447	81.339	47.9879	239.538	135.389	735.254	71.6635	51.3445
ENSG00000275663	HIST1H4G	0	0	0	0	0	0	0	0
ENSG00000277775	HIST1H3F	9.6307	8.60887	5.76571	13.2343	11.4919	218.351	9.51282	8.10193

ENSG00000275713	HIST1H2BH	6.26255	8.23261	4.33993	9.39993	8.17624	53.5451	5.81246	7.12113
ENSG00000273983	HIST1H3G	3.16186	2.12541	1.56366	3.11332	3.40055	53.205	1.46961	1.0825
ENSG00000218690	HIST1H2APS4	0.21527	0.17916	0.15817	0.31949	0	1.13931	0	0
ENSG00000278588	HIST1H2BI	32.5138	38.7481	11.874	34.8343	39.2955	323.449	18.2789	22.204
ENSG00000158406	HIST1H4H	9.8276	11.1686	6.57339	19.1117	10.0529	54.9411	6.94784	11.6085
ENSG00000220875	HIST1H3PS1	0	0	0	0	0	0	0	0.21582
ENSG00000186470	BTN3A2	19.9749	18.0535	2.18732	3.95472	13.5788	10.5771	3.7997	9.43167
ENSG00000124508	BTN2A2	2.52049	1.50651	0.42089	0.95217	1.51824	2.28387	0.61109	1.29411
ENSG00000026950	BTN3A1	19.9273	18.938	5.26239	7.19747	10.5873	19.6287	5.22025	11.6978
ENSG00000124549	BTN2A3P	0.31587	0.37972	0.12894	0.02604	0.21335	0.21671	0.14625	0.25855
ENSG00000111801	BTN3A3	10.1569	9.05683	2.1322	4.2876	7.85953	14.1242	2.74247	4.58097
ENSG00000112763	BTN2A1	12.0393	10.8896	1.76347	2.90134	7.48326	5.05364	3.22631	5.77483
ENSG00000124557	BTN1A1	0.07596	0.04214	0	0	0.06157	0	0	0
ENSG00000199289	RNU6-502P	0	0	0	0	0	0	0	0
ENSG00000228223	HCG11	2.7634	2.1829	0.41296	1.63352	1.65136	1.44598	1.13202	1.35139
ENSG00000261353		0.85731	1.427	0	1.27235	0.78173	0	0.7145	0.5263
ENSG00000182952	HMGN4	33.4282	22.9843	8.53933	12.3814	23.2934	25.8827	14.0977	19.2851
ENSG00000146109	ABT1	5.86602	5.00611	1.02272	1.54934	4.80487	5.48117	2.73445	2.89919
ENSG00000275846	AL513548.3	0.07462	0	0.05483	0.08306	0.04536	0	0.09329	0
ENSG00000271071	AL513548.2	0	0	0	0	0	0.10713	0	0
ENSG00000181315	ZNF322	1.333	1.61585	0.14904	0.90314	0.916	0.89463	0.60378	1.38758
ENSG00000261584	AL513548.1	0	0	0	0	0.05169	0	0	0
ENSG00000241549	GUSBP2	0.35394	0.41239	0.05201	0.21011	0.5594	0.3122	0.53096	0.43456
ENSG00000243307	POM121L6P	0	0	0	0	0	0	0	0
ENSG00000224843	LINC00240	0.01131	0	0	0	0.01375	0	0	0.01389
ENSG00000272312	AL133255.1	0	0	0	0	0	0	0	0
ENSG00000220581	VN1R12P	0	0	0	0	0	0	0	0
ENSG00000216762	VN1R13P	0	0	0	0	0	0	0	0
ENSG00000219770	VN1R11P	0	0	0	0	0	0	0	0
ENSG00000222800	RNU2-62P	0	0	0	0	0	0	0	0
ENSG00000272468	AL021807.1	0.64868	0.64783	0	0.19254	0.94637	0.45774	0.64875	1.11501
ENSG00000124635	HIST1H2BJ	14.8144	17.0812	14.2863	24.1619	11.6321	74.048	15.3047	19.7994
ENSG00000196787	HIST1H2AG	7.75355	11.6586	3.63834	5.12502	13.0704	77.4721	4.99582	6.63978
ENSG00000276180	HIST1H4I	3.96524	5.65727	2.74696	5.88485	7.16239	45.5689	3.77683	3.82522
ENSG00000197903	HIST1H2BK	143.138	145.706	45.7996	67.9554	143.616	579.018	67.3435	109.603
ENSG00000274997	HIST1H2AH	10.7962	12.926	5.56661	8.7141	13.5863	171.413	5.36709	10.2322
ENSG00000265565	MIR3143	0	1.93664	1.70975	5.18027	0	0	0	2.85705
ENSG00000174572	RPL10P2	0	0	0	0	0	0	0	0
ENSG00000112812	PRSS16	0.02771	0	0	0	0.01685	0	0	0
ENSG00000270935	AL021808.1	0	0	0	0	0	0	0	0
ENSG00000158553	POM121L2	0	0	0	0	0	0	0	0
ENSG00000220758	VN1R10P	0	0	0	0	0	0	0	0
ENSG00000204789	ZNF204P	0.42812	0.3054	0.40443	0.2723	0.66921	0.10789	0.45875	0.63828
ENSG00000124613	ZNF391	0.09098	0.05048	0.04457	0.09002	0.18435	0.02675	0.12638	0.11171
ENSG00000271755	AL031118.1	0.02481	0	0	0	0	0	0	0
ENSG00000237154	MCFD2P1	0	0	0	0	0	0	0	0
ENSG00000096654	ZNF184	4.69573	3.67354	1.20756	1.74224	4.96678	5.13602	3.28736	2.59441
ENSG00000278332	AL021918.2	0	0	0	0	0	0	0	0
ENSG00000270666	AL021918.1	0	0	0	0	0	0	0	0
ENSG00000218347	HNRNPA1P1	0	0	0	0	0	0	0	0
ENSG00000219738	CD83P1	0	0	0	0	0	0	0	0
ENSG00000206671	RNU6-471P	0	0	0	0	0	0	0	0
ENSG00000216676	AL031229.1	0	0	0	0	0	0	0	0
ENSG00000281706	LINC01012	0.08401	0	0	0.12468	0.05107	0.03705	0	0.02579
ENSG00000216915	GPR89P	0	0.08161	0.07205	0.14553	0.05961	0	0	0
ENSG00000218069	RSL24D1P1	0	0	0	0	0	0	0	0
ENSG00000217862	HIST1H4PS1	0	0	0	0	0	0	0	0
ENSG00000185130	HIST1H2BL	11.716	14.501	6.40103	13.3753	20.8182	182.308	4.75702	8.11449

ENSG00000196747	HIST1H2AI	18.5072	24.0136	10.0647	22.0599	26.044	274.048	16.0316	11.9877
ENSG00000278828	HIST1H3H	18.5473	34.62	23.9461	25.5035	31.3385	110.6	31.0142	44.7439
ENSG00000276368	HIST1H2AJ	20.2665	16.3939	7.2366	9.5574	22.3367	438.056	8.2085	3.95336
ENSG00000273703	HIST1H2BM	19.8161	29.7816	29.6849	33.6922	36.7226	255.569	27.8994	19.6057
ENSG00000197238	HIST1H4J	2.94773	5.88781	2.02144	3.20816	5.49513	16.9873	0.98268	2.89535
ENSG00000273542	HIST1H4K	6.57824	8.60316	2.7619	3.13805	8.56891	29.0123	4.69924	4.03833
ENSG00000233822	HIST1H2BN	1.87366	2.04944	0.72766	1.96637	2.14778	12.1355	1.45001	1.1338
ENSG00000275221	HIST1H2AK	13.8021	22.6632	4.11122	22.4215	20.4084	92.4596	10.8812	15.343
ENSG00000217646	HIST1H2BPS2	0.64677	0	0	0	0	0	0	0
ENSG00000276903	HIST1H2AL	16.2268	17.075	6.30387	13.5636	26.9844	211.242	7.77229	9.16001
ENSG00000184357	HIST1H1B	17.0065	14.3329	3.32158	6.54951	19.7601	217.039	6.10005	10.0437
ENSG00000275379	HIST1H3I	18.1913	19.8895	4.7174	19.5867	25.369	204.193	13.972	12.4813
ENSG00000275126	HIST1H4L	19.7347	35.5858	13.8095	46.3734	38.8457	179.047	25.8458	25.9607
ENSG00000197153	HIST1H3J	6.90442	5.98235	2.50174	5.05327	9.6591	152.504	2.99539	3.25151
ENSG00000278677	HIST1H2AM	0.18651	1.24182	0.27408	0.55362	1.58732	1.64519	0	0.229
ENSG00000274641	HIST1H2BO	12.1205	20.8151	7.63327	13.1342	16.8409	183.616	10.2619	10.8658
ENSG00000238610	RNU7-26P	0	0	0	0	0	0	0	0
ENSG00000168131	OR2B2	0	0	0	0	0	0	0	0
ENSG00000168126	OR2W6P	0.15612	0	0	0	0	0	0.13012	0
ENSG00000124657	OR2B6	0.23344	0.6476	0.34304	0.6929	0	0.82364	0.38911	0.66877
ENSG00000214374	RPLP2P1	0	0	0	0	0.26211	0	0	0
ENSG00000216629	OR2W4P	0	0	0	0	0	0	0	0
ENSG00000217539	IQCB2P	0	0	0	0	0	0	0	0
ENSG00000199851	RF00012	0	0	0	0	0	0	0	0
ENSG00000217315	OR2W2P	0	0	0	0	0	0	0	0
ENSG00000187763	OR2B7P	0	0	0	0	0	0	0	0
ENSG00000182477	OR2B8P	0	0	0	0	0	0	0	0
ENSG00000220721	OR1F12	0	0	0	0	0	0	0	0
ENSG00000272009	AL121944.1	0	0.17785	0.15702	0.15858	0.12991	0.1885	0.17811	0.39357
ENSG00000197279	ZNF165	0.38268	0.11581	0	0.05163	0.16918	0.12274	0.34793	0.04271
ENSG00000219891	ZSCAN12P1	0	0	0	0	0	0.07804	0	0
ENSG00000219392	ZNF602P	0	0	0	0	0	0	0	0
ENSG00000269293	ZSCAN16-AS1	0.5761	0.22563	0.39839	0.45265	0.61801	1.31524	0.50838	0.74894
ENSG00000196812	ZSCAN16	5.46441	5.60431	1.70331	1.14684	4.42899	5.55028	2.11607	2.50745
ENSG00000261839	AL358933.1	0.15541	0.12934	0	0.11532	0.12596	0.09139	0.04317	0
ENSG00000198315	ZKSCAN8	2.558	1.85472	0.64073	1.61058	3.35734	2.27343	3.35935	1.42758
ENSG00000226314	ZNF192P1	0.02892	0	0	0.12874	0.07031	0.20404	0.14459	0.071
ENSG00000280107	AL022393.1	0	0	0	0	0	0	0	0
ENSG00000216901	ZNF603P	0	0	0	0	0	0	0	0
ENSG00000218016	ZNF192P2	0	0	0	0	0	0	0	0
ENSG00000176933	TOB2P1	0	0	0	0	0.35934	0	0	0
ENSG00000137185	ZSCAN9	1.20683	0.95499	0.14536	0.52851	0.88996	0.31412	0.49466	0.5344
ENSG00000187626	ZKSCAN4	1.41971	1.79802	0.31747	0.91609	2.43898	1.03449	1.02889	1.28838
ENSG00000189134	NKAPL	0.35778	0.22332	0	0	0.05437	0	0	0.05491
ENSG00000197062	ZSCAN26	2.42418	1.91353	0.55087	0.96435	1.9142	2.11625	1.37468	1.68764
ENSG00000276302	AL021997.3	0	0.21905	0	0.19531	0.15999	0	0	0.16157
ENSG00000137338	PGBD1	0.18916	0	0.06949	0.14037	0.17248	0.20857	0.07883	0.05806
ENSG00000273712	AL021997.2	0	0	0	0	0	0	0	0
ENSG00000270326	AL021997.1	0	0	0	0	0.46174	0	0	0
ENSG00000235109	ZSCAN31	0.1183	0.12307	0.06519	0.06584	0.1618	0.07826	0.07394	0.01816
ENSG00000189298	ZKSCAN3	0.65095	0.50396	0.15572	0.38194	0.57055	0.66766	0.27757	0.33456
ENSG00000158691	ZSCAN12	0.96044	0.6439	0.45085	0.53452	1.16768	1.52962	1.08951	0.78614
ENSG00000251877	RNU2-45P	0	0	0	0	0	0	0	0
ENSG00000187987	ZSCAN23	0.04465	0	0	0	0.05429	0.07878	0	0
ENSG00000231162	COX11P1	0	0	0	0	0	0	0	0
ENSG00000219262	OR2E1P	0	0	0	0	0	0	0	0
ENSG00000198704	GPX6	0	0	0	0	0	0	0	0
ENSG00000224586	GPX5	0	0	0	0	0	0	0	0

ENSG00000232040	ZBED9	0	0	0	0	0	0	0	0
ENSG00000246350	AL049543.1	0	0	0	0	0	0	0	0
ENSG00000271440	AL121932.1	0	0	0	0	0	0	0	0
ENSG00000235570	LINC00533	0	0	0	0	0	0	0	0
ENSG00000237425	RPSAP2	0	0	0	0	0	0	0	0
ENSG00000235559	NOP56P1	0	0	0	0	0	0	0	0
ENSG00000225173	AL662890.1	0	0	0	0	0	0	0	0
ENSG00000225595	LINC01623	0	0	0	0	0	0	0	0
ENSG00000213916	RPL13P	0	0	0	0	0	0	0	0
ENSG00000233366	ZNF90P2	0	0	0	0	0	0	0	0
ENSG00000224157	HCG14	0	0	0	0	0	0	0	0
ENSG00000204713	TRIM27	2.55165	3.07084	0.17534	0.66748	2.37689	1.32777	0.33658	1.2509
ENSG00000212240	RNU6-930P	0	0	0	0	0	0	0	0
ENSG00000204709	LINC01556	0	0	0	0	0	0	0	0
ENSG00000228666	KRT18P1	0	0	0	0	0	0	0	0
ENSG00000263426	RN7SL471P	0	0	0	0	0	0	0	0
ENSG00000227214	HCG15	0	0	0	0	0	0	0	0
ENSG00000244349	AL662791.1	0	0	0	0	0	0	0	0
ENSG00000197935	ZNF311	0	0	0	0.03425	0	0.04072	0	0.02834
ENSG00000223677	OR2AD1P	0	0	0	0	0	0	0	0
ENSG00000277661	AL662791.2	0	0	0	0	0	0	0	0
ENSG00000204704	OR2W1	0	0	0	0	0	0	0	0
ENSG00000236909	OR2P1P	0	0	0	0	0	0	0	0
ENSG00000228364	SAR1AP1	0	0	0	0	0	0	0	0
ENSG00000204703	OR2B3	0	0	0	0	0	0	0	0
ENSG00000204702	OR2J1	0	0	0	0	0	0	0	0
ENSG00000204701	OR2J3	0	0	0	0	0	0	0	0
ENSG00000227206	AL645937.2	0	0	0	0	0	0	0	0
ENSG00000203492	AL645937.1	0	0	0	0	0	0	0	0
ENSG00000204700	OR2J2	0	0	0	0	0	0	0	0
ENSG00000224233	OR2J4P	0	0	0	0	0	0	0	0
ENSG00000230598	AL645937.3	0	0	0	0	0	0	0	0
ENSG00000232505		0	0	0	0	0	0	0	0
ENSG00000213911	OR2G1P	0	0	0	0	0	0	0	0
ENSG00000204697	OR2U1P	0	0	0	0	0	0	0	0
ENSG00000242524	OR2U2P	0	0	0	0	0	0	0	0
ENSG00000197171	AL672167.1	0	0	0	0	0	0	0	0
ENSG00000204695	OR14J1	0	0	0	0	0	0	0	0
ENSG00000230056	DDX6P1	0	0	0	0	0	0	0	0
ENSG00000243729	OR5V1	0	0	0	0	0	0	0	0
ENSG00000112462	OR12D3	0	0	0	0	0	0	0	0
ENSG00000280236	OR12D2	0	0	0	0	0	0	0	0
ENSG00000251608	OR12D1	0	0	0	0	0	0	0	0
ENSG00000204694	OR11A1	0	0	0	0	0	0	0	0
ENSG00000206474	OR10C1	0	0	0	0	0	0	0	0
ENSG00000277881	AL645927.1	0	0	0	0	0	0	0	0
ENSG00000204688	OR2H1	0	0	0	0	0	0	0	0
ENSG00000225797	UBDP1	0	0	0	0	0	0	0	0
ENSG00000230164	MAS1LP1	0	0	0	0	0	0	0	0
ENSG00000204687	MAS1L	0	0	0	0	0	0	0	0
ENSG00000213900	RPS17P1	0	0	0	0	0	0	0	0
ENSG00000229274	AL662860.1	0	0	0	0	0	0	0	0
ENSG00000224582	LINC01015	0	0	0	0	0	0	0	0
ENSG00000229281	GPR53P	0	0	0	0	0	0	0	0
ENSG00000237988	OR2I1P	0	0	0	0	0	0	0	0
ENSG00000213886	UBD	0	0	0	0	0	0	0	0
ENSG00000204681	GABBR1	0.4154	0.15845	0.17804	0.11559	0.24199	0.10687	0.0577	0.10625
ENSG00000232173	OR2H5P	0	0	0	0	0	0	0	0

ENSG00000227609	TMEM183AP1	0	0	0	0	0	0	0	0
ENSG00000201330	SNORD32B	0	0	0	0	0	0	0	0
ENSG00000231301	RPL13AP	0	0	0	0	0	0	0	0
ENSG00000204657	OR2H2	0	0	0	0	0	0	0	0
ENSG00000235238	SUMO2P1	0	0	0	0	0	0	0	0
ENSG00000204655	MOG	0	0	0	0	0	0	0	0
ENSG00000204644	ZFP57	0.3752	0	0.85209	1.873	0.08294	0.48138	0.68225	0
ENSG00000233916	ZDHHC20P1	0	0	0	0	0	0	0	0
ENSG00000204642	HLA-F	21.0346	11.8018	2.48074	4.20912	11.4526	11.9722	4.13646	5.76213
ENSG00000225864	AL645939.1	0	0	0	0	0	0	0	0
ENSG00000214922	HLA-F-AS1	0.12278	0	0	0	0.02132	0	0.02924	0.04307
ENSG00000239257	RPL23AP1	0	0	0	0	0	0	0	0
ENSG00000273340	MICE	0	0	0	0	0	0	0	0
ENSG00000227758	AL645939.2	0	0	0	0	0	0	0	0
ENSG00000199290	RF00019	0	0	0	0	0	0	0	0
ENSG00000235821	IFITM4P	0	0	0	0	0	0.30789	0	0
ENSG00000272236	AL645939.4	0	0	0	0	0	0	0	0
ENSG00000270896	AL645939.3	0	0	0	0	0	0	0	0
ENSG00000181126	HLA-V	0	0	0	0	0	0	0	0
ENSG00000176998	HCG4	0	0	0	0	0	0	0	0
ENSG00000261548	HLA-P	0	0	0	0	0	0	0	0
ENSG00000213880	RPL7AP7	0	0	0	0	0	0	0	0
ENSG00000237042	MICG	0	0	0	0	0	0	0	0
ENSG00000204632	HLA-G	0.04158	0.48444	0.0611	0	0	0.07335	0.0693	0.20419
ENSG00000229142	HCG4P8	0	0	0	0	0	0	0	0
ENSG00000233265		0	0	0	0	0	0	0	0
ENSG00000281831	HCP5B	0	0	0	0	0	0	0	0
ENSG00000230521	AL645929.1	0	0	0	0.11158	0.27421	0	0.50125	0
ENSG00000206341	HLA-H	4.88223	8.90573	1.76902	0.79406	3.98423	7.78702	4.23618	4.18782
ENSG00000231130	HLA-T	0	0	0	0	0	0	0	0
ENSG00000233677	DDX39BP1	0	0	0	0	0	0	0	0
ENSG00000235963	MCCD1P1	0	0	0	0	0	0	0	0
ENSG00000227262	HCG4B	0.07397	0	0	0	0	0	0.24658	0
ENSG00000230795	HLA-K	0.63069	0	0	0.104	0.34079	0	0.23361	0
ENSG00000228078	HLA-U	0	0	0	0	0.95824	0	0	0
ENSG00000206503	HLA-A	286.656	154.784	24.5635	46.1637	173.695	161.234	67.357	40.0171
ENSG00000227766	AL671277.1	0	0	0	0	0.08974	0.13022	0.12304	0
ENSG00000235290	HLA-W	0	0	0	0	0	0	0	0
ENSG00000229390	MICD	0	0	0	0	0	0	0	0
ENSG00000204625	HCG9	0	0	0	0	0	0	0.17454	0
ENSG00000238024	DDX39BP2	0	0	0	0	0	0	0	0
ENSG00000224312	MCCD1P2	0	0	0	0	0	0	0	0
ENSG00000204623	ZNRD1ASP	0.04597	0.03478	0.03685	0.13646	0.03049	0.11797	0.26473	0.06671
ENSG00000204622	HLA-J	0	0.12539	0	0.1118	0.18318	0.06645	0.06279	0.27748
ENSG00000237669	AL671277.2	0	0	0	0	0	0	0	0
ENSG00000232757	ETF1P1	0	0	0	0	0	0	0	0
ENSG00000278104	RF02218	0	0	0	0	0	0	0	0
ENSG00000275856	RF02219	0	0	0	0	0	0	0	0
ENSG00000066379	ZNRD1	0.97814	0.73099	0.176	0.79989	1.06785	0.77474	0.59892	0.8088
ENSG00000278773	RF02220	0	0	0	0	0	0	0	0
ENSG00000204619	PPP1R11	3.69679	2.98051	0.46685	0.90012	2.80904	2.49656	0.77025	1.0638
ENSG00000204618	RNF39	0	0	0	0	0	0	0	0
ENSG00000204616	TRIM31	0	0	0	0	0	0	0	0.02546
ENSG00000231226	TRIM31-AS1	0	0	0	0	0	0	0	0
ENSG00000252228	RF00554	0	0	0	0	0	0	0	0
ENSG00000204614	TRIM40	0.2642	0.34203	0	0.08713	0.14276	0.20715	0.34252	0.14417
ENSG00000204613	TRIM10	1.509	1.27307	1.12392	1.44189	2.11105	1.82335	3.37667	1.31975
ENSG00000204610	TRIM15	0.02915	0	0	0.04325	0.03543	0	0.04858	0

ENSG00000234127	TRIM26	1.94262	2.34149	0.29531	0.54679	1.5881	1.86124	0.53037	0.86358
ENSG00000233892	PAIP1P1	0	0	0	0	0	0	0	0
ENSG00000270604	HCG17	0	0	0	0.14333	0	0	0.16098	0.11857
ENSG00000236475	TRIM26BP	0	0	0	0	0	0	0	0
ENSG00000243753	HLA-L	0.01332	0	0	0	0.01619	0.09396	0.02219	0.01635
ENSG00000280128	AL662795.2	0	0	0	0	0	0.06979	0.0989	0
ENSG00000231074	HCG18	0.15273	0.06136	0.03096	0.12506	0.08964	0.2044	0.07023	0.09053
ENSG00000204599	TRIM39	0.12142	0.11548	0.02549	0	0.06326	0.1224	0	0
ENSG00000248167	TRIM39-RPP21	0	0	0	0	0	0	0	0
ENSG00000241370	RPP21	0.04564	0	0	0	0.05549	0	0.07608	0
ENSG00000224372	HLA-N	0	0	0	0	0	0	0	0
ENSG00000224486	AL662795.1	0	0	0	0	0	0	0	0
ENSG00000236405	UBQLN1P1	0	0	0	0	0	0	0	0
ENSG00000226577	MICC	0	0	0	0	0	0	0	0
ENSG00000229068	TMPOP1	0	0	0	0	0	0	0	0
ENSG00000224936	SUCLA2P1	0	0	0	0	0	0	0	0
ENSG00000236603	RANP1	0	0	0	0	0	0	0	0
ENSG00000204592	HLA-E	245.92	232.698	33.5633	44.3553	157.265	141.96	62.9588	88.9988
ENSG00000235781	LINC02569	0	0	0	0	0	0	0	0
ENSG00000204590	GNL1	0.94953	0.84669	0.12458	0.31456	0.80397	1.07685	0.33915	0.53086
ENSG00000204576	PRR3	0.25683	0.171	0.10064	0.20329	0.18735	0.30206	0.2854	0.12613
ENSG00000204574	ABCF1	7.34138	5.6378	1.04785	2.1406	6.00942	7.69066	1.78287	2.40762
ENSG00000216101	MIR877	0	0	0	0	0	0	0	0
ENSG00000204569	PPP1R10	6.92945	6.97191	1.25993	1.91913	4.66517	5.20719	1.33543	2.51957
ENSG00000204568	MRPS18B	2.40711	1.09859	0.34231	1.32525	2.73769	3.42457	1.03543	1.71605
ENSG00000137343	ATAT1	0.0545	0.12095	0.05339	0.08088	0.19877	0.03205	0.09084	0.15613
ENSG00000228415	PTMAP1	0	0	0	0	0	0	0	0
ENSG00000204564	C6orf136	1.34972	1.32493	0.8137	1.54087	1.47266	1.5874	1.03836	0.59488
ENSG00000204560	DHX16	5.55971	4.96192	1.07499	1.38425	4.66929	4.16198	1.1584	2.38016
ENSG00000146112	PPP1R18	6.12032	6.63234	0.60895	0.99348	4.70871	3.54279	1.06267	2.05473
ENSG00000137404	NRM	0.60934	0.23865	0.05267	0.0532	0.34862	0.25293	0.11949	0.13203
ENSG00000230449	RPL7P4	0	0	0	0	0	0	0	0
ENSG00000137337	MDC1	0.32723	0.24208	0.06679	0.14839	0.4973	0.33675	0.07576	0.10044
ENSG00000224328	MDC1-AS1	0	0	0	0	0	0	0	0
ENSG00000196230	TUBB	5.34641	3.63229	1.15442	1.84602	5.86329	10.9717	2.03694	2.2506
ENSG00000272540	AL662797.1	0	0	0	0	0	0	0	0
ENSG00000137312	FLOT1	22.892	32.436	2.23136	3.3462	16.6989	7.7928	3.10628	6.21449
ENSG00000201988	RF00019	0	0	0	0	0	0	0	0
ENSG00000272273	IER3-AS1	0.13909	0	0	0.20642	0	0	0	0
ENSG00000137331	IER3	2.86498	0.71981	0	0.08023	0.85436	1.43044	0.45052	0.26548
ENSG00000263608	RN7SL353P	0	0	0	0	0	0	0	0
ENSG00000228022	HCG20	0	0	0	0	0	0	0	0
ENSG00000214894	LINC00243	0.23634	0	0.09923	0.05011	0.41049	0.17869	0	0.04145
ENSG00000237923	LINC02570	0	0	0	0	0	0	0	0
ENSG00000202241	RN7SKP186	0	0	0	0	0	0	0	0
ENSG00000237775	DDR1-DT	0	0	0	0	0	0	0	0
ENSG00000204580	DDR1	0.06428	0.02675	0	0.0477	0.06839	0	0	0.06906
ENSG00000284370	MIR4640	0	0	0	0	0	0	0	0
ENSG00000213780	GTF2H4	0	0	0	0	0	0	0	0
ENSG00000137411	VARS2	0.60737	0.30329	0.08925	0.24466	0.52744	0.4592	0.07231	0.27698
ENSG00000196260	SFTA2	0	0	0	0	0	0	0	0
ENSG00000252761	RF00019	0	0	0	0	0	0	0	0
ENSG00000168631	MUCL3	0	0	0	0	0	0	0	0
ENSG00000233529	HCG21	0	0	0	0	0	0	0	0
ENSG00000275906	NAPGP2	0	0	0	0	0	0	0	0
ENSG00000204544	MUC21	0	0	0	0	0	0	0	0
ENSG00000261272	MUC22	0	0	0	0	0	0	0	0
ENSG00000228789	HCG22	0	0	0	0	0	0	0	0

ENSG00000222895	RNU6-1133P	0	0	0	0	0	0	0	0
ENSG00000204542	C6orf15	0	0	0	0	0	0	0	0
ENSG00000204540	PSORS1C1	0	0	0	0	0	0	0	0
ENSG00000204539	CDSN	0	0	0	0	0	0	0	0
ENSG00000204538	PSORS1C2	0	0	0	0	0	0	0	0
ENSG00000238211	POLR2LP1	0	0	0	0	0	0	0	0
ENSG00000204536	CCHCR1	0.43237	0.47978	0.02118	0.04278	0.35044	0.27968	0.12011	0.19465
ENSG00000137310	TCF19	0.35221	0.20691	0	0	0.10075	0.18275	0.13813	0.10175
ENSG00000204531	POU5F1	0.02531	0	0	0	0	0.0893	0	0
ENSG00000204528	PSORS1C3	0	0	0	0	0	0	0	0
ENSG00000272501	AL662844.4	1.23975	0	0	0	0	0	0.25831	0
ENSG00000206344	HCG27	0.25452	0	0	0	0	0	0.18855	0
ENSG00000271821	AL662844.3	0	0	0	0	0	0	0	0
ENSG00000255726	AL662844.1	0	0	0	0	0	0	0	0
ENSG00000255899	AL662844.2	0	0	0	0	0	0	0	0
ENSG00000204525	HLA-C	310.16	189.364	23.2012	29.9844	26.4471	124.102	59.9449	52.0166
ENSG00000234745	HLA-B	509.979	185.872	39.3998	23.9607	88.4741	116.554	101.917	91.7669
ENSG00000214892	USP8P1	0	0	0	0	0.056	0	0.07677	0
ENSG00000227939	RPL3P2	0	0	0	0	0	0	0	0
ENSG00000231402	WASF5P	0	0	0	0	0	0	0	0
ENSG00000256166	LINC02571	0	0	0	0	0	0	0	0
ENSG00000229836	AL671883.1	0	0	0	0	0	0	0	0
ENSG00000277402	MIR6891	0	0	0	0	0	0	0	0
ENSG00000271581	AL671883.2	0	0.24623	0.10869	0	0	0	0.24658	0.27244
ENSG00000228432	DHFRP2	0	0	0	0	0	0	0	0
ENSG00000201658	RNU6-283P	0	0	0	0	0	0	0	0
ENSG00000230994	FGFR3P1	0	0	0	0	0	0	0	0
ENSG00000223702	ZDHHC20P2	0	0	0	0	0	0	0	0
ENSG00000225851	HLA-S	0	0	0	0	0	0	0	0
ENSG00000272221	AL645933.2	0.06073	0	0	0	0	0	0	0
ENSG00000204520	MICA	0.67586	1.07384	0.22572	0.13678	0.33615	0.81294	0.10241	0.33947
ENSG00000206337	HCP5	1.12123	1.04979	0.1373	0.58934	1.05077	1.978	0.15574	0.97511
ENSG00000199332	RF00019	0	0	0	0	0	0	0	0
ENSG00000230174	LINC01149	0	0	0	0	0	0	0	0
ENSG00000233902	AL645933.1	0	0	0	0	0	0	0	0
ENSG00000204516	MICB	1.36227	1.83144	0.26948	0.50544	1.84731	2.58808	0.393	1.25443
ENSG00000201680	RF00019	0	0	0	0	0	0	0	0
ENSG00000256851	AL663061.1	0	0	0	0	0	0	0	0
ENSG00000219797	PPIAP9	0	0	0	0	0	0	0	0
ENSG00000225499	RPL15P4	0	0	0	0	0	0	0	0
ENSG00000204511	MCCD1	0	0	0	0	0	0	0	0
ENSG00000198563	DDX39B	0.03138	0.01741	0.01537	0.0621	0.01272	0	0	0.01284
ENSG00000254870	ATP6V1G2-DDX39	0	0	0	0	0	0	0	0
ENSG00000201785	SNORD117	0	0	0	0	0	0	0	0
ENSG00000265236	SNORD84	0	0	0	0	0	0	0	0
ENSG00000234006	DDX39B-AS1	0	0	0	0	0	0	0	0
ENSG00000213760	ATP6V1G2	0	0	0	0	0	0	0	0
ENSG00000204498	NFKBIL1	0.82406	0.4115	0.06055	0.18345	0.50094	0.79957	0.41208	0.15177
ENSG00000226979	LTA	0.211	0.07024	0	0	0.25652	0.22334	0	0.05181
ENSG00000232810	TNF	1.79315	0.65518	0.19281	0.12982	0.74441	1.15733	0.0729	0.21479
ENSG00000227507	LTB	13.3707	8.74328	2.87705	6.44919	12.0757	6.14968	4.3778	6.33204
ENSG00000204482	LST1	34.5132	30.8097	3.77778	5.81844	17.4639	9.01384	6.3206	12.1522
ENSG00000204475	NCR3	2.2982	1.71134	0.44436	0.6283	1.98527	1.70709	0.80647	0.89106
ENSG00000230622	UQCRHP1	0	0	0	0	0	0	0	0
ENSG00000204472	AIF1	109.778	114.168	11.2272	14.4081	76.1263	22.9032	16.5652	49.442
ENSG00000204469	PRRC2A	8.86198	6.33132	0.79175	1.31283	5.82705	5.68887	1.63533	2.18205
ENSG00000200816	SNORA38	0	0	0	0	0	0	0	0
ENSG00000274494	MIR6832	0	0	0	0	0	0	0	0

ENSG00000204463	BAG6	35.0765	35.0631	19.8591	15.0784	37.2914	55.3532	36.9762	22.1349
ENSG00000204444	APOM	0.57404	0.3675	0	0.26213	0.32211	0.38949	0.73603	0.10843
ENSG00000204439	C6orf47	0	0	0	0	0	0	0	0
ENSG00000227198	C6orf47-AS1	0	0	0	0	0	0	0	0
ENSG00000204438	GPANK1	0.93495	0.58791	0.09159	0.21584	0.90935	0.43984	0.17316	0.2806
ENSG00000201207	RF00019	0	0	0	0	0	0	0	0
ENSG00000204435	CSNK2B	1.27457	0.62398	0.25707	0.29672	0.78999	1.49901	0.45823	0.3989
ENSG00000263020	AL662899.2	0	0.04834	0	0	0	0	0	0
ENSG00000240053	LY6G5B	0.02418	0.04025	0	0	0	0	0	0
ENSG00000204428	LY6G5C	0.0839	0	0.0411	0	0.102	0	0.09323	0.03434
ENSG00000204427	ABHD16A	0.26475	0.47005	0.12968	0.20956	0.23604	0.37364	0.1471	0.17336
ENSG00000204422	AL662899.1	0	0	0	0	0	0	0	0
ENSG00000266776	MIR4646	0	0	0	0	0	0	0	0
ENSG00000204424	LY6G6F	1.75283	0.66309	0	0.23649	1.06553	1.12445	1.19524	0.19565
ENSG00000250641	LY6G6F-LY6G6D	0	0	0	0	0	0	0	0
ENSG00000255552	LY6G6E	0.13279	0	0	0	0	0	0	0
ENSG00000244355	LY6G6D	0	0	0	0	0	0	0	0
ENSG00000204420	MPIG6B	2.02156	0.70621	0.25672	0.29632	1.21371	2.99394	0.70721	0.88863
ENSG00000204421	LY6G6C	0.13291	0	0	0	0	0	0	0
ENSG00000213722	DDAH2	3.55238	2.98051	0.84881	0.94298	1.12361	0.66235	0.86653	0.74466
ENSG00000213719	CLIC1	77.5655	94.8676	15.3085	21.9028	52.4513	42.9486	22.0201	43.2839
ENSG00000204410	MSH5	0	0	0	0	0.02859	0	0	0
ENSG00000255152	MSH5-SAPCD1	0	0	0	0	0	0	0	0
ENSG00000252743	RNU6-850P	0	0	0	0	0	0	0	0
ENSG00000228727	SAPCD1	0	0	0	0	0	0	0	0
ENSG00000235663	SAPCD1-AS1	0	0	0	0	0	0	0	0
ENSG00000204396	VWA7	0.20149	0.03354	0	0.05981	0.12248	0	0.06717	0
ENSG00000204394	VARS	2.44696	1.06339	0.4074	0.85869	2.49134	2.76444	0.54249	0.56239
ENSG00000201555	RF00019	0	0	0	0	0	0	0	0
ENSG00000204392	LSM2	3.74329	2.83214	0.40005	1.01008	2.15138	3.2418	0.45378	1.00275
ENSG00000204390	HSPA1L	0	0.09592	0	0	0	0.05083	0	0
ENSG00000204389	HSPA1A	0	0.04914	0	0	0	0	0	0.03625
ENSG00000204388	HSPA1B	0	0	0	0	0	0	0	0
ENSG00000204387	C6orf48	11.8843	7.2487	2.45212	5.85909	11.8261	9.1904	7.59811	5.69665
ENSG00000201823	SNORD48	0	0	0	0	0	0	0	0
ENSG00000201754	SNORD52	0	0	0	0	0	0	0	0
ENSG00000204386	NEU1	2.18898	1.18922	0.33507	0.27072	0.8871	1.07268	0.15203	0.59724
ENSG00000204385	SLC44A4	0.36987	0.07243	0.44761	0	0.02645	0.46059	0.50772	0
ENSG00000204371	EHMT2	1.89058	1.12149	0.32511	0.61191	1.7728	2.43047	0.33525	0.51857
ENSG00000237080	EHMT2-AS1	0	0	0	0	0	0	0	0
ENSG00000166278	C2	0.13241	0.24243	0.11674	0.0393	0.08049	0.2803	0.02207	0.01626
ENSG00000204366	ZBTB12	0	0	0	0	0	0	0	0
ENSG00000244255	AL645922.1	0	0	0	0	0	0	0	0
ENSG00000281756	C2-AS1	0	0	0	0	0	0	0	0
ENSG00000243649	CFB	0	0	0	0	0.01783	0	0	0
ENSG00000204356	NELFE	4.85254	4.21802	1.545	1.60038	4.85078	4.61318	1.70756	2.48245
ENSG00000284446	MIR1236	0	0	0	0	0	0	0	0
ENSG00000204351	SKIV2L	4.51788	2.95977	0.46453	1.27063	3.04262	2.69545	1.31731	1.69806
ENSG00000204348	DXO	0.38377	0.24569	0.08676	0.39431	0.71781	0.46871	0.09841	0.28996
ENSG00000204344	STK19	0.40784	0.33943	0.08173	0.11005	0.31554	0.22893	0.0927	0.09104
ENSG00000244731	C4A	0	0.03393	0.01498	0.01513	0.03718	0	0.01699	0.05006
ENSG00000233627	C4A-AS1	0	0	0	0	0	0	0	0
ENSG00000204338	CYP21A1P	0	0	0	0	0	0	0	0
ENSG00000248290	TNXA	0	0.05987	0	0	0.08746	0	0.05995	0.04416
ENSG00000250535	STK19B	0.34739	0	0	1.03114	0.42235	1.2257	1.15811	0
ENSG00000224389	C4B	0.0206	0	0.01514	0	0	0.01817	0	0
ENSG00000229776	C4B-AS1	0	0	0	0	0	0	0	0
ENSG00000231852	CYP21A2	0	0	0	0.07479	0	0	0	0

ENSG00000168477	TNXB	0.0563	0.09371	0.04137	0.03482	0.05134	0.09105	0.06256	0.03456
ENSG00000252512	RNA5SP206	0	0	0	0	0	0	0	0
ENSG00000213676	ATF6B	11.3555	7.26717	1.20663	2.55617	8.83862	10.1047	2.13649	2.38517
ENSG00000204315	FKBPL	0.1635	0.09071	0	0	0.33129	0.57686	0.09084	0.13382
ENSG00000204314	PRRT1	0	0	0	0.05106	0	0	0	0
ENSG00000221988	PPT2	0.02493	0	0	0.037	0	0	0	0
ENSG00000258388	PPT2-EGFL8	0.0133	0	0	0	0	0	0	0
ENSG00000241404	EGFL8	0	0	0	0	0	0	0	0
ENSG00000204310	AGPAT1	2.01874	2.04972	0.53397	1.25833	1.52168	1.99434	0.94218	1.06579
ENSG00000284469	MIR6721	0	0	0	0	0	0	0	0
ENSG00000204308	RNF5	5.16463	5.2822	2.56026	2.95513	5.67381	6.25702	1.6595	1.83356
ENSG00000277264	MIR6833	0	0	0	0	0	0	0	0
ENSG00000204305	AGER	1.87045	0.99863	0.05186	0.10475	0.90104	0.62259	0.52943	0.60662
ENSG00000273333	AL662884.1	0	0	0	0	0	0	0	0
ENSG00000204304	PBX2	11.103	9.07199	0.96705	2.05351	6.15447	3.51261	2.44698	3.58414
ENSG00000213654	GPSM3	46.7715	45.2761	3.36969	6.33703	32.3973	14.7167	6.12879	9.02883
ENSG00000204301	NOTCH4	0.09023	0.03755	0	0.01116	0.02743	0	0	0.00923
ENSG00000277427		0	0	0	0	0	0	0	0
ENSG00000225914	TSBP1-AS1	0	0	0	0	0	0	0	0
ENSG00000204296	TSBP1	0	0	0	0	0	0	0	0
ENSG00000237285	HNRNPA1P2	0	0	0	0	0	0	0	0
ENSG00000223335	RNU6-603P	0	0	0	0	0	0	0	0
ENSG00000228962		0	0	0	0	0	0	0	0
ENSG00000204290	BTNL2	0	0	0	0	0	0	0	0
ENSG00000204287	HLA-DRA	169.736	125.726	18.2609	25.7516	128.593	117.795	31.3087	55.8263
ENSG00000196301	HLA-DRB9	0	0	0	0	0	0	0	0
ENSG00000198502	HLA-DRB5	3.72319	3.48596	0	0.51803	70.5153	0.10263	0.58181	0.78569
ENSG00000251916	RNU1-61P	0	0	0	0	0	0	0	0
ENSG00000229391	HLA-DRB6	0.49751	0	0	0	0.06721	0.19504	0.09214	0
ENSG00000196126	HLA-DRB1	16.5805	19.2593	3.06752	5.22242	53.5135	4.41912	5.07014	7.32279
ENSG00000196735	HLA-DQA1	0.03175	3.17042	0	1.30348	4.99167	0.056	0.05291	0
ENSG00000179344	HLA-DQB1	0.02989	17.1668	0	2.04084	4.92468	0.02637	0	0.01835
ENSG00000223534	HLA-DQB1-AS1	0	0	0	0	0	0	0	0
ENSG00000235040	MTCO3P1	0	0	0	0	0	0	0	0
ENSG00000232080	AL662789.1	0	0	0	0	0	0	0	0
ENSG00000226030	HLA-DQB3	0	0	0	0	0	0	0	0
ENSG00000237541	HLA-DQA2	0.14429	0	0.07068	0	0.2339	0	0	0
ENSG00000263649	MIR3135B	0	0	0	0	0	0	0	0
ENSG00000232629	HLA-DQB2	0	0	0	0	0	0	0	0
ENSG00000241106	HLA-DOB	0.1578	0	0	0.17565	0.5276	0.41758	0	0.04844
ENSG00000250264	AL669918.1	0	0	0	0	0	0	0	0
ENSG00000204267	TAP2	0.09177	0	0.08428	0.03405	0.20919	0.28331	0.01912	0.05634
ENSG00000204264	PSMB8	17.7952	21.166	4.34937	6.23647	14.2604	20.4346	6.578	12.517
ENSG00000204261	PSMB8-AS1	1.84096	1.75102	0.71772	0.27879	1.27897	0.59652	0.37575	0.9687
ENSG00000240065	PSMB9	15.9629	22.7574	3.75176	4.03697	12.2129	26.056	4.49426	7.85131
ENSG00000168394	TAP1	15.451	26.1713	6.11744	5.08522	11.4656	28.3593	4.75057	17.85
ENSG00000234515	PPP1R2P1	0	0	0	0	0	0	0	0
ENSG00000235301	HLA-Z	0	0	0	0	0	0	0	0
ENSG00000242574	HLA-DMB	4.47203	2.59481	0.30081	0.63098	3.00565	3.27794	0.76117	1.08267
ENSG00000248993	AL645941.2	0	0	0	0	0	0	0	0
ENSG00000204257	HLA-DMA	13.2484	6.88525	1.06269	2.4041	9.10861	8.87934	1.78401	2.87678
ENSG00000204256	BRD2	13.2874	10.0607	1.27724	2.4168	10.3607	9.21767	2.94751	3.81479
ENSG00000223837	AL645941.1	0	0	0	0	0	0	0	0
ENSG00000263756	AL645941.3	0	0	0	0	0	0	0	0
ENSG00000204252	HLA-DOA	0.60292	0.36493	0	0.29827	1.71036	0.35454	0.48726	0.35891
ENSG00000231389	HLA-DPA1	48.3058	30.7781	4.25384	8.17596	16.4497	26.7712	5.63288	8.31392
ENSG00000223865	HLA-DPB1	47.2211	26.3164	2.00685	6.97694	23.3408	24.2778	4.99053	4.12742
ENSG00000224796	RPL32P1	0	0	0	0	0	0	0	0

ENSG00000231461	HLA-DPA2	0	0	0	0	0	0	0	0
ENSG00000228688	COL11A2P1	0	0	0	0	0	0	0	0
ENSG00000224557	HLA-DPB2	0.04581	0	0.06732	0	0.0557	0	0	0.05625
ENSG00000237398	HLA-DPA3	0	0	0	0	0	0	0	0
ENSG00000230313	HCG24	0	0	0	0	0	0	0	0
ENSG00000204248	COL11A2	0.02175	0.0362	0.03196	0	0.01322	0	0	0.01335
ENSG00000204231	RXRB	1.9821	1.6496	0.16487	0.38852	1.70504	1.08859	0.31168	0.73467
ENSG00000202441	RNY4P10	0	0	0	0	0	0	0	0
ENSG00000112473	SLC39A7	3.37517	2.78754	0.41647	0.57356	2.31797	3.31802	0.9448	1.10717
ENSG00000204228	HSD17B8	1.25193	0.72934	0.09198	0	0.60882	0.22086	0.10434	0.30742
ENSG00000199036	MIR219A1	0	0	0	0	0	0	0	0
ENSG00000204227	RING1	5.32214	2.28179	0.47399	0.59838	2.74507	3.05853	0.73926	1.4851
ENSG00000225463	ZNF70P1	0	0	0	0	0	0	0	0
ENSG00000223457	HTATSF1P1	0	0	0	0	0	0	0	0
ENSG00000272217	AL645940.1	0	0	0.18134	0.36628	0	0.43539	0	0.45453
ENSG00000232940	HCG25	0	0	0	0	0.12972	0.09411	0.17785	0
ENSG00000223501	VPS52	2.44172	2.35726	0.40665	0.86971	1.84052	2.35478	1.08532	1.1392
ENSG00000231500	RPS18	149.459	75.0821	46.7631	61.7293	167.763	139.463	123.146	134
ENSG00000235863	B3GALT4	0	0	0	0	0	0	0	0
ENSG00000227057	WDR46	3.38852	2.78093	0.55327	0.76831	2.03123	2.65681	0.66679	1.24234
ENSG00000284517	MIR6873	0	0	0	0	0	0	0	0
ENSG00000204220	PFDN6	1.68792	1.73531	0.54714	0.6631	1.78052	2.01434	0.78612	0.73144
ENSG00000284064	MIR6834	0	0	0	0	0	0	0	0
ENSG00000237441	RGL2	7.50598	4.83628	0.80649	1.55716	4.51373	1.79402	1.42602	2.55662
ENSG00000231925	TAPBP	18.847	15.3476	1.76825	3.85139	13.0961	19.1052	2.75486	4.59241
ENSG00000236104	ZBTB22	0	0	0.11845	0	0	0.0474	0	0
ENSG00000204209	DAXX	4.40081	3.97746	0.81934	1.50722	4.45462	5.26943	0.79661	1.85813
ENSG00000229596	MYL8P	0	0	0	0	0	0	0	0
ENSG00000228285	LYPLA2P1	0	0	0	0	0	0	0	0
ENSG00000225644	RPL35AP4	0	0	0	0	0	0	0	0
ENSG00000237649	KIFC1	0.24105	0.40122	0.64403	0.26018	0.69269	2.24218	0.62095	0.37667
ENSG00000204194	RPL12P1	0	0	0	0	0	0	0	0
ENSG00000112511	PHF1	3.70986	2.54467	0.53917	0.93781	2.45343	1.2586	1.69884	1.67681
ENSG00000112514	CUTA	6.56421	4.0973	1.0335	3.07337	5.27289	5.65222	2.40974	2.87837
ENSG00000197283	SYNGAP1	0.23579	0.1107	0.04442	0.08973	0.27932	0.22398	0.04031	0.02969
ENSG00000274259	SYNGAP1-AS1	0	0	0	0	0	0	0	0
ENSG00000284256	MIR5004	0	0	0	0	0	0	0	0
ENSG00000213588	ZBTB9	0	0	0	0	0	0	0	0
ENSG00000204188	GGNBP1	0	0	0	0	0	0	0	0
ENSG00000242014	RN7SL26P	0	0	0	0	0	0	0	0
ENSG00000276746	RF00017	0.29203	0	0.42914	0	0	0	0	0
ENSG00000030110	BAK1	5.26888	4.24713	1.16869	1.37703	3.70648	8.82734	1.82276	3.62104
ENSG00000197251	LINC00336	0	0	0	0.10326	0.0423	0	0	0
ENSG00000096433	ITPR3	2.50276	1.80479	0.19644	0.89277	2.37464	2.52859	0.54467	0.88447
ENSG00000137288	UQCC2	0.65656	0.28622	0.18377	0.3016	0.79823	0.85491	0.3648	0.46064
ENSG00000266509	MIR3934	0	0	0	0	0	0	0	0
ENSG00000161896	IP6K3	0	0	0	0	0	0	0	0
ENSG00000161904	LEMD2	4.00625	2.76802	0.6506	1.08978	2.92767	2.20981	1.54795	1.72357
ENSG00000096395	MLN	0	0	0	0	0	0	0	0
ENSG00000249346	LINC01016	0	0	0	0	0	0	0	0.02371
ENSG00000271362	AL138889.2	0	0	0	0	0	0	0	0
ENSG00000233183	AL138889.1	0	0	0	0	0	0	0	0
ENSG00000276824	MIR7159	0	0	0	0	0	0	0	0
ENSG00000221697	MIR1275	0	0	0	0	0	0	0	0
ENSG00000124493	GRM4	0	0	0	0	0	0	0	0
ENSG00000271231	KRT18P9	0	0	0	0	0	0	0	0
ENSG00000214810	CYCSP55	0.23569	0	0	0.34979	0	0	0	0
ENSG00000137309	HMGA1	9.54448	7.18314	1.71268	4.90868	9.22953	26.8404	2.78279	2.97799

ENSG00000276404	MIR6835	2.29064	5.71915	1.68303	0	0	4.04099	0	0
ENSG00000186577	SMIM29	2.86098	2.40842	0.38659	0.58566	1.95905	2.72663	1.04147	1.45352
ENSG00000225339	AL354740.1	0.10614	0.02945	0.05199	0.02625	0.08603	0.03121	0.04423	0.02172
ENSG00000220583	RPL35P2	0.20193	0	0	0	0.2455	0	0	0
ENSG00000272325	NUDT3	7.48174	2.90702	0.72861	1.83415	4.94843	4.30822	2.18333	2.95296
ENSG00000270800	RPS10-NUDT3	0	0	0	0	0	0	0	0
ENSG00000124614	RPS10	1.56243	1.4861	0.59039	0.99378	2.4423	1.73256	1.26496	1.09619
ENSG00000124507	PACSIN1	0.77768	0.17767	0.06722	0.20368	0.31516	1.12983	0.07625	0.11233
ENSG00000124664	SPDEF	0	0	0	0	0	0	0	0
ENSG00000196114	AL031577.1	0.55113	1.22314	0	0	1.11675	0	0	0.22556
ENSG00000196821	C6orf106	21.6954	17.0774	8.09452	12.7646	20.4042	22.93	16.135	13.7636
ENSG00000216636	RPL7P25	0	0	0	0	0	0	0	0
ENSG00000265123	RN7SL200P	0	0.84728	0	0	0	0	0.42424	0
ENSG00000220643	AL031577.2	0	0	0	0	0	0	0	0
ENSG00000272288	AL451165.2	1.61622	2.05721	0.55883	0.98768	1.04027	3.01895	0.95082	0.70037
ENSG00000186328	AL451165.1	0	0	0	0	0	0	0	0
ENSG00000217130	AL139100.1	0	0	0	0	0	0	0	0
ENSG00000124562	SNRPC	13.2595	6.8791	1.60264	2.30009	8.65346	13.5691	3.54007	3.52377
ENSG00000065060	UHRF1BP1	2.61893	1.3915	0.33701	1.03208	1.9248	2.37533	1.25781	0.92649
ENSG00000206717	RF00019	0	0	0	0	0	0	0	0.91834
ENSG00000252106	RNY3P15	0	0	0	0	0	0	0	0
ENSG00000064995	TAF11	5.93826	3.78381	1.63616	2.61637	5.58389	5.07426	4.09845	3.81633
ENSG00000064999	ANKS1A	2.40116	2.23241	0.57219	0.69024	2.14343	1.24026	0.70312	0.84991
ENSG00000187762	HSPE1P11	0.23645	0.39358	0	0.35092	0.28747	0.83427	0	0
ENSG00000273870	AL138721.1	0	0.68161	0	0	0	0	0	0
ENSG00000124678	TCP11	0	0	0	0	0	0.03911	0	0
ENSG00000146197	SCUBE3	0.06975	0.01659	0	0.01479	0	0.05274	0	0
ENSG00000272374	Z97832.2	0.0172	0.02863	0	0	0.02091	0.0607	0	0.02112
ENSG00000065029	ZNF76	1.28483	0.8806	0.11106	0.35252	0.87948	1.25712	0.39593	0.517
ENSG00000217004	Z97832.1	0	0	0	0	0	0	0	0
ENSG00000023892	DEF6	20.5644	21.8609	4.14773	8.67721	21.395	18.9522	6.38506	12.0939
ENSG00000112033	PPARD	1.75973	0.84861	0.26584	0.41493	1.63957	1.16053	0.54826	0.64615
ENSG00000227154	AL022721.1	0	0	0	0	0	0	0	0
ENSG00000112039	FANCE	0.574	0.42994	0.1687	0.21297	1.08168	0.6582	0	0.2819
ENSG00000198755	RPL10A	109.584	61.2075	17.7251	35.1506	111.915	95.2824	50.549	61.3971
ENSG00000276712	MIR7111	1.01806	0	0	0	0	7.18399	0	0
ENSG00000007866	TEAD3	0.07219	0	0	0.03571	0.05851	0	0	0
ENSG00000112041	TULP1	0	0	0	0	0	0	0	0
ENSG00000228559	AL033519.3	0	0	0	0	0	0	0	0
ENSG00000219023	AL033519.2	0.37398	0	1.09912	0.27751	0	0.32988	0.62337	0.68875
ENSG00000096060	FKBP5	7.17278	8.31146	1.0743	3.64393	10.3556	6.88657	4.04662	3.22628
ENSG00000218749	AL033519.1	0	0	0	0	0	0	0	0
ENSG00000212579	RF00561	0	0	0	0	0	0	0	0.7031
ENSG00000265527	MIR5690	6.02469	3.3427	0	5.96086	0	3.54279	5.02111	1.23284
ENSG00000232909	AL157823.2	0	0	0	0	0	0	0	0
ENSG00000157343	ARMC12	0.10339	0.08604	0	0.07672	0.31423	0.09119	0	0
ENSG00000220734	AL157823.1	0	0	0.17152	0.17323	0	0.41182	0	0.42992
ENSG00000196748	CLPSL2	0	0	0	0	0	0	0	0
ENSG00000204140	CLPSL1	0	0	0	0	0	0	0	0
ENSG00000137392	CLPS	0	0	0	0	0	0	0	0
ENSG00000197753	LHFPL5	0	0	0	0.03674	0	0	0	0
ENSG00000096063	SRPK1	20.8225	26.9287	4.7322	7.96548	16.9125	12.1737	9.29315	14.8067
ENSG00000112053	SLC26A8	1.95516	3.31501	0.26768	1.15346	0.60533	0.29993	0.34411	1.10334
ENSG00000271304	DPRXP2	0.65799	1.31427	0	0	0	0	0	0
ENSG00000112062	MAPK14	21.2528	33.9881	4.99315	7.56425	14.134	7.9736	6.82144	16.7925
ENSG00000237719	Z95152.1	0	0	0	0	0	0	0	0
ENSG00000156711	MAPK13	3.2286	2.00042	0.38913	0.63485	2.22885	2.04829	0.93371	1.50058
ENSG00000246982	Z84485.1	0.95195	0.0834	0.36813	1.71023	0.79188	1.06066	1.8373	0.67667

ENSG00000096070	BRPF3	2.70456	2.17708	0.42349	0.85541	1.81926	2.01408	0.97922	0.95264
ENSG00000180316	PNPLA1	1.06302	1.1662	0.10651	0.28684	0.58745	0.38359	0.08054	0.32629
ENSG00000189325	C6orf222	0	0	0	0	0.02385	0	0	0
ENSG00000010030	ETV7	0.40139	6.32142	1.3158	0.73318	0.60062	3.86737	0.10293	3.29812
ENSG00000224666	Z84484.1	0	0.33797	0	0	0	0	0	0.2493
ENSG00000179165	PXT1	0.31295	0.46303	0.1022	0.41285	0.21138	0.12269	0.05796	0.25616
ENSG00000112078	KCTD20	22.9335	14.9211	5.00674	8.26486	15.0837	13.6587	15.8625	13.8768
ENSG00000240733	RN7SL502P	0	0	0	0	0	0	0	0
ENSG00000112079	STK38	61.9445	61.6866	14.9703	26.6184	55.8973	41.5128	15.3409	31.056
ENSG00000239964	RN7SL748P	0	0	0	0	0	0	0	0
ENSG00000112081	SRSF3	20.6987	14.6897	6.24977	12.8684	17.8033	23.3081	10.7968	12.062
ENSG00000263894	MIR3925	0	0	0	0	0	0	0	0
ENSG00000238554	RNU1-88P	0	0.95319	0	0	0	0	0	0
ENSG00000251864	RF00019	0	0	0	0	0	0	0	0
ENSG00000281450	PANDAR	0	0	0	0	0	0	0	0
ENSG00000213500	LAP3P2	0	0	0	0	0	0	0	0
ENSG00000124762	CDKN1A	10.7908	8.6006	2.68402	3.56674	6.45731	7.50432	3.44508	4.66214
ENSG00000255587	RAB44	2.53712	0.80439	0.05072	0.48668	0.77639	0.27403	0.31645	0.72049
ENSG00000220349	Z85996.1	0	0	0	0	0.09511	0	0.1304	0
ENSG00000124772	CPNE5	2.86022	0.94446	0.25525	1.04833	2.18216	1.5117	0.57905	0.90992
ENSG00000137168	PPIL1	2.30835	2.15706	1.19021	3.48595	2.60949	3.28638	2.2951	1.59111
ENSG00000198663	C6orf89	12.493	8.9761	4.31443	10.8786	11.1335	9.84813	12.8173	8.48474
ENSG00000232598	AL122034.1	0	0	0	0	0	0	0	0
ENSG00000164530	PI16	0.34576	0.15696	0.13857	0.27989	0.57322	0	0	0.15437
ENSG00000137409	MTCH1	14.2802	10.5772	2.03299	4.48924	9.35069	8.14921	7.50436	8.66574
ENSG00000146192	FGD2	5.13326	3.73214	0.80518	1.16629	2.79613	1.86966	0.87727	1.434
ENSG00000226976	COX6A1P2	0.22212	0	0	0	0.5401	0.39185	0	0
ENSG00000216412	RPL12P2	0	0	0	0	0	0	0	0
ENSG00000137193	PIM1	130.153	81.8035	47.0005	50.1723	113.364	112.371	107.816	74.1259
ENSG00000172738	TMEM217	0.21425	0.31204	0.23613	0.35772	0.42328	1.37013	0.2232	0.13153
ENSG00000065491	TBC1D22B	14.461	13.7092	10.6096	12.6634	17.7358	15.5757	25.0219	11.88
ENSG00000275550	AL096712.1	0	0	0	0	0	0	0	0
ENSG00000112130	RNF8	1.67225	1.0206	0.45871	0.64527	1.38249	1.35704	0.72472	0.87602
ENSG00000239953	RN7SL273P	0	0	0	0	0	0	0	0
ENSG00000137200	CMTR1	6.49057	7.2872	2.00151	2.26895	5.103	5.66383	2.27032	3.8224
ENSG00000198937	CCDC167	3.07885	0.78842	0.34803	1.05447	3.74319	7.31165	0.59215	1.30852
ENSG00000204110	LINC02520	0.819	0.2272	0.40117	2.43097	0.33191	0.2408	0	0
ENSG00000229559	AL353597.2	0	0	0	0	0	0	0	0
ENSG00000227920	AL353597.1	0.44068	0	0.10793	0.32701	0.17859	0	0	0
ENSG00000263926	MIR4462	0	0	0	0	0	0	0	0
ENSG00000279942	AL353597.3	0	0	0	0	0.07139	0	0	0
ENSG00000112139	MDGA1	0.36167	0.1634	0.05315	0.06901	0.13819	0	0.02584	0.08247
ENSG00000225945	AL121574.1	1.92435	2.03833	0.51415	0.7789	0.63807	1.5431	0.2916	0.85916
ENSG00000156639	ZFAND3	10.6972	12.5757	3.09584	6.36111	8.43473	8.52253	7.14434	7.29163
ENSG00000243313	RN7SL285P	0	0	0	0	0	0	0	0
ENSG00000200597	RNU1-87P	0.86236	0	0.63361	0	0.52422	0	0.71871	1.05879
ENSG00000218521	AL589655.1	0	0	0	0	0	0	0	0
ENSG00000183826	BTBD9	2.69006	1.34328	0.42918	0.88972	1.98099	1.50504	0.93522	1.32113
ENSG00000200706	RF00279	0	0	0	0	0	0	0	0
ENSG00000226533	BTBD9-AS1	0	0	0	0	0	0	0	0
ENSG00000199938	RF00019	0	0	0	0	0	0	0	0
ENSG00000217227	AL079341.1	0	0	0	0	0	0	0	0
ENSG00000124767	GLO1	9.97284	6.84742	2.88519	6.89161	8.44941	9.73137	5.66227	6.88753
ENSG00000236355	AL391415.1	0	0	0	0	0	0	0	0
ENSG00000124721	DNAH8	0.04869	0	0.00715	0.00723	0.02368	0	0.01623	0.01793
ENSG00000244297	RN7SL465P	0	0	0	0	0	0	0	0
ENSG00000219273	AL035555.1	0	0	0	0	0	0	0	0
ENSG00000212586	RF00393	0	0	0	0	0	0	0	0

ENSG00000231150	AL034345.2	0.06043	0	0	0	0	0	0	0
ENSG00000220076	AL034345.1	0	0	0	0	0	0	0	0
ENSG00000220556	AL035690.1	0	0	0	0	0	0	0	0
ENSG00000112164	GLP1R	0	0	0	0	0	0	0	0
ENSG00000112167	SAYSD1	2.15516	1.45993	0.4419	1.19013	2.34598	1.72416	1.21136	1.07689
ENSG00000217165	ANKRD18EP	0	0	0	0	0.03386	0	0	0
ENSG00000164626	KCNK5	0.17564	0.25987	0	0.02896	0.21354	0.8607	0.03253	0.04792
ENSG00000124780	KCNK17	0.37123	0	0	0.05009	0.08206	0	0	0.04144
ENSG00000095981	KCNK16	0	0	0	0	0	0	0	0
ENSG00000164627	KIF6	0.01853	0	0	0	0.03754	0	0	0
ENSG00000218986	AL136087.1	0	0	0	0	0	0	0	0
ENSG00000231276	E2F4P1	0	0	0	0	0	0	0	0
ENSG00000252990	RNU1-54P	0	0	0	0	0	0	0	0
ENSG00000146122	DAAM2	1.06938	0.16952	0.01663	0.03359	0.5228	0	0.01886	0.09031
ENSG00000281969	AL592158.1	0.13098	0.0545	0	0	0	0	0.05458	0
ENSG00000235033	AL590999.1	0	0	0	0	0	0	0	0
ENSG00000124615	MOCS1	0	0	0	0.04205	0.03445	0	0	0.03479
ENSG00000180211	FO393411.1	0	0.87149	0	1.03605	0.42437	0.92366	0	0.21428
ENSG00000220586	TUBBP9	0	0	0	0	0	0	0	0
ENSG00000227131	AL139275.2	0	0	0	0	0	0	0	0
ENSG00000204091	TDRG1	0	0	0	0	0	0	0	0
ENSG00000204092	LINC00951	0	0	0	0	0	0	0	0.16245
ENSG00000226070	AL139275.1	0	0	0.13072	0	0	0	0	0
ENSG00000156564	LRFN2	0.08966	0.03731	0.13176	0.06654	0.10901	0.07909	0	0.02752
ENSG00000252767	RNU6-250P	0	0	0	0	0	0	0	0
ENSG00000236075	AL359475.1	0	0	0	0	0	0	0	0
ENSG00000226454	AL033380.1	0.34822	0.28981	0	0.7752	0	0	0	0
ENSG00000237947	AL583854.1	0	0	0	0	0	0	0	0
ENSG00000124602	UNC5CL	0.35061	0.07781	0	0.17345	0.22734	0.08247	0.11688	0.22958
ENSG00000124596	OARD1	4.00045	2.95529	0.94124	2.31814	3.7297	2.61676	1.94799	1.83499
ENSG00000112212	TSPO2	0.44514	0.37047	2.61653	1.32128	1.35299	1.04706	1.48397	0.72872
ENSG00000124701	APOBEC2	0.06368	0.106	0.09358	0.47257	0.69683	0	0.10615	0.15638
ENSG00000001167	NFYA	8.86682	8.38788	1.32841	3.02579	8.41826	5.25934	3.30217	4.60494
ENSG00000161912	ADCY10P1	0.62563	0.87134	0.09381	0.24634	0.45016	0.40544	0.31923	0.36055
ENSG00000161911	TREML1	23.1813	13.4102	1.67055	4.18124	15.5639	22.8454	6.92053	8.67807
ENSG00000095970	TREM2	0	0.11142	0	0	0	0	0.33474	0.08219
ENSG00000112195	TREML2	22.1544	26.4561	2.64229	3.78872	10.7415	3.21126	3.9592	7.76777
ENSG00000184106	TREML3P	8.61916	2.99407	0.44055	1.11233	2.5514	1.45443	2.6235	1.10426
ENSG00000188056	TREML4	1.86494	0	0	0.3209	0	0.28609	0	0
ENSG00000212176	RNA5SP207	0	0	0	0	0	0	0	0
ENSG00000225690	TREML5P	0.25452	0	0	0	0	0	0	0
ENSG00000274256	AL391903.2	0	0	0	0	0	0	0	0
ENSG00000278745	AL391903.3	0	0	0	0	0	0	0	0
ENSG00000124731	TREM1	64.0045	76.7732	10.8485	11.8863	47.3683	5.40666	14.5916	31.4543
ENSG00000218809	AL391903.1	6.62715	9.69383	2.36085	1.78826	7.81297	0.70856	1.00422	7.64359
ENSG00000206745	RNU6-643P	0	0	0	0	0	0	0	0
ENSG00000213461	AL672212.1	0	0	0	0	0	0	0	0
ENSG00000096264	NCR2	0.06013	0	0	0	0	0	0	0
ENSG00000279284	AL136967.1	0	0	0	0	0	0	0	0
ENSG00000280371	AL136967.2	0	0	0	0	0	0	0	0
ENSG00000234753	FOXP4-AS1	0	0	0	0	0	0	0	0
ENSG00000226917	LINC01276	0	0	0	0	0	0	0	0
ENSG00000137166	FOXP4	0.57025	0.49436	0.03492	0.12342	0.27443	0.48204	0.17822	0.14586
ENSG00000266494	MIR4641	0	0	0	0	0	0	0	0
ENSG00000112559	MDFI	0	0	0	0	0	0	0	0
ENSG00000230913	NPM1P51	0	0	0	0	0	0	0	0
ENSG00000112561	TFEB	5.55463	5.91897	0.82006	1.30974	3.64482	1.69424	0.9302	1.92805
ENSG00000231102	AL035588.1	0	0	0	0	0	0	0.29946	0

ENSG00000096088	PGC	0.0362	0	0	0	0	0	0	0
ENSG00000269387	AL365205.3	0	0	0	0	0	0	0	0
ENSG00000137218	FRS3	0.76942	0.38421	0.11307	0.38064	0.40536	0.27147	0.29925	0.12596
ENSG00000124593	AL365205.1	0.09819	0.16344	0.02405	0.07286	0.11938	0.05774	0.05456	0.06028
ENSG00000278224	PRICKLE4	0	0	0	0	0	0	0	0
ENSG00000214736	TOMM6	0	0	0	0	0	0	0	0
ENSG00000164663	USP49	0.89268	1.77633	2.39671	4.73155	2.17875	1.50376	4.15062	2.30741
ENSG00000268745	AL365205.2	2.15589	1.79424	1.58403	0	2.62108	0	0	0
ENSG00000206977	RF00393	0	0	0	0	0	0	0	0
ENSG00000227516	AL160163.1	0	0	0	0	0	0	0	0
ENSG00000124641	MED20	2.50447	2.08434	0.95293	1.12834	1.54962	2.84028	0.74546	1.72966
ENSG00000207371	RF00019	0	0	0	0	0	0	0	0
ENSG00000112578	BYSL	0.55432	0.81411	0.43124	0.43553	0.39643	1.4956	0.65221	0.48041
ENSG00000112576	CCND3	19.519	18.1898	4.38133	9.65609	14.6531	15.7572	8.44438	8.34513
ENSG00000206875	RNU6-761P	2.05515	0	0	1.01669	0	0	2.28374	0
ENSG00000223946	AL513008.1	0	0	0	0	0	0	0	0
ENSG00000137413	TAF8	2.64437	2.68035	0.61478	1.34723	2.62955	3.62062	1.2368	1.56036
ENSG00000261068	AL512274.1	0	0	0	0	0	0	0	0
ENSG00000188112	C6orf132	0.0107	0	0	0	0.02601	0	0	0
ENSG00000048545	GUCA1A	0	0	0	0	0	0	0	0
ENSG00000214732	AL096814.1	0	0	0	0	0	0	0	0
ENSG00000112599	GUCA1B	0	0.21924	0.19356	0.09774	0.24021	0.11618	0.10978	0.16172
ENSG00000048544	MRPS10	10.665	5.60892	1.83777	2.68098	9.79861	8.57993	4.11128	4.52118
ENSG00000124496	TRERF1	6.81621	3.69245	0.57117	1.82904	5.41708	6.23814	2.19646	2.02528
ENSG00000237107	AL591473.1	0	0	0	0	0	0	0	0
ENSG00000024048	UBR2	39.3699	46.4985	28.0739	58.0232	42.7841	38.4278	68.6919	50.8728
ENSG00000206848	RNU6-890P	3.42525	2.28053	2.01334	1.01669	1.66573	0	2.28374	6.72875
ENSG00000112619	PRPH2	0.02463	0	0	0	0	0	0	0
ENSG00000213435	ATP6V0CP3	0.31392	0	0	0	0	0.2769	0	0
ENSG00000124659	TBCC	12.882	12.08	1.99964	3.16394	12.408	12.7231	4.46079	7.74108
ENSG00000112624	BICRAL	10.3582	9.46756	2.70741	5.26203	10.1449	11.0169	5.69568	6.9441
ENSG00000146223	RPL7L1	6.6082	4.43724	1.02072	2.53541	6.12827	6.55749	3.64554	3.35371
ENSG00000221821	C6orf226	2.12465	0.44206	0.39027	0.19708	2.58309	1.40556	1.10671	0.65215
ENSG00000171611	PTCRA	4.64403	3.3427	1.10665	1.58335	2.06006	6.64273	2.09213	3.31325
ENSG00000137161	CNPY3	54.4615	50.9556	5.97712	13.5982	39.6652	20.9225	13.3456	19.8708
ENSG00000231113	AL035587.1	3.49284	2.53843	0.86749	1.09516	2.33258	2.08287	1.722	2.11402
ENSG00000181524	RPL24P4	0.52233	0.86942	0	0	0.42336	0	0.58043	0.21377
ENSG00000124713	GNMT	0.20513	0.11381	0.20096	0	0	0.24125	0.11397	0.08395
ENSG00000124587	PEX6	1.84351	2.82443	0.30784	1.83434	2.19035	3.54785	0.80313	1.13171
ENSG00000112640	PPP2R5D	5.61807	3.23982	1.00758	1.24739	4.84037	6.2822	1.36411	1.60224
ENSG00000124733	MEA1	12.7889	11.6519	2.86842	3.49633	11.5385	11.6369	4.26341	5.86758
ENSG00000124702	KLHDC3	14.5947	8.8228	2.24071	3.77167	12.3589	12.2971	5.1438	6.28508
ENSG00000124541	RRP36	9.54363	7.39111	1.55825	5.1147	9.22591	8.30121	4.75023	3.62104
ENSG00000272223	AL136304.1	0.72004	0	0	0.21372	0	0	0.24004	0
ENSG00000240625	RN7SL403P	0.24033	0	0	0	0	0	0	0
ENSG00000044090	CUL7	1.32847	0.7154	0.13397	0.28994	1.02924	1.12585	0.2388	0.49572
ENSG00000137171	KLC4	1.1644	0.60805	0.0671	0.22025	0.68007	0.54375	0.30445	0.40647
ENSG00000112651	MRPL2	2.16523	1.53772	0.50909	0.557	1.26357	3.00488	0.91431	0.92159
ENSG00000272170	AL355385.1	0	0	0	0	0	0	0	0
ENSG00000112655	PTK7	0.21932	0.16225	0.04775	0.02411	0.21726	0.10031	0.12186	0.11968
ENSG00000112658	SRF	5.39024	4.32634	0.51268	1.26856	4.687	6.24709	1.33753	1.86334
ENSG00000112659	CUL9	1.69923	1.08554	0.20222	0.63934	0.94565	1.04497	0.55849	0.58034
ENSG00000245261	AL133375.1	0	0	0	0	0.09094	0.5278	0.24935	0
ENSG00000112667	DNPH1	1.40737	1.07367	0.43086	0.26109	1.49716	1.96554	0.48872	0.79197
ENSG00000146216	TTBK1	0	0	0	0	0	0	0	0
ENSG00000137204	SLC22A7	0	0	0	0	0	0	0	0
ENSG00000146215	CRIP3	0.08965	0	0.04391	0	0.07266	0.05272	0.09962	0.03669
ENSG00000171467	ZNF318	3.91845	2.34853	0.45073	1.24047	4.36307	2.94905	1.06088	1.37458

ENSG00000219222	RPL12P47	0	0	0	0	0	0	0.96205	0
ENSG00000220614	AL583834.1	0	0	0	0	0	0	0	0
ENSG00000179157	RPS2P28	0	0	0	0	0	0	0	0
ENSG00000226558	AL590383.1	0	0	0	0	0	0	0	0
ENSG00000124574	ABCC10	1.11542	0.86038	0.17324	0.43067	1.16867	1.24784	0.37789	0.36743
ENSG00000276770	MIR6780B	0.92785	0	0	0	0	0	1.54659	0
ENSG00000171462	DLK2	0	0	0	0	0.03986	0	0	0
ENSG00000207076	RNU6-1113P	0	0	0	0	0	0	0	0
ENSG00000137221	TJAP1	1.3701	0.83258	0.33556	0.45186	0.88575	1.05506	0.41687	0.60078
ENSG00000204052	LRRC73	0.03456	0.05752	0	0	0	0	0.05761	0
ENSG00000171453	POLR1C	1.61072	0.51067	0.18785	0.41739	0.96359	1.48842	1.10802	0.91033
ENSG00000137207	YIPF3	18.3191	19.0226	4.12792	5.73682	15.9669	12.749	5.80286	9.13825
ENSG00000271754	AL355802.2	0.1345	0.44774	0	0.19961	0.49055	0.23727	0	0
ENSG00000124571	XPO5	2.15033	1.90415	0.88477	2.33605	2.46791	3.53551	1.70611	1.73193
ENSG00000219470	AL355802.1	0	0	0.12212	0	0	0	0	0.10204
ENSG00000252218	RF00426	0	0	0	0	0	0	0	0
ENSG00000271970		0	0	0	0	0.32524	0.47194	0	0.16423
ENSG00000170734	POLH	2.98171	1.93008	0.60855	1.90529	4.30479	3.4337	0.89737	1.24572
ENSG00000203362	POLH-AS1	0	0	0	0	0	0	0	0
ENSG00000172432	GTPBP2	10.9855	9.02398	1.91015	2.44674	6.22506	7.27099	2.61588	4.00938
ENSG00000124688	MAD2L1BP	3.78199	3.3747	1.71884	2.141	2.27532	2.88888	3.1195	2.68076
ENSG00000172426	RSPH9	0	0.13912	0.2047	0	0.03387	0	0.04644	0.20524
ENSG00000096080	MRPS18A	6.33671	4.276	1.00667	3.05007	5.48304	7.25113	1.61765	2.66346
ENSG00000218107	AL136131.1	0	0	0	0	0	0	0	0
ENSG00000236961	AL136131.2	0	0	0	0	0	0	0	0
ENSG00000112715	VEGFA	1.22921	0.64255	0.0821	0.3543	0.41992	0.19714	0.1778	0.36171
ENSG00000272114	AL136131.3	0	0	0	0	0	0	0	0
ENSG00000283573	AL157371.2	0	0.25418	0	0	0	0	0	0
ENSG00000283215	LINC02537	0.10724	0	0	0	0	0	0	0
ENSG00000223469	AL157371.1	0	0	0	0	0	0	0	0
ENSG00000237686	AL109615.3	0	0	0	0	0	0	0	0
ENSG00000181577	C6orf223	0	0	0	0	0	0	0	0
ENSG00000231881	AL109615.2	0	0	0	0	0	0	0	0
ENSG00000183239	AL109615.1	0	0	0	0	0	0.5684	0.26853	0
ENSG00000180992	MRPL14	4.35675	3.56229	1.68479	3.17623	3.99585	4.58457	2.67548	2.81534
ENSG00000137216	TMEM63B	11.7446	16.5711	14.0941	10.4483	20.1394	22.9401	6.73717	12.9962
ENSG00000137225	CAPN11	0.16523	0.24065	0.03035	0	0.12555	0.10931	0.06885	0
ENSG00000265439	RN7SL811P	0	0	0	0	0	0	0	0
ENSG00000262179	MYMX	0.08695	0	0	0	0	0.15339	0	0
ENSG00000112759	SLC29A1	8.33508	1.57407	0.77562	0.78334	3.52937	1.82348	1.57628	1.37709
ENSG00000096384	HSP90AB1	67.9873	54.1669	14.0606	27.7075	65.9404	70.0367	66.5432	28.9621
ENSG00000157593	SLC35B2	2.6367	1.85878	0.63817	0.78263	2.33823	2.84563	0.82729	1.37109
ENSG00000284427	MIR4647	0	0	0	0	0	0	0	0
ENSG00000146232	NFKBIE	7.46919	4.20719	0.79293	0.80082	2.69318	2.60527	1.37281	1.4645
ENSG00000178233	TMEM151B	0	0	0	0	0	0	0	0
ENSG00000272442	AL353588.1	0	0	0	0	0	0	0	0
ENSG00000146221	TCTE1	0	0	0	0	0	0	0.03984	0
ENSG00000124608	AARS2	2.16592	0.90839	0.25061	0.65808	1.70022	1.71493	0.6254	0.75381
ENSG00000249481	SPATS1	0	0	0	0	0	0	0	0
ENSG00000096401	CDC5L	12.5077	12.2521	2.6329	5.57224	12.5288	8.4557	4.05175	8.26689
ENSG00000266619	MIR4642	0	0	0	0	0	1.57697	0	0
ENSG00000237530	AL136140.1	0	0	0	0	0	0	0	0
ENSG00000279076	AL136140.2	0	0	0	0	0	0	0	0
ENSG00000196284	SUPT3H	0.80323	0.99318	0.43841	0.81743	1.25556	0.97166	0.5738	0.70442
ENSG00000270900	NUDT19P4	0	0	0	0	0	0	0	0
ENSG00000276156	AL138880.2	0	0	0	0	0	0	0	0
ENSG00000277769	RBM22P4	0	0	0	0	0	0	0	0
ENSG00000219384	AL138880.1	0	0	0	0	0	0	0	0

ENSG00000207769	MIR586	0.75567	0	0	0	0	0	0	0
ENSG00000124813	RUNX2	3.69544	2.18936	0.43391	1.0358	2.20834	1.73636	0.76064	1.13155
ENSG00000271857	AL096865.1	0.07907	0.39485	0.34859	0.35206	0.09613	0.27899	0.1318	0.09708
ENSG00000261080	RUNX2-AS1	0	0	0	0	0	0	0	0
ENSG00000112782	CLIC5	0.19553	0.27122	0.08381	0.22974	0.1882	0.12935	0	0.11003
ENSG00000212468	RNU6-754P	0	0	0	0	0	0	0	0
ENSG00000231769	AL035701.1	1.48783	0.4953	0	0.73603	0.12059	0.34996	0	0.48713
ENSG00000001561	ENPP4	5.20084	6.87298	2.33909	5.4732	4.71355	6.83954	2.81086	4.39246
ENSG00000112796	ENPP5	0.48133	0.65811	0.25261	0.66333	0.50159	1.1524	0.3725	0.48544
ENSG00000229349	ACTG1P9	0	0.0459	0	0	0	0	0	0
ENSG00000172348	RCAN2	0.01975	0.29582	0.05804	0.02931	0	0.45287	0	0.02424
ENSG00000226541	AL359633.1	0	0	0	0	0	0	0	0
ENSG00000236466	AL359633.2	0	0	0	0	0	0	0	0
ENSG00000146233	CYP39A1	0.02356	0	0	0	0	0	0	0
ENSG00000153291	SLC25A27	0	0	0	0.06617	0	0.07866	0	0.05474
ENSG00000242973	AL591242.1	0	0	0	0	0	0	0	0
ENSG00000180113	TDRD6	0	0	0	0	0	0	0	0
ENSG00000146070	PLA2G7	9.87709	6.44369	0.1595	0.91281	3.21102	2.42539	0.66337	2.70968
ENSG00000230062	ANKRD66	0	0	0	0	0	0	0	0
ENSG00000236164	AL161618.1	0	0	0	0	0	0	0	0
ENSG00000112818	MEP1A	0.0246	0.08188	0	0	0	0	0	0
ENSG00000069122	ADGRF5	0.01102	0	0	0	0	0	0	0
ENSG00000225730	AL096772.1	0	0	0	0	0	0	0	0
ENSG00000153292	ADGRF1	0	0	0	0	0.02156	0	0	0
ENSG00000146072	TNFRSF21	1.52921	0.61089	0.26966	0.57495	0.39662	0.14388	0.84965	0.25034
ENSG00000271328	B3GNTL1P2	0	0	0	0	0	0	0	0
ENSG00000270761	AL355353.1	0.44424	0	0	0.65931	0	0	0.37024	0
ENSG00000198087	CD2AP	14.3865	14.8196	9.82844	15.9851	17.7415	19.844	18.7134	11.5579
ENSG00000199762	RF00019	0	0	0	0	0	0	0	0
ENSG00000216813	AL358178.1	0.36927	0.61465	0	0.27402	0.44895	0	0.30776	0
ENSG00000164393	ADGRF2	0	0	0	0	0	0	0	0
ENSG00000153294	ADGRF4	0	0.03598	0	0	0	0	0	0
ENSG00000252711	RN7SKP116	0	0	0	0	0	0	0	0
ENSG00000226594	AL356421.2	0	0	0	0	0	0	0	0
ENSG00000216616	AL356421.1	0	0	0	0	0	0	0	0
ENSG00000124818	OPN5	0	0	0	0	0	0	0	0
ENSG00000200340	RNU1-105P	0	0	0	0	0	0	0	0
ENSG00000244694	PTCHD4	0	0	0	0	0	0	0	0
ENSG00000226068	HNRNPA3P4	0	0	0	0	0	0	0	0
ENSG00000216835	RBMXP1	0	0	0	0	0	0	0	0
ENSG00000278736	FO393412.1	0	0	0	0	0	0	0	0
ENSG00000271162	AL022099.1	0	0	0	0	0	0	0	0
ENSG00000218337	AL589994.1	0	0	0	0	0	0	0	0
ENSG00000274867	AL589994.2	0	0	0	0	0	0	0	0
ENSG00000217631	FO393413.1	0	0	0	0	0	0	0	0
ENSG00000252457	RNU7-65P	0	0	0	0	0	0	0	0
ENSG00000277043	EEF1A1P42	0	0	0	0	0	0	0	0
ENSG00000146085	MUT	3.85174	2.44082	1.14926	1.53791	2.90004	2.93185	1.46655	2.16051
ENSG00000031691	CENPQ	1.69187	1.40806	1.67817	2.25983	2.05693	4.10395	1.62155	2.70043
ENSG00000203972	GLYATL3	0	0	0	0	0	0	0	0
ENSG00000197261	C6orf141	0	0	0	0	0	0	0	0
ENSG00000216998	CYP2AC1P	0	0	0	0	0	0	0	0
ENSG00000112077	RHAG	7.74407	7.72125	20.7879	47.4515	10.5803	10.9563	27.4139	14.1679
ENSG00000124490	CRISP2	0.24144	0.16075	0.28383	0.28666	0.41095	0.25556	0.16098	0.41501
ENSG00000279127	AL121950.1	0	0	0	0	0	0	0	0
ENSG00000096006	CRISP3	2.87069	2.71492	1.77366	1.98496	2.06234	6.84829	0.92437	3.56464
ENSG00000170950	PGK2	0	0	0	0	0	0	0	0
ENSG00000235122	AL121974.1	0	0	0	0	0	0	0	0

ENSG00000226707	AL359458.1	0	0	0	0	0	0	0	0
ENSG00000124812	CRISP1	0	0	0	0	0	0	0	0
ENSG00000214643	DEFB133	0	0	0	0	0	0	0	0
ENSG00000177684	DEFB114	0	0	0	0	0	0	0	0
ENSG00000214642	DEFB113	0	0	0	0	0	0	0	0
ENSG00000203970	DEFB110	0	0	0	0	0	0	0	0
ENSG00000180872	DEFB112	0	0	0	0	0	0	0	0
ENSG00000226733	AL138826.1	0	0	0	0	0	0	0	0
ENSG00000233470	AL360175.1	0	0	0	0	0	0	0	0
ENSG00000230472	AL135908.1	0	0	0	0	0	0	0	0
ENSG00000008197	TFAP2D	0	0	0	0	0	0	0	0
ENSG00000008196	TFAP2B	0	0	0	0	0	0	0	0
ENSG00000060303	RPS17P5	0	0	0	0	0.21842	0	0	0
ENSG00000216913	AL049693.1	0	0	0	0	0	0	0	0
ENSG00000230204	FTH1P5	0	0	0	0	0	0	0	0
ENSG00000270306	AL158050.2	0	0	0	0	0	0	0	0
ENSG00000232702	AL158050.1	0.16039	0	0	0.23804	0.195	0.28296	0	0.19693
ENSG00000212532	RF00572	0	0	0	0	0	0	0	0
ENSG00000214641	AL445529.1	0	0	0	0	0	0	0	0
ENSG00000228689	AL355997.1	0	0	0	0	0	0	0	0
ENSG00000170927	PKHD1	0	0.00699	0	0	0	0	0	0
ENSG00000240641	RN7SL580P	0	0	0	0	0	0	0	0
ENSG00000207604	MIR206	0	0	0	0	0	0	0	0
ENSG00000225613	LINCMD1	0	0	0	0	0	0	0	0
ENSG00000199080	MIR133B	0	0	0	0	0	0	0	0
ENSG00000112115	IL17A	0	0	0	0	0	0	0	0
ENSG00000112116	IL17F	0	0	0	0	0	0	0	0
ENSG00000218363	SLC25A20P1	0	0	0	0	0	0	0	0
ENSG00000112118	MCM3	6.06485	5.01405	1.06238	3.06984	6.66544	13.6043	2.04192	3.25469
ENSG00000170915	PAQR8	3.30482	1.64061	0.5112	0.94652	2.76672	1.68768	0.62818	1.69067
ENSG00000096093	EFHC1	0.12574	0.17172	0.13739	0.21053	0.14895	0.11375	0.17734	0.12271
ENSG00000065308	TRAM2	2.2283	1.44828	0.48337	0.86613	1.19975	1.23546	0.49523	0.7947
ENSG00000225791	TRAM2-AS1	0.48944	0.62298	0.16923	0.34182	0.35003	0.35553	0.33592	0.31814
ENSG00000270382	AL109918.2	0	0	0	0	0	0	0	0
ENSG00000216775	AL109918.1	0.86832	0.44162	0.38988	0.28637	0.67446	1.82968	0.68347	0.59228
ENSG00000096092	TMEM14A	1.99507	1.35292	1.73732	1.75461	1.70687	1.04284	0.86216	0.63506
ENSG00000220377	GSTA8P	0	0	0	0	0	0	0	0
ENSG00000271227	AL109918.3	0	0	0	0	0	0	0	0
ENSG00000237674	GSTA7P	0.9817	0.27234	0.48087	0.48565	0	0	2.45452	0
ENSG00000244067	GSTA2	0	0	0	0	0	0	0	0
ENSG00000217228	GSTA12P	0	0	0	0	0	0	0	0
ENSG00000243955	GSTA1	0.6474	0.11973	1.16276	0.10676	0.34982	0.2538	0.95922	0.17664
ENSG00000223622	GSTA6P	0.88162	0.20964	0.18508	0	0.15312	0	0.6298	0.4639
ENSG00000182793	GSTA5	0	0	0	0	0.19124	0	0	0
ENSG00000230375	GSTA11P	0	0	0	0	0	0	0	0
ENSG00000230845	GSTA10P	0	0	0	0	0	0	0	0
ENSG00000174156	GSTA3	0	0	0	0	0	0	0	0
ENSG00000243236	GSTA9P	0	0	0	0	0	0	0.13744	0
ENSG00000170899	GSTA4	0.30331	0.11219	0.04952	0.75025	0.20487	0	0.28087	0.08276
ENSG00000202198	RF00100	0	0	0	0	0	0	0	0
ENSG00000283293	RN7SK	0	0	0	0	0	0	0	0
ENSG00000112144	ICK	3.94667	2.53413	0.89489	1.84235	3.51683	3.22298	1.6983	2.18558
ENSG00000112146	FBXO9	18.7112	14.1377	16.7033	24.3848	18.2403	17.6766	26.8635	22.8079
ENSG00000242865	RN7SL244P	0	0	0	0	0	0	0	0
ENSG00000261745	AL512347.1	0	0	0	0	0	0	0.2429	0
ENSG00000137270	GCM1	0.02651	0.08825	0	0	0.25784	0.04677	0.13256	0.09765
ENSG00000253026	RNU6-464P	0	0	0	0	0	0	0	0
ENSG00000219074	SOD1P1	0.16659	0	0	0.24724	0	0	0	0

ENSG00000218713	AL512378.1	0.06981	0	0	0.0518	0	0.12315	0.11636	0.04286
ENSG00000206908	RNU1-136P	0	0	0	0	0	0	0	0
ENSG00000219163	HMGB1P20	0.32482	0.72088	0.1591	0.16069	0	0	0	0.53174
ENSG00000012660	ELOVL5	91.5245	99.1939	16.6105	30.6985	70.3491	32.7264	24.2887	44.6091
ENSG00000264056	MIR5685	0	0	0	0	0	0	0	0
ENSG00000217527	RPS16P5	3.08442	5.13402	0	0	2.64703	2.88071	0.30243	1.33659
ENSG00000271367	AL034374.1	0	0	0	0	0	0	0	0
ENSG00000219863	RPL31P28	0	0	0	0	0	0	0	0
ENSG00000218049	RPL31P33	0	0	0	0	0	0	0	0
ENSG00000218261	AL591034.1	0	0	0	0	0	0	0	0
ENSG00000271338	AL591034.2	0	0	0	0	0	0	0	0
ENSG00000253010	RN7SKP256	0	0	0	0	0	0	0	0
ENSG00000271373	NANOGP3	0	0	0	0	0	0	0	0
ENSG00000001084	GCLC	5.65581	6.58843	6.32563	12.2116	6.79195	8.52605	17.7142	7.07173
ENSG00000249379	AL033397.2	0	0	0	0	0	0	0	0
ENSG00000231683	AL033397.1	0	0	0	0	0	0	0	0
ENSG00000235899	LINC01564	0	0	0	0	0	0	0	0
ENSG00000124743	KLHL31	0	0	0	0.01894	0.03103	0	0.02127	0.01567
ENSG00000227885	AL590652.1	0	0	0	0.25597	0	0	0	0
ENSG00000228614		0	0	0	0	0	0	0	0
ENSG00000137269	LRRC1	0.35924	0.19932	0.13198	0.1555	0.43676	0.05281	0.0499	0.1654
ENSG00000271218	AL033384.2	0	0	0	0	0	0	0	0
ENSG00000146147	MLIP	0	0	0	0	0	0	0	0
ENSG00000236740	AL033384.1	0	0	0	0	0	0	0	0
ENSG00000235050	MLIP-AS1	0	0	0	0	0	0	0	0
ENSG00000236996	MLIP-IT1	0	0	0	0	0	0	0	0
ENSG00000218143	ERHP2	0	0	0	0	0	0	0	0
ENSG00000137251	TINAG	0	0	0	0	0	0	0	0
ENSG00000228231	AL589946.1	0	0	0	0	0	0	0	0
ENSG00000213335	CLNS1AP1	0.21496	0	0	0.15951	0	0	0	0
ENSG00000216347	RPL10P10	0	0	0	0	0	0	0	0
ENSG00000220773	RPSAP44	0	0	0	0	0	0	0	0
ENSG00000220635	KRASP1	0	0	0	0	0	0	0	0
ENSG00000251946	RNU6-1023P	0	0	0	0	0	0	0	0
ENSG00000224984	AL512363.1	0	0	0	0	0	0	0	0
ENSG00000168143	FAM83B	0	0	0	0	0	0	0	0
ENSG00000261116	AL049555.1	0	0	0	0	0	0	0	0
ENSG00000137252	HCRTR2	0	0	0	0.05539	0	0	0	0
ENSG00000187871	GFRAL	0	0	0	0	0	0	0	0
ENSG00000146151	HMGCLL1	0	0	0	0	0	0	0	0
ENSG00000220725	AL590290.1	0	0	0	0	0	0	0	0
ENSG00000112175	BMP5	0	0	0	0	0	0	0	0
ENSG00000214604	NPM1P36	0	0	0	0	0	0	0	0
ENSG00000124749	COL21A1	0	0	0	0	0	0	0	0
ENSG00000219095	DHFRP6	0	0	0	0	0	0	0	0
ENSG00000227602	AL031779.1	0	0	0	0	0	0	0	0
ENSG00000220666	RCC2P7	0	0	0	0	0	0	0	0
ENSG00000151914	DST	0.65612	0.41206	0.08904	0.22353	0.4883	0.18324	0.25105	0.18067
ENSG00000231441	AL512422.1	0	0	0	0	0	0	0	0
ENSG00000216854	RPL17P26	0	0	0	0	0	0	0	0
ENSG00000200224	RNU6-626P	0	0	0	0	0	0	0	0
ENSG00000151917	BEND6	0.11806	0.08734	0.03855	0	0.04784	0	0.02186	0.01611
ENSG00000219790	OSTCP6	0	0	0	0	0	0	0	0
ENSG00000218980	FTH1P15	0	0	0	0	0	0	0	0
ENSG00000276576	MRPL30P1	0	0	0	0.69734	0	0	0.26107	0
ENSG00000168116	KIAA1586	26.7833	35.0053	41.1246	93.8098	41.4483	32.8583	71.2096	58.0103
ENSG00000112200	ZNF451	8.76514	10.2863	11.1152	24.3535	13.1373	15.0612	22.6483	15.8164
ENSG00000226803	ZNF451-AS1	0	0	0	0.02212	0	0.02629	0	0

ENSG00000112208	BAG2	0.67228	0.73378	0.3239	1.03045	1.3131	2.00257	1.12058	0.92013
ENSG00000112210	RAB23	3.13974	4.35922	3.97969	12.8308	6.00624	5.14517	12.4264	7.03388
ENSG00000146143	PRIM2	1.90044	1.83	2.56187	6.59661	3.24616	4.82114	3.95312	2.04407
ENSG00000212017	MIR548U	0	4.51883	0	1.34303	0	0	0	0
ENSG00000266579	FO680682.1	0.42493	0.35365	0	0.63064	0.25831	0	0	0.26086
ENSG00000272541	AL021368.3	0	0	0	0	0	0	0	0.31141
ENSG00000271761	AL021368.1	0	0	0	0.20146	0	0	0	0
ENSG00000272316	AL021368.2	0.6574	0.25076	0.10063	0.36587	0.38298	0.21745	0.34243	0.3195
ENSG00000239650	GUSBP4	0.10908	0	0	0.16188	0.13261	0.19243	0.18182	0
ENSG00000283352	AL021368.4	0	0	0	0	0.13202	0.19157	0	0
ENSG00000218189	POM121L14P	0	0	0	0	0	0	0	0
ENSG00000215190	LINC00680	0.69128	0.65751	0.10884	0.25648	0.78041	0.30488	0.16461	0.51531
ENSG00000225096	AL445250.1	0	0	0	0	0	0	0	0
ENSG00000214563	GAPDHP15	0	0	0	0	0	0	0	0
ENSG00000216687	AL390237.1	0	0	0	0	0	0	0	0
ENSG00000214561	RBBP4P4	0	0	0	0	0	0	0	0
ENSG00000223633		0	0	0	0	0	0	0	0
ENSG00000275046	AL603755.1	0	0	0	0	0	0	0	0
ENSG00000283453	AC244258.1	0	0	0	0.20315	0	0	0.22816	0
ENSG00000277172	GAPDHP41	0	0	0	0	0	0	0	0
ENSG00000275773	AL590227.1	0	0	0	0	0	0	0	0
ENSG00000274844	AL512427.1	0	0	0	0	0	0	0	0
ENSG00000276290	RBBP4P3	0	0	0	0	0	0	0	0
ENSG00000220030	AL356131.1	0	0	0	0	0	0	0	0
ENSG00000255633	MTRNR2L9	0	0	0	0	0	0	0	0
ENSG00000250686	KHDRBS2-OT	0.10892	0	0.16005	0.16164	1.05934	0.19214	0.36309	0.5349
ENSG00000112232	KHDRBS2	0.18859	0.10464	0.09238	0.32654	0.2675	0.1109	0.36675	0.11578
ENSG00000216859	DHFRP5	0	0	0	0	0	0	0	0
ENSG00000217067	AL355375.1	0	0	0	0	0	0	0	0
ENSG00000262566	AL355375.2	0	0	0	0	0	0	0	0
ENSG00000217477	AL450346.1	0	0	0	0	0	0	0	0
ENSG00000198225	FKBP1C	0.09284	0	0.06822	0.13779	0.05644	0.24568	0	0.11399
ENSG00000219940	SPTLC1P3	0	0	0	0	0	0	0	0
ENSG00000218520	AL450346.2	0	0	0	0	0	0	0	0
ENSG00000146166	LGSN	0.16364	0.18858	0.20348	0.14946	0.09183	0	0.04196	0.1391
ENSG00000218617	AL121949.3	0	0	0	0	0	0	0	0
ENSG00000218274	AL121949.2	0.0947	0	0	0	0	0	0	0.05814
ENSG00000218048	AL121949.1	0	0	0	0	0	0	0	0
ENSG00000225121	EEF1B2P5	0	0	0	0	0	0	0	0
ENSG00000112245	PTP4A1	19.1197	19.4326	5.73061	6.80364	11.1916	10.0068	7.35609	7.75992
ENSG00000213312	RPL7AP34	0	0	0	0	0	0	0	0
ENSG00000266680	AL135905.1	0.26104	0.0869	0.07672	0.07748	0.06347	0	0	0.3205
ENSG00000262651	AL513043.1	0	0	0	0	0	0	0	0
ENSG00000213309	RPL9P18	0	0	0	0	0	0	0	0
ENSG00000118482	PHF3	31.9532	30.0025	7.91923	13.2916	32.3904	20.9082	17.012	19.8934
ENSG00000188107	EYS	0.08159	0.04289	0.00631	0.01275	0.03132	0	0.00716	0.02636
ENSG00000218813	AL354719.1	0	0	0	0	0.10571	0	0	0
ENSG00000236345	SCAT8	3.02477	2.93693	1.33346	0.67336	1.53227	0.71148	0.25209	1.29982
ENSG00000219653	GCNT1P4	0	0	0	0	0	0	0	0
ENSG00000232120	AL357375.1	0	0	0	0	0	0	0	0
ENSG00000217811	HNRNPDP2	0	0	0	0	0	0	0	0
ENSG00000243828	AL355357.1	0	0	0	0	0	0	0	0
ENSG00000214558	AL365217.1	0.06018	0.10017	0.17687	0.04466	0.10975	0.21233	0.45141	0.18472
ENSG00000220483	SLC25A51P1	0	0	0	0	0	0	0	0
ENSG00000233859	ADH5P4	0	0	0	0	0	0	0	0
ENSG00000218890	NUFIP1P	0	0	0	0	0	0	0.16522	0.06085
ENSG00000270521	AL450336.1	0	0	0	0	0	0	0	0
ENSG00000238949	RNU7-66P	0	0	0	0	0	0	0	0

ENSG00000212229	RF00571	0	0	0	0	0	0	0	0
ENSG00000271111	AL365503.1	0	0	0	0	0	0	0	0
ENSG00000251965	RNA5SP208	0	0	0	0	0	0	0	0
ENSG00000201015	RNU6-280P	0	0	0	0	0	0	0	0
ENSG00000206672	RF00019	0	0	0	0	0	0	0	0
ENSG00000227706	AL713998.1	0	0	0	0	0	0	0	0
ENSG00000270509	AL713998.2	0	0	0	0	0	0	0	0
ENSG00000223504	AL646090.1	0.14898	0	0	0	0	0	0	0
ENSG00000226497	LINC02549	0	0	0	0	0	0	0	0
ENSG00000218834	AL606923.1	0	0	0	0	0	0	0	0
ENSG00000230910	AL391807.1	0.14919	0.08277	0	0	0.06046	0	0	0
ENSG00000135298	ADGRB3	0.04518	0.1316	0	0	0.02746	0	0	0
ENSG00000168216	LMBRD1	32.9882	30.5275	10.4485	19.5651	33.5764	13.9971	16.7858	23.7544
ENSG00000219085	NPM1P37	1.09028	1.81477	0.36973	1.36916	1.22357	1.03568	1.39794	0.92674
ENSG00000219881	GAPDHP42	0	0	0	0	0	0	0	0
ENSG00000082293	COL19A1	0.37033	0.07168	0.02531	0.30676	0.92143	0.12155	0.21533	0.15861
ENSG00000216365	RPL37P15	0	0	0	0	0	0	0	0
ENSG00000112280	COL9A1	0	0	0	0	0	0	0	0
ENSG00000253809	AL160262.1	0	0	0	0	0	0	0	0
ENSG00000237643	AL365226.2	0	0	0	0	0	0	0	0
ENSG00000224349	AL365226.1	0.21003	0	0	0	0	0	0	0
ENSG00000082269	FAM135A	1.15603	0.80655	0.38655	0.92461	1.16982	0.98916	0.61154	0.9264
ENSG00000238386	RNU7-48P	0	1.93664	1.70975	0	0	0	0	1.42852
ENSG00000154079	SDHAF4	1.93431	1.18619	0.37401	1.96419	1.73282	1.7062	0.59393	0.93747
ENSG00000271967	AL583856.2	0.13499	0	0	0	0.16412	0.23814	0.22501	0.16574
ENSG00000232389	AL583856.1	0	0	0	0	0	0	0	0.08174
ENSG00000218870	SLC25A6P6	0	0	0	0	0	0	0.15704	0
ENSG00000112305	SMAP1	7.11688	5.86438	1.9156	4.78438	6.59648	7.14773	3.49422	3.78502
ENSG00000262803	AL354943.1	0.46927	0	0.13792	0	0.34232	0.16557	0.31288	0.11523
ENSG00000217767	NDUFAB1P1	0.15935	0.26524	0	0.23649	0	0	0	0.19565
ENSG00000112309	B3GAT2	0	0	0	0	0	0	0	0
ENSG00000219669	BECN1P2	0	0	0	0	0	0	0	0
ENSG00000221345	RF00012	0	0	0	0	0	0	0	0
ENSG00000218350	LYPLA1P3	0.10547	0	0	0.15653	0	0.18606	0.1758	0
ENSG00000207180	RNU6-411P	0	0	0	0	0	0	0	0
ENSG00000232295	AL589935.1	0	0	0	0.02105	0	0	0	0.01741
ENSG00000276127	AL589935.2	0	0	0	0	0	0	0	0
ENSG00000119900	OGFRL1	76.2806	60.3774	13.5074	28.6778	51.2586	16.5729	24.1094	35.5573
ENSG00000269966	AL136164.2	0	0	0	0	0	0	0	0
ENSG00000230597	AL136164.1	0	0	0	0	0	0	0	0
ENSG00000233237	LINC00472	0	0	0	0	0.02588	0	0	0
ENSG00000279312	AL136164.4	0	0	0	0	0	0	0	0
ENSG00000199094	MIR30C2	0	0	0	0	0	0	0	0
ENSG00000279289	AL136164.3	0	0	0	0	0	0	0	0
ENSG00000207827	MIR30A	0	0	0	0	0	0	0	0
ENSG00000223765	LINC01626	0	0	0	0	0	0	0	0
ENSG00000219575	AL035467.1	0	0	0	0	0	0	0	0
ENSG00000218014	KRT19P1	0	0	0	0	0	0	0	0
ENSG00000202069	RNU4-66P	0	0	0	0	0	0	0	0
ENSG00000079841	RIMS1	0	0	0.00842	0	0	0	0	0
ENSG00000218732	AL035633.1	0	0	0	0	0	0	0	0
ENSG00000217483	FO393414.2	0	0	0	0	0	0	0	0
ENSG00000181514	FO393414.1	0	0	0	0	0	0	0	0
ENSG00000185760	KCNQ5	0.40669	0.19341	0.12806	0.15808	0.37672	0.39289	0.14526	0.107
ENSG00000233844	KCNQ5-IT1	1.17176	0.65013	0.19132	0.57968	2.21605	0.22968	0.65105	0.31971
ENSG00000266180	MIR4282	0	0	0	0	0	0	0	0
ENSG00000232422	KNOP1P4	0	0	0	0	0	0	0	0
ENSG00000220515	PGAM1P10	0.19264	0	0	0	0	0.33985	0	0.11826

ENSG00000229154	KCNQ5-AS1	0	0	0	0	0	0	0	0
ENSG00000234025	RBPMS2P1	0	0	0	0	0	0	0	0
ENSG00000251360	KHDC1P1	0	0	0	0	0	0	0	0
ENSG00000243501	AL365232.1	0	0	0	0	0	0	0	0
ENSG00000256980	KHDC1L	0	0	0	0	0	0	0	0
ENSG00000135314	KHDC1	0.06564	0.10926	0.03215	0.06495	0.0266	0.2316	0	0
ENSG00000229852	AC019205.1	0.12107	0.10992	0.03235	0.01633	0.12043	0.05825	0.01835	0.05405
ENSG00000239626	RPSAP41	0	0	0	0	0	0	0	0
ENSG00000234882	EIF3EP1	0.1671	0	0.08185	0.08266	0.54174	0.09826	0	0.47871
ENSG00000223389	SDCBP2P1	0	0	0	0	0	0	0	0
ENSG00000226840	PAICSP3	0	0	0	0	0	0	0	0
ENSG00000203909	DPPA5	0	0	0	0	0	0	0	0
ENSG00000203908	KHDC3L	0	0	0	0	0	0	0	0
ENSG00000203907	OOEP	0	0.16634	0	0.07416	0	0.17629	0	0
ENSG00000231332	OOEP-AS1	0	0	0	0	0	0	0	0
ENSG00000235174	RPL39P3	0.93975	1.56421	0.69047	2.78938	1.71378	4.97353	3.13283	1.15381
ENSG00000224221	RPS6P8	0	0	0	0	0	0	0	0
ENSG00000080007	DDX43	0.3917	0.65199	0.03837	0.3488	0.53972	0.23034	0.04353	0.25649
ENSG00000164430	CGAS	3.45773	6.54823	1.65913	2.4611	3.2601	4.07698	2.70532	3.03239
ENSG00000135297	MTO1	1.48775	1.32409	0.50863	1.12652	1.65373	2.03539	1.06279	1.13326
ENSG00000207023	RNU6-975P	1.3962	0	0	0	0.84873	0	1.16362	0
ENSG00000271986	RN7SL827P	0	0	0	0	0	0	0	0
ENSG00000223821	AL603910.1	0.19811	0	0	0	0	0	0	0
ENSG00000156508	EEF1A1	808.115	437.101	95.7075	221.668	741.955	603.926	318.145	368.749
ENSG00000229862	AL121972.1	0.09117	0.15175	0	0	0.22168	0.16084	0	0.11194
ENSG00000223967	AL603910.2	0	0	0	0	0	0	0	0
ENSG00000119899	SLC17A5	3.7423	4.06244	2.122	5.04085	3.95634	3.33685	3.69524	2.89669
ENSG00000218459	RPS27P15	0	0	0	0	0	0	0	0
ENSG00000231652	AL590428.1	0.40867	0.06184	0.05459	0.16541	0.22584	0.06554	0	0
ENSG00000156535	CD109	0.80482	0.54861	0.66455	1.11482	1.17418	0.47327	0.4855	0.70582
ENSG00000279790	AL590552.1	0	0	0	0	0	0	0	0
ENSG00000217268	TXNP7	0	0	0	0	0	0	0	0
ENSG00000223786	AL357507.1	0	0	0	0	0	0	0	0
ENSG00000217178	AL357563.1	0	0	0	0	0	0	0	0
ENSG00000272243	AL356277.3	0	0	0	0	0	0	0	0
ENSG00000218483	AL356277.1	0	0	0	0	0	0	0	0
ENSG00000224583	AL356277.2	0	0	0	0	0	0	0	0
ENSG00000219736	AL356473.1	0	0	0	0	0	0	0	0
ENSG00000111799	COL12A1	0.01762	0.00978	0	0	0.11424	0.06216	0.00979	0
ENSG00000112695	COX7A2	11.2506	9.07763	2.80213	6.28263	11.0816	12.4468	6.48408	9.64589
ENSG00000112697	TMEM30A	29.7179	26.2927	7.96869	15.3527	24.0578	13.8828	14.2858	23.1536
ENSG00000225793	AL080250.1	0.96511	0.24096	0.14182	0.71617	0.58668	0.59591	0.16087	0.47398
ENSG00000118407	FILIP1	0	0	0	0	0.01001	0	0.01372	0
ENSG00000203489	HMGB1P39	0	0	0	0	0	0	0	0
ENSG00000237174	AL445465.1	0	0	0	0	0	0	0	0
ENSG00000221332	RF00012	0	0	0	0	0	0	0	0
ENSG00000263533	MIR4463	0	0	0	0	0	0	0	0
ENSG00000238156	AL445465.2	0	0	0	0	0	0	0	0
ENSG00000217680	UBE2V1P15	0	0	0	0	0	0	0	0
ENSG00000201291	RNU1-34P	0	0	0	0	0	0	0	0
ENSG00000219722	RPL26P20	0	0	0	0	0	0	0	0
ENSG00000220730	AL356057.2	0	0	0	0	0	0	0	0
ENSG00000252137	RNU6-1338P	0	0	0	0	0	0	0	0
ENSG00000112701	SENP6	20.454	24.2114	12.5418	29.136	26.2806	19.5542	21.1091	23.0867
ENSG00000217488	AL356057.1	0	0	0	0	0	0	0	0
ENSG00000252498	RNU6-1016P	17.1554	18.1715	2.29179	3.47188	7.5844	0	2.59958	9.57416
ENSG00000200091	RN7SKP163	0	0	0	0	0	0	0.74049	0.27272
ENSG00000196586	MYO6	0.30038	0.76386	0.26974	0.35911	0.15216	0.25023	0.06954	0.20489

ENSG00000252156	RNU6-155P	0	0	0	0	0	0	0	0
ENSG00000223169	RNA5SP209	0	0	0	0	0	0	0	0
ENSG00000112706	IMPG1	0	0	0	0	0	0	0	0
ENSG00000200040	RF00019	0	2.15944	0	0	0	0	0	0
ENSG00000222652	RNU6-248P	0	0	0	0	0	0	0	0
ENSG00000238697	RNU6-261P	0	0	0	0	0	0	0	0
ENSG00000236635	LINC02540	0	0	0	0	0	0	0	0
ENSG00000272445	RNU6-84P	0	0	0	0	0	0	0	0
ENSG00000280511	AL591030.1	0	0	0	0	0	0	0	0
ENSG00000231762	AL590426.1	0	0	0	0	0	0	0	0
ENSG00000271945	AL590426.2	0	0	0	0	0	0	0	0
ENSG00000276064	AL365222.1	0	0	0	0	0	0	0	0
ENSG00000135312	HTR1B	0	0	0	0	0	0	0	0
ENSG00000219253	RPS6P7	0	0	0	0	0	0	0	0
ENSG00000269964	MEI4	0	0	0	0	0	0	0	0
ENSG00000230309	AL121718.1	0	0	0	0	0	0	0	0
ENSG00000229495	AL450327.1	0	0	0	0	0	0	0	0
ENSG00000146243	IRAK1BP1	0.53454	0.53385	0.19995	0.40387	0.74441	0.63439	0.2592	0.42958
ENSG00000146247	PHIP	16.8316	14.9979	4.885	8.89035	13.9942	10.6947	9.36728	12.068
ENSG00000217512	AL356776.1	2.62373	1.0918	0.24097	0.48674	1.39556	1.73573	0.82	1.00668
ENSG00000118418	HMGN3	27.0358	16.8454	2.65567	5.79332	15.9953	12.115	4.57875	10.118
ENSG00000270362	HMGN3-AS1	0.19905	0.33132	0.03656	0.03693	0.15125	0.04389	0	0.0611
ENSG00000220154	AL355796.1	0	0	0	0	0	0	0	0
ENSG00000201464	RF00019	0	0	0	0	0	0	0	0
ENSG00000217786	AL355379.1	0	0	0	0	0	0	0	0
ENSG00000218029	AL078601.1	0	0	0	0	0	0	0	0
ENSG00000262048	AL078601.3	0	0	0	0	0	0	0	0
ENSG00000231533	AL078601.2	0	0	0	0	0	0	0	0
ENSG00000218748	DBIP1	0	0	0	0	0	0	0	0
ENSG00000135338	LCA5	0.65139	0.45006	0.63212	0.60193	0.5977	1.0841	0.86042	0.63378
ENSG00000261970	AL391840.1	0.39946	0	0	0.29642	0	0	0	0
ENSG00000272137	AL451064.1	0	0	0.22869	0.46194	0	0	0	0
ENSG00000279659	AL451064.2	0	0	0	0	0	0	0	0
ENSG00000198478	SH3BGRL2	58.1299	31.6493	9.37549	17.9322	19.5739	28.7637	18.8535	29.2834
ENSG00000235357	LINC01621	0	0	0	0	0	0	0	0
ENSG00000220918	AL132875.1	0	0	0	0	0	0	0	0
ENSG00000233835	AL132875.2	0	0	0	0	0	0	0	0
ENSG00000118402	ELOVL4	0.6265	0.08022	0.07082	0.25033	0.49802	0.17004	0.08033	0.3846
ENSG00000218582	GAPDHP63	0	0	0	0.10803	0	0	0	0
ENSG00000220311	RPL35AP18	0	0	0	0	0	0	0	0
ENSG00000112742	TTK	1.31265	1.67865	2.04654	7.22229	2.41329	4.68787	5.22982	3.59674
ENSG00000218418	AL591135.1	0.01239	0.08252	0.07285	0.27592	0.07534	0.0656	0.18594	0.13696
ENSG00000217377	AK4P5	0	0	0	0	0	0	0	0
ENSG00000083123	BCKDHB	1.38642	1.01899	0.74085	1.30938	1.61991	1.6835	1.29053	0.81795
ENSG00000272129	AL359715.3	0.15313	0.08496	0	0	0.49647	0.1801	0.17017	0.18802
ENSG00000219547	RPL17P25	0	0	0	0	0	0	0	0
ENSG00000279022	AL359715.4	0	0	0	0	0.20117	0	0.2758	0.20315
ENSG00000233967	AL359715.1	0.0784	0	0.1152	0.11635	0	0.1383	0	0.09625
ENSG00000260645	AL359715.2	0.10358	0	0	0	0.08395	0	0	0
ENSG00000219361	RPSAP72	0	0	0	0	0	0	0	0
ENSG00000181705	AL596028.1	0	0	0	0	0	0	0	0
ENSG00000216352	AL590824.1	0	0	0	0	0	0	0	0
ENSG00000112773	TENT5A	6.99944	6.75249	1.79731	1.79722	3.44512	2.9054	2.82591	3.3156
ENSG00000226089	AL122017.1	0.09326	0	0	0	0.22676	0	0.15545	0.1145
ENSG00000277797	AL359693.1	0	0	0	0	0	0	0	0
ENSG00000212336	RNA5SP210	0	0	0	0	0	0	0	0
ENSG00000232031	AL078599.2	1.55627	1.29521	0.22869	0.23097	1.13524	0.54909	0.77822	0
ENSG00000206886	RF00156	0	0	0	0	0	0	0	0

ENSG00000219702	AL078599.1	0	0	0	0	0	0	0	0
ENSG00000224995	LINC01526	0	0	0.20714	0	0	0	0	0
ENSG00000226453	LINC02542	0.08917	0	0	0	0	0	0	0
ENSG00000260574	AL360157.1	0	0	0	0	0	0	0	0
ENSG00000005700	IBTK	5.17691	3.8049	2.16119	5.21353	5.45612	4.96669	4.49666	4.07577
ENSG00000223044	RNU6-130P	0	0	0	0	0	0	0	0
ENSG00000220537	AL121977.1	0	0	0	0	0	0	0	0
ENSG00000146242	TPBG	0.01062	0	0	0	0	0	0	0
ENSG00000118420	UBE3D	0.22928	0.36255	0.33692	0.57847	0.51569	0.52582	0.45861	0.42226
ENSG00000220091	LAP3P1	0.24932	0	0	0	0	0	0	0
ENSG00000227215	AL355613.1	0	0	0	0	0	0	0	0
ENSG00000083097	DOP1A	3.94049	3.85034	2.23041	5.16583	4.93269	3.88772	4.41528	3.78086
ENSG00000013375	PGM3	1.10925	0.54891	0.33775	0.65257	0.86262	0.75807	0.61632	0.80979
ENSG00000013392	RWDD2A	0.31325	0.35847	0.05754	0.14528	0.57126	0.449	0.1958	0.31249
ENSG00000065833	ME1	1.30466	0.43432	0.35148	1.32311	0.68734	0.15344	0.3262	0.53395
ENSG00000219951	AL391416.1	0	0	0	0	0	0	0	0
ENSG00000146250	PRSS35	0.78107	0.15001	0.08829	0.08917	0.10957	0	0	0
ENSG00000065609	SNAP91	0	0	0	0	0	0	0	0
ENSG00000237874	AL136972.1	0	0	0	0	0	0	0	0
ENSG00000203877	RIPPLY2	0	0	0	0	0	0	0	0
ENSG00000065615	CYB5R4	14.5231	15.1323	2.92901	3.9969	10.7291	6.45552	5.47814	10.6764
ENSG00000228679	AL034347.1	0.35114	0	0	0	0.10673	0.15486	0	0.10778
ENSG00000230852	AL161621.1	0	0	0	0.31996	0	0	0	0
ENSG00000135324	MRAP2	0	0	0	0	0	0	0	0
ENSG00000135315	CEP162	7.77996	10.0522	10.1338	18.4026	11.4123	9.44916	15.7134	14.4022
ENSG00000231776	LINC01611	0	0	0	0	0	0	0	0
ENSG00000226309	SMARCE1P2	0	0	0	0	0	0	0	0
ENSG00000112837	TBX18	0.01731	0	0	0	0	0	0	0
ENSG00000228290	TBX18-AS1	0	0	0	0	0	0	0	0
ENSG00000219604	AL590143.1	0	0	0	0	0	0	0	0
ENSG00000216523	AL109941.1	0	0	0	0	0	0	0	0
ENSG00000220291	AL139806.1	0	0	0	0	0	0	0	0
ENSG00000216902	AL122016.1	0	0	0	0	0	0	0	0
ENSG00000217060	AL135903.1	0	0	0	0.06842	0	0	0	0.0566
ENSG00000213211	KRT18P64	0	0	0	0	0	0	0	0
ENSG00000234155	LINC02535	0	0	0	0	0	0	0	0
ENSG00000216439	DUTP5	0	0	0	0	0	0	0	0
ENSG00000214460	TPT1P6	0.28522	0.94948	0	1.48152	0.52014	0	0.95082	0.87546
ENSG00000135318	NT5E	1.35831	0.53831	0.04752	1.22393	3.59769	0.59906	0.61993	0.95298
ENSG00000280232	AL589666.2	0.11861	0.39485	0.17429	0.35206	0	0.41848	0.1977	0
ENSG00000135317	SNX14	6.82967	6.3022	2.8653	5.64981	7.26229	5.60563	4.60863	5.68736
ENSG00000271793	AL589666.1	0	0	0	0	0	0	0	0
ENSG00000135316	SYNCRIP	19.2966	12.6098	3.95148	9.28093	19.8109	15.9808	9.29196	8.28127
ENSG00000220563	PKMP3	0.10252	0.34128	0	0.15215	0.24928	0	0.17088	0.12587
ENSG00000203875	SNHG5	10.0742	7.03055	2.46732	11.2913	14.8315	4.3505	3.58582	8.9224
ENSG00000275072	SNORD50B	0	0	0	0	0	0	0	0
ENSG00000217334	AL355615.1	0	0	0	0	0	0.20173	0	0
ENSG00000198738	SMIM11P1	0	0	0	0	0	0	0	0
ENSG00000222686	RNU4-72P	0	0.70525	0	0	0	0	0	0
ENSG00000200427	RF00019	0	0	0	0	0	0	0	0
ENSG00000218766	AL450338.1	0	0	0	0	0	0	0	0
ENSG00000202103	RNU4-12P	0	0	0	0	0	0	0	0
ENSG00000220069	RPL7P27	0	0	0	0	0	0	0	0
ENSG00000217272	AL049545.1	0	0	0	0	0	0	0	0
ENSG00000220831	NDUFA5P9	0	0	0	0	0	0	0	0
ENSG00000219500	AL391417.1	0	0	0	0	0	0	0	0
ENSG00000243124	RN7SL643P	0	0	0	0	0	0	0	0
ENSG00000218561	AL353133.1	0	0	0	0	0	0	0	0

ENSG00000219190	AL353133.2	0	0	0	0	0	0	0	0
ENSG00000217769	AL157777.1	0.96448	1.60537	1.77161	2.50493	0.87944	1.2761	2.41145	0.88813
ENSG00000220240	AL157777.2	0	0	0	0	0	0	0	0
ENSG00000168830	HTR1E	0	0	0	0	0	0	0	0
ENSG00000214457	RPL7P29	0	0	0	0	0	0	0	0
ENSG00000217169	MTHFD2P2	0	0	0	0	0	0	0	0
ENSG00000217776	AL138827.1	0	0	0	0	0	0	0	0
ENSG00000252697	RN7SKP209	0	0	0	0	0	0	0	0
ENSG00000135346	CGA	0	0	0	0	0	0	0	0
ENSG00000215477	RCN1P1	0	0	0	0	0	0	0	0
ENSG00000272008	AL139274.2	0.05328	0.05913	0	0.02636	0.06478	0.21933	0.05921	0.04361
ENSG00000188994	ZNF292	11.0498	12.9648	3.11786	6.51322	11.4586	8.53017	6.76608	6.91019
ENSG00000218313	AL139274.1	0	0	0	0	0	0	0	0
ENSG00000164411	GJB7	0	0	0	0	0	0	0	0
ENSG00000216990	HSPD1P10	0.04374	0.14559	0	0	0	0	0	0
ENSG00000111850	SMIM8	0.84619	0.6136	0.35704	0.94499	0.90655	0.59121	0.93566	0.87436
ENSG00000203872	C6orf163	1.62664	2.03065	1.34456	3.47028	2.28663	1.38997	3.09264	2.37163
ENSG00000279616	AL096817.1	0.08259	0.20621	0	0.12258	0.05021	0	0.06883	0.10141
ENSG00000272514	CFAP206	0	0	0	0	0	0	0	0
ENSG00000213204	AL049697.1	0.43606	0.31107	0.06103	0.27736	0.17672	0.21979	0.2769	0.10198
ENSG00000216324	AL049697.2	0	0	0	0	0	0	0	0
ENSG00000218793	AL049697.3	0	0	0	0	0	0	0	0
ENSG00000218698	ST13P16	0	0	0	0	0	0	0	0
ENSG00000164414	SLC35A1	4.5583	4.28315	1.45851	1.74581	3.79886	2.46432	1.04166	3.20451
ENSG00000206715	RNU6-444P	0.67871	0	0	1.00728	0	0	1.1313	0
ENSG00000146282	RARS2	8.20849	8.43752	4.19005	8.84819	8.89416	8.94258	7.63327	8.41623
ENSG00000135336	ORC3	6.45774	6.58165	4.81778	10.4687	9.80791	8.20248	9.73733	8.14828
ENSG00000135334	AKIRIN2	18.6276	26.1839	6.30442	8.76163	12.1259	14.0373	8.8625	8.91417
ENSG00000238628	RF01210	0	0	0	0	0	0	0	0
ENSG00000207131	RF00019	0	0	0	0	0	0	0	0
ENSG00000240997	RN7SL183P	0	0	0	0	0	0	0	0
ENSG00000271114	AL590392.1	0	0	0	0	0	0	0	0
ENSG00000118434	SPACA1	0	0	0	0	0	0	0	0
ENSG00000118432	CNR1	0.01153	0.09596	0.05083	0.08556	0.08411	0	0.01922	0.12741
ENSG00000279565	AL121835.2	0	0	0	0	0	0	0	0
ENSG00000220131	AL121835.1	0	0	0	0	0	0	0	0
ENSG00000220267	ACTBP8	0	0.22023	0	0	0	0	0.11027	0
ENSG00000234426	AL139042.1	0	22.4016	0.70632	4.28009	0	0	1.20177	3.54087
ENSG00000111880	RNGTT	5.6511	5.68347	2.36638	5.00113	9.11832	6.91802	4.27482	4.70489
ENSG00000222145	RF00045	0	0	0	0	0	0	0	0
ENSG00000280277	AL079342.3	0	0	0	0	0	0	0	0
ENSG00000220130	AL079342.1	0	0	0	0	0	0	0	0
ENSG00000220960	AL079342.2	0	0	0	0	0	0	0	0
ENSG00000223001	RNU2-61P	0	0	0	0	0	0	0	0
ENSG00000271931	AL353135.1	0.14544	0	0	0.21584	0.17682	0.25657	0.24242	0
ENSG00000146278	PNRC1	219.117	199.854	51.5529	79.6986	193.573	129.096	86.3266	115.865
ENSG00000264017	RN7SL336P	0.74041	2.05401	0	0	0.30006	0.43539	0.41138	0.30302
ENSG00000154548	SRSF12	0	0	0	0	0	0	0	0
ENSG00000146281	PM20D2	1.82161	1.47716	0.45758	1.54814	2.91503	1.67545	0.88236	1.29987
ENSG00000146276	GABRR1	0	0	0	0	0	0.03423	0	0
ENSG00000111886	GABRR2	0.13148	0.10942	0.0483	0	0.11989	0	0.05479	0.04036
ENSG00000220418	TUBB3P1	0	0	0	0	0	0	0	0
ENSG00000198833	UBE2J1	38.4442	37.1429	9.18958	10.2446	32.6346	19.3214	13.9268	17.391
ENSG00000025039	RRAGD	4.82695	4.88323	1.59277	2.08048	4.46286	1.81016	1.83078	4.0811
ENSG00000218965	NACAP7	0	0	0	0.17863	0	0	0	0
ENSG00000135299	ANKRD6	0.52804	0.29297	0.0388	0.15673	0.60988	0.17078	0.24938	0.28094
ENSG00000239316	RN7SL11P	0	0	0	0.37128	0	0	0	0
ENSG00000237027	AL159174.1	0	0	0	0	0	0	0	0

ENSG00000083099	LYRM2	2.00163	1.39173	0.48402	0.92754	1.86879	1.47504	1.09806	1.18213
ENSG00000112159	MDN1	5.1817	3.05644	1.01333	2.66087	4.9488	4.35859	2.50903	2.58835
ENSG00000228124	AL096678.1	0.1553	0	0	0	0.56642	0.27397	0.25886	0.19067
ENSG00000220871	DNAJC19P6	0	0	0	0	0	0	0	0
ENSG00000217653	AL353692.1	0	0	0	0	0	0	0	0
ENSG00000118412	CASP8AP2	10.7291	8.2424	8.85862	14.9246	13.1314	13.3885	11.6184	11.8623
ENSG00000199630	RF00019	0	0	0	0	0	0	0	0.93747
ENSG00000218300	RPL22P14	0	0	0	0	0	0	0	0
ENSG00000219240	AL353692.2	0	0	0	0	0	0	0	0
ENSG00000135355	GJA10	0	0	0	0	0	0	0	0
ENSG00000252891	RF00019	0	0	0	0	0	0	0	0
ENSG00000112182	BACH2	2.21714	0.51117	0.34122	1.25616	4.52595	2.43137	2.25982	1.48983
ENSG00000226455	AL121787.1	0	0	0	0	0	0	0	0
ENSG00000222078	RN7SKP110	0.77158	0.4281	0	0	1.25076	0	0	0.63156
ENSG00000218730	AL117342.1	0.36109	0	0.26531	0.26795	0.2195	0	0.30094	0.44334
ENSG00000266760	MIR4464	0	0	0	0	0.96866	0	0	0
ENSG00000260271	AL132996.1	0.05122	0	0	0	0.06228	0	0	0
ENSG00000135341	MAP3K7	7.28236	6.3984	1.83324	4.01907	6.86841	6.36986	4.31105	4.55776
ENSG00000224605	AL080284.1	0	0	0	0	0	0	0	0
ENSG00000263734	MIR4643	0	0	0	0	0	0	0	0
ENSG00000224944	CASC6	0	0	0	0	0	0	0	0
ENSG00000262095	MTATP6P25	0	0	0	0	0	0	0	0
ENSG00000219087	MTND4LP19	0	0	0	0	0	0	0	0
ENSG00000220340	MTCO1P56	0	0	0	0	0	0	0	0
ENSG00000240961	RN7SL415P	0	0	0	0	0	0	0	0
ENSG00000219867	AL590635.1	0	0	0	0	0	0	0	0
ENSG00000220553	RPL5P19	0	0	0	0	0	0	0	0
ENSG00000200492	RF00012	0	0	0	0	0	0	0	0
ENSG00000261038	AL133457.1	0	0	0	0	0	0	0	0
ENSG00000220908	LINC02531	0	0	0	0	0	0	0	0
ENSG00000221455	RF00012	0	0	0	0	0	0	0	0
ENSG00000219387	ATF1P1	0	0	0	0	0	0	0	0
ENSG00000235099	AL138731.1	0	0	0	0	0	0	0	0
ENSG00000213187	COPS5P1	0	0	0	0	0	0	0	0
ENSG00000135333	EPHA7	0	0	0	0	0	0	0	0
ENSG00000280451	AL356094.1	0	0	0	0	0	0	0	0
ENSG00000252249	RF00425	0	0	0	0	0	0	0	0
ENSG00000229600	AL391336.1	0	0	0	0	0	0	0	0
ENSG00000231143	AL157378.1	0	0	0	0	0	0	0	0
ENSG00000220370	AL078595.1	0	0	0	0	0	0	0	0
ENSG00000279403	AL078595.3	0	0	0	0	0	0	0	0
ENSG00000270484	AL078595.2	0	0	0	0	0	0	0	0
ENSG00000216853	MTCYBP36	0	0	0	0	0	0	0	0
ENSG00000219627	CYCSP17	0	0	0	0	0	0	0	0
ENSG00000261366	MANEA-DT	1.32509	0.96832	0.52242	0.71948	1.21809	0.91225	1.23904	1.2698
ENSG00000172469	MANEA	3.02415	2.39924	1.88972	3.7751	4.27801	3.14118	1.29553	3.3313
ENSG00000219549	AL607077.1	0	0	0	0	0	0	0	0
ENSG00000219941	KRT18P50	0	0	0	0	0	0	0	0
ENSG00000172461	FUT9	0	0	0	0	0	0	0	0
ENSG00000242560	RN7SL797P	0	0	0	0	0	0	0	0
ENSG00000233797	UFL1-AS1	0.6688	0	0.19656	1.3896	0.32524	0.23597	0	0.82114
ENSG00000014123	UFL1	11.2559	7.06945	2.80023	5.03305	9.46337	9.77174	4.73755	6.32498
ENSG00000112214	FHL5	0	0	0	0	0	0	0	0
ENSG00000213183	RPS7P8	0	0	0	0	0	0	0	0
ENSG00000207309	RNU4-70P	0	0	0	0	0	0	0	0
ENSG00000219139	TYMSP1	0	0	0	0	0	0	0	0
ENSG00000217331	AL355143.1	0	0	0	0	0	0	0	0
ENSG00000216518	EEF1GP6	0	0	0	0	0	0	0	0

ENSG00000224384	AL033379.1	0	0	0	0	0	0	0	0
ENSG00000112218	GPR63	0.04926	0	0.0181	0.05483	0.01497	0	0	0.01512
ENSG00000123545	NDUFAF4	1.15434	1.82532	0.84814	1.88448	2.66648	1.9855	0.91395	2.01962
ENSG00000263479	RN7SL509P	0	0	0	0	0	0	0	0
ENSG00000186231	KLHL32	0.19551	0.02958	0.10448	0.10551	0.17287	0.03136	0.17776	0.13094
ENSG00000146263	MMS22L	0.76687	1.1253	1.45807	3.30955	1.25131	1.50121	2.79199	1.6312
ENSG00000271860	AL589740.1	0.02438	0	0	0	0	0.02151	0.04065	0.01497
ENSG00000226207	AL080316.1	0	0	0	0	0	0	0	0
ENSG00000238367	MIR2113	0	0	0	0	0	0	0	0
ENSG00000220505	EIF4EBP2P3	0	0	0	0	0	0	0	0
ENSG00000236920	AL590727.1	0	0	0	0	0	0	0	0
ENSG00000187472	AL589826.1	0	0	0	0	0	0	0.49068	0
ENSG00000283010	AL589826.2	0	0	0	0	0	0	0	0
ENSG00000184486	POU3F2	0	0.02501	0	0	0.03653	0	0	0
ENSG00000112234	FBXL4	6.74743	6.91607	4.8979	7.42665	6.28154	7.15477	7.70873	6.39905
ENSG00000271042	AL078603.1	0	0	0	0	0	0	0	0
ENSG00000265226	MIR548AI	0	0	0	0	0	0	0	0
ENSG00000232699	BDH2P1	0	0	0	0	0	0	0.33201	0
ENSG00000146267	FAXC	0	0	0	0	0	0	0	0
ENSG00000132423	COQ3	0.45247	0.58577	0.44327	0.67152	0.48898	0.79822	0.419	0.43208
ENSG00000132424	PNISR	17.526	11.707	2.85387	6.06478	12.2648	7.93477	7.6995	8.70993
ENSG00000228506	AL513550.1	1.5215	0.89384	0.52608	0.73055	1.8498	1.10523	0.67132	1.09887
ENSG00000123552	USP45	3.0318	3.90985	4.30802	7.58027	5.89979	3.50142	7.75483	4.9073
ENSG00000228439	TSTD3	3.42881	3.55118	5.82238	10.5167	5.83612	6.18331	8.50944	7.10995
ENSG00000274603	RF00019	0	0	0	0	0	0	1.12092	0
ENSG00000112237	CCNC	5.16218	3.53433	2.22341	4.54768	4.85388	5.04686	3.77244	3.98079
ENSG00000272017	AL137784.2	0	0	0	0	0.08912	0	0	0
ENSG00000219755	AL137784.1	0	0	0	0	0	0.17357	0	0
ENSG00000279170	AL137784.3	0	0	0	0.04755	0	0	0	0
ENSG00000112238	PRDM13	0	0	0	0	0	0	0	0
ENSG00000199303	RF00019	0	0	0	0.98005	0	0	0	0
ENSG00000152034	MCHR2	0	0	0	0	0	0	0	0
ENSG00000229315	MCHR2-AS1	0	0	0	0	0	0	0	0
ENSG00000219257	NPM1P38	0	0	0	0	0	0	0	0
ENSG00000275716	AL080285.2	0	0	0	0	0	0	0	0
ENSG00000213177	PRDX2P4	0	0	0	0	0	0	0	0
ENSG00000216378	AL080285.1	0	0	0	0	0	0	0	0
ENSG00000112246	SIM1	0	0	0	0	0	0	0	0
ENSG00000228082	Z86062.2	0	0	0	0	0	0	0	0
ENSG00000112249	ASCC3	7.21694	6.13631	2.46996	4.29356	6.404	10.2515	4.64516	4.84086
ENSG00000220695	Z86062.1	0.37735	0.6281	0.13863	1.8201	0.57347	0.16642	0.47174	0
ENSG00000260000	AL133338.1	0.28992	0.24128	0	0.28684	0.29373	0.34097	0.4027	0.05933
ENSG00000270987	AL133338.2	0	0.16577	0	0.14781	0.12108	0	0	0
ENSG00000164418	GRIK2	0	0	0	0	0	0	0	0
ENSG00000218475	ACTG1P18	0	0	0	0	0	0	0	0
ENSG00000218536	AP002530.1	0	0	0	0	0	0	0	0
ENSG00000219757	AL357139.1	0	0	0	0	0	0	0	0
ENSG00000217120	Z98755.1	0	0	0	0	0	0	0	0
ENSG00000202283	RF00438	0	0	0	0	0	0	0	0
ENSG00000216817	R3HDM2P2	0	0	0	0	0	0	0	0
ENSG00000214428	NPM1P10	0	0	0	0	0	0	0	0
ENSG00000218173	AL356967.1	0	0	0	0	0	0	0	0
ENSG00000085382	HACE1	0.9839	1.24148	0.39644	1.33068	1.33125	1.58175	1.38871	1.13982
ENSG00000252944	RNU6-897P	0	0	0	0	0	0	0	0
ENSG00000227535	AL357315.1	0	0	0	0.42829	0	0	0	0
ENSG00000271099	AL357315.2	0	0	0	0	0	0	0	0
ENSG00000203809	LIN28B-AS1	0	0	0	0	0	0	0	0
ENSG00000187772	LIN28B	0	0	0	0	0	0	0	0

ENSG00000212147	RNU6-1106P	0	0	0	0	0	0	0	0
ENSG00000112276	BVES	0	0	0	0	0.01534	0	0	0.0155
ENSG00000203808	BVES-AS1	0	0	0	0	0	0	0	0
ENSG00000132429	POPDC3	0	0	0	0	0	0	0	0
ENSG00000272102	AL133406.3	0.13549	0.67657	0	0.20108	0.49418	0.23902	0	0
ENSG00000085377	PREP	4.26709	4.3783	0.51538	1.76394	3.17428	5.84344	1.26662	1.91381
ENSG00000231628	AL133406.2	0	0	0	0	0	0	0	0
ENSG00000216639	AL133406.1	0	0	0	0	0	0	0	0
ENSG00000219902	RPL35P3	0.39515	0	0	0.29322	0	0.6971	0.32933	0
ENSG00000237567	Z97206.2	0	0	0	0	0	0	0	0
ENSG00000219088	Z97206.1	0	0	0	0	0	0	0	0
ENSG00000200198	RN7SKP211	0	0	0	0	0	0	0	0
ENSG00000057663	ATG5	5.55366	4.97588	4.11957	6.82252	5.16899	4.64088	5.09362	5.93782
ENSG00000057657	PRDM1	6.74423	9.29096	1.30224	5.36328	4.11372	12.9535	2.51305	5.12937
ENSG00000269919	AL022067.1	2.74473	0.53748	0.23726	3.59424	1.37405	1.13931	0.80736	2.77524
ENSG00000273744	RF00015	0	0	0	0	0	0	0	0
ENSG00000244710	RN7SL47P	0.24515	0	0	0.36383	0	0	0.40863	0.30099
ENSG00000252444	RNU6-344P	0	0	0	0	0	0	0	0
ENSG00000233941	AL356859.1	0	0	0	0	0	0	0	0
ENSG00000112297	CRYBG1	12.1107	11.9374	3.40565	5.8514	10.0765	8.71303	5.18212	6.98358
ENSG00000207499	RF00019	0	0	2.11204	3.19958	0	0	0	0
ENSG00000202386	RNA5SP211	0	0	0	0	0	0	0	0
ENSG00000282408	AL109920.1	0	0	0	0	0	0	0	0
ENSG00000200314	RF00019	0	0	0	1.06653	0	0	2.39569	0.88232
ENSG00000130347	RTN4IP1	1.00259	1.17435	0.60023	0.82664	0.99319	1.70319	1.36169	0.77505
ENSG00000200295	RNU6-527P	0	0	0	0	0	0	0	0
ENSG00000130348	QRSL1	1.7206	1.00686	0.57284	0.69824	2.22261	2.2291	0.94106	1.41936
ENSG00000216977	RPL21P65	0.1573	0.26182	0	0	0	0	0	0.19313
ENSG00000229654	LINC02526	0	0	0	0	0	0	0	0.21479
ENSG00000235142	LINC02532	0	0	0	0	0	0	0	0
ENSG00000202285	RNU6-117P	0	0	0	0	0	0	0	0
ENSG00000207577	MIR587	0	0	0	0	0	0	0	0
ENSG00000272398	CD24	4.80464	1.57583	3.23455	2.1427	7.97072	5.38625	2.24872	3.31278
ENSG00000130349	C6orf203	1.87211	2.45695	0.74067	1.87009	2.75754	2.03241	1.92032	2.03333
ENSG00000178409	BEND3	0.12862	0.14272	0.07875	0.15907	0.28667	0.20799	0.10719	0.0658
ENSG00000252125	RNU6-1299P	0	0	0	0	0	0	0	0
ENSG00000164494	PDSS2	2.44583	1.52278	0.82308	0.96982	2.02022	2.30561	1.43156	1.35248
ENSG00000276620	AL591516.1	0	0	0	0	0	0	0	0
ENSG00000220237	RPS24P12	0	0	0	0	0	0	0	0
ENSG00000112320	SOBP	0	0.05302	0.0312	0.03151	0.01291	0.05619	0.0177	0.01304
ENSG00000234206	AL121957.1	0	0	0	0	0	0	0	0
ENSG00000280135	AL096816.1	1.55638	0.62676	0.11067	0.26079	1.22078	2.21424	0.41843	0.43149
ENSG00000146285	SCML4	1.64812	1.85173	0.5752	1.16185	2.09141	2.68945	1.11604	0.99912
ENSG00000025796	SEC63	7.08505	7.13267	4.03912	7.32248	8.28172	5.78266	5.76255	6.39318
ENSG00000238420	RNU6-437P	0	0	0	0	0	0	0	0
ENSG00000232778	RPL23AP50	0	0	0	0	0	0	0	0
ENSG00000272476	AL024507.2	0.02848	0.0948	0	0	0.17311	0.20095	0	0.03496
ENSG00000224987	AL024507.1	0	0	0	0	0	0	0	0
ENSG00000253090	RF00045	0	0	0	0	0	0	0	0
ENSG00000231112	MTHFD2P3	0	0	0	0	0	0	0	0
ENSG00000225093	RPL3P7	0	0	0	0	0	0.10669	0	0
ENSG00000271734	Z98200.1	0.15596	0	0.22918	0.23146	0.18961	0	0	0.19148
ENSG00000081087	OSTM1	17.8949	15.0764	4.14673	7.08895	13.4386	8.08654	3.94648	10.225
ENSG00000238490	RF00019	0	0	1.90644	1.92541	0	0	0	0.79643
ENSG00000225174	OSTM1-AS1	0	0	0	0	0	0	0	0
ENSG00000112333	NR2E1	0	0	0	0	0.02387	0	0	0
ENSG00000279398	AL078596.1	0	0	0	0	0	0	0	0
ENSG00000112335	SNX3	136.413	186.788	122.859	146.465	109.243	167.08	109.047	76.4144

ENSG00000233511	Z98742.2	0	0	0	0	0	0	0	0
ENSG00000212525	RNA5SP212	0	0	0	0	0	0	0	0
ENSG00000230248	Z98742.1	0	0	0	0	0	0	0	0
ENSG00000206974	RNU6-1144P	0	0	0	0	0	0	0	0
ENSG00000135537	AFG1L	0.72867	0.38985	0.52264	1.0428	0.48513	0.505	0.82417	0.45797
ENSG00000238055	Z98742.3	0	0	0	0.3337	1.09346	0.39666	0	0.55213
ENSG00000279498	AL356121.2	0	0	0	0	0	0	0	0
ENSG00000238079	AL356121.1	0	0	0	0	0	0	0	0
ENSG00000227072	AL353706.1	0	0	0	0	0	0	0.24733	0
ENSG00000200799	RNU6-770P	0	0	0	0	0	0	0	0
ENSG00000271608	AL391646.1	0	0	0	0	0	0	0	0
ENSG00000118689	FOXO3	29.7246	41.4649	24.7347	28.4328	24.8963	35.6064	30.0506	21.424
ENSG00000228976	SUMO2P8	0	0	0	0	0	0	0	0
ENSG00000203801	LINC00222	0.02214	0.03685	0	0	0.02692	0	0	0.05436
ENSG00000219565	ZNF259P1	0	0	0	0	0	0	0	0
ENSG00000231559	Z95118.1	0	0	0	0	0	0	0	0
ENSG00000118690	ARMC2	1.15556	1.20573	0.63361	1.61259	1.0694	1.67345	1.17868	1.12232
ENSG00000218872	AL445189.1	0	0	0	0	0	0	0	0
ENSG00000228834	AL445189.2	0	0	0	0	0.34408	0	0	0
ENSG00000230290	ARMC2-AS1	0	0	0	0	0	0	0	0
ENSG00000080546	SESN1	2.90568	1.78688	0.84135	2.52792	2.80175	2.34837	2.12342	2.79428
ENSG00000271730	AL390208.1	0.22764	0	0	0	0	0	0	0
ENSG00000199944	RNU6-653P	0	0	0	0	0	0	0	0
ENSG00000183137	CEP57L1	6.32489	10.1863	25.8065	41.7249	9.5878	10.1125	26.0431	28.9216
ENSG00000203799	CCDC162P	0.05857	0.04874	0.03228	0.05433	0.0089	0.03875	0.09763	0.02697
ENSG00000233908	AL359711.1	0	0	0	0	0	0	0	0
ENSG00000219700	PTCHD3P3	0	0	0	0	0	0	0	0
ENSG00000201023	RNY3P11	0	0	0	0	0	0	0	0
ENSG00000218632	RPL7P28	0	0	0	0	0	0	0	0
ENSG00000135535	CD164	30.0936	32.3478	19.1593	30.0027	23.9398	23.8703	20.0539	23.8475
ENSG00000260273	AL359711.2	0.4203	0	0.24705	0.99803	0.61319	0.88976	0.42035	0.20642
ENSG00000185250	PPIL6	0.03272	0.02723	0.02404	0.04855	0.19888	0.02886	0.02727	0.08034
ENSG00000135587	SMPD2	3.38477	1.93857	0.21393	0.48613	2.07968	2.24723	0.90998	0.9384
ENSG00000135596	MICAL1	7.15759	5.72347	1.20598	1.83468	4.42047	3.26212	1.33332	2.46165
ENSG00000112365	ZBTB24	5.47196	3.4929	0.66358	2.06967	5.63539	3.6317	2.10312	2.21772
ENSG00000223537	AL109947.1	0	0	0	0	0	0	0	0
ENSG00000155085	AK9	1.42274	1.47406	1.20536	2.00378	1.90624	1.28057	1.53663	1.4438
ENSG00000112367	FIG4	8.22384	6.04608	2.21905	4.42169	6.12801	4.64399	2.99328	4.71031
ENSG00000232311	AL512303.1	0	0.52477	0	0.23395	0.19165	0	0.52551	0.38708
ENSG00000146360	GPR6	0	0	0	0	0	0.06648	0	0
ENSG00000112290	WASF1	0.92188	0.71109	0.82603	1.90208	1.2028	1.11065	0.97444	1.40793
ENSG00000168438	CDC40	8.71566	7.9205	3.20642	6.40346	9.59211	7.41596	7.64398	7.14407
ENSG00000053328	METTL24	0.04132	0	0.06072	0.06132	0.05023	0	0	0.05073
ENSG00000203797	DDO	0.06883	0.17184	0.10114	0.20429	0.04184	0.06071	0	0.04225
ENSG00000004809	SLC22A16	0.9193	0.46842	1.54389	2.0326	1.09486	1.3239	2.43923	1.45119
ENSG00000241162	RN7SL617P	0	0.39613	0	0.3532	0	0	0	0.2922
ENSG00000260188	AC002464.1	0.43118	1.11642	0.91522	1.56424	1.04843	1.4368	0.47914	0.52939
ENSG00000219559	AL512430.3	0	0	0	0	0	0	0	0
ENSG00000270934	AL512430.4	0	0	0	0	0	0	0	0
ENSG00000155111	CDK19	10.6839	10.4062	2.90419	8.78475	8.05285	5.78209	7.4528	7.57984
ENSG00000213150	AL512430.1	0	0	0	0	0	0	0	0
ENSG00000217041	AL512430.2	0	0	0	0	0	0	0	0
ENSG00000219150	AL603914.1	0.35932	0	0	0	0	0	0	0.44116
ENSG00000200522	RNU6-957P	0.74041	1.23241	0	1.09885	0	0	0	0
ENSG00000212587	SNORA40C	0	0	0	0	0	0	0.95453	0
ENSG00000199360	RNU6-1115P	0	0	0	0	0	0	0	0
ENSG00000213149	CNN2P9	0.72575	0.93956	0.1185	0	0.19608	0.28451	0.26882	0.39603
ENSG00000271607	Z84480.1	0.47138	0	0	0.34979	0.28655	0.41579	0	0.57876

ENSG00000123505	AMD1	31.6693	31.5482	11.7264	23.6087	25.5797	22.1562	20.6387	27.5614
ENSG00000219329	AL357515.1	0	0	0	0.20071	0.16442	0	0	0
ENSG00000155115	GTF3C6	12.3656	7.20388	3.63421	8.36029	8.60264	14.9066	7.32853	8.01286
ENSG00000197498	RPF2	2.07454	1.33768	0.65914	1.49781	2.31767	2.24202	1.2461	1.58332
ENSG00000218803	GSTM2P1	0	0	0	0	0	0	0	0
ENSG00000112394	SLC16A10	0.28702	0.06515	0.12461	0.25171	0.53928	0.26468	0.41318	0.25629
ENSG00000200926	RNU6-960P	0	0	0	0	0	0	0	0
ENSG00000231046	AL360227.1	0	0	0	0	0	0	0	0
ENSG00000173214	MFSD4B	1.96845	2.08877	2.87454	5.89758	2.64747	1.71461	4.45001	3.32315
ENSG00000230177	AL080317.1	1.18439	0.65714	0.77353	0.78123	0.79997	0	1.75483	0.6463
ENSG00000271789	AL080317.2	0.05292	0.17619	0	0.07855	0.51475	0	0.08822	0.12996
ENSG00000009413	REV3L	6.67225	6.42202	3.51967	7.82732	7.12216	6.95213	6.7654	5.42517
ENSG00000272356	AL080317.3	0.15289	0.05655	0	0.12606	0.10326	0.05994	0.08495	0.20857
ENSG00000220392	FCF1P5	0	0	0	0	0	0	0.25349	0
ENSG00000229276	REV3L-IT1	0.95943	0	0	0	0.23329	0	0	0.23559
ENSG00000218676	BRD7P4	0	0	0	0	0	0	0.08028	0
ENSG00000231889	TRAF3IP2-AS1	0.31942	0.50636	0.15646	0.31604	0.42533	0.322	0.60849	0.33616
ENSG00000220506	AL136310.1	0	0	0	0	0	0	0	0
ENSG00000056972	TRAF3IP2	1.10739	0.85646	0.36162	0.54783	1.34634	0.63146	0.33561	0.53562
ENSG00000255389	Z97989.1	0.51471	0.10079	0	0.08987	0.3681	0.37389	0.05047	0.14869
ENSG00000010810	FYN	18.1396	16.4632	3.09581	8.91012	14.6223	28.9124	7.53426	7.55339
ENSG00000227012	LINC02527	0	0	0	0	0	0	0	0
ENSG00000112761	WISP3	0	0	0	0	0	0	0	0
ENSG00000074935	TUBE1	1.0512	0.40244	0.2935	0.39002	0.89462	1.01996	0.43805	0.43882
ENSG00000203778	FAM229B	0.17295	0	0	0.08556	0.14018	0	0.09609	0
ENSG00000112769	LAMA4	0	0	0	0.0094	0.0231	0.02234	0	0
ENSG00000237234	Z99289.1	0	0	0	0.07209	0	0	0	0
ENSG00000207044	RNU6-1226P	0	0	0	0	0	0	0	0
ENSG00000270661	Z99289.2	0	0	0	0	0	0	0	0
ENSG00000271208	Z99289.3	0	0	0	0	0	0	0	0
ENSG00000226440	AL365214.2	0	0	0	0	0	0	0	0
ENSG00000281613	AL365214.3	0	0	0	0	0	0	0	0
ENSG00000216663	AL365214.1	0	0	0	0	0	0	0	0
ENSG00000251258	RFPL4B	0	0	0	0	0	0	0	0
ENSG00000219773	RPSAP45	0	0	0	0	0	0	0	0
ENSG00000215807	KRT18P65	0	0	0	0	0	0	0	0
ENSG00000217770	FEM1AP3	0	0	0	0	0	0	0	0
ENSG00000232299	AL357514.1	0	0	0	0	0	0	0	0
ENSG00000225580	AL358942.1	0	0	0	0	0	0	0	0
ENSG00000271498	AL360085.1	0	0	0	0	0	0	0	0
ENSG00000201386	RNU6-1163P	0	0	0	0	0	0	0	0
ENSG00000218490	FCF1P10	0	0	0	0	0	0	0	0
ENSG00000216904	SOCS5P5	0	0	0	0	0	0	0	0
ENSG00000223811	AL589684.1	0	0	0	0	0	0	0	0
ENSG00000232316	LINC02518	0.24152	0	0	0	0	0	0	0
ENSG00000236347	AL513123.1	0	0	0	0	0	0	0	0
ENSG00000218208	RPS27AP11	0.16398	0	0	0	0.19937	0	0.27333	0
ENSG00000230943	LINC02541	0	0	0	0	0	0	0	0.14128
ENSG00000231912	FO393415.3	0	0	0	0	0	0	0	0
ENSG00000219758	FO393415.2	0	0	0	0	0	0	0	0
ENSG00000277443	MARCKS	50.1186	78.1425	11.0612	8.47384	26.0784	7.77383	6.88783	22.2519
ENSG00000227502	LINC01268	0.05321	0.04429	0.15639	0.11846	0.03235	0	0	0.16333
ENSG00000175967	FO393415.1	0	0	0	0	0	0	0	0
ENSG00000196591	HDAC2	2.67391	2.58048	0.56431	1.25243	2.56496	2.04075	1.05893	1.34463
ENSG00000228624	HDAC2-AS2	0.04545	0	0	0.02698	0.06632	0.03208	0.01515	0.01116
ENSG00000216723	NUDT19P3	0	0	0	0	0	0	0	0
ENSG00000249853	HS3ST5	0.01786	0.05946	0	0.02651	0.04343	0	0.05954	0.06579
ENSG00000216471	RPSAP43	0	0	0	0	0	0	0	0

ENSG00000212333	RNA5SP213	0	0	0	0	0	0	0	0
ENSG00000218089	DNAJA1P4	0	0	0	0	0	0	0	0
ENSG00000232395	AL138830.2	0	0	0	0	0	0	0	0
ENSG00000226079	AL138830.1	0	0	0	0	0	0	0	0
ENSG00000251882	RNU6-475P	0	0	0	0	0	0	0	0
ENSG00000219619	AL606845.1	0	0	0	0	0	0	0	0
ENSG00000228777	LINC02534	0	0	0	0	0	0	0	0
ENSG00000111816	FRK	0	0.01844	0.00814	0.02467	0.02694	0	0.01847	0.02041
ENSG00000186743	TPI1P3	0	0	0	0	0	0.17475	0	0
ENSG00000178425	NT5DC1	3.45993	1.78614	0.44211	1.4735	3.54808	2.52997	0.71881	1.39139
ENSG00000123500	COL10A1	0.07683	0.03197	0	0	0.02335	0.13555	0.06404	0.04717
ENSG00000218428	NIP7P3	0	0	0	0	0	0	0	0
ENSG00000236326	AL050331.2	0.17411	0	0.25585	0	0	0	0	0
ENSG00000187189	TSPYL4	2.38489	1.547	0.30923	0.54653	2.57969	2.56767	0.96458	0.79662
ENSG00000111817	DSE	5.42821	5.42766	0.9397	1.44893	2.75967	2.27346	1.51342	2.22955
ENSG00000237021		0.14089	0	0.05176	0.15683	0.1713	0.18642	0.11742	0.04325
ENSG00000233558	AL050331.1	0	0	0	0.17462	0.14304	0	0	0
ENSG00000189241	TSPYL1	21.0469	13.7929	4.53396	10.8917	19.5596	17.7677	10.4695	11.7976
ENSG00000240050		0.09186	0	0	0.06816	0	0.08102	0	0
ENSG00000217241	CBX3P9	0	0	0.19513	0.19708	0	0	0	0.16304
ENSG00000218153	KRT18P22	0	0	0	0	0	0.096	0	0
ENSG00000244158	Z84488.1	0	0	0	0	0	0.24398	0	0
ENSG00000188820	CALHM6	2.14867	8.27696	0.90213	2.73331	2.0152	5.73997	1.94424	10.1002
ENSG00000234117	AL445224.1	0	0	0	0	0	0	0	0
ENSG00000173626	TRAPPC3L	5.15788	3.23055	1.17208	2.05182	2.0364	1.92303	2.21582	2.48088
ENSG00000178033	CALHM5	0.23974	0.36164	0.09908	0.15566	0.20949	0.14538	0.08741	0.20236
ENSG00000164451	CALHM4	0	0	0	0	0	0	0	0
ENSG00000111832	RWDD1	6.15281	5.4717	1.36716	1.84102	5.62541	5.71169	1.81957	3.15271
ENSG00000111834	RSPH4A	0.07743	0.17184	0	0.07661	0.18827	0.18213	0.04302	0.12676
ENSG00000153975	ZUP1	9.7145	8.13084	6.97545	19.5372	17.6489	17.6246	22.1728	11.7579
ENSG00000196911	KPNA5	3.56968	4.19982	5.12295	13.3493	5.60443	5.6971	9.98461	6.82644
ENSG00000183807	FAM162B	0	0	0	0	0	0	0	0
ENSG00000173612	GPRC6A	0	0	0	0	0	0	0	0
ENSG00000185002	RFX6	0	0	0	0	0	0	0	0
ENSG00000217089	RPS29P13	0	0	0	0	0	0	0	0
ENSG00000200248	RNA5SP214	0	0	0	0	0	0	0	0
ENSG00000170162	VGLL2	0	0	0	0	0	0	0	0
ENSG00000047936	ROS1	0	0	0	0	0	0	0	0
ENSG00000251814	RN7SKP18	0	0	0	0	0	0	0	0
ENSG00000222713	RN7SKP51	0	0	0	0	0	0	0	0
ENSG00000282218	AL132671.2	0	0	0	0	0	0	0	0
ENSG00000219703	RAP1BP3	0	0	0	0	0	0	0	0
ENSG00000226181	AL132671.1	0	0	0	0	0	0	0	0
ENSG00000164465	DCBLD1	0.49447	0.2326	0.09478	0.22335	0.35286	0.39823	0.21501	0.27716
ENSG00000207461	RNU6-253P	0	0	0	0	0	0	0	0
ENSG00000047932	GOPC	7.04811	6.61714	1.34992	3.43187	7.5676	7.57206	2.40242	4.0837
ENSG00000218233	NEPNP	0	0	0	0.07935	0	0	0	0
ENSG00000153989	NUS1	5.56493	4.80102	2.37266	6.02556	5.94619	4.39704	4.12842	3.9263
ENSG00000196376	SLC35F1	0	0.02513	0	0	0	0	0	0
ENSG00000230202	AL450405.1	0.30928	0.2574	0.45449	0.68852	0.37602	0.54562	0.77329	9.49336
ENSG00000216809	AL589993.1	0.26137	0.14502	0.08535	0.0431	0.52962	0.25616	0.29044	0.17828
ENSG00000111860	CEP85L	9.06377	6.09913	1.77588	3.90902	8.77793	6.94255	4.45489	5.05056
ENSG00000169075	Z99496.1	0	0	0	0	0	0	0	0
ENSG00000198523	PLN	0	0	0	0	0.08907	0	0	0
ENSG00000217330	SSXP10	0	0	0	0	0	0	0	0
ENSG00000225549	SELENOKP3	0	0	0	0	0	0	0	0
ENSG00000111877	MCM9	1.90642	1.62079	0.44823	0.7661	1.6474	1.68677	0.7822	1.26036
ENSG00000111875	ASF1A	9.43556	9.21002	5.64888	11.4102	9.24094	8.57969	7.76673	8.76014

ENSG00000253194	AL137009.1	0.93376	0	0.22869	0.46194	0	0	0.25941	0
ENSG00000111879	FAM184A	0.10349	0.04698	0	0.08378	0.28596	0.28216	0.09409	0.18482
ENSG00000222649	RF00019	0	0	0	0	0	0	0	0
ENSG00000207982	MIR548B	0	0	0	0	0	1.33311	0	0
ENSG00000220139	AL022722.2	0	0	0	0	0	0	0	0
ENSG00000111885	MAN1A1	30.3055	32.753	14.8875	27.229	21.666	19.5489	19.5186	21.8262
ENSG00000216316	AL022722.1	0.88162	0.83855	0.7403	0.56075	0.61249	1.33311	1.67945	0.61854
ENSG00000200732	RNU6-194P	0	0	0	0	0	0	0	0
ENSG00000265725	MIR3144	0	0	0	0	0	0	0	0
ENSG00000206857	RNU6-214P	0	0	0	0	0	0	0	0
ENSG00000200310	RNA5SP215	0	0	0	0	0.73046	0	0	0.73768
ENSG00000216710	COX6A1P3	0	0	0	0	0	0	0	0
ENSG00000146350	TBC1D32	1.7604	2.93018	3.0388	6.05939	2.60439	3.91004	5.356	2.98167
ENSG00000206598	RNU6-1286P	0	0	0	0	0	0	0	0
ENSG00000207403	RF00019	0	0	0	0	0	0	0	0
ENSG00000220447	AL139098.1	0	0	0	0	0	0	0	0
ENSG00000272714	AL139098.2	0	0	0	0	0	0	0	0
ENSG00000152661	GJA1	0	0.12052	0	0.03582	0.08803	0	0	0
ENSG00000199458	RNU4-35P	0	0	0	0	0	0	0	0
ENSG00000219784	AL138729.1	0	0	0	0	0	0	0	0
ENSG00000201379	RNU4-76P	0	0	0	0	0	0	0	0
ENSG00000222659	RNU2-8P	0	0	0	0	0	0	0	0
ENSG00000220326	AL603865.2	0	0	0	0	0	0	0	0
ENSG00000218180	SLC25A5P7	0	0	0	0	0	0	0	0
ENSG00000219135	RPL23AP48	0	0	0	0	0	0	0	0
ENSG00000217139	AL603865.1	0	0	0	0.0807	0	0	0	0
ENSG00000220184	HMGB3P18	0	0	0	0	0	0	0	0
ENSG00000199932	RNU1-18P	0	0	0	0	0	0	0	0
ENSG00000025156	HSF2	3.30964	2.3029	1.19594	1.9728	4.35359	3.92434	3.57226	2.43145
ENSG00000279453	Z99129.4	4.63583	2.00425	0.57506	1.74236	3.62322	2.17732	2.20778	1.62623
ENSG00000111897	SERINC1	109.588	88.4435	27.8127	52.1214	87.1849	47.9818	54.8	80.3576
ENSG00000275339	Z99129.1	0	0	0	0	0	0	0	0
ENSG00000135549	PKIB	0.08543	0	0.03139	0.25358	0.10387	0.07536	0.0712	0.07867
ENSG00000279114	Z99129.3	0.0114	0	0	0	0.01386	0	0.019	0.014
ENSG00000277408	Z99129.2	0	0	0	0	0	0	0	0
ENSG00000272472	AL512283.1	0	0	0	0	0	0	0	0
ENSG00000240606	RN7SL564P	0.2932	0	0.86171	2.61086	0.35647	0.51725	0.48872	3.59988
ENSG00000164434	FABP7	0	0	0	0	0	0	0	0
ENSG00000172594	SMPDL3A	3.1829	2.70091	0.77954	1.01885	1.40371	1.59644	0.41611	1.26433
ENSG00000232362	ATP5MGP2	0	0	0	0	0	0	0	0
ENSG00000146352	CLVS2	0	0	0	0	0	0	0	0
ENSG00000186439	TRDN	0	0	0	0	0	0	0	0
ENSG00000235535	TRDN-AS1	0	0	0	0	0	0	0	0
ENSG00000188580	NKAIN2	0.05239	0.08721	0.02566	0.10368	0.04247	0.21567	0.05822	0.02144
ENSG00000237321	AL354936.1	0	0	0	0.16762	0	0	0	0
ENSG00000236548	RNF217-AS1	0.08132	0.16243	0.1434	0.12069	0.17796	0.11477	0.35242	0.17971
ENSG00000146373	RNF217	0.90055	1.11707	0.60689	0.85129	1.03908	0.99167	1.0804	0.83807
ENSG00000111907	TPD52L1	0	0	0	0.01432	0.02347	0	0	0
ENSG00000111906	HDDC2	1.26981	1.09824	0.53052	0.81294	1.37731	2.15227	0.93378	1.25336
ENSG00000226409	AL450332.1	0	0	0	0	0	0	0	0
ENSG00000237742	AL365259.1	0	0	0	0.03511	0	0	0	0
ENSG00000224506	LINC02523	0	0	0	0	0	0	0	0
ENSG00000135547	HEY2	0.08023	0	0.0393	0.03969	0	0	0	0
ENSG00000111912	NCOA7	7.74247	8.36273	4.40773	6.71912	6.245	8.38377	4.12476	4.76455
ENSG00000232131	NCOA7-AS1	0	0	0	0	0	0	0	0
ENSG00000222445	RN7SKP56	0	0.73721	0	0.65732	0	2.34402	0	0
ENSG00000111911	HINT3	8.13957	8.4677	2.99025	3.18413	6.50761	4.60434	3.17064	4.77957
ENSG00000251920	RNA5SP216	0	0	0	0	0	0	0	0

ENSG00000066651	TRMT11	2.97016	3.17494	1.56165	3.55879	3.08099	2.16321	4.2695	3.17833
ENSG00000231842		0.25146	0.41855	0.36952	0.1866	0.15286	0.2218	0.20957	0.15437
ENSG00000213133	PPP1R14BP5	0	0	0	0	0	0	0	0
ENSG00000203760	CENPW	0.55321	0.46041	0	0.13684	1.12096	2.6025	1.22949	0.45282
ENSG00000207632	MIR588	0	0	0	0	0	0	0	0
ENSG00000201613	RNU6-200P	0	0	0	0	0	0	0	0
ENSG00000220548	VIM2P	0	0	0	0	0	0	0	0
ENSG00000275953	AL359535.1	0	0	0	0	0	0	0	0
ENSG00000217325	PRELID1P1	0	0	0	0	0	0	0	0
ENSG00000217404	RPS4XP9	0	0	0	0	0	0	0	0
ENSG00000146374	RSPO3	0	0	0	0	0	0	0	0
ENSG00000118518	RNF146	5.41186	6.60821	1.90372	3.1631	6.17313	2.54348	3.72671	6.49067
ENSG00000093144	ECHDC1	6.91951	4.79181	1.59351	2.95051	6.56054	5.10142	2.45307	5.11959
ENSG00000251768	RNA5SP217	1.611	0	0	0	0	1.42101	0	0
ENSG00000213131	YWHAZP4	0	0	0	0	0	0.18264	0	0
ENSG00000220744	RPL5P18	0	0	0	0	0	0	0	0
ENSG00000220522	AL590002.1	0	0	0	0	0	0	0	0
ENSG00000220694	AL096711.1	0	0	0	0	0	0	0	0
ENSG00000255330	AL096711.2	0	0	0	0	0	0	0	0
ENSG00000189367	KIAA0408	0.01014	0	0	0.01505	0	0	0	0
ENSG00000214338	SOGA3	0	0.02821	0	0	0	0	0	0
ENSG00000184530	C6orf58	0	0	0	0	0	0	0	0
ENSG00000219284	AL592291.1	0.20824	0.34662	0	0	0	0.36736	0	0
ENSG00000218187	AL356432.1	0	0	0	0	0	0	0.2916	0
ENSG00000260212	AL356432.3	0	0	0	0	0	0	0	0
ENSG00000219699	AL356432.2	0	0	0	0	0	0	0	0
ENSG00000281248	LINC02536	0	0	0	0	0	0	0	0
ENSG00000172673	THEMIS	3.13002	1.69889	1.09989	1.41379	3.43313	8.91288	1.13419	1.35758
ENSG00000274493	MRPS17P5	0	0	0	0	0	0	0	0
ENSG00000152894	PTPRK	0.24433	0.10845	0.01197	0.0967	0.63372	0.22989	0.04073	0.13
ENSG00000227945	AL590006.1	0.09747	0	0	0	0	0	0	0
ENSG00000224733	AL034349.1	0	0	0	0	0	0	0	0
ENSG00000213130	EEF1DP5	0	0	0	0	0.11544	0	0	0
ENSG00000206982	RF00019	0	0	0	0	0	0	0	0
ENSG00000220110	AL080315.1	0	0	0	0	0	0	0	0
ENSG00000202200	RF00019	0	0	0	0	0	0	0	0
ENSG00000196569	LAMA2	0.21785	0.15713	0.05336	0.10777	0.13243	0.19216	0.04842	0.18723
ENSG00000220884	MESTP1	0	0	0	0	0	0	0	0
ENSG00000219642	BMPR1AP1	0	0	0	0	0	0	0	0
ENSG00000252554	RNU6-861P	0	0	0	0	0	0	0	0
ENSG00000233351	AL356124.2	0	0	0	0	0.22792	0	0	0
ENSG00000226149	AL356124.1	0.84838	0.98849	0.24934	0.50364	0.51572	1.49666	0.28282	0.83331
ENSG00000146376	ARHGAP18	19.1466	13.016	3.31963	6.98667	9.77595	11.259	7.6626	8.90273
ENSG00000218689	RPL5P21	0	0	0	0	0	0	0	0
ENSG00000219666	AL451046.2	0	0	0	0	0	0	0	0
ENSG00000218725	B3GALNT2P1	0	0	0	0	0	0	0	0
ENSG00000219302	AL451046.1	0	0	0	0	0	0.24216	0	0
ENSG00000203756	TMEM244	0.32821	0.3642	0	0.32473	0	0.193	0	0
ENSG00000198945	L3MBTL3	6.26486	4.05661	1.11653	1.85104	6.43146	4.60292	1.52934	2.76345
ENSG00000227678	AL355581.1	0.30269	0.8397	0.07413	0.18717	0.24533	0.53398	0.04204	0.34066
ENSG00000164483	SAMD3	4.9589	6.13312	2.63706	7.40682	5.91271	9.17902	2.70804	4.53701
ENSG00000164484	TMEM200A	0.23888	0.19881	0.07021	0.08863	0.21782	0.04214	0.01991	0.01466
ENSG00000202438	RF00019	0	0	0	0	0	0	0	0
ENSG00000229923	AL583803.1	0	0	0	0	0	0	0	0
ENSG00000256162	SMLR1	0	0	0	0	0	0	0	0
ENSG00000079819	EPB41L2	1.71176	0.91033	0.15656	0.62193	1.05351	0.61398	0.3315	0.2965
ENSG00000118507	AKAP7	27.7921	47.7316	41.1098	111.162	77.6171	49.0879	73.3805	59.684
ENSG00000218857	AL137222.1	0	0	0	0	0	0	0	0

ENSG00000219776	RPL21P67	0	0	0	0	0	0	0	0
ENSG00000118520	ARG1	2.38739	4.28427	2.0282	3.93068	4.03633	5.33041	6.40436	4.7632
ENSG00000112282	MED23	6.52701	6.56792	1.96187	3.74263	7.14184	7.25765	3.51112	5.46393
ENSG00000154269	ENPP3	1.05658	0.21984	0.1109	0.28001	0.48171	0.06657	0.25159	0.27798
ENSG00000252560	RNU4-18P	0	0	0	0	0	0	0	0
ENSG00000180658	OR2A4	0	0	0	0	0	0	0	0
ENSG00000236761	CTAGE9	0	0	0	0	0	0	0	0
ENSG00000216917	AC005587.1	0	0	0	0	0	0	0	0
ENSG00000266807	MIR548H5	0	0	0	0	0	0	0	0
ENSG00000197594	ENPP1	0.03535	0.01471	0	0.06558	0.03223	0	0	0.0217
ENSG00000219532	SELENOKP2	0	0	0	0	0	0	0	0
ENSG00000200895	RN7SKP245	0	0	0	0	0	0	0	0
ENSG00000237115	AL139805.1	0	0	0	0	0	0	0	0
ENSG00000236673	AL117378.1	0	0	0	0	0	0	0	0
ENSG00000280058	AL117378.2	0	0	0	0	0	0	0	0
ENSG00000118523	CTGF	0.06268	0	0	0	0.1143	0.05529	0	0
ENSG00000227220	AL133346.1	0	0.24648	0	0.21977	0	0	0.24683	0
ENSG00000236166	AL021408.1	0.09826	0	0.14439	0.14583	1.19459	0.17334	0	0.24128
ENSG00000265669	MIR548AJ1	0	0	0	0	0	0	0	0
ENSG00000280155	AL021408.2	0	0	0	0.14091	0.05772	0	0	0
ENSG00000228495	LINC01013	0.15685	0	0	0.15519	0.21188	0.03074	0.02905	0.0214
ENSG00000223912	EEF1A1P36	0	0	0	0	0	0	0	0
ENSG00000079931	MOXD1	0	0.02305	0	0.06166	0.06735	0.07329	0.11542	0.03401
ENSG00000079950	STX7	8.04707	10.714	2.79358	6.29333	8.85348	3.62202	4.65996	7.90296
ENSG00000225343	RPL21P66	0	0	0	0	0	0	0	0
ENSG00000237110	TAAR9	0	0	0	0	0	0	0	0
ENSG00000146385	TAAR8	0	0	0	0	0	0	0	0
ENSG00000235998	TAAR7P	0	0	0	0	0	0	0	0
ENSG00000146383	TAAR6	0	0	0	0	0	0	0	0
ENSG00000135569	TAAR5	0	0	0	0	0	0	0	0
ENSG00000237449	TAAR4P	0	0	0	0	0	0	0	0
ENSG00000179073	TAAR3P	0	0	0	0	0	0	0	0
ENSG00000146378	TAAR2	0	0	0	0	0	0	0	0
ENSG00000146399	TAAR1	0	0	0	0	0	0	0	0
ENSG00000218194	HLFP1	0	0	0	0	0	0	0	0
ENSG00000112299	VNN1	15.4105	8.7205	2.21948	1.64615	2.4388	3.1641	4.24838	2.5788
ENSG00000218991	CCNG1P1	0	0	0	0	0	0	0	0
ENSG00000093134	VNN3	13.0172	18.6534	2.38057	6.30765	10.9832	2.42081	6.60778	20.4752
ENSG00000112303	VNN2	226.749	469.73	19.1455	41.8947	215.633	35.5079	58.7932	196.196
ENSG00000234484	AL032821.1	0.25232	0	0	0	0	0	0	0.1549
ENSG00000271488	RBM11P1	0	0	0	0	0	0	0	0
ENSG00000146409	SLC18B1	7.91788	8.48668	4.14284	6.23159	5.39659	4.44443	2.89953	3.24048
ENSG00000112306	RPS12	456.813	677.33	605.136	573.572	629.394	566.308	896.361	1130.3
ENSG00000206754	SNORD101	13.0535	6.6854	1.47553	10.4315	7.32466	3.54279	5.02111	2.46567
ENSG00000221500	SNORD100	1.92896	1.60537	2.83458	5.72556	1.17259	0	6.43054	2.36834
ENSG00000200534	SNORA33	6.76619	6.56969	0.82857	6.69451	5.4841	5.96824	9.39849	6.23057
ENSG00000220557	HMGB1P13	0	0	0	0	0	0	0	0
ENSG00000279960	AL137783.1	0	0	0	0	0	0	0	0
ENSG00000213122	RPL23AP46	0	0	0	0	0	0	0	0
ENSG00000231023	LINC00326	0	0	0	0.04325	0	0	0	0
ENSG00000220113	MTCYBP4	0	0	0	0	0	0	0	0
ENSG00000112319	EYA4	0	0	0	0	0	0	0	0
ENSG00000223542	AL024497.1	0	0	0	0	0	0	0	0
ENSG00000234567	AL024497.2	0	0	0	0	0	0	0	0
ENSG00000227954	TARID	0	0	0	0	0	0	0	0
ENSG00000238059	HSPE1P21	0	0	0	0	0	0	0	0
ENSG00000272428	AL450270.1	0	0	0	0	0	0	0	0
ENSG00000218213	FTH1P26	0	0	0	0	0	0	0	0

ENSG00000223586	LINC01312	0	0	0	0	0	0	0	0
ENSG00000118526	TCF21	0	0	0	0	0	0	0	0
ENSG00000028839	TBPL1	7.37513	13.0991	13.6379	24.5248	8.55973	12.4462	15.1301	11.5555
ENSG00000146411	SLC2A12	0.09843	0.02731	0	0	0	0.02894	0	0.02014
ENSG00000278744	AL449363.1	0	0	0	0	0	0	0	0
ENSG00000216753	HMGA1P7	0	0	0	0	0	0	0	0
ENSG00000266875	RN7SL408P	0	0	0	0.36752	0	0	0	0
ENSG00000118515	SGK1	21.8316	10.1104	4.64619	4.05007	17.1687	6.85901	4.16968	9.14202
ENSG00000276703	RPS29P32	0	0	0	0	0	0	0	0
ENSG00000200058	RNA5SP218	0	0	0	0	0	0	0	0
ENSG00000201309	RF00019	0	0	0	0	0	0	0	0
ENSG00000220378	KRT8P42	0	0	0	0	0	0	0	0
ENSG00000213118	CHCHD2P4	0	0	0	0	0	0	0	0
ENSG00000231971	AL078590.2	0	0	0	0	0	0	0	0
ENSG00000236700	LINC01010	0.02838	0	0	0	0	0	0	0.06968
ENSG00000232310	AL078590.3	0	0	0	0	0	0	0	0
ENSG00000218772	FAM8A6P	0	0	0	0	0	0	0	0
ENSG00000229722	AL078590.1	0	0	0	0	0	0	0	0
ENSG00000227723	AL596188.1	0	0	0	0	0	0	0	0
ENSG00000236389	AL121970.1	0	0	0	0	0	0	0	0
ENSG00000213117	AL021939.1	0	0	0	0	0	0	0	0
ENSG00000118514	ALDH8A1	0.12779	0.17017	0	0.03793	0.12429	0	0.0852	0.1569
ENSG00000240056	AL445190.1	0	0	0	0	0	0	0	0
ENSG00000112339	HBS1L	4.64938	3.14393	2.01861	3.59711	4.8577	6.47782	5.07479	2.99206
ENSG00000283409	MIR3662	0.77158	0	0	0	1.87614	0	1.28611	2.84201
ENSG00000232876	AL353596.1	0.09279	0.15444	0	0.55081	0.11281	0	0.46398	0
ENSG00000118513	MYB	1.85486	1.46597	1.00007	1.70318	2.4011	2.94265	1.26785	1.58924
ENSG00000236703	MYB-AS1	0	0	0	0	0	0	0	0
ENSG00000207689	MIR548A2	0	0	0	0	0	0	0	0
ENSG00000224374	AL023693.1	0	0	0	1.00728	0	0	0	0
ENSG00000135541	AHI1	0.9852	0.98111	0.59394	1.39966	1.30015	1.26267	0.75793	1.07521
ENSG00000234084	AL049552.1	0	0	0	0	0	0	0	0
ENSG00000201012	RF00019	0	0	0	0	0	0	0	0
ENSG00000231028	LINC00271	0.01916	0	0	0	0.0699	0	0	0.02353
ENSG00000226540	GAPDHP73	0	0	0	0	0	0	0	0
ENSG00000233534	AL035604.1	0	0	0	0	0	0	0	0
ENSG00000217482	HMGB1P17	0	0.19428	0	0	0	0	0	0
ENSG00000236378	AL133319.1	0	0	0	0	0	0	0	0.11583
ENSG00000171408	PDE7B	0.25888	0.22571	0.09058	0.10977	0.46462	0.21748	0.20548	0.07568
ENSG00000237596	AL138828.1	0	0.01853	0	0	0	0.01964	0.01855	0
ENSG00000213111	COX5BP2	0	0	0	0	0	0	0	0
ENSG00000277973	AL138828.2	0	0	0	0	0	0	0	0
ENSG00000146410	MTFR2	0.26927	0.1992	0.13189	0.31082	0.10912	0.68614	0.29922	0.29387
ENSG00000029363	BCLAF1	35.493	24.0997	6.21448	12.7466	29.9517	22.9243	17.3024	19.1644
ENSG00000216519	AL023284.1	0	0	0	0	0	0	0	0
ENSG00000260418	AL023284.4	0	0	0	0	0	0	0	0
ENSG00000135525	MAP7	1.09002	1.03036	0.43505	0.55921	1.60335	0.68846	0.78508	0.5948
ENSG00000220660	AL023284.3	0	0	0	0	0	0	0	0
ENSG00000216613	AL023284.2	0	0	0	0	0	0	0	0
ENSG00000218424	NDUFS5P1	0	0	0	0	0	0	0	0
ENSG00000271765	RN7SKP299	0	0	0	0	0	0	0	0
ENSG00000272189	AL024508.2	0	0	0	0	0	0	0	0
ENSG00000197442	MAP3K5	21.328	24.5489	8.93723	15.7342	18.6871	18.8249	11.5199	17.9044
ENSG00000234263	AL024508.1	0	0	0	0	0	0	0	0.17821
ENSG00000212242	RNA5SP219	0	0	0	0	0	0	0	0
ENSG00000112357	PEX7	1.31821	0.6649	0.99789	1.48209	1.89403	1.05704	2.397	1.27516
ENSG00000201807	RF00443	0	0	0	0	0	0	0	0
ENSG00000213109	AL365223.1	0	0	0	0	0	0	0	0

ENSG00000182747	SLC35D3	0.3418	0	0.09132	0.18446	0.49111	0	0.05179	0.34335
ENSG00000207247	RF00019	0	0	0	0	0	0	0	0
ENSG00000230350	RPL35AP3	0	0	0	0	0	0	0	0
ENSG00000225391	NHEG1	0	0	0	0	0	0	0	0
ENSG00000235399	AL135902.1	0.19089	0	0.28051	0.2833	0.23207	0	0	0.46873
ENSG00000016402	IL20RA	0	0	0	0	0	0	0	0
ENSG00000164485	IL22RA2	0	0	0	0	0	0	0	0
ENSG00000027697	IFNGR1	86.5007	91.1623	18.895	32.6653	62.5654	25.3493	46.3344	61.9933
ENSG00000177468	OLIG3	0	0	0	0	0	0	0	0
ENSG00000213108	BTF3L4P3	0	0	0	0	0	0	0	0
ENSG00000230533	AL356234.2	0	0	0	0	0	0.09856	0	0
ENSG00000283265	AL356234.3	0	0	0	0	0	0	0	0
ENSG00000220412	AL356234.1	0.0412	0.13717	0	0	0	0	0	0
ENSG00000234956	LINC02539	0	0	0	0.14761	0	0	0	0
ENSG00000207300	RF00019	0	0	0	0	0	0	0	0
ENSG00000237499	AL357060.1	1.03998	0.92521	0.26349	1.01122	0.58859	1.45507	0.56786	0.46231
ENSG00000118503	TNFAIP3	37.0057	14.2275	4.67023	12.5133	15.0674	67.792	15.1183	5.80856
ENSG00000226004	LINC02528	0	0	1.31588	0	0.31105	2.7081	0	0.47119
ENSG00000229922	LINC02528	0	0	0	0	0	0	0	0
ENSG00000219463	RPSAP42	0	0	0	0	0	0	0	0
ENSG00000112378	PERP	0.71763	0.4266	0.17576	0.40573	0.5401	0.87414	0.05696	0.18881
ENSG00000112379	ARFGEF3	0.01478	0.0082	0.00724	0.00731	0.00599	0	0.00821	0
ENSG00000254440	PBOV1	0	0	0	0	0	0	0	0
ENSG00000262543	SMIM28	0	0	0	0	0	0	0	0
ENSG00000218499	AL031003.1	0	0	0	0	0	0	0	0
ENSG00000215878	MARCKSL1P2	0	0	0	0	0	0	0	0
ENSG00000051620	HEBP2	5.42179	4.85395	0.83209	1.44964	3.02047	1.51089	1.55734	1.95532
ENSG00000135540	NHSL1	0.31027	0.1675	0.02465	0.08712	0.09176	0.07397	0.02796	0.07207
ENSG00000266555	MIR3145	0	0	0	0	0	0	0	0
ENSG00000274594	AL391669.1	0	0	0	0	0	0	0	0
ENSG00000216802	AL590617.1	0.19599	0.16311	0	0	0	0	0	0
ENSG00000225177	AL590617.2	0.06008	0.30002	0	0	0.14609	0.31798	0.2003	0
ENSG00000279968	GVQW2	0.02449	0.04076	0.07198	0.03635	0.14888	0.0432	0	0
ENSG00000024862	CCDC28A	9.72798	11.9823	4.14559	3.24841	8.16065	5.57748	6.08064	8.53987
ENSG00000203734	ECT2L	0.0317	0	0	0.02353	0.01927	0	0.02642	0.05839
ENSG00000225415	CCRL1P1	0	0	0	0	0	0	0	0
ENSG00000220600	AL121834.1	0	0.2397	0	0	0	0	0	0
ENSG00000135597	REPS1	4.38184	3.71032	2.53524	6.52578	5.49438	5.66968	5.76418	4.2646
ENSG00000146386	ABRACL	15.6985	15.0477	4.93689	13.5982	23.3189	20.1511	8.55262	18.0744
ENSG00000112406	HECA	43.0349	36.5547	13.8911	16.2578	29.0018	28.7692	26.138	25.8898
ENSG00000231329	AL031772.1	0	0	0	0.02322	0	0.0276	0	0
ENSG00000272446	AL158850.1	0	0	0	0	0	0	0	0
ENSG00000164440	TXLNB	0.20786	0.12357	0.10909	0.13221	0.19856	0.18335	0.0495	0.10937
ENSG00000226571	AL592429.2	0.0802	0.13349	0	0	0.58501	0	0	0.2954
ENSG00000218565	AL592429.1	0.09953	0	0	0	0.363	0	0.08295	0
ENSG00000164442	CITED2	9.59989	8.37455	1.65714	4.54883	8.191	12.3956	4.77154	4.33122
ENSG00000238099	LINC01625	0	0	0	0	0	0	0	0
ENSG00000219806	ATP5PBP6	0	0	0.14044	0	0	0	0	0
ENSG00000205695	AL390205.1	0	0	0	0	0	0	0	0
ENSG00000231426	FILNC1	0.27897	0.11609	0	0.10351	0	0	0	0
ENSG00000225148	AL050338.1	0	0	0	0	0	0	0	0
ENSG00000236013	AL357146.1	0	0	0	0	0	0	0	0
ENSG00000252107	RNA5SP220	0	0	0	0	0	0	0	0
ENSG00000263514	MIR3668	0	0	0	0	0	0	0	0
ENSG00000264390	MIR4465	0	0	0	0	0	0	0	0
ENSG00000217684	RPS3AP24	0	0	0	0	0	0	0	0
ENSG00000234147	AL035446.1	0	0.26933	0.23778	0	0	0	0	0
ENSG00000216548	AL357084.1	0	0	0	0	0	0	0	0

ENSG00000275138	AL357080.1	0	0	0	0	0	0	0	0
ENSG00000259828	AL355596.1	0	0	0	0	0	0	0	0
ENSG00000222764	RN7SKP106	1.34743	0.44856	0	0	0.32763	0	0.44919	0.66174
ENSG00000218351	RPS3AP23	0.2575	0	0	0	0	0	0	0
ENSG00000135577	NMBR	0	0	0	0	0	0	0	0
ENSG00000270983	AL589674.1	0	0	0	0	0	0	0	0
ENSG00000236822	NMBR-AS1	0	0	0	0	0	0	0	0
ENSG00000203733	GJE1	0	0	0	0	0	0	0	0
ENSG00000009844	VTA1	4.04757	5.14109	1.92823	3.02599	5.08046	4.38043	2.2545	3.2965
ENSG00000237494	AL360007.1	0	0	0	0	0	0	0	0
ENSG00000112414	ADGRG6	0.08651	0.0432	0	0.05136	0.13673	0	0.04326	0
ENSG00000236366	AL359313.1	0	0	0	0	0	0	0	0
ENSG00000233138	AL023584.1	0	0	0	0	0	0	0	0
ENSG00000010818	HIVEP2	19.3304	14.3687	2.19824	4.82723	13.7404	14.1877	4.11767	5.85545
ENSG00000237851	AL023584.2	0	0	0	0	0	0	0	0
ENSG00000232618	AL355304.1	1.32311	0.22023	0.19443	0	1.44774	0.46683	0.22054	0.6498
ENSG00000229017	LINC01277	0.20656	0.14735	0	0	0.07175	0	0.04919	0.03623
ENSG00000227192	AL023581.2	0	0	0	0	0	0	0	0
ENSG00000146416	AIG1	4.19138	4.07641	1.50398	1.3562	1.6739	2.08498	1.29979	2.10931
ENSG00000225752	AL023581.1	0	0	0	0	0	0	0	0
ENSG00000217495	AL136116.2	0	0	0	0	0	0	0	0
ENSG00000216642	AL136116.1	0	0	0	0	0	0	0	0
ENSG00000217648	AL136116.3	0	0	0	0.09559	0	0	0	0.15817
ENSG00000270655	AL031320.1	0	0	0	0	0	0	0	0
ENSG00000189007	ADAT2	0.48016	0.44212	0.13511	0.36388	0.52166	0.5587	0.63006	0.36375
ENSG00000218896	TUBB8P2	0	0	0	0	0	0	0	0
ENSG00000223203	RNA5SP221	0	1.18455	0	0	0	1.25545	0	1.74752
ENSG00000034693	PEX3	2.83701	1.64728	0.77562	2.30105	3.16841	3.78275	1.42965	2.10614
ENSG00000278206	AL031320.2	0.10577	0	0	0.05233	0	0.2488	0.05877	0
ENSG00000229036	VDAC1P8	0.12845	0.14253	0.02097	0.06354	0.06941	0.27695	0.11895	0.08761
ENSG00000001036	FUCA2	6.48234	3.84416	1.15697	1.75272	1.97824	3.37979	1.8373	2.22334
ENSG00000112419	PHACTR2	7.78457	5.33733	2.12088	4.03197	4.66068	9.21681	2.06827	3.3356
ENSG00000235740	PHACTR2-AS1	0	0	0.1902	0	0	0	0.07191	0
ENSG00000257065	AL049844.1	0.09619	0	0.14136	0.28553	0	0.1697	0	0
ENSG00000135521	LTV1	4.72265	3.96346	0.87477	1.35467	4.43895	3.01051	2.84448	1.90032
ENSG00000280148	AL049844.3	0	0	0	0	0	0	0	0
ENSG00000270890	AL049844.2	0	0	0	0	0	0	0	0
ENSG00000118491	ZC2HC1B	0.06756	0	0	0	0.08214	0.11918	0	0
ENSG00000118495	PLAGL1	1.11813	1.34337	0.29649	0.34935	0.94033	0.53392	0.43441	0.90833
ENSG00000283122	HYMAI	0.1971	0.10936	0.06436	0	0.07988	0.03864	0.0365	0.02689
ENSG00000276680	RF02143	0	0	0	0	0	0	0	0
ENSG00000217231	AL109755.1	0	0	0	0	0	0	0	0.13867
ENSG00000169976	SF3B5	17.1351	11.0917	4.50751	5.33725	12.4738	18.2865	7.40487	8.70101
ENSG00000219297	MRPL42P3	0	0	0	0	0	0	0	0
ENSG00000135604	STX11	64.3186	72.597	8.61264	7.22979	50.4347	36.5242	13.4487	28.1299
ENSG00000217027	TPT1P4	0	0	0	0.62882	0.68683	0.24916	0.23541	0.1734
ENSG00000216475	AL024474.1	0	0	0	0	0	0	0	0
ENSG00000152818	UTRN	27.5654	26.7259	8.2681	15.9484	23.4142	24.1109	14.2811	18.5429
ENSG00000225311	AL024474.2	0	0	0	0	0	0	0	0
ENSG00000219409	AL590704.1	0.09232	0.15366	0.13566	0.27402	0.11224	0	0.30776	0.34004
ENSG00000217195	AL513475.1	0	0	0	0	0	0	0	0
ENSG00000220739	AL513475.2	0	0	0	0	0	0	0	0
ENSG00000112425	EPM2A	0.24017	0.22282	0.12729	0.18114	0.4356	0.26394	0.27564	0.23687
ENSG00000201119	RNU1-33P	0	0	0	0	0	0	0	0
ENSG00000217612	AL031119.1	0	0	0	0	0.04743	0	0	0
ENSG00000270638	AL023806.1	0.17781	0.29596	0.06532	0.06597	0.27021	0.15684	0.14819	0.10915
ENSG00000270828	AL023806.2	0.55742	0	0	0.82727	0	0	0	0
ENSG00000280184	AL023806.3	0.24397	0.13536	0	0.08046	0.03296	0.04782	0.27111	0.13313

ENSG00000118496	FBXO30	4.03563	4.52335	2.4869	4.03313	4.32279	3.87548	8.5712	3.76611
ENSG00000235652	AL356599.1	0.11985	0.01995	0.03522	0.1423	0.26228	0.08457	0.05993	0.10301
ENSG00000146414	SHPRH	2.69312	2.35204	0.87945	2.19582	3.25402	2.68346	2.14753	1.91863
ENSG00000152822	GRM1	0	0	0	0	0	0	0	0
ENSG00000187103	FUNDC2P3	0	0	0	0	0	0	0	0
ENSG00000222971	RNA5SP222	0	0	0	0	0	0	0	0
ENSG00000216811	AL035698.1	0	0	0	0	0	0	0	0
ENSG00000118508	RAB32	17.4668	13.2335	3.71733	4.73759	7.83524	8.07535	4.81894	7.83869
ENSG00000237468	AL359547.1	0	0	0	0	0	0	0	0.04109
ENSG00000118492	ADGB	0.02829	0.03139	0.04157	0.014	0.04586	0.01664	0	0.03473
ENSG00000273719	AL359547.2	0	0	0	0	0	0	0	0
ENSG00000207431	RNU6-906P	0	0	0	0	0	0	0	0
ENSG00000228283	KATNBL1P6	0.05	0.08323	0.2939	0.14841	0.06079	0	0	0.49112
ENSG00000233452	STXBP5-AS1	0.89019	0.54803	0.39423	0.57913	0.38547	0.60235	0.44717	0.68871
ENSG00000227748	AL138916.1	0	0	0	0	0	0	0	0
ENSG00000272397	AL138916.2	0	0	0	0.05074	0	0	0.05699	0
ENSG00000196634	LUADT1	0	0	0	0	0	0	0	0
ENSG00000164506	STXBP5	16.6433	11.8942	3.03535	6.3486	10.902	6.0815	5.87405	9.35613
ENSG00000273997	AL355365.1	0.74796	0	0	0	0.90935	0	0	0
ENSG00000220494	YAP1P1	0	0	0	0	0	0	0.08771	0.06461
ENSG00000203727	SAMD5	0	0	0	0	0	0	0	0
ENSG00000227681	AL033504.1	0	0	0	0	0	0	0	0
ENSG00000226249	AL365271.1	0	0	0	0	0	0	0	0
ENSG00000234675	AL445123.1	0	0	0	0	0	0	0	0
ENSG00000230205	AL359382.1	0	0	0	0	0	0	0	0
ENSG00000224658	AL513164.1	0	0	0	0	0	0	0	0
ENSG00000111961	SASH1	0.32233	0.32848	0.12566	0.13668	0.41587	0.18568	0.12061	0.29076
ENSG00000207345	RNU6-1222P	0	0	0	0	0	0	0	0
ENSG00000215223	CYP51A1P3	0	0.08544	0	0	0.12481	0	0	0
ENSG00000217824	SNRPEP6	0	0.44046	0	0	0	0	0	0
ENSG00000231368	RPSAP40	0	0	0	0	0	0	0	0
ENSG00000111962	UST	0.96384	0.56151	0.14164	0.66754	1.40618	0.48177	0.32132	0.45363
ENSG00000227660	UST-AS1	0	0	0	0	0	0	0	0
ENSG00000236591	AL357992.1	0	0	0	0	0	0	0	0
ENSG00000219487	AL603766.1	0	0	0	0	0	0.38949	0	0
ENSG00000228408	AL031056.1	0.07708	0	0.03775	0	0	0.04532	0	0.03154
ENSG00000055208	TAB2	26.4174	22.4325	7.97549	11.9465	20.7148	18.3963	15.3691	13.3723
ENSG00000225924	TAB2-AS1	0	0	0	0	0	0	0	0
ENSG00000283608	AL031056.2	0	0	0	0	0	0	0	0
ENSG00000241391	RN7SL234P	0	0	0	0	0	0	0	0
ENSG00000216265	FABP12P1	0	0	0	0	0	0	0	0
ENSG00000177688	SUMO4	1.78781	2.518	0.40418	0.51025	1.50478	0.60653	0.80231	1.26638
ENSG00000178199	ZC3H12D	0.89271	0.52551	0.07999	0.29083	0.5162	0.88346	0.14517	0.29406
ENSG00000219553	AL031133.1	0	0	0	0	0	0	0	0
ENSG00000131013	PPIL4	15.0978	17.8448	10.9912	22.5712	24.7883	20.1347	23.5033	18.0266
ENSG00000252244	RNU7-3P	0	0	0	0	0	0	0	0
ENSG00000235168	AL078581.2	0	0	0	0	0	0	0	0
ENSG00000055211	GINM1	11.8689	8.19541	8.86436	17.8083	13.201	9.14616	17.5553	12.9711
ENSG00000281021	AL078581.3	0	0	0	0	0	0.17381	0	0
ENSG00000233330	AL078581.1	0.21559	0	0	0.31996	0	0	0	0
ENSG00000220848	RPS18P9	0.47805	0.79571	0.23416	0.23649	1.54986	0.56223	0	0
ENSG00000202343	RF00410	1.08593	0	0	0	0	0.95787	0	1.33329
ENSG00000186625	KATNA1	6.5424	7.50659	6.25376	14.2345	12.8192	9.69278	17.2577	12.205
ENSG00000207292	RF00019	0	0	2.85966	1.92541	0	0	1.08124	1.59287
ENSG00000131023	LATS1	5.98751	4.75724	2.18095	4.4053	5.67097	5.71527	3.91576	3.86312
ENSG00000278899	AL358852.1	0	0	0	0	0.07028	0	0	0
ENSG00000120253	NUP43	3.44009	4.15688	2.67341	10.2109	5.26818	6.03961	7.14005	4.85318
ENSG00000120265	PCMT1	18.8945	22.5319	15.7463	45.4426	19.6044	17.5795	35.2476	28.4419

ENSG00000219433	BTBD10P2	0.65059	1.08292	0	0	0	0	0	0.13313
ENSG00000231760	AL355312.2	0	0	0	0	0	0	0	0
ENSG00000120256	LRP11	0.64262	0.58658	0.79201	1.72285	0.52926	0.69483	1.00204	0.43268
ENSG00000273132	AL355312.3	0	0	0	0	0	0	0	0
ENSG00000268592	RAET1E-AS1	0	0	0	0	0	0	0	0
ENSG00000217733	CCT7P1	0	0	0	0	0	0	0	0
ENSG00000164520	RAET1E	0	0	0	0	0	0	0	0
ENSG00000223701	RAET1E-AS1	0	0	0	0	0	0	0	0
ENSG00000216906	AL355312.1	0.03369	0	0	0	0	0	0	0
ENSG00000203722	RAET1G	0	0	0	0	0	0	0	0
ENSG00000216621	AL583835.2	0	0	0	0	0	0	0	0
ENSG00000131015	ULBP2	0.16301	0	0	0.08064	0	0.19172	0.27171	0
ENSG00000215094	AL583835.1	0	0	0	0	0	0	0	0
ENSG00000111981	ULBP1	0.04666	0.03883	0	0	0	0	0.07777	0
ENSG00000219298	AL355497.1	0	0	0	0	0	0	0	0
ENSG00000231120	BTF3P10	0	0	0	0.21977	0	0	0	0
ENSG00000218358	RAET1K	0.03866	0	0	0	0	0	0	0.04747
ENSG00000155918	RAET1L	0	0	0	0	0	0	0	0
ENSG00000216444	RAET1M	0	0	0	0	0	0	0	0
ENSG00000235972	AL355497.2	0	0	0	0	0	0	0	0
ENSG00000213091	PHBP1	0	0	0	0	0	0	0	0
ENSG00000131019	ULBP3	0.59353	0	0	0	0	1.04706	0	0.09109
ENSG00000198729	PPP1R14C	0.13625	0.0567	0.05005	0.05055	0	0	0	0
ENSG00000238594	RF01210	0	0	0	0	0	0	0	0
ENSG00000201628	RNU4-7P	0	0	0	0	0	0	0	0
ENSG00000009765	IYD	0	0	0	0	0	0	0	0
ENSG00000220598	SSR1P1	0	0	0	0	0	0	0	0
ENSG00000219622	AL451061.1	0	0	0	0	0	0	0	0
ENSG00000120278	PLEKHG1	1.5896	2.0671	0.29199	0.57504	1.21995	0.45569	0.4968	0.31715
ENSG00000232891	AL450344.3	0.38783	0.32277	0.28496	0.28779	1.41455	0.34209	0	0
ENSG00000224029	AL450344.1	0.22942	0	0	0	0	0	0	0
ENSG00000226599	AL450344.2	0	0	0	0	0	0	0	0
ENSG00000213089	PDCL3P5	0.10152	0.50696	0	0.15067	0	0	0	0
ENSG00000120254	MTHFD1L	0.72994	0.51029	0.25743	0.38999	0.83419	1.28771	0.46234	0.4481
ENSG00000218996	ARL4AP5	0.12237	0	0.17982	0.36322	0.14878	0.21588	0.20397	0
ENSG00000202119	RNU6-302P	0	0	0	0	0	0	0	0
ENSG00000232290	AL133260.2	0	0	0	0	0	0	0	0
ENSG00000223598	AL138733.1	0	0	0	0	0	0	0	0
ENSG00000219747	AL133260.1	0	0.60251	0	0.80582	0.22004	0.31929	0.30168	0.44443
ENSG00000231883	AL138733.2	0	0	0	0	0	0	0	0
ENSG00000238616	RNU6-300P	0	0	0	0	0	0	0	0
ENSG00000213087	AL356535.1	0	0.30655	0	0.27333	0.22391	0	0	0.45225
ENSG00000131016	AKAP12	3.16145	1.29811	0.30807	0.98319	1.82495	0.42902	0.47525	0.43243
ENSG00000252431	RNU6-1247P	0	0	0	0	0	0	0	0
ENSG00000252487	RNY4P20	0	0	0	0	0	0	1.31377	0
ENSG00000223039	RN7SKP268	0	0	0	0	0	0	0	0
ENSG00000181472	ZBTB2	10.0818	8.33132	1.81266	2.85166	7.70037	7.28163	2.84692	4.74742
ENSG00000252615	RF00019	1.46601	1.22008	0	0	0	1.29312	0	0
ENSG00000155906	RMND1	3.24338	2.51935	1.0894	1.78788	2.25327	3.54204	1.54463	2.19968
ENSG00000219395	HSPA8P15	0	0.15523	0	0	0.22676	0.16452	0	0.1145
ENSG00000146476	ARMT1	9.2828	8.87256	2.75112	4.09056	7.27096	6.78898	2.47048	6.19348
ENSG00000120262	CCDC170	2.28599	0.36024	0.13914	0.28105	0.77293	0.23863	0.22547	0.56466
ENSG00000207422	RNU6-813P	0	0	0	0	0	0	0	0
ENSG00000091831	ESR1	0.08067	0.05371	0.01581	0.02395	0.09808	0.08539	0.01793	0.01321
ENSG00000233823	AL356311.1	0	0	0	0	0	0	0	0
ENSG00000131018	SYNE1	9.49989	7.14347	1.68595	3.7904	6.11149	6.13378	3.66032	4.79448
ENSG00000234577	SYNE1-AS1	0	0	0	0	0	0	0	0
ENSG00000226193	AL049548.1	0	0	0	0	0	0	0	0

ENSG00000202044	RNA5SP223	0	0	0	0	0	0	0	0
ENSG00000233464	NANOGP11	0	0	0	0	0	0	0	0
ENSG00000120279	MYCT1	1.91113	1.16774	0.99538	1.2566	1.35293	0.76819	1.49197	2.13854
ENSG00000218792	HSPD1P16	0	0	0	0	0	0	0.3101	0
ENSG00000146469	VIP	0	0	0	0	0	0	0	0.05682
ENSG00000224893	AL160162.1	0	0	0	0	0	0	0	0
ENSG00000217372	TUBB4BP7	0	0	0	0	0	0	0	0
ENSG00000220745	AL080276.1	0	0	0	0	0	0	0	0
ENSG00000112029	FBXO5	1.47251	1.36874	1.01166	2.07184	1.67399	4.18332	1.11566	1.22094
ENSG00000227627	AL080276.2	0.51235	0.28427	0.15058	0.15208	0.74748	0.4218	0.45547	0.29356
ENSG00000112031	MTRF1L	4.46012	2.42704	0.78145	1.2984	2.39841	2.81442	1.68702	2.00087
ENSG00000091844	RGS17	0.0464	0.03089	0.04091	0.01377	0	0.01637	0.03094	0.02279
ENSG00000201939	RNA5SP224	0	0	0	0	0	0	0	0
ENSG00000213121	AL590867.1	0	0	0	0	0	0	0	0
ENSG00000218426	AL590867.2	0.81991	1.0918	0.24097	0.24337	0.79746	2.89288	1.09334	0.40267
ENSG00000237312	AL590867.3	0	0	0	0	0	0	0	0
ENSG00000222806	RNA5SP225	0	0	2.39364	1.61164	1.32025	0.95787	0	0
ENSG00000217160	MTCO2P31	0	0	0	0	0	0	0	0
ENSG00000242246	AL603908.1	0	0	0	0	0	0	0	0
ENSG00000218776	MTATP6P31	0	0	0	0	0	0	0	0
ENSG00000217044	MTCO3P31	0	0	0	0	0	0	0	0
ENSG00000220733	MTND3P20	0	0	0	0	0	0	0	0
ENSG00000220514	MTND4LP20	0	0	0	0	0	0	0	0
ENSG00000216713	MTND4P13	0	0	0	0	0	0	0	0
ENSG00000199246	RNU6-896P	0	0	0	0	0	0	0	0
ENSG00000217085	HMGB3P19	0	0	0	0	0	0	0	0
ENSG00000112038	OPRM1	0	0	0	0	0	0	0	0
ENSG00000074706	IPCEF1	10.3454	9.15712	3.96587	5.39179	12.1045	10.4988	5.11848	6.94573
ENSG00000237593	AL445220.1	0	0	0	0	0	0	0	0
ENSG00000153721	CNKSR3	0.0102	0.05092	0.00999	0.03531	0.04133	0.06596	0.03966	0.02921
ENSG00000275419	AL357075.2	0	0	0	0	0	0	0	0
ENSG00000220181	AL357075.1	0	0	0	0	0	0	0	0
ENSG00000219146	RPS4XP8	0	0	0	0.1411	0	0	0	0
ENSG00000231316	AL591419.1	0	0	0	0	0	0	0	0
ENSG00000205625	AL591499.1	0	0	0	0	0	0	0	0
ENSG00000213079	SCAF8	18.509	19.2774	8.74525	15.9179	20.8606	22.248	20.1293	17.208
ENSG00000200594	RNU6-824P	0	0	0	0	0	0	0	0
ENSG00000146426	TIAM2	0.83903	0.81466	0.44834	1.26407	1.02779	0.85221	1.33496	0.90528
ENSG00000218757	AL121952.1	0	0	0	0	0	0	0	0
ENSG00000264814	MIR1273C	0	0	0	0	0	0	0	0
ENSG00000238963	RF00096	0	0	0	0	0	0	0	0
ENSG00000274451	AL136228.1	0	0	0	0	0	0	0	0
ENSG00000235381	AL596202.1	0.16616	0.15804	0.03488	0.21137	0.17315	0.20938	0	0.23315
ENSG00000029639	TFB1M	1.07164	0.7135	0.27558	0.51689	1.62859	1.08705	0.62519	0.88813
ENSG00000171217	CLDN20	0	0	0	0	0	0	0	0
ENSG00000232529	AL031773.1	0	0	0	0	0	0	0	0
ENSG00000074771	NOX3	0	0	0	0	0	0	0	0
ENSG00000238789	RNU7-152P	0	0	0	0	0	0	0	0
ENSG00000221456	MIR1202	0	0	0	0	0	0	0	0
ENSG00000212295	SNORD28B	0	0	0	0	0	0	0	0
ENSG00000227360	AL512658.1	0	0	0	0.13414	0	0	0.15065	0
ENSG00000220347	AL355297.1	0	0	0	0	0	0	0	0
ENSG00000271265	AL355297.3	0	0	0	0	0	0	0.27395	0
ENSG00000271551	AL355297.4	0.17606	0.09768	0.08624	0	0	0.10353	0.19565	0.07206
ENSG00000049618	ARID1B	1.71031	1.26279	0.39606	0.80494	1.48265	1.63775	0.92901	0.82933
ENSG00000271899	MIR4466	0	0	0	0	0	0	0	0
ENSG00000270487	AL355297.2	0	0	0	0.8433	0	0	0.94713	0
ENSG00000218596	AL162578.1	1.1635	0.96832	0	0.28779	1.17879	1.71047	0.96969	0.71426

ENSG00000233044	AL049820.1	0	0	0	0	0	0	0	0
ENSG00000215712	TMEM242	1.59417	1.12971	0.13917	0.4685	1.42003	0.94673	1.15761	0.67827
ENSG00000217783	LDHAL6FP	0.06775	0	0	0	0	0	0.11292	0
ENSG00000271040	AL390955.2	0	0	0	0	0	0	0	0.17509
ENSG00000213078	AL390955.1	0	0.13063	0	0	0	0	0	0
ENSG00000218631	AL117344.1	0.11899	0	0	0.1766	0	0	0.19834	0.2922
ENSG00000175048	ZDHHC14	0.23728	0.25233	0.04843	0.26411	0.38464	0.22093	0.30762	0.22659
ENSG00000266617	MIR3692	0	0	0	0	0	0	0	0
ENSG00000130340	SNX9	4.94781	2.73644	0.81302	2.04105	4.18966	2.59349	3.60981	1.86321
ENSG00000234361	AL391863.2	0	0	0	0	0	0	0	0
ENSG00000229502	AL391863.1	0	0	0	0	0	0	0	0
ENSG00000236324	AL035634.1	0	0	0	0	0	0	0	0
ENSG00000220867	HSPE1P26	0	0	0	0	0	0	0	0
ENSG00000252658	RNU6-786P	0	0	0	1.20873	0	0	1.35756	0.99997
ENSG00000078269	SYNJ2	1.27907	0.89849	0.23279	1.14071	1.391	0.95227	0.25428	0.61232
ENSG00000233496	SYNJ2-IT1	0	0	0	0	0	0	0	0
ENSG00000122335	SERAC1	0.60306	0.60502	0.15781	0.31877	0.35153	0.40806	0.28917	0.36514
ENSG00000272047	GTF2H5	1.43054	0.63606	0.31676	0.50896	0.70283	0.84698	0.4573	0.6015
ENSG00000130338	TULP4	4.02152	3.15822	0.82418	1.64706	4.20013	4.84196	1.63106	1.78749
ENSG00000188451	SRP72P2	0.03704	0	0	0	0	0.06534	0	0.04548
ENSG00000274023	AL360169.2	0	0.43497	0	0	0	0	0	0.16042
ENSG00000238019	AL360169.1	0	0	0	0	0	0	0	0
ENSG00000243373	RN7SL173P	0	0	0	0.37255	0	0	0	0
ENSG00000219188	CACYBPP3	0	0	0	0	0	0	0	0
ENSG00000146433	TMEM181	7.9692	10.1636	8.03358	19.4967	9.30848	10.676	13.5554	9.20007
ENSG00000218226	TATDN2P2	0	0.05481	0	0	0.04003	0.05809	0	0
ENSG00000278571	MIR7161	9.59885	4.35745	2.56462	2.59014	2.12183	4.61828	4.36358	4.28557
ENSG00000146425	DYNLT1	8.94816	15.5148	5.47884	9.92315	6.98065	8.63833	8.1205	12.3292
ENSG00000164674	SYTL3	11.4911	13.2288	3.01272	6.9118	6.92377	5.9387	5.86432	5.9045
ENSG00000219249	AMZ2P2	0	0	0	0	0	0	0	0
ENSG00000265558	MIR3918	2.36453	1.31192	1.15821	1.16974	0.95824	1.39045	1.31377	0
ENSG00000092820	EZR	43.7346	26.4315	6.18823	12.3043	33.7054	39.8537	16.3053	12.4412
ENSG00000233893	EZR-AS1	0	0	0	0	0.82135	0	0	0
ENSG00000243775	OSTCP1	0	0	0	0	0	0	0	0
ENSG00000203711	C6orf99	0	0.14204	0	0.12664	0.10374	0.15054	0	0
ENSG00000213076	AL627422.1	0	0	0	0	0	0	0	0
ENSG00000223191	RNU6-293P	0	0	0	0	0	0	0	0
ENSG00000130363	RSPH3	2.37966	2.15714	0.3776	0.514	1.29036	0.80808	0.31658	0.57611
ENSG00000271913	AL035530.2	0.11271	0.25015	0	0	0.31974	0.26512	0.18787	0.18451
ENSG00000164691	TAGAP	61.003	106.672	18.8875	28.5466	96.0472	53.6348	14.731	55.9483
ENSG00000226032	AL035530.1	0	0	0	0	0	0	0	0
ENSG00000224478	AL356417.1	0.34698	0	0	0.12874	0.21093	0.30606	0	0
ENSG00000233682	AL356417.2	0	0	0	0	0	0	0	0
ENSG00000164694	FNDC1	0	0	0	0	0	0	0	0
ENSG00000235086	FNDC1-IT1	0	0	0	0	0	0	0	0
ENSG00000231178	AL357832.1	0	0	0	0	0	0	0	0
ENSG00000234777	LINC02529	0	0	0	0	0	0	0	0
ENSG00000216480	AL078604.1	0	0	0	0	0	0	0	0
ENSG00000276413	AL078604.3	0	0	0	0	0	0	0	0
ENSG00000237927	AL078604.2	0.14645	0.18283	0	0.05434	0.08903	0	0.06103	0.22477
ENSG00000112096	SOD2	134.462	154.842	18.4109	27.1377	92.947	23.3967	46.7355	91.0684
ENSG00000220305	HNRNPH1P1	0.06186	0.30888	0	0	0.0752	0.10912	0.10311	0.07595
ENSG00000251988	RNU4ATAC18P	0.58175	0	0	0	0	1.02628	0	1.42852
ENSG00000146457	WTAP	15.2384	16.5329	3.01156	6.195	13.9713	11.4229	8.23617	10.8521
ENSG00000120437	ACAT2	1.94376	1.44741	0.35078	0.70854	1.78275	2.88763	0.76736	0.96298
ENSG00000120438	TCP1	14.0831	13.4905	5.04807	10.3732	13.8855	15.2687	14.3027	9.16586
ENSG00000207392	SNORA20	3.88714	0.92431	12.2402	25.5482	2.02538	5.87781	7.40487	8.18155
ENSG00000206910	SNORA29	0	0	0	0	0	0	0	0

ENSG00000112110	MRPL18	9.64899	9.13955	2.89848	7.0414	9.91626	11.2854	3.99863	5.95617
ENSG00000146453	PNLDC1	0	0.05531	0.24414	0	0.12119	0.23447	0.05539	0.08159
ENSG00000236823	AL035691.1	0	0	0	0	0	0	0	0
ENSG00000130368	MAS1	0	0	0	0.01105	0.00906	0	0	0
ENSG00000197081	IGF2R	54.7381	51.9585	5.62011	10.7903	33.2536	16.3819	10.9963	30.4014
ENSG00000268257	AIRN	0	0	0	0	0	0	0	0
ENSG00000213073	AL353625.1	3.74579	2.96156	0.44723	0.97285	1.85007	0.78471	0.74143	1.72463
ENSG00000175003	SLC22A1	0.53897	0.84435	0	0.04705	0.424	0.16779	0.42277	0.38926
ENSG00000216516	AL645733.1	0	0	0	0	0	0	0	0
ENSG00000112499	SLC22A2	0	0	0	0	0	0	0	0
ENSG00000230234	AL162582.1	0	0	0	0	0	0	0	0
ENSG00000146477	SLC22A3	0	0	0	0	0	0	0	0
ENSG00000213071	LPAL2	0.12348	0.25691	0.02268	0.41233	0.22518	0	0.05146	0.20846
ENSG00000198670	LPA	0	0	0	0	0	0	0.01903	0
ENSG00000243831	AL109933.2	0	0	0	0	0	0	0	0
ENSG00000224477	AL109933.1	0	0	0	0	0	0	0	0
ENSG00000122194	PLG	0	0	0	0	0	0	0	0
ENSG00000276960	AL109933.4	0	0	0	0	0	0	0	0
ENSG00000274903	AL109933.3	0	0	0	0	0	0	0	0
ENSG00000224371	AL391361.2	0	0	0	0	0	0	0	0
ENSG00000220913	AL391361.1	0	0	0	0	0	0	0	0
ENSG00000233342	AL391361.3	0	0	0	0	0	0	0	0
ENSG00000231863	AL139393.1	0	0	0	0	0	0	0	0
ENSG00000272841	AL139393.2	0.28958	0.36151	0.05319	0.16116	0.13202	0.12772	0.06034	0.04444
ENSG00000085511	MAP3K4	3.78098	3.47113	1.24583	2.42967	4.17031	4.0743	2.51767	2.50058
ENSG00000026652	AGPAT4	1.60493	2.39314	0.9704	2.19581	1.46343	1.38617	1.81132	1.16999
ENSG00000279355	AGPAT4-IT1	0.19516	0.58471	0.05736	0.34756	0.14236	0.34428	0.19518	0.23961
ENSG00000185345	PRKN	0.01496	0	0.02199	0	0.10914	0.05279	0.02494	0.03674
ENSG00000227913	KRT8P44	0	0	0	0	0	0	0	0
ENSG00000112530	PACRG	0	0	0	0.04165	0.03412	0	0	0
ENSG00000217557	AL590286.1	0	0	0	0	0	0	0	0
ENSG00000225437	PACRG-AS2	0	0	0	0	0	0	0	0
ENSG00000225683	PACRG-AS3	0	0	0	0	0	0	0	0
ENSG00000281692	PACRG-AS1	0	0	0	0	0	0	0	0
ENSG00000217514	AL031121.1	0	0	0	0	0	0	0	0
ENSG00000270419	CAHM	0.57266	0.13617	0.12022	1.09271	0.29838	0.14432	0.40909	0.40177
ENSG00000112531	QKI	19.3	14.7312	4.10563	7.4975	11.0062	6.67853	7.45688	9.28056
ENSG00000228692	AL445307.1	0.36528	0	0	0	0	0	0	0
ENSG00000235538	AL137005.1	0	0	0	0	0.12395	0	0	0
ENSG00000266128	RN7SL366P	0	0	0	0.37255	0	0	0	0
ENSG00000260422	Z97205.2	0	0	0	0	0	0	0	0
ENSG00000230627	Z97205.1	0	0	0	0	0	0	0	0
ENSG00000226739	AL358972.1	0	0	0	0	0	0	0	0
ENSG00000227455	AL450345.1	0	0	0	0	0	0	0	0
ENSG00000217878	AL136100.1	0	0	0	0	0	0	0	0
ENSG00000112539	C6orf118	0	0	0	0	0	0	0	0
ENSG00000112541	PDE10A	0	0	0	0	0	0	0	0
ENSG00000199523	RNA5SP226	0	0	0	0	0	0	0	0
ENSG00000206681	RNU6-730P	0	0	0	0	0	0	0	0
ENSG00000236627	AL590302.2	0	0	0	0	0	0	0	0
ENSG00000223942	AL590302.1	0	0	0	0	0	0	0	0
ENSG00000223414		0	0	0	0	0	0	0	0
ENSG00000281832	LINC00602	0	0	0	0	0	0	0	0
ENSG00000216624	GAPDHP72	0	0	0	0	0	0	0	0
ENSG00000231297	AL627443.1	0	0	0	0	0	0	0	0
ENSG00000276643	RF00279	0	0	0	0	0	0	0	0
ENSG00000164458	TBXT	0.05576	0	0	0	0	0	0	0
ENSG00000201292	RNU6-153P	0	0	0	0	1.71378	0	0	0

ENSG00000280991	AL121956.5	0	0	0	0	0	0	0	0
ENSG00000233365	AL121956.1	0.04735	0	0	0	0	0	0	0
ENSG00000213536	GNG5P1	0	0	0	0	0	0	0	0
ENSG00000280850	AL121956.4	0	0	0	0	0	0	0	0
ENSG00000176381	PRR18	0	0	0	0	0	0	0.0388	0
ENSG00000198818	SFT2D1	7.3442	6.80009	2.74143	5.11685	4.73719	2.91618	3.34579	5.97274
ENSG00000233231	HNRNPA1P49	0	0	0	0	0	0	0	0
ENSG00000060762	MPC1	10.0041	4.81405	2.15985	3.65903	5.99491	6.27321	4.82083	3.55098
ENSG00000261420	AL022069.1	0.0448	0	0	0	0	0	0	0
ENSG00000280057	AL022069.2	0	0	0	0	0.04993	0	0	0
ENSG00000071242	RPS6KA2	0.78253	0.25162	0.06534	0.15837	0.57299	0.10981	0.29645	0.28387
ENSG00000232082	RPS6KA2-IT1	0.37979	0	0	0	0	0.22334	0	0
ENSG00000222958	MIR1913	0.91625	0	0	0	0	0	0	0
ENSG00000235272	RAMACL	0.41413	0	0	0	0	0	0.34514	0
ENSG00000235008	AL023775.1	0.33585	0	0.06169	0	0	0	0	0.05154
ENSG00000249141	AL159163.1	0.24433	0.61004	0.17952	0.72524	0.29706	1.29312	0.20363	0
ENSG00000231654	RPS6KA2-AS1	0	0	0	0	0	0	0	0
ENSG00000026297	RNASET2	9.96455	5.90647	1.73346	3.33061	9.66606	3.18077	4.12437	4.58051
ENSG00000227598	Z94721.1	1.90391	1.90143	0	0.56512	1.38883	1.3435	1.90411	0.46752
ENSG00000265828	MIR3939	2.76605	0	0	1.02628	2.52217	0	3.45793	0
ENSG00000213066	FGFR1OP	0.51256	0.49096	0.22738	0.47364	0.57612	0.17914	0.61255	0.56994
ENSG00000272980	Z94721.2	0.06902	0.04595	0.04057	0.22536	0.05035	0.02435	0.16107	0.11864
ENSG00000112486	CCR6	0.27752	0.08661	0.05098	0.18019	0.35849	0.0306	0.11565	0.10648
ENSG00000166984	TCP10L2	0	0	0	0	0	0	0	0
ENSG00000120436	GPR31	0	0	0	0	0	0	0	0
ENSG00000216966	AL353747.1	0	0	0	0	0	0	0	0
ENSG00000280707	HPAT5	0	0	0	0	0	0	0	0
ENSG00000231720	AL353747.3	0	0	0	0	0	0	0	0
ENSG00000228648	AL353747.2	0	0	0	0	0	0	0	0
ENSG00000112494	UNC93A	0	0	0	0	0	0	0	0
ENSG00000120440	TTLL2	0	0	0	0	0	0	0	0
ENSG00000217447	AL021331.1	0	0	0	0	0	0	0	0
ENSG00000203690	TCP10	0	0	0	0	0	0	0	0
ENSG00000272549	LINC02538	0	0	0	0	0	0	0	0
ENSG00000203688	LINC02487	0	0	0	0	0	0	0	0
ENSG00000213065	AL009178.1	0	0	0	0	0	0	0	0
ENSG00000146521	LINC01558	0	0	0	0	0	0	0	0
ENSG00000269155	AL009178.2	0	0	0	0	0	0	0	0
ENSG00000198221	AFDN-DT	0	0	0	0	0	0	0	0
ENSG00000130396	AFDN	0.55563	0.4891	0.02355	0.28544	1.79922	0.1508	0.18701	0.30174
ENSG00000235994	HGC6.3	0	0	0	0	0	0	0	0
ENSG00000229921	KIF25-AS1	0	0	0	0	0	0	0	0
ENSG00000125337	KIF25	0	0	0	0	0	0	0	0
ENSG00000153303	FRMD1	0	0	0	0	0	0	0	0
ENSG00000234768	AL611929.1	0	0	0	0	0	0	0	0
ENSG00000237987	AL606970.4	0	0	0	0	0	0	0	0
ENSG00000228361	AL606970.2	0	0	0	0	0	0	0	0
ENSG00000234647	AL606970.3	0	0	0	0	0	0	0	0
ENSG00000230711	CTAGE13P	0	0	0	0	0	0	0	0
ENSG00000164488	DACT2	0	0	0	0	0	0	0	0
ENSG00000224417	AL606970.1	0	0	0	0	0	0	0.04775	0
ENSG00000260771	AL138918.1	0	0	0	0	0	0	0	0
ENSG00000112562	SMOC2	0	0	0	0	0.08841	0	0	0.02232
ENSG00000235815	AL136099.1	0	0	0	0	0	0	0	0
ENSG00000280665	AL513210.1	0	0	0	0	0	0	0	0
ENSG00000225879	AL109924.1	0	0	0	0	0	0	0	0
ENSG00000279810	AL109924.4	0	0	0	0	0	0	0	0
ENSG00000229720	AL109924.2	0	0	0	0	0	0	0	0

ENSG00000234519	AL109924.3	0	0	0	0	0	0	0	0
ENSG00000223485	LINC01615	0	0	0	0	0	0	0	0
ENSG00000261039	LINC02544	0	0	0	0	0	0	0	0
ENSG00000226445	BX322234.1	0	0	0.10991	0	0.18187	0.2639	0.12467	0.09183
ENSG00000186340	THBS2	0.01146	0.03815	0	0	0.15324	0.06064	0	0
ENSG00000233085	BX322234.2	0	0	0	0	0	0	0	0
ENSG00000224999	VTA1P1	0	0	0	0	0	0	0	0
ENSG00000226194	LINC02519	0	0	0	0	0	0	0	0
ENSG00000272848	AL135910.1	0	0	0	0	0	0	0	0
ENSG00000184465	WDR27	0.30768	0.20247	0.06309	0.12743	0.26968	0.15148	0.3101	0.2987
ENSG00000185127	C6orf120	8.46513	6.72488	1.979	4.36448	7.75218	6.64256	5.03931	5.50038
ENSG00000130024	PHF10	4.29153	2.97635	1.22623	3.71531	4.89546	3.94899	3.09094	2.84595
ENSG00000232640	AL354892.2	0	0	0	0	0	0	0	0
ENSG00000227704	AL354892.1	0	0	0	0	0	0	0	0
ENSG00000184786	TCTE3	0	0	0	0	0	0	0	0
ENSG00000130023	ERMARD	2.55198	2.12388	2.66054	7.0886	3.71057	3.16359	2.84541	2.83689
ENSG00000266896	AL354892.3	0	0.07485	0	0	0	0	0	0
ENSG00000229214	LINC00242	0	0	0	0	0	0	0	0
ENSG00000231690	LINC00574	0	0	0	0	0	0	0	0
ENSG00000236173	AL049612.1	0	0	0	0	0	0	0	0
ENSG00000232197	AL596442.1	0	0	0	0	0	0	0	0
ENSG00000273100	AL596442.2	0	0	0	0	0	0	0	0
ENSG00000218716	RPL12P23	0	0	0	0	0	0	0	0
ENSG00000230960	AL109910.1	0	0	0	0	0	0	0	0
ENSG00000271820	AL109910.2	0	0	0	0	0.04563	0	0	0
ENSG00000227508	LINC01624	0	0	0	0.03013	0.02468	0	0.06767	0.02492
ENSG00000198719	DLL1	0.15905	0.11346	0.03339	0	0.05525	0.16034	0	0.08369
ENSG00000112584	FAM120B	1.37385	1.59872	0.30372	1.02849	2.12851	1.41559	0.64849	1.05984
ENSG00000271234	AL078605.1	0	0	0	0	0	0	0	0
ENSG00000266245	MIR4644	0	0	0	0	0	0	0	0
ENSG00000261003	AL008628.1	0	0	0	0	0	0	0	0
ENSG00000008018	PSMB1	20.8269	17.9011	6.58888	14.1286	18.645	19.7178	16.7348	13.3639
ENSG00000112592	TBP	2.81438	2.05277	0.55762	1.17327	2.88341	3.1798	1.63399	1.82479
ENSG00000071994	PDCD2	4.62938	2.48689	0.69858	1.59584	3.59164	3.17489	2.0942	2.44587
ENSG00000217874	OR4F7P	0	0	0	0	0	0	0	0
ENSG00000273640	WBP1LP8	0	0	0	0	0	0	0	0
ENSG00000234986	AL731661.2	0	0	0	0	0	0	0	0
ENSG00000213863	AL731661.1	0	0	0	0	0	0	0	0
ENSG00000230423	AL731684.2	0	0	0	0	0	0	0	0
ENSG00000225532	AL731684.1	0	0	0	0	0	0	0	0
ENSG00000219814	RPL23AP47	0	0	0	0	0	0.56223	0	0.19565
ENSG00000283061	AC215522.3	0	0	0	0	0	0	0	0
ENSG00000282572	AC215522.2	0.65951	0.54888	0.41535	0.20974	0.91637	0	0.54965	0.7519
ENSG00000273644	AC215522.1	0	0	0	0	0	0	0	0
ENSG00000232325	AC093627.1	0	0	0	0	0	0	0	0
ENSG00000242611	AC093627.6	0	0	0	0	0	0	0.17902	0
ENSG00000242474	AC093627.5	0	0	0	0	0.10092	0	0	0
ENSG00000240859	AC093627.4	0	0	0	0	0.05192	0	0	0
ENSG00000261795	AC093627.7	0	0	0	0	0	0	0	0
ENSG00000239715	AC093627.2	0	0	0	0	0	0	0	0
ENSG00000240093	AC093627.3	0	0	0	0	0	0	0	0
ENSG00000177706	FAM20C	0.38004	0.26357	0.09308	0.094	0.26953	0.36316	0.13197	0.21386
ENSG00000249852	AC145676.1	0	0	0	0	0	0	0	0
ENSG00000248767	AC187653.1	0	0	0	0	0	0	0	0
ENSG00000249574	AC226118.1	0.06831	0	0	0	0.04153	0	0	0
ENSG00000234471	AC147651.2	0	0	0	0	0	0	0	0
ENSG00000197461	PDGFA	2.24263	1.67538	0.38923	0.57656	0.96609	1.83797	1.14792	1.40925
ENSG00000223855	HRAT92	1.50531	1.1136	0.3994	0.62057	0.63546	0.77454	0.66213	1.64284

ENSG00000188191	PRKAR1B	0.96297	0.48901	0.21586	0.24223	0.63499	1.75641	0.54411	0.44087
ENSG00000229380	AC147651.1	0	0	0	0	0	0	0	0
ENSG00000237181	AC147651.3	1.60439	1.16834	0	0.44645	0.24382	1.41518	0.83571	0.36934
ENSG00000164818	DNAAF5	0.65731	0.41028	0.18111	0.35058	0.59936	0.88781	0.18831	0.31525
ENSG00000164828	SUN1	1.84942	0.95037	0.34655	0.83816	1.87122	0.93062	0.51723	0.89913
ENSG00000239857	GET4	0.16313	0.04526	0.03995	0	0.04958	0.09593	0.06798	0.01669
ENSG00000273151	AC073957.3	0.10846	0.2347	0.01594	0.06439	0.10549	0.38269	0.03616	0.06659
ENSG00000105963	ADAP1	0.83987	1.01232	0.14895	0.21491	0.54576	0.61309	0.21723	0.40892
ENSG00000240230	COX19	3.06851	1.95936	0.23323	1.02072	2.63716	1.93664	0.79367	0.94187
ENSG00000073067	CYP2W1	0	0	0	0	0	0	0	0
ENSG00000146540	C7orf50	2.5709	1.47561	0.23883	0.35085	1.70653	1.40755	0.93587	0.63493
ENSG00000199023	MIR339	1.55958	0	0	0	0.94805	0	0	0
ENSG00000225146	AC073957.1	0	0	0	0.20334	0	0.48341	0	0
ENSG00000164849	GPR146	5.07999	10.695	7.70359	5.26716	4.82245	7.20218	10.2848	7.74658
ENSG00000257607	AC073957.2	0	0	0	0	0.05705	0	0	0
ENSG00000224079	AC091729.1	0	0	0	0	0	0	0	0
ENSG00000226291	AC091729.2	0	0	0	0	0	0	0	0
ENSG00000164850	GPER1	0.06983	0.23247	0.03421	0.03455	0.0283	0	0	0
ENSG00000178381	ZFAND2A	3.59914	2.78143	0.99167	1.33538	2.46135	1.81411	1.4998	2.40676
ENSG00000229043	AC091729.3	0.10984	0.06094	0.0538	0.21735	0.17806	0.19377	0.18309	0.13486
ENSG00000164853	UNCX	0	0	0	0	0	0	0	0
ENSG00000233082	AC073094.1	0	0	0	0	0	0	0	0
ENSG00000164877	MICALL2	0.10776	0.15177	0.17053	0.14762	0.24186	0.05849	0.11053	0.05089
ENSG00000225981	AC102953.1	0.29438	0.48999	0	0	0	0.25966	0	0.18072
ENSG00000273230	AC102953.2	0.07267	0.08064	0	0.03595	0.0589	0.1282	0	0.05948
ENSG00000164880	INTS1	4.29513	2.38308	0.49923	0.93636	2.18317	3.52461	1.41568	1.25134
ENSG00000231927	AC093734.1	0	0	0	0	0	0	0	0
ENSG00000198517	MAFK	4.2257	2.21684	0.96648	0.92729	2.33886	4.23498	2.24737	1.27183
ENSG00000164855	TMEM184A	0.02817	0.01563	0	0	0.04567	0.03314	0.01565	0
ENSG00000157778	PSMG3	1.45853	1.92973	1.15408	1.22106	2.04605	1.64939	0.99739	1.102
ENSG00000230487	PSMG3-AS1	0.25375	0.0768	0.01695	0.13695	0.23839	0.38661	0.0769	0.05665
ENSG00000230444	TFAMP1	0.09959	0	0	0	0	0	0	0
ENSG00000231476	AC074389.2	0.16774	0	0	0	0	0	0	0
ENSG00000225968	ELFN1	0	0	0	0.02829	0.02318	0	0	0
ENSG00000234977	AC074389.3	0	0	0	0	0	0	0	0
ENSG00000205971	AC074389.1	0	0	0	0	0	0	0	0
ENSG00000236081	ELFN1-AS1	0	0	0	0.11	0	0	0	0.091
ENSG00000002822	MAD1L1	3.39635	2.68317	1.46002	2.37734	2.90893	3.88114	1.8758	1.48128
ENSG00000176349	AC104129.1	0.01539	0	0	0	0	0	0	0
ENSG00000265089	MIR4655	0	0	0	0	0	0	0	1.21618
ENSG00000122687	MRM2	3.40332	2.10285	0.49254	0.84182	3.29133	2.22873	0.98844	1.10795
ENSG00000106268	NUDT1	1.64602	2.04028	0.51464	0.93557	1.74572	3.45985	1.34264	1.28997
ENSG00000106266	SNX8	0.64755	0.35928	0.13061	0.22613	0.41679	0.49279	0.23281	0.24943
ENSG00000284047	MIR6836	0	0	0	0	0	0	0	0
ENSG00000271098	IMMP1LP3	0	0	0	0	0	0	0	0
ENSG00000106263	EIF3B	7.83673	7.18654	3.01666	5.30751	9.23058	11.4969	3.44897	4.71089
ENSG00000230914	AC004840.1	0.098	0	0	0	0.11914	0	0	0
ENSG00000271163	AC004840.2	0	0	0	0	0	0	0	0
ENSG00000136213	CHST12	0.73105	0.99765	0.22686	0.64019	0.72318	1.81836	0.51467	0.60767
ENSG00000275572	GRIFIN	0	0	0	0	0	0	0	0
ENSG00000106003	LFNG	15.8696	8.92397	2.07991	3.94659	7.30037	4.16158	5.86237	6.71423
ENSG00000264357	MIR4648	5.0903	0	0	3.02183	0	0	0	1.24996
ENSG00000106009	BRAT1	2.61669	1.41507	0.37316	0.44242	1.77186	1.42186	0.88336	0.96246
ENSG00000106012	IQCE	2.28469	1.2148	0.20983	0.47093	1.71675	1.0636	0.54214	0.6818
ENSG00000136295	TTYH3	6.35054	3.48368	0.38664	0.81647	2.50091	2.21533	0.99674	0.95445
ENSG00000174945	AMZ1	0.04182	0	0	0	0.05084	0.05533	0	0.06418
ENSG00000146535	GNA12	5.43764	4.64509	2.23524	3.43066	8.76605	14.6427	3.77322	3.8234
ENSG00000231357	AC006028.1	0	0	0	0	0	0	0	0

ENSG00000198286	CARD11	9.37472	6.32464	1.39591	3.14316	10.0912	10.3021	2.5438	3.21213
ENSG00000237286	AC004906.1	0	0	0	0	0	0	0	0
ENSG00000201794	RN7SKP130	0	0	0	0	0	0	0	0
ENSG00000228334	AC024028.1	0.33547	0	0.49297	0.49788	0.61178	0	0	0.20594
ENSG00000217455	AC073316.1	0	0	0	0	0	0	0	0
ENSG00000231892	AC073316.2	0	0	0	0	0	0	0	0
ENSG00000236708	AC073316.3	0	0	0	0	0	0	0	0
ENSG00000146555	SDK1	0.06077	0.07081	0.02679	0.05411	0.18471	0.11793	0.02026	0.14176
ENSG00000224593	AC092427.1	0	0	0	0	0	0	0	0
ENSG00000236510	AC011284.1	0	0	0	0	0	0	0	0
ENSG00000283991	AC017000.1	0	0	0	0	0	0	0	0
ENSG00000261809	CYP3A54P	0	0	0	0	0	0	0	0
ENSG00000164916	FOXK1	3.2079	1.33256	0.36198	1.01366	2.68174	2.71601	1.1758	1.0173
ENSG00000242802	AP5Z1	2.34629	2.02371	0.23508	0.69643	1.64671	2.05078	1.08439	1.04754
ENSG00000264474	MIR4656	0	0	0	0	0	0	0	0
ENSG00000157927	RADIL	0.01125	0	0	0	0	0.01985	0	0
ENSG00000206895	RF00019	0	0	0	0	0	0	0	0
ENSG00000218823	PAPOLB	0	0	0	0.02552	0	0	0	0
ENSG00000213935	RPL22P16	0.19652	0	0	0.5833	0	0	0	0
ENSG00000136297	MMD2	0	0	0	0	0	0	0	0
ENSG00000196204	RNF216P1	1.55647	1.30644	0.43007	0.96742	1.4718	1.24383	0.70958	0.84934
ENSG00000272968	RBAK-RBAKDN	0	0	0	0	0	0	0	0
ENSG00000270822	AC092032.1	0	0	0	0	0	0	0	0
ENSG00000275328	SPDYE19P	0	0	0	0	0	0	0	0
ENSG00000146587	RBAK	4.98147	2.82232	1.11428	2.1257	3.55955	3.5858	2.52787	2.39216
ENSG00000273313	RBAKDN	0	0	0	0	0	0	0	0
ENSG00000272595	OR10AH1P	0	0	0	0	0	0	0	0
ENSG00000159904	ZNF890P	0.42199	0.07024	0.06201	0.06263	0.0513	0.37223	0.14068	0
ENSG00000202039	RNU6-215P	0	0	0	0	0	0	0	0
ENSG00000157954	WIPI2	6.49962	5.77627	2.43577	2.85991	6.1251	5.08489	2.98067	3.58906
ENSG00000164638	SLC29A4	0.05046	0	0	0	0	0	0	0
ENSG00000182095	TNRC18	8.0052	6.26929	0.65972	0.90857	4.51538	4.60799	1.28284	1.91133
ENSG00000241269	AC093620.1	0	0	0	0	0	0	0	0
ENSG00000188365	AC092171.1	0	0	0	0	0	0	0	0
ENSG00000272953	AC092171.4	0.11598	0.57915	0.17043	0.17213	0	0.20461	0.19332	0.712
ENSG00000234432	AC092171.3	0.08295	0.23012	0	0.08207	0.10085	0	0.18435	0.03395
ENSG00000273084	AC092171.5	0	0	0	0	0.0946	0.41182	0.1297	0.09554
ENSG00000155034	FBXL18	0.72033	0.40765	0.04234	0.12829	0.38533	0.68621	0.3602	0.21226
ENSG00000230733	AC092171.2	0	0	0	0	0	0	0	0
ENSG00000207973	MIR589	0	0	0	0	0	0	0	0
ENSG00000075624	ACTB	1250.23	1069.15	206.959	370.927	959.537	1345.66	332.394	548.883
ENSG00000272719	AC006483.2	0.14233	0	0	0	0	0	0.23724	0
ENSG00000228974	AC006483.1	0	0	0	0	0	0	0	0
ENSG00000075618	FSCN1	0.49635	0.26437	0.05835	0.20626	0.43448	0.2802	0.06619	0.17063
ENSG00000011275	RNF216	8.54321	6.94995	1.95098	5.1116	7.69639	6.78891	3.91285	4.64219
ENSG00000237738	RNF216-IT1	1.08754	0	0	0.32281	0	0	0.36255	0.26705
ENSG00000283419	MIR6874	2.0648	0	0	0	0	0	0	0
ENSG00000235944	ZNF815P	2.01716	0.67151	0.22801	0.36845	2.1883	1.14968	1.29319	0.95255
ENSG00000277265	RN7SL556P	0	0	0	0	0	0	0	0
ENSG00000122543	OCM	0.88101	1.02651	0.64732	0.13075	1.17823	0	0.44055	1.4062
ENSG00000122674	CCZ1	2.67852	3.32009	4.32129	8.25493	5.05794	2.43302	8.16942	3.49856
ENSG00000155026	RSPH10B	0.08808	0	0	0	0.07139	0	0.02447	0
ENSG00000122512	PMS2	2.56998	2.07687	0.82099	1.93471	3.21508	3.12109	1.89355	2.03499
ENSG00000201990	RF00019	0	1.18455	0	0	0	0	0	0
ENSG00000106305	AIMP2	2.31307	1.85867	0.70325	1.65724	2.52126	3.30667	0.93064	1.27308
ENSG00000207217	SNORA80D	5.42966	6.32637	2.39364	12.8931	3.96074	12.4522	4.5252	1.33329
ENSG00000086232	EIF2AK1	75.9814	67.6652	45.1622	75.3856	67.5842	101.469	82.136	49.5551
ENSG00000157999	ANKRD61	0	0	0	0	0.0709	0	0	0

ENSG00000240718	RN7SL851P	0.48383	0.40267	0.35549	0	0	1.28031	0.40324	0.29702
ENSG00000252929	RNU6-218P	0	0	0	0	0	0	0	0
ENSG00000231704	AC004895.1	0.17878	0	0	0	0	0	0	0
ENSG00000106346	USP42	2.79851	3.05366	1.09664	2.55351	3.21334	4.53469	2.50511	1.88342
ENSG00000008256	CYTH3	1.57757	1.68056	0.72329	2.07907	1.38094	0.84605	1.0939	1.17765
ENSG00000202273	RF00019	0	0	0	0	0	0	0	0
ENSG00000178397	FAM220A	2.66268	1.88489	0.76457	1.18097	3.05118	1.88973	0.96928	1.50308
ENSG00000274319	RPSAP73	0	0	0	0	0	0	0	0
ENSG00000136238	RAC1	40.6989	31.227	6.53755	14.9519	25.7849	21.4274	10.5152	17.3694
ENSG00000164535	DAGLB	2.38137	1.99249	0.58011	0.79378	1.53276	1.79725	1.10378	1.21956
ENSG00000136240	KDELR2	11.1696	8.34795	3.07754	6.81615	8.59898	6.28734	3.88895	5.21036
ENSG00000231359	AC072052.1	0	0	0	0	0	0	0	0
ENSG00000215045	GRID2IP	0.03546	0	0	0.02631	0.02156	0.03128	0	0
ENSG00000136247	ZDHHC4	2.84381	2.19526	2.45286	3.9147	3.2069	3.34458	2.81664	2.53014
ENSG00000232581	AC079742.1	0	0	0	0	0	0	0	0
ENSG00000146576	C7orf26	4.59471	3.17544	0.5133	1.75461	3.65875	3.60253	1.16448	1.61652
ENSG00000236609	ZNF853	1.02624	0.2847	0	0.11282	0.97042	0.23469	0.12671	0.21
ENSG00000205903	ZNF316	3.50671	2.37256	0.50048	0.78627	1.85564	2.18079	0.84103	1.08412
ENSG00000228010	AC073343.2	0	0	0	0.15835	0	0	0.08892	0.1965
ENSG00000198580	AC073343.1	0.67558	0	0	0.16711	0.68446	0.79454	0.18768	0.13824
ENSG00000164631	ZNF12	5.28615	4.3303	1.32177	2.77253	4.77801	4.49186	3.41376	4.57036
ENSG00000187953	PMS2CL	2.38099	1.63559	0.36099	1.43028	2.64203	1.93351	1.07093	1.34566
ENSG00000277327	SPDYE20P	0	0	0	0	0	0	0	0
ENSG00000169402	RSPH10B2	0.05873	0	0.02158	0.15254	0.01785	0.10362	0.02448	0
ENSG00000146574	CCZ1B	3.94756	5.06979	6.37509	13.2488	5.45712	5.00208	10.705	7.28243
ENSG00000273252	OR7E39P	0	0	0	0	0	0	0	0
ENSG00000155070	UNC93B2	0	0	0	0	0	0	0	0
ENSG00000205897	OR7E136P	0	0	0	0	0	0	0	0
ENSG00000250561	OR7E59P	0	0	0	0	0	0	0	0
ENSG00000243531	AC079804.2	0	0	0	0	0	0	0	0
ENSG00000226943	ALG1L5P	0	0	0	0	0	0	0	0
ENSG00000241539	AC079804.1	0	0	0	0	0	0	0	0
ENSG00000242731	FAM86LP	0	0.14303	0.50511	0	0.20895	0	0	0.10551
ENSG00000266287	MIR3683	0	0	0	0	0	0	0	0
ENSG00000239696	AC092104.1	0	0	0	0	0.06184	0.17948	0.84789	0.12491
ENSG00000201218	RF00019	0	0	0	0	0	0	0	0
ENSG00000106392	C1GALT1	11.9459	14.491	7.38334	15.5687	11.0411	10.5703	13.9791	11.7267
ENSG00000244167	AC005532.2	0.19652	0.3271	0.28878	0	0.23892	0	0	0
ENSG00000230825	AC005532.1	0.29628	0.49316	0.17415	0.17589	0.21613	0.20907	0.19754	0.36377
ENSG00000236712	AC079448.1	0	0	0	0	0	0	0	0
ENSG00000215018	COL28A1	0.01107	0.01843	0	0	0.01346	0.01954	0.01846	0.0136
ENSG00000272732	AC004982.1	0.08858	0	0	0	0	0	0	0.05438
ENSG00000272894	AC004982.2	0.12711	0.07053	0.12452	0	0.05151	0	0.07062	0.10404
ENSG00000164654	MIOS	3.52636	2.74401	1.05885	1.68509	3.59705	3.3705	1.91077	2.68088
ENSG00000272745	AC004948.1	0	0	0	0	0	0	0	0
ENSG00000106399	RPA3	1.17934	1.58551	0.96649	2.22149	1.57167	3.12077	1.1719	1.69858
ENSG00000219545	UMAD1	2.71555	3.47945	1.26317	4.08816	4.01405	4.44595	4.33102	3.14222
ENSG00000283549	AC007161.3	0.11507	0	0	0	0	0	0	0
ENSG00000270314	AC007161.2	0	0	0	0	0	0	0	0
ENSG00000234718	AC007161.1	0	0	0	0	0	0	0	0
ENSG00000269721		0	0	0	0	0	0	0	0
ENSG00000201747	RNU6-534P	0.69151	0	0	0	0	0	0	0
ENSG00000214998	CCNB2P1	0	0	0	0	0	0	0	0.07826
ENSG00000234141	AC006042.4	0.21185	1.05788	0.15566	0.94323	0.12878	0.18687	0.17656	0.26011
ENSG00000233108	AC006042.2	0.2581	0.2148	0	0.19152	0	0	0	0.31689
ENSG00000106415	GLCCI1	3.54525	3.27837	1.70528	5.73554	3.8313	5.05227	3.95733	3.21558
ENSG00000233264	AC006042.3	0.74847	0.99667	0.21997	0.29622	0.97064	0.79224	0.24952	0.73517
ENSG00000003147	ICA1	0.95915	0.8232	0.4845	0.64502	1.09323	1.05755	0.99923	0.62562

ENSG00000227719	AC006042.1	0	0	0	0.20761	0	0	0	0
ENSG00000244239	AC007009.1	0	0	0	0	0	0	0	0
ENSG00000229970	AC007128.1	0	0	0	0	0	0	0	0
ENSG00000272328	AC007128.2	0	0	0	0	0	0	0	0
ENSG00000122584	NXPH1	0	0	0	0	0	0	0	0
ENSG00000236748	AC009500.1	0	0	0	0	0	0	0	0
ENSG00000278254	AC004852.2	0	0	0	0	0	0	0	0
ENSG00000271526	AC004852.1	0	0	0	0	0	0	0	0
ENSG00000278446	AC004852.3	0	0	0	0	0	0	0	0
ENSG00000233876	GAPDHP68	0	0	0	0	0	0	0	0
ENSG00000271077	AC096553.1	0	0	0	0	0	0	0	0
ENSG00000197320	AC060834.1	0	0	0	0	0	0	0	0
ENSG00000234710	AC060834.2	0	0	0	0	0	0	0	0
ENSG00000270611	AC006363.1	0	0	0	0	0	0	0	0
ENSG00000235431	AC004936.1	0	0	0	0	0	0	0	0
ENSG00000212422	RF00012	0	0	0	0	0	0	0	0
ENSG00000283117	MGC4859	0	0	0	0	0	0	0	0
ENSG00000229091	HSPA8P8	0	0	0	0	0	0	0	0.13903
ENSG00000234356	AC009945.1	0	0	0	0	0	0	0	0
ENSG00000236414	AC004415.1	0	0	0	0	0	0	0	0
ENSG00000201774	RF00019	0	0	0	2.243	0	0	0	0
ENSG00000189043	NDUFA4	9.52327	7.30816	4.02867	8.87173	6.8667	8.90928	5.87538	6.22589
ENSG00000271185	AC007029.1	0	0	0	0.69512	0.28472	0.41314	0	0
ENSG00000106443	PHF14	3.96127	2.19319	0.93729	1.93059	4.40788	4.67856	2.12633	2.00932
ENSG00000230370	RPL23AP52	0	0	0	0	0	0	0	0
ENSG00000230333	AC004160.1	0	0.06467	0	0.05766	0.04723	0	0.02159	0
ENSG00000226827	NPM1P11	0	0	0	0	0	0	0	0
ENSG00000005108	THSD7A	0.05366	0.03349	0.00986	0.01991	0.08154	0.0355	0	0.01647
ENSG00000230435	AC004160.2	0	0	0	0	0	0	0	0
ENSG00000235920	AC073109.1	0.22485	0	0	0	0	0	0	0
ENSG00000106460	TMEM106B	4.86308	3.27303	2.19048	6.04845	5.80958	4.38282	2.72256	3.23852
ENSG00000146530	VWDE	0.10165	0.0752	0.06639	0.06705	0.10985	0.09962	0.11296	0.22187
ENSG00000274970	AC013470.4	0	0	0	0	0	0	0	0
ENSG00000236048	AC013470.3	0	0.10218	0	0	0	0	0	0
ENSG00000226690	AC013470.2	0	0	0	0	0	0	0.04877	0
ENSG00000203523	TAS2R2P	0	0	0	0	0	0	0	0
ENSG00000224667	AC013470.1	0	0	0	0	0	0	0	0
ENSG00000225606	AC005281.1	0	0	0	0	0	0	0	0
ENSG00000006747	SCIN	0	0	0	0	0	0	0	0
ENSG00000229233	AC011891.1	0	0	0	0	0	0	0	0
ENSG00000271253	AC011891.2	0	0	0	0	0.22448	0	0	0
ENSG00000122644	ARL4A	23.0955	20.7973	20.7805	73.2264	37.1632	8.93308	129.66	37.0249
ENSG00000199470	RF00264	0	0	0	0	0	0	0	0
ENSG00000229618	AC011287.1	0	0	0	0.04037	0	0.024	0.02267	0
ENSG00000222974	RN7SKP228	0	0	0	0	0	0	0.46456	0.34219
ENSG00000230879	RBMX2P4	0	0	0	0	0	0	0	0
ENSG00000237713	AC011287.2	0	0	0	0	0	0	0	0
ENSG00000224330	AC005019.2	0	0	0	0	0	0	0	0
ENSG00000213875	AC005019.1	0	0	0	0	0	0	0	0
ENSG00000006468	ETV1	0.00762	0.01269	0	0	0.00927	0	0.01271	0.01872
ENSG00000252374	RF00019	0	0	0	0	0	0	0	0
ENSG00000223445	RPL6P21	0	0	0	0	0	0	0	0
ENSG00000136267	DGKB	0	0	0	0	0	0	0	0
ENSG00000233167	EEF1A1P26	0	0	0	0	0	0	0	0
ENSG00000226323	AC006150.1	0	0	0	0	0	0	0	0
ENSG00000225816	GTF3AP5	0	0	0	0	0	0	0	0
ENSG00000227489	AC006458.1	0	0	0	0	0	0	0	0
ENSG00000187546	AGMO	0	0	0	0	0	0	0	0

ENSG00000106511	MEOX2	0	0	0	0	0	0	0	0
ENSG00000237070	AC005550.2	0	0	0	0	0	0	0	0
ENSG00000229108	LINC02587	0	0	0	0	0	0	0	0
ENSG00000225974	AC005550.1	0	0	0	0	0	0	0	0
ENSG00000235828	RPL36AP26	0	0	0	0	0	0	0	0
ENSG00000229379	AC006041.1	0	0	0	0	0	0	0	0.27438
ENSG00000214960	ISPD	3.59008	12.2415	28.5632	55.6775	7.42992	8.48551	37.8606	26.5755
ENSG00000224683	RPL36AP29	0	0	0	0	0	0	0.38062	0
ENSG00000229688	ISPD-AS1	0	0	0	0.27471	0	0	0	0.12987
ENSG00000171243	SOSTDC1	0	0	0	0.08798	0	0	0	0
ENSG00000272537	AC005014.3	0	0	0	0	0	0	0	0
ENSG00000205858	LRRC72	0	0	0	0	0	0	0	0
ENSG00000224280	AC005014.1	0	0	0	0	0	0	0	0
ENSG00000272361	AC005014.2	0.12237	0	0	0	0	0	0	0
ENSG00000106524	ANKMY2	3.36452	2.052	1.05676	3.69734	2.99762	4.62151	2.91109	2.39656
ENSG00000136261	BZW2	3.30272	1.79914	1.11773	2.82213	4.08837	4.2021	2.73588	1.79404
ENSG00000235837	AC073333.1	0	0	0	0	0	0	0	0
ENSG00000106537	TSPAN13	4.35056	3.34646	4.74639	11.8373	3.96697	1.68617	5.60359	4.81549
ENSG00000106541	AGR2	0	0	0	0	0.15662	0	0	0
ENSG00000270593	AC073333.2	0	0	0	0	0	0	0	0
ENSG00000173467	AGR3	0	0	0	0	0	0	0	0
ENSG00000232400	RAD17P1	0	0	0	0	0	0	0	0
ENSG00000227965	AC098592.2	0	0	0	0	0	0	0	0
ENSG00000223867	AC098592.1	0	0	0	0	0	0	0	0
ENSG00000232841	BRWD1P3	0	0	0	0	0	0	0	0
ENSG00000278569	RF00017	0	0	0	0	0	0	0	0
ENSG00000237773	AC073332.1	0.45613	0.22777	0.20108	0.06769	0.22182	0.08047	0.22809	0.056
ENSG00000106546	AHR	16.0004	8.38911	1.8273	3.47866	6.62254	4.29033	2.14609	2.36443
ENSG00000283321	AC019117.3	0	0	0	0	0	0	0	0
ENSG00000199473	RF00092	0	0	0	0	0	0	0	0
ENSG00000236318	AC019117.2	0	0	0	0	0	0	0	0
ENSG00000236039	AC019117.1	0	0.10933	0	0	0	0.05794	0	0.04032
ENSG00000228675	AC006482.1	0	0	0	0	0.32643	0	0	0
ENSG00000071189	SNX13	7.25949	9.03656	5.08456	11.7347	8.5925	6.1277	12.0856	9.30777
ENSG00000279048	AC080080.1	0.12116	0	0	0.04495	0.03683	0.05343	0.05049	0.03719
ENSG00000236195	RNMTL1P2	0	0	0	0	0	0	0	0
ENSG00000280340	AC080080.2	0	0	0	0	0	0	0	0
ENSG00000229937	PRPS1L1	0	0.11193	0	0	0	0	0	0
ENSG00000048052	HDAC9	1.48979	1.08761	0.44169	1.02793	2.10783	0.78385	0.66801	0.88248
ENSG00000221393	MIR1302-6	0	0	0	0	0	0	0	0
ENSG00000225000	AC010082.1	0	0	0	0	0	0	0	0
ENSG00000222164	RN7SKP266	0	0	0	0	0	0	0	0
ENSG00000226522	AC004994.1	0	0	0	0	0	0	0	0
ENSG00000232492	NPM1P13	0	0	0	0	0	0	0	0
ENSG00000122691	TWIST1	0	0.06001	0	0	0	0	0	0
ENSG00000232821	AC003986.2	0	0	0	0	0	0	0	0
ENSG00000236536	AC003986.3	0	0	0	0	0	0	0	0
ENSG00000229533	AC003986.1	0	0	0	0	0	0	0	0
ENSG00000146618	FERD3L	0	0	0	0	0	0	0	0
ENSG00000223838	AC007091.1	0	0	0	0	0	0	0.21587	0
ENSG00000105849	TWISTNB	3.33767	2.9304	0.9971	2.15013	3.05454	4.52931	2.01749	2.20658
ENSG00000265932	MIR3146	2.78356	1.54441	0	0	0	0	0	0
ENSG00000173452	TMEM196	0	0	0	0	0	0	0	0
ENSG00000237921	AC004543.1	0	0	0	0	0	0	0	0
ENSG00000243004	AC005062.1	0	0	0	0.04243	0	0	0.04765	0
ENSG00000213860	RPL21P75	1.36584	1.51563	0.44602	0.45046	1.47605	1.60636	0.75888	1.67696
ENSG00000267055	AC007001.1	0	0	0	0	0	0	0	0
ENSG00000183742	MACC1	0.41471	0.03698	0.08706	0.09892	0.15306	0.14371	0.17282	0.08183

ENSG00000228598	MACC1-AS1	0	0	0	0	0	0	0	0
ENSG00000233834	AC005083.1	0.24406	0	0.11955	0.24148	0.29673	0.14352	0	0
ENSG00000226097	AC099342.1	0	0	0	0	0	0	0	0
ENSG00000271133	AC004130.1	0	0.40851	0	0	0.19892	0.14432	0	0
ENSG00000105855	ITGB8	0.25633	0.15295	0.07818	0.15073	0.05292	0.08532	0.03225	0.07126
ENSG00000200753	RF00275	0	0	0	0	0	0	0	0
ENSG00000179460	EEF1A1P27	0	0	0	0	0	0	0	0
ENSG00000231525	AC002486.2	0	0	0	0	0	0	0	0
ENSG00000004846	ABCB5	0.24547	0.38222	0.24435	0.78737	0.44284	0.30732	0.76552	0.43749
ENSG00000223866	AC002486.1	0	0	0	0	0	0.33072	0.31248	0.23017
ENSG00000164651	SP8	0	0	0	0	0	0	0	0
ENSG00000219451	RPL23P8	0	0.57013	0	0.76252	0	0	0	0.21027
ENSG00000232790	LINC01162	0	0	0	0	0	0	0	0
ENSG00000234839	RPS26P30	0	0	0	0	0	0	0	0
ENSG00000243633	RN7SL542P	0	0	0	0	0	0	0	0
ENSG00000225308	ASS1P11	0	0	0	0	0	0	0	0
ENSG00000195024	RNU1-15P	0	0	0	0	0	0	0	0
ENSG00000229949	AC005094.1	0	0	0	0	0	0	0	0
ENSG00000105866	SP4	10.8528	8.68973	2.92006	6.80565	10.3069	15.495	4.58617	6.58304
ENSG00000221783	MIR1183	2.4708	1.37088	0	0	0	0	1.37281	0
ENSG00000105877	DNAH11	0	0.00763	0	0.0068	0.00557	0.01617	0.00764	0.01125
ENSG00000164649	CDCA7L	4.33613	1.58725	0.55523	1.0681	6.12486	4.69763	1.55949	1.83352
ENSG00000136237	RAPGEF5	0.11448	0.04764	0.05257	0.05309	0.08699	0.02524	0	0.01757
ENSG00000251993	RNA5SP227	0	0	0	0	0	0	0	0
ENSG00000105889	STEAP1B	0.60134	0	0.04651	0.3288	0.30783	0.1675	0.10551	0.15544
ENSG00000233476	EEF1A1P6	0.31778	0	0.07783	0.23581	0.19317	0.8409	0.17656	0.13005
ENSG00000232759	AC002480.1	0	0	0	0	0	0	0.05024	0
ENSG00000225541		0.22871	0.09517	0	0	0.06951	0.10087	0.0953	0
ENSG00000232949	AC002480.2	0	0	0	0	0	0	0	0
ENSG00000238033		0.05656	0	0	0	0	0.04989	0	0
ENSG00000179428	AC073072.1	0	0	0	0	0.39201	0.0948	0	0
ENSG00000136244	IL6	0.07086	0.05897	0	0.05258	0.21536	0	0.05905	0.0435
ENSG00000228803	MTCYBP42	0	0	0	0	0	0	0	0
ENSG00000234513	AC073072.2	0	0	0	0	0	0	0	0
ENSG00000196683	TOMM7	31.3128	25.642	12.1801	18.0792	34.5066	21.8597	21.4915	32.6374
ENSG00000228649	SNHG26	0.69757	0.18578	0	0.28987	0.61062	0.88603	0.27906	0.23981
ENSG00000221740	SNORD93	0.99055	0	0	0	1.20428	0	3.30217	1.21618
ENSG00000226329	AC005682.1	0	0	0	0	0.30374	0	0	0
ENSG00000226279	RPL12P10	0	0	0	0	0.3579	0.25966	0	0
ENSG00000122591	FAM126A	5.18193	5.5118	2.10242	4.32922	5.99889	4.03124	2.75557	2.97321
ENSG00000230658	KLHL7-DT	0.20012	0.09517	0	0.04243	0.17378	0.20173	0.19061	0
ENSG00000122550	KLHL7	2.23034	1.86483	1.72257	3.87971	2.28278	1.39084	2.43807	1.92323
ENSG00000230042	AK3P3	0.88314	0.55124	0	0.16383	0.13421	0.77899	0.36801	0.13554
ENSG00000136243	NUPL2	6.24547	5.64492	6.19244	14.8752	8.96065	7.16753	13.1247	7.99791
ENSG00000226816	AC005082.1	0.50477	0.12003	0.05298	0.10702	0.70136	0.19082	0.1803	0.08854
ENSG00000136235	GPNMB	0.11076	0	0.03617	0	0	0	0	0
ENSG00000156928	MALSU1	1.90341	0.63365	0.36361	0.53673	1.78187	2.0483	0.88835	0.81794
ENSG00000136231	IGF2BP3	3.8842	1.8636	1.2909	2.14734	2.3978	1.35222	1.56476	2.13599
ENSG00000232627	AC021876.1	0	0	0	0	0	0	0	0
ENSG00000212264	SNORD65C	0	0	0	0	0	0	0	0
ENSG00000252590	RNU7-143P	0	0	0	0	0	0	0	0
ENSG00000236654	AC079780.1	0	0	0	0	0	0	0	0
ENSG00000232818	RPS2P32	0.08218	0.13678	0	0	0.09991	0.14497	0	0.40357
ENSG00000200847	RF00019	0	0	0	0	0	0	0	0
ENSG00000164548	TRA2A	24.4334	25.9216	9.40506	18.2547	30.3899	17.6535	13.6219	16.6633
ENSG00000224785	AC006026.1	0	0	0	0.06034	0	0	0	0
ENSG00000232553	CLK2P1	0	0	0	0	0	0	0	0
ENSG00000169193	CCDC126	12.5953	17.528	13.4111	26.1699	21.0115	8.23224	14.2142	22.1824

ENSG00000227436	FCF1P1	0.77023	0	0	0.19052	0.15607	0.6794	0	0.15761
ENSG00000228953	AC006026.2	0	0	0	0	0	0	0	0
ENSG00000188732	FAM221A	0.23588	0.14723	0.24553	0.84601	0.38237	0.3121	0.31127	0.42235
ENSG00000234286	AC006026.3	1.02518	0	0	0	0	0	0	0
ENSG00000196335	STK31	0.07113	0.0592	0.10453	0.36948	0.05765	0.04183	0.07904	0.07278
ENSG00000234800	PCMTD1P3	0	0	0	0	0	0	0	0
ENSG00000231328	TPT1P7	0	0	0	0	0	0	0	0
ENSG00000227930	AC003087.1	0	0	0	0	0	0	0	0
ENSG00000252041	RNA5SP228	0	0	0	0	0	0	0	0
ENSG00000228944	AC004485.1	0	0	0	0	0	0	0	0
ENSG00000122585	NPY	0	0	0	0	0	0	0	0
ENSG00000206877	RNU6-1103P	0	0	0	0	0	0	0	0
ENSG00000105926	MPP6	6.86139	9.27207	10.784	23.9075	17.2926	12.5461	25.4559	23.1501
ENSG00000271096	SUMO2P14	0	0	0	0	0	0	0	0
ENSG00000271330	AC005084.1	0	0	0	0	0	0	0	0
ENSG00000105928	GSDME	0.04119	0.01371	0	0	0.0601	0.07267	0.02747	0.03035
ENSG00000070882	OSBPL3	2.63338	2.16904	0.54522	1.74594	1.92536	2.72992	1.11622	1.39995
ENSG00000234999	SNRPCP19	0	0	0	0	0.19373	0	0	0
ENSG00000172115	CYCS	8.4533	6.6794	2.56174	6.15077	6.73824	9.37126	4.3861	6.70384
ENSG00000153790	C7orf31	1.41366	1.04933	0.22458	0.36857	1.46321	1.17954	0.31843	0.44565
ENSG00000234406	AC004129.2	0	0.85023	0.37531	2.27427	1.24204	0.45056	2.12858	0.62716
ENSG00000213798	AC004129.1	0	0	0	0	0	0	0	0
ENSG00000105954	NPVF	0	0	0	0	0	0	0	0
ENSG00000202233	RF00012	0	0	0.47451	0	0	0	0	0
ENSG00000234922	TSEN15P3	0	0	0	0	0	0	0	0
ENSG00000233824	AC003985.1	0	0	0	0	0	0	0	0
ENSG00000260951	AC003985.2	0	0	0	0	0	0	0	0
ENSG00000227386	AC091705.1	0	0	0	0	0	0	0	0
ENSG00000223561	AC005165.1	0	0	0	0	0.03003	0.04357	0.04117	0
ENSG00000239622	AC005165.2	0	1.60537	0	0	0.23452	0	0.64305	0
ENSG00000270933	AC010719.1	0	0	0	0	0	0	0	0
ENSG00000199085	MIR148A	0	0	0	0	0	0	0	0
ENSG00000050344	NFE2L3	2.96721	2.09617	0.73516	1.20333	1.53107	2.67814	0.48884	1.12258
ENSG00000273237		0.41501	0	0	0.23097	0.18921	0.27455	0.08647	0.31846
ENSG00000122566	HNRNPA2B1	45.7823	39.7365	16.8691	45.5858	41.7398	39.0456	39.9059	30.3014
ENSG00000122565	CBX3	17.8947	17.2983	7.68225	14.6501	16.9554	16.7735	12.8736	15.4358
ENSG00000232383	AC010677.1	0	0	0.26144	0	0	0	0	0
ENSG00000086300	SNX10	39.9631	53.7906	10.2264	13.6059	31.7152	16.2834	13.6489	38.3217
ENSG00000225792	AC004540.2	0	0	0.12212	0.12334	0	0	0	0
ENSG00000214870	AC004540.1	0.04307	0.07169	0	0	0	0	0.07179	0
ENSG00000122548	KIAA0087	0.01711	0.08546	0	0	0	0	0	0
ENSG00000280255	AC004947.2	0	0	0	0	0	0	0	0
ENSG00000233760	AC004947.1	0	0	0	0	0	0	0	0
ENSG00000222004	C7orf71	0	0	0	0	0	0	0	0
ENSG00000005020	SKAP2	44.8301	41.3326	9.21032	14.958	25.169	11.549	12.8157	18.2603
ENSG00000213787	RPL7AP38	0	0	0	0	0	0	0	0
ENSG00000226059	HMGB3P20	0	0	0	0	0	0	0	0
ENSG00000214869	TPM3P4	0	0	0	0	0	0	0	0
ENSG00000213786	NHP2P2	0	0	0	0	0	0	0	0
ENSG00000105991	HOXA1	0.75061	0.38443	0.04242	0.12854	0.24569	0.30558	0.04812	0.14178
ENSG00000233429	HOTAIRM1	6.85966	2.93603	0.72001	0.21815	1.96581	0.77795	1.38841	1.08285
ENSG00000276528	RF01975	0	0	0	0	0	0	0	0
ENSG00000276771	RF01976	0	0	0	0	0	0	0	0
ENSG00000274864	RF01977	0	0	0	0	0	0	0	0
ENSG00000274396	RF01978	0	0	0	0	0	0	0	0
ENSG00000277694	RF01979	0	0	0	0	0	0	0	0
ENSG00000105996	HOXA2	0	0	0	0	0	0	0	0
ENSG00000105997	HOXA3	0.03405	0	0	0	0	0	0	0.0418

ENSG00000253552	HOXA-AS2	0.16656	0.0231	0	0	0.05062	0	0.04627	0
ENSG00000197576	HOXA4	0	0	0.06166	0	0	0	0	0
ENSG00000273433	AC004080.6	0	0	0	0	0	0	0	0
ENSG00000254369	HOXA-AS3	0	0	0	0	0	0	0	0
ENSG00000106004	HOXA5	0.08588	0.14295	0	0.06373	0	0.15151	0.14315	0
ENSG00000106006	HOXA6	0.05704	0	0	0	0	0	0	0
ENSG00000272801	AC004080.5	0	0	0	0	0	0	0	0
ENSG00000122592	HOXA7	0	0	0	0	0	0	0	0
ENSG00000270182	AC004080.4	0	0	0	0	0	0	0	0
ENSG00000078399	HOXA9	0.0968	0.12084	0	0.14366	0.02942	0.04269	0	0.08914
ENSG00000257184	AC004080.3	0	0	0	0	0	0	0	0
ENSG00000253187	HOXA10-AS	0	0	0	0	0	0	0	0
ENSG00000283745	MIR196B	0	0	0	0	0	0	0	0
ENSG00000253293	HOXA10	0.07909	0.09874	0	0.14673	0.09616	0.24418	0.03296	0.04856
ENSG00000005073	HOXA11	0	0	0	0	0	0	0	0
ENSG00000240990	HOXA11-AS	0	0	0	0	0	0	0	0
ENSG00000273961	RF02137	0	0	0	0	0	0	0	0
ENSG00000278334	RF02138	0	0	0	0	0	0	0	0
ENSG00000276496	RF02139	0	0	0	0	0	0	0	0
ENSG00000253508	AC004080.2	0	0	0	0	0	0	0	0
ENSG00000277966	RF02140	0	0	0	0	0	0	0	0
ENSG00000278592	RF02141	0	0	0	0	0	0	0	0
ENSG00000278020	RF02142	0	0	0	0	0	0	0	0
ENSG00000106031	HOXA13	0	0	0	0	0	0	0	0
ENSG00000243766	HOTTIP	0	0	0	0	0	0	0	0
ENSG00000276609	RF02040	0	0	0	0	0	0	0	0
ENSG00000278708	RF02041	0	0	0	0	0	0	0	0
ENSG00000277469	RF02042	0	0	0	0	0	0	0	0
ENSG00000277553	RF02043	0	0	0	0	0	0	0	0
ENSG00000253308	AC004080.1	0	0	0	0	0	0	0	0
ENSG00000253405	EVX1-AS	0	0	0	0	0	0	0	0
ENSG00000106038	EVX1	0	0	0	0	0	0	0	0
ENSG00000213783	RPL35P4	0	0	0	0	0	0	0	0
ENSG00000236569	HNRNPA1P73	0	0	0	0	0	0	0	0
ENSG00000224322	AC004009.1	0	0	0	0	0	0	0	0
ENSG00000279655	AC004009.3	0	0	0	0	0	0	0	0
ENSG00000279429	AC004009.2	0	0	0	0	0	0	0	0
ENSG00000233830	EIF4HP1	0	0	0	0.15835	0	0	0	0
ENSG00000213781	PSMC1P2	0	0	0	0	0	0	0	0
ENSG00000235574	AC073150.1	0	0	0	0	0	0	0	0
ENSG00000106049	HIBADH	6.37394	8.6136	4.86869	27.1145	15.0766	14.6958	16.0418	10.6927
ENSG00000229893	AC005091.1	0.0616	0.20506	0	0.09142	0	0	0	0.07563
ENSG00000106052	TAX1BP1	27.4246	29.5004	11.8468	21.4011	23.9881	18.8077	22.1031	22.5459
ENSG00000153814	JAZF1	19.7656	26.7083	18.9705	56.1789	22.5452	25.5729	24.8008	20.6635
ENSG00000206623	RNU6-979P	0	0	0	0	0	0	1.14187	0.84109
ENSG00000234336	JAZF1-AS1	0.1433	0.23853	0.05265	0.3456	0.06533	0.0632	0.05972	0.08797
ENSG00000174495	PPIAP80	0	0	0	0	0	0	0	0
ENSG00000146592	CREB5	15.3018	20.3278	2.0299	3.45721	11.1071	3.38953	3.04561	8.54178
ENSG00000225286	AC005105.1	0	0	0	0	0	0	0	0
ENSG00000255690	TRIL	0	0	0	0	0	0	0	0
ENSG00000228421	AC005013.1	0	0	0	0	0	0	0	0
ENSG00000272568	AC005162.3	0.01491	0	0	0	0	0	0	0
ENSG00000233517	AC005162.2	0	0	0	0	0	0	0	0
ENSG00000106066	CPVL	36.0137	25.1688	4.25696	11.1717	15.7155	13.8991	7.57225	13.7151
ENSG00000229452	AC005162.1	0	0	0	0	0	0	0	0
ENSG00000106069	CHN2	1.58182	1.11042	0.26277	0.57159	0.79434	0.76437	0.45855	0.50665
ENSG00000235669	AC004593.1	0	0	0	0	0	0	0	0
ENSG00000237065	NANOGP4	0.32114	0.66817	0.11798	0	0.09761	0	0.40147	0.09857

ENSG00000223813	AC007255.1	0.15271	0	0.0748	0	0	0.0898	0.08485	0.0625
ENSG00000270196	AC007255.2	0	0	0	0	0	0	0	0
ENSG00000176532	PRR15	0	0	0	0	0	0	0	0
ENSG00000225264	ZNRF2P2	0.24792	0.13755	0	0.12264	0	0.29157	0.27549	0.10146
ENSG00000223437	TMSB4XP3	0	0	0	0	0	0	0	0
ENSG00000227855	DPY19L2P3	0.03439	0	0.05053	0	0	0	0.1433	0.10556
ENSG00000212024	MIR550A3	0	0	0	0	0	0	0	0
ENSG00000122574	WIPF3	0	0	0	0	0	0.02132	0	0.01484
ENSG00000238906	RF01210	0	0	0	0	0	0	0	0
ENSG00000136193	SCRN1	6.94692	3.22881	0.76608	1.85328	3.90604	1.88214	1.69751	1.66716
ENSG00000227014	AC007285.1	0.07692	0	0	0.11415	0.09351	0	0	0
ENSG00000106080	FKBP14	0.53675	0.2749	0.04045	0.32681	0.25099	0.26707	0.32116	0.32106
ENSG00000106086	PLEKHA8	1.15788	0.77323	0.22415	0.63798	0.91039	0.77059	0.52006	0.664
ENSG00000231519	AC007285.2	0	0	0	0	0	0	0	0
ENSG00000283618	RF01699	0	0	0	0	0	0	0	0
ENSG00000180354	MTURN	21.7702	13.2618	4.30394	6.91152	15.0893	13.4221	20.7437	16.2303
ENSG00000230751	AC007036.2	0.67094	0	0	0	0	0.88772	0.27959	0.61783
ENSG00000251660	AC007036.3	0	0	0	0	0	0	0	0
ENSG00000225907	RPS27P16	1.09403	0	0	0	0.44337	1.93003	0.60786	0.89549
ENSG00000227017	AC007036.1	0	0	0	0	0	0	0	0
ENSG00000180233	ZNRF2	5.92987	6.27208	2.82691	5.38631	4.50888	4.86319	4.72721	4.84562
ENSG00000207771	MIR550A1	1.51135	0	1.11045	0	0	0	3.77877	0
ENSG00000283237	MIR550B1	0	0	0	0	0	0	0	0
ENSG00000281593	AC006978.2	0.33018	0.1832	0	0.32668	0.13381	0.19416	0	0.13513
ENSG00000235859	AC006978.1	7.60328	5.04249	0.43644	1.14604	4.18863	2.93414	2.67331	2.26087
ENSG00000281404	LINC01176	0.71687	1.19324	0.52672	0.93093	0.43578	0.15808	0.59746	0.88017
ENSG00000106100	NOD1	1.20376	1.46696	0.31588	0.59019	1.03229	1.38413	0.46579	0.85774
ENSG00000272638	AC006027.1	0.19784	0.49396	0	0.29362	0	0.17451	0.65954	0.36436
ENSG00000006625	GGCT	2.11134	1.22238	0.74192	1.90733	1.72988	2.18624	1.45362	1.52155
ENSG00000281039	AC005154.5	0	0	0	0	0	0	0	0
ENSG00000196295	GARS-DT	2.01724	1.35476	0.42213	1.03741	1.64535	0.93472	1.32475	1.10121
ENSG00000244480	AC005154.2	0.2631	0.26276	0	0.07809	0.06397	0.09283	0	0.19382
ENSG00000264520	AC005154.4	0	0	0	0	0	0	0.33201	0
ENSG00000226874	AC005154.1	0	0	0	0	0	0	0	0
ENSG00000263683	AC005154.3	0	0	0	0	0	0	0	0
ENSG00000106105	GARS	10.4866	9.96168	4.90316	6.4192	9.29372	17.8146	5.5911	5.4189
ENSG00000106113	CRHR2	0	0	0	0	0	0	0	0
ENSG00000241644	INMT	0	0	0	0	0.0264	0	0	0
ENSG00000223419	AC006022.1	0	0	0	0	0	0	0	0
ENSG00000254959	INMT-MINDY4	0	0	0	0	0	0	0	0
ENSG00000106125	MINDY4	0.01998	0	0	0.01483	0	0.05288	0.01665	0
ENSG00000229263	AC004691.1	0	0	0	0	0	0	0	0
ENSG00000250424	AC004691.2	0	0	0	0	0	0	0	0
ENSG00000240583	AQP1	0.29875	0.39071	0.56444	0.15835	0.49293	0.86584	0.35569	0.4192
ENSG00000106128	GHRHR	0	0	0	0	0	0	0	0
ENSG00000078549	ADCYAP1R1	0	0	0	0	0	0	0	0
ENSG00000232887	AC006466.1	0	0	0	0	0	0	0	0
ENSG00000271027	AC006380.1	0	0	0	0	0	0	0	0
ENSG00000164600	NEUROD6	0	0	0	0	0	0	0	0
ENSG00000231418	AC005090.1	0	0	0	0	0	0	0	0
ENSG00000180347	ITPRID1	0	0	0	0	0	0	0	0
ENSG00000106341	PPP1R17	0.21523	0.25589	0.09036	0.09126	0.07476	0.10848	0.05125	0.11325
ENSG00000154678	PDE1C	0.0053	0.01766	0	0	0.00645	0	0	0
ENSG00000236460	SNX2P2	0	0	0	0	0	0	0	0
ENSG00000226468	AC018641.1	0	0	0	0	0	0	0	0
ENSG00000226421	SLC25A5P5	0	0	0	0	0	0	0	0
ENSG00000106355	LSM5	1.71518	1.21983	1.12274	2.29096	1.32699	2.20058	1.92328	1.74213
ENSG00000105778	AVL9	4.22208	3.67919	1.02188	1.83558	3.94947	2.44481	2.23546	3.34203

ENSG00000229358	DPY19L1P1	12.829	18.2495	34.5897	51.8766	20.9909	18.2452	44.6519	34.4862
ENSG00000237004	ZNRF2P1	0	0	0	0	0.20117	0	0	0.20315
ENSG00000283461	MIR550B2	0	0	0	0	0	0	0	0
ENSG00000207573	MIR550A2	0	0	0	0	0	0	0	0
ENSG00000273014	AC018645.2	0.31059	0.51699	0	1.15239	0.75523	0.27397	0	0
ENSG00000281332	LINC00997	0.0277	0.04611	0	0	0	0.14661	0	0
ENSG00000250756	AC018645.1	0	0	0	0	0	0	0	0
ENSG00000251999	RF00322	0	0	0	0	0	0	0	0
ENSG00000231952	DPY19L1P2	5.84801	10.134	13.0669	25.0861	27.9524	20.0681	30.0443	15.8355
ENSG00000272905	AC018648.1	0	0	0	0	0.13341	0.19358	0	0
ENSG00000170852	KBTBD2	16.5503	18.0309	3.67182	6.58779	12.5803	9.77553	7.52143	10.9902
ENSG00000205763	RP9P	0.86958	0.68114	0.15033	0.07591	0.74627	0	0.17052	0.37682
ENSG00000224573	AC083863.1	0	0	0	0	0	0	0	0.3896
ENSG00000122642	FKBP9	0.18431	0.23599	0.04167	0.06313	0.17237	0.17508	0.0709	0.05222
ENSG00000252821	RNU6-388P	0	1.16199	0	1.03605	0	0	0	0
ENSG00000122643	NT5C3A	38.8221	55.6076	37.4054	68.9215	25.3343	29.9124	84.5783	55.7554
ENSG00000229054	RPS29P14	1.28597	2.854	0.62991	0	1.0423	0.75621	1.42901	1.5789
ENSG00000241420	RN7SL505P	0.25017	0	0	0	0	0	0.417	0
ENSG00000164610	RP9	1.54479	0.96424	0.22701	0.63048	1.50249	1.36261	0.57936	0.61642
ENSG00000228015	AC074338.1	0	0	0.69047	0	0	0	0	0
ENSG00000122507	BBS9	4.05844	3.80107	2.1734	6.83094	4.14295	5.1349	3.68809	3.13791
ENSG00000222741	RNA5SP229	0	0	0	0	0	0	0	0
ENSG00000224946	AC007312.1	0	0	0	0	0	0	0	0
ENSG00000238090	AC006195.1	0.07942	0	0	0.35358	0	0.1401	0.13237	0
ENSG00000173862	AC008080.1	0	0	0	0	0	0	0	0.08506
ENSG00000227481	AC008080.2	0	0	0	0	0	0	0	0
ENSG00000236494	AC008080.4	0	0	0	0	0	0	0	0
ENSG00000233219	AC008080.3	0	0	0	0	0	0	0	0
ENSG00000164619	BMPER	0	0.02072	0	0	0	0	0	0
ENSG00000236212	AC009262.1	0	0	0	0	0	0	0	0
ENSG00000202431	RNU6-438P	0	0	0	0	0	0	0	0
ENSG00000197085	NPSR1-AS1	0	0	0	0	0	0	0	0
ENSG00000187258	NPSR1	0	0	0	0	0	0	0	0
ENSG00000235379	RPL7P31	0	0	0	0	0	0	0	0
ENSG00000230356	NCAPD2P1	0	0	0	0	0	0	0.06784	0
ENSG00000242653	RN7SL132P	0	0	0	0	0	0	0	0
ENSG00000251802	RF00322	0	0	0	0	0	0	0	0
ENSG00000173852	DPY19L1	2.80218	3.43987	1.4069	3.22302	2.98097	3.52219	2.93871	2.58036
ENSG00000221669	MIR548N	0	0	0	1.45048	0	0	0	0
ENSG00000189212	DPY19L2P1	0.09747	0.0295	0.01302	0.23672	0.11851	0.09379	0.01477	0
ENSG00000236592	S100A11P2	0	0.39105	0	0	0	0	0	0
ENSG00000164532	TBX20	0	0	0	0	0	0	0	0
ENSG00000226063	AC009531.1	0	0	0	0	0	0	0	0
ENSG00000235464	AC007652.1	0	0	0	0	0	0	0	0
ENSG00000122557	HERPUD2	27.0755	30.9344	11.3488	22.1191	32.3137	34.9771	20.5892	19.0474
ENSG00000271122	AC018647.2	0.92498	0.58099	0.12823	0.46622	0.97604	1.01602	0.40727	0.25713
ENSG00000227544	AC018647.1	0	0	0	0	0	0	0	0
ENSG00000228878	SEPT7-AS1	1.26661	1.29508	0.55838	3.81327	1.6499	0.79802	1.80963	1.13302
ENSG00000232736	AC007551.1	0	0	0	0.20961	0	0	0	0.1734
ENSG00000122545	SEPT7	25.4259	21.2335	6.63706	13.9847	21.2128	17.9988	11.7072	16.2813
ENSG00000274328	AC007551.2	0	0	0	0	0	0	0	0
ENSG00000200446	RNU6-1085P	0	0	0	0	0	0	0	0
ENSG00000273580	AC087072.2	0	0.1789	0	0	0	0	0	0
ENSG00000226102	SEPT7P3	0.47214	0.39294	0.17345	0.35036	0.28701	0.20823	0.19675	0.14492
ENSG00000270889	AC087072.1	0	0	0	0	0	0	0	0
ENSG00000232930	AC083864.2	0	0	0	0.2257	0	0	0	0
ENSG00000205745	AC083864.1	0	0	0	0	0	0	0	0
ENSG00000261184	AC083864.3	0	0	0	0	0	0	0	0

ENSG00000228815	MARK2P13	0	0	0	0	0	0	0	0
ENSG00000271117	AC078841.1	0	0	0	0	0	0	0	0
ENSG00000122547	EEPD1	5.64081	3.42339	0.35556	1.05486	2.4821	0.8804	0.98309	1.57824
ENSG00000228770	AC007327.1	0	0	0	0	0	0	0	0
ENSG00000229679	AC007327.2	0	0	0	0	0	0	0	0
ENSG00000234457	AC006960.1	0	0	0	0	0	0	0	0
ENSG00000260070	AC006960.3	0.20794	0.69225	0.15279	0.15431	0.12641	0	0	0.25531
ENSG00000164542	KIAA0895	0.05447	0	0.03202	0.01617	0.05298	0.07688	0.01816	0.01338
ENSG00000201432	RF00019	0	0	0	0	0	0	0	0
ENSG00000011426	ANLN	3.03589	4.1806	3.29663	7.2741	5.89961	6.86135	6.7065	5.89801
ENSG00000237400	AC006960.2	0	0	0	0	0	0	0	0
ENSG00000136250	AOAH	35.5851	44.215	6.9968	17.3871	27.5982	25.3097	9.36368	20.7173
ENSG00000230539	AOAH-IT1	3.96564	2.12929	0.93991	2.27823	1.55526	2.7081	1.06615	1.72769
ENSG00000272984	AC007349.4	0	0	0	0	0	0	0	0
ENSG00000229424	AC007349.1	0	0	0.1835	0	0	0	0	0
ENSG00000235728	AC007349.3	0.50436	0.41975	0.24705	0.49902	0.40879	1.33464	0	0.10321
ENSG00000225739	NPM1P18	0.10014	0	0	0	0	0	0	0
ENSG00000230831	AC007349.2	0	0	0	0	0	0.23384	0	0
ENSG00000155849	ELMO1	7.42195	5.4566	1.14805	2.27347	5.07509	4.60766	2.5534	3.15037
ENSG00000221325	MIR1200	1.92896	3.21075	1.41729	0	0	0	1.60764	2.36834
ENSG00000224101	ELMO1-AS1	0	0	0	0	0	0	0	0
ENSG00000224706	RPS17P13	0	0	0	0	0	0	0	0
ENSG00000222869	RNU6-565P	0	0	0	0	0	0	0	0
ENSG00000213744	RPS10P14	0	0	0	0	0	0	0	0
ENSG00000200113	RF00432	0	0	0	0	0	0	0	0
ENSG00000201566	RF00019	0	0	0	0	0	0	0	0
ENSG00000178503	NECAP1P1	0	0	0	0	0	0	0	0
ENSG00000187037	GPR141	2.2193	5.91045	0.88519	1.12926	2.42835	1.28641	0.84554	3.46442
ENSG00000086289	EPDR1	0.56306	0.38341	0.3761	2.24105	0.31116	0.85786	3.66882	1.1941
ENSG00000283444	GPR141BP	0.08821	0.29364	0.12962	0.39273	0.10724	0.15561	0.14703	0.2166
ENSG00000225314	AC018634.1	0	0	0.26861	0.27129	0.88894	0.32247	0	0
ENSG00000086288	NME8	1.53273	2.70129	0.8391	1.7395	2.63075	0.37113	1.65312	2.54604
ENSG00000106483	SFRP4	0	0	0	0	0.02471	0	0	0
ENSG00000010270	STARD3NL	4.43376	3.76015	2.65073	2.72715	3.6073	3.09301	2.16373	5.02973
ENSG00000227191	TRGC2	22.2868	31.556	11.2712	23.5183	19.8819	28.8494	6.99552	22.2994
ENSG00000211687	TRGJ2	5.86403	19.5214	6.46284	6.52714	0	10.3449	2.44361	5.39982
ENSG00000211688	TRGJP2	18.3251	30.5021	17.9523	21.7571	29.7056	43.1039	24.4361	20.9993
ENSG00000211689	TRGC1	11.0622	7.10599	2.20952	6.05693	12.8615	11.8891	3.08807	6.72506
ENSG00000211690	TRGJ1	13.1941	17.0812	0	6.52714	3.56467	10.3449	0	1.79994
ENSG00000211691	TRGJP	7.20987	2.00014	1.7658	5.35012	1.46093	2.11987	0	2.95072
ENSG00000211692	TRGJP1	7.33003	0	0	9.06548	0	6.46559	4.07268	4.49985
ENSG00000211693	TRGV11	0	0	0	0.23496	0.19248	0	0.26389	0
ENSG00000231202	TRGVB	0	0	0	0	0	0	0	0
ENSG00000211694	TRGV10	8.16039	3.81277	2.10379	5.3118	7.65846	26.519	3.5795	5.27327
ENSG00000231916	AC006033.1	0.5162	0.57281	0.25285	0	0	0	0	0.42252
ENSG00000211695	TRGV9	1.90116	1.6174	0.99333	2.57073	3.13321	2.75766	1.19716	2.38609
ENSG00000225992	TRGVA	0	0.4281	0	0	0	0	0	0
ENSG00000272908	AC006033.2	2.45824	1.48791	0.43786	1.65832	1.26792	1.11702	0.68292	2.19505
ENSG00000211696	TRGV8	5.95174	1.82491	0.46032	2.09203	2.66588	6.63137	1.30535	2.88452
ENSG00000249978	TRGV7	4.77594	2.76506	0.30514	3.08175	2.52455	4.76219	1.38448	2.03959
ENSG00000226212	TRGV6	0	0	0	0	0	0	0	0
ENSG00000281103	TRG-AS1	2.43151	2.27473	0.62594	1.00093	2.50303	3.50676	0.76917	1.50358
ENSG00000228668	TRGV5P	0.62119	0	0	0.3073	0	0.73058	0	0.25423
ENSG00000211697	TRGV5	1.06232	3.53648	0	0.7883	0.32289	0.46852	0.22134	0.97823
ENSG00000211698	TRGV4	0.85233	1.4187	0.15656	0.79059	0.77718	0.93977	0.35518	0.52324
ENSG00000211699	TRGV3	1.638	1.36322	0.80234	1.21548	2.32334	1.44482	0.22752	1.67592
ENSG00000233306	TRGV2	0.53897	5.83129	1.18802	2.19971	1.96581	1.18853	0.67379	1.15805
ENSG00000211701	TRGV1	0	0	0	0	0	0	0	0

ENSG00000078053	AMPH	0	0	0	0	0	0	0	0.01597
ENSG00000241756	RN7SL83P	0	0	0	0	0	0	0	0
ENSG00000227404	KRT8P20	0	0	0	0	0	0	0	0
ENSG00000164556	FAM183BP	0	0.15299	0	0	0	0	0	0
ENSG00000006715	VPS41	9.80971	7.53853	2.154	3.89068	8.31649	7.16097	4.11914	6.26438
ENSG00000106536	POU6F2	0	0	0	0	0	0	0	0
ENSG00000233854	POU6F2-AS2	0	0	0	0	0	0	0	0
ENSG00000206947	SNORA20B	0	0	0	0	0	0	0	0
ENSG00000224122	POU6F2-AS1	0	0	0	0	0	0	0	0
ENSG00000227172	AC011290.1	0	0	0	0	0	0	0	0
ENSG00000241127	YAE1	1.05571	1.09233	0.20964	0.63518	1.38757	1.20805	0.66583	0.8758
ENSG00000236015	AC011290.2	0	0	0	0	0	0	0	0
ENSG00000231951	AC004837.3	0	0	0	0	0	0	0	0
ENSG00000106540	AC004837.1	0	0	0	0	0	0	0	0
ENSG00000006451	RALA	4.76002	3.92824	1.1756	2.04808	4.79017	4.30451	1.56684	2.16091
ENSG00000228554	AC004837.2	0	0	0	0	0	0	0	0
ENSG00000188185	LINC00265	0.42357	0.23501	0.13832	0.17462	0.25748	0.41513	0.03922	0.28892
ENSG00000252480	RNU6-719P	0	1.17316	0	0	0	0	0	0
ENSG00000270797	AC004987.4	0	0	0	0	0	0	0	0
ENSG00000226220	CICP22	0	0	0	0	0	0	0	0
ENSG00000237251	AC004987.3	0	0	0.13971	0	0	0	0	0
ENSG00000231198	AC004987.1	0	0	0	0	0	0	0	0
ENSG00000233225	AC004987.2	0.70236	0.5314	0.28148	0.28428	0.46577	0.67585	0.31929	0.31358
ENSG00000175741	RWDD4P2	4.03608	1.51698	2.86982	1.5458	3.32407	2.52652	2.17017	2.07808
ENSG00000242855	RN7SL496P	0.25276	0	0	0	0	0	0	0
ENSG00000259826	AC072061.1	0.68904	0.3823	0.19286	0.09739	0.51858	0.23153	0.10938	0.36257
ENSG00000065883	CDK13	10.6734	10.1206	3.0427	5.49965	12.387	10.1611	5.86181	8.29252
ENSG00000273444	AC006023.2	0	0	0	0	1.06727	1.54864	0	0.5389
ENSG00000270577	AC006023.1	0	0	0	0	0	0	0.47174	0
ENSG00000168303	MPLKIP	0.80298	0.66828	0.35118	0.49654	0.83678	0.94437	0.47801	0.5751
ENSG00000199273	RF00019	0	0	0	0	0	0	0	0
ENSG00000175600	SUGCT	0.05131	0.17082	0.0754	0	0.15596	0	0.1283	0.0315
ENSG00000248711	THUMPD3P1	0	0	0	0	0	0	0	0
ENSG00000203446	AC004988.1	0	0	0	0	0	0	0	0
ENSG00000227658	AC005160.1	0	0	0	0	0	0	0	0
ENSG00000232458	LINC01450	0	0	0	0	0	0	0	0
ENSG00000224017	LINC01449	0	0	0	0	0	0	0	0
ENSG00000122641	INHBA	0.07946	0.0147	0	0.03931	0.06441	0.09346	0.02943	0.07588
ENSG00000224116	INHBA-AS1	0.06766	0	0	0.02008	0.01645	0.04774	0.02255	0
ENSG00000106571	GLI3	0.00793	0	0	0	0	0	0	0
ENSG00000213721	HMGN2P30	0	0	0	0	0.34811	0	0	0
ENSG00000238284	LINC01448	0	0	0	0	0	0	0	0
ENSG00000229161	TCP1P1	0	0	0	0.06757	0	0	0	0
ENSG00000231539	AC010132.1	0	0	0	0	0	0	0	0
ENSG00000261019	AC010132.4	0	0	0	0	0.13029	0.09453	0.08931	0.26315
ENSG00000136197	C7orf25	0.20311	0.15026	0.03316	0.13397	0.2195	0.23888	0.07523	0.22167
ENSG00000256646	AC010132.3	0	0.07954	0	0	0.05809	0	0	0
ENSG00000106588	PSMA2	0.64564	0.64022	0.60559	0.75432	0.40082	0.87242	0.52664	0.80957
ENSG00000234983	AC010132.2	0	0	0	0	0.17089	0	0	0
ENSG00000106591	MRPL32	2.75862	2.86551	0.67055	1.50835	3.37907	3.76885	1.45206	1.70623
ENSG00000232006	AC005537.1	0	0	0	0	0	0	0.05406	0
ENSG00000201933	RF00019	0	0	0	0	0	0	0	0
ENSG00000002746	HECW1	0	0	0	0	0	0	0	0
ENSG00000181211	HECW1-IT1	0	0	0	0	0	0	0	0
ENSG00000264069	MIR3943	0	0	0	0	0	0	0	0
ENSG00000252983	RNU7-35P	0	0	0	0	0	0	0	0
ENSG00000223258	RNU6-575P	0	0	0	0	0	0	0	0
ENSG00000228680	AC004692.2	0	0	0	0	0	0	0	0

ENSG00000213717	AC004692.1	0	0	0	0	0	0	0	0
ENSG00000231638	LUARIS	0	0	0	0	0	0	0	0
ENSG00000164543	STK17A	21.3539	22.7331	8.1717	11.6202	23.2933	34.5843	11.3702	15.1482
ENSG00000106603	COA1	1.68057	2.05412	0.31895	0.97557	1.48528	1.58631	1.4058	1.21823
ENSG00000106605	BLVRA	14.4105	11.3901	2.07028	2.32984	5.38321	6.249	2.28124	3.50895
ENSG00000229497	AC005189.1	0	0	0.34746	0	0	0	0	0
ENSG00000062582	MRPS24	0.16831	0.23346	0	0	0.20463	0.04949	0.09352	0.20665
ENSG00000270617	URGCP-MRPS24	0	0	0	0	0	0	0	0
ENSG00000106608	URGCP	0.92679	0.95051	0.15132	0.55574	1.09262	1.00741	0.3745	0.42527
ENSG00000237972	TUBG1P	0	0	0	0	0	0	0	0
ENSG00000078967	UBE2D4	1.18201	0.44009	0.15998	0.20774	0.73744	0.46643	0.31109	0.30553
ENSG00000214783	POLR2J4	0.03387	0	0.03318	0.03351	0.19218	0.03984	0.13174	0.01386
ENSG00000273432	AC004951.4	0	0	0	0.0524	0	0.06229	0	0.04335
ENSG00000241057	AC004951.3	0.1277	0	0	0.09476	0	0.11264	0	0
ENSG00000239556	AC004951.2	0.46451	0.46391	0.27304	0.27576	0.56474	0	0.15485	0
ENSG00000136206	SPDYE1	0	0	0	0	0	0	0	0
ENSG00000228434	AC004951.1	0.27497	0.13077	0.05772	0.1166	0.04776	0.0693	0.06548	0.04823
ENSG00000272655	POLR2J4	1.70106	1.11541	0.07575	0.153	0.6267	2.27341	0.17184	0.31645
ENSG00000228903	RASA4CP	0.88553	0.1504	0.05311	0.26821	0.35155	0.3507	0.24099	0.13313
ENSG00000235314	LINC00957	1.11184	0.63193	0.3188	0.24148	0.92315	1.00464	0.63282	0.19977
ENSG00000136279	DBNL	12.8874	10.3717	1.25796	2.83481	8.57395	5.4141	3.56242	5.1051
ENSG00000284067	MIR6837	0	0	0	0	0	0	0	0
ENSG00000164708	PGAM2	0.25659	0	0	0	0	0	0	0
ENSG00000239775	AC017116.1	0	0	0	0	0	0	0	0
ENSG00000122678	POLM	2.69547	1.98498	0.36009	0.50914	1.8868	1.67151	0.98027	1.10315
ENSG00000283969	MIR6838	0	0	0	0	0	0	0	0
ENSG00000106624	AEBP1	0.48818	0.04925	0.02174	0.13173	0.66545	0.52194	0.19726	0.12714
ENSG00000284412	MIR4649	0	0	0	0	0	0	0	0
ENSG00000106628	POLD2	2.49955	1.05664	0.11661	0.70659	2.05005	2.9747	0.42986	0.73069
ENSG00000252848	RNA5SP230	0	0	0	0	0	0	0	0
ENSG00000106631	MYL7	0	0	0	0	0	0	0	0
ENSG00000106633	GCK	0	0	0	0	0.02162	0	0	0
ENSG00000106636	YKT6	6.99982	5.5623	1.22039	1.99553	5.07246	6.03478	2.60379	2.96187
ENSG00000058404	CAMK2B	0	0	0	0	0.02184	0	0	0
ENSG00000279853	AC004453.2	0	0	0	0	0.04139	0	0	0
ENSG00000015676	NUDCD3	2.60933	1.77348	0.66036	1.25857	2.52026	2.28882	1.3048	1.37047
ENSG00000146677	AC004453.1	0.35932	1.79424	0.52801	0.79989	1.31054	0.31694	0.29946	0.44116
ENSG00000200456	RNU6-1097P	0	0	0	0	0	0	0	0
ENSG00000225443	AC004938.1	0	0	0	0	0	0	0	0
ENSG00000015520	NPC1L1	0	0	0	0	0	0	0	0
ENSG00000136271	DDX56	3.13527	1.5596	0.34422	1.39057	2.51928	3.24233	1.6519	1.54862
ENSG00000158604	TMED4	7.33198	4.95285	1.54326	3.43473	5.62743	4.87192	2.98238	3.89215
ENSG00000271586	AC011894.1	0	0	0	0	0	0	0	0
ENSG00000105953	OGDH	19.6546	15.6667	3.92815	8.24767	15.2918	19.7318	6.47251	7.42778
ENSG00000122515	ZMIZ2	2.29719	1.18035	0.30822	0.66703	1.7	1.79722	0.54939	0.56409
ENSG00000228596	AC013436.1	0	0	0	0.25779	0	0	0	0
ENSG00000196262	PPIA	54.2145	36.6411	9.8916	20.3237	50.0678	66.8442	18.1947	20.2859
ENSG00000105968	H2AFV	12.2707	6.32009	2.97126	6.8034	7.91898	10.2382	6.66345	6.84122
ENSG00000234183	LINC01952	0	0	0	0	0	0	0	0
ENSG00000146676	PURB	6.96713	4.49342	1.34212	2.67496	5.20805	4.29186	3.03127	3.68165
ENSG00000284261	MIR4657	0	0	0	0	0	0	0	0
ENSG00000272768	AC004854.2	0.25434	0.16934	0.07475	0.22648	0.68028	0.26921	0.33915	0.37473
ENSG00000230160	AC004854.1	0	0	0	0	0	0	0	0
ENSG00000260997	AC004847.1	1.41921	0.7023	0.11273	0.17078	0.5596	1.08267	0.19181	0.32966
ENSG00000136286	MYO1G	18.8312	10.6597	1.73029	3.23101	9.03216	14.2866	3.3888	4.23308
ENSG00000232956	SNHG15	2.11037	0.90327	0.38395	0.59658	1.14847	1.38282	0.73704	0.71563
ENSG00000277184	SNORA9	0	0	0	0	0	0	0	0
ENSG00000136280	CCM2	5.32134	5.04647	0.95938	2.08387	4.83857	3.64459	1.84851	3.43693

ENSG00000136274	NACAD	0	0	0	0	0	0.0263	0	0
ENSG00000136270	TBRG4	2.08419	1.21945	0.59398	1.06855	1.9657	2.29521	1.51594	1.36477
ENSG00000206838	SNORA5A	2.18807	2.73153	4.82301	6.49467	5.3204	4.82507	10.9415	12.0892
ENSG00000201772	SNORA5C	10.7008	8.01516	7.0761	13.499	8.45633	12.2705	2.67548	11.1675
ENSG00000200656	SNORA5B	0	0	0	0	0	0	0	0
ENSG00000122679	RAMP3	0	0	0	0	0	0	0.08562	0
ENSG00000275295	AC073968.1	0	0	0	0	0	0	0	0
ENSG00000234359	ELK1P1	0	0	0	0	0	0	0	0
ENSG00000225537	AC073325.1	0	0	0	0	0	0	0	0
ENSG00000226999	AC073325.2	0	0	0	0	0	0	0	0
ENSG00000164742	ADCY1	0	0.02705	0	0	0	0	0	0
ENSG00000214765	SEPT7P2	2.05424	1.84532	1.48538	3.00032	1.56588	2.01331	3.91325	2.38204
ENSG00000272556	GTF2IP13	0.02837	0.01181	0.01042	0.02105	0.01725	0.03754	0.05912	0.01742
ENSG00000212450	RNU6-241P	0	0	0	0	0	0	0	0
ENSG00000235955	CICP20	0	0	0	0	0	0	0	0
ENSG00000226838	AC096582.1	0	0	0	0	0	0	0	0
ENSG00000251378	AC096582.2	0	0	0	0	0	0	0	0
ENSG00000202350	RNU6-326P	0	0	0	0	0	0	0	0
ENSG00000283247	AC096582.3	0	0	0	0	0	0	0	0
ENSG00000146678	IGFBP1	0	0	0	0	0	0	0	0
ENSG00000146674	IGFBP3	0.57729	0.48045	0.02356	0.21419	0.07798	0	0	0.05907
ENSG00000237471	AC073115.2	0.26415	0	0.38816	0	0	0	0	0.16216
ENSG00000229628	AC073115.1	0	0.22934	0.20247	0	0.16751	0	0.22966	0.16917
ENSG00000233952	FTLP15	0	0	0	0	0	0	0	0
ENSG00000221971	TTC4P1	0	0	0	0	0	0	0	0
ENSG00000224547	ZNF619P1	0	0	0	0	0	0	0	0
ENSG00000229459	AC023669.1	0	0	0	0	0	0	0	0
ENSG00000237760	AC023669.2	0	0	0	0	0	0	0	0
ENSG00000240355	AC004869.2	0	0	0	0	0	0	0	0
ENSG00000230680	AC004869.1	0	0	0	0	0	0	0	0
ENSG00000224519	HMGN1P19	0	0	0	0	0	0	0	0
ENSG00000233539	AC011294.1	0	0	0	0	0	0	0	0
ENSG00000242948	EPS15P1	0	0	0	0	0	0	0	0
ENSG00000232072	AC004870.4	0	0	0	0	0	0	0	0
ENSG00000223829	AC004870.2	0	0	0	0	0	0	0	0
ENSG00000229192	AC004870.3	0.27661	0	0	0	0	0	0	0
ENSG00000242552	MRPL42P4	0	0	0	0	0	0	0	0
ENSG00000214754	AC004870.1	0	0	0	0	0	0	0	0
ENSG00000136205	TNS3	2.21821	1.47895	0.46566	0.44263	1.05759	1.18383	0.56963	0.6103
ENSG00000236078	LINC01447	0	0	0	0	0	0	0	0.0326
ENSG00000221845	C7orf65	0	0	0	0	0	0	0	0
ENSG00000136273	HUS1	2.31641	2.30756	1.16043	2.68253	3.09359	2.41473	2.36931	1.98222
ENSG00000146666	LINC00525	0	0	0	0	0	0	0	0
ENSG00000158683	PKD1L1	0.0538	0	0	0.04562	0.02803	0.01356	0.01281	0.06605
ENSG00000136275	C7orf69	0	0	0	0	0	0	0	0
ENSG00000225507	AC069282.1	0	0	0	0	0	0	0	0
ENSG00000164744	SUN3	0	0	0	0.06281	0	0	0	0.05196
ENSG00000164746	C7orf57	0	0	0	0.09564	0.03917	0	0	0
ENSG00000183696	UPP1	5.33297	4.98483	0.68248	1.16463	3.01793	2.17544	1.22794	2.37921
ENSG00000179869	ABCA13	1.00685	1.86141	1.1012	1.03231	0.99984	1.36945	0.52526	1.54761
ENSG00000228173	AC091770.1	0	0	0	0	0	0	0	0
ENSG00000225705	AC004899.1	0	0	0	0	0	0	0	0
ENSG00000279578	AC004899.2	0	0	0	0	0	0	0	0
ENSG00000229165	GDI2P1	0	0.18265	0.08062	0.08143	0.13341	0	0.09145	0.06736
ENSG00000218305	CDC14C	0	0	0	0.06328	0	0	0	0
ENSG00000234686	AC091730.1	0	0	0	0	0	0	0	0
ENSG00000270763	DDX43P2	0	0	0	0	0	0	0	0
ENSG00000279104	AC093775.1	0	0	0	0	0	0	0	0

ENSG00000188730	VWC2	0	0	0	0	0	0	0	0
ENSG00000042813	ZPBP	0.02773	0.13849	0.04075	0	0	0	0.18491	0.03405
ENSG00000223893	GNL2P1	0.03347	0	0	0	0	0	0	0
ENSG00000164500	SPATA48	0	0	0	0	0	0	0	0
ENSG00000228005	AC020743.1	0	0	0	0	0.11619	0	0.1593	0
ENSG00000231681	AC020743.2	0	0	0	0.07629	0	0	0	0
ENSG00000235620	AC020743.3	0	0	0	0	0	0	0	0
ENSG00000185811	IKZF1	32.8218	30.1779	6.64955	14.3654	32.3804	35.262	11.1016	17.9737
ENSG00000200815	RNU6-1091P	0	0	0	0	0	0	0	0
ENSG00000132436	FIGNL1	1.77645	1.26032	0.23537	1.14534	2.17746	2.38895	0.72813	1.01904
ENSG00000132437	DDC	0	0	0	0	0	0	0	0
ENSG00000226122	DDC-AS1	0	0	0	0	0	0	0	0
ENSG00000106070	GRB10	0.70027	1.30057	0.83407	0.92989	0.57356	1.78155	0.58977	0.56112
ENSG00000236046	AC004920.1	0	0	0	0	0	0	0	0
ENSG00000228085	AC009296.1	0	0	0	0	0	0	0	0
ENSG00000228204	AC004830.1	0	0	0	0	0	0	0	0
ENSG00000201133	RF00394	0	0	0	0	0	0	0	0
ENSG00000106078	COBL	0.00511	0.0085	0	0.03791	0.00621	0	0	0
ENSG00000231490	RPL7L1P2	0	0	0	0	0	0	0	0
ENSG00000227233	CICP17	0	0	0	0	0	0	0	0
ENSG00000228897	AC012441.2	0	0	0	0	0	0	0	0
ENSG00000227080	AC012441.1	0	0	0	0	0	0	0	0
ENSG00000229478	ROBO2P1	0	0	0	0	0	0	0	0
ENSG00000229403	AC005999.1	0	0	0	0	0	0	0	0
ENSG00000242650	RN7SL292P	0	0	0	0	0	0	0	0
ENSG00000233960	AC079763.1	0	0	0	0	0	0	0	0
ENSG00000253028	RF00322	0	0	0	0	0	0	0	0
ENSG00000234304	SGO1P2	0	0	0	0	0	0	0	0
ENSG00000223970	AC074397.1	0	0	0	0	0	0	0	0
ENSG00000221900	POM121L12	0	0	0	0	0	0	0	0
ENSG00000227344	HAUS6P1	0.2363	0.39332	0.69448	2.94584	0.4022	0.33349	1.73305	0.63828
ENSG00000271194	RNF138P2	0	0	0	0	0	0	0	0
ENSG00000199629	RNU1-14P	0	0	0	0	0	0	0	0
ENSG00000222154	RN7SKP218	0	0	0	0	0	0	0	0
ENSG00000234105	AC009468.2	0	0	0	0	0	0	0	0
ENSG00000228627	AC009468.1	0	0	0	0	0	0	0	0
ENSG00000205628	LINC01446	0.02104	0	0	0	0.02558	0.03712	0	0
ENSG00000222355	RNU2-29P	0	0	0	0	0	0	0	0
ENSG00000271306	RAC1P9	0	0	0	0	0	0	0	0
ENSG00000232418	AC074348.1	0	0	0	0	0	0	0	0
ENSG00000235454	HAUS6P3	0.06622	0	0	0	0.0805	0.35044	0.11037	0
ENSG00000238131	AC073521.1	0	0	0	0	0	0	0	0
ENSG00000280920	AC092848.1	0	0	0	0	0	0	0	0
ENSG00000231427	LINC01445	0	0	0	0	0	0	0	0
ENSG00000213673	SLC25A5P3	0	0	0	0	0	0	0	0
ENSG00000170419	VSTM2A	0	0	0	0	0	0	0	0
ENSG00000224223	VSTM2A-OT1	0	0	0	0	0	0	0	0
ENSG00000228735	AC011228.1	0	0	0	0	0	0	0	0
ENSG00000200471	RNU6-1125P	0	0	0	0	0	0	0	0
ENSG00000232612	RPL31P35	0	0	0	0	0	0	0	0
ENSG00000132432	SEC61G	11.5004	5.23272	2.68992	6.43737	9.19227	10.6005	3.91348	4.6904
ENSG00000234707	AC074351.1	0.76196	0	0	0.45233	0.37055	0.53768	0	0.37421
ENSG00000218586	AC006971.1	0	0	0	0.02425	0	0.02883	0	0
ENSG00000146648	EGFR	0	0.00974	0	0	0.02135	0	0	0
ENSG00000224057	EGFR-AS1	0	0	0	0	0	0	0	0
ENSG00000280890	ELDR	0	0	0	0	0	0	0	0
ENSG00000237210	AC073324.1	0	0	0	0	0	0	0	0
ENSG00000230564	CALM1P2	0	0	0	0	0	0	0	0

ENSG00000230936	AC073347.1	0	0	0	0	0	0	0	0
ENSG00000132434	LANCL2	1.47021	0.60596	0.20576	0.41561	1.03842	1.43268	0.67683	0.46417
ENSG00000154978	VOPP1	7.77552	5.64096	1.20485	3.5694	6.19696	5.54469	3.30279	3.40593
ENSG00000223475	AC099681.1	0	0	0	0	0	0	0	0
ENSG00000233977	AC099681.3	0	0	0	0	0	0	0	0
ENSG00000231394	AC099681.2	0	0	0	0	0	0	0	0
ENSG00000234844	CDC42P2	0	0	0	0	0	0	0	0
ENSG00000235207	TUBBP6	0	0	0	0	0	0	0	0
ENSG00000231317	AC091812.1	0	0	0	0	0	0	0	0
ENSG00000176826	FKBP9P1	0	0	0	0	0	0	0	0
ENSG00000252857	RNU6-389P	0	0	0	0	0	0	0	0
ENSG00000275853	RF00017	0	0	0	0	0	0	0	0
ENSG00000224971	SUMO2P3	0	0	0.74801	0	0	0	0	0
ENSG00000237799	CICP11	0	0	0	0	0	0	0	0
ENSG00000275875	AC091812.3	0	0	0	0	0	0	0	0
ENSG00000273720	AC091812.2	0	0	0	0	0	0	0	0
ENSG00000226278	PSPHP1	0	4.20719	0	2.50082	0	0	0	0
ENSG00000206729	RNU6-1126P	0	0	0	0	0	0	0	0
ENSG00000154997	SEPT14	0	0	0	0	0	0	0	0
ENSG00000235738	AC092647.4	0	0	0	0	0	0	0	0
ENSG00000225270	CICP12	0	0	0	0	0	0	0	0
ENSG00000229762	AC092647.3	0	0	0	0	0	0	0	0
ENSG00000225018	AC092647.1	0	0	0	0	0	0	0	0
ENSG00000227499	AC092647.2	0	0	0	0	0	0	0	0
ENSG00000249773	AC092647.5	0	0	0	0	0	0	0	0
ENSG00000178665	ZNF713	0.41675	0.31711	0.10498	0.22973	0.2461	0.16805	0.15878	0.0731
ENSG00000146729	NIPSNAP2	5.01986	4.05903	1.98235	3.88864	4.39985	4.25624	3.45936	3.08962
ENSG00000239789	MRPS17	0.88273	0.39558	0.39913	0.15116	0.74298	0.17968	0.11318	0.66695
ENSG00000146733	PSPH	0.87578	0.70678	0.117	0.31509	0.48398	0.18727	0.30965	0.13034
ENSG00000146731	CCT6A	16.9299	13.136	3.2582	8.08712	13.3184	15.6129	6.9531	8.78977
ENSG00000206603	SNORA22B	0	0.88412	0.78054	3.94151	0.64577	0.93704	0	2.60861
ENSG00000207168	SNORA15	0.55113	0	0.80988	0	0	0	0	0
ENSG00000129103	SUMF2	5.57662	3.09409	0.78796	2.62614	4.88936	4.54057	3.12825	2.74314
ENSG00000164776	PHKG1	0.58386	0.57426	0.77997	1.29976	0.96796	1.45136	1.41556	0.87977
ENSG00000106153	CHCHD2	32.247	24.0352	9.13475	14.8956	24.326	38.8392	18.2407	18.6036
ENSG00000185290	NUPR2	0	0	0	0	0	0	0	0
ENSG00000230191	AC006970.1	0	0	0	0	0	0	0	0
ENSG00000233028	AC006970.2	0	0	0	0	0	0	0	0
ENSG00000227491	CCNJP1	0	0	0	0	0	0	0	0
ENSG00000223559	AC073136.1	0	0	0	0	0.1236	0	0.04236	0.03121
ENSG00000224155	AC073136.2	0	0	0	0	0	0	0	0
ENSG00000203462	AC093392.1	0	0	0	0	0	0	0	0
ENSG00000202296	RNU6-1335P	0	0	0	0	0	0	0	0
ENSG00000231299	SEPT14P24	0	0	0	0	0	0	0	0
ENSG00000225371	CICP8	0	0	0	0	0	0	0	0
ENSG00000224370	AC093392.2	0	0	0	0	0	0	0	0
ENSG00000223330	RNU6-1052P	0	0	0	0	0	0	0	0
ENSG00000232944	AC092423.1	0	0	0	0	0	0	0	0
ENSG00000237268	AC092447.7	0	0	0	0	0	0	0	0
ENSG00000278205	AC092447.10	0	0	0	0	0	0	0	0
ENSG00000225488	AC092447.4	0	0	0	0	0	0	0	0
ENSG00000226485	RBM22P3	0	0	0	0	0	0	0	0
ENSG00000261275	AC092447.8	0	0	0	0	0	0	0	0
ENSG00000231504	NMD3P2	0	0	0	0	0	0	0	0
ENSG00000223740	AC092447.3	0	0	0	0	0	0	0	0
ENSG00000233288	AC092447.5	0	0	0	0	0	0	0	0
ENSG00000224947	VN1R25P	0	0	0	0	0	0	0	0
ENSG00000213650	AC092447.2	0	0	0	0.16261	0	0	0	0

ENSG00000234716	AC092447.6	0	0	0	0	0	0	0	0
ENSG00000213067	AC092447.1	0	0	0	0	0	0	0	0
ENSG00000271047	AC092447.9	0	0	0	0	0	0	0	0
ENSG00000229909	TRIM60P16	0	0	0	0	0	0	0	0
ENSG00000228303	AC095038.1	0	0	0	0.13675	0	0	0	0.22627
ENSG00000277174	AC095038.2	0	0	0	0	0	0	0	0
ENSG00000282879	AC095038.3	0	0	0	0	0	0	0	0
ENSG00000277253	AC118758.2	0	0	0	0	0	0	0	0
ENSG00000279072	AC118758.3	0	0	0	0	0	0	0	0
ENSG00000233437	AC118758.1	0	0	0	0	0	0	0	0
ENSG00000225244	AC069152.1	0	0	0	0	0	0	0	0
ENSG00000264426	MIR4283-1	0	0	0	0	0	0	0	0
ENSG00000189166	TNRC18P3	0	0	0	0	0.03312	0	0	0
ENSG00000214668	SLC29A4P1	0	0	0	0	0	0	0	0
ENSG00000229508	PHKG1P4	0	0	0	0	0	0	0	0
ENSG00000275061	AC122133.1	0	0	0	0	0	0	0	0
ENSG00000185177	ZNF479	0	0	0	0	0	0	0.05888	0
ENSG00000235095	AC099654.2	0.07404	0	0.0544	0.32965	0.09002	0	0.18512	0.09091
ENSG00000283431	AC099654.6	0	0	0	0	0	0	0	0
ENSG00000284474	AC099654.12	0	0	0	0	0	0	0	0
ENSG00000283547	AC099654.7	0	0	0	0	0	0	0	0
ENSG00000231537	MTCO3P10	0	0	0	0	0	0	0	0
ENSG00000283230	AC099654.5	0	0	0	0	0	0	0	0
ENSG00000283481	MTND4LP32	0	0	0	0	0	0	0	0
ENSG00000225295	MTND4P4	0	0	0	0	0	0	0	0
ENSG00000228440	MTND5P6	0	0	0	0	0	0	0	0
ENSG00000283311	MTND6P29	0	0	0	0	0	0	0	0
ENSG00000224880	MTCYBP29	0	0	0	0	0	0	0	0
ENSG00000224723	GUSBP10	0	0	0	0	0	0	0	0
ENSG00000230764	MTND1P4	0	0	0	0	0	0	0	0
ENSG00000237309	MTND2P6	0	0	0	0	0	0	0	0
ENSG00000236692	AC099654.3	0	0	0	0	0	0	0	0
ENSG00000284291	AC099654.11	0	0	0	0	0	0	0	0
ENSG00000284098	AC099654.9	0	0	0	0	0	0	0	0
ENSG00000283626	AC099654.8	0	0	0	0	0	0	0	0
ENSG00000228851	MTCO3P4	0	0	0	0	0	0	0	0
ENSG00000284558	AC099654.13	0	0	0	0	0	0	0	0
ENSG00000284221	AC099654.10	0	0	0	0	0	0	0	0
ENSG00000227584	MTND4P5	0	0	0	0	0	0	0	0
ENSG00000230531	MTND5P7	0	0	0	0	0	0	0	0
ENSG00000284572	AC099654.14	0	0	0	0	0	0	0	0
ENSG00000234671	MTCYBP5	0	0	0	0	0	0	0	0
ENSG00000169325	GUSBP12	0	0	0	0	0	0	0	0
ENSG00000234089	AC099654.1	0	0	0	0	0	0	0	0
ENSG00000238431	RNU7-157P	0	0	0	0	0	0	0	0
ENSG00000274299	AC099654.4	0	0	0	0	0	0	0	0
ENSG00000280225	AC237221.2	0	0	0	0	0	0	0	0
ENSG00000260653	AC237221.1	0	0	0	0	0	0	0	0
ENSG00000266168	MIR3147	0	0	0	0	0	0	0	0
ENSG00000227182	VN1R28P	0	0	0	0	0	0	0	0
ENSG00000235368	SAPCD2P2	0	0	0	0	0	0	0	0
ENSG00000182111	ZNF716	0	0	0	0	0	0	0	0
ENSG00000270957	AC092175.1	0	0	0	0	0	0	0	0
ENSG00000233601	NCOR1P3	0	0	0	0	0	0	0	0
ENSG00000243981	AC064862.6	0	0	0	0	0	0	0	0
ENSG00000270749	AC064862.7	0	0	0	0	0	0	0	0
ENSG00000229627	BSNDP4	0	0	0	0	0	0	0	0
ENSG00000230796	AC064862.1	0	0	0	0	0	0	0	0

ENSG00000237639	AC064862.5	0	0	0	0	0	0	0	0
ENSG00000233962	AC064862.2	0	0	0	0	0	0	0	0
ENSG00000236261	AC064862.4	0	0	0	0	0	0	0	0
ENSG00000234085	AC064862.3	0	0	0	0	0	0	0	0
ENSG00000227015	AC023141.4	0	0	0	0	0	0	0	0
ENSG00000232161	AC023141.8	0	0	0	0	0	0	0	0
ENSG00000230271	AC023141.5	0	0	0	0	0	0	0	0
ENSG00000250923	AC023141.12	0	0	0	0	0	0	0	0
ENSG00000224484	AC023141.2	0	0	0	0	0	0	0	0
ENSG00000236907	AC023141.9	0	0	0	0	0	0	0	0
ENSG00000250618	AC023141.11	0	0	0	0	0	0	0	0
ENSG00000224653	AC023141.3	0	0	0	0	0	0	0	0
ENSG00000223836	AC023141.1	0	0	0	0	0	0	0	0
ENSG00000237236	AC023141.10	0	0	0	0	0	0	0	0
ENSG00000231232	AC023141.6	0	0	0	0	0	0	0	0
ENSG00000231484	AC023141.7	0	0	0	0	0	0	0	0
ENSG00000271696	AC023141.13	0	0	0	0	0	0	0	0
ENSG00000233918	AC128676.1	0	0	0	0	0	0	0	0
ENSG00000207443	RNU6-417P	0	0	0	0	0	0	0	0
ENSG00000227148	AC069285.1	0	0	0	0	0	0	0	0
ENSG00000233454	AC069285.2	0	0	0	0	0	0	0	0
ENSG00000233782	ZNF90P3	0	0	0	0	0	0	0	0
ENSG00000234312	SAPCD2P4	0	0	0	0	0	0	0	0
ENSG00000231523	SEPT14P1	0	0	0	0	0	0	0	0
ENSG00000199231	RF00494	0	0	0	0	0	0	0	0
ENSG00000237572	AC092001.1	0	0	0	0	0	0	0	0
ENSG00000226075	PHKG1P1	0	0	0	0	0	0	0	0
ENSG00000244550	AC006455.8	0	0	0	0	0	0	0	0
ENSG00000240651	SEPT7P4	0	0	0	0	0	0	0	0
ENSG00000236638	AC006455.7	0	0	0	0	0	0	0	0
ENSG00000185037	ZNF733P	0	0	0	0	0	0	0	0
ENSG00000226587	AC006455.1	0	0	0	0	0	0	0	0
ENSG00000227298	ARAFP3	0	0	0	0	0	0	0	0
ENSG00000232165	AC006455.6	0	0	0	0	0	0	0	0
ENSG00000230000	AC006455.5	0	0	0	0	0	0	0	0
ENSG00000227545	AC006455.2	0	0	0	0	0	0	0	0
ENSG00000227923	AC006455.3	0	0	0	0	0	0	0	0
ENSG00000229301	AC006455.4	0	0	0	0	0	0	0	0
ENSG00000236284	VN1R31P	0	0	0	0	0	0	0	0
ENSG00000230361	VN1R32P	0	0	0	0	0	0	0	0
ENSG00000230600	AC073188.3	0	0	0	0	0	0	0	0
ENSG00000232817	AC073188.4	0	0	0	0	0	0	0	0
ENSG00000223889	AC073188.1	0	0	0	0	0	0	0	0
ENSG00000227305	AC073188.2	0	0	0	0	0	0	0	0
ENSG00000234387	AC073188.5	0	0	0	0	0	0	0	0
ENSG00000268181	AC073188.6	0	0	0	0	0	0	0	0
ENSG00000234467	SLC25A1P2	0	0	0	0	0	0	0	0
ENSG00000227426	VN1R33P	0	0	0	0	0	0	0	0
ENSG00000224368	ARAFP2	0	0	0	0	0	0	0	0
ENSG00000197990	ZNF734P	0	0	0	0	0	0	0	0
ENSG00000230132	AC006457.1	0	0	0	0	0	0	0	0
ENSG00000164740	SEPT7P5	0	0	0	0	0	0	0	0
ENSG00000228645	PHKG1P2	0	0	0	0	0	0	0	0
ENSG00000214660	SLC29A4P2	0	0	0	0	0	0	0	0
ENSG00000223566	TNRC18P2	0	0	0	0	0	0	0	0
ENSG00000265214	MIR4283-2	0	0	0	0	0	0	0	0
ENSG00000226401	AC006015.1	0	0	0	0	0	0	0	0
ENSG00000226411	AC079355.1	0	0	0	0	0	0	0	0

ENSG00000271466	CICP24	0	0	0	0	0	0	0	0
ENSG00000227397	AC079355.2	0	0	0	0	0	0	0	0
ENSG00000236574	AC092634.6	0	0	0	0	0	0	0	0
ENSG00000225451	AC092634.2	0	0	0	0	0	0	0	0
ENSG00000236299	AC092634.5	0	0	0	0	0	0	0	0
ENSG00000235720	GABPAP	0.05406	0.08998	0.15887	0	0.26288	0	0	0
ENSG00000226581	AC092634.3	0	0	0	0	0	0	0.19181	0
ENSG00000226342	NMD3P1	0	0	0	0	0	0	0.16422	0
ENSG00000227910	AC092634.4	0	0	0	0.12418	0.10173	0	0	0
ENSG00000213645	SLC25A1P3	0	0	0	0	0	0	0	0
ENSG00000233173	VN1R34P	0	0	0	0	0	0	0	0
ENSG00000231264	ARAFP1	0	0	0	0	0	0	0	0
ENSG00000284534	AC092634.8	0	0	0	0	0	0	0	0
ENSG00000228660	VN1R35P	0	0	0	0	0	0	0	0
ENSG00000230639	VN1R36P	0	0	0	0	0	0	0	0
ENSG00000176232	AC092634.1	0	0	0	0	0	0	0	0
ENSG00000278577	AC092634.7	0	0	0	0	0	0	0	0
ENSG00000241149	AC115220.1	0	0	0	0	0	0	0	0
ENSG00000228564	LINC01005	0	0	0	0	0	0	0	0
ENSG00000214652	ZNF727	0	0	0	0.25917	0	0	0.29108	0
ENSG00000223558	TRIM60P17	0	0	0	0	0	0	0	0
ENSG00000224458	GUSBP6	0	0	0	0	0	0	0	0
ENSG00000248704	MTND4P2	0	0	0	0	0	0	0	0
ENSG00000248315	MTND4LP2	0	0	0	0	0	0	0	0
ENSG00000230052	MTND3P2	0	0	0	0	0	0	0	0
ENSG00000233385	MTCO3P8	0	0	0	0	0	0	0	0
ENSG00000227035	MTATP6P18	0	0	0	0	0	0	0	0
ENSG00000234234	MTCO2P8	0	0	0	0	0	0	0	0
ENSG00000234700	MTCO1P8	0	0	0	0	0	0	0	0
ENSG00000230252	MTND2P4	0	0	0	0	0	0	0	0
ENSG00000235646	MTND1P2	0	0	0	0	0	0	0	0
ENSG00000229881	AC091685.1	0	0	0	0	0	0	0	0
ENSG00000236976	AC091685.2	0	0	0	0	0.10314	0	0	0
ENSG00000213643	VN1R37P	0	0	0	0	0	0	0	0
ENSG00000235227	VN1R38P	0	0	0	0	0	0	0	0
ENSG00000213644	SAPCD2P1	0	0	0	0	0	0	0	0
ENSG00000223614	ZNF735	0	0	0	0	0	0	0	0
ENSG00000197123	ZNF679	0	0	0	0	0	0	0	0
ENSG00000234444	ZNF736	1.9554	1.64684	0.69833	1.09838	1.36389	1.42932	2.37634	1.30084
ENSG00000227986	TRIM60P18	0.9738	1.17883	0	0.65692	1.07629	0.15617	1.32805	1.08692
ENSG00000276650	AC022202.1	0	0	0	0	0	0	0	0
ENSG00000232727	YWHAEP1	0	0	0.14117	0	0.2336	0	0	0
ENSG00000223845	VN1R40P	0	0	0	0	0	0	0	0
ENSG00000228014	ZNF680P1	0	0.16611	0	0	0	0	0	0
ENSG00000228653	HNRNPCP7	0	0	0	0	0	0	0	0
ENSG00000173041	ZNF680	4.81583	4.63047	1.94757	5.41397	5.20087	4.81505	3.76209	3.28668
ENSG00000223974	BNIP3P42	0	0	0	0	0	0	0	0
ENSG00000235349	AC016769.3	0	0	0	0	0	0	0	0
ENSG00000213642	AC016769.1	0	0	0	0.21542	0	0.12803	0	0
ENSG00000224669	AC016769.2	0	0	0	0	0	0	0	0
ENSG00000276475	AC016769.4	0	0	0	0	0	0	0	0
ENSG00000196247	ZNF107	12.5988	9.64211	1.57916	4.13149	6.60723	4.85687	4.06017	3.2797
ENSG00000271550	BNIP3P11	0.1302	0	0	0	0	0	0	0
ENSG00000275667	MIR6839	0	0	0	0	0	0	0	0.79643
ENSG00000224172	AC091799.1	0	0	0	0	0	0	0	0
ENSG00000277206	AC073349.3	0	0	0	0	0	0	0	0
ENSG00000197008	ZNF138	6.94424	3.63885	0.90706	2.55742	4.97177	4.21965	2.05777	1.89467
ENSG00000234338	AC073349.2	0	0	0	0	0	0	0	0

ENSG00000182722	SEPHS1P1	0	0	0	0	0	0	0	0
ENSG00000213640	EEF1DP4	0	0	0	0	0	0	0	0
ENSG00000198039	ZNF273	1.48261	1.81913	0.46064	1.38308	1.49352	1.53943	1.20034	1.05059
ENSG00000189316	AC073349.1	0.06902	0	0.10143	0	0	0	0.23009	0
ENSG00000223476	VN1R42P	0.42616	0.35468	0.31312	0	0.51812	0.75181	1.06553	0.52324
ENSG00000270948	MTDHP1	0	0	0	0	0	0	0	0
ENSG00000152926	ZNF117	4.88166	7.20114	1.72286	2.58456	3.95944	1.80221	1.95425	5.48013
ENSG00000213462	ERV3-1	5.65698	5.68164	0.68293	1.49836	3.77976	1.75281	1.76299	5.98144
ENSG00000201165	RNU6-1229P	0	0	0	0	0	0	0	0
ENSG00000234585	CCT6P3	1.36503	1.30723	0.76939	0.94355	0.86389	0.56079	1.34024	1.46934
ENSG00000239985	AC073210.2	0	0	0	0	0	0	0	0
ENSG00000207344	SNORA22C	5.47017	11.8366	7.23452	14.613	7.31555	6.75509	5.47076	7.38782
ENSG00000207062	SNORA15B-1	0	0	0	0.80582	0	0	0	0
ENSG00000227113	AC073210.1	0.94785	0	0.46428	0	0.38412	0	0.52664	0.77584
ENSG00000226002	GTF2IP14	0.25017	0.83282	0.36762	0.18564	1.06453	1.76535	0.6255	1.68936
ENSG00000164669	INTS4P1	0.05758	0.14376	0.08461	0.12818	0.17501	0.15237	0.33592	0.14139
ENSG00000237026	AC104073.2	0	0	0	0	0	0	0	0
ENSG00000229064	AC104073.1	0	0	0	0	0	0	0	0
ENSG00000282381	AC104073.4	0	0	0	0	0	0	0	0
ENSG00000277544	AC104073.3	0	0	0	0	0	0	0	0
ENSG00000231556	RSL24D1P3	0	0	0	0	0	0	0	0
ENSG00000232906	AC092685.1	0	0	0	0	0	0	0	0
ENSG00000146757	ZNF92	10.8114	7.33024	2.97501	6.44289	13.2965	10.1991	6.36646	7.38017
ENSG00000238124	AC114501.3	0	0	0	0	0	0	0	0
ENSG00000226767	AC114501.1	0	0	0	0	0	0	0	0
ENSG00000235421	AC114501.2	0	0	0	0	0	0	0	0
ENSG00000230386	AC104057.1	0	0	0	0	0	0	0	0
ENSG00000272693	AC073107.1	0.26152	0.06219	0.3294	0.33268	0.40879	0.39545	0.62273	0.13761
ENSG00000273024	INTS4P2	0.09819	0.16344	0.07215	0.21859	0.41783	0.43306	0.32734	0.24112
ENSG00000228409	CCT6P1	4.43313	2.41095	3.09597	4.55988	2.56144	1.47121	6.1456	2.91008
ENSG00000206634	SNORA22	53.0607	55.5412	52.2493	93.3609	51.8739	43.4256	20.0595	44.9985
ENSG00000206785	SNORA15B-2	0	0	0	0	0	0	0	0.66664
ENSG00000275833	AC073107.2	0	0	0	0	0	0.54333	0.51336	0
ENSG00000224316	GTF2IP5	0.10027	0.16691	0.14735	0.74409	0	1.41518	0.83571	0.61557
ENSG00000200670	RNU6-912P	0	0	0	0	0	0	0	0.86536
ENSG00000236529	AC093582.1	0	0	0	0	0	0	0	0
ENSG00000201560	RNU6-973P	0	0	0	0	0	0	0	0
ENSG00000196715	VKORC1L1	3.74019	2.37853	1.09558	2.23141	2.84013	2.23594	1.53269	1.38833
ENSG00000169919	GUSB	7.56507	5.31801	1.13326	1.77131	3.92899	4.50259	2.4485	1.96136
ENSG00000224365	AC073261.1	0	0	0	3.59622	0	0	2.01951	0
ENSG00000229886	AC068533.2	0.23954	0	0.35201	0.71102	0.58246	0.42259	0	0.88232
ENSG00000205596	AC068533.1	0	0	0	0	0	0	0	0
ENSG00000126522	ASL	1.72067	1.33656	0.16857	0.44263	1.08781	1.45703	0.34417	0.42252
ENSG00000249319	AC068533.4	0	0	0	0	0	0	0	0
ENSG00000241258	CRCP	5.9013	3.48103	1.03454	1.47506	3.62508	4.63915	1.86377	2.18637
ENSG00000234185	AC068533.3	0	0	0	0	0	0	0	0
ENSG00000169902	TPST1	2.80844	1.61488	0.4127	1.17463	2.17282	1.2161	3.48965	1.78678
ENSG00000252126	RNU6-313P	0	0	0	0	0	0	0	0
ENSG00000179406	LINC00174	0.59394	0.6555	0.80638	1.21682	0.66716	0.58084	0.87164	0.76094
ENSG00000251451	GTF2IP9	0.24352	0	0	0	0	0	0	0.29899
ENSG00000231234	SKP1P1	0	0	0	0	0	0	0	0
ENSG00000230189	AC008267.2	0.14944	0.12437	0	0	0.09084	0	0	0
ENSG00000236928	AC008267.4	0.14069	0	0	0.2088	0	0	0.23451	0.17274
ENSG00000279785	AC008267.7	0	0	0	0	0	0	0	0
ENSG00000244510	AC008267.5	0	0	0	0	0	0	0	0
ENSG00000244657	AC008267.6	0	0	0	0	0.64113	0	0	0
ENSG00000237310	GS1-124K5.4	0.27148	1.12971	0.19947	1.00728	0.33006	0.95787	0.90504	1.33329
ENSG00000179342	AC008267.1	0	0	0	0	0	0	0	0

ENSG00000234500	AC008267.3	0	0	0.15128	0	0	0	0	0
ENSG00000229180	AC006001.3	0.87376	0.64775	0.25895	0.47947	0.79449	1.06216	0.78329	0.61302
ENSG00000275400	AC006001.4	0	0	0	0	0	0	0	0
ENSG00000232491	SAPCD2P3	0	0	0	0	0	0	0	0
ENSG00000177418	AC006001.1	0	0	0	0	0	0	0	0
ENSG00000225573	RPL35P5	0	0	0.29112	0	0.48171	0	0	0
ENSG00000243335	KCTD7	0.93018	0.66125	0.25629	0.69025	0.87173	0.7692	0.29071	0.73758
ENSG00000284461	AC027644.4	0	0.02334	0	0.04162	0.01705	0	0	0
ENSG00000226824	AC006001.2	0	1.82102	0.11483	0.34793	0.28502	0.6893	0.52102	0.28784
ENSG00000154710	RABGEF1	0.51901	0.24366	0.15645	0.27651	0.14562	0.28173	0.11091	0.17973
ENSG00000272831	AC027644.3	7.23792	4.81901	0.58015	1.75776	4.95981	4.41099	1.75483	2.58519
ENSG00000271064	AC027644.2	0	0	0	0	0	0	0	0
ENSG00000230583	GTF2IRD1P1	0.02709	0	0.03981	0.0402	0	0.14336	0	0.03326
ENSG00000232546	AC027644.1	1.47585	2.04712	1.80728	1.46021	3.88764	5.20719	2.05001	1.20801
ENSG00000230295	GTF2IP23	0.83533	1.04281	1.53439	1.54965	0.76168	1.10523	4.5252	0.5128
ENSG00000212568	RNU6-1254P	0	0	0	0	0	0	0	0
ENSG00000273142	LINC02604	0.0213	0.07091	0	0	0.0777	0	0.07101	0.05231
ENSG00000179131	AC073335.1	0	1.34445	0	0	0	0	0	0
ENSG00000274135	RF00017	0	0	0	0	0	0	0	0
ENSG00000106609	TMEM248	16.747	11.5627	4.00274	8.08514	15.1039	11.009	7.08675	9.69689
ENSG00000275878	RN7SL43P	0	0.41927	0.37015	0	0	0.44437	0.41986	0
ENSG00000126524	SBDS	47.3151	49.5924	31.5928	103.196	53.4597	33.5972	68.0739	63.0811
ENSG00000198874	TYW1	4.88041	2.97964	1.21836	2.82451	4.4902	5.41846	2.57552	2.70685
ENSG00000233383	AC073089.1	0	0	0	0	0	0	0	0
ENSG00000266525	MIR4650-1	0	0	0	0	0	0	0	0
ENSG00000230358	SPDYE21P	0	0	0	0	0	0	0	0
ENSG00000067601	PMS2P4	1.86708	1.49633	1.52425	0.7184	0.9248	2.31785	2.88161	2.29238
ENSG00000201565	RF00019	0	0	0	0	0	0	0	0
ENSG00000106610	STAG3L4	1.87574	1.02446	0.21534	0.73945	1.1046	1.39601	0.58623	1.00756
ENSG00000228019	AC006480.1	0	0	0	0	0	0	0	0
ENSG00000273448	AC006480.2	0.27412	0.18251	0	0.08137	0.06665	0.09672	0.18277	0.06731
ENSG00000235475		0.2998	0.74852	0.22027	0.44493	0.18224	0	0	0
ENSG00000226392	MTATP6P21	0	0	0	0	0	0	0	0
ENSG00000237466	MTCO3P41	0	0	0	0	0	0	0	0
ENSG00000233423	AC005482.1	0	0	0	0	0	0	0	0
ENSG00000223948	AC092648.1	0	0	0	0	0	0	0	0
ENSG00000225209	AC092637.1	0	0	0	0	0	0	0	0
ENSG00000236531	AC006013.1	0	0	0	0	0	0	0	0
ENSG00000232975	MTCO1P57	0	0	0	0	0	0	0	0
ENSG00000226829	AC093655.1	0	0	0	0	0	0	0	0
ENSG00000237754	AC093655.2	0	0	0	0	0	0	0	0
ENSG00000225959	MTND4P3	0	0	0	0	0	0	0	0
ENSG00000228429	AC004910.1	0	0	0	0	0	0	0	0
ENSG00000222428	RNA5SP231	0	0	0	0	0	0	0	0
ENSG00000233689	AC104688.1	0	0	0	0	0	0	0	0
ENSG00000200189	RNU6-832P	0	0	0	0	0	0	0	0
ENSG00000236839	AC069280.1	0	0	0	0	0	0	0	0
ENSG00000225718	AC092100.1	0	0	0	0	0	0	0	0
ENSG00000230654	MTCO2P25	0	0	0	0	0	0	0	0
ENSG00000229031	MTCO1P25	0	0	0	0	0	0	0	0
ENSG00000252423	RNU6-229P	0	0	0	0	0	0	0	0
ENSG00000200857	RF00019	0	0	0	0	0	0	0	0
ENSG00000234215	AC006012.1	0	0	0	0	0	0	0	0
ENSG00000158321	AUTS2	1.12553	1.04211	0.17573	0.84565	0.31644	1.38992	0.57455	0.38866
ENSG00000236978	AC073873.1	0	0	0	0	0	0	0	0
ENSG00000185274	GALNT17	0	0	0	0	0	0	0	0
ENSG00000265878	MIR3914-1	0	0	0	0	0	0	0	0
ENSG00000283278	MIR3914-2	0	0	0	0	0	0	0	0

ENSG00000199940	RN7SKP75	0	0	0	0	0	0	0	0
ENSG00000183166	CALN1	0	0	0	0	0.00888	0	0.03654	0
ENSG00000201014	RNA5SP232	0	0	0	0	0	0	0	0
ENSG00000235581	AC005011.1	0	0	0	0	0	0	0	0
ENSG00000228769	ABCF2P2	0	0	0	0	0	0	0	0
ENSG00000277149	TYW1B	2.11487	2.24693	1.81838	4.94177	2.43443	2.69896	2.81262	2.15463
ENSG00000264494	MIR4650-2	0	0	0	0	0	0	0	0
ENSG00000270694	AC211469.2	0	0	0	0	0	0	0	0
ENSG00000270555	AC211469.1	0	0	0	0	0	0	0	0
ENSG00000266569	RN7SL377P	0	0	0	0	0	0	0	0
ENSG00000225648	SBDSP1	21.8304	22.9443	32.6011	75.9481	37.9771	23.7959	62.1256	33.7761
ENSG00000274656	RN7SL625P	0	0	0	0	0	0	0	0
ENSG00000179994	SPDYE7P	0.05808	0	0	0	0	0	0	0
ENSG00000196313	POM121	4.36578	3.72659	2.90615	4.16447	4.29161	4.56844	3.53281	3.20698
ENSG00000272843	AC211476.2	0.41413	0.34466	0.30428	0	0	0.18264	0	0.12711
ENSG00000106133	NSUN5P2	0.5691	0.09473	0.04181	0.33784	0.72649	0.60238	0.4743	0.52405
ENSG00000155428	TRIM74	0.04983	0	0	0	0.06058	0	0	0
ENSG00000174353	STAG3L3	1.44831	1.10491	0.59119	1.07472	1.07605	1.41945	0.53647	1.03729
ENSG00000205584	AC211476.1	0	0	0	0	0	0	0	0
ENSG00000276178	RF00019	0	0	0	0	0	0	0	0
ENSG00000229018	PMS2P7	0.21946	0.18265	0	0.16285	0.0667	0.09679	0	0.13473
ENSG00000201282	RF00019	0	0	0	0	0	0	0	0
ENSG00000273520	SPDYE8P	0	0	0	0	0	0	0	0
ENSG00000273897	AC211476.3	0	0	0	0	0	0	0	0
ENSG00000273927	RF00019	0	0	0	0	0	0	0	0
ENSG00000275976	SPDYE11	0	0	0	0	0	0	0	0
ENSG00000277125	AC211476.4	0	0	0	0	0	0	0	0
ENSG00000274984	RF00019	0	0	0	0	0	0	0	0
ENSG00000262461	SPDYE9P	0	0	0	0	0	0	0	0
ENSG00000174384	PMS2P6	1.85832	1.37474	0.30342	1.53219	3.26343	2.73194	1.37668	0.38027
ENSG00000199913	RF00019	0	0	0	0	0	0	0	0
ENSG00000274570	SPDYE10P	0	0	0	0	0	0	0	0
ENSG00000233369	GTF2IP4	13.3236	7.06561	1.91014	4.94344	10.7908	6.52113	4.36721	6.33396
ENSG00000233523	PHBP5	0	0	0	0	0	0	0	0
ENSG00000182487	NCF1B	29.6747	39.9639	2.43517	4.40405	13.5877	10.742	7.90125	11.8766
ENSG00000214544	GTF2IRD2P1	0.66486	0.69166	0.8854	1.48008	0.65676	0.69641	0.48484	0.73977
ENSG00000205578	POM121B	0.02947	0.09812	0.04331	0	0	0.10399	0	0.07237
ENSG00000130305	NSUN5	1.71764	1.53672	0.1893	0.66916	1.48789	1.55295	0.71576	0.79084
ENSG00000146755	TRIM50	0	0	0	0	0	0	0	0
ENSG00000225005	AC211485.1	0	0	0	0	0	0	0	0
ENSG00000077800	FKBP6	0	0	0	0	0	0	0	0
ENSG00000206709	RNU6-1080P	0	0	0	0	0	0	0	0
ENSG00000202035	RF00019	0	0	0	0	0	0	0	0
ENSG00000188763	FZD9	0	0	0	0.04653	0	0	0	0
ENSG00000009954	BAZ1B	15.1319	11.9535	4.8107	10.6426	16.1226	19.7644	8.90411	9.86357
ENSG00000252713	RNU6-1198P	0	0	0	0	0	0	0	0
ENSG00000106635	BCL7B	7.40867	3.23794	0.54739	1.75065	4.42816	6.60797	1.89721	2.10891
ENSG00000106638	TBL2	1.33089	1.37251	0.42516	0.47233	1.33666	1.14842	0.40992	0.85255
ENSG00000009950	MLXIPL	0	0	0	0	0	0	0	0
ENSG00000274080	AC005089.1	0	0	0	0	0	0	0	0
ENSG00000176428	VPS37D	0	0	0	0	0.10961	0	0	0
ENSG00000176410	DNAJC30	0.88491	0.39278	0	0.30644	1.00413	0.88463	0.4425	0.32595
ENSG00000071462	BUD23	1.78084	0.95223	0.23051	0.38343	1.82855	1.38362	0.78439	0.71372
ENSG00000106089	STX1A	0.51606	0.49557	0.0875	0.2062	0.31371	0.49022	0.19851	0.12185
ENSG00000265724	MIR4284	0	0	0	0	0	0	0	0
ENSG00000241709	RN7SL265P	0	0	0	0	0	0	0	0
ENSG00000225969	ABHD11-AS1	0.13136	0	0	0	0	0	0	0
ENSG00000106077	ABHD11	1.02473	0.90614	0.09411	0.14258	0.97331	0.50843	0.21351	0.47181

ENSG00000165215	CLDN3	0	0	0	0	0	0	0	0
ENSG00000189143	CLDN4	0	0	0	0	0	0	0	0.01914
ENSG00000165171	METTL27	0.24949	0	0	0.07405	0.12133	0	0.08317	0
ENSG00000175877	TMEM270	0	0	0	0	0	0	0	0
ENSG00000261467	AC099398.1	0.02641	0	0	0	0	0	0	0
ENSG00000049540	ELN	0	0	0	0	0	0	0	0.01376
ENSG00000232415	ELN-AS1	0.13778	0	0	0.20448	0	0	0	0
ENSG00000106683	LIMK1	3.16481	4.10113	0.54622	1.08754	2.45323	3.63467	0.92051	0.88667
ENSG00000106682	EIF4H	34.0889	23.7823	6.44731	12.8184	25.3023	26.7458	12.7502	17.8246
ENSG00000207741	MIR590	2.26702	3.77346	0	0	2.75619	2.66622	2.51918	0.9278
ENSG00000086730	LAT2	23.8529	24.4285	3.36532	5.33756	15.2743	5.34887	5.40335	11.5244
ENSG00000049541	RFC2	5.56963	6.78745	3.31278	4.32978	6.56989	7.13525	5.08394	4.55887
ENSG00000106665	CLIP2	2.83582	2.20166	0.36813	0.56512	1.96142	1.53795	0.6013	0.67667
ENSG00000006704	GTF2IRD1	0.02256	0	0	0	0	0	0	0
ENSG00000238391	RNA5SP233	0	0	0	0	0	0	0	0
ENSG00000273069	AC211433.2	0	0	0	0	0	0	0	0
ENSG00000279005	AC211433.3	0	0	0	0	0	0	0	0
ENSG00000263001	GTF2I	3.94662	2.87546	1.99165	3.3038	4.57622	4.91826	2.56347	2.84528
ENSG00000232729	AC211433.1	0	0	0	0	0	0	0	0
ENSG00000262648	PHBP15	0	0	0	0	0	0	0	0
ENSG00000158517	NCF1	45.9239	40.6695	3.85804	6.88159	51.1996	20.8049	10.5523	18.301
ENSG00000196275	GTF2IRD2	0.28159	0.21011	0.25684	0.34585	0.38957	0.22269	0.12948	0.4411
ENSG00000277072	STAG3L2	1.03393	1.08694	0.51978	0.80761	1.6209	1.104	0.72564	0.76835
ENSG00000123965	PMS2P5	0.56822	0	0.20875	0.8433	1.72707	2.50604	0.71035	1.04648
ENSG00000239069	RF00019	0	0	0	0	0	0	0	0
ENSG00000184616	SPDYE12P	0	0	0	0	0	0	0	0
ENSG00000274070	CASTOR2	1.66714	1.96932	1.02735	0.87795	5.16524	4.07954	1.52389	0.79234
ENSG00000274523	RCC1L	1.99148	1.77372	0.35939	0.5963	2.08137	2.34216	0.69884	1.02952
ENSG00000277127	RF00017	0	0	0	0	0	0	0	0
ENSG00000174428	GTF2IRD2B	0.90462	0.51626	0.17091	0.59456	0.75416	0.54716	0.4739	0.50774
ENSG00000165178	NCF1C	31.231	26.2485	4.378	8.00456	18.7976	16.6737	11.9012	17.2174
ENSG00000277053	GTF2IP1	1.68179	1.03133	0.70817	1.40124	1.90119	1.96058	1.27869	1.13508
ENSG00000276592	PHBP6	0	0	0	0	0	0	0	0
ENSG00000277675	AC211486.5	0	0	0	0	0	0	0	0
ENSG00000263081	AC211486.2	0	0	0	0	0	0	0	0
ENSG00000275121	AC211486.4	0	0	0	0	0	0	0	0
ENSG00000274959	SPDYE13P	0	0	0	0	0	0	0	0
ENSG00000276840	PMS2P10	0	0	0	0.35551	0	0	0.19964	0.14705
ENSG00000267828	RF00019	0	0	0	0	0	0	0	0
ENSG00000278807	SPDYE14P	0	0	0	0	0	0	0	0
ENSG00000273598	AC211486.3	0	0	0	0.17518	0	0	0	0
ENSG00000202021	RF00019	0	0	0	0	0	0	0	0
ENSG00000278080	SPDYE15P	0	0	0	0	0	0	0	0
ENSG00000278416	PMS2P2	0	0	0	0.38576	0	0	0	0.07978
ENSG00000199870	RF00019	0	0	0	0	0	0	0	0
ENSG00000205583	STAG3L1	0.21003	0.46613	0.2572	0.31171	0.42558	0.74104	0.17504	0.04298
ENSG00000275930	RF00019	0	0	0	0	0	0	0	0
ENSG00000146722	AC211486.1	0	0	0	0	0	0	0	0
ENSG00000178809	TRIM73	0.01322	0	0	0	0.04822	0	0	0.01623
ENSG00000223705	NSUN5P1	0.39523	0.07591	0.02234	0.18048	0.35114	0.37544	0.35473	0.31729
ENSG00000272391	POM121C	2.30762	1.71177	0.31944	0.60802	1.92119	1.93223	0.80831	0.82123
ENSG00000242073	AC211429.1	0	0	0	0	0	0	0	0
ENSG00000170092	SPDYE5	0.02205	0	0	0	0	0	0	0
ENSG00000127957	PMS2P3	4.71126	5.15728	2.7443	6.99202	6.24384	5.69062	7.4992	5.83652
ENSG00000200874	RF00019	0	0	0	0	0	0	0	0
ENSG00000127946	HIP1	8.35098	6.85472	0.68577	2.05348	5.38502	2.49871	1.35104	2.94529
ENSG00000279996	AC004491.1	0.42843	0.06483	0	0.0578	0.04735	0	0.06492	0.14346
ENSG00000006606	CCL26	0	0	0	0	0	0	0	0

ENSG00000106178	CCL24	0	0	0	0.17631	0	0	0	0
ENSG00000219039	AC005102.1	0	0	0	0	0	0	0	0
ENSG00000005486	RHBDD2	6.78651	4.89833	0.52952	2.00547	4.38099	5.40344	2.05219	1.91718
ENSG00000127948	POR	7.80735	9.46205	0.51108	1.24591	5.39482	2.24265	1.07947	1.95838
ENSG00000284334	MIR4651	0	0	0	0	0	0	0	0
ENSG00000235090	RPL7L1P3	0.42555	0.53124	0.15633	0.15789	0.12934	0.18768	0	0.39186
ENSG00000201643	SNORA14A	1.6289	0.90377	0	0	0	0	0	1.33329
ENSG00000189077	TMEM120A	6.90058	6.72055	0.80907	1.03502	4.55178	2.39586	1.77428	2.5237
ENSG00000127952	STYXL1	4.37344	4.99344	1.69963	2.46753	3.42756	3.50698	2.16888	2.57388
ENSG00000280388	AC006330.1	0.77469	0.49123	0	0	0.0897	0.26032	0.12298	0.13588
ENSG00000146701	MDH2	12.824	7.40562	1.4496	2.74877	9.7414	15.4137	2.38253	3.55935
ENSG00000230882	AC005077.4	0	0	0	0	0	0	0	0
ENSG00000251798	RNU6-863P	0	0	0	0	0	0	0	0
ENSG00000227038	GTF2IP7	0	0.0474	0	0.21132	0	0	0	0
ENSG00000224273	AC005077.2	0.21246	0	0	0.15766	0	0	0	0
ENSG00000213549	AC005077.1	0	0	0	0	0	0	0	0
ENSG00000228829	AC005077.3	0	0	0	0	0	0	0	0
ENSG00000177679	SRRM3	0	0	0	0	0	0	0	0
ENSG00000106211	HSPB1	5.74201	7.76553	1.25249	3.52855	5.99929	11.0793	5.68281	2.86404
ENSG00000170027	YWHAG	17.9244	11.4851	3.73408	6.64318	12.0486	12.0001	7.03759	10.2957
ENSG00000213548	PPIAP81	0	0	0	0	0	0	0	0
ENSG00000146700	SSC4D	0.052	0	0	0	0	0	0	0
ENSG00000188372	ZP3	0.56676	0.31445	0.03966	0.08011	0.16406	0	0.26991	0.13254
ENSG00000091073	DTX2	1.51195	1.5245	0.19227	0.28049	1.29027	1.33364	0.36349	0.44624
ENSG00000233980	FDPSP2	0	0	0	0	0	0	0	0
ENSG00000250614	AC005522.1	0	0	0	0	0	0	0	0
ENSG00000243566	UPK3B	0	0.04587	0	0	0.0335	0	0.04593	0
ENSG00000250778	AC004980.4	0	0	0	0	0	0	0	0
ENSG00000238560	RF00019	0	0	0	0	0	0	0	0
ENSG00000230305	AC004980.3	0.20082	0	0	0	0	0	0.33474	0.24657
ENSG00000185040	SPDYE16	0	0	0	0	0	0	0	0.04985
ENSG00000205485	AC004980.1	0.07071	0	0.03463	0	0.02865	0.04158	0	0.02894
ENSG00000146707	POMZP3	0.6302	0.85229	0.17364	1.0522	1.00562	1.45919	0.91914	0.38688
ENSG00000214243	AC004980.2	0.58994	0	0.43346	0.65665	0	0.52037	1.22918	0.72432
ENSG00000231183	AC007003.1	0	0	0	0	0	0	0	0
ENSG00000265479	DTX2P1-UPK3BP1-	0.10154	0.07244	0.1279	0.1507	0.15873	0.17914	0.1209	0.21373
ENSG00000283398	AC114737.3	0	0	0	0	0	0	0	0
ENSG00000231087	FDPSP7	0	0	0	0	0	0	0	0
ENSG00000236280	AC114737.2	0	0	0	0	0	0	0	0
ENSG00000225703	AC114737.1	0	0	0	0	0	0	0	0
ENSG00000186704	DTX2P1	0.13466	0	0	0	0	0	0.07482	0.05511
ENSG00000242435	UPK3BP1	0.28802	0.2397	0	0	0.17508	0.5081	0.24004	0.17681
ENSG00000241350	PMS2P11	0.1277	0.42512	0	0	0	0	0	0
ENSG00000201885	RF00019	0	0	0	0	0	0	0	0
ENSG00000186645	SPDYE17	0	0	0	0	0	0	0	0
ENSG00000233448	PMS2P9	0.42699	0.23691	0	0.84494	0.69217	1.00436	0.71173	0.3495
ENSG00000201959	RF00019	0	0	0	0	0	0	0	0
ENSG00000259628	AC007000.3	0	0.59323	0	0.17631	0	0.20958	0.59407	0.58345
ENSG00000205482	SPDYE18	0.6981	0.81339	0	0.20721	1.2731	0.12315	0	0.51427
ENSG00000225726	AC007000.2	0.15051	0.25053	0	0	0.36598	0	0	0
ENSG00000214439	FAM185BP	22.4201	31.6949	14.5925	53.9371	48.7901	34.3146	48.2919	39.8451
ENSG00000213542	AC007000.1	0.55391	0.15366	0	0	0.44895	0.48858	0.30776	0
ENSG00000135205	CCDC146	0.97032	4.37993	1.20837	1.34243	2.2794	3.36553	1.01429	3.10961
ENSG00000127951	FGL2	273.857	301.152	59.6357	74.4298	158.471	141.108	56.1807	156.654
ENSG00000250990	AC098851.1	0	0	0	0	0	0	0	0
ENSG00000186088	GSAP	4.36496	3.93095	2.22584	6.24844	3.53457	4.29555	4.04508	5.25924
ENSG00000273341	AC004921.1	0.1	0.3329	0	0	0.12158	0	0.16669	0.12278
ENSG00000237620	GCNT1P5	0	0	0	0	0	0	0	0

ENSG00000127947	PTPN12	32.296	28.2421	11.3598	26.9268	28.4364	20.5146	21.4537	18.8366
ENSG00000214293	APTR	0.61478	0.51165	0.10424	0.14037	0.4887	0.33371	0.15765	0.40644
ENSG00000187257	RSBN1L	12.6948	15.0183	7.45896	17.6026	15.1743	10.444	14.446	15.0994
ENSG00000135211	TMEM60	4.80071	8.22931	3.79052	6.27405	5.13967	4.80337	2.62753	7.03789
ENSG00000006576	PHTF2	4.00159	3.95123	1.45761	2.64226	3.34986	3.43994	1.41314	2.30041
ENSG00000222432	RF00019	0	0	0	1.92541	0	0	0	0.79643
ENSG00000232756	AC004990.1	0	0	0	0	0	0	0	0
ENSG00000187391	MAGI2	0.01162	0.20123	0.041	0.20702	0.02261	0.02461	0.0465	0.13416
ENSG00000281008	AC004808.2	0	0	0	0	0	0	0	0
ENSG00000280958	AC004808.1	0	0.16922	0	0	0	0	0	0
ENSG00000231322	RPL13AP17	0	0	0	0	0	0	0	0
ENSG00000226230	AC007237.1	0	0	0	0	0	0	0	0
ENSG00000251276	MAGI2-AS1	0	0	0	0	0	0	0	0
ENSG00000226978	MAGI2-AS2	0	0	0	0	0	0	0	0
ENSG00000212545	RNU6-337P	0	0	0	0	0	0	0	0
ENSG00000229110	AC006355.1	0	0	0	0	0	0	0	0
ENSG00000281120	AC006355.2	0	0	0	0	0	0	0	0
ENSG00000212482	RNU6-530P	0	0	0	0	0	0	0	0
ENSG00000222024	AC004945.1	0	0	0	0	0	0	0	0
ENSG00000234456	MAGI2-AS3	0.44768	1.65827	0.40769	0.45854	0.65927	0.16685	0.09459	0.86707
ENSG00000226285	NUP35P2	0	0	0	0	0	0	0	0
ENSG00000200786	RNA5SP234	0	0	0	0	0	0	0	0
ENSG00000206818	RNU6-849P	0	0	0	0	0	0	0	0
ENSG00000230853	RPL10P11	0	0	0	0	0	0	0	0
ENSG00000127955	GNAI1	0.25793	0.161	0.04738	0.02392	0.35278	0.34127	0.18809	0.23751
ENSG00000244392	RN7SL869P	0	0	0	0	0	0	0	0
ENSG00000234223	AC003988.1	0	0	0	0	0	0	0	0
ENSG00000232667	AC004862.1	0	0	0	0	0	0	0	0
ENSG00000240347	RN7SL35P	0	0	0	0	0	0	0	0
ENSG00000135218	CD36	26.0881	21.0846	19.0083	31.8667	21.0039	21.4774	38.0855	22.3413
ENSG00000223550	SNRPBP1	0	0	0	0	0	0	0	0
ENSG00000214415	GNAT3	0	0	0	0	0	0	0	0
ENSG00000229436	AC073850.1	0	0	0	0	0	0	0	0
ENSG00000075223	SEMA3C	1.6606	1.81027	0.30933	0.72894	1.72034	0.57765	0.48732	0.74663
ENSG00000279326	AC004972.1	0	0	0	0	0	0	0	0
ENSG00000237896	AC005008.2	0	0	0	0	0	0	0	0
ENSG00000226671	AC005008.1	0	0	0	0	0	0	0	0
ENSG00000223514	AC004866.1	0	0	0	0	0	0	0	0
ENSG00000224134	AC004866.2	0	0	0	0	0	0	0	0
ENSG00000232968	EIF4EP4	0	0	0	0	0	0	0	0
ENSG00000233491	AC008163.1	0	0	0	0	0	0	0	0
ENSG00000230196	DDX43P3	0	0	0	0	0	0	0	0
ENSG00000019991	HGF	1.70543	1.82389	0.27846	0.52577	1.00165	0.61044	0.23346	0.69797
ENSG00000153956	CACNA2D1	0	0	0	0	0.01978	0	0	0
ENSG00000223770	AC006145.1	0.02765	0	0	0	0	0	0	0
ENSG00000213530	MTHFD2P5	0	0	0	0	0	0	0	0
ENSG00000228711	AC004006.1	0	0	0	0	0	0	0	0
ENSG00000186472	PCLO	0	0	0	0	0.0039	0	0	0
ENSG00000202281	RNA5SP235	0	0	0	0	0	0	0	0
ENSG00000235503	AC079799.1	0	0	0	0	0	0	0	0
ENSG00000170381	SEMA3E	0	0	0	0	0	0	0	0
ENSG00000230617	AC079987.1	0	0	0	0	0	0	0	0
ENSG00000230244	RAD23BP2	0	0	0	0	0	0	0	0
ENSG00000075213	SEMA3A	0.08604	0.03906	0.01149	0.01161	0.02853	0.0276	0.02608	0
ENSG00000227982	RPL7P30	0	0	0	0	0	0	0	0
ENSG00000235139	AC003984.1	0	0	0	0	0	0	0	0
ENSG00000235243	AC093716.1	0	0	0	0	0	0	0	0
ENSG00000232605	HMGN2P11	0	0	0	0	0	0	0	0

ENSG00000280325	AC074183.2	0	0	0	0	0	0	0	0
ENSG00000232019	AC074183.1	0	0	0	0	0	0	0	0
ENSG00000229251	HNRNPA1P8	0	0	0	0	0	0	0	0
ENSG00000153993	SEMA3D	0.02728	0	0	0	0.01105	0	0.03031	0.03349
ENSG00000224059	HSPA8P16	0.0393	0	0	0	0	0	0	0
ENSG00000230614	DYNLL1P7	0	0	0	0	0	0	0	0
ENSG00000225128	LINC00972	0	0	0	0	0	0	0	0
ENSG00000227785	AC092013.1	0	0	0	0	0	0	0	0
ENSG00000236610	SOCS5P1	0	0	0	0	0	0	0	0
ENSG00000198822	GRM3	0	0	0	0	0	0	0	0
ENSG00000231255	AC005009.1	0	0	0	0	0	0	0	0
ENSG00000233073	AC005009.2	0	0	0	0	0	0	0	0
ENSG00000164659	KIAA1324L	0.69552	0.3458	0.10619	0.46919	0.52712	0.66927	0.43663	0.26617
ENSG00000261462	AC004023.1	0	0	0	0	0	0	0	0
ENSG00000224046	AC005076.1	1.40327	0.54959	0.1213	0.36752	0.90321	0	0	0.2027
ENSG00000135164	DMTF1	6.5462	4.74197	1.54416	3.71976	5.45091	5.36911	4.07397	4.33882
ENSG00000278236	RF02271	4.26165	0	0	0.63248	2.07248	3.75906	2.8414	0.52324
ENSG00000135185	TMEM243	3.78715	2.984	1.44892	2.95993	3.59627	2.2929	3.5485	3.4392
ENSG00000200397	RF00019	2.87452	2.39232	1.05602	1.06653	6.11585	3.80329	5.98923	0.88232
ENSG00000182165	TP53TG1	1.5047	1.31192	0.42117	0.42536	2.09071	1.76966	0.95547	0.79176
ENSG00000273623	RF02195	0	0	0	0	0	0	0	0
ENSG00000276185	RF02196	0	0	0	0	0	0	0	0
ENSG00000005469	CROT	1.01581	0.90667	0.34614	1.13614	1.07391	0.75317	0.61348	1.04837
ENSG00000005471	ABCB4	0.29344	0.73264	0.10061	0.18871	0.76107	0.51766	0.50542	0.37229
ENSG00000085563	ABCB1	2.61336	2.31156	0.66788	1.79873	2.48655	2.65038	0.8628	1.62757
ENSG00000231956	HNRNPA1P9	0	0	0	0	0	0	0	0
ENSG00000105784	RUNDC3B	0.03113	0.0259	0.16008	0	0.01892	0	0.02594	0.03822
ENSG00000075303	SLC25A40	5.8571	5.52292	1.97154	3.63975	5.73532	4.22471	2.98177	4.07387
ENSG00000006634	DBF4	10.9559	13.2045	7.2389	21.197	20.4173	15.9136	14.9826	14.0013
ENSG00000008277	ADAM22	0.29443	0.32005	0.18542	0.87392	0.33604	0.2438	0.27042	0.42789
ENSG00000075142	SRI	5.98582	5.09689	2.38969	3.8256	5.63683	3.99808	3.57573	3.80209
ENSG00000254003	AC003991.2	0	0	0	0	0.15472	0	0.21212	0
ENSG00000228113	AC003991.1	0.16666	0.12609	0.11132	0.35977	0.05526	0.24056	0.07576	0.13021
ENSG00000127954	STEAP4	20.0313	34.7092	5.63235	6.92669	18.9725	4.5423	4.90025	21.6009
ENSG00000225701	EIF4A1P13	0	0	0	0	0	0	0	0
ENSG00000207094	RF00272	0	0	0	0	0	0	0	0
ENSG00000270812	AC002069.2	0	0	0	0	0	0	0	0
ENSG00000235639	AC002069.1	0	0	0	0	0	0	0	0
ENSG00000228928	KPNA2P2	0	0	0	0	0	0	0	0
ENSG00000231763	PQLC1P1	0	0	0	0	0	0	0	0
ENSG00000237709	EEF1A1P28	0	0	0	0	0	0	0	0
ENSG00000233420	AC002127.2	0.09255	0	0	0	0	0	0	0
ENSG00000227979	AC002127.1	0	0	0	0	0	0	0	0
ENSG00000182348	ZNF804B	0	0	0	0	0	0	0	0
ENSG00000164645	TEX47	0	0	0	0	0	0	0	0
ENSG00000227863	AC002383.1	0	0	0	0	0	0	0	0
ENSG00000239075	RNU6-274P	0	0	0	0	0	0	0	0
ENSG00000227646	STEAP2-AS1	0	0	0	0	0	0	0	0
ENSG00000238358	AC004969.1	0	0	0	0	0	0	0	0
ENSG00000235436	DPY19L2P4	0	0	0	0	0	0	0	0.0446
ENSG00000164647	STEAP1	0	0	0	0	0	0	0	0
ENSG00000157214	STEAP2	0	0	0	0	0	0	0	0
ENSG00000105792	CFAP69	0.00802	0	0	0	0.00975	0	0	0.00985
ENSG00000207488	RF00019	0	0	0	0	0	0	0	0
ENSG00000234459	AC002064.2	0	0	0	0	0	0	0	0
ENSG00000225498	AC002064.1	0	0	0	0	0	0	0	0
ENSG00000283267	FAM237B	0	0	0	0	0	0	0	0
ENSG00000105793	GTPBP10	1.57333	1.73029	0.97021	2.36627	2.48495	1.92059	1.63906	2.05244

ENSG00000280440	AC002064.3	0.21185	0	0	0	0.25756	0	0	0.78032
ENSG00000157224	CLDN12	0.11961	0.0905	0.04794	0.17752	0.11898	0.03837	0.09062	0.09345
ENSG00000273299	AC006153.1	0	0	0	0	0	0	0	0
ENSG00000058091	CDK14	3.69663	4.20903	1.29112	1.94005	4.58545	1.79573	2.21464	3.52569
ENSG00000251154	AC002456.2	0	0	0	0	0	0	0	0
ENSG00000223969	AC002456.1	0.59497	1.18839	0	0	0.14467	0.62977	0.19834	0.1461
ENSG00000223584	TVP23CP1	0.11471	0	0	0	0	0.40473	0	0
ENSG00000226254	PTP4A1P3	0	0.2905	0	0	0	0	0	0.21428
ENSG00000157240	FZD1	1.06324	0.98125	0.21657	0.21873	0.51194	0.09286	0.17547	0.47823
ENSG00000243144	AC079760.2	0	0	0	0	0	0	0	0
ENSG00000225843	NIPA2P1	0	0	0	0	0	0	0	0
ENSG00000235450	AC079760.1	0	0	0	0	0	0	0	0
ENSG00000223665	AC000058.1	0	0	0	0	0	0	0	0
ENSG00000127989	MTERF1	2.72815	2.0709	0.70488	1.17907	2.4056	1.93042	1.32424	1.45394
ENSG00000127914	AKAP9	13.1667	11.8323	4.09325	7.43757	13.1644	10.8932	10.8493	11.5993
ENSG00000001630	CYP51A1	0.08996	0.08984	0	0.0267	0.02187	0.1587	0.05998	0.06627
ENSG00000240720	LRRD1	0	0	0	0.01491	0	0	0.01675	0
ENSG00000188693	CYP51A1-AS1	0.02479	0	0	0	0.06028	0.08746	0	0
ENSG00000001631	KRIT1	9.65287	12.7043	13.5424	29.0902	14.6804	11.7973	23.1008	17.7122
ENSG00000243107	AC000120.1	0	0.61427	0	0.06846	0.28042	0	0.07689	0.16991
ENSG00000200769	RF00019	0	1.32618	1.1708	1.18245	0	4.21669	0	0.97823
ENSG00000221520	MIR1285-1	0.87262	0	0	0	0	0	0	1.07139
ENSG00000001629	ANKIB1	9.86933	11.0543	6.60182	9.42911	14.1737	14.2914	11.2755	11.1707
ENSG00000230927	TMBIM7P	0	0	0	0	0	0	0	0
ENSG00000270453	AC007566.2	0	0	0	0	0	0	0	0
ENSG00000157259	GATAD1	3.47301	2.49569	0.53958	1.33967	2.67852	1.37652	0.84157	1.69061
ENSG00000244055	AC007566.1	4.81001	4.00313	2.30486	6.8282	4.06809	4.61169	5.92601	4.36505
ENSG00000242950	ERVW-1	0	0	0	0	0	0	0	0
ENSG00000127980	PEX1	3.32462	3.24879	1.68796	2.81354	4.29177	4.29996	3.44016	2.67159
ENSG00000127993	RBM48	3.58672	2.91497	0.86607	1.59942	3.13229	2.55475	1.71215	2.81176
ENSG00000278819	AC005156.1	0.25585	0	0	0	0	0	0	0.15706
ENSG00000234545	FAM133B	10.7601	12.5782	5.50398	5.9976	14.3801	10.0273	9.3374	12.4245
ENSG00000105810	CDK6	6.18808	3.66225	1.8538	4.09943	5.16819	6.28628	3.43819	3.44279
ENSG00000206763	RNU6-10P	1.3701	0	0	0	0	0	0	0
ENSG00000237819	AC002454.1	0.1099	0	0	0.1631	0	0	0	0.26986
ENSG00000266794	RN7SL7P	0	0	0	0	0	0	0	0
ENSG00000205413	SAMD9	67.74	106.737	33.5162	34.0878	46.5409	57.2141	39.4791	53.5387
ENSG00000177409	SAMD9L	24.6663	66.8781	18.7348	10.9319	20.721	39.0745	6.06192	24.7981
ENSG00000188175	HEPACAM2	1.65317	2.55515	2.86312	3.98691	4.271	5.88491	10.0874	3.1171
ENSG00000004766	VPS50	7.1331	8.50472	9.69585	22.5534	11.3022	9.72506	12.9366	11.8993
ENSG00000004948	CALCR	0	0	0	0	0	0	0	0.02227
ENSG00000208014	MIR653	0	0	0	0	0	0	0	0
ENSG00000207656	MIR489	0	0	0	0	0	0	0	0
ENSG00000127928	GNGT1	0	0	0	0	0	0	0	0
ENSG00000237729	AC002075.2	0	0	0	0	0	0	0	0.35713
ENSG00000265423	MIR4652	0	0	0	0	0	0	0	0
ENSG00000225898	AC002075.1	0	0	0	0	0	0	0	0
ENSG00000213483	NDUFAF4P2	0	0	0	0	0	0	0	0
ENSG00000105825	TFPI2	0	0.04582	0	0	0	0	0.04588	0
ENSG00000234695	AC002076.1	0	0.23152	0	0	0	0	0	0
ENSG00000278388	AC002076.2	0	0	0	0	0	0	0	0
ENSG00000127920	GNG11	33.2551	20.408	15.7957	28.2024	22.1698	26.0317	40.3135	42.0674
ENSG00000278959	AC006378.2	0.08881	0.04928	0	0.04394	0.10798	0	0.04935	0
ENSG00000105829	BET1	2.82193	2.53458	1.35489	3.44167	2.0551	1.89766	2.88743	2.60712
ENSG00000236861	AC006378.1	0	0	0	0	0	0.20332	0	0
ENSG00000236453	AC003092.1	0	0	0	0	0	0	0	0
ENSG00000236938	AC003092.2	0	0	0	0	0	0	0	0
ENSG00000164692	COL1A2	0.01971	0.13124	0	0	0.47131	0.10432	0	0

ENSG00000222870	RNU6-1328P	0	0	0	0	0	0	0	0
ENSG00000127995	CASD1	3.65737	2.8008	1.26698	2.82748	4.1253	3.48364	1.90074	3.0221
ENSG00000127990	SGCE	0.05756	0	0	0.08542	0.27991	0	0	0.21201
ENSG00000242265	PEG10	0.25204	0.01824	0	0.09758	0.57288	0.09666	0.09133	0.08073
ENSG00000232385	RPS3AP25	0	0	0	0	0	0	0	0
ENSG00000239030	RNU6-956P	0	0	0	0	0	0	0	0
ENSG00000214342	ATP5PBP2	0	0	0	0	0	0	0	0
ENSG00000230682	GRPEL2P3	0	0	0	0	0	0	0	0
ENSG00000222220	RN7SKP129	0	0	0	0	0	0	0	0
ENSG00000219807	ARF1P1	0	0	0	0	0	0	0	0
ENSG00000158528	PPP1R9A	0.10517	0.18601	0	0.48778	0.32766	0.17395	0.23009	0.08071
ENSG00000271159	HINT1P2	0	0	0	0	0	0	0	0
ENSG00000231153	AC002429.1	0	0	0	0	0	0	0	0
ENSG00000236197	AC002429.2	0	0	0	0	0	0	0	0
ENSG00000201607	RNU4-16P	1.03972	0	0	0	0	0	0	0
ENSG00000005421	PON1	0	0	0	0	0	0	0	0
ENSG00000237160	AC004022.2	0	0	0	0	0	0	0	0
ENSG00000228751	AC004022.1	0	0	0	0	0	0	0	0
ENSG00000105852	PON3	0	0	0	0	0	0	0	0
ENSG00000105854	PON2	0.93461	0.57147	0.25226	0.99076	0.37103	0.60568	0.22255	0.53862
ENSG00000273138	AC005021.1	0	0	0	0	0	0	0	0
ENSG00000233942	AC004012.1	0	0	0	0	0	0	0	0
ENSG00000005981	ASB4	0	0	0	0	0	0	0	0
ENSG00000279525	AC002451.2	0	0	0	0	0	0	0	0
ENSG00000004799	PDK4	25.9203	9.82764	2.24056	3.61094	8.79532	2.94736	4.94778	4.22204
ENSG00000231170	AC002451.1	0.49195	0.11698	0.10327	0	0	0	0	0
ENSG00000158560	DYNC1I1	0.19256	0.08013	0.02358	0	0.19509	0.08492	0.05349	0.21672
ENSG00000279067	AC002540.1	0	0	0	0	0	0	0	0
ENSG00000004864	SLC25A13	3.1612	3.15709	1.68394	3.48935	2.35403	2.30043	2.79928	2.86244
ENSG00000208025	MIR591	0.77158	0	0	2.29023	0	0	0	0.94734
ENSG00000237551	AC096775.1	0	0	0	0	0	0	0	0
ENSG00000275834	AC084368.1	0	0	0	0	0	0	0	0
ENSG00000207045	RNU6-532P	0	0	0	0	0	0	0	0
ENSG00000207115	RNU6-364P	0	0	0	0	0	0	0	0
ENSG00000252872	RNU7-188P	0	0	0	0	0	0	0	0
ENSG00000127922	SEM1	1.33969	1.11496	0.49685	0.72903	0.99277	1.87947	0.52105	0.73627
ENSG00000227284	MARK2P10	0	0	0	0	0	0	0	0
ENSG00000244318	RN7SL252P	0	0	0	0	0	0	0	0
ENSG00000231764	DLX6-AS1	0	0	0	0	0	0	0	0
ENSG00000274409	RF01887	0	0	0	0	0	0	0	0
ENSG00000277124	RF01888	0	0	0	0	0	0	0	0
ENSG00000006377	DLX6	0	0	0	0	0	0	0	0
ENSG00000274709	RF01778	0	0	0	0	0	0	0	0
ENSG00000105880	DLX5	0	0	0	0	0	0	0	0
ENSG00000196636	SDHAF3	2.06883	2.54295	1.63699	2.31459	2.05088	2.41442	1.85684	1.64128
ENSG00000224551	HMGB3P21	0	0	0	0	0	0	0	0
ENSG00000224475	AC073900.1	0	0	0	0	0	0	0	0
ENSG00000226046	AP1S2P1	0	0	0	0	0	0	0	0
ENSG00000199475	RN7SKP104	0	0	0	0	0	0	0	0
ENSG00000006128	TAC1	0	0	0	0	0	0	0	0
ENSG00000070669	ASNS	1.08593	0.73945	0.26596	0.61047	1.24023	0.87079	0.41138	0.76765
ENSG00000226744	AC005326.1	0	0	0	0	0	0	0	0
ENSG00000231812	SNRPCP9	0	0	0	0	0	0	0	0
ENSG00000237818	RPS3AP29	0.38128	0	0	0.14146	0	0	0	0
ENSG00000231859	AC079781.1	0	0	0	0	0	0	0	0
ENSG00000232032	AC079781.2	0	0	0	0	0	0	0	0
ENSG00000232097	AC079781.3	0.4118	0	0	0	0.25033	0	0	0
ENSG00000238228	OR7E7P	0.07243	0.36169	0.10644	0	0.08806	0.12778	0	0.26679

ENSG00000266318	MIR5692A1	0	0	0	0	0	0	0	0
ENSG00000266668	MIR5692C2	0.95195	0	0	1.4128	1.15736	3.35875	3.17351	0
ENSG00000183444	OR7E38P	2.38133	1.858	0.32806	0.7731	2.35232	3.54459	1.24041	1.27915
ENSG00000243554	AC004967.1	4.91949	2.7295	0.48194	1.70358	2.79113	4.33932	0.82	1.00668
ENSG00000135175	OCM2	0	0	0	0	0	0	0	0
ENSG00000277946	RN7SL478P	0	0	0	0	0	0	0	0
ENSG00000164715	LMTK2	6.89772	9.02481	1.69721	2.89254	6.66985	6.87663	3.77009	4.93364
ENSG00000180535	BHLHA15	0.64299	0	0	0	0.22335	0	0	0
ENSG00000205356	TECPR1	4.18004	2.48969	0.41584	1.01994	3.43059	3.0381	1.18596	1.32027
ENSG00000164713	BRI3	6.01921	6.31529	1.38337	1.4183	3.00004	1.7614	1.94956	3.95784
ENSG00000006453	BAIAP2L1	0.01528	0.05086	0	0.02267	0.05572	0	0	0
ENSG00000272950	AC093799.1	0	0	0	0	0	0	0	0
ENSG00000214389	RPS3AP26	0.28049	0	0.13739	0.13876	0.11367	0.82469	0.46753	0.22958
ENSG00000213455	PPIAP82	0	0	0	0	0	0	0	0
ENSG00000223402	AC074121.1	0	0	0	0	0	0	0	0
ENSG00000106236	NPTX2	0.04861	0	0	0	0	0.08575	0	0
ENSG00000207204	RNU6-393P	0	0	0	0	0	0	0	0
ENSG00000166448	TMEM130	0	0	0	0	0	0	0	0
ENSG00000196367	TRRAP	5.51777	2.73222	0.8475	1.72009	4.1194	5.42957	2.05205	1.8043
ENSG00000266019	MIR3609	171.34	221.14	406.62	1453.65	218.336	193.968	449.013	604.105
ENSG00000238109	AC004893.1	0	0	0	0	0	0	0	0
ENSG00000242687		0.96126	0.93092	0.1541	0.49283	1.06243	0.67832	0.7283	0.49355
ENSG00000198742	SMURF1	3.5272	2.09957	0.6007	0.78001	1.95954	2.24585	1.75211	1.21889
ENSG00000185467	KPNA7	0	0	0	0	0	0	0	0
ENSG00000002079	MYH16	0	0	0	0	0	0	0	0
ENSG00000284523	AC004834.1	0.03326	0	0	0.04936	0.02022	0.05867	0	0
ENSG00000284292	AC004922.1	0.10839	0	0.07964	0.16087	0.03295	0.09561	0.27101	0.09981
ENSG00000241685	ARPC1A	2.28506	2.0206	0.52467	0.90081	1.95336	0.9448	0.95221	1.79731
ENSG00000130429	ARPC1B	7.57136	6.92986	1.19111	2.1872	4.54654	4.15982	2.82498	4.47837
ENSG00000106244	PDAP1	15.7953	17.6557	4.98186	7.92832	17.3612	17.4893	9.03295	10.7845
ENSG00000106245	BUD31	15.4073	13.7221	2.67697	3.0702	11.3742	13.9443	3.19089	5.23151
ENSG00000106246	PTCD1	0.21466	0.27323	0.05567	0.0937	0.21492	0.24504	0.16838	0.15503
ENSG00000248919	ATP5MF-PTCD1	0	0	0	0	0.09351	0.09046	0	0
ENSG00000160917	CPSF4	1.29354	0.77511	0.2281	0.56313	0.69196	0.76066	0.66121	0.59292
ENSG00000228335	AC073063.1	2.41517	3.61804	3.54906	3.94281	4.55126	2.76945	5.83728	4.89276
ENSG00000241468	ATP5MF	5.99291	4.77308	1.60979	2.24744	4.58314	9.1513	2.63158	2.92738
ENSG00000198556	ZNF789	0.36195	0.14605	0.06447	0.13022	0.50672	0.30959	0.23767	0.33666
ENSG00000160908	ZNF394	20.2439	13.9936	4.73265	6.11435	16.1198	18.8157	11.9566	10.1166
ENSG00000196652	ZKSCAN5	2.2513	2.29673	0.40909	0.98798	1.81	1.87904	1.19033	1.57525
ENSG00000221909	FAM200A	1.24101	1.5717	0.47573	0.76074	2.09918	1.71337	0.89938	1.42432
ENSG00000197343	ZNF655	6.47409	4.73453	1.65398	3.0819	6.81025	5.98765	4.07219	4.84212
ENSG00000272647	AC005020.2	0	0	0	0	0	0	0	0
ENSG00000244219	TMEM225B	0	0	0.07639	0	0.12641	0.09171	0	0
ENSG00000197037	ZSCAN25	2.02521	1.75428	0.42515	0.72074	2.41195	1.9322	1.18839	1.19252
ENSG00000224448	AC005020.1	0	0	0	0	0	0	0	0
ENSG00000106258	CYP3A5	0	0	0	0	0.01324	0	0.14522	0
ENSG00000282301	CYP3A7-CYP3A51F	0	0	0	0	0	0	0	0
ENSG00000282277	CYP3A51P	0	0	0	0	0	0	0	0
ENSG00000160870	CYP3A7	0	0	0	0	0	0	0	0
ENSG00000160868	CYP3A4	0	0	0	0.03463	0.02837	0	0.0389	0.02865
ENSG00000273407	AC069294.1	0	0	0	0	0	0	0	0
ENSG00000261511	CYP3A137P	0	0	0	0	0	0	0	0
ENSG00000021461	CYP3A43	0.0278	0.04627	0.08169	0.08251	0.03379	0	0	0.03413
ENSG00000260445	AC011904.1	0	0	0	0	0	0	0	0
ENSG00000260524	CYP3A52P	0	0	0	0	0	0	0	0
ENSG00000244623	OR2AE1	0	0	0	0	0.16642	0	0	0
ENSG00000146833	TRIM4	4.31619	4.4403	0.83687	2.35765	4.59158	3.46347	2.17332	2.68648
ENSG00000176402	GJC3	0.06568	0	0	0	0.07985	0	0	0

ENSG00000237640	AC004522.2	0	0	0	0	0	0	0	0
ENSG00000199711	RF00019	0	0	0	0	0	0	0	0
ENSG00000222966	RF00561	0	0	0	0	0	0	0	0
ENSG00000160862	AZGP1	0	0	0	0	0.09906	0	0	0
ENSG00000214313	AZGP1P1	0	0	0	0	0	0	0	0
ENSG00000235713	AC004522.1	0	0	0	0	0	0	0	0
ENSG00000106261	ZKSCAN1	10.5202	9.10423	4.06054	5.85096	14.0338	14.4863	8.95129	8.35519
ENSG00000166529	ZSCAN21	1.57561	1.19728	0.25167	0.96585	2.58188	1.02724	0.9135	1.47191
ENSG00000166526	ZNF3	2.32442	1.5296	0.53619	1.24349	2.48094	2.2887	2.27511	1.47672
ENSG00000168090	COPS6	4.88321	3.47726	0.53723	1.08514	4.12724	3.409	1.39286	1.47484
ENSG00000166508	MCM7	3.76028	2.78177	1.12969	2.72832	3.47445	11.7342	2.6464	2.66749
ENSG00000207547	MIR25	3.49049	0	0	0	4.24365	0	0	0
ENSG00000207757	MIR93	0	0	0	0	0	0	0	0
ENSG00000208036	MIR106B	0	0	0	0	0	0	0	0
ENSG00000221838	AP4M1	0.46569	0.5426	0.27373	0.27646	0.64167	0.5477	0.36225	0.34306
ENSG00000106290	TAF6	3.60582	1.79161	0.31634	0.69222	2.81352	3.29134	0.71765	0.6828
ENSG00000242798	AC073842.2	0.51259	0	0	0	0.1558	0.22607	0	0.15734
ENSG00000166997	CNPY4	2.31007	1.47889	0.30465	0.74722	2.37644	2.35112	0.78985	0.8727
ENSG00000214309	MBLAC1	0.23329	0.07766	0.06856	0.06925	0	0.41156	0	0.17186
ENSG00000235077	AC073842.1	0	0	0	0	0	0	0	0
ENSG00000214142	RPL7P60	0	0.166	0	0	0	0	0.16623	0.12244
ENSG00000188186	LAMTOR4	14.1224	14.6209	1.94731	2.80955	11.0475	5.27667	4.35457	5.90373
ENSG00000146826	C7orf43	6.89091	4.79788	0.86039	1.20316	3.47706	2.18499	2.02695	3.12432
ENSG00000284518	MIR4658	0	0	0	1.67363	0	0	0	0
ENSG00000197093	GAL3ST4	0.12947	0.07183	0.06342	0.06405	0.34104	0.19033	0.03597	0.05299
ENSG00000213420	GPC2	0.09574	0.03187	0.05628	0.02842	0.06984	0.03378	0.09575	0.04702
ENSG00000066923	STAG3	0.02562	0.05686	0.08785	0.06337	0.15574	0.13559	0.14235	0.09437
ENSG00000239521	CASTOR3	1.46299	1.35565	0.2438	0.51483	1.17355	1.30376	0.70392	0.68516
ENSG00000213413	PVRIG	0	0	0	0	0	0.06518	0	0
ENSG00000214300	SPDYE3	0	0	0	0	0	0	0	0
ENSG00000078319	PMS2P1	4.77527	4.76906	3.85946	11.2506	5.87822	7.4765	5.77073	5.34999
ENSG00000201913	RF00019	0	0	0	0	1.76469	0	0	0
ENSG00000242294	STAG3L5P	0.86266	1.30537	0.11524	0.15519	0.76277	0.5534	0.39216	0.73821
ENSG00000272752	STAG3L5P-PVRIG2	0.01921	0.03197	0	0.02851	0.02335	0.06777	0	0.02358
ENSG00000121716	PILRB	0.78845	0.59269	0.20556	0.75493	0.41744	0.85251	0.21197	0.65577
ENSG00000235333	PVRIG2P	0	0	0	0	0	0	0	0
ENSG00000284012	MIR6840	0	0	0	0	0	0	0	0
ENSG00000085514	PILRA	75.712	78.6426	8.37656	14.6964	46.7796	20.8859	11.1461	21.6244
ENSG00000078487	ZCWPW1	1.5906	1.4341	0.29217	0.4918	1.47722	1.13021	0.40506	0.86796
ENSG00000146834	MEPCE	9.45189	4.8518	1.07084	2.00395	5.99323	6.65464	3.64397	2.65781
ENSG00000241357	AC092849.2	1.32073	0.73278	0	0.32668	0.80285	0.38832	1.83454	1.08105
ENSG00000160813	PPP1R35	3.33464	4.0087	0.54447	1.64966	3.3034	3.70396	2.16157	2.50202
ENSG00000240211	AC092849.1	1.69341	0	0	0.35903	0.58823	0	0	0.29702
ENSG00000185955	C7orf61	0.06155	0.10244	0	0	0.22448	0	0	0
ENSG00000166925	TSC22D4	23.1641	20.0337	3.45033	6.06155	14.8008	14.3757	5.78835	8.26083
ENSG00000166924	NYAP1	0	0	0	0	0	0	0	0
ENSG00000225807	AC069281.1	0	0.4281	0	0.19085	0	0	0.21435	0
ENSG00000242101	RN7SL416P	0	0	0	0	0	0	0	0
ENSG00000106351	AGFG2	1.5388	0.97999	0.35393	0.77449	0.99235	2.36057	0.13382	0.50929
ENSG00000184414	IRS3P	0	0.12128	0	0	0	0	0	0
ENSG00000205307	SAP25	0	0	0	0.07838	0	0	0	0
ENSG00000274272	AC069281.2	0.51632	0.22178	0.02447	0.02472	0.22274	0.26444	0.16657	0.0818
ENSG00000077454	LRCH4	4.70589	3.15816	0.78761	1.22499	2.85413	2.32602	1.03633	1.50039
ENSG00000106336	FBXO24	0.06558	0.03639	0.03212	0.03244	0.02658	0	0.03644	0.08052
ENSG00000224729	PCOLCE-AS1	0	0	0	0.04022	0	0	0	0
ENSG00000106333	PCOLCE	0.06226	0.20726	0	0.0616	0.07569	0.10983	0	0
ENSG00000106330	MOSPD3	1.48639	0.84826	0.24963	0.50422	0.77448	1.27364	0.49552	0.31285
ENSG00000106327	TFR2	0.62229	0.6263	0.0638	0.47251	1.09087	2.11903	0.57894	0.62189

ENSG00000077080	ACTL6B	0	0	0	0	0	0	0	0
ENSG00000172354	GNB2	33.0198	35.1463	4.93893	6.73961	23.1136	14.2573	8.93794	14.1752
ENSG00000146830	GIGYF1	4.66526	3.67353	0.69037	1.10261	3.10826	2.89116	1.56618	1.90484
ENSG00000172336	POP7	5.26753	4.07518	1.19924	2.31225	4.05896	6.675	1.60764	2.09507
ENSG00000130427	EPO	0	0	0	0	0	0	0	0
ENSG00000146839	ZAN	0.00829	0	0	0.0123	0	0	0	0
ENSG00000196411	EPHB4	2.71103	2.3321	0.41847	0.92979	1.77264	0.54257	0.96833	1.59435
ENSG00000263672	RN7SL750P	0	0	0	0	0	0	0	0
ENSG00000146828	SLC12A9	6.2883	5.3912	0.70243	1.50446	4.0881	1.86102	1.71717	3.14696
ENSG00000236305	SLC12A9-AS1	0.19865	0	0	0	0.24151	0.35044	0	0
ENSG00000087077	TRIP6	0.41856	0.39189	0.07688	0.07765	0.25444	0.41535	0.0436	0.16059
ENSG00000283821	MIR6875	0	0	0	0	0	0	0	0
ENSG00000087087	SRRT	8.83502	5.50474	0.96257	1.99172	6.27391	7.13075	1.75761	2.53043
ENSG00000176125	UFSP1	0.44157	0.3675	0	0.10922	1.07369	0.64916	0.24534	0.27108
ENSG00000087085	ACHE	0.17682	0.65403	0.37531	0.34989	0.28663	0.51988	0.03275	0.16885
ENSG00000239825	RN7SL549P	0	0	0	0	0	0	0	0
ENSG00000235926	RPS29P15	0	0	0	0	0	0	0	0
ENSG00000169894	MUC3A	0	0	0	0	0.01567	0	0	0
ENSG00000274993	AC254629.1	0	0	0	0	0	0	0	0
ENSG00000205277	MUC12	0	0	0	0	0	0	0	0
ENSG00000227053	AC105446.1	0.5403	0.29978	0.26465	0	0	0.31772	0.3002	0
ENSG00000169876	MUC17	0	0	0	0	0	0	0	0
ENSG00000222636	RN7SKP54	0	0	0	0	0	0	0	0
ENSG00000169871	TRIM56	5.47801	5.91861	0.6652	1.32416	4.22732	4.36325	1.27943	2.71449
ENSG00000106366	SERPINE1	1.28678	1.22391	0.47273	0.23871	0.47492	0.4459	0.1149	0.36676
ENSG00000106367	AP1S1	1.38123	1.26844	0.41994	0.70686	1.50554	2.85679	0.63512	0.87716
ENSG00000264425	MIR4653	0	0	0	0	0	0	0	0
ENSG00000128564	VGF	0	0	0	0	0	0	0	0
ENSG00000167011	NAT16	0	0	0	0	0	0	0	0
ENSG00000106384	MOGAT3	0	0	0	0	0	0	0	0
ENSG00000205267	DGAT2L7P	0	0	0	0	0	0	0	0
ENSG00000213394	RPSAP46	0.16621	0	0	0	0	0	0	0
ENSG00000106397	PLOD3	2.45584	1.89327	0.32289	0.46039	1.47716	1.0261	0.36626	0.79348
ENSG00000106400	ZNHIT1	2.38126	1.72716	0.41053	0.88845	1.58502	2.27645	0.55436	0.98
ENSG00000106404	CLDN15	1.34287	0.50608	0.18616	0.78966	0.89332	0.98336	0.29563	0.68439
ENSG00000214253	FIS1	32.0153	26.5088	11.2815	14.424	41.7533	42.7918	29.8814	21.9101
ENSG00000207454	RNU6-1104P	0	0	0	0	0	0	0	0
ENSG00000278683	AC006329.2	0	0	0	0	0	0	0	0
ENSG00000214252	AZGP1P2	0	0	0	0	0	0	0	0
ENSG00000232301	LNCPRESS1	0	0	0	0	0	0	0	0
ENSG00000232445	AC006329.1	0	0	0	0	0	0.18342	0	0
ENSG00000128581	IFT22	0.15966	0.11811	0	0.26328	0.36665	0.21907	0.14785	0.1089
ENSG00000160963	COL26A1	0.11613	0	0	0.17235	0.11295	0	0.07743	0.05703
ENSG00000233683	AC004965.1	0	0	0	0	0	0	0	0
ENSG00000233123	LINC01007	0	0	0	0	0	0	0	0
ENSG00000106436	MYL10	0	0	0	0	0	0	0	0
ENSG00000257923	CUX1	6.91466	5.2403	0.95676	2.10379	4.69431	3.58392	2.18424	2.82316
ENSG00000259294	AC005096.1	0	0	0	0	0	0	0	0
ENSG00000272219	AC005072.1	0.16481	0.13717	0.06055	0.06115	0.25047	0.07269	0.20604	0
ENSG00000261535	AC005086.1	0	0	0	0	0	0	0	0
ENSG00000280404	AC005086.4	0	0	0	0	0	0	0	0
ENSG00000280004	AC005086.3	0	0	0	0	0	0	0	0
ENSG00000279724	AC005086.2	0	0	0	0	0	0	0	0
ENSG00000252824	RF00554	0	0	0	0	0	0	0	0
ENSG00000278586	AC005088.1	0	0	0	0	0	0	0	0
ENSG00000160999	SH2B2	7.10251	5.88548	0.58737	0.95826	3.1774	1.24756	1.1275	1.66102
ENSG00000264675	MIR4285	0.86236	0	0	0	0	0	0	0
ENSG00000170409	AC091390.1	0	0	0.32542	0.65732	0.26923	0.39067	0	0.27189

ENSG00000200552	RF00019	0	0	0	0	0	0	0	0
ENSG00000228546	AC091390.2	0	0.32623	0	0.29087	0.23828	0	0	0.48127
ENSG00000260097	SPDYE6	0	0	0	0	0	0	0	0
ENSG00000128563	PRKRIP1	5.41423	4.95164	1.83603	6.32817	4.24365	4.33838	3.5702	3.60378
ENSG00000239969	AC091390.4	0.29716	1.15413	0.14556	0.73504	0.72257	0.69898	0.82554	0.36485
ENSG00000239486	AC091390.3	0	0	0	0	0	0	0	0
ENSG00000221510	MIR548O	0	0	0.94486	0	0.78173	0	0	1.5789
ENSG00000239480	AC073517.1	0.13549	0	0	0	0.32945	0.23902	0	0.33271
ENSG00000160991	ORAI2	5.3673	5.16473	0.68879	1.51867	4.79976	2.93493	2.36592	2.91803
ENSG00000160993	ALKBH4	1.4901	1.05544	0.04659	0.42347	1.73454	0.78303	0.47562	0.35033
ENSG00000161036	LRWD1	1.06376	1.07061	0.25447	0.36715	0.72184	0.63281	0.26803	0.39486
ENSG00000284176	MIR5090	0	0	0	0	0	0	0	0
ENSG00000284596	MIR4467	0	0	0	0	0	0	0	0
ENSG00000005075	POLR2J	15.2551	12.7861	2.4643	5.13822	12.9564	13.1698	3.15595	6.04408
ENSG00000170667	RASA4B	0	0	0	0	0	0	0	0
ENSG00000272949	AC093668.2	0	0	0	0	0	0	0	0
ENSG00000168255	POLR2J3	0.16633	0.0969	0.07332	0.19748	0.21233	0.27875	0.13862	0.16337
ENSG00000270249	AC093668.1	0	0	0	0	0	0	0	0
ENSG00000205238	SPDYE2	0	0	0	0	0	0.06241	0	0
ENSG00000105808	RASA4	0.00908	0.04532	0.01334	0.02694	0.05517	0.06404	0.03025	0.04457
ENSG00000279168	AC105052.4	0.29533	0	0.17359	0	0.07181	0	0	0
ENSG00000205236	AC105052.1	0.02054	0	0	0	0	0	0	0
ENSG00000267368	UPK3BL1	0	0	0	0	0	0	0	0
ENSG00000267645	AC105052.3	0.04587	0	0	0	0.05577	0	0	0
ENSG00000173678	SPDYE2B	0	0	0	0	0	0	0	0
ENSG00000228049	POLR2J2	0.21298	0	0	0	0.04316	0.06262	0.1775	0.08716
ENSG00000233297	RASA4DP	0	0	0.08984	0	0	0.10785	0	0
ENSG00000222011	FAM185A	8.08479	10.8641	6.82833	18.2173	23.0547	17.011	12.8493	12.3668
ENSG00000213385	AC105052.2	0.27695	0.30733	0	0	0.22448	0.32572	0	0
ENSG00000161040	FBXL13	4.58927	4.13898	1.47774	3.28338	2.93425	1.03217	4.26671	4.57955
ENSG00000252643	RNU6-1136P	0	0	0	0	0	0	0	0.86536
ENSG00000238324	RN7SKP198	0	0	0	0	0	0	0	0
ENSG00000128606	LRRC17	0.16447	0	0.04028	0.04068	0.03333	0	0.09138	0.03366
ENSG00000230257	NFE4	2.89813	4.60129	0.78623	0.33086	3.08981	0.62925	0.52023	3.55828
ENSG00000236226	AC073127.1	0.22012	0	0	0	0	0	0	0
ENSG00000170632	ARMC10	1.47241	0.8791	0.56444	0.90259	1.28421	0.84702	0.72028	0.96285
ENSG00000233025	CRYZP1	0	0	0	0	0	0	0	0
ENSG00000161048	NAPEPLD	0.349	0.43569	0.45587	0.9352	0.80147	0.87223	1.01803	0.79749
ENSG00000224415	AC007683.1	0	0	0	0	0	0	0.41347	0
ENSG00000236848	RPL23AP95	0	0	0	0.41051	0	0	0	0.33961
ENSG00000279482	AC007683.2	0	0	0	0	0	0	0	0
ENSG00000170629	DPY19L2P2	0.98522	0.87461	1.31264	4.9324	1.98037	0.78792	1.83927	1.96768
ENSG00000237632	S100A11P1	0	0	0	0	0	0	0	0
ENSG00000105819	PMPCB	10.8093	8.04978	2.91841	8.43736	13.068	12.1689	7.4041	6.51478
ENSG00000105821	DNAJC2	9.75998	10.3161	3.26555	3.89467	11.8388	10.579	5.08154	7.40378
ENSG00000161057	PSMC2	8.80348	6.56995	2.67235	4.84583	7.91422	10.2443	4.51244	5.78498
ENSG00000235354	RPS29P16	0.42866	0	0	0.63617	0.52115	0	0	1.5789
ENSG00000170615	SLC26A5	0	0	0	0.03221	0	0	0	0
ENSG00000251852	RF00019	0	0	0	0	0	0	0	0
ENSG00000252047	RF00019	0	0	0	0	0	0	0	0
ENSG00000234715	AC005064.1	0	0	0	0	0	0	0	0
ENSG00000189056	RELN	0	0.0193	0.17893	0.30978	0.16213	0.13297	1.44003	0.19933
ENSG00000222386	RN7SKP86	0	0.3675	0	0.32767	0	0	0	0.27108
ENSG00000164815	ORC5	5.96402	8.05964	8.12772	26.0864	10.3618	10.6255	17.9793	12.9776
ENSG00000187416	LHFPL3	0	0	0	0	0	0	0	0
ENSG00000197258	EIF4BP6	0.80197	0.6007	0.17677	0.47609	1.26752	0.99035	0.93574	0.78772
ENSG00000226869	LHFPL3-AS1	0	0	0	0	0	0	0	0
ENSG00000237606	AC091286.1	0	0	0	0	0	0	0	0

ENSG00000225329	LHFPL3-AS2	0	0	0	0	0	0	0	0.03789
ENSG00000243352	RN7SL8P	0	0	0.40494	0.40897	0	0.48613	0	0.67667
ENSG00000237513	AC007384.1	6.16744	3.89389	0.31252	1.57813	4.82643	6.12793	1.77244	1.74076
ENSG00000228393	LINC01004	1.36101	0.43151	0.57143	0.38474	1.02433	0.91467	0.54014	1.11402
ENSG00000239569	KMT2E-AS1	5.37801	4.14431	1.31715	0.44342	4.48005	4.39238	0.99604	1.83418
ENSG00000005483	KMT2E	38.0676	40.9471	16.3245	26.9519	47.1987	38.5512	34.8368	30.9935
ENSG00000272918	AC005070.3	0.96958	0.92221	0.20354	0.45225	0.5052	0.39097	0.23088	0.5442
ENSG00000135250	SRPK2	19.0389	14.1808	3.22751	5.91879	13.1124	10.6451	5.37586	8.87055
ENSG00000270764	AC005070.1	0	0	0	0	0	0	0	0
ENSG00000271482	AC005070.2	0	0	0	0	0	0	0	0
ENSG00000213361	AC004884.1	0	0	0	0	0	0	0	0
ENSG00000244490	RWDD4P1	0.51894	0.43189	0.38129	0.77016	1.1041	0	0.86499	0
ENSG00000242154	AC004884.2	0	0	0	0	0	0	0	0
ENSG00000201179	RNU6-1322P	0	0	0	0	0	0	0	0
ENSG00000091127	PUS7	3.87973	5.16073	5.04338	11.885	4.93861	5.46965	7.71054	5.80167
ENSG00000223886	AC073073.1	0	0	0	0	0	0	0	0
ENSG00000135249	RINT1	3.04833	2.37007	1.53246	3.60139	3.77923	4.17477	2.94169	3.1025
ENSG00000185055	EFCAB10	0.02843	0	0	0	0	0	0.14218	0.03491
ENSG00000272604	AC073073.2	0.1706	0.09465	0.04178	0.0844	0.24198	0.40128	0.09479	0.10473
ENSG00000231167	YBX1P2	0.44024	0.61065	0.10782	0.10889	0.62444	0.38832	0.36691	0.7207
ENSG00000146776	ATXN7L1	2.33289	1.76504	0.37658	0.7213	2.01976	1.79275	0.94268	0.91136
ENSG00000226624	AC005099.1	0	0	0	0	0	1.05993	0	0.14754
ENSG00000128536	CDHR3	0.20686	0.03443	0.0152	0.04605	0.21377	0.18246	0.18964	0.16509
ENSG00000280439	AC004836.1	0	0	0	0	0	0	0	0
ENSG00000008282	SYPL1	8.44366	6.96197	4.45607	9.54397	9.72529	6.04131	6.66676	5.96985
ENSG00000242562	DCAF13P1	0	0	0	0	0.06701	0	0	0
ENSG00000201796	RNU6-392P	0	0	0	0	0	0	0	0
ENSG00000105835	NAMPT	480.976	764.262	123.593	120.82	418.058	56.9321	163.713	356.219
ENSG00000273320	AC007032.1	22.5872	52.0013	10.9367	9.37586	31.4591	2.90074	4.03902	34.745
ENSG00000243438	LARP1BP2	2.11851	2.58592	0.41508	0.20961	1.37367	0	0	1.21383
ENSG00000243797	AC004917.1	0.10562	0.1172	0.20694	0.05225	0.08561	0	0.05868	0.17291
ENSG00000267052	AC005050.1	0	0	0.19584	0.39558	0	0	0	0
ENSG00000253276	CCDC71L	8.539	8.93729	2.18889	2.3392	3.74829	2.75001	3.11802	5.89064
ENSG00000228742	LINC02577	0.02069	0.10334	0.03041	0.06143	0	0	0	0
ENSG00000251978	RNA5SP236	0	0	0	0	0	0	0	0
ENSG00000283585		0	0	0	0	0	0	0	0
ENSG00000105851	PIK3CG	29.8022	25.0601	4.5229	6.97109	19.777	16.9872	6.96407	13.067
ENSG00000005249	PRKAR2B	32.0328	17.0948	8.10723	17.4087	18.5539	16.3811	18.477	19.8419
ENSG00000243193	AC006387.1	0	0.11142	0.04918	0.1987	0.08139	0	0.27895	0.08219
ENSG00000105856	HBP1	13.8268	12.66	3.03587	4.89887	10.0889	9.24383	9.08032	8.96995
ENSG00000272072	AC004492.1	0.09879	0	0	0	0	0	0	0
ENSG00000164597	COG5	4.31128	3.42955	1.56476	4.84377	3.96799	4.84136	3.53539	2.90176
ENSG00000228341	AC002381.1	0	0	0	0	0	0	0	0
ENSG00000172209	GPR22	0	0	0	0	0	0	0	0
ENSG00000105865	DUS4L	0.08086	0.04486	0.0198	0.14001	0.13108	0.0951	0	0.26475
ENSG00000272854	AC004839.1	0	0	0	0	0	0	0	0
ENSG00000075790	BCAP29	1.69108	1.94424	1.35779	2.96253	1.58967	1.55316	2.28116	1.50905
ENSG00000238832	RF01233	0	0	0	0	0	0	0	0
ENSG00000250474	WBP1LP2	0.14689	0	0	0.109	0.17859	0	0	0.09018
ENSG00000225935	BANF1P5	0	0	0	0	0	0	0	0
ENSG00000233705	SLC26A4-AS1	0.01458	0	0	0	0	0	0	0
ENSG00000091137	SLC26A4	0.02528	0	0.01857	0.05627	0.01536	0.04459	0.04213	0.01552
ENSG00000241764	AC002467.1	2.30926	1.85989	0.10947	0.22111	1.99245	1.05132	1.98667	1.28045
ENSG00000105879	CBLL1	8.4387	7.45719	2.6334	6.39099	8.0483	7.00704	4.72582	4.90953
ENSG00000091138	SLC26A3	0	0	0.0637	0	0	0	0	0
ENSG00000229626	PIGCP2	0	0	0	0	0	0	0	0
ENSG00000091140	DLD	10.6236	7.39097	2.3173	4.08537	8.52654	8.98032	5.81042	5.53671
ENSG00000091136	LAMB1	0.009	0.01499	0	0	0.06569	0	0	0

ENSG00000273055	AC005046.1	0	0.19521	0	0	0	0	0	0
ENSG00000238297	RF00012	0	0	0	0	0	0	0	0
ENSG00000091128	LAMB4	0	0	0	0	0	0	0	0
ENSG00000091129	NRCAM	0.77836	0.02978	0.01315	0.07967	0.83754	0.12627	0.43247	0.1428
ENSG00000135241	PNPLA8	12.6995	11.8607	4.16185	8.7328	12.7826	7.41467	10.929	11.8292
ENSG00000231006	RPL7P32	0	0.16399	0	0	0	0	0	0
ENSG00000177683	THAP5	8.79131	11.2392	2.25806	4.62685	10.4727	5.73445	4.58084	7.94572
ENSG00000128590	DNAJB9	7.46697	5.84884	2.99944	6.74878	6.65941	5.78872	6.97681	6.0273
ENSG00000225647	AC005487.1	0	0	0	0	0	0	0	0
ENSG00000270425	AC002487.1	0	0	0	0	0	0	0	0
ENSG00000205174	C7orf66	0	0	0	0	0	0	0	0
ENSG00000279043	AC004014.2	0	0	0	0	0	0	0	0
ENSG00000229603	AC004014.1	0	0	0	0	0	0	0	0
ENSG00000227968	BUB3P1	0	0	0	0	0	0	0	0
ENSG00000230941	AC002386.1	0	0	0	0	0	0	0	0
ENSG00000234273	AC073071.1	0	0	0	0	0	0	0	0
ENSG00000230192	AC073387.1	0	0	0	0	0	0	0	0
ENSG00000271368	AC073387.2	0	0	0	0	0	0	0	0
ENSG00000237064	EIF3IP1	0	0	0	0	0	0	0	0
ENSG00000232199	RPL3P8	0	0	0	0	0	0	0	0
ENSG00000278424	AC073114.1	0	0	0	0	0	0	0	0
ENSG00000226965	AC092167.1	0	0	0	0	0	0	0	0
ENSG00000184903	IMMP2L	3.69618	5.61575	2.99995	5.45364	3.42254	2.2367	5.58784	3.35081
ENSG00000228540	AC073326.1	0.3948	0	0	0	0.15999	0	0	0.16157
ENSG00000173114	LRRN3	2.04295	0.15743	0.58374	0.98258	9.86608	1.63517	1.29275	2.67088
ENSG00000243621	AC003989.2	0	0	0	0	0	0	0	0
ENSG00000227948	AC003989.1	0	0	0	0	0	0	0	0
ENSG00000128512	DOCK4	3.95856	9.43261	1.43319	1.15417	1.30974	0.92213	1.0094	1.61226
ENSG00000225572	DOCK4-AS1	0	0	0	0	0	0	0	0
ENSG00000222346	RNA5SP237	1.29735	3.23916	0	0	0	0	2.16248	0
ENSG00000234358	AC003080.1	0.19114	0.63629	0.14044	0.14183	0.11619	0	0.47789	0.35201
ENSG00000198839	ZNF277	6.31837	5.80559	3.99835	6.55858	6.0832	6.63633	6.05727	5.47067
ENSG00000202406	RN7SKP187	0	0	0	0	0	0	0	0
ENSG00000226851	AC004112.1	0	0	0	0	0	0	0	0
ENSG00000230999	MTND5P8	0	0	0	0	0	0	0	0
ENSG00000232922	MTND6P24	0	0	0	0	0	0	0	0
ENSG00000236232	MTCYBP24	0	0	0	0	0	0	0	0
ENSG00000006652	IFRD1	13.9867	29.9115	3.75395	9.06427	12.6018	4.23039	5.33489	15.2859
ENSG00000180019	AC079741.1	0	0	0	0	0.42036	0	0	0
ENSG00000181016	LSMEM1	0.90494	1.64749	0.37401	0.54561	0.65325	0.64855	0.70706	0.83331
ENSG00000182965	NPM1P14	0	0	0	0	0	0	0	0
ENSG00000223646	AC002463.1	0	0	0	0	0.07501	0	0	0
ENSG00000225358	MIPEPP1	0.07232	0	0	0	0	0	0.06028	0.0444
ENSG00000146802	TMEM168	2.04452	1.70919	0.66016	1.78248	1.85033	1.69828	1.20729	1.56468
ENSG00000164603	BMT2	7.82957	8.31474	3.38396	4.88984	6.09474	5.65622	3.74986	7.0684
ENSG00000234520	HRAT17	0	0	0	0	0	0	0	0
ENSG00000279288	AC073346.2	0	0	0	0	0	0	0	0
ENSG00000164604	GPR85	0.00991	0.0165	0	0	0.01205	0	0	0.02434
ENSG00000225457	AC073346.1	0	0	0	0	0	0	0	0
ENSG00000214194	SMIM30	4.32998	2.70272	2.72694	4.53274	2.96116	3.75113	1.80435	2.84801
ENSG00000270997	AC073137.1	0	0	0	0	0	0	0	0
ENSG00000278894	AC073348.1	0	0	0	0	0	0	0	0
ENSG00000154415	PPP1R3A	0	0	0	0	0	0	0	0
ENSG00000128573	FOXP2	0	0	0	0	0	0.00801	0	0
ENSG00000236580	AC073626.2	0	0	0	0	0	0	0	0
ENSG00000224595	AC073626.1	0	0	0	0	0	0	0	0
ENSG00000282859	AC003992.1	0	0	0	0	0	0	0	0
ENSG00000202334	RNA5SP238	0	0	0	0	0	0	0	0

ENSG00000272230	MIR3666	0	0	0	0	0	0	0	0
ENSG00000135272	MDFIC	10.942	7.61972	3.5891	7.47746	8.90827	6.94325	3.93155	5.67194
ENSG00000233607	LINC01392	0	0	0	0	0	0	0	0
ENSG00000225535	LINC01393	0	0.06667	0	0	0	0	0	0
ENSG00000226962	RAC1P6	0	0	0	0	0	0	0	0
ENSG00000199764	RF00019	0	0	0	0	0	0	0	0
ENSG00000244565	AC073901.1	0	0	0	0	0	0	0	0
ENSG00000202377	SNORA25B	0	0	0	0	0	0	0	0
ENSG00000242072	AC092590.1	0	0	0	0	0	0	0	0
ENSG00000240973	AC093714.1	0	0	0	0	0	0	0	0
ENSG00000243345	AC093714.2	0	0	0	0	0	0	0	0
ENSG00000199224	RF00019	0	0	0	0	0	0	0	0
ENSG00000105967	TFEC	6.11402	6.33755	3.09036	2.57995	3.08259	3.11162	1.03183	4.35201
ENSG00000279086	AC073130.3	0.09653	0.16068	0.09457	0.14326	0.1956	0.34059	0.21454	0.39507
ENSG00000135269	TES	22.2641	11.7162	5.3857	10.4973	17.74	18.4602	6.65141	12.4061
ENSG00000243243	AC073130.2	0	0	0	0	0	0	0	0
ENSG00000237813	AC002066.1	0	0	0	0.08968	0	0	0	0
ENSG00000237870	AC073130.1	0.1718	0	0	0	0	0	0	0
ENSG00000105971	CAV2	0.45508	0.1057	0.07776	0.10995	0.20587	0.20538	0.26461	0.37683
ENSG00000213167	RF00017	0	0	0	0	0	0	0	0
ENSG00000105974	CAV1	0.30527	0.05978	0	0.0533	0.19648	0.09504	0.02993	0.08819
ENSG00000235427	AC006159.1	0	0	0	0	0	0	0	0
ENSG00000243220	AC006159.2	0	0	0	0	0	0	0	0
ENSG00000231210	LINC01510	0	0	0	0	0	0	0	0
ENSG00000105976	MET	0.01041	0.03467	0	0	0.20257	0.03674	0	0
ENSG00000198898	CAPZA2	28.7372	32.6128	13.1174	21.925	21.0812	15.5788	17.1705	27.1967
ENSG00000235945	AC002543.1	0	0	0	0	0	0	0	0
ENSG00000252115	RF00019	0	0	0	0	0	0	0	0
ENSG00000222150	RNA5SP239	0	0	0	0	0	0	0	0
ENSG00000227199	ST7-AS1	0.07761	0.06459	0	0.05759	0	0	0.06468	0.04764
ENSG00000275359	RF02179	0	0	0	0	0.76825	0	0	0
ENSG00000277583	RF02180	0	0	0	0	0	0	0	0
ENSG00000004866	ST7	1.60908	1.64681	0.86273	2.25897	2.12812	2.47422	3.57009	2.18919
ENSG00000214188	ST7-OT4	0.08255	0.1374	0.04043	0.04084	0	0	0.09173	0.06757
ENSG00000273596	RF02187	0	0	0	0	0	0	0	0
ENSG00000273571	RF02188	0	0	0	0	0	0	0	0
ENSG00000276100	RF02189	0	0	0	0	0	0	0	0
ENSG00000274606	RF02190	0	0	0	0	0	0	0	0
ENSG00000228368	AC106873.1	0	0	0	0	0	0	0	0
ENSG00000213050	TPM3P1	0	0	0	0	0	0	0	0
ENSG00000283923	MIR6132	0	0	0	0	0	0	0	0
ENSG00000226367	ST7-AS2	0	0	0	0	0	0	0	0
ENSG00000274344	RF02181	0	0	0	0	0	0	0	0
ENSG00000275054	RF02183	0	0	0	0	0	0	0	0
ENSG00000227532	AC002542.1	0	0	0	0	0	0	0	0
ENSG00000278526	RF02184	0	0	0	0	0	0	0	0
ENSG00000278482	RF02185	0	0	0	0	0	0	0	0
ENSG00000230785	AC006326.1	0	0	0	0	0	0	0	0
ENSG00000224042	MTND4P6	0	0	0	0	0	0	0	0
ENSG00000230605	MTCYBP6	0	0	0	0	0	0	0	0
ENSG00000105989	WNT2	0	0	0	0	0	0	0	0
ENSG00000238202	AC002465.1	0	0	0	0	0	0	0	0
ENSG00000154438	ASZ1	0	0	0	0	0	0	0	0
ENSG00000214684	AC003045.1	0	0	0	0	0	0	0	0
ENSG00000001626	CFTR	0	0	0	0	0	0	0	0
ENSG00000237974	AC000111.2	0	0	0	0	0	0	0	0
ENSG00000232661	CFTR-AS1	0	0	0	0	0	0	0	0
ENSG00000234001	AC000111.1	0	0	0	0	0	0	0	0

ENSG00000083622	AC000061.1	0	0	0	0	0	0	0	0
ENSG00000077063	CTTNBP2	0.01042	0.05203	0	0.01546	0	0	0	0.01279
ENSG00000224136	AC007568.1	0	0	0	0	0	0	0	0
ENSG00000234826	AC003084.1	0	0	0	0	0	0	0	0
ENSG00000128534	LSM8	8.67564	13.7726	3.20887	11.772	12.6715	13.9723	4.2891	7.44903
ENSG00000106013	ANKRD7	0.04818	0	0	0.03575	0.02929	0.04249	0.12045	0.02958
ENSG00000229982	GTF3AP6	0	0	0	0	0	0	0	0
ENSG00000230520	AC092098.1	0	0	0	0	0	0	0	0
ENSG00000270516	AC079791.1	0	0	0	0	0	0	0	0
ENSG00000225546	LINC02476	0.03676	0.06119	0	0	0	0	0	0.04513
ENSG00000271739	RNU1-29P	0	0	0	0	0	0	0	0
ENSG00000233417	AC004872.1	0	0	0	0	0	0	0	0
ENSG00000233969	AC004946.1	0	0	0	0	0	0	0	0
ENSG00000184408	KCND2	0	0	0	0	0	0	0	0
ENSG00000252023	RNU6-581P	0	0	0	0	0	0	0	0
ENSG00000231295	AC004888.1	0	0	0	0	0	0	0	0
ENSG00000106025	TSPAN12	0	0.03623	0	0	0.02646	0	0	0
ENSG00000071243	ING3	4.1191	3.27602	1.13622	2.2116	3.86275	3.54653	2.69481	3.67652
ENSG00000202225	RNA5SP240	0	0	0	0	0	0	0	0
ENSG00000106034	CPED1	2.7779	1.44098	0.36347	0.77654	0.74023	1.39298	0.26957	0.47889
ENSG00000234927	HMGN1P18	0	0	0	0	0	0	0	0
ENSG00000212628	RNA5SP241	0	0	0.94486	0	0	0	0	0.78945
ENSG00000207090	RNU6-517P	0	0	0	0	0	0	0	0
ENSG00000002745	WNT16	0.02248	0	0.03303	0	0.1913	0.03965	0	0.11039
ENSG00000196937	FAM3C	3.7914	2.42448	1.03874	2.95648	5.13033	3.58989	1.6424	2.28806
ENSG00000213310	CYCSP19	0	0	0	0	0	0	0	0
ENSG00000227371	AC074085.1	0	0	0	0	0	0	0	0
ENSG00000234985	AC074085.2	0	0	0	0	0	0	0.28547	0
ENSG00000227743	AC004875.1	0	0	0	0	0	0	0	0
ENSG00000252704	RN7SKP277	0	0	0	0	0	0	0	0
ENSG00000233272	PNPT1P2	0	0	0	0	0	0	0	0
ENSG00000106278	PTPRZ1	0	0.0123	0	0	0.00899	0	0	0
ENSG00000008311	AASS	0.20756	0.15945	0.11731	0.18956	0.1747	1.52098	0.45242	0.13722
ENSG00000252003	RNU7-154P	0	0	0	0	0	0	0	0
ENSG00000226636	AC006020.1	0	0	0	0	0	0	0	0
ENSG00000234418	AC015983.2	0	0	0	0	0	0	0	0
ENSG00000213302	AC015983.1	0	0	0	0	0	0	0	0
ENSG00000128610	FEZF1	0	0	0	0	0	0	0	0
ENSG00000230316	FEZF1-AS1	0	0	0	0	0	0	0	0
ENSG00000081803	CADPS2	0	0	0.01696	0	0	0	0	0.01417
ENSG00000240499	AC004594.1	0	0	0	0	0	0	0	0
ENSG00000225795	AC006463.1	0	0	0	0	0	0	0	0
ENSG00000235459	RPS26P31	0	0	0	0	0	0	0	0
ENSG00000188050	RNF133	0	0	0	0	0	0	0	0
ENSG00000235631	RNF148	0	0	0	0	0	0	0	0
ENSG00000227573	AC004986.1	0	0	0	0	0	0	0	0
ENSG00000128519	TAS2R16	0	0	0	0	0	0	0	0
ENSG00000226680	AC073311.1	0	0	0	0	0	0	0	0
ENSG00000081800	SLC13A1	0	0	0	0	0	0	0	0
ENSG00000223595	LYPLA1P1	0	0	0	0	0	0	0	0
ENSG00000164675	IQUB	0.09951	0.04141	0.01828	0.03692	0.09073	0.02194	0.1244	0.12217
ENSG00000232524	AC073323.1	0	0	0	0	0	0	0	0
ENSG00000207338	RNU6-296P	0	0	0	0	0	0	0	0
ENSG00000128609	NDUFA5	5.32769	3.61653	2.67891	6.13997	5.16553	3.43921	4.13014	4.2847
ENSG00000146809	ASB15	0	0	0	0	0	0	0	0
ENSG00000230442	AC006333.1	0	0	0	0.06299	0	0	0	0
ENSG00000170807	LMOD2	0	0	0	0	0	0	0	0
ENSG00000106299	WASL	3.71873	3.05365	1.16579	2.47742	3.3155	3.49885	2.9753	2.39451

ENSG00000272686	AC006333.2	0.59367	0.75565	0.20527	0.15548	0.25474	0.24643	0.52388	0.64314
ENSG00000201104	RNU6-11P	0	0	2.01334	0	0	0	1.14187	0
ENSG00000228211	HYAL6P	0	0.19079	0	0	0	0	0	0
ENSG00000106302	HYAL4	0	0	0	0	0.03255	0	0.04462	0
ENSG00000106304	SPAM1	0	0	0	0	0	0	0	0
ENSG00000225583	AC004690.1	0	0	0	0	0	0	0	0
ENSG00000241345	AC004690.2	0	0	0	0	0	0	0	0
ENSG00000234224	TMEM229A	0	0	0	0	0	0	0	0
ENSG00000242593	AC006148.1	0	0	0	0	0	0	0	0
ENSG00000243574	AC006148.2	0	0	0	0	0	0	0	0
ENSG00000241324	AC004930.1	0	0	0	0	0	0	0	0
ENSG00000230268	SSU72P8	0	0	0	0	0	0	0	0
ENSG00000213296	AC005521.1	0	0	0.12353	0	0	0	0	0
ENSG00000207214	RNU6-102P	0	0	0	0	0	0	0	0
ENSG00000279419	AC004925.1	0	0	0	0	0.06587	0	0	0
ENSG00000170775	GPR37	0	0	0	0	0	0	0	0
ENSG00000275356	C7orf77	0	0	0	0	0	0	0	0
ENSG00000128513	POT1	3.91548	3.12418	1.36994	3.43126	3.97451	4.62689	2.50701	2.51815
ENSG00000224897	POT1-AS1	0.23255	0.09677	0.05126	0.08628	0.07068	0.10256	0.05814	0.09993
ENSG00000186676	EEF1GP1	0.05643	0	0	0	0	0.09955	0.09406	0
ENSG00000224899	AC019155.3	0	0	0	0	0	0	0	0
ENSG00000205898	AC019155.1	0	0	0	0	0	0	0.21286	0
ENSG00000237764	AC010099.4	0.70627	0	0	0	0.73599	1.51294	0	0
ENSG00000219445	AC019155.2	0	0	0	0	0	0	0	0
ENSG00000213291	AC010099.1	0	0	0	0	0	0	0	0
ENSG00000234071	AC010099.3	0	0	0	0	0	0	0	0
ENSG00000227847	AC010099.2	0	0	0	0	0	0	0	0
ENSG00000227869	AC073486.1	0	0	0	0	0	0	0	0
ENSG00000197462	AC003975.1	0	0	0	0	0	0	0	0
ENSG00000241921	AC000372.1	0	0	0	0	0	0	0	0
ENSG00000179603	GRM8	0	0	0	0	0	0.02003	0	0
ENSG00000227249	AC000374.1	0	0	0	0	0	0	0	0
ENSG00000224981	AC000367.1	0	0	0	0	0	0	0	0
ENSG00000230820	AC000362.1	0	0	0	0	0	0	0	0
ENSG00000207692	MIR592	0	0	0	0	0	0	0	0
ENSG00000236340	AC000099.1	0	0	0	0	0	0	0	0
ENSG00000236105	PRELID3BP10	0	0	0	0	0	0	0	0
ENSG00000048405	ZNF800	9.38872	9.24594	3.84892	8.43169	9.90698	10.7816	7.06549	7.55025
ENSG00000279265	AC000123.2	0.79501	0.79398	0.93461	0.94391	0.96656	1.96352	0.53007	1.36655
ENSG00000224138	AC000123.1	0	0	0	0	0	0	0	0
ENSG00000280347	AC000123.3	1.14923	0.85835	0.21651	0.89653	0.82399	1.29962	0.81044	0.74168
ENSG00000226770	AC000124.1	0	0	0	0	0	0	0	0
ENSG00000179562	GCC1	5.70293	6.04293	1.16634	1.5706	4.94993	4.40806	2.98897	4.13261
ENSG00000004059	ARF5	18.5308	16.9547	2.30281	4.53516	14.2892	15.2741	5.87719	7.35946
ENSG00000106328	FSCN3	0.11403	0	0	0.03385	0.11091	0	0.03802	0.11201
ENSG00000106331	PAX4	0	0	0	0	0	0	0	0
ENSG00000240790	AC073934.1	0.29616	0.49296	0.43521	0.43954	0.18003	0.26124	0.24683	0.18181
ENSG00000197157	SND1	11.7055	8.16518	2.27028	5.22171	9.55605	11.081	4.77443	5.80135
ENSG00000240579	AC006529.1	0	0	0	0	0	0	0	0
ENSG00000279078	SND1-IT1	0.2659	0.19671	0	0.0877	0.14368	0.20848	0.09849	0.03627
ENSG00000128594	LRRC4	8.93552	9.31086	1.62799	1.99458	7.63983	3.52435	1.5439	5.10637
ENSG00000207588	MIR593	1.46601	0	0	0	0	0	1.2218	0
ENSG00000207705	MIR129-1	0	0	0	0	0	0	0	0
ENSG00000174697	LEP	0.1929	0.03567	0	0	0.07817	0	0	0.02631
ENSG00000272915	AC018635.2	0	0	0	0	0	0	0	0
ENSG00000106344	RBM28	1.15037	1.11504	0.31908	0.62394	1.20762	1.24699	0.43124	0.70337
ENSG00000242261	AC018635.1	0	0	0	0	0	0	0	0
ENSG00000238750	RNU7-27P	0	0	0	0	0	0	0	0

ENSG00000224940	PRRT4	0	0	0	0.02954	0.07259	0.03511	0	0
ENSG00000106348	IMPDH1	32.9471	25.9543	2.67667	4.78277	21.6099	7.80391	5.30493	11.5015
ENSG00000228700	AC010655.1	0	0	0	0	0	0	0	0
ENSG00000238590	RNU7-54P	0	0	0	0	0	0	0	0
ENSG00000273184	AC010655.4	0	0	0	0	0	0	0	0
ENSG00000135245	HILPDA	0.60198	0.17682	0.05204	0.05255	0.51662	0.18741	0.11805	0.17391
ENSG00000240758	AC010655.2	0.11073	0	0	0	0.13462	0	0	0.13595
ENSG00000272601	AC010655.3	0	0	0	0	0	0	0	0
ENSG00000165055	METTL2B	1.57483	1.26546	0.14364	0.43521	1.5053	1.32205	0.43448	0.48006
ENSG00000276988	RF00017	0	0	0	0	0	0	0	0
ENSG00000273270	AC090114.2	0.48839	0.13837	0.01527	0.01542	0.3032	0.25664	0.12125	0.24241
ENSG00000280828	AC090114.3	4.42101	5.73984	1.42926	1.83715	6.01994	4.99153	3.24242	2.82259
ENSG00000213280	AC090114.1	0	0	0	0	0	0	0	0
ENSG00000242588	AC108010.1	1.58917	1.4328	0.87572	0.78617	1.20754	0.58406	1.43482	0.65039
ENSG00000281896	AC018638.8	6.52138	7.81549	3.83324	4.64565	8.87995	5.52221	10.4353	6.08521
ENSG00000223189	RNU6-177P	0.70481	0	0	1.04602	0	0	1.17481	0.86536
ENSG00000243302	AC018638.4	5.48159	2.54626	0.37466	0.56758	3.2547	3.03602	1.16868	0.9391
ENSG00000230715	AC018638.2	6.54792	2.11925	0	0.80982	4.20153	3.85048	1.21271	1.11659
ENSG00000229413	AC018638.1	0	0	0	0	0	0	0	0
ENSG00000243679	AC018638.5	3.88861	2.75085	0.28571	1.00995	2.9548	2.57252	1.94451	0.5968
ENSG00000281490	CICP14	0	0	0	0	0	0	0	0
ENSG00000271553	AC018638.7	0.2632	0	0	0.09765	0.55998	0	0.21935	0.32315
ENSG00000241493	AC018638.3	0	0	0	0	0	0	0	0
ENSG00000205085	FAM71F2	0.29992	0.19969	0	0	0.07293	1.48148	0.09998	0.29459
ENSG00000271344	AC018638.6	0.05785	0	0	0	0	0	0	0
ENSG00000239722	IMP3P2	0	0	0	0	0	0	0	0
ENSG00000201041	RNA5SP242	0	0	0	0	0	0	1.03543	0
ENSG00000252866	RNA5SP243	0	0	0	6.21633	0	0	0	0
ENSG00000135248	FAM71F1	0	0	0	0	0	0	0.04847	0
ENSG00000128595	CALU	9.85524	6.18513	1.68563	3.33287	5.9123	6.12785	3.09692	3.8284
ENSG00000244218	RN7SL81P	0	2.33733	0	0.4168	0.68289	0	0	0
ENSG00000128617	OPN1SW	0	0	0	0	0	0	0	0
ENSG00000128596	CCDC136	0.18953	0.19717	0.03481	0.0879	0.15842	0.14628	0.07898	0.05818
ENSG00000128591	FLNC	0	0	0	0	0	0	0	0
ENSG00000242902	FLNC-AS1	0	0	0	0	0	0	0	0
ENSG00000135253	KCP	0.00888	0.01477	0	0	0.01079	0	0	0.0109
ENSG00000128524	ATP6V1F	23.9689	17.474	5.73382	8.27268	20.1049	22.2895	10.3753	11.0643
ENSG00000272899	ATP6V1FNB	0.11271	0.0938	0	0.05576	0.06852	0	0.03131	0.02306
ENSG00000224163	AC025594.1	0	0	0	0	0	0	0	0
ENSG00000128604	IRF5	7.1206	5.46598	1.08329	1.31786	3.74799	2.00987	1.00537	1.8308
ENSG00000275106	AC025594.2	0.37114	0.46332	0.27269	0.41311	0.78964	0	0	0.45568
ENSG00000064419	TNPO3	11.992	12.3157	3.9621	7.35164	10.787	10.0662	7.26393	7.96809
ENSG00000242241	RN7SL306P	0	0	0	0	0	0	0	0
ENSG00000244556	ODCP	0	0	0	0	0	0	0	0
ENSG00000230359	TPI1P2	0.4361	0.12098	0	0.05393	0.22091	0.38467	0.12115	0.13386
ENSG00000242162	AC018639.1	0	0	0	0	0	0	0	0
ENSG00000241102	AC011005.2	0	0	0	0	0	0	0	0
ENSG00000241573	AC011005.3	0	0	0	0	0	0	0	0
ENSG00000240416	CYCSP20	0	0	0	0	0	0	0	0
ENSG00000230626	AC011005.1	0	0	0.08528	0	0	0	0	0.07126
ENSG00000158457	TSPAN33	11.1417	5.98951	2.31095	4.43055	5.99512	6.11292	4.79835	6.02162
ENSG00000200629	RNY1P11	0	0	0.95322	0.96271	0	0	0	1.59287
ENSG00000128602	SMO	0	0.0566	0	0	0.08269	0.08999	0	0
ENSG00000243230	AC011005.4	0	0	0	0	0.16657	0	0	0
ENSG00000158467	AHCYL2	1.94527	1.44105	0.34554	0.6345	1.11753	1.28218	0.48102	0.83987
ENSG00000238072	AC009244.1	0.28411	1.8916	0.83499	0	1.38165	0.50121	0.47357	2.09295
ENSG00000128578	STRIP2	0.46156	0.2284	0.42162	0.66649	0.34882	0.24207	0.93569	0.32164
ENSG00000239919	SNRPGP3	0	0	0	0	0	0	0	0

ENSG00000199846	RNU1-72P	0	0	0.70401	0.71102	2.32985	0.84518	0.79856	0.58822
ENSG00000240204	SMKR1	0.28329	0	0	0.10511	0	0.24988	0	0.26086
ENSG00000273329	AC078846.1	0.49674	0.24116	0.04562	0.06143	0.21389	0.38339	0.25875	0.33036
ENSG00000106459	NRF1	3.28741	3.4606	1.48841	1.97792	4.36402	4.85899	3.25814	2.57446
ENSG00000212238	RNA5SP244	0	0	0	0	0	0	0	0
ENSG00000207990	MIR182	0	0	0	0	0	0	0	0
ENSG00000199158	MIR96	0	0	0	0	0	0	0	0
ENSG00000207691	MIR183	0	0	0	0	0	0	0	0
ENSG00000242078	AC084864.1	0	0	0	0	0	0	0	0
ENSG00000186591	UBE2H	27.4991	39.626	24.3165	30.3109	26.6012	25.1867	58.3568	23.7383
ENSG00000244036	AC073320.1	0.09563	0	0	0	0	0	0	0
ENSG00000199516	RF00019	0	0	0	0	0	0	0	0
ENSG00000091732	ZC3HC1	1.14418	0.9725	0.50083	1.04775	1.53905	2.19027	1.37965	1.04613
ENSG00000201109	RNA5SP245	0	0	0	0	0	0	0	0
ENSG00000128607	KLHDC10	4.77083	5.38002	4.31051	9.03536	6.44622	6.7175	6.53154	5.88474
ENSG00000229196	AC087071.1	0	0	0	0	0	0	0.2429	0.17892
ENSG00000146842	TMEM209	5.48328	4.94802	3.6893	10.7208	7.77126	10.4341	7.00887	5.55981
ENSG00000240571	AC087071.2	0	0	0	0	0	0	0	0
ENSG00000165120	SSMEM1	0	0	0	0	0	0	0	0
ENSG00000270992	AC024085.1	0	0	0	0	0	0	0	0
ENSG00000158516	CPA2	0	0	0	0	0	0	0	0
ENSG00000128510	CPA4	0	0.03083	0	0	0	0	0	0
ENSG00000158525	CPA5	0	0	0.03633	0	0.06011	1.13393	0	0
ENSG00000091704	CPA1	0	0	0	0	0	0	0	0
ENSG00000106477	CEP41	0.29166	0.26215	0.14572	0.25971	0.46806	0.83354	0.1264	0.27215
ENSG00000259920	AC007938.1	0	0	0	0	0	0	0.0421	0
ENSG00000272701	MESTIT1	0.0534	0.02963	0	0	0	0	0	0
ENSG00000106484	MEST	0.30074	0.26346	0.16282	0.30538	1.01991	0.86562	0.60681	0.66074
ENSG00000277269	RF02148	0	0	0	0	0	0	0	0
ENSG00000278055	RF02149	0	0	0	0	0	0	0	0
ENSG00000274500	RF02150	0	0	0	0	0	0	0	0
ENSG00000270823	AC007938.2	0	0	0	0	0	0.04909	0	0
ENSG00000199043	MIR335	0	0	0	0	0.94805	0	0	0
ENSG00000158623	COPG2	2.08974	0.58956	0.36434	1.10389	1.61482	2.24944	1.21029	1.17416
ENSG00000270953	AC007938.3	0	0	0	0	0	0	0	0
ENSG00000239021	RNA5SP246	0	0	0	0	0	0	0	0
ENSG00000226045	AC234644.1	0.39622	0	0	0	0	0	0	0.24324
ENSG00000213265	TSGA13	0	0	0	0	0	0	0	0
ENSG00000266265	KLF14	0.05186	0	0	0	0	0	0	0
ENSG00000271522	AC016831.6	0	0	0	0	0	0	0	0
ENSG00000229858	AC016831.2	0	0	0	0	0	0	0	0
ENSG00000240680	AC016831.4	0	0	0	0	0.58056	0	0	0
ENSG00000232716	AC016831.3	0	0	0	0	0	0	0	0.05223
ENSG00000233559	LINC00513	0.04536	0.0755	0	0	0.05515	0	0.07561	0.05569
ENSG00000284032	MIR29A	4.58127	0	0	1.69978	0	0	1.90907	0
ENSG00000226380	AC016831.1	2.50278	1.75114	0.45566	2.23521	2.32923	0.89868	0.95987	2.69216
ENSG00000283797	MIR29B1	0	0	0	0	0	0	0	0
ENSG00000271204	AC016831.5	1.83434	1.16024	0.4852	1.74231	1.33809	1.22969	1.28418	1.35131
ENSG00000273319	AC058791.1	0	0	0	0.13607	0	0	0	0.02814
ENSG00000231721	LINC-PINT	9.35127	7.48535	1.57456	3.54186	5.13186	5.35577	4.05915	3.76732
ENSG00000199627	RNU6-1010P	2.05515	3.4208	0	0	2.4986	0	1.14187	1.68219
ENSG00000128585	MKLN1	18.1344	23.3349	23.4364	47.2801	22.6632	23.346	37.1626	27.1011
ENSG00000233287	AC009362.1	0	0	0	0	0	0	0	0
ENSG00000236753	MKLN1-AS	0.62096	0.22469	0.31739	0.40068	0.59083	1.09546	1.03505	0.46407
ENSG00000273489	AC008264.2	0	0	0.05774	0.02916	0	0	0	0
ENSG00000128567	PODXL	0.15995	0.12424	0.01567	0.0633	0.07779	0.09406	0.01777	0.02618
ENSG00000224545	AC008264.1	0	0.33245	0	0	0	0	0	0
ENSG00000213261	EEF1B2P6	0	0	0	0	0	0.19214	0.18155	0.40118

ENSG00000236238	AC093106.1	0	0	0	0	0	0	0	0
ENSG00000236395	AC093106.2	0	0	0	0	0	0	0	0
ENSG00000236281	NDUFB9P2	0	0	0	0	0	0	0	0
ENSG00000233263	AC009518.2	0	0	0	0.14882	0	0	0	0
ENSG00000236386	AC009518.3	0	0	0	0	0	0	0	0
ENSG00000224865	AC009518.1	0	0	0	0	0	0	0	0
ENSG00000229532	AC105443.1	0	0	0	0	0	0	0	0
ENSG00000221866	PLXNA4	0.44324	0.09688	0.0329	0.12625	0.09798	0.22115	0.13433	0.08246
ENSG00000225144	AC018643.1	0	0	0	0	0	0	0	0
ENSG00000223436	AC011625.1	0	0	0	0	0	0	0	0
ENSG00000183470	FLJ40288	0	0	0	0	0	0	0	0
ENSG00000201009	RF00218	0	0	0	0	0	0	0	0
ENSG00000225881	AC009365.1	0	0	0	0	0	0	0	0
ENSG00000106554	CHCHD3	5.22447	4.627	1.65134	3.15999	4.43426	7.16467	3.31905	2.61422
ENSG00000227197	AC009365.2	0	0	0	0	0	0	0	0
ENSG00000283041	AC008038.1	0.39049	0.92853	0.16395	0.33116	1.08513	0.8857	0.46492	0.34245
ENSG00000284135	MIR3654	0	0	0	0	0	0	0	0
ENSG00000272393	RNU6-92P	0	0	0	0	0	0	0	0
ENSG00000237821	AC083873.1	0.20009	0.22204	0.09801	0	0.16218	0.23533	0	0.08189
ENSG00000131558	EXOC4	5.59858	4.60142	1.45652	2.65471	5.06494	5.35498	2.56855	2.80468
ENSG00000276137	MIR6133	0	1.12971	0	0	0	2.39466	0	0
ENSG00000235429	AC083875.1	0	0	0	0	0	0	0	0
ENSG00000235967	COX5BP3	0	0	0	0	0	0	0	0
ENSG00000226205	AC007790.1	0	0	0	0	0	0	0	0
ENSG00000155530	LRGUK	0.20305	0.18776	0.16576	0.40179	0.21943	0.3582	0.30084	0.277
ENSG00000231098	AC008154.2	0	0	0	0	0	0	0	0
ENSG00000205060	SLC35B4	1.58788	1.28719	0.39395	0.84164	1.5795	1.85537	0.70466	0.92415
ENSG00000229177	AC008154.1	0	0	0	0	0	0	0	0
ENSG00000273297	AC009275.1	0	0	0	0	0	0	0	0
ENSG00000085662	AKR1B1	1.30178	1.06674	0.44145	0.86196	1.41223	2.19053	1.26854	1.20488
ENSG00000198074	AKR1B10	0	0	0	0	0.61319	0	0	0
ENSG00000227471	AKR1B15	0	0	0	0	0	0	0	0
ENSG00000172331	BPGM	67.5841	100.961	81.2415	200.305	67.8817	122.487	151.39	46.4341
ENSG00000224375	AC009276.1	0.28972	0	0	0.85997	0	0	0.48293	0
ENSG00000230101	TUBB3P2	0	0	0	0	0	0.55738	0	0
ENSG00000122786	CALD1	3.85007	1.79734	0.29547	0.70733	1.09551	0.80138	0.70754	1.04233
ENSG00000146856	AGBL3	0.1605	0.13358	0.11793	0.14888	0.23173	0.12388	0.20065	0.23401
ENSG00000122783	CYREN	2.76891	1.628	0.36438	1.55379	1.94278	1.53104	1.07921	1.58987
ENSG00000223500	AC083862.1	0	0	0	0	0.07175	0	0	0
ENSG00000146859	TMEM140	20.0918	51.3248	7.77701	9.40646	12.7915	19.9027	11.1457	23.7346
ENSG00000272941	AC083862.2	6.01767	9.16396	1.97554	1.80518	2.87975	4.96919	1.60062	4.0872
ENSG00000105875	WDR91	1.79848	0.91021	0.26786	0.8296	1.24101	0.81463	1.11404	1.0444
ENSG00000231794	AC009542.1	0.20499	0.14623	0.08607	0	0.17802	0.05166	0.14644	0.14382
ENSG00000277138	MIR6509	0	0	0	0	0	0	0	1.05879
ENSG00000146857	STRA8	0	0	0	0	0	0	0	0
ENSG00000276067	AC009542.2	0	0	0	0	0	0.04811	0	0
ENSG00000080802	CNOT4	5.1527	4.49753	1.80062	3.09306	4.71113	5.35797	2.9502	2.66172
ENSG00000227073	SDHDP2	0	0	0	0	0	0	0	0
ENSG00000155561	NUP205	5.38761	4.48384	2.40338	4.48336	6.49163	7.82422	3.97699	3.95708
ENSG00000271414	AC093107.2	0	0.27418	0.24205	0	0.20026	0	0	0.20224
ENSG00000223718	AC093107.1	0	0	0	0	0	0	0	0
ENSG00000243317	STMP1	24.2352	27.0498	24.7108	59.9535	19.3779	20.9837	34.9936	20.2083
ENSG00000212303	RNU6-1154P	0	0	0	0	0	0	0	0
ENSG00000164707	SLC13A4	0	0	0	0	0	0	0	0
ENSG00000273219	AC091736.1	0.48066	0	0	0	0	0	0	0
ENSG00000189320	FAM180A	0	0	0	0	0	0	0	0
ENSG00000224746	AC015987.1	0.18234	0	0	0	0	0	0	0
ENSG00000105887	MTPN	110.484	102.196	38.8341	78.2681	82.129	85.4657	80.9141	96.8154

ENSG00000267697	LUZP6	0	0	0	0	0	0	0	0
ENSG00000199700	RNU6-223P	0	0	0	0	0	0	0	0
ENSG00000230649	AC024084.1	3.22199	5.09486	13.0204	5.02088	1.17516	0.5684	5.10204	4.94489
ENSG00000232053	AC078845.1	0.05704	0.28484	0.3353	0.25397	0.06935	0.3019	0.09508	0.07004
ENSG00000206705	RF00019	0	0	0	0	0	0	0	0
ENSG00000236503	AC009784.1	0	0	0	0	0	0	0	0
ENSG00000226066	AC009541.1	0	0	0	0.04087	0	0	0	0
ENSG00000234352	AC009264.1	0	0	0	0	0	0	0	0
ENSG00000231244	PSMC1P3	0	0	0	0	0	0	0	0
ENSG00000181072	CHRM2	0	0	0	0	0	0	0	0
ENSG00000207597	MIR490	0	0	0	0	0	0	0	0
ENSG00000223657	KRT8P51	0	0	0	0	0	0	0	0
ENSG00000224469	AC009517.1	0	0	0	0	0	0	0	0
ENSG00000105894	PTN	0.04279	0	0	0	0	0.07549	0	0
ENSG00000202023	RF00136	0	0	0	0	0	0	0	0
ENSG00000231114	AC078842.2	0	0	0	0	0	0	0	0
ENSG00000228031	AC078842.1	0	0	0	0	0	0	0	0
ENSG00000157680	DGKI	0.03771	0	0	0	0.01146	0	0.06286	0.01736
ENSG00000279483	AC090498.1	3.90935	6.50712	21.5428	13.0543	3.56467	6.89663	21.1779	20.3993
ENSG00000230383	AC009245.1	0.08504	0.28308	0	0.2524	0.20677	0	0	0
ENSG00000182158	CREB3L2	2.5857	2.3602	0.7109	1.51023	1.80505	2.23662	0.63954	0.84999
ENSG00000237243	AC022173.1	0	0	0	0	0	0	0	0
ENSG00000122787	AKR1D1	0	0	0	0	0	0	0	0
ENSG00000234639	AC009263.1	0	0	0	0	0	0	0	0
ENSG00000231923	AC024082.1	0	0	0	0	0	0	0	0
ENSG00000231931	AC024082.2	0	0	0	0	0	0	0	0
ENSG00000222514	RN7SKP223	0	0	0	0	0	0	0	0
ENSG00000233951	RCC2P3	0	0	0	0	0	0	0	0
ENSG00000284028	MIR4468	0	0	0	0	0	0	0	0
ENSG00000225559	AC083867.1	0	0	0	0	0	0	0	0
ENSG00000252820	RF00019	0	0	0	0	0	0	0	0
ENSG00000201465	RF00432	0	0	0	0	0	0	0	0
ENSG00000213238	AC008155.1	0	0	0	0	0	0	0	0
ENSG00000213237	PTMAP10	0	0	0	0	0	0	0	0
ENSG00000214815	IMPDH1P3	0	0	0	0	0	0	0	0
ENSG00000122779	TRIM24	6.86669	6.95831	2.86132	5.79137	8.44497	9.6742	6.37198	6.39142
ENSG00000234995	AC013429.2	0	0	0	0	0	0	0	0
ENSG00000157703	SVOPL	0.02326	0	0	0.01726	0	0	0.01939	0
ENSG00000252188	RF00561	0	0	0	0	0	0	0	0
ENSG00000234658	AC013429.1	0	0	0	0	0	0	0	0
ENSG00000243099	AC020983.1	0	0	0	0	0	0	0	0
ENSG00000223721	UQCRFS1P2	0	0	0	0	0	0	0	0
ENSG00000105929	ATP6V0A4	0	0	0	0	0	0	0	0
ENSG00000214128	TMEM213	0	0	0	0	0	0	0	0
ENSG00000122778	KIAA1549	0.0118	0	0	0.00875	0.02868	0	0.00983	0.02897
ENSG00000199646	RNU6-1272P	0	0	0	0	0	0	0	0
ENSG00000146858	ZC3HAV1L	0.5854	0.8352	0.18434	0.62057	0.30502	1.10649	0.27879	0.46205
ENSG00000105939	ZC3HAV1	35.5598	45.9143	13.4855	26.5128	34.1425	35.284	24.2877	25.3653
ENSG00000229677	AC018644.1	1.72941	8.19295	1.36842	1.38203	3.71993	2.58154	1.5522	2.77668
ENSG00000105948	TTC26	0.19062	1.36677	0.53868	0.69637	0.49915	0.36215	0.61102	1.04417
ENSG00000239254	AC009220.2	0	0	0	0	0	0	0	0
ENSG00000271537	AC009220.3	0	0	0	0	0	0	0	0
ENSG00000228360	AC009220.1	0	0	0	0	0	0	0	0
ENSG00000271404	MZT1P2	0	0	0	0	0	0	0	0
ENSG00000157741	UBN2	3.89182	3.70806	2.70172	4.85971	4.49227	4.49032	5.44316	3.98742
ENSG00000206843	RNU6-206P	0	0	0	0	0	0	0	0
ENSG00000164898	FMC1	0.19239	0.08006	0.07068	0.14276	0.58476	0	0	0.17716
ENSG00000146963	LUC7L2	2.70408	1.97037	1.00619	1.68794	2.6244	2.12926	2.10856	2.05186

ENSG00000269955	C7orf55-LUC7L2	0	0	0	0	0	0	0	0
ENSG00000273391	AC083880.1	0.41103	0.22805	0	0.20334	0	0.2417	0.45675	0.50466
ENSG00000252332	RNU6-911P	0	0	0	0	0	0	0	0
ENSG00000188883	KLRG2	0	0	0	0	0	0	0	0
ENSG00000253183	AC005531.1	0	0	0	0.07999	0.06553	0	0	0
ENSG00000236279	CLEC2L	0.58014	0.24141	0	0	0.28213	0.20469	0.0967	0.21369
ENSG00000254270	ERHP1	0	0	0	0	0	0	0	0
ENSG00000064393	HIPK2	7.31956	4.951	0.69935	1.68808	4.46683	4.23987	1.8166	2.24963
ENSG00000059377	TBXAS1	18.5852	13.0864	2.36276	4.31634	11.3121	4.04621	4.47338	10.1319
ENSG00000059378	PARP12	4.58346	8.34744	2.72498	1.88666	2.72242	5.94608	1.90512	3.49392
ENSG00000006459	KDM7A	25.3714	23.4701	9.94898	20.0267	29.3622	21.3508	28.5044	21.8742
ENSG00000251728	RF00019	3.05418	0	0	9.06548	6.49809	0	2.54542	2.81241
ENSG00000260231	KDM7A-DT	8.43878	4.25492	2.67022	3.8852	7.00202	12.7138	13.0394	4.0839
ENSG00000199971	RNU6-797P	0	0	0	0	0	0	0	0
ENSG00000199283	RNU1-58P	0	0	0	0	0	0	0	0
ENSG00000270512	AC005377.1	0	0	0	0	0	0	0	0
ENSG00000241661	PPP1R2P6	0	0	0	0	0	0	0	0
ENSG00000157800	SLC37A3	1.37767	3.19195	0.8342	1.52062	1.59077	1.16024	0.9924	2.68597
ENSG00000223113	RNA5SP247	0	0	0	0	0	0	0	0
ENSG00000202472	RNA5SP248	0	0	0	0	0	0	0.96205	0
ENSG00000146955	RAB19	0.3676	0.12238	0.21608	0.10911	0.26815	0.5188	0.36764	0.63188
ENSG00000103200	AC069335.1	0	0	0	0	0	0	0	0
ENSG00000133606	MKRN1	324.899	302.641	250.482	380.036	225.365	283.873	269.259	166.178
ENSG00000146966	DENND2A	0	0	0	0	0	0	0	0
ENSG00000201354	RF00019	0	0	0	0	0	0	0	0
ENSG00000240173	RN7SL771P	0	0	0	0	0	0	0	0
ENSG00000133597	ADCK2	1.98789	1.21464	0.40675	0.7469	2.75335	2.21956	0.67109	0.92685
ENSG00000090266	NDUFB2	1.61534	1.61873	0.70243	1.34545	1.1623	1.77378	1.09899	1.53807
ENSG00000240889	NDUFB2-AS1	0.04448	0.07403	0	0	0.16223	0.07847	0	0.05461
ENSG00000157764	BRAF	11.108	11.2723	4.06781	7.01133	12.9897	14.2766	9.4166	8.31476
ENSG00000271932	RNU6-85P	0	0	0	0	0	0	0	0
ENSG00000271611	AC006006.1	0	0	0	0	0	0	0	0
ENSG00000242703	CCT4P1	0	0	0	0	0	0	0	0
ENSG00000090263	MRPS33	1.14227	0.51011	0.53222	0.90965	1.03309	1.81852	0.58048	0.92357
ENSG00000223212	RNU4-74P	0	0	0	0	0	0	0	0
ENSG00000261115	TMEM178B	0.02752	0.01145	0.01011	0.01021	0.00836	0.02427	0	0.02534
ENSG00000261778	AC006362.1	0	0	0	0	0	0	0	0
ENSG00000261624	NDUFB10P2	0	0	0	0	0	0	0	0
ENSG00000204990	AC005692.1	0	0	0	0	0	0	0	0
ENSG00000261629	AC005692.2	0	0	0	0	0	0	0	0
ENSG00000261797	AC073878.2	0	0	0	0	0	0	0	0
ENSG00000261570	AC073878.1	0	0.19336	0	0	0	0	0	0
ENSG00000006530	AGK	1.64581	1.41632	0.57583	2.20994	1.91927	4.24653	1.30634	1.15469
ENSG00000244701	AC004918.1	0	0	0	0.14128	0	0	0	0.11688
ENSG00000257093	KIAA1147	0.61781	0.34831	0.1025	0.3993	0.67843	0.4219	0.28236	0.29363
ENSG00000270157	AC004918.3	0	0	0	0	0	0	0	0
ENSG00000228775	WEE2-AS1	0	0	0	0.03105	0.02543	0	0	0.02568
ENSG00000214102	WEE2	0	0	0	0	0	0	0	0
ENSG00000212153	RNU1-82P	0	0	0	0	0	0	0	0
ENSG00000106028	SSBP1	4.52616	3.17985	1.03654	1.57028	4.03776	5.10714	1.17575	2.2373
ENSG00000127362	TAS2R3	0.06658	0	0.19567	0	0	0.11745	0.22194	0
ENSG00000127364	TAS2R4	0.03082	0.10261	0	0.13724	0.1499	0	0	0.07569
ENSG00000270923	TAS2R6P	0	0	0	0	0	0	0	0.10896
ENSG00000127366	TAS2R5	0	0.106	0	0.18903	0.15485	0	0	0.07819
ENSG00000241926	MTCO1P55	0	0	0	0	0	0	0	0
ENSG00000224354	MTND2P5	0	0	0	0	0	0	0	0
ENSG00000239648	MTND1P3	0	0	0	0	0	0	0	0
ENSG00000240677	MYL6P4	0.16181	0	0	0	0	0	0	0.19867

ENSG00000165076	PRSS37	0	0	0	0	0	0	0	0
ENSG00000240031	OR9A3P	0	0	0	0	0	0	0	0
ENSG00000237621	OR9A1P	0	0	0	0	0	0	0	0
ENSG00000257335	MGAM	31.7315	37.372	3.29134	6.71891	26.85	2.54612	6.76321	20.3448
ENSG00000244292	OR9N1P	0	0	0	0	0	0	0	0
ENSG00000258083	OR9A4	0	0	0	0	0	0	0	0
ENSG00000258227	CLEC5A	1.50897	1.53656	0.36522	0.31616	0.66908	0.50109	0.14795	0.76287
ENSG00000257138	TAS2R38	0.06413	0.10674	0	0	0	0	0	0
ENSG00000257743	MGAM2	0.34127	0.70257	0.0132	0.14661	0.42582	0.09506	0.02994	0.24258
ENSG00000240268	MOXD2P	0	0	0	0	0	0	0	0
ENSG00000258223	PRSS58	0	0	0	0	0	0	0	0
ENSG00000186163	TRY2P	0	0	0	0	0	0	0	0
ENSG00000241881	AC245088.1	0	0	0	0	0	0	0	0
ENSG00000277083	PRSS3P3	0	0	0	0	0	0	0	0
ENSG00000242736	TRBV1	0	0	0	0	0	0	0	0
ENSG00000226660	TRBV2	3.83042	2.60826	1.02341	1.29199	5.71532	3.993	1.45107	2.56524
ENSG00000237702	TRBV3-1	3.93685	2.80838	0	1.1129	6.38176	1.32288	0.62496	0.46034
ENSG00000211710	TRBV4-1	6.48502	0.6542	0.28878	1.45825	7.16756	5.89357	2.29293	0.72384
ENSG00000211734	TRBV5-1	2.02363	0.18713	1.48685	1.0011	2.32359	1.78498	0.56218	2.34655
ENSG00000211706	TRBV6-1	0.91397	0.30426	0	0	1.55565	0.96742	1.21876	0.89773
ENSG00000211707	TRBV7-1	0	0	0	0	0	0	0	0
ENSG00000211745	TRBV4-2	1.1277	0.5363	0.23673	0.95636	1.37103	1.13681	0	1.38457
ENSG00000283063	TRBV6-2	0.34576	0	0.50808	0.51314	0.42036	1.21992	0.57632	1.27354
ENSG00000282939	TRBV7-2	2.60458	2.47733	1.09354	1.65664	5.8808	5.25124	1.24041	3.19787
ENSG00000243889	TRBV8-1	0	0	0	0	0	0	0	0
ENSG00000242771	TRBV5-2	0	0	0	0	0	0	0	0
ENSG00000211713	TRBV6-4	0.20082	0	0	0	0	0	0	0
ENSG00000211714	TRBV7-3	1.10781	0	0.54264	0	0.67343	1.62861	0.30776	0.22669
ENSG00000239944	TRBV8-2	0	0	0	0	0	0	0	0
ENSG00000211715	TRBV5-3	0	0	0	0	0	0	0	0
ENSG00000211716	TRBV9	0.7518	0	0	0	2.28504	0.33157	0.93985	0.69229
ENSG00000211717	TRBV10-1	0	0	0	0	0.64891	0	0	0
ENSG00000211720	TRBV11-1	0.20137	0	0	0	0.24483	0	0	0
ENSG00000244661	TRBV12-1	0	0	0	0	0	0	0	0
ENSG00000229769	TRBV10-2	0.55671	0.61776	0	0.27541	0.45122	0.32737	0	0
ENSG00000241657	TRBV11-2	0.35932	4.18657	0.264	1.59979	2.40266	0.63388	0.59892	0.66174
ENSG00000211739	TRBV12-2	0	0.35468	0	0	0	0	0	0
ENSG00000211721	TRBV6-5	1.07269	0.59516	0.78815	0.53066	2.60829	2.20776	1.788	1.31703
ENSG00000253409	TRBV7-4	1.47868	1.05483	0	0	1.2841	0.74531	2.11263	1.29679
ENSG00000230099	TRBV5-4	2.42538	0.67284	0.59401	0.99987	2.12963	2.37705	0.67379	0.49631
ENSG00000211724	TRBV6-6	0.37784	0.31445	0.55523	0.84113	1.60777	0.99983	0.3149	0.69585
ENSG00000227550	TRBV7-5	0.63739	0	0	0	0.25831	0.74963	0.70829	0
ENSG00000211725	TRBV5-5	0.41886	0	0	0	0.76386	0.36946	0	0
ENSG00000253188	TRBV6-7	0	0	0	0	0	0	0	0
ENSG00000211727	TRBV7-6	0.18699	1.24498	0	0.55503	0.68202	3.29877	0.93505	1.14792
ENSG00000211728	TRBV5-6	1.14532	0	0	0.56659	1.62452	0.6735	0.95453	0.7031
ENSG00000253534	TRBV6-8	0	0	0	0	0	0	0	0
ENSG00000253291	TRBV7-7	0.21124	0	0	0	0	0	0	0
ENSG00000211731	TRBV5-7	0	0	0	0	0	0	0	0
ENSG00000278030	TRBV7-9	2.53488	8.08702	1.24166	0.3135	1.79774	14.9063	2.81684	3.37165
ENSG00000276405	TRBV13	0.58797	0	0.28801	0.58174	0.23828	0.34575	0	0
ENSG00000275791	TRBV10-3	2.7886	0.33154	0	0.59123	1.93732	2.81113	0.33201	0.73367
ENSG00000276597	TRBV11-3	0.37398	0.31125	0	0	0	0.32988	0	0
ENSG00000274752	TRBV12-3	1.25453	1.19324	0.26336	1.06392	1.74311	2.21316	0.59746	0.44008
ENSG00000276953	TRBV12-4	1.62488	3.90668	1.32653	0.53589	2.41449	0.95551	0.60187	2.21668
ENSG00000275158	TRBV12-5	0.35669	0	0.26208	0	0.86732	0.31463	0	0
ENSG00000275743	TRBV14	0	0	0	0.53589	0.878	0.637	0.60187	0.22167
ENSG00000276819	TRBV15	1.39288	0.28981	0.25585	1.03359	3.59854	2.15008	0.87064	0.42754

ENSG00000275243	TRBV16	0	0	0	0	0	0	0	0.25936
ENSG00000277880	TRBV17	0	0	0	0	0	0	0	0
ENSG00000276557	TRBV18	1.3701	0	0	0.76252	1.66573	1.81278	0.57094	0.63082
ENSG00000211746	TRBV19	0.66395	3.09442	0	1.37953	2.74454	2.57687	1.10671	1.79342
ENSG00000211747	TRBV20-1	0.88741	1.4771	0.78243	1.05361	4.0998	8.7669	1.18334	3.26866
ENSG00000251578	TRBV21-1	0.21063	0	0	0	0.76825	0.74317	0	0.51722
ENSG00000240578	TRBV22-1	0	0	0	0	0	0	0	0
ENSG00000211749	TRBV23-1	0	0.35161	0	0	0	0.37266	0	0.25936
ENSG00000211750	TRBV24-1	0.57717	0.32023	0	0.28553	0.4678	0	0.32068	0.23621
ENSG00000270672	MTRNR2L6	0	0	0	0	0	0	0	0
ENSG00000282499	TRBV25-1	0.76956	0	0	0	0.70171	0.6788	0.32068	1.18106
ENSG00000239992	TRBVA	0	0	0	0	0	0	0	0
ENSG00000249912	TRBV26	0	0	0	0	0	0	0	0
ENSG00000241911	TRBVB	0	0	0	0	0	0	0	0
ENSG00000211752	TRBV27	2.08347	0.9458	0	0.8433	2.99358	5.68036	1.57856	1.8604
ENSG00000211753	TRBV28	6.24261	2.01113	1.18367	2.09203	9.54821	3.55252	4.69924	0.98898
ENSG00000244273	PGBD4P1	0.1801	0	0	0.26729	0	0	0	0.22112
ENSG00000232869	TRBV29-1	3.2821	2.42803	0.26795	0.27061	5.98545	5.14674	1.51966	1.79099
ENSG00000204983	PRSS1	0	0	0	0	0	0	0	0
ENSG00000275896	PRSS2	0.23288	0	0	0.08641	0	0	0	0
ENSG00000250591	PRSS3P1	0	0	0	0	0	0	0	0
ENSG00000243661	WBP1LP1	0.08181	0	0	0	0	0.14432	0	0
ENSG00000282431	TRBD1	0	0	0	0	0	0	0	0
ENSG00000282320	TRBJ1-1	1.52709	0	0	0	0	0	0	0
ENSG00000282420	TRBJ1-2	0	0	0	0	0	0	0	0
ENSG00000282133	TRBJ1-3	2.93201	2.44017	0	0	0	2.58624	0	3.59988
ENSG00000281958	TRBJ1-4	4.31178	0	0	2.13305	0	0	4.79139	0
ENSG00000282173	TRBJ1-5	0	0	0	0	0	0	0	0
ENSG00000282780	TRBJ1-6	5.5321	6.90614	0	4.10512	11.7701	2.43985	20.7476	0
ENSG00000211751	TRBC1	42.5335	18.4618	4.81878	18.0355	49.4832	71.1215	16.3979	18.1178
ENSG00000211764	TRBJ2-1	0	0	0	0	0	0	0	0
ENSG00000211765	TRBJ2-2	0	7.17697	0	0	3.49477	0	0	1.76465
ENSG00000211766	TRBJ2-2P	0	0	0	0	0	0	0	1.95646
ENSG00000211767	TRBJ2-3	1.49593	2.48997	0	2.22012	0	0	2.49348	0
ENSG00000211768	TRBJ2-4	0	0	0	0	0	0	0	0
ENSG00000211769	TRBJ2-5	0	0	0	0	0	0	0	0
ENSG00000211770	TRBJ2-6	0	0	0	0	0	0	0	0
ENSG00000211771	TRBJ2-7	20.2746	5.19185	0	2.31459	30.3376	30.2645	0	3.82966
ENSG00000211772	TRBC2	59.9554	29.134	9.09458	23.3932	91.7032	143.301	19.0202	21.3713
ENSG00000237254	TRBV30	0.65593	0	0.24097	0.73011	0.99683	1.15715	0	0.40267
ENSG00000106123	EPHB6	0.79839	0.27495	0.04046	0.16343	1.17149	0.26712	0.34417	0.28731
ENSG00000165125	TRPV6	0	0	0	0	0	0	0	0
ENSG00000224970	AC245427.1	0	0	0	0	0	0	0	0
ENSG00000127412	TRPV5	0	0	0	0	0	0	0	0
ENSG00000165131	LLCFC1	0	0.13219	0	0	0	0	0	0
ENSG00000197993	KEL	1.21451	0.71532	1.18065	1.44197	1.90818	4.4829	1.7441	1.65174
ENSG00000179468	OR9A2	0	0	0	0	0	0	0	0
ENSG00000239293	OR9P1P	0	0	0	0	0	0	0	0
ENSG00000225781	OR6V1	0	0	0	0	0	0	0	0
ENSG00000179420	OR6W1P	0	0	0	0	0	0	0	0
ENSG00000159763	PIP	0	0	0	0	0	0	0	0
ENSG00000236398	TAS2R39	0	0	0	0	0	0	0	0
ENSG00000268170	AC073342.2	0.40722	1.22008	0.17952	0.06044	0.89117	0	0.20363	0.74998
ENSG00000221937	TAS2R40	0	0	0	0	0	0	0	0
ENSG00000279223	AC073342.3	0	0	0	0	0	0	0	0
ENSG00000197448	GSTK1	14.2509	11.1966	2.77692	5.32607	12.3094	11.8057	4.73926	8.15252
ENSG00000231840	AC073342.1	0	0	0	0	0	0.27809	0	0.09677
ENSG00000178826	TMEM139	0.05718	0.04759	0	0.04243	0	0	0	0

ENSG00000106144	CASP2	2.60481	2.12982	0.56409	1.19366	2.34459	2.48304	0.83789	1.35781
ENSG00000239419	RN7SL535P	0	0	0	0.73504	0	0.43686	0.41277	0.30404
ENSG00000240322	RN7SL481P	0.4903	0	0	0.36383	0.29805	0.43248	0.81726	0.30099
ENSG00000231531	HINT1P1	0	0	0	0	0	0	0	0
ENSG00000188037	CLCN1	0.1552	0.11072	0.03258	0	0.08087	0	0	0
ENSG00000159784	FAM131B	0.0114	0.0759	0.01675	0.03384	0.08316	0.02011	0	0.13996
ENSG00000272619	AC093673.2	0	0	0	0	0	0	0	0
ENSG00000232533	AC093673.1	6.56083	6.2134	1.16358	1.34303	4.67587	3.79155	0.7542	4.72207
ENSG00000159840	ZYX	61.9958	77.543	6.37251	11.5284	44.2256	30.2668	8.98282	16.6709
ENSG00000278449	MIR6892	0	0	0	0	0	0	0	0
ENSG00000146904	EPHA1	0.41815	0.12889	0	0.09194	0.37658	0.27321	0.12907	0.17113
ENSG00000229153	EPHA1-AS1	0.6067	0.11476	0.06079	0.12278	0.46939	0.07298	0.09193	0.01693
ENSG00000234066	TAS2R62P	0.07798	0	0	0	0.0948	0	0	0
ENSG00000185899	TAS2R60	0.30638	0	0	0	0.4656	0	0	0
ENSG00000221855	TAS2R41	0.07933	0	0	0	0	0	0	0
ENSG00000236853	OR2R1P	0	0.13349	0	0	0	0	0	0
ENSG00000176510	OR10AC1	0	0	0	0	0	0	0	0
ENSG00000229977	AC073264.1	0	0	0	0	0	0	0	0
ENSG00000229877	PAICSP5	0	0	0	0	0	0	0	0
ENSG00000271079	CTAGE15	0.02833	0	0	0	0	0	0	0
ENSG00000252037	RNU6-162P	0	0	0	0	0	0	0	0
ENSG00000230556	AC073264.2	0	0	0	0	0	0	0	0
ENSG00000223459	TCAF1P1	0.26273	0.10933	0.04826	0.04874	0.11978	0.05794	0.05474	0
ENSG00000170379	TCAF2	0.51013	0.30191	0.16659	0.43743	0.63399	0.95994	0.66135	0.32013
ENSG00000283537	AC073264.3	0	0	0	0	0	0	0	0
ENSG00000283528	TCAF2C	0	0	0.0418	0	0	0	0	0.03492
ENSG00000252523	RNU6-267P	0	0	0	0	1.73042	0	0	0
ENSG00000271321	CTAGE6	0	0	0	0	0	0.14875	0.04685	0
ENSG00000253882	AC099548.2	0.1011	0.06732	0.02971	0.06002	0.07375	0.07134	0.06741	0.04965
ENSG00000241136	PAICSP6	0	0	0	0	0	0	0	0
ENSG00000232145	AC099548.1	0	0	0	0	0	0	0	0
ENSG00000159860	TCAF2P1	0.12111	0	0	0	0	0	0.10093	0.03717
ENSG00000198420	TCAF1	1.23135	0.77343	0.20484	0.49997	0.83326	0.73775	0.23236	0.51345
ENSG00000221910	OR2F2	0	0	0	0	0	0	0	0
ENSG00000243583		0	0	0	0	0	0	0	0
ENSG00000213215	OR2F1	0	0	0	0	0	0	0	0
ENSG00000270729	AC004853.1	0	0	0	0	0	0	0	0
ENSG00000273377	OR2Q1P	0	0	0	0	0	0	0	0
ENSG00000242280	SLC16A1P1	0	0	0	0	0	0	0	0
ENSG00000221813	OR6B1	0	0	0	0	0	0	0	0
ENSG00000221836	OR2A5	0	0	0	0	0	0	0	0
ENSG00000221933	OR2A25	0	0	0	0	0	0	0	0
ENSG00000239967	OR2A41P	0	0	0	0	0	0	0	0
ENSG00000221858	OR2A12	0	0	0	0	0	0	0	0
ENSG00000221989	OR2A2	0	0	0	0	0	0	0	0
ENSG00000239981	OR2A15P	0	0	0	0	0	0	0	0
ENSG00000221938	OR2A14	0	0	0	0	0	0	0	0
ENSG00000273234	OR2A13P	0	0	0	0	0	0	0	0
ENSG00000183122	OR2A3P	0	0	0	0	0	0	0	0
ENSG00000240621	OR2AO1P	0	0	0	0	0	0	0	0
ENSG00000225932	CTAGE4	0.08497	0.04714	0	0	0.03443	0.09993	0	0
ENSG00000213214	ARHGEF35	0.18091	0	0	0	0.21995	0.15958	0.05026	0.03702
ENSG00000244198	AC004889.1	0.13037	0.16275	0	0.14511	0.27738	0.17249	0.21731	0.04002
ENSG00000212807	OR2A42	0	0	0	0	0	0	0	0
ENSG00000170356	OR2A20P	0.05033	0	0	0	0.03059	0.04439	0	0
ENSG00000244479	OR2A1-AS1	0.26746	0.10475	0.09248	0.02335	0.30605	0.19429	0.02622	0.17385
ENSG00000243896	OR2A7	0	0	0	0	0	0	0	0
ENSG00000244693	CTAGE8	0.02832	0	0	0	0	0	0	0

ENSG00000204959	ARHGEF34P	0.09704	0.16153	0	0	0.05899	0.19973	0.10784	0.01986
ENSG00000228960	OR2A9P	0	0	0	0	0.02671	0	0.07323	0.02697
ENSG00000221970	OR2A1	0	0	0	0	0	0	0	0
ENSG00000050327	ARHGEF5	0.25198	0.23068	0.03703	0.05609	0.21444	0.31117	0.021	0.06187
ENSG00000106410	NOBOX	0	0	0	0	0	0	0	0
ENSG00000242978	PPIAP83	0	0	0	0	0	0	0	0
ENSG00000221507	RNU6ATAC40P	0	0	0	0	0	0	0	0
ENSG00000196511	TPK1	1.78866	1.84993	0.38278	1.13399	1.82625	1.302	1.02758	1.36456
ENSG00000243746	EEF1A1P10	0.10547	0.78998	0	0.23479	0.32056	0.37212	0	0.06475
ENSG00000200673	RN7SKP174	0	0	0	0	0	0	0	0
ENSG00000236343	EI24P4	0	0	0	0	0	0	0	0
ENSG00000214035	AC073310.1	0	0	0	0	0	0	0	0
ENSG00000225918	RPL7P59	0	0	0	0	0.0945	0	0	0
ENSG00000226592	AC004911.1	0	0	0	0	0	0	0	0
ENSG00000231839	DPY19L4P2	0	0	0	0	0	0	0	0
ENSG00000230746	AC006007.1	0	0	0	0	0	0	0	0
ENSG00000280144	AC073308.1	0	0	0	0	0	0	0	0
ENSG00000174469	CNTNAP2	0.0044	0.00732	0	0.00653	0.01069	0.03879	0.00733	0
ENSG00000274127	RF02271	0	0	0	0	0	0	0	0
ENSG00000274118	RF00019	0	0	0	0	0	0	0	0
ENSG00000236795	AC006004.1	0	0	0	0	0	0	0	0
ENSG00000226857	DUTP3	0	0	0	0	0	0	0	0
ENSG00000232391	RANP2	0	0	0	0	0	0	0	0
ENSG00000221442	MIR548F4	0	0	0	0	0	0	0	0
ENSG00000230190	AC005518.1	0	0	0	0	0	0	0	0
ENSG00000270634	AC005518.2	0	0	0	0	0	0	0	0
ENSG00000207318	RNU6-1184P	0	0	0	0	0	0	0	0
ENSG00000251936	RNA5SP249	0	0	0	0	0	0	0	0
ENSG00000242266	RN7SL456P	0	0	0	0	0	0	0	0
ENSG00000199370	RF00012	0	0	0	0	0	0	0	0
ENSG00000243374	RN7SL72P	0	0	0	0	0	0	0	0
ENSG00000274133	AC083849.1	0	0	0	0	0	0	0	0
ENSG00000283504	AC006974.1	0	0	0	0	0	0	0	0
ENSG00000283648	AC006974.2	0	0	0	0	0	0.10539	0	0
ENSG00000213210	AC005229.2	0	0	0	0	0	0	0	0
ENSG00000170279	C7orf33	0	0	0	0	0	0	0	0
ENSG00000213209	AC005229.1	0	0	0	0	0	0	0	0
ENSG00000270200	AC005229.3	0	0	0	0	0	0	0	0
ENSG00000273314	AC005229.4	0.23344	0.33305	0	0.04949	0.20272	0.23533	0.05559	0.08189
ENSG00000055130	CUL1	14.5857	12.3109	5.06996	8.21719	14.8946	20.082	7.02502	10.3745
ENSG00000106462	EZH2	13.9404	22.3134	28.6554	61.2972	30.5464	34.4298	61.3604	37.6149
ENSG00000251712	RNU7-20P	0	0	0	0	0	0	0	0
ENSG00000275586	RF00017	0	0	0	0	0	0	0	0
ENSG00000239468	RN7SL569P	0.25452	0	0	0.37773	0	0	0	0
ENSG00000252316	RNY4	5.34482	3.81277	273.773	239.102	12.0679	30.9809	156.544	196.869
ENSG00000202354	RNY3	0	2.39232	2.11204	8.53221	0.87369	0	3.59354	2.64697
ENSG00000201098	RNY1	520.238	639.195	5555.37	10716.8	654.574	1277.1	4346.59	2992.2
ENSG00000281189	GHET1	0	0.12803	0	0	0.04676	0.06784	0	0.18887
ENSG00000155660	PDIA4	18.23	11.5046	2.89253	5.77623	14.9026	13.8901	3.98941	5.24548
ENSG00000202528	RNU6-650P	0	0	0	0	0	0	0	0
ENSG00000234565	COX6B1P1	0	0	0	0	0	0	0	0
ENSG00000197362	ZNF786	1.14287	1.5666	0.26344	0.53212	1.63467	0.83019	1.15793	0.82541
ENSG00000204947	ZNF425	0.15905	0.56256	0.32136	0.26555	0.26588	0.24551	0.23197	0.09764
ENSG00000240877	RN7SL521P	0.2468	0	0	1.83141	0.60011	0.43539	0	0
ENSG00000197024	ZNF398	3.84642	2.94872	0.71322	1.54868	3.28969	2.39744	1.67869	1.74303
ENSG00000239719	AC004890.1	0	0	0	0	0	0	0	0.99444
ENSG00000170265	ZNF282	2.71624	1.87852	0.59029	0.56778	1.90699	3.27329	0.73334	0.93943
ENSG00000170260	ZNF212	0.76019	1.38036	0.0677	0.30769	0.75618	0.65022	0.57596	0.5091

ENSG00000204946	ZNF783	1.07891	0.63489	0.09609	0.29113	1.05997	0.80748	0.58129	0.53522
ENSG00000271664	AC004890.3	0	0	0	0	0	0	0	0
ENSG00000244560	AC004890.2	0.1373	0.02857	0	0.07641	0.04173	0.09083	0.05721	0.06321
ENSG00000231397	AC004941.2	0	0	0	0	0	0	0	0
ENSG00000215311	NPM1P12	0	0	0	0	0	0	0	0
ENSG00000228151	AC004941.1	0	0	0	0	0	0	0	0
ENSG00000261842	AC073314.1	0	0	0	0	0	0	0.05514	0
ENSG00000196453	ZNF777	1.51906	1.54728	0.26654	0.40378	2.28787	2.55984	0.64246	0.69593
ENSG00000181220	ZNF746	4.65892	4.56424	0.41729	1.51457	5.22185	2.31697	1.64189	3.09433
ENSG00000133624	ZNF767P	1.99146	2.28082	0.72489	1.68114	2.53223	1.80495	2.00995	1.86185
ENSG00000133619	KRBA1	0.38456	0.29719	0.04036	0.1223	0.55103	0.36344	0.11447	0.1349
ENSG00000181444	ZNF467	11.7967	6.78321	0.74856	0.83559	6.12799	2.12839	1.16192	2.89676
ENSG00000197558	SSPO	0.03813	0.02885	0	0.01029	0.01264	0.00612	0.03467	0.01277
ENSG00000106479	ZNF862	1.49557	0.98417	0.16611	0.60652	1.05714	0.69028	0.36234	0.49109
ENSG00000280149	AC004877.2	0.8566	1.01843	0	0.18161	0.44633	0	0.61192	0.60098
ENSG00000273419	AC004877.1	0.01791	0	0	0	0.06532	0	0.0597	0
ENSG00000204934	ATP6V0E2-AS1	0.15591	0.11122	0	0.13222	0.18955	0.03929	0.1485	0.21877
ENSG00000171130	ATP6V0E2	2.32979	0.76827	0.38758	0.97858	2.61872	5.38961	0.84262	0.9445
ENSG00000273293	AC093458.1	0	0	0	0	0	0	0	0
ENSG00000279536	AC093458.2	0.11653	0	0	0	0	0	0	0
ENSG00000273011	AC092681.3	0	0	0	0	0	0	0	0
ENSG00000224016	AC092681.1	0.25189	2.09637	0	0.74767	0	0.44437	1.25959	0.61854
ENSG00000260555	AC092681.2	0	0	0	0	0	0	0	0
ENSG00000241449	AC092666.1	0	0	0	0	0	0	0	0
ENSG00000276538	AC092666.2	0	0	0	0	0	0	0	0
ENSG00000106526	ACTR3C	0.1929	0.24553	0.11672	0.1684	0.31729	0.48042	0.18913	0.12538
ENSG00000127399	LRRC61	0.75042	0.80298	0.11815	0.39775	1.36852	0.70921	0.40206	0.69102
ENSG00000188707	ZBED6CL	1.89936	0.79037	0.2938	0.51926	1.7319	2.11625	0.66651	1.01258
ENSG00000106538	RARRES2	0	0	0	0	0.04639	0	0	0
ENSG00000261305	AC005586.2	0	0	0	0	0.21294	0	0	0
ENSG00000240449	AC005586.1	0.12704	0	0	0	0.46334	0	0	0
ENSG00000214022	REPIN1	5.17154	2.6244	0.78776	1.75499	3.96798	3.61595	1.86595	1.45188
ENSG00000284041	AC073111.3	0	0	0	0	0	0	0	0
ENSG00000284048	AC073111.4	1.8517	0.82692	0.33184	0.93839	2.03162	2.31056	0.90337	0.60996
ENSG00000196456	ZNF775	0.15367	0.30694	0.04516	0.18245	0.41102	0.27109	0	0.07547
ENSG00000239377	AC073111.1	0	0	0	0	0	0	0	0
ENSG00000242258	LINC00996	0.16882	0.0562	0	0.20043	0.16419	0.35738	0.28139	0.12436
ENSG00000171115	GIMAP8	17.0637	24.67	3.26952	6.65611	15.3995	10.8481	3.65022	10.6689
ENSG00000271582	AC073111.2	0.34093	0	0	0.20239	0.0829	0	0	0.33487
ENSG00000213205	STRADBP1	0.05722	0	0.25226	0.08492	0.13914	0.20189	0.66765	0.28102
ENSG00000179144	GIMAP7	49.5478	70.1355	12.0921	20.4406	100.256	84.5263	25.6812	46.6466
ENSG00000243853	ALDH7A1P3	0	0	0	0.31532	0	0.37482	0	0
ENSG00000133574	GIMAP4	129.721	198.618	29.5685	46.1831	159.215	172.946	31.4783	80.1889
ENSG00000271568	AC069304.3	0	0	0	0.10036	0	0.11929	0	0
ENSG00000232361	AC069304.1	0.10412	0	0	0	0	0	0	0.25567
ENSG00000133561	GIMAP6	17.9458	19.2382	4.48693	8.36813	19.8366	25.2807	8.27441	14.5357
ENSG00000270990	AC069304.2	1.85357	0	0.49524	0.50016	0.81946	0	0.28087	0.20689
ENSG00000106560	GIMAP2	20.134	28.4512	12.6873	15.6668	24.8609	33.7308	11.7694	24.4627
ENSG00000213203	GIMAP1	8.62179	7.80492	0.95578	2.94076	9.80173	9.55296	1.9918	3.84428
ENSG00000281887	GIMAP1-GIMAP5	0	0	0	0	0	0	0	0
ENSG00000196329	GIMAP5	2.74679	0.82944	0.41078	1.11834	3.14738	4.09527	3.50476	1.62654
ENSG00000177590	GIMAP3P	0	0	0	0	0.2132	0.15468	0	0.10765
ENSG00000241134	BET1P1	0	0	0	0.30302	0	0	0	0
ENSG00000106565	TMEM176B	0.8519	30.0243	2.23157	4.12275	13.4643	7.25296	0.74086	0.72762
ENSG00000002933	TMEM176A	0.25283	5.92683	0.52634	0.813	3.1251	0.92923	0.14048	0.07761
ENSG00000002726	AOC1	0.80508	0.86146	0.14084	0.25603	0.69914	0.03382	0.03195	0.14121
ENSG00000055118	KCNH2	0.57373	0.41521	0.3299	0.61084	0.45491	0.7041	0.41579	0.2297
ENSG00000164867	NOS3	0.09851	0.06149	0.03619	0.01827	0.31437	0.08689	0.06157	0.06047

ENSG00000181652	ATG9B	0.09175	0.05727	0.03371	0	0.25099	0.26303	0.05735	0.04225
ENSG00000197150	ABCB8	0.5496	0.33978	0.12691	0.2447	0.54409	0.49863	0.20939	0.29883
ENSG00000243433	AC010973.1	0	0	0	0	0	0	0	0
ENSG00000213199	ASIC3	0.18672	0.0888	0	0.07917	0.09729	0	0.04446	0
ENSG00000164885	CDK5	1.17261	1.12125	0.07332	0.25919	1.00097	1.10034	0.20793	0.30632
ENSG00000164889	SLC4A2	1.8639	1.67179	0.31221	0.58764	1.30329	1.43112	0.35415	0.85373
ENSG00000244151	AC010973.2	0.29797	0	0	0	0	0.21026	0	0
ENSG00000164896	FASTK	2.25391	1.44294	0.28309	0.80053	1.85025	1.90314	0.86698	1.20627
ENSG00000164897	TMUB1	5.48382	2.98175	0.64467	0.81386	3.51133	7.2234	0.85313	1.03239
ENSG00000133612	AGAP3	0.98336	0.68569	0.22457	0.26624	0.46852	0.52747	0.29903	0.20395
ENSG00000164900	GBX1	0	0	0	0	0	0	0	0
ENSG00000146926	ASB10	0	0	0	0	0	0	0	0
ENSG00000278685	IQCA1L	0	0	0	0.03849	0	0	0	0
ENSG00000033050	ABCF2	4.33408	2.66071	1.13816	2.12778	4.04712	4.448	1.20862	1.98285
ENSG00000033100	CHPF2	8.69842	6.44135	1.33202	2.61293	5.00155	4.73579	3.22521	3.95944
ENSG00000284191	MIR671	0	0	0	0	0	0	0	0
ENSG00000082014	SMARCD3	1.21274	1.38486	0.10361	0.293	0.85722	0.72144	0.4466	0.6233
ENSG00000272661	AC021097.1	0.3831	0.63768	0.18765	0.18952	0.15526	0.22528	0	0.47037
ENSG00000243018	AC005486.1	0	0	0	0	0	0	0	0
ENSG00000013374	NUB1	22.8124	28.1627	5.79278	8.14234	18.3306	19.5246	6.68368	13.8214
ENSG00000187260	WDR86	0.19938	0.03017	0	0	0	0.03198	0	0.02225
ENSG00000243836	WDR86-AS1	1.40754	0.45055	0	0.40172	0.32909	0.76403	0.36095	0.26587
ENSG00000241456	AC005996.1	0	0	0	0	0	0	0	0
ENSG00000127377	CRYGN	0	0	0	0	0.03281	0	0	0
ENSG00000265810	MIR3907	0	0	0	0	0	0	0	0
ENSG00000241959	RN7SL76P	0	0	0	0	0	0	0	0.31141
ENSG00000106615	RHEB	35.4371	45.9387	28.7034	85.3668	57.7557	50.3347	73.2736	49.9248
ENSG00000240132	ETF1P2	0	0	0	0	0	0	0	0
ENSG00000106617	PRKAG2	4.65468	4.68846	0.97486	1.01684	2.82952	2.30229	1.1783	1.69579
ENSG00000242048	AC093583.1	0.09201	0.05105	0	0	0	0.05411	0	0
ENSG00000239911	PRKAG2-AS1	0.6933	0.20982	0	0.18708	0.07663	0.11119	0.31517	0.23215
ENSG00000212219	RNU6-604P	0	0	0	0	0	0	0	0
ENSG00000222941	RF00019	0	0	0	0	0	0	0	0
ENSG00000227562	AC074257.1	0	0	0	0	0	0	0	0
ENSG00000106648	GALNTL5	0	0	0	0	0	0	0	0
ENSG00000276213	RF00017	0	0	0	0	0.31941	0.46348	0	0
ENSG00000178234	GALNT11	2.05011	1.70621	0.40255	0.9836	1.14955	1.87069	0.97214	0.87881
ENSG00000229591	AC006017.1	0.43566	0.54387	0.3201	0	0.26483	0.76857	0.54464	0.26745
ENSG00000213188	YBX1P4	0	0.151	0	0	0	0	0	0.22277
ENSG00000055609	KMT2C	18.6403	17.2331	5.70326	9.55134	16.021	12.8772	10.9278	11.159
ENSG00000270405	AC104692.2	0	0	0	0	0	0	0	0
ENSG00000204894	AC104692.1	0	0	0	0	0	0	0	0
ENSG00000241007	SEPT7P6	0	0.10674	0	0	0.2339	0	0	0.07874
ENSG00000253088	RF00019	0	0	0	0	0	0	0	0
ENSG00000241735	FABP5P3	0.07137	0.1188	0	0	0.08677	0	0	0
ENSG00000020219	CCT8L1P	0.04379	0	0	0	0	0	0	0
ENSG00000261455	LINC01003	2.03612	1.38332	0.30531	0.49336	1.41455	1.68604	0.90042	0.8163
ENSG00000231139	AC104843.2	0	0	0	0	0	0	0	0
ENSG00000199404	RNA5SP250	0	0	0	0	0	0	0	0
ENSG00000225416	AC104843.1	0	0	0.33766	0	0.27936	0.40537	0	0
ENSG00000212590	RF00568	0	0	0	0	0	0	0	0
ENSG00000196584	XRCC2	0.25677	0.42739	0.19976	0.51557	0.44072	0.74608	0.47835	0.42653
ENSG00000214003	ATP5PBP3	0	0	0	0	0	0	0	0
ENSG00000244328	RN7SL845P	0	0	0	0	0	0	0	0
ENSG00000133627	ACTR3B	1.02212	0.81015	0.35762	0.81264	0.90241	1.00891	0.97355	1.06072
ENSG00000224104	AC079809.1	0	0	0	0	0	0	0	0
ENSG00000234722	LINC01287	0	0	0	0	0	0	0	0
ENSG00000233489	PAXBP1P1	0	0	0	0	0	0	0	0

ENSG00000130226	DPP6	0	0	0	0	0	0	0	0
ENSG00000226509	AC005588.1	0	0	0	0	0	0	0	0
ENSG00000270114	AC006019.3	0	0	0	0	0	0	0	0
ENSG00000203335	AC006019.1	0	0	0	0	0	0	0	0
ENSG00000233363	AC006019.2	0	0	0	0	0	0	0	0
ENSG00000236408	AC073336.1	0	0	0	0	0	0	0	0
ENSG00000214106	PAXIP1-AS2	2.24288	2.1333	0.43462	0.95105	1.76795	1.26094	0.65733	1.42228
ENSG00000157212	PAXIP1	1.42906	1.16403	0.32393	0.58663	1.50638	1.95785	0.73488	0.53197
ENSG00000272760	AC093726.1	2.2835	1.64705	0.55926	1.01669	2.40606	1.47708	0.25375	1.96255
ENSG00000273183	AC093726.2	0.21591	0.17969	0.31727	0.16021	0.39374	0.19044	0.35988	0.53017
ENSG00000273344	PAXIP1-AS1	0.7473	0.5949	0	0.67509	0.39502	0.68783	0.43326	0.27925
ENSG00000220575	HTR5A-AS1	0	0	0	0	0	0	0	0
ENSG00000157219	HTR5A	0	0	0	0	0	0	0	0
ENSG00000283339	AC093726.3	0	0	0	0	0	0	0	0
ENSG00000274637	AC092628.2	0	0	0	0	0	0	0	0
ENSG00000228806	AC092628.1	0	0	0	0	0	0	0	0
ENSG00000201066	RN7SKP280	0.24112	0	0	0	0	0	0	0
ENSG00000217825	AC099552.1	0	0.16399	0	0	0	0	0	0
ENSG00000228521	AC099552.3	0	0	0	0	0	0	0	0
ENSG00000278998	AC099552.4	0	0	0	0	0	0	0	0
ENSG00000225612	AC099552.2	0.32149	0.80269	0	0	0	0	0	0.19736
ENSG00000273117	AC144652.1	1.35407	1.5777	0.39796	0.20096	0.439	1.99064	0.2257	0.16625
ENSG00000186480	INSIG1	15.9654	11.3652	4.13149	5.63301	8.42336	11.4786	8.36852	6.16418
ENSG00000204960	BLACE	0	0	0	0	0	0	0	0
ENSG00000218672	AC008060.1	0	0	0	0	0	0	0	0
ENSG00000260426	AC008060.4	0	0	0	0	0	0	0	0
ENSG00000236544	AC008060.3	0	0	0	0	0	0	0	0
ENSG00000164778	EN2	0	0	0	0	0	0	0	0
ENSG00000146910	CNPY1	0	0.03375	0	0	0	0	0	0
ENSG00000283128	AC009403.2	0	0	0	0	0	0.02379	0	0.03311
ENSG00000227365	AC008060.2	0	0	0	0	0	0	0	0
ENSG00000216895	AC009403.1	0.75791	0.18022	0.1591	0.64275	0.70205	0.57302	2.16568	0.57605
ENSG00000184863	RBM33	43.7949	69.5054	55.5959	101.695	92.4419	70.0422	87.6438	58.9381
ENSG00000164690	SHH	0	0	0	0	0.01769	0	0	0.01786
ENSG00000204876	AC021218.1	0	0	0	0	0	0	0	0
ENSG00000207061	RF00019	0	0	0	0	0	0	0	0
ENSG00000270584	AC093813.1	0	0	0	0	0	0	0	0
ENSG00000228569	AC073133.1	0	0	0	0	0	0	0	0
ENSG00000233878	AC073133.2	0	0	0	0	0	0	0	0
ENSG00000182648	LINC01006	0.24039	0.09234	0.10869	0.05489	0.26978	0.06524	0.21576	0.06811
ENSG00000279418	LINC00244	0	0	0	0	0	0	0	0
ENSG00000234450	AC005534.2	0	0	0	0	0	0	0	0
ENSG00000105982	RNF32	0.45278	0.36287	0.44357	0.54754	0.46893	0.38459	1.28581	0.55592
ENSG00000224903	AC005534.1	0.39515	0.16443	0	0	0.48041	0.17427	0.16466	0.12129
ENSG00000105983	LMBR1	3.65292	3.77825	2.46979	6.8296	3.83038	3.85124	4.99307	3.99005
ENSG00000206938	RNU4-31P	0	0	0	0	0	0	0	0
ENSG00000230033	AC006357.1	0	0	0	0	0	0	0	0
ENSG00000146909	NOM1	1.71298	1.33163	0.52557	2.23494	2.46021	1.49436	2.33755	1.6756
ENSG00000130675	MNX1	0	0	0	0	0	0	0	0
ENSG00000235029	MNX1-AS2	0	0	0	0	0	0	0	0
ENSG00000243479	MNX1-AS1	0	0	0	0	0	0	0	0
ENSG00000236102	AC006967.2	0	0	0	0	0	0	0	0
ENSG00000229365	AC006967.1	0	0	0	0	0	0	0	0
ENSG00000269446	AC006967.3	0	0	0	0	0	0	0	0
ENSG00000009335	UBE3C	8.79286	7.63546	4.48613	10.8432	8.46705	7.60163	7.76547	6.66681
ENSG00000225224	RPS27AP12	0.31527	0.78715	0.23164	0.4679	0.57495	0	0.52551	0.77417
ENSG00000236018	AC004898.1	0	0	0	0	0	0	0	0
ENSG00000224629	AC004975.1	0	0	0	0	0	0	0	0

ENSG00000229660	AC004975.2	0	0	0	0	0	0	0	0
ENSG00000105993	DNAJB6	10.3338	11.3367	3.07015	6.44102	8.72072	8.44182	10.8471	6.30178
ENSG00000234210	AC006372.3	0	0	0	0	0	0	0	0
ENSG00000223872	AC006372.1	0.13425	0	0	0	0	0	0	0
ENSG00000233191	AC006372.2	0.25103	0	0	0	0	0	0	0
ENSG00000280266	AC006372.4	0	0	0	0	0	0	0	0
ENSG00000155093	PTPRN2	2.89683	2.09532	0.42346	0.54021	0.18439	0.40134	0.20224	0.39105
ENSG00000207960	MIR153-2	0	0	0	0	0	0	0	0
ENSG00000222012	AC005481.1	0	0	0	0.03148	0	0	0	0
ENSG00000231515	AC006003.1	0	0	0	0	0	0	0	0
ENSG00000233038	AC011899.2	1.65091	1.04683	0.13863	0.37335	0.6117	0.33285	0.07862	0.28957
ENSG00000272839	AC011899.3	0.71281	0.19774	0	0	0	0	0	0.29172
ENSG00000231980	AC011899.1	0	0	0	0	0	0	0	0
ENSG00000207637	MIR595	0	0	0	0	0	0	0	0
ENSG00000225365	AC078942.1	0	0	0	0	0	0	0	0
ENSG00000232715	LINC01022	0	0	0	0	0	0	0	0
ENSG00000265598	MIR5707	0	0	0	0	0	0	0	0
ENSG00000224324	THAP5P1	0	0	0	0	1.02042	0.49356	0	0
ENSG00000146918	NCAPG2	1.32167	0.96028	1.49517	3.93858	2.07871	2.99778	5.66491	1.99621
ENSG00000228030	AC019084.1	0	0	0	0	0	0	0	0
ENSG00000117868	ESYT2	24.5884	24.1335	17.3301	37.2275	31.8274	21.6795	29.452	21.6941
ENSG00000126870	WDR60	3.5952	2.69015	1.034	0.84849	5.34673	2.73481	2.16248	2.25431
ENSG00000231419	LINC00689	0	0	0	0.01188	0.00973	0.04236	0	0
ENSG00000106018	VIPR2	0.07018	0.02336	0	0.04166	0.06826	0.04953	0.0234	0.01723
ENSG00000229435	PIP5K1P2	0	0	0	0	0	0	0	0
ENSG00000228572	AL954722.1	0	0	0	0	0	0	0	0
ENSG00000182378	PLCXD1	0.91167	0.72917	0.13919	0.35143	0.5326	0.93992	0.45391	0.34888
ENSG00000178605	GTPBP6	2.57194	2.34632	0.43609	0.69734	2.55564	3.27206	1.27786	1.36635
ENSG00000226179	LINC00685	0	0	0	0	0.20822	0.60426	0	0.21027
ENSG00000167393	PPP2R3B	0.58543	0.48722	0.72885	1.64114	1.44327	0.83196	1.76189	1.03824
ENSG00000281849	AL732314.6	0	0	0	0	0	0	0	0
ENSG00000275287	RF00017	0	0	0	0	0	0	0	0
ENSG00000280767	AL732314.4	0	0	0	0	0	0	0	0
ENSG00000234958	FABP5P13	0	0	0	0	0	0	0	0
ENSG00000229232	KRT18P53	0	0	0	0	0	0	0	0
ENSG00000185960	SHOX	0	0	0	0	0	0	0	0
ENSG00000237531	AL672277.1	0	0	0.18319	0.18501	0	0	0	0
ENSG00000225661	RPL14P5	0	0	0	0.14902	0	0	0	0
ENSG00000205755	CRLF2	0.21881	0.1821	0	0	0.13301	0	0.06079	0.04477
ENSG00000198223	CSF2RA	12.8419	18.9862	2.36239	6.02156	13.5187	3.51134	4.56182	10.2451
ENSG00000265658	MIR3690	0.97734	0	0	0	1.18822	0	1.62907	0
ENSG00000223274	RNA5SP498	0	0	0	0	0	0	0	0
ENSG00000185291	IL3RA	2.78627	2.4259	0.25196	1.14511	2.34518	0.68059	0.78596	2.05256
ENSG00000169100	SLC25A6	51.8928	30.5585	4.67889	10.406	44.3524	55.0352	15.5266	17.9246
ENSG00000236871	LINC00106	0	0	0	0	0	0.21843	0	0
ENSG00000236017	ASMTL-AS1	0.27453	0	0	0.08149	0	0.09686	0.09152	0.13483
ENSG00000169093	ASMTL	3.2406	2.36639	0.09217	0.52748	2.59267	2.65558	0.87122	1.36048
ENSG00000182162	P2RY8	28.4555	16.6636	3.06788	9.12444	20.5054	35.2062	9.42589	7.06413
ENSG00000197976	AKAP17A	11.6201	6.73667	0.81942	2.37593	8.26653	5.17247	2.72844	3.37903
ENSG00000196433	ASMT	0	0	0	0	0	0	0	0
ENSG00000223511	AL683807.1	1.57002	1.46998	0.1442	1.01941	1.19299	3.28905	0.16356	1.56621
ENSG00000234622	AL683807.2	0	0.66793	0	0	0.16262	0.23597	0.22296	0.16423
ENSG00000169084	DHRSX	2.47034	2.22446	0.62486	1.26215	2.3387	1.8218	0.81004	2.28722
ENSG00000223571	DHRSX-IT1	0.1126	0.18742	0	0.16711	0	0	0	0
ENSG00000214717	ZBED1	7.18359	5.43971	0.70224	1.71586	7.15932	5.46618	2.72368	4.06927
ENSG00000277120	MIR6089	0	0	0	1.69978	0	0	0	0
ENSG00000223773	CD99P1	0.65613	0.57697	0.12734	0.36746	0.78265	0.48047	0.49524	0.24319
ENSG00000230542	LINC00102	0	0	0	0	0	0	0	0

ENSG00000002586	CD99	26.6729	23.406	3.77196	6.92333	19.6469	33.7455	7.03169	10.0027
ENSG00000124343	XG	0.23782	0	0.54362	1.05884	0.54614	0.51277	0.44045	0.35687
ENSG00000056998	GYG2	0	0	0	0	0	0	0	0
ENSG00000235483	GYG2-AS1	0	0	0	0	0	0	0	0.17475
ENSG00000006756	ARSD	3.05505	1.71818	0.42902	0.80467	1.25499	1.45315	0.57353	0.94734
ENSG00000229851	ARSD-AS1	0	0	0	0	0	0	0	0
ENSG00000157399	ARSE	0	0.04239	0	0	0	0	0	0
ENSG00000205667	ARSH	0	0	0	0	0	0	0	0
ENSG00000062096	ARSF	0	0	0	0	0	0	0	0
ENSG00000239228	RN7SL578P	0	0	0	0	0	0	0	0
ENSG00000230249	AC112653.1	0	0	0	0	0	0	0	0
ENSG00000228459	LINC01546	0	0	0	0	0	0	0	0
ENSG00000101825	MXRA5	0	0	0	0	0.0455	0	0	0
ENSG00000213487	ASS1P4	0	0	0	0	0	0	0	0
ENSG00000212214	SNORA48B	0	0	0	0	0	0	0	0
ENSG00000207435	RNU6-114P	0	0	0	0	0	0	0	0
ENSG00000183943	PRKX	8.12302	4.93556	1.75294	3.59135	5.93927	9.88077	3.02989	2.90133
ENSG00000207332	RNU6-146P	0	0	0	0	0	0	0	0
ENSG00000207320	RF00019	0	2.15944	0	0	0	2.2887	0	0
ENSG00000236188	PRKX-AS1	0	0	0	0	0	0	0	0
ENSG00000182888	AC007685.1	0.16217	0	0	0	0	0	0	0
ENSG00000205664		2.52015	3.27593	3.06847	7.12415	3.91016	8.38367	5.56092	2.59324
ENSG00000234449		1.22139	1.67097	0.86053	2.06099	0	2.56794	0.11155	0
ENSG00000205663		0.03962	0	0	1.11726	0	0.1398	1.02367	0.02432
ENSG00000205662		0	0	0	0	0	0.12211	0.05769	0.04249
ENSG00000225722	BX890604.1	0	0	0	0	0	0	0	0
ENSG00000236513	AC074035.1	0	0	0	0	0	0	0	0
ENSG00000235039	MTND6P12	0	0	0	0	0	0	0	0
ENSG00000226313	MTCYBP12	0	0	0	0	0	0	0	0
ENSG00000238193	AC112656.1	0	0	0.07612	0.07688	0.06298	0.27416	0	0.0636
ENSG00000201660	RF00401	0	0	0	0	0	0	0	0
ENSG00000236120	AC110995.1	0	0	0	0	0	0	0	0
ENSG00000146938	NLGN4X	0.02955	0	0	0	0	0	0.01642	0.02419
ENSG00000252291	RF00322	0	0	0	0	0	0	0	0
ENSG00000265284	MIR4770	0	0	0	0	0	0	0	0
ENSG00000237730	AC078991.1	0	0	0	0	0	0	0	0
ENSG00000169059	VCX3A	0	0	0	0	0	0	0	0
ENSG00000231217	AC097347.1	0	0	0	0	0	0	0.47265	0
ENSG00000237490	RPS27AP17	0	0	0	0	0	0	0	0
ENSG00000130021	PUDP	1.80474	1.46349	0.51001	1.06451	2.25042	1.91845	0.69421	0.7102
ENSG00000228550	AC073583.1	0	0	0	0	0	0	0	0
ENSG00000264268	MIR4767	0.93975	0	0	0	0	0	1.56641	0
ENSG00000101846	STS	1.77602	1.57165	0.3634	0.58388	0.90196	1.74504	0.59957	0.88327
ENSG00000279682	AC120338.1	0	0	0	0	0	0	0	0
ENSG00000182583	VCX	0.06948	0	0	0	0	0	0	0
ENSG00000274923	AC005296.1	0	0	0	0	0	0	0	0
ENSG00000006757	PNPLA4	0.83078	0.56732	0.25042	0.47422	0.51797	0.78917	0.4971	0.15693
ENSG00000230394	AC078960.1	0	0	0.60855	0.61461	0	0	0	0
ENSG00000207628	MIR651	0	0	0	0	0	0	0	0
ENSG00000177504	VCX2	0	0	0	0	0	0	0	0
ENSG00000205642	VCX3B	0	0	0	0	0	0	0	0
ENSG00000011201	ANOS1	0	0.01711	0	0	0.08748	0.01813	0	0
ENSG00000225574	DRAXINP1	0	0	0	0	0	0	0	0
ENSG00000183304	FAM9A	0	0	0	0	0	0	0	0
ENSG00000231619	AC003685.2	0	0	0	0	0	0	0	0
ENSG00000229012	AC003685.1	0	0	0	0	0	0	0	0
ENSG00000225271	AC074281.1	0	0	0	0	0	0	0	0
ENSG00000177138	FAM9B	0	0	0.01574	0	0	0	0.01785	0

ENSG00000200852	RNA5SP499	0	0	0	0	0	0	0	0
ENSG00000219870	AC119618.1	0	0	0	0	0	0	0	0
ENSG00000228543	AC003684.1	0	0	0	0	0	0	0	0
ENSG00000232111	AC073488.1	0	0	0	0	0	0	0	0
ENSG00000282306	AC073488.7	0	0	0	0	0	0	0	0
ENSG00000282501	AC073488.9	0.09722	0	0	0	0	0	0	0
ENSG00000282358	AC073488.8	0	0	0	0	0	0	0	0
ENSG00000282668	AC073488.10	0	0	0	0	0	0	0	0
ENSG00000282299	AC073488.5	0	0	0	0	0	0	0	0
ENSG00000282697	AC073488.11	0	0	0	0	0	0	0	0
ENSG00000282222	AC073488.4	0	0	0	0	0	0	0	0
ENSG00000281955	AC073488.3	0	0	0	0	0	0	0	0
ENSG00000281941	AC073488.2	0	0	0	0	0	0	0	0
ENSG00000282300	AC073488.6	0	0	0	0	0	0	0	0
ENSG00000101849	TBL1X	10.0188	9.48703	0.99892	2.06427	8.0102	3.78213	2.8414	4.58396
ENSG00000101850	GPR143	0	0	0	0	0	0	0	0
ENSG00000146950	SHROOM2	0.0179	0	0	0	0.02176	0	0.01492	0
ENSG00000215319	EIF5P1	0	0.09911	0	0	0	0	0	0
ENSG00000230326	HMGN1P33	0	0	0	0	0	0	0	0.19955
ENSG00000206844	RF00019	0	0	0	0	0	0	0	0
ENSG00000234469	CLDN34	0	0	0	0	0	0	0	0
ENSG00000047644	WWC3	11.0765	14.9895	2.81625	2.73723	11.5874	16.214	5.03217	9.1591
ENSG00000225076	WWC3-AS1	0	0	0	0	0	0	0	0
ENSG00000202469	RF00019	0	0	0	0	0	0	0	0
ENSG00000073464	CLCN4	1.81388	1.04235	0.44425	1.49022	1.70646	1.0095	0.62989	1.12679
ENSG00000227042	AC003666.1	0.23994	0.19969	0.52887	0	0	0.21164	0.99984	0.58918
ENSG00000101871	MID1	0.00789	0.01313	0.02318	0.02341	0.06711	0	0.05258	0.04841
ENSG00000207202	RNU6-800P	0	0	0	0	0	0	0	0
ENSG00000234129	AC073529.1	3.9982	7.36805	7.58894	22.4282	8.10152	10.1182	29.1963	12.3015
ENSG00000004961	HCCS	3.29616	3.50089	0.87647	3.12147	3.35857	3.54431	2.56396	2.73653
ENSG00000207151	RF00019	0	0	0	0	0	0	0	0
ENSG00000047648	ARHGAP6	2.72378	1.36167	0.28687	0.64844	1.6275	1.60717	0.92972	1.3354
ENSG00000278218	RF02271	0	0	0	0	0	0	0	0
ENSG00000275194	AC004467.1	0	0	0	0	0	0	0	0
ENSG00000125363	AMELX	0	0	0	0	0	0	0	0
ENSG00000263652	MIR548AX	0	0	0	0	0	0	0	0
ENSG00000232392	AC002366.1	0	0	0	0	0	0	0	0
ENSG00000282840	AC004554.2	0	0.45117	0	0	0.09415	0	0	0.14263
ENSG00000223418	AC004554.1	0	0	0	0	0	0	0	0
ENSG00000005302	MSL3	6.24001	9.63521	1.50892	2.52873	7.53157	7.12648	2.76486	5.76336
ENSG00000206792	RF00019	0	0	0	0	0	0	0	0
ENSG00000169933	FRMPD4	0	0	0	0	0	0	0	0
ENSG00000206795	RF00019	0	0	0	0	0	0	0	0
ENSG00000223487	FRMPD4-AS1	0	0	0	0	0	0	0	0
ENSG00000231211	RPL17P49	0	0	0	0	0	0	0	0
ENSG00000273800	AC119619.1	0	0	0	0	0	0	0	0
ENSG00000271814	RN7SKP290	0	0	0	0	0	0	0	0
ENSG00000101911	PRPS2	6.32541	4.24685	1.71842	4.29936	5.29918	10.8307	3.41113	4.24207
ENSG00000229083	PSMA6P2	0	0	0	0	0	0	0	0
ENSG00000230691	MRPL35P4	0.12951	0	0	0	0	0	0	0
ENSG00000196664	TLR7	3.77126	4.6611	1.0546	0.89802	3.64405	3.22721	0.86786	2.26332
ENSG00000233338	TLR8-AS1	0.81505	0.09044	0	0.16128	0	0.09586	0	0.33357
ENSG00000101916	TLR8	68.9559	81.6193	9.00709	14.8196	55.7465	20.9133	14.2586	49.1645
ENSG00000205542	TMSB4X	865.245	647.897	669.595	1007.27	576.828	538.18	1040.36	1318.55
ENSG00000187268	FAM9C	0.01251	0	0.01839	0.05571	0.89756	0	0.02086	0.03073
ENSG00000261030	AC079171.1	0.0802	0	0	0	0.0975	0	0	0
ENSG00000235385	LINC02154	0	0	0	0	0	0	0	0
ENSG00000231216	GS1-600G8.3	0	0	0	0	0	0	0	0

ENSG00000123594	ATXN3L	0	0	0	0	0	0	0	0
ENSG00000235592	AC004674.1	0	0	0	0	0	0	0	0
ENSG00000226985	LINC01203	0	0	0	0	0	0	0	0
ENSG00000226434		0	0	0	0	0	0	0	0
ENSG00000197582	GPX1P1	19.7161	0	0.17775	0	16.1763	0.42677	0	9.35613
ENSG00000198759	EGFL6	0	0	0	0	0	0	0	0
ENSG00000233357	RPL30P15	0	0	0.33556	0	0	0	0	0
ENSG00000283493	MIR6086	0	0	0	0	0	0	0	0
ENSG00000199622	RN7SKP20	0	0	0	0	0	0	0	0
ENSG00000176896	TCEANC	5.10062	3.45197	3.8712	5.1367	6.89968	10.605	36.577	6.62376
ENSG00000123595	RAB9A	7.40617	7.71914	1.9834	4.05764	5.84854	6.89907	3.40351	4.07919
ENSG00000196459	TRAPPC2	3.08469	2.21761	1.32283	2.67199	2.17429	2.75267	2.1207	3.06523
ENSG00000046651	OFD1	11.7632	8.86991	4.62345	10.2223	11.9408	8.7758	9.59111	9.50085
ENSG00000046653	GPM6B	0.01292	0.34399	0.03796	0.03834	0.18844	0	0.08612	0.04758
ENSG00000233535	AC003035.2	0	0	0	0	0	0	0	0
ENSG00000212663	AC003035.1	0.13474	0	0	0	0.16382	0	0.44919	0
ENSG00000046647	GEMIN8	1.1491	1.15767	0.22218	0.89757	1.83822	2.08051	0.80647	1.26234
ENSG00000233247	AC002524.1	0.67002	0.4461	0.39384	0.99439	0	0	0.89346	0.82264
ENSG00000101958	GLRA2	0	0	0	0	0.024	0	0	0
ENSG00000231066	NPM1P9	0.1737	0.14456	0	0.12889	0	0.15321	0.14476	0.21326
ENSG00000181544	FANCB	2.80434	2.84623	3.65189	7.78249	3.71435	4.78635	14.1372	7.0542
ENSG00000130150	MOSPD2	13.5429	15.2511	3.12325	4.23141	9.29612	4.13816	2.44101	9.51525
ENSG00000235929	TPT1P14	0	0	0	0	0	0	0	0
ENSG00000236929	AC112498.1	0	0	0	0	0	0	0	0
ENSG00000102048	ASB9	0.09306	0	0	0.04604	0	0.10945	0.05171	0
ENSG00000165192	ASB11	0	0	0	0	0	0	0	0.02866
ENSG00000165195	PIGA	2.80631	2.16183	0.61346	1.25635	2.12887	2.08666	1.68165	1.32411
ENSG00000165197	VEGFD	0	0	0	0	0	0	0	0
ENSG00000087842	PIR	0.02918	0.04857	0.08576	0.08661	0.14191	0	0.29183	0.10748
ENSG00000102010	BMX	1.93308	3.17413	0.11516	0.73661	2.15963	1.47469	0.13063	2.21304
ENSG00000130234	ACE2	0	0	0	0	0	0	0.03112	0
ENSG00000225833	GS1-594A7.3	0	0	0	0	0	0	0	0
ENSG00000147003	CLTRN	0.13935	0	0	0.06894	0.11295	0	0.07743	0
ENSG00000237539	AC003669.1	0	0	0	0	0	0	0	0
ENSG00000186312	CA5BP1	1.6431	0.861	0.6707	0.5419	1.29476	1.87875	1.1158	0.48566
ENSG00000169239	CA5B	2.66703	2.39148	0.67005	1.8131	2.65676	2.86854	1.07553	1.03518
ENSG00000279413	AC112497.1	0	0	0	0	0	0	0	0
ENSG00000200620	RF00409	0	0	0	0	1.28225	0	0	0.64746
ENSG00000281371	INE2	0	0	0	0	0	0	0	0
ENSG00000169249	ZRSR2	8.85815	8.51768	1.60767	6.33754	6.82212	7.28429	4.00013	3.46643
ENSG00000182287	AP1S2	47.5095	34.2939	9.06503	14.7202	19.5749	16.1462	12.4331	17.8047
ENSG00000200566	RNU5F-7P	0	0	0	0	0	0	0	0
ENSG00000226536	SETP15	0	0	0	0	0.26444	0	0	0
ENSG00000126010	GRPR	0.038	0.06325	0.05584	0	0.23099	0.06704	0	0.09331
ENSG00000238178	AC078993.1	0	0	0	0	0	0	0	0
ENSG00000182798	MAGEB17	0	0	0	0	0	0	0	0
ENSG00000215313	RPL6P30	0	0	0	0	0	0	0	0
ENSG00000235071	AC073909.1	0	0	0	0	0	0	0.1223	0
ENSG00000239333	RN7SL658P	0	0	0	0	0	0	0	0
ENSG00000047230	CTPS2	1.2638	1.46144	1.09473	1.89536	1.94083	1.61933	1.59655	1.09434
ENSG00000265144	MIR548AM	0	0	0	0	0	0	0	0
ENSG00000169906	S100G	0	0	0	0	0	0	0	0
ENSG00000169895	SYAP1	8.48585	5.64608	1.99719	4.93248	5.70691	6.10493	6.26322	4.50126
ENSG00000238709	RNU7-56P	0	1.96788	0	0	0	0	0	0
ENSG00000086712	TXLNG	4.32242	3.09125	2.24605	6.29299	5.19514	6.17565	4.24618	2.4618
ENSG00000230180	RPL12P49	0	0	0	0	0	1.06869	0	0
ENSG00000102054	RBBP7	4.32345	4.10986	1.38431	3.64974	4.30393	6.64752	3.80161	3.25071
ENSG00000222736	RNU4-6P	0	0	0	0	0	0	0	0.73768

ENSG00000223498	AL929302.1	0	0	0	0	0	0	0	0
ENSG00000199410	RF00019	0	0	0	0	0	0	0	0
ENSG00000169891	REPS2	7.36179	6.17	1.52367	2.18322	5.45998	3.02958	3.12175	4.86148
ENSG00000201467	RF00190	0	0	0	0	0	0	0	0
ENSG00000214322	CBX1P2	0	0	0	0	0	0	0	0
ENSG00000214321	CBX1P4	0	0	0	0	0	0	0	0
ENSG00000238764	RF01210	0	0	0	0	0	0	0	0
ENSG00000188158	NHS	1.91036	1.5599	0.10593	0.26747	0.67486	0.2162	0.40855	0.72578
ENSG00000265465	MIR4768	0	0	0	0	0	0	0	0
ENSG00000235834	Z93022.1	1.32073	0.6281	0	0.21001	0.40143	0.08321	0.39312	0.28957
ENSG00000230020	NHS-AS1	0	0	0	0	0	0	0	0
ENSG00000226153	Z93242.1	0	0	0	0	0	0	0	0
ENSG00000047634	SCML1	0.76007	0.26635	0.19595	0.35622	0.63226	0.5881	0.48899	0.54028
ENSG00000227873	Z93242.2	0	0	0	0	0	0	0	0
ENSG00000131831	RAI2	0.05591	0.1396	0	0.04149	0.03399	0	0	0
ENSG00000276467	Z93929.2	0	0	0	0	0	0	0	0
ENSG00000225066	Z93929.1	0	0	0	0	0	0	0	0
ENSG00000225882	LINC01456	0	0	0	0	0	0	0	0
ENSG00000177324	BEND2	19.6174	17.2606	4.64585	5.53665	7.16854	3.45798	5.53329	6.17189
ENSG00000102098	SCML2	0.12208	0.26126	0.1794	0.1553	0.38166	0.21537	0.40698	0.27836
ENSG00000235211	TMSB10P2	0	0	0	0	0	0	0	0
ENSG00000207025	RF00019	0	0	0	0	0	0	0	0
ENSG00000008086	CDKL5	1.36942	1.02894	0.30911	0.34404	1.01773	0.3938	0.5152	0.89602
ENSG00000236187	GJA6P	0	0	0	0	0	0	0	0.17274
ENSG00000102104	RS1	0	0	0	0	0.02557	0	0	0
ENSG00000086717	PPEF1	0.25039	0.31258	0.03066	0.15483	0.30441	0.11043	0.03478	0.15371
ENSG00000237221	PPEF1-AS1	0	0	0	0	0	0	0	0
ENSG00000196564	AL096700.1	0	0	0	0	0	0	0	0
ENSG00000224224	HAUS1P2	0	0	0	0	0	0	0	0
ENSG00000237836	PHKA2-AS1	0.15764	0	0	0	0.19165	0	0	0.09677
ENSG00000044446	PHKA2	3.45841	2.41882	0.42947	0.89159	2.45764	1.96209	0.73073	1.43533
ENSG00000266710	RN7SL48P	0.25017	0	0	0	0.30415	0	0	0
ENSG00000173698	ADGRG2	0	0	0	0	0	0	0	0
ENSG00000131828	PDHA1	5.82422	4.50777	0.95895	1.67065	4.52228	4.37467	1.03335	1.9029
ENSG00000180815	MAP3K15	0.07892	0.08757	0.01933	0.01952	0.09594	0	0.02192	0.0646
ENSG00000202144	RF00019	0	0	0	0	0	0	0	0
ENSG00000275800	EIF5P2	0	0	0	0	0	0	0	0
ENSG00000147010	SH3KBP1	31.0322	27.7158	3.74576	7.69781	23.1888	22.775	6.84886	10.5412
ENSG00000173681	BCLAF3	1.95354	2.72858	0.84873	1.48745	2.30278	1.54335	1.98207	1.9084
ENSG00000206663	RF00019	0	0	0	0	0	0	0	0
ENSG00000184368	MAP7D2	0	0.05576	0.02461	0.09944	0	0.0591	0.08376	0.04113
ENSG00000264566	MIR23C	0	0	0	0	0	0	0	0
ENSG00000173674	EIF1AX	7.05352	5.20886	4.54992	8.60063	6.54234	10.2234	7.36891	5.42788
ENSG00000201882	RF00493	2.0943	0	1.53877	4.66225	3.81929	3.69462	5.2363	1.28567
ENSG00000201592	RF00494	11.9113	18.3013	10.7714	17.6777	26.735	51.7247	9.16352	6.74978
ENSG00000225037	EIF1AX-AS1	0	0	0	0	0	0	0	0
ENSG00000177189	RPS6KA3	26.2693	20.0885	5.46066	13.0242	21.1674	16.0524	11.4894	13.4532
ENSG00000283631	AL928596.1	0	0	0	0	0	0	0	0
ENSG00000283400	AL807740.1	0	0	0	0	0	0	0	0
ENSG00000252978	RN7SKP183	0	0	0	0	0	0	0	0
ENSG00000283380	AL807742.1	0	0	0	0	0	0	0.01964	0
ENSG00000283546	BX088723.1	0	0	0	0	0	0	0	0
ENSG00000206716	RNU6-133P	0	0	0	0	0	0	0	0
ENSG00000149970	CNKSR2	0.55282	0.03408	0.12035	0.28868	1.08284	0.32509	0.49486	0.49021
ENSG00000231913	RARRES2P3	0	0	0	0	0	0	0	0
ENSG00000185915	KLHL34	0	0.03351	0.05917	0	0.12238	0.03552	0.10067	0.04944
ENSG00000091482	SMPX	0	0	0	0	0	0	0	0
ENSG00000012174	MBTPS2	0.47624	0.34168	0.33785	0.62149	0.64888	0.60839	0.43797	0.40326

ENSG00000275681	U73479.1	0	0	0	0	0	0	0	0
ENSG00000230797	YY2	0.05348	0	0.07859	0	0.03251	0	0.04458	0.03283
ENSG00000206639	RF00019	0	0	0	0	0	0	0	0
ENSG00000271286	U73023.1	0	0	0	0	0	0	0	0
ENSG00000102172	SMS	17.2731	14.6297	3.24139	5.99329	13.4913	17.1218	5.43024	7.12477
ENSG00000102174	PHEX	0.34446	0.26756	0.08436	0.25561	0.60025	0.42537	0.13397	0.14097
ENSG00000224204	PHEX-AS1	0	0	0	0	0	0	0	0
ENSG00000233067	PTCHD1-AS	0	0	0	0	0	0	0	0
ENSG00000175809	CBLL2	0	0	0	0	0	0	0	0
ENSG00000231846	AC092832.2	0	0	0	0	0	0	0	0
ENSG00000224396	METTL15P3	0	0	0	0	0	0	0	0
ENSG00000201095	RNU6-266P	0	0	0	0	0	0	0	0
ENSG00000231011	AC092832.1	0	0	0	0	0	0	0	0
ENSG00000237019	AC004470.1	0	0	0	0	0	0	0	0
ENSG00000184735	DDX53	0	0	0	0	0	0	0	0
ENSG00000174028	FAM3C2	0.64582	0.8958	0.63268	0.31949	1.17775	0.18989	0.71765	0.39646
ENSG00000228610	PDCL2P1	0	0	0	0	0	0	0	0
ENSG00000165186	PTCHD1	0	0	0	0	0	0	0	0
ENSG00000226774	AC073910.1	0	0	0	0	0	0	0	0
ENSG00000123131	PRDX4	2.27139	1.9224	0.50915	0.22854	1.96581	1.35832	0.6417	0.89808
ENSG00000123130	ACOT9	5.3312	7.03616	1.66502	1.7247	2.94936	2.97268	1.88864	3.10335
ENSG00000233785	AC131011.1	0.43631	0.72624	0.48087	0.16188	0.13261	0	0	0.40177
ENSG00000202272	RF00019	0.72575	1.208	2.13295	0	0.88234	0	0	1.78212
ENSG00000130066	SAT1	212.33	439.631	98.9174	86.4886	120.654	56.4251	68.8671	225.641
ENSG00000184831	APOO	3.48901	0.72593	0.50355	2.12671	0.94684	5.33075	2.64819	0.8032
ENSG00000238103	RPL9P7	0.25276	0	0	0	0	0.22295	0.21066	0
ENSG00000165182	CXorf58	0	0	0.05596	0.05651	0	0.06717	0	0.04675
ENSG00000174010	KLHL15	6.28756	3.63769	1.32238	3.22611	3.82213	3.48433	3.62333	2.53978
ENSG00000130741	EIF2S3	60.3166	41.6769	12.5956	25.1912	47.5614	49.6092	28.504	26.2059
ENSG00000201407	RF00263	0	0	0	0	0	0	0	0
ENSG00000234230	ZFX-AS1	0.52734	0	0.25831	0	0.42742	0	0	0
ENSG00000005889	ZFX	9.94425	8.04659	3.01556	6.57265	8.7174	12.2486	6.47608	5.54701
ENSG00000223611	SUPT20HL2	0	0	0	0	0	0	0	0
ENSG00000223731	SUPT20HL1	0	0	0	0	0	0	0	0
ENSG00000225071	AC004552.1	0	0	0	0	0.25608	0	0	0
ENSG00000067992	PDK3	8.94835	7.01719	1.95844	3.63292	6.38201	5.75785	4.08023	6.17791
ENSG00000214124	SNRPEP9	0	0	0	0	0.37444	0	0	0.75628
ENSG00000260822	AC004656.1	7.35816	4.46042	1.10956	4.08688	4.94993	4.10058	3.30684	4.81705
ENSG00000102230	PCYT1B	1.64942	2.32815	0.32964	0.50917	0.73796	1.93211	1.03375	0.76145
ENSG00000226222	AC079168.1	0	0	0	0	0	0	0	0
ENSG00000236836	PCYT1B-AS1	0	0	0	0	0	0	0	0
ENSG00000236487	AC079168.2	0	0	0	0	0	0	0	0
ENSG00000101868	POLA1	5.77286	6.00799	6.23911	8.79077	11.3224	12.6944	11.8979	8.52245
ENSG00000251869	SCARNA23	0	0	4.97141	2.51044	0	0	0.93985	1.38457
ENSG00000232472	EEF1B2P3	1.08113	0.89977	0.47661	1.12316	1.18296	0.7629	0.18021	0.26548
ENSG00000004848	ARX	0	0	0	0	0	0	0	0
ENSG00000214111	AC002504.1	0	0	0	0	0	0	0	0
ENSG00000240439	RN7SL91P	0	0	0	0	0	0	0	0
ENSG00000176812	AC112778.1	0	0	0	0	0	0	0	0
ENSG00000229310	RPP40P1	0	0	0	0	0	0	0	0
ENSG00000228519	AC097263.1	0	0	0	0	0	0	0	0.14851
ENSG00000233403	AC121342.1	0	0	0	0	0	0	0	0
ENSG00000176774	MAGEB18	0	0	0	0	0	0	0	0
ENSG00000232446	AC129850.1	0	0	0	0	0	0	0	0
ENSG00000232030	MAGEB6B	0	0	0	0	0	0	0	0
ENSG00000227511	AC005297.1	0	0	0	0	0	0	0	0
ENSG00000176746	MAGEB6	0	0	0	0	0	0	0	0
ENSG00000232644	AC005297.2	0	0	0	0	0	0	0	0

ENSG00000188408	MAGEB5	0	0	0	0	0	0	0	0
ENSG00000237171	AC005297.3	0	0	0	0	0	0	0	0
ENSG00000228464	AC073614.1	0	0	0	0	0	0	0	0
ENSG00000231388	AC079178.2	0	0	0	0	0	0	0	0
ENSG00000230265	AC079178.1	0	0	0	0	0	0	0	0
ENSG00000259849	VENTXP1	0	0	0	0	0	0	0	0
ENSG00000236160	AC005345.1	0	0	0	0	0	0	0	0
ENSG00000216938	RPL7P58	0	0	0	0.14352	0	0	0	0
ENSG00000236683	HMGA1P1	0	0	0	0	0	0	0	0
ENSG00000252491	RNU1-142P	0	0	0	0	0	0	0	0
ENSG00000242021	AC112493.1	0	0	0	0	0	0	0	0
ENSG00000228933	AC107419.1	0	0	0	0	0	0	0	0
ENSG00000233807	AC097262.1	0	0	0	0	0	0	0	0
ENSG00000252486	RF00019	0	0	0	0	0	0	0	0
ENSG00000235642	PTP4A1P5	0	0	0	0	0	0	0	0
ENSG00000224960	PPP4R3C	0	0	0	0	0	0	0	0
ENSG00000223391	AC006210.1	0	0	0	0	0	0	0	0
ENSG00000238247	AC006210.2	0	0.07897	0	0.07041	0.11536	0	0	0
ENSG00000189186	DCAF8L2	0	0	0	0	0	0	0	0
ENSG00000177689	MAGEB10	0	0	0	0	0	0	0	0
ENSG00000229731	AC107613.1	0	0	0	0	0	0	0	0
ENSG00000226199	AC112492.2	0	0	0	0	0	0	0	0
ENSG00000232834	AC112492.4	0	0	0	0	0	0	0	0
ENSG00000228345	AC112492.3	0	0	0	0	0	0	0	0
ENSG00000233243	AC112492.5	0	0	0	0	0	0	0	0
ENSG00000224589	AC112492.1	0	0	0	0	0	0	0	0
ENSG00000226372	DCAF8L1	0	0	0	0	0	0	0	0
ENSG00000277569	MIR6134	0	0	0	0	0	0	0	0
ENSG00000223742	AC112495.1	0	0	0	0	0	0	0	0
ENSG00000169306	IL1RAPL1	0	0	0	0	0.0243	0	0	0
ENSG00000226436	AC002367.1	0	0	0	0	0	0	0	0
ENSG00000201666	RF00284	0	0	0	0	0	0	0	0
ENSG00000237994	AC003659.1	0	0	0	0	0	0	0	0
ENSG00000201356	RNA5SP500	0	0	0	0	0	0	0	0
ENSG00000227393	AL031575.1	0	0	0	0	0	0	0	0
ENSG00000264090	MIR4666B	0	0	0	0	0	0	0	0
ENSG00000227592	PIGFP3	0	0	0	0	0	0	0	0
ENSG00000270794	AC005185.1	0	0	0	0	0	0	0	0
ENSG00000099399	MAGEB2	0	0	0	0	0	0	0	0
ENSG00000198798	MAGEB3	0	0	0	0	0	0	0	0
ENSG00000120289	MAGEB4	0	0	0	0	0	0	0	0
ENSG00000214107	MAGEB1	0	0	0	0	0	0	0	0
ENSG00000276940	PLCE1P1	0	0	0	0	0	0	0	0
ENSG00000169297	NR0B1	0	0	0	0	0	0	0	0
ENSG00000120280	CXorf21	11.4989	19.9949	3.77434	2.63901	10.8573	5.36766	3.425	10.3824
ENSG00000178556	CKS1BP6	0	0	0	0	0	0	0	0
ENSG00000232368	FTLP2	0	0	0.40801	0.20603	0.16878	0.73473	0	0.3409
ENSG00000198814	GK	21.7206	36.5411	5.3465	6.73948	16.6626	6.34615	7.68329	22.789
ENSG00000229331	GK-IT1	3.13881	1.2293	0.27132	0.82206	1.12238	0	0.30776	1.36016
ENSG00000241886	AC112496.1	3.52155	3.90775	0.38332	0.58071	0.63428	0	1.30442	4.64398
ENSG00000243055	GK-AS1	0	0	0	0	0	0	0	0
ENSG00000277876	AC108359.1	0.06053	0.2015	0	0	0	0	0	0
ENSG00000157625	TAB3	4.63642	4.85692	1.66939	4.24496	3.828	3.96756	5.28669	2.90998
ENSG00000231542	TAB3-AS1	0	0	0	0	0	0	0	0
ENSG00000235512	TAB3-AS2	0	0	0	0	0	0	0	0
ENSG00000132446	FTHL17	0	0	0	0	0	0	0	0
ENSG00000198947	DMD	0.02713	0.05807	0.0057	0.01151	0.07069	0.04103	0.00646	0.04759
ENSG00000252903	RNU6-894P	0	0	0	0	0	0	0	0

ENSG00000222922	RNA5SP501	0	0	0	0	0	0	0	0
ENSG00000236285	NPM1P8	0	0	0	0	0	0	0	0
ENSG00000263600	MIR3915	0	0	0	0	0	0	0	0
ENSG00000221348	MIR548F5	0	0	0	0	0	0	0	0
ENSG00000236828	DMD-AS3	0	0	0	0	0	0	0	0
ENSG00000226781	TBCAP1	0	0	0.3294	0	1.09011	0.39545	0.37364	0.82566
ENSG00000215310	AL050305.1	0	0	0	0	0	0	0	0
ENSG00000233928	AL591501.1	0	0	0	0	0	0	0	0
ENSG00000185448	FAM47A	0	0	0	0	0	0	0	0
ENSG00000185638	FTH1P14	0	0	0	0	0	0	0	0
ENSG00000233571	AL592043.1	0	0	0	0	0	0	0	0
ENSG00000236072	AL592043.2	0	0	0	0	0	0	0	0
ENSG00000271136	AL592504.1	0	0	0	0	0	0	0	0
ENSG00000147027	TMEM47	0	0.15048	0	0	0	0	0	0
ENSG00000225384	AL606662.1	0	0	0	0	0	0	0	0
ENSG00000273704	AL596245.1	0	0	0	0	0	0	0	0
ENSG00000189132	FAM47B	0	0	0	0	0	0	0	0
ENSG00000229347	AL596268.1	0	0	0	0	0	0	0	0
ENSG00000252411	RNU6-1087P	0	0	0	0	0	0	0	0
ENSG00000230227	SIAH1P1	0	0	0	0	0	0	0	0
ENSG00000233663	AL161722.1	0	0	0	0	0	0	0	0
ENSG00000271088	AL161722.3	0	0	0	0	0	0	0	0
ENSG00000233880	AL161722.2	0	0	0	0	0	0	0	0
ENSG00000189023	MAGEB16	0	0	0	0	0	0	0	0
ENSG00000271519	AL590065.1	0	0	0	0	0	0	0	0
ENSG00000165164	CFAP47	0	0	0	0.00951	0	0.0113	0.01068	0.02359
ENSG00000206733	RNU6-641P	0	0	0	0	0	0	0	0
ENSG00000229384	HMGB1P16	0	0	0	0	0	0	0	0
ENSG00000226484	AL606516.1	0	0	0	0	0	0	0	0
ENSG00000233921	RPS15AP40	0	0	0	0	0.45584	0	0	0
ENSG00000249467	AL592156.1	0	0	0	0	0	0	0	0
ENSG00000275314	AC006924.1	0	0	0	0	0	0	0	0
ENSG00000235510	BX842568.4	0	0	0	0	0	0	0	0
ENSG00000223723	BX842568.2	0	0.09919	0.17514	0.26533	0.21736	0.10513	0.19867	0.07317
ENSG00000231069	BX842568.3	0	0	0	0	0	0	0	0
ENSG00000198173	FAM47C	0	0	0	0	0	0	0	0
ENSG00000243048	FTH1P18	0	0	0	0	0	0	0	0
ENSG00000214071	BX842568.1	0	0	0	0	0	0	0	0
ENSG00000233810	MOB1AP2	0	0	0	0	0	0	0	0
ENSG00000130962	PRRG1	0.08303	0.07897	0.12201	0.05281	0.04326	0.04185	0	0.02913
ENSG00000250349	AF241726.2	0	0	0	0	0	0	0	0
ENSG00000241607	AC245096.1	0	0	0	0	0	0	0	0
ENSG00000219186	FTH1P19	0	0	0	0	0	0	0	0
ENSG00000234355	FTH1P27	0	0	0	0	0	0	0	0
ENSG00000174678	FAM47DP	0	0	0	0	0	0	0	0
ENSG00000147036	LANCL3	0.66197	0.80565	0.23011	0.44368	0.25096	0.2637	0.30848	0.35831
ENSG00000047597	XK	14.3757	10.8744	14.8143	30.9264	11.6916	18.3063	19.9253	14.5904
ENSG00000165168	CYBB	241.976	152.131	34.0457	43.0175	107.089	87.6209	45.5241	83.4248
ENSG00000165169	DYNLT3	8.5356	6.357	2.46802	4.26812	5.28658	2.39466	2.1859	4.15241
ENSG00000206983	RNU6-49P	0	0	0	0	0	0	0	0
ENSG00000259977	AL121578.3	0	0	0.15171	0	0	0	0	0.25351
ENSG00000224339	AL121578.1	0	0	0	0	0	0	0	0
ENSG00000187516	HYPM	0	0	0	0	0	0	0	0
ENSG00000229674	AL121578.2	0	0	0	0	0	0	0	0
ENSG00000147041	SYTL5	0.0306	0.0764	0	0	0	0.02699	0	0.01878
ENSG00000221466	MIR548AJ2	0	0	0	0	0	0	0	0
ENSG00000101955	SRPX	0	0	0	0	0	0	0.06149	0
ENSG00000226679	AL606748.1	0	0	0	0	0	0	0	0

ENSG00000156313	RPGR	4.00212	3.22828	1.29685	3.09163	3.76778	1.41206	2.18943	2.83485
ENSG00000252050	RF00322	7.33003	6.56969	2.48571	0.83681	2.74205	0.99471	1.8797	0
ENSG00000036473	OTC	0	0	0	0	0	0	0	0
ENSG00000227988	TDGF1P1	0	0	0	0	0	0	0	0
ENSG00000232370	AF241726.1	0	0	0	0	0	0	0	0
ENSG00000229735	FTLP16	0	0	0	0	0	0	0	0
ENSG00000233007	UBTFL11	0	0	0	0	0	0	0	0
ENSG00000156298	TSPAN7	0.44782	0.19616	0.51952	0.80452	0.48713	1.2058	1.29645	0.40513
ENSG00000238606	RNU7-7P	0	0	0	0	0	0	0	0
ENSG00000235806	AF241728.1	0	0.1034	0.45642	0.55315	0.07552	0	0	0.07627
ENSG00000165175	MID1IP1	6.63378	7.40421	1.9326	1.54998	7.78301	3.61664	1.83754	3.39567
ENSG00000238123	MID1IP1-AS1	0.19495	0	0	0.28932	0.71104	0.34391	0	0
ENSG00000207122	RNU6-591P	0	0	0	0	0	0	0	0
ENSG00000224610	AC108879.1	0	0.14735	0	0	0.21526	0	0	0.21738
ENSG00000235304	LINC01281	0.04134	0	0.06075	0.06136	0	0.14587	0.06891	0
ENSG00000236747	LINC01282	0	0	0	0	0	0	0	0
ENSG00000234191	AC091808.1	0	0	0	0	0	0	0	0
ENSG00000263730	MIR3937	0	0	0	0	0	0	0	0
ENSG00000264618	RN7SL732P	0.24433	0.40669	0.71809	1.45048	0	0.86208	0.81454	0.29999
ENSG00000228232	GAPDHP1	0.14587	0.1214	0	0	0	0.12867	0	0.08955
ENSG00000270759	AC091810.1	0	0	0	0	0	0	0	0
ENSG00000261435	AL592164.1	0	0	0	0	0	0	0	0
ENSG00000263972	MIR1587	0	0	0	0	0	0	0	0
ENSG00000214047	RPS11P7	0	0	0	0	0.3833	0	0	0.19354
ENSG00000223486	AC092198.1	0.10896	0	0	0	0	0.09611	0.0454	0
ENSG00000183337	BCOR	4.5298	3.74137	0.7312	1.14592	4.28686	5.28205	0.87231	1.47467
ENSG00000236393	AC091806.1	0	0	0	0	0	0	0	0
ENSG00000214045	AC091807.1	0	0	0	0	0	0	0	0
ENSG00000206677	RF00019	0	0	0	0	0	0	0	0
ENSG00000235685	AC092473.1	0	0	0	0	0	0	0	0
ENSG00000238730	RNU7-164P	0	0	0	0	0	0	0	0
ENSG00000182220	ATP6AP2	10.7957	9.21445	2.94073	5.08653	7.9422	4.84243	5.84063	7.94535
ENSG00000238205	MPC1L	0	0	0	0	0	0	0	0
ENSG00000185753	CXorf38	8.63641	5.97626	1.56856	2.66429	4.95502	6.1628	3.78086	3.90191
ENSG00000180182	MED14	3.58303	2.56501	1.08205	2.25323	3.21174	4.08452	2.01188	2.17164
ENSG00000234636	MED14OS	1.14023	0.40669	0.11968	0.24175	0.09902	0.14368	0.40727	0.29999
ENSG00000270791	DPRXP6	0	0	0	0	0	0	0	0
ENSG00000214031	AC092474.1	0	0	0	0	0	0	0	0
ENSG00000237661	AC092474.2	0	0	0	0	0	0	0	0
ENSG00000238222	MKRN4P	0.09638	0.08022	0.07082	0.57218	0	0.08502	0.24099	0.17751
ENSG00000232801	SDCBPP3	0	0	0	0	0	0	0.13713	0
ENSG00000189145	AC092268.1	0	0	0	0	0	0	0	0
ENSG00000216866	RPS2P55	0.16603	0	0.12199	0.3696	0.20185	0.14645	0.13837	0
ENSG00000237931	CLIC4P3	0	0	0	0	0	0	0	0
ENSG00000124486	USP9X	23.0055	21.1387	5.62408	12.0302	18.9186	15.6245	15.1916	14.1551
ENSG00000223714	LINC02601	0	0.14612	0	0	0	0	0	0
ENSG00000199564	RNA5SP502	0	0	0	0	0	0	0	0
ENSG00000238426	RF00019	0	0	0	0	0	0	0	0
ENSG00000226641	AL391647.1	0	0	0	0	0	0.25306	0	0
ENSG00000215301	DDX3X	95.3939	73.5358	18.9899	33.4838	69.7023	49.8969	38.7151	44.807
ENSG00000264573	RN7SL15P	0	0	0	0	0	0	0	0
ENSG00000188937	NYX	0	0	0	0	0	0	0	0
ENSG00000225238	Z93015.1	0	0	0	0	0	0	0	0
ENSG00000147044	CASK	1.59424	1.21672	0.15345	0.71291	1.55736	1.1913	0.48738	0.46157
ENSG00000233033	CASK-AS1	0	0	0	0	0	0	0	0
ENSG00000212207	RNU6-1321P	0	3.4208	0	0	1.66573	0	0	0
ENSG00000217624	AL627402.1	0.09973	0.166	0.14655	0.14801	0	0	0	0.12244
ENSG00000242559	RN7SL144P	0	0	0.36513	0.36877	0	0	0	0.30507

ENSG00000171659	GPR34	11.4817	4.74618	1.56431	2.36981	4.25246	1.34141	0.4436	2.38063
ENSG00000171657	GPR82	0.89738	0.32788	0.6111	0.42228	0.63864	0.61779	0.07297	0.72557
ENSG00000229822	AL353691.1	0	0	0	0	0	0	0	0
ENSG00000237875	AL353691.2	0	0	0	0	0	0	0	0
ENSG00000251807	RNU6-202P	0.76355	0	1.12202	0	0	2.694	0	0
ENSG00000233103	AL133329.1	0	0	0	0	0	0	0	0
ENSG00000228847	ATP5MC2P4	0	0	0	0	0	0	0	0
ENSG00000212560	RNU6-630P	0	0	0	0	0	0	0	0
ENSG00000228780	Z92545.1	0	0	0	0	0	0	0	0
ENSG00000206896	RNU6-1124P	0	0	0	0	0	0	0	0
ENSG00000231772	Z93403.1	0	0	0	0	0	0	0	0
ENSG00000283737	AL023875.1	0	0	0	0	0	0	0	0
ENSG00000102055	PPP1R2C	0	0	0	0.12362	0	0	0	0
ENSG00000270280	AL451086.1	0	0	0	0	0	0	0	0
ENSG00000224294	PINCR	0	0	0	0	0	0	0	0
ENSG00000237206	IMPDH1P4	0	0	0	0	0	0	0	0
ENSG00000231750	NANOGP10	0	0	0	0	0	0	0	0
ENSG00000189221	MAOA	0.18564	0.08828	0.05846	0.03936	0	0.25731	0.41994	0.09768
ENSG00000069535	MAOB	0.17748	0.12661	0	0	0.33908	0.17892	0.08452	0.12452
ENSG00000124479	NDP	0	0	0	0	0	0	0	0
ENSG00000236276	NDP-AS1	0	0	0	0	0	0	0	0
ENSG00000214019	AL034370.1	0	0	0	0.13684	0.05605	0.08133	0	0.16981
ENSG00000183690	EFHC2	3.00357	1.98507	0.87625	1.11441	1.74528	1.55844	1.80381	1.41002
ENSG00000198414	TATDN2P1	0	0	0	0	0.03823	0	0	0
ENSG00000214018	RRM2P3	2.39502	1.15399	0.18523	0.74831	1.1494	2.11257	1.05056	1.54767
ENSG00000200702	RF00019	0	0	0	0	0	0	0	0
ENSG00000224054	AL022163.1	0	0	0	0	0	0	0	0
ENSG00000215035	FDPSP5	0	0	0	0	0	0	0	0
ENSG00000069509	FUNDC1	7.17809	9.3316	10.4711	23.6389	20.5752	17.4696	34.6716	13.1232
ENSG00000233543	CHTF8P1	0	0	0	0	0	0	0	0
ENSG00000229168	RPL19P20	0	0	0	0	0	0	0	0
ENSG00000214016	RPSAP61	0	0	0	0	0.1015	0	0	0.1025
ENSG00000242065	RN7SL291P	0	0	0	0	0	0	0	0
ENSG00000189037	DUSP21	0	0	0.12171	0	0	0	0	0.10169
ENSG00000147050	KDM6A	8.69665	7.09252	3.87764	7.31435	7.32756	11.3933	10.6966	5.7233
ENSG00000147113	DIPK2B	0	0	0	0	0.01326	0	0.03636	0.01339
ENSG00000229491	AC136489.1	0	0	0	0.06306	0	0	0	0
ENSG00000237782	AL020989.1	0	0	0	0	0	0	0	0
ENSG00000229563	LINC01204	0	0	0.04096	0.04136	0	0.04917	0	0
ENSG00000214282	KRT8P14	0	0	0	0	0	0	0	0
ENSG00000204915	AC234772.1	0	0	0	0	0	0	0	0
ENSG00000270069	MIR222HG	1.10543	0.61333	0.38676	0.39061	1.08796	1.30008	0.08774	0.2262
ENSG00000207870	MIR221	0	0	0	0	0	0	0	0
ENSG00000207725	MIR222	0.66637	0	0	0	0	1.17556	0	0
ENSG00000269902	AC234772.2	0.83296	0.23108	0.204	0	0.67513	0.73473	0	0.17045
ENSG00000231566	LINC02595	0	0	0	0.2901	0.23764	0	0	0
ENSG00000212347	RF00591	0	0	0	0	0	0	0	0
ENSG00000215286	AL031584.1	0	0	0	0	0	0	0	0
ENSG00000234613	AL031584.2	0	0	0	0	0	0	0	0
ENSG00000224701	MED28P4	0	0	0	0	0	0	0	0
ENSG00000213100	KRT18P68	0	0	0	0	0	0	0	0
ENSG00000214009	PCNAP3	0	0	0	0	0	0	0	0
ENSG00000278838	RF00017	0	0	0	0	0	0	0	0
ENSG00000229145	ACTBP1	0	0	0	0	0	0	0	0
ENSG00000236751	LINC01186	0.11709	0	0	0	0	0	0	0
ENSG00000231235	AL139811.2	0	0	0	0	0	0	0	0
ENSG00000235587	GAPDHP65	0	0	0	0	0	0	0	0.08946
ENSG00000147121	KRBOX4	1.72576	1.5103	0.28759	0.68651	1.77368	1.31823	0.62276	0.78638

ENSG00000224072	AL139811.1	0	0	0	0.04732	0.03876	0	0	0
ENSG00000251192	ZNF674	1.26441	1.75057	0.39939	0.59628	1.02004	0.39609	0.29545	0.87052
ENSG00000199226	RNU6-50P	0	0	0	1.05617	0	0	0	0
ENSG00000230844	ZNF674-AS1	1.26988	1.05686	0.20734	0.36646	1.15792	0.74675	0.35278	0.56302
ENSG00000277721	RF00017	0	0	0	0	0	0	0	0
ENSG00000270499	MKI67P1	0	0	0	0	0	0	0	0.06164
ENSG00000147119	CHST7	2.91408	1.97218	0.42351	0.33268	0.97331	1.07336	0.90741	0.70771
ENSG00000065923	SLC9A7	1.87762	0.80261	0.28987	0.85658	2.40712	1.23731	0.99858	0.82525
ENSG00000213997	PGAM1P7	0.09946	0	0	0	0.12092	0	0	0
ENSG00000226048	YBX1P8	0	0	0	0	0.0945	0	0	0
ENSG00000215284	AL512633.1	0.22382	0	0.16445	0	0.13606	0.19742	0.18653	0.1374
ENSG00000102218	RP2	30.2415	36.702	8.49702	9.32531	21.0431	12.2069	8.48162	20.399
ENSG00000204904	LINC01545	0	0	0	0	0	0	0	0
ENSG00000102221	JADE3	0.19977	0.33251	0.0629	0.10588	0.32961	0.02517	0.23784	0.17519
ENSG00000130988	RGN	0	0	0	0	0	0	0	0
ENSG00000206685	RNU6-1189P	0	0	0	0	0	0	0	0
ENSG00000201659	RNU12-2P	0	0	0	0	0	0	0	0
ENSG00000147123	NDUFB11	7.70222	4.81839	1.89905	2.1481	7.73015	8.75454	2.92957	4.3158
ENSG00000182872	RBM10	8.76438	8.59612	0.9417	2.06998	7.83695	7.8139	1.6651	3.2398
ENSG00000237351	AL513366.1	0	0	0	0	0	0	0	0
ENSG00000130985	UBA1	17.3315	12.9837	2.17536	4.64752	12.8615	17.4371	4.68829	6.16571
ENSG00000224975	INE1	0.38783	0	0	0.11512	0.37721	0	0.25858	0.47617
ENSG00000102225	CDK16	2.65158	2.59951	0.75948	1.33398	2.44511	2.71547	1.27349	1.28291
ENSG00000102226	USP11	2.991	2.52399	0.36797	1.48654	3.2812	3.43588	1.27537	1.00775
ENSG00000237623	AL591503.3	0	0	0	0	0	0	0	0
ENSG00000266158	RN7SL785P	0	0	0	0	0	0	0	0
ENSG00000203402	AL591503.1	0	0	0	0.22806	0	0	0.25614	0
ENSG00000147117	ZNF157	0.15811	0.21054	0	0	0.19223	0.11157	0.05271	0.07765
ENSG00000221459	SNORA11C	0	0	0	0	0	0	0	0
ENSG00000232009	AL591503.2	0	0	0	0	0	0	0	0
ENSG00000226556	NPM1P49	0	0	0	0.15236	0.12481	0	0	0
ENSG00000147124	ZNF41	3.70792	3.06041	0.33773	1.47807	2.88735	3.00034	1.35359	1.41091
ENSG00000277605	RF00017	0	0	0	0	0	0	0	0
ENSG00000196741	LINC01560	0.4095	0.48687	0	0	0.42674	0.41281	0.19502	0.35913
ENSG00000235636	NUS1P1	0.24932	0.13833	0.24425	1.11006	0	0.29322	0	0
ENSG00000078061	ARAF	12.2547	8.55958	2.01107	3.20049	9.32763	9.84013	2.79961	3.5897
ENSG00000008056	SYN1	0.09589	0.03192	0	0	0	0	0.03197	0.02355
ENSG00000102265	TIMP1	69.4474	51.9387	5.522	14.1188	29.7248	33.9853	14.5094	13.082
ENSG00000263858	MIR4769	0	0	0	0	0	3.35875	0	0
ENSG00000283743	AL009172.2	0.40893	0.34033	0	0	0.24858	0	0	0
ENSG00000126759	CFP	25.4992	18.2459	2.66552	4.64813	11.677	5.50214	5.28571	7.88295
ENSG00000126767	ELK1	3.51617	1.68758	0.53889	1.02447	2.22923	2.58776	0.75509	0.82104
ENSG00000126756	UXT	21.937	16.5884	4.07285	7.52661	21.6518	24.3435	9.63288	12.1637
ENSG00000267064	UXT-AS1	0.14233	0.15794	0	0	0.05768	0	0	0
ENSG00000187893	CXXC1P1	0	0	0	0	0	0	0	0
ENSG00000224656	AL009172.1	0	0	0	0	0	0	0	0
ENSG00000229968	AL022578.1	0	0	0	0	0	0	0	0
ENSG00000188459	WASF4P	0.14719	0.08167	0.21629	0.07282	0.05965	0.17311	0.32712	0.12048
ENSG00000197779	ZNF81	2.38749	1.53179	0.39549	1.03078	1.63604	1.3478	0.62226	0.99132
ENSG00000224401	RPL7P57	0	0	0	0	0	0	0	0
ENSG00000147118	ZNF182	3.31047	2.75514	0.42041	0.97049	3.0807	2.19906	1.32842	1.98209
ENSG00000221994	ZNF630	0.61815	0.66381	0.11721	0.26634	0.3394	0	0.29914	0.14689
ENSG00000171489	SPACA5	0	0	0	0	0	0	0	0
ENSG00000277541	ZNF630-AS1	0	0	0	0	0	0	0	0
ENSG00000171483	SSX6P	0	0	0	0	0	0	0	0
ENSG00000171478	SPACA5B	0	0	0	0	0	0	0	0
ENSG00000230241	AC244636.1	0	0	0	0	0	0	0	0
ENSG00000233139	AC244636.2	0	0	0	0	0	0	0	0

ENSG00000201517	RNU6-707P	0	0	0	0	0	0	0	0
ENSG00000225198	SSXP3	0	0	0	0	0	0	0	0
ENSG00000238032	AC245047.7	0	0	0	0	0	0	0	0
ENSG00000165583	SSX5	0	0	0	0	0	0	0	0
ENSG00000234780	AC245047.5	0	0	0	0	0	0	0	0
ENSG00000252856	RNA5SP503	0	0	0	0	0	0	0	0
ENSG00000204368	AC245047.1	0	0	0	0	0	0	0	0
ENSG00000231568	AC245047.3	0	0	0	0	0	0	0	0
ENSG00000229662	AC245047.2	0	0	0	0	0	0	0	0
ENSG00000126752	SSX1	0	0	0	0	0	0	0	0
ENSG00000232813	AC245047.4	0	0	0	0	0	0	0	0
ENSG00000241207	AC245047.8	0	0	0	0	0	0	0	0
ENSG00000237345	AC245047.6	0	0	0	0	0	0	0	0
ENSG00000204648	SSX9P	0	0	0	0	0	0	0	0
ENSG00000234391	AL606490.6	0	0	0	0	0	0	0	0
ENSG00000227302	SSXP9	0	0	0	0	0	0	0	0
ENSG00000230100	AL606490.3	0	0	0	0	0	0	0	0
ENSG00000270747	AL606490.9	0	0	0	0	0	0	0	0
ENSG00000165584	SSX3	0	0	0	0	0	0	0	0
ENSG00000225055	AL606490.1	0	0	0	0	0	0	0	0
ENSG00000234448	AL606490.7	0	0	0	0	0	0	0	0
ENSG00000268009	SSX4	0	0	0	0	0	0	0	0
ENSG00000269791	SSX4B	0	0	0	0	0	0	0	0
ENSG00000231603	AL606490.5	0	0	0	0	0	0	0	0
ENSG00000231489	AL606490.4	0	0	0	0	0	0	0	0
ENSG00000235350	AL606490.8	0	0	0	0	0	0	0	0
ENSG00000226971	AL606490.2	0	0.09174	0.08099	0	0	0.09723	0	0.06767
ENSG00000017483	SLC38A5	2.42874	5.07609	4.77655	3.14378	8.79178	15.5277	3.59175	4.91015
ENSG00000068438	FTSJ1	5.66597	3.35608	1.01263	2.15905	4.62339	4.90772	0.5105	1.94283
ENSG00000224292	AF196972.1	0.11225	0.18684	0	0	0	0.19803	0	0
ENSG00000102312	PORCN	0.87639	0.81042	0.14309	0.46968	0.50315	1.2025	0.28405	0.44834
ENSG00000147155	EBP	2.64721	2.17782	0.44713	1.26443	2.29358	3.54279	1.26796	1.12076
ENSG00000276823	RF00017	0	0	0	0	0	0	0	0
ENSG00000068354	TBC1D25	3.71449	2.3691	0.68868	0.79857	3.29202	4.01133	2.05418	1.64096
ENSG00000204620	AC115618.1	0.13352	0	0	0	0	0	0	0
ENSG00000102317	RBM3	32.2003	16.0236	3.78197	7.98909	28.9253	29.9798	18.797	16.6197
ENSG00000228343	AC115618.2	0	0	0	0	0	0	0	0
ENSG00000279528	AC115618.3	2.24213	0.28708	0.25344	1.02387	1.04843	2.12984	0.86245	0.42352
ENSG00000220125	MRPL32P1	0	0	0	0	0	0	0	0
ENSG00000101940	WDR13	2.8264	2.16656	0.4372	0.7865	2.47546	1.77139	0.74387	1.03878
ENSG00000231725	VN1R110P	0.45294	0.37696	0	0.22407	0.27533	0	0.25166	0.55611
ENSG00000015285	WAS	45.9012	45.0422	5.24772	11.7421	28.7449	25.7429	10.1603	17.8785
ENSG00000101945	SUV39H1	1.41464	0.9781	0.19189	0.7752	0.79379	2.34205	0.58043	0.48098
ENSG00000232828	AC231533.1	0	0	0	0.11761	0.19268	0.1398	0.26417	0
ENSG00000206723	RNU6-1056P	0	0	0	0	0	0	0	0
ENSG00000171433	GLOD5	0.06909	0	0	0	0	0	0	0
ENSG00000233585	AC231533.2	0.2998	0.24951	0	0	0	0.13222	1.37422	0
ENSG00000207367	RNU6-29P	0	0	0	0	0	0	0	0
ENSG00000102145	GATA1	11.4837	14.3157	12.7821	9.86324	17.6451	13.276	9.85588	11.9396
ENSG00000094631	HDAC6	2.5344	2.00379	0.90003	1.27467	2.04561	2.04922	1.2908	1.37433
ENSG00000187682	ERAS	0	0	0	0	0	0	0	0
ENSG00000102109	PCSK1N	1.88225	0.26854	0.39514	0.71832	0.52306	0.37949	0.26892	0
ENSG00000126768	TIMM17B	5.69351	4.33363	1.55559	2.33537	4.73063	4.74446	0.61996	1.89689
ENSG00000102103	PQBP1	8.24292	5.38556	1.24525	2.68677	6.88395	7.95033	1.73351	2.64836
ENSG00000102100	SLC35A2	1.64412	1.92216	0.17257	0.58097	1.54675	1.0704	0.45675	0.79303
ENSG00000102096	PIM2	30.0794	29.6515	6.35906	14.3813	35.3755	28.4758	14.736	22.9801
ENSG00000068308	OTUD5	13.6724	12.5337	1.49936	3.30113	11.3133	10.368	4.11576	5.83778
ENSG00000279155	AC233300.1	0.17944	0.44801	0	0.13315	0	0	0.14955	0.44062

ENSG00000223309	RNU6-722P	0	0	0	0	0	0	0	0
ENSG00000102057	KCND1	0.13885	0.12606	0.01855	0	0.07673	0.02227	0	0.031
ENSG00000068400	GRIPAP1	6.3432	6.47407	0.6613	1.30397	5.01534	3.95062	1.4288	2.2496
ENSG00000068323	TFE3	13.0068	15.7247	3.52089	4.4195	11.111	9.51044	6.93201	7.06024
ENSG00000147144	CCDC120	0.12405	0.1606	0.04051	0.10228	0.26812	0.17021	0.06892	0.08462
ENSG00000243279	PRAF2	0.50668	0.42169	0.12409	0.18799	0.92402	0.44693	0.3519	0.15552
ENSG00000196998	WDR45	10.7154	7.56399	6.97414	6.2454	11.0333	8.94177	3.67527	8.41865
ENSG00000206936	RNU4-52P	0.53116	0	0	0	0	0	0	0
ENSG00000068394	GPKOW	11.277	7.8444	1.42217	2.18571	8.23639	7.49741	3.01593	3.7714
ENSG00000265033	RN7SL262P	0	0	0	0	0	0	0	0
ENSG00000280116	AC231657.2	0	0	0	0	0	0	0	0
ENSG00000269313	MAGIX	0.244	0.09556	0.04218	0.08521	0.0698	0.05064	0.0957	0.07049
ENSG00000279422	AC231657.1	0	0	0	0	0.67343	0.65144	0	0
ENSG00000102007	PLP2	50.548	48.3646	6.73739	15.0548	34.7687	17.8954	10.4126	15.1989
ENSG00000012211	PRICKLE3	0.68848	1.14598	0.16862	0.20436	0.64173	1.01215	0.15301	0.16906
ENSG00000102003	SYP	0.09839	0.11698	0	0	0.05127	0.09918	0	0.01726
ENSG00000237341	SYP-AS1	0	0	0	0	0	0	0	0
ENSG00000102001	CACNA1F	0.08464	0.01761	0	0	0	0.03733	0.01764	0.03897
ENSG00000230216	HSPB1P2	0	0	0	0	0	0	0	0
ENSG00000101997	CCDC22	2.73857	2.42465	0.81342	1.34036	1.62932	2.467	0.77698	1.68119
ENSG00000049768	FOXP3	0.63691	0.18437	0.04069	0.0411	0.43767	0.39082	0.18463	0.068
ENSG00000049769	PPP1R3F	0.52696	0.38009	0	0.15641	0.51253	0.68173	0.17567	0.2588
ENSG00000270012	AC232271.1	0.51297	0.22469	0.11902	0.16027	0.16412	0.3334	0.13501	0.19889
ENSG00000215274	GAGE10	0	0	0	0.40441	0	0	0	0.16728
ENSG00000224659	GAGE12J	0	0	0	0	0	0	0	0
ENSG00000274274	GAGE13	0	0	0	0	0	0	0	0
ENSG00000275113	GAGE2E	0	0	0	0	0	0	0	0
ENSG00000236737	GAGE12B	0	0	0	0	0	0	0	0
ENSG00000237671	GAGE12C	0	0	0	0	0	0	0	0
ENSG00000227488	GAGE12D	0	0	0	0	0	0	0	0
ENSG00000236362	GAGE12F	0	0	0	0	0	0	0	0
ENSG00000216649	GAGE12E	0	0	0	0	0	0	0	0
ENSG00000215269	GAGE12G	0	0	0	0	0	0	0	0
ENSG00000224902	GAGE12H	0	0	0	0	0	0	0	0
ENSG00000205777	GAGE1	0	0	0	0	0	0	0	0
ENSG00000189064	GAGE2A	0	0	0	0	0	0	0	0
ENSG00000213856	VDAC1P2	0	0	0	0	0	0.15213	0	0
ENSG00000227391	SALL1P1	0	0	0	0	0	0	0	0
ENSG00000068985	PAGE1	0	0	0	0	0	0.19445	0.36746	0
ENSG00000101951	PAGE4	0	0	0	0	0	0	0	0
ENSG00000234390	USP27X-AS1	0.37071	0.16829	0.09905	0.05002	0.32779	0.17836	0.11235	0.08276
ENSG00000273820	USP27X	0.64397	0.69906	0.16457	0.16621	1.12332	0.54333	0.23335	0.20626
ENSG00000220132	AC236430.1	0	0	0	0	0	0	0	0
ENSG00000171365	CLCN5	1.22869	0.86172	0.20287	0.37904	0.77208	0.58452	0.24162	0.39833
ENSG00000201341	RNU6-421P	0	0	0	0	0	0	0	0
ENSG00000207758	MIR532	0	0	0	0	0	0	0	0
ENSG00000207768	MIR188	0	0	0	0	0	0	0	0
ENSG00000207785	MIR500A	0	0	0	0	3.18274	0	0	0
ENSG00000208015	MIR362	0	0	0	0	0	0	0	0
ENSG00000211538	MIR501	0	0	0	0	0	0	0	0
ENSG00000239057	MIR500B	0	0	0	0	0	0	0	1.1392
ENSG00000207970	MIR660	0	0	0	0	0	0	0	0
ENSG00000272080	MIR502	0	0	0	0	0	0	0	0
ENSG00000181977	PYY3	0	0	0	0	0	0	0	0
ENSG00000202495	RF00019	0	0	0	0	0	0	0	0
ENSG00000147081	AKAP4	0	0	0	0	0	0	0	0
ENSG00000147082	CCNB3	0.08132	0.09024	0	0.06035	0.03296	0.02391	0.02259	0.01664
ENSG00000274588	DGKK	0.05938	0.13178	0	0.07343	0.06016	0	0.03299	0.20655

ENSG00000158352	SHROOM4	0.09222	0.12988	0.01042	0.0737	0.13799	0.22526	0.08277	0.07839
ENSG00000222303	RNU6-935P	0	0	0	0	0	0	0	0
ENSG00000226881	H3F3AP5	0	0.2844	0	0	0	0	0	0
ENSG00000130385	BMP15	0	0	0	0	0	0	0	0
ENSG00000238190	HMGB1P15	0	0	0	0	0	0	0	0
ENSG00000227493	AC239367.1	0	0	0	0	0	0	0	0
ENSG00000230926	AC239367.2	0	0	0	0	0	0	0	0
ENSG00000230317	LINC01284	0	0	0	0	0	0	0	0
ENSG00000237926	AC239367.3	0	0	0	0	0	0	0	0
ENSG00000122824	NUDT10	0.03569	0	0.05244	0.05296	0.21693	0.31478	0	0
ENSG00000229151	AC233976.1	2.43793	0.54106	0.47767	0.24121	5.13755	0.86017	1.89637	0.7982
ENSG00000226530	AL158055.1	0.26148	0.14508	0	0	0.42386	0.15376	0.14528	0.42805
ENSG00000187690	CXorf67	0.038	0	0	0	0	0	0	0
ENSG00000196368	NUDT11	0.06147	0.15347	0.09033	0.09122	0.22419	0	0.25614	0.03773
ENSG00000234766	LINC01496	0	0	0	0	0	0	0	0
ENSG00000224109	CENPVL3	0	0	0	0	0	0	0	0
ENSG00000283093	CENPVL2	0	0	0	0	0	0	0	0
ENSG00000223591	CENPVL1	0	0	0	0	0	0	0	0
ENSG00000189369	GSPT2	1.1772	1.13213	0.38442	0.93178	3.05325	2.12289	0.74128	1.0278
ENSG00000179222	MAGED1	2.32719	0.93291	0.17905	0.5244	2.459	2.88028	0.8733	0.56847
ENSG00000236576	AC241520.1	0.13339	0.33305	0.09801	0.09899	0.32436	0.23533	0	0.40945
ENSG00000252377	RNU6-504P	0	0	0	0	0	0	0	0
ENSG00000228827	AL929410.1	0	0	0	0	0	0	0	0
ENSG00000232951	IPO7P1	0	0	0	0	0.28024	0.60996	0	0
ENSG00000237528	AL929410.2	0	0	0	0	0	0	0	0
ENSG00000232562	TPMTP3	0	0	0	0	0	0	0	0
ENSG00000182776	AC239585.1	0	0	0	0	0	0	0	0
ENSG00000279750	AC239585.2	0	0	0	0	0	0	0	0
ENSG00000187243	MAGED4B	0	0	0	0	0	0	0	0
ENSG00000221705	SNORA11E	0	0	0	0	0	0	0	0
ENSG00000154545	MAGED4	0	0	0	0	0	0	0	0
ENSG00000221475	SNORA11D	0	0	0	0	0	0	0	0
ENSG00000179028	AC245177.1	0	0	0	0	0	0	0	0
ENSG00000276474	AC231759.1	0	0	0	0	0	0	0.20466	0
ENSG00000275335	MIR8088	0	0	0	0	0	0	0	0
ENSG00000155622	XAGE2	0	0	0	0	0	0	0	0
ENSG00000225957	AC231532.1	0	0	0	0	0	0	0	0
ENSG00000276897	AC231532.2	0	0	0	0	0	0	0	0
ENSG00000224556	BX510359.1	0	0	0	0	0	0	0	0
ENSG00000250084	BX510359.5	0	0	0	0	0	0	0	0
ENSG00000278358	BX510359.8	0	0	0	0	0	0	0	0
ENSG00000278160	BX510359.7	0	0	0	0	0	0	0	0
ENSG00000275643	RBM22P6	0	0	0	0	0	0	0	0
ENSG00000204379	XAGE1A	0	0	0	0	0	0	0	0
ENSG00000223395	RBM22P7	0	0	0	0	0	0	0	0
ENSG00000204382	XAGE1B	0	0	0	0	0	0	0	0
ENSG00000277289	BX510359.6	0	0	0	0	0	0	0	0
ENSG00000227058	BX510359.2	0	0	0	0	0	0	0	0
ENSG00000231593	BX510359.4	0	0	0	0	0	0	0	0
ENSG00000228771	BX510359.3	0	0	0	0	0	0	0	0
ENSG00000185319	SSXP4	0	0	0	0	0	0	0	0
ENSG00000197185	SSXP1	0	0	0	0	0	0	0	0
ENSG00000234792	AL450023.2	0	0	0	0	0	0	0	0
ENSG00000157965	SSX8P	0	0	0	0	0	0	0	0
ENSG00000187754	SSX7	0	0	0	0	0	0	0	0
ENSG00000228354	AL450023.1	0	0	0	0	0	0	0	0
ENSG00000222608	RNA5SP504	0	0	0	0	0	0	0	0
ENSG00000225508	SSXP5	0	0	0	0	0	0	0	0

ENSG00000237275	AL450023.3	0	0	0	0	0	0	0	0
ENSG00000241476	SSX2	0	0	0	0	0	0	0	0
ENSG00000223958	AC244505.2	0	0	0	0	0	0	0	0
ENSG00000225397	AC244505.3	0	0	0	0	0	0	0	0
ENSG00000229826	AC244505.5	0	0	0	0	0	0	0	0
ENSG00000268447	SSX2B	0	0	0	0	0	0	0	0
ENSG00000269281	AC244505.7	0	0	0	0	0	0	0	0
ENSG00000226867	AC244505.4	0	0	0	0	0	0	0	0
ENSG00000204363	SPANXN5	0	0	0	0	0	0	0	0
ENSG00000229885	AC244505.6	0	0	0	0	0	0	0	0
ENSG00000171405	XAGE5	0	0	0	0	0	0	0	0
ENSG00000196395	AC244505.1	0	0	0	0	0	0	0	0
ENSG00000224781	EIF4A2P4	0	0	0	0	0.07451	0	0	0
ENSG00000171402	XAGE3	0	0	0	0	0	0	0	0
ENSG00000179304	FAM156B	0.01645	0	0	0	0	0	0.02742	0
ENSG00000268350	FAM156A	0.04437	0.05539	0	0	0.02697	0.01957	0	0.04086
ENSG00000184194	GPR173	0	0	0	0.02361	0.01934	0	0	0
ENSG00000270497	BX322635.1	0	0	0	0	0	0	0	0
ENSG00000184205	TSPYL2	3.25911	1.75073	0.283	0.63759	2.82767	3.24063	1.95074	0.78211
ENSG00000234019	AL591212.1	0	0	0	0	0	0	0	0
ENSG00000232593	KANTR	0.29536	0.17207	0.0217	0.19725	0.24238	0.15632	0.14769	0.21758
ENSG00000235224	AL591212.2	0	0	0	0	0	0	0	0
ENSG00000231340	ACTG1P10	0	0.11276	0	0.10054	0	0	0.11292	0
ENSG00000227329	AL139396.1	0	0	0	0	0	0	0	0
ENSG00000126012	KDM5C	7.20657	6.86446	1.46877	3.37813	5.25898	9.64615	3.73807	3.11437
ENSG00000276575	MIR6895	0.93975	0	0	0	0	0	1.56641	2.30762
ENSG00000277474	MIR6894	0	0	0	0	0	0	0	0
ENSG00000235262	KDM5C-IT1	0.19417	0.1616	0.28533	0.14409	0.23607	0	0	0
ENSG00000124313	IQSEC2	0.24633	0.15842	0.03291	0.06647	0.09529	0.02963	0.028	0.08936
ENSG00000226299	RPSAP62	0	0	0	0	0	0	0	0
ENSG00000207408	RF00019	0	0	0	0.96271	0	0	0	0
ENSG00000236571	AC245102.2	0	0	0	0	0	0	0	0
ENSG00000234413	AC245102.1	0	0	0	0	0	0	0	0
ENSG00000072501	SMC1A	18.437	17.887	12.144	30.5065	16.1675	24.027	16.5603	13.709
ENSG00000278204	MIR6857	0	0	0	0	0	0	0	0
ENSG00000158423	RIBC1	0.17143	0.16306	0	0.03635	0.08933	0.17282	0.04082	0.03007
ENSG00000072506	HSD17B10	7.62587	5.25238	0.77284	1.17079	6.23417	10.6697	1.42452	1.69501
ENSG00000233250	AC233728.1	0	0	0	0.49902	0	0	0	0.41283
ENSG00000235416	AC233728.2	0.09683	0	0.14229	0.14371	0	0	0.1614	0.23777
ENSG00000199422	VTRNA3-1P	0	0	0	0	0	0	0	0
ENSG00000086758	HUWE1	12.4881	11.0776	3.81151	8.01184	13.2239	14.3523	8.4289	5.75815
ENSG00000271886	MIR98	0.61597	0	0.90516	0	0.74888	0	1.02673	1.51256
ENSG00000208012	MIRLET7F2	0	0	0	0	0	0	0	1.0843
ENSG00000224735	BX323845.1	0	0	0	0	0	0	0	0
ENSG00000226110	BX323845.2	0	0	0	0	0	0	0	0
ENSG00000229037	BX323845.3	0	0	0	0	0	0	0	0.17077
ENSG00000235916	AC233279.1	0	0	0	0	0	0	0	0
ENSG00000201618	RNA5SP505	0	0	0	0	0	0	0	0
ENSG00000172943	PHF8	4.4964	4.37146	1.14704	2.43757	4.08266	4.70485	2.43954	2.37596
ENSG00000184083	FAM120C	0.37487	0.41599	0.13466	0.24727	0.59755	0.67602	0.22217	0.35798
ENSG00000224949	AL589872.1	0	0	0	0	0	0	0	0
ENSG00000196632	WNK3	0.00639	0	0	0	0.00777	0.01127	0	0
ENSG00000234566	RPL7AP71	0	0	0	0	0	0	0	0
ENSG00000207104	RNU6-434P	0	0	0	0	0	0	0	0
ENSG00000158526	TSR2	8.69665	5.7266	1.1937	2.41116	8.83034	7.58674	3.02663	3.22675
ENSG00000102302	FGD1	0	0.08562	0	0	0	0	0	0
ENSG00000130119	GNL3L	10.1358	6.69185	3.16194	4.36992	9.29374	7.3921	5.14394	7.85606
ENSG00000215197	PGAM4P1	0	0	0	0	0	0	0	0

ENSG00000102313	ITIH6	0	0	0	0	0	0	0	0
ENSG00000102316	MAGED2	7.82868	5.85143	1.06248	3.18217	7.24452	4.26641	2.65971	4.01007
ENSG00000221716	SNORA11	1.14532	0	0.84152	4.24944	2.08867	0	0	2.10931
ENSG00000275387	AL049732.1	0	0	0	0	0	0	0	0
ENSG00000067445	TRO	0.08731	0.03633	0.01604	0	0.01327	0.05776	0	0.0536
ENSG00000221750	SNORA11G	0	0	0	0	0	0	0	0
ENSG00000158571	PFKFB1	0.17809	0.29642	0.20936	0.58146	0.25982	0.1885	0.23747	0.34984
ENSG00000169188	APEX2	2.25887	1.95514	0.5311	0.70401	2.91105	2.39097	0.60243	0.74882
ENSG00000158578	ALAS2	318.516	1303.84	1270.61	721.075	467.132	2080.32	574.66	417.681
ENSG00000278283	AL020991.1	0	0	0	0	0	0	0	0
ENSG00000234466	AL590240.2	0	0	0	0	0	0	0	0
ENSG00000238269	PAGE2B	10.5541	12.273	10.4102	24.8898	13.886	8.92685	24.8216	14.7332
ENSG00000234068	PAGE2	0.11089	0.36916	0	0.65831	0.26964	0.19563	0	0.40846
ENSG00000278319	AL590240.4	0	0.62568	0	0	0	0	0	0
ENSG00000276929	AL590240.3	0	0	0	0	0	0	0	0
ENSG00000182518	FAM104B	2.52254	2.58762	2.58617	7.53098	2.9242	2.48378	3.56909	1.87269
ENSG00000186678	AL590240.1	0	0	0	0	0	0	0	0
ENSG00000229760	MTCO1P52	0	0	0	0	0	0	0	0
ENSG00000231947	MTND2P24	0	0	0	0	0	0	0	0
ENSG00000238047	MTND1P30	0	0	0	0	0	0	0	0
ENSG00000256045	MTRNR2L10	0	0	0	0	0	0	0.07986	0
ENSG00000158639	PAGE5	0	0	0	0	0	0	0	0.12113
ENSG00000204279	PAGE3	0	0	0	0	0	0	0	0
ENSG00000232765	AL158819.1	0	0	0	0	0	0	0	0
ENSG00000266328	MIR4536-2	0	0	0	0	0	0	0	0
ENSG00000283334	MIR4536-1	0	0	0	0	0	0	0	0
ENSG00000187601	MAGEH1	4.4622	2.25323	0.58941	1.7114	4.87643	5.13002	1.50427	1.78517
ENSG00000247746	USP51	0.29939	0.08306	0.04888	0	0.42465	0.02934	0.16634	0.10211
ENSG00000277516	AL590410.1	0	0	0	0	0	0	0	0
ENSG00000200635	RF00019	0	0	0	0	0	0	0	0
ENSG00000235136	AL627224.1	0	0	0	0	0	0	0	0
ENSG00000189299	FOXR2	0	0	0	0	0	0	0	0
ENSG00000229594	AL159987.2	0	0	0	0	0	0	0	0
ENSG00000169164	AL159987.1	0	0	0	0	0	0	0	0
ENSG00000083750	RRAGB	2.36713	2.28346	0.83009	2.15575	2.25653	2.27779	1.7038	1.6183
ENSG00000227486	AL445472.1	0.21837	0.48464	1.28358	2.37665	1.41596	0.25683	1.9413	0.71497
ENSG00000214745	AL391419.1	0	0	0.25344	0.08532	0.13979	0	0.09583	0
ENSG00000102349	KLF8	0.30061	0.12171	0.04776	0.15675	0.52352	0.18633	0.12188	0.10973
ENSG00000234135	RPL23AP83	0	0	0	0	0	0	0	0
ENSG00000188021	UBQLN2	5.19861	5.45144	1.29868	3.3433	4.25569	5.01351	1.96413	3.82966
ENSG00000230105	AL354793.1	0.10281	0.34224	0	0	0.62494	0.36273	0.17136	0
ENSG00000204272	NBDY	5.0241	3.14492	0.88342	1.21085	4.17653	5.07551	0.93049	1.26534
ENSG00000237748	UQCRBP1	0	0	0.32058	0	0	0.38486	0	0
ENSG00000204271	SPIN3	0.17584	0.10013	0.0204	0.06867	0.15752	0.0653	0.13883	0.1818
ENSG00000224799	AL139397.1	0	0	0	0	0	0	0	0
ENSG00000237708	AL139397.2	0	0	0	0	0	0	0	0
ENSG00000186787	SPIN2B	0.99256	0.66084	0.29171	0.14731	0.78437	1.4008	0.16544	0.1828
ENSG00000226310	AL022157.1	0.55113	0.55041	3.88742	3.59893	0.40203	0	1.28611	0.67667
ENSG00000147059	SPIN2A	0	0	0	0	0	0	0	0
ENSG00000231765	PPP1R11P2	0	0	0.28197	0.56956	0	0	0.31984	0.23559
ENSG00000165591	FAAH2	0.65349	0.30215	0.16005	0.53881	0.70622	1.02476	0.84721	0.57948
ENSG00000231831	MTHFD1P1	0	0.04414	0	0	0	0	0	0
ENSG00000198455	ZXDB	1.25608	0.76592	0.31068	0.66445	1.57247	1.57965	0.9121	0.82454
ENSG00000215174	NLRP2B	0	0	0	0	0	0	0	0
ENSG00000227427	Z93019.1	0	0	0	0	0	0	0	0
ENSG00000198205	ZXDA	1.35444	0.94925	0.26189	0.47609	1.30002	1.28903	0.83176	0.59079
ENSG00000228004	MYCLP2	0	0	0	0	0	0	0	0
ENSG00000235124	KRT8P17	0	0	0	0	0	0	0	0

ENSG00000236852	BX322784.1	0	0	0	0	0	0	0.11345	0
ENSG00000235170	MTND1P31	0	0	0	0	0	0	0	0
ENSG00000228182	MTND2P25	0	0	0	0	0	0	0	0
ENSG00000234570	ZFRP1	0	0	0	0	0	0	0	0
ENSG00000277499	AL359854.1	0	0	0	0	0	0	0	0
ENSG00000230823	CBX1P1	0	0	0	0	0	0	0	0
ENSG00000235437	LINC01278	0.47113	0.22952	0.06754	0.34107	0.47499	0.24326	0.32561	0.25395
ENSG00000186767	SPIN4	1.06826	0.94833	0.83722	3.09155	0.77926	1.28778	1.30579	1.11485
ENSG00000233661	SPIN4-AS1	0	0	0	0	0	0	0	0
ENSG00000131089	ARHGEF9	0.9581	0.59361	0.15644	0.56877	0.73126	0.67609	0.32827	0.42479
ENSG00000231729	ARHGEF9-IT1	0.55812	0	0.27339	0	0.22618	0	0	0.22842
ENSG00000236813	BTF3P8	0	0	0	0	0	0	0	0
ENSG00000222532	MIR1468	0	0	0	0	0	0	0	0
ENSG00000232794	HNRNPDP1	0	0	0	0	0	0	0.16204	0
ENSG00000226010	AL355852.1	0	0.06889	0	0	0.10064	0	0	0
ENSG00000184675	AMER1	0.68586	0.41907	0.15309	0.28346	0.52775	0.93427	0.11577	0.25582
ENSG00000244006	RN7SL799P	0	0	0	0	0	0	0	0
ENSG00000198881	ASB12	0	0.09465	0	0	0	0.20064	0	0
ENSG00000102043	MTMR8	0.57966	0.39471	0.11615	0.74297	0.60863	1.11556	0.79053	0.5823
ENSG00000213754	AL356317.1	0.20418	0.25489	0.07501	0.15151	0.06206	0.45025	0.08508	0.18802
ENSG00000283349	PFN5P	0	0	0	0	0	0	0	0
ENSG00000230889	SHC1P1	0	0	0	0	0	0	0	0
ENSG00000227261	YWHAZP7	0	0	0	0	0	0	0	0
ENSG00000226502	KRT8P27	0	0	0	0	0	0	0	0
ENSG00000225814	GRPEL2P2	0	0	0	0	0	0	0	0
ENSG00000236982	BLOC1S2P1	0	0	0	0	0	0	0	0
ENSG00000126970	ZC4H2	0.45343	0.3302	0.12493	0.23132	0.31009	0.77492	0.21257	0.10438
ENSG00000237971	AL445523.1	0	0	0	0	0	0	0	0
ENSG00000225569	CCT4P2	0.04735	0	0	0	0.05757	0	0.07893	0.05814
ENSG00000231857	MORF4L1P5	0	0	0	0	0	0	0	0
ENSG00000237963	AL441964.1	0	0	0	0	0	0	0	0
ENSG00000102053	ZC3H12B	0.24343	0.09351	0.04127	0.04169	0.27319	0.13214	0.10924	0.04598
ENSG00000228158	TLE1P1	0.12445	0.62144	0	0	0.4539	0.21954	0.20744	0.1528
ENSG00000227534	Z83841.1	0	0	0	0	0	0	0	0
ENSG00000001497	LAS1L	2.11153	1.17883	0.53963	0.89536	1.78584	2.59133	0.96188	0.78902
ENSG00000227942	FRMD8P1	0.0481	0	0	0	0	0	0	0
ENSG00000147065	MSN	259.258	204.928	28.4303	58.8474	163.905	173.643	54.4706	80.411
ENSG00000231809	NANOGP9	0	0	0	0	0	0	0	0
ENSG00000276689	AL450487.2	0	0	0	0	0	0	0	0
ENSG00000233833	ETF1P3	0	0	0	0	0	0	0	0
ENSG00000230039	AL450487.1	0.14734	0.36786	0.32477	3.06131	0.53739	1.42958	0.49118	0.27135
ENSG00000217835	AL034397.1	0	0	0	0	0	0	0	0
ENSG00000237311	AL034397.2	1.11191	0	0.12569	0	0.62392	0.15089	0	0.21003
ENSG00000274536	AL034397.3	49.8765	30.6534	9.4361	11.448	20.5238	15.0329	15.1463	31.0403
ENSG00000284567	MIR223	0	0	0	0	0	0	0	0
ENSG00000155659	VSIG4	1.97296	0.33396	0.09828	0.19851	0.24393	0.23597	0.11148	0.08211
ENSG00000215168	AL139276.1	0	0	0	0	0	0	0	0
ENSG00000237294	EIF4BP9	0.09689	0	0	0.1438	0.0589	0.08547	0	0
ENSG00000089472	HEPH	0.01031	0	0.01514	0.01529	0	0	0.05153	0
ENSG00000231356	AL157698.1	0	0	0	0	0	0	0	0
ENSG00000226820	AL592049.1	0	0	0	0	0	0	0	0
ENSG00000235892	PKMP2	0	0	0	0	0	0	0	0
ENSG00000131080	EDA2R	0	0	0	0	0	0	0	0
ENSG00000222667	RNU6-394P	0	0	0	0	0	0	0	0
ENSG00000226280	AL049641.1	0	0	0	0	0	0	0	0
ENSG00000233710	AL049564.1	0	0	0	0	0	0	0	0
ENSG00000169083	AR	0.0459	0	0	0.02555	0.03488	0.0911	0.05738	0.07045
ENSG00000234201	BMI1P1	0	0	0	0	0	0	0	0

ENSG00000260118	AL157700.1	0	0	0	0	0	0	0	0
ENSG00000079482	OPHN1	1.03391	0.80113	0.58939	1.045	1.0186	0.36165	0.6834	0.64565
ENSG00000228328	AL158201.1	0	0	0	0.08717	0	0	0	0.07211
ENSG00000252145	RNU6-1225P	0	0	0	0	0	0	0	0
ENSG00000228125	AKIRIN1P2	0	0	0	0	0	0	0	0
ENSG00000206646	RF00019	0	0	0	0	0	0	0	0
ENSG00000225925	AL672138.1	0	0	0	0	0	0	0	0
ENSG00000181704	YIPF6	7.83527	7.16886	7.87468	17.4549	7.42135	5.20169	14.2199	9.16241
ENSG00000206747	RNU6-245P	0	0	0	0	0	0	0	0
ENSG00000236735	RPL31P63	0	0	0	0	0	0	0	0
ENSG00000234463	COX6CP12	0	0	0	0	0	0	0	0
ENSG00000130052	STARD8	1.5694	1.05369	0.1938	0.56761	0.57722	0.30246	0.43966	0.421
ENSG00000232267	ACTR3P2	0	0	0	0	0	0	0	0
ENSG00000213740	SERBP1P1	0	0.21036	0	0.09378	0.38412	0	0	0
ENSG00000090776	EFNB1	2.14548	1.61991	0.32503	0.755	1.21009	1.32667	0.29494	0.46166
ENSG00000181191	PJA1	2.51625	2.38772	0.55291	0.90742	2.91623	2.73807	0.78396	0.77957
ENSG00000172186	HMGN1P35	0	0	0	0	0	0	0	0
ENSG00000215162	LINC00269	0	0	0	0	0	0	0	0
ENSG00000231706	CYCSP43	0	0	0	0	0	0	0	0
ENSG00000130054	FAM155B	0	0	0	0.02711	0	0	0	0
ENSG00000228160	AL158069.1	0	0	0	0	0	0	0	0
ENSG00000158813	EDA	0.03142	0.01743	0.01539	0.04663	0.02547	0.01848	0.03491	0
ENSG00000272351	RNA5SP506	0	0	5.43096	8.22749	4.49328	0	0	10.5879
ENSG00000212434	RF00012	0	0	0	0	0	0	0	0
ENSG00000233229	CNOT7P1	0	0	0	0	0	0	0	0
ENSG00000211991	MIR676	0	0	0	0	0	0	0	0
ENSG00000147160	AWAT2	0	0	0	0	0	0	0	0
ENSG00000189401	OTUD6A	0	0	0	0	0	0	0	0
ENSG00000226579	MTCYBP31	0	0	0	0	0	0	0	0
ENSG00000235382	MTND4P31	0	0	0	0	0	0	0	0
ENSG00000089289	IGBP1	17.7149	13.9569	4.16509	8.06253	15.0283	15.9035	7.7429	10.5367
ENSG00000220925	IGBP1-AS2	0	0	0	0	0	0	0	0
ENSG00000203588	IGBP1-AS1	0	0	0	0	0	0	0	0
ENSG00000184210	DGAT2L6	0	0	0	0	0	0	0	0
ENSG00000242657	RN7SL581P	0	0	0	0	0	0	0	0
ENSG00000204195	AWAT1	0	0	0	0	0	0	0	0
ENSG00000200473	RNA5SP507	0	0	0	0	0	0	0	0
ENSG00000186912	P2RY4	0	0	0	0	0	0	0	0
ENSG00000120500	ARR3	0	0	0	0	0	0	0	0
ENSG00000147127	RAB41	0	0	0	0	0	0	0	0
ENSG00000120509	PDZD11	2.52578	2.18967	0.46395	1.95236	2.36706	6.49808	1.14023	1.48595
ENSG00000090889	KIF4A	0.8732	0.88822	0.92672	1.7759	2.35914	4.45017	3.09966	1.96552
ENSG00000231053	AL357752.1	0	0	0	0	0	0	0	0
ENSG00000201377	RNY4P23	0	0	0	0	0	0	0	0
ENSG00000130055	GDPD2	0	0.04625	0	0	0	0	0	0
ENSG00000284391	AL139398.1	0	0	0	0	0	0	0	0
ENSG00000082458	DLG3	0.79431	0.82438	0.23344	0.36058	0.64758	1.86285	0.43614	0.58514
ENSG00000200431	RNU4-81P	0	0	0	0	0	0	0	0
ENSG00000231651	DLG3-AS1	0	0	0	0	0	0	0	0
ENSG00000120498	TEX11	0	0	0.12781	0.09681	0	0	0.10873	0.0267
ENSG00000230187	AL139109.1	0	0	0	0	0	0	0	0
ENSG00000277492	RF01848	0	0	0	0	0	0	0	0
ENSG00000280704	SNORD3E	0	0	0	0	0	0	0	0
ENSG00000213747	AL627390.1	0	0	0	0	0	0	0	0
ENSG00000165349	SLC7A3	0	0	0	0.04803	0.07869	0	0	0
ENSG00000230629	RPS23P8	0.44424	0	0.97922	1.64827	0.81015	0.39185	0.37024	1.09087
ENSG00000229324	NUTF2P7	0	0	0	0	0	0	0	0
ENSG00000212605	RNU1-56P	0	0	0	0	0	0	0	0

ENSG00000227536	SOCS5P4	0.23404	0	0	0	0	0.49545	0	0.05747
ENSG00000147164	SNX12	5.81883	4.13747	1.28676	2.51528	4.63613	5.73058	2.35415	2.9132
ENSG00000184481	FOXO4	27.0065	21.5559	13.8119	16.0322	47.0273	26.7477	72.0251	28.5891
ENSG00000204165	CXorf65	2.01446	1.40613	0.19098	0.86797	1.34307	1.37566	0.6499	0.71806
ENSG00000147168	IL2RG	38.7217	37.7077	6.32234	14.3865	31.9658	55.3323	9.73901	13.2061
ENSG00000184634	MED12	8.2432	4.86391	1.07351	1.91932	5.25698	5.8691	1.59616	2.41204
ENSG00000196338	NLGN3	0.43914	0.30456	0	0.08147	0.37818	0.06456	0.0305	0.04493
ENSG00000228427	AL590764.1	1.41351	2.01667	0.14837	0.29969	2.2095	1.60304	0.67317	1.11567
ENSG00000169562	GJB1	0	0	0	0	0	0	0	0
ENSG00000147130	ZMYM3	2.08794	1.51717	0.26477	0.66064	1.7911	2.31849	0.54766	0.84584
ENSG00000147140	NONO	26.525	16.692	3.84693	9.2916	22.6833	27.7341	7.94218	10.4588
ENSG00000147166	ITGB1BP2	0.23312	0	0	0.09885	0.20245	0	0.05551	0.08178
ENSG00000224208	AL590762.3	0	0	0	0.16998	0	0	0	0
ENSG00000213706	AL590762.2	0	0	0	0	0	0	0	0
ENSG00000147133	TAF1	7.76876	6.47117	2.11262	3.95675	7.40998	6.09647	5.44524	5.05508
ENSG00000275975	RF00017	0	0	0	0	0.29411	0	0	0
ENSG00000229601	AL590762.4	0	0	0	0	0	0	0	0
ENSG00000213018	AL590762.1	0	0	0	0	0	0	0	0
ENSG00000199751	RF00019	0	0	0	1.06653	0	0	0	0
ENSG00000243468	INGX	0	0.09784	0	0	0	0	0	0
ENSG00000215120	AL590763.1	0	0	0	0	0	0	0	0
ENSG00000147162	OGT	14.9111	10.5073	3.00791	8.84743	13.6236	13.8906	10.2357	10.4929
ENSG00000147174	GCNA	0.76924	0.40434	0.14874	0.33047	0.63989	1.14277	0.40491	0.64621
ENSG00000186810	CXCR3	2.1582	1.50225	0.23065	1.68886	2.52847	7.68394	0.52326	0.77085
ENSG00000237265	BX276092.7	0	0	0	0	0	0	0	0
ENSG00000283599	BX276092.9	0	0	0	0	0	0	0	0
ENSG00000230934	BX276092.4	0	0	0	0	0	0	0	0
ENSG00000281852	LINC00891	0.07129	0.02966	0.02619	0	0.08667	0.06288	0	0.08752
ENSG00000215115	CXorf49	0	0	0	0	0	0	0	0
ENSG00000226870	BX276092.2	0	0	0	0	0	0	0	0
ENSG00000237182	BX276092.6	0	0	0	0	0	0	0	0
ENSG00000237717	BX276092.8	0	0	0	0	0	0	0	0
ENSG00000234442	BX276092.5	0	0	0	0	0	0	0	0
ENSG00000215113	CXorf49B	0	0	0	0	0	0	0	0
ENSG00000224617	BX276092.1	0	0	0	0	0	0	0	0
ENSG00000229030	BX276092.3	0	0	0	0	0	0	0	0
ENSG00000283178	BX119927.1	0	0	0	0	0	0	0	0
ENSG00000204131	NHSL2	9.58791	9.2888	1.42962	1.97223	6.17566	2.9067	1.09718	3.45634
ENSG00000201392	RNU6-1078P	0	0	0	0	0	0	0	0
ENSG00000196933	RPS26P11	0	0	0	0	0	0	0	0
ENSG00000242732	RTL5	0.24541	0.28083	0.04508	0.04553	0.14918	0.13529	0.05113	0.0565
ENSG00000225471	BX119917.1	0	0	0	0	0	0	0	0
ENSG00000102309	PIN4	1.57164	1.1559	0.56395	0.37971	0.88872	1.48301	0.51784	0.83019
ENSG00000239577	RN7SL388P	0.43245	0	0	0	0	0	0	0
ENSG00000186871	ERCC6L	0.06719	0.19571	0.09873	0.27421	0.14295	0.80005	0.11199	0.08249
ENSG00000198034	RPS4X	195.246	116.812	31.4256	76.3543	180.983	233.472	85.8376	69.8368
ENSG00000238926	RF00019	0	0	0	0	0	0	0	0
ENSG00000125931	CITED1	0.0833	0	0	0.06181	0	0.07347	0	0
ENSG00000147099	HDAC8	1.3082	0.97534	0.36045	0.80897	1.07689	1.44241	0.59057	0.68598
ENSG00000222810	RNU2-68P	0	0	0	0	0	0	0	0
ENSG00000067177	PHKA1	0.10389	0.03843	0	0.01713	0.07017	0	0	0.01417
ENSG00000225858	BX295541.1	0	0	0	0	0	0	0	0
ENSG00000231944	PHKA1-AS1	0	0	0	0	0	0	0	0
ENSG00000273788	RF00012	0	0	0	0	0	0	0	0
ENSG00000213698	AC240504.1	0	0	0	0	0	0	0	0
ENSG00000201271	RNU1-112P	0	0	0	0	0	0	0	0
ENSG00000184911	DMRTC1B	0	0	0	0	0	0	0	0
ENSG00000269911	FAM226B	0	0	0	0	0	0	0	0

ENSG00000268994	FAM236B	0	0	0	0	0	0	0	0
ENSG00000225396	FAM236D	0	0	0	0	0	0	0	0
ENSG00000269502	DMRTC1	0	0	0	0	0	0	0.06594	0
ENSG00000283594	FAM236C	0	0	0	0	0	0	0	0
ENSG00000275520	FAM236A	0	0	0	0	0	0	0	0
ENSG00000226725	PABPC1L2B-AS1	0	0	0	0	0	0	0	0
ENSG00000184388	PABPC1L2B	0	0	0	0	0	0	0	0
ENSG00000186288	PABPC1L2A	0	0	0	0	0	0	0	0
ENSG00000231963	AL662864.1	0	0	0	0	0	0	0	0
ENSG00000204118	NAP1L6	0	0	0	0	0	0	0	0
ENSG00000186462	NAP1L2	0.51741	0.28708	0.04224	0.38395	0.27958	0.20284	0.3354	0.38822
ENSG00000230311	TOMM20P4	0	0	0	0.25008	0	0	0	0
ENSG00000237014	AC004074.1	0	0	0	0	0	0	0	0
ENSG00000225664	YWHAZP8	0	0	0	0	0	0	0	0
ENSG00000207486	RNU6-1044P	0	0	0	0	0	0	0	0
ENSG00000131264	CDX4	0	0	0	0	0	0	0	0
ENSG00000233162	MORF4L1P6	0	0	0	0	0	0	0	0
ENSG00000269904	MAP2K4P1	0.20209	0.33638	0.25454	0.85692	0.31589	0.25465	0.76994	0.4608
ENSG00000237475	RPL7P53	0	0	0	0	0	0	0	0
ENSG00000204116	CHIC1	2.47187	1.88138	0.82272	2.44568	2.47867	2.73942	1.77838	1.78982
ENSG00000230146	SEPHS1P4	0	0	0	0	0	0	0	0
ENSG00000270641	TSIX	0	0	0.00291	0.00294	0	0	0	0
ENSG00000229807	XIST	0.00734	0.00611	2.54162	5.47716	0	8.81038	7.79809	0.01353
ENSG00000274655	RF02266	0	0	0	0	0	0	0	0
ENSG00000277577	RF01881	0	0	0	0	0	0	0	0
ENSG00000278039	RF01880	0	0	0	0	0	0	0	0
ENSG00000182707	FXYD6P3	0	0	0	0	0	0	0	0
ENSG00000225470	JPX	1.29764	1.20596	0.31781	0.60985	1.05833	1.04922	0.64889	0.79661
ENSG00000274430	RF02124	0	0	0	0	0	0	0	0
ENSG00000276784	RF02125	0	0	0	0	0	0	0	0
ENSG00000230590	FTX	1.17971	0.97534	0.27813	0.59259	0.83219	0.54431	0.62665	0.75446
ENSG00000228906	AL353804.1	1.30023	1.35264	0.47767	0.96484	0.98799	1.72033	1.08364	0.19955
ENSG00000271199	AL353804.2	0	0	0	0	0	0	0	0
ENSG00000233984	RPSAP14	0	0	0	0	0	0	0	0
ENSG00000203397	AL139400.1	0	0	0	0	0	0	0	0
ENSG00000234969	AL627389.1	0	0	0	0.09204	0	0	0	0
ENSG00000223650	UHRF2P1	0	0	0	0	0	0	0	0
ENSG00000232928	DDX3P1	0	0	0	0	0	0	0	0
ENSG00000280375	AL359740.1	0	0	0	0	0	0	0	0
ENSG00000230986	DDX3P2	0	0	0	0	0	1.16497	0	0
ENSG00000224430	MKRN5P	0.1499	0.08317	0	0	0.06075	0	0	0
ENSG00000231549	ATP5MDP1	0	0	0	0	0	0.73058	0	0
ENSG00000204113	BMP2KL	0	0	0	0	0	0	0	0
ENSG00000228492	RAB11FIP1P1	0	0	0	0	0	0	0	0
ENSG00000271533	Z83843.1	1.31284	0.82774	0.58461	0.9742	0.91898	0.84219	1.061	0.92806
ENSG00000202566	MIR421	6.89886	1.43539	0	1.27983	2.09686	3.04263	1.43742	3.17637
ENSG00000212027	MIR374B	0	0	0	0	0	3.59199	0	2.49992
ENSG00000283534	MIR374C	0	0	0	0	0	0	0	0
ENSG00000265727	RN7SL648P	2.43523	1.21603	0.35785	0	0.29607	1.28882	0.81183	2.09295
ENSG00000278421	RF02119	0	0	0	0	0	0	0	0
ENSG00000273974	RF02120	0	0	0	0	0	0	0	0
ENSG00000275254	RF02121	0	0	0	0	0	0	0	0
ENSG00000275625	RF02123	0	0	0	0	0	0	0	0
ENSG00000207820	MIR545	0	0	1.01617	1.02628	4.20362	0	2.30529	2.54709
ENSG00000199168	MIR374A	0	0	0	0	0	0	0	0
ENSG00000277922	RF02122	0	0	0	0.49902	0	0	0	0
ENSG00000187969	ZCCHC13	0	0	0	0	0	0	0	0
ENSG00000226515	AC004386.2	0	0	0	0	0	0	0	0

ENSG00000213269	AC004386.1	0	0	0	0	0	0	0	0
ENSG00000239745	RN7SL790P	0	0	0	0	0	0	0	0
ENSG00000230665	THAP12P1	0	0.05538	0	0	0	0	0	0
ENSG00000213695	RPS7P14	0	0	0	0	0	0	0	0
ENSG00000147100	SLC16A2	0.0339	0	0	0	0.04122	0.08972	0	0.02081
ENSG00000231206	HNRNPA1P25	0	0	0	0	0	0	0	0
ENSG00000230673	PABPC1P3	4.95057	5.05046	0.46934	0.47401	3.10646	2.53553	0.26619	1.56858
ENSG00000131263	RLIM	14.6706	16.5561	3.95941	6.28384	13.4967	8.07138	7.37836	10.0749
ENSG00000199885	RNU6-330P	0	0	0	0	0	0	0	0
ENSG00000050030	NEXMIF	0.0187	0	0	0	0.04548	0.011	0.03118	0.01531
ENSG00000271589	AL139395.1	0	0	0	0	0	0	0	0
ENSG00000131269	ABCB7	9.20573	6.73717	3.33659	7.65529	8.52159	8.31601	10.5314	5.9998
ENSG00000229661	BUD31P2	0	0	0	0.36262	0.29706	0	0	0
ENSG00000199601	RNU6-562P	0	0	0	0	0	0	0	0
ENSG00000094841	UPRT	4.59262	4.53483	1.25825	4.2359	5.26814	5.26402	3.15723	3.09016
ENSG00000232186	AL137013.1	0	0	0	0	0	0	0	0
ENSG00000102383	ZDHHC15	0.03224	0.03578	0.07897	0.14356	0.14374	0	0.10749	0.0132
ENSG00000233254	RPL21P134	0	0	0.22301	0	0	0	0.25296	0
ENSG00000237089	PCNPP4	0	0	0	0	0	0	0	0
ENSG00000224775	BRAFP1	0	0	0	0	0	0	0	0.0333
ENSG00000215105	TTC3P1	0.10849	0.08026	0	0.12523	0.17586	0.04253	0.02009	0.1184
ENSG00000186675	MAGEE2	0	0	0	0	0	0	0	0
ENSG00000233063	SAR1AP4	0	0	0	0.18407	0	0	0	0
ENSG00000226753	AL451105.1	0	0	0	0	0	0	0	0
ENSG00000226854	AL451105.2	0	0	0	0	0	0	0	0
ENSG00000232332	AC233982.1	0	0	0	0	0	0	0	0
ENSG00000102390	PBDC1	5.00469	5.48581	0.80718	3.0797	6.15877	4.73748	2.64504	2.9974
ENSG00000237252	AC233981.1	0	0	0	0	0	0	0	0
ENSG00000201826	RNU6-867P	0	0	0	0	0	0	0	0
ENSG00000274398	AC233981.2	0	0	0	0	0	0	0	0
ENSG00000213684	LDHBP2	0	0	0	0	0	0	0	0
ENSG00000198934	MAGEE1	0.68656	0.16806	0.05935	0.59937	0.90835	0.78371	0.33658	0.52064
ENSG00000223259	RNA5SP508	0	0	0	0	0	0	0	0
ENSG00000235447	TRAPPC13P1	0	0	0	0	0	0	0	0
ENSG00000280870	MIR325HG	0	0	0	0	0	0	0	0
ENSG00000283242	MIR384	0	0	0	0	0	0	0	0
ENSG00000207995	MIR325	0	0	0	0	0	0	0	0
ENSG00000201447	RNA5SP509	0	0	0	0	0	0	0	0
ENSG00000234404	SPRYD7P1	0	0	0	0	0	0	0	0
ENSG00000196468	FGF16	0	0	0	0	0	0	0	0
ENSG00000085224	ATRX	14.7953	13.6281	4.06257	7.5184	14.9266	12.1326	7.34272	9.53031
ENSG00000237106	FABP5P15	0	0	0	0	0	0	0	0
ENSG00000102158	MAGT1	13.5082	9.93734	4.96255	10.8741	9.69616	10.4258	8.44356	9.21813
ENSG00000200906	RNU6-854P	0	0	0	0	0	0	0	0
ENSG00000263410	RN7SL460P	0	0	0	0	0	0	0	0
ENSG00000131174	COX7B	7.68393	7.22206	3.63321	5.89975	6.45389	10.0918	6.46457	7.38071
ENSG00000165240	ATP7A	10.0811	12.5313	10.76	27.7417	18.327	17.6176	22.9634	17.8327
ENSG00000248503	AL356235.1	1.416	0.9528	0.39845	1.52023	0.73257	0.42519	7.13095	0.99873
ENSG00000226784	PGAM4	0.09582	0	0	0	0	0	0	0
ENSG00000102144	PGK1	54.092	49.6145	8.15563	13.2592	38.3786	27.3907	16.8548	25.9105
ENSG00000187325	TAF9B	5.09504	2.09832	2.00685	4.32648	3.41651	4.07719	2.62659	3.32128
ENSG00000223592	FNDC3CP	0	0	0	0	0	0	0	0
ENSG00000173198	CYSLTR1	11.0523	8.84786	3.78961	3.33914	6.19066	3.5978	2.63178	3.82863
ENSG00000224755	HMGN1P34	0	0	0	0	0	0	0	0
ENSG00000235061	UBE2V1P7	0	0	0	0	0	0	0	0
ENSG00000179300	RTL3	0	0	0	0	0	0	0	0
ENSG00000271433	PPATP2	0	0	0	0	0	0	0	0
ENSG00000147145	LPAR4	0.49448	0.19597	0.13841	0.13978	0	0.08308	0.27474	0.08673

ENSG00000227645	RPL7P54	0	0	0	0	0	0	0	0
ENSG00000283622	MIR4328	0	0	0	0	0	0	0	0
ENSG00000078589	P2RY10	29.434	12.6542	4.18191	9.95543	26.4928	23.5243	12.9431	15.9729
ENSG00000232168	P2RY10BP	0	0	0	0	0	0	0	0
ENSG00000147138	GPR174	7.34169	7.46793	3.3393	7.95571	8.28828	18.0913	7.38132	5.86626
ENSG00000271005	CTHRC1P1	0	0	0	0	0	0	0	0
ENSG00000225025	KIF4CP	0	0	0	0	0	0	0	0.07037
ENSG00000078596	ITM2A	8.32715	4.58052	4.09639	5.67532	11.8185	16.6447	9.29309	8.86368
ENSG00000270268	AL009028.1	0	0	0	0	0	0	0	0
ENSG00000122145	TBX22	0	0	0	0	0	0	0	0
ENSG00000278530	CHMP1B2P	0.03121	0.24242	0.16816	1.08076	0.39208	0.29364	1.54329	0.24268
ENSG00000174016	TENT5D	0	0	0	0	0	0	0	0
ENSG00000230247	HNRNPH3P1	0	0	0	0	0	0	0	0
ENSG00000228051	AL512504.2	0	0	0	0	0	0	0	0
ENSG00000228612	HK2P1	0	0	0	0	0	0	0	0
ENSG00000165288	BRWD3	14.1473	13.6387	2.43532	6.29027	11.7732	5.888	5.5729	7.37332
ENSG00000225715	AL512504.1	0	0	0	0	0	0	0	0
ENSG00000239008	RF01225	0	0	0	0	0	0	0	0
ENSG00000212623	RNU6-493P	0	0	0	0	0	0	0	0
ENSG00000073905	VDAC1P1	0.08644	0	0	0	0	0	0	0
ENSG00000212546	RNU6-995P	0	0	0	0	0	0	0	0
ENSG00000198157	HMGN5	0.57645	0.404	0.2675	0.45027	1.03281	0.26762	0.354	0.52151
ENSG00000131171	SH3BGRL	161.299	102.945	45.4886	132.677	101.262	78.5749	81.2414	103.434
ENSG00000231875	AL359885.1	1.13644	0.27023	0.47714	0.72283	0.78952	0.1432	0.1353	0.79732
ENSG00000224523	AL445213.1	0.29438	1.30665	1.58615	2.18445	2.26669	2.76973	0.32712	0.36143
ENSG00000233446	AL691517.1	0	0	0	0	0	0	0	0
ENSG00000206826	RNU6-974P	0	0	0	0	0	0	0	0
ENSG00000228041	AL031313.1	0	0	0	0	0	0	0	0
ENSG00000213653	RPL22P22	0	0	0	0	0	0	0	0
ENSG00000202183	RF00394	0	0	0	0	0	0	0	0
ENSG00000236365	AL008987.1	0	0	0	0	0	0	0	0
ENSG00000232735	ATG4AP1	0	0	0	0	0	0	0	0
ENSG00000279437	Z82170.1	0	0	0	0	0	0	0	0
ENSG00000196767	POU3F4	0	0	0	0	0	0	0	0
ENSG00000236870	AL121882.1	0	0	0	0	0	0	0	0
ENSG00000215102	TERF1P4	0	0	0	0.09757	0.07993	0	0	0
ENSG00000183035	CYLC1	0	0	0	0	0	0	0	0
ENSG00000072133	RPS6KA6	0.02586	0	0.01267	0.03839	0.04193	0	0	0
ENSG00000221494	MIR548I4	0	0	0	0	0	0	0	0
ENSG00000165259	HDX	0.59586	0.6556	0.23745	0.32974	0.38063	0.30288	0.18517	0.27279
ENSG00000233484	AL450471.1	0	0.9607	0.84814	0	0	0	0	0
ENSG00000237211	SETP4	0	0	0	0.12533	0	0	0	0
ENSG00000229547	UBE2DNL	0	0	0	0	0	0	0	0
ENSG00000230781	Z83820.1	0	0	0	0	0	0	0	0
ENSG00000155008	APOOL	1.93662	2.98127	2.91163	7.7087	2.89888	2.13282	3.47061	4.0408
ENSG00000184788	SATL1	0	0	0	0	0	0	0	0
ENSG00000283446	Z99571.1	0	0	0	0	0	0	0	0
ENSG00000276391	AC003001.2	0	0	0	0	0	0	0	0
ENSG00000235461	AC003001.1	0	0	0	0	0	0	0	0
ENSG00000147180	ZNF711	0.11719	0.19506	0.02153	0.06522	0.64113	0.20673	0.07325	0.17985
ENSG00000124429	POF1B	0.01819	0.06057	0.02673	0.081	0.02212	0.0321	0.03033	0.06701
ENSG00000283435	MIR1321	0	0	0	0	0	0	0	0
ENSG00000223707	SFR1P2	0	0	0	0	0	0	0	0
ENSG00000276892	AL022401.1	0	0	0	0	0	0	0	0
ENSG00000188419	CHM	3.0185	2.54037	0.49839	0.98153	2.41218	2.73731	0.89038	1.36375
ENSG00000199051	MIR361	0	0	0	0	0	0	0	0
ENSG00000199567	RF00019	0	0	0	0	0	0	0	0
ENSG00000234039	NDUFA5P7	0	0	0	0	0	0	0	0

ENSG00000225536	STIP1P3	0	0	0	0	0	0	0	0
ENSG00000223574	TPMTP4	0	0	0	0	0	0	0	0
ENSG00000126733	DACH2	0	0	0	0	0	0	0	0
ENSG00000215093	EEF1A1P29	0	0	0	0	0	0	0	0
ENSG00000223772	GEMIN8P3	0	0	0	0	0	0	0	0
ENSG00000277717	RF00026	0	0	0	0	0	0	0	0
ENSG00000231478	FCF1P9	0	0	0	0	0	0	0	0
ENSG00000271290	AL390058.1	0	0	0	0	0	0	0	0
ENSG00000236190	Z82210.1	0	0	0	0	0	0	0	0
ENSG00000271457	AL590229.1	0	0	0	0	0	0	0	0
ENSG00000200422	RF00279	0	0	0	0	0	0	0	0
ENSG00000230478	Z96811.1	0	0	0	0	0	0	0	0
ENSG00000102271	KLHL4	0	0.02097	0	0	0.03063	0	0.021	0.01547
ENSG00000237506	RPSAP15	0.08513	0	0	0	0	0	0	0.10453
ENSG00000282914	Z83818.2	0	0	0	0	0	0	0	0
ENSG00000271205	Z83818.1	0	0	0	0	0	0	0	0
ENSG00000270446	AL136363.1	0	0	0	0	0	0	0	0
ENSG00000271056	AL136363.2	0	0	0	0	0	0	0	0
ENSG00000230706	AL035246.1	0	0	0	0	0	0	0	0
ENSG00000230956	AL024493.1	0	0	0	0.1306	0	0	0.29335	0
ENSG00000252016	RF01225	0	0	0	0	0	0	0	0
ENSG00000271693	AL929325.1	0	0	0	0	0	0	0	0
ENSG00000147183	CPXCR1	0	0	0	0	0	0	0	0
ENSG00000271157	AL022154.1	0	0	0	0	0	0	0	0
ENSG00000232576	AL645638.1	0	0	0	0	0	0	0	0
ENSG00000270745	AL731768.1	0	0	0	0	0	0	0	0
ENSG00000224065	SRIP2	0	0	0	0	0	0	0	0
ENSG00000225012	AL121872.1	0.46887	1.04058	0	0	0.76006	0	0	2.68648
ENSG00000153779	TGIF2LX	0	0	0	0	0	0	0	0
ENSG00000227581	AL121877.1	0	0	0	0	0	0	0	0
ENSG00000226081	USP12PX	0	0.11543	0.20381	0	0	0	0	0.08514
ENSG00000274022	AL121873.1	0	0	0	0	0	0	0	0
ENSG00000227434	RNF19BPX	0	0	0	0	0	0	0	0
ENSG00000229667	UBE2V1P9	0	0	0	0	0	0	0	0
ENSG00000207515	RNU6-555P	0	0	0	0	0	0	0	0
ENSG00000206062	AL121823.1	0	0	0	0	0	0	0	0
ENSG00000234161	PABPC5-AS1	0	0	0	0	0	0	0	0
ENSG00000236413	AL662874.1	0	0	0	0	0	0	0	0
ENSG00000174740	PABPC5	0	0	0	0	0	0	0	0
ENSG00000230275	SERBP1P4	0	0	0	0	0	0	0	0
ENSG00000102290	PCDH11X	0	0	0	0	0	0	0	0
ENSG00000222440	RNU2-26P	0	0	0	0	0	0	0	0
ENSG00000234702	VDAC1P3	0	0	0	0	0	0	0	0
ENSG00000229132	EIF4A1P10	0	0.20067	0	0.08946	0.21986	0.21268	0	0
ENSG00000215089	KRT18P11	0	0	0	0	0	0	0	0
ENSG00000230543	SNX3P1X	0	0	0	0	0	0	0	0
ENSG00000225912	AL121871.1	0.67248	0	0	0	0	0	0	0
ENSG00000237873	AL158053.1	0	0	0	0	0	0	0	0
ENSG00000234130	AL359263.1	0	0	0	0.07782	0	0	0	0
ENSG00000234322	ST13P18	0	0	0	0	0	0	0	0
ENSG00000237381	AL023280.1	0	0	0	0	0	0	0	0
ENSG00000228301	RPL7P55	0	0	0	0	0	0	0	0
ENSG00000186310	NAP1L3	0.13888	0.23116	0.12245	0.24733	0.37146	0.098	0.37038	0.40923
ENSG00000179083	FAM133A	0	0	0	0	0	0	0	0
ENSG00000270826	Z94722.1	0	0	0	0	0	0	0	0
ENSG00000212495	RNU6-332P	0	0	0	0	0	0	0	0
ENSG00000202410	RF00019	0	0	0	0	0	0	0	0
ENSG00000227521	AL390840.1	0	0	0	0	0	0	0	0

ENSG00000232293	PAICSP7	0	0	0	0	0	0	0	0
ENSG00000231001	CCNB1IP1P3	0	0	0	0	0	0	0	0
ENSG00000221187	MIR548M	0	0	0	0	0	0	0	0
ENSG00000271094	AL022161.1	0	0	0	0	0	0	0	0
ENSG00000223467	CALM1P1	0	0	0	0	0	0	0	0
ENSG00000201567	RNA5SP510	0	0	0	0	0	0	0	0
ENSG00000215085	AL365400.1	0	0	0	0	0	0	0	0
ENSG00000236185	HNRNPDLP3	0	0	0	0	0	0	0	0
ENSG00000226307	HNRNPDLP1	0	0	0	0	0	0	0	0
ENSG00000235425	RPS7P13	0	0	0	0	0	0	0	0
ENSG00000270583	AL365400.2	0	0	0	0	0	0	0	0
ENSG00000232842	AL137845.2	0	0	0	0	0	0	0	0
ENSG00000242998	RN7SL379P	0	0	0	0	0	0	0	0
ENSG00000203262	AL137845.1	0	0	0	0	0	0	0	0
ENSG00000232069	RPS29P28	0	0	0	0	0	0	0	0
ENSG00000271209	BRDTP1	0	0	0	0	0	0	0	0
ENSG00000223260	RN7SKP194	0	0	0	0	0	0	0	0
ENSG00000229693	AL606530.1	0	0	0	0	0	0	0	0
ENSG00000147202	DIAPH2	6.28618	4.22878	0.92487	1.74285	3.15408	3.18202	1.35614	1.97899
ENSG00000207426	RF00019	0	0	0	0	0	0	0	0
ENSG00000204086	RPA4	0.3759	0.07821	0.20714	0.13947	0.05713	0.16578	0	0.05769
ENSG00000214628	NDUFB5P2	0	0	0	0	0	0	0	0
ENSG00000277900	RF00017	0	0	0	0	0	0	0	0
ENSG00000265260	RN7SL74P	0	0	0	0	0	0	0	0
ENSG00000236256	DIAPH2-AS1	0	0	0	0	0	0	0	0
ENSG00000233887	AL354685.1	0	0	0	0	0	0	0	0
ENSG00000237752	AL354685.2	0	0	0	0	0	0	0	0
ENSG00000234889	AL391666.1	0	0	0	0	0	0	0	0
ENSG00000236243	RPL6P29	0	0	0	0	0	0	0	0
ENSG00000271322	AL357312.1	0	0	0	0	0	0	0	0
ENSG00000213704	EEF1A1P15	0	0	0	0	0	0	0	0
ENSG00000226837	HMGB1P32	0	0	0	0	0	0	0	0
ENSG00000281566	AL157778.1	0	0	0	0	0	0	0	0
ENSG00000270308	AL137843.1	0	0	0	0	0	0	0	0
ENSG00000215070	XRCC6P5	0	0	0	0	0	0	0	0
ENSG00000229946	B3GNT2P1	0	0	0	0	0	0	0	0
ENSG00000252296	RF00402	0	0	0	0	0	0	0	0
ENSG00000236988	AL359641.1	0	0	0	0	0	0	0	0
ENSG00000230592	RPSAP8	0	0	0	0	0	0	0	0
ENSG00000165194	PCDH19	0	0	0	0	0	0	0	0
ENSG00000000005	TNMD	0	0	0	0	0	0	0	0
ENSG00000000003	TSPAN6	0.04849	0	0.0475	0.11994	0.05895	0.08554	0.18859	0.15876
ENSG00000102359	SRPX2	0	0	0	0	0	0	0	0
ENSG00000102362	SYTL4	1.15457	1.03481	0.66705	0.74691	1.21174	0.64412	0.83888	0.72696
ENSG00000223819	PPIAP89	0	0	0	0	0	0	0	0
ENSG00000238116	Z95327.1	0	0.06514	0	0	0	0.06904	0	0
ENSG00000275320	Z95327.2	0	0	0	0	0	0.22607	0	0
ENSG00000101811	CSTF2	1.6423	1.72943	0.49252	0.89536	1.83368	3.42939	0.5028	0.98762
ENSG00000202231	RF00411	0	0	0	0	0	0	0	0
ENSG00000007952	NOX1	0	0	0	0	0	0	0	0
ENSG00000239345	HNRNPA1P26	0	0	0	0	0.08161	0	0	0
ENSG00000233680	HNRNPA1P27	0	0	0	0.11391	0	0	0	0.09424
ENSG00000179031	Z73417.1	0	0	0	0	0	0.16621	0.15704	0
ENSG00000182489	XKRX	0.13043	0	0	0.12905	0.13214	0.15339	0.25364	0.02669
ENSG00000182574	Z97985.1	0	0	0	0	0	0	0	0
ENSG00000174225	ARL13A	0	0	0	0	0	0	0	0
ENSG00000188917	TRMT2B	4.91682	3.55043	2.57663	5.36551	5.86785	5.22987	4.39909	3.63983
ENSG00000225839	TRMT2B-AS1	0	0	0	0.53066	0	0	0	0

ENSG00000126950	TMEM35A	0	0	0	0	0	0	0	0
ENSG00000102384	CENPI	2.08654	2.28713	4.91082	10.8257	3.09361	3.95028	8.85037	5.70683
ENSG00000231643	Z70280.1	0	0	0	0	0	0	0	0
ENSG00000102385	DRP2	0	0.05961	0	0.05315	0	0.07897	0.04477	0.03298
ENSG00000102387	TAF7L	0	0	0.03969	0	0	0	0.09004	0
ENSG00000202024	RNU6-934P	0	0	0	0	0	0	0	0
ENSG00000270813	NANOGNBP3	0	0	0	0	0	0	0	0
ENSG00000183911	RPL21P132	0	0	0	0	0	0	0	0
ENSG00000126953	TIMM8A	0.61339	0.74254	0.12291	0.45517	0.64405	0.59024	0.60416	0.37656
ENSG00000010671	BTK	21.9981	15.4677	3.39034	5.49279	14.0834	6.10521	3.97921	6.96371
ENSG00000241343	RPL36A	0.66569	0.49247	0.10869	0.57631	0.56204	0.68505	0.30822	0.54489
ENSG00000257529	RPL36A-HNRNPH2	0	0.17555	0	0	0	0	0	0
ENSG00000102393	GLA	7.84473	6.02657	2.29512	3.58229	5.30816	4.32083	2.48502	3.83523
ENSG00000126945	HNRNPH2	73.5641	74.3138	9.59395	16.198	55.5225	28.1543	17.7527	35.9907
ENSG00000196440	ARMCX4	0.14824	0.07311	0.05647	0.07333	0.1335	0.12591	0.11897	0.12134
ENSG00000234557	PPIAP90	0	0	0	0	0	0	0	0
ENSG00000237682	AC234775.2	0	0	0	0	0	0	0	0
ENSG00000126947	ARMCX1	0.68473	0.34192	0.10062	0.20324	0.37461	0	0.11413	0.12611
ENSG00000204072	ARMCX7P	0	0	0	0	0	0	0	0
ENSG00000198960	ARMCX6	3.53397	2.64703	1.03862	1.09266	2.21986	2.28595	1.57082	1.44632
ENSG00000102401	ARMCX3	6.29425	5.95795	1.60084	3.20788	4.73018	3.4776	2.73818	4.03384
ENSG00000228275	ARMCX3-AS1	0	0	0	0	0	0	0	0
ENSG00000261101	AC234775.3	1.4526	0.74395	0	0.16583	0.54339	0.19712	0.3725	0
ENSG00000207291	RNU6-30P	0	0	0	0	0	0	0	0
ENSG00000184867	ARMCX2	0.28593	0.034	0.06002	0.09093	0.64559	0.18015	0.2383	0.27583
ENSG00000223753	AC234775.1	0	0	0	0	0	0	0	0
ENSG00000212584	RNU6-587P	0	0	0	0	0	0	0	0
ENSG00000229090	FO082842.1	0	0	0	0	0	0	0	0
ENSG00000178146	AL672207.1	0.18675	0.23314	0.13722	0.27716	0.22705	0	0.15564	0.17197
ENSG00000126952	NXF5	0.06793	0	0	0	0	0	0	0
ENSG00000166432	ZMAT1	0.89748	0.60079	0.20069	0.31851	0.69974	0.20651	0.35773	0.51502
ENSG00000235196	Z68868.1	0	0	0	0	0	0	0	0
ENSG00000252810	RNU6-345P	0	0	0	0	0	0	0	0
ENSG00000235976	Z80107.2	0	0	0	0	0	0	0	0
ENSG00000226119	Z80107.1	0	0	0	0	0	0	0	0
ENSG00000234901	MTND6P13	0	0	0	0	0	0	0	0
ENSG00000184905	TCEAL2	0	0	0	0	0	0	0	0
ENSG00000204071	TCEAL6	0	0	0	0	0	0	0	0
ENSG00000184515	BEX5	0.41374	0.22956	0.10133	0	0.41918	0	0	0.25399
ENSG00000251525	TCP11X3P	0	0	0	0	0	0	0	0
ENSG00000268235	TCP11X1	0	0	0	0	0	0	0	0
ENSG00000269405	NXF2	0	0	0	0	0	0	0	0
ENSG00000273746	AC234791.1	0	0	0	0	0	0	0	0
ENSG00000269437	NXF2B	0	0	0	0	0	0	0	0
ENSG00000215029	TCP11X2	0	0	0	0	0	0	0	0
ENSG00000231590	AC235565.1	0	0	0	0	0	0	0	0
ENSG00000158164	TMSB15A	0	0	0.15725	0.31762	0	0	0	0.52553
ENSG00000176318	FOXN3P1	0	0	0	0	0.06021	0	0	0
ENSG00000196970	NXF4	0	0	0	0	0	0	0	0
ENSG00000125962	ARMCX5	1.25376	0.79501	0.15353	0.44303	1.19766	0.60561	0.87076	0.62307
ENSG00000271147	ARMCX5-GPRASP2	1.37894	0.67507	0.27813	0.52166	1.1834	0.52469	0.67603	0.09959
ENSG00000270050	AL035427.1	0	0	0	0	0	0	0	0
ENSG00000227211	AL035551.1	0	0	0	0	0	0	0	0
ENSG00000198932	GPRASP1	1.27177	0.53884	0.20387	0.7893	2.20683	0.63228	1.42608	0.90849
ENSG00000252294	RNU6-589P	0	0	0	0	0	0	0	0
ENSG00000230320	BEND7P1	0	0	0	0	0	0	0	0.19693
ENSG00000158301	GPRASP2	0.0922	0.18416	0	0.05473	0.33629	0.32531	0.06147	0.22641
ENSG00000198908	BHLHB9	0.19926	0.11055	0.03904	0.09857	0.37145	0.11717	0.17714	0.09786

ENSG00000223546	LINC00630	0.53937	0.74815	0.60766	0.80048	1.3115	1.77617	1.46844	0.72845
ENSG00000232282	MTND1P32	0.31258	0	0	0	0	0.13786	0.13026	0
ENSG00000229954	MTND2P2	0.15513	0	0.22797	0	0	0	0	0
ENSG00000237300	MTCO1P19	0	0	0	0	0	0	0.24436	0
ENSG00000237628	MTCO2P19	0	0	0	0	0	0	0	0
ENSG00000228316	MTATP6P19	0	0.18514	0	0	0	0	0	0
ENSG00000224471	MTCO3P19	0	0	0	0	0	0	0	0
ENSG00000226056	MTND4P32	0	0	0	0	0.07204	0	0	0
ENSG00000234886	MTND5P26	0	0	0	0.12418	0	0.07381	0	0.05137
ENSG00000227649	MTND6P32	0	0	0	0	0	0	0	0
ENSG00000229794	MTCYBP32	0	0.11255	0	0	0	0	0	0.08302
ENSG00000239407	Z68871.1	1.09983	2.04604	1.14084	1.05617	1.73042	1.36959	2.2646	1.11206
ENSG00000102128	RAB40AL	0	0	0	0	0	0	0	0
ENSG00000234050	Z95624.1	0	0	0	0	0	0	0	0
ENSG00000224632	Z73361.1	0	0	0	0	0	0	0	0
ENSG00000133169	BEX1	0.16968	0.42364	0	0.25182	0	0	0	0.10416
ENSG00000147206	NXF3	0.31183	0.0346	0	0	0.05055	0.1467	0	0
ENSG00000226241	Z75746.1	0	0.05866	0	0	0	0	0	0
ENSG00000233071	RPSAP59	0	0	0	0	0	0	0	0
ENSG00000102409	BEX4	15.3997	10.3817	1.45998	4.42352	13.3541	8.08199	4.69217	4.87936
ENSG00000180964	TCEAL8	16.8555	10.5834	3.43768	6.32061	11.5954	12.0635	4.49928	8.69039
ENSG00000224692	Z68694.1	0	0	0	0	0	0	0	0
ENSG00000204065	TCEAL5	0	0	0	0	0	0.11071	0	0
ENSG00000133134	BEX2	1.55826	0.93374	0.09159	0.92505	3.3343	1.31951	1.55842	1.22445
ENSG00000182916	TCEAL7	0	0	0	0	0	0	0	0
ENSG00000238049	Z92846.1	0	0	0	0	0	0	0	0
ENSG00000185222	TCEAL9	2.53232	2.96192	0.40229	0.71102	0.99851	0.84518	0.11408	1.0924
ENSG00000166681	BEX3	27.284	31.2929	5.48543	8.86402	13.285	33.4055	11.6524	11.8329
ENSG00000281091	AL606763.1	0	0	0	0	0	0	0	0
ENSG00000234405	Z69733.1	0	0	0	0	0	0	0	0
ENSG00000172476	RAB40A	0.03649	0	0.13404	0.1083	0	0.03218	0	0.0448
ENSG00000133142	TCEAL4	2.03774	1.88973	0.17111	0.38883	2.33586	1.07845	0.82489	0.64335
ENSG00000279894	Z73965.1	0	0	0	0	0	0	0	0
ENSG00000196507	TCEAL3	2.16244	0.95278	0.18692	0.42476	1.39184	0.72931	0.26503	0.58566
ENSG00000224031	TCEAL3-AS1	0	0	0	0	0	0	0	0
ENSG00000172465	TCEAL1	1.14389	0.53312	0.06724	0.74697	0.83443	0.40359	0.76267	1.06738
ENSG00000180284	AL049610.1	1.57635	0.65596	3.47464	3.50922	0.23956	0	0.98533	1.45157
ENSG00000123562	MORF4L2	12.3736	10.6896	3.41691	4.8477	10.8703	7.71573	4.79862	5.30194
ENSG00000231154	MORF4L2-AS1	0.07028	0.05849	0	0.05215	0.12816	0.12398	0.05857	0.12943
ENSG00000188828	GLRA4	0.05386	0	0	0	0	0.09501	0	0.03306
ENSG00000179363	TMEM31	0.10069	0	0	0	0.12241	0	0	0
ENSG00000123560	PLP1	0	0	0	0	0	0	0	0
ENSG00000123570	RAB9B	0.09779	0.06511	0.02874	0.02903	0.11889	0.17251	0.0978	0.09605
ENSG00000222419	RNA5SP511	0	0	0	0	0	0	0	0
ENSG00000231787	AC234782.2	0	0	0	0	0	0	0	0
ENSG00000276298	AC234782.4	0	0	0	0	0	0	0	0
ENSG00000234345	AC234782.3	0.06994	0	0	0	0	0	0	0
ENSG00000231728	TMSB15B-AS1	0.27956	0.18613	0	0	0.13595	0.19727	0.0932	0
ENSG00000158427	TMSB15B	0.0398	0	0	0	0.04839	0	0.02211	0.01629
ENSG00000230312	AC234782.1	0.07976	0	0	0	0.09697	0	0	0.09793
ENSG00000227067	DPPA3P1	0	0	0	0	0	0	0	0
ENSG00000123569	H2BFWT	0	0	0	0	0	0	0	0
ENSG00000101812	H2BFM	0	0	0	0	0	0	0	0
ENSG00000235946	AC234783.1	0	0	0	0	0	0	0	0
ENSG00000269226	TMSB15B	0	0	0	0	0.02911	0	0	0
ENSG00000269743	SLC25A53	1.19936	0.65951	0.33046	0.39732	0.76813	0.64231	0.39269	0.56536
ENSG00000166707	ZCCHC18	0.25955	0.072	0	0.0642	0.18407	0.11447	0.14421	0.21245
ENSG00000123575	FAM199X	7.22486	4.66306	1.87781	4.2744	5.48539	3.57224	3.29331	4.61028

ENSG00000123576	ESX1	0	0	0	0	0	0	0	0
ENSG00000189108	IL1RAPL2	0	0	0	0	0	0	0	0
ENSG00000232665	PHBP10	0	0	0	0	0	0	0	0
ENSG00000223733	RPL18AP14	0	0	0	0	0	0	0	0
ENSG00000268629	TEX13A	0	0	0	0	0	0	0	0
ENSG00000181819	KCTD9P2	0.07236	0	0.21266	0.10739	0.26392	0	0	0
ENSG00000206864	RNU6-207P	0	0	0	0	0	0	0	0
ENSG00000123572	NRK	0	0	0	0	0	0	0	0
ENSG00000123561	SERPINA7	0	0	0	0	0	0	0	0
ENSG00000230788	AL512661.1	0	0	0	0	0	0	0	0
ENSG00000157502	MUM1L1	0	0	0	0	0	0	0	0
ENSG00000256709	AL133271.1	0	0	0	0	0	0	0	0
ENSG00000202407	RF00019	0	0	0	0	0	0	0	0
ENSG00000224816	NAP1L4P2	0	0	0	0	0	0	0	0
ENSG00000227657	SERPINA7P1	0	0	0	0	0	0	0	0
ENSG00000277673	AL590808.1	0	0	0	0	0	0	0	0
ENSG00000147231	RADX	0.15104	0.35197	0.08878	0.1345	0.23872	0.18652	0.22659	0.31526
ENSG00000263515	MIR548AN	0	0	0	0	1.07369	0	0	0
ENSG00000133135	RNF128	0	0	0	0	0.02597	0	0	0
ENSG00000133138	TBC1D8B	0.17741	0.12839	0.04534	0.03434	0.07502	0.01361	0.03857	0.05682
ENSG00000133131	MORC4	0.49634	0.26195	0.08895	0.26949	0.55928	0.32034	0.24214	0.41617
ENSG00000147223	RIPPLY1	0	0	0	0	0	0	0	0
ENSG00000165376	CLDN2	0	0	0	0	0.20907	0	0	0
ENSG00000231144	EEF1A1P40	0	0	0	0	0	0	0	0
ENSG00000089682	RBM41	4.10895	2.91972	2.5953	5.56321	4.39668	3.43362	6.30828	3.51079
ENSG00000198088	NUP62CL	6.9492	11.8284	17.6542	42.5937	19.5226	14.6142	37.4329	14.7987
ENSG00000213569	AL390039.1	0	0	0	0	0.14585	0	0.19997	0
ENSG00000199832	RF00019	0	0	0	0	0	0	0	0
ENSG00000080572	PIH1D3	0	0	0	0	0	0	0	0
ENSG00000204053	MYCLP1	0	0	0	0	0	0	0	0
ENSG00000215007	DNAJA1P3	0	0	0	0	0	0	0	0
ENSG00000214720	KRT18P49	0	0	0	0	0	0	0	0
ENSG00000227610	FRMPD3-AS1	0	0	0	0	0	0	0	0
ENSG00000147234	FRMPD3	0.2001	0.2931	0.03529	0.11879	0.1557	0.40948	0.05337	0.09827
ENSG00000147224	PRPS1	7.00854	4.01344	1.37005	3.91247	7.3873	8.96108	4.01909	4.26302
ENSG00000157514	TSC22D3	63.5034	59.1099	12.4755	39.4422	55.1603	25.1244	36.044	31.7803
ENSG00000170935	NCBP2L	0.35258	0.17606	0.10362	0.05233	0.08573	0.37319	0.29384	0.17315
ENSG00000080561	MID2	0.35502	0.11819	0.20868	0.24588	0.28775	0.12526	0.1578	0.31965
ENSG00000236064	AL034399.2	0	0	0	0	0	0	0	0
ENSG00000207363	RF00019	0	0	0	0	0	0	0	0
ENSG00000225728	AL034399.1	0	0	0	0	0	0	0	0
ENSG00000277883	NLRP3P1	0	0	0	0	0	0	0	0
ENSG00000170925	TEX13B	0	0	0	0	0	0	0	0
ENSG00000101842	VSIG1	1.6142	0.58269	0.22863	0.46181	2.05708	0.7548	0.45384	0.78798
ENSG00000101843	PSMD10	4.73963	3.72128	2.01498	2.78711	3.51538	5.57424	2.58372	3.11092
ENSG00000101844	ATG4A	4.33241	3.41807	1.104	1.96981	4.07982	3.26924	3.58986	2.12156
ENSG00000197565	COL4A6	0	0	0	0	0	0	0	0
ENSG00000189372	AL034369.1	0	0	0	0	0	0	0	0
ENSG00000188153	COL4A5	0	0.01396	0	0	0.0102	0	0	0
ENSG00000207146	RF00019	0	0	0	1.01669	0	0	0	0
ENSG00000261409	AL035425.3	0	0	0.02156	0	0	0	0	0
ENSG00000260548	AL035425.2	0	0	0	0	0	0	0	0
ENSG00000280322	AL035425.4	0	0	0	0	0	0	0	0
ENSG00000133124	IRS4	0	0	0.02742	0	0	0	0	0
ENSG00000237863	AL035425.1	0	0	0	0	0	0	0	0
ENSG00000201443	RNU6-309P	0	0	0	0	0	0	0	0
ENSG00000275110	MIR6087	0	0	0	0	0	0	0	0
ENSG00000215000	AL034403.1	0	0	0	0	0	0	0	0

ENSG00000101890	GUCY2F	0	0	0	0	0	0	0	0
ENSG00000101888	NXT2	6.24393	5.1766	2.53114	5.60971	4.62453	6.54156	4.18699	5.16954
ENSG00000233874	AL118496.1	0	0	0	0	0	0	0	0
ENSG00000176076	KCNE5	0.14929	0.24849	0	0.07385	0	0.17558	0	0
ENSG00000068366	ACSL4	40.6894	53.6913	8.49622	11.1584	34.8826	16.2673	10.1325	25.6874
ENSG00000237319	AL118496.2	0	0	0	0	0	0	0	0
ENSG00000252069	RF00019	0	0	4.02669	0	0	0	0	0
ENSG00000225381	RPS5P7	0	0	0	0	0	0	0	0
ENSG00000157600	TMEM164	13.7292	15.3831	2.55254	4.46182	9.36788	7.71981	7.86943	5.97428
ENSG00000208013	MIR652	1.49593	0	0	1.11006	0	0	1.24674	0
ENSG00000265584	MIR3978	1.45149	0	0	0	0	0	0	0
ENSG00000101935	AMMECR1	2.99231	3.68872	2.23164	8.26411	5.6074	4.74302	9.05664	4.75123
ENSG00000208883	SNORD96B	0	0	0	0	0	0	0	0
ENSG00000224142	AMMECR1-IT1	0.13066	0.21748	0.192	0	0	0	0.43558	0
ENSG00000133136	GNG5P2	0	0	0	0	0	0	0	0
ENSG00000243978	RTL9	0	0	0	0	0	0	0	0
ENSG00000225366	TDGF1P3	0	0	0	0	0	0	0	0
ENSG00000228042	AL512882.1	0	0	0	0	0	0	0	0
ENSG00000101938	CHRDL1	0	0	0	0	0	0	0	0
ENSG00000077264	PAK3	0.04265	0.0355	0.01045	0.01055	0	0	0	0.00873
ENSG00000226112	FCF1P4	0	0	0	0	0	0	0	0
ENSG00000234349	GLUD1P9	0	0	0	0	0	0	0	0
ENSG00000077274	CAPN6	0	0	0	0	0	0	0	0
ENSG00000077279	DCX	0	0	0	0	0.00873	0	0	0
ENSG00000251954	RNU6-496P	0	0	0	0	0	0	0	0
ENSG00000223904	HMGB1P12	0	0	0	0	0	0	0	0
ENSG00000260802	SERTM2	0	0	0	0	0	0	0	0
ENSG00000229939	AL589880.1	0	0	0	0	0	0	0	0
ENSG00000225031	EIF4BP7	0.44473	0.0673	0.11882	0.12001	0.19662	0.2853	0.06739	0.09928
ENSG00000236189	RPL18AP15	0	0	0	0	0	0	0	0
ENSG00000101901	ALG13	2.46185	1.51989	0.94717	1.82018	2.16588	2.93747	2.02938	1.49482
ENSG00000201420	RNA5SP512	0	0	0	0	0	0	0	0
ENSG00000229487	ALG13-AS1	0	0	0	0	0	0	0	0
ENSG00000072315	TRPC5	0	0	0	0	0	0	0	0
ENSG00000204025	TRPC5OS	0	0	0	0	0	0	0	0
ENSG00000271421	AC005191.1	0	0	0	0	0	0	0	0
ENSG00000271464	DPRXP7	0	0	0	0	0	0	0	0
ENSG00000271400	AL392112.1	0	0	0	0	0	0	0	0
ENSG00000187823	RTL4	0	0	0	0	0	0	0	0
ENSG00000182508	LHFPL1	0	0	0	0	0	0	0	0.10285
ENSG00000200436	RF00019	0	0	0	0	0	0	0	0
ENSG00000213652	HMGB3P30	0	0	0	0	0	0	0	0
ENSG00000126016	AMOT	0.38797	0.31571	0.11402	0.16634	0.44024	0.13689	0.04311	0.17995
ENSG00000283775	MIR4329	0	0	0	0	0	0	0	0
ENSG00000243011	RN7SL266P	0	0	0	0	0	0	0	0
ENSG00000252930	RNU6-1015P	0	0	0	0	0	0	0	0
ENSG00000235329	AL031183.1	0	0	0	0	0	0	0	0
ENSG00000220541	AC233289.1	0	0	0	0	0	0	0	0
ENSG00000241743	XACT	0	0	0.21673	0	0	0	0	0
ENSG00000270524	QTRT1P1	0	0	0	0	0	0	0	0
ENSG00000201674	RF00012	0	0	0	0	0	0	0	0.84109
ENSG00000207220	RNU1-57P	0	0	0	0	0	0	0	0
ENSG00000240327	RN7SL93P	0	0	0	0	0	0	0	0
ENSG00000212611	RF00088	0	0	0	0	0	0	0	0
ENSG00000230502	AL953862.1	0	0	0	0	0	0	0	0
ENSG00000271686	AL953862.2	0	0	0	0	0	0	0	0
ENSG00000226547	SSU72P1	0	0	0	0	0	0	0	0
ENSG00000147246	HTR2C	0	0	0	0	0	0.02671	0	0

ENSG00000224664	RPL36AP53	0	0	0	0	0	0	0	0
ENSG00000208839	SNORA35	0	0	0	0	0	0	0	0
ENSG00000212100	MIR764	0	0	0	0	0	0	0	0
ENSG00000222321	MIR1912	0	0	0	0	0	0	0	0
ENSG00000276158	MIR1264	0	0	0	0	0	0	0	0
ENSG00000221710	MIR1298	0	0	0	0	0	0	0	0
ENSG00000222715	MIR1911	0	0	0	0	0	0	0	0
ENSG00000224773	HSPA8P7	0	0	0	0	0	0	0	0
ENSG00000199001	MIR448	0	0	0	0	0	0	0	0
ENSG00000270413	AL355812.1	0	0	0	0	0	0	0	0
ENSG00000123496	IL13RA2	0	0	0	0	0	0	0	0
ENSG00000222122	RNU6-648P	0	0	0	0	0	0	0	0
ENSG00000238223	AL121878.1	0	0	0	0	0	0	0	0
ENSG00000236080	YAP1P2	0	0	0	0	0	0	0	0
ENSG00000130224	LRCH2	0	0	0	0.04414	0	0	0	0
ENSG00000271907	SNORA35B	0	0	0	0	0	0	0	0
ENSG00000212241	RF00019	0	0	0	0	0	0	0	0
ENSG00000175718	RBMXL3	0	0	0	0	0	0	0	0
ENSG00000235656	RPL36P18	0	0	0	0	0	0	0	0
ENSG00000102021	LUZP4	0	0	0	0	0	0	0	0
ENSG00000274374	AC239600.2	0	0	0	0	0	0	0	0
ENSG00000271021	AC239600.1	0	0	0	0	0	0	0	0
ENSG00000271826	PLS3-AS1	0	0	0	0	0	0	0	0
ENSG00000252441	RF00264	0	0	0	0	0	0	0	0
ENSG00000102024	PLS3	0.53647	0.47625	0.07883	0.21232	0.4783	0.50475	0.11923	0.1976
ENSG00000270431	AC005000.2	0	0	0	0	0	0	0	0
ENSG00000227881	ASS1P5	0	0	0	0	0	0	0	0
ENSG00000234785	EEF1GP5	0	0	0	0	0	0	0	0
ENSG00000228532	AC005000.1	1.01806	0.42364	0	0	0	0	0	0.31249
ENSG00000229335	DANT1	0	0	0	0	0	0	0	0
ENSG00000235244	DANT2	0	0	0	0	0	0	0	0
ENSG00000266828	RN7SL712P	0	0.39613	0	0	0	0	0	0
ENSG00000231371	AKR1B1P8	0	0	0	0	0	0	0	0
ENSG00000234558	API5P1	0	0	0	0.07204	0	0.08564	0	0
ENSG00000180772	AGTR2	0	0	0	0	0	0	0	0
ENSG00000207033	RNU6-154P	0	0	0	0	0	0	0	0
ENSG00000268104	SLC6A14	0	0	0	0	0	0	0	0
ENSG00000204019	CT83	0	0	0	0	0	0	0	0
ENSG00000236077	BX119904.3	0	0	0	0	0	0	0	0
ENSG00000237860	BX119904.4	0	0	0	0	0	0	0	0
ENSG00000230159	BX119904.2	0	0	0	0	0	0	0	0
ENSG00000224217	BX119904.1	0	0	0	0	0	0	0	0
ENSG00000206752	RNU6-1323P	0	0	0	0	0	0	0	0
ENSG00000213526	SETP8	0.11672	0	0	0.17323	0	0.41182	0	0
ENSG00000238066	AC005002.2	0	0	0	0	0	0	0	0
ENSG00000224314	AC005002.1	0	0	0	0	0	0	0	0
ENSG00000279587	AC006963.1	0	0	0	0	0	0	0	0
ENSG00000003096	KLHL13	0.08398	0.10483	0.01543	0.01558	0.06381	0.01852	0.17497	0.09022
ENSG00000200291	RF00019	0	0	1.05602	2.13305	0	0	0	0
ENSG00000237458	TUBB4BP3	0	0	0	0	0	0	0	0
ENSG00000234524	RPL12P43	0	0	0	0	0	0	0	0
ENSG00000270619	AL391358.1	0	0	0	0	0	0	0	0
ENSG00000131725	WDR44	14.9148	13.821	6.99807	13.3329	14.3579	13.479	12.3576	10.6874
ENSG00000221463	MIR1277	0	0	0	0	0	0	0	0
ENSG00000147251	DOCK11	58.3371	47.8159	13.5424	27.8074	46.6334	40.4232	20.0695	33.61
ENSG00000206862	RF00019	0	0	0	0	0	0	0	0
ENSG00000230399	RBBP8P1	0	0	0	0	0	0	0	0
ENSG00000131724	IL13RA1	50.7503	49.8694	6.38104	10.7243	34.6316	7.1577	14.8394	23.0962

ENSG00000239182	RF00566	1.14532	0	0	0	0	0	0	0
ENSG00000250995	AL391280.1	0.62119	1.37863	0.10143	0.10243	0.25174	0.36529	0.23009	0.42371
ENSG00000174460	ZCCHC12	0.03286	0	0	0	0	0	0	0
ENSG00000203650	LINC01285	0	0	0	0	0	0	0	0
ENSG00000224341	AC006965.1	0	0	0	0	0	0	0	0
ENSG00000231270	AL772284.1	0	0	0	0	0	0	0	0
ENSG00000175556	LONRF3	0.79812	0.8152	0.07997	0.21536	0.50722	0.352	0.27212	0.26725
ENSG00000277535	AL772284.2	0	0	0	0	0	0	0	0
ENSG00000250423	KIAA1210	0	0	0	0	0	0	0	0
ENSG00000227864	ARL5AP1	0	0	0	0	0	0	0	0
ENSG00000230170	HNRNPA1P28	0	0.12696	0	0	0	0	0	0
ENSG00000270625	AC004835.2	0	0	0	0	0	0	0	0
ENSG00000101856	PGRMC1	55.4241	29.31	13.4151	19.1041	25.0307	34.7033	26.2315	34.5169
ENSG00000214992	AKAP17BP	0	0	0	0	0	0	0	0
ENSG00000230392	AC004835.1	0	0	0	0	0	0	0	0
ENSG00000226661	AC004973.1	0	0	0	0	0	0	0	0
ENSG00000227814	MRPS17P9	0	0	0	0	0	0	0	0
ENSG00000077713	SLC25A43	2.4002	1.72339	0.41495	0.97785	1.85958	0.91328	0.70602	0.86675
ENSG00000237903	AC004000.1	0	0	0	0	0.61744	0	0	0
ENSG00000207175	RNU1-67P	0	0	0.65679	0	0	0	0	0
ENSG00000266420	RN7SL118P	0.24272	0	0	0.72044	0	0.42818	1.21371	0.298
ENSG00000252147	RNY3P16	0	0	0	0	0.88234	0	0	0
ENSG00000224281	SLC25A5-AS1	0.28698	0.23884	0.07029	0.03549	0.17445	0.4219	0.11959	0.05873
ENSG00000005022	SLC25A5	35.5262	22.5309	5.09846	11.1687	26.3191	47.3281	13.3584	16.8594
ENSG00000018610	CXorf56	6.22201	4.63443	0.75149	2.10825	4.76671	5.21257	2.93612	2.47666
ENSG00000077721	UBE2A	6.12689	5.43442	1.57202	2.32994	4.74636	4.87738	1.31999	2.88277
ENSG00000186416	NKRF	1.611	0.94462	0.32282	1.11394	2.2702	2.48677	1.15955	1.41604
ENSG00000125354	SEPT6	20.5405	10.4718	2.91492	7.12464	16.6675	32.6541	7.70841	7.78197
ENSG00000211578	MIR766	0	0	0	0	0	0	1.10072	0
ENSG00000279601	AC005052.1	0	0	0	0	0	0	0	0
ENSG00000187808	SOWAHD	0.70844	1.65089	0.0694	0.14019	0.34452	0.58324	0.23617	0.57988
ENSG00000198918	RPL39	26.7387	24.6182	42.9145	50.0994	32.7163	35.0308	60.2627	93.2758
ENSG00000206622	SNORA69	0	0	0	0	0	0	0	0
ENSG00000125351	UPF3B	3.18801	2.45522	0.24472	1.16518	2.97923	2.09854	1.50693	1.48973
ENSG00000125352	RNF113A	7.9639	7.93551	1.51261	2.09049	8.03565	5.63887	2.52849	2.32808
ENSG00000125356	NDUFA1	25.8369	27.3962	11.9526	20.3086	18.4981	25.1533	19.938	27.7276
ENSG00000186471	AKAP14	0	0	0	0	0	0	0	0
ENSG00000101882	NKAP	1.87569	1.79288	0.87328	1.59857	2.1901	1.49067	1.37751	1.74431
ENSG00000282933	RHOXF1P3	0.02746	0.04571	0	0	0	0	0	0.03372
ENSG00000282850	RHOXF1P2	0	0	0	0	0	0	0	0
ENSG00000234493	RHOXF1P1	2.84846	0.79021	0.13953	0.14091	1.38523	0.1675	0	0.69946
ENSG00000258545	RHOXF1-AS1	0	0.0489	0	0	0.03572	0	0	0.07214
ENSG00000203989	RHOXF2B	0	0	0	0	0	0	0	0
ENSG00000101883	RHOXF1	0	0	0	0	0	0	0	0
ENSG00000228139	LINC01402	0	0	0	0	0	0	0	0
ENSG00000233382	NKAPP1	0.75075	1.43896	0.60175	1.51935	1.63187	1.12375	0.75841	1.4804
ENSG00000131721	RHOXF2	0	0	0	0	0	0	0	0
ENSG00000227541	SFR1P1	0.10654	0	0	0	0.12953	0.56386	0.17759	0.39243
ENSG00000237757	EEF1A1P30	0.05574	0.09278	0	0	0.06777	0.09834	0.27874	0.13688
ENSG00000177485	ZBTB33	11.1337	8.74934	2.33147	5.67168	11.2214	9.44344	4.94424	6.03029
ENSG00000125355	TMEM255A	0.095	0.18975	0.19544	0.08459	0	0.13407	0	0.02333
ENSG00000232042	AL512286.1	0	0	0	0	0	0	0	0
ENSG00000101892	ATP1B4	0	0	0	0	0	0	0	0
ENSG00000005893	LAMP2	54.1197	52.1285	9.72661	15.0599	38.3011	19.1651	19.0147	32.3116
ENSG00000251707	RNU7-37P	0	0	0	0	0	0	0	0
ENSG00000158290	CUL4B	15.7318	14.5244	3.81465	6.52931	13.6128	9.87632	6.6034	9.47203
ENSG00000232119	MCTS1	2.81626	3.13945	1.02512	1.6105	2.82711	2.96273	1.46427	2.22058
ENSG00000171155	C1GALT1C1	10.8	12.5084	2.35341	3.59572	8.0879	8.69323	1.57431	6.60483

ENSG00000278317	AC011890.2	0	0	0	0	0	0	0	0
ENSG00000181511	AC011890.1	0	0	0	0	0	0	0	0
ENSG00000237828	PA2G4P1	0	0	0	0	0	0	0	0
ENSG00000236446	CT47B1	0	0	0	0	0	0	0	0
ENSG00000271346	AC008162.1	0	0	0	0	0	0	0	0
ENSG00000278646	AC008162.2	0	0	0	0	0	0	0	0
ENSG00000226685	CT47A12	0	0	0	0	0	0	0	0
ENSG00000270678	AL670379.6	0	0	0	0	0	0	0	0
ENSG00000226929	CT47A11	0	0	0	0	0	0	0	0
ENSG00000270493	AL670379.4	0	0	0	0	0	0	0	0
ENSG00000224089	CT47A10	0	0	0	0	0	0	0	0
ENSG00000271154	AL670379.10	0	0	0	0	0	0	0	0
ENSG00000226600	CT47A9	0	0	0	0	0	0	0	0
ENSG00000271041	AL670379.9	0	0	0	0	0	0	0	0
ENSG00000230347	CT47A8	0	0	0	0	0	0	0	0
ENSG00000270397	AL670379.2	0	0	0	0	0	0	0	0
ENSG00000228517	CT47A7	0	0	0	0	0	0	0	0
ENSG00000271502	AL670379.12	0	0	0	0	0	0	0	0
ENSG00000226023	CT47A6	0	0	0	0	0	0	0	0
ENSG00000271211	AL670379.11	0	0	0	0	0	0	0	0
ENSG00000237957	CT47A5	0	0	0	0	0	0	0	0
ENSG00000270736	AL670379.7	0	0	0	0	0	0	0	0
ENSG00000230594	CT47A4	0	0	0	0	0	0	0	0
ENSG00000270771	AL670379.8	0	0	0	0	0	0	0	0
ENSG00000236126	CT47A3	0	0	0	0	0	0	0	0
ENSG00000270646	AL670379.5	0	0	0	0	0	0	0	0
ENSG00000242362	CT47A2	0	0	0	0	0	0	0	0
ENSG00000270456	AL670379.3	0	0	0	0	0	0	0	0
ENSG00000236371	CT47A1	0	0	0	0	0	0	0	0
ENSG00000270251	AL670379.1	0	0	0	0	0	0	0	0
ENSG00000233433	AC006144.1	0	0	0	0	0	0	0	0
ENSG00000182890	GLUD2	0.0294	0.09788	0	0.04364	0	0.05187	0.04901	0
ENSG00000234176	HSPA8P1	0.03999	0	0	0	0	0	0	0
ENSG00000265456	MIR3672	0	0	0	0	0	0	0	0
ENSG00000213498	TPT1P13	0	0	0	0	0	0	0	0
ENSG00000260585	AL359851.1	0	0	0.02156	0	0	0	0	0
ENSG00000237891	AL441985.1	0	0	0	0	0	0	0	0
ENSG00000280142	AL357562.1	0	0	0	0	0	0	0	0
ENSG00000232496	RPL3P12	0	0	0	0	0	0	0	0
ENSG00000212321	RF00012	0	0	0	0	0	0	0	0
ENSG00000224236	MRRFP1	0	0	0	0	0	0.0772	0	0
ENSG00000125675	GRIA3	0	0.28225	0	0	0.06872	0	0	0.01388
ENSG00000179101	AL590139.1	0.18325	0	0	0.27196	0	0.32328	0	0
ENSG00000229733	AL030996.1	0	0	0	0	0	0	0	0
ENSG00000228466	TUBB4AP1	0	0	0	0	0	0	0	0
ENSG00000125676	THOC2	9.97832	8.904	5.00995	10.4795	11.0561	9.12241	7.95355	8.44188
ENSG00000232149	FERP1	0	0	0	0	0	0	0	0
ENSG00000230376	MEMO1P4	0	0	0	0	0	0	0	0
ENSG00000252178	RNU7-69P	0	0	0	0	0	0	0	0
ENSG00000226902	CHCHD2P1	0	0	0	0	0	0	0	0
ENSG00000264933	RN7SL190P	0	0	0	0	0	0	0	0
ENSG00000199705	RF00019	0	0	0	0	0	0	0	0
ENSG00000101966	XIAP	10.7209	9.7742	2.93603	4.96617	8.0674	7.04946	4.11555	7.34934
ENSG00000237331	XIAP-AS1	0	0	0	0	0	0	0	0
ENSG00000252627	RNU6-122P	0	0	0	1.01669	0	0	1.14187	2.52328
ENSG00000237025	AL121601.2	0	0	0	0	0	0	0	0
ENSG00000232412	AL121601.1	0.22485	0.37426	0.33041	0.3337	1.36682	0.39666	0.74957	0.55213
[truncated: 576,781 more chars]
